# Genes Possessing the Most Frequent DNA DSBs Are Highly Associated with Development and Cancers, and Essentially Overlap with the rDNA-Contacting Genes

**DOI:** 10.3390/ijms23137201

**Published:** 2022-06-28

**Authors:** Nickolai A. Tchurikov, Ildar R. Alembekov, Elena S. Klushevskaya, Antonina N. Kretova, Ann M. Keremet, Anastasia E. Sidorova, Polina B. Meilakh, Vladimir R. Chechetkin, Galina I. Kravatskaya, Yuri V. Kravatsky

**Affiliations:** 1 Department of Epigenetic Mechanisms of Gene Expression Regulation, Engelhardt Institute of Molecular Biology Russian Academy of Sciences, 119334 Moscow, Russia; alembeki@gmail.com (I.R.A.); giedre@inbox.ru (E.S.K.); tonya_kretova@mail.ru (A.N.K.); annakeremet@yandex.ru (A.M.K.); sidorova.anastasiya.2003@mail.ru (A.E.S.); polina.meylakh@gmail.com (P.B.M.); vladimir_chechet@mail.ru (V.R.C.); galina.kravatskaya@gmail.com (G.I.K.); jiri@eimb.ru (Y.V.K.); 2 Center for Precision Genome Editing and Genetic Technologies for Biomedicine, Engelhardt Institute of Molecular Biology Russian Academy of Sciences, 119334 Moscow, Russia

**Keywords:** DNA double strand breaks (DSBs), whole-genome analysis, 4C-rDNA, rDNA-contacting genes, HEK293T, epigenetics, differentiation, morphogenesis, cancers

## Abstract

Double-strand DNA breakes (DSBs) are the most deleterious and widespread examples of DNA damage. They inevitably originate from endogenous mechanisms in the course of transcription, replication, and recombination, as well as from different exogenous factors. If not properly repaired, DSBs result in cell death or diseases. Genome-wide analysis of DSBs has revealed the numerous endogenous DSBs in human chromosomes. However, until now, it has not been clear what kind of genes are preferentially subjected to breakage. We performed a genetic and epigenetic analysis of the most frequent DSBs in HEK293T cells. Here, we show that they predominantly occur in the active genes controlling differentiation, development, and morphogenesis. These genes are highly associated with cancers and other diseases. About one-third of the genes possessing frequent DSBs correspond to rDNA-contacting genes. Our data suggest that a specific set of active genes controlling morphogenesis are the main targets of DNA breakage in human cells, although there is a specific set of silent genes controlling metabolism that also are enriched in DSBs. We detected this enrichment by different activators and repressors of transcription at DSB target sites, as well breakage at promoters. We propose that both active transcription and silencing of genes give a propensity for DNA breakage. These results have implications for medicine and gene therapy.

## 1. Introduction

The purpose of DNA is the storage and realization of genetic information. For several normal cellular DNA-based processes there is a need for DNA breakage, either to overcome the topological constraints of DNA, e.g., to reduce the torsional stress of DNA for transcription or replication [1], or for recombination. Therefore, both integrity and physiological breakage are natural attributes of DNA. DNA is continuously damaged during transcription at R-loops, when single DNA strands are targeted by DNA deaminases, resulting in DSB formation, or when DSBs are formed at stalled replication forks [2,3]. Endogenous DSBs are usually repaired with high fidelity. If they are not correctly repaired, deletions, translocations, or DNA fusions are formed, leading either to apoptosis or to the transformation of healthy cells to cancer cells [4]. Even a single unrepaired DSB can lead to cell death.

It has been estimated that the spontaneous rate of DSB production is about 50 per cell, per cell cycle, or about one DSB in 100 Mb [4]. The ligation-mediated PCR approach for the genome-wide mapping and analysis of endogenous DSBs revealed the hot spots of DSBs in different genomic regions, including nine in vivo hot spots of DSBs (Pleiades) in the intergenic spacer of human rDNA clusters [5,6,7]. This approach detects DSBs by taking a “snapshot” of all existing DSBs and includes DSBs that should be repaired, providing data on all sites of DNA breakage. Detected by this approach, hot spots of DSBs in eukaryotic cells delimit 50–100 kb of DNA fragments, which can be observed in the pulsed-field gels or visualized in a single-cell comet assay [8]. The data suggest that unrepaired DSBs occur at high frequencies. We suppose that the spread of DNA breakage sites reflects the expression patterns and organization of human chromosomes and could be used for the analysis of genes that are subjected to the most frequent DNA breakages and their possible relation to diseases.

The data suggest a strong link between DSBs, cancers, and neurodegenerative disorders, as well as several other diseases [9,10,11]. Genome-wide analysis of genes that are the main targets of DSBs was therefore important, including their roles in different biological processes and associations with diseases.

Here, we report that DSBs preferentially target actively expressed genes that are involved in differentiation, development, and morphogenesis. There are extremely high associations of these genes with different cancer types and several neurological and mental diseases. About one-third of these genes shape frequent contacts with rDNA clusters. Surprisingly, breakages also occur in specific sets of silenced genes, suggesting mechanisms of breakage of compact chromatin regions. Taken together, our data demonstrate that the formation of DSBs is tightly linked with the mechanisms of gene expression regulation and diseases.

## 2. Results

### 2.1. Nature of the Genes That Are the Main Targets of DSBs in HEK293T Cells

**Figure 1:**
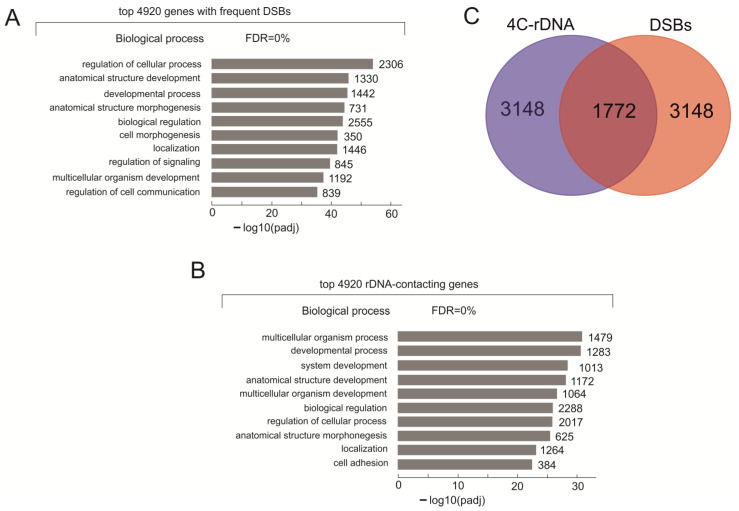
**The characterization of the genes frequently targeted by DSBs in HEK293T cells**. (**A**) The top ten Gene Ontology (GO) biological process associations of genes subjected to breakage were determined using Profiler (https://biit.cs.ut.ee/gprofiler/gost, accessed 28 June 2022). The values to the right of the bars show the number of genes associated with a process. The complete list of the corresponding genes is shown in Table S1. (**B**) The top ten Gene Ontology biological process associations of 4920 rDNA-contacting genes (4C-rDNA) selected after DFAM filtering of 4C-rDNA reads and selection of about 16% of top genes that form the most frequent contacts with rDNA (see Methods). The values to the right of the bars show the number of genes associated with a process. The complete list of the corresponding genes is shown in Table S2. (**C**) A Venn diagram showing the intersections between 4920 genes targeted by DSBs and 4920 rDNA-contacting genes. Table S3 shows the list of overlapping genes.

To identify genes subjected to the most frequent breakage, we performed genome-wide mapping of DSBs. The previously described technique using the ligation of the biotinylated adapter at DSBs sites was utilized (Figure S1, see Supplementary Materials). About 200 million paired-end reads were processed and DSB sites were mapped in two human genome versions (hg19 and 38), as described in the Methods section. The reads that mapped completely to low complexity and/or repeat regions present in the DFAM database were removed from the genome-wide mapping. The genes most frequently targeted by DSBs were selected (4920 genes). They corresponded to the top 2.5% of genes possessing DSBs. To uncover the nature of these genes we used the Gene Ontology (GO) search and revealed that these genes were extremely highly associated with GO Biological Process items (padj up to 10^−53^, Table S1) relating to development and morphogenesis (Figure 1A). Among these genes were 12 *HOX* genes and 86 *ZNF* genes, which are members of the *PAX* family genes, and others involved in development and transcriptional regulation. Previously, it was described that rDNA-contacting genes are mainly involved in development and morphogenesis [12]. For this study, we also performed the DFAM filtering to remove 4C-rDNA reads that were mapped completely to low complexity and/or repeat regions and performed a new genome-wide mapping. Figure 1B shows that the top 16% of rDNA-contacting genes (4920 genes) are also highly associated with similar biological processes (Table S2 shows the names of the genes). For this reason, we decided to compare two gene lists. Of the ten top processes shown in Figure 1A,B, six are common to the two sets of genes. Direct comparison of the two gene lists revealed that about 36% of the genes are common to both (1772 genes, Figure 1C). The names of the overlapping genes are shown in Table S3. This was unexpected because the genes were selected using different and independent procedures by mapping of DSBs and rDNA contacts. According to hypergeometric tests, two randomly generated gene lists of 4920 genes intersected in 464 genes from a total of 57,736 human genes, with a *p*-value < 0.01 (in 450 genes with a *p*-value < 0.05). The results shown in Figure 1C indicate that specific sets of genes targeted by DSBs shape the frequent contacts with rDNA clusters. GO search for this group of 1772 genes also revealed that they are involved in development and morphogenesis (Figure S2, Table S4).

### 2.2. Associations of the Genes That Are Targeted by DSBs with Diseases

Next, we searched for possible associations of the genes subjected frequently to breakage with diseases. It was described previously that DSBs can lead to cancers and other diseases [9,10,11]. Nevertheless, the genome-wide identification and analysis of the corresponding genes affected by DSBs have not been performed yet. We performed a search in the Jensen Diseases library (Enrichr databases) [13]. Table 1 shows 32 diseases that are associated with the selected genes with frequent DSBs. They include 14 cancers and six diseases of the nervous system (schizophrenia, cognitive disorders, Alzheimer’s disease, Parkinson’s disease, and some others). Table S5 shows the genes associated with these diseases.

### 2.3. Frequent DSBs and rDNA Contacts in Both Silent and Active Genes

**Figure 2:**
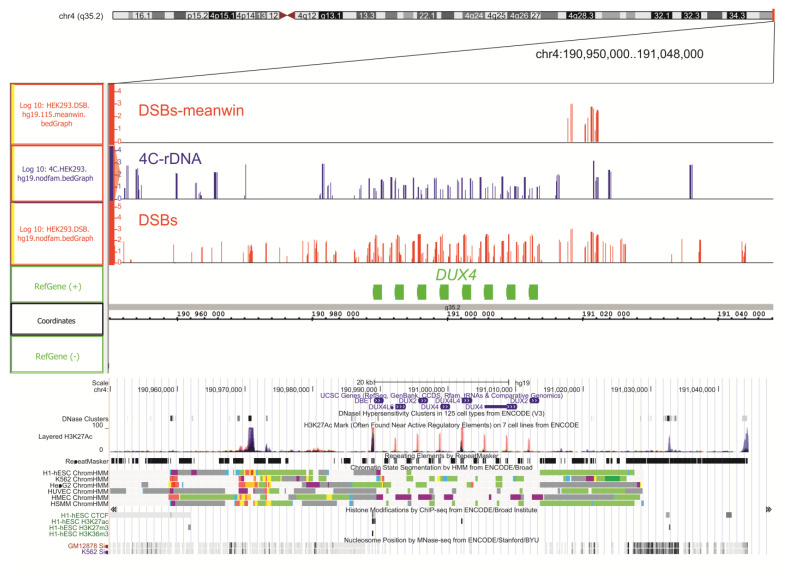
**The targets of DSBs and rDNA contacts at the cluster of *DUX4* genes in chr4.** The top half shows the sites of DSBs and rDNA-contacting sites inside and around the cluster of *DUX4* genes (shown in red and in blue, respectively) in IGB Browser. The upper lane, designated “DSBs meanwin”, shows the log10 values of the top 2.5% DSBs that delimit about 115 kb regions (see Methods). The lower lane, designated “DSBs”, shows the log10 values of all DSBs. The lane designated “4C-rDNA” shows the log10 values of all rDNA-contacting sites The distribution of DNaseI hypersensitive sites, layered H3K27ac marks, genome segmentation from ENCODE, histone modifications, and nucleosome positions inside the same region of chr4 are shown as in the UCSC Browser.

We selected two genes that are targeted by multiple DSBs for a more detailed analysis. The first example is the cluster of *DUX4* genes from the sub-telomeric region of chromosome 4q. The genes encode two homeoboxes that function as transcriptional activators of paired-like homeodomain transcription factor 1. The genes are associated with autosomal dominant facioscapulohumeral muscular dystrophy. Our RNA-Seq analysis revealed that, in HEK293T cells, the genes are completely silent. We observed that numerous DSBs (from a single up to one hundred DSBs at a particular site) are located inside the cluster and around it (Figure 2). At the same time, in the DSBs meanwin lane only, the most frequent DSBs (up to one thousand in a 1 kb window, see Methods) located downstream from the cluster are shown. The log10 presentation of DSBs allows observation of the much less frequent DSBs, which in a non-logarithmic scale is difficult to see. It follows that the hot spots of DSBs delimit 50–150 kb DNA domains (Figure S1), while rarer DSBs are scattered inside these domains non-randomly, at specific sites that possess particular structural or functional properties (Figure 2). We suppose that these rarer DSBs reflect the sum of events occurring in different cells.

We did not expect to detect frequent DSBs inside the silenced genes, because it was shown previously that more compact chromatin is more protected from DSBs [14,15]. Of note, this occurred practically in the same regions where numerous rDNA contacts were detected. The data suggest a strong link between a specific set of rDNA contacts and DNA breakage.

**Figure 3:**
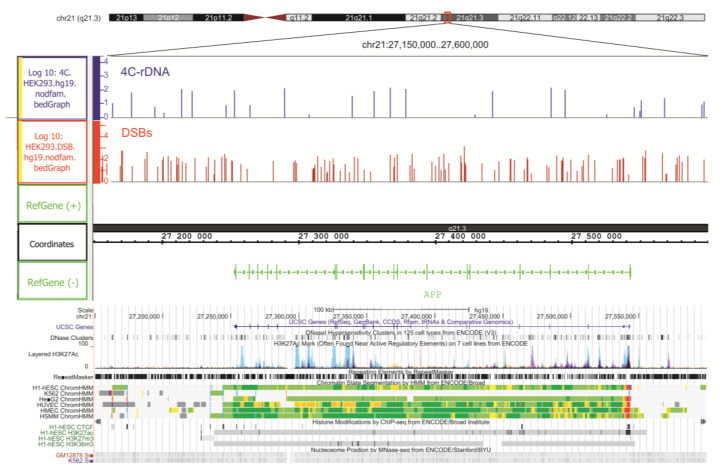
**The targets of DSBs and rDNA contacts at the *APP* gene in chr21.** The top half shows the sites of all DSBs and rDNA contacts inside and around the *APP* gene in IGB Browser. The distribution of DNaseI hypersensitive sites, layered H3K27ac marks, genome segmentation from ENCODE, histone modifications, and nucleosome positions inside the same region of chr21 are shown, as in the UCSC Browser.

Another example is actively transcribed in HEK293T cells: gene–*APP* (Figure 3). The gene encodes a protein that is cleaved to give a number of peptides. Some of them are involved in transcriptional activation, while others are involved in the formation of amyloid plaques found in the brains of patients with Alzheimer’s disease [16]. Inside the gene, we observed a high density of DSBs and many contact sites of rDNA. The data on genome segmentation from ENCODE showed that in five cell lines (H1-hESC, HepG2, HUVEC, HMEC, and HSMM) the gene is actively transcribed, while in K562 erythroleukemia cells are silenced (Figure 3). Again, we observed the link between DSBs and rDNA contacts in HEK293T cells, but now in the active genes. These data suggest the possible involvement of rDNA clusters in activations or silencing of particular genes coupled with DNA breakage inside the corresponding genes.

### 2.4. Expression Patterns of Genes Frequently Targeted by DSBs in HEK293T Cells

**Figure 4:**
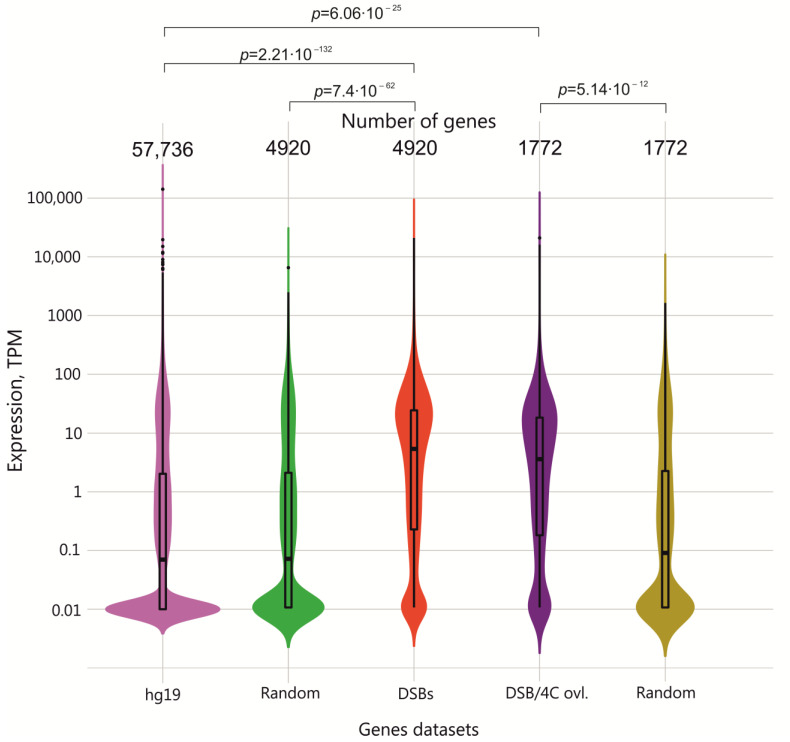
**Analysis of expression patterns of genes possessing the most frequent DSBs**. Violin plots showing the distribution of genes along with their expression levels for all HEK293T genes (light violet), random genes (green or olive), DSBs (red), and overlapping genes between 4C-rDNA and DSBs (dark violet). The numbers of corresponding genes are shown at the top. TPM, transcripts per million.

The results observed with *DUX* and *APP* genes raised a question—how many genes with the most frequent breakage are actively transcribed or silenced? To check this, we used the violin presentations for our RNA-Seq data. Figure 4 shows that, for all human genes, the mean value of expression is below 0.1 TPM, (transcripts per million, see Table S6.) because there are so many silenced genes in HEK293T cells, while for the set of 4920 genes with the most frequent breakage, the value is about 8 TPM. The data indicated that genes with frequent DSBs are much more actively transcribed, probably because they reside in open chromatin regions. The 1772 overlapping genes between genes with DSBs and rDNA-contacting genes had a value of about 5 TPM, which was much higher than the same amount of randomly generated genes.

At the same time, we detected a fraction of silenced genes (between 0 and 0.01 TPM): 719 genes among the DSB genes (see Table S7) that were mainly associated with metabolic processes and detection of stimulus. Thereafter, we concluded that the high level of DNA breakage is characteristic of specific functional sets of active and silenced genes. The data also suggest that different chromatin states are characteristic of these sets of active and silent genes that are subjected to frequent DSB formation. Following this, we undertook a made search for genetic and epigenetic properties at the sites of these DSBs.

### 2.5. Genetic Annotations of DSBs Mapped in HEK293T Cells

**Figure 5:**
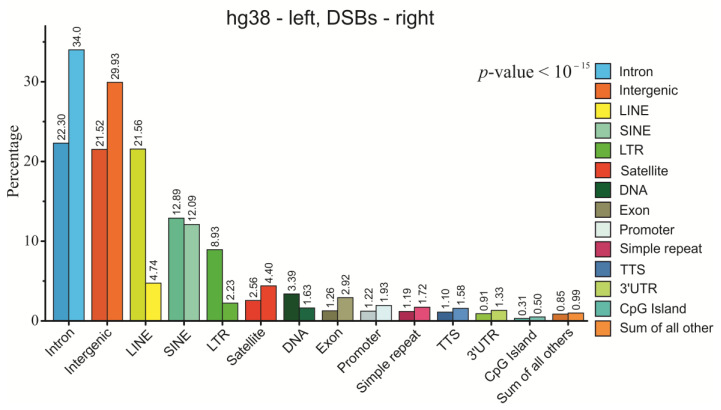
**Distribution of DSBs in different portions of the human genome.** Percentages of DSB targets in different portions of the whole genome (left darker bars, hg38) and at DSB targets (right brighter bars). The values in the labels represent a percentage of the corresponding portion. The statistical significance of differences for each annotation column pair was assessed by T-test for two independent means. The differences in all column pairs are significant at the level *p* < 10^−15^.

Our data demonstrate that the most frequent DSBs are not randomly distributed in the human genome and attack specific sets of both active and silent genes. For this reason, we studied the preferences in the distribution of all mapped DSBs in different portions of the genome, not only in genes. Figure 5 shows that DSBs are enriched in introns (34%) and intergenic sequences (about 30%). LINEs, LTR-element, and transposons are depleted with DSBs. The data suggest that DSBs occur preferentially in genes, including their introns, promoters, transcription start sites (TSS), exons, and 3′ UTRs. We suppose that such a distribution indicates a link between frequent DSBs and genic transcription, which makes the corresponding areas vulnerable to breakage.

### 2.6. Epigenetic Profiles at the Targets of DSBs in HEK293T Cells

Our data on the distribution of DSBs in active and silent chromatin regions led us to suppose that breakage should occur at particular chromatin regions, decorated by specific active or repressive epigenetic marks. We, therefore, performed a study of the distribution of binding sites of different factors around DSBs. For this study, we used the top 2.5% of DSBs that mainly delimit the 50–150 kb DNA fragments used for ligation of biotinylated adapters in the DBS library preparation procedure (Figure S1) and Q1 DSBs (25%).

**Figure 6:**
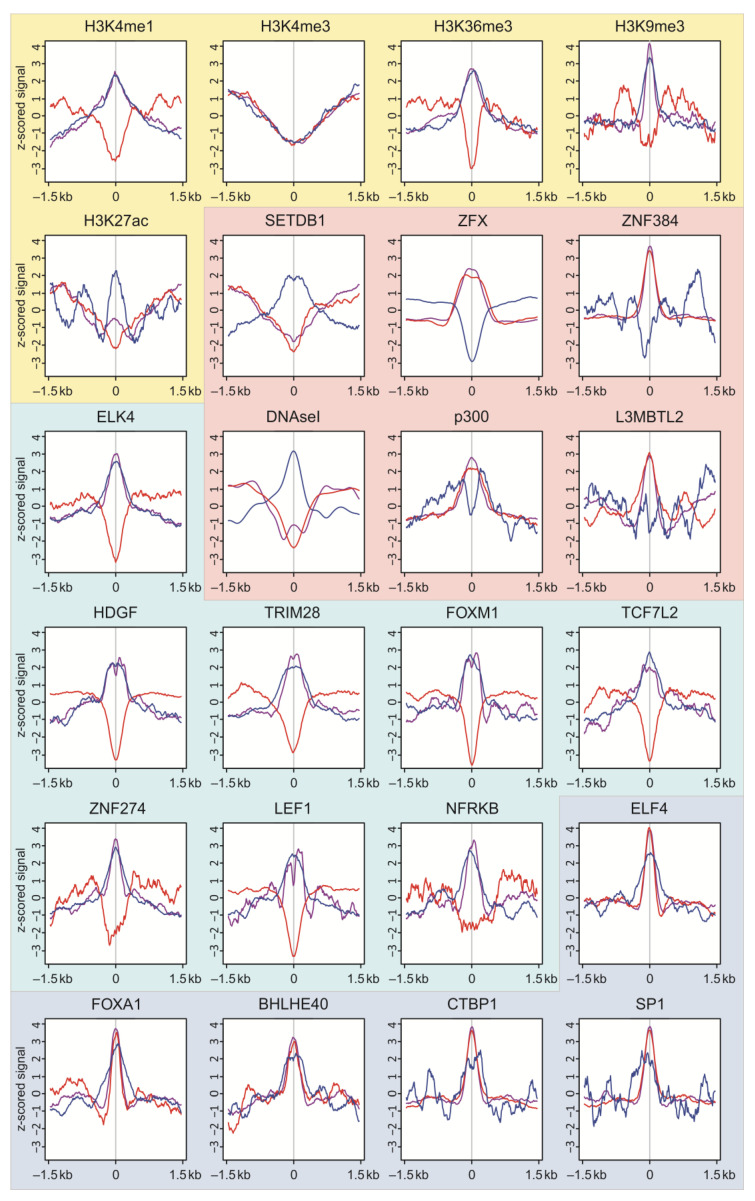
**Profiles of histone marks, binding sites of different factors, and DNaseI sites around DSB targets and rDNA contacting sites.** The profiles of the most frequent DSBs (top 2.5%) are shown in red, and Q1 DSBs (25%) in violet. The profiles of all rDNA-contacting sites are shown in blue. The z-scored signals ±1.5 kb around DSBs are indicated. Profiles for several histone marks are shown on a yellow background. Opposite profiles for DSBs and rDNA contacts are shown on a pink background. Opposite profiles for 2.5% of DSBs are shown on a cyan background. The same profiles for 4C and both DSB sets are shown on a blue background.

Figure 6 shows that the H3K4me1 mark, which is characteristic of enhancers, is depleted at the sites of the most frequent DSBs (top DBSs, 2.5%), while it is enriched at rDNA contacts and Q1 DSBs. The same is true for the H3K36me3 mark that positively correlates with exonic expression [17]. All three profiles are depleted at promoter regions that are marked by H3K4me3. The regions of constitutive heterochromatin marked by H3K9me3 are rather depleted with the top 2.5% DSBs and are significantly enriched with both Q1 DSBs and all rDNA contacts. The results indicate that silenced regions could possess both DSBs and rDNA contacts, suggesting a role of rDNA contacts in silencing. Interestingly, the regions with an active H3K27ac mark are characteristic of rDNA contacts [7]. This suggests the role of rDNA clusters in the inactivation of gene expression. Interestingly, the regions of this mark tend to escape DSBs.

The binding regions of SETDB1 and histone lysine methyltransferase 1 that are associated with gene silencing are enriched with rDNA contacts and depleted of DSBs. The same is true for DNAseI hypersensitive sites that mark the regions of open chromatin. The latter fact additionally demonstrates the opposite effects of exogenous and endogenous DNA breakage; the sites not accessible for exogenous DNaseI are targeted by endogenous enzymes producing DSBs [6,7].

On the contrary, the binding regions of ZFX, a transcriptional regulator for self-renewal of hematopoietic stem cell types, transcriptional activator P300, histone acetyltransferase binding sites, ZNF384, transcription factor, L3MBTL2, and PcG protein maintain the transcriptionally repressive state of genes and are depleted of rDNA contacts but are enriched with DSBs. The data suggest that DSBs could target the binding sites of both repressors (L3MBTL2) and activators (p300).

We observed similar profiles for eight transcription factors (ELK4, HDGF, TRIN28, FOXM1, TCF7L2, ZNF274M LEF1, and NFRKB) that are characterized by enrichment with both 2.5% and Q1 DSBs and by depletion of rDNA contacts. The data showed examples of opposite distribution of DSBs and rDNA contacts.

At the same time, we revealed examples of a similar distribution of both sets of DSBs and rDNA contacts at the binding sites of five transcription factors (ELK4, FOXA1, BHLHE40, and CTBP1). Taken together, the data on profiles demonstrate both the different and the same behavior of two sets of DSBs and rDNA contacts, suggesting their complex roles in both the activation of transcription and gene silencing in the course of development and morphogenesis.

### 2.7. DSBs at Bi- and Unidirectional Promoters in HEK293T Cells

**Figure 7:**
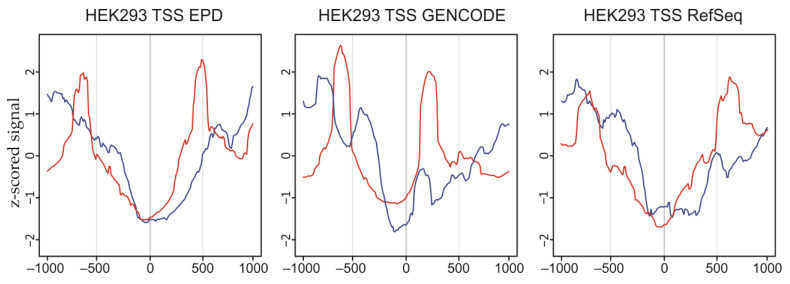
**DSB signal profiles around TSSs.** The data for bidirectional (red) and unidirectional (blue) promoter TSSs are shown. Data from EPD, GENCODE, and RefSeq TSS databases were used. Data are shown for ± 1000 bp span regions.

We detected that DSBs are enriched at promoters and in the vicinity of TSSs (Figure 5). To elucidate the patterns of DSBs at promoters in more detail, we used the available TSS databases (EPD, GENCODE, and known RefSeq) [18,19,20]. We built the profiles of all mapped DSBs ±1000 bp around unidirectional and bidirectional promoter TSSs and observed that the profiles of DSBs around TSSs in these databases are quite similar, especially for DSB signals around TSSs of bidirectional promoters (Figure 7). The differences in the DSB signals around unidirectional promoter TSSs can be accounted for by the alternative start sites in the RefSeq database, and especially for a large number of alternative TSSs in the GENCODE database. Alternative TSSs are generally located near each other and present (by bidirectional divergent TSS definition) among unidirectional promoter TSSs only. The DSB profiles for the most robust EPD database (that contains only experimentally validated promoters and TSSs) are quite similar to transcription factor occupancy profiles around TSSs [21]. The data indicate that TSSs are protected from DSBs, while the adjacent regions at a distance of a few nucleosomes are vulnerable to breakage. We assume that these DSBs are involved in facilitating nucleosome breathing in promoters. We studied this recently, and the results will be published separately (Kravatsky et al., in preparation).

We also performed an assessment of the coordinate correlations between bi- and unidirectional promoter TSS and DSB subsets, including the top 2.5% DSBs, which should delimit 50–150 kb DNA fragments observed in the pulsed-field gels (Figure S1), Q1 DSBs quartile, and the sum of Q1 + Q2 + Q3 DSBs quartiles using the Genome Track Analyzer [22]. As expected, no correlation (and even an anti-correlation) was detected for the top 2.5% DSBs, as far as these hot spots of DSBs separate very long DNA fragments possessing several genes. Q1 subsets showed statistically significant anti-correlations with unidirectional active promoter TSSs and statistically significant correlations with bidirectional active promoter TSSs. This result is in good agreement with Figure 7. For the Q1 + Q2 + Q3 DSBs subset, we detected a statistically significant correlation with all types of silent promoter TSSs. Moreover, the correlation pairs number reached almost the complete silent TSS amount (Table S8).

Taken together, these data indicate that the promoter regions of 200–600 bp around promoter TSSs are targeted by frequent DSBs. The described results were consistent for all three TSS databases: EPD, GENCODE, and RefSeq.

## 3. Discussion

For many years, endogenous DSBs were studied by artificially inducing them, either by ionizing radiation; by using the rare-cutting endonuclease, I-*Sce*I; or, more recently, by using the programmable nucleases Cas9 and Cas12a to study different aspects of the DNA damage response [23,24,25]. In our study, we used the genome-wide analysis of physiological DSBs to study the organization of chromosomes. We consider DSBs as a sensor of the epigenome. Our data indicate that endogenous DSBs are numerous and are distributed non-randomly. Previously, it was described that the hot spots of DSBs in rDNA genes are produced in vivo and correspond to the regions of 50–100 bp that are enriched with breakages [7,26]. The separation of damaged DNA in comet assays or pulsed-field gels reflects the abundance of endogenous breakages [27,28]. These data reflect snapshots of pre-existing DSBs that normally are successfully repaired. Nevertheless, the detected patterns of these endogenous DSBs could provide data on the organization and functioning of chromosomes, similarly to treatment with exogenous nucleases, which revealed nuclease-resistant DNA stretches and organization of chromatin at the nucleosome level. Previously, it was found that 50–150 kb DNA stretches observed in pulsed-field gels (Figure S1) contain coordinately expressed genes [6]. Mostly, these stretches are delimited by the hot spots of DSBs containing silent genes [29].

Here, we used the study of the genome-wide distribution of the most frequent DSBs (top 2.5%) to understand what groups of genes are the main targets of breakage and found that this DNA damage is non-random. The GO data on extremely high associations of the target genes with different cancers and other diseases argue in favor of a specific pattern of DSB distribution (Table 1). The data on the expression of these genes revealed that they preferentially correspond to active genes, suggesting a link between transcription and DSBs (Figure 4).

Although active genes are the main targets of DSBs, there is a group of silenced genes, including *DUX4* genes, which are also hot spots for DSBs (Figure 2). *DUX4* genes, located in the facultative heterochromatin, are required for early embryonic development and later epigenetically silenced in most somatic tissues [30]. We suggest that the silent genes that are preferentially located in the compact chromatin regions could be enriched by breakage, due to replication stress and late replication. We observed frequent DSBs in the regions of constitutive chromatin marked by H3K9me3 (Figure 6). Our data are consistent with the observations suggesting that DNA compaction may be problematic to the replication machinery and that the compact state of chromatin hampers DNA repair [31,32]. Previously, nine hot spots of DSBs (Pleiades) were described to reside in the non-transcribed IGS in active rDNA clusters [26]. It follows that both active transcription and silencing make the corresponding genes vulnerable to breakage, although the immediate causes are different—active transcription or problems with replication and reparation.

Surprisingly, we observed that 36% of genes frequently targeted by breaks overlap with rDNA-contacting genes. Among these genes are active and silent genes (Figure 4). rDNA contacts can be involved in dynamic regulatory contacts with the genes involved in development and cancers via the formation of phase-separated condensates [33]. It is also possible that, as far as nucleoli possess different factors capable of DNA repair, these contacts are a part of the DNA damage response [33].

It was described recently that, in *Arabidopsis*, important genomic regions crucial for viability and reproduction mutate less often than in other regions [34,35]. The sites of frequent DSBs could potentially produce mutations. We observed a link between epigenome-associated DSBs and gene transcription or silencing (Figure 4, Figure 5 and Figure 6) and found that the genes most vulnerable to breakage are very important genes that are involved in development and morphogenesis (Figure 1). We concluded that mutations associated with these DSBs should happen more frequently in very important genomic regions. This suggests that damage to key genes should lead to a negative selection of organisms starting from early development or later, inducing fewer diseases and keeping the most important genes in new generations at a lower mutation rate. Our data on the high level of DSBs in human cells also suggest that DNA repair mechanisms should be powerful and not be exposed to additional external or internal risks to be able to heal all DSBs.

## 4. Methods

### 4.1. DSB Library Preparation

The procedure was performed as described previously [36]. About 6 million HEK293T cells in 2 mL of culture medium were pelleted by centrifugation at 2000 rpm, resuspended in 0.3 mL of the same medium, gently mixed at 42 °C with an equal volume of a 1% agarose L (LKB) in PBS solution, and distributed on a mold containing 100 µL wells. The mold was placed on ice for 2–5 min covered with parafilm. The agarose plugs were then placed in Petri dishes with 5 mL of solution containing 0.5 M EDTA (pH 9.5), 1% sodium laurylsarcosine, and 1–2 mg of proteinase K (Merck, Darmstadtm Germany) solution per ml for 40–48 h at 50 °C and stored at 4 °C in the same solution. Each DNA-agarose plus usually contained about 15 µg of DNA, corresponding to about 1 million cells.

To test the quality of isolated DNA, we used fractionation in the pulsed-field gels (Figure S1). Portions of the original agarose-DNA plugs (5–50 µL) containing 1–10 µg of DNA were used for electrophoresis without any restriction enzyme digestion. The DNA samples were run in 0.8% agarose gels on an LKB Pulsaphor system, using a hexagonal electrode and switching times of 25 or 450 s.

For elution of the DNA preparations, fractionation in 1% agarose conventional mini-gel was performed (Figure S1). One-half of the DNA-agarose plug was washed in 1xTE three times (for 15 min each), followed by washing three times in the same solution containing 17.4 µg/mL PMSF in ethanol. After fractionation in the mini-gel, the ethidium bromide-stained DNA band was excised and electroeluted inside the dialysis cellulose membrane bag. After overnight dialysis without stirring against 1 L of 0.01 x TE at 4 °C, the DNA was concentrated with PEG (4 °C) and redialyzed.

Library preparation was performed as described below. About 1.5 µg of isolated DNA was ligated with 70 ng of a double-stranded oligonucleotide containing EcoRI and PstI sites (25 bp long 5′-phosphorylated 5′ pCCCCTGCAGTATAAGGAGAATTCGGG 3′ oligonucleotide annealed with 26 bp long 5′ biotinylated 5′ bio-CCGAATTCTCCTTATACTGCAGGGG 3′ oligonucleotide) in 150 µL of solution containing 0.1 M NaCl, 50 mM Tris HCl (pH 7.4), 8 mM MgCl2, 9 mM 2-mercaptoethanol, 7 µM ATP, 7.5% PEG, and 40 units of T4 DNA ligase at 20 °C for 16 hr. After heating at 65 °C for 10 min, the DNA preparation was digested with the Sau3A enzyme to shorten the forum domain to the termini attached to the ligated oligonucleotide. The selection of such termini was performed in 0.5 mL Eppendorf tubes, using 300 µL of suspension containing streptavidin magnetosphere paramagnetic particles, SA-PMP (Promega), according to the manufacturer’s recommendations. After extensive washing with 0.5 × SSC, removing DNA fragments corresponding to internal parts of large DNA fragments, the fragments attached to DSBs were eluted from the SA-PMP using digestion with EcoRI enzyme at a final volume of 50 µL. This DNA was then ligated with 100x molar excess of double-stranded Sau3A adaptor (5′-phosphorylated 5′ pGATCGTTTGCGGCCGCTTAAGCTTGGG 3′ oligonucleotide annealed with 5′ CCCAAGCTTAAGCGGCCGCAAAC 3′ oligonucleotide). The final DNA sample was used for PCR amplification. Twenty cycle PCR amplification in 60 µL of a solution containing 67 mM Tris-HCl (pH 8.4); 6 mM MgCl2; 10 mM 2-mercaptoethanol; 16.6 mM ammonium sulfate; 6.7 µM EDTA; 5 µg/mL BSA; 1 mM dNTPs; 1 µg of primer corresponding to Sau3A adaptor (5′ CCCAAGCTTAAGCGGCCGCAAAC 3′); 1 µg of primer corresponding to biotinylated oligonucleotide (5′ CCGAATTCTCCTTATACTGCAGGGG 3′), and 1 u of Taq polymerase was performed using Eppendorf Mastercycler Personal. Amplification conditions were 90 °C for melting, 65 °C for annealing, and 72 °C for extension, for 1 min each.

Paired-end sequencing of two biological replicates (about 200 million reads for each replicate, 150—nt long) was performed. Both raw reads and the processed mappings were uploaded to the GEO database under accession no. GSE201829.

### 4.2. C-rDNA Procedure

The DNA samples for the 4C experiments were isolated according to procedures described previously [37,38]. The HEK 293 cells were seeded in 10 cm culture plates 1–2 days before the experiment in DMEM containing 10% FBS and used at approximately 60–80% confluency. The cells were fixed in 1.5% formaldehyde, and nuclei were isolated, followed by digestion with *EcoR*I enzyme and ligation of extensively diluted DNA to favor intramolecular ligations. Then, to shorten the ligated DNA fragments, digestion with *Fae*I endonuclease was performed, followed by ligation of diluted DNA samples to favor circularization and minimize dimerization. The primers 5′ TCTTTGAAAAAAATCCCAGAAGTGGT 3′ and 5′ AAGTCCAGAAATCAACTCGCCAGT 3′for 4C-rDNA were selected inside the IGS (intergenic spacer in rDNA genes), as described previously [7]. The final DNA samples were used for the preparation of DNA libraries that were subjected to deep sequencing using a HiSeq1500 (Illumina, San Diego, CA, USA), using up to 150 nt long reads. The 4C-rDNA raw data corresponding to two biological replicates, corresponding to HEK293T cells, were deposited under accession number GSE121413 {12].

### 4.3. DSBs Mapping and Processing

As described in Section 4.1, the DSB sites are adjacent to the EcoRI/PstI primer (CCGAATTCTCCTTATACTGCAGGGG). Consequently, we set the adapter removal program cutadapt [39] 3.5 to remove all reads that lacked this mandatory adapter (--discard-untrimmed). We also set the minimal trimmed FastQ sequence length to 20 and the minimal FastQ quality to 26. At the next adapter removal steps, the 3′ HindIII/NotI primer (CCCAAGCTTAAGCGGCCGCAAAC) and all 5′/3′ incomplete adapters were eliminated. We removed primers from PE reads simultaneously to maintain the data integrity. The complete adapter removal script was uploaded to the Github repository (https://github.com/lokapal/IJMS2022.DSB/blob/main/DSB/01.adapter.removal.sh accessed on 28 June 2022).

DSB reads were aligned to the GRCh38/hg38 p.12 and GRCh37/hg19 p.13 human genomes with the rDNA sequence at the very beginning of chr14 by the bwa [40] mem v. 0.7.17-r1188 algorithm. All unaligned reads were removed subsequently from the alignment file by samtools 1.14 [41] using the -F4 option. The alignment file was sorted by coordinate (samtools sort) and converted to the resulting table (with genome coordinates, number of reads, coverage, and sequence per mapping) by ad hoc in-house bash and Perl scripts. The complete mapping procedure was uploaded to the Github repository as 02.DSB.mapping.sh. F-seq [42] 1.85 and bedGraphToBigWig [43] tools were used to obtain a genome-wide DSB profile from the alignment BAM files. The complete profile creation script was deposited in Github as 03.DSB.profile.sh.

The deepTools [44] 3.2.0 package was used to perform the replicate consistency check. High correlation coefficients (Pearson’s *r* = 0.88 and Spearman’s *ρ* = 0.93) suggest that the replicates were consistent with the high extent of similarity.

The following procedure was applied to assign genes to the DSB table. At the first step, bedtools intersectBed [45] 2.29.1 was applied to find and remove exact intersections (parameters –v –f 1.0) between each replicate and low complexity and/or repeat regions from the DFAM [46] database. At the next step, intersections between replicates were found by intersectBed again. In-house Perl scripts were used to convert the resulting file to the bedGraph format, and to add sequences by coordinates to it from the reference genome. BEDOPS [47] 2.4.40 bedmap and partition tools, as well as Linux awk utility, were used to merge the bedGraph genome track with intersected segments. Thus, we obtained the genome track that presented intersected segments of both replicates with low complexity and/or repeat regions removed.

We used the Ensembl genome annotation GRCh38/hg38 p.12 v.97 to obtain the list of DSB-associated genes. The gene names, IDs, and chromosome coordinates were extracted from the GTF file by an R script, with the help of the refGenome and dplyr libraries. The intersectBed tool was applied to find intersections between DSB processed mapping files and the hg38 gene list. The complete script was deposited in Github as 04.DSB.genes.sh.

### 4.4. 4C Mapping and Processing

The HEK293T 4C-rDNA-contacting region data were obtained in the following way. The HEK293T line was provided by Dr. V. S. Prassolov (Engelhardt Institute of Molecular Biology). Raw data for HEK293T cells were downloaded from GEO GSE121413, and adapters were removed by cutadapt [39] 3.5 with a two-step procedure: in the first step, both “external” and “internal” 4C adapters we removed at the 5′ and 3′ ends by the options -g GCCTAAGCCTGCTGAGAACTTTC -g CAGCATTCTGTAGGGAGATCAAATC -a GAAAGTTCTCAGCAGGCTTAGGC -a GATTTGATCTCCCTACAGAATGCTG. Low-quality and short reads were trimmed with the options --minimum-length 20 -q 26 too. All the untrimmed reads (i.e., the reads with no adapters found) were trimmed again from the reverse complement adapters by using the options -g TCTTTGAAAAAAATCCCAGAAGTGGT -g AAGTCCAGAAATCAACTCGCCAGT -a ACTGGCGAGTTGATTTCTGGACTT -a ACCACTTCTGGGATTTTTTTCAAAGA. All the trimmed reads after both steps were combined. Thus, we ensured that only the reads with initially present 4C adapters were selected for further processing. The complete adapter removal script was deposited in the Github repository (/4C/01.adapter.removal.sh).

Before the alignment of the reads, we performed the DFAM filtering to remove 4C-rDNA reads that were mapped completely to low complexity and/or repeat regions. The alignment to the genome and gene assigning procedure coincided with the equivalent procedures for DSB processing completely and were deposited in Github as 02.4C.mapping.sh and 03.4C.genes.sh.

A HEK293T 4C-rDNA-associated genome-wide average profile was created as follows: an intersection of 4C mapped replicates was created by intersectBed [45], then the margins of intersected segments were identified by an in-house Perl script, and finally, all non-intersected alignments from BAM files were removed by samtools [41]. The deepTools module bamCoverage [44] 3.2.0 was used to generate RPKM normalized profiles for each replicate. An average profile was created by WiggleTools [48] and converted to bigWig format by bedGraphToBigWig [43]. The 04.4C.profile.sh script was uploaded to Github.

### 4.5. Genome-Wide Profiles

The following profiles of genome-wide HEK293 fold change over the control were downloaded from the Encode project: H3K4me1 (ENCFF274LAP), H3K4me3 (ENCFF439DDQ), H3K36me3 (ENCFF185KBK), H3K9me3 (ENCFF526FQB), H3K27ac (ENCFF885SUR), SETDB1 (ENCFF818NCB), ZFX (ENCFF625XHP), ZNF384 (ENCFF681CQH), HDGF (ENCFF347GJE), DNAseI (HEK293T) (ENCFF716SFD), L3MBTL2(ENCFF472YKH), TRIM28 (ENCFF340FXG), FOXM1 (ENCFF784PBW), TCF7L2 (ENCFF851WEQ), ELK4 (ENCFF658HSX), ZNF274 (ENCFF235YDG), LEF1 (ENCFF112HIM), NFRKB (ENCFF070ECI), ELF4 (ENCFF956HGR), FOXA1 (ENCFF395RZM), BHLHE40 (ENCFF249NAE), CTBP1 (ENCFF678YHO), and SP1 (ENCFF466TSP).

The p300 HEK293 data were downloaded from the NCBI GEO/SRA database (SRR1001893 and SRR1001894/SRR1001900). Raw reads were trimmed for short and low-quality ends by Trimmomatic [49] (using the following options: LEADING:18, TRAILING:18, SLIDINGWINDOW:4:22, and MINLEN:20). Trimmed data were aligned to the hg38 genome by bowtie [50] 1.2.3 using the options --best, --strata, and -m 1. The alignment SAM files were sorted and converted to BAM files by using samtools [41] 1.14, and unaligned reads were removed by using the –F4 option. Samtools [3] fixmate/markdup procedures were used to find, mark, and remove the complete duplicates. MACS2 [51] 2.1.2, with the options --bdg --gsize hs --call-summits, was used as a peak caller. We applied it to generate the profile of the fold change over control (options bdgcmp -m FE) too. The profile was converted to bigWig format by the bedGraphToBigWig tool [43].

All epigenetic plots were created using SeqPlots [52] interactively. Profile plots were created at the 10 bp binning size with the mean values from z-score-normalized (in the plot range) data, and the midpoints of the appropriate tracks were applied as the plot center.

### 4.6. RNA-Seq Analysis

RNA-Seq expression data for HEK293T cells [12] were used (two replicates, GSE130262). Trimmomatic [49] 0.36 was applied to remove low-quality reads with the following options: LEADING:18, TRAILING:18, SLIDINGWINDOW:4:22, and MINLEN:20. We applied the RSEM [53] 1.3.1 software package to accurately quantify transcripts from the RNA-Seq data. The resulting gene tables were combined for each replicate. Gene expression values (in TPM) were assigned to the previously obtained DSB mappings and were used to create DSB-associated gene expression violin plots. All the charts were created by using R scripts with the help of the ggplot2 library.

### 4.7. Transcription Start-Site Analysis

*H. sapiens* transcription start sites (TSSs) were obtained from the following databases: EPD [18], as the database of experimentally validated promoters, GENCODE [19], as the most complete database with biological evidence, and NCBI RefSeq Curated [20], as the reference database that included automated computational methods, collaboration, and manual data review by NCBI staff.

All three TSSs datasets were processed uniformly. We removed all exact gene duplicates and converted the list of genes to the TSS list. All silent (not expressed) TSSs were omitted from future consideration. The selection was performed according to the CAGE/Fantom5 [54] genome-wide expression data (downloaded from the EPD [18] server ftp://ccg.epfl.ch/mga/hg38/fantom5/ accessed on 28 June 2022).

Some genes had multiple TSSs in the databases, so the minor TSSs, with expression levels of less than 5% of the major TSSs, were excluded from further consideration. The list was then divided into a list of bidirectional promoter TSSs (i.e., the distance between TSSs should be less than 1000 bp, the TSSs should be located on the opposite strands, and transcription from these TSSs should not intersect), and a list of unidirectional promoter TSSs. Bidirectional TSSs were removed from the unidirectional TSSs lists (to avoid the presence of genes in both lists). The lists were converted to GFF format, and DSB profiles around bidirectional and unidirectional promoter TSSs were created by the Seqplots [52] software package. All the TSS list processing scripts were deposited in Github (TSS directory).

### 4.8. Analysis of the Distribution of Genes and Their Expression Levels by Violin Plots

Non-parametric statistical tests should be used for expression datasets since gene expression datasets do not follow a normal distribution [55]. We tested and affirmed the applicability of the non-parametric independent two-group Mann–Whitney U test for this task [56].

Accordingly, we applied the Mann–Whitney U test to find out whether the DSBs and 4C-rDNA associated gene expression subsets and the full expression dataset originated from the same distribution. We found that all three datasets did not originate from the same distribution (*p*_DSB_ = 2.21 × 10^−132^, *p*_4C_ = 6.96 × 10^−47^) and thus were statistically independent.

We also tested whether the difference in expression distributions could be obtained by chance using Monte Carlo (MC) simulations. The test was repeated 10,000 times. In all cases, the results were negative, i.e., the DSB and/or 4C-rDNA associated gene expression datasets and the randomly picked gene datasets did not originate from the same distribution and were independent. The maximum *p* was 6.49 × 10^−42^ and 1.04 × 10^−12^ for DSB and 4C-rDNA, respectively, i.e., *p* ≪ 0.01 in both cases. We can conclude that, at the level of *p* = 0.0001, the expression of the DSB and 4C-rDNA associated gene datasets cannot be obtained from the full gene expression dataset by chance.

Thus, we can conclude that the DSB-associated and 4C-rDNA-associated gene expression datasets significantly differ from the full expression set.

We created an averaged random dataset for presentation purposes. The random violin plot dataset in Figure 4 represents the dataset that was obtained by averaging 10,000 random datasets of the full dataset. It should be noted that the conclusion of independence of the full expression dataset and the DSB and 4C-rDNA associated gene datasets was made solely on a statistical basis.

## Figures and Tables

**Table 1:** Associations of the 4020 top genes possessing the most frequent DSBs. The search was performed in Jensen_DISEASES library (Enrichr) [13]. The corresponding genes are shown in Table S5.

Index	Name	Adjusted *p*-Value
1	Kidney cancer	3.358 × 10^−56^
2	Liver cancer	1.896 × 10^−26^
3	Skin cancer	8.424 × 10^−16^
4	Melanoma	2.769 × 10^−15^
5	Breast cancer	3.143 × 10^−12^
6	Pancreatic cancer	3.595 × 10^−11^
7	Endometrial cancer	8.648 × 10^−11^
8	Acquired metabolic disease	2.167 × 10^−11^
9	Carcinoma	3.577 × 10^−8^
10	Ovarian cancer	0.00003196
11	Attention deficit hyperactivity disorder	0.00003272
12	Type 2 diabetes mellitus	0.00005111
13	Bipolar disorder	0.0001426
14	Schizophrenia	0.0002438
15	Anorexia nervosa	0.0006728
16	Retinal disease	0.0008184
17	Refractive error	0.001096
18	Immune system cancer	0.001685
19	Cognitive disorder	0.001689
20	Obesity	0.003255
21	Alzheimer’s disease	0.003277
22	Large intestine cancer	0.003464
23	Anemia	0.003785
24	Osteoporosis	0.004880
25	Globe disease	0.008163
26	DOID:2627	0.008163
27	Pericholangitis	0.01312
28	Lymphoid leukemia	0.02868
29	Parkinson’s disease	0.04334
30	Chronic obstructive pulmonary disease	0.04528
31	Restless legs syndrome	0.04648
32	Lung cancer	0.04799

## Data Availability

Not applicable.

## Supplementary Materials

The following supporting information can be downloaded at: https://www.mdpi.com/article/10.3390/ijms23137201/s1.
