# Supplementary material for: Genes Possessing the Most Frequent DNA DSBs Are Highly Associated with Development and Cancers, and Essentially Overlap with the rDNA-Contacting Genes
Source: Int J Mol Sci. 2022 Jun 28;23(13):7201. doi: 10.3390/ijms23137201 (PMC9266645; doi:10.3390/ijms23137201)
Supplement: Supplementary file 1 [file ijms-23-07201-s001.zip › ijms-1745547-supplementary.pdf]

Supplemental Information for:

Genes possessing the most frequent DNA DSBs are highly associated with development and cancers, and essentially overlap with the rDNA-contacting genes

Nickolai A. Tchurikov,[1]* Ildar R. Alembekov[1], Elena S. Klushevskaya[1], Antonina N. Kretova[1], Ann M. Keremet[1], Anastasia E. Sidorova[1], Polina B. .Meilakh[1], Vladimir R. Chechetkin[1], Galina I. Kravatskaya[1] and Yuri V. Kravatsky[1,2]

[1] Department of Epigenetic Mechanisms of Gene Expression Regulation, Engelhardt Institute of Molecular Biology Russian Academy of Sciences, Moscow, 119334, Russia
[2] Center for Precision Genome Editing and Genetic Technologies for Biomedicine, Engelhardt Institute of Molecular Biology Russian Academy of Sciences, Moscow, 119334, Russia

This PDF file includes:

Figures S1–S2

Tables S1–S8

Figures S1–S2

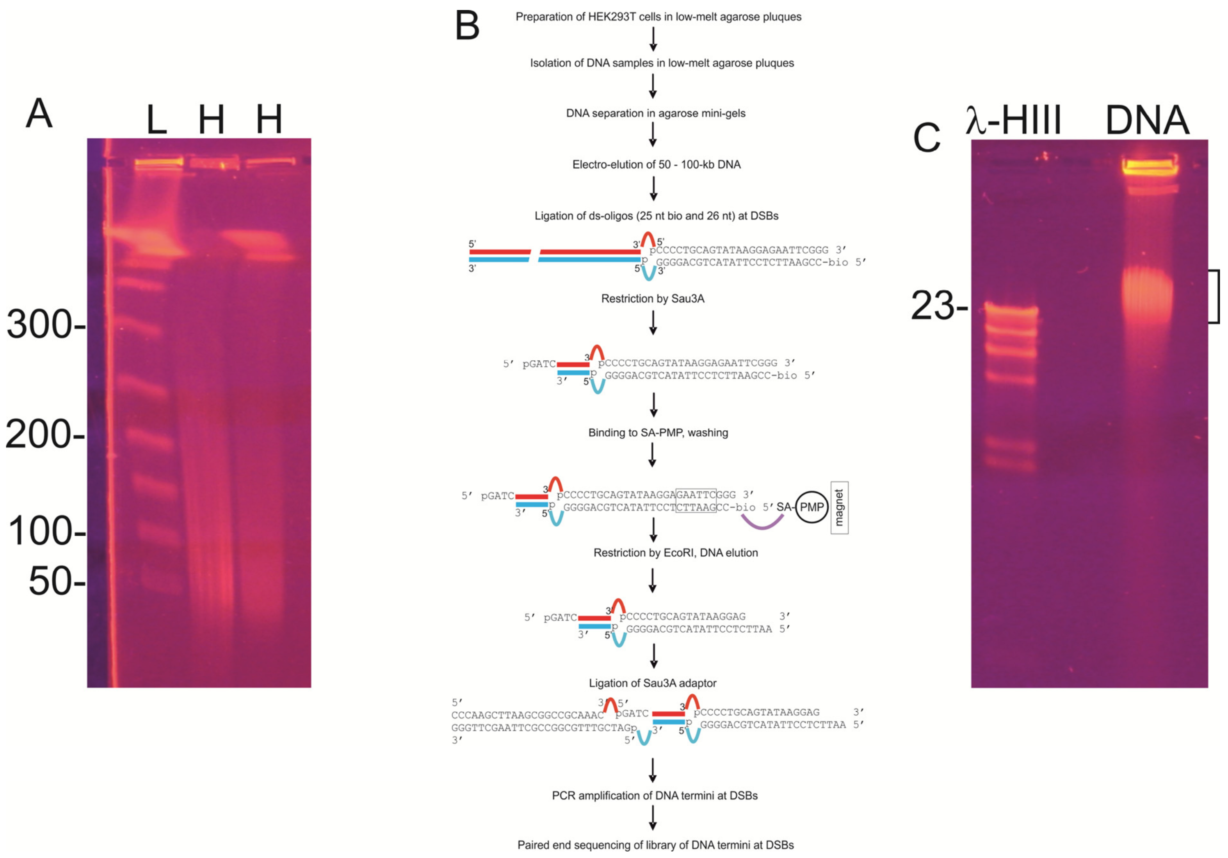

Figure S1. **Separation of DNA isolated in agarose plugs and preparation of library of DNA termini at DSBs.** (**A**) Pulsed-field gel separation of DNA samples from HEK293T cells (H). λ-ladder (L) was used as the size marker. The values on the left show the length of DNA fragments in kb. (**B**) The steps of the procedure used for preparation of the library of DNA fragments with DSBs. (**C**) Isolation of DNA fragments by separation of DNA-agarose plugs in mini-gel. The value to the left indicates the 23 kb DNA fragment in the λ-HindIII size marker (λ-HIII). The bracket on the right indicates the region that was used for electro-elution.

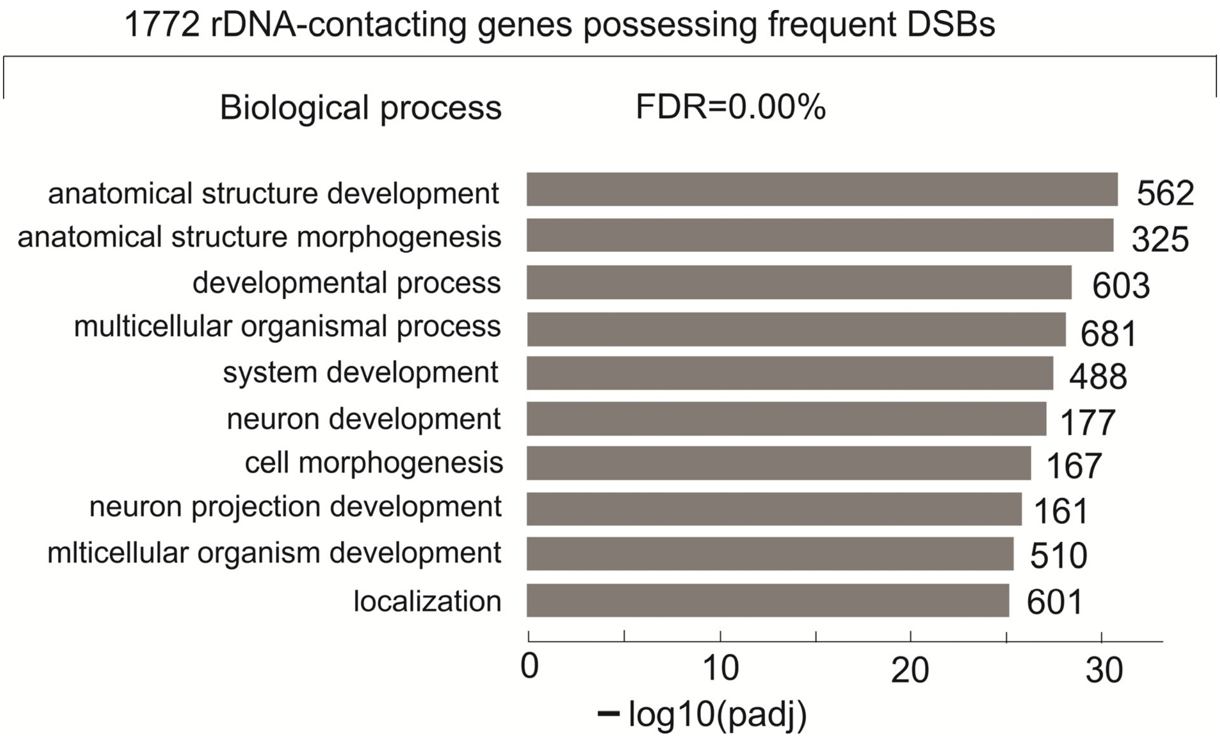

Figure S2. **The characterization of 1772 rDNA-contacting genes frequently targeted by DSBs in HEK293T cells**. Related to the Venn diagram in Figure 1C. The top ten Gene Ontology (GO) biological process associations of the genes. The values to the right of the bars show the number of genes associated with a process. The complete list of the corresponding genes is shown in Table S4.

Tables S1–S8

Table S1. GO associations with Biological Processes (GO Profiler) of the top 4920 genes possessing frequent DSDs. Related to Figure 1A.

GO.ID	Description	padj	Genes
GO:0050794	regulation of cellular process	9.841549568240879e-54	*CD247,POLDIP3,CLRN1,DNAH11,RIC8B,ENPP1,PRDX2,ADAMTS16,LDB2,GPC5,NRXN1,ASPH,B4GALNT2,PRMT3,GP6,PRKCI,SLCO3A1,MAP4K4,PDE4D,PDCL,DNMT1,S1PR2,HLX,TMBIM4,SLC9A1,PDE7B,C6ORF89,NEGR1,PBX3,ADCY8,MED13L,LMBR1,TRPS1,CBFB,PDE8A,PDE4DIP,ZNF823,CLASP2,SEMA3A,IL31RA,GRID2,TAS1R2,RPS6KA2,PRDM12,SETD4,PTGFR,TENM3,CHERP,MED26,DOCK1,WWC1,CLDN18,C12ORF49,CTDSPL2,FBXL2,RYR1,NRG3,ASH1L,NOS1AP,LATS2,NOX5,ANP32A,ASTN2,EPB41,SEMA3D,PHACTR1,DPP6,PTH2R,NREP,SCAF8,TIAM2,STOX2,FHOD3,KSR2,MAPRE2,NLGN4X,PTGIS,PRKAG2,WWC3,TJP1,GRM8,UNC5C,NUDT6,HPSE2,OR5P2,OR5P3,CLCN1,PRLR,MVP,PAGR1,ADCY7,HIVEP3,FTO,NPAS3,UTRN,RGS7BP,KALRN,PITPNC1,DCAF12,NTRK3,CBL,ARNT,ATRNL1,PLEKHG4B,EGLN2,MIA,RAB4B,RAB4B-EGLN2,SEMA5A,LRRFIP2,FLT3,RAD51B,STAT5B,TOX,ENPP2,KCNQ5,GRM5,PLCE1,LHFPL4,SAMHD1,ZNF566,FER,CASK,MARVELD3,MAPK4,MAP2K5,KITLG,KCNIP4,MAPK10,PTPRR,SPNS2,LRP2,PIK3CD,ZNF536,SP3,DEPDC5,HDGF,PRCC,ANK2,EIF4G3,SAAL1,OLFM1,SH2D1A,EZR,IKZF2,MEGF10,NRXN3,ROBO1,MALRD1,TOM1L1,TNFAIP8L1,CHD7,SLC26A6,HTR2B,ITGB6,CDKL5,MECOM,TACC1,TENM1,CAMKMT,RYR3,LMNA,NUCB1,GOT1,NMT2,INPP4B,TRAF6,ESRP1,ROBO2,ITPKB,CTNNA3,DNAJA3,OXR1,SLC24A2,VAV2,GRK5,ABI1,ULK4,NHLH1,ITGB1,TRIOBP,HSP90AA1,CDC6,PSMB7,TSC22D3,CNIH2,SRGAP3,MCC,TSSK1B,PCDH17,KNDC1,ZC4H2,KIF26B,CCDC22,DSCAM,STK38L,SRGAP2B,CDAN1,GMDS,SAMD12,CNGB1,TRIO,ARHGAP24,KLHL12,AKAP13,GFI1B,PDE1A,PARD6G,PTPRK,RUNX1,DAB1,OMA1,CDC42EP3,MYO10,ABCC8,ERC1,HMGN3,NRIP1,RGS6,DGKI,ANKS1B,THRB,STXBP4,PDE4A,ERC2,GABRA3,EFCAB7,ITGB3BP,LAMA2,GFRA2,MAST4,IL17RB,TCIRG1,DACH1,PTPN11,ZNF569,KCNS3,NETO2,HERC4,MGAT5,CCT2,CACNB2,DOCK10,ORC2,CYTH3,HDAC6,GOLGA4,SERTAD2,DDB1,MYT1,ADORA1,GPRASP1,ADAMTS3,EPHA1,IKBKB,KIF18B,PEX14,ERBB4,GBE1,ADRA1D,SORCS1,MRE11A,GBF1,LIMK1,ZNF609,TLK1,BRD8,ATP8A2,KAT6B,ABR,HIF3A,SNIP1,CUL4B,KLHL6,ESR1,KCNJ16,NPHP3,GRM7,MIER1,PCBP3,PMEPA1,MAML3,DPP10,PTPRO,CDON,NTRK2,TNRC6A,FRMD4A,STX18,EP300,CELA1,ZYG11B,TENM2,ZNF76,RNF220,RGS22,ZNF471,FNTA,HOOK3,PDGFB,CACNA1B,RIMS1,TNIK,CCND3,CDH8,USP46,TOB2,MID1,ZNF19,ZNF23,BRMS1,CHFR,ZNF605,SCML4,BPI,STK39,DAP3,HNF4G,INSR,FMN2,RERE,PRKAR1A,ASB5,AFAP1,FUBP1,ATP8B1,UNC5D,H2AFY2,LAMA3,TCF7L2,PIP4K2A,CDH4,JMJD1C,CAB39,USP34,ZNF443,ZNF490,ZNF564,ZNF709,ZNF799,PAK1,LITAF,FBXW11,ESRRB,MYOM1,MAP3K4,ZDHHC13,PTPRD,RNF144A,BASP1,EGLN3,RBM20,SLC4A10,TBR1,CATSPER2,SAMD4A,BDNF,TFAP2A,AMIGO1,CDK14,FANCA,KCNMA1,PEG3,ZIM2,CHRM3,TGIF2,TGIF2-C20ORF24,FUT8,OR2T3,NPRL3,PHACTR4,MEIS1,TENM4,GRIK4,VRTN,KCNRG,TRIM13,RASGEF1B,UBR2,ARHGAP6,LCK,MDM4,EDARADD,IL5RA,ENTPD5,SHC4,ECT2,TMC2,ZNF148,MTA3,SNX6,TFDP2,CHML,VAMP7,LAMC1,PPM1L,ARHGAP42,CST2,PTCD3,PTPRU,RFTN1,ERN2,CDC45,LSP1,C1D,AP3S1,NPLOC4,AFF3,SGCD,DENND5A,DMD,CENPF,ATG10,LRRN2,SLC30A9,TOX3,PDS5A,LARP4B,USP13,KAT7,ZNF667,SLC8A1,GSN,KHDRBS2,RBM14,RBM4,GPR171,MED12L,SATB2,PAFAH1B2,RIT2,HIRA,TANC2,JPH2,DRG2,DEPDC1B,CASS4,KCTD16,FYN,MKRN2,ARNTL,ADAMTS9,NF1,SMG7,PLCB1,DST,MGMT,LMTK2,ARID4B,SNCB,PPP4R2,FAT3,RTN4,AFF2,RXFP1,DPEP1,CHRDL1,DGKH,APOD,RTN4R,BCR,PPP1R12B,RWDD3,BRPF1,SHISA9,CHI3L1,TTN,NDRG4,ICA1,BMP2K,PAQR3,IFI30,PIK3R2,CACNA1E,RANBP9,KLHL21,NTN1,EGFLAM,DCTN1,NOMO3,NRG1,ARHGAP10,SH2D3C,CDK19,SLC43A2,UBP1,BDKRB1,BDKRB2,DOCK8,BID,FRY,MAP2K1,KCNIP1,MDFI,FNIP1,RAPGEF6,STIM1,VAX2,NALCN,RALGPS2,MYH9,FRMPD4,MAD1L1,LRFN5,DAGLA,TMBIM6,VGLL4,PPP2CB,CNTN4,PPP3R1,HDAC5,CSRNP3,RBM5,NTNG1,VSTM1,ZNF692,OR6N2,NFIA,RNF4,TMEM30A,CSNK2A3,JDP2,CMKLR1,INPP4A,ITIH2,CDKL2,ROR2,DCN,ZDHHC3,SLC39A14,SCFD1,CATSPER3,PCBD2,HNRNPLL,DOCK2,CDH13,CREBRF,TRDMT1,SOX13,FHIT,WDR70,PTPRT,AUNIP,UST,COPS5,CSPP1,PPP1R42,FGF14,EXOC4,IPO5,ARHGAP32,MAD2L2,TLE6,RAB27A,RGS8,CAPRIN2,CCNYL1,ZNF418,NLN,NCKAP1,PHF20L1,DEFA1B,DEFA3,CD99,RAB3C,PDF,TERF2,PTGER2,RALGPS1,SLX1B,ZNF286A,GOLPH3L,FOXN3,RCC2,BOC,ANO6,ARL3,CERKL,NEUROD1,GNAQ,KIF2A,FBXO9,ICK,TMEM117,RAD51D,RFFL,RANBP1,CCBE1,DENND1A,HERC5,USP22,ZDHHC15,JAK2,YTHDC2,FAM168A,TRAPPC9,FBXW7,LINC00473,KCNB2,OAZ2,SYN3,SKAP1,SMC3,CLSTN2,UIMC1,SYT1,ITCH,MLIP,CROCC,NPSR1,ZFYVE28,BBS12,LZTS1,BCL11B,SMG1,SCN4A,RBFOX1,TMC1,PKNOX1,DTHD1,CLDN1,HUNK,MLLT3,TSHZ2,ATP9A,ARAP2,SLC9A7,CDC42BPB,PLS1,TEX11,DOCK9,SH2D6,TCF7,PDE2A,SEPT7,KLF15,TBX15,ANXA4,LAMTOR3,MAGI1,KCNC4,CYFIP2,WNT11,ARHGAP23,IFT80,TRIM59,APBB1IP,FIG4,MTA1,KREMEN1,NRG4,KLF8,CLPB,NOX4,LCOR,SIPA1L3,RPRD1B,MKLN*

			1,CACNA1H,SIAH1,QKI,IL1RAPL1,CCDC62,CAMK2B,ERCC8,PRKCD,SOX6,TAB2,ACVR2A,RUNX2,SEMA5B,CD4,PPM1E,TGFB1,BANP,PLCXD3,SGK1,SPSB4,NUP93,MMP28,TRIM65,NSD1,IGSF1,PIBF1,ZHX2,PKNOX2,ASCC2,KEL,BTRC,DUS2,NFATC3,JPH3,CRADD,DLGAP1,F2RL1,BCAS3,C9ORF47,GRIK3,CDYL2,RPGRIP1L,DNAJB2,CELSR1,SLIT3,AAK1,CC2D1B,ZFYVE9,SLIT2,ASB15,COLGALT1,TP73,GABRR3,CADPS2,ITIH4,GABRB1,SYT13,HHAT,GPR176,HP,HPR,CORO1C,SAP18,RRAS2,CTNND2,ZBTB22,C12ORF4,OR9A4,ILF2,DISC1,BLID,KCNJ3,CEP135,KANK1,CACNA1D,ZFAND2A,PTPDC1,CACNA2D3,LAMC2,CLN6,MTDH,ANK1,CLMN,TTBK2,FANK1,XPR1,MYT1L,KDM4B,CHRM1,RAB11FIP4,OR51A7,OR51F2,OR51T1,SMAD6,RXFP2,BNC2,ZNF398,CLOCK,ITSN2,TCF12,ZNF675,SMOC1,PLCB4,ETV6,NELL1,SUCO,KCNH1,TFAP2D,BMPER,TIMP2,BCL3,DCLK1,ANKRD54,DAPK2,TNFRSF10B,SND1,DAPP1,ADAM12,DUSP22,GPR52,NAIP,HNRNPC,TRRAP,ANKFN1,HOMER2,SLC17A7,DOCK3,SBNO2,LINGO2,YTHDF1,FGF10,CNTNAP2,CIZ1,FBXL17,RASA4,RASA4B,ARHGEF28,SMYD3,STXBP5,KCNH7,KANK4,KCTD10,IL1RAPL2,IQCJ-SCHIP1,SH3RF3,LOXL3,RHPN2,MAST2,FANCI,SVIL,GLP2R,ADCY2,CAPN3,SLC16A1,LUM,VMP1,SMURF2,EPHA4,RORA,HIVEP2,HSD17B12,PRKCA,AUTS2,CNR1,CD6,TNFSF11,SMG6,PPP3CA,NSUN2,OR51B2,OR51B4,OR51I1,UBQLN3,GNG4,NFYB,MAGEA4,MAG,KLF12,CAMK4,GATAD2B,PIP5KL1,UFL1,TRAK1,CTNNB1,PARK2,SOD2,DACH2,METTL13,FCGR2A,FCGR2B,FCGR3A,FCGR3B,ARHGAP22,SMARCC1,KLF17,CDHR2,IGF1R,PPARG,NGRN,AXIN1,GARNL3,PRKAR1B,OTUB1,DLG5,IL18R1,IL1RL1,ADD2,CIPC,MTF1,MSR1,CELF2,CBX5,P2RY10,TRPM1,ANKRD17,CYBB,XK,CASKIN1,APOL3,SCAMP5,BRIP1,ANXA13,ANKRD26,ECM2,LRPPRC,SREBF2,KCNJ12,RYR2,CDK11A,KCNA6,FBLIM1,CDK11B,DRAXIN,LEPR,LEPROT,FGF1,NIN,PROS1,RGS10,NPAT,NR4A3,FOXK2,NOL3,DCT,PRKAR2A,RIOK2,ESCO1,MYOCD,OR52E6,OR52E8,OR52N1,TRIM5,PER2,KIR2DL1,KIR2DL4,KIR3DL2,AJUBA,CACNA1C,CPNE6,GLG1,ZNF626,ZNF737,SCN3B,CHEK2,SUPT3H,PHLDB1,UBQLN4,PRDM16,MYO1E,ASB8,PPP1CB,SPDYA,HCK,CSTL1,SORBS1,TBC1D14,RAB3GAP2,CAPN2,TBCK,TRIM8,DIO2,CSPG4,GRM1,BRMS1L,RIMS4,DCC,ZBTB8A,ZBTB8B,ARHGAP31,P2RY14,ANKFY1,CTDP1,BAZ1B,OBSCN,RGL1,NF2,FLT4,MEF2B,SGMS1,BICC1,HDAC4,PAX2,PHF5A,TNS3,SECISBP2L,SPTBN1,TPTE,ELN,TRABD2B,SFRP1,MED13,ST8SIA1,PPP6R2,ZNF395,FOXO3,DGKB,TRHDE,ARL13B,CNTN6,NFIB,MUC12,SP4,ARHGAP29,ZCCHC17,IFT122,BCL2L13,DNM3,SSH1,CELF5,SYNCRIP,SMAD3,RNFT2,CUX2,ITPR1,PTPRM,WWP2,ARNT2,SBNO1,ACKR2,KRBOX1,ZNF662,ZNF777,SIMC1,WIPI1,EBF3,MTBP,RNF168,CASZ1,ABI2,C10ORF90,GRIK5,PSAP,SLC16A2,LZTFL1,SHANK1,SYT3,IFNAR1,DCP1B,OR14K1,MIER3,NEDD4,NRP2,ARHGEF18,VANGL1,PREX1,PLA2G4C,DOCK4,ESRRG,HOXD3,HOXD4,KPNB1,NAV3,SLC4A4,ZNF114,TAB1,SLC6A1,KTI12,TXNDC12,NFATC1,RAB6C,PRTG,CDC73,APP,SSBP3,GSX2,PDGFRA,BET1L,SLK,FCRL4,ADD3,RBM8A,CCNI2,DIP2B,NOX1,ARIH1,YAP1,HEG1,AMFR,RAB11FIP5,SESN1,CDK3,TEN1,FSTL4,NLGN2,TNFRSF19,VDAC1,EYA2,SH3D19,BORA,IBTK,NVL,JAK1,ANGPTL4,LRP5,MTCP1,MUC20,POLR3G,PTPRG,ISLR2,SLC9B2,ZNF787,GNG2,SOX2,SETD2,ZDHHC6,GPC6,KIF24,CHST11,TEAD1,PRICKLE1,RCAN1,ZNF653,SPTBN4,GRIA3,VASH2,ZNF521,DRD1,TMEM14A,GLRA2,ARID3A,ZNF761,CHUK,ERLIN1,FRS2,PALB2,SFMBT1,VILL,MMP2,ZNF584,SERGEF,TPH1,ESR2,SYNE2,S100A12,DCUN1D3,CPNE9,KDM6A,PRKD1,STAT1,CELF6,PARP6,ST18,DGKG,ETV5,RHOXF2B,RND3,SLC6A3,TAF3,PLAGL1,HNF4A,ZBTB7C,LRRC2,TASP1,SHANK2,VPS4A,EREG,CCNY,TCTN3,MAPKAPK3,FXYD2,FXYD6,SEMA6D,RBFOX3,ATF2,POU2F2,TCF3,ZNF730,GRAMD4,TMEM237,KCNG4,RAF1,CELF4,RASGRF2,ZNF766,ACOX2,CARD16,CASP1,PTPN1,ADAMTS12,SRRM4,BMPR2,USP33,DPYSL2,VBP1,CAMK1D,BMPR1A,ZKSCAN1,PABPC4,TSPAN12,NLGN1,BTBD10,COL16A1,CTNNA2,IKZF4,PIK3R3,CDK12,TAF8,CAND2,SPAG9,MORC2,DENND4A,RAB11A,CRMP1,EVC,PDE11A,EPG5,MYB,FGF2,ZNF71,LRRC4C,POU6F2,BACH1,GRIK1,OR6C70,MXD3,PPM1F,TICRR,ADCY5,GLI4,ZFP41,NEDD9,AGBL4,BEND5,PEAK1,SEMA4D,NFX1,CDC27,DOK6,RORC,MCTP1,ELP3,PLXNA2,PTAFR,ADCYAP1R1,SP140,SP140L,SETD1A,RHOXF2,YME1L1,ABCA13,JARID2,RILPL1,ANKDD1A,DDX58,DKK2,SORCS2,RAB11FIP3,BRDT,PHC2,ITGA11,PI4KA,RARB,SPEN,PIK3C2B,PRKCG,SIN3B,VIPR1,NCOA1,SPOCK1,AREL1,BLOC1S5,EEF1E1,EEF1E1-BLOC1S5,TXNDC5,GRIA2,EHMT1,GAS8,LMO7,AP2M1,CHRD,DVL3,ECE2,EIF2B5,EIF4G1,EPHB3,PTPRE,RBX1,TCF20,ANAPC5,ARHGAP39,ATF3,LIN28B,ATP6V0B,SHOC2,SRSF5,CKS1B,PAWR,EBF2,AGO3,DEPTOR,DLEC1,PKN3,FBXL20,MAML2,TSG101,DESI1,CCDC3,VCL,LAMB1,TERF2IP,CLIC4,MYO9A,CRYM,IDE,WDR59,KCNQ1,RFX2,WNT3,RCE1,ZNF322,RHOJ,P4HB,CCL14,CCL15,CCL15-CCL14,SUFU,TG,MAGEA11,NLGN3,PTPN13,SCUBE2,PAFAH1B1,KIF3A,PRDM15,ZNF670,ZNF695,CCDC169-SOHLH2,SOHLH2,FAM13A,CNTFR,COL4A3,TRPA1,DDAH1,NMUR2,ZNF354C,HIP1,AKAP6,RASAL1,TCEA3,PADI6,ZNF704,BTBD9,NR2C1,TRPC5,UBA2,IRS4,GLI2,NEO1,TNKS,SCUBE1,WBP2NL,ERCC1,RUFY3,TNRC6B,GLIS3,GPR39,WDTC1,ZNF664,STON2,MGLL,DAB2,THEMIS,BLM,PKHD1,USH2A,UBR1,CACUL1,LDLRAD4,MYSM1,SETD5,DZIP1,SMG5,DLG3,WNK1,RELN,NEK10,SIN3A,RUVBL2,ABCB1,OR5H2,OR5H6,OR5K4,RSPO2,COMMD6,GUCY1A2,CSF3R,KCNQ2,DOCK11,GMEB1,PELI1,PPP2R5C,CFDP1,IQGAP1,MAP3K7,APOLD1,DDX47,TAS2R41,ZNF423,SP1,OR56B1,TRIM22,MACF1,ARHGAP12,ALK,SLC8A2,INPP5D,STAU1,CLASP1,DSC

			2,TEAD4,FASTKD5,HOXC13,APBB3,SLC35A4,SRA1,UBE2V1,MBOAT1,TPCN1,GPM6B,ADIRF,CLIC5,XDH,AHNAK,LTBP1,OVOL2,SNX5,NFATC2,RBBP6,OR6C74,PRKG1,PAX7,RGS7,BNC1,ATP2B4,NSF,PACRG,PACSIN1,SPTBN5,ACTL6B,ASXL3,PAK3,SSH2,TET1,CAMTA1,CCPG1,DYX1C1,P2RX6,ASGR2,ATAD1,ADNP,GPR161,MAPKAPK2,ARID4A,MAP1B,CDC42BPA,COL4A6,MPHOSPH9,SCARB1,MARK1,CDK6,ATP6V0A2,PHF2,KCNAB2,CELF1,P2RY8,RNF34,TFRC,UVRAG,EPHA5,SORCS3,WWOX,GABRG3,MEF2A,SYT12,CR1L,ADCY9,CACNA1A,DCAF6,DCUN1D5,DROSHA,FNBP1,GFRAL,GPC3,SGIP1,STOM,XRN1,ADRBK1,SATB1,TBC1D5,PSMB2,ZNF207,EYA1,GATAD2A,PTGER3,SCOC,BRINP1,DIS3L2,GNB3,SHPK,TRPV1,RBM12B,TSPAN6,HOXB3,HOXB4,HOXB5,HOXB6,TFEB,CLEC16A,GHR,MNAT1,FLRT2,FGD1,SNX9,ACTR2,L3MBTL4,ELAVL4,LGI4,HDAC1,INVS,RECQL5,C2,SMOC2,TRIM24,ADIPOR2,PLCG2,ROCK1,EPS8,NID1,SCMH1,C1QTNF1,PAXIP1,ZNF713,EGR2,HIPK3,RNF10,RYBP,GAPVD1,CAPN10,GPR35,CUL2,AP3B1,PTPN2,INSRR,NTRK1,KPNA6,TRIM44,ZFAND1,MAP1A,PKN2,PPP1R9A,TMIGD1,SLC39A10,EIF3A,ANK3,DNMBP,UNC13A,EPHB1,PDE6A,EPHA10,PTPN9,AXIN2,SARM1,EPCAM,PARD3,RCAN2,ZNF425,GNL3L,MUC1,PTPN14,TRIM46,TUB,SH3GL3,JAG2,KCND3,BCOR,CACNG8,AP3D1,ZNF41,CARM1,VWF,AP2B1,NETO1,ZNF383,ANXA8L1,ADAM19,ARHGAP44,SNX33,PIFO,CD84,HTR1D,LPA,ZNF616,ZNF836,SMARCA2,ARHGEF3,CUX1,RAD51C,SPI1,DGKK,DNMT3B,EPHA7,CHRNB4,FOXP2,MCU,RANBP10,PTPRA,CTNNA1,LIMA1,AIFM2,FBXW4,PSPC1,GLIS1,SFMBT2,TRDN,ATG14,EIF3H,GABRA6,LY86,STAC,BCAR3,JAZF1,STK38,TRPC6,ABCB7,ACOT8,RBPMS,RENBP,CHURC1,CREB5,FCHSD2,MAP3K13,OTUD3,RAB15,RAPGEF3,PDXP,SH3BP1,MST1,ERCC3,PTK7,SMYD1,SNCA,BMPR1B,CACNG2,CD53,GRM4,MAGI2,PRIM2,USP42,PNPT1,USP50,ZEB2,DHRS4,CABLES1,FOXJ3,SYT9,HEY2,RC3H1,CHMP3,EIF3E,CNGA3,RNF19B,WNT7B,PTPRS,ZMYND11,KMT2D,PRKAG1,CDKL1,CREM,PSMF1,RAB5B,KMT2C,CAST,TRIP12,DFFA,SPPL2A,ADTRP,ABCA12,GNG7,PLD1,RASA1,MACC1,MITF,SRSF6,OPRD1,CABIN1,HIPK1,NUGGC,PKP2,RPTOR,RTN4RL1,NFIX,BTK,SIGLEC9,TBC1D16,XPO4,ZNRF3,KCTD13,MLLT1,SLC4A8,TROVE2,CBFA2T2,IGF1,MLXIP,PLA2G4E,STX8,ATF7IP,CTNNBL1,HTT,DPYSL3,MAPK1,PTEN,ARHGEF17,MIB1,SPRED2,BMP7,SCN8A,ATXN2,MXI1,PIK3C3,PUM1,RASA2,SOX5,CIR1,PCBP2,PRCP,RAB30,ZNF780A,ZNF780B,LRRK2,ATP6V1A,MLYCD,OSGIN1,GNA14,TMEM59,ZRANB1,EBAG9,SPAG5,UNC119,ZBTB20,CHMP5,CNIH3,RAPH1,RFC3,BAG6,LILRB4,MORC3,IFT81,TMCO1,TMEM108,ITGAM,ARHGAP25,IL11RA,NSG2,PKD2,ZNF652,EFNA5,SHANK3,KCTD1,STMN4,RIPPLY1,RCOR3,TADA2A,DAZL,BTBD11,RGS9,PDGFC,SERPINA3,SERPINA4,SERPINA5,AMOTL1,FAM13B,NCOA3,PKIB,MTMR3,ZNF146,ZNF565,PPP2R3C,TBCD,WNT7A,ZBED6,BRWD3,NLRP1,RPS6KC1,UACA,LPGAT1,MAP4,CRTC3,DNAJC1,GPR141,ARNTL2,ELOVL5,PLCL2,KAT6A,MTIF2,GPR21,RABEP1,TTC28,RHOT1,SIK3,ZKSCAN7,ZNF197,ZNF660,BMP6,TAF15,MYCBP2,NFE2L1,ANO1,GPI,IL17RD,SH3RF2,SOS1,TSHR,WDR43,ZNF30,EXT1,N4BP1,PDCD1LG2,ATRX,DPH6,PRICKLE2,GABRR2,IKZF1,PRMT2,RAPGEF2,ASCC1,KCNC2,SMO,VRK3,RALY,ENPP3,BLOC1S3,MARK4,PRKAR2B,STXBP5L,CPT1A,PSEN2,TCF4,TNFRSF11B,PAG1,SYT17,SOX30,DNAJC6,TFE3,UBR5,GRB14,GRIN1,JADE1,KCTD8,ARRDC4,RHOA,SPRTN,WSB2,OR4M1,OR4N2,SYNGAP1,VIPR2,GRIN3A,ROR1,RQCD1,TF,ONECUT2,RHOBTB1,FAM19A4,CUL3,SH3KBP1,TFEC,HSPD1,ITPR2,NFKBID,EPM2A,GABRB3,GSK3B,ABCG8,PLD2,PRR16,DNAJC3,GNG12,RDX,STAT6,ACTA2,SYN1,MLF1,EEF2K,FAM49B,IWS1,PPP1R16A,PTPRB,RBBP8,NECAB2,CDK13,STK32B,CD27,DUSP26,ITGBL1,MCTP2,SCN9A,BOK,RNF144B,SULF1,ZNF362,KRT8,NACC2,RNF43,SUPT4H1,TNFRSF8,ZNF354A,AGT,HDGFRP3,METTL16,CCNJL,POLH,PRKAA2,SYT7,ADAR,BLOC1S6,CUL9,FBN2,PITPNM2,STAT2,ZMPSTE24,BRINP3,HRH4,NDRG2,PTK2,TPPP2,LAT2,PEX5L,SNX3,PDE4B,RIMS2,PBX1,TCP11,TEC,EXOSC3,FBXO10,SHB,VEPH1,CDC14A,GAP43,MAP3K5,CPE,SEC16B,TCFL5,CREBBP,IQGAP2,PRKD3,UTP20,APCDD1L,DLX1,FMOD,INSL6,PCSK6,CEP70,GTF2IRD2,NCF1,SLC29A1,TAOK2,UGT1A1,UGT1A10,UGT1A4,UGT1A8,PYGO2,SHC1,ZNF813,ARHGAP15,CD160,ECE1,KCNJ15,LBX2,PARP10,RNFT1,RPS6KB1,STC2,YLPM1,DPRX,GPR173,GUCY2F,PRG3,SCEL,SERPINB11,SETDB2,ANKS1A,ITGAL,ALOX5,CPLX2,DNAJB6,SP7,CD44,FGD3,LARP4,ADAM8,SLC5A3,CLDN4,PACSIN2,RPS6KA5,ZNF484,BCL2L14,CDS1,ZNF93,CSNK1A1,GRID1,HUS1,PRDM2,CXCL17,FCRL6,FGD4,NOS1,ZNF44,GCKR,MED15,PLEKHA1,PLVAP,RGS14,SIPA1L2,ACTN4,ARHGAP11A,MC1R,STK4,TCF25,ZFAND6,DRAM1,ZNF282,MARK3,SPEF1,EPHB2,SLC1A1,WLS,EDRF1,SIGMAR1,TMEFF2,XCR1,BCO2,CLPTM1,IL18,DTNA,LRRC52,MFSD8,UBE2K,GPSM2,DNAJC7,HCAR1,HCAR2,HCAR3,DIS3,DLC1,PPARA,PPP1R10,ARID5B,CALCRL,MCOLN1,EPB41L4B,SLC25A33,SMARCAL1,PHLPP1,MAGI3,CPEB4,GPSM3,MOB3B,NOTCH4,SYTL4,ZMYND8,ZNF366,CRX,FBN1,PAX6,PRKCZ,ZC3HAV1,FAM20C,ADORA2A,GRIN2B,PPP1CC,RABGEF1,CCNG2,CPEB1,PHLDB2,PLCXD2,TYK2,OTUD7B,DENND4B,NIPBL,TMIGD2,YEATS4,CNRIP1,COL28A1,PHF20,ZNF143,ASPN,ANGPT1,BRF1,CADPS,TBL1X,EYA3,FHL2,FRMPD1,LRRK1,MBTD1,OSBPL8,ATF6,ZBTB5,ZNF708,IGF2BP3,EPB41L2,GCNT2,ETS1,PPP1CA,TBC1D10C,VWC2,ARHGAP21,GRIA4,MAP2,MTF2,RBMS3,CCL22,STPG1,BTN3A2,TAGLN3,FBXO31,ATG3,CDK5RAP1,CNTN1,NCOR2,PRKCQ,BRAF,CSRNP1,DIAPH1,HDAC2,HTR2C,IMPACT,ATP8A1,PHKG2,SRCAP,CAPN6,POU3F3,NOTO,PPP1R14A,SPINT2,TNR,MBNL3,UBE2E2,ZKSCAN5,HIGD2A,CD300A,ELF2,IRAK1BP1,PHIP,CDKL3,PPP2CA,SKP1,ANGPT4,ARHGEF6,MAOA,RPL23,SH3BP4,RFC5,ATXN1,CAMSAP3,DCDC1,SP100,ZNF347,ZNF415,OPN1LW,

			ANXA2,TRIM29,KCND2,PARN,ATP6V0A1,PIGU,RAB27B,C1QTNF9,CR2,DONSON,FCRL2,ITSN1,NUP214,SRGAP2,ADCY1,MADD,NUMB,RBM42,SQLE,SYK,CNOT1,RBM39,WWTR1,ARHGAP19,GRIK2,GTF2H5,SCN1A,SLIT1,ASB1,FRMD5,NFXL1,CIT,MYRIP,RADIL,ZBTB38,BRCA2,HRK,RAP1GAP2,ATF7,CACNG3,DVL2,MORC1,MTRF1,NEB,YBX1,ANKRD13A,GRIP1,PTPRJ,SLC10A1,TAF1,EIF4E3,TICAM1,TANK,THAP3,GPM6A,UCN2,ITGAE,TAC3,PLEKHB2,CADM1,HSF1,MAX,SAP130,DHFR,EZH1,ETF1,INPP5A,NRF1,SRPK2,ANKRD13C,PLEKHM1,SASH1,ASAP1,NCOA2,ADAMTS7,RALGAPA1,GBP5,SULT2B1,WDR18,BMP1,CGNL1,EEFSEC,FCHO1,QRICH1,VAV3,ZP3,CHD6,DLGAP2,KCNQ3,RNF2,ZNF554,MOB3A,CAV2,GNA12,SH2D3A,SKA2,AKTIP,COL4A5,MYEF2,INPP5F,NUAK2,AXL,REPS2,TNFSF9,TRIM37,WIF1,CD109,DENND4C,MET,SESTD1,TBC1D10A,ZNF461,CAMK2D,CCR3,KCNJ6,RAD9B,TIMELESS,UCHL5,ANKRD6,BRD9,MALT1,SERPINE3,SETD3,TRA2B,SPTAN1,EHD2,KDM2B,MLXIPL,SEMA3C,THEM4,CCT3,NEDD4L,PHB,PTPRC,EDA,GPR89A,ZNF555,CTCF,PBLD,SH3GL2,TYRO3,NR6A1,RNF213,SYNDIG1,TSPAN5,ADCY10,NCAM1,TRAF3IP2,ATF6B,CREB1,RASSF8,RGMB,SGTB,SHISA6,TNXB,TRAP1,CLNK,EBF4,EDNRA,GNGT1,PRAP1,STXBP6,ZNF511,BMP4,ABLIM3,CASP12,CPNE1
GO:0048856	anatomical structure development	1.8234538370754043e-44	*CLRN1,DNAH11,RCN1,ENPP1,PRDX2,ADAMTS16,LDB2,NRXN1,ASPH,PRMT3,GSS,RDH13,PRKCI,MOV10L1,TACC2,MAP4K4,PDE4D,DNMT1,S1PR2,HLX,LLPH,SLC9A1,NEGR1,PBX3,SPAG16,LMBR1,TRPS1,CBFB,CLASP2,SEMA3A,IL31RA,LSAMP,GRID2,RPS6KA2,PRDM12,TENM3,CHERP,DOCK1,CLDN18,RYR1,NRG3,ASH1L,NOX5,IQCG,ASTN2,EPB41,SEMA3D,PHACTR1,MYOT,NREP,TIAM2,STOX2,FHOD3,NLGN4X,PTGIS,DCHS2,TJP1,UNC5C,ZSWIM6,PRLR,HIVEP3,FTO,UTRN,KALRN,SPRR2D,NTRK3,CBL,ARNT,ATRNL1,VWDE,SEMA5A,FLT3,RAD51B,STAT5B,TOX,ENPP2,ATP2B2,GRM5,PLCE1,LHFPL4,SAMHD1,FER,MAP2K5,SPATA5,KITLG,PTPRR,SPNS2,NDUFV2,LRP2,PIK3CD,ZNF536,SP3,ANK2,OLFM1,MFAP5,EZR,MEGF10,NRXN3,XRCC4,ROBO1,CHD7,SLC26A6,HTR2B,ITGB6,CDKL5,MECOM,TACC1,TENM1,KIF5C,LMNA,KIAA1217,TRAF6,ESRP1,ROBO2,NFASC,ITPKB,DNAJA3,VAV2,ABI1,ULK4,NHLH1,ITGB1,TRIOBP,HSP90AA1,SLC24A3,GAS7,PSMB7,TSSK1B,PCDH17,KNDC1,ZC4H2,KIF26B,DSCAM,FAM126A,SRGAP2B,CNGB1,TRIO,ARHGAP24,KLHL12,AKAP13,TTLL5,GFI1B,RUNX1,DAB1,OMA1,CDC42EP3,MYO10,ABCC8,CDH10,NRIP1,THRB,XIRP2,IMPG2,LAMA2,GFRA2,TCIRG1,DACH1,PTPN11,RBM19,DOCK10,HDAC6,GOLGA4,MYT1,ADORA1,ADAMTS3,EPHA1,IKBKB,ERBB4,CDH12,DPY19L2,LIMK1,ZNF609,ATP8A2,ACTG2,HIF3A,CUL4B,KLHL6,LARGE,CECR2,ESR1,MYO18B,NPHP3,GRM7,ANKRD11,PTPRO,CDON,NTRK2,DNAI2,EP300,CELA1,MEGF9,DYM,TENM2,RNF220,HOOK3,PDGFB,RIMS1,TNIK,SNX2,KIRREL3,CDH8,CSGALNACT1,TOB2,MID1,HNF4G,INSR,FMN2,RERE,PRKAR1A,ATP8B1,UNC5D,H2AFY2,PLLP,LAMA3,TCF7L2,PIP4K2A,CDH4,PAK1,FBXW11,ESRRB,MAP3K4,PTPRD,CNTN5,BASP1,RBM20,SLC4A10,TBR1,CATSPER2,STRC,BDNF,TFAP2A,ATRN,AMIGO1,FANCA,CHRM3,TGIF2,POMGNT2,FUT8,NPRL3,FMNL2,PHACTR4,MEIS1,TENM4,LHFPL2,TRIM13,LCK,MDM4,ECT2,ZNF148,MYO3B,TFDP2,NAV2,LAMC1,PTPRU,MB,AFF3,SGCD,DENND5A,DMD,CENPF,CRISPLD2,KAT7,WDR7,SLC8A1,GSN,RBM4,PRPS2,GPR171,SATB2,PAFAH1B2,RIT2,HIRA,MACROD2,TANC2,JPH2,CASS4,FYN,ARNTL,ADAMTS9,NF1,PLCB1,MGMT,PCDH15,LMTK2,ARID4B,FAT3,RTN4,AFF2,RXFP1,MAL2,CHRDL1,APOD,B4GALT6,RTN4R,PLEKHM3,BCR,CHI3L1,CCDC141,TTN,NDRG4,BMP2K,PAQR3,ANKH,ADAMTSL1,TLL1,NTN1,EGFLAM,DCTN1,SLC4A5,NRG1,UBP1,STRBP,FRY,MAP2K1,MDFI,FNIP1,STIM1,VAX2,MYH9,MAD1L1,LRFN5,DAGLA,PLEKHA5,VGLL4,CNTN4,HDAC5,NTNG1,PCSK2,NAV1,NFIA,TMEM30A,SYNE1,CMKLR1,SBF2,ROR2,DCN,SLC39A14,PRPSAP2,LIN7A,SCFD1,CATSPER3,DOCK2,CDH13,PLXDC1,SOX13,ACSBG1,UST,FGF14,DMC1,PCDHB16,EXOC4,MAD2L2,TLE6,CAPRIN2,NLN,NCKAP1,TERF2,FOXN3,RCC2,BOC,ANO6,ARL3,NEUROD1,KIF2A,ACTL8,COL12A1,CCBE1,USP22,ZDHHC15,JAK2,MYPN,YTHDC2,TRAPPC9,FBXW7,CLSTN2,SYT1,ITCH,LZTS1,BCL11B,RBFOX1,TMC1,LRIG3,PKNOX1,CLDN1,HUNK,MLLT3,PNPLA1,PLS1,TEX11,TCF7,PDE2A,MSI2,SEPT7,KLF15,TBX15,ANXA4,CYFIP2,WNT11,PALMD,IFT80,FIG4,KREMEN1,IMMP2L,NRG4,ASTN1,NOX4,SIPA1L3,MKLN1,CACNA1H,SIAH1,QKI,IL1RAPL1,CCDC62,CAMK2B,ADAM23,SOX6,TAB2,ACVR2A,RUNX2,SEMA5B,CD4,TGFB1,SGK1,PCSK5,PDLIM4,MYO3A,ZHX2,KEL,BTRC,NFATC3,F2RL1,BCAS3,C9ORF47,C2ORF49,RPGRIP1L,CELSR1,SLIT3,SDK2,SLIT2,TP73,MUSTN1,GABRB1,CDH23,CORO1C,CTNND2,DISC1,KANK1,LRRC10,LAMC2,MTDH,CLMN,TTBK2,MYT1L,OPCML,CHRM1,SMAD6,RXFP2,BNC2,CLOCK,ITSN2,TCF12,ZNF675,SMOC1,ETV6,SYNJ2,NELL1,KCNH1,PRR14,TFAP2D,BMPER,BCL3,DCLK1,ANKRD54,PARVA,ADAM12,NAIP,STRIP1,SLC17A7,SBNO2,LINGO2,YTHDF1,FGF10,CNTNAP2,FBXL17,ARHGEF28,SMYD3,ENAH,GREB1L,IL1RAPL2,PPL,LOXL3,SVIL,CAPN3,VMP1,SMURF2,EPHA4,RORA,PRKCA,AUTS2,CNR1,TNFSF11,PPP3CA,NSUN2,MAG,CAMK4,UFL1,TRAK1,CTNNB1,PARK2,SOD2,DACH2,FCGR2B,ARHGAP22,SMARCC1,CDHR2,IGF1R,PPARG,NGRN,AXIN1,DLG5,IL18R1,BFSP1,ADD2,MTF1,CPQ,ANKRD17,CYBB,OTC,XK,BRIP1,PFAS,RYR2,FBLIM1,DRAXIN,LEPR,FGF1,NIN,NPAT,NR4A3,DCT,MYOCD,PER2,AJUBA,CACNA1C,CPNE6,GLG1,SCN3B,PHLDB1,KRT6B,MYO1E,CSMD1,HCK,CAPN2,CSPG4,DCC,CTDP1,MMP16,OBSCN,NF2,FLT4,MEF2B,BICC1,HDAC4,PAX2,ELN,SPG11,SFRP1,FOXO3,ARL13B,CNTN6,NFIB,IFT122,DDX10,DNM3,SSH1,SMAD3,CUX2,ITPR1,PTPRM,ARNT2,ACKR2,NHS,RNF168,EMG1,CASZ1,ABI2,PSAP,SHANK1,SYT3,NEDD4,NRP2,ARHGEF18,VANGL1,BTBD3,PREX1,CRTAC1,HOXD3,HOXD4,NAV3,TAB1,NFATC1,PRTG,CDC73,APP,SSBP3,GS*

			X2,PDGFRA,CEP85L,DIP2B,NOX1,YAP1,HEG1,FSTL4,NLGN2,TNFRSF19,VDAC1,EYA2,SH3D19,ALPL,JAK1,ANGPTL4,PEMT,LRP5,PTPRG,BDH2,ISLR2,SLC9B2,SOX2,SETD2,ZDHHC6,PLAC1,GPC6,TTC39C,ADAMTS2,CHST11,TEAD1,PRICKLE1,RCAN1,ELAVL3,SPTBN4,VASH2,ZNF521,PAQR5,DRD1,CHUK,FRS2,PALB2,SFMBT1,MMP2,TPH1,SYNE2,CPNE9,KDM6A,PRKD1,STAT1,PARP6,DGKG,SYBU,RND3,SLC6A3,HNF4A,SHANK2,STRA6,VPS4A,EREG,ABLIM1,MYOF,MYH15,DSCAML1,SEMA6D,RBFOX3,ATF2,POU2F2,TCF3,RAF1,CELF4,ADAMTS12,SRRM4,BMPR2,USP33,DPYSL2,CAMK1D,BMPR1A,AP1B1,PABPC4,TSPAN12,NLGN1,CTNNA2,PIK3R3,TAF8,CERS3,SPAG9,RAB11A,CRMP1,EVC,MYB,FGF2,LRRC4C,POU6F2,GRIK1,NEDD9,AGBL4,PEAK1,ADAMTS4,SEMA4D,SH3PXD2A,ISM1,FBN3,ARMC2,RORC,ELP3,PLXNA2,ADCYAP1R1,FNDC3A,SETD1A,JARID2,RILPL1,KLHL3,DKK2,ITGA11,RARB,SPEN,PRKCG,NCOA1,SPOCK1,BLOC1S5,EHMT1,GAS8,OC90,NTM,CHRD,DVL3,EIF2B5,EIF4G1,EPHB3,ATF3,FBXO45,MATN3,LMBRD1,SRSF5,EBF2,ADAM20,ADAM21,TSG101,VCL,LAMB1,CLIC4,MYO9A,IDE,COL11A1,KCNQ1,RFX2,WNT3,ADAMTS6,RHOJ,P4HB,SUFU,TG,NLGN3,SCUBE2,PAFAH1B1,KIF3A,CCDC169-SOHLH2,SOHLH2,POLE,CNTFR,COL4A3,DDAH1,NMUR2,AKAP6,RASAL1,PADI6,NR2C1,TRPC5,TTLL7,GLI2,NEO1,SCUBE1,ERCC1,RUFY3,WDTC1,TPD52,COL19A1,DAB2,THEMIS,PKHD1,XKR4,USH2A,LDLRAD4,MYSM1,SETD5,DZIP1,DLG3,WNK1,RELN,SIN3A,RSPO2,CSF3R,KCNQ2,EYS,DOCK11,CRELD1,CFDP1,IQGAP1,APOLD1,ZNF423,SP1,LUZP1,MACF1,ARHGAP12,MYO7A,ALK,GSTM3,INPP5D,CLASP1,DSC2,TEAD4,HOXC13,AKAP2,SRA1,TOP1,MBOAT1,GPM6B,XDH,OVOL2,NFATC2,RBBP6,PRKG1,PAX7,RGS7,BNC1,ATP2B4,PACRG,SYNE3,GALNTL5,PACSIN1,TMEM2,ACTL6B,ASXL3,PAK3,TET1,DYX1C1,ASGR2,ADNP,GPR161,MAPKAPK2,ARID4A,MAP1B,MARK1,CDK6,PHF2,CELF1,TYR,TFRC,EPHA5,WWOX,MEF2A,ADCY9,DHX30,DROSHA,GFRAL,GPC3,ADRBK1,PLS3,PSMB2,CALD1,EYA1,BRINP1,TRPV1,HOXB3,HOXB4,HOXB5,HOXB6,TFEB,GHR,MNAT1,FLRT2,FGD1,DIAPH2,ACTR2,ELAVL4,LGI4,HDAC1,SMOC2,ADIPOR2,PLCG2,ROCK1,EPS8,NID1,SCMH1,PAXIP1,EGR2,RNF10,RYBP,AP3B1,PTPN2,INSRR,NTRK1,MAP1A,PPP1R9A,ANK3,DNMBP,ACO2,UNC13A,PTPRQ,EPHB1,PDE6A,EPHA10,PTPN9,AXIN2,SARM1,SNTG2,EPCAM,PARD3,PTPN14,TRIM46,TUB,SH3GL3,JAG2,BCOR,AP3D1,CARM1,AP2B1,ADAM19,ARHGAP44,SRD5A2,PIFO,COCH,SMARCA2,CUX1,SPI1,EPHA7,FOXP2,MCU,CTNNA1,FBXW4,BCAR3,ILDR2,SHROOM3,CHURC1,MAP3K13,RAPGEF3,SDK1,SH3BP1,MST1,KAZN,ERCC3,PTK7,SMYD1,TTC9,SNCA,BMPR1B,CD53,MAGI2,PNPT1,ZEB2,RAI2,OCA2,HEY2,RC3H1,COL13A1,HYDIN,RNF103,WNT7B,HMGCS2,PTPRS,KMT2D,TLL2,TRIP12,ADTRP,ABCA12,CSRP1,RASA1,MITF,SRSF6,RBM45,HIPK1,PKP2,RTN4RL1,BTK,SGCZ,ZNRF3,TROVE2,CBFA2T2,IGF1,CTNNBL1,DPYSL3,MAPK1,PTEN,CLDN11,MIB1,SPRED2,BMP7,SCN8A,SOX5,CIR1,LHFPL5,TTLL4,PRCP,LRRK2,MEGF11,ZRANB1,ALDOC,UNC119,RAPH1,BAG6,LILRB4,MORC3,TMEM108,ITGAM,IL11RA,PKD2,EFNA5,SHANK3,STMN4,RIPPLY1,DAZL,ALPK3,PDGFC,AMOTL1,NCOA3,PPP2R3C,TBCD,WNT7A,ZBED6,BRWD3,MAP4,PLCL2,KAT6A,SH3PXD2B,BMP6,MYCBP2,NFE2L1,GPI,IL17RD,SOS1,TSHR,EXT1,ATRX,PRICKLE2,SERINC5,IKZF1,PARVB,PRMT2,RAPGEF2,CDH9,KCNC2,SMO,SUN1,BLOC1S3,MARK4,CPT1A,MYLPF,TCF4,TNFRSF11B,SYT17,BPGM,SOX30,TFE3,GRIN1,ADAM29,RHOA,SYNGAP1,GRIN3A,NDE1,ROR1,TF,ONECUT2,RHOBTB1,SHROOM1,CUL3,SH3KBP1,HSPD1,NFKBID,EPM2A,GABRB3,GSK3B,EMB,RDX,STAT6,ACTA2,SYN1,MLF1,EEF2K,FAM49B,PTPRB,RBBP8,CCDC14,CDK13,ALAS2,UPF2,CD27,SCN9A,BOK,SULF1,MTHFD1L,KRT8,AGT,HDGFRP3,CDH20,ADAR,BLOC1S6,FBN2,STAT2,ZMPSTE24,BRINP3,NDRG2,PTK2,PHEX,SNX3,RIMS2,PBX1,TCP11,TEC,EXOSC3,SHB,GAP43,MAP3K5,CPE,TRPC4AP,CREBBP,FAM171A1,ARVCF,COL9A1,DLX1,PCSK6,SLC29A1,TAOK2,UGT1A1,PYGO2,SHC1,NDUFS2,ALDOA,ARHGAP15,CD160,ECE1,LBX2,RPS6KB1,STC2,GPR173,PGM5,SCEL,SETDB2,ANKS1A,ALOX5,CPLX2,DNAJB6,MDGA2,SP7,CD44,FGD3,LARP4,ADAM8,SLC5A3,CLDN4,PACSIN2,RPS6KA5,THSD7A,HUS1,PKD1L1,PRDM2,CXCL17,FGD4,NOS1,PEX7,TBC1D23,PLEKHA1,RGS14,ACTN4,MC1R,STK4,TCF25,TUBB3,SPEF1,EPHB2,SLC1A1,WLS,SIGMAR1,TMEFF2,CLPTM1,IL18,MFSD8,ASF1B,DLC1,PPARA,ARID5B,CALCRL,PHLPP1,PRRC2C,NOTCH4,SOBP,CRX,FBN1,PAX6,PRKCZ,FAM20C,ADORA2A,GRIN2B,PPP1CC,COL22A1,PHLDB2,OTUD7B,KLC3,KLHL1,NIPBL,TMIGD2,PCDH10,UPB1,ASPN,ANGPT1,EYA3,FHL2,LRRK1,MBTD1,ATF6,SHROOM4,IGF2BP3,GCNT2,ETS1,PPP1CA,VWC2,MAP2,MTF2,NEBL,TAGLN3,FBXO31,CDK5RAP1,CNTN1,NCOR2,PRKCQ,LGI2,BRAF,CSRNP1,DIAPH1,HDAC2,IMPACT,POU3F3,NOTO,SPINT2,TNR,MBNL3,PHIP,CDKL3,PPP2CA,ANGPT4,ACAN,ATXN1,CAMSAP3,SP100,ANXA2,CR2,SRGAP2,TMEFF1,ADCY1,NUMB,SYK,CNOT1,WWTR1,SLIT1,ASB1,CIT,RADIL,BRCA2,DVL2,NEB,YBX1,GRIP1,PTPRJ,TAF1,GPRIN3,RNF38,GPM6A,OSBP2,POTEE,CADM1,HSF1,MAX,DHFR,EZH1,SRPK2,SASH1,ASAP1,ADAMTS7,SULT2B1,BMP1,VAV3,ZP3,RNF2,CAV2,GNA12,MYEF2,INPP5F,AXL,TNFSF9,WIF1,CD109,MET,FMNL1,CCR3,SNX1,TIMELESS,UCHL5,ANKRD6,MALT1,SETD3,TRA2B,CCDC136,EHD2,KDM2B,MLXIPL,SEMA3C,NEDD4L,PTPRC,EDA,GPR89A,SH3GL2,TYRO3,RNF213,SYNDIG1,VPS52,NCAM1,TANC1,TRAF3IP2,CREB1,LIG1,AFF4,EDNRA,GNGT1,BMP4,CPNE1
GO:0032502	developmental process	2.614587173958802e-	*RDH14,CLRN1,DNAH11,RCN1,ENPP1,PRDX2,ADAMTS16,LDB2,NRXN1,ASPH,PRMT3,GSS,RDH13,PRKCI,MOV10L1,TACC2,MAP4K4,PDE4D,DNMT1,S1PR2,HLX,LLPH,SLC9A1,NEGR1,PBX3,SPAG16,LMBR1,TRPS1,CBFB,CLASP2,SEMA3A,IL31R*

		44	A,LSAMP,GRID2,RPS6KA2,PRDM12,TENM3,CALR3,CHERP,DOCK1,WWC1,CLDN18,RYR1,NRG3,ASH1L,LATS2,NOX5,IQCG,ASTN2,EPB41,SEMA3D,PHACTR1,MYOT,NREP,TIAM2,STOX2,FHOD3,NLGN4X,PTGIS,DCHS2,WWC3,TJP1,UNC5C,ZSWIM6,PRLR,HIVEP3,FTO,UTRN,KALRN,WIPF3,SPRR2D,NTRK3,CBL,ARNT,ATRNL1,VWDE,SEMA5A,FLT3,RAD51B,STAT5B,TOX,ENPP2,ATP2B2,GRM5,NOC3L,PLCE1,LHFPL4,SAMHD1,FER,MAP2K5,SPATA5,KITLG,MAPK10,PTPRR,SPNS2,NDUFV2,LRP2,PIK3CD,ZNF536,SP3,ANK2,OLFM1,MFAP5,EZR,MEGF10,NRXN3,XRCC4,ROBO1,CHD7,SLC26A6,HTR2B,ITGB6,CDKL5,MECOM,TACC1,TENM1,KIF5C,LMNA,KIAA1217,TRAF6,ESRP1,ROBO2,NFASC,ITPKB,DNAJA3,VAV2,GRK5,ABI1,ULK4,NHLH1,ITGB1,TRIOBP,HSP90AA1,SLC24A3,GAS7,PSMB7,TSSK1B,PCDH17,KNDC1,ZC4H2,KIF26B,DSCAM,FAM126A,SRGAP2B,CNGB1,TRIO,ARHGAP24,KLHL12,AKAP13,TTLL5,GFI1B,RUNX1,DAB1,OMA1,CDC42EP3,MYO10,ABCC8,CDH10,NRIP1,THRB,XIRP2,IMPG2,LAMA2,GFRA2,TCIRG1,DACH1,PTPN11,RBM19,HERC4,DOCK10,HDAC6,GOLGA4,MYT1,ADORA1,ADAMTS3,EPHA1,IKBKB,ERBB4,CDH12,DPY19L2,LIMK1,ZNF609,ATP8A2,ACTG2,HIF3A,CUL4B,KLHL6,LARGE,CECR2,ESR1,MYO18B,NPHP3,GRM7,ANKRD11,PTPRO,CDON,NTRK2,DNAI2,EP300,CELA1,MEGF9,DYM,TENM2,RNF220,HOOK3,PDGFB,RIMS1,TNIK,SNX2,KIRREL3,CDH8,CSGALNACT1,TOB2,MID1,GOLGA3,HNF4G,INSR,FMN2,RERE,PRKAR1A,ATP8B1,UNC5D,H2AFY2,PLLP,LAMA3,TCF7L2,PIP4K2A,CDH4,PAK1,FBXW11,ESRRB,MAP3K4,PTPRD,CNTN5,BASP1,CATSPERB,RBM20,SLC4A10,TBR1,CATSPER2,STRC,BDNF,TFAP2A,ATRN,AMIGO1,FANCA,CHRM3,TGIF2,POMGNT2,FUT8,NPRL3,FMNL2,PHACTR4,MEIS1,TENM4,LHFPL2,TRIM13,UBR2,LCK,MDM4,EDARADD,SHC4,ECT2,ZNF148,MYO3B,TFDP2,NAV2,ACOX1,LAMC1,PTPRU,MB,AFF3,SGCD,DENND5A,DMD,CENPF,CRISPLD2,USP13,KAT7,WDR7,SLC8A1,GSN,RBM4,PRPS2,GPR171,SATB2,PAFAH1B2,RIT2,HIRA,MACROD2,TANC2,JPH2,CASS4,FYN,ARNTL,ADAMTS9,NF1,PLCB1,MGMT,PCDH15,LMTK2,ARID4B,FAT3,RTN4,AFF2,RXFP1,MAL2,CHRDL1,APOD,SLC2A14,B4GALT6,RTN4R,PLEKHM3,BCR,CHI3L1,CCDC141,TTN,NDRG4,BMP2K,PAQR3,ANKH,ADAMTSL1,TLL1,NTN1,EGFLAM,DCTN1,SLC4A5,NRG1,UBP1,STRBP,FRY,MAP2K1,MDFI,FNIP1,STIM1,VAX2,MYH9,MAD1L1,LRFN5,DAGLA,PLEKHA5,VGLL4,CNTN4,HDAC5,NTNG1,PCSK2,NAV1,NFIA,TMEM30A,SYNE1,JDP2,CMKLR1,SBF2,CDKL2,TSPAN8,LRGUK,ROR2,DCN,SLC39A14,PRPSAP2,LIN7A,SCFD1,CATSPER3,DOCK2,CDH13,PLXDC1,SOX13,ACSBG1,UST,FGF14,DMC1,PCDHB16,EXOC4,MAD2L2,TLE6,RAB27A,CAPRIN2,CCNYL1,NLN,NCKAP1,TERF2,FOXN3,RCC2,BOC,ANO6,ARL3,NEUROD1,KIF2A,FBXO9,ACTL8,PARP11,COL12A1,CCBE1,USP22,ZDHHC15,JAK2,MYPN,YTHDC2,TRAPPC9,FBXW7,SMC3,CLSTN2,SYT1,ITCH,BBS12,LZTS1,BCL11B,RBFOX1,TMC1,LRIG3,PKNOX1,CLDN1,HUNK,MLLT3,PNPLA1,PLS1,TEX11,TCF7,PDE2A,MSI2,SEPT7,KLF15,TBX15,ANXA4,CYFIP2,WNT11,PALMD,IFT80,FIG4,KREMEN1,IMMP2L,NRG4,ASTN1,NOX4,SIPA1L3,MKLN1,CACNA1H,SIAH1,QKI,IL1RAPL1,CCDC62,CAMK2B,ADAM23,PRKCD,SOX6,TAB2,ACVR2A,RUNX2,SEMA5B,CD4,TGFB1,SGK1,PCSK5,PDLIM4,MYO3A,ZHX2,KEL,BTRC,NFATC3,F2RL1,BCAS3,C9ORF47,C2ORF49,RPGRIP1L,CELSR1,SLIT3,SDK2,SLIT2,TP73,MUSTN1,GABRB1,CDH23,CORO1C,RRAS2,CTNND2,DISC1,TSNAX,KANK1,LRRC10,LAMC2,MTDH,CLMN,TTBK2,MYT1L,OPCML,FSTL5,CHRM1,SMAD6,RXFP2,BNC2,CLOCK,ITSN2,TCF12,ZNF675,SMOC1,ETV6,SYNJ2,NELL1,SUCO,KCNH1,PRR14,TFAP2D,BMPER,BCL3,DCLK1,ANKRD54,PARVA,SND1,ADAM12,NAIP,STRIP1,HNRNPC,SLC17A7,SBNO2,LINGO2,YTHDF1,FGF10,CNTNAP2,FBXL17,ARHGEF28,SMYD3,ENAH,GREB1L,IL1RAPL2,PPL,LOXL3,MAST2,SVIL,CAPN3,VMP1,SMURF2,EPHA4,RORA,PRKCA,AUTS2,CNR1,TNFSF11,PPP3CA,NSUN2,MAG,CAMK4,UFL1,TRAK1,CTNNB1,PARK2,SOD2,DACH2,FCGR2B,ARHGAP22,SMARCC1,CDHR2,IGF1R,PPARG,NGRN,AXIN1,DLG5,IL18R1,BFSP1,ADD2,MTF1,CPQ,MSR1,ANKRD17,CYBB,OTC,XK,BRIP1,SPATA6L,ANXA13,ANKRD26,PFAS,RYR2,FBLIM1,DRAXIN,LEPR,FGF1,NIN,NPAT,NR4A3,DCT,MYOCD,PER2,KIR2DL4,AJUBA,CACNA1C,CPNE6,GLG1,SCN3B,CHEK2,PHLDB1,KRT6B,PRDM16,MYO1E,CSMD1,HCK,CAPN2,TRIM8,DIO2,MRC2,CSPG4,DCC,CTDP1,MMP16,OBSCN,NF2,FLT4,MEF2B,BICC1,HDAC4,PAX2,PHF5A,ELN,SPG11,SFRP1,FOXO3,ARL13B,CNTN6,NFIB,IFT122,DDX10,DNM3,SSH1,SYNCRIP,SMAD3,CUX2,ITPR1,PTPRM,ARNT2,ACKR2,NHS,RNF168,EMG1,CASZ1,ABI2,PSAP,SHANK1,SYT3,NEDD4,NRP2,ARHGEF18,VANGL1,BTBD3,PREX1,CRTAC1,HOXD3,HOXD4,NAV3,TAB1,NFATC1,PRTG,CDC73,APP,SSBP3,GSX2,PDGFRA,ODF3,CEP85L,DIP2B,NOX1,YAP1,HEG1,AMFR,FSTL4,NLGN2,TNFRSF19,VDAC1,EYA2,SH3D19,ALPL,JAK1,ANGPTL4,PEMT,LRP5,PTPRG,BDH2,ISLR2,SLC9B2,SOX2,SETD2,ZDHHC6,PLAC1,GPC6,TTC39C,ADAMTS2,CHST11,TEAD1,DEFB118,PRICKLE1,RCAN1,ELAVL3,SPTBN4,VASH2,ZNF521,PAQR5,DRD1,CHUK,FRS2,PALB2,SFMBT1,MMP2,TPH1,SYNE2,CPNE9,KDM6A,PRKD1,STAT1,PARP6,DGKG,ETV5,SYBU,RND3,SLC6A3,MORN2,HNF4A,ZBTB7C,SHANK2,STRA6,VPS4A,EREG,ABLIM1,MYOF,MYH15,DSCAML1,SEMA6D,RBFOX3,ATF2,POU2F2,TCF3,RAF1,CELF4,ADAMTS12,PSMD11,SRRM4,GGT7,BMPR2,USP33,DPYSL2,CAMK1D,BMPR1A,AP1B1,PABPC4,TSPAN12,NLGN1,CTNNA2,PIK3R3,CDK12,TAF8,CERS3,SPAG9,RAB11A,CRMP1,EVC,MYB,FGF2,LRRC4C,POU6F2,GRIK1,ZFP41,NEDD9,AGBL4,PEAK1,ADAMTS4,SEMA4D,SH3PXD2A,ISM1,FBN3,ARMC2,RORC,ELP3,PLXNA2,ADCYAP1R1,FNDC3A,SETD1A,JARID2,RILPL1,KLHL3,DKK2,BRDT,PHC2,ITGA11,RARB,SPEN,PRKCG,NCOA1,SPOCK1,BLOC1S5,EEF1E1,EHMT1,GAS8,OC90,NTM,CHRD,DVL3,EIF2B5,EIF4G1,EPHB3,ATF3,FBXO45,MATN3,LMBRD1,SRSF5,PAWR,EBF2,DLEC

			1,ADAM20,ADAM21,TSG101,CCDC3,VCL,LAMB1,CLIC4,MYO9A,ANKS4B,IDE,COL11A1,KCNQ1,RFX2,WNT3,ADAMTS6,ZNF322,RHOJ,P4HB,SUFU,TG,NLGN3,SCUBE2,PAFAH1B1,KIF3A,CCDC169-SOHLH2,SOHLH2,POLE,CNTFR,COL4A3,DDAH1,NMUR2,HIP1,AKAP6,RASAL1,PADI6,NR2C1,TRPC5,TTLL7,GLI2,NEO1,SCUBE1,ERCC1,RUFY3,WDTC1,ALDH6A1,TPD52,COL19A1,DAB2,THEMIS,PKHD1,XKR4,USH2A,LDLRAD4,MYSM1,SETD5,DZIP1,DLG3,WNK1,RELN,SIN3A,RSPO2,CSF3R,KCNQ2,EYS,DOCK11,CRELD1,CFDP1,IQGAP1,APOLD1,ZNF423,SP1,LUZP1,MACF1,ARHGAP12,MYO7A,ALK,GSTM3,INPP5D,CLASP1,DSC2,TEAD4,HOXC13,SLC9C1,AKAP2,SRA1,TOP1,UBE2V1,MBOAT1,GPM6B,ADIRF,XDH,OVOL2,NFATC2,RBBP6,PRKG1,PAX7,RGS7,BNC1,ATP2B4,PACRG,SYNE3,GALNTL5,PACSIN1,TMEM2,ACTL6B,ASXL3,PAK3,TET1,DYX1C1,ASGR2,ADNP,GPR161,MAPKAPK2,ARID4A,MAP1B,MARK1,CDK6,PHF2,CELF1,TYR,TFRC,EPHA5,WWOX,MEF2A,ADCY9,DHX30,DROSHA,GFRAL,GPC3,ADRBK1,PLS3,PSMB2,CALD1,EYA1,BRINP1,DIS3L2,GNB3,TRPV1,HOXB3,HOXB4,HOXB5,HOXB6,TFEB,GHR,MNAT1,FLRT2,FGD1,DIAPH2,ACTR2,ELAVL4,LGI4,HDAC1,SMOC2,ADIPOR2,PLCG2,ROCK1,EPS8,NID1,SCMH1,PAXIP1,EGR2,RNF10,RYBP,AP3B1,PTPN2,INSRR,NTRK1,MAP1A,PPP1R9A,ANK3,DNMBP,ACO2,UNC13A,PTPRQ,EPHB1,PDE6A,EPHA10,PTPN9,AXIN2,SARM1,SNTG2,EPCAM,PARD3,PTPN14,TRIM46,TUB,SH3GL3,JAG2,BCOR,AP3D1,CARM1,AP2B1,ADAM19,ARHGAP44,SRD5A2,PIFO,COCH,SMARCA2,CUX1,RAD51C,SPI1,EPHA7,FOXP2,MCU,CTNNA1,FBXW4,GLIS1,LRRC8C,BCAR3,ILDR2,SHROOM3,CHURC1,MAP3K13,RAPGEF3,SDK1,SH3BP1,MST1,KAZN,ERCC3,PTK7,SMYD1,TTC9,NHSL2,SNCA,BMPR1B,CD53,MAGI2,USP42,PNPT1,ZEB2,RAI2,FOXJ3,OCA2,HEY2,RC3H1,COL13A1,HYDIN,RNF103,WNT7B,HMGCS2,PTPRS,KMT2D,PRKAG1,CREM,TLL2,TRIP12,ADTRP,ABCA12,CSRP1,RASA1,MITF,SRSF6,RBM45,HIPK1,PKP2,RTN4RL1,BTK,SGCZ,ZNRF3,TROVE2,CBFA2T2,IGF1,CTNNBL1,DPYSL3,MAPK1,PTEN,CLDN11,MIB1,SPRED2,BMP7,SCN8A,PUM1,SOX5,CIR1,LHFPL5,TTLL4,PRCP,LRRK2,OSGIN1,SEPT6,MEGF11,ZRANB1,ALDOC,UNC119,RAPH1,BAG6,LILRB4,MORC3,IFT81,TMEM108,ITGAM,IL11RA,PKD2,EFNA5,HSF2BP,SHANK3,STMN4,RIPPLY1,DAZL,ALPK3,PDGFC,SERPINA5,AMOTL1,NCOA3,STEAP4,PPP2R3C,TBCD,WNT7A,ZBED6,BRWD3,MAP4,NME8,PLCL2,SPANXA2,KAT6A,GPR21,SH3PXD2B,BMP6,MYCBP2,NFE2L1,GPI,IL17RD,SOS1,TSHR,WDR43,EXT1,ATRX,PRICKLE2,SERINC5,IKZF1,PARVB,PNPLA3,PRMT2,RAPGEF2,CDH9,KCNC2,SMO,SUN1,BLOC1S3,MARK4,CPT1A,MYLPF,TCF4,TNFRSF11B,SYT17,BPGM,SOX30,TFE3,GRIN1,ADAM29,BBS9,RHOA,SYNGAP1,GRIN3A,NDE1,ROR1,RQCD1,TF,ONECUT2,RHOBTB1,SHROOM1,CUL3,SH3KBP1,HSPD1,NFKBID,EPM2A,GABRB3,GSK3B,EMB,RDX,STAT6,ACTA2,SYN1,MLF1,EEF2K,FAM49B,PTPRB,RBBP8,CCDC14,CDK13,ALAS2,UPF2,CD27,SCN9A,BOK,SULF1,MTHFD1L,KRT8,AGT,HDGFRP3,CDH20,ADAR,BLOC1S6,FBN2,STAT2,ZMPSTE24,BRINP3,NDRG2,PTK2,TPPP2,PHEX,SNX3,RIMS2,PBX1,TCP11,TEC,EXOSC3,SHB,GAP43,MAP3K5,CPE,TCFL5,TRPC4AP,CREBBP,FAM171A1,ARVCF,COL9A1,DLX1,PCSK6,SLC29A1,TAOK2,UGT1A1,PYGO2,SHC1,NDUFS2,ALDOA,ARHGAP15,CD160,ECE1,LBX2,RPS6KB1,STC2,GPR173,PGM5,SCEL,SETDB2,ANKS1A,ALOX5,CPLX2,DNAJB6,MDGA2,SP7,CD44,FGD3,LARP4,ADAM8,SLC5A3,CLDN4,PACSIN2,RPS6KA5,CDS1,THSD7A,HUS1,PKD1L1,PRDM2,CXCL17,FGD4,NOS1,PEX7,TBC1D23,MED15,PLEKHA1,PLVAP,RGS14,ACTN4,MC1R,SCNN1B,STK4,TCF25,TUBB3,SPEF1,EPHB2,SLC1A1,WLS,SIGMAR1,TMEFF2,CLPTM1,IL18,MFSD8,ASF1B,DLC1,PPARA,ARID5B,CALCRL,PHLPP1,PRRC2C,NOTCH4,SOBP,CRX,FBN1,PAX6,PRKCZ,FAM20C,ADORA2A,GRIN2B,PPP1CC,COL22A1,PHLDB2,OTUD7B,KLC3,KLHL1,NIPBL,TMIGD2,PCDH10,UPB1,ASPN,ANGPT1,EYA3,FHL2,LRRK1,MBTD1,OSBPL8,ATF6,SHROOM4,IGF2BP3,GCNT2,ETS1,PPP1CA,VWC2,MAP2,MTF2,NEBL,TAGLN3,FBXO31,CDK5RAP1,CNTN1,NCOR2,PRKCQ,LGI2,BRAF,CSRNP1,DIAPH1,HDAC2,HTR2C,IMPACT,POU3F3,NOTO,SPINT2,TNR,MBNL3,ELF2,PHIP,CDKL3,PPP2CA,ANGPT4,ACAN,ATXN1,CAMSAP3,SP100,ANXA2,PRMT7,CR2,SRGAP2,TMEFF1,ADCY1,NUMB,SYK,CNOT1,WWTR1,SLIT1,ASB1,CIT,RADIL,BRCA2,DVL2,MORC1,NEB,YBX1,GRIP1,PTPRJ,TAF1,GPRIN3,RNF38,GPM6A,OSBP2,PLEKHB2,POTEE,CADM1,HSF1,MAX,DHFR,EZH1,SRPK2,SASH1,ASAP1,ADAMTS7,SULT2B1,BMP1,VAV3,ZP3,RNF2,CAV2,GNA12,MYEF2,INPP5F,AXL,TNFSF9,WIF1,CD109,MET,FMNL1,CCR3,SNX1,TIMELESS,UCHL5,ANKRD6,MALT1,SETD3,TRA2B,TMEM120B,CCDC136,EHD2,KDM2B,MLXIPL,SEMA3C,NEDD4L,NDC1,PHB,PTPRC,EDA,GPR89A,SH3GL2,TYRO3,RNF213,SYNDIG1,VPS52,ADCY10,NCAM1,TANC1,TRAF3IP2,CREB1,LIG1,SHISA6,AFF4,EDNRA,GNGT1,BMP4,CPNE1
GO:0009653	anatomical structure morphogenesis	1.360511596905982e-43	*CLRN1,DNAH11,ADAMTS16,NRXN1,ASPH,PRMT3,RDH13,PRKCI,MAP4K4,HLX,LLPH,PBX3,LMBR1,CLASP2,SEMA3A,GRID2,TENM3,DOCK1,RYR1,NRG3,ASH1L,NOX5,ASTN2,EPB41,SEMA3D,PHACTR1,MYOT,TIAM2,FHOD3,PTGIS,TJP1,UNC5C,KALRN,ATRNL1,SEMA5A,ENPP2,FER,MAP2K5,LRP2,PIK3CD,SP3,ANK2,OLFM1,MFAP5,EZR,NRXN3,ROBO1,CHD7,HTR2B,ITGB6,CDKL5,KIF5C,TRAF6,ROBO2,NFASC,VAV2,ABI1,ITGB1,TRIOBP,HSP90AA1,GAS7,PSMB7,KNDC1,KIF26B,DSCAM,TRIO,ARHGAP24,KLHL12,AKAP13,RUNX1,DAB1,OMA1,CDC42EP3,MYO10,ABCC8,CDH10,THRB,XIRP2,IMPG2,LAMA2,TCIRG1,PTPN11,DOCK10,HDAC6,GOLGA4,EPHA1,ERBB4,CDH12,LIMK1,ATP8A2,ACTG2,HIF3A,KLHL6,CECR2,ESR1,MYO18B,NPHP3,ANKRD11,PTPRO,CDON,NTRK2,EP300,CELA1,MEGF9,RIMS1,TNIK,SNX2,KIRREL3,CDH8,CSGALNACT1,INSR,RERE,PRKAR1A,ATP8B1,UNC5D,LAMA3,CDH4,PAK1,FBXW11,PTPRD,BASP1,SLC4A10,TBR1,STRC,BDNF,TFAP2A,ATR*

			N,AMIGO1,TGIF2,NPRL3,FMNL2,PHACTR4,MEIS1,TENM4,TRIM13,MDM4,ECT2,MYO3B,LAMC1,AFF3,SGCD,DMD,CRISPLD2,SATB2,HIRA,TANC2,CASS4,FYN,ADAMTS9,NF1,PCDH15,LMTK2,FAT3,RTN4,RXFP1,APOD,B4GALT6,RTN4R,BCR,CHI3L1,CCDC141,TTN,NDRG4,ADAMTSL1,NTN1,EGFLAM,NRG1,UBP1,FRY,MAP2K1,MDFI,STIM1,VAX2,MYH9,CNTN4,HDAC5,NTNG1,ROR2,DCN,SCFD1,CDH13,PLXDC1,SOX13,UST,EXOC4,CAPRIN2,NCKAP1,FOXN3,RCC2,BOC,NEUROD1,COL12A1,CCBE1,ZDHHC15,MYPN,FBXW7,SYT1,LZTS1,BCL11B,LRIG3,PKNOX1,MLLT3,PLS1,SEPT7,TBX15,CYFIP2,WNT11,PALMD,IFT80,FIG4,NOX4,SIPA1L3,MKLN1,CACNA1H,SIAH1,QKI,IL1RAPL1,CAMK2B,SOX6,ACVR2A,RUNX2,SEMA5B,TGFB1,SGK1,PCSK5,MYO3A,ZHX2,KEL,BTRC,BCAS3,C9ORF47,C2ORF49,RPGRIP1L,CELSR1,SLIT3,SDK2,SLIT2,CDH23,CORO1C,CTNND2,DISC1,KANK1,LAMC2,MTDH,SMAD6,ITSN2,KCNH1,BMPER,BCL3,DCLK1,PARVA,ADAM12,STRIP1,SBNO2,YTHDF1,FGF10,CNTNAP2,ARHGEF28,ENAH,GREB1L,CAPN3,SMURF2,EPHA4,RORA,PRKCA,AUTS2,TNFSF11,PPP3CA,MAG,CTNNB1,PARK2,ARHGAP22,SMARCC1,IGF1R,PPARG,AXIN1,DLG5,CYBB,XK,RYR2,FBLIM1,DRAXIN,LEPR,FGF1,NIN,NR4A3,MYOCD,AJUBA,CACNA1C,CPNE6,GLG1,PHLDB1,MYO1E,CSMD1,HCK,CAPN2,CSPG4,DCC,MMP16,OBSCN,NF2,FLT4,PAX2,ELN,SPG11,SFRP1,FOXO3,ARL13B,CNTN6,NFIB,IFT122,DNM3,SSH1,SMAD3,CUX2,PTPRM,ABI2,SHANK1,SYT3,NEDD4,NRP2,ARHGEF18,VANGL1,BTBD3,PREX1,HOXD3,HOXD4,TAB1,NFATC1,PRTG,CDC73,APP,SSBP3,GSX2,PDGFRA,DIP2B,NOX1,YAP1,HEG1,FSTL4,EYA2,SH3D19,ALPL,JAK1,ANGPTL4,LRP5,ISLR2,SOX2,SETD2,ZDHHC6,GPC6,TTC39C,CHST11,PRICKLE1,SPTBN4,VASH2,CHUK,FRS2,PALB2,MMP2,CPNE9,KDM6A,PRKD1,STAT1,PARP6,RND3,SLC6A3,HNF4A,STRA6,EREG,ABLIM1,MYOF,DSCAML1,SEMA6D,ATF2,ADAMTS12,BMPR2,USP33,DPYSL2,BMPR1A,TSPAN12,NLGN1,CTNNA2,PIK3R3,SPAG9,RAB11A,CRMP1,FGF2,LRRC4C,NEDD9,PEAK1,SEMA4D,SH3PXD2A,ISM1,FBN3,PLXNA2,RILPL1,KLHL3,RARB,NCOA1,DVL3,EPHB3,FBXO45,LMBRD1,VCL,LAMB1,CLIC4,MYO9A,COL11A1,KCNQ1,RFX2,WNT3,RHOJ,P4HB,SUFU,NLGN3,SCUBE2,PAFAH1B1,KIF3A,COL4A3,DDAH1,RASAL1,TRPC5,GLI2,NEO1,ERCC1,RUFY3,TPD52,DAB2,PKHD1,USH2A,DLG3,WNK1,RELN,SIN3A,RSPO2,CFDP1,IQGAP1,APOLD1,SP1,LUZP1,MACF1,ARHGAP12,MYO7A,CLASP1,TEAD4,HOXC13,AKAP2,XDH,OVOL2,NFATC2,PAX7,ATP2B4,SYNE3,PACSIN1,TMEM2,ASXL3,PAK3,TET1,ADNP,MAP1B,TFRC,EPHA5,WWOX,MEF2A,GPC3,PSMB2,CALD1,EYA1,HOXB3,HOXB4,HOXB5,HOXB6,GHR,FLRT2,FGD1,ACTR2,ELAVL4,HDAC1,SMOC2,ADIPOR2,ROCK1,EPS8,PAXIP1,EGR2,AP3B1,NTRK1,MAP1A,ANK3,DNMBP,UNC13A,PTPRQ,EPHB1,EPHA10,AXIN2,SARM1,PARD3,PTPN14,TRIM46,JAG2,BCOR,ARHGAP44,COCH,CUX1,SPI1,EPHA7,MCU,CTNNA1,FBXW4,BCAR3,SHROOM3,MAP3K13,RAPGEF3,SDK1,SH3BP1,MST1,PTK7,BMPR1B,CD53,MAGI2,PNPT1,HEY2,RC3H1,COL13A1,WNT7B,PTPRS,ADTRP,CSRP1,RASA1,SRSF6,HIPK1,PKP2,ZNRF3,MAPK1,PTEN,MIB1,BMP7,SOX5,LHFPL5,PRCP,LRRK2,MEGF11,ZRANB1,RAPH1,TMEM108,PKD2,EFNA5,SHANK3,RIPPLY1,PDGFC,AMOTL1,TBCD,WNT7A,BRWD3,BMP6,MYCBP2,NFE2L1,GPI,SOS1,TSHR,EXT1,ATRX,PRICKLE2,PARVB,RAPGEF2,CDH9,SMO,MYLPF,TNFRSF11B,SYT17,SOX30,RHOA,SYNGAP1,ROR1,ONECUT2,RHOBTB1,SHROOM1,CUL3,SH3KBP1,GSK3B,EMB,RDX,STAT6,ACTA2,EEF2K,FAM49B,PTPRB,SULF1,MTHFD1L,KRT8,AGT,CDH20,FBN2,STAT2,ZMPSTE24,PTK2,PHEX,RIMS2,PBX1,SHB,GAP43,CPE,CREBBP,FAM171A1,COL9A1,DLX1,TAOK2,SHC1,ALDOA,ARHGAP15,CD160,ECE1,LBX2,PGM5,SETDB2,ALOX5,DNAJB6,CD44,FGD3,LARP4,ADAM8,CLDN4,PACSIN2,RPS6KA5,THSD7A,CXCL17,FGD4,NOS1,PEX7,PLEKHA1,ACTN4,STK4,TUBB3,SPEF1,EPHB2,SLC1A1,WLS,TMEFF2,IL18,DLC1,PPARA,ARID5B,CALCRL,NOTCH4,SOBP,CRX,FBN1,PAX6,PRKCZ,FAM20C,ADORA2A,COL22A1,PHLDB2,NIPBL,TMIGD2,ASPN,ANGPT1,EYA3,FHL2,IGF2BP3,ETS1,PPP1CA,MAP2,NEBL,FBXO31,PRKCQ,BRAF,CSRNP1,DIAPH1,HDAC2,IMPACT,NOTO,SPINT2,TNR,PHIP,CDKL3,ANGPT4,ACAN,CAMSAP3,SP100,ANXA2,SRGAP2,TMEFF1,ADCY1,NUMB,SYK,CNOT1,WWTR1,SLIT1,RADIL,DVL2,NEB,YBX1,GRIP1,GPM6A,EZH1,SRPK2,SASH1,BMP1,VAV3,ZP3,RNF2,GNA12,AXL,CD109,MET,FMNL1,CCR3,SNX1,TIMELESS,UCHL5,ANKRD6,CCDC136,EHD2,KDM2B,MLXIPL,SEMA3C,NEDD4L,EDA,SH3GL2,TYRO3,RNF213,VPS52,NCAM1,TANC1,CREB1,LIG1,EDNRA,GNGT1,BMP4,CPNE1
GO:0065007	biological regulation	1.98768953900901e-43	*RDH14,CD247,POLDIP3,CLRN1,DNAH11,RIC8B,ENPP1,PRDX2,ADAMTS16,LDB2,GPC5,NRXN1,ASPH,B4GALNT2,F8,PRMT3,GP6,RDH13,PRKCI,MOV10L1,SLCO3A1,MAP4K4,PDE4D,PDCL,DNMT1,S1PR2,HLX,LLPH,TMBIM4,SLC9A1,PDE7B,FAM155A,C6ORF89,NEGR1,PBX3,ADCY8,MED13L,LMBR1,TRPS1,CBFB,PDE8A,PDE4DIP,ZNF823,CLASP2,SEMA3A,IL31RA,GRID2,TAS1R2,RPS6KA2,PRDM12,SETD4,PTGFR,TENM3,CHERP,MED26,DOCK1,WWC1,CLDN18,C12ORF49,CTDSPL2,FBXL2,RYR1,NRG3,ASH1L,NOS1AP,LATS2,TBC1D19,NOX5,ANP32A,ASTN2,EPB41,SEMA3D,PHACTR1,DPP6,PTH2R,NREP,SCAF8,TIAM2,STOX2,FHOD3,KSR2,MAPRE2,NLGN4X,PTGIS,PRKAG2,WWC3,TJP1,GRM8,UNC5C,NUDT6,HPSE2,OR5P2,OR5P3,CLCN1,PRLR,MVP,PAGR1,ADCY7,HIVEP3,FTO,NPAS3,UTRN,RGS7BP,KALRN,UBAP2,PITPNC1,SEC23B,PHACTR2,DCAF12,ACOXL,NTRK3,CBL,ARNT,ATRNL1,PLEKHG4B,EGLN2,MIA,RAB4B,RAB4B-EGLN2,SEMA5A,LRRFIP2,FLT3,RAD51B,SUSD4,STAT5B,TOX,ENPP2,ATP2B2,KCNQ5,EXD1,GRM5,PLCE1,LHFPL4,SAMHD1,ZNF566,FER,CASK,MARVELD3,MAPK4,MAP2K5,KITLG,KCNIP4,MAPK10,PTPRR,SPNS2,DENND2D,LRP2,PIK3CD,ZNF536,SP3,DEPDC5,HDGF,PRCC,ZSWIM7,ANK2,EIF4G3,SAAL1,OLFM1,SH2D1A,E*

			ZR,IKZF2,MEGF10,NRXN3,XRCC4,ROBO1,MALRD1,SLC9A9,TOM1L1,TNFAIP8L1,CHD7,SLC26A6,HTR2B,PSMD1,ITGB6,CDKL5,MECOM,TACC1,MIR1185-1,TENM1,CAMKMT,RYR3,LMNA,NUCB1,GOT1,NMT2,INPP4B,AFM,TRAF6,ESRP1,ROBO2,ITPKB,CTNNA3,DNAJA3,OXR1,SLC24A2,NKAIN2,VAV2,GRK5,ABI1,ULK4,NHLH1,ITGB1,TRIOBP,HSP90AA1,SLC24A3,CDC6,PSMB7,TSC22D3,CNIH2,SRGAP3,MCC,TSSK1B,NKAIN3,PCDH17,KNDC1,ZC4H2,KIF26B,CCDC22,DSCAM,STK38L,SRGAP2B,CDAN1,GMDS,SAMD12,CNGB1,TRIO,ARHGAP24,KLHL12,AKAP13,GFI1B,PDE1A,PARD6G,PTPRK,RUNX1,DAB1,OMA1,CDC42EP3,MYO10,ABCC8,ERC1,HMGN3,NCF4,NRIP1,RGS6,DGKI,ANKS1B,THRB,STXBP4,PDE4A,ERC2,GABRA3,MIR105-2,MIR767,EFCAB7,ITGB3BP,LAMA2,GFRA2,MAST4,IL17RB,TCIRG1,DACH1,PTPN11,ZNF569,KCNS3,NETO2,RBM19,HERC4,MGAT5,CCT2,CACNB2,DOCK10,ORC2,CYTH3,HDAC6,GOLGA4,SERTAD2,DDB1,MYT1,ADORA1,GPRASP1,GPRASP2,ADAMTS3,EPHA1,IKBKB,KIF18B,PEX14,ERBB4,GBE1,ADRA1D,CNNM1,SORCS1,MRE11A,GBF1,LIMK1,ZNF609,TLK1,BRD8,ATP8A2,KAT6B,ABR,ACTG2,HIF3A,SNIP1,CUL4B,KLHL6,LARGE,ESR1,KCNJ16,NPHP3,GRM7,MIER1,PCBP3,PMEPA1,MAML3,DPP10,PTPRO,CDON,NTRK2,CYB561A3,TNRC6A,FRMD4A,STX18,EP300,CELA1,ZYG11B,TENM2,ZNF76,RNF220,MCF2L2,RGS22,ZNF471,FNTA,HOOK3,PDGFB,CACNA1B,RIMS1,TNIK,CCND3,CDH8,USP46,TOB2,MID1,ZNF19,ZNF23,BRMS1,CHFR,ZNF605,ALOX5AP,SCML4,BPI,STK39,DAP3,HNF4G,INSR,FMN2,RERE,PRKAR1A,ASB5,AFAP1,FUBP1,ATP8B1,UNC5D,H2AFY2,LAMA3,TCF7L2,PIP4K2A,CDH4,HPS1,JMJD1C,CAB39,USP34,ZNF443,ZNF490,ZNF564,ZNF709,ZNF799,PAK1,LITAF,FBXW11,ESRRB,MYOM1,MAP3K4,ZDHHC13,PTPRD,RNF144A,BASP1,EGLN3,RBM20,SLC4A10,TBR1,CATSPER2,SAMD4A,BDNF,TFAP2A,ATRN,AMIGO1,CDK14,CLDN12,FANCA,KCNMA1,PEG3,ZIM2,CHRM3,TGIF2,TGIF2-C20ORF24,FUT8,OR2T3,NPRL3,FMNL2,PHACTR4,MEIS1,TENM4,GRIK4,LHFPL2,VRTN,KCNRG,TRIM13,RASGEF1B,UBR2,ARHGAP6,LCK,MDM4,EDARADD,IL5RA,ENTPD5,SHC4,ECT2,TMC2,SLC12A8,ZNF148,MTA3,SNX6,TFDP2,NAV2,CHML,ACOX1,VAMP7,LAMC1,PPM1L,ARHGAP42,CST2,PTCD3,PTPRU,RFTN1,ERN2,CDC45,LSP1,C1D,MB,AP3S1,NPLOC4,AFF3,SGCD,DENND5A,MICU3,DMD,CENPF,ATG10,LRRN2,SLC30A9,TOX3,PDS5A,LARP4B,USP13,KAT7,ZNF667,SLC8A1,GSN,KHDRBS2,RBM14,RBM4,GPR171,MED12L,SATB2,PAFAH1B2,RIT2,HIRA,TANC2,JPH2,DRG2,DEPDC1B,CASS4,KCTD16,FYN,MKRN2,ARNTL,ADAMTS9,NF1,NCF2,SMG7,PLCB1,DST,MGMT,PCDH15,LMTK2,STX6,ARID4B,SNCB,PPP4R2,FAT3,RTN4,AFF2,RXFP1,DPEP1,CHRDL1,DGKH,APOD,RTN4R,BCR,PPP1R12B,NOSIP,RWDD3,BRPF1,SHISA9,CHI3L1,TTN,NDRG4,ICA1,BMP2K,PAQR3,IFI30,PIK3R2,ANKH,CACNA1E,RANBP9,KLHL21,NTN1,EGFLAM,DCTN1,SLC4A5,NOMO3,NRG1,ARHGAP10,SMAP2,SH2D3C,CNNM2,CDK19,SLC16A10,SLC43A2,UBP1,BDKRB1,BDKRB2,DOCK8,BID,MIR600HG,FRY,MAP2K1,KCNIP1,MDFI,FNIP1,RAPGEF6,STIM1,VAX2,NALCN,RALGPS2,MYH9,FRMPD4,MAD1L1,LRFN5,DAGLA,TMBIM6,VGLL4,PPP2CB,CNTN4,PPP3R1,HDAC5,CSRNP3,RBM5,NTNG1,PCSK2,VSTM1,ZNF692,OR6N2,NFIA,RNF4,TMEM30A,CSNK2A3,JDP2,CMKLR1,SBF2,INPP4A,ITIH2,CDKL2,TSPAN8,ROR2,DCN,ZDHHC3,SLC39A14,PRPSAP2,LIN7A,SCFD1,CATSPER3,PCBD2,SYN2,HNRNPLL,DOCK2,CDH13,CREBRF,TRDMT1,SOX13,FHIT,WDR70,PTPRT,ENTPD1,AUNIP,UST,COPS5,CSPP1,PPP1R42,FGF14,EXOC4,IPO5,ARHGAP32,MAD2L2,TLE6,RAB27A,RGS8,CAPRIN2,CCNYL1,ZNF418,NLN,NCKAP1,PHF20L1,DEFA1B,DEFA3,CD99,RAB3C,F5,PDF,TERF2,PTGER2,RALGPS1,SLX1B,ZNF286A,GOLPH3L,CLDN16,FOXN3,RCC2,BOC,ANO6,ARL3,CERKL,NEUROD1,GNAQ,KIF2A,FBXO9,ICK,TMEM117,RAD51D,RFFL,RANBP1,CCBE1,DENND1A,HERC5,USP22,ZDHHC15,JAK2,YTHDC2,FAM168A,TRAPPC9,FBXW7,LINC00473,KCNB2,OAZ2,SYN3,SKAP1,SMC3,CLSTN2,UIMC1,SYT1,ITCH,MLIP,CROCC,NPSR1,ZFYVE28,BBS12,ABCC2,LZTS1,BCL11B,SMG1,SCN4A,RBFOX1,TMC1,PKNOX1,DTHD1,CLDN1,HUNK,MLLT3,TSHZ2,ATP9A,ARAP2,SLC9A7,CDC42BPB,PNPLA1,PLS1,TEX11,DOCK9,DENND2A,SH2D6,TCF7,PDE2A,SEPT7,KLF15,TBX15,ANXA4,LAMTOR3,MAGI1,KCNC4,CYFIP2,WNT11,PALMD,ARHGAP23,IFT80,TRIM59,APBB1IP,FIG4,MTA1,KREMEN1,NRG4,KLF8,CLPB,NOX4,LCOR,SIPA1L3,RPRD1B,MKLN1,CACNA1H,SIAH1,QKI,IL1RAPL1,CCDC62,CAMK2B,ERCC8,PRKCD,SOX6,TAB2,ACVR2A,RUNX2,SEMA5B,CD4,PPM1E,TGFB1,BANP,PLCXD3,SGK1,PCSK5,SPSB4,NUP93,MMP28,TRIM65,NSD1,IGSF1,PIBF1,ZHX2,PKNOX2,ASCC2,KEL,BTRC,DUS2,NFATC3,JPH3,CRADD,DLGAP1,MIR153-2,PTPRN2,CLDN10,F2RL1,BCAS3,C9ORF47,GRIK3,CDYL2,RPGRIP1L,DNAJB2,CELSR1,SLIT3,AAK1,CC2D1B,ZFYVE9,MIR218-1,SLIT2,ASB15,COLGALT1,TP73,GABRR3,CADPS2,ITIH4,GABRB1,SYT13,HHAT,CDH23,GPR176,HP,HPR,CORO1C,SAP18,RRAS2,CTNND2,ZBTB22,C12ORF4,OR9A4,ILF2,DISC1,BLID,KCNJ3,CEP135,KANK1,CACNA1D,ZFAND2A,CPT1C,PTPDC1,CACNA2D3,ANO4,LAMC2,CALML4,CLN6,MTDH,ANK1,CLMN,TTBK2,FANK1,XPR1,MYT1L,KDM4B,CHRM1,RAB11FIP4,MMP26,OR51A7,OR51F2,OR51T1,SMAD6,RXFP2,BNC2,ZNF398,TMTC2,CLOCK,ITSN2,CHST9,TCF12,ZNF675,ANO3,SMOC1,PLCB4,ETV6,NELL1,SUCO,KCNH1,ENOX2,TFAP2D,BMPER,TIMP2,BCL3,DCLK1,ANKRD54,DAPK2,TNFRSF10B,PARVA,SND1,DAPP1,ADAM12,DUSP22,GPR52,RABGAP1L,NAIP,STRIP1,HNRNPC,TRRAP,ANKFN1,HOMER2,SLC17A7,DOCK3,SBNO2,LINGO2,YTHDF1,FGF10,CNTNAP2,CIZ1,FBXL17,RASA4,RASA4B,ARHGEF28,SMYD3,STXBP5,KCNH7,KANK4,KCTD10,IL1RAPL2,IQCJ-

			SCHIP1,SH3RF3,PLIN3,LOXL3,RHPN2,MAST2,FANCI,SVIL,GLP2R,ADCY2,CAPN3,SLC16A1,LUM,VMP1,SMURF2,EPHA4,RORA,HIVEP2,HSD17B12,PRKCA,AUTS2,CNR1,CD6,TNFSF11,SMG6,PPP3CA,NSUN2,OR51B2,OR51B4,OR51I1,UBQLN3,GNG4,NFYB,MAGEA4,MAG,KLF12,CAMK4,GATAD2B,PIP5KL1,UFL1,TRAK1,CTNNB1,PARK2,SOD2,DACH2,METTL13,FCGR2A,FCGR2B,FCGR3A,FCGR3B,ARHGAP22,SMARCC1,KLF17,CDHR2,IGF1R,PPARG,IARS2,NGRN,AXIN1,GARNL3,PRKAR1B,OTUB1,DLG5,IL18R1,IL1RL1,ADD2,CIPC,MTF1,CPQ,MSR1,CELF2,CBX5,P2RY10,TRPM1,ANKRD17,CYBB,OTC,XK,CASKIN1,APOL3,SCAMP5,BRIP1,ANXA13,ANKRD26,ECM2,LRPPRC,SREBF2,AARS2,KCNJ12,RYR2,CDK11A,KCNA6,FBLIM1,CDK11B,DRAXIN,LEPR,LEPROT,FGF1,NIN,PROS1,RGS10,NPAT,NR4A3,FOXK2,NOL3,DCT,PRKAR2A,RIOK2,AGAP1,ESCO1,MYOCD,OR52E6,OR52E8,OR52N1,TRIM5,PER2,KIR2DL1,KIR2DL4,KIR3DL2,AJUBA,CACNA1C,CPNE6,GLG1,ZNF626,ZNF737,SCN3B,CHEK2,SUPT3H,PHLDB1,UBQLN4,PRDM16,MYO1E,ASB8,CSMD1,PPP1CB,SPDYA,HCK,CSTL1,SORBS1,TBC1D14,RAB3GAP2,CAPN2,TBCK,TRIM8,DIO2,PPP2R2B,CSPG4,GRM1,BRMS1L,RIMS4,AGFG2,DCC,ZBTB8A,ZBTB8B,ARHGAP31,P2RY14,ANKFY1,CTDP1,ZUFSP,HS3ST5,BAZ1B,MMP16,OBSCN,RGL1,NF2,FLT4,MEF2B,SGMS1,BICC1,HDAC4,PAX2,PHF5A,TNS3,SECISBP2L,SPTBN1,TPTE,ELN,TRABD2B,SFRP1,MED13,ST8SIA1,PPP6R2,ZNF395,FOXO3,DGKB,TRHDE,ARL13B,CNTN6,NFIB,MUC12,SP4,ARHGAP29,ZCCHC17,IFT122,RANBP3,BCL2L13,DNM3,SSH1,CELF5,ATP10D,SYNCRIP,SMAD3,RNFT2,CUX2,ITPR1,PTPRM,WWP2,ARNT2,SBNO1,ACKR2,KRBOX1,ZNF662,CYP4F11,ZNF777,SIMC1,WIPI1,EBF3,MTBP,RNF168,C1S,CASZ1,ABI2,C10ORF90,GRIK5,PSAP,SLC16A2,LZTFL1,SHANK1,SYT3,IFNAR1,DCP1B,OR14K1,MIER3,NEDD4,NRP2,ARHGEF18,VANGL1,PREX1,PLA2G4C,DOCK4,ESRRG,HOXD3,HOXD4,KPNB1,NAV3,SLC4A4,ZNF114,TAB1,SLC6A1,KTI12,TXNDC12,NFATC1,RAB6C,KCNK1,PRTG,CDC73,APP,SSBP3,GSX2,PDGFRA,BET1L,SLK,FCRL4,ADD3,RBM8A,CCNI2,DIP2B,NOX1,ARIH1,YAP1,HEG1,AMFR,RAB11FIP5,SESN1,CDK3,TEN1,FSTL4,NLGN2,TNFRSF19,VDAC1,EYA2,SH3D19,BORA,RNLS,IBTK,NVL,ALPL,JAK1,PDE6D,ANGPTL4,TNNI3K,PEMT,LRP5,MTCP1,MUC20,POLR3G,PTPRG,BDH2,ISLR2,SLC9B2,ZNF787,GNG2,SOX2,SETD2,ZDHHC6,GPC6,KIF24,CHST11,TEAD1,PRICKLE1,RCAN1,ECSIT,ZNF653,SPTBN4,GRIA3,VASH2,ZNF521,DRD1,TMEM14A,GLRA2,ARID3A,ZNF761,CHUK,ERLIN1,FRS2,PALB2,RFT1,SFMBT1,VILL,MMP2,ZNF584,SERGEF,TPH1,ESR2,SYNE2,S100A12,DCUN1D3,CPNE9,KDM6A,PRKD1,STAT1,CELF6,PARP6,ST18,DGKG,ETV5,RHOXF2B,RND3,SLC6A3,USP53,TAF3,ARHGEF33,PLAGL1,HNF4A,ZBTB7C,LRRC2,TASP1,SHANK2,TMEM183A,STRA6,VPS4A,EREG,CCNY,TCTN3,MAPKAPK3,FXYD2,FXYD6,FXYD6-FXYD2,PITRM1,SEMA6D,RBFOX3,ATF2,POU2F2,TCF3,ZNF730,GRAMD4,TMEM237,KCNG4,RAF1,CELF4,RASGRF2,ZNF766,ACOX2,CARD16,CASP1,PTPN1,ADAMTS12,SRRM4,POLA1,GGT7,BMPR2,USP33,DPYSL2,VBP1,CAMK1D,BMPR1A,ZKSCAN1,PABPC4,TSPAN12,NLGN1,BTBD10,COL16A1,CTNNA2,ATP9B,IKZF4,PIK3R3,CDK12,TAF8,CAND2,SPAG9,MORC2,DENND4A,RAB11A,CRMP1,EVC,PDE11A,EPG5,MYB,FGF2,ZNF71,LRRC4C,POU6F2,PPA2,BACH1,GRIK1,OR6C70,MXD3,PPM1F,TICRR,ADCY5,GLI4,ZFP41,NEDD9,AGBL4,BEND5,PEAK1,SEMA4D,SH3PXD2A,ISM1,NFX1,CDC27,DOK6,RORC,MCTP1,ELP3,PLXNA2,PTAFR,ADCYAP1R1,SP140,SP140L,SETD1A,RHOXF2,YME1L1,ABCA13,JARID2,RILPL1,ANKDD1A,KLHL3,DDX58,DKK2,SORCS2,RAB11FIP3,BRDT,PHC2,ITGA11,PI4KA,RARB,SPEN,PIK3C2B,PRKCG,SIN3B,SLC9B1,VIPR1,NCOA1,SPOCK1,AREL1,BLOC1S5,EEF1E1,EEF1E1-BLOC1S5,TXNDC5,GRIA2,EHMT1,GAS8,LMO7,AP2M1,CHRD,DVL3,ECE2,EIF2B5,EIF4G1,EPHB3,PSMD2,PTPRE,RBX1,TCF20,ANAPC5,ARHGAP39,ATF3,TRAPPC11,LIN28B,AKR1D1,ATP6V0B,SHOC2,SRSF5,LINC00461,CKS1B,PAWR,EBF2,AGO3,DEPTOR,DLEC1,PKN3,FBXL20,MAML2,TSG101,DESI1,CCDC3,VCL,LAMB1,TERF2IP,CLIC4,MYO9A,CRYM,IDE,WDR59,KCNQ1,RFX2,WNT3,RCE1,ZNF322,RHOJ,P4HB,CCL14,CCL15,CCL15-CCL14,SUFU,TG,MAGEA11,NLGN3,PTPN13,SCUBE2,PAFAH1B1,KIF3A,PRDM15,ZNF670,ZNF695,CCDC169-SOHLH2,SOHLH2,FAM13A,CNTFR,COL4A3,TRPA1,DDAH1,NMUR2,ZNF354C,HIP1,AKAP6,RASAL1,TSPAN1,TCEA3,PADI6,ZNF704,BTBD9,NR2C1,TRPC5,UBA2,IRS4,ME1,GLI2,NEO1,TNKS,SCUBE1,WBP2NL,ERCC1,RUFY3,TNRC6B,GLIS3,GPR39,WDTC1,ZNF664,STON2,MGLL,C9,DAB2,THEMIS,BLM,PKHD1,XKR4,USH2A,UBR1,CACUL1,LDLRAD4,MYSM1,SETD5,DZIP1,SMG5,DLG3,WNK1,RELN,NEK10,SIN3A,RUVBL2,ABCB1,OR5H2,OR5H6,OR5K4,RSPO2,COMMD6,GUCY1A2,CSF3R,KCNQ2,DOCK11,GMEB1,PELI1,SLC22A3,PPP2R5C,CFDP1,IQGAP1,MAP3K7,APOLD1,DDX47,TAS2R41,ZNF423,SP1,OR56B1,TRIM22,MACF1,ARHGAP12,ALK,SLC8A2,INPP5D,KLK2,STAU1,CLASP1,DSC2,EEPD1,TEAD4,FASTKD5,HOXC13,SLC9C1,AKAP2,APBB3,SLC35A4,SRA1,TOP1,UBE2V1,MBOAT1,TPCN1,GPM6B,ADIRF,CLIC5,XDH,AHNAK,LTBP1,OVOL2,SNX5,NFATC2,RBBP6,OR6C74,PRKG1,PAX7,RGS7,BNC1,ATP2B4,NSF,PACRG,SYNE3,PACSIN1,SPTBN5,TMEM2,ACTL6B,ASXL3,PAK3,MASP1,SLCO4A1,SSH2,TET1,CAMTA1,CCPG1,DYX1C1,P2RX6,ASGR2,ATAD1,ADNP,GPR161,MAPKAPK2,ARID4A,MAP1B,CDC42BPA,COL4A6,MPHOSPH9,SCARB1,MARK1,CDK6,ATP6V0A2,PHF2,KCNAB2,CELF1,P2RY8,RNF34,TFRC,UVRAG,EPHA5,SORCS3,WWOX,GABRG3,MEF2A,SYT12,CR1L,ADCY9,CACNA1A,MIR1226,DCAF6,DCUN1D5,DROSHA,FNBP1,GFRAL,GPC3,SGIP1,STOM,XRN1,ADRB

			K1,SATB1,TBC1D5,PSMB2,ZNF207,EYA1,GATAD2A,PTGER3,SCOC,BRINP1,DIS3L2,GNB3,SHPK,TRPV1,RBM12B,RNF216,TSPAN6,HOXB3,HOXB4,HOXB5,HOXB6,MIR10A,TFEB,CLEC16A,GHR,MNAT1,FLRT2,FGD1,SNX9,RPGR,ACTR2,L3MBTL4,ACACA,ELAVL4,LGI4,HDAC1,INVS,RECQL5,C2,CFB,SMOC2,TRIM24,ADIPOR2,PLCG2,ROCK1,EPS8,NID1,SCMH1,C1QTNF1,PAXIP1,ZNF713,EGR2,HIPK3,RNF10,RYBP,GAPVD1,CAPN10,GPR35,CUL2,ABCC1,AP3B1,PTPN2,INSRR,NTRK1,KPNA6,TRIM44,ZFAND1,MAP1A,PKN2,PPP1R9A,TMIGD1,SLC39A10,EIF3A,ANK3,DNMBP,UNC13A,EPHB1,PDE6A,EPHA10,PTPN9,AXIN2,SARM1,TMEM199,ATP13A3,EPCAM,PARD3,RCAN2,ZNF425,GNL3L,KRTCAP2,MUC1,PTPN14,TRIM46,TUB,SH3GL3,JAG2,KCND3,BCOR,CACNG8,AP3D1,ZNF41,CARM1,SLC9C2,VWF,AP2B1,NETO1,ZNF383,ANXA8L1,ADAM19,ARHGAP44,DTD2,SNX33,SRD5A2,PIFO,CD84,COCH,HTR1D,LPA,ZNF616,ZNF836,SMARCA2,ARHGEF3,CUX1,MIR1302-10,RAD51C,SPI1,DGKK,DNMT3B,EPHA7,CHRNB4,FOXP2,MCU,RANBP10,PTPRA,CTNNA1,LIMA1,AIFM2,FBXW4,PSPC1,ACAP2,GLIS1,SFMBT2,TRDN,ATG14,EIF3H,GABRA6,LRRC8D,LY86,STAC,BCAR3,JAZF1,ILDR2,SHROOM3,STK38,TRPC6,ABCB7,ACOT8,RBPMS,RENBP,CHURC1,CREB5,FCHSD2,MAP3K13,OTUD3,RAB15,RAPGEF3,SDK1,PDXP,SH3BP1,MST1,ATP13A5,ERCC3,PTK7,SMYD1,CHID1,SNCA,BMPR1B,CACNG2,CD53,GRM4,MAGI2,PRIM2,TBC1D9,USP42,PNPT1,USP50,ZEB2,DHRS4,CABLES1,FOXJ3,SYT9,HEY2,RC3H1,CHMP3,EIF3E,CNGA3,RNF19B,WNT7B,PTPRS,ZMYND11,KMT2D,PRKAG1,CDKL1,CREM,PSMF1,RAB5B,KMT2C,SULT2A1,TLL2,CAST,TRIP12,DFFA,SPPL2A,ADTRP,ABCA12,GNG7,CSRP1,PLD1,RASA1,MACC1,MITF,SRSF6,OPRD1,CABIN1,HIPK1,NUGGC,PKP2,RPTOR,RTN4RL1,NFIX,BTK,MIR383,SGCZ,SIGLEC9,TBC1D16,XPO4,DSG1,ZNRF3,KCTD13,MLLT1,SLC4A8,TROVE2,CBFA2T2,IGF1,MLXIP,PLA2G4E,STX8,ATF7IP,CTNNBL1,HTT,DPYSL3,LARS2,MAPK1,PTEN,ARHGEF17,MIB1,SPRED2,SLC30A7,BMP7,SCN8A,ATXN2,MXI1,PIK3C3,PUM1,RASA2,SOX5,CIR1,PCBP2,TTLL4,PRCP,RAB30,ZNF780A,ZNF780B,LRRK2,ATP6V1A,MLYCD,OSGIN1,GNA14,TMEM59,ZRANB1,EBAG9,SPAG5,UNC119,ZBTB20,CHMP5,CNIH3,RAPH1,RFC3,BAG6,LILRB4,MORC3,IFT81,TMCO1,TMEM108,ITGAM,ARHGAP25,IL11RA,NSG2,PKD2,ZNF652,EFNA5,SHANK3,KCTD1,STMN4,RIPPLY1,MIA2,NT5E,RCOR3,TADA2A,DAZL,BTBD11,RGS9,PDGFC,SERPINA3,SERPINA4,SERPINA5,AMOTL1,ATP8B4,FAM13B,NCOA3,BEST3,PKIB,MTMR3,ZNF146,ZNF565,SGSM1,STEAP4,CUEDC2,PPP2R3C,TBCD,WNT7A,ZBED6,SNAP23,BRWD3,NLRP1,RPS6KC1,UACA,LPGAT1,MAP4,CRTC3,DNAJC1,AGAP5,GPR141,ARNTL2,ELOVL5,PLCL2,KAT6A,MTIF2,GPR21,RABEP1,SH3PXD2B,TTC28,RHOT1,SIK3,ZKSCAN7,ZNF197,ZNF660,BMP6,TAF15,MYCBP2,NFE2L1,ANO1,GPI,IL17RD,SH3RF2,SOS1,TSHR,WDR43,ZNF30,EXT1,N4BP1,PDCD1LG2,ATRX,DPH6,PLIN2,PRICKLE2,SERINC5,GABRR2,IKZF1,PARVB,PNPLA3,PRMT2,RAPGEF2,ASCC1,KCNC2,SMO,VRK3,RALY,ENPP3,GET4,BLOC1S3,MARK4,PPP1R37,PRKAR2B,STXBP5L,CPT1A,PSEN2,TCF4,TNFRSF11B,PAG1,SYT17,AFF1,BPGM,DECR1,SOX30,DNAJC6,TFE3,UBR5,GRB14,GRIN1,JADE1,KCTD8,ARRDC4,RHOA,SPRTN,WSB2,OR4M1,OR4N2,SYNGAP1,VIPR2,GRIN3A,PPP3R2,ROR1,RQCD1,TF,ONECUT2,RHOBTB1,FAM19A4,CUL3,SH3KBP1,TFEC,HSPD1,ITPR2,NFKBID,EPM2A,GABRB3,GSK3B,ABCG8,PLD2,PRR16,DNAJC3,GNG12,RDX,STAT6,ACTA2,SYN1,MLF1,EEF2K,FAM49B,IWS1,PPP1R16A,PTPRB,RBBP8,NECAB2,CDK13,ALAS2,STK32B,UPF2,CD27,DUSP26,ITGBL1,MCTP2,SCN9A,TAPBPL,BOK,RNF144B,SULF1,ZNF362,KRT8,NACC2,RNF43,SUPT4H1,TNFRSF8,ZNF354A,AGT,HDGFRP3,METTL16,CCNJL,ELOVL3,POLH,PRKAA2,SYT7,ADAR,BLOC1S6,CUL9,FBN2,PITPNM2,STAT2,ZMPSTE24,BRINP3,FGGY,HRH4,NDRG2,PTK2,TPPP2,UPK3B,HEPHL1,LAT2,PEX5L,SNX3,PDE4B,RIMS2,PBX1,TCP11,TEC,EXOSC3,FBXO10,SHB,VEPH1,CDC14A,GAP43,MAP3K5,CPE,SEC16B,TCFL5,CREBBP,IQGAP2,PRKD3,FAM171A1,UTP20,APCDD1L,DLX1,FMOD,INSL6,PCSK6,CEP70,GTF2IRD2,NCF1,SLC29A1,TAOK2,UGT1A1,UGT1A10,UGT1A3,UGT1A4,UGT1A7,UGT1A8,UGT1A9,PYGO2,SHC1,ZNF813,ALDOA,ARHGAP15,CD160,ECE1,KCNJ15,LBX2,PARP10,RNFT1,RPS6KB1,STC2,YLPM1,DPRX,GPR173,GUCY2F,PRG3,SCEL,SERPINB11,SETDB2,ANKS1A,ITGAL,ALOX5,CPLX2,DNAJB6,SP7,CD44,FGD3,LARP4,ADAM8,SLC5A3,ARFGAP3,CLDN4,PACSIN2,RPS6KA5,ZNF484,BCL2L14,CDS1,ZNF93,CSNK1A1,GRID1,HUS1,MIR346,PRDM2,CXCL17,FCRL6,FGD4,NOS1,ZNF44,GCKR,MED15,PLEKHA1,PLVAP,RGS14,SIPA1L2,ACTN4,ARHGAP11A,MC1R,SCNN1B,STK4,TCF25,ZFAND6,DRAM1,ZNF282,MARK3,SPEF1,COX6A1,EPHB2,GATC,SLC1A1,WLS,EDRF1,SIGMAR1,TMEFF2,XCR1,BCO2,CLPTM1,IL18,DTNA,LRRC52,MFSD8,NKAIN1,UBE2K,GPSM2,DNAJC7,HCAR1,HCAR2,HCAR3,DIS3,DLC1,PPARA,PPP1R10,ARID5B,GRTP1,PDP1,CALCRL,MCOLN1,STEAP3,EPB41L4B,SLC25A33,SMARCAL1,PHLPP1,MAGI3,CPEB4,GPSM3,MOB3B,NOTCH4,SYTL4,ZMYND8,ZNF366,CRX,CYP2C18,FBN1,PAX6,PRKCZ,ZC3HAV1,ATP1A3,CD96,FAM20C,ADORA2A,GRIN2B,KCTD7,PAH,PPP1CC,RABGEF1,CCNG2,CPEB1,PHLDB2,PLCXD2,TYK2,OTUD7B,DENND4B,NIPBL,TMIGD2,YEATS4,CNRIP1,COL28A1,PHF20,ZNF143,ASPN,ANGPT1,BRF1,CADPS,PACS2,TBL1X,EYA3,FHL2,FRMPD1,LRRK1,MBTD1,OSBPL8,ATF6,ZBTB5,ZNF708,IGF2BP3,EPB41L2,GCNT2,ETS1,PPP1CA,TBC1D10C,VWC2,ARHGAP21,CFI,GRIA4,MAP2,MTF2,RBMS3,CCL22,STPG1,BTN3A2,TAGLN3,FBXO31,ATG3,CDK5RAP1,CNTN1,NCOR2,PRKCQ,BRAF,CSRNP1,DIAPH1,HDAC2,HTR2C,IMPACT,MIR1912,ATP8A1,PHKG2,SRCAP,CAPN6,POU3F3,NOTO,PPP1R14A,SPINT2,TNR,MBNL3,UBE2E2,ZKSCAN5,HIGD2A,CD300A,ELF2,IRAK1BP1,MOXD1,PHIP,CDKL3,PPP2CA,SKP1,ANGPT4,ARHGEF6,DNAJC9,MAOA,RPL23,SH3BP4,RFC5,A

			TXN1,CAMSAP3,DCDC1,SP100,ZNF347,ZNF415,OPN1LW,ANXA2,TRIM29,KCND2,PARN,ATP6V0A1,PIGU,PRMT7,RAB27B,C1QTNF9,CR2,DONSON,FCRL2,ITSN1,NUP214,SRGAP2,ADCY1,MADD,NUMB,RBM42,SQLE,SYK,CNOT1,RBM39,WWTR1,ARHGAP19,CYP39A1,GRIK2,GTF2H5,SCN1A,SLIT1,ASB1,FRMD5,NFXL1,CIT,MYRIP,RADIL,UBAP2L,ZBTB38,BRCA2,HRK,RAP1GAP2,ALDH8A1,ATF7,CACNG3,DVL2,MORC1,MTRF1,NEB,YBX1,ANKRD13A,GRIP1,PTPRJ,SLC10A1,TAF1,ATP2B3,EIF4E3,PPME1,TICAM1,TANK,THAP3,GPM6A,UCN2,ITGAE,TAC3,PLEKHB2,POTEE,CADM1,HSF1,MAX,SAP130,DHFR,EZH1,ETF1,INPP5A,MIR663B,NRF1,SRPK2,ANKRD13C,ATP10B,PLEKHM1,SASH1,DENND2C,ASAP1,NCOA2,ADAMTS7,RALGAPA1,GBP5,MCM8,SULT2B1,WDR18,BMP1,CGNL1,EEFSEC,FCHO1,QRICH1,VAV3,ZP3,CHD6,DLGAP2,KCNQ3,CIB2,RNF2,ZNF554,MOB3A,CAV2,GNA12,SH2D3A,SKA2,AKTIP,COL4A5,MYEF2,POC1B,CA12,INPP5F,NUAK2,TMPRSS3,AXL,REPS2,TNFSF9,TRIM37,WIF1,CD109,DENND4C,MET,SESTD1,TBC1D10A,FMNL1,ZNF461,CAMK2D,CCR3,KCNJ6,NBEA,RAD9B,SNX1,TIMELESS,UCHL5,ANKRD6,BRD9,MALT1,SERPINE3,SETD3,TRA2B,ZDHHC11,SPTAN1,EHD2,KDM2B,MLXIPL,SEMA3C,THEM4,CCT3,NEDD4L,PHB,PTPRC,RPH3A,EDA,GPR89A,ZNF555,CTCF,PBLD,SH3GL2,TYRO3,MTMR12,NR6A1,RNF213,SPON2,SYNDIG1,TSPAN5,ADCY10,NCAM1,TRAF3IP2,TRIM60,ATF6B,CREB1,RASSF8,RGMB,SGTB,SHISA6,TNXB,TRAP1,AFF4,CLNK,EBF4,EDNRA,GNGT1,PRAP1,STXBP6,ZNF511,BMP4,ABLIM3,CASP12,CPNE1
GO:0000902	cell morphogenesis	3.8022776620538266e-41	*CLRN1,NRXN1,PRMT3,MAP4K4,LLPH,CLASP2,SEMA3A,DOCK1,EPB41,SEMA3D,PHACTR1,MYOT,TIAM2,UNC5C,KALRN,ATRNL1,SEMA5A,ENPP2,FER,LRP2,OLFM1,EZR,NRXN3,ROBO1,ITGB6,CDKL5,KIF5C,ROBO2,NFASC,ABI1,ITGB1,TRIOBP,HSP90AA1,GAS7,KNDC1,DSCAM,TRIO,DAB1,CDC42EP3,MYO10,CDH10,LAMA2,PTPN11,DOCK10,HDAC6,GOLGA4,EPHA1,CDH12,LIMK1,ATP8A2,PTPRO,NTRK2,EP300,MEGF9,RIMS1,TNIK,SNX2,KIRREL3,CDH8,RERE,UNC5D,LAMA3,CDH4,PAK1,PTPRD,TBR1,STRC,BDNF,ATRN,AMIGO1,FMNL2,ECT2,LAMC1,DMD,TANC2,CASS4,FYN,PCDH15,LMTK2,FAT3,RTN4,B4GALT6,RTN4R,CCDC141,ADAMTSL1,NTN1,NRG1,FRY,MAP2K1,VAX2,MYH9,CNTN4,NTNG1,SCFD1,UST,CAPRIN2,NCKAP1,RCC2,BOC,ZDHHC15,MYPN,SYT1,LZTS1,BCL11B,PLS1,SEPT7,CYFIP2,PALMD,NOX4,SIPA1L3,MKLN1,SIAH1,IL1RAPL1,CAMK2B,SEMA5B,TGFB1,SGK1,KEL,SLIT3,SLIT2,CDH23,CORO1C,CTNND2,DISC1,KANK1,LAMC2,ITSN2,DCLK1,PARVA,STRIP1,YTHDF1,CNTNAP2,ARHGEF28,ENAH,EPHA4,AUTS2,PPP3CA,MAG,CTNNB1,PARK2,IGF1R,XK,FBLIM1,DRAXIN,NIN,CPNE6,HCK,DCC,PAX2,SPG11,ARL13B,CNTN6,NFIB,DNM3,SSH1,CUX2,PTPRM,ABI2,SHANK1,SYT3,NEDD4,NRP2,ARHGEF18,BTBD3,PREX1,PRTG,APP,DIP2B,YAP1,HEG1,FSTL4,SH3D19,ISLR2,SPTBN4,CPNE9,PARP6,RND3,DSCAML1,SEMA6D,BMPR2,USP33,DPYSL2,NLGN1,CTNNA2,SPAG9,RAB11A,CRMP1,LRRC4C,NEDD9,PEAK1,SEMA4D,PLXNA2,RILPL1,DVL3,EPHB3,FBXO45,VCL,LAMB1,CLIC4,MYO9A,WNT3,RHOJ,P4HB,NLGN3,PAFAH1B1,KIF3A,RASAL1,TRPC5,GLI2,NEO1,RUFY3,DAB2,PKHD1,USH2A,RELN,SIN3A,CFDP1,IQGAP1,MACF1,MYO7A,AKAP2,SYNE3,PACSIN1,PAK3,ADNP,MAP1B,EPHA5,MEF2A,FLRT2,FGD1,ACTR2,ELAVL4,ROCK1,EPS8,EGR2,AP3B1,NTRK1,MAP1A,ANK3,DNMBP,UNC13A,PTPRQ,EPHB1,EPHA10,SARM1,PARD3,TRIM46,ARHGAP44,COCH,CUX1,SPI1,EPHA7,SHROOM3,MAP3K13,PTK7,BMPR1B,WNT7B,PTPRS,RASA1,PTEN,BMP7,LHFPL5,LRRK2,ZRANB1,RAPH1,TMEM108,EFNA5,SHANK3,TBCD,WNT7A,BRWD3,MYCBP2,SOS1,EXT1,PARVB,RAPGEF2,CDH9,SMO,SYT17,RHOA,SYNGAP1,RHOBTB1,SHROOM1,CUL3,SH3KBP1,GSK3B,EMB,RDX,EEF2K,CDH20,ZMPSTE24,PTK2,RIMS2,GAP43,FAM171A1,TAOK2,ALDOA,ARHGAP15,ECE1,CD44,FGD3,LARP4,ADAM8,CLDN4,PACSIN2,RPS6KA5,FGD4,ACTN4,STK4,TUBB3,EPHB2,TMEFF2,DLC1,NOTCH4,PAX6,PRKCZ,ADORA2A,COL22A1,MAP2,FBXO31,PRKCQ,BRAF,DIAPH1,IMPACT,SPINT2,TNR,PHIP,CDKL3,SRGAP2,TMEFF1,ADCY1,NUMB,SLIT1,RADIL,DVL2,GRIP1,GPM6A,GNA12,AXL,MET,FMNL1,SNX1,SEMA3C,NEDD4L,SH3GL2,TYRO3,NCAM1,CREB1,EDNRA,CPNE1*
GO:0051179	localization	6.19580958582497e-41	*CD247,POLDIP3,CLRN1,DNAH11,ZDHHC14,SLC39A11,ENPP1,SLC35E3,HOOK2,RTBDN,BLZF1,LDB2,GPC5,NRXN1,ASPH,PRKCI,SLCO3A1,MAP4K4,PDE4D,S1PR2,SLC9A1,ANTXR2,FAM155A,CD302,LY75,ADCY8,SPAG16,CLASP2,SEMA3A,GRID2,CHERP,SLC35E1,DOCK1,WWC1,CLDN18,KIF22,CTDSPL2,SLC25A17,CACHD1,RYR1,NRG3,ASH1L,NOS1AP,LATS2,TUBA1C,LRP1B,NOX5,ANP32A,IQCG,ASTN2,EPB41,SEMA3D,PHACTR1,DPP6,SLC14A2,MAPRE2,NLGN4X,PRKAG2,WWC3,TJP1,UNC5C,CLCN1,SLC35F3,PRLR,MVP,FTO,UTRN,KALRN,PITPNC1,SEC23B,LTV1,SNX31,NTRK3,CBL,ATRNL1,RAB4B,RAB4B-EGLN2,SEMA5A,STAT5B,ENPP2,ATP2B2,KCNQ5,GRM5,LHFPL4,FAM53A,FER,CASK,MARVELD3,MAP2K5,CCDC93,KITLG,KCNIP4,PTPRR,SPNS2,CEP128,LRP2,PIK3CD,SLC38A11,TNPO3,HDGF,ANK2,EZR,MEGF10,NRXN3,XRCC4,ROBO1,SLC9A9,TOM1L1,CHD7,SLC26A6,HTR2B,ITGB6,CDKL5,TENM1,RYR3,KIF5C,LMNA,SRP72,AFM,NFASC,CTNNA3,DNAJA3,SLC24A2,NKAIN2,VAV2,ULK4,ITGB1,HSP90AA1,APOL4,SLC24A3,ELMO2,PSMB7,CNIH2,SRGAP3,MCC,NKAIN3,PCDH17,CCDC22,SLC22A8,FAM126A,SRGAP2B,CDAN1,CNGB1,ARHGAP24,KLHL12,AKAP13,DLG2,FLVCR2,IFT43,PARD6G,PTPRK,DAB1,MYO10,ABCC8,ERC1,HMGN3,AFTPH,NCF4,NRIP1,DGKI,ANKS1B,STXBP4,ERC2,GABRA3,IMPG2,EFCAB7,LAMA2,TCIRG1,DACH1,PTPN11,KCNS3,NIPAL2,NETO2,MGAT5,CCT2,CACNB2,DOCK10,CYTH3,HDAC6,GOLGA4,SV2B,ADORA1,GPRASP1,EPHA1,IKBKB,PEX14,ERBB4,CNNM1,SORCS1,GBF1,DNAH2,LIMK1,ZNF609,TLK1,ATP8A2,OSBPL10,CECR2,ESR1,K*

			CNJ16,NPHP3,GRM7,DPP10,PTPRO,NTRK2,CYB561A3,FRMD4A,STX18,MEGF9,FNTA,HOOK3,VPS45,PDGFB,CACNA1B,AVL9,RIMS1,TNIK,SNX2,KIRREL3,MID1,STK39,MSTO1,INSR,FMN2,RERE,ATP8B1,UNC5D,H2AFY2,PLLP,LAMA3,TCF7L2,PIP4K2A,HPS1,CAB39,PAK1,FBXW11,SCAMP4,MYOM1,ZDHHC13,SPTSSA,SLC20A2,SLC4A10,TBR1,CATSPER2,BDNF,GLTP,ATRN,AMIGO1,SFT2D1,KCNMA1,CHRM3,POMGNT2,FUT8,FMNL2,PHACTR4,GRIK4,KCNRG,LCK,ECT2,TMC2,TMEM144,SLC12A8,SNX6,CHML,VAMP7,LAMC1,SPNS3,SLC6A16,PTPRU,RFTN1,SHFM1,MB,AP3S1,NPLOC4,TMPRSS15,RHCE,DENND5A,MICU3,DMD,CENPF,RANBP17,ATG10,SLC30A9,KAT7,SLC8A1,PRELID2,GSN,RBM4,SATB2,RIT2,TANC2,JPH2,DEPDC1B,CASS4,FYN,ARNTL,ADAMTS9,NF1,NCF2,SMG7,PLCB1,SLC16A6,DST,LMTK2,STX6,SNCB,SLC25A21,FAT3,RTN4,MAL2,DPEP1,GOLGA2P5,SLC5A6,APOD,SLC2A14,VTA1,BCR,SHISA9,CCDC141,TTN,NDRG4,ICA1,BMP2K,PAQR3,PIK3R2,ANKH,CACNA1E,KLHL21,NTN1,DCTN1,SLC4A5,NRG1,KCNN3,CNNM2,SLC16A10,SLC43A2,BDKRB1,BDKRB2,DOCK8,BID,MAP2K1,KCNIP1,SLC35A2,MDFI,SLCO1A2,RAPGEF6,STIM1,NALCN,MYH9,MAD1L1,TMBIM6,HDAC5,NTNG1,NAV1,TMEM30A,SYNE1,CMKLR1,PARD3B,ROR2,SCFD2,DCN,ZDHHC3,SLC39A14,LIN7A,SCFD1,CATSPER3,SYN2,DOCK2,CDH13,PITPNB,CREBRF,PTPRT,COPS5,FGF14,EXOC4,IPO5,MAN1A1,TLE6,RAB27A,MTX3,CCNYL1,NCKAP1,DEFA1B,CD99,RAB3C,COG8,TERF2,TMED6,GOLPH3L,CLDN16,RCC2,ANO6,ARL3,NEUROD1,IPO11,KIF2A,ICK,RFFL,TTLL8,PARP11,RANBP1,CCBE1,DENND1A,ZDHHC15,JAK2,FBXW7,KCNB2,OAZ2,SYN3,SKAP1,STARD6,SYT1,CROCC,NPSR1,BBS12,SNAP25-AS1,ABCC2,BCL11B,SMG1,DNAH14,SCN4A,RBFOX1,TMC1,SLC1A4,DYNC1I1,CLDN1,SLC5A9,ATP9A,SLC9A7,CDC42BPB,PLS1,DENND2A,SEPT7,KLF15,TTC7B,LAMTOR3,KCNC4,KPNA3,CTAGE6,CENPC,WNT11,IFT80,VPS13B,CLCA2,CYB561D1,IMMP2L,ASTN1,MKLN1,CACNA1H,QKI,IL1RAPL1,CAMK2B,PRKCD,SLC35F1,SEMA5B,CD4,TGFB1,BANP,SGK1,PCSK5,NUP93,MMP28,PIBF1,KEL,JPH3,PTPRN2,ARMC1,CLDN10,F2RL1,BCAS3,GRIK3,DNAJB2,CELSR1,AAK1,ZFYVE9,SLIT2,GABRR3,CADPS2,GABRB1,SYT13,CDH23,CORO1C,RRAS2,C12ORF4,DISC1,KCNJ3,CEP135,KANK1,CACNA1D,ZFAND2A,ABCC11,CACNA2D3,GRPEL2,ANO4,LAMC2,ANK1,CLMN,TTBK2,XPR1,SPAG17,CHRM1,RAB11FIP4,CLOCK,ITSN2,ANO3,SYNJ2,KCNH1,BMPER,BCL3,DCLK1,ANKRD54,DAPK2,PARVA,DUSP22,RABGAP1L,ANKFN1,HOMER2,SLC17A7,YTHDF1,FGF10,CNTNAP2,CIZ1,SMYD3,STXBP5,KCNH7,ENAH,PLIN3,LOXL3,CAPN3,SLC16A1,VMP1,SMURF2,EPHA4,SNX8,PRKCA,AUTS2,CNR1,CD6,ANO2,TNFSF11,SMG6,PPP3CA,NSUN2,HBE1,PIP5KL1,UFL1,TRAK1,CTNNB1,PARK2,SOD2,RN7SL832P,FCGR2B,HSPA6,TSNARE1,IGF1R,PPARG,AXIN1,PRKAR1B,COX8A,DLG5,MYO1D,ADD2,TMEM63C,MSR1,TRPM1,CYBB,SYTL5,XK,APOL3,SCAMP5,ANXA13,LRPPRC,SREBF2,KCNJ12,RYR2,KCNA6,NDUFA9,LEPR,LEPROT,FGF1,NIN,PROS1,NR4A3,WDR45B,NOL3,RIOK2,AGAP1,SCARA3,MYOCD,TRIM5,PER2,AJUBA,CACNA1C,CPNE6,SCN3B,MYO1E,HCK,SLC35E4,SORBS1,TBC1D14,RAB3GAP2,TBCK,TRIM8,MRC2,CSPG4,GRM1,RIMS4,DCC,ANKFY1,OBSCN,NF2,FLT4,HDAC4,DNAH9,SPTBN1,TPTE,AP3B2,SPG11,SFRP1,FOXO3,ARL13B,CCDC91,IFT122,RANBP3,DNM3,ATP10D,SNX14,SMAD3,ITPR1,RAB6A,PTPRM,WWP2,ACKR2,WIPI1,MTBP,VPS39,ABI2,GRIK5,PSAP,SLC16A2,LZTFL1,SHANK1,SYT3,NEDD4,NRP2,ARHGEF18,SVOPL,PREX1,PLA2G4C,DOCK4,SLC35F4,KPNB1,NAV3,SLC4A4,VPS53,SLC6A1,RAB6C,SEC61B,KCNK1,THOC3,APP,GSX2,PDGFRA,BET1L,SLK,CEP85L,RBM8A,NOX1,YAP1,HEG1,RAB11FIP5,SFXN5,MYO1F,NLGN2,VDAC1,EYA2,SSR2,BORA,IBTK,SCRN1,NVL,JAK1,LRP5,SEC22C,PTPRG,SLC9B2,SETD2,ZDHHC6,GPC6,CHST11,PRICKLE1,SPTBN4,GRIA3,DRD1,TMEM14A,GLRA2,RFT1,MMP2,SERGEF,TPH1,SYNE2,S100A12,PRKD1,OSBPL2,SYBU,RND3,SLC6A3,TAF3,HNF4A,STRA6,VPS4A,MAPKAPK3,FXYD2,FXYD6,FXYD6-FXYD2,PITRM1,SEMA6D,SLC16A12,ATF2,KCNG4,RAF1,RASGRF2,PTPN1,ADAMTS12,BMPR2,USP33,DPYSL2,CAMK1D,BMPR1A,AP1B1,NLGN1,CTNNA2,ATP9B,PIK3R3,SNX16,TAF8,SPAG9,RAB11A,EPG5,IPO9,FGF2,GRIK1,RAB24,PPM1F,ADCY5,NEDD9,AGBL4,PEAK1,SEMA4D,ARMC2,MCTP1,XPO6,ELP3,PLXNA2,PTAFR,HHIPL1,ADCYAP1R1,EEA1,ABCA13,RILPL1,KLHL3,DDX58,MRS2,SORCS2,RAB11FIP3,SLC5A8,ITGA11,PIK3C2B,PRKCG,SLC9B1,SPOCK1,BLOC1S5,TXNDC5,TANGO6,GRIA2,GAS8,OC90,AP2M1,CHRD,EPHB3,SLC35D1,TNPO1,FBXO45,TRAPPC11,TECPR2,ATP6V0B,LMBRD1,PKN3,FBXL20,TSG101,DESI1,VCL,LAMB1,TERF2IP,CLIC4,ANKS4B,CRYM,KCNQ1,SLC2A11,RHOJ,CCL14,CCL15,CCL15-CCL14,SUFU,TG,NLGN3,ZFYVE1,PAFAH1B1,KIF3A,ATG4C,TRPA1,TRPM3,NMUR2,HIP1,AKAP6,CEP350,PADI6,BTBD9,TRPC5,ENTHD1,TNKS,RUFY3,STON2,TPD52,DAB2,PKHD1,XKR4,TRAM2,USH2A,LDLRAD4,MYSM1,DZIP1,SMG5,DLG3,WNK1,RELN,NEK10,SIN3A,RUVBL2,ABCB1,CSF3R,KCNQ2,SLC22A3,SLC25A16,IQGAP1,SLC47A1,EML4,FAM91A1,PLEKHF2,APOLD1,ZNF423,SP1,TRIM22,MACF1,ARHGAP12,MYO7A,SLC8A2,CLASP1,DSC2,EEPD1,SLC9C1,APBB3,SLC35A4,TPCN1,NBEAL1,GPM6B,CLIC5,XDH,AHNAK,LTBP1,OVOL2,SNX5,NFATC2,PRKG1,RGS7,TTLL9,ATP2B4,NSF,PACRG,SYNE3,PACSIN1,SPTBN5,PAK3,SLCO4A1,SSH2,DYX1C1,P2RX6,ASGR2,ATAD1,MAPKAPK2,NXT2,CEP41,MAP1B,CDC42BPA,SCARB1,MARK1,CDK6,ATP6V0A2,KCNAB2,TFRC,UVRAG,EPHA5,GABRG3,MEF2A,SYT12,CACNA1A,FNBP1,GPC3,SGIP1,STOM,ADRBK1,TBC1D5,PSMB2,PTGER3,TRPV1,TFEB,CLEC16A,GHR,SLC38A6,FLRT2,PRSS12,SNX9,RPGR,ARL5A,ACTR2,C2,SMOC2,PLCG2,ROCK1,EPS8,C1QTNF1,PAXIP1,EGR2,GAPVD1,CAPN10,GPR35,ABCC1,AP3B1,LRBA,PTPN2,KPNA6,ZFAND1,MAP1A,PKN2,TMIGD1,SLC39A10,ANK3,UNC

			13A,EPHB1,PTPN9,SNUPN,AXIN2,CEP112,ATP13A3,PARD3,GNL3L,PTPN14,TRIM46,TUB,SH3GL3,TEKT1,KCND3,CACNG8,AP3D1,KIF3C,SLC9C2,AP2B1,NETO1,ANXA8L1,ARHGAP44,HEATR5A,SNX33,CD84,LPA,CUX1,SPI1,CHRNB4,MCU,VPS16,CTNNA1,LIMA1,ACAP2,TRDN,ATG14,GABRA6,LRRC8C,LRRC8D,STAC,ILDR2,SHROOM3,TRPC6,ABCB7,SLC41A2,FCHSD2,RAB15,RAPGEF3,SLC48A1,SH3BP1,MST1,ATP13A5,ERCC3,PTK7,RSRC1,SNCA,CACNG2,GRM4,MAGI2,TBC1D9,PNPT1,ZEB2,OCA2,SLC39A9,SYT9,HEY2,CHMP3,RNF103-CHMP3,CNGA3,SLC22A10,PRKAG1,RAB5B,OSBPL1A,ADTRP,ABCA12,PLD1,CLCC1,MITF,OPRD1,PKP2,BTK,TBC1D16,XPO4,VPS41,KCTD13,SLC4A8,IGF1,PLA2G4E,STX8,HTT,DPYSL3,MAPK1,PTEN,MIB1,SLC7A14,IFFO1,SLC30A7,BMP7,SCN8A,ATXN2,PIK3C3,LHFPL5,NUP88,PRCP,LRRK2,ATP6V1A,OSGIN1,SEPT6,TMEM59,ZRANB1,SPAG5,UNC119,CHMP5,CNIH3,BAG6,LILRB4,MORC3,SLC44A1,IFT81,TMCO1,TMEM108,IPCEF1,ITGAM,ARHGAP25,NSG2,PKD2,EFNA5,SHANK3,MIA2,PDGFC,SERPINA5,AMOTL1,ATP8B4,BEST3,SGSM1,STEAP4,TMCC1,WNT7A,ZBED6,ZC3H11A,SNAP23,MAP4,DNAJC1,NME8,RABEP1,SH3PXD2B,POM121C,RHOT1,BMP6,MYCBP2,ANO1,GPI,ANO8,SH3RF2,SOS1,EXT1,ATRX,PLIN2,SERINC5,GABRR2,SAMM50,RAPGEF2,ABCG2,KCNC2,SMO,SLC6A14,GET4,SUN1,LSG1,BLOC1S3,MARK4,STXBP5L,CPT1A,PSEN2,SYT17,SOX30,DNAJC6,UBR5,GRIN1,ARRDC4,BBS9,RHOA,SYNGAP1,GRIN3A,NDE1,TF,ONECUT2,RHOBTB1,ESYT2,FAM19A4,CUL3,SH3KBP1,HSPD1,ITPR2,TSPAN33,EPM2A,GABRB3,GSK3B,ABCG8,PLD2,EMB,RDX,ACTA2,SYN1,EEF2K,FAM49B,IWS1,CCDC14,NECAB2,UPF2,ITGBL1,MCTP2,SCN9A,VAMP1,BOK,SULF1,AGT,PRKAA2,SYT7,ADAR,BLOC1S6,FBN2,PITPNM2,ZMPSTE24,PTK2,TPPP2,HEPHL1,LAT2,PEX5L,SNX3,EXOC6B,PDE4B,RIMS2,COX5A,SLC25A51,TOMM5,COPG2,CPE,SEC16B,TRPC4AP,STOML1,TIMM44,PCSK6,CHKA,SLC25A42,GTF2IRD2,NCF1,SLC29A1,TAOK2,UGT1A3,SLC44A3,CD160,KCNJ15,RPS6KB1,SLC5A10,STC2,ZDHHC23,GPR173,ANKS1A,ITGAL,ALOX5,CPLX2,DNAJB6,CD44,ADAM8,SLC5A3,ARFGAP3,CLDN4,PACSIN2,CDS1,GRID1,PKD1L1,SLC25A18,COX5B,CXCL17,MON2,NOS1,PEX7,GCKR,TBC1D23,PLEKHA1,PLVAP,RGS14,ACTN4,COG4,SCNN1B,STK4,ZFAND6,SPEF1,COX6A1,EPHB2,SLC1A1,WLS,SIGMAR1,TMEFF2,XCR1,LRRC52,MFSD8,NKAIN1,GPSM2,HCAR2,DLC1,PPARA,PPP1R10,ABCD3,ARID5B,GRTP1,CALCRL,MCOLN1,STEAP3,EPB41L4B,SLC25A33,KPNA4,GPSM3,LDHC,SYTL4,ZMYND8,FBN1,PAX6,PRKCZ,ATP1A3,ADORA2A,GRIN2B,KCTD7,PPP1CC,RABGEF1,WDR83OS,PHLDB2,OTUD7B,GPR107,KLC3,NIPBL,ANGPT1,CADPS,PACS2,FRMPD1,OSBPL8,IGF2BP3,EPB41L2,SLC25A26,SLCO2B1,GCNT2,ETS1,HACL1,TBC1D10C,ARHGAP21,CFI,GRIA4,MAP2,BIN2,CCL22,STPG1,FBXO31,ATG3,CNTN1,NCOR2,PRKCQ,BRAF,DIAPH1,HTR2C,SIL1,TNS4,ATP8A1,POU3F3,SPINT2,TNR,TMEM163,CLTB,UQCR10,CD300A,EMC3,SKP1,SLC26A2,ANGPT4,FAM149B1,RPL23,SH3BP4,ATXN1,CAMSAP3,SP100,VTI1A,ANXA2,FAT2,TRIM29,KCND2,PARN,ATP6V0A1,PIGU,RAB27B,ITSN1,NUP214,PITPNM3,SRGAP2,WIPI2,ADCY1,NUMB,SLC18B1,SQLE,SYK,MOGAT2,WWTR1,GRIK2,SCN1A,SLIT1,FRMD5,MYRIP,RAB2A,RADIL,SEC31B,SLC44A5,BRCA2,CACNG3,DNAH3,DVL2,YBX1,ANKRD13A,GRIP1,PTPRJ,SLC10A1,ATP2B3,ERGIC1,GPM6A,OSBP2,MYO1A,SLC13A3,SLC2A12,HSF1,CNIH4,ANKRD13C,ATP10B,PLEKHM1,SASH1,HGSNAT,SLC5A4,GBP5,MCM8,CGNL1,FCHO1,VAV3,ZP3,KCNQ3,MLPH,RAB37,CAV2,GNA12,AKTIP,EPS15,INPP5F,TMPRSS3,AXL,REPS2,DENND4C,MET,SESTD1,TBC1D10A,FMNL1,MICAL3,CAMK2D,CCR3,KCNJ6,NBEA,SNX1,MALT1,ZDHHC11,ZDHHC11B,EHD2,SEMA3C,THEM4,TRAPPC8,CCT3,NEDD4L,NDC1,PTPRC,RPH3A,GPR89A,CTCF,RAB28,SH3GL2,TYRO3,MTMR12,RNF213,SPON2,SYNDIG1,TSPAN5,VPS52,ADCY10,TRAF3IP2,CREB1,SGTB,SHISA6,CLNK,EDNRA,GNGT1,PRAP1,SLC25A13,STXBP6,BMP4,ABLIM3,CPNE1
GO:0023051	regulation of signaling	1.722211415775773e-38	*RIC8B,ENPP1,PRDX2,GPC5,NRXN1,PRKCI,MAP4K4,PDE4D,PDCL,S1PR2,TMBIM4,SLC9A1,ADCY8,PDE8A,SEMA3A,GRID2,CHERP,WWC1,C12ORF49,CTDSPL2,FBXL2,NRG3,ASH1L,NOS1AP,LATS2,NREP,TIAM2,MAPRE2,NLGN4X,PTGIS,WWC3,GRM8,PRLR,MVP,PAGR1,RGS7BP,KALRN,NTRK3,CBL,ARNT,PLEKHG4B,SEMA5A,FLT3,ATP2B2,GRM5,PLCE1,SAMHD1,FER,CASK,MARVELD3,MAP2K5,KITLG,PTPRR,LRP2,PIK3CD,ZNF536,DEPDC5,ANK2,SH2D1A,EZR,NRXN3,ROBO1,TNFAIP8L1,CHD7,HTR2B,MECOM,TENM1,CAMKMT,RYR3,LMNA,NMT2,TRAF6,ROBO2,ITPKB,DNAJA3,SLC24A2,VAV2,GRK5,ULK4,ITGB1,HSP90AA1,PSMB7,CNIH2,SRGAP3,MCC,PCDH17,CCDC22,TRIO,ARHGAP24,AKAP13,DAB1,ABCC8,ERC1,HMGN3,RGS6,DGKI,STXBP4,PDE4A,ERC2,LAMA2,TCIRG1,PTPN11,NETO2,HERC4,MGAT5,CACNB2,CYTH3,HDAC6,ADORA1,GPRASP1,ADAMTS3,IKBKB,ERBB4,ADRA1D,GBF1,ABR,ESR1,NPHP3,GRM7,MIER1,PMEPA1,PTPRO,CDON,NTRK2,EP300,RNF220,RGS22,PDGFB,CACNA1B,RIMS1,TNIK,CCND3,USP46,MID1,STK39,INSR,AFAP1,TCF7L2,PIP4K2A,USP34,PAK1,LITAF,FBXW11,MAP3K4,ZDHHC13,PTPRD,SLC4A10,BDNF,CDK14,FANCA,NPRL3,PHACTR4,GRIK4,TRIM13,UBR2,ARHGAP6,LCK,ENTPD5,ECT2,SNX6,VAMP7,ARHGAP42,PTPRU,ERN2,NPLOC4,DMD,SLC8A1,RIT2,DEPDC1B,CASS4,KCTD16,FYN,MKRN2,ARNTL,NF1,PLCB1,RTN4,CHRDL1,APOD,RTN4R,BCR,RWDD3,SHISA9,CHI3L1,NDRG4,ICA1,BMP2K,PAQR3,PIK3R2,RANBP9,NOMO3,NRG1,ARHGAP10,BDKRB2,DOCK8,BID,MAP2K1,MDFI,FNIP1,RALGPS2,MAD1L1,TMBIM6,VGLL4,PPP2CB,CNTN4,NTNG1,CMKLR1,ROR2,DCN,ZDHHC3,SLC39A14,DOCK2,CDH13,CREBRF,SOX13,PTPRT,COPS5,ARHGAP32,MAD2L2,TLE6,RGS8,CAPRIN2,CCNYL1,RALGPS1,NEUROD1,GNAQ,FBXO9,RFFL,CCBE1,DENND1A,JAK2,FBXW7,LINC00473,SYN3,CLSTN2,SYT1,ITCH,NPSR1,ZFY*

			VE28,LZTS1,MLLT3,PDE2A,KLF15,LAMTOR3,CYFIP2,WNT11,ARHGAP23,IFT80,TRIM59,KREMEN1,NOX4,SIPA1L3,SIAH1,CAMK2B,PRKCD,TAB2,ACVR2A,RUNX2,CD4,SGK1,NUP93,IGSF1,PIBF1,BTRC,JPH3,CRADD,DLGAP1,F2RL1,GRIK3,RPGRIP1L,SLIT3,AAK1,SLIT2,TP73,CTNND2,DISC1,KANK1,MTDH,SMAD6,CLOCK,ZNF675,PLCB4,BMPER,BCL3,ANKRD54,DAPK2,TNFRSF10B,DUSP22,NAIP,HOMER2,DOCK3,YTHDF1,FGF10,FBXL17,RASA4,RASA4B,ARHGEF28,STXBP5,KCTD10,IQCJ-SCHIP1,SH3RF3,LOXL3,CAPN3,SLC16A1,SMURF2,EPHA4,RORA,PRKCA,AUTS2,CNR1,TNFSF11,PPP3CA,GNG4,UFL1,CTNNB1,PARK2,SOD2,FCGR2B,ARHGAP22,IGF1R,PPARG,AXIN1,GARNL3,PRKAR1B,DLG5,IL18R1,P2RY10,ANKRD17,APOL3,SREBF2,RYR2,DRAXIN,LEPROT,FGF1,RGS10,NOL3,MYOCD,TRIM5,PER2,AJUBA,GLG1,CHEK2,PRDM16,SORBS1,TBCK,TRIM8,CSPG4,GRM1,RIMS4,DCC,ARHGAP31,OBSCN,NF2,FLT4,SGMS1,BICC1,SPTBN1,TPTE,TRABD2B,SFRP1,FOXO3,DGKB,CNTN6,ARHGAP29,IFT122,SMAD3,CUX2,ITPR1,WWP2,GRIK5,SHANK1,NEDD4,ARHGEF18,PREX1,TAB1,SLC6A1,TXNDC12,NFATC1,CDC73,APP,GSX2,PDGFRA,NOX1,YAP1,HEG1,AMFR,RAB11FIP5,SESN1,FSTL4,NLGN2,TNFRSF19,EYA2,TNNI3K,LRP5,SLC9B2,SOX2,GPC6,CHST11,PRICKLE1,RCAN1,ZNF653,DRD1,TMEM14A,CHUK,FRS2,ESR2,S100A12,PRKD1,STAT1,DGKG,HNF4A,SHANK2,EREG,CCNY,GRAMD4,TMEM237,RAF1,CELF4,RASGRF2,CARD16,CASP1,PTPN1,ADAMTS12,BMPR2,USP33,BMPR1A,TSPAN12,NLGN1,SPAG9,DENND4A,EVC,PDE11A,FGF2,LRRC4C,GRIK1,ADCY5,SEMA4D,DOK6,MCTP1,ADCYAP1R1,DKK2,SORCS2,RAB11FIP3,PRKCG,NCOA1,EEF1E1,EEF1E1-BLOC1S5,GAS8,LMO7,CHRD,DVL3,PTPRE,RBX1,ARHGAP39,ATF3,SHOC2,PAWR,AGO3,DEPTOR,FBXL20,TSG101,CCDC3,TERF2IP,MYO9A,WDR59,WNT3,P4HB,CCL14,CCL15,SUFU,NLGN3,PTPN13,SCUBE2,PAFAH1B1,PRDM15,FAM13A,HIP1,AKAP6,RASAL1,BTBD9,NR2C1,GLI2,NEO1,TNKS,SCUBE1,MGLL,DAB2,PKHD1,UBR1,LDLRAD4,WNK1,RELN,NEK10,RUVBL2,RSPO2,GUCY1A2,PELI1,IQGAP1,MAP3K7,ZNF423,TRIM22,MACF1,ARHGAP12,ALK,SLC8A2,INPP5D,STAU1,UBE2V1,XDH,LTBP1,OVOL2,SNX5,RGS7,ATP2B4,NSF,PAK3,CAMTA1,DYX1C1,ATAD1,ADNP,GPR161,MAP1B,P2RY8,RNF34,TFRC,EPHA5,SORCS3,WWOX,SYT12,CACNA1A,GFRAL,GPC3,ADRBK1,PSMB2,EYA1,TSPAN6,CLEC16A,GHR,FGD1,ELAVL4,HDAC1,INVS,SMOC2,TRIM24,PLCG2,ROCK1,EPS8,C1QTNF1,EGR2,HIPK3,CAPN10,GPR35,PTPN2,NTRK1,TRIM44,MAP1A,PPP1R9A,SLC39A10,EIF3A,DNMBP,UNC13A,EPHB1,AXIN2,SARM1,MUC1,TUB,JAG2,CACNG8,CARM1,VWF,NETO1,ARHGAP44,ARHGEF3,SPI1,EPHA7,CHRNB4,MCU,PTPRA,CTNNA1,LY86,BCAR3,STK38,RBPMS,MAP3K13,OTUD3,SH3BP1,MST1,PTK7,SNCA,BMPR1B,CACNG2,GRM4,MAGI2,ZEB2,SYT9,HEY2,RC3H1,WNT7B,PTPRS,ZMYND11,KMT2D,SPPL2A,ABCA12,GNG7,RASA1,HIPK1,RPTOR,TBC1D16,ZNRF3,KCTD13,SLC4A8,CBFA2T2,IGF1,HTT,MAPK1,PTEN,ARHGEF17,SPRED2,BMP7,PUM1,RASA2,PRCP,LRRK2,ZRANB1,CHMP5,CNIH3,LILRB4,IFT81,TMEM108,ARHGAP25,PKD2,EFNA5,SHANK3,RGS9,PDGFC,FAM13B,NCOA3,WNT7A,ZBED6,UACA,CRTC3,PLCL2,KAT6A,GPR21,SIK3,BMP6,ANO1,IL17RD,SH3RF2,SOS1,PRMT2,RAPGEF2,SMO,VRK3,PRKAR2B,STXBP5L,CPT1A,SOX30,UBR5,GRB14,GRIN1,JADE1,KCTD8,RHOA,SYNGAP1,GRIN3A,ROR1,RQCD1,ONECUT2,FAM19A4,CUL3,GSK3B,RDX,ACTA2,SYN1,MLF1,FAM49B,NECAB2,CD27,DUSP26,MCTP2,BOK,SULF1,NACC2,RNF43,AGT,PRKAA2,SYT7,ADAR,FBN2,STAT2,ZMPSTE24,HRH4,NDRG2,PTK2,PEX5L,SNX3,PDE4B,RIMS2,VEPH1,MAP3K5,CREBBP,APCDD1L,DLX1,PCSK6,NCF1,TAOK2,SHC1,ARHGAP15,CD160,ECE1,LBX2,RPS6KB1,GUCY2F,SCEL,ANKS1A,ALOX5,CPLX2,CD44,FGD3,ADAM8,PACSIN2,BCL2L14,CSNK1A1,GRID1,CXCL17,FGD4,PLEKHA1,RGS14,SIPA1L2,ACTN4,ARHGAP11A,MC1R,STK4,ZFAND6,MARK3,SPEF1,EPHB2,SLC1A1,WLS,IL18,UBE2K,HCAR2,DLC1,PPARA,PPP1R10,PHLPP1,MAGI3,MOB3B,SYTL4,ZNF366,FBN1,PRKCZ,ZC3HAV1,FAM20C,ADORA2A,GRIN2B,RABGEF1,OTUD7B,DENND4B,CNRIP1,ASPN,ANGPT1,TBL1X,EYA3,FHL2,FRMPD1,LRRK1,OSBPL8,ATF6,GCNT2,PPP1CA,TBC1D10C,VWC2,ARHGAP21,RBMS3,CCL22,NCOR2,PRKCQ,BRAF,HDAC2,HTR2C,TNR,CD300A,PHIP,PPP2CA,MAOA,RPL23,SH3BP4,SP100,ANXA2,PIGU,ITSN1,SRGAP2,ADCY1,MADD,SYK,CNOT1,WWTR1,ARHGAP19,GRIK2,CIT,MYRIP,RAP1GAP2,CACNG3,DVL2,PTPRJ,TAF1,ATP2B3,TICAM1,TANK,ANKRD13C,SASH1,RALGAPA1,CGNL1,VAV3,DLGAP2,CAV2,GNA12,INPP5F,AXL,WIF1,CD109,DENND4C,MET,UCHL5,ANKRD6,MALT1,PHB,PTPRC,EDA,GPR89A,PBLD,TYRO3,RNF213,TSPAN5,NCAM1,TRAF3IP2,ATF6B,CREB1,SHISA6,TRAP1,PRAP1,BMP4,CPNE1
GO:0007275	multicellular organism development	1.8565882910257026e-38	*CLRN1,DNAH11,RCN1,ENPP1,PRDX2,ADAMTS16,LDB2,NRXN1,ASPH,PRMT3,GSS,RDH13,PRKCI,TACC2,MAP4K4,DNMT1,S1PR2,HLX,LLPH,SLC9A1,NEGR1,PBX3,LMBR1,TRPS1,CBFB,CLASP2,SEMA3A,IL31RA,LSAMP,GRID2,RPS6KA2,PRDM12,TENM3,CHERP,DOCK1,CLDN18,RYR1,NRG3,ASH1L,NOX5,ASTN2,SEMA3D,PHACTR1,MYOT,NREP,TIAM2,STOX2,FHOD3,NLGN4X,PTGIS,DCHS2,TJP1,UNC5C,ZSWIM6,PRLR,HIVEP3,FTO,UTRN,KALRN,SPRR2D,NTRK3,CBL,ARNT,ATRNL1,SEMA5A,FLT3,RAD51B,STAT5B,TOX,ENPP2,ATP2B2,GRM5,PLCE1,LHFPL4,SAMHD1,MAP2K5,SPATA5,KITLG,PTPRR,SPNS2,NDUFV2,LRP2,PIK3CD,ZNF536,SP3,ANK2,OLFM1,MFAP5,MEGF10,NRXN3,XRCC4,ROBO1,CHD7,HTR2B,ITGB6,CDKL5,MECOM,TACC1,TENM1,KIF5C,LMNA,KIAA1217,TRAF6,ESRP1,ROBO2,NFASC,ITPKB,DNAJA3,VAV2,ABI1,ULK4,NHLH1,ITGB1,TRIOBP,HSP90AA1,SLC24A3,GAS7,PSMB7,PCDH17,KNDC1,ZC4H2,KIF26B,DSCAM,FAM126A,SRGAP2B,CNGB1,TRIO,*

			ARHGAP24,KLHL12,AKAP13,TTLL5,GFI1B,RUNX1,DAB1,ABCC8,NRIP1,THRB,XIRP2,IMPG2,LAMA2,GFRA2,TCIRG1,DACH1,PTPN11,RBM19,DOCK10,HDAC6,GOLGA4,MYT1,ADORA1,ADAMTS3,EPHA1,IKBKB,ERBB4,LIMK1,ZNF609,ATP8A2,ACTG2,HIF3A,CUL4B,LARGE,CECR2,ESR1,MYO18B,NPHP3,GRM7,ANKRD11,PTPRO,CDON,NTRK2,DNAI2,EP300,CELA1,MEGF9,DYM,TENM2,RNF220,HOOK3,PDGFB,RIMS1,TNIK,KIRREL3,CSGALNACT1,TOB2,MID1,INSR,RERE,PRKAR1A,ATP8B1,UNC5D,H2AFY2,PLLP,LAMA3,TCF7L2,PIP4K2A,CDH4,PAK1,FBXW11,ESRRB,MAP3K4,PTPRD,CNTN5,BASP1,RBM20,SLC4A10,TBR1,STRC,BDNF,TFAP2A,ATRN,AMIGO1,FANCA,CHRM3,TGIF2,POMGNT2,FUT8,NPRL3,PHACTR4,MEIS1,TENM4,LHFPL2,LCK,MDM4,ECT2,ZNF148,MYO3B,TFDP2,NAV2,LAMC1,PTPRU,MB,AFF3,SGCD,DENND5A,DMD,CENPF,CRISPLD2,KAT7,WDR7,SLC8A1,GSN,PRPS2,GPR171,SATB2,PAFAH1B2,RIT2,HIRA,MACROD2,TANC2,JPH2,FYN,ARNTL,ADAMTS9,NF1,PLCB1,MGMT,PCDH15,LMTK2,ARID4B,FAT3,RTN4,AFF2,RXFP1,MAL2,CHRDL1,APOD,B4GALT6,RTN4R,BCR,CHI3L1,CCDC141,TTN,NDRG4,BMP2K,PAQR3,ANKH,ADAMTSL1,TLL1,NTN1,EGFLAM,DCTN1,SLC4A5,NRG1,UBP1,FRY,MAP2K1,MDFI,FNIP1,STIM1,VAX2,MYH9,MAD1L1,LRFN5,DAGLA,PLEKHA5,VGLL4,CNTN4,HDAC5,NTNG1,PCSK2,NAV1,NFIA,TMEM30A,CMKLR1,SBF2,ROR2,DCN,SLC39A14,PRPSAP2,LIN7A,DOCK2,CDH13,PLXDC1,SOX13,ACSBG1,UST,FGF14,DMC1,PCDHB16,EXOC4,MAD2L2,TLE6,CAPRIN2,NLN,NCKAP1,TERF2,FOXN3,BOC,ANO6,ARL3,NEUROD1,KIF2A,COL12A1,CCBE1,USP22,ZDHHC15,JAK2,MYPN,TRAPPC9,FBXW7,CLSTN2,SYT1,ITCH,LZTS1,BCL11B,RBFOX1,TMC1,LRIG3,PKNOX1,CLDN1,HUNK,MLLT3,PNPLA1,PLS1,TEX11,TCF7,PDE2A,SEPT7,KLF15,TBX15,CYFIP2,WNT11,IFT80,FIG4,KREMEN1,IMMP2L,NRG4,ASTN1,NOX4,SIPA1L3,CACNA1H,SIAH1,QKI,IL1RAPL1,CCDC62,CAMK2B,ADAM23,SOX6,TAB2,ACVR2A,RUNX2,SEMA5B,CD4,TGFB1,SGK1,PCSK5,PDLIM4,MYO3A,ZHX2,KEL,BTRC,NFATC3,F2RL1,BCAS3,C9ORF47,C2ORF49,RPGRIP1L,CELSR1,SLIT3,SDK2,SLIT2,TP73,MUSTN1,GABRB1,CDH23,CORO1C,CTNND2,DISC1,KANK1,LRRC10,LAMC2,MTDH,CLMN,TTBK2,MYT1L,OPCML,CHRM1,SMAD6,RXFP2,BNC2,CLOCK,ITSN2,TCF12,ZNF675,SMOC1,ETV6,SYNJ2,NELL1,PRR14,TFAP2D,BMPER,BCL3,DCLK1,ANKRD54,PARVA,ADAM12,NAIP,SLC17A7,SBNO2,LINGO2,YTHDF1,FGF10,CNTNAP2,FBXL17,ENAH,GREB1L,IL1RAPL2,PPL,LOXL3,SVIL,CAPN3,VMP1,SMURF2,EPHA4,RORA,PRKCA,AUTS2,CNR1,TNFSF11,PPP3CA,NSUN2,MAG,CAMK4,UFL1,TRAK1,CTNNB1,PARK2,SOD2,DACH2,FCGR2B,ARHGAP22,SMARCC1,IGF1R,PPARG,NGRN,AXIN1,DLG5,IL18R1,BFSP1,ADD2,MTF1,ANKRD17,CYBB,OTC,XK,BRIP1,RYR2,DRAXIN,LEPR,FGF1,NIN,NPAT,NR4A3,DCT,MYOCD,PER2,CACNA1C,CPNE6,GLG1,SCN3B,PHLDB1,MYO1E,CSMD1,CAPN2,CSPG4,DCC,CTDP1,MMP16,NF2,FLT4,MEF2B,BICC1,HDAC4,PAX2,ELN,SPG11,SFRP1,FOXO3,ARL13B,CNTN6,NFIB,IFT122,DNM3,SMAD3,CUX2,ITPR1,PTPRM,ARNT2,ACKR2,NHS,RNF168,EMG1,CASZ1,ABI2,PSAP,SHANK1,SYT3,NEDD4,NRP2,VANGL1,BTBD3,PREX1,CRTAC1,HOXD3,HOXD4,NAV3,TAB1,NFATC1,PRTG,CDC73,APP,SSBP3,GSX2,PDGFRA,CEP85L,DIP2B,NOX1,YAP1,HEG1,FSTL4,NLGN2,TNFRSF19,EYA2,ALPL,JAK1,ANGPTL4,PEMT,LRP5,PTPRG,ISLR2,SLC9B2,SOX2,SETD2,PLAC1,GPC6,TTC39C,ADAMTS2,CHST11,TEAD1,PRICKLE1,RCAN1,ELAVL3,SPTBN4,VASH2,ZNF521,DRD1,FRS2,PALB2,SFMBT1,MMP2,TPH1,SYNE2,CPNE9,KDM6A,PRKD1,STAT1,PARP6,DGKG,SYBU,SLC6A3,HNF4A,SHANK2,STRA6,VPS4A,EREG,ABLIM1,MYH15,DSCAML1,SEMA6D,RBFOX3,ATF2,POU2F2,TCF3,RAF1,CELF4,ADAMTS12,SRRM4,BMPR2,USP33,DPYSL2,CAMK1D,BMPR1A,AP1B1,PABPC4,TSPAN12,NLGN1,CTNNA2,PIK3R3,TAF8,CERS3,SPAG9,RAB11A,CRMP1,EVC,MYB,FGF2,LRRC4C,POU6F2,GRIK1,AGBL4,ADAMTS4,SEMA4D,SH3PXD2A,ISM1,RORC,ELP3,PLXNA2,ADCYAP1R1,FNDC3A,SETD1A,JARID2,KLHL3,DKK2,ITGA11,RARB,SPEN,PRKCG,NCOA1,SPOCK1,BLOC1S5,EHMT1,GAS8,OC90,NTM,CHRD,DVL3,EIF2B5,EIF4G1,EPHB3,ATF3,FBXO45,MATN3,LMBRD1,SRSF5,EBF2,ADAM20,ADAM21,TSG101,VCL,LAMB1,CLIC4,MYO9A,IDE,COL11A1,KCNQ1,WNT3,ADAMTS6,RHOJ,SUFU,TG,NLGN3,SCUBE2,PAFAH1B1,POLE,CNTFR,COL4A3,DDAH1,NMUR2,AKAP6,RASAL1,PADI6,TRPC5,TTLL7,GLI2,NEO1,SCUBE1,ERCC1,RUFY3,WDTC1,TPD52,COL19A1,DAB2,THEMIS,PKHD1,USH2A,LDLRAD4,MYSM1,SETD5,DZIP1,WNK1,RELN,SIN3A,RSPO2,CSF3R,KCNQ2,DOCK11,CRELD1,IQGAP1,APOLD1,ZNF423,SP1,LUZP1,MACF1,MYO7A,ALK,GSTM3,INPP5D,CLASP1,DSC2,TEAD4,HOXC13,TOP1,MBOAT1,GPM6B,XDH,OVOL2,NFATC2,RBBP6,PRKG1,PAX7,RGS7,ATP2B4,PACSIN1,TMEM2,ACTL6B,ASXL3,PAK3,TET1,DYX1C1,ASGR2,ADNP,GPR161,MAPKAPK2,ARID4A,MAP1B,MARK1,CDK6,PHF2,CELF1,TYR,TFRC,EPHA5,WWOX,MEF2A,ADCY9,DHX30,DROSHA,GFRAL,GPC3,ADRBK1,PLS3,PSMB2,CALD1,EYA1,BRINP1,TRPV1,HOXB3,HOXB4,HOXB5,HOXB6,TFEB,GHR,MNAT1,FLRT2,FGD1,ACTR2,ELAVL4,LGI4,HDAC1,SMOC2,ADIPOR2,PLCG2,ROCK1,NID1,SCMH1,PAXIP1,EGR2,RNF10,RYBP,AP3B1,PTPN2,INSRR,NTRK1,MAP1A,PPP1R9A,ANK3,ACO2,UNC13A,PTPRQ,EPHB1,PDE6A,EPHA10,PTPN9,AXIN2,SARM1,SNTG2,EPCAM,PARD3,PTPN14,TRIM46,TUB,SH3GL3,JAG2,BCOR,AP3D1,CARM1,AP2B1,ADAM19,ARHGAP44,SRD5A2,PIFO,SMARCA2,CUX1,SPI1,EPHA7,FOXP2,CTNNA1,FBXW4,BCAR3,ILDR2,SHROOM3,CHURC1,MAP3K13,RAPGEF3,SDK1,MST1,KAZN,ERCC3,PTK7,SMYD1,TTC9,SNCA,BMPR1B,MAGI2,PNPT1,ZEB2,RAI2,HEY2,RC3H1,COL13A1,HYDIN,RNF103,WNT7B,HMGCS2,PTPRS,TLL2,TRIP12,ADTRP,ABCA12,RASA1,MITF,SRSF6,RBM45,HIPK1,PKP2,RTN4RL1,BTK,SGCZ,ZNRF3,TROVE2,CBFA2T2,IGF1,CTNNBL1,DPYSL3,MAPK1,PTEN,CLDN11,MIB1,SPRED2,BMP7,SCN8A,SOX5,CIR

			1,LHFPL5,TTLL4,PRCP,LRRK2,MEGF11,UNC119,RAPH1,BAG6,LILRB4,MORC3,TMEM108,ITGAM,PKD2,EFNA5,SHANK3,STMN4,RIPPLY1,ALPK3,PDGFC,AMOTL1,NCOA3,PPP2R3C,TBCD,WNT7A,ZBED6,MAP4,PLCL2,KAT6A,SH3PXD2B,BMP6,MYCBP2,GPI,IL17RD,SOS1,TSHR,EXT1,ATRX,PRICKLE2,SERINC5,IKZF1,RAPGEF2,KCNC2,SMO,SUN1,BLOC1S3,MARK4,CPT1A,MYLPF,TCF4,TNFRSF11B,SYT17,BPGM,TFE3,GRIN1,ADAM29,RHOA,SYNGAP1,GRIN3A,NDE1,ROR1,TF,ONECUT2,CUL3,HSPD1,NFKBID,EPM2A,GABRB3,GSK3B,EMB,STAT6,ACTA2,SYN1,MLF1,EEF2K,PTPRB,RBBP8,CCDC14,CDK13,ALAS2,UPF2,CD27,SCN9A,BOK,SULF1,MTHFD1L,KRT8,AGT,HDGFRP3,ADAR,BLOC1S6,FBN2,ZMPSTE24,BRINP3,NDRG2,PTK2,PHEX,SNX3,RIMS2,PBX1,EXOSC3,SHB,GAP43,CPE,TRPC4AP,CREBBP,ARVCF,COL9A1,DLX1,PCSK6,SLC29A1,TAOK2,UGT1A1,PYGO2,SHC1,NDUFS2,CD160,ECE1,LBX2,RPS6KB1,STC2,GPR173,SCEL,SETDB2,ANKS1A,ALOX5,CPLX2,DNAJB6,MDGA2,SP7,CD44,ADAM8,SLC5A3,CLDN4,RPS6KA5,THSD7A,HUS1,PKD1L1,PRDM2,CXCL17,PEX7,TBC1D23,PLEKHA1,RGS14,MC1R,STK4,TCF25,TUBB3,SPEF1,EPHB2,SLC1A1,WLS,SIGMAR1,TMEFF2,CLPTM1,IL18,MFSD8,ASF1B,DLC1,PPARA,ARID5B,CALCRL,PHLPP1,PRRC2C,NOTCH4,SOBP,CRX,FBN1,PAX6,PRKCZ,FAM20C,ADORA2A,GRIN2B,PPP1CC,COL22A1,PHLDB2,OTUD7B,KLHL1,NIPBL,TMIGD2,PCDH10,UPB1,ASPN,ANGPT1,EYA3,FHL2,LRRK1,MBTD1,ATF6,SHROOM4,IGF2BP3,GCNT2,ETS1,PPP1CA,VWC2,MAP2,MTF2,NEBL,TAGLN3,FBXO31,CDK5RAP1,CNTN1,NCOR2,PRKCQ,LGI2,BRAF,CSRNP1,HDAC2,IMPACT,POU3F3,NOTO,SPINT2,TNR,CDKL3,PPP2CA,ANGPT4,ACAN,ATXN1,CAMSAP3,SP100,ANXA2,CR2,SRGAP2,TMEFF1,ADCY1,NUMB,SYK,CNOT1,WWTR1,SLIT1,ASB1,CIT,RADIL,BRCA2,DVL2,NEB,YBX1,GRIP1,PTPRJ,TAF1,GPRIN3,RNF38,GPM6A,POTEE,CADM1,HSF1,MAX,DHFR,EZH1,SRPK2,SASH1,ASAP1,ADAMTS7,SULT2B1,BMP1,VAV3,ZP3,RNF2,CAV2,GNA12,MYEF2,INPP5F,AXL,TNFSF9,CD109,MET,CCR3,TIMELESS,UCHL5,ANKRD6,MALT1,TRA2B,KDM2B,SEMA3C,NEDD4L,PTPRC,EDA,GPR89A,SH3GL2,TYRO3,RNF213,SYNDIG1,VPS52,NCAM1,TRAF3IP2,CREB1,EDNRA,GNGT1,BMP4,CPNE1
GO:0010646	regulation of cell communication	1.2763780440980062e-37	RIC8B,ENPP1,PRDX2,GPC5,NRXN1,ASPH,PRKCI,MAP4K4,PDE4D,PDCL,S1PR2,TMBIM4,SLC9A1,ADCY8,PDE8A,SEMA3A,GRID2,CHERP,WWC1,C12ORF49,CTDSPL2,FBXL2,NRG3,ASH1L,NOS1AP,LATS2,NREP,TIAM2,MAPRE2,NLGN4X,PTGIS,WWC3,GRM8,PRLR,MVP,PAGR1,RGS7BP,KALRN,NTRK3,CBL,ARNT,PLEKHG4B,SEMA5A,FLT3,GRM5,PLCE1,SAMHD1,FER,CASK,MARVELD3,MAP2K5,KITLG,PTPRR,LRP2,PIK3CD,ZNF536,DEPDC5,ANK2,SH2D1A,EZR,NRXN3,ROBO1,TNFAIP8L1,CHD7,HTR2B,MECOM,TENM1,CAMKMT,LMNA,NMT2,TRAF6,ROBO2,ITPKB,DNAJA3,SLC24A2,VAV2,GRK5,ULK4,ITGB1,HSP90AA1,PSMB7,CNIH2,SRGAP3,MCC,PCDH17,CCDC22,TRIO,ARHGAP24,AKAP13,DAB1,ABCC8,ERC1,HMGN3,RGS6,DGKI,STXBP4,PDE4A,ERC2,LAMA2,TCIRG1,PTPN11,NETO2,HERC4,MGAT5,CACNB2,CYTH3,HDAC6,ADORA1,GPRASP1,ADAMTS3,IKBKB,ERBB4,ADRA1D,GBF1,ABR,ESR1,NPHP3,GRM7,MIER1,PMEPA1,PTPRO,CDON,NTRK2,EP300,RNF220,RGS22,PDGFB,CACNA1B,RIMS1,TNIK,CCND3,USP46,MID1,STK39,INSR,AFAP1,TCF7L2,PIP4K2A,USP34,PAK1,LITAF,FBXW11,MAP3K4,ZDHHC13,PTPRD,SLC4A10,BDNF,CDK14,FANCA,NPRL3,PHACTR4,GRIK4,TRIM13,UBR2,ARHGAP6,LCK,ENTPD5,ECT2,SNX6,VAMP7,ARHGAP42,PTPRU,ERN2,NPLOC4,DMD,SLC8A1,RIT2,DEPDC1B,CASS4,KCTD16,FYN,MKRN2,ARNTL,NF1,PLCB1,RTN4,CHRDL1,APOD,RTN4R,BCR,RWDD3,SHISA9,CHI3L1,NDRG4,ICA1,BMP2K,PAQR3,PIK3R2,RANBP9,NOMO3,NRG1,ARHGAP10,BDKRB2,DOCK8,BID,MAP2K1,MDFI,FNIP1,RALGPS2,MAD1L1,TMBIM6,VGLL4,PPP2CB,CNTN4,NTNG1,CMKLR1,ROR2,DCN,ZDHHC3,SLC39A14,DOCK2,CDH13,CREBRF,SOX13,PTPRT,COPS5,ARHGAP32,MAD2L2,TLE6,RGS8,CAPRIN2,CCNYL1,RALGPS1,NEUROD1,GNAQ,FBXO9,RFFL,CCBE1,DENND1A,JAK2,FBXW7,LINC00473,SYN3,CLSTN2,SYT1,ITCH,NPSR1,ZFYVE28,LZTS1,MLLT3,PDE2A,KLF15,LAMTOR3,CYFIP2,WNT11,ARHGAP23,IFT80,TRIM59,KREMEN1,NOX4,SIPA1L3,SIAH1,CAMK2B,PRKCD,TAB2,ACVR2A,RUNX2,CD4,SGK1,NUP93,IGSF1,PIBF1,BTRC,JPH3,CRADD,DLGAP1,F2RL1,GRIK3,RPGRIP1L,SLIT3,AAK1,SLIT2,TP73,CTNND2,DISC1,KANK1,MTDH,SMAD6,CLOCK,ZNF675,PLCB4,BMPER,BCL3,ANKRD54,DAPK2,TNFRSF10B,DUSP22,NAIP,HOMER2,DOCK3,YTHDF1,FGF10,FBXL17,RASA4,RASA4B,ARHGEF28,STXBP5,KCTD10,IQCJ-SCHIP1,SH3RF3,LOXL3,CAPN3,SLC16A1,SMURF2,EPHA4,RORA,PRKCA,AUTS2,CNR1,TNFSF11,PPP3CA,GNG4,UFL1,CTNNB1,PARK2,SOD2,FCGR2B,ARHGAP22,IGF1R,PPARG,AXIN1,GARNL3,PRKAR1B,DLG5,IL18R1,P2RY10,ANKRD17,APOL3,SREBF2,RYR2,DRAXIN,LEPROT,FGF1,RGS10,NOL3,MYOCD,TRIM5,PER2,AJUBA,GLG1,CHEK2,PRDM16,SORBS1,TBCK,TRIM8,CSPG4,GRM1,RIMS4,DCC,ARHGAP31,OBSCN,NF2,FLT4,SGMS1,BICC1,SPTBN1,TPTE,TRABD2B,SFRP1,FOXO3,DGKB,CNTN6,ARHGAP29,IFT122,SMAD3,CUX2,ITPR1,WWP2,GRIK5,SHANK1,NEDD4,ARHGEF18,PREX1,TAB1,SLC6A1,TXNDC12,NFATC1,CDC73,APP,GSX2,PDGFRA,NOX1,YAP1,HEG1,AMFR,RAB11FIP5,SESN1,FSTL4,NLGN2,TNFRSF19,EYA2,LRP5,SLC9B2,SOX2,GPC6,CHST11,PRICKLE1,RCAN1,ZNF653,DRD1,TMEM14A,CHUK,FRS2,ESR2,S100A12,PRKD1,STAT1,DGKG,HNF4A,SHANK2,EREG,CCNY,GRAMD4,TMEM237,RAF1,CELF4,RASGRF2,CARD16,CASP1,PTPN1,ADAMTS12,BMPR2,USP33,BMPR1A,TSPAN12,NLGN1,SPAG9,DENND4A,EVC,PDE11A,FGF2,LRRC4C,GRIK1,ADCY5,SEMA4D,DOK6,MCTP1,ADCYAP1R1,DKK2,SORCS2,RAB11FIP3,PRKCG,NCOA1,EEF1E1,EEF1E1-BLOC1S5,GAS8,CHRD,DVL3,PTPRE,RBX1,ARHGAP39,ATF3,SHOC2,PAWR,AGO3,

			DEPTOR,FBXL20,TSG101,CCDC3,TERF2IP,MYO9A,WDR59,WNT3,P4HB,CCL14,CCL15,SUFU,NLGN3,PTPN13,SCUBE2,PAFAH1B1,PRDM15,FAM13A,HIP1,AKAP6,RASAL1,BTBD9,NR2C1,GLI2,NEO1,TNKS,SCUBE1,MGLL,DAB2,PKHD1,UBR1,LDLRAD4,WNK1,RELN,NEK10,RUVBL2,RSPO2,GUCY1A2,PELI1,IQGAP1,MAP3K7,ZNF423,TRIM22,MACF1,ARHGAP12,ALK,SLC8A2,INPP5D,STAU1,UBE2V1,XDH,LTBP1,OVOL2,SNX5,RGS7,ATP2B4,PAK3,CAMTA1,DYX1C1,ATAD1,ADNP,GPR161,MAP1B,P2RY8,RNF34,TFRC,EPHA5,SORCS3,WWOX,SYT12,CACNA1A,GFRAL,GPC3,ADRBK1,PSMB2,EYA1,TSPAN6,CLEC16A,GHR,FGD1,ELAVL4,HDAC1,INVS,SMOC2,TRIM24,PLCG2,ROCK1,EPS8,C1QTNF1,EGR2,HIPK3,CAPN10,GPR35,PTPN2,NTRK1,TRIM44,MAP1A,PPP1R9A,SLC39A10,EIF3A,ANK3,DNMBP,UNC13A,EPHB1,AXIN2,SARM1,MUC1,TUB,JAG2,CACNG8,CARM1,VWF,NETO1,ARHGAP44,ARHGEF3,SPI1,EPHA7,CHRNB4,MCU,PTPRA,CTNNA1,TRDN,LY86,BCAR3,STK38,RBPMS,MAP3K13,OTUD3,SH3BP1,MST1,PTK7,SNCA,BMPR1B,CACNG2,GRM4,MAGI2,ZEB2,SYT9,HEY2,RC3H1,WNT7B,PTPRS,ZMYND11,KMT2D,SPPL2A,ABCA12,GNG7,RASA1,HIPK1,RPTOR,ZNRF3,KCTD13,SLC4A8,CBFA2T2,IGF1,HTT,MAPK1,PTEN,ARHGEF17,SPRED2,BMP7,PUM1,RASA2,PRCP,LRRK2,ZRANB1,CNIH3,LILRB4,IFT81,TMEM108,ARHGAP25,PKD2,EFNA5,SHANK3,RGS9,PDGFC,FAM13B,NCOA3,WNT7A,ZBED6,UACA,CRTC3,PLCL2,KAT6A,GPR21,SIK3,BMP6,ANO1,IL17RD,SH3RF2,SOS1,PRMT2,RAPGEF2,SMO,VRK3,PRKAR2B,STXBP5L,CPT1A,SOX30,UBR5,GRB14,GRIN1,JADE1,KCTD8,RHOA,SYNGAP1,GRIN3A,ROR1,RQCD1,ONECUT2,FAM19A4,CUL3,GSK3B,RDX,ACTA2,SYN1,MLF1,FAM49B,NECAB2,CD27,DUSP26,MCTP2,BOK,SULF1,NACC2,RNF43,AGT,PRKAA2,SYT7,ADAR,FBN2,STAT2,ZMPSTE24,HRH4,NDRG2,PTK2,PEX5L,SNX3,PDE4B,RIMS2,VEPH1,MAP3K5,CREBBP,APCDD1L,DLX1,PCSK6,NCF1,TAOK2,SHC1,ARHGAP15,CD160,LBX2,RPS6KB1,GUCY2F,SCEL,ANKS1A,ALOX5,CPLX2,CD44,FGD3,ADAM8,PACSIN2,BCL2L14,CSNK1A1,GRID1,CXCL17,FGD4,PLEKHA1,RGS14,SIPA1L2,ACTN4,ARHGAP11A,MC1R,STK4,ZFAND6,MARK3,SPEF1,EPHB2,SLC1A1,WLS,IL18,UBE2K,HCAR2,DLC1,PPARA,PPP1R10,PHLPP1,MAGI3,MOB3B,SYTL4,ZNF366,FBN1,PRKCZ,ZC3HAV1,FAM20C,ADORA2A,GRIN2B,RABGEF1,OTUD7B,DENND4B,CNRIP1,ASPN,ANGPT1,TBL1X,EYA3,FHL2,FRMPD1,LRRK1,OSBPL8,ATF6,GCNT2,PPP1CA,TBC1D10C,VWC2,ARHGAP21,RBMS3,CCL22,NCOR2,PRKCQ,BRAF,HDAC2,HTR2C,TNR,CD300A,PHIP,PPP2CA,MAOA,RPL23,SH3BP4,SP100,PIGU,ITSN1,SRGAP2,ADCY1,MADD,SYK,CNOT1,WWTR1,ARHGAP19,GRIK2,CIT,MYRIP,RAP1GAP2,CACNG3,DVL2,PTPRJ,TAF1,TICAM1,TANK,ANKRD13C,SASH1,RALGAPA1,CGNL1,VAV3,DLGAP2,CAV2,GNA12,INPP5F,AXL,WIF1,CD109,DENND4C,MET,CAMK2D,UCHL5,ANKRD6,MALT1,PHB,PTPRC,EDA,GPR89A,PBLD,TYRO3,RNF213,TSPAN5,NCAM1,TRAF3IP2,ATF6B,CREB1,SHISA6,TRAP1,PRAP1,BMP4,CPNE1
GO:0051716	cellular response to stimulus	3.2067752355721965e-37	*CD247,RIC8B,ENPP1,PRDX2,GPC5,NRXN1,ASPH,GP6,PRKCI,MAP4K4,PDE4D,PDCL,DNMT1,S1PR2,TMBIM4,SLC9A1,PDE7B,ADCY8,LMBR1,PDE8A,CLASP2,SEMA3A,IL31RA,GRID2,TAS1R2,RPS6KA2,PTGFR,TENM3,CALR3,CHERP,DOCK1,WWC1,CLDN18,C12ORF49,KIF22,CTDSPL2,PMS1,FBXL2,RYR1,NRG3,ASH1L,NOS1AP,LATS2,ANP32A,SEMA3D,PTH2R,NREP,TIAM2,KSR2,MAPRE2,NLGN4X,PTGIS,PRKAG2,WWC3,TJP1,GRM8,UNC5C,OR5P2,OR5P3,PRLR,MVP,PAGR1,ADCY7,FTO,RGS7BP,KALRN,PITPNC1,NTRK3,CBL,ARNT,ATRNL1,PLEKHG4B,EGLN2,MIA,RAB4B,RAB4B-EGLN2,SEMA5A,LRRFIP2,FLT3,RAD51B,STAT5B,GRM5,PLCE1,SAMHD1,FER,CASK,MARVELD3,MAPK4,MAP2K5,KITLG,MAPK10,PTPRR,SPNS2,LRP2,PIK3CD,ZNF536,DEPDC5,HDGF,ZSWIM7,ANK2,OLFM1,SH2D1A,EZR,NRXN3,XRCC4,ROBO1,TOM1L1,TNFAIP8L1,SLC26A6,HTR2B,ITGB6,MECOM,TENM1,CAMKMT,RYR3,LMNA,NUCB1,GOT1,LPO,NMT2,INPP4B,TRAF6,ROBO2,ITPKB,DNAJA3,OXR1,SLC24A2,VAV2,GRK5,ABI1,ULK4,ERCC6L2,ITGB1,HSP90AA1,ELMO2,CDC6,PSMB7,CNIH2,SRGAP3,MCC,TSSK1B,KNDC1,CCDC22,DSCAM,STK38L,GMDS,SAMD12,CNGB1,TRIO,ARHGAP24,KLHL12,AKAP13,DLG2,PDE1A,PTPRK,DAB1,OMA1,CDC42EP3,MYO10,ABCC8,ERC1,HMGN3,NRIP1,RGS6,DGKI,DNAJB14,ANKS1B,THRB,STXBP4,PDE4A,GABRA3,ITGB3BP,GFRA2,MAST4,IL17RB,TCIRG1,PTPN11,NETO2,HERC4,MGAT5,DOCK10,CYTH3,HDAC6,DDB1,ADORA1,MSRA,GPRASP1,ADAMTS3,EPHA1,IKBKB,ERBB4,ADRA1D,SORCS1,MRE11A,GBF1,LIMK1,TLK1,BRD8,TEX264,ABR,HIF3A,SNIP1,CUL4B,KLHL6,ESR1,NPHP3,GRM7,MIER1,PMEPA1,MAML3,PTPRO,CDON,NTRK2,TNRC6A,EP300,CELA1,TENM2,RNF220,RGS22,CPNE4,FNTA,PDGFB,RIMS1,TNIK,CCND3,MID1,CHFR,ALOX5AP,BPI,STK39,DAP3,HNF4G,INSR,FMN2,PRKAR1A,ASB5,AFAP1,UNC5D,LAMA3,TCF7L2,PIP4K2A,CAB39,USP34,PAK1,LITAF,FBXW11,ESRRB,MYOM1,MAP3K4,ZDHHC13,PTPRD,EGLN3,BDNF,TFAP2A,AMIGO1,CDK14,FANCA,CHRM3,TGIF2,FUT8,OR2T3,NPRL3,PHACTR4,TENM4,GRIK4,TRIM13,RASGEF1B,UBR2,ARHGAP6,LCK,MDM4,EDARADD,IL5RA,ENTPD5,SHC4,ECT2,SNX6,CHML,PPM1L,ARHGAP42,PTPRU,RFTN1,ERN2,CDC45,LSP1,SHFM1,AP3S1,NPLOC4,SGCD,DMD,CENPF,ATG10,LRRN2,SLC30A9,PDS5A,USP13,KAT7,SLC8A1,GSN,RBM14,RBM4,GPR171,SATB2,RIT2,MACROD2,DRG2,DEPDC1B,CASS4,KCTD16,FYN,MKRN2,ARNTL,NF1,PLCB1,DST,MGMT,LMTK2,PPP4R2,RTN4,RXFP1,DPEP1,CHRDL1,DGKH,APOD,NEIL2,RTN4R,BCR,PPP1R12B,RWDD3,SHISA9,CHI3L1,TTN,NDRG4,BMP2K,PAQR3,PIK3R2,RANBP9,NTN1,NOMO3,NRG1,ARHGAP10,SH2D3C,CDK19,BDKRB1,BDKRB2,DOCK8,BID,MAP2K1,MDFI,FNIP1,RAPGEF6,VAX2,RALGPS2,MYH9,MAD1L1,DAGLA,TMBIM6,VGLL4,PPP2CB,PPP3R1,HDAC5,VSTM1,OR6N2,CMKLR1,INPP4A,CDKL2,ROR2,DCN*

			,ZDHHC3,SLC39A14,DOCK2,CDH13,CREBRF,SOX13,FHIT,WDR70,PTPRT,AUNIP,COPS5,FGF14,DMC1,IPO5,ARHGAP32,MAD2L2,MAN1A1,TLE6,RGS8,CAPRIN2,CCNYL1,NLN,NCKAP1,DEFA1B,DEFA3,TERF2,PTGER2,RALGPS1,SLX1B,STIP1,FOXN3,RCC2,BOC,ANO6,ARL3,NEUROD1,GNAQ,FBXO9,ICK,TMEM117,RAD51D,RFFL,RANBP1,CCBE1,DENND1A,JAK2,FAM168A,FBXW7,LINC00473,SKAP1,SMC3,UIMC1,SYT1,ITCH,NPSR1,ZFYVE28,SMG1,DTHD1,CLDN1,HUNK,MLLT3,ARAP2,CDC42BPB,DOCK9,SH2D6,TCF7,PDE2A,KLF15,ANXA4,LAMTOR3,MAGI1,CYFIP2,WNT11,ARHGAP23,IFT80,TRIM59,APBB1IP,MTA1,KREMEN1,IMMP2L,NRG4,CLPB,NOX4,LCOR,SIPA1L3,MKLN1,CACNA1H,SIAH1,IL1RAPL1,CCDC62,CAMK2B,ADAM23,ERCC8,PRKCD,SOX6,TAB2,ACVR2A,RUNX2,SEMA5B,CD4,PPM1E,TGFB1,PLCXD3,SGK1,SPSB4,NUP93,MMP28,IGSF1,PIBF1,ASCC2,BTRC,NFATC3,POLK,CRADD,DLGAP1,PTPRN2,F2RL1,BCAS3,C9ORF47,GRIK3,RPGRIP1L,DNAJB2,CELSR1,SLIT3,AAK1,ZFYVE9,SLIT2,ASB15,TP73,GABRR3,GABRB1,SYT13,HHAT,GPR176,HP,CORO1C,RRAS2,CTNND2,OR9A4,DISC1,KANK1,CACNA1D,ZFAND2A,PTPDC1,MTDH,ANK1,TTBK2,XPR1,CHRM1,OR51A7,OR51F2,OR51T1,SMAD6,RXFP2,CLOCK,ZNF675,PLCB4,KCNH1,BMPER,BCL3,DCLK1,ANKRD54,DAPK2,TNFRSF10B,PARVA,DAPP1,ADAM12,DUSP22,GPR52,NAIP,TRRAP,HOMER2,DOCK3,SBNO2,FGF10,FBXL17,RASA4,RASA4B,ARHGEF28,SMYD3,KCTD10,IL1RAPL2,IQCJ-SCHIP1,SH3RF3,LOXL3,RHPN2,MAST2,FANCI,GLP2R,ADCY2,CAPN3,SLC16A1,SMURF2,EPHA4,RORA,PRKCA,AUTS2,CNR1,CD6,TNFSF11,PPP3CA,NSUN2,HBE1,OR51B2,OR51B4,OR51I1,UBQLN3,GNG4,NFYB,MAG,CAMK4,UFL1,CTNNB1,PARK2,SOD2,FCGR2A,FCGR2B,FCGR3A,FCGR3B,HSPA6,ARHGAP22,SMARCC1,IGF1R,PPARG,AXIN1,GARNL3,MACROD1,OTUB1,DLG5,IL18R1,IL1RL1,MTF1,MSR1,CBX5,P2RY10,TRPM1,ANKRD17,CYBB,CASKIN1,APOL3,SCAMP5,BRIP1,SREBF2,TDP1,RYR2,DRAXIN,LEPR,LEPROT,FGF1,RGS10,NR4A3,WDR45B,NOL3,PRKAR2A,MYOCD,OR52E6,OR52E8,OR52N1,TRIM5,KIR2DL1,KIR2DL4,AJUBA,CACNA1C,CPNE6,GLG1,CHEK2,UBQLN4,PRDM16,MYO1E,ASB8,PPP1CB,SPDYA,HCK,SORBS1,CAPN2,TBCK,TRIM8,CSPG4,GRM1,DCC,ARHGAP31,P2RY14,BAZ1B,OBSCN,RGL1,NF2,FLT4,SGMS1,BICC1,HDAC4,PAX2,TNS3,SPTBN1,TPTE,TRABD2B,SFRP1,ST8SIA1,FOXO3,DGKB,TPH2,TRHDE,ARL13B,CNTN6,ARHGAP29,IFT122,SSH1,SYNCRIP,SMAD3,RNFT2,CUX2,ITPR1,PTPRM,WWP2,ACKR2,WIPI1,RNF168,ABI2,GRIK5,PSAP,SHANK1,SYT3,IFNAR1,OR14K1,NEDD4,NRP2,ARHGEF18,VANGL1,PREX1,PLA2G4C,DOCK4,ESRRG,HOXD3,KPNB1,TAB1,TXNDC12,NFATC1,RAB6C,SEC61B,CDC73,APP,GSX2,PDGFRA,AOC2,FCRL4,NOX1,YAP1,HEG1,AMFR,RAB11FIP5,SESN1,CDK3,FSTL4,NLGN2,TNFRSF19,EYA2,ALPL,JAK1,LRP5,MUC20,PTPRG,SLC9B2,GNG2,SOX2,SETD2,GPC6,CHST11,TEAD1,PRICKLE1,RCAN1,ZNF653,GRIA3,DRD1,TMEM14A,GLRA2,CHUK,ERLIN1,FRS2,PALB2,MMP2,SERGEF,ESR2,S100A12,CPNE9,KDM6A,PRKD1,STAT1,ST18,DGKG,ETV5,RND3,HNF4A,LRRC2,SHANK2,SHPRH,VPS4A,EREG,CCNY,TCTN3,MAPKAPK3,SEMA6D,ATF2,GRAMD4,TMEM237,RAF1,CELF4,RASGRF2,EPHX1,CARD16,CASP1,PTPN1,ADAMTS12,POLA1,BMPR2,USP33,DPYSL2,CAMK1D,BMPR1A,TSPAN12,NLGN1,COL16A1,PIK3R3,SPAG9,MORC2,DENND4A,EVC,PDE11A,EPG5,FGF2,BACH1,GRIK1,OR6C70,PPM1F,TICRR,ADCY5,NEDD9,SEMA4D,DOK6,HELQ,RORC,MCTP1,PLXNA2,PTAFR,ADCYAP1R1,JARID2,ANKDD1A,DDX58,DKK2,SORCS2,CHCHD6,ITGA11,PI4KA,RARB,SPEN,PIK3C2B,PRKCG,VIPR1,NCOA1,BLOC1S5,EEF1E1,EEF1E1-BLOC1S5,GRIA2,GAS8,ALKBH3,CHRD,DVL3,ECE2,EIF2B5,EIF4G1,EPHB3,PTPRE,RBX1,ARHGAP39,ATF3,FBXO45,SHOC2,SRSF5,STT3B,PAWR,AGO3,ACAA1,DEPTOR,PKN3,MAML2,TSG101,CCDC3,TERF2IP,CLIC4,MYO9A,ANKS4B,IDE,WDR59,KCNQ1,RFX2,WNT3,RCE1,RHOJ,P4HB,CCL14,CCL15,CCL15-CCL14,SUFU,TG,NLGN3,PTPN13,SCUBE2,ZFYVE1,PAFAH1B1,PRDM15,FAM13A,POLE,CNTFR,COL4A3,TRPA1,DDAH1,NMUR2,HIP1,AKAP6,RASAL1,NR2C1,EDEM3,IRS4,GLI2,NEO1,TNKS,SCUBE1,ERCC1,PXDNL,GPR39,WDTC1,MGLL,DAB2,THEMIS,BLM,PKHD1,UBR1,LDLRAD4,DZIP1,WNK1,RELN,NEK10,SIN3A,RUVBL2,OR5H2,OR5H6,OR5K4,RSPO2,GUCY1A2,CSF3R,DOCK11,PELI1,PPP2R5C,IQGAP1,SPATA18,MAP3K7,DDX47,TAS2R41,ZNF423,SP1,OR56B1,TRIM22,MACF1,ARHGAP12,ALK,SLC8A2,GSTM3,INPP5D,STAU1,DSC2,EEPD1,TEAD4,SLC35A4,SRA1,UBE2V1,XDH,LTBP1,OVOL2,SNX5,NFATC2,RBBP6,CHD1L,OR6C74,PRKG1,RGS7,ATP2B4,PACRG,APEX2,PAK3,CAMTA1,DYX1C1,P2RX6,ASGR2,ADNP,GPR161,MAPKAPK2,MAP1B,CDC42BPA,COL4A6,SCARB1,MARK1,CDK6,ATP6V0A2,P2RY8,RNF34,TFRC,UVRAG,EPHA5,SORCS3,WWOX,GABRG3,MEF2A,SYT12,ADCY9,CACNA1A,NFRKB,DCUN1D5,FNBP1,GFRAL,GPC3,XRN1,ADRBK1,PSMB2,ZNF207,EYA1,PTGER3,BRINP1,GNB3,SHPK,TRPV1,TSPAN6,TFEB,CLEC16A,GHR,MNAT1,FLRT2,FGD1,ACTR2,ACACA,ELAVL4,HDAC1,INVS,RECQL5,SMOC2,TRIM24,ADIPOR2,PLCG2,ROCK1,EPS8,C1QTNF1,PAXIP1,EGR2,HIPK3,RNF10,GAPVD1,CAPN10,GPR35,CUL2,ABCC1,AP3B1,PTPN2,INSRR,NTRK1,TRIM44,ZFAND1,PKN2,PPP1R9A,SLC39A10,EIF3A,ANK3,DNMBP,UNC13A,EPHB1,MAN1B1,PDE6A,EPHA10,AXIN2,SARM1,TMEM199,EPCAM,PARD3,RCAN2,MUC1,TUB,SH3GL3,JAG2,CACNG8,AP3D1,CARM1,VWF,NETO1,ARHGAP44,HTR1D,ARHGEF3,RAD51C,SPI1,DGKK,EPHA7,CHRNB4,MCU,RANBP10,PTPRA,CTNNA1,AIFM2,FBXW4,ACAP2,ATG14,GABRA6,LRRC8C,LRRC8D,LY86,STAC,BCAR3,SHROOM3,STK38,RBPMS,CHURC1,MAP3K13,OTUD3,RAB15,RAPGEF3,PDXP,SH3BP1,MST1,ERCC3,PTK7,SNCA,BMPR1B,POLN,CACNG2,CD53,GRM4,MAGI2,PNPT1,USP50,ZEB2,SYT9,HEY2,RC3H1,RNF103,RNF103-

			CHMP3,CNGA3,WNT7B,HMGCS2,PTPRS,ZMYND11,KMT2D,PRKAG1,CREM,SULT2A1,TRIP12,DFFA,SPPL2A,ADTRP,ABCA12,GNG7,PLD1,RASA1,MACC1,MITF,OPRD1,CABIN1,HIPK1,NUGGC,RPTOR,RTN4RL1,BTK,SIGLEC9,VPS41,ZNRF3,KCTD13,TROVE2,CBFA2T2,IGF1,STX8,HTT,DPYSL3,MAPK1,PTEN,ARHGEF17,MIB1,SPRED2,IFFO1,BMP7,PIK3C3,PUM1,RASA2,SOX5,PRCP,RAB30,LRRK2,ATP6V1A,OSGIN1,GNA14,ZRANB1,UNC119,ZBTB20,CHMP5,CNIH3,RAPH1,RFC3,BAG6,CHAF1B,LILRB4,IFT81,TMCO1,TMEM108,IPCEF1,ITGAM,ARHGAP25,IL11RA,NSG2,PKD2,EFNA5,HSF2BP,SHANK3,SEL1L2,BTBD11,RGS9,AOX1,PDGFC,AMOTL1,FAM13B,NCOA3,MTMR3,WNT7A,ZBED6,RPS6KC1,UACA,CRTC3,GPR141,NME8,GLDC,PLCL2,KAT6A,GPR21,RABEP1,RHOT1,SIK3,BMP6,NFE2L1,ANO1,GPI,IL17RD,SH3RF2,SOS1,TSHR,EXT1,N4BP1,PDCD1LG2,ATRX,PRICKLE2,GABRR2,PNPLA3,PRMT2,RAPGEF2,ASCC1,ABCG2,KCNC2,SMO,VRK3,GET4,BLOC1S3,MARK4,PRKAR2B,CPT1A,PSEN2,TNFRSF11B,PAG1,SYT17,SOX30,UBR5,GRB14,GRIN1,JADE1,KCTD8,RHOA,SPRTN,WSB2,OR4M1,OR4N2,SYNGAP1,VIPR2,GRIN3A,MCM3,ROR1,RQCD1,TF,ONECUT2,RHOBTB1,FAM19A4,CUL3,TFEC,HSPD1,ITPR2,NFKBID,EPM2A,GABRB3,GSK3B,ABCG8,PLD2,DNAJC3,GNG12,RDX,STAT6,ACTA2,MLF1,EEF2K,FAM49B,RBBP8,NECAB2,STK32B,CD27,DUSP26,ITGBL1,MCTP2,BOK,SULF1,KRT8,NACC2,RNF43,TNFRSF8,AGT,HDGFRP3,POLH,PRKAA2,SYT7,ADAR,BLOC1S6,FBN2,PITPNM2,STAT2,ZMPSTE24,BRINP3,HRH4,NDRG2,PTK2,LAT2,PEX5L,PHEX,RNF121,SNX3,PDE4B,RIMS2,TCP11,TEC,SHB,VEPH1,GAP43,MAP3K5,CPE,CREBBP,IQGAP2,PRKD3,APCDD1L,DLX1,FMOD,INSL6,PCSK6,NCF1,SLC29A1,TAOK2,UGT1A1,UGT1A10,UGT1A3,UGT1A4,UGT1A5,UGT1A6,UGT1A7,UGT1A8,UGT1A9,PYGO2,SHC1,NDUFS2,ARHGAP15,CD160,ECE1,LBX2,PARP10,RNFT1,RPS6KB1,STC2,GPR173,GUCY2F,SCEL,ANKS1A,ITGAL,ALOX5,DNAJB6,CD44,FGD3,ADAM8,RPS6KA5,BCL2L14,CDS1,CSNK1A1,GRID1,HUS1,CXCL17,FCRL6,FGD4,NOS1,PLEKHA1,PLVAP,RGS14,SIPA1L2,ACTN4,ARHGAP11A,MC1R,SCNN1B,STK4,ZFAND6,MARK3,SPEF1,EPHB2,SLC1A1,WLS,SIGMAR1,XCR1,IL18,TEX12,DTNA,MFSD8,TPMT,UBE2K,GPSM2,DNAJC7,HCAR1,HCAR2,HCAR3,DLC1,PPARA,PPP1R10,ARID5B,CALCRL,MCOLN1,SLC25A33,SMARCAL1,PHLPP1,MAGI3,CPEB4,GPSM3,MOB3B,NOTCH4,ZNF366,CYP2C18,CYP2C19,FBN1,PAX6,PRKCZ,ZC3HAV1,ATP1A3,FAM20C,ADORA2A,GRIN2B,PPP1CC,RABGEF1,CPEB1,PLCXD2,TYK2,OTUD7B,DENND4B,KIN,NIPBL,CNRIP1,ASPN,ANGPT1,TBL1X,EYA3,FHL2,FRMPD1,LRRK1,OSBPL8,ATF6,GCNT2,PPP1CA,TBC1D10C,VWC2,ARHGAP21,GRIA4,MTF2,RBMS3,BIN2,CCL22,BTN3A2,FBXO31,CNTN1,NCOR2,PRKCQ,BRAF,CSRNP1,DIAPH1,HDAC2,HTR2C,IMPACT,SPINT2,TNR,UBE2E2,CD300A,IRAK1BP1,PHIP,PPP2CA,ANGPT4,ARHGEF6,MAOA,RPL23,SH3BP4,RFC5,DCDC1,SP100,OPN1LW,REV3L,KCND2,PIGU,C1QTNF9,CR2,DONSON,FCRL2,ITSN1,SRGAP2,WIPI2,ADCY1,MADD,SYK,CNOT1,WWTR1,ARHGAP19,GRIK2,GTF2H5,ASB1,CIT,RADIL,ZBTB38,BRCA2,HRK,RAP1GAP2,CACNG3,DVL2,YBX1,GRIP1,PTPRJ,SLC10A1,TAF1,TICAM1,TANK,UCN2,ITGAE,TAC3,A3GALT2,HSF1,MAX,DHFR,INPP5A,SRPK2,ANKRD13C,PLEKHM1,SASH1,NCOA2,ADAMTS7,RALGAPA1,GBP5,MCM8,BMP1,CGNL1,FCHO1,QRICH1,VAV3,ZP3,CHD6,DLGAP2,CIB2,MOB3A,CAV2,GNA12,SH2D3A,UGGT1,COL4A5,MYEF2,INPP5F,NUAK2,AXL,REPS2,TNFSF9,WIF1,CD109,DENND4C,MET,CAMK2D,CCR3,RAD9B,TIMELESS,UCHL5,ANKRD6,MALT1,PFKP,TRA2B,INO80C,MLXIPL,SEMA3C,THEM4,PHB,PTPRC,EDA,GPR89A,EXD2,PBLD,SH3GL2,TYRO3,NR6A1,RNF213,SPON2,TSPAN5,ADCY10,NCAM1,TRAF3IP2,ATF6B,CREB1,LIG1,RASSF8,RGMB,SGTB,SHISA6,TRAP1,CLNK,EDNRA,GNGT1,PRAP1,BMP4,CASP12,CPNE1
GO:0023052	signaling	7.605238647256185e-36	CD247,RIC8B,ENPP1,PRDX2,GPC5,NRXN1,ASPH,GP6,PRKCI,MAP4K4,PDE4D,PDCL,DNMT1,S1PR2,TMBIM4,SLC9A1,PDE7B,ADCY8,LMBR1,PDE8A,CLASP2,SEMA3A,IL31RA,GRID2,TAS1R2,RPS6KA2,PTGFR,TENM3,CHERP,DOCK1,WWC1,CLDN18,C12ORF49,CTDSPL2,FBXL2,NRG3,ASH1L,NOS1AP,LATS2,ANP32A,SEMA3D,PTH2R,NREP,TIAM2,KSR2,MAPRE2,NLGN4X,PTGIS,PRKAG2,WWC3,GRM8,UNC5C,OR5P2,OR5P3,CLCN1,PRLR,MVP,PAGR1,ADCY7,RGS7BP,KALRN,PITPNC1,NTRK3,CBL,ARNT,ATRNL1,PLEKHG4B,EGLN2,MIA,RAB4B,RAB4B-EGLN2,SEMA5A,LRRFIP2,FLT3,STAT5B,ATP2B2,GRM5,PLCE1,SAMHD1,FER,CASK,MARVELD3,MAPK4,MAP2K5,KITLG,MAPK10,PTPRR,SPNS2,LRP2,PIK3CD,ZNF536,DEPDC5,HDGF,ANK2,OLFM1,SH2D1A,EZR,NRXN3,ROBO1,TOM1L1,TNFAIP8L1,CHD7,HTR2B,ITGB6,MECOM,TENM1,CAMKMT,RYR3,LMNA,NUCB1,GOT1,NMT2,INPP4B,TRAF6,ROBO2,NFASC,ITPKB,CTNNA3,DNAJA3,SLC24A2,VAV2,GRK5,ABI1,ULK4,ITGB1,HSP90AA1,CDC6,PSMB7,CNIH2,SRGAP3,MCC,TSSK1B,PCDH17,KNDC1,CCDC22,DSCAM,STK38L,GMDS,SAMD12,CNGB1,TRIO,ARHGAP24,KLHL12,AKAP13,DLG2,PDE1A,PTPRK,DAB1,CDC42EP3,MYO10,ABCC8,ERC1,HMGN3,RGS6,DGKI,ANKS1B,THRB,STXBP4,PDE4A,ERC2,GABRA3,ITGB3BP,LAMA2,GFRA2,MAST4,IL17RB,TCIRG1,PTPN11,NETO2,HERC4,MGAT5,CACNB2,DOCK10,CYTH3,HDAC6,DDB1,SV2B,ADORA1,GPRASP1,ADAMTS3,EPHA1,IKBKB,ERBB4,ADRA1D,SORCS1,MRE11A,GBF1,LIMK1,TLK1,BRD8,ABR,SNIP1,KLHL6,ESR1,NPHP3,GRM7,MIER1,PMEPA1,MAML3,PTPRO,CDON,NTRK2,EP300,CELA1,TENM2,RNF220,RGS22,FNTA,PDGFB,CACNA1B,RIMS1,TNIK,CCND3,CDH8,USP46,MID1,CHFR,BPI,STK39,DAP3,HNF4G,INSR,FMN2,PRKAR1A,ASB5,AFAP1,UNC5D,LAMA3,TCF7L2,PIP4K2A,CAB39,USP34,PAK1,LITAF,FBXW11,ESRRB,MYOM1,MAP3K4,ZDHHC13,PTPRD,SLC4A10,BDNF,CDK14,FANCA,CHRM3,TGIF2,FUT8,OR2T3,NPRL3,PHACTR4,TENM4,GRIK4,TRIM13,RASGEF1B,UBR2,ARHGAP6,LCK,MDM4

			,EDARADD,IL5RA,ENTPD5,SHC4,ECT2,SNX6,CHML,VAMP7,PPM1L,ARHGAP42,PTPRU,RFTN1,ERN2,CDC45,LSP1,AP3S1,NPLOC4,SGCD,DMD,CENPF,ATG10,LRRN2,KAT7,SLC8A1,RBM14,GPR171,RIT2,DRG2,DEPDC1B,CASS4,KCTD16,FYN,MKRN2,ARNTL,NF1,PLCB1,DST,LMTK2,SNCB,RTN4,RXFP1,CHRDL1,DGKH,APOD,RTN4R,BCR,PPP1R12B,RWDD3,SHISA9,CHI3L1,TTN,NDRG4,ICA1,BMP2K,PAQR3,PIK3R2,CACNA1E,RANBP9,NTN1,NOMO3,NRG1,ARHGAP10,SH2D3C,SLC16A10,BDKRB1,BDKRB2,DOCK8,BID,MAP2K1,MDFI,FNIP1,RAPGEF6,VAX2,RALGPS2,MYH9,MAD1L1,DAGLA,TMBIM6,VGLL4,PPP2CB,CNTN4,PPP3R1,NTNG1,VSTM1,OR6N2,CMKLR1,INPP4A,CDKL2,ROR2,DCN,ZDHHC3,SLC39A14,LIN7A,SYN2,DOCK2,CDH13,CREBRF,SOX13,FHIT,PTPRT,COPS5,FGF14,PCDHB16,EXOC4,ARHGAP32,MAD2L2,TLE6,RGS8,CAPRIN2,CCNYL1,NLN,NCKAP1,DEFA1B,DEFA3,PTGER2,RALGPS1,FOXN3,RCC2,BOC,ANO6,ARL3,NEUROD1,GNAQ,FBXO9,ICK,TMEM117,RFFL,RANBP1,CCBE1,DENND1A,JAK2,FBXW7,LINC00473,SYN3,SKAP1,CLSTN2,UIMC1,SYT1,ITCH,NPSR1,ZFYVE28,LZTS1,SMG1,SCN4A,SLC1A4,DTHD1,HUNK,MLLT3,ARAP2,CDC42BPB,DOCK9,SH2D6,TCF7,PDE2A,KLF15,ANXA4,LAMTOR3,MAGI1,KCNC4,CYFIP2,WNT11,ARHGAP23,IFT80,TRIM59,APBB1IP,MTA1,KREMEN1,NRG4,CLPB,NOX4,SIPA1L3,MKLN1,CACNA1H,SIAH1,IL1RAPL1,CAMK2B,PRKCD,TAB2,ACVR2A,RUNX2,SEMA5B,CD4,TGFB1,PLCXD3,SGK1,PCSK5,PDLIM4,SPSB4,NUP93,IGSF1,PIBF1,BTRC,NFATC3,JPH3,CRADD,DLGAP1,PTPRN2,F2RL1,C9ORF47,GRIK3,RPGRIP1L,CELSR1,SLIT3,AAK1,ZFYVE9,SLIT2,ASB15,TP73,GABRR3,CADPS2,GABRB1,SYT13,HHAT,GPR176,CORO1C,RRAS2,CTNND2,OR9A4,DISC1,KCNJ3,KANK1,CACNA1D,PTPDC1,MTDH,ANK1,TTBK2,XPR1,CHRM1,OR51A7,OR51F2,OR51T1,SMAD6,RXFP2,CLOCK,ZNF675,PLCB4,KCNH1,BMPER,BCL3,DCLK1,ANKRD54,DAPK2,TNFRSF10B,DAPP1,ADAM12,DUSP22,GPR52,NAIP,HOMER2,SLC17A7,DOCK3,OTOA,YTHDF1,FGF10,CNTNAP2,FBXL17,RASA4,RASA4B,ARHGEF28,STXBP5,KCTD10,IL1RAPL2,IQCJ-SCHIP1,SH3RF3,LOXL3,RHPN2,MAST2,GLP2R,ADCY2,CAPN3,SLC16A1,SMURF2,EPHA4,RORA,PRKCA,AUTS2,CNR1,CD6,TNFSF11,PPP3CA,NSUN2,OR51B2,OR51B4,OR51I1,GNG4,MAG,CAMK4,UFL1,CTNNB1,PARK2,SOD2,FCGR2A,FCGR2B,FCGR3A,FCGR3B,ARHGAP22,SMARCC1,IGF1R,PPARG,AXIN1,GARNL3,PRKAR1B,DLG5,IL18R1,IL1RL1,P2RY10,TRPM1,ANKRD17,CYBB,CASKIN1,APOL3,BRIP1,SREBF2,RYR2,DRAXIN,LEPR,LEPROT,FGF1,RGS10,NR4A3,NOL3,PRKAR2A,MYOCD,OR52E6,OR52E8,OR52N1,TRIM5,PER2,KIR2DL1,KIR2DL4,AJUBA,CACNA1C,CPNE6,GLG1,SCN3B,CHEK2,PRDM16,MYO1E,ASB8,PPP1CB,HCK,SORBS1,TBCK,TRIM8,CSPG4,GRM1,RIMS4,DCC,ARHGAP31,P2RY14,OBSCN,RGL1,NF2,FLT4,SGMS1,BICC1,HDAC4,PAX2,TNS3,SPTBN1,TPTE,TRABD2B,SPG11,SFRP1,FOXO3,DGKB,TRHDE,ARL13B,CNTN6,ARHGAP29,IFT122,SMAD3,CUX2,ITPR1,PTPRM,WWP2,ACKR2,ABI2,GRIK5,PSAP,SLC16A2,SHANK1,SYT3,IFNAR1,OR14K1,NEDD4,NRP2,ARHGEF18,VANGL1,PREX1,PLA2G4C,DOCK4,ESRRG,HOXD3,KPNB1,TAB1,SLC6A1,TXNDC12,NFATC1,RAB6C,CDC73,APP,GSX2,PDGFRA,FCRL4,NOX1,YAP1,HEG1,AMFR,RAB11FIP5,SESN1,FSTL4,NLGN2,TNFRSF19,EYA2,JAK1,TNNI3K,LRP5,MUC20,PTPRG,SLC9B2,GNG2,SOX2,GPC6,CHST11,TEAD1,PRICKLE1,RCAN1,ZNF653,SPTBN4,GRIA3,DRD1,TMEM14A,GLRA2,CHUK,ERLIN1,FRS2,MMP2,SERGEF,ESR2,S100A12,KDM6A,PRKD1,STAT1,ST18,DGKG,RND3,SLC6A3,HNF4A,LRRC2,SHANK2,VPS4A,EREG,CCNY,TCTN3,MAPKAPK3,SEMA6D,ATF2,GRAMD4,TMEM237,RAF1,CELF4,RASGRF2,CARD16,CASP1,PTPN1,ADAMTS12,BMPR2,USP33,DPYSL2,BMPR1A,TSPAN12,NLGN1,COL16A1,PIK3R3,SPAG9,DENND4A,EVC,PDE11A,EPG5,FGF2,LRRC4C,GRIK1,OR6C70,PPM1F,TICRR,ADCY5,NEDD9,SEMA4D,DOK6,RORC,MCTP1,PLXNA2,PTAFR,ADCYAP1R1,ANKDD1A,DDX58,DKK2,SORCS2,RAB11FIP3,ITGA11,PI4KA,RARB,SPEN,PIK3C2B,PRKCG,VIPR1,NCOA1,EEF1E1,EEF1E1-BLOC1S5,GRIA2,GAS8,LMO7,CHRD,DVL3,ECE2,EIF2B5,EPHB3,PTPRE,RBX1,ARHGAP39,ATF3,SHOC2,PAWR,AGO3,DEPTOR,PKN3,FBXL20,MAML2,TSG101,CCDC3,TERF2IP,MYO9A,IDE,WDR59,KCNQ1,WNT3,RCE1,RHOJ,P4HB,CCL14,CCL15,CCL15-CCL14,SUFU,TG,NLGN3,PTPN13,SCUBE2,PAFAH1B1,PRDM15,FAM13A,CNTFR,COL4A3,TRPA1,DDAH1,NMUR2,HIP1,AKAP6,RASAL1,BTBD9,NR2C1,IRS4,GLI2,NEO1,TNKS,SCUBE1,GPR39,MGLL,DAB2,THEMIS,BLM,PKHD1,UBR1,LDLRAD4,DZIP1,WNK1,RELN,NEK10,RUVBL2,OR5H2,OR5H6,OR5K4,RSPO2,GUCY1A2,CSF3R,KCNQ2,DOCK11,PELI1,PPP2R5C,IQGAP1,MAP3K7,DDX47,TAS2R41,ZNF423,OR56B1,TRIM22,MACF1,ARHGAP12,ALK,SLC8A2,INPP5D,STAU1,DSC2,TEAD4,UBE2V1,XDH,LTBP1,OVOL2,SNX5,NFATC2,OR6C74,PRKG1,RGS7,ATP2B4,NSF,PAK3,CAMTA1,DYX1C1,P2RX6,ASGR2,ATAD1,ADNP,GPR161,MAPKAPK2,MAP1B,CDC42BPA,COL4A6,SCARB1,MARK1,CDK6,P2RY8,RNF34,TFRC,EPHA5,SORCS3,WWOX,GABRG3,MEF2A,SYT12,ADCY9,CACNA1A,FNBP1,GFRAL,GPC3,ADRBK1,PSMB2,ZNF207,EYA1,PTGER3,GNB3,TRPV1,TSPAN6,CLEC16A,GHR,FLRT2,FGD1,ELAVL4,HDAC1,INVS,SMOC2,TRIM24,ADIPOR2,PLCG2,ROCK1,EPS8,C1QTNF1,PAXIP1,EGR2,HIPK3,RNF10,GAPVD1,CAPN10,GPR35,CUL2,AP3B1,PTPN2,INSRR,NTRK1,TRIM44,MAP1A,PKN2,PPP1R9A,SLC39A10,EIF3A,ANK3,DNMBP,UNC13A,EPHB1,PDE6A,EPHA10,AXIN2,SARM1,EPCAM,PARD3,RCAN2,MUC1,TUB,SH3GL3,JAG2,KCND3,CACNG8,CARM1,VWF,NETO1,ARHGAP44,SRD5A2,HTR1D,ARHGEF3,SPI1,DGKK,EPHA7,CHRNB4,MCU,RANBP10,PTPRA,CTNNA1,FBXW4,TRDN,GABRA6,LY86,STAC,BCAR3,ILDR2,STK38,RBPMS,CHURC1,FCHSD2,MAP3K13,OT

			UD3,RAB15,RAPGEF3,SH3BP1,MST1,PTK7,SNCA,BMPR1B,CACNG2,CD53,GRM4,MAGI2,USP50,ZEB2,SYT9,HEY2,RC3H1,CNGA3,WNT7B,PTPRS,ZMYND11,KMT2D,PRKAG1,CREM,DFFA,SPPL2A,ADTRP,ABCA12,GNG7,PLD1,RASA1,MACC1,MITF,OPRD1,CABIN1,HIPK1,PKP2,RPTOR,BTK,SIGLEC9,TBC1D16,ZNRF3,KCTD13,SLC4A8,TROVE2,CBFA2T2,IGF1,HTT,MAPK1,PTEN,ARHGEF17,MIB1,SPRED2,BMP7,SCN8A,PIK3C3,PUM1,RASA2,PRCP,RAB30,LRRK2,OSGIN1,GNA14,ZRANB1,UNC119,CHMP5,CNIH3,RAPH1,BAG6,LILRB4,IFT81,TMCO1,TMEM108,ITGAM,ARHGAP25,IL11RA,NSG2,PKD2,EFNA5,SHANK3,BTBD11,RGS9,PDGFC,AMOTL1,FAM13B,NCOA3,WNT7A,ZBED6,SNAP23,RPS6KC1,UACA,CRTC3,GPR141,CEP89,PLCL2,KAT6A,GPR21,RABEP1,RHOT1,SIK3,BMP6,ANO1,GPI,IL17RD,SH3RF2,SOS1,TSHR,EXT1,PDCD1LG2,ATRX,PRICKLE2,GABRR2,PRMT2,RAPGEF2,KCNC2,SMO,VRK3,MARK4,PRKAR2B,STXBP5L,CPT1A,PSEN2,TNFRSF11B,PAG1,SYT17,SOX30,UBR5,GRB14,GRIN1,JADE1,KCTD8,RHOA,WSB2,OR4M1,OR4N2,SYNGAP1,VIPR2,GRIN3A,ROR1,RQCD1,TF,ONECUT2,RHOBTB1,FAM19A4,CUL3,SH3KBP1,HSPD1,ITPR2,NFKBID,EPM2A,GABRB3,GSK3B,ABCG8,PLD2,GNG12,RDX,STAT6,ACTA2,SYN1,MLF1,FAM49B,NECAB2,STK32B,CD27,DUSP26,ITGBL1,MCTP2,SCN9A,BOK,SULF1,KRT8,NACC2,RNF43,TNFRSF8,AGT,HDGFRP3,PRKAA2,SYT7,ADAR,BLOC1S6,FBN2,PITPNM2,STAT2,ZMPSTE24,HRH4,NDRG2,PTK2,LAT2,PEX5L,PHEX,SNX3,PDE4B,RIMS2,TCP11,TEC,SHB,VEPH1,GAP43,MAP3K5,CPE,CREBBP,IQGAP2,PRKD3,APCDD1L,DLX1,FMOD,INSL6,PCSK6,NCF1,SLC29A1,TAOK2,PYGO2,SHC1,ARHGAP15,CD160,ECE1,LBX2,RPS6KB1,STC2,GPR173,GUCY2F,SCEL,ANKS1A,ITGAL,ALOX5,CPLX2,CD44,FGD3,ADAM8,PACSIN2,RPS6KA5,BCL2L14,CDS1,CSNK1A1,GRID1,HUS1,CXCL17,FCRL6,FGD4,NOS1,PLEKHA1,PLVAP,RGS14,SIPA1L2,ACTN4,ARHGAP11A,MC1R,STK4,ZFAND6,MARK3,SPEF1,EPHB2,SLC1A1,WLS,SIGMAR1,XCR1,IL18,DTNA,MFSD8,UBE2K,GPSM2,HCAR1,HCAR2,HCAR3,DLC1,PPARA,PPP1R10,ARID5B,CALCRL,MCOLN1,SLC25A33,PHLPP1,MAGI3,CPEB4,MOB3B,NOTCH4,SYTL4,ZNF366,FBN1,PAX6,PRKCZ,ZC3HAV1,ATP1A3,FAM20C,ADORA2A,GRIN2B,PPP1CC,RABGEF1,PLCXD2,TYK2,OTUD7B,DENND4B,CNRIP1,ASPN,ANGPT1,CADPS,TBL1X,EYA3,FHL2,FRMPD1,LRRK1,OSBPL8,ATF6,GCNT2,PPP1CA,TBC1D10C,VWC2,ARHGAP21,GRIA4,RBMS3,CCL22,BTN3A2,FBXO31,CNTN1,NCOR2,PRKCQ,BRAF,CSRNP1,HDAC2,HTR2C,TNR,CD300A,IRAK1BP1,PHIP,PPP2CA,ANGPT4,ARHGEF6,MAOA,RPL23,SH3BP4,DCDC1,SP100,OPN1LW,ANXA2,KCND2,PIGU,C1QTNF9,CR2,DONSON,FCRL2,ITSN1,SRGAP2,ADCY1,MADD,SYK,CNOT1,WWTR1,ARHGAP19,GRIK2,SCN1A,ASB1,CIT,MYRIP,RADIL,BRCA2,HRK,RAP1GAP2,CACNG3,DVL2,GRIP1,PTPRJ,SLC10A1,TAF1,ATP2B3,TICAM1,TANK,UCN2,ITGAE,TAC3,HSF1,INPP5A,SRPK2,ANKRD13C,PLEKHM1,SASH1,RALGAPA1,BMP1,CGNL1,FCHO1,QRICH1,VAV3,ZP3,DLGAP2,KCNQ3,MOB3A,CAV2,GNA12,SH2D3A,COL4A5,INPP5F,NUAK2,AXL,REPS2,TNFSF9,WIF1,CD109,DENND4C,MET,CAMK2D,CCR3,RAD9B,TIMELESS,UCHL5,ANKRD6,MALT1,MLXIPL,SEMA3C,THEM4,PHB,PTPRC,RPH3A,EDA,GPR89A,PBLD,SH3GL2,TYRO3,NR6A1,RNF213,TSPAN5,ADCY10,NCAM1,TRAF3IP2,ATF6B,CREB1,RASSF8,RGMB,SHISA6,TRAP1,CLNK,EDNRA,GNGT1,PRAP1,BMP4,CASP12,CPNE1
GO:0050789	regulation of biological process	1.9413868053455873e-35	CD247,POLDIP3,CLRN1,DNAH11,RIC8B,ENPP1,PRDX2,ADAMTS16,LDB2,GPC5,NRXN1,ASPH,B4GALNT2,PRMT3,GP6,PRKCI,MOV10L1,SLCO3A1,MAP4K4,PDE4D,PDCL,DNMT1,S1PR2,HLX,LLPH,TMBIM4,SLC9A1,PDE7B,C6ORF89,NEGR1,PBX3,ADCY8,MED13L,LMBR1,TRPS1,CBFB,PDE8A,PDE4DIP,ZNF823,CLASP2,SEMA3A,IL31RA,GRID2,TAS1R2,RPS6KA2,PRDM12,SETD4,PTGFR,TENM3,CHERP,MED26,DOCK1,WWC1,CLDN18,C12ORF49,CTDSPL2,FBXL2,RYR1,NRG3,ASH1L,NOS1AP,LATS2,NOX5,ANP32A,ASTN2,EPB41,SEMA3D,PHACTR1,DPP6,PTH2R,NREP,SCAF8,TIAM2,STOX2,FHOD3,KSR2,MAPRE2,NLGN4X,PTGIS,PRKAG2,WWC3,TJP1,GRM8,UNC5C,NUDT6,HPSE2,OR5P2,OR5P3,CLCN1,PRLR,MVP,PAGR1,ADCY7,HIVEP3,FTO,NPAS3,UTRN,RGS7BP,KALRN,UBAP2,PITPNC1,DCAF12,NTRK3,CBL,ARNT,ATRNL1,PLEKHG4B,EGLN2,MIA,RAB4B,RAB4B-EGLN2,SEMA5A,LRRFIP2,FLT3,RAD51B,SUSD4,STAT5B,TOX,ENPP2,ATP2B2,KCNQ5,EXD1,GRM5,PLCE1,LHFPL4,SAMHD1,ZNF566,FER,CASK,MARVELD3,MAPK4,MAP2K5,KITLG,KCNIP4,MAPK10,PTPRR,SPNS2,LRP2,PIK3CD,ZNF536,SP3,DEPDC5,HDGF,PRCC,ANK2,EIF4G3,SAAL1,OLFM1,SH2D1A,EZR,IKZF2,MEGF10,NRXN3,ROBO1,MALRD1,TOM1L1,TNFAIP8L1,CHD7,SLC26A6,HTR2B,PSMD1,ITGB6,CDKL5,MECOM,TACC1,MIR1185-1,TENM1,CAMKMT,RYR3,LMNA,NUCB1,GOT1,NMT2,INPP4B,TRAF6,ESRP1,ROBO2,ITPKB,CTNNA3,DNAJA3,OXR1,SLC24A2,NKAIN2,VAV2,GRK5,ABI1,ULK4,NHLH1,ITGB1,TRIOBP,HSP90AA1,SLC24A3,CDC6,PSMB7,TSC22D3,CNIH2,SRGAP3,MCC,TSSK1B,NKAIN3,PCDH17,KNDC1,ZC4H2,KIF26B,CCDC22,DSCAM,STK38L,SRGAP2B,CDAN1,GMDS,SAMD12,CNGB1,TRIO,ARHGAP24,KLHL12,AKAP13,GFI1B,PDE1A,PARD6G,PTPRK,RUNX1,DAB1,OMA1,CDC42EP3,MYO10,ABCC8,ERC1,HMGN3,NRIP1,RGS6,DGKI,ANKS1B,THRB,STXBP4,PDE4A,ERC2,GABRA3,MIR105-2,MIR767,EFCAB7,ITGB3BP,LAMA2,GFRA2,MAST4,IL17RB,TCIRG1,DACH1,PTPN11,ZNF569,KCNS3,NETO2,RBM19,HERC4,MGAT5,CCT2,CACNB2,DOCK10,ORC2,CYTH3,HDAC6,GOLGA4,SERTAD2,DDB1,MYT1,ADORA1,GPRASP1,ADAMTS3,EPHA1,IKBKB,KIF18B,PEX14,ERBB4,GBE1,ADRA1D,SORCS1,MRE11A,GBF1,LIMK1,ZNF609,TLK1,BRD8,ATP8A2,KAT6B,ABR,ACTG2,HIF3A,SNIP1,CUL4B,KLHL6,ESR1,KCNJ16,NPHP3,GRM7,MIER1,PCBP3,PMEPA1,MAML3,DPP10,PTPRO,CDON,NTRK2,TNRC6A,FRMD4A,STX18,EP300,CELA1,ZYG11B,TENM2,ZNF76,RNF220,R

			GS22,ZNF471,FNTA,HOOK3,PDGFB,CACNA1B,RIMS1,TNIK,CCND3,CDH8,USP46,TOB2,MID1,ZNF19,ZNF23,BRMS1,CHFR,ZNF605,ALOX5AP,SCML4,BPI,STK39,DAP3,HNF4G,INSR,FMN2,RERE,PRKAR1A,ASB5,AFAP1,FUBP1,ATP8B1,UNC5D,H2AFY2,LAMA3,TCF7L2,PIP4K2A,CDH4,JMJD1C,CAB39,USP34,ZNF443,ZNF490,ZNF564,ZNF709,ZNF799,PAK1,LITAF,FBXW11,ESRRB,MYOM1,MAP3K4,ZDHHC13,PTPRD,RNF144A,BASP1,EGLN3,RBM20,SLC4A10,TBR1,CATSPER2,SAMD4A,BDNF,TFAP2A,ATRN,AMIGO1,CDK14,FANCA,KCNMA1,PEG3,ZIM2,CHRM3,TGIF2,TGIF2-C20ORF24,FUT8,OR2T3,NPRL3,FMNL2,PHACTR4,MEIS1,TENM4,GRIK4,LHFPL2,VRTN,KCNRG,TRIM13,RASGEF1B,UBR2,ARHGAP6,LCK,MDM4,EDARADD,IL5RA,ENTPD5,SHC4,ECT2,TMC2,ZNF148,MTA3,SNX6,TFDP2,CHML,VAMP7,LAMC1,PPM1L,ARHGAP42,CST2,PTCD3,PTPRU,RFTN1,ERN2,CDC45,LSP1,C1D,AP3S1,NPLOC4,AFF3,SGCD,DENND5A,DMD,CENPF,ATG10,LRRN2,SLC30A9,TOX3,PDS5A,LARP4B,USP13,KAT7,ZNF667,SLC8A1,GSN,KHDRBS2,RBM14,RBM4,GPR171,MED12L,SATB2,PAFAH1B2,RIT2,HIRA,TANC2,JPH2,DRG2,DEPDC1B,CASS4,KCTD16,FYN,MKRN2,ARNTL,ADAMTS9,NF1,SMG7,PLCB1,DST,MGMT,LMTK2,STX6,ARID4B,SNCB,PPP4R2,FAT3,RTN4,AFF2,RXFP1,DPEP1,CHRDL1,DGKH,APOD,RTN4R,BCR,PPP1R12B,RWDD3,BRPF1,SHISA9,CHI3L1,TTN,NDRG4,ICA1,BMP2K,PAQR3,IFI30,PIK3R2,ANKH,CACNA1E,RANBP9,KLHL21,NTN1,EGFLAM,DCTN1,SLC4A5,NOMO3,NRG1,ARHGAP10,SH2D3C,CDK19,SLC43A2,UBP1,BDKRB1,BDKRB2,DOCK8,BID,MIR600HG,FRY,MAP2K1,KCNIP1,MDFI,FNIP1,RAPGEF6,STIM1,VAX2,NALCN,RALGPS2,MYH9,FRMPD4,MAD1L1,LRFN5,DAGLA,TMBIM6,VGLL4,PPP2CB,CNTN4,PPP3R1,HDAC5,CSRNP3,RBM5,NTNG1,VSTM1,ZNF692,OR6N2,NFIA,RNF4,TMEM30A,CSNK2A3,JDP2,CMKLR1,INPP4A,ITIH2,CDKL2,TSPAN8,ROR2,DCN,ZDHHC3,SLC39A14,SCFD1,CATSPER3,PCBD2,HNRNPLL,DOCK2,CDH13,CREBRF,TRDMT1,SOX13,FHIT,WDR70,PTPRT,AUNIP,UST,COPS5,CSPP1,PPP1R42,FGF14,EXOC4,IPO5,ARHGAP32,MAD2L2,TLE6,RAB27A,RGS8,CAPRIN2,CCNYL1,ZNF418,NLN,NCKAP1,PHF20L1,DEFA1B,DEFA3,CD99,RAB3C,PDF,TERF2,PTGER2,RALGPS1,SLX1B,ZNF286A,GOLPH3L,FOXN3,RCC2,BOC,ANO6,ARL3,CERKL,NEUROD1,GNAQ,KIF2A,FBXO9,ICK,TMEM117,RAD51D,RFFL,RANBP1,CCBE1,DENND1A,HERC5,USP22,ZDHHC15,JAK2,YTHDC2,FAM168A,TRAPPC9,FBXW7,LINC00473,KCNB2,OAZ2,SYN3,SKAP1,SMC3,CLSTN2,UIMC1,SYT1,ITCH,MLIP,CROCC,NPSR1,ZFYVE28,BBS12,ABCC2,LZTS1,BCL11B,SMG1,SCN4A,RBFOX1,TMC1,PKNOX1,DTHD1,CLDN1,HUNK,MLLT3,TSHZ2,ATP9A,ARAP2,SLC9A7,CDC42BPB,PLS1,TEX11,DOCK9,SH2D6,TCF7,PDE2A,SEPT7,KLF15,TBX15,ANXA4,LAMTOR3,MAGI1,KCNC4,CYFIP2,WNT11,PALMD,ARHGAP23,IFT80,TRIM59,APBB1IP,FIG4,MTA1,KREMEN1,NRG4,KLF8,CLPB,NOX4,LCOR,SIPA1L3,RPRD1B,MKLN1,CACNA1H,SIAH1,QKI,IL1RAPL1,CCDC62,CAMK2B,ERCC8,PRKCD,SOX6,TAB2,ACVR2A,RUNX2,SEMA5B,CD4,PPM1E,TGFB1,BANP,PLCXD3,SGK1,SPSB4,NUP93,MMP28,TRIM65,NSD1,IGSF1,PIBF1,ZHX2,PKNOX2,ASCC2,KEL,BTRC,DUS2,NFATC3,JPH3,CRADD,DLGAP1,MIR153-2,CLDN10,F2RL1,BCAS3,C9ORF47,GRIK3,CDYL2,RPGRIP1L,DNAJB2,CELSR1,SLIT3,AAK1,CC2D1B,ZFYVE9,MIR218-1,SLIT2,ASB15,COLGALT1,TP73,GABRR3,CADPS2,ITIH4,GABRB1,SYT13,HHAT,GPR176,HP,HPR,CORO1C,SAP18,RRAS2,CTNND2,ZBTB22,C12ORF4,OR9A4,ILF2,DISC1,BLID,KCNJ3,CEP135,KANK1,CACNA1D,ZFAND2A,PTPDC1,CACNA2D3,LAMC2,CLN6,MTDH,ANK1,CLMN,TTBK2,FANK1,XPR1,MYT1L,KDM4B,CHRM1,RAB11FIP4,MMP26,OR51A7,OR51F2,OR51T1,SMAD6,RXFP2,BNC2,ZNF398,CLOCK,ITSN2,TCF12,ZNF675,SMOC1,PLCB4,ETV6,NELL1,SUCO,KCNH1,ENOX2,TFAP2D,BMPER,TIMP2,BCL3,DCLK1,ANKRD54,DAPK2,TNFRSF10B,PARVA,SND1,DAPP1,ADAM12,DUSP22,GPR52,RABGAP1L,NAIP,STRIP1,HNRNPC,TRRAP,ANKFN1,HOMER2,SLC17A7,DOCK3,SBNO2,LINGO2,YTHDF1,FGF10,CNTNAP2,CIZ1,FBXL17,RASA4,RASA4B,ARHGEF28,SMYD3,STXBP5,KCNH7,KANK4,KCTD10,IL1RAPL2,IQCJ-SCHIP1,SH3RF3,PLIN3,LOXL3,RHPN2,MAST2,FANCI,SVIL,GLP2R,ADCY2,CAPN3,SLC16A1,LUM,VMP1,SMURF2,EPHA4,RORA,HIVEP2,HSD17B12,PRKCA,AUTS2,CNR1,CD6,TNFSF11,SMG6,PPP3CA,NSUN2,OR51B2,OR51B4,OR51I1,UBQLN3,GNG4,NFYB,MAGEA4,MAG,KLF12,CAMK4,GATAD2B,PIP5KL1,UFL1,TRAK1,CTNNB1,PARK2,SOD2,DACH2,METTL13,FCGR2A,FCGR2B,FCGR3A,FCGR3B,ARHGAP22,SMARCC1,KLF17,CDHR2,IGF1R,PPARG,NGRN,AXIN1,GARNL3,PRKAR1B,OTUB1,DLG5,IL18R1,IL1RL1,ADD2,CIPC,MTF1,MSR1,CELF2,CBX5,P2RY10,TRPM1,ANKRD17,CYBB,XK,CASKIN1,APOL3,SCAMP5,BRIP1,ANXA13,ANKRD26,ECM2,LRPPRC,SREBF2,KCNJ12,RYR2,CDK11A,KCNA6,FBLIM1,CDK11B,DRAXIN,LEPR,LEPROT,FGF1,NIN,PROS1,RGS10,NPAT,NR4A3,FOXK2,NOL3,DCT,PRKAR2A,RIOK2,ESCO1,MYOCD,OR52E6,OR52E8,OR52N1,TRIM5,PER2,KIR2DL1,KIR2DL4,KIR3DL2,AJUBA,CACNA1C,CPNE6,GLG1,ZNF626,ZNF737,SCN3B,CHEK2,SUPT3H,PHLDB1,UBQLN4,PRDM16,MYO1E,ASB8,PPP1CB,SPDYA,HCK,CSTL1,SORBS1,TBC1D14,RAB3GAP2,CAPN2,TBCK,TRIM8,DIO2,CSPG4,GRM1,BRMS1L,RIMS4,DCC,ZBTB8A,ZBTB8B,ARHGAP31,P2RY14,ANKFY1,CTDP1,ZUFSP,HS3ST5,BAZ1B,OBSCN,RGL1,NF2,FLT4,MEF2B,SGMS1,BICC1,HDAC4,PAX2,PHF5A,TNS3,SECISBP2L,SPTBN1,TPTE,ELN,TRABD2B,SFRP1,MED13,ST8SIA1,PPP6R2,ZNF395,FOXO3,DGKB,TRHDE,ARL13B,CNTN6,NFIB,MUC12,SP4,ARHGAP29,ZCCHC17,IFT122,BCL2L13,DNM3,SSH1,CELF5,SYNCRIP,SMAD3,RNFT2,CUX2,ITPR1,PTPRM,WWP2,AR

			NT2,SBNO1,ACKR2,KRBOX1,ZNF662,ZNF777,SIMC1,WIPI1,EBF3,MTBP,RNF168,C1S,CASZ1,ABI2,C10ORF90,GRIK5,PSAP,SLC16A2,LZTFL1,SHANK1,SYT3,IFNAR1,DCP1B,OR14K1,MIER3,NEDD4,NRP2,ARHGEF18,VANGL1,PREX1,PLA2G4C,DOCK4,ESRRG,HOXD3,HOXD4,KPNB1,NAV3,SLC4A4,ZNF114,TAB1,SLC6A1,KTI12,TXNDC12,NFATC1,RAB6C,PRTG,CDC73,APP,SSBP3,GSX2,PDGFRA,BET1L,SLK,FCRL4,ADD3,RBM8A,CCNI2,DIP2B,NOX1,ARIH1,YAP1,HEG1,AMFR,RAB11FIP5,SESN1,CDK3,TEN1,FSTL4,NLGN2,TNFRSF19,VDAC1,EYA2,SH3D19,BORA,RNLS,IBTK,NVL,ALPL,JAK1,ANGPTL4,TNNI3K,PEMT,LRP5,MTCP1,MUC20,POLR3G,PTPRG,ISLR2,SLC9B2,ZNF787,GNG2,SOX2,SETD2,ZDHHC6,GPC6,KIF24,CHST11,TEAD1,PRICKLE1,RCAN1,ZNF653,SPTBN4,GRIA3,VASH2,ZNF521,DRD1,TMEM14A,GLRA2,ARID3A,ZNF761,CHUK,ERLIN1,FRS2,PALB2,SFMBT1,VILL,MMP2,ZNF584,SERGEF,TPH1,ESR2,SYNE2,S100A12,DCUN1D3,CPNE9,KDM6A,PRKD1,STAT1,CELF6,PARP6,ST18,DGKG,ETV5,RHOXF2B,RND3,SLC6A3,TAF3,PLAGL1,HNF4A,ZBTB7C,LRRC2,TASP1,SHANK2,STRA6,VPS4A,EREG,CCNY,TCTN3,MAPKAPK3,FXYD2,FXYD6,FXYD6-FXYD2,SEMA6D,RBFOX3,ATF2,POU2F2,TCF3,ZNF730,GRAMD4,TMEM237,KCNG4,RAF1,CELF4,RASGRF2,ZNF766,ACOX2,CARD16,CASP1,PTPN1,ADAMTS12,SRRM4,POLA1,GGT7,BMPR2,USP33,DPYSL2,VBP1,CAMK1D,BMPR1A,ZKSCAN1,PABPC4,TSPAN12,NLGN1,BTBD10,COL16A1,CTNNA2,IKZF4,PIK3R3,CDK12,TAF8,CAND2,SPAG9,MORC2,DENND4A,RAB11A,CRMP1,EVC,PDE11A,EPG5,MYB,FGF2,ZNF71,LRRC4C,POU6F2,BACH1,GRIK1,OR6C70,MXD3,PPM1F,TICRR,ADCY5,GLI4,ZFP41,NEDD9,AGBL4,BEND5,PEAK1,SEMA4D,ISM1,NFX1,CDC27,DOK6,RORC,MCTP1,ELP3,PLXNA2,PTAFR,ADCYAP1R1,SP140,SP140L,SETD1A,RHOXF2,YME1L1,ABCA13,JARID2,RILPL1,ANKDD1A,DDX58,DKK2,SORCS2,RAB11FIP3,BRDT,PHC2,ITGA11,PI4KA,RARB,SPEN,PIK3C2B,PRKCG,SIN3B,VIPR1,NCOA1,SPOCK1,AREL1,BLOC1S5,EEF1E1,EEF1E1-BLOC1S5,TXNDC5,GRIA2,EHMT1,GAS8,LMO7,AP2M1,CHRD,DVL3,ECE2,EIF2B5,EIF4G1,EPHB3,PSMD2,PTPRE,RBX1,TCF20,ANAPC5,ARHGAP39,ATF3,LIN28B,ATP6V0B,SHOC2,SRSF5,LINC00461,CKS1B,PAWR,EBF2,AGO3,DEPTOR,DLEC1,PKN3,FBXL20,MAML2,TSG101,DESI1,CCDC3,VCL,LAMB1,TERF2IP,CLIC4,MYO9A,CRYM,IDE,WDR59,KCNQ1,RFX2,WNT3,RCE1,ZNF322,RHOJ,P4HB,CCL14,CCL15,CCL15-CCL14,SUFU,TG,MAGEA11,NLGN3,PTPN13,SCUBE2,PAFAH1B1,KIF3A,PRDM15,ZNF670,ZNF695,CCDC169-SOHLH2,SOHLH2,FAM13A,CNTFR,COL4A3,TRPA1,DDAH1,NMUR2,ZNF354C,HIP1,AKAP6,RASAL1,TCEA3,PADI6,ZNF704,BTBD9,NR2C1,TRPC5,UBA2,IRS4,ME1,GLI2,NEO1,TNKS,SCUBE1,WBP2NL,ERCC1,RUFY3,TNRC6B,GLIS3,GPR39,WDTC1,ZNF664,STON2,MGLL,C9,DAB2,THEMIS,BLM,PKHD1,USH2A,UBR1,CACUL1,LDLRAD4,MYSM1,SETD5,DZIP1,SMG5,DLG3,WNK1,RELN,NEK10,SIN3A,RUVBL2,ABCB1,OR5H2,OR5H6,OR5K4,RSPO2,COMMD6,GUCY1A2,CSF3R,KCNQ2,DOCK11,GMEB1,PELI1,PPP2R5C,CFDP1,IQGAP1,MAP3K7,APOLD1,DDX47,TAS2R41,ZNF423,SP1,OR56B1,TRIM22,MACF1,ARHGAP12,ALK,SLC8A2,INPP5D,STAU1,CLASP1,DSC2,EEPD1,TEAD4,FASTKD5,HOXC13,AKAP2,APBB3,SLC35A4,SRA1,TOP1,UBE2V1,MBOAT1,TPCN1,GPM6B,ADIRF,CLIC5,XDH,AHNAK,LTBP1,OVOL2,SNX5,NFATC2,RBBP6,OR6C74,PRKG1,PAX7,RGS7,BNC1,ATP2B4,NSF,PACRG,SYNE3,PACSIN1,SPTBN5,TMEM2,ACTL6B,ASXL3,PAK3,MASP1,SSH2,TET1,CAMTA1,CCPG1,DYX1C1,P2RX6,ASGR2,ATAD1,ADNP,GPR161,MAPKAPK2,ARID4A,MAP1B,CDC42BPA,COL4A6,MPHOSPH9,SCARB1,MARK1,CDK6,ATP6V0A2,PHF2,KCNAB2,CELF1,P2RY8,RNF34,TFRC,UVRAG,EPHA5,SORCS3,WWOX,GABRG3,MEF2A,SYT12,CR1L,ADCY9,CACNA1A,MIR1226,DCAF6,DCUN1D5,DROSHA,FNBP1,GFRAL,GPC3,SGIP1,STOM,XRN1,ADRBK1,SATB1,TBC1D5,PSMB2,ZNF207,EYA1,GATAD2A,PTGER3,SCOC,BRINP1,DIS3L2,GNB3,SHPK,TRPV1,RBM12B,RNF216,TSPAN6,HOXB3,HOXB4,HOXB5,HOXB6,MIR10A,TFEB,CLEC16A,GHR,MNAT1,FLRT2,FGD1,SNX9,ACTR2,L3MBTL4,ELAVL4,LGI4,HDAC1,INVS,RECQL5,C2,CFB,SMOC2,TRIM24,ADIPOR2,PLCG2,ROCK1,EPS8,NID1,SCMH1,C1QTNF1,PAXIP1,ZNF713,EGR2,HIPK3,RNF10,RYBP,GAPVD1,CAPN10,GPR35,CUL2,ABCC1,AP3B1,PTPN2,INSRR,NTRK1,KPNA6,TRIM44,ZFAND1,MAP1A,PKN2,PPP1R9A,TMIGD1,SLC39A10,EIF3A,ANK3,DNMBP,UNC13A,EPHB1,PDE6A,EPHA10,PTPN9,AXIN2,SARM1,EPCAM,PARD3,RCAN2,ZNF425,GNL3L,MUC1,PTPN14,TRIM46,TUB,SH3GL3,JAG2,KCND3,BCOR,CACNG8,AP3D1,ZNF41,CARM1,VWF,AP2B1,NETO1,ZNF383,ANXA8L1,ADAM19,ARHGAP44,SNX33,PIFO,CD84,COCH,HTR1D,LPA,ZNF616,ZNF836,SMARCA2,ARHGEF3,CUX1,MIR1302-10,RAD51C,SPI1,DGKK,DNMT3B,EPHA7,CHRNB4,FOXP2,MCU,RANBP10,PTPRA,CTNNA1,LIMA1,AIFM2,FBXW4,PSPC1,GLIS1,SFMBT2,TRDN,ATG14,EIF3H,GABRA6,LY86,STAC,BCAR3,JAZF1,SHROOM3,STK38,TRPC6,ABCB7,ACOT8,RBPMS,RENBP,CHURC1,CREB5,FCHSD2,MAP3K13,OTUD3,RAB15,RAPGEF3,SDK1,PDXP,SH3BP1,MST1,ERCC3,PTK7,SMYD1,CHID1,SNCA,BMPR1B,CACNG2,CD53,GRM4,MAGI2,PRIM2,USP42,PNPT1,USP50,ZEB2,DHRS4,CABLES1,FOXJ3,SYT9,HEY2,RC3H1,CHMP3,EIF3E,CNGA3,RNF19B,WNT7B,PTPRS,ZMYND11,KMT2D,PRKAG1,CDKL1,CREM,PSMF1,RAB5B,KMT2C,TLL2,CAST,TRIP12,DFFA,SPPL2A,ADTRP,ABCA12,GNG7,PLD1,RASA1,MACC1,MITF,SRSF6,OPRD1,CABIN1,HIPK1,NUGGC,PKP2,RPTOR,RTN4RL1,NFIX,BTK,MIR383,SIGLEC9,TBC1D16,XPO4,ZNRF3,KCTD13,MLLT1,SLC4A8,TROVE2,CBFA2T2,IGF1,MLXIP,PLA2G4E,STX8,ATF7IP,CTN

			NBL1,HTT,DPYSL3,MAPK1,PTEN,ARHGEF17,MIB1,SPRED2,BMP7,SCN8A,ATXN2,MXI1,PIK3C3,PUM1,RASA2,SOX5,CIR1,PCBP2,TTLL4,PRCP,RAB30,ZNF780A,ZNF780B,LRRK2,ATP6V1A,MLYCD,OSGIN1,GNA14,TMEM59,ZRANB1,EBAG9,SPAG5,UNC119,ZBTB20,CHMP5,CNIH3,RAPH1,RFC3,BAG6,LILRB4,MORC3,IFT81,TMCO1,TMEM108,ITGAM,ARHGAP25,IL11RA,NSG2,PKD2,ZNF652,EFNA5,SHANK3,KCTD1,STMN4,RIPPLY1,NT5E,RCOR3,TADA2A,DAZL,BTBD11,RGS9,PDGFC,SERPINA3,SERPINA4,SERPINA5,AMOTL1,FAM13B,NCOA3,BEST3,PKIB,MTMR3,ZNF146,ZNF565,CUEDC2,PPP2R3C,TBCD,WNT7A,ZBED6,BRWD3,NLRP1,RPS6KC1,UACA,LPGAT1,MAP4,CRTC3,DNAJC1,GPR141,ARNTL2,ELOVL5,PLCL2,KAT6A,MTIF2,GPR21,RABEP1,TTC28,RHOT1,SIK3,ZKSCAN7,ZNF197,ZNF660,BMP6,TAF15,MYCBP2,NFE2L1,ANO1,GPI,IL17RD,SH3RF2,SOS1,TSHR,WDR43,ZNF30,EXT1,N4BP1,PDCD1LG2,ATRX,DPH6,PLIN2,PRICKLE2,GABRR2,IKZF1,PARVB,PRMT2,RAPGEF2,ASCC1,KCNC2,SMO,VRK3,RALY,ENPP3,BLOC1S3,MARK4,PRKAR2B,STXBP5L,CPT1A,PSEN2,TCF4,TNFRSF11B,PAG1,SYT17,AFF1,DECR1,SOX30,DNAJC6,TFE3,UBR5,GRB14,GRIN1,JADE1,KCTD8,ARRDC4,RHOA,SPRTN,WSB2,OR4M1,OR4N2,SYNGAP1,VIPR2,GRIN3A,ROR1,RQCD1,TF,ONECUT2,RHOBTB1,FAM19A4,CUL3,SH3KBP1,TFEC,HSPD1,ITPR2,NFKBID,EPM2A,GABRB3,GSK3B,ABCG8,PLD2,PRR16,DNAJC3,GNG12,RDX,STAT6,ACTA2,SYN1,MLF1,EEF2K,FAM49B,IWS1,PPP1R16A,PTPRB,RBBP8,NECAB2,CDK13,STK32B,UPF2,CD27,DUSP26,ITGBL1,MCTP2,SCN9A,TAPBPL,BOK,RNF144B,SULF1,ZNF362,KRT8,NACC2,RNF43,SUPT4H1,TNFRSF8,ZNF354A,AGT,HDGFRP3,METTL16,CCNJL,ELOVL3,POLH,PRKAA2,SYT7,ADAR,BLOC1S6,CUL9,FBN2,PITPNM2,STAT2,ZMPSTE24,BRINP3,HRH4,NDRG2,PTK2,TPPP2,UPK3B,LAT2,PEX5L,SNX3,PDE4B,RIMS2,PBX1,TCP11,TEC,EXOSC3,FBXO10,SHB,VEPH1,CDC14A,GAP43,MAP3K5,CPE,SEC16B,TCFL5,CREBBP,IQGAP2,PRKD3,FAM171A1,UTP20,APCDD1L,DLX1,FMOD,INSL6,PCSK6,CEP70,GTF2IRD2,NCF1,SLC29A1,TAOK2,UGT1A1,UGT1A10,UGT1A4,UGT1A8,PYGO2,SHC1,ZNF813,ALDOA,ARHGAP15,CD160,ECE1,KCNJ15,LBX2,PARP10,RNFT1,RPS6KB1,STC2,YLPM1,DPRX,GPR173,GUCY2F,PRG3,SCEL,SERPINB11,SETDB2,ANKS1A,ITGAL,ALOX5,CPLX2,DNAJB6,SP7,CD44,FGD3,LARP4,ADAM8,SLC5A3,CLDN4,PACSIN2,RPS6KA5,ZNF484,BCL2L14,CDS1,ZNF93,CSNK1A1,GRID1,HUS1,MIR346,PRDM2,CXCL17,FCRL6,FGD4,NOS1,ZNF44,GCKR,MED15,PLEKHA1,PLVAP,RGS14,SIPA1L2,ACTN4,ARHGAP11A,MC1R,STK4,TCF25,ZFAND6,DRAM1,ZNF282,MARK3,SPEF1,EPHB2,SLC1A1,WLS,EDRF1,SIGMAR1,TMEFF2,XCR1,BCO2,CLPTM1,IL18,DTNA,LRRC52,MFSD8,NKAIN1,UBE2K,GPSM2,DNAJC7,HCAR1,HCAR2,HCAR3,DIS3,DLC1,PPARA,PPP1R10,ARID5B,CALCRL,MCOLN1,EPB41L4B,SLC25A33,SMARCAL1,PHLPP1,MAGI3,CPEB4,GPSM3,MOB3B,NOTCH4,SYTL4,ZMYND8,ZNF366,CRX,FBN1,PAX6,PRKCZ,ZC3HAV1,ATP1A3,CD96,FAM20C,ADORA2A,GRIN2B,KCTD7,PPP1CC,RABGEF1,CCNG2,CPEB1,PHLDB2,PLCXD2,TYK2,OTUD7B,DENND4B,NIPBL,TMIGD2,YEATS4,CNRIP1,COL28A1,PHF20,ZNF143,ASPN,ANGPT1,BRF1,CADPS,TBL1X,EYA3,FHL2,FRMPD1,LRRK1,MBTD1,OSBPL8,ATF6,ZBTB5,ZNF708,IGF2BP3,EPB41L2,GCNT2,ETS1,PPP1CA,TBC1D10C,VWC2,ARHGAP21,CFI,GRIA4,MAP2,MTF2,RBMS3,CCL22,STPG1,BTN3A2,TAGLN3,FBXO31,ATG3,CDK5RAP1,CNTN1,NCOR2,PRKCQ,BRAF,CSRNP1,DIAPH1,HDAC2,HTR2C,IMPACT,MIR1912,ATP8A1,PHKG2,SRCAP,CAPN6,POU3F3,NOTO,PPP1R14A,SPINT2,TNR,MBNL3,UBE2E2,ZKSCAN5,HIGD2A,CD300A,ELF2,IRAK1BP1,PHIP,CDKL3,PPP2CA,SKP1,ANGPT4,ARHGEF6,MAOA,RPL23,SH3BP4,RFC5,ATXN1,CAMSAP3,DCDC1,SP100,ZNF347,ZNF415,OPN1LW,ANXA2,TRIM29,KCND2,PARN,ATP6V0A1,PIGU,PRMT7,RAB27B,C1QTNF9,CR2,DONSON,FCRL2,ITSN1,NUP214,SRGAP2,ADCY1,MADD,NUMB,RBM42,SQLE,SYK,CNOT1,RBM39,WWTR1,ARHGAP19,GRIK2,GTF2H5,SCN1A,SLIT1,ASB1,FRMD5,NFXL1,CIT,MYRIP,RADIL,ZBTB38,BRCA2,HRK,RAP1GAP2,ATF7,CACNG3,DVL2,MORC1,MTRF1,NEB,YBX1,ANKRD13A,GRIP1,PTPRJ,SLC10A1,TAF1,ATP2B3,EIF4E3,TICAM1,TANK,THAP3,GPM6A,UCN2,ITGAE,TAC3,PLEKHB2,CADM1,HSF1,MAX,SAP130,DHFR,EZH1,ETF1,INPP5A,MIR663B,NRF1,SRPK2,ANKRD13C,PLEKHM1,SASH1,ASAP1,NCOA2,ADAMTS7,RALGAPA1,GBP5,SULT2B1,WDR18,BMP1,CGNL1,EEFSEC,FCHO1,QRICH1,VAV3,ZP3,CHD6,DLGAP2,KCNQ3,RNF2,ZNF554,MOB3A,CAV2,GNA12,SH2D3A,SKA2,AKTIP,COL4A5,MYEF2,INPP5F,NUAK2,AXL,REPS2,TNFSF9,TRIM37,WIF1,CD109,DENND4C,MET,SESTD1,TBC1D10A,FMNL1,ZNF461,CAMK2D,CCR3,KCNJ6,RAD9B,SNX1,TIMELESS,UCHL5,ANKRD6,BRD9,MALT1,SERPINE3,SETD3,TRA2B,ZDHHC11,SPTAN1,EHD2,KDM2B,MLXIPL,SEMA3C,THEM4,CCT3,NEDD4L,PHB,PTPRC,EDA,GPR89A,ZNF555,CTCF,PBLD,SH3GL2,TYRO3,NR6A1,RNF213,SPON2,SYNDIG1,TSPAN5,ADCY10,NCAM1,TRAF3IP2,TRIM60,ATF6B,CREB1,RASSF8,RGMB,SGTB,SHISA6,TNXB,TRAP1,AFF4,CLNK,EBF4,EDNRA,GNGT1,PRAP1,STXBP6,ZNF511,BMP4,ABLIM3,CASP12,CPNE1
GO:0120036	plasma membrane bounded cell projection organization	2.943940985078052e-35	*CLRN1,ADAMTS16,NRXN1,PRMT3,PRKCI,MAP4K4,S1PR2,LLPH,NEGR1,SPAG16,CLASP2,SEMA3A,GRID2,PRDM12,TENM3,IQCG,SEMA3D,PHACTR1,MYOT,NREP,TIAM2,UNC5C,KALRN,NTRK3,SEMA5A,TOX,ENPP2,PLCE1,FER,LRP2,OLFM1,EZR,NRXN3,ROBO1,CDKL5,TENM1,KIF5C,ROBO2,NFASC,VAV2,ABI1,ULK4,ITGB1,TRIOBP,HSP90AA1,GAS7,KNDC1,DSCAM,TRIO,ARHGAP24,IFT43,DAB1,CDC42EP3,MYO10,LAMA2,TCIRG1,PTPN11,DOCK10,HDAC6,GOLGA4,EPHA1,GBF1,DNAH2,LIMK1,ATP8A2,CUL4B,CECR2,NPHP3,GRM7,PTPRO,NTRK2,DNAI2,EP300,TENM2,RIMS1,TNIK,SNX2,KIRREL3,INSR,RERE,ATP8B1,UNC5D,LAMA3,CDH4,PAK1,PTPRD,TBR1,STRC,BDNF,AMIGO1,LRRC49,DENND5A,DMD,GSN,RIT2,TANC2,*

			FYN,PCDH15,LMTK2,FAT3,RTN4,APOD,B4GALT6,RTN4R,CCDC141,NDRG4,PAQR3,ADAMTSL1,NTN1,DCTN1,FRY,MAP2K1,RAPGEF6,VAX2,MYH9,CNTN4,WDR92,NTNG1,TMEM30A,LRGUK,ROR2,CDH13,UST,CAPRIN2,NCKAP1,RCC2,BOC,ANO6,ARL3,ICK,TTLL8,ZDHHC15,JAK2,MYPN,SYT1,CROCC,BBS12,LZTS1,BCL11B,PLS1,SEPT7,CYFIP2,IFT80,FIG4,KREMEN1,SIAH1,IL1RAPL1,CAMK2B,PRKCD,SEMA5B,SGK1,PIBF1,KEL,F2RL1,BCAS3,RPGRIP1L,SLIT3,SLIT2,CDH23,CORO1C,CTNND2,DISC1,CEP135,KANK1,PTPDC1,LAMC2,CLMN,TTBK2,SPAG17,ITSN2,DCLK1,PARVA,YTHDF1,CNTNAP2,ENAH,EPHA4,AUTS2,CNR1,PPP3CA,MAG,CTNNB1,PARK2,FCGR2B,CDHR2,IGF1R,DLG5,XK,DRAXIN,NIN,AJUBA,CPNE6,CSPG4,DCC,HDAC4,PAX2,SPG11,SFRP1,ARL13B,CNTN6,NFIB,IFT122,DNM3,CUX2,PTPRM,ABI2,SHANK1,SYT3,NEDD4,NRP2,BTBD3,CRTAC1,PRTG,APP,DIP2B,YAP1,FSTL4,NLGN2,PTPRG,ISLR2,KIF24,TTC39C,SPTBN4,VASH2,MMP2,SYNE2,CPNE9,PRKD1,PARP6,DGKG,TCTN3,ABLIM1,DSCAML1,SEMA6D,TMEM237,ABLIM2,BMPR2,USP33,DPYSL2,CAMK1D,NLGN1,CTNNA2,SPAG9,RAB11A,CRMP1,LRRC4C,SEMA4D,ARMC2,PLXNA2,RILPL1,SPOCK1,BLOC1S5,GAS8,DVL3,EPHB3,FBXO45,VCL,LAMB1,WDR90,MYO9A,KCNQ1,RFX2,WNT3,NLGN3,PAFAH1B1,KIF3A,CEP350,RASAL1,TRPC5,GLI2,NEO1,RUFY3,DAB2,PKHD1,DZIP1,RELN,SIN3A,DOCK11,IQGAP1,ZNF423,MACF1,MYO7A,ALK,MBOAT1,GPM6B,PRKG1,PACSIN1,ACTL6B,PAK3,DYX1C1,ADNP,CEP41,MAP1B,MPHOSPH9,MARK1,AIF1L,EPHA5,MEF2A,FLRT2,FGD1,RPGR,ACTR2,ELAVL4,ROCK1,EPS8,EGR2,NTRK1,MAP1A,PPP1R9A,ANK3,UNC13A,EPHB1,EPHA10,PTPN9,SARM1,PARD3,TRIM46,TUB,TEKT1,CARM1,ARHGAP44,PIFO,CUX1,EPHA7,CTNNA1,LIMA1,MAP3K13,SDK1,PTK7,BMPR1B,MAGI2,HYDIN,WNT7B,PTPRS,CDKL1,PLD1,RTN4RL1,TROVE2,CBFA2T2,HTT,DPYSL3,PTEN,BMP7,LHFPL5,LRRK2,SEPT6,RAPH1,IFT81,TMEM108,PKD2,EFNA5,SHANK3,STMN4,WNT7A,MAP4,NME8,CEP89,MYCBP2,SOS1,TSHR,EXT1,PARVB,RAPGEF2,SMO,BLOC1S3,MARK4,SYT17,BBS9,RHOA,SYNGAP1,GRIN3A,ROR1,ONECUT2,GSK3B,EMB,RDX,EEF2K,AGT,HDGFRP3,BLOC1S6,PTK2,SNX3,RIMS2,CDC14A,GAP43,CEP70,TAOK2,ECE1,CD44,FGD3,RPS6KA5,FGD4,TBC1D23,PLEKHA1,TUBB3,SPEF1,EPHB2,PAX6,PRKCZ,ATP1A3,ADORA2A,GRIN2B,KLC3,KLHL1,MAP2,FBXO31,ATG3,CNTN1,PRKCQ,BRAF,HDAC2,IMPACT,NOTO,TNR,CDKL3,ARHGEF6,FAM149B1,CAMSAP3,SRGAP2,TMEFF1,ADCY1,NUMB,WWTR1,SLIT1,DVL2,GRIP1,GPRIN3,GPM6A,MYO1A,DHFR,PLEKHM1,ASAP1,VAV3,POC1B,INPP5F,SNX1,EHD2,SEMA3C,NEDD4L,SH3GL2,NCAM1,TANC1,CREB1,EDNRA,ABLIM3,CPNE1
GO:0048731	system development	3.01825245 11189066e-35	*CLRN1,DNAH11,RCN1,ENPP1,PRDX2,ADAMTS16,LDB2,NRXN1,PRMT3,GSS,RDH13,PRKCI,TACC2,MAP4K4,DNMT1,HLX,LLPH,SLC9A1,NEGR1,PBX3,TRPS1,CBFB,CLASP2,SEMA3A,IL31RA,LSAMP,GRID2,RPS6KA2,PRDM12,TENM3,CHERP,DOCK1,CLDN18,RYR1,NRG3,ASH1L,NOX5,ASTN2,SEMA3D,PHACTR1,MYOT,NREP,TIAM2,STOX2,FHOD3,NLGN4X,PTGIS,DCHS2,TJP1,UNC5C,ZSWIM6,PRLR,HIVEP3,FTO,UTRN,KALRN,SPRR2D,NTRK3,CBL,ARNT,ATRNL1,SEMA5A,FLT3,STAT5B,TOX,ENPP2,ATP2B2,GRM5,PLCE1,LHFPL4,SAMHD1,MAP2K5,SPATA5,KITLG,SPNS2,NDUFV2,LRP2,PIK3CD,ZNF536,SP3,ANK2,OLFM1,MFAP5,MEGF10,NRXN3,XRCC4,ROBO1,CHD7,HTR2B,ITGB6,CDKL5,MECOM,TACC1,TENM1,KIF5C,LMNA,KIAA1217,TRAF6,ESRP1,ROBO2,NFASC,ITPKB,DNAJA3,VAV2,ABI1,ULK4,NHLH1,ITGB1,TRIOBP,HSP90AA1,SLC24A3,GAS7,PSMB7,PCDH17,KNDC1,ZC4H2,KIF26B,DSCAM,FAM126A,SRGAP2B,CNGB1,TRIO,ARHGAP24,KLHL12,AKAP13,TTLL5,GFI1B,RUNX1,DAB1,ABCC8,NRIP1,THRB,XIRP2,IMPG2,LAMA2,GFRA2,TCIRG1,PTPN11,DOCK10,HDAC6,GOLGA4,MYT1,ADORA1,EPHA1,ERBB4,LIMK1,ZNF609,ATP8A2,ACTG2,HIF3A,CUL4B,LARGE,CECR2,ESR1,MYO18B,NPHP3,GRM7,ANKRD11,PTPRO,CDON,NTRK2,EP300,CELA1,MEGF9,DYM,TENM2,RNF220,HOOK3,PDGFB,RIMS1,TNIK,KIRREL3,CSGALNACT1,TOB2,INSR,RERE,PRKAR1A,ATP8B1,UNC5D,H2AFY2,PLLP,LAMA3,TCF7L2,PIP4K2A,CDH4,PAK1,FBXW11,ESRRB,MAP3K4,PTPRD,CNTN5,BASP1,RBM20,SLC4A10,TBR1,STRC,BDNF,TFAP2A,ATRN,AMIGO1,FANCA,CHRM3,TGIF2,POMGNT2,NPRL3,PHACTR4,MEIS1,TENM4,LCK,MDM4,ECT2,ZNF148,MYO3B,TFDP2,NAV2,LAMC1,PTPRU,MB,SGCD,DENND5A,DMD,CENPF,CRISPLD2,KAT7,WDR7,SLC8A1,GSN,PRPS2,GPR171,SATB2,PAFAH1B2,RIT2,MACROD2,TANC2,JPH2,FYN,ARNTL,ADAMTS9,NF1,PLCB1,MGMT,PCDH15,LMTK2,ARID4B,FAT3,RTN4,AFF2,RXFP1,MAL2,CHRDL1,APOD,B4GALT6,RTN4R,BCR,CHI3L1,CCDC141,TTN,NDRG4,BMP2K,PAQR3,ANKH,ADAMTSL1,TLL1,NTN1,EGFLAM,DCTN1,SLC4A5,NRG1,UBP1,FRY,MAP2K1,MDFI,FNIP1,STIM1,VAX2,MYH9,MAD1L1,LRFN5,DAGLA,PLEKHA5,VGLL4,CNTN4,HDAC5,NTNG1,PCSK2,NAV1,NFIA,TMEM30A,CMKLR1,SBF2,ROR2,DCN,SLC39A14,PRPSAP2,LIN7A,DOCK2,CDH13,PLXDC1,SOX13,ACSBG1,UST,FGF14,DMC1,PCDHB16,EXOC4,MAD2L2,CAPRIN2,NLN,NCKAP1,FOXN3,BOC,ANO6,ARL3,NEUROD1,KIF2A,CCBE1,ZDHHC15,JAK2,MYPN,TRAPPC9,FBXW7,CLSTN2,SYT1,ITCH,LZTS1,BCL11B,RBFOX1,TMC1,LRIG3,PKNOX1,CLDN1,MLLT3,PNPLA1,PLS1,TEX11,TCF7,PDE2A,KLF15,TBX15,CYFIP2,WNT11,IFT80,FIG4,KREMEN1,IMMP2L,NRG4,ASTN1,NOX4,SIPA1L3,CACNA1H,SIAH1,QKI,IL1RAPL1,CAMK2B,ADAM23,SOX6,TAB2,ACVR2A,RUNX2,SEMA5B,CD4,TGFB1,SGK1,PCSK5,PDLIM4,MYO3A,ZHX2,KEL,BTRC,NFATC3,F2RL1,BCAS3,RPGRIP1L,CELSR1,SLIT3,SDK2,SLIT2,TP73,MUSTN1,GABRB1,CDH23,CORO1C,CTNND2,DISC1,KANK1,LRRC10,LAMC2,MTDH,CLMN,TTBK2,MYT1L,OPCML,CHRM1,SMAD6,RXFP2,BNC2,CLOCK,ITSN2,TCF12,ZNF675,SMOC1,ETV6,SYNJ2,NELL1,PRR14,TFAP2D,BMPER,BCL3,DCLK1,ANKRD54,PARVA,ADA*

			M12,NAIP,SLC17A7,SBNO2,LINGO2,YTHDF1,FGF10,CNTNAP2,FBXL17,ENAH,GREB1L,IL1RAPL2,PPL,LOXL3,SVIL,CAPN3,SMURF2,EPHA4,RORA,PRKCA,AUTS2,CNR1,TNFSF11,PPP3CA,NSUN2,MAG,CAMK4,UFL1,TRAK1,CTNNB1,PARK2,SOD2,FCGR2B,ARHGAP22,SMARCC1,IGF1R,PPARG,NGRN,DLG5,IL18R1,BFSP1,ADD2,MTF1,ANKRD17,CYBB,OTC,XK,BRIP1,RYR2,DRAXIN,LEPR,FGF1,NIN,NR4A3,DCT,MYOCD,PER2,CACNA1C,CPNE6,GLG1,SCN3B,PHLDB1,MYO1E,CSMD1,CSPG4,DCC,CTDP1,MMP16,NF2,FLT4,MEF2B,BICC1,HDAC4,PAX2,ELN,SPG11,SFRP1,FOXO3,ARL13B,CNTN6,NFIB,IFT122,DNM3,SMAD3,CUX2,PTPRM,ARNT2,NHS,RNF168,CASZ1,ABI2,PSAP,SHANK1,SYT3,NEDD4,NRP2,VANGL1,BTBD3,PREX1,CRTAC1,HOXD3,HOXD4,NAV3,TAB1,NFATC1,PRTG,CDC73,APP,SSBP3,GSX2,PDGFRA,CEP85L,DIP2B,NOX1,YAP1,HEG1,FSTL4,NLGN2,TNFRSF19,ALPL,JAK1,ANGPTL4,LRP5,PTPRG,ISLR2,SLC9B2,SOX2,SETD2,PLAC1,GPC6,TTC39C,ADAMTS2,CHST11,TEAD1,PRICKLE1,RCAN1,ELAVL3,SPTBN4,VASH2,ZNF521,DRD1,FRS2,PALB2,SFMBT1,MMP2,TPH1,SYNE2,CPNE9,KDM6A,PRKD1,STAT1,PARP6,DGKG,SYBU,SLC6A3,HNF4A,SHANK2,STRA6,EREG,ABLIM1,MYH15,DSCAML1,SEMA6D,RBFOX3,ATF2,POU2F2,TCF3,RAF1,CELF4,ADAMTS12,SRRM4,BMPR2,USP33,DPYSL2,CAMK1D,BMPR1A,AP1B1,PABPC4,TSPAN12,NLGN1,CTNNA2,PIK3R3,CERS3,SPAG9,RAB11A,CRMP1,EVC,MYB,FGF2,LRRC4C,POU6F2,GRIK1,AGBL4,ADAMTS4,SEMA4D,SH3PXD2A,ISM1,RORC,ELP3,PLXNA2,FNDC3A,SETD1A,JARID2,KLHL3,ITGA11,RARB,SPEN,PRKCG,NCOA1,SPOCK1,BLOC1S5,GAS8,OC90,NTM,CHRD,DVL3,EIF2B5,EIF4G1,EPHB3,ATF3,FBXO45,MATN3,SRSF5,EBF2,ADAM20,ADAM21,TSG101,VCL,LAMB1,CLIC4,MYO9A,COL11A1,KCNQ1,WNT3,ADAMTS6,RHOJ,SUFU,TG,NLGN3,SCUBE2,PAFAH1B1,POLE,CNTFR,COL4A3,DDAH1,NMUR2,AKAP6,RASAL1,TRPC5,TTLL7,GLI2,NEO1,SCUBE1,ERCC1,RUFY3,TPD52,COL19A1,DAB2,THEMIS,PKHD1,USH2A,LDLRAD4,MYSM1,SETD5,DZIP1,WNK1,RELN,SIN3A,RSPO2,CSF3R,KCNQ2,DOCK11,CRELD1,IQGAP1,APOLD1,ZNF423,SP1,LUZP1,MACF1,MYO7A,ALK,GSTM3,INPP5D,CLASP1,DSC2,TEAD4,HOXC13,MBOAT1,GPM6B,XDH,OVOL2,NFATC2,RBBP6,PRKG1,PAX7,RGS7,ATP2B4,PACSIN1,TMEM2,ACTL6B,ASXL3,PAK3,DYX1C1,ASGR2,ADNP,GPR161,MAPKAPK2,ARID4A,MAP1B,MARK1,CDK6,PHF2,CELF1,TYR,TFRC,EPHA5,WWOX,MEF2A,DHX30,DROSHA,GFRAL,GPC3,ADRBK1,PLS3,PSMB2,CALD1,EYA1,BRINP1,TRPV1,HOXB3,HOXB4,HOXB5,HOXB6,TFEB,GHR,MNAT1,FLRT2,FGD1,ACTR2,ELAVL4,LGI4,HDAC1,SMOC2,ADIPOR2,PLCG2,ROCK1,NID1,PAXIP1,EGR2,RNF10,AP3B1,PTPN2,NTRK1,MAP1A,PPP1R9A,ANK3,ACO2,UNC13A,PTPRQ,EPHB1,PDE6A,EPHA10,PTPN9,AXIN2,SARM1,SNTG2,EPCAM,PARD3,PTPN14,TRIM46,TUB,SH3GL3,JAG2,BCOR,AP3D1,CARM1,AP2B1,ADAM19,ARHGAP44,SRD5A2,PIFO,SMARCA2,CUX1,SPI1,EPHA7,FOXP2,CTNNA1,FBXW4,BCAR3,ILDR2,SHROOM3,MAP3K13,RAPGEF3,SDK1,MST1,KAZN,ERCC3,PTK7,SMYD1,TTC9,SNCA,BMPR1B,MAGI2,PNPT1,ZEB2,HEY2,RC3H1,COL13A1,HYDIN,RNF103,WNT7B,HMGCS2,PTPRS,TLL2,ADTRP,ABCA12,RASA1,MITF,SRSF6,RBM45,HIPK1,PKP2,RTN4RL1,BTK,SGCZ,ZNRF3,TROVE2,CBFA2T2,IGF1,CTNNBL1,DPYSL3,MAPK1,PTEN,CLDN11,MIB1,SPRED2,BMP7,SCN8A,SOX5,LHFPL5,PRCP,LRRK2,MEGF11,UNC119,RAPH1,BAG6,LILRB4,TMEM108,ITGAM,PKD2,EFNA5,SHANK3,STMN4,ALPK3,PDGFC,AMOTL1,NCOA3,PPP2R3C,TBCD,WNT7A,ZBED6,MAP4,PLCL2,KAT6A,SH3PXD2B,BMP6,MYCBP2,IL17RD,SOS1,TSHR,EXT1,ATRX,PRICKLE2,SERINC5,IKZF1,RAPGEF2,KCNC2,SMO,SUN1,BLOC1S3,MARK4,CPT1A,MYLPF,TCF4,TNFRSF11B,SYT17,BPGM,TFE3,GRIN1,ADAM29,RHOA,SYNGAP1,GRIN3A,NDE1,ROR1,TF,ONECUT2,CUL3,HSPD1,NFKBID,EPM2A,GABRB3,GSK3B,EMB,STAT6,ACTA2,SYN1,MLF1,EEF2K,PTPRB,CCDC14,CDK13,ALAS2,UPF2,CD27,BOK,SULF1,MTHFD1L,KRT8,AGT,HDGFRP3,ADAR,BLOC1S6,FBN2,ZMPSTE24,BRINP3,NDRG2,PTK2,PHEX,SNX3,RIMS2,PBX1,EXOSC3,SHB,GAP43,CPE,TRPC4AP,COL9A1,DLX1,SLC29A1,TAOK2,UGT1A1,PYGO2,SHC1,NDUFS2,CD160,ECE1,RPS6KB1,STC2,GPR173,SCEL,SETDB2,ANKS1A,ALOX5,CPLX2,DNAJB6,MDGA2,SP7,CD44,ADAM8,SLC5A3,CLDN4,RPS6KA5,THSD7A,CXCL17,PEX7,TBC1D23,PLEKHA1,RGS14,STK4,TCF25,TUBB3,SPEF1,EPHB2,SLC1A1,WLS,SIGMAR1,TMEFF2,CLPTM1,IL18,MFSD8,DLC1,PPARA,ARID5B,CALCRL,PHLPP1,PRRC2C,NOTCH4,SOBP,CRX,FBN1,PAX6,PRKCZ,FAM20C,ADORA2A,GRIN2B,PPP1CC,COL22A1,PHLDB2,KLHL1,NIPBL,TMIGD2,PCDH10,UPB1,ASPN,ANGPT1,FHL2,LRRK1,MBTD1,ATF6,SHROOM4,IGF2BP3,GCNT2,ETS1,PPP1CA,VWC2,MAP2,NEBL,TAGLN3,FBXO31,CDK5RAP1,CNTN1,NCOR2,PRKCQ,LGI2,BRAF,CSRNP1,HDAC2,IMPACT,POU3F3,NOTO,SPINT2,TNR,CDKL3,PPP2CA,ANGPT4,ACAN,ATXN1,CAMSAP3,SP100,ANXA2,CR2,SRGAP2,TMEFF1,ADCY1,NUMB,SYK,WWTR1,SLIT1,ASB1,CIT,RADIL,BRCA2,DVL2,NEB,GRIP1,PTPRJ,TAF1,GPRIN3,RNF38,GPM6A,POTEE,CADM1,HSF1,MAX,DHFR,EZH1,SRPK2,SASH1,ASAP1,ADAMTS7,BMP1,VAV3,ZP3,CAV2,MYEF2,INPP5F,AXL,TNFSF9,CD109,MET,CCR3,TIMELESS,UCHL5,ANKRD6,MALT1,TRA2B,KDM2B,SEMA3C,NEDD4L,PTPRC,EDA,GPR89A,SH3GL2,TYRO3,RNF213,SYNDIG1,VPS52,NCAM1,TRAF3IP2,CREB1,EDNRA,GNGT1,BMP4,CPNE1
GO:0007154	cell communication	5.221525592385442e-35	*CD247,RIC8B,ENPP1,PRDX2,GPC5,NRXN1,ASPH,GP6,PRKCI,MAP4K4,PDE4D,PDCL,DNMT1,S1PR2,TMBIM4,SLC9A1,PDE7B,ADCY8,LMBR1,PDE8A,CLASP2,SEMA3A,IL31RA,GRID2,TAS1R2,RPS6KA2,PTGFR,TENM3,CHERP,DOCK1,WWC1,CLDN18,C12ORF49,CTDSPL2,FBXL2,NRG3,ASH1L,NOS1AP,LATS2,ANP32A,SEMA3D,PTH2R,NREP,TIAM2,KSR2,MAPRE2,NLGN4X,PTGIS,PRKAG2,WWC3,GRM8,UNC5C,OR5P2,OR5P3,CLCN1,PRLR,MVP,PAGR1,ADCY7,RGS7BP,KALRN,PITPNC1,NT*

			RK3,CBL,ARNT,ATRNL1,PLEKHG4B,EGLN2,MIA,RAB4B,RAB4B-EGLN2,SEMA5A,LRRFIP2,FLT3,STAT5B,GRM5,PLCE1,SAMHD1,FER,CASK,MARVELD3,MAPK4,MAP2K5,KITLG,MAPK10,PTPRR,SPNS2,LRP2,PIK3CD,ZNF536,DEPDC5,HDGF,ANK2,OLFM1,SH2D1A,EZR,NRXN3,ROBO1,TOM1L1,TNFAIP8L1,CHD7,HTR2B,ITGB6,MECOM,TENM1,CAMKMT,LMNA,NUCB1,GOT1,NMT2,INPP4B,TRAF6,ROBO2,NFASC,ITPKB,CTNNA3,DNAJA3,SLC24A2,VAV2,GRK5,ABI1,ULK4,ITGB1,HSP90AA1,CDC6,PSMB7,CNIH2,SRGAP3,MCC,TSSK1B,PCDH17,KNDC1,CCDC22,DSCAM,STK38L,GMDS,SAMD12,CNGB1,TRIO,ARHGAP24,KLHL12,AKAP13,DLG2,PDE1A,PTPRK,DAB1,CDC42EP3,MYO10,ABCC8,ERC1,HMGN3,RGS6,DGKI,ANKS1B,THRB,STXBP4,PDE4A,ERC2,GABRA3,ITGB3BP,LAMA2,GFRA2,MAST4,IL17RB,TCIRG1,PTPN11,NETO2,HERC4,MGAT5,CACNB2,DOCK10,CYTH3,HDAC6,DDB1,SV2B,ADORA1,GPRASP1,ADAMTS3,EPHA1,IKBKB,ERBB4,ADRA1D,SORCS1,MRE11A,GBF1,LIMK1,TLK1,BRD8,ABR,SNIP1,KLHL6,ESR1,NPHP3,GRM7,MIER1,PMEPA1,MAML3,PTPRO,CDON,NTRK2,TNRC6A,EP300,CELA1,TENM2,RNF220,RGS22,FNTA,PDGFB,CACNA1B,RIMS1,TNIK,CCND3,CDH8,USP46,MID1,CHFR,BPI,STK39,DAP3,HNF4G,INSR,FMN2,PRKAR1A,ASB5,AFAP1,UNC5D,LAMA3,TCF7L2,PIP4K2A,CAB39,USP34,PAK1,LITAF,FBXW11,ESRRB,MYOM1,MAP3K4,ZDHHC13,PTPRD,SLC4A10,BDNF,CDK14,FANCA,CHRM3,TGIF2,FUT8,OR2T3,NPRL3,PHACTR4,TENM4,GRIK4,TRIM13,RASGEF1B,UBR2,ARHGAP6,LCK,MDM4,EDARADD,IL5RA,ENTPD5,SHC4,ECT2,SNX6,CHML,VAMP7,PPM1L,ARHGAP42,PTPRU,RFTN1,ERN2,CDC45,LSP1,AP3S1,NPLOC4,SGCD,DMD,CENPF,ATG10,LRRN2,KAT7,SLC8A1,RBM14,GPR171,RIT2,DRG2,DEPDC1B,CASS4,KCTD16,FYN,MKRN2,ARNTL,NF1,PLCB1,DST,LMTK2,SNCB,RTN4,RXFP1,CHRDL1,DGKH,APOD,RTN4R,BCR,PPP1R12B,RWDD3,SHISA9,CHI3L1,TTN,NDRG4,ICA1,BMP2K,PAQR3,PIK3R2,CACNA1E,RANBP9,NTN1,NOMO3,NRG1,ARHGAP10,SH2D3C,SLC16A10,BDKRB1,BDKRB2,DOCK8,BID,MAP2K1,MDFI,FNIP1,RAPGEF6,VAX2,RALGPS2,MYH9,MAD1L1,DAGLA,TMBIM6,VGLL4,PPP2CB,CNTN4,PPP3R1,NTNG1,VSTM1,OR6N2,CMKLR1,INPP4A,CDKL2,ROR2,DCN,ZDHHC3,SLC39A14,LIN7A,SYN2,DOCK2,CDH13,CREBRF,SOX13,FHIT,PTPRT,COPS5,FGF14,PCDHB16,EXOC4,ARHGAP32,MAD2L2,TLE6,RGS8,CAPRIN2,CCNYL1,NLN,NCKAP1,DEFA1B,DEFA3,PTGER2,RALGPS1,FOXN3,RCC2,BOC,ANO6,ARL3,NEUROD1,GNAQ,FBXO9,ICK,TMEM117,RFFL,RANBP1,CCBE1,DENND1A,JAK2,FBXW7,LINC00473,SYN3,SKAP1,CLSTN2,UIMC1,SYT1,ITCH,NPSR1,ZFYVE28,LZTS1,SMG1,SCN4A,SLC1A4,DTHD1,HUNK,MLLT3,ARAP2,CDC42BPB,DOCK9,SH2D6,TCF7,PDE2A,KLF15,ANXA4,LAMTOR3,MAGI1,KCNC4,CYFIP2,WNT11,ARHGAP23,IFT80,TRIM59,APBB1IP,MTA1,KREMEN1,NRG4,CLPB,NOX4,SIPA1L3,MKLN1,CACNA1H,SIAH1,IL1RAPL1,CAMK2B,PRKCD,TAB2,ACVR2A,RUNX2,SEMA5B,CD4,TGFB1,PLCXD3,SGK1,PCSK5,PDLIM4,SPSB4,NUP93,IGSF1,PIBF1,BTRC,NFATC3,JPH3,CRADD,DLGAP1,PTPRN2,F2RL1,C9ORF47,GRIK3,RPGRIP1L,CELSR1,SLIT3,AAK1,ZFYVE9,SLIT2,ASB15,TP73,GABRR3,CADPS2,GABRB1,SYT13,HHAT,GPR176,CORO1C,RRAS2,CTNND2,OR9A4,DISC1,KCNJ3,KANK1,CACNA1D,PTPDC1,MTDH,ANK1,TTBK2,XPR1,CHRM1,OR51A7,OR51F2,OR51T1,SMAD6,RXFP2,CLOCK,ZNF675,PLCB4,KCNH1,BMPER,BCL3,DCLK1,ANKRD54,DAPK2,TNFRSF10B,DAPP1,ADAM12,DUSP22,GPR52,NAIP,HOMER2,SLC17A7,DOCK3,OIP5,OTOA,YTHDF1,FGF10,CNTNAP2,FBXL17,RASA4,RASA4B,ARHGEF28,STXBP5,KCTD10,IL1RAPL2,IQCJ-SCHIP1,SH3RF3,LOXL3,RHPN2,MAST2,GLP2R,ADCY2,CAPN3,SLC16A1,SMURF2,EPHA4,RORA,PRKCA,AUTS2,CNR1,CD6,TNFSF11,PPP3CA,NSUN2,OR51B2,OR51B4,OR51I1,GNG4,MAG,CAMK4,UFL1,CTNNB1,PARK2,SOD2,FCGR2A,FCGR2B,FCGR3A,FCGR3B,ARHGAP22,SMARCC1,IGF1R,PPARG,AXIN1,GARNL3,PRKAR1B,DLG5,IL18R1,IL1RL1,P2RY10,TRPM1,ANKRD17,CYBB,CASKIN1,APOL3,BRIP1,SREBF2,RYR2,DRAXIN,LEPR,LEPROT,FGF1,RGS10,NR4A3,WDR45B,NOL3,PRKAR2A,MYOCD,OR52E6,OR52E8,OR52N1,TRIM5,PER2,KIR2DL1,KIR2DL4,AJUBA,CACNA1C,CPNE6,GLG1,SCN3B,CHEK2,PRDM16,MYO1E,ASB8,PPP1CB,HCK,SORBS1,TBCK,TRIM8,CSPG4,GRM1,RIMS4,DCC,ARHGAP31,P2RY14,OBSCN,RGL1,NF2,FLT4,SGMS1,BICC1,HDAC4,PAX2,TNS3,SPTBN1,TPTE,TRABD2B,SPG11,SFRP1,FOXO3,DGKB,TRHDE,ARL13B,CNTN6,ARHGAP29,IFT122,SMAD3,CUX2,ITPR1,PTPRM,WWP2,ACKR2,WIPI1,ABI2,GRIK5,PSAP,SLC16A2,SHANK1,SYT3,IFNAR1,OR14K1,NEDD4,NRP2,ARHGEF18,VANGL1,PREX1,PLA2G4C,DOCK4,ESRRG,HOXD3,KPNB1,TAB1,SLC6A1,TXNDC12,NFATC1,RAB6C,CDC73,APP,GSX2,PDGFRA,FCRL4,NOX1,YAP1,HEG1,AMFR,RAB11FIP5,SESN1,FSTL4,NLGN2,TNFRSF19,EYA2,JAK1,TNNI3K,LRP5,MUC20,PTPRG,SLC9B2,GNG2,SOX2,GPC6,CHST11,TEAD1,PRICKLE1,RCAN1,ZNF653,SPTBN4,GRIA3,DRD1,TMEM14A,GLRA2,CHUK,ERLIN1,FRS2,MMP2,SERGEF,ESR2,S100A12,KDM6A,PRKD1,STAT1,ST18,DGKG,RND3,SLC6A3,HNF4A,LRRC2,SHANK2,VPS4A,EREG,CCNY,TCTN3,MAPKAPK3,SEMA6D,ATF2,GRAMD4,TMEM237,RAF1,CELF4,RASGRF2,CARD16,CASP1,PTPN1,ADAMTS12,BMPR2,USP33,DPYSL2,BMPR1A,TSPAN12,NLGN1,COL16A1,PIK3R3,SPAG9,DENND4A,EVC,PDE11A,EPG5,FGF2,LRRC4C,GRIK1,OR6C70,PPM1F,TICRR,ADCY5,NEDD9,SEMA4D,DOK6,RORC,MCTP1,PLXNA2,PTAFR,ADCYAP1R1,ANKDD1A,DDX58,DKK2,SORCS2,RAB11FIP3,ITGA11,PI4KA,RARB,SPEN,PIK3C2B,PRKCG,VIPR1,NCOA1,EEF1E1,EEF1E1-BLOC1S5,GRIA2,GAS8,CHRD,DVL3,ECE2,EIF2B5,EIF4G1,EPHB3,PTPRE,RBX1,ARHGAP39,ATF3,SHOC2,PAWR,AGO3,DEPTOR,PKN3,FBXL20,MAML2,TSG101,CCDC3,TERF2IP,MYO9A,IDE,WDR59,KCNQ1,WNT3,RCE1,RHOJ,P4HB,CCL14,CCL1

			5,CCL15-CCL14,SUFU,TG,NLGN3,PTPN13,SCUBE2,ZFYVE1,PAFAH1B1,PRDM15,FAM13A,CNTFR,COL4A3,TRPA1,DDAH1,NMUR2,HIP1,AKAP6,RASAL1,BTBD9,NR2C1,IRS4,GLI2,NEO1,TNKS,SCUBE1,GPR39,MGLL,DAB2,THEMIS,BLM,PKHD1,UBR1,LDLRAD4,DZIP1,WNK1,RELN,NEK10,RUVBL2,OR5H2,OR5H6,OR5K4,RSPO2,GUCY1A2,CSF3R,KCNQ2,DOCK11,PELI1,PPP2R5C,IQGAP1,MAP3K7,DDX47,TAS2R41,ZNF423,OR56B1,TRIM22,MACF1,ARHGAP12,ALK,SLC8A2,INPP5D,STAU1,DSC2,TEAD4,UBE2V1,XDH,LTBP1,OVOL2,SNX5,NFATC2,OR6C74,PRKG1,RGS7,ATP2B4,PAK3,CAMTA1,DYX1C1,P2RX6,ASGR2,ATAD1,ADNP,GPR161,MAPKAPK2,MAP1B,CDC42BPA,COL4A6,SCARB1,MARK1,CDK6,P2RY8,RNF34,TFRC,EPHA5,SORCS3,WWOX,GABRG3,MEF2A,SYT12,ADCY9,CACNA1A,FNBP1,GFRAL,GPC3,ADRBK1,PSMB2,ZNF207,EYA1,PTGER3,GNB3,TRPV1,TSPAN6,TFEB,CLEC16A,GHR,FLRT2,FGD1,ELAVL4,HDAC1,INVS,SMOC2,TRIM24,ADIPOR2,PLCG2,ROCK1,EPS8,C1QTNF1,PAXIP1,EGR2,HIPK3,RNF10,GAPVD1,CAPN10,GPR35,CUL2,AP3B1,PTPN2,INSRR,NTRK1,TRIM44,MAP1A,PKN2,PPP1R9A,SLC39A10,EIF3A,ANK3,DNMBP,UNC13A,EPHB1,PDE6A,EPHA10,AXIN2,SARM1,EPCAM,PARD3,RCAN2,MUC1,TUB,SH3GL3,JAG2,CACNG8,CARM1,VWF,NETO1,ARHGAP44,SRD5A2,HTR1D,ARHGEF3,SPI1,DGKK,EPHA7,CHRNB4,MCU,RANBP10,PTPRA,CTNNA1,FBXW4,TRDN,ATG14,GABRA6,LY86,STAC,BCAR3,ILDR2,STK38,RBPMS,CHURC1,FCHSD2,MAP3K13,OTUD3,RAB15,RAPGEF3,SH3BP1,MST1,PTK7,SNCA,BMPR1B,CACNG2,CD53,GRM4,MAGI2,USP50,ZEB2,SYT9,HEY2,RC3H1,CNGA3,WNT7B,PTPRS,ZMYND11,KMT2D,PRKAG1,CREM,DFFA,SPPL2A,ADTRP,ABCA12,GNG7,PLD1,RASA1,MACC1,MITF,OPRD1,CABIN1,HIPK1,PKP2,RPTOR,BTK,SIGLEC9,VPS41,ZNRF3,KCTD13,SLC4A8,TROVE2,CBFA2T2,IGF1,HTT,MAPK1,PTEN,ARHGEF17,MIB1,SPRED2,BMP7,SCN8A,PIK3C3,PUM1,RASA2,PRCP,RAB30,LRRK2,OSGIN1,GNA14,ZRANB1,UNC119,CNIH3,RAPH1,BAG6,LILRB4,IFT81,TMCO1,TMEM108,ITGAM,ARHGAP25,IL11RA,NSG2,PKD2,EFNA5,SHANK3,BTBD11,RGS9,PDGFC,AMOTL1,FAM13B,NCOA3,MTMR3,WNT7A,ZBED6,SNAP23,RPS6KC1,UACA,CRTC3,GPR141,CEP89,PLCL2,KAT6A,GPR21,RABEP1,RHOT1,SIK3,BMP6,ANO1,GPI,IL17RD,SH3RF2,SOS1,TSHR,EXT1,PDCD1LG2,ATRX,PRICKLE2,GABRR2,PRMT2,RAPGEF2,KCNC2,SMO,VRK3,MARK4,PRKAR2B,STXBP5L,CPT1A,PSEN2,TNFRSF11B,PAG1,SYT17,SOX30,UBR5,GRB14,GRIN1,JADE1,KCTD8,RHOA,WSB2,OR4M1,OR4N2,SYNGAP1,VIPR2,GRIN3A,ROR1,RQCD1,TF,ONECUT2,RHOBTB1,FAM19A4,CUL3,SH3KBP1,HSPD1,ITPR2,NFKBID,EPM2A,GABRB3,GSK3B,ABCG8,PLD2,GNG12,RDX,STAT6,ACTA2,SYN1,MLF1,FAM49B,NECAB2,STK32B,CD27,DUSP26,ITGBL1,MCTP2,SCN9A,BOK,SULF1,KRT8,NACC2,RNF43,TNFRSF8,AGT,HDGFRP3,PRKAA2,SYT7,ADAR,BLOC1S6,FBN2,PITPNM2,STAT2,ZMPSTE24,HRH4,NDRG2,PTK2,LAT2,PEX5L,PHEX,SNX3,FREM3,PDE4B,RIMS2,TCP11,TEC,SHB,VEPH1,GAP43,MAP3K5,CPE,CREBBP,IQGAP2,PRKD3,APCDD1L,DLX1,FMOD,INSL6,PCSK6,NCF1,SLC29A1,TAOK2,PYGO2,SHC1,ARHGAP15,CD160,ECE1,LBX2,RPS6KB1,STC2,GPR173,GUCY2F,SCEL,ANKS1A,ITGAL,ALOX5,CPLX2,CD44,FGD3,ADAM8,PACSIN2,RPS6KA5,BCL2L14,CDS1,CSNK1A1,GRID1,HUS1,CXCL17,FCRL6,FGD4,NOS1,PLEKHA1,PLVAP,RGS14,SIPA1L2,ACTN4,ARHGAP11A,MC1R,STK4,ZFAND6,MARK3,SPEF1,EPHB2,SLC1A1,WLS,SIGMAR1,XCR1,IL18,DTNA,MFSD8,UBE2K,GPSM2,HCAR1,HCAR2,HCAR3,DLC1,PPARA,PPP1R10,ARID5B,CALCRL,MCOLN1,SLC25A33,PHLPP1,MAGI3,CPEB4,MOB3B,NOTCH4,SYTL4,ZNF366,FBN1,PAX6,PRKCZ,ZC3HAV1,ATP1A3,FAM20C,ADORA2A,GRIN2B,PPP1CC,RABGEF1,PLCXD2,TYK2,OTUD7B,DENND4B,CNRIP1,ASPN,ANGPT1,CADPS,TBL1X,EYA3,FHL2,FRMPD1,LRRK1,OSBPL8,ATF6,GCNT2,PPP1CA,TBC1D10C,VWC2,ARHGAP21,GRIA4,RBMS3,CCL22,BTN3A2,FBXO31,CNTN1,NCOR2,PRKCQ,BRAF,CSRNP1,HDAC2,HTR2C,IMPACT,TNR,CD300A,IRAK1BP1,PHIP,PPP2CA,ANGPT4,ARHGEF6,MAOA,RPL23,SH3BP4,DCDC1,SP100,OPN1LW,SNTG1,KCND2,PIGU,C1QTNF9,CR2,DONSON,FCRL2,ITSN1,SRGAP2,WIPI2,ADCY1,MADD,SYK,CNOT1,WWTR1,ARHGAP19,GRIK2,SCN1A,ASB1,CIT,MYRIP,RADIL,BRCA2,HRK,RAP1GAP2,CACNG3,DVL2,GRIP1,PTPRJ,SLC10A1,TAF1,TICAM1,TANK,UCN2,ITGAE,TAC3,HSF1,MAX,INPP5A,SRPK2,ANKRD13C,PLEKHM1,SASH1,RALGAPA1,BMP1,CGNL1,FCHO1,QRICH1,VAV3,ZP3,DLGAP2,KCNQ3,MOB3A,CAV2,GNA12,SH2D3A,COL4A5,INPP5F,NUAK2,AXL,REPS2,TNFSF9,WIF1,CD109,DENND4C,MET,CAMK2D,CCR3,RAD9B,TIMELESS,UCHL5,ANKRD6,MALT1,MLXIPL,SEMA3C,THEM4,PHB,PTPRC,RPH3A,EDA,GPR89A,PBLD,SH3GL2,TYRO3,NR6A1,RNF213,TSPAN5,ADCY10,NCAM1,TRAF3IP2,ATF6B,CREB1,RASSF8,RGMB,SHISA6,TRAP1,CLNK,EDNRA,GNGT1,PRAP1,BMP4,CASP12,CPNE1
GO:0032501	multicellular organismal process	6.776815889445187e-35	RDH14,CLRN1,DNAH11,RCN1,ENPP1,PRDX2,ADAMTS16,LDB2,NRXN1,ASPH,F8,PRMT3,GSS,GP6,RDH13,PRKCI,MOV10L1,TACC2,SLCO3A1,MAP4K4,PDE4D,PDCL,DNMT1,S1PR2,HLX,LLPH,SLC9A1,NEGR1,PBX3,ADCY8,SPAG16,LMBR1,TRPS1,CBFB,CLASP2,SEMA3A,IL31RA,LSAMP,GRID2,TAS1R2,RPS6KA2,PRDM12,SETD4,PTGFR,TENM3,CALR3,CHERP,DOCK1,WWC1,CLDN18,RYR1,NRG3,ASH1L,NOS1AP,LATS2,NOX5,SPESP1,IQCG,ASTN2,EPB41,SEMA3D,PHACTR1,MYOT,PTH2R,NREP,TIAM2,STOX2,FHOD3,KSR2,MAPRE2,NLGN4X,PTGIS,DCHS2,WWC3,TJP1,GRM8,UNC5C,ZSWIM6,OR5P2,OR5P3,CLCN1,PRLR,ADCY7,HIVEP3,FTO,UTRN,KALRN,WIPF3,SPRR2D,NTRK3,CBL,ARNT,ATRNL1,SEMA5A,FLT3,RAD51B,STAT5B,TOX,ENPP2,ATP2B2,GRM5,PLCE1,LHFPL4,SAMHD1,MARVELD3,MAP2K5,SPATA5,KITLG,PTPRR,SPNS2,NDUFV2,LRP2,PIK3CD,ZNF536,SP3,ANK2,OLFM1,MFAP5,EZR,MEGF10,NRXN3,XRCC4,ROBO1,CHD7,SLC26A6,HTR2B,ITGB6,CDKL5,M

			ECOM,TACC1,TENM1,RYR3,KIF5C,LMNA,LPO,KIAA1217,TRAF6,ESRP1,ROBO2,NFASC,ITPKB,CTNNA3,DNAJA3,OXR1,SLC24A2,VAV2,ABI1,ULK4,NHLH1,ITGB1,TRIOBP,HSP90AA1,SLC24A3,GAS7,PSMB7,CNIH2,MCC,TSSK1B,PCDH17,KNDC1,ZC4H2,KIF26B,SLC22A8,DSCAM,FAM126A,SRGAP2B,CNGB1,TRIO,ARHGAP24,KLHL12,AKAP13,TTLL5,GFI1B,RUNX1,DAB1,OMA1,ABCC8,NRIP1,DGKI,THRB,PDE4A,GABRA3,XIRP2,IMPG2,LAMA2,GFRA2,TCIRG1,DACH1,PTPN11,RBM19,HERC4,CACNB2,DOCK10,HDAC6,GOLGA4,MYT1,ADORA1,ADAMTS3,EPHA1,IKBKB,ERBB4,ADRA1D,DPY19L2,LIMK1,ZNF609,ATP8A2,ACTG2,HIF3A,CUL4B,LARGE,CECR2,ESR1,MYO18B,NPHP3,GRM7,ANKRD11,PTPRO,CDON,NTRK2,DNAI2,EP300,CELA1,MEGF9,DYM,TENM2,RNF220,HOOK3,PDGFB,RIMS1,TNIK,KIRREL3,USP46,CSGALNACT1,TOB2,MID1,GOLGA3,ALOX5AP,BPI,STK39,INSR,FMN2,RERE,PRKAR1A,ATP8B1,UNC5D,H2AFY2,PLLP,LAMA3,TCF7L2,PIP4K2A,CDH4,HPS1,JMJD1C,PAK1,LITAF,FBXW11,ESRRB,MYOM1,MAP3K4,PTPRD,CNTN5,BASP1,CATSPERB,RBM20,SLC4A10,TBR1,CATSPER2,STRC,BDNF,TFAP2A,ATRN,AMIGO1,CLDN12,FANCA,KCNMA1,CHRM3,TGIF2,POMGNT2,FUT8,OR2T3,NPRL3,PHACTR4,MEIS1,TENM4,LHFPL2,UBR2,PKHD1L1,LCK,MDM4,IL5RA,ECT2,TMC2,ZNF148,MYO3B,TFDP2,NAV2,ACOX1,VAMP7,LAMC1,ARHGAP42,CST2,PTPRU,RFTN1,MB,NPLOC4,AFF3,SGCD,DENND5A,DMD,CENPF,CRISPLD2,KAT7,WDR7,SLC8A1,GSN,RBM4,PRPS2,GPR171,SATB2,PAFAH1B2,RIT2,HIRA,MACROD2,TANC2,JPH2,FYN,ARNTL,ADAMTS9,NF1,PLCB1,MGMT,PCDH15,LMTK2,ARID4B,FAT3,RTN4,AFF2,RXFP1,MAL2,CHRDL1,SLC5A6,DGKH,APOD,SLC2A14,B4GALT6,RTN4R,BCR,PPP1R12B,SHISA9,CHI3L1,CCDC141,TTN,NDRG4,BMP2K,PAQR3,ANKH,ADAMTSL1,TLL1,NTN1,EGFLAM,DCTN1,SLC4A5,NRG1,UBP1,BDKRB1,BDKRB2,STRBP,FRY,MAP2K1,MDFI,FNIP1,STIM1,VAX2,MYH9,MAD1L1,LRFN5,DAGLA,PLEKHA5,VGLL4,CNTN4,VWA1,HDAC5,NTNG1,PCSK2,NAV1,OR6N2,NFIA,TMEM30A,SYNE1,CMKLR1,SBF2,PPIP5K2,TSPAN8,LRGUK,ROR2,DCN,SLC39A14,PRPSAP2,LIN7A,CATSPER3,DOCK2,CDH13,PLXDC1,SOX13,ENTPD1,ACSBG1,UST,LHFPL3,FGF14,DMC1,PCDHB16,EXOC4,MAD2L2,TLE6,RAB27A,CAPRIN2,CCNYL1,NLN,NCKAP1,F5,TERF2,CLDN16,FOXN3,BOC,ANO6,ARL3,NEUROD1,GNAQ,KIF2A,PARP11,COL12A1,CCBE1,USP22,ZDHHC15,JAK2,MYPN,YTHDC2,TRAPPC9,FBXW7,KCNB2,SMC3,CLSTN2,SYT1,ITCH,MLIP,CROCC,NPSR1,BBS12,ABCC2,LZTS1,BCL11B,SCN4A,RBFOX1,TMC1,SLC1A4,LRIG3,PKNOX1,CLDN1,HUNK,MLLT3,PNPLA1,PLS1,TEX11,TCF7,PDE2A,SEPT7,KLF15,TBX15,ANXA4,CYFIP2,WNT11,IFT80,FIG4,MTA1,KREMEN1,IMMP2L,NRG4,ASTN1,NOX4,SIPA1L3,CACNA1H,SIAH1,QKI,IL1RAPL1,CCDC62,CAMK2B,ADAM23,PRKCD,SOX6,TAB2,ACVR2A,RUNX2,SEMA5B,CD4,TGFB1,SGK1,PCSK5,PDLIM4,MYO3A,PIBF1,ZHX2,KEL,BTRC,NFATC3,JPH3,DLGAP1,F2RL1,BCAS3,C9ORF47,C2ORF49,RPGRIP1L,CELSR1,SLIT3,SDK2,SLIT2,TP73,GABRR3,MUSTN1,GABRB1,CDH23,CORO1C,RRAS2,CTNND2,OR9A4,DISC1,TSNAX,KCNJ3,KANK1,CACNA1D,LRRC10,LAMC2,CLN6,MTDH,CLMN,TTBK2,MYT1L,OPCML,CHRM1,OR51A7,OR51F2,OR51T1,SMAD6,RXFP2,BNC2,CLOCK,ITSN2,TCF12,ZNF675,SMOC1,OTOGL,ETV6,SYNJ2,NELL1,SUCO,PRR14,TFAP2D,BMPER,BCL3,DCLK1,ANKRD54,PARVA,SND1,ADAM12,NAIP,HNRNPC,ANKFN1,HOMER2,SLC17A7,OTOA,SBNO2,LINGO2,YTHDF1,FGF10,CNTNAP2,FBXL17,ENAH,GREB1L,IL1RAPL2,PPL,LOXL3,RHPN2,MAST2,SVIL,CAPN3,SLC16A1,LUM,VMP1,SMURF2,EPHA4,RORA,PRKCA,AUTS2,CNR1,CD6,TNFSF11,PPP3CA,NSUN2,OR51B2,OR51B4,OR51I1,MAG,CAMK4,UFL1,TRAK1,CTNNB1,PARK2,SOD2,DACH2,FCGR2B,ARHGAP22,SMARCC1,IGF1R,PPARG,IGDCC3,NGRN,AXIN1,PRKAR1B,DLG5,IL18R1,IL1RL1,BFSP1,ADD2,TMEM63C,MTF1,MSR1,CELF2,TRPM1,ANKRD17,CYBB,OTC,XK,SCAMP5,BRIP1,SPATA6L,KCNJ12,RYR2,DRAXIN,LEPR,FGF1,NIN,PROS1,NPAT,NR4A3,NOL3,DCT,MYOCD,OR52E6,OR52E8,OR52N1,PER2,KIR2DL4,CACNA1C,CPNE6,GLG1,SCN3B,PHLDB1,PRDM16,MYO1E,CSMD1,PPP1CB,SPDYA,CAPN2,TRIM8,DIO2,MRC2,CSPG4,GRM1,DCC,CTDP1,HS3ST5,MMP16,NF2,FLT4,MEF2B,BICC1,HDAC4,PAX2,DNAH9,SPTBN1,ELN,SPG11,SFRP1,FOXO3,DGKB,TRHDE,ARL13B,CNTN6,NFIB,IFT122,DNM3,SYNCRIP,SMAD3,CUX2,ITPR1,PTPRM,ARNT2,ACKR2,CYP4F11,NHS,SNTB1,RNF168,EMG1,CASZ1,ABI2,GRIK5,PSAP,SLC16A2,SHANK1,SYT3,OR14K1,NEDD4,NRP2,VANGL1,BTBD3,PREX1,PLA2G4C,DOCK4,CRTAC1,ESRRG,HOXD3,HOXD4,NAV3,SLC4A4,TAB1,SLC6A1,NFATC1,PRTG,CDC73,APP,SSBP3,GSX2,PDGFRA,AOC2,ODF3,CEP85L,DIP2B,NOX1,YAP1,HEG1,AMFR,RAB11FIP5,PPCS,FSTL4,NLGN2,TNFRSF19,EYA2,RNLS,ALPL,JAK1,PDE6D,ANGPTL4,TNNI3K,PEMT,LRP5,POLR3G,PTPRG,ISLR2,SLC9B2,SOX2,SETD2,PLAC1,GPC6,TTC39C,ADAMTS2,CHST11,TEAD1,DEFB118,PRICKLE1,RCAN1,SSPN,ELAVL3,SPTBN4,VASH2,ZNF521,PAQR5,DRD1,GLRA2,CHUK,FRS2,PALB2,SFMBT1,MMP2,TPH1,SYNE2,CPNE9,KDM6A,PRKD1,STAT1,PARP6,DGKG,SYBU,SLC6A3,USP53,MORN2,HNF4A,SHANK2,STRA6,VPS4A,EREG,ABLIM1,MYOF,MYH15,DSCAML1,SEMA6D,SLC16A12,RBFOX3,ATF2,POU2F2,TCF3,RAF1,CELF4,CARD16,CASP1,ADAMTS12,SRRM4,POLA1,GGT7,BMPR2,USP33,DPYSL2,CAMK1D,BMPR1A,AP1B1,PABPC4,TSPAN12,NLGN1,COL16A1,CTNNA2,PIK3R3,TAF8,CERS3,SPAG9,RAB11A,CRMP1,EVC,MYB,FGF2,LRRC4C,POU6F2,GRIK1,OR6C70,PPM1F,ADCY5,ZFP41,AGBL4,ADAMTS4,SEMA4D,SH3PXD2A,ISM1,ARMC2,RORC,ELP3,PLXNA2,PTAFR,ADCYAP1R1,FNDC3A,SETD1A,JARID2,KLHL3,DDX58,DKK2,RAB11FIP3,BRDT,PHC2,ITGA11,RARB,SPEN,PRKCG,NCOA1,SPOCK1,BLOC1S5,EHMT1,GAS8,OC90,NTM,CHRD,DVL3,EIF2B5,EIF4G1,EPHB3,XPNPEP3,ATF3,FBXO45,AKR1D1,MATN3,LMBRD1,SRSF5,EBF2,DLEC1,PKN3,FBXL20,ADAM20,ADA

			M21,TSG101,VCL,LAMB1,CLIC4,MYO9A,CRYM,IDE,COL11A1,KCNQ1,RFX2,WNT3,ADAMTS6,ZNF322,RHOJ,SUFU,TG,NLGN3,SCUBE2,PAFAH1B1,CCDC169-SOHLH2,SOHLH2,POLE,CNTFR,COL4A3,TRPA1,DDAH1,TRPM3,NMUR2,AKAP6,RASAL1,PADI6,BTBD9,TRPC5,TTLL7,GLI2,NEO1,SCUBE1,ERCC1,RUFY3,WDTC1,TPD52,MGLL,COL19A1,DAB2,THEMIS,PKHD1,USH2A,LDLRAD4,MYSM1,SETD5,DZIP1,WNK1,RELN,NEK10,SIN3A,ABCB1,OR5H2,OR5H6,OR5K4,RSPO2,CSF3R,KCNQ2,EYS,DOCK11,PELI1,SLC22A3,CRELD1,IQGAP1,MAP3K7,APOLD1,TAS2R41,ZNF423,SP1,OR56B1,LUZP1,MACF1,MYO7A,ALK,SLC8A2,GSTM3,INPP5D,KLK2,CLASP1,DSC2,TEAD4,HOXC13,SLC9C1,TOP1,MBOAT1,GPM6B,CLIC5,XDH,LTBP1,OVOL2,SNX5,NFATC2,RBBP6,OR6C74,PRKG1,PAX7,RGS7,BNC1,ATP2B4,PACRG,GALNTL5,PACSIN1,TMEM2,ACTL6B,ASXL3,PAK3,TET1,CAMTA1,DYX1C1,P2RX6,ASGR2,ATAD1,ADNP,GPR161,MAPKAPK2,ARID4A,MAP1B,SCARB1,MARK1,CDK6,PHF2,CELF1,TYR,TFRC,EPHA5,SORCS3,WWOX,GABRG3,MEF2A,ADCY9,DHX30,DROSHA,GFRAL,GPC3,SGIP1,ADRBK1,PLS3,PSMB2,CALD1,EYA1,PTGER3,BRINP1,DIS3L2,GNB3,TRPV1,RNF216,HOXB3,HOXB4,HOXB5,HOXB6,TFEB,GHR,MNAT1,FLRT2,FGD1,DIAPH2,RPGR,ACTR2,ACACA,ELAVL4,LGI4,HDAC1,SMOC2,ADIPOR2,PLCG2,ROCK1,EPS8,NID1,SCMH1,C1QTNF1,PAXIP1,EGR2,RNF10,RYBP,GPR35,ABCC1,AP3B1,PTPN2,INSRR,NTRK1,KPNA6,MYBPC2,MAP1A,PKN2,PPP1R9A,ANK3,ACO2,UNC13A,PTPRQ,EPHB1,PDE6A,EPHA10,PTPN9,AXIN2,SARM1,SNTG2,EPCAM,PARD3,PTPN14,TRIM46,TUB,SH3GL3,JAG2,KCND3,BCOR,CACNG8,AP3D1,CARM1,VWF,AP2B1,NETO1,ADAM19,ARHGAP44,SRD5A2,PIFO,CD84,COCH,HTR1D,LPA,SMARCA2,CUX1,HMCN1,RAD51C,SPI1,DGKK,EPHA7,CHRNB4,FOXP2,CTNNA1,LIMA1,FBXW4,GLIS1,TRDN,GABRA6,STAC,BCAR3,ILDR2,SHROOM3,RENBP,CHURC1,MAP3K13,RAPGEF3,SDK1,SH3BP1,MST1,KAZN,ERCC3,PTK7,SMYD1,TTC9,CHID1,SNCA,BMPR1B,CACNG2,MAGI2,USP42,PNPT1,USP50,ZEB2,RAI2,FOXJ3,OCA2,HEY2,RC3H1,COL13A1,HYDIN,RNF103,CNGA3,WNT7B,HMGCS2,PTPRS,KMT2D,PRKAG1,CREM,TLL2,TRIP12,ADTRP,ABCA12,CSRP1,RASA1,MITF,SRSF6,OPRD1,RBM45,HIPK1,PKP2,RTN4RL1,BTK,SGCZ,DSG1,ZNRF3,SLC4A8,TROVE2,CBFA2T2,IGF1,CTNNBL1,HTT,DPYSL3,MAPK1,PTEN,CLDN11,MIB1,SPRED2,BMP7,SCN8A,PUM1,SOX5,CIR1,LHFPL5,TTLL4,PRCP,LRRK2,SEPT6,MEGF11,UNC119,ZBTB20,RAPH1,BAG6,LILRB4,MORC3,SLC44A1,IFT81,TMEM108,ITGAM,PKD2,EFNA5,HSF2BP,SHANK3,STMN4,RIPPLY1,DAZL,RGS9,ALPK3,PDGFC,SERPINA3,SERPINA5,AMOTL1,NCOA3,CUEDC2,PPP2R3C,TBCD,WNT7A,ZBED6,SNAP23,NLRP1,MAP4,CRTC3,NME8,PLCL2,SPANXA2,KAT6A,GPR21,SH3PXD2B,BMP6,MYCBP2,ANO1,GPI,IL17RD,SOS1,TSHR,WDR43,EXT1,N4BP1,PDCD1LG2,ATRX,PRICKLE2,SERINC5,GABRR2,IKZF1,RAPGEF2,ABCG2,KCNC2,SMO,SUN1,BLOC1S3,MARK4,PRKAR2B,CPT1A,MYLPF,TCF4,TNFRSF11B,SYT17,BPGM,DECR1,SOX30,TFE3,GRIN1,ADAM29,BBS9,RHOA,OR4M1,OR4N2,SYNGAP1,GRIN3A,NDE1,ROR1,TF,ONECUT2,FAM19A4,CUL3,HSPD1,NFKBID,EPM2A,GABRB3,GSK3B,ABCG8,EMB,STAT6,ACTA2,SYN1,MLF1,EEF2K,FAM49B,PTPRB,RBBP8,CCDC14,CDK13,ALAS2,UPF2,CD27,SCN9A,BOK,SULF1,MTHFD1L,KRT8,TNFRSF8,ZNF354A,AGT,HDGFRP3,ELOVL3,PRKAA2,SYT7,ADAR,BLOC1S6,FBN2,ESPNL,ZMPSTE24,BRINP3,NDRG2,PTK2,TPPP2,PHEX,SNX3,PDE4B,RIMS2,PBX1,TCP11,TEC,EXOSC3,SHB,CDC14A,GAP43,CPE,TCFL5,TRPC4AP,CREBBP,ARVCF,COL9A1,DLX1,PCSK6,NCF1,SLC29A1,TAOK2,UGT1A1,UGT1A7,PYGO2,SHC1,NDUFS2,ALDOA,CD160,ECE1,LBX2,RPS6KB1,STC2,GPR173,GUCY2F,PRG3,SCEL,SETDB2,ANKS1A,ALOX5,CPLX2,DNAJB6,MDGA2,SP7,CD44,ADAM8,SLC5A3,CLDN4,RPS6KA5,THSD7A,HUS1,PKD1L1,PRDM2,COX5B,CXCL17,NOS1,PEX7,TBC1D23,MED15,PLEKHA1,PLVAP,RGS14,MC1R,SCNN1B,STK4,TCF25,TUBB3,SPEF1,EPHB2,SLC1A1,WLS,SIGMAR1,TMEFF2,CLPTM1,IL18,DTNA,MFSD8,ASF1B,HCAR2,DLC1,PPARA,ARID5B,CALCRL,EPB41L4B,PHLPP1,PRRC2C,GPSM3,NOTCH4,SOBP,CRX,FBN1,PAX6,PRKCZ,ZC3HAV1,ATP1A3,CD96,FAM20C,ADORA2A,GRIN2B,PPP1CC,RABGEF1,COL22A1,PHLDB2,OTUD7B,KLC3,KLHL1,NIPBL,TMIGD2,PCDH10,UPB1,ASPN,ANGPT1,TBL1X,EYA3,FHL2,LRRK1,MBTD1,ATF6,SHROOM4,IGF2BP3,SLCO2B1,GCNT2,ETS1,PPP1CA,VWC2,MAP2,MTF2,BTN3A2,KIFC3,NEBL,TAGLN3,FBXO31,CDK5RAP1,CNTN1,NCOR2,PRKCQ,LGI2,BRAF,CSRNP1,DIAPH1,HDAC2,HTR2C,IMPACT,MYOM3,ATP8A1,POU3F3,NOTO,SPINT2,TNR,CDKL3,PPP2CA,SLC26A2,ANGPT4,ACAN,ATXN1,CAMSAP3,SP100,OPN1LW,VTI1A,ANXA2,FAT2,KCND2,PARN,PRMT7,CR2,SRGAP2,TMEFF1,ADCY1,NUMB,SYK,CNOT1,MOGAT2,WWTR1,CYP39A1,GRIK2,SCN1A,SLIT1,ASB1,CIT,RADIL,BRCA2,CACNG3,DVL2,MORC1,NEB,YBX1,GRIP1,PTPRJ,TAF1,ATP2B3,GPRIN3,TICAM1,RNF38,GPM6A,OSBP2,UCN2,MYO1A,TAC3,POTEE,SLC13A3,CADM1,HSF1,MAX,DHFR,EZH1,SRPK2,PLEKHM1,SASH1,ASAP1,NCOA2,ADAMTS7,GBP5,MCM8,SULT2B1,BMP1,VAV3,ZP3,DLGAP2,CIB2,RNF2,CAV2,GNA12,SYNM,MYEF2,POC1B,INPP5F,TMPRSS3,AXL,TNFSF9,CD109,MET,CAMK2D,CCR3,TIMELESS,UCHL5,ANKRD6,MALT1,SETD3,TRA2B,CCDC136,KDM2B,MLXIPL,SEMA3C,NEDD4L,NDC1,PHB,PTPRC,EDA,GPR89A,PBLD,SH3GL2,TYRO3,NR6A1,RNF213,SPON2,SYNDIG1,VPS52,ADCY10,NCAM1,TANC1,TRAF3IP2,CREB1,SHISA6,AFF4,CLNK,EDNRA,GNGT1,PRAP1,BMP4,CPNE1
GO:0030030	cell projection organization	9.332718703266314e-35	*CLRN1,ADAMTS16,NRXN1,PRMT3,PRKCI,MAP4K4,PDCL,S1PR2,LLPH,NEGR1,SPAG16,CLASP2,SEMA3A,GRID2,PRDM12,TENM3,IQCG,SEMA3D,PHACTR1,MYOT,NREP,TIAM2,UNC5C,KALRN,NTRK3,SEMA5A,TOX,ENPP2,PLCE1,FER,LRP2,OLFM1,EZR,NRXN3,ROBO1,CDKL5,TENM1,KIF5C,ROBO2,NFASC,VAV2,ABI1,ULK4,I*

			TGB1,TRIOBP,HSP90AA1,GAS7,KNDC1,DSCAM,TRIO,ARHGAP24,IFT43,DAB1,CDC42EP3,MYO10,LAMA2,TCIRG1,PTPN11,DOCK10,HDAC6,GOLGA4,EPHA1,GBF1,DNAH2,LIMK1,ATP8A2,CUL4B,CECR2,NPHP3,GRM7,PTPRO,NTRK2,DNAI2,EP300,TENM2,RIMS1,TNIK,SNX2,KIRREL3,INSR,RERE,ATP8B1,UNC5D,LAMA3,CDH4,PAK1,PTPRD,TBR1,STRC,BDNF,AMIGO1,LRRC49,DENND5A,DMD,GSN,RIT2,TANC2,FYN,PCDH15,LMTK2,FAT3,RTN4,APOD,B4GALT6,RTN4R,CCDC141,NDRG4,PAQR3,ADAMTSL1,NTN1,DCTN1,FRY,MAP2K1,RAPGEF6,VAX2,MYH9,CNTN4,WDR92,NTNG1,TMEM30A,LRGUK,ROR2,CDH13,UST,CAPRIN2,NCKAP1,RCC2,BOC,ANO6,ARL3,ICK,TTLL8,ZDHHC15,JAK2,MYPN,SYT1,CROCC,BBS12,LZTS1,BCL11B,PLS1,SEPT7,CYFIP2,IFT80,FIG4,KREMEN1,SIAH1,IL1RAPL1,CAMK2B,PRKCD,SEMA5B,SGK1,PIBF1,KEL,F2RL1,BCAS3,RPGRIP1L,SLIT3,SLIT2,CDH23,CORO1C,CTNND2,DISC1,CEP135,KANK1,PTPDC1,LAMC2,CLMN,TTBK2,SPAG17,ITSN2,DCLK1,PARVA,YTHDF1,CNTNAP2,ENAH,EPHA4,AUTS2,CNR1,PPP3CA,MAG,CTNNB1,PARK2,FCGR2B,CDHR2,IGF1R,DLG5,XK,DRAXIN,NIN,AJUBA,CPNE6,CSPG4,DCC,HDAC4,PAX2,DNAH9,SPG11,SFRP1,ARL13B,CNTN6,NFIB,IFT122,DNM3,CUX2,PTPRM,ABI2,SHANK1,SYT3,NEDD4,NRP2,BTBD3,CRTAC1,SPAG6,PRTG,APP,DIP2B,YAP1,FSTL4,NLGN2,PTPRG,ISLR2,KIF24,TTC39C,SPTBN4,VASH2,MMP2,SYNE2,CPNE9,PRKD1,PARP6,DGKG,TCTN3,ABLIM1,DSCAML1,SEMA6D,TMEM237,ABLIM2,BMPR2,USP33,DPYSL2,CAMK1D,NLGN1,CTNNA2,SPAG9,RAB11A,CRMP1,LRRC4C,SEMA4D,ARMC2,PLXNA2,POC1A,RILPL1,SPOCK1,BLOC1S5,GAS8,DVL3,EPHB3,FBXO45,VCL,LAMB1,WDR90,MYO9A,KCNQ1,RFX2,WNT3,RHOJ,NLGN3,PAFAH1B1,KIF3A,CEP350,RASAL1,TRPC5,GLI2,NEO1,RUFY3,DAB2,PKHD1,DZIP1,RELN,SIN3A,DOCK11,IQGAP1,ZNF423,MACF1,MYO7A,ALK,MBOAT1,GPM6B,PRKG1,PACSIN1,ACTL6B,PAK3,DYX1C1,ADNP,CEP41,MAP1B,MPHOSPH9,MARK1,AIF1L,EPHA5,MEF2A,FLRT2,FGD1,RPGR,ACTR2,ELAVL4,ROCK1,EPS8,EGR2,NTRK1,MAP1A,PKN2,PPP1R9A,ANK3,UNC13A,EPHB1,EPHA10,PTPN9,SARM1,PARD3,TRIM46,TUB,TEKT1,CARM1,ARHGAP44,PIFO,CUX1,EPHA7,CTNNA1,LIMA1,MAP3K13,SDK1,PTK7,BMPR1B,MAGI2,HYDIN,WNT7B,PTPRS,CDKL1,PLD1,RTN4RL1,TROVE2,CBFA2T2,HTT,DPYSL3,PTEN,BMP7,LHFPL5,LRRK2,SEPT6,RAPH1,IFT81,TMEM108,PKD2,EFNA5,SHANK3,STMN4,WNT7A,MAP4,NME8,CEP89,MYCBP2,SOS1,TSHR,EXT1,PARVB,RAPGEF2,SMO,BLOC1S3,MARK4,SYT17,BBS9,RHOA,SYNGAP1,GRIN3A,ROR1,ONECUT2,CUL3,GSK3B,EMB,RDX,EEF2K,AGT,HDGFRP3,BLOC1S6,PTK2,SNX3,RIMS2,CDC14A,GAP43,CEP70,TAOK2,ECE1,CD44,FGD3,PACSIN2,RPS6KA5,FGD4,TBC1D23,PLEKHA1,TUBB3,SPEF1,EPHB2,PAX6,PRKCZ,ATP1A3,ADORA2A,GRIN2B,KLC3,KLHL1,MAP2,FBXO31,ATG3,CNTN1,PRKCQ,BRAF,HDAC2,IMPACT,NOTO,TNR,CDKL3,ARHGEF6,FAM149B1,CAMSAP3,SRGAP2,TMEFF1,ADCY1,NUMB,WWTR1,SLIT1,DVL2,GRIP1,GPRIN3,GPM6A,MYO1A,DHFR,PLEKHM1,ASAP1,VAV3,POC1B,INPP5F,SNX1,EHD2,SEMA3C,NEDD4L,SH3GL2,NCAM1,TANC1,CREB1,EDNRA,ABLIM3,CPNE1
GO:0120039	plasma membrane bounded cell projection morphogenesis	4.255970818953704e-34	*NRXN1,PRMT3,MAP4K4,LLPH,CLASP2,SEMA3A,SEMA3D,PHACTR1,MYOT,TIAM2,UNC5C,KALRN,SEMA5A,ENPP2,LRP2,OLFM1,NRXN3,ROBO1,CDKL5,KIF5C,ROBO2,NFASC,ABI1,ITGB1,HSP90AA1,GAS7,KNDC1,DSCAM,TRIO,DAB1,LAMA2,PTPN11,DOCK10,HDAC6,GOLGA4,EPHA1,LIMK1,ATP8A2,PTPRO,NTRK2,RIMS1,TNIK,SNX2,KIRREL3,RERE,UNC5D,LAMA3,CDH4,PAK1,PTPRD,TBR1,BDNF,AMIGO1,DMD,TANC2,FYN,LMTK2,RTN4,B4GALT6,RTN4R,CCDC141,ADAMTSL1,NTN1,MAP2K1,VAX2,CNTN4,NTNG1,UST,CAPRIN2,NCKAP1,BOC,ZDHHC15,MYPN,SYT1,LZTS1,BCL11B,CYFIP2,SIAH1,IL1RAPL1,CAMK2B,SEMA5B,SGK1,KEL,SLIT3,SLIT2,CORO1C,CTNND2,DISC1,KANK1,LAMC2,ITSN2,DCLK1,YTHDF1,CNTNAP2,ENAH,EPHA4,AUTS2,PPP3CA,MAG,CTNNB1,PARK2,IGF1R,XK,DRAXIN,NIN,CPNE6,DCC,PAX2,SPG11,CNTN6,NFIB,DNM3,CUX2,PTPRM,ABI2,SHANK1,SYT3,NEDD4,NRP2,BTBD3,PRTG,APP,DIP2B,FSTL4,ISLR2,SPTBN4,CPNE9,PARP6,DSCAML1,SEMA6D,BMPR2,USP33,DPYSL2,NLGN1,CTNNA2,SPAG9,RAB11A,CRMP1,LRRC4C,SEMA4D,PLXNA2,DVL3,EPHB3,FBXO45,VCL,MYO9A,WNT3,NLGN3,PAFAH1B1,RASAL1,TRPC5,GLI2,NEO1,RUFY3,RELN,SIN3A,IQGAP1,MACF1,PACSIN1,PAK3,ADNP,MAP1B,EPHA5,MEF2A,FLRT2,ACTR2,ELAVL4,ROCK1,EGR2,NTRK1,MAP1A,ANK3,UNC13A,EPHB1,EPHA10,SARM1,PARD3,TRIM46,ARHGAP44,CUX1,EPHA7,MAP3K13,PTK7,BMPR1B,WNT7B,PTPRS,PTEN,BMP7,LRRK2,RAPH1,TMEM108,EFNA5,SHANK3,WNT7A,MYCBP2,SOS1,EXT1,RAPGEF2,SMO,SYT17,RHOA,SYNGAP1,GSK3B,EMB,EEF2K,PTK2,RIMS2,GAP43,TAOK2,ECE1,CD44,RPS6KA5,TUBB3,EPHB2,PAX6,PRKCZ,ADORA2A,MAP2,FBXO31,PRKCQ,BRAF,IMPACT,TNR,CDKL3,SRGAP2,TMEFF1,ADCY1,NUMB,SLIT1,DVL2,GRIP1,GPM6A,SNX1,SEMA3C,NEDD4L,SH3GL2,NCAM1,CREB1,EDNRA,CPNE1*
GO:0048858	cell projection morphogenesis	4.470128094284913e-34	*NRXN1,PRMT3,MAP4K4,LLPH,CLASP2,SEMA3A,SEMA3D,PHACTR1,MYOT,TIAM2,UNC5C,KALRN,SEMA5A,ENPP2,LRP2,OLFM1,NRXN3,ROBO1,CDKL5,KIF5C,ROBO2,NFASC,ABI1,ITGB1,HSP90AA1,GAS7,KNDC1,DSCAM,TRIO,DAB1,LAMA2,PTPN11,DOCK10,HDAC6,GOLGA4,EPHA1,LIMK1,ATP8A2,PTPRO,NTRK2,RIMS1,TNIK,SNX2,KIRREL3,RERE,UNC5D,LAMA3,CDH4,PAK1,PTPRD,TBR1,BDNF,AMIGO1,DMD,TANC2,FYN,LMTK2,RTN4,B4GALT6,RTN4R,CCDC141,ADAMTSL1,NTN1,MAP2K1,VAX2,CNTN4,NTNG1,UST,CAPRIN2,NCKAP1,BOC,ZDHHC15,MYPN,SYT1,LZTS1,BCL11B,CYFIP2,SIAH1,IL1RAPL1,CAMK2B,SEMA5B,SGK1,KEL,SLIT3,SLIT2,CORO1C,CTNND2,DISC1,KANK1,LAMC2,ITSN2,DCLK1,YTHDF1,CNTNAP2,ENAH,EPHA4,AUTS2,PPP3CA,MAG,CTNNB1,PARK2,IGF1R,XK,DRAXIN,NIN,CPNE6,DCC,PAX2,SPG11,CNTN6,NFIB,DNM3,CUX2,PTPRM,ABI2,SHANK1,SYT3,NEDD*

			4,NRP2,BTBD3,PRTG,APP,DIP2B,FSTL4,ISLR2,SPTBN4,CPNE9,PARP6,DSCAML1,SEMA6D,BMPR2,USP33,DPYSL2,NLGN1,CTNNA2,SPAG9,RAB11A,CRMP1,LRRC4C,SEMA4D,PLXNA2,DVL3,EPHB3,FBXO45,VCL,MYO9A,WNT3,NLGN3,PAFAH1B1,RASAL1,TRPC5,GLI2,NEO1,RUFY3,RELN,SIN3A,IQGAP1,MACF1,PACSIN1,PAK3,ADNP,MAP1B,EPHA5,MEF2A,FLRT2,ACTR2,ELAVL4,ROCK1,EGR2,NTRK1,MAP1A,ANK3,UNC13A,EPHB1,EPHA10,SARM1,PARD3,TRIM46,ARHGAP44,CUX1,EPHA7,MAP3K13,PTK7,BMPR1B,WNT7B,PTPRS,PTEN,BMP7,LRRK2,RAPH1,TMEM108,EFNA5,SHANK3,WNT7A,MYCBP2,SOS1,EXT1,RAPGEF2,SMO,SYT17,RHOA,SYNGAP1,GSK3B,EMB,EEF2K,PTK2,RIMS2,GAP43,TAOK2,ECE1,CD44,PACSIN2,RPS6KA5,TUBB3,EPHB2,PAX6,PRKCZ,ADORA2A,MAP2,FBXO31,PRKCQ,BRAF,IMPACT,TNR,CDKL3,SRGAP2,TMEFF1,ADCY1,NUMB,SLIT1,DVL2,GRIP1,GPM6A,SNX1,SEMA3C,NEDD4L,SH3GL2,NCAM1,CREB1,EDNRA,CPNE1*
GO:0031175	neuron projection development	2.3989330253712672e-33	*CLRN1,NRXN1,PRMT3,PRKCI,MAP4K4,LLPH,NEGR1,CLASP2,SEMA3A,GRID2,PRDM12,TENM3,SEMA3D,PHACTR1,MYOT,NREP,TIAM2,UNC5C,KALRN,NTRK3,SEMA5A,TOX,LRP2,OLFM1,NRXN3,ROBO1,CDKL5,KIF5C,ROBO2,NFASC,ABI1,ULK4,ITGB1,TRIOBP,HSP90AA1,GAS7,KNDC1,DSCAM,TRIO,DAB1,LAMA2,PTPN11,DOCK10,HDAC6,GOLGA4,EPHA1,LIMK1,ATP8A2,CUL4B,CECR2,GRM7,PTPRO,NTRK2,EP300,RIMS1,TNIK,KIRREL3,RERE,UNC5D,LAMA3,CDH4,PAK1,PTPRD,TBR1,STRC,BDNF,AMIGO1,DENND5A,DMD,RIT2,TANC2,FYN,PCDH15,LMTK2,FAT3,RTN4,APOD,B4GALT6,RTN4R,CCDC141,NDRG4,PAQR3,ADAMTSL1,NTN1,FRY,MAP2K1,VAX2,CNTN4,NTNG1,TMEM30A,ROR2,UST,CAPRIN2,NCKAP1,BOC,ZDHHC15,JAK2,MYPN,SYT1,LZTS1,BCL11B,PLS1,CYFIP2,FIG4,KREMEN1,SIAH1,IL1RAPL1,CAMK2B,SEMA5B,SGK1,KEL,SLIT3,SLIT2,CDH23,CTNND2,DISC1,KANK1,LAMC2,CLMN,ITSN2,DCLK1,YTHDF1,CNTNAP2,ENAH,EPHA4,AUTS2,CNR1,PPP3CA,MAG,CTNNB1,PARK2,IGF1R,DLG5,XK,DRAXIN,NIN,CPNE6,DCC,PAX2,SPG11,SFRP1,CNTN6,NFIB,DNM3,CUX2,PTPRM,ABI2,SHANK1,SYT3,NEDD4,NRP2,BTBD3,CRTAC1,PRTG,APP,DIP2B,FSTL4,NLGN2,PTPRG,ISLR2,SPTBN4,VASH2,MMP2,CPNE9,PRKD1,PARP6,DGKG,DSCAML1,SEMA6D,BMPR2,USP33,DPYSL2,CAMK1D,NLGN1,CTNNA2,SPAG9,RAB11A,CRMP1,LRRC4C,SEMA4D,PLXNA2,SPOCK1,BLOC1S5,DVL3,EPHB3,FBXO45,VCL,LAMB1,MYO9A,WNT3,NLGN3,PAFAH1B1,RASAL1,TRPC5,GLI2,NEO1,RUFY3,DAB2,RELN,SIN3A,IQGAP1,MACF1,MYO7A,ALK,MBOAT1,GPM6B,PRKG1,PACSIN1,ACTL6B,PAK3,ADNP,MAP1B,MARK1,EPHA5,MEF2A,FLRT2,ACTR2,ELAVL4,ROCK1,EGR2,NTRK1,MAP1A,PPP1R9A,ANK3,UNC13A,EPHB1,EPHA10,PTPN9,SARM1,PARD3,TRIM46,CARM1,ARHGAP44,CUX1,EPHA7,CTNNA1,MAP3K13,SDK1,PTK7,BMPR1B,MAGI2,WNT7B,PTPRS,RTN4RL1,CBFA2T2,DPYSL3,PTEN,BMP7,LHFPL5,LRRK2,RAPH1,TMEM108,EFNA5,SHANK3,STMN4,WNT7A,MAP4,MYCBP2,SOS1,TSHR,EXT1,RAPGEF2,SMO,BLOC1S3,SYT17,RHOA,SYNGAP1,GRIN3A,ROR1,GSK3B,EMB,EEF2K,AGT,HDGFRP3,BLOC1S6,PTK2,SNX3,RIMS2,GAP43,TAOK2,ECE1,RPS6KA5,TBC1D23,TUBB3,EPHB2,PAX6,PRKCZ,ADORA2A,KLHL1,MAP2,FBXO31,CNTN1,PRKCQ,BRAF,HDAC2,IMPACT,TNR,CDKL3,CAMSAP3,SRGAP2,TMEFF1,ADCY1,NUMB,SLIT1,DVL2,GRIP1,GPRIN3,GPM6A,DHFR,ASAP1,INPP5F,SEMA3C,NEDD4L,SH3GL2,NCAM1,CREB1,EDNRA,CPNE1*
GO:0050896	response to stimulus	2.411365338360672e-33	*CD247,CLRN1,RIC8B,ENPP1,PRDX2,GPC5,NRXN1,ASPH,F8,GSS,GP6,RDH13,PRKCI,MAP4K4,PDE4D,PDCL,DNMT1,S1PR2,HLX,TMBIM4,SLC9A1,PDE7B,C6ORF89,LY75,ADCY8,LMBR1,PDE8A,CLASP2,SEMA3A,IL31RA,GRID2,TAS1R2,RNASET2,RPS6KA2,PRDM12,SETD4,PTGFR,TENM3,CALR3,CHERP,DOCK1,WWC1,CLDN18,C12ORF49,KIF22,CTDSPL2,PMS1,FBXL2,RYR1,NRG3,ASH1L,NOS1AP,LATS2,ANP32A,SEMA3D,MYOT,PTH2R,NREP,TIAM2,KSR2,MAPRE2,NLGN4X,PTGIS,PRKAG2,WWC3,TJP1,GRM8,UNC5C,OR5P2,OR5P3,PRLR,MVP,PAGR1,ADCY7,FTO,RGS7BP,KALRN,PITPNC1,NTRK3,CBL,ARNT,ATRNL1,PLEKHG4B,EGLN2,MIA,RAB4B,RAB4B-EGLN2,SEMA5A,LRRFIP2,FLT3,RAD51B,SUSD4,STAT5B,ENPP2,GRM5,PLCE1,SAMHD1,FER,CASK,MARVELD3,MAPK4,MAP2K5,KITLG,MAPK10,PTPRR,SPNS2,LRP2,PIK3CD,NAALADL2,ZNF536,DEPDC5,HDGF,ZSWIM7,ANK2,OLFM1,SH2D1A,EZR,NRXN3,XRCC4,ROBO1,TOM1L1,TNFAIP8L1,CHD7,SLC26A6,HTR2B,ITGB6,MECOM,TENM1,CAMKMT,RYR3,KIF5C,LMNA,NUCB1,GOT1,LPO,NMT2,INPP4B,TRAF6,ROBO2,NFASC,ITPKB,DNAJA3,OXR1,SLC24A2,VAV2,GRK5,ABI1,ULK4,ERCC6L2,ITGB1,HSP90AA1,ELMO2,CDC6,PSMB7,TSC22D3,CNIH2,SRGAP3,MCC,TSSK1B,KNDC1,CCDC22,SLC22A8,DSCAM,STK38L,GMDS,SAMD12,CNGB1,TRIO,ARHGAP24,KLHL12,AKAP13,DLG2,PDE1A,PTPRK,DAB1,OMA1,CDC42EP3,MYO10,ABCC8,ERC1,HMGN3,NRIP1,RGS6,DGKI,DNAJB14,ANKS1B,THRB,STXBP4,PDE4A,GABRA3,ITGB3BP,LAMA2,GFRA2,MAST4,IL17RB,TCIRG1,DACH1,PTPN11,NETO2,HERC4,MGAT5,DOCK10,CYTH3,HDAC6,DDB1,ADORA1,MSRA,GPRASP1,ADAMTS3,EPHA1,IKBKB,ERBB4,ADRA1D,SORCS1,MRE11A,GBF1,LIMK1,TLK1,BRD8,ATP8A2,TEX264,ABR,HIF3A,SNIP1,CUL4B,KLHL6,ESR1,NPHP3,GRM7,MIER1,PMEPA1,MAML3,PTPRO,CDON,NTRK2,TNRC6A,EP300,CELA1,TENM2,RNF220,RGS22,CPNE4,FNTA,PDGFB,CACNA1B,RIMS1,TNIK,CCND3,CDH8,USP46,MID1,CHFR,ALOX5AP,BPI,STK39,DAP3,HNF4G,INSR,FMN2,PRKAR1A,ASB5,AFAP1,UNC5D,PLLP,LAMA3,TCF7L2,PIP4K2A,CDH4,HPS1,JMJD1C,CAB39,USP34,PAK1,LITAF,FBXW11,ESRRB,MYOM1,MAP3K4,ZDHHC13,PTPRD,EGLN3,SLC4A10,TBR1,STRC,BDNF,TFAP2A,ATRN,TTC4,AMIGO1,CDK14,FANCA,KCNMA1,CHRM3,TGIF2,FUT8,OR2T3,NPRL3,PHACTR4,TENM4,GRIK4,TRIM13,RASGEF1B,UBR2,HLCS,PKH*

			D1L1,ARHGAP6,LCK,MDM4,EDARADD,IL5RA,ENTPD5,SHC4,ECT2,TMC2,ZNF148,MYO3B,SNX6,CHML,VAMP7,PPM1L,ARHGAP42,CST2,PTPRU,RFTN1,ERN2,CDC45,LSP1,SHFM1,MB,AP3S1,NPLOC4,AFF3,SGCD,DMD,CENPF,ATG10,LRRN2,SLC30A9,CERS6,PDS5A,USP13,KAT7,SLC8A1,GSN,RBM14,RBM4,GPR171,SATB2,RIT2,MACROD2,DRG2,DEPDC1B,CASS4,KCTD16,FYN,MKRN2,ARNTL,ADAMTS9,NF1,NCF2,PLCB1,DST,MGMT,PCDH15,LMTK2,PPP4R2,RTN4,RXFP1,DPEP1,CHRDL1,DGKH,APOD,NEIL2,RTN4R,BCR,PPP1R12B,RWDD3,SHISA9,CHI3L1,CCDC141,TTN,NDRG4,BMP2K,PAQR3,PIK3R2,ADAMTSL1,RANBP9,NTN1,NOMO3,NRG1,ARHGAP10,SH2D3C,CDK19,BDKRB1,BDKRB2,DOCK8,BID,STRBP,MAP2K1,MDFI,FNIP1,RAPGEF6,STIM1,VAX2,RALGPS2,MYH9,MAD1L1,LRFN5,DAGLA,TMBIM6,VGLL4,PPP2CB,CNTN4,PPP3R1,VWA1,HDAC5,VSTM1,OR6N2,RNF4,CMKLR1,INPP4A,CDKL2,TSPAN8,ROR2,DCN,ZDHHC3,SLC39A14,SCFD1,DOCK2,CDH13,CREBRF,TRDMT1,SOX13,FHIT,WDR70,PTPRT,ENTPD1,ACSBG1,AUNIP,COPS5,FGF14,DMC1,IPO5,ARHGAP32,MAD2L2,MAN1A1,TLE6,RAB27A,RGS8,CAPRIN2,CCNYL1,NLN,NCKAP1,DEFA1B,DEFA3,F5,TERF2,PTGER2,RALGPS1,SLX1B,STIP1,FOXN3,RCC2,BOC,ANO6,ARL3,NEUROD1,GNAQ,FBXO9,ICK,TMEM117,RAD51D,RFFL,RANBP1,CCBE1,DENND1A,HERC5,JAK2,MYPN,YTHDC2,FAM168A,FBXW7,LINC00473,SKAP1,SMC3,UIMC1,SYT1,ITCH,MLIP,NPSR1,ZFYVE28,BCL11B,SMG1,TMC1,DTHD1,CLDN1,HUNK,B3GALT5,MLLT3,ARAP2,CDC42BPB,DOCK9,SH2D6,TCF7,PDE2A,KLF15,ANXA4,LAMTOR3,MAGI1,CYFIP2,WNT11,TPST1,ARHGAP23,IFT80,TRIM59,APBB1IP,MTA1,KREMEN1,IMMP2L,NRG4,WDFY4,CLPB,NOX4,LCOR,SIPA1L3,MKLN1,CACNA1H,SIAH1,IL1RAPL1,CCDC62,CAMK2B,ADAM23,ERCC8,PRKCD,SOX6,TAB2,ACVR2A,RUNX2,SEMA5B,CD4,PPM1E,TGFB1,PLCXD3,SGK1,SPSB4,NUP93,MYO3A,MMP28,IGSF1,PIBF1,ASCC2,BTRC,NFATC3,POLK,CRADD,DLGAP1,PTPRN2,F2RL1,BCAS3,C9ORF47,GRIK3,RPGRIP1L,DNAJB2,CELSR1,SLIT3,AAK1,ZFYVE9,SLIT2,ASB15,TP73,GABRR3,ITIH4,GABRB1,SYT13,HHAT,CDH23,GPR176,HP,HPR,CORO1C,RRAS2,CTNND2,C12ORF4,OR9A4,DISC1,KCNJ3,KANK1,CACNA1D,ZFAND2A,PTPDC1,LAMC2,MTDH,ANK1,TTBK2,XPR1,CHRM1,MMP26,OR51A7,OR51F2,OR51T1,SMAD6,RXFP2,CLOCK,TCF12,ZNF675,ANO3,PLCB4,BCKDHB,KCNH1,BMPER,TIMP2,BCL3,DCLK1,ANKRD54,DAPK2,TNFRSF10B,PARVA,DAPP1,ADAM12,DUSP22,GPR52,NAIP,TRRAP,ANKFN1,HOMER2,DOCK3,SBNO2,YTHDF1,FGF10,CNTNAP2,FBXL17,RASA4,RASA4B,ARHGEF28,SMYD3,KCTD10,ENAH,IL1RAPL2,PPL,IQCJ-SCHIP1,SH3RF3,LOXL3,RHPN2,MAST2,FANCI,GLP2R,ADCY2,CAPN3,SLC16A1,SMURF2,EPHA4,RORA,PRKCA,AUTS2,CNR1,CD6,TNFSF11,PPP3CA,NSUN2,HBE1,OR51B2,OR51B4,OR51I1,UBQLN3,GNG4,NFYB,MAG,CAMK4,UFL1,CTNNB1,PARK2,SOD2,FCGR2A,FCGR2B,FCGR3A,FCGR3B,HSPA6,ARHGAP22,SMARCC1,CDHR2,IGF1R,PPARG,AXIN1,GARNL3,MACROD1,OTUB1,DLG5,IL18R1,IL1RL1,MTF1,MSR1,CBX5,P2RY10,TRPM1,ANKRD17,CYBB,OTC,CASKIN1,APOL3,SCAMP5,BRIP1,SREBF2,TDP1,RYR2,DRAXIN,LEPR,LEPROT,FGF1,PROS1,RGS10,NR4A3,FOXK2,WDR45B,NOL3,DCT,PRKAR2A,SCARA3,MYOCD,OR52E6,OR52E8,OR52N1,TRIM5,PER2,KIR2DL1,KIR2DL3,KIR2DL4,KIR3DL1,KIR3DL2,AJUBA,CACNA1C,CPNE6,GLG1,CHEK2,UBQLN4,PRDM16,MYO1E,ASB8,CSMD1,PPP1CB,SPDYA,HCK,SORBS1,CAPN2,TBCK,TRIM8,DIO2,CSPG4,GRM1,DCC,ARHGAP31,P2RY14,CTDP1,BAZ1B,OBSCN,RGL1,NF2,FLT4,SGMS1,BICC1,HDAC4,PAX2,TNS3,CPOX,SPTBN1,TPTE,TRABD2B,SFRP1,ST8SIA1,FOXO3,DGKB,TPH2,TRHDE,ARL13B,CNTN6,NFIB,ARHGAP29,IFT122,SSH1,SYNCRIP,SMAD3,RNFT2,CUX2,ITPR1,PTPRM,WWP2,ARNT2,ACKR2,CYP4F11,WIPI1,RNF168,C1S,ABI2,GRIK5,PSAP,SHANK1,SYT3,IFNAR1,OR14K1,NEDD4,NRP2,ARHGEF18,VANGL1,PREX1,PLA2G4C,DOCK4,ESRRG,HOXD3,KPNB1,TAB1,SLC6A1,TXNDC12,NFATC1,RAB6C,SEC61B,KCNK1,PRTG,CDC73,APP,GSX2,PDGFRA,AOC2,FCRL4,NOX1,YAP1,HEG1,AMFR,RAB11FIP5,SESN1,CDK3,FSTL4,NLGN2,TNFRSF19,EYA2,RNLS,ALPL,JAK1,PDE6D,ANGPTL4,LRP5,MUC20,POLR3G,PTPRG,SLC9B2,GNG2,SOX2,SETD2,GPC6,CHST11,TEAD1,DEFB118,PRICKLE1,RCAN1,ECSIT,ZNF653,GRIA3,DRD1,TMEM14A,GLRA2,CHUK,ERLIN1,FRS2,PALB2,MMP2,SERGEF,TPH1,ESR2,S100A12,DCUN1D3,CPNE9,KDM6A,PRKD1,STAT1,ST18,DGKG,ETV5,RND3,SLC6A3,USP53,HNF4A,LRRC2,SHANK2,SHPRH,STRA6,VPS4A,EREG,CCNY,TCTN3,MAPKAPK3,MYOF,DSCAML1,SEMA6D,ATF2,POU2F2,GRAMD4,TMEM237,RAF1,CELF4,RASGRF2,EPHX1,ACOX2,CARD16,CASP1,PTPN1,ADAMTS12,POLA1,GGT7,BMPR2,USP33,DPYSL2,CAMK1D,BMPR1A,PABPC4,TSPAN12,NLGN1,COL16A1,CTNNA2,PIK3R3,SPAG9,MORC2,DENND4A,CRMP1,EVC,PDE11A,EPG5,MYB,FGF2,BACH1,GRIK1,OR6C70,PPM1F,TICRR,ADCY5,NEDD9,AGBL4,SEMA4D,NFX1,DOK6,HELQ,RORC,MCTP1,PLXNA2,PTAFR,ADCYAP1R1,SP140,JARID2,ANKDD1A,DDX58,DKK2,SORCS2,CHCHD6,ITGA11,PI4KA,RARB,SPEN,PIK3C2B,PRKCG,VIPR1,NCOA1,AREL1,BLOC1S5,EEF1E1,EEF1E1-BLOC1S5,GRIA2,GAS8,ALKBH3,ABCF3,CHRD,DVL3,ECE2,EIF2B5,EIF4G1,EPHB3,PTPRE,RBX1,ARHGAP39,ATF3,FBXO45,SHOC2,SRSF5,STT3B,PAWR,AGO3,ACAA1,DEPTOR,DLEC1,PKN3,FBXL20,MAML2,TSG101,CCDC3,VCL,TERF2IP,CLIC4,MYO9A,ANKS4B,IDE,WDR59,COL11A1,KCNQ1,RFX2,WNT3,FKBP5,RCE1,RHOJ,P4HB,CCL14,CCL15,CCL15-CCL14,SUFU,TG,NLGN3,PTPN13,SCUBE2,ZFYVE1,PAFAH1B1,PRDM15,FAM13A,POLE,CNTFR,COL4A3,TRPA1,DDAH1,TRPM3,NMUR2,HIP1,AKAP6,RASAL1,NR2C1,EDEM3,IRS4,ME1,GLI2,NEO1,TNKS,SCUBE1,ERCC1,PXDNL,GPR39,WDTC1,

			MGLL,C9,DAB2,THEMIS,BLM,PKHD1,USH2A,UBR1,LDLRAD4,DZIP1,WNK1,RELN,NEK10,SIN3A,RUVBL2,ABCB1,OR5H2,OR5H6,OR5K4,RSPO2,GUCY1A2,CSF3R,EYS,DOCK11,PELI1,SLC22A3,PPP2R5C,IQGAP1,SPATA18,SLC47A1,MAP3K7,DDX47,TAS2R41,ZNF423,SP1,OR56B1,TRIM22,MACF1,ARHGAP12,ALK,SLC8A2,GSTM3,INPP5D,STAU1,CLASP1,DSC2,EEPD1,TEAD4,SLC35A4,SRA1,TOP1,UBE2V1,CLIC5,XDH,LTBP1,OVOL2,SNX5,NFATC2,RBBP6,CHD1L,OR6C74,PRKG1,RGS7,ATP2B4,PACRG,APEX2,PAK3,MASP1,ZPLD1,CAMTA1,DYX1C1,P2RX6,ASGR2,ADNP,GPR161,MAPKAPK2,MAP1B,CDC42BPA,COL4A6,SCARB1,MARK1,CDK6,ATP6V0A2,CELF1,P2RY8,TYR,RNF34,TFRC,UVRAG,EPHA5,SORCS3,WWOX,GABRG3,MEF2A,SYT12,CR1L,ADCY9,CACNA1A,NFRKB,DCUN1D5,DROSHA,FNBP1,GFRAL,GPC3,SGIP1,XRN1,ADRBK1,TBC1D5,PSMB2,ZNF207,EYA1,PTGER3,BRINP1,GNB3,SHPK,TRPV1,RNF216,TSPAN6,TFEB,CLEC16A,GHR,MNAT1,FLRT2,FGD1,RPGR,ACTR2,ACACA,ELAVL4,HDAC1,INVS,RECQL5,C2,CFB,SMOC2,TRIM24,ADIPOR2,PLCG2,ROCK1,EPS8,C1QTNF1,PAXIP1,EGR2,HIPK3,RNF10,GAPVD1,CAPN10,GPR35,CUL2,ABCC1,AP3B1,PTPN2,INSRR,NTRK1,CRISP3,KPNA6,TRIM44,ZFAND1,PKN2,PPP1R9A,SLC39A10,EIF3A,ANK3,DNMBP,ACO2,UNC13A,PTPRQ,EPHB1,MAN1B1,PDE6A,EPHA10,AXIN2,SARM1,TMEM199,EPCAM,PARD3,RCAN2,MUC1,TUB,SH3GL3,JAG2,CACNG8,AP3D1,CARM1,VWF,NETO1,ARHGAP44,SRD5A2,CD84,COCH,HTR1D,ARHGEF3,HMCN1,RAD51C,SPI1,DGKK,EPHA7,CHRNB4,MCU,RANBP10,PTPRA,CTNNA1,AIFM2,FBXW4,PSPC1,ACAP2,TRDN,ATG14,GABRA6,LRRC8C,LRRC8D,LY86,STAC,BCAR3,ILDR2,SHROOM3,STK38,LAIR1,RBPMS,CHURC1,MAP3K13,OTUD3,RAB15,RAPGEF3,SDK1,PDXP,SH3BP1,MST1,ERCC3,PTK7,RSRC1,CHID1,SNCA,BMPR1B,POLN,CACNG2,CD53,GRM4,MAGI2,PNPT1,USP50,ZEB2,SYT9,HEY2,RC3H1,RNF103,RNF103-CHMP3,CNGA3,RNF19B,WNT7B,HMGCS2,PTPRS,ZMYND11,KMT2D,PRKAG1,CREM,SULT2A1,TRIP12,DFFA,SPPL2A,ADTRP,ABCA12,GNG7,CSRP1,PLD1,RASA1,MACC1,MITF,SRSF6,OPRD1,CABIN1,HIPK1,NUGGC,RPTOR,RTN4RL1,BTK,SIGLEC9,DSG1,VPS41,ZNRF3,KCTD13,TROVE2,CBFA2T2,IGF1,STX8,HTT,DPYSL3,MAPK1,PTEN,ARHGEF17,MIB1,SPRED2,IFFO1,BMP7,PIK3C3,PUM1,RASA2,SOX5,LHFPL5,PCBP2,PRCP,RAB30,LRRK2,ATP6V1A,MLYCD,OSGIN1,GNA14,ZRANB1,UNC119,ZBTB20,CHMP5,CNIH3,RAPH1,RFC3,BAG6,CHAF1B,LILRB4,IFT81,TMCO1,TMEM108,IPCEF1,ITGAM,ARHGAP25,IL11RA,MSRB3,NSG2,PKD2,EFNA5,HSF2BP,SHANK3,SEL1L2,NT5E,BTBD11,RGS9,AOX1,PDGFC,SERPINA3,AMOTL1,FAM13B,NCOA3,MTMR3,CUEDC2,PPP2R3C,WNT7A,ZBED6,SNAP23,NLRP1,RPS6KC1,UACA,CRTC3,GPR141,NME8,ARNTL2,GLDC,PLCL2,KAT6A,GPR21,RABEP1,RHOT1,SIK3,BMP6,MYCBP2,NFE2L1,ANO1,GPI,IL17RD,SH3RF2,SOS1,TSHR,EXT1,N4BP1,PDCD1LG2,ATRX,PLIN2,PRICKLE2,SERINC5,GABRR2,PNPLA3,PRMT2,RAPGEF2,ASCC1,ABCG2,KCNC2,SMO,SLC6A14,VRK3,ENPP3,GET4,BLOC1S3,MARK4,PRKAR2B,CPT1A,MYLPF,PSEN2,TNFRSF11B,PAG1,SYT17,SOX30,TFE3,UBR5,GRB14,GRIN1,JADE1,KCTD8,BBS9,RHOA,SPRTN,WSB2,OR4M1,OR4N2,SYNGAP1,VIPR2,GRIN3A,MCM3,ROR1,RQCD1,TF,ONECUT2,RHOBTB1,FAM19A4,CUL3,TFEC,HSPD1,ITPR2,NFKBID,EPM2A,GABRB3,GSK3B,ABCG8,PLD2,DNAJC3,EMB,GNG12,RDX,STAT6,ACTA2,MLF1,EEF2K,FAM49B,RBBP8,NECAB2,ALAS2,STK32B,CD27,DUSP26,ITGBL1,MCTP2,SCN9A,BOK,SULF1,KRT8,NACC2,RNF43,TNFRSF8,AGT,HDGFRP3,POLH,PRKAA2,SYT7,ADAR,BLOC1S6,FBN2,PITPNM2,STAT2,ZMPSTE24,BRINP3,HRH4,NDRG2,PTK2,LAT2,PEX5L,PHEX,RNF121,SNX3,PDE4B,RIMS2,TCP11,TEC,EXOSC3,SHB,VEPH1,GAP43,MAP3K5,CPE,CREBBP,IQGAP2,PRKD3,APCDD1L,DLX1,FMOD,INSL6,PCSK6,NCF1,SLC29A1,TAOK2,UGT1A1,UGT1A10,UGT1A3,UGT1A4,UGT1A5,UGT1A6,UGT1A7,UGT1A8,UGT1A9,PYGO2,SHC1,NDUFS2,ARHGAP15,CD160,ECE1,LBX2,PARP10,RNFT1,RPS6KB1,STC2,GPR173,GUCY2F,PRG3,SCEL,ANKS1A,ITGAL,ALOX5,CPLX2,DNAJB6,CD44,FGD3,ADAM8,CLDN4,RPS6KA5,BCL2L14,CDS1,MUC7,CSNK1A1,GRID1,HUS1,PKD1L1,CXCL17,FCRL6,FGD4,NOS1,GCKR,PLEKHA1,PLVAP,RGS14,SIPA1L2,ACTN4,ARHGAP11A,MC1R,SCNN1B,STK4,TUBB3,ZFAND6,MARK3,SPEF1,EPHB2,SLC1A1,WLS,MARCH8,SIGMAR1,TMEFF2,XCR1,IL18,TEX12,DTNA,MFSD8,TPMT,UBE2K,GPSM2,DNAJC7,HCAR1,HCAR2,HCAR3,DLC1,PPARA,PPP1R10,ABCD3,ARID5B,CALCRL,MCOLN1,EPB41L4B,SLC25A33,SMARCAL1,TAF2,PHLPP1,MAGI3,CPEB4,GPSM3,MOB3B,NOTCH4,SYTL4,ZNF366,CRX,CYP2C18,CYP2C19,FBN1,PAX6,PRKCZ,ZC3HAV1,ATP1A3,CD96,FAM20C,ADORA2A,GRIN2B,PPP1CC,RABGEF1,CPEB1,PHLDB2,PLCXD2,TYK2,OTUD7B,DENND4B,KIN,NIPBL,TMIGD2,CNRIP1,ASPN,ANGPT1,TBL1X,EYA3,FHL2,FRMPD1,LRRK1,OSBPL8,ATF6,GCNT2,ETS1,PPP1CA,TBC1D10C,VWC2,ARHGAP21,CFI,GRIA4,MTF2,RBMS3,BIN2,CCL22,BTN3A2,FBXO31,CNTN1,NCOR2,PRKCQ,BRAF,CSRNP1,DIAPH1,HDAC2,HTR2C,IMPACT,SPINT2,TNR,UBE2E2,CD300A,IRAK1BP1,PHIP,PPP2CA,ANGPT4,ARHGEF6,MAOA,RPL23,SH3BP4,RFC5,DCDC1,SP100,OPN1LW,REV3L,TRIM29,KCND2,PIGU,C1QTNF9,CR2,DONSON,FCRL2,ITSN1,SRGAP2,TMEFF1,WIPI2,ADCY1,ALG2,MADD,SQLE,SYK,CNOT1,WWTR1,ARHGAP19,GRIK2,GTF2H5,SCN1A,SLIT1,ASB1,CIT,RADIL,ZBTB38,BRCA2,HRK,RAP1GAP2,CACNG3,DVL2,MORC1,YBX1,GRIP1,PTPRJ,SLC10A1,TAF1,TICAM1,TANK,GPM6A,UCN2,ITGAE,TAC3,A3GALT2,CADM1,HSF1,MAX,DHFR,INPP5A,SRPK2,ANKRD13C,PLEKHM1,SASH1,NCOA2,ADAMTS7,RALGAPA1,GBP5,MCM8,BMP1,CGNL1,FCHO1,QRICH1,VAV3,ZP3,CHD6,DLGAP2,CIB2,MOB3A,CAV2,GNA12,SH2D3A,UGGT1,COL4A5,MYEF2,INPP5F,NUAK2,AXL,REPS2,TNFSF9,WIF1,CD109,DENND4C,MET,CAMK2D,CCR3,RAD9B,TIMELESS,UCHL5,ANK

			RD6,IFI44L,MALT1,PFKP,TRA2B,ZDHHC11,INO80C,MLXIPL,SEMA3C,THEM4,NEDD4L,PHB,PTPRC,EDA,GPR89A,EXD2,PBLD,SH3GL2,TYRO3,NR6A1,RNF213,SPON2,TSPAN5,ADCY10,NCAM1,TANC1,TRAF3IP2,TRIM60,ATF6B,CREB1,LIG1,RASSF8,RGMB,SGTB,SHISA6,TRAP1,CLNK,EDNRA,GNGT1,HADHA,PRAP1,SLC25A13,BMP4,CASP12,CPNE1
GO:0048812	neuron projection morphogenesis	4.610860855348641e-33	*NRXN1,PRMT3,MAP4K4,LLPH,CLASP2,SEMA3A,SEMA3D,PHACTR1,MYOT,TIAM2,UNC5C,KALRN,SEMA5A,LRP2,OLFM1,NRXN3,ROBO1,CDKL5,KIF5C,ROBO2,NFASC,ABI1,ITGB1,HSP90AA1,GAS7,KNDC1,DSCAM,TRIO,DAB1,LAMA2,PTPN11,DOCK10,HDAC6,GOLGA4,EPHA1,LIMK1,ATP8A2,PTPRO,NTRK2,RIMS1,TNIK,KIRREL3,RERE,UNC5D,LAMA3,CDH4,PAK1,PTPRD,TBR1,BDNF,AMIGO1,DMD,TANC2,FYN,LMTK2,RTN4,B4GALT6,RTN4R,CCDC141,ADAMTSL1,NTN1,MAP2K1,VAX2,CNTN4,NTNG1,UST,CAPRIN2,NCKAP1,BOC,ZDHHC15,MYPN,SYT1,LZTS1,BCL11B,CYFIP2,SIAH1,IL1RAPL1,CAMK2B,SEMA5B,SGK1,KEL,SLIT3,SLIT2,CTNND2,DISC1,LAMC2,ITSN2,DCLK1,YTHDF1,CNTNAP2,ENAH,EPHA4,AUTS2,PPP3CA,MAG,CTNNB1,PARK2,IGF1R,XK,DRAXIN,NIN,CPNE6,DCC,PAX2,SPG11,CNTN6,NFIB,DNM3,CUX2,PTPRM,ABI2,SHANK1,SYT3,NEDD4,NRP2,BTBD3,PRTG,APP,DIP2B,FSTL4,ISLR2,SPTBN4,CPNE9,PARP6,DSCAML1,SEMA6D,BMPR2,USP33,DPYSL2,NLGN1,CTNNA2,SPAG9,RAB11A,CRMP1,LRRC4C,SEMA4D,PLXNA2,DVL3,EPHB3,FBXO45,VCL,MYO9A,WNT3,NLGN3,PAFAH1B1,RASAL1,TRPC5,GLI2,NEO1,RUFY3,RELN,SIN3A,IQGAP1,MACF1,PACSIN1,PAK3,ADNP,MAP1B,EPHA5,MEF2A,FLRT2,ACTR2,ELAVL4,ROCK1,EGR2,NTRK1,MAP1A,ANK3,UNC13A,EPHB1,EPHA10,SARM1,PARD3,TRIM46,ARHGAP44,CUX1,EPHA7,MAP3K13,PTK7,BMPR1B,WNT7B,PTPRS,PTEN,BMP7,LRRK2,RAPH1,TMEM108,EFNA5,SHANK3,WNT7A,MYCBP2,SOS1,EXT1,RAPGEF2,SMO,SYT17,RHOA,SYNGAP1,GSK3B,EMB,EEF2K,PTK2,RIMS2,GAP43,TAOK2,ECE1,RPS6KA5,TUBB3,EPHB2,PAX6,PRKCZ,ADORA2A,MAP2,FBXO31,PRKCQ,BRAF,IMPACT,TNR,CDKL3,SRGAP2,TMEFF1,ADCY1,NUMB,SLIT1,DVL2,GRIP1,GPM6A,SEMA3C,NEDD4L,SH3GL2,NCAM1,CREB1,EDNRA,CPNE1*
GO:0048666	neuron development	5.154869252684276e-33	*CLRN1,NRXN1,PRMT3,RDH13,PRKCI,MAP4K4,LLPH,NEGR1,PBX3,CLASP2,SEMA3A,GRID2,PRDM12,TENM3,SEMA3D,PHACTR1,MYOT,NREP,TIAM2,UNC5C,KALRN,NTRK3,SEMA5A,TOX,LRP2,OLFM1,NRXN3,ROBO1,CDKL5,TENM1,KIF5C,ROBO2,NFASC,ABI1,ULK4,ITGB1,TRIOBP,HSP90AA1,GAS7,KNDC1,ZC4H2,DSCAM,CNGB1,TRIO,RUNX1,DAB1,THRB,LAMA2,PTPN11,DOCK10,HDAC6,GOLGA4,EPHA1,LIMK1,ATP8A2,CUL4B,CECR2,GRM7,PTPRO,NTRK2,EP300,TENM2,RNF220,RIMS1,TNIK,KIRREL3,RERE,ATP8B1,UNC5D,LAMA3,CDH4,PAK1,PTPRD,SLC4A10,TBR1,STRC,BDNF,AMIGO1,TENM4,DENND5A,DMD,RIT2,TANC2,FYN,PCDH15,LMTK2,FAT3,RTN4,APOD,B4GALT6,RTN4R,CCDC141,NDRG4,PAQR3,ADAMTSL1,NTN1,FRY,MAP2K1,VAX2,CNTN4,NTNG1,TMEM30A,ROR2,UST,CAPRIN2,NCKAP1,BOC,ARL3,NEUROD1,ZDHHC15,JAK2,MYPN,SYT1,LZTS1,BCL11B,TMC1,PLS1,CYFIP2,FIG4,KREMEN1,SIAH1,IL1RAPL1,CAMK2B,SEMA5B,SGK1,KEL,RPGRIP1L,SLIT3,SLIT2,GABRB1,CDH23,CTNND2,DISC1,KANK1,LAMC2,CLMN,MYT1L,OPCML,ITSN2,DCLK1,YTHDF1,CNTNAP2,ENAH,EPHA4,AUTS2,CNR1,PPP3CA,MAG,CTNNB1,PARK2,SOD2,IGF1R,DLG5,XK,DRAXIN,NIN,CPNE6,DCC,PAX2,SPG11,SFRP1,CNTN6,NFIB,DNM3,CUX2,PTPRM,ABI2,SHANK1,SYT3,NEDD4,NRP2,BTBD3,CRTAC1,PRTG,APP,DIP2B,FSTL4,NLGN2,PTPRG,ISLR2,SPTBN4,VASH2,DRD1,MMP2,CPNE9,PRKD1,PARP6,DGKG,DSCAML1,SEMA6D,SRRM4,BMPR2,USP33,DPYSL2,CAMK1D,NLGN1,CTNNA2,SPAG9,RAB11A,CRMP1,LRRC4C,AGBL4,SEMA4D,PLXNA2,SPOCK1,BLOC1S5,NTM,DVL3,EPHB3,FBXO45,VCL,LAMB1,MYO9A,KCNQ1,WNT3,NLGN3,PAFAH1B1,RASAL1,TRPC5,GLI2,NEO1,RUFY3,DAB2,WNK1,RELN,SIN3A,IQGAP1,MACF1,MYO7A,ALK,MBOAT1,GPM6B,PRKG1,PACSIN1,ACTL6B,PAK3,ADNP,MAP1B,MARK1,EPHA5,MEF2A,FLRT2,ACTR2,ELAVL4,LGI4,ROCK1,EGR2,NTRK1,MAP1A,PPP1R9A,ANK3,UNC13A,PTPRQ,EPHB1,EPHA10,PTPN9,SARM1,PARD3,TRIM46,CARM1,ARHGAP44,CUX1,EPHA7,CTNNA1,MAP3K13,SDK1,PTK7,BMPR1B,MAGI2,WNT7B,PTPRS,RTN4RL1,CBFA2T2,DPYSL3,PTEN,BMP7,LHFPL5,LRRK2,RAPH1,TMEM108,EFNA5,SHANK3,STMN4,TBCD,WNT7A,MAP4,MYCBP2,SOS1,TSHR,EXT1,RAPGEF2,SMO,BLOC1S3,SYT17,RHOA,SYNGAP1,GRIN3A,ROR1,ONECUT2,GSK3B,EMB,SYN1,EEF2K,AGT,HDGFRP3,BLOC1S6,PTK2,SNX3,RIMS2,PBX1,GAP43,TAOK2,ECE1,ANKS1A,RPS6KA5,TBC1D23,TUBB3,EPHB2,MFSD8,PAX6,PRKCZ,ADORA2A,KLHL1,MAP2,FBXO31,CNTN1,PRKCQ,BRAF,HDAC2,IMPACT,TNR,CDKL3,CAMSAP3,SRGAP2,TMEFF1,ADCY1,NUMB,SLIT1,DVL2,GRIP1,GPRIN3,GPM6A,DHFR,ASAP1,INPP5F,SEMA3C,NEDD4L,SH3GL2,NCAM1,CREB1,EDNRA,GNGT1,CPNE1*
GO:0048468	cell development	9.865867175843609e-33	*CLRN1,NRXN1,PRMT3,RDH13,PRKCI,MOV10L1,MAP4K4,PDE4D,S1PR2,LLPH,NEGR1,PBX3,SPAG16,CBFB,CLASP2,SEMA3A,GRID2,RPS6KA2,PRDM12,TENM3,DOCK1,CLDN18,RYR1,IQCG,SEMA3D,PHACTR1,MYOT,NREP,TIAM2,FHOD3,TJP1,UNC5C,KALRN,NTRK3,ATRNL1,SEMA5A,TOX,GRM5,FER,KITLG,LRP2,ANK2,OLFM1,EZR,MEGF10,NRXN3,ROBO1,CHD7,SLC26A6,HTR2B,CDKL5,TENM1,KIF5C,LMNA,TRAF6,ROBO2,NFASC,ABI1,ULK4,ITGB1,TRIOBP,HSP90AA1,GAS7,TSSK1B,KNDC1,ZC4H2,DSCAM,CNGB1,TRIO,KLHL12,AKAP13,RUNX1,DAB1,ABCC8,THRB,LAMA2,PTPN11,DOCK10,HDAC6,GOLGA4,EPHA1,IKBKB,ERBB4,DPY19L2,LIMK1,ATP8A2,CUL4B,CECR2,ESR1,MYO18B,GRM7,PTPRO,NTRK2,EP300,MEGF9,TENM2,RNF220,HOOK3,PDGFB,RIMS1,TNIK,KIRREL3,FMN2,RERE,PRKAR1A,ATP8B1,UNC5D,LAMA3,PIP4K2A,CDH4,PAK1,FBXW11,PTPRD,SLC4A10,TBR1,CATSP*

			ER2,STRC,BDNF,ATRN,AMIGO1,PHACTR4,MEIS1,TENM4,LAMC1,SGCD,DENND5A,DMD,SLC8A1,SATB2,RIT2,TANC2,CASS4,FYN,ARNTL,NF1,PLCB1,PCDH15,LMTK2,ARID4B,FAT3,RTN4,APOD,B4GALT6,RTN4R,CCDC141,TTN,NDRG4,PAQR3,ADAMTSL1,NTN1,SLC4A5,NRG1,STRBP,FRY,MAP2K1,VAX2,MYH9,CNTN4,NTNG1,TMEM30A,ROR2,CATSPER3,UST,DMC1,CAPRIN2,NCKAP1,RCC2,BOC,ARL3,NEUROD1,ZDHHC15,JAK2,MYPN,YTHDC2,FBXW7,SYT1,LZTS1,BCL11B,TMC1,CLDN1,PLS1,PDE2A,MSI2,CYFIP2,FIG4,KREMEN1,SIPA1L3,SIAH1,QKI,IL1RAPL1,CAMK2B,RUNX2,SEMA5B,TGFB1,SGK1,KEL,F2RL1,C9ORF47,RPGRIP1L,SLIT3,SLIT2,TP73,GABRB1,CDH23,CORO1C,CTNND2,DISC1,KANK1,LRRC10,LAMC2,CLMN,MYT1L,OPCML,CLOCK,ITSN2,DCLK1,PARVA,YTHDF1,CNTNAP2,SMYD3,ENAH,CAPN3,EPHA4,AUTS2,CNR1,TNFSF11,PPP3CA,NSUN2,MAG,UFL1,CTNNB1,PARK2,SOD2,IGF1R,PPARG,DLG5,BFSP1,XK,BRIP1,DRAXIN,NIN,DCT,PER2,CPNE6,MYO1E,DCC,CTDP1,OBSCN,NF2,HDAC4,PAX2,SPG11,SFRP1,FOXO3,ARL13B,CNTN6,NFIB,DNM3,SMAD3,CUX2,PTPRM,ABI2,SHANK1,SYT3,NEDD4,NRP2,BTBD3,PREX1,CRTAC1,PRTG,APP,GSX2,PDGFRA,DIP2B,YAP1,HEG1,FSTL4,NLGN2,LRP5,PTPRG,ISLR2,SLC9B2,SETD2,CHST11,RCAN1,SPTBN4,VASH2,PAQR5,DRD1,FRS2,MMP2,CPNE9,PRKD1,PARP6,DGKG,HNF4A,EREG,MYOF,DSCAML1,SEMA6D,POU2F2,CELF4,SRRM4,BMPR2,USP33,DPYSL2,CAMK1D,BMPR1A,PABPC4,NLGN1,CTNNA2,SPAG9,RAB11A,CRMP1,FGF2,LRRC4C,NEDD9,AGBL4,PEAK1,SEMA4D,ARMC2,PLXNA2,FNDC3A,RILPL1,RARB,SPEN,SPOCK1,BLOC1S5,NTM,DVL3,EIF2B5,EPHB3,FBXO45,VCL,LAMB1,CLIC4,MYO9A,COL11A1,KCNQ1,RFX2,WNT3,P4HB,NLGN3,PAFAH1B1,CCDC169-SOHLH2,SOHLH2,AKAP6,RASAL1,TRPC5,GLI2,NEO1,ERCC1,RUFY3,DAB2,PKHD1,USH2A,DZIP1,WNK1,RELN,SIN3A,IQGAP1,MACF1,MYO7A,ALK,GSTM3,MBOAT1,GPM6B,OVOL2,NFATC2,PRKG1,BNC1,PACRG,GALNTL5,PACSIN1,ACTL6B,PAK3,ADNP,ARID4A,MAP1B,MARK1,CDK6,CELF1,EPHA5,MEF2A,BRINP1,TRPV1,HOXB3,FLRT2,DIAPH2,ACTR2,ELAVL4,LGI4,HDAC1,ROCK1,EGR2,RNF10,NTRK1,MAP1A,PPP1R9A,ANK3,UNC13A,PTPRQ,EPHB1,EPHA10,PTPN9,AXIN2,SARM1,PARD3,TRIM46,CARM1,ARHGAP44,SMARCA2,CUX1,SPI1,EPHA7,CTNNA1,SHROOM3,MAP3K13,RAPGEF3,SDK1,PTK7,BMPR1B,MAGI2,OCA2,HEY2,HYDIN,WNT7B,PTPRS,KMT2D,ABCA12,CSRP1,RTN4RL1,BTK,SGCZ,CBFA2T2,IGF1,DPYSL3,MAPK1,PTEN,BMP7,LHFPL5,LRRK2,RAPH1,TMEM108,EFNA5,SHANK3,STMN4,DAZL,ALPK3,TBCD,WNT7A,MAP4,BMP6,MYCBP2,SOS1,TSHR,EXT1,ATRX,PARVB,PRMT2,RAPGEF2,SMO,BLOC1S3,SYT17,BPGM,SOX30,RHOA,SYNGAP1,GRIN3A,ROR1,ONECUT2,CUL3,GSK3B,EMB,RDX,ACTA2,SYN1,EEF2K,ALAS2,SULF1,KRT8,AGT,HDGFRP3,BLOC1S6,ZMPSTE24,PTK2,SNX3,RIMS2,PBX1,TCP11,GAP43,DLX1,TAOK2,PYGO2,ECE1,RPS6KB1,PGM5,ANKS1A,RPS6KA5,TBC1D23,RGS14,ACTN4,TUBB3,EPHB2,TMEFF2,MFSD8,PPARA,ARID5B,NOTCH4,FBN1,PAX6,PRKCZ,FAM20C,ADORA2A,PPP1CC,COL22A1,KLC3,KLHL1,FHL2,LRRK1,MAP2,NEBL,FBXO31,CNTN1,PRKCQ,BRAF,HDAC2,IMPACT,SPINT2,TNR,CDKL3,ACAN,CAMSAP3,ANXA2,SRGAP2,TMEFF1,ADCY1,NUMB,SLIT1,RADIL,BRCA2,DVL2,NEB,GRIP1,GPRIN3,GPM6A,OSBP2,DHFR,ASAP1,ZP3,RNF2,CAV2,INPP5F,AXL,MET,CCDC136,SEMA3C,NEDD4L,PTPRC,SH3GL2,TYRO3,NCAM1,CREB1,AFF4,EDNRA,GNGT1,BMP4,CPNE1
GO:0030154	cell differentiation	2.6947440733255574e-32	RDH14,CLRN1,ENPP1,PRDX2,NRXN1,PRMT3,RDH13,PRKCI,MOV10L1,MAP4K4,PDE4D,DNMT1,S1PR2,HLX,LLPH,SLC9A1,NEGR1,PBX3,SPAG16,TRPS1,CBFB,CLASP2,SEMA3A,IL31RA,GRID2,RPS6KA2,PRDM12,TENM3,CALR3,DOCK1,CLDN18,RYR1,NRG3,IQCG,ASTN2,SEMA3D,PHACTR1,MYOT,NREP,TIAM2,FHOD3,NLGN4X,TJP1,UNC5C,ZSWIM6,PRLR,HIVEP3,FTO,KALRN,WIPF3,SPRR2D,NTRK3,ARNT,ATRNL1,SEMA5A,FLT3,STAT5B,TOX,ATP2B2,GRM5,NOC3L,FER,SPATA5,KITLG,LRP2,PIK3CD,ZNF536,SP3,ANK2,OLFM1,EZR,MEGF10,NRXN3,ROBO1,CHD7,SLC26A6,HTR2B,ITGB6,CDKL5,MECOM,TENM1,KIF5C,LMNA,TRAF6,ESRP1,ROBO2,NFASC,ITPKB,DNAJA3,GRK5,ABI1,ULK4,NHLH1,ITGB1,TRIOBP,HSP90AA1,GAS7,PSMB7,TSSK1B,KNDC1,ZC4H2,DSCAM,CNGB1,TRIO,ARHGAP24,KLHL12,AKAP13,RUNX1,DAB1,ABCC8,THRB,LAMA2,TCIRG1,PTPN11,HERC4,DOCK10,HDAC6,GOLGA4,MYT1,EPHA1,IKBKB,ERBB4,DPY19L2,LIMK1,ZNF609,ATP8A2,CUL4B,CECR2,ESR1,MYO18B,NPHP3,GRM7,PTPRO,CDON,NTRK2,EP300,CELA1,MEGF9,TENM2,RNF220,HOOK3,PDGFB,RIMS1,TNIK,KIRREL3,TOB2,HNF4G,FMN2,RERE,PRKAR1A,ATP8B1,UNC5D,H2AFY2,LAMA3,TCF7L2,PIP4K2A,CDH4,PAK1,FBXW11,ESRRB,MAP3K4,PTPRD,BASP1,CATSPERB,SLC4A10,TBR1,CATSPER2,STRC,BDNF,TFAP2A,ATRN,AMIGO1,FANCA,TGIF2,POMGNT2,PHACTR4,MEIS1,TENM4,LCK,EDARADD,SHC4,ECT2,NAV2,LAMC1,PTPRU,MB,SGCD,DENND5A,DMD,CENPF,USP13,KAT7,WDR7,SLC8A1,RBM4,GPR171,SATB2,RIT2,HIRA,TANC2,CASS4,FYN,ARNTL,ADAMTS9,NF1,PLCB1,MGMT,PCDH15,LMTK2,ARID4B,FAT3,RTN4,RXFP1,CHRDL1,APOD,SLC2A14,B4GALT6,RTN4R,PLEKHM3,BCR,CCDC141,TTN,NDRG4,PAQR3,ADAMTSL1,TLL1,NTN1,SLC4A5,NRG1,STRBP,FRY,MAP2K1,MDFI,FNIP1,VAX2,MYH9,DAGLA,CNTN4,HDAC5,NTNG1,NAV1,TMEM30A,SYNE1,JDP2,CMKLR1,LRGUK,ROR2,SLC39A14,CATSPER3,DOCK2,SOX13,UST,DMC1,MAD2L2,RAB27A,CAPRIN2,NLN,NCKAP1,RCC2,BOC,ARL3,NEUROD1,KIF2A,FBXO9,ACTL8,PARP11,COL12A1,ZDHHC15,JAK2,MYPN,YTHDC2,TRAPPC9,FBXW7,SYT1,ITCH,BBS12,LZTS1,BCL11B,RBFOX1,TMC1,PKNOX1,CLDN1,MLLT3,PNPLA1,PLS1,TCF7,PDE2A,MSI2,SEPT7,KLF15,TBX15,ANXA4,CYFIP2,WNT11,IFT80,FIG4,KREMEN1,ASTN1,NOX4,SIPA1L3,CACNA1H,SIAH1,QKI,IL1RAPL1,CAMK2B,SOX6,ACVR

			2A,RUNX2,SEMA5B,CD4,TGFB1,SGK1,ZHX2,KEL,NFATC3,F2RL1,C9ORF47,RPGRIP1L,CELSR1,SLIT3,SDK2,SLIT2,TP73,MUSTN1,GABRB1,CDH23,CORO1C,RRAS2,CTNND2,DISC1,TSNAX,KANK1,LRRC10,LAMC2,CLMN,MYT1L,OPCML,FSTL5,CHRM1,SMAD6,CLOCK,ITSN2,TCF12,ZNF675,SMOC1,ETV6,NELL1,SUCO,KCNH1,BCL3,DCLK1,ANKRD54,PARVA,SND1,ADAM12,HNRNPC,SBNO2,YTHDF1,FGF10,CNTNAP2,FBXL17,ARHGEF28,SMYD3,ENAH,PPL,LOXL3,MAST2,CAPN3,EPHA4,RORA,PRKCA,AUTS2,CNR1,TNFSF11,PPP3CA,NSUN2,MAG,CAMK4,UFL1,TRAK1,CTNNB1,PARK2,SOD2,FCGR2B,ARHGAP22,CDHR2,IGF1R,PPARG,NGRN,AXIN1,DLG5,IL18R1,BFSP1,MSR1,ANKRD17,XK,BRIP1,ANXA13,ANKRD26,DRAXIN,LEPR,FGF1,NIN,NR4A3,DCT,MYOCD,PER2,CPNE6,GLG1,PHLDB1,PRDM16,MYO1E,HCK,CAPN2,DIO2,MRC2,CSPG4,DCC,CTDP1,OBSCN,NF2,FLT4,MEF2B,HDAC4,PAX2,PHF5A,SPG11,SFRP1,FOXO3,ARL13B,CNTN6,NFIB,DNM3,SYNCRIP,SMAD3,CUX2,PTPRM,NHS,CASZ1,ABI2,PSAP,SHANK1,SYT3,NEDD4,NRP2,BTBD3,PREX1,CRTAC1,HOXD3,NAV3,NFATC1,PRTG,CDC73,APP,SSBP3,GSX2,PDGFRA,ODF3,CEP85L,DIP2B,YAP1,HEG1,FSTL4,NLGN2,VDAC1,EYA2,ALPL,LRP5,PTPRG,BDH2,ISLR2,SLC9B2,SOX2,SETD2,CHST11,PRICKLE1,RCAN1,ELAVL3,SPTBN4,VASH2,ZNF521,PAQR5,DRD1,FRS2,SFMBT1,MMP2,TPH1,SYNE2,CPNE9,KDM6A,PRKD1,STAT1,PARP6,DGKG,ETV5,MORN2,HNF4A,ZBTB7C,EREG,MYOF,DSCAML1,SEMA6D,ATF2,POU2F2,TCF3,RAF1,CELF4,ADAMTS12,PSMD11,SRRM4,BMPR2,USP33,DPYSL2,CAMK1D,BMPR1A,PABPC4,NLGN1,CTNNA2,CDK12,TAF8,CERS3,SPAG9,RAB11A,CRMP1,MYB,FGF2,LRRC4C,POU6F2,ZFP41,NEDD9,AGBL4,PEAK1,SEMA4D,SH3PXD2A,ARMC2,RORC,ELP3,PLXNA2,ADCYAP1R1,FNDC3A,SETD1A,JARID2,RILPL1,BRDT,ITGA11,RARB,SPEN,NCOA1,SPOCK1,BLOC1S5,NTM,CHRD,DVL3,EIF2B5,EIF4G1,EPHB3,ATF3,FBXO45,EBF2,DLEC1,TSG101,CCDC3,VCL,LAMB1,CLIC4,MYO9A,ANKS4B,COL11A1,KCNQ1,RFX2,WNT3,P4HB,SUFU,NLGN3,SCUBE2,PAFAH1B1,CCDC169-SOHLH2,SOHLH2,HIP1,AKAP6,RASAL1,NR2C1,TRPC5,TTLL7,GLI2,NEO1,SCUBE1,ERCC1,RUFY3,ALDH6A1,TPD52,COL19A1,DAB2,THEMIS,PKHD1,USH2A,LDLRAD4,DZIP1,WNK1,RELN,SIN3A,RSPO2,CSF3R,DOCK11,IQGAP1,APOLD1,ZNF423,MACF1,MYO7A,ALK,GSTM3,INPP5D,CLASP1,TEAD4,SLC9C1,SRA1,UBE2V1,MBOAT1,GPM6B,ADIRF,XDH,OVOL2,NFATC2,PRKG1,BNC1,PACRG,GALNTL5,PACSIN1,ACTL6B,PAK3,TET1,DYX1C1,ADNP,ARID4A,MAP1B,MARK1,CDK6,CELF1,TFRC,EPHA5,WWOX,MEF2A,DROSHA,GPC3,PSMB2,EYA1,BRINP1,GNB3,TRPV1,HOXB3,HOXB4,HOXB5,GHR,FLRT2,DIAPH2,ACTR2,ELAVL4,LGI4,HDAC1,PLCG2,ROCK1,PAXIP1,EGR2,RNF10,AP3B1,PTPN2,NTRK1,MAP1A,PPP1R9A,ANK3,UNC13A,PTPRQ,EPHB1,EPHA10,PTPN9,AXIN2,SARM1,EPCAM,PARD3,TRIM46,SH3GL3,JAG2,AP3D1,CARM1,ARHGAP44,SRD5A2,SMARCA2,CUX1,SPI1,EPHA7,CTNNA1,GLIS1,LRRC8C,ILDR2,SHROOM3,MAP3K13,RAPGEF3,SDK1,SH3BP1,KAZN,ERCC3,PTK7,SMYD1,NHSL2,BMPR1B,CD53,MAGI2,USP42,ZEB2,FOXJ3,OCA2,HEY2,RC3H1,COL13A1,HYDIN,WNT7B,PTPRS,KMT2D,CREM,TLL2,ABCA12,CSRP1,RASA1,MITF,SRSF6,RBM45,HIPK1,PKP2,RTN4RL1,BTK,SGCZ,CBFA2T2,IGF1,DPYSL3,MAPK1,PTEN,MIB1,SPRED2,BMP7,PUM1,SOX5,LHFPL5,LRRK2,OSGIN1,SEPT6,ALDOC,RAPH1,BAG6,LILRB4,TMEM108,ITGAM,EFNA5,SHANK3,STMN4,DAZL,ALPK3,NCOA3,STEAP4,PPP2R3C,TBCD,WNT7A,ZBED6,MAP4,NME8,PLCL2,KAT6A,SH3PXD2B,BMP6,MYCBP2,IL17RD,SOS1,TSHR,EXT1,ATRX,IKZF1,PARVB,PNPLA3,PRMT2,RAPGEF2,SMO,SUN1,BLOC1S3,CPT1A,TCF4,SYT17,BPGM,SOX30,TFE3,BBS9,RHOA,SYNGAP1,GRIN3A,NDE1,ROR1,TF,ONECUT2,CUL3,NFKBID,EPM2A,GSK3B,EMB,RDX,STAT6,ACTA2,SYN1,MLF1,EEF2K,CDK13,ALAS2,CD27,BOK,SULF1,KRT8,AGT,HDGFRP3,ADAR,BLOC1S6,FBN2,ZMPSTE24,BRINP3,NDRG2,PTK2,TPPP2,SNX3,RIMS2,PBX1,TCP11,SHB,GAP43,MAP3K5,TCFL5,DLX1,TAOK2,PYGO2,NDUFS2,ECE1,LBX2,RPS6KB1,GPR173,PGM5,SCEL,ANKS1A,ALOX5,CPLX2,DNAJB6,MDGA2,SP7,ADAM8,RPS6KA5,CDS1,THSD7A,CXCL17,NOS1,PEX7,TBC1D23,PLEKHA1,RGS14,ACTN4,STK4,TUBB3,EPHB2,SLC1A1,TMEFF2,CLPTM1,IL18,MFSD8,ASF1B,PPARA,ARID5B,PRRC2C,NOTCH4,CRX,FBN1,PAX6,PRKCZ,FAM20C,ADORA2A,PPP1CC,COL22A1,PHLDB2,KLC3,KLHL1,NIPBL,ANGPT1,EYA3,FHL2,LRRK1,OSBPL8,GCNT2,ETS1,VWC2,MAP2,MTF2,NEBL,FBXO31,CDK5RAP1,CNTN1,PRKCQ,BRAF,HDAC2,HTR2C,IMPACT,POU3F3,NOTO,SPINT2,TNR,MBNL3,ELF2,CDKL3,PPP2CA,ACAN,CAMSAP3,ANXA2,PRMT7,CR2,SRGAP2,TMEFF1,ADCY1,NUMB,SYK,CNOT1,WWTR1,SLIT1,CIT,RADIL,BRCA2,DVL2,MORC1,NEB,YBX1,GRIP1,PTPRJ,GPRIN3,GPM6A,OSBP2,PLEKHB2,CADM1,HSF1,DHFR,SRPK2,ASAP1,ADAMTS7,SULT2B1,BMP1,ZP3,RNF2,CAV2,GNA12,MYEF2,INPP5F,AXL,TNFSF9,WIF1,CD109,MET,MALT1,SETD3,TMEM120B,CCDC136,EHD2,SEMA3C,NEDD4L,PHB,PTPRC,EDA,GPR89A,SH3GL2,TYRO3,VPS52,NCAM1,TANC1,TRAF3IP2,CREB1,AFF4,EDNRA,GNGT1,BMP4,CPNE1
GO:0032990	cell part morphogenesis	3.568483364954669e-32	*NRXN1,PRMT3,MAP4K4,LLPH,CLASP2,SEMA3A,SEMA3D,PHACTR1,MYOT,TIAM2,UNC5C,KALRN,SEMA5A,ENPP2,LRP2,OLFM1,NRXN3,ROBO1,CDKL5,KIF5C,ROBO2,NFASC,ABI1,ITGB1,HSP90AA1,GAS7,KNDC1,DSCAM,TRIO,DAB1,LAMA2,PTPN11,DOCK10,HDAC6,GOLGA4,EPHA1,LIMK1,ATP8A2,PTPRO,NTRK2,RIMS1,TNIK,SNX2,KIRREL3,RERE,UNC5D,LAMA3,CDH4,PAK1,PTPRD,TBR1,BDNF,AMIGO1,DMD,TANC2,FYN,LMTK2,RTN4,B4GALT6,RTN4R,CCDC141,ADAMTSL1,NTN1,MAP2K1,VAX2,CNTN4,NTNG1,UST,CAPRIN2,NCKAP1,BOC,ZDHHC15,MYPN,SYT1,LZTS1,BCL11B,CYFIP2,SIAH1,IL1RAPL1,CAMK2B,SEMA5B,SGK1,KEL,SLIT3,SLIT2,CORO1C,CTNND2,DISC1,KANK1,LAMC2,ITSN2,DCLK1,YTHDF1,CNTNAP2,E*

			NAH,EPHA4,AUTS2,PPP3CA,MAG,CTNNB1,PARK2,IGF1R,XK,DRAXIN,NIN,CPNE6,DCC,PAX2,SPG11,CNTN6,NFIB,DNM3,CUX2,PTPRM,ABI2,SHANK1,SYT3,NEDD4,NRP2,BTBD3,PRTG,APP,DIP2B,FSTL4,ISLR2,SPTBN4,CPNE9,PARP6,DSCAML1,SEMA6D,BMPR2,USP33,DPYSL2,NLGN1,CTNNA2,SPAG9,RAB11A,CRMP1,LRRC4C,SEMA4D,PLXNA2,DVL3,EPHB3,FBXO45,VCL,MYO9A,WNT3,NLGN3,PAFAH1B1,RASAL1,TRPC5,GLI2,NEO1,RUFY3,RELN,SIN3A,IQGAP1,MACF1,PACSIN1,PAK3,ADNP,MAP1B,EPHA5,MEF2A,FLRT2,ACTR2,ELAVL4,ROCK1,EGR2,NTRK1,MAP1A,ANK3,UNC13A,EPHB1,EPHA10,SARM1,PARD3,TRIM46,ARHGAP44,CUX1,EPHA7,MAP3K13,PTK7,BMPR1B,PNPT1,WNT7B,PTPRS,PTEN,BMP7,LRRK2,RAPH1,TMEM108,EFNA5,SHANK3,WNT7A,MYCBP2,SOS1,EXT1,RAPGEF2,SMO,SYT17,RHOA,SYNGAP1,GSK3B,EMB,EEF2K,PTK2,RIMS2,GAP43,TAOK2,ECE1,CD44,PACSIN2,RPS6KA5,TUBB3,EPHB2,PAX6,PRKCZ,ADORA2A,MAP2,FBXO31,PRKCQ,BRAF,IMPACT,TNR,CDKL3,SRGAP2,TMEFF1,ADCY1,NUMB,SLIT1,DVL2,GRIP1,GPM6A,SNX1,SEMA3C,NEDD4L,SH3GL2,NCAM1,CREB1,EDNRA,CPNE1
GO:0048699	generation of neurons	3.7485556565278417e-32	*CLRN1,NRXN1,PRMT3,RDH13,PRKCI,MAP4K4,LLPH,NEGR1,PBX3,CLASP2,SEMA3A,GRID2,PRDM12,TENM3,NRG3,ASTN2,SEMA3D,PHACTR1,MYOT,NREP,TIAM2,NLGN4X,UNC5C,ZSWIM6,KALRN,NTRK3,SEMA5A,TOX,ATP2B2,GRM5,LRP2,ZNF536,OLFM1,NRXN3,ROBO1,CHD7,CDKL5,TENM1,KIF5C,ESRP1,ROBO2,NFASC,ABI1,ULK4,ITGB1,TRIOBP,HSP90AA1,GAS7,KNDC1,ZC4H2,DSCAM,CNGB1,TRIO,RUNX1,DAB1,ABCC8,THRB,LAMA2,PTPN11,DOCK10,HDAC6,GOLGA4,EPHA1,ERBB4,LIMK1,ZNF609,ATP8A2,CUL4B,CECR2,GRM7,PTPRO,CDON,NTRK2,EP300,TENM2,RNF220,HOOK3,RIMS1,TNIK,KIRREL3,RERE,ATP8B1,UNC5D,LAMA3,CDH4,PAK1,PTPRD,SLC4A10,TBR1,STRC,BDNF,AMIGO1,TGIF2,POMGNT2,MEIS1,TENM4,ECT2,DENND5A,DMD,SATB2,RIT2,TANC2,FYN,ARNTL,NF1,PCDH15,LMTK2,FAT3,RTN4,APOD,B4GALT6,RTN4R,CCDC141,NDRG4,PAQR3,ADAMTSL1,NTN1,NRG1,FRY,MAP2K1,VAX2,DAGLA,CNTN4,NTNG1,NAV1,TMEM30A,ROR2,UST,CAPRIN2,NCKAP1,BOC,ARL3,NEUROD1,ZDHHC15,JAK2,MYPN,TRAPPC9,SYT1,LZTS1,BCL11B,TMC1,PLS1,CYFIP2,WNT11,FIG4,KREMEN1,ASTN1,SIAH1,IL1RAPL1,CAMK2B,RUNX2,SEMA5B,TGFB1,SGK1,ZHX2,KEL,RPGRIP1L,CELSR1,SLIT3,SDK2,SLIT2,TP73,GABRB1,CDH23,CTNND2,DISC1,KANK1,LAMC2,CLMN,MYT1L,OPCML,ITSN2,TCF12,DCLK1,YTHDF1,CNTNAP2,ENAH,EPHA4,RORA,AUTS2,CNR1,PPP3CA,MAG,UFL1,CTNNB1,PARK2,SOD2,IGF1R,PPARG,NGRN,DLG5,XK,DRAXIN,NIN,DCT,PER2,CPNE6,DCC,NF2,PAX2,SPG11,SFRP1,FOXO3,CNTN6,NFIB,DNM3,CUX2,PTPRM,CASZ1,ABI2,SHANK1,SYT3,NEDD4,NRP2,BTBD3,CRTAC1,HOXD3,PRTG,APP,GSX2,CEP85L,DIP2B,YAP1,FSTL4,NLGN2,PTPRG,ISLR2,SOX2,SPTBN4,VASH2,ZNF521,DRD1,FRS2,MMP2,CPNE9,PRKD1,PARP6,DGKG,DSCAML1,SEMA6D,TCF3,SRRM4,BMPR2,USP33,DPYSL2,CAMK1D,BMPR1A,NLGN1,CTNNA2,SPAG9,RAB11A,CRMP1,LRRC4C,AGBL4,SEMA4D,ELP3,PLXNA2,SPEN,NCOA1,SPOCK1,BLOC1S5,NTM,DVL3,EIF4G1,EPHB3,FBXO45,VCL,LAMB1,MYO9A,KCNQ1,WNT3,SUFU,NLGN3,PAFAH1B1,RASAL1,TRPC5,GLI2,NEO1,RUFY3,DAB2,USH2A,WNK1,RELN,SIN3A,RSPO2,IQGAP1,MACF1,MYO7A,ALK,MBOAT1,GPM6B,PRKG1,PACSIN1,ACTL6B,PAK3,DYX1C1,ADNP,MAP1B,MARK1,CDK6,EPHA5,MEF2A,EYA1,BRINP1,HOXB3,FLRT2,ACTR2,ELAVL4,LGI4,HDAC1,ROCK1,EGR2,RNF10,NTRK1,MAP1A,PPP1R9A,ANK3,UNC13A,PTPRQ,EPHB1,EPHA10,PTPN9,SARM1,PARD3,TRIM46,SH3GL3,JAG2,CARM1,ARHGAP44,CUX1,EPHA7,CTNNA1,MAP3K13,SDK1,ERCC3,PTK7,BMPR1B,MAGI2,HEY2,WNT7B,PTPRS,HIPK1,RTN4RL1,CBFA2T2,DPYSL3,PTEN,MIB1,BMP7,SOX5,LHFPL5,LRRK2,RAPH1,TMEM108,EFNA5,SHANK3,STMN4,TBCD,WNT7A,MAP4,BMP6,MYCBP2,SOS1,TSHR,EXT1,RAPGEF2,SMO,BLOC1S3,TCF4,SYT17,RHOA,SYNGAP1,GRIN3A,NDE1,ROR1,ONECUT2,GSK3B,EMB,SYN1,EEF2K,AGT,HDGFRP3,BLOC1S6,BRINP3,PTK2,SNX3,RIMS2,PBX1,GAP43,DLX1,TAOK2,ECE1,GPR173,ANKS1A,MDGA2,RPS6KA5,PEX7,TBC1D23,RGS14,TUBB3,EPHB2,MFSD8,PAX6,PRKCZ,ADORA2A,PPP1CC,KLHL1,NIPBL,VWC2,MAP2,FBXO31,CDK5RAP1,CNTN1,PRKCQ,BRAF,HDAC2,IMPACT,NOTO,TNR,CDKL3,CAMSAP3,SRGAP2,TMEFF1,ADCY1,NUMB,SLIT1,CIT,DVL2,GRIP1,GPRIN3,GPM6A,DHFR,ASAP1,MYEF2,INPP5F,AXL,MET,SEMA3C,NEDD4L,SH3GL2,NCAM1,CREB1,EDNRA,GNGT1,BMP4,CPNE1*
GO:0035556	intracellular signal transduction	5.036055204645524e-32	*PRDX2,NRXN1,ASPH,PRKCI,MAP4K4,PDE4D,DNMT1,TMBIM4,SLC9A1,PDE7B,ADCY8,PDE8A,SEMA3A,IL31RA,RPS6KA2,PTGFR,CHERP,DOCK1,WWC1,FBXL2,NRG3,ASH1L,NOS1AP,LATS2,ANP32A,TIAM2,KSR2,MAPRE2,PRKAG2,WWC3,ADCY7,KALRN,NTRK3,CBL,PLEKHG4B,RAB4B,RAB4B-EGLN2,SEMA5A,FLT3,GRM5,PLCE1,FER,MARVELD3,MAPK4,MAP2K5,KITLG,MAPK10,PTPRR,LRP2,PIK3CD,DEPDC5,ANK2,EZR,ROBO1,TNFAIP8L1,HTR2B,MECOM,TENM1,NUCB1,TRAF6,ITPKB,DNAJA3,VAV2,ULK4,ITGB1,HSP90AA1,CDC6,PSMB7,SRGAP3,TSSK1B,KNDC1,CCDC22,STK38L,TRIO,ARHGAP24,AKAP13,DAB1,CDC42EP3,ERC1,RGS6,DGKI,PDE4A,MAST4,PTPN11,DOCK10,CYTH3,ADORA1,IKBKB,ERBB4,ADRA1D,MRE11A,GBF1,LIMK1,TLK1,ABR,SNIP1,ESR1,MIER1,CDON,NTRK2,EP300,PDGFB,TNIK,MID1,CHFR,STK39,INSR,FMN2,PRKAR1A,ASB5,TCF7L2,CAB39,PAK1,LITAF,FBXW11,MYOM1,MAP3K4,ZDHHC13,CHRM3,NPRL3,PHACTR4,TRIM13,RASGEF1B,UBR2,ARHGAP6,LCK,MDM4,ENTPD5,SHC4,ECT2,CHML,PPM1L,ARHGAP42,ERN2,CDC45,NPLOC4,SGCD,DMD,CENPF,KAT7,SLC8A1,RIT2,DEPDC1B,CASS4,FYN,MKRN2,ARNTL,NF1,PLCB1,RTN4,DGKH,RTN4R,BCR,CHI3L1,TTN,NDRG4,PAQR3,PIK3R2,RANBP9,NTN1,NRG1,ARHGAP10,SH2D3C,BDKRB2,DOCK8,BID,MAP2K1,FNIP1,RAPGEF6,RALGPS2,MAD1L1,TMBIM6,VGL*

			L4,PPP2CB,PPP3R1,CMKLR1,ROR2,DCN,DOCK2,CDH13,FHIT,COPS5,FGF14,ARHGAP32,MAD2L2,NCKAP1,RALGPS1,FOXN3,ARL3,NEUROD1,FBXO9,ICK,TMEM117,RFFL,DENND1A,JAK2,FBXW7,LINC00473,UIMC1,ITCH,NPSR1,SMG1,HUNK,CDC42BPB,DOCK9,SH2D6,PDE2A,LAMTOR3,WNT11,ARHGAP23,TRIM59,CLPB,NOX4,SIPA1L3,SIAH1,PRKCD,TAB2,CD4,SGK1,SPSB4,BTRC,NFATC3,CRADD,F2RL1,CELSR1,SLIT2,ASB15,TP73,RRAS2,DISC1,KANK1,MTDH,CLOCK,ZNF675,PLCB4,KCNH1,BMPER,BCL3,DCLK1,ANKRD54,DAPK2,TNFRSF10B,DUSP22,NAIP,HOMER2,DOCK3,FGF10,RASA4,RASA4B,ARHGEF28,KCTD10,IQCJ-SCHIP1,SH3RF3,MAST2,ADCY2,CAPN3,EPHA4,RORA,PRKCA,AUTS2,TNFSF11,PPP3CA,NSUN2,CAMK4,UFL1,CTNNB1,PARK2,SOD2,FCGR2B,ARHGAP22,IGF1R,PPARG,AXIN1,GARNL3,DLG5,IL18R1,P2RY10,ANKRD17,APOL3,BRIP1,RYR2,FGF1,NOL3,PRKAR2A,TRIM5,AJUBA,CACNA1C,CHEK2,ASB8,PPP1CB,TBCK,TRIM8,CSPG4,GRM1,ARHGAP31,OBSCN,RGL1,NF2,FLT4,SGMS1,HDAC4,PAX2,TNS3,TPTE,SFRP1,FOXO3,DGKB,ARHGAP29,SMAD3,ITPR1,ACKR2,ABI2,NEDD4,ARHGEF18,PREX1,PLA2G4C,DOCK4,KPNB1,TAB1,TXNDC12,NFATC1,RAB6C,APP,PDGFRA,NOX1,YAP1,HEG1,SESN1,TNFRSF19,JAK1,SOX2,TEAD1,RCAN1,CHUK,FRS2,S100A12,PRKD1,STAT1,DGKG,RND3,SHANK2,VPS4A,MAPKAPK3,ATF2,RAF1,RASGRF2,CARD16,CASP1,PTPN1,NLGN1,PIK3R3,SPAG9,DENND4A,PDE11A,FGF2,PPM1F,TICRR,ADCY5,SEMA4D,DOK6,MCTP1,PTAFR,ADCYAP1R1,DDX58,PI4KA,PIK3C2B,PRKCG,EEF1E1,EEF1E1-BLOC1S5,DVL3,RBX1,ARHGAP39,ATF3,SHOC2,AGO3,DEPTOR,PKN3,TERF2IP,MYO9A,WDR59,RCE1,RHOJ,P4HB,CCL14,CCL15,PTPN13,PAFAH1B1,PRDM15,FAM13A,DDAH1,NMUR2,HIP1,AKAP6,RASAL1,DAB2,BLM,PKHD1,UBR1,WNK1,RELN,NEK10,GUCY1A2,DOCK11,PELI1,PPP2R5C,IQGAP1,MAP3K7,TRIM22,ARHGAP12,ALK,SLC8A2,INPP5D,TEAD4,UBE2V1,XDH,NFATC2,PRKG1,RGS7,ATP2B4,PAK3,CAMTA1,ADNP,MAPKAPK2,CDC42BPA,MARK1,P2RY8,RNF34,TFRC,EPHA5,WWOX,MEF2A,ADCY9,GFRAL,PSMB2,ZNF207,TSPAN6,CLEC16A,GHR,FGD1,HDAC1,TRIM24,PLCG2,ROCK1,EPS8,C1QTNF1,PAXIP1,HIPK3,GPR35,CUL2,PTPN2,NTRK1,TRIM44,PKN2,PPP1R9A,EIF3A,DNMBP,UNC13A,EPHB1,RCAN2,MUC1,VWF,ARHGAP44,ARHGEF3,SPI1,DGKK,EPHA7,MCU,STAC,BCAR3,STK38,MAP3K13,OTUD3,RAB15,RAPGEF3,SH3BP1,GRM4,MAGI2,USP50,RC3H1,WNT7B,ZMYND11,ADTRP,PLD1,RASA1,HIPK1,RPTOR,BTK,KCTD13,IGF1,HTT,MAPK1,PTEN,ARHGEF17,SPRED2,BMP7,PIK3C3,PUM1,RASA2,RAB30,LRRK2,BAG6,LILRB4,ARHGAP25,SHANK3,RGS9,PDGFC,AMOTL1,FAM13B,WNT7A,UACA,PLCL2,KAT6A,RHOT1,SIK3,SH3RF2,SOS1,ATRX,RAPGEF2,KCNC2,SMO,VRK3,MARK4,PRKAR2B,PSEN2,PAG1,GRIN1,RHOA,WSB2,SYNGAP1,ROR1,TF,RHOBTB1,CUL3,ITPR2,GSK3B,PLD2,RDX,ACTA2,MLF1,FAM49B,NECAB2,STK32B,CD27,DUSP26,MCTP2,BOK,NACC2,AGT,PRKAA2,PITPNM2,ZMPSTE24,HRH4,NDRG2,PTK2,LAT2,PEX5L,TCP11,TEC,MAP3K5,PRKD3,NCF1,TAOK2,SHC1,ARHGAP15,CD160,RPS6KB1,GUCY2F,CD44,FGD3,ADAM8,RPS6KA5,HUS1,CXCL17,FGD4,NOS1,PLEKHA1,PLVAP,RGS14,SIPA1L2,ACTN4,ARHGAP11A,MC1R,STK4,ZFAND6,MARK3,EPHB2,WLS,XCR1,IL18,MFSD8,GPSM2,DLC1,PPARA,PPP1R10,MCOLN1,PHLPP1,MAGI3,MOB3B,PRKCZ,ZC3HAV1,GRIN2B,PPP1CC,RABGEF1,TYK2,OTUD7B,DENND4B,ANGPT1,FHL2,LRRK1,OSBPL8,GCNT2,TBC1D10C,ARHGAP21,CCL22,FBXO31,PRKCQ,BRAF,HTR2C,CD300A,IRAK1BP1,PPP2CA,ARHGEF6,RPL23,SH3BP4,DCDC1,SP100,DONSON,ITSN1,SRGAP2,ADCY1,MADD,SYK,WWTR1,ARHGAP19,GRIK2,ASB1,CIT,BRCA2,RAP1GAP2,DVL2,GRIP1,PTPRJ,TAF1,TICAM1,TANK,HSF1,INPP5A,SRPK2,PLEKHM1,SASH1,RALGAPA1,CGNL1,QRICH1,VAV3,CAV2,GNA12,SH2D3A,INPP5F,NUAK2,AXL,DENND4C,MET,CAMK2D,CCR3,RAD9B,TIMELESS,ANKRD6,MALT1,THEM4,PHB,PTPRC,EDA,GPR89A,TYRO3,ADCY10,TRAF3IP2,TRAP1,CLNK,EDNRA,PRAP1,BMP4,CPNE1
GO:0048869	cellular developmental process	7.030347516629712e-32	*RDH14,CLRN1,ENPP1,PRDX2,NRXN1,PRMT3,RDH13,PRKCI,MOV10L1,MAP4K4,PDE4D,DNMT1,S1PR2,HLX,LLPH,SLC9A1,NEGR1,PBX3,SPAG16,TRPS1,CBFB,CLASP2,SEMA3A,IL31RA,GRID2,RPS6KA2,PRDM12,TENM3,CALR3,DOCK1,CLDN18,RYR1,NRG3,IQCG,ASTN2,SEMA3D,PHACTR1,MYOT,NREP,TIAM2,FHOD3,NLGN4X,TJP1,UNC5C,ZSWIM6,PRLR,HIVEP3,FTO,KALRN,WIPF3,SPRR2D,NTRK3,ARNT,ATRNL1,SEMA5A,FLT3,STAT5B,TOX,ENPP2,ATP2B2,GRM5,NOC3L,FER,SPATA5,KITLG,MAPK10,LRP2,PIK3CD,ZNF536,SP3,ANK2,OLFM1,EZR,MEGF10,NRXN3,ROBO1,CHD7,SLC26A6,HTR2B,ITGB6,CDKL5,MECOM,TENM1,KIF5C,LMNA,TRAF6,ESRP1,ROBO2,NFASC,ITPKB,DNAJA3,GRK5,ABI1,ULK4,NHLH1,ITGB1,TRIOBP,HSP90AA1,GAS7,PSMB7,TSSK1B,KNDC1,ZC4H2,DSCAM,CNGB1,TRIO,ARHGAP24,KLHL12,AKAP13,RUNX1,DAB1,ABCC8,THRB,LAMA2,TCIRG1,PTPN11,HERC4,DOCK10,HDAC6,GOLGA4,MYT1,EPHA1,IKBKB,ERBB4,DPY19L2,LIMK1,ZNF609,ATP8A2,CUL4B,CECR2,ESR1,MYO18B,NPHP3,GRM7,PTPRO,CDON,NTRK2,EP300,CELA1,MEGF9,TENM2,RNF220,HOOK3,PDGFB,RIMS1,TNIK,SNX2,KIRREL3,TOB2,HNF4G,FMN2,RERE,PRKAR1A,ATP8B1,UNC5D,H2AFY2,LAMA3,TCF7L2,PIP4K2A,CDH4,PAK1,FBXW11,ESRRB,MAP3K4,PTPRD,BASP1,CATSPERB,SLC4A10,TBR1,CATSPER2,STRC,BDNF,TFAP2A,ATRN,AMIGO1,FANCA,TGIF2,POMGNT2,PHACTR4,MEIS1,TENM4,LCK,EDARADD,SHC4,ECT2,NAV2,LAMC1,PTPRU,MB,SGCD,DENND5A,DMD,CENPF,USP13,KAT7,WDR7,SLC8A1,RBM4,GPR171,SATB2,RIT2,HIRA,TANC2,CASS4,FYN,ARNTL,ADAMTS9,NF1,PLCB1,MGMT,PCDH15,LMTK2,ARID4B,FAT3,RTN4,RXFP1,CHRDL1,APOD,SLC2A14,B4GALT6,RTN4R,PLEKHM3,BCR,CCDC141,TTN,NDRG4,PAQR3,ADAMTSL1,TLL1,NTN1,SLC4A5,NRG1,STRB*

			P,FRY,MAP2K1,MDFI,FNIP1,VAX2,MYH9,DAGLA,CNTN4,HDAC5,NTNG1,NAV1,TMEM30A,SYNE1,JDP2,CMKLR1,LRGUK,ROR2,SLC39A14,CATSPER3,DOCK2,SOX13,UST,DMC1,MAD2L2,RAB27A,CAPRIN2,NLN,NCKAP1,TERF2,RCC2,BOC,ARL3,NEUROD1,KIF2A,FBXO9,ACTL8,PARP11,COL12A1,ZDHHC15,JAK2,MYPN,YTHDC2,TRAPPC9,FBXW7,SYT1,ITCH,BBS12,LZTS1,BCL11B,RBFOX1,TMC1,PKNOX1,CLDN1,MLLT3,PNPLA1,PLS1,TCF7,PDE2A,MSI2,SEPT7,KLF15,TBX15,ANXA4,CYFIP2,WNT11,IFT80,FIG4,KREMEN1,ASTN1,NOX4,SIPA1L3,CACNA1H,SIAH1,QKI,IL1RAPL1,CAMK2B,PRKCD,SOX6,ACVR2A,RUNX2,SEMA5B,CD4,TGFB1,SGK1,ZHX2,KEL,NFATC3,F2RL1,C9ORF47,RPGRIP1L,CELSR1,SLIT3,SDK2,SLIT2,TP73,MUSTN1,GABRB1,CDH23,CORO1C,RRAS2,CTNND2,DISC1,TSNAX,KANK1,LRRC10,LAMC2,CLMN,MYT1L,OPCML,FSTL5,CHRM1,SMAD6,CLOCK,ITSN2,TCF12,ZNF675,SMOC1,ETV6,NELL1,SUCO,KCNH1,BCL3,DCLK1,ANKRD54,PARVA,SND1,ADAM12,HNRNPC,SBNO2,YTHDF1,FGF10,CNTNAP2,FBXL17,ARHGEF28,SMYD3,ENAH,PPL,LOXL3,MAST2,CAPN3,EPHA4,RORA,PRKCA,AUTS2,CNR1,TNFSF11,PPP3CA,NSUN2,MAG,CAMK4,UFL1,TRAK1,CTNNB1,PARK2,SOD2,FCGR2B,ARHGAP22,CDHR2,IGF1R,PPARG,NGRN,AXIN1,DLG5,IL18R1,BFSP1,MSR1,ANKRD17,XK,BRIP1,ANXA13,ANKRD26,DRAXIN,LEPR,FGF1,NIN,NR4A3,DCT,MYOCD,PER2,KIR2DL4,CPNE6,GLG1,CHEK2,PHLDB1,PRDM16,MYO1E,HCK,CAPN2,DIO2,MRC2,CSPG4,DCC,CTDP1,OBSCN,NF2,FLT4,MEF2B,HDAC4,PAX2,PHF5A,SPG11,SFRP1,FOXO3,ARL13B,CNTN6,NFIB,DNM3,SYNCRIP,SMAD3,CUX2,PTPRM,NHS,CASZ1,ABI2,PSAP,SHANK1,SYT3,NEDD4,NRP2,BTBD3,PREX1,CRTAC1,HOXD3,NAV3,NFATC1,PRTG,CDC73,APP,SSBP3,GSX2,PDGFRA,ODF3,CEP85L,DIP2B,YAP1,HEG1,FSTL4,NLGN2,VDAC1,EYA2,ALPL,LRP5,PTPRG,BDH2,ISLR2,SLC9B2,SOX2,SETD2,CHST11,PRICKLE1,RCAN1,ELAVL3,SPTBN4,VASH2,ZNF521,PAQR5,DRD1,FRS2,SFMBT1,MMP2,TPH1,SYNE2,CPNE9,KDM6A,PRKD1,STAT1,PARP6,DGKG,ETV5,MORN2,HNF4A,ZBTB7C,EREG,MYOF,DSCAML1,SEMA6D,ATF2,POU2F2,TCF3,RAF1,CELF4,ADAMTS12,PSMD11,SRRM4,BMPR2,USP33,DPYSL2,CAMK1D,BMPR1A,PABPC4,NLGN1,CTNNA2,CDK12,TAF8,CERS3,SPAG9,RAB11A,CRMP1,MYB,FGF2,LRRC4C,POU6F2,ZFP41,NEDD9,AGBL4,PEAK1,SEMA4D,SH3PXD2A,ARMC2,RORC,ELP3,PLXNA2,ADCYAP1R1,FNDC3A,SETD1A,JARID2,RILPL1,BRDT,ITGA11,RARB,SPEN,NCOA1,SPOCK1,BLOC1S5,EEF1E1,NTM,CHRD,DVL3,EIF2B5,EIF4G1,EPHB3,ATF3,FBXO45,PAWR,EBF2,DLEC1,TSG101,CCDC3,VCL,LAMB1,CLIC4,MYO9A,ANKS4B,COL11A1,KCNQ1,RFX2,WNT3,P4HB,SUFU,NLGN3,SCUBE2,PAFAH1B1,CCDC169-SOHLH2,SOHLH2,HIP1,AKAP6,RASAL1,NR2C1,TRPC5,TTLL7,GLI2,NEO1,SCUBE1,ERCC1,RUFY3,ALDH6A1,TPD52,COL19A1,DAB2,THEMIS,PKHD1,USH2A,LDLRAD4,DZIP1,WNK1,RELN,SIN3A,RSPO2,CSF3R,DOCK11,IQGAP1,APOLD1,ZNF423,MACF1,MYO7A,ALK,GSTM3,INPP5D,CLASP1,TEAD4,SLC9C1,SRA1,UBE2V1,MBOAT1,GPM6B,ADIRF,XDH,OVOL2,NFATC2,PRKG1,BNC1,PACRG,GALNTL5,PACSIN1,ACTL6B,PAK3,TET1,DYX1C1,ADNP,ARID4A,MAP1B,MARK1,CDK6,CELF1,TFRC,EPHA5,WWOX,MEF2A,DROSHA,GPC3,PSMB2,EYA1,BRINP1,GNB3,TRPV1,HOXB3,HOXB4,HOXB5,GHR,FLRT2,DIAPH2,ACTR2,ELAVL4,LGI4,HDAC1,PLCG2,ROCK1,PAXIP1,EGR2,RNF10,AP3B1,PTPN2,NTRK1,MAP1A,PPP1R9A,ANK3,UNC13A,PTPRQ,EPHB1,EPHA10,PTPN9,AXIN2,SARM1,EPCAM,PARD3,TRIM46,SH3GL3,JAG2,AP3D1,CARM1,ARHGAP44,SRD5A2,SMARCA2,CUX1,SPI1,EPHA7,CTNNA1,GLIS1,LRRC8C,ILDR2,SHROOM3,MAP3K13,RAPGEF3,SDK1,SH3BP1,KAZN,ERCC3,PTK7,SMYD1,NHSL2,BMPR1B,CD53,MAGI2,USP42,PNPT1,ZEB2,FOXJ3,OCA2,HEY2,RC3H1,COL13A1,HYDIN,WNT7B,PTPRS,KMT2D,CREM,TLL2,ABCA12,CSRP1,RASA1,MITF,SRSF6,RBM45,HIPK1,PKP2,RTN4RL1,BTK,SGCZ,CBFA2T2,IGF1,DPYSL3,MAPK1,PTEN,MIB1,SPRED2,BMP7,PUM1,SOX5,LHFPL5,LRRK2,OSGIN1,SEPT6,ALDOC,RAPH1,BAG6,LILRB4,MORC3,TMEM108,ITGAM,EFNA5,SHANK3,STMN4,DAZL,ALPK3,NCOA3,STEAP4,PPP2R3C,TBCD,WNT7A,ZBED6,MAP4,NME8,PLCL2,KAT6A,SH3PXD2B,BMP6,MYCBP2,IL17RD,SOS1,TSHR,EXT1,ATRX,IKZF1,PARVB,PNPLA3,PRMT2,RAPGEF2,SMO,SUN1,BLOC1S3,CPT1A,TCF4,SYT17,BPGM,SOX30,TFE3,BBS9,RHOA,SYNGAP1,GRIN3A,NDE1,ROR1,TF,ONECUT2,CUL3,NFKBID,EPM2A,GSK3B,EMB,RDX,STAT6,ACTA2,SYN1,MLF1,EEF2K,CDK13,ALAS2,CD27,BOK,SULF1,KRT8,AGT,HDGFRP3,ADAR,BLOC1S6,FBN2,ZMPSTE24,BRINP3,NDRG2,PTK2,TPPP2,SNX3,RIMS2,PBX1,TCP11,SHB,GAP43,MAP3K5,TCFL5,DLX1,TAOK2,PYGO2,NDUFS2,ECE1,LBX2,RPS6KB1,GPR173,PGM5,SCEL,ANKS1A,ALOX5,CPLX2,DNAJB6,MDGA2,SP7,CD44,ADAM8,PACSIN2,RPS6KA5,CDS1,THSD7A,CXCL17,NOS1,PEX7,TBC1D23,PLEKHA1,RGS14,ACTN4,STK4,TUBB3,EPHB2,SLC1A1,TMEFF2,CLPTM1,IL18,MFSD8,ASF1B,PPARA,ARID5B,PRRC2C,NOTCH4,CRX,FBN1,PAX6,PRKCZ,FAM20C,ADORA2A,PPP1CC,COL22A1,PHLDB2,KLC3,KLHL1,NIPBL,ANGPT1,EYA3,FHL2,LRRK1,OSBPL8,GCNT2,ETS1,VWC2,MAP2,MTF2,NEBL,FBXO31,CDK5RAP1,CNTN1,PRKCQ,BRAF,HDAC2,HTR2C,IMPACT,POU3F3,NOTO,SPINT2,TNR,MBNL3,ELF2,CDKL3,PPP2CA,ACAN,CAMSAP3,ANXA2,PRMT7,CR2,SRGAP2,TMEFF1,ADCY1,NUMB,SYK,CNOT1,WWTR1,SLIT1,CIT,RADIL,BRCA2,DVL2,MORC1,NEB,YBX1,GRIP1,PTPRJ,GPRIN3,GPM6A,OSBP2,PLEKHB2,CADM1,HSF1,DHFR,SRPK2,ASAP1,ADAMTS7,SULT2B1,BMP1,ZP3,RNF2,CAV2,GNA12,MYEF2,INPP5F,AXL,TNFSF9,WIF1,CD109,MET,SNX1,MALT1,SETD3,TMEM120B,CCDC136,EHD2,SEMA3C,NEDD4L,PHB,PTPRC,EDA,GPR89A,SH3GL2,TYRO3,VPS52,NCAM1,TANC1,TRAF3IP2,CREB1,AFF4,EDNRA,GNGT1,BMP4,CPNE1
GO:00	nervous system	2.72358206	*CLRN1,DNAH11,LDB2,NRXN1,PRMT3,GSS,RDH13,PRKCI,TACC2,MAP4K4,HLX,L*

07399	development	6127127e-31	LPH,NEGR1,PBX3,CLASP2,SEMA3A,LSAMP,GRID2,PRDM12,TENM3,CHERP,NRG3,ASTN2,SEMA3D,PHACTR1,MYOT,NREP,TIAM2,NLGN4X,UNC5C,ZSWIM6,KALRN,NTRK3,SEMA5A,TOX,ATP2B2,GRM5,LHFPL4,SPATA5,NDUFV2,LRP2,ZNF536,ANK2,OLFM1,NRXN3,ROBO1,CHD7,CDKL5,TACC1,TENM1,KIF5C,TRAF6,ESRP1,ROBO2,NFASC,ABI1,ULK4,NHLH1,ITGB1,TRIOBP,HSP90AA1,GAS7,PCDH17,KNDC1,ZC4H2,DSCAM,FAM126A,SRGAP2B,CNGB1,TRIO,RUNX1,DAB1,ABCC8,THRB,LAMA2,GFRA2,TCIRG1,PTPN11,DOCK10,HDAC6,GOLGA4,MYT1,ADORA1,EPHA1,ERBB4,LIMK1,ZNF609,ATP8A2,CUL4B,CECR2,NPHP3,GRM7,PTPRO,CDON,NTRK2,EP300,TENM2,RNF220,HOOK3,RIMS1,TNIK,KIRREL3,RERE,ATP8B1,UNC5D,H2AFY2,PLLP,LAMA3,CDH4,PAK1,FBXW11,PTPRD,CNTN5,BASP1,SLC4A10,TBR1,STRC,BDNF,TFAP2A,ATRN,AMIGO1,CHRM3,TGIF2,POMGNT2,PHACTR4,MEIS1,TENM4,ECT2,ZNF148,NAV2,DENND5A,DMD,CENPF,SLC8A1,GSN,SATB2,PAFAH1B2,RIT2,MACROD2,TANC2,FYN,ARNTL,NF1,PLCB1,PCDH15,LMTK2,FAT3,RTN4,AFF2,MAL2,CHRDL1,APOD,B4GALT6,RTN4R,BCR,CCDC141,NDRG4,PAQR3,ADAMTSL1,NTN1,DCTN1,NRG1,FRY,MAP2K1,VAX2,LRFN5,DAGLA,CNTN4,NTNG1,PCSK2,NAV1,NFIA,TMEM30A,SBF2,ROR2,PLXDC1,SOX13,ACSBG1,UST,FGF14,PCDHB16,CAPRIN2,NCKAP1,BOC,ARL3,NEUROD1,KIF2A,ZDHHC15,JAK2,MYPN,TRAPPC9,CLSTN2,SYT1,LZTS1,BCL11B,RBFOX1,TMC1,CLDN1,PLS1,KLF15,CYFIP2,WNT11,FIG4,KREMEN1,IMMP2L,NRG4,ASTN1,SIAH1,QKI,IL1RAPL1,CAMK2B,ADAM23,SOX6,RUNX2,SEMA5B,TGFB1,SGK1,ZHX2,KEL,RPGRIP1L,CELSR1,SLIT3,SDK2,SLIT2,TP73,GABRB1,CDH23,CTNND2,DISC1,KANK1,LAMC2,CLMN,TTBK2,MYT1L,OPCML,CHRM1,ITSN2,TCF12,ETV6,SYNJ2,NELL1,TFAP2D,DCLK1,NAIP,SLC17A7,LINGO2,YTHDF1,FGF10,CNTNAP2,FBXL17,ENAH,IL1RAPL2,LOXL3,EPHA4,RORA,AUTS2,CNR1,PPP3CA,MAG,UFL1,TRAK1,CTNNB1,PARK2,SOD2,FCGR2B,SMARCC1,IGF1R,PPARG,NGRN,DLG5,ADD2,MTF1,XK,DRAXIN,LEPR,NIN,DCT,PER2,CPNE6,SCN3B,CSPG4,DCC,NF2,HDAC4,PAX2,SPG11,SFRP1,FOXO3,ARL13B,CNTN6,NFIB,IFT122,DNM3,CUX2,PTPRM,ARNT2,CASZ1,ABI2,SHANK1,SYT3,NEDD4,NRP2,BTBD3,CRTAC1,HOXD3,NAV3,PRTG,APP,SSBP3,GSX2,CEP85L,DIP2B,YAP1,FSTL4,NLGN2,PTPRG,ISLR2,SOX2,SETD2,GPC6,PRICKLE1,ELAVL3,SPTBN4,VASH2,ZNF521,DRD1,FRS2,MMP2,SYNE2,CPNE9,KDM6A,PRKD1,PARP6,DGKG,SYBU,SLC6A3,SHANK2,DSCAML1,SEMA6D,RBFOX3,ATF2,TCF3,SRRM4,BMPR2,USP33,DPYSL2,CAMK1D,BMPR1A,NLGN1,CTNNA2,SPAG9,RAB11A,CRMP1,FGF2,LRRC4C,POU6F2,GRIK1,AGBL4,SEMA4D,ELP3,PLXNA2,SETD1A,JARID2,RARB,SPEN,PRKCG,NCOA1,SPOCK1,BLOC1S5,GAS8,NTM,CHRD,DVL3,EIF2B5,EIF4G1,EPHB3,FBXO45,VCL,LAMB1,MYO9A,KCNQ1,WNT3,SUFU,TG,NLGN3,PAFAH1B1,CNTFR,NMUR2,RASAL1,TRPC5,TTLL7,GLI2,NEO1,RUFY3,DAB2,USH2A,SETD5,WNK1,RELN,SIN3A,RSPO2,KCNQ2,IQGAP1,ZNF423,LUZP1,MACF1,MYO7A,ALK,GSTM3,MBOAT1,GPM6B,OVOL2,PRKG1,RGS7,ATP2B4,PACSIN1,ACTL6B,PAK3,DYX1C1,ADNP,GPR161,MAP1B,MARK1,CDK6,CELF1,EPHA5,MEF2A,DHX30,GFRAL,EYA1,BRINP1,TRPV1,HOXB3,MNAT1,FLRT2,ACTR2,ELAVL4,LGI4,HDAC1,ROCK1,EGR2,RNF10,NTRK1,MAP1A,PPP1R9A,ANK3,UNC13A,PTPRQ,EPHB1,EPHA10,PTPN9,SARM1,SNTG2,PARD3,TRIM46,SH3GL3,JAG2,CARM1,ARHGAP44,SRD5A2,SMARCA2,CUX1,EPHA7,FOXP2,CTNNA1,SHROOM3,MAP3K13,SDK1,ERCC3,PTK7,SNCA,BMPR1B,MAGI2,ZEB2,HEY2,HYDIN,RNF103,WNT7B,HMGCS2,PTPRS,RBM45,HIPK1,RTN4RL1,CBFA2T2,DPYSL3,MAPK1,PTEN,CLDN11,MIB1,BMP7,SCN8A,SOX5,LHFPL5,LRRK2,UNC119,RAPH1,BAG6,TMEM108,ITGAM,PKD2,EFNA5,SHANK3,STMN4,PDGFC,TBCD,WNT7A,MAP4,BMP6,MYCBP2,SOS1,TSHR,EXT1,ATRX,SERINC5,RAPGEF2,KCNC2,SMO,SUN1,BLOC1S3,MARK4,TCF4,SYT17,GRIN1,RHOA,SYNGAP1,GRIN3A,NDE1,ROR1,ONECUT2,EPM2A,GABRB3,GSK3B,EMB,SYN1,EEF2K,CCDC14,BOK,SULF1,MTHFD1L,AGT,HDGFRP3,BLOC1S6,BRINP3,NDRG2,PTK2,SNX3,RIMS2,PBX1,GAP43,DLX1,TAOK2,PYGO2,NDUFS2,ECE1,GPR173,ANKS1A,CPLX2,MDGA2,SLC5A3,RPS6KA5,PEX7,TBC1D23,RGS14,STK4,TUBB3,EPHB2,SLC1A1,WLS,SIGMAR1,MFSD8,DLC1,CRX,PAX6,PRKCZ,ADORA2A,GRIN2B,PPP1CC,KLHL1,NIPBL,PCDH10,SHROOM4,IGF2BP3,VWC2,MAP2,TAGLN3,FBXO31,CDK5RAP1,CNTN1,PRKCQ,LGI2,BRAF,HDAC2,IMPACT,POU3F3,NOTO,SPINT2,TNR,CDKL3,ACAN,ATXN1,CAMSAP3,SRGAP2,TMEFF1,ADCY1,NUMB,SLIT1,CIT,BRCA2,DVL2,GRIP1,TAF1,GPRIN3,GPM6A,POTEE,CADM1,DHFR,EZH1,ASAP1,MYEF2,INPP5F,AXL,MET,UCHL5,TRA2B,KDM2B,SEMA3C,NEDD4L,SH3GL2,TYRO3,SYNDIG1,NCAM1,CREB1,EDNRA,GNGT1,BMP4,CPNE1
GO:0032989	cellular component morphogenesis	3.831472861119627e-31	NRXN1,PRMT3,MAP4K4,LLPH,CLASP2,SEMA3A,SEMA3D,PHACTR1,MYOT,TIAM2,FHOD3,UNC5C,KALRN,SEMA5A,ENPP2,LRP2,ANK2,OLFM1,NRXN3,ROBO1,CDKL5,KIF5C,ROBO2,NFASC,ABI1,ITGB1,HSP90AA1,GAS7,KNDC1,DSCAM,TRIO,AKAP13,DAB1,LAMA2,PTPN11,DOCK10,HDAC6,GOLGA4,EPHA1,LIMK1,ATP8A2,PTPRO,NTRK2,RIMS1,TNIK,SNX2,KIRREL3,RERE,PRKAR1A,UNC5D,LAMA3,CDH4,PAK1,PTPRD,TBR1,BDNF,AMIGO1,TENM4,DMD,TANC2,FYN,LMTK2,RTN4,B4GALT6,RTN4R,CCDC141,TTN,ADAMTSL1,NTN1,MAP2K1,VAX2,CNTN4,NTNG1,UST,CAPRIN2,NCKAP1,BOC,ZDHHC15,MYPN,SYT1,LZTS1,BCL11B,CYFIP2,FIG4,SIAH1,IL1RAPL1,CAMK2B,SEMA5B,SGK1,KEL,SLIT3,SLIT2,CORO1C,CTNND2,DISC1,KANK1,LAMC2,ITSN2,DCLK1,YTHDF1,CNTNAP2,ENAH,CAPN3,EPHA4,AUTS2,PPP3CA,MAG,CTNNB1,PARK2,IGF1R,XK,DRAXIN,NIN,CPNE6,PHLDB1,DCC,OBSCN,PAX2,SPG11,CNTN6,NFIB,DNM3,CUX2,PTPRM,ABI2,SHANK1,SYT3,NEDD4,NRP2,BTBD3,PRTG,APP,PDGFRA,DIP2B,FSTL4,ISLR2,SPTBN4,CPNE9,PARP6,DSCAML1,SEMA6D,BMPR2,USP33,DPYSL2,NLGN1,CTNNA2,SPAG9,RAB11A,CRMP1,LRR

			C4C,SEMA4D,PLXNA2,DVL3,EPHB3,FBXO45,VCL,MYO9A,RFX2,WNT3,NLGN3,PAFAH1B1,RASAL1,TRPC5,GLI2,NEO1,RUFY3,RELN,SIN3A,IQGAP1,MACF1,CLASP1,PACSIN1,PAK3,ADNP,MAP1B,EPHA5,MEF2A,FLRT2,ACTR2,ELAVL4,ROCK1,EGR2,NTRK1,MAP1A,ANK3,UNC13A,EPHB1,EPHA10,SARM1,PARD3,TRIM46,ARHGAP44,CUX1,EPHA7,MAP3K13,PTK7,BMPR1B,PNPT1,WNT7B,PTPRS,CSRP1,PTEN,BMP7,LRRK2,RAPH1,TMEM108,EFNA5,SHANK3,WNT7A,MYCBP2,SOS1,EXT1,RAPGEF2,SMO,SYT17,SOX30,RHOA,SYNGAP1,GSK3B,EMB,EEF2K,KRT8,PTK2,RIMS2,GAP43,TAOK2,ECE1,PGM5,CD44,PACSIN2,RPS6KA5,TUBB3,EPHB2,PAX6,PRKCZ,ADORA2A,PHLDB2,MAP2,NEBL,FBXO31,PRKCQ,BRAF,IMPACT,TNR,CDKL3,SRGAP2,TMEFF1,ADCY1,NUMB,SLIT1,DVL2,NEB,GRIP1,GPM6A,SNX1,CCDC136,SEMA3C,NEDD4L,SH3GL2,NCAM1,CREB1,EDNRA,CPNE1
GO:0030182	neuron differentiation	5.676183490736121e-31	CLRN1,NRXN1,PRMT3,RDH13,PRKCI,MAP4K4,LLPH,NEGR1,PBX3,CLASP2,SEMA3A,GRID2,PRDM12,TENM3,SEMA3D,PHACTR1,MYOT,NREP,TIAM2,NLGN4X,UNC5C,ZSWIM6,KALRN,NTRK3,SEMA5A,TOX,ATP2B2,LRP2,ZNF536,OLFM1,NRXN3,ROBO1,CDKL5,TENM1,KIF5C,ESRP1,ROBO2,NFASC,ABI1,ULK4,ITGB1,TRIOBP,HSP90AA1,GAS7,KNDC1,ZC4H2,DSCAM,CNGB1,TRIO,RUNX1,DAB1,THRB,LAMA2,PTPN11,DOCK10,HDAC6,GOLGA4,EPHA1,ERBB4,LIMK1,ATP8A2,CUL4B,CECR2,GRM7,PTPRO,CDON,NTRK2,EP300,TENM2,RNF220,RIMS1,TNIK,KIRREL3,RERE,ATP8B1,UNC5D,LAMA3,CDH4,PAK1,PTPRD,SLC4A10,TBR1,STRC,BDNF,AMIGO1,TGIF2,MEIS1,TENM4,ECT2,DENND5A,DMD,SATB2,RIT2,TANC2,FYN,PCDH15,LMTK2,FAT3,RTN4,APOD,B4GALT6,RTN4R,CCDC141,NDRG4,PAQR3,ADAMTSL1,NTN1,NRG1,FRY,MAP2K1,VAX2,CNTN4,NTNG1,TMEM30A,ROR2,UST,CAPRIN2,NCKAP1,BOC,ARL3,NEUROD1,ZDHHC15,JAK2,MYPN,TRAPPC9,SYT1,LZTS1,BCL11B,TMC1,PLS1,CYFIP2,WNT11,FIG4,KREMEN1,SIAH1,IL1RAPL1,CAMK2B,RUNX2,SEMA5B,SGK1,ZHX2,KEL,RPGRIP1L,SLIT3,SDK2,SLIT2,TP73,GABRB1,CDH23,CTNND2,DISC1,KANK1,LAMC2,CLMN,MYT1L,OPCML,ITSN2,TCF12,DCLK1,YTHDF1,CNTNAP2,ENAH,EPHA4,RORA,AUTS2,CNR1,PPP3CA,MAG,CTNNB1,PARK2,SOD2,IGF1R,NGRN,DLG5,XK,DRAXIN,NIN,CPNE6,DCC,PAX2,SPG11,SFRP1,FOXO3,CNTN6,NFIB,DNM3,CUX2,PTPRM,CASZ1,ABI2,SHANK1,SYT3,NEDD4,NRP2,BTBD3,CRTAC1,HOXD3,PRTG,APP,GSX2,DIP2B,FSTL4,NLGN2,PTPRG,ISLR2,SOX2,SPTBN4,VASH2,ZNF521,DRD1,MMP2,CPNE9,PRKD1,PARP6,DGKG,DSCAML1,SEMA6D,TCF3,SRRM4,BMPR2,USP33,DPYSL2,CAMK1D,NLGN1,CTNNA2,SPAG9,RAB11A,CRMP1,LRRC4C,AGBL4,SEMA4D,PLXNA2,NCOA1,SPOCK1,BLOC1S5,NTM,DVL3,EIF4G1,EPHB3,FBXO45,VCL,LAMB1,MYO9A,KCNQ1,WNT3,SUFU,NLGN3,PAFAH1B1,RASAL1,TRPC5,GLI2,NEO1,RUFY3,DAB2,USH2A,WNK1,RELN,SIN3A,RSPO2,IQGAP1,MACF1,MYO7A,ALK,MBOAT1,GPM6B,PRKG1,PACSIN1,ACTL6B,PAK3,ADNP,MAP1B,MARK1,EPHA5,MEF2A,EYA1,BRINP1,FLRT2,ACTR2,ELAVL4,LGI4,HDAC1,ROCK1,EGR2,NTRK1,MAP1A,PPP1R9A,ANK3,UNC13A,PTPRQ,EPHB1,EPHA10,PTPN9,SARM1,PARD3,TRIM46,SH3GL3,JAG2,CARM1,ARHGAP44,CUX1,EPHA7,CTNNA1,MAP3K13,SDK1,ERCC3,PTK7,BMPR1B,MAGI2,HEY2,WNT7B,PTPRS,HIPK1,RTN4RL1,CBFA2T2,DPYSL3,PTEN,MIB1,BMP7,LHFPL5,LRRK2,RAPH1,TMEM108,EFNA5,SHANK3,STMN4,TBCD,WNT7A,MAP4,BMP6,MYCBP2,SOS1,TSHR,EXT1,RAPGEF2,SMO,BLOC1S3,TCF4,SYT17,RHOA,SYNGAP1,GRIN3A,ROR1,ONECUT2,GSK3B,EMB,SYN1,EEF2K,AGT,HDGFRP3,BLOC1S6,BRINP3,PTK2,SNX3,RIMS2,PBX1,GAP43,DLX1,TAOK2,ECE1,ANKS1A,MDGA2,RPS6KA5,TBC1D23,TUBB3,EPHB2,MFSD8,PAX6,PRKCZ,ADORA2A,PPP1CC,KLHL1,VWC2,MAP2,FBXO31,CDK5RAP1,CNTN1,PRKCQ,BRAF,HDAC2,IMPACT,NOTO,TNR,CDKL3,CAMSAP3,SRGAP2,TMEFF1,ADCY1,NUMB,SLIT1,DVL2,GRIP1,GPRIN3,GPM6A,DHFR,ASAP1,MYEF2,INPP5F,MET,SEMA3C,NEDD4L,SH3GL2,NCAM1,CREB1,EDNRA,GNGT1,BMP4,CPNE1
GO:0048522	positive regulation of cellular process	1.1053077774697584e-30	CD247,POLDIP3,CLRN1,PRDX2,LDB2,GPC5,NRXN1,ASPH,GP6,PRKCI,SLCO3A1,MAP4K4,PDCL,DNMT1,S1PR2,HLX,SLC9A1,C6ORF89,NEGR1,PBX3,ADCY8,CBFB,PDE8A,PDE4DIP,CLASP2,SEMA3A,IL31RA,GRID2,TAS1R2,RPS6KA2,PRDM12,PTGFR,TENM3,CHERP,MED26,DOCK1,WWC1,C12ORF49,CTDSPL2,NRG3,ASH1L,NOS1AP,LATS2,NOX5,EPB41,SEMA3D,SCAF8,TIAM2,STOX2,MAPRE2,PTGIS,PRKAG2,TJP1,UNC5C,HPSE2,PRLR,PAGR1,HIVEP3,FTO,NPAS3,UTRN,KALRN,NTRK3,CBL,ARNT,SEMA5A,FLT3,RAD51B,STAT5B,TOX,ENPP2,GRM5,PLCE1,FER,CASK,MAP2K5,KITLG,LRP2,PIK3CD,SP3,HDGF,ANK2,SAAL1,OLFM1,SH2D1A,EZR,MEGF10,ROBO1,TOM1L1,CHD7,SLC26A6,HTR2B,CDKL5,MECOM,TACC1,TENM1,LMNA,TRAF6,ROBO2,ITPKB,DNAJA3,SLC24A2,VAV2,GRK5,ABI1,NHLH1,ITGB1,TRIOBP,HSP90AA1,CDC6,PSMB7,KNDC1,ZC4H2,KIF26B,CCDC22,DSCAM,AKAP13,GFI1B,RUNX1,DAB1,OMA1,CDC42EP3,MYO10,ABCC8,HMGN3,NRIP1,DGKI,THRB,STXBP4,EFCAB7,LAMA2,TCIRG1,PTPN11,MGAT5,CCT2,CACNB2,CYTH3,HDAC6,GOLGA4,SERTAD2,DDB1,ADORA1,ADAMTS3,EPHA1,IKBKB,ERBB4,ADRA1D,MRE11A,LIMK1,ZNF609,BRD8,ATP8A2,KAT6B,CUL4B,ESR1,MIER1,MAML3,DPP10,CDON,NTRK2,TNRC6A,FRMD4A,STX18,EP300,CELA1,ZYG11B,TENM2,ZNF76,RNF220,FNTA,PDGFB,RIMS1,TNIK,CCND3,MID1,BRMS1,CHFR,STK39,HNF4G,INSR,FMN2,RERE,H2AFY2,TCF7L2,PIP4K2A,CDH4,CAB39,USP34,PAK1,LITAF,FBXW11,ESRRB,MYOM1,MAP3K4,ZDHHC13,PTPRD,RNF144A,EGLN3,RBM20,TBR1,SAMD4A,BDNF,TFAP2A,AMIGO1,KCNMA1,PEG3,TGIF2,MEIS1,TENM4,TRIM13,LCK,IL5RA,ENTPD5,SHC4,ECT2,ZNF148,MTA3,TFDP2,VAMP7,LAMC1,PTPRU,ERN2,DMD,ATG10,SLC30A9,TOX3,LARP4B,USP13,KAT7,SLC8A1,GSN,RBM14,RBM4,MED12L,SATB2,PAFAH1B2,RIT2,JPH2,DEPDC1B,CASS4,FYN,MKRN2,ARNTL,ADAMTS

			9,NF1,PLCB1,MGMT,LMTK2,ARID4B,RTN4,RTN4R,RWDD3,BRPF1,CHI3L1,TTN,NDRG4,BMP2K,PIK3R2,NTN1,EGFLAM,DCTN1,NRG1,SH2D3C,CDK19,UBP1,BDKRB1,DOCK8,BID,MAP2K1,FNIP1,STIM1,MYH9,FRMPD4,MAD1L1,VGLL4,PPP3R1,HDAC5,CSRNP3,RBM5,NFIA,RNF4,TMEM30A,CSNK2A3,JDP2,CMKLR1,ROR2,DCN,ZDHHC3,SLC39A14,PCBD2,HNRNPLL,CDH13,CREBRF,SOX13,COPS5,CSPP1,IPO5,MAD2L2,RAB27A,CAPRIN2,CCNYL1,NCKAP1,CD99,PDF,TERF2,SLX1B,GOLPH3L,RCC2,BOC,ANO6,ARL3,NEUROD1,RANBP1,CCBE1,USP22,ZDHHC15,JAK2,YTHDC2,FAM168A,FBXW7,OAZ2,SKAP1,CLSTN2,UIMC1,SYT1,ITCH,MLIP,CROCC,NPSR1,BCL11B,PKNOX1,CLDN1,MLLT3,PLS1,SEPT7,KLF15,LAMTOR3,MAGI1,CYFIP2,WNT11,APBB1IP,FIG4,MTA1,NOX4,RPRD1B,CACNA1H,SIAH1,IL1RAPL1,CCDC62,CAMK2B,ERCC8,PRKCD,SOX6,TAB2,ACVR2A,RUNX2,SEMA5B,CD4,PPM1E,TGFB1,BANP,SPSB4,NUP93,TRIM65,NSD1,PIBF1,BTRC,NFATC3,CRADD,F2RL1,BCAS3,C9ORF47,DNAJB2,AAK1,SLIT2,COLGALT1,TP73,CADPS2,HP,HPR,CORO1C,SAP18,RRAS2,ILF2,DISC1,BLID,CEP135,KANK1,ZFAND2A,LAMC2,CLN6,MTDH,FANK1,CHRM1,SMAD6,ZNF398,CLOCK,ITSN2,TCF12,ETV6,NELL1,SUCO,TFAP2D,BMPER,BCL3,ANKRD54,DAPK2,TNFRSF10B,DUSP22,NAIP,DOCK3,SBNO2,LINGO2,YTHDF1,FGF10,CNTNAP2,CIZ1,SMYD3,STXBP5,IQCJ-SCHIP1,SH3RF3,LOXL3,FANCI,SVIL,GLP2R,CAPN3,LUM,VMP1,SMURF2,EPHA4,RORA,HSD17B12,PRKCA,AUTS2,CNR1,CD6,TNFSF11,PPP3CA,NFYB,MAGEA4,MAG,KLF12,CAMK4,PIP5KL1,UFL1,CTNNB1,PARK2,SOD2,FCGR2B,SMARCC1,IGF1R,PPARG,NGRN,AXIN1,DLG5,IL18R1,IL1RL1,MTF1,MSR1,P2RY10,ANKRD17,APOL3,SCAMP5,ANXA13,ECM2,SREBF2,RYR2,LEPR,LEPROT,FGF1,NIN,NPAT,NR4A3,FOXK2,DCT,RIOK2,MYOCD,TRIM5,PER2,KIR2DL4,AJUBA,CPNE6,CHEK2,SUPT3H,PHLDB1,PRDM16,SPDYA,HCK,SORBS1,RAB3GAP2,CAPN2,TRIM8,CSPG4,GRM1,ANKFY1,NF2,FLT4,MEF2B,HDAC4,PAX2,PHF5A,SPTBN1,TRABD2B,SFRP1,MED13,ST8SIA1,ZNF395,FOXO3,CNTN6,NFIB,BCL2L13,DNM3,SMAD3,RNFT2,CUX2,WWP2,ARNT2,WIPI1,EBF3,MTBP,RNF168,CASZ1,ABI2,GRIK5,SHANK1,SYT3,IFNAR1,DCP1B,NEDD4,NRP2,PREX1,DOCK4,ESRRG,HOXD3,HOXD4,NAV3,SLC4A4,TAB1,NFATC1,CDC73,APP,SSBP3,GSX2,PDGFRA,DIP2B,NOX1,ARIH1,YAP1,SESN1,NLGN2,TNFRSF19,VDAC1,EYA2,SH3D19,BORA,NVL,JAK1,LRP5,MTCP1,ISLR2,SLC9B2,SOX2,ZDHHC6,TEAD1,PRICKLE1,VASH2,ZNF521,DRD1,ARID3A,CHUK,FRS2,MMP2,TPH1,ESR2,SYNE2,S100A12,DCUN1D3,CPNE9,PRKD1,STAT1,PARP6,ST18,ETV5,PLAGL1,HNF4A,ZBTB7C,TASP1,SHANK2,VPS4A,EREG,CCNY,TCTN3,FXYD2,SEMA6D,ATF2,POU2F2,TCF3,GRAMD4,RAF1,CELF4,RASGRF2,ACOX2,CARD16,CASP1,PTPN1,BMPR2,CAMK1D,BMPR1A,NLGN1,BTBD10,COL16A1,IKZF4,PIK3R3,CDK12,CAND2,SPAG9,MORC2,RAB11A,EVC,MYB,FGF2,ZNF71,BACH1,PPM1F,NEDD9,AGBL4,SEMA4D,CDC27,DOK6,RORC,ELP3,PLXNA2,PTAFR,ADCYAP1R1,ABCA13,JARID2,DDX58,DKK2,BRDT,RARB,SPEN,PRKCG,VIPR1,NCOA1,BLOC1S5,EEF1E1,EEF1E1-BLOC1S5,GAS8,LMO7,AP2M1,CHRD,DVL3,EIF2B5,EIF4G1,EPHB3,RBX1,TCF20,ANAPC5,ATF3,LIN28B,SHOC2,SRSF5,CKS1B,PAWR,EBF2,AGO3,MAML2,TSG101,CCDC3,LAMB1,TERF2IP,WDR59,KCNQ1,RFX2,WNT3,RHOJ,P4HB,CCL14,CCL15,NLGN3,SCUBE2,PAFAH1B1,KIF3A,PRDM15,CNTFR,COL4A3,DDAH1,HIP1,AKAP6,RASAL1,NR2C1,TRPC5,UBA2,GLI2,NEO1,TNKS,SCUBE1,WBP2NL,ERCC1,RUFY3,TNRC6B,GLIS3,DAB2,BLM,PKHD1,CACUL1,MYSM1,DZIP1,DLG3,WNK1,RELN,NEK10,SIN3A,RUVBL2,ABCB1,RSPO2,GUCY1A2,DOCK11,GMEB1,PELI1,IQGAP1,MAP3K7,ZNF423,SP1,TRIM22,MACF1,ALK,SLC8A2,INPP5D,STAU1,CLASP1,TEAD4,HOXC13,APBB3,SLC35A4,SRA1,UBE2V1,TPCN1,ADIRF,XDH,OVOL2,SNX5,NFATC2,RGS7,BNC1,ATP2B4,NSF,PACSIN1,ACTL6B,ASXL3,PAK3,SSH2,TET1,CAMTA1,CCPG1,ATAD1,ADNP,ARID4A,MAP1B,SCARB1,CDK6,PHF2,CELF1,P2RY8,TFRC,UVRAG,EPHA5,WWOX,MEF2A,SYT12,DCAF6,DCUN1D5,GFRAL,GPC3,SGIP1,STOM,ADRBK1,TBC1D5,PSMB2,EYA1,SCOC,BRINP1,DIS3L2,TRPV1,TSPAN6,HOXB3,HOXB4,HOXB5,TFEB,CLEC16A,GHR,MNAT1,FLRT2,SNX9,ACTR2,ELAVL4,HDAC1,SMOC2,TRIM24,PLCG2,ROCK1,EPS8,NID1,C1QTNF1,PAXIP1,EGR2,RNF10,RYBP,CAPN10,GPR35,AP3B1,PTPN2,INSRR,NTRK1,KPNA6,TRIM44,ZFAND1,MAP1A,PKN2,SLC39A10,ANK3,UNC13A,EPHB1,EPHA10,PTPN9,AXIN2,SARM1,EPCAM,PARD3,GNL3L,MUC1,TRIM46,SH3GL3,JAG2,CACNG8,AP3D1,CARM1,VWF,AP2B1,NETO1,ADAM19,SNX33,PIFO,ZNF836,SMARCA2,ARHGEF3,CUX1,RAD51C,SPI1,DNMT3B,EPHA7,CHRNB4,MCU,CTNNA1,AIFM2,FBXW4,GLIS1,TRDN,ATG14,LY86,STAC,BCAR3,TRPC6,ABCB7,RBPMS,CHURC1,CREB5,FCHSD2,MAP3K13,RAB15,RAPGEF3,PDXP,MST1,ERCC3,PTK7,SMYD1,SNCA,BMPR1B,CACNG2,CD53,GRM4,MAGI2,PRIM2,PNPT1,USP50,ZEB2,DHRS4,FOXJ3,SYT9,HEY2,RC3H1,EIF3E,RNF19B,WNT7B,KMT2D,PRKAG1,CREM,KMT2C,DFFA,ADTRP,ABCA12,PLD1,MACC1,MITF,SRSF6,OPRD1,HIPK1,RPTOR,NFIX,BTK,XPO4,KCTD13,SLC4A8,CBFA2T2,IGF1,MLXIP,PLA2G4E,ATF7IP,CTNNBL1,HTT,DPYSL3,MAPK1,PTEN,MIB1,SPRED2,BMP7,PUM1,SOX5,PCBP2,ZNF780B,LRRK2,MLYCD,OSGIN1,TMEM59,ZRANB1,SPAG5,UNC119,ZBTB20,RFC3,BAG6,LILRB4,TMEM108,ITGAM,IL11RA,PKD2,EFNA5,SHANK3,TADA2A,DAZL,PDGFC,NCOA3,PKIB,PPP2R3C,WNT7A,ZBED6,NLRP1,UACA,LPGAT1,CRTC3,ARNTL2,ELOVL5,KAT6A,SIK3,ZNF197,BMP6,TAF15,MYCBP2,ANO1,GPI,SH3RF2,SOS1,TSHR,WDR43,PDCD1LG2,ATRX,PRMT2,RAPGEF2,KCNC2,SMO,VRK3,BLOC1S3,MARK4,CPT1A,TCF4,SYT17,SOX30,TFE3,UBR5,GRIN1,JADE1,ARRDC4,RHOA,SPRTN,ROR1,RQCD1,TF,ONECUT2,CUL3,SH3KBP1,TFEC,HSPD1,NFKBID,EPM2A,GSK3B,PRR16,DNAJC3,RDX,STAT6,ACTA2,EEF2K,F

			AM49B,NECAB2,CDK13,CD27,DUSP26,BOK,RNF144B,SULF1,NACC2,SUPT4H1,TNFRSF8,AGT,METTL16,PRKAA2,SYT7,BLOC1S6,FBN2,BRINP3,PTK2,TPPP2,SNX3,RIMS2,PBX1,TEC,EXOSC3,CDC14A,MAP3K5,SEC16B,CREBBP,DLX1,NCF1,TAOK2,SHC1,CD160,ECE1,LBX2,RNFT1,RPS6KB1,SCEL,SETDB2,ALOX5,SP7,CD44,LARP4,ADAM8,SLC5A3,CLDN4,RPS6KA5,ZNF484,BCL2L14,CDS1,CSNK1A1,PRDM2,CXCL17,NOS1,PLVAP,RGS14,ACTN4,MC1R,STK4,EPHB2,SLC1A1,WLS,EDRF1,IL18,LRRC52,UBE2K,GPSM2,HCAR2,DIS3,DLC1,PPARA,PPP1R10,ARID5B,CALCRL,EPB41L4B,SLC25A33,GPSM3,MOB3B,NOTCH4,SYTL4,CRX,PAX6,PRKCZ,ZC3HAV1,FAM20C,ADORA2A,GRIN2B,PPP1CC,CPEB1,PHLDB2,NIPBL,TMIGD2,YEATS4,ZNF143,ANGPT1,BRF1,CADPS,TBL1X,EYA3,LRRK1,OSBPL8,ATF6,EPB41L2,GCNT2,ETS1,PPP1CA,VWC2,MAP2,MTF2,CCL22,STPG1,FBXO31,CDK5RAP1,CNTN1,PRKCQ,BRAF,CSRNP1,DIAPH1,HDAC2,HTR2C,IMPACT,ATP8A1,PHKG2,SRCAP,POU3F3,TNR,UBE2E2,CD300A,ELF2,PHIP,CDKL3,PPP2CA,SKP1,ANGPT4,MAOA,RPL23,SH3BP4,RFC5,SP100,OPN1LW,ANXA2,PARN,RAB27B,ADCY1,MADD,NUMB,SYK,CNOT1,WWTR1,GRIK2,FRMD5,CIT,MYRIP,ZBTB38,BRCA2,HRK,ATF7,CACNG3,DVL2,YBX1,GRIP1,PTPRJ,TAF1,TICAM1,TANK,THAP3,GPM6A,CADM1,HSF1,MAX,EZH1,NRF1,SRPK2,PLEKHM1,SASH1,ASAP1,NCOA2,GBP5,SULT2B1,FCHO1,QRICH1,VAV3,ZP3,CHD6,MOB3A,CAV2,GNA12,SH2D3A,AKTIP,INPP5F,AXL,TNFSF9,TRIM37,WIF1,MET,TBC1D10A,CAMK2D,CCR3,TIMELESS,UCHL5,ANKRD6,MALT1,SETD3,TRA2B,EHD2,KDM2B,MLXIPL,SEMA3C,CCT3,NEDD4L,PHB,PTPRC,EDA,GPR89A,CTCF,TYRO3,NR6A1,SYNDIG1,TSPAN5,ADCY10,TRAF3IP2,ATF6B,CREB1,RGMB,SGTB,EDNRA,PRAP1,BMP4,ABLIM3,CPNE1
GO:0048518	positive regulation of biological process	1.7406069590670796e-30	*CD247,POLDIP3,CLRN1,PRDX2,LDB2,GPC5,NRXN1,ASPH,GP6,PRKCI,SLCO3A1,MAP4K4,PDE4D,PDCL,DNMT1,S1PR2,HLX,LLPH,SLC9A1,C6ORF89,NEGR1,PBX3,ADCY8,CBFB,PDE8A,PDE4DIP,CLASP2,SEMA3A,IL31RA,GRID2,TAS1R2,RPS6KA2,PRDM12,SETD4,PTGFR,TENM3,CHERP,MED26,DOCK1,WWC1,C12ORF49,CTDSPL2,NRG3,ASH1L,NOS1AP,LATS2,NOX5,EPB41,SEMA3D,PTH2R,SCAF8,TIAM2,STOX2,KSR2,MAPRE2,PTGIS,PRKAG2,TJP1,UNC5C,HPSE2,PRLR,PAGR1,HIVEP3,FTO,NPAS3,UTRN,KALRN,NTRK3,CBL,ARNT,EGLN2,SEMA5A,FLT3,RAD51B,SUSD4,STAT5B,TOX,ENPP2,GRM5,PLCE1,FER,CASK,MAP2K5,KITLG,LRP2,PIK3CD,SP3,HDGF,ANK2,SAAL1,OLFM1,SH2D1A,EZR,MEGF10,ROBO1,TOM1L1,CHD7,SLC26A6,HTR2B,CDKL5,MECOM,TACC1,TENM1,LMNA,TRAF6,ROBO2,ITPKB,DNAJA3,SLC24A2,VAV2,GRK5,ABI1,NHLH1,ITGB1,TRIOBP,HSP90AA1,SLC24A3,CDC6,PSMB7,KNDC1,ZC4H2,KIF26B,CCDC22,DSCAM,CNGB1,AKAP13,GFI1B,RUNX1,DAB1,OMA1,CDC42EP3,MYO10,ABCC8,HMGN3,NRIP1,DGKI,THRB,STXBP4,EFCAB7,LAMA2,TCIRG1,PTPN11,RBM19,MGAT5,CCT2,CACNB2,CYTH3,HDAC6,GOLGA4,SERTAD2,DDB1,ADORA1,ADAMTS3,EPHA1,IKBKB,ERBB4,ADRA1D,MRE11A,LIMK1,ZNF609,BRD8,ATP8A2,KAT6B,ACTG2,CUL4B,KLHL6,ESR1,MIER1,MAML3,DPP10,CDON,NTRK2,TNRC6A,FRMD4A,STX18,EP300,CELA1,ZYG11B,TENM2,ZNF76,RNF220,FNTA,PDGFB,RIMS1,TNIK,CCND3,TOB2,MID1,BRMS1,CHFR,ALOX5AP,STK39,HNF4G,INSR,FMN2,RERE,ASB5,FUBP1,H2AFY2,TCF7L2,PIP4K2A,CDH4,CAB39,USP34,PAK1,LITAF,FBXW11,ESRRB,MYOM1,MAP3K4,ZDHHC13,PTPRD,RNF144A,BASP1,EGLN3,RBM20,TBR1,SAMD4A,BDNF,TFAP2A,AMIGO1,KCNMA1,PEG3,CHRM3,TGIF2,MEIS1,TENM4,LHFPL2,TRIM13,LCK,IL5RA,ENTPD5,SHC4,ECT2,ZNF148,MTA3,TFDP2,VAMP7,LAMC1,PTPRU,RFTN1,ERN2,DMD,ATG10,SLC30A9,TOX3,LARP4B,USP13,KAT7,SLC8A1,GSN,RBM14,RBM4,MED12L,SATB2,PAFAH1B2,RIT2,JPH2,DEPDC1B,CASS4,FYN,MKRN2,ARNTL,ADAMTS9,NF1,PLCB1,MGMT,LMTK2,ARID4B,RTN4,RTN4R,BCR,RWDD3,BRPF1,CHI3L1,TTN,NDRG4,BMP2K,PIK3R2,RANBP9,NTN1,EGFLAM,DCTN1,NRG1,SH2D3C,CDK19,UBP1,BDKRB1,DOCK8,BID,MAP2K1,FNIP1,STIM1,MYH9,FRMPD4,MAD1L1,VGLL4,PPP3R1,HDAC5,CSRNP3,RBM5,NFIA,RNF4,TMEM30A,CSNK2A3,JDP2,CMKLR1,ROR2,DCN,ZDHHC3,SLC39A14,PCBD2,HNRNPLL,DOCK2,CDH13,CREBRF,SOX13,COPS5,CSPP1,IPO5,MAD2L2,TLE6,RAB27A,CAPRIN2,CCNYL1,NCKAP1,CD99,PDF,TERF2,SLX1B,GOLPH3L,RCC2,BOC,ANO6,ARL3,NEUROD1,RANBP1,CCBE1,USP22,ZDHHC15,JAK2,YTHDC2,FAM168A,FBXW7,OAZ2,SKAP1,CLSTN2,UIMC1,SYT1,ITCH,MLIP,CROCC,NPSR1,BCL11B,PKNOX1,CLDN1,MLLT3,PLS1,PDE2A,SEPT7,KLF15,LAMTOR3,MAGI1,CYFIP2,WNT11,APBB1IP,FIG4,MTA1,NOX4,RPRD1B,CACNA1H,SIAH1,QKI,IL1RAPL1,CCDC62,CAMK2B,ERCC8,PRKCD,SOX6,TAB2,ACVR2A,RUNX2,SEMA5B,CD4,PPM1E,TGFB1,BANP,SGK1,SPSB4,NUP93,TRIM65,NSD1,PIBF1,BTRC,NFATC3,CRADD,F2RL1,BCAS3,C9ORF47,DNAJB2,AAK1,SLIT2,COLGALT1,TP73,CADPS2,HP,HPR,CORO1C,SAP18,RRAS2,C12ORF4,ILF2,DISC1,BLID,CEP135,KANK1,CACNA1D,ZFAND2A,LAMC2,CLN6,MTDH,FANK1,CHRM1,SMAD6,ZNF398,CLOCK,ITSN2,TCF12,ETV6,NELL1,SUCO,TFAP2D,BMPER,BCL3,ANKRD54,DAPK2,TNFRSF10B,ADAM12,DUSP22,NAIP,HNRNPC,DOCK3,SBNO2,LINGO2,YTHDF1,FGF10,CNTNAP2,CIZ1,SMYD3,STXBP5,IQCJ-SCHIP1,SH3RF3,PLIN3,LOXL3,FANCI,SVIL,GLP2R,CAPN3,LUM,VMP1,SMURF2,EPHA4,RORA,HSD17B12,PRKCA,AUTS2,CNR1,CD6,TNFSF11,PPP3CA,NFYB,MAGEA4,MAG,KLF12,CAMK4,PIP5KL1,UFL1,CTNNB1,PARK2,SOD2,FCGR2B,SMARCC1,IGF1R,PPARG,NGRN,AXIN1,DLG5,IL18R1,IL1RL1,MTF1,MSR1,P2RY10,ANKRD17,CYBB,APOL3,SCAMP5,ANXA13,ECM2,SREBF2,RYR2,LEPR,LEPROT,FGF1,NIN,NPAT,NR4A3,FOXK2,DCT,RIOK2,MYOCD,TRIM5,PER2,KIR2DL4,AJUBA,CPNE6,SCN3B,CHEK2,SUPT3H,PHLDB1,PRDM16,SPDYA,HCK,SORBS1,RAB3GAP2,CAPN2,TRIM8,DIO2,CSPG4,GRM1,ANKFY1,NF2,FLT4,MEF2B,HDAC4,PAX2,PHF5A,*

			SPTBN1,TRABD2B,SFRP1,MED13,ST8SIA1,ZNF395,FOXO3,CNTN6,NFIB,BCL2L13,DNM3,SYNCRIP,SMAD3,RNFT2,CUX2,WWP2,ARNT2,WIPI1,EBF3,MTBP,RNF168,C1S,CASZ1,ABI2,GRIK5,SHANK1,SYT3,IFNAR1,DCP1B,NEDD4,NRP2,PREX1,DOCK4,ESRRG,HOXD3,HOXD4,NAV3,SLC4A4,TAB1,SLC6A1,NFATC1,CDC73,APP,SSBP3,GSX2,PDGFRA,DIP2B,NOX1,ARIH1,YAP1,HEG1,SESN1,NLGN2,TNFRSF19,VDAC1,EYA2,SH3D19,BORA,NVL,ALPL,JAK1,ANGPTL4,PEMT,LRP5,MTCP1,POLR3G,ISLR2,SLC9B2,SOX2,SETD2,ZDHHC6,TEAD1,PRICKLE1,SPTBN4,VASH2,ZNF521,DRD1,ARID3A,CHUK,FRS2,MMP2,TPH1,ESR2,SYNE2,S100A12,DCUN1D3,CPNE9,KDM6A,PRKD1,STAT1,PARP6,ST18,ETV5,RHOXF2B,SLC6A3,PLAGL1,HNF4A,ZBTB7C,TASP1,SHANK2,STRA6,VPS4A,EREG,CCNY,TCTN3,FXYD2,SEMA6D,ATF2,POU2F2,TCF3,GRAMD4,RAF1,CELF4,RASGRF2,ACOX2,CARD16,CASP1,PTPN1,BMPR2,CAMK1D,BMPR1A,NLGN1,BTBD10,COL16A1,IKZF4,PIK3R3,CDK12,CAND2,SPAG9,MORC2,RAB11A,EVC,MYB,FGF2,ZNF71,BACH1,PPM1F,NEDD9,AGBL4,SEMA4D,CDC27,DOK6,RORC,ELP3,PLXNA2,PTAFR,ADCYAP1R1,RHOXF2,ABCA13,JARID2,DDX58,DKK2,BRDT,RARB,SPEN,PRKCG,VIPR1,NCOA1,AREL1,BLOC1S5,EEF1E1,EEF1E1-BLOC1S5,EHMT1,GAS8,LMO7,AP2M1,CHRD,DVL3,EIF2B5,EIF4G1,EPHB3,RBX1,TCF20,ANAPC5,ATF3,LIN28B,SHOC2,SRSF5,CKS1B,PAWR,EBF2,AGO3,MAML2,TSG101,CCDC3,LAMB1,TERF2IP,IDE,WDR59,KCNQ1,RFX2,WNT3,ZNF322,RHOJ,P4HB,CCL14,CCL15,NLGN3,SCUBE2,PAFAH1B1,KIF3A,PRDM15,CNTFR,COL4A3,DDAH1,HIP1,AKAP6,RASAL1,NR2C1,TRPC5,UBA2,GLI2,NEO1,TNKS,SCUBE1,WBP2NL,ERCC1,RUFY3,TNRC6B,GLIS3,C9,DAB2,THEMIS,BLM,PKHD1,CACUL1,MYSM1,DZIP1,DLG3,WNK1,RELN,NEK10,SIN3A,RUVBL2,ABCB1,RSPO2,GUCY1A2,DOCK11,GMEB1,PELI1,IQGAP1,MAP3K7,ZNF423,SP1,TRIM22,MACF1,ALK,SLC8A2,INPP5D,STAU1,CLASP1,EEPD1,TEAD4,HOXC13,APBB3,SLC35A4,SRA1,UBE2V1,TPCN1,GPM6B,ADIRF,XDH,OVOL2,SNX5,NFATC2,RGS7,BNC1,ATP2B4,NSF,PACSIN1,ACTL6B,ASXL3,PAK3,MASP1,SSH2,TET1,CAMTA1,CCPG1,ATAD1,ADNP,MAPKAPK2,ARID4A,MAP1B,SCARB1,MARK1,CDK6,PHF2,CELF1,P2RY8,TFRC,UVRAG,EPHA5,WWOX,MEF2A,SYT12,CR1L,DCAF6,DCUN1D5,DROSHA,GFRAL,GPC3,SGIP1,STOM,ADRBK1,TBC1D5,PSMB2,EYA1,PTGER3,SCOC,BRINP1,DIS3L2,TRPV1,TSPAN6,HOXB3,HOXB4,HOXB5,TFEB,CLEC16A,GHR,MNAT1,FLRT2,SNX9,ACTR2,ELAVL4,HDAC1,C2,CFB,SMOC2,TRIM24,ADIPOR2,PLCG2,ROCK1,EPS8,NID1,C1QTNF1,PAXIP1,EGR2,RNF10,RYBP,CAPN10,GPR35,ABCC1,AP3B1,PTPN2,INSRR,NTRK1,KPNA6,TRIM44,ZFAND1,MAP1A,PKN2,SLC39A10,ANK3,UNC13A,EPHB1,EPHA10,PTPN9,AXIN2,SARM1,EPCAM,PARD3,GNL3L,MUC1,TRIM46,TUB,SH3GL3,JAG2,CACNG8,AP3D1,CARM1,VWF,AP2B1,NETO1,ADAM19,SNX33,PIFO,CD84,COCH,ZNF836,SMARCA2,ARHGEF3,CUX1,RAD51C,SPI1,DNMT3B,EPHA7,CHRNB4,MCU,CTNNA1,AIFM2,FBXW4,PSPC1,GLIS1,TRDN,ATG14,LY86,STAC,BCAR3,TRPC6,ABCB7,RBPMS,CHURC1,CREB5,FCHSD2,MAP3K13,RAB15,RAPGEF3,PDXP,MST1,ERCC3,PTK7,SMYD1,SNCA,BMPR1B,CACNG2,CD53,GRM4,MAGI2,PRIM2,PNPT1,USP50,ZEB2,DHRS4,FOXJ3,SYT9,HEY2,RC3H1,EIF3E,RNF19B,WNT7B,KMT2D,PRKAG1,CREM,KMT2C,DFFA,ADTRP,ABCA12,PLD1,MACC1,MITF,SRSF6,OPRD1,HIPK1,PKP2,RPTOR,NFIX,BTK,XPO4,KCTD13,SLC4A8,CBFA2T2,IGF1,MLXIP,PLA2G4E,ATF7IP,CTNNBL1,HTT,DPYSL3,MAPK1,PTEN,MIB1,SPRED2,BMP7,PUM1,SOX5,PCBP2,ZNF780B,LRRK2,MLYCD,OSGIN1,TMEM59,ZRANB1,SPAG5,UNC119,ZBTB20,RFC3,BAG6,LILRB4,TMEM108,ITGAM,IL11RA,PKD2,EFNA5,SHANK3,TADA2A,DAZL,PDGFC,NCOA3,PKIB,PPP2R3C,WNT7A,ZBED6,NLRP1,UACA,LPGAT1,CRTC3,ARNTL2,ELOVL5,PLCL2,KAT6A,GPR21,SIK3,ZNF197,BMP6,TAF15,MYCBP2,ANO1,GPI,SH3RF2,SOS1,TSHR,WDR43,PDCD1LG2,ATRX,PLIN2,PRMT2,RAPGEF2,KCNC2,SMO,VRK3,ENPP3,BLOC1S3,MARK4,CPT1A,TCF4,SYT17,DECR1,SOX30,TFE3,UBR5,GRIN1,JADE1,ARRDC4,RHOA,SPRTN,ROR1,RQCD1,TF,ONECUT2,CUL3,SH3KBP1,TFEC,HSPD1,NFKBID,EPM2A,GSK3B,PLD2,PRR16,DNAJC3,RDX,STAT6,ACTA2,EEF2K,FAM49B,NECAB2,CDK13,CD27,DUSP26,BOK,RNF144B,SULF1,NACC2,SUPT4H1,TNFRSF8,AGT,METTL16,ELOVL3,PRKAA2,SYT7,ADAR,BLOC1S6,FBN2,ZMPSTE24,BRINP3,PTK2,TPPP2,LAT2,SNX3,PDE4B,RIMS2,PBX1,TEC,EXOSC3,CDC14A,MAP3K5,SEC16B,CREBBP,DLX1,NCF1,TAOK2,SHC1,CD160,ECE1,LBX2,RNFT1,RPS6KB1,PRG3,SCEL,SETDB2,ALOX5,SP7,CD44,LARP4,ADAM8,SLC5A3,CLDN4,RPS6KA5,ZNF484,BCL2L14,CDS1,CSNK1A1,PRDM2,CXCL17,NOS1,PLEKHA1,PLVAP,RGS14,ACTN4,MC1R,STK4,EPHB2,SLC1A1,WLS,EDRF1,IL18,LRRC52,UBE2K,GPSM2,HCAR2,DIS3,DLC1,PPARA,PPP1R10,ARID5B,CALCRL,EPB41L4B,SLC25A33,GPSM3,MOB3B,NOTCH4,SYTL4,CRX,PAX6,PRKCZ,ZC3HAV1,FAM20C,ADORA2A,GRIN2B,KCTD7,PPP1CC,RABGEF1,CPEB1,PHLDB2,NIPBL,TMIGD2,YEATS4,ZNF143,ANGPT1,BRF1,CADPS,TBL1X,EYA3,LRRK1,OSBPL8,ATF6,EPB41L2,GCNT2,ETS1,PPP1CA,VWC2,CFI,MAP2,MTF2,RBMS3,CCL22,STPG1,BTN3A2,FBXO31,CDK5RAP1,CNTN1,NCOR2,PRKCQ,BRAF,CSRNP1,DIAPH1,HDAC2,HTR2C,IMPACT,ATP8A1,PHKG2,SRCAP,POU3F3,TNR,UBE2E2,CD300A,ELF2,PHIP,CDKL3,PPP2CA,SKP1,ANGPT4,MAOA,RPL23,SH3BP4,RFC5,SP100,OPN1LW,ANXA2,PARN,RAB27B,CR2,ADCY1,MADD,NUMB,SYK,CNOT1,WWTR1,GRIK2,FRMD5,CIT,MYRIP,ZBTB38,BRCA2,HRK,ATF7,CACNG3,DVL2,YBX1,GRIP1,PTPRJ,TAF1,TICAM1,TANK,THAP3,GPM6A,CADM1,HSF1,MAX,EZH1,NRF1,SRPK2,PLEKHM1,SASH1,ASAP1,NCOA2,GBP5,SULT2B1,BMP1,FCHO1,QRICH1,VAV3,ZP3,CHD6,MOB3A,CAV2,GNA12,SH2D3A,AKTIP,INPP5F,AXL,TNFSF9,TRIM37,WIF1,MET,TBC1D10A,CAMK2D,CCR3,SNX1,TIMELESS,UCHL5,ANKRD6,MA

			LT1,SETD3,TRA2B,EHD2,KDM2B,MLXIPL,SEMA3C,CCT3,NEDD4L,PHB,PTPRC,EDA,GPR89A,CTCF,TYRO3,NR6A1,SPON2,SYNDIG1,TSPAN5,ADCY10,TRAF3IP2,ATF6B,CREB1,RGMB,SGTB,CLNK,EDNRA,PRAP1,BMP4,ABLIM3,CPNE1
GO:0022008	neurogenesis	2.195213545909502e-30	*CLRN1,NRXN1,PRMT3,RDH13,PRKCI,MAP4K4,LLPH,NEGR1,PBX3,CLASP2,SEMA3A,GRID2,PRDM12,TENM3,NRG3,ASTN2,SEMA3D,PHACTR1,MYOT,NREP,TIAM2,NLGN4X,UNC5C,ZSWIM6,KALRN,NTRK3,SEMA5A,TOX,ATP2B2,GRM5,LRP2,ZNF536,OLFM1,NRXN3,ROBO1,CHD7,CDKL5,TENM1,KIF5C,ESRP1,ROBO2,NFASC,ABI1,ULK4,ITGB1,TRIOBP,HSP90AA1,GAS7,KNDC1,ZC4H2,DSCAM,CNGB1,TRIO,RUNX1,DAB1,ABCC8,THRB,LAMA2,PTPN11,DOCK10,HDAC6,GOLGA4,EPHA1,ERBB4,LIMK1,ZNF609,ATP8A2,CUL4B,CECR2,GRM7,PTPRO,CDON,NTRK2,EP300,TENM2,RNF220,HOOK3,RIMS1,TNIK,KIRREL3,RERE,ATP8B1,UNC5D,LAMA3,CDH4,PAK1,PTPRD,SLC4A10,TBR1,STRC,BDNF,AMIGO1,TGIF2,POMGNT2,MEIS1,TENM4,ECT2,NAV2,DENND5A,DMD,SATB2,RIT2,TANC2,FYN,ARNTL,NF1,PCDH15,LMTK2,FAT3,RTN4,APOD,B4GALT6,RTN4R,CCDC141,NDRG4,PAQR3,ADAMTSL1,NTN1,NRG1,FRY,MAP2K1,VAX2,DAGLA,CNTN4,NTNG1,NAV1,TMEM30A,ROR2,SOX13,UST,CAPRIN2,NCKAP1,BOC,ARL3,NEUROD1,ZDHHC15,JAK2,MYPN,TRAPPC9,SYT1,LZTS1,BCL11B,TMC1,PLS1,KLF15,CYFIP2,WNT11,FIG4,KREMEN1,ASTN1,SIAH1,IL1RAPL1,CAMK2B,SOX6,RUNX2,SEMA5B,TGFB1,SGK1,ZHX2,KEL,RPGRIP1L,CELSR1,SLIT3,SDK2,SLIT2,TP73,GABRB1,CDH23,CTNND2,DISC1,KANK1,LAMC2,CLMN,MYT1L,OPCML,CHRM1,ITSN2,TCF12,ETV6,DCLK1,YTHDF1,FGF10,CNTNAP2,ENAH,EPHA4,RORA,AUTS2,CNR1,PPP3CA,MAG,UFL1,TRAK1,CTNNB1,PARK2,SOD2,IGF1R,PPARG,NGRN,DLG5,XK,DRAXIN,LEPR,NIN,DCT,PER2,CPNE6,CSPG4,DCC,NF2,PAX2,SPG11,SFRP1,FOXO3,CNTN6,NFIB,DNM3,CUX2,PTPRM,CASZ1,ABI2,SHANK1,SYT3,NEDD4,NRP2,BTBD3,CRTAC1,HOXD3,NAV3,PRTG,APP,GSX2,CEP85L,DIP2B,YAP1,FSTL4,NLGN2,PTPRG,ISLR2,SOX2,SPTBN4,VASH2,ZNF521,DRD1,FRS2,MMP2,SYNE2,CPNE9,PRKD1,PARP6,DGKG,DSCAML1,SEMA6D,TCF3,SRRM4,BMPR2,USP33,DPYSL2,CAMK1D,BMPR1A,NLGN1,CTNNA2,SPAG9,RAB11A,CRMP1,FGF2,LRRC4C,AGBL4,SEMA4D,ELP3,PLXNA2,RARB,SPEN,NCOA1,SPOCK1,BLOC1S5,NTM,DVL3,EIF2B5,EIF4G1,EPHB3,FBXO45,VCL,LAMB1,MYO9A,KCNQ1,WNT3,SUFU,NLGN3,PAFAH1B1,RASAL1,TRPC5,GLI2,NEO1,RUFY3,DAB2,USH2A,WNK1,RELN,SIN3A,RSPO2,IQGAP1,MACF1,MYO7A,ALK,MBOAT1,GPM6B,PRKG1,PACSIN1,ACTL6B,PAK3,DYX1C1,ADNP,MAP1B,MARK1,CDK6,EPHA5,MEF2A,EYA1,BRINP1,HOXB3,FLRT2,ACTR2,ELAVL4,LGI4,HDAC1,ROCK1,EGR2,RNF10,NTRK1,MAP1A,PPP1R9A,ANK3,UNC13A,PTPRQ,EPHB1,EPHA10,PTPN9,SARM1,PARD3,TRIM46,SH3GL3,JAG2,CARM1,ARHGAP44,CUX1,EPHA7,CTNNA1,MAP3K13,SDK1,ERCC3,PTK7,BMPR1B,MAGI2,HEY2,WNT7B,PTPRS,HIPK1,RTN4RL1,CBFA2T2,DPYSL3,MAPK1,PTEN,MIB1,BMP7,SOX5,LHFPL5,LRRK2,RAPH1,TMEM108,EFNA5,SHANK3,STMN4,TBCD,WNT7A,MAP4,BMP6,MYCBP2,SOS1,TSHR,EXT1,RAPGEF2,SMO,SUN1,BLOC1S3,TCF4,SYT17,RHOA,SYNGAP1,GRIN3A,NDE1,ROR1,ONECUT2,EPM2A,GSK3B,EMB,SYN1,EEF2K,BOK,AGT,HDGFRP3,BLOC1S6,BRINP3,PTK2,SNX3,RIMS2,PBX1,GAP43,DLX1,TAOK2,NDUFS2,ECE1,GPR173,ANKS1A,MDGA2,RPS6KA5,PEX7,TBC1D23,RGS14,TUBB3,EPHB2,SLC1A1,MFSD8,PAX6,PRKCZ,ADORA2A,PPP1CC,KLHL1,NIPBL,VWC2,MAP2,FBXO31,CDK5RAP1,CNTN1,PRKCQ,BRAF,HDAC2,IMPACT,NOTO,TNR,CDKL3,CAMSAP3,SRGAP2,TMEFF1,ADCY1,NUMB,SLIT1,CIT,DVL2,GRIP1,GPRIN3,GPM6A,DHFR,ASAP1,MYEF2,INPP5F,AXL,MET,SEMA3C,NEDD4L,SH3GL2,NCAM1,CREB1,EDNRA,GNGT1,BMP4,CPNE1*
GO:0009966	regulation of signal transduction	1.814793478085714e-29	*RIC8B,ENPP1,PRDX2,GPC5,NRXN1,PRKCI,MAP4K4,PDE4D,PDCL,S1PR2,TMBIM4,SLC9A1,PDE8A,SEMA3A,CHERP,WWC1,C12ORF49,CTDSPL2,FBXL2,ASH1L,NOS1AP,LATS2,NREP,TIAM2,MAPRE2,NLGN4X,PTGIS,WWC3,PRLR,MVP,PAGR1,RGS7BP,KALRN,NTRK3,CBL,ARNT,PLEKHG4B,SEMA5A,FLT3,GRM5,PLCE1,SAMHD1,FER,MARVELD3,MAP2K5,KITLG,PTPRR,LRP2,PIK3CD,ZNF536,DEPDC5,SH2D1A,EZR,ROBO1,TNFAIP8L1,HTR2B,MECOM,TENM1,CAMKMT,LMNA,NMT2,TRAF6,ROBO2,ITPKB,DNAJA3,VAV2,GRK5,ULK4,ITGB1,HSP90AA1,PSMB7,CNIH2,SRGAP3,MCC,CCDC22,TRIO,ARHGAP24,AKAP13,DAB1,RGS6,DGKI,PDE4A,PTPN11,NETO2,HERC4,MGAT5,CYTH3,HDAC6,ADORA1,GPRASP1,ADAMTS3,IKBKB,ERBB4,ADRA1D,GBF1,ABR,ESR1,NPHP3,MIER1,PMEPA1,PTPRO,CDON,NTRK2,EP300,RNF220,RGS22,PDGFB,RIMS1,TNIK,CCND3,MID1,STK39,INSR,AFAP1,TCF7L2,PIP4K2A,USP34,PAK1,LITAF,FBXW11,MAP3K4,ZDHHC13,PTPRD,BDNF,CDK14,FANCA,NPRL3,PHACTR4,TRIM13,UBR2,ARHGAP6,LCK,ENTPD5,ECT2,SNX6,ARHGAP42,PTPRU,ERN2,NPLOC4,DMD,RIT2,DEPDC1B,CASS4,KCTD16,FYN,MKRN2,ARNTL,NF1,PLCB1,RTN4,CHRDL1,APOD,RTN4R,BCR,RWDD3,SHISA9,CHI3L1,NDRG4,BMP2K,PAQR3,PIK3R2,RANBP9,NOMO3,NRG1,ARHGAP10,BDKRB2,DOCK8,BID,MAP2K1,MDFI,FNIP1,RALGPS2,MAD1L1,TMBIM6,VGLL4,PPP2CB,CMKLR1,ROR2,DCN,ZDHHC3,SLC39A14,DOCK2,CDH13,CREBRF,SOX13,PTPRT,COPS5,ARHGAP32,MAD2L2,TLE6,RGS8,CAPRIN2,CCNYL1,RALGPS1,NEUROD1,GNAQ,FBXO9,RFFL,CCBE1,DENND1A,JAK2,FBXW7,LINC00473,ITCH,NPSR1,ZFYVE28,MLLT3,PDE2A,KLF15,LAMTOR3,CYFIP2,WNT11,ARHGAP23,IFT80,TRIM59,KREMEN1,NOX4,SIPA1L3,SIAH1,PRKCD,TAB2,ACVR2A,RUNX2,CD4,SGK1,NUP93,IGSF1,PIBF1,BTRC,CRADD,DLGAP1,F2RL1,RPGRIP1L,SLIT3,AAK1,SLIT2,TP73,CTNND2,DISC1,KANK1,MTDH,SMAD6,CLOCK,ZNF675,BMPER,BCL3,ANKRD54,DAPK2,TNFRSF10B,DUSP22,NAIP,HOMER2,DOCK3,FGF10,FBXL17,RASA4,RASA4B,ARHGEF28,KCTD10,IQCJ-*

			SCHIP1,SH3RF3,LOXL3,CAPN3,SMURF2,EPHA4,RORA,PRKCA,AUTS2,TNFSF11,GNG4,UFL1,CTNNB1,PARK2,SOD2,FCGR2B,ARHGAP22,IGF1R,PPARG,AXIN1,GARNL3,DLG5,IL18R1,P2RY10,ANKRD17,APOL3,SREBF2,DRAXIN,LEPROT,FGF1,RGS10,NOL3,MYOCD,TRIM5,AJUBA,GLG1,CHEK2,PRDM16,SORBS1,TBCK,TRIM8,CSPG4,GRM1,ARHGAP31,OBSCN,NF2,FLT4,SGMS1,BICC1,SPTBN1,TPTE,TRABD2B,SFRP1,FOXO3,CNTN6,ARHGAP29,IFT122,SMAD3,CUX2,ITPR1,WWP2,SHANK1,NEDD4,ARHGEF18,PREX1,TAB1,TXNDC12,NFATC1,CDC73,APP,GSX2,PDGFRA,NOX1,YAP1,HEG1,AMFR,SESN1,FSTL4,NLGN2,TNFRSF19,EYA2,SOX2,GPC6,CHST11,PRICKLE1,RCAN1,ZNF653,TMEM14A,CHUK,FRS2,ESR2,S100A12,PRKD1,STAT1,DGKG,HNF4A,SHANK2,EREG,CCNY,GRAMD4,TMEM237,RAF1,CELF4,RASGRF2,CARD16,CASP1,PTPN1,ADAMTS12,BMPR2,USP33,BMPR1A,TSPAN12,NLGN1,SPAG9,DENND4A,EVC,PDE11A,FGF2,SEMA4D,DOK6,ADCYAP1R1,DKK2,NCOA1,EEF1E1,EEF1E1-BLOC1S5,GAS8,CHRD,DVL3,PTPRE,RBX1,ARHGAP39,ATF3,SHOC2,PAWR,AGO3,DEPTOR,TSG101,CCDC3,TERF2IP,MYO9A,WDR59,WNT3,P4HB,CCL14,CCL15,SUFU,NLGN3,PTPN13,SCUBE2,PAFAH1B1,PRDM15,FAM13A,HIP1,AKAP6,RASAL1,NR2C1,GLI2,NEO1,TNKS,SCUBE1,MGLL,DAB2,PKHD1,UBR1,LDLRAD4,WNK1,RELN,NEK10,RUVBL2,RSPO2,GUCY1A2,PELI1,IQGAP1,MAP3K7,ZNF423,TRIM22,MACF1,ARHGAP12,ALK,SLC8A2,INPP5D,UBE2V1,XDH,LTBP1,OVOL2,SNX5,RGS7,ATP2B4,PAK3,CAMTA1,DYX1C1,GPR161,P2RY8,RNF34,TFRC,WWOX,GFRAL,GPC3,ADRBK1,PSMB2,EYA1,TSPAN6,CLEC16A,GHR,FGD1,HDAC1,INVS,SMOC2,TRIM24,PLCG2,ROCK1,EPS8,C1QTNF1,HIPK3,GPR35,PTPN2,NTRK1,TRIM44,SLC39A10,EIF3A,DNMBP,EPHB1,AXIN2,SARM1,MUC1,TUB,JAG2,CACNG8,CARM1,VWF,NETO1,ARHGAP44,ARHGEF3,SPI1,EPHA7,CTNNA1,LY86,BCAR3,STK38,RBPMS,MAP3K13,OTUD3,SH3BP1,MST1,PTK7,SNCA,BMPR1B,CACNG2,GRM4,MAGI2,ZEB2,HEY2,RC3H1,WNT7B,PTPRS,ZMYND11,KMT2D,SPPL2A,GNG7,RASA1,HIPK1,RPTOR,ZNRF3,KCTD13,CBFA2T2,IGF1,HTT,MAPK1,PTEN,ARHGEF17,SPRED2,BMP7,PUM1,RASA2,PRCP,LRRK2,ZRANB1,CNIH3,LILRB4,IFT81,TMEM108,ARHGAP25,PKD2,SHANK3,RGS9,PDGFC,FAM13B,NCOA3,WNT7A,UACA,CRTC3,PLCL2,KAT6A,GPR21,SIK3,BMP6,IL17RD,SH3RF2,SOS1,PRMT2,RAPGEF2,SMO,VRK3,SOX30,UBR5,GRB14,GRIN1,JADE1,KCTD8,RHOA,SYNGAP1,ROR1,RQCD1,ONECUT2,FAM19A4,CUL3,GSK3B,RDX,ACTA2,MLF1,FAM49B,NECAB2,CD27,DUSP26,BOK,SULF1,NACC2,RNF43,AGT,PRKAA2,ADAR,FBN2,STAT2,ZMPSTE24,HRH4,NDRG2,PTK2,PEX5L,SNX3,PDE4B,RIMS2,VEPH1,MAP3K5,CREBBP,APCDD1L,DLX1,PCSK6,NCF1,TAOK2,SHC1,ARHGAP15,CD160,LBX2,RPS6KB1,GUCY2F,SCEL,ANKS1A,CD44,FGD3,ADAM8,BCL2L14,CSNK1A1,CXCL17,FGD4,PLEKHA1,RGS14,SIPA1L2,ACTN4,ARHGAP11A,MC1R,STK4,ZFAND6,MARK3,SPEF1,EPHB2,WLS,IL18,UBE2K,DLC1,PPARA,PPP1R10,PHLPP1,MAGI3,MOB3B,ZNF366,FBN1,PRKCZ,ZC3HAV1,FAM20C,ADORA2A,RABGEF1,OTUD7B,DENND4B,CNRIP1,ASPN,ANGPT1,TBL1X,EYA3,FHL2,FRMPD1,LRRK1,OSBPL8,ATF6,GCNT2,PPP1CA,TBC1D10C,VWC2,ARHGAP21,RBMS3,CCL22,NCOR2,PRKCQ,BRAF,HDAC2,HTR2C,CD300A,PHIP,PPP2CA,MAOA,RPL23,SH3BP4,SP100,PIGU,ITSN1,SRGAP2,MADD,SYK,CNOT1,WWTR1,ARHGAP19,GRIK2,CIT,RAP1GAP2,CACNG3,DVL2,PTPRJ,TAF1,TICAM1,TANK,ANKRD13C,SASH1,RALGAPA1,CGNL1,VAV3,DLGAP2,CAV2,GNA12,INPP5F,AXL,WIF1,CD109,DENND4C,MET,UCHL5,ANKRD6,MALT1,PHB,PTPRC,EDA,GPR89A,PBLD,TYRO3,RNF213,TSPAN5,NCAM1,TRAF3IP2,ATF6B,SHISA6,TRAP1,PRAP1,BMP4,CPNE1
GO:0036211	protein modification process	4.857697077765972e-29	*LNX2,ZDHHC14,ENPP1,NRXN1,ASPH,B4GALNT2,PRMT3,PRKCI,SLCO3A1,MAP4K4,PDE4D,DNMT1,S1PR2,C6ORF89,ADCY8,MGAT4C,CBFB,PDE8A,IL31RA,RPS6KA2,PRDM12,SETD4,CTDSPL2,FBXL2,NRG3,ASH1L,NOS1AP,LATS2,RNF145,KSR2,PRKAG2,PRLR,MVP,PAGR1,KALRN,DCAF12,NTRK3,CBL,ARNT,EGLN2,FLT3,ENPP2,GRM5,PLCE1,FER,CASK,MAPK4,MAP2K5,KITLG,USP32,MAPK10,PTPRR,PIK3CD,CAMK1G,B3GALT1,ROBO1,TOM1L1,HTR2B,CDKL5,MECOM,TENM1,CAMKMT,LMNA,NMT2,TRAF6,SENP5,ITPKB,DNAJA3,OXR1,GRK5,ABI1,ULK4,TRIOBP,HSP90AA1,CDC6,PSMB7,TSSK1B,KNDC1,ZC4H2,STK38L,TRIO,KLHL12,AKAP13,TTLL5,PTPRK,DAB1,ERC1,KLHL7,MAST4,PTPN11,HERC4,MGAT5,HDAC6,DDB1,ADORA1,MSRA,EPHA1,IKBKB,ERBB4,DPY19L2,MRE11A,LIMK1,TLK1,BRD8,KAT6B,ABR,CUL4B,LARGE,MIER1,PMEPA1,PTPRO,CDON,KANSL1,FBXL18,NTRK2,EP300,RNF220,FNTA,TPST2,PDGFB,FKBP9,TNIK,CCND3,GALNTL6,FBXL7,USP46,BRMS1,CHFR,STK39,INSR,PRKAR1A,ASB5,ST6GALNAC3,JMJD1C,CAB39,USP34,PAK1,FBXW11,MAP3K4,ZDHHC13,PTPRD,RNF144A,EGLN3,BDNF,CDK14,CHRM3,POMGNT2,FUT8,TRIM13,UBR2,HLCS,LCK,ENTPD5,ECT2,MYO3B,MTA3,SNX6,CHML,PPM1L,PTPRU,ERN2,DMD,ATG10,USP13,KAT7,SLC8A1,RBM14,RIT2,MACROD2,CASS4,FYN,MKRN2,ARNTL,NF1,LMTK2,ARID4B,GXYLT2,PPP4R2,FBXO21,B4GALT6,ASPHD2,BCR,NOSIP,RWDD3,BRPF1,CHI3L1,TTN,BMP2K,PAQR3,TPGS2,KLHL21,EGFLAM,PPIG,NRG1,SH2D3C,CDK19,BDKRB1,BDKRB2,FRY,MAP2K1,FNIP1,GALNT16,PPP2CB,HDAC5,RNF4,CSNK2A3,GALNT18,UBE3D,JDP2,WDSUB1,CDKL2,ROR2,DCN,ZDHHC3,WDR70,PTPRT,UST,COPS5,IPO5,MAD2L2,MAN1A1,CAPRIN2,CCNYL1,PHF20L1,PDF,GNAQ,FBXO9,ICK,RFFL,TTLL8,PARP11,HERC5,USP22,ZDHHC15,JAK2,FBXW7,UBR7,UIMC1,ITCH,USP12,ZFYVE28,SMG1,HUNK,B3GALT5,CDC42BPB,PHKA1,DHDDS,KLF15,SPSB1,WNT11,TPST1,TRIM59,MTA1,NOX4,RPRD1B,SIAH1,CAMK2B,ERCC8,PRKCD,TAB2,ACVR2A,CD4,PPM1E,TGFB1,SGK1,P4HA2,SPSB4,MYO3A,NSD1,PIBF1,BTRC,PTPRN2,PDZRN3,DNAJB*

			2,AAK1,KLHL2,SLIT2,ASB15,RNF133,RNF148,HHAT,CORO1C,PTPDC1,TMTC1,TTBK2,KDM4B,SMAD6,TMTC2,CLOCK,ZNF675,BMPER,DCLK1,RNF212,ANKRD54,DAPK2,TNFRSF10B,DAPP1,DUSP22,TRRAP,DOCK3,FGF10,FBXL17,SMYD3,UNKL,KCTD10,SH3RF3,LOXL3,MAST2,FANCI,CAPN3,SMURF2,EPHA4,PRKCA,AUTS2,CD6,TNFSF11,PPP3CA,CAMK4,PIP5KL1,UFL1,TRAK1,CTNNB1,PARK2,IGF1R,PPARG,AXIN1,PRKAR1B,MACROD1,OTUB1,RSPRY1,CDK11A,DYRK4,GALNT8,CDK11B,LEPR,FGF1,WDR45B,PRKAR2A,RIOK2,ESCO1,MYOCD,TRIM5,PER2,AJUBA,CHEK2,SUPT3H,PRDM16,ASB8,PPP1CB,SPDYA,HCK,RAB3GAP2,TBCK,TRIM8,PPP2R2B,CSPG4,BRMS1L,CTDP1,HS3ST5,BAZ1B,OBSCN,NF2,FLT4,HDAC4,SPTBN1,TPTE,TRABD2B,SFRP1,ST8SIA1,PPP6R2,USP54,SSH1,RNFT2,RAB6A,PTPRM,WWP2,WIPI1,MTBP,RNF168,ABI2,C10ORF90,NEDD4,GALNT14,TAB1,ST3GAL3,CDC73,APP,USP49,PDGFRA,SLK,CCNI2,DIP2B,KANSL2,ARIH1,HEG1,AMFR,ST6GALNAC5,CDK3,EYA2,BORA,IBTK,SBK3,JAK1,FPGT-TNNI3K,TNNI3K,LRP5,MTCP1,PTPRG,SETD2,ZDHHC6,PRICKLE1,RCAN1,SPTBN4,DRD1,CHUK,RFT1,S100A12,DCUN1D3,KDM6A,PRKD1,NAA25,PARP6,USP53,LRRC2,SHPRH,EREG,CCNY,MAPKAPK3,ATF2,RAF1,PTPN1,BMPR2,USP33,CAMK1D,BMPR1A,PIK3R3,CDK12,CAND2,SPAG9,MYB,FGF2,PPA2,PPM1F,UBE2R2,NEDD9,AGBL4,PEAK1,SEMA4D,NFX1,CDC27,SETD1A,JARID2,KLHL3,PRKCG,SIN3B,NCOA1,AREL1,EHMT1,LMO7,UCHL3,DVL3,EIF4G1,EPHB3,PTPRE,RBX1,ANAPC5,FBXO45,STT3B,CKS1B,DEPTOR,PKN3,FBXL20,TSG101,DESI1,TERF2IP,FKBP5,RCE1,B3GALNT1,P4HB,SUMF1,PTPN13,QPCTL,PADI6,TRPC5,UBA2,EDEM3,TTLL7,TNKS,WDTC1,DAB2,BLM,UBR1,CACUL1,LDLRAD4,MYSM1,SETD5,DLG3,WNK1,RELN,NEK10,SIN3A,RUVBL2,JOSD1,PELI1,PPP2R5C,IQGAP1,DCAF5,MAP3K7,TRIM22,ZNRF1,ALK,SLC8A2,UBOX5,TOP1,AGBL1,UBE2V1,PADI3,XDH,RBBP6,PRKG1,TTLL9,ATP2B4,PARP12,ACTL6B,PAK3,SSH2,TET1,HECTD4,MKRN3,CAMTA1,ADNP,MAPKAPK2,CEP41,ARID4A,UBE3C,CDC42BPA,MARK1,CDK6,PHF2,RNF34,TFRC,UVRAG,EPHA5,ALG14,DCAF6,DCUN1D5,ADRBK1,PSMB2,EYA1,CDCA3,RNF216,GHR,MNAT1,SNX9,HDAC1,TRIM24,PLCG2,ROCK1,PAXIP1,EGR2,HIPK3,RNF10,RYBP,CUL2,AP3B1,PTPN2,INSRR,NTRK1,TRIM44,PKN2,SLC39A10,PTPRQ,EPHB1,MAN1B1,EPHA10,PTPN9,AXIN2,PARD3,GNL3L,KRTCAP2,MUC1,PTPN14,NUDT14,BCOR,MGAT4A,CARM1,FOLH1,SPI1,DNMT3B,EPHA7,PTPRA,FBXW4,ATG14,BCAR3,STK38,RBPMS,NTMT1,MAP3K13,OTUD3,RAPGEF3,PDXP,PTK7,RSRC1,SMYD1,SNCA,BMPR1B,MAGI2,USP42,USP50,RC3H1,RNF103,RNF103-CHMP3,RNF19B,PTPRS,KMT2D,PRKAG1,CDKL1,KMT2C,UBE2QL1,TRIP12,ADTRP,OPRD1,HIPK1,RPTOR,BTK,ZNRF3,ASPHD1,KCTD13,MLLT1,ZZZ3,IGF1,HTT,MAPK1,PTEN,MIB1,SPRED2,BMP7,PCMTD2,PIK3C3,TTLL4,LRRK2,TMEM59,ZRANB1,PIGS,UNC119,BAG6,LILRB4,MORC3,PKD2,EFNA5,SEL1L2,RCOR3,TADA2A,ALPK3,PDGFC,NCOA3,PKIB,MTMR3,PPP2R3C,RPS6KC1,PLCL2,WDR5B,KAT6A,SIK3,BMP6,MYCBP2,SH3RF2,EXT1,DPY19L4,N4BP1,ATRX,DPH6,C8ORF44-SGK3,PRMT2,RAPGEF2,VRK3,ALG9,MARK4,PRKAR2B,DPY19L1,GALNT10,UBE2H,DNAJC6,UBR5,JADE1,ARRDC4,RHOA,SPRTN,WSB2,ROR1,RQCD1,CUL3,EPM2A,GSK3B,DNAJC3,EEF2K,IWS1,PPP1R16A,PTPRB,CDK13,PGGT1B,STK32B,DUSP26,RNF144B,RNF43,AGT,CCNJL,PRKAA2,ADAR,CUL9,STAT2,ZMPSTE24,PTK2,PHEX,RNF121,TEC,DCAF10,FBXO10,CDC14A,MAP3K5,CPE,TRPC4AP,CREBBP,PRKD3,LMTK3,NCF1,TAOK2,PYGO2,SHC1,PARP10,RNFT1,RPS6KB1,ZDHHC23,GALNT13,GUCY2F,SETDB2,FBXO28,CD44,ADAM8,FKBP14,RPS6KA5,CSNK1A1,HUS1,PRDM2,NOS1,RGS14,STK4,MARK3,EPHB2,SLC1A1,TTLL11,MARCH8,IL18,PCMT1,UBE2K,DLC1,FBXL13,PDP1,PHLPP1,PPIL6,MOB3B,ACP1,RNF150,PAX6,PRKCZ,FAM20C,ADORA2A,MAN2B1,PPP1CC,RABGEF1,CCNG2,TYK2,OTUD7B,NIPBL,YEATS4,PHF20,ANGPT1,TBL1X,EYA3,LRRK1,OSBPL8,GCNT2,PPP1CA,MTF2,FBXO31,ATG3,CDK5RAP1,CNTN1,PRKCQ,BRAF,HDAC2,IMPACT,FKBP3,PHKG2,SRCAP,UBE2E2,C18ORF25,CD300A,PHIP,CDKL3,PPP2CA,SKP1,ANGPT4,RPL23,OPN1LW,PIGU,PRMT7,WIPI2,ALG2,MADD,SYK,WWTR1,GTF2H5,ASB1,CIT,BRCA2,DVL2,PTPRJ,TAF1,PPME1,TICAM1,RNF38,TANK,RIMKLA,GALNT7,HSF1,EZH1,ETF1,RNGTT,SRPK2,SASH1,RNF2,MOB3A,GNA12,SH2D3A,UGGT1,AKTIP,GALNT4,POC1B-GALNT4,INPP5F,NUAK2,AXL,AGBL3,TRIM37,CD109,MET,CAMK2D,UCHL5,MALT1,SETD3,TRIM69,ZDHHC11,ZDHHC11B,KDM2B,MLXIPL,NEDD4L,PHKB,PHB,PTPRC,CTCF,SH3GL2,TYRO3,RNF213,TRAF3IP2,TRIM60,CREB1,METTL21A,TNXB,EDNRA,BMP4*
GO:0006464	cellular protein modification process	4.857697077765972e-29	*LNX2,ZDHHC14,ENPP1,NRXN1,ASPH,B4GALNT2,PRMT3,PRKCI,SLCO3A1,MAP4K4,PDE4D,DNMT1,S1PR2,C6ORF89,ADCY8,MGAT4C,CBFB,PDE8A,IL31RA,RPS6KA2,PRDM12,SETD4,CTDSPL2,FBXL2,NRG3,ASH1L,NOS1AP,LATS2,RNF145,KSR2,PRKAG2,PRLR,MVP,PAGR1,KALRN,DCAF12,NTRK3,CBL,ARNT,EGLN2,FLT3,ENPP2,GRM5,PLCE1,FER,CASK,MAPK4,MAP2K5,KITLG,USP32,MAPK10,PTPRR,PIK3CD,CAMK1G,B3GALT1,ROBO1,TOM1L1,HTR2B,CDKL5,MECOM,TENM1,CAMKMT,LMNA,NMT2,TRAF6,SENP5,ITPKB,DNAJA3,OXR1,GRK5,ABI1,ULK4,TRIOBP,HSP90AA1,CDC6,PSMB7,TSSK1B,KNDC1,ZC4H2,STK38L,TRIO,KLHL12,AKAP13,TTLL5,PTPRK,DAB1,ERC1,KLHL7,MAST4,PTPN11,HERC4,MGAT5,HDAC6,DDB1,ADORA1,MSRA,EPHA1,IKBKB,ERBB4,DPY19L2,MRE11A,LIMK1,TLK1,BRD8,KAT6B,ABR,CUL4B,LARGE,MIER1,PMEPA1,PTPRO,CDON,KANSL1,FBXL18,NTRK2,EP300,RNF220,FNTA,TPST2,PDGFB,FKBP9,TNIK,CCND3,GALNTL6,FBXL7,USP46,BRMS1,CHFR,STK39,INSR,PRKAR1A,ASB5,ST6GALNAC3,JMJD1C,CAB39,USP34,*

			PAK1,FBXW11,MAP3K4,ZDHHC13,PTPRD,RNF144A,EGLN3,BDNF,CDK14,CHRM3,POMGNT2,FUT8,TRIM13,UBR2,HLCS,LCK,ENTPD5,ECT2,MYO3B,MTA3,SNX6,CHML,PPM1L,PTPRU,ERN2,DMD,ATG10,USP13,KAT7,SLC8A1,RBM14,RIT2,MACROD2,CASS4,FYN,MKRN2,ARNTL,NF1,LMTK2,ARID4B,GXYLT2,PPP4R2,FBXO21,B4GALT6,ASPHD2,BCR,NOSIP,RWDD3,BRPF1,CHI3L1,TTN,BMP2K,PAQR3,TPGS2,KLHL21,EGFLAM,PPIG,NRG1,SH2D3C,CDK19,BDKRB1,BDKRB2,FRY,MAP2K1,FNIP1,GALNT16,PPP2CB,HDAC5,RNF4,CSNK2A3,GALNT18,UBE3D,JDP2,WDSUB1,CDKL2,ROR2,DCN,ZDHHC3,WDR70,PTPRT,UST,COPS5,IPO5,MAD2L2,MAN1A1,CAPRIN2,CCNYL1,PHF20L1,PDF,GNAQ,FBXO9,ICK,RFFL,TTLL8,PARP11,HERC5,USP22,ZDHHC15,JAK2,FBXW7,UBR7,UIMC1,ITCH,USP12,ZFYVE28,SMG1,HUNK,B3GALT5,CDC42BPB,PHKA1,DHDDS,KLF15,SPSB1,WNT11,TPST1,TRIM59,MTA1,NOX4,RPRD1B,SIAH1,CAMK2B,ERCC8,PRKCD,TAB2,ACVR2A,CD4,PPM1E,TGFB1,SGK1,P4HA2,SPSB4,MYO3A,NSD1,PIBF1,BTRC,PTPRN2,PDZRN3,DNAJB2,AAK1,KLHL2,SLIT2,ASB15,RNF133,RNF148,HHAT,CORO1C,PTPDC1,TMTC1,TTBK2,KDM4B,SMAD6,TMTC2,CLOCK,ZNF675,BMPER,DCLK1,RNF212,ANKRD54,DAPK2,TNFRSF10B,DAPP1,DUSP22,TRRAP,DOCK3,FGF10,FBXL17,SMYD3,UNKL,KCTD10,SH3RF3,LOXL3,MAST2,FANCI,CAPN3,SMURF2,EPHA4,PRKCA,AUTS2,CD6,TNFSF11,PPP3CA,CAMK4,PIP5KL1,UFL1,TRAK1,CTNNB1,PARK2,IGF1R,PPARG,AXIN1,PRKAR1B,MACROD1,OTUB1,RSPRY1,CDK11A,DYRK4,GALNT8,CDK11B,LEPR,FGF1,WDR45B,PRKAR2A,RIOK2,ESCO1,MYOCD,TRIM5,PER2,AJUBA,CHEK2,SUPT3H,PRDM16,ASB8,PPP1CB,SPDYA,HCK,RAB3GAP2,TBCK,TRIM8,PPP2R2B,CSPG4,BRMS1L,CTDP1,HS3ST5,BAZ1B,OBSCN,NF2,FLT4,HDAC4,SPTBN1,TPTE,TRABD2B,SFRP1,ST8SIA1,PPP6R2,USP54,SSH1,RNFT2,RAB6A,PTPRM,WWP2,WIPI1,MTBP,RNF168,ABI2,C10ORF90,NEDD4,GALNT14,TAB1,ST3GAL3,CDC73,APP,USP49,PDGFRA,SLK,CCNI2,DIP2B,KANSL2,ARIH1,HEG1,AMFR,ST6GALNAC5,CDK3,EYA2,BORA,IBTK,SBK3,JAK1,FPGT-TNNI3K,TNNI3K,LRP5,MTCP1,PTPRG,SETD2,ZDHHC6,PRICKLE1,RCAN1,SPTBN4,DRD1,CHUK,RFT1,S100A12,DCUN1D3,KDM6A,PRKD1,NAA25,PARP6,USP53,LRRC2,SHPRH,EREG,CCNY,MAPKAPK3,ATF2,RAF1,PTPN1,BMPR2,USP33,CAMK1D,BMPR1A,PIK3R3,CDK12,CAND2,SPAG9,MYB,FGF2,PPA2,PPM1F,UBE2R2,NEDD9,AGBL4,PEAK1,SEMA4D,NFX1,CDC27,SETD1A,JARID2,KLHL3,PRKCG,SIN3B,NCOA1,AREL1,EHMT1,LMO7,UCHL3,DVL3,EIF4G1,EPHB3,PTPRE,RBX1,ANAPC5,FBXO45,STT3B,CKS1B,DEPTOR,PKN3,FBXL20,TSG101,DESI1,TERF2IP,FKBP5,RCE1,B3GALNT1,P4HB,SUMF1,PTPN13,QPCTL,PADI6,TRPC5,UBA2,EDEM3,TTLL7,TNKS,WDTC1,DAB2,BLM,UBR1,CACUL1,LDLRAD4,MYSM1,SETD5,DLG3,WNK1,RELN,NEK10,SIN3A,RUVBL2,JOSD1,PELI1,PPP2R5C,IQGAP1,DCAF5,MAP3K7,TRIM22,ZNRF1,ALK,SLC8A2,UBOX5,TOP1,AGBL1,UBE2V1,PADI3,XDH,RBBP6,PRKG1,TTLL9,ATP2B4,PARP12,ACTL6B,PAK3,SSH2,TET1,HECTD4,MKRN3,CAMTA1,ADNP,MAPKAPK2,CEP41,ARID4A,UBE3C,CDC42BPA,MARK1,CDK6,PHF2,RNF34,TFRC,UVRAG,EPHA5,ALG14,DCAF6,DCUN1D5,ADRBK1,PSMB2,EYA1,CDCA3,RNF216,GHR,MNAT1,SNX9,HDAC1,TRIM24,PLCG2,ROCK1,PAXIP1,EGR2,HIPK3,RNF10,RYBP,CUL2,AP3B1,PTPN2,INSRR,NTRK1,TRIM44,PKN2,SLC39A10,PTPRQ,EPHB1,MAN1B1,EPHA10,PTPN9,AXIN2,PARD3,GNL3L,KRTCAP2,MUC1,PTPN14,NUDT14,BCOR,MGAT4A,CARM1,FOLH1,SPI1,DNMT3B,EPHA7,PTPRA,FBXW4,ATG14,BCAR3,STK38,RBPMS,NTMT1,MAP3K13,OTUD3,RAPGEF3,PDXP,PTK7,RSRC1,SMYD1,SNCA,BMPR1B,MAGI2,USP42,USP50,RC3H1,RNF103,RNF103-CHMP3,RNF19B,PTPRS,KMT2D,PRKAG1,CDKL1,KMT2C,UBE2QL1,TRIP12,ADTRP,OPRD1,HIPK1,RPTOR,BTK,ZNRF3,ASPHD1,KCTD13,MLLT1,ZZZ3,IGF1,HTT,MAPK1,PTEN,MIB1,SPRED2,BMP7,PCMTD2,PIK3C3,TTLL4,LRRK2,TMEM59,ZRANB1,PIGS,UNC119,BAG6,LILRB4,MORC3,PKD2,EFNA5,SEL1L2,RCOR3,TADA2A,ALPK3,PDGFC,NCOA3,PKIB,MTMR3,PPP2R3C,RPS6KC1,PLCL2,WDR5B,KAT6A,SIK3,BMP6,MYCBP2,SH3RF2,EXT1,DPY19L4,N4BP1,ATRX,DPH6,C8ORF44-SGK3,PRMT2,RAPGEF2,VRK3,ALG9,MARK4,PRKAR2B,DPY19L1,GALNT10,UBE2H,DNAJC6,UBR5,JADE1,ARRDC4,RHOA,SPRTN,WSB2,ROR1,RQCD1,CUL3,EPM2A,GSK3B,DNAJC3,EEF2K,IWS1,PPP1R16A,PTPRB,CDK13,PGGT1B,STK32B,DUSP26,RNF144B,RNF43,AGT,CCNJL,PRKAA2,ADAR,CUL9,STAT2,ZMPSTE24,PTK2,PHEX,RNF121,TEC,DCAF10,FBXO10,CDC14A,MAP3K5,CPE,TRPC4AP,CREBBP,PRKD3,LMTK3,NCF1,TAOK2,PYGO2,SHC1,PARP10,RNFT1,RPS6KB1,ZDHHC23,GALNT13,GUCY2F,SETDB2,FBXO28,CD44,ADAM8,FKBP14,RPS6KA5,CSNK1A1,HUS1,PRDM2,NOS1,RGS14,STK4,MARK3,EPHB2,SLC1A1,TTLL11,MARCH8,IL18,PCMT1,UBE2K,DLC1,FBXL13,PDP1,PHLPP1,PPIL6,MOB3B,ACP1,RNF150,PAX6,PRKCZ,FAM20C,ADORA2A,MAN2B1,PPP1CC,RABGEF1,CCNG2,TYK2,OTUD7B,NIPBL,YEATS4,PHF20,ANGPT1,TBL1X,EYA3,LRRK1,OSBPL8,GCNT2,PPP1CA,MTF2,FBXO31,ATG3,CDK5RAP1,CNTN1,PRKCQ,BRAF,HDAC2,IMPACT,FKBP3,PHKG2,SRCAP,UBE2E2,C18ORF25,CD300A,PHIP,CDKL3,PPP2CA,SKP1,ANGPT4,RPL23,OPN1LW,PIGU,PRMT7,WIPI2,ALG2,MADD,SYK,WWTR1,GTF2H5,ASB1,CIT,BRCA2,DVL2,PTPRJ,TAF1,PPME1,TICAM1,RNF38,TANK,RIMKLA,GALNT7,HSF1,EZH1,ETF1,RNGTT,SRPK2,SASH1,RNF2,MOB3A,GNA12,SH2D3A,UGGT1,AKTIP,GALNT4,POC1B-GALNT4,INPP5F,NUAK2,AXL,AGBL3,TRIM37,CD109,MET,CAMK2D,UCHL5,MALT1,SETD3,TRIM69,ZDHHC11,ZDHHC11B,KDM2B,MLXIPL,NEDD4L,PHKB,PHB,PTPRC,CTCF,SH3GL2,TYRO3,RNF213,TRAF3IP2,TRIM60,CREB1,METTL21A,TNXB,EDNRA,BMP4

GO:0007165	signal transduction	7.864492541437179e-29	*CD247,RIC8B,ENPP1,PRDX2,GPC5,NRXN1,ASPH,GP6,PRKCI,MAP4K4,PDE4D,PDCL,DNMT1,S1PR2,TMBIM4,SLC9A1,PDE7B,ADCY8,LMBR1,PDE8A,CLASP2,SEMA3A,IL31RA,GRID2,TAS1R2,RPS6KA2,PTGFR,TENM3,CHERP,DOCK1,WWC1,CLDN18,C12ORF49,CTDSPL2,FBXL2,NRG3,ASH1L,NOS1AP,LATS2,ANP32A,SEMA3D,PTH2R,NREP,TIAM2,KSR2,MAPRE2,NLGN4X,PTGIS,PRKAG2,WWC3,GRM8,UNC5C,OR5P2,OR5P3,PRLR,MVP,PAGR1,ADCY7,RGS7BP,KALRN,PITPNC1,NTRK3,CBL,ARNT,ATRNL1,PLEKHG4B,EGLN2,MIA,RAB4B,RAB4B-EGLN2,SEMA5A,LRRFIP2,FLT3,STAT5B,GRM5,PLCE1,SAMHD1,FER,MARVELD3,MAPK4,MAP2K5,KITLG,MAPK10,PTPRR,SPNS2,LRP2,PIK3CD,ZNF536,DEPDC5,HDGF,ANK2,OLFM1,SH2D1A,EZR,NRXN3,ROBO1,TOM1L1,TNFAIP8L1,HTR2B,ITGB6,MECOM,TENM1,CAMKMT,LMNA,NUCB1,GOT1,NMT2,INPP4B,TRAF6,ROBO2,ITPKB,DNAJA3,SLC24A2,VAV2,GRK5,ABI1,ULK4,ITGB1,HSP90AA1,CDC6,PSMB7,CNIH2,SRGAP3,MCC,TSSK1B,KNDC1,CCDC22,DSCAM,STK38L,GMDS,SAMD12,CNGB1,TRIO,ARHGAP24,KLHL12,AKAP13,PDE1A,PTPRK,DAB1,CDC42EP3,MYO10,ERC1,RGS6,DGKI,ANKS1B,THRB,STXBP4,PDE4A,GABRA3,ITGB3BP,GFRA2,MAST4,IL17RB,PTPN11,NETO2,HERC4,MGAT5,DOCK10,CYTH3,HDAC6,DDB1,ADORA1,GPRASP1,ADAMTS3,EPHA1,IKBKB,ERBB4,ADRA1D,SORCS1,MRE11A,GBF1,LIMK1,TLK1,BRD8,ABR,SNIP1,KLHL6,ESR1,NPHP3,GRM7,MIER1,PMEPA1,MAML3,PTPRO,CDON,NTRK2,EP300,CELA1,TENM2,RNF220,RGS22,FNTA,PDGFB,RIMS1,TNIK,CCND3,MID1,CHFR,BPI,STK39,DAP3,HNF4G,INSR,FMN2,PRKAR1A,ASB5,AFAP1,UNC5D,LAMA3,TCF7L2,PIP4K2A,CAB39,USP34,PAK1,LITAF,FBXW11,ESRRB,MYOM1,MAP3K4,ZDHHC13,PTPRD,BDNF,CDK14,FANCA,CHRM3,TGIF2,FUT8,OR2T3,NPRL3,PHACTR4,TENM4,GRIK4,TRIM13,RASGEF1B,UBR2,ARHGAP6,LCK,MDM4,EDARADD,IL5RA,ENTPD5,SHC4,ECT2,SNX6,CHML,PPM1L,ARHGAP42,PTPRU,RFTN1,ERN2,CDC45,LSP1,AP3S1,NPLOC4,SGCD,DMD,CENPF,ATG10,LRRN2,KAT7,SLC8A1,RBM14,GPR171,RIT2,DRG2,DEPDC1B,CASS4,KCTD16,FYN,MKRN2,ARNTL,NF1,PLCB1,DST,LMTK2,RTN4,RXFP1,CHRDL1,DGKH,APOD,RTN4R,BCR,PPP1R12B,RWDD3,SHISA9,CHI3L1,TTN,NDRG4,BMP2K,PAQR3,PIK3R2,RANBP9,NTN1,NOMO3,NRG1,ARHGAP10,SH2D3C,BDKRB1,BDKRB2,DOCK8,BID,MAP2K1,MDFI,FNIP1,RAPGEF6,VAX2,RALGPS2,MYH9,MAD1L1,DAGLA,TMBIM6,VGLL4,PPP2CB,PPP3R1,VSTM1,OR6N2,CMKLR1,INPP4A,CDKL2,ROR2,DCN,ZDHHC3,SLC39A14,DOCK2,CDH13,CREBRF,SOX13,FHIT,PTPRT,COPS5,FGF14,ARHGAP32,MAD2L2,TLE6,RGS8,CAPRIN2,CCNYL1,NLN,NCKAP1,DEFA1B,DEFA3,PTGER2,RALGPS1,FOXN3,RCC2,BOC,ANO6,ARL3,NEUROD1,GNAQ,FBXO9,ICK,TMEM117,RFFL,RANBP1,CCBE1,DENND1A,JAK2,FBXW7,LINC00473,SKAP1,UIMC1,ITCH,NPSR1,ZFYVE28,SMG1,DTHD1,HUNK,MLLT3,ARAP2,CDC42BPB,DOCK9,SH2D6,TCF7,PDE2A,KLF15,ANXA4,LAMTOR3,MAGI1,CYFIP2,WNT11,ARHGAP23,IFT80,TRIM59,APBB1IP,MTA1,KREMEN1,NRG4,CLPB,NOX4,SIPA1L3,MKLN1,SIAH1,IL1RAPL1,CAMK2B,PRKCD,TAB2,ACVR2A,RUNX2,SEMA5B,CD4,TGFB1,PLCXD3,SGK1,SPSB4,NUP93,IGSF1,PIBF1,BTRC,NFATC3,CRADD,DLGAP1,F2RL1,C9ORF47,GRIK3,RPGRIP1L,CELSR1,SLIT3,AAK1,ZFYVE9,SLIT2,ASB15,TP73,GABRR3,GABRB1,HHAT,GPR176,CORO1C,RRAS2,CTNND2,OR9A4,DISC1,KANK1,CACNA1D,PTPDC1,MTDH,ANK1,TTBK2,XPR1,CHRM1,OR51A7,OR51F2,OR51T1,SMAD6,RXFP2,CLOCK,ZNF675,PLCB4,KCNH1,BMPER,BCL3,DCLK1,ANKRD54,DAPK2,TNFRSF10B,DAPP1,ADAM12,DUSP22,GPR52,NAIP,HOMER2,DOCK3,FGF10,FBXL17,RASA4,RASA4B,ARHGEF28,KCTD10,IL1RAPL2,IQCJ-SCHIP1,SH3RF3,LOXL3,RHPN2,MAST2,GLP2R,ADCY2,CAPN3,SMURF2,EPHA4,RORA,PRKCA,AUTS2,CNR1,CD6,TNFSF11,PPP3CA,NSUN2,OR51B2,OR51B4,OR51I1,GNG4,CAMK4,UFL1,CTNNB1,PARK2,SOD2,FCGR2A,FCGR2B,FCGR3A,FCGR3B,ARHGAP22,SMARCC1,IGF1R,PPARG,AXIN1,GARNL3,DLG5,IL18R1,IL1RL1,P2RY10,TRPM1,ANKRD17,CYBB,CASKIN1,APOL3,BRIP1,SREBF2,RYR2,DRAXIN,LEPR,LEPROT,FGF1,RGS10,NR4A3,NOL3,PRKAR2A,MYOCD,OR52E6,OR52E8,OR52N1,TRIM5,KIR2DL1,KIR2DL4,AJUBA,CACNA1C,GLG1,CHEK2,PRDM16,MYO1E,ASB8,PPP1CB,HCK,SORBS1,TBCK,TRIM8,CSPG4,GRM1,DCC,ARHGAP31,P2RY14,OBSCN,RGL1,NF2,FLT4,SGMS1,BICC1,HDAC4,PAX2,TNS3,SPTBN1,TPTE,TRABD2B,SFRP1,FOXO3,DGKB,TRHDE,ARL13B,CNTN6,ARHGAP29,IFT122,SMAD3,CUX2,ITPR1,PTPRM,WWP2,ACKR2,ABI2,GRIK5,PSAP,SHANK1,IFNAR1,OR14K1,NEDD4,NRP2,ARHGEF18,VANGL1,PREX1,PLA2G4C,DOCK4,ESRRG,HOXD3,KPNB1,TAB1,TXNDC12,NFATC1,RAB6C,CDC73,APP,GSX2,PDGFRA,FCRL4,NOX1,YAP1,HEG1,AMFR,SESN1,FSTL4,NLGN2,TNFRSF19,EYA2,JAK1,LRP5,MUC20,PTPRG,GNG2,SOX2,GPC6,CHST11,TEAD1,PRICKLE1,RCAN1,ZNF653,GRIA3,DRD1,TMEM14A,GLRA2,CHUK,ERLIN1,FRS2,MMP2,SERGEF,ESR2,S100A12,KDM6A,PRKD1,STAT1,ST18,DGKG,RND3,HNF4A,LRRC2,SHANK2,VPS4A,EREG,CCNY,TCTN3,MAPKAPK3,SEMA6D,ATF2,GRAMD4,TMEM237,RAF1,CELF4,RASGRF2,CARD16,CASP1,PTPN1,ADAMTS12,BMPR2,USP33,DPYSL2,BMPR1A,TSPAN12,NLGN1,COL16A1,PIK3R3,SPAG9,DENND4A,EVC,PDE11A,EPG5,FGF2,GRIK1,OR6C70,PPM1F,TICRR,ADCY5,NEDD9,SEMA4D,DOK6,RORC,MCTP1,PLXNA2,PTAFR,ADCYAP1R1,ANKDD1A,DDX58,DKK2,SORCS2,ITGA11,PI4KA,RARB,SPEN,PIK3C2B,PRKCG,VIPR1,NCOA1,EEF1E1,EEF1E1-BLOC1S5,GRIA2,GAS8,CHRD,DVL3,ECE2,EIF2B5,EPHB3,PTPRE,RBX1,ARHGAP39,ATF3,SHOC2,PAWR,AGO3,DEPTOR,PKN3,MAML2,TSG101,CCDC3,TERF2IP,MYO9A,IDE,WDR59,KCNQ1,WNT3,RCE1,RHOJ,P4HB,CCL14,CCL15,CCL15-CCL14,SUFU,TG,NLGN3,PTPN13,SCUBE2,PAFAH1B1,PRDM15,FAM13A,CNTFR,*

			COL4A3,TRPA1,DDAH1,NMUR2,HIP1,AKAP6,RASAL1,NR2C1,IRS4,GLI2,NEO1,TNKS,SCUBE1,GPR39,MGLL,DAB2,THEMIS,BLM,PKHD1,UBR1,LDLRAD4,DZIP1,WNK1,RELN,NEK10,RUVBL2,OR5H2,OR5H6,OR5K4,RSPO2,GUCY1A2,CSF3R,DOCK11,PELI1,PPP2R5C,IQGAP1,MAP3K7,DDX47,TAS2R41,ZNF423,OR56B1,TRIM22,MACF1,ARHGAP12,ALK,SLC8A2,INPP5D,TEAD4,UBE2V1,XDH,LTBP1,OVOL2,SNX5,NFATC2,OR6C74,PRKG1,RGS7,ATP2B4,PAK3,CAMTA1,DYX1C1,P2RX6,ASGR2,ADNP,GPR161,MAPKAPK2,CDC42BPA,COL4A6,SCARB1,MARK1,CDK6,P2RY8,RNF34,TFRC,EPHA5,SORCS3,WWOX,GABRG3,MEF2A,ADCY9,FNBP1,GFRAL,GPC3,ADRBK1,PSMB2,ZNF207,EYA1,PTGER3,GNB3,TRPV1,TSPAN6,CLEC16A,GHR,FLRT2,FGD1,HDAC1,INVS,SMOC2,TRIM24,ADIPOR2,PLCG2,ROCK1,EPS8,C1QTNF1,PAXIP1,HIPK3,RNF10,GAPVD1,GPR35,CUL2,AP3B1,PTPN2,INSRR,NTRK1,TRIM44,PKN2,PPP1R9A,SLC39A10,EIF3A,ANK3,DNMBP,UNC13A,EPHB1,PDE6A,EPHA10,AXIN2,SARM1,EPCAM,PARD3,RCAN2,MUC1,TUB,SH3GL3,JAG2,CACNG8,CARM1,VWF,NETO1,ARHGAP44,HTR1D,ARHGEF3,SPI1,DGKK,EPHA7,CHRNB4,MCU,RANBP10,PTPRA,CTNNA1,FBXW4,GABRA6,LY86,STAC,BCAR3,STK38,RBPMS,CHURC1,MAP3K13,OTUD3,RAB15,RAPGEF3,SH3BP1,MST1,PTK7,SNCA,BMPR1B,CACNG2,CD53,GRM4,MAGI2,USP50,ZEB2,HEY2,RC3H1,CNGA3,WNT7B,PTPRS,ZMYND11,KMT2D,PRKAG1,CREM,DFFA,SPPL2A,ADTRP,GNG7,PLD1,RASA1,MACC1,MITF,OPRD1,CABIN1,HIPK1,RPTOR,BTK,SIGLEC9,ZNRF3,KCTD13,TROVE2,CBFA2T2,IGF1,HTT,MAPK1,PTEN,ARHGEF17,MIB1,SPRED2,BMP7,PIK3C3,PUM1,RASA2,PRCP,RAB30,LRRK2,OSGIN1,GNA14,ZRANB1,UNC119,CNIH3,RAPH1,BAG6,LILRB4,IFT81,TMCO1,TMEM108,ITGAM,ARHGAP25,IL11RA,NSG2,PKD2,EFNA5,SHANK3,BTBD11,RGS9,PDGFC,AMOTL1,FAM13B,NCOA3,WNT7A,RPS6KC1,UACA,CRTC3,GPR141,PLCL2,KAT6A,GPR21,RABEP1,RHOT1,SIK3,BMP6,ANO1,GPI,IL17RD,SH3RF2,SOS1,TSHR,EXT1,PDCD1LG2,ATRX,PRICKLE2,GABRR2,PRMT2,RAPGEF2,KCNC2,SMO,VRK3,MARK4,PRKAR2B,PSEN2,TNFRSF11B,PAG1,SOX30,UBR5,GRB14,GRIN1,JADE1,KCTD8,RHOA,WSB2,OR4M1,OR4N2,SYNGAP1,VIPR2,GRIN3A,ROR1,RQCD1,TF,ONECUT2,RHOBTB1,FAM19A4,CUL3,HSPD1,ITPR2,NFKBID,EPM2A,GABRB3,GSK3B,ABCG8,PLD2,GNG12,RDX,STAT6,ACTA2,MLF1,FAM49B,NECAB2,STK32B,CD27,DUSP26,ITGBL1,MCTP2,BOK,SULF1,KRT8,NACC2,RNF43,TNFRSF8,AGT,HDGFRP3,PRKAA2,ADAR,FBN2,PITPNM2,STAT2,ZMPSTE24,HRH4,NDRG2,PTK2,LAT2,PEX5L,SNX3,PDE4B,RIMS2,TCP11,TEC,SHB,VEPH1,GAP43,MAP3K5,CPE,CREBBP,IQGAP2,PRKD3,APCDD1L,DLX1,FMOD,INSL6,PCSK6,NCF1,SLC29A1,TAOK2,PYGO2,SHC1,ARHGAP15,CD160,ECE1,LBX2,RPS6KB1,STC2,GPR173,GUCY2F,SCEL,ANKS1A,ITGAL,CD44,FGD3,ADAM8,RPS6KA5,BCL2L14,CDS1,CSNK1A1,GRID1,HUS1,CXCL17,FCRL6,FGD4,NOS1,PLEKHA1,PLVAP,RGS14,SIPA1L2,ACTN4,ARHGAP11A,MC1R,STK4,ZFAND6,MARK3,SPEF1,EPHB2,SLC1A1,WLS,SIGMAR1,XCR1,IL18,DTNA,MFSD8,UBE2K,GPSM2,HCAR1,HCAR2,HCAR3,DLC1,PPARA,PPP1R10,ARID5B,CALCRL,MCOLN1,SLC25A33,PHLPP1,MAGI3,CPEB4,MOB3B,NOTCH4,ZNF366,FBN1,PAX6,PRKCZ,ZC3HAV1,FAM20C,ADORA2A,GRIN2B,PPP1CC,RABGEF1,PLCXD2,TYK2,OTUD7B,DENND4B,CNRIP1,ASPN,ANGPT1,TBL1X,EYA3,FHL2,FRMPD1,LRRK1,OSBPL8,ATF6,GCNT2,PPP1CA,TBC1D10C,VWC2,ARHGAP21,GRIA4,RBMS3,CCL22,BTN3A2,FBXO31,CNTN1,NCOR2,PRKCQ,BRAF,CSRNP1,HDAC2,HTR2C,CD300A,IRAK1BP1,PHIP,PPP2CA,ANGPT4,ARHGEF6,MAOA,RPL23,SH3BP4,DCDC1,SP100,OPN1LW,PIGU,C1QTNF9,CR2,DONSON,FCRL2,ITSN1,SRGAP2,ADCY1,MADD,SYK,CNOT1,WWTR1,ARHGAP19,GRIK2,ASB1,CIT,RADIL,BRCA2,HRK,RAP1GAP2,CACNG3,DVL2,GRIP1,PTPRJ,SLC10A1,TAF1,TICAM1,TANK,UCN2,ITGAE,TAC3,HSF1,INPP5A,SRPK2,ANKRD13C,PLEKHM1,SASH1,RALGAPA1,BMP1,CGNL1,FCHO1,QRICH1,VAV3,ZP3,DLGAP2,MOB3A,CAV2,GNA12,SH2D3A,COL4A5,INPP5F,NUAK2,AXL,REPS2,TNFSF9,WIF1,CD109,DENND4C,MET,CAMK2D,CCR3,RAD9B,TIMELESS,UCHL5,ANKRD6,MALT1,MLXIPL,SEMA3C,THEM4,PHB,PTPRC,EDA,GPR89A,PBLD,SH3GL2,TYRO3,NR6A1,RNF213,TSPAN5,ADCY10,NCAM1,TRAF3IP2,ATF6B,CREB1,RASSF8,RGMB,SHISA6,TRAP1,CLNK,EDNRA,GNGT1,PRAP1,BMP4,CASP12,CPNE1
GO:0048583	regulation of response to stimulus	1.1384268334153873e-28	*CD247,RIC8B,ENPP1,PRDX2,GPC5,NRXN1,PRKCI,MAP4K4,PDE4D,PDCL,S1PR2,HLX,TMBIM4,SLC9A1,ADCY8,PDE8A,CLASP2,SEMA3A,GRID2,SETD4,CHERP,WWC1,C12ORF49,CTDSPL2,FBXL2,ASH1L,NOS1AP,LATS2,SEMA3D,NREP,TIAM2,MAPRE2,NLGN4X,PTGIS,WWC3,PRLR,MVP,PAGR1,ADCY7,RGS7BP,KALRN,NTRK3,CBL,ARNT,PLEKHG4B,SEMA5A,FLT3,SUSD4,STAT5B,GRM5,PLCE1,SAMHD1,FER,CASK,MARVELD3,MAP2K5,KITLG,MAPK10,PTPRR,SPNS2,LRP2,PIK3CD,ZNF536,DEPDC5,SH2D1A,EZR,ROBO1,TNFAIP8L1,HTR2B,MECOM,TENM1,CAMKMT,LMNA,NMT2,TRAF6,ROBO2,ITPKB,DNAJA3,OXR1,VAV2,GRK5,ULK4,ITGB1,HSP90AA1,PSMB7,CNIH2,SRGAP3,MCC,CCDC22,DSCAM,TRIO,ARHGAP24,AKAP13,DAB1,ABCC8,RGS6,DGKI,PDE4A,PTPN11,NETO2,HERC4,MGAT5,CYTH3,HDAC6,ADORA1,GPRASP1,ADAMTS3,IKBKB,ERBB4,ADRA1D,GBF1,LIMK1,ABR,KLHL6,ESR1,NPHP3,MIER1,PMEPA1,PTPRO,CDON,NTRK2,EP300,RNF220,RGS22,PDGFB,RIMS1,TNIK,CCND3,MID1,ALOX5AP,STK39,INSR,FMN2,AFAP1,TCF7L2,PIP4K2A,USP34,PAK1,LITAF,FBXW11,MAP3K4,ZDHHC13,PTPRD,TBR1,BDNF,CDK14,FANCA,FUT8,NPRL3,PHACTR4,TRIM13,UBR2,ARHGAP6,LCK,ENTPD5,ECT2,SNX6,VAMP7,ARHGAP42,PTPRU,RFTN1,ERN2,NPLOC4,DMD,USP13,KAT7,RBM14,RIT2,DEPDC1B,CASS4,KCTD16,FYN,MKRN2,ARNTL,NF1,PLCB1,MGMT,PPP4R2,RTN4,CHRDL1,APOD,RTN4R,BCR,RWDD3,SHISA9,CHI3L1,NDRG4,BMP2K,PAQR3,PIK3R2,RANBP9,NOMO3,NRG1,ARHGAP10,CDK19,BDKRB2,DOCK8,BID,MAP2K1,*

			MDFI,FNIP1,RALGPS2,MAD1L1,LRFN5,DAGLA,TMBIM6,VGLL4,PPP2CB,CMKLR1,TSPAN8,ROR2,DCN,ZDHHC3,SLC39A14,DOCK2,CDH13,CREBRF,SOX13,PTPRT,AUNIP,COPS5,ARHGAP32,MAD2L2,TLE6,RGS8,CAPRIN2,CCNYL1,TERF2,RALGPS1,ANO6,NEUROD1,GNAQ,FBXO9,RFFL,CCBE1,DENND1A,HERC5,JAK2,FAM168A,FBXW7,LINC00473,SKAP1,UIMC1,ITCH,MLIP,NPSR1,ZFYVE28,SMG1,CLDN1,MLLT3,PDE2A,KLF15,LAMTOR3,CYFIP2,WNT11,ARHGAP23,IFT80,TRIM59,KREMEN1,CLPB,NOX4,SIPA1L3,SIAH1,CAMK2B,ERCC8,PRKCD,TAB2,ACVR2A,RUNX2,SEMA5B,CD4,TGFB1,SGK1,NUP93,MMP28,IGSF1,PIBF1,BTRC,CRADD,DLGAP1,F2RL1,RPGRIP1L,SLIT3,AAK1,SLIT2,TP73,CTNND2,C12ORF4,DISC1,KANK1,MTDH,MMP26,SMAD6,CLOCK,ZNF675,BMPER,BCL3,ANKRD54,DAPK2,TNFRSF10B,DUSP22,NAIP,HOMER2,DOCK3,SBNO2,YTHDF1,FGF10,FBXL17,RASA4,RASA4B,ARHGEF28,KCTD10,IQCJ-SCHIP1,SH3RF3,LOXL3,CAPN3,SMURF2,EPHA4,RORA,PRKCA,AUTS2,CNR1,TNFSF11,PPP3CA,GNG4,UFL1,CTNNB1,PARK2,SOD2,FCGR2A,FCGR2B,FCGR3A,FCGR3B,ARHGAP22,IGF1R,PPARG,AXIN1,GARNL3,OTUB1,DLG5,IL18R1,IL1RL1,P2RY10,ANKRD17,APOL3,SREBF2,DRAXIN,LEPROT,FGF1,PROS1,RGS10,NR4A3,NOL3,MYOCD,TRIM5,KIR2DL1,KIR2DL4,AJUBA,GLG1,CHEK2,UBQLN4,PRDM16,HCK,SORBS1,TBCK,TRIM8,CSPG4,GRM1,ARHGAP31,CTDP1,OBSCN,NF2,FLT4,SGMS1,BICC1,HDAC4,SPTBN1,TPTE,TRABD2B,SFRP1,FOXO3,CNTN6,ARHGAP29,IFT122,SMAD3,RNFT2,CUX2,ITPR1,WWP2,RNF168,C1S,SHANK1,NEDD4,ARHGEF18,PREX1,TAB1,TXNDC12,NFATC1,CDC73,APP,GSX2,PDGFRA,NOX1,YAP1,HEG1,AMFR,SESN1,FSTL4,NLGN2,TNFRSF19,EYA2,POLR3G,SOX2,SETD2,GPC6,CHST11,PRICKLE1,RCAN1,ZNF653,DRD1,TMEM14A,CHUK,FRS2,ESR2,S100A12,PRKD1,STAT1,DGKG,SLC6A3,HNF4A,SHANK2,EREG,CCNY,MAPKAPK3,SEMA6D,GRAMD4,TMEM237,RAF1,CELF4,RASGRF2,ACOX2,CARD16,CASP1,PTPN1,ADAMTS12,GGT7,BMPR2,USP33,CAMK1D,BMPR1A,TSPAN12,NLGN1,CTNNA2,SPAG9,DENND4A,EVC,PDE11A,EPG5,MYB,FGF2,PPM1F,ADCY5,SEMA4D,DOK6,MCTP1,PTAFR,ADCYAP1R1,JARID2,DDX58,DKK2,PRKCG,NCOA1,AREL1,EEF1E1,EEF1E1-BLOC1S5,GAS8,CHRD,DVL3,EIF2B5,EIF4G1,PTPRE,RBX1,ARHGAP39,ATF3,SHOC2,PAWR,AGO3,DEPTOR,TSG101,CCDC3,TERF2IP,MYO9A,WDR59,WNT3,P4HB,CCL14,CCL15,SUFU,NLGN3,PTPN13,SCUBE2,PAFAH1B1,PRDM15,FAM13A,DDAH1,HIP1,AKAP6,RASAL1,NR2C1,GLI2,NEO1,TNKS,SCUBE1,ERCC1,MGLL,C9,DAB2,THEMIS,PKHD1,UBR1,LDLRAD4,WNK1,RELN,NEK10,SIN3A,RUVBL2,ABCB1,RSPO2,GUCY1A2,PELI1,IQGAP1,MAP3K7,ZNF423,TRIM22,MACF1,ARHGAP12,ALK,SLC8A2,INPP5D,CLASP1,UBE2V1,XDH,LTBP1,OVOL2,SNX5,NFATC2,PRKG1,RGS7,ATP2B4,PAK3,MASP1,CAMTA1,DYX1C1,GPR161,MAPKAPK2,CDK6,CELF1,P2RY8,RNF34,TFRC,WWOX,MEF2A,CR1L,DROSHA,GFRAL,GPC3,ADRBK1,PSMB2,EYA1,PTGER3,SHPK,RNF216,TSPAN6,CLEC16A,GHR,FGD1,ACTR2,HDAC1,INVS,RECQL5,C2,CFB,SMOC2,TRIM24,PLCG2,ROCK1,EPS8,C1QTNF1,PAXIP1,HIPK3,GPR35,ABCC1,AP3B1,PTPN2,NTRK1,TRIM44,SLC39A10,EIF3A,DNMBP,EPHB1,AXIN2,SARM1,MUC1,TUB,JAG2,CACNG8,CARM1,VWF,NETO1,ARHGAP44,CD84,COCH,ARHGEF3,SPI1,EPHA7,MCU,CTNNA1,AIFM2,PSPC1,LY86,BCAR3,STK38,RBPMS,MAP3K13,OTUD3,SH3BP1,MST1,PTK7,SNCA,BMPR1B,CACNG2,GRM4,MAGI2,PNPT1,ZEB2,HEY2,RC3H1,WNT7B,PTPRS,ZMYND11,KMT2D,TRIP12,SPPL2A,ADTRP,GNG7,RASA1,SRSF6,HIPK1,RPTOR,RTN4RL1,BTK,ZNRF3,KCTD13,CBFA2T2,IGF1,HTT,MAPK1,PTEN,ARHGEF17,SPRED2,BMP7,PUM1,RASA2,PCBP2,PRCP,LRRK2,ZRANB1,CNIH3,BAG6,LILRB4,IFT81,TMEM108,ITGAM,ARHGAP25,PKD2,SHANK3,NT5E,RGS9,PDGFC,FAM13B,NCOA3,PPP2R3C,WNT7A,NLRP1,UACA,CRTC3,PLCL2,KAT6A,GPR21,SIK3,BMP6,MYCBP2,IL17RD,SH3RF2,SOS1,N4BP1,PRMT2,RAPGEF2,SMO,VRK3,ENPP3,SOX30,UBR5,GRB14,GRIN1,JADE1,KCTD8,RHOA,SYNGAP1,GRIN3A,ROR1,RQCD1,ONECUT2,FAM19A4,CUL3,HSPD1,NFKBID,GSK3B,PLD2,RDX,STAT6,ACTA2,MLF1,FAM49B,NECAB2,CD27,DUSP26,BOK,SULF1,NACC2,RNF43,AGT,POLH,PRKAA2,ADAR,FBN2,STAT2,ZMPSTE24,HRH4,NDRG2,PTK2,LAT2,PEX5L,SNX3,PDE4B,RIMS2,TEC,EXOSC3,VEPH1,MAP3K5,CREBBP,APCDD1L,DLX1,PCSK6,NCF1,TAOK2,SHC1,ARHGAP15,CD160,LBX2,RNFT1,RPS6KB1,GUCY2F,SCEL,ANKS1A,ALOX5,DNAJB6,CD44,FGD3,ADAM8,CLDN4,BCL2L14,CSNK1A1,CXCL17,FGD4,PLEKHA1,RGS14,SIPA1L2,ACTN4,ARHGAP11A,MC1R,STK4,ZFAND6,MARK3,SPEF1,EPHB2,WLS,IL18,UBE2K,DNAJC7,DLC1,PPARA,PPP1R10,CALCRL,PHLPP1,MAGI3,GPSM3,MOB3B,ZNF366,FBN1,PRKCZ,ZC3HAV1,CD96,FAM20C,ADORA2A,RABGEF1,PHLDB2,OTUD7B,DENND4B,CNRIP1,ASPN,ANGPT1,TBL1X,EYA3,FHL2,FRMPD1,LRRK1,OSBPL8,ATF6,GCNT2,ETS1,PPP1CA,TBC1D10C,VWC2,ARHGAP21,CFI,RBMS3,CCL22,BTN3A2,NCOR2,PRKCQ,BRAF,HDAC2,HTR2C,TNR,CD300A,PHIP,PPP2CA,MAOA,RPL23,SH3BP4,SP100,PIGU,CR2,ITSN1,SRGAP2,MADD,SYK,CNOT1,WWTR1,ARHGAP19,GRIK2,SLIT1,CIT,RAP1GAP2,CACNG3,DVL2,YBX1,PTPRJ,TAF1,TICAM1,TANK,CADM1,HSF1,DHFR,ANKRD13C,SASH1,RALGAPA1,GBP5,CGNL1,FCHO1,VAV3,ZP3,DLGAP2,CAV2,GNA12,INPP5F,AXL,WIF1,CD109,DENND4C,MET,CAMK2D,TIMELESS,UCHL5,ANKRD6,MALT1,ZDHHC11,SEMA3C,PHB,PTPRC,EDA,GPR89A,PBLD,TYRO3,RNF213,TSPAN5,NCAM1,TRAF3IP2,ATF6B,SHISA6,TRAP1,CLNK,PRAP1,BMP4,CASP12,CPNE1
GO:0065009	regulation of molecular function	2.547050164640545e-	*RIC8B,ENPP1,PRDX2,LDB2,NRXN1,ASPH,PRKCI,SLCO3A1,MAP4K4,PDE4D,SLC9A1,C6ORF89,ADCY8,DOCK1,NRG3,NOS1AP,LATS2,TBC1D19,EPB41,PHACTR1,TIAM2,MAPRE2,PTGIS,PRKAG2,PRLR,MVP,UTRN,KALRN,SEC23B,PHACTR2,NTR*

		28	*K3,CBL,PLEKHG4B,FLT3,GRM5,PLCE1,FER,MAP2K5,KITLG,MAPK10,DENND2D,DEPDC5,ANK2,XRCC4,ROBO1,TOM1L1,HTR2B,PSMD1,CDKL5,TENM1,NUCB1,TRAF6,DNAJA3,VAV2,ABI1,ITGB1,HSP90AA1,CDC6,CNIH2,SRGAP3,KNDC1,ZC4H2,CCDC22,TRIO,ARHGAP24,AKAP13,DAB1,ABCC8,ERC1,NCF4,RGS6,DGKI,NETO2,MGAT5,CCT2,CACNB2,DOCK10,CYTH3,HDAC6,ADORA1,EPHA1,IKBKB,PEX14,ERBB4,MRE11A,GBF1,LIMK1,ABR,ESR1,GRM7,PTPRO,CDON,NTRK2,EP300,RNF220,MCF2L2,FNTA,PDGFB,RIMS1,CCND3,BRMS1,ALOX5AP,STK39,INSR,PRKAR1A,TCF7L2,PIP4K2A,HPS1,CAB39,PAK1,MAP3K4,EGLN3,BDNF,AMIGO1,FANCA,CHRM3,NPRL3,PHACTR4,KCNRG,TRIM13,RASGEF1B,ARHGAP6,LCK,ECT2,SNX6,CHML,ARHGAP42,CST2,ERN2,DENND5A,DMD,SLC8A1,GSN,JPH2,DEPDC1B,CASS4,FYN,NF1,NCF2,PLCB1,MGMT,LMTK2,SNCB,PPP4R2,RXFP1,DPEP1,DGKH,RTN4R,BCR,PPP1R12B,NOSIP,RWDD3,BRPF1,SHISA9,CHI3L1,TTN,BMP2K,PAQR3,PIK3R2,NRG1,ARHGAP10,SMAP2,SH2D3C,DOCK8,BID,FRY,MAP2K1,MDFI,FNIP1,RAPGEF6,STIM1,RALGPS2,TMBIM6,PPP2CB,PPP3R1,HDAC5,CMKLR1,SBF2,ITIH2,ROR2,SLC39A14,PRPSAP2,DOCK2,PTPRT,COPS5,PPP1R42,FGF14,IPO5,ARHGAP32,MAD2L2,RGS8,CAPRIN2,CCNYL1,TERF2,RALGPS1,RCC2,NEUROD1,GNAQ,RFFL,RANBP1,DENND1A,HERC5,JAK2,TRAPPC9,FBXW7,OAZ2,ITCH,ZFYVE28,ARAP2,DOCK9,DENND2A,ANXA4,LAMTOR3,CYFIP2,WNT11,ARHGAP23,NOX4,SIPA1L3,PRKCD,TAB2,ACVR2A,CD4,PPM1E,TGFB1,SGK1,PCSK5,NSD1,PIBF1,BTRC,DUS2,JPH3,CRADD,DLGAP1,F2RL1,BCAS3,DNAJB2,SLIT2,ITIH4,HP,CORO1C,DISC1,BLID,CACNA1D,CALML4,MTDH,TTBK2,FANK1,RXFP2,CLOCK,ITSN2,ZNF675,TIMP2,BCL3,ANKRD54,TNFRSF10B,PARVA,DUSP22,RABGAP1L,NAIP,DOCK3,FGF10,RASA4,RASA4B,ARHGEF28,SMYD3,STXBP5,CAPN3,VMP1,EPHA4,CNR1,TNFSF11,SMG6,PPP3CA,UFL1,CTNNB1,PARK2,SOD2,FCGR2B,ARHGAP22,IGF1R,PPARG,AXIN1,GARNL3,PRKAR1B,IL18R1,ADD2,RYR2,LEPR,FGF1,PROS1,RGS10,NOL3,PRKAR2A,AGAP1,MYOCD,TRIM5,PER2,AJUBA,CACNA1C,SCN3B,SPDYA,HCK,CSTL1,TBC1D14,RAB3GAP2,TBCK,TRIM8,PPP2R2B,AGFG2,ARHGAP31,MMP16,OBSCN,RGL1,NF2,FLT4,HDAC4,PAX2,SFRP1,MED13,PPP6R2,ARHGAP29,RANBP3,BCL2L13,SMAD3,WWP2,SIMC1,PSAP,SHANK1,DCP1B,NEDD4,ARHGEF18,PREX1,DOCK4,TAB1,APP,PDGFRA,CCNI2,HEG1,AMFR,TEN1,NLGN2,BORA,IBTK,NVL,PDE6D,ANGPTL4,LRP5,MTCP1,SOX2,RCAN1,ECSIT,DRD1,CHUK,FRS2,SERGEF,ESR2,S100A12,DCUN1D3,PRKD1,ST18,TAF3,ARHGEF33,ZBTB7C,EREG,CCNY,FXYD2,FXYD6,PITRM1,ATF2,TCF3,GRAMD4,RAF1,RASGRF2,CARD16,CASP1,PTPN1,BMPR2,USP33,CAMK1D,BMPR1A,NLGN1,PIK3R3,CDK12,DENND4A,CRMP1,FGF2,PPM1F,ADCY5,NEDD9,SEMA4D,SH3PXD2A,ELP3,PLXNA2,PTAFR,ADCYAP1R1,DDX58,DKK2,SPOCK1,GAS8,DVL3,EIF2B5,EIF4G1,EPHB3,PSMD2,ARHGAP39,SHOC2,CKS1B,EBF2,DEPTOR,TSG101,TERF2IP,MYO9A,IDE,KCNQ1,CCL14,CCL15,SUFU,NLGN3,PAFAH1B1,FAM13A,COL4A3,DDAH1,NMUR2,HIP1,AKAP6,RASAL1,TNKS,WDTC1,DAB2,BLM,PKHD1,CACUL1,DZIP1,DLG3,WNK1,RELN,NEK10,SIN3A,ABCB1,COMMD6,DOCK11,PELI1,PPP2R5C,IQGAP1,MAP3K7,TRIM22,ARHGAP12,ALK,SLC8A2,UBE2V1,XDH,AHNAK,PRKG1,RGS7,ATP2B4,CCPG1,ADNP,SCARB1,RNF34,TFRC,UVRAG,EPHA5,DCUN1D5,GPC3,STOM,TBC1D5,GHR,MNAT1,FGD1,SNX9,RPGR,HDAC1,PLCG2,ROCK1,PAXIP1,HIPK3,GAPVD1,GPR35,AP3B1,PTPN2,INSRR,NTRK1,SLC39A10,ANK3,DNMBP,EPHB1,EPHA10,AXIN2,RCAN2,GNL3L,KRTCAP2,CACNG8,CARM1,NETO1,ANXA8L1,ARHGAP44,PIFO,LPA,ARHGEF3,SPI1,EPHA7,ACAP2,TRDN,ATG14,STAC,BCAR3,STK38,TRPC6,RENBP,MAP3K13,RAPGEF3,SH3BP1,SNCA,BMPR1B,CACNG2,MAGI2,PRIM2,TBC1D9,USP50,ZEB2,HEY2,EIF3E,PRKAG1,PSMF1,CAST,DFFA,RASA1,OPRD1,CABIN1,RPTOR,BTK,TBC1D16,MLLT1,IGF1,HTT,MAPK1,PTEN,ARHGEF17,SPRED2,BMP7,RASA2,LRRK2,UNC119,CNIH3,RFC3,LILRB4,ITGAM,ARHGAP25,PKD2,EFNA5,SHANK3,MIA2,RGS9,PDGFC,SERPINA3,SERPINA4,SERPINA5,FAM13B,NCOA3,PKIB,SGSM1,PPP2R3C,TBCD,NLRP1,UACA,CRTC3,AGAP5,PLCL2,RABEP1,SH3PXD2B,MYCBP2,GPI,SH3RF2,SOS1,TSHR,SERINC5,PRMT2,RAPGEF2,KCNC2,SMO,VRK3,PPP1R37,PRKAR2B,STXBP5L,PSEN2,GRIN1,ARRDC4,RHOA,SYNGAP1,VIPR2,PPP3R2,ROR1,FAM19A4,HSPD1,EPM2A,GSK3B,DNAJC3,RDX,PPP1R16A,PTPRB,CD27,BOK,AGT,CCNJL,ADAR,ZMPSTE24,PTK2,PDE4B,PBX1,MAP3K5,IQGAP2,PCSK6,NCF1,TAOK2,UGT1A1,UGT1A10,UGT1A4,UGT1A7,UGT1A8,PYGO2,SHC1,ARHGAP15,PARP10,SERPINB11,DNAJB6,CD44,FGD3,ADAM8,SLC5A3,ARFGAP3,CLDN4,RPS6KA5,CSNK1A1,FGD4,NOS1,GCKR,RGS14,SIPA1L2,ACTN4,ARHGAP11A,STK4,MARK3,COX6A1,EPHB2,SLC1A1,IL18,LRRC52,GPSM2,DIS3,DLC1,PPARA,PPP1R10,ARID5B,GRTP1,PDP1,GPSM3,MOB3B,PAX6,PRKCZ,ADORA2A,GRIN2B,KCTD7,RABGEF1,CCNG2,DENND4B,CNRIP1,COL28A1,ANGPT1,OSBPL8,PPP1CA,TBC1D10C,ARHGAP21,MAP2,CCL22,CDK5RAP1,PRKCQ,HDAC2,PPP1R14A,SPINT2,CD300A,PPP2CA,SKP1,ANGPT4,ARHGEF6,DNAJC9,RPL23,SH3BP4,RFC5,SP100,ANXA2,PARN,PRMT7,ITSN1,SRGAP2,ADCY1,MADD,SYK,WWTR1,ARHGAP19,CIT,RAP1GAP2,CACNG3,DVL2,PTPRJ,TAF1,PPME1,TICAM1,TANK,HSF1,DHFR,ANKRD13C,SASH1,DENND2C,ASAP1,RALGAPA1,VAV3,DLGAP2,RNF2,MOB3A,CAV2,GNA12,SH2D3A,AKTIP,AXL,TRIM37,CD109,DENND4C,MET,TBC1D10A,CAMK2D,UCHL5,MALT1,SERPINE3,NEDD4L,PHB,PTPRC,EDA,TYRO3,MTMR12,SHISA6,TNXB,EDNRA,BMP4,CPNE1*
GO:0048523	negative regulation of	3.10552679 94434715e-	*ENPP1,PRDX2,LDB2,NRXN1,ASPH,B4GALNT2,PRMT3,PRKCI,MAP4K4,PDE4D,DNMT1,S1PR2,HLX,TMBIM4,SLC9A1,ADCY8,TRPS1,CBFB,PDE8A,CLASP2,SEMA3A,IL31RA,GRID2,RPS6KA2,PRDM12,PTGFR,CHERP,WWC1,CLDN18,CTDSPL2,RYR*

	cellular process	28	*1,NRG3,ASH1L,LATS2,ASTN2,SEMA3D,SCAF8,FHOD3,NLGN4X,PTGIS,PRKAG2,WWC3,TJP1,NUDT6,PRLR,MVP,FTO,RGS7BP,KALRN,NTRK3,CBL,SEMA5A,STAT5B,GRM5,SAMHD1,ZNF566,FER,CASK,MARVELD3,MAP2K5,KITLG,PTPRR,LRP2,ZNF536,SP3,DEPDC5,HDGF,ANK2,SH2D1A,EZR,ROBO1,MALRD1,TOM1L1,TNFAIP8L1,CHD7,HTR2B,MECOM,TENM1,RYR3,LMNA,TRAF6,ROBO2,ITPKB,DNAJA3,OXR1,SLC24A2,GRK5,ABI1,ITGB1,TRIOBP,CDC6,PSMB7,TSC22D3,CNIH2,SRGAP3,MCC,PCDH17,CCDC22,DSCAM,SRGAP2B,CDAN1,TRIO,ARHGAP24,GFI1B,PTPRK,RUNX1,DAB1,OMA1,ABCC8,NRIP1,RGS6,DGKI,THRB,DACH1,PTPN11,HERC4,MGAT5,ORC2,HDAC6,SERTAD2,DDB1,ADORA1,GPRASP1,EPHA1,IKBKB,PEX14,ERBB4,GBE1,MRE11A,LIMK1,ATP8A2,KAT6B,ESR1,NPHP3,GRM7,MIER1,PCBP3,PMEPA1,PTPRO,NTRK2,TNRC6A,FRMD4A,EP300,CELA1,TENM2,RGS22,HOOK3,PDGFB,CCND3,TOB2,MID1,ZNF19,BRMS1,CHFR,SCML4,BPI,STK39,FMN2,RERE,PRKAR1A,ATP8B1,H2AFY2,TCF7L2,PIP4K2A,CAB39,PAK1,LITAF,FBXW11,ESRRB,PTPRD,BASP1,TBR1,SAMD4A,BDNF,TFAP2A,PEG3,ZIM2,TGIF2,NPRL3,PHACTR4,MEIS1,KCNRG,UBR2,ARHGAP6,LCK,MDM4,ZNF148,MTA3,SNX6,TFDP2,ARHGAP42,CST2,PTPRU,ERN2,CDC45,C1D,NPLOC4,DENND5A,DMD,CENPF,TOX3,PDS5A,LARP4B,KAT7,SLC8A1,GSN,RBM14,RBM4,GPR171,SATB2,RIT2,HIRA,JPH2,FYN,MKRN2,ARNTL,ADAMTS9,NF1,PLCB1,MGMT,SNCB,FAT3,RTN4,DPEP1,CHRDL1,APOD,RTN4R,BCR,NDRG4,PAQR3,IFI30,PIK3R2,RANBP9,NTN1,NOMO3,NRG1,ARHGAP10,SLC43A2,BDKRB1,BDKRB2,DOCK8,BID,FRY,MAP2K1,MDFI,FNIP1,VAX2,MYH9,MAD1L1,LRFN5,TMBIM6,VGLL4,PPP2CB,CNTN4,HDAC5,RBM5,ZNF692,JDP2,ITIH2,ROR2,DCN,SCFD1,CDH13,CREBRF,TRDMT1,SOX13,FHIT,PTPRT,AUNIP,COPS5,IPO5,MAD2L2,TLE6,RGS8,CAPRIN2,TERF2,SLX1B,FOXN3,RCC2,CERKL,NEUROD1,GNAQ,RFFL,JAK2,YTHDC2,FBXW7,LINC00473,OAZ2,UIMC1,ITCH,MLIP,NPSR1,ZFYVE28,BBS12,LZTS1,BCL11B,SMG1,MLLT3,ATP9A,TEX11,TCF7,PDE2A,KLF15,TBX15,ANXA4,WNT11,IFT80,TRIM59,FIG4,MTA1,KREMEN1,KLF8,NOX4,LCOR,IL1RAPL1,PRKCD,SOX6,TAB2,RUNX2,SEMA5B,PPM1E,TGFB1,MMP28,NSD1,IGSF1,PIBF1,ZHX2,KEL,BTRC,DUS2,NFATC3,JPH3,CRADD,F2RL1,BCAS3,C9ORF47,GRIK3,CDYL2,RPGRIP1L,DNAJB2,SLIT3,CC2D1B,SLIT2,TP73,ITIH4,HP,CORO1C,SAP18,KANK1,MTDH,CLMN,TTBK2,FANK1,MYT1L,KDM4B,SMAD6,ZNF398,CLOCK,ZNF675,ETV6,NELL1,TFAP2D,BMPER,TIMP2,BCL3,DCLK1,DUSP22,NAIP,HNRNPC,HOMER2,SBNO2,YTHDF1,FGF10,RASA4,RASA4B,SMYD3,KANK4,KCTD10,IQCJ-SCHIP1,LOXL3,RHPN2,SVIL,CAPN3,SMURF2,EPHA4,RORA,PRKCA,CNR1,SMG6,PPP3CA,NSUN2,GNG4,MAGEA4,MAG,KLF12,GATAD2B,PIP5KL1,UFL1,CTNNB1,PARK2,SOD2,METTL13,FCGR2B,SMARCC1,CDHR2,IGF1R,PPARG,AXIN1,PRKAR1B,OTUB1,DLG5,ADD2,CIPC,CBX5,ANKRD17,SCAMP5,BRIP1,ANXA13,ANKRD26,LRPPRC,SREBF2,RYR2,DRAXIN,LEPR,LEPROT,PROS1,RGS10,NPAT,NR4A3,FOXK2,NOL3,PRKAR2A,MYOCD,PER2,KIR2DL4,KIR3DL2,AJUBA,CACNA1C,GLG1,CHEK2,UBQLN4,PRDM16,HCK,CSTL1,TBC1D14,BRMS1L,DCC,CTDP1,BAZ1B,NF2,FLT4,BICC1,HDAC4,PAX2,SPTBN1,TPTE,TRABD2B,SFRP1,FOXO3,NFIB,ZCCHC17,IFT122,DNM3,SSH1,SYNCRIP,SMAD3,CUX2,ITPR1,PTPRM,WWP2,SIMC1,MTBP,RNF168,SLC16A2,LZTFL1,SHANK1,DCP1B,MIER3,NEDD4,ARHGEF18,NAV3,SLC6A1,TXNDC12,NFATC1,PRTG,CDC73,APP,PDGFRA,ADD3,DIP2B,YAP1,HEG1,AMFR,RAB11FIP5,SESN1,TEN1,FSTL4,VDAC1,EYA2,IBTK,ANGPTL4,LRP5,PTPRG,SOX2,KIF24,CHST11,PRICKLE1,RCAN1,ZNF653,SPTBN4,DRD1,TMEM14A,ERLIN1,FRS2,PALB2,SFMBT1,VILL,MMP2,SERGEF,ESR2,DCUN1D3,PRKD1,STAT1,ST18,DGKG,ETV5,TAF3,PLAGL1,HNF4A,ZBTB7C,SHANK2,VPS4A,EREG,SEMA6D,ATF2,TCF3,GRAMD4,RAF1,CELF4,CARD16,PTPN1,ADAMTS12,BMPR2,VBP1,CAMK1D,BMPR1A,NLGN1,BTBD10,CTNNA2,IKZF4,CDK12,SPAG9,CRMP1,PDE11A,MYB,FGF2,BACH1,MXD3,PPM1F,TICRR,AGBL4,BEND5,SEMA4D,NFX1,RORC,MCTP1,PLXNA2,ADCYAP1R1,YME1L1,JARID2,DKK2,SORCS2,RAB11FIP3,PHC2,RARB,SPEN,PRKCG,SIN3B,SPOCK1,AREL1,EEF1E1,TXNDC5,EHMT1,GAS8,AP2M1,CHRD,EIF4G1,PTPRE,RBX1,ATF3,LIN28B,PAWR,AGO3,DEPTOR,DLEC1,TSG101,CCDC3,VCL,TERF2IP,CLIC4,CRYM,KCNQ1,WNT3,SUFU,MAGEA11,PTPN13,PAFAH1B1,PRDM15,CNTFR,COL4A3,TRPA1,DDAH1,AKAP6,RASAL1,NR2C1,TRPC5,GLI2,TNKS,ERCC1,RUFY3,TNRC6B,GLIS3,WDTC1,DAB2,BLM,PKHD1,USH2A,UBR1,LDLRAD4,DLG3,WNK1,SIN3A,RUVBL2,PELI1,PPP2R5C,CFDP1,IQGAP1,ZNF423,TRIM22,ARHGAP12,INPP5D,CLASP1,SRA1,TPCN1,GPM6B,XDH,LTBP1,OVOL2,SNX5,NFATC2,PRKG1,PAX7,RGS7,ATP2B4,PACRG,PACSIN1,SPTBN5,SSH2,TET1,ATAD1,ADNP,GPR161,MAPKAPK2,ARID4A,MAP1B,MPHOSPH9,MARK1,CDK6,PHF2,CELF1,RNF34,TFRC,SORCS3,WWOX,MEF2A,GFRAL,GPC3,XRN1,ADRBK1,SATB1,PSMB2,ZNF207,EYA1,GATAD2A,BRINP1,DIS3L2,TRPV1,TSPAN6,HOXB3,HOXB4,CLEC16A,GHR,MNAT1,L3MBTL4,ELAVL4,HDAC1,INVS,RECQL5,TRIM24,PLCG2,ROCK1,EPS8,SCMH1,C1QTNF1,HIPK3,RNF10,RYBP,GPR35,PTPN2,NTRK1,TRIM44,MAP1A,TMIGD1,SLC39A10,EIF3A,ANK3,EPHB1,PTPN9,AXIN2,SARM1,EPCAM,PARD3,ZNF425,GNL3L,MUC1,PTPN14,TRIM46,SH3GL3,BCOR,CARM1,AP2B1,ANXA8L1,ARHGAP44,SNX33,CD84,LPA,SMARCA2,CUX1,SPI1,DNMT3B,EPHA7,FOXP2,CTNNA1,LIMA1,AIFM2,PSPC1,GLIS1,SFMBT2,TRDN,ATG14,EIF3H,JAZF1,STK38,ABCB7,ACOT8,RENBP,OTUD3,RAPGEF3,SH3BP1,MST1,SMYD1,SNCA,BMPR1B,MAGI2,PNPT1,ZEB2,HEY2,RC3H1,EIF3E,PTPRS,ZMYND11,CREM,PSMF1,CAST,TRIP12,DFFA,ADTRP,ABCA12,RASA1,MITF,SRSF6,OPRD1,NUGGC,PKP2,RTN4RL1,NFIX,BTK,ZNRF3,KCTD13,MLLT1,CBFA2T2,IGF1,ATF7IP,HTT,DPYSL3,MAPK1,PTEN,*

			MIB1,SPRED2,BMP7,ATXN2,MXI1,PUM1,RASA2,CIR1,LRRK2,OSGIN1,TMEM59,UNC119,ZBTB20,BAG6,LILRB4,MORC3,ITGAM,ARHGAP25,PKD2,EFNA5,SHANK3,KCTD1,RIPPLY1,RCOR3,DAZL,RGS9,SERPINA3,SERPINA4,SERPINA5,PKIB,TBCD,WNT7A,ZBED6,UACA,MAP4,CRTC3,DNAJC1,PLCL2,KAT6A,GPR21,BMP6,TAF15,GPI,IL17RD,SH3RF2,N4BP1,PDCD1LG2,ATRX,IKZF1,PRMT2,RAPGEF2,SMO,VRK3,ENPP3,PRKAR2B,STXBP5L,PSEN2,PAG1,SOX30,UBR5,GRB14,JADE1,RHOA,SYNGAP1,VIPR2,RQCD1,ONECUT2,CUL3,TFEC,HSPD1,ITPR2,EPM2A,GSK3B,DNAJC3,RDX,STAT6,EEF2K,FAM49B,PTPRB,RBBP8,NECAB2,CDK13,CD27,DUSP26,BOK,RNF144B,SULF1,NACC2,RNF43,SUPT4H1,TNFRSF8,AGT,HDGFRP3,METTL16,PRKAA2,ADAR,FBN2,STAT2,ZMPSTE24,BRINP3,NDRG2,PTK2,SNX3,PDE4B,PBX1,EXOSC3,VEPH1,TCFL5,CREBBP,UTP20,APCDD1L,DLX1,TAOK2,UGT1A1,UGT1A10,UGT1A4,UGT1A8,SHC1,CD160,PARP10,RPS6KB1,GPR173,PRG3,SERPINB11,SETDB2,ALOX5,DNAJB6,CD44,ADAM8,PACSIN2,RPS6KA5,ZNF93,CSNK1A1,HUS1,PRDM2,NOS1,GCKR,PLEKHA1,RGS14,ACTN4,STK4,TCF25,ZFAND6,ZNF282,MARK3,SPEF1,EPHB2,SLC1A1,TMEFF2,XCR1,IL18,DIS3,DLC1,PPARA,PPP1R10,ARID5B,MCOLN1,SMARCAL1,PHLPP1,CPEB4,NOTCH4,SYTL4,ZMYND8,ZNF366,FBN1,PAX6,PRKCZ,ADORA2A,GRIN2B,RABGEF1,CPEB1,PHLDB2,OTUD7B,NIPBL,COL28A1,ASPN,ANGPT1,TBL1X,EYA3,FHL2,LRRK1,MBTD1,OSBPL8,ZBTB5,IGF2BP3,GCNT2,ETS1,TBC1D10C,VWC2,GRIA4,MAP2,MTF2,RBMS3,TAGLN3,FBXO31,CDK5RAP1,NCOR2,PRKCQ,BRAF,DIAPH1,HDAC2,HTR2C,IMPACT,POU3F3,SPINT2,TNR,MBNL3,HIGD2A,CD300A,ELF2,PHIP,CDKL3,PPP2CA,ANGPT4,RPL23,SH3BP4,ATXN1,CAMSAP3,SP100,ANXA2,TRIM29,PARN,DONSON,SRGAP2,NUMB,RBM42,CNOT1,WWTR1,GRIK2,SLIT1,FRMD5,CIT,ZBTB38,BRCA2,MORC1,YBX1,ANKRD13A,PTPRJ,TAF1,TANK,HSF1,MAX,SAP130,DHFR,EZH1,INPP5A,NCOA2,ADAMTS7,SULT2B1,CGNL1,ZP3,RNF2,ZNF554,CAV2,GNA12,INPP5F,NUAK2,AXL,TRIM37,WIF1,CD109,MET,SESTD1,CAMK2D,RAD9B,TIMELESS,UCHL5,ANKRD6,MALT1,SERPINE3,SPTAN1,KDM2B,MLXIPL,SEMA3C,NEDD4L,PHB,PTPRC,ZNF555,CTCF,PBLD,SH3GL2,TYRO3,NR6A1,RNF213,TRAF3IP2,ATF6B,CREB1,SHISA6,TRAP1,CLNK,PRAP1,STXBP6,BMP4,CPNE1
GO:0065008	regulation of biological quality	1.3144890316240835e-27	RDH14,CLRN1,ENPP1,PRDX2,ADAMTS16,LDB2,NRXN1,ASPH,F8,PRMT3,GP6,RDH13,PRKCI,PDE4D,S1PR2,SLC9A1,FAM155A,NEGR1,ADCY8,CLASP2,SEMA3A,IL31RA,GRID2,RPS6KA2,PTGFR,CHERP,CLDN18,RYR1,NOS1AP,NOX5,EPB41,SEMA3D,PTH2R,FHOD3,KSR2,NLGN4X,TJP1,CLCN1,PRLR,FTO,RGS7BP,KALRN,ACOXL,NTRK3,ARNT,EGLN2,SEMA5A,FLT3,STAT5B,ATP2B2,GRM5,PLCE1,LHFPL4,FER,CASK,KITLG,SPNS2,PIK3CD,SP3,ZSWIM7,ANK2,OLFM1,EZR,NRXN3,MALRD1,SLC9A9,CHD7,SLC26A6,HTR2B,ITGB6,CDKL5,TENM1,RYR3,LMNA,GOT1,AFM,TRAF6,ROBO2,ITPKB,CTNNA3,DNAJA3,SLC24A2,VAV2,ITGB1,TRIOBP,HSP90AA1,SLC24A3,PSMB7,TSC22D3,CNIH2,CCDC22,DSCAM,CNGB1,OMA1,CDC42EP3,MYO10,ABCC8,ERC1,HMGN3,DGKI,THRB,STXBP4,ERC2,GABRA3,LAMA2,TCIRG1,PTPN11,CCT2,CACNB2,DOCK10,HDAC6,GOLGA4,DDB1,ADORA1,GPRASP2,ERBB4,ADRA1D,CNNM1,LIMK1,ATP8A2,LARGE,ESR1,NPHP3,PTPRO,NTRK2,CYB561A3,TNRC6A,EP300,PDGFB,RIMS1,CDH8,CHFR,STK39,INSR,ATP8B1,TCF7L2,CDH4,JMJD1C,FBXW11,ESRRB,PTPRD,SLC4A10,SAMD4A,BDNF,AMIGO1,CLDN12,KCNMA1,CHRM3,FMNL2,GRIK4,LCK,MDM4,SLC12A8,NAV2,ACOX1,VAMP7,LAMC1,ARHGAP42,MB,SGCD,MICU3,DMD,SLC30A9,LARP4B,USP13,KAT7,SLC8A1,GSN,TANC2,JPH2,FYN,ARNTL,NF1,PCDH15,RTN4,DGKH,RTN4R,BCR,SHISA9,ICA1,IFI30,PIK3R2,NTN1,DCTN1,SLC4A5,NRG1,CNNM2,SLC16A10,BDKRB1,BDKRB2,BID,STIM1,NALCN,MYH9,FRMPD4,LRFN5,DAGLA,TMBIM6,CNTN4,PCSK2,TMEM30A,CMKLR1,TSPAN8,DCN,SLC39A14,LIN7A,SYN2,TRDMT1,ENTPD1,RAB27A,CAPRIN2,NCKAP1,F5,PTGER2,CLDN16,ANO6,NEUROD1,GNAQ,ZDHHC15,JAK2,FBXW7,SYN3,CLSTN2,SYT1,CROCC,NPSR1,BBS12,LZTS1,SCN4A,PKNOX1,CLDN1,ATP9A,SLC9A7,PNPLA1,PLS1,PDE2A,SEPT7,KLF15,CYFIP2,PALMD,NOX4,MKLN1,CACNA1H,IL1RAPL1,CAMK2B,PRKCD,ACVR2A,SEMA5B,TGFB1,SGK1,PCSK5,KEL,BTRC,JPH3,PTPRN2,F2RL1,C9ORF47,GRIK3,DNAJB2,AAK1,SLIT2,GABRR3,CADPS2,GABRB1,CDH23,DISC1,KCNJ3,KANK1,CACNA1D,CPT1C,ANO4,CLN6,XPR1,CHRM1,TMTC2,CLOCK,CHST9,ZNF675,ANO3,KCNH1,ANKRD54,PARVA,HNRNPC,HOMER2,SLC17A7,LINGO2,YTHDF1,FGF10,STXBP5,KCNH7,KANK4,IL1RAPL2,SVIL,CAPN3,SLC16A1,VMP1,EPHA4,RORA,HSD17B12,PRKCA,CNR1,TNFSF11,PPP3CA,NSUN2,MAG,PIP5KL1,UFL1,CTNNB1,PARK2,SOD2,FCGR2B,ARHGAP22,CDHR2,IGF1R,PPARG,IARS2,DLG5,IL18R1,ADD2,MTF1,CPQ,P2RY10,TRPM1,OTC,XK,SREBF2,AARS2,RYR2,FBLIM1,DRAXIN,LEPR,PROS1,NR4A3,FOXK2,NOL3,PER2,CACNA1C,SCN3B,CHEK2,PRDM16,CSMD1,HCK,DIO2,GRM1,RIMS4,DCC,NF2,PAX2,SPTBN1,ELN,SFRP1,MED13,FOXO3,DGKB,TRHDE,ZCCHC17,DNM3,SSH1,ATP10D,SYNCRIP,SMAD3,CUX2,ITPR1,WWP2,ACKR2,CYP4F11,ABI2,C10ORF90,GRIK5,SLC16A2,SHANK1,DCP1B,NEDD4,NRP2,ARHGEF18,DOCK4,ESRRG,SLC4A4,SLC6A1,KCNK1,CDC73,APP,PDGFRA,ADD3,DIP2B,NOX1,YAP1,HEG1,RAB11FIP5,FSTL4,NLGN2,EYA2,RNLS,IBTK,ALPL,ANGPTL4,TNNI3K,PEMT,LRP5,BDH2,ISLR2,SLC9B2,GPC6,ECSIT,SPTBN4,DRD1,TMEM14A,GLRA2,RFT1,VILL,MMP2,TPH1,PRKD1,STAT1,DGKG,RND3,SLC6A3,USP53,HNF4A,SHANK2,TMEM183A,SEMA6D,ATF2,RAF1,CELF4,RASGRF2,ACOX2,BMPR2,USP33,DPYSL2,PABPC4,NLGN1,CTNNA2,ATP9B,RAB11A,FGF2,PPA2,GRIK1,ADCY5,SEMA4D,MCTP1,PLXNA2,PTAFR,ADCYAP1R1,KLHL3,SORCS2,RAB11FIP3,PRKCG,SLC9B1,EHMT1,AP2M1,DVL3,ECE2,EIF4G1,EPHB3,TRAPPC11,LIN28B,AKR1D1,EBF2,DEPTOR,FBXL20,VCL,T

			ERF2IP,CLIC4,CRYM,IDE,KCNQ1,WNT3,RHOJ,CCL14,CCL15,TG,NLGN3,PAFAH1B1,KIF3A,TRPA1,DDAH1,NMUR2,HIP1,AKAP6,TSPAN1,BTBD9,TRPC5,NEO1,SCUBE1,RUFY3,TNRC6B,WDTC1,DAB2,PKHD1,XKR4,USH2A,SETD5,DLG3,WNK1,RELN,SIN3A,ABCB1,DOCK11,SLC22A3,CFDP1,ZNF423,MACF1,ALK,SLC8A2,INPP5D,KLK2,STAU1,DSC2,FASTKD5,SLC9C1,AKAP2,TPCN1,GPM6B,XDH,SNX5,PRKG1,ATP2B4,SYNE3,SPTBN5,PAK3,SLCO4A1,SSH2,P2RX6,ASGR2,ATAD1,ADNP,MAPKAPK2,ARID4A,MAP1B,SCARB1,CDK6,ATP6V0A2,KCNAB2,CELF1,P2RY8,TFRC,EPHA5,SORCS3,GABRG3,SYT12,CACNA1A,SGIP1,ADRBK1,PSMB2,ZNF207,PTGER3,SCOC,DIS3L2,GNB3,TRPV1,HOXB6,GHR,FLRT2,FGD1,SNX9,ACTR2,ACACA,ELAVL4,TRIM24,ADIPOR2,PLCG2,ROCK1,EPS8,C1QTNF1,EGR2,CAPN10,GPR35,ABCC1,AP3B1,PTPN2,NTRK1,TRIM44,MAP1A,TMIGD1,SLC39A10,ANK3,DNMBP,UNC13A,EPHB1,AXIN2,TMEM199,ATP13A3,GNL3L,TRIM46,TUB,KCND3,CACNG8,AP3D1,SLC9C2,VWF,AP2B1,NETO1,ARHGAP44,DTD2,SRD5A2,COCH,HTR1D,SPI1,DGKK,EPHA7,CHRNB4,MCU,LIMA1,TRDN,ATG14,GABRA6,LRRC8D,ILDR2,SHROOM3,TRPC6,ABCB7,RENBP,FCHSD2,MAP3K13,OTUD3,PDXP,SH3BP1,ATP13A5,SNCA,CACNG2,GRM4,PNPT1,DHRS4,SYT9,HEY2,RC3H1,WNT7B,PTPRS,SULT2A1,ADTRP,ABCA12,CSRP1,RASA1,OPRD1,PKP2,RPTOR,BTK,SGCZ,DSG1,SLC4A8,IGF1,ATF7IP,HTT,LARS2,MAPK1,PTEN,SLC30A7,SCN8A,PUM1,PRCP,LRRK2,ATP6V1A,GNA14,ZBTB20,BAG6,MORC3,TMCO1,TMEM108,PKD2,EFNA5,SHANK3,TADA2A,DAZL,PDGFC,SERPINA3,ATP8B4,STEAP4,PPP2R3C,WNT7A,ZBED6,SNAP23,BRWD3,CRTC3,PLCL2,GPR21,RHOT1,BMP6,TAF15,ANO1,GPI,SOS1,TSHR,EXT1,GABRR2,IKZF1,PARVB,PNPLA3,RAPGEF2,KCNC2,SMO,RALY,GET4,BLOC1S3,STXBP5L,CPT1A,TNFRSF11B,BPGM,DECR1,TFE3,GRIN1,RHOA,SYNGAP1,GRIN3A,TF,RHOBTB1,FAM19A4,CUL3,SH3KBP1,HSPD1,ITPR2,GABRB3,GSK3B,ABCG8,PRR16,RDX,STAT6,ACTA2,SYN1,EEF2K,FAM49B,ALAS2,MCTP2,SCN9A,BOK,AGT,METTL16,ELOVL3,PRKAA2,SYT7,ADAR,BLOC1S6,ZMPSTE24,FGGY,HRH4,PTK2,HEPHL1,PDE4B,RIMS2,TEC,EXOSC3,CPE,CREBBP,FAM171A1,PCSK6,SLC29A1,TAOK2,UGT1A1,UGT1A3,UGT1A7,UGT1A8,UGT1A9,ALDOA,ARHGAP15,ECE1,STC2,ALOX5,CPLX2,FGD3,ADAM8,CLDN4,GRID1,FGD4,NOS1,GCKR,PLEKHA1,PLVAP,RGS14,SCNN1B,STK4,EPHB2,GATC,SLC1A1,XCR1,BCO2,IL18,UBE2K,HCAR2,DIS3,DLC1,PPARA,MCOLN1,STEAP3,SLC25A33,SYTL4,CYP2C18,PAX6,PRKCZ,ATP1A3,ADORA2A,GRIN2B,KCTD7,PAH,ANGPT1,CADPS,PACS2,TBL1X,LRRK1,GCNT2,ETS1,PPP1CA,MAP2,STPG1,CDK5RAP1,NCOR2,PRKCQ,BRAF,DIAPH1,HTR2C,ATP8A1,POU3F3,TNR,MOXD1,PHIP,CDKL3,MAOA,RPL23,KCND2,PARN,ATP6V0A1,ADCY1,NUMB,SYK,CNOT1,WWTR1,CYP39A1,GRIK2,SCN1A,SLIT1,CIT,MYRIP,UBAP2L,RAP1GAP2,ALDH8A1,CACNG3,NEB,YBX1,GRIP1,PTPRJ,TAF1,ATP2B3,GPM6A,TAC3,POTEE,HSF1,ATP10B,PLEKHM1,MCM8,VAV3,KCNQ3,CIB2,CAV2,GNA12,MYEF2,POC1B,CA12,TMPRSS3,AXL,MET,FMNL1,CAMK2D,CCR3,NBEA,TRA2B,SPTAN1,MLXIPL,SEMA3C,THEM4,CCT3,NEDD4L,PHB,PTPRC,RPH3A,GPR89A,PBLD,SH3GL2,TYRO3,SYNDIG1,TRAF3IP2,CREB1,SHISA6,EDNRA,BMP4
GO:0000904	cell morphogenesis involved in differentiation	1.717623606990732e-27	CLRN1,NRXN1,PRMT3,CLASP2,SEMA3A,DOCK1,SEMA3D,PHACTR1,MYOT,TIAM2,UNC5C,KALRN,ATRNL1,SEMA5A,FER,OLFM1,NRXN3,ROBO1,CDKL5,KIF5C,ROBO2,NFASC,ABI1,ITGB1,TRIOBP,HSP90AA1,KNDC1,DSCAM,TRIO,DAB1,LAMA2,PTPN11,DOCK10,HDAC6,GOLGA4,EPHA1,LIMK1,ATP8A2,PTPRO,NTRK2,EP300,MEGF9,TNIK,RERE,UNC5D,LAMA3,CDH4,PAK1,PTPRD,TBR1,STRC,BDNF,ATRN,AMIGO1,LAMC1,TANC2,CASS4,FYN,PCDH15,LMTK2,FAT3,RTN4,B4GALT6,RTN4R,CCDC141,ADAMTSL1,NTN1,MAP2K1,VAX2,MYH9,CNTN4,NTNG1,UST,CAPRIN2,RCC2,BOC,ZDHHC15,MYPN,LZTS1,BCL11B,PLS1,SIPA1L3,SIAH1,IL1RAPL1,CAMK2B,SEMA5B,KEL,SLIT3,SLIT2,CDH23,CORO1C,CTNND2,DISC1,KANK1,LAMC2,DCLK1,PARVA,YTHDF1,ENAH,EPHA4,AUTS2,PPP3CA,MAG,CTNNB1,IGF1R,XK,DRAXIN,NIN,DCC,PAX2,SPG11,ARL13B,CNTN6,NFIB,DNM3,CUX2,PTPRM,ABI2,SHANK1,NEDD4,NRP2,BTBD3,PREX1,PRTG,APP,DIP2B,HEG1,FSTL4,ISLR2,SPTBN4,PARP6,DSCAML1,SEMA6D,BMPR2,USP33,DPYSL2,NLGN1,CTNNA2,RAB11A,CRMP1,LRRC4C,NEDD9,PEAK1,SEMA4D,PLXNA2,RILPL1,EPHB3,FBXO45,VCL,LAMB1,CLIC4,WNT3,P4HB,NLGN3,PAFAH1B1,TRPC5,GLI2,NEO1,RUFY3,DAB2,PKHD1,USH2A,RELN,SIN3A,MACF1,MYO7A,PAK3,ADNP,MAP1B,EPHA5,MEF2A,FLRT2,ACTR2,ELAVL4,ROCK1,EGR2,NTRK1,MAP1A,ANK3,PTPRQ,EPHB1,EPHA10,SARM1,PARD3,TRIM46,ARHGAP44,CUX1,SPI1,EPHA7,MAP3K13,PTK7,BMPR1B,PTPRS,PTEN,BMP7,LHFPL5,LRRK2,RAPH1,EFNA5,SHANK3,TBCD,WNT7A,MYCBP2,SOS1,EXT1,PARVB,RAPGEF2,SMO,RHOA,SYNGAP1,CUL3,GSK3B,EMB,EEF2K,PTK2,GAP43,TAOK2,ECE1,RPS6KA5,ACTN4,TUBB3,EPHB2,TMEFF2,NOTCH4,PAX6,COL22A1,MAP2,FBXO31,PRKCQ,BRAF,SPINT2,TNR,CDKL3,SRGAP2,TMEFF1,ADCY1,NUMB,SLIT1,RADIL,AXL,MET,SEMA3C,NEDD4L,TYRO3,NCAM1,CREB1,EDNRA
GO:0050793	regulation of developmental process	1.5068637197883785e-26	ENPP1,PRDX2,NRXN1,PRKCI,DNMT1,S1PR2,HLX,LLPH,TRPS1,CBFB,CLASP2,SEMA3A,GRID2,DOCK1,WWC1,CLDN18,LATS2,EPB41,SEMA3D,NREP,TIAM2,PTGIS,WWC3,TJP1,PRLR,FTO,KALRN,NTRK3,ARNT,SEMA5A,STAT5B,TOX,ENPP2,GRM5,KITLG,LRP2,PIK3CD,ZNF536,OLFM1,EZR,MEGF10,ROBO1,CHD7,CDKL5,LMNA,TRAF6,ESRP1,ROBO2,ITPKB,ITGB1,TRIOBP,PSMB7,KNDC1,ZC4H2,DSCAM,TRIO,GFI1B,RUNX1,DAB1,OMA1,CDC42EP3,MYO10,ABCC8,LAMA2,TCIRG1,PTPN11,RBM19,HDAC6,GOLGA4,EPHA1,IKBKB,ERBB4,LIMK1,ATP8A2,ESR1,NPHP3,CDON,NTRK2,CELA1,HOOK3,PDGFB,RIMS1,TNIK,TOB2,INSR,H2AFY2,LAMA3,TCF7L2,CDH4,PAK1,ESRRB,PTPRD,BASP1,BDNF,TFAP2A,ATRN,AMIGO1,FANCA,T

			GIF2,FMNL2,MEIS1,TENM4,ECT2,DMD,CENPF,KAT7,SLC8A1,RBM4,GPR171,TANC2,JPH2,CASS4,FYN,ARNTL,ADAMTS9,NF1,PLCB1,FAT3,RTN4,RTN4R,BCR,CHI3L1,BMP2K,ANKH,NTN1,NRG1,MAP2K1,FNIP1,STIM1,MYH9,VGLL4,CNTN4,HDAC5,NTNG1,JDP2,CMKLR1,ROR2,DCN,SOX13,UST,MAD2L2,TLE6,CAPRIN2,NLN,TERF2,RCC2,BOC,ANO6,NEUROD1,CCBE1,ZDHHC15,JAK2,FBXW7,CLSTN2,SYT1,ITCH,BBS12,LZTS1,BCL11B,RBFOX1,MLLT3,PLS1,TCF7,SEPT7,WNT11,PALMD,FIG4,KREMEN1,MKLN1,IL1RAPL1,CAMK2B,SOX6,ACVR2A,RUNX2,SEMA5B,CD4,TGFB1,ZHX2,KEL,NFATC3,C9ORF47,CELSR1,SLIT2,TP73,CORO1C,DISC1,KANK1,MTDH,SMAD6,CLOCK,ITSN2,TCF12,ZNF675,SMOC1,NELL1,SUCO,BMPER,ANKRD54,PARVA,ADAM12,STRIP1,LINGO2,FGF10,LOXL3,CAPN3,SMURF2,EPHA4,RORA,PRKCA,TNFSF11,PPP3CA,NSUN2,MAG,CAMK4,UFL1,CTNNB1,PARK2,SOD2,FCGR2B,IGF1R,PPARG,AXIN1,DLG5,MSR1,ANKRD17,CYBB,XK,ANKRD26,FBLIM1,DRAXIN,FGF1,NIN,DCT,MYOCD,PER2,KIR2DL4,CPNE6,GLG1,PHLDB1,HCK,CAPN2,DCC,CTDP1,NF2,FLT4,HDAC4,PAX2,SFRP1,FOXO3,NFIB,DNM3,SMAD3,CUX2,PTPRM,CASZ1,ABI2,SHANK1,SYT3,NEDD4,ARHGEF18,VANGL1,PREX1,HOXD3,NFATC1,PRTG,CDC73,APP,SSBP3,GSX2,PDGFRA,DIP2B,YAP1,FSTL4,NLGN2,SH3D19,JAK1,ANGPTL4,LRP5,ISLR2,SLC9B2,SOX2,ZDHHC6,GPC6,PRICKLE1,SPTBN4,VASH2,FRS2,SFMBT1,TPH1,CPNE9,PRKD1,STAT1,PARP6,DGKG,RND3,SLC6A3,HNF4A,ZBTB7C,EREG,SEMA6D,ATF2,TCF3,RAF1,CELF4,ADAMTS12,BMPR2,DPYSL2,CAMK1D,BMPR1A,TSPAN12,NLGN1,CDK12,TAF8,SPAG9,RAB11A,MYB,FGF2,LRRC4C,NEDD9,AGBL4,SEMA4D,ISM1,RORC,PLXNA2,SETD1A,JARID2,RARB,SPEN,NCOA1,BLOC1S5,EEF1E1,EHMT1,CHRD,DVL3,EIF4G1,EPHB3,PAWR,CCDC3,VCL,MYO9A,WNT3,ZNF322,RHOJ,P4HB,SUFU,TG,NLGN3,SCUBE2,PAFAH1B1,KIF3A,COL4A3,DDAH1,AKAP6,RASAL1,TRPC5,GLI2,RUFY3,DAB2,PKHD1,USH2A,LDLRAD4,MYSM1,RELN,SIN3A,RSPO2,CSF3R,CFDP1,APOLD1,SP1,MACF1,ALK,INPP5D,CLASP1,TEAD4,AKAP2,SRA1,GPM6B,ADIRF,XDH,OVOL2,NFATC2,BNC1,ATP2B4,SYNE3,PACSIN1,TMEM2,PAK3,ADNP,MAP1B,MARK1,CDK6,CELF1,TFRC,DROSHA,GPC3,PSMB2,EYA1,BRINP1,GNB3,TRPV1,HOXB3,HOXB4,GHR,FLRT2,FGD1,ACTR2,ELAVL4,LGI4,HDAC1,SMOC2,ROCK1,EPS8,PAXIP1,EGR2,RNF10,AP3B1,PTPN2,NTRK1,DNMBP,UNC13A,EPHB1,AXIN2,SARM1,PARD3,TRIM46,SH3GL3,BCOR,AP3D1,CARM1,ARHGAP44,COCH,CUX1,SPI1,EPHA7,MCU,CTNNA1,GLIS1,SHROOM3,MAP3K13,RAPGEF3,SDK1,MST1,PTK7,SMYD1,BMPR1B,CD53,MAGI2,PNPT1,ZEB2,HEY2,RC3H1,WNT7B,PTPRS,TLL2,TRIP12,ABCA12,RASA1,MITF,SRSF6,HIPK1,PKP2,RTN4RL1,BTK,ZNRF3,IGF1,MAPK1,PTEN,MIB1,SPRED2,BMP7,SOX5,TTLL4,LRRK2,ZRANB1,BAG6,LILRB4,EFNA5,SHANK3,NCOA3,PPP2R3C,WNT7A,ZBED6,BRWD3,GPR21,BMP6,IL17RD,SOS1,TSHR,WDR43,PRICKLE2,PARVB,RAPGEF2,SMO,TCF4,TNFRSF11B,SYT17,TFE3,RHOA,SYNGAP1,GRIN3A,ROR1,RHOBTB1,SH3KBP1,NFKBID,GSK3B,RDX,STAT6,EEF2K,FAM49B,CDK13,CD27,SULF1,AGT,BLOC1S6,FBN2,STAT2,ZMPSTE24,BRINP3,PTK2,RIMS2,PBX1,TCP11,EXOSC3,MAP3K5,TCFL5,FAM171A1,DLX1,TAOK2,SHC1,ALDOA,ARHGAP15,CD160,LBX2,RPS6KB1,STC2,ALOX5,SP7,CD44,FGD3,LARP4,ADAM8,CLDN4,CDS1,FGD4,RGS14,ACTN4,STK4,SPEF1,EPHB2,CLPTM1,IL18,DLC1,PPARA,NOTCH4,FBN1,PAX6,PRKCZ,FAM20C,PPP1CC,PHLDB2,NIPBL,TMIGD2,ASPN,GCNT2,ETS1,VWC2,MAP2,FBXO31,CDK5RAP1,BRAF,DIAPH1,HDAC2,HTR2C,IMPACT,TNR,MBNL3,PHIP,CDKL3,PPP2CA,ANGPT4,SP100,NUMB,SYK,CNOT1,WWTR1,SLIT1,DVL2,YBX1,GRIP1,PLEKHB2,HSF1,SASH1,ASAP1,ADAMTS7,SULT2B1,BMP1,ZP3,GNA12,INPP5F,AXL,TNFSF9,WIF1,CD109,FMNL1,CCR3,ANKRD6,MALT1,SETD3,EHD2,KDM2B,SEMA3C,NEDD4L,PTPRC,SYNDIG1,CREB1,BMP4,CPNE1
GO:0048667	cell morphogenesis involved in neuron differentiation	4.4875636103668245e-26	CLRN1,NRXN1,PRMT3,CLASP2,SEMA3A,SEMA3D,PHACTR1,MYOT,TIAM2,UNC5C,KALRN,SEMA5A,OLFM1,NRXN3,ROBO1,CDKL5,KIF5C,ROBO2,NFASC,ABI1,ITGB1,TRIOBP,HSP90AA1,KNDC1,DSCAM,TRIO,DAB1,LAMA2,PTPN11,DOCK10,HDAC6,GOLGA4,EPHA1,LIMK1,ATP8A2,PTPRO,NTRK2,TNIK,RERE,UNC5D,LAMA3,CDH4,PAK1,PTPRD,TBR1,STRC,BDNF,AMIGO1,TANC2,FYN,PCDH15,LMTK2,RTN4,B4GALT6,RTN4R,CCDC141,ADAMTSL1,NTN1,MAP2K1,VAX2,CNTN4,NTNG1,UST,CAPRIN2,BOC,ZDHHC15,MYPN,LZTS1,BCL11B,PLS1,SIAH1,IL1RAPL1,CAMK2B,SEMA5B,KEL,SLIT3,SLIT2,CDH23,CTNND2,DISC1,LAMC2,DCLK1,YTHDF1,ENAH,EPHA4,AUTS2,PPP3CA,MAG,IGF1R,XK,DRAXIN,NIN,DCC,PAX2,SPG11,CNTN6,NFIB,DNM3,CUX2,PTPRM,ABI2,SHANK1,NEDD4,NRP2,BTBD3,PRTG,APP,DIP2B,FSTL4,ISLR2,SPTBN4,PARP6,DSCAML1,SEMA6D,BMPR2,USP33,DPYSL2,NLGN1,CTNNA2,RAB11A,CRMP1,LRRC4C,SEMA4D,PLXNA2,EPHB3,FBXO45,VCL,WNT3,NLGN3,PAFAH1B1,TRPC5,GLI2,NEO1,RUFY3,RELN,SIN3A,MACF1,MYO7A,PAK3,ADNP,MAP1B,EPHA5,MEF2A,FLRT2,ACTR2,ELAVL4,EGR2,NTRK1,MAP1A,ANK3,PTPRQ,EPHB1,EPHA10,SARM1,PARD3,TRIM46,ARHGAP44,CUX1,EPHA7,MAP3K13,PTK7,BMPR1B,PTPRS,PTEN,BMP7,LHFPL5,LRRK2,RAPH1,EFNA5,SHANK3,TBCD,WNT7A,MYCBP2,SOS1,EXT1,RAPGEF2,SMO,SYNGAP1,GSK3B,EMB,EEF2K,PTK2,GAP43,TAOK2,ECE1,RPS6KA5,TUBB3,EPHB2,PAX6,MAP2,FBXO31,PRKCQ,BRAF,TNR,CDKL3,TMEFF1,ADCY1,NUMB,SLIT1,SEMA3C,NEDD4L,NCAM1,CREB1,EDNRA
GO:0043412	macromolecule modification	5.209842937996584e-26	LNX2,ZDHHC14,ENPP1,NRXN1,ASPH,B4GALNT2,PRMT3,PRKCI,MOV10L1,SLCO3A1,MAP4K4,PDE4D,DNMT1,S1PR2,C6ORF89,ADCY8,MGAT4C,CBFB,PDE8A,IL31RA,RPS6KA2,PRDM12,SETD4,CTDSPL2,FBXL2,NRG3,ASH1L,NOS1AP,LATS2,RNF145,KSR2,PRKAG2,PRLR,MVP,PAGR1,FTO,KALRN,DCAF12,MRM1,NTRK3,CBL,ARNT,EGLN2,FLT3,TOX,ENPP2,GRM5,PLCE1,FER,CASK,MAPK4,MAP2K5,KITLG

			,USP32,MAPK10,PTPRR,PIK3CD,CAMK1G,B3GALT1,ROBO1,TOM1L1,HTR2B,CDKL5,MECOM,TENM1,CAMKMT,LMNA,NMT2,TRAF6,SENP5,ITPKB,DNAJA3,OXR1,GRK5,ABI1,ULK4,TRIOBP,HSP90AA1,CDC6,PSMB7,THUMPD3,TSSK1B,KNDC1,ZC4H2,STK38L,TRIO,KLHL12,AKAP13,TTLL5,PTPRK,DAB1,ERC1,KLHL7,MAST4,PTPN11,HERC4,MGAT5,HDAC6,DDB1,ADORA1,MSRA,EPHA1,IKBKB,ERBB4,DPY19L2,MRE11A,LIMK1,TLK1,BRD8,KAT6B,ABR,CUL4B,LARGE,MIER1,PMEPA1,PTPRO,CDON,KANSL1,FBXL18,NTRK2,EP300,RNF220,FNTA,TPST2,PDGFB,FKBP9,TNIK,CCND3,GALNTL6,FBXL7,USP46,BRMS1,CHFR,STK39,INSR,PRKAR1A,ASB5,ST6GALNAC3,JMJD1C,CAB39,USP34,PAK1,FBXW11,MAP3K4,ZDHHC13,PTPRD,RNF144A,EGLN3,BDNF,CDK14,CHRM3,POMGNT2,FUT8,TRIM13,UBR2,HLCS,LCK,ENTPD5,ECT2,MYO3B,MTA3,SNX6,CHML,PPM1L,PTPRU,ERN2,DMD,ATG10,USP13,KAT7,SLC8A1,RBM14,RIT2,MACROD2,CASS4,FYN,MKRN2,ARNTL,NF1,MGMT,LMTK2,ARID4B,GXYLT2,PPP4R2,FBXO21,NEIL2,B4GALT6,ASPHD2,BCR,NOSIP,RWDD3,BRPF1,CHI3L1,TTN,BMP2K,PAQR3,TPGS2,KLHL21,EGFLAM,PPIG,NRG1,SH2D3C,CDK19,BDKRB1,BDKRB2,FRY,MAP2K1,FNIP1,GALNT16,PPP2CB,HDAC5,RNF4,CSNK2A3,GALNT18,UBE3D,JDP2,WDSUB1,CDKL2,ROR2,DCN,ZDHHC3,TRDMT1,WDR70,PTPRT,UST,COPS5,IPO5,MAD2L2,MAN1A1,CAPRIN2,CCNYL1,PHF20L1,PDF,GNAQ,FBXO9,ICK,RFFL,TTLL8,PARP11,HERC5,USP22,ZDHHC15,JAK2,FBXW7,UBR7,UIMC1,ITCH,USP12,ZFYVE28,SMG1,HUNK,B3GALT5,CDC42BPB,PHKA1,DHDDS,KLF15,SPSB1,WNT11,TPST1,TRIM59,MTA1,NOX4,RPRD1B,SIAH1,CAMK2B,ERCC8,PRKCD,TAB2,ACVR2A,CD4,PPM1E,TGFB1,SGK1,P4HA2,SPSB4,MYO3A,NSD1,PIBF1,BTRC,DUS2,PTPRN2,PDZRN3,DNAJB2,AAK1,KLHL2,SLIT2,ASB15,RNF133,RNF148,HHAT,CORO1C,CDKAL1,PTPDC1,TMTC1,TTBK2,KDM4B,SMAD6,TMTC2,CLOCK,ZNF675,BMPER,DCLK1,RNF212,ANKRD54,DAPK2,TNFRSF10B,DAPP1,DUSP22,TRRAP,DOCK3,FGF10,FBXL17,SMYD3,UNKL,KCTD10,SH3RF3,LOXL3,MAST2,FANCI,CAPN3,SMURF2,EPHA4,PRKCA,AUTS2,CD6,TNFSF11,PPP3CA,NSUN2,CAMK4,PIP5KL1,UFL1,TRAK1,CTNNB1,PARK2,IGF1R,PPARG,AXIN1,PRKAR1B,MACROD1,OTUB1,RSPRY1,AARS2,CDK11A,DYRK4,GALNT8,CDK11B,LEPR,FGF1,WDR45B,PRKAR2A,RIOK2,ESCO1,MYOCD,TRIM5,PER2,AJUBA,CHEK2,SUPT3H,PRDM16,ASB8,PPP1CB,SPDYA,HCK,RAB3GAP2,TBCK,TRIM8,PPP2R2B,CSPG4,BRMS1L,CTDP1,HS3ST5,BAZ1B,OBSCN,NF2,FLT4,HDAC4,SPTBN1,TPTE,TRABD2B,SFRP1,ST8SIA1,PPP6R2,USP54,SSH1,TYW1B,RNFT2,RAB6A,PTPRM,WWP2,WIPI1,MTBP,RNF168,EMG1,ABI2,C10ORF90,NEDD4,GALNT14,TAB1,KTI12,ST3GAL3,CDC73,APP,USP49,PDGFRA,SLK,CCNI2,DIP2B,KANSL2,ARIH1,HEG1,AMFR,ST6GALNAC5,CDK3,EYA2,BORA,IBTK,SBK3,JAK1,FPGT-TNNI3K,TNNI3K,LRP5,MTCP1,PTPRG,SETD2,ZDHHC6,PRICKLE1,RCAN1,SPTBN4,DRD1,CHUK,RFT1,S100A12,DCUN1D3,KDM6A,PRKD1,NAA25,PARP6,USP53,LRRC2,SHPRH,EREG,CCNY,MAPKAPK3,ATF2,RAF1,PTPN1,BMPR2,USP33,CAMK1D,BMPR1A,PIK3R3,CDK12,CAND2,SPAG9,MYB,FGF2,PPA2,PPM1F,UBE2R2,NEDD9,AGBL4,PEAK1,SEMA4D,NFX1,CDC27,ELP3,SETD1A,JARID2,KLHL3,PRKCG,SIN3B,NCOA1,AREL1,EHMT1,LMO7,UCHL3,ALKBH3,DVL3,EIF4G1,EPHB3,PTPRE,RBX1,ANAPC5,FBXO45,STT3B,CKS1B,TRMT61B,DEPTOR,PKN3,FBXL20,TSG101,DESI1,TERF2IP,FKBP5,RCE1,B3GALNT1,P4HB,SUMF1,PTPN13,QPCTL,PADI6,TRPC5,UBA2,EDEM3,TTLL7,TNKS,WDTC1,DAB2,BLM,UBR1,CACUL1,LDLRAD4,MYSM1,SETD5,DLG3,WNK1,RELN,NEK10,SIN3A,RUVBL2,TYW1,JOSD1,PELI1,PPP2R5C,IQGAP1,DCAF5,MAP3K7,TRIM22,ZNRF1,ALK,SLC8A2,UBOX5,TOP1,AGBL1,UBE2V1,PADI3,XDH,RBBP6,PRKG1,TTLL9,ATP2B4,PARP12,ACTL6B,PAK3,SSH2,TET1,HECTD4,MKRN3,CAMTA1,ADNP,MAPKAPK2,CEP41,ARID4A,UBE3C,CDC42BPA,MARK1,CDK6,PHF2,RNF34,TFRC,UVRAG,EPHA5,ALG14,DCAF6,DCUN1D5,ADRBK1,PSMB2,EYA1,GATAD2A,CDCA3,RNF216,GHR,MNAT1,SNX9,HDAC1,TRIM24,PLCG2,ROCK1,PAXIP1,EGR2,HIPK3,RNF10,RYBP,CUL2,AP3B1,PTPN2,INSRR,NTRK1,TRIM44,PKN2,SLC39A10,PTPRQ,EPHB1,MAN1B1,EPHA10,PTPN9,AXIN2,PARD3,GNL3L,KRTCAP2,MUC1,PTPN14,NUDT14,BCOR,MGAT4A,CARM1,FOLH1,SPI1,DNMT3B,EPHA7,PTPRA,FBXW4,ATG14,BCAR3,STK38,RBPMS,NTMT1,MAP3K13,OTUD3,RAPGEF3,PDXP,PTK7,RSRC1,SMYD1,SNCA,BMPR1B,MAGI2,USP42,USP50,RC3H1,RNF103,RNF103-CHMP3,RNF19B,PTPRS,KMT2D,PRKAG1,CDKL1,KMT2C,UBE2QL1,TRIP12,ADTRP,OPRD1,HIPK1,RPTOR,BTK,ZNRF3,ASPHD1,KCTD13,MLLT1,ZZZ3,IGF1,ATF7IP,HTT,MAPK1,PTEN,MIB1,SPRED2,BMP7,PCMTD2,PIK3C3,TTLL4,LRRK2,TMEM59,ZRANB1,PIGS,UNC119,BAG6,LILRB4,MORC3,PKD2,EFNA5,SEL1L2,RCOR3,TADA2A,ALPK3,PDGFC,NCOA3,PKIB,MTMR3,PPP2R3C,RPS6KC1,PLCL2,WDR5B,KAT6A,SIK3,BMP6,MYCBP2,SH3RF2,EXT1,DPY19L4,N4BP1,ATRX,DPH6,C8ORF44-SGK3,PRMT2,RAPGEF2,VRK3,ALG9,FDXACB1,MARK4,PRKAR2B,DPY19L1,GALNT10,UBE2H,DNAJC6,UBR5,JADE1,ARRDC4,RHOA,SPRTN,WSB2,ROR1,RQCD1,CUL3,EPM2A,GSK3B,DNAJC3,EEF2K,IWS1,PPP1R16A,PTPRB,CDK13,PGGT1B,STK32B,DUSP26,RNF144B,RNF43,AGT,METTL16,CCNJL,PRKAA2,ADAR,CUL9,STAT2,ZMPSTE24,PTK2,PHEX,RNF121,TEC,DCAF10,EXOSC3,FBXO10,CDC14A,MAP3K5,CPE,TRPC4AP,CREBBP,PRKD3,LMTK3,NCF1,TAOK2,PYGO2,SHC1,PARP10,RNFT1,RPS6KB1,ZDHHC23,GALNT13,GUCY2F,SETDB2,FBXO28,CD44,ADAM8,FKBP14,RPS6KA5,CSNK1A1,HUS1,PRDM2,NOS1,RGS14,STK4,MARK3,EPHB2,SLC1A1,TTLL11,MARCH8,IL18,PCMT1,UBE2K,DLC1,FBXL13,PDP1,PHLPP1,PPIL6,MOB3B,ACP1,RNF150,PAX6,PRKCZ,FAM20C,ADORA2A,MAN2B1,PPP1CC,RABGEF1,CCNG2,TYK2,OTUD7B,NIPBL,YEATS4,PHF20,ANGPT1,TBL1X,EYA3,LRRK1,OSB

			PL8,TRMT2B,GCNT2,PPP1CA,MTF2,FBXO31,ATG3,CDK5RAP1,CNTN1,PRKCQ,BRAF,HDAC2,IMPACT,FKBP3,PHKG2,SRCAP,UBE2E2,C18ORF25,CD300A,PHIP,CDKL3,PPP2CA,SKP1,ANGPT4,RPL23,OPN1LW,PARN,PIGU,PRMT7,WIPI2,ALG2,MADD,SYK,WWTR1,GTF2H5,ASB1,CIT,BRCA2,DVL2,MORC1,PTPRJ,TAF1,PPME1,TICAM1,RNF38,TANK,RIMKLA,GALNT7,NSUN6,HSF1,EZH1,ETF1,RNGTT,SRPK2,SASH1,RNF2,MOB3A,GNA12,SH2D3A,UGGT1,AKTIP,GALNT4,POC1B-GALNT4,INPP5F,NUAK2,AXL,AGBL3,TRIM37,CD109,MET,CAMK2D,UCHL5,MALT1,SETD3,TRIM69,ZDHHC11,ZDHHC11B,KDM2B,MLXIPL,NEDD4L,PHKB,PHB,PTPRC,CTCF,SH3GL2,TYRO3,RNF213,TRAF3IP2,TRIM60,CREB1,METTL21A,TNXB,EDNRA,BMP4
GO:0006928	movement of cell or subcellular component	2.690705470199556e-25	CLRN1,DNAH11,LDB2,GPC5,NRXN1,PRKCI,MAP4K4,PDE4D,S1PR2,SLC9A1,SPAG16,CLASP2,SEMA3A,DOCK1,WWC1,KIF22,NRG3,ASH1L,NOS1AP,IQCG,ASTN2,SEMA3D,PHACTR1,MYOT,MAPRE2,WWC3,TJP1,UNC5C,KALRN,WIPF3,NTRK3,ATRNL1,SEMA5A,STAT5B,ENPP2,FER,MARVELD3,MAP2K5,KITLG,PTPRR,SPNS2,PIK3CD,ANK2,MEGF10,NRXN3,ROBO1,HTR2B,ITGB6,CDKL5,KIF5C,LMNA,ROBO2,NFASC,CTNNA3,VAV2,ULK4,ITGB1,ELMO2,CNIH2,SRGAP3,MCC,KIF26B,DSCAM,SRGAP2B,TRIO,ARHGAP24,DLG2,IFT43,PTPRK,DAB1,ABCC8,LAMA2,DACH1,PTPN11,MGAT5,CACNB2,DOCK10,HDAC6,ADORA1,EPHA1,KIF18B,PEX14,ERBB4,GBF1,DNAH2,ZNF609,NPHP3,PTPRO,NTRK2,DNAI2,MEGF9,PDGFB,AVL9,KIRREL3,STK39,INSR,FMN2,RERE,UNC5D,LAMA3,CDH4,PAK1,FBXW11,TBR1,CATSPER2,BDNF,ATRN,POMGNT2,FUT8,FMNL2,PHACTR4,LCK,LAMC1,PTPRU,AP3S1,STARD9,SGCD,SLC8A1,GSN,SATB2,DEPDC1B,CASS4,FYN,ADAMTS9,NF1,PLCB1,DST,FAT3,RTN4,DPEP1,APOD,BCR,CCDC141,NDRG4,ADAMTSL1,NTN1,NRG1,BDKRB1,DOCK8,MAP2K1,MYH9,CNTN4,HDAC5,NTNG1,NAV1,CMKLR1,ROR2,DCN,CATSPER3,CDH13,PTPRT,CCNYL1,NCKAP1,DEFA1B,CD99,TERF2,RCC2,BOC,ANO6,ARL3,KIF2A,ICK,RFFL,TTLL8,CCBE1,JAK2,MYPN,FBXW7,BBS12,BCL11B,DNAH14,KIF21A,DYNC1I1,CLDN1,CDC42BPB,WNT11,ASTN1,SIAH1,CAMK2B,PRKCD,SEMA5B,TGFB1,SGK1,MMP28,F2RL1,BCAS3,CELSR1,SLIT3,SLIT2,CORO1C,RRAS2,DISC1,KCNJ3,KANK1,CACNA1D,LAMC2,TTBK2,SPAG17,BMPER,DCLK1,DAPK2,PARVA,DUSP22,YTHDF1,FGF10,ENAH,SMURF2,EPHA4,PRKCA,AUTS2,TNFSF11,PPP3CA,PIP5KL1,TRAK1,CTNNB1,SOD2,IGF1R,PPARG,DNAH10,DLG5,MYO1D,ADD2,LRPPRC,RYR2,DRAXIN,FGF1,NR4A3,DNHD1,MYOCD,AJUBA,CACNA1C,SCN3B,MYO1E,HCK,CSPG4,DCC,NF2,FLT4,HDAC4,DNAH9,TPTE,AP3B2,SPG11,SFRP1,FOXO3,ARL13B,CNTN6,NFIB,IFT122,SMAD3,RAB6A,PTPRM,ACKR2,ABI2,NRP2,PREX1,DOCK4,KPNB1,NAV3,PRTG,APP,GSX2,PDGFRA,SLK,CEP85L,NOX1,MYO1F,LRP5,PTPRG,SLC9B2,SETD2,GPC6,KIF24,DRD1,MMP2,SYNE2,S100A12,PRKD1,SYBU,RND3,DSCAML1,SEMA6D,RAF1,ADAMTS12,BMPR2,USP33,DPYSL2,CAMK1D,BMPR1A,CTNNA2,PIK3R3,SPAG9,RAB11A,CRMP1,FGF2,PPM1F,NEDD9,AGBL4,PEAK1,SEMA4D,ARMC2,MCTP1,ELP3,PLXNA2,PTAFR,DDX58,ITGA11,PIK3C2B,SLC9B1,SPOCK1,BLOC1S5,GAS8,CHRD,EPHB3,FBXO45,PKN3,VCL,LAMB1,CLIC4,KCNQ1,WNT3,RHOJ,CCL14,CCL15,CCL15-CCL14,PAFAH1B1,KIF3A,GLI2,NEO1,RUFY3,DAB2,PKHD1,USH2A,LDLRAD4,MYSM1,DZIP1,WNK1,RELN,NEK10,CSF3R,IQGAP1,SP1,MACF1,MYO7A,CLASP1,DSC2,SLC9C1,OVOL2,NFATC2,PRKG1,TTLL9,ATP2B4,PAK3,SSH2,DYX1C1,MAP1B,CDC42BPA,SCARB1,MARK1,CDK6,EPHA5,GPC3,FLRT2,RPGR,ACTR2,SMOC2,PLCG2,ROCK1,EPS8,PAXIP1,EGR2,ABCC1,AP3B1,NTRK1,MAP1A,PKN2,TMIGD1,EPHB1,EPHA10,TRIM46,TUB,DNAH12,TEKT1,KCND3,AP3D1,KIF3C,SPI1,EPHA7,MCU,CTNNA1,LIMA1,SH3BP1,MST1,PTK7,BMPR1B,MAGI2,ZEB2,HYDIN,ADTRP,PLD1,MITF,PKP2,KCTD13,IGF1,HTT,DPYSL3,MAPK1,PTEN,BMP7,PRCP,LRRK2,OSGIN1,ZRANB1,IFT81,TMEM108,EFNA5,PDGFC,AMOTL1,WNT7A,MAP4,EPDR1,NME8,RHOT1,MYCBP2,GPI,SH3RF2,SOS1,EXT1,RAPGEF2,SMO,SUN1,BLOC1S3,MYLPF,RHOA,NDE1,TF,ONECUT2,FAM19A4,CUL3,SH3KBP1,PLD2,EMB,RDX,ACTA2,FAM49B,ITGBL1,SULF1,AGT,BLOC1S6,PTK2,TPPP2,PDE4B,COPG2,GAP43,TAOK2,ECE1,RPS6KB1,KIF6,GPR173,ANKS1A,ITGAL,ALOX5,CD44,ADAM8,CLDN4,RPS6KA5,CXCL17,PEX7,PLVAP,ACTN4,STK4,TUBB3,SPEF1,EPHB2,TMEFF2,XCR1,DLC1,ARID5B,EPB41L4B,GPSM3,LDHC,ZMYND8,PAX6,PRKCZ,RABGEF1,PHLDB2,KLC3,NIPBL,ANGPT1,OSBPL8,GCNT2,ETS1,ARHGAP21,MAP2,BIN2,CCL22,KIFC3,FBXO31,PRKCQ,BRAF,DIAPH1,ATP8A1,POU3F3,SPINT2,TNR,CD300A,ANGPT4,CAMSAP3,SP100,FAT2,RAB27B,SRGAP2,TMEFF1,NUMB,SYK,SCN1A,SLIT1,FRMD5,RADIL,DNAH3,PTPRJ,GPM6A,MYO1A,SASH1,VAV3,ZP3,GNA12,INPP5F,AXL,MET,FMNL1,CAMK2D,CCR3,SEMA3C,NEDD4L,PTPRC,TYRO3,ADCY10,NCAM1,EDNRA,BMP4
GO:0032879	regulation of localization	3.7833534397362676e-25	CD247,DNAH11,ENPP1,LDB2,GPC5,NRXN1,ASPH,PRKCI,MAP4K4,PDE4D,S1PR2,SLC9A1,ADCY8,CLASP2,SEMA3A,CHERP,DOCK1,WWC1,CLDN18,CTDSPL2,RYR1,NRG3,NOS1AP,LATS2,ASTN2,EPB41,SEMA3D,PHACTR1,DPP6,MAPRE2,PRKAG2,TJP1,UNC5C,CLCN1,FTO,UTRN,KALRN,NTRK3,CBL,RAB4B,RAB4B-EGLN2,SEMA5A,ENPP2,KCNQ5,GRM5,FER,CASK,MARVELD3,MAP2K5,KITLG,KCNIP4,PTPRR,PIK3CD,ANK2,EZR,NRXN3,ROBO1,CHD7,SLC26A6,HTR2B,TENM1,RYR3,LMNA,CTNNA3,NKAIN2,ULK4,ITGB1,CNIH2,SRGAP3,MCC,NKAIN3,PCDH17,SRGAP2B,PARD6G,PTPRK,ABCC8,HMGN3,DGKI,STXBP4,EFCAB7,LAMA2,TCIRG1,DACH1,PTPN11,KCNS3,NETO2,MGAT5,CCT2,CACNB2,DOCK10,HDAC6,ADORA1,EPHA1,ERBB4,GBF1,ZNF609,ATP8A2,KCNJ16,GRM7,DPP10,PTPRO,FRMD4A,STX18,PDGFB,CACNA1B,RIMS1,STK39,INSR,ATP8B1,UNC5D,LAMA3,TCF7L2,

			CAB39,PAK1,MYOM1,TBR1,CATSPER2,BDNF,AMIGO1,KCNMA1,CHRM3,KCNRG,LCK,ECT2,TMC2,VAMP7,PTPRU,DMD,KAT7,SLC8A1,GSN,RBM4,RIT2,JPH2,CASS4,FYN,ARNTL,ADAMTS9,NF1,PLCB1,STX6,RTN4,DPEP1,APOD,BCR,SHISA9,TTN,NDRG4,ICA1,BMP2K,PIK3R2,CACNA1E,NTN1,NRG1,SLC43A2,BDKRB1,DOCK8,MAP2K1,KCNIP1,STIM1,NALCN,MAD1L1,TMBIM6,HDAC5,NTNG1,TMEM30A,CMKLR1,ROR2,DCN,ZDHHC3,SCFD1,CATSPER3,DOCK2,CDH13,CREBRF,PTPRT,FGF14,IPO5,RAB27A,CD99,RAB3C,GOLPH3L,RCC2,ANO6,NEUROD1,KIF2A,RFFL,CCBE1,JAK2,FBXW7,KCNB2,OAZ2,SYT1,CROCC,NPSR1,SCN4A,TMC1,CLDN1,ATP9A,PLS1,KLF15,KCNC4,WNT11,MKLN1,CACNA1H,IL1RAPL1,CAMK2B,PRKCD,SEMA5B,CD4,TGFB1,SGK1,MMP28,KEL,JPH3,CLDN10,F2RL1,BCAS3,DNAJB2,AAK1,SLIT2,CADPS2,SYT13,CORO1C,RRAS2,C12ORF4,KCNJ3,CEP135,KANK1,CACNA1D,CACNA2D3,LAMC2,TTBK2,CHRM1,CLOCK,KCNH1,BMPER,DCLK1,DAPK2,DUSP22,RABGAP1L,HOMER2,SLC17A7,YTHDF1,FGF10,STXBP5,KCNH7,PLIN3,CAPN3,SLC16A1,VMP1,SMURF2,EPHA4,PRKCA,CNR1,TNFSF11,PPP3CA,NSUN2,PIP5KL1,UFL1,CTNNB1,PARK2,SOD2,FCGR2B,IGF1R,PPARG,PRKAR1B,DLG5,MSR1,CYBB,SCAMP5,ANXA13,SREBF2,KCNJ12,RYR2,KCNA6,LEPR,LEPROT,FGF1,NR4A3,NOL3,RIOK2,MYOCD,TRIM5,PER2,AJUBA,CACNA1C,SCN3B,HCK,SORBS1,TRIM8,RIMS4,ANKFY1,NF2,FLT4,HDAC4,SPTBN1,SFRP1,FOXO3,DNM3,SMAD3,ITPR1,PTPRM,WWP2,GRIK5,LZTFL1,SHANK1,SYT3,NEDD4,NRP2,DOCK4,NAV3,SLC6A1,APP,GSX2,PDGFRA,BET1L,SLK,NOX1,YAP1,RAB11FIP5,NLGN2,VDAC1,BORA,IBTK,NVL,LRP5,PTPRG,SLC9B2,SETD2,GPC6,SPTBN4,DRD1,TMEM14A,MMP2,SERGEF,SYNE2,PRKD1,HNF4A,VPS4A,FXYD2,FXYD6,FXYD6-FXYD2,SEMA6D,KCNG4,RAF1,RASGRF2,PTPN1,BMPR2,CAMK1D,BMPR1A,NLGN1,CTNNA2,PIK3R3,SPAG9,RAB11A,FGF2,PPM1F,ADCY5,NEDD9,SEMA4D,MCTP1,ELP3,PLXNA2,PTAFR,ADCYAP1R1,ABCA13,DDX58,RAB11FIP3,PRKCG,GAS8,AP2M1,CHRD,FBXL20,TSG101,VCL,LAMB1,CLIC4,KCNQ1,RHOJ,SUFU,NLGN3,KIF3A,TRPA1,HIP1,AKAP6,BTBD9,RUFY3,STON2,DAB2,PKHD1,LDLRAD4,MYSM1,DZIP1,WNK1,RELN,SIN3A,RUVBL2,ABCB1,KCNQ2,SP1,TRIM22,MACF1,CLASP1,DSC2,EEPD1,APBB3,TPCN1,GPM6B,CLIC5,AHNAK,SNX5,PRKG1,RGS7,ATP2B4,NSF,PACSIN1,PAK3,SSH2,ATAD1,MAP1B,SCARB1,CDK6,KCNAB2,TFRC,EPHA5,MEF2A,SYT12,CACNA1A,GPC3,SGIP1,STOM,ADRBK1,TBC1D5,PTGER3,TRPV1,FLRT2,C2,SMOC2,PLCG2,ROCK1,C1QTNF1,GAPVD1,CAPN10,GPR35,PTPN2,ZFAND1,MAP1A,PKN2,TMIGD1,ANK3,PTPN9,GNL3L,PTPN14,TRIM46,TUB,SH3GL3,KCND3,CACNG8,AP3D1,AP2B1,NETO1,ARHGAP44,SNX33,CD84,SPI1,CHRNB4,MCU,CTNNA1,TRDN,STAC,TRPC6,RAB15,RAPGEF3,SH3BP1,MST1,SNCA,CACNG2,MAGI2,SYT9,CHMP3,RAB5B,ADTRP,ABCA12,PLD1,MITF,OPRD1,PKP2,XPO4,SLC4A8,IGF1,PLA2G4E,STX8,HTT,DPYSL3,MAPK1,PTEN,MIB1,BMP7,SCN8A,ATXN2,PIK3C3,PRCP,LRRK2,OSGIN1,TMEM59,SPAG5,UNC119,CNIH3,LILRB4,ITGAM,PKD2,EFNA5,SHANK3,PDGFC,AMOTL1,BEST3,WNT7A,ZBED6,DNAJC1,BMP6,MYCBP2,ANO1,GPI,SH3RF2,PLIN2,RAPGEF2,KCNC2,SMO,MARK4,STXBP5L,CPT1A,PSEN2,SYT17,DNAJC6,UBR5,GRIN1,RHOA,GRIN3A,TF,ONECUT2,ITPR2,EPM2A,GSK3B,ABCG8,RDX,ACTA2,SYN1,EEF2K,FAM49B,IWS1,NECAB2,MCTP2,SCN9A,BOK,SULF1,AGT,PRKAA2,SYT7,ZMPSTE24,PTK2,TPPP2,SNX3,PDE4B,RIMS2,SEC16B,CD160,KCNJ15,RPS6KB1,STC2,GPR173,ALOX5,CPLX2,DNAJB6,ADAM8,SLC5A3,CLDN4,PACSIN2,CXCL17,NOS1,PLVAP,ACTN4,STK4,EPHB2,SLC1A1,WLS,TMEFF2,XCR1,LRRC52,NKAIN1,GPSM2,HCAR2,DLC1,PPARA,MCOLN1,EPB41L4B,GPSM3,SYTL4,ZMYND8,PAX6,PRKCZ,ADORA2A,KCTD7,PPP1CC,RABGEF1,PHLDB2,OTUD7B,NIPBL,ANGPT1,CADPS,OSBPL8,EPB41L2,GCNT2,ETS1,MAP2,FBXO31,ATG3,CNTN1,BRAF,DIAPH1,HTR2C,ATP8A1,SPINT2,CD300A,ANGPT4,CAMSAP3,SP100,ANXA2,TRIM29,KCND2,PARN,RAB27B,NUP214,SRGAP2,ADCY1,NUMB,SYK,WWTR1,SCN1A,FRMD5,MYRIP,CACNG3,ANKRD13A,PTPRJ,SLC10A1,SASH1,ZP3,KCNQ3,GNA12,INPP5F,AXL,MET,SESTD1,CAMK2D,KCNJ6,EHD2,SEMA3C,CCT3,NEDD4L,PTPRC,GPR89A,CREB1,SHISA6,EDNRA,PRAP1,STXBP6,BMP4,ABLIM3
GO:0051234	establishment of localization	9.815696773218974e-25	*POLDIP3,DNAH11,ZDHHC14,SLC39A11,ENPP1,SLC35E3,HOOK2,RTBDN,BLZF1,NRXN1,ASPH,PRKCI,SLCO3A1,PDE4D,SLC9A1,ANTXR2,FAM155A,CD302,LY75,ADCY8,SPAG16,CLASP2,GRID2,CHERP,SLC35E1,DOCK1,WWC1,KIF22,CTDSPL2,SLC25A17,CACHD1,RYR1,NOS1AP,TUBA1C,LRP1B,NOX5,ANP32A,ASTN2,EPB41,DPP6,SLC14A2,NLGN4X,PRKAG2,CLCN1,SLC35F3,PRLR,MVP,UTRN,KALRN,PITPNC1,SEC23B,LTV1,SNX31,CBL,RAB4B,RAB4B-EGLN2,STAT5B,ENPP2,ATP2B2,KCNQ5,GRM5,FAM53A,FER,CASK,CCDC93,KCNIP4,SPNS2,LRP2,PIK3CD,SLC38A11,TNPO3,ANK2,EZR,MEGF10,NRXN3,SLC9A9,TOM1L1,CHD7,SLC26A6,HTR2B,TENM1,RYR3,KIF5C,LMNA,SRP72,AFM,SLC24A2,NKAIN2,VAV2,ITGB1,HSP90AA1,APOL4,SLC24A3,ELMO2,PSMB7,CNIH2,MCC,NKAIN3,PCDH17,CCDC22,SLC22A8,CNGB1,KLHL12,AKAP13,DLG2,FLVCR2,IFT43,MYO10,ABCC8,ERC1,HMGN3,AFTPH,NCF4,DGKI,STXBP4,ERC2,GABRA3,EFCAB7,TCIRG1,PTPN11,KCNS3,NIPAL2,NETO2,CCT2,CACNB2,CYTH3,HDAC6,GOLGA4,SV2B,ADORA1,GPRASP1,PEX14,ERBB4,CNNM1,SORCS1,GBF1,LIMK1,TLK1,ATP8A2,OSBPL10,CECR2,KCNJ16,NPHP3,GRM7,DPP10,NTRK2,CYB561A3,FRMD4A,STX18,HOOK3,VPS45,PDGFB,CACNA1B,RIMS1,SNX2,STK39,INSR,FMN2,ATP8B1,H2AFY2,PLLP,TCF7L2,PIP4K2A,HPS1,CAB39,PAK1,FBXW11,SCAMP4,MYOM1,ZDHHC13,SLC20A2,SLC4A10,CATSPER2,GLTP,AMIGO1,SFT2D1,KCNMA1,CHRM3,GRIK4,KCNRG,LCK,ECT2,TMC2,TMEM144,SLC12A8,SNX6,CHML,VAMP7,SPNS3,SLC6A16,RFTN1,SHFM1,MB,AP3S1,NPLOC4,TMPRSS15,RHCE,DENND5A,MICU3,*

			DMD,CENPF,RANBP17,ATG10,SLC30A9,SLC8A1,PRELID2,GSN,RBM4,RIT2,TANC2,JPH2,FYN,ARNTL,ADAMTS9,NF1,NCF2,SMG7,SLC16A6,DST,LMTK2,STX6,SNCB,SLC25A21,MAL2,GOLGA2P5,SLC5A6,APOD,SLC2A14,VTA1,BCR,SHISA9,TTN,NDRG4,ICA1,BMP2K,PIK3R2,ANKH,CACNA1E,NTN1,DCTN1,SLC4A5,NRG1,KCNN3,CNNM2,SLC16A10,SLC43A2,BDKRB1,BDKRB2,BID,MAP2K1,KCNIP1,SLC35A2,SLCO1A2,STIM1,NALCN,MYH9,MAD1L1,TMBIM6,TMEM30A,PARD3B,SCFD2,ZDHHC3,SLC39A14,LIN7A,SCFD1,CATSPER3,SYN2,DOCK2,CDH13,PITPNB,CREBRF,COPS5,FGF14,EXOC4,IPO5,MAN1A1,TLE6,RAB27A,MTX3,RAB3C,COG8,TERF2,TMED6,GOLPH3L,CLDN16,RCC2,ANO6,ARL3,NEUROD1,IPO11,ICK,PARP11,RANBP1,DENND1A,ZDHHC15,JAK2,FBXW7,KCNB2,OAZ2,SYN3,STARD6,SYT1,CROCC,NPSR1,BBS12,SNAP25-AS1,ABCC2,SMG1,SCN4A,RBFOX1,TMC1,SLC1A4,DYNC1I1,CLDN1,SLC5A9,ATP9A,SLC9A7,PLS1,DENND2A,KLF15,KCNC4,KPNA3,CTAGE6,CENPC,VPS13B,CLCA2,CYB561D1,IMMP2L,MKLN1,CACNA1H,QKI,IL1RAPL1,CAMK2B,PRKCD,SLC35F1,CD4,TGFB1,SGK1,PCSK5,NUP93,PIBF1,KEL,JPH3,PTPRN2,CLDN10,F2RL1,BCAS3,GRIK3,AAK1,ZFYVE9,GABRR3,CADPS2,GABRB1,SYT13,CDH23,CORO1C,C12ORF4,KCNJ3,CEP135,CACNA1D,ZFAND2A,ABCC11,CACNA2D3,GRPEL2,ANO4,ANK1,CLMN,XPR1,SPAG17,CHRM1,RAB11FIP4,CLOCK,ITSN2,ANO3,SYNJ2,KCNH1,BCL3,DCLK1,ANKRD54,RABGAP1L,ANKFN1,HOMER2,SLC17A7,FGF10,SMYD3,STXBP5,KCNH7,PLIN3,LOXL3,CAPN3,SLC16A1,VMP1,SNX8,CNR1,CD6,ANO2,TNFSF11,SMG6,PPP3CA,NSUN2,HBE1,TRAK1,CTNNB1,PARK2,RN7SL832P,FCGR2B,HSPA6,TSNARE1,IGF1R,PPARG,AXIN1,PRKAR1B,COX8A,MYO1D,TMEM63C,MSR1,TRPM1,CYBB,SYTL5,XK,APOL3,SCAMP5,ANXA13,LRPPRC,SREBF2,KCNJ12,RYR2,KCNA6,NDUFA9,LEPR,LEPROT,PROS1,NR4A3,NOL3,RIOK2,AGAP1,SCARA3,PER2,CACNA1C,CPNE6,SCN3B,MYO1E,HCK,SLC35E4,SORBS1,TBC1D14,RAB3GAP2,TBCK,MRC2,GRM1,RIMS4,ANKFY1,DNAH9,SPTBN1,AP3B2,SPG11,SFRP1,CCDC91,IFT122,RANBP3,DNM3,ATP10D,SNX14,SMAD3,ITPR1,RAB6A,WWP2,ACKR2,WIPI1,VPS39,GRIK5,PSAP,SLC16A2,SHANK1,SYT3,NEDD4,SVOPL,SLC35F4,KPNB1,SLC4A4,VPS53,SLC6A1,RAB6C,SEC61B,KCNK1,THOC3,APP,BET1L,RBM8A,NOX1,RAB11FIP5,SFXN5,MYO1F,NLGN2,VDAC1,EYA2,SSR2,IBTK,SCRN1,LRP5,SEC22C,SLC9B2,SETD2,ZDHHC6,PRICKLE1,SPTBN4,GRIA3,DRD1,TMEM14A,GLRA2,RFT1,SERGEF,TPH1,SYNE2,PRKD1,OSBPL2,SYBU,SLC6A3,HNF4A,STRA6,VPS4A,MAPKAPK3,FXYD2,FXYD6,FXYD6-FXYD2,PITRM1,SLC16A12,ATF2,KCNG4,RAF1,RASGRF2,PTPN1,USP33,DPYSL2,CAMK1D,AP1B1,NLGN1,ATP9B,SNX16,SPAG9,RAB11A,EPG5,IPO9,FGF2,GRIK1,RAB24,PPM1F,ADCY5,AGBL4,MCTP1,XPO6,PTAFR,HHIPL1,ADCYAP1R1,EEA1,ABCA13,RILPL1,KLHL3,MRS2,SORCS2,RAB11FIP3,SLC5A8,PRKCG,SLC9B1,BLOC1S5,TXNDC5,TANGO6,GRIA2,GAS8,OC90,AP2M1,SLC35D1,TNPO1,TRAPPC11,TECPR2,ATP6V0B,LMBRD1,FBXL20,TSG101,DESI1,CLIC4,CRYM,KCNQ1,SLC2A11,RHOJ,SUFU,TG,NLGN3,PAFAH1B1,KIF3A,ATG4C,TRPA1,TRPM3,NMUR2,HIP1,AKAP6,BTBD9,TRPC5,ENTHD1,TNKS,RUFY3,STON2,TPD52,DAB2,PKHD1,XKR4,TRAM2,USH2A,DZIP1,SMG5,WNK1,RELN,NEK10,RUVBL2,ABCB1,KCNQ2,SLC22A3,SLC25A16,SLC47A1,EML4,FAM91A1,PLEKHF2,APOLD1,MACF1,ARHGAP12,MYO7A,SLC8A2,CLASP1,EEPD1,SLC9C1,APBB3,SLC35A4,TPCN1,GPM6B,CLIC5,XDH,AHNAK,SNX5,RGS7,ATP2B4,NSF,SYNE3,PACSIN1,SPTBN5,SLCO4A1,DYX1C1,P2RX6,ASGR2,ATAD1,MAPKAPK2,NXT2,CEP41,MAP1B,SCARB1,MARK1,ATP6V0A2,KCNAB2,TFRC,UVRAG,EPHA5,GABRG3,MEF2A,SYT12,CACNA1A,FNBP1,GPC3,SGIP1,STOM,ADRBK1,TBC1D5,PSMB2,PTGER3,TRPV1,CLEC16A,GHR,SLC38A6,PRSS12,SNX9,RPGR,ARL5A,ACTR2,C2,PLCG2,ROCK1,C1QTNF1,EGR2,GAPVD1,CAPN10,GPR35,ABCC1,AP3B1,KPNA6,ZFAND1,MAP1A,SLC39A10,ANK3,UNC13A,SNUPN,ATP13A3,PARD3,PTPN14,TRIM46,TUB,SH3GL3,KCND3,CACNG8,AP3D1,KIF3C,SLC9C2,AP2B1,NETO1,ANXA8L1,ARHGAP44,HEATR5A,SNX33,CD84,LPA,CUX1,SPI1,CHRNB4,MCU,VPS16,LIMA1,ACAP2,TRDN,ATG14,GABRA6,LRRC8C,LRRC8D,STAC,ILDR2,TRPC6,ABCB7,SLC41A2,FCHSD2,RAB15,RAPGEF3,SLC48A1,SH3BP1,ATP13A5,RSRC1,SNCA,CACNG2,GRM4,MAGI2,TBC1D9,PNPT1,OCA2,SLC39A9,SYT9,CHMP3,RNF103-CHMP3,CNGA3,SLC22A10,PRKAG1,RAB5B,OSBPL1A,ADTRP,ABCA12,PLD1,CLCC1,OPRD1,PKP2,BTK,TBC1D16,XPO4,VPS41,SLC4A8,IGF1,PLA2G4E,STX8,HTT,MAPK1,PTEN,MIB1,SLC7A14,SLC30A7,SCN8A,ATXN2,PIK3C3,LHFPL5,NUP88,LRRK2,ATP6V1A,SPAG5,UNC119,CHMP5,CNIH3,BAG6,LILRB4,SLC44A1,IFT81,TMCO1,TMEM108,IPCEF1,ITGAM,ARHGAP25,NSG2,PKD2,EFNA5,SHANK3,MIA2,SERPINA5,ATP8B4,BEST3,SGSM1,STEAP4,TMCC1,WNT7A,ZBED6,ZC3H11A,SNAP23,MAP4,DNAJC1,RABEP1,POM121C,RHOT1,BMP6,ANO1,ANO8,EXT1,PLIN2,SERINC5,GABRR2,SAMM50,ABCG2,KCNC2,SMO,SLC6A14,GET4,SUN1,LSG1,BLOC1S3,STXBP5L,CPT1A,PSEN2,SYT17,SOX30,DNAJC6,UBR5,GRIN1,ARRDC4,BBS9,RHOA,GRIN3A,NDE1,TF,RHOBTB1,ESYT2,FAM19A4,CUL3,SH3KBP1,HSPD1,ITPR2,EPM2A,GABRB3,GSK3B,ABCG8,PLD2,EMB,RDX,SYN1,EEF2K,IWS1,NECAB2,UPF2,MCTP2,SCN9A,VAMP1,BOK,AGT,SYT7,ADAR,BLOC1S6,PITPNM2,ZMPSTE24,PTK2,HEPHL1,LAT2,PEX5L,SNX3,EXOC6B,PDE4B,RIMS2,COX5A,SLC25A51,TOMM5,COPG2,CPE,SEC16B,TRPC4AP,STOML1,TIMM44,PCSK6,CHKA,SLC25A42,GTF2IRD2,NCF1,SLC29A1,TAOK2,UGT1A3,SLC44A3,CD160,KCNJ15,RPS6KB1,SLC5A10,STC2,ZDHHC23,ITGAL,ALOX5,CPLX2,ADAM8,SLC5A3,ARFGAP3,CLDN4,PACSIN2,GRID1,PKD1L1,SLC25A18,COX5B,MON2,NOS1,PEX7,GCKR,TBC1D23,PLEKHA1,

			RGS14,ACTN4,COG4,SCNN1B,STK4,ZFAND6,COX6A1,EPHB2,SLC1A1,WLS,SIGMAR1,XCR1,LRRC52,MFSD8,NKAIN1,GPSM2,HCAR2,PPARA,PPP1R10,ABCD3,GRTP1,CALCRL,MCOLN1,STEAP3,SLC25A33,KPNA4,SYTL4,PAX6,PRKCZ,ATP1A3,ADORA2A,GRIN2B,KCTD7,PPP1CC,RABGEF1,WDR83OS,PHLDB2,GPR107,KLC3,NIPBL,ANGPT1,CADPS,FRMPD1,OSBPL8,IGF2BP3,SLC25A26,SLCO2B1,HACL1,TBC1D10C,ARHGAP21,CFI,GRIA4,MAP2,BIN2,STPG1,ATG3,CNTN1,NCOR2,BRAF,DIAPH1,HTR2C,SIL1,ATP8A1,TMEM163,CLTB,UQCR10,CD300A,EMC3,SLC26A2,RPL23,SH3BP4,ATXN1,CAMSAP3,SP100,VTI1A,ANXA2,KCND2,ATP6V0A1,RAB27B,ITSN1,NUP214,PITPNM3,ADCY1,NUMB,SLC18B1,SYK,MOGAT2,GRIK2,SCN1A,SLIT1,MYRIP,RAB2A,SEC31B,SLC44A5,BRCA2,CACNG3,YBX1,ANKRD13A,GRIP1,PTPRJ,SLC10A1,ATP2B3,ERGIC1,GPM6A,OSBP2,MYO1A,SLC13A3,SLC2A12,HSF1,CNIH4,ATP10B,PLEKHM1,HGSNAT,SLC5A4,FCHO1,VAV3,ZP3,KCNQ3,MLPH,RAB37,CAV2,AKTIP,EPS15,INPP5F,TMPRSS3,AXL,REPS2,DENND4C,MET,SESTD1,TBC1D10A,MICAL3,CAMK2D,KCNJ6,SNX1,MALT1,ZDHHC11,ZDHHC11B,EHD2,THEM4,TRAPPC8,CCT3,NEDD4L,NDC1,PTPRC,RPH3A,GPR89A,RAB28,SH3GL2,TYRO3,MTMR12,SPON2,SYNDIG1,VPS52,ADCY10,TRAF3IP2,CREB1,SGTB,SHISA6,CLNK,EDNRA,PRAP1,SLC25A13,STXBP6,BMP4,ABLIM3,CPNE1
GO:0006810	transport	1.049781333710413e-24	POLDIP3,DNAH11,ZDHHC14,SLC39A11,ENPP1,SLC35E3,HOOK2,RTBDN,BLZF1,NRXN1,ASPH,PRKCI,SLCO3A1,PDE4D,SLC9A1,ANTXR2,FAM155A,CD302,LY75,ADCY8,SPAG16,CLASP2,GRID2,CHERP,SLC35E1,DOCK1,WWC1,CTDSPL2,SLC25A17,CACHD1,RYR1,NOS1AP,TUBA1C,LRP1B,NOX5,ANP32A,ASTN2,EPB41,DPP6,SLC14A2,NLGN4X,PRKAG2,CLCN1,SLC35F3,PRLR,MVP,UTRN,KALRN,PITPNC1,SEC23B,LTV1,SNX31,CBL,RAB4B,RAB4B-EGLN2,STAT5B,ENPP2,ATP2B2,KCNQ5,GRM5,FAM53A,FER,CASK,CCDC93,KCNIP4,SPNS2,LRP2,PIK3CD,SLC38A11,TNPO3,ANK2,EZR,MEGF10,NRXN3,SLC9A9,TOM1L1,CHD7,SLC26A6,HTR2B,TENM1,RYR3,KIF5C,LMNA,SRP72,AFM,SLC24A2,NKAIN2,VAV2,ITGB1,HSP90AA1,APOL4,SLC24A3,ELMO2,PSMB7,CNIH2,NKAIN3,PCDH17,CCDC22,SLC22A8,CNGB1,KLHL12,AKAP13,DLG2,FLVCR2,IFT43,MYO10,ABCC8,ERC1,HMGN3,AFTPH,NCF4,DGKI,STXBP4,ERC2,GABRA3,EFCAB7,TCIRG1,PTPN11,KCNS3,NIPAL2,NETO2,CCT2,CACNB2,CYTH3,HDAC6,GOLGA4,SV2B,ADORA1,GPRASP1,PEX14,ERBB4,CNNM1,SORCS1,GBF1,LIMK1,TLK1,ATP8A2,OSBPL10,CECR2,KCNJ16,NPHP3,GRM7,DPP10,NTRK2,CYB561A3,FRMD4A,STX18,HOOK3,VPS45,PDGFB,CACNA1B,RIMS1,SNX2,STK39,INSR,FMN2,ATP8B1,PLLP,TCF7L2,PIP4K2A,HPS1,CAB39,PAK1,FBXW11,SCAMP4,MYOM1,ZDHHC13,SLC20A2,SLC4A10,CATSPER2,GLTP,AMIGO1,SFT2D1,KCNMA1,CHRM3,GRIK4,KCNRG,LCK,ECT2,TMC2,TMEM144,SLC12A8,SNX6,CHML,VAMP7,SPNS3,SLC6A16,RFTN1,SHFM1,MB,AP3S1,NPLOC4,TMPRSS15,RHCE,DENND5A,MICU3,DMD,CENPF,RANBP17,ATG10,SLC30A9,SLC8A1,PRELID2,GSN,RBM4,RIT2,TANC2,JPH2,FYN,ARNTL,ADAMTS9,NF1,NCF2,SMG7,SLC16A6,DST,LMTK2,STX6,SNCB,SLC25A21,MAL2,GOLGA2P5,SLC5A6,APOD,SLC2A14,VTA1,BCR,SHISA9,TTN,NDRG4,ICA1,BMP2K,PIK3R2,ANKH,CACNA1E,NTN1,DCTN1,SLC4A5,NRG1,KCNN3,CNNM2,SLC16A10,SLC43A2,BDKRB1,BDKRB2,BID,MAP2K1,KCNIP1,SLC35A2,SLCO1A2,STIM1,NALCN,MYH9,TMBIM6,TMEM30A,SCFD2,ZDHHC3,SLC39A14,LIN7A,SCFD1,CATSPER3,SYN2,DOCK2,CDH13,PITPNB,CREBRF,COPS5,FGF14,EXOC4,IPO5,MAN1A1,RAB27A,MTX3,RAB3C,COG8,TERF2,TMED6,GOLPH3L,CLDN16,ANO6,ARL3,NEUROD1,IPO11,ICK,PARP11,RANBP1,DENND1A,ZDHHC15,JAK2,FBXW7,KCNB2,OAZ2,SYN3,STARD6,SYT1,NPSR1,BBS12,SNAP25-AS1,ABCC2,SMG1,SCN4A,RBFOX1,TMC1,SLC1A4,DYNC1I1,CLDN1,SLC5A9,ATP9A,SLC9A7,PLS1,DENND2A,KLF15,KCNC4,KPNA3,CTAGE6,VPS13B,CLCA2,CYB561D1,IMMP2L,MKLN1,CACNA1H,QKI,IL1RAPL1,CAMK2B,PRKCD,SLC35F1,CD4,TGFB1,SGK1,PCSK5,NUP93,KEL,JPH3,PTPRN2,CLDN10,F2RL1,BCAS3,GRIK3,AAK1,ZFYVE9,GABRR3,CADPS2,GABRB1,SYT13,CDH23,CORO1C,C12ORF4,KCNJ3,CACNA1D,ZFAND2A,ABCC11,CACNA2D3,GRPEL2,ANO4,ANK1,CLMN,XPR1,SPAG17,CHRM1,RAB11FIP4,CLOCK,ITSN2,ANO3,SYNJ2,KCNH1,BCL3,DCLK1,ANKRD54,RABGAP1L,HOMER2,SLC17A7,FGF10,STXBP5,KCNH7,PLIN3,LOXL3,CAPN3,SLC16A1,VMP1,SNX8,CNR1,CD6,ANO2,TNFSF11,SMG6,PPP3CA,NSUN2,HBE1,TRAK1,CTNNB1,PARK2,RN7SL832P,FCGR2B,HSPA6,TSNARE1,IGF1R,PPARG,AXIN1,PRKAR1B,COX8A,MYO1D,TMEM63C,MSR1,TRPM1,CYBB,SYTL5,XK,APOL3,SCAMP5,ANXA13,LRPPRC,SREBF2,KCNJ12,RYR2,KCNA6,NDUFA9,LEPR,LEPROT,PROS1,NR4A3,NOL3,RIOK2,AGAP1,SCARA3,PER2,CACNA1C,CPNE6,SCN3B,MYO1E,HCK,SLC35E4,SORBS1,TBC1D14,RAB3GAP2,TBCK,MRC2,GRM1,RIMS4,ANKFY1,DNAH9,SPTBN1,AP3B2,SPG11,SFRP1,CCDC91,IFT122,RANBP3,DNM3,ATP10D,SNX14,SMAD3,ITPR1,RAB6A,WWP2,ACKR2,WIPI1,VPS39,GRIK5,PSAP,SLC16A2,SHANK1,SYT3,NEDD4,SVOPL,SLC35F4,KPNB1,SLC4A4,VPS53,SLC6A1,RAB6C,SEC61B,KCNK1,THOC3,APP,BET1L,RBM8A,NOX1,RAB11FIP5,SFXN5,MYO1F,NLGN2,VDAC1,EYA2,SSR2,IBTK,SCRN1,LRP5,SEC22C,SLC9B2,SETD2,ZDHHC6,PRICKLE1,SPTBN4,GRIA3,DRD1,TMEM14A,GLRA2,RFT1,SERGEF,TPH1,SYNE2,PRKD1,OSBPL2,SYBU,SLC6A3,HNF4A,STRA6,VPS4A,MAPKAPK3,FXYD2,FXYD6,FXYD6-FXYD2,PITRM1,SLC16A12,ATF2,KCNG4,RAF1,RASGRF2,PTPN1,USP33,DPYSL2,CAMK1D,AP1B1,NLGN1,ATP9B,SNX16,SPAG9,RAB11A,EPG5,IPO9,FGF2,GRIK1,RAB24,PPM1F,ADCY5,AGBL4,MCTP1,XPO6,PTAFR,HHIPL1,ADCYAP1R1,EEA1,ABCA13,RILPL1,KLHL3,MRS2,SORCS2,RAB11FIP3,SLC5A8,PRKCG,SLC9B1,BLOC1S5,TXNDC5,TANGO6,GRIA2,GAS8,OC90,AP2M1,SLC35D1,TNPO1,TRAPPC11,TECPR

			2,ATP6V0B,LMBRD1,FBXL20,TSG101,DESI1,CLIC4,CRYM,KCNQ1,SLC2A11,RHOJ,SUFU,TG,NLGN3,PAFAH1B1,KIF3A,ATG4C,TRPA1,TRPM3,NMUR2,HIP1,AKAP6,BTBD9,TRPC5,ENTHD1,TNKS,RUFY3,STON2,TPD52,DAB2,XKR4,TRAM2,DZIP1,SMG5,WNK1,RELN,NEK10,ABCB1,KCNQ2,SLC22A3,SLC25A16,SLC47A1,FAM91A1,PLEKHF2,APOLD1,MACF1,ARHGAP12,MYO7A,SLC8A2,CLASP1,EEPD1,SLC9C1,APBB3,SLC35A4,TPCN1,GPM6B,CLIC5,XDH,AHNAK,SNX5,RGS7,ATP2B4,NSF,SYNE3,PACSIN1,SPTBN5,SLCO4A1,DYX1C1,P2RX6,ASGR2,ATAD1,MAPKAPK2,NXT2,CEP41,MAP1B,SCARB1,ATP6V0A2,KCNAB2,TFRC,UVRAG,EPHA5,GABRG3,MEF2A,SYT12,CACNA1A,FNBP1,GPC3,SGIP1,STOM,ADRBK1,TBC1D5,PSMB2,PTGER3,TRPV1,CLEC16A,GHR,SLC38A6,PRSS12,SNX9,RPGR,ARL5A,ACTR2,C2,PLCG2,ROCK1,C1QTNF1,EGR2,GAPVD1,CAPN10,GPR35,ABCC1,AP3B1,KPNA6,ZFAND1,MAP1A,SLC39A10,ANK3,UNC13A,SNUPN,ATP13A3,PARD3,PTPN14,TRIM46,TUB,SH3GL3,KCND3,CACNG8,AP3D1,KIF3C,SLC9C2,AP2B1,NETO1,ANXA8L1,ARHGAP44,HEATR5A,SNX33,CD84,LPA,CUX1,SPI1,CHRNB4,MCU,VPS16,LIMA1,ACAP2,TRDN,ATG14,GABRA6,LRRC8C,LRRC8D,STAC,ILDR2,TRPC6,ABCB7,SLC41A2,FCHSD2,RAB15,RAPGEF3,SLC48A1,SH3BP1,ATP13A5,RSRC1,SNCA,CACNG2,GRM4,MAGI2,TBC1D9,PNPT1,OCA2,SLC39A9,SYT9,CHMP3,RNF103-CHMP3,CNGA3,SLC22A10,PRKAG1,RAB5B,OSBPL1A,ADTRP,ABCA12,PLD1,CLCC1,OPRD1,PKP2,BTK,TBC1D16,XPO4,VPS41,SLC4A8,IGF1,PLA2G4E,STX8,HTT,MAPK1,PTEN,MIB1,SLC7A14,SLC30A7,SCN8A,ATXN2,PIK3C3,LHFPL5,NUP88,LRRK2,ATP6V1A,SPAG5,UNC119,CHMP5,CNIH3,LILRB4,SLC44A1,IFT81,TMCO1,TMEM108,IPCEF1,ITGAM,ARHGAP25,NSG2,PKD2,EFNA5,SHANK3,MIA2,SERPINA5,ATP8B4,BEST3,SGSM1,STEAP4,TMCC1,WNT7A,ZBED6,ZC3H11A,SNAP23,DNAJC1,RABEP1,POM121C,RHOT1,BMP6,ANO1,ANO8,EXT1,PLIN2,SERINC5,GABRR2,SAMM50,ABCG2,KCNC2,SMO,SLC6A14,SUN1,LSG1,BLOC1S3,STXBP5L,CPT1A,PSEN2,SYT17,SOX30,DNAJC6,UBR5,GRIN1,ARRDC4,BBS9,RHOA,GRIN3A,NDE1,TF,RHOBTB1,ESYT2,FAM19A4,CUL3,SH3KBP1,HSPD1,ITPR2,EPM2A,GABRB3,GSK3B,ABCG8,PLD2,EMB,RDX,SYN1,EEF2K,IWS1,NECAB2,UPF2,MCTP2,SCN9A,VAMP1,BOK,AGT,SYT7,ADAR,BLOC1S6,PITPNM2,ZMPSTE24,PTK2,HEPHL1,LAT2,PEX5L,SNX3,EXOC6B,PDE4B,RIMS2,COX5A,SLC25A51,TOMM5,COPG2,CPE,SEC16B,TRPC4AP,STOML1,TIMM44,PCSK6,CHKA,SLC25A42,GTF2IRD2,NCF1,SLC29A1,TAOK2,UGT1A3,SLC44A3,CD160,KCNJ15,RPS6KB1,SLC5A10,STC2,ZDHHC23,ITGAL,ALOX5,CPLX2,ADAM8,SLC5A3,ARFGAP3,CLDN4,PACSIN2,GRID1,PKD1L1,SLC25A18,COX5B,MON2,NOS1,PEX7,GCKR,TBC1D23,RGS14,ACTN4,COG4,SCNN1B,STK4,ZFAND6,COX6A1,EPHB2,SLC1A1,WLS,SIGMAR1,XCR1,LRRC52,MFSD8,NKAIN1,HCAR2,PPARA,PPP1R10,ABCD3,GRTP1,CALCRL,MCOLN1,STEAP3,SLC25A33,KPNA4,SYTL4,PRKCZ,ATP1A3,ADORA2A,GRIN2B,KCTD7,PPP1CC,RABGEF1,GPR107,KLC3,ANGPT1,CADPS,OSBPL8,IGF2BP3,SLC25A26,SLCO2B1,HACL1,TBC1D10C,ARHGAP21,CFI,GRIA4,MAP2,BIN2,STPG1,ATG3,CNTN1,NCOR2,BRAF,DIAPH1,HTR2C,SIL1,ATP8A1,TMEM163,CLTB,UQCR10,CD300A,SLC26A2,RPL23,SH3BP4,ATXN1,CAMSAP3,SP100,VTI1A,ANXA2,KCND2,ATP6V0A1,RAB27B,ITSN1,NUP214,PITPNM3,ADCY1,NUMB,SLC18B1,SYK,MOGAT2,GRIK2,SCN1A,SLIT1,MYRIP,RAB2A,SEC31B,SLC44A5,CACNG3,YBX1,ANKRD13A,GRIP1,PTPRJ,SLC10A1,ATP2B3,ERGIC1,GPM6A,OSBP2,MYO1A,SLC13A3,SLC2A12,HSF1,CNIH4,ATP10B,PLEKHM1,HGSNAT,SLC5A4,FCHO1,VAV3,ZP3,KCNQ3,MLPH,RAB37,CAV2,AKTIP,EPS15,INPP5F,TMPRSS3,AXL,REPS2,DENND4C,MET,SESTD1,TBC1D10A,MICAL3,CAMK2D,KCNJ6,SNX1,MALT1,ZDHHC11,ZDHHC11B,EHD2,THEM4,TRAPPC8,CCT3,NEDD4L,NDC1,PTPRC,RPH3A,GPR89A,RAB28,SH3GL2,TYRO3,MTMR12,SPON2,SYNDIG1,VPS52,ADCY10,TRAF3IP2,CREB1,SGTB,SHISA6,CLNK,EDNRA,PRAP1,SLC25A13,STXBP6,BMP4,ABLIM3,CPNE1
GO:0019538	protein metabolic process	1.4424249254899502e-24	*POLDIP3,LNX2,ZDHHC14,ENPP1,ADAMTS16,NRXN1,ASPH,B4GALNT2,F8,PRMT3,PRKCI,SLCO3A1,MAP4K4,PDE4D,DNMT1,S1PR2,C6ORF89,ADCY8,MGAT4C,CBFB,PDE8A,IL31RA,RPS6KA2,PRDM12,SETD4,CALR3,CTDSPL2,FBXL2,NRG3,ASH1L,NOS1AP,LATS2,CPA6,DPP6,RNF145,KSR2,PRKAG2,OVCH2,PRLR,MVP,PAGR1,FTO,KALRN,DCAF12,NTRK3,CBL,ARNT,EGLN2,FLT3,ENPP2,GRM5,PLCE1,FER,CASK,MAPK4,MAP2K5,ISCA1,KITLG,USP32,MAPK10,PTPRR,LRP2,PIK3CD,NAALADL2,CAMK1G,EIF4G3,EZR,B3GALT1,ROBO1,TOM1L1,HTR2B,PSMD1,CDKL5,MECOM,TENM1,CAMKMT,LMNA,NMT2,TRAF6,SENP5,ITPKB,DNAJA3,OXR1,GRK5,ABI1,ULK4,TRIOBP,HSP90AA1,APOL4,CDC6,PSMB7,TSSK1B,KNDC1,ZC4H2,PGPEP1L,CCDC22,ADGB,MOCOS,STK38L,TRIO,KLHL12,AKAP13,TTLL5,PTPRK,RUNX1,DAB1,OMA1,ERC1,DNAJB14,KLHL7,MAST4,TCIRG1,PTPN11,HERC4,MGAT5,HDAC6,DDB1,ADORA1,MSRA,ADAMTS3,EPHA1,IKBKB,ERBB4,DPY19L2,MRE11A,LIMK1,TLK1,BRD8,KAT6B,ABR,CUL4B,LARGE,MIER1,PMEPA1,DPP10,PTPRO,CDON,KANSL1,FBXL18,NTRK2,TNRC6A,EP300,CELA1,ZYG11B,RNF220,FNTA,TPST2,PDGFB,FKBP9,TNIK,CCND3,GALNTL6,FBXL7,USP46,CSGALNACT1,CAPN14,BRMS1,CHFR,STK39,INSR,FMN2,PRKAR1A,ASB5,TCF7L2,ST6GALNAC3,JMJD1C,CAB39,USP34,PAK1,FBXW11,MAP3K4,ZDHHC13,PTPRD,RNF144A,EGLN3,SAMD4A,BDNF,CDK14,CHRM3,POMGNT2,FUT8,TRIM13,UBR2,HLCS,LCK,MDM4,ENTPD5,ECT2,MYO3B,MTA3,SNX6,CHML,PPM1L,CST2,PTCD3,PTPRU,ERN2,NPLOC4,TMPRSS15,DMD,ATG10,LARP4B,USP13,KAT7,SLC8A1,GSN,RBM14,RBM4,RIT2,MACROD2,DRG2,CASS4,FYN,MKRN2,ARNTL,ADAMTS9,LINC01138,NF1,PLCB1,MGMT,LMTK2,ARID4B,GXYLT2,PPP4R2,RTN4,FBXO21,DPEP1,APOD,B4GALT6,ADAMTS19,ASPHD2,BCR,NOSIP,RWDD3,BRPF1,CHI3L1,TTN,BMP2K,PAQR3,RAN*

			BP9,TPGS2,TLL1,KLHL21,EGFLAM,PPIG,NRG1,SH2D3C,CDK19,BDKRB1,BDKRB2,BID,FRY,MAP2K1,FNIP1,MYH9,GALNT16,VGLL4,PPP2CB,HDAC5,PCSK2,RNF4,CSNK2A3,GALNT18,UBE3D,JDP2,WDSUB1,ITIH2,CDKL2,ROR2,CPB1,DCN,ZDHHC3,CREBRF,FHIT,WDR70,PTPRT,UST,COPS5,IPO5,MAD2L2,MAN1A1,SRBD1,CAPRIN2,CCNYL1,NLN,PHF20L1,PDF,GNAQ,FBXO9,ICK,RFFL,TTLL8,PARP11,CCBE1,HERC5,USP22,ZDHHC15,JAK2,FBXW7,OAZ2,UBR7,UIMC1,ITCH,USP12,ZFYVE28,SMG1,HUNK,B3GALT5,CDC42BPB,PHKA1,DHDDS,KLF15,SPSB1,CYFIP2,WNT11,TPST1,TRIM59,CLCA2,MTA1,IMMP2L,NOX4,RPRD1B,SIAH1,QKI,CAMK2B,ADAM23,ERCC8,PRKCD,TAB2,ACVR2A,CD4,PPM1E,TGFB1,BANP,MRPL33,SGK1,PCSK5,P4HA2,SPSB4,MYO3A,MMP28,NSD1,PIBF1,ASCC2,KEL,BTRC,CRADD,PTPRN2,PDZRN3,DNAJB2,AAK1,KLHL2,HS6ST2,SLIT2,ASB15,RNF133,RNF148,ITIH4,HHAT,HP,HPR,CORO1C,CDKAL1,DISC1,BLID,ZFAND2A,PTPDC1,CLN6,TMTC1,TTBK2,KDM4B,MMP26,SMAD6,TMTC2,CLOCK,CHST9,ZNF675,RPL36,NELL1,BMPER,TIMP2,BCL3,DCLK1,RNF212,ANKRD54,EIF3L,DAPK2,TNFRSF10B,DAPP1,ADAM12,DUSP22,NAIP,TRRAP,DOCK3,YTHDF1,FGF10,FBXL17,SMYD3,UNKL,KCTD10,SH3RF3,LOXL3,MAST2,FANCI,CAPN3,GANC,SMURF2,EPHA4,PRKCA,AUTS2,CD6,TNFSF11,PPP3CA,UBQLN3,UBQLNL,CAMK4,PIP5KL1,UFL1,TRAK1,CTNNB1,PARK2,SMARCC1,IGF1R,PPARG,IARS2,NGRN,AXIN1,PRKAR1B,MACROD1,OTUB1,CPQ,APOL3,LRPPRC,RSPRY1,AARS2,CDK11A,DYRK4,GALNT8,CDK11B,LEPR,FGF1,PROS1,WDR45B,NOL3,PRKAR2A,RIOK2,ESCO1,MYOCD,TRIM5,PER2,AJUBA,GLG1,CHEK2,SUPT3H,UBQLN4,PRDM16,ASB8,PPP1CB,SPDYA,HCK,CSTL1,RAB3GAP2,CAPN2,TBCK,TRIM8,DIO2,PPP2R2B,CSPG4,BRMS1L,CTDP1,ZUFSP,HS3ST5,BAZ1B,MMP16,OBSCN,NF2,FLT4,HDAC4,PAX2,SECISBP2L,SPTBN1,TPTE,TRABD2B,SFRP1,ST8SIA1,PPP6R2,FOXO3,TRHDE,USP54,BCL2L13,SSH1,SYNCRIP,SMAD3,RNFT2,RAB6A,PTPRM,WWP2,SIMC1,WIPI1,MTBP,RNF168,C1S,ABI2,C10ORF90,DCP1B,NEDD4,GALNT14,TAB1,SEC61B,ST3GAL3,CDC73,APP,USP49,PDGFRA,SLK,RBM8A,CCNI2,DIP2B,KANSL2,ARIH1,HEG1,AMFR,ST6GALNAC5,CDK3,NLGN2,EYA2,SH3D19,BORA,IBTK,SCRN1,SBK3,JAK1,FPGT-TNNI3K,TNNI3K,LRP5,MTCP1,PTPRG,SOX2,SETD2,ZDHHC6,ADAMTS2,CHST11,PRICKLE1,RCAN1,SPTBN4,VASH2,DRD1,CHUK,ERLIN1,RFT1,MMP2,S100A12,DCUN1D3,KDM6A,PRKD1,NAA25,PARP6,ST18,USP53,LRRC2,TASP1,SHPRH,VPS4A,EREG,CCNY,MAPKAPK3,PITRM1,ATF2,GRAMD4,RAF1,CELF4,CARD16,CASP1,PTPN1,ADAMTS12,PSMD11,GGT7,BMPR2,USP33,VBP1,CAMK1D,BMPR1A,PABPC4,PIK3R3,CDK12,CAND2,SPAG9,MYB,FGF2,PPA2,PPM1F,UBE2R2,NEDD9,AGBL4,PEAK1,ADAMTS4,SEMA4D,NFX1,CDC27,PTAFR,SETD1A,YME1L1,JARID2,KLHL3,DDX58,PRKCG,SIN3B,NCOA1,SPOCK1,AREL1,EEF1E1,EHMT1,LMO7,UCHL3,DVL3,ECE2,EIF2B5,EIF4G1,EPHB3,PSMD2,PTPRE,RBX1,XPNPEP3,ANAPC5,FBXO45,STT3B,CKS1B,PAWR,AGO3,DEPTOR,PKN3,FBXL20,ADAM20,ADAM21,TSG101,DESI1,TERF2IP,IDE,COL11A1,FKBP5,ADAMTS6,RCE1,B3GALNT1,P4HB,SUFU,SUMF1,PTPN13,QPCTL,ADAMTS14,ATG4C,COL4A3,HIP1,PADI6,TRPC5,UBA2,EDEM3,TTLL7,TNKS,TNRC6B,GLCE,WDTC1,MRPS28,DAB2,BLM,UBR1,CACUL1,LDLRAD4,MYSM1,SETD5,DLG3,WNK1,GTPBP2,RELN,NEK10,SIN3A,RUVBL2,PSMD7,JOSD1,PELI1,PPP2R5C,IQGAP1,SPATA18,DCAF5,MAP3K7,APOLD1,SP1,TRIM22,ZNRF1,ALK,SLC8A2,KLK2,TMPRSS12,UBOX5,SLC35A4,TOP1,AGBL1,UBE2V1,PADI3,XDH,SNX5,RBBP6,PRKG1,TTLL9,ATP2B4,NSF,PARP12,ACTL6B,PAK3,MASP1,SSH2,TET1,HECTD4,MKRN3,CAMTA1,DYX1C1,ASGR2,ADNP,MAPKAPK2,CEP41,ARID4A,UBE3C,C12ORF65,CDC42BPA,MARK1,CDK6,PHF2,CELF1,RNF34,TFRC,UVRAG,EPHA5,ALG14,DCAF6,DCUN1D5,GPC3,XRN1,ADRBK1,PSMB2,RHBDL3,EYA1,CDCA3,DIS3L2,RNF216,GHR,MNAT1,PRSS12,SNX9,ACACA,ELAVL4,HDAC1,HS6ST3,C2,CFB,TRIM24,PLCG2,ROCK1,MRPS17,PAXIP1,EGR2,HIPK3,RNF10,RYBP,CAPN10,CUL2,AP3B1,PTPN2,INSRR,NTRK1,TRIM44,MAP1A,PKN2,SLC39A10,EIF3A,PTPRQ,EPHB1,MAN1B1,EPHA10,PTPN9,AXIN2,TMEM199,PARD3,GNL3L,KRTCAP2,MUC1,PTPN14,FBXO39,NUDT14,BCOR,MGAT4A,CARM1,ANXA8L1,ADAM19,SNX33,FOLH1,CD84,LPA,SPI1,DNMT3B,EPHA7,LRRC47,XPNPEP2,CUZD1,PTPRA,FBXW4,MRPS24,ATG14,EIF3H,BCAR3,STK38,ACOT8,RBPMS,RENBP,NTMT1,MAP3K13,OTUD3,RAPGEF3,ADAMTS17,PDXP,MST1,PTK7,RSRC1,SMYD1,SNCA,BMPR1B,MAGI2,USP42,PNPT1,USP50,RC3H1,EIF3E,RNF103,RNF103-CHMP3,RNF19B,PTPRS,KMT2D,PRKAG1,CDKL1,PSMF1,KMT2C,TLL2,UBE2QL1,CAST,TRIP12,SPPL2A,ADTRP,PLD1,OPRD1,HIPK1,RPTOR,BTK,ZNRF3,ASPHD1,KCTD13,MLLT1,ZZZ3,IGF1,HTT,LARS2,MAPK1,PTEN,MIB1,SPRED2,BMP7,PCMTD2,ATXN2,PIK3C3,PUM1,PCBP2,TTLL4,PRCP,LRRK2,TMEM59,ZRANB1,PIGS,UNC119,BAG6,LILRB4,MORC3,MSRB3,PKD2,EFNA5,SEL1L2,RCOR3,TADA2A,DAZL,AMZ2,ALPK3,PDGFC,SERPINA3,SERPINA4,SERPINA5,NCOA3,PKIB,MTMR3,HS2ST1,PPP2R3C,WNT7A,NLRP1,PEPD,RPS6KC1,UACA,DNAJC1,PLCL2,WDR5B,KAT6A,MTIF2,SIK3,BMP6,MYCBP2,GPI,SH3RF2,EXT1,DPY19L4,N4BP1,ATRX,DPH6,C8ORF44-SGK3,PRMT2,RAPGEF2,VRK3,GET4,ALG9,MARK4,PRKAR2B,DPY19L1,PSEN2,GALNT10,UBE2H,DNAJC6,UBR5,GRIN1,JADE1,ADAM29,ARRDC4,RHOA,SPRTN,WSB2,ROR1,RQCD1,CPVL,CUL3,HSPD1,TSPAN33,EPM2A,GSK3B,PRR16,DNAJC3,RDX,EEF2K,IWS1,PPP1R16A,PTPRB,NECAB2,CDK13,PGGT1B,ALAS2,STK32B,CD27,DUSP26,BOK,RNF144B,SULF1,RNF43,AGT,METTL16,CCNJL,PRKAA2,ADAR,CUL9,STAT2,ZMPSTE24,PTK2,ARMC8,PHEX,RNF121,SNX3,TEC,DCAF10,EXOSC3,FBXO10,CDC14A,MAP3K5,CPE,TRPC4AP,CREBBP,PRKD3,LMTK3,PCSK6,NCF1,T

			AOK2,PYGO2,SHC1,ECE1,PARP10,RNFT1,RPS6KB1,ZDHHC23,GALNT13,GUCY2F,PRG3,SERPINB11,SETDB2,DNAJB6,FBXO28,CD44,LARP4,ADAM8,FKBP14,MRPS6,CLDN4,RPS6KA5,CSNK1A1,HUS1,PRDM2,NOS1,RGS14,STK4,MARK3,EPHB2,GATC,SLC1A1,TTLL11,MARCH8,IL18,PCMT1,MFSD8,UBE2K,DIS3,DLC1,FBXL13,PPARA,PDP1,PHLPP1,PPIL6,CPEB4,MOB3B,ACP1,RNF150,PAX6,PRKCZ,FAM20C,ADORA2A,GRIN2B,MAN2B1,PPP1CC,RABGEF1,CCNG2,CPEB1,TYK2,OTUD7B,NIPBL,YEATS4,COL28A1,PHF20,ANGPT1,BRF1,TBL1X,EYA3,LRRK1,OSBPL8,IGF2BP3,L2HGDH,GCNT2,PPP1CA,CFI,MTF2,FBXO31,ATG3,CDK5RAP1,CNTN1,PRKCQ,BRAF,HDAC2,IMPACT,FKBP3,PHKG2,SRCAP,CAPN6,SPINT2,UBE2E2,UQCRC2,C18ORF25,CD300A,PHIP,CDKL3,PPP2CA,SKP1,ANGPT4,MRPS16,RPL23,ACAN,OPN1LW,ANXA2,PARN,PIGU,PRMT7,FARSB,WIPI2,ALG2,MADD,SYK,CNOT1,WWTR1,GTF2H5,ASB1,CIT,BRCA2,DVL2,MTRF1,YBX1,PTPRJ,TAF1,EIF4E3,PPME1,TICAM1,RNF38,TANK,RIMKLA,GALNT7,HSF1,DHFR,EZH1,ETF1,RNGTT,SRPK2,SASH1,ADAMTS7,BMP1,EEFSEC,RNF2,MOB3A,GNA12,SH2D3A,UGGT1,AKTIP,GALNT4,POC1B-GALNT4,INPP5F,NUAK2,TMPRSS3,AXL,AGBL3,TRIM37,CD109,MET,TBC1D10A,CAMK2D,MRPL1,SNX1,UCHL5,MALT1,SERPINE3,SETD3,TRIM69,ZDHHC11,ZDHHC11B,KDM2B,MLXIPL,FBXL4,NEDD4L,PHKB,PHB,PTPRC,CTCF,SH3GL2,TYRO3,RNF213,TSPAN5,TRAF3IP2,TRIM60,CREB1,METTL21A,SGTB,TNXB,TRAP1,EDNRA,BMP4,CASP12,CPNE1
GO:1901564	organonitrogen compound metabolic process	2.271630663374417e-24	*NT5C1B,NT5C1B-RDH14,SUCLG2,POLDIP3,LNX2,ZDHHC14,ENPP1,ADAMTS16,GPC5,NRXN1,ASPH,B4GALNT2,F8,PRMT3,GSS,PRKCI,SLCO3A1,MAP4K4,PDE4D,DNMT1,S1PR2,PDE7B,C6ORF89,ADCY8,MGAT4C,CBFB,PDE8A,IL31RA,ALDH4A1,RPS6KA2,PRDM12,SETD4,CALR3,CTDSPL2,FBXL2,NRG3,ASH1L,NOS1AP,LATS2,CPA6,DPP6,RNF145,KSR2,PTGIS,PRKAG2,HPSE2,OVCH2,PRLR,MVP,PAGR1,ADCY7,FTO,KALRN,DCAF12,NTRK3,CBL,ARNT,EGLN2,FLT3,STAT5B,ENPP2,GRM5,PLCE1,SAMHD1,FER,CASK,MAPK4,MAP2K5,ISCA1,KITLG,USP32,MAPK10,PTPRR,SPNS2,CHI3L2,LRP2,PIK3CD,NAALADL2,CAMK1G,EIF4G3,ADPGK,EZR,B3GALT1,ROBO1,TOM1L1,HTR2B,PSMD1,CDKL5,MECOM,TENM1,CAMKMT,NADK2,LMNA,GOT1,NMT2,TRAF6,SENP5,ITPKB,DNAJA3,OXR1,GRK5,ABI1,ULK4,TRIOBP,HSP90AA1,APOL4,CDC6,PSMB7,TSSK1B,KNDC1,ZC4H2,PGPEP1L,CCDC22,ADGB,MOCOS,STK38L,AMDHD1,TRIO,KLHL12,AKAP13,DLG2,TTLL5,PTPRK,RUNX1,DAB1,OMA1,ERC1,MMAA,DNAJB14,KLHL7,PDE4A,MAST4,TCIRG1,PTPN11,HERC4,MGAT5,HDAC6,DDB1,ADORA1,MSRA,ADAMTS3,EPHA1,IKBKB,ERBB4,DPY19L2,MRE11A,LIMK1,TLK1,BRD8,KAT6B,ABR,OSBPL10,CUL4B,LARGE,MIER1,PMEPA1,DPP10,PTPRO,CDON,KANSL1,FBXL18,NTRK2,TNRC6A,EP300,CELA1,ZYG11B,RNF220,FNTA,TPST2,PDGFB,FKBP9,TNIK,CCND3,GALNTL6,FBXL7,USP46,CSGALNACT1,CAPN14,BRMS1,CHFR,STK39,INSR,FMN2,PRKAR1A,ASB5,TCF7L2,ST6GALNAC3,JMJD1C,CAB39,USP34,PAK1,FBXW11,MAP3K4,ZDHHC13,PTPRD,RNF144A,EGLN3,SPTSSA,SAMD4A,BDNF,GLTP,CDK14,CHRM3,POMGNT2,FUT8,TRIM13,UBR2,HLCS,LCK,MDM4,ENTPD5,ECT2,MYO3B,MTA3,SNX6,PIPOX,CHML,PPM1L,CST2,PTCD3,PTPRU,ERN2,NPLOC4,TMPRSS15,DMD,ATG10,CERS6,LARP4B,USP13,KAT7,SLC8A1,GSN,RBM14,RBM4,PRPS2,RIT2,MACROD2,DRG2,CASS4,FYN,MKRN2,ARNTL,ADAMTS9,LINC01138,NF1,PLCB1,MGMT,DMGDH,LMTK2,ARID4B,SNCB,SLC25A21,GXYLT2,PPP4R2,RTN4,FBXO21,DPEP1,APOD,NEIL2,HS3ST2,B4GALT6,ADAMTS19,ASPHD2,BCR,NOSIP,RWDD3,BRPF1,CHI3L1,TTN,BMP2K,PAQR3,BLVRB,RANBP9,TPGS2,TLL1,KLHL21,EGFLAM,PPIG,NRG1,SH2D3C,CDK19,SLC16A10,BDKRB1,BDKRB2,BID,FRY,MAP2K1,FNIP1,MYH9,GALNT16,VGLL4,PPP2CB,HDAC5,PCSK2,ALDH3B2,RNF4,CSNK2A3,GALNT18,UBE3D,JDP2,WDSUB1,ITIH2,CDKL2,LRGUK,ROR2,CPB1,DCN,ZDHHC3,PRPSAP2,PCBD2,CREBRF,FHIT,WDR70,PTPRT,ACSBG1,UST,COPS5,AK2,IPO5,MAD2L2,MAN1A1,SRBD1,CAPRIN2,CCNYL1,NLN,PHF20L1,BBOX1,PDF,SULT1A4,NME7,AK5,CERKL,GNAQ,FBXO9,ICK,RFFL,TTLL8,PARP11,CCBE1,HERC5,USP22,ZDHHC15,JAK2,FBXW7,LINC00473,OAZ2,UBR7,UIMC1,ITCH,USP12,ZFYVE28,ABCC2,SMG1,IMPDH1,HUNK,B3GALT5,DPYS,CDC42BPB,PNPLA1,PHKA1,DHDDS,PDE2A,KLF15,SPSB1,CYFIP2,WNT11,TPST1,TRIM59,CLCA2,MTA1,IMMP2L,NOX4,RPRD1B,SIAH1,QKI,CAMK2B,ADAM23,ERCC8,PRKCD,TAB2,ACVR2A,CD4,PPM1E,TGFB1,BANP,MRPL33,SGK1,PCSK5,P4HA2,SPSB4,MYO3A,MMP28,NSD1,PIBF1,ASCC2,KEL,BTRC,CRADD,PTPRN2,PDZRN3,DNAJB2,AAK1,KLHL2,HS6ST2,SLIT2,ASB15,RNF133,RNF148,ITIH4,HHAT,HP,HPR,CORO1C,CDKAL1,DISC1,BLID,ZFAND2A,CPT1C,PTPDC1,CLN6,TMTC1,TTBK2,KDM4B,MMP26,SMAD6,TMTC2,CLOCK,CHST9,ZNF675,RPL36,BCKDHB,NELL1,BMPER,TIMP2,BCL3,DCLK1,RNF212,ANKRD54,EIF3L,DAPK2,TNFRSF10B,DAPP1,ADAM12,DUSP22,NAIP,TRRAP,DOCK3,YTHDF1,FGF10,FBXL17,ADK,SMYD3,UNKL,KCTD10,SH3RF3,LOXL3,MAST2,FANCI,ADCY2,CAPN3,GANC,SMURF2,EPHA4,RORA,HSD17B12,PRKCA,AUTS2,CD6,TNFSF11,PPP3CA,UBQLN3,UBQLNL,CAMK4,PIP5KL1,UFL1,TRAK1,CTNNB1,PARK2,SOD2,SMARCC1,IGF1R,PPARG,IARS2,NGRN,AXIN1,PRKAR1B,MACROD1,OTUB1,CPQ,OTC,APOL3,LRPPRC,RSPRY1,AARS2,PFAS,CDK11A,DYRK4,GALNT8,CDK11B,LEPR,FGF1,PROS1,FOXK2,WDR45B,NOL3,DCT,PRKAR2A,RIOK2,ESCO1,MYOCD,TRIM5,PER2,AJUBA,GLG1,CHEK2,SUPT3H,UBQLN4,PRDM16,ASB8,PPP1CB,SPDYA,HCK,CSTL1,RAB3GAP2,CAPN2,TBCK,TRIM8,DIO2,PPP2R2B,CSPG4,BRMS1L,CTDP1,ZUFSP,HS3ST5,BAZ1B,MMP16,OBSCN,NF2,FLT4,SGMS1,HDAC4,PAX2,CPOX,SECISBP2L,SPTBN*

			1,TPTE,TRABD2B,SFRP1,ST8SIA1,PPP6R2,FOXO3,TPH2,TRHDE,USP54,BCL2L13,SSH1,SYNCRIP,SMAD3,TYW1B,RNFT2,RAB6A,PTPRM,WWP2,SIMC1,WIPI1,MTBP,RNF168,C1S,ABI2,C10ORF90,PSAP,SLC16A2,DCP1B,NEDD4,PLA2G4C,OGDHL,SLC4A4,GALNT14,TAB1,SEC61B,ST3GAL3,UCK2,CDC73,APP,USP49,PDGFRA,AOC2,SLK,RBM8A,CCNI2,DIP2B,KANSL2,ARIH1,HEG1,AMFR,ST6GALNAC5,PPCS,CDK3,NLGN2,EYA2,SH3D19,BORA,IBTK,SCRN1,SBK3,ALPL,JAK1,FPGT-TNNI3K,TNNI3K,PEMT,LRP5,MTCP1,PTPRG,BDH2,SOX2,SETD2,ZDHHC6,GPC6,ADAMTS2,CHST11,PRICKLE1,RCAN1,SPTBN4,VASH2,ELOVL2,DRD1,CHUK,ERLIN1,RFT1,MMP2,TPH1,S100A12,DCUN1D3,NUDT10,KDM6A,PRKD1,NAA25,HEXA,PARP6,ST18,SLC6A3,USP53,HNF4A,LRRC2,TASP1,SHPRH,VPS4A,EREG,CCNY,MAPKAPK3,PITRM1,ATF2,GRAMD4,RAF1,CELF4,CARD16,CASP1,PTPN1,ADAMTS12,PSMD11,GGT7,BMPR2,USP33,VBP1,CAMK1D,BMPR1A,IDO2,PABPC4,PIK3R3,CDK12,CAND2,CERS3,SPAG9,MYB,FGF2,PPA2,PPM1F,UBE2R2,ADCY5,NEDD9,AGBL4,PEAK1,ADAMTS4,SEMA4D,NFX1,CDC27,PTAFR,HIBADH,SETD1A,YME1L1,JARID2,KLHL3,DDX58,SLC5A8,PRKCG,SIN3B,NCOA1,SPOCK1,AREL1,EEF1E1,EHMT1,LMO7,UCHL3,DVL3,ECE2,EIF2B5,EIF4G1,EPHB3,PSMD2,PTPRE,RBX1,XPNPEP3,ANAPC5,FBXO45,STT3B,CKS1B,PAWR,AGO3,DEPTOR,PKN3,FBXL20,ADAM20,ADAM21,TSG101,DESI1,TERF2IP,CRYM,IDE,COL11A1,FKBP5,ADAMTS6,RCE1,B3GALNT1,P4HB,SUFU,TG,SUMF1,PTPN13,PAFAH1B1,QPCTL,ADAMTS14,ATG4C,COL4A3,DDAH1,HIP1,PADI6,CMPK1,BTBD9,CERS4,TRPC5,UBA2,EDEM3,TTLL7,TNKS,TNRC6B,GLCE,WDTC1,ALDH6A1,MRPS28,TPK1,DAB2,BLM,UBR1,CACUL1,LDLRAD4,MYSM1,SETD5,DLG3,WNK1,GTPBP2,RELN,NEK10,SIN3A,RUVBL2,TYW1,PSMD7,GUCY1A2,JOSD1,PELI1,SLC22A3,PPP2R5C,SLC25A16,IQGAP1,SPATA18,DCAF5,MAP3K7,APOLD1,SP1,TRIM22,ZNRF1,ALK,SLC8A2,GSTM3,GSTM5,KLK2,TMPRSS12,UBOX5,SLC35A4,TOP1,AGBL1,UBE2V1,MBOAT1,PADI3,CLIC5,XDH,SNX5,RBBP6,PRKG1,TTLL9,ATP2B4,NSF,PARP12,TMEM2,ACTL6B,PAK3,MASP1,SSH2,TET1,HECTD4,MKRN3,CAMTA1,DYX1C1,ASGR2,ADNP,MAPKAPK2,CEP41,ARID4A,UBE3C,C12ORF65,CDC42BPA,SCARB1,MARK1,CDK6,PHF2,CELF1,TYR,RNF34,TFRC,UVRAG,EPHA5,ALG14,ADCY9,DCAF6,DCUN1D5,GPC3,XRN1,ADRBK1,PSMB2,RHBDL3,EYA1,CDCA3,DIS3L2,RNF216,GHR,MNAT1,PRSS12,SNX9,ACACA,ELAVL4,HDAC1,HS6ST3,C2,CFB,TRIM24,PLCG2,ROCK1,MRPS17,PAXIP1,EGR2,HIPK3,RNF10,RYBP,CAPN10,CUL2,ABCC1,AP3B1,PTPN2,INSRR,NTRK1,TRIM44,MAP1A,PKN2,SLC39A10,EIF3A,HYKK,PTPRQ,EPHB1,MAN1B1,EPHA10,PTPN9,AXIN2,SARM1,TMEM199,PARD3,GNL3L,KRTCAP2,MUC1,PTPN14,FBXO39,NUDT14,BCOR,MGAT4A,CARM1,ANXA8L1,ADAM19,SNX33,FOLH1,CD84,LPA,SPI1,DNMT3B,EPHA7,LRRC47,XPNPEP2,CUZD1,PTPRA,FBXW4,MRPS24,ATG14,EIF3H,BCAR3,STK38,ABCB7,ACOT8,RBPMS,RENBP,NTMT1,MAP3K13,OTUD3,RAPGEF3,ADAMTS17,PDXP,MST1,PTK7,RSRC1,SMYD1,SNCA,BMPR1B,MAGI2,USP42,PNPT1,USP50,NADK,RC3H1,EIF3E,RNF103,RNF103-CHMP3,RNF19B,HMGCS2,PTPRS,KMT2D,PRKAG1,CDKL1,CREM,PSMF1,KMT2C,SULT2A1,TLL2,UBE2QL1,CAST,TRIP12,SPPL2A,ADTRP,ABCA12,PLD1,OPRD1,HIPK1,RPTOR,BTK,ZNRF3,ASPHD1,KCTD13,MLLT1,ZZZ3,IGF1,PLA2G4E,HTT,LARS2,MAPK1,PTEN,MIB1,SPRED2,BMP7,PCMTD2,ATXN2,PIK3C3,PUM1,PCBP2,TTLL4,PRCP,LRRK2,ATP6V1A,MLYCD,TMEM59,ZRANB1,ALDOC,PIGS,UNC119,ZBTB20,BAG6,LILRB4,MORC3,SLC44A1,ITGAM,MSRB3,PKD2,BLVRA,EFNA5,SEL1L2,NT5E,RCOR3,TADA2A,DAZL,AMZ2,ALPK3,PDGFC,SERPINA3,SERPINA4,SERPINA5,SPTLC3,NCOA3,PKIB,MTMR3,HS2ST1,PPP2R3C,WNT7A,NLRP1,PEPD,RPS6KC1,UACA,DNAJC1,ELOVL5,GLDC,PLCL2,WDR5B,KAT6A,MTIF2,SIK3,BMP6,MYCBP2,NFE2L1,GPI,SH3RF2,EXT1,DPY19L4,N4BP1,ATRX,DPH6,SERINC5,C8ORF44-SGK3,PRMT2,RAPGEF2,MTHFD2L,VRK3,FAR1,AMPD1,ENPP3,GET4,ACADSB,ALG9,MARK4,PRKAR2B,DPY19L1,CPT1A,PSEN2,GALNT10,UBE2H,BPGM,DNAJC6,UBR5,GRIN1,JADE1,ADAM29,ARRDC4,RHOA,SPRTN,WSB2,ROR1,RQCD1,CKMT1B,CPVL,CUL3,HSPD1,TSPAN33,EPM2A,GSK3B,PRR16,DNAJC3,RDX,EEF2K,IWS1,PPP1R16A,PTPRB,NECAB2,CDK13,PGGT1B,ALAS2,STK32B,CD27,DUSP26,BOK,RNF144B,SULF1,MTHFD1L,RNF43,AGT,METTL16,CCNJL,ELOVL3,PRKAA2,ADAR,CUL9,STAT2,ZMPSTE24,PTK2,ARMC8,PHEX,PPCDC,RNF121,SNX3,PDE4B,TEC,DCAF10,EXOSC3,FBXO10,CDC14A,MAP3K5,CPE,TRPC4AP,CREBBP,PRKD3,LMTK3,PCSK6,CHKA,NCF1,TAOK2,UGT1A1,UGT1A4,PYGO2,SHC1,SLC44A3,ALDOA,ECE1,PARP10,RNFT1,RPS6KB1,ZDHHC23,GALNT13,GUCY2F,PRG3,SERPINB11,SETDB2,DNAJB6,FBXO28,CD44,LARP4,ADAM8,FKBP14,MRPS6,CLDN4,RPS6KA5,CDS1,CSNK1A1,HUS1,PRDM2,NOS1,RGS14,STK4,MARK3,EPHB2,GATC,SLC1A1,TTLL11,MARCH8,IL18,PCMT1,MFSD8,UBE2K,DIS3,DLC1,FBXL13,PPARA,PDP1,PNPLA6,PHLPP1,MAGI3,PPIL6,CPEB4,LDHC,MOB3B,ACP1,RNF150,PAX6,PRKCZ,FAM20C,ADORA2A,GRIN2B,MAN2B1,PAH,PPP1CC,RABGEF1,CCNG2,CPEB1,TYK2,OTUD7B,NIPBL,YEATS4,COL28A1,PHF20,UPB1,ANGPT1,BRF1,TBL1X,EYA3,LRRK1,OSBPL8,IGF2BP3,L2HGDH,NT5M,SLCO2B1,GCNT2,PPP1CA,CFI,MTF2,FBXO31,ATG3,CDK5RAP1,CNTN1,PRKCQ,BRAF,HDAC2,IMPACT,FKBP3,PHKG2,SRCAP,CAPN6,SPINT2,UBE2E2,WSCD1,UQCRC2,C18ORF25,CD300A,MOXD1,PHIP,CDKL3,PPP2CA,SKP1,SLC26A2,ANGPT4,MAOA,MRPS16,RPL23,ACAN,OPN1LW,ANXA2,PARN,PIGU,PRMT7,FARSB,WIPI2,ADCY1,ALG2,MADD,SYK,CNOT1,WWTR1,GTF2H5,ASB1,CIT,SLC44A5,BRCA2,ALDH8A1,DVL2,MTRF1,YBX1,PTPRJ,TAF1,EIF4E3,PPME1,TICAM1,RNF38,TANK,RIMKLA,GALNT7,A3GALT2,HSF1,DHFR,EZH1,ETF1,RNGTT,SRPK2,SASH1,ADAMTS7,CEPT1,SULT2B1,BMP1,EEFSEC,RN

			F2,MOB3A,GNA12,SH2D3A,UGGT1,AKTIP,GALNT4,POC1B-GALNT4,INPP5F,NUAK2,TMPRSS3,AXL,AGBL3,TRIM37,CD109,MET,TBC1D10A,CAMK2D,MRPL1,SNX1,UCHL5,MALT1,PFKP,SERPINE3,SETD3,TRIM69,ZDHHC11,ZDHHC11B,KDM2B,MLXIPL,FBXL4,GNS,NEDD4L,PHKB,PHB,PTPRC,CTCF,SH3GL2,TYRO3,RNF213,TSPAN5,ADCY10,TRAF3IP2,TRIM60,CREB1,METTL21A,SGTB,TNXB,TRAP1,EDNRA,SLC25A13,BMP4,CASP12,CPNE1
GO:0051128	regulation of cellular component organization	3.834830473423998e-24	*CLRN1,ENPP1,ADAMTS16,NRXN1,ASPH,PRKCI,MAP4K4,DNMT1,SLC9A1,NEGR1,PDE4DIP,CLASP2,SEMA3A,GRID2,RPS6KA2,TENM3,NRG3,NOX5,SEMA3D,SCAF8,TIAM2,FHOD3,MAPRE2,TJP1,KALRN,NTRK3,CBL,EGLN2,RAB4B,RAB4B-EGLN2,SEMA5A,TOX,ENPP2,PLCE1,LHFPL4,FER,MAP2K5,OLFM1,EZR,ROBO1,TOM1L1,CDKL5,TENM1,LMNA,ROBO2,ULK4,TRIOBP,HSP90AA1,CDC6,KNDC1,DSCAM,STK38L,ARHGAP24,AKAP13,RUNX1,DAB1,OMA1,CDC42EP3,MYO10,ABCC8,IL17RB,PTPN11,CCT2,HDAC6,GOLGA4,SERTAD2,EPHA1,IKBKB,MRE11A,LIMK1,TLK1,ATP8A2,ESR1,PMEPA1,PTPRO,NTRK2,STX18,EP300,TENM2,PDGFB,RIMS1,TNIK,CDH8,MID1,CHFR,INSR,AFAP1,ATP8B1,PIP4K2A,CDH4,PAK1,MAP3K4,PTPRD,TBR1,BDNF,AMIGO1,NPRL3,ARHGAP6,ECT2,DENND5A,DMD,CENPF,GSN,RBM14,RIT2,TANC2,FYN,PLCB1,FAT3,RTN4,APOD,RTN4R,NDRG4,BMP2K,PAQR3,NTN1,DCTN1,NRG1,BDKRB1,BID,MAP2K1,FNIP1,MYH9,FRMPD4,MAD1L1,LRFN5,VGLL4,NTNG1,RNF4,TMEM30A,CSNK2A3,ROR2,DCN,SCFD1,CDH13,UST,MAD2L2,CAPRIN2,NCKAP1,TERF2,SLX1B,RCC2,ANO6,ARL3,ZDHHC15,FBXW7,SKAP1,CLSTN2,SYT1,ITCH,CROCC,LZTS1,SMG1,CLDN1,MLLT3,PLS1,PDE2A,SEPT7,CYFIP2,WNT11,FIG4,KREMEN1,MKLN1,IL1RAPL1,CAMK2B,PRKCD,SEMA5B,PPM1E,TGFB1,SGK1,KEL,F2RL1,BCAS3,DNAJB2,CELSR1,SLIT3,AAK1,SLIT2,COLGALT1,CORO1C,DISC1,CEP135,KANK1,TTBK2,SMAD6,ITSN2,DUSP22,HNRNPC,SLC17A7,LINGO2,CNTNAP2,STXBP5,KANK4,IL1RAPL2,IQCJ-SCHIP1,RHPN2,SVIL,EPHA4,AUTS2,CNR1,SMG6,PPP3CA,GNG4,MAG,CTNNB1,PARK2,FCGR2B,ARHGAP22,CDHR2,IGF1R,PPARG,DLG5,ADD2,XK,SCAMP5,SREBF2,CDK11A,FBLIM1,CDK11B,DRAXIN,NIN,NOL3,RIOK2,MYOCD,AJUBA,CPNE6,PHLDB1,UBQLN4,HCK,TBC1D14,RAB3GAP2,CAPN2,DCC,ANKFY1,CTDP1,BAZ1B,NF2,HDAC4,SPTBN1,ELN,TRABD2B,SFRP1,DGKB,MUC12,DNM3,SSH1,SMAD3,CUX2,WIPI1,MTBP,ABI2,C10ORF90,GRIK5,SHANK1,SYT3,NEDD4,NRP2,ARHGEF18,NAV3,RAB6C,CDC73,APP,PDGFRA,SLK,ADD3,DIP2B,NOX1,YAP1,TEN1,FSTL4,NLGN2,VDAC1,BORA,LRP5,PTPRG,ISLR2,ZDHHC6,GPC6,KIF24,SPTBN4,TMEM14A,VILL,ESR2,SYNE2,DCUN1D3,CPNE9,PRKD1,PARP6,DGKG,RND3,HNF4A,SHANK2,EREG,SEMA6D,RAF1,PTPN1,BMPR2,DPYSL2,VBP1,CAMK1D,NLGN1,COL16A1,CTNNA2,SPAG9,MORC2,RAB11A,CRMP1,LRRC4C,PPM1F,PEAK1,SEMA4D,CDC27,MCTP1,PLXNA2,SETD1A,ABCA13,SPOCK1,AP2M1,DVL3,EIF4G1,EPHB3,ANAPC5,TSG101,VCL,TERF2IP,CLIC4,MYO9A,WNT3,NLGN3,PAFAH1B1,KIF3A,HIP1,AKAP6,RASAL1,BTBD9,TRPC5,TNKS,ERCC1,RUFY3,STON2,DAB2,PKHD1,LDLRAD4,SETD5,DZIP1,SMG5,RELN,SIN3A,DOCK11,IQGAP1,MACF1,ALK,CLASP1,MBOAT1,GPM6B,NFATC2,PACSIN1,SPTBN5,PAK3,SSH2,TET1,ATAD1,ADNP,MAP1B,MPHOSPH9,MARK1,PHF2,TFRC,UVRAG,EPHA5,DCUN1D5,GPC3,SGIP1,XRN1,TBC1D5,ZNF207,CLEC16A,MNAT1,FLRT2,SNX9,ACTR2,ELAVL4,PLCG2,ROCK1,EPS8,NTRK1,MAP1A,UNC13A,EPHB1,PTPN9,AXIN2,SARM1,GNL3L,TRIM46,SH3GL3,CARM1,AP2B1,ARHGAP44,SNX33,PIFO,SMARCA2,CUX1,EPHA7,MCU,PTPRA,LIMA1,ABCB7,FCHSD2,MAP3K13,RAPGEF3,PDXP,SH3BP1,PTK7,SNCA,CD53,MAGI2,USP50,ZEB2,SYT9,CHMP3,PTPRS,CDKL1,RAB5B,ADTRP,PLD1,RASA1,OPRD1,RPTOR,RTN4RL1,CBFA2T2,IGF1,ATF7IP,HTT,DPYSL3,MAPK1,PTEN,MIB1,BMP7,ATXN2,LRRK2,OSGIN1,EBAG9,SPAG5,UNC119,CHMP5,EFNA5,SHANK3,STMN4,PKIB,MTMR3,TBCD,WNT7A,MAP4,RHOT1,BMP6,MYCBP2,ATRX,RAPGEF2,MARK4,SYT17,DNAJC6,JADE1,RHOA,SYNGAP1,ROR1,TF,RHOBTB1,CUL3,EPM2A,GSK3B,RDX,EEF2K,FAM49B,NECAB2,CDK13,BOK,AGT,HDGFRP3,PRKAA2,CUL9,STAT2,ZMPSTE24,PTK2,TPPP2,SNX3,RIMS2,GAP43,IQGAP2,CEP70,TAOK2,PARP10,YLPM1,SETDB2,CPLX2,DNAJB6,CD44,PACSIN2,SPEF1,EPHB2,TMEFF2,GPSM2,DLC1,PPARA,PPP1R10,SLC25A33,SYTL4,PRKCZ,GRIN2B,RABGEF1,PHLDB2,ANGPT1,MAP2,FBXO31,ATG3,CNTN1,PRKCQ,BRAF,DIAPH1,HDAC2,IMPACT,ATP8A1,CAPN6,NOTO,TNR,CD300A,PHIP,CDKL3,PPP2CA,SH3BP4,CAMSAP3,ANXA2,PARN,NUMB,SYK,CNOT1,SLIT1,CIT,HRK,DVL2,MTRF1,NEB,ANKRD13A,GRIP1,PTPRJ,TICAM1,GPM6A,HSF1,ETF1,PLEKHM1,ASAP1,GBP5,CGNL1,CAV2,SKA2,INPP5F,AXL,TRIM37,MET,CAMK2D,SPTAN1,EHD2,KDM2B,SEMA3C,CCT3,NEDD4L,PHB,CTCF,SYNDIG1,RASSF8,PRAP1,STXBP6,BMP4*
GO:0018193	peptidyl-amino acid modification	4.030262157598333e-24	*ZDHHC14,NRXN1,ASPH,B4GALNT2,PRMT3,PRKCI,PDE4D,DNMT1,S1PR2,IL31RA,RPS6KA2,PRDM12,SETD4,ASH1L,NOS1AP,LATS2,PRKAG2,PRLR,MVP,PAGR1,NTRK3,CBL,ARNT,EGLN2,FLT3,ENPP2,GRM5,FER,MAP2K5,KITLG,CAMK1G,MECOM,TENM1,CAMKMT,LMNA,NMT2,SENP5,OXR1,ABI1,HSP90AA1,TSSK1B,STK38L,TTLL5,MAST4,PTPN11,MGAT5,HDAC6,ADORA1,EPHA1,IKBKB,ERBB4,DPY19L2,TLK1,BRD8,KAT6B,KANSL1,NTRK2,EP300,TPST2,PDGFB,FKBP9,GALNTL6,STK39,INSR,CAB39,PAK1,EGLN3,BDNF,FUT8,LCK,SNX6,DMD,KAT7,CASS4,FYN,LMTK2,ARID4B,ASPHD2,BCR,RWDD3,BRPF1,CHI3L1,TTN,PAQR3,TPGS2,EGFLAM,PPIG,NRG1,SH2D3C,BDKRB2,MAP2K1,FNIP1,GALNT16,CSNK2A3,ROR2,DCN,ZDHHC3,MAD2L2,CAPRIN2,PHF20L1,PDF,TTLL8,USP22,ZDHHC15,JAK2,FBXW7,ZFYVE28,SMG1,CDC42BPB,KLF15,TPST1,NOX4,PRKCD,CD4,TGFB1,SGK1,P4HA2,NSD1,PIBF1,HHAT,TTBK2,KDM4B,CLOCK,DCLK1,RNF212,DUSP22,TRRAP,DOCK3,*

			FGF10,SMYD3,LOXL3,MAST2,CAPN3,EPHA4,PRKCA,AUTS2,CAMK4,CTNNB1,IGF1R,AXIN1,DYRK4,ESCO1,MYOCD,CHEK2,SUPT3H,PRDM16,HCK,CSPG4,BAZ1B,NF2,FLT4,HDAC4,SFRP1,RAB6A,ABI2,APP,PDGFRA,DIP2B,KANSL2,IBTK,JAK1,MTCP1,SETD2,ZDHHC6,SPTBN4,DRD1,CHUK,KDM6A,PRKD1,NAA25,EREG,MAPKAPK3,ATF2,RAF1,PTPN1,CAMK1D,MYB,PPM1F,NEDD9,AGBL4,PEAK1,SEMA4D,SETD1A,JARID2,PRKCG,NCOA1,EHMT1,EIF4G1,EPHB3,STT3B,PKN3,TSG101,DESI1,TERF2IP,FKBP5,P4HB,QPCTL,PADI6,TRPC5,UBA2,TTLL7,TNKS,SETD5,DLG3,WNK1,RELN,SIN3A,RUVBL2,MAP3K7,ALK,TOP1,AGBL1,PADI3,TTLL9,ATP2B4,ACTL6B,ADNP,MAPKAPK2,CEP41,ARID4A,CDC42BPA,MARK1,TFRC,EPHA5,ADRBK1,EYA1,GHR,PLCG2,ROCK1,PAXIP1,EGR2,HIPK3,PTPN2,INSRR,NTRK1,PKN2,EPHB1,EPHA10,PARD3,GNL3L,KRTCAP2,MUC1,NUDT14,BCOR,CARM1,FOLH1,SPI1,DNMT3B,EPHA7,BCAR3,STK38,NTMT1,MAP3K13,RAPGEF3,SMYD1,SNCA,KMT2D,KMT2C,OPRD1,HIPK1,RPTOR,BTK,ASPHD1,ZZZ3,IGF1,MAPK1,PTEN,SPRED2,BMP7,TTLL4,LRRK2,UNC119,BAG6,LILRB4,MORC3,EFNA5,TADA2A,PDGFC,NCOA3,PLCL2,WDR5B,KAT6A,BMP6,DPY19L4,ATRX,DPH6,PRMT2,VRK3,DPY19L1,JADE1,ROR1,RQCD1,EPM2A,GSK3B,IWS1,STK32B,AGT,PRKAA2,ZMPSTE24,PTK2,TEC,CREBBP,LMTK3,NCF1,PYGO2,SHC1,RPS6KB1,ZDHHC23,GALNT13,SETDB2,CD44,FKBP14,RPS6KA5,CSNK1A1,NOS1,STK4,MARK3,EPHB2,SLC1A1,TTLL11,IL18,UBE2K,PPIL6,PRKCZ,TYK2,YEATS4,PHF20,ANGPT1,LRRK1,MTF2,CNTN1,PRKCQ,BRAF,HDAC2,FKBP3,SRCAP,CD300A,PPP2CA,ANGPT4,PRMT7,SYK,CIT,BRCA2,DVL2,PTPRJ,TAF1,HSF1,EZH1,SRPK2,SH2D3A,UGGT1,GALNT4,INPP5F,AXL,AGBL3,MET,CAMK2D,SETD3,ZDHHC11,ZDHHC11B,MLXIPL,PTPRC,CTCF,TYRO3,METTL21A
GO:0044260	cellular macromolecule metabolic process	4.280725526118149e-24	*POLDIP3,LNX2,ZDHHC14,ENPP1,NRXN1,ASPH,B4GALNT2,PRMT3,PRKCI,MOV10L1,SLCO3A1,MAP4K4,PDE4D,DNMT1,S1PR2,C6ORF89,ADCY8,MGAT4C,CBFB,PDE8A,IL31RA,RNASET2,RPS6KA2,PRDM12,SETD4,CALR3,KIF22,CTDSPL2,PMS1,FBXL2,NRG3,ASH1L,NOS1AP,LATS2,RNF145,KSR2,PRKAG2,PRLR,MVP,PAGR1,FTO,KALRN,DCAF12,MRM1,NTRK3,CBL,ARNT,EGLN2,FLT3,RAD51B,TOX,ENPP2,GRM5,NOC3L,PLCE1,SAMHD1,FER,CASK,MAPK4,MAP2K5,KITLG,USP32,MAPK10,PTPRR,LRP2,PIK3CD,CAMK1G,ZSWIM7,EIF4G3,EZR,XRCC4,B3GALT1,ROBO1,TOM1L1,HTR2B,PSMD1,CDKL5,MECOM,TENM1,CAMKMT,LMNA,NMT2,TRAF6,SENP5,ITPKB,DNAJA3,OXR1,GRK5,ABI1,ULK4,ERCC6L2,ITGB1,TRIOBP,HSP90AA1,CDC6,PSMB7,THUMPD3,TSSK1B,KNDC1,ZC4H2,CCDC22,MOCOS,STK38L,CDAN1,TRIO,KLHL12,AKAP13,TTLL5,PTPRK,DAB1,OMA1,ERC1,DNAJB14,KLHL7,MAST4,TCIRG1,DACH1,PTPN11,HERC4,MGAT5,CCT2,ORC2,HDAC6,DDB1,ADORA1,MSRA,GPRASP1,EPHA1,IKBKB,ERBB4,GBE1,DPY19L2,MRE11A,LIMK1,TLK1,BRD8,TEX264,KAT6B,ABR,CUL4B,LARGE,CECR2,MIER1,PMEPA1,PTPRO,CDON,KANSL1,FBXL18,NTRK2,TNRC6A,EP300,CELA1,ZYG11B,RNF220,FNTA,TPST2,PDGFB,FKBP9,TNIK,CCND3,GALNTL6,FBXL7,USP46,CSGALNACT1,BRMS1,CHFR,STK39,INSR,FMN2,PRKAR1A,ASB5,TCF7L2,ST6GALNAC3,JMJD1C,CAB39,USP34,PAK1,FBXW11,MAP3K4,ZDHHC13,PTPRD,RNF144A,EGLN3,SAMD4A,BDNF,CDK14,FANCA,CHRM3,POMGNT2,FUT8,VRTN,TRIM13,UBR2,HLCS,LCK,ENTPD5,ECT2,MYO3B,MTA3,SNX6,CHML,PPM1L,CST2,PTCD3,PTPRU,ERN2,CDC45,SHFM1,NPLOC4,DMD,CENPF,ATG10,SLC30A9,PDS5A,LARP4B,USP13,KAT7,SLC8A1,GSN,HFM1,RBM14,RBM4,RIT2,MACROD2,DRG2,CASS4,FYN,MKRN2,ARNTL,ADAMTS9,LINC01138,NF1,SMG7,PLCB1,MGMT,LMTK2,ARID4B,GXYLT2,PPP4R2,FBXO21,DPEP1,POLA2,NEIL2,B4GALT6,ASPHD2,BCR,NOSIP,RWDD3,BRPF1,CHI3L1,TTN,BMP2K,PAQR3,TPGS2,KLHL21,EGFLAM,PPIG,NRG1,SH2D3C,CDK19,BDKRB1,BDKRB2,BID,FRY,MAP2K1,FNIP1,MYH9,GALNT16,VGLL4,PPP2CB,HDAC5,NFIA,RNF4,CSNK2A3,GALNT18,UBE3D,JDP2,WDSUB1,ITIH2,CDKL2,ROR2,DCN,ZDHHC3,TRDMT1,FHIT,WDR70,PTPRT,AUNIP,UST,COPS5,DMC1,IPO5,MAD2L2,MAN1A1,SRBD1,CAPRIN2,CCNYL1,PHF20L1,PDF,TERF2,SLX1B,GNAQ,FBXO9,ICK,RAD51D,RFFL,TTLL8,PARP11,CCBE1,HERC5,USP22,ZDHHC15,JAK2,FAM168A,FBXW7,OAZ2,UBR7,SMC3,UIMC1,ITCH,USP12,ZFYVE28,BCL11B,SMG1,HUNK,B3GALT5,CDC42BPB,PHKA1,TEX11,DHDDS,TCF7,KLF15,SPSB1,CYFIP2,WNT11,TPST1,TRIM59,MTA1,IMMP2L,NOX4,RPRD1B,MKLN1,SIAH1,QKI,CAMK2B,ERCC8,PRKCD,TAB2,ACVR2A,CD4,PPM1E,TGFB1,MRPL33,SGK1,PCSK5,P4HA2,SPSB4,MYO3A,NSD1,PIBF1,ZHX2,ASCC2,BTRC,POLK,CRADD,PTPRN2,PDZRN3,DNAJB2,AAK1,KLHL2,HS6ST2,SLIT2,ASB15,TP73,RNF133,RNF148,ITIH4,HHAT,CORO1C,CDKAL1,MGAM,DISC1,BLID,ZFAND2A,PTPDC1,CLN6,TMTC1,TTBK2,KDM4B,SMAD6,TMTC2,CLOCK,CHST9,ZNF675,RPL36,NELL1,BMPER,TIMP2,BCL3,DCLK1,RNF212,ANKRD54,EIF3L,DAPK2,TNFRSF10B,SND1,DAPP1,DUSP22,NAIP,HNRNPC,TRRAP,DOCK3,YTHDF1,FGF10,CIZ1,FBXL17,SMYD3,UNKL,KCTD10,SH3RF3,LOXL3,MAST2,FANCI,CAPN3,GANC,SMURF2,EPHA4,PRKCA,AUTS2,CD6,TNFSF11,SMG6,PPP3CA,NSUN2,UBQLN3,UBQLNL,CAMK4,PIP5KL1,UFL1,TRAK1,CTNNB1,PARK2,SMARCC1,IGF1R,PPARG,IARS2,NGRN,AXIN1,PRKAR1B,MACROD1,OTUB1,ANKRD17,BRIP1,LRPPRC,RSPRY1,AARS2,TDP1,CDK11A,DYRK4,GALNT8,CDK11B,LEPR,FGF1,PROS1,WDR45B,NOL3,PRKAR2A,RIOK2,ESCO1,MYOCD,TRIM5,PER2,AJUBA,GLG1,CHEK2,SUPT3H,UBQLN4,PRDM16,ASB8,PPP1CB,SPDYA,HCK,CSTL1,SORBS1,RAB3GAP2,CAPN2,TBCK,TRIM8,DIO2,PPP2R2B,CSPG4,BRMS1L,CTDP1,HS3ST5,BAZ1B,OBSCN,NF2,FLT4,HDAC4,PAX2,SECISBP2L,SPTBN1,TPTE,TRABD2B,SFRP1,ST8SIA1,PPP6R2,FOXO3,USP54,NFIB,ZCCHC17,BCL2L13,DNM3,SSH1,SYNCRIP,SMAD3,RNFT2,RAB6A,PTPRM,WWP2,SIMC1,WIPI1,MTB*

			P,RNF168,EMG1,ABI2,C10ORF90,DCP1B,NEDD4,GALNT14,TAB1,SEC61B,ST3GAL3,CDC73,APP,USP49,PDGFRA,SLK,RBM8A,CCNI2,DIP2B,KANSL2,ARIH1,HEG1,AMFR,ST6GALNAC5,CDK3,TEN1,EYA2,SH3D19,BORA,IBTK,SBK3,NVL,JAK1,FPGT-TNNI3K,TNNI3K,LRP5,MTCP1,PTPRG,SETD2,ZDHHC6,CHST11,PRICKLE1,RCAN1,SPTBN4,DRD1,CHUK,ERLIN1,PALB2,RFT1,MMP2,S100A12,DCUN1D3,KDM6A,PRKD1,NAA25,PARP6,ST18,USP53,LRRC2,SHPRH,VPS4A,EREG,CCNY,MAPKAPK3,ATF2,TCF3,GRAMD4,RAF1,CELF4,CARD16,CASP1,PTPN1,ADAMTS12,PSMD11,POLA1,GGT7,BMPR2,USP33,CAMK1D,BMPR1A,PABPC4,PIK3R3,CDK12,CAND2,SPAG9,MYB,FGF2,PPA2,BACH1,PPM1F,UBE2R2,TICRR,NEDD9,AGBL4,PEAK1,SEMA4D,NFX1,CDC27,HELQ,PTAFR,SETD1A,YME1L1,JARID2,KLHL3,PRKCG,SIN3B,NCOA1,SPOCK1,AREL1,EEF1E1,EHMT1,LMO7,UCHL3,ALKBH3,AP2M1,DVL3,EIF2B5,EIF4G1,EPHB3,PSMD2,PTPRE,RBX1,ANAPC5,FBXO45,LIN28B,LMBRD1,STT3B,CKS1B,PAWR,TRMT61B,AGO3,DEPTOR,PKN3,FBXL20,TSG101,DESI1,TERF2IP,IDE,COL11A1,FKBP5,C11ORF80,RCE1,B3GALNT1,P4HB,SUFU,SUMF1,PTPN13,QPCTL,POLE,COL4A3,HIP1,PADI6,TRPC5,UBA2,EDEM3,TTLL7,GLI2,TNKS,ERCC1,TNRC6B,GLCE,WDTC1,MRPS28,DAB2,BLM,UBR1,CACUL1,LDLRAD4,MYSM1,SETD5,SMG5,DLG3,WNK1,GTPBP2,RELN,NEK10,SIN3A,RUVBL2,PSMD7,JOSD1,PELI1,PPP2R5C,IQGAP1,SPATA18,DCAF5,MAP3K7,TRIM22,ZNRF1,ALK,SLC8A2,EEPD1,FASTKD5,UBOX5,SLC35A4,TOP1,AGBL1,UBE2V1,PADI3,XDH,SNX5,RBBP6,CHD1L,PRKG1,TTLL9,ATP2B4,NSF,PARP12,APEX2,ACTL6B,PAK3,SSH2,TET1,HECTD4,MKRN3,CAMTA1,DYX1C1,ASGR2,ATAD1,ADNP,MAPKAPK2,CEP41,ARID4A,UBE3C,C12ORF65,CDC42BPA,MARK1,CDK6,PHF2,CELF1,RNF34,TFRC,UVRAG,EPHA5,ALG14,NFRKB,DCAF6,DCUN1D5,DROSHA,GPC3,XRN1,ADRBK1,TBC1D5,PSMB2,EYA1,GATAD2A,CDCA3,DIS3L2,RNF216,GHR,MNAT1,SNX9,ACTR2,ELAVL4,HDAC1,HS6ST3,RECQL5,SMOC2,TRIM24,PLCG2,ROCK1,MRPS17,PAXIP1,EGR2,HIPK3,RNF10,RYBP,CUL2,AP3B1,PTPN2,INSRR,NTRK1,TRIM44,MAP1A,PKN2,SLC39A10,EIF3A,PTPRQ,EPHB1,MAN1B1,EPHA10,PTPN9,AXIN2,TMEM199,PARD3,GNL3L,KRTCAP2,MUC1,PTPN14,FBXO39,NUDT14,BCOR,CACNG8,MGAT4A,CARM1,AP2B1,PGBD5,ANXA8L1,SNX33,FOLH1,LPA,RAD51C,SPI1,DNMT3B,EPHA7,LRRC47,PTPRA,FBXW4,MRPS24,ATG14,EIF3H,BCAR3,STK38,ACOT8,RBPMS,RENBP,NTMT1,MAP3K13,OTUD3,RAPGEF3,PDXP,ERCC3,PTK7,RSRC1,SMYD1,SNCA,BMPR1B,POLN,CACNG2,MAGI2,PRIM2,USP42,PNPT1,USP50,MCMBP,RC3H1,EIF3E,RNF103,RNF103-CHMP3,RNF19B,PTPRS,KMT2D,PRKAG1,CDKL1,PSMF1,KMT2C,UBE2QL1,CAST,TRIP12,DFFA,ADTRP,PLD1,OPRD1,HIPK1,NUGGC,RPTOR,NFIX,BTK,TBC1D16,ZNRF3,ASPHD1,KCTD13,MLLT1,ZZZ3,IGF1,ATF7IP,HTT,LARS2,MAPK1,PTEN,MIB1,SPRED2,IFFO1,BMP7,PCMTD2,ATXN2,PIK3C3,PUM1,PCBP2,TTLL4,LRRK2,TMEM59,ZRANB1,PIGS,UNC119,CHMP5,RFC3,BAG6,CHAF1B,LILRB4,MORC3,MSRB3,PKD2,EFNA5,HSF2BP,SEL1L2,NT5E,RCOR3,TADA2A,DAZL,ALPK3,PDGFC,SERPINA3,SERPINA4,SERPINA5,NCOA3,PKIB,MTMR3,HS2ST1,PPP2R3C,WNT7A,NLRP1,RPS6KC1,UACA,DNAJC1,PLCL2,WDR5B,KAT6A,MTIF2,SIK3,BMP6,TAF15,MYCBP2,GPI,SH3RF2,EXT1,DPY19L4,N4BP1,ATRX,DPH6,C8ORF44-SGK3,PRMT2,RAPGEF2,ASCC1,VRK3,GET4,ALG9,FDXACB1,MARK4,PRKAR2B,DPY19L1,PSEN2,GALNT10,UBE2H,DNAJC6,UBR5,GRIN1,JADE1,ARRDC4,RHOA,SPRTN,WSB2,MCM3,ROR1,RQCD1,CUL3,HSPD1,EPM2A,GSK3B,PRR16,DNAJC3,RDX,STAT6,EEF2K,IWS1,PPP1R16A,PTPRB,RBBP8,NECAB2,CDK13,PGGT1B,ALAS2,STK32B,UPF2,CD27,DUSP26,BOK,RNF144B,SULF1,RNF43,AGT,METTL16,CCNJL,POLH,PRKAA2,ADAR,CUL9,STAT2,ZMPSTE24,PTK2,ARMC8,PHEX,RNF121,TEC,DCAF10,EXOSC3,FBXO10,CDC14A,MAP3K5,CPE,TRPC4AP,CREBBP,PRKD3,LMTK3,PCSK6,NCF1,TAOK2,PYGO2,SHC1,ECE1,PARP10,RNFT1,RPS6KB1,YLPM1,ZDHHC23,GALNT13,GUCY2F,PRG3,SERPINB11,SETDB2,DNAJB6,FBXO28,CD44,LARP4,ADAM8,FKBP14,MRPS6,CLDN4,RPS6KA5,ZNF93,CSNK1A1,HUS1,PRDM2,NOS1,RGS14,MC1R,STK4,MARK3,EPHB2,GATC,SLC1A1,TTLL11,MARCH8,IL18,PCMT1,TEX12,MFSD8,UBE2K,DIS3,DLC1,FBXL13,PPP1R10,PDP1,CALCRL,SMARCAL1,PHLPP1,PPIL6,CPEB4,MOB3B,ACP1,RNF150,PAX6,PRKCZ,ZC3HAV1,FAM20C,ADORA2A,RBMS1,GRIN2B,MAN2B1,PPP1CC,RABGEF1,CCNG2,CPEB1,TMEM161B,TYK2,OTUD7B,KIN,NIPBL,YEATS4,COL28A1,PHF20,ANGPT1,BRF1,TBL1X,EYA3,LRRK1,OSBPL8,TRMT2B,IGF2BP3,L2HGDH,NT5M,GCNT2,PPP1CA,MTF2,FBXO31,ATG3,CDK5RAP1,CNTN1,PRKCQ,BRAF,HDAC2,IMPACT,FKBP3,PHKG2,SRCAP,SPINT2,UBE2E2,C18ORF25,CD300A,PHIP,CDKL3,PPP2CA,SKP1,ANGPT4,MRPS16,RPL23,RFC5,ACAN,SP100,OPN1LW,REV3L,ANXA2,PARN,PIGU,PRMT7,DONSON,FARSB,WIPI2,ALG2,MADD,NUMB,SYK,CNOT1,WWTR1,GTF2H5,ASB1,CIT,ZBTB38,BRCA2,CACNG3,DVL2,MORC1,MTRF1,YBX1,ANKRD13A,PTPRJ,TAF1,EIF4E3,PPME1,TICAM1,RNF38,TANK,RIMKLA,GALNT7,NSUN6,HSF1,DHFR,EZH1,POP1,ETF1,RNGTT,SRPK2,SASH1,ADAMTS7,MCM8,WDR18,EEFSEC,RNF2,MOB3A,GNA12,SH2D3A,UGGT1,AKTIP,GALNT4,MYEF2,POC1B-GALNT4,INPP5F,NUAK2,AXL,AGBL3,ORC4,TRIM37,CD109,MET,TBC1D10A,CAMK2D,MRPL1,RAD9B,SNX1,TIMELESS,UCHL5,MALT1,SERPINE3,SETD3,TRIM69,ZDHHC11,ZDHHC11B,INO80C,KDM2B,MLXIPL,CCT3,FBXL4,NEDD4L,PHKB,PHB,PTPRC,CTCF,EXD2,SH3GL2,TYRO3,RNF213,TRAF3IP2,TRIM60,CREB1,LIG1,METTL21A,SGTB,TNXB,TRAP1,EDNRA,BMP4
GO:00	cellular protein	5.44873981	*POLDIP3,LNX2,ZDHHC14,ENPP1,NRXN1,ASPH,B4GALNT2,PRMT3,PRKCI,SLCO*

44267	metabolic process	7684358e-23	3A1,MAP4K4,PDE4D,DNMT1,S1PR2,C6ORF89,ADCY8,MGAT4C,CBFB,PDE8A,IL31RA,RPS6KA2,PRDM12,SETD4,CALR3,CTDSPL2,FBXL2,NRG3,ASH1L,NOS1AP,LATS2,RNF145,KSR2,PRKAG2,PRLR,MVP,PAGR1,FTO,KALRN,DCAF12,NTRK3,CBL,ARNT,EGLN2,FLT3,ENPP2,GRM5,PLCE1,FER,CASK,MAPK4,MAP2K5,KITLG,USP32,MAPK10,PTPRR,LRP2,PIK3CD,CAMK1G,EIF4G3,EZR,B3GALT1,ROBO1,TOM1L1,HTR2B,PSMD1,CDKL5,MECOM,TENM1,CAMKMT,LMNA,NMT2,TRAF6,SENP5,ITPKB,DNAJA3,OXR1,GRK5,ABI1,ULK4,TRIOBP,HSP90AA1,CDC6,PSMB7,TSSK1B,KNDC1,ZC4H2,CCDC22,MOCOS,STK38L,TRIO,KLHL12,AKAP13,TTLL5,PTPRK,DAB1,OMA1,ERC1,DNAJB14,KLHL7,MAST4,TCIRG1,PTPN11,HERC4,MGAT5,HDAC6,DDB1,ADORA1,MSRA,EPHA1,IKBKB,ERBB4,DPY19L2,MRE11A,LIMK1,TLK1,BRD8,KAT6B,ABR,CUL4B,LARGE,MIER1,PMEPA1,PTPRO,CDON,KANSL1,FBXL18,NTRK2,TNRC6A,EP300,ZYG11B,RNF220,FNTA,TPST2,PDGFB,FKBP9,TNIK,CCND3,GALNTL6,FBXL7,USP46,BRMS1,CHFR,STK39,INSR,PRKAR1A,ASB5,ST6GALNAC3,JMJD1C,CAB39,USP34,PAK1,FBXW11,MAP3K4,ZDHHC13,PTPRD,RNF144A,EGLN3,SAMD4A,BDNF,CDK14,CHRM3,POMGNT2,FUT8,TRIM13,UBR2,HLCS,LCK,ENTPD5,ECT2,MYO3B,MTA3,SNX6,CHML,PPM1L,CST2,PTCD3,PTPRU,ERN2,NPLOC4,DMD,ATG10,LARP4B,USP13,KAT7,SLC8A1,GSN,RBM14,RBM4,RIT2,MACROD2,DRG2,CASS4,FYN,MKRN2,ARNTL,LINC01138,NF1,LMTK2,ARID4B,GXYLT2,PPP4R2,FBXO21,DPEP1,B4GALT6,ASPHD2,BCR,NOSIP,RWDD3,BRPF1,CHI3L1,TTN,BMP2K,PAQR3,TPGS2,KLHL21,EGFLAM,PPIG,NRG1,SH2D3C,CDK19,BDKRB1,BDKRB2,BID,FRY,MAP2K1,FNIP1,MYH9,GALNT16,VGLL4,PPP2CB,HDAC5,RNF4,CSNK2A3,GALNT18,UBE3D,JDP2,WDSUB1,ITIH2,CDKL2,ROR2,DCN,ZDHHC3,FHIT,WDR70,PTPRT,UST,COPS5,IPO5,MAD2L2,MAN1A1,SRBD1,CAPRIN2,CCNYL1,PHF20L1,PDF,GNAQ,FBXO9,ICK,RFFL,TTLL8,PARP11,CCBE1,HERC5,USP22,ZDHHC15,JAK2,FBXW7,OAZ2,UBR7,UIMC1,ITCH,USP12,ZFYVE28,SMG1,HUNK,B3GALT5,CDC42BPB,PHKA1,DHDDS,KLF15,SPSB1,CYFIP2,WNT11,TPST1,TRIM59,MTA1,IMMP2L,NOX4,RPRD1B,SIAH1,QKI,CAMK2B,ERCC8,PRKCD,TAB2,ACVR2A,CD4,PPM1E,TGFB1,MRPL33,SGK1,PCSK5,P4HA2,SPSB4,MYO3A,NSD1,PIBF1,ASCC2,BTRC,CRADD,PTPRN2,PDZRN3,DNAJB2,AAK1,KLHL2,SLIT2,ASB15,RNF133,RNF148,ITIH4,HHAT,CORO1C,CDKAL1,DISC1,BLID,ZFAND2A,PTPDC1,CLN6,TMTC1,TTBK2,KDM4B,SMAD6,TMTC2,CLOCK,ZNF675,RPL36,NELL1,BMPER,TIMP2,BCL3,DCLK1,RNF212,ANKRD54,EIF3L,DAPK2,TNFRSF10B,DAPP1,DUSP22,NAIP,TRRAP,DOCK3,YTHDF1,FGF10,FBXL17,SMYD3,UNKL,KCTD10,SH3RF3,LOXL3,MAST2,FANCI,CAPN3,SMURF2,EPHA4,PRKCA,AUTS2,CD6,TNFSF11,PPP3CA,UBQLN3,UBQLNL,CAMK4,PIP5KL1,UFL1,TRAK1,CTNNB1,PARK2,SMARCC1,IGF1R,PPARG,IARS2,NGRN,AXIN1,PRKAR1B,MACROD1,OTUB1,LRPPRC,RSPRY1,AARS2,CDK11A,DYRK4,GALNT8,CDK11B,LEPR,FGF1,PROS1,WDR45B,NOL3,PRKAR2A,RIOK2,ESCO1,MYOCD,TRIM5,PER2,AJUBA,GLG1,CHEK2,SUPT3H,UBQLN4,PRDM16,ASB8,PPP1CB,SPDYA,HCK,CSTL1,RAB3GAP2,CAPN2,TBCK,TRIM8,DIO2,PPP2R2B,CSPG4,BRMS1L,CTDP1,HS3ST5,BAZ1B,OBSCN,NF2,FLT4,HDAC4,PAX2,SECISBP2L,SPTBN1,TPTE,TRABD2B,SFRP1,ST8SIA1,PPP6R2,FOXO3,USP54,BCL2L13,SSH1,SYNCRIP,SMAD3,RNFT2,RAB6A,PTPRM,WWP2,SIMC1,WIPI1,MTBP,RNF168,ABI2,C10ORF90,DCP1B,NEDD4,GALNT14,TAB1,SEC61B,ST3GAL3,CDC73,APP,USP49,PDGFRA,SLK,RBM8A,CCNI2,DIP2B,KANSL2,ARIH1,HEG1,AMFR,ST6GALNAC5,CDK3,EYA2,SH3D19,BORA,IBTK,SBK3,JAK1,FPGT-TNNI3K,TNNI3K,LRP5,MTCP1,PTPRG,SETD2,ZDHHC6,PRICKLE1,RCAN1,SPTBN4,DRD1,CHUK,ERLIN1,RFT1,MMP2,S100A12,DCUN1D3,KDM6A,PRKD1,NAA25,PARP6,ST18,USP53,LRRC2,SHPRH,VPS4A,EREG,CCNY,MAPKAPK3,ATF2,GRAMD4,RAF1,CELF4,CARD16,CASP1,PTPN1,ADAMTS12,PSMD11,GGT7,BMPR2,USP33,CAMK1D,BMPR1A,PABPC4,PIK3R3,CDK12,CAND2,SPAG9,MYB,FGF2,PPA2,PPM1F,UBE2R2,NEDD9,AGBL4,PEAK1,SEMA4D,NFX1,CDC27,PTAFR,SETD1A,YME1L1,JARID2,KLHL3,PRKCG,SIN3B,NCOA1,SPOCK1,AREL1,EEF1E1,EHMT1,LMO7,UCHL3,DVL3,EIF2B5,EIF4G1,EPHB3,PSMD2,PTPRE,RBX1,ANAPC5,FBXO45,STT3B,CKS1B,AGO3,DEPTOR,PKN3,FBXL20,TSG101,DESI1,TERF2IP,IDE,FKBP5,RCE1,B3GALNT1,P4HB,SUFU,SUMF1,PTPN13,QPCTL,COL4A3,HIP1,PADI6,TRPC5,UBA2,EDEM3,TTLL7,TNKS,TNRC6B,WDTC1,MRPS28,DAB2,BLM,UBR1,CACUL1,LDLRAD4,MYSM1,SETD5,DLG3,WNK1,GTPBP2,RELN,NEK10,SIN3A,RUVBL2,PSMD7,JOSD1,PELI1,PPP2R5C,IQGAP1,SPATA18,DCAF5,MAP3K7,TRIM22,ZNRF1,ALK,SLC8A2,UBOX5,SLC35A4,TOP1,AGBL1,UBE2V1,PADI3,XDH,SNX5,RBBP6,PRKG1,TTLL9,ATP2B4,PARP12,ACTL6B,PAK3,SSH2,TET1,HECTD4,MKRN3,CAMTA1,DYX1C1,ADNP,MAPKAPK2,CEP41,ARID4A,UBE3C,C12ORF65,CDC42BPA,MARK1,CDK6,PHF2,CELF1,RNF34,TFRC,UVRAG,EPHA5,ALG14,DCAF6,DCUN1D5,GPC3,XRN1,ADRBK1,PSMB2,EYA1,CDCA3,DIS3L2,RNF216,GHR,MNAT1,SNX9,ELAVL4,HDAC1,TRIM24,PLCG2,ROCK1,MRPS17,PAXIP1,EGR2,HIPK3,RNF10,RYBP,CUL2,AP3B1,PTPN2,INSRR,NTRK1,TRIM44,MAP1A,PKN2,SLC39A10,EIF3A,PTPRQ,EPHB1,MAN1B1,EPHA10,PTPN9,AXIN2,TMEM199,PARD3,GNL3L,KRTCAP2,MUC1,PTPN14,FBXO39,NUDT14,BCOR,MGAT4A,CARM1,ANXA8L1,SNX33,FOLH1,LPA,SPI1,DNMT3B,EPHA7,LRRC47,PTPRA,FBXW4,MRPS24,ATG14,EIF3H,BCAR3,STK38,RBPMS,RENBP,NTMT1,MAP3K13,OTUD3,RAPGEF3,PDXP,PTK7,RSRC1,SMYD1,SNCA,BMPR1B,MAGI2,USP42,PNPT1,USP50,RC3H1,EIF3E,RNF103,RNF103-CHMP3,RNF19B,PTPRS,KMT2D,PRKAG1,CDKL1,PSMF1,KMT2C,UBE2QL1,CAST,TRIP12,ADTRP,PLD1,OPRD1,HIPK1,RPTOR,BTK,ZNRF3,ASPHD1,KCTD13,MLLT1,ZZZ3,IGF1,HTT,LARS2,MAPK1,PTEN,MIB1,SPRED2,BMP7,PCMTD2,ATXN2,PIK

			3C3,PUM1,PCBP2,TTLL4,LRRK2,TMEM59,ZRANB1,PIGS,UNC119,BAG6,LILRB4,MORC3,MSRB3,PKD2,EFNA5,SEL1L2,RCOR3,TADA2A,DAZL,ALPK3,PDGFC,SERPINA3,SERPINA4,SERPINA5,NCOA3,PKIB,MTMR3,PPP2R3C,WNT7A,NLRP1,RPS6KC1,UACA,DNAJC1,PLCL2,WDR5B,KAT6A,MTIF2,SIK3,BMP6,MYCBP2,GPI,SH3RF2,EXT1,DPY19L4,N4BP1,ATRX,DPH6,C8ORF44-SGK3,PRMT2,RAPGEF2,VRK3,GET4,ALG9,MARK4,PRKAR2B,DPY19L1,PSEN2,GALNT10,UBE2H,DNAJC6,UBR5,GRIN1,JADE1,ARRDC4,RHOA,SPRTN,WSB2,ROR1,RQCD1,CUL3,HSPD1,EPM2A,GSK3B,PRR16,DNAJC3,RDX,EEF2K,IWS1,PPP1R16A,PTPRB,CDK13,PGGT1B,STK32B,CD27,DUSP26,BOK,RNF144B,RNF43,AGT,METTL16,CCNJL,PRKAA2,ADAR,CUL9,STAT2,ZMPSTE24,PTK2,ARMC8,PHEX,RNF121,TEC,DCAF10,EXOSC3,FBXO10,CDC14A,MAP3K5,CPE,TRPC4AP,CREBBP,PRKD3,LMTK3,NCF1,TAOK2,PYGO2,SHC1,PARP10,RNFT1,RPS6KB1,ZDHHC23,GALNT13,GUCY2F,PRG3,SERPINB11,SETDB2,DNAJB6,FBXO28,CD44,LARP4,ADAM8,FKBP14,MRPS6,CLDN4,RPS6KA5,CSNK1A1,HUS1,PRDM2,NOS1,RGS14,STK4,MARK3,EPHB2,GATC,SLC1A1,TTLL11,MARCH8,IL18,PCMT1,MFSD8,UBE2K,DIS3,DLC1,FBXL13,PDP1,PHLPP1,PPIL6,CPEB4,MOB3B,ACP1,RNF150,PAX6,PRKCZ,FAM20C,ADORA2A,GRIN2B,MAN2B1,PPP1CC,RABGEF1,CCNG2,CPEB1,TYK2,OTUD7B,NIPBL,YEATS4,COL28A1,PHF20,ANGPT1,BRF1,TBL1X,EYA3,LRRK1,OSBPL8,IGF2BP3,L2HGDH,GCNT2,PPP1CA,MTF2,FBXO31,ATG3,CDK5RAP1,CNTN1,PRKCQ,BRAF,HDAC2,IMPACT,FKBP3,PHKG2,SRCAP,SPINT2,UBE2E2,C18ORF25,CD300A,PHIP,CDKL3,PPP2CA,SKP1,ANGPT4,MRPS16,RPL23,OPN1LW,ANXA2,PARN,PIGU,PRMT7,FARSB,WIPI2,ALG2,MADD,SYK,CNOT1,WWTR1,GTF2H5,ASB1,CIT,BRCA2,DVL2,MTRF1,YBX1,PTPRJ,TAF1,EIF4E3,PPME1,TICAM1,RNF38,TANK,RIMKLA,GALNT7,HSF1,DHFR,EZH1,ETF1,RNGTT,SRPK2,SASH1,ADAMTS7,EEFSEC,RNF2,MOB3A,GNA12,SH2D3A,UGGT1,AKTIP,GALNT4,POC1B-GALNT4,INPP5F,NUAK2,AXL,AGBL3,TRIM37,CD109,MET,TBC1D10A,CAMK2D,MRPL1,UCHL5,MALT1,SERPINE3,SETD3,TRIM69,ZDHHC11,ZDHHC11B,KDM2B,MLXIPL,FBXL4,NEDD4L,PHKB,PHB,PTPRC,CTCF,SH3GL2,TYRO3,RNF213,TRAF3IP2,TRIM60,CREB1,METTL21A,SGTB,TNXB,TRAP1,EDNRA,BMP4
GO:0007166	cell surface receptor signaling pathway	8.882147723665408e-22	CD247,ENPP1,PRDX2,GPC5,NRXN1,GP6,PRKCI,PDE4D,PDCL,S1PR2,CLASP2,SEMA3A,IL31RA,GRID2,DOCK1,CLDN18,CTDSPL2,NRG3,LATS2,SEMA3D,PTH2R,NREP,NLGN4X,GRM8,UNC5C,PRLR,MVP,KALRN,NTRK3,CBL,ARNT,SEMA5A,LRRFIP2,FLT3,STAT5B,GRM5,PLCE1,SAMHD1,FER,MAP2K5,KITLG,MAPK10,PTPRR,LRP2,PIK3CD,SH2D1A,EZR,ROBO1,ITGB6,LMNA,GOT1,TRAF6,ROBO2,ITPKB,DNAJA3,VAV2,GRK5,ABI1,ITGB1,PSMB7,MCC,DSCAM,GMDS,SAMD12,TRIO,KLHL12,PTPRK,DAB1,DGKI,ANKS1B,STXBP4,GFRA2,IL17RB,PTPN11,HERC4,MGAT5,DDB1,ADORA1,ADAMTS3,EPHA1,IKBKB,ERBB4,LIMK1,BRD8,KLHL6,NPHP3,GRM7,PMEPA1,MAML3,PTPRO,CDON,NTRK2,EP300,CELA1,RNF220,FNTA,PDGFB,RIMS1,TNIK,CCND3,BPI,STK39,INSR,UNC5D,LAMA3,TCF7L2,PIP4K2A,USP34,PAK1,FBXW11,PTPRD,BDNF,CDK14,FANCA,TGIF2,FUT8,PHACTR4,GRIK4,LCK,IL5RA,SHC4,SNX6,PPM1L,PTPRU,RFTN1,AP3S1,RBM14,GPR171,DEPDC1B,CASS4,FYN,ARNTL,NF1,PLCB1,DST,LMTK2,RTN4,CHRDL1,APOD,RTN4R,NDRG4,BMP2K,PIK3R2,RANBP9,NOMO3,NRG1,BDKRB2,BID,MDFI,VAX2,MYH9,DAGLA,VGLL4,PPP2CB,CMKLR1,ROR2,DCN,ZDHHC3,SLC39A14,CDH13,SOX13,PTPRT,MAD2L2,TLE6,CAPRIN2,CCNYL1,RCC2,BOC,ANO6,ARL3,NEUROD1,GNAQ,RFFL,CCBE1,JAK2,FBXW7,SKAP1,ZFYVE28,MLLT3,SH2D6,TCF7,KLF15,ANXA4,MAGI1,CYFIP2,WNT11,IFT80,KREMEN1,PRKCD,ACVR2A,RUNX2,SEMA5B,CD4,TGFB1,NUP93,IGSF1,PIBF1,BTRC,CRADD,F2RL1,C9ORF47,GRIK3,RPGRIP1L,CELSR1,SLIT3,AAK1,ZFYVE9,SLIT2,HHAT,CTNND2,DISC1,KANK1,PTPDC1,MTDH,TTBK2,SMAD6,ZNF675,BMPER,BCL3,TNFRSF10B,ADAM12,DUSP22,NAIP,HOMER2,FGF10,FBXL17,ARHGEF28,IL1RAPL2,LOXL3,GLP2R,SMURF2,EPHA4,RORA,PRKCA,CD6,TNFSF11,PPP3CA,CTNNB1,PARK2,FCGR2A,FCGR2B,FCGR3A,FCGR3B,SMARCC1,IGF1R,PPARG,AXIN1,DLG5,IL18R1,IL1RL1,P2RY10,TRPM1,SREBF2,DRAXIN,LEPR,LEPROT,FGF1,NR4A3,NOL3,MYOCD,TRIM5,KIR2DL1,GLG1,PRDM16,MYO1E,HCK,SORBS1,CSPG4,GRM1,DCC,P2RY14,NF2,FLT4,BICC1,SPTBN1,TRABD2B,SFRP1,FOXO3,ARL13B,CNTN6,IFT122,SMAD3,CUX2,WWP2,ACKR2,GRIK5,SHANK1,IFNAR1,NEDD4,NRP2,VANGL1,HOXD3,TAB1,NFATC1,CDC73,APP,GSX2,PDGFRA,FCRL4,YAP1,AMFR,FSTL4,NLGN2,TNFRSF19,EYA2,JAK1,LRP5,MUC20,PTPRG,SOX2,GPC6,CHST11,PRICKLE1,GRIA3,GLRA2,CHUK,FRS2,MMP2,KDM6A,PRKD1,STAT1,ST18,HNF4A,EREG,CCNY,TCTN3,MAPKAPK3,SEMA6D,ATF2,TMEM237,RAF1,CELF4,CARD16,CASP1,PTPN1,ADAMTS12,BMPR2,BMPR1A,TSPAN12,NLGN1,COL16A1,PIK3R3,EVC,FGF2,GRIK1,ADCY5,NEDD9,SEMA4D,DOK6,PLXNA2,PTAFR,ADCYAP1R1,DKK2,ITGA11,SPEN,VIPR1,GRIA2,GAS8,CHRD,DVL3,EIF2B5,EPHB3,PTPRE,RBX1,ATF3,SHOC2,PAWR,DEPTOR,MAML2,TSG101,CCDC3,IDE,WNT3,P4HB,CCL14,CCL15,SUFU,NLGN3,SCUBE2,PAFAH1B1,PRDM15,CNTFR,COL4A3,TRPA1,HIP1,IRS4,GLI2,NEO1,TNKS,SCUBE1,DAB2,THEMIS,LDLRAD4,DZIP1,WNK1,RELN,RUVBL2,RSPO2,CSF3R,PELI1,IQGAP1,MAP3K7,DDX47,ZNF423,MACF1,ALK,SLC8A2,INPP5D,XDH,LTBP1,OVOL2,SNX5,NFATC2,PAK3,P2RX6,ASGR2,GPR161,MAPKAPK2,COL4A6,SCARB1,MARK1,CDK6,P2RY8,RNF34,EPHA5,WWOX,GFRAL,GPC3,PSMB2,EYA1,TRPV1,GHR,FLRT2,HDAC1,INVS,SMOC2,ADIPOR2,PLCG2,GPR35,PTPN2,INSRR,NTRK1,TRIM44,SLC39A10,EPHB1,EPHA10,AXIN2,JAG2,NETO1,SPI1,EPHA7,CHRNB4,RANBP10,PTPRA,CTNNA1,FBXW4,LY86,BCAR3,RBPMS,CHURC1,SH3BP1,MST1,PTK7,SNCA,BMPR1B,GRM4,MAGI2,ZEB2,HEY2,RC3H1,W

			NT7B,ZMYND11,SPPL2A,RASA1,MITF,CABIN1,HIPK1,BTK,SIGLEC9,ZNRF3,TROVE2,CBFA2T2,IGF1,HTT,MAPK1,PTEN,MIB1,SPRED2,BMP7,LRRK2,ZRANB1,BAG6,LILRB4,IFT81,TMEM108,ITGAM,IL11RA,PKD2,EFNA5,SHANK3,BTBD11,PDGFC,AMOTL1,WNT7A,PLCL2,GPR21,BMP6,IL17RD,SOS1,TSHR,EXT1,PDCD1LG2,PRICKLE2,RAPGEF2,SMO,PSEN2,SOX30,UBR5,GRB14,GRIN1,JADE1,RHOA,VIPR2,GRIN3A,ROR1,RQCD1,TF,ONECUT2,CUL3,NFKBID,EPM2A,GSK3B,PLD2,STAT6,NECAB2,CD27,ITGBL1,BOK,SULF1,KRT8,RNF43,AGT,PRKAA2,ADAR,FBN2,STAT2,NDRG2,PTK2,LAT2,SNX3,PDE4B,RIMS2,TEC,VEPH1,CPE,CREBBP,APCDD1L,DLX1,FMOD,PCSK6,NCF1,SLC29A1,PYGO2,SHC1,CD160,ECE1,LBX2,RPS6KB1,GUCY2F,SCEL,ANKS1A,ITGAL,CD44,RPS6KA5,BCL2L14,CSNK1A1,GRID1,FCRL6,PLEKHA1,PLVAP,RGS14,ACTN4,STK4,SPEF1,EPHB2,SLC1A1,WLS,XCR1,IL18,UBE2K,PPARA,ARID5B,CALCRL,CPEB4,NOTCH4,FBN1,PAX6,PRKCZ,FAM20C,ADORA2A,GRIN2B,RABGEF1,TYK2,ASPN,ANGPT1,TBL1X,EYA3,LRRK1,OSBPL8,GCNT2,PPP1CA,VWC2,GRIA4,RBMS3,CCL22,BTN3A2,CNTN1,PRKCQ,BRAF,CSRNP1,HDAC2,CD300A,PHIP,PPP2CA,ANGPT4,SP100,PIGU,CR2,FCRL2,MADD,SYK,WWTR1,GRIK2,DVL2,PTPRJ,TICAM1,ITGAE,HSF1,SASH1,FCHO1,VAV3,CAV2,COL4A5,INPP5F,AXL,REPS2,TNFSF9,WIF1,CD109,MET,CCR3,UCHL5,ANKRD6,MALT1,SEMA3C,PHB,PTPRC,EDA,PBLD,TYRO3,RNF213,TSPAN5,NCAM1,TRAF3IP2,RGMB,SHISA6,CLNK,EDNRA,BMP4,CPNE1
GO:0050790	regulation of catalytic activity	1.8962112664254195e-21	RIC8B,LDB2,NRXN1,ASPH,MAP4K4,C6ORF89,ADCY8,DOCK1,NRG3,NOS1AP,LATS2,TBC1D19,PHACTR1,TIAM2,MAPRE2,PRKAG2,PRLR,MVP,KALRN,SEC23B,PHACTR2,NTRK3,CBL,PLEKHG4B,FLT3,GRM5,PLCE1,MAP2K5,KITLG,DENND2D,DEPDC5,XRCC4,ROBO1,TOM1L1,HTR2B,PSMD1,CDKL5,TENM1,NUCB1,TRAF6,DNAJA3,VAV2,ABI1,ITGB1,HSP90AA1,CDC6,SRGAP3,KNDC1,TRIO,ARHGAP24,AKAP13,DAB1,NCF4,RGS6,DGKI,MGAT5,CCT2,DOCK10,CYTH3,HDAC6,ADORA1,EPHA1,IKBKB,ERBB4,MRE11A,GBF1,LIMK1,ABR,ESR1,GRM7,PTPRO,NTRK2,MCF2L2,FNTA,PDGFB,RIMS1,CCND3,ALOX5AP,INSR,PRKAR1A,PIP4K2A,HPS1,CAB39,PAK1,MAP3K4,EGLN3,NPRL3,PHACTR4,RASGEF1B,ARHGAP6,LCK,ECT2,SNX6,CHML,ARHGAP42,CST2,ERN2,DENND5A,SLC8A1,GSN,DEPDC1B,CASS4,FYN,NF1,NCF2,PLCB1,MGMT,LMTK2,SNCB,PPP4R2,RXFP1,DPEP1,DGKH,RTN4R,BCR,PPP1R12B,NOSIP,BRPF1,CHI3L1,TTN,BMP2K,PAQR3,PIK3R2,NRG1,ARHGAP10,SMAP2,SH2D3C,DOCK8,BID,FRY,MAP2K1,FNIP1,RAPGEF6,STIM1,RALGPS2,TMBIM6,PPP3R1,SBF2,ITIH2,ROR2,SLC39A14,PRPSAP2,DOCK2,PTPRT,PPP1R42,IPO5,ARHGAP32,MAD2L2,RGS8,CCNYL1,TERF2,RALGPS1,RCC2,GNAQ,RFFL,RANBP1,DENND1A,HERC5,JAK2,FBXW7,OAZ2,ZFYVE28,ARAP2,DOCK9,DENND2A,ANXA4,LAMTOR3,CYFIP2,WNT11,ARHGAP23,NOX4,SIPA1L3,PRKCD,TAB2,ACVR2A,CD4,PPM1E,TGFB1,SGK1,PCSK5,PIBF1,BTRC,DUS2,CRADD,F2RL1,BCAS3,SLIT2,ITIH4,HP,CORO1C,BLID,CACNA1D,CALML4,RXFP2,ITSN2,ZNF675,TIMP2,ANKRD54,TNFRSF10B,PARVA,DUSP22,RABGAP1L,NAIP,DOCK3,RASA4,RASA4B,ARHGEF28,SMYD3,STXBP5,CAPN3,EPHA4,CNR1,TNFSF11,SMG6,CTNNB1,PARK2,SOD2,ARHGAP22,IGF1R,PPARG,AXIN1,GARNL3,PRKAR1B,LEPR,FGF1,PROS1,RGS10,NOL3,PRKAR2A,AGAP1,MYOCD,AJUBA,CACNA1C,SPDYA,CSTL1,TBC1D14,RAB3GAP2,TBCK,PPP2R2B,AGFG2,ARHGAP31,MMP16,OBSCN,RGL1,NF2,FLT4,PAX2,SFRP1,PPP6R2,ARHGAP29,RANBP3,BCL2L13,SMAD3,SIMC1,PSAP,DCP1B,ARHGEF18,PREX1,DOCK4,TAB1,APP,PDGFRA,CCNI2,HEG1,TEN1,BORA,IBTK,NVL,PDE6D,ANGPTL4,LRP5,MTCP1,SOX2,RCAN1,ECSIT,DRD1,FRS2,SERGEF,S100A12,DCUN1D3,PRKD1,ST18,ARHGEF33,EREG,CCNY,PITRM1,GRAMD4,RAF1,RASGRF2,CARD16,CASP1,PTPN1,BMPR2,BMPR1A,PIK3R3,CDK12,DENND4A,FGF2,PPM1F,ADCY5,NEDD9,SEMA4D,SH3PXD2A,ELP3,PLXNA2,PTAFR,ADCYAP1R1,SPOCK1,DVL3,EIF2B5,EPHB3,PSMD2,ARHGAP39,SHOC2,CKS1B,DEPTOR,TSG101,MYO9A,CCL14,CCL15,PAFAH1B1,FAM13A,COL4A3,DDAH1,NMUR2,HIP1,RASAL1,TNKS,WDTC1,DAB2,BLM,CACUL1,DLG3,WNK1,RELN,NEK10,DOCK11,PPP2R5C,IQGAP1,MAP3K7,ARHGAP12,ALK,SLC8A2,XDH,PRKG1,RGS7,ATP2B4,CCPG1,ADNP,SCARB1,RNF34,UVRAG,EPHA5,DCUN1D5,GPC3,TBC1D5,GHR,MNAT1,FGD1,SNX9,RPGR,HDAC1,ROCK1,HIPK3,GAPVD1,AP3B1,PTPN2,INSRR,NTRK1,SLC39A10,DNMBP,EPHB1,EPHA10,AXIN2,RCAN2,KRTCAP2,ANXA8L1,ARHGAP44,PIFO,LPA,ARHGEF3,EPHA7,ACAP2,ATG14,BCAR3,STK38,RENBP,MAP3K13,RAPGEF3,SH3BP1,SNCA,BMPR1B,MAGI2,PRIM2,TBC1D9,USP50,ZEB2,PRKAG1,PSMF1,CAST,DFFA,RASA1,CABIN1,RPTOR,BTK,TBC1D16,MLLT1,IGF1,HTT,MAPK1,PTEN,ARHGEF17,SPRED2,BMP7,RASA2,LRRK2,UNC119,RFC3,LILRB4,ITGAM,ARHGAP25,PKD2,EFNA5,MIA2,RGS9,PDGFC,SERPINA3,SERPINA4,SERPINA5,FAM13B,PKIB,SGSM1,PPP2R3C,TBCD,NLRP1,UACA,AGAP5,RABEP1,SH3PXD2B,MYCBP2,GPI,SH3RF2,SOS1,TSHR,SERINC5,RAPGEF2,VRK3,PPP1R37,PRKAR2B,STXBP5L,PSEN2,GRIN1,ARRDC4,RHOA,SYNGAP1,VIPR2,PPP3R2,ROR1,HSPD1,EPM2A,GSK3B,DNAJC3,RDX,PPP1R16A,PTPRB,CD27,BOK,AGT,CCNJL,ADAR,PTK2,MAP3K5,IQGAP2,PCSK6,NCF1,TAOK2,UGT1A1,UGT1A10,UGT1A4,UGT1A7,UGT1A8,SHC1,ARHGAP15,SERPINB11,DNAJB6,CD44,FGD3,ADAM8,SLC5A3,ARFGAP3,CLDN4,FGD4,NOS1,GCKR,RGS14,SIPA1L2,ARHGAP11A,STK4,COX6A1,EPHB2,SLC1A1,IL18,GPSM2,DIS3,DLC1,PPP1R10,GRTP1,PDP1,GPSM3,MOB3B,PRKCZ,ADORA2A,GRIN2B,RABGEF1,CCNG2,DENND4B,COL28A1,ANGPT1,OSBPL8,TBC1D10C,ARHGAP21,CCL22,CDK5RAP1,PRKCQ,PPP1R14A,SPINT2,CD300A,PPP2CA,SKP1,ANGPT4,ARHGEF6,RPL23,SH3BP4,RFC5,ANXA2,PARN,ITSN1,SRGAP2,MADD,SYK,WWTR1,ARHGAP19,CIT,RAP1GAP2,DVL2,PTPRJ,PPME1,TANK,HSF1,DHFR,SASH1,

			DENND2C,ASAP1,RALGAPA1,VAV3,MOB3A,CAV2,GNA12,SH2D3A,AXL,CD109,DENND4C,MET,TBC1D10A,CAMK2D,UCHL5,MALT1,SERPINE3,PHB,PTPRC,TYRO3,MTMR12,TNXB,EDNRA,BMP4
GO:0031323	regulation of cellular metabolic process	7.370991750191733e-21	*POLDIP3,ENPP1,PRDX2,LDB2,NRXN1,ASPH,PRMT3,PRKCI,SLCO3A1,PDE4D,DNMT1,S1PR2,HLX,SLC9A1,C6ORF89,PBX3,ADCY8,MED13L,TRPS1,CBFB,PDE8A,ZNF823,IL31RA,PRDM12,MED26,WWC1,FBXL2,NRG3,ASH1L,NOS1AP,LATS2,NOX5,SCAF8,STOX2,PTGIS,PRKAG2,WWC3,PRLR,MVP,PAGR1,HIVEP3,FTO,NPAS3,DCAF12,NTRK3,CBL,ARNT,LRRFIP2,FLT3,STAT5B,TOX,ENPP2,GRM5,PLCE1,ZNF566,FER,CASK,MAP2K5,KITLG,MAPK10,LRP2,ZNF536,SP3,DEPDC5,HDGF,EIF4G3,EZR,IKZF2,ROBO1,MALRD1,TOM1L1,CHD7,HTR2B,MECOM,TACC1,TENM1,LMNA,TRAF6,ESRP1,ITPKB,DNAJA3,OXR1,VAV2,ABI1,NHLH1,HSP90AA1,CDC6,PSMB7,TSC22D3,KNDC1,ZC4H2,CCDC22,DSCAM,CDAN1,AKAP13,GFI1B,PTPRK,RUNX1,DAB1,ERC1,HMGN3,NRIP1,THRB,EFCAB7,ITGB3BP,DACH1,PTPN11,ZNF569,MGAT5,CCT2,ORC2,HDAC6,SERTAD2,DDB1,MYT1,ADORA1,EPHA1,IKBKB,PEX14,ERBB4,MRE11A,LIMK1,ZNF609,BRD8,KAT6B,HIF3A,SNIP1,ESR1,MIER1,PCBP3,PMEPA1,MAML3,PTPRO,CDON,NTRK2,TNRC6A,EP300,CELA1,ZYG11B,TENM2,ZNF76,RNF220,ZNF471,FNTA,PDGFB,TNIK,CCND3,TOB2,ZNF19,ZNF23,BRMS1,CHFR,ZNF605,SCML4,HNF4G,INSR,FMN2,RERE,PRKAR1A,FUBP1,ATP8B1,H2AFY2,TCF7L2,PIP4K2A,JMJD1C,CAB39,ZNF443,ZNF490,ZNF564,ZNF709,ZNF799,PAK1,LITAF,FBXW11,ESRRB,MAP3K4,RNF144A,BASP1,EGLN3,RBM20,TBR1,SAMD4A,BDNF,TFAP2A,CDK14,FANCA,PEG3,ZIM2,TGIF2,TGIF2-C20ORF24,MEIS1,VRTN,TRIM13,LCK,MDM4,ENTPD5,ECT2,ZNF148,MTA3,SNX6,TFDP2,CST2,PTCD3,ERN2,C1D,AFF3,DMD,CENPF,ATG10,SLC30A9,TOX3,PDS5A,LARP4B,USP13,KAT7,ZNF667,SLC8A1,GSN,KHDRBS2,RBM14,RBM4,MED12L,SATB2,PAFAH1B2,RIT2,HIRA,CASS4,FYN,MKRN2,ARNTL,NF1,SMG7,PLCB1,MGMT,LMTK2,ARID4B,PPP4R2,RTN4,AFF2,DPEP1,APOD,BCR,RWDD3,BRPF1,CHI3L1,TTN,PAQR3,PIK3R2,NRG1,SH2D3C,UBP1,BDKRB1,BDKRB2,BID,FRY,MAP2K1,MDFI,FNIP1,VAX2,MYH9,TMBIM6,VGLL4,PPP2CB,PPP3R1,HDAC5,CSRNP3,RBM5,ZNF692,NFIA,RNF4,JDP2,CMKLR1,ITIH2,ROR2,DCN,SCFD1,PCBD2,HNRNPLL,CDH13,CREBRF,TRDMT1,SOX13,FHIT,WDR70,PTPRT,AUNIP,COPS5,PPP1R42,EXOC4,IPO5,MAD2L2,TLE6,RAB27A,CAPRIN2,CCNYL1,ZNF418,NLN,PHF20L1,TERF2,SLX1B,ZNF286A,FOXN3,NEUROD1,GNAQ,RFFL,CCBE1,HERC5,USP22,JAK2,FAM168A,TRAPPC9,FBXW7,SKAP1,SMC3,UIMC1,ITCH,MLIP,ZFYVE28,LZTS1,BCL11B,SMG1,RBFOX1,PKNOX1,MLLT3,TSHZ2,TCF7,PDE2A,KLF15,TBX15,ANXA4,LAMTOR3,CYFIP2,WNT11,MTA1,KLF8,NOX4,LCOR,RPRD1B,MKLN1,QKI,CCDC62,ERCC8,PRKCD,SOX6,TAB2,ACVR2A,RUNX2,CD4,PPM1E,TGFB1,BANP,SGK1,SPSB4,TRIM65,NSD1,IGSF1,PIBF1,ZHX2,PKNOX2,ASCC2,BTRC,NFATC3,CRADD,F2RL1,BCAS3,CDYL2,DNAJB2,CC2D1B,SLIT2,TP73,ITIH4,HP,CORO1C,SAP18,ZBTB22,ILF2,DISC1,BLID,ZFAND2A,CLN6,MTDH,FANK1,MYT1L,KDM4B,SMAD6,BNC2,ZNF398,CLOCK,TCF12,ZNF675,ETV6,NELL1,TFAP2D,BMPER,TIMP2,BCL3,ANKRD54,DAPK2,TNFRSF10B,SND1,DUSP22,NAIP,HNRNPC,TRRAP,DOCK3,SBNO2,YTHDF1,FGF10,CIZ1,SMYD3,LOXL3,FANCI,CAPN3,LUM,SMURF2,EPHA4,RORA,HIVEP2,PRKCA,AUTS2,CNR1,CD6,TNFSF11,SMG6,PPP3CA,NSUN2,UBQLN3,NFYB,MAGEA4,KLF12,CAMK4,GATAD2B,PIP5KL1,UFL1,TRAK1,CTNNB1,PARK2,SOD2,DACH2,METTL13,SMARCC1,KLF17,IGF1R,PPARG,NGRN,AXIN1,PRKAR1B,OTUB1,IL18R1,CIPC,MTF1,CELF2,CBX5,ANKRD17,BRIP1,LRPPRC,SREBF2,CDK11A,CDK11B,LEPR,FGF1,PROS1,NPAT,NR4A3,FOXK2,NOL3,PRKAR2A,RIOK2,ESCO1,MYOCD,TRIM5,PER2,AJUBA,GLG1,ZNF626,ZNF737,CHEK2,SUPT3H,UBQLN4,PRDM16,PPP1CB,SPDYA,HCK,CSTL1,SORBS1,TBC1D14,RAB3GAP2,CAPN2,TRIM8,DIO2,CSPG4,BRMS1L,ZBTB8A,ZBTB8B,CTDP1,BAZ1B,NF2,FLT4,MEF2B,HDAC4,PAX2,PHF5A,SECISBP2L,TRABD2B,SFRP1,MED13,PPP6R2,ZNF395,FOXO3,NFIB,SP4,ZCCHC17,BCL2L13,SSH1,CELF5,SYNCRIP,SMAD3,RNFT2,CUX2,ITPR1,WWP2,ARNT2,SBNO1,KRBOX1,ZNF662,ZNF777,SIMC1,WIPI1,EBF3,MTBP,RNF168,CASZ1,PSAP,IFNAR1,DCP1B,MIER3,NEDD4,ESRRG,HOXD3,HOXD4,SLC4A4,ZNF114,TAB1,KTI12,NFATC1,CDC73,APP,SSBP3,GSX2,PDGFRA,RBM8A,CCNI2,DIP2B,ARIH1,YAP1,HEG1,SESN1,TEN1,VDAC1,EYA2,SH3D19,BORA,IBTK,NVL,LRP5,MTCP1,POLR3G,ZNF787,SOX2,SETD2,TEAD1,PRICKLE1,RCAN1,ZNF653,SPTBN4,ZNF521,DRD1,ARID3A,ZNF761,CHUK,ERLIN1,SFMBT1,ZNF584,ESR2,S100A12,DCUN1D3,PRKD1,STAT1,CELF6,ST18,ETV5,RHOXF2B,SLC6A3,TAF3,PLAGL1,HNF4A,ZBTB7C,TASP1,EREG,CCNY,RBFOX3,ATF2,POU2F2,TCF3,ZNF730,GRAMD4,RAF1,CELF4,ZNF766,CARD16,CASP1,PTPN1,SRRM4,BMPR2,USP33,CAMK1D,BMPR1A,ZKSCAN1,PABPC4,BTBD10,IKZF4,PIK3R3,CDK12,CAND2,SPAG9,DENND4A,MYB,FGF2,ZNF71,POU6F2,BACH1,MXD3,PPM1F,TICRR,GLI4,ZFP41,NEDD9,AGBL4,BEND5,SEMA4D,NFX1,RORC,ELP3,PTAFR,ADCYAP1R1,SP140,SP140L,RHOXF2,JARID2,DDX58,BRDT,PHC2,RARB,SPEN,PRKCG,SIN3B,NCOA1,SPOCK1,EHMT1,LMO7,AP2M1,DVL3,EIF2B5,EIF4G1,EPHB3,RBX1,TCF20,ATF3,LIN28B,ATP6V0B,SRSF5,CKS1B,PAWR,EBF2,AGO3,DEPTOR,MAML2,TSG101,DESI1,TERF2IP,CRYM,IDE,RFX2,ZNF322,SUFU,MAGEA11,PTPN13,PRDM15,ZNF670,ZNF695,CCDC169-SOHLH2,SOHLH2,COL4A3,DDAH1,ZNF354C,HIP1,TCEA3,PADI6,ZNF704,NR2C1,TRPC5,UBA2,GLI2,TNKS,WBP2NL,ERCC1,TNRC6B,GLIS3,WDTC1,ZNF664,DAB2,BLM,PKHD1,CACUL1,LDLRAD4,MYSM1,SETD5,SMG5,DLG3,WNK1,RELN,NEK10,SIN3A,RUVBL2,COMMD6,GMEB1,PELI1,IQGAP1,MAP3K7,ZNF423,SP1,TRIM22,ALK,SLC8A2,TEAD4,FASTKD5,HOXC13,APBB3,SLC35A4,SRA1,UBE2V1,TPCN1,A*

			DIRF,XDH,AHNAK,OVOL2,SNX5,NFATC2,RBBP6,PAX7,BNC1,ATP2B4,NSF,ACTL6B,ASXL3,PAK3,TET1,CAMTA1,CCPG1,DYX1C1,ATAD1,ADNP,MAPKAPK2,ARID4A,SCARB1,CDK6,ATP6V0A2,PHF2,CELF1,RNF34,TFRC,UVRAG,EPHA5,WWOX,MEF2A,DCAF6,DCUN1D5,DROSHA,GPC3,XRN1,SATB1,TBC1D5,PSMB2,EYA1,GATAD2A,SCOC,DIS3L2,GNB3,TRPV1,RBM12B,HOXB3,HOXB4,HOXB5,HOXB6,TFEB,CLEC16A,GHR,MNAT1,SNX9,ACTR2,L3MBTL4,ELAVL4,HDAC1,RECQL5,SMOC2,TRIM24,PLCG2,ROCK1,SCMH1,C1QTNF1,PAXIP1,ZNF713,EGR2,HIPK3,RNF10,RYBP,AP3B1,PTPN2,INSRR,NTRK1,KPNA6,TRIM44,MAP1A,SLC39A10,EPHB1,EPHA10,AXIN2,EPCAM,PARD3,ZNF425,GNL3L,MUC1,PTPN14,BCOR,AP3D1,ZNF41,CARM1,ZNF383,ANXA8L1,SNX33,PIFO,LPA,ZNF616,ZNF836,SMARCA2,CUX1,SPI1,DNMT3B,EPHA7,FOXP2,PSPC1,GLIS1,SFMBT2,ATG14,EIF3H,BCAR3,JAZF1,STK38,ABCB7,ACOT8,RBPMS,RENBP,CHURC1,CREB5,MAP3K13,RAPGEF3,SMYD1,SNCA,BMPR1B,MAGI2,PRIM2,PNPT1,USP50,ZEB2,DHRS4,FOXJ3,HEY2,RC3H1,EIF3E,RNF19B,ZMYND11,KMT2D,PRKAG1,CREM,PSMF1,KMT2C,CAST,TRIP12,DFFA,ADTRP,PLD1,RASA1,MACC1,MITF,SRSF6,OPRD1,NUGGC,RPTOR,NFIX,BTK,TBC1D16,KCTD13,MLLT1,CBFA2T2,IGF1,MLXIP,ATF7IP,HTT,MAPK1,PTEN,SPRED2,BMP7,ATXN2,MXI1,PUM1,SOX5,CIR1,PCBP2,PRCP,ZNF780A,ZNF780B,LRRK2,ATP6V1A,MLYCD,TMEM59,UNC119,ZBTB20,CHMP5,RFC3,BAG6,LILRB4,ITGAM,PKD2,ZNF652,EFNA5,KCTD1,RIPPLY1,RCOR3,TADA2A,DAZL,PDGFC,SERPINA3,SERPINA4,SERPINA5,NCOA3,PKIB,MTMR3,ZNF146,ZNF565,PPP2R3C,WNT7A,ZBED6,BRWD3,NLRP1,UACA,LPGAT1,CRTC3,DNAJC1,ARNTL2,ELOVL5,PLCL2,KAT6A,MTIF2,ZKSCAN7,ZNF197,ZNF660,BMP6,TAF15,MYCBP2,NFE2L1,GPI,SH3RF2,WDR43,ZNF30,N4BP1,ATRX,DPH6,IKZF1,PRMT2,RAPGEF2,ASCC1,SMO,VRK3,RALY,PRKAR2B,CPT1A,TCF4,SOX30,TFE3,UBR5,GRIN1,JADE1,ARRDC4,RHOA,SPRTN,ROR1,RQCD1,TF,ONECUT2,CUL3,TFEC,HSPD1,EPM2A,GSK3B,PRR16,DNAJC3,RDX,STAT6,MLF1,EEF2K,IWS1,PPP1R16A,PTPRB,RBBP8,NECAB2,CDK13,CD27,DUSP26,BOK,RNF144B,ZNF362,NACC2,SUPT4H1,ZNF354A,AGT,HDGFRP3,METTL16,CCNJL,POLH,PRKAA2,ADAR,STAT2,ZMPSTE24,PTK2,PBX1,TEC,EXOSC3,MAP3K5,TCFL5,CREBBP,DLX1,GTF2IRD2,NCF1,TAOK2,UGT1A1,UGT1A10,UGT1A4,UGT1A8,PYGO2,SHC1,ZNF813,ECE1,LBX2,PARP10,RNFT1,RPS6KB1,STC2,YLPM1,DPRX,PRG3,SERPINB11,SETDB2,ALOX5,DNAJB6,SP7,CD44,LARP4,ADAM8,SLC5A3,CLDN4,RPS6KA5,ZNF484,ZNF93,CSNK1A1,HUS1,PRDM2,NOS1,ZNF44,GCKR,MED15,RGS14,ACTN4,MC1R,STK4,TCF25,DRAM1,ZNF282,EPHB2,SLC1A1,EDRF1,BCO2,IL18,MFSD8,UBE2K,DIS3,DLC1,PPARA,PPP1R10,ARID5B,SLC25A33,SMARCAL1,CPEB4,MOB3B,NOTCH4,ZMYND8,ZNF366,CRX,PAX6,PRKCZ,ZC3HAV1,FAM20C,ADORA2A,GRIN2B,RABGEF1,CCNG2,CPEB1,OTUD7B,NIPBL,YEATS4,COL28A1,PHF20,ZNF143,ANGPT1,BRF1,TBL1X,EYA3,FHL2,LRRK1,MBTD1,OSBPL8,ATF6,ZBTB5,ZNF708,IGF2BP3,ETS1,PPP1CA,MTF2,TAGLN3,CDK5RAP1,CNTN1,NCOR2,PRKCQ,BRAF,CSRNP1,HDAC2,HTR2C,IMPACT,PHKG2,SRCAP,POU3F3,NOTO,PPP1R14A,SPINT2,MBNL3,ZKSCAN5,CD300A,ELF2,PHIP,PPP2CA,SKP1,ANGPT4,RPL23,SH3BP4,RFC5,ATXN1,SP100,ZNF347,ZNF415,ANXA2,TRIM29,PARN,ATP6V0A1,DONSON,ADCY1,MADD,NUMB,RBM42,SYK,CNOT1,RBM39,WWTR1,GTF2H5,NFXL1,ZBTB38,BRCA2,ATF7,DVL2,MORC1,MTRF1,YBX1,ANKRD13A,PTPRJ,TAF1,EIF4E3,TICAM1,TANK,THAP3,HSF1,MAX,SAP130,DHFR,EZH1,ETF1,NRF1,SRPK2,SASH1,NCOA2,WDR18,EEFSEC,QRICH1,VAV3,ZP3,CHD6,RNF2,ZNF554,MOB3A,GNA12,SH2D3A,AKTIP,MYEF2,INPP5F,AXL,TRIM37,CD109,MET,TBC1D10A,ZNF461,CAMK2D,TIMELESS,UCHL5,BRD9,MALT1,SERPINE3,SETD3,TRA2B,KDM2B,MLXIPL,CCT3,PHB,PTPRC,EDA,ZNF555,CTCF,SH3GL2,TYRO3,NR6A1,TRAF3IP2,ATF6B,CREB1,RGMB,TNXB,TRAP1,EBF4,ZNF511,BMP4,ABLIM3
GO:0016310	phosphorylation	7.690378350133354e-21	ENPP1,LDB2,NRXN1,PRKCI,SLCO3A1,MAP4K4,PDE4D,S1PR2,ADCY8,PDE8A,IL31RA,RPS6KA2,GK5,NRG3,LATS2,KSR2,PRKAG2,PRLR,MVP,KALRN,NTRK3,CBL,ARNT,FLT3,ENPP2,GRM5,PLCE1,FER,CASK,MAPK4,MAP2K5,KITLG,MAPK10,PIK3CD,CAMK1G,ADPGK,ROBO1,TOM1L1,HTR2B,CDKL5,TENM1,NADK2,TRAF6,ITPKB,DNAJA3,VAV2,GRK5,ABI1,ULK4,HSP90AA1,CDC6,TSSK1B,KNDC1,DSCAM,STK38L,TRIO,AKAP13,DAB1,ERC1,DGKI,MAST4,PTPN11,HDAC6,ADORA1,EPHA1,IKBKB,ERBB4,MRE11A,LIMK1,TLK1,ABR,PMEPA1,PTPRO,CDON,NTRK2,EP300,PDGFB,TNIK,CCND3,STK39,INSR,PRKAR1A,PIP4K2A,CAB39,PAK1,MAP3K4,PPIP5K1,BDNF,CDK14,LCK,ENTPD5,ECT2,MYO3B,SNX6,ERN2,DMD,SLC8A1,PRPS2,RIT2,CASS4,FYN,NF1,LMTK2,DGKH,BCR,CHI3L1,TTN,BMP2K,PAQR3,PIK3R2,NRG1,SH2D3C,CDK19,BDKRB1,BDKRB2,MAP2K1,FNIP1,CSNK2A3,PPIP5K2,CDKL2,LRGUK,ROR2,PTPRT,AK2,IPO5,MAD2L2,GALK2,CAPRIN2,CCNYL1,NME7,AK5,CERKL,GNAQ,ICK,HERC5,JAK2,FBXW7,ZFYVE28,SMG1,HUNK,IDNK,CDC42BPB,PHKA1,LAMTOR3,WNT11,NOX4,CAMK2B,PRKCD,TAB2,ACVR2A,CD4,PPM1E,TGFB1,SGK1,MYO3A,NSD1,PIBF1,AAK1,SLIT2,CORO1C,TTBK2,SMAD6,ZNF675,BMPER,DCLK1,ANKRD54,DAPK2,TNFRSF10B,DUSP22,TRRAP,DOCK3,FGF10,ADK,SMYD3,MAST2,EPHA4,PRKCA,CD6,TNFSF11,CAMK4,PIP5KL1,PARK2,IGF1R,PPARG,AXIN1,PRKAR1B,CDK11A,DYRK4,CDK11B,LEPR,FGF1,FOXK2,PRKAR2A,RIOK2,MYOCD,AJUBA,CHEK2,SPDYA,HCK,TBCK,CSPG4,BAZ1B,OBSCN,NF2,FLT4,SGMS1,HDAC4,SPTBN1,SFRP1,DGKB,ABI2,OGDHL,SLC4A4,TAB1,UCK2,APP,PDGFRA,SLK,CCNI2,HEG1,CDK3,BORA,IBTK,SBK3,JAK1,FPGT-TNNI3K,TNNI3K,LRP5,MTCP1,SPTBN4,DRD1,CHUK,S100A12,PRKD1,DGKG,EREG,CCNY,MAPKAPK3,ATF2,RAF1,PTPN1,BMPR2,CAMK1D,BMPR1A,BTBD10,PIK3R3,CDK12,SPAG9,FGF2,PPM1F,NEDD9,PEAK1,SEMA4D,PI4KA,PIK3C2B,PRKCG

			,DVL3,EIF4G1,EPHB3,CKS1B,DEPTOR,PKN3,TSG101,TERF2IP,PTPN13,CMPK1,TRPC5,TNKS,TPK1,DAB2,BLM,CACUL1,LDLRAD4,DLG3,WNK1,RELN,NEK10,IQGAP1,MAP3K7,ALK,SLC8A2,TOP1,XDH,PRKG1,ATP2B4,PACSIN1,PAK3,ADNP,MAPKAPK2,CDC42BPA,MARK1,CDK6,TFRC,UVRAG,EPHA5,RBKS,ADRBK1,SHPK,GHR,MNAT1,SNX9,TRIM24,PLCG2,ROCK1,HIPK3,PTPN2,INSRR,NTRK1,PKN2,HYKK,EPHB1,EPHA10,AXIN2,PARD3,PIFO,DGKK,EPHA7,PTPRA,ATG14,BCAR3,STK38,RBPMS,MAP3K13,RAPGEF3,PTK7,RSRC1,N4BP2,SNCA,BMPR1B,MAGI2,NADK,PRKAG1,CDKL1,ADTRP,OPRD1,HIPK1,RPTOR,BTK,MLLT1,IGF1,HTT,MAPK1,PTEN,SPRED2,BMP7,PIK3C3,LRRK2,ALDOC,UNC119,ZBTB20,LILRB4,MORC3,PKD2,EFNA5,TADA2A,ALPK3,PDGFC,PKIB,PPP2R3C,RPS6KC1,PLCL2,SIK3,BMP6,GPI,C8ORF44-SGK3,RAPGEF2,VRK3,MARK4,PRKAR2B,BPGM,RHOA,ROR1,RQCD1,TF,CKMT1B,SH3KBP1,EPM2A,GSK3B,DNAJC3,EEF2K,PTPRB,CDK13,STK32B,AGT,CCNJL,PRKAA2,ADAR,STAT2,ZMPSTE24,FGGY,PTK2,TEC,MAP3K5,PRKD3,LMTK3,XYLB,CHKA,NCF1,TAOK2,SHC1,ALDOA,RPS6KB1,GUCY2F,CD44,ADAM8,RPS6KA5,CSNK1A1,HUS1,NOS1,GCKR,RGS14,STK4,MARK3,EPHB2,SLC1A1,IL18,UBE2K,PPARA,MOB3B,PAX6,PRKCZ,FAM20C,ADORA2A,RABGEF1,CCNG2,TYK2,ANGPT1,LRRK1,OSBPL8,CDK5RAP1,CNTN1,PRKCQ,BRAF,HDAC2,IMPACT,PHKG2,PPP1R14A,CD300A,PHIP,CDKL3,PPP2CA,ANGPT4,MADD,SYK,WWTR1,GTF2H5,CIT,DVL2,PTPRJ,TAF1,PFKFB4,HSF1,SRPK2,SASH1,VAV3,MOB3A,SH2D3A,AKTIP,INPP5F,NUAK2,AXL,CD109,MET,CAMK2D,MALT1,PFKP,MLXIPL,PHKB,PHB,PTPRC,SH3GL2,TYRO3,CREB1,TNXB,EDNRA,BMP4
GO:0007155	cell adhesion	9.369331095705121e-21	PRDX2,NRXN1,B4GALNT2,GP6,MAP4K4,HLX,SLC9A1,NEGR1,CBFB,CLASP2,LSAMP,GRID2,TENM3,DOCK1,CLDN18,ASTN2,TLN2,MYOT,NLGN4X,DCHS2,TJP1,PRLR,UTRN,PCDH9,ATRNL1,SEMA5A,STAT5B,FER,CASK,MAP2K5,KITLG,EZR,MEGF10,NRXN3,ROBO1,ITGB6,TENM1,TRAF6,ROBO2,NFASC,ITPKB,CTNNA3,DNAJA3,ITGB1,TRIOBP,ELMO2,PCDH17,KIF26B,DSCAM,DLG2,PTPRK,RUNX1,DAB1,MYO10,PDZD2,CDH10,ITGB3BP,LAMA2,COL14A1,PTPN11,CYTH3,EPHA1,CDH12,PTPRO,CDON,MEGF9,TENM2,CNTNAP3B,PDGFB,KIRREL3,CDH8,PRKAR1A,UNC5D,LAMA3,CDH4,PTPRD,CNTN5,STRC,ATRN,AMIGO1,CLDN12,PCDH7,TENM4,ARHGAP6,LCK,LAMC1,PTPRU,DMD,LRRN2,CASS4,FYN,ADAMTS9,NF1,DST,PCDH15,FAT3,RTN4,APOD,BCR,CCDC141,ADAMTSL1,NTN1,EGFLAM,NRG1,DOCK8,MYH9,MAD1L1,LRFN5,CNTN4,NTNG1,PARD3B,CDH13,SOX13,PTPRT,ENTPD1,PCDHB16,MAD2L2,CD99,CLDN16,RCC2,BOC,COL12A1,JAK2,MYPN,SKAP1,CLSTN2,ITCH,CLDN1,MAGI1,CYFIP2,APBB1IP,CLCA2,ASTN1,MKLN1,IL1RAPL1,ADAM23,PRKCD,CD4,TGFB1,CLDN10,BCAS3,CELSR1,SDK2,CDH23,CORO1C,CTNND2,DISC1,KANK1,LAMC2,OPCML,SMAD6,PARVA,ADAM12,DUSP22,OTOA,CNTNAP2,LOXL3,VMP1,ZAN,EPHA4,HSD17B12,PRKCA,CD6,TNFSF11,PPP3CA,MAG,CTNNB1,FCGR2B,CDHR2,CADM3,DLG5,ADD2,ECM2,FBLIM1,NR4A3,AJUBA,PPP1CB,HCK,SORBS1,OBSCN,NF2,SFRP1,FOXO3,CNTN6,CDH26,SMAD3,PTPRM,NRP2,PREX1,HOXD3,PRTG,APP,PDGFRA,SLK,NLGN2,JAK1,SOX2,GPC6,DEFB118,PLEKHA7,SSPN,MMP2,RND3,CNTNAP3,DSCAML1,ADAMTS12,NLGN1,COL16A1,CTNNA2,MYB,LRRC4C,PPM1F,NEDD9,PEAK1,SEMA4D,PLXNA2,PTAFR,FNDC3A,ITGA11,SPOCK1,LMO7,NTM,CHRD,EPHB3,MUC16,PAWR,VCL,LAMB1,P4HB,NLGN3,COL4A3,GLI2,NEO1,LPP,COL19A1,DAB2,PKHD1,USH2A,DLG3,WNK1,RELN,CSF3R,PELI1,CFDP1,COL6A5,MACF1,CLASP1,DSC2,GPM6B,PRKG1,COL4A6,CDK6,TFRC,FLRT2,ROCK1,NID1,C1QTNF1,AP3B1,PTPN2,MYBPC2,PKN2,ANK3,EPHB1,EPCAM,PARD3,MUC1,JAG2,AP3D1,VWF,ADAM19,CD84,CNTNAP5,HMCN1,SPI1,EPHA7,CUZD1,PTPRA,CTNNA1,SDK1,PTK7,RC3H1,COL13A1,WNT7B,PTPRS,ADTRP,ABCA12,CSRP1,RASA1,PKP2,SIGLEC9,PCDH11X,DSG1,IGF1,CADM2,PTEN,CLDN11,BMP7,MEGF11,LILRB4,ITGAM,EFNA5,NT5E,TBCD,EPDR1,BMP6,EXT1,PDCD1LG2,PARVB,CDH9,PAG1,TFE3,RHOA,ONECUT2,HSPD1,NFKBID,GSK3B,EMB,RDX,FAM49B,CD27,DUSP26,ITGBL1,CDH20,PTK2,FREM3,ARVCF,VEZT,TAOK2,SHC1,CD160,PGM5,ITGAL,ALOX5,DNAJB6,CD44,ADAM8,CLDN4,PKD1L1,ACTN4,STK4,EPHB2,TMEFF2,IL18,DLC1,PPARA,EPB41L4B,NOTCH4,FBN1,PRKCZ,CD96,ADORA2A,SPECC1L,PHLDB2,CNTN3,TMIGD2,COL28A1,PCDH10,THSD1,ANGPT1,GCNT2,ETS1,PPP1CA,VWC2,KIFC3,CNTN1,PRKCQ,BRAF,SPINT2,TNR,CD300A,PPP2CA,ACAN,CAMSAP3,ANXA2,FAT2,TRIM29,SRGAP2,SYK,FRMD5,RADIL,PTPRJ,ITGAE,CADM1,FCHO1,VAV3,ZP3,AXL,TNFSF9,CCR3,VSIG10,MALT1,PTPRC,EDA,TYRO3,SPON2,NCAM1,RGMB,TNXB,STXBP6,BMP4
GO:0022610	biological adhesion	1.2886155620487679e-20	PRDX2,NRXN1,B4GALNT2,GP6,MAP4K4,HLX,SLC9A1,NEGR1,CBFB,CLASP2,LSAMP,GRID2,TENM3,DOCK1,CLDN18,ASTN2,TLN2,MYOT,NLGN4X,DCHS2,TJP1,PRLR,UTRN,PCDH9,ATRNL1,SEMA5A,STAT5B,FER,CASK,MAP2K5,KITLG,EZR,MEGF10,NRXN3,ROBO1,ITGB6,TENM1,TRAF6,ROBO2,NFASC,ITPKB,CTNNA3,DNAJA3,ITGB1,TRIOBP,ELMO2,PCDH17,KIF26B,DSCAM,DLG2,PTPRK,RUNX1,DAB1,MYO10,PDZD2,CDH10,ITGB3BP,LAMA2,COL14A1,PTPN11,CYTH3,EPHA1,CDH12,PTPRO,CDON,MEGF9,TENM2,CNTNAP3B,PDGFB,KIRREL3,CDH8,PRKAR1A,UNC5D,LAMA3,CDH4,PTPRD,CNTN5,STRC,ATRN,AMIGO1,CLDN12,PCDH7,TENM4,ARHGAP6,LCK,LAMC1,PTPRU,DMD,LRRN2,CASS4,FYN,ADAMTS9,NF1,DST,PCDH15,FAT3,RTN4,APOD,BCR,CCDC141,ADAMTSL1,NTN1,EGFLAM,NRG1,DOCK8,MYH9,MAD1L1,LRFN5,CNTN4,NTNG1,PARD3B,CDH13,SOX13,PTPRT,ENTPD1,PCDHB16,MAD2L2,CD99,CLDN16,RCC2,BOC,COL12A1,JAK2,MYPN,SKAP1,CLSTN2,ITCH,CLDN1,MAGI1,CYFIP2,APBB1IP,CLCA2,ASTN1,MKLN1,IL1RAPL1,ADAM2

			3,PRKCD,CD4,TGFB1,CLDN10,BCAS3,CELSR1,SDK2,CDH23,CORO1C,CTNND2,DISC1,KANK1,LAMC2,OPCML,SMAD6,PARVA,ADAM12,DUSP22,OTOA,CNTNAP2,LOXL3,VMP1,ZAN,EPHA4,HSD17B12,PRKCA,CD6,TNFSF11,PPP3CA,MAG,CTNNB1,FCGR2B,CDHR2,CADM3,DLG5,ADD2,ECM2,FBLIM1,NR4A3,AJUBA,PPP1CB,HCK,SORBS1,OBSCN,NF2,SFRP1,FOXO3,CNTN6,CDH26,SMAD3,PTPRM,NRP2,PREX1,HOXD3,PRTG,APP,PDGFRA,SLK,NLGN2,JAK1,SOX2,GPC6,DEFB118,PLEKHA7,SSPN,MMP2,RND3,CNTNAP3,DSCAML1,ADAMTS12,NLGN1,COL16A1,CTNNA2,MYB,LRRC4C,PPM1F,NEDD9,PEAK1,SEMA4D,PLXNA2,PTAFR,FNDC3A,ITGA11,SPOCK1,LMO7,NTM,CHRD,EPHB3,MUC16,PAWR,VCL,LAMB1,P4HB,NLGN3,COL4A3,GLI2,NEO1,LPP,COL19A1,DAB2,PKHD1,USH2A,DLG3,WNK1,RELN,CSF3R,PELI1,CFDP1,COL6A5,MACF1,CLASP1,DSC2,GPM6B,PRKG1,COL4A6,SCARB1,CDK6,TFRC,FLRT2,ROCK1,NID1,C1QTNF1,AP3B1,PTPN2,MYBPC2,PKN2,ANK3,EPHB1,EPCAM,PARD3,MUC1,JAG2,AP3D1,VWF,ADAM19,CD84,CNTNAP5,HMCN1,SPI1,EPHA7,CUZD1,PTPRA,CTNNA1,SDK1,PTK7,RC3H1,COL13A1,WNT7B,PTPRS,ADTRP,ABCA12,CSRP1,RASA1,PKP2,SIGLEC9,PCDH11X,DSG1,IGF1,CADM2,PTEN,CLDN11,BMP7,MEGF11,LILRB4,ITGAM,EFNA5,NT5E,TBCD,EPDR1,BMP6,EXT1,PDCD1LG2,PARVB,CDH9,PAG1,TFE3,RHOA,ONECUT2,HSPD1,NFKBID,GSK3B,EMB,RDX,FAM49B,CD27,DUSP26,ITGBL1,CDH20,PTK2,FREM3,ARVCF,VEZT,TAOK2,SHC1,CD160,PGM5,ITGAL,ALOX5,DNAJB6,CD44,ADAM8,CLDN4,PKD1L1,ACTN4,STK4,EPHB2,TMEFF2,IL18,DLC1,PPARA,EPB41L4B,NOTCH4,FBN1,PRKCZ,CD96,ADORA2A,SPECC1L,PHLDB2,CNTN3,TMIGD2,COL28A1,PCDH10,THSD1,ANGPT1,GCNT2,ETS1,PPP1CA,VWC2,KIFC3,CNTN1,PRKCQ,BRAF,SPINT2,TNR,CD300A,PPP2CA,ACAN,CAMSAP3,ANXA2,FAT2,TRIM29,SRGAP2,SYK,FRMD5,RADIL,PTPRJ,ITGAE,CADM1,FCHO1,VAV3,ZP3,AXL,TNFSF9,CCR3,VSIG10,MALT1,PTPRC,EDA,TYRO3,SPON2,NCAM1,RGMB,TNXB,STXBP6,BMP4
GO:0009893	positive regulation of metabolic process	1.4123181505804303e-20	*POLDIP3,LDB2,NRXN1,ASPH,SLCO3A1,PDE4D,DNMT1,S1PR2,SLC9A1,C6ORF89,PBX3,ADCY8,CBFB,PDE8A,IL31RA,RPS6KA2,PRDM12,SETD4,PTGFR,MED26,WWC1,NRG3,ASH1L,NOS1AP,NOX5,PTH2R,SCAF8,STOX2,KSR2,PRKAG2,PRLR,PAGR1,HIVEP3,FTO,NPAS3,NTRK3,ARNT,EGLN2,FLT3,STAT5B,TOX,ENPP2,GRM5,CASK,MAP2K5,KITLG,LRP2,PIK3CD,SP3,HDGF,ANK2,OLFM1,EZR,ROBO1,TOM1L1,CHD7,HTR2B,MECOM,TACC1,TENM1,LMNA,TRAF6,DNAJA3,VAV2,ABI1,NHLH1,HSP90AA1,SLC24A3,CDC6,KNDC1,CCDC22,DSCAM,CNGB1,AKAP13,GFI1B,RUNX1,DAB1,OMA1,ABCC8,HMGN3,NRIP1,THRB,EFCAB7,PTPN11,CCT2,HDAC6,SERTAD2,DDB1,ADORA1,EPHA1,IKBKB,ERBB4,MRE11A,ZNF609,BRD8,KAT6B,ACTG2,CUL4B,ESR1,MAML3,CDON,NTRK2,TNRC6A,EP300,CELA1,ZYG11B,ZNF76,FNTA,PDGFB,RIMS1,TNIK,CCND3,BRMS1,CHFR,HNF4G,INSR,FMN2,RERE,ASB5,FUBP1,TCF7L2,PIP4K2A,CAB39,PAK1,LITAF,FBXW11,ESRRB,MYOM1,MAP3K4,RNF144A,EGLN3,RBM20,TBR1,SAMD4A,BDNF,TFAP2A,PEG3,MEIS1,TRIM13,LCK,ENTPD5,ECT2,ZNF148,MTA3,TFDP2,RFTN1,ERN2,ATG10,SLC30A9,TOX3,LARP4B,USP13,KAT7,GSN,RBM14,MED12L,SATB2,PAFAH1B2,RIT2,CASS4,FYN,MKRN2,ARNTL,PLCB1,MGMT,LMTK2,ARID4B,RTN4,RWDD3,BRPF1,CHI3L1,TTN,PIK3R2,RANBP9,NRG1,SH2D3C,UBP1,BID,MAP2K1,FNIP1,MYH9,VGLL4,PPP3R1,HDAC5,CSRNP3,NFIA,RNF4,CSNK2A3,JDP2,CMKLR1,ROR2,DCN,PCBD2,HNRNPLL,CDH13,CREBRF,COPS5,MAD2L2,RAB27A,CAPRIN2,CCNYL1,TERF2,SLX1B,NEUROD1,CCBE1,USP22,JAK2,FAM168A,FBXW7,OAZ2,SKAP1,UIMC1,ITCH,MLIP,BCL11B,PKNOX1,MLLT3,PDE2A,KLF15,LAMTOR3,CYFIP2,WNT11,MTA1,NOX4,RPRD1B,QKI,CCDC62,ERCC8,PRKCD,TAB2,ACVR2A,RUNX2,CD4,TGFB1,BANP,SPSB4,TRIM65,NSD1,PIBF1,BTRC,NFATC3,CRADD,F2RL1,BCAS3,DNAJB2,TP73,ILF2,DISC1,BLID,ZFAND2A,CLN6,MTDH,FANK1,SMAD6,ZNF398,CLOCK,TCF12,ETV6,SUCO,TFAP2D,BCL3,TNFRSF10B,HNRNPC,DOCK3,SBNO2,YTHDF1,FGF10,CIZ1,SMYD3,FANCI,CAPN3,LUM,SMURF2,EPHA4,RORA,AUTS2,CD6,TNFSF11,PPP3CA,NFYB,KLF12,CAMK4,UFL1,CTNNB1,PARK2,SOD2,SMARCC1,IGF1R,PPARG,NGRN,AXIN1,IL18R1,IL1RL1,MTF1,CYBB,SCAMP5,SREBF2,LEPR,FGF1,NPAT,NR4A3,FOXK2,RIOK2,MYOCD,TRIM5,PER2,KIR2DL4,AJUBA,CHEK2,SUPT3H,PRDM16,SPDYA,SORBS1,RAB3GAP2,CAPN2,TRIM8,DIO2,CSPG4,FLT4,MEF2B,HDAC4,PAX2,PHF5A,SPTBN1,TRABD2B,SFRP1,MED13,ZNF395,FOXO3,NFIB,BCL2L13,SYNCRIP,SMAD3,RNFT2,CUX2,WWP2,ARNT2,WIPI1,EBF3,RNF168,CASZ1,IFNAR1,DCP1B,NEDD4,ESRRG,HOXD3,HOXD4,SLC4A4,TAB1,NFATC1,CDC73,APP,SSBP3,PDGFRA,DIP2B,NOX1,ARIH1,YAP1,HEG1,SESN1,VDAC1,EYA2,SH3D19,BORA,NVL,ALPL,PEMT,LRP5,MTCP1,POLR3G,SOX2,SETD2,TEAD1,PRICKLE1,ZNF521,DRD1,ARID3A,CHUK,ESR2,S100A12,DCUN1D3,KDM6A,PRKD1,STAT1,ST18,ETV5,RHOXF2B,PLAGL1,HNF4A,ZBTB7C,TASP1,EREG,CCNY,ATF2,POU2F2,TCF3,GRAMD4,RAF1,CELF4,CASP1,PTPN1,BMPR2,CAMK1D,BMPR1A,BTBD10,IKZF4,PIK3R3,CDK12,CAND2,MYB,FGF2,ZNF71,BACH1,PPM1F,NEDD9,AGBL4,SEMA4D,RORC,PTAFR,ADCYAP1R1,RHOXF2,JARID2,DDX58,BRDT,RARB,PRKCG,NCOA1,AREL1,EHMT1,LMO7,AP2M1,DVL3,EIF2B5,EIF4G1,EPHB3,RBX1,TCF20,ATF3,LIN28B,SRSF5,CKS1B,PAWR,EBF2,AGO3,MAML2,TSG101,CCDC3,TERF2IP,IDE,RFX2,WNT3,PRDM15,COL4A3,DDAH1,HIP1,TRPC5,UBA2,GLI2,TNKS,WBP2NL,ERCC1,TNRC6B,GLIS3,DAB2,BLM,CACUL1,MYSM1,DLG3,WNK1,RELN,NEK10,SIN3A,RUVBL2,GMEB1,PELI1,IQGAP1,MAP3K7,ZNF423,SP1,TRIM22,ALK,SLC8A2,TEAD4,HOXC13,SLC35A4,SRA1,UBE2V1,TPCN1,ADIRF,XDH,OVOL2,SNX5,NFATC2,BNC1,ATP2B4,NSF,ACTL6B,ASXL3,PAK3,TET1,CAMTA1,CCPG1,ATAD1,ADNP,MAPKAPK2,ARID4A,SCARB1,MARK1,CDK6,PHF2,CELF1,TFRC,UVRAG,EPHA5,WWOX,MEF2A,DCAF*

			6,DCUN1D5,DROSHA,GPC3,TBC1D5,EYA1,SCOC,DIS3L2,TRPV1,HOXB3,HOXB4,HOXB5,TFEB,GHR,MNAT1,SNX9,ACTR2,ELAVL4,HDAC1,SMOC2,TRIM24,ADIPOR2,PLCG2,ROCK1,C1QTNF1,PAXIP1,EGR2,RNF10,RYBP,AP3B1,PTPN2,INSRR,NTRK1,KPNA6,TRIM44,SLC39A10,ANK3,EPHB1,EPHA10,AXIN2,EPCAM,MUC1,AP3D1,CARM1,ADAM19,SNX33,PIFO,CD84,ZNF836,SMARCA2,SPI1,DNMT3B,EPHA7,GLIS1,ATG14,BCAR3,ABCB7,RBPMS,CHURC1,CREB5,MAP3K13,RAPGEF3,MST1,SNCA,BMPR1B,MAGI2,PRIM2,PNPT1,USP50,ZEB2,DHRS4,FOXJ3,HEY2,RC3H1,EIF3E,RNF19B,KMT2D,PRKAG1,CREM,KMT2C,ADTRP,PLD1,MACC1,MITF,OPRD1,RPTOR,NFIX,KCTD13,IGF1,MLXIP,ATF7IP,HTT,MAPK1,PTEN,BMP7,PUM1,PCBP2,ZNF780B,LRRK2,MLYCD,TMEM59,UNC119,ZBTB20,RFC3,BAG6,ITGAM,PKD2,EFNA5,TADA2A,DAZL,PDGFC,NCOA3,PKIB,PPP2R3C,WNT7A,NLRP1,UACA,LPGAT1,CRTC3,ARNTL2,ELOVL5,KAT6A,ZNF197,BMP6,TAF15,MYCBP2,GPI,SH3RF2,TSHR,WDR43,ATRX,PRMT2,RAPGEF2,SMO,VRK3,CPT1A,TCF4,DECR1,SOX30,TFE3,UBR5,GRIN1,JADE1,ARRDC4,RHOA,SPRTN,ROR1,RQCD1,TF,ONECUT2,CUL3,TFEC,HSPD1,EPM2A,GSK3B,PRR16,DNAJC3,RDX,STAT6,ACTA2,FAM49B,CDK13,BOK,RNF144B,SULF1,SUPT4H1,TNFRSF8,AGT,METTL16,ELOVL3,PRKAA2,ADAR,ZMPSTE24,PTK2,PDE4B,PBX1,TEC,EXOSC3,MAP3K5,SEC16B,CREBBP,DLX1,NCF1,TAOK2,SHC1,CD160,ECE1,RNFT1,RPS6KB1,PRG3,SP7,CD44,LARP4,ADAM8,SLC5A3,CLDN4,RPS6KA5,ZNF484,CSNK1A1,PRDM2,CXCL17,NOS1,ACTN4,MC1R,STK4,EPHB2,SLC1A1,EDRF1,IL18,UBE2K,DIS3,DLC1,PPARA,PPP1R10,ARID5B,EPB41L4B,GPSM3,MOB3B,NOTCH4,CRX,PAX6,PRKCZ,ZC3HAV1,GRIN2B,CPEB1,NIPBL,TMIGD2,YEATS4,ZNF143,ANGPT1,BRF1,TBL1X,EYA3,LRRK1,OSBPL8,ATF6,ETS1,MTF2,RBMS3,BTN3A2,CDK5RAP1,CNTN1,NCOR2,PRKCQ,BRAF,CSRNP1,HDAC2,HTR2C,IMPACT,PHKG2,SRCAP,POU3F3,CD300A,ELF2,PHIP,PPP2CA,SKP1,ANGPT4,RPL23,SH3BP4,RFC5,SP100,ANXA2,PARN,MADD,SYK,CNOT1,WWTR1,ZBTB38,BRCA2,ATF7,DVL2,YBX1,PTPRJ,TAF1,TICAM1,TANK,THAP3,CADM1,HSF1,MAX,EZH1,NRF1,SRPK2,SASH1,NCOA2,GBP5,QRICH1,VAV3,ZP3,CHD6,MOB3A,GNA12,SH2D3A,AKTIP,INPP5F,AXL,TRIM37,MET,TBC1D10A,SNX1,TIMELESS,MALT1,SETD3,TRA2B,MLXIPL,CCT3,NEDD4L,PHB,PTPRC,EDA,CTCF,TYRO3,NR6A1,SPON2,TRAF3IP2,ATF6B,CREB1,RGMB,CLNK,BMP4,ABLIM3
GO:0061564	axon development	1.7426114904699122e-20	*NRXN1,CLASP2,SEMA3A,SEMA3D,MYOT,NREP,TIAM2,UNC5C,KALRN,SEMA5A,OLFM1,NRXN3,ROBO1,CDKL5,KIF5C,ROBO2,NFASC,ITGB1,HSP90AA1,DSCAM,TRIO,DAB1,LAMA2,PTPN11,HDAC6,GOLGA4,EPHA1,LIMK1,ATP8A2,GRM7,PTPRO,NTRK2,UNC5D,LAMA3,CDH4,PAK1,TBR1,BDNF,AMIGO1,FYN,LMTK2,RTN4,APOD,B4GALT6,RTN4R,CCDC141,ADAMTSL1,NTN1,MAP2K1,VAX2,CNTN4,NTNG1,UST,BOC,JAK2,MYPN,BCL11B,KREMEN1,SIAH1,SEMA5B,KEL,SLIT3,SLIT2,DISC1,LAMC2,DCLK1,YTHDF1,ENAH,EPHA4,AUTS2,CNR1,MAG,IGF1R,XK,DRAXIN,NIN,DCC,PAX2,SPG11,CNTN6,NFIB,PTPRM,NRP2,CRTAC1,PRTG,APP,DIP2B,FSTL4,ISLR2,SPTBN4,VASH2,MMP2,DSCAML1,SEMA6D,BMPR2,USP33,DPYSL2,CTNNA2,RAB11A,CRMP1,LRRC4C,SEMA4D,PLXNA2,EPHB3,FBXO45,VCL,WNT3,NLGN3,PAFAH1B1,TRPC5,GLI2,NEO1,RUFY3,RELN,SIN3A,MACF1,PAK3,ADNP,MAP1B,EPHA5,FLRT2,EGR2,NTRK1,MAP1A,ANK3,EPHB1,EPHA10,PARD3,TRIM46,EPHA7,CTNNA1,MAP3K13,PTK7,BMPR1B,PTPRS,RTN4RL1,PTEN,BMP7,RAPH1,EFNA5,WNT7A,MYCBP2,SOS1,EXT1,SMO,SYNGAP1,GSK3B,EMB,PTK2,GAP43,TAOK2,ECE1,RPS6KA5,TUBB3,EPHB2,PAX6,MAP2,PRKCQ,BRAF,TNR,CDKL3,TMEFF1,ADCY1,NUMB,SLIT1,DHFR,INPP5F,SEMA3C,NCAM1,CREB1,EDNRA*
GO:0040011	locomotion	2.348938988688033e-20	*CLRN1,DNAH11,LDB2,GPC5,NRXN1,PRKCI,MAP4K4,S1PR2,SLC9A1,SPAG16,CLASP2,SEMA3A,DOCK1,WWC1,NRG3,ASH1L,IQCG,ASTN2,SEMA3D,PHACTR1,MYOT,MAPRE2,WWC3,TJP1,UNC5C,KALRN,NTRK3,ATRNL1,SEMA5A,STAT5B,ENPP2,GRM5,FER,MARVELD3,MAP2K5,KITLG,PTPRR,SPNS2,PIK3CD,MEGF10,NRXN3,ROBO1,HTR2B,ITGB6,CDKL5,KIF5C,LMNA,ROBO2,NFASC,CTNNA3,VAV2,ULK4,ITGB1,ELMO2,SRGAP3,MCC,DSCAM,SRGAP2B,TRIO,ARHGAP24,PTPRK,DAB1,ABCC8,LAMA2,DACH1,PTPN11,MGAT5,DOCK10,HDAC6,ADORA1,EPHA1,ERBB4,GBF1,DNAH2,ZNF609,PTPRO,NTRK2,EP300,MEGF9,PDGFB,AVL9,KIRREL3,STK39,INSR,RERE,UNC5D,LAMA3,CDH4,PAK1,TBR1,CATSPER2,BDNF,ATRN,POMGNT2,FUT8,FMNL2,PHACTR4,LCK,LAMC1,PTPRU,LSP1,SLC8A1,SATB2,DEPDC1B,CASS4,FYN,ADAMTS9,NF1,PLCB1,DST,FAT3,RTN4,DPEP1,APOD,BCR,CCDC141,NDRG4,ADAMTSL1,NTN1,NRG1,BDKRB1,DOCK8,MAP2K1,MYH9,CNTN4,HDAC5,NTNG1,NAV1,CMKLR1,ROR2,DCN,CATSPER3,DOCK2,CDH13,PTPRT,CCNYL1,NCKAP1,DEFA1B,CD99,RCC2,BOC,ANO6,KIF2A,RFFL,TTLL8,CCBE1,JAK2,MYPN,FBXW7,BCL11B,DNAH14,CLDN1,CDC42BPB,WNT11,ASTN1,SIAH1,CAMK2B,PRKCD,SEMA5B,TGFB1,SGK1,MMP28,JPH3,F2RL1,BCAS3,CELSR1,SLIT3,SLIT2,CORO1C,RRAS2,DISC1,KANK1,LAMC2,CLN6,TTBK2,CHRM1,BMPER,DCLK1,DAPK2,PARVA,DUSP22,YTHDF1,FGF10,ENAH,SMURF2,EPHA4,PRKCA,AUTS2,TNFSF11,PPP3CA,PIP5KL1,CTNNB1,SOD2,IGF1R,PPARG,DLG5,ADD2,DRAXIN,FGF1,NR4A3,MYOCD,AJUBA,HCK,CSPG4,DCC,NF2,FLT4,HDAC4,TPTE,SFRP1,FOXO3,ARL13B,CNTN6,NFIB,SMAD3,PTPRM,ACKR2,ABI2,NRP2,PREX1,DOCK4,NAV3,PRTG,APP,GSX2,PDGFRA,SLK,CEP85L,NOX1,NLGN2,LRP5,PTPRG,SLC9B2,SETD2,GPC6,RCAN1,DRD1,MMP2,SYNE2,S100A12,PRKD1,RND3,DSCAML1,SEMA6D,RAF1,ADAMTS12,BMPR2,USP33,DPYSL2,CAMK1D,BMPR1A,CTNNA2,PIK3R3,SPAG9,RAB11A,CRMP1,FGF2,PPM1F,NEDD9,PEAK1,SEMA4D,ARMC2,MCTP1,ELP3,PLXNA2,PTAFR,DDX58,ITGA11,PIK3C2B,SLC9B1,SPOCK1,GAS8,CHRD,EPHB3,FBXO45,PKN3,VCL,LAMB1,CLIC4,WNT3,RHOJ,CCL14,CCL15,CCL15-*

			CCL14,PAFAH1B1,GLI2,NEO1,RUFY3,DAB2,USH2A,LDLRAD4,MYSM1,DZIP1,WNK1,RELN,CSF3R,IQGAP1,SP1,MACF1,CLASP1,SLC9C1,OVOL2,NFATC2,PRKG1,TTLL9,ATP2B4,PAK3,SSH2,DYX1C1,MAP1B,CDC42BPA,SCARB1,MARK1,CDK6,EPHA5,GPC3,FLRT2,SMOC2,PLCG2,ROCK1,EPS8,PAXIP1,EGR2,ABCC1,PTPN2,NTRK1,PKN2,TMIGD1,EPHB1,EPHA10,TRIM46,TEKT1,HTR1D,SPI1,EPHA7,MCU,CTNNA1,LIMA1,SH3BP1,MST1,PTK7,SNCA,BMPR1B,MAGI2,ZEB2,ADTRP,PLD1,MITF,KCTD13,IGF1,DPYSL3,MAPK1,PTEN,BMP7,PRCP,LRRK2,OSGIN1,ZRANB1,EFNA5,SHANK3,PDGFC,AMOTL1,WNT7A,NME8,MYCBP2,GPI,SH3RF2,SOS1,TSHR,EXT1,RAPGEF2,SMO,SUN1,RHOA,NDE1,TF,ONECUT2,FAM19A4,CUL3,SH3KBP1,PLD2,EMB,RDX,ACTA2,FAM49B,ITGBL1,SULF1,AGT,PTK2,TPPP2,PDE4B,GAP43,TAOK2,ECE1,RPS6KB1,GPR173,ANKS1A,ITGAL,ALOX5,CD44,ADAM8,CLDN4,RPS6KA5,CXCL17,PEX7,PLVAP,ACTN4,STK4,TUBB3,SPEF1,EPHB2,TMEFF2,XCR1,DLC1,ARID5B,EPB41L4B,GPSM3,LDHC,ZMYND8,PAX6,PRKCZ,ADORA2A,RABGEF1,PHLDB2,KLC3,NIPBL,ANGPT1,OSBPL8,GCNT2,ETS1,BIN2,CCL22,FBXO31,PRKCQ,BRAF,DIAPH1,ATP8A1,POU3F3,SPINT2,TNR,CD300A,ANGPT4,CAMSAP3,SP100,FAT2,SRGAP2,TMEFF1,NUMB,SYK,SLIT1,FRMD5,RADIL,DNAH3,PTPRJ,GPM6A,SASH1,VAV3,ZP3,GNA12,INPP5F,AXL,MET,FMNL1,CCR3,SEMA3C,PTPRC,TYRO3,NCAM1,EDNRA,BMP4
GO:0006468	protein phosphorylation	4.853035178501259e-20	*ENPP1,NRXN1,PRKCI,SLCO3A1,MAP4K4,PDE4D,S1PR2,ADCY8,PDE8A,IL31RA,RPS6KA2,NRG3,LATS2,KSR2,PRKAG2,PRLR,MVP,KALRN,NTRK3,CBL,FLT3,ENPP2,GRM5,PLCE1,FER,CASK,MAPK4,MAP2K5,KITLG,MAPK10,PIK3CD,CAMK1G,ROBO1,TOM1L1,HTR2B,CDKL5,TENM1,TRAF6,ITPKB,DNAJA3,GRK5,ABI1,ULK4,HSP90AA1,CDC6,TSSK1B,KNDC1,STK38L,TRIO,AKAP13,DAB1,ERC1,MAST4,PTPN11,HDAC6,ADORA1,EPHA1,IKBKB,ERBB4,MRE11A,LIMK1,TLK1,ABR,PMEPA1,PTPRO,CDON,NTRK2,PDGFB,TNIK,CCND3,STK39,INSR,PRKAR1A,CAB39,PAK1,MAP3K4,BDNF,CDK14,LCK,ECT2,MYO3B,SNX6,ERN2,DMD,SLC8A1,RIT2,CASS4,FYN,NF1,LMTK2,BCR,CHI3L1,TTN,BMP2K,PAQR3,NRG1,SH2D3C,CDK19,BDKRB1,BDKRB2,MAP2K1,FNIP1,CSNK2A3,CDKL2,ROR2,PTPRT,IPO5,MAD2L2,CAPRIN2,CCNYL1,GNAQ,ICK,HERC5,JAK2,FBXW7,ZFYVE28,SMG1,HUNK,CDC42BPB,PHKA1,WNT11,NOX4,CAMK2B,PRKCD,TAB2,ACVR2A,CD4,PPM1E,TGFB1,SGK1,MYO3A,NSD1,PIBF1,AAK1,SLIT2,CORO1C,TTBK2,SMAD6,ZNF675,BMPER,DCLK1,ANKRD54,DAPK2,TNFRSF10B,DUSP22,DOCK3,FGF10,SMYD3,MAST2,EPHA4,PRKCA,CD6,TNFSF11,CAMK4,PIP5KL1,PARK2,IGF1R,PPARG,AXIN1,PRKAR1B,CDK11A,DYRK4,CDK11B,LEPR,FGF1,PRKAR2A,RIOK2,MYOCD,AJUBA,CHEK2,SPDYA,HCK,TBCK,CSPG4,BAZ1B,OBSCN,NF2,FLT4,SPTBN1,SFRP1,ABI2,TAB1,APP,PDGFRA,SLK,CCNI2,HEG1,CDK3,BORA,IBTK,SBK3,JAK1,FPGT-TNNI3K,TNNI3K,LRP5,MTCP1,SPTBN4,DRD1,CHUK,S100A12,PRKD1,EREG,CCNY,MAPKAPK3,ATF2,RAF1,PTPN1,BMPR2,CAMK1D,BMPR1A,PIK3R3,CDK12,SPAG9,FGF2,PPM1F,NEDD9,PEAK1,SEMA4D,PRKCG,DVL3,EIF4G1,EPHB3,CKS1B,DEPTOR,PKN3,TSG101,TERF2IP,PTPN13,TRPC5,TNKS,DAB2,BLM,CACUL1,LDLRAD4,DLG3,WNK1,RELN,NEK10,IQGAP1,MAP3K7,ALK,SLC8A2,TOP1,XDH,PRKG1,ATP2B4,PAK3,ADNP,MAPKAPK2,CDC42BPA,MARK1,CDK6,TFRC,UVRAG,EPHA5,ADRBK1,GHR,MNAT1,SNX9,TRIM24,PLCG2,ROCK1,HIPK3,PTPN2,INSRR,NTRK1,PKN2,EPHB1,EPHA10,AXIN2,PARD3,EPHA7,PTPRA,ATG14,BCAR3,STK38,RBPMS,MAP3K13,RAPGEF3,PTK7,RSRC1,SNCA,BMPR1B,PRKAG1,CDKL1,ADTRP,OPRD1,HIPK1,RPTOR,BTK,MLLT1,IGF1,HTT,MAPK1,PTEN,SPRED2,BMP7,PIK3C3,LRRK2,UNC119,LILRB4,MORC3,PKD2,EFNA5,TADA2A,ALPK3,PDGFC,PKIB,PPP2R3C,RPS6KC1,PLCL2,SIK3,BMP6,C8ORF44-SGK3,RAPGEF2,VRK3,MARK4,PRKAR2B,RHOA,ROR1,RQCD1,EPM2A,GSK3B,DNAJC3,EEF2K,PTPRB,CDK13,STK32B,AGT,CCNJL,PRKAA2,ADAR,STAT2,ZMPSTE24,PTK2,TEC,MAP3K5,PRKD3,LMTK3,NCF1,TAOK2,SHC1,RPS6KB1,GUCY2F,CD44,ADAM8,RPS6KA5,CSNK1A1,HUS1,NOS1,RGS14,STK4,MARK3,EPHB2,SLC1A1,IL18,UBE2K,MOB3B,PAX6,PRKCZ,FAM20C,ADORA2A,RABGEF1,CCNG2,TYK2,ANGPT1,LRRK1,OSBPL8,CDK5RAP1,CNTN1,PRKCQ,BRAF,HDAC2,IMPACT,PHKG2,CD300A,PHIP,CDKL3,PPP2CA,ANGPT4,MADD,SYK,WWTR1,GTF2H5,CIT,DVL2,PTPRJ,TAF1,HSF1,SRPK2,SASH1,MOB3A,SH2D3A,AKTIP,INPP5F,NUAK2,AXL,CD109,MET,CAMK2D,MALT1,MLXIPL,PHKB,PHB,PTPRC,SH3GL2,TYRO3,CREB1,TNXB,EDNRA,BMP4*
GO:0051641	cellular localization	5.981514951848912e-20	*CD247,POLDIP3,DNAH11,ZDHHC14,HOOK2,BLZF1,GPC5,NRXN1,ASPH,PRKCI,PDE4D,SLC9A1,FAM155A,ADCY8,CLASP2,GRID2,CHERP,WWC1,CLDN18,KIF22,CTDSPL2,RYR1,NOS1AP,LATS2,TUBA1C,NOX5,ANP32A,ASTN2,EPB41,DPP6,MAPRE2,NLGN4X,SEC23B,LTV1,SNX31,CBL,LHFPL4,FAM53A,FER,CASK,CCDC93,KCNIP4,PIK3CD,TNPO3,HDGF,ANK2,EZR,NRXN3,XRCC4,TOM1L1,CHD7,HTR2B,TENM1,RYR3,KIF5C,LMNA,SRP72,NFASC,DNAJA3,SLC24A2,ITGB1,HSP90AA1,CNIH2,PCDH17,CCDC22,FAM126A,CNGB1,KLHL12,AKAP13,DLG2,IFT43,PARD6G,PTPRK,DAB1,MYO10,ERC1,HMGN3,AFTPH,DGKI,STXBP4,ERC2,EFCAB7,TCIRG1,PTPN11,CCT2,CACNB2,HDAC6,GOLGA4,ADORA1,GPRASP1,IKBKB,PEX14,ERBB4,GBF1,TLK1,ESR1,DPP10,STX18,FNTA,HOOK3,VPS45,RIMS1,TNIK,SNX2,MID1,MSTO1,FMN2,H2AFY2,TCF7L2,PIP4K2A,FBXW11,BDNF,LCK,ECT2,SNX6,CHML,VAMP7,PTPRU,RFTN1,SHFM1,AP3S1,NPLOC4,DENND5A,DMD,CENPF,RANBP17,KAT7,SLC8A1,GSN,RBM4,RIT2,TANC2,JPH2,FYN,NF1,SMG7,DST,LMTK2,STX6,SNCB,RTN4,GOLGA2P5,APOD,VTA1,BCR,NDRG4,PAQR3,PIK3R2,KLHL21,NTN1,DCTN1,SLC4A5,NRG1,BDKRB1,BID,MAP2K1,RAPGEF6,MYH9,MAD1L1,TMBIM6,TME*

			M30A,SYNE1,PARD3B,SCFD2,ZDHHC3,SLC39A14,LIN7A,SCFD1,SYN2,DOCK2,CDH13,CREBRF,COPS5,EXOC4,IPO5,MAN1A1,TLE6,RAB27A,RAB3C,TERF2,TMED6,GOLPH3L,RCC2,ARL3,NEUROD1,IPO11,ICK,RANBP1,DENND1A,ZDHHC15,JAK2,FBXW7,KCNB2,OAZ2,SYN3,SKAP1,SYT1,CROCC,NPSR1,BBS12,SNAP25-AS1,SMG1,DYNC1I1,ATP9A,PLS1,DENND2A,SEPT7,TTC7B,LAMTOR3,KPNA3,CTAGE6,CENPC,WNT11,IFT80,IMMP2L,PRKCD,CD4,TGFB1,BANP,NUP93,PIBF1,JPH3,PTPRN2,ARMC1,F2RL1,BCAS3,CELSR1,ZFYVE9,CADPS2,CORO1C,C12ORF4,DISC1,KCNJ3,CACNA1D,ZFAND2A,GRPEL2,ANK1,CLMN,TTBK2,CHRM1,RAB11FIP4,ITSN2,SYNJ2,BCL3,DCLK1,ANKRD54,RABGAP1L,ANKFN1,SLC17A7,FGF10,CNTNAP2,CIZ1,STXBP5,CAPN3,VMP1,SNX8,CNR1,SMG6,PPP3CA,NSUN2,TRAK1,CTNNB1,PARK2,RN7SL832P,FCGR2B,TSNARE1,AXIN1,PRKAR1B,MYO1D,TRPM1,SYTL5,ANXA13,LRPPRC,SREBF2,RYR2,LEPROT,PROS1,NR4A3,WDR45B,NOL3,RIOK2,PER2,AJUBA,CACNA1C,SCN3B,MYO1E,HCK,SORBS1,TBC1D14,RAB3GAP2,TBCK,TRIM8,RIMS4,ANKFY1,OBSCN,NF2,SPTBN1,AP3B2,SPG11,ARL13B,CCDC91,IFT122,RANBP3,DNM3,SMAD3,ITPR1,RAB6A,WIPI1,MTBP,VPS39,GRIK5,PSAP,LZTFL1,NEDD4,KPNB1,VPS53,SLC6A1,RAB6C,SEC61B,THOC3,APP,BET1L,YAP1,RAB11FIP5,MYO1F,NLGN2,EYA2,SSR2,IBTK,NVL,LRP5,SEC22C,SLC9B2,SETD2,ZDHHC6,GPC6,PRICKLE1,SPTBN4,DRD1,TMEM14A,TPH1,SYNE2,PRKD1,OSBPL2,SYBU,SLC6A3,TAF3,VPS4A,PITRM1,ATF2,RAF1,PTPN1,AP1B1,NLGN1,SNX16,TAF8,SPAG9,RAB11A,EPG5,IPO9,FGF2,RAB24,ADCY5,AGBL4,MCTP1,XPO6,PTAFR,ADCYAP1R1,EEA1,ABCA13,RILPL1,SORCS2,RAB11FIP3,PRKCG,BLOC1S5,GAS8,AP2M1,TNPO1,TRAPPC11,TECPR2,LMBRD1,FBXL20,TSG101,DESI1,VCL,TERF2IP,KCNQ1,SUFU,NLGN3,PAFAH1B1,KIF3A,TRPA1,AKAP6,CEP350,PADI6,BTBD9,TNKS,RUFY3,STON2,DAB2,PKHD1,TRAM2,DZIP1,SMG5,DLG3,RELN,SIN3A,RUVBL2,SLC22A3,SLC25A16,EML4,FAM91A1,ZNF423,MACF1,MYO7A,SLC8A2,CLASP1,TPCN1,GPM6B,SNX5,ATP2B4,NSF,PACRG,SYNE3,PACSIN1,SPTBN5,ATAD1,NXT2,MAP1B,MARK1,KCNAB2,TFRC,UVRAG,EPHA5,MEF2A,SYT12,GPC3,STOM,TBC1D5,TRPV1,TFEB,CLEC16A,SNX9,RPGR,ARL5A,ACTR2,PLCG2,ROCK1,EGR2,CAPN10,AP3B1,KPNA6,ZFAND1,MAP1A,ANK3,UNC13A,PTPN9,SNUPN,AXIN2,PARD3,GNL3L,PTPN14,TRIM46,TUB,KCND3,CACNG8,AP3D1,KIF3C,AP2B1,ANXA8L1,ARHGAP44,HEATR5A,SNX33,CD84,SPI1,CHRNB4,VPS16,CTNNA1,ACAP2,TRDN,ATG14,STAC,RAB15,RAPGEF3,RSRC1,SNCA,CACNG2,GRM4,MAGI2,TBC1D9,SYT9,HEY2,CHMP3,RNF103-CHMP3,PRKAG1,RAB5B,ABCA12,PLD1,PKP2,BTK,TBC1D16,XPO4,VPS41,SLC4A8,PLA2G4E,STX8,HTT,MAPK1,PTEN,IFFO1,SLC30A7,BMP7,PIK3C3,NUP88,LRRK2,SEPT6,TMEM59,SPAG5,CHMP5,BAG6,LILRB4,MORC3,IFT81,TMEM108,ITGAM,NSG2,PKD2,EFNA5,SHANK3,MIA2,SGSM1,STEAP4,TMCC1,WNT7A,ZBED6,ZC3H11A,SNAP23,MAP4,RABEP1,SH3PXD2B,POM121C,RHOT1,ANO1,ATRX,SAMM50,RAPGEF2,SMO,GET4,SUN1,LSG1,BLOC1S3,MARK4,STXBP5L,CPT1A,PSEN2,DNAJC6,UBR5,GRIN1,BBS9,SYNGAP1,GRIN3A,NDE1,ESYT2,CUL3,HSPD1,ITPR2,TSPAN33,EPM2A,GSK3B,PLD2,RDX,SYN1,IWS1,CCDC14,NECAB2,UPF2,MCTP2,BOK,AGT,SYT7,ADAR,BLOC1S6,ZMPSTE24,LAT2,PEX5L,SNX3,EXOC6B,RIMS2,TOMM5,COPG2,CPE,SEC16B,TIMM44,GTF2IRD2,NCF1,SLC29A1,TAOK2,CD160,ZDHHC23,ITGAL,CPLX2,DNAJB6,SLC5A3,ARFGAP3,PACSIN2,MON2,NOS1,PEX7,GCKR,TBC1D23,RGS14,ACTN4,STK4,ZFAND6,EPHB2,SLC1A1,WLS,XCR1,GPSM2,PPP1R10,ABCD3,GRTP1,MCOLN1,KPNA4,SYTL4,PAX6,PRKCZ,ADORA2A,GRIN2B,PPP1CC,RABGEF1,WDR83OS,OTUD7B,KLC3,NIPBL,ANGPT1,CADPS,PACS2,FRMPD1,OSBPL8,EPB41L2,HACL1,TBC1D10C,ARHGAP21,MAP2,STPG1,ATG3,BRAF,DIAPH1,HTR2C,SIL1,CLTB,CD300A,EMC3,SKP1,FAM149B1,RPL23,SH3BP4,ATXN1,CAMSAP3,SP100,VTI1A,ANXA2,TRIM29,PIGU,RAB27B,ITSN1,NUP214,WIPI2,ADCY1,NUMB,SYK,WWTR1,GRIK2,SLIT1,MYRIP,RAB2A,SEC31B,BRCA2,CACNG3,DVL2,YBX1,ANKRD13A,GRIP1,ERGIC1,MYO1A,CNIH4,ANKRD13C,PLEKHM1,HGSNAT,GBP5,MCM8,MLPH,CAV2,AKTIP,EPS15,INPP5F,REPS2,DENND4C,TBC1D10A,CAMK2D,SNX1,MALT1,ZDHHC11,ZDHHC11B,EHD2,THEM4,TRAPPC8,CCT3,NEDD4L,NDC1,PTPRC,RPH3A,CTCF,RAB28,SYNDIG1,TSPAN5,VPS52,TRAF3IP2,SGTB,SHISA6,CLNK,EDNRA,SLC25A13,STXBP6,BMP4,ABLIM3
GO:0007409	axonogenesis	6.647892184849242e-20	*NRXN1,CLASP2,SEMA3A,SEMA3D,MYOT,TIAM2,UNC5C,KALRN,SEMA5A,OLFM1,NRXN3,ROBO1,CDKL5,KIF5C,ROBO2,NFASC,ITGB1,HSP90AA1,DSCAM,TRIO,DAB1,LAMA2,PTPN11,HDAC6,GOLGA4,EPHA1,LIMK1,ATP8A2,PTPRO,NTRK2,UNC5D,LAMA3,CDH4,PAK1,TBR1,BDNF,AMIGO1,FYN,LMTK2,RTN4,B4GALT6,RTN4R,CCDC141,ADAMTSL1,NTN1,MAP2K1,VAX2,CNTN4,NTNG1,UST,BOC,MYPN,BCL11B,SIAH1,SEMA5B,KEL,SLIT3,SLIT2,DISC1,LAMC2,DCLK1,YTHDF1,ENAH,EPHA4,AUTS2,MAG,IGF1R,XK,DRAXIN,NIN,DCC,PAX2,SPG11,CNTN6,NFIB,PTPRM,NRP2,PRTG,APP,DIP2B,FSTL4,ISLR2,SPTBN4,DSCAML1,SEMA6D,BMPR2,USP33,DPYSL2,CTNNA2,RAB11A,CRMP1,LRRC4C,SEMA4D,PLXNA2,EPHB3,FBXO45,VCL,WNT3,NLGN3,PAFAH1B1,TRPC5,GLI2,NEO1,RUFY3,RELN,SIN3A,MACF1,PAK3,ADNP,MAP1B,EPHA5,FLRT2,EGR2,NTRK1,MAP1A,ANK3,EPHB1,EPHA10,PARD3,TRIM46,EPHA7,MAP3K13,PTK7,BMPR1B,PTPRS,PTEN,BMP7,RAPH1,EFNA5,WNT7A,MYCBP2,SOS1,EXT1,SMO,SYNGAP1,GSK3B,EMB,PTK2,GAP43,TAOK2,ECE1,RPS6KA5,TUBB3,EPHB2,PAX6,MAP2,PRKCQ,BRAF,TNR,CDKL3,TMEFF1,ADCY1,NUMB,SLIT1,SEMA3C,NCAM1,CREB1,EDNRA*
GO:00	regulation of	7.31131209	*CLASP2,SEMA3A,DOCK1,EPB41,SEMA3D,TIAM2,PTGIS,TJP1,KALRN,SEMA5A,ENPP2,PIK3CD,EZR,ROBO1,CDKL5,ROBO2,ITGB1,TRIOBP,PSMB7,KNDC1,DSCA*

22603	anatomical structure morphogenesis	9839326e-20	*M,RUNX1,DAB1,OMA1,CDC42EP3,MYO10,ABCC8,GOLGA4,EPHA1,LIMK1,ESR1,NPHP3,NTRK2,CELA1,RIMS1,TNIK,CDH4,PAK1,PTPRD,BDNF,AMIGO1,TGIF2,FMNL2,TENM4,TANC2,CASS4,FYN,ADAMTS9,NF1,RTN4,RTN4R,BCR,CHI3L1,NTN1,MAP2K1,STIM1,MYH9,NTNG1,ROR2,DCN,UST,CAPRIN2,RCC2,CCBE1,ZDHHC15,SYT1,LZTS1,MLLT3,SEPT7,WNT11,PALMD,MKLN1,IL1RAPL1,CAMK2B,RUNX2,SEMA5B,TGFB1,KEL,CELSR1,SLIT2,CORO1C,DISC1,KANK1,MTDH,ITSN2,BMPER,PARVA,ADAM12,STRIP1,FGF10,SMURF2,EPHA4,PRKCA,PPP3CA,MAG,CTNNB1,PARK2,PPARG,CYBB,XK,FBLIM1,DRAXIN,FGF1,NIN,CPNE6,PHLDB1,HCK,DCC,FLT4,PAX2,SFRP1,DNM3,CUX2,PTPRM,ABI2,SYT3,NEDD4,ARHGEF18,VANGL1,PREX1,DIP2B,FSTL4,SH3D19,JAK1,ANGPTL4,ISLR2,ZDHHC6,GPC6,PRICKLE1,VASH2,FRS2,CPNE9,PRKD1,STAT1,PARP6,RND3,HNF4A,SEMA6D,ATF2,ADAMTS12,BMPR2,BMPR1A,TSPAN12,NLGN1,SPAG9,RAB11A,FGF2,LRRC4C,NEDD9,SEMA4D,ISM1,PLXNA2,DVL3,EPHB3,MYO9A,WNT3,RHOJ,P4HB,PAFAH1B1,KIF3A,COL4A3,DDAH1,RASAL1,TRPC5,RUFY3,DAB2,PKHD1,RELN,RSPO2,CFDP1,SP1,MACF1,CLASP1,AKAP2,ATP2B4,SYNE3,TMEM2,PAK3,ADNP,MAP1B,TFRC,GPC3,PSMB2,FGD1,ACTR2,SMOC2,ROCK1,EPS8,DNMBP,UNC13A,SARM1,TRIM46,ARHGAP44,COCH,CUX1,EPHA7,MCU,SHROOM3,MAP3K13,RAPGEF3,PTK7,MAGI2,RC3H1,PTPRS,RASA1,HIPK1,ZNRF3,PTEN,BMP7,LRRK2,ZRANB1,EFNA5,SHANK3,WNT7A,BRWD3,PRICKLE2,PARVB,RAPGEF2,SMO,TNFRSF11B,SYT17,RHOA,SYNGAP1,ROR1,RHOBTB1,SH3KBP1,GSK3B,RDX,EEF2K,FAM49B,SULF1,AGT,STAT2,ZMPSTE24,PTK2,RIMS2,FAM171A1,TAOK2,SHC1,ALDOA,ARHGAP15,CD160,LBX2,ALOX5,CD44,FGD3,LARP4,CLDN4,FGD4,ACTN4,SPEF1,EPHB2,DLC1,PHLDB2,TMIGD2,ETS1,MAP2,FBXO31,BRAF,DIAPH1,TNR,PHIP,CDKL3,ANGPT4,SP100,SLIT1,DVL2,GRIP1,SASH1,GNA12,FMNL1,CCR3,ANKRD6,SEMA3C,NEDD4L,BMP4*
GO:1902531	regulation of intracellular signal transduction	1.0009170622441729e-19	*PRDX2,NRXN1,MAP4K4,PDE4D,TMBIM4,SLC9A1,PDE8A,SEMA3A,CHERP,WWC1,FBXL2,ASH1L,NOS1AP,TIAM2,MAPRE2,WWC3,KALRN,NTRK3,CBL,PLEKHG4B,SEMA5A,FLT3,GRM5,PLCE1,MARVELD3,MAP2K5,KITLG,PTPRR,LRP2,PIK3CD,DEPDC5,EZR,ROBO1,TNFAIP8L1,HTR2B,MECOM,TENM1,TRAF6,ITPKB,DNAJA3,VAV2,ULK4,ITGB1,HSP90AA1,SRGAP3,CCDC22,TRIO,ARHGAP24,AKAP13,DGKI,PDE4A,PTPN11,CYTH3,ADORA1,IKBKB,ERBB4,ADRA1D,GBF1,ABR,ESR1,MIER1,CDON,NTRK2,EP300,PDGFB,TNIK,MID1,STK39,INSR,TCF7L2,PAK1,LITAF,FBXW11,MAP3K4,ZDHHC13,NPRL3,TRIM13,UBR2,ARHGAP6,LCK,ENTPD5,ECT2,ARHGAP42,ERN2,NPLOC4,DMD,RIT2,CASS4,FYN,MKRN2,ARNTL,NF1,PLCB1,RTN4,RTN4R,BCR,CHI3L1,NDRG4,PAQR3,PIK3R2,RANBP9,NRG1,ARHGAP10,BDKRB2,DOCK8,BID,MAP2K1,FNIP1,RALGPS2,MAD1L1,TMBIM6,VGLL4,PPP2CB,CMKLR1,ROR2,DCN,DOCK2,CDH13,COPS5,ARHGAP32,RALGPS1,FBXO9,RFFL,DENND1A,JAK2,FBXW7,LINC00473,ITCH,NPSR1,PDE2A,LAMTOR3,WNT11,ARHGAP23,TRIM59,NOX4,SIPA1L3,SIAH1,PRKCD,TAB2,CD4,SGK1,BTRC,F2RL1,SLIT2,TP73,KANK1,MTDH,ZNF675,BMPER,BCL3,ANKRD54,DAPK2,TNFRSF10B,DUSP22,NAIP,HOMER2,DOCK3,FGF10,RASA4,RASA4B,ARHGEF28,KCTD10,IQCJ-SCHIP1,SH3RF3,CAPN3,EPHA4,RORA,PRKCA,AUTS2,TNFSF11,UFL1,CTNNB1,PARK2,SOD2,FCGR2B,ARHGAP22,IGF1R,PPARG,AXIN1,GARNL3,DLG5,IL18R1,P2RY10,ANKRD17,APOL3,FGF1,NOL3,TRIM5,AJUBA,CHEK2,TBCK,TRIM8,CSPG4,GRM1,ARHGAP31,OBSCN,NF2,FLT4,SGMS1,TPTE,SFRP1,ARHGAP29,ITPR1,NEDD4,ARHGEF18,PREX1,TAB1,TXNDC12,APP,PDGFRA,NOX1,HEG1,SESN1,TNFRSF19,SOX2,RCAN1,CHUK,FRS2,S100A12,PRKD1,STAT1,DGKG,SHANK2,RAF1,RASGRF2,CARD16,CASP1,PTPN1,NLGN1,SPAG9,DENND4A,PDE11A,FGF2,SEMA4D,DOK6,ADCYAP1R1,EEF1E1,EEF1E1-BLOC1S5,DVL3,ARHGAP39,ATF3,SHOC2,AGO3,DEPTOR,TERF2IP,MYO9A,WDR59,P4HB,CCL14,CCL15,PTPN13,PAFAH1B1,PRDM15,FAM13A,HIP1,AKAP6,RASAL1,DAB2,PKHD1,UBR1,RELN,NEK10,GUCY1A2,PELI1,IQGAP1,MAP3K7,TRIM22,ARHGAP12,ALK,SLC8A2,UBE2V1,XDH,ATP2B4,PAK3,CAMTA1,P2RY8,RNF34,TFRC,GFRAL,TSPAN6,CLEC16A,GHR,FGD1,HDAC1,TRIM24,PLCG2,ROCK1,EPS8,C1QTNF1,HIPK3,GPR35,PTPN2,NTRK1,TRIM44,EIF3A,DNMBP,EPHB1,MUC1,VWF,ARHGAP44,ARHGEF3,SPI1,EPHA7,BCAR3,STK38,MAP3K13,OTUD3,SH3BP1,GRM4,MAGI2,RC3H1,WNT7B,ZMYND11,RASA1,RPTOR,KCTD13,IGF1,HTT,MAPK1,PTEN,ARHGEF17,SPRED2,BMP7,PUM1,RASA2,LRRK2,LILRB4,ARHGAP25,PDGFC,FAM13B,WNT7A,UACA,KAT6A,SIK3,SH3RF2,SOS1,RAPGEF2,VRK3,RHOA,SYNGAP1,ROR1,CUL3,GSK3B,RDX,ACTA2,MLF1,FAM49B,NECAB2,CD27,DUSP26,BOK,NACC2,AGT,PRKAA2,ZMPSTE24,HRH4,NDRG2,PTK2,PEX5L,MAP3K5,NCF1,TAOK2,SHC1,ARHGAP15,CD44,FGD3,ADAM8,CXCL17,FGD4,PLEKHA1,RGS14,SIPA1L2,ACTN4,ARHGAP11A,MC1R,STK4,ZFAND6,MARK3,EPHB2,WLS,IL18,DLC1,PPARA,PPP1R10,PHLPP1,MAGI3,MOB3B,PRKCZ,ZC3HAV1,RABGEF1,OTUD7B,DENND4B,ANGPT1,FHL2,LRRK1,OSBPL8,GCNT2,TBC1D10C,ARHGAP21,CCL22,BRAF,HTR2C,CD300A,PPP2CA,RPL23,SH3BP4,ITSN1,SRGAP2,MADD,SYK,ARHGAP19,GRIK2,CIT,RAP1GAP2,DVL2,PTPRJ,TAF1,TICAM1,TANK,SASH1,RALGAPA1,CGNL1,VAV3,CAV2,GNA12,INPP5F,AXL,DENND4C,MET,ANKRD6,MALT1,PHB,PTPRC,EDA,GPR89A,TRAF3IP2,TRAP1,PRAP1,BMP4,CPNE1*
GO:0051171	regulation of nitrogen compound metabolic process	1.0934174761913844e-19	*POLDIP3,ENPP1,PRDX2,LDB2,NRXN1,ASPH,PRMT3,PRKCI,SLCO3A1,PDE4D,DNMT1,S1PR2,HLX,SLC9A1,C6ORF89,PBX3,ADCY8,MED13L,TRPS1,CBFB,PDE8A,ZNF823,IL31RA,RPS6KA2,PRDM12,MED26,WWC1,NRG3,ASH1L,NOS1AP,LATS2,SCAF8,STOX2,PTGIS,PRKAG2,WWC3,PRLR,MVP,PAGR1,HIVEP3,FTO,NPAS3,NTRK3,CBL,ARNT,EGLN2,LRRFIP2,FLT3,STAT5B,TOX,ENPP2,GRM5,PLCE1,ZNF566,F*

			ER,CASK,MAP2K5,KITLG,MAPK10,LRP2,ZNF536,SP3,HDGF,EIF4G3,EZR,IKZF2,ROBO1,TOM1L1,CHD7,HTR2B,PSMD1,MECOM,TACC1,TENM1,LMNA,TRAF6,ESRP1,ITPKB,DNAJA3,OXR1,ABI1,NHLH1,HSP90AA1,CDC6,PSMB7,TSC22D3,KNDC1,ZC4H2,CCDC22,AKAP13,GFI1B,PTPRK,RUNX1,DAB1,ERC1,HMGN3,NRIP1,THRB,EFCAB7,ITGB3BP,DACH1,PTPN11,ZNF569,MGAT5,CCT2,ORC2,HDAC6,SERTAD2,DDB1,MYT1,ADORA1,EPHA1,IKBKB,PEX14,ERBB4,MRE11A,LIMK1,ZNF609,BRD8,KAT6B,HIF3A,SNIP1,CUL4B,ESR1,MIER1,PCBP3,PMEPA1,MAML3,PTPRO,CDON,NTRK2,TNRC6A,EP300,CELA1,ZYG11B,TENM2,ZNF76,RNF220,ZNF471,FNTA,PDGFB,TNIK,CCND3,TOB2,ZNF19,ZNF23,BRMS1,CHFR,ZNF605,SCML4,HNF4G,INSR,FMN2,RERE,PRKAR1A,ASB5,FUBP1,ATP8B1,H2AFY2,TCF7L2,JMJD1C,CAB39,ZNF443,ZNF490,ZNF564,ZNF709,ZNF799,PAK1,LITAF,FBXW11,ESRRB,MAP3K4,RNF144A,BASP1,EGLN3,RBM20,TBR1,SAMD4A,BDNF,TFAP2A,CDK14,FANCA,PEG3,ZIM2,TGIF2,TGIF2-C20ORF24,MEIS1,VRTN,TRIM13,LCK,MDM4,ENTPD5,ECT2,ZNF148,MTA3,SNX6,TFDP2,CST2,PTCD3,ERN2,C1D,AFF3,DMD,CENPF,ATG10,SLC30A9,TOX3,LARP4B,USP13,KAT7,ZNF667,SLC8A1,GSN,KHDRBS2,RBM14,RBM4,MED12L,SATB2,RIT2,HIRA,CASS4,FYN,MKRN2,ARNTL,NF1,PLCB1,MGMT,ARID4B,PPP4R2,RTN4,AFF2,DPEP1,APOD,BCR,RWDD3,BRPF1,CHI3L1,TTN,PAQR3,PIK3R2,RANBP9,NRG1,SH2D3C,UBP1,BDKRB1,BDKRB2,BID,FRY,MAP2K1,MDFI,FNIP1,VAX2,MYH9,TMBIM6,VGLL4,PPP2CB,PPP3R1,HDAC5,CSRNP3,RBM5,ZNF692,NFIA,RNF4,CSNK2A3,JDP2,CMKLR1,ITIH2,ROR2,DCN,PCBD2,HNRNPLL,CDH13,CREBRF,TRDMT1,SOX13,FHIT,WDR70,PTPRT,AUNIP,COPS5,IPO5,MAD2L2,TLE6,CAPRIN2,CCNYL1,ZNF418,PHF20L1,TERF2,SLX1B,ZNF286A,FOXN3,NEUROD1,GNAQ,RFFL,CCBE1,HERC5,USP22,JAK2,FAM168A,TRAPPC9,FBXW7,OAZ2,SKAP1,UIMC1,ITCH,MLIP,ZFYVE28,BCL11B,SMG1,RBFOX1,PKNOX1,MLLT3,TSHZ2,TCF7,PDE2A,KLF15,TBX15,ANXA4,CYFIP2,WNT11,MTA1,KLF8,NOX4,LCOR,RPRD1B,QKI,CCDC62,ERCC8,PRKCD,SOX6,TAB2,ACVR2A,RUNX2,CD4,PPM1E,TGFB1,BANP,SGK1,SPSB4,NSD1,IGSF1,PIBF1,ZHX2,PKNOX2,ASCC2,BTRC,NFATC3,CRADD,F2RL1,BCAS3,CDYL2,DNAJB2,CC2D1B,SLIT2,TP73,ITIH4,CORO1C,SAP18,ZBTB22,ILF2,DISC1,BLID,ZFAND2A,CLN6,MTDH,FANK1,MYT1L,KDM4B,SMAD6,BNC2,ZNF398,CLOCK,TCF12,ZNF675,ETV6,NELL1,TFAP2D,BMPER,TIMP2,BCL3,ANKRD54,TNFRSF10B,SND1,DUSP22,NAIP,HNRNPC,TRRAP,DOCK3,SBNO2,YTHDF1,FGF10,CIZ1,SMYD3,LOXL3,FANCI,CAPN3,LUM,SMURF2,EPHA4,RORA,HIVEP2,PRKCA,AUTS2,CD6,TNFSF11,SMG6,PPP3CA,NSUN2,UBQLN3,NFYB,MAGEA4,KLF12,CAMK4,GATAD2B,PIP5KL1,UFL1,TRAK1,CTNNB1,PARK2,SOD2,DACH2,METTL13,SMARCC1,KLF17,IGF1R,PPARG,NGRN,AXIN1,PRKAR1B,OTUB1,IL18R1,CIPC,MTF1,CELF2,CBX5,BRIP1,LRPPRC,SREBF2,CDK11A,CDK11B,LEPR,FGF1,PROS1,NPAT,NR4A3,FOXK2,NOL3,PRKAR2A,RIOK2,MYOCD,TRIM5,PER2,AJUBA,GLG1,ZNF626,ZNF737,CHEK2,SUPT3H,UBQLN4,PRDM16,SPDYA,HCK,CSTL1,RAB3GAP2,CAPN2,TRIM8,DIO2,CSPG4,BRMS1L,ZBTB8A,ZBTB8B,CTDP1,BAZ1B,NF2,FLT4,MEF2B,HDAC4,PAX2,PHF5A,SECISBP2L,TRABD2B,SFRP1,MED13,PPP6R2,ZNF395,FOXO3,NFIB,SP4,ZCCHC17,BCL2L13,SSH1,CELF5,SYNCRIP,SMAD3,RNFT2,CUX2,WWP2,ARNT2,SBNO1,KRBOX1,ZNF662,ZNF777,SIMC1,EBF3,MTBP,RNF168,CASZ1,DCP1B,MIER3,NEDD4,ESRRG,HOXD3,HOXD4,SLC4A4,ZNF114,TAB1,KTI12,NFATC1,CDC73,APP,SSBP3,GSX2,RBM8A,CCNI2,DIP2B,ARIH1,YAP1,HEG1,TEN1,EYA2,SH3D19,BORA,IBTK,NVL,LRP5,MTCP1,POLR3G,ZNF787,SOX2,SETD2,TEAD1,PRICKLE1,RCAN1,ZNF653,SPTBN4,ZNF521,DRD1,ARID3A,ZNF761,CHUK,SFMBT1,ZNF584,ESR2,S100A12,DCUN1D3,PRKD1,STAT1,CELF6,ST18,ETV5,RHOXF2B,SLC6A3,TAF3,PLAGL1,HNF4A,ZBTB7C,TASP1,EREG,CCNY,RBFOX3,ATF2,POU2F2,TCF3,ZNF730,GRAMD4,RAF1,CELF4,ZNF766,CARD16,CASP1,PTPN1,SRRM4,BMPR2,VBP1,CAMK1D,BMPR1A,ZKSCAN1,PABPC4,IKZF4,PIK3R3,CDK12,CAND2,SPAG9,DENND4A,MYB,FGF2,ZNF71,POU6F2,BACH1,MXD3,PPM1F,TICRR,GLI4,ZFP41,NEDD9,AGBL4,BEND5,SEMA4D,NFX1,RORC,ELP3,PTAFR,SP140,SP140L,RHOXF2,JARID2,DDX58,BRDT,PHC2,RARB,SPEN,PRKCG,SIN3B,NCOA1,SPOCK1,AREL1,EHMT1,LMO7,DVL3,EIF2B5,EIF4G1,PSMD2,RBX1,TCF20,ATF3,LIN28B,SRSF5,CKS1B,PAWR,EBF2,AGO3,DEPTOR,FBXL20,MAML2,TSG101,DESI1,TERF2IP,CRYM,IDE,RFX2,ZNF322,SUFU,MAGEA11,PTPN13,PRDM15,ZNF670,ZNF695,CCDC169-SOHLH2,SOHLH2,COL4A3,DDAH1,ZNF354C,HIP1,TCEA3,PADI6,ZNF704,NR2C1,TRPC5,UBA2,GLI2,TNKS,WBP2NL,ERCC1,TNRC6B,GLIS3,WDTC1,ZNF664,DAB2,BLM,PKHD1,CACUL1,LDLRAD4,MYSM1,SETD5,SMG5,DLG3,WNK1,RELN,NEK10,SIN3A,RUVBL2,COMMD6,GMEB1,PELI1,IQGAP1,MAP3K7,ZNF423,SP1,TRIM22,ALK,SLC8A2,TEAD4,FASTKD5,HOXC13,APBB3,SLC35A4,SRA1,UBE2V1,ADIRF,XDH,AHNAK,OVOL2,SNX5,NFATC2,PAX7,BNC1,ATP2B4,NSF,ACTL6B,ASXL3,PAK3,TET1,CAMTA1,CCPG1,DYX1C1,ADNP,MAPKAPK2,ARID4A,SCARB1,CDK6,PHF2,CELF1,RNF34,TFRC,UVRAG,EPHA5,WWOX,MEF2A,DCAF6,DCUN1D5,DROSHA,GPC3,XRN1,SATB1,PSMB2,EYA1,GATAD2A,DIS3L2,TRPV1,RBM12B,HOXB3,HOXB4,HOXB5,HOXB6,TFEB,GHR,MNAT1,SNX9,ACTR2,L3MBTL4,ELAVL4,HDAC1,RECQL5,SMOC2,TRIM24,PLCG2,ROCK1,SCMH1,PAXIP1,ZNF713,EGR2,HIPK3,RNF10,RYBP,AP3B1,PTPN2,NTRK1,KPNA6,TRIM44,MAP1A,SLC39A10,AXIN2,EPCAM,PARD3,ZNF425,GNL3L,MUC1,PTPN14,BCOR,AP3D1,ZNF41,CARM1,ZNF383,ANXA8L1,SNX33,CD84,LPA,ZNF616,ZNF836,SMARCA2,CUX1,SPI1,DNMT3B,EPHA7,FOXP2,PSPC1,GLIS1,SFMBT2,ATG14,EIF3H,BCAR3,JAZF1,STK38,ABCB7,ACOT8,R

			BPMS,RENBP,CHURC1,CREB5,MAP3K13,RAPGEF3,SMYD1,SNCA,BMPR1B,MAGI2,PRIM2,PNPT1,USP50,ZEB2,FOXJ3,HEY2,RC3H1,EIF3E,RNF19B,ZMYND11,KMT2D,PRKAG1,CREM,PSMF1,KMT2C,CAST,TRIP12,DFFA,ADTRP,PLD1,RASA1,MACC1,MITF,SRSF6,OPRD1,RPTOR,NFIX,BTK,MLLT1,CBFA2T2,IGF1,MLXIP,ATF7IP,HTT,MAPK1,PTEN,SPRED2,BMP7,ATXN2,MXI1,PUM1,SOX5,CIR1,PCBP2,ZNF780A,ZNF780B,LRRK2,TMEM59,UNC119,ZBTB20,RFC3,BAG6,LILRB4,ITGAM,PKD2,ZNF652,EFNA5,KCTD1,RIPPLY1,RCOR3,TADA2A,DAZL,PDGFC,SERPINA3,SERPINA4,SERPINA5,NCOA3,PKIB,ZNF146,ZNF565,PPP2R3C,WNT7A,ZBED6,BRWD3,NLRP1,UACA,CRTC3,DNAJC1,ARNTL2,PLCL2,KAT6A,MTIF2,ZKSCAN7,ZNF197,ZNF660,BMP6,TAF15,MYCBP2,NFE2L1,GPI,SH3RF2,WDR43,ZNF30,N4BP1,ATRX,DPH6,IKZF1,PRMT2,RAPGEF2,ASCC1,SMO,VRK3,RALY,PRKAR2B,TCF4,SOX30,TFE3,UBR5,GRIN1,JADE1,ARRDC4,RHOA,SPRTN,ROR1,RQCD1,TF,ONECUT2,CUL3,TFEC,HSPD1,EPM2A,GSK3B,PRR16,DNAJC3,RDX,STAT6,MLF1,EEF2K,IWS1,PPP1R16A,PTPRB,RBBP8,NECAB2,CDK13,CD27,DUSP26,BOK,RNF144B,ZNF362,NACC2,SUPT4H1,ZNF354A,AGT,HDGFRP3,METTL16,CCNJL,POLH,PRKAA2,ADAR,STAT2,ZMPSTE24,PTK2,SNX3,PBX1,TEC,EXOSC3,MAP3K5,TCFL5,CREBBP,DLX1,GTF2IRD2,NCF1,TAOK2,PYGO2,SHC1,ZNF813,LBX2,PARP10,RNFT1,RPS6KB1,YLPM1,DPRX,PRG3,SERPINB11,SETDB2,DNAJB6,SP7,CD44,LARP4,ADAM8,CLDN4,RPS6KA5,ZNF484,ZNF93,CSNK1A1,HUS1,PRDM2,NOS1,ZNF44,MED15,RGS14,ACTN4,MC1R,STK4,TCF25,ZNF282,EPHB2,SLC1A1,EDRF1,IL18,MFSD8,UBE2K,DIS3,DLC1,PPARA,PPP1R10,ARID5B,SMARCAL1,CPEB4,MOB3B,NOTCH4,ZMYND8,ZNF366,CRX,PAX6,PRKCZ,ZC3HAV1,ADORA2A,GRIN2B,RABGEF1,CCNG2,CPEB1,OTUD7B,NIPBL,YEATS4,COL28A1,PHF20,ZNF143,ANGPT1,BRF1,TBL1X,EYA3,FHL2,LRRK1,MBTD1,OSBPL8,ATF6,ZBTB5,ZNF708,IGF2BP3,ETS1,PPP1CA,MTF2,TAGLN3,CDK5RAP1,CNTN1,NCOR2,PRKCQ,BRAF,CSRNP1,HDAC2,IMPACT,SRCAP,POU3F3,NOTO,SPINT2,MBNL3,ZKSCAN5,CD300A,ELF2,PHIP,PPP2CA,SKP1,ANGPT4,RPL23,RFC5,ATXN1,SP100,ZNF347,ZNF415,ANXA2,TRIM29,PARN,ADCY1,MADD,RBM42,SYK,CNOT1,RBM39,WWTR1,GTF2H5,NFXL1,ZBTB38,BRCA2,ATF7,DVL2,MORC1,MTRF1,YBX1,PTPRJ,TAF1,EIF4E3,TICAM1,TANK,THAP3,HSF1,MAX,SAP130,DHFR,EZH1,ETF1,NRF1,SRPK2,SASH1,NCOA2,WDR18,EEFSEC,QRICH1,ZP3,CHD6,RNF2,ZNF554,MOB3A,GNA12,SH2D3A,AKTIP,MYEF2,INPP5F,TRIM37,CD109,MET,TBC1D10A,ZNF461,CAMK2D,SNX1,TIMELESS,UCHL5,BRD9,MALT1,SERPINE3,SETD3,TRA2B,KDM2B,MLXIPL,CCT3,NEDD4L,PHB,PTPRC,EDA,ZNF555,CTCF,SH3GL2,NR6A1,TRAF3IP2,ATF6B,CREB1,RGMB,TNXB,TRAP1,EBF4,ZNF511,BMP4,ABLIM3
GO:0006793	phosphorus metabolic process	2.508804720652927e-19	NT5C1B,NT5C1B-RDH14,SUCLG2,ENPP1,LDB2,NRXN1,B4GALNT2,PRKCI,SLCO3A1,MAP4K4,PDE4D,S1PR2,PDE7B,ADCY8,PDE8A,IL31RA,RPS6KA2,GK5,CTDSPL2,NRG3,LATS2,KSR2,PTGIS,PRKAG2,PRLR,MVP,ADCY7,KALRN,NTRK3,CBL,ARNT,FLT3,ENPP2,GRM5,PLCE1,SAMHD1,FER,CASK,MAPK4,MAP2K5,KITLG,MAPK10,PTPRR,PIK3CD,CAMK1G,ADPGK,ROBO1,TOM1L1,HTR2B,CDKL5,TENM1,NADK2,INPP4B,TRAF6,ITPKB,DNAJA3,VAV2,GRK5,ABI1,ULK4,HSP90AA1,CDC6,TSSK1B,KNDC1,DSCAM,MOCOS,FAM126A,STK38L,GMDS,TRIO,AKAP13,DLG2,PTPRK,DAB1,ERC1,DGKI,PDE4A,MAST4,PTPN11,MGAT5,HDAC6,ADORA1,EPHA1,IKBKB,ERBB4,MRE11A,LIMK1,TLK1,ABR,OSBPL10,PMEPA1,PTPRO,CDON,NTRK2,EP300,TPST2,PDGFB,TNIK,CCND3,CSGALNACT1,STK39,INSR,PRKAR1A,PIP4K2A,CAB39,PAK1,FBXW11,MAP3K4,PTPRD,PPIP5K1,BDNF,CDK14,FUT8,LCK,ENTPD5,ECT2,MYO3B,SNX6,PIPOX,PPM1L,PTPRU,ERN2,DMD,SLC8A1,PRPS2,RIT2,CASS4,FYN,NF1,SMG7,PLCB1,LMTK2,PPP4R2,DGKH,NEIL2,BCR,CHI3L1,TTN,BMP2K,PAQR3,PIK3R2,NRG1,SH2D3C,ACYP1,CDK19,BDKRB1,BDKRB2,MAP2K1,FNIP1,PPP2CB,CSNK2A3,INPP4A,PPIP5K2,CDKL2,LRGUK,ROR2,PRPSAP2,FHIT,PTPRT,ENTPD1,ACSBG1,PPP1R42,AK2,IPO5,MAD2L2,GALK2,CAPRIN2,CCNYL1,NME7,AK5,CERKL,GNAQ,ICK,HERC5,JAK2,FBXW7,LINC00473,ZFYVE28,SMG1,IMPDH1,HUNK,DPYS,IDNK,CDC42BPB,PHKA1,DHDDS,PDE2A,TTC7B,LAMTOR3,WNT11,TPST1,FIG4,NOX4,RPRD1B,CAMK2B,PRKCD,TAB2,ACVR2A,CD4,PPM1E,TGFB1,SGK1,MYO3A,NSD1,PIBF1,BTRC,PTPRN2,AAK1,SLIT2,CORO1C,PTPDC1,TTBK2,SMAD6,ZNF675,SYNJ2,BMPER,DCLK1,ANKRD54,DAPK2,TNFRSF10B,DAPP1,DUSP22,TRRAP,DOCK3,FGF10,ADK,SMYD3,MAST2,ADCY2,EPHA4,RORA,HSD17B12,PRKCA,CD6,TNFSF11,SMG6,PPP3CA,CAMK4,PIP5KL1,PARK2,IGF1R,PPARG,AXIN1,PRKAR1B,PFAS,CDK11A,DYRK4,CDK11B,LEPR,FGF1,FOXK2,PRKAR2A,RIOK2,MYOCD,AJUBA,CHEK2,PPP1CB,SPDYA,HCK,AGPAT4,CAPN2,TBCK,PPP2R2B,CSPG4,CTDP1,BAZ1B,OBSCN,NF2,FLT4,SGMS1,HDAC4,TNS3,SPTBN1,TPTE,SFRP1,PPP6R2,DGKB,SSH1,SMAD3,PTPRM,ABI2,PLA2G4C,OGDHL,SLC4A4,TAB1,UCK2,APP,PDGFRA,SLK,CCNI2,HEG1,PPCS,CDK3,EYA2,BORA,IBTK,SBK3,ALPL,JAK1,FPGT-TNNI3K,TNNI3K,PEMT,LRP5,MTCP1,PTPRG,RCAN1,SPTBN4,ELOVL2,DRD1,CHUK,S100A12,NUDT10,PRKD1,DGKG,LRRC2,EREG,CCNY,MAPKAPK3,ATF2,RAF1,PTPN1,BMPR2,CAMK1D,BMPR1A,IDO2,BTBD10,PIK3R3,CDK12,SPAG9,FGF2,PPA2,PPM1F,ADCY5,NEDD9,PEAK1,SEMA4D,PTAFR,ADCYAP1R1,SLC5A8,PI4KA,PIK3C2B,PRKCG,OC90,DVL3,EIF4G1,EPHB3,PTPRE,CKS1B,DEPTOR,PKN3,TSG101,TERF2IP,PTPN13,PAFAH1B1,CMPK1,TRPC5,ME1,TNKS,TPK1,DAB2,BLM,CACUL1,LDLRAD4,SMG5,DLG3,WNK1,RELN,NEK10,TMEM68,GUCY1A2,PPP2R5C,SLC25A16,IQGAP1,MAP3K7,ALK,SLC8A2,INPP5D,TOP1,MBOAT1,XDH,PRKG1,ATP2B4,PACSIN1,PAK3,SSH2,CAMTA1,PHOSPHO2,ADNP,MAPKAPK2,CDC42BPA,SC

			ARB1,MARK1,CDK6,TFRC,UVRAG,EPHA5,ADCY9,RBKS,ADRBK1,EYA1,GNB3,SHPK,GHR,MNAT1,SNX9,ACACA,TRIM24,PLCG2,ROCK1,HIPK3,PTPN2,INSRR,NTRK1,PKN2,SLC39A10,HYKK,PTPRQ,EPHB1,EPHA10,PTPN9,AXIN2,SARM1,PARD3,PTPN14,NUDT14,NUDT5,PIFO,DGKK,EPHA7,PTPRA,AGPAT3,ATG14,BCAR3,STK38,ACOT8,RBPMS,MAP3K13,RAPGEF3,PDXP,PTK7,RSRC1,N4BP2,SNCA,BMPR1B,MAGI2,NADK,HMGCS2,PTPRS,PRKAG1,CDKL1,SULT2A1,GGPS1,ADTRP,PLD1,OPRD1,HIPK1,RPTOR,BTK,MLLT1,IGF1,PLA2G4E,HTT,MAPK1,PTEN,SPRED2,BMP7,PIK3C3,LRRK2,ATP6V1A,MLYCD,ALDOC,PIGS,UNC119,ZBTB20,LILRB4,MORC3,SLC44A1,GALT,PKD2,EFNA5,NT5E,TADA2A,NT5DC4,ALPK3,PDGFC,PKIB,MTMR3,PPP2R3C,RPS6KC1,LPGAT1,ELOVL5,PLCL2,SIK3,BMP6,GPI,SERINC5,PNPLA3,C8ORF44-SGK3,RAPGEF2,MTHFD2L,VRK3,FAR1,AMPD1,ENPP3,MARK4,PRKAR2B,BPGM,DNAJC6,RHOA,ROR1,RQCD1,TF,CKMT1B,SH3KBP1,EPM2A,GSK3B,PLD2,DNAJC3,EEF2K,PPP1R16A,PTPRB,CDK13,STK32B,DUSP26,AGT,CCNJL,ELOVL3,PRKAA2,ADAR,PITPNM2,STAT2,ZMPSTE24,FGGY,PTK2,PHEX,PPCDC,PDE4B,TEC,CDC14A,MAP3K5,PRKD3,LMTK3,XYLB,CHKA,NCF1,TAOK2,SHC1,SLC44A3,ALDOA,PARP10,RPS6KB1,GUCY2F,CD44,ADAM8,RPS6KA5,CDS1,CSNK1A1,HUS1,NOS1,GCKR,RGS14,STK4,MARK3,EPHB2,SLC1A1,IL18,UBE2K,DLC1,PPARA,PDP1,PNPLA6,PHLPP1,MAGI3,LDHC,MOB3B,ACP1,PAX6,PRKCZ,FAM20C,ADORA2A,PPP1CC,RABGEF1,CCNG2,TYK2,UPB1,ANGPT1,EYA3,LRRK1,OSBPL8,NT5M,PPP1CA,RPIA,CDK5RAP1,CNTN1,PRKCQ,BRAF,HDAC2,HTR2C,IMPACT,PHKG2,PPP1R14A,CD300A,PHIP,CDKL3,PPP2CA,SLC26A2,ANGPT4,PIGU,PITPNM3,ADCY1,MADD,SYK,WWTR1,GTF2H5,CIT,SLC44A5,DVL2,PTPRJ,TAF1,PFKFB4,HSF1,INPP5A,RNGTT,SRPK2,SASH1,CEPT1,SULT2B1,VAV3,MOB3A,GNA12,SH2D3A,UGGT1,AKTIP,INPP5F,NUAK2,AXL,CD109,MET,CAMK2D,MALT1,PFKP,MLXIPL,PHKB,PHB,PTPRC,SH3GL2,TYRO3,MTMR12,ADCY10,CREB1,TNXB,EDNRA,HADHA,SLC25A13,BMP4
GO:0031325	positive regulation of cellular metabolic process	3.068362827460258e-19	POLDIP3,LDB2,NRXN1,ASPH,SLCO3A1,DNMT1,S1PR2,SLC9A1,C6ORF89,PBX3,ADCY8,CBFB,PDE8A,IL31RA,PRDM12,MED26,WWC1,NRG3,ASH1L,NOS1AP,NOX5,SCAF8,STOX2,PRKAG2,PRLR,PAGR1,HIVEP3,FTO,NPAS3,NTRK3,ARNT,FLT3,STAT5B,TOX,ENPP2,GRM5,CASK,MAP2K5,KITLG,LRP2,SP3,HDGF,EZR,ROBO1,TOM1L1,CHD7,HTR2B,MECOM,TACC1,TENM1,LMNA,TRAF6,DNAJA3,VAV2,ABI1,NHLH1,HSP90AA1,CDC6,KNDC1,CCDC22,DSCAM,AKAP13,GFI1B,RUNX1,DAB1,HMGN3,NRIP1,THRB,EFCAB7,PTPN11,CCT2,HDAC6,SERTAD2,DDB1,ADORA1,EPHA1,IKBKB,ERBB4,MRE11A,ZNF609,BRD8,KAT6B,ESR1,MAML3,CDON,NTRK2,TNRC6A,EP300,CELA1,ZYG11B,ZNF76,FNTA,PDGFB,TNIK,CCND3,BRMS1,CHFR,HNF4G,INSR,FMN2,RERE,TCF7L2,PIP4K2A,CAB39,PAK1,LITAF,FBXW11,ESRRB,MAP3K4,RNF144A,EGLN3,RBM20,TBR1,SAMD4A,BDNF,TFAP2A,PEG3,MEIS1,TRIM13,LCK,ENTPD5,ECT2,ZNF148,MTA3,TFDP2,ERN2,ATG10,SLC30A9,TOX3,LARP4B,USP13,KAT7,GSN,RBM14,MED12L,SATB2,PAFAH1B2,RIT2,CASS4,FYN,MKRN2,ARNTL,PLCB1,MGMT,LMTK2,ARID4B,RWDD3,BRPF1,CHI3L1,PIK3R2,NRG1,SH2D3C,UBP1,BID,MAP2K1,FNIP1,MYH9,VGLL4,PPP3R1,HDAC5,CSRNP3,NFIA,RNF4,JDP2,ROR2,DCN,PCBD2,HNRNPLL,CDH13,CREBRF,COPS5,MAD2L2,RAB27A,CAPRIN2,CCNYL1,TERF2,SLX1B,NEUROD1,CCBE1,USP22,JAK2,FAM168A,FBXW7,SKAP1,UIMC1,ITCH,MLIP,BCL11B,PKNOX1,MLLT3,KLF15,LAMTOR3,CYFIP2,WNT11,MTA1,NOX4,RPRD1B,CCDC62,ERCC8,PRKCD,TAB2,ACVR2A,RUNX2,CD4,TGFB1,BANP,SPSB4,TRIM65,NSD1,PIBF1,BTRC,NFATC3,CRADD,F2RL1,BCAS3,DNAJB2,TP73,ILF2,DISC1,BLID,ZFAND2A,CLN6,MTDH,FANK1,SMAD6,ZNF398,CLOCK,TCF12,ETV6,TFAP2D,BCL3,TNFRSF10B,DOCK3,SBNO2,YTHDF1,FGF10,CIZ1,SMYD3,FANCI,CAPN3,LUM,EPHA4,RORA,AUTS2,CD6,TNFSF11,PPP3CA,NFYB,KLF12,CAMK4,UFL1,CTNNB1,PARK2,SOD2,SMARCC1,IGF1R,PPARG,NGRN,AXIN1,MTF1,SREBF2,LEPR,FGF1,NPAT,NR4A3,FOXK2,RIOK2,MYOCD,TRIM5,PER2,AJUBA,CHEK2,SUPT3H,PRDM16,SPDYA,SORBS1,RAB3GAP2,CAPN2,TRIM8,CSPG4,FLT4,MEF2B,HDAC4,PAX2,PHF5A,TRABD2B,SFRP1,MED13,ZNF395,FOXO3,NFIB,BCL2L13,SMAD3,RNFT2,WWP2,ARNT2,WIPI1,EBF3,RNF168,CASZ1,IFNAR1,DCP1B,ESRRG,HOXD3,HOXD4,SLC4A4,TAB1,NFATC1,CDC73,APP,SSBP3,PDGFRA,DIP2B,ARIH1,YAP1,SESN1,VDAC1,EYA2,SH3D19,BORA,NVL,LRP5,MTCP1,SOX2,TEAD1,PRICKLE1,ZNF521,ARID3A,CHUK,ESR2,S100A12,DCUN1D3,PRKD1,STAT1,ST18,ETV5,PLAGL1,HNF4A,ZBTB7C,TASP1,EREG,CCNY,ATF2,POU2F2,TCF3,GRAMD4,RAF1,CELF4,CASP1,PTPN1,BMPR2,BMPR1A,BTBD10,IKZF4,PIK3R3,CDK12,CAND2,MYB,FGF2,ZNF71,BACH1,PPM1F,NEDD9,AGBL4,SEMA4D,RORC,PTAFR,ADCYAP1R1,JARID2,DDX58,BRDT,RARB,PRKCG,NCOA1,LMO7,AP2M1,DVL3,EIF2B5,EIF4G1,EPHB3,RBX1,TCF20,ATF3,LIN28B,SRSF5,CKS1B,PAWR,EBF2,MAML2,TSG101,TERF2IP,RFX2,PRDM15,COL4A3,DDAH1,HIP1,TRPC5,UBA2,GLI2,TNKS,WBP2NL,ERCC1,TNRC6B,GLIS3,DAB2,BLM,CACUL1,MYSM1,DLG3,WNK1,RELN,NEK10,SIN3A,RUVBL2,GMEB1,PELI1,IQGAP1,MAP3K7,ZNF423,SP1,TRIM22,ALK,SLC8A2,TEAD4,HOXC13,SLC35A4,SRA1,UBE2V1,TPCN1,ADIRF,XDH,OVOL2,SNX5,NFATC2,BNC1,ATP2B4,NSF,ACTL6B,ASXL3,PAK3,TET1,CAMTA1,CCPG1,ATAD1,ADNP,ARID4A,SCARB1,PHF2,CELF1,TFRC,UVRAG,EPHA5,WWOX,MEF2A,DCAF6,DCUN1D5,TBC1D5,EYA1,SCOC,DIS3L2,TRPV1,HOXB3,HOXB4,HOXB5,TFEB,GHR,MNAT1,SNX9,ACTR2,HDAC1,SMOC2,TRIM24,PLCG2,ROCK1,PAXIP1,EGR2,RNF10,RYBP,AP3B1,PTPN2,INSRR,NTRK1,KPNA6,TRIM44,SLC39A10,EPHB1,EPHA10,AXIN2,EPCAM,MUC1,AP3D1,CARM1,SNX33,PIFO,ZNF836,SMARC

			A2,SPI1,DNMT3B,EPHA7,GLIS1,ATG14,BCAR3,ABCB7,RBPMS,CHURC1,CREB5,MAP3K13,RAPGEF3,SNCA,BMPR1B,MAGI2,PRIM2,PNPT1,USP50,ZEB2,DHRS4,FOXJ3,HEY2,RC3H1,EIF3E,RNF19B,KMT2D,PRKAG1,CREM,KMT2C,ADTRP,PLD1,MACC1,MITF,OPRD1,RPTOR,NFIX,KCTD13,IGF1,MLXIP,ATF7IP,HTT,MAPK1,PTEN,BMP7,PUM1,PCBP2,ZNF780B,LRRK2,MLYCD,TMEM59,UNC119,ZBTB20,RFC3,BAG6,ITGAM,PKD2,EFNA5,TADA2A,DAZL,PDGFC,NCOA3,PKIB,PPP2R3C,WNT7A,NLRP1,UACA,LPGAT1,CRTC3,ARNTL2,ELOVL5,KAT6A,ZNF197,BMP6,TAF15,MYCBP2,SH3RF2,WDR43,ATRX,PRMT2,RAPGEF2,SMO,VRK3,CPT1A,TCF4,SOX30,TFE3,GRIN1,JADE1,ARRDC4,RHOA,SPRTN,ROR1,RQCD1,TF,ONECUT2,CUL3,TFEC,HSPD1,EPM2A,GSK3B,PRR16,DNAJC3,RDX,STAT6,CDK13,BOK,RNF144B,SUPT4H1,AGT,METTL16,PRKAA2,PTK2,PBX1,TEC,EXOSC3,MAP3K5,CREBBP,DLX1,NCF1,TAOK2,SHC1,ECE1,RNFT1,RPS6KB1,SP7,CD44,LARP4,ADAM8,SLC5A3,CLDN4,RPS6KA5,ZNF484,CSNK1A1,PRDM2,NOS1,ACTN4,MC1R,STK4,EPHB2,SLC1A1,EDRF1,IL18,UBE2K,DIS3,DLC1,PPARA,PPP1R10,ARID5B,MOB3B,NOTCH4,CRX,PAX6,PRKCZ,ZC3HAV1,GRIN2B,CPEB1,NIPBL,YEATS4,ZNF143,ANGPT1,BRF1,TBL1X,EYA3,LRRK1,OSBPL8,ATF6,ETS1,MTF2,CDK5RAP1,CNTN1,PRKCQ,BRAF,CSRNP1,HDAC2,HTR2C,IMPACT,PHKG2,SRCAP,POU3F3,CD300A,ELF2,PHIP,PPP2CA,SKP1,ANGPT4,SH3BP4,RFC5,SP100,ANXA2,PARN,MADD,SYK,CNOT1,WWTR1,ZBTB38,BRCA2,ATF7,DVL2,YBX1,PTPRJ,TAF1,TICAM1,TANK,THAP3,HSF1,MAX,EZH1,NRF1,SASH1,NCOA2,QRICH1,VAV3,ZP3,CHD6,MOB3A,GNA12,SH2D3A,AKTIP,INPP5F,AXL,TRIM37,MET,TBC1D10A,TIMELESS,MALT1,SETD3,TRA2B,MLXIPL,CCT3,PHB,PTPRC,CTCF,TYRO3,NR6A1,ATF6B,CREB1,RGMB,BMP4,ABLIM3
GO:0080090	regulation of primary metabolic process	3.620346785799433e-19	POLDIP3,ENPP1,PRDX2,LDB2,NRXN1,ASPH,PRMT3,PRKCI,SLCO3A1,PDE4D,DNMT1,S1PR2,HLX,SLC9A1,C6ORF89,PBX3,ADCY8,MED13L,TRPS1,CBFB,PDE8A,ZNF823,IL31RA,RPS6KA2,PRDM12,MED26,WWC1,NRG3,ASH1L,NOS1AP,LATS2,SCAF8,STOX2,PTGIS,PRKAG2,WWC3,PRLR,MVP,PAGR1,HIVEP3,FTO,NPAS3,NTRK3,CBL,ARNT,EGLN2,LRRFIP2,FLT3,STAT5B,TOX,ENPP2,GRM5,PLCE1,ZNF566,FER,CASK,MAP2K5,KITLG,MAPK10,LRP2,ZNF536,SP3,HDGF,EIF4G3,EZR,IKZF2,ROBO1,MALRD1,TOM1L1,CHD7,HTR2B,PSMD1,MECOM,TACC1,TENM1,LMNA,TRAF6,ESRP1,ITPKB,DNAJA3,OXR1,VAV2,ABI1,NHLH1,HSP90AA1,CDC6,PSMB7,TSC22D3,KNDC1,ZC4H2,CCDC22,AKAP13,GFI1B,PTPRK,RUNX1,DAB1,ERC1,HMGN3,NRIP1,THRB,EFCAB7,ITGB3BP,DACH1,PTPN11,ZNF569,MGAT5,CCT2,ORC2,HDAC6,SERTAD2,DDB1,MYT1,ADORA1,EPHA1,IKBKB,PEX14,ERBB4,MRE11A,LIMK1,ZNF609,BRD8,KAT6B,HIF3A,SNIP1,CUL4B,ESR1,MIER1,PCBP3,PMEPA1,MAML3,PTPRO,CDON,NTRK2,TNRC6A,EP300,CELA1,ZYG11B,TENM2,ZNF76,RNF220,ZNF471,FNTA,PDGFB,TNIK,CCND3,TOB2,ZNF19,ZNF23,BRMS1,CHFR,ZNF605,SCML4,HNF4G,INSR,FMN2,RERE,PRKAR1A,ASB5,FUBP1,ATP8B1,H2AFY2,TCF7L2,PIP4K2A,JMJD1C,CAB39,ZNF443,ZNF490,ZNF564,ZNF709,ZNF799,PAK1,LITAF,FBXW11,ESRRB,MAP3K4,RNF144A,BASP1,EGLN3,RBM20,TBR1,SAMD4A,BDNF,TFAP2A,CDK14,FANCA,PEG3,ZIM2,TGIF2,TGIF2-C20ORF24,MEIS1,VRTN,TRIM13,LCK,MDM4,ENTPD5,ECT2,ZNF148,MTA3,SNX6,TFDP2,CST2,PTCD3,ERN2,C1D,AFF3,DMD,CENPF,ATG10,SLC30A9,TOX3,LARP4B,USP13,KAT7,ZNF667,SLC8A1,GSN,KHDRBS2,RBM14,RBM4,MED12L,SATB2,RIT2,HIRA,CASS4,FYN,MKRN2,ARNTL,NF1,PLCB1,MGMT,ARID4B,PPP4R2,RTN4,AFF2,DPEP1,APOD,RWDD3,BRPF1,CHI3L1,TTN,PAQR3,PIK3R2,RANBP9,NRG1,SH2D3C,UBP1,BDKRB1,BDKRB2,BID,FRY,MAP2K1,MDFI,FNIP1,VAX2,MYH9,TMBIM6,VGLL4,PPP2CB,PPP3R1,HDAC5,CSRNP3,RBM5,ZNF692,NFIA,RNF4,CSNK2A3,JDP2,CMKLR1,ITIH2,ROR2,DCN,PCBD2,HNRNPLL,CDH13,CREBRF,TRDMT1,SOX13,FHIT,WDR70,PTPRT,AUNIP,COPS5,IPO5,MAD2L2,TLE6,CAPRIN2,CCNYL1,ZNF418,NLN,PHF20L1,TERF2,SLX1B,ZNF286A,FOXN3,NEUROD1,GNAQ,RFFL,CCBE1,HERC5,USP22,JAK2,FAM168A,TRAPPC9,FBXW7,OAZ2,SKAP1,UIMC1,ITCH,MLIP,ZFYVE28,BCL11B,SMG1,RBFOX1,PKNOX1,MLLT3,TSHZ2,TCF7,PDE2A,KLF15,TBX15,ANXA4,CYFIP2,WNT11,MTA1,KLF8,NOX4,LCOR,RPRD1B,QKI,CCDC62,ERCC8,PRKCD,SOX6,TAB2,ACVR2A,RUNX2,CD4,PPM1E,TGFB1,BANP,SGK1,SPSB4,NSD1,IGSF1,PIBF1,ZHX2,PKNOX2,ASCC2,BTRC,NFATC3,CRADD,F2RL1,BCAS3,CDYL2,DNAJB2,CC2D1B,SLIT2,TP73,ITIH4,CORO1C,SAP18,ZBTB22,ILF2,DISC1,BLID,ZFAND2A,CLN6,MTDH,FANK1,MYT1L,KDM4B,SMAD6,BNC2,ZNF398,CLOCK,TCF12,ZNF675,ETV6,NELL1,TFAP2D,BMPER,TIMP2,BCL3,ANKRD54,TNFRSF10B,SND1,DUSP22,NAIP,HNRNPC,TRRAP,DOCK3,SBNO2,YTHDF1,FGF10,CIZ1,SMYD3,LOXL3,FANCI,CAPN3,LUM,SMURF2,EPHA4,RORA,HIVEP2,PRKCA,AUTS2,CNR1,CD6,TNFSF11,SMG6,PPP3CA,NSUN2,UBQLN3,NFYB,MAGEA4,KLF12,CAMK4,GATAD2B,PIP5KL1,UFL1,TRAK1,CTNNB1,PARK2,SOD2,DACH2,METTL13,SMARCC1,KLF17,IGF1R,PPARG,NGRN,AXIN1,PRKAR1B,OTUB1,IL18R1,CIPC,MTF1,CELF2,CBX5,BRIP1,LRPPRC,SREBF2,CDK11A,CDK11B,LEPR,FGF1,PROS1,NPAT,NR4A3,FOXK2,NOL3,PRKAR2A,RIOK2,MYOCD,TRIM5,PER2,AJUBA,GLG1,ZNF626,ZNF737,CHEK2,SUPT3H,UBQLN4,PRDM16,PPP1CB,SPDYA,HCK,CSTL1,SORBS1,RAB3GAP2,CAPN2,TRIM8,DIO2,CSPG4,BRMS1L,ZBTB8A,ZBTB8B,CTDP1,BAZ1B,NF2,FLT4,MEF2B,HDAC4,PAX2,PHF5A,SECISBP2L,TRABD2B,SFRP1,MED13,PPP6R2,ZNF395,FOXO3,NFIB,SP4,ZCCHC17,BCL2L13,SSH1,CELF5,SYNCRIP,SMAD3,RNFT2,CUX2,WWP2,ARNT2,SBNO1,KRBOX1,ZNF662,ZNF777,SIMC1,EBF3,MTBP,RNF168,CASZ1,PSAP,DCP1B,MIER3,NEDD4,ESRRG,HOXD3,HOXD4,SLC4A4,ZNF114,TAB1,KTI12,NFATC1,CDC73,APP,SSBP3,GSX2,PDGFRA,RBM8A,CCNI2,DIP2B,ARIH1,YAP1,HEG1,TEN1,EYA2,SH3D19,BORA,IBTK,NVL,LRP5,MTC

			P1,POLR3G,ZNF787,SOX2,SETD2,TEAD1,PRICKLE1,RCAN1,ZNF653,SPTBN4,ZNF521,DRD1,ARID3A,ZNF761,CHUK,ERLIN1,SFMBT1,ZNF584,ESR2,S100A12,DCUN1D3,PRKD1,STAT1,CELF6,ST18,ETV5,RHOXF2B,SLC6A3,TAF3,PLAGL1,HNF4A,ZBTB7C,TASP1,EREG,CCNY,RBFOX3,ATF2,POU2F2,TCF3,ZNF730,GRAMD4,RAF1,CELF4,ZNF766,CARD16,CASP1,PTPN1,SRRM4,BMPR2,VBP1,CAMK1D,BMPR1A,ZKSCAN1,PABPC4,IKZF4,PIK3R3,CDK12,CAND2,SPAG9,DENND4A,MYB,FGF2,ZNF71,POU6F2,BACH1,MXD3,PPM1F,TICRR,GLI4,ZFP41,NEDD9,AGBL4,BEND5,SEMA4D,NFX1,RORC,ELP3,PTAFR,ADCYAP1R1,SP140,SP140L,RHOXF2,JARID2,DDX58,BRDT,PHC2,RARB,SPEN,PRKCG,SIN3B,NCOA1,SPOCK1,AREL1,EHMT1,LMO7,DVL3,EIF2B5,EIF4G1,PSMD2,RBX1,TCF20,ATF3,LIN28B,SRSF5,CKS1B,PAWR,EBF2,AGO3,DEPTOR,FBXL20,MAML2,TSG101,DESI1,CCDC3,TERF2IP,CRYM,IDE,RFX2,ZNF322,SUFU,MAGEA11,PTPN13,PRDM15,ZNF670,ZNF695,CCDC169-SOHLH2,SOHLH2,COL4A3,ZNF354C,HIP1,TCEA3,PADI6,ZNF704,NR2C1,TRPC5,UBA2,GLI2,TNKS,WBP2NL,ERCC1,TNRC6B,GLIS3,WDTC1,ZNF664,DAB2,BLM,PKHD1,CACUL1,LDLRAD4,MYSM1,SETD5,SMG5,DLG3,WNK1,RELN,NEK10,SIN3A,RUVBL2,COMMD6,GMEB1,PELI1,IQGAP1,MAP3K7,ZNF423,SP1,TRIM22,ALK,SLC8A2,TEAD4,FASTKD5,HOXC13,APBB3,SLC35A4,SRA1,UBE2V1,ADIRF,XDH,AHNAK,OVOL2,SNX5,NFATC2,PAX7,BNC1,ATP2B4,NSF,ACTL6B,ASXL3,PAK3,TET1,CAMTA1,CCPG1,DYX1C1,ADNP,MAPKAPK2,ARID4A,SCARB1,CDK6,PHF2,CELF1,RNF34,TFRC,UVRAG,EPHA5,WWOX,MEF2A,DCAF6,DCUN1D5,DROSHA,GPC3,XRN1,SATB1,PSMB2,EYA1,GATAD2A,DIS3L2,GNB3,TRPV1,RBM12B,HOXB3,HOXB4,HOXB5,HOXB6,TFEB,GHR,MNAT1,SNX9,ACTR2,L3MBTL4,ELAVL4,HDAC1,RECQL5,SMOC2,TRIM24,PLCG2,ROCK1,SCMH1,C1QTNF1,PAXIP1,ZNF713,EGR2,HIPK3,RNF10,RYBP,AP3B1,PTPN2,NTRK1,KPNA6,TRIM44,MAP1A,SLC39A10,AXIN2,EPCAM,PARD3,ZNF425,GNL3L,MUC1,PTPN14,BCOR,AP3D1,ZNF41,CARM1,ZNF383,ANXA8L1,SNX33,CD84,LPA,ZNF616,ZNF836,SMARCA2,CUX1,SPI1,DNMT3B,EPHA7,FOXP2,PSPC1,GLIS1,SFMBT2,ATG14,EIF3H,BCAR3,JAZF1,STK38,ACOT8,RBPMS,RENBP,CHURC1,CREB5,MAP3K13,RAPGEF3,SMYD1,SNCA,BMPR1B,MAGI2,PRIM2,PNPT1,USP50,ZEB2,FOXJ3,HEY2,RC3H1,EIF3E,RNF19B,ZMYND11,KMT2D,PRKAG1,CREM,PSMF1,KMT2C,CAST,TRIP12,DFFA,ADTRP,PLD1,RASA1,MACC1,MITF,SRSF6,OPRD1,RPTOR,NFIX,BTK,MLLT1,CBFA2T2,IGF1,MLXIP,ATF7IP,HTT,MAPK1,PTEN,SPRED2,BMP7,ATXN2,MXI1,PUM1,SOX5,CIR1,PCBP2,ZNF780A,ZNF780B,LRRK2,MLYCD,TMEM59,UNC119,ZBTB20,RFC3,BAG6,LILRB4,ITGAM,PKD2,ZNF652,EFNA5,KCTD1,RIPPLY1,RCOR3,TADA2A,DAZL,PDGFC,SERPINA3,SERPINA4,SERPINA5,NCOA3,PKIB,MTMR3,ZNF146,ZNF565,PPP2R3C,WNT7A,ZBED6,BRWD3,NLRP1,UACA,LPGAT1,CRTC3,DNAJC1,ARNTL2,ELOVL5,PLCL2,KAT6A,MTIF2,ZKSCAN7,ZNF197,ZNF660,BMP6,TAF15,MYCBP2,NFE2L1,GPI,SH3RF2,WDR43,ZNF30,N4BP1,ATRX,DPH6,IKZF1,PRMT2,RAPGEF2,ASCC1,SMO,VRK3,RALY,PRKAR2B,CPT1A,TCF4,SOX30,TFE3,UBR5,GRIN1,JADE1,ARRDC4,RHOA,SPRTN,ROR1,RQCD1,TF,ONECUT2,CUL3,TFEC,HSPD1,EPM2A,GSK3B,PRR16,DNAJC3,RDX,STAT6,MLF1,EEF2K,IWS1,PPP1R16A,PTPRB,RBBP8,NECAB2,CDK13,CD27,DUSP26,BOK,RNF144B,ZNF362,NACC2,SUPT4H1,ZNF354A,AGT,HDGFRP3,METTL16,CCNJL,POLH,PRKAA2,ADAR,STAT2,ZMPSTE24,PTK2,SNX3,PBX1,TEC,EXOSC3,MAP3K5,TCFL5,CREBBP,DLX1,GTF2IRD2,NCF1,TAOK2,UGT1A1,UGT1A10,UGT1A4,UGT1A8,PYGO2,SHC1,ZNF813,LBX2,PARP10,RNFT1,RPS6KB1,YLPM1,DPRX,PRG3,SERPINB11,SETDB2,DNAJB6,SP7,CD44,LARP4,ADAM8,CLDN4,RPS6KA5,ZNF484,ZNF93,CSNK1A1,HUS1,PRDM2,NOS1,ZNF44,GCKR,MED15,RGS14,ACTN4,MC1R,STK4,TCF25,ZNF282,EPHB2,SLC1A1,EDRF1,IL18,MFSD8,UBE2K,HCAR1,HCAR2,DIS3,DLC1,PPARA,PPP1R10,ARID5B,SMARCAL1,CPEB4,MOB3B,NOTCH4,ZMYND8,ZNF366,CRX,PAX6,PRKCZ,ZC3HAV1,ADORA2A,GRIN2B,RABGEF1,CCNG2,CPEB1,OTUD7B,NIPBL,YEATS4,COL28A1,PHF20,ZNF143,ANGPT1,BRF1,TBL1X,EYA3,FHL2,LRRK1,MBTD1,OSBPL8,ATF6,ZBTB5,ZNF708,IGF2BP3,ETS1,PPP1CA,MTF2,TAGLN3,CDK5RAP1,CNTN1,NCOR2,PRKCQ,BRAF,CSRNP1,HDAC2,HTR2C,IMPACT,PHKG2,SRCAP,POU3F3,NOTO,SPINT2,MBNL3,ZKSCAN5,CD300A,ELF2,PHIP,PPP2CA,SKP1,ANGPT4,RPL23,RFC5,ATXN1,SP100,ZNF347,ZNF415,ANXA2,TRIM29,PARN,ADCY1,MADD,RBM42,SYK,CNOT1,RBM39,WWTR1,GTF2H5,NFXL1,ZBTB38,BRCA2,ATF7,DVL2,MORC1,MTRF1,YBX1,PTPRJ,TAF1,EIF4E3,TICAM1,TANK,THAP3,HSF1,MAX,SAP130,DHFR,EZH1,ETF1,NRF1,SRPK2,SASH1,NCOA2,WDR18,EEFSEC,QRICH1,VAV3,ZP3,CHD6,RNF2,ZNF554,MOB3A,GNA12,SH2D3A,AKTIP,MYEF2,INPP5F,TRIM37,CD109,MET,TBC1D10A,ZNF461,CAMK2D,SNX1,TIMELESS,UCHL5,BRD9,MALT1,SERPINE3,SETD3,TRA2B,KDM2B,MLXIPL,CCT3,NEDD4L,PHB,PTPRC,EDA,ZNF555,CTCF,SH3GL2,NR6A1,RNF213,TRAF3IP2,ATF6B,CREB1,RGMB,TNXB,TRAP1,EBF4,ZNF511,BMP4,ABLIM3
GO:0010604	positive regulation of macromolecule metabolic process	7.371942056106729e-19	*POLDIP3,LDB2,NRXN1,ASPH,SLCO3A1,PDE4D,DNMT1,S1PR2,SLC9A1,C6ORF89,PBX3,ADCY8,CBFB,PDE8A,IL31RA,RPS6KA2,PRDM12,SETD4,PTGFR,MED26,WWC1,NRG3,ASH1L,NOS1AP,NOX5,SCAF8,STOX2,PRKAG2,PRLR,PAGR1,HIVEP3,FTO,NPAS3,NTRK3,ARNT,EGLN2,FLT3,STAT5B,TOX,ENPP2,GRM5,CASK,MAP2K5,KITLG,LRP2,PIK3CD,SP3,HDGF,ANK2,OLFM1,EZR,ROBO1,TOM1L1,CHD7,HTR2B,MECOM,TACC1,TENM1,LMNA,TRAF6,DNAJA3,ABI1,NHLH1,HSP90AA1,SLC24A3,CDC6,KNDC1,CCDC22,CNGB1,AKAP13,GFI1B,RUNX1,DAB1,ABCC8,HMGN3,NRIP1,THRB,EFCAB7,PTPN11,CCT2,HDAC6,SERTAD2,DDB1,ADORA1,IKBKB,ERBB4,MRE11A,ZNF609,BRD8,KAT6B,ACTG2,CUL4B,ESR1,MAML3,CDON,NTRK2,TNRC6A,EP300,CELA1,ZYG11B,ZNF76,FNTA,PDGFB,RIMS1,TNIK,CCND3,BRMS1,CH*

			FR,HNF4G,INSR,FMN2,RERE,ASB5,FUBP1,TCF7L2,CAB39,PAK1,LITAF,FBXW11,ESRRB,MYOM1,MAP3K4,RNF144A,EGLN3,RBM20,TBR1,SAMD4A,BDNF,TFAP2A,PEG3,MEIS1,TRIM13,LCK,ECT2,ZNF148,MTA3,TFDP2,RFTN1,ERN2,ATG10,SLC30A9,TOX3,LARP4B,USP13,KAT7,GSN,RBM14,MED12L,SATB2,RIT2,CASS4,FYN,MKRN2,ARNTL,PLCB1,MGMT,ARID4B,RTN4,RWDD3,BRPF1,CHI3L1,TTN,PIK3R2,RANBP9,NRG1,SH2D3C,UBP1,BID,MAP2K1,FNIP1,MYH9,VGLL4,PPP3R1,HDAC5,CSRNP3,NFIA,RNF4,CSNK2A3,JDP2,ROR2,DCN,PCBD2,HNRNPLL,CDH13,CREBRF,COPS5,MAD2L2,RAB27A,CAPRIN2,CCNYL1,TERF2,SLX1B,NEUROD1,CCBE1,USP22,JAK2,FAM168A,FBXW7,OAZ2,SKAP1,UIMC1,ITCH,MLIP,BCL11B,PKNOX1,MLLT3,PDE2A,KLF15,CYFIP2,WNT11,MTA1,NOX4,RPRD1B,QKI,CCDC62,ERCC8,PRKCD,TAB2,ACVR2A,RUNX2,CD4,TGFB1,BANP,SPSB4,NSD1,PIBF1,BTRC,NFATC3,CRADD,F2RL1,BCAS3,DNAJB2,TP73,ILF2,DISC1,BLID,ZFAND2A,CLN6,MTDH,FANK1,SMAD6,ZNF398,CLOCK,TCF12,ETV6,TFAP2D,BCL3,TNFRSF10B,HNRNPC,DOCK3,SBNO2,YTHDF1,FGF10,CIZ1,SMYD3,FANCI,CAPN3,LUM,SMURF2,EPHA4,RORA,AUTS2,CD6,TNFSF11,PPP3CA,NFYB,KLF12,CAMK4,CTNNB1,PARK2,SMARCC1,IGF1R,PPARG,NGRN,AXIN1,IL18R1,IL1RL1,MTF1,CYBB,SCAMP5,SREBF2,LEPR,FGF1,NPAT,NR4A3,FOXK2,RIOK2,MYOCD,TRIM5,PER2,KIR2DL4,AJUBA,CHEK2,SUPT3H,PRDM16,SPDYA,SORBS1,RAB3GAP2,TRIM8,CSPG4,FLT4,MEF2B,HDAC4,PAX2,PHF5A,SPTBN1,TRABD2B,SFRP1,MED13,ZNF395,FOXO3,NFIB,BCL2L13,SYNCRIP,SMAD3,RNFT2,CUX2,WWP2,ARNT2,EBF3,RNF168,CASZ1,DCP1B,NEDD4,ESRRG,HOXD3,HOXD4,TAB1,NFATC1,CDC73,APP,SSBP3,DIP2B,NOX1,ARIH1,YAP1,HEG1,EYA2,SH3D19,BORA,NVL,LRP5,MTCP1,POLR3G,SOX2,SETD2,TEAD1,PRICKLE1,ZNF521,DRD1,ARID3A,CHUK,ESR2,S100A12,DCUN1D3,KDM6A,PRKD1,STAT1,ST18,ETV5,RHOXF2B,PLAGL1,HNF4A,ZBTB7C,TASP1,EREG,CCNY,ATF2,POU2F2,TCF3,GRAMD4,RAF1,CELF4,CASP1,PTPN1,BMPR2,BMPR1A,IKZF4,PIK3R3,CDK12,CAND2,MYB,FGF2,ZNF71,BACH1,PPM1F,NEDD9,AGBL4,SEMA4D,RORC,PTAFR,RHOXF2,JARID2,DDX58,BRDT,RARB,PRKCG,NCOA1,AREL1,LMO7,AP2M1,DVL3,EIF2B5,EIF4G1,RBX1,TCF20,ATF3,LIN28B,SRSF5,CKS1B,PAWR,EBF2,AGO3,MAML2,TSG101,TERF2IP,IDE,RFX2,WNT3,PRDM15,COL4A3,HIP1,TRPC5,UBA2,GLI2,TNKS,WBP2NL,ERCC1,TNRC6B,GLIS3,DAB2,BLM,CACUL1,MYSM1,DLG3,WNK1,RELN,NEK10,SIN3A,RUVBL2,GMEB1,PELI1,IQGAP1,MAP3K7,ZNF423,SP1,TRIM22,SLC8A2,TEAD4,HOXC13,SLC35A4,SRA1,UBE2V1,ADIRF,XDH,OVOL2,SNX5,NFATC2,BNC1,ATP2B4,NSF,ACTL6B,ASXL3,PAK3,TET1,CAMTA1,CCPG1,ATAD1,ADNP,MAPKAPK2,ARID4A,MARK1,CDK6,PHF2,CELF1,TFRC,WWOX,MEF2A,DCAF6,DCUN1D5,DROSHA,GPC3,TBC1D5,EYA1,DIS3L2,HOXB3,HOXB4,HOXB5,TFEB,GHR,MNAT1,SNX9,ACTR2,ELAVL4,HDAC1,SMOC2,TRIM24,PLCG2,ROCK1,C1QTNF1,PAXIP1,EGR2,RNF10,RYBP,AP3B1,NTRK1,KPNA6,TRIM44,SLC39A10,ANK3,AXIN2,EPCAM,MUC1,AP3D1,CARM1,ADAM19,SNX33,CD84,ZNF836,SMARCA2,SPI1,DNMT3B,EPHA7,GLIS1,ATG14,BCAR3,RBPMS,CHURC1,CREB5,MAP3K13,RAPGEF3,MST1,SNCA,BMPR1B,MAGI2,PRIM2,PNPT1,USP50,ZEB2,FOXJ3,HEY2,RC3H1,EIF3E,RNF19B,KMT2D,PRKAG1,CREM,KMT2C,ADTRP,PLD1,MACC1,MITF,OPRD1,RPTOR,NFIX,KCTD13,IGF1,MLXIP,ATF7IP,MAPK1,PTEN,BMP7,PUM1,PCBP2,ZNF780B,LRRK2,UNC119,ZBTB20,RFC3,BAG6,PKD2,EFNA5,TADA2A,DAZL,PDGFC,NCOA3,PKIB,PPP2R3C,WNT7A,NLRP1,UACA,CRTC3,ARNTL2,KAT6A,ZNF197,BMP6,TAF15,MYCBP2,GPI,SH3RF2,WDR43,ATRX,PRMT2,RAPGEF2,SMO,VRK3,TCF4,SOX30,TFE3,UBR5,GRIN1,JADE1,ARRDC4,RHOA,SPRTN,RQCD1,TF,ONECUT2,CUL3,TFEC,HSPD1,GSK3B,PRR16,DNAJC3,RDX,STAT6,ACTA2,FAM49B,CDK13,BOK,RNF144B,SULF1,SUPT4H1,TNFRSF8,AGT,METTL16,PRKAA2,ADAR,ZMPSTE24,PTK2,PDE4B,PBX1,TEC,EXOSC3,MAP3K5,SEC16B,CREBBP,DLX1,NCF1,TAOK2,SHC1,CD160,ECE1,RNFT1,RPS6KB1,PRG3,SP7,CD44,LARP4,ADAM8,CLDN4,RPS6KA5,ZNF484,CSNK1A1,PRDM2,CXCL17,NOS1,ACTN4,MC1R,STK4,EPHB2,SLC1A1,EDRF1,IL18,UBE2K,DIS3,DLC1,PPARA,PPP1R10,ARID5B,EPB41L4B,GPSM3,MOB3B,NOTCH4,CRX,PAX6,PRKCZ,ZC3HAV1,GRIN2B,CPEB1,NIPBL,TMIGD2,YEATS4,ZNF143,ANGPT1,BRF1,TBL1X,EYA3,LRRK1,OSBPL8,ATF6,ETS1,MTF2,RBMS3,BTN3A2,CDK5RAP1,CNTN1,NCOR2,PRKCQ,BRAF,CSRNP1,HDAC2,IMPACT,PHKG2,SRCAP,POU3F3,CD300A,ELF2,PHIP,PPP2CA,SKP1,ANGPT4,RPL23,RFC5,SP100,ANXA2,PARN,MADD,SYK,CNOT1,WWTR1,ZBTB38,BRCA2,ATF7,DVL2,YBX1,PTPRJ,TAF1,TICAM1,TANK,THAP3,CADM1,HSF1,MAX,EZH1,NRF1,SRPK2,SASH1,NCOA2,GBP5,QRICH1,ZP3,CHD6,MOB3A,GNA12,SH2D3A,AKTIP,INPP5F,TRIM37,MET,TBC1D10A,SNX1,TIMELESS,MALT1,SETD3,TRA2B,MLXIPL,CCT3,NEDD4L,PHB,PTPRC,EDA,CTCF,NR6A1,SPON2,TRAF3IP2,ATF6B,CREB1,RGMB,CLNK,BMP4,ABLIM3
GO:0006796	phosphate-containing compound metabolic process	1.0442363168093072e-18	*NT5C1B,NT5C1B-RDH14,SUCLG2,ENPP1,LDB2,NRXN1,PRKCI,SLCO3A1,MAP4K4,PDE4D,S1PR2,PDE7B,ADCY8,PDE8A,IL31RA,RPS6KA2,GK5,CTDSPL2,NRG3,LATS2,KSR2,PTGIS,PRKAG2,PRLR,MVP,ADCY7,KALRN,NTRK3,CBL,ARNT,FLT3,ENPP2,GRM5,PLCE1,SAMHD1,FER,CASK,MAPK4,MAP2K5,KITLG,MAPK10,PTPRR,PIK3CD,CAMK1G,ADPGK,ROBO1,TOM1L1,HTR2B,CDKL5,TENM1,NADK2,INPP4B,TRAF6,ITPKB,DNAJA3,VAV2,GRK5,ABI1,ULK4,HSP90AA1,CDC6,TSSK1B,KNDC1,DSCAM,MOCOS,FAM126A,STK38L,TRIO,AKAP13,DLG2,PTPRK,DAB1,ERC1,DGKI,PDE4A,MAST4,PTPN11,MGAT5,HDAC6,ADORA1,EPHA1,IKBKB,ERBB4,MRE11A,LIMK1,TLK1,ABR,OSBPL10,PMEPA1,PTPRO,CDON,NTRK2,EP300,TPST2,PDGFB,TNIK,CCND3,STK39,INSR,PRKAR1A,PIP4K2A,CAB39,PAK1,FBXW11,MAP3K4,PTPRD,PPIP5K1,B*

			DNF,CDK14,LCK,ENTPD5,ECT2,MYO3B,SNX6,PIPOX,PPM1L,PTPRU,ERN2,DMD,SLC8A1,PRPS2,RIT2,CASS4,FYN,NF1,SMG7,PLCB1,LMTK2,PPP4R2,DGKH,NEIL2,BCR,CHI3L1,TTN,BMP2K,PAQR3,PIK3R2,NRG1,SH2D3C,ACYP1,CDK19,BDKRB1,BDKRB2,MAP2K1,FNIP1,PPP2CB,CSNK2A3,INPP4A,PPIP5K2,CDKL2,LRGUK,ROR2,PRPSAP2,FHIT,PTPRT,ENTPD1,ACSBG1,PPP1R42,AK2,IPO5,MAD2L2,GALK2,CAPRIN2,CCNYL1,NME7,AK5,CERKL,GNAQ,ICK,HERC5,JAK2,FBXW7,LINC00473,ZFYVE28,SMG1,IMPDH1,HUNK,DPYS,IDNK,CDC42BPB,PHKA1,DHDDS,PDE2A,TTC7B,LAMTOR3,WNT11,TPST1,FIG4,NOX4,RPRD1B,CAMK2B,PRKCD,TAB2,ACVR2A,CD4,PPM1E,TGFB1,SGK1,MYO3A,NSD1,PIBF1,BTRC,PTPRN2,AAK1,SLIT2,CORO1C,PTPDC1,TTBK2,SMAD6,ZNF675,SYNJ2,BMPER,DCLK1,ANKRD54,DAPK2,TNFRSF10B,DAPP1,DUSP22,TRRAP,DOCK3,FGF10,ADK,SMYD3,MAST2,ADCY2,EPHA4,RORA,HSD17B12,PRKCA,CD6,TNFSF11,SMG6,PPP3CA,CAMK4,PIP5KL1,PARK2,IGF1R,PPARG,AXIN1,PRKAR1B,PFAS,CDK11A,DYRK4,CDK11B,LEPR,FGF1,FOXK2,PRKAR2A,RIOK2,MYOCD,AJUBA,CHEK2,PPP1CB,SPDYA,HCK,AGPAT4,CAPN2,TBCK,PPP2R2B,CSPG4,CTDP1,BAZ1B,OBSCN,NF2,FLT4,SGMS1,HDAC4,TNS3,SPTBN1,TPTE,SFRP1,PPP6R2,DGKB,SSH1,SMAD3,PTPRM,ABI2,PLA2G4C,OGDHL,SLC4A4,TAB1,UCK2,APP,PDGFRA,SLK,CCNI2,HEG1,PPCS,CDK3,EYA2,BORA,IBTK,SBK3,ALPL,JAK1,FPGT-TNNI3K,TNNI3K,PEMT,LRP5,MTCP1,PTPRG,RCAN1,SPTBN4,ELOVL2,DRD1,CHUK,S100A12,NUDT10,PRKD1,DGKG,LRRC2,EREG,CCNY,MAPKAPK3,ATF2,RAF1,PTPN1,BMPR2,CAMK1D,BMPR1A,IDO2,BTBD10,PIK3R3,CDK12,SPAG9,FGF2,PPA2,PPM1F,ADCY5,NEDD9,PEAK1,SEMA4D,PTAFR,ADCYAP1R1,SLC5A8,PI4KA,PIK3C2B,PRKCG,OC90,DVL3,EIF4G1,EPHB3,PTPRE,CKS1B,DEPTOR,PKN3,TSG101,TERF2IP,PTPN13,PAFAH1B1,CMPK1,TRPC5,ME1,TNKS,TPK1,DAB2,BLM,CACUL1,LDLRAD4,SMG5,DLG3,WNK1,RELN,NEK10,TMEM68,GUCY1A2,PPP2R5C,SLC25A16,IQGAP1,MAP3K7,ALK,SLC8A2,INPP5D,TOP1,MBOAT1,XDH,PRKG1,ATP2B4,PACSIN1,PAK3,SSH2,CAMTA1,PHOSPHO2,ADNP,MAPKAPK2,CDC42BPA,SCARB1,MARK1,CDK6,TFRC,UVRAG,EPHA5,ADCY9,RBKS,ADRBK1,EYA1,GNB3,SHPK,GHR,MNAT1,SNX9,ACACA,TRIM24,PLCG2,ROCK1,HIPK3,PTPN2,INSRR,NTRK1,PKN2,SLC39A10,HYKK,PTPRQ,EPHB1,EPHA10,PTPN9,AXIN2,SARM1,PARD3,PTPN14,NUDT14,NUDT5,PIFO,DGKK,EPHA7,PTPRA,AGPAT3,ATG14,BCAR3,STK38,ACOT8,RBPMS,MAP3K13,RAPGEF3,PDXP,PTK7,RSRC1,N4BP2,SNCA,BMPR1B,MAGI2,NADK,HMGCS2,PTPRS,PRKAG1,CDKL1,SULT2A1,GGPS1,ADTRP,PLD1,OPRD1,HIPK1,RPTOR,BTK,MLLT1,IGF1,PLA2G4E,HTT,MAPK1,PTEN,SPRED2,BMP7,PIK3C3,LRRK2,ATP6V1A,MLYCD,ALDOC,PIGS,UNC119,ZBTB20,LILRB4,MORC3,SLC44A1,PKD2,EFNA5,NT5E,TADA2A,NT5DC4,ALPK3,PDGFC,PKIB,MTMR3,PPP2R3C,RPS6KC1,LPGAT1,ELOVL5,PLCL2,SIK3,BMP6,GPI,SERINC5,PNPLA3,C8ORF44-SGK3,RAPGEF2,MTHFD2L,VRK3,FAR1,AMPD1,ENPP3,MARK4,PRKAR2B,BPGM,DNAJC6,RHOA,ROR1,RQCD1,TF,CKMT1B,SH3KBP1,EPM2A,GSK3B,PLD2,DNAJC3,EEF2K,PPP1R16A,PTPRB,CDK13,STK32B,DUSP26,AGT,CCNJL,ELOVL3,PRKAA2,ADAR,PITPNM2,STAT2,ZMPSTE24,FGGY,PTK2,PPCDC,PDE4B,TEC,CDC14A,MAP3K5,PRKD3,LMTK3,XYLB,CHKA,NCF1,TAOK2,SHC1,SLC44A3,ALDOA,PARP10,RPS6KB1,GUCY2F,CD44,ADAM8,RPS6KA5,CDS1,CSNK1A1,HUS1,NOS1,GCKR,RGS14,STK4,MARK3,EPHB2,SLC1A1,IL18,UBE2K,DLC1,PPARA,PDP1,PNPLA6,PHLPP1,MAGI3,LDHC,MOB3B,ACP1,PAX6,PRKCZ,FAM20C,ADORA2A,PPP1CC,RABGEF1,CCNG2,TYK2,UPB1,ANGPT1,EYA3,LRRK1,OSBPL8,NT5M,PPP1CA,RPIA,CDK5RAP1,CNTN1,PRKCQ,BRAF,HDAC2,HTR2C,IMPACT,PHKG2,PPP1R14A,CD300A,PHIP,CDKL3,PPP2CA,SLC26A2,ANGPT4,PIGU,PITPNM3,ADCY1,MADD,SYK,WWTR1,GTF2H5,CIT,SLC44A5,DVL2,PTPRJ,TAF1,PFKFB4,HSF1,INPP5A,RNGTT,SRPK2,SASH1,CEPT1,SULT2B1,VAV3,MOB3A,GNA12,SH2D3A,AKTIP,INPP5F,NUAK2,AXL,CD109,MET,CAMK2D,MALT1,PFKP,MLXIPL,PHKB,PHB,PTPRC,SH3GL2,TYRO3,MTMR12,ADCY10,CREB1,TNXB,EDNRA,HADHA,SLC25A13,BMP4
GO:0051173	positive regulation of nitrogen compound metabolic process	2.771797209383728e-18	*POLDIP3,LDB2,NRXN1,ASPH,SLCO3A1,DNMT1,S1PR2,SLC9A1,C6ORF89,PBX3,ADCY8,CBFB,PDE8A,IL31RA,PRDM12,MED26,WWC1,NRG3,ASH1L,NOS1AP,SCAF8,STOX2,PRKAG2,PRLR,PAGR1,HIVEP3,FTO,NPAS3,NTRK3,ARNT,EGLN2,FLT3,STAT5B,TOX,ENPP2,GRM5,CASK,MAP2K5,KITLG,LRP2,SP3,HDGF,EZR,ROBO1,TOM1L1,CHD7,HTR2B,MECOM,TACC1,TENM1,LMNA,TRAF6,DNAJA3,ABI1,NHLH1,HSP90AA1,CDC6,KNDC1,CCDC22,AKAP13,GFI1B,RUNX1,DAB1,HMGN3,NRIP1,THRB,EFCAB7,PTPN11,CCT2,HDAC6,SERTAD2,DDB1,ADORA1,IKBKB,ERBB4,MRE11A,ZNF609,BRD8,KAT6B,CUL4B,ESR1,MAML3,CDON,NTRK2,TNRC6A,EP300,CELA1,ZYG11B,ZNF76,FNTA,PDGFB,TNIK,CCND3,BRMS1,CHFR,HNF4G,INSR,FMN2,RERE,ASB5,TCF7L2,CAB39,PAK1,LITAF,FBXW11,ESRRB,MAP3K4,RNF144A,EGLN3,RBM20,TBR1,SAMD4A,BDNF,TFAP2A,PEG3,MEIS1,TRIM13,LCK,ENTPD5,ECT2,ZNF148,MTA3,TFDP2,ERN2,ATG10,SLC30A9,TOX3,LARP4B,USP13,KAT7,GSN,RBM14,MED12L,SATB2,RIT2,CASS4,FYN,MKRN2,ARNTL,PLCB1,MGMT,ARID4B,RWDD3,BRPF1,CHI3L1,PIK3R2,RANBP9,NRG1,SH2D3C,UBP1,BID,MAP2K1,FNIP1,MYH9,VGLL4,PPP3R1,HDAC5,CSRNP3,NFIA,RNF4,CSNK2A3,JDP2,ROR2,DCN,PCBD2,HNRNPLL,CDH13,CREBRF,COPS5,MAD2L2,CAPRIN2,CCNYL1,TERF2,SLX1B,NEUROD1,CCBE1,USP22,JAK2,FAM168A,FBXW7,OAZ2,SKAP1,UIMC1,ITCH,MLIP,BCL11B,PKNOX1,MLLT3,KLF15,CYFIP2,WNT11,MTA1,NOX4,RPRD1B,CCDC62,ERCC8,PRKCD,TAB2,ACVR2A,RUNX2,CD4,TGFB1,BANP,SPSB4,NSD1,PIBF1,BTRC,NFATC3,CRADD,F2RL1,BCAS3,DNAJB2,TP73,ILF2,DISC1,BLID,ZFAND*

			2A,CLN6,MTDH,FANK1,SMAD6,ZNF398,CLOCK,TCF12,ETV6,TFAP2D,BCL3,TNFRSF10B,DOCK3,SBNO2,YTHDF1,FGF10,CIZ1,SMYD3,FANCI,CAPN3,LUM,SMURF2,EPHA4,RORA,AUTS2,CD6,TNFSF11,PPP3CA,NFYB,KLF12,CAMK4,CTNNB1,PARK2,SOD2,SMARCC1,IGF1R,PPARG,NGRN,AXIN1,MTF1,SREBF2,LEPR,FGF1,NPAT,NR4A3,FOXK2,RIOK2,MYOCD,TRIM5,PER2,AJUBA,CHEK2,SUPT3H,PRDM16,SPDYA,RAB3GAP2,CAPN2,TRIM8,CSPG4,FLT4,MEF2B,HDAC4,PAX2,PHF5A,TRABD2B,SFRP1,MED13,ZNF395,FOXO3,NFIB,BCL2L13,SMAD3,RNFT2,WWP2,ARNT2,EBF3,RNF168,CASZ1,DCP1B,NEDD4,ESRRG,HOXD3,HOXD4,SLC4A4,TAB1,NFATC1,CDC73,APP,SSBP3,DIP2B,ARIH1,YAP1,EYA2,SH3D19,BORA,NVL,LRP5,MTCP1,SOX2,TEAD1,PRICKLE1,ZNF521,ARID3A,CHUK,ESR2,S100A12,DCUN1D3,PRKD1,STAT1,ST18,ETV5,PLAGL1,HNF4A,ZBTB7C,TASP1,EREG,CCNY,ATF2,POU2F2,TCF3,GRAMD4,RAF1,CELF4,CASP1,PTPN1,BMPR2,BMPR1A,IKZF4,PIK3R3,CDK12,CAND2,MYB,FGF2,ZNF71,BACH1,PPM1F,NEDD9,AGBL4,SEMA4D,RORC,PTAFR,JARID2,DDX58,BRDT,RARB,PRKCG,NCOA1,AREL1,LMO7,DVL3,EIF2B5,EIF4G1,RBX1,TCF20,ATF3,LIN28B,SRSF5,CKS1B,PAWR,EBF2,MAML2,TSG101,TERF2IP,IDE,RFX2,PRDM15,COL4A3,DDAH1,HIP1,TRPC5,UBA2,GLI2,TNKS,WBP2NL,ERCC1,TNRC6B,GLIS3,DAB2,BLM,CACUL1,MYSM1,DLG3,WNK1,RELN,NEK10,SIN3A,RUVBL2,GMEB1,PELI1,IQGAP1,MAP3K7,ZNF423,SP1,TRIM22,SLC8A2,TEAD4,HOXC13,SLC35A4,SRA1,UBE2V1,ADIRF,XDH,OVOL2,SNX5,NFATC2,BNC1,ATP2B4,NSF,ACTL6B,ASXL3,PAK3,TET1,CAMTA1,CCPG1,ADNP,ARID4A,PHF2,CELF1,TFRC,WWOX,MEF2A,DCAF6,DCUN1D5,GPC3,EYA1,DIS3L2,TRPV1,HOXB3,HOXB4,HOXB5,TFEB,GHR,MNAT1,SNX9,ACTR2,HDAC1,SMOC2,TRIM24,PLCG2,ROCK1,PAXIP1,EGR2,RNF10,RYBP,AP3B1,NTRK1,KPNA6,TRIM44,SLC39A10,AXIN2,EPCAM,MUC1,AP3D1,CARM1,SNX33,ZNF836,SMARCA2,SPI1,DNMT3B,EPHA7,GLIS1,ATG14,BCAR3,ABCB7,RBPMS,CHURC1,CREB5,MAP3K13,RAPGEF3,SNCA,BMPR1B,MAGI2,PRIM2,PNPT1,USP50,ZEB2,FOXJ3,HEY2,RC3H1,EIF3E,RNF19B,KMT2D,PRKAG1,CREM,KMT2C,ADTRP,PLD1,MACC1,MITF,OPRD1,RPTOR,NFIX,IGF1,MLXIP,ATF7IP,MAPK1,PTEN,BMP7,PUM1,PCBP2,ZNF780B,LRRK2,UNC119,ZBTB20,RFC3,BAG6,PKD2,EFNA5,TADA2A,DAZL,PDGFC,NCOA3,PKIB,PPP2R3C,WNT7A,NLRP1,UACA,CRTC3,ARNTL2,KAT6A,ZNF197,BMP6,TAF15,MYCBP2,SH3RF2,WDR43,ATRX,PRMT2,RAPGEF2,SMO,VRK3,TCF4,SOX30,TFE3,GRIN1,JADE1,ARRDC4,RHOA,SPRTN,RQCD1,TF,ONECUT2,CUL3,TFEC,HSPD1,GSK3B,PRR16,DNAJC3,RDX,STAT6,CDK13,BOK,RNF144B,SUPT4H1,AGT,METTL16,PRKAA2,PTK2,PBX1,TEC,EXOSC3,MAP3K5,CREBBP,DLX1,NCF1,TAOK2,SHC1,RNFT1,RPS6KB1,SP7,CD44,LARP4,ADAM8,CLDN4,RPS6KA5,ZNF484,CSNK1A1,PRDM2,NOS1,ACTN4,MC1R,STK4,SLC1A1,EDRF1,IL18,UBE2K,DIS3,DLC1,PPARA,PPP1R10,ARID5B,MOB3B,NOTCH4,CRX,PAX6,PRKCZ,ZC3HAV1,GRIN2B,CPEB1,NIPBL,YEATS4,ZNF143,ANGPT1,BRF1,TBL1X,EYA3,LRRK1,OSBPL8,ATF6,ETS1,MTF2,CDK5RAP1,CNTN1,PRKCQ,BRAF,CSRNP1,HDAC2,IMPACT,SRCAP,POU3F3,CD300A,ELF2,PHIP,PPP2CA,SKP1,ANGPT4,RFC5,SP100,PARN,MADD,SYK,CNOT1,WWTR1,ZBTB38,BRCA2,ATF7,DVL2,YBX1,PTPRJ,TAF1,TICAM1,TANK,THAP3,HSF1,MAX,EZH1,NRF1,SASH1,NCOA2,QRICH1,ZP3,CHD6,MOB3A,GNA12,SH2D3A,AKTIP,TRIM37,MET,TBC1D10A,SNX1,TIMELESS,MALT1,SETD3,TRA2B,MLXIPL,CCT3,NEDD4L,PHB,PTPRC,CTCF,NR6A1,ATF6B,CREB1,RGMB,BMP4,ABLIM3
GO:0007010	cytoskeleton organization	4.047869140070285e-18	*CLRN1,HOOK2,PRKCI,TACC2,S1PR2,SLC9A1,SPAG16,PDE4DIP,CLASP2,NOS1AP,TUBA1C,IQCG,TLN2,EPB41,PHACTR1,FHOD3,MAPRE2,TJP1,UTRN,PHACTR2,NTRK3,SEMA5A,PLCE1,FER,ANK2,STAG2,EZR,TACC1,TENM1,LMNA,CTNNA3,ABI1,ULK4,ITGB1,TRIOBP,ELMO2,GAS7,AKAP13,TTLL5,PARD6G,CDC42EP3,XIRP2,MAST4,HDAC6,TBCEL,EPHA1,IKBKB,KIF18B,PEX14,DNAH2,LIMK1,ATP8A2,CECR2,DNAI2,HOOK3,TNIK,MID1,FMN2,PRKAR1A,AFAP1,PAK1,FBXW11,FMNL2,PHACTR4,ARHGAP6,ECT2,LRRC49,STARD9,DMD,GSN,RBM14,CASS4,NF1,DST,PCDH15,BCR,TTN,RANBP9,DCTN1,ARHGAP10,MYH9,FRMPD4,WDR92,NAV1,RNF4,PARD3B,LRGUK,DOCK2,AUNIP,MAD2L2,TLE6,NCKAP1,ARL3,KIF2A,RANBP1,JAK2,MYPN,SMC3,CROCC,CDC42BPB,PLS1,CYFIP2,WNT11,SIPA1L3,MKLN1,PRKCD,PPM1E,TGFB1,LRCH3,PDLIM4,PIBF1,F2RL1,BCAS3,CELSR1,SLIT2,CORO1C,DISC1,CEP135,KANK1,ANK1,TTBK2,SPAG17,PARVA,STRIP1,ANKFN1,FGF10,KANK4,ENAH,PPL,IQCJ-SCHIP1,RHPN2,MAST2,SVIL,CAPN3,SLC16A1,AUTS2,CTNNB1,MPRIP,PARK2,AXIN1,MYO1D,BFSP1,ADD2,NIN,TUBGCP6,AJUBA,HAUS4,CHEK2,PHLDB1,KRT6B,MYO1E,HCK,SORBS1,CAPN2,TBCK,OBSCN,NF2,SPTBN1,HEPACAM2,ELN,SFRP1,SSH1,SMAD3,ABI2,C10ORF90,SHANK1,ARHGEF18,PREX1,KPNB1,NAV3,RAB6C,PDGFRA,SLK,ADD3,MYO1F,SH3D19,BORA,SETD2,KIF24,SPTBN4,VILL,SYNE2,RND3,VPS4A,ABLIM1,ATF2,RAF1,ABLIM2,PTPN1,USP33,DPYSL2,NLGN1,CTNNA2,RAB11A,CRMP1,PPM1F,NEDD9,ARMC2,GAS8,PAWR,CLIC4,RHOJ,PAFAH1B1,KIF3A,HIP1,CEP350,PADI6,TTLL7,TNKS,RUFY3,PKHD1,DZIP1,IQGAP1,EML4,MACF1,ARHGAP12,MYO7A,CLASP1,GPM6B,PRKG1,TTLL9,SYNE3,PACSIN1,SPTBN5,ACTL6B,PAK3,SSH2,DYX1C1,MAP1B,CDC42BPA,MARK1,AIF1L,UVRAG,EPHA5,MEF2A,PLS3,ZNF207,CALD1,FGD1,SNX9,DIAPH2,ACTR2,ROCK1,EPS8,CAPN10,GPR35,INSRR,MAP1A,PPP1R9A,ANK3,NCKAP5,PARD3,TRIM46,ARHGAP44,MCU,RANBP10,CTNNA1,LIMA1,TRDN,SHROOM3,NTMT1,FCHSD2,RAPGEF3,PDXP,SH3BP1,PTK7,SNCA,HAUS3,CHMP3,HYDIN,CSRP1,RASA1,PKP2,KCTD13,HTT,DPYSL3,MAPK1,ARHGEF17,TTLL4,ZRANB1,SPAG5,CHMP5,ARHGAP25,PKD2,EFNA5,SHANK3,STMN4,AMOTL1,PPP2R3C,TBCD,BRWD3,MAP4,SIK3,MYCBP2,ATRX,PAR*

			VB,SUN1,MARK4,RHOA,NDE1,TF,RHOBTB1,SHROOM1,CUL3,SH3KBP1,GSK3B,PLD2,RDX,FAM49B,KRT8,HDGFRP3,PRKAA2,CUL9,ESPNL,PTK2,CDC14A,IQGAP2,FAM171A1,CEP70,TAOK2,SHC1,ALDOA,PGM5,DNAJB6,FGD3,LARP4,PACSIN2,THSD7A,CSNK1A1,FGD4,RGS14,ACTN4,TUBB3,MARK3,SPEF1,THSD7B,TTLL11,TMEFF2,GPSM2,DLC1,EPB41L4B,CCDC6,PAX6,PRKCZ,SPECC1L,STAG1,PHLDB2,KLHL1,YEATS4,SHROOM4,EPB41L2,MAP2,NEBL,BRAF,DIAPH1,CAPN6,PHIP,CAMSAP3,SRGAP2,FRMD5,CIT,BRCA2,NEB,MYO1A,CGNL1,EPB41L1,SKA2,SYNM,POC1B,NUAK2,TRIM37,MET,FMNL1,MICAL3,SETD3,SPTAN1,EHD2,RASSF8,TNXB,CCDC170,ABLIM3
GO:0034330	cell junction organization	4.6919483142519284e-18	NRXN1,PRMT3,PRKCI,MAP4K4,SLC9A1,NEGR1,CLASP2,GRID2,CLDN18,NOS1AP,TLN2,MYOT,MAPRE2,NLGN4X,TJP1,KALRN,NTRK3,GRM5,LHFPL4,MARVELD3,ANK2,NRXN3,CDKL5,ROBO2,NFASC,DNAJA3,PCDH17,ZC4H2,DSCAM,PTPRK,ABCC8,ERC1,CDH10,ERC2,XIRP2,CACNB2,DOCK10,HDAC6,IKBKB,ERBB4,CDH12,PTPRO,NTRK2,FNTA,KIRREL3,CDH8,INSR,PTPRD,CNTN5,BDNF,AMIGO1,ARHGAP6,ECT2,LAMC1,TANC2,FYN,DST,SNCB,APOD,BCR,NTN1,DCTN1,NRG1,FRMPD4,LRFN5,NTNG1,NFIA,PCDHB16,CAPRIN2,CLDN16,RCC2,ZDHHC15,CLSTN2,CLDN1,WNT11,IL1RAPL1,CAMK2B,CLDN10,F2RL1,BCAS3,PDZRN3,SDK2,CORO1C,CTNND2,DISC1,PPFIBP2,DUSP22,LINGO2,CNTNAP2,IL1RAPL2,VMP1,EPHA4,PRKCA,CTNNB1,FCGR2B,ARHGAP22,IGF1R,DLG5,ADD2,AJUBA,SORBS1,NF2,SFRP1,DGKB,DNM3,SMAD3,CUX2,ABI2,SHANK1,NEDD4,NRP2,SLC6A1,APP,SLK,HEG1,NLGN2,GPC6,PLEKHA7,DRD1,SYBU,SHANK2,NLGN1,COL16A1,CTNNA2,LRRC4C,PPM1F,PEAK1,SEMA4D,EIF4G1,EPHB3,ARHGAP39,FBXO45,VCL,MYO9A,NLGN3,PAFAH1B1,PKHD1,SETD5,DLG3,RELN,MACF1,SLC8A2,STAU1,CLASP1,GPM6B,PAK3,ADNP,MAP1B,FLRT2,ACTR2,ROCK1,NTRK1,PKN2,ANK3,UNC13A,EPHB1,PARD3,ARHGAP44,EPHA7,PTPRA,PPFIA4,CTNNA1,SDK1,SH3BP1,SNCA,CACNG2,WNT7B,PTPRS,CAST,HIPK1,PKP2,DSG1,PTEN,CLDN11,LRRK2,TMEM108,ITGAM,EFNA5,SHANK3,TBCD,WNT7A,BMP6,EXT1,RAPGEF2,CDH9,RHOA,SYNGAP1,GABRB3,RDX,SYN1,EEF2K,AGT,CDH20,PTK2,GAP43,ARVCF,TAOK2,CLDN4,EPHB2,SLC1A1,DLC1,GRIN2B,PHLDB2,THSD1,KIFC3,LGI2,TNR,CAMSAP3,NUMB,SLIT1,PTPRJ,GPM6A,COL4A5,SYNDIG1,TANC1,SHISA6
GO:0031344	regulation of cell projection organization	1.788991714268238e-17	CLRN1,ADAMTS16,NRXN1,PRKCI,NEGR1,SEMA3A,GRID2,TENM3,SEMA3D,TIAM2,KALRN,NTRK3,SEMA5A,TOX,ENPP2,PLCE1,FER,EZR,ROBO1,CDKL5,TENM1,ROBO2,ULK4,KNDC1,DSCAM,ARHGAP24,DAB1,CDC42EP3,MYO10,GOLGA4,LIMK1,ATP8A2,PTPRO,NTRK2,EP300,TENM2,TNIK,ATP8B1,CDH4,PAK1,PTPRD,TBR1,BDNF,AMIGO1,DENND5A,DMD,RIT2,TANC2,FYN,FAT3,RTN4,RTN4R,NDRG4,PAQR3,NTN1,MAP2K1,NTNG1,TMEM30A,ROR2,UST,CAPRIN2,NCKAP1,RCC2,ZDHHC15,CROCC,LZTS1,PLS1,SEPT7,FIG4,KREMEN1,IL1RAPL1,CAMK2B,PRKCD,SEMA5B,KEL,F2RL1,BCAS3,SLIT2,CORO1C,DISC1,CEP135,KANK1,EPHA4,AUTS2,CNR1,PPP3CA,MAG,FCGR2B,CDHR2,IGF1R,XK,DRAXIN,NIN,DCC,HDAC4,SFRP1,DNM3,CUX2,ABI2,NEDD4,DIP2B,YAP1,FSTL4,PTPRG,ISLR2,KIF24,SYNE2,PRKD1,PARP6,DGKG,SEMA6D,BMPR2,DPYSL2,CAMK1D,NLGN1,CTNNA2,RAB11A,CRMP1,LRRC4C,SEMA4D,PLXNA2,SPOCK1,DVL3,EPHB3,MYO9A,WNT3,PAFAH1B1,TRPC5,RUFY3,DAB2,DZIP1,RELN,DOCK11,MACF1,ALK,MBOAT1,PACSIN1,PAK3,ADNP,MAP1B,MPHOSPH9,MARK1,ACTR2,ELAVL4,EPS8,NTRK1,PTPN9,SARM1,TRIM46,CARM1,ARHGAP44,PIFO,CUX1,EPHA7,MAP3K13,PTK7,MAGI2,PTPRS,CDKL1,PLD1,RTN4RL1,CBFA2T2,HTT,DPYSL3,PTEN,BMP7,LRRK2,EFNA5,SHANK3,WNT7A,MAP4,RAPGEF2,MARK4,RHOA,SYNGAP1,ROR1,GSK3B,RDX,EEF2K,AGT,SNX3,GAP43,CD44,EPHB2,GRIN2B,MAP2,FBXO31,ATG3,CNTN1,BRAF,HDAC2,NOTO,TNR,CDKL3,SLIT1,DVL2,GRIP1,GPM6A,PLEKHM1,INPP5F,SEMA3C,NEDD4L
GO:0044093	positive regulation of molecular function	4.059184180730585e-17	NRXN1,ASPH,PRKCI,SLCO3A1,MAP4K4,SLC9A1,C6ORF89,ADCY8,NRG3,NOS1AP,EPB41,TIAM2,MAPRE2,PRKAG2,PRLR,KALRN,NTRK3,FLT3,GRM5,FER,MAP2K5,KITLG,ANK2,XRCC4,ROBO1,TOM1L1,HTR2B,CDKL5,TENM1,TRAF6,DNAJA3,VAV2,ABI1,ITGB1,HSP90AA1,CDC6,ZC4H2,ARHGAP24,AKAP13,DAB1,ABCC8,ERC1,RGS6,CCT2,CACNB2,DOCK10,HDAC6,ADORA1,EPHA1,IKBKB,ERBB4,MRE11A,ABR,ESR1,NTRK2,EP300,RNF220,FNTA,PDGFB,CCND3,STK39,INSR,TCF7L2,CAB39,PAK1,MAP3K4,EGLN3,BDNF,AMIGO1,PHACTR4,TRIM13,ARHGAP6,LCK,ECT2,ARHGAP42,ERN2,DMD,GSN,JPH2,CASS4,FYN,NF1,LMTK2,RTN4R,BCR,CHI3L1,NRG1,DOCK8,BID,MAP2K1,RAPGEF6,STIM1,PPP2CB,HDAC5,ROR2,COPS5,RGS8,CAPRIN2,CCNYL1,TERF2,RCC2,NEUROD1,GNAQ,DENND1A,JAK2,TRAPPC9,FBXW7,ARAP2,DOCK9,LAMTOR3,CYFIP2,WNT11,NOX4,SIPA1L3,PRKCD,TAB2,CD4,TGFB1,SGK1,PIBF1,BTRC,CRADD,F2RL1,BCAS3,DNAJB2,CORO1C,BLID,CACNA1D,MTDH,FANK1,CLOCK,TNFRSF10B,RABGAP1L,DOCK3,FGF10,CAPN3,VMP1,EPHA4,TNFSF11,PPP3CA,CTNNB1,PARK2,ARHGAP22,IGF1R,PPARG,AXIN1,GARNL3,IL18R1,ADD2,RYR2,FGF1,RGS10,MYOCD,TRIM5,AJUBA,CACNA1C,SPDYA,TBC1D14,TBCK,TRIM8,FLT4,HDAC4,SFRP1,ARHGAP29,BCL2L13,SMAD3,PSAP,DCP1B,PREX1,TAB1,APP,PDGFRA,AMFR,BORA,NVL,LRP5,MTCP1,CHUK,ESR2,S100A12,DCUN1D3,PRKD1,ST18,EREG,CCNY,FXYD2,ATF2,TCF3,GRAMD4,CARD16,CASP1,PTPN1,BMPR2,USP33,CAMK1D,BMPR1A,FGF2,PPM1F,NEDD9,SEMA4D,PTAFR,ADCYAP1R1,DDX58,DVL3,EIF4G1,EPHB3,CKS1B,EBF2,TERF2IP,MYO9A,IDE,CCL14,CCL15,NLGN3,COL4A3,NMUR2,HIP1,AKAP6,TNKS,CACUL1,DLG3,WNK1,RELN,NEK10,ABCB1,DOCK11,IQGAP1,MAP3K7,TRIM22,ALK,SLC8A2,UBE2V1,XDH,RGS7,ATP2B4,ADNP,SCARB1,TFRC,EPHA5,DCUN1D5,TBC1D5,GHR,MN

			AT1,SNX9,HDAC1,PLCG2,ROCK1,INSRR,NTRK1,SLC39A10,ANK3,EPHB1,EPHA10,AXIN2,PIFO,EPHA7,TRDN,ATG14,STAC,BCAR3,TRPC6,MAP3K13,RAPGEF3,SH3BP1,SNCA,BMPR1B,CACNG2,MAGI2,PRIM2,TBC1D9,USP50,EIF3E,PRKAG1,RASA1,OPRD1,RPTOR,BTK,TBC1D16,IGF1,HTT,MAPK1,PTEN,LRRK2,UNC119,RFC3,ITGAM,ARHGAP25,PKD2,EFNA5,SHANK3,PDGFC,NCOA3,PKIB,SGSM1,PPP2R3C,NLRP1,UACA,CRTC3,PLCL2,SERINC5,RAPGEF2,KCNC2,SMO,VRK3,PSEN2,GRIN1,ARRDC4,RHOA,ROR1,HSPD1,GSK3B,BOK,AGT,PTK2,MAP3K5,NCF1,TAOK2,PYGO2,CD44,ADAM8,SLC5A3,CLDN4,RPS6KA5,NOS1,RGS14,SIPA1L2,ACTN4,ARHGAP11A,STK4,MARK3,EPHB2,SLC1A1,IL18,LRRC52,DLC1,ARID5B,GRTP1,PDP1,MOB3B,PAX6,PRKCZ,GRIN2B,KCTD7,ANGPT1,OSBPL8,TBC1D10C,CCL22,PRKCQ,HDAC2,CD300A,PPP2CA,SKP1,ANGPT4,DNAJC9,RFC5,SP100,ANXA2,PARN,SRGAP2,ADCY1,MADD,SYK,RAP1GAP2,CACNG3,DVL2,TAF1,TICAM1,TANK,HSF1,DHFR,SASH1,ASAP1,RALGAPA1,VAV3,MOB3A,CAV2,GNA12,AKTIP,AXL,TRIM37,MET,TBC1D10A,MALT1,PHB,PTPRC,EDA,TYRO3,EDNRA,BMP4
GO:0048513	animal organ development	4.356071687392164e-17	CLRN1,DNAH11,RCN1,ENPP1,PRDX2,ADAMTS16,LDB2,NRXN1,RDH13,PRKCI,TACC2,HLX,SLC9A1,NEGR1,PBX3,TRPS1,CBFB,CLASP2,SEMA3A,IL31RA,GRID2,RPS6KA2,TENM3,DOCK1,CLDN18,RYR1,NRG3,ASH1L,SEMA3D,PHACTR1,STOX2,FHOD3,NLGN4X,DCHS2,UNC5C,ZSWIM6,PRLR,HIVEP3,FTO,UTRN,KALRN,SPRR2D,NTRK3,CBL,ARNT,ATRNL1,SEMA5A,FLT3,STAT5B,TOX,ATP2B2,PLCE1,MAP2K5,SPATA5,KITLG,SPNS2,NDUFV2,LRP2,PIK3CD,SP3,ANK2,OLFM1,MFAP5,MEGF10,ROBO1,CHD7,HTR2B,ITGB6,MECOM,TACC1,LMNA,TRAF6,ESRP1,ROBO2,ITPKB,DNAJA3,ABI1,ITGB1,TRIOBP,SLC24A3,PSMB7,KNDC1,ZC4H2,KIF26B,DSCAM,KLHL12,AKAP13,TTLL5,GFI1B,RUNX1,DAB1,NRIP1,THRB,XIRP2,IMPG2,LAMA2,TCIRG1,PTPN11,DOCK10,ERBB4,ZNF609,ATP8A2,ACTG2,LARGE,CECR2,ESR1,MYO18B,NPHP3,ANKRD11,PTPRO,CDON,NTRK2,EP300,CELA1,MEGF9,DYM,HOOK3,PDGFB,KIRREL3,CSGALNACT1,TOB2,INSR,RERE,PRKAR1A,ATP8B1,H2AFY2,LAMA3,TCF7L2,PIP4K2A,FBXW11,ESRRB,MAP3K4,BASP1,RBM20,SLC4A10,TBR1,STRC,TFAP2A,ATRN,AMIGO1,FANCA,TGIF2,NPRL3,PHACTR4,MEIS1,TENM4,LCK,MDM4,ZNF148,MYO3B,TFDP2,LAMC1,PTPRU,MB,SGCD,DMD,CENPF,CRISPLD2,KAT7,WDR7,SLC8A1,PRPS2,GPR171,SATB2,PAFAH1B2,MACROD2,JPH2,FYN,ARNTL,ADAMTS9,NF1,PLCB1,MGMT,PCDH15,ARID4B,FAT3,RTN4,AFF2,RXFP1,CHRDL1,APOD,RTN4R,BCR,CHI3L1,CCDC141,TTN,NDRG4,BMP2K,ANKH,NTN1,EGFLAM,DCTN1,SLC4A5,NRG1,MAP2K1,MDFI,FNIP1,STIM1,VAX2,MYH9,MAD1L1,VGLL4,CNTN4,HDAC5,NTNG1,NFIA,ROR2,DCN,SLC39A14,PRPSAP2,LIN7A,DOCK2,PLXDC1,SOX13,ACSBG1,DMC1,EXOC4,MAD2L2,NLN,FOXN3,ANO6,ARL3,NEUROD1,CCBE1,JAK2,TRAPPC9,FBXW7,SYT1,ITCH,BCL11B,RBFOX1,TMC1,LRIG3,PKNOX1,CLDN1,MLLT3,PNPLA1,PLS1,TEX11,TCF7,PDE2A,KLF15,TBX15,WNT11,IFT80,IMMP2L,NOX4,SIPA1L3,CACNA1H,SOX6,TAB2,ACVR2A,RUNX2,SEMA5B,CD4,TGFB1,PCSK5,PDLIM4,MYO3A,ZHX2,KEL,BTRC,F2RL1,RPGRIP1L,CELSR1,SLIT3,SDK2,SLIT2,TP73,MUSTN1,CDH23,CORO1C,DISC1,LRRC10,LAMC2,TTBK2,SMAD6,RXFP2,BNC2,CLOCK,TCF12,ZNF675,SMOC1,ETV6,SYNJ2,NELL1,PRR14,TFAP2D,BMPER,BCL3,DCLK1,ANKRD54,PARVA,SLC17A7,SBNO2,FGF10,CNTNAP2,FBXL17,GREB1L,PPL,LOXL3,SVIL,CAPN3,SMURF2,EPHA4,RORA,PRKCA,TNFSF11,PPP3CA,NSUN2,MAG,CAMK4,UFL1,CTNNB1,SOD2,FCGR2B,SMARCC1,IGF1R,PPARG,DLG5,IL18R1,BFSP1,ADD2,OTC,XK,BRIP1,RYR2,DRAXIN,LEPR,FGF1,NIN,NR4A3,DCT,MYOCD,CACNA1C,GLG1,PHLDB1,MYO1E,CSMD1,DCC,CTDP1,MMP16,NF2,FLT4,MEF2B,BICC1,HDAC4,PAX2,ELN,SFRP1,FOXO3,ARL13B,NFIB,IFT122,SMAD3,PTPRM,ARNT2,NHS,ABI2,PSAP,NRP2,VANGL1,BTBD3,PREX1,HOXD3,HOXD4,TAB1,NFATC1,CDC73,APP,SSBP3,GSX2,PDGFRA,YAP1,HEG1,NLGN2,TNFRSF19,ALPL,LRP5,PTPRG,SLC9B2,SOX2,SETD2,PLAC1,GPC6,TTC39C,ADAMTS2,CHST11,TEAD1,PRICKLE1,RCAN1,VASH2,DRD1,FRS2,PALB2,SFMBT1,MMP2,TPH1,SYNE2,KDM6A,STAT1,SLC6A3,HNF4A,SHANK2,STRA6,EREG,ABLIM1,MYH15,DSCAML1,SEMA6D,ATF2,POU2F2,TCF3,RAF1,CELF4,ADAMTS12,BMPR2,DPYSL2,BMPR1A,AP1B1,PABPC4,TSPAN12,CTNNA2,CERS3,EVC,MYB,FGF2,SEMA4D,SH3PXD2A,RORC,PLXNA2,FNDC3A,SETD1A,JARID2,KLHL3,ITGA11,RARB,NCOA1,GAS8,OC90,CHRD,DVL3,EIF2B5,EPHB3,ATF3,FBXO45,MATN3,SRSF5,EBF2,ADAM20,ADAM21,TSG101,LAMB1,CLIC4,COL11A1,KCNQ1,WNT3,ADAMTS6,RHOJ,SUFU,TG,SCUBE2,PAFAH1B1,POLE,CNTFR,COL4A3,AKAP6,GLI2,SCUBE1,ERCC1,TPD52,COL19A1,DAB2,THEMIS,PKHD1,USH2A,LDLRAD4,MYSM1,DZIP1,RELN,SIN3A,RSPO2,CSF3R,DOCK11,CRELD1,IQGAP1,ZNF423,LUZP1,MYO7A,ALK,INPP5D,CLASP1,TEAD4,HOXC13,GPM6B,XDH,OVOL2,RBBP6,PRKG1,PAX7,RGS7,ATP2B4,ACTL6B,ASXL3,DYX1C1,ASGR2,MAPKAPK2,ARID4A,MARK1,CDK6,PHF2,CELF1,TYR,TFRC,EPHA5,WWOX,MEF2A,DROSHA,GPC3,ADRBK1,PLS3,PSMB2,EYA1,HOXB3,HOXB4,HOXB5,HOXB6,TFEB,GHR,MNAT1,FLRT2,FGD1,ELAVL4,HDAC1,PLCG2,ROCK1,NID1,PAXIP1,EGR2,AP3B1,PTPN2,NTRK1,ACO2,PTPRQ,EPHB1,PDE6A,AXIN2,EPCAM,TUB,JAG2,BCOR,AP3D1,AP2B1,ADAM19,SRD5A2,PIFO,SPI1,EPHA7,FOXP2,CTNNA1,FBXW4,BCAR3,ILDR2,SDK1,MST1,KAZN,ERCC3,PTK7,SMYD1,TTC9,BMPR1B,MAGI2,PNPT1,HEY2,RC3H1,COL13A1,HYDIN,WNT7B,HMGCS2,PTPRS,TLL2,ABCA12,MITF,SRSF6,HIPK1,PKP2,RTN4RL1,BTK,SGCZ,ZNRF3,IGF1,MAPK1,PTEN,MIB1,SPRED2,BMP7,SOX5,LHFPL5,LRRK2,MEGF11,BAG6,LILRB4,TMEM108,ITGAM,PKD2,SHANK3,ALPK3,PDGFC,NCOA3,PPP2R3C,WNT7A,ZBED6,PLCL2,KAT6A,SH3PXD2B,BMP6,IL17RD,SOS1,TSHR,EXT1,ATRX,PRICKLE2,IKZF1,RAPGEF2,KCNC2,SMO,SUN1,BLOC1S3,CPT1A,MYLP

			F,TNFRSF11B,BPGM,TFE3,GRIN1,ADAM29,RHOA,NDE1,ROR1,TF,ONECUT2,CUL3,NFKBID,GSK3B,STAT6,ACTA2,MLF1,CCDC14,CDK13,ALAS2,UPF2,CD27,BOK,SULF1,MTHFD1L,KRT8,AGT,ADAR,FBN2,ZMPSTE24,NDRG2,PTK2,PHEX,PBX1,CPE,TRPC4AP,COL9A1,DLX1,SLC29A1,UGT1A1,PYGO2,SHC1,ECE1,RPS6KB1,STC2,SCEL,SETDB2,ALOX5,DNAJB6,MDGA2,SP7,CD44,ADAM8,CLDN4,PEX7,TBC1D23,PLEKHA1,STK4,TCF25,TUBB3,SPEF1,EPHB2,SLC1A1,WLS,TMEFF2,CLPTM1,IL18,DLC1,PPARA,ARID5B,CALCRL,PRRC2C,NOTCH4,SOBP,CRX,FBN1,PAX6,PRKCZ,FAM20C,GRIN2B,PHLDB2,KLHL1,NIPBL,UPB1,ASPN,ANGPT1,FHL2,LRRK1,ATF6,SHROOM4,GCNT2,ETS1,PPP1CA,NEBL,CDK5RAP1,CNTN1,NCOR2,BRAF,CSRNP1,HDAC2,POU3F3,NOTO,SPINT2,TNR,PPP2CA,ACAN,ATXN1,ANXA2,CR2,SRGAP2,TMEFF1,ADCY1,NUMB,SYK,WWTR1,SLIT1,ASB1,CIT,RADIL,BRCA2,DVL2,NEB,PTPRJ,TAF1,RNF38,GPM6A,POTEE,CADM1,HSF1,MAX,EZH1,ADAMTS7,BMP1,ZP3,CAV2,AXL,TNFSF9,CD109,MET,TIMELESS,UCHL5,ANKRD6,MALT1,TRA2B,KDM2B,SEMA3C,PTPRC,EDA,GPR89A,TYRO3,VPS52,TRAF3IP2,CREB1,EDNRA,GNGT1,BMP4
GO:0051239	regulation of multicellular organismal process	4.3952382214084724e-17	ENPP1,PRDX2,NRXN1,ASPH,PRKCI,MAP4K4,PDE4D,S1PR2,HLX,SLC9A1,PBX3,TRPS1,CBFB,CLASP2,SEMA3A,GRID2,SETD4,DOCK1,WWC1,CLDN18,NOS1AP,LATS2,NOX5,EPB41,SEMA3D,PTH2R,TIAM2,KSR2,MAPRE2,NLGN4X,PTGIS,WWC3,TJP1,PRLR,ADCY7,FTO,KALRN,NTRK3,ARNT,SEMA5A,STAT5B,TOX,ENPP2,ATP2B2,GRM5,PLCE1,MARVELD3,MAP2K5,KITLG,PTPRR,LRP2,PIK3CD,ANK2,OLFM1,EZR,ROBO1,CHD7,HTR2B,ITGB6,CDKL5,RYR3,LMNA,TRAF6,ESRP1,ROBO2,ITPKB,CTNNA3,ITGB1,HSP90AA1,CNIH2,MCC,DSCAM,GFI1B,RUNX1,DAB1,OMA1,ABCC8,THRB,LAMA2,PTPN11,RBM19,CACNB2,HDAC6,GOLGA4,ADORA1,EPHA1,IKBKB,ERBB4,ADRA1D,LIMK1,ATP8A2,ESR1,NPHP3,PTPRO,CDON,NTRK2,CELA1,HOOK3,PDGFB,RIMS1,TOB2,BPI,STK39,INSR,H2AFY2,LAMA3,TCF7L2,CDH4,LITAF,ESRRB,PTPRD,BASP1,BDNF,TFAP2A,ATRN,AMIGO1,FANCA,KCNMA1,CHRM3,TGIF2,MEIS1,TENM4,IL5RA,VAMP7,ARHGAP42,RFTN1,NPLOC4,DMD,KAT7,SLC8A1,GPR171,ARNTL,ADAMTS9,NF1,PLCB1,RTN4,APOD,RTN4R,BCR,PPP1R12B,SHISA9,CHI3L1,BMP2K,ANKH,NTN1,NRG1,BDKRB2,MAP2K1,STIM1,VGLL4,HDAC5,CMKLR1,TSPAN8,ROR2,DCN,SOX13,MAD2L2,TLE6,CAPRIN2,ANO6,NEUROD1,CCBE1,JAK2,FBXW7,KCNB2,CLSTN2,ITCH,MLIP,NPSR1,SCN4A,PLS1,TCF7,SEPT7,ANXA4,WNT11,FIG4,KREMEN1,CACNA1H,IL1RAPL1,CAMK2B,PRKCD,SOX6,ACVR2A,RUNX2,SEMA5B,CD4,TGFB1,SGK1,PIBF1,DLGAP1,F2RL1,BCAS3,C9ORF47,SLIT2,TP73,CORO1C,DISC1,KCNJ3,CACNA1D,MTDH,SMAD6,CLOCK,ZNF675,NELL1,SUCO,BMPER,BCL3,ANKRD54,ADAM12,HOMER2,LINGO2,FGF10,LOXL3,MAST2,CAPN3,LUM,SMURF2,EPHA4,RORA,PRKCA,CNR1,CD6,TNFSF11,PPP3CA,MAG,CAMK4,UFL1,CTNNB1,FCGR2B,IGF1R,PPARG,DLG5,IL18R1,IL1RL1,CELF2,CYBB,SCAMP5,KCNJ12,RYR2,DRAXIN,LEPR,FGF1,NIN,PROS1,NR4A3,NOL3,DCT,MYOCD,PER2,KIR2DL4,CACNA1C,GLG1,SCN3B,PHLDB1,PRDM16,CAPN2,DIO2,GRM1,DCC,CTDP1,HS3ST5,NF2,FLT4,HDAC4,PAX2,SPTBN1,SFRP1,FOXO3,NFIB,SMAD3,CUX2,ITPR1,PTPRM,SHANK1,NRP2,DOCK4,ESRRG,NAV3,PRTG,CDC73,APP,GSX2,PDGFRA,DIP2B,NOX1,YAP1,HEG1,RAB11FIP5,FSTL4,NLGN2,RNLS,ALPL,JAK1,ANGPTL4,TNNI3K,PEMT,POLR3G,PTPRG,ISLR2,SLC9B2,SETD2,PRICKLE1,SPTBN4,VASH2,CHUK,FRS2,SFMBT1,MMP2,TPH1,PRKD1,STAT1,PARP6,SLC6A3,HNF4A,EREG,SEMA6D,ATF2,POU2F2,CELF4,CARD16,CASP1,ADAMTS12,POLA1,BMPR2,BMPR1A,TSPAN12,NLGN1,RAB11A,MYB,FGF2,PPM1F,SEMA4D,ISM1,PLXNA2,PTAFR,JARID2,DDX58,RAB11FIP3,RARB,SPEN,EHMT1,EPHB3,EBF2,VCL,KCNQ1,WNT3,ZNF322,RHOJ,TG,NLGN3,SCUBE2,PAFAH1B1,COL4A3,DDAH1,NMUR2,AKAP6,TRPC5,GLI2,RUFY3,MGLL,DAB2,LDLRAD4,MYSM1,WNK1,RELN,RSPO2,CSF3R,PELI1,IQGAP1,MAP3K7,APOLD1,ZNF423,SP1,MACF1,SLC8A2,INPP5D,CLASP1,DSC2,TEAD4,GPM6B,XDH,LTBP1,OVOL2,SNX5,PRKG1,ATP2B4,TMEM2,PAK3,ADNP,MAPKAPK2,MAP1B,SCARB1,MARK1,CDK6,CELF1,TFRC,MEF2A,DROSHA,ADRBK1,PTGER3,BRINP1,TRPV1,RNF216,HOXB3,GHR,FLRT2,ACTR2,LGI4,HDAC1,SMOC2,ADIPOR2,PLCG2,ROCK1,C1QTNF1,PAXIP1,EGR2,RNF10,GPR35,AP3B1,PTPN2,NTRK1,KPNA6,EPHB1,AXIN2,PARD3,TRIM46,KCND3,BCOR,AP3D1,NETO1,CD84,CUX1,SPI1,EPHA7,CHRNB4,CTNNA1,TRDN,MAP3K13,RAPGEF3,SH3BP1,MST1,CHID1,BMPR1B,USP50,HEY2,RC3H1,PTPRS,TRIP12,ADTRP,ABCA12,MITF,SRSF6,OPRD1,HIPK1,PKP2,BTK,IGF1,MAPK1,PTEN,SPRED2,BMP7,SOX5,PRCP,LRRK2,ZBTB20,BAG6,LILRB4,TMEM108,EFNA5,SHANK3,PDGFC,NCOA3,CUEDC2,PPP2R3C,WNT7A,NLRP1,PLCL2,GPR21,BMP6,GPI,IL17RD,SOS1,TSHR,WDR43,N4BP1,PDCD1LG2,RAPGEF2,SMO,TNFRSF11B,DECR1,TFE3,GRIN1,RHOA,SYNGAP1,TF,FAM19A4,HSPD1,NFKBID,GSK3B,ABCG8,STAT6,EEF2K,FAM49B,CD27,SULF1,TNFRSF8,AGT,ELOVL3,SYT7,FBN2,ZMPSTE24,NDRG2,PTK2,PDE4B,RIMS2,PBX1,TCP11,EXOSC3,DLX1,NCF1,SHC1,CD160,ECE1,RPS6KB1,STC2,PRG3,ALOX5,ADAM8,SLC5A3,RPS6KA5,CXCL17,NOS1,RGS14,MC1R,STK4,EPHB2,SLC1A1,CLPTM1,IL18,HCAR2,PPARA,CALCRL,EPB41L4B,GPSM3,NOTCH4,FBN1,PAX6,PRKCZ,ZC3HAV1,ATP1A3,CD96,FAM20C,ADORA2A,PPP1CC,RABGEF1,PHLDB2,OTUD7B,NIPBL,TMIGD2,ASPN,ANGPT1,IGF2BP3,GCNT2,ETS1,MAP2,BTN3A2,FBXO31,PRKCQ,BRAF,HDAC2,HTR2C,TNR,CDKL3,PPP2CA,ANGPT4,SP100,ANXA2,NUMB,SYK,CNOT1,WWTR1,SLIT1,ASB1,BRCA2,PTPRJ,ATP2B3,TICAM1,CADM1,HSF1,PLEKHM1,SASH1,ADAMTS7,GBP5,SULT2B1,BMP1,ZP3,DLGAP2,AXL,TNFSF9,CD109,MET,CAMK2D,CCR3,MALT1,SETD3,KDM2B,SEMA3C,PHB,PTPRC,SPON2,SYNDIG1,CREB1,SHISA6,CLNK,EDNRA,PRAP1,BMP4

GO:0120035	regulation of plasma membrane bounded cell projection organization	5.1886809225935036e-17	CLRN1,ADAMTS16,NRXN1,PRKCI,NEGR1,SEMA3A,GRID2,TENM3,SEMA3D,TIAM2,KALRN,NTRK3,SEMA5A,TOX,ENPP2,PLCE1,FER,EZR,ROBO1,CDKL5,TENM1,ROBO2,ULK4,KNDC1,DSCAM,ARHGAP24,DAB1,CDC42EP3,MYO10,GOLGA4,LIMK1,ATP8A2,PTPRO,NTRK2,EP300,TENM2,TNIK,ATP8B1,CDH4,PAK1,PTPRD,TBR1,BDNF,AMIGO1,DENND5A,DMD,RIT2,TANC2,FYN,FAT3,RTN4,RTN4R,NDRG4,PAQR3,NTN1,MAP2K1,NTNG1,TMEM30A,ROR2,UST,CAPRIN2,NCKAP1,RCC2,ZDHHC15,CROCC,LZTS1,PLS1,SEPT7,FIG4,KREMEN1,IL1RAPL1,CAMK2B,PRKCD,SEMA5B,KEL,F2RL1,BCAS3,SLIT2,CORO1C,DISC1,CEP135,KANK1,EPHA4,AUTS2,CNR1,PPP3CA,MAG,FCGR2B,CDHR2,IGF1R,XK,DRAXIN,NIN,DCC,HDAC4,SFRP1,DNM3,CUX2,ABI2,NEDD4,DIP2B,YAP1,FSTL4,PTPRG,ISLR2,KIF24,SYNE2,PRKD1,PARP6,DGKG,SEMA6D,BMPR2,DPYSL2,CAMK1D,NLGN1,CTNNA2,RAB11A,CRMP1,LRRC4C,SEMA4D,PLXNA2,SPOCK1,EPHB3,WNT3,PAFAH1B1,TRPC5,RUFY3,DAB2,DZIP1,RELN,DOCK11,MACF1,ALK,MBOAT1,PACSIN1,PAK3,ADNP,MAP1B,MPHOSPH9,MARK1,ACTR2,ELAVL4,EPS8,NTRK1,PTPN9,SARM1,TRIM46,CARM1,ARHGAP44,CUX1,EPHA7,MAP3K13,PTK7,MAGI2,PTPRS,CDKL1,PLD1,RTN4RL1,CBFA2T2,HTT,DPYSL3,PTEN,BMP7,LRRK2,EFNA5,SHANK3,WNT7A,MAP4,RAPGEF2,MARK4,RHOA,SYNGAP1,ROR1,GSK3B,RDX,EEF2K,AGT,SNX3,GAP43,CD44,EPHB2,GRIN2B,MAP2,FBXO31,ATG3,CNTN1,BRAF,HDAC2,NOTO,TNR,CDKL3,SLIT1,GPM6A,PLEKHM1,INPP5F,SEMA3C,NEDD4L
GO:0048589	developmental growth	3.008893756787155e-16	HLX,LLPH,CLASP2,SEMA3A,WWC1,LATS2,SEMA3D,NLGN4X,WWC3,PRLR,FTO,SEMA5A,RAD51B,STAT5B,OLFM1,EZR,CHD7,CDKL5,ITGB1,HSP90AA1,KIF26B,DSCAM,AKAP13,RUNX1,PTPN11,HDAC6,GOLGA4,ERBB4,LIMK1,ATP8A2,LARGE,ESR1,EP300,CELA1,RIMS1,INSR,PRKAR1A,CDH4,BASP1,SLC4A10,BDNF,ATRN,TENM4,DMD,PLCB1,PCDH15,RTN4,APOD,RTN4R,NDRG4,NTN1,NRG1,VGLL4,SYT1,PLS1,CYFIP2,WNT11,IFT80,SEMA5B,TGFB1,SLIT3,SLIT2,TP73,MUSTN1,DISC1,BNC2,ITSN2,DCLK1,FGF10,CAPN3,AUTS2,PPP3CA,MAG,CTNNB1,PARK2,CPQ,DRAXIN,LEPR,FGF1,NIN,CPNE6,DCC,CTDP1,SPG11,SFRP1,FOXO3,SMAD3,PSAP,SYT3,NRP2,APP,DIP2B,YAP1,HEG1,FSTL4,ISLR2,SOX2,CHST11,SPTBN4,PALB2,CPNE9,KDM6A,SLC6A3,STRA6,EREG,SEMA6D,ATF2,BMPR2,DPYSL2,BMPR1A,TAF8,SPAG9,RAB11A,EVC,FGF2,SEMA4D,JARID2,RARB,VCL,CLIC4,WNT3,NLGN3,PAFAH1B1,AKAP6,RASAL1,TRPC5,GLI2,ERCC1,RUFY3,WDTC1,SIN3A,RSPO2,EYS,IQGAP1,MACF1,RBBP6,ADNP,MAP1B,CELF1,GHR,UNC13A,TRIM46,EPHA7,MAP3K13,MST1,PTK7,BMPR1B,MAGI2,HEY2,WNT7B,PTPRS,KMT2D,TLL2,IGF1,PTEN,RAPH1,TMEM108,EFNA5,WNT7A,GPR21,SOS1,TSHR,EXT1,ATRX,PRMT2,SMO,SYT17,GSK3B,AGT,ZMPSTE24,RIMS2,TEC,GAP43,PYGO2,RPS6KB1,STC2,PLEKHA1,SCNN1B,STK4,PPARA,ARID5B,PRKCZ,NIPBL,MAP2,IMPACT,TNR,CDKL3,WWTR1,SLIT1,BRCA2,ZP3,SEMA3C,NEDD4L,SH3GL2,CREB1,EDNRA,BMP4,CPNE1
GO:0048585	negative regulation of response to stimulus	3.0655584307168257e-16	ENPP1,PRDX2,NRXN1,PDE4D,S1PR2,HLX,ADCY8,PDE8A,CLASP2,SEMA3A,GRID2,WWC1,CTDSPL2,ASH1L,LATS2,SEMA3D,NLGN4X,PTGIS,WWC3,MVP,RGS7BP,CBL,SEMA5A,SUSD4,GRM5,SAMHD1,FER,CASK,MARVELD3,MAP2K5,PTPRR,LRP2,ZNF536,DEPDC5,SH2D1A,EZR,ROBO1,TNFAIP8L1,HTR2B,MECOM,LMNA,ROBO2,DNAJA3,OXR1,ITGB1,PSMB7,MCC,CCDC22,ARHGAP24,DAB1,ABCC8,RGS6,HERC4,ADORA1,GPRASP1,ESR1,NPHP3,PMEPA1,PTPRO,RGS22,PDGFB,TCF7L2,PIP4K2A,LITAF,FBXW11,PTPRD,BDNF,NPRL3,PHACTR4,UBR2,SNX6,ARHGAP42,PTPRU,NPLOC4,DMD,FYN,MKRN2,ARNTL,NF1,CHRDL1,APOD,RTN4R,BCR,NDRG4,PAQR3,PIK3R2,RANBP9,NOMO3,NRG1,BDKRB2,BID,MDFI,FNIP1,MAD1L1,LRFN5,TMBIM6,VGLL4,PPP2CB,TSPAN8,ROR2,DCN,CREBRF,SOX13,PTPRT,AUNIP,MAD2L2,TLE6,RGS8,TERF2,NEUROD1,RFFL,FBXW7,LINC00473,ITCH,MLIP,ZFYVE28,MLLT3,PDE2A,WNT11,IFT80,TRIM59,KREMEN1,PRKCD,RUNX2,SEMA5B,TGFB1,MMP28,IGSF1,PIBF1,BTRC,F2RL1,RPGRIP1L,SLIT3,SLIT2,KANK1,MMP26,SMAD6,CLOCK,ZNF675,BMPER,BCL3,DUSP22,NAIP,HOMER2,FGF10,RASA4,RASA4B,KCTD10,LOXL3,SMURF2,EPHA4,RORA,UFL1,CTNNB1,PARK2,SOD2,FCGR2B,IGF1R,PPARG,AXIN1,OTUB1,DLG5,IL1RL1,DRAXIN,LEPROT,PROS1,RGS10,NR4A3,NOL3,MYOCD,KIR2DL4,AJUBA,GLG1,CHEK2,UBQLN4,PRDM16,HCK,NF2,BICC1,TRABD2B,SFRP1,FOXO3,IFT122,SMAD3,ITPR1,WWP2,NEDD4,TXNDC12,NFATC1,PDGFRA,YAP1,HEG1,AMFR,SESN1,FSTL4,EYA2,SOX2,CHST11,PRICKLE1,RCAN1,ZNF653,DRD1,TMEM14A,STAT1,DGKG,SLC6A3,SHANK2,SEMA6D,GRAMD4,RAF1,CELF4,CARD16,PTPN1,ADAMTS12,GGT7,CTNNA2,PDE11A,FGF2,ADCY5,SEMA4D,MCTP1,DKK2,CHRD,PTPRE,RBX1,ATF3,PAWR,DEPTOR,TSG101,CCDC3,WNT3,SUFU,PAFAH1B1,PRDM15,DDAH1,RASAL1,GLI2,ERCC1,DAB2,PKHD1,UBR1,LDLRAD4,RUVBL2,ARHGAP12,INPP5D,CLASP1,XDH,LTBP1,OVOL2,SNX5,PRKG1,RGS7,ATP2B4,MASP1,GPR161,CDK6,RNF34,WWOX,CR1L,GFRAL,GPC3,ADRBK1,PSMB2,EYA1,TSPAN6,HDAC1,INVS,RECQL5,C1QTNF1,HIPK3,PTPN2,EIF3A,AXIN2,SARM1,MUC1,ARHGAP44,CD84,SPI1,CTNNA1,STK38,OTUD3,SH3BP1,SNCA,MAGI2,HEY2,RC3H1,PTPRS,ZMYND11,TRIP12,ADTRP,RASA1,RTN4RL1,ZNRF3,KCTD13,CBFA2T2,IGF1,HTT,PTEN,SPRED2,BMP7,RASA2,PCBP2,LRRK2,LILRB4,ARHGAP25,NT5E,RGS9,UACA,CRTC3,PLCL2,GPR21,IL17RD,SH3RF2,VRK3,ENPP3,SOX30,UBR5,GRB14,JADE1,RHOA,SYNGAP1,GRIN3A,RQCD1,ONECUT2,CUL3,GSK3B,STAT6,FAM49B,NECAB2,DUSP26,BOK,SULF1,RNF43,AGT,PRKAA2,ADAR,FBN2,STAT2,NDRG2,PDE4B,VEPH1,APCDD1L,DLX1,CD160,RPS6KB1,ALOX5,CD44,CSNK1A1,CXCL17,PLEKHA1,RGS14,STK4,MARK3,EPHB2,DLC1,PPARA,PPP1R10,CALCRL,PHLPP1,ZNF366,FBN1,PRKCZ,CD96,ADORA2A,RABGEF1,PHLDB2,OTUD7B,ASPN,EYA3,FHL2,TBC1D10C,VWC2,RBMS3,NCOR2,PRKCQ,TNR,C

			D300A,PHIP,PPP2CA,SH3BP4,CR2,SYK,CNOT1,WWTR1,SLIT1,CIT,YBX1,PTPRJ,TANK,HSF1,CGNL1,CAV2,INPP5F,WIF1,CD109,MET,ANKRD6,SEMA3C,PHB,PTPRC,PBLD,TYRO3,RNF213,ATF6B,SHISA6,TRAP1,PRAP1,BMP4,CPNE1
GO:0010975	regulation of neuron projection development	3.3913711848970854e-16	PRKCI,NEGR1,SEMA3A,GRID2,TENM3,SEMA3D,TIAM2,KALRN,NTRK3,SEMA5A,TOX,ROBO1,CDKL5,ROBO2,ULK4,KNDC1,DSCAM,DAB1,GOLGA4,LIMK1,ATP8A2,PTPRO,NTRK2,EP300,TNIK,CDH4,PAK1,PTPRD,TBR1,BDNF,AMIGO1,DENND5A,DMD,RIT2,TANC2,FYN,FAT3,RTN4,RTN4R,NDRG4,PAQR3,NTN1,MAP2K1,NTNG1,TMEM30A,ROR2,UST,CAPRIN2,ZDHHC15,LZTS1,FIG4,KREMEN1,IL1RAPL1,CAMK2B,SEMA5B,KEL,SLIT2,DISC1,KANK1,EPHA4,CNR1,PPP3CA,MAG,IGF1R,XK,DRAXIN,NIN,DCC,SFRP1,DNM3,CUX2,ABI2,NEDD4,DIP2B,FSTL4,PTPRG,ISLR2,PRKD1,PARP6,DGKG,SEMA6D,BMPR2,DPYSL2,CAMK1D,NLGN1,CTNNA2,RAB11A,CRMP1,LRRC4C,SEMA4D,PLXNA2,SPOCK1,EPHB3,WNT3,PAFAH1B1,TRPC5,RUFY3,DAB2,RELN,MACF1,ALK,MBOAT1,PACSIN1,PAK3,ADNP,MAP1B,MARK1,ACTR2,ELAVL4,NTRK1,PTPN9,SARM1,TRIM46,CARM1,ARHGAP44,CUX1,EPHA7,MAP3K13,PTK7,MAGI2,PTPRS,RTN4RL1,CBFA2T2,DPYSL3,PTEN,BMP7,LRRK2,EFNA5,SHANK3,WNT7A,RAPGEF2,RHOA,SYNGAP1,ROR1,GSK3B,EEF2K,AGT,SNX3,EPHB2,MAP2,FBXO31,CNTN1,BRAF,HDAC2,TNR,CDKL3,SLIT1,INPP5F,SEMA3C,NEDD4L
GO:0010648	negative regulation of cell communication	7.619089021861706e-16	ENPP1,PRDX2,PDE4D,S1PR2,ADCY8,GRID2,WWC1,CTDSPL2,ASH1L,LATS2,NLGN4X,WWC3,MVP,RGS7BP,KALRN,CBL,GRM5,SAMHD1,MARVELD3,MAP2K5,PTPRR,LRP2,ZNF536,DEPDC5,SH2D1A,EZR,ROBO1,TNFAIP8L1,HTR2B,MECOM,LMNA,DNAJA3,SLC24A2,ITGB1,PSMB7,MCC,PCDH17,CCDC22,ARHGAP24,DAB1,ABCC8,RGS6,DGKI,PTPN11,HERC4,ADORA1,GPRASP1,ESR1,NPHP3,PMEPA1,PTPRO,RGS22,TCF7L2,PIP4K2A,LITAF,FBXW11,PTPRD,BDNF,NPRL3,PHACTR4,UBR2,SNX6,ARHGAP42,PTPRU,NPLOC4,DMD,MKRN2,ARNTL,NF1,CHRDL1,APOD,NDRG4,PAQR3,PIK3R2,RANBP9,NOMO3,NRG1,BDKRB2,BID,MDFI,FNIP1,MAD1L1,TMBIM6,VGLL4,PPP2CB,ROR2,DCN,CREBRF,SOX13,PTPRT,MAD2L2,TLE6,RGS8,NEUROD1,RFFL,FBXW7,LINC00473,ITCH,ZFYVE28,MLLT3,PDE2A,WNT11,IFT80,TRIM59,KREMEN1,PRKCD,RUNX2,IGSF1,PIBF1,BTRC,F2RL1,GRIK3,RPGRIP1L,SLIT3,SLIT2,KANK1,SMAD6,CLOCK,ZNF675,BMPER,BCL3,DUSP22,NAIP,HOMER2,FGF10,RASA4,RASA4B,KCTD10,SMURF2,EPHA4,RORA,PPP3CA,UFL1,CTNNB1,PARK2,SOD2,FCGR2B,IGF1R,PPARG,AXIN1,DLG5,DRAXIN,LEPROT,RGS10,NOL3,MYOCD,AJUBA,GLG1,CHEK2,PRDM16,NF2,BICC1,TRABD2B,SFRP1,FOXO3,IFT122,SMAD3,ITPR1,WWP2,NEDD4,SLC6A1,TXNDC12,NFATC1,YAP1,HEG1,AMFR,RAB11FIP5,SESN1,FSTL4,EYA2,SOX2,CHST11,PRICKLE1,RCAN1,ZNF653,DRD1,TMEM14A,STAT1,DGKG,SHANK2,GRAMD4,RAF1,CELF4,CARD16,PTPN1,ADAMTS12,PDE11A,FGF2,DKK2,SORCS2,RAB11FIP3,CHRD,PTPRE,RBX1,ATF3,PAWR,DEPTOR,TSG101,CCDC3,SUFU,PAFAH1B1,PRDM15,RASAL1,GLI2,DAB2,PKHD1,UBR1,LDLRAD4,RUVBL2,ARHGAP12,INPP5D,XDH,LTBP1,OVOL2,SNX5,RGS7,ATP2B4,ATAD1,ADNP,GPR161,RNF34,SORCS3,WWOX,GFRAL,GPC3,ADRBK1,PSMB2,EYA1,TSPAN6,HDAC1,INVS,HIPK3,GPR35,PTPN2,EIF3A,AXIN2,SARM1,MUC1,ARHGAP44,SPI1,CTNNA1,STK38,OTUD3,SH3BP1,SNCA,MAGI2,HEY2,PTPRS,ZMYND11,RASA1,ZNRF3,KCTD13,CBFA2T2,IGF1,HTT,PTEN,SPRED2,BMP7,RASA2,LRRK2,LILRB4,ARHGAP25,SHANK3,RGS9,UACA,CRTC3,PLCL2,GPR21,IL17RD,SH3RF2,VRK3,STXBP5L,SOX30,GRB14,JADE1,RHOA,SYNGAP1,RQCD1,ONECUT2,CUL3,GSK3B,FAM49B,NECAB2,DUSP26,BOK,SULF1,RNF43,AGT,PRKAA2,ADAR,FBN2,STAT2,NDRG2,PDE4B,VEPH1,APCDD1L,DLX1,CD160,RPS6KB1,CD44,CSNK1A1,PLEKHA1,RGS14,STK4,MARK3,EPHB2,DLC1,PPARA,PPP1R10,PHLPP1,SYTL4,ZNF366,FBN1,PRKCZ,RABGEF1,OTUD7B,ASPN,EYA3,FHL2,TBC1D10C,VWC2,RBMS3,NCOR2,PRKCQ,BRAF,TNR,CD300A,PHIP,PPP2CA,SH3BP4,CNOT1,WWTR1,GRIK2,CIT,PTPRJ,TANK,CGNL1,CAV2,INPP5F,WIF1,CD109,MET,ANKRD6,PHB,PTPRC,PBLD,TYRO3,RNF213,ATF6B,SHISA6,TRAP1,PRAP1,BMP4,CPNE1
GO:0016477	cell migration	9.46329616726348e-16	LDB2,GPC5,PRKCI,MAP4K4,S1PR2,SLC9A1,CLASP2,SEMA3A,DOCK1,WWC1,NRG3,ASTN2,SEMA3D,PHACTR1,MAPRE2,WWC3,TJP1,UNC5C,NTRK3,ATRNL1,SEMA5A,STAT5B,ENPP2,FER,MARVELD3,MAP2K5,KITLG,PTPRR,SPNS2,PIK3CD,MEGF10,ROBO1,HTR2B,ITGB6,CDKL5,LMNA,CTNNA3,VAV2,ULK4,ITGB1,ELMO2,SRGAP3,MCC,SRGAP2B,ARHGAP24,PTPRK,DAB1,ABCC8,LAMA2,DACH1,PTPN11,MGAT5,DOCK10,HDAC6,ADORA1,EPHA1,ERBB4,GBF1,ZNF609,PTPRO,NTRK2,MEGF9,PDGFB,AVL9,KIRREL3,STK39,INSR,RERE,UNC5D,LAMA3,PAK1,ATRN,POMGNT2,FUT8,FMNL2,PHACTR4,LCK,LAMC1,PTPRU,SLC8A1,SATB2,DEPDC1B,CASS4,FYN,ADAMTS9,NF1,PLCB1,FAT3,RTN4,DPEP1,APOD,BCR,CCDC141,NDRG4,NTN1,NRG1,BDKRB1,DOCK8,MYH9,HDAC5,NTNG1,NAV1,CMKLR1,ROR2,DCN,CDH13,PTPRT,NCKAP1,DEFA1B,CD99,RCC2,ANO6,KIF2A,RFFL,CCBE1,JAK2,FBXW7,BCL11B,CLDN1,CDC42BPB,WNT11,ASTN1,CAMK2B,PRKCD,SEMA5B,TGFB1,SGK1,MMP28,F2RL1,BCAS3,CELSR1,SLIT2,CORO1C,RRAS2,DISC1,KANK1,LAMC2,TTBK2,BMPER,DCLK1,DAPK2,PARVA,DUSP22,FGF10,SMURF2,EPHA4,PRKCA,AUTS2,TNFSF11,PPP3CA,PIP5KL1,CTNNB1,SOD2,IGF1R,PPARG,DLG5,ADD2,FGF1,NR4A3,MYOCD,AJUBA,HCK,CSPG4,DCC,NF2,FLT4,HDAC4,SFRP1,FOXO3,ARL13B,SMAD3,PTPRM,ACKR2,ABI2,NRP2,PREX1,DOCK4,NAV3,APP,GSX2,PDGFRA,SLK,CEP85L,NOX1,LRP5,PTPRG,SETD2,GPC6,DRD1,MMP2,SYNE2,S100A12,PRKD1,RND3,SEMA6D,ADAMTS12,BMPR2,USP33,CAMK1D,BMPR1A,CTNNA2,PIK3R3,SPAG9,RAB11A,FGF2,PPM1F,NEDD9,PEAK1,SEMA4D,MCTP1,ELP3,PLXNA2,PTAFR,DDX58,ITGA11,PIK3C2B,SPOCK1,CHRD,EPHB3,FBXO45,PKN3,VC

			L,LAMB1,CLIC4,RHOJ,CCL14,CCL15,CCL15-CCL14,PAFAH1B1,RUFY3,DAB2,USH2A,LDLRAD4,MYSM1,WNK1,RELN,CSF3R,IQGAP1,SP1,MACF1,CLASP1,OVOL2,NFATC2,PRKG1,ATP2B4,PAK3,SSH2,DYX1C1,MAP1B,CDC42BPA,SCARB1,MARK1,GPC3,FLRT2,SMOC2,PLCG2,ROCK1,EPS8,PAXIP1,ABCC1,PKN2,TMIGD1,EPHB1,TRIM46,SPI1,MCU,CTNNA1,LIMA1,SH3BP1,MST1,PTK7,MAGI2,ZEB2,ADTRP,MITF,KCTD13,IGF1,DPYSL3,MAPK1,PTEN,BMP7,PRCP,LRRK2,OSGIN1,ZRANB1,PDGFC,AMOTL1,WNT7A,GPI,SH3RF2,SOS1,EXT1,RAPGEF2,SMO,SUN1,RHOA,NDE1,ONECUT2,FAM19A4,CUL3,SH3KBP1,RDX,ACTA2,FAM49B,ITGBL1,SULF1,AGT,PTK2,PDE4B,TAOK2,RPS6KB1,GPR173,ANKS1A,ITGAL,ALOX5,CD44,ADAM8,CLDN4,CXCL17,PEX7,PLVAP,ACTN4,STK4,SPEF1,EPHB2,TMEFF2,XCR1,DLC1,ARID5B,EPB41L4B,GPSM3,ZMYND8,PAX6,PRKCZ,RABGEF1,PHLDB2,NIPBL,ANGPT1,OSBPL8,GCNT2,ETS1,BIN2,CCL22,FBXO31,PRKCQ,BRAF,DIAPH1,ATP8A1,POU3F3,TNR,CD300A,ANGPT4,CAMSAP3,SP100,FAT2,SRGAP2,NUMB,SYK,SLIT1,FRMD5,RADIL,PTPRJ,GPM6A,SASH1,VAV3,ZP3,GNA12,AXL,MET,FMNL1,CCR3,SEMA3C,PTPRC,TYRO3,EDNRA,BMP4
GO:0023057	negative regulation of signaling	1.1165852649432197e-15	*ENPP1,PRDX2,PDE4D,S1PR2,ADCY8,GRID2,WWC1,CTDSPL2,ASH1L,LATS2,NLGN4X,WWC3,MVP,RGS7BP,KALRN,CBL,GRM5,SAMHD1,MARVELD3,MAP2K5,PTPRR,LRP2,ZNF536,DEPDC5,SH2D1A,EZR,ROBO1,TNFAIP8L1,HTR2B,MECOM,LMNA,DNAJA3,SLC24A2,ITGB1,PSMB7,MCC,PCDH17,CCDC22,ARHGAP24,DAB1,ABCC8,RGS6,DGKI,PTPN11,HERC4,ADORA1,GPRASP1,ESR1,NPHP3,PMEPA1,PTPRO,RGS22,TCF7L2,PIP4K2A,LITAF,FBXW11,PTPRD,BDNF,NPRL3,PHACTR4,UBR2,SNX6,ARHGAP42,PTPRU,NPLOC4,DMD,MKRN2,ARNTL,NF1,CHRDL1,APOD,NDRG4,PAQR3,PIK3R2,RANBP9,NOMO3,NRG1,BDKRB2,BID,MDFI,FNIP1,MAD1L1,TMBIM6,VGLL4,PPP2CB,ROR2,DCN,CREBRF,SOX13,PTPRT,MAD2L2,TLE6,RGS8,NEUROD1,RFFL,FBXW7,LINC00473,ITCH,ZFYVE28,MLLT3,PDE2A,WNT11,IFT80,TRIM59,KREMEN1,PRKCD,RUNX2,IGSF1,PIBF1,BTRC,F2RL1,GRIK3,RPGRIP1L,SLIT3,SLIT2,KANK1,SMAD6,CLOCK,ZNF675,BMPER,BCL3,DUSP22,NAIP,HOMER2,FGF10,RASA4,RASA4B,KCTD10,SMURF2,EPHA4,RORA,PPP3CA,UFL1,CTNNB1,PARK2,SOD2,FCGR2B,IGF1R,PPARG,AXIN1,DLG5,DRAXIN,LEPROT,RGS10,NOL3,MYOCD,AJUBA,GLG1,CHEK2,PRDM16,NF2,BICC1,TRABD2B,SFRP1,FOXO3,IFT122,SMAD3,ITPR1,WWP2,NEDD4,SLC6A1,TXNDC12,NFATC1,YAP1,HEG1,AMFR,RAB11FIP5,SESN1,FSTL4,EYA2,SOX2,CHST11,PRICKLE1,RCAN1,ZNF653,DRD1,TMEM14A,STAT1,DGKG,SHANK2,GRAMD4,RAF1,CELF4,CARD16,PTPN1,ADAMTS12,PDE11A,FGF2,DKK2,SORCS2,RAB11FIP3,CHRD,PTPRE,RBX1,ATF3,PAWR,DEPTOR,TSG101,CCDC3,SUFU,PAFAH1B1,PRDM15,RASAL1,GLI2,DAB2,PKHD1,UBR1,LDLRAD4,RUVBL2,ARHGAP12,INPP5D,XDH,LTBP1,OVOL2,SNX5,RGS7,ATP2B4,ATAD1,ADNP,GPR161,RNF34,SORCS3,WWOX,GFRAL,GPC3,ADRBK1,PSMB2,EYA1,TSPAN6,HDAC1,INVS,HIPK3,GPR35,PTPN2,EIF3A,AXIN2,SARM1,MUC1,ARHGAP44,SPI1,CTNNA1,STK38,OTUD3,SH3BP1,SNCA,MAGI2,HEY2,PTPRS,ZMYND11,RASA1,ZNRF3,KCTD13,CBFA2T2,IGF1,HTT,PTEN,SPRED2,BMP7,RASA2,LRRK2,LILRB4,ARHGAP25,SHANK3,RGS9,UACA,CRTC3,PLCL2,GPR21,IL17RD,SH3RF2,VRK3,STXBP5L,SOX30,GRB14,JADE1,RHOA,SYNGAP1,RQCD1,ONECUT2,CUL3,GSK3B,FAM49B,NECAB2,DUSP26,BOK,SULF1,RNF43,AGT,PRKAA2,ADAR,FBN2,STAT2,NDRG2,PDE4B,VEPH1,APCDD1L,DLX1,CD160,RPS6KB1,CD44,CSNK1A1,PLEKHA1,RGS14,STK4,MARK3,EPHB2,DLC1,PPARA,PPP1R10,PHLPP1,SYTL4,ZNF366,FBN1,PRKCZ,RABGEF1,OTUD7B,ASPN,EYA3,FHL2,TBC1D10C,VWC2,RBMS3,NCOR2,PRKCQ,BRAF,TNR,CD300A,PHIP,PPP2CA,SH3BP4,CNOT1,WWTR1,GRIK2,CIT,PTPRJ,TANK,CGNL1,CAV2,INPP5F,WIF1,CD109,MET,ANKRD6,PHB,PTPRC,PBLD,TYRO3,RNF213,ATF6B,SHISA6,TRAP1,PRAP1,BMP4,CPNE1*
GO:0060560	developmental growth involved in morphogenesis	2.7334162056036948e-15	*LLPH,CLASP2,SEMA3A,SEMA3D,SEMA5A,OLFM1,CDKL5,ITGB1,HSP90AA1,KIF26B,DSCAM,HDAC6,GOLGA4,LIMK1,ESR1,RIMS1,CDH4,BDNF,RTN4,RTN4R,NTN1,SYT1,CYFIP2,WNT11,SEMA5B,TGFB1,SLIT3,SLIT2,DISC1,ITSN2,DCLK1,FGF10,AUTS2,MAG,CTNNB1,PARK2,DRAXIN,FGF1,NIN,CPNE6,DCC,SPG11,SFRP1,SYT3,NRP2,APP,DIP2B,YAP1,FSTL4,ISLR2,CPNE9,SEMA6D,BMPR2,DPYSL2,SPAG9,RAB11A,SEMA4D,VCL,WNT3,NLGN3,PAFAH1B1,RASAL1,TRPC5,RUFY3,SIN3A,IQGAP1,MACF1,ADNP,MAP1B,UNC13A,TRIM46,EPHA7,MAP3K13,MST1,PTK7,MAGI2,WNT7B,PTPRS,RAPH1,TMEM108,EFNA5,EXT1,SYT17,GSK3B,RIMS2,PRKCZ,MAP2,IMPACT,TNR,CDKL3,SLIT1,SEMA3C,NEDD4L,SH3GL2,EDNRA,BMP4,CPNE1*
GO:0048588	developmental cell growth	7.788142840315632e-15	*LLPH,CLASP2,SEMA3A,SEMA3D,SEMA5A,OLFM1,CDKL5,ITGB1,HSP90AA1,DSCAM,AKAP13,HDAC6,GOLGA4,LIMK1,RIMS1,CDH4,BDNF,RTN4,RTN4R,NTN1,SYT1,CYFIP2,SEMA5B,SLIT3,SLIT2,DISC1,ITSN2,DCLK1,AUTS2,MAG,CTNNB1,PARK2,DRAXIN,NIN,CPNE6,DCC,CTDP1,SPG11,SYT3,NRP2,APP,DIP2B,FSTL4,ISLR2,CPNE9,SEMA6D,BMPR2,DPYSL2,SPAG9,RAB11A,SEMA4D,VCL,WNT3,NLGN3,PAFAH1B1,AKAP6,RASAL1,TRPC5,RUFY3,SIN3A,IQGAP1,MACF1,ADNP,MAP1B,UNC13A,TRIM46,EPHA7,MAP3K13,PTPRS,KMT2D,IGF1,RAPH1,TMEM108,EFNA5,EXT1,PRMT2,SYT17,GSK3B,AGT,RIMS2,PPARA,PRKCZ,MAP2,IMPACT,TNR,CDKL3,SLIT1,SEMA3C,NEDD4L,SH3GL2,EDNRA,CPNE1*
GO:0009968	negative regulation of signal transduction	8.259926716251854e-15	*ENPP1,PRDX2,PDE4D,S1PR2,WWC1,CTDSPL2,ASH1L,LATS2,NLGN4X,WWC3,MVP,RGS7BP,CBL,GRM5,SAMHD1,MARVELD3,MAP2K5,PTPRR,LRP2,ZNF536,DEPDC5,SH2D1A,EZR,ROBO1,TNFAIP8L1,HTR2B,MECOM,LMNA,DNAJA3,ITGB1,PSMB7,MCC,CCDC22,ARHGAP24,DAB1,RGS6,HERC4,GPRASP1,ESR1,NPHP3,PMEPA1,PTPRO,RGS22,TCF7L2,PIP4K2A,LITAF,FBXW11,PTPRD,BDNF,NPRL3,PHACTR4,UBR2,SNX6,ARHGAP42,PTPRU,NPLOC4,DMD,MKRN2,ARNTL,NF1,CHRDL1,AP*

			OD,NDRG4,PAQR3,PIK3R2,RANBP9,NOMO3,NRG1,BDKRB2,BID,MDFI,FNIP1,MAD1L1,TMBIM6,VGLL4,PPP2CB,ROR2,DCN,CREBRF,SOX13,PTPRT,MAD2L2,TLE6,RGS8,NEUROD1,RFFL,FBXW7,LINC00473,ITCH,ZFYVE28,MLLT3,PDE2A,WNT11,IFT80,TRIM59,KREMEN1,PRKCD,RUNX2,IGSF1,PIBF1,BTRC,F2RL1,RPGRIP1L,SLIT3,SLIT2,KANK1,SMAD6,CLOCK,ZNF675,BMPER,BCL3,DUSP22,NAIP,HOMER2,FGF10,RASA4,RASA4B,KCTD10,SMURF2,EPHA4,RORA,UFL1,CTNNB1,PARK2,SOD2,FCGR2B,IGF1R,PPARG,AXIN1,DLG5,DRAXIN,LEPROT,RGS10,NOL3,MYOCD,AJUBA,GLG1,CHEK2,PRDM16,NF2,BICC1,TRABD2B,SFRP1,FOXO3,IFT122,SMAD3,ITPR1,WWP2,NEDD4,TXNDC12,NFATC1,YAP1,HEG1,AMFR,SESN1,FSTL4,EYA2,SOX2,CHST11,PRICKLE1,RCAN1,ZNF653,TMEM14A,STAT1,DGKG,SHANK2,GRAMD4,RAF1,CELF4,CARD16,PTPN1,ADAMTS12,PDE11A,FGF2,DKK2,CHRD,PTPRE,RBX1,ATF3,PAWR,DEPTOR,TSG101,CCDC3,SUFU,PAFAH1B1,PRDM15,RASAL1,GLI2,DAB2,PKHD1,UBR1,LDLRAD4,RUVBL2,ARHGAP12,INPP5D,XDH,LTBP1,OVOL2,SNX5,RGS7,ATP2B4,GPR161,RNF34,WWOX,GFRAL,GPC3,ADRBK1,PSMB2,EYA1,TSPAN6,HDAC1,INVS,HIPK3,PTPN2,EIF3A,AXIN2,SARM1,MUC1,ARHGAP44,SPI1,CTNNA1,STK38,OTUD3,SH3BP1,SNCA,MAGI2,HEY2,PTPRS,ZMYND11,RASA1,ZNRF3,KCTD13,CBFA2T2,IGF1,HTT,PTEN,SPRED2,BMP7,RASA2,LRRK2,LILRB4,ARHGAP25,RGS9,UACA,CRTC3,PLCL2,GPR21,IL17RD,SH3RF2,VRK3,SOX30,GRB14,JADE1,RHOA,SYNGAP1,RQCD1,ONECUT2,CUL3,GSK3B,FAM49B,NECAB2,DUSP26,BOK,SULF1,RNF43,AGT,PRKAA2,ADAR,FBN2,STAT2,NDRG2,PDE4B,VEPH1,APCDD1L,DLX1,CD160,RPS6KB1,CD44,CSNK1A1,PLEKHA1,RGS14,STK4,MARK3,EPHB2,DLC1,PPARA,PPP1R10,PHLPP1,ZNF366,FBN1,PRKCZ,RABGEF1,OTUD7B,ASPN,EYA3,FHL2,TBC1D10C,VWC2,RBMS3,NCOR2,PRKCQ,CD300A,PHIP,PPP2CA,SH3BP4,CNOT1,WWTR1,CIT,PTPRJ,TANK,CGNL1,CAV2,INPP5F,WIF1,CD109,MET,ANKRD6,PHB,PTPRC,PBLD,TYRO3,RNF213,ATF6B,SHISA6,TRAP1,PRAP1,BMP4,CPNE1
GO:0048870	cell motility	1.0932566888605246e-14	*CLRN1,DNAH11,LDB2,GPC5,PRKCI,MAP4K4,S1PR2,SLC9A1,SPAG16,CLASP2,SEMA3A,DOCK1,WWC1,NRG3,ASH1L,IQCG,ASTN2,SEMA3D,PHACTR1,MAPRE2,WWC3,TJP1,UNC5C,NTRK3,ATRNL1,SEMA5A,STAT5B,ENPP2,FER,MARVELD3,MAP2K5,KITLG,PTPRR,SPNS2,PIK3CD,MEGF10,ROBO1,HTR2B,ITGB6,CDKL5,LMNA,CTNNA3,VAV2,ULK4,ITGB1,ELMO2,SRGAP3,MCC,SRGAP2B,ARHGAP24,PTPRK,DAB1,ABCC8,LAMA2,DACH1,PTPN11,MGAT5,DOCK10,HDAC6,ADORA1,EPHA1,ERBB4,GBF1,DNAH2,ZNF609,PTPRO,NTRK2,MEGF9,PDGFB,AVL9,KIRREL3,STK39,INSR,RERE,UNC5D,LAMA3,PAK1,CATSPER2,ATRN,POMGNT2,FUT8,FMNL2,PHACTR4,LCK,LAMC1,PTPRU,SLC8A1,SATB2,DEPDC1B,CASS4,FYN,ADAMTS9,NF1,PLCB1,DST,FAT3,RTN4,DPEP1,APOD,BCR,CCDC141,NDRG4,NTN1,NRG1,BDKRB1,DOCK8,MAP2K1,MYH9,HDAC5,NTNG1,NAV1,CMKLR1,ROR2,DCN,CATSPER3,CDH13,PTPRT,CCNYL1,NCKAP1,DEFA1B,CD99,RCC2,ANO6,KIF2A,RFFL,TTLL8,CCBE1,JAK2,FBXW7,BCL11B,DNAH14,CLDN1,CDC42BPB,WNT11,ASTN1,CAMK2B,PRKCD,SEMA5B,TGFB1,SGK1,MMP28,F2RL1,BCAS3,CELSR1,SLIT2,CORO1C,RRAS2,DISC1,KANK1,LAMC2,TTBK2,BMPER,DCLK1,DAPK2,PARVA,DUSP22,FGF10,ENAH,SMURF2,EPHA4,PRKCA,AUTS2,TNFSF11,PPP3CA,PIP5KL1,CTNNB1,SOD2,IGF1R,PPARG,DLG5,ADD2,FGF1,NR4A3,MYOCD,AJUBA,HCK,CSPG4,DCC,NF2,FLT4,HDAC4,TPTE,SFRP1,FOXO3,ARL13B,SMAD3,PTPRM,ACKR2,ABI2,NRP2,PREX1,DOCK4,NAV3,APP,GSX2,PDGFRA,SLK,CEP85L,NOX1,LRP5,PTPRG,SLC9B2,SETD2,GPC6,DRD1,MMP2,SYNE2,S100A12,PRKD1,RND3,SEMA6D,RAF1,ADAMTS12,BMPR2,USP33,CAMK1D,BMPR1A,CTNNA2,PIK3R3,SPAG9,RAB11A,FGF2,PPM1F,NEDD9,PEAK1,SEMA4D,ARMC2,MCTP1,ELP3,PLXNA2,PTAFR,DDX58,ITGA11,PIK3C2B,SLC9B1,SPOCK1,GAS8,CHRD,EPHB3,FBXO45,PKN3,VCL,LAMB1,CLIC4,RHOJ,CCL14,CCL15,CCL15-CCL14,PAFAH1B1,RUFY3,DAB2,USH2A,LDLRAD4,MYSM1,DZIP1,WNK1,RELN,CSF3R,IQGAP1,SP1,MACF1,CLASP1,SLC9C1,OVOL2,NFATC2,PRKG1,TTLL9,ATP2B4,PAK3,SSH2,DYX1C1,MAP1B,CDC42BPA,SCARB1,MARK1,CDK6,GPC3,FLRT2,SMOC2,PLCG2,ROCK1,EPS8,PAXIP1,ABCC1,PKN2,TMIGD1,EPHB1,TRIM46,TEKT1,SPI1,MCU,CTNNA1,LIMA1,SH3BP1,MST1,PTK7,MAGI2,ZEB2,ADTRP,PLD1,MITF,KCTD13,IGF1,DPYSL3,MAPK1,PTEN,BMP7,PRCP,LRRK2,OSGIN1,ZRANB1,PDGFC,AMOTL1,WNT7A,NME8,GPI,SH3RF2,SOS1,EXT1,RAPGEF2,SMO,SUN1,RHOA,NDE1,TF,ONECUT2,FAM19A4,CUL3,SH3KBP1,PLD2,RDX,ACTA2,FAM49B,ITGBL1,SULF1,AGT,PTK2,TPPP2,PDE4B,TAOK2,RPS6KB1,GPR173,ANKS1A,ITGAL,ALOX5,CD44,ADAM8,CLDN4,CXCL17,PEX7,PLVAP,ACTN4,STK4,SPEF1,EPHB2,TMEFF2,XCR1,DLC1,ARID5B,EPB41L4B,GPSM3,LDHC,ZMYND8,PAX6,PRKCZ,RABGEF1,PHLDB2,KLC3,NIPBL,ANGPT1,OSBPL8,GCNT2,ETS1,BIN2,CCL22,FBXO31,PRKCQ,BRAF,DIAPH1,ATP8A1,POU3F3,SPINT2,TNR,CD300A,ANGPT4,CAMSAP3,SP100,FAT2,SRGAP2,NUMB,SYK,SLIT1,FRMD5,RADIL,DNAH3,PTPRJ,GPM6A,SASH1,VAV3,ZP3,GNA12,INPP5F,AXL,MET,FMNL1,CCR3,SEMA3C,PTPRC,TYRO3,EDNRA,BMP4*
GO:0051674	localization of cell	1.0932566888605246e-14	*CLRN1,DNAH11,LDB2,GPC5,PRKCI,MAP4K4,S1PR2,SLC9A1,SPAG16,CLASP2,SEMA3A,DOCK1,WWC1,NRG3,ASH1L,IQCG,ASTN2,SEMA3D,PHACTR1,MAPRE2,WWC3,TJP1,UNC5C,NTRK3,ATRNL1,SEMA5A,STAT5B,ENPP2,FER,MARVELD3,MAP2K5,KITLG,PTPRR,SPNS2,PIK3CD,MEGF10,ROBO1,HTR2B,ITGB6,CDKL5,LMNA,CTNNA3,VAV2,ULK4,ITGB1,ELMO2,SRGAP3,MCC,SRGAP2B,ARHGAP24,PTPRK,DAB1,ABCC8,LAMA2,DACH1,PTPN11,MGAT5,DOCK10,HDAC6,ADORA1,EPHA1,ERBB4,GBF1,DNAH2,ZNF609,PTPRO,NTRK2,MEGF9,PDGFB,AVL9,KIRREL3,ST*

			K39,INSR,RERE,UNC5D,LAMA3,PAK1,CATSPER2,ATRN,POMGNT2,FUT8,FMNL2,PHACTR4,LCK,LAMC1,PTPRU,SLC8A1,SATB2,DEPDC1B,CASS4,FYN,ADAMTS9,NF1,PLCB1,DST,FAT3,RTN4,DPEP1,APOD,BCR,CCDC141,NDRG4,NTN1,NRG1,BDKRB1,DOCK8,MAP2K1,MYH9,HDAC5,NTNG1,NAV1,CMKLR1,ROR2,DCN,CATSPER3,CDH13,PTPRT,CCNYL1,NCKAP1,DEFA1B,CD99,RCC2,ANO6,KIF2A,RFFL,TTLL8,CCBE1,JAK2,FBXW7,BCL11B,DNAH14,CLDN1,CDC42BPB,WNT11,ASTN1,CAMK2B,PRKCD,SEMA5B,TGFB1,SGK1,MMP28,F2RL1,BCAS3,CELSR1,SLIT2,CORO1C,RRAS2,DISC1,KANK1,LAMC2,TTBK2,BMPER,DCLK1,DAPK2,PARVA,DUSP22,FGF10,ENAH,SMURF2,EPHA4,PRKCA,AUTS2,TNFSF11,PPP3CA,PIP5KL1,CTNNB1,SOD2,IGF1R,PPARG,DLG5,ADD2,FGF1,NR4A3,MYOCD,AJUBA,HCK,CSPG4,DCC,NF2,FLT4,HDAC4,TPTE,SFRP1,FOXO3,ARL13B,SMAD3,PTPRM,ACKR2,ABI2,NRP2,PREX1,DOCK4,NAV3,APP,GSX2,PDGFRA,SLK,CEP85L,NOX1,LRP5,PTPRG,SLC9B2,SETD2,GPC6,DRD1,MMP2,SYNE2,S100A12,PRKD1,RND3,SEMA6D,RAF1,ADAMTS12,BMPR2,USP33,CAMK1D,BMPR1A,CTNNA2,PIK3R3,SPAG9,RAB11A,FGF2,PPM1F,NEDD9,PEAK1,SEMA4D,ARMC2,MCTP1,ELP3,PLXNA2,PTAFR,DDX58,ITGA11,PIK3C2B,SLC9B1,SPOCK1,GAS8,CHRD,EPHB3,FBXO45,PKN3,VCL,LAMB1,CLIC4,RHOJ,CCL14,CCL15,CCL15-CCL14,PAFAH1B1,RUFY3,DAB2,USH2A,LDLRAD4,MYSM1,DZIP1,WNK1,RELN,CSF3R,IQGAP1,SP1,MACF1,CLASP1,SLC9C1,OVOL2,NFATC2,PRKG1,TTLL9,ATP2B4,PAK3,SSH2,DYX1C1,MAP1B,CDC42BPA,SCARB1,MARK1,CDK6,GPC3,FLRT2,SMOC2,PLCG2,ROCK1,EPS8,PAXIP1,ABCC1,PKN2,TMIGD1,EPHB1,TRIM46,TEKT1,SPI1,MCU,CTNNA1,LIMA1,SH3BP1,MST1,PTK7,MAGI2,ZEB2,ADTRP,PLD1,MITF,KCTD13,IGF1,DPYSL3,MAPK1,PTEN,BMP7,PRCP,LRRK2,OSGIN1,ZRANB1,PDGFC,AMOTL1,WNT7A,NME8,GPI,SH3RF2,SOS1,EXT1,RAPGEF2,SMO,SUN1,RHOA,NDE1,TF,ONECUT2,FAM19A4,CUL3,SH3KBP1,PLD2,RDX,ACTA2,FAM49B,ITGBL1,SULF1,AGT,PTK2,TPPP2,PDE4B,TAOK2,RPS6KB1,GPR173,ANKS1A,ITGAL,ALOX5,CD44,ADAM8,CLDN4,CXCL17,PEX7,PLVAP,ACTN4,STK4,SPEF1,EPHB2,TMEFF2,XCR1,DLC1,ARID5B,EPB41L4B,GPSM3,LDHC,ZMYND8,PAX6,PRKCZ,RABGEF1,PHLDB2,KLC3,NIPBL,ANGPT1,OSBPL8,GCNT2,ETS1,BIN2,CCL22,FBXO31,PRKCQ,BRAF,DIAPH1,ATP8A1,POU3F3,SPINT2,TNR,CD300A,ANGPT4,CAMSAP3,SP100,FAT2,SRGAP2,NUMB,SYK,SLIT1,FRMD5,RADIL,DNAH3,PTPRJ,GPM6A,SASH1,VAV3,ZP3,GNA12,INPP5F,AXL,MET,FMNL1,CCR3,SEMA3C,PTPRC,TYRO3,EDNRA,BMP4
GO:0051094	positive regulation of developmental process	1.611014102795239e-14	*NRXN1,PRKCI,S1PR2,HLX,LLPH,CBFB,CLASP2,SEMA3A,GRID2,DOCK1,TIAM2,PTGIS,TJP1,KALRN,ARNT,SEMA5A,STAT5B,TOX,ENPP2,GRM5,KITLG,LRP2,PIK3CD,OLFM1,EZR,ROBO1,CHD7,CDKL5,LMNA,TRAF6,ROBO2,ITPKB,ITGB1,TRIOBP,ZC4H2,DSCAM,RUNX1,DAB1,RBM19,GOLGA4,EPHA1,ERBB4,LIMK1,ATP8A2,CDON,NTRK2,CELA1,PDGFB,RIMS1,INSR,H2AFY2,TCF7L2,CDH4,ESRRB,PTPRD,BASP1,BDNF,TFAP2A,AMIGO1,TGIF2,TENM4,ECT2,DMD,KAT7,SLC8A1,RBM4,CASS4,ARNTL,ADAMTS9,PLCB1,RTN4,CHI3L1,NTN1,NRG1,MAP2K1,STIM1,CMKLR1,ROR2,DCN,SOX13,MAD2L2,TLE6,CAPRIN2,BOC,ANO6,NEUROD1,CCBE1,ZDHHC15,JAK2,CLSTN2,SYT1,PLS1,WNT11,IL1RAPL1,CAMK2B,SOX6,ACVR2A,RUNX2,CD4,TGFB1,SLIT2,TP73,CORO1C,DISC1,MTDH,ITSN2,TCF12,NELL1,SUCO,BMPER,ANKRD54,ADAM12,LINGO2,FGF10,CAPN3,SMURF2,EPHA4,PRKCA,TNFSF11,MAG,UFL1,CTNNB1,PARK2,SOD2,IGF1R,PPARG,DLG5,MSR1,CYBB,FGF1,NIN,DCT,MYOCD,KIR2DL4,CPNE6,PHLDB1,CAPN2,NF2,PAX2,SFRP1,FOXO3,SMAD3,CUX2,SHANK1,SYT3,PREX1,HOXD3,SSBP3,GSX2,YAP1,NLGN2,JAK1,ANGPTL4,LRP5,ISLR2,SLC9B2,SOX2,ZDHHC6,SPTBN4,VASH2,FRS2,TPH1,CPNE9,PRKD1,STAT1,PARP6,SLC6A3,ZBTB7C,TCF3,BMPR2,BMPR1A,NLGN1,SPAG9,RAB11A,MYB,FGF2,NEDD9,SEMA4D,PLXNA2,SPEN,NCOA1,BLOC1S5,EEF1E1,DVL3,EIF4G1,EPHB3,PAWR,CCDC3,WNT3,ZNF322,P4HB,NLGN3,SCUBE2,PAFAH1B1,DDAH1,AKAP6,RASAL1,TRPC5,GLI2,RUFY3,DAB2,RELN,SIN3A,SP1,MACF1,ALK,INPP5D,CLASP1,TEAD4,GPM6B,ADIRF,OVOL2,NFATC2,BNC1,PACSIN1,PAK3,ADNP,MAP1B,CELF1,TFRC,BRINP1,HOXB4,GHR,FLRT2,ACTR2,ELAVL4,HDAC1,SMOC2,PAXIP1,EGR2,AP3B1,NTRK1,UNC13A,EPHB1,AXIN2,SH3GL3,AP3D1,CARM1,CUX1,MCU,MAP3K13,RAPGEF3,MST1,SMYD1,BMPR1B,CD53,ZEB2,HEY2,WNT7B,HIPK1,BTK,IGF1,BMP7,SOX5,LILRB4,EFNA5,SHANK3,NCOA3,PPP2R3C,WNT7A,GPR21,BMP6,TSHR,RAPGEF2,SMO,TCF4,SYT17,TFE3,RHOA,NFKBID,GSK3B,STAT6,EEF2K,CD27,AGT,BLOC1S6,FBN2,BRINP3,RIMS2,EXOSC3,MAP3K5,DLX1,LBX2,RPS6KB1,ALOX5,SP7,ADAM8,CDS1,RGS14,STK4,EPHB2,IL18,PAX6,PRKCZ,FAM20C,PPP1CC,PHLDB2,NIPBL,TMIGD2,GCNT2,ETS1,VWC2,FBXO31,BRAF,HDAC2,HTR2C,IMPACT,CDKL3,ANGPT4,NUMB,SYK,WWTR1,DVL2,GRIP1,HSF1,SASH1,SULT2B1,BMP1,ZP3,AXL,TNFSF9,WIF1,CCR3,MALT1,SETD3,EHD2,KDM2B,NEDD4L,PTPRC,SYNDIG1,CREB1,BMP4,CPNE1*
GO:0006996	organelle organization	1.8525372609123947e-14	*CLRN1,HOOK2,ADAMTS16,BLZF1,NRXN1,PRKCI,MOV10L1,TACC2,DNMT1,S1PR2,SLC9A1,SPAG16,PDE4DIP,CLASP2,GRID2,RPS6KA2,KIF22,ASH1L,NOS1AP,TUBA1C,IQCG,TLN2,EPB41,PHACTR1,FHOD3,MAPRE2,TJP1,UTRN,SEC23B,PHACTR2,NTRK3,SEMA5A,RAD51B,TOX,PLCE1,FER,ANK2,STAG2,EZR,TOM1L1,CHD7,CDKL5,MECOM,TACC1,TENM1,CAMKMT,KIF5C,LMNA,CTNNA3,DNAJA3,ABI1,ULK4,ITGB1,TRIOBP,HSP90AA1,ELMO2,GAS7,CDC6,BCORL1,CDAN1,KLHL12,AKAP13,IFT43,TTLL5,GFI1B,PARD6G,OMA1,CDC42EP3,HMGN3,XIRP2,ITGB3BP,CLVS1,MAST4,TCIRG1,CCT2,HDAC6,TBCEL,EPHA1,IKBKB,KIF18B,PEX14,ERBB4,MRE11A,GBF1,DNAH2,LIMK1,TLK1,BRD8,ATP8A2,RAD54L2,TEX264,KAT6B,CECR*

			2,ESR1,NPHP3,KANSL1,STX18,DNAI2,EP300,DYM,HOOK3,PDGFB,TNIK,MID1,CHFR,MSTO1,INSR,FMN2,RERE,PRKAR1A,AFAP1,ATP8B1,H2AFY2,TCF7L2,PIP4K2A,HPS1,JMJD1C,PAK1,FBXW11,MAP3K4,PTPRD,FANCA,TGIF2-C20ORF24,NPRL3,FMNL2,PHACTR4,UBR2,ARHGAP6,ECT2,NAV2,ACOX1,VAMP7,ERN2,LRRC49,NPLOC4,PYROXD2,STARD9,DMD,CENPF,PDS5A,KAT7,GSN,HFM1,RBM14,SATB2,HIRA,CASS4,NF1,PLCB1,DST,PCDH15,STX6,ARID4B,RTN4,AFF2,VTA1,BCR,BRPF1,TTN,RANBP9,DCTN1,SLC4A5,ARHGAP10,BID,MAP2K1,MYH9,FRMPD4,MAD1L1,PPP2CB,WDR92,HDAC5,NAV1,RNF4,SYNE1,PARD3B,LRGUK,DCN,SCFD1,DOCK2,PITPNB,AUNIP,DMC1,MAD2L2,TLE6,RAB27A,MTX3,NCKAP1,TERF2,TMED6,SLX1B,GOLPH3L,ARL3,DNAJC11,KIF2A,ICK,RAD51D,TTLL8,PARP11,RANBP1,USP22,ZDHHC15,JAK2,MYPN,FBXW7,SMC3,UIMC1,SYT1,CROCC,BBS12,SMG1,MLLT3,CDC42BPB,PLS1,TEX11,PDE2A,SEPT7,CYFIP2,CENPC,WNT11,IFT80,FIG4,MTA1,IMMP2L,SIPA1L3,MKLN1,PRKCD,PPM1E,TGFB1,BANP,LRCH3,PDLIM4,ATL2,NUP93,NSD1,PIBF1,ARMC1,F2RL1,BCAS3,RPGRIP1L,CELSR1,SLIT2,SYT13,CORO1C,DISC1,CEP135,KANK1,PTPDC1,GRPEL2,CLN6,ANK1,TTBK2,KDM4B,SPAG17,RNF212,PARVA,STRIP1,HNRNPC,TRRAP,ANKFN1,OIP5,YTHDF1,FGF10,SMYD3,KANK4,ENAH,PPL,IQCJ-SCHIP1,RHPN2,MAST2,SVIL,CAPN3,SLC16A1,VMP1,PRKCA,AUTS2,SMG6,GATAD2B,UFL1,TRAK1,CTNNB1,MPRIP,PARK2,SOD2,SMARCC1,TSNARE1,PPARG,NGRN,AXIN1,MYO1D,BFSP1,ADD2,BRIP1,CENPP,SREBF2,NDUFA9,LEPROT,NIN,WDR45B,TUBGCP6,NOL3,RIOK2,ESCO1,AJUBA,HAUS4,CHEK2,PHLDB1,KRT6B,UBQLN4,PRDM16,MYO1E,SPDYA,HCK,SORBS1,TBC1D14,RAB3GAP2,CAPN2,TBCK,ANKFY1,CTDP1,BAZ1B,OBSCN,NF2,SMC2,HDAC4,SPTBN1,HEPACAM2,ELN,SPG11,SFRP1,ARL13B,IFT122,SSH1,SMAD3,WIPI1,MTBP,VPS39,RNF168,EMG1,ABI2,C10ORF90,GRIK5,SHANK1,ARHGEF18,PREX1,PLA2G4C,KPNB1,NAV3,RAB6C,USP49,PDGFRA,SLK,ADD3,KANSL2,YAP1,MYO1F,TEN1,NLGN2,VDAC1,EYA2,SH3D19,BORA,LRP5,SOX2,SETD2,ZDHHC6,KIF24,TTC39C,SPTBN4,TMEM14A,SFMBT1,VILL,SYNE2,KDM6A,GTF2F2,PRKD1,RND3,SHPRH,VPS4A,EREG,TCTN3,ABLIM1,PITRM1,ATF2,TMEM237,RAF1,ABLIM2,SMIM20,PTPN1,USP33,DPYSL2,NLGN1,CTNNA2,MORC2,RAB11A,CRMP1,MYB,PPM1F,NEDD9,ARMC2,CDC27,HELQ,EEA1,SETD1A,YME1L1,JARID2,RILPL1,BRDT,CHCHD6,PIK3C2B,BLOC1S5,EHMT1,GAS8,AP2M1,ANAPC5,TRAPPC11,PAWR,TSG101,TERF2IP,WDR90,CLIC4,KCNQ1,RFX2,C11ORF80,RHOJ,ZFYVE1,PAFAH1B1,KIF3A,HIP1,CEP350,PADI6,TTLL7,TNKS,WBP2NL,ERCC1,RUFY3,BLM,PKHD1,TRAM2,MYSM1,SETD5,DZIP1,SMG5,DLG3,RELN,SIN3A,RUVBL2,IQGAP1,TMEM170A,SPATA18,EML4,ZNF423,MACF1,ARHGAP12,MYO7A,CLASP1,TOP1,GPM6B,CHD1L,PRKG1,TTLL9,SYNE3,PACSIN1,SPTBN5,ACTL6B,PAK3,HORMAD2,SSH2,TET1,DYX1C1,CEP41,ARID4A,MAP1B,CDC42BPA,MPHOSPH9,MARK1,PHF2,AIF1L,TFRC,UVRAG,EPHA5,MEF2A,DHX30,XRN1,PLS3,SATB1,ZNF207,CALD1,EYA1,DIS3L2,TFEB,CLEC16A,MNAT1,FGD1,SNX9,DIAPH2,RPGR,ACTR2,L3MBTL4,FAM174B,HDAC1,RECQL5,ROCK1,EPS8,SCMH1,CAPN10,GPR35,AP3B1,INSRR,ZFAND1,MAP1A,PPP1R9A,ANK3,UNC13A,AXIN2,NCKAP5,TMEM199,PARD3,GNL3L,TRIM46,TUB,TEKT1,BCOR,AP3D1,CARM1,ANXA8L1,NUDT5,ARHGAP44,SNX33,PIFO,SMARCA2,RAD51C,SPI1,MCU,RANBP10,VPS16,CTNNA1,LIMA1,AIFM2,TRDN,ATG14,SHROOM3,ACOT8,NTMT1,FCHSD2,RAPGEF3,PDXP,SH3BP1,ERCC3,PTK7,SMYD1,SNCA,HAUS3,PNPT1,USP50,ZEB2,MCMBP,FOXJ3,SYT9,RC3H1,CHMP3,HYDIN,RNF103-CHMP3,PTPRS,ZMYND11,KMT2D,CDKL1,RAB5B,KMT2C,CSRP1,RASA1,CABIN1,PKP2,VPS41,KCTD13,TROVE2,IGF1,STX8,ATF7IP,HTT,DPYSL3,MAPK1,PTEN,ARHGEF17,BMP7,ATXN2,PIK3C3,TTLL4,RAB30,LRRK2,SEPT6,ZRANB1,SPAG5,CHMP5,RFC3,BAG6,CHAF1B,IFT81,TMEM108,ARHGAP25,PKD2,EFNA5,HSF2BP,SHANK3,STMN4,AMOTL1,ATP8B4,PKIB,MTMR3,PPP2R3C,TBCD,TMCC1,SNAP23,BRWD3,MAP4,NME8,CEP89,KAT6A,MTIF2,SH3PXD2B,RHOT1,SIK3,MYCBP2,EXT1,ATRX,IKZF1,PARVB,PNPLA3,SAMM50,GET4,SUN1,BLOC1S3,MARK4,SOX30,BBS9,RHOA,SYNGAP1,MCM3,NDE1,TF,ONECUT2,RHOBTB1,ESYT2,SHROOM1,CUL3,SH3KBP1,HSPD1,EPM2A,GSK3B,PLD2,RDX,FAM49B,CDK13,VAMP1,BOK,KRT8,SUPT4H1,HDGFRP3,PRKAA2,SYT7,BLOC1S6,CUL9,ESPNL,STAT2,ZMPSTE24,PTK2,PEX5L,SNX3,TOMM5,CDC14A,GAP43,SEC16B,TIMM44,IQGAP2,FAM171A1,CEP70,TAOK2,PYGO2,SHC1,NDUFS2,ALDOA,PARP10,YLPM1,RSBN1,PGM5,SETDB2,CPLX2,DNAJB6,FGD3,LARP4,ARFGAP3,PACSIN2,CDS1,THSD7A,CSNK1A1,HUS1,FGD4,PEX7,RGS14,ACTN4,COG4,TUBB3,ZFAND6,MARK3,SPEF1,THSD7B,TTLL11,TMEFF2,TEX12,MFSD8,ASF1B,GPSM2,DLC1,PPP1R10,ABCD3,MCOLN1,EPB41L4B,SLC25A33,SMARCAL1,CCDC6,PRRC2C,PAX6,PRKCZ,SPECC1L,STAG1,WDR83OS,PHLDB2,KLC3,KLHL1,NIPBL,YEATS4,PHF20,PACS2,EYA3,MBTD1,SHROOM4,EPB41L2,ETS1,HACL1,ARHGAP21,MAP2,MTF2,BIN2,STPG1,KIFC3,NEBL,ATG3,PRKCQ,MDN1,BRAF,DIAPH1,HDAC2,SRCAP,CAPN6,NOTO,HIGD2A,PHIP,EMC3,DNAJC9,FAM149B1,RFC5,CAMSAP3,SP100,VTI1A,ANXA2,CHCHD3,PARN,PRMT7,SRGAP2,WIPI2,SQLE,CNOT1,WWTR1,FRMD5,CIT,RAB2A,SEC31B,UBAP2L,BRCA2,HRK,MORC1,NEB,MYO1A,EZH1,SRPK2,ATP10B,PLEKHM1,ASAP1,MCM8,CGNL1,VAV3,CHD6,EPB41L1,MTFR2,CAV2,SKA2,SYNM,AKTIP,EPS15,POC1B,NUAK2,TRIM37,CENPK,MET,FMNL1,MICAL3,BRD9,SETD3,INO80C,SPTAN1,CCDC136,EHD2,KDM2B,THEM4,TRAPPC8,CCT3,UQCC1,NDC1,PHB,CTCF,RNF213,CREB1,RASSF8,TNXB,CCDC170,EDNRA,PRAP1,STXBP6,BMP4,ABLIM3
GO:00	actin filament-	2.22839135	*CLRN1,PRKCI,PDE4D,S1PR2,SLC9A1,CLASP2,NOS1AP,EPB41,PHACTR1,FHOD3,*

30029	based process	2101679e-14	*TJP1,UTRN,PHACTR2,WIPF3,NTRK3,SEMA5A,FER,ANK2,EZR,TENM1,CTNNA3,ABI1,ITGB1,TRIOBP,ELMO2,GAS7,AKAP13,CDC42EP3,XIRP2,CACNB2,ADORA1,EPHA1,IKBKB,LIMK1,TNIK,FMN2,PRKAR1A,PAK1,FMNL2,PHACTR4,ARHGAP6,ECT2,SGCD,DMD,GSN,CASS4,NF1,PCDH15,BCR,TTN,MYH9,FRMPD4,DOCK2,MAD2L2,TLE6,NCKAP1,JAK2,MYPN,CDC42BPB,PLS1,CYFIP2,WNT11,MKLN1,PRKCD,PPM1E,TGFB1,PDLIM4,F2RL1,BCAS3,CELSR1,SLIT2,CORO1C,KCNJ3,KANK1,CACNA1D,PARVA,STRIP1,FGF10,KANK4,ENAH,RHPN2,SVIL,CAPN3,AUTS2,MPRIP,PARK2,MYO1D,ADD2,RYR2,CACNA1C,SCN3B,MYO1E,HCK,SORBS1,TBCK,OBSCN,NF2,SPTBN1,ELN,SFRP1,SSH1,SMAD3,ABI2,SHANK1,ARHGEF18,PREX1,PDGFRA,ADD3,MYO1F,SPTBN4,VILL,SYNE2,RND3,VPS4A,ABLIM2,PTPN1,CTNNA2,PPM1F,NEDD9,PAWR,KCNQ1,RHOJ,PAFAH1B1,HIP1,RUFY3,IQGAP1,ARHGAP12,MYO7A,CLASP1,DSC2,GPM6B,PRKG1,PACSIN1,SPTBN5,PAK3,SSH2,CDC42BPA,AIF1L,EPHA5,MEF2A,PLS3,CALD1,FGD1,SNX9,DIAPH2,ACTR2,ROCK1,EPS8,CAPN10,INSRR,PPP1R9A,KCND3,ARHGAP44,MCU,CTNNA1,LIMA1,ACAP2,SHROOM3,FCHSD2,RAPGEF3,PDXP,SH3BP1,PTK7,CSRP1,RASA1,PKP2,KCTD13,DPYSL3,ARHGEF17,ARHGAP25,EFNA5,SHANK3,AMOTL1,EPDR1,PARVB,MYLPF,RHOA,TF,RHOBTB1,SHROOM1,CUL3,SH3KBP1,RDX,ACTA2,FAM49B,KRT8,ESPNL,PDE4B,IQGAP2,FAM171A1,TAOK2,SHC1,ALDOA,PGM5,DNAJB6,FGD3,PACSIN2,THSD7A,FGD4,ACTN4,THSD7B,TMEFF2,DLC1,EPB41L4B,SPECC1L,PHLDB2,KLHL1,SHROOM4,EPB41L2,NEBL,BRAF,DIAPH1,SRGAP2,SCN1A,FRMD5,CIT,NEB,MYO1A,CGNL1,EPB41L1,NUAK2,MET,FMNL1,MICAL3,CAMK2D,SETD3,SPTAN1,EHD2,NEDD4L,TNXB,ABLIM3*
GO:0031399	regulation of protein modification process	2.6009758018573292e-14	*ENPP1,NRXN1,PRMT3,SLCO3A1,PDE4D,DNMT1,S1PR2,C6ORF89,ADCY8,PDE8A,IL31RA,PRDM12,NRG3,NOS1AP,LATS2,PRKAG2,PRLR,MVP,NTRK3,CBL,ARNT,FLT3,ENPP2,GRM5,PLCE1,FER,MAP2K5,KITLG,ROBO1,TOM1L1,HTR2B,TENM1,LMNA,TRAF6,ITPKB,DNAJA3,OXR1,ABI1,HSP90AA1,CDC6,KNDC1,AKAP13,DAB1,PTPN11,MGAT5,HDAC6,ADORA1,EPHA1,IKBKB,ERBB4,MRE11A,LIMK1,PMEPA1,PTPRO,CDON,NTRK2,EP300,FNTA,PDGFB,TNIK,CCND3,BRMS1,CHFR,INSR,PRKAR1A,CAB39,PAK1,MAP3K4,BDNF,ECT2,SNX6,ERN2,DMD,ATG10,KAT7,SLC8A1,RIT2,CASS4,FYN,ARNTL,NF1,RWDD3,CHI3L1,TTN,PAQR3,NRG1,SH2D3C,BDKRB1,BDKRB2,FRY,MAP2K1,FNIP1,JDP2,ROR2,WDR70,PTPRT,IPO5,MAD2L2,CAPRIN2,CCNYL1,GNAQ,HERC5,JAK2,FBXW7,ITCH,ZFYVE28,KLF15,WNT11,MTA1,NOX4,PRKCD,TAB2,ACVR2A,CD4,PPM1E,TGFB1,SPSB4,NSD1,PIBF1,BTRC,DNAJB2,SLIT2,CORO1C,KDM4B,SMAD6,ZNF675,BMPER,ANKRD54,TNFRSF10B,DUSP22,DOCK3,FGF10,SMYD3,FANCI,CAPN3,EPHA4,AUTS2,CD6,TNFSF11,PIP5KL1,UFL1,CTNNB1,PARK2,PPARG,AXIN1,PRKAR1B,OTUB1,LEPR,FGF1,PRKAR2A,MYOCD,PER2,AJUBA,CHEK2,SPDYA,RAB3GAP2,CSPG4,NF2,FLT4,HDAC4,TRABD2B,SFRP1,PPP6R2,MTBP,TAB1,APP,CCNI2,DIP2B,HEG1,BORA,IBTK,LRP5,MTCP1,PRICKLE1,RCAN1,SPTBN4,DRD1,S100A12,DCUN1D3,PRKD1,EREG,CCNY,ATF2,RAF1,PTPN1,BMPR2,BMPR1A,PIK3R3,CDK12,SPAG9,MYB,FGF2,PPM1F,NEDD9,SEMA4D,JARID2,PRKCG,DVL3,EIF4G1,RBX1,CKS1B,DEPTOR,TSG101,TERF2IP,PTPN13,TRPC5,UBA2,DAB2,BLM,CACUL1,LDLRAD4,SETD5,DLG3,WNK1,RELN,NEK10,SIN3A,RUVBL2,PELI1,IQGAP1,MAP3K7,SLC8A2,XDH,ATP2B4,TET1,CAMTA1,ADNP,TFRC,UVRAG,DCUN1D5,GHR,MNAT1,SNX9,PLCG2,ROCK1,PAXIP1,HIPK3,PTPN2,NTRK1,TRIM44,SLC39A10,AXIN2,PARD3,GNL3L,MUC1,BCOR,SPI1,DNMT3B,EPHA7,ATG14,BCAR3,STK38,RBPMS,MAP3K13,RAPGEF3,SNCA,BMPR1B,MAGI2,PRKAG1,TRIP12,ADTRP,OPRD1,RPTOR,MLLT1,IGF1,HTT,MAPK1,PTEN,SPRED2,BMP7,LRRK2,TMEM59,UNC119,LILRB4,PKD2,EFNA5,TADA2A,PDGFC,PKIB,PPP2R3C,PLCL2,BMP6,MYCBP2,SH3RF2,N4BP1,ATRX,RAPGEF2,VRK3,PRKAR2B,UBR5,ARRDC4,RHOA,SPRTN,RQCD1,CUL3,EPM2A,GSK3B,DNAJC3,EEF2K,IWS1,PPP1R16A,PTPRB,AGT,CCNJL,PRKAA2,ADAR,STAT2,ZMPSTE24,PTK2,TEC,MAP3K5,NCF1,TAOK2,PYGO2,SHC1,PARP10,CD44,ADAM8,RPS6KA5,HUS1,NOS1,RGS14,STK4,EPHB2,SLC1A1,IL18,UBE2K,DLC1,MOB3B,PAX6,PRKCZ,ADORA2A,RABGEF1,CCNG2,NIPBL,ANGPT1,LRRK1,OSBPL8,MTF2,CDK5RAP1,CNTN1,BRAF,HDAC2,IMPACT,CD300A,PHIP,PPP2CA,SKP1,ANGPT4,RPL23,MADD,SYK,WWTR1,DVL2,PTPRJ,TAF1,TICAM1,TANK,HSF1,SASH1,MOB3A,GNA12,SH2D3A,AKTIP,INPP5F,CD109,CAMK2D,MALT1,MLXIPL,PHB,PTPRC,CTCF,SH3GL2,TNXB,BMP4*
GO:0051049	regulation of transport	5.49609858637286e-14	*ENPP1,NRXN1,ASPH,PRKCI,PDE4D,SLC9A1,ADCY8,CLASP2,WWC1,CTDSPL2,NOS1AP,EPB41,DPP6,PRKAG2,CLCN1,UTRN,KALRN,CBL,RAB4B,RAB4B-EGLN2,KCNQ5,GRM5,FER,CASK,KCNIP4,ANK2,EZR,NRXN3,CHD7,SLC26A6,TENM1,NKAIN2,ITGB1,CNIH2,NKAIN3,ABCC8,HMGN3,DGKI,STXBP4,EFCAB7,TCIRG1,PTPN11,KCNS3,NETO2,CACNB2,ADORA1,ATP8A2,KCNJ16,GRM7,DPP10,FRMD4A,STX18,PDGFB,CACNA1B,RIMS1,STK39,INSR,ATP8B1,TCF7L2,CAB39,MYOM1,CATSPER2,AMIGO1,KCNMA1,CHRM3,KCNRG,ECT2,TMC2,VAMP7,DMD,SLC8A1,RBM4,RIT2,JPH2,FYN,ARNTL,NF1,APOD,BCR,SHISA9,TTN,NDRG4,ICA1,BMP2K,PIK3R2,CACNA1E,NRG1,SLC43A2,BDKRB1,MAP2K1,KCNIP1,STIM1,NALCN,TMBIM6,TMEM30A,SCFD1,CATSPER3,DOCK2,CDH13,CREBRF,FGF14,IPO5,RAB27A,RAB3C,GOLPH3L,ANO6,NEUROD1,JAK2,FBXW7,KCNB2,OAZ2,SYT1,NPSR1,SCN4A,TMC1,ATP9A,KLF15,KCNC4,MKLN1,CACNA1H,IL1RAPL1,CAMK2B,PRKCD,CD4,TGFB1,SGK1,KEL,JPH3,CLDN10,F2RL1,BCAS3,AAK1,CADPS2,SYT13,C12ORF4,KCNJ3,CACNA1D,CACNA2D3,CHRM1,CLOCK,KCNH1,HOMER2,SLC17A7,FGF10,STXBP5,KCNH7,CAPN3,SLC16A1,VMP1,CNR1,TNFSF11,PPP3CA,NSUN2,*

			CTNNB1,PARK2,FCGR2B,PPARG,PRKAR1B,CYBB,SCAMP5,ANXA13,SREBF2,KCNJ12,RYR2,KCNA6,LEPR,LEPROT,NR4A3,RIOK2,PER2,CACNA1C,SCN3B,HCK,SORBS1,RIMS4,ANKFY1,SFRP1,DNM3,SMAD3,WWP2,GRIK5,SHANK1,SYT3,NEDD4,SLC6A1,APP,BET1L,NOX1,RAB11FIP5,NLGN2,VDAC1,LRP5,SLC9B2,SETD2,SPTBN4,DRD1,TMEM14A,SERGEF,PRKD1,HNF4A,VPS4A,FXYD2,FXYD6,FXYD6-FXYD2,KCNG4,RASGRF2,PTPN1,CAMK1D,NLGN1,RAB11A,PPM1F,ADCY5,MCTP1,PTAFR,ADCYAP1R1,ABCA13,RAB11FIP3,PRKCG,AP2M1,FBXL20,TSG101,CLIC4,KCNQ1,SUFU,NLGN3,KIF3A,HIP1,AKAP6,BTBD9,RUFY3,STON2,DAB2,WNK1,RELN,ABCB1,KCNQ2,CLASP1,EEPD1,APBB3,TPCN1,GPM6B,CLIC5,AHNAK,SNX5,RGS7,ATP2B4,NSF,PACSIN1,ATAD1,MAP1B,SCARB1,KCNAB2,EPHA5,MEF2A,SYT12,CACNA1A,GPC3,SGIP1,STOM,ADRBK1,TBC1D5,PTGER3,TRPV1,C2,PLCG2,ROCK1,C1QTNF1,GAPVD1,CAPN10,GPR35,ZFAND1,ANK3,PTPN14,TRIM46,TUB,SH3GL3,KCND3,CACNG8,AP2B1,NETO1,ARHGAP44,SNX33,CD84,SPI1,CHRNB4,MCU,TRDN,STAC,TRPC6,RAB15,RAPGEF3,SNCA,CACNG2,MAGI2,SYT9,CHMP3,RAB5B,ADTRP,ABCA12,PLD1,OPRD1,PKP2,XPO4,SLC4A8,IGF1,PLA2G4E,HTT,MAPK1,PTEN,MIB1,SCN8A,ATXN2,PIK3C3,LRRK2,SPAG5,UNC119,CNIH3,ITGAM,PKD2,EFNA5,SHANK3,BEST3,WNT7A,ZBED6,DNAJC1,BMP6,ANO1,KCNC2,SMO,STXBP5L,CPT1A,PSEN2,SYT17,DNAJC6,UBR5,GRIN1,RHOA,GRIN3A,TF,EPM2A,GSK3B,ABCG8,RDX,SYN1,EEF2K,IWS1,NECAB2,MCTP2,SCN9A,BOK,AGT,SYT7,ZMPSTE24,SNX3,PDE4B,RIMS2,SEC16B,CD160,KCNJ15,RPS6KB1,STC2,ALOX5,CPLX2,ADAM8,PACSIN2,NOS1,ACTN4,EPHB2,SLC1A1,WLS,LRRC52,NKAIN1,HCAR2,PPARA,SYTL4,PRKCZ,ADORA2A,KCTD7,PPP1CC,RABGEF1,ANGPT1,CADPS,OSBPL8,MAP2,ATG3,CNTN1,BRAF,DIAPH1,HTR2C,ATP8A1,CD300A,SP100,ANXA2,KCND2,RAB27B,NUP214,ADCY1,NUMB,SYK,SCN1A,MYRIP,CACNG3,ANKRD13A,PTPRJ,SLC10A1,ZP3,KCNQ3,INPP5F,AXL,SESTD1,CAMK2D,KCNJ6,EHD2,NEDD4L,PTPRC,GPR89A,CREB1,SHISA6,EDNRA,PRAP1,STXBP6,BMP4,ABLIM3
GO:0051246	regulation of protein metabolic process	9.655302892875581e-14	*POLDIP3,ENPP1,NRXN1,ASPH,PRMT3,SLCO3A1,PDE4D,DNMT1,S1PR2,C6ORF89,ADCY8,PDE8A,IL31RA,RPS6KA2,PRDM12,NRG3,NOS1AP,LATS2,PRKAG2,PRLR,MVP,FTO,NTRK3,CBL,ARNT,EGLN2,FLT3,ENPP2,GRM5,PLCE1,FER,MAP2K5,KITLG,LRP2,EIF4G3,EZR,ROBO1,TOM1L1,HTR2B,PSMD1,TENM1,LMNA,TRAF6,ITPKB,DNAJA3,OXR1,ABI1,HSP90AA1,CDC6,KNDC1,CCDC22,AKAP13,RUNX1,DAB1,PTPN11,MGAT5,HDAC6,DDB1,ADORA1,EPHA1,IKBKB,ERBB4,MRE11A,LIMK1,CUL4B,PMEPA1,PTPRO,CDON,NTRK2,TNRC6A,EP300,ZYG11B,FNTA,PDGFB,TNIK,CCND3,BRMS1,CHFR,INSR,FMN2,PRKAR1A,ASB5,TCF7L2,CAB39,PAK1,FBXW11,MAP3K4,RNF144A,EGLN3,SAMD4A,BDNF,LCK,MDM4,ECT2,SNX6,CST2,PTCD3,ERN2,DMD,ATG10,LARP4B,USP13,KAT7,SLC8A1,GSN,RBM4,RIT2,CASS4,FYN,MKRN2,ARNTL,NF1,PLCB1,MGMT,RTN4,DPEP1,APOD,RWDD3,CHI3L1,TTN,PAQR3,RANBP9,NRG1,SH2D3C,BDKRB1,BDKRB2,BID,FRY,MAP2K1,FNIP1,MYH9,VGLL4,CSNK2A3,JDP2,ITIH2,ROR2,CREBRF,FHIT,WDR70,PTPRT,IPO5,MAD2L2,CAPRIN2,CCNYL1,GNAQ,RFFL,CCBE1,HERC5,JAK2,FBXW7,OAZ2,ITCH,ZFYVE28,KLF15,CYFIP2,WNT11,MTA1,NOX4,QKI,PRKCD,TAB2,ACVR2A,CD4,PPM1E,TGFB1,BANP,SPSB4,NSD1,PIBF1,BTRC,CRADD,DNAJB2,SLIT2,ITIH4,CORO1C,DISC1,BLID,ZFAND2A,CLN6,KDM4B,SMAD6,ZNF675,NELL1,BMPER,TIMP2,BCL3,ANKRD54,TNFRSF10B,DUSP22,NAIP,DOCK3,YTHDF1,FGF10,SMYD3,FANCI,CAPN3,SMURF2,EPHA4,AUTS2,CD6,TNFSF11,UBQLN3,PIP5KL1,UFL1,CTNNB1,PARK2,SMARCC1,PPARG,NGRN,AXIN1,PRKAR1B,OTUB1,LRPPRC,LEPR,FGF1,PROS1,NOL3,PRKAR2A,MYOCD,PER2,AJUBA,GLG1,CHEK2,UBQLN4,SPDYA,CSTL1,RAB3GAP2,DIO2,CSPG4,NF2,FLT4,HDAC4,PAX2,SECISBP2L,TRABD2B,SFRP1,PPP6R2,FOXO3,BCL2L13,SSH1,SYNCRIP,SMAD3,RNFT2,WWP2,SIMC1,MTBP,DCP1B,NEDD4,TAB1,APP,RBM8A,CCNI2,DIP2B,ARIH1,HEG1,SH3D19,BORA,IBTK,LRP5,MTCP1,SOX2,PRICKLE1,RCAN1,SPTBN4,DRD1,S100A12,DCUN1D3,PRKD1,ST18,EREG,CCNY,ATF2,GRAMD4,RAF1,CELF4,CARD16,CASP1,PTPN1,BMPR2,VBP1,BMPR1A,PIK3R3,CDK12,SPAG9,MYB,FGF2,PPM1F,NEDD9,AGBL4,SEMA4D,PTAFR,JARID2,DDX58,PRKCG,SPOCK1,AREL1,DVL3,EIF2B5,EIF4G1,PSMD2,RBX1,CKS1B,PAWR,AGO3,DEPTOR,FBXL20,TSG101,DESI1,TERF2IP,IDE,SUFU,PTPN13,COL4A3,HIP1,PADI6,TRPC5,UBA2,TNRC6B,DAB2,BLM,CACUL1,LDLRAD4,SETD5,DLG3,WNK1,RELN,NEK10,SIN3A,RUVBL2,PELI1,IQGAP1,MAP3K7,SP1,SLC8A2,SLC35A4,XDH,ATP2B4,NSF,TET1,CAMTA1,DYX1C1,ADNP,CELF1,RNF34,TFRC,UVRAG,DCUN1D5,GPC3,XRN1,DIS3L2,GHR,MNAT1,SNX9,ELAVL4,HDAC1,PLCG2,ROCK1,PAXIP1,HIPK3,RYBP,PTPN2,NTRK1,TRIM44,MAP1A,SLC39A10,AXIN2,PARD3,GNL3L,MUC1,BCOR,ANXA8L1,SNX33,CD84,LPA,SPI1,DNMT3B,EPHA7,ATG14,EIF3H,BCAR3,STK38,ACOT8,RBPMS,RENBP,MAP3K13,RAPGEF3,SNCA,BMPR1B,MAGI2,PNPT1,USP50,RC3H1,EIF3E,RNF19B,PRKAG1,PSMF1,CAST,TRIP12,ADTRP,PLD1,OPRD1,RPTOR,MLLT1,IGF1,HTT,MAPK1,PTEN,SPRED2,BMP7,ATXN2,PUM1,LRRK2,TMEM59,UNC119,BAG6,LILRB4,PKD2,EFNA5,TADA2A,DAZL,PDGFC,SERPINA3,SERPINA4,SERPINA5,PKIB,PPP2R3C,WNT7A,NLRP1,UACA,DNAJC1,PLCL2,MTIF2,BMP6,MYCBP2,GPI,SH3RF2,N4BP1,ATRX,DPH6,RAPGEF2,VRK3,PRKAR2B,UBR5,GRIN1,ARRDC4,RHOA,SPRTN,RQCD1,CUL3,HSPD1,EPM2A,GSK3B,PRR16,DNAJC3,RDX,EEF2K,IWS1,PPP1R16A,PTPRB,NECAB2,CD27,BOK,RNF144B,AGT,METTL16,CCNJL,PRKAA2,ADAR,STAT2,ZMPSTE24,PTK2,SNX3,TEC,EXOSC3,MAP3K5,NCF1,TAOK2,PYGO2,SHC1,PARP10,RNFT1,RPS6KB1,PRG3,SERPINB11,DNAJB6,CD44,LARP4,ADAM8,CLDN4,RPS6KA5,CSNK1A1,HUS1,NOS1,RGS14,STK4,EPHB2,SLC1A1,IL18,MFSD8,UBE2K,DIS3,DLC1,CPEB4,MOB3B,PAX6,PRKC*

			Z,ADORA2A,GRIN2B,RABGEF1,CCNG2,CPEB1,NIPBL,COL28A1,ANGPT1,LRRK1,OSBPL8,IGF2BP3,PPP1CA,MTF2,CDK5RAP1,CNTN1,BRAF,HDAC2,IMPACT,SPINT2,CD300A,PHIP,PPP2CA,SKP1,ANGPT4,RPL23,ANXA2,PARN,MADD,SYK,CNOT1,WWTR1,DVL2,MTRF1,YBX1,PTPRJ,TAF1,EIF4E3,TICAM1,TANK,HSF1,DHFR,ETF1,SASH1,EEFSEC,MOB3A,GNA12,SH2D3A,AKTIP,INPP5F,CD109,TBC1D10A,CAMK2D,SNX1,UCHL5,MALT1,SERPINE3,MLXIPL,NEDD4L,PHB,PTPRC,CTCF,SH3GL2,TNXB,TRAP1,BMP4
GO:0007267	cell-cell signaling	1.0405302228102364e-13	GPC5,NRXN1,S1PR2,PDE7B,ADCY8,GRID2,RPS6KA2,NRG3,LATS2,NLGN4X,GRM8,KALRN,SEMA5A,LRRFIP2,GRM5,CASK,ANK2,SH2D1A,NRXN3,CHD7,HTR2B,SLC24A2,GRK5,ITGB1,PSMB7,CNIH2,MCC,PCDH17,KLHL12,DLG2,ABCC8,ERC1,HMGN3,DGKI,STXBP4,ERC2,GABRA3,LAMA2,TCIRG1,PTPN11,CACNB2,DDB1,SV2B,ADORA1,ADRA1D,ABR,NPHP3,GRM7,PTPRO,NTRK2,CELA1,RNF220,PDGFB,CACNA1B,RIMS1,TNIK,CDH8,USP46,TCF7L2,USP34,FBXW11,PTPRD,SLC4A10,BDNF,CDK14,CHRM3,GRIK4,VAMP7,PTPRU,RIT2,DEPDC1B,FYN,ARNTL,NF1,PLCB1,SNCB,BCR,SHISA9,ICA1,CACNA1E,NRG1,SLC16A10,MDFI,VAX2,DAGLA,VGLL4,CNTN4,NTNG1,ROR2,ZDHHC3,LIN7A,SYN2,SOX13,FGF14,PCDHB16,EXOC4,MAD2L2,TLE6,RGS8,CAPRIN2,CCNYL1,NEUROD1,GNAQ,JAK2,SYN3,CLSTN2,SYT1,LZTS1,SLC1A4,MLLT3,TCF7,KLF15,KCNC4,WNT11,IFT80,KREMEN1,IL1RAPL1,CAMK2B,PCSK5,PDLIM4,BTRC,JPH3,DLGAP1,PTPRN2,F2RL1,GRIK3,CELSR1,GABRR3,CADPS2,GABRB1,SYT13,GPR176,CTNND2,DISC1,KCNJ3,KANK1,CACNA1D,CHRM1,CLOCK,PLCB4,SLC17A7,YTHDF1,FGF10,STXBP5,SLC16A1,SMURF2,EPHA4,CNR1,TNFSF11,PPP3CA,CTNNB1,PARK2,PPARG,AXIN1,PRKAR1B,RYR2,DRAXIN,RGS10,PER2,CACNA1C,CPNE6,SCN3B,GRM1,RIMS4,DCC,BICC1,TRABD2B,SPG11,SFRP1,FOXO3,DGKB,TRHDE,SMAD3,CUX2,GRIK5,SLC16A2,SHANK1,SYT3,VANGL1,SLC6A1,NFATC1,CDC73,APP,YAP1,AMFR,RAB11FIP5,NLGN2,LRP5,SLC9B2,SOX2,GPC6,PRICKLE1,DRD1,GLRA2,ESR2,KDM6A,SLC6A3,HNF4A,SHANK2,EREG,CCNY,TMEM237,RAF1,CELF4,RASGRF2,NLGN1,FGF2,LRRC4C,GRIK1,ADCY5,MCTP1,DKK2,SORCS2,RAB11FIP3,PRKCG,GRIA2,DVL3,ECE2,RBX1,FBXL20,KCNQ1,WNT3,CCL15,NLGN3,PAFAH1B1,PRDM15,NMUR2,BTBD9,GLI2,TNKS,DAB2,WNK1,RELN,RUVBL2,RSPO2,KCNQ2,ZNF423,MACF1,SLC8A2,STAU1,P2RX6,ATAD1,ADNP,MAP1B,MARK1,EPHA5,SORCS3,WWOX,GABRG3,SYT12,CACNA1A,GPC3,ADRBK1,PSMB2,TRPV1,ELAVL4,HDAC1,INVS,PLCG2,C1QTNF1,EGR2,RNF10,CAPN10,NTRK1,MAP1A,PPP1R9A,UNC13A,EPHB1,AXIN2,CACNG8,NETO1,SRD5A2,HTR1D,CHRNB4,MCU,PTPRA,FBXW4,GABRA6,ILDR2,FCHSD2,PTK7,SNCA,CACNG2,GRM4,MAGI2,ZEB2,SYT9,WNT7B,PTPRS,ABCA12,MITF,PKP2,BTK,ZNRF3,KCTD13,SLC4A8,MAPK1,PTEN,LRRK2,ZRANB1,UNC119,TMEM108,ITGAM,PKD2,EFNA5,SHANK3,AMOTL1,WNT7A,ZBED6,SNAP23,CEP89,PLCL2,BMP6,ANO1,TSHR,EXT1,PRICKLE2,GABRR2,RAPGEF2,SMO,PRKAR2B,STXBP5L,CPT1A,SYT17,SOX30,UBR5,GRIN1,JADE1,RHOA,SYNGAP1,VIPR2,GRIN3A,ROR1,CUL3,SH3KBP1,EPM2A,GABRB3,GSK3B,SYN1,MCTP2,SULF1,RNF43,AGT,PRKAA2,SYT7,BLOC1S6,HRH4,NDRG2,PHEX,SNX3,RIMS2,CPE,APCDD1L,SLC29A1,PYGO2,LBX2,SCEL,ALOX5,CPLX2,PACSIN2,CSNK1A1,GRID1,NOS1,RGS14,STK4,SPEF1,EPHB2,SLC1A1,WLS,IL18,DTNA,HCAR2,SYTL4,PRKCZ,ADORA2A,GRIN2B,CADPS,TBL1X,LRRK1,PPP1CA,RBMS3,CCL22,BRAF,HTR2C,TNR,PPP2CA,KCND2,FCRL2,ADCY1,SYK,WWTR1,GRIK2,MYRIP,CACNG3,DVL2,DLGAP2,KCNQ3,TNFSF9,WIF1,ANKRD6,RPH3A,EDA,RNF213,CREB1,SHISA6,EDNRA,BMP4
GO:0019219	regulation of nucleobase-containing compound metabolic process	1.3347414789210667e-13	ENPP1,PRDX2,LDB2,ASPH,PRKCI,SLCO3A1,DNMT1,HLX,SLC9A1,PBX3,ADCY8,MED13L,TRPS1,CBFB,PDE8A,ZNF823,IL31RA,PRDM12,MED26,WWC1,ASH1L,SCAF8,STOX2,PTGIS,PRKAG2,WWC3,PAGR1,HIVEP3,FTO,NPAS3,ARNT,LRRFIP2,STAT5B,TOX,GRM5,ZNF566,FER,CASK,MAP2K5,MAPK10,ZNF536,SP3,HDGF,EZR,IKZF2,CHD7,MECOM,TACC1,TENM1,LMNA,TRAF6,ESRP1,DNAJA3,NHLH1,HSP90AA1,PSMB7,TSC22D3,ZC4H2,CCDC22,GFI1B,PTPRK,RUNX1,ERC1,HMGN3,NRIP1,THRB,EFCAB7,ITGB3BP,DACH1,ZNF569,CCT2,ORC2,HDAC6,SERTAD2,MYT1,IKBKB,PEX14,ERBB4,MRE11A,ZNF609,BRD8,KAT6B,HIF3A,SNIP1,ESR1,MIER1,PCBP3,MAML3,CDON,TNRC6A,EP300,CELA1,TENM2,ZNF76,RNF220,ZNF471,PDGFB,CCND3,TOB2,ZNF19,ZNF23,BRMS1,ZNF605,SCML4,HNF4G,INSR,FMN2,RERE,PRKAR1A,FUBP1,ATP8B1,H2AFY2,TCF7L2,JMJD1C,ZNF443,ZNF490,ZNF564,ZNF709,ZNF799,LITAF,FBXW11,ESRRB,MAP3K4,BASP1,RBM20,TBR1,SAMD4A,TFAP2A,CDK14,FANCA,PEG3,ZIM2,TGIF2,TGIF2-C20ORF24,MEIS1,VRTN,TRIM13,MDM4,ENTPD5,ZNF148,MTA3,SNX6,TFDP2,ERN2,C1D,AFF3,DMD,CENPF,SLC30A9,TOX3,LARP4B,USP13,KAT7,ZNF667,KHDRBS2,RBM14,RBM4,MED12L,SATB2,RIT2,HIRA,MKRN2,ARNTL,PLCB1,MGMT,ARID4B,PPP4R2,AFF2,RWDD3,BRPF1,PIK3R2,NRG1,UBP1,MAP2K1,MDFI,FNIP1,VAX2,TMBIM6,VGLL4,PPP2CB,PPP3R1,HDAC5,CSRNP3,RBM5,ZNF692,NFIA,RNF4,JDP2,CMKLR1,ROR2,DCN,PCBD2,HNRNPLL,CDH13,CREBRF,TRDMT1,SOX13,WDR70,AUNIP,COPS5,MAD2L2,TLE6,CAPRIN2,ZNF418,PHF20L1,TERF2,SLX1B,ZNF286A,FOXN3,NEUROD1,USP22,JAK2,FAM168A,TRAPPC9,SKAP1,UIMC1,ITCH,MLIP,BCL11B,SMG1,RBFOX1,PKNOX1,MLLT3,TSHZ2,TCF7,PDE2A,KLF15,TBX15,ANXA4,WNT11,MTA1,KLF8,NOX4,LCOR,RPRD1B,QKI,CCDC62,ERCC8,PRKCD,SOX6,ACVR2A,RUNX2,CD4,TGFB1,BANP,SGK1,NSD1,IGSF1,ZHX2,PKNOX2,ASCC2,BTRC,NFATC3,F2RL1,BCAS3,CDYL2,CC2D1B,TP73,SAP18,ZBTB22,ILF2,MTDH,FANK1,MYT1L,SMAD6,BNC2,ZNF398,CLOCK,TCF12,ZNF675,ETV6,TFAP2D,BCL3,SND1,DUSP22,HNRNPC,TRRAP,SBNO2,YTHDF1,FGF10,CIZ1,SMYD3,LOXL3,CAPN

			3,LUM,SMURF2,RORA,HIVEP2,PRKCA,AUTS2,TNFSF11,SMG6,PPP3CA,NSUN2,NFYB,MAGEA4,KLF12,CAMK4,GATAD2B,UFL1,TRAK1,CTNNB1,PARK2,SOD2,DACH2,METTL13,SMARCC1,KLF17,IGF1R,PPARG,AXIN1,OTUB1,IL18R1,CIPC,MTF1,CELF2,CBX5,BRIP1,LRPPRC,SREBF2,CDK11A,CDK11B,FGF1,NPAT,NR4A3,FOXK2,RIOK2,MYOCD,TRIM5,PER2,AJUBA,ZNF626,ZNF737,CHEK2,SUPT3H,UBQLN4,PRDM16,HCK,TRIM8,BRMS1L,ZBTB8A,ZBTB8B,CTDP1,BAZ1B,MEF2B,HDAC4,PAX2,PHF5A,SFRP1,MED13,ZNF395,FOXO3,NFIB,SP4,ZCCHC17,CELF5,SYNCRIP,SMAD3,CUX2,WWP2,ARNT2,SBNO1,KRBOX1,ZNF662,ZNF777,EBF3,RNF168,CASZ1,DCP1B,MIER3,NEDD4,ESRRG,HOXD3,HOXD4,SLC4A4,ZNF114,KTI12,NFATC1,CDC73,APP,SSBP3,GSX2,RBM8A,YAP1,TEN1,EYA2,NVL,LRP5,POLR3G,ZNF787,SOX2,SETD2,TEAD1,PRICKLE1,ZNF653,ZNF521,ARID3A,ZNF761,CHUK,SFMBT1,ZNF584,ESR2,S100A12,PRKD1,STAT1,CELF6,ST18,ETV5,RHOXF2B,TAF3,PLAGL1,HNF4A,ZBTB7C,TASP1,EREG,RBFOX3,ATF2,POU2F2,TCF3,ZNF730,RAF1,CELF4,ZNF766,CARD16,SRRM4,BMPR2,CAMK1D,BMPR1A,ZKSCAN1,PABPC4,IKZF4,CDK12,CAND2,DENND4A,MYB,FGF2,ZNF71,POU6F2,BACH1,MXD3,PPM1F,TICRR,GLI4,ZFP41,BEND5,SEMA4D,NFX1,RORC,ELP3,PTAFR,SP140,SP140L,RHOXF2,JARID2,DDX58,BRDT,PHC2,RARB,SPEN,PRKCG,SIN3B,NCOA1,EHMT1,LMO7,DVL3,TCF20,ATF3,LIN28B,SRSF5,CKS1B,PAWR,EBF2,MAML2,TSG101,TERF2IP,CRYM,RFX2,ZNF322,SUFU,MAGEA11,PRDM15,ZNF670,ZNF695,CCDC169-SOHLH2,SOHLH2,ZNF354C,TCEA3,ZNF704,NR2C1,GLI2,TNKS,WBP2NL,ERCC1,TNRC6B,GLIS3,WDTC1,ZNF664,DAB2,BLM,PKHD1,MYSM1,SETD5,SMG5,RELN,SIN3A,RUVBL2,COMMD6,GMEB1,PELI1,MAP3K7,ZNF423,SP1,TRIM22,ALK,TEAD4,FASTKD5,HOXC13,APBB3,SRA1,UBE2V1,ADIRF,AHNAK,OVOL2,SNX5,NFATC2,PAX7,BNC1,ATP2B4,ACTL6B,ASXL3,PAK3,TET1,CAMTA1,CCPG1,ADNP,MAPKAPK2,ARID4A,CDK6,PHF2,CELF1,TFRC,EPHA5,WWOX,MEF2A,DCAF6,DROSHA,XRN1,SATB1,PSMB2,EYA1,GATAD2A,DIS3L2,TRPV1,RBM12B,HOXB3,HOXB4,HOXB5,HOXB6,TFEB,MNAT1,ACTR2,L3MBTL4,ELAVL4,HDAC1,RECQL5,SMOC2,TRIM24,PLCG2,ROCK1,SCMH1,PAXIP1,ZNF713,EGR2,RNF10,RYBP,AP3B1,PTPN2,NTRK1,KPNA6,TRIM44,AXIN2,EPCAM,ZNF425,GNL3L,MUC1,PTPN14,BCOR,AP3D1,ZNF41,CARM1,ZNF383,ZNF616,ZNF836,SMARCA2,CUX1,SPI1,DNMT3B,FOXP2,PSPC1,GLIS1,SFMBT2,JAZF1,RBPMS,CHURC1,CREB5,MAP3K13,SMYD1,SNCA,BMPR1B,PRIM2,PNPT1,ZEB2,FOXJ3,HEY2,RC3H1,ZMYND11,KMT2D,PRKAG1,CREM,KMT2C,TRIP12,DFFA,RASA1,MACC1,MITF,SRSF6,OPRD1,RPTOR,NFIX,BTK,MLLT1,CBFA2T2,IGF1,MLXIP,ATF7IP,MAPK1,PTEN,BMP7,MXI1,PUM1,SOX5,CIR1,PCBP2,ZNF780A,ZNF780B,ZBTB20,RFC3,LILRB4,PKD2,ZNF652,KCTD1,RIPPLY1,RCOR3,TADA2A,DAZL,NCOA3,PKIB,ZNF146,ZNF565,WNT7A,ZBED6,BRWD3,CRTC3,ARNTL2,KAT6A,ZKSCAN7,ZNF197,ZNF660,BMP6,TAF15,NFE2L1,WDR43,ZNF30,ATRX,IKZF1,PRMT2,ASCC1,SMO,RALY,TCF4,SOX30,TFE3,UBR5,GRIN1,JADE1,RHOA,ROR1,RQCD1,TF,ONECUT2,CUL3,TFEC,STAT6,MLF1,IWS1,RBBP8,CDK13,DUSP26,ZNF362,NACC2,SUPT4H1,ZNF354A,AGT,HDGFRP3,METTL16,POLH,PRKAA2,STAT2,ZMPSTE24,PBX1,EXOSC3,MAP3K5,TCFL5,CREBBP,DLX1,GTF2IRD2,NCF1,SHC1,ZNF813,LBX2,PARP10,YLPM1,DPRX,SETDB2,DNAJB6,SP7,ADAM8,RPS6KA5,ZNF484,ZNF93,PRDM2,NOS1,ZNF44,MED15,RGS14,ACTN4,MC1R,TCF25,ZNF282,EDRF1,IL18,DIS3,PPARA,PPP1R10,ARID5B,SMARCAL1,NOTCH4,ZMYND8,ZNF366,CRX,PAX6,PRKCZ,ZC3HAV1,ADORA2A,CPEB1,OTUD7B,NIPBL,YEATS4,PHF20,ZNF143,BRF1,TBL1X,EYA3,FHL2,MBTD1,ATF6,ZBTB5,ZNF708,IGF2BP3,ETS1,MTF2,TAGLN3,NCOR2,PRKCQ,CSRNP1,HDAC2,IMPACT,SRCAP,POU3F3,NOTO,MBNL3,ZKSCAN5,ELF2,PHIP,PPP2CA,RPL23,RFC5,ATXN1,SP100,ZNF347,ZNF415,TRIM29,PARN,ADCY1,RBM42,SYK,CNOT1,RBM39,WWTR1,GTF2H5,NFXL1,ZBTB38,BRCA2,ATF7,DVL2,MORC1,YBX1,TAF1,TICAM1,THAP3,HSF1,MAX,SAP130,EZH1,NRF1,SRPK2,NCOA2,WDR18,QRICH1,ZP3,CHD6,RNF2,ZNF554,MYEF2,TRIM37,MET,ZNF461,CAMK2D,TIMELESS,BRD9,MALT1,SETD3,TRA2B,KDM2B,MLXIPL,CCT3,PHB,PTPRC,EDA,ZNF555,CTCF,NR6A1,TRAF3IP2,ATF6B,CREB1,RGMB,EBF4,ZNF511,BMP4,ABLIM3
GO:0016358	dendrite development	2.6216539784641236e-13	PRMT3,LLPH,SEMA3A,PHACTR1,KALRN,CDKL5,ABI1,ITGB1,KNDC1,DSCAM,DAB1,DOCK10,HDAC6,TNIK,RERE,PTPRD,TANC2,FYN,FAT3,NTN1,CAPRIN2,ZDHHC15,LZTS1,IL1RAPL1,CAMK2B,CTNND2,DISC1,LAMC2,DCLK1,EPHA4,PPP3CA,DLG5,DCC,DNM3,CUX2,ABI2,SHANK1,NEDD4,BTBD3,APP,FSTL4,NLGN2,PARP6,DGKG,CAMK1D,NLGN1,CTNNA2,SEMA4D,EPHB3,PAFAH1B1,TRPC5,RELN,ALK,PRKG1,PACSIN1,ACTL6B,PAK3,MAP1B,MARK1,MEF2A,ACTR2,ELAVL4,MAP1A,EPHB1,SARM1,CARM1,ARHGAP44,CUX1,SDK1,PTPRS,PTEN,BMP7,LRRK2,SHANK3,WNT7A,RAPGEF2,RHOA,SYNGAP1,GRIN3A,GSK3B,EEF2K,TAOK2,EPHB2,KLHL1,MAP2,FBXO31,HDAC2,CDKL3,SRGAP2,TMEFF1,GRIP1,ASAP1,NEDD4L
GO:0016192	vesicle-mediated transport	3.0794658208255997e-13	ENPP1,HOOK2,BLZF1,NRXN1,PRKCI,CD302,LY75,CLASP2,DOCK1,LRP1B,NLGN4X,KALRN,SEC23B,CBL,RAB4B,RAB4B-EGLN2,ENPP2,FER,CASK,CCDC93,LRP2,PIK3CD,ANK2,EZR,MEGF10,NRXN3,HTR2B,VAV2,ITGB1,ELMO2,CNIH2,PCDH17,CCDC22,KLHL12,ERC1,NCF4,DGKI,ERC2,CACNB2,CYTH3,GOLGA4,ADORA1,GPRASP1,SORCS1,GBF1,LIMK1,CECR2,STX18,HOOK3,VPS45,RIMS1,SNX2,INSR,FMN2,PIP4K2A,HPS1,PAK1,SFT2D1,SNX6,CHML,VAMP7,AP3S1,TMPRSS15,DENND5A,GSN,RIT2,FYN,ADAMTS9,NCF2,LMTK2,STX6,SNCB,MAL2,VTA1,BCR,NDRG4,BMP2K,DCTN1,NRG1,MAP2K1,MYH9,SCFD2,LIN7A,SCFD1,SYN2,DOCK2,CDH13,PITPNB,COPS5,EXOC4,RAB27A,RAB3C,COG8,TMED6,GOLPH3L,ANO6,ARL3,DENND1A,SYN3,SYT1,ATP9A,DENND2A,CT

			AGE6,MKLN1,CACNA1H,IL1RAPL1,PRKCD,TGFB1,F2RL1,AAK1,ZFYVE9,CADPS2,SYT13,CORO1C,C12ORF4,ANK1,RAB11FIP4,ITSN2,SYNJ2,DCLK1,RABGAP1L,SLC17A7,STXBP5,PLIN3,LOXL3,SNX8,CNR1,CD6,PPP3CA,TRAK1,CTNNB1,PARK2,FCGR2B,HSPA6,TSNARE1,IGF1R,PPARG,PRKAR1B,MYO1D,MSR1,SYTL5,SCAMP5,ANXA13,LEPR,LEPROT,PROS1,NR4A3,SCARA3,CPNE6,MYO1E,HCK,TBC1D14,MRC2,RIMS4,ANKFY1,SPTBN1,AP3B2,SPG11,CCDC91,DNM3,RAB6A,ACKR2,WIPI1,VPS39,GRIK5,SYT3,NEDD4,VPS53,RAB6C,APP,BET1L,RAB11FIP5,NLGN2,SCRN1,LRP5,SEC22C,SLC9B2,SPTBN4,DRD1,TPH1,PRKD1,VPS4A,MAPKAPK3,PTPN1,USP33,DPYSL2,CAMK1D,AP1B1,NLGN1,ATP9B,SNX16,SPAG9,RAB11A,EPG5,MCTP1,PTAFR,HHIPL1,EEA1,ABCA13,RAB11FIP3,PRKCG,TXNDC5,AP2M1,TRAPPC11,LMBRD1,FBXL20,TSG101,RHOJ,NLGN3,KIF3A,HIP1,BTBD9,ENTHD1,STON2,DAB2,XKR4,FAM91A1,MACF1,ARHGAP12,MYO7A,CLASP1,TPCN1,SNX5,NSF,PACSIN1,ASGR2,ATAD1,MAPKAPK2,SCARB1,TFRC,UVRAG,SYT12,FNBP1,GPC3,SGIP1,ADRBK1,TBC1D5,CLEC16A,GHR,PRSS12,SNX9,ARL5A,C2,PLCG2,ROCK1,GAPVD1,AP3B1,ANK3,UNC13A,TUB,SH3GL3,CACNG8,AP3D1,AP2B1,ANXA8L1,ARHGAP44,HEATR5A,SNX33,CD84,CUX1,SPI1,VPS16,ACAP2,ATG14,FCHSD2,RAB15,SH3BP1,SNCA,CACNG2,MAGI2,SYT9,CHMP3,RAB5B,OSBPL1A,ABCA12,PLD1,BTK,VPS41,SLC4A8,PLA2G4E,STX8,HTT,MAPK1,PTEN,MIB1,ATXN2,PIK3C3,LRRK2,UNC119,CHMP5,CNIH3,LILRB4,TMEM108,ITGAM,ARHGAP25,NSG2,MIA2,TMCC1,WNT7A,SNAP23,RABEP1,STXBP5L,SYT17,SOX30,DNAJC6,GRIN3A,TF,RHOBTB1,ESYT2,FAM19A4,CUL3,SH3KBP1,GSK3B,PLD2,RDX,SYN1,EEF2K,NECAB2,VAMP1,SYT7,BLOC1S6,PTK2,LAT2,SNX3,EXOC6B,RIMS2,COPG2,SEC16B,CD160,ITGAL,CPLX2,ARFGAP3,PACSIN2,MON2,TBC1D23,COG4,EPHB2,SLC1A1,CALCRL,SYTL4,ADORA2A,RABGEF1,GPR107,ANGPT1,CADPS,TBC1D10C,CFI,BIN2,ATG3,BRAF,CLTB,CD300A,SH3BP4,VTI1A,ANXA2,RAB27B,ITSN1,ADCY1,NUMB,SYK,RAB2A,SEC31B,CACNG3,ANKRD13A,GRIP1,PTPRJ,ERGIC1,CNIH4,PLEKHM1,FCHO1,VAV3,ZP3,CAV2,AKTIP,EPS15,INPP5F,TMPRSS3,AXL,REPS2,MET,TBC1D10A,MICAL3,SNX1,EHD2,TRAPPC8,NEDD4L,PTPRC,SH3GL2,TYRO3,SPON2,SYNDIG1,VPS52,CLNK,STXBP6,CPNE1
GO:0070887	cellular response to chemical stimulus	3.7585758658740223e-13	ENPP1,PRDX2,NRXN1,ASPH,PRKCI,PDE4D,DNMT1,SLC9A1,ADCY8,PDE8A,IL31RA,RPS6KA2,PTGFR,CLDN18,CTDSPL2,RYR1,LATS2,NREP,PTGIS,TJP1,UNC5C,PRLR,PAGR1,ADCY7,NTRK3,CBL,ARNT,EGLN2,SEMA5A,FLT3,STAT5B,GRM5,SAMHD1,FER,CASK,MAP2K5,LRP2,PIK3CD,HDGF,EZR,ROBO1,SLC26A6,HTR2B,ITGB6,RYR3,LMNA,GOT1,LPO,TRAF6,ROBO2,ITPKB,DNAJA3,OXR1,ERCC6L2,ELMO2,CDC6,PSMB7,DLG2,PTPRK,ABCC8,HMGN3,NRIP1,DNAJB14,THRB,STXBP4,PDE4A,IL17RB,TCIRG1,PTPN11,HDAC6,MSRA,ADAMTS3,IKBKB,ERBB4,GBF1,BRD8,ESR1,PMEPA1,PTPRO,NTRK2,EP300,CPNE4,FNTA,PDGFB,CCND3,ALOX5AP,BPI,STK39,INSR,FMN2,PIP4K2A,CAB39,PAK1,LITAF,ESRRB,EGLN3,BDNF,TFAP2A,AMIGO1,CHRM3,FUT8,UBR2,MDM4,IL5RA,ECT2,SNX6,ERN2,LSP1,AP3S1,DMD,SLC8A1,GSN,SATB2,FYN,ARNTL,NF1,PLCB1,MGMT,RTN4,RXFP1,DPEP1,CHRDL1,BCR,RWDD3,CHI3L1,PIK3R2,NTN1,CDK19,BDKRB2,DOCK8,TMBIM6,PPP2CB,HDAC5,CMKLR1,ROR2,DCN,SLC39A14,CREBRF,PTPRT,COPS5,IPO5,RGS8,DEFA1B,DEFA3,PTGER2,STIP1,ANO6,NEUROD1,GNAQ,RFFL,CCBE1,JAK2,FBXW7,SYT1,CLDN1,TCF7,PDE2A,KLF15,LAMTOR3,CYFIP2,WNT11,NOX4,LCOR,CACNA1H,IL1RAPL1,CCDC62,ADAM23,PRKCD,SOX6,ACVR2A,RUNX2,CD4,PPM1E,TGFB1,SGK1,MMP28,BTRC,PTPRN2,F2RL1,BCAS3,SLIT3,ZFYVE9,SLIT2,GABRB1,SYT13,HP,KANK1,ZFAND2A,MTDH,CHRM1,SMAD6,RXFP2,CLOCK,ZNF675,KCNH1,BMPER,DAPK2,PARVA,DUSP22,NAIP,SBNO2,FGF10,RASA4,RASA4B,SMYD3,IL1RAPL2,GLP2R,ADCY2,CAPN3,SLC16A1,SMURF2,EPHA4,RORA,PRKCA,CD6,TNFSF11,PPP3CA,HBE1,NFYB,UFL1,CTNNB1,PARK2,SOD2,FCGR2B,HSPA6,SMARCC1,IGF1R,PPARG,AXIN1,IL18R1,IL1RL1,MTF1,MSR1,CYBB,BRIP1,RYR2,LEPR,LEPROT,FGF1,RGS10,NR4A3,NOL3,MYOCD,TRIM5,AJUBA,CPNE6,GLG1,CHEK2,PRDM16,HCK,SORBS1,CAPN2,FLT4,HDAC4,PAX2,SFRP1,FOXO3,TPH2,SSH1,SYNCRIP,SMAD3,CUX2,ACKR2,GRIK5,SYT3,IFNAR1,NEDD4,NRP2,PREX1,DOCK4,ESRRG,TAB1,CDC73,APP,PDGFRA,AOC2,NOX1,YAP1,AMFR,RAB11FIP5,SESN1,TNFRSF19,ALPL,JAK1,LRP5,SLC9B2,GNG2,CHST11,DRD1,GLRA2,CHUK,FRS2,MMP2,ESR2,S100A12,CPNE9,PRKD1,STAT1,ST18,ETV5,HNF4A,EREG,ATF2,RAF1,EPHX1,CARD16,CASP1,PTPN1,ADAMTS12,BMPR2,CAMK1D,BMPR1A,TSPAN12,NLGN1,COL16A1,PIK3R3,EPG5,FGF2,BACH1,PPM1F,ADCY5,RORC,PTAFR,JARID2,DDX58,RARB,NCOA1,EEF1E1,CHRD,PTPRE,ATF3,SHOC2,SRSF5,PAWR,ACAA1,CCDC3,CLIC4,IDE,KCNQ1,RFX2,WNT3,P4HB,CCL14,CCL15,CCL15-CCL14,PAFAH1B1,CNTFR,TRPA1,DDAH1,AKAP6,RASAL1,NR2C1,IRS4,GLI2,NEO1,PXDNL,WDTC1,DAB2,BLM,UBR1,LDLRAD4,WNK1,SIN3A,RUVBL2,CSF3R,IQGAP1,MAP3K7,ZNF423,SP1,ALK,GSTM3,STAU1,SRA1,XDH,LTBP1,OVOL2,SNX5,ATP2B4,PACRG,PAK3,DYX1C1,MAPKAPK2,MAP1B,COL4A6,SCARB1,ATP6V0A2,TFRC,EPHA5,WWOX,MEF2A,SYT12,CACNA1A,GPC3,XRN1,ADRBK1,PSMB2,BRINP1,SHPK,TRPV1,GHR,FLRT2,ACTR2,ACACA,ELAVL4,HDAC1,RECQL5,SMOC2,TRIM24,ADIPOR2,PLCG2,ROCK1,EPS8,EGR2,CAPN10,GPR35,ABCC1,PTPN2,INSRR,NTRK1,TRIM44,ZFAND1,ANK3,EPHB1,AXIN2,TMEM199,CARM1,HTR1D,SPI1,MCU,PTPRA,CTNNA1,AIFM2,ACAP2,LRRC8C,LRRC8D,LY86,BCAR3,CHURC1,RAB15,RAPGEF3,PDXP,MST1,PTK7,SNCA,BMPR1B,MAGI2,PNPT1,ZEB2,SYT9,RC3H1,WNT7B,HMGCS2,KMT2D,SULT2A1,SPPL2A,ADTRP,ABCA12,OPRD1,HIPK1,NUGGC,RPTOR,BTK,TROVE2,IGF1,STX8,DPYSL3,MAPK1,PTEN,SPRED2,BMP7,SOX5,PRCP

			,LRRK2,ATP6V1A,GNA14,ZBTB20,CHMP5,BAG6,LILRB4,TMEM108,IPCEF1,IL11RA,NSG2,PKD2,EFNA5,AOX1,PDGFC,NCOA3,WNT7A,ZBED6,NME8,GLDC,GPR21,BMP6,NFE2L1,ANO1,IL17RD,SOS1,TSHR,EXT1,PDCD1LG2,ATRX,PNPLA3,PRMT2,RAPGEF2,ABCG2,KCNC2,SMO,CPT1A,SYT17,UBR5,GRB14,GRIN1,RHOA,RQCD1,TF,ONECUT2,FAM19A4,CUL3,HSPD1,ITPR2,GABRB3,GSK3B,RDX,STAT6,EEF2K,BOK,SULF1,KRT8,AGT,PRKAA2,SYT7,ADAR,FBN2,STAT2,BRINP3,HRH4,PTK2,PHEX,PDE4B,VEPH1,MAP3K5,CREBBP,DLX1,FMOD,PCSK6,NCF1,SLC29A1,UGT1A1,UGT1A10,UGT1A3,UGT1A4,UGT1A5,UGT1A6,UGT1A7,UGT1A8,UGT1A9,SHC1,NDUFS2,RPS6KB1,STC2,GPR173,ALOX5,CD44,ADAM8,RPS6KA5,CXCL17,NOS1,PLEKHA1,PLVAP,ACTN4,SCNN1B,ZFAND6,EPHB2,SLC1A1,SIGMAR1,XCR1,IL18,TPMT,UBE2K,PPARA,ARID5B,CALCRL,MCOLN1,SLC25A33,CPEB4,GPSM3,ZNF366,CYP2C18,CYP2C19,FBN1,PAX6,PRKCZ,ATP1A3,FAM20C,RABGEF1,CPEB1,TYK2,ASPN,ANGPT1,OSBPL8,ATF6,GCNT2,VWC2,MTF2,BIN2,CCL22,NCOR2,PRKCQ,BRAF,DIAPH1,HDAC2,HTR2C,IMPACT,SPINT2,PHIP,ANGPT4,RPL23,SH3BP4,SP100,KCND2,ADCY1,SYK,CNOT1,YBX1,PTPRJ,TAF1,TICAM1,TANK,UCN2,A3GALT2,HSF1,MAX,DHFR,SASH1,NCOA2,ADAMTS7,GBP5,QRICH1,VAV3,CHD6,CIB2,CAV2,UGGT1,AXL,CD109,DENND4C,MET,CAMK2D,CCR3,TIMELESS,MALT1,PFKP,TRA2B,MLXIPL,PHB,PTPRC,EDA,PBLD,SH3GL2,SPON2,ADCY10,TRAF3IP2,ATF6B,CREB1,RGMB,TRAP1,EDNRA,GNGT1,BMP4,CPNE1*
GO:0051252	regulation of RNA metabolic process	6.803765571201209e-13	*ENPP1,PRDX2,LDB2,ASPH,PRKCI,SLCO3A1,DNMT1,HLX,SLC9A1,PBX3,ADCY8,MED13L,TRPS1,CBFB,PDE8A,ZNF823,IL31RA,PRDM12,MED26,WWC1,ASH1L,SCAF8,STOX2,PTGIS,WWC3,PAGR1,HIVEP3,FTO,NPAS3,ARNT,LRRFIP2,STAT5B,TOX,GRM5,ZNF566,FER,CASK,MAP2K5,MAPK10,ZNF536,SP3,HDGF,EZR,IKZF2,CHD7,MECOM,TACC1,TENM1,TRAF6,ESRP1,DNAJA3,NHLH1,PSMB7,TSC22D3,ZC4H2,CCDC22,GFI1B,PTPRK,RUNX1,ERC1,HMGN3,NRIP1,THRB,EFCAB7,ITGB3BP,DACH1,ZNF569,ORC2,HDAC6,SERTAD2,MYT1,IKBKB,PEX14,ERBB4,ZNF609,BRD8,KAT6B,HIF3A,SNIP1,ESR1,MIER1,PCBP3,MAML3,CDON,TNRC6A,EP300,CELA1,TENM2,ZNF76,RNF220,ZNF471,PDGFB,CCND3,TOB2,ZNF19,ZNF23,BRMS1,ZNF605,SCML4,HNF4G,INSR,RERE,PRKAR1A,FUBP1,ATP8B1,H2AFY2,TCF7L2,JMJD1C,ZNF443,ZNF490,ZNF564,ZNF709,ZNF799,LITAF,FBXW11,ESRRB,BASP1,RBM20,TBR1,SAMD4A,TFAP2A,CDK14,FANCA,PEG3,ZIM2,TGIF2,TGIF2-C20ORF24,MEIS1,VRTN,TRIM13,MDM4,ZNF148,MTA3,SNX6,TFDP2,ERN2,C1D,AFF3,DMD,CENPF,SLC30A9,TOX3,LARP4B,USP13,KAT7,ZNF667,KHDRBS2,RBM14,RBM4,MED12L,SATB2,RIT2,HIRA,MKRN2,ARNTL,PLCB1,ARID4B,AFF2,RWDD3,BRPF1,PIK3R2,NRG1,UBP1,MAP2K1,MDFI,FNIP1,VAX2,TMBIM6,VGLL4,PPP2CB,PPP3R1,HDAC5,CSRNP3,RBM5,ZNF692,NFIA,RNF4,JDP2,CMKLR1,ROR2,DCN,PCBD2,HNRNPLL,CDH13,CREBRF,TRDMT1,SOX13,COPS5,MAD2L2,TLE6,CAPRIN2,ZNF418,PHF20L1,ZNF286A,FOXN3,NEUROD1,USP22,JAK2,TRAPPC9,SKAP1,UIMC1,ITCH,MLIP,BCL11B,RBFOX1,PKNOX1,MLLT3,TSHZ2,TCF7,PDE2A,KLF15,TBX15,ANXA4,WNT11,MTA1,KLF8,LCOR,RPRD1B,QKI,CCDC62,PRKCD,SOX6,ACVR2A,RUNX2,CD4,TGFB1,BANP,SGK1,NSD1,IGSF1,ZHX2,PKNOX2,ASCC2,BTRC,NFATC3,F2RL1,BCAS3,CDYL2,CC2D1B,TP73,SAP18,ZBTB22,ILF2,MTDH,FANK1,MYT1L,SMAD6,BNC2,ZNF398,CLOCK,TCF12,ZNF675,ETV6,TFAP2D,BCL3,SND1,DUSP22,HNRNPC,TRRAP,SBNO2,YTHDF1,FGF10,SMYD3,LOXL3,CAPN3,LUM,SMURF2,RORA,HIVEP2,PRKCA,AUTS2,TNFSF11,PPP3CA,NSUN2,NFYB,MAGEA4,KLF12,CAMK4,GATAD2B,UFL1,TRAK1,CTNNB1,PARK2,SOD2,DACH2,METTL13,SMARCC1,KLF17,PPARG,AXIN1,IL18R1,CIPC,MTF1,CELF2,CBX5,BRIP1,LRPPRC,SREBF2,CDK11A,CDK11B,FGF1,NPAT,NR4A3,FOXK2,RIOK2,MYOCD,TRIM5,PER2,AJUBA,ZNF626,ZNF737,CHEK2,SUPT3H,PRDM16,HCK,TRIM8,BRMS1L,ZBTB8A,ZBTB8B,CTDP1,BAZ1B,MEF2B,HDAC4,PAX2,PHF5A,SFRP1,MED13,ZNF395,FOXO3,NFIB,SP4,ZCCHC17,CELF5,SYNCRIP,SMAD3,CUX2,WWP2,ARNT2,SBNO1,KRBOX1,ZNF662,ZNF777,EBF3,RNF168,CASZ1,DCP1B,MIER3,NEDD4,ESRRG,HOXD3,HOXD4,ZNF114,KTI12,NFATC1,CDC73,APP,SSBP3,GSX2,RBM8A,YAP1,LRP5,POLR3G,ZNF787,SOX2,SETD2,TEAD1,PRICKLE1,ZNF653,ZNF521,ARID3A,ZNF761,CHUK,SFMBT1,ZNF584,ESR2,S100A12,PRKD1,STAT1,CELF6,ST18,ETV5,RHOXF2B,TAF3,PLAGL1,HNF4A,ZBTB7C,TASP1,EREG,RBFOX3,ATF2,POU2F2,TCF3,ZNF730,RAF1,CELF4,ZNF766,CARD16,SRRM4,BMPR2,CAMK1D,BMPR1A,ZKSCAN1,PABPC4,IKZF4,CDK12,CAND2,DENND4A,MYB,FGF2,ZNF71,POU6F2,BACH1,MXD3,PPM1F,GLI4,ZFP41,BEND5,SEMA4D,NFX1,RORC,ELP3,PTAFR,SP140,SP140L,RHOXF2,JARID2,DDX58,BRDT,PHC2,RARB,SPEN,SIN3B,NCOA1,EHMT1,LMO7,DVL3,TCF20,ATF3,LIN28B,SRSF5,CKS1B,PAWR,EBF2,MAML2,TSG101,TERF2IP,CRYM,RFX2,ZNF322,SUFU,MAGEA11,PRDM15,ZNF670,ZNF695,CCDC169-SOHLH2,SOHLH2,ZNF354C,TCEA3,ZNF704,NR2C1,GLI2,TNKS,WBP2NL,ERCC1,TNRC6B,GLIS3,WDTC1,ZNF664,DAB2,BLM,PKHD1,MYSM1,SETD5,RELN,SIN3A,RUVBL2,COMMD6,GMEB1,PELI1,MAP3K7,ZNF423,SP1,TRIM22,ALK,TEAD4,FASTKD5,HOXC13,APBB3,SRA1,UBE2V1,ADIRF,AHNAK,OVOL2,SNX5,NFATC2,PAX7,BNC1,ATP2B4,ACTL6B,ASXL3,TET1,CAMTA1,CCPG1,ADNP,MAPKAPK2,ARID4A,CDK6,PHF2,CELF1,TFRC,EPHA5,WWOX,MEF2A,DCAF6,DROSHA,SATB1,PSMB2,EYA1,GATAD2A,DIS3L2,TRPV1,RBM12B,HOXB3,HOXB4,HOXB5,HOXB6,TFEB,MNAT1,ACTR2,L3MBTL4,ELAVL4,HDAC1,RECQL5,TRIM24,PLCG2,ROCK1,SCMH1,PAXIP1,ZNF713,EGR2,RNF10,RYBP,AP3B1,PTPN2,NTRK1,KPNA6,TRIM44,AXIN2,EPCAM,ZNF425,MUC1,PTPN14,BCOR,AP3D1,ZNF41,CARM1,ZNF383,ZNF616,ZNF836,SMARCA2,CUX1,SPI1,DNMT3B,FOXP2,PSPC1,GLIS1,SFMBT2,JAZF1,RBPMS,*

			CHURC1,CREB5,MAP3K13,SMYD1,SNCA,BMPR1B,PRIM2,PNPT1,ZEB2,FOXJ3,HEY2,RC3H1,ZMYND11,KMT2D,CREM,KMT2C,RASA1,MACC1,MITF,SRSF6,OPRD1,RPTOR,NFIX,BTK,MLLT1,CBFA2T2,IGF1,MLXIP,ATF7IP,PTEN,BMP7,MXI1,PUM1,SOX5,CIR1,PCBP2,ZNF780A,ZNF780B,ZBTB20,LILRB4,PKD2,ZNF652,KCTD1,RIPPLY1,RCOR3,TADA2A,DAZL,NCOA3,ZNF146,ZNF565,WNT7A,ZBED6,BRWD3,CRTC3,ARNTL2,KAT6A,ZKSCAN7,ZNF197,ZNF660,BMP6,TAF15,NFE2L1,WDR43,ZNF30,ATRX,IKZF1,PRMT2,ASCC1,SMO,RALY,TCF4,SOX30,TFE3,GRIN1,JADE1,RHOA,ROR1,RQCD1,TF,ONECUT2,CUL3,TFEC,STAT6,MLF1,IWS1,RBBP8,CDK13,DUSP26,ZNF362,NACC2,SUPT4H1,ZNF354A,AGT,HDGFRP3,METTL16,STAT2,ZMPSTE24,PBX1,EXOSC3,MAP3K5,TCFL5,CREBBP,DLX1,GTF2IRD2,NCF1,SHC1,ZNF813,LBX2,PARP10,DPRX,SETDB2,DNAJB6,SP7,ADAM8,RPS6KA5,ZNF484,ZNF93,PRDM2,NOS1,ZNF44,MED15,RGS14,ACTN4,MC1R,TCF25,ZNF282,EDRF1,IL18,DIS3,PPARA,ARID5B,SMARCAL1,NOTCH4,ZMYND8,ZNF366,CRX,PAX6,PRKCZ,ZC3HAV1,ADORA2A,CPEB1,OTUD7B,NIPBL,YEATS4,PHF20,ZNF143,BRF1,TBL1X,FHL2,MBTD1,ATF6,ZBTB5,ZNF708,IGF2BP3,ETS1,MTF2,TAGLN3,NCOR2,PRKCQ,CSRNP1,HDAC2,IMPACT,SRCAP,POU3F3,NOTO,MBNL3,ZKSCAN5,ELF2,PHIP,PPP2CA,RPL23,ATXN1,SP100,ZNF347,ZNF415,TRIM29,PARN,ADCY1,RBM42,SYK,CNOT1,RBM39,WWTR1,GTF2H5,NFXL1,ZBTB38,BRCA2,ATF7,DVL2,MORC1,YBX1,TAF1,TICAM1,THAP3,HSF1,MAX,SAP130,EZH1,NRF1,SRPK2,NCOA2,QRICH1,ZP3,CHD6,RNF2,ZNF554,MYEF2,TRIM37,MET,ZNF461,CAMK2D,TIMELESS,BRD9,MALT1,SETD3,TRA2B,KDM2B,MLXIPL,PHB,EDA,ZNF555,CTCF,NR6A1,TRAF3IP2,ATF6B,CREB1,RGMB,EBF4,ZNF511,BMP4,ABLIM3
GO:1990138	neuron projection extension	6.820684042697838e-13	*LLPH,CLASP2,SEMA3A,SEMA3D,SEMA5A,OLFM1,CDKL5,ITGB1,HSP90AA1,DSCAM,GOLGA4,LIMK1,RIMS1,CDH4,RTN4,RTN4R,NTN1,SYT1,CYFIP2,SEMA5B,SLIT3,SLIT2,DISC1,ITSN2,DCLK1,AUTS2,MAG,CTNNB1,PARK2,DRAXIN,CPNE6,SPG11,SYT3,NRP2,DIP2B,ISLR2,CPNE9,SEMA6D,BMPR2,DPYSL2,SPAG9,RAB11A,SEMA4D,VCL,WNT3,NLGN3,PAFAH1B1,RASAL1,TRPC5,RUFY3,SIN3A,IQGAP1,MACF1,ADNP,MAP1B,UNC13A,TRIM46,MAP3K13,PTPRS,RAPH1,TMEM108,SYT17,GSK3B,RIMS2,PRKCZ,MAP2,IMPACT,TNR,CDKL3,SLIT1,SEMA3C,NEDD4L,SH3GL2,EDNRA,CPNE1*
GO:0030036	actin cytoskeleton organization	9.595622508125728e-13	*CLRN1,PRKCI,S1PR2,SLC9A1,CLASP2,NOS1AP,EPB41,PHACTR1,FHOD3,TJP1,UTRN,PHACTR2,NTRK3,SEMA5A,FER,EZR,TENM1,CTNNA3,ABI1,ITGB1,TRIOBP,ELMO2,GAS7,AKAP13,CDC42EP3,XIRP2,EPHA1,IKBKB,LIMK1,TNIK,FMN2,PRKAR1A,PAK1,FMNL2,PHACTR4,ARHGAP6,ECT2,DMD,GSN,CASS4,NF1,PCDH15,BCR,TTN,MYH9,FRMPD4,DOCK2,MAD2L2,TLE6,NCKAP1,JAK2,MYPN,CDC42BPB,PLS1,CYFIP2,WNT11,MKLN1,PRKCD,PPM1E,TGFB1,PDLIM4,F2RL1,BCAS3,CELSR1,SLIT2,CORO1C,KANK1,PARVA,STRIP1,FGF10,KANK4,ENAH,RHPN2,SVIL,CAPN3,AUTS2,MPRIP,PARK2,MYO1D,ADD2,MYO1E,HCK,SORBS1,TBCK,OBSCN,NF2,SPTBN1,ELN,SFRP1,SSH1,SMAD3,ABI2,SHANK1,ARHGEF18,PREX1,PDGFRA,ADD3,MYO1F,SPTBN4,VILL,RND3,VPS4A,ABLIM2,PTPN1,CTNNA2,PPM1F,NEDD9,PAWR,RHOJ,PAFAH1B1,HIP1,RUFY3,IQGAP1,ARHGAP12,MYO7A,CLASP1,GPM6B,PRKG1,PACSIN1,SPTBN5,PAK3,SSH2,CDC42BPA,AIF1L,EPHA5,MEF2A,PLS3,CALD1,FGD1,SNX9,DIAPH2,ACTR2,ROCK1,EPS8,CAPN10,INSRR,PPP1R9A,ARHGAP44,MCU,CTNNA1,LIMA1,SHROOM3,FCHSD2,RAPGEF3,PDXP,SH3BP1,PTK7,CSRP1,RASA1,KCTD13,DPYSL3,ARHGEF17,ARHGAP25,EFNA5,SHANK3,AMOTL1,PARVB,RHOA,TF,RHOBTB1,SHROOM1,CUL3,SH3KBP1,RDX,FAM49B,KRT8,ESPNL,IQGAP2,FAM171A1,TAOK2,SHC1,ALDOA,PGM5,DNAJB6,FGD3,PACSIN2,THSD7A,FGD4,ACTN4,THSD7B,TMEFF2,DLC1,EPB41L4B,SPECC1L,PHLDB2,KLHL1,SHROOM4,EPB41L2,NEBL,BRAF,DIAPH1,FRMD5,CIT,NEB,MYO1A,CGNL1,EPB41L1,NUAK2,MET,FMNL1,MICAL3,SETD3,SPTAN1,EHD2,TNXB,ABLIM3*
GO:0006811	ion transport	1.0001287497797018e-12	*SLC39A11,ENPP1,RTBDN,NRXN1,ASPH,SLCO3A1,PDE4D,SLC9A1,FAM155A,GRID2,CHERP,SLC25A17,CACHD1,RYR1,NOS1AP,NOX5,EPB41,DPP6,CLCN1,SLC35F3,UTRN,ATP2B2,KCNQ5,GRM5,CASK,KCNIP4,LRP2,SLC38A11,ANK2,SLC9A9,CHD7,SLC26A6,HTR2B,RYR3,SLC24A2,NKAIN2,ITGB1,SLC24A3,CNIH2,NKAIN3,SLC22A8,CNGB1,FLVCR2,ABCC8,GABRA3,TCIRG1,KCNS3,NIPAL2,NETO2,CACNB2,ADORA1,CNNM1,KCNJ16,GRM7,DPP10,NTRK2,PDGFB,CACNA1B,STK39,ATP8B1,PLLP,CAB39,ZDHHC13,SLC20A2,SLC4A10,CATSPER2,GLTP,AMIGO1,KCNMA1,CHRM3,GRIK4,KCNRG,LCK,TMC2,SLC12A8,VAMP7,SLC6A16,RHCE,MICU3,DMD,SLC30A9,SLC8A1,JPH2,FYN,NF1,SLC16A6,LMTK2,SLC25A21,SLC5A6,SLC2A14,SHISA9,ANKH,CACNA1E,SLC4A5,KCNN3,CNNM2,SLC43A2,BDKRB1,BDKRB2,KCNIP1,SLC35A2,SLCO1A2,STIM1,NALCN,TMBIM6,SLC39A14,CATSPER3,FGF14,CLDN16,ANO6,KCNB2,SYT1,NPSR1,ABCC2,SCN4A,TMC1,SLC1A4,SLC5A9,SLC9A7,KCNC4,CLCA2,CACNA1H,CAMK2B,CD4,TGFB1,SGK1,KEL,JPH3,CLDN10,GRIK3,GABRR3,GABRB1,SYT13,CDH23,KCNJ3,CACNA1D,ABCC11,CACNA2D3,ANO4,XPR1,CHRM1,ANO3,KCNH1,HOMER2,SLC17A7,KCNH7,CAPN3,SLC16A1,VMP1,CNR1,ANO2,TNFSF11,PPP3CA,CTNNB1,PARK2,COX8A,TMEM63C,TRPM1,CYBB,KCNJ12,RYR2,KCNA6,NDUFA9,NOL3,PER2,CACNA1C,SCN3B,GRM1,ATP10D,ITPR1,WWP2,GRIK5,PSAP,SLC16A2,SHANK1,SYT3,NEDD4,SLC4A4,SLC6A1,KCNK1,APP,NOX1,SFXN5,NLGN2,VDAC1,IBTK,SLC9B2,SPTBN4,GRIA3,DRD1,GLRA2,PRKD1,SLC6A3,FXYD2,FXYD6,FXYD6-FXYD2,SLC16A12,KCNG4,RASGRF2,NLGN1,FGF2,GRIK1,PTAFR,ADCYAP1R1,KLHL3,MRS2,SLC5A8,SLC9B1,GRIA2,OC90,SLC35D1,ATP6V0B,CLIC4,KCNQ1,TG,NLGN3,TRPA1,TRPM3,NMUR2,AKAP6,TRPC5,WNK1,RELN,ABCB1,KCNQ2,SLC22A*

			3,SLC47A1,SLC8A2,SLC9C1,TPCN1,GPM6B,CLIC5,AHNAK,RGS7,ATP2B4,NSF,SLCO4A1,P2RX6,ATP6V0A2,KCNAB2,TFRC,GABRG3,SYT12,CACNA1A,STOM,TRPV1,SLC38A6,PLCG2,GPR35,ABCC1,SLC39A10,ANK3,ATP13A3,KCND3,CACNG8,SLC9C2,NETO1,CD84,CHRNB4,MCU,TRDN,GABRA6,LRRC8C,LRRC8D,STAC,TRPC6,ABCB7,SLC41A2,SLC48A1,ATP13A5,SNCA,CACNG2,SLC39A9,SYT9,CNGA3,SLC22A10,CLCC1,OPRD1,PKP2,BTK,SLC4A8,HTT,SLC30A7,SCN8A,LHFPL5,ATP6V1A,CNIH3,SLC44A1,TMCO1,PKD2,SHANK3,BEST3,STEAP4,SNAP23,ANO1,ANO8,SERINC5,GABRR2,ABCG2,KCNC2,SLC6A14,PSEN2,SYT17,GRIN1,RHOA,GRIN3A,TF,ITPR2,EPM2A,GABRB3,EMB,SCN9A,AGT,SYT7,ZMPSTE24,HEPHL1,PEX5L,PDE4B,COX5A,TRPC4AP,SLC25A42,SLC29A1,UGT1A3,SLC44A3,KCNJ15,SLC5A10,STC2,SLC5A3,CLDN4,GRID1,PKD1L1,SLC25A18,COX5B,NOS1,ACTN4,SCNN1B,COX6A1,EPHB2,SLC1A1,XCR1,LRRC52,NKAIN1,ABCD3,CALCRL,MCOLN1,STEAP3,ATP1A3,ADORA2A,GRIN2B,SLC25A26,SLCO2B1,GRIA4,CNTN1,DIAPH1,HTR2C,ATP8A1,TMEM163,UQCR10,SLC26A2,ANXA2,KCND2,ATP6V0A1,SYK,GRIK2,SCN1A,SLC44A5,CACNG3,SLC10A1,ATP2B3,GPM6A,SLC13A3,SLC5A4,KCNQ3,SESTD1,CAMK2D,KCNJ6,NEDD4L,PTPRC,GPR89A,SHISA6,EDNRA,SLC25A13
GO:0043549	regulation of kinase activity	1.0431542402185251e-12	*LDB2,NRXN1,ADCY8,NRG3,LATS2,PRKAG2,PRLR,MVP,NTRK3,CBL,FLT3,GRM5,PLCE1,MAP2K5,KITLG,ROBO1,TOM1L1,HTR2B,TENM1,TRAF6,DNAJA3,VAV2,ABI1,HSP90AA1,CDC6,AKAP13,DAB1,ADORA1,EPHA1,ERBB4,MRE11A,PTPRO,NTRK2,PDGFB,CCND3,INSR,PRKAR1A,PIP4K2A,CAB39,PAK1,MAP3K4,ECT2,SNX6,ERN2,SLC8A1,CASS4,NF1,LMTK2,CHI3L1,TTN,PAQR3,PIK3R2,NRG1,MAP2K1,ROR2,PTPRT,IPO5,CCNYL1,GNAQ,HERC5,JAK2,FBXW7,ZFYVE28,LAMTOR3,WNT11,NOX4,PRKCD,TAB2,CD4,PPM1E,TGFB1,PIBF1,CORO1C,ZNF675,ANKRD54,TNFRSF10B,DUSP22,DOCK3,SMYD3,EPHA4,TNFSF11,PARK2,IGF1R,PPARG,AXIN1,PRKAR1B,FGF1,PRKAR2A,MYOCD,AJUBA,SPDYA,NF2,FLT4,SFRP1,TAB1,APP,PDGFRA,CCNI2,HEG1,BORA,IBTK,LRP5,MTCP1,S100A12,PRKD1,EREG,CCNY,PTPN1,BMPR2,BMPR1A,PIK3R3,CDK12,FGF2,PPM1F,NEDD9,DVL3,EPHB3,CKS1B,DEPTOR,TSG101,DAB2,BLM,CACUL1,DLG3,WNK1,RELN,NEK10,IQGAP1,MAP3K7,ALK,SLC8A2,ATP2B4,ADNP,UVRAG,EPHA5,GHR,MNAT1,SNX9,HIPK3,PTPN2,INSRR,NTRK1,EPHB1,EPHA10,AXIN2,PIFO,EPHA7,ATG14,STK38,MAP3K13,SNCA,BMPR1B,PRKAG1,RPTOR,MLLT1,IGF1,HTT,PTEN,BMP7,LRRK2,UNC119,LILRB4,PKD2,EFNA5,PDGFC,PKIB,PPP2R3C,RAPGEF2,PRKAR2B,RHOA,ROR1,EPM2A,PTPRB,AGT,CCNJL,ADAR,PTK2,MAP3K5,NCF1,TAOK2,SHC1,CD44,ADAM8,GCKR,RGS14,STK4,EPHB2,SLC1A1,IL18,MOB3B,PRKCZ,ADORA2A,CCNG2,ANGPT1,OSBPL8,CDK5RAP1,CD300A,PPP2CA,ANGPT4,MADD,SYK,WWTR1,DVL2,PTPRJ,SASH1,VAV3,MOB3A,AXL,MET,MALT1,PHB,PTPRC,TYRO3,TNXB,BMP4*
GO:0051056	regulation of small GTPase mediated signal transduction	1.214675892252613e-12	*MAP4K4,TIAM2,MAPRE2,KALRN,CBL,PLEKHG4B,PLCE1,KITLG,ROBO1,ITPKB,VAV2,ITGB1,SRGAP3,TRIO,ARHGAP24,AKAP13,DGKI,CYTH3,GBF1,ABR,CDON,ARHGAP6,ECT2,ARHGAP42,RIT2,NF1,RTN4,RTN4R,BCR,NRG1,ARHGAP10,DOCK8,RALGPS2,PPP2CB,DOCK2,ARHGAP32,RALGPS1,DENND1A,ARHGAP23,SIPA1L3,F2RL1,SLIT2,KANK1,DOCK3,FGF10,RASA4,RASA4B,ARHGEF28,KCTD10,AUTS2,ARHGAP22,GARNL3,P2RY10,ARHGAP31,OBSCN,ARHGAP29,ARHGEF18,PREX1,HEG1,RAF1,RASGRF2,DENND4A,ADCYAP1R1,ARHGAP39,SHOC2,MYO9A,FAM13A,RASAL1,RELN,ARHGAP12,P2RY8,FGD1,EPS8,GPR35,NTRK1,DNMBP,ARHGAP44,ARHGEF3,SH3BP1,RASA1,KCTD13,IGF1,ARHGEF17,RASA2,ARHGAP25,FAM13B,SOS1,SYNGAP1,CUL3,RDX,FAM49B,ARHGAP15,FGD3,FGD4,SIPA1L2,ARHGAP11A,EPHB2,DLC1,RABGEF1,DENND4B,ARHGAP21,ITSN1,SRGAP2,MADD,ARHGAP19,RAP1GAP2,RALGAPA1,CGNL1,VAV3,DENND4C,MET*
GO:0051649	establishment of localization in cell	1.373770641003123e-12	*POLDIP3,ZDHHC14,HOOK2,NRXN1,ASPH,PRKCI,PDE4D,SLC9A1,FAM155A,ADCY8,CLASP2,CHERP,WWC1,KIF22,CTDSPL2,RYR1,NOS1AP,TUBA1C,NOX5,ANP32A,NLGN4X,SEC23B,LTV1,SNX31,CBL,FAM53A,FER,CASK,CCDC93,PIK3CD,TNPO3,ANK2,EZR,NRXN3,TOM1L1,CHD7,HTR2B,TENM1,RYR3,KIF5C,LMNA,SRP72,SLC24A2,ITGB1,HSP90AA1,CNIH2,PCDH17,CCDC22,KLHL12,AKAP13,DLG2,IFT43,MYO10,ERC1,HMGN3,AFTPH,DGKI,STXBP4,ERC2,EFCAB7,TCIRG1,PTPN11,CACNB2,HDAC6,ADORA1,GPRASP1,PEX14,GBF1,TLK1,STX18,HOOK3,VPS45,RIMS1,SNX2,FMN2,TCF7L2,PIP4K2A,FBXW11,LCK,ECT2,SNX6,CHML,VAMP7,RFTN1,SHFM1,AP3S1,NPLOC4,DENND5A,DMD,CENPF,RANBP17,SLC8A1,RBM4,TANC2,JPH2,FYN,NF1,SMG7,DST,LMTK2,STX6,SNCB,GOLGA2P5,APOD,VTA1,BCR,NDRG4,PIK3R2,NTN1,DCTN1,NRG1,BDKRB1,BID,MAP2K1,MYH9,MAD1L1,TMBIM6,TMEM30A,PARD3B,SCFD2,ZDHHC3,SLC39A14,LIN7A,SCFD1,SYN2,CREBRF,COPS5,EXOC4,IPO5,MAN1A1,TLE6,RAB27A,TERF2,TMED6,ARL3,NEUROD1,IPO11,ICK,RANBP1,DENND1A,ZDHHC15,JAK2,FBXW7,OAZ2,SYN3,SYT1,NPSR1,BBS12,SNAP25-AS1,SMG1,DYNC1I1,ATP9A,DENND2A,KPNA3,CTAGE6,CENPC,IMMP2L,PRKCD,TGFB1,NUP93,PIBF1,JPH3,PTPRN2,F2RL1,BCAS3,ZFYVE9,CADPS2,CORO1C,C12ORF4,KCNJ3,CACNA1D,ZFAND2A,GRPEL2,ANK1,CLMN,CHRM1,RAB11FIP4,ITSN2,SYNJ2,BCL3,DCLK1,ANKRD54,RABGAP1L,ANKFN1,SLC17A7,FGF10,STXBP5,CAPN3,SNX8,CNR1,SMG6,PPP3CA,NSUN2,TRAK1,CTNNB1,PARK2,RN7SL832P,FCGR2B,TSNARE1,AXIN1,PRKAR1B,MYO1D,TRPM1,SYTL5,LRPPRC,SREBF2,RYR2,LEPROT,PROS1,NR4A3,NOL3,RIOK2,PER2,CACNA1C,MYO1E,HCK,TBC1D14,RAB3GAP2,TBCK,RIMS4,ANKFY1,AP3B2,SPG11,CCDC91,IFT122,RANBP3,DNM3,SMAD3,ITPR1,RAB6A,WIPI1,VPS39,GRIK5,PSAP,NEDD4,KPNB1,VPS53,SLC6A1,RAB6C,SEC61B,THOC3,APP,BET1L,RAB11FIP5,MYO1F,NLGN2,EYA2,SSR2,IBTK,LRP5,*

			SEC22C,SLC9B2,SETD2,ZDHHC6,PRICKLE1,DRD1,TMEM14A,TPH1,SYNE2,PRKD1,OSBPL2,SYBU,SLC6A3,VPS4A,PITRM1,ATF2,RAF1,PTPN1,AP1B1,NLGN1,SNX16,SPAG9,RAB11A,EPG5,IPO9,FGF2,RAB24,ADCY5,AGBL4,MCTP1,XPO6,PTAFR,ADCYAP1R1,EEA1,ABCA13,RILPL1,SORCS2,RAB11FIP3,PRKCG,BLOC1S5,AP2M1,TNPO1,TRAPPC11,TECPR2,FBXL20,TSG101,DESI1,KCNQ1,SUFU,NLGN3,PAFAH1B1,KIF3A,TRPA1,AKAP6,BTBD9,RUFY3,STON2,DAB2,PKHD1,TRAM2,DZIP1,SMG5,SLC22A3,SLC25A16,EML4,FAM91A1,MYO7A,SLC8A2,CLASP1,TPCN1,GPM6B,SNX5,ATP2B4,NSF,SYNE3,PACSIN1,SPTBN5,ATAD1,NXT2,MAP1B,EPHA5,SYT12,STOM,TBC1D5,TRPV1,CLEC16A,SNX9,RPGR,ARL5A,ACTR2,PLCG2,ROCK1,EGR2,CAPN10,AP3B1,KPNA6,ZFAND1,MAP1A,ANK3,UNC13A,SNUPN,PARD3,PTPN14,TRIM46,TUB,KCND3,CACNG8,AP3D1,KIF3C,AP2B1,ANXA8L1,ARHGAP44,HEATR5A,SNX33,CD84,SPI1,CHRNB4,VPS16,ACAP2,TRDN,ATG14,RAPGEF3,RSRC1,SNCA,CACNG2,GRM4,TBC1D9,SYT9,CHMP3,RNF103-CHMP3,PRKAG1,RAB5B,ABCA12,PLD1,BTK,TBC1D16,XPO4,VPS41,SLC4A8,PLA2G4E,STX8,HTT,MAPK1,PTEN,PIK3C3,NUP88,LRRK2,SPAG5,CHMP5,IFT81,TMEM108,ITGAM,NSG2,PKD2,EFNA5,MIA2,SGSM1,STEAP4,TMCC1,WNT7A,ZBED6,ZC3H11A,SNAP23,MAP4,POM121C,RHOT1,ANO1,SAMM50,SMO,SUN1,LSG1,BLOC1S3,STXBP5L,CPT1A,DNAJC6,UBR5,GRIN1,GRIN3A,NDE1,CUL3,HSPD1,ITPR2,EPM2A,GSK3B,PLD2,RDX,SYN1,IWS1,UPF2,MCTP2,BOK,AGT,SYT7,ADAR,BLOC1S6,ZMPSTE24,LAT2,PEX5L,SNX3,EXOC6B,RIMS2,TOMM5,COPG2,SEC16B,TIMM44,GTF2IRD2,NCF1,SLC29A1,TAOK2,CD160,ZDHHC23,CPLX2,ARFGAP3,MON2,NOS1,PEX7,GCKR,TBC1D23,RGS14,ACTN4,STK4,ZFAND6,SLC1A1,WLS,XCR1,GPSM2,PPP1R10,ABCD3,GRTP1,MCOLN1,KPNA4,SYTL4,PAX6,PRKCZ,ADORA2A,GRIN2B,PPP1CC,RABGEF1,KLC3,ANGPT1,CADPS,HACL1,TBC1D10C,ARHGAP21,MAP2,STPG1,ATG3,BRAF,DIAPH1,HTR2C,SIL1,CLTB,CD300A,RPL23,ATXN1,CAMSAP3,SP100,VTI1A,ANXA2,RAB27B,ITSN1,NUP214,ADCY1,NUMB,SYK,SLIT1,MYRIP,RAB2A,SEC31B,CACNG3,GRIP1,ERGIC1,MYO1A,CNIH4,PLEKHM1,HGSNAT,MLPH,AKTIP,EPS15,INPP5F,REPS2,TBC1D10A,CAMK2D,SNX1,MALT1,ZDHHC11,ZDHHC11B,EHD2,THEM4,TRAPPC8,NDC1,PTPRC,RPH3A,RAB28,SYNDIG1,VPS52,TRAF3IP2,SGTB,CLNK,SLC25A13,BMP4,ABLIM3
GO:0051338	regulation of transferase activity	1.4023391870652485e-12	LDB2,NRXN1,ADCY8,NRG3,LATS2,PRKAG2,PRLR,MVP,NTRK3,CBL,FLT3,GRM5,PLCE1,MAP2K5,KITLG,ROBO1,TOM1L1,HTR2B,TENM1,TRAF6,DNAJA3,VAV2,ABI1,HSP90AA1,CDC6,AKAP13,DAB1,CCT2,ADORA1,EPHA1,ERBB4,MRE11A,LIMK1,PTPRO,NTRK2,PDGFB,CCND3,INSR,PRKAR1A,PIP4K2A,CAB39,PAK1,MAP3K4,ECT2,SNX6,ERN2,SLC8A1,CASS4,NF1,LMTK2,CHI3L1,TTN,PAQR3,PIK3R2,NRG1,MAP2K1,ROR2,PTPRT,IPO5,MAD2L2,CCNYL1,GNAQ,HERC5,JAK2,FBXW7,ZFYVE28,LAMTOR3,WNT11,NOX4,PRKCD,TAB2,CD4,PPM1E,TGFB1,PIBF1,BTRC,CORO1C,ZNF675,ANKRD54,TNFRSF10B,DUSP22,DOCK3,SMYD3,EPHA4,TNFSF11,SMG6,CTNNB1,PARK2,IGF1R,PPARG,AXIN1,PRKAR1B,FGF1,PRKAR2A,MYOCD,AJUBA,SPDYA,NF2,FLT4,SFRP1,TAB1,APP,PDGFRA,CCNI2,HEG1,TEN1,BORA,IBTK,NVL,LRP5,MTCP1,S100A12,DCUN1D3,PRKD1,EREG,CCNY,PTPN1,BMPR2,BMPR1A,PIK3R3,CDK12,FGF2,PPM1F,NEDD9,DVL3,EPHB3,CKS1B,DEPTOR,TSG101,TNKS,DAB2,BLM,CACUL1,DLG3,WNK1,RELN,NEK10,IQGAP1,MAP3K7,ALK,SLC8A2,ATP2B4,ADNP,UVRAG,EPHA5,DCUN1D5,GHR,MNAT1,SNX9,HIPK3,PTPN2,INSRR,NTRK1,EPHB1,EPHA10,AXIN2,PIFO,EPHA7,ATG14,STK38,MAP3K13,SNCA,BMPR1B,PRIM2,PRKAG1,RPTOR,MLLT1,IGF1,HTT,MAPK1,PTEN,BMP7,LRRK2,UNC119,RFC3,LILRB4,PKD2,EFNA5,PDGFC,PKIB,PPP2R3C,SERINC5,RAPGEF2,PRKAR2B,ARRDC4,RHOA,ROR1,EPM2A,GSK3B,PTPRB,AGT,CCNJL,ADAR,PTK2,MAP3K5,NCF1,TAOK2,UGT1A1,UGT1A10,UGT1A4,SHC1,CD44,ADAM8,GCKR,RGS14,STK4,EPHB2,SLC1A1,IL18,MOB3B,PRKCZ,ADORA2A,CCNG2,ANGPT1,OSBPL8,CDK5RAP1,PRKCQ,CD300A,PPP2CA,SKP1,ANGPT4,RPL23,RFC5,PARN,MADD,SYK,WWTR1,DVL2,PTPRJ,SASH1,VAV3,MOB3A,AXL,MET,MALT1,PHB,PTPRC,TYRO3,TNXB,BMP4
GO:0007264	small GTPase mediated signal transduction	1.561296285805527e-12	MAP4K4,DNMT1,DOCK1,TIAM2,KSR2,MAPRE2,KALRN,CBL,PLEKHG4B,RAB4B,RAB4B-EGLN2,PLCE1,KITLG,ROBO1,NUCB1,ITPKB,DNAJA3,VAV2,ITGB1,SRGAP3,KNDC1,TRIO,ARHGAP24,AKAP13,DAB1,CDC42EP3,DGKI,DOCK10,CYTH3,GBF1,LIMK1,ABR,CDON,PHACTR4,RASGEF1B,ARHGAP6,ECT2,CHML,ARHGAP42,RIT2,NF1,RTN4,RTN4R,BCR,NTN1,NRG1,ARHGAP10,SH2D3C,DOCK8,RAPGEF6,RALGPS2,PPP2CB,DOCK2,CDH13,ARHGAP32,NCKAP1,RALGPS1,ARL3,DENND1A,DOCK9,ARHGAP23,SIPA1L3,F2RL1,CELSR1,SLIT2,RRAS2,KANK1,DOCK3,FGF10,RASA4,RASA4B,ARHGEF28,KCTD10,AUTS2,ARHGAP22,GARNL3,P2RY10,ARHGAP31,OBSCN,RGL1,ARHGAP29,ABI2,ARHGEF18,PREX1,DOCK4,KPNB1,RAB6C,HEG1,PRKD1,RND3,RAF1,RASGRF2,DENND4A,FGF2,ADCYAP1R1,ARHGAP39,SHOC2,MYO9A,RHOJ,FAM13A,RASAL1,RELN,DOCK11,ARHGAP12,P2RY8,FGD1,ROCK1,EPS8,GPR35,NTRK1,DNMBP,ARHGAP44,ARHGEF3,BCAR3,RAB15,RAPGEF3,SH3BP1,USP50,PLD1,RASA1,KCTD13,IGF1,ARHGEF17,RASA2,RAB30,ARHGAP25,FAM13B,RHOT1,SOS1,RAPGEF2,RHOA,SYNGAP1,RHOBTB1,CUL3,PLD2,RDX,FAM49B,ARHGAP15,FGD3,FGD4,SIPA1L2,ARHGAP11A,EPHB2,GPSM2,DLC1,RABGEF1,DENND4B,ARHGAP21,ITSN1,SRGAP2,MADD,ARHGAP19,RAP1GAP2,RALGAPA1,CGNL1,VAV3,GNA12,SH2D3A,DENND4C,MET
GO:19	cell surface	1.84511473	GPC5,NRXN1,S1PR2,GRID2,LATS2,NLGN4X,SEMA5A,LRRFIP2,GRK5,PSMB7,MCC,KLHL12,DGKI,DDB1,ADORA1,NPHP3,PTPRO,CELA1,RNF220,RIMS1,TNIK,TCF

05114	receptor signaling pathway involved in cell-cell signaling	13622103e-12	*7L2,USP34,FBXW11,CDK14,PTPRU,DEPDC1B,ARNTL,MDFI,VAX2,VGLL4,ROR2,SOX13,MAD2L2,TLE6,CAPRIN2,CCNYL1,GNAQ,MLLT3,TCF7,KLF15,WNT11,IFT80,KREMEN1,BTRC,CELSR1,CTNND2,DISC1,KANK1,FGF10,SMURF2,PPP3CA,CTNNB1,PARK2,AXIN1,DRAXIN,BICC1,TRABD2B,SFRP1,FOXO3,SMAD3,CUX2,GRIK5,SHANK1,VANGL1,NFATC1,CDC73,APP,YAP1,AMFR,NLGN2,LRP5,SOX2,GPC6,PRICKLE1,GLRA2,KDM6A,CCNY,TMEM237,CELF4,NLGN1,FGF2,DKK2,DVL3,RBX1,WNT3,NLGN3,PRDM15,GLI2,TNKS,DAB2,WNK1,RELN,RUVBL2,RSPO2,ZNF423,MACF1,SLC8A2,P2RX6,MARK1,WWOX,GPC3,PSMB2,TRPV1,HDAC1,INVS,PLCG2,AXIN2,NETO1,CHRNB4,FBXW4,PTK7,SNCA,MAGI2,ZEB2,WNT7B,MITF,ZNRF3,PTEN,LRRK2,ZRANB1,TMEM108,ITGAM,PKD2,SHANK3,AMOTL1,WNT7A,EXT1,PRICKLE2,SMO,SOX30,UBR5,GRIN1,JADE1,RHOA,ROR1,CUL3,EPM2A,GSK3B,SULF1,RNF43,PRKAA2,NDRG2,SNX3,RIMS2,CPE,APCDD1L,SLC29A1,PYGO2,LBX2,SCEL,CSNK1A1,STK4,SPEF1,WLS,PRKCZ,ADORA2A,GRIN2B,TBL1X,LRRK1,PPP1CA,RBMS3,PPP2CA,WWTR1,GRIK2,DVL2,WIF1,ANKRD6,EDA,RNF213,SHISA6,EDNRA,BMP4*
GO:0032268	regulation of cellular protein metabolic process	2.4732301266042e-12	*POLDIP3,ENPP1,NRXN1,ASPH,PRMT3,SLCO3A1,PDE4D,DNMT1,S1PR2,C6ORF89,ADCY8,PDE8A,IL31RA,PRDM12,NRG3,NOS1AP,LATS2,PRKAG2,PRLR,MVP,FTO,NTRK3,CBL,ARNT,FLT3,ENPP2,GRM5,PLCE1,FER,MAP2K5,KITLG,LRP2,EIF4G3,EZR,ROBO1,TOM1L1,HTR2B,TENM1,LMNA,TRAF6,ITPKB,DNAJA3,OXR1,ABI1,HSP90AA1,CDC6,KNDC1,CCDC22,AKAP13,DAB1,PTPN11,MGAT5,HDAC6,ADORA1,EPHA1,IKBKB,ERBB4,MRE11A,LIMK1,PMEPA1,PTPRO,CDON,NTRK2,TNRC6A,EP300,ZYG11B,FNTA,PDGFB,TNIK,CCND3,BRMS1,CHFR,INSR,PRKAR1A,CAB39,PAK1,FBXW11,MAP3K4,RNF144A,EGLN3,SAMD4A,BDNF,LCK,ECT2,SNX6,CST2,PTCD3,ERN2,DMD,ATG10,LARP4B,USP13,KAT7,SLC8A1,GSN,RBM4,RIT2,CASS4,FYN,MKRN2,ARNTL,NF1,DPEP1,RWDD3,CHI3L1,TTN,PAQR3,NRG1,SH2D3C,BDKRB1,BDKRB2,BID,FRY,MAP2K1,FNIP1,MYH9,VGLL4,JDP2,ITIH2,ROR2,FHIT,WDR70,PTPRT,IPO5,MAD2L2,CAPRIN2,CCNYL1,GNAQ,RFFL,CCBE1,HERC5,JAK2,FBXW7,ITCH,ZFYVE28,KLF15,CYFIP2,WNT11,MTA1,NOX4,QKI,PRKCD,TAB2,ACVR2A,CD4,PPM1E,TGFB1,SPSB4,NSD1,PIBF1,BTRC,CRADD,DNAJB2,SLIT2,ITIH4,CORO1C,DISC1,BLID,ZFAND2A,CLN6,KDM4B,SMAD6,ZNF675,NELL1,BMPER,TIMP2,BCL3,ANKRD54,TNFRSF10B,DUSP22,NAIP,DOCK3,YTHDF1,FGF10,SMYD3,FANCI,CAPN3,EPHA4,AUTS2,CD6,TNFSF11,UBQLN3,PIP5KL1,UFL1,CTNNB1,PARK2,SMARCC1,PPARG,NGRN,AXIN1,PRKAR1B,OTUB1,LRPPRC,LEPR,FGF1,PROS1,NOL3,PRKAR2A,MYOCD,PER2,AJUBA,GLG1,CHEK2,UBQLN4,SPDYA,CSTL1,RAB3GAP2,DIO2,CSPG4,NF2,FLT4,HDAC4,PAX2,SECISBP2L,TRABD2B,SFRP1,PPP6R2,FOXO3,BCL2L13,SSH1,SYNCRIP,SMAD3,RNFT2,SIMC1,MTBP,DCP1B,TAB1,APP,RBM8A,CCNI2,DIP2B,ARIH1,HEG1,SH3D19,BORA,IBTK,LRP5,MTCP1,PRICKLE1,RCAN1,SPTBN4,DRD1,S100A12,DCUN1D3,PRKD1,ST18,EREG,CCNY,ATF2,GRAMD4,RAF1,CELF4,CARD16,CASP1,PTPN1,BMPR2,BMPR1A,PIK3R3,CDK12,SPAG9,MYB,FGF2,PPM1F,NEDD9,AGBL4,SEMA4D,PTAFR,JARID2,PRKCG,SPOCK1,DVL3,EIF2B5,EIF4G1,RBX1,CKS1B,AGO3,DEPTOR,TSG101,DESI1,TERF2IP,SUFU,PTPN13,COL4A3,HIP1,PADI6,TRPC5,UBA2,TNRC6B,DAB2,BLM,CACUL1,LDLRAD4,SETD5,DLG3,WNK1,RELN,NEK10,SIN3A,RUVBL2,PELI1,IQGAP1,MAP3K7,SLC8A2,SLC35A4,XDH,ATP2B4,TET1,CAMTA1,DYX1C1,ADNP,CELF1,RNF34,TFRC,UVRAG,DCUN1D5,GPC3,XRN1,DIS3L2,GHR,MNAT1,SNX9,ELAVL4,PLCG2,ROCK1,PAXIP1,HIPK3,RYBP,PTPN2,NTRK1,TRIM44,MAP1A,SLC39A10,AXIN2,PARD3,GNL3L,MUC1,BCOR,ANXA8L1,SNX33,LPA,SPI1,DNMT3B,EPHA7,ATG14,EIF3H,BCAR3,STK38,RBPMS,RENBP,MAP3K13,RAPGEF3,SNCA,BMPR1B,MAGI2,PNPT1,USP50,RC3H1,EIF3E,RNF19B,PRKAG1,PSMF1,CAST,TRIP12,ADTRP,PLD1,OPRD1,RPTOR,MLLT1,IGF1,HTT,MAPK1,PTEN,SPRED2,BMP7,ATXN2,PUM1,LRRK2,TMEM59,UNC119,BAG6,LILRB4,PKD2,EFNA5,TADA2A,DAZL,PDGFC,SERPINA3,SERPINA4,SERPINA5,PKIB,PPP2R3C,WNT7A,NLRP1,UACA,DNAJC1,PLCL2,MTIF2,BMP6,MYCBP2,GPI,SH3RF2,N4BP1,ATRX,DPH6,RAPGEF2,VRK3,PRKAR2B,UBR5,GRIN1,ARRDC4,RHOA,SPRTN,RQCD1,CUL3,HSPD1,EPM2A,GSK3B,PRR16,DNAJC3,RDX,EEF2K,IWS1,PPP1R16A,PTPRB,CD27,BOK,RNF144B,AGT,METTL16,CCNJL,PRKAA2,ADAR,STAT2,ZMPSTE24,PTK2,TEC,EXOSC3,MAP3K5,NCF1,TAOK2,PYGO2,SHC1,PARP10,RNFT1,RPS6KB1,PRG3,SERPINB11,DNAJB6,CD44,LARP4,ADAM8,CLDN4,RPS6KA5,CSNK1A1,HUS1,NOS1,RGS14,STK4,EPHB2,SLC1A1,IL18,MFSD8,UBE2K,DIS3,DLC1,CPEB4,MOB3B,PAX6,PRKCZ,ADORA2A,GRIN2B,RABGEF1,CCNG2,CPEB1,NIPBL,COL28A1,ANGPT1,LRRK1,OSBPL8,IGF2BP3,PPP1CA,MTF2,CDK5RAP1,CNTN1,BRAF,HDAC2,IMPACT,SPINT2,CD300A,PHIP,PPP2CA,SKP1,ANGPT4,RPL23,ANXA2,PARN,MADD,SYK,CNOT1,WWTR1,DVL2,MTRF1,YBX1,PTPRJ,TAF1,EIF4E3,TICAM1,TANK,HSF1,DHFR,ETF1,SASH1,EEFSEC,MOB3A,GNA12,SH2D3A,AKTIP,INPP5F,CD109,TBC1D10A,CAMK2D,UCHL5,MALT1,SERPINE3,MLXIPL,PHB,PTPRC,CTCF,SH3GL2,TNXB,TRAP1,BMP4*
GO:0010556	regulation of macromolecule biosynthetic process	3.0577207354622652e-12	*POLDIP3,ENPP1,PRDX2,LDB2,ASPH,PRKCI,SLCO3A1,DNMT1,HLX,SLC9A1,PBX3,ADCY8,MED13L,TRPS1,CBFB,PDE8A,ZNF823,IL31RA,PRDM12,MED26,WWC1,ASH1L,SCAF8,STOX2,PTGIS,WWC3,PAGR1,HIVEP3,FTO,NPAS3,ARNT,LRRFIP2,STAT5B,TOX,GRM5,ZNF566,FER,CASK,MAP2K5,MAPK10,ZNF536,SP3,HDGF,EIF4G3,EZR,IKZF2,CHD7,MECOM,TACC1,TENM1,TRAF6,DNAJA3,NHLH1,HSP90AA1,CDC6,PSMB7,TSC22D3,ZC4H2,CCDC22,CDAN1,GFI1B,PTPRK,RUNX1,ERC1,HMGN3,NRIP1,THRB,EFCAB7,ITGB3BP,DACH1,ZNF569,CCT2,ORC2,HDAC6,SERTAD2,MYT1,IKBKB,PEX14,ERBB4,ZNF609,BRD8,KAT6B,HIF3A,SNIP1,ESR1,MIER1,PC*

			BP3,MAML3,CDON,TNRC6A,EP300,CELA1,TENM2,ZNF76,RNF220,ZNF471,PDGFB,CCND3,TOB2,ZNF19,ZNF23,BRMS1,ZNF605,SCML4,HNF4G,INSR,RERE,PRKAR1A,FUBP1,ATP8B1,H2AFY2,TCF7L2,JMJD1C,ZNF443,ZNF490,ZNF564,ZNF709,ZNF799,LITAF,FBXW11,ESRRB,MAP3K4,BASP1,TBR1,SAMD4A,TFAP2A,CDK14,FANCA,PEG3,ZIM2,TGIF2,TGIF2-C20ORF24,MEIS1,VRTN,TRIM13,MDM4,ZNF148,MTA3,SNX6,TFDP2,PTCD3,ERN2,C1D,AFF3,DMD,CENPF,SLC30A9,TOX3,PDS5A,LARP4B,USP13,KAT7,ZNF667,RBM14,RBM4,MED12L,SATB2,RIT2,HIRA,MKRN2,ARNTL,PLCB1,ARID4B,RWDD3,BRPF1,PIK3R2,NRG1,UBP1,MAP2K1,MDFI,FNIP1,VAX2,TMBIM6,VGLL4,PPP2CB,PPP3R1,HDAC5,CSRNP3,ZNF692,NFIA,RNF4,JDP2,CMKLR1,ROR2,DCN,PCBD2,CDH13,CREBRF,SOX13,COPS5,MAD2L2,TLE6,CAPRIN2,ZNF418,PHF20L1,TERF2,ZNF286A,FOXN3,NEUROD1,USP22,JAK2,TRAPPC9,SKAP1,SMC3,UIMC1,ITCH,MLIP,BCL11B,PKNOX1,MLLT3,TSHZ2,TCF7,PDE2A,KLF15,TBX15,ANXA4,WNT11,MTA1,KLF8,NOX4,LCOR,RPRD1B,QKI,CCDC62,PRKCD,SOX6,ACVR2A,RUNX2,CD4,TGFB1,BANP,SGK1,NSD1,IGSF1,ZHX2,PKNOX2,ASCC2,BTRC,NFATC3,F2RL1,BCAS3,CDYL2,CC2D1B,TP73,SAP18,ZBTB22,ILF2,MTDH,FANK1,MYT1L,SMAD6,BNC2,ZNF398,CLOCK,TCF12,ZNF675,ETV6,TFAP2D,BCL3,SND1,DUSP22,HNRNPC,TRRAP,SBNO2,YTHDF1,FGF10,CIZ1,SMYD3,LOXL3,CAPN3,LUM,SMURF2,RORA,HIVEP2,AUTS2,TNFSF11,SMG6,PPP3CA,NFYB,MAGEA4,KLF12,CAMK4,GATAD2B,UFL1,TRAK1,CTNNB1,PARK2,SOD2,DACH2,METTL13,SMARCC1,KLF17,PPARG,NGRN,AXIN1,IL18R1,CIPC,MTF1,CBX5,ANKRD17,BRIP1,LRPPRC,SREBF2,CDK11A,CDK11B,FGF1,NPAT,NR4A3,FOXK2,ESCO1,MYOCD,TRIM5,PER2,AJUBA,ZNF626,ZNF737,CHEK2,SUPT3H,PRDM16,PPP1CB,HCK,SORBS1,RAB3GAP2,TRIM8,DIO2,BRMS1L,ZBTB8A,ZBTB8B,CTDP1,BAZ1B,MEF2B,HDAC4,PAX2,PHF5A,SECISBP2L,SFRP1,MED13,ZNF395,FOXO3,NFIB,SP4,SYNCRIP,SMAD3,CUX2,WWP2,ARNT2,SBNO1,KRBOX1,ZNF662,ZNF777,EBF3,RNF168,CASZ1,DCP1B,MIER3,NEDD4,ESRRG,HOXD3,HOXD4,ZNF114,KTI12,NFATC1,CDC73,APP,SSBP3,GSX2,RBM8A,NOX1,YAP1,TEN1,NVL,LRP5,POLR3G,ZNF787,SOX2,SETD2,TEAD1,PRICKLE1,ZNF653,ZNF521,ARID3A,ZNF761,CHUK,SFMBT1,ZNF584,ESR2,S100A12,PRKD1,STAT1,ST18,ETV5,RHOXF2B,TAF3,PLAGL1,HNF4A,ZBTB7C,TASP1,EREG,ATF2,POU2F2,TCF3,ZNF730,RAF1,CELF4,ZNF766,CARD16,BMPR2,CAMK1D,BMPR1A,ZKSCAN1,IKZF4,CDK12,CAND2,DENND4A,MYB,FGF2,ZNF71,POU6F2,BACH1,MXD3,PPM1F,TICRR,GLI4,ZFP41,BEND5,SEMA4D,NFX1,RORC,ELP3,PTAFR,SP140,SP140L,RHOXF2,JARID2,DDX58,BRDT,PHC2,RARB,SPEN,SIN3B,NCOA1,EHMT1,LMO7,DVL3,EIF2B5,EIF4G1,TCF20,ATF3,CKS1B,PAWR,EBF2,AGO3,MAML2,TSG101,TERF2IP,CRYM,RFX2,ZNF322,SUFU,MAGEA11,PRDM15,ZNF670,ZNF695,CCDC169-SOHLH2,SOHLH2,ZNF354C,TCEA3,PADI6,ZNF704,NR2C1,GLI2,TNKS,WBP2NL,ERCC1,TNRC6B,GLIS3,WDTC1,ZNF664,DAB2,BLM,PKHD1,MYSM1,SETD5,SMG5,RELN,SIN3A,RUVBL2,COMMD6,GMEB1,PELI1,MAP3K7,ZNF423,SP1,TRIM22,ALK,TEAD4,HOXC13,APBB3,SLC35A4,SRA1,UBE2V1,ADIRF,OVOL2,SNX5,NFATC2,RBBP6,PAX7,BNC1,ATP2B4,ACTL6B,ASXL3,PAK3,TET1,CAMTA1,CCPG1,ADNP,ARID4A,CDK6,PHF2,CELF1,TFRC,EPHA5,WWOX,MEF2A,DCAF6,XRN1,SATB1,PSMB2,EYA1,GATAD2A,DIS3L2,TRPV1,HOXB3,HOXB4,HOXB5,HOXB6,TFEB,MNAT1,ACTR2,L3MBTL4,ELAVL4,HDAC1,RECQL5,SMOC2,TRIM24,PLCG2,ROCK1,SCMH1,PAXIP1,ZNF713,EGR2,RNF10,RYBP,AP3B1,PTPN2,NTRK1,KPNA6,TRIM44,EPCAM,ZNF425,GNL3L,MUC1,PTPN14,BCOR,AP3D1,ZNF41,CARM1,ZNF383,ZNF616,ZNF836,SMARCA2,CUX1,SPI1,DNMT3B,FOXP2,PSPC1,GLIS1,SFMBT2,EIF3H,BCAR3,JAZF1,ACOT8,RBPMS,CHURC1,CREB5,MAP3K13,SMYD1,SNCA,BMPR1B,PRIM2,PNPT1,ZEB2,FOXJ3,HEY2,RC3H1,EIF3E,ZMYND11,KMT2D,CREM,KMT2C,PLD1,MACC1,MITF,OPRD1,NUGGC,RPTOR,NFIX,BTK,KCTD13,MLLT1,CBFA2T2,IGF1,MLXIP,ATF7IP,MAPK1,PTEN,BMP7,ATXN2,MXI1,PUM1,SOX5,CIR1,PCBP2,ZNF780A,ZNF780B,LRRK2,TMEM59,ZBTB20,RFC3,LILRB4,PKD2,ZNF652,KCTD1,RIPPLY1,RCOR3,TADA2A,DAZL,NCOA3,PKIB,ZNF146,ZNF565,WNT7A,ZBED6,BRWD3,CRTC3,DNAJC1,ARNTL2,KAT6A,MTIF2,ZKSCAN7,ZNF197,ZNF660,BMP6,TAF15,NFE2L1,WDR43,ZNF30,ATRX,DPH6,IKZF1,PRMT2,ASCC1,SMO,RALY,TCF4,SOX30,TFE3,GRIN1,JADE1,RHOA,ROR1,RQCD1,TF,ONECUT2,CUL3,TFEC,GSK3B,PRR16,DNAJC3,STAT6,MLF1,RBBP8,NECAB2,CDK13,DUSP26,ZNF362,NACC2,SUPT4H1,ZNF354A,AGT,HDGFRP3,METTL16,STAT2,ZMPSTE24,PBX1,EXOSC3,MAP3K5,TCFL5,CREBBP,DLX1,GTF2IRD2,NCF1,SHC1,ZNF813,LBX2,PARP10,RPS6KB1,DPRX,PRG3,SETDB2,DNAJB6,SP7,LARP4,ADAM8,RPS6KA5,ZNF484,ZNF93,PRDM2,NOS1,ZNF44,MED15,RGS14,ACTN4,MC1R,TCF25,ZNF282,EDRF1,TMEFF2,IL18,DIS3,PPARA,ARID5B,SMARCAL1,CPEB4,NOTCH4,ZMYND8,ZNF366,CRX,PAX6,PRKCZ,ADORA2A,CPEB1,OTUD7B,NIPBL,YEATS4,PHF20,ZNF143,BRF1,TBL1X,FHL2,MBTD1,ATF6,ZBTB5,ZNF708,IGF2BP3,ETS1,PPP1CA,MTF2,TAGLN3,CDK5RAP1,NCOR2,PRKCQ,CSRNP1,HDAC2,IMPACT,SRCAP,POU3F3,NOTO,ZKSCAN5,ELF2,PHIP,PPP2CA,RPL23,RFC5,ATXN1,SP100,ZNF347,ZNF415,TRIM29,PARN,DONSON,ADCY1,SYK,CNOT1,WWTR1,GTF2H5,NFXL1,ZBTB38,BRCA2,ATF7,DVL2,MORC1,MTRF1,YBX1,TAF1,EIF4E3,TICAM1,THAP3,HSF1,MAX,SAP130,DHFR,EZH1,ETF1,NRF1,NCOA2,WDR18,EEFSEC,QRICH1,ZP3,CHD6,RNF2,ZNF554,MYEF2,TRIM37,MET,ZNF461,CAMK2D,TIMELESS,BRD9,MALT1,SETD3,KDM2B,MLXIPL,CCT3,PHB,EDA,ZNF555,CTCF,NR6A1,ATF6B,CREB1,RGMB,TRAP1,EBF4,ZNF511,BMP4,ABLIM3

GO:0042325	regulation of phosphorylation	3.1074059974566237e-12	*ENPP1,LDB2,NRXN1,SLCO3A1,PDE4D,S1PR2,ADCY8,PDE8A,IL31RA,NRG3,LATS2,PRKAG2,PRLR,MVP,NTRK3,CBL,ARNT,FLT3,ENPP2,GRM5,PLCE1,FER,MAP2K5,KITLG,ROBO1,TOM1L1,HTR2B,TENM1,TRAF6,ITPKB,DNAJA3,VAV2,ABI1,HSP90AA1,CDC6,KNDC1,DSCAM,AKAP13,DAB1,PTPN11,HDAC6,ADORA1,EPHA1,IKBKB,ERBB4,MRE11A,PMEPA1,PTPRO,CDON,NTRK2,EP300,PDGFB,TNIK,CCND3,INSR,PRKAR1A,PIP4K2A,CAB39,PAK1,MAP3K4,BDNF,ENTPD5,ECT2,SNX6,ERN2,DMD,SLC8A1,RIT2,CASS4,FYN,NF1,LMTK2,CHI3L1,TTN,PAQR3,PIK3R2,NRG1,SH2D3C,BDKRB1,BDKRB2,MAP2K1,FNIP1,ROR2,PTPRT,IPO5,MAD2L2,CAPRIN2,CCNYL1,GNAQ,HERC5,JAK2,FBXW7,ZFYVE28,LAMTOR3,WNT11,NOX4,PRKCD,TAB2,ACVR2A,CD4,PPM1E,TGFB1,NSD1,PIBF1,SLIT2,CORO1C,SMAD6,ZNF675,BMPER,ANKRD54,TNFRSF10B,DUSP22,DOCK3,FGF10,SMYD3,EPHA4,CD6,TNFSF11,PIP5KL1,PARK2,IGF1R,PPARG,AXIN1,PRKAR1B,LEPR,FGF1,PRKAR2A,MYOCD,AJUBA,CHEK2,SPDYA,CSPG4,NF2,FLT4,HDAC4,SFRP1,SLC4A4,TAB1,APP,PDGFRA,CCNI2,HEG1,BORA,IBTK,LRP5,MTCP1,SPTBN4,DRD1,S100A12,PRKD1,EREG,CCNY,ATF2,RAF1,PTPN1,BMPR2,BMPR1A,BTBD10,PIK3R3,CDK12,SPAG9,FGF2,PPM1F,NEDD9,SEMA4D,DVL3,EIF4G1,EPHB3,CKS1B,DEPTOR,TSG101,TERF2IP,PTPN13,TRPC5,DAB2,BLM,CACUL1,LDLRAD4,DLG3,WNK1,RELN,NEK10,IQGAP1,MAP3K7,ALK,SLC8A2,XDH,ATP2B4,ADNP,TFRC,UVRAG,EPHA5,GHR,MNAT1,SNX9,PLCG2,ROCK1,HIPK3,PTPN2,INSRR,NTRK1,EPHB1,EPHA10,AXIN2,PARD3,PIFO,EPHA7,ATG14,BCAR3,STK38,RBPMS,MAP3K13,RAPGEF3,SNCA,BMPR1B,PRKAG1,ADTRP,OPRD1,RPTOR,MLLT1,IGF1,HTT,MAPK1,PTEN,SPRED2,BMP7,LRRK2,UNC119,ZBTB20,LILRB4,PKD2,EFNA5,TADA2A,PDGFC,PKIB,PPP2R3C,PLCL2,BMP6,RAPGEF2,PRKAR2B,RHOA,ROR1,RQCD1,TF,EPM2A,DNAJC3,EEF2K,PTPRB,AGT,CCNJL,PRKAA2,ADAR,STAT2,PTK2,TEC,MAP3K5,NCF1,TAOK2,SHC1,CD44,ADAM8,RPS6KA5,HUS1,NOS1,GCKR,RGS14,STK4,EPHB2,SLC1A1,IL18,UBE2K,PPARA,MOB3B,PAX6,PRKCZ,ADORA2A,RABGEF1,CCNG2,ANGPT1,LRRK1,OSBPL8,CDK5RAP1,CNTN1,BRAF,HDAC2,IMPACT,PPP1R14A,CD300A,PHIP,PPP2CA,ANGPT4,MADD,SYK,WWTR1,DVL2,PTPRJ,HSF1,SASH1,VAV3,MOB3A,SH2D3A,AKTIP,INPP5F,AXL,CD109,MET,MALT1,MLXIPL,PHB,PTPRC,SH3GL2,TYRO3,TNXB,BMP4*
GO:0009887	animal organ morphogenesis	3.3350068505609647e-12	*CLRN1,DNAH11,ADAMTS16,RDH13,PRKCI,HLX,PBX3,SEMA3A,TENM3,RYR1,NRG3,ASH1L,ATRNL1,LRP2,SP3,OLFM1,MFAP5,ROBO1,CHD7,HTR2B,ITGB6,TRAF6,ROBO2,TRIOBP,PSMB7,KIF26B,DSCAM,THRB,XIRP2,IMPG2,LAMA2,TCIRG1,ERBB4,ATP8A2,ACTG2,ESR1,NPHP3,ANKRD11,CDON,NTRK2,EP300,CELA1,MEGF9,CSGALNACT1,INSR,LAMA3,FBXW11,BASP1,SLC4A10,TBR1,STRC,TFAP2A,ATRN,PHACTR4,MEIS1,MDM4,MYO3B,LAMC1,SATB2,NF1,PCDH15,FAT3,RTN4,BCR,TTN,NDRG4,NTN1,EGFLAM,NRG1,MAP2K1,MDFI,STIM1,VAX2,NTNG1,ROR2,DCN,EXOC4,FOXN3,NEUROD1,FBXW7,BCL11B,LRIG3,MLLT3,PLS1,TBX15,WNT11,IFT80,SOX6,ACVR2A,RUNX2,TGFB1,MYO3A,ZHX2,BTRC,RPGRIP1L,CELSR1,SLIT3,SDK2,SLIT2,CDH23,LAMC2,SMAD6,PARVA,FGF10,GREB1L,SMURF2,TNFSF11,CTNNB1,SMARCC1,DLG5,RYR2,FGF1,GLG1,CSMD1,MMP16,NF2,PAX2,ELN,SFRP1,FOXO3,ARL13B,NFIB,IFT122,SMAD3,PTPRM,ABI2,NRP2,VANGL1,HOXD3,HOXD4,TAB1,GSX2,PDGFRA,YAP1,HEG1,ALPL,LRP5,SETD2,GPC6,TTC39C,CHST11,PRICKLE1,FRS2,PALB2,MMP2,KDM6A,STAT1,STRA6,EREG,ABLIM1,DSCAML1,ATF2,BMPR2,BMPR1A,TSPAN12,CTNNA2,FGF2,KLHL3,RARB,DVL3,LAMB1,COL11A1,KCNQ1,SUFU,SCUBE2,PAFAH1B1,GLI2,DAB2,PKHD1,USH2A,RSPO2,MYO7A,HOXC13,OVOL2,ASXL3,WWOX,GPC3,PSMB2,EYA1,HOXB3,HOXB4,HOXB5,HOXB6,GHR,FLRT2,FGD1,HDAC1,AP3B1,PTPRQ,EPHB1,AXIN2,JAG2,BCOR,CTNNA1,BCAR3,SDK1,MST1,PTK7,BMPR1B,MAGI2,HEY2,COL13A1,WNT7B,SRSF6,HIPK1,PKP2,ZNRF3,MAPK1,PTEN,MIB1,BMP7,SOX5,LHFPL5,LRRK2,MEGF11,PKD2,SHANK3,PDGFC,WNT7A,BMP6,SOS1,TSHR,EXT1,PRICKLE2,SMO,MYLPF,TNFRSF11B,RHOA,ROR1,ONECUT2,CUL3,STAT6,ACTA2,SULF1,MTHFD1L,AGT,FBN2,ZMPSTE24,PTK2,PHEX,PBX1,CPE,COL9A1,DLX1,SETDB2,PEX7,PLEKHA1,SPEF1,EPHB2,SLC1A1,TMEFF2,DLC1,PPARA,ARID5B,SOBP,CRX,FBN1,PAX6,FAM20C,NIPBL,ASPN,FHL2,BRAF,CSRNP1,HDAC2,NOTO,ACAN,TMEFF1,SYK,WWTR1,SLIT1,DVL2,BMP1,UCHL5,ANKRD6,KDM2B,SEMA3C,EDA,EDNRA,GNGT1,BMP4*
GO:0050804	modulation of chemical synaptic transmission	3.886812577237111e-12	*NRXN1,S1PR2,ADCY8,GRID2,NRG3,NLGN4X,GRM8,GRM5,CASK,NRXN3,SLC24A2,ITGB1,CNIH2,PCDH17,ERC1,DGKI,ERC2,LAMA2,CACNB2,ADORA1,ABR,GRM7,NTRK2,CACNA1B,RIMS1,USP46,PTPRD,SLC4A10,BDNF,GRIK4,FYN,NF1,PLCB1,BCR,SHISA9,CNTN4,NTNG1,ROR2,ZDHHC3,JAK2,SYN3,CLSTN2,SYT1,LZTS1,CAMK2B,JPH3,DLGAP1,GRIK3,DISC1,PLCB4,YTHDF1,STXBP5,EPHA4,CNR1,PPP3CA,PARK2,PRKAR1B,GRM1,RIMS4,DCC,DGKB,CUX2,GRIK5,SHANK1,SLC6A1,APP,NLGN2,DRD1,SHANK2,CELF4,RASGRF2,NLGN1,LRRC4C,GRIK1,MCTP1,SORCS2,PRKCG,FBXL20,NLGN3,BTBD9,RELN,SLC8A2,STAU1,ATAD1,ADNP,MAP1B,SORCS3,SYT12,CACNA1A,ELAVL4,EGR2,NTRK1,MAP1A,PPP1R9A,UNC13A,EPHB1,CACNG8,NETO1,CHRNB4,PTPRA,SNCA,CACNG2,GRM4,PTPRS,KCTD13,SLC4A8,MAPK1,PTEN,LRRK2,TMEM108,SHANK3,WNT7A,PLCL2,RAPGEF2,PRKAR2B,STXBP5L,GRIN1,SYNGAP1,GRIN3A,GSK3B,SYN1,MCTP2,AGT,SYT7,RIMS2,CPLX2,PACSIN2,GRID1,RGS14,EPHB2,SLC1A1,PRKCZ,ADORA2A,GRIN2B,BRAF,TNR,ADCY1,GRIK2,CACNG3,DLGAP2,SHISA6*
GO:0040007	growth	4.745928962149846e-	*ENPP1,HLX,LLPH,SLC9A1,CLASP2,SEMA3A,WWC1,NRG3,LATS2,SEMA3D,NLGN4X,WWC3,PRLR,FTO,EGLN2,SEMA5A,RAD51B,STAT5B,PLCE1,MAP2K5,OLFM1,EZR,CHD7,CDKL5,ITGB1,HSP90AA1,KIF26B,DSCAM,AKAP13,RUNX1,IL17RB,PTP*

		12	*N11,HDAC6,GOLGA4,SERTAD2,ERBB4,LIMK1,BRD8,ATP8A2,LARGE,ESR1,EP300,CELA1,RIMS1,INSR,PRKAR1A,CDH4,BASP1,SLC4A10,BDNF,ATRN,TENM4,RFTN1,DMD,PLCB1,PCDH15,RTN4,APOD,RTN4R,NDRG4,NTN1,NRG1,BDKRB1,VGLL4,CSNK2A3,MAD2L2,CAPRIN2,SYT1,ITCH,PLS1,CYFIP2,WNT11,IFT80,SEMA5B,TGFB1,SGK1,DNAJB2,SLIT3,SLIT2,TP73,MUSTN1,DISC1,BNC2,ITSN2,ENOX2,DCLK1,FGF10,CAPN3,AUTS2,PPP3CA,GNG4,MAG,CTNNB1,PARK2,CDHR2,PPARG,CPQ,CDK11A,CDK11B,DRAXIN,LEPR,FGF1,NIN,MYOCD,CPNE6,BRMS1L,DCC,CTDP1,SPG11,SFRP1,FOXO3,MUC12,SMAD3,PSAP,SYT3,NRP2,CDC73,APP,DIP2B,YAP1,HEG1,FSTL4,ISLR2,SOX2,CHST11,SPTBN4,PALB2,ESR2,DCUN1D3,CPNE9,KDM6A,SLC6A3,HNF4A,STRA6,EREG,SEMA6D,ATF2,BMPR2,DPYSL2,BMPR1A,TAF8,SPAG9,RAB11A,EVC,FGF2,PPM1F,NEDD9,SEMA4D,JARID2,RARB,SPOCK1,EIF4G1,TSG101,VCL,CLIC4,WNT3,NLGN3,PAFAH1B1,AKAP6,RASAL1,TRPC5,GLI2,ERCC1,RUFY3,WDTC1,DAB2,SIN3A,RUVBL2,RSPO2,EYS,IQGAP1,MACF1,RBBP6,ADNP,MAP1B,CELF1,DCUN1D5,GPC3,GHR,UNC13A,TRIM46,SMARCA2,EPHA7,MAP3K13,MST1,PTK7,BMPR1B,MAGI2,PNPT1,HEY2,WNT7B,PTPRS,KMT2D,TLL2,RPTOR,IGF1,PTEN,OSGIN1,EBAG9,RAPH1,TMEM108,EFNA5,WNT7A,GPR21,SOS1,TSHR,EXT1,ATRX,PRMT2,SMO,SYT17,JADE1,RHOA,EPM2A,GSK3B,AGT,ZMPSTE24,RIMS2,TEC,GAP43,TAOK2,PYGO2,SHC1,RPS6KB1,STC2,PLEKHA1,SCNN1B,STK4,PPARA,ARID5B,SLC25A33,PRKCZ,NIPBL,YEATS4,MAP2,PRKCQ,IMPACT,TNR,CDKL3,PPP2CA,SH3BP4,WWTR1,SLIT1,BRCA2,PTPRJ,ZP3,CAMK2D,KDM2B,SEMA3C,NEDD4L,PHB,SH3GL2,CREB1,EDNRA,BMP4,CPNE1*
GO:0099177	regulation of trans-synaptic signaling	4.795084714962361e-12	*NRXN1,S1PR2,ADCY8,GRID2,NRG3,NLGN4X,GRM8,GRM5,CASK,NRXN3,SLC24A2,ITGB1,CNIH2,PCDH17,ERC1,DGKI,ERC2,LAMA2,CACNB2,ADORA1,ABR,GRM7,NTRK2,CACNA1B,RIMS1,USP46,PTPRD,SLC4A10,BDNF,GRIK4,FYN,NF1,PLCB1,BCR,SHISA9,CNTN4,NTNG1,ROR2,ZDHHC3,JAK2,SYN3,CLSTN2,SYT1,LZTS1,CAMK2B,JPH3,DLGAP1,GRIK3,DISC1,PLCB4,YTHDF1,STXBP5,EPHA4,CNR1,PPP3CA,PARK2,PRKAR1B,GRM1,RIMS4,DCC,DGKB,CUX2,GRIK5,SHANK1,SLC6A1,APP,NLGN2,DRD1,SHANK2,CELF4,RASGRF2,NLGN1,LRRC4C,GRIK1,MCTP1,SORCS2,PRKCG,FBXL20,NLGN3,BTBD9,RELN,SLC8A2,STAU1,ATAD1,ADNP,MAP1B,SORCS3,SYT12,CACNA1A,ELAVL4,EGR2,NTRK1,MAP1A,PPP1R9A,UNC13A,EPHB1,CACNG8,NETO1,CHRNB4,PTPRA,SNCA,CACNG2,GRM4,PTPRS,KCTD13,SLC4A8,MAPK1,PTEN,LRRK2,TMEM108,SHANK3,WNT7A,PLCL2,RAPGEF2,PRKAR2B,STXBP5L,GRIN1,SYNGAP1,GRIN3A,GSK3B,SYN1,MCTP2,AGT,SYT7,RIMS2,CPLX2,PACSIN2,GRID1,RGS14,EPHB2,SLC1A1,PRKCZ,ADORA2A,GRIN2B,BRAF,TNR,ADCY1,GRIK2,CACNG3,DLGAP2,SHISA6*
GO:0045595	regulation of cell differentiation	5.838672773267638e-12	*ENPP1,PRDX2,PRKCI,DNMT1,S1PR2,HLX,TRPS1,CBFB,CLASP2,SEMA3A,DOCK1,CLDN18,SEMA3D,NREP,TIAM2,PRLR,FTO,KALRN,NTRK3,ARNT,SEMA5A,STAT5B,TOX,GRM5,KITLG,LRP2,ZNF536,OLFM1,MEGF10,ROBO1,CHD7,CDKL5,TRAF6,ESRP1,ROBO2,ITPKB,ITGB1,TRIOBP,PSMB7,ZC4H2,DSCAM,TRIO,RUNX1,DAB1,ABCC8,TCIRG1,PTPN11,HDAC6,GOLGA4,IKBKB,LIMK1,NPHP3,CDON,NTRK2,CELA1,HOOK3,PDGFB,TOB2,H2AFY2,TCF7L2,CDH4,ESRRB,PTPRD,BDNF,TFAP2A,AMIGO1,FANCA,TGIF2,MEIS1,TENM4,ECT2,DMD,KAT7,RBM4,GPR171,CASS4,ARNTL,ADAMTS9,NF1,PLCB1,RTN4,RTN4R,NTN1,NRG1,MAP2K1,FNIP1,CNTN4,HDAC5,JDP2,CMKLR1,ROR2,SOX13,MAD2L2,CAPRIN2,NLN,RCC2,BOC,NEUROD1,JAK2,FBXW7,ITCH,BBS12,BCL11B,RBFOX1,TCF7,IL1RAPL1,CAMK2B,SOX6,ACVR2A,RUNX2,SEMA5B,CD4,TGFB1,ZHX2,NFATC3,C9ORF47,SLIT2,TP73,CORO1C,DISC1,KANK1,SMAD6,CLOCK,TCF12,ZNF675,SMOC1,NELL1,SUCO,ANKRD54,FGF10,LOXL3,CAPN3,EPHA4,RORA,PRKCA,TNFSF11,PPP3CA,NSUN2,MAG,CAMK4,UFL1,CTNNB1,SOD2,FCGR2B,PPARG,AXIN1,MSR1,ANKRD17,ANKRD26,DRAXIN,NIN,DCT,MYOCD,PER2,GLG1,PHLDB1,DCC,CTDP1,NF2,HDAC4,PAX2,SFRP1,FOXO3,SMAD3,CUX2,CASZ1,PREX1,HOXD3,NFATC1,PRTG,CDC73,APP,GSX2,PDGFRA,DIP2B,YAP1,FSTL4,LRP5,ISLR2,SLC9B2,SOX2,PRICKLE1,FRS2,TPH1,PRKD1,STAT1,PARP6,ZBTB7C,EREG,SEMA6D,TCF3,RAF1,ADAMTS12,BMPR2,DPYSL2,BMPR1A,NLGN1,CDK12,TAF8,SPAG9,RAB11A,MYB,FGF2,NEDD9,SEMA4D,RORC,PLXNA2,SETD1A,RARB,SPEN,NCOA1,BLOC1S5,CHRD,EIF4G1,CCDC3,VCL,WNT3,P4HB,SUFU,SCUBE2,PAFAH1B1,AKAP6,TRPC5,GLI2,RUFY3,DAB2,USH2A,LDLRAD4,RELN,SIN3A,CSF3R,APOLD1,MACF1,ALK,INPP5D,CLASP1,SRA1,ADIRF,XDH,OVOL2,NFATC2,BNC1,PAK3,ADNP,MAP1B,MARK1,CDK6,DROSHA,PSMB2,EYA1,BRINP1,GNB3,TRPV1,HOXB3,HOXB4,GHR,ACTR2,HDAC1,ROCK1,EGR2,RNF10,AP3B1,PTPN2,EPHB1,AXIN2,TRIM46,SH3GL3,AP3D1,CARM1,CUX1,SPI1,EPHA7,CTNNA1,GLIS1,MAP3K13,SMYD1,BMPR1B,ZEB2,HEY2,RC3H1,WNT7B,PTPRS,ABCA12,MITF,SRSF6,PKP2,BTK,IGF1,MAPK1,PTEN,MIB1,SPRED2,BMP7,SOX5,LRRK2,LILRB4,EFNA5,SHANK3,NCOA3,PPP2R3C,WNT7A,ZBED6,BMP6,IL17RD,SOS1,RAPGEF2,SMO,TCF4,TFE3,RHOA,SYNGAP1,NFKBID,GSK3B,EEF2K,CDK13,CD27,AGT,BLOC1S6,FBN2,BRINP3,PTK2,PBX1,TCP11,MAP3K5,TCFL5,DLX1,ALOX5,SP7,ADAM8,CDS1,RGS14,ACTN4,STK4,EPHB2,CLPTM1,IL18,PPARA,NOTCH4,FBN1,PAX6,PRKCZ,FAM20C,PPP1CC,PHLDB2,GCNT2,ETS1,VWC2,MAP2,FBXO31,CDK5RAP1,BRAF,HDAC2,HTR2C,IMPACT,TNR,MBNL3,CDKL3,PPP2CA,NUMB,SYK,WWTR1,SLIT1,YBX1,PLEKHB2,HSF1,ADAMTS7,SULT2B1,AXL,TNFSF9,WIF1,CD109,MALT1,SETD3,SEMA3C,PTPRC,CREB1,BMP4,CPNE1*
GO:0007167	enzyme linked receptor protein	8.893811871406031e-	*ENPP1,NRXN1,GP6,CLASP2,IL31RA,CTDSPL2,NRG3,LATS2,NREP,PRLR,MVP,KALRN,NTRK3,CBL,ARNT,FLT3,STAT5B,PLCE1,FER,PTPRR,LRP2,PIK3CD,ROBO1,ITGB6,VAV2,ABI1,SAMD12,TRIO,PTPRK,ANKS1B,STXBP4,GFRA2,PTPN11,ADORA*

	signaling pathway	12	1,ADAMTS3,EPHA1,ERBB4,PMEPA1,NTRK2,EP300,FNTA,PDGFB,CCND3,INSR,PIP4K2A,PAK1,PTPRD,BDNF,TGIF2,FUT8,LCK,SHC4,SNX6,PPM1L,PTPRU,AP3S1,RBM14,CASS4,FYN,NF1,PLCB1,LMTK2,RTN4,CHRDL1,APOD,NDRG4,PIK3R2,NOMO3,NRG1,BDKRB2,ROR2,DCN,SLC39A14,CDH13,PTPRT,CCBE1,JAK2,FBXW7,ZFYVE28,SH2D6,CYFIP2,PRKCD,ACVR2A,RUNX2,CD4,TGFB1,NUP93,IGSF1,ZFYVE9,KANK1,SMAD6,BMPER,DUSP22,FGF10,ARHGEF28,SMURF2,EPHA4,CTNNB1,SMARCC1,IGF1R,PPARG,AXIN1,LEPROT,FGF1,NR4A3,MYOCD,GLG1,PRDM16,MYO1E,HCK,SORBS1,CSPG4,FLT4,SPTBN1,SFRP1,SMAD3,NEDD4,NRP2,TAB1,PDGFRA,FSTL4,MUC20,PTPRG,CHST11,FRS2,MMP2,PRKD1,HNF4A,EREG,MAPKAPK3,ATF2,RAF1,PTPN1,ADAMTS12,BMPR2,BMPR1A,TSPAN12,PIK3R3,FGF2,NEDD9,DOK6,CHRD,EPHB3,PTPRE,SHOC2,TSG101,IDE,COL4A3,HIP1,IRS4,NEO1,DAB2,LDLRAD4,IQGAP1,MAP3K7,ZNF423,ALK,XDH,LTBP1,OVOL2,SNX5,PAK3,MAPKAPK2,COL4A6,EPHA5,GFRAL,GPC3,GHR,FLRT2,SMOC2,PTPN2,INSRR,NTRK1,EPHB1,EPHA10,SPI1,EPHA7,PTPRA,BCAR3,RBPMS,CHURC1,SNCA,BMPR1B,MAGI2,ZEB2,RASA1,IGF1,MAPK1,SPRED2,BMP7,TMEM108,EFNA5,BTBD11,PDGFC,GPR21,BMP6,IL17RD,SOS1,EXT1,RAPGEF2,GRB14,ROR1,RQCD1,TF,ONECUT2,GSK3B,STAT6,SULF1,AGT,FBN2,PTK2,VEPH1,CREBBP,DLX1,FMOD,PCSK6,NCF1,SHC1,RPS6KB1,GUCY2F,ANKS1A,RPS6KA5,PLEKHA1,RGS14,EPHB2,PPARA,ARID5B,FBN1,PRKCZ,FAM20C,RABGEF1,ASPN,ANGPT1,OSBPL8,GCNT2,VWC2,PRKCQ,BRAF,CSRNP1,PHIP,ANGPT4,SYK,WWTR1,PTPRJ,CAV2,COL4A5,AXL,REPS2,CD109,MET,PBLD,TYRO3,RGMB,CLNK,BMP4
GO:0033036	macromolecule localization	1.0401112367034777e-11	CD247,POLDIP3,DNAH11,ZDHHC14,ENPP1,HOOK2,BLZF1,GPC5,NRXN1,ASPH,PRKCI,SLCO3A1,ADCY8,CLASP2,GRID2,CLDN18,CTDSPL2,SLC25A17,LATS2,LRP1B,ASTN2,EPB41,DPP6,MAPRE2,TJP1,MVP,FTO,PITPNC1,SEC23B,SNX31,RAB4B,RAB4B-EGLN2,STAT5B,LHFPL4,FAM53A,MARVELD3,CCDC93,KCNIP4,SPNS2,CEP128,LRP2,TNPO3,HDGF,ANK2,EZR,XRCC4,TOM1L1,TENM1,KIF5C,LMNA,SRP72,AFM,NFASC,DNAJA3,ITGB1,HSP90AA1,APOL4,MCC,CCDC22,FAM126A,CDAN1,CNGB1,DLG2,PTPRK,ABCC8,ERC1,HMGN3,AFTPH,NRIP1,STXBP4,IMPG2,EFCAB7,TCIRG1,PTPN11,CCT2,CACNB2,HDAC6,GOLGA4,ADORA1,IKBKB,PEX14,ERBB4,GBF1,TLK1,ATP8A2,OSBPL10,ESR1,DPP10,FRMD4A,STX18,FNTA,HOOK3,VPS45,RIMS1,TNIK,SNX2,MID1,FMN2,ATP8B1,H2AFY2,TCF7L2,PIP4K2A,SCAMP4,MYOM1,SPTSSA,BDNF,GLTP,SFT2D1,ECT2,SNX6,CHML,VAMP7,SPNS3,PTPRU,RFTN1,SHFM1,AP3S1,NPLOC4,DMD,CENPF,RANBP17,ATG10,KAT7,PRELID2,GSN,RIT2,FYN,ARNTL,ADAMTS9,NF1,SMG7,LMTK2,STX6,RTN4,GOLGA2P5,APOD,VTA1,BCR,TTN,ICA1,PAQR3,PIK3R2,KLHL21,BDKRB2,BID,MDFI,SLCO1A2,RAPGEF6,MYH9,MAD1L1,TMEM30A,SYNE1,PARD3B,SCFD2,ZDHHC3,LIN7A,SCFD1,PITPNB,CREBRF,EXOC4,IPO5,MAN1A1,RAB27A,MTX3,RAB3C,COG8,TERF2,TMED6,GOLPH3L,RCC2,ANO6,ARL3,NEUROD1,IPO11,PARP11,DENND1A,ZDHHC15,JAK2,FBXW7,KCNB2,OAZ2,SKAP1,STARD6,CROCC,SNAP25-AS1,ABCC2,SMG1,RBFOX1,ATP9A,PLS1,DENND2A,SEPT7,TTC7B,LAMTOR3,KPNA3,CTAGE6,WNT11,IFT80,VPS13B,IMMP2L,QKI,PRKCD,CD4,TGFB1,BANP,PCSK5,NUP93,PIBF1,PTPRN2,F2RL1,BCAS3,DNAJB2,CELSR1,AAK1,CADPS2,CORO1C,DISC1,CEP135,ZFAND2A,ABCC11,GRPEL2,ANO4,ANK1,TTBK2,CHRM1,CLOCK,ANO3,BCL3,DCLK1,RABGAP1L,FGF10,CNTNAP2,CIZ1,SMYD3,STXBP5,PLIN3,CAPN3,SLC16A1,VMP1,SNX8,CNR1,TNFSF11,SMG6,PPP3CA,NSUN2,UFL1,TRAK1,CTNNB1,PARK2,RN7SL832P,TSNARE1,PPARG,DLG5,MYO1D,MSR1,SYTL5,APOL3,SCAMP5,ANXA13,LRPPRC,SREBF2,RYR2,LEPROT,NIN,WDR45B,AGAP1,TRIM5,PER2,AJUBA,SCN3B,SORBS1,TBC1D14,RAB3GAP2,TBCK,TRIM8,OBSCN,NF2,SPTBN1,AP3B2,SFRP1,ARL13B,CCDC91,IFT122,RANBP3,ATP10D,SNX14,SMAD3,RAB6A,WWP2,WIPI1,MTBP,VPS39,GRIK5,PSAP,LZTFL1,SHANK1,NEDD4,ARHGEF18,PLA2G4C,KPNB1,VPS53,RAB6C,SEC61B,THOC3,BET1L,RBM8A,YAP1,HEG1,RAB11FIP5,NLGN2,SSR2,BORA,NVL,JAK1,LRP5,SEC22C,SLC9B2,SETD2,ZDHHC6,GPC6,CHST11,PRICKLE1,SPTBN4,DRD1,RFT1,SERGEF,PRKD1,OSBPL2,TAF3,HNF4A,STRA6,VPS4A,PITRM1,ATF2,RAF1,PTPN1,AP1B1,NLGN1,ATP9B,SNX16,TAF8,RAB11A,IPO9,RAB24,PPM1F,ADCY5,XPO6,ABCA13,RILPL1,SORCS2,RAB11FIP3,SLC5A8,TANGO6,GAS8,OC90,AP2M1,TNPO1,TECPR2,LMBRD1,TSG101,DESI1,VCL,TERF2IP,ANKS4B,KCNQ1,SUFU,ZFYVE1,PAFAH1B1,KIF3A,ATG4C,NMUR2,HIP1,AKAP6,CEP350,PADI6,TRPC5,TNKS,RUFY3,DAB2,XKR4,TRAM2,USH2A,DZIP1,SMG5,DLG3,RELN,SIN3A,RUVBL2,ABCB1,FAM91A1,PLEKHF2,APOLD1,ZNF423,TRIM22,MACF1,MYO7A,EEPD1,APBB3,NBEAL1,GPM6B,LTBP1,SNX5,ATP2B4,NSF,PACRG,SYNE3,PACSIN1,ATAD1,NXT2,CEP41,SCARB1,KCNAB2,TFRC,EPHA5,GPC3,STOM,TBC1D5,SNX9,RPGR,ARL5A,ROCK1,C1QTNF1,EGR2,GAPVD1,CAPN10,ABCC1,AP3B1,LRBA,PTPN2,KPNA6,ZFAND1,MAP1A,ANK3,PTPN9,SNUPN,AXIN2,PARD3,GNL3L,PTPN14,TRIM46,TUB,CACNG8,AP3D1,AP2B1,ARHGAP44,HEATR5A,SNX33,LPA,SPI1,MCU,VPS16,CTNNA1,LIMA1,STAC,ILDR2,SHROOM3,FCHSD2,RAB15,RAPGEF3,ERCC3,CACNG2,MAGI2,TBC1D9,PNPT1,SYT9,HEY2,CHMP3,RAB5B,OSBPL1A,ADTRP,ABCA12,PKP2,TBC1D16,XPO4,VPS41,IGF1,STX8,IFFO1,BMP7,ATXN2,PIK3C3,NUP88,LRRK2,SEPT6,TMEM59,SPAG5,UNC119,CHMP5,BAG6,LILRB4,MORC3,ITGAM,PKD2,EFNA5,SHANK3,MIA2,SERPINA5,ATP8B4,SGSM1,WNT7A,ZBED6,ZC3H11A,SNAP23,DNAJC1,RABEP1,SH3PXD2B,POM121C,BMP6,MYCBP2,ANO1,ATRX,PLIN2,SAMM50,RAPGEF2,SMO,GET4,SUN1,LSG1,BLOC1S3,MARK4,STXBP5L,CPT1A,UBR5,ARRDC4,BBS9,SYNGAP1,ESYT2,HSPD1,TSPAN33,

			EPM2A,GSK3B,ABCG8,RDX,IWS1,CCDC14,NECAB2,UPF2,AGT,PRKAA2,SYT7,ADAR,BLOC1S6,FBN2,PITPNM2,PEX5L,SNX3,EXOC6B,RIMS2,TOMM5,COPG2,CPE,SEC16B,STOML1,TIMM44,CHKA,GTF2IRD2,NCF1,TAOK2,RPS6KB1,ZDHHC23,ITGAL,ALOX5,DNAJB6,ADAM8,SLC5A3,ARFGAP3,PACSIN2,CDS1,MON2,PEX7,GCKR,PLEKHA1,ACTN4,COG4,STK4,ZFAND6,EPHB2,SLC1A1,WLS,SIGMAR1,GPSM2,HCAR2,PPARA,PPP1R10,ABCD3,GRTP1,CALCRL,MCOLN1,STEAP3,KPNA4,SYTL4,FBN1,PAX6,PRKCZ,ADORA2A,RABGEF1,WDR83OS,PHLDB2,OTUD7B,NIPBL,ANGPT1,CADPS,PACS2,FRMPD1,OSBPL8,IGF2BP3,EPB41L2,SLCO2B1,HACL1,TBC1D10C,ATG3,BRAF,DIAPH1,SIL1,TNS4,ATP8A1,CLTB,EMC3,SKP1,FAM149B1,RPL23,SH3BP4,CAMSAP3,SP100,VTI1A,ANXA2,TRIM29,PARN,PIGU,RAB27B,ITSN1,NUP214,PITPNM3,WIPI2,NUMB,SQLE,SYK,WWTR1,GRIK2,MYRIP,RAB2A,SEC31B,BRCA2,CACNG3,DVL2,YBX1,ANKRD13A,GRIP1,SLC10A1,OSBP2,HSF1,CNIH4,ANKRD13C,ATP10B,PLEKHM1,GBP5,MCM8,CGNL1,MLPH,RAB37,AKTIP,EPS15,DENND4C,TBC1D10A,NBEA,SNX1,ZDHHC11,ZDHHC11B,EHD2,TRAPPC8,CCT3,NEDD4L,NDC1,RPH3A,GPR89A,CTCF,RAB28,RNF213,SYNDIG1,TSPAN5,VPS52,TRAF3IP2,SGTB,SHISA6,EDNRA,GNGT1,PRAP1,BMP4,ABLIM3
GO:0043085	positive regulation of catalytic activity	1.0898925163235999e-11	NRXN1,ASPH,MAP4K4,C6ORF89,ADCY8,NRG3,NOS1AP,TIAM2,MAPRE2,PRKAG2,PRLR,KALRN,NTRK3,FLT3,GRM5,MAP2K5,KITLG,XRCC4,ROBO1,TOM1L1,HTR2B,CDKL5,TENM1,TRAF6,DNAJA3,VAV2,ABI1,ITGB1,HSP90AA1,CDC6,ARHGAP24,AKAP13,DAB1,RGS6,CCT2,DOCK10,ADORA1,EPHA1,ERBB4,MRE11A,ABR,ESR1,NTRK2,FNTA,PDGFB,CCND3,INSR,CAB39,PAK1,MAP3K4,EGLN3,PHACTR4,ARHGAP6,LCK,ECT2,ARHGAP42,ERN2,GSN,CASS4,FYN,NF1,LMTK2,RTN4R,BCR,CHI3L1,NRG1,DOCK8,BID,MAP2K1,RAPGEF6,STIM1,ROR2,RGS8,CCNYL1,TERF2,RCC2,GNAQ,DENND1A,JAK2,FBXW7,ARAP2,DOCK9,LAMTOR3,CYFIP2,WNT11,NOX4,SIPA1L3,PRKCD,TAB2,CD4,TGFB1,PIBF1,BTRC,CRADD,F2RL1,BCAS3,CORO1C,BLID,CACNA1D,TNFRSF10B,RABGAP1L,DOCK3,EPHA4,TNFSF11,CTNNB1,ARHGAP22,IGF1R,PPARG,AXIN1,GARNL3,FGF1,RGS10,AJUBA,CACNA1C,SPDYA,TBC1D14,TBCK,FLT4,SFRP1,ARHGAP29,BCL2L13,SMAD3,PSAP,DCP1B,PREX1,TAB1,APP,PDGFRA,BORA,NVL,MTCP1,S100A12,DCUN1D3,PRKD1,ST18,EREG,CCNY,GRAMD4,CASP1,PTPN1,BMPR2,BMPR1A,FGF2,PPM1F,NEDD9,SEMA4D,PTAFR,ADCYAP1R1,DVL3,EPHB3,CKS1B,MYO9A,CCL14,CCL15,COL4A3,NMUR2,HIP1,TNKS,CACUL1,DLG3,WNK1,RELN,NEK10,DOCK11,IQGAP1,MAP3K7,ALK,SLC8A2,XDH,RGS7,ATP2B4,ADNP,SCARB1,EPHA5,DCUN1D5,TBC1D5,GHR,MNAT1,SNX9,ROCK1,INSRR,NTRK1,SLC39A10,EPHB1,EPHA10,AXIN2,PIFO,EPHA7,ATG14,BCAR3,MAP3K13,RAPGEF3,SH3BP1,SNCA,BMPR1B,MAGI2,PRIM2,TBC1D9,USP50,PRKAG1,RASA1,RPTOR,TBC1D16,IGF1,MAPK1,PTEN,LRRK2,UNC119,RFC3,ITGAM,ARHGAP25,PKD2,EFNA5,PDGFC,PKIB,SGSM1,PPP2R3C,NLRP1,UACA,SERINC5,RAPGEF2,VRK3,PSEN2,GRIN1,ARRDC4,RHOA,ROR1,HSPD1,GSK3B,BOK,AGT,PTK2,MAP3K5,NCF1,TAOK2,CD44,ADAM8,SLC5A3,CLDN4,NOS1,RGS14,SIPA1L2,ARHGAP11A,STK4,EPHB2,SLC1A1,IL18,DLC1,GRTP1,PDP1,MOB3B,PRKCZ,GRIN2B,ANGPT1,OSBPL8,TBC1D10C,CCL22,PRKCQ,CD300A,PPP2CA,SKP1,ANGPT4,RFC5,PARN,SRGAP2,MADD,SYK,RAP1GAP2,DVL2,TANK,HSF1,DHFR,SASH1,ASAP1,RALGAPA1,VAV3,MOB3A,CAV2,GNA12,AXL,MET,TBC1D10A,MALT1,PHB,PTPRC,TYRO3,EDNRA
GO:0007411	axon guidance	1.4786693665678427e-11	NRXN1,SEMA3A,SEMA3D,MYOT,UNC5C,KALRN,SEMA5A,NRXN3,ROBO1,KIF5C,ROBO2,NFASC,DSCAM,TRIO,LAMA2,EPHA1,PTPRO,UNC5D,LAMA3,CDH4,TBR1,BDNF,FYN,CCDC141,ADAMTSL1,NTN1,CNTN4,BOC,MYPN,BCL11B,SIAH1,SEMA5B,SLIT3,SLIT2,LAMC2,YTHDF1,ENAH,EPHA4,DRAXIN,DCC,CNTN6,NFIB,PTPRM,NRP2,PRTG,APP,DSCAML1,SEMA6D,BMPR2,USP33,DPYSL2,CRMP1,SEMA4D,PLXNA2,EPHB3,WNT3,GLI2,NEO1,RELN,EPHA5,FLRT2,EGR2,NTRK1,EPHB1,EPHA10,EPHA7,PTK7,BMPR1B,BMP7,EFNA5,MYCBP2,SOS1,EXT1,SMO,EMB,PTK2,GAP43,ECE1,RPS6KA5,TUBB3,EPHB2,PAX6,PRKCQ,TNR,TMEFF1,SLIT1,SEMA3C,NCAM1,EDNRA
GO:0009888	tissue development	1.6356056823161933e-11	CLRN1,ENPP1,ADAMTS16,LDB2,PDE4D,S1PR2,HLX,SLC9A1,TRPS1,CLASP2,SEMA3A,IL31RA,RYR1,ASH1L,ASTN2,SEMA3D,FHOD3,TJP1,UNC5C,PRLR,HIVEP3,FTO,SPRR2D,ATRNL1,SEMA5A,RAD51B,STAT5B,TOX,KITLG,NDUFV2,LRP2,PIK3CD,OLFM1,EZR,MEGF10,ROBO1,CHD7,HTR2B,ITGB6,LMNA,TRAF6,ESRP1,ROBO2,ABI1,ITGB1,TRIOBP,SLC24A3,PSMB7,KIF26B,ARHGAP24,KLHL12,AKAP13,RUNX1,THRB,XIRP2,LAMA2,TCIRG1,PTPN11,IKBKB,ERBB4,ACTG2,CECR2,ESR1,MYO18B,NPHP3,PTPRO,CDON,EP300,MEGF9,RNF220,PDGFB,CSGALNACT1,INSR,PRKAR1A,H2AFY2,LAMA3,TCF7L2,PAK1,BASP1,STRC,TFAP2A,ATRN,NPRL3,PHACTR4,TENM4,MDM4,LAMC1,SGCD,DMD,CENPF,SLC8A1,SATB2,JPH2,ARNTL,ADAMTS9,NF1,PLCB1,MGMT,PCDH15,ARID4B,RTN4,RXFP1,BCR,CHI3L1,TTN,NDRG4,BMP2K,ANKH,NTN1,EGFLAM,SLC4A5,NRG1,MAP2K1,STIM1,VAX2,VGLL4,NTNG1,ROR2,SLC39A14,EXOC4,MAD2L2,NLN,ANO6,NEUROD1,ACTL8,COL12A1,JAK2,BCL11B,RBFOX1,TMC1,CLDN1,MLLT3,PNPLA1,PLS1,PDE2A,KLF15,ANXA4,WNT11,IFT80,NOX4,SIPA1L3,SOX6,ACVR2A,RUNX2,SEMA5B,TGFB1,KEL,BTRC,F2RL1,C9ORF47,RPGRIP1L,CELSR1,SLIT2,TP73,MUSTN1,CDH23,CORO1C,LRRC10,LAMC2,SMAD6,BNC2,CLOCK,NELL1,BMPER,SBNO2,FGF10,FBXL17,GREB1L,PPL,LOXL3,SVIL,SMURF2,EPHA4,TNFSF11,PPP3CA,NSUN2,CTNNB1,CDHR2,PPARG,AXIN1,DLG5,BFSP1,XK,RYR2,FGF1,DCT,MYOCD,AJUBA,GLG1,PHLDB1,KRT6B,MYO1E,CSMD1,HCK,CTDP1,NF2,HDAC4,PAX2,ELN,SFRP1,ARL13B,NFIB,IFT122,SMAD3,ABI2,PSAP,NRP2,VANGL1,HOXD3,CDC73,SSBP3,PDGFRA,YAP1,HEG1,TN

			FRSF19,VDAC1,EYA2,ALPL,LRP5,BDH2,SOX2,SETD2,GPC6,CHST11,PRICKLE1,RCAN1,FRS2,PALB2,MMP2,KDM6A,STAT1,HNF4A,STRA6,EREG,MYH15,SEMA6D,ATF2,ADAMTS12,BMPR2,BMPR1A,CERS3,EVC,FGF2,SEMA4D,RORC,PLXNA2,FNDC3A,JARID2,RILPL1,KLHL3,RARB,OC90,CHRD,DVL3,ATF3,MATN3,EBF2,TSG101,VCL,LAMB1,CLIC4,MYO9A,COL11A1,KCNQ1,WNT3,SUFU,SCUBE2,PAFAH1B1,AKAP6,GLI2,SCUBE1,COL19A1,DAB2,PKHD1,USH2A,LDLRAD4,MYSM1,DLG3,RSPO2,CRELD1,IQGAP1,APOLD1,LUZP1,ARHGAP12,MYO7A,GSTM3,CLASP1,HOXC13,GPM6B,XDH,OVOL2,RBBP6,BNC1,ASGR2,GPR161,ARID4A,MARK1,CDK6,MEF2A,GPC3,PSMB2,EYA1,HOXB3,HOXB4,HOXB5,GHR,HDAC1,ROCK1,PAXIP1,EGR2,AP3B1,NTRK1,PTPRQ,EPHB1,AXIN2,EPCAM,JAG2,BCOR,PIFO,SPI1,EPHA7,FBXW4,SHROOM3,RAPGEF3,SH3BP1,MST1,KAZN,ERCC3,PTK7,SMYD1,BMPR1B,MAGI2,HEY2,HYDIN,WNT7B,HMGCS2,PTPRS,TLL2,ABCA12,CSRP1,SRSF6,PKP2,BTK,SGCZ,ZNRF3,CBFA2T2,IGF1,MAPK1,PTEN,MIB1,SPRED2,BMP7,SOX5,LHFPL5,ALDOC,ITGAM,PKD2,SHANK3,RIPPLY1,ALPK3,NCOA3,WNT7A,ZBED6,SH3PXD2B,BMP6,GPI,IL17RD,SOS1,EXT1,ATRX,PRICKLE2,IKZF1,RAPGEF2,SMO,CPT1A,MYLPF,RHOA,ROR1,ONECUT2,GSK3B,RDX,STAT6,ACTA2,SULF1,MTHFD1L,KRT8,AGT,FBN2,ZMPSTE24,PHEX,PBX1,TRPC4AP,PYGO2,ECE1,LBX2,RPS6KB1,STC2,PGM5,SCEL,SETDB2,ALOX5,CD44,STK4,TUBB3,SPEF1,WLS,TMEFF2,DLC1,PPARA,ARID5B,NOTCH4,PAX6,FAM20C,COL22A1,PHLDB2,ASPN,FHL2,GCNT2,PPP1CA,NEBL,HDAC2,POU3F3,NOTO,SPINT2,PPP2CA,ACAN,CAMSAP3,TMEFF1,WWTR1,RADIL,BRCA2,DVL2,NEB,YBX1,ADAMTS7,SULT2B1,BMP1,CAV2,CD109,MET,TIMELESS,ANKRD6,KDM2B,SEMA3C,PTPRC,EDA,VPS52,CREB1,EDNRA,BMP4
GO:0050808	synapse organization	1.678645815875542e-11	NRXN1,PRMT3,NEGR1,GRID2,NOS1AP,MYOT,NLGN4X,KALRN,NTRK3,GRM5,LHFPL4,NRXN3,CDKL5,ROBO2,NFASC,DNAJA3,PCDH17,ZC4H2,DSCAM,ERC1,ERC2,CACNB2,DOCK10,HDAC6,ERBB4,PTPRO,NTRK2,FNTA,KIRREL3,CDH8,INSR,PTPRD,CNTN5,BDNF,AMIGO1,TANC2,FYN,SNCB,NTN1,DCTN1,NRG1,FRMPD4,LRFN5,NTNG1,NFIA,PCDHB16,CAPRIN2,ZDHHC15,CLSTN2,IL1RAPL1,CAMK2B,PDZRN3,SDK2,CTNND2,DISC1,PPFIBP2,LINGO2,IL1RAPL2,EPHA4,CTNNB1,FCGR2B,ARHGAP22,IGF1R,DLG5,ADD2,DGKB,DNM3,CUX2,ABI2,SHANK1,NEDD4,NRP2,SLC6A1,APP,NLGN2,GPC6,DRD1,SYBU,SHANK2,NLGN1,CTNNA2,LRRC4C,SEMA4D,EIF4G1,EPHB3,ARHGAP39,FBXO45,NLGN3,PAFAH1B1,SETD5,DLG3,RELN,SLC8A2,STAU1,PAK3,ADNP,MAP1B,FLRT2,ACTR2,NTRK1,ANK3,UNC13A,EPHB1,ARHGAP44,EPHA7,PPFIA4,SDK1,SNCA,CACNG2,WNT7B,PTPRS,CAST,PTEN,LRRK2,TMEM108,ITGAM,EFNA5,SHANK3,WNT7A,RHOA,SYNGAP1,GABRB3,SYN1,EEF2K,GAP43,EPHB2,SLC1A1,GRIN2B,LGI2,TNR,SLIT1,GPM6A,COL4A5,SYNDIG1,TANC1,SHISA6
GO:0033554	cellular response to stress	1.7417017951964385e-11	PRDX2,MAP4K4,SLC9A1,ADCY8,PDE8A,SEMA3A,CALR3,C12ORF49,KIF22,PMS1,NREP,PTGIS,PRKAG2,PAGR1,FTO,CBL,ARNT,EGLN2,RAD51B,SAMHD1,FER,MARVELD3,MAP2K5,MAPK10,DEPDC5,ZSWIM7,EZR,XRCC4,MECOM,LMNA,TRAF6,OXR1,ULK4,ERCC6L2,HSP90AA1,PSMB7,PTPRK,OMA1,DNAJB14,STXBP4,PTPN11,HDAC6,DDB1,MSRA,IKBKB,MRE11A,TLK1,TEX264,HIF3A,CUL4B,TNRC6A,EP300,TNIK,MID1,STK39,FMN2,CAB39,PAK1,MAP3K4,EGLN3,FANCA,FUT8,NPRL3,TRIM13,MDM4,ECT2,ERN2,CDC45,SHFM1,NPLOC4,ATG10,SLC30A9,PDS5A,USP13,KAT7,SLC8A1,RBM4,MACROD2,FYN,ARNTL,NF1,PLCB1,MGMT,PPP4R2,RTN4,APOD,NEIL2,RTN4R,RWDD3,PIK3R2,SH2D3C,BDKRB2,BID,MAP2K1,FNIP1,TMBIM6,PPP2CB,ROR2,CREBRF,WDR70,AUNIP,COPS5,FGF14,DMC1,MAD2L2,MAN1A1,TERF2,SLX1B,FOXN3,TMEM117,RAD51D,JAK2,FAM168A,FBXW7,SMC3,UIMC1,ITCH,SMG1,MTA1,KREMEN1,IMMP2L,CLPB,ERCC8,PRKCD,SGK1,ASCC2,POLK,CRADD,F2RL1,DNAJB2,TP73,XPR1,CLOCK,ZNF675,BCL3,TNFRSF10B,DUSP22,NAIP,TRRAP,FGF10,SH3RF3,FANCI,CAPN3,EPHA4,RORA,TNFSF11,UBQLN3,MAG,UFL1,CTNNB1,PARK2,SOD2,FCGR2B,HSPA6,IGF1R,PPARG,AXIN1,MACROD1,OTUB1,CBX5,CYBB,SCAMP5,BRIP1,SREBF2,TDP1,FGF1,NR4A3,WDR45B,NOL3,KIR2DL4,AJUBA,CHEK2,UBQLN4,SPDYA,BAZ1B,FLT4,PAX2,SFRP1,ST8SIA1,FOXO3,SMAD3,RNFT2,ITPR1,WIPI1,RNF168,NEDD4,TXNDC12,SEC61B,APP,PDGFRA,NOX1,YAP1,AMFR,SESN1,CDK3,TNFRSF19,EYA2,SETD2,CHUK,ERLIN1,PALB2,MMP2,PRKD1,ETV5,SHPRH,ATF2,RASGRF2,CARD16,PTPN1,POLA1,BMPR2,USP33,BMPR1A,MORC2,BACH1,TICRR,HELQ,CHCHD6,PIK3C2B,PRKCG,EEF1E1,EEF1E1-BLOC1S5,ALKBH3,EIF2B5,EIF4G1,RBX1,ATF3,FBXO45,STT3B,PAWR,TERF2IP,ANKS4B,WDR59,P4HB,ZFYVE1,PAFAH1B1,POLE,TRPA1,DDAH1,EDEM3,ERCC1,BLM,SIN3A,RUVBL2,PPP2R5C,SPATA18,MAP3K7,GSTM3,STAU1,DSC2,EEPD1,SLC35A4,UBE2V1,XDH,NFATC2,RBBP6,CHD1L,PACRG,APEX2,MAPKAPK2,MAP1B,CDK6,RNF34,UVRAG,NFRKB,DCUN1D5,GFRAL,PSMB2,EYA1,TRPV1,TFEB,CLEC16A,MNAT1,ACTR2,ELAVL4,RECQL5,PAXIP1,HIPK3,ABCC1,PTPN2,EPHB1,MAN1B1,AXIN2,MUC1,RAD51C,SPI1,CTNNA1,AIFM2,ATG14,LRRC8C,LRRC8D,STAC,MAP3K13,MST1,ERCC3,SNCA,POLN,PNPT1,RNF103,RNF103-CHMP3,WNT7B,PTPRS,ZMYND11,PRKAG1,TRIP12,OPRD1,HIPK1,RPTOR,RTN4RL1,BTK,VPS41,MAPK1,PTEN,SPRED2,IFFO1,BMP7,PIK3C3,LRRK2,RFC3,BAG6,CHAF1B,TMCO1,PKD2,HSF2BP,SEL1L2,MTMR3,WNT7A,NME8,KAT6A,NFE2L1,ANO1,SH3RF2,ATRX,ASCC1,GET4,UBR5,RHOA,SPRTN,MCM3,CUL3,TFEC,HSPD1,GSK3B,DNAJC3,STAT6,EEF2K,RBBP8,CD27,BOK,NACC2,AGT,POLH,PRKAA2,ZMPSTE24,RNF121,GAP43,MAP3K5,CREBBP,NCF1,SLC29A1,TAOK2,PARP10,RNFT1,STC2,ALOX5,DNAJB6,CD44,ADAM8,HUS1,PLEKHA1,RGS14,MC1R,SLC1A1,SIGMA

			R1,TEX12,DNAJC7,PPARA,PPP1R10,SMARCAL1,PHLPP1,MAGI3,CPEB4,CPEB1,KIN,NIPBL,EYA3,ATF6,PPP1CA,FBXO31,BRAF,HDAC2,IMPACT,TNR,UBE2E2,ANGPT4,ARHGEF6,RFC5,SP100,REV3L,KCND2,DONSON,WIPI2,GRIK2,GTF2H5,ZBTB38,BRCA2,YBX1,TAF1,TANK,HSF1,MAX,DHFR,SASH1,MCM8,QRICH1,VAV3,CHD6,SH2D3A,UGGT1,MYEF2,INPP5F,NUAK2,AXL,MET,RAD9B,TIMELESS,UCHL5,ANKRD6,INO80C,EXD2,ATF6B,LIG1,SGTB,TRAP1,EDNRA,GNGT1,PRAP1,BMP4
GO:0060322	head development	1.80291686834093e-11	*NRXN1,ASPH,TACC2,NEGR1,PBX3,SEMA3A,GRID2,NRG3,PHACTR1,NLGN4X,UNC5C,ZSWIM6,SEMA5A,TOX,ATP2B2,SPATA5,LRP2,ROBO1,CHD7,TACC1,ROBO2,ITGB1,KNDC1,DAB1,PTPN11,ERBB4,ANKRD11,CDON,NTRK2,EP300,HOOK3,KIRREL3,RERE,H2AFY2,FBXW11,BASP1,SLC4A10,TBR1,ATRN,AMIGO1,MEIS1,ZNF148,DMD,CENPF,CRISPLD2,SLC8A1,SATB2,PAFAH1B2,MACROD2,FYN,NF1,PLCB1,RTN4,AFF2,APOD,RTN4R,BCR,CCDC141,NDRG4,NRG1,MAP2K1,VAX2,CNTN4,NEUROD1,TRAPPC9,SYT1,BCL11B,IMMP2L,SOX6,TGFB1,RPGRIP1L,SLIT2,DISC1,TTBK2,SYNJ2,TFAP2D,DCLK1,SLC17A7,FGF10,CNTNAP2,RORA,PPP3CA,MAG,CTNNB1,FCGR2B,IGF1R,AXIN1,DLG5,PFAS,DRAXIN,NIN,DCT,NF2,PAX2,SFRP1,FOXO3,ARL13B,NFIB,DDX10,ARNT2,NRP2,BTBD3,APP,SSBP3,GSX2,PDGFRA,NLGN2,PTPRG,SOX2,SETD2,DRD1,FRS2,MMP2,SYNE2,SLC6A3,SHANK2,STRA6,DSCAML1,SEMA6D,ATF2,RAF1,DPYSL2,BMPR1A,CTNNA2,FGF2,PLXNA2,SETD1A,RARB,GAS8,EIF2B5,EPHB3,FBXO45,LAMB1,WNT3,PAFAH1B1,GLI2,RELN,SIN3A,ZNF423,ALK,PRKG1,RGS7,ATP2B4,CDK6,CELF1,EPHA5,HOXB3,MNAT1,ELAVL4,HDAC1,EGR2,EPHB1,SRD5A2,EPHA7,FOXP2,CTNNA1,HYDIN,WNT7B,HMGCS2,PTPRS,RTN4RL1,MAPK1,PTEN,BMP7,LRRK2,BAG6,TMEM108,ITGAM,IL11RA,SHANK3,WNT7A,SOS1,EXT1,ATRX,RAPGEF2,KCNC2,SMO,SUN1,GRIN1,RHOA,NDE1,GSK3B,CCDC14,BOK,NDRG2,PBX1,DLX1,PYGO2,TBC1D23,PLEKHA1,EPHB2,SLC1A1,WLS,DLC1,ARID5B,PAX6,GRIN2B,KLHL1,NIPBL,SHROOM4,CDK5RAP1,CNTN1,BRAF,CSRNP1,POU3F3,TNR,ATXN1,SRGAP2,ADCY1,NUMB,SLIT1,BRCA2,TAF1,POTEE,CADM1,EZH1,AXL,UCHL5,TRA2B,KDM2B,TYRO3,CREB1,EDNRA,BMP4*
GO:0097485	neuron projection guidance	1.993648954605532e-11	*NRXN1,SEMA3A,SEMA3D,MYOT,UNC5C,KALRN,SEMA5A,NRXN3,ROBO1,KIF5C,ROBO2,NFASC,DSCAM,TRIO,LAMA2,EPHA1,PTPRO,UNC5D,LAMA3,CDH4,TBR1,BDNF,FYN,CCDC141,ADAMTSL1,NTN1,CNTN4,BOC,MYPN,BCL11B,SIAH1,SEMA5B,SLIT3,SLIT2,LAMC2,YTHDF1,ENAH,EPHA4,DRAXIN,DCC,CNTN6,NFIB,PTPRM,NRP2,PRTG,APP,DSCAML1,SEMA6D,BMPR2,USP33,DPYSL2,CRMP1,SEMA4D,PLXNA2,EPHB3,WNT3,GLI2,NEO1,RELN,EPHA5,FLRT2,EGR2,NTRK1,EPHB1,EPHA10,EPHA7,PTK7,BMPR1B,BMP7,EFNA5,MYCBP2,SOS1,EXT1,SMO,EMB,PTK2,GAP43,ECE1,RPS6KA5,TUBB3,EPHB2,PAX6,PRKCQ,TNR,TMEFF1,SLIT1,SEMA3C,NCAM1,EDNRA*
GO:0034329	cell junction assembly	2.6551326248932206e-11	*NRXN1,MAP4K4,SLC9A1,NEGR1,CLASP2,GRID2,CLDN18,TLN2,NLGN4X,TJP1,NTRK3,LHFPL4,MARVELD3,ANK2,NRXN3,ROBO2,PCDH17,DSCAM,PTPRK,CDH10,IKBKB,ERBB4,CDH12,PTPRO,NTRK2,KIRREL3,CDH8,PTPRD,CNTN5,BDNF,AMIGO1,ARHGAP6,ECT2,LAMC1,DST,APOD,BCR,NTN1,NRG1,LRFN5,PCDHB16,CLDN16,RCC2,CLSTN2,CLDN1,WNT11,IL1RAPL1,CLDN10,BCAS3,SDK2,CORO1C,CTNND2,DUSP22,LINGO2,CNTNAP2,IL1RAPL2,VMP1,PRKCA,CTNNB1,DLG5,ADD2,AJUBA,SORBS1,SFRP1,DNM3,SMAD3,CUX2,APP,SLK,HEG1,NLGN2,GPC6,DRD1,SHANK2,NLGN1,COL16A1,PPM1F,PEAK1,SEMA4D,EIF4G1,EPHB3,FBXO45,VCL,MYO9A,NLGN3,SETD5,MACF1,CLASP1,GPM6B,ADNP,MAP1B,FLRT2,ROCK1,NTRK1,PKN2,EPHB1,PARD3,EPHA7,PTPRA,CTNNA1,SDK1,SH3BP1,SNCA,PTPRS,HIPK1,PKP2,DSG1,PTEN,CLDN11,EFNA5,SHANK3,TBCD,WNT7A,RAPGEF2,CDH9,RHOA,GABRB3,EEF2K,AGT,CDH20,PTK2,GAP43,ARVCF,TAOK2,CLDN4,EPHB2,DLC1,PHLDB2,THSD1,LGI2,CAMSAP3,SLIT1,PTPRJ,GPM6A,SYNDIG1*
GO:0001932	regulation of protein phosphorylation	3.78995734296513e-11	*ENPP1,NRXN1,SLCO3A1,PDE4D,S1PR2,ADCY8,PDE8A,IL31RA,NRG3,LATS2,PRKAG2,PRLR,MVP,NTRK3,CBL,FLT3,ENPP2,GRM5,PLCE1,FER,MAP2K5,KITLG,ROBO1,TOM1L1,HTR2B,TENM1,TRAF6,ITPKB,DNAJA3,ABI1,HSP90AA1,CDC6,KNDC1,AKAP13,DAB1,PTPN11,HDAC6,ADORA1,EPHA1,ERBB4,MRE11A,PMEPA1,PTPRO,CDON,NTRK2,PDGFB,TNIK,CCND3,INSR,PRKAR1A,CAB39,PAK1,MAP3K4,BDNF,ECT2,SNX6,ERN2,DMD,SLC8A1,RIT2,CASS4,FYN,NF1,CHI3L1,TTN,PAQR3,NRG1,SH2D3C,BDKRB1,BDKRB2,MAP2K1,FNIP1,ROR2,PTPRT,IPO5,MAD2L2,CAPRIN2,CCNYL1,GNAQ,HERC5,JAK2,FBXW7,ZFYVE28,WNT11,NOX4,PRKCD,TAB2,ACVR2A,CD4,PPM1E,TGFB1,NSD1,PIBF1,SLIT2,CORO1C,SMAD6,ZNF675,BMPER,ANKRD54,TNFRSF10B,DUSP22,DOCK3,FGF10,SMYD3,EPHA4,CD6,TNFSF11,PIP5KL1,PARK2,PPARG,AXIN1,PRKAR1B,LEPR,FGF1,PRKAR2A,MYOCD,AJUBA,CHEK2,SPDYA,CSPG4,NF2,FLT4,SFRP1,TAB1,APP,CCNI2,HEG1,BORA,IBTK,LRP5,MTCP1,SPTBN4,DRD1,S100A12,PRKD1,EREG,CCNY,ATF2,RAF1,PTPN1,BMPR2,BMPR1A,PIK3R3,CDK12,SPAG9,FGF2,PPM1F,NEDD9,SEMA4D,DVL3,EIF4G1,CKS1B,DEPTOR,TSG101,TERF2IP,PTPN13,TRPC5,DAB2,BLM,CACUL1,LDLRAD4,DLG3,WNK1,RELN,NEK10,IQGAP1,MAP3K7,SLC8A2,XDH,ATP2B4,ADNP,TFRC,UVRAG,GHR,MNAT1,SNX9,PLCG2,HIPK3,PTPN2,NTRK1,AXIN2,PARD3,EPHA7,ATG14,BCAR3,STK38,RBPMS,MAP3K13,RAPGEF3,SNCA,BMPR1B,PRKAG1,ADTRP,OPRD1,RPTOR,MLLT1,IGF1,HTT,MAPK1,PTEN,SPRED2,BMP7,LRRK2,UNC119,LILRB4,PKD2,EFNA5,TADA2A,PDGFC,PKIB,PPP2R3C,PLCL2,BMP6,RAPGEF2,PRKAR2B,RHOA,RQCD1,EPM2A,DNAJC3,EEF2K,PTPRB,AGT,CCNJL,ADAR,STAT2,PTK2,TEC,MAP3K5,NCF1,TAOK2,SHC1,CD44,ADAM8,RPS6KA5,HUS1,NOS1,RGS14,STK4,EPHB2,SLC1A1,IL18,UBE2K,MOB3B,PAX6,PRKCZ,ADORA2A,RABGEF1,CCNG2,ANGPT1,LRRK1,OSBPL8,CDK5RAP1,CNTN1,BRAF,HDAC2,IMPACT,CD300A,PH*

			IP,PPP2CA,ANGPT4,MADD,SYK,WWTR1,DVL2,PTPRJ,HSF1,SASH1,MOB3A,SH2D3A,AKTIP,INPP5F,CD109,MALT1,MLXIPL,PHB,PTPRC,SH3GL2,TNXB,BMP4
GO:0048519	negative regulation of biological process	4.067282416802779e-11	*ENPP1,PRDX2,LDB2,NRXN1,ASPH,B4GALNT2,PRMT3,PRKCI,MOV10L1,MAP4K4,PDE4D,DNMT1,S1PR2,HLX,TMBIM4,SLC9A1,ADCY8,TRPS1,CBFB,PDE8A,CLASP2,SEMA3A,IL31RA,GRID2,RPS6KA2,PRDM12,PTGFR,CHERP,WWC1,CLDN18,CTDSPL2,RYR1,NRG3,ASH1L,LATS2,ASTN2,SEMA3D,SCAF8,FHOD3,NLGN4X,PTGIS,PRKAG2,WWC3,TJP1,NUDT6,PRLR,MVP,ADCY7,FTO,RGS7BP,KALRN,NTRK3,CBL,SEMA5A,SUSD4,STAT5B,EXD1,GRM5,SAMHD1,ZNF566,FER,CASK,MARVELD3,MAP2K5,KITLG,PTPRR,LRP2,ZNF536,SP3,DEPDC5,HDGF,ANK2,OLFM1,SH2D1A,EZR,ROBO1,MALRD1,TOM1L1,TNFAIP8L1,CHD7,HTR2B,MECOM,MIR1185-1,TENM1,RYR3,LMNA,TRAF6,ROBO2,ITPKB,DNAJA3,OXR1,SLC24A2,GRK5,ABI1,ITGB1,TRIOBP,SLC24A3,CDC6,PSMB7,TSC22D3,CNIH2,SRGAP3,MCC,PCDH17,CCDC22,DSCAM,SRGAP2B,CDAN1,TRIO,ARHGAP24,GFI1B,PTPRK,RUNX1,DAB1,OMA1,ABCC8,NRIP1,RGS6,DGKI,THRB,MIR105-2,MIR767,DACH1,PTPN11,HERC4,MGAT5,ORC2,HDAC6,SERTAD2,DDB1,ADORA1,GPRASP1,EPHA1,IKBKB,PEX14,ERBB4,GBE1,MRE11A,LIMK1,ATP8A2,KAT6B,SNIP1,ESR1,NPHP3,GRM7,MIER1,PCBP3,PMEPA1,PTPRO,NTRK2,TNRC6A,FRMD4A,EP300,CELA1,TENM2,RGS22,HOOK3,PDGFB,CCND3,TOB2,MID1,ZNF19,BRMS1,CHFR,SCML4,BPI,STK39,FMN2,RERE,PRKAR1A,ATP8B1,H2AFY2,TCF7L2,PIP4K2A,CAB39,PAK1,LITAF,FBXW11,ESRRB,PTPRD,BASP1,TBR1,SAMD4A,BDNF,TFAP2A,KCNMA1,PEG3,ZIM2,TGIF2,NPRL3,PHACTR4,MEIS1,KCNRG,TRIM13,UBR2,ARHGAP6,LCK,MDM4,ZNF148,MTA3,SNX6,TFDP2,ARHGAP42,CST2,PTPRU,ERN2,CDC45,C1D,NPLOC4,DENND5A,DMD,CENPF,TOX3,PDS5A,LARP4B,KAT7,SLC8A1,GSN,RBM14,RBM4,GPR171,SATB2,RIT2,HIRA,JPH2,FYN,MKRN2,ARNTL,ADAMTS9,NF1,SMG7,PLCB1,MGMT,SNCB,FAT3,RTN4,AFF2,DPEP1,CHRDL1,APOD,RTN4R,BCR,NDRG4,PAQR3,IFI30,PIK3R2,RANBP9,NTN1,NOMO3,NRG1,ARHGAP10,SLC43A2,UBP1,BDKRB1,BDKRB2,DOCK8,BID,MIR600HG,FRY,MAP2K1,MDFI,FNIP1,VAX2,MYH9,MAD1L1,LRFN5,TMBIM6,VGLL4,PPP2CB,CNTN4,HDAC5,RBM5,ZNF692,JDP2,CMKLR1,ITIH2,TSPAN8,ROR2,DCN,SCFD1,CDH13,CREBRF,TRDMT1,SOX13,FHIT,PTPRT,AUNIP,COPS5,IPO5,MAD2L2,TLE6,RGS8,CAPRIN2,TERF2,SLX1B,FOXN3,RCC2,CERKL,NEUROD1,GNAQ,RFFL,JAK2,YTHDC2,FBXW7,LINC00473,OAZ2,UIMC1,ITCH,MLIP,NPSR1,ZFYVE28,BBS12,ABCC2,LZTS1,BCL11B,SMG1,MLLT3,ATP9A,TEX11,TCF7,PDE2A,KLF15,TBX15,ANXA4,WNT11,IFT80,TRIM59,FIG4,MTA1,KREMEN1,KLF8,NOX4,LCOR,IL1RAPL1,PRKCD,SOX6,TAB2,RUNX2,SEMA5B,PPM1E,TGFB1,BANP,MMP28,NSD1,IGSF1,PIBF1,ZHX2,KEL,BTRC,DUS2,NFATC3,JPH3,CRADD,MIR153-2,F2RL1,BCAS3,C9ORF47,GRIK3,CDYL2,RPGRIP1L,DNAJB2,SLIT3,CC2D1B,MIR218-1,SLIT2,TP73,ITIH4,HP,CORO1C,SAP18,KANK1,MTDH,CLMN,TTBK2,FANK1,MYT1L,KDM4B,MMP26,SMAD6,ZNF398,CLOCK,ZNF675,ETV6,NELL1,TFAP2D,BMPER,TIMP2,BCL3,DCLK1,SND1,DUSP22,NAIP,HNRNPC,HOMER2,SBNO2,YTHDF1,FGF10,RASA4,RASA4B,SMYD3,KANK4,KCTD10,IQCJ-SCHIP1,LOXL3,RHPN2,SVIL,CAPN3,SMURF2,EPHA4,RORA,PRKCA,CNR1,SMG6,PPP3CA,NSUN2,GNG4,MAGEA4,MAG,KLF12,GATAD2B,PIP5KL1,UFL1,CTNNB1,PARK2,SOD2,METTL13,FCGR2B,SMARCC1,CDHR2,IGF1R,PPARG,AXIN1,PRKAR1B,OTUB1,DLG5,IL18R1,IL1RL1,ADD2,CIPC,MSR1,CBX5,ANKRD17,SCAMP5,BRIP1,ANXA13,ANKRD26,LRPPRC,SREBF2,RYR2,DRAXIN,LEPR,LEPROT,PROS1,RGS10,NPAT,NR4A3,FOXK2,NOL3,PRKAR2A,MYOCD,TRIM5,PER2,KIR2DL4,KIR3DL2,AJUBA,CACNA1C,GLG1,CHEK2,UBQLN4,PRDM16,HCK,CSTL1,TBC1D14,TRIM8,BRMS1L,DCC,CTDP1,HS3ST5,BAZ1B,NF2,FLT4,BICC1,HDAC4,PAX2,SPTBN1,TPTE,TRABD2B,SFRP1,FOXO3,NFIB,ZCCHC17,IFT122,DNM3,SSH1,SYNCRIP,SMAD3,CUX2,ITPR1,PTPRM,WWP2,SIMC1,MTBP,RNF168,SLC16A2,LZTFL1,SHANK1,DCP1B,MIER3,NEDD4,ARHGEF18,DOCK4,NAV3,SLC6A1,TXNDC12,NFATC1,PRTG,CDC73,APP,PDGFRA,ADD3,RBM8A,DIP2B,YAP1,HEG1,AMFR,RAB11FIP5,SESN1,TEN1,FSTL4,VDAC1,EYA2,RNLS,IBTK,ANGPTL4,LRP5,PTPRG,SOX2,KIF24,CHST11,PRICKLE1,RCAN1,ZNF653,SPTBN4,DRD1,TMEM14A,ERLIN1,FRS2,PALB2,SFMBT1,VILL,MMP2,SERGEF,TPH1,ESR2,DCUN1D3,PRKD1,STAT1,ST18,DGKG,ETV5,SLC6A3,TAF3,PLAGL1,HNF4A,ZBTB7C,SHANK2,VPS4A,EREG,SEMA6D,ATF2,TCF3,GRAMD4,RAF1,CELF4,CARD16,PTPN1,ADAMTS12,GGT7,BMPR2,VBP1,CAMK1D,BMPR1A,PABPC4,NLGN1,BTBD10,CTNNA2,IKZF4,CDK12,SPAG9,MORC2,CRMP1,PDE11A,MYB,FGF2,BACH1,MXD3,PPM1F,TICRR,ADCY5,AGBL4,BEND5,SEMA4D,ISM1,NFX1,RORC,MCTP1,PLXNA2,ADCYAP1R1,YME1L1,JARID2,DKK2,SORCS2,RAB11FIP3,PHC2,RARB,SPEN,PRKCG,SIN3B,SPOCK1,AREL1,EEF1E1,TXNDC5,EHMT1,GAS8,AP2M1,CHRD,EIF4G1,PTPRE,RBX1,ATF3,LIN28B,LINC00461,PAWR,AGO3,DEPTOR,DLEC1,TSG101,CCDC3,VCL,TERF2IP,CLIC4,CRYM,KCNQ1,WNT3,SUFU,MAGEA11,PTPN13,PAFAH1B1,PRDM15,CNTFR,COL4A3,TRPA1,DDAH1,AKAP6,RASAL1,NR2C1,TRPC5,GLI2,TNKS,ERCC1,RUFY3,TNRC6B,GLIS3,WDTC1,DAB2,BLM,PKHD1,USH2A,UBR1,LDLRAD4,SMG5,DLG3,WNK1,SIN3A,RUVBL2,RSPO2,PELI1,PPP2R5C,CFDP1,IQGAP1,ZNF423,TRIM22,ARHGAP12,ALK,INPP5D,CLASP1,FASTKD5,SRA1,TPCN1,GPM6B,XDH,LTBP1,OVOL2,SNX5,NFATC2,PRKG1,PAX7,RGS7,ATP2B4,PACRG,PACSIN1,SPTBN5,ASXL3,MASP1,SSH2,TET1,ATAD1,ADNP,GPR161,MAPKAPK2,ARID4A,MAP1B,MPHOSPH9,MARK1,CDK6,PHF2,CELF1,RNF34,TFRC,SORCS3,WWOX,MEF2A,CR1L,MIR1226,DROSHA,GFR*

			AL,GPC3,XRN1,ADRBK1,SATB1,PSMB2,ZNF207,EYA1,GATAD2A,PTGER3,BRINP1,DIS3L2,TRPV1,RNF216,TSPAN6,HOXB3,HOXB4,MIR10A,CLEC16A,GHR,MNAT1,L3MBTL4,ELAVL4,HDAC1,INVS,RECQL5,TRIM24,PLCG2,ROCK1,EPS8,SCMH1,C1QTNF1,HIPK3,RNF10,RYBP,GPR35,PTPN2,NTRK1,TRIM44,MAP1A,TMIGD1,SLC39A10,EIF3A,ANK3,EPHB1,PTPN9,AXIN2,SARM1,EPCAM,PARD3,ZNF425,GNL3L,MUC1,PTPN14,TRIM46,SH3GL3,BCOR,CARM1,AP2B1,ANXA8L1,ARHGAP44,SNX33,CD84,LPA,SMARCA2,CUX1,MIR1302-10,SPI1,DNMT3B,EPHA7,FOXP2,CTNNA1,LIMA1,AIFM2,PSPC1,GLIS1,SFMBT2,TRDN,ATG14,EIF3H,JAZF1,STK38,ABCB7,ACOT8,RENBP,OTUD3,RAPGEF3,SH3BP1,MST1,SMYD1,CHID1,SNCA,BMPR1B,MAGI2,PNPT1,ZEB2,HEY2,RC3H1,EIF3E,PTPRS,ZMYND11,CREM,PSMF1,TLL2,CAST,TRIP12,DFFA,ADTRP,ABCA12,RASA1,MITF,SRSF6,OPRD1,NUGGC,PKP2,RTN4RL1,NFIX,BTK,MIR383,ZNRF3,KCTD13,MLLT1,CBFA2T2,IGF1,ATF7IP,HTT,DPYSL3,MAPK1,PTEN,MIB1,SPRED2,BMP7,ATXN2,MXI1,PUM1,RASA2,CIR1,PCBP2,LRRK2,OSGIN1,TMEM59,UNC119,ZBTB20,BAG6,LILRB4,MORC3,ITGAM,ARHGAP25,PKD2,EFNA5,SHANK3,KCTD1,RIPPLY1,NT5E,RCOR3,DAZL,RGS9,SERPINA3,SERPINA4,SERPINA5,BEST3,PKIB,CUEDC2,TBCD,WNT7A,ZBED6,UACA,MAP4,CRTC3,DNAJC1,PLCL2,KAT6A,GPR21,BMP6,TAF15,MYCBP2,GPI,IL17RD,SH3RF2,N4BP1,PDCD1LG2,ATRX,IKZF1,PRMT2,RAPGEF2,SMO,VRK3,ENPP3,PRKAR2B,STXBP5L,PSEN2,TNFRSF11B,PAG1,SOX30,TFE3,UBR5,GRB14,JADE1,RHOA,SYNGAP1,VIPR2,GRIN3A,RQCD1,ONECUT2,CUL3,TFEC,HSPD1,ITPR2,EPM2A,GSK3B,ABCG8,DNAJC3,RDX,STAT6,EEF2K,FAM49B,PTPRB,RBBP8,NECAB2,CDK13,UPF2,CD27,DUSP26,TAPBPL,BOK,RNF144B,SULF1,NACC2,RNF43,SUPT4H1,TNFRSF8,AGT,HDGFRP3,METTL16,PRKAA2,ADAR,FBN2,STAT2,ZMPSTE24,BRINP3,NDRG2,PTK2,UPK3B,SNX3,PDE4B,PBX1,EXOSC3,VEPH1,TCFL5,CREBBP,UTP20,APCDD1L,DLX1,TAOK2,UGT1A1,UGT1A10,UGT1A4,UGT1A8,SHC1,CD160,PARP10,RPS6KB1,STC2,GPR173,PRG3,SERPINB11,SETDB2,ALOX5,DNAJB6,CD44,ADAM8,PACSIN2,RPS6KA5,ZNF93,CSNK1A1,HUS1,MIR346,PRDM2,CXCL17,NOS1,GCKR,PLEKHA1,RGS14,ACTN4,MC1R,STK4,TCF25,ZFAND6,ZNF282,MARK3,SPEF1,EPHB2,SLC1A1,TMEFF2,XCR1,IL18,HCAR1,HCAR2,DIS3,DLC1,PPARA,PPP1R10,ARID5B,CALCRL,MCOLN1,SMARCAL1,PHLPP1,CPEB4,NOTCH4,SYTL4,ZMYND8,ZNF366,FBN1,PAX6,PRKCZ,ZC3HAV1,CD96,ADORA2A,GRIN2B,RABGEF1,CPEB1,PHLDB2,OTUD7B,NIPBL,COL28A1,ASPN,ANGPT1,TBL1X,EYA3,FHL2,LRRK1,MBTD1,OSBPL8,ZBTB5,IGF2BP3,GCNT2,ETS1,TBC1D10C,VWC2,GRIA4,MAP2,MTF2,RBMS3,TAGLN3,FBXO31,ATG3,CDK5RAP1,NCOR2,PRKCQ,BRAF,DIAPH1,HDAC2,HTR2C,IMPACT,MIR1912,POU3F3,SPINT2,TNR,MBNL3,HIGD2A,CD300A,ELF2,PHIP,CDKL3,PPP2CA,ANGPT4,RPL23,SH3BP4,ATXN1,CAMSAP3,SP100,ANXA2,TRIM29,PARN,CR2,DONSON,SRGAP2,NUMB,RBM42,SYK,CNOT1,WWTR1,GRIK2,SLIT1,ASB1,FRMD5,CIT,ZBTB38,BRCA2,MORC1,YBX1,ANKRD13A,PTPRJ,TAF1,TANK,HSF1,MAX,SAP130,DHFR,EZH1,ETF1,INPP5A,MIR663B,SRPK2,ASAP1,NCOA2,ADAMTS7,SULT2B1,CGNL1,ZP3,RNF2,ZNF554,CAV2,GNA12,MYEF2,INPP5F,NUAK2,AXL,TRIM37,WIF1,CD109,MET,SESTD1,CAMK2D,RAD9B,TIMELESS,UCHL5,ANKRD6,MALT1,SERPINE3,SPTAN1,KDM2B,MLXIPL,SEMA3C,NEDD4L,PHB,PTPRC,ZNF555,CTCF,PBLD,SH3GL2,TYRO3,NR6A1,RNF213,TRAF3IP2,ATF6B,CREB1,SHISA6,TRAP1,CLNK,PRAP1,STXBP6,BMP4,CPNE1
GO:0006355	regulation of transcription, DNA-templated	6.186948329460267e-11	ENPP1,PRDX2,LDB2,ASPH,PRKCI,SLCO3A1,DNMT1,HLX,SLC9A1,PBX3,ADCY8,MED13L,TRPS1,CBFB,PDE8A,ZNF823,IL31RA,PRDM12,MED26,WWC1,ASH1L,SCAF8,STOX2,PTGIS,WWC3,PAGR1,HIVEP3,NPAS3,ARNT,LRRFIP2,STAT5B,TOX,GRM5,ZNF566,FER,CASK,MAP2K5,MAPK10,ZNF536,SP3,HDGF,EZR,IKZF2,CHD7,MECOM,TACC1,TENM1,TRAF6,DNAJA3,NHLH1,PSMB7,TSC22D3,ZC4H2,CCDC22,GFI1B,PTPRK,RUNX1,ERC1,HMGN3,NRIP1,THRB,EFCAB7,ITGB3BP,DACH1,ZNF569,ORC2,HDAC6,SERTAD2,MYT1,IKBKB,PEX14,ERBB4,ZNF609,BRD8,KAT6B,HIF3A,SNIP1,ESR1,MIER1,PCBP3,MAML3,CDON,EP300,CELA1,TENM2,ZNF76,RNF220,ZNF471,PDGFB,CCND3,TOB2,ZNF19,ZNF23,BRMS1,ZNF605,SCML4,HNF4G,INSR,RERE,PRKAR1A,FUBP1,ATP8B1,H2AFY2,TCF7L2,JMJD1C,ZNF443,ZNF490,ZNF564,ZNF709,ZNF799,LITAF,FBXW11,ESRRB,BASP1,TBR1,TFAP2A,CDK14,FANCA,PEG3,ZIM2,TGIF2,TGIF2-C20ORF24,MEIS1,VRTN,TRIM13,MDM4,ZNF148,MTA3,SNX6,TFDP2,ERN2,C1D,AFF3,DMD,CENPF,SLC30A9,TOX3,USP13,KAT7,ZNF667,RBM14,MED12L,SATB2,RIT2,HIRA,MKRN2,ARNTL,PLCB1,ARID4B,RWDD3,BRPF1,PIK3R2,NRG1,UBP1,MAP2K1,MDFI,FNIP1,VAX2,TMBIM6,VGLL4,PPP2CB,PPP3R1,HDAC5,CSRNP3,ZNF692,NFIA,RNF4,JDP2,CMKLR1,ROR2,DCN,PCBD2,CDH13,CREBRF,SOX13,COPS5,MAD2L2,TLE6,CAPRIN2,ZNF418,PHF20L1,ZNF286A,FOXN3,NEUROD1,USP22,JAK2,TRAPPC9,SKAP1,UIMC1,ITCH,MLIP,BCL11B,PKNOX1,MLLT3,TSHZ2,TCF7,PDE2A,KLF15,TBX15,ANXA4,WNT11,MTA1,KLF8,LCOR,RPRD1B,CCDC62,SOX6,ACVR2A,RUNX2,CD4,TGFB1,BANP,SGK1,NSD1,IGSF1,ZHX2,PKNOX2,ASCC2,BTRC,NFATC3,F2RL1,BCAS3,CDYL2,CC2D1B,TP73,SAP18,ZBTB22,ILF2,MTDH,FANK1,MYT1L,SMAD6,BNC2,ZNF398,CLOCK,TCF12,ZNF675,ETV6,TFAP2D,BCL3,SND1,DUSP22,TRRAP,SBNO2,FGF10,SMYD3,LOXL3,CAPN3,LUM,SMURF2,RORA,HIVEP2,AUTS2,TNFSF11,PPP3CA,NFYB,MAGEA4,KLF12,CAMK4,GATAD2B,UFL1,TRAK1,CTNNB1,PARK2,SOD2,DACH2,METTL13,SMARCC1,KLF17,PPARG,AXIN1,IL18R1,CIPC,MTF1,CBX5,BRIP1,SREBF2,CDK11A,CDK11B,FGF1,NPAT,NR4A3,FOXK2,MYOCD,TRIM5,PER2,AJUBA,ZNF626,ZNF737,CHEK2,SUPT3H,PRDM16,HCK,

			TRIM8,BRMS1L,ZBTB8A,ZBTB8B,CTDP1,BAZ1B,MEF2B,HDAC4,PAX2,PHF5A,SFRP1,MED13,ZNF395,FOXO3,NFIB,SP4,SMAD3,CUX2,WWP2,ARNT2,SBNO1,KRBOX1,ZNF662,ZNF777,EBF3,RNF168,CASZ1,MIER3,NEDD4,ESRRG,HOXD3,HOXD4,ZNF114,KTI12,NFATC1,CDC73,APP,SSBP3,GSX2,YAP1,LRP5,POLR3G,ZNF787,SOX2,SETD2,TEAD1,PRICKLE1,ZNF653,ZNF521,ARID3A,ZNF761,CHUK,SFMBT1,ZNF584,ESR2,S100A12,PRKD1,STAT1,ST18,ETV5,RHOXF2B,TAF3,PLAGL1,HNF4A,ZBTB7C,TASP1,EREG,ATF2,POU2F2,TCF3,ZNF730,RAF1,ZNF766,CARD16,BMPR2,CAMK1D,BMPR1A,ZKSCAN1,IKZF4,CDK12,CAND2,DENND4A,MYB,FGF2,ZNF71,POU6F2,BACH1,MXD3,PPM1F,GLI4,ZFP41,BEND5,SEMA4D,NFX1,RORC,ELP3,PTAFR,SP140,SP140L,RHOXF2,JARID2,DDX58,BRDT,PHC2,RARB,SPEN,SIN3B,NCOA1,EHMT1,LMO7,DVL3,TCF20,ATF3,CKS1B,PAWR,EBF2,MAML2,TSG101,TERF2IP,CRYM,RFX2,ZNF322,SUFU,MAGEA11,PRDM15,ZNF670,ZNF695,CCDC169-SOHLH2,SOHLH2,ZNF354C,TCEA3,ZNF704,NR2C1,GLI2,TNKS,WBP2NL,ERCC1,GLIS3,WDTC1,ZNF664,DAB2,BLM,PKHD1,MYSM1,SETD5,RELN,SIN3A,RUVBL2,COMMD6,GMEB1,PELI1,MAP3K7,ZNF423,SP1,TRIM22,ALK,TEAD4,HOXC13,APBB3,SRA1,UBE2V1,ADIRF,OVOL2,SNX5,NFATC2,PAX7,BNC1,ATP2B4,ACTL6B,ASXL3,TET1,CAMTA1,CCPG1,ADNP,ARID4A,CDK6,PHF2,TFRC,EPHA5,WWOX,MEF2A,DCAF6,SATB1,PSMB2,EYA1,GATAD2A,TRPV1,HOXB3,HOXB4,HOXB5,HOXB6,TFEB,MNAT1,ACTR2,L3MBTL4,HDAC1,RECQL5,TRIM24,PLCG2,SCMH1,PAXIP1,ZNF713,EGR2,RNF10,RYBP,AP3B1,PTPN2,NTRK1,KPNA6,TRIM44,EPCAM,ZNF425,MUC1,PTPN14,BCOR,AP3D1,ZNF41,CARM1,ZNF383,ZNF616,ZNF836,SMARCA2,CUX1,SPI1,DNMT3B,FOXP2,PSPC1,GLIS1,SFMBT2,JAZF1,RBPMS,CHURC1,CREB5,MAP3K13,SMYD1,SNCA,BMPR1B,ZEB2,FOXJ3,HEY2,ZMYND11,KMT2D,CREM,KMT2C,MACC1,MITF,OPRD1,RPTOR,NFIX,BTK,MLLT1,CBFA2T2,IGF1,MLXIP,ATF7IP,PTEN,BMP7,MXI1,SOX5,CIR1,PCBP2,ZNF780A,ZNF780B,ZBTB20,LILRB4,PKD2,ZNF652,KCTD1,RIPPLY1,RCOR3,TADA2A,NCOA3,ZNF146,ZNF565,WNT7A,ZBED6,BRWD3,CRTC3,ARNTL2,KAT6A,ZKSCAN7,ZNF197,ZNF660,BMP6,TAF15,NFE2L1,WDR43,ZNF30,ATRX,IKZF1,PRMT2,ASCC1,SMO,RALY,TCF4,SOX30,TFE3,GRIN1,JADE1,RHOA,ROR1,RQCD1,TF,ONECUT2,CUL3,TFEC,STAT6,MLF1,RBBP8,CDK13,DUSP26,ZNF362,NACC2,SUPT4H1,ZNF354A,AGT,HDGFRP3,STAT2,ZMPSTE24,PBX1,MAP3K5,TCFL5,CREBBP,DLX1,GTF2IRD2,NCF1,SHC1,ZNF813,LBX2,PARP10,DPRX,SETDB2,DNAJB6,SP7,ADAM8,RPS6KA5,ZNF484,ZNF93,PRDM2,NOS1,ZNF44,MED15,RGS14,ACTN4,MC1R,TCF25,ZNF282,EDRF1,IL18,PPARA,ARID5B,SMARCAL1,NOTCH4,ZMYND8,ZNF366,CRX,PAX6,PRKCZ,ADORA2A,OTUD7B,NIPBL,YEATS4,PHF20,ZNF143,BRF1,TBL1X,FHL2,MBTD1,ATF6,ZBTB5,ZNF708,ETS1,MTF2,TAGLN3,NCOR2,PRKCQ,CSRNP1,HDAC2,IMPACT,SRCAP,POU3F3,NOTO,ZKSCAN5,ELF2,PHIP,PPP2CA,RPL23,ATXN1,SP100,ZNF347,ZNF415,TRIM29,ADCY1,SYK,CNOT1,WWTR1,GTF2H5,NFXL1,ZBTB38,BRCA2,ATF7,DVL2,MORC1,YBX1,TAF1,TICAM1,THAP3,HSF1,MAX,SAP130,EZH1,NRF1,NCOA2,QRICH1,ZP3,CHD6,RNF2,ZNF554,MYEF2,TRIM37,MET,ZNF461,CAMK2D,TIMELESS,BRD9,MALT1,SETD3,KDM2B,MLXIPL,PHB,EDA,ZNF555,CTCF,NR6A1,ATF6B,CREB1,RGMB,EBF4,ZNF511,BMP4,ABLIM3
GO:2001141	regulation of RNA biosynthetic process	6.465542349358198e-11	*ENPP1,PRDX2,LDB2,ASPH,PRKCI,SLCO3A1,DNMT1,HLX,SLC9A1,PBX3,ADCY8,MED13L,TRPS1,CBFB,PDE8A,ZNF823,IL31RA,PRDM12,MED26,WWC1,ASH1L,SCAF8,STOX2,PTGIS,WWC3,PAGR1,HIVEP3,NPAS3,ARNT,LRRFIP2,STAT5B,TOX,GRM5,ZNF566,FER,CASK,MAP2K5,MAPK10,ZNF536,SP3,HDGF,EZR,IKZF2,CHD7,MECOM,TACC1,TENM1,TRAF6,DNAJA3,NHLH1,PSMB7,TSC22D3,ZC4H2,CCDC22,GFI1B,PTPRK,RUNX1,ERC1,HMGN3,NRIP1,THRB,EFCAB7,ITGB3BP,DACH1,ZNF569,ORC2,HDAC6,SERTAD2,MYT1,IKBKB,PEX14,ERBB4,ZNF609,BRD8,KAT6B,HIF3A,SNIP1,ESR1,MIER1,PCBP3,MAML3,CDON,EP300,CELA1,TENM2,ZNF76,RNF220,ZNF471,PDGFB,CCND3,TOB2,ZNF19,ZNF23,BRMS1,ZNF605,SCML4,HNF4G,INSR,RERE,PRKAR1A,FUBP1,ATP8B1,H2AFY2,TCF7L2,JMJD1C,ZNF443,ZNF490,ZNF564,ZNF709,ZNF799,LITAF,FBXW11,ESRRB,BASP1,TBR1,TFAP2A,CDK14,FANCA,PEG3,ZIM2,TGIF2,TGIF2-C20ORF24,MEIS1,VRTN,TRIM13,MDM4,ZNF148,MTA3,SNX6,TFDP2,ERN2,C1D,AFF3,DMD,CENPF,SLC30A9,TOX3,USP13,KAT7,ZNF667,RBM14,MED12L,SATB2,RIT2,HIRA,MKRN2,ARNTL,PLCB1,ARID4B,RWDD3,BRPF1,PIK3R2,NRG1,UBP1,MAP2K1,MDFI,FNIP1,VAX2,TMBIM6,VGLL4,PPP2CB,PPP3R1,HDAC5,CSRNP3,ZNF692,NFIA,RNF4,JDP2,CMKLR1,ROR2,DCN,PCBD2,CDH13,CREBRF,SOX13,COPS5,MAD2L2,TLE6,CAPRIN2,ZNF418,PHF20L1,ZNF286A,FOXN3,NEUROD1,USP22,JAK2,TRAPPC9,SKAP1,UIMC1,ITCH,MLIP,BCL11B,PKNOX1,MLLT3,TSHZ2,TCF7,PDE2A,KLF15,TBX15,ANXA4,WNT11,MTA1,KLF8,LCOR,RPRD1B,CCDC62,SOX6,ACVR2A,RUNX2,CD4,TGFB1,BANP,SGK1,NSD1,IGSF1,ZHX2,PKNOX2,ASCC2,BTRC,NFATC3,F2RL1,BCAS3,CDYL2,CC2D1B,TP73,SAP18,ZBTB22,ILF2,MTDH,FANK1,MYT1L,SMAD6,BNC2,ZNF398,CLOCK,TCF12,ZNF675,ETV6,TFAP2D,BCL3,SND1,DUSP22,TRRAP,SBNO2,FGF10,SMYD3,LOXL3,CAPN3,LUM,SMURF2,RORA,HIVEP2,AUTS2,TNFSF11,PPP3CA,NFYB,MAGEA4,KLF12,CAMK4,GATAD2B,UFL1,TRAK1,CTNNB1,PARK2,SOD2,DACH2,METTL13,SMARCC1,KLF17,PPARG,AXIN1,IL18R1,CIPC,MTF1,CBX5,BRIP1,SREBF2,CDK11A,CDK11B,FGF1,NPAT,NR4A3,FOXK2,MYOCD,TRIM5,PER2,AJUBA,ZNF626,ZNF737,CHEK2,SUPT3H,PRDM16,HCK,TRIM8,BRMS1L,ZBTB8A,ZBTB8B,CTDP1,BAZ1B,MEF2B,HDAC4,PAX2,PHF5A,SFRP1,MED13,ZNF395,FOXO3,NFIB,SP4,SMAD3,CUX2,WWP2,ARNT2,SBNO1,KRBOX1,ZNF662,ZNF777,EBF3,RNF168,CASZ1,MIER3,NEDD4,ESRRG,HOXD3,HOXD4,*

			ZNF114,KTI12,NFATC1,CDC73,APP,SSBP3,GSX2,YAP1,LRP5,POLR3G,ZNF787,SOX2,SETD2,TEAD1,PRICKLE1,ZNF653,ZNF521,ARID3A,ZNF761,CHUK,SFMBT1,ZNF584,ESR2,S100A12,PRKD1,STAT1,ST18,ETV5,RHOXF2B,TAF3,PLAGL1,HNF4A,ZBTB7C,TASP1,EREG,ATF2,POU2F2,TCF3,ZNF730,RAF1,ZNF766,CARD16,BMPR2,CAMK1D,BMPR1A,ZKSCAN1,IKZF4,CDK12,CAND2,DENND4A,MYB,FGF2,ZNF71,POU6F2,BACH1,MXD3,PPM1F,GLI4,ZFP41,BEND5,SEMA4D,NFX1,RORC,ELP3,PTAFR,SP140,SP140L,RHOXF2,JARID2,DDX58,BRDT,PHC2,RARB,SPEN,SIN3B,NCOA1,EHMT1,LMO7,DVL3,TCF20,ATF3,CKS1B,PAWR,EBF2,MAML2,TSG101,TERF2IP,CRYM,RFX2,ZNF322,SUFU,MAGEA11,PRDM15,ZNF670,ZNF695,CCDC169-SOHLH2,SOHLH2,ZNF354C,TCEA3,ZNF704,NR2C1,GLI2,TNKS,WBP2NL,ERCC1,GLIS3,WDTC1,ZNF664,DAB2,BLM,PKHD1,MYSM1,SETD5,RELN,SIN3A,RUVBL2,COMMD6,GMEB1,PELI1,MAP3K7,ZNF423,SP1,TRIM22,ALK,TEAD4,HOXC13,APBB3,SRA1,UBE2V1,ADIRF,OVOL2,SNX5,NFATC2,PAX7,BNC1,ATP2B4,ACTL6B,ASXL3,TET1,CAMTA1,CCPG1,ADNP,ARID4A,CDK6,PHF2,TFRC,EPHA5,WWOX,MEF2A,DCAF6,SATB1,PSMB2,EYA1,GATAD2A,TRPV1,HOXB3,HOXB4,HOXB5,HOXB6,TFEB,MNAT1,ACTR2,L3MBTL4,HDAC1,RECQL5,TRIM24,PLCG2,SCMH1,PAXIP1,ZNF713,EGR2,RNF10,RYBP,AP3B1,PTPN2,NTRK1,KPNA6,TRIM44,EPCAM,ZNF425,MUC1,PTPN14,BCOR,AP3D1,ZNF41,CARM1,ZNF383,ZNF616,ZNF836,SMARCA2,CUX1,SPI1,DNMT3B,FOXP2,PSPC1,GLIS1,SFMBT2,JAZF1,RBPMS,CHURC1,CREB5,MAP3K13,SMYD1,SNCA,BMPR1B,PRIM2,ZEB2,FOXJ3,HEY2,ZMYND11,KMT2D,CREM,KMT2C,MACC1,MITF,OPRD1,RPTOR,NFIX,BTK,MLLT1,CBFA2T2,IGF1,MLXIP,ATF7IP,PTEN,BMP7,MXI1,SOX5,CIR1,PCBP2,ZNF780A,ZNF780B,ZBTB20,LILRB4,PKD2,ZNF652,KCTD1,RIPPLY1,RCOR3,TADA2A,NCOA3,ZNF146,ZNF565,WNT7A,ZBED6,BRWD3,CRTC3,ARNTL2,KAT6A,ZKSCAN7,ZNF197,ZNF660,BMP6,TAF15,NFE2L1,WDR43,ZNF30,ATRX,IKZF1,PRMT2,ASCC1,SMO,RALY,TCF4,SOX30,TFE3,GRIN1,JADE1,RHOA,ROR1,RQCD1,TF,ONECUT2,CUL3,TFEC,STAT6,MLF1,RBBP8,CDK13,DUSP26,ZNF362,NACC2,SUPT4H1,ZNF354A,AGT,HDGFRP3,STAT2,ZMPSTE24,PBX1,MAP3K5,TCFL5,CREBBP,DLX1,GTF2IRD2,NCF1,SHC1,ZNF813,LBX2,PARP10,DPRX,SETDB2,DNAJB6,SP7,ADAM8,RPS6KA5,ZNF484,ZNF93,PRDM2,NOS1,ZNF44,MED15,RGS14,ACTN4,MC1R,TCF25,ZNF282,EDRF1,IL18,PPARA,ARID5B,SMARCAL1,NOTCH4,ZMYND8,ZNF366,CRX,PAX6,PRKCZ,ADORA2A,OTUD7B,NIPBL,YEATS4,PHF20,ZNF143,BRF1,TBL1X,FHL2,MBTD1,ATF6,ZBTB5,ZNF708,ETS1,MTF2,TAGLN3,NCOR2,PRKCQ,CSRNP1,HDAC2,IMPACT,SRCAP,POU3F3,NOTO,ZKSCAN5,ELF2,PHIP,PPP2CA,RPL23,ATXN1,SP100,ZNF347,ZNF415,TRIM29,ADCY1,SYK,CNOT1,WWTR1,GTF2H5,NFXL1,ZBTB38,BRCA2,ATF7,DVL2,MORC1,YBX1,TAF1,TICAM1,THAP3,HSF1,MAX,SAP130,EZH1,NRF1,NCOA2,QRICH1,ZP3,CHD6,RNF2,ZNF554,MYEF2,TRIM37,MET,ZNF461,CAMK2D,TIMELESS,BRD9,MALT1,SETD3,KDM2B,MLXIPL,PHB,EDA,ZNF555,CTCF,NR6A1,ATF6B,CREB1,RGMB,EBF4,ZNF511,BMP4,ABLIM3
GO:0045859	regulation of protein kinase activity	6.548248045301779e-11	NRXN1,ADCY8,NRG3,LATS2,PRKAG2,PRLR,MVP,NTRK3,CBL,FLT3,GRM5,PLCE1,MAP2K5,KITLG,ROBO1,TOM1L1,HTR2B,TENM1,TRAF6,DNAJA3,ABI1,HSP90AA1,CDC6,AKAP13,DAB1,ADORA1,EPHA1,PTPRO,PDGFB,CCND3,INSR,PRKAR1A,CAB39,PAK1,MAP3K4,ECT2,SNX6,ERN2,SLC8A1,CASS4,NF1,CHI3L1,TTN,PAQR3,NRG1,MAP2K1,ROR2,PTPRT,IPO5,CCNYL1,GNAQ,HERC5,JAK2,FBXW7,ZFYVE28,WNT11,NOX4,PRKCD,TAB2,CD4,PPM1E,TGFB1,PIBF1,CORO1C,ZNF675,ANKRD54,TNFRSF10B,DUSP22,DOCK3,SMYD3,EPHA4,TNFSF11,PPARG,AXIN1,PRKAR1B,FGF1,PRKAR2A,MYOCD,AJUBA,SPDYA,NF2,SFRP1,TAB1,APP,CCNI2,HEG1,BORA,IBTK,LRP5,MTCP1,S100A12,EREG,CCNY,PTPN1,BMPR2,BMPR1A,CDK12,FGF2,PPM1F,NEDD9,DVL3,CKS1B,DEPTOR,TSG101,DAB2,BLM,CACUL1,DLG3,WNK1,RELN,NEK10,IQGAP1,MAP3K7,SLC8A2,ATP2B4,ADNP,UVRAG,GHR,MNAT1,SNX9,HIPK3,PTPN2,AXIN2,STK38,MAP3K13,SNCA,BMPR1B,PRKAG1,RPTOR,MLLT1,IGF1,HTT,PTEN,BMP7,LRRK2,UNC119,LILRB4,PKD2,EFNA5,PDGFC,PKIB,PPP2R3C,RAPGEF2,PRKAR2B,RHOA,EPM2A,PTPRB,AGT,CCNJL,ADAR,PTK2,MAP3K5,NCF1,TAOK2,SHC1,ADAM8,RGS14,STK4,EPHB2,SLC1A1,IL18,MOB3B,PRKCZ,ADORA2A,CCNG2,ANGPT1,OSBPL8,CDK5RAP1,CD300A,PPP2CA,ANGPT4,MADD,SYK,WWTR1,DVL2,PTPRJ,SASH1,MOB3A,MALT1,PHB,PTPRC,TNXB,BMP4
GO:0045935	positive regulation of nucleobase-containing compound metabolic process	6.61346904646165e-11	LDB2,ASPH,SLC9A1,PBX3,CBFB,IL31RA,MED26,WWC1,ASH1L,SCAF8,STOX2,PAGR1,HIVEP3,FTO,NPAS3,ARNT,STAT5B,TOX,CASK,MAP2K5,SP3,HDGF,CHD7,MECOM,TACC1,TRAF6,NHLH1,HSP90AA1,GFI1B,RUNX1,HMGN3,NRIP1,THRB,EFCAB7,CCT2,SERTAD2,IKBKB,ERBB4,MRE11A,ZNF609,BRD8,KAT6B,ESR1,MAML3,CDON,TNRC6A,EP300,CELA1,ZNF76,PDGFB,HNF4G,INSR,FMN2,RERE,TCF7L2,LITAF,FBXW11,ESRRB,MAP3K4,RBM20,TBR1,SAMD4A,TFAP2A,PEG3,MEIS1,TRIM13,ENTPD5,ZNF148,MTA3,TFDP2,SLC30A9,TOX3,KAT7,RBM14,MED12L,SATB2,RIT2,MKRN2,ARNTL,PLCB1,MGMT,ARID4B,BRPF1,PIK3R2,UBP1,MAP2K1,PPP3R1,HDAC5,CSRNP3,NFIA,RNF4,ROR2,DCN,PCBD2,HNRNPLL,CDH13,CREBRF,COPS5,MAD2L2,CAPRIN2,TERF2,SLX1B,NEUROD1,USP22,JAK2,FAM168A,SKAP1,UIMC1,MLIP,BCL11B,PKNOX1,MLLT3,KLF15,WNT11,MTA1,NOX4,RPRD1B,CCDC62,ERCC8,PRKCD,ACVR2A,RUNX2,CD4,TGFB1,BANP,NSD1,BTRC,NFATC3,F2RL1,BCAS3,TP73,ILF2,MTDH,FANK1,SMAD6,ZNF398,CLOCK,TCF12,ETV6,TFAP2D,BCL3,SBNO2,YTHDF1,FGF10,CIZ1,SMYD3,CAPN3,LUM,RORA,AUTS2,TNFSF11,PPP3CA,NFYB,KLF12,CAMK4,CTNNB1,PARK2,SMARCC1,IGF1R,PPARG,AXIN1,MTF1,SREBF2,FGF1,NPAT,NR4A3,FOXK2,RIOK2,MYOCD,TRIM5,PER2,CHEK2,SUPT3H,PRDM16,TRIM8,MEF2B,HDAC4,PAX2,PHF5A,SFRP1,MED13,ZNF395,FO

			XO3,NFIB,SMAD3,WWP2,ARNT2,EBF3,RNF168,CASZ1,DCP1B,ESRRG,HOXD3,HOXD4,SLC4A4,NFATC1,CDC73,APP,SSBP3,YAP1,EYA2,NVL,LRP5,SOX2,TEAD1,ZNF521,ARID3A,CHUK,ESR2,PRKD1,STAT1,ST18,ETV5,PLAGL1,HNF4A,ZBTB7C,TASP1,ATF2,POU2F2,TCF3,RAF1,CELF4,BMPR2,BMPR1A,IKZF4,CDK12,CAND2,MYB,FGF2,ZNF71,BACH1,RORC,DDX58,BRDT,RARB,PRKCG,NCOA1,LMO7,DVL3,TCF20,ATF3,LIN28B,SRSF5,EBF2,MAML2,TSG101,RFX2,PRDM15,GLI2,TNKS,WBP2NL,ERCC1,TNRC6B,GLIS3,DAB2,BLM,MYSM1,SIN3A,RUVBL2,GMEB1,ZNF423,SP1,TRIM22,TEAD4,HOXC13,SRA1,UBE2V1,ADIRF,OVOL2,SNX5,NFATC2,BNC1,ACTL6B,ASXL3,PAK3,TET1,CAMTA1,CCPG1,ARID4A,PHF2,CELF1,TFRC,WWOX,MEF2A,DCAF6,EYA1,DIS3L2,HOXB3,HOXB4,HOXB5,TFEB,ACTR2,HDAC1,SMOC2,TRIM24,ROCK1,PAXIP1,EGR2,RNF10,RYBP,AP3B1,KPNA6,TRIM44,EPCAM,MUC1,AP3D1,CARM1,ZNF836,SMARCA2,SPI1,GLIS1,RBPMS,CHURC1,CREB5,BMPR1B,PRIM2,PNPT1,ZEB2,FOXJ3,HEY2,RC3H1,KMT2D,CREM,KMT2C,MACC1,MITF,RPTOR,NFIX,IGF1,MLXIP,ATF7IP,MAPK1,BMP7,PUM1,PCBP2,ZNF780B,ZBTB20,RFC3,PKD2,TADA2A,NCOA3,PKIB,WNT7A,CRTC3,ARNTL2,KAT6A,ZNF197,BMP6,TAF15,WDR43,ATRX,PRMT2,SMO,TCF4,SOX30,TFE3,GRIN1,JADE1,RQCD1,TF,ONECUT2,TFEC,STAT6,CDK13,SUPT4H1,AGT,METTL16,PRKAA2,PBX1,EXOSC3,MAP3K5,CREBBP,DLX1,NCF1,SHC1,SP7,RPS6KA5,ZNF484,PRDM2,NOS1,ACTN4,MC1R,EDRF1,IL18,DIS3,PPARA,PPP1R10,ARID5B,NOTCH4,CRX,PAX6,ZC3HAV1,CPEB1,NIPBL,YEATS4,ZNF143,BRF1,TBL1X,EYA3,ATF6,ETS1,MTF2,PRKCQ,CSRNP1,HDAC2,SRCAP,POU3F3,ELF2,PHIP,RFC5,SP100,PARN,CNOT1,WWTR1,ZBTB38,BRCA2,ATF7,DVL2,YBX1,THAP3,HSF1,MAX,EZH1,NRF1,NCOA2,QRICH1,ZP3,CHD6,TRIM37,MET,TIMELESS,SETD3,TRA2B,MLXIPL,CCT3,PHB,PTPRC,CTCF,NR6A1,ATF6B,CREB1,RGMB,BMP4,ABLIM3
GO:1903506	regulation of nucleic acid-templated transcription	6.621098957375006e-11	*ENPP1,PRDX2,LDB2,ASPH,PRKCI,SLCO3A1,DNMT1,HLX,SLC9A1,PBX3,ADCY8,MED13L,TRPS1,CBFB,PDE8A,ZNF823,IL31RA,PRDM12,MED26,WWC1,ASH1L,SCAF8,STOX2,PTGIS,WWC3,PAGR1,HIVEP3,NPAS3,ARNT,LRRFIP2,STAT5B,TOX,GRM5,ZNF566,FER,CASK,MAP2K5,MAPK10,ZNF536,SP3,HDGF,EZR,IKZF2,CHD7,MECOM,TACC1,TENM1,TRAF6,DNAJA3,NHLH1,PSMB7,TSC22D3,ZC4H2,CCDC22,GFI1B,PTPRK,RUNX1,ERC1,HMGN3,NRIP1,THRB,EFCAB7,ITGB3BP,DACH1,ZNF569,ORC2,HDAC6,SERTAD2,MYT1,IKBKB,PEX14,ERBB4,ZNF609,BRD8,KAT6B,HIF3A,SNIP1,ESR1,MIER1,PCBP3,MAML3,CDON,EP300,CELA1,TENM2,ZNF76,RNF220,ZNF471,PDGFB,CCND3,TOB2,ZNF19,ZNF23,BRMS1,ZNF605,SCML4,HNF4G,INSR,RERE,PRKAR1A,FUBP1,ATP8B1,H2AFY2,TCF7L2,JMJD1C,ZNF443,ZNF490,ZNF564,ZNF709,ZNF799,LITAF,FBXW11,ESRRB,BASP1,TBR1,TFAP2A,CDK14,FANCA,PEG3,ZIM2,TGIF2,TGIF2-C20ORF24,MEIS1,VRTN,TRIM13,MDM4,ZNF148,MTA3,SNX6,TFDP2,ERN2,C1D,AFF3,DMD,CENPF,SLC30A9,TOX3,USP13,KAT7,ZNF667,RBM14,MED12L,SATB2,RIT2,HIRA,MKRN2,ARNTL,PLCB1,ARID4B,RWDD3,BRPF1,PIK3R2,NRG1,UBP1,MAP2K1,MDFI,FNIP1,VAX2,TMBIM6,VGLL4,PPP2CB,PPP3R1,HDAC5,CSRNP3,ZNF692,NFIA,RNF4,JDP2,CMKLR1,ROR2,DCN,PCBD2,CDH13,CREBRF,SOX13,COPS5,MAD2L2,TLE6,CAPRIN2,ZNF418,PHF20L1,ZNF286A,FOXN3,NEUROD1,USP22,JAK2,TRAPPC9,SKAP1,UIMC1,ITCH,MLIP,BCL11B,PKNOX1,MLLT3,TSHZ2,TCF7,PDE2A,KLF15,TBX15,ANXA4,WNT11,MTA1,KLF8,LCOR,RPRD1B,CCDC62,SOX6,ACVR2A,RUNX2,CD4,TGFB1,BANP,SGK1,NSD1,IGSF1,ZHX2,PKNOX2,ASCC2,BTRC,NFATC3,F2RL1,BCAS3,CDYL2,CC2D1B,TP73,SAP18,ZBTB22,ILF2,MTDH,FANK1,MYT1L,SMAD6,BNC2,ZNF398,CLOCK,TCF12,ZNF675,ETV6,TFAP2D,BCL3,SND1,DUSP22,TRRAP,SBNO2,FGF10,SMYD3,LOXL3,CAPN3,LUM,SMURF2,RORA,HIVEP2,AUTS2,TNFSF11,PPP3CA,NFYB,MAGEA4,KLF12,CAMK4,GATAD2B,UFL1,TRAK1,CTNNB1,PARK2,SOD2,DACH2,METTL13,SMARCC1,KLF17,PPARG,AXIN1,IL18R1,CIPC,MTF1,CBX5,BRIP1,SREBF2,CDK11A,CDK11B,FGF1,NPAT,NR4A3,FOXK2,MYOCD,TRIM5,PER2,AJUBA,ZNF626,ZNF737,CHEK2,SUPT3H,PRDM16,HCK,TRIM8,BRMS1L,ZBTB8A,ZBTB8B,CTDP1,BAZ1B,MEF2B,HDAC4,PAX2,PHF5A,SFRP1,MED13,ZNF395,FOXO3,NFIB,SP4,SMAD3,CUX2,WWP2,ARNT2,SBNO1,KRBOX1,ZNF662,ZNF777,EBF3,RNF168,CASZ1,MIER3,NEDD4,ESRRG,HOXD3,HOXD4,ZNF114,KTI12,NFATC1,CDC73,APP,SSBP3,GSX2,YAP1,LRP5,POLR3G,ZNF787,SOX2,SETD2,TEAD1,PRICKLE1,ZNF653,ZNF521,ARID3A,ZNF761,CHUK,SFMBT1,ZNF584,ESR2,S100A12,PRKD1,STAT1,ST18,ETV5,RHOXF2B,TAF3,PLAGL1,HNF4A,ZBTB7C,TASP1,EREG,ATF2,POU2F2,TCF3,ZNF730,RAF1,ZNF766,CARD16,BMPR2,CAMK1D,BMPR1A,ZKSCAN1,IKZF4,CDK12,CAND2,DENND4A,MYB,FGF2,ZNF71,POU6F2,BACH1,MXD3,PPM1F,GLI4,ZFP41,BEND5,SEMA4D,NFX1,RORC,ELP3,PTAFR,SP140,SP140L,RHOXF2,JARID2,DDX58,BRDT,PHC2,RARB,SPEN,SIN3B,NCOA1,EHMT1,LMO7,DVL3,TCF20,ATF3,CKS1B,PAWR,EBF2,MAML2,TSG101,TERF2IP,CRYM,RFX2,ZNF322,SUFU,MAGEA11,PRDM15,ZNF670,ZNF695,CCDC169-SOHLH2,SOHLH2,ZNF354C,TCEA3,ZNF704,NR2C1,GLI2,TNKS,WBP2NL,ERCC1,GLIS3,WDTC1,ZNF664,DAB2,BLM,PKHD1,MYSM1,SETD5,RELN,SIN3A,RUVBL2,COMMD6,GMEB1,PELI1,MAP3K7,ZNF423,SP1,TRIM22,ALK,TEAD4,HOXC13,APBB3,SRA1,UBE2V1,ADIRF,OVOL2,SNX5,NFATC2,PAX7,BNC1,ATP2B4,ACTL6B,ASXL3,TET1,CAMTA1,CCPG1,ADNP,ARID4A,CDK6,PHF2,TFRC,EPHA5,WWOX,MEF2A,DCAF6,SATB1,PSMB2,EYA1,GATAD2A,TRPV1,HOXB3,HOXB4,HOXB5,HOXB6,TFEB,MNAT1,ACTR2,L3MBTL4,HDAC1,RECQL5,TRIM24,PLCG2,SCMH1,PAXIP1,ZNF713,EGR2,RNF10,RYBP,AP3B1,PTPN2,NTRK1,KPNA6,TRIM44,EPCAM,ZNF425,MUC1,PTPN14,BCOR,AP3D1,ZNF41,CARM1,ZNF383,ZNF616,ZNF836,SMARCA2,*

			CUX1,SPI1,DNMT3B,FOXP2,PSPC1,GLIS1,SFMBT2,JAZF1,RBPMS,CHURC1,CREB5,MAP3K13,SMYD1,SNCA,BMPR1B,ZEB2,FOXJ3,HEY2,ZMYND11,KMT2D,CREM,KMT2C,MACC1,MITF,OPRD1,RPTOR,NFIX,BTK,MLLT1,CBFA2T2,IGF1,MLXIP,ATF7IP,PTEN,BMP7,MXI1,SOX5,CIR1,PCBP2,ZNF780A,ZNF780B,ZBTB20,LILRB4,PKD2,ZNF652,KCTD1,RIPPLY1,RCOR3,TADA2A,NCOA3,ZNF146,ZNF565,WNT7A,ZBED6,BRWD3,CRTC3,ARNTL2,KAT6A,ZKSCAN7,ZNF197,ZNF660,BMP6,TAF15,NFE2L1,WDR43,ZNF30,ATRX,IKZF1,PRMT2,ASCC1,SMO,RALY,TCF4,SOX30,TFE3,GRIN1,JADE1,RHOA,ROR1,RQCD1,TF,ONECUT2,CUL3,TFEC,STAT6,MLF1,RBBP8,CDK13,DUSP26,ZNF362,NACC2,SUPT4H1,ZNF354A,AGT,HDGFRP3,STAT2,ZMPSTE24,PBX1,MAP3K5,TCFL5,CREBBP,DLX1,GTF2IRD2,NCF1,SHC1,ZNF813,LBX2,PARP10,DPRX,SETDB2,DNAJB6,SP7,ADAM8,RPS6KA5,ZNF484,ZNF93,PRDM2,NOS1,ZNF44,MED15,RGS14,ACTN4,MC1R,TCF25,ZNF282,EDRF1,IL18,PPARA,ARID5B,SMARCAL1,NOTCH4,ZMYND8,ZNF366,CRX,PAX6,PRKCZ,ADORA2A,OTUD7B,NIPBL,YEATS4,PHF20,ZNF143,BRF1,TBL1X,FHL2,MBTD1,ATF6,ZBTB5,ZNF708,ETS1,MTF2,TAGLN3,NCOR2,PRKCQ,CSRNP1,HDAC2,IMPACT,SRCAP,POU3F3,NOTO,ZKSCAN5,ELF2,PHIP,PPP2CA,RPL23,ATXN1,SP100,ZNF347,ZNF415,TRIM29,ADCY1,SYK,CNOT1,WWTR1,GTF2H5,NFXL1,ZBTB38,BRCA2,ATF7,DVL2,MORC1,YBX1,TAF1,TICAM1,THAP3,HSF1,MAX,SAP130,EZH1,NRF1,NCOA2,QRICH1,ZP3,CHD6,RNF2,ZNF554,MYEF2,TRIM37,MET,ZNF461,CAMK2D,TIMELESS,BRD9,MALT1,SETD3,KDM2B,MLXIPL,PHB,EDA,ZNF555,CTCF,NR6A1,ATF6B,CREB1,RGMB,EBF4,ZNF511,BMP4,ABLIM3
GO:0071310	cellular response to organic substance	6.772802716229501e-11	*ENPP1,PRDX2,NRXN1,PRKCI,PDE4D,DNMT1,SLC9A1,ADCY8,PDE8A,IL31RA,RPS6KA2,PTGFR,CLDN18,CTDSPL2,RYR1,LATS2,NREP,PTGIS,TJP1,PRLR,PAGR1,ADCY7,NTRK3,CBL,ARNT,EGLN2,FLT3,STAT5B,GRM5,SAMHD1,FER,CASK,MAP2K5,LRP2,PIK3CD,HDGF,EZR,ROBO1,SLC26A6,HTR2B,ITGB6,RYR3,GOT1,TRAF6,ROBO2,DNAJA3,CDC6,PSMB7,PTPRK,ABCC8,HMGN3,NRIP1,DNAJB14,THRB,STXBP4,IL17RB,TCIRG1,PTPN11,HDAC6,ADAMTS3,IKBKB,ERBB4,BRD8,ESR1,PMEPA1,NTRK2,EP300,FNTA,PDGFB,CCND3,BPI,STK39,INSR,PIP4K2A,PAK1,LITAF,ESRRB,BDNF,AMIGO1,CHRM3,FUT8,UBR2,IL5RA,SNX6,ERN2,LSP1,AP3S1,DMD,SLC8A1,GSN,SATB2,FYN,ARNTL,NF1,PLCB1,MGMT,RXFP1,CHRDL1,BCR,CHI3L1,PIK3R2,CDK19,DOCK8,TMBIM6,PPP2CB,HDAC5,CMKLR1,ROR2,DCN,SLC39A14,CREBRF,PTPRT,COPS5,IPO5,RGS8,DEFA1B,DEFA3,PTGER2,STIP1,NEUROD1,RFFL,CCBE1,JAK2,CLDN1,TCF7,PDE2A,KLF15,LAMTOR3,CYFIP2,WNT11,NOX4,LCOR,CACNA1H,IL1RAPL1,CCDC62,ADAM23,PRKCD,SOX6,ACVR2A,RUNX2,CD4,TGFB1,SGK1,BTRC,PTPRN2,F2RL1,BCAS3,SLIT3,ZFYVE9,SLIT2,GABRB1,KANK1,MTDH,CHRM1,SMAD6,RXFP2,CLOCK,ZNF675,BMPER,DUSP22,NAIP,SBNO2,FGF10,SMYD3,IL1RAPL2,GLP2R,ADCY2,SLC16A1,SMURF2,EPHA4,RORA,PRKCA,CD6,TNFSF11,PPP3CA,NFYB,UFL1,CTNNB1,PARK2,SOD2,FCGR2B,HSPA6,SMARCC1,IGF1R,PPARG,AXIN1,IL18R1,IL1RL1,MSR1,CYBB,BRIP1,RYR2,LEPR,LEPROT,FGF1,NR4A3,NOL3,MYOCD,TRIM5,GLG1,CHEK2,PRDM16,HCK,SORBS1,CAPN2,FLT4,HDAC4,PAX2,SFRP1,FOXO3,SSH1,SYNCRIP,SMAD3,CUX2,ACKR2,GRIK5,IFNAR1,NEDD4,NRP2,ESRRG,TAB1,CDC73,APP,PDGFRA,YAP1,AMFR,RAB11FIP5,SESN1,TNFRSF19,ALPL,JAK1,LRP5,SLC9B2,GNG2,CHST11,DRD1,GLRA2,CHUK,FRS2,MMP2,ESR2,PRKD1,STAT1,ST18,HNF4A,EREG,ATF2,RAF1,CARD16,CASP1,PTPN1,ADAMTS12,BMPR2,BMPR1A,TSPAN12,COL16A1,PIK3R3,EPG5,FGF2,ADCY5,RORC,PTAFR,JARID2,DDX58,RARB,NCOA1,EEF1E1,CHRD,PTPRE,ATF3,SHOC2,SRSF5,CCDC3,IDE,KCNQ1,RFX2,WNT3,P4HB,CCL14,CCL15,PAFAH1B1,CNTFR,TRPA1,AKAP6,NR2C1,IRS4,GLI2,NEO1,WDTC1,DAB2,BLM,UBR1,LDLRAD4,WNK1,SIN3A,RUVBL2,CSF3R,IQGAP1,MAP3K7,ZNF423,SP1,ALK,SRA1,XDH,LTBP1,OVOL2,SNX5,ATP2B4,PACRG,PAK3,DYX1C1,MAPKAPK2,MAP1B,COL4A6,SCARB1,TFRC,EPHA5,WWOX,CACNA1A,GPC3,XRN1,PSMB2,BRINP1,SHPK,TRPV1,GHR,FLRT2,ACTR2,ACACA,ELAVL4,HDAC1,RECQL5,SMOC2,TRIM24,ADIPOR2,PLCG2,ROCK1,EPS8,EGR2,CAPN10,GPR35,ABCC1,PTPN2,INSRR,NTRK1,TRIM44,AXIN2,CARM1,HTR1D,SPI1,PTPRA,CTNNA1,ACAP2,LY86,BCAR3,CHURC1,RAB15,RAPGEF3,PDXP,PTK7,SNCA,BMPR1B,MAGI2,PNPT1,ZEB2,RC3H1,WNT7B,HMGCS2,KMT2D,SPPL2A,ADTRP,ABCA12,OPRD1,HIPK1,NUGGC,RPTOR,BTK,TROVE2,IGF1,STX8,DPYSL3,MAPK1,SPRED2,BMP7,SOX5,PRCP,LRRK2,GNA14,ZBTB20,CHMP5,BAG6,LILRB4,TMEM108,IL11RA,NSG2,PKD2,EFNA5,PDGFC,NCOA3,WNT7A,ZBED6,GLDC,GPR21,BMP6,ANO1,IL17RD,SOS1,TSHR,EXT1,PDCD1LG2,PNPLA3,PRMT2,RAPGEF2,SMO,CPT1A,UBR5,GRB14,RHOA,RQCD1,ONECUT2,CUL3,HSPD1,ITPR2,GABRB3,GSK3B,RDX,STAT6,EEF2K,BOK,SULF1,KRT8,AGT,PRKAA2,ADAR,FBN2,STAT2,BRINP3,HRH4,PTK2,PHEX,PDE4B,VEPH1,MAP3K5,CREBBP,DLX1,FMOD,PCSK6,SLC29A1,UGT1A1,SHC1,RPS6KB1,STC2,GPR173,CD44,RPS6KA5,NOS1,PLVAP,ACTN4,SCNN1B,ZFAND6,EPHB2,SLC1A1,XCR1,IL18,UBE2K,PPARA,ARID5B,CALCRL,SLC25A33,CPEB4,ZNF366,FBN1,PAX6,PRKCZ,ATP1A3,FAM20C,RABGEF1,CPEB1,TYK2,ASPN,ANGPT1,OSBPL8,ATF6,GCNT2,VWC2,MTF2,CCL22,NCOR2,PRKCQ,DIAPH1,HDAC2,HTR2C,IMPACT,SPINT2,PHIP,RPL23,SH3BP4,SP100,ADCY1,SYK,CNOT1,YBX1,PTPRJ,TAF1,TICAM1,TANK,UCN2,HSF1,MAX,SASH1,NCOA2,ADAMTS7,GBP5,QRICH1,CIB2,CAV2,UGGT1,AXL,CD109,DENND4C,CCR3,TIMELESS,MALT1,PFKP,TRA2B,MLXIPL,PHB,PTPRC,EDA,PBLD,SH3GL2,SPON2,TRAF3IP2,ATF6B,CREB1,RGMB,EDNRA,BMP4,CPNE1*
GO:00	transmembrane	7.23311520	*SLC39A11,ENPP1,SLC35E3,NRXN1,ASPH,PRKCI,SLCO3A1,PDE4D,SLC9A1,FAM155A,GRID2,CHERP,SLC35E1,SLC25A17,CACHD1,RYR1,NOS1AP,NOX5,DPP6,SLC*

55085	transport	1061069e-11	*14A2,PRKAG2,CLCN1,UTRN,RAB4B,ATP2B2,KCNQ5,GRM5,KCNIP4,SPNS2,LRP2,SLC38A11,ANK2,SLC9A9,CHD7,SLC26A6,HTR2B,RYR3,SLC24A2,ITGB1,SLC24A3,PSMB7,CNIH2,SLC22A8,CNGB1,FLVCR2,ABCC8,STXBP4,GABRA3,TCIRG1,PTPN11,KCNS3,NIPAL2,NETO2,CACNB2,SV2B,PEX14,CNNM1,KCNJ16,DPP10,CYB561A3,CACNA1B,STK39,INSR,ATP8B1,CAB39,ZDHHC13,SLC20A2,SLC4A10,CATSPER2,AMIGO1,KCNMA1,CHRM3,GRIK4,KCNRG,LCK,TMC2,TMEM144,SLC12A8,SPNS3,SLC6A16,RHCE,MICU3,DMD,SLC30A9,SLC8A1,JPH2,FYN,SLC16A6,SLC25A21,SLC5A6,SLC2A14,BCR,SHISA9,ANKH,CACNA1E,SLC4A5,KCNN3,CNNM2,SLC16A10,SLC43A2,BDKRB1,KCNIP1,SLC35A2,SLCO1A2,STIM1,NALCN,TMEM30A,SLC39A14,CATSPER3,FGF14,CLDN16,ANO6,KCNB2,OAZ2,NPSR1,ABCC2,SCN4A,TMC1,SLC1A4,SLC5A9,SLC9A7,KLF15,KCNC4,CLCA2,CYB561D1,CACNA1H,SLC35F1,TGFB1,KEL,JPH3,GRIK3,GABRR3,GABRB1,KCNJ3,CACNA1D,ABCC11,CACNA2D3,GRPEL2,ANO4,XPR1,ANO3,KCNH1,SLC17A7,KCNH7,CAPN3,SLC16A1,VMP1,ANO2,COX8A,TMEM63C,TRPM1,CYBB,KCNJ12,RYR2,KCNA6,NR4A3,NOL3,PER2,CACNA1C,SCN3B,SLC35E4,SORBS1,GRM1,ATP10D,ITPR1,WWP2,GRIK5,SLC16A2,SHANK1,NEDD4,SVOPL,SLC35F4,SLC4A4,SLC6A1,SEC61B,KCNK1,APP,NOX1,SFXN5,NLGN2,VDAC1,IBTK,SLC9B2,GRIA3,DRD1,GLRA2,PRKD1,SLC6A3,STRA6,FXYD2,FXYD6,SLC16A12,KCNG4,RASGRF2,NLGN1,FGF2,GRIK1,PTAFR,ABCA13,MRS2,SLC5A8,SLC9B1,GRIA2,SLC35D1,ATP6V0B,CLIC4,KCNQ1,SLC2A11,NLGN3,TRPA1,TRPM3,AKAP6,TRPC5,TRAM2,WNK1,RELN,ABCB1,KCNQ2,SLC22A3,SLC25A16,SLC47A1,SLC8A2,SLC9C1,SLC35A4,TPCN1,CLIC5,AHNAK,RGS7,ATP2B4,SLCO4A1,P2RX6,SCARB1,ATP6V0A2,KCNAB2,GABRG3,MEF2A,CACNA1A,GPC3,STOM,PSMB2,TRPV1,SLC38A6,PLCG2,CAPN10,GPR35,ABCC1,SLC39A10,ANK3,ATP13A3,KCND3,CACNG8,SLC9C2,NETO1,CHRNB4,MCU,TRDN,GABRA6,LRRC8C,LRRC8D,STAC,TRPC6,ABCB7,SLC41A2,ATP13A5,SNCA,CACNG2,PNPT1,OCA2,SLC39A9,CNGA3,SLC22A10,ABCA12,SLC4A8,IGF1,HTT,SLC7A14,SLC30A7,SCN8A,ATP6V1A,CNIH3,SLC44A1,TMCO1,PKD2,SHANK3,ANO1,ANO8,GABRR2,ABCG2,KCNC2,SLC6A14,BLOC1S3,CPT1A,PSEN2,GRIN1,GRIN3A,HSPD1,ITPR2,EPM2A,GABRB3,ABCG8,EMB,SCN9A,AGT,ZMPSTE24,PEX5L,PDE4B,COX5A,SLC25A51,TRPC4AP,TIMM44,SLC25A42,SLC29A1,SLC44A3,KCNJ15,RPS6KB1,SLC5A10,SLC5A3,GRID1,PKD1L1,SLC25A18,COX5B,NOS1,PEX7,ACTN4,SCNN1B,COX6A1,EPHB2,SLC1A1,XCR1,LRRC52,MFSD8,ABCD3,MCOLN1,SLC25A33,ATP1A3,GRIN2B,OSBPL8,SLC25A26,SLCO2B1,GRIA4,BRAF,DIAPH1,HTR2C,ATP8A1,TMEM163,UQCR10,SLC26A2,ANXA2,KCND2,ATP6V0A1,SLC18B1,GRIK2,SCN1A,SLC44A5,CACNG3,SLC10A1,ATP2B3,GPM6A,SLC13A3,SLC2A12,SLC5A4,KCNQ3,SESTD1,CAMK2D,KCNJ6,NEDD4L,PTPRC,GPR89A,SHISA6,EDNRA,SLC25A13*
GO:0051174	regulation of phosphorus metabolic process	8.175555337923619e-11	*ENPP1,LDB2,NRXN1,SLCO3A1,PDE4D,S1PR2,ADCY8,PDE8A,IL31RA,NRG3,LATS2,PRKAG2,PRLR,MVP,NTRK3,CBL,ARNT,FLT3,ENPP2,GRM5,PLCE1,FER,MAP2K5,KITLG,ROBO1,TOM1L1,HTR2B,TENM1,TRAF6,ITPKB,DNAJA3,VAV2,ABI1,HSP90AA1,CDC6,KNDC1,DSCAM,AKAP13,DAB1,PTPN11,MGAT5,HDAC6,ADORA1,EPHA1,IKBKB,ERBB4,MRE11A,PMEPA1,PTPRO,CDON,NTRK2,EP300,PDGFB,TNIK,CCND3,INSR,PRKAR1A,PIP4K2A,CAB39,PAK1,MAP3K4,BDNF,ENTPD5,ECT2,SNX6,ERN2,DMD,SLC8A1,RIT2,CASS4,FYN,NF1,SMG7,LMTK2,CHI3L1,TTN,PAQR3,PIK3R2,NRG1,SH2D3C,BDKRB1,BDKRB2,MAP2K1,FNIP1,ROR2,PTPRT,PPP1R42,IPO5,MAD2L2,CAPRIN2,CCNYL1,GNAQ,HERC5,JAK2,FBXW7,ZFYVE28,LAMTOR3,WNT11,NOX4,PRKCD,TAB2,ACVR2A,CD4,PPM1E,TGFB1,NSD1,PIBF1,SLIT2,CORO1C,SMAD6,ZNF675,BMPER,ANKRD54,TNFRSF10B,DUSP22,DOCK3,FGF10,SMYD3,EPHA4,CD6,TNFSF11,SMG6,PIP5KL1,PARK2,IGF1R,PPARG,AXIN1,PRKAR1B,LEPR,FGF1,PRKAR2A,MYOCD,AJUBA,CHEK2,SPDYA,CAPN2,CSPG4,NF2,FLT4,HDAC4,SFRP1,PPP6R2,SMAD3,SLC4A4,TAB1,APP,PDGFRA,CCNI2,HEG1,BORA,IBTK,LRP5,MTCP1,RCAN1,SPTBN4,DRD1,S100A12,PRKD1,EREG,CCNY,ATF2,RAF1,PTPN1,BMPR2,BMPR1A,BTBD10,PIK3R3,CDK12,SPAG9,FGF2,PPM1F,NEDD9,SEMA4D,PTAFR,ADCYAP1R1,DVL3,EIF4G1,EPHB3,CKS1B,DEPTOR,TSG101,TERF2IP,PTPN13,TRPC5,DAB2,BLM,CACUL1,LDLRAD4,SMG5,DLG3,WNK1,RELN,NEK10,IQGAP1,MAP3K7,ALK,SLC8A2,XDH,ATP2B4,CAMTA1,ADNP,SCARB1,TFRC,UVRAG,EPHA5,GNB3,GHR,MNAT1,SNX9,PLCG2,ROCK1,HIPK3,PTPN2,INSRR,NTRK1,SLC39A10,EPHB1,EPHA10,AXIN2,PARD3,PIFO,EPHA7,ATG14,BCAR3,STK38,RBPMS,MAP3K13,RAPGEF3,SNCA,BMPR1B,MAGI2,PRKAG1,ADTRP,OPRD1,RPTOR,MLLT1,IGF1,HTT,MAPK1,PTEN,SPRED2,BMP7,LRRK2,UNC119,ZBTB20,LILRB4,PKD2,EFNA5,TADA2A,PDGFC,PKIB,MTMR3,PPP2R3C,PLCL2,BMP6,RAPGEF2,VRK3,PRKAR2B,RHOA,ROR1,RQCD1,TF,EPM2A,GSK3B,DNAJC3,EEF2K,PPP1R16A,PTPRB,AGT,CCNJL,PRKAA2,ADAR,STAT2,PTK2,TEC,MAP3K5,NCF1,TAOK2,SHC1,CD44,ADAM8,RPS6KA5,HUS1,NOS1,GCKR,RGS14,STK4,EPHB2,SLC1A1,IL18,UBE2K,DLC1,PPARA,MOB3B,PAX6,PRKCZ,FAM20C,ADORA2A,RABGEF1,CCNG2,ANGPT1,LRRK1,OSBPL8,CDK5RAP1,CNTN1,BRAF,HDAC2,HTR2C,IMPACT,PPP1R14A,CD300A,PHIP,PPP2CA,ANGPT4,MADD,SYK,WWTR1,DVL2,PTPRJ,HSF1,SASH1,VAV3,MOB3A,GNA12,SH2D3A,AKTIP,INPP5F,AXL,CD109,MET,MALT1,MLXIPL,PHB,PTPRC,SH3GL2,TYRO3,TNXB,BMP4*
GO:0040012	regulation of locomotion	8.707747052340582e-11	*LDB2,MAP4K4,S1PR2,CLASP2,SEMA3A,DOCK1,NRG3,SEMA3D,PHACTR1,MAPRE2,TJP1,UNC5C,NTRK3,SEMA5A,ENPP2,GRM5,FER,MARVELD3,MAP2K5,KITLG,PTPRR,PIK3CD,ROBO1,LMNA,ROBO2,ULK4,ITGB1,SRGAP3,MCC,DSCAM,SRGAP2B,PTPRK,ABCC8,LAMA2,DACH1,MGAT5,DOCK10,HDAC6,ADORA1,EPHA1,ERBB4,ZNF609,PTPRO,PDGFB,STK39,INSR,UNC5D,LAMA3,PAK1,TBR1,PTPRU,SLC*

			8A1,CASS4,ADAMTS9,NF1,PLCB1,RTN4,DPEP1,APOD,BCR,NDRG4,NTN1,NRG1,BDKRB1,DOCK8,HDAC5,NTNG1,CMKLR1,ROR2,DCN,CDH13,PTPRT,CD99,RCC2,ANO6,KIF2A,RFFL,CCBE1,JAK2,FBXW7,CLDN1,WNT11,CAMK2B,SEMA5B,TGFB1,SGK1,MMP28,F2RL1,BCAS3,SLIT2,CORO1C,RRAS2,KANK1,LAMC2,TTBK2,CHRM1,BMPER,DAPK2,DUSP22,YTHDF1,FGF10,SMURF2,EPHA4,PRKCA,PPP3CA,PIP5KL1,SOD2,IGF1R,PPARG,DLG5,FGF1,NR4A3,MYOCD,AJUBA,NF2,FLT4,HDAC4,SFRP1,FOXO3,SMAD3,PTPRM,NRP2,DOCK4,NAV3,APP,GSX2,PDGFRA,SLK,PTPRG,DRD1,MMP2,SYNE2,PRKD1,SEMA6D,RAF1,BMPR2,CAMK1D,BMPR1A,CTNNA2,PIK3R3,SPAG9,RAB11A,FGF2,PPM1F,NEDD9,SEMA4D,MCTP1,ELP3,PLXNA2,PTAFR,DDX58,CHRD,VCL,LAMB1,CLIC4,WNT3,RHOJ,RUFY3,DAB2,LDLRAD4,MYSM1,WNK1,RELN,SP1,MACF1,CLASP1,PRKG1,ATP2B4,PAK3,SSH2,SCARB1,CDK6,FLRT2,SMOC2,PLCG2,ROCK1,PTPN2,PKN2,TMIGD1,HTR1D,SPI1,MCU,CTNNA1,SH3BP1,MST1,SNCA,MAGI2,ADTRP,MITF,IGF1,DPYSL3,MAPK1,PTEN,BMP7,PRCP,LRRK2,OSGIN1,PDGFC,AMOTL1,WNT7A,MYCBP2,GPI,SH3RF2,TSHR,RAPGEF2,SMO,RHOA,TF,ONECUT2,RDX,ACTA2,FAM49B,SULF1,AGT,PTK2,TPPP2,RPS6KB1,GPR173,ADAM8,CLDN4,CXCL17,PLVAP,ACTN4,STK4,EPHB2,TMEFF2,DLC1,EPB41L4B,GPSM3,ZMYND8,PAX6,ADORA2A,RABGEF1,PHLDB2,NIPBL,ANGPT1,OSBPL8,GCNT2,ETS1,FBXO31,BRAF,DIAPH1,ATP8A1,SPINT2,CD300A,ANGPT4,CAMSAP3,SP100,SRGAP2,NUMB,SLIT1,FRMD5,PTPRJ,SASH1,ZP3,GNA12,INPP5F,MET,SEMA3C,PTPRC,BMP4
GO:0099536	synaptic signaling	9.289218374991936e-11	NRXN1,S1PR2,PDE7B,ADCY8,GRID2,RPS6KA2,NRG3,NLGN4X,GRM8,GRM5,CASK,NRXN3,HTR2B,SLC24A2,ITGB1,CNIH2,PCDH17,DLG2,ERC1,DGKI,ERC2,GABRA3,LAMA2,CACNB2,SV2B,ADORA1,ABR,GRM7,NTRK2,CACNA1B,RIMS1,CDH8,USP46,PTPRD,SLC4A10,BDNF,CHRM3,GRIK4,RIT2,FYN,NF1,PLCB1,SNCB,BCR,SHISA9,CACNA1E,NRG1,DAGLA,CNTN4,NTNG1,ROR2,ZDHHC3,LIN7A,SYN2,PCDHB16,EXOC4,RGS8,GNAQ,JAK2,SYN3,CLSTN2,SYT1,LZTS1,SLC1A4,KCNC4,IL1RAPL1,CAMK2B,PDLIM4,JPH3,DLGAP1,PTPRN2,GRIK3,GABRR3,CADPS2,GABRB1,GPR176,DISC1,CHRM1,PLCB4,SLC17A7,YTHDF1,STXBP5,EPHA4,CNR1,PPP3CA,CTNNB1,PARK2,PRKAR1B,RGS10,CPNE6,GRM1,RIMS4,DCC,SPG11,DGKB,CUX2,GRIK5,SHANK1,SLC6A1,APP,NLGN2,DRD1,GLRA2,SLC6A3,SHANK2,CELF4,RASGRF2,NLGN1,LRRC4C,GRIK1,MCTP1,SORCS2,PRKCG,GRIA2,FBXL20,NLGN3,PAFAH1B1,BTBD9,RELN,KCNQ2,SLC8A2,STAU1,P2RX6,ATAD1,ADNP,MAP1B,SORCS3,GABRG3,SYT12,CACNA1A,ADRBK1,TRPV1,ELAVL4,EGR2,RNF10,NTRK1,MAP1A,PPP1R9A,UNC13A,EPHB1,CACNG8,NETO1,HTR1D,CHRNB4,PTPRA,GABRA6,FCHSD2,SNCA,CACNG2,GRM4,SYT9,PTPRS,KCTD13,SLC4A8,MAPK1,PTEN,LRRK2,UNC119,TMEM108,SHANK3,WNT7A,SNAP23,CEP89,PLCL2,EXT1,GABRR2,RAPGEF2,PRKAR2B,STXBP5L,GRIN1,SYNGAP1,GRIN3A,GABRB3,GSK3B,SYN1,MCTP2,AGT,SYT7,BLOC1S6,HRH4,RIMS2,SLC29A1,CPLX2,PACSIN2,GRID1,NOS1,RGS14,EPHB2,SLC1A1,DTNA,PRKCZ,ADORA2A,GRIN2B,CADPS,BRAF,HTR2C,TNR,KCND2,ADCY1,GRIK2,CACNG3,DLGAP2,KCNQ3,RPH3A,SHISA6
GO:0019220	regulation of phosphate metabolic process	1.1115695246836619e-10	ENPP1,LDB2,NRXN1,SLCO3A1,PDE4D,S1PR2,ADCY8,PDE8A,IL31RA,NRG3,LATS2,PRKAG2,PRLR,MVP,NTRK3,CBL,ARNT,FLT3,ENPP2,GRM5,PLCE1,FER,MAP2K5,KITLG,ROBO1,TOM1L1,HTR2B,TENM1,TRAF6,ITPKB,DNAJA3,VAV2,ABI1,HSP90AA1,CDC6,KNDC1,DSCAM,AKAP13,DAB1,PTPN11,MGAT5,HDAC6,ADORA1,EPHA1,IKBKB,ERBB4,MRE11A,PMEPA1,PTPRO,CDON,NTRK2,EP300,PDGFB,TNIK,CCND3,INSR,PRKAR1A,PIP4K2A,CAB39,PAK1,MAP3K4,BDNF,ENTPD5,ECT2,SNX6,ERN2,DMD,SLC8A1,RIT2,CASS4,FYN,NF1,SMG7,LMTK2,CHI3L1,TTN,PAQR3,PIK3R2,NRG1,SH2D3C,BDKRB1,BDKRB2,MAP2K1,FNIP1,ROR2,PTPRT,PPP1R42,IPO5,MAD2L2,CAPRIN2,CCNYL1,GNAQ,HERC5,JAK2,FBXW7,ZFYVE28,LAMTOR3,WNT11,NOX4,PRKCD,TAB2,ACVR2A,CD4,PPM1E,TGFB1,NSD1,PIBF1,SLIT2,CORO1C,SMAD6,ZNF675,BMPER,ANKRD54,TNFRSF10B,DUSP22,DOCK3,FGF10,SMYD3,EPHA4,CD6,TNFSF11,SMG6,PIP5KL1,PARK2,IGF1R,PPARG,AXIN1,PRKAR1B,LEPR,FGF1,PRKAR2A,MYOCD,AJUBA,CHEK2,SPDYA,CAPN2,CSPG4,NF2,FLT4,HDAC4,SFRP1,PPP6R2,SMAD3,SLC4A4,TAB1,APP,PDGFRA,CCNI2,HEG1,BORA,IBTK,LRP5,MTCP1,RCAN1,SPTBN4,DRD1,S100A12,PRKD1,EREG,CCNY,ATF2,RAF1,PTPN1,BMPR2,BMPR1A,BTBD10,PIK3R3,CDK12,SPAG9,FGF2,PPM1F,NEDD9,SEMA4D,PTAFR,ADCYAP1R1,DVL3,EIF4G1,EPHB3,CKS1B,DEPTOR,TSG101,TERF2IP,PTPN13,TRPC5,DAB2,BLM,CACUL1,LDLRAD4,SMG5,DLG3,WNK1,RELN,NEK10,IQGAP1,MAP3K7,ALK,SLC8A2,XDH,ATP2B4,CAMTA1,ADNP,SCARB1,TFRC,UVRAG,EPHA5,GNB3,GHR,MNAT1,SNX9,PLCG2,ROCK1,HIPK3,PTPN2,INSRR,NTRK1,SLC39A10,EPHB1,EPHA10,AXIN2,PARD3,PIFO,EPHA7,ATG14,BCAR3,STK38,RBPMS,MAP3K13,RAPGEF3,SNCA,BMPR1B,MAGI2,PRKAG1,ADTRP,OPRD1,RPTOR,MLLT1,IGF1,HTT,MAPK1,PTEN,SPRED2,BMP7,LRRK2,UNC119,ZBTB20,LILRB4,PKD2,EFNA5,TADA2A,PDGFC,PKIB,MTMR3,PPP2R3C,PLCL2,BMP6,RAPGEF2,VRK3,PRKAR2B,RHOA,ROR1,RQCD1,TF,EPM2A,GSK3B,DNAJC3,EEF2K,PPP1R16A,PTPRB,AGT,CCNJL,PRKAA2,ADAR,STAT2,PTK2,TEC,MAP3K5,NCF1,TAOK2,SHC1,CD44,ADAM8,RPS6KA5,HUS1,NOS1,GCKR,RGS14,STK4,EPHB2,SLC1A1,IL18,UBE2K,DLC1,PPARA,MOB3B,PAX6,PRKCZ,ADORA2A,RABGEF1,CCNG2,ANGPT1,LRRK1,OSBPL8,CDK5RAP1,CNTN1,BRAF,HDAC2,HTR2C,IMPACT,PPP1R14A,CD300A,PHIP,PPP2CA,ANGPT4,MADD,SYK,WWTR1,DVL2,PTPRJ,HSF1,SASH1,VAV3,MOB3A,GNA12,SH2D3A,AKTIP,INPP5F,AXL,CD109,MET,MALT1,MLXIPL,PHB,PTPRC,SH3GL2,TYRO3,TNXB,BMP4
GO:00	regulation of	1.25428112	POLDIP3,ENPP1,PRDX2,LDB2,ASPH,PRMT3,PRKCI,SLCO3A1,DNMT1,HLX,SLC9

31326	cellular biosynthetic process	19437656e-10	*A1,PBX3,ADCY8,MED13L,TRPS1,CBFB,PDE8A,ZNF823,IL31RA,PRDM12,MED26,WWC1,ASH1L,NOS1AP,SCAF8,STOX2,PTGIS,WWC3,PAGR1,HIVEP3,FTO,NPAS3,ARNT,LRRFIP2,STAT5B,TOX,GRM5,ZNF566,FER,CASK,MAP2K5,MAPK10,ZNF536,SP3,HDGF,EIF4G3,EZR,IKZF2,MALRD1,CHD7,HTR2B,MECOM,TACC1,TENM1,TRAF6,DNAJA3,NHLH1,HSP90AA1,CDC6,PSMB7,TSC22D3,ZC4H2,CCDC22,CDAN1,GFI1B,PTPRK,RUNX1,ERC1,HMGN3,NRIP1,THRB,EFCAB7,ITGB3BP,DACH1,ZNF569,CCT2,ORC2,HDAC6,SERTAD2,DDB1,MYT1,IKBKB,PEX14,ERBB4,ZNF609,BRD8,KAT6B,HIF3A,SNIP1,ESR1,MIER1,PCBP3,MAML3,CDON,TNRC6A,EP300,CELA1,TENM2,ZNF76,RNF220,ZNF471,PDGFB,CCND3,TOB2,ZNF19,ZNF23,BRMS1,ZNF605,SCML4,HNF4G,INSR,RERE,PRKAR1A,FUBP1,ATP8B1,H2AFY2,TCF7L2,JMJD1C,ZNF443,ZNF490,ZNF564,ZNF709,ZNF799,LITAF,FBXW11,ESRRB,MAP3K4,BASP1,TBR1,SAMD4A,TFAP2A,CDK14,FANCA,PEG3,ZIM2,TGIF2,TGIF2-C20ORF24,MEIS1,VRTN,TRIM13,MDM4,ZNF148,MTA3,SNX6,TFDP2,PTCD3,ERN2,C1D,AFF3,DMD,CENPF,SLC30A9,TOX3,PDS5A,LARP4B,USP13,KAT7,ZNF667,RBM14,RBM4,MED12L,SATB2,RIT2,HIRA,FYN,MKRN2,ARNTL,PLCB1,ARID4B,RWDD3,BRPF1,PIK3R2,NRG1,UBP1,MAP2K1,MDFI,FNIP1,VAX2,TMBIM6,VGLL4,PPP2CB,PPP3R1,HDAC5,CSRNP3,ZNF692,NFIA,RNF4,JDP2,CMKLR1,ROR2,DCN,PCBD2,CDH13,CREBRF,SOX13,COPS5,MAD2L2,TLE6,CAPRIN2,ZNF418,PHF20L1,TERF2,ZNF286A,FOXN3,NEUROD1,USP22,JAK2,TRAPPC9,FBXW7,SKAP1,SMC3,UIMC1,ITCH,MLIP,BCL11B,PKNOX1,MLLT3,TSHZ2,TCF7,PDE2A,KLF15,TBX15,ANXA4,WNT11,MTA1,KLF8,NOX4,LCOR,RPRD1B,QKI,CCDC62,PRKCD,SOX6,ACVR2A,RUNX2,CD4,TGFB1,BANP,SGK1,NSD1,IGSF1,PIBF1,ZHX2,PKNOX2,ASCC2,BTRC,NFATC3,F2RL1,BCAS3,CDYL2,CC2D1B,TP73,SAP18,ZBTB22,ILF2,MTDH,FANK1,MYT1L,SMAD6,BNC2,ZNF398,CLOCK,TCF12,ZNF675,ETV6,TFAP2D,BCL3,SND1,DUSP22,HNRNPC,TRRAP,SBNO2,YTHDF1,FGF10,CIZ1,SMYD3,LOXL3,CAPN3,LUM,SMURF2,RORA,HIVEP2,AUTS2,TNFSF11,SMG6,PPP3CA,NFYB,MAGEA4,KLF12,CAMK4,GATAD2B,UFL1,TRAK1,CTNNB1,PARK2,SOD2,DACH2,METTL13,SMARCC1,KLF17,IGF1R,PPARG,NGRN,AXIN1,IL18R1,CIPC,MTF1,CBX5,ANKRD17,BRIP1,LRPPRC,SREBF2,CDK11A,CDK11B,LEPR,FGF1,NPAT,NR4A3,FOXK2,ESCO1,MYOCD,TRIM5,PER2,AJUBA,ZNF626,ZNF737,CHEK2,SUPT3H,PRDM16,PPP1CB,HCK,SORBS1,RAB3GAP2,CAPN2,TRIM8,DIO2,BRMS1L,ZBTB8A,ZBTB8B,CTDP1,BAZ1B,MEF2B,HDAC4,PAX2,PHF5A,SECISBP2L,SFRP1,MED13,ZNF395,FOXO3,NFIB,SP4,SYNCRIP,SMAD3,CUX2,WWP2,ARNT2,SBNO1,KRBOX1,ZNF662,ZNF777,EBF3,RNF168,CASZ1,DCP1B,MIER3,NEDD4,ESRRG,HOXD3,HOXD4,ZNF114,KTI12,NFATC1,CDC73,APP,SSBP3,GSX2,RBM8A,YAP1,TEN1,NVL,LRP5,POLR3G,ZNF787,SOX2,SETD2,TEAD1,PRICKLE1,ZNF653,ZNF521,ARID3A,ZNF761,CHUK,ERLIN1,SFMBT1,ZNF584,ESR2,S100A12,PRKD1,STAT1,ST18,ETV5,RHOXF2B,TAF3,PLAGL1,HNF4A,ZBTB7C,TASP1,EREG,ATF2,POU2F2,TCF3,ZNF730,RAF1,CELF4,ZNF766,CARD16,BMPR2,CAMK1D,BMPR1A,ZKSCAN1,IKZF4,CDK12,CAND2,DENND4A,MYB,FGF2,ZNF71,POU6F2,BACH1,MXD3,PPM1F,TICRR,GLI4,ZFP41,BEND5,SEMA4D,NFX1,RORC,ELP3,PTAFR,ADCYAP1R1,SP140,SP140L,RHOXF2,JARID2,DDX58,BRDT,PHC2,RARB,SPEN,SIN3B,NCOA1,EHMT1,LMO7,DVL3,EIF2B5,EIF4G1,TCF20,ATF3,CKS1B,PAWR,EBF2,AGO3,MAML2,TSG101,TERF2IP,CRYM,RFX2,ZNF322,SUFU,MAGEA11,PRDM15,ZNF670,ZNF695,CCDC169-SOHLH2,SOHLH2,DDAH1,ZNF354C,TCEA3,PADI6,ZNF704,NR2C1,GLI2,TNKS,WBP2NL,ERCC1,TNRC6B,GLIS3,WDTC1,ZNF664,DAB2,BLM,PKHD1,MYSM1,SETD5,SMG5,RELN,SIN3A,RUVBL2,COMMD6,GMEB1,PELI1,MAP3K7,ZNF423,SP1,TRIM22,ALK,TEAD4,HOXC13,APBB3,SLC35A4,SRA1,UBE2V1,ADIRF,OVOL2,SNX5,NFATC2,RBBP6,PAX7,BNC1,ATP2B4,ACTL6B,ASXL3,PAK3,TET1,CAMTA1,CCPG1,ADNP,ARID4A,SCARB1,CDK6,PHF2,CELF1,TFRC,EPHA5,WWOX,MEF2A,DCAF6,XRN1,SATB1,PSMB2,EYA1,GATAD2A,DIS3L2,TRPV1,HOXB3,HOXB4,HOXB5,HOXB6,TFEB,MNAT1,ACTR2,L3MBTL4,ELAVL4,HDAC1,RECQL5,SMOC2,TRIM24,PLCG2,ROCK1,SCMH1,PAXIP1,ZNF713,EGR2,RNF10,RYBP,AP3B1,PTPN2,NTRK1,KPNA6,TRIM44,EPCAM,ZNF425,GNL3L,MUC1,PTPN14,BCOR,AP3D1,ZNF41,CARM1,ZNF383,ZNF616,ZNF836,SMARCA2,CUX1,SPI1,DNMT3B,FOXP2,PSPC1,GLIS1,SFMBT2,EIF3H,BCAR3,JAZF1,ABCB7,ACOT8,RBPMS,CHURC1,CREB5,MAP3K13,SMYD1,SNCA,BMPR1B,PRIM2,PNPT1,ZEB2,FOXJ3,HEY2,RC3H1,EIF3E,ZMYND11,KMT2D,CREM,KMT2C,PLD1,MACC1,MITF,OPRD1,NUGGC,RPTOR,NFIX,BTK,KCTD13,MLLT1,CBFA2T2,IGF1,MLXIP,ATF7IP,MAPK1,PTEN,BMP7,ATXN2,MXI1,PUM1,SOX5,CIR1,PCBP2,ZNF780A,ZNF780B,TMEM59,ZBTB20,RFC3,LILRB4,PKD2,ZNF652,KCTD1,RIPPLY1,RCOR3,TADA2A,DAZL,NCOA3,PKIB,ZNF146,ZNF565,WNT7A,ZBED6,BRWD3,LPGAT1,CRTC3,DNAJC1,ARNTL2,ELOVL5,KAT6A,MTIF2,ZKSCAN7,ZNF197,ZNF660,BMP6,TAF15,NFE2L1,WDR43,ZNF30,ATRX,DPH6,IKZF1,PRMT2,RAPGEF2,ASCC1,SMO,RALY,TCF4,SOX30,TFE3,GRIN1,JADE1,RHOA,ROR1,RQCD1,TF,ONECUT2,CUL3,TFEC,GSK3B,PRR16,DNAJC3,STAT6,MLF1,RBBP8,NECAB2,CDK13,DUSP26,ZNF362,NACC2,SUPT4H1,ZNF354A,AGT,HDGFRP3,METTL16,STAT2,ZMPSTE24,PBX1,EXOSC3,MAP3K5,TCFL5,CREBBP,DLX1,GTF2IRD2,NCF1,SHC1,ZNF813,LBX2,PARP10,RPS6KB1,STC2,DPRX,PRG3,SETDB2,DNAJB6,SP7,LARP4,ADAM8,RPS6KA5,ZNF484,ZNF93,PRDM2,NOS1,ZNF44,MED15,RGS14,ACTN4,MC1R,TCF25,ZNF282,EDRF1,IL18,DIS3,PPARA,ARID5B,SMARCAL1,CPEB4,NOTCH4,ZMYND8,ZNF366,CRX,PAX6,PRKCZ,ADORA2A,CPEB1,OTUD7B,NIPBL,YEATS4,PHF20,ZNF143,BRF1,TBL1X,FHL2,MBTD1,ATF6,ZBTB5,ZNF708,IGF2BP3,ETS1,PPP1CA,MTF2,TAGLN3,CDK5RAP1,NCOR2,PRKCQ,CSRNP1,HDAC2,HT*

			R2C,IMPACT,SRCAP,POU3F3,NOTO,ZKSCAN5,ELF2,PHIP,PPP2CA,RPL23,RFC5,ATXN1,SP100,ZNF347,ZNF415,TRIM29,PARN,DONSON,ADCY1,SYK,CNOT1,WWTR1,GTF2H5,NFXL1,ZBTB38,BRCA2,ATF7,DVL2,MORC1,MTRF1,YBX1,TAF1,EIF4E3,TICAM1,THAP3,HSF1,MAX,SAP130,DHFR,EZH1,ETF1,NRF1,NCOA2,WDR18,EEFSEC,QRICH1,ZP3,CHD6,RNF2,ZNF554,MYEF2,TRIM37,MET,ZNF461,CAMK2D,TIMELESS,BRD9,MALT1,SETD3,KDM2B,MLXIPL,CCT3,PHB,EDA,ZNF555,CTCF,NR6A1,ATF6B,CREB1,RGMB,TRAP1,EBF4,ZNF511,BMP4,ABLIM3
GO:0033674	positive regulation of kinase activity	1.3909236799501242e-10	*NRXN1,ADCY8,NRG3,PRKAG2,PRLR,NTRK3,FLT3,GRM5,MAP2K5,KITLG,ROBO1,TOM1L1,HTR2B,TENM1,TRAF6,VAV2,ABI1,HSP90AA1,CDC6,AKAP13,DAB1,ADORA1,EPHA1,ERBB4,MRE11A,NTRK2,PDGFB,CCND3,INSR,CAB39,PAK1,MAP3K4,ECT2,ERN2,CASS4,LMTK2,CHI3L1,NRG1,MAP2K1,ROR2,CCNYL1,JAK2,FBXW7,LAMTOR3,WNT11,NOX4,PRKCD,TAB2,CD4,TGFB1,PIBF1,TNFRSF10B,DOCK3,EPHA4,TNFSF11,IGF1R,AXIN1,FGF1,AJUBA,SPDYA,FLT4,TAB1,PDGFRA,BORA,MTCP1,S100A12,PRKD1,EREG,CCNY,PTPN1,BMPR2,BMPR1A,FGF2,NEDD9,DVL3,EPHB3,CKS1B,CACUL1,DLG3,WNK1,RELN,NEK10,IQGAP1,MAP3K7,ALK,SLC8A2,ATP2B4,ADNP,EPHA5,GHR,MNAT1,SNX9,INSRR,NTRK1,EPHB1,EPHA10,AXIN2,PIFO,EPHA7,ATG14,MAP3K13,SNCA,BMPR1B,PRKAG1,RPTOR,IGF1,LRRK2,UNC119,PKD2,EFNA5,PDGFC,PPP2R3C,RAPGEF2,RHOA,ROR1,AGT,PTK2,MAP3K5,NCF1,TAOK2,CD44,ADAM8,STK4,EPHB2,SLC1A1,IL18,MOB3B,PRKCZ,ANGPT1,OSBPL8,CD300A,PPP2CA,ANGPT4,MADD,SYK,DVL2,SASH1,VAV3,MOB3A,AXL,MET,MALT1,PHB,PTPRC,TYRO3*
GO:0099537	trans-synaptic signaling	1.5451618668284143e-10	*NRXN1,S1PR2,PDE7B,ADCY8,GRID2,RPS6KA2,NRG3,NLGN4X,GRM8,GRM5,CASK,NRXN3,HTR2B,SLC24A2,ITGB1,CNIH2,PCDH17,DLG2,ERC1,DGKI,ERC2,GABRA3,LAMA2,CACNB2,SV2B,ADORA1,ABR,GRM7,NTRK2,CACNA1B,RIMS1,CDH8,USP46,PTPRD,SLC4A10,BDNF,CHRM3,GRIK4,RIT2,FYN,NF1,PLCB1,SNCB,BCR,SHISA9,CACNA1E,DAGLA,CNTN4,NTNG1,ROR2,ZDHHC3,LIN7A,SYN2,PCDHB16,EXOC4,JAK2,SYN3,CLSTN2,SYT1,LZTS1,SLC1A4,KCNC4,IL1RAPL1,CAMK2B,PDLIM4,JPH3,DLGAP1,PTPRN2,GRIK3,GABRR3,CADPS2,GABRB1,GPR176,DISC1,CHRM1,PLCB4,SLC17A7,YTHDF1,STXBP5,EPHA4,CNR1,PPP3CA,CTNNB1,PARK2,PRKAR1B,CPNE6,GRM1,RIMS4,DCC,SPG11,DGKB,CUX2,GRIK5,SHANK1,SLC6A1,APP,NLGN2,DRD1,GLRA2,SLC6A3,SHANK2,CELF4,RASGRF2,NLGN1,LRRC4C,GRIK1,MCTP1,SORCS2,PRKCG,GRIA2,FBXL20,NLGN3,PAFAH1B1,BTBD9,RELN,KCNQ2,SLC8A2,STAU1,P2RX6,ATAD1,ADNP,MAP1B,SORCS3,GABRG3,SYT12,CACNA1A,TRPV1,ELAVL4,EGR2,NTRK1,MAP1A,PPP1R9A,UNC13A,EPHB1,CACNG8,NETO1,HTR1D,CHRNB4,PTPRA,GABRA6,FCHSD2,SNCA,CACNG2,GRM4,SYT9,PTPRS,KCTD13,SLC4A8,MAPK1,PTEN,LRRK2,UNC119,TMEM108,SHANK3,WNT7A,SNAP23,CEP89,PLCL2,EXT1,GABRR2,RAPGEF2,PRKAR2B,STXBP5L,GRIN1,SYNGAP1,GRIN3A,GABRB3,GSK3B,SYN1,MCTP2,AGT,SYT7,BLOC1S6,HRH4,RIMS2,SLC29A1,CPLX2,PACSIN2,GRID1,NOS1,RGS14,EPHB2,SLC1A1,DTNA,PRKCZ,ADORA2A,GRIN2B,CADPS,BRAF,HTR2C,TNR,KCND2,ADCY1,GRIK2,CACNG3,DLGAP2,KCNQ3,RPH3A,SHISA6*
GO:0009889	regulation of biosynthetic process	1.7707255364732847e-10	*POLDIP3,ENPP1,PRDX2,LDB2,ASPH,PRMT3,PRKCI,SLCO3A1,DNMT1,HLX,SLC9A1,PBX3,ADCY8,MED13L,TRPS1,CBFB,PDE8A,ZNF823,IL31RA,PRDM12,MED26,WWC1,ASH1L,NOS1AP,SCAF8,STOX2,PTGIS,WWC3,PAGR1,HIVEP3,FTO,NPAS3,ARNT,LRRFIP2,STAT5B,TOX,GRM5,ZNF566,FER,CASK,MAP2K5,MAPK10,ZNF536,SP3,HDGF,EIF4G3,EZR,IKZF2,MALRD1,CHD7,HTR2B,MECOM,TACC1,TENM1,TRAF6,DNAJA3,NHLH1,HSP90AA1,CDC6,PSMB7,TSC22D3,ZC4H2,CCDC22,CDAN1,GFI1B,PTPRK,RUNX1,ERC1,HMGN3,NRIP1,THRB,EFCAB7,ITGB3BP,DACH1,ZNF569,CCT2,ORC2,HDAC6,SERTAD2,DDB1,MYT1,IKBKB,PEX14,ERBB4,ZNF609,BRD8,KAT6B,HIF3A,SNIP1,ESR1,MIER1,PCBP3,MAML3,CDON,TNRC6A,EP300,CELA1,TENM2,ZNF76,RNF220,ZNF471,PDGFB,CCND3,TOB2,ZNF19,ZNF23,BRMS1,ZNF605,SCML4,HNF4G,INSR,RERE,PRKAR1A,FUBP1,ATP8B1,H2AFY2,TCF7L2,JMJD1C,ZNF443,ZNF490,ZNF564,ZNF709,ZNF799,LITAF,FBXW11,ESRRB,MAP3K4,BASP1,TBR1,SAMD4A,TFAP2A,CDK14,FANCA,PEG3,ZIM2,TGIF2,TGIF2-C20ORF24,MEIS1,VRTN,TRIM13,MDM4,ZNF148,MTA3,SNX6,TFDP2,PTCD3,ERN2,C1D,AFF3,DMD,CENPF,SLC30A9,TOX3,PDS5A,LARP4B,USP13,KAT7,ZNF667,RBM14,RBM4,MED12L,SATB2,RIT2,HIRA,FYN,MKRN2,ARNTL,PLCB1,ARID4B,RWDD3,BRPF1,PIK3R2,NRG1,UBP1,MAP2K1,MDFI,FNIP1,VAX2,TMBIM6,VGLL4,PPP2CB,PPP3R1,HDAC5,CSRNP3,ZNF692,NFIA,RNF4,JDP2,CMKLR1,ROR2,DCN,PCBD2,CDH13,CREBRF,SOX13,COPS5,MAD2L2,TLE6,RAB27A,CAPRIN2,ZNF418,NLN,PHF20L1,TERF2,ZNF286A,FOXN3,NEUROD1,USP22,JAK2,TRAPPC9,FBXW7,SKAP1,SMC3,UIMC1,ITCH,MLIP,BCL11B,PKNOX1,MLLT3,TSHZ2,TCF7,PDE2A,KLF15,TBX15,ANXA4,WNT11,MTA1,KLF8,NOX4,LCOR,RPRD1B,QKI,CCDC62,PRKCD,SOX6,ACVR2A,RUNX2,CD4,TGFB1,BANP,SGK1,NSD1,IGSF1,PIBF1,ZHX2,PKNOX2,ASCC2,BTRC,NFATC3,F2RL1,BCAS3,CDYL2,CC2D1B,TP73,SAP18,ZBTB22,ILF2,MTDH,FANK1,MYT1L,SMAD6,BNC2,ZNF398,CLOCK,TCF12,ZNF675,ETV6,SUCO,TFAP2D,BCL3,SND1,DUSP22,HNRNPC,TRRAP,SBNO2,YTHDF1,FGF10,CIZ1,SMYD3,LOXL3,CAPN3,LUM,SMURF2,RORA,HIVEP2,AUTS2,TNFSF11,SMG6,PPP3CA,NFYB,MAGEA4,KLF12,CAMK4,GATAD2B,UFL1,TRAK1,CTNNB1,PARK2,SOD2,DACH2,METTL13,SMARCC1,KLF17,IGF1R,PPARG,NGRN,AXIN1,IL18R1,CIPC,MTF1,CBX5,ANKRD17,BRIP1,LRPPRC,SREBF2,CDK11A,CDK11B,LEPR,FGF1,NPAT,NR4A3,FOXK2,ESCO1,MYOCD,TRIM5,PER2,AJUBA,ZNF626,ZNF737,CHEK2,SUPT3H,PRDM16,PPP1CB,HCK,SORBS1,RAB3GAP2,CAPN2,TRIM8,DIO2,BRMS1L,ZBTB*

			8A,ZBTB8B,CTDP1,BAZ1B,MEF2B,HDAC4,PAX2,PHF5A,SECISBP2L,SFRP1,MED13,ZNF395,FOXO3,NFIB,SP4,SYNCRIP,SMAD3,CUX2,WWP2,ARNT2,SBNO1,KRBOX1,ZNF662,ZNF777,EBF3,RNF168,CASZ1,DCP1B,MIER3,NEDD4,ESRRG,HOXD3,HOXD4,ZNF114,KTI12,NFATC1,CDC73,APP,SSBP3,GSX2,RBM8A,NOX1,YAP1,TEN1,NVL,LRP5,POLR3G,ZNF787,SOX2,SETD2,TEAD1,PRICKLE1,ZNF653,ZNF521,ARID3A,ZNF761,CHUK,ERLIN1,SFMBT1,ZNF584,ESR2,S100A12,PRKD1,STAT1,ST18,ETV5,RHOXF2B,TAF3,PLAGL1,HNF4A,ZBTB7C,TASP1,EREG,ATF2,POU2F2,TCF3,ZNF730,RAF1,CELF4,ZNF766,CARD16,BMPR2,CAMK1D,BMPR1A,ZKSCAN1,IKZF4,CDK12,CAND2,DENND4A,MYB,FGF2,ZNF71,POU6F2,BACH1,MXD3,PPM1F,TICRR,GLI4,ZFP41,BEND5,SEMA4D,NFX1,RORC,ELP3,PTAFR,ADCYAP1R1,SP140,SP140L,RHOXF2,JARID2,DDX58,BRDT,PHC2,RARB,SPEN,SIN3B,NCOA1,EHMT1,LMO7,DVL3,EIF2B5,EIF4G1,TCF20,ATF3,CKS1B,PAWR,EBF2,AGO3,MAML2,TSG101,CCDC3,TERF2IP,CRYM,RFX2,ZNF322,SUFU,MAGEA11,PRDM15,ZNF670,ZNF695,CCDC169-SOHLH2,SOHLH2,DDAH1,ZNF354C,TCEA3,PADI6,ZNF704,NR2C1,GLI2,TNKS,WBP2NL,ERCC1,TNRC6B,GLIS3,WDTC1,ZNF664,DAB2,BLM,PKHD1,MYSM1,SETD5,SMG5,RELN,SIN3A,RUVBL2,COMMD6,GMEB1,PELI1,MAP3K7,ZNF423,SP1,TRIM22,ALK,TEAD4,HOXC13,APBB3,SLC35A4,SRA1,UBE2V1,ADIRF,OVOL2,SNX5,NFATC2,RBBP6,PAX7,BNC1,ATP2B4,ACTL6B,ASXL3,PAK3,TET1,CAMTA1,CCPG1,ADNP,ARID4A,SCARB1,CDK6,PHF2,CELF1,TFRC,EPHA5,WWOX,MEF2A,DCAF6,XRN1,SATB1,PSMB2,EYA1,GATAD2A,DIS3L2,TRPV1,HOXB3,HOXB4,HOXB5,HOXB6,TFEB,MNAT1,ACTR2,L3MBTL4,ELAVL4,HDAC1,RECQL5,SMOC2,TRIM24,PLCG2,ROCK1,SCMH1,PAXIP1,ZNF713,EGR2,RNF10,RYBP,AP3B1,PTPN2,NTRK1,KPNA6,TRIM44,EPCAM,ZNF425,GNL3L,MUC1,PTPN14,BCOR,AP3D1,ZNF41,CARM1,ZNF383,ZNF616,ZNF836,SMARCA2,CUX1,SPI1,DNMT3B,FOXP2,PSPC1,GLIS1,SFMBT2,EIF3H,BCAR3,JAZF1,ABCB7,ACOT8,RBPMS,CHURC1,CREB5,MAP3K13,SMYD1,SNCA,BMPR1B,PRIM2,PNPT1,ZEB2,FOXJ3,HEY2,RC3H1,EIF3E,ZMYND11,KMT2D,CREM,KMT2C,PLD1,MACC1,MITF,OPRD1,NUGGC,RPTOR,NFIX,BTK,KCTD13,MLLT1,CBFA2T2,IGF1,MLXIP,ATF7IP,MAPK1,PTEN,BMP7,ATXN2,MXI1,PUM1,SOX5,CIR1,PCBP2,ZNF780A,ZNF780B,LRRK2,TMEM59,ZBTB20,RFC3,LILRB4,PKD2,ZNF652,KCTD1,RIPPLY1,RCOR3,TADA2A,DAZL,NCOA3,PKIB,ZNF146,ZNF565,WNT7A,ZBED6,BRWD3,LPGAT1,CRTC3,DNAJC1,ARNTL2,ELOVL5,KAT6A,MTIF2,ZKSCAN7,ZNF197,ZNF660,BMP6,TAF15,NFE2L1,WDR43,ZNF30,ATRX,DPH6,IKZF1,PRMT2,RAPGEF2,ASCC1,SMO,RALY,TCF4,SOX30,TFE3,GRIN1,JADE1,RHOA,ROR1,RQCD1,TF,ONECUT2,CUL3,TFEC,GSK3B,PRR16,DNAJC3,STAT6,MLF1,RBBP8,NECAB2,CDK13,DUSP26,ZNF362,NACC2,SUPT4H1,ZNF354A,AGT,HDGFRP3,METTL16,STAT2,ZMPSTE24,PBX1,EXOSC3,MAP3K5,TCFL5,CREBBP,DLX1,GTF2IRD2,NCF1,SHC1,ZNF813,LBX2,PARP10,RPS6KB1,STC2,DPRX,PRG3,SETDB2,ALOX5,DNAJB6,SP7,LARP4,ADAM8,SLC5A3,RPS6KA5,ZNF484,ZNF93,PRDM2,NOS1,ZNF44,MED15,RGS14,ACTN4,MC1R,TCF25,ZNF282,EDRF1,TMEFF2,IL18,DIS3,PPARA,ARID5B,SLC25A33,SMARCAL1,CPEB4,NOTCH4,ZMYND8,ZNF366,CRX,PAX6,PRKCZ,ADORA2A,CPEB1,OTUD7B,NIPBL,YEATS4,PHF20,ZNF143,BRF1,TBL1X,FHL2,MBTD1,ATF6,ZBTB5,ZNF708,IGF2BP3,ETS1,PPP1CA,MTF2,TAGLN3,CDK5RAP1,NCOR2,PRKCQ,CSRNP1,HDAC2,HTR2C,IMPACT,SRCAP,POU3F3,NOTO,ZKSCAN5,ELF2,PHIP,PPP2CA,RPL23,RFC5,ATXN1,SP100,ZNF347,ZNF415,TRIM29,PARN,DONSON,ADCY1,SYK,CNOT1,WWTR1,GTF2H5,NFXL1,ZBTB38,BRCA2,ATF7,DVL2,MORC1,MTRF1,YBX1,TAF1,EIF4E3,TICAM1,THAP3,HSF1,MAX,SAP130,DHFR,EZH1,ETF1,NRF1,NCOA2,WDR18,EEFSEC,QRICH1,ZP3,CHD6,RNF2,ZNF554,MYEF2,TRIM37,MET,ZNF461,CAMK2D,TIMELESS,BRD9,MALT1,SETD3,KDM2B,MLXIPL,CCT3,PHB,EDA,ZNF555,CTCF,NR6A1,ATF6B,CREB1,RGMB,TRAP1,EBF4,ZNF511,BMP4,ABLIM3
GO:0007169	transmembrane receptor protein tyrosine kinase signaling pathway	2.332114652275598e-10	ENPP1,NRXN1,CLASP2,IL31RA,NRG3,PRLR,MVP,KALRN,NTRK3,CBL,ARNT,FLT3,STAT5B,PLCE1,FER,PTPRR,PIK3CD,ROBO1,VAV2,ABI1,SAMD12,ANKS1B,STXBP4,GFRA2,PTPN11,ADORA1,ADAMTS3,EPHA1,ERBB4,NTRK2,PDGFB,CCND3,INSR,PIP4K2A,PAK1,BDNF,LCK,SHC4,SNX6,AP3S1,CASS4,FYN,NF1,PLCB1,LMTK2,RTN4,APOD,NDRG4,PIK3R2,NRG1,BDKRB2,ROR2,DCN,SLC39A14,CDH13,PTPRT,CCBE1,JAK2,FBXW7,ZFYVE28,SH2D6,CYFIP2,PRKCD,RUNX2,CD4,KANK1,FGF10,ARHGEF28,EPHA4,CTNNB1,SMARCC1,IGF1R,LEPROT,FGF1,NR4A3,MYOCD,MYO1E,HCK,SORBS1,CSPG4,FLT4,NEDD4,NRP2,PDGFRA,FSTL4,MUC20,PTPRG,FRS2,MMP2,PRKD1,HNF4A,EREG,MAPKAPK3,RAF1,PTPN1,ADAMTS12,TSPAN12,PIK3R3,FGF2,NEDD9,DOK6,EPHB3,PTPRE,SHOC2,TSG101,IDE,COL4A3,HIP1,IRS4,IQGAP1,ALK,XDH,SNX5,PAK3,MAPKAPK2,COL4A6,EPHA5,GFRAL,GHR,FLRT2,SMOC2,PTPN2,INSRR,NTRK1,EPHB1,EPHA10,EPHA7,PTPRA,BCAR3,CHURC1,SNCA,RASA1,IGF1,MAPK1,TMEM108,EFNA5,PDGFC,GPR21,SOS1,EXT1,RAPGEF2,GRB14,ROR1,RQCD1,GSK3B,STAT6,SULF1,AGT,PTK2,NCF1,SHC1,RPS6KB1,ANKS1A,RPS6KA5,PLEKHA1,RGS14,EPHB2,ARID5B,PRKCZ,FAM20C,RABGEF1,ANGPT1,OSBPL8,PRKCQ,BRAF,CSRNP1,PHIP,ANGPT4,SYK,PTPRJ,CAV2,COL4A5,AXL,REPS2,MET,TYRO3,CLNK
GO:0006812	cation transport	2.4837390042857737e-10	SLC39A11,NRXN1,ASPH,PDE4D,SLC9A1,FAM155A,CHERP,SLC25A17,CACHD1,RYR1,NOS1AP,NOX5,EPB41,DPP6,SLC35F3,UTRN,ATP2B2,KCNQ5,CASK,KCNIP4,LRP2,SLC38A11,ANK2,SLC9A9,CHD7,HTR2B,RYR3,SLC24A2,NKAIN2,ITGB1,SLC24A3,CNIH2,NKAIN3,CNGB1,FLVCR2,ABCC8,TCIRG1,KCNS3,NIPAL2,NETO2,CACNB2,ADORA1,KCNJ16,DPP10,PDGFB,CACNA1B,STK39,CAB39,ZDHHC13,SLC20A

			2,SLC4A10,CATSPER2,AMIGO1,KCNMA1,KCNRG,LCK,TMC2,SLC12A8,VAMP7,SLC6A16,RHCE,MICU3,DMD,SLC30A9,SLC8A1,JPH2,FYN,LMTK2,SLC5A6,SHISA9,CACNA1E,SLC4A5,KCNN3,CNNM2,SLC43A2,BDKRB1,KCNIP1,STIM1,NALCN,TMBIM6,SLC39A14,CATSPER3,FGF14,CLDN16,ANO6,KCNB2,SYT1,NPSR1,SCN4A,TMC1,SLC1A4,SLC5A9,SLC9A7,KCNC4,CACNA1H,CAMK2B,CD4,TGFB1,SGK1,KEL,JPH3,SYT13,CDH23,KCNJ3,CACNA1D,CACNA2D3,KCNH1,HOMER2,SLC17A7,KCNH7,CAPN3,VMP1,CNR1,PPP3CA,CTNNB1,PARK2,COX8A,TMEM63C,TRPM1,KCNJ12,RYR2,KCNA6,NDUFA9,NOL3,CACNA1C,SCN3B,ATP10D,ITPR1,WWP2,SHANK1,SYT3,NEDD4,SLC4A4,SLC6A1,KCNK1,APP,NLGN2,VDAC1,IBTK,SLC9B2,SPTBN4,DRD1,PRKD1,SLC6A3,FXYD2,FXYD6,KCNG4,RASGRF2,NLGN1,FGF2,ADCYAP1R1,KLHL3,MRS2,SLC5A8,SLC9B1,ATP6V0B,KCNQ1,NLGN3,TRPA1,TRPM3,NMUR2,AKAP6,TRPC5,WNK1,RELN,KCNQ2,SLC22A3,SLC47A1,SLC8A2,SLC9C1,TPCN1,GPM6B,AHNAK,RGS7,ATP2B4,NSF,P2RX6,ATP6V0A2,KCNAB2,TFRC,SYT12,CACNA1A,STOM,TRPV1,SLC38A6,PLCG2,GPR35,SLC39A10,ANK3,ATP13A3,KCND3,CACNG8,SLC9C2,NETO1,CD84,MCU,TRDN,STAC,TRPC6,ABCB7,SLC41A2,SLC48A1,ATP13A5,SNCA,CACNG2,SLC39A9,SYT9,CNGA3,OPRD1,PKP2,BTK,SLC4A8,HTT,SLC30A7,SCN8A,ATP6V1A,CNIH3,SLC44A1,TMCO1,PKD2,SHANK3,STEAP4,SNAP23,ANO1,SERINC5,KCNC2,SLC6A14,PSEN2,SYT17,GRIN1,RHOA,GRIN3A,TF,ITPR2,EPM2A,SCN9A,AGT,SYT7,ZMPSTE24,HEPHL1,PEX5L,PDE4B,COX5A,TRPC4AP,SLC25A42,SLC29A1,SLC44A3,KCNJ15,SLC5A10,STC2,SLC5A3,PKD1L1,SLC25A18,COX5B,NOS1,ACTN4,SCNN1B,COX6A1,EPHB2,SLC1A1,XCR1,LRRC52,NKAIN1,CALCRL,MCOLN1,STEAP3,ATP1A3,ADORA2A,GRIN2B,SLC25A26,CNTN1,DIAPH1,HTR2C,ATP8A1,TMEM163,UQCR10,ANXA2,KCND2,ATP6V0A1,SYK,SCN1A,SLC44A5,CACNG3,SLC10A1,ATP2B3,GPM6A,SLC13A3,SLC5A4,KCNQ3,SESTD1,CAMK2D,KCNJ6,NEDD4L,PTPRC,SHISA6,EDNRA
GO:0051347	positive regulation of transferase activity	2.502882729194088e-10	*NRXN1,ADCY8,NRG3,PRKAG2,PRLR,NTRK3,FLT3,GRM5,MAP2K5,KITLG,ROBO1,TOM1L1,HTR2B,TENM1,TRAF6,VAV2,ABI1,HSP90AA1,CDC6,AKAP13,DAB1,CCT2,ADORA1,EPHA1,ERBB4,MRE11A,NTRK2,PDGFB,CCND3,INSR,CAB39,PAK1,MAP3K4,ECT2,ERN2,CASS4,LMTK2,CHI3L1,NRG1,MAP2K1,ROR2,CCNYL1,JAK2,FBXW7,LAMTOR3,WNT11,NOX4,PRKCD,TAB2,CD4,TGFB1,PIBF1,BTRC,TNFRSF10B,DOCK3,EPHA4,TNFSF11,CTNNB1,IGF1R,AXIN1,FGF1,AJUBA,SPDYA,FLT4,TAB1,PDGFRA,BORA,NVL,MTCP1,S100A12,DCUN1D3,PRKD1,EREG,CCNY,PTPN1,BMPR2,BMPR1A,FGF2,NEDD9,DVL3,EPHB3,CKS1B,TNKS,CACUL1,DLG3,WNK1,RELN,NEK10,IQGAP1,MAP3K7,ALK,SLC8A2,ATP2B4,ADNP,EPHA5,DCUN1D5,GHR,MNAT1,SNX9,INSRR,NTRK1,EPHB1,EPHA10,AXIN2,PIFO,EPHA7,ATG14,MAP3K13,SNCA,BMPR1B,PRIM2,PRKAG1,RPTOR,IGF1,MAPK1,PTEN,LRRK2,UNC119,RFC3,PKD2,EFNA5,PDGFC,PKIB,PPP2R3C,SERINC5,RAPGEF2,ARRDC4,RHOA,ROR1,AGT,PTK2,MAP3K5,NCF1,TAOK2,CD44,ADAM8,STK4,EPHB2,SLC1A1,IL18,MOB3B,PRKCZ,ANGPT1,OSBPL8,PRKCQ,CD300A,PPP2CA,SKP1,ANGPT4,RFC5,PARN,MADD,SYK,DVL2,SASH1,VAV3,MOB3A,AXL,MET,MALT1,PHB,PTPRC,TYRO3*
GO:0034220	ion transmembrane transport	2.5095008381161527e-10	*SLC39A11,NRXN1,ASPH,PDE4D,SLC9A1,FAM155A,GRID2,CHERP,SLC25A17,CACHD1,RYR1,NOS1AP,NOX5,DPP6,CLCN1,UTRN,ATP2B2,KCNQ5,GRM5,KCNIP4,LRP2,ANK2,SLC9A9,CHD7,SLC26A6,HTR2B,RYR3,SLC24A2,ITGB1,SLC24A3,CNIH2,SLC22A8,CNGB1,ABCC8,GABRA3,TCIRG1,KCNS3,NIPAL2,NETO2,CACNB2,KCNJ16,DPP10,CACNA1B,STK39,ATP8B1,CAB39,ZDHHC13,SLC20A2,SLC4A10,CATSPER2,AMIGO1,KCNMA1,CHRM3,GRIK4,KCNRG,LCK,TMC2,SLC12A8,SLC6A16,RHCE,MICU3,DMD,SLC30A9,SLC8A1,JPH2,FYN,SLC25A21,SLC5A6,SHISA9,ANKH,CACNA1E,SLC4A5,KCNN3,CNNM2,SLC43A2,BDKRB1,KCNIP1,SLC35A2,STIM1,NALCN,SLC39A14,CATSPER3,FGF14,CLDN16,ANO6,KCNB2,NPSR1,SCN4A,TMC1,SLC1A4,SLC9A7,KCNC4,CLCA2,CACNA1H,TGFB1,KEL,JPH3,GRIK3,GABRR3,GABRB1,KCNJ3,CACNA1D,CACNA2D3,ANO4,XPR1,ANO3,KCNH1,SLC17A7,KCNH7,CAPN3,SLC16A1,VMP1,ANO2,COX8A,TMEM63C,TRPM1,CYBB,KCNJ12,RYR2,KCNA6,NOL3,PER2,CACNA1C,SCN3B,GRM1,ATP10D,ITPR1,WWP2,GRIK5,SHANK1,NEDD4,SLC4A4,SLC6A1,KCNK1,APP,NOX1,SFXN5,NLGN2,VDAC1,IBTK,SLC9B2,GRIA3,DRD1,GLRA2,PRKD1,SLC6A3,FXYD2,FXYD6,SLC16A12,KCNG4,RASGRF2,NLGN1,FGF2,GRIK1,PTAFR,MRS2,SLC5A8,SLC9B1,GRIA2,SLC35D1,ATP6V0B,CLIC4,KCNQ1,NLGN3,TRPA1,TRPM3,AKAP6,TRPC5,WNK1,RELN,ABCB1,KCNQ2,SLC47A1,SLC8A2,SLC9C1,TPCN1,CLIC5,AHNAK,RGS7,ATP2B4,P2RX6,ATP6V0A2,KCNAB2,GABRG3,CACNA1A,STOM,TRPV1,PLCG2,GPR35,ABCC1,SLC39A10,ANK3,ATP13A3,KCND3,CACNG8,SLC9C2,NETO1,CHRNB4,MCU,TRDN,GABRA6,LRRC8C,LRRC8D,STAC,TRPC6,ABCB7,SLC41A2,ATP13A5,SNCA,CACNG2,CNGA3,SLC4A8,HTT,SLC30A7,SCN8A,ATP6V1A,CNIH3,TMCO1,PKD2,SHANK3,ANO1,ANO8,GABRR2,KCNC2,SLC6A14,PSEN2,GRIN1,GRIN3A,ITPR2,EPM2A,GABRB3,EMB,SCN9A,AGT,ZMPSTE24,PEX5L,PDE4B,COX5A,TRPC4AP,SLC25A42,KCNJ15,SLC5A10,GRID1,PKD1L1,SLC25A18,COX5B,NOS1,ACTN4,SCNN1B,COX6A1,EPHB2,SLC1A1,XCR1,LRRC52,MCOLN1,ATP1A3,GRIN2B,SLC25A26,GRIA4,DIAPH1,HTR2C,ATP8A1,TMEM163,UQCR10,SLC26A2,ANXA2,KCND2,ATP6V0A1,GRIK2,SCN1A,CACNG3,ATP2B3,GPM6A,SLC13A3,KCNQ3,SESTD1,CAMK2D,KCNJ6,NEDD4L,PTPRC,GPR89A,SHISA6,EDNRA,SLC25A13*
GO:0018209	peptidyl-serine modification	3.309682358903556e-10	*NRXN1,PRKCI,PDE4D,RPS6KA2,LATS2,NTRK3,CAMK1G,TENM1,HSP90AA1,TSSK1B,STK38L,MAST4,HDAC6,IKBKB,TLK1,NTRK2,PDGFB,STK39,CAB39,PAK1,BDNF,DMD,LMTK2,PAQR3,EGFLAM,SH2D3C,BDKRB2,FNIP1,GALNT16,CSNK2A3,DCN,MAD2L2,CAPRIN2,SMG1,PRKCD,SGK1,NSD1,TTBK2,DCLK1,SMYD3,MAST2,PR*

			KCA,CAMK4,AXIN1,DYRK4,CHEK2,APP,MTCP1,SPTBN4,DRD1,CHUK,PRKD1,MAPKAPK3,RAF1,CAMK1D,PPM1F,PRKCG,EIF4G1,PKN3,TERF2IP,TNKS,WNK1,TOP1,ATP2B4,MAPKAPK2,MARK1,TFRC,ADRBK1,ROCK1,HIPK3,PKN2,BCAR3,STK38,NTMT1,MAP3K13,RAPGEF3,SNCA,OPRD1,HIPK1,RPTOR,MAPK1,PTEN,LRRK2,MORC3,PLCL2,VRK3,RQCD1,EPM2A,GSK3B,STK32B,PRKAA2,RPS6KB1,GALNT13,CD44,RPS6KA5,CSNK1A1,NOS1,STK4,MARK3,SLC1A1,PRKCZ,ANGPT1,PRKCQ,BRAF,SYK,TAF1,SRPK2,SH2D3A,GALNT4,INPP5F,CAMK2D,MLXIPL
GO:0030334	regulation of cell migration	3.3182115260434347e-10	LDB2,MAP4K4,S1PR2,CLASP2,SEMA3A,DOCK1,NRG3,SEMA3D,PHACTR1,MAPRE2,TJP1,UNC5C,NTRK3,SEMA5A,ENPP2,FER,MARVELD3,MAP2K5,KITLG,PTPRR,PIK3CD,ROBO1,LMNA,ULK4,ITGB1,SRGAP3,MCC,SRGAP2B,PTPRK,ABCC8,LAMA2,DACH1,MGAT5,DOCK10,HDAC6,ADORA1,EPHA1,ERBB4,ZNF609,PDGFB,STK39,INSR,UNC5D,LAMA3,PAK1,PTPRU,SLC8A1,CASS4,ADAMTS9,NF1,PLCB1,RTN4,DPEP1,APOD,BCR,NDRG4,NTN1,NRG1,BDKRB1,DOCK8,HDAC5,NTNG1,CMKLR1,ROR2,DCN,CDH13,PTPRT,CD99,RCC2,ANO6,KIF2A,RFFL,CCBE1,JAK2,FBXW7,CLDN1,WNT11,CAMK2B,SEMA5B,TGFB1,SGK1,MMP28,F2RL1,BCAS3,SLIT2,CORO1C,RRAS2,KANK1,LAMC2,TTBK2,BMPER,DAPK2,DUSP22,FGF10,SMURF2,EPHA4,PRKCA,PPP3CA,PIP5KL1,SOD2,IGF1R,PPARG,DLG5,FGF1,NR4A3,MYOCD,AJUBA,NF2,FLT4,HDAC4,SFRP1,FOXO3,SMAD3,PTPRM,NRP2,DOCK4,NAV3,APP,GSX2,PDGFRA,SLK,PTPRG,DRD1,MMP2,SYNE2,PRKD1,SEMA6D,BMPR2,CAMK1D,BMPR1A,CTNNA2,PIK3R3,SPAG9,RAB11A,FGF2,PPM1F,NEDD9,SEMA4D,MCTP1,ELP3,PLXNA2,PTAFR,DDX58,CHRD,VCL,LAMB1,CLIC4,RHOJ,RUFY3,DAB2,LDLRAD4,MYSM1,WNK1,RELN,SP1,MACF1,CLASP1,PRKG1,ATP2B4,PAK3,SSH2,SCARB1,FLRT2,SMOC2,PLCG2,ROCK1,TMIGD1,SPI1,MCU,SH3BP1,MST1,MAGI2,ADTRP,MITF,IGF1,DPYSL3,MAPK1,PTEN,BMP7,PRCP,OSGIN1,PDGFC,AMOTL1,WNT7A,GPI,SH3RF2,RAPGEF2,SMO,RHOA,ONECUT2,RDX,ACTA2,FAM49B,SULF1,AGT,PTK2,RPS6KB1,GPR173,ADAM8,CLDN4,CXCL17,PLVAP,ACTN4,STK4,EPHB2,TMEFF2,DLC1,EPB41L4B,GPSM3,ZMYND8,PAX6,RABGEF1,PHLDB2,NIPBL,ANGPT1,OSBPL8,GCNT2,ETS1,FBXO31,BRAF,DIAPH1,ATP8A1,CD300A,ANGPT4,CAMSAP3,SP100,SRGAP2,NUMB,FRMD5,PTPRJ,SASH1,ZP3,GNA12,MET,SEMA3C,PTPRC,BMP4
GO:0007268	chemical synaptic transmission	3.5939564209462613e-10	NRXN1,S1PR2,PDE7B,ADCY8,GRID2,RPS6KA2,NRG3,NLGN4X,GRM8,GRM5,CASK,NRXN3,HTR2B,SLC24A2,ITGB1,CNIH2,PCDH17,DLG2,ERC1,DGKI,ERC2,GABRA3,LAMA2,CACNB2,SV2B,ADORA1,ABR,GRM7,NTRK2,CACNA1B,RIMS1,CDH8,USP46,PTPRD,SLC4A10,BDNF,CHRM3,GRIK4,RIT2,FYN,NF1,PLCB1,SNCB,BCR,SHISA9,CACNA1E,CNTN4,NTNG1,ROR2,ZDHHC3,LIN7A,SYN2,PCDHB16,EXOC4,JAK2,SYN3,CLSTN2,SYT1,LZTS1,SLC1A4,KCNC4,CAMK2B,PDLIM4,JPH3,DLGAP1,PTPRN2,GRIK3,GABRR3,CADPS2,GABRB1,GPR176,DISC1,CHRM1,PLCB4,SLC17A7,YTHDF1,STXBP5,EPHA4,CNR1,PPP3CA,CTNNB1,PARK2,PRKAR1B,CPNE6,GRM1,RIMS4,DCC,SPG11,DGKB,CUX2,GRIK5,SHANK1,SLC6A1,APP,NLGN2,DRD1,GLRA2,SLC6A3,SHANK2,CELF4,RASGRF2,NLGN1,LRRC4C,GRIK1,MCTP1,SORCS2,PRKCG,GRIA2,FBXL20,NLGN3,PAFAH1B1,BTBD9,RELN,KCNQ2,SLC8A2,STAU1,P2RX6,ATAD1,ADNP,MAP1B,SORCS3,GABRG3,SYT12,CACNA1A,TRPV1,ELAVL4,EGR2,NTRK1,MAP1A,PPP1R9A,UNC13A,EPHB1,CACNG8,NETO1,HTR1D,CHRNB4,PTPRA,GABRA6,FCHSD2,SNCA,CACNG2,GRM4,SYT9,PTPRS,KCTD13,SLC4A8,MAPK1,PTEN,LRRK2,UNC119,TMEM108,SHANK3,WNT7A,SNAP23,CEP89,PLCL2,EXT1,GABRR2,RAPGEF2,PRKAR2B,STXBP5L,GRIN1,SYNGAP1,GRIN3A,GABRB3,GSK3B,SYN1,MCTP2,AGT,SYT7,BLOC1S6,HRH4,RIMS2,SLC29A1,CPLX2,PACSIN2,GRID1,RGS14,EPHB2,SLC1A1,DTNA,PRKCZ,ADORA2A,GRIN2B,CADPS,BRAF,HTR2C,TNR,KCND2,ADCY1,GRIK2,CACNG3,DLGAP2,KCNQ3,RPH3A,SHISA6
GO:0098916	anterograde trans-synaptic signaling	3.5939564209462613e-10	NRXN1,S1PR2,PDE7B,ADCY8,GRID2,RPS6KA2,NRG3,NLGN4X,GRM8,GRM5,CASK,NRXN3,HTR2B,SLC24A2,ITGB1,CNIH2,PCDH17,DLG2,ERC1,DGKI,ERC2,GABRA3,LAMA2,CACNB2,SV2B,ADORA1,ABR,GRM7,NTRK2,CACNA1B,RIMS1,CDH8,USP46,PTPRD,SLC4A10,BDNF,CHRM3,GRIK4,RIT2,FYN,NF1,PLCB1,SNCB,BCR,SHISA9,CACNA1E,CNTN4,NTNG1,ROR2,ZDHHC3,LIN7A,SYN2,PCDHB16,EXOC4,JAK2,SYN3,CLSTN2,SYT1,LZTS1,SLC1A4,KCNC4,CAMK2B,PDLIM4,JPH3,DLGAP1,PTPRN2,GRIK3,GABRR3,CADPS2,GABRB1,GPR176,DISC1,CHRM1,PLCB4,SLC17A7,YTHDF1,STXBP5,EPHA4,CNR1,PPP3CA,CTNNB1,PARK2,PRKAR1B,CPNE6,GRM1,RIMS4,DCC,SPG11,DGKB,CUX2,GRIK5,SHANK1,SLC6A1,APP,NLGN2,DRD1,GLRA2,SLC6A3,SHANK2,CELF4,RASGRF2,NLGN1,LRRC4C,GRIK1,MCTP1,SORCS2,PRKCG,GRIA2,FBXL20,NLGN3,PAFAH1B1,BTBD9,RELN,KCNQ2,SLC8A2,STAU1,P2RX6,ATAD1,ADNP,MAP1B,SORCS3,GABRG3,SYT12,CACNA1A,TRPV1,ELAVL4,EGR2,NTRK1,MAP1A,PPP1R9A,UNC13A,EPHB1,CACNG8,NETO1,HTR1D,CHRNB4,PTPRA,GABRA6,FCHSD2,SNCA,CACNG2,GRM4,SYT9,PTPRS,KCTD13,SLC4A8,MAPK1,PTEN,LRRK2,UNC119,TMEM108,SHANK3,WNT7A,SNAP23,CEP89,PLCL2,EXT1,GABRR2,RAPGEF2,PRKAR2B,STXBP5L,GRIN1,SYNGAP1,GRIN3A,GABRB3,GSK3B,SYN1,MCTP2,AGT,SYT7,BLOC1S6,HRH4,RIMS2,SLC29A1,CPLX2,PACSIN2,GRID1,RGS14,EPHB2,SLC1A1,DTNA,PRKCZ,ADORA2A,GRIN2B,CADPS,BRAF,HTR2C,TNR,KCND2,ADCY1,GRIK2,CACNG3,DLGAP2,KCNQ3,RPH3A,SHISA6
GO:0097435	supramolecular fiber organization	3.810214352300162e-10	CLRN1,HOOK2,PRKCI,SLC9A1,PDE4DIP,CLASP2,PHACTR1,FHOD3,TJP1,SEMA5A,FER,MFAP5,EZR,TENM1,CTNNA3,ABI1,ITGB1,TRIOBP,ELMO2,GAS7,AKAP13,CDC42EP3,XIRP2,COL14A1,HDAC6,ADAMTS3,EPHA1,KIF18B,LIMK1,HOOK3,MID1,FMN2,PRKAR1A,PAK1,ARHGAP6,DMD,GSN,CASS4,NF1,DST,PCDH15,TTN,RANBP9,TLL1,DCTN1,BID,MAD2L2,TLE6,NCKAP1,ARL3,KIF2A,COL12A1,JAK2,MYPN

			,PLS1,CYFIP2,WNT11,SIAH1,PRKCD,PPM1E,F2RL1,SLIT2,COLGALT1,CORO1C,KANK1,TTBK2,COL24A1,KANK4,ENAH,LOXL3,RHPN2,SVIL,CAPN3,LUM,MPRIP,PARK2,AXIN1,MYO1D,BFSP1,ADD2,NIN,TUBGCP6,MYO1E,HCK,SORBS1,OBSCN,NF2,SPTBN1,ELN,SFRP1,SSH1,SMAD3,THSD4,ABI2,SHANK1,ARHGEF18,PREX1,KPNB1,NAV3,APP,PDGFRA,SLK,ADD3,MYO1F,KIF24,ADAMTS2,SPTBN4,VILL,RND3,VBP1,COL16A1,CTNNA2,RAB11A,PPM1F,NEDD9,PAWR,COL11A1,PAFAH1B1,ADAMTS14,COL4A3,HIP1,RUFY3,ARHGAP12,MYO7A,CLASP1,PACSIN1,SPTBN5,PAK3,SSH2,MAP1B,COL4A6,AIF1L,MEF2A,PLS3,ZNF207,CALD1,SNX9,DIAPH2,ACTR2,ROCK1,EPS8,MAP1A,PPP1R9A,PTPRQ,NCKAP5,MCU,CTNNA1,LIMA1,TRDN,SHROOM3,FCHSD2,RAPGEF3,PDXP,SH3BP1,SNCA,COL13A1,TLL2,CSRP1,RASA1,PKP2,KCTD13,DPYSL3,ARHGAP25,SHANK3,STMN4,TBCD,EXT1,RHOA,NDE1,TF,RHOBTB1,SHROOM1,CUL3,SH3KBP1,RDX,FAM49B,KRT8,HDGFRP3,ESPNL,IQGAP2,FAM171A1,FMOD,COL21A1,ALDOA,PGM5,DNAJB6,SPEF1,TMEFF2,DLC1,PHLDB2,SHROOM4,MAP2,NEBL,BRAF,DIAPH1,ACAN,CAMSAP3,CIT,NEB,MYO1A,BMP1,CGNL1,COL4A5,MET,MICAL3,SPTAN1,TNXB
GO:0051254	positive regulation of RNA metabolic process	3.820991234350505e-10	LDB2,ASPH,SLC9A1,PBX3,CBFB,IL31RA,MED26,WWC1,ASH1L,SCAF8,STOX2,PAGR1,HIVEP3,FTO,NPAS3,ARNT,STAT5B,CASK,MAP2K5,SP3,HDGF,CHD7,MECOM,TACC1,TRAF6,NHLH1,GFI1B,RUNX1,HMGN3,NRIP1,THRB,EFCAB7,SERTAD2,IKBKB,ERBB4,ZNF609,BRD8,KAT6B,ESR1,MAML3,CDON,TNRC6A,EP300,CELA1,ZNF76,PDGFB,HNF4G,INSR,RERE,TCF7L2,LITAF,FBXW11,ESRRB,RBM20,TBR1,SAMD4A,TFAP2A,PEG3,MEIS1,TRIM13,ZNF148,MTA3,TFDP2,SLC30A9,TOX3,KAT7,RBM14,MED12L,SATB2,RIT2,MKRN2,ARNTL,PLCB1,ARID4B,BRPF1,PIK3R2,UBP1,MAP2K1,PPP3R1,HDAC5,CSRNP3,NFIA,RNF4,ROR2,DCN,PCBD2,HNRNPLL,CDH13,CREBRF,COPS5,MAD2L2,CAPRIN2,NEUROD1,USP22,JAK2,SKAP1,MLIP,BCL11B,PKNOX1,MLLT3,KLF15,WNT11,MTA1,RPRD1B,CCDC62,ACVR2A,RUNX2,CD4,TGFB1,BANP,NSD1,BTRC,NFATC3,F2RL1,BCAS3,TP73,ILF2,MTDH,FANK1,SMAD6,ZNF398,CLOCK,TCF12,ETV6,TFAP2D,BCL3,SBNO2,YTHDF1,FGF10,SMYD3,CAPN3,LUM,RORA,AUTS2,TNFSF11,PPP3CA,NFYB,KLF12,CAMK4,CTNNB1,PARK2,SMARCC1,PPARG,AXIN1,MTF1,SREBF2,FGF1,NPAT,NR4A3,FOXK2,RIOK2,MYOCD,TRIM5,PER2,CHEK2,SUPT3H,PRDM16,TRIM8,MEF2B,HDAC4,PAX2,PHF5A,SFRP1,MED13,ZNF395,FOXO3,NFIB,SMAD3,WWP2,ARNT2,EBF3,CASZ1,DCP1B,ESRRG,HOXD3,HOXD4,NFATC1,CDC73,APP,SSBP3,YAP1,LRP5,SOX2,TEAD1,ZNF521,ARID3A,CHUK,ESR2,PRKD1,STAT1,ST18,ETV5,PLAGL1,HNF4A,ZBTB7C,TASP1,ATF2,POU2F2,TCF3,RAF1,CELF4,BMPR2,BMPR1A,IKZF4,CDK12,CAND2,MYB,FGF2,ZNF71,BACH1,RORC,DDX58,BRDT,RARB,NCOA1,LMO7,DVL3,TCF20,ATF3,LIN28B,SRSF5,EBF2,MAML2,TSG101,RFX2,PRDM15,GLI2,TNKS,WBP2NL,ERCC1,TNRC6B,GLIS3,DAB2,BLM,MYSM1,SIN3A,RUVBL2,GMEB1,ZNF423,SP1,TRIM22,TEAD4,HOXC13,SRA1,UBE2V1,ADIRF,OVOL2,SNX5,NFATC2,BNC1,ACTL6B,ASXL3,TET1,CAMTA1,CCPG1,ARID4A,PHF2,CELF1,WWOX,MEF2A,DCAF6,EYA1,DIS3L2,HOXB3,HOXB4,HOXB5,TFEB,ACTR2,HDAC1,TRIM24,ROCK1,PAXIP1,EGR2,RNF10,RYBP,AP3B1,KPNA6,TRIM44,EPCAM,MUC1,AP3D1,CARM1,ZNF836,SMARCA2,SPI1,GLIS1,RBPMS,CHURC1,CREB5,BMPR1B,PRIM2,PNPT1,ZEB2,FOXJ3,HEY2,RC3H1,KMT2D,CREM,KMT2C,MACC1,MITF,RPTOR,NFIX,IGF1,MLXIP,ATF7IP,BMP7,PUM1,PCBP2,ZNF780B,PKD2,TADA2A,NCOA3,WNT7A,CRTC3,ARNTL2,KAT6A,ZNF197,BMP6,TAF15,WDR43,ATRX,PRMT2,SMO,TCF4,SOX30,TFE3,GRIN1,JADE1,RQCD1,TF,ONECUT2,TFEC,STAT6,CDK13,SUPT4H1,AGT,METTL16,PBX1,EXOSC3,MAP3K5,CREBBP,DLX1,NCF1,SHC1,SP7,RPS6KA5,ZNF484,PRDM2,NOS1,ACTN4,MC1R,EDRF1,IL18,DIS3,PPARA,ARID5B,NOTCH4,CRX,PAX6,ZC3HAV1,CPEB1,NIPBL,YEATS4,ZNF143,BRF1,TBL1X,ATF6,ETS1,MTF2,CSRNP1,HDAC2,SRCAP,POU3F3,ELF2,PHIP,SP100,PARN,CNOT1,WWTR1,ZBTB38,BRCA2,ATF7,DVL2,YBX1,THAP3,HSF1,MAX,EZH1,NRF1,NCOA2,QRICH1,ZP3,CHD6,TRIM37,MET,SETD3,TRA2B,MLXIPL,PHB,CTCF,NR6A1,ATF6B,CREB1,RGMB,BMP4,ABLIM3
GO:0019438	aromatic compound biosynthetic process	4.097855368354485e-10	ENPP1,PRDX2,LDB2,ASPH,PRKCI,SLCO3A1,DNMT1,HLX,SLC9A1,PBX3,ADCY8,MED13L,TRPS1,CBFB,PDE8A,ZNF823,IL31RA,PRDM12,MED26,WWC1,ASH1L,SCAF8,STOX2,PTGIS,PRKAG2,WWC3,PAGR1,ADCY7,HIVEP3,NPAS3,ARNT,LRRFIP2,STAT5B,TOX,GRM5,ZNF566,FER,CASK,MAP2K5,MAPK10,ZNF536,SP3,HDGF,GTF3C1,EZR,IKZF2,CHD7,MECOM,TACC1,TENM1,NADK2,TRAF6,GTF2E2,DNAJA3,NHLH1,HSP90AA1,PSMB7,TSC22D3,ZC4H2,CCDC22,GMDS,GFI1B,PTPRK,RUNX1,ERC1,HMGN3,NRIP1,THRB,EFCAB7,ITGB3BP,DACH1,ZNF569,CCT2,ORC2,HDAC6,SERTAD2,MYT1,IKBKB,PEX14,ERBB4,MRE11A,ZNF609,BRD8,KAT6B,HIF3A,SNIP1,ESR1,MIER1,PCBP3,MAML3,CDON,EP300,CELA1,TENM2,ZNF76,RNF220,ZNF471,PDGFB,CCND3,TOB2,ZNF19,ZNF23,BRMS1,ZNF605,SCML4,HNF4G,INSR,RERE,PRKAR1A,FUBP1,ATP8B1,H2AFY2,TCF7L2,JMJD1C,ZNF443,ZNF490,ZNF564,ZNF709,ZNF799,LITAF,FBXW11,ESRRB,MAP3K4,BASP1,TBR1,TFAP2A,CDK14,FANCA,PEG3,ZIM2,TGIF2,TGIF2-C20ORF24,MEIS1,VRTN,TRIM13,MDM4,ZNF148,MTA3,SNX6,TFDP2,ERN2,C1D,AFF3,DMD,CENPF,SLC30A9,TOX3,USP13,KAT7,ZNF667,RBM14,PRPS2,MED12L,SATB2,RIT2,HIRA,MKRN2,ARNTL,PLCB1,ARID4B,POLA2,RWDD3,BRPF1,PIK3R2,NRG1,UBP1,MAP2K1,MDFI,FNIP1,VAX2,TMBIM6,VGLL4,PPP2CB,PPP3R1,HDAC5,CSRNP3,ZNF692,NFIA,RNF4,JDP2,CMKLR1,ROR2,DCN,PRPSAP2,PCBD2,CDH13,CREBRF,SOX13,ACSBG1,COPS5,AK2,MAD2L2,TLE6,CAPRIN2,ZNF418,PHF20L1,TERF2,ZNF286A,NME7,FOXN3,AK5,NEUROD1,USP22,JAK2,TRAPPC9,SKAP1,UI

			MC1,ITCH,MLIP,BCL11B,IMPDH1,PKNOX1,MLLT3,TSHZ2,TCF7,PDE2A,KLF15,TBX15,ANXA4,WNT11,MTA1,KLF8,NOX4,LCOR,RPRD1B,CCDC62,SOX6,ACVR2A,RUNX2,CD4,TGFB1,BANP,SGK1,NSD1,IGSF1,ZHX2,PKNOX2,ASCC2,BTRC,NFATC3,POLK,F2RL1,BCAS3,CDYL2,CC2D1B,TP73,SAP18,ZBTB22,ILF2,MTDH,FANK1,MYT1L,SMAD6,BNC2,ZNF398,CLOCK,TCF12,ZNF675,ETV6,PHRF1,TFAP2D,BCL3,SND1,DUSP22,HNRNPC,TRRAP,SBNO2,FGF10,POLR2J2,ADK,SMYD3,LOXL3,ADCY2,CAPN3,LUM,SMURF2,RORA,HIVEP2,HSD17B12,AUTS2,TNFSF11,SMG6,PPP3CA,NFYB,MAGEA4,KLF12,CAMK4,GATAD2B,UFL1,TRAK1,CTNNB1,PARK2,SOD2,DACH2,METTL13,SMARCC1,KLF17,PPARG,AXIN1,IL18R1,CIPC,MTF1,CBX5,BRIP1,SREBF2,PFAS,CDK11A,CDK11B,FGF1,NPAT,NR4A3,FOXK2,DCT,MYOCD,TRIM5,PER2,AJUBA,ZNF626,ZNF737,CHEK2,SUPT3H,PRDM16,HCK,TRIM8,BRMS1L,ZBTB8A,ZBTB8B,CTDP1,BAZ1B,MEF2B,HDAC4,PAX2,PHF5A,CPOX,SNAPC4,SFRP1,MED13,ZNF395,FOXO3,TPH2,NFIB,SP4,SMAD3,CUX2,WWP2,ARNT2,SBNO1,KRBOX1,ZNF662,ZNF777,EBF3,RNF168,CASZ1,MIER3,NEDD4,ESRRG,HOXD3,HOXD4,ZNF114,KTI12,NFATC1,UCK2,CDC73,APP,SSBP3,GSX2,YAP1,PPCS,TEN1,NVL,LRP5,POLR3G,ZNF787,SOX2,SETD2,TEAD1,PRICKLE1,ZNF653,ZNF521,ELOVL2,ARID3A,ZNF761,CHUK,SFMBT1,ZNF584,TPH1,ESR2,S100A12,GTF2F2,PRKD1,STAT1,ST18,ETV5,RHOXF2B,SLC6A3,TAF3,PLAGL1,HNF4A,ZBTB7C,TASP1,EREG,ATF2,POU2F2,TCF3,ZNF730,RAF1,ABLIM2,ZNF766,CARD16,POLA1,BMPR2,CAMK1D,BMPR1A,ZKSCAN1,IDO2,IKZF4,CDK12,TAF8,CAND2,DENND4A,MYB,FGF2,ZNF71,POU6F2,BACH1,MXD3,PPM1F,ADCY5,GLI4,ZFP41,BEND5,SEMA4D,NFX1,RORC,ELP3,PTAFR,SP140,SP140L,RHOXF2,JARID2,DDX58,SLC5A8,BRDT,PHC2,RARB,SPEN,SIN3B,NCOA1,EHMT1,LMO7,DVL3,POLR2H,TCF20,ATF3,TCEANC,CKS1B,PAWR,EBF2,MAML2,TSG101,TERF2IP,CRYM,RFX2,ZNF322,SUFU,MAGEA11,PRDM15,ZNF670,ZNF695,CCDC169-SOHLH2,SOHLH2,POLE,ZNF354C,TCEA3,CMPK1,ZNF704,NR2C1,ME1,GLI2,TNKS,WBP2NL,ERCC1,GLIS3,WDTC1,ZNF664,TPK1,DAB2,BLM,PKHD1,MYSM1,SETD5,SMG5,RELN,SIN3A,RUVBL2,COMMD6,GUCY1A2,GMEB1,PELI1,SLC25A16,MAP3K7,ZNF423,SP1,TRIM22,ALK,TEAD4,HOXC13,APBB3,SRA1,UBE2V1,ADIRF,OVOL2,SNX5,NFATC2,PAX7,BNC1,ATP2B4,ACTL6B,ASXL3,PAK3,TET1,CAMTA1,CCPG1,ADNP,ARID4A,CDK6,PHF2,TYR,TFRC,EPHA5,WWOX,MEF2A,ADCY9,DCAF6,XRN1,SATB1,PSMB2,EYA1,GATAD2A,TRPV1,HOXB3,HOXB4,HOXB5,HOXB6,TFEB,MNAT1,ACTR2,L3MBTL4,ACACA,HDAC1,RECQL5,SMOC2,TRIM24,PLCG2,SCMH1,PAXIP1,ZNF713,EGR2,HIPK3,RNF10,RYBP,AP3B1,PTPN2,NTRK1,KPNA6,TRIM44,EPCAM,ZNF425,GNL3L,MUC1,PTPN14,BCOR,AP3D1,ZNF41,CARM1,ZNF383,ZNF616,ZNF836,SMARCA2,CUX1,SPI1,DNMT3B,FOXP2,PSPC1,GLIS1,SFMBT2,JAZF1,ABCB7,RBPMS,CHURC1,CREB5,MAP3K13,ERCC3,SMYD1,SNCA,BMPR1B,POLN,PRIM2,ZEB2,FOXJ3,NADK,OCA2,HEY2,ZMYND11,KMT2D,CREM,KMT2C,MACC1,MITF,OPRD1,RPTOR,NFIX,BTK,MLLT1,TROVE2,CBFA2T2,IGF1,MLXIP,ATF7IP,MAPK1,PTEN,BMP7,MXI1,SOX5,CIR1,PCBP2,ZNF780A,ZNF780B,ATP6V1A,MLYCD,ZBTB20,RFC3,LILRB4,PKD2,ZNF652,HSF2BP,KCTD1,RIPPLY1,NT5E,RCOR3,TADA2A,NCOA3,PKIB,ZNF146,ZNF565,WNT7A,ZBED6,BRWD3,CRTC3,ARNTL2,ELOVL5,KAT6A,ZKSCAN7,ZNF197,ZNF660,BMP6,TAF15,NFE2L1,WDR43,ZNF30,ATRX,IKZF1,PRMT2,RAPGEF2,ASCC1,MTHFD2L,SMO,RALY,AMPD1,TCF4,SOX30,TFE3,GRIN1,JADE1,RHOA,SPRTN,ROR1,RQCD1,TF,ONECUT2,CUL3,TFEC,STAT6,MLF1,RBBP8,CDK13,ALAS2,DUSP26,MTHFD1L,ZNF362,NACC2,SUPT4H1,ZNF354A,AGT,HDGFRP3,ELOVL3,POLH,POLR2F,STAT2,ZMPSTE24,GTF2E1,PPCDC,PBX1,MAP3K5,TCFL5,CREBBP,DLX1,GTF2IRD2,NCF1,SHC1,ZNF813,ALDOA,LBX2,PARP10,DPRX,GUCY2F,PRG3,SETDB2,DNAJB6,SP7,ADAM8,RPS6KA5,ZNF484,ZNF93,PRDM2,NOS1,ZNF44,MED15,RGS14,ACTN4,MC1R,TCF25,ZNF282,EDRF1,IL18,TEX12,PPARA,ARID5B,SLC25A33,SMARCAL1,TAF2,LDHC,NOTCH4,ZMYND8,ZNF366,CRX,PAX6,PRKCZ,ADORA2A,PAH,OTUD7B,NIPBL,YEATS4,PHF20,ZNF143,BRF1,TBL1X,FHL2,MBTD1,ATF6,ZBTB5,ZNF708,ETS1,MTF2,TAGLN3,NCOR2,PRKCQ,CSRNP1,HDAC2,IMPACT,SRCAP,POU3F3,NOTO,ZKSCAN5,ELF2,MOXD1,PHIP,PPP2CA,SLC26A2,RPL23,RFC5,ATXN1,SP100,ZNF347,ZNF415,REV3L,TRIM29,PARN,ADCY1,SYK,CNOT1,WWTR1,GTF2H5,NFXL1,ZBTB38,BRCA2,ATF7,DVL2,MORC1,YBX1,TAF1,TICAM1,THAP3,SNAPC3,HSF1,MAX,SAP130,DHFR,EZH1,NRF1,NCOA2,QRICH1,ZP3,CHD6,RNF2,ZNF554,MYEF2,TBP,TRIM37,MET,ZNF461,CAMK2D,TIMELESS,BRD9,MALT1,SETD3,KDM2B,MLXIPL,CCT3,PHB,EDA,ZNF555,CTCF,NR6A1,ADCY10,ATF6B,CREB1,LIG1,RGMB,EBF4,SLC25A13,ZNF511,BMP4,ABLIM3
GO:0018105	peptidyl-serine phosphorylation	4.722979079886677e-10	NRXN1,PRKCI,PDE4D,RPS6KA2,LATS2,NTRK3,CAMK1G,TENM1,HSP90AA1,TSSK1B,STK38L,MAST4,HDAC6,IKBKB,TLK1,NTRK2,PDGFB,STK39,CAB39,PAK1,BDNF,DMD,LMTK2,PAQR3,SH2D3C,BDKRB2,FNIP1,CSNK2A3,MAD2L2,CAPRIN2,SMG1,PRKCD,SGK1,NSD1,TTBK2,DCLK1,SMYD3,MAST2,PRKCA,CAMK4,AXIN1,DYRK4,CHEK2,APP,MTCP1,SPTBN4,DRD1,CHUK,PRKD1,MAPKAPK3,RAF1,CAMK1D,PPM1F,PRKCG,EIF4G1,PKN3,TERF2IP,TNKS,WNK1,TOP1,ATP2B4,MAPKAPK2,MARK1,TFRC,ADRBK1,ROCK1,HIPK3,PKN2,BCAR3,STK38,MAP3K13,RAPGEF3,SNCA,OPRD1,HIPK1,RPTOR,MAPK1,PTEN,LRRK2,MORC3,PLCL2,VRK3,RQCD1,EPM2A,GSK3B,STK32B,PRKAA2,RPS6KB1,CD44,RPS6KA5,CSNK1A1,NOS1,STK4,MARK3,SLC1A1,PRKCZ,ANGPT1,PRKCQ,BRAF,SYK,TAF1,SRPK2,SH2D3A,INPP5F,CAMK2D,MLXIPL
GO:00	regulation of cell	4.86637745	DOCK1,EPB41,KALRN,ENPP2,EZR,CDKL5,TRIOBP,CDC42EP3,MYO10,RIMS1,PT

22604	morphogenesis	8622225e-10	*PRD,FMNL2,CASS4,FYN,MYH9,NTNG1,CAPRIN2,RCC2,SYT1,SEPT7,PALMD,MKLN1,IL1RAPL1,CAMK2B,CORO1C,KANK1,ITSN2,PARVA,STRIP1,EPHA4,PARK2,FBLIM1,CPNE6,HCK,CUX2,SYT3,ARHGEF18,PREX1,SH3D19,CPNE9,PARP6,RND3,SPAG9,NEDD9,SEMA4D,PLXNA2,DVL3,MYO9A,RHOJ,P4HB,PAFAH1B1,KIF3A,RASAL1,DAB2,RELN,CFDP1,MACF1,AKAP2,SYNE3,PAK3,FGD1,ACTR2,EPS8,DNMBP,UNC13A,COCH,CUX1,SHROOM3,MAP3K13,RASA1,ZRANB1,EFNA5,BRWD3,PARVB,SYT17,RHOA,RHOBTB1,SH3KBP1,RDX,EEF2K,ZMPSTE24,PTK2,RIMS2,FAM171A1,TAOK2,ALDOA,ARHGAP15,CD44,FGD3,LARP4,CLDN4,FGD4,ACTN4,DLC1,FBXO31,BRAF,DIAPH1,PHIP,CDKL3,DVL2,GRIP1,GNA12,FMNL1,NEDD4L*
GO:0006351	transcription, DNA-templated	4.995620495571218e-10	*ENPP1,PRDX2,LDB2,ASPH,PRKCI,SLCO3A1,DNMT1,HLX,SLC9A1,PBX3,ADCY8,MED13L,TRPS1,CBFB,PDE8A,ZNF823,IL31RA,PRDM12,MED26,WWC1,ASH1L,SCAF8,STOX2,PTGIS,WWC3,PAGR1,HIVEP3,NPAS3,ARNT,LRRFIP2,STAT5B,TOX,GRM5,ZNF566,FER,CASK,MAP2K5,MAPK10,ZNF536,SP3,HDGF,GTF3C1,EZR,IKZF2,CHD7,MECOM,TACC1,TENM1,TRAF6,GTF2E2,DNAJA3,NHLH1,PSMB7,TSC22D3,ZC4H2,CCDC22,GFI1B,PTPRK,RUNX1,ERC1,HMGN3,NRIP1,THRB,EFCAB7,ITGB3BP,DACH1,ZNF569,ORC2,HDAC6,SERTAD2,MYT1,IKBKB,PEX14,ERBB4,ZNF609,BRD8,KAT6B,HIF3A,SNIP1,ESR1,MIER1,PCBP3,MAML3,CDON,EP300,CELA1,TENM2,ZNF76,RNF220,ZNF471,PDGFB,CCND3,TOB2,ZNF19,ZNF23,BRMS1,ZNF605,SCML4,HNF4G,INSR,RERE,PRKAR1A,FUBP1,ATP8B1,H2AFY2,TCF7L2,JMJD1C,ZNF443,ZNF490,ZNF564,ZNF709,ZNF799,LITAF,FBXW11,ESRRB,BASP1,TBR1,TFAP2A,CDK14,FANCA,PEG3,ZIM2,TGIF2,TGIF2-C20ORF24,MEIS1,VRTN,TRIM13,MDM4,ZNF148,MTA3,SNX6,TFDP2,ERN2,C1D,AFF3,DMD,CENPF,SLC30A9,TOX3,USP13,KAT7,ZNF667,RBM14,MED12L,SATB2,RIT2,HIRA,MKRN2,ARNTL,PLCB1,ARID4B,RWDD3,BRPF1,PIK3R2,NRG1,UBP1,MAP2K1,MDFI,FNIP1,VAX2,TMBIM6,VGLL4,PPP2CB,PPP3R1,HDAC5,CSRNP3,ZNF692,NFIA,RNF4,JDP2,CMKLR1,ROR2,DCN,PCBD2,CDH13,CREBRF,SOX13,COPS5,MAD2L2,TLE6,CAPRIN2,ZNF418,PHF20L1,ZNF286A,FOXN3,NEUROD1,USP22,JAK2,TRAPPC9,SKAP1,UIMC1,ITCH,MLIP,BCL11B,PKNOX1,MLLT3,TSHZ2,TCF7,PDE2A,KLF15,TBX15,ANXA4,WNT11,MTA1,KLF8,LCOR,RPRD1B,CCDC62,SOX6,ACVR2A,RUNX2,CD4,TGFB1,BANP,SGK1,NSD1,IGSF1,ZHX2,PKNOX2,ASCC2,BTRC,NFATC3,F2RL1,BCAS3,CDYL2,CC2D1B,TP73,SAP18,ZBTB22,ILF2,MTDH,FANK1,MYT1L,SMAD6,BNC2,ZNF398,CLOCK,TCF12,ZNF675,ETV6,PHRF1,TFAP2D,BCL3,SND1,DUSP22,TRRAP,SBNO2,FGF10,POLR2J2,SMYD3,LOXL3,CAPN3,LUM,SMURF2,RORA,HIVEP2,AUTS2,TNFSF11,PPP3CA,NFYB,MAGEA4,KLF12,CAMK4,GATAD2B,UFL1,TRAK1,CTNNB1,PARK2,SOD2,DACH2,METTL13,SMARCC1,KLF17,PPARG,AXIN1,IL18R1,CIPC,MTF1,CBX5,BRIP1,SREBF2,CDK11A,CDK11B,FGF1,NPAT,NR4A3,FOXK2,MYOCD,TRIM5,PER2,AJUBA,ZNF626,ZNF737,CHEK2,SUPT3H,PRDM16,HCK,TRIM8,BRMS1L,ZBTB8A,ZBTB8B,CTDP1,BAZ1B,MEF2B,HDAC4,PAX2,PHF5A,SNAPC4,SFRP1,MED13,ZNF395,FOXO3,NFIB,SP4,SMAD3,CUX2,WWP2,ARNT2,SBNO1,KRBOX1,ZNF662,ZNF777,EBF3,RNF168,CASZ1,MIER3,NEDD4,ESRRG,HOXD3,HOXD4,ZNF114,KTI12,NFATC1,CDC73,APP,SSBP3,GSX2,YAP1,LRP5,POLR3G,ZNF787,SOX2,SETD2,TEAD1,PRICKLE1,ZNF653,ZNF521,ARID3A,ZNF761,CHUK,SFMBT1,ZNF584,ESR2,S100A12,GTF2F2,PRKD1,STAT1,ST18,ETV5,RHOXF2B,TAF3,PLAGL1,HNF4A,ZBTB7C,TASP1,EREG,ATF2,POU2F2,TCF3,ZNF730,RAF1,ABLIM2,ZNF766,CARD16,BMPR2,CAMK1D,BMPR1A,ZKSCAN1,IKZF4,CDK12,TAF8,CAND2,DENND4A,MYB,FGF2,ZNF71,POU6F2,BACH1,MXD3,PPM1F,GLI4,ZFP41,BEND5,SEMA4D,NFX1,RORC,ELP3,PTAFR,SP140,SP140L,RHOXF2,JARID2,DDX58,BRDT,PHC2,RARB,SPEN,SIN3B,NCOA1,EHMT1,LMO7,DVL3,POLR2H,TCF20,ATF3,TCEANC,CKS1B,PAWR,EBF2,MAML2,TSG101,TERF2IP,CRYM,RFX2,ZNF322,SUFU,MAGEA11,PRDM15,ZNF670,ZNF695,CCDC169-SOHLH2,SOHLH2,ZNF354C,TCEA3,ZNF704,NR2C1,GLI2,TNKS,WBP2NL,ERCC1,GLIS3,WDTC1,ZNF664,DAB2,BLM,PKHD1,MYSM1,SETD5,RELN,SIN3A,RUVBL2,COMMD6,GMEB1,PELI1,MAP3K7,ZNF423,SP1,TRIM22,ALK,TEAD4,HOXC13,APBB3,SRA1,UBE2V1,ADIRF,OVOL2,SNX5,NFATC2,PAX7,BNC1,ATP2B4,ACTL6B,ASXL3,TET1,CAMTA1,CCPG1,ADNP,ARID4A,CDK6,PHF2,TFRC,EPHA5,WWOX,MEF2A,DCAF6,SATB1,PSMB2,EYA1,GATAD2A,TRPV1,HOXB3,HOXB4,HOXB5,HOXB6,TFEB,MNAT1,ACTR2,L3MBTL4,HDAC1,RECQL5,TRIM24,PLCG2,SCMH1,PAXIP1,ZNF713,EGR2,HIPK3,RNF10,RYBP,AP3B1,PTPN2,NTRK1,KPNA6,TRIM44,EPCAM,ZNF425,MUC1,PTPN14,BCOR,AP3D1,ZNF41,CARM1,ZNF383,ZNF616,ZNF836,SMARCA2,CUX1,SPI1,DNMT3B,FOXP2,PSPC1,GLIS1,SFMBT2,JAZF1,RBPMS,CHURC1,CREB5,MAP3K13,ERCC3,SMYD1,SNCA,BMPR1B,ZEB2,FOXJ3,HEY2,ZMYND11,KMT2D,CREM,KMT2C,MACC1,MITF,OPRD1,RPTOR,NFIX,BTK,MLLT1,TROVE2,CBFA2T2,IGF1,MLXIP,ATF7IP,PTEN,BMP7,MXI1,SOX5,CIR1,PCBP2,ZNF780A,ZNF780B,ZBTB20,LILRB4,PKD2,ZNF652,HSF2BP,KCTD1,RIPPLY1,RCOR3,TADA2A,NCOA3,ZNF146,ZNF565,WNT7A,ZBED6,BRWD3,CRTC3,ARNTL2,KAT6A,ZKSCAN7,ZNF197,ZNF660,BMP6,TAF15,NFE2L1,WDR43,ZNF30,ATRX,IKZF1,PRMT2,ASCC1,SMO,RALY,TCF4,SOX30,TFE3,GRIN1,JADE1,RHOA,ROR1,RQCD1,TF,ONECUT2,CUL3,TFEC,STAT6,MLF1,RBBP8,CDK13,DUSP26,ZNF362,NACC2,SUPT4H1,ZNF354A,AGT,HDGFRP3,POLR2F,STAT2,ZMPSTE24,GTF2E1,PBX1,MAP3K5,TCFL5,CREBBP,DLX1,GTF2IRD2,NCF1,SHC1,ZNF813,LBX2,PARP10,DPRX,SETDB2,DNAJB6,SP7,ADAM8,RPS6KA5,ZNF484,ZNF93,PRDM2,NOS1,ZNF44,MED15,RGS14,ACTN4,MC1R,TCF25,ZNF282,EDRF1,IL18,PPARA,ARID5B,SLC25A33,SMARCAL1,TAF2,NOTCH4,ZMYND8,ZNF366,CRX,PAX6,PRKCZ,ADORA2A,OTUD7B,NIPBL,YEATS*

			4,PHF20,ZNF143,BRF1,TBL1X,FHL2,MBTD1,ATF6,ZBTB5,ZNF708,ETS1,MTF2,TAGLN3,NCOR2,PRKCQ,CSRNP1,HDAC2,IMPACT,SRCAP,POU3F3,NOTO,ZKSCAN5,ELF2,PHIP,PPP2CA,RPL23,ATXN1,SP100,ZNF347,ZNF415,TRIM29,ADCY1,SYK,CNOT1,WWTR1,GTF2H5,NFXL1,ZBTB38,BRCA2,ATF7,DVL2,MORC1,YBX1,TAF1,TICAM1,THAP3,SNAPC3,HSF1,MAX,SAP130,EZH1,NRF1,NCOA2,QRICH1,ZP3,CHD6,RNF2,ZNF554,MYEF2,TBP,TRIM37,MET,ZNF461,CAMK2D,TIMELESS,BRD9,MALT1,SETD3,KDM2B,MLXIPL,PHB,EDA,ZNF555,CTCF,NR6A1,ATF6B,CREB1,RGMB,EBF4,ZNF511,BMP4,ABLIM3
GO:0098609	cell-cell adhesion	5.156421946358689e-10	PRDX2,NRXN1,B4GALNT2,GP6,HLX,NEGR1,CBFB,GRID2,TENM3,CLDN18,ASTN2,TLN2,MYOT,NLGN4X,DCHS2,TJP1,PCDH9,STAT5B,FER,MAP2K5,EZR,MEGF10,NRXN3,ROBO1,TENM1,TRAF6,ROBO2,ITPKB,CTNNA3,DNAJA3,ITGB1,ELMO2,PCDH17,KIF26B,DSCAM,DLG2,RUNX1,DAB1,MYO10,CDH10,COL14A1,PTPN11,CDH12,TENM2,KIRREL3,CDH8,PRKAR1A,UNC5D,LAMA3,CDH4,PTPRD,AMIGO1,CLDN12,PCDH7,TENM4,LCK,PTPRU,FYN,PCDH15,FAT3,NTN1,NRG1,DOCK8,MYH9,MAD1L1,LRFN5,CNTN4,NTNG1,CDH13,SOX13,PTPRT,PCDHB16,MAD2L2,CD99,CLDN16,JAK2,MYPN,SKAP1,CLSTN2,ITCH,CLDN1,MAGI1,CYFIP2,ASTN1,IL1RAPL1,PRKCD,CD4,TGFB1,CLDN10,CELSR1,SDK2,CDH23,CTNND2,PARVA,DUSP22,LOXL3,VMP1,ZAN,PRKCA,CD6,TNFSF11,MAG,CTNNB1,FCGR2B,CDHR2,CADM3,DLG5,ADD2,FBLIM1,NR4A3,AJUBA,OBSCN,NF2,FOXO3,CNTN6,CDH26,PTPRM,PDGFRA,NLGN2,JAK1,SOX2,GPC6,PLEKHA7,DSCAML1,NLGN1,CTNNA2,MYB,LRRC4C,PPM1F,SEMA4D,PTAFR,FNDC3A,EPHB3,PAWR,VCL,LAMB1,NLGN3,GLI2,LPP,COL19A1,PKHD1,DLG3,WNK1,PELI1,DSC2,PRKG1,TFRC,ROCK1,C1QTNF1,AP3B1,PTPN2,ANK3,EPCAM,AP3D1,ADAM19,CD84,HMCN1,SPI1,EPHA7,CTNNA1,SDK1,RC3H1,COL13A1,WNT7B,PTPRS,ADTRP,ABCA12,CSRP1,PKP2,PCDH11X,DSG1,IGF1,CLDN11,BMP7,MEGF11,LILRB4,ITGAM,EFNA5,NT5E,BMP6,EXT1,PDCD1LG2,CDH9,PAG1,RHOA,HSPD1,NFKBID,EMB,RDX,FAM49B,CD27,CDH20,PTK2,ARVCF,VEZT,SHC1,CD160,ITGAL,ALOX5,DNAJB6,CD44,ADAM8,CLDN4,PKD1L1,IL18,PPARA,NOTCH4,PRKCZ,ADORA2A,TMIGD2,PCDH10,GCNT2,ETS1,PPP1CA,KIFC3,PRKCQ,SPINT2,TNR,CD300A,CAMSAP3,ANXA2,FAT2,TRIM29,SYK,CADM1,FCHO1,ZP3,TNFSF9,VSIG10,MALT1,PTPRC,TYRO3,TNXB,STXBP6,BMP4
GO:0032774	RNA biosynthetic process	5.27204118064551e-10	ENPP1,PRDX2,LDB2,ASPH,PRKCI,SLCO3A1,DNMT1,HLX,SLC9A1,PBX3,ADCY8,MED13L,TRPS1,CBFB,PDE8A,ZNF823,IL31RA,PRDM12,MED26,WWC1,ASH1L,SCAF8,STOX2,PTGIS,WWC3,PAGR1,HIVEP3,NPAS3,ARNT,LRRFIP2,STAT5B,TOX,GRM5,ZNF566,FER,CASK,MAP2K5,MAPK10,ZNF536,SP3,HDGF,GTF3C1,EZR,IKZF2,CHD7,MECOM,TACC1,TENM1,TRAF6,GTF2E2,DNAJA3,NHLH1,PSMB7,TSC22D3,ZC4H2,CCDC22,GFI1B,PTPRK,RUNX1,ERC1,HMGN3,NRIP1,THRB,EFCAB7,ITGB3BP,DACH1,ZNF569,ORC2,HDAC6,SERTAD2,MYT1,IKBKB,PEX14,ERBB4,ZNF609,BRD8,KAT6B,HIF3A,SNIP1,ESR1,MIER1,PCBP3,MAML3,CDON,EP300,CELA1,TENM2,ZNF76,RNF220,ZNF471,PDGFB,CCND3,TOB2,ZNF19,ZNF23,BRMS1,ZNF605,SCML4,HNF4G,INSR,RERE,PRKAR1A,FUBP1,ATP8B1,H2AFY2,TCF7L2,JMJD1C,ZNF443,ZNF490,ZNF564,ZNF709,ZNF799,LITAF,FBXW11,ESRRB,BASP1,TBR1,TFAP2A,CDK14,FANCA,PEG3,ZIM2,TGIF2,TGIF2-C20ORF24,MEIS1,VRTN,TRIM13,MDM4,ZNF148,MTA3,SNX6,TFDP2,ERN2,C1D,AFF3,DMD,CENPF,SLC30A9,TOX3,USP13,KAT7,ZNF667,RBM14,MED12L,SATB2,RIT2,HIRA,MKRN2,ARNTL,PLCB1,ARID4B,POLA2,RWDD3,BRPF1,PIK3R2,NRG1,UBP1,MAP2K1,MDFI,FNIP1,VAX2,TMBIM6,VGLL4,PPP2CB,PPP3R1,HDAC5,CSRNP3,ZNF692,NFIA,RNF4,JDP2,CMKLR1,ROR2,DCN,PCBD2,CDH13,CREBRF,SOX13,COPS5,MAD2L2,TLE6,CAPRIN2,ZNF418,PHF20L1,ZNF286A,FOXN3,NEUROD1,USP22,JAK2,TRAPPC9,SKAP1,UIMC1,ITCH,MLIP,BCL11B,PKNOX1,MLLT3,TSHZ2,TCF7,PDE2A,KLF15,TBX15,ANXA4,WNT11,MTA1,KLF8,LCOR,RPRD1B,CCDC62,SOX6,ACVR2A,RUNX2,CD4,TGFB1,BANP,SGK1,NSD1,IGSF1,ZHX2,PKNOX2,ASCC2,BTRC,NFATC3,F2RL1,BCAS3,CDYL2,CC2D1B,TP73,SAP18,ZBTB22,ILF2,MTDH,FANK1,MYT1L,SMAD6,BNC2,ZNF398,CLOCK,TCF12,ZNF675,ETV6,PHRF1,TFAP2D,BCL3,SND1,DUSP22,TRRAP,SBNO2,FGF10,POLR2J2,SMYD3,LOXL3,CAPN3,LUM,SMURF2,RORA,HIVEP2,AUTS2,TNFSF11,PPP3CA,NFYB,MAGEA4,KLF12,CAMK4,GATAD2B,UFL1,TRAK1,CTNNB1,PARK2,SOD2,DACH2,METTL13,SMARCC1,KLF17,PPARG,AXIN1,IL18R1,CIPC,MTF1,CBX5,BRIP1,SREBF2,CDK11A,CDK11B,FGF1,NPAT,NR4A3,FOXK2,MYOCD,TRIM5,PER2,AJUBA,ZNF626,ZNF737,CHEK2,SUPT3H,PRDM16,HCK,TRIM8,BRMS1L,ZBTB8A,ZBTB8B,CTDP1,BAZ1B,MEF2B,HDAC4,PAX2,PHF5A,SNAPC4,SFRP1,MED13,ZNF395,FOXO3,NFIB,SP4,SMAD3,CUX2,WWP2,ARNT2,SBNO1,KRBOX1,ZNF662,ZNF777,EBF3,RNF168,CASZ1,MIER3,NEDD4,ESRRG,HOXD3,HOXD4,ZNF114,KTI12,NFATC1,CDC73,APP,SSBP3,GSX2,YAP1,LRP5,POLR3G,ZNF787,SOX2,SETD2,TEAD1,PRICKLE1,ZNF653,ZNF521,ARID3A,ZNF761,CHUK,SFMBT1,ZNF584,ESR2,S100A12,GTF2F2,PRKD1,STAT1,ST18,ETV5,RHOXF2B,TAF3,PLAGL1,HNF4A,ZBTB7C,TASP1,EREG,ATF2,POU2F2,TCF3,ZNF730,RAF1,ABLIM2,ZNF766,CARD16,POLA1,BMPR2,CAMK1D,BMPR1A,ZKSCAN1,IKZF4,CDK12,TAF8,CAND2,DENND4A,MYB,FGF2,ZNF71,POU6F2,BACH1,MXD3,PPM1F,GLI4,ZFP41,BEND5,SEMA4D,NFX1,RORC,ELP3,PTAFR,SP140,SP140L,RHOXF2,JARID2,DDX58,BRDT,PHC2,RARB,SPEN,SIN3B,NCOA1,EHMT1,LMO7,DVL3,POLR2H,TCF20,ATF3,TCEANC,CKS1B,PAWR,EBF2,MAML2,TSG101,TERF2IP,CRYM,RFX2,ZNF322,SUFU,MAGEA11,PRDM15,ZNF670,ZNF695,CCDC169-SOHLH2,SOHLH2,ZNF354C,TCEA3,ZNF704,NR2C1,GLI2,TNKS,WBP2NL,ERCC1,GLIS3,WDTC1,ZNF664,DAB2,BLM,PKHD1,MYSM1,SETD5,RELN,SIN3A,RUVBL2,C

			OMMD6,GMEB1,PELI1,MAP3K7,ZNF423,SP1,TRIM22,ALK,TEAD4,HOXC13,APBB3,SRA1,UBE2V1,ADIRF,OVOL2,SNX5,NFATC2,PAX7,BNC1,ATP2B4,ACTL6B,ASXL3,TET1,CAMTA1,CCPG1,ADNP,ARID4A,CDK6,PHF2,TFRC,EPHA5,WWOX,MEF2A,DCAF6,SATB1,PSMB2,EYA1,GATAD2A,TRPV1,HOXB3,HOXB4,HOXB5,HOXB6,TFEB,MNAT1,ACTR2,L3MBTL4,HDAC1,RECQL5,TRIM24,PLCG2,SCMH1,PAXIP1,ZNF713,EGR2,HIPK3,RNF10,RYBP,AP3B1,PTPN2,NTRK1,KPNA6,TRIM44,EPCAM,ZNF425,MUC1,PTPN14,BCOR,AP3D1,ZNF41,CARM1,ZNF383,ZNF616,ZNF836,SMARCA2,CUX1,SPI1,DNMT3B,FOXP2,PSPC1,GLIS1,SFMBT2,JAZF1,RBPMS,CHURC1,CREB5,MAP3K13,ERCC3,SMYD1,SNCA,BMPR1B,PRIM2,ZEB2,FOXJ3,HEY2,ZMYND11,KMT2D,CREM,KMT2C,MACC1,MITF,OPRD1,RPTOR,NFIX,BTK,MLLT1,TROVE2,CBFA2T2,IGF1,MLXIP,ATF7IP,PTEN,BMP7,MXI1,SOX5,CIR1,PCBP2,ZNF780A,ZNF780B,ZBTB20,LILRB4,PKD2,ZNF652,HSF2BP,KCTD1,RIPPLY1,RCOR3,TADA2A,NCOA3,ZNF146,ZNF565,WNT7A,ZBED6,BRWD3,CRTC3,ARNTL2,KAT6A,ZKSCAN7,ZNF197,ZNF660,BMP6,TAF15,NFE2L1,WDR43,ZNF30,ATRX,IKZF1,PRMT2,ASCC1,SMO,RALY,TCF4,SOX30,TFE3,GRIN1,JADE1,RHOA,ROR1,RQCD1,TF,ONECUT2,CUL3,TFEC,STAT6,MLF1,RBBP8,CDK13,DUSP26,ZNF362,NACC2,SUPT4H1,ZNF354A,AGT,HDGFRP3,POLR2F,STAT2,ZMPSTE24,GTF2E1,PBX1,MAP3K5,TCFL5,CREBBP,DLX1,GTF2IRD2,NCF1,SHC1,ZNF813,LBX2,PARP10,DPRX,SETDB2,DNAJB6,SP7,ADAM8,RPS6KA5,ZNF484,ZNF93,PRDM2,NOS1,ZNF44,MED15,RGS14,ACTN4,MC1R,TCF25,ZNF282,EDRF1,IL18,PPARA,ARID5B,SLC25A33,SMARCAL1,TAF2,NOTCH4,ZMYND8,ZNF366,CRX,PAX6,PRKCZ,ADORA2A,OTUD7B,NIPBL,YEATS4,PHF20,ZNF143,BRF1,TBL1X,FHL2,MBTD1,ATF6,ZBTB5,ZNF708,ETS1,MTF2,TAGLN3,NCOR2,PRKCQ,CSRNP1,HDAC2,IMPACT,SRCAP,POU3F3,NOTO,ZKSCAN5,ELF2,PHIP,PPP2CA,RPL23,ATXN1,SP100,ZNF347,ZNF415,TRIM29,ADCY1,SYK,CNOT1,WWTR1,GTF2H5,NFXL1,ZBTB38,BRCA2,ATF7,DVL2,MORC1,YBX1,TAF1,TICAM1,THAP3,SNAPC3,HSF1,MAX,SAP130,EZH1,NRF1,NCOA2,QRICH1,ZP3,CHD6,RNF2,ZNF554,MYEF2,TBP,TRIM37,MET,ZNF461,CAMK2D,TIMELESS,BRD9,MALT1,SETD3,KDM2B,MLXIPL,PHB,EDA,ZNF555,CTCF,NR6A1,ATF6B,CREB1,RGMB,EBF4,ZNF511,BMP4,ABLIM3
GO:0097659	nucleic acid-templated transcription	5.32653192753716e-10	ENPP1,PRDX2,LDB2,ASPH,PRKCI,SLCO3A1,DNMT1,HLX,SLC9A1,PBX3,ADCY8,MED13L,TRPS1,CBFB,PDE8A,ZNF823,IL31RA,PRDM12,MED26,WWC1,ASH1L,SCAF8,STOX2,PTGIS,WWC3,PAGR1,HIVEP3,NPAS3,ARNT,LRRFIP2,STAT5B,TOX,GRM5,ZNF566,FER,CASK,MAP2K5,MAPK10,ZNF536,SP3,HDGF,GTF3C1,EZR,IKZF2,CHD7,MECOM,TACC1,TENM1,TRAF6,GTF2E2,DNAJA3,NHLH1,PSMB7,TSC22D3,ZC4H2,CCDC22,GFI1B,PTPRK,RUNX1,ERC1,HMGN3,NRIP1,THRB,EFCAB7,ITGB3BP,DACH1,ZNF569,ORC2,HDAC6,SERTAD2,MYT1,IKBKB,PEX14,ERBB4,ZNF609,BRD8,KAT6B,HIF3A,SNIP1,ESR1,MIER1,PCBP3,MAML3,CDON,EP300,CELA1,TENM2,ZNF76,RNF220,ZNF471,PDGFB,CCND3,TOB2,ZNF19,ZNF23,BRMS1,ZNF605,SCML4,HNF4G,INSR,RERE,PRKAR1A,FUBP1,ATP8B1,H2AFY2,TCF7L2,JMJD1C,ZNF443,ZNF490,ZNF564,ZNF709,ZNF799,LITAF,FBXW11,ESRRB,BASP1,TBR1,TFAP2A,CDK14,FANCA,PEG3,ZIM2,TGIF2,TGIF2-C20ORF24,MEIS1,VRTN,TRIM13,MDM4,ZNF148,MTA3,SNX6,TFDP2,ERN2,C1D,AFF3,DMD,CENPF,SLC30A9,TOX3,USP13,KAT7,ZNF667,RBM14,MED12L,SATB2,RIT2,HIRA,MKRN2,ARNTL,PLCB1,ARID4B,RWDD3,BRPF1,PIK3R2,NRG1,UBP1,MAP2K1,MDFI,FNIP1,VAX2,TMBIM6,VGLL4,PPP2CB,PPP3R1,HDAC5,CSRNP3,ZNF692,NFIA,RNF4,JDP2,CMKLR1,ROR2,DCN,PCBD2,CDH13,CREBRF,SOX13,COPS5,MAD2L2,TLE6,CAPRIN2,ZNF418,PHF20L1,ZNF286A,FOXN3,NEUROD1,USP22,JAK2,TRAPPC9,SKAP1,UIMC1,ITCH,MLIP,BCL11B,PKNOX1,MLLT3,TSHZ2,TCF7,PDE2A,KLF15,TBX15,ANXA4,WNT11,MTA1,KLF8,LCOR,RPRD1B,CCDC62,SOX6,ACVR2A,RUNX2,CD4,TGFB1,BANP,SGK1,NSD1,IGSF1,ZHX2,PKNOX2,ASCC2,BTRC,NFATC3,F2RL1,BCAS3,CDYL2,CC2D1B,TP73,SAP18,ZBTB22,ILF2,MTDH,FANK1,MYT1L,SMAD6,BNC2,ZNF398,CLOCK,TCF12,ZNF675,ETV6,PHRF1,TFAP2D,BCL3,SND1,DUSP22,TRRAP,SBNO2,FGF10,POLR2J2,SMYD3,LOXL3,CAPN3,LUM,SMURF2,RORA,HIVEP2,AUTS2,TNFSF11,PPP3CA,NFYB,MAGEA4,KLF12,CAMK4,GATAD2B,UFL1,TRAK1,CTNNB1,PARK2,SOD2,DACH2,METTL13,SMARCC1,KLF17,PPARG,AXIN1,IL18R1,CIPC,MTF1,CBX5,BRIP1,SREBF2,CDK11A,CDK11B,FGF1,NPAT,NR4A3,FOXK2,MYOCD,TRIM5,PER2,AJUBA,ZNF626,ZNF737,CHEK2,SUPT3H,PRDM16,HCK,TRIM8,BRMS1L,ZBTB8A,ZBTB8B,CTDP1,BAZ1B,MEF2B,HDAC4,PAX2,PHF5A,SNAPC4,SFRP1,MED13,ZNF395,FOXO3,NFIB,SP4,SMAD3,CUX2,WWP2,ARNT2,SBNO1,KRBOX1,ZNF662,ZNF777,EBF3,RNF168,CASZ1,MIER3,NEDD4,ESRRG,HOXD3,HOXD4,ZNF114,KTI12,NFATC1,CDC73,APP,SSBP3,GSX2,YAP1,LRP5,POLR3G,ZNF787,SOX2,SETD2,TEAD1,PRICKLE1,ZNF653,ZNF521,ARID3A,ZNF761,CHUK,SFMBT1,ZNF584,ESR2,S100A12,GTF2F2,PRKD1,STAT1,ST18,ETV5,RHOXF2B,TAF3,PLAGL1,HNF4A,ZBTB7C,TASP1,EREG,ATF2,POU2F2,TCF3,ZNF730,RAF1,ABLIM2,ZNF766,CARD16,BMPR2,CAMK1D,BMPR1A,ZKSCAN1,IKZF4,CDK12,TAF8,CAND2,DENND4A,MYB,FGF2,ZNF71,POU6F2,BACH1,MXD3,PPM1F,GLI4,ZFP41,BEND5,SEMA4D,NFX1,RORC,ELP3,PTAFR,SP140,SP140L,RHOXF2,JARID2,DDX58,BRDT,PHC2,RARB,SPEN,SIN3B,NCOA1,EHMT1,LMO7,DVL3,POLR2H,TCF20,ATF3,TCEANC,CKS1B,PAWR,EBF2,MAML2,TSG101,TERF2IP,CRYM,RFX2,ZNF322,SUFU,MAGEA11,PRDM15,ZNF670,ZNF695,CCDC169-SOHLH2,SOHLH2,ZNF354C,TCEA3,ZNF704,NR2C1,GLI2,TNKS,WBP2NL,ERCC1,GLIS3,WDTC1,ZNF664,DAB2,BLM,PKHD1,MYSM1,SETD5,RELN,SIN3A,RUVBL2,COMMD6,GMEB1,PELI1,MAP3K7,ZNF423,SP1,TRIM22,ALK,TEAD4,HOXC13,APBB

			3,SRA1,UBE2V1,ADIRF,OVOL2,SNX5,NFATC2,PAX7,BNC1,ATP2B4,ACTL6B,ASXL3,TET1,CAMTA1,CCPG1,ADNP,ARID4A,CDK6,PHF2,TFRC,EPHA5,WWOX,MEF2A,DCAF6,SATB1,PSMB2,EYA1,GATAD2A,TRPV1,HOXB3,HOXB4,HOXB5,HOXB6,TFEB,MNAT1,ACTR2,L3MBTL4,HDAC1,RECQL5,TRIM24,PLCG2,SCMH1,PAXIP1,ZNF713,EGR2,HIPK3,RNF10,RYBP,AP3B1,PTPN2,NTRK1,KPNA6,TRIM44,EPCAM,ZNF425,MUC1,PTPN14,BCOR,AP3D1,ZNF41,CARM1,ZNF383,ZNF616,ZNF836,SMARCA2,CUX1,SPI1,DNMT3B,FOXP2,PSPC1,GLIS1,SFMBT2,JAZF1,RBPMS,CHURC1,CREB5,MAP3K13,ERCC3,SMYD1,SNCA,BMPR1B,ZEB2,FOXJ3,HEY2,ZMYND11,KMT2D,CREM,KMT2C,MACC1,MITF,OPRD1,RPTOR,NFIX,BTK,MLLT1,TROVE2,CBFA2T2,IGF1,MLXIP,ATF7IP,PTEN,BMP7,MXI1,SOX5,CIR1,PCBP2,ZNF780A,ZNF780B,ZBTB20,LILRB4,PKD2,ZNF652,HSF2BP,KCTD1,RIPPLY1,RCOR3,TADA2A,NCOA3,ZNF146,ZNF565,WNT7A,ZBED6,BRWD3,CRTC3,ARNTL2,KAT6A,ZKSCAN7,ZNF197,ZNF660,BMP6,TAF15,NFE2L1,WDR43,ZNF30,ATRX,IKZF1,PRMT2,ASCC1,SMO,RALY,TCF4,SOX30,TFE3,GRIN1,JADE1,RHOA,ROR1,RQCD1,TF,ONECUT2,CUL3,TFEC,STAT6,MLF1,RBBP8,CDK13,DUSP26,ZNF362,NACC2,SUPT4H1,ZNF354A,AGT,HDGFRP3,POLR2F,STAT2,ZMPSTE24,GTF2E1,PBX1,MAP3K5,TCFL5,CREBBP,DLX1,GTF2IRD2,NCF1,SHC1,ZNF813,LBX2,PARP10,DPRX,SETDB2,DNAJB6,SP7,ADAM8,RPS6KA5,ZNF484,ZNF93,PRDM2,NOS1,ZNF44,MED15,RGS14,ACTN4,MC1R,TCF25,ZNF282,EDRF1,IL18,PPARA,ARID5B,SLC25A33,SMARCAL1,TAF2,NOTCH4,ZMYND8,ZNF366,CRX,PAX6,PRKCZ,ADORA2A,OTUD7B,NIPBL,YEATS4,PHF20,ZNF143,BRF1,TBL1X,FHL2,MBTD1,ATF6,ZBTB5,ZNF708,ETS1,MTF2,TAGLN3,NCOR2,PRKCQ,CSRNP1,HDAC2,IMPACT,SRCAP,POU3F3,NOTO,ZKSCAN5,ELF2,PHIP,PPP2CA,RPL23,ATXN1,SP100,ZNF347,ZNF415,TRIM29,ADCY1,SYK,CNOT1,WWTR1,GTF2H5,NFXL1,ZBTB38,BRCA2,ATF7,DVL2,MORC1,YBX1,TAF1,TICAM1,THAP3,SNAPC3,HSF1,MAX,SAP130,EZH1,NRF1,NCOA2,QRICH1,ZP3,CHD6,RNF2,ZNF554,MYEF2,TBP,TRIM37,MET,ZNF461,CAMK2D,TIMELESS,BRD9,MALT1,SETD3,KDM2B,MLXIPL,PHB,EDA,ZNF555,CTCF,NR6A1,ATF6B,CREB1,RGMB,EBF4,ZNF511,BMP4,ABLIM3
GO:0034654	nucleobase-containing compound biosynthetic process	5.554707638777302e-10	ENPP1,PRDX2,LDB2,ASPH,PRKCI,SLCO3A1,DNMT1,HLX,SLC9A1,PBX3,ADCY8,MED13L,TRPS1,CBFB,PDE8A,ZNF823,IL31RA,PRDM12,MED26,WWC1,ASH1L,SCAF8,STOX2,PTGIS,PRKAG2,WWC3,PAGR1,ADCY7,HIVEP3,NPAS3,ARNT,LRRFIP2,STAT5B,TOX,GRM5,ZNF566,FER,CASK,MAP2K5,MAPK10,ZNF536,SP3,HDGF,GTF3C1,EZR,IKZF2,CHD7,MECOM,TACC1,TENM1,NADK2,TRAF6,GTF2E2,DNAJA3,NHLH1,HSP90AA1,PSMB7,TSC22D3,ZC4H2,CCDC22,GMDS,GFI1B,PTPRK,RUNX1,ERC1,HMGN3,NRIP1,THRB,EFCAB7,ITGB3BP,DACH1,ZNF569,CCT2,ORC2,HDAC6,SERTAD2,MYT1,IKBKB,PEX14,ERBB4,MRE11A,ZNF609,BRD8,KAT6B,HIF3A,SNIP1,ESR1,MIER1,PCBP3,MAML3,CDON,EP300,CELA1,TENM2,ZNF76,RNF220,ZNF471,PDGFB,CCND3,TOB2,ZNF19,ZNF23,BRMS1,ZNF605,SCML4,HNF4G,INSR,RERE,PRKAR1A,FUBP1,ATP8B1,H2AFY2,TCF7L2,JMJD1C,ZNF443,ZNF490,ZNF564,ZNF709,ZNF799,LITAF,FBXW11,ESRRB,MAP3K4,BASP1,TBR1,TFAP2A,CDK14,FANCA,PEG3,ZIM2,TGIF2,TGIF2-C20ORF24,MEIS1,VRTN,TRIM13,MDM4,ZNF148,MTA3,SNX6,TFDP2,ERN2,C1D,AFF3,DMD,CENPF,SLC30A9,TOX3,USP13,KAT7,ZNF667,RBM14,PRPS2,MED12L,SATB2,RIT2,HIRA,MKRN2,ARNTL,PLCB1,ARID4B,POLA2,RWDD3,BRPF1,PIK3R2,NRG1,UBP1,MAP2K1,MDFI,FNIP1,VAX2,TMBIM6,VGLL4,PPP2CB,PPP3R1,HDAC5,CSRNP3,ZNF692,NFIA,RNF4,JDP2,CMKLR1,ROR2,DCN,PRPSAP2,PCBD2,CDH13,CREBRF,SOX13,ACSBG1,COPS5,AK2,MAD2L2,TLE6,CAPRIN2,ZNF418,PHF20L1,TERF2,ZNF286A,NME7,FOXN3,AK5,NEUROD1,USP22,JAK2,TRAPPC9,SKAP1,UIMC1,ITCH,MLIP,BCL11B,IMPDH1,PKNOX1,MLLT3,TSHZ2,TCF7,PDE2A,KLF15,TBX15,ANXA4,WNT11,MTA1,KLF8,NOX4,LCOR,RPRD1B,CCDC62,SOX6,ACVR2A,RUNX2,CD4,TGFB1,BANP,SGK1,NSD1,IGSF1,ZHX2,PKNOX2,ASCC2,BTRC,NFATC3,POLK,F2RL1,BCAS3,CDYL2,CC2D1B,TP73,SAP18,ZBTB22,ILF2,MTDH,FANK1,MYT1L,SMAD6,BNC2,ZNF398,CLOCK,TCF12,ZNF675,ETV6,PHRF1,TFAP2D,BCL3,SND1,DUSP22,HNRNPC,TRRAP,SBNO2,FGF10,POLR2J2,ADK,SMYD3,LOXL3,ADCY2,CAPN3,LUM,SMURF2,RORA,HIVEP2,HSD17B12,AUTS2,TNFSF11,SMG6,PPP3CA,NFYB,MAGEA4,KLF12,CAMK4,GATAD2B,UFL1,TRAK1,CTNNB1,PARK2,SOD2,DACH2,METTL13,SMARCC1,KLF17,PPARG,AXIN1,IL18R1,CIPC,MTF1,CBX5,BRIP1,SREBF2,PFAS,CDK11A,CDK11B,FGF1,NPAT,NR4A3,FOXK2,MYOCD,TRIM5,PER2,AJUBA,ZNF626,ZNF737,CHEK2,SUPT3H,PRDM16,HCK,TRIM8,BRMS1L,ZBTB8A,ZBTB8B,CTDP1,BAZ1B,MEF2B,HDAC4,PAX2,PHF5A,SNAPC4,SFRP1,MED13,ZNF395,FOXO3,NFIB,SP4,SMAD3,CUX2,WWP2,ARNT2,SBNO1,KRBOX1,ZNF662,ZNF777,EBF3,RNF168,CASZ1,MIER3,NEDD4,ESRRG,HOXD3,HOXD4,ZNF114,KTI12,NFATC1,UCK2,CDC73,APP,SSBP3,GSX2,YAP1,PPCS,TEN1,NVL,LRP5,POLR3G,ZNF787,SOX2,SETD2,TEAD1,PRICKLE1,ZNF653,ZNF521,ELOVL2,ARID3A,ZNF761,CHUK,SFMBT1,ZNF584,ESR2,S100A12,GTF2F2,PRKD1,STAT1,ST18,ETV5,RHOXF2B,TAF3,PLAGL1,HNF4A,ZBTB7C,TASP1,EREG,ATF2,POU2F2,TCF3,ZNF730,RAF1,ABLIM2,ZNF766,CARD16,POLA1,BMPR2,CAMK1D,BMPR1A,ZKSCAN1,IDO2,IKZF4,CDK12,TAF8,CAND2,DENND4A,MYB,FGF2,ZNF71,POU6F2,BACH1,MXD3,PPM1F,ADCY5,GLI4,ZFP41,BEND5,SEMA4D,NFX1,RORC,ELP3,PTAFR,SP140,SP140L,RHOXF2,JARID2,DDX58,SLC5A8,BRDT,PHC2,RARB,SPEN,SIN3B,NCOA1,EHMT1,LMO7,DVL3,POLR2H,TCF20,ATF3,TCEANC,CKS1B,PAWR,EBF2,MAML2,TSG101,TERF2IP,CRYM,RFX2,ZNF322,SUFU,MAGEA11,PRDM15,ZNF670,ZNF695,CCDC169-

			SOHLH2,SOHLH2,POLE,ZNF354C,TCEA3,CMPK1,ZNF704,NR2C1,ME1,GLI2,TNKS,WBP2NL,ERCC1,GLIS3,WDTC1,ZNF664,DAB2,BLM,PKHD1,MYSM1,SETD5,SMG5,RELN,SIN3A,RUVBL2,COMMD6,GUCY1A2,GMEB1,PELI1,SLC25A16,MAP3K7,ZNF423,SP1,TRIM22,ALK,TEAD4,HOXC13,APBB3,SRA1,UBE2V1,ADIRF,OVOL2,SNX5,NFATC2,PAX7,BNC1,ATP2B4,ACTL6B,ASXL3,PAK3,TET1,CAMTA1,CCPG1,ADNP,ARID4A,CDK6,PHF2,TFRC,EPHA5,WWOX,MEF2A,ADCY9,DCAF6,XRN1,SATB1,PSMB2,EYA1,GATAD2A,TRPV1,HOXB3,HOXB4,HOXB5,HOXB6,TFEB,MNAT1,ACTR2,L3MBTL4,ACACA,HDAC1,RECQL5,SMOC2,TRIM24,PLCG2,SCMH1,PAXIP1,ZNF713,EGR2,HIPK3,RNF10,RYBP,AP3B1,PTPN2,NTRK1,KPNA6,TRIM44,EPCAM,ZNF425,GNL3L,MUC1,PTPN14,BCOR,AP3D1,ZNF41,CARM1,ZNF383,ZNF616,ZNF836,SMARCA2,CUX1,SPI1,DNMT3B,FOXP2,PSPC1,GLIS1,SFMBT2,JAZF1,RBPMS,CHURC1,CREB5,MAP3K13,ERCC3,SMYD1,SNCA,BMPR1B,POLN,PRIM2,ZEB2,FOXJ3,NADK,HEY2,ZMYND11,KMT2D,CREM,KMT2C,MACC1,MITF,OPRD1,RPTOR,NFIX,BTK,MLLT1,TROVE2,CBFA2T2,IGF1,MLXIP,ATF7IP,MAPK1,PTEN,BMP7,MXI1,SOX5,CIR1,PCBP2,ZNF780A,ZNF780B,ATP6V1A,MLYCD,ZBTB20,RFC3,LILRB4,PKD2,ZNF652,HSF2BP,KCTD1,RIPPLY1,NT5E,RCOR3,TADA2A,NCOA3,PKIB,ZNF146,ZNF565,WNT7A,ZBED6,BRWD3,CRTC3,ARNTL2,ELOVL5,KAT6A,ZKSCAN7,ZNF197,ZNF660,BMP6,TAF15,NFE2L1,WDR43,ZNF30,ATRX,IKZF1,PRMT2,ASCC1,MTHFD2L,SMO,RALY,AMPD1,TCF4,SOX30,TFE3,GRIN1,JADE1,RHOA,SPRTN,ROR1,RQCD1,TF,ONECUT2,CUL3,TFEC,STAT6,MLF1,RBBP8,CDK13,DUSP26,ZNF362,NACC2,SUPT4H1,ZNF354A,AGT,HDGFRP3,ELOVL3,POLH,POLR2F,STAT2,ZMPSTE24,GTF2E1,PPCDC,PBX1,MAP3K5,TCFL5,CREBBP,DLX1,GTF2IRD2,NCF1,SHC1,ZNF813,ALDOA,LBX2,PARP10,DPRX,GUCY2F,SETDB2,DNAJB6,SP7,ADAM8,RPS6KA5,ZNF484,ZNF93,PRDM2,NOS1,ZNF44,MED15,RGS14,ACTN4,MC1R,TCF25,ZNF282,EDRF1,IL18,TEX12,PPARA,ARID5B,SLC25A33,SMARCAL1,TAF2,LDHC,NOTCH4,ZMYND8,ZNF366,CRX,PAX6,PRKCZ,ADORA2A,OTUD7B,NIPBL,YEATS4,PHF20,ZNF143,BRF1,TBL1X,FHL2,MBTD1,ATF6,ZBTB5,ZNF708,ETS1,MTF2,TAGLN3,NCOR2,PRKCQ,CSRNP1,HDAC2,IMPACT,SRCAP,POU3F3,NOTO,ZKSCAN5,ELF2,PHIP,PPP2CA,SLC26A2,RPL23,RFC5,ATXN1,SP100,ZNF347,ZNF415,REV3L,TRIM29,PARN,ADCY1,SYK,CNOT1,WWTR1,GTF2H5,NFXL1,ZBTB38,BRCA2,ATF7,DVL2,MORC1,YBX1,TAF1,TICAM1,THAP3,SNAPC3,HSF1,MAX,SAP130,EZH1,NRF1,NCOA2,QRICH1,ZP3,CHD6,RNF2,ZNF554,MYEF2,TBP,TRIM37,MET,ZNF461,CAMK2D,TIMELESS,BRD9,MALT1,SETD3,KDM2B,MLXIPL,CCT3,PHB,EDA,ZNF555,CTCF,NR6A1,ADCY10,ATF6B,CREB1,LIG1,RGMB,EBF4,SLC25A13,ZNF511,BMP4,ABLIM3
GO:0050770	regulation of axonogenesis	6.116196991327511e-10	*SEMA3A,SEMA3D,TIAM2,SEMA5A,ROBO1,CDKL5,ROBO2,DSCAM,DAB1,GOLGA4,LIMK1,NTRK2,CDH4,PAK1,BDNF,AMIGO1,RTN4,RTN4R,NTN1,MAP2K1,UST,SEMA5B,KEL,SLIT2,DISC1,EPHA4,MAG,XK,DRAXIN,NIN,DCC,DIP2B,FSTL4,ISLR2,SEMA6D,BMPR2,RAB11A,LRRC4C,SEMA4D,PLXNA2,EPHB3,WNT3,PAFAH1B1,TRPC5,RUFY3,MACF1,PAK3,ADNP,MAP1B,TRIM46,EPHA7,MAP3K13,PTPRS,PTEN,EFNA5,WNT7A,SYNGAP1,GSK3B,EPHB2,MAP2,BRAF,TNR,CDKL3,SLIT1,SEMA3C*
GO:0030001	metal ion transport	6.195733274045206e-10	*SLC39A11,ASPH,PDE4D,SLC9A1,FAM155A,CHERP,CACHD1,RYR1,NOS1AP,NOX5,EPB41,DPP6,UTRN,ATP2B2,KCNQ5,CASK,KCNIP4,LRP2,SLC38A11,ANK2,SLC9A9,CHD7,HTR2B,RYR3,SLC24A2,NKAIN2,ITGB1,SLC24A3,NKAIN3,FLVCR2,ABCC8,KCNS3,NIPAL2,NETO2,CACNB2,ADORA1,KCNJ16,DPP10,PDGFB,CACNA1B,STK39,CAB39,ZDHHC13,SLC20A2,SLC4A10,CATSPER2,AMIGO1,KCNMA1,KCNRG,LCK,TMC2,SLC12A8,SLC6A16,MICU3,DMD,SLC30A9,SLC8A1,JPH2,FYN,LMTK2,SLC5A6,CACNA1E,SLC4A5,KCNN3,CNNM2,BDKRB1,KCNIP1,STIM1,NALCN,TMBIM6,SLC39A14,CATSPER3,FGF14,CLDN16,ANO6,KCNB2,NPSR1,SCN4A,TMC1,SLC5A9,SLC9A7,KCNC4,CACNA1H,CAMK2B,CD4,TGFB1,SGK1,KEL,JPH3,CDH23,KCNJ3,CACNA1D,CACNA2D3,KCNH1,HOMER2,SLC17A7,KCNH7,CAPN3,VMP1,PPP3CA,CTNNB1,TRPM1,KCNJ12,RYR2,KCNA6,NDUFA9,NOL3,CACNA1C,SCN3B,ITPR1,WWP2,NEDD4,SLC4A4,SLC6A1,KCNK1,VDAC1,IBTK,SLC9B2,SPTBN4,DRD1,PRKD1,SLC6A3,FXYD2,FXYD6,KCNG4,FGF2,ADCYAP1R1,KLHL3,MRS2,SLC5A8,SLC9B1,KCNQ1,TRPA1,TRPM3,NMUR2,AKAP6,TRPC5,WNK1,KCNQ2,SLC8A2,SLC9C1,TPCN1,AHNAK,RGS7,ATP2B4,NSF,KCNAB2,TFRC,CACNA1A,STOM,TRPV1,SLC38A6,PLCG2,GPR35,SLC39A10,ANK3,KCND3,CACNG8,SLC9C2,NETO1,CD84,MCU,TRDN,STAC,TRPC6,ABCB7,SLC41A2,SLC48A1,SNCA,CACNG2,SLC39A9,OPRD1,PKP2,SLC4A8,HTT,SLC30A7,SCN8A,TMCO1,PKD2,STEAP4,KCNC2,SLC6A14,PSEN2,GRIN1,RHOA,GRIN3A,TF,ITPR2,EPM2A,SCN9A,AGT,ZMPSTE24,HEPHL1,PDE4B,TRPC4AP,KCNJ15,SLC5A10,STC2,SLC5A3,PKD1L1,NOS1,ACTN4,SCNN1B,SLC1A1,XCR1,LRRC52,NKAIN1,CALCRL,MCOLN1,STEAP3,ATP1A3,ADORA2A,GRIN2B,CNTN1,DIAPH1,HTR2C,TMEM163,ANXA2,KCND2,SCN1A,CACNG3,SLC10A1,ATP2B3,GPM6A,SLC13A3,SLC5A4,KCNQ3,SESTD1,CAMK2D,KCNJ6,NEDD4L,PTPRC,EDNRA*
GO:0010033	response to organic substance	6.426784015110434e-10	*ENPP1,PRDX2,NRXN1,ASPH,PRKCI,PDE4D,DNMT1,SLC9A1,ADCY8,PDE8A,IL31RA,RPS6KA2,PTGFR,CALR3,CLDN18,CTDSPL2,RYR1,LATS2,NREP,PTGIS,TJP1,PRLR,PAGR1,ADCY7,NTRK3,CBL,ARNT,EGLN2,FLT3,STAT5B,GRM5,SAMHD1,FER,CASK,MAP2K5,LRP2,PIK3CD,HDGF,ANK2,EZR,ROBO1,SLC26A6,HTR2B,ITGB6,RYR3,GOT1,TRAF6,ROBO2,DNAJA3,ITGB1,HSP90AA1,CDC6,PSMB7,PTPRK,ABCC8,HMGN3,NRIP1,DNAJB14,THRB,STXBP4,IL17RB,TCIRG1,PTPN11,HDAC6,ADAMTS3,IKBKB,ERBB4,BRD8,ESR1,PMEPA1,NTRK2,EP300,FNTA,PDGFB,CACNA1B,CCND3,USP46,BPI,STK39,INSR,TCF7L2,PIP4K2A,CAB39,PAK1,LITAF,ESRRB,BDN*

			F,AMIGO1,KCNMA1,CHRM3,FUT8,TRIM13,UBR2,HLCS,IL5RA,SNX6,PTPRU,RFTN1,ERN2,LSP1,AP3S1,NPLOC4,AFF3,DMD,USP13,KAT7,SLC8A1,GSN,RBM14,SATB2,FYN,ARNTL,NF1,PLCB1,MGMT,RXFP1,CHRDL1,BCR,CHI3L1,PIK3R2,CDK19,BDKRB1,DOCK8,TMBIM6,PPP2CB,HDAC5,CMKLR1,ROR2,DCN,SLC39A14,CDH13,CREBRF,TRDMT1,PTPRT,ACSBG1,COPS5,IPO5,MAN1A1,RGS8,DEFA1B,DEFA3,F5,PTGER2,STIP1,NEUROD1,GNAQ,RFFL,CCBE1,JAK2,YTHDC2,CLDN1,TCF7,PDE2A,KLF15,LAMTOR3,CYFIP2,WNT11,NOX4,LCOR,CACNA1H,IL1RAPL1,CCDC62,ADAM23,PRKCD,SOX6,TAB2,ACVR2A,RUNX2,CD4,TGFB1,SGK1,BTRC,PTPRN2,F2RL1,BCAS3,DNAJB2,SLIT3,ZFYVE9,SLIT2,TP73,ITIH4,GABRB1,KANK1,MTDH,CHRM1,SMAD6,RXFP2,CLOCK,ZNF675,BCKDHB,BMPER,TIMP2,DUSP22,NAIP,HOMER2,SBNO2,FGF10,SMYD3,IL1RAPL2,GLP2R,ADCY2,SLC16A1,SMURF2,EPHA4,RORA,PRKCA,CNR1,CD6,TNFSF11,PPP3CA,HBE1,NFYB,UFL1,CTNNB1,PARK2,SOD2,FCGR2B,HSPA6,SMARCC1,IGF1R,PPARG,AXIN1,IL18R1,IL1RL1,MSR1,CYBB,OTC,BRIP1,SREBF2,RYR2,LEPR,LEPROT,FGF1,RGS10,NR4A3,NOL3,MYOCD,TRIM5,GLG1,CHEK2,PRDM16,HCK,SORBS1,CAPN2,DIO2,FLT4,HDAC4,PAX2,CPOX,SFRP1,FOXO3,TPH2,SSH1,SYNCRIP,SMAD3,RNFT2,CUX2,ACKR2,GRIK5,IFNAR1,NEDD4,NRP2,ESRRG,TAB1,SLC6A1,SEC61B,CDC73,APP,PDGFRA,YAP1,AMFR,RAB11FIP5,SESN1,TNFRSF19,RNLS,ALPL,JAK1,LRP5,SLC9B2,GNG2,SOX2,SETD2,CHST11,DRD1,GLRA2,CHUK,ERLIN1,FRS2,MMP2,ESR2,PRKD1,STAT1,ST18,SLC6A3,HNF4A,STRA6,EREG,MAPKAPK3,ATF2,RAF1,EPHX1,CARD16,CASP1,PTPN1,ADAMTS12,GGT7,BMPR2,BMPR1A,TSPAN12,COL16A1,PIK3R3,EPG5,FGF2,ADCY5,RORC,PTAFR,ADCYAP1R1,JARID2,DDX58,RARB,PRKCG,NCOA1,EEF1E1,CHRD,EIF2B5,PTPRE,ATF3,SHOC2,SRSF5,STT3B,CCDC3,IDE,KCNQ1,RFX2,WNT3,P4HB,CCL14,CCL15,PAFAH1B1,CNTFR,COL4A3,TRPA1,AKAP6,NR2C1,EDEM3,IRS4,ME1,GLI2,NEO1,ERCC1,WDTC1,DAB2,BLM,UBR1,LDLRAD4,WNK1,SIN3A,RUVBL2,CSF3R,PELI1,IQGAP1,MAP3K7,ZNF423,SP1,ALK,GSTM3,SRA1,XDH,LTBP1,OVOL2,SNX5,RGS7,ATP2B4,PACRG,PAK3,DYX1C1,P2RX6,ADNP,MAPKAPK2,MAP1B,COL4A6,SCARB1,TYR,TFRC,EPHA5,WWOX,CACNA1A,GPC3,XRN1,ADRBK1,PSMB2,BRINP1,SHPK,TRPV1,GHR,FLRT2,ACTR2,ACACA,ELAVL4,HDAC1,RECQL5,C2,SMOC2,TRIM24,ADIPOR2,PLCG2,ROCK1,EPS8,EGR2,CAPN10,GPR35,ABCC1,PTPN2,INSRR,NTRK1,TRIM44,MAN1B1,AXIN2,SARM1,CARM1,SRD5A2,HTR1D,SPI1,PTPRA,CTNNA1,ACAP2,LY86,BCAR3,ILDR2,CHURC1,RAB15,RAPGEF3,SDK1,PDXP,PTK7,SNCA,BMPR1B,MAGI2,PNPT1,ZEB2,RC3H1,RNF103,RNF103-CHMP3,CNGA3,WNT7B,HMGCS2,KMT2D,SPPL2A,ADTRP,ABCA12,SRSF6,OPRD1,HIPK1,NUGGC,RPTOR,BTK,DSG1,TROVE2,IGF1,STX8,DPYSL3,MAPK1,SPRED2,BMP7,PIK3C3,SOX5,PRCP,LRRK2,GNA14,ZBTB20,CHMP5,RFC3,BAG6,LILRB4,TMEM108,IL11RA,NSG2,PKD2,EFNA5,SEL1L2,PDGFC,NCOA3,WNT7A,ZBED6,NLRP1,GLDC,GPR21,BMP6,ANO1,GPI,IL17RD,SOS1,TSHR,EXT1,PDCD1LG2,ATRX,PLIN2,PNPLA3,PRMT2,RAPGEF2,KCNC2,SMO,GET4,PRKAR2B,CPT1A,TNFRSF11B,SOX30,UBR5,GRB14,GRIN1,RHOA,GRIN3A,RQCD1,ONECUT2,CUL3,HSPD1,ITPR2,GABRB3,GSK3B,DNAJC3,RDX,STAT6,EEF2K,FAM49B,CD27,BOK,SULF1,KRT8,AGT,PRKAA2,ADAR,FBN2,STAT2,BRINP3,HRH4,PTK2,PHEX,RNF121,PDE4B,VEPH1,MAP3K5,CREBBP,DLX1,FMOD,PCSK6,SLC29A1,UGT1A1,SHC1,RNFT1,RPS6KB1,STC2,GPR173,CD44,CLDN4,RPS6KA5,NOS1,GCKR,PLVAP,ACTN4,SCNN1B,ZFAND6,EPHB2,SLC1A1,XCR1,IL18,UBE2K,PPARA,ABCD3,ARID5B,CALCRL,SLC25A33,TAF2,CPEB4,ZNF366,FBN1,PAX6,PRKCZ,ATP1A3,CD96,FAM20C,ADORA2A,GRIN2B,RABGEF1,CPEB1,TYK2,ASPN,ANGPT1,FHL2,OSBPL8,ATF6,GCNT2,VWC2,MTF2,CCL22,NCOR2,PRKCQ,DIAPH1,HDAC2,HTR2C,IMPACT,SPINT2,PHIP,PPP2CA,RPL23,SH3BP4,SP100,ADCY1,SQLE,SYK,CNOT1,YBX1,PTPRJ,SLC10A1,TAF1,TICAM1,TANK,UCN2,HSF1,MAX,DHFR,SASH1,NCOA2,ADAMTS7,GBP5,QRICH1,CIB2,CAV2,UGGT1,AXL,CD109,DENND4C,CCR3,TIMELESS,MALT1,PFKP,TRA2B,MLXIPL,PHB,PTPRC,EDA,PBLD,SH3GL2,SPON2,TRAF3IP2,ATF6B,CREB1,RGMB,SGTB,EDNRA,HADHA,BMP4,CPNE1
GO:0071495	cellular response to endogenous stimulus	6.447936657781169e-10	ENPP1,NRXN1,PRKCI,PDE4D,DNMT1,SLC9A1,ADCY8,PDE8A,PTGFR,CTDSPL2,RYR1,LATS2,NREP,PRLR,PAGR1,NTRK3,CBL,EGLN2,FLT3,STAT5B,GRM5,FER,LRP2,EZR,SLC26A6,HTR2B,ITGB6,RYR3,GOT1,ROBO2,CDC6,PTPRK,THRB,STXBP4,PTPN11,HDAC6,ERBB4,BRD8,ESR1,PMEPA1,NTRK2,EP300,FNTA,PDGFB,CCND3,INSR,PIP4K2A,PAK1,ESRRB,BDNF,AMIGO1,CHRM3,FUT8,UBR2,SNX6,AP3S1,SLC8A1,FYN,ARNTL,PLCB1,RXFP1,CHRDL1,PIK3R2,HDAC5,ROR2,SLC39A14,IPO5,RGS8,DEFA1B,DEFA3,PTGER2,JAK2,CLDN1,PDE2A,KLF15,LAMTOR3,CACNA1H,PRKCD,SOX6,ACVR2A,RUNX2,TGFB1,SGK1,BCAS3,SLIT3,ZFYVE9,SLIT2,GABRB1,KANK1,CHRM1,SMAD6,RXFP2,CLOCK,BMPER,DUSP22,FGF10,SMYD3,GLP2R,SMURF2,EPHA4,UFL1,CTNNB1,PARK2,FCGR2B,SMARCC1,IGF1R,PPARG,AXIN1,CYBB,BRIP1,RYR2,LEPR,LEPROT,FGF1,NR4A3,MYOCD,GLG1,PRDM16,SORBS1,CAPN2,HDAC4,PAX2,SFRP1,FOXO3,SSH1,SMAD3,NEDD4,ESRRG,TAB1,APP,PDGFRA,YAP1,SESN1,GNG2,CHST11,DRD1,GLRA2,FRS2,MMP2,ESR2,STAT1,HNF4A,ATF2,PTPN1,ADAMTS12,BMPR2,BMPR1A,COL16A1,PIK3R3,FGF2,ADCY5,PTAFR,RARB,NCOA1,CHRD,PTPRE,SHOC2,SRSF5,IDE,KCNQ1,AKAP6,NR2C1,IRS4,NEO1,WDTC1,DAB2,BLM,UBR1,LDLRAD4,SIN3A,IQGAP1,MAP3K7,ZNF423,SP1,ALK,SRA1,LTBP1,OVOL2,SNX5,ATP2B4,DYX1C1,MAP1B,COL4A6,WWOX,CACNA1A,GPC3,XRN1,TRPV1,GHR,FLRT2,ACTR2,ACACA,ELAVL4,HDAC1,RECQL5,SMOC2,TRIM24,ADIPOR2,ROCK1,CAPN10,ABCC1,PTPN2,INSRR,NTRK1,CARM1,HTR1D,SPI1,PTPRA,CTNNA1,ACAP2,BCAR3,CHURC1,RAB15,RAPGEF3,PDXP,SNCA,BMPR1B,

			MAGI2,ZEB2,HMGCS2,KMT2D,ADTRP,RPTOR,IGF1,MAPK1,SPRED2,BMP7,SOX5,PRCP,LRRK2,GNA14,CHMP5,TMEM108,NSG2,PKD2,EFNA5,PDGFC,NCOA3,WNT7A,GPR21,BMP6,ANO1,IL17RD,TSHR,EXT1,PNPLA3,PRMT2,RAPGEF2,UBR5,GRB14,RHOA,RQCD1,ONECUT2,ITPR2,GABRB3,GSK3B,RDX,STAT6,EEF2K,SULF1,AGT,PRKAA2,FBN2,ZMPSTE24,HRH4,PTK2,PHEX,PDE4B,VEPH1,CREBBP,DLX1,FMOD,PCSK6,UGT1A1,SHC1,RPS6KB1,GPR173,CD44,SCNN1B,EPHB2,SLC1A1,PPARA,SLC25A33,CPEB4,ZNF366,FBN1,PRKCZ,ATP1A3,FAM20C,CPEB1,ASPN,OSBPL8,GCNT2,VWC2,NCOR2,PRKCQ,DIAPH1,HDAC2,HTR2C,IMPACT,SPINT2,PHIP,RPL23,SH3BP4,CNOT1,TAF1,UCN2,HSF1,MAX,NCOA2,ADAMTS7,CIB2,CAV2,CD109,DENND4C,TIMELESS,PHB,PBLD,SH3GL2,RGMB,EDNRA,BMP4
GO:0051270	regulation of cellular component movement	7.400912813234606e-10	DNAH11,LDB2,MAP4K4,PDE4D,S1PR2,CLASP2,SEMA3A,DOCK1,NRG3,SEMA3D,PHACTR1,MAPRE2,TJP1,UNC5C,NTRK3,SEMA5A,ENPP2,FER,MARVELD3,MAP2K5,KITLG,PTPRR,PIK3CD,ANK2,ROBO1,LMNA,CTNNA3,ULK4,ITGB1,CNIH2,SRGAP3,MCC,SRGAP2B,PTPRK,ABCC8,LAMA2,DACH1,MGAT5,DOCK10,HDAC6,ADORA1,EPHA1,ERBB4,ZNF609,PTPRO,PDGFB,STK39,INSR,UNC5D,LAMA3,PAK1,TBR1,PTPRU,SLC8A1,CASS4,ADAMTS9,NF1,PLCB1,RTN4,DPEP1,APOD,BCR,NDRG4,NTN1,NRG1,BDKRB1,DOCK8,HDAC5,NTNG1,CMKLR1,ROR2,DCN,CDH13,PTPRT,CD99,RCC2,ANO6,KIF2A,RFFL,CCBE1,JAK2,FBXW7,CLDN1,WNT11,CAMK2B,SEMA5B,TGFB1,SGK1,MMP28,F2RL1,BCAS3,SLIT2,CORO1C,RRAS2,KANK1,LAMC2,TTBK2,BMPER,DAPK2,DUSP22,YTHDF1,FGF10,SMURF2,EPHA4,PRKCA,PPP3CA,PIP5KL1,SOD2,IGF1R,PPARG,DLG5,RYR2,FGF1,NR4A3,MYOCD,AJUBA,CACNA1C,NF2,FLT4,HDAC4,SFRP1,FOXO3,SMAD3,PTPRM,NRP2,DOCK4,NAV3,APP,GSX2,PDGFRA,SLK,PTPRG,DRD1,MMP2,SYNE2,PRKD1,SEMA6D,RAF1,BMPR2,CAMK1D,BMPR1A,CTNNA2,PIK3R3,SPAG9,RAB11A,FGF2,PPM1F,NEDD9,SEMA4D,MCTP1,ELP3,PLXNA2,PTAFR,DDX58,CHRD,VCL,LAMB1,CLIC4,RHOJ,KIF3A,RUFY3,DAB2,PKHD1,LDLRAD4,MYSM1,WNK1,RELN,SP1,MACF1,CLASP1,DSC2,PRKG1,ATP2B4,PAK3,SSH2,SCARB1,CDK6,FLRT2,SMOC2,PLCG2,ROCK1,PKN2,TMIGD1,TRIM46,SPI1,MCU,CTNNA1,SH3BP1,MST1,MAGI2,ADTRP,MITF,PKP2,IGF1,DPYSL3,MAPK1,PTEN,BMP7,PRCP,OSGIN1,PDGFC,AMOTL1,WNT7A,MYCBP2,GPI,SH3RF2,RAPGEF2,SMO,RHOA,TF,ONECUT2,RDX,ACTA2,FAM49B,SULF1,AGT,PTK2,TPPP2,PDE4B,RPS6KB1,GPR173,ADAM8,CLDN4,CXCL17,PLVAP,ACTN4,STK4,EPHB2,TMEFF2,DLC1,EPB41L4B,GPSM3,ZMYND8,PAX6,RABGEF1,PHLDB2,NIPBL,ANGPT1,OSBPL8,GCNT2,ETS1,MAP2,FBXO31,BRAF,DIAPH1,ATP8A1,SPINT2,CD300A,ANGPT4,CAMSAP3,SP100,SRGAP2,NUMB,FRMD5,PTPRJ,SASH1,ZP3,GNA12,INPP5F,MET,CAMK2D,SEMA3C,PTPRC,BMP4
GO:1902680	positive regulation of RNA biosynthetic process	8.035261678670152e-10	LDB2,ASPH,SLC9A1,PBX3,CBFB,IL31RA,MED26,WWC1,ASH1L,SCAF8,STOX2,PAGR1,HIVEP3,NPAS3,ARNT,STAT5B,CASK,MAP2K5,SP3,HDGF,CHD7,MECOM,TACC1,TRAF6,NHLH1,GFI1B,RUNX1,HMGN3,NRIP1,THRB,EFCAB7,SERTAD2,IKBKB,ERBB4,ZNF609,BRD8,KAT6B,ESR1,MAML3,CDON,EP300,CELA1,ZNF76,PDGFB,HNF4G,INSR,RERE,TCF7L2,LITAF,FBXW11,ESRRB,TBR1,TFAP2A,PEG3,MEIS1,TRIM13,ZNF148,MTA3,TFDP2,SLC30A9,TOX3,KAT7,RBM14,MED12L,SATB2,RIT2,MKRN2,ARNTL,PLCB1,ARID4B,BRPF1,PIK3R2,UBP1,MAP2K1,PPP3R1,HDAC5,CSRNP3,NFIA,RNF4,ROR2,DCN,PCBD2,CDH13,CREBRF,COPS5,MAD2L2,CAPRIN2,NEUROD1,USP22,JAK2,SKAP1,MLIP,BCL11B,PKNOX1,MLLT3,KLF15,WNT11,MTA1,RPRD1B,CCDC62,ACVR2A,RUNX2,CD4,TGFB1,BANP,NSD1,BTRC,NFATC3,F2RL1,BCAS3,TP73,ILF2,MTDH,FANK1,SMAD6,ZNF398,CLOCK,TCF12,ETV6,TFAP2D,BCL3,SBNO2,FGF10,SMYD3,CAPN3,LUM,RORA,AUTS2,TNFSF11,PPP3CA,NFYB,KLF12,CAMK4,CTNNB1,PARK2,SMARCC1,PPARG,AXIN1,MTF1,SREBF2,FGF1,NPAT,NR4A3,FOXK2,MYOCD,TRIM5,PER2,CHEK2,SUPT3H,PRDM16,TRIM8,MEF2B,HDAC4,PAX2,PHF5A,SFRP1,MED13,ZNF395,FOXO3,NFIB,SMAD3,WWP2,ARNT2,EBF3,CASZ1,ESRRG,HOXD3,HOXD4,NFATC1,CDC73,APP,SSBP3,YAP1,LRP5,SOX2,TEAD1,ZNF521,ARID3A,CHUK,ESR2,PRKD1,STAT1,ST18,ETV5,PLAGL1,HNF4A,ZBTB7C,TASP1,ATF2,POU2F2,TCF3,RAF1,BMPR2,BMPR1A,IKZF4,CDK12,CAND2,MYB,FGF2,ZNF71,BACH1,RORC,DDX58,BRDT,RARB,NCOA1,LMO7,DVL3,TCF20,ATF3,EBF2,MAML2,TSG101,RFX2,PRDM15,GLI2,TNKS,WBP2NL,ERCC1,GLIS3,DAB2,BLM,MYSM1,SIN3A,RUVBL2,GMEB1,ZNF423,SP1,TRIM22,TEAD4,HOXC13,SRA1,UBE2V1,ADIRF,OVOL2,SNX5,NFATC2,BNC1,ACTL6B,ASXL3,TET1,CAMTA1,CCPG1,ARID4A,PHF2,WWOX,MEF2A,DCAF6,EYA1,HOXB3,HOXB4,HOXB5,TFEB,ACTR2,HDAC1,TRIM24,PAXIP1,EGR2,RNF10,RYBP,AP3B1,KPNA6,TRIM44,EPCAM,MUC1,AP3D1,CARM1,ZNF836,SMARCA2,SPI1,GLIS1,RBPMS,CHURC1,CREB5,BMPR1B,PRIM2,ZEB2,FOXJ3,HEY2,KMT2D,CREM,KMT2C,MACC1,MITF,RPTOR,NFIX,IGF1,MLXIP,ATF7IP,BMP7,PCBP2,ZNF780B,PKD2,TADA2A,NCOA3,WNT7A,CRTC3,ARNTL2,KAT6A,ZNF197,BMP6,TAF15,WDR43,ATRX,PRMT2,SMO,TCF4,SOX30,TFE3,GRIN1,JADE1,RQCD1,TF,ONECUT2,TFEC,STAT6,CDK13,SUPT4H1,AGT,PBX1,MAP3K5,CREBBP,DLX1,NCF1,SHC1,SP7,RPS6KA5,ZNF484,PRDM2,NOS1,ACTN4,MC1R,EDRF1,IL18,PPARA,ARID5B,NOTCH4,CRX,PAX6,NIPBL,YEATS4,ZNF143,BRF1,TBL1X,ATF6,ETS1,MTF2,CSRNP1,HDAC2,SRCAP,POU3F3,ELF2,PHIP,SP100,WWTR1,ZBTB38,BRCA2,ATF7,DVL2,YBX1,THAP3,HSF1,MAX,EZH1,NRF1,NCOA2,QRICH1,ZP3,CHD6,TRIM37,MET,SETD3,MLXIPL,PHB,CTCF,NR6A1,ATF6B,CREB1,RGMB,BMP4,ABLIM3
GO:0072359	circulatory system development	9.504822648122226e-	DNAH11,NRXN1,DNMT1,SLC9A1,RPS6KA2,RYR1,NOX5,FHOD3,PTGIS,TJP1,NTRK3,SEMA5A,ENPP2,PLCE1,MAP2K5,NDUFV2,LRP2,PIK3CD,ANK2,OLFM1,NRXN3,ROBO1,CHD7,HTR2B,LMNA,ROBO2,VAV2,ITGB1,ARHGAP24,AKAP13,RUNX1,A

		10	*BCC8,XIRP2,PTPN11,EPHA1,ERBB4,HIF3A,MYO18B,NPHP3,NTRK2,EP300,CELA1,PDGFB,INSR,PRKAR1A,TCF7L2,BASP1,RBM20,NPRL3,MEIS1,TENM4,MDM4,TFDP2,MB,SGCD,SLC8A1,JPH2,ADAMTS9,NF1,RTN4,APOD,CHI3L1,TTN,NDRG4,NRG1,UBP1,MAP2K1,STIM1,MYH9,VGLL4,HDAC5,DCN,CDH13,PLXDC1,CCBE1,FBXW7,PKNOX1,PDE2A,WNT11,IMMP2L,NOX4,QKI,SOX6,TAB2,TGFB1,PCSK5,PDLIM4,NFATC3,BCAS3,RPGRIP1L,SLIT3,SLIT2,TP73,LRRC10,MTDH,SMAD6,BMPER,PARVA,ADAM12,FGF10,GREB1L,RORA,PRKCA,CTNNB1,SOD2,ARHGAP22,IGF1R,PPARG,ANKRD17,CYBB,RYR2,LEPR,FGF1,MYOCD,CACNA1C,MYO1E,CSPG4,CTDP1,FLT4,BICC1,ELN,SFRP1,ARL13B,IFT122,SMAD3,PTPRM,NRP2,TAB1,NFATC1,PDGFRA,NOX1,YAP1,HEG1,JAK1,ANGPTL4,LRP5,SETD2,PRICKLE1,VASH2,FRS2,MMP2,KDM6A,PRKD1,STAT1,STRA6,EREG,ATF2,BMPR2,BMPR1A,AP1B1,TSPAN12,PIK3R3,FGF2,ISM1,JARID2,RARB,EPHB3,CLIC4,COL11A1,KCNQ1,ADAMTS6,RHOJ,SUFU,COL4A3,DDAH1,AKAP6,GLI2,SCUBE1,DZIP1,CRELD1,APOLD1,SP1,LUZP1,XDH,OVOL2,NFATC2,ATP2B4,TMEM2,DYX1C1,MEF2A,GPC3,ADRBK1,CALD1,EYA1,HOXB3,MNAT1,FLRT2,SMOC2,ADIPOR2,ROCK1,PAXIP1,EPHB1,AXIN2,PTPN14,BCOR,AP2B1,PIFO,SPI1,RAPGEF3,PTK7,SMYD1,HEY2,WNT7B,ADTRP,RASA1,HIPK1,PKP2,SGCZ,IGF1,MAPK1,PTEN,MIB1,BMP7,PRCP,PKD2,ALPK3,AMOTL1,WNT7A,SH3PXD2B,SOS1,EXT1,RAPGEF2,SMO,RHOA,ACTA2,PTPRB,SULF1,AGT,ZMPSTE24,PTK2,SHB,CPE,SHC1,CD160,ECE1,SETDB2,ALOX5,ADAM8,THSD7A,CXCL17,STK4,TCF25,EPHB2,SLC1A1,IL18,DLC1,PPARA,CALCRL,NOTCH4,FBN1,PAX6,COL22A1,NIPBL,TMIGD2,ANGPT1,FHL2,ETS1,NEBL,NOTO,ANGPT4,ACAN,SP100,ANXA2,SYK,DVL2,NEB,SRPK2,SASH1,VAV3,CCR3,SEMA3C,RNF213,TRAF3IP2,CREB1,EDNRA,BMP4*
GO:0045893	positive regulation of transcription, DNA-templated	1.064358907878083e-9	*LDB2,ASPH,SLC9A1,PBX3,CBFB,IL31RA,MED26,WWC1,ASH1L,SCAF8,STOX2,PAGR1,HIVEP3,NPAS3,ARNT,STAT5B,CASK,MAP2K5,SP3,HDGF,CHD7,MECOM,TACC1,TRAF6,NHLH1,GFI1B,RUNX1,HMGN3,NRIP1,THRB,EFCAB7,SERTAD2,IKBKB,ERBB4,ZNF609,BRD8,KAT6B,ESR1,MAML3,CDON,EP300,CELA1,ZNF76,PDGFB,HNF4G,INSR,RERE,TCF7L2,LITAF,FBXW11,ESRRB,TBR1,TFAP2A,PEG3,MEIS1,TRIM13,ZNF148,MTA3,TFDP2,SLC30A9,TOX3,KAT7,RBM14,MED12L,SATB2,RIT2,MKRN2,ARNTL,PLCB1,ARID4B,BRPF1,PIK3R2,UBP1,MAP2K1,PPP3R1,HDAC5,CSRNP3,NFIA,RNF4,ROR2,DCN,PCBD2,CDH13,CREBRF,COPS5,MAD2L2,CAPRIN2,NEUROD1,USP22,JAK2,SKAP1,MLIP,BCL11B,PKNOX1,MLLT3,KLF15,WNT11,MTA1,RPRD1B,CCDC62,ACVR2A,RUNX2,CD4,TGFB1,BANP,NSD1,BTRC,NFATC3,F2RL1,BCAS3,TP73,ILF2,MTDH,FANK1,SMAD6,ZNF398,CLOCK,TCF12,ETV6,TFAP2D,BCL3,SBNO2,FGF10,SMYD3,CAPN3,LUM,RORA,AUTS2,TNFSF11,PPP3CA,NFYB,KLF12,CAMK4,CTNNB1,PARK2,SMARCC1,PPARG,AXIN1,MTF1,SREBF2,FGF1,NPAT,NR4A3,FOXK2,MYOCD,TRIM5,PER2,CHEK2,SUPT3H,PRDM16,TRIM8,MEF2B,HDAC4,PAX2,PHF5A,SFRP1,MED13,ZNF395,FOXO3,NFIB,SMAD3,WWP2,ARNT2,EBF3,CASZ1,ESRRG,HOXD3,HOXD4,NFATC1,CDC73,APP,SSBP3,YAP1,LRP5,SOX2,TEAD1,ZNF521,ARID3A,CHUK,ESR2,PRKD1,STAT1,ST18,ETV5,PLAGL1,HNF4A,ZBTB7C,TASP1,ATF2,POU2F2,TCF3,RAF1,BMPR2,BMPR1A,IKZF4,CDK12,CAND2,MYB,FGF2,ZNF71,BACH1,RORC,DDX58,BRDT,RARB,NCOA1,LMO7,DVL3,TCF20,ATF3,EBF2,MAML2,TSG101,RFX2,PRDM15,GLI2,TNKS,WBP2NL,ERCC1,GLIS3,DAB2,BLM,MYSM1,SIN3A,RUVBL2,GMEB1,ZNF423,SP1,TRIM22,TEAD4,HOXC13,SRA1,UBE2V1,ADIRF,OVOL2,SNX5,NFATC2,BNC1,ACTL6B,ASXL3,TET1,CAMTA1,CCPG1,ARID4A,PHF2,WWOX,MEF2A,DCAF6,EYA1,HOXB3,HOXB4,HOXB5,TFEB,ACTR2,HDAC1,TRIM24,PAXIP1,EGR2,RNF10,RYBP,AP3B1,KPNA6,TRIM44,EPCAM,MUC1,AP3D1,CARM1,ZNF836,SMARCA2,SPI1,GLIS1,RBPMS,CHURC1,CREB5,BMPR1B,ZEB2,FOXJ3,HEY2,KMT2D,CREM,KMT2C,MACC1,MITF,RPTOR,NFIX,IGF1,MLXIP,ATF7IP,BMP7,PCBP2,ZNF780B,PKD2,TADA2A,NCOA3,WNT7A,CRTC3,ARNTL2,KAT6A,ZNF197,BMP6,TAF15,WDR43,ATRX,PRMT2,SMO,TCF4,SOX30,TFE3,GRIN1,JADE1,RQCD1,TF,ONECUT2,TFEC,STAT6,CDK13,SUPT4H1,AGT,PBX1,MAP3K5,CREBBP,DLX1,NCF1,SHC1,SP7,RPS6KA5,ZNF484,PRDM2,NOS1,ACTN4,MC1R,EDRF1,IL18,PPARA,ARID5B,NOTCH4,CRX,PAX6,NIPBL,YEATS4,ZNF143,BRF1,TBL1X,ATF6,ETS1,MTF2,CSRNP1,HDAC2,SRCAP,POU3F3,ELF2,PHIP,SP100,WWTR1,ZBTB38,BRCA2,ATF7,DVL2,YBX1,THAP3,HSF1,MAX,EZH1,NRF1,NCOA2,QRICH1,ZP3,CHD6,TRIM37,MET,SETD3,MLXIPL,PHB,CTCF,NR6A1,ATF6B,CREB1,RGMB,BMP4,ABLIM3*
GO:1903508	positive regulation of nucleic acid-templated transcription	1.064358907878083e-9	*LDB2,ASPH,SLC9A1,PBX3,CBFB,IL31RA,MED26,WWC1,ASH1L,SCAF8,STOX2,PAGR1,HIVEP3,NPAS3,ARNT,STAT5B,CASK,MAP2K5,SP3,HDGF,CHD7,MECOM,TACC1,TRAF6,NHLH1,GFI1B,RUNX1,HMGN3,NRIP1,THRB,EFCAB7,SERTAD2,IKBKB,ERBB4,ZNF609,BRD8,KAT6B,ESR1,MAML3,CDON,EP300,CELA1,ZNF76,PDGFB,HNF4G,INSR,RERE,TCF7L2,LITAF,FBXW11,ESRRB,TBR1,TFAP2A,PEG3,MEIS1,TRIM13,ZNF148,MTA3,TFDP2,SLC30A9,TOX3,KAT7,RBM14,MED12L,SATB2,RIT2,MKRN2,ARNTL,PLCB1,ARID4B,BRPF1,PIK3R2,UBP1,MAP2K1,PPP3R1,HDAC5,CSRNP3,NFIA,RNF4,ROR2,DCN,PCBD2,CDH13,CREBRF,COPS5,MAD2L2,CAPRIN2,NEUROD1,USP22,JAK2,SKAP1,MLIP,BCL11B,PKNOX1,MLLT3,KLF15,WNT11,MTA1,RPRD1B,CCDC62,ACVR2A,RUNX2,CD4,TGFB1,BANP,NSD1,BTRC,NFATC3,F2RL1,BCAS3,TP73,ILF2,MTDH,FANK1,SMAD6,ZNF398,CLOCK,TCF12,ETV6,TFAP2D,BCL3,SBNO2,FGF10,SMYD3,CAPN3,LUM,RORA,AUTS2,TNFSF11,PPP3CA,NFYB,KLF12,CAMK4,CTNNB1,PARK2,SMARCC1,PPARG,AXIN1,MTF1,SREBF2,FGF1,NPAT,NR4A3,FOXK2,MYOCD,TRIM5,PER2,CHEK2,SUPT3H,PRDM16,TRIM8,MEF2B,HDAC4,PAX2,PHF5A,SFRP1,MED13,ZNF395,FOXO3,NFIB,SMAD3,WWP2,ARN*

			T2,EBF3,CASZ1,ESRRG,HOXD3,HOXD4,NFATC1,CDC73,APP,SSBP3,YAP1,LRP5,SOX2,TEAD1,ZNF521,ARID3A,CHUK,ESR2,PRKD1,STAT1,ST18,ETV5,PLAGL1,HNF4A,ZBTB7C,TASP1,ATF2,POU2F2,TCF3,RAF1,BMPR2,BMPR1A,IKZF4,CDK12,CAND2,MYB,FGF2,ZNF71,BACH1,RORC,DDX58,BRDT,RARB,NCOA1,LMO7,DVL3,TCF20,ATF3,EBF2,MAML2,TSG101,RFX2,PRDM15,GLI2,TNKS,WBP2NL,ERCC1,GLIS3,DAB2,BLM,MYSM1,SIN3A,RUVBL2,GMEB1,ZNF423,SP1,TRIM22,TEAD4,HOXC13,SRA1,UBE2V1,ADIRF,OVOL2,SNX5,NFATC2,BNC1,ACTL6B,ASXL3,TET1,CAMTA1,CCPG1,ARID4A,PHF2,WWOX,MEF2A,DCAF6,EYA1,HOXB3,HOXB4,HOXB5,TFEB,ACTR2,HDAC1,TRIM24,PAXIP1,EGR2,RNF10,RYBP,AP3B1,KPNA6,TRIM44,EPCAM,MUC1,AP3D1,CARM1,ZNF836,SMARCA2,SPI1,GLIS1,RBPMS,CHURC1,CREB5,BMPR1B,ZEB2,FOXJ3,HEY2,KMT2D,CREM,KMT2C,MACC1,MITF,RPTOR,NFIX,IGF1,MLXIP,ATF7IP,BMP7,PCBP2,ZNF780B,PKD2,TADA2A,NCOA3,WNT7A,CRTC3,ARNTL2,KAT6A,ZNF197,BMP6,TAF15,WDR43,ATRX,PRMT2,SMO,TCF4,SOX30,TFE3,GRIN1,JADE1,RQCD1,TF,ONECUT2,TFEC,STAT6,CDK13,SUPT4H1,AGT,PBX1,MAP3K5,CREBBP,DLX1,NCF1,SHC1,SP7,RPS6KA5,ZNF484,PRDM2,NOS1,ACTN4,MC1R,EDRF1,IL18,PPARA,ARID5B,NOTCH4,CRX,PAX6,NIPBL,YEATS4,ZNF143,BRF1,TBL1X,ATF6,ETS1,MTF2,CSRNP1,HDAC2,SRCAP,POU3F3,ELF2,PHIP,SP100,WWTR1,ZBTB38,BRCA2,ATF7,DVL2,YBX1,THAP3,HSF1,MAX,EZH1,NRF1,NCOA2,QRICH1,ZP3,CHD6,TRIM37,MET,SETD3,MLXIPL,PHB,CTCF,NR6A1,ATF6B,CREB1,RGMB,BMP4,ABLIM3
GO:0009987	cellular process	1.0739315806161925e-9	NT5C1B,NT5C1B-RDH14,RDH14,CD247,SUCLG2,POLDIP3,LNX2,CLRN1,DNAH11,ZDHHC14,RIC8B,SLC39A11,ENPP1,SLC35E3,HOOK2,PRDX2,ADAMTS16,BLZF1,LDB2,GPC5,NRXN1,ASPH,B4GALNT2,PRMT3,GSS,GP6,RDH13,PRKCI,MOV10L1,TACC2,SLCO3A1,MAP4K4,PDE4D,PDCL,DNMT1,S1PR2,HLX,LLPH,TMBIM4,SLC9A1,PDE7B,FAM155A,C6ORF89,NEGR1,PBX3,ADCY8,MGAT4C,MED13L,SPAG16,LMBR1,TRPS1,CBFB,PDE8A,PDE4DIP,ZNF823,CLASP2,SEMA3A,IL31RA,LSAMP,GRID2,ALDH4A1,TAS1R2,RNASET2,RPS6KA2,PRDM12,SETD4,GK5,PTGFR,TENM3,CALR3,CHERP,MED26,SLC35E1,DOCK1,WWC1,CLDN18,C12ORF49,KIF22,CTDSPL2,SLC25A17,PMS1,CACHD1,FBXL2,RYR1,NRG3,ASH1L,NOS1AP,LATS2,TUBA1C,NOX5,SPESP1,ANP32A,IQCG,ASTN2,TLN2,EPB41,SEMA3D,PHACTR1,MYOT,DPP6,PTH2R,RNF145,NREP,SCAF8,TIAM2,SLC14A2,STOX2,FHOD3,KSR2,MAPRE2,NLGN4X,PTGIS,PRKAG2,DCHS2,WWC3,TJP1,GRM8,UNC5C,ZSWIM6,NUDT6,HPSE2,OR5P2,OR5P3,CLCN1,PRLR,MVP,PAGR1,ADCY7,HIVEP3,FTO,NPAS3,UTRN,RGS7BP,KALRN,PITPNC1,SEC23B,PCDH9,LTV1,PHACTR2,DCAF12,MRM1,WIPF3,SNX31,ACOXL,SPRR2D,NTRK3,CBL,ARNT,ATRNL1,PLEKHG4B,EGLN2,MIA,RAB4B,RAB4B-EGLN2,SEMA5A,LRRFIP2,FLT3,RAD51B,PTGES3L,STAT5B,TOX,ENPP2,ATP2B2,KCNQ5,EXD1,GRM5,NOC3L,PLCE1,LHFPL4,SAMHD1,FAM53A,ZNF566,FER,CASK,RNU6-202P,MARVELD3,MAPK4,MAP2K5,ISCA1,CCDC93,SPATA5,KITLG,KCNIP4,USP32,MAPK10,PTPRR,SPNS2,NDUFV2,LRP2,PIK3CD,ZNF536,SLC38A11,SP3,DEPDC5,TNPO3,CAMK1G,HDGF,PRCC,GTF3C1,ZSWIM7,ANK2,EIF4G3,SAAL1,OLFM1,MFAP5,SH2D1A,STAG2,ADPGK,EZR,IKZF2,MEGF10,NRXN3,XRCC4,B3GALT1,ROBO1,MALRD1,SLC9A9,TOM1L1,TNFAIP8L1,CHD7,SLC26A6,HTR2B,PSMD1,ITGB6,CDKL5,MECOM,TACC1,TENM1,CAMKMT,RYR3,NADK2,KIF5C,LMNA,SRP72,NUCB1,GOT1,LPO,NMT2,INPP4B,TRAF6,ESRP1,ROBO2,NFASC,SENP5,GTF2E2,ITPKB,CTNNA3,SCD5,DNAJA3,OXR1,SLC24A2,VAV2,GRK5,ABI1,ULK4,ERCC6L2,NHLH1,ITGB1,TRIOBP,HSP90AA1,SLC24A3,ELMO2,GAS7,CDC6,PSMB7,TSC22D3,CNIH2,SRGAP3,THUMPD3,MCC,TSSK1B,PCDH17,KNDC1,ZC4H2,KIF26B,CCDC22,SLC22A8,DSCAM,BCORL1,MOCOS,FAM126A,STK38L,SRGAP2B,CDAN1,GMDS,AMDHD1,SAMD12,CNGB1,TRIO,ARHGAP24,KLHL12,AKAP13,DLG2,FLVCR2,IFT43,TTLL5,GFI1B,PDE1A,PARD6G,PTPRK,RUNX1,DAB1,OMA1,CDC42EP3,SREK1,MYO10,ABCC8,ERC1,MMAA,PDZD2,HMGN3,AFTPH,NCF4,CDH10,NRIP1,RGS6,DGKI,DNAJB14,ANKS1B,THRB,STXBP4,KLHL7,PDE4A,ERC2,GABRA3,XIRP2,IMPG2,EFCAB7,ITGB3BP,LAMA2,CLVS1,GFRA2,MAST4,COL14A1,IL17RB,TCIRG1,DACH1,PTPN11,ZNF569,KCNS3,NIPAL2,NETO2,RBM19,HERC4,MGAT5,CCT2,CACNB2,DOCK10,ORC2,CYTH3,HDAC6,GOLGA4,SERTAD2,DDB1,MYT1,SV2B,TBCEL,ADORA1,MSRA,GPRASP1,ADAMTS3,EPHA1,IKBKB,KIF18B,PEX14,ERBB4,CDH12,GBE1,ADRA1D,CNNM1,SORCS1,DPY19L2,MRE11A,GBF1,DNAH2,LIMK1,ZNF609,TLK1,BRD8,ATP8A2,RNU6-78P,RAD54L2,TEX264,KAT6B,ABR,HIF3A,OSBPL10,SNIP1,CUL4B,KLHL6,LARGE,CECR2,ESR1,KCNJ16,MYO18B,ACAD11,NPHP3,RNU6-179P,GRM7,MIER1,PCBP3,PMEPA1,SF3B2,MAML3,DPP10,PTPRO,CDON,KANSL1,FBXL18,NTRK2,CYB561A3,TNRC6A,FRMD4A,STX18,DNAI2,EP300,CELA1,ZYG11B,MEGF9,DYM,TENM2,ZNF76,RNF220,CNTNAP3B,RGS22,ZNF471,CPNE4,FNTA,HOOK3,RNU1-124P,TPST2,VPS45,PDGFB,CACNA1B,AVL9,FKBP9,RIMS1,TNIK,CCND3,GALNTL6,FBXL7,SNX2,KIRREL3,CDH8,USP46,CSGALNACT1,TOB2,MID1,RNU6-1061P,ZNF19,ZNF23,BRMS1,CHFR,ZNF605,ALOX5AP,SCML4,BPI,STK39,DAP3,MSTO1,HNF4G,INSR,FMN2,RERE,ARSG,PRKAR1A,ASB5,AFAP1,FUBP1,ATP8B1,UNC5D,PRUNE2,H2AFY2,PLLP,LAMA3,TCF7L2,PIP4K2A,ST6GALNAC3,CDH4,HPS1,JMJD1C,CAB39,USP34,ZNF443,ZNF490,ZNF564,ZNF709,ZNF799,PAK1,LITAF,FBXW11,ESRRB,MYOM1,MAP3K4,ZDHHC13,PTPRD,RNF144A,CNTN5,BASP1,EGLN

			3,SPTSSA,CATSPERB,RBM20,SLC20A2,SLC4A10,TBR1,PPIP5K1,CATSPER2,STRC,SAMD4A,BDNF,TFAP2A,GLTP,ATRN,AMIGO1,CDK14,CLDN12,GTPBP10,FANCA,KCNMA1,PEG3,ZIM2,CHRM3,TGIF2,TGIF2-C20ORF24,POMGNT2,FUT8,OR2T3,NPRL3,PCDH7,FMNL2,PHACTR4,RNU6ATAC27P,MEIS1,TENM4,GRIK4,VRTN,KCNRG,TRIM13,RASGEF1B,UBR2,HLCS,ARHGAP6,LCK,MDM4,EDARADD,IL5RA,ENTPD5,SHC4,ECT2,TMC2,TMEM144,FAM135B,SLC12A8,ZNF148,MYO3B,MTA3,SNX6,PIPOX,TFDP2,NAV2,CHML,ACOX1,VAMP7,LAMC1,PPM1L,SPNS3,ARHGAP42,SLC6A16,CST2,PTCD3,PTPRU,RFTN1,ERN2,CDC45,LSP1,SHFM1,C1D,MB,AP3S1,LRRC49,NPLOC4,AFF3,RHCE,PYROXD2,STARD9,SGCD,DENND5A,MICU3,DMD,CENPF,RANBP17,ATG10,LRRN2,SLC30A9,RNU6-1269P,CERS6,CRISPLD2,TOX3,PDS5A,LARP4B,USP13,KAT7,WDR7,ZNF667,SLC8A1,PRELID2,GSN,HFM1,KHDRBS2,RBM14,RBM4,PRPS2,GPR171,MED12L,SATB2,PAFAH1B2,RIT2,HIRA,MACROD2,TANC2,JPH2,DRG2,DEPDC1B,CASS4,KCTD16,FYN,MKRN2,ARNTL,ADAMTS9,LINC01138,NF1,NCF2,SMG7,PLCB1,RNU105B,SLC16A6,DST,MGMT,DMGDH,PCDH15,LMTK2,STX6,ARID4B,SNCB,SLC25A21,GXYLT2,PPP4R2,FAT3,SH3BGR,RTN4,AFF2,FBXO21,RXFP1,MAL2,DPEP1,CHRDL1,GOLGA2P5,SLC5A6,POLA2,DGKH,APOD,SLC2A14,NEIL2,B4GALT6,ASPHD2,RTN4R,PLEKHM3,VTA1,BCR,PPP1R12B,NOSIP,RWDD3,BRPF1,SHISA9,CHI3L1,CCDC141,TTN,NDRG4,ICA1,BMP2K,PAQR3,IFI30,PIK3R2,ANKH,COA6,CACNA1E,BLVRB,ADAMTSL1,RANBP9,TPGS2,TLL1,KLHL21,NTN1,EGFLAM,DCTN1,SLC4A5,NOMO3,PPIG,NRG1,ARHGAP10,KCNN3,SH2D3C,ACYP1,CNNM2,CDK19,SLC16A10,SLC43A2,UBP1,BDKRB1,BDKRB2,DOCK8,BID,STRBP,FRY,MAP2K1,KCNIP1,SLC35A2,MDFI,SLCO1A2,FNIP1,RAPGEF6,STIM1,VAX2,NALCN,RALGPS2,MYH9,FRMPD4,MAD1L1,LRFN5,DAGLA,GALNT16,TMBIM6,VGLL4,PPP2CB,CNTN4,PPP3R1,WDR92,VWA1,HDAC5,CSRNP3,RBM5,RBM6,NTNG1,PCSK2,VSTM1,ALDH3B2,NAV1,ZNF692,OR6N2,NFIA,RNF4,TMEM30A,CSNK2A3,GALNT18,SYNE1,UBE3D,JDP2,WDSUB1,CMKLR1,SBF2,INPP4A,PPIP5K2,ITIH2,PARD3B,CDKL2,LRGUK,ROR2,SCFD2,DCN,ZDHHC3,SLC39A14,PRPSAP2,LIN7A,SCFD1,CATSPER3,PCBD2,SYN2,HNRNPLL,DOCK2,CDH13,PITPNB,CREBRF,TRDMT1,SOX13,FHIT,WDR70,PTPRT,ENTPD1,ACSBG1,AUNIP,UST,COPS5,CSPP1,PPP1R42,FGF14,DMC1,PCDHB16,AK2,EXOC4,IPO5,ARHGAP32,MAD2L2,MAN1A1,TLE6,RAB27A,GALK2,SRBD1,RGS8,CAPRIN2,MTX3,CCNYL1,ZNF418,NLN,NCKAP1,PHF20L1,BBOX1,DEFA1B,DEFA3,CD99,RAB3C,PDF,TERF2,TMED6,PTGER2,RALGPS1,SLX1B,SULT1A4,ZNF286A,STIP1,NME7,GOLPH3L,CLDN16,FOXN3,RCC2,BOC,ANO6,ARL3,DNAJC11,AK5,CERKL,NEUROD1,GNAQ,IPO11,KIF2A,FBXO9,ICK,TMEM117,RAD51D,RFFL,ACTL8,TTLL8,PARP11,COL12A1,RANBP1,CCBE1,DENND1A,HERC5,USP22,ZDHHC15,JAK2,MYPN,YTHDC2,FAM168A,TRAPPC9,FBXW7,LINC00473,KCNB2,OAZ2,SYN3,SKAP1,UBR7,SMC3,CLSTN2,UIMC1,SYT1,ITCH,MLIP,CROCC,RNU1-2,RNU1-4,USP12,NPSR1,ZFYVE28,BBS12,SNAP25-AS1,ABCC2,LZTS1,BCL11B,SMG1,DNAH14,IMPDH1,SCN4A,RBFOX1,TMC1,SLC1A4,PKNOX1,KIF21A,DYNC1I1,DTHD1,CLDN1,HUNK,B3GALT5,MLLT3,SLC5A9,TSHZ2,ATP9A,ARAP2,DPYS,SLC9A7,IDNK,CDC42BPB,PNPLA1,PHKA1,PLS1,TEX11,DOCK9,DHDDS,DENND2A,SH2D6,TCF7,CA13,PDE2A,MSI2,SEPT7,KLF15,SPSB1,TBX15,ANXA4,TTC7B,LAMTOR3,MAGI1,KCNC4,KPNA3,CYFIP2,CTAGE6,CENPC,WNT11,TPST1,ARHGAP23,HADHB,IFT80,TRIM59,APBB1IP,CLCA2,FIG4,CYB561D1,MTA1,KREMEN1,IMMP2L,NRG4,KLF8,ASTN1,WDFY4,CLPB,NOX4,LCOR,SIPA1L3,RPRD1B,MKLN1,CACNA1H,SIAH1,QKI,IL1RAPL1,CCDC62,CAMK2B,ADAM23,ERCC8,PRKCD,SOX6,TAB2,ACVR2A,SLC35F1,RUNX2,HAO2,SEMA5B,CD4,PPM1E,TGFB1,BANP,LRCH3,MRPL33,PLCXD3,SGK1,PCSK5,P4HA2,PDLIM4,ATL2,SPSB4,NUP93,MYO3A,MMP28,TRIM65,NSD1,IGSF1,PIBF1,ZHX2,PKNOX2,ASCC2,KEL,BTRC,DUS2,NFATC3,JPH3,POLK,CRADD,DLGAP1,PTPRN2,ARMC1,CLDN10,F2RL1,BCAS3,C9ORF47,GRIK3,CDYL2,PDZRN3,RPGRIP1L,DNAJB2,CELSR1,SLIT3,AAK1,KLHL2,HS6ST2,CC2D1B,SDK2,ZFYVE9,SLIT2,ASB15,COLGALT1,TP73,GABRR3,CADPS2,RNF133,RNF148,ITIH4,MUSTN1,GABRB1,SYT13,HHAT,CDH23,GPR176,HP,HPR,TXNL4B,CORO1C,SAP18,RRAS2,QSOX2,CDKAL1,CTNND2,ZBTB22,C12ORF4,SRRM2,MGAM,OR9A4,ILF2,DISC1,TSNAX,BLID,KCNJ3,CEP135,KANK1,CACNA1D,ZFAND2A,ABCC11,LRRC10,CPT1C,PTPDC1,CACNA2D3,GRPEL2,ANO4,LAMC2,COL23A1,CLN6,TMTC1,MTDH,ANK1,CLMN,TTBK2,FANK1,XPR1,MYT1L,OPCML,FSTL5,KDM4B,SPAG17,CHRM1,RAB11FIP4,MMP26,OR51A7,OR51F2,OR51T1,SMAD6,RXFP2,BNC2,ZNF398,TMTC2,COL24A1,CLOCK,ITSN2,CHST9,TCF12,ZNF675,PPFIBP2,ANO3,SMOC1,RPL36,PLCB4,BCKDHB,ETV6,SYNJ2,NELL1,SUCO,PHRF1,KCNH1,ENOX2,TFAP2D,BMPER,TIMP2,BCL3,DCLK1,RNF212,ANKRD54,EIF3L,LYRM4,DAPK2,TNFRSF10B,PARVA,SND1,DAPP1,ADAM12,DUSP22,GPR52,RABGAP1L,NAIP,STRIP1,HNRNPC,RNU6-816P,PDCD11,TRRAP,ANKFN1,HOMER2,SLC17A7,DOCK3,OIP5,OTOA,SBNO2,LINGO2,YTHDF1,FGF10,CNTNAP2,CIZ1,FBXL17,POLR2J2,RASA4,RASA4B,ARHGEF28,ADK,SMYD3,UNKL,STXBP5,KCNH7,KANK4,USB1,KCTD10,ENAH,IL1RAPL2,PPL,IQCJ-SCHIP1,SH3RF3,LOXL3,RHPN2,MAST2,FANCI,SVIL,GLP2R,ADCY2,CAPN3,GANC,SLC16A1,LUM,VMP1,ZAN,SMURF2,EPHA4,RORA,AGPS,HIVEP2,HSD17B12,SNX8,PRKCA,AUTS2,CNR1,CD6,ANO2,TNFSF11,SMG6,PPP3CA,NSUN2,HBE1,OR51B2,OR51B4,OR51I1,UBQLN3,UBQLNL,GNG4,NFYB,MAGEA4,MAG,KLF12,CAMK4,G

			ATAD2B,PIP5KL1,LIX1L,WDFY3,UFL1,TRAK1,IDH2,CTNNB1,MPRIP,PARK2,SOD2,DACH2,METTL13,RN7SL832P,FCGR2A,FCGR2B,FCGR3A,FCGR3B,HSPA6,ARHGAP22,SMARCC1,TSNARE1,KLF17,CDHR2,IGF1R,PPARG,IARS2,NGRN,AXIN1,GARNL3,PRKAR1B,DNAH10,COX8A,MACROD1,OTUB1,CADM3,DLG5,IL18R1,IL1RL1,MYO1D,BFSP1,ADD2,CIPC,TMEM63C,MTF1,CPQ,MSR1,CELF2,CBX5,P2RY10,TRPM1,ANKRD17,CYBB,OTC,SYTL5,XK,CASKIN1,APOL3,SCAMP5,BRIP1,ANXA13,ANKRD26,CENPP,ECM2,LRPPRC,RSPRY1,SREBF2,AARS2,PFAS,TDP1,KCNJ12,RYR2,CDK11A,DYRK4,GALNT8,KCNA6,NDUFA9,FBLIM1,CDK11B,DRAXIN,LEPR,LEPROT,FGF1,NIN,PROS1,RGS10,NPAT,NR4A3,DNHD1,FOXK2,WDR45B,TUBGCP6,NOL3,DCT,PRKAR2A,RIOK2,ESCO1,MPP6,MYOCD,OR52E6,OR52E8,OR52N1,TRIM5,PER2,KIR2DL1,KIR2DL4,KIR3DL1,KIR3DL2,AJUBA,HAUS4,CACNA1C,CPNE6,GLG1,ZNF626,ZNF737,SCN3B,CHEK2,SUPT3H,PHLDB1,KRT6B,UBQLN4,PRDM16,MYO1E,ASB8,PPP1CB,SPDYA,HCK,CSTL1,SLC35E4,SORBS1,TBC1D14,AGPAT4,RAB3GAP2,CAPN2,TBCK,TRIM8,DIO2,RPRD2,MRC2,PPP2R2B,CSPG4,GRM1,BRMS1L,RIMS4,DCC,ZBTB8A,ZBTB8B,ARHGAP31,P2RY14,ANKFY1,CTDP1,HS3ST5,BAZ1B,MMP16,OBSCN,RGL1,NF2,FLT4,MEF2B,SGMS1,SMC2,BICC1,HDAC4,PAX2,PHF5A,TNS3,CPOX,DNAH9,SECISBP2L,SPTBN1,TPTE,HEPACAM2,ELN,CDCA4,TRABD2B,SNAPC4,AP3B2,SPG11,SFRP1,MED13,ST8SIA1,PPP6R2,ZNF395,FOXO3,DGKB,TPH2,TRHDE,USP54,ARL13B,CNTN6,CCDC91,NFIB,MUC12,SP4,ARHGAP29,ZCCHC17,IFT122,DDX10,RANBP3,BCL2L13,DNM3,CDH26,SSH1,CELF5,ATP10D,SNX14,SYNCRIP,SMAD3,TYW1B,RNFT2,CUX2,ITPR1,RAB6A,EVA1A,THSD4,PTPRM,WWP2,ARNT2,SBNO1,ACKR2,KRBOX1,ZNF662,CYP4F11,NHS,ZNF777,SIMC1,WIPI1,EBF3,MTBP,VPS39,RNF168,EMG1,CASZ1,ABI2,C10ORF90,GRIK5,PSAP,SLC16A2,LZTFL1,SHANK1,SYT3,IFNAR1,DCP1B,OR14K1,MIER3,NEDD4,NRP2,ARHGEF18,VANGL1,SVOPL,BTBD3,PRPF38B,PREX1,PLA2G4C,DOCK4,CRTAC1,ESRRG,SPAG6,HOXD3,HOXD4,SLC35F4,OGDHL,KPNB1,NAV3,SLC4A4,ZNF114,GALNT14,TAB1,VPS53,SLC6A1,KTI12,TXNDC12,NFATC1,RAB6C,SEC61B,ST3GAL3,KCNK1,PRTG,UCK2,CDC73,THOC3,APP,SSBP3,USP49,FIP1L1,GSX2,PDGFRA,AOC2,BET1L,ODF3,SLK,FCRL4,CEP85L,ADD3,RBM8A,CCNI2,DIP2B,KANSL2,NOX1,ARIH1,YAP1,HEG1,AMFR,ST6GALNAC5,RAB11FIP5,SFXN5,MYO1F,SESN1,PPCS,CDK3,TEN1,FSTL4,NLGN2,TNFRSF19,VDAC1,EYA2,SSR2,SH3D19,BORA,IBTK,SCRN1,SBK3,NVL,ALPL,JAK1,ANGPTL4,FPGT-TNNI3K,TNNI3K,PEMT,LRP5,MTCP1,MUC20,POLR3G,SEC22C,PTPRG,BDH2,ISLR2,SLC9B2,ZNF787,GNG2,SOX2,SETD2,ZDHHC6,GPC6,KIF24,TTC39C,ACBD5,ADAMTS2,CHST11,TEAD1,DEFB118,PRICKLE1,RCAN1,PLEKHA7,SSPN,ELAVL3,ZNF653,SPTBN4,GRIA3,VASH2,ZNF521,PAQR5,ELOVL2,DRD1,TMEM14A,GLRA2,ARID3A,ZNF761,CHUK,ERLIN1,FRS2,PALB2,RFT1,SFMBT1,VILL,MMP2,ZNF584,SERGEF,TPH1,ESR2,SYNE2,S100A12,DCUN1D3,ERI2,NUDT10,CPNE9,KDM6A,GTF2F2,PRKD1,STAT1,NAA25,CELF6,HEXA,PARP6,ST18,OSBPL2,DGKG,ETV5,SYBU,RHOXF2B,RND3,SLC6A3,USP53,TAF3,MORN2,PLAGL1,HNF4A,ZBTB7C,LRRC2,TBCA,TASP1,SHANK2,SHPRH,CNTNAP3,STRA6,VPS4A,EREG,CCNY,TCTN3,ABLIM1,MAPKAPK3,MYOF,DSCAML1,FXYD2,FXYD6,RNU6-167P,PITRM1,SEMA6D,SLC16A12,RBFOX3,ATF2,POU2F2,TCF3,ZNF730,GRAMD4,TMEM237,KCNG4,RAF1,CELF4,RASGRF2,EPHX1,ABLIM2,SMIM20,ZNF766,ACOX2,CARD16,CASP1,PTPN1,ADAMTS12,PSMD11,RNU6-923P,SRRM4,POLA1,GGT7,BMPR2,USP33,DPYSL2,VBP1,CAMK1D,BMPR1A,ZKSCAN1,AP1B1,IDO2,PABPC4,TSPAN12,NLGN1,BTBD10,COL16A1,CTNNA2,ATP9B,IKZF4,PIK3R3,CDK12,SNX16,TAF8,CAND2,CERS3,SPAG9,MORC2,DENND4A,RAB11A,CRMP1,EVC,PDE11A,EPG5,IPO9,MYB,FGF2,ZNF71,LRRC4C,POU6F2,PPA2,BACH1,GRIK1,OR6C70,MXD3,RAB24,PPM1F,UBE2R2,TICRR,ADCY5,GLI4,ZFP41,NEDD9,AGBL4,BEND5,PEAK1,ADAMTS4,SEMA4D,SH3PXD2A,ARMC2,NFX1,CDC27,DOK6,HELQ,RORC,MCTP1,XPO6,ELP3,PLXNA2,PTAFR,POC1A,ADCYAP1R1,HIBADH,EEA1,FNDC3A,SP140,SP140L,SETD1A,RHOXF2,YME1L1,ABCA13,JARID2,RILPL1,ANKDD1A,KLHL3,PLEKHO2,DDX58,DKK2,MRS2,SORCS2,RAB11FIP3,SLC5A8,BRDT,CHCHD6,PHC2,ITGA11,PI4KA,RARB,SPEN,PIK3C2B,PRKCG,SIN3B,SLC9B1,VIPR1,NCOA1,SPOCK1,AREL1,BLOC1S5,EEF1E1,EEF1E1-BLOC1S5,TXNDC5,TANGO6,GRIA2,EHMT1,GAS8,OC90,LMO7,UCHL3,ALKBH3,NTM,AP2M1,CHRD,DVL3,ECE2,EIF2B5,EIF4G1,EPHB3,POLR2H,PSMD2,SNORD66,PTPRE,RBX1,TCF20,RNU6-229P,SLC35D1,ANAPC5,ARHGAP39,TNPO1,ATF3,FBXO45,MUC16,TRAPPC11,LIN28B,TCEANC,TECPR2,AKR1D1,ATP6V0B,SHOC2,ZBTB8OS,LMBRD1,SRSF5,STT3B,CKS1B,PAWR,TRMT61B,EBF2,AGO3,ACAA1,DEPTOR,DLEC1,PKN3,FBXL20,MAML2,TSG101,DESI1,CCDC3,VCL,LAMB1,TERF2IP,WDR90,CLIC4,MYO9A,ANKS4B,CRYM,IDE,WDR59,COL11A1,KCNQ1,RFX2,WNT3,FKBP5,SUGP2,ADAMTS6,SLC2A11,C11ORF80,RCE1,B3GALNT1,ZNF322,RHOJ,P4HB,CCL14,CCL15,CCL15-CCL14,SUFU,TG,MAGEA11,SUMF1,NLGN3,PTPN13,CPSF4,PTCD1,SCUBE2,ZFYVE1,PAFAH1B1,QPCTL,KIF3A,PRDM15,ZNF670,ZNF695,ADAMTS14,CCDC169-SOHLH2,SOHLH2,FAM13A,POLE,ATG4C,CNTFR,COL4A3,TRPA1,DDAH1,TRPM3,NMUR2,ZNF354C,HIP1,AKAP6,CEP350,RASAL1,TCEA3,PADI6,FDXR,CMPK1,ZNF704,BTBD9,CERS4,NR2C1,TRPC5,UBA2,EDEM3,IRS4,ME1,TTLL7,GLI2,NEO1,TNKS,SCUBE1,WBP2NL,ERCC1,LPP,PXDNL,RUFY3,TNRC6B,GLIS3,GPR39,GLCE,WDTC1,ALDH6A1,ZNF664,MRPS28,STON2,TPD52,MGLL,TPK1,C9,COL19A1,DAB2,THEMIS,BLM,PKHD1,XKR4,TRAM2,USH2A,UBR1,CACUL1,LDLRAD4,MYSM1,SET

			D5,DZIP1,SMG5,DLG3,WNK1,GTPBP2,RELN,NEK10,SIN3A,RUVBL2,TMEM68,ABCB1,TYW1,PSMD7,OR5H2,OR5H6,OR5K4,RSPO2,COMMD6,GUCY1A2,JOSD1,CSF3R,KCNQ2,DOCK11,GMEB1,PELI1,SLC22A3,PPP2R5C,SLC25A16,CFDP1,IQGAP1,TMEM170A,SPATA18,COL6A5,SLC47A1,DCAF5,EML4,FAM91A1,MAP3K7,APOLD1,DDX47,TAS2R41,ZNF423,SP1,OR56B1,TRIM22,MACF1,ZNRF1,ARHGAP12,MYO7A,ALK,SLC8A2,GSTM3,GSTM5,INPP5D,STAU1,TMPRSS12,CLASP1,DSC2,EEPD1,TEAD4,FASTKD5,HOXC13,UBOX5,SLC9C1,APBB3,SLC35A4,SRA1,TOP1,AGBL1,UBE2V1,MBOAT1,PADI3,TPCN1,GPM6B,ADIRF,CLIC5,XDH,AHNAK,LTBP1,OVOL2,SNORD17,SNX5,NFATC2,RBBP6,CHD1L,OR6C74,PRKG1,PAX7,RGS7,BNC1,TTLL9,ATP2B4,NSF,PACRG,PARP12,SYNE3,GALNTL5,APEX2,PACSIN1,SPTBN5,ACTL6B,ASXL3,PAK3,HORMAD2,SLCO4A1,SSH2,TET1,HECTD4,MKRN3,CAMTA1,CCPG1,DYX1C1,P2RX6,PHOSPHO2,ASGR2,ATAD1,ADNP,GPR161,MAPKAPK2,NXT2,CEP41,ARID4A,MAP1B,UBE3C,C12ORF65,CDC42BPA,COL4A6,MPHOSPH9,SCARB1,MARK1,CDK6,BCL7C,ATP6V0A2,PHF2,KCNAB2,CELF1,P2RY8,TYR,AIF1L,RNF34,TFRC,UVRAG,EPHA5,SORCS3,WWOX,GABRG3,ALG14,MEF2A,SYT12,CR1L,ADCY9,CACNA1A,DHX30,RBKS,NFRKB,DCAF6,DCUN1D5,DROSHA,FNBP1,GFRAL,GPC3,SGIP1,STOM,XRN1,ADRBK1,PLS3,SATB1,TBC1D5,PSMB2,ZNF207,CALD1,EYA1,GATAD2A,PTGER3,SCOC,BRINP1,CDCA3,DIS3L2,GNB3,SHPK,TRPV1,RBM12B,RNF216,TSPAN6,HOXB3,HOXB4,HOXB5,HOXB6,TFEB,CLEC16A,GHR,MNAT1,SLC38A6,FLRT2,FGD1,PRSS12,SNX9,DIAPH2,RPGR,ARL5A,ACTR2,L3MBTL4,ACACA,ELAVL4,FAM174B,LGI4,HDAC1,HS6ST3,INVS,RECQL5,C2,SMOC2,TRIM24,ADIPOR2,PLCG2,ROCK1,EPS8,NID1,SCMH1,C1QTNF1,MRPS17,PAXIP1,ZNF713,EGR2,HIPK3,RNF10,RYBP,GAPVD1,CAPN10,GPR35,CUL2,ABCC1,AP3B1,PTPN2,INSRR,NTRK1,KPNA6,TRIM44,MYBPC2,ZFAND1,MAP1A,PKN2,PPP1R9A,TMIGD1,SLC39A10,EIF3A,ANK3,DNMBP,ACO2,HYKK,UNC13A,PTPRQ,EPHB1,MAN1B1,PDE6A,EPHA10,PTPN9,SNUPN,AXIN2,NCKAP5,SARM1,TMEM199,ATP13A3,EPCAM,PARD3,RCAN2,ZNF425,GNL3L,KRTCAP2,MUC1,PTPN14,TRIM46,TUB,DNAH12,FBXO39,SH3GL3,TEKT1,JAG2,KCND3,NUDT14,BCOR,CACNG8,AP3D1,ZNF41,KIF3C,MGAT4A,CARM1,SLC9C2,VWF,AP2B1,LDHB,NETO1,RBM26,ZNF383,PGBD5,ANXA8L1,NUDT5,ADAM19,ARHGAP44,DTD2,HEATR5A,SNX33,SRD5A2,PIFO,CA14,FOLH1,CD84,CNTNAP5,HTR1D,LPA,ZNF616,ZNF836,SMARCA2,ARHGEF3,CUX1,HMCN1,RAD51C,SPI1,DGKK,DNMT3B,EPHA7,LRRC47,CHRNB4,FOXP2,MCU,RANBP10,CUZD1,PTPRA,VPS16,AGPAT3,PPFIA4,CTNNA1,LIMA1,AIFM2,FBXW4,MRPS24,PSPC1,URGCP,ACAP2,GLIS1,SFMBT2,TRDN,ATG14,EIF3H,GABRA6,LRRC8C,LRRC8D,LY86,SNORA35,STAC,BCAR3,JAZF1,RNU6-979P,ILDR2,SHROOM3,STK38,TRPC6,ABCB7,ACOT8,RBPMS,RENBP,SLC41A2,NTMT1,CHURC1,CREB5,FCHSD2,MAP3K13,OTUD3,RAB15,RAPGEF3,SDK1,ADAMTS17,PDXP,SH3BP1,MST1,RNU2-47P,KAZN,ATP13A5,ERCC3,PTK7,RSRC1,SMYD1,N4BP2,NHSL2,SNCA,BMPR1B,HAUS3,POLN,CACNG2,CD53,GRM4,MAGI2,PRIM2,TBC1D9,USP42,PNPT1,USP50,ZEB2,DHRS4,MCMBP,CABLES1,FOXJ3,NADK,OCA2,SLC39A9,SYT9,HEY2,RC3H1,CHMP3,COL13A1,EIF3E,HYDIN,RNF103,RNF103-CHMP3,RNU6-640P,CNGA3,RNF19B,SLC22A10,WNT7B,HMGCS2,PTPRS,ADAMTSL3,ZMYND11,KMT2D,PRKAG1,CDKL1,CREM,PSMF1,RAB5B,OSBPL1A,KMT2C,SULT2A1,TLL2,UBE2QL1,CAST,TRIP12,DFFA,GGPS1,SPPL2A,ADTRP,ABCA12,GNG7,CSRP1,PLD1,RASA1,MACC1,MITF,SRSF6,OPRD1,CABIN1,RBM45,HIPK1,NUGGC,PKP2,RPTOR,RTN4RL1,NFIX,BTK,SGCZ,SIGLEC9,PCDH11X,TBC1D16,XPO4,DSG1,VPS41,ZNRF3,ASPHD1,KCTD13,MLLT1,SLC4A8,TROVE2,ZZZ3,CBFA2T2,IGF1,MLXIP,PLA2G4E,STX8,ATF7IP,CADM2,CTNNBL1,HTT,DPYSL3,LARS2,MAPK1,PTEN,ARHGEF17,CLDN11,MIB1,SLC7A14,SPRED2,IFFO1,SLC30A7,BMP7,SCN8A,PCMTD2,ATXN2,MXI1,PIK3C3,PUM1,RASA2,SOX5,CIR1,LHFPL5,NUP88,PCBP2,TTLL4,PRCP,RAB30,ZNF780A,ZNF780B,LRRK2,ATP6V1A,MLYCD,OSGIN1,SEPT6,GNA14,MEGF11,TMEM59,ZRANB1,ALDOC,EBAG9,PIGS,SPAG5,UNC119,ZBTB20,CHMP5,CNIH3,RAPH1,RFC3,BAG6,CHAF1B,LILRB4,MORC3,SLC44A1,IFT81,TMCO1,TMEM108,IPCEF1,ITGAM,ARHGAP25,GALT,IL11RA,MSRB3,NSG2,PKD2,ZNF652,BLVRA,EFNA5,HSF2BP,SHANK3,SRRM1,KCTD1,STMN4,RIPPLY1,SEL1L2,MIA2,NT5E,RCOR3,IDH3B,TADA2A,NT5DC4,DAZL,BTBD11,RGS9,ALPK3,AOX1,PDGFC,SERPINA3,SERPINA4,SERPINA5,SPTLC3,AMOTL1,ATP8B4,FAM13B,NCOA3,PKIB,MTMR3,ZNF146,ZNF565,HS2ST1,SGSM1,STEAP4,PPP2R3C,TBCD,TMCC1,WNT7A,ZBED6,ZC3H11A,SNAP23,BRWD3,NLRP1,PEPD,RPS6KC1,UACA,LPGAT1,MAP4,CRTC3,DNAJC1,EPDR1,GPR141,NME8,ARNTL2,CEP89,ELOVL5,GLDC,PLCL2,WDR5B,KAT6A,MTIF2,GPR21,RABEP1,SH3PXD2B,TTC28,POM121C,RHOT1,SIK3,ZKSCAN7,ZNF197,ZNF660,BMP6,TAF15,MYCBP2,NFE2L1,ANO1,GPI,PDCD2L,ANO8,IL17RD,SH3RF2,SNRNP40,SOS1,TSHR,WDR43,ZNF30,EXT1,DPY19L4,N4BP1,PDCD1LG2,ATRX,DPH6,PRICKLE2,SERINC5,GABRR2,IKZF1,PARVB,PNPLA3,SAMM50,C8ORF44-SGK3,PRMT2,RAPGEF2,ASCC1,ABCG2,CDH9,KCNC2,MTHFD2L,RNU6ATAC31P,SMO,SLC6A14,VRK3,FAR1,RALY,AMPD1,ENPP3,GET4,SUN1,LSG1,ACADSB,ALG9,BLOC1S3,FDXACB1,GEMIN7,MARK4,PRKAR2B,STXBP5L,DPY19L1,CPT1A,MYLPF,PSEN2,TCF4,TNFRSF11B,PAG1,GALNT10,SYT17,UBE2H,BPGM,DECR1,SOX30,DNAJC6,TFE3,UBR5,GRB14,GRIN1,JADE1,KCTD8,ARRDC4,BBS9,RHOA,SPRTN,WSB2,OR4M1,OR4N2,SYNGAP1,VIPR2,GRIN3A,MCM3,NDE1,ROR1,RQCD1,TF,ONECUT2,RHOBTB1,ESYT2,FAM19A4,SHROOM1,CKMT1B,CUL3,SH3KBP1,TFEC,H

			SPD1,ITPR2,NFKBID,PCCA,TSPAN33,EPM2A,ESF1,GABRB3,GSK3B,ABCG8,PLD2,PRR16,DNAJC3,EMB,GNG12,RDX,STAT6,ACTA2,SYN1,MLF1,EEF2K,FAM49B,IWS1,PPP1R16A,PTPRB,RBBP8,CCDC14,NECAB2,CDK13,PGGT1B,ALAS2,STK32B,UPF2,CD27,DUSP26,ITGBL1,MCTP2,SCN9A,TAPBPL,VAMP1,BOK,RNF144B,SULF1,MTHFD1L,ZNF362,KRT8,NACC2,RNF43,SUPT4H1,TNFRSF8,ZNF354A,AGT,HDGFRP3,METTL16,CCNJL,CDH20,ELOVL3,POLH,PRKAA2,SYT7,ADAR,BLOC1S6,CUL9,FBN2,POLR2F,ESPNL,PITPNM2,STAT2,ZMPSTE24,BRINP3,FGGY,HRH4,NDRG2,PTK2,TPPP2,ARMC8,GTF2E1,HEPHL1,LAT2,PEX5L,PHEX,PPCDC,RNF121,SNX3,EXOC6B,FREM3,PDE4B,RIMS2,COX5A,PBX1,TCP11,TEC,DCAF10,EXOSC3,FBXO10,SHB,SLC25A51,TOMM5,VEPH1,CDC14A,COPG2,GAP43,MAP3K5,RNU6-1056P,CPE,SEC16B,TCFL5,TRPC4AP,CREBBP,TIMM44,IQGAP2,PRKD3,FAM171A1,LMTK3,UTP20,XYLB,ACSS3,APCDD1L,ARVCF,COL9A1,DLX1,FMOD,INSL6,PCSK6,CHKA,COL21A1,SLC25A42,VEZT,CEP70,GTF2IRD2,NCF1,SLC29A1,TAOK2,UGT1A1,UGT1A10,UGT1A3,UGT1A4,UGT1A5,UGT1A6,UGT1A7,UGT1A8,UGT1A9,PYGO2,SHC1,NDUFS2,SLC44A3,ZNF813,ALDOA,ARHGAP15,CD160,ECE1,KCNJ15,LBX2,PARP10,PDXDC1,RNFT1,RPS6KB1,SLC5A10,STC2,YLPM1,ZDHHC23,DPRX,KIF6,RSBN1,SF3A1,GALNT13,GPR173,GUCY2F,PGM5,PRG3,SCEL,SERPINB11,SETDB2,ANKS1A,ITGAL,ALOX5,CPLX2,DNAJB6,FBXO28,MDGA2,SP7,CD44,FGD3,LARP4,ADAM8,DBT,FKBP14,MRPS6,NOL10,SLC5A3,ARFGAP3,CLDN4,PACSIN2,RPS6KA5,SNHG14,SNORD109B,SNORD115-16,SNORD115-19,SNORD115-35,SNORD115-48,SNORD115-5,SNRPN,ZNF484,BCL2L14,CDS1,MUC7,THSD7A,ZNF93,CSNK1A1,GRID1,HUS1,PKD1L1,PRDM2,SLC25A18,COX5B,CXCL17,FCRL6,FGD4,MON2,NOS1,PEX7,ZNF44,GCKR,TBC1D23,MED15,PLEKHA1,PLVAP,RGS14,SIPA1L2,ACTN4,ARHGAP11A,COG4,MC1R,SCNN1B,STK4,TCF25,TUBB3,ZFAND6,DRAM1,ZNF282,MARK3,SPEF1,THSD7B,COX6A1,EPHB2,GATC,SLC1A1,TTLL11,WLS,EDRF1,MARCH8,SIGMAR1,TMEFF2,XCR1,BCO2,CLPTM1,FRMD4B,IL18,PCMT1,SDHD,TEX12,DTNA,LRRC52,MFSD8,ASF1B,TPMT,UBE2K,GPSM2,DNAJC7,HCAR1,HCAR2,HCAR3,DIS3,DLC1,FBXL13,PPARA,PPP1R10,ABCD3,ARID5B,GRTP1,PDP1,CALCRL,MCOLN1,PNPLA6,STEAP3,EPB41L4B,SLC25A33,SMARCAL1,TAF2,PHLPP1,CCDC6,KPNA4,MAGI3,PPIL6,PRRC2C,CPEB4,GPSM3,LDHC,MOB3B,NOTCH4,SYTL4,ZMYND8,ZNF366,ACP1,CRX,CYP2C18,CYP2C19,FBN1,RNF150,PAX6,PRKCZ,ZC3HAV1,ATP1A3,CD96,FAM20C,ADORA2A,SPECC1L,SPECC1L-ADORA2A,RBMS1,GRIN2B,KCTD7,MAN2B1,PAH,PPP1CC,RABGEF1,STAG1,WDR83OS,CCNG2,COL22A1,CPEB1,PHLDB2,PLCXD2,TMEM161B,TYK2,OTUD7B,CNTN3,DENND4B,KIN,KLC3,KLHL1,NIPBL,TMIGD2,YEATS4,CNRIP1,COL28A1,PCDH10,PHF20,THSD1,UPB1,ZNF143,ASPN,ANGPT1,BRF1,CADPS,PACS2,TBL1X,EYA3,FHL2,FRMPD1,LRRK1,MBTD1,OSBPL8,TRMT2B,ATF6,SHROOM4,ZBTB5,ZNF708,IGF2BP3,EPB41L2,L2HGDH,NT5M,SLC25A26,SLCO2B1,GCNT2,ETS1,HACL1,PPP1CA,TBC1D10C,VWC2,ARHGAP21,GRIA4,MAP2,MTF2,RBMS3,BIN2,CCL22,STPG1,BTN3A2,KIFC3,NEBL,RPIA,TAGLN3,FBXO31,ATG3,CDK5RAP1,CNTN1,NCOR2,PRKCQ,LGI2,MDN1,BRAF,CSRNP1,DIAPH1,HDAC2,HTR2C,IMPACT,SIL1,TNS4,ATP8A1,FKBP3,PHKG2,SRCAP,CAPN6,POU3F3,NOTO,PPP1R14A,SPINT2,TNR,MBNL3,TMEM163,UBE2E2,ZKSCAN5,CLTB,HIGD2A,UQCR10,UQCRC2,C18ORF25,CD300A,ELF2,IRAK1BP1,MOXD1,PHIP,CDKL3,EMC3,PPP2CA,SKP1,SLC26A2,ANGPT4,ARHGEF6,DNAJC9,FAM149B1,MAOA,MRPS16,RPL23,SH3BP4,RFC5,ACAN,ATXN1,CAMSAP3,DCDC1,SP100,ZNF347,ZNF415,OPN1LW,REV3L,VTI1A,ANXA2,CHCHD3,FAT2,SNTG1,TRIM29,KCND2,PARN,ATP6V0A1,PIGU,PRMT7,RAB27B,C1QTNF9,CR2,CRYZL1,DONSON,FCRL2,ITSN1,NUP214,PITPNM3,FARSB,SRGAP2,TMEFF1,WIPI2,ADCY1,ALG2,MADD,NUMB,RBM42,SLC18B1,SQLE,SYK,CNOT1,MOGAT2,RBM39,WWTR1,ARHGAP19,CYP39A1,GRIK2,GTF2H5,SCN1A,SLIT1,ASB1,FRMD5,NFXL1,CIT,MYRIP,PAPOLB,RAB2A,RADIL,SEC31B,SLC44A5,UBAP2L,ZBTB38,BRCA2,HRK,RAP1GAP2,ALDH8A1,ATF7,CACNG3,DNAH3,DVL2,MORC1,MTRF1,NEB,YBX1,ANKRD13A,GRIP1,PTPRJ,SLC10A1,TAF1,ATP2B3,EIF4E3,GPRIN3,PPME1,TICAM1,RNF38,TANK,THAP3,ERGIC1,GPM6A,OSBP2,PFKFB4,RIMKLA,UCN2,GALNT7,ITGAE,MYO1A,NSUN6,SNAPC3,TAC3,A3GALT2,PLEKHB2,SLC13A3,SLC2A12,CADM1,HSF1,MAX,SAP130,CNIH4,DHFR,EZH1,POP1,ANKRD30BL,ETF1,INPP5A,NRF1,RNGTT,SRPK2,ANKRD13C,ATP10B,PLEKHM1,SASH1,HGSNAT,ASAP1,NCOA2,ADAMTS7,CEPT1,RALGAPA1,SLC5A4,GBP5,MCM8,SULT2B1,WDR18,BMP1,BMS1,CGNL1,EEFSEC,FCHO1,GEMIN6,QRICH1,VAV3,ZP3,CHD6,DLGAP2,KCNQ3,MLPH,CIB2,EPB41L1,MTFR2,RNF2,ZNF554,MOB3A,SNORD114-13,SNORD114-15,SNORD114-16,SNORD114-20,SNORD114-21,SNORD114-25,CAV2,GNA12,SH2D3A,SKA2,SYNM,UGGT1,AKTIP,COL4A5,EPS15,GALNT4,MYEF2,POC1B,POC1B-GALNT4,CA12,INPP5F,NUAK2,TBP,TMPRSS3,AXL,AGBL3,ORC4,REPS2,TNFSF9,TRIM37,WIF1,CD109,CENPK,DENND4C,MECR,MET,SESTD1,TBC1D10A,FMNL1,MICAL3,ZNF461,CAMK2D,CCR3,KCNJ6,MRPL1,RAD9B,SNX1,TIMELESS,UCHL5,VSIG10,ANKRD6,BRD9,MALT1,PFKP,SERPINE3,SETD3,TRA2B,TRIM69,ZDHHC11,ZDHHC11B,INO80C,SPTAN1,TMEM120B,CCDC136,EHD2,KDM2B,MLXIPL,SEMA3C,THEM4,TRAPPC8,CCT3,FBXL4,GNS,NEDD4L,PHKB,UQCC1,NDC1,PHB,PTPRC,RPH3A,EDA,GPR89A,ZNF555,CTCF,EXD2,FAM135A,PBLD,RAB28,SH3GL2,TYRO3,MTMR12,NR6A1,RNF213,SPON2,SYNDIG1,TSPAN5,VPS52,ADCY10,NCAM1,TANC1,TRAF3IP2,TRIM60,ATF6B,CREB1,LIG1,METTL21A,RASSF8,RGMB,SGTB,SHI

			SA6,TNXB,TRAP1,AFF4,CCDC170,CLNK,EBF4,EDNRA,GNGT1,HADHA,PRAP1,SLC25A13,STXBP6,ZNF511,BMP4,ABLIM3,CASP12,CPNE1
GO:0018130	heterocycle biosynthetic process	1.0918870064896365e-9	*ENPP1,PRDX2,LDB2,ASPH,PRKCI,SLCO3A1,DNMT1,HLX,SLC9A1,PBX3,ADCY8,MED13L,TRPS1,CBFB,PDE8A,ZNF823,IL31RA,PRDM12,MED26,WWC1,ASH1L,SCAF8,STOX2,PTGIS,PRKAG2,WWC3,PAGR1,ADCY7,HIVEP3,NPAS3,ARNT,LRRFIP2,STAT5B,TOX,GRM5,ZNF566,FER,CASK,MAP2K5,MAPK10,ZNF536,SP3,HDGF,GTF3C1,EZR,IKZF2,CHD7,MECOM,TACC1,TENM1,NADK2,TRAF6,GTF2E2,DNAJA3,NHLH1,HSP90AA1,PSMB7,TSC22D3,ZC4H2,CCDC22,MOCOS,GMDS,GFI1B,PTPRK,RUNX1,ERC1,HMGN3,NRIP1,THRB,EFCAB7,ITGB3BP,DACH1,ZNF569,CCT2,ORC2,HDAC6,SERTAD2,MYT1,IKBKB,PEX14,ERBB4,MRE11A,ZNF609,BRD8,KAT6B,HIF3A,SNIP1,ESR1,MIER1,PCBP3,MAML3,CDON,EP300,CELA1,TENM2,ZNF76,RNF220,ZNF471,PDGFB,CCND3,TOB2,ZNF19,ZNF23,BRMS1,ZNF605,SCML4,HNF4G,INSR,RERE,PRKAR1A,FUBP1,ATP8B1,H2AFY2,TCF7L2,JMJD1C,ZNF443,ZNF490,ZNF564,ZNF709,ZNF799,LITAF,FBXW11,ESRRB,MAP3K4,BASP1,TBR1,TFAP2A,CDK14,FANCA,PEG3,ZIM2,TGIF2,TGIF2-C20ORF24,MEIS1,VRTN,TRIM13,MDM4,ZNF148,MTA3,SNX6,TFDP2,ERN2,C1D,AFF3,DMD,CENPF,SLC30A9,TOX3,USP13,KAT7,ZNF667,RBM14,PRPS2,MED12L,SATB2,RIT2,HIRA,MKRN2,ARNTL,PLCB1,ARID4B,POLA2,RWDD3,BRPF1,PIK3R2,NRG1,UBP1,MAP2K1,MDFI,FNIP1,VAX2,TMBIM6,VGLL4,PPP2CB,PPP3R1,HDAC5,CSRNP3,ZNF692,NFIA,RNF4,JDP2,CMKLR1,ROR2,DCN,PRPSAP2,PCBD2,CDH13,CREBRF,SOX13,ACSBG1,COPS5,AK2,MAD2L2,TLE6,CAPRIN2,ZNF418,PHF20L1,TERF2,ZNF286A,NME7,FOXN3,AK5,NEUROD1,USP22,JAK2,TRAPPC9,SKAP1,UIMC1,ITCH,MLIP,BCL11B,IMPDH1,PKNOX1,MLLT3,TSHZ2,TCF7,PDE2A,KLF15,TBX15,ANXA4,WNT11,MTA1,KLF8,NOX4,LCOR,RPRD1B,CCDC62,SOX6,ACVR2A,RUNX2,CD4,TGFB1,BANP,SGK1,NSD1,IGSF1,ZHX2,PKNOX2,ASCC2,BTRC,NFATC3,POLK,F2RL1,BCAS3,CDYL2,CC2D1B,TP73,SAP18,ZBTB22,ILF2,MTDH,FANK1,MYT1L,SMAD6,BNC2,ZNF398,CLOCK,TCF12,ZNF675,ETV6,PHRF1,TFAP2D,BCL3,SND1,DUSP22,HNRNPC,TRRAP,SBNO2,FGF10,POLR2J2,ADK,SMYD3,LOXL3,ADCY2,CAPN3,LUM,SMURF2,RORA,HIVEP2,HSD17B12,AUTS2,TNFSF11,SMG6,PPP3CA,NFYB,MAGEA4,KLF12,CAMK4,GATAD2B,UFL1,TRAK1,CTNNB1,PARK2,SOD2,DACH2,METTL13,SMARCC1,KLF17,PPARG,AXIN1,IL18R1,CIPC,MTF1,CBX5,BRIP1,SREBF2,PFAS,CDK11A,CDK11B,FGF1,NPAT,NR4A3,FOXK2,MYOCD,TRIM5,PER2,AJUBA,ZNF626,ZNF737,CHEK2,SUPT3H,PRDM16,HCK,TRIM8,BRMS1L,ZBTB8A,ZBTB8B,CTDP1,BAZ1B,MEF2B,HDAC4,PAX2,PHF5A,CPOX,SNAPC4,SFRP1,MED13,ZNF395,FOXO3,TPH2,NFIB,SP4,SMAD3,CUX2,WWP2,ARNT2,SBNO1,KRBOX1,ZNF662,ZNF777,EBF3,RNF168,CASZ1,MIER3,NEDD4,ESRRG,HOXD3,HOXD4,ZNF114,KTI12,NFATC1,UCK2,CDC73,APP,SSBP3,GSX2,YAP1,PPCS,TEN1,NVL,LRP5,POLR3G,ZNF787,SOX2,SETD2,TEAD1,PRICKLE1,ZNF653,ZNF521,ELOVL2,ARID3A,ZNF761,CHUK,SFMBT1,ZNF584,TPH1,ESR2,S100A12,GTF2F2,PRKD1,STAT1,ST18,ETV5,RHOXF2B,TAF3,PLAGL1,HNF4A,ZBTB7C,TASP1,EREG,ATF2,POU2F2,TCF3,ZNF730,RAF1,ABLIM2,ZNF766,CARD16,POLA1,BMPR2,CAMK1D,BMPR1A,ZKSCAN1,IDO2,IKZF4,CDK12,TAF8,CAND2,DENND4A,MYB,FGF2,ZNF71,POU6F2,BACH1,MXD3,PPM1F,ADCY5,GLI4,ZFP41,BEND5,SEMA4D,NFX1,RORC,ELP3,PTAFR,SP140,SP140L,RHOXF2,JARID2,DDX58,SLC5A8,BRDT,PHC2,RARB,SPEN,SIN3B,NCOA1,EHMT1,LMO7,DVL3,POLR2H,TCF20,ATF3,TCEANC,CKS1B,PAWR,EBF2,MAML2,TSG101,TERF2IP,CRYM,RFX2,ZNF322,SUFU,MAGEA11,PRDM15,ZNF670,ZNF695,CCDC169-SOHLH2,SOHLH2,POLE,ZNF354C,TCEA3,CMPK1,ZNF704,NR2C1,ME1,GLI2,TNKS,WBP2NL,ERCC1,GLIS3,WDTC1,ZNF664,TPK1,DAB2,BLM,PKHD1,MYSM1,SETD5,SMG5,RELN,SIN3A,RUVBL2,COMMD6,GUCY1A2,GMEB1,PELI1,SLC25A16,MAP3K7,ZNF423,SP1,TRIM22,ALK,TEAD4,HOXC13,APBB3,SRA1,UBE2V1,ADIRF,OVOL2,SNX5,NFATC2,PAX7,BNC1,ATP2B4,ACTL6B,ASXL3,PAK3,TET1,CAMTA1,CCPG1,ADNP,ARID4A,CDK6,PHF2,TFRC,EPHA5,WWOX,MEF2A,ADCY9,DCAF6,XRN1,SATB1,PSMB2,EYA1,GATAD2A,TRPV1,HOXB3,HOXB4,HOXB5,HOXB6,TFEB,MNAT1,ACTR2,L3MBTL4,ACACA,HDAC1,RECQL5,SMOC2,TRIM24,PLCG2,SCMH1,PAXIP1,ZNF713,EGR2,HIPK3,RNF10,RYBP,AP3B1,PTPN2,NTRK1,KPNA6,TRIM44,EPCAM,ZNF425,GNL3L,MUC1,PTPN14,BCOR,AP3D1,ZNF41,CARM1,ZNF383,ZNF616,ZNF836,SMARCA2,CUX1,SPI1,DNMT3B,FOXP2,PSPC1,GLIS1,SFMBT2,JAZF1,ABCB7,RBPMS,CHURC1,CREB5,MAP3K13,ERCC3,SMYD1,SNCA,BMPR1B,POLN,PRIM2,ZEB2,FOXJ3,NADK,HEY2,ZMYND11,KMT2D,CREM,KMT2C,MACC1,MITF,OPRD1,RPTOR,NFIX,BTK,MLLT1,TROVE2,CBFA2T2,IGF1,MLXIP,ATF7IP,MAPK1,PTEN,BMP7,MXI1,SOX5,CIR1,PCBP2,ZNF780A,ZNF780B,ATP6V1A,MLYCD,ZBTB20,RFC3,LILRB4,PKD2,ZNF652,HSF2BP,KCTD1,RIPPLY1,NT5E,RCOR3,TADA2A,NCOA3,PKIB,ZNF146,ZNF565,WNT7A,ZBED6,BRWD3,CRTC3,ARNTL2,ELOVL5,KAT6A,ZKSCAN7,ZNF197,ZNF660,BMP6,TAF15,NFE2L1,WDR43,ZNF30,ATRX,IKZF1,PRMT2,ASCC1,MTHFD2L,SMO,RALY,AMPD1,TCF4,SOX30,TFE3,GRIN1,JADE1,RHOA,SPRTN,ROR1,RQCD1,TF,ONECUT2,CUL3,TFEC,STAT6,MLF1,RBBP8,CDK13,ALAS2,DUSP26,MTHFD1L,ZNF362,NACC2,SUPT4H1,ZNF354A,AGT,HDGFRP3,ELOVL3,POLH,POLR2F,STAT2,ZMPSTE24,GTF2E1,PPCDC,PBX1,MAP3K5,TCFL5,CREBBP,DLX1,GTF2IRD2,NCF1,SHC1,ZNF813,ALDOA,LBX2,PARP10,DPRX,GUCY2F,PRG3,SETDB2,ALOX5,DNAJB6,SP7,ADAM8,RPS6KA5,ZNF484,ZNF93,PRDM2,NOS1,ZNF44,MED15,RGS14,ACTN4,MC1R,TCF25,ZNF282,EDRF1,IL18,TEX12,PPARA,ARID5B,SLC25A33,SMARCAL1,TAF2,LDHC,NOTCH4,ZMYND8,ZNF366,CRX*

			,PAX6,PRKCZ,ADORA2A,OTUD7B,NIPBL,YEATS4,PHF20,ZNF143,BRF1,TBL1X,FHL2,MBTD1,ATF6,ZBTB5,ZNF708,ETS1,MTF2,TAGLN3,NCOR2,PRKCQ,CSRNP1,HDAC2,IMPACT,SRCAP,POU3F3,NOTO,ZKSCAN5,ELF2,PHIP,PPP2CA,SLC26A2,RPL23,RFC5,ATXN1,SP100,ZNF347,ZNF415,REV3L,TRIM29,PARN,ADCY1,SYK,CNOT1,WWTR1,GTF2H5,NFXL1,ZBTB38,BRCA2,ATF7,DVL2,MORC1,YBX1,TAF1,TICAM1,THAP3,SNAPC3,HSF1,MAX,SAP130,DHFR,EZH1,NRF1,NCOA2,QRICH1,ZP3,CHD6,RNF2,ZNF554,MYEF2,TBP,TRIM37,MET,ZNF461,CAMK2D,TIMELESS,BRD9,MALT1,SETD3,KDM2B,MLXIPL,CCT3,PHB,EDA,ZNF555,CTCF,NR6A1,ADCY10,ATF6B,CREB1,LIG1,RGMB,EBF4,SLC25A13,ZNF511,BMP4,ABLIM3
GO:0008104	protein localization	1.179043935706782e-9	CD247,DNAH11,ZDHHC14,HOOK2,BLZF1,GPC5,NRXN1,ASPH,PRKCI,ADCY8,CLASP2,GRID2,CLDN18,CTDSPL2,LATS2,LRP1B,ASTN2,EPB41,DPP6,MAPRE2,TJP1,MVP,SEC23B,SNX31,RAB4B,RAB4B-EGLN2,LHFPL4,FAM53A,MARVELD3,CCDC93,KCNIP4,CEP128,LRP2,TNPO3,HDGF,ANK2,EZR,XRCC4,TOM1L1,TENM1,KIF5C,LMNA,SRP72,AFM,NFASC,DNAJA3,ITGB1,HSP90AA1,MCC,CCDC22,FAM126A,CDAN1,CNGB1,DLG2,PTPRK,ABCC8,ERC1,HMGN3,AFTPH,STXBP4,IMPG2,EFCAB7,TCIRG1,PTPN11,CCT2,CACNB2,HDAC6,GOLGA4,ADORA1,IKBKB,PEX14,ERBB4,GBF1,TLK1,ESR1,DPP10,FRMD4A,STX18,FNTA,HOOK3,VPS45,RIMS1,TNIK,SNX2,MID1,FMN2,ATP8B1,H2AFY2,TCF7L2,SCAMP4,MYOM1,SPTSSA,BDNF,SFT2D1,ECT2,SNX6,CHML,VAMP7,PTPRU,RFTN1,AP3S1,NPLOC4,DMD,CENPF,RANBP17,ATG10,KAT7,GSN,RIT2,FYN,ARNTL,ADAMTS9,NF1,LMTK2,STX6,RTN4,GOLGA2P5,APOD,VTA1,BCR,TTN,ICA1,PAQR3,PIK3R2,KLHL21,BID,MDFI,RAPGEF6,MYH9,MAD1L1,TMEM30A,SYNE1,PARD3B,SCFD2,ZDHHC3,LIN7A,SCFD1,CREBRF,EXOC4,IPO5,MAN1A1,RAB27A,MTX3,RAB3C,COG8,TERF2,TMED6,GOLPH3L,RCC2,ARL3,NEUROD1,IPO11,PARP11,DENND1A,ZDHHC15,JAK2,FBXW7,KCNB2,OAZ2,SKAP1,CROCC,SNAP25-AS1,PLS1,DENND2A,SEPT7,TTC7B,LAMTOR3,KPNA3,CTAGE6,WNT11,IFT80,VPS13B,IMMP2L,PRKCD,CD4,TGFB1,BANP,PCSK5,NUP93,PIBF1,PTPRN2,F2RL1,BCAS3,DNAJB2,CELSR1,AAK1,CADPS2,CORO1C,DISC1,CEP135,ZFAND2A,GRPEL2,ANK1,TTBK2,CHRM1,CLOCK,BCL3,DCLK1,RABGAP1L,FGF10,CNTNAP2,CIZ1,SMYD3,STXBP5,CAPN3,SLC16A1,VMP1,SNX8,CNR1,PPP3CA,UFL1,TRAK1,CTNNB1,PARK2,RN7SL832P,TSNARE1,PPARG,DLG5,MYO1D,MSR1,SYTL5,SCAMP5,ANXA13,SREBF2,RYR2,LEPROT,NIN,WDR45B,AGAP1,TRIM5,PER2,AJUBA,SCN3B,SORBS1,TBC1D14,RAB3GAP2,TBCK,TRIM8,OBSCN,NF2,SPTBN1,AP3B2,SFRP1,ARL13B,CCDC91,IFT122,RANBP3,SNX14,SMAD3,RAB6A,WWP2,WIPI1,MTBP,VPS39,GRIK5,LZTFL1,SHANK1,NEDD4,ARHGEF18,KPNB1,VPS53,RAB6C,SEC61B,BET1L,YAP1,HEG1,RAB11FIP5,NLGN2,SSR2,BORA,NVL,JAK1,LRP5,SEC22C,SLC9B2,SETD2,ZDHHC6,GPC6,PRICKLE1,SPTBN4,DRD1,SERGEF,PRKD1,TAF3,HNF4A,VPS4A,PITRM1,ATF2,RAF1,PTPN1,AP1B1,NLGN1,SNX16,TAF8,RAB11A,IPO9,RAB24,PPM1F,ADCY5,XPO6,RILPL1,SORCS2,RAB11FIP3,TANGO6,GAS8,AP2M1,TNPO1,TECPR2,LMBRD1,TSG101,DESI1,VCL,TERF2IP,ANKS4B,SUFU,PAFAH1B1,KIF3A,ATG4C,HIP1,AKAP6,CEP350,PADI6,TNKS,RUFY3,DAB2,TRAM2,USH2A,DZIP1,DLG3,RELN,SIN3A,RUVBL2,FAM91A1,PLEKHF2,ZNF423,TRIM22,MACF1,MYO7A,APBB3,NBEAL1,GPM6B,LTBP1,SNX5,ATP2B4,NSF,PACRG,SYNE3,PACSIN1,ATAD1,NXT2,CEP41,KCNAB2,TFRC,EPHA5,GPC3,STOM,TBC1D5,SNX9,RPGR,ARL5A,ROCK1,EGR2,GAPVD1,CAPN10,AP3B1,LRBA,KPNA6,ZFAND1,MAP1A,ANK3,PTPN9,SNUPN,AXIN2,PARD3,GNL3L,PTPN14,TRIM46,TUB,CACNG8,AP3D1,AP2B1,ARHGAP44,HEATR5A,SNX33,SPI1,MCU,VPS16,CTNNA1,STAC,ILDR2,SHROOM3,FCHSD2,RAB15,RAPGEF3,ERCC3,CACNG2,MAGI2,TBC1D9,SYT9,HEY2,CHMP3,RAB5B,ADTRP,ABCA12,PKP2,TBC1D16,XPO4,VPS41,IGF1,STX8,IFFO1,BMP7,PIK3C3,NUP88,LRRK2,SEPT6,TMEM59,SPAG5,UNC119,CHMP5,BAG6,LILRB4,MORC3,ITGAM,PKD2,EFNA5,SHANK3,MIA2,SGSM1,WNT7A,ZBED6,SNAP23,DNAJC1,RABEP1,SH3PXD2B,POM121C,BMP6,MYCBP2,ANO1,ATRX,SAMM50,RAPGEF2,SMO,GET4,SUN1,LSG1,BLOC1S3,MARK4,STXBP5L,CPT1A,UBR5,ARRDC4,BBS9,SYNGAP1,HSPD1,TSPAN33,EPM2A,GSK3B,RDX,CCDC14,NECAB2,AGT,PRKAA2,SYT7,ADAR,BLOC1S6,FBN2,PEX5L,SNX3,EXOC6B,RIMS2,TOMM5,COPG2,CPE,SEC16B,TIMM44,GTF2IRD2,NCF1,TAOK2,ZDHHC23,ITGAL,ALOX5,DNAJB6,ADAM8,SLC5A3,ARFGAP3,PACSIN2,MON2,PEX7,GCKR,PLEKHA1,ACTN4,COG4,STK4,ZFAND6,EPHB2,SLC1A1,WLS,GPSM2,HCAR2,PPP1R10,GRTP1,CALCRL,MCOLN1,STEAP3,KPNA4,SYTL4,FBN1,PAX6,PRKCZ,ADORA2A,RABGEF1,WDR83OS,PHLDB2,OTUD7B,NIPBL,ANGPT1,CADPS,PACS2,FRMPD1,OSBPL8,EPB41L2,HACL1,TBC1D10C,ATG3,BRAF,DIAPH1,SIL1,TNS4,CLTB,EMC3,SKP1,FAM149B1,RPL23,SH3BP4,CAMSAP3,SP100,VTI1A,TRIM29,PIGU,RAB27B,ITSN1,NUP214,WIPI2,NUMB,SYK,WWTR1,GRIK2,MYRIP,RAB2A,SEC31B,BRCA2,CACNG3,DVL2,YBX1,ANKRD13A,GRIP1,CNIH4,ANKRD13C,PLEKHM1,GBP5,MCM8,CGNL1,MLPH,RAB37,AKTIP,EPS15,DENND4C,TBC1D10A,NBEA,SNX1,ZDHHC11,ZDHHC11B,EHD2,TRAPPC8,CCT3,NEDD4L,NDC1,RPH3A,GPR89A,CTCF,RAB28,SYNDIG1,TSPAN5,VPS52,TRAF3IP2,SGTB,SHISA6,EDNRA,GNGT1,BMP4,ABLIM3
GO:0016049	cell growth	1.4793833386135852e-9	ENPP1,LLPH,SLC9A1,CLASP2,SEMA3A,NRG3,SEMA3D,EGLN2,SEMA5A,PLCE1,MAP2K5,OLFM1,CDKL5,ITGB1,HSP90AA1,DSCAM,AKAP13,IL17RB,HDAC6,GOLGA4,SERTAD2,LIMK1,RIMS1,CDH4,BDNF,RTN4,RTN4R,NTN1,NRG1,BDKRB1,VGLL4,CSNK2A3,MAD2L2,CAPRIN2,SYT1,ITCH,CYFIP2,WNT11,SEMA5B,TGFB1,SGK1,DNAJB2,SLIT3,SLIT2,DISC1,ITSN2,DCLK1,AUTS2,GNG4,MAG,CTNNB1,PARK2,CDHR2,PPARG,CDK11A,CDK11B,DRAXIN,NIN,MYOCD,CPNE6,DCC,CTDP1,SPG1

			1,SFRP1,MUC12,SMAD3,SYT3,NRP2,CDC73,APP,DIP2B,FSTL4,ISLR2,ESR2,DCUN1D3,CPNE9,HNF4A,SEMA6D,BMPR2,DPYSL2,SPAG9,RAB11A,SEMA4D,SPOCK1,EIF4G1,TSG101,VCL,WNT3,NLGN3,PAFAH1B1,AKAP6,RASAL1,TRPC5,RUFY3,DAB2,SIN3A,IQGAP1,MACF1,ADNP,MAP1B,DCUN1D5,UNC13A,TRIM46,SMARCA2,EPHA7,MAP3K13,PTPRS,KMT2D,RPTOR,IGF1,OSGIN1,EBAG9,RAPH1,TMEM108,EFNA5,EXT1,PRMT2,SYT17,JADE1,RHOA,EPM2A,GSK3B,AGT,RIMS2,TAOK2,PPARA,SLC25A33,PRKCZ,MAP2,PRKCQ,IMPACT,TNR,CDKL3,PPP2CA,SH3BP4,SLIT1,PTPRJ,CAMK2D,KDM2B,SEMA3C,NEDD4L,PHB,SH3GL2,EDNRA,CPNE1
GO:0042221	response to chemical	1.739492151853541e-9	ENPP1,PRDX2,NRXN1,ASPH,GSS,PRKCI,PDE4D,DNMT1,SLC9A1,ADCY8,PDE8A,SEMA3A,IL31RA,TAS1R2,RPS6KA2,PTGFR,CALR3,CLDN18,CTDSPL2,RYR1,NRG3,LATS2,SEMA3D,MYOT,NREP,PTGIS,TJP1,UNC5C,OR5P2,OR5P3,PRLR,PAGR1,ADCY7,KALRN,NTRK3,CBL,ARNT,EGLN2,SEMA5A,FLT3,STAT5B,ENPP2,GRM5,SAMHD1,FER,CASK,MAP2K5,LRP2,PIK3CD,HDGF,ANK2,EZR,NRXN3,ROBO1,CHD7,SLC26A6,HTR2B,ITGB6,RYR3,KIF5C,LMNA,GOT1,LPO,TRAF6,ROBO2,NFASC,ITPKB,DNAJA3,OXR1,ERCC6L2,ITGB1,HSP90AA1,ELMO2,CDC6,PSMB7,SLC22A8,DSCAM,CNGB1,TRIO,DLG2,PTPRK,ABCC8,HMGN3,NRIP1,DNAJB14,THRB,STXBP4,PDE4A,LAMA2,IL17RB,TCIRG1,PTPN11,HDAC6,MSRA,ADAMTS3,EPHA1,IKBKB,ERBB4,GBF1,BRD8,ESR1,PMEPA1,PTPRO,NTRK2,EP300,CPNE4,FNTA,PDGFB,CACNA1B,CCND3,USP46,ALOX5AP,BPI,STK39,INSR,FMN2,UNC5D,LAMA3,TCF7L2,PIP4K2A,CDH4,CAB39,PAK1,LITAF,ESRRB,EGLN3,TBR1,BDNF,TFAP2A,AMIGO1,KCNMA1,CHRM3,FUT8,OR2T3,TRIM13,UBR2,HLCS,LCK,MDM4,IL5RA,ECT2,SNX6,CST2,PTPRU,RFTN1,ERN2,LSP1,AP3S1,NPLOC4,AFF3,DMD,CENPF,USP13,KAT7,SLC8A1,GSN,RBM14,RBM4,SATB2,FYN,ARNTL,NF1,PLCB1,MGMT,RTN4,RXFP1,DPEP1,CHRDL1,APOD,BCR,RWDD3,CHI3L1,CCDC141,TTN,PIK3R2,ADAMTSL1,NTN1,NRG1,CDK19,BDKRB1,BDKRB2,DOCK8,MAP2K1,STIM1,TMBIM6,PPP2CB,CNTN4,HDAC5,OR6N2,RNF4,CMKLR1,ROR2,DCN,SLC39A14,SCFD1,DOCK2,CDH13,CREBRF,TRDMT1,PTPRT,ACSBG1,COPS5,IPO5,MAN1A1,RGS8,DEFA1B,DEFA3,F5,PTGER2,STIP1,BOC,ANO6,NEUROD1,GNAQ,RFFL,CCBE1,JAK2,MYPN,YTHDC2,FBXW7,SYT1,BCL11B,CLDN1,TCF7,PDE2A,KLF15,LAMTOR3,CYFIP2,WNT11,NOX4,LCOR,CACNA1H,SIAH1,IL1RAPL1,CCDC62,ADAM23,PRKCD,SOX6,TAB2,ACVR2A,RUNX2,SEMA5B,CD4,PPM1E,TGFB1,SGK1,MMP28,BTRC,PTPRN2,F2RL1,BCAS3,DNAJB2,SLIT3,ZFYVE9,SLIT2,TP73,ITIH4,GABRB1,SYT13,HP,OR9A4,KANK1,ZFAND2A,LAMC2,MTDH,CHRM1,OR51A7,OR51F2,OR51T1,SMAD6,RXFP2,CLOCK,ZNF675,BCKDHB,KCNH1,BMPER,TIMP2,DAPK2,PARVA,DUSP22,GPR52,NAIP,HOMER2,SBNO2,YTHDF1,FGF10,RASA4,RASA4B,SMYD3,ENAH,IL1RAPL2,GLP2R,ADCY2,CAPN3,SLC16A1,SMURF2,EPHA4,RORA,PRKCA,CNR1,CD6,TNFSF11,PPP3CA,HBE1,OR51B2,OR51B4,OR51I1,NFYB,UFL1,CTNNB1,PARK2,SOD2,FCGR2B,HSPA6,SMARCC1,IGF1R,PPARG,AXIN1,IL18R1,IL1RL1,MTF1,MSR1,CYBB,OTC,BRIP1,SREBF2,RYR2,DRAXIN,LEPR,LEPROT,FGF1,RGS10,NR4A3,NOL3,MYOCD,OR52E6,OR52E8,OR52N1,TRIM5,AJUBA,CPNE6,GLG1,CHEK2,PRDM16,HCK,SORBS1,CAPN2,DIO2,DCC,FLT4,HDAC4,PAX2,CPOX,SFRP1,FOXO3,TPH2,ARL13B,CNTN6,NFIB,SSH1,SYNCRIP,SMAD3,RNFT2,CUX2,PTPRM,ACKR2,GRIK5,SHANK1,SYT3,IFNAR1,OR14K1,NEDD4,NRP2,PREX1,DOCK4,ESRRG,TAB1,SLC6A1,RAB6C,SEC61B,KCNK1,PRTG,CDC73,APP,PDGFRA,AOC2,NOX1,YAP1,AMFR,RAB11FIP5,SESN1,TNFRSF19,RNLS,ALPL,JAK1,LRP5,SLC9B2,GNG2,SOX2,SETD2,CHST11,DRD1,GLRA2,CHUK,ERLIN1,FRS2,MMP2,ESR2,S100A12,CPNE9,PRKD1,STAT1,ST18,ETV5,SLC6A3,HNF4A,SHANK2,STRA6,EREG,MAPKAPK3,DSCAML1,SEMA6D,ATF2,RAF1,EPHX1,CARD16,CASP1,PTPN1,ADAMTS12,GGT7,BMPR2,USP33,DPYSL2,CAMK1D,BMPR1A,TSPAN12,NLGN1,COL16A1,PIK3R3,CRMP1,EPG5,FGF2,BACH1,OR6C70,PPM1F,ADCY5,SEMA4D,RORC,PLXNA2,PTAFR,ADCYAP1R1,JARID2,DDX58,RARB,PRKCG,NCOA1,EEF1E1,CHRD,EIF2B5,EPHB3,PTPRE,RBX1,ATF3,SHOC2,SRSF5,STT3B,PAWR,ACAA1,CCDC3,CLIC4,IDE,KCNQ1,RFX2,WNT3,P4HB,CCL14,CCL15,CCL15-CCL14,PAFAH1B1,CNTFR,COL4A3,TRPA1,DDAH1,AKAP6,RASAL1,NR2C1,EDEM3,IRS4,ME1,GLI2,NEO1,ERCC1,PXDNL,WDTC1,DAB2,BLM,UBR1,LDLRAD4,WNK1,RELN,SIN3A,RUVBL2,ABCB1,OR5H2,OR5H6,OR5K4,CSF3R,PELI1,IQGAP1,SLC47A1,MAP3K7,ZNF423,SP1,OR56B1,ALK,GSTM3,STAU1,SRA1,TOP1,XDH,LTBP1,OVOL2,SNX5,NFATC2,OR6C74,RGS7,ATP2B4,PACRG,PAK3,DYX1C1,P2RX6,ADNP,MAPKAPK2,MAP1B,COL4A6,SCARB1,ATP6V0A2,TYR,TFRC,EPHA5,WWOX,MEF2A,SYT12,CACNA1A,GPC3,XRN1,ADRBK1,PSMB2,BRINP1,SHPK,TRPV1,GHR,MNAT1,FLRT2,ACTR2,ACACA,ELAVL4,HDAC1,RECQL5,C2,SMOC2,TRIM24,ADIPOR2,PLCG2,ROCK1,EPS8,EGR2,CAPN10,GPR35,ABCC1,PTPN2,INSRR,NTRK1,TRIM44,ZFAND1,ANK3,EPHB1,MAN1B1,EPHA10,AXIN2,SARM1,TMEM199,CARM1,SRD5A2,HTR1D,SPI1,EPHA7,CHRNB4,MCU,PTPRA,CTNNA1,AIFM2,ACAP2,LRRC8C,LRRC8D,LY86,BCAR3,ILDR2,CHURC1,RAB15,RAPGEF3,SDK1,PDXP,MST1,PTK7,RSRC1,SNCA,BMPR1B,CACNG2,MAGI2,PNPT1,ZEB2,SYT9,RC3H1,RNF103,RNF103-CHMP3,CNGA3,WNT7B,HMGCS2,KMT2D,SULT2A1,SPPL2A,ADTRP,ABCA12,PLD1,SRSF6,OPRD1,HIPK1,NUGGC,RPTOR,BTK,DSG1,TROVE2,IGF1,STX8,DPYSL3,MAPK1,PTEN,SPRED2,BMP7,PIK3C3,SOX5,PRCP,LRRK2,ATP6V1A,GNA14,ZBTB20,CHMP5,RFC3,BAG6,LILRB4,TMEM108,IPCEF1,IL11RA,NSG2,PKD2,EFNA5,SEL1L2,AOX1,PDGFC,NCOA3,WNT7A,ZBED6,NLRP1,NME8,GLDC,GPR21,BMP6,MYCBP2,NFE2L1,ANO1,GPI,IL17RD,SOS1,TSHR,EXT1,PDCD1LG2,ATRX,PLIN2,PNP

			LA3,PRMT2,RAPGEF2,ABCG2,KCNC2,SMO,SLC6A14,GET4,BLOC1S3,PRKAR2B,CPT1A,TNFRSF11B,SYT17,SOX30,UBR5,GRB14,GRIN1,RHOA,OR4M1,OR4N2,GRIN3A,RQCD1,TF,ONECUT2,FAM19A4,CUL3,HSPD1,ITPR2,GABRB3,GSK3B,ABCG8,DNAJC3,EMB,RDX,STAT6,EEF2K,FAM49B,CD27,SCN9A,BOK,SULF1,KRT8,AGT,PRKAA2,SYT7,ADAR,FBN2,STAT2,BRINP3,HRH4,PTK2,PHEX,RNF121,PDE4B,VEPH1,GAP43,MAP3K5,CREBBP,DLX1,FMOD,PCSK6,NCF1,SLC29A1,UGT1A1,UGT1A10,UGT1A3,UGT1A4,UGT1A5,UGT1A6,UGT1A7,UGT1A8,UGT1A9,SHC1,NDUFS2,ECE1,RNFT1,RPS6KB1,STC2,GPR173,ALOX5,CD44,ADAM8,CLDN4,RPS6KA5,CXCL17,NOS1,GCKR,PLEKHA1,PLVAP,ACTN4,SCNN1B,TUBB3,ZFAND6,EPHB2,SLC1A1,SIGMAR1,XCR1,IL18,TPMT,UBE2K,PPARA,ABCD3,ARID5B,CALCRL,MCOLN1,SLC25A33,TAF2,CPEB4,GPSM3,ZNF366,CYP2C18,CYP2C19,FBN1,PAX6,PRKCZ,ATP1A3,CD96,FAM20C,ADORA2A,GRIN2B,RABGEF1,CPEB1,TYK2,ASPN,ANGPT1,FHL2,OSBPL8,ATF6,GCNT2,ETS1,PPP1CA,VWC2,MTF2,BIN2,CCL22,NCOR2,PRKCQ,BRAF,DIAPH1,HDAC2,HTR2C,IMPACT,SPINT2,TNR,PHIP,PPP2CA,ANGPT4,RPL23,SH3BP4,SP100,KCND2,TMEFF1,ADCY1,ALG2,SQLE,SYK,CNOT1,SLIT1,YBX1,PTPRJ,SLC10A1,TAF1,TICAM1,TANK,UCN2,A3GALT2,HSF1,MAX,DHFR,SASH1,NCOA2,ADAMTS7,GBP5,QRICH1,VAV3,CHD6,CIB2,CAV2,UGGT1,AXL,CD109,DENND4C,MET,CAMK2D,CCR3,TIMELESS,MALT1,PFKP,TRA2B,MLXIPL,SEMA3C,NEDD4L,PHB,PTPRC,EDA,PBLD,SH3GL2,SPON2,ADCY10,NCAM1,TRAF3IP2,ATF6B,CREB1,RGMB,SGTB,TRAP1,EDNRA,GNGT1,HADHA,SLC25A13,BMP4,CPNE1
GO:0048638	regulation of developmental growth	1.8557971631442532e-9	*HLX,CLASP2,SEMA3A,WWC1,LATS2,SEMA3D,WWC3,FTO,SEMA5A,STAT5B,OLFM1,EZR,CHD7,CDKL5,DSCAM,RUNX1,GOLGA4,ERBB4,LIMK1,ATP8A2,RIMS1,INSR,CDH4,BASP1,BDNF,ATRN,PLCB1,RTN4,RTN4R,NTN1,NRG1,VGLL4,SYT1,PLS1,SEMA5B,TP73,DISC1,ITSN2,CAPN3,MAG,PARK2,DRAXIN,CPNE6,DCC,CTDP1,SYT3,APP,DIP2B,YAP1,FSTL4,ISLR2,SPTBN4,CPNE9,SLC6A3,SEMA6D,BMPR2,DPYSL2,BMPR1A,SPAG9,RAB11A,FGF2,SEMA4D,JARID2,WNT3,PAFAH1B1,AKAP6,RASAL1,TRPC5,RUFY3,SIN3A,MACF1,ADNP,MAP1B,CELF1,GHR,UNC13A,TRIM46,EPHA7,MAP3K13,HEY2,PTPRS,TLL2,IGF1,PTEN,EFNA5,GPR21,TSHR,SMO,SYT17,GSK3B,ZMPSTE24,RIMS2,RPS6KB1,STC2,STK4,PPARA,NIPBL,MAP2,TNR,CDKL3,SLIT1,ZP3,SEMA3C,NEDD4L,CREB1,BMP4*
GO:0009059	macromolecule biosynthetic process	1.977869143812523e-9	*POLDIP3,ZDHHC14,ENPP1,PRDX2,LDB2,GPC5,ASPH,B4GALNT2,PRKCI,SLCO3A1,DNMT1,HLX,SLC9A1,PBX3,ADCY8,MGAT4C,MED13L,TRPS1,CBFB,PDE8A,ZNF823,IL31RA,PRDM12,MED26,WWC1,ASH1L,SCAF8,STOX2,PTGIS,WWC3,PAGR1,HIVEP3,FTO,NPAS3,ARNT,LRRFIP2,STAT5B,TOX,GRM5,NOC3L,SAMHD1,ZNF566,FER,CASK,MAP2K5,MAPK10,ZNF536,SP3,HDGF,GTF3C1,EIF4G3,EZR,IKZF2,B3GALT1,CHD7,MECOM,TACC1,TENM1,NMT2,TRAF6,GTF2E2,DNAJA3,NHLH1,HSP90AA1,CDC6,PSMB7,TSC22D3,ZC4H2,CCDC22,CDAN1,GFI1B,PTPRK,RUNX1,ERC1,HMGN3,NRIP1,THRB,EFCAB7,ITGB3BP,DACH1,ZNF569,MGAT5,CCT2,ORC2,HDAC6,SERTAD2,MYT1,IKBKB,PEX14,ERBB4,GBE1,DPY19L2,MRE11A,ZNF609,BRD8,KAT6B,HIF3A,SNIP1,LARGE,ESR1,MIER1,PCBP3,MAML3,CDON,TNRC6A,EP300,CELA1,TENM2,ZNF76,RNF220,ZNF471,PDGFB,CCND3,GALNTL6,CSGALNACT1,TOB2,ZNF19,ZNF23,BRMS1,ZNF605,SCML4,HNF4G,INSR,RERE,PRKAR1A,FUBP1,ATP8B1,H2AFY2,TCF7L2,ST6GALNAC3,JMJD1C,ZNF443,ZNF490,ZNF564,ZNF709,ZNF799,LITAF,FBXW11,ESRRB,MAP3K4,ZDHHC13,BASP1,TBR1,SAMD4A,TFAP2A,CDK14,FANCA,PEG3,ZIM2,TGIF2,TGIF2-C20ORF24,POMGNT2,FUT8,MEIS1,VRTN,TRIM13,MDM4,ENTPD5,ZNF148,MTA3,SNX6,TFDP2,PTCD3,ERN2,CDC45,C1D,AFF3,DMD,CENPF,ATG10,SLC30A9,TOX3,PDS5A,LARP4B,USP13,KAT7,ZNF667,RBM14,RBM4,MED12L,SATB2,RIT2,HIRA,DRG2,MKRN2,ARNTL,LINC01138,PLCB1,ARID4B,GXYLT2,POLA2,HS3ST2,B4GALT6,RWDD3,BRPF1,PIK3R2,NRG1,UBP1,MAP2K1,MDFI,FNIP1,VAX2,GALNT16,TMBIM6,VGLL4,PPP2CB,PPP3R1,HDAC5,CSRNP3,ZNF692,NFIA,RNF4,GALNT18,JDP2,CMKLR1,ROR2,DCN,ZDHHC3,PCBD2,CDH13,CREBRF,SOX13,UST,COPS5,MAD2L2,MAN1A1,TLE6,SRBD1,CAPRIN2,ZNF418,PHF20L1,PDF,TERF2,ZNF286A,FOXN3,NEUROD1,USP22,ZDHHC15,JAK2,TRAPPC9,OAZ2,SKAP1,SMC3,UIMC1,ITCH,MLIP,BCL11B,PKNOX1,B3GALT5,MLLT3,TSHZ2,DHDDS,TCF7,PDE2A,KLF15,TBX15,ANXA4,WNT11,MTA1,KLF8,NOX4,LCOR,RPRD1B,QKI,CCDC62,PRKCD,SOX6,ACVR2A,RUNX2,CD4,TGFB1,BANP,MRPL33,SGK1,NSD1,IGSF1,ZHX2,PKNOX2,ASCC2,BTRC,NFATC3,POLK,F2RL1,BCAS3,CDYL2,HS6ST2,CC2D1B,TP73,HHAT,SAP18,CDKAL1,ZBTB22,ILF2,TMTC1,MTDH,FANK1,MYT1L,SMAD6,BNC2,ZNF398,TMTC2,CLOCK,CHST9,TCF12,ZNF675,RPL36,ETV6,PHRF1,TFAP2D,BCL3,EIF3L,SND1,DUSP22,HNRNPC,TRRAP,SBNO2,YTHDF1,FGF10,CIZ1,POLR2J2,SMYD3,LOXL3,CAPN3,LUM,SMURF2,RORA,HIVEP2,AUTS2,TNFSF11,SMG6,PPP3CA,NFYB,MAGEA4,KLF12,CAMK4,GATAD2B,UFL1,TRAK1,CTNNB1,PARK2,SOD2,DACH2,METTL13,SMARCC1,KLF17,PPARG,IARS2,NGRN,AXIN1,IL18R1,CIPC,MTF1,CBX5,ANKRD17,BRIP1,LRPPRC,SREBF2,AARS2,CDK11A,GALNT8,CDK11B,FGF1,NPAT,NR4A3,FOXK2,WDR45B,ESCO1,MYOCD,TRIM5,PER2,AJUBA,ZNF626,ZNF737,CHEK2,SUPT3H,PRDM16,PPP1CB,HCK,SORBS1,RAB3GAP2,TRIM8,DIO2,BRMS1L,ZBTB8A,ZBTB8B,CTDP1,HS3ST5,BAZ1B,MEF2B,HDAC4,PAX2,PHF5A,SECISBP2L,SNAPC4,SFRP1,MED13,ST8SIA1,ZNF395,FOXO3,NFIB,SP4,SYNCRIP,SMAD3,CUX2,WWP2,ARNT2,SBNO1,KRBOX1,ZNF662,ZNF777,WIPI1,EBF3,RNF168,CASZ1,DCP1B,MIER3,NEDD4,ESRRG,HOXD3,HOXD4,ZNF114,GALNT14,KTI12,NFATC1,ST3GAL3,CDC73,APP,SSBP3,GSX2,RBM8A,NOX1,YAP1,ST6GALNAC5,TEN1,NVL,L*

			RP5,POLR3G,ZNF787,SOX2,SETD2,ZDHHC6,GPC6,CHST11,TEAD1,PRICKLE1,ZNF653,ZNF521,ARID3A,ZNF761,CHUK,RFT1,SFMBT1,ZNF584,ESR2,S100A12,GTF2F2,PRKD1,STAT1,HEXA,ST18,ETV5,RHOXF2B,TAF3,PLAGL1,HNF4A,ZBTB7C,TASP1,EREG,ATF2,POU2F2,TCF3,ZNF730,RAF1,CELF4,ABLIM2,ZNF766,CARD16,POLA1,GGT7,BMPR2,CAMK1D,BMPR1A,ZKSCAN1,PABPC4,IKZF4,CDK12,TAF8,CAND2,DENND4A,MYB,FGF2,ZNF71,POU6F2,BACH1,MXD3,PPM1F,TICRR,GLI4,ZFP41,BEND5,SEMA4D,NFX1,RORC,ELP3,PTAFR,SP140,SP140L,RHOXF2,JARID2,DDX58,BRDT,PHC2,RARB,SPEN,SIN3B,NCOA1,EEF1E1,EHMT1,LMO7,DVL3,EIF2B5,EIF4G1,POLR2H,TCF20,ATF3,TCEANC,STT3B,CKS1B,PAWR,EBF2,AGO3,MAML2,TSG101,TERF2IP,CRYM,RFX2,B3GALNT1,ZNF322,SUFU,MAGEA11,PRDM15,ZNF670,ZNF695,CCDC169-SOHLH2,SOHLH2,POLE,ZNF354C,TCEA3,PADI6,ZNF704,NR2C1,EDEM3,GLI2,TNKS,WBP2NL,ERCC1,TNRC6B,GLIS3,GLCE,WDTC1,ZNF664,MRPS28,DAB2,BLM,PKHD1,MYSM1,SETD5,SMG5,GTPBP2,RELN,SIN3A,RUVBL2,COMMD6,GMEB1,PELI1,MAP3K7,ZNF423,SP1,TRIM22,ALK,TEAD4,HOXC13,APBB3,SLC35A4,SRA1,TOP1,UBE2V1,ADIRF,OVOL2,SNX5,NFATC2,RBBP6,PAX7,BNC1,ATP2B4,ACTL6B,ASXL3,PAK3,TET1,CAMTA1,CCPG1,ADNP,ARID4A,C12ORF65,CDK6,PHF2,CELF1,TFRC,EPHA5,WWOX,ALG14,MEF2A,DCAF6,GPC3,XRN1,SATB1,PSMB2,EYA1,GATAD2A,DIS3L2,TRPV1,HOXB3,HOXB4,HOXB5,HOXB6,TFEB,MNAT1,ACTR2,L3MBTL4,ELAVL4,HDAC1,HS6ST3,RECQL5,SMOC2,TRIM24,PLCG2,ROCK1,SCMH1,MRPS17,PAXIP1,ZNF713,EGR2,HIPK3,RNF10,RYBP,AP3B1,PTPN2,NTRK1,KPNA6,TRIM44,EIF3A,MAN1B1,EPCAM,ZNF425,GNL3L,KRTCAP2,MUC1,PTPN14,NUDT14,BCOR,AP3D1,ZNF41,MGAT4A,CARM1,ZNF383,ZNF616,ZNF836,SMARCA2,CUX1,SPI1,DNMT3B,LRRC47,FOXP2,MRPS24,PSPC1,GLIS1,SFMBT2,EIF3H,BCAR3,JAZF1,ACOT8,RBPMS,CHURC1,CREB5,MAP3K13,ERCC3,SMYD1,SNCA,BMPR1B,POLN,PRIM2,PNPT1,ZEB2,MCMBP,FOXJ3,HEY2,RC3H1,EIF3E,ZMYND11,KMT2D,CREM,KMT2C,PLD1,MACC1,MITF,OPRD1,NUGGC,RPTOR,NFIX,BTK,KCTD13,MLLT1,TROVE2,CBFA2T2,IGF1,MLXIP,ATF7IP,LARS2,MAPK1,PTEN,BMP7,ATXN2,MXI1,PIK3C3,PUM1,SOX5,CIR1,PCBP2,ZNF780A,ZNF780B,LRRK2,TMEM59,PIGS,ZBTB20,RFC3,CHAF1B,LILRB4,PKD2,ZNF652,HSF2BP,KCTD1,RIPPLY1,RCOR3,TADA2A,DAZL,NCOA3,PKIB,ZNF146,ZNF565,HS2ST1,WNT7A,ZBED6,BRWD3,CRTC3,DNAJC1,ARNTL2,KAT6A,MTIF2,ZKSCAN7,ZNF197,ZNF660,BMP6,TAF15,NFE2L1,WDR43,ZNF30,EXT1,DPY19L4,ATRX,DPH6,IKZF1,PRMT2,ASCC1,SMO,RALY,ALG9,DPY19L1,TCF4,GALNT10,SOX30,TFE3,GRIN1,JADE1,RHOA,SPRTN,MCM3,ROR1,RQCD1,TF,ONECUT2,CUL3,TFEC,EPM2A,GSK3B,PRR16,DNAJC3,STAT6,MLF1,EEF2K,RBBP8,NECAB2,CDK13,ALAS2,DUSP26,ZNF362,NACC2,SUPT4H1,ZNF354A,AGT,HDGFRP3,METTL16,POLH,POLR2F,STAT2,ZMPSTE24,GTF2E1,PBX1,EXOSC3,MAP3K5,TCFL5,CREBBP,DLX1,GTF2IRD2,NCF1,SHC1,ZNF813,LBX2,PARP10,RPS6KB1,ZDHHC23,DPRX,GALNT13,PRG3,SETDB2,DNAJB6,SP7,LARP4,ADAM8,MRPS6,RPS6KA5,ZNF484,ZNF93,PRDM2,NOS1,ZNF44,MED15,RGS14,ACTN4,MC1R,TCF25,ZNF282,GATC,EDRF1,TMEFF2,IL18,TEX12,DIS3,PPARA,ARID5B,SLC25A33,SMARCAL1,TAF2,CPEB4,NOTCH4,ZMYND8,ZNF366,CRX,PAX6,PRKCZ,ADORA2A,RBMS1,CPEB1,OTUD7B,KIN,NIPBL,YEATS4,PHF20,ZNF143,ANGPT1,BRF1,TBL1X,FHL2,MBTD1,ATF6,ZBTB5,ZNF708,IGF2BP3,NT5M,GCNT2,ETS1,PPP1CA,MTF2,TAGLN3,CDK5RAP1,NCOR2,PRKCQ,CSRNP1,HDAC2,IMPACT,PHKG2,SRCAP,POU3F3,NOTO,ZKSCAN5,ELF2,PHIP,PPP2CA,MRPS16,RPL23,RFC5,ACAN,ATXN1,SP100,ZNF347,ZNF415,REV3L,TRIM29,PARN,PIGU,DONSON,FARSB,WIPI2,ADCY1,ALG2,SYK,CNOT1,WWTR1,GTF2H5,NFXL1,ZBTB38,BRCA2,ATF7,DVL2,MORC1,MTRF1,YBX1,TAF1,EIF4E3,TICAM1,THAP3,GALNT7,SNAPC3,HSF1,MAX,SAP130,DHFR,EZH1,ETF1,NRF1,SRPK2,NCOA2,WDR18,EEFSEC,QRICH1,ZP3,CHD6,RNF2,ZNF554,UGGT1,GALNT4,MYEF2,POC1B-GALNT4,TBP,ORC4,TRIM37,MET,ZNF461,CAMK2D,MRPL1,TIMELESS,BRD9,MALT1,SETD3,ZDHHC11,ZDHHC11B,KDM2B,MLXIPL,CCT3,PHB,EDA,ZNF555,CTCF,EXD2,NR6A1,ATF6B,CREB1,LIG1,RGMB,TRAP1,EBF4,ZNF511,BMP4,ABLIM3
GO:0048813	dendrite morphogenesis	2.4163383591164583e-9	*PRMT3,SEMA3A,PHACTR1,KALRN,CDKL5,ABI1,ITGB1,KNDC1,DSCAM,DOCK10,HDAC6,TNIK,RERE,PTPRD,TANC2,FYN,CAPRIN2,ZDHHC15,LZTS1,IL1RAPL1,CAMK2B,CTNND2,DCLK1,EPHA4,PPP3CA,DNM3,CUX2,ABI2,SHANK1,NEDD4,BTBD3,PARP6,NLGN1,CTNNA2,SEMA4D,EPHB3,PAFAH1B1,TRPC5,RELN,PAK3,MEF2A,ACTR2,ELAVL4,EPHB1,SARM1,ARHGAP44,CUX1,PTEN,LRRK2,SHANK3,WNT7A,RAPGEF2,GSK3B,EEF2K,TAOK2,EPHB2,MAP2,FBXO31,CDKL3,NEDD4L*
GO:0007417	central nervous system development	2.642570653957871e-9	*NRXN1,TACC2,NEGR1,PBX3,SEMA3A,GRID2,NRG3,PHACTR1,NLGN4X,UNC5C,ZSWIM6,KALRN,NTRK3,SEMA5A,TOX,ATP2B2,SPATA5,LRP2,ROBO1,CHD7,TACC1,ROBO2,NHLH1,ITGB1,HSP90AA1,KNDC1,ZC4H2,TRIO,DAB1,PTPN11,ERBB4,CDON,NTRK2,HOOK3,KIRREL3,RERE,H2AFY2,FBXW11,BASP1,SLC4A10,TBR1,TFAP2A,ATRN,AMIGO1,MEIS1,TENM4,ZNF148,NAV2,DMD,CENPF,SLC8A1,GSN,SATB2,PAFAH1B2,MACROD2,FYN,NF1,PLCB1,RTN4,AFF2,APOD,B4GALT6,RTN4R,BCR,CCDC141,NDRG4,DCTN1,NRG1,MAP2K1,VAX2,CNTN4,ROR2,PLXDC1,SOX13,NCKAP1,NEUROD1,TRAPPC9,SYT1,BCL11B,IMMP2L,ADAM23,SOX6,TGFB1,RPGRIP1L,CELSR1,SLIT2,TP73,GABRB1,DISC1,TTBK2,SYNJ2,TFAP2D,DCLK1,SLC17A7,FGF10,CNTNAP2,IL1RAPL2,LOXL3,EPHA4,RORA,PPP3CA,MAG,CTNNB1,PARK2,FCGR2B,IGF1R,PPARG,DLG5,MTF1,DRAXIN,NIN,DCT,DCC,NF2,PAX2,SFRP1,FOXO3,ARL13B,CNTN6,NFIB,ARNT2,NRP2,BTBD3,APP,SSBP3,GSX2,NLGN2,PTPRG,SOX2,SETD2,SPTBN4,DRD1,FRS2,SYNE2,SLC6A3,SHANK2,DSCAML1,SEMA6D,*

			ATF2,DPYSL2,BMPR1A,CTNNA2,FGF2,POU6F2,GRIK1,AGBL4,ELP3,PLXNA2,SETD1A,JARID2,RARB,SPOCK1,GAS8,CHRD,EIF2B5,EPHB3,FBXO45,LAMB1,WNT3,SUFU,PAFAH1B1,NMUR2,GLI2,RELN,SIN3A,ZNF423,ALK,PRKG1,RGS7,ATP2B4,ACTL6B,CDK6,CELF1,EPHA5,DHX30,TRPV1,HOXB3,MNAT1,ELAVL4,HDAC1,EGR2,EPHB1,SNTG2,SH3GL3,SRD5A2,EPHA7,FOXP2,CTNNA1,HYDIN,RNF103,WNT7B,HMGCS2,PTPRS,RTN4RL1,MAPK1,PTEN,BMP7,LRRK2,BAG6,TMEM108,ITGAM,PKD2,SHANK3,PDGFC,WNT7A,MYCBP2,SOS1,EXT1,ATRX,RAPGEF2,KCNC2,SMO,SUN1,GRIN1,RHOA,NDE1,ROR1,GSK3B,CCDC14,BOK,NDRG2,PBX1,DLX1,PYGO2,MDGA2,TBC1D23,STK4,EPHB2,SLC1A1,WLS,DLC1,PAX6,ADORA2A,GRIN2B,KLHL1,NIPBL,SHROOM4,MAP2,TAGLN3,CDK5RAP1,CNTN1,HDAC2,POU3F3,NOTO,TNR,ACAN,ATXN1,SRGAP2,ADCY1,NUMB,SLIT1,BRCA2,TAF1,POTEE,CADM1,EZH1,AXL,UCHL5,TRA2B,KDM2B,SH3GL2,TYRO3,CREB1,BMP4
GO:2000145	regulation of cell motility	4.41053137804538e-9	*LDB2,MAP4K4,S1PR2,CLASP2,SEMA3A,DOCK1,NRG3,SEMA3D,PHACTR1,MAPRE2,TJP1,UNC5C,NTRK3,SEMA5A,ENPP2,FER,MARVELD3,MAP2K5,KITLG,PTPRR,PIK3CD,ROBO1,LMNA,ULK4,ITGB1,SRGAP3,MCC,SRGAP2B,PTPRK,ABCC8,LAMA2,DACH1,MGAT5,DOCK10,HDAC6,ADORA1,EPHA1,ERBB4,ZNF609,PDGFB,STK39,INSR,UNC5D,LAMA3,PAK1,PTPRU,SLC8A1,CASS4,ADAMTS9,NF1,PLCB1,RTN4,DPEP1,APOD,BCR,NDRG4,NTN1,NRG1,BDKRB1,DOCK8,HDAC5,NTNG1,CMKLR1,ROR2,DCN,CDH13,PTPRT,CD99,RCC2,ANO6,KIF2A,RFFL,CCBE1,JAK2,FBXW7,CLDN1,WNT11,CAMK2B,SEMA5B,TGFB1,SGK1,MMP28,F2RL1,BCAS3,SLIT2,CORO1C,RRAS2,KANK1,LAMC2,TTBK2,BMPER,DAPK2,DUSP22,FGF10,SMURF2,EPHA4,PRKCA,PPP3CA,PIP5KL1,SOD2,IGF1R,PPARG,DLG5,FGF1,NR4A3,MYOCD,AJUBA,NF2,FLT4,HDAC4,SFRP1,FOXO3,SMAD3,PTPRM,NRP2,DOCK4,NAV3,APP,GSX2,PDGFRA,SLK,PTPRG,DRD1,MMP2,SYNE2,PRKD1,SEMA6D,RAF1,BMPR2,CAMK1D,BMPR1A,CTNNA2,PIK3R3,SPAG9,RAB11A,FGF2,PPM1F,NEDD9,SEMA4D,MCTP1,ELP3,PLXNA2,PTAFR,DDX58,CHRD,VCL,LAMB1,CLIC4,RHOJ,RUFY3,DAB2,LDLRAD4,MYSM1,WNK1,RELN,SP1,MACF1,CLASP1,PRKG1,ATP2B4,PAK3,SSH2,SCARB1,CDK6,FLRT2,SMOC2,PLCG2,ROCK1,PKN2,TMIGD1,SPI1,MCU,CTNNA1,SH3BP1,MST1,MAGI2,ADTRP,MITF,IGF1,DPYSL3,MAPK1,PTEN,BMP7,PRCP,OSGIN1,PDGFC,AMOTL1,WNT7A,GPI,SH3RF2,RAPGEF2,SMO,RHOA,TF,ONECUT2,RDX,ACTA2,FAM49B,SULF1,AGT,PTK2,TPPP2,RPS6KB1,GPR173,ADAM8,CLDN4,CXCL17,PLVAP,ACTN4,STK4,EPHB2,TMEFF2,DLC1,EPB41L4B,GPSM3,ZMYND8,PAX6,RABGEF1,PHLDB2,NIPBL,ANGPT1,OSBPL8,GCNT2,ETS1,FBXO31,BRAF,DIAPH1,ATP8A1,SPINT2,CD300A,ANGPT4,CAMSAP3,SP100,SRGAP2,NUMB,FRMD5,PTPRJ,SASH1,ZP3,GNA12,INPP5F,MET,SEMA3C,PTPRC,BMP4*
GO:2000026	regulation of multicellular organismal development	4.826892982295191e-9	*ENPP1,PRDX2,NRXN1,PRKCI,S1PR2,HLX,TRPS1,CBFB,CLASP2,SEMA3A,GRID2,CLDN18,SEMA3D,TIAM2,PTGIS,TJP1,PRLR,KALRN,NTRK3,ARNT,SEMA5A,STAT5B,TOX,ENPP2,GRM5,KITLG,LRP2,PIK3CD,ROBO1,CHD7,CDKL5,TRAF6,ESRP1,ROBO2,ITPKB,ITGB1,DSCAM,GFI1B,RUNX1,DAB1,ABCC8,LAMA2,PTPN11,RBM19,GOLGA4,EPHA1,IKBKB,ERBB4,LIMK1,NPHP3,NTRK2,CELA1,HOOK3,TOB2,INSR,H2AFY2,LAMA3,CDH4,PTPRD,BASP1,BDNF,TFAP2A,AMIGO1,FANCA,TGIF2,MEIS1,TENM4,KAT7,SLC8A1,GPR171,ARNTL,ADAMTS9,NF1,PLCB1,RTN4,RTN4R,CHI3L1,BMP2K,ANKH,NTN1,NRG1,MAP2K1,STIM1,VGLL4,ROR2,DCN,SOX13,TLE6,CAPRIN2,ANO6,CCBE1,FBXW7,CLSTN2,TCF7,SEPT7,WNT11,FIG4,IL1RAPL1,CAMK2B,SOX6,ACVR2A,RUNX2,SEMA5B,CD4,TGFB1,C9ORF47,SLIT2,TP73,DISC1,MTDH,ZNF675,NELL1,BMPER,ANKRD54,ADAM12,LINGO2,LOXL3,CAPN3,EPHA4,PRKCA,TNFSF11,PPP3CA,MAG,CAMK4,UFL1,CTNNB1,FCGR2B,PPARG,DLG5,CYBB,DRAXIN,FGF1,NIN,DCT,PER2,GLG1,PHLDB1,DCC,CTDP1,NF2,PAX2,SFRP1,FOXO3,SMAD3,CUX2,PTPRM,PRTG,CDC73,GSX2,DIP2B,YAP1,FSTL4,NLGN2,JAK1,ANGPTL4,ISLR2,SLC9B2,VASH2,PRKD1,STAT1,PARP6,HNF4A,SEMA6D,ATF2,ADAMTS12,BMPR2,BMPR1A,TSPAN12,NLGN1,RAB11A,MYB,FGF2,SEMA4D,ISM1,PLXNA2,JARID2,RARB,SPEN,EHMT1,EPHB3,VCL,WNT3,RHOJ,TG,NLGN3,PAFAH1B1,COL4A3,DDAH1,AKAP6,TRPC5,GLI2,RUFY3,MYSM1,RELN,CSF3R,APOLD1,SP1,MACF1,INPP5D,CLASP1,GPM6B,XDH,OVOL2,ATP2B4,TMEM2,PAK3,ADNP,MAP1B,CDK6,DROSHA,BRINP1,HOXB3,FLRT2,ACTR2,LGI4,HDAC1,SMOC2,ROCK1,EGR2,RNF10,AP3B1,PTPN2,NTRK1,EPHB1,AXIN2,PARD3,TRIM46,BCOR,AP3D1,CUX1,SPI1,EPHA7,CTNNA1,MAP3K13,RAPGEF3,BMPR1B,HEY2,RC3H1,PTPRS,TRIP12,ABCA12,MITF,SRSF6,HIPK1,BTK,IGF1,PTEN,SPRED2,BMP7,SOX5,BAG6,LILRB4,EFNA5,SHANK3,NCOA3,PPP2R3C,WNT7A,BMP6,SOS1,RAPGEF2,SMO,TFE3,RHOA,SYNGAP1,NFKBID,EEF2K,CD27,SULF1,AGT,FBN2,ZMPSTE24,DLX1,SHC1,CD160,ALOX5,ADAM8,RGS14,EPHB2,CLPTM1,IL18,PPARA,NOTCH4,FBN1,PAX6,PRKCZ,FAM20C,PPP1CC,PHLDB2,NIPBL,TMIGD2,ASPN,ETS1,MAP2,FBXO31,BRAF,HDAC2,TNR,CDKL3,ANGPT4,SP100,NUMB,SYK,WWTR1,SLIT1,HSF1,SASH1,ADAMTS7,SULT2B1,BMP1,AXL,TNFSF9,CD109,CCR3,MALT1,SEMA3C,PTPRC,SYNDIG1,CREB1,BMP4*
GO:0007420	brain development	5.325954710638395e-9	*NRXN1,TACC2,NEGR1,PBX3,SEMA3A,GRID2,NRG3,PHACTR1,NLGN4X,UNC5C,ZSWIM6,SEMA5A,TOX,ATP2B2,SPATA5,LRP2,ROBO1,CHD7,TACC1,ROBO2,ITGB1,KNDC1,DAB1,PTPN11,ERBB4,CDON,NTRK2,HOOK3,KIRREL3,RERE,H2AFY2,FBXW11,BASP1,SLC4A10,TBR1,ATRN,AMIGO1,MEIS1,ZNF148,DMD,CENPF,SLC8A1,SATB2,PAFAH1B2,MACROD2,FYN,NF1,PLCB1,RTN4,AFF2,APOD,RTN4R,BCR,CCDC141,NDRG4,NRG1,MAP2K1,VAX2,CNTN4,NEUROD1,TRAPPC9,SYT1,BCL11B,IMMP2L,SOX6,RPGRIP1L,SLIT2,DISC1,TTBK2,SYNJ2,TFAP2D,DCLK1,SLC17A7,FGF10,CNTNAP2,RORA,PPP3CA,MAG,CTNNB1,FCGR2B,IGF1R,DLG5,DRAXIN,NI*

			N,DCT,NF2,PAX2,SFRP1,FOXO3,ARL13B,NFIB,ARNT2,NRP2,BTBD3,APP,SSBP3,GSX2,NLGN2,PTPRG,SOX2,SETD2,DRD1,FRS2,SYNE2,SLC6A3,SHANK2,DSCAML1,SEMA6D,ATF2,DPYSL2,BMPR1A,CTNNA2,FGF2,PLXNA2,SETD1A,RARB,GAS8,EIF2B5,EPHB3,FBXO45,LAMB1,WNT3,PAFAH1B1,GLI2,RELN,SIN3A,ZNF423,ALK,PRKG1,RGS7,ATP2B4,CDK6,CELF1,EPHA5,HOXB3,MNAT1,ELAVL4,HDAC1,EGR2,EPHB1,SRD5A2,EPHA7,FOXP2,CTNNA1,HYDIN,WNT7B,HMGCS2,PTPRS,RTN4RL1,PTEN,BMP7,LRRK2,BAG6,TMEM108,ITGAM,SHANK3,WNT7A,SOS1,EXT1,ATRX,RAPGEF2,KCNC2,SMO,SUN1,GRIN1,RHOA,NDE1,GSK3B,CCDC14,BOK,NDRG2,PBX1,DLX1,PYGO2,TBC1D23,EPHB2,SLC1A1,WLS,DLC1,PAX6,GRIN2B,KLHL1,NIPBL,SHROOM4,CDK5RAP1,CNTN1,POU3F3,TNR,ATXN1,SRGAP2,ADCY1,NUMB,SLIT1,BRCA2,TAF1,POTEE,CADM1,EZH1,AXL,UCHL5,TRA2B,KDM2B,TYRO3,CREB1,BMP4
GO:1901362	organic cyclic compound biosynthetic process	6.820638062279643e-9	ENPP1,PRDX2,LDB2,ASPH,PRKCI,SLCO3A1,DNMT1,HLX,SLC9A1,PBX3,ADCY8,MED13L,TRPS1,CBFB,PDE8A,ZNF823,IL31RA,PRDM12,MED26,WWC1,ASH1L,SCAF8,STOX2,PTGIS,PRKAG2,WWC3,PRLR,PAGR1,ADCY7,HIVEP3,NPAS3,ARNT,LRRFIP2,STAT5B,TOX,GRM5,ZNF566,FER,CASK,MAP2K5,MAPK10,ZNF536,SP3,HDGF,GTF3C1,EZR,IKZF2,MALRD1,CHD7,MECOM,TACC1,TENM1,NADK2,TRAF6,GTF2E2,DNAJA3,NHLH1,HSP90AA1,PSMB7,TSC22D3,ZC4H2,CCDC22,MOCOS,GMDS,GFI1B,PTPRK,RUNX1,ERC1,HMGN3,NRIP1,THRB,EFCAB7,ITGB3BP,DACH1,ZNF569,CCT2,ORC2,HDAC6,SERTAD2,MYT1,IKBKB,PEX14,ERBB4,MRE11A,ZNF609,BRD8,KAT6B,HIF3A,SNIP1,ESR1,MIER1,PCBP3,MAML3,CDON,EP300,CELA1,TENM2,ZNF76,RNF220,ZNF471,PDGFB,CCND3,TOB2,ZNF19,ZNF23,BRMS1,ZNF605,SCML4,HNF4G,INSR,RERE,PRKAR1A,FUBP1,ATP8B1,H2AFY2,TCF7L2,JMJD1C,ZNF443,ZNF490,ZNF564,ZNF709,ZNF799,LITAF,FBXW11,ESRRB,MAP3K4,BASP1,TBR1,TFAP2A,CDK14,FANCA,PEG3,ZIM2,TGIF2,TGIF2-C20ORF24,MEIS1,VRTN,TRIM13,MDM4,ZNF148,MTA3,SNX6,TFDP2,ERN2,C1D,AFF3,DMD,CENPF,SLC30A9,TOX3,USP13,KAT7,ZNF667,RBM14,PRPS2,MED12L,SATB2,RIT2,HIRA,MKRN2,ARNTL,PLCB1,ARID4B,POLA2,RWDD3,BRPF1,PIK3R2,NRG1,UBP1,MAP2K1,MDFI,FNIP1,VAX2,TMBIM6,VGLL4,PPP2CB,PPP3R1,HDAC5,CSRNP3,ZNF692,NFIA,RNF4,JDP2,CMKLR1,ROR2,DCN,PRPSAP2,PCBD2,CDH13,CREBRF,SOX13,ACSBG1,COPS5,AK2,MAD2L2,TLE6,CAPRIN2,ZNF418,PHF20L1,TERF2,ZNF286A,NME7,FOXN3,AK5,NEUROD1,USP22,JAK2,TRAPPC9,SKAP1,UIMC1,ITCH,MLIP,BCL11B,IMPDH1,PKNOX1,MLLT3,TSHZ2,TCF7,PDE2A,KLF15,TBX15,ANXA4,WNT11,MTA1,KLF8,NOX4,LCOR,RPRD1B,CACNA1H,CCDC62,SOX6,ACVR2A,RUNX2,CD4,TGFB1,BANP,SGK1,NSD1,IGSF1,ZHX2,PKNOX2,ASCC2,BTRC,NFATC3,POLK,F2RL1,BCAS3,CDYL2,CC2D1B,TP73,SAP18,ZBTB22,ILF2,MTDH,FANK1,MYT1L,SMAD6,BNC2,ZNF398,CLOCK,TCF12,ZNF675,ETV6,PHRF1,TFAP2D,BCL3,SND1,DUSP22,HNRNPC,TRRAP,SBNO2,FGF10,POLR2J2,ADK,SMYD3,LOXL3,ADCY2,CAPN3,LUM,SMURF2,RORA,HIVEP2,HSD17B12,AUTS2,TNFSF11,SMG6,PPP3CA,NFYB,MAGEA4,KLF12,CAMK4,GATAD2B,UFL1,TRAK1,CTNNB1,PARK2,SOD2,DACH2,METTL13,SMARCC1,KLF17,IGF1R,PPARG,AXIN1,IL18R1,CIPC,MTF1,CBX5,BRIP1,SREBF2,PFAS,CDK11A,CDK11B,FGF1,NPAT,NR4A3,FOXK2,DCT,MYOCD,TRIM5,PER2,AJUBA,ZNF626,ZNF737,CHEK2,SUPT3H,PRDM16,HCK,TRIM8,BRMS1L,ZBTB8A,ZBTB8B,CTDP1,BAZ1B,MEF2B,HDAC4,PAX2,PHF5A,CPOX,SNAPC4,SFRP1,MED13,ZNF395,FOXO3,TPH2,NFIB,SP4,SMAD3,CUX2,WWP2,ARNT2,SBNO1,KRBOX1,ZNF662,ZNF777,EBF3,RNF168,CASZ1,MIER3,NEDD4,ESRRG,HOXD3,HOXD4,ZNF114,KTI12,NFATC1,UCK2,CDC73,APP,SSBP3,GSX2,YAP1,PPCS,TEN1,NVL,LRP5,POLR3G,ZNF787,SOX2,SETD2,TEAD1,PRICKLE1,ZNF653,ZNF521,ELOVL2,ARID3A,ZNF761,CHUK,ERLIN1,SFMBT1,ZNF584,TPH1,ESR2,S100A12,GTF2F2,PRKD1,STAT1,ST18,OSBPL2,ETV5,RHOXF2B,SLC6A3,TAF3,PLAGL1,HNF4A,ZBTB7C,TASP1,EREG,ATF2,POU2F2,TCF3,ZNF730,RAF1,ABLIM2,ZNF766,CARD16,POLA1,BMPR2,CAMK1D,BMPR1A,ZKSCAN1,IDO2,IKZF4,CDK12,TAF8,CAND2,DENND4A,MYB,FGF2,ZNF71,POU6F2,BACH1,MXD3,PPM1F,ADCY5,GLI4,ZFP41,BEND5,SEMA4D,NFX1,RORC,ELP3,PTAFR,SP140,SP140L,RHOXF2,JARID2,DDX58,SLC5A8,BRDT,PHC2,RARB,SPEN,SIN3B,NCOA1,EHMT1,LMO7,DVL3,POLR2H,TCF20,ATF3,TCEANC,AKR1D1,CKS1B,PAWR,EBF2,MAML2,TSG101,TERF2IP,CRYM,RFX2,ZNF322,SUFU,MAGEA11,PRDM15,ZNF670,ZNF695,CCDC169-SOHLH2,SOHLH2,POLE,ZNF354C,TCEA3,FDXR,CMPK1,ZNF704,NR2C1,ME1,GLI2,TNKS,WBP2NL,ERCC1,GLIS3,WDTC1,ZNF664,TPK1,DAB2,BLM,PKHD1,MYSM1,SETD5,SMG5,RELN,SIN3A,RUVBL2,COMMD6,GUCY1A2,GMEB1,PELI1,SLC25A16,MAP3K7,ZNF423,SP1,TRIM22,ALK,TEAD4,HOXC13,APBB3,SRA1,UBE2V1,ADIRF,OVOL2,SNX5,NFATC2,PAX7,BNC1,ATP2B4,ACTL6B,ASXL3,PAK3,TET1,CAMTA1,CCPG1,ADNP,ARID4A,CDK6,PHF2,TYR,TFRC,EPHA5,WWOX,MEF2A,ADCY9,DCAF6,XRN1,SATB1,PSMB2,EYA1,GATAD2A,TRPV1,HOXB3,HOXB4,HOXB5,HOXB6,TFEB,MNAT1,ACTR2,L3MBTL4,ACACA,HDAC1,RECQL5,SMOC2,TRIM24,PLCG2,SCMH1,PAXIP1,ZNF713,EGR2,HIPK3,RNF10,RYBP,AP3B1,PTPN2,NTRK1,KPNA6,TRIM44,EPCAM,ZNF425,GNL3L,MUC1,PTPN14,BCOR,AP3D1,ZNF41,CARM1,ZNF383,SRD5A2,ZNF616,ZNF836,SMARCA2,CUX1,SPI1,DNMT3B,FOXP2,PSPC1,GLIS1,SFMBT2,JAZF1,ABCB7,ACOT8,RBPMS,CHURC1,CREB5,MAP3K13,ERCC3,SMYD1,SNCA,BMPR1B,POLN,PRIM2,ZEB2,FOXJ3,NADK,OCA2,HEY2,HMGCS2,ZMYND11,KMT2D,CREM,OSBPL1A,KMT2C,MACC1,MITF,OPRD1,RPTOR,NFIX,BTK,MLLT1,TROVE2,CBFA2T2,IGF1,MLXIP,ATF7IP,MAPK1,PTEN,BMP7,MXI1,SOX5,CIR

			1,PCBP2,ZNF780A,ZNF780B,ATP6V1A,MLYCD,ZBTB20,RFC3,LILRB4,PKD2,ZNF652,HSF2BP,KCTD1,RIPPLY1,NT5E,RCOR3,TADA2A,NCOA3,PKIB,ZNF146,ZNF565,WNT7A,ZBED6,BRWD3,CRTC3,ARNTL2,ELOVL5,KAT6A,ZKSCAN7,ZNF197,ZNF660,BMP6,TAF15,NFE2L1,WDR43,ZNF30,ATRX,IKZF1,PRMT2,RAPGEF2,ASCC1,MTHFD2L,SMO,RALY,AMPD1,TCF4,SOX30,TFE3,GRIN1,JADE1,RHOA,SPRTN,ROR1,RQCD1,TF,ONECUT2,CUL3,TFEC,STAT6,MLF1,RBBP8,CDK13,ALAS2,DUSP26,MTHFD1L,ZNF362,NACC2,SUPT4H1,ZNF354A,AGT,HDGFRP3,ELOVL3,POLH,PRKAA2,POLR2F,STAT2,ZMPSTE24,GTF2E1,PPCDC,PBX1,MAP3K5,TCFL5,CREBBP,DLX1,GTF2IRD2,NCF1,SHC1,ZNF813,ALDOA,LBX2,PARP10,DPRX,GUCY2F,PRG3,SETDB2,ALOX5,DNAJB6,SP7,ADAM8,RPS6KA5,ZNF484,ZNF93,PRDM2,NOS1,ZNF44,MED15,RGS14,ACTN4,MC1R,TCF25,ZNF282,EDRF1,IL18,TEX12,PPARA,ABCD3,ARID5B,SLC25A33,SMARCAL1,TAF2,LDHC,NOTCH4,ZMYND8,ZNF366,CRX,PAX6,PRKCZ,ADORA2A,PAH,OTUD7B,NIPBL,YEATS4,PHF20,ZNF143,BRF1,TBL1X,FHL2,MBTD1,ATF6,ZBTB5,ZNF708,ETS1,MTF2,TAGLN3,NCOR2,PRKCQ,CSRNP1,HDAC2,IMPACT,SRCAP,POU3F3,NOTO,ZKSCAN5,ELF2,MOXD1,PHIP,PPP2CA,SLC26A2,RPL23,RFC5,ATXN1,SP100,ZNF347,ZNF415,REV3L,TRIM29,PARN,ADCY1,SQLE,SYK,CNOT1,WWTR1,CYP39A1,GTF2H5,NFXL1,ZBTB38,BRCA2,ATF7,DVL2,MORC1,YBX1,TAF1,TICAM1,THAP3,SNAPC3,HSF1,MAX,SAP130,DHFR,EZH1,NRF1,NCOA2,QRICH1,ZP3,CHD6,RNF2,ZNF554,MYEF2,TBP,TRIM37,MET,ZNF461,CAMK2D,TIMELESS,BRD9,MALT1,SETD3,KDM2B,MLXIPL,CCT3,PHB,EDA,ZNF555,CTCF,NR6A1,ADCY10,ATF6B,CREB1,LIG1,RGMB,EBF4,SLC25A13,ZNF511,BMP4,ABLIM3
GO:0010557	positive regulation of macromolecule biosynthetic process	7.121109779052223e-9	POLDIP3,LDB2,ASPH,SLC9A1,PBX3,CBFB,IL31RA,MED26,WWC1,ASH1L,SCAF8,STOX2,PAGR1,HIVEP3,NPAS3,ARNT,STAT5B,CASK,MAP2K5,SP3,HDGF,CHD7,MECOM,TACC1,TRAF6,NHLH1,HSP90AA1,GFI1B,RUNX1,HMGN3,NRIP1,THRB,EFCAB7,CCT2,SERTAD2,IKBKB,ERBB4,ZNF609,BRD8,KAT6B,ESR1,MAML3,CDON,EP300,CELA1,ZNF76,PDGFB,HNF4G,INSR,RERE,TCF7L2,LITAF,FBXW11,ESRRB,MAP3K4,TBR1,SAMD4A,TFAP2A,PEG3,MEIS1,TRIM13,ZNF148,MTA3,TFDP2,SLC30A9,TOX3,LARP4B,KAT7,RBM14,MED12L,SATB2,RIT2,MKRN2,ARNTL,PLCB1,ARID4B,BRPF1,PIK3R2,UBP1,MAP2K1,PPP3R1,HDAC5,CSRNP3,NFIA,RNF4,ROR2,DCN,PCBD2,CDH13,CREBRF,COPS5,MAD2L2,CAPRIN2,NEUROD1,USP22,JAK2,SKAP1,MLIP,BCL11B,PKNOX1,MLLT3,KLF15,WNT11,MTA1,NOX4,RPRD1B,CCDC62,ACVR2A,RUNX2,CD4,TGFB1,BANP,NSD1,BTRC,NFATC3,F2RL1,BCAS3,TP73,ILF2,MTDH,FANK1,SMAD6,ZNF398,CLOCK,TCF12,ETV6,TFAP2D,BCL3,SBNO2,YTHDF1,FGF10,CIZ1,SMYD3,CAPN3,LUM,RORA,AUTS2,TNFSF11,PPP3CA,NFYB,KLF12,CAMK4,CTNNB1,PARK2,SMARCC1,PPARG,NGRN,AXIN1,MTF1,SREBF2,FGF1,NPAT,NR4A3,FOXK2,MYOCD,TRIM5,PER2,CHEK2,SUPT3H,PRDM16,SORBS1,TRIM8,MEF2B,HDAC4,PAX2,PHF5A,SFRP1,MED13,ZNF395,FOXO3,NFIB,SMAD3,WWP2,ARNT2,EBF3,CASZ1,ESRRG,HOXD3,HOXD4,NFATC1,CDC73,APP,SSBP3,NOX1,YAP1,NVL,LRP5,SOX2,TEAD1,ZNF521,ARID3A,CHUK,ESR2,PRKD1,STAT1,ST18,ETV5,PLAGL1,HNF4A,ZBTB7C,TASP1,EREG,ATF2,POU2F2,TCF3,RAF1,BMPR2,BMPR1A,IKZF4,CDK12,CAND2,MYB,FGF2,ZNF71,BACH1,RORC,PTAFR,DDX58,BRDT,RARB,NCOA1,LMO7,DVL3,EIF2B5,EIF4G1,TCF20,ATF3,PAWR,EBF2,MAML2,TSG101,RFX2,PRDM15,GLI2,TNKS,WBP2NL,ERCC1,GLIS3,DAB2,BLM,MYSM1,SIN3A,RUVBL2,GMEB1,ZNF423,SP1,TRIM22,TEAD4,HOXC13,SLC35A4,SRA1,UBE2V1,ADIRF,OVOL2,SNX5,NFATC2,BNC1,ACTL6B,ASXL3,PAK3,TET1,CAMTA1,CCPG1,ARID4A,PHF2,WWOX,MEF2A,DCAF6,EYA1,HOXB3,HOXB4,HOXB5,TFEB,ACTR2,HDAC1,SMOC2,TRIM24,PAXIP1,EGR2,RNF10,RYBP,AP3B1,KPNA6,TRIM44,EPCAM,MUC1,AP3D1,CARM1,ZNF836,SMARCA2,SPI1,GLIS1,BCAR3,RBPMS,CHURC1,CREB5,BMPR1B,PRIM2,ZEB2,FOXJ3,HEY2,EIF3E,KMT2D,CREM,KMT2C,PLD1,MACC1,MITF,RPTOR,NFIX,KCTD13,IGF1,MLXIP,ATF7IP,MAPK1,BMP7,PCBP2,ZNF780B,LRRK2,RFC3,PKD2,TADA2A,DAZL,NCOA3,PKIB,WNT7A,CRTC3,ARNTL2,KAT6A,ZNF197,BMP6,TAF15,WDR43,ATRX,PRMT2,SMO,TCF4,SOX30,TFE3,GRIN1,JADE1,RHOA,RQCD1,TF,ONECUT2,TFEC,PRR16,DNAJC3,STAT6,CDK13,SUPT4H1,AGT,PBX1,MAP3K5,CREBBP,DLX1,NCF1,SHC1,RPS6KB1,SP7,LARP4,RPS6KA5,ZNF484,PRDM2,NOS1,ACTN4,MC1R,EDRF1,IL18,PPARA,ARID5B,NOTCH4,CRX,PAX6,NIPBL,YEATS4,ZNF143,BRF1,TBL1X,ATF6,ETS1,MTF2,CDK5RAP1,PRKCQ,CSRNP1,HDAC2,IMPACT,SRCAP,POU3F3,ELF2,PHIP,RFC5,SP100,PARN,WWTR1,ZBTB38,BRCA2,ATF7,DVL2,YBX1,THAP3,HSF1,MAX,EZH1,NRF1,NCOA2,QRICH1,ZP3,CHD6,TRIM37,MET,SETD3,MLXIPL,CCT3,PHB,CTCF,NR6A1,ATF6B,CREB1,RGMB,BMP4,ABLIM3
GO:0051130	positive regulation of cellular component organization	8.19113864288807e-9	CLRN1,NRXN1,PRKCI,MAP4K4,DNMT1,NEGR1,PDE4DIP,CLASP2,GRID2,TENM3,TIAM2,MAPRE2,KALRN,NTRK3,CBL,SEMA5A,TOX,ENPP2,PLCE1,FER,ROBO1,CDKL5,TENM1,ROBO2,HSP90AA1,DSCAM,RUNX1,CDC42EP3,ABCC8,CCT2,GOLGA4,EPHA1,MRE11A,LIMK1,ATP8A2,ESR1,NTRK2,STX18,EP300,TENM2,PDGFB,INSR,PIP4K2A,CDH4,PAK1,MAP3K4,PTPRD,BDNF,AMIGO1,DMD,GSN,RIT2,FYN,PLCB1,NDRG4,NTN1,DCTN1,NRG1,BID,MAP2K1,FNIP1,FRMPD4,MAD1L1,TMEM30A,ROR2,DCN,CAPRIN2,NCKAP1,TERF2,SLX1B,ANO6,ARL3,ZDHHC15,SKAP1,CLSTN2,SYT1,CROCC,CLDN1,SEPT7,WNT11,FIG4,IL1RAPL1,CAMK2B,PPM1E,F2RL1,BCAS3,SLIT2,COLGALT1,CORO1C,DISC1,CEP135,LINGO2,CNTNAP2,EPHA4,AUTS2,CNR1,PPP3CA,CTNNB1,PARK2,IGF1R,PPARG,DLG5,NIN,AJUBA,PHLDB1,HCK,RAB3GAP2,CAPN2,ANKFY1,NF2,HDAC4,TRABD2B,SFRP1,DNM3,SMAD3,CUX2,WIPI1,ABI2,NAV3,APP,NOX1,NLGN2,VDAC1,LRP5,ISLR2,ZDHHC6,PRKD1,PA

			RP6,EREG,BMPR2,CAMK1D,NLGN1,COL16A1,MORC2,RAB11A,PPM1F,SEMA4D,CDC27,PLXNA2,ABCA13,AP2M1,DVL3,EIF4G1,EPHB3,ANAPC5,TSG101,WNT3,NLGN3,PAFAH1B1,KIF3A,HIP1,TRPC5,TNKS,ERCC1,RUFY3,DAB2,DZIP1,RELN,SIN3A,DOCK11,MACF1,ALK,CLASP1,NFATC2,PACSIN1,PAK3,ATAD1,ADNP,MAP1B,TFRC,UVRAG,GPC3,SGIP1,TBC1D5,FLRT2,SNX9,ACTR2,ELAVL4,PLCG2,ROCK1,EPS8,NTRK1,EPHB1,GNL3L,AP2B1,CUX1,MCU,ABCB7,FCHSD2,MAP3K13,RAPGEF3,PDXP,PTK7,SNCA,CD53,MAGI2,USP50,SYT9,CBFA2T2,IGF1,ATF7IP,HTT,DPYSL3,MAPK1,MIB1,BMP7,LRRK2,SPAG5,EFNA5,SHANK3,PKIB,WNT7A,ATRX,RAPGEF2,MARK4,RHOA,ROR1,TF,CUL3,GSK3B,EEF2K,BOK,AGT,TPPP2,SNX3,SETDB2,EPHB2,GPSM2,PPP1R10,PHLDB2,ANGPT1,FBXO31,CNTN1,PRKCQ,BRAF,ATP8A1,PHIP,CDKL3,ANXA2,PARN,SYK,CNOT1,HRK,DVL2,GRIP1,PTPRJ,GPM6A,HSF1,PLEKHM1,ASAP1,GBP5,AXL,MET,EHD2,CCT3,NEDD4L,SYNDIG1,PRAP1
GO:0051129	negative regulation of cellular component organization	8.406168700526965e-9	*NRXN1,CLASP2,SEMA3A,RPS6KA2,SEMA3D,SCAF8,FHOD3,TJP1,SEMA5A,TOM1L1,LMNA,ROBO2,TRIOBP,ARHGAP24,DAB1,OMA1,HDAC6,IKBKB,PMEPA1,PTPRO,EP300,MID1,ARHGAP6,DENND5A,CENPF,GSN,RBM14,RIT2,FYN,FAT3,RTN4,APOD,RTN4R,PAQR3,NTN1,MAD1L1,SCFD1,MAD2L2,TERF2,SLX1B,RCC2,KREMEN1,PRKCD,SEMA5B,BCAS3,DNAJB2,SLIT2,CORO1C,KANK1,TTBK2,SMAD6,DUSP22,HNRNPC,KANK4,IQCJ-SCHIP1,RHPN2,SVIL,EPHA4,SMG6,PPP3CA,MAG,PARK2,PPARG,ADD2,SCAMP5,DRAXIN,NOL3,UBQLN4,DCC,BAZ1B,SPTBN1,DNM3,SSH1,MTBP,SHANK1,ARHGEF18,NAV3,ADD3,DIP2B,YAP1,TEN1,FSTL4,PTPRG,KIF24,SPTBN4,TMEM14A,VILL,PRKD1,SEMA6D,RAF1,VBP1,NLGN1,CTNNA2,CRMP1,SEMA4D,MCTP1,SPOCK1,TERF2IP,WNT3,PAFAH1B1,TRPC5,TNKS,ERCC1,RUFY3,DAB2,LDLRAD4,CLASP1,PACSIN1,SPTBN5,SSH2,TET1,MAP1B,MPHOSPH9,PHF2,TFRC,XRN1,ZNF207,CLEC16A,MNAT1,ROCK1,EPS8,PTPN9,GNL3L,TRIM46,SH3GL3,CARM1,ARHGAP44,SNX33,EPHA7,LIMA1,RAPGEF3,SNCA,PTPRS,ADTRP,OPRD1,RTN4RL1,CBFA2T2,IGF1,DPYSL3,PTEN,BMP7,ATXN2,LRRK2,UNC119,SHANK3,TBCD,MAP4,BMP6,ATRX,RAPGEF2,RHOA,SYNGAP1,GSK3B,RDX,FAM49B,NECAB2,BOK,HDGFRP3,DNAJB6,PACSIN2,SPEF1,EPHB2,TMEFF2,DLC1,PRKCZ,GRIN2B,RABGEF1,PHLDB2,MAP2,HDAC2,IMPACT,TNR,CD300A,CDKL3,CAMSAP3,SLIT1,ANKRD13A,HSF1,CGNL1,INPP5F,TRIM37,MET,SPTAN1,SEMA3C,PRAP1,BMP4*
GO:0009891	positive regulation of biosynthetic process	8.621898013909877e-9	*POLDIP3,LDB2,ASPH,SLC9A1,PBX3,CBFB,IL31RA,MED26,WWC1,ASH1L,NOS1AP,SCAF8,STOX2,PAGR1,HIVEP3,NPAS3,ARNT,STAT5B,CASK,MAP2K5,SP3,HDGF,CHD7,HTR2B,MECOM,TACC1,TRAF6,NHLH1,HSP90AA1,GFI1B,RUNX1,HMGN3,NRIP1,THRB,EFCAB7,CCT2,SERTAD2,DDB1,IKBKB,ERBB4,ZNF609,BRD8,KAT6B,ESR1,MAML3,CDON,EP300,CELA1,ZNF76,PDGFB,HNF4G,INSR,RERE,TCF7L2,LITAF,FBXW11,ESRRB,MAP3K4,TBR1,SAMD4A,TFAP2A,PEG3,MEIS1,TRIM13,ZNF148,MTA3,TFDP2,SLC30A9,TOX3,LARP4B,KAT7,RBM14,MED12L,SATB2,RIT2,MKRN2,ARNTL,PLCB1,ARID4B,BRPF1,PIK3R2,UBP1,MAP2K1,PPP3R1,HDAC5,CSRNP3,NFIA,RNF4,ROR2,DCN,PCBD2,CDH13,CREBRF,COPS5,MAD2L2,RAB27A,CAPRIN2,NEUROD1,USP22,JAK2,SKAP1,MLIP,BCL11B,PKNOX1,MLLT3,KLF15,WNT11,MTA1,NOX4,RPRD1B,CCDC62,PRKCD,ACVR2A,RUNX2,CD4,TGFB1,BANP,NSD1,BTRC,NFATC3,F2RL1,BCAS3,TP73,ILF2,MTDH,FANK1,SMAD6,ZNF398,CLOCK,TCF12,ETV6,SUCO,TFAP2D,BCL3,SBNO2,YTHDF1,FGF10,CIZ1,SMYD3,CAPN3,LUM,RORA,AUTS2,TNFSF11,PPP3CA,NFYB,KLF12,CAMK4,CTNNB1,PARK2,SOD2,SMARCC1,IGF1R,PPARG,NGRN,AXIN1,MTF1,SREBF2,FGF1,NPAT,NR4A3,FOXK2,MYOCD,TRIM5,PER2,AJUBA,CHEK2,SUPT3H,PRDM16,SORBS1,RAB3GAP2,CAPN2,TRIM8,MEF2B,HDAC4,PAX2,PHF5A,SFRP1,MED13,ZNF395,FOXO3,NFIB,SMAD3,WWP2,ARNT2,EBF3,CASZ1,ESRRG,HOXD3,HOXD4,NFATC1,CDC73,APP,SSBP3,NOX1,YAP1,NVL,LRP5,SOX2,TEAD1,ZNF521,ARID3A,CHUK,ESR2,PRKD1,STAT1,ST18,ETV5,PLAGL1,HNF4A,ZBTB7C,TASP1,EREG,ATF2,POU2F2,TCF3,RAF1,BMPR2,BMPR1A,IKZF4,CDK12,CAND2,MYB,FGF2,ZNF71,BACH1,RORC,PTAFR,ADCYAP1R1,DDX58,BRDT,RARB,NCOA1,LMO7,DVL3,EIF2B5,EIF4G1,TCF20,ATF3,PAWR,EBF2,MAML2,TSG101,CCDC3,RFX2,PRDM15,DDAH1,GLI2,TNKS,WBP2NL,ERCC1,GLIS3,DAB2,BLM,MYSM1,SIN3A,RUVBL2,GMEB1,ZNF423,SP1,TRIM22,TEAD4,HOXC13,SLC35A4,SRA1,UBE2V1,ADIRF,OVOL2,SNX5,NFATC2,BNC1,ACTL6B,ASXL3,PAK3,TET1,CAMTA1,CCPG1,ARID4A,SCARB1,PHF2,WWOX,MEF2A,DCAF6,EYA1,TRPV1,HOXB3,HOXB4,HOXB5,TFEB,ACTR2,HDAC1,SMOC2,TRIM24,PLCG2,PAXIP1,EGR2,RNF10,RYBP,AP3B1,PTPN2,KPNA6,TRIM44,EPCAM,MUC1,AP3D1,CARM1,ZNF836,SMARCA2,SPI1,GLIS1,BCAR3,ABCB7,RBPMS,CHURC1,CREB5,SNCA,BMPR1B,PRIM2,ZEB2,FOXJ3,HEY2,EIF3E,KMT2D,CREM,KMT2C,PLD1,MACC1,MITF,RPTOR,NFIX,KCTD13,IGF1,MLXIP,ATF7IP,MAPK1,BMP7,PCBP2,ZNF780B,LRRK2,ZBTB20,RFC3,PKD2,TADA2A,DAZL,NCOA3,PKIB,WNT7A,LPGAT1,CRTC3,ARNTL2,ELOVL5,KAT6A,ZNF197,BMP6,TAF15,WDR43,ATRX,PRMT2,SMO,TCF4,SOX30,TFE3,GRIN1,JADE1,RHOA,RQCD1,TF,ONECUT2,TFEC,PRR16,DNAJC3,STAT6,CDK13,SUPT4H1,AGT,PBX1,MAP3K5,CREBBP,DLX1,NCF1,SHC1,RPS6KB1,SP7,LARP4,SLC5A3,RPS6KA5,ZNF484,PRDM2,NOS1,ACTN4,MC1R,EDRF1,IL18,PPARA,ARID5B,NOTCH4,CRX,PAX6,NIPBL,YEATS4,ZNF143,BRF1,TBL1X,ATF6,ETS1,MTF2,CDK5RAP1,PRKCQ,CSRNP1,HDAC2,HTR2C,IMPACT,SRCAP,POU3F3,ELF2,PHIP,RFC5,SP100,PARN,WWTR1,ZBTB38,BRCA2,ATF7,DVL2,YBX1,TICAM1,THAP3,HSF1,MAX,EZH1,NRF1,NCOA2,QRICH1,ZP3,CHD6,TRIM37,MET,SETD3,MLXIPL,CCT3,PHB,CTCF,NR6A1,ATF6B,CREB1,RGMB,BMP4,ABLIM3*
GO:00	positive regulation	1.13243039	*NRXN1,SLCO3A1,S1PR2,ADCY8,PDE8A,IL31RA,NRG3,PRKAG2,PRLR,NTRK3,ARN*

42327	of phosphorylation	44557889e-8	T,FLT3,ENPP2,GRM5,MAP2K5,KITLG,ROBO1,TOM1L1,HTR2B,TENM1,TRAF6,VAV2,ABI1,HSP90AA1,CDC6,KNDC1,DSCAM,AKAP13,DAB1,PTPN11,HDAC6,ADORA1,EPHA1,ERBB4,MRE11A,CDON,NTRK2,PDGFB,TNIK,CCND3,INSR,CAB39,PAK1,MAP3K4,BDNF,ENTPD5,ECT2,ERN2,CASS4,FYN,LMTK2,CHI3L1,NRG1,SH2D3C,MAP2K1,FNIP1,ROR2,MAD2L2,CAPRIN2,CCNYL1,JAK2,FBXW7,LAMTOR3,WNT11,NOX4,PRKCD,TAB2,ACVR2A,CD4,TGFB1,PIBF1,TNFRSF10B,DOCK3,FGF10,SMYD3,EPHA4,CD6,TNFSF11,IGF1R,PPARG,AXIN1,LEPR,FGF1,AJUBA,CHEK2,SPDYA,CSPG4,FLT4,SLC4A4,TAB1,APP,PDGFRA,BORA,MTCP1,S100A12,PRKD1,EREG,CCNY,ATF2,RAF1,PTPN1,BMPR2,BMPR1A,BTBD10,PIK3R3,FGF2,NEDD9,SEMA4D,DVL3,EIF4G1,EPHB3,CKS1B,TERF2IP,TRPC5,DAB2,CACUL1,DLG3,WNK1,RELN,NEK10,IQGAP1,MAP3K7,ALK,SLC8A2,ATP2B4,ADNP,TFRC,EPHA5,GHR,MNAT1,SNX9,PLCG2,INSRR,NTRK1,EPHB1,EPHA10,AXIN2,PIFO,EPHA7,ATG14,BCAR3,RBPMS,MAP3K13,RAPGEF3,SNCA,BMPR1B,PRKAG1,ADTRP,OPRD1,RPTOR,IGF1,MAPK1,BMP7,LRRK2,UNC119,ZBTB20,PKD2,EFNA5,PDGFC,PPP2R3C,BMP6,RAPGEF2,RHOA,ROR1,RQCD1,TF,AGT,PRKAA2,PTK2,TEC,MAP3K5,NCF1,TAOK2,CD44,ADAM8,RPS6KA5,NOS1,STK4,EPHB2,SLC1A1,IL18,UBE2K,MOB3B,PRKCZ,ANGPT1,LRRK1,OSBPL8,CNTN1,BRAF,HDAC2,CD300A,PPP2CA,ANGPT4,MADD,SYK,DVL2,PTPRJ,HSF1,SASH1,VAV3,MOB3A,SH2D3A,AKTIP,AXL,MET,MALT1,MLXIPL,PHB,PTPRC,TYRO3,BMP4
GO:0031346	positive regulation of cell projection organization	1.2012751634556175e-8	CLRN1,PRKCI,NEGR1,TENM3,TIAM2,KALRN,NTRK3,SEMA5A,TOX,ENPP2,PLCE1,ROBO1,CDKL5,TENM1,ROBO2,DSCAM,CDC42EP3,GOLGA4,LIMK1,ATP8A2,NTRK2,EP300,TENM2,CDH4,PTPRD,BDNF,AMIGO1,DMD,RIT2,FYN,NDRG4,NTN1,MAP2K1,TMEM30A,ROR2,CAPRIN2,NCKAP1,ZDHHC15,CROCC,SEPT7,FIG4,IL1RAPL1,CAMK2B,F2RL1,BCAS3,SLIT2,CORO1C,DISC1,CEP135,EPHA4,AUTS2,CNR1,IGF1R,NIN,HDAC4,DNM3,CUX2,ABI2,ISLR2,PRKD1,PARP6,BMPR2,CAMK1D,NLGN1,RAB11A,SEMA4D,PLXNA2,DVL3,WNT3,PAFAH1B1,TRPC5,RUFY3,DZIP1,RELN,DOCK11,MACF1,ALK,PACSIN1,PAK3,ADNP,MAP1B,ACTR2,ELAVL4,EPS8,NTRK1,CUX1,MAP3K13,PTK7,MAGI2,CBFA2T2,HTT,DPYSL3,BMP7,EFNA5,RAPGEF2,MARK4,ROR1,EEF2K,AGT,SNX3,FBXO31,CNTN1,BRAF,CDKL3,DVL2,GRIP1,GPM6A,PLEKHM1
GO:0051172	negative regulation of nitrogen compound metabolic process	1.4361940014910841e-8	ENPP1,LDB2,PRMT3,PDE4D,DNMT1,TRPS1,CBFB,PRDM12,WWC1,LATS2,SCAF8,PTGIS,PRKAG2,WWC3,MVP,FTO,NTRK3,CBL,ZNF566,MAP2K5,ZNF536,SP3,HDGF,EZR,MECOM,TRAF6,DNAJA3,OXR1,GFI1B,PTPRK,RUNX1,NRIP1,THRB,DACH1,MGAT5,ORC2,HDAC6,EPHA1,IKBKB,PEX14,LIMK1,KAT6B,ESR1,MIER1,PCBP3,PMEPA1,PTPRO,NTRK2,TNRC6A,EP300,CELA1,TENM2,PDGFB,CCND3,TOB2,ZNF19,BRMS1,SCML4,FMN2,RERE,PRKAR1A,ATP8B1,H2AFY2,TCF7L2,FBXW11,BASP1,TBR1,SAMD4A,TFAP2A,PEG3,ZIM2,TGIF2,MDM4,ZNF148,MTA3,SNX6,TFDP2,CST2,ERN2,C1D,DMD,CENPF,LARP4B,KAT7,SLC8A1,RBM4,SATB2,HIRA,FYN,ARNTL,NF1,PLCB1,RTN4,DPEP1,APOD,PAQR3,NRG1,BDKRB1,BDKRB2,FRY,MDFI,FNIP1,VAX2,TMBIM6,VGLL4,HDAC5,ZNF692,JDP2,ITIH2,CREBRF,TRDMT1,SOX13,FHIT,PTPRT,AUNIP,IPO5,MAD2L2,TLE6,CAPRIN2,TERF2,SLX1B,FOXN3,GNAQ,RFFL,UIMC1,MLIP,ZFYVE28,TCF7,PDE2A,KLF15,TBX15,WNT11,MTA1,KLF8,LCOR,PRKCD,SOX6,RUNX2,PPM1E,TGFB1,BANP,NSD1,PIBF1,ZHX2,BTRC,NFATC3,CDYL2,DNAJB2,CC2D1B,SLIT2,ITIH4,CORO1C,SAP18,MTDH,MYT1L,KDM4B,SMAD6,ZNF398,CLOCK,ZNF675,ETV6,NELL1,TIMP2,BCL3,DUSP22,NAIP,HNRNPC,SBNO2,YTHDF1,SMYD3,LOXL3,CAPN3,SMURF2,EPHA4,SMG6,PPP3CA,NSUN2,MAGEA4,KLF12,GATAD2B,PIP5KL1,UFL1,CTNNB1,PARK2,METTL13,SMARCC1,PPARG,AXIN1,PRKAR1B,OTUB1,CIPC,CBX5,LRPPRC,SREBF2,PROS1,NPAT,NR4A3,FOXK2,NOL3,PRKAR2A,MYOCD,PER2,AJUBA,GLG1,UBQLN4,PRDM16,CSTL1,BRMS1L,NF2,HDAC4,PAX2,SFRP1,FOXO3,NFIB,ZCCHC17,SYNCRIP,SMAD3,CUX2,WWP2,SIMC1,RNF168,DCP1B,MIER3,NEDD4,CDC73,APP,YAP1,HEG1,TEN1,IBTK,LRP5,SOX2,PRICKLE1,ZNF653,SFMBT1,ESR2,DCUN1D3,STAT1,ETV5,TAF3,PLAGL1,HNF4A,EREG,ATF2,TCF3,RAF1,CELF4,CARD16,PTPN1,BMPR2,VBP1,IKZF4,SPAG9,MYB,BACH1,MXD3,PPM1F,BEND5,SEMA4D,NFX1,RORC,JARID2,PHC2,RARB,SPEN,PRKCG,SIN3B,SPOCK1,EHMT1,EIF4G1,ATF3,LIN28B,PAWR,AGO3,DEPTOR,TSG101,TERF2IP,CRYM,SUFU,MAGEA11,PTPN13,COL4A3,NR2C1,GLI2,TNKS,ERCC1,TNRC6B,GLIS3,WDTC1,DAB2,BLM,LDLRAD4,SIN3A,RUVBL2,ZNF423,TRIM22,XDH,OVOL2,NFATC2,ATP2B4,MAPKAPK2,ARID4A,CDK6,CELF1,RNF34,MEF2A,GPC3,XRN1,SATB1,GATAD2A,DIS3L2,TRPV1,HOXB3,HOXB4,L3MBTL4,ELAVL4,HDAC1,RECQL5,TRIM24,ROCK1,SCMH1,HIPK3,RYBP,PTPN2,TRIM44,MAP1A,AXIN2,PARD3,ZNF425,GNL3L,MUC1,BCOR,ANXA8L1,CD84,LPA,SMARCA2,CUX1,SPI1,DNMT3B,FOXP2,PSPC1,GLIS1,SFMBT2,ATG14,EIF3H,JAZF1,STK38,ACOT8,RENBP,SMYD1,SNCA,PNPT1,ZEB2,HEY2,RC3H1,EIF3E,ZMYND11,CREM,PSMF1,CAST,TRIP12,DFFA,MITF,SRSF6,NFIX,MLLT1,CBFA2T2,IGF1,ATF7IP,PTEN,SPRED2,BMP7,MXI1,PUM1,CIR1,LRRK2,TMEM59,ZBTB20,BAG6,LILRB4,ITGAM,KCTD1,RIPPLY1,RCOR3,DAZL,SERPINA3,SERPINA4,SERPINA5,PKIB,ZBED6,DNAJC1,KAT6A,BMP6,TAF15,MYCBP2,GPI,SH3RF2,N4BP1,ATRX,IKZF1,PRMT2,SMO,PRKAR2B,SOX30,UBR5,RQCD1,CUL3,TFEC,EPM2A,GSK3B,DNAJC3,STAT6,PTPRB,RBBP8,CD27,DUSP26,NACC2,SUPT4H1,AGT,METTL16,PRKAA2,ADAR,ZMPSTE24,SNX3,EXOSC3,TCFL5,CREBBP,DLX1,SHC1,PARP10,PRG3,SERPINB11,SETDB2,DNAJB6,CD44,RPS6KA5,ZNF93,PRDM2,RGS14,TCF25,ZNF282,EPHB2,DIS3,PPARA,ARID5B,CPEB4,NOTCH4,ZMYND8,ZNF366,PAX6,PRKCZ,ADORA2A,RABGEF1,CPEB1,OTUD7B,NIPBL,COL28A1,ANGPT1,TBL1X,FHL2,LRRK1,MBTD1,ZB

			TB5,IGF2BP3,MTF2,TAGLN3,CDK5RAP1,NCOR2,HDAC2,IMPACT,POU3F3,SPINT2,CD300A,ELF2,PPP2CA,RPL23,ATXN1,SP100,ANXA2,TRIM29,PARN,RBM42,CNOT1,WWTR1,ZBTB38,MORC1,YBX1,PTPRJ,TAF1,HSF1,MAX,SAP130,DHFR,EZH1,NCOA2,ZP3,RNF2,ZNF554,INPP5F,TRIM37,CD109,TIMELESS,UCHL5,SERPINE3,KDM2B,MLXIPL,PHB,PTPRC,ZNF555,CTCF,SH3GL2,NR6A1,TRAF3IP2,CREB1,BMP4
GO:0031328	positive regulation of cellular biosynthetic process	1.6644730240675658e-8	*POLDIP3,LDB2,ASPH,SLC9A1,PBX3,CBFB,IL31RA,MED26,WWC1,ASH1L,NOS1AP,SCAF8,STOX2,PAGR1,HIVEP3,NPAS3,ARNT,STAT5B,CASK,MAP2K5,SP3,HDGF,CHD7,HTR2B,MECOM,TACC1,TRAF6,NHLH1,HSP90AA1,GFI1B,RUNX1,HMGN3,NRIP1,THRB,EFCAB7,CCT2,SERTAD2,DDB1,IKBKB,ERBB4,ZNF609,BRD8,KAT6B,ESR1,MAML3,CDON,EP300,CELA1,ZNF76,PDGFB,HNF4G,INSR,RERE,TCF7L2,LITAF,FBXW11,ESRRB,MAP3K4,TBR1,SAMD4A,TFAP2A,PEG3,MEIS1,TRIM13,ZNF148,MTA3,TFDP2,SLC30A9,TOX3,LARP4B,KAT7,RBM14,MED12L,SATB2,RIT2,MKRN2,ARNTL,PLCB1,ARID4B,BRPF1,PIK3R2,UBP1,MAP2K1,PPP3R1,HDAC5,CSRNP3,NFIA,RNF4,ROR2,DCN,PCBD2,CDH13,CREBRF,COPS5,MAD2L2,CAPRIN2,NEUROD1,USP22,JAK2,SKAP1,MLIP,BCL11B,PKNOX1,MLLT3,KLF15,WNT11,MTA1,NOX4,RPRD1B,CCDC62,PRKCD,ACVR2A,RUNX2,CD4,TGFB1,BANP,NSD1,BTRC,NFATC3,F2RL1,BCAS3,TP73,ILF2,MTDH,FANK1,SMAD6,ZNF398,CLOCK,TCF12,ETV6,TFAP2D,BCL3,SBNO2,YTHDF1,FGF10,CIZ1,SMYD3,CAPN3,LUM,RORA,AUTS2,TNFSF11,PPP3CA,NFYB,KLF12,CAMK4,CTNNB1,PARK2,SOD2,SMARCC1,IGF1R,PPARG,NGRN,AXIN1,MTF1,SREBF2,FGF1,NPAT,NR4A3,FOXK2,MYOCD,TRIM5,PER2,AJUBA,CHEK2,SUPT3H,PRDM16,SORBS1,RAB3GAP2,CAPN2,TRIM8,MEF2B,HDAC4,PAX2,PHF5A,SFRP1,MED13,ZNF395,FOXO3,NFIB,SMAD3,WWP2,ARNT2,EBF3,CASZ1,ESRRG,HOXD3,HOXD4,NFATC1,CDC73,APP,SSBP3,YAP1,NVL,LRP5,SOX2,TEAD1,ZNF521,ARID3A,CHUK,ESR2,PRKD1,STAT1,ST18,ETV5,PLAGL1,HNF4A,ZBTB7C,TASP1,EREG,ATF2,POU2F2,TCF3,RAF1,BMPR2,BMPR1A,IKZF4,CDK12,CAND2,MYB,FGF2,ZNF71,BACH1,RORC,PTAFR,ADCYAP1R1,DDX58,BRDT,RARB,NCOA1,LMO7,DVL3,EIF2B5,EIF4G1,TCF20,ATF3,PAWR,EBF2,MAML2,TSG101,RFX2,PRDM15,DDAH1,GLI2,TNKS,WBP2NL,ERCC1,GLIS3,DAB2,BLM,MYSM1,SIN3A,RUVBL2,GMEB1,ZNF423,SP1,TRIM22,TEAD4,HOXC13,SLC35A4,SRA1,UBE2V1,ADIRF,OVOL2,SNX5,NFATC2,BNC1,ACTL6B,ASXL3,PAK3,TET1,CAMTA1,CCPG1,ARID4A,SCARB1,PHF2,WWOX,MEF2A,DCAF6,EYA1,TRPV1,HOXB3,HOXB4,HOXB5,TFEB,ACTR2,HDAC1,SMOC2,TRIM24,PAXIP1,EGR2,RNF10,RYBP,AP3B1,PTPN2,KPNA6,TRIM44,EPCAM,MUC1,AP3D1,CARM1,ZNF836,SMARCA2,SPI1,GLIS1,BCAR3,ABCB7,RBPMS,CHURC1,CREB5,SNCA,BMPR1B,PRIM2,ZEB2,FOXJ3,HEY2,EIF3E,KMT2D,CREM,KMT2C,PLD1,MACC1,MITF,RPTOR,NFIX,KCTD13,IGF1,MLXIP,ATF7IP,MAPK1,BMP7,PCBP2,ZNF780B,RFC3,PKD2,TADA2A,DAZL,NCOA3,PKIB,WNT7A,LPGAT1,CRTC3,ARNTL2,ELOVL5,KAT6A,ZNF197,BMP6,TAF15,WDR43,ATRX,PRMT2,SMO,TCF4,SOX30,TFE3,GRIN1,JADE1,RHOA,RQCD1,TF,ONECUT2,TFEC,PRR16,DNAJC3,STAT6,CDK13,SUPT4H1,AGT,PBX1,MAP3K5,CREBBP,DLX1,NCF1,SHC1,RPS6KB1,SP7,LARP4,RPS6KA5,ZNF484,PRDM2,NOS1,ACTN4,MC1R,EDRF1,IL18,PPARA,ARID5B,NOTCH4,CRX,PAX6,NIPBL,YEATS4,ZNF143,BRF1,TBL1X,ATF6,ETS1,MTF2,CDK5RAP1,PRKCQ,CSRNP1,HDAC2,HTR2C,IMPACT,SRCAP,POU3F3,ELF2,PHIP,RFC5,SP100,PARN,WWTR1,ZBTB38,BRCA2,ATF7,DVL2,YBX1,TICAM1,THAP3,HSF1,MAX,EZH1,NRF1,NCOA2,QRICH1,ZP3,CHD6,TRIM37,MET,SETD3,MLXIPL,CCT3,PHB,CTCF,NR6A1,ATF6B,CREB1,RGMB,BMP4,ABLIM3*
GO:1901888	regulation of cell junction assembly	1.703121817309274e-8	*NRXN1,MAP4K4,SLC9A1,NEGR1,CLASP2,GRID2,TJP1,NTRK3,LHFPL4,ROBO2,IKBKB,NTRK2,PTPRD,BDNF,AMIGO1,ARHGAP6,APOD,NTN1,LRFN5,RCC2,CLSTN2,CLDN1,IL1RAPL1,BCAS3,CORO1C,DUSP22,LINGO2,CNTNAP2,IL1RAPL2,DLG5,SFRP1,SMAD3,CUX2,APP,SLK,NLGN2,GPC6,NLGN1,COL16A1,PPM1F,PEAK1,SEMA4D,EIF4G1,EPHB3,VCL,NLGN3,SETD5,MACF1,CLASP1,GPM6B,ADNP,FLRT2,ROCK1,NTRK1,EPHB1,EPHA7,PTPRA,SNCA,PTPRS,PTEN,EFNA5,WNT7A,RAPGEF2,RHOA,EEF2K,AGT,PTK2,EPHB2,DLC1,PHLDB2,CAMSAP3,SLIT1,PTPRJ,SYNDIG1*
GO:0000165	MAPK cascade	1.750909136710196e-8	*PRDX2,NRXN1,MAP4K4,PDE8A,SEMA3A,IL31RA,WWC1,ASH1L,NTRK3,FLT3,GRM5,PLCE1,MARVELD3,MAPK4,MAP2K5,KITLG,MAPK10,PTPRR,EZR,ROBO1,HTR2B,MECOM,TENM1,TRAF6,ITPKB,ULK4,PSMB7,AKAP13,PTPN11,ADORA1,IKBKB,ERBB4,ADRA1D,CDON,NTRK2,PDGFB,TNIK,MID1,STK39,INSR,PAK1,MAP3K4,PPM1L,ERN2,DMD,RIT2,NF1,PLCB1,CHI3L1,NDRG4,PAQR3,PIK3R2,RANBP9,NRG1,SH2D3C,MAP2K1,ROR2,COPS5,FGF14,JAK2,FBXW7,ITCH,NPSR1,LAMTOR3,NOX4,PRKCD,CD4,F2RL1,TP73,ZNF675,BMPER,DUSP22,NAIP,FGF10,RASA4,RASA4B,SH3RF3,EPHA4,PRKCA,TNFSF11,CTNNB1,PARK2,FCGR2B,IGF1R,PPARG,AXIN1,FGF1,TRIM5,AJUBA,PPP1CB,CSPG4,GRM1,NF2,FLT4,SFRP1,SMAD3,TAB1,APP,PDGFRA,NOX1,TNFRSF19,SOX2,FRS2,S100A12,MAPKAPK3,ATF2,RAF1,PTPN1,SPAG9,FGF2,DOK6,DVL3,RBX1,ATF3,RCE1,CCL14,CCL15,PAFAH1B1,PRDM15,DAB2,PKHD1,NEK10,IQGAP1,MAP3K7,ALK,XDH,PAK3,MAPKAPK2,MEF2A,GFRAL,PSMB2,GHR,PLCG2,ROCK1,C1QTNF1,HIPK3,PTPN2,NTRK1,EIF3A,EPHB1,SPI1,EPHA7,BCAR3,STK38,MAP3K13,GRM4,WNT7B,ZMYND11,RASA1,IGF1,MAPK1,PTEN,SPRED2,BMP7,LRRK2,LILRB4,SHANK3,PDGFC,WNT7A,SH3RF2,RAPGEF2,VRK3,SYNGAP1,ROR1,TF,ACTA2,NECAB2,CD27,DUSP26,AGT,HRH4,NDRG2,MAP3K5,NCF1,TAOK2,SHC1,CD44,ADAM8,CXCL17,PLVAP,RGS14,STK4,EPHB2,IL18,PHLPP1,MAGI3,PRKCZ,PPP1CC,ANGPT1,GCNT2,TBC1D10C,CCL22,BRAF,H*

			TR2C,CD300A,ARHGEF6,MADD,SYK,GRIK2,DVL2,PTPRJ,HSF1,SASH1,CAV2,SH2D3A,ANKRD6,PHB,PTPRC,BMP4
GO:1901576	organic substance biosynthetic process	2.0981338757800087e-8	*POLDIP3,ZDHHC14,ENPP1,PRDX2,LDB2,GPC5,ASPH,B4GALNT2,PRMT3,GSS,PRKCI,SLCO3A1,DNMT1,HLX,SLC9A1,PBX3,ADCY8,MGAT4C,MED13L,TRPS1,CBFB,PDE8A,ZNF823,IL31RA,PRDM12,GK5,MED26,WWC1,ASH1L,SCAF8,STOX2,PTGIS,PRKAG2,WWC3,PRLR,PAGR1,ADCY7,HIVEP3,FTO,NPAS3,ARNT,LRRFIP2,STAT5B,TOX,GRM5,NOC3L,PLCE1,SAMHD1,ZNF566,FER,CASK,MAP2K5,MAPK10,SPNS2,PIK3CD,ZNF536,SP3,HDGF,GTF3C1,EIF4G3,EZR,IKZF2,B3GALT1,MALRD1,CHD7,HTR2B,MECOM,TACC1,TENM1,NADK2,GOT1,NMT2,INPP4B,TRAF6,GTF2E2,ITPKB,SCD5,DNAJA3,NHLH1,HSP90AA1,CDC6,PSMB7,TSC22D3,ZC4H2,CCDC22,MOCOS,FAM126A,CDAN1,GMDS,GFI1B,PTPRK,RUNX1,ERC1,HMGN3,NRIP1,THRB,EFCAB7,ITGB3BP,DACH1,ZNF569,MGAT5,CCT2,ORC2,HDAC6,SERTAD2,DDB1,MYT1,IKBKB,PEX14,ERBB4,GBE1,DPY19L2,MRE11A,ZNF609,BRD8,KAT6B,HIF3A,SNIP1,LARGE,ESR1,MIER1,PCBP3,MAML3,CDON,TNRC6A,EP300,CELA1,TENM2,ZNF76,RNF220,ZNF471,PDGFB,CCND3,GALNTL6,CSGALNACT1,TOB2,ZNF19,ZNF23,BRMS1,ZNF605,ALOX5AP,SCML4,HNF4G,INSR,RERE,PRKAR1A,FUBP1,ATP8B1,H2AFY2,TCF7L2,PIP4K2A,ST6GALNAC3,JMJD1C,ZNF443,ZNF490,ZNF564,ZNF709,ZNF799,LITAF,FBXW11,ESRRB,MAP3K4,ZDHHC13,BASP1,SPTSSA,TBR1,PPIP5K1,SAMD4A,TFAP2A,CDK14,FANCA,PEG3,ZIM2,TGIF2,TGIF2-C20ORF24,POMGNT2,FUT8,MEIS1,VRTN,TRIM13,MDM4,ENTPD5,ZNF148,MTA3,SNX6,TFDP2,PPM1L,PTCD3,ERN2,CDC45,C1D,AFF3,DMD,CENPF,ATG10,SLC30A9,CERS6,TOX3,PDS5A,LARP4B,USP13,KAT7,ZNF667,RBM14,RBM4,PRPS2,MED12L,SATB2,RIT2,HIRA,DRG2,MKRN2,ARNTL,LINC01138,PLCB1,ARID4B,GXYLT2,POLA2,DGKH,HS3ST2,B4GALT6,RWDD3,BRPF1,PIK3R2,NRG1,UBP1,MAP2K1,MDFI,FNIP1,VAX2,GALNT16,TMBIM6,VGLL4,PPP2CB,PPP3R1,HDAC5,CSRNP3,ALDH3B2,ZNF692,NFIA,RNF4,GALNT18,JDP2,CMKLR1,INPP4A,PPIP5K2,ROR2,DCN,ZDHHC3,SLC39A14,PRPSAP2,PCBD2,CDH13,CREBRF,SOX13,ACSBG1,UST,COPS5,AK2,MAD2L2,MAN1A1,TLE6,SRBD1,CAPRIN2,ZNF418,NLN,PHF20L1,BBOX1,PDF,TERF2,ZNF286A,NME7,FOXN3,AK5,CERKL,NEUROD1,USP22,ZDHHC15,JAK2,TRAPPC9,FBXW7,OAZ2,SKAP1,SMC3,UIMC1,ITCH,MLIP,BCL11B,SMG1,IMPDH1,PKNOX1,B3GALT5,MLLT3,TSHZ2,PNPLA1,DHDDS,TCF7,PDE2A,KLF15,TBX15,ANXA4,TTC7B,WNT11,FIG4,MTA1,KLF8,NOX4,LCOR,RPRD1B,CACNA1H,QKI,CCDC62,PRKCD,SOX6,ACVR2A,RUNX2,CD4,TGFB1,BANP,MRPL33,SGK1,PCSK5,NSD1,IGSF1,PIBF1,ZHX2,PKNOX2,ASCC2,BTRC,NFATC3,POLK,F2RL1,BCAS3,CDYL2,HS6ST2,CC2D1B,TP73,HHAT,SAP18,CDKAL1,ZBTB22,ILF2,TMTC1,MTDH,FANK1,MYT1L,SMAD6,BNC2,ZNF398,TMTC2,CLOCK,CHST9,TCF12,ZNF675,RPL36,ETV6,SYNJ2,PHRF1,TFAP2D,BCL3,EIF3L,SND1,DUSP22,HNRNPC,TRRAP,SBNO2,YTHDF1,FGF10,CIZ1,POLR2J2,ADK,SMYD3,LOXL3,ADCY2,CAPN3,LUM,SMURF2,RORA,AGPS,HIVEP2,HSD17B12,AUTS2,TNFSF11,SMG6,PPP3CA,NFYB,MAGEA4,KLF12,CAMK4,GATAD2B,PIP5KL1,UFL1,TRAK1,CTNNB1,PARK2,SOD2,DACH2,METTL13,SMARCC1,KLF17,IGF1R,PPARG,IARS2,NGRN,AXIN1,IL18R1,CIPC,MTF1,CBX5,ANKRD17,OTC,BRIP1,LRPPRC,SREBF2,AARS2,PFAS,CDK11A,GALNT8,NDUFA9,CDK11B,LEPR,FGF1,NPAT,NR4A3,FOXK2,WDR45B,DCT,ESCO1,MYOCD,TRIM5,PER2,AJUBA,ZNF626,ZNF737,CHEK2,SUPT3H,PRDM16,PPP1CB,HCK,SORBS1,AGPAT4,RAB3GAP2,CAPN2,TRIM8,DIO2,BRMS1L,ZBTB8A,ZBTB8B,CTDP1,HS3ST5,BAZ1B,MEF2B,SGMS1,HDAC4,PAX2,PHF5A,CPOX,SECISBP2L,SNAPC4,SFRP1,MED13,ST8SIA1,ZNF395,FOXO3,DGKB,TPH2,NFIB,SP4,SYNCRIP,SMAD3,TYW1B,CUX2,WWP2,ARNT2,SBNO1,KRBOX1,ZNF662,CYP4F11,ZNF777,WIPI1,EBF3,RNF168,CASZ1,DCP1B,MIER3,NEDD4,PLA2G4C,ESRRG,HOXD3,HOXD4,ZNF114,GALNT14,KTI12,NFATC1,ST3GAL3,UCK2,CDC73,APP,SSBP3,GSX2,RBM8A,NOX1,YAP1,ST6GALNAC5,PPCS,TEN1,NVL,PEMT,LRP5,POLR3G,BDH2,ZNF787,SOX2,SETD2,ZDHHC6,GPC6,CHST11,TEAD1,PRICKLE1,ZNF653,ZNF521,ELOVL2,ARID3A,ZNF761,CHUK,ERLIN1,RFT1,SFMBT1,ZNF584,TPH1,ESR2,S100A12,GTF2F2,PRKD1,STAT1,HEXA,ST18,OSBPL2,DGKG,ETV5,RHOXF2B,SLC6A3,TAF3,PLAGL1,HNF4A,ZBTB7C,TASP1,EREG,ATF2,POU2F2,TCF3,ZNF730,RAF1,CELF4,ABLIM2,ZNF766,CARD16,POLA1,GGT7,BMPR2,CAMK1D,BMPR1A,ZKSCAN1,IDO2,PABPC4,IKZF4,PIK3R3,CDK12,TAF8,CAND2,CERS3,DENND4A,MYB,FGF2,ZNF71,POU6F2,BACH1,MXD3,PPM1F,TICRR,ADCY5,GLI4,ZFP41,BEND5,SEMA4D,NFX1,RORC,ELP3,PTAFR,ADCYAP1R1,SP140,SP140L,RHOXF2,JARID2,DDX58,SLC5A8,BRDT,PHC2,PI4KA,RARB,SPEN,PIK3C2B,SIN3B,NCOA1,EEF1E1,EHMT1,LMO7,DVL3,EIF2B5,EIF4G1,POLR2H,TCF20,ATF3,TCEANC,AKR1D1,STT3B,CKS1B,PAWR,EBF2,AGO3,MAML2,TSG101,CCDC3,TERF2IP,CRYM,RFX2,B3GALNT1,ZNF322,SUFU,MAGEA11,PRDM15,ZNF670,ZNF695,CCDC169-SOHLH2,SOHLH2,POLE,ZNF354C,TCEA3,PADI6,FDXR,CMPK1,ZNF704,CERS4,NR2C1,EDEM3,ME1,GLI2,TNKS,WBP2NL,ERCC1,TNRC6B,GLIS3,GLCE,WDTC1,ZNF664,MRPS28,MGLL,TPK1,DAB2,BLM,PKHD1,MYSM1,SETD5,SMG5,GTPBP2,RELN,SIN3A,RUVBL2,TYW1,COMMD6,GUCY1A2,GMEB1,PELI1,SLC25A16,MAP3K7,ZNF423,SP1,TRIM22,ALK,GSTM3,GSTM5,INPP5D,TEAD4,HOXC13,APBB3,SLC35A4,SRA1,TOP1,UBE2V1,MBOAT1,ADIRF,OVOL2,SNX5,NFATC2,RBBP6,PAX7,BNC1,ATP2B4,ACTL6B,ASXL3,PAK3,TET1,CAMTA1,CCPG1,ADNP,ARID4A,C12ORF65,SCARB1,CDK6,PHF2,CELF1,TYR,TFRC,EPHA5,WWOX,ALG14,MEF2A,ADCY9,DCAF6,GPC3,XRN1,SATB1,PSMB2,EYA1,GATAD2A,DIS3L2,TRPV1,HOXB3,HOXB4,HOXB5,HOXB6,TFEB,MNAT1,ACTR2,L3MBTL4,ACACA,ELAVL4,HDAC1,HS6ST3,REC*

			QL5,SMOC2,TRIM24,PLCG2,ROCK1,SCMH1,MRPS17,PAXIP1,ZNF713,EGR2,HIPK3,RNF10,RYBP,AP3B1,PTPN2,NTRK1,KPNA6,TRIM44,EIF3A,MAN1B1,SARM1,EPCAM,ZNF425,GNL3L,KRTCAP2,MUC1,PTPN14,NUDT14,BCOR,AP3D1,ZNF41,MGAT4A,CARM1,ZNF383,SRD5A2,ZNF616,ZNF836,SMARCA2,CUX1,SPI1,DNMT3B,LRRC47,FOXP2,AGPAT3,MRPS24,PSPC1,GLIS1,SFMBT2,EIF3H,BCAR3,JAZF1,ABCB7,ACOT8,RBPMS,CHURC1,CREB5,MAP3K13,ERCC3,SMYD1,SNCA,BMPR1B,POLN,PRIM2,PNPT1,ZEB2,MCMBP,FOXJ3,NADK,OCA2,HEY2,RC3H1,EIF3E,HMGCS2,ZMYND11,KMT2D,PRKAG1,CREM,OSBPL1A,KMT2C,GGPS1,PLD1,MACC1,MITF,OPRD1,NUGGC,RPTOR,NFIX,BTK,KCTD13,MLLT1,TROVE2,CBFA2T2,IGF1,MLXIP,ATF7IP,LARS2,MAPK1,PTEN,BMP7,ATXN2,MXI1,PIK3C3,PUM1,SOX5,CIR1,PCBP2,ZNF780A,ZNF780B,LRRK2,ATP6V1A,MLYCD,TMEM59,PIGS,ZBTB20,RFC3,CHAF1B,LILRB4,SLC44A1,PKD2,ZNF652,HSF2BP,KCTD1,RIPPLY1,NT5E,RCOR3,TADA2A,DAZL,SPTLC3,NCOA3,PKIB,MTMR3,ZNF146,ZNF565,HS2ST1,WNT7A,ZBED6,BRWD3,LPGAT1,CRTC3,DNAJC1,ARNTL2,ELOVL5,KAT6A,MTIF2,ZKSCAN7,ZNF197,ZNF660,BMP6,TAF15,NFE2L1,GPI,WDR43,ZNF30,EXT1,DPY19L4,ATRX,DPH6,SERINC5,IKZF1,PNPLA3,PRMT2,RAPGEF2,ASCC1,MTHFD2L,SMO,FAR1,RALY,AMPD1,ALG9,DPY19L1,TCF4,GALNT10,SOX30,TFE3,GRIN1,JADE1,RHOA,SPRTN,MCM3,ROR1,RQCD1,TF,ONECUT2,CKMT1B,CUL3,TFEC,EPM2A,GSK3B,PLD2,PRR16,DNAJC3,STAT6,MLF1,EEF2K,RBBP8,NECAB2,CDK13,ALAS2,DUSP26,MTHFD1L,ZNF362,NACC2,SUPT4H1,ZNF354A,AGT,HDGFRP3,METTL16,ELOVL3,POLH,PRKAA2,POLR2F,PITPNM2,STAT2,ZMPSTE24,GTF2E1,PPCDC,PBX1,EXOSC3,MAP3K5,TCFL5,CREBBP,PRKD3,XYLB,ACSS3,DLX1,CHKA,GTF2IRD2,NCF1,SHC1,SLC44A3,ZNF813,ALDOA,LBX2,PARP10,RPS6KB1,ZDHHC23,DPRX,GALNT13,GUCY2F,PRG3,SETDB2,ALOX5,DNAJB6,SP7,LARP4,ADAM8,MRPS6,RPS6KA5,ZNF484,CDS1,ZNF93,PRDM2,NOS1,PEX7,ZNF44,MED15,RGS14,ACTN4,MC1R,TCF25,ZNF282,GATC,SLC1A1,EDRF1,TMEFF2,IL18,TEX12,DIS3,PPARA,ABCD3,ARID5B,SLC25A33,SMARCAL1,TAF2,CPEB4,LDHC,NOTCH4,ZMYND8,ZNF366,CRX,PAX6,PRKCZ,ADORA2A,RBMS1,PAH,CPEB1,OTUD7B,KIN,NIPBL,YEATS4,PHF20,UPB1,ZNF143,ANGPT1,BRF1,TBL1X,FHL2,MBTD1,ATF6,ZBTB5,ZNF708,IGF2BP3,NT5M,GCNT2,ETS1,PPP1CA,MTF2,TAGLN3,CDK5RAP1,NCOR2,PRKCQ,CSRNP1,HDAC2,HTR2C,IMPACT,PHKG2,SRCAP,POU3F3,NOTO,ZKSCAN5,ELF2,MOXD1,PHIP,PPP2CA,SLC26A2,MRPS16,RPL23,RFC5,ACAN,ATXN1,SP100,ZNF347,ZNF415,REV3L,TRIM29,PARN,PIGU,DONSON,PITPNM3,FARSB,WIPI2,ADCY1,ALG2,SQLE,SYK,CNOT1,MOGAT2,WWTR1,CYP39A1,GTF2H5,NFXL1,SLC44A5,ZBTB38,BRCA2,ALDH8A1,ATF7,DVL2,MORC1,MTRF1,YBX1,TAF1,EIF4E3,TICAM1,THAP3,GALNT7,SNAPC3,A3GALT2,HSF1,MAX,SAP130,DHFR,EZH1,ETF1,NRF1,SRPK2,NCOA2,CEPT1,WDR18,EEFSEC,QRICH1,ZP3,CHD6,RNF2,ZNF554,UGGT1,GALNT4,MYEF2,POC1B-GALNT4,INPP5F,TBP,ORC4,TRIM37,MECR,MET,ZNF461,CAMK2D,MRPL1,TIMELESS,BRD9,MALT1,SETD3,ZDHHC11,ZDHHC11B,KDM2B,MLXIPL,CCT3,PHB,EDA,ZNF555,CTCF,EXD2,NR6A1,ADCY10,ATF6B,CREB1,LIG1,RGMB,TRAP1,EBF4,SLC25A13,ZNF511,BMP4,ABLIM3
GO:0031589	cell-substrate adhesion	2.2432745720921826e-8	MAP4K4,SLC9A1,CLASP2,DOCK1,UTRN,ATRNL1,FER,CASK,ITGB6,ITGB1,TRIOBP,PTPRK,EPHA1,PTPRO,MEGF9,PDGFB,STRC,ATRN,ARHGAP6,LAMC1,DMD,CASS4,ADAMTS9,NF1,APOD,BCR,EGFLAM,NTNG1,CDH13,RCC2,JAK2,SKAP1,MKLN1,BCAS3,CORO1C,DISC1,KANK1,SMAD6,PARVA,DUSP22,OTOA,HSD17B12,CTNNB1,ECM2,AJUBA,SORBS1,NF2,SFRP1,SMAD3,PREX1,HOXD3,SLK,DEFB118,ADAMTS12,COL16A1,PPM1F,NEDD9,PEAK1,ITGA11,SPOCK1,EPHB3,VCL,LAMB1,P4HB,DAB2,PKHD1,USH2A,MACF1,CLASP1,GPM6B,CDK6,ROCK1,NID1,EPHB1,VWF,PTPRA,COL13A1,RASA1,PTEN,EFNA5,TBCD,EPDR1,PARVB,RHOA,ONECUT2,GSK3B,ITGBL1,PTK2,TAOK2,ITGAL,CD44,ACTN4,STK4,TMEFF2,DLC1,PRKCZ,CD96,PHLDB2,THSD1,ANGPT1,GCNT2,VWC2,BRAF,CAMSAP3,FAT2,SRGAP2,RADIL,PTPRJ,AXL,EDA,TYRO3,TNXB
GO:0051240	positive regulation of multicellular organismal process	2.2714773880982437e-8	PRDX2,NRXN1,PRKCI,PDE4D,HLX,SLC9A1,CBFB,SEMA3A,GRID2,SETD4,NOX5,PTH2R,TIAM2,KSR2,PTGIS,TJP1,PRLR,KALRN,ARNT,SEMA5A,STAT5B,TOX,GRM5,KITLG,LRP2,PIK3CD,OLFM1,EZR,ROBO1,CHD7,HTR2B,CDKL5,TRAF6,ROBO2,ITPKB,ITGB1,HSP90AA1,DSCAM,RUNX1,OMA1,ABCC8,PTPN11,RBM19,GOLGA4,EPHA1,ERBB4,ADRA1D,LIMK1,ATP8A2,CDON,NTRK2,CELA1,PDGFB,RIMS1,TOB2,H2AFY2,TCF7L2,CDH4,ESRRB,PTPRD,BASP1,BDNF,TFAP2A,AMIGO1,CHRM3,TENM4,VAMP7,RFTN1,SLC8A1,PLCB1,RTN4,CHI3L1,NTN1,NRG1,MAP2K1,STIM1,CMKLR1,ROR2,SOX13,MAD2L2,TLE6,CAPRIN2,ANO6,CCBE1,JAK2,CLSTN2,ITCH,PLS1,WNT11,IL1RAPL1,CAMK2B,SOX6,ACVR2A,RUNX2,CD4,TGFB1,PIBF1,F2RL1,SLIT2,TP73,DISC1,MTDH,NELL1,BMPER,BCL3,ADAM12,LINGO2,FGF10,LUM,SMURF2,EPHA4,RORA,PRKCA,CNR1,CD6,TNFSF11,PPP3CA,MAG,UFL1,CTNNB1,IGF1R,PPARG,DLG5,IL18R1,IL1RL1,CYBB,SCAMP5,RYR2,LEPR,FGF1,NIN,NR4A3,DCT,MYOCD,PER2,KIR2DL4,SCN3B,PRDM16,DIO2,FLT4,HDAC4,PAX2,SPTBN1,FOXO3,SMAD3,CUX2,ESRRG,APP,GSX2,NOX1,YAP1,HEG1,NLGN2,ALPL,JAK1,ANGPTL4,PEMT,POLR3G,ISLR2,SLC9B2,SETD2,SPTBN4,VASH2,CHUK,FRS2,PRKD1,STAT1,PARP6,SLC6A3,EREG,ATF2,POU2F2,CASP1,BMPR2,BMPR1A,NLGN1,RAB11A,MYB,FGF2,SEMA4D,PLXNA2,PTAFR,DDX58,SPEN,EHMT1,EPHB3,EBF2,KCNQ1,WNT3,ZNF322,NLGN3,SCUBE2,PAFAH1B1,DDAH1,AKAP6,TRPC5,GLI2,RUFY3,DAB2,RELN,PELI1,MAP3K7,SP1,MACF1,INPP5D,TEAD4,GPM6B,OVOL2,SNX5,PAK3,ADNP,MAPKAPK2,MAP1B,CELF1,TFRC,MEF2A,PTGER3,TRPV1,GHR,FLR

			T2,ACTR2,HDAC1,SMOC2,ADIPOR2,PLCG2,ROCK1,C1QTNF1,PAXIP1,EGR2,RNF10,AP3B1,NTRK1,KPNA6,EPHB1,AXIN2,PARD3,AP3D1,CD84,CUX1,CHRNB4,MAP3K13,RAPGEF3,MST1,BMPR1B,USP50,HEY2,HIPK1,BTK,IGF1,BMP7,SOX5,LRRK2,ZBTB20,LILRB4,EFNA5,SHANK3,PDGFC,NCOA3,PPP2R3C,WNT7A,NLRP1,GPR21,BMP6,TSHR,SMO,DECR1,RHOA,HSPD1,NFKBID,STAT6,EEF2K,FAM49B,CD27,SULF1,TNFRSF8,AGT,ELOVL3,FBN2,PDE4B,RIMS2,EXOSC3,CD160,RPS6KB1,PRG3,ALOX5,ADAM8,CXCL17,RGS14,EPHB2,SLC1A1,IL18,HCAR2,GPSM3,PAX6,PRKCZ,ZC3HAV1,FAM20C,ADORA2A,PPP1CC,NIPBL,TMIGD2,GCNT2,ETS1,BTN3A2,FBXO31,PRKCQ,BRAF,HDAC2,TNR,CDKL3,ANGPT4,ANXA2,NUMB,SYK,WWTR1,PTPRJ,TICAM1,CADM1,HSF1,SASH1,GBP5,BMP1,ZP3,AXL,TNFSF9,CAMK2D,CCR3,MALT1,KDM2B,PHB,PTPRC,SPON2,SYNDIG1,CREB1,CLNK,PRAP1,BMP4
GO:0009058	biosynthetic process	2.37529819786792e-8	*POLDIP3,ZDHHC14,ENPP1,PRDX2,LDB2,GPC5,ASPH,B4GALNT2,PRMT3,GSS,PRKCI,SLCO3A1,DNMT1,HLX,SLC9A1,PBX3,ADCY8,MGAT4C,MED13L,TRPS1,CBFB,PDE8A,ZNF823,IL31RA,PRDM12,GK5,MED26,WWC1,ASH1L,NOS1AP,SCAF8,STOX2,PTGIS,PRKAG2,WWC3,PRLR,PAGR1,ADCY7,HIVEP3,FTO,NPAS3,ARNT,LRRFIP2,STAT5B,TOX,GRM5,NOC3L,PLCE1,SAMHD1,ZNF566,FER,CASK,MAP2K5,MAPK10,SPNS2,PIK3CD,ZNF536,SP3,HDGF,GTF3C1,EIF4G3,EZR,IKZF2,B3GALT1,MALRD1,CHD7,HTR2B,MECOM,TACC1,TENM1,NADK2,GOT1,NMT2,INPP4B,TRAF6,GTF2E2,ITPKB,SCD5,DNAJA3,NHLH1,HSP90AA1,CDC6,PSMB7,TSC22D3,ZC4H2,CCDC22,MOCOS,FAM126A,CDAN1,GMDS,GFI1B,PTPRK,RUNX1,ERC1,HMGN3,NRIP1,THRB,EFCAB7,ITGB3BP,DACH1,ZNF569,MGAT5,CCT2,ORC2,HDAC6,SERTAD2,DDB1,MYT1,ADAMTS3,IKBKB,PEX14,ERBB4,GBE1,DPY19L2,MRE11A,GBF1,ZNF609,BRD8,KAT6B,HIF3A,SNIP1,LARGE,ESR1,MIER1,PCBP3,MAML3,CDON,TNRC6A,EP300,CELA1,TENM2,ZNF76,RNF220,ZNF471,PDGFB,CCND3,GALNTL6,CSGALNACT1,TOB2,ZNF19,ZNF23,BRMS1,ZNF605,ALOX5AP,SCML4,HNF4G,INSR,RERE,PRKAR1A,FUBP1,ATP8B1,H2AFY2,TCF7L2,PIP4K2A,ST6GALNAC3,JMJD1C,ZNF443,ZNF490,ZNF564,ZNF709,ZNF799,LITAF,FBXW11,ESRRB,MAP3K4,ZDHHC13,BASP1,SPTSSA,TBR1,PPIP5K1,SAMD4A,TFAP2A,CDK14,FANCA,PEG3,ZIM2,TGIF2,TGIF2-C20ORF24,POMGNT2,FUT8,MEIS1,VRTN,TRIM13,MDM4,ENTPD5,ZNF148,MTA3,SNX6,TFDP2,ACOX1,PPM1L,PTCD3,ERN2,CDC45,C1D,AFF3,DMD,CENPF,ATG10,SLC30A9,CERS6,TOX3,PDS5A,LARP4B,USP13,KAT7,ZNF667,RBM14,RBM4,PRPS2,MED12L,SATB2,RIT2,HIRA,DRG2,FYN,MKRN2,ARNTL,LINC01138,PLCB1,ARID4B,GXYLT2,POLA2,DGKH,HS3ST2,B4GALT6,RWDD3,BRPF1,PIK3R2,NRG1,UBP1,MAP2K1,MDFI,FNIP1,VAX2,DAGLA,GALNT16,TMBIM6,VGLL4,PPP2CB,PPP3R1,HDAC5,CSRNP3,ALDH3B2,ZNF692,NFIA,RNF4,GALNT18,JDP2,CMKLR1,INPP4A,PPIP5K2,ROR2,DCN,ZDHHC3,SLC39A14,PRPSAP2,PCBD2,CDH13,CREBRF,SOX13,ACSBG1,UST,COPS5,AK2,MAD2L2,MAN1A1,TLE6,RAB27A,SRBD1,CAPRIN2,ZNF418,NLN,PHF20L1,BBOX1,PDF,TERF2,ZNF286A,NME7,FOXN3,AK5,CERKL,NEUROD1,USP22,ZDHHC15,JAK2,TRAPPC9,FBXW7,OAZ2,SKAP1,SMC3,UIMC1,ITCH,MLIP,BCL11B,SMG1,IMPDH1,PKNOX1,B3GALT5,MLLT3,TSHZ2,PNPLA1,DHDDS,TCF7,PDE2A,KLF15,TBX15,ANXA4,TTC7B,WNT11,FIG4,MTA1,KLF8,NOX4,LCOR,RPRD1B,CACNA1H,QKI,CCDC62,PRKCD,SOX6,ACVR2A,RUNX2,CD4,TGFB1,BANP,MRPL33,SGK1,PCSK5,NSD1,IGSF1,PIBF1,ZHX2,PKNOX2,ASCC2,BTRC,NFATC3,POLK,F2RL1,BCAS3,CDYL2,HS6ST2,CC2D1B,TP73,HHAT,SAP18,CDKAL1,ZBTB22,ILF2,TMTC1,MTDH,FANK1,MYT1L,SMAD6,BNC2,ZNF398,TMTC2,CLOCK,CHST9,TCF12,ZNF675,RPL36,ETV6,SYNJ2,SUCO,PHRF1,TFAP2D,BCL3,EIF3L,SND1,DUSP22,HNRNPC,TRRAP,SBNO2,YTHDF1,FGF10,CIZ1,POLR2J2,ADK,SMYD3,LOXL3,ADCY2,CAPN3,LUM,SMURF2,RORA,AGPS,HIVEP2,HSD17B12,AUTS2,TNFSF11,SMG6,PPP3CA,NFYB,MAGEA4,KLF12,CAMK4,GATAD2B,PIP5KL1,UFL1,TRAK1,CTNNB1,PARK2,SOD2,DACH2,METTL13,SMARCC1,KLF17,IGF1R,PPARG,IARS2,NGRN,AXIN1,IL18R1,CIPC,MTF1,CBX5,ANKRD17,CYBB,OTC,BRIP1,LRPPRC,SREBF2,AARS2,PFAS,CDK11A,GALNT8,NDUFA9,CDK11B,LEPR,FGF1,NPAT,NR4A3,FOXK2,WDR45B,DCT,ESCO1,MYOCD,TRIM5,PER2,AJUBA,ZNF626,ZNF737,CHEK2,SUPT3H,PRDM16,PPP1CB,HCK,SORBS1,AGPAT4,RAB3GAP2,CAPN2,TRIM8,DIO2,BRMS1L,ZBTB8A,ZBTB8B,CTDP1,HS3ST5,BAZ1B,MEF2B,SGMS1,HDAC4,PAX2,PHF5A,CPOX,SECISBP2L,SNAPC4,SFRP1,MED13,ST8SIA1,ZNF395,FOXO3,DGKB,TPH2,NFIB,SP4,SYNCRIP,SMAD3,TYW1B,CUX2,WWP2,ARNT2,SBNO1,KRBOX1,ZNF662,CYP4F11,ZNF777,WIPI1,EBF3,RNF168,CASZ1,DCP1B,MIER3,NEDD4,PLA2G4C,ESRRG,HOXD3,HOXD4,ZNF114,GALNT14,KTI12,NFATC1,ST3GAL3,UCK2,CDC73,APP,SSBP3,GSX2,RBM8A,NOX1,YAP1,ST6GALNAC5,PPCS,TEN1,NVL,PEMT,LRP5,POLR3G,BDH2,ZNF787,SOX2,SETD2,ZDHHC6,GPC6,CHST11,TEAD1,PRICKLE1,ZNF653,ZNF521,ELOVL2,ARID3A,ZNF761,CHUK,ERLIN1,RFT1,SFMBT1,ZNF584,TPH1,ESR2,S100A12,GTF2F2,PRKD1,STAT1,HEXA,ST18,OSBPL2,DGKG,ETV5,RHOXF2B,SLC6A3,TAF3,PLAGL1,HNF4A,ZBTB7C,TASP1,EREG,ATF2,POU2F2,TCF3,ZNF730,RAF1,CELF4,ABLIM2,ZNF766,CARD16,POLA1,GGT7,BMPR2,CAMK1D,BMPR1A,ZKSCAN1,IDO2,PABPC4,IKZF4,PIK3R3,CDK12,TAF8,CAND2,CERS3,DENND4A,MYB,FGF2,ZNF71,POU6F2,BACH1,MXD3,PPM1F,TICRR,ADCY5,GLI4,ZFP41,BEND5,SEMA4D,NFX1,RORC,ELP3,PTAFR,ADCYAP1R1,SP140,SP140L,RHOXF2,JARID2,DDX58,SLC5A8,BRDT,PHC2,PI4KA,RARB,SPEN,PIK3C2B,SIN3B,NCOA1,EEF1E1,EHMT1,LMO7,DVL3,EIF2B5,EIF4G1,POLR2H,TCF20,ATF3,TCEANC,AKR1D1,STT3B,CKS1B,PAWR,EBF2,AGO3,MAML2,TSG101,CCDC3,TERF2IP,CRYM,RFX2,B3GALNT1,ZNF322,SUFU,TG,MAGEA11,PRDM15,ZNF670,Z*

			NF695,CCDC169-SOHLH2,SOHLH2,POLE,DDAH1,ZNF354C,TCEA3,PADI6,FDXR,CMPK1,ZNF704,CERS4,NR2C1,EDEM3,ME1,GLI2,TNKS,WBP2NL,ERCC1,TNRC6B,GLIS3,GLCE,WDTC1,ZNF664,MRPS28,MGLL,TPK1,DAB2,BLM,PKHD1,TRAM2,MYSM1,SETD5,SMG5,GTPBP2,RELN,SIN3A,RUVBL2,TYW1,COMMD6,GUCY1A2,GMEB1,PELI1,SLC25A16,MAP3K7,ZNF423,SP1,TRIM22,ALK,GSTM3,GSTM5,INPP5D,TEAD4,HOXC13,APBB3,SLC35A4,SRA1,TOP1,UBE2V1,MBOAT1,ADIRF,OVOL2,SNX5,NFATC2,RBBP6,PAX7,BNC1,ATP2B4,ACTL6B,ASXL3,PAK3,TET1,CAMTA1,CCPG1,ADNP,ARID4A,C12ORF65,SCARB1,CDK6,PHF2,CELF1,TYR,TFRC,EPHA5,WWOX,ALG14,MEF2A,ADCY9,DCAF6,GPC3,XRN1,SATB1,PSMB2,EYA1,GATAD2A,DIS3L2,TRPV1,HOXB3,HOXB4,HOXB5,HOXB6,TFEB,MNAT1,ACTR2,L3MBTL4,ACACA,ELAVL4,HDAC1,HS6ST3,RECQL5,SMOC2,TRIM24,PLCG2,ROCK1,SCMH1,MRPS17,PAXIP1,ZNF713,EGR2,HIPK3,RNF10,RYBP,AP3B1,PTPN2,NTRK1,KPNA6,TRIM44,EIF3A,MAN1B1,SARM1,EPCAM,ZNF425,GNL3L,KRTCAP2,MUC1,PTPN14,NUDT14,BCOR,AP3D1,ZNF41,MGAT4A,CARM1,ZNF383,SRD5A2,ZNF616,ZNF836,SMARCA2,CUX1,SPI1,DNMT3B,LRRC47,FOXP2,AGPAT3,MRPS24,PSPC1,GLIS1,SFMBT2,EIF3H,BCAR3,JAZF1,ABCB7,ACOT8,RBPMS,CHURC1,CREB5,MAP3K13,ERCC3,SMYD1,SNCA,BMPR1B,POLN,PRIM2,PNPT1,ZEB2,MCMBP,FOXJ3,NADK,OCA2,HEY2,RC3H1,EIF3E,HMGCS2,ZMYND11,KMT2D,PRKAG1,CREM,OSBPL1A,KMT2C,GGPS1,PLD1,MACC1,MITF,OPRD1,NUGGC,RPTOR,NFIX,BTK,KCTD13,MLLT1,TROVE2,CBFA2T2,IGF1,MLXIP,ATF7IP,LARS2,MAPK1,PTEN,BMP7,ATXN2,MXI1,PIK3C3,PUM1,SOX5,CIR1,PCBP2,ZNF780A,ZNF780B,LRRK2,ATP6V1A,MLYCD,TMEM59,PIGS,ZBTB20,RFC3,CHAF1B,LILRB4,SLC44A1,PKD2,ZNF652,HSF2BP,KCTD1,RIPPLY1,NT5E,RCOR3,TADA2A,DAZL,SPTLC3,NCOA3,PKIB,MTMR3,ZNF146,ZNF565,HS2ST1,WNT7A,ZBED6,BRWD3,LPGAT1,CRTC3,DNAJC1,ARNTL2,ELOVL5,KAT6A,MTIF2,ZKSCAN7,ZNF197,ZNF660,BMP6,TAF15,NFE2L1,GPI,WDR43,ZNF30,EXT1,DPY19L4,ATRX,DPH6,SERINC5,IKZF1,PNPLA3,PRMT2,RAPGEF2,ASCC1,MTHFD2L,SMO,FAR1,RALY,AMPD1,ALG9,DPY19L1,TCF4,GALNT10,SOX30,TFE3,GRIN1,JADE1,RHOA,SPRTN,MCM3,ROR1,RQCD1,TF,ONECUT2,CKMT1B,CUL3,TFEC,EPM2A,GSK3B,PLD2,PRR16,DNAJC3,STAT6,MLF1,EEF2K,RBBP8,NECAB2,CDK13,ALAS2,DUSP26,MTHFD1L,ZNF362,NACC2,SUPT4H1,ZNF354A,AGT,HDGFRP3,METTL16,ELOVL3,POLH,PRKAA2,POLR2F,PITPNM2,STAT2,ZMPSTE24,GTF2E1,PPCDC,PBX1,EXOSC3,MAP3K5,TCFL5,CREBBP,PRKD3,XYLB,ACSS3,DLX1,CHKA,GTF2IRD2,NCF1,SHC1,SLC44A3,ZNF813,ALDOA,LBX2,PARP10,RPS6KB1,STC2,ZDHHC23,DPRX,GALNT13,GUCY2F,PRG3,SETDB2,ALOX5,DNAJB6,SP7,LARP4,ADAM8,MRPS6,SLC5A3,RPS6KA5,ZNF484,CDS1,ZNF93,PRDM2,NOS1,PEX7,ZNF44,MED15,RGS14,ACTN4,MC1R,TCF25,ZNF282,GATC,SLC1A1,EDRF1,TMEFF2,IL18,TEX12,DIS3,PPARA,ABCD3,ARID5B,SLC25A33,SMARCAL1,TAF2,CPEB4,LDHC,NOTCH4,ZMYND8,ZNF366,CRX,PAX6,PRKCZ,ADORA2A,RBMS1,PAH,CPEB1,OTUD7B,KIN,NIPBL,YEATS4,PHF20,UPB1,ZNF143,ANGPT1,BRF1,TBL1X,FHL2,MBTD1,ATF6,ZBTB5,ZNF708,IGF2BP3,NT5M,GCNT2,ETS1,PPP1CA,MTF2,TAGLN3,CDK5RAP1,NCOR2,PRKCQ,CSRNP1,HDAC2,HTR2C,IMPACT,PHKG2,SRCAP,POU3F3,NOTO,ZKSCAN5,ELF2,MOXD1,PHIP,PPP2CA,SLC26A2,MRPS16,RPL23,RFC5,ACAN,ATXN1,SP100,ZNF347,ZNF415,REV3L,TRIM29,PARN,PIGU,DONSON,PITPNM3,FARSB,WIPI2,ADCY1,ALG2,SQLE,SYK,CNOT1,MOGAT2,WWTR1,CYP39A1,GTF2H5,NFXL1,SLC44A5,ZBTB38,BRCA2,ALDH8A1,ATF7,DVL2,MORC1,MTRF1,YBX1,TAF1,EIF4E3,TICAM1,THAP3,GALNT7,SNAPC3,A3GALT2,HSF1,MAX,SAP130,DHFR,EZH1,ETF1,NRF1,SRPK2,NCOA2,CEPT1,WDR18,EEFSEC,QRICH1,ZP3,CHD6,RNF2,ZNF554,UGGT1,GALNT4,MYEF2,POC1B-GALNT4,INPP5F,TBP,ORC4,TRIM37,MECR,MET,ZNF461,CAMK2D,MRPL1,TIMELESS,BRD9,MALT1,SETD3,ZDHHC11,ZDHHC11B,KDM2B,MLXIPL,CCT3,PHB,EDA,ZNF555,CTCF,EXD2,PBLD,NR6A1,ADCY10,ATF6B,CREB1,LIG1,RGMB,TRAP1,EBF4,SLC25A13,ZNF511,BMP4,ABLIM3
GO:0051640	organelle localization	2.4325647856291906e-8	*NRXN1,CLASP2,KIF22,LTV1,CBL,FER,PIK3CD,EZR,KIF5C,LMNA,ITGB1,CNIH2,PCDH17,KLHL12,DAB1,TCIRG1,HDAC6,PEX14,GBF1,HOOK3,MSTO1,FMN2,FBXW11,VAMP7,AP3S1,DMD,CENPF,TANC2,STX6,NDRG4,NTN1,DCTN1,SLC4A5,MYH9,MAD1L1,PARD3B,SCFD2,LIN7A,SYN2,COPS5,EXOC4,TLE6,RAB27A,RAB3C,SYN3,SYT1,CROCC,DYNC1I1,ATP9A,CENPC,PIBF1,ARMC1,C12ORF4,CLMN,ANKFN1,FGF10,VMP1,TRAK1,CTNNB1,PARK2,TSNARE1,MYO1D,LRPPRC,NR4A3,RIOK2,MYO1E,AP3B2,SPG11,RAB6A,WIPI1,KPNB1,MYO1F,NLGN2,SYNE2,SYBU,VPS4A,NLGN1,SPAG9,RAB11A,AGBL4,BLOC1S5,TSG101,PAFAH1B1,KIF3A,PKHD1,EML4,FAM91A1,MYO7A,CLASP1,NSF,SYNE3,MAP1B,MARK1,UVRAG,MEF2A,TFEB,ACTR2,AP3B1,UNC13A,PARD3,TRIM46,AP3D1,KIF3C,CD84,ATG14,RAB15,SNCA,BTK,STX8,HTT,PTEN,IFFO1,NUP88,LRRK2,SPAG5,CHMP5,SNAP23,MAP4,RHOT1,SUN1,LSG1,BLOC1S3,PSEN2,NDE1,ESYT2,CUL3,SYN1,BLOC1S6,LAT2,EXOC6B,COPG2,SEC16B,CPLX2,ARFGAP3,TBC1D23,ACTN4,GPSM2,SYTL4,PAX6,PRKCZ,RABGEF1,PACS2,ARHGAP21,MAP2,CD300A,RAB27B,SYK,SLIT1,MYO1A,PLEKHM1,MLPH,CAV2,SYNDIG1,CLNK*
GO:0044249	cellular biosynthetic process	2.517822626543307e-8	*POLDIP3,ZDHHC14,ENPP1,PRDX2,LDB2,ASPH,B4GALNT2,PRMT3,GSS,PRKCI,SLCO3A1,DNMT1,HLX,SLC9A1,PBX3,ADCY8,MGAT4C,MED13L,TRPS1,CBFB,PDE8A,ZNF823,IL31RA,PRDM12,MED26,WWC1,ASH1L,NOS1AP,SCAF8,STOX2,PTGIS,PRKAG2,WWC3,PAGR1,ADCY7,HIVEP3,FTO,NPAS3,ARNT,LRRFIP2,STAT5B,TOX,GRM5,NOC3L,PLCE1,SAMHD1,ZNF566,FER,CASK,MAP2K5,MAPK10,SPNS2,PIK3*

			CD,ZNF536,SP3,HDGF,GTF3C1,EIF4G3,EZR,IKZF2,B3GALT1,MALRD1,CHD7,HTR2B,MECOM,TACC1,TENM1,NADK2,GOT1,NMT2,INPP4B,TRAF6,GTF2E2,ITPKB,SCD5,DNAJA3,NHLH1,HSP90AA1,CDC6,PSMB7,TSC22D3,ZC4H2,CCDC22,MOCOS,FAM126A,CDAN1,GMDS,GFI1B,PTPRK,RUNX1,ERC1,HMGN3,NRIP1,THRB,EFCAB7,ITGB3BP,DACH1,ZNF569,MGAT5,CCT2,ORC2,HDAC6,SERTAD2,DDB1,MYT1,IKBKB,PEX14,ERBB4,GBE1,DPY19L2,MRE11A,ZNF609,BRD8,KAT6B,HIF3A,SNIP1,LARGE,ESR1,MIER1,PCBP3,MAML3,CDON,TNRC6A,EP300,CELA1,TENM2,ZNF76,RNF220,ZNF471,PDGFB,CCND3,GALNTL6,CSGALNACT1,TOB2,ZNF19,ZNF23,BRMS1,ZNF605,ALOX5AP,SCML4,HNF4G,INSR,RERE,PRKAR1A,FUBP1,ATP8B1,H2AFY2,TCF7L2,PIP4K2A,ST6GALNAC3,JMJD1C,ZNF443,ZNF490,ZNF564,ZNF709,ZNF799,LITAF,FBXW11,ESRRB,MAP3K4,ZDHHC13,BASP1,SPTSSA,TBR1,SAMD4A,TFAP2A,CDK14,FANCA,PEG3,ZIM2,TGIF2,TGIF2-C20ORF24,POMGNT2,FUT8,MEIS1,VRTN,TRIM13,MDM4,ENTPD5,ZNF148,MTA3,SNX6,TFDP2,ACOX1,PPM1L,PTCD3,ERN2,CDC45,C1D,AFF3,DMD,CENPF,ATG10,SLC30A9,CERS6,TOX3,PDS5A,LARP4B,USP13,KAT7,ZNF667,RBM14,RBM4,PRPS2,MED12L,SATB2,RIT2,HIRA,DRG2,FYN,MKRN2,ARNTL,LINC01138,PLCB1,ARID4B,GXYLT2,POLA2,DGKH,B4GALT6,RWDD3,BRPF1,PIK3R2,NRG1,UBP1,MAP2K1,MDFI,FNIP1,VAX2,DAGLA,GALNT16,TMBIM6,VGLL4,PPP2CB,PPP3R1,HDAC5,CSRNP3,ALDH3B2,ZNF692,NFIA,RNF4,GALNT18,JDP2,CMKLR1,INPP4A,ROR2,DCN,ZDHHC3,PRPSAP2,PCBD2,CDH13,CREBRF,SOX13,ACSBG1,UST,COPS5,AK2,MAD2L2,MAN1A1,TLE6,SRBD1,CAPRIN2,ZNF418,PHF20L1,BBOX1,PDF,TERF2,ZNF286A,NME7,FOXN3,AK5,CERKL,NEUROD1,USP22,ZDHHC15,JAK2,TRAPPC9,FBXW7,OAZ2,SKAP1,SMC3,UIMC1,ITCH,MLIP,BCL11B,SMG1,IMPDH1,PKNOX1,B3GALT5,MLLT3,TSHZ2,PNPLA1,DHDDS,TCF7,PDE2A,KLF15,TBX15,ANXA4,TTC7B,WNT11,FIG4,MTA1,KLF8,NOX4,LCOR,RPRD1B,CACNA1H,QKI,CCDC62,PRKCD,SOX6,ACVR2A,RUNX2,CD4,TGFB1,BANP,MRPL33,SGK1,PCSK5,NSD1,IGSF1,PIBF1,ZHX2,PKNOX2,ASCC2,BTRC,NFATC3,POLK,F2RL1,BCAS3,CDYL2,HS6ST2,CC2D1B,TP73,HHAT,SAP18,CDKAL1,ZBTB22,ILF2,TMTC1,MTDH,FANK1,MYT1L,SMAD6,BNC2,ZNF398,TMTC2,CLOCK,CHST9,TCF12,ZNF675,RPL36,ETV6,SYNJ2,PHRF1,TFAP2D,BCL3,EIF3L,SND1,DUSP22,HNRNPC,TRRAP,SBNO2,YTHDF1,FGF10,CIZ1,POLR2J2,ADK,SMYD3,LOXL3,ADCY2,CAPN3,LUM,SMURF2,RORA,AGPS,HIVEP2,HSD17B12,AUTS2,TNFSF11,SMG6,PPP3CA,NFYB,MAGEA4,KLF12,CAMK4,GATAD2B,PIP5KL1,UFL1,TRAK1,CTNNB1,PARK2,SOD2,DACH2,METTL13,SMARCC1,KLF17,IGF1R,PPARG,IARS2,NGRN,AXIN1,IL18R1,CIPC,MTF1,CBX5,ANKRD17,CYBB,OTC,BRIP1,LRPPRC,SREBF2,AARS2,PFAS,CDK11A,GALNT8,NDUFA9,CDK11B,LEPR,FGF1,NPAT,NR4A3,FOXK2,WDR45B,DCT,ESCO1,MYOCD,TRIM5,PER2,AJUBA,ZNF626,ZNF737,CHEK2,SUPT3H,PRDM16,PPP1CB,HCK,SORBS1,AGPAT4,RAB3GAP2,CAPN2,TRIM8,DIO2,BRMS1L,ZBTB8A,ZBTB8B,CTDP1,HS3ST5,BAZ1B,MEF2B,SGMS1,HDAC4,PAX2,PHF5A,CPOX,SECISBP2L,SNAPC4,SFRP1,MED13,ST8SIA1,ZNF395,FOXO3,DGKB,TPH2,NFIB,SP4,SYNCRIP,SMAD3,CUX2,WWP2,ARNT2,SBNO1,KRBOX1,ZNF662,CYP4F11,ZNF777,WIPI1,EBF3,RNF168,CASZ1,DCP1B,MIER3,NEDD4,PLA2G4C,ESRRG,HOXD3,HOXD4,ZNF114,GALNT14,KTI12,NFATC1,ST3GAL3,UCK2,CDC73,APP,SSBP3,GSX2,RBM8A,YAP1,ST6GALNAC5,PPCS,TEN1,NVL,PEMT,LRP5,POLR3G,BDH2,ZNF787,SOX2,SETD2,ZDHHC6,CHST11,TEAD1,PRICKLE1,ZNF653,ZNF521,ELOVL2,ARID3A,ZNF761,CHUK,ERLIN1,RFT1,SFMBT1,ZNF584,TPH1,ESR2,S100A12,GTF2F2,PRKD1,STAT1,ST18,OSBPL2,DGKG,ETV5,RHOXF2B,SLC6A3,TAF3,PLAGL1,HNF4A,ZBTB7C,TASP1,EREG,ATF2,POU2F2,TCF3,ZNF730,RAF1,CELF4,ABLIM2,ZNF766,CARD16,POLA1,GGT7,BMPR2,CAMK1D,BMPR1A,ZKSCAN1,IDO2,PABPC4,IKZF4,PIK3R3,CDK12,TAF8,CAND2,CERS3,DENND4A,MYB,FGF2,ZNF71,POU6F2,BACH1,MXD3,PPM1F,TICRR,ADCY5,GLI4,ZFP41,BEND5,SEMA4D,NFX1,RORC,ELP3,PTAFR,ADCYAP1R1,SP140,SP140L,RHOXF2,JARID2,DDX58,SLC5A8,BRDT,PHC2,PI4KA,RARB,SPEN,PIK3C2B,SIN3B,NCOA1,EEF1E1,EHMT1,LMO7,DVL3,EIF2B5,EIF4G1,POLR2H,TCF20,ATF3,TCEANC,AKR1D1,STT3B,CKS1B,PAWR,EBF2,AGO3,MAML2,TSG101,TERF2IP,CRYM,RFX2,B3GALNT1,ZNF322,SUFU,MAGEA11,PRDM15,ZNF670,ZNF695,CCDC169-SOHLH2,SOHLH2,POLE,DDAH1,ZNF354C,TCEA3,PADI6,FDXR,CMPK1,ZNF704,CERS4,NR2C1,EDEM3,ME1,GLI2,TNKS,WBP2NL,ERCC1,TNRC6B,GLIS3,GLCE,WDTC1,ZNF664,MRPS28,MGLL,TPK1,DAB2,BLM,PKHD1,MYSM1,SETD5,SMG5,GTPBP2,RELN,SIN3A,RUVBL2,COMMD6,GUCY1A2,GMEB1,PELI1,SLC25A16,MAP3K7,ZNF423,SP1,TRIM22,ALK,GSTM3,GSTM5,INPP5D,TEAD4,HOXC13,APBB3,SLC35A4,SRA1,TOP1,UBE2V1,MBOAT1,ADIRF,OVOL2,SNX5,NFATC2,RBBP6,PAX7,BNC1,ATP2B4,ACTL6B,ASXL3,PAK3,TET1,CAMTA1,CCPG1,ADNP,ARID4A,C12ORF65,SCARB1,CDK6,PHF2,CELF1,TYR,TFRC,EPHA5,WWOX,ALG14,MEF2A,ADCY9,DCAF6,XRN1,SATB1,PSMB2,EYA1,GATAD2A,DIS3L2,TRPV1,HOXB3,HOXB4,HOXB5,HOXB6,TFEB,MNAT1,ACTR2,L3MBTL4,ACACA,ELAVL4,HDAC1,HS6ST3,RECQL5,SMOC2,TRIM24,PLCG2,ROCK1,SCMH1,MRPS17,PAXIP1,ZNF713,EGR2,HIPK3,RNF10,RYBP,AP3B1,PTPN2,NTRK1,KPNA6,TRIM44,EIF3A,MAN1B1,SARM1,EPCAM,ZNF425,GNL3L,KRTCAP2,MUC1,PTPN14,NUDT14,BCOR,AP3D1,ZNF41,MGAT4A,CARM1,ZNF383,SRD5A2,ZNF616,ZNF836,SMARCA2,CUX1,SPI1,DNMT3B,LRRC47,FOXP2,AGPAT3,MRPS24,PSPC1,GLIS1,SFMBT2,EIF3H,BCAR3,JAZF1,ABCB7,ACOT8,RBPMS,CHURC1,CREB5,MAP3K13,ERCC3,SMYD1,SNCA,BMPR1B,POLN,PRIM2,PNPT1,ZEB2,MCMBP,FOXJ3,NADK,OCA2,HEY2,RC3H1,EIF3E,HMGCS2,Z

			MYND11,KMT2D,PRKAG1,CREM,OSBPL1A,KMT2C,GGPS1,PLD1,MACC1,MITF,OPRD1,NUGGC,RPTOR,NFIX,BTK,KCTD13,MLLT1,TROVE2,CBFA2T2,IGF1,MLXIP,ATF7IP,LARS2,MAPK1,PTEN,BMP7,ATXN2,MXI1,PIK3C3,PUM1,SOX5,CIR1,PCBP2,ZNF780A,ZNF780B,ATP6V1A,MLYCD,TMEM59,PIGS,ZBTB20,RFC3,CHAF1B,LILRB4,SLC44A1,PKD2,ZNF652,HSF2BP,KCTD1,RIPPLY1,NT5E,RCOR3,TADA2A,DAZL,SPTLC3,NCOA3,PKIB,MTMR3,ZNF146,ZNF565,HS2ST1,WNT7A,ZBED6,BRWD3,LPGAT1,CRTC3,DNAJC1,ARNTL2,ELOVL5,KAT6A,MTIF2,ZKSCAN7,ZNF197,ZNF660,BMP6,TAF15,NFE2L1,WDR43,ZNF30,EXT1,DPY19L4,ATRX,DPH6,SERINC5,IKZF1,PNPLA3,PRMT2,RAPGEF2,ASCC1,MTHFD2L,SMO,FAR1,RALY,AMPD1,ALG9,DPY19L1,TCF4,GALNT10,SOX30,TFE3,GRIN1,JADE1,RHOA,SPRTN,MCM3,ROR1,RQCD1,TF,ONECUT2,CUL3,TFEC,EPM2A,GSK3B,PLD2,PRR16,DNAJC3,STAT6,MLF1,EEF2K,RBBP8,NECAB2,CDK13,ALAS2,DUSP26,MTHFD1L,ZNF362,NACC2,SUPT4H1,ZNF354A,AGT,HDGFRP3,METTL16,ELOVL3,POLH,PRKAA2,POLR2F,PITPNM2,STAT2,ZMPSTE24,GTF2E1,PPCDC,PBX1,EXOSC3,MAP3K5,TCFL5,CREBBP,PRKD3,ACSS3,DLX1,CHKA,GTF2IRD2,NCF1,SHC1,SLC44A3,ZNF813,ALDOA,LBX2,PARP10,RPS6KB1,STC2,ZDHHC23,DPRX,GALNT13,GUCY2F,PRG3,SETDB2,ALOX5,DNAJB6,SP7,LARP4,ADAM8,MRPS6,RPS6KA5,ZNF484,CDS1,ZNF93,PRDM2,NOS1,PEX7,ZNF44,MED15,RGS14,ACTN4,MC1R,TCF25,ZNF282,GATC,SLC1A1,EDRF1,IL18,TEX12,DIS3,PPARA,ABCD3,ARID5B,SLC25A33,SMARCAL1,TAF2,CPEB4,LDHC,NOTCH4,ZMYND8,ZNF366,CRX,PAX6,PRKCZ,ADORA2A,RBMS1,PAH,CPEB1,OTUD7B,KIN,NIPBL,YEATS4,PHF20,UPB1,ZNF143,ANGPT1,BRF1,TBL1X,FHL2,MBTD1,ATF6,ZBTB5,ZNF708,IGF2BP3,NT5M,GCNT2,ETS1,PPP1CA,MTF2,TAGLN3,CDK5RAP1,NCOR2,PRKCQ,CSRNP1,HDAC2,HTR2C,IMPACT,PHKG2,SRCAP,POU3F3,NOTO,ZKSCAN5,ELF2,MOXD1,PHIP,PPP2CA,SLC26A2,MRPS16,RPL23,RFC5,ACAN,ATXN1,SP100,ZNF347,ZNF415,REV3L,TRIM29,PARN,PIGU,DONSON,PITPNM3,FARSB,WIPI2,ADCY1,ALG2,SYK,CNOT1,MOGAT2,WWTR1,CYP39A1,GTF2H5,NFXL1,SLC44A5,ZBTB38,BRCA2,ALDH8A1,ATF7,DVL2,MORC1,MTRF1,YBX1,TAF1,EIF4E3,TICAM1,THAP3,GALNT7,SNAPC3,A3GALT2,HSF1,MAX,SAP130,DHFR,EZH1,ETF1,NRF1,SRPK2,NCOA2,CEPT1,WDR18,EEFSEC,QRICH1,ZP3,CHD6,RNF2,ZNF554,UGGT1,GALNT4,MYEF2,POC1B-GALNT4,INPP5F,TBP,ORC4,TRIM37,MECR,MET,ZNF461,CAMK2D,MRPL1,TIMELESS,BRD9,MALT1,SETD3,ZDHHC11,ZDHHC11B,KDM2B,MLXIPL,CCT3,PHB,EDA,ZNF555,CTCF,EXD2,NR6A1,ADCY10,ATF6B,CREB1,LIG1,RGMB,TRAP1,EBF4,SLC25A13,ZNF511,BMP4,ABLIM3
GO:0031324	negative regulation of cellular metabolic process	3.043482512244192e-8	ENPP1,LDB2,PRMT3,PDE4D,DNMT1,TRPS1,CBFB,PRDM12,WWC1,LATS2,SCAF8,PTGIS,PRKAG2,WWC3,MVP,FTO,NTRK3,CBL,ZNF566,MAP2K5,ZNF536,SP3,HDGF,EZR,MALRD1,HTR2B,MECOM,TRAF6,DNAJA3,OXR1,CDC6,CDAN1,GFI1B,PTPRK,RUNX1,NRIP1,THRB,DACH1,MGAT5,ORC2,HDAC6,EPHA1,IKBKB,PEX14,LIMK1,KAT6B,ESR1,MIER1,PCBP3,PMEPA1,PTPRO,NTRK2,TNRC6A,EP300,CELA1,TENM2,PDGFB,CCND3,TOB2,ZNF19,BRMS1,SCML4,RERE,PRKAR1A,ATP8B1,H2AFY2,TCF7L2,PIP4K2A,FBXW11,BASP1,TBR1,SAMD4A,TFAP2A,PEG3,ZIM2,TGIF2,MDM4,ZNF148,MTA3,SNX6,TFDP2,CST2,ERN2,C1D,DMD,CENPF,PDS5A,LARP4B,KAT7,SLC8A1,RBM4,SATB2,HIRA,FYN,ARNTL,NF1,PLCB1,RTN4,DPEP1,APOD,BCR,PAQR3,NRG1,BDKRB1,BDKRB2,FRY,MDFI,FNIP1,VAX2,TMBIM6,VGLL4,HDAC5,ZNF692,JDP2,ITIH2,SCFD1,CREBRF,TRDMT1,SOX13,FHIT,PTPRT,AUNIP,IPO5,MAD2L2,TLE6,CAPRIN2,TERF2,SLX1B,FOXN3,GNAQ,RFFL,FBXW7,UIMC1,MLIP,ZFYVE28,LZTS1,SMG1,TCF7,PDE2A,KLF15,TBX15,WNT11,MTA1,KLF8,LCOR,PRKCD,SOX6,TAB2,RUNX2,PPM1E,TGFB1,NSD1,PIBF1,ZHX2,BTRC,NFATC3,CDYL2,DNAJB2,CC2D1B,SLIT2,ITIH4,HP,CORO1C,SAP18,MTDH,MYT1L,KDM4B,SMAD6,ZNF398,CLOCK,ZNF675,ETV6,NELL1,TIMP2,BCL3,DUSP22,NAIP,HNRNPC,SBNO2,YTHDF1,SMYD3,LOXL3,CAPN3,SMURF2,EPHA4,CNR1,SMG6,PPP3CA,NSUN2,MAGEA4,KLF12,GATAD2B,PIP5KL1,UFL1,CTNNB1,PARK2,METTL13,SMARCC1,PPARG,AXIN1,PRKAR1B,OTUB1,CIPC,CBX5,LRPPRC,SREBF2,LEPR,PROS1,NPAT,NR4A3,FOXK2,NOL3,PRKAR2A,MYOCD,PER2,AJUBA,GLG1,UBQLN4,PRDM16,CSTL1,TBC1D14,BRMS1L,NF2,HDAC4,PAX2,SFRP1,FOXO3,NFIB,ZCCHC17,SYNCRIP,SMAD3,CUX2,WWP2,SIMC1,RNF168,DCP1B,MIER3,NEDD4,CDC73,APP,YAP1,HEG1,TEN1,IBTK,LRP5,SOX2,PRICKLE1,ZNF653,ERLIN1,SFMBT1,ESR2,DCUN1D3,STAT1,ETV5,TAF3,PLAGL1,HNF4A,EREG,ATF2,TCF3,RAF1,CELF4,CARD16,PTPN1,BMPR2,IKZF4,SPAG9,MYB,BACH1,MXD3,PPM1F,BEND5,SEMA4D,NFX1,RORC,JARID2,PHC2,RARB,SPEN,PRKCG,SIN3B,SPOCK1,EHMT1,EIF4G1,ATF3,LIN28B,PAWR,AGO3,DEPTOR,TSG101,TERF2IP,CRYM,SUFU,MAGEA11,PTPN13,COL4A3,NR2C1,GLI2,TNKS,ERCC1,TNRC6B,GLIS3,WDTC1,DAB2,BLM,LDLRAD4,SIN3A,RUVBL2,IQGAP1,ZNF423,TRIM22,XDH,OVOL2,NFATC2,ATP2B4,MAPKAPK2,ARID4A,CDK6,CELF1,RNF34,MEF2A,GPC3,XRN1,SATB1,GATAD2A,DIS3L2,TRPV1,HOXB3,HOXB4,CLEC16A,L3MBTL4,ELAVL4,HDAC1,RECQL5,TRIM24,ROCK1,SCMH1,HIPK3,RYBP,PTPN2,TRIM44,MAP1A,AXIN2,PARD3,ZNF425,GNL3L,MUC1,BCOR,ANXA8L1,LPA,SMARCA2,CUX1,SPI1,DNMT3B,FOXP2,PSPC1,GLIS1,SFMBT2,ATG14,EIF3H,JAZF1,STK38,ABCB7,ACOT8,RENBP,SMYD1,SNCA,PNPT1,ZEB2,HEY2,RC3H1,EIF3E,ZMYND11,CREM,PSMF1,CAST,TRIP12,DFFA,MITF,SRSF6,NFIX,MLLT1,CBFA2T2,IGF1,ATF7IP,PTEN,SPRED2,BMP7,ATXN2,MXI1,PUM1,CIR1,LRRK2,TMEM59,ZBTB20,BAG6,LILRB4,ITGAM,KCTD1,RIPPLY1,RCOR3,DAZL,SERPINA3,SERPINA4,SERPINA5,PKIB,ZBED6,DNAJC1,KAT6A,BMP6,TAF15,GPI,SH3RF2,N4BP1,ATRX,IKZF1,PRMT2,RAPGEF2,SMO,PRKAR2B,SOX30,UBR5,RHOA

			,RQCD1,CUL3,TFEC,EPM2A,GSK3B,DNAJC3,STAT6,PTPRB,RBBP8,NECAB2,CD27,DUSP26,NACC2,SUPT4H1,AGT,METTL16,PRKAA2,ADAR,ZMPSTE24,EXOSC3,TCFL5,CREBBP,DLX1,UGT1A1,UGT1A10,UGT1A4,UGT1A8,SHC1,PARP10,PRG3,SERPINB11,SETDB2,DNAJB6,CD44,RPS6KA5,ZNF93,PRDM2,GCKR,RGS14,TCF25,ZNF282,EPHB2,DIS3,PPARA,ARID5B,SMARCAL1,CPEB4,NOTCH4,ZMYND8,ZNF366,PAX6,PRKCZ,ADORA2A,RABGEF1,CPEB1,OTUD7B,NIPBL,COL28A1,ANGPT1,TBL1X,FHL2,LRRK1,MBTD1,ZBTB5,IGF2BP3,MTF2,TAGLN3,CDK5RAP1,NCOR2,HDAC2,IMPACT,POU3F3,SPINT2,CD300A,ELF2,PPP2CA,RPL23,ATXN1,SP100,ANXA2,TRIM29,PARN,DONSON,RBM42,CNOT1,WWTR1,ZBTB38,BRCA2,MORC1,YBX1,ANKRD13A,PTPRJ,TAF1,HSF1,MAX,SAP130,DHFR,EZH1,NCOA2,ZP3,RNF2,ZNF554,INPP5F,TRIM37,CD109,MET,TIMELESS,UCHL5,SERPINE3,KDM2B,MLXIPL,PHB,PTPRC,ZNF555,CTCF,SH3GL2,NR6A1,TRAF3IP2,CREB1,TRAP1,BMP4
GO:0048675	axon extension	4.231823860436558e-8	CLASP2,SEMA3A,SEMA3D,SEMA5A,OLFM1,CDKL5,ITGB1,HSP90AA1,DSCAM,GOLGA4,LIMK1,CDH4,RTN4,RTN4R,NTN1,SEMA5B,SLIT3,SLIT2,DISC1,DCLK1,AUTS2,MAG,DRAXIN,SPG11,NRP2,DIP2B,ISLR2,SEMA6D,BMPR2,DPYSL2,RAB11A,SEMA4D,VCL,WNT3,NLGN3,PAFAH1B1,TRPC5,RUFY3,SIN3A,MACF1,ADNP,MAP1B,TRIM46,MAP3K13,PTPRS,RAPH1,GSK3B,MAP2,TNR,CDKL3,SLIT1,SEMA3C,EDNRA
GO:0009719	response to endogenous stimulus	5.0129533085166376e-8	ENPP1,NRXN1,PRKCI,PDE4D,DNMT1,SLC9A1,ADCY8,PDE8A,PTGFR,CTDSPL2,RYR1,LATS2,NREP,PRLR,PAGR1,NTRK3,CBL,EGLN2,FLT3,STAT5B,GRM5,FER,LRP2,EZR,SLC26A6,HTR2B,ITGB6,RYR3,GOT1,ROBO2,ITGB1,CDC6,PTPRK,ABCC8,THRB,STXBP4,PTPN11,HDAC6,ERBB4,BRD8,ESR1,PMEPA1,NTRK2,EP300,FNTA,PDGFB,CCND3,INSR,PIP4K2A,CAB39,PAK1,ESRRB,BDNF,AMIGO1,CHRM3,FUT8,UBR2,SNX6,PTPRU,AP3S1,SLC8A1,RBM14,FYN,ARNTL,PLCB1,RXFP1,CHRDL1,PIK3R2,HDAC5,ROR2,SLC39A14,CDH13,ACSBG1,IPO5,RGS8,DEFA1B,DEFA3,PTGER2,JAK2,CLDN1,PDE2A,KLF15,LAMTOR3,CACNA1H,PRKCD,SOX6,ACVR2A,RUNX2,TGFB1,SGK1,BCAS3,SLIT3,ZFYVE9,SLIT2,GABRB1,KANK1,CHRM1,SMAD6,RXFP2,CLOCK,BCKDHB,BMPER,TIMP2,DUSP22,FGF10,SMYD3,GLP2R,SMURF2,EPHA4,UFL1,CTNNB1,PARK2,FCGR2B,SMARCC1,IGF1R,PPARG,AXIN1,CYBB,OTC,BRIP1,SREBF2,RYR2,LEPR,LEPROT,FGF1,NR4A3,MYOCD,GLG1,PRDM16,SORBS1,CAPN2,HDAC4,PAX2,SFRP1,FOXO3,TPH2,SSH1,SMAD3,NEDD4,ESRRG,TAB1,APP,PDGFRA,YAP1,SESN1,ALPL,GNG2,CHST11,DRD1,GLRA2,FRS2,MMP2,ESR2,STAT1,HNF4A,EREG,ATF2,PTPN1,ADAMTS12,BMPR2,BMPR1A,COL16A1,PIK3R3,FGF2,ADCY5,PTAFR,RARB,NCOA1,CHRD,EIF2B5,PTPRE,SHOC2,SRSF5,IDE,KCNQ1,AKAP6,NR2C1,IRS4,ME1,NEO1,WDTC1,DAB2,BLM,UBR1,LDLRAD4,SIN3A,IQGAP1,MAP3K7,ZNF423,SP1,ALK,GSTM3,SRA1,LTBP1,OVOL2,SNX5,ATP2B4,DYX1C1,MAP1B,COL4A6,WWOX,CACNA1A,GPC3,XRN1,TRPV1,GHR,FLRT2,ACTR2,ACACA,ELAVL4,HDAC1,RECQL5,C2,SMOC2,TRIM24,ADIPOR2,ROCK1,EGR2,CAPN10,ABCC1,PTPN2,INSRR,NTRK1,CARM1,SRD5A2,HTR1D,SPI1,PTPRA,CTNNA1,ACAP2,BCAR3,CHURC1,RAB15,RAPGEF3,PDXP,SNCA,BMPR1B,MAGI2,PNPT1,ZEB2,CNGA3,WNT7B,HMGCS2,KMT2D,ADTRP,SRSF6,RPTOR,DSG1,IGF1,MAPK1,SPRED2,BMP7,SOX5,PRCP,LRRK2,GNA14,CHMP5,TMEM108,NSG2,PKD2,EFNA5,PDGFC,NCOA3,WNT7A,GPR21,BMP6,ANO1,GPI,IL17RD,TSHR,EXT1,PNPLA3,PRMT2,RAPGEF2,KCNC2,TNFRSF11B,SOX30,UBR5,GRB14,RHOA,RQCD1,ONECUT2,ITPR2,GABRB3,GSK3B,RDX,STAT6,EEF2K,SULF1,AGT,PRKAA2,FBN2,STAT2,ZMPSTE24,HRH4,PTK2,PHEX,PDE4B,VEPH1,CREBBP,DLX1,FMOD,PCSK6,UGT1A1,SHC1,RPS6KB1,STC2,GPR173,CD44,CLDN4,PKD1L1,NOS1,SCNN1B,EPHB2,SLC1A1,PPARA,SLC25A33,CPEB4,ZNF366,FBN1,PRKCZ,ATP1A3,FAM20C,CPEB1,ASPN,FHL2,OSBPL8,GCNT2,VWC2,NCOR2,PRKCQ,DIAPH1,HDAC2,HTR2C,IMPACT,SPINT2,PHIP,RPL23,SH3BP4,SYK,CNOT1,TAF1,TICAM1,UCN2,HSF1,MAX,NCOA2,ADAMTS7,CIB2,CAV2,CD109,DENND4C,TIMELESS,PHB,PBLD,SH3GL2,CREB1,RGMB,EDNRA,HADHA,BMP4
GO:0048639	positive regulation of developmental growth	7.070765002426305e-8	HLX,SEMA5A,STAT5B,EZR,CHD7,CDKL5,DSCAM,GOLGA4,ERBB4,LIMK1,ATP8A2,RIMS1,INSR,CDH4,BASP1,BDNF,PLCB1,NTN1,NRG1,SYT1,PLS1,DISC1,ITSN2,CAPN3,PARK2,CPNE6,SYT3,YAP1,ISLR2,SPTBN4,CPNE9,SLC6A3,BMPR2,BMPR1A,RAB11A,FGF2,SEMA4D,WNT3,PAFAH1B1,AKAP6,RASAL1,TRPC5,RUFY3,MACF1,ADNP,MAP1B,CELF1,GHR,UNC13A,MAP3K13,HEY2,IGF1,EFNA5,GPR21,TSHR,SMO,SYT17,RIMS2,RPS6KB1,NIPBL,ZP3,NEDD4L,CREB1
GO:0034765	regulation of ion transmembrane transport	7.178109931935118e-8	NRXN1,ASPH,PDE4D,SLC9A1,NOS1AP,DPP6,CLCN1,UTRN,KCNQ5,GRM5,KCNIP4,ANK2,CHD7,ITGB1,CNIH2,ABCC8,TCIRG1,KCNS3,NETO2,CACNB2,KCNJ16,DPP10,CACNA1B,STK39,CAB39,CATSPER2,AMIGO1,KCNMA1,CHRM3,KCNRG,TMC2,DMD,SLC8A1,JPH2,FYN,SHISA9,CACNA1E,SLC43A2,BDKRB1,KCNIP1,STIM1,NALCN,CATSPER3,FGF14,ANO6,KCNB2,NPSR1,SCN4A,TMC1,KCNC4,CACNA1H,TGFB1,KEL,JPH3,KCNJ3,CACNA1D,CACNA2D3,KCNH1,KCNH7,CAPN3,VMP1,CYBB,KCNJ12,RYR2,KCNA6,PER2,CACNA1C,SCN3B,WWP2,SHANK1,NEDD4,APP,NOX1,NLGN2,VDAC1,DRD1,PRKD1,FXYD2,FXYD6,KCNG4,RASGRF2,NLGN1,PTAFR,CLIC4,KCNQ1,NLGN3,AKAP6,WNK1,RELN,ABCB1,KCNQ2,TPCN1,CLIC5,AHNAK,RGS7,ATP2B4,KCNAB2,CACNA1A,STOM,PLCG2,GPR35,ANK3,KCND3,CACNG8,NETO1,TRDN,STAC,TRPC6,SNCA,CACNG2,HTT,SCN8A,CNIH3,PKD2,SHANK3,KCNC2,PSEN2,SCN9A,AGT,ZMPSTE24,PDE4B,KCNJ15,NOS1,ACTN4,EPHB2,LRRC52,DIAPH1,KCND2,SCN1A,CACNG3,KCNQ3,SESTD1,CAMK2D,KCNJ6,NEDD4L,GPR89A,SHISA6,EDNRA
GO:00	regulation of	7.18697518	POLDIP3,ENPP1,PRDX2,LDB2,NRXN1,ASPH,PRMT3,PRKCI,MOV10L1,SLCO3A1,

60255	macromolecule metabolic process	881273e-8	*PDE4D,DNMT1,S1PR2,HLX,SLC9A1,C6ORF89,PBX3,ADCY8,MED13L,TRPS1,CBFB,PDE8A,ZNF823,IL31RA,RPS6KA2,PRDM12,SETD4,PTGFR,MED26,WWC1,NRG3,ASH1L,NOS1AP,LATS2,NOX5,SCAF8,STOX2,PTGIS,PRKAG2,WWC3,PRLR,MVP,PAGR1,ADCY7,HIVEP3,FTO,NPAS3,UBAP2,NTRK3,CBL,ARNT,EGLN2,LRRFIP2,FLT3,STAT5B,TOX,ENPP2,EXD1,GRM5,PLCE1,ZNF566,FER,CASK,MAP2K5,KITLG,MAPK10,LRP2,PIK3CD,ZNF536,SP3,HDGF,ANK2,EIF4G3,OLFM1,EZR,IKZF2,ROBO1,TOM1L1,CHD7,HTR2B,PSMD1,ITGB6,MECOM,TACC1,MIR1185-1,TENM1,LMNA,TRAF6,ESRP1,ITPKB,DNAJA3,OXR1,ABI1,NHLH1,HSP90AA1,SLC24A3,CDC6,PSMB7,TSC22D3,KNDC1,ZC4H2,CCDC22,CDAN1,CNGB1,AKAP13,GFI1B,PTPRK,RUNX1,DAB1,ABCC8,ERC1,HMGN3,NRIP1,THRB,MIR105-2,MIR767,EFCAB7,ITGB3BP,TCIRG1,DACH1,PTPN11,ZNF569,MGAT5,CCT2,ORC2,HDAC6,SERTAD2,DDB1,MYT1,ADORA1,EPHA1,IKBKB,PEX14,ERBB4,MRE11A,LIMK1,ZNF609,BRD8,KAT6B,ACTG2,HIF3A,SNIP1,CUL4B,ESR1,MIER1,PCBP3,PMEPA1,MAML3,PTPRO,CDON,NTRK2,TNRC6A,EP300,CELA1,ZYG11B,TENM2,ZNF76,RNF220,ZNF471,FNTA,PDGFB,RIMS1,TNIK,CCND3,TOB2,ZNF19,ZNF23,BRMS1,CHFR,ZNF605,SCML4,BPI,HNF4G,INSR,FMN2,RERE,PRKAR1A,ASB5,FUBP1,ATP8B1,H2AFY2,TCF7L2,JMJD1C,CAB39,ZNF443,ZNF490,ZNF564,ZNF709,ZNF799,PAK1,LITAF,FBXW11,ESRRB,MYOM1,MAP3K4,RNF144A,BASP1,EGLN3,RBM20,TBR1,SAMD4A,BDNF,TFAP2A,CDK14,FANCA,PEG3,ZIM2,TGIF2,TGIF2-C20ORF24,FUT8,MEIS1,VRTN,TRIM13,LCK,MDM4,IL5RA,SHC4,ECT2,ZNF148,MTA3,SNX6,TFDP2,CST2,PTCD3,RFTN1,ERN2,C1D,NPLOC4,AFF3,DMD,CENPF,ATG10,SLC30A9,TOX3,PDS5A,LARP4B,USP13,KAT7,ZNF667,SLC8A1,GSN,KHDRBS2,RBM14,RBM4,MED12L,SATB2,RIT2,HIRA,CASS4,FYN,MKRN2,ARNTL,NF1,SMG7,PLCB1,MGMT,ARID4B,PPP4R2,RTN4,AFF2,DPEP1,APOD,RWDD3,BRPF1,CHI3L1,TTN,PAQR3,PIK3R2,RANBP9,SLC4A5,NRG1,SH2D3C,UBP1,BDKRB1,BDKRB2,BID,MIR600HG,FRY,MAP2K1,MDFI,FNIP1,VAX2,MYH9,TMBIM6,VGLL4,PPP2CB,PPP3R1,HDAC5,CSRNP3,RBM5,ZNF692,NFIA,RNF4,CSNK2A3,JDP2,CMKLR1,ITIH2,TSPAN8,ROR2,DCN,PCBD2,HNRNPLL,CDH13,CREBRF,TRDMT1,SOX13,FHIT,WDR70,PTPRT,AUNIP,COPS5,IPO5,MAD2L2,TLE6,RAB27A,CAPRIN2,CCNYL1,ZNF418,PHF20L1,TERF2,SLX1B,ZNF286A,FOXN3,NEUROD1,GNAQ,RFFL,CCBE1,HERC5,USP22,JAK2,FAM168A,TRAPPC9,FBXW7,OAZ2,SKAP1,SMC3,UIMC1,ITCH,MLIP,ZFYVE28,ABCC2,BCL11B,SMG1,RBFOX1,PKNOX1,MLLT3,TSHZ2,TCF7,PDE2A,KLF15,TBX15,ANXA4,CYFIP2,WNT11,MTA1,KLF8,NOX4,LCOR,RPRD1B,MKLN1,QKI,CCDC62,ERCC8,PRKCD,SOX6,TAB2,ACVR2A,RUNX2,CD4,PPM1E,TGFB1,BANP,SGK1,SPSB4,NSD1,IGSF1,PIBF1,ZHX2,PKNOX2,ASCC2,BTRC,NFATC3,CRADD,MIR153-2,F2RL1,BCAS3,C9ORF47,CDYL2,DNAJB2,SLIT3,CC2D1B,MIR218-1,SLIT2,TP73,ITIH4,CORO1C,SAP18,ZBTB22,ILF2,DISC1,BLID,ZFAND2A,CLN6,MTDH,FANK1,MYT1L,KDM4B,SMAD6,BNC2,ZNF398,CLOCK,TCF12,ZNF675,ETV6,NELL1,TFAP2D,BMPER,TIMP2,BCL3,ANKRD54,TNFRSF10B,SND1,DUSP22,NAIP,HNRNPC,TRRAP,HOMER2,DOCK3,SBNO2,YTHDF1,FGF10,CIZ1,SMYD3,LOXL3,MAST2,FANCI,CAPN3,LUM,SMURF2,EPHA4,RORA,HIVEP2,PRKCA,AUTS2,CD6,TNFSF11,SMG6,PPP3CA,NSUN2,UBQLN3,NFYB,MAGEA4,KLF12,CAMK4,GATAD2B,PIP5KL1,UFL1,TRAK1,CTNNB1,PARK2,SOD2,DACH2,METTL13,FCGR2B,SMARCC1,KLF17,IGF1R,PPARG,NGRN,AXIN1,PRKAR1B,OTUB1,IL18R1,IL1RL1,CIPC,MTF1,MSR1,CELF2,CBX5,ANKRD17,CYBB,SCAMP5,BRIP1,LRPPRC,SREBF2,CDK11A,CDK11B,LEPR,FGF1,PROS1,NPAT,NR4A3,FOXK2,NOL3,PRKAR2A,RIOK2,ESCO1,MYOCD,TRIM5,PER2,KIR2DL4,AJUBA,GLG1,ZNF626,ZNF737,CHEK2,SUPT3H,UBQLN4,PRDM16,PPP1CB,SPDYA,HCK,CSTL1,SORBS1,RAB3GAP2,CAPN2,TRIM8,DIO2,CSPG4,BRMS1L,ZBTB8A,ZBTB8B,CTDP1,ZUFSP,BAZ1B,NF2,FLT4,MEF2B,HDAC4,PAX2,PHF5A,SECISBP2L,SPTBN1,TRABD2B,SFRP1,MED13,PPP6R2,ZNF395,FOXO3,NFIB,SP4,ZCCHC17,BCL2L13,SSH1,CELF5,SYNCRIP,SMAD3,RNFT2,CUX2,WWP2,ARNT2,SBNO1,KRBOX1,ZNF662,ZNF777,SIMC1,EBF3,MTBP,RNF168,CASZ1,DCP1B,MIER3,NEDD4,ESRRG,HOXD3,HOXD4,NAV3,ZNF114,TAB1,KTI12,NFATC1,CDC73,APP,SSBP3,GSX2,PDGFRA,RBM8A,CCNI2,DIP2B,NOX1,ARIH1,YAP1,HEG1,TEN1,EYA2,SH3D19,BORA,IBTK,NVL,LRP5,MTCP1,POLR3G,ZNF787,SOX2,SETD2,TEAD1,PRICKLE1,RCAN1,ZNF653,SPTBN4,ZNF521,DRD1,ARID3A,ZNF761,CHUK,SFMBT1,ZNF584,ESR2,S100A12,DCUN1D3,KDM6A,PRKD1,STAT1,CELF6,ST18,ETV5,RHOXF2B,TAF3,PLAGL1,HNF4A,ZBTB7C,TASP1,EREG,CCNY,RBFOX3,ATF2,POU2F2,TCF3,ZNF730,GRAMD4,RAF1,CELF4,ZNF766,CARD16,CASP1,PTPN1,SRRM4,POLA1,BMPR2,VBP1,CAMK1D,BMPR1A,ZKSCAN1,PABPC4,IKZF4,PIK3R3,CDK12,CAND2,SPAG9,MORC2,DENND4A,MYB,FGF2,ZNF71,POU6F2,BACH1,MXD3,PPM1F,TICRR,GLI4,ZFP41,NEDD9,AGBL4,BEND5,SEMA4D,NFX1,RORC,ELP3,PTAFR,SP140,SP140L,RHOXF2,JARID2,DDX58,BRDT,PHC2,RARB,SPEN,PRKCG,SIN3B,NCOA1,SPOCK1,AREL1,EHMT1,LMO7,AP2M1,DVL3,EIF2B5,EIF4G1,PSMD2,RBX1,TCF20,ATF3,LIN28B,SRSF5,LINC00461,CKS1B,PAWR,EBF2,AGO3,DEPTOR,FBXL20,MAML2,TSG101,DESI1,CCDC3,TERF2IP,CRYM,IDE,KCNQ1,RFX2,WNT3,ZNF322,SUFU,MAGEA11,PTPN13,PRDM15,ZNF670,ZNF695,CCDC169-SOHLH2,SOHLH2,COL4A3,ZNF354C,HIP1,TCEA3,PADI6,ZNF704,NR2C1,TRPC5,UBA2,GLI2,TNKS,WBP2NL,ERCC1,TNRC6B,GLIS3,WDTC1,ZNF664,DAB2,BLM,PKHD1,CACUL1,LDLRAD4,MYSM1,SETD5,SMG5,DLG3,WNK1,RELN,NEK10,SIN3A,RUVBL2,COMMD6,GMEB1,PELI1,IQGAP1,MAP3K7,ZNF423,SP1,TRIM22,ALK,SLC8A2,INPP5D,TEAD4,FASTKD5,HOXC13,APBB3,SLC35A4,SRA1,TOP1,UBE2V1,ADI*

			RF,XDH,AHNAK,LTBP1,OVOL2,SNX5,NFATC2,RBBP6,PAX7,BNC1,ATP2B4,NSF,ACTL6B,ASXL3,PAK3,TET1,CAMTA1,CCPG1,DYX1C1,ATAD1,ADNP,MAPKAPK2,ARID4A,MARK1,CDK6,PHF2,CELF1,RNF34,TFRC,UVRAG,EPHA5,WWOX,MEF2A,MIR1226,DCAF6,DCUN1D5,DROSHA,GPC3,XRN1,SATB1,TBC1D5,PSMB2,EYA1,GATAD2A,DIS3L2,GNB3,TRPV1,RBM12B,RNF216,HOXB3,HOXB4,HOXB5,HOXB6,MIR10A,TFEB,GHR,MNAT1,SNX9,ACTR2,L3MBTL4,ELAVL4,HDAC1,RECQL5,SMOC2,TRIM24,PLCG2,ROCK1,SCMH1,C1QTNF1,PAXIP1,ZNF713,EGR2,HIPK3,RNF10,RYBP,AP3B1,PTPN2,NTRK1,KPNA6,TRIM44,MAP1A,SLC39A10,ANK3,AXIN2,EPCAM,PARD3,ZNF425,GNL3L,MUC1,PTPN14,BCOR,AP3D1,ZNF41,CARM1,ZNF383,ANXA8L1,ADAM19,SNX33,CD84,LPA,ZNF616,ZNF836,SMARCA2,CUX1,MIR1302-10,SPI1,DNMT3B,EPHA7,FOXP2,PSPC1,GLIS1,SFMBT2,ATG14,EIF3H,BCAR3,JAZF1,STK38,ACOT8,RBPMS,RENBP,CHURC1,CREB5,MAP3K13,RAPGEF3,MST1,SMYD1,CHID1,SNCA,BMPR1B,MAGI2,PRIM2,PNPT1,USP50,ZEB2,FOXJ3,HEY2,RC3H1,EIF3E,RNF19B,PTPRS,ZMYND11,KMT2D,PRKAG1,CREM,PSMF1,KMT2C,CAST,TRIP12,DFFA,ADTRP,PLD1,RASA1,MACC1,MITF,SRSF6,OPRD1,NUGGC,RPTOR,NFIX,BTK,MIR383,TBC1D16,KCTD13,MLLT1,TROVE2,CBFA2T2,IGF1,MLXIP,ATF7IP,HTT,MAPK1,PTEN,SPRED2,BMP7,ATXN2,MXI1,PUM1,SOX5,CIR1,PCBP2,ZNF780A,ZNF780B,LRRK2,TMEM59,UNC119,ZBTB20,CHMP5,RFC3,BAG6,LILRB4,PKD2,ZNF652,EFNA5,KCTD1,RIPPLY1,RCOR3,TADA2A,DAZL,PDGFC,SERPINA3,SERPINA4,SERPINA5,NCOA3,PKIB,MTMR3,ZNF146,ZNF565,CUEDC2,PPP2R3C,WNT7A,ZBED6,BRWD3,NLRP1,UACA,CRTC3,DNAJC1,ARNTL2,PLCL2,KAT6A,MTIF2,ZKSCAN7,ZNF197,ZNF660,BMP6,TAF15,MYCBP2,NFE2L1,GPI,SH3RF2,WDR43,ZNF30,N4BP1,PDCD1LG2,ATRX,DPH6,IKZF1,PRMT2,RAPGEF2,ASCC1,SMO,VRK3,RALY,PRKAR2B,TCF4,AFF1,SOX30,TFE3,UBR5,GRIN1,JADE1,ARRDC4,RHOA,SPRTN,ROR1,RQCD1,TF,ONECUT2,CUL3,TFEC,HSPD1,NFKBID,EPM2A,GSK3B,PRR16,DNAJC3,RDX,STAT6,ACTA2,MLF1,EEF2K,FAM49B,IWS1,PPP1R16A,PTPRB,RBBP8,NECAB2,CDK13,UPF2,CD27,DUSP26,BOK,RNF144B,SULF1,ZNF362,NACC2,SUPT4H1,TNFRSF8,ZNF354A,AGT,HDGFRP3,METTL16,CCNJL,POLH,PRKAA2,ADAR,STAT2,ZMPSTE24,NDRG2,PTK2,UPK3B,SNX3,PDE4B,PBX1,TEC,EXOSC3,MAP3K5,SEC16B,TCFL5,CREBBP,DLX1,GTF2IRD2,NCF1,TAOK2,PYGO2,SHC1,ZNF813,CD160,ECE1,LBX2,PARP10,RNFT1,RPS6KB1,STC2,YLPM1,DPRX,PRG3,SERPINB11,SETDB2,ALOX5,DNAJB6,SP7,CD44,LARP4,ADAM8,CLDN4,RPS6KA5,ZNF484,ZNF93,CSNK1A1,HUS1,MIR346,PRDM2,CXCL17,NOS1,ZNF44,MED15,RGS14,ACTN4,MC1R,STK4,TCF25,ZNF282,EPHB2,SLC1A1,EDRF1,TMEFF2,IL18,MFSD8,UBE2K,DIS3,DLC1,PPARA,PPP1R10,ARID5B,EPB41L4B,SMARCAL1,CPEB4,GPSM3,MOB3B,NOTCH4,ZMYND8,ZNF366,CRX,PAX6,PRKCZ,ZC3HAV1,CD96,ADORA2A,GRIN2B,PPP1CC,RABGEF1,CCNG2,CPEB1,OTUD7B,NIPBL,TMIGD2,YEATS4,COL28A1,PHF20,ZNF143,ANGPT1,BRF1,TBL1X,EYA3,FHL2,LRRK1,MBTD1,OSBPL8,ATF6,ZBTB5,ZNF708,IGF2BP3,GCNT2,ETS1,PPP1CA,MTF2,RBMS3,BTN3A2,TAGLN3,CDK5RAP1,CNTN1,NCOR2,PRKCQ,BRAF,CSRNP1,HDAC2,IMPACT,MIR1912,PHKG2,SRCAP,POU3F3,NOTO,SPINT2,MBNL3,ZKSCAN5,CD300A,ELF2,PHIP,PPP2CA,SKP1,ANGPT4,RPL23,RFC5,ATXN1,SP100,ZNF347,ZNF415,ANXA2,TRIM29,PARN,PRMT7,DONSON,ADCY1,MADD,NUMB,RBM42,SYK,CNOT1,RBM39,WWTR1,GTF2H5,ASB1,NFXL1,ZBTB38,BRCA2,ATF7,DVL2,MORC1,MTRF1,YBX1,ANKRD13A,PTPRJ,SLC10A1,TAF1,EIF4E3,TICAM1,TANK,THAP3,CADM1,HSF1,MAX,SAP130,DHFR,EZH1,ETF1,MIR663B,NRF1,SRPK2,SASH1,NCOA2,GBP5,WDR18,EEFSEC,QRICH1,ZP3,CHD6,RNF2,ZNF554,MOB3A,GNA12,SH2D3A,AKTIP,MYEF2,INPP5F,AXL,TRIM37,CD109,MET,TBC1D10A,ZNF461,CAMK2D,SNX1,TIMELESS,UCHL5,BRD9,MALT1,SERPINE3,SETD3,TRA2B,KDM2B,MLXIPL,CCT3,NEDD4L,PHB,PTPRC,EDA,ZNF555,CTCF,SH3GL2,NR6A1,SPON2,TRAF3IP2,TRIM60,ATF6B,CREB1,RGMB,TNXB,TRAP1,AFF4,CLNK,EBF4,ZNF511,BMP4,ABLIM3,CPNE1
GO:0007507	heart development	7.290619566890709e-8	*DNAH11,SLC9A1,RPS6KA2,RYR1,FHOD3,NTRK3,PLCE1,MAP2K5,NDUFV2,LRP2,ANK2,OLFM1,ROBO1,CHD7,HTR2B,LMNA,ROBO2,ITGB1,AKAP13,RUNX1,XIRP2,PTPN11,ERBB4,MYO18B,NPHP3,EP300,PDGFB,INSR,PRKAR1A,BASP1,RBM20,NPRL3,TENM4,MDM4,TFDP2,MB,SGCD,SLC8A1,JPH2,ADAMTS9,NF1,RTN4,TTN,NDRG4,NRG1,MAP2K1,VGLL4,PDE2A,WNT11,NOX4,SOX6,TAB2,TGFB1,PCSK5,PDLIM4,RPGRIP1L,SLIT3,SLIT2,TP73,LRRC10,SMAD6,PARVA,GREB1L,CTNNB1,SOD2,IGF1R,PPARG,RYR2,MYOCD,CACNA1C,CTDP1,BICC1,ELN,ARL13B,IFT122,SMAD3,NRP2,TAB1,NFATC1,PDGFRA,YAP1,HEG1,SETD2,PRICKLE1,FRS2,MMP2,KDM6A,STRA6,ATF2,BMPR2,BMPR1A,AP1B1,FGF2,JARID2,RARB,COL11A1,KCNQ1,ADAMTS6,SUFU,AKAP6,GLI2,SCUBE1,DZIP1,CRELD1,LUZP1,OVOL2,DYX1C1,MEF2A,GPC3,ADRBK1,EYA1,MNAT1,FLRT2,ROCK1,AXIN2,BCOR,AP2B1,PIFO,PTK7,SMYD1,HEY2,PKP2,SGCZ,IGF1,MAPK1,PTEN,MIB1,BMP7,PKD2,ALPK3,SH3PXD2B,SOS1,EXT1,SMO,AGT,ZMPSTE24,PTK2,CPE,SHC1,ECE1,SETDB2,STK4,TCF25,DLC1,PPARA,CALCRL,FBN1,NIPBL,FHL2,NEBL,NOTO,ACAN,DVL2,NEB,SEMA3C,TRAF3IP2,CREB1,EDNRA,BMP4*
GO:0043408	regulation of MAPK cascade	7.360455127912465e-8	*PRDX2,NRXN1,MAP4K4,PDE8A,SEMA3A,WWC1,ASH1L,NTRK3,FLT3,GRM5,PLCE1,MARVELD3,MAP2K5,KITLG,PTPRR,EZR,ROBO1,HTR2B,MECOM,TENM1,TRAF6,ULK4,AKAP13,PTPN11,ADORA1,ERBB4,ADRA1D,CDON,NTRK2,PDGFB,TNIK,MID1,STK39,INSR,PAK1,MAP3K4,ERN2,DMD,RIT2,NF1,PLCB1,CHI3L1,NDRG4,PAQR3,PIK3R2,RANBP9,NRG1,MAP2K1,ROR2,COPS5,JAK2,FBXW7,ITCH,NPSR1,LAMTOR3,NOX4,PRKCD,CD4,F2RL1,TP73,ZNF675,BMPER,DUSP22,NAIP,FGF10,SH3RF3,EPHA4,PRKCA,TNFSF11,CTNNB1,PARK2,FCGR2B,IGF1R,PPARG,AXIN1,*

			FGF1,TRIM5,AJUBA,CSPG4,GRM1,NF2,FLT4,SFRP1,TAB1,APP,PDGFRA,NOX1,TNFRSF19,SOX2,FRS2,S100A12,RAF1,PTPN1,SPAG9,FGF2,DOK6,DVL3,ATF3,CCL14,CCL15,PAFAH1B1,PRDM15,DAB2,PKHD1,NEK10,IQGAP1,MAP3K7,ALK,XDH,PAK3,GFRAL,GHR,PLCG2,ROCK1,C1QTNF1,HIPK3,PTPN2,NTRK1,EIF3A,EPHB1,SPI1,EPHA7,BCAR3,STK38,MAP3K13,GRM4,WNT7B,ZMYND11,IGF1,MAPK1,PTEN,SPRED2,BMP7,LRRK2,LILRB4,PDGFC,WNT7A,SH3RF2,RAPGEF2,VRK3,SYNGAP1,ROR1,ACTA2,NECAB2,CD27,DUSP26,AGT,HRH4,NDRG2,MAP3K5,NCF1,TAOK2,SHC1,CD44,ADAM8,CXCL17,RGS14,STK4,EPHB2,PHLPP1,MAGI3,PRKCZ,ANGPT1,GCNT2,TBC1D10C,CCL22,BRAF,HTR2C,CD300A,MADD,SYK,GRIK2,DVL2,PTPRJ,SASH1,CAV2,ANKRD6,PHB,PTPRC,BMP4
GO:0050767	regulation of neurogenesis	7.575571764350656e-8	PRKCI,SEMA3A,SEMA3D,TIAM2,KALRN,NTRK3,SEMA5A,GRM5,LRP2,ROBO1,CHD7,CDKL5,ROBO2,DSCAM,DAB1,ABCC8,GOLGA4,LIMK1,NTRK2,HOOK3,CDH4,PTPRD,BDNF,AMIGO1,TENM4,ARNTL,NF1,RTN4,RTN4R,NTN1,MAP2K1,CAPRIN2,IL1RAPL1,CAMK2B,SEMA5B,TGFB1,SLIT2,TP73,DISC1,EPHA4,PPP3CA,MAG,UFL1,CTNNB1,PPARG,DRAXIN,NIN,DCT,PER2,DCC,NF2,CUX2,PRTG,GSX2,DIP2B,YAP1,FSTL4,ISLR2,PARP6,SEMA6D,BMPR2,BMPR1A,RAB11A,SEMA4D,PLXNA2,SPEN,WNT3,PAFAH1B1,TRPC5,RUFY3,RELN,MACF1,PAK3,ADNP,MAP1B,BRINP1,HOXB3,ACTR2,HDAC1,EGR2,RNF10,TRIM46,CUX1,EPHA7,CTNNA1,MAP3K13,HEY2,PTPRS,PTEN,BMP7,EFNA5,SHANK3,WNT7A,RAPGEF2,SMO,SYNGAP1,EEF2K,DLX1,RGS14,EPHB2,PAX6,PPP1CC,MAP2,FBXO31,BRAF,HDAC2,TNR,CDKL3,NUMB,SLIT1,SEMA3C
GO:0060284	regulation of cell development	7.905018524405166e-8	PRKCI,S1PR2,SEMA3A,DOCK1,CLDN18,SEMA3D,TIAM2,KALRN,NTRK3,SEMA5A,GRM5,LRP2,ROBO1,CHD7,CDKL5,ROBO2,TRIOBP,DSCAM,DAB1,ABCC8,GOLGA4,IKBKB,LIMK1,NTRK2,HOOK3,CDH4,PTPRD,BDNF,AMIGO1,TENM4,CASS4,ARNTL,NF1,PLCB1,RTN4,RTN4R,NTN1,MAP2K1,CAPRIN2,RCC2,FBXW7,IL1RAPL1,CAMK2B,SEMA5B,TGFB1,C9ORF47,SLIT2,TP73,CORO1C,DISC1,KANK1,CLOCK,EPHA4,TNFSF11,PPP3CA,MAG,UFL1,CTNNB1,PPARG,DRAXIN,NIN,DCT,PER2,DCC,NF2,HDAC4,CUX2,PREX1,PRTG,GSX2,DIP2B,YAP1,FSTL4,ISLR2,SLC9B2,PARP6,SEMA6D,BMPR2,BMPR1A,RAB11A,NEDD9,SEMA4D,PLXNA2,SPEN,VCL,WNT3,P4HB,PAFAH1B1,TRPC5,RUFY3,DAB2,RELN,MACF1,BNC1,PAK3,ADNP,MAP1B,BRINP1,TRPV1,HOXB3,ACTR2,HDAC1,ROCK1,EGR2,RNF10,AXIN2,TRIM46,CUX1,EPHA7,CTNNA1,MAP3K13,HEY2,PTPRS,IGF1,PTEN,BMP7,LRRK2,EFNA5,SHANK3,WNT7A,RAPGEF2,SMO,SYNGAP1,GSK3B,EEF2K,TCP11,DLX1,RGS14,ACTN4,EPHB2,FBN1,PAX6,PPP1CC,MAP2,FBXO31,BRAF,HDAC2,TNR,CDKL3,NUMB,SLIT1,SEMA3C,BMP4
GO:0045937	positive regulation of phosphate metabolic process	7.957406667419123e-8	NRXN1,SLCO3A1,S1PR2,ADCY8,PDE8A,IL31RA,NRG3,PRKAG2,PRLR,NTRK3,ARNT,FLT3,ENPP2,GRM5,MAP2K5,KITLG,ROBO1,TOM1L1,HTR2B,TENM1,TRAF6,VAV2,ABI1,HSP90AA1,CDC6,KNDC1,DSCAM,AKAP13,DAB1,PTPN11,HDAC6,ADORA1,EPHA1,ERBB4,MRE11A,CDON,NTRK2,PDGFB,TNIK,CCND3,INSR,CAB39,PAK1,MAP3K4,BDNF,ENTPD5,ECT2,ERN2,CASS4,FYN,LMTK2,CHI3L1,NRG1,SH2D3C,MAP2K1,FNIP1,ROR2,MAD2L2,CAPRIN2,CCNYL1,JAK2,FBXW7,LAMTOR3,WNT11,NOX4,PRKCD,TAB2,ACVR2A,CD4,TGFB1,PIBF1,TNFRSF10B,DOCK3,FGF10,SMYD3,EPHA4,CD6,TNFSF11,IGF1R,PPARG,AXIN1,LEPR,FGF1,AJUBA,CHEK2,SPDYA,CAPN2,CSPG4,FLT4,SMAD3,SLC4A4,TAB1,APP,PDGFRA,BORA,MTCP1,S100A12,PRKD1,EREG,CCNY,ATF2,RAF1,PTPN1,BMPR2,BMPR1A,BTBD10,PIK3R3,FGF2,NEDD9,SEMA4D,PTAFR,ADCYAP1R1,DVL3,EIF4G1,EPHB3,CKS1B,TERF2IP,TRPC5,DAB2,CACUL1,DLG3,WNK1,RELN,NEK10,IQGAP1,MAP3K7,ALK,SLC8A2,ATP2B4,CAMTA1,ADNP,TFRC,EPHA5,GHR,MNAT1,SNX9,PLCG2,ROCK1,INSRR,NTRK1,SLC39A10,EPHB1,EPHA10,AXIN2,PIFO,EPHA7,ATG14,BCAR3,RBPMS,MAP3K13,RAPGEF3,SNCA,BMPR1B,MAGI2,PRKAG1,ADTRP,OPRD1,RPTOR,IGF1,MAPK1,BMP7,LRRK2,UNC119,ZBTB20,PKD2,EFNA5,PDGFC,PPP2R3C,BMP6,RAPGEF2,VRK3,RHOA,ROR1,RQCD1,TF,AGT,PRKAA2,PTK2,TEC,MAP3K5,NCF1,TAOK2,CD44,ADAM8,RPS6KA5,NOS1,STK4,EPHB2,SLC1A1,IL18,UBE2K,DLC1,PPARA,MOB3B,PRKCZ,ANGPT1,LRRK1,OSBPL8,CNTN1,BRAF,HDAC2,HTR2C,CD300A,PPP2CA,ANGPT4,MADD,SYK,DVL2,PTPRJ,HSF1,SASH1,VAV3,MOB3A,GNA12,SH2D3A,AKTIP,AXL,MET,MALT1,MLXIPL,PHB,PTPRC,TYRO3,BMP4
GO:0010562	positive regulation of phosphorus metabolic process	7.957406667419123e-8	NRXN1,SLCO3A1,S1PR2,ADCY8,PDE8A,IL31RA,NRG3,PRKAG2,PRLR,NTRK3,ARNT,FLT3,ENPP2,GRM5,MAP2K5,KITLG,ROBO1,TOM1L1,HTR2B,TENM1,TRAF6,VAV2,ABI1,HSP90AA1,CDC6,KNDC1,DSCAM,AKAP13,DAB1,PTPN11,HDAC6,ADORA1,EPHA1,ERBB4,MRE11A,CDON,NTRK2,PDGFB,TNIK,CCND3,INSR,CAB39,PAK1,MAP3K4,BDNF,ENTPD5,ECT2,ERN2,CASS4,FYN,LMTK2,CHI3L1,NRG1,SH2D3C,MAP2K1,FNIP1,ROR2,MAD2L2,CAPRIN2,CCNYL1,JAK2,FBXW7,LAMTOR3,WNT11,NOX4,PRKCD,TAB2,ACVR2A,CD4,TGFB1,PIBF1,TNFRSF10B,DOCK3,FGF10,SMYD3,EPHA4,CD6,TNFSF11,IGF1R,PPARG,AXIN1,LEPR,FGF1,AJUBA,CHEK2,SPDYA,CAPN2,CSPG4,FLT4,SMAD3,SLC4A4,TAB1,APP,PDGFRA,BORA,MTCP1,S100A12,PRKD1,EREG,CCNY,ATF2,RAF1,PTPN1,BMPR2,BMPR1A,BTBD10,PIK3R3,FGF2,NEDD9,SEMA4D,PTAFR,ADCYAP1R1,DVL3,EIF4G1,EPHB3,CKS1B,TERF2IP,TRPC5,DAB2,CACUL1,DLG3,WNK1,RELN,NEK10,IQGAP1,MAP3K7,ALK,SLC8A2,ATP2B4,CAMTA1,ADNP,TFRC,EPHA5,GHR,MNAT1,SNX9,PLCG2,ROCK1,INSRR,NTRK1,SLC39A10,EPHB1,EPHA10,AXIN2,PIFO,EPHA7,ATG14,BCAR3,RBPMS,MAP3K13,RAPGEF3,SNCA,BMPR1B,MAGI2,PRKAG1,ADTRP,OPRD1,RPTOR,IGF1,MAPK1,BMP7,LRRK2,UNC119,ZBTB20,PKD2,EFNA5,PDGFC,PPP2R3C,BMP6,RAPGEF2,VRK3,RHOA,ROR1,RQCD1,TF,AGT,PRKAA2,PTK2,TEC,MAP3K5,NCF1,TAOK2,C

			D44,ADAM8,RPS6KA5,NOS1,STK4,EPHB2,SLC1A1,IL18,UBE2K,DLC1,PPARA,MOB3B,PRKCZ,ANGPT1,LRRK1,OSBPL8,CNTN1,BRAF,HDAC2,HTR2C,CD300A,PPP2CA,ANGPT4,MADD,SYK,DVL2,PTPRJ,HSF1,SASH1,VAV3,MOB3A,GNA12,SH2D3A,AKTIP,AXL,MET,MALT1,MLXIPL,PHB,PTPRC,TYRO3,BMP4
GO:0044087	regulation of cellular component biogenesis	8.22499987296928e-8	*CLRN1,ADAMTS16,LDB2,NRXN1,MAP4K4,DNMT1,SLC9A1,NEGR1,PDE4DIP,CLASP2,GRID2,FHOD3,TJP1,NTRK3,PLCE1,LHFPL4,FER,EZR,CDKL5,TENM1,ROBO2,TRIOBP,HSP90AA1,ARHGAP24,CDC42EP3,MYO10,PTPN11,HDAC6,EPHA1,IKBKB,LIMK1,ESR1,PMEPA1,NTRK2,STX18,EP300,TENM2,ATP8B1,PIP4K2A,PAK1,PTPRD,BDNF,AMIGO1,NPRL3,ARHGAP6,ECT2,GSN,RBM14,APOD,NTN1,DCTN1,NRG1,BID,FNIP1,LRFN5,RNF4,SCFD1,NCKAP1,TERF2,SLX1B,RCC2,ARL3,SKAP1,CLSTN2,CROCC,CLDN1,SEPT7,CYFIP2,WNT11,IL1RAPL1,PRKCD,PPM1E,F2RL1,BCAS3,DNAJB2,SLIT2,CORO1C,CEP135,KANK1,SMAD6,DUSP22,LINGO2,CNTNAP2,STXBP5,KANK4,IL1RAPL2,RHPN2,SVIL,PRKCA,AUTS2,PARK2,DLG5,ADD2,FBLIM1,NIN,AJUBA,PHLDB1,HCK,TBC1D14,RAB3GAP2,NF2,HDAC4,SPTBN1,ELN,TRABD2B,SFRP1,DNM3,SSH1,SMAD3,CUX2,WIPI1,ABI2,C10ORF90,SHANK1,ARHGEF18,NAV3,APP,SLK,ADD3,YAP1,NLGN2,GPC6,KIF24,SPTBN4,VILL,SYNE2,VPS4A,RAF1,NLGN1,COL16A1,MORC2,PPM1F,PEAK1,SEMA4D,EIF4G1,EPHB3,TSG101,VCL,NLGN3,ERCC1,LDLRAD4,SETD5,DZIP1,SIN3A,DOCK11,MACF1,CLASP1,GPM6B,SPTBN5,PAK3,SSH2,TET1,ADNP,MAP1B,MPHOSPH9,PHF2,TFRC,FLRT2,SNX9,ACTR2,PLCG2,ROCK1,EPS8,NTRK1,EPHB1,GNL3L,ARHGAP44,EPHA7,PTPRA,ABCB7,FCHSD2,RAPGEF3,SNCA,USP50,PTPRS,CDKL1,PLD1,RASA1,OPRD1,ATF7IP,HTT,DPYSL3,PTEN,BMP7,LRRK2,SPAG5,CHMP5,EFNA5,SHANK3,MTMR3,TBCD,WNT7A,MAP4,RAPGEF2,MARK4,RHOA,GSK3B,RDX,EEF2K,FAM49B,AGT,PRKAA2,PTK2,TPPP2,GAP43,PARP10,SETDB2,DNAJB6,EPHB2,TMEFF2,GPSM2,DLC1,PRKCZ,PHLDB2,MAP2,ATG3,BRAF,IMPACT,NOTO,CAMSAP3,SYK,CNOT1,SLIT1,HRK,PTPRJ,TICAM1,GPM6A,HSF1,PLEKHM1,ASAP1,GBP5,CGNL1,TRIM37,MET,SPTAN1,SYNDIG1,STXBP6*
GO:0016055	Wnt signaling pathway	8.339196570084625e-8	*GPC5,LATS2,SEMA5A,LRRFIP2,GRK5,PSMB7,MCC,KLHL12,DDB1,NPHP3,PTPRO,CELA1,RNF220,TNIK,TCF7L2,USP34,FBXW11,CDK14,PTPRU,DEPDC1B,ARNTL,MDFI,VAX2,VGLL4,ROR2,SOX13,MAD2L2,TLE6,CAPRIN2,CCNYL1,GNAQ,MLLT3,TCF7,KLF15,WNT11,IFT80,KREMEN1,BTRC,CELSR1,CTNND2,DISC1,KANK1,FGF10,SMURF2,CTNNB1,PARK2,AXIN1,DRAXIN,BICC1,TRABD2B,SFRP1,FOXO3,SMAD3,VANGL1,NFATC1,CDC73,APP,YAP1,AMFR,LRP5,SOX2,GPC6,PRICKLE1,KDM6A,CCNY,TMEM237,FGF2,DKK2,DVL3,RBX1,WNT3,PRDM15,TNKS,DAB2,WNK1,RUVBL2,RSPO2,MACF1,MARK1,WWOX,GPC3,PSMB2,HDAC1,INVS,PLCG2,AXIN2,FBXW4,PTK7,MAGI2,ZEB2,WNT7B,MITF,ZNRF3,PTEN,LRRK2,ZRANB1,PKD2,AMOTL1,WNT7A,EXT1,PRICKLE2,SOX30,UBR5,JADE1,RHOA,ROR1,CUL3,EPM2A,GSK3B,SULF1,RNF43,PRKAA2,NDRG2,SNX3,CPE,APCDD1L,PYGO2,LBX2,SCEL,CSNK1A1,STK4,SPEF1,WLS,TBL1X,LRRK1,PPP1CA,RBMS3,PPP2CA,WWTR1,DVL2,WIF1,ANKRD6,EDA,RNF213,SHISA6,EDNRA*
GO:0051960	regulation of nervous system development	9.978896570223035e-8	*NRXN1,PRKCI,SEMA3A,GRID2,SEMA3D,TIAM2,KALRN,NTRK3,SEMA5A,GRM5,LRP2,ROBO1,CHD7,CDKL5,ROBO2,DSCAM,DAB1,ABCC8,GOLGA4,LIMK1,NPHP3,NTRK2,HOOK3,CDH4,PTPRD,BDNF,AMIGO1,TENM4,ARNTL,NF1,RTN4,RTN4R,NTN1,MAP2K1,CAPRIN2,CLSTN2,FIG4,IL1RAPL1,CAMK2B,SEMA5B,TGFB1,SLIT2,TP73,DISC1,LINGO2,EPHA4,PPP3CA,MAG,UFL1,CTNNB1,PPARG,DLG5,DRAXIN,NIN,DCT,PER2,DCC,NF2,CUX2,PRTG,GSX2,DIP2B,YAP1,FSTL4,NLGN2,ISLR2,PARP6,SEMA6D,BMPR2,BMPR1A,NLGN1,RAB11A,SEMA4D,PLXNA2,SPEN,EPHB3,WNT3,TG,NLGN3,PAFAH1B1,TRPC5,RUFY3,RELN,MACF1,PAK3,ADNP,MAP1B,BRINP1,HOXB3,FLRT2,ACTR2,LGI4,HDAC1,EGR2,RNF10,NTRK1,EPHB1,PARD3,TRIM46,CUX1,EPHA7,CTNNA1,MAP3K13,HEY2,PTPRS,PTEN,BMP7,EFNA5,SHANK3,WNT7A,RAPGEF2,SMO,SYNGAP1,EEF2K,DLX1,RGS14,EPHB2,PAX6,PPP1CC,MAP2,FBXO31,BRAF,HDAC2,TNR,CDKL3,NUMB,SLIT1,SEMA3C,SYNDIG1*
GO:0010243	response to organonitrogen compound	1.1006182852847169e-7	*ENPP1,ASPH,PRKCI,PDE4D,DNMT1,SLC9A1,ADCY8,CALR3,RYR1,FLT3,STAT5B,GRM5,FER,EZR,SLC26A6,HTR2B,RYR3,GOT1,ITGB1,CDC6,ABCC8,DNAJB14,STXBP4,PTPN11,NTRK2,CACNA1B,CCND3,INSR,PIP4K2A,AMIGO1,CHRM3,TRIM13,UBR2,HLCS,SNX6,AP3S1,NPLOC4,USP13,KAT7,SLC8A1,FYN,PLCB1,MGMT,PIK3R2,TMBIM6,HDAC5,SLC39A14,CDH13,TRDMT1,IPO5,MAN1A1,RGS8,JAK2,PDE2A,KLF15,LAMTOR3,PRKCD,TGFB1,DNAJB2,SLIT2,TP73,GABRB1,KANK1,CHRM1,BCKDHB,HOMER2,GLP2R,EPHA4,CNR1,PPP3CA,UFL1,CTNNB1,PARK2,FCGR2B,SMARCC1,IGF1R,PPARG,CYBB,OTC,BRIP1,SREBF2,RYR2,LEPROT,NR4A3,SORBS1,CAPN2,SFRP1,FOXO3,SSH1,RNFT2,SLC6A1,SEC61B,APP,PDGFRA,AMFR,SESN1,RNLS,GNG2,DRD1,GLRA2,ERLIN1,MMP2,STAT1,SLC6A3,HNF4A,EREG,ATF2,PTPN1,COL16A1,PIK3R3,ADCY5,PTAFR,PRKCG,EIF2B5,PTPRE,SRSF5,STT3B,IDE,KCNQ1,AKAP6,EDEM3,IRS4,WDTC1,BLM,UBR1,SIN3A,SP1,ALK,SNX5,RGS7,ATP2B4,P2RX6,MAP1B,COL4A6,TYR,CACNA1A,XRN1,PSMB2,TRPV1,GHR,ACTR2,ELAVL4,RECQL5,ROCK1,EGR2,CAPN10,ABCC1,PTPN2,INSRR,MAN1B1,CARM1,SRD5A2,HTR1D,PTPRA,CTNNA1,BCAR3,RAB15,RAPGEF3,SDK1,PDXP,SNCA,PNPT1,RNF103,RNF103-CHMP3,CNGA3,HMGCS2,ADTRP,SRSF6,RPTOR,IGF1,MAPK1,BMP7,PIK3C3,LRRK2,GNA14,CHMP5,BAG6,NSG2,PKD2,SEL1L2,PDGFC,NLRP1,GLDC,GPR21,ANO1,GPI,TSHR,EXT1,PNPLA3,RAPGEF2,KCNC2,GET4,PRKAR2B,CPT1A,GRB14,GRIN1,RHOA,ITPR2,GABRB3,GSK3B,STAT6,EEF2K,AGT,STAT2,HRH4,PTK2,PHEX,RNF121,PDE4B,SHC1,RNFT1,RPS6KB1,STC2,GPR173,EPHB2,SLC1A1,PPARA,SLC25*

			A33,CPEB4,FBN1,PRKCZ,ATP1A3,ADORA2A,CPEB1,OSBPL8,NCOR2,PRKCQ,DIAPH1,HDAC2,HTR2C,IMPACT,PHIP,RPL23,SH3BP4,SYK,TAF1,TICAM1,HSF1,MAX,DHFR,CIB2,CAV2,UGGT1,DENND4C,TIMELESS,CREB1,SGTB,EDNRA,HADHA
GO:0023056	positive regulation of signaling	1.103098537731916e-7	*PRDX2,GPC5,NRXN1,PRKCI,MAP4K4,PDCL,SLC9A1,ADCY8,PDE8A,SEMA3A,CHERP,WWC1,C12ORF49,NOS1AP,MAPRE2,PTGIS,PRLR,PAGR1,NTRK3,CBL,ARNT,SEMA5A,FLT3,GRM5,PLCE1,MAP2K5,KITLG,LRP2,PIK3CD,ROBO1,HTR2B,TENM1,TRAF6,ROBO2,ITPKB,SLC24A2,ITGB1,HSP90AA1,PSMB7,CCDC22,AKAP13,DGKI,LAMA2,PTPN11,MGAT5,CACNB2,ADORA1,ADAMTS3,IKBKB,ERBB4,ADRA1D,MIER1,CDON,NTRK2,EP300,RNF220,PDGFB,RIMS1,TNIK,MID1,STK39,INSR,TCF7L2,USP34,PAK1,LITAF,MAP3K4,ZDHHC13,BDNF,TRIM13,LCK,ECT2,VAMP7,ERN2,RIT2,DEPDC1B,CASS4,FYN,ARNTL,NF1,PLCB1,RTN4,RTN4R,RWDD3,CHI3L1,NDRG4,BMP2K,NRG1,BID,MAP2K1,FNIP1,ROR2,DCN,ZDHHC3,SLC39A14,CDH13,CAPRIN2,CCBE1,JAK2,FBXW7,CLSTN2,SYT1,NPSR1,MLLT3,LAMTOR3,CYFIP2,WNT11,NOX4,SIAH1,PRKCD,TAB2,ACVR2A,CD4,NUP93,PIBF1,CRADD,F2RL1,AAK1,TP73,DISC1,KANK1,MTDH,BMPER,TNFRSF10B,DUSP22,NAIP,YTHDF1,FGF10,IQCJ-SCHIP1,SH3RF3,LOXL3,SMURF2,EPHA4,PRKCA,AUTS2,CNR1,TNFSF11,CTNNB1,PARK2,FCGR2B,IGF1R,PPARG,AXIN1,DLG5,IL18R1,P2RY10,ANKRD17,APOL3,FGF1,MYOCD,TRIM5,AJUBA,SORBS1,TRIM8,CSPG4,GRM1,FLT4,SFRP1,CNTN6,SMAD3,CUX2,SHANK1,NEDD4,TAB1,CDC73,APP,GSX2,PDGFRA,NOX1,YAP1,NLGN2,TNFRSF19,SOX2,DRD1,CHUK,FRS2,S100A12,PRKD1,SHANK2,EREG,RAF1,RASGRF2,CARD16,CASP1,PTPN1,BMPR2,BMPR1A,NLGN1,SPAG9,EVC,FGF2,SEMA4D,DOK6,ADCYAP1R1,DKK2,PRKCG,EEF1E1,EEF1E1-BLOC1S5,GAS8,DVL3,ATF3,SHOC2,AGO3,TERF2IP,WDR59,WNT3,CCL14,CCL15,NLGN3,SCUBE2,PAFAH1B1,PRDM15,HIP1,AKAP6,NR2C1,NEO1,TNKS,SCUBE1,DAB2,WNK1,RELN,NEK10,RSPO2,GUCY1A2,PELI1,IQGAP1,MAP3K7,ZNF423,TRIM22,MACF1,ALK,SLC8A2,STAU1,UBE2V1,XDH,SNX5,NSF,CAMTA1,P2RY8,TFRC,WWOX,SYT12,GFRAL,GPC3,PSMB2,TSPAN6,CLEC16A,GHR,SMOC2,PLCG2,ROCK1,C1QTNF1,CAPN10,GPR35,PTPN2,NTRK1,TRIM44,SLC39A10,JAG2,CACNG8,VWF,NETO1,ARHGEF3,SPI1,CHRNB4,MCU,CTNNA1,LY86,BCAR3,RBPMS,MAP3K13,PTK7,SNCA,BMPR1B,CACNG2,GRM4,ZEB2,RC3H1,WNT7B,KMT2D,RPTOR,KCTD13,SLC4A8,IGF1,HTT,MAPK1,PTEN,SPRED2,BMP7,PUM1,LRRK2,ZRANB1,TMEM108,PKD2,SHANK3,PDGFC,WNT7A,SIK3,BMP6,ANO1,SH3RF2,SOS1,RAPGEF2,SMO,UBR5,GRIN1,RHOA,ROR1,RQCD1,GSK3B,ACTA2,NECAB2,CD27,BOK,SULF1,NACC2,AGT,PTK2,RIMS2,MAP3K5,CREBBP,NCF1,TAOK2,SHC1,ECE1,LBX2,SCEL,CD44,ADAM8,BCL2L14,CXCL17,RGS14,ACTN4,MC1R,STK4,EPHB2,SLC1A1,WLS,IL18,UBE2K,HCAR2,PRKCZ,ZC3HAV1,ADORA2A,GRIN2B,ANGPT1,TBL1X,LRRK1,OSBPL8,ATF6,GCNT2,PPP1CA,CCL22,BRAF,HTR2C,TNR,CD300A,PHIP,MAOA,RPL23,ANXA2,ADCY1,MADD,SYK,GRIK2,MYRIP,CACNG3,DVL2,PTPRJ,TICAM1,SASH1,CAV2,INPP5F,AXL,MET,UCHL5,ANKRD6,MALT1,PHB,PTPRC,EDA,GPR89A,TSPAN5,TRAF3IP2,CREB1,BMP4,CPNE1*
GO:0198738	cell-cell signaling by wnt	1.1536263217103031e-7	*GPC5,LATS2,SEMA5A,LRRFIP2,GRK5,PSMB7,MCC,KLHL12,DDB1,NPHP3,PTPRO,CELA1,RNF220,TNIK,TCF7L2,USP34,FBXW11,CDK14,PTPRU,DEPDC1B,ARNTL,MDFI,VAX2,VGLL4,ROR2,SOX13,MAD2L2,TLE6,CAPRIN2,CCNYL1,GNAQ,MLLT3,TCF7,KLF15,WNT11,IFT80,KREMEN1,BTRC,CELSR1,CTNND2,DISC1,KANK1,FGF10,SMURF2,CTNNB1,PARK2,AXIN1,DRAXIN,BICC1,TRABD2B,SFRP1,FOXO3,SMAD3,VANGL1,NFATC1,CDC73,APP,YAP1,AMFR,LRP5,SOX2,GPC6,PRICKLE1,KDM6A,CCNY,TMEM237,FGF2,DKK2,DVL3,RBX1,WNT3,PRDM15,TNKS,DAB2,WNK1,RUVBL2,RSPO2,MACF1,MARK1,WWOX,GPC3,PSMB2,HDAC1,INVS,PLCG2,AXIN2,FBXW4,PTK7,MAGI2,ZEB2,WNT7B,MITF,ZNRF3,PTEN,LRRK2,ZRANB1,PKD2,AMOTL1,WNT7A,EXT1,PRICKLE2,SOX30,UBR5,JADE1,RHOA,ROR1,CUL3,EPM2A,GSK3B,SULF1,RNF43,PRKAA2,NDRG2,SNX3,CPE,APCDD1L,PYGO2,LBX2,SCEL,CSNK1A1,STK4,SPEF1,WLS,TBL1X,LRRK1,PPP1CA,RBMS3,PPP2CA,WWTR1,DVL2,WIF1,ANKRD6,EDA,RNF213,SHISA6,EDNRA*
GO:0007610	behavior	1.195576246334924e-7	*DNAH11,NRXN1,NEGR1,PBX3,ADCY8,NLGN4X,KALRN,GRM5,NRXN3,CHD7,HTR2B,OXR1,SLC24A2,ITGB1,PCDH17,DSCAM,DAB1,ABCC8,DGKI,THRB,DACH1,ADORA1,ATP8A2,NTRK2,EP300,KIRREL3,USP46,INSR,SLC4A10,TBR1,BDNF,MEIS1,NAV2,FYN,NF1,PLCB1,PCDH15,AFF2,NDRG4,ANKH,DCTN1,NRG1,STRBP,VWA1,NPSR1,BBS12,FIG4,MTA1,ASTN1,SGK1,JPH3,CELSR1,CDH23,GPR176,CLN6,GPR52,ANKFN1,HOMER2,YTHDF1,CNTNAP2,SLC16A1,EPHA4,CNR1,CAMK4,PARK2,SOD2,PRKAR1B,LEPR,NR4A3,CSMD1,CAPN2,GRM1,HDAC4,SPG11,CUX2,SHANK1,SLC6A1,APP,AMFR,NLGN2,RCAN1,SPTBN4,DRD1,SLC6A3,SHANK2,STRA6,NLGN1,ADCY5,PRKCG,EIF4G1,FBXL20,KCNQ1,NLGN3,PAFAH1B1,CNTFR,NMUR2,BTBD9,RELN,ALK,SLC8A2,ATAD1,ADNP,SORCS3,GFRAL,SGIP1,BRINP1,TRPV1,ACTR2,ELAVL4,LGI4,EPS8,EGR2,NTRK1,MAP1A,NETO1,HTR1D,CHRNB4,SDK1,SNCA,OPRD1,RPTOR,HTT,MAPK1,PTEN,PUM1,LRRK2,SHANK3,GPI,TSHR,EXT1,PRKAR2B,CPT1A,GRIN1,SYNGAP1,EPM2A,SCN9A,AGT,ZMPSTE24,RPS6KB1,GRID1,RGS14,MC1R,EPHB2,SLC1A1,PPARA,SOBP,PRKCZ,ADORA2A,GRIN2B,KLHL1,BRAF,HDAC2,HTR2C,ATP8A1,TNR,ATXN1,KCND2,ADCY1,GRIK2,SCN1A,MORC1,NCOA2,INPP5F,TANC1,CREB1*
GO:0034762	regulation of transmembrane	1.2545554660829968e-	*ENPP1,NRXN1,ASPH,PRKCI,PDE4D,SLC9A1,NOS1AP,DPP6,PRKAG2,CLCN1,UTRN,KCNQ5,GRM5,KCNIP4,ANK2,CHD7,SLC26A6,ITGB1,CNIH2,ABCC8,STXBP4,TCIRG1,PTPN11,KCNS3,NETO2,CACNB2,KCNJ16,DPP10,CACNA1B,STK39,INSR,CA*

	transport	7	B39,CATSPER2,AMIGO1,KCNMA1,CHRM3,KCNRG,TMC2,DMD,SLC8A1,JPH2,FYN,SHISA9,CACNA1E,SLC43A2,BDKRB1,KCNIP1,STIM1,NALCN,CATSPER3,FGF14,ANO6,KCNB2,OAZ2,NPSR1,SCN4A,TMC1,KLF15,KCNC4,CACNA1H,TGFB1,KEL,JPH3,KCNJ3,CACNA1D,CACNA2D3,KCNH1,KCNH7,CAPN3,VMP1,CYBB,KCNJ12,RYR2,KCNA6,NR4A3,PER2,CACNA1C,SCN3B,SORBS1,WWP2,SHANK1,NEDD4,APP,NOX1,NLGN2,VDAC1,DRD1,PRKD1,FXYD2,FXYD6,KCNG4,RASGRF2,NLGN1,PTAFR,CLIC4,KCNQ1,NLGN3,AKAP6,WNK1,RELN,ABCB1,KCNQ2,TPCN1,CLIC5,AHNAK,RGS7,ATP2B4,KCNAB2,MEF2A,CACNA1A,GPC3,STOM,PLCG2,CAPN10,GPR35,ANK3,KCND3,CACNG8,NETO1,TRDN,STAC,TRPC6,SNCA,CACNG2,IGF1,HTT,SCN8A,CNIH3,PKD2,SHANK3,KCNC2,PSEN2,SCN9A,AGT,ZMPSTE24,PDE4B,KCNJ15,RPS6KB1,NOS1,ACTN4,EPHB2,LRRC52,OSBPL8,BRAF,DIAPH1,KCND2,SCN1A,CACNG3,KCNQ3,SESTD1,CAMK2D,KCNJ6,NEDD4L,GPR89A,SHISA6,EDNRA
GO:0043269	regulation of ion transport	1.3633330680417653e-7	NRXN1,ASPH,PDE4D,SLC9A1,NOS1AP,EPB41,DPP6,CLCN1,UTRN,KCNQ5,GRM5,CASK,KCNIP4,ANK2,CHD7,NKAIN2,ITGB1,CNIH2,NKAIN3,ABCC8,TCIRG1,KCNS3,NETO2,CACNB2,ADORA1,KCNJ16,GRM7,DPP10,PDGFB,CACNA1B,STK39,ATP8B1,CAB39,CATSPER2,AMIGO1,KCNMA1,CHRM3,KCNRG,TMC2,VAMP7,DMD,SLC8A1,JPH2,FYN,SHISA9,CACNA1E,SLC43A2,BDKRB1,KCNIP1,STIM1,NALCN,TMBIM6,CATSPER3,FGF14,ANO6,KCNB2,SYT1,NPSR1,SCN4A,TMC1,KCNC4,CACNA1H,CAMK2B,CD4,TGFB1,KEL,JPH3,CLDN10,SYT13,KCNJ3,CACNA1D,CACNA2D3,CHRM1,KCNH1,HOMER2,KCNH7,CAPN3,VMP1,CNR1,TNFSF11,CTNNB1,PARK2,CYBB,KCNJ12,RYR2,KCNA6,PER2,CACNA1C,SCN3B,WWP2,SHANK1,SYT3,NEDD4,SLC6A1,APP,NOX1,NLGN2,VDAC1,SPTBN4,DRD1,PRKD1,FXYD2,FXYD6,FXYD6-FXYD2,KCNG4,RASGRF2,NLGN1,PTAFR,ADCYAP1R1,CLIC4,KCNQ1,NLGN3,AKAP6,WNK1,RELN,ABCB1,KCNQ2,TPCN1,GPM6B,CLIC5,AHNAK,RGS7,ATP2B4,KCNAB2,SYT12,CACNA1A,STOM,PLCG2,GPR35,ANK3,KCND3,CACNG8,NETO1,CD84,TRDN,STAC,TRPC6,SNCA,CACNG2,SYT9,OPRD1,PKP2,HTT,SCN8A,CNIH3,PKD2,SHANK3,BEST3,KCNC2,PSEN2,SYT17,GRIN1,RHOA,TF,SCN9A,AGT,SYT7,ZMPSTE24,PDE4B,KCNJ15,STC2,NOS1,ACTN4,EPHB2,LRRC52,NKAIN1,ADORA2A,CNTN1,DIAPH1,KCND2,SYK,SCN1A,CACNG3,SLC10A1,KCNQ3,SESTD1,CAMK2D,KCNJ6,NEDD4L,GPR89A,SHISA6,EDNRA
GO:0009790	embryo development	1.6067959134292532e-7	CLRN1,RCN1,DNMT1,HLX,PBX3,LMBR1,CLASP2,SEMA3A,STOX2,TJP1,ARNT,RAD51B,MAP2K5,KITLG,PTPRR,LRP2,SP3,MFAP5,CHD7,HTR2B,KIAA1217,TRAF6,ABI1,ITGB1,TRIOBP,KLHL12,LAMA2,RBM19,ADAMTS3,ERBB4,ATP8A2,CECR2,MYO18B,NPHP3,CDON,EP300,RNF220,PDGFB,INSR,PRKAR1A,LAMA3,BASP1,STRC,TFAP2A,TGIF2,FUT8,PHACTR4,MEIS1,TENM4,MYO3B,AFF3,SATB2,HIRA,TANC2,NF1,PLCB1,PCDH15,RTN4,BCR,NDRG4,NTN1,MAP2K1,MDFI,VAX2,MYH9,ROR2,EXOC4,TLE6,TERF2,NEUROD1,COL12A1,USP22,LRIG3,PLS1,SEPT7,TBX15,WNT11,CCDC62,ACVR2A,RUNX2,TGFB1,PCSK5,MYO3A,C2ORF49,RPGRIP1L,CELSR1,CDH23,SMAD6,FGF10,LOXL3,NSUN2,CTNNB1,AXIN1,RYR2,NPAT,NR4A3,CACNA1C,PHLDB1,MYO1E,CAPN2,MMP16,NF2,PAX2,SFRP1,ARL13B,IFT122,SMAD3,ARNT2,EMG1,NRP2,HOXD3,HOXD4,TAB1,CDC73,SSBP3,PDGFRA,YAP1,HEG1,EYA2,PEMT,LRP5,SOX2,SETD2,TTC39C,CHST11,TEAD1,PRICKLE1,VASH2,FRS2,PALB2,MMP2,KDM6A,HNF4A,STRA6,DSCAML1,ATF2,CELF4,BMPR2,BMPR1A,TAF8,FGF2,AGBL4,SH3PXD2A,PLXNA2,RARB,NCOA1,EHMT1,CHRD,DVL3,LMBRD1,LAMB1,COL11A1,KCNQ1,WNT3,SUFU,PAFAH1B1,POLE,PADI6,GLI2,ERCC1,WDTC1,WNK1,SIN3A,RSPO2,LUZP1,MYO7A,CLASP1,TEAD4,TOP1,OVOL2,RBBP6,TET1,GPR161,CELF1,ADCY9,GPC3,EYA1,HOXB3,HOXB4,HOXB5,HOXB6,TFEB,HDAC1,ROCK1,PTPRQ,AXIN2,JAG2,BCOR,PIFO,FBXW4,SHROOM3,ERCC3,PTK7,RAI2,HEY2,COL13A1,WNT7B,TRIP12,HIPK1,IGF1,MAPK1,MIB1,BMP7,CIR1,LHFPL5,TTLL4,BAG6,PKD2,SHANK3,RIPPLY1,PDGFC,WNT7A,ZBED6,GPI,SOS1,TSHR,EXT1,SMO,CUL3,RBBP8,SULF1,MTHFD1L,KRT8,FBN2,PBX1,CREBBP,DLX1,PCSK6,ECE1,LBX2,SCEL,SETDB2,DNAJB6,HUS1,TBC1D23,STK4,EPHB2,WLS,ASF1B,DLC1,SOBP,FBN1,PAX6,PHLDB2,OTUD7B,NIPBL,UPB1,ANGPT1,MBTD1,HDAC2,NOTO,SPINT2,CAMSAP3,CNOT1,BRCA2,DVL2,YBX1,ZP3,RNF2,GNA12,TRA2B,KDM2B,SEMA3C,VPS52,EDNRA,BMP4
GO:0019222	regulation of metabolic process	1.6160231729071815e-7	POLDIP3,ENPP1,PRDX2,LDB2,NRXN1,ASPH,PRMT3,PRKCI,MOV10L1,SLCO3A1,PDE4D,DNMT1,S1PR2,HLX,SLC9A1,C6ORF89,PBX3,ADCY8,MED13L,TRPS1,CBFB,PDE8A,ZNF823,IL31RA,RPS6KA2,PRDM12,SETD4,PTGFR,MED26,WWC1,FBXL2,NRG3,ASH1L,NOS1AP,LATS2,NOX5,PTH2R,SCAF8,STOX2,KSR2,PTGIS,PRKAG2,WWC3,PRLR,MVP,PAGR1,ADCY7,HIVEP3,FTO,NPAS3,UBAP2,DCAF12,NTRK3,CBL,ARNT,EGLN2,LRRFIP2,FLT3,STAT5B,TOX,ENPP2,EXD1,GRM5,PLCE1,ZNF566,FER,CASK,MAP2K5,KITLG,MAPK10,LRP2,PIK3CD,ZNF536,SP3,DEPDC5,HDGF,ANK2,EIF4G3,OLFM1,EZR,IKZF2,ROBO1,MALRD1,TOM1L1,CHD7,HTR2B,PSMD1,ITGB6,MECOM,TACC1,MIR1185-1,TENM1,LMNA,TRAF6,ESRP1,ITPKB,DNAJA3,OXR1,VAV2,ABI1,NHLH1,ITGB1,HSP90AA1,SLC24A3,CDC6,PSMB7,TSC22D3,KNDC1,ZC4H2,CCDC22,DSCAM,CDAN1,CNGB1,AKAP13,GFI1B,PTPRK,RUNX1,DAB1,OMA1,ABCC8,ERC1,HMGN3,NRIP1,THRB,MIR105-2,MIR767,EFCAB7,ITGB3BP,TCIRG1,DACH1,PTPN11,ZNF569,MGAT5,CCT2,ORC2,HDAC6,SERTAD2,DDB1,MYT1,ADORA1,EPHA1,IKBKB,PEX14,ERBB4,MRE11A,LIMK1,ZNF609,BRD8,KAT6B,ACTG2,HIF3A,SNIP1,CUL4B,ESR1,MIER1,PCBP3,PMEPA1,MAML3,PTPRO,CDON,NTRK2,TNRC6A,EP300,CELA1,ZYG11B,TENM2,ZN

			F76,RNF220,ZNF471,FNTA,PDGFB,RIMS1,TNIK,CCND3,TOB2,ZNF19,ZNF23,BRMS1,CHFR,ZNF605,SCML4,BPI,HNF4G,INSR,FMN2,RERE,PRKAR1A,ASB5,FUBP1,ATP8B1,H2AFY2,TCF7L2,PIP4K2A,JMJD1C,CAB39,ZNF443,ZNF490,ZNF564,ZNF709,ZNF799,PAK1,LITAF,FBXW11,ESRRB,MYOM1,MAP3K4,RNF144A,BASP1,EGLN3,RBM20,TBR1,SAMD4A,BDNF,TFAP2A,CDK14,FANCA,PEG3,ZIM2,TGIF2,TGIF2-C20ORF24,FUT8,MEIS1,VRTN,TRIM13,LCK,MDM4,IL5RA,ENTPD5,SHC4,ECT2,ZNF148,MTA3,SNX6,TFDP2,CST2,PTCD3,RFTN1,ERN2,C1D,NPLOC4,AFF3,DMD,CENPF,ATG10,SLC30A9,TOX3,PDS5A,LARP4B,USP13,KAT7,ZNF667,SLC8A1,GSN,KHDRBS2,RBM14,RBM4,MED12L,SATB2,PAFAH1B2,RIT2,HIRA,CASS4,FYN,MKRN2,ARNTL,NF1,SMG7,PLCB1,MGMT,LMTK2,ARID4B,PPP4R2,RTN4,AFF2,DPEP1,APOD,BCR,RWDD3,BRPF1,CHI3L1,TTN,PAQR3,PIK3R2,RANBP9,SLC4A5,NRG1,SH2D3C,UBP1,BDKRB1,BDKRB2,BID,MIR600HG,FRY,MAP2K1,MDFI,FNIP1,VAX2,MYH9,TMBIM6,VGLL4,PPP2CB,PPP3R1,HDAC5,CSRNP3,RBM5,ZNF692,NFIA,RNF4,CSNK2A3,JDP2,CMKLR1,ITIH2,TSPAN8,ROR2,DCN,SCFD1,PCBD2,HNRNPLL,CDH13,CREBRF,TRDMT1,SOX13,FHIT,WDR70,PTPRT,AUNIP,COPS5,PPP1R42,EXOC4,IPO5,MAD2L2,TLE6,RAB27A,CAPRIN2,CCNYL1,ZNF418,NLN,PHF20L1,TERF2,SLX1B,ZNF286A,FOXN3,NEUROD1,GNAQ,RFFL,CCBE1,HERC5,USP22,JAK2,FAM168A,TRAPPC9,FBXW7,OAZ2,SKAP1,SMC3,UIMC1,ITCH,MLIP,ZFYVE28,ABCC2,LZTS1,BCL11B,SMG1,RBFOX1,PKNOX1,MLLT3,TSHZ2,TCF7,PDE2A,KLF15,TBX15,ANXA4,LAMTOR3,CYFIP2,WNT11,MTA1,KLF8,NOX4,LCOR,RPRD1B,MKLN1,QKI,CCDC62,ERCC8,PRKCD,SOX6,TAB2,ACVR2A,RUNX2,CD4,PPM1E,TGFB1,BANP,SGK1,SPSB4,TRIM65,NSD1,IGSF1,PIBF1,ZHX2,PKNOX2,ASCC2,BTRC,NFATC3,CRADD,MIR153-2,F2RL1,BCAS3,C9ORF47,CDYL2,DNAJB2,SLIT3,CC2D1B,MIR218-1,SLIT2,TP73,ITIH4,HP,CORO1C,SAP18,ZBTB22,ILF2,DISC1,BLID,ZFAND2A,CLN6,MTDH,FANK1,MYT1L,KDM4B,SMAD6,BNC2,ZNF398,CLOCK,TCF12,ZNF675,ETV6,NELL1,SUCO,TFAP2D,BMPER,TIMP2,BCL3,ANKRD54,DAPK2,TNFRSF10B,SND1,DUSP22,NAIP,HNRNPC,TRRAP,HOMER2,DOCK3,SBNO2,YTHDF1,FGF10,CIZ1,SMYD3,LOXL3,MAST2,FANCI,CAPN3,LUM,SMURF2,EPHA4,RORA,HIVEP2,PRKCA,AUTS2,CNR1,CD6,TNFSF11,SMG6,PPP3CA,NSUN2,UBQLN3,NFYB,MAGEA4,KLF12,CAMK4,GATAD2B,PIP5KL1,UFL1,TRAK1,CTNNB1,PARK2,SOD2,DACH2,METTL13,FCGR2B,SMARCC1,KLF17,IGF1R,PPARG,NGRN,AXIN1,PRKAR1B,OTUB1,IL18R1,IL1RL1,CIPC,MTF1,MSR1,CELF2,CBX5,ANKRD17,CYBB,SCAMP5,BRIP1,LRPPRC,SREBF2,CDK11A,CDK11B,LEPR,FGF1,PROS1,NPAT,NR4A3,FOXK2,NOL3,PRKAR2A,RIOK2,ESCO1,MYOCD,TRIM5,PER2,KIR2DL4,AJUBA,GLG1,ZNF626,ZNF737,CHEK2,SUPT3H,UBQLN4,PRDM16,PPP1CB,SPDYA,HCK,CSTL1,SORBS1,TBC1D14,RAB3GAP2,CAPN2,TRIM8,DIO2,CSPG4,BRMS1L,ZBTB8A,ZBTB8B,CTDP1,ZUFSP,BAZ1B,NF2,FLT4,MEF2B,HDAC4,PAX2,PHF5A,SECISBP2L,SPTBN1,TRABD2B,SFRP1,MED13,PPP6R2,ZNF395,FOXO3,NFIB,SP4,ZCCHC17,BCL2L13,SSH1,CELF5,SYNCRIP,SMAD3,RNFT2,CUX2,ITPR1,WWP2,ARNT2,SBNO1,KRBOX1,ZNF662,ZNF777,SIMC1,WIPI1,EBF3,MTBP,RNF168,CASZ1,PSAP,IFNAR1,DCP1B,MIER3,NEDD4,ESRRG,HOXD3,HOXD4,NAV3,SLC4A4,ZNF114,TAB1,KTI12,NFATC1,CDC73,APP,SSBP3,GSX2,PDGFRA,RBM8A,CCNI2,DIP2B,NOX1,ARIH1,YAP1,HEG1,SESN1,TEN1,VDAC1,EYA2,SH3D19,BORA,IBTK,NVL,ALPL,PEMT,LRP5,MTCP1,POLR3G,ZNF787,SOX2,SETD2,TEAD1,PRICKLE1,RCAN1,ZNF653,SPTBN4,ZNF521,DRD1,ARID3A,ZNF761,CHUK,ERLIN1,SFMBT1,ZNF584,ESR2,S100A12,DCUN1D3,KDM6A,PRKD1,STAT1,CELF6,ST18,ETV5,RHOXF2B,SLC6A3,TAF3,PLAGL1,HNF4A,ZBTB7C,TASP1,EREG,CCNY,RBFOX3,ATF2,POU2F2,TCF3,ZNF730,GRAMD4,RAF1,CELF4,ZNF766,CARD16,CASP1,PTPN1,SRRM4,POLA1,BMPR2,USP33,VBP1,CAMK1D,BMPR1A,ZKSCAN1,PABPC4,BTBD10,IKZF4,PIK3R3,CDK12,CAND2,SPAG9,MORC2,DENND4A,MYB,FGF2,ZNF71,POU6F2,BACH1,MXD3,PPM1F,TICRR,GLI4,ZFP41,NEDD9,AGBL4,BEND5,SEMA4D,NFX1,RORC,ELP3,PTAFR,ADCYAP1R1,SP140,SP140L,RHOXF2,JARID2,DDX58,BRDT,PHC2,RARB,SPEN,PRKCG,SIN3B,NCOA1,SPOCK1,AREL1,EHMT1,LMO7,AP2M1,DVL3,EIF2B5,EIF4G1,EPHB3,PSMD2,RBX1,TCF20,ATF3,LIN28B,ATP6V0B,SRSF5,LINC00461,CKS1B,PAWR,EBF2,AGO3,DEPTOR,FBXL20,MAML2,TSG101,DESI1,CCDC3,TERF2IP,CRYM,IDE,KCNQ1,RFX2,WNT3,ZNF322,SUFU,MAGEA11,PTPN13,PRDM15,ZNF670,ZNF695,CCDC169-SOHLH2,SOHLH2,COL4A3,DDAH1,ZNF354C,HIP1,TCEA3,PADI6,ZNF704,NR2C1,TRPC5,UBA2,ME1,GLI2,TNKS,WBP2NL,ERCC1,TNRC6B,GLIS3,WDTC1,ZNF664,DAB2,BLM,PKHD1,CACUL1,LDLRAD4,MYSM1,SETD5,SMG5,DLG3,WNK1,RELN,NEK10,SIN3A,RUVBL2,COMMD6,GMEB1,PELI1,IQGAP1,MAP3K7,ZNF423,SP1,TRIM22,ALK,SLC8A2,INPP5D,TEAD4,FASTKD5,HOXC13,APBB3,SLC35A4,SRA1,TOP1,UBE2V1,TPCN1,ADIRF,XDH,AHNAK,LTBP1,OVOL2,SNX5,NFATC2,RBBP6,PAX7,BNC1,ATP2B4,NSF,ACTL6B,ASXL3,PAK3,TET1,CAMTA1,CCPG1,DYX1C1,ATAD1,ADNP,MAPKAPK2,ARID4A,SCARB1,MARK1,CDK6,ATP6V0A2,PHF2,CELF1,RNF34,TFRC,UVRAG,EPHA5,WWOX,MEF2A,MIR1226,DCAF6,DCUN1D5,DROSHA,GPC3,XRN1,SATB1,TBC1D5,PSMB2,EYA1,GATAD2A,SCOC,DIS3L2,GNB3,TRPV1,RBM12B,RNF216,HOXB3,HOXB4,HOXB5,HOXB6,MIR10A,TFEB,CLEC16A,GHR,MNAT1,SNX9,ACTR2,L3MBTL4,ELAVL4,HDAC1,RECQL5,SMOC2,TRIM24,ADIPOR2,PLCG2,ROCK1,SCMH1,C1QTNF1,PAXIP1,ZNF713,EGR2,HIPK3,RNF10,RYBP,AP3B1,PTPN2,INSRR,NTRK1,KPNA6,TRIM44,MAP1A,SLC39A10,ANK3,EPHB1,EPHA10,AXIN2,EPCAM,PARD3,ZNF425,GNL3L,MUC1,PTPN14,BCOR,AP3D1,ZNF41,CARM1

			,ZNF383,ANXA8L1,ADAM19,SNX33,PIFO,CD84,LPA,ZNF616,ZNF836,SMARCA2,CUX1,MIR1302-10,SPI1,DNMT3B,EPHA7,FOXP2,PSPC1,GLIS1,SFMBT2,ATG14,EIF3H,BCAR3,JAZF1,STK38,ABCB7,ACOT8,RBPMS,RENBP,CHURC1,CREB5,MAP3K13,RAPGEF3,MST1,SMYD1,CHID1,SNCA,BMPR1B,MAGI2,PRIM2,PNPT1,USP50,ZEB2,DHRS4,FOXJ3,HEY2,RC3H1,EIF3E,RNF19B,PTPRS,ZMYND11,KMT2D,PRKAG1,CREM,PSMF1,KMT2C,CAST,TRIP12,DFFA,ADTRP,PLD1,RASA1,MACC1,MITF,SRSF6,OPRD1,NUGGC,RPTOR,NFIX,BTK,MIR383,TBC1D16,KCTD13,MLLT1,TROVE2,CBFA2T2,IGF1,MLXIP,ATF7IP,HTT,MAPK1,PTEN,SPRED2,BMP7,ATXN2,MXI1,PUM1,SOX5,CIR1,PCBP2,PRCP,ZNF780A,ZNF780B,LRRK2,ATP6V1A,MLYCD,TMEM59,UNC119,ZBTB20,CHMP5,RFC3,BAG6,LILRB4,ITGAM,PKD2,ZNF652,EFNA5,KCTD1,RIPPLY1,RCOR3,TADA2A,DAZL,PDGFC,SERPINA3,SERPINA4,SERPINA5,NCOA3,PKIB,MTMR3,ZNF146,ZNF565,CUEDC2,PPP2R3C,WNT7A,ZBED6,BRWD3,NLRP1,UACA,LPGAT1,CRTC3,DNAJC1,ARNTL2,ELOVL5,PLCL2,KAT6A,MTIF2,ZKSCAN7,ZNF197,ZNF660,BMP6,TAF15,MYCBP2,NFE2L1,GPI,SH3RF2,TSHR,WDR43,ZNF30,N4BP1,PDCD1LG2,ATRX,DPH6,IKZF1,PRMT2,RAPGEF2,ASCC1,SMO,VRK3,RALY,PRKAR2B,CPT1A,TCF4,AFF1,DECR1,SOX30,TFE3,UBR5,GRIN1,JADE1,ARRDC4,RHOA,SPRTN,ROR1,RQCD1,TF,ONECUT2,CUL3,TFEC,HSPD1,NFKBID,EPM2A,GSK3B,PRR16,DNAJC3,RDX,STAT6,ACTA2,MLF1,EEF2K,FAM49B,IWS1,PPP1R16A,PTPRB,RBBP8,NECAB2,CDK13,UPF2,CD27,DUSP26,BOK,RNF144B,SULF1,ZNF362,NACC2,SUPT4H1,TNFRSF8,ZNF354A,AGT,HDGFRP3,METTL16,CCNJL,ELOVL3,POLH,PRKAA2,ADAR,STAT2,ZMPSTE24,NDRG2,PTK2,UPK3B,SNX3,PDE4B,PBX1,TEC,EXOSC3,MAP3K5,SEC16B,TCFL5,CREBBP,DLX1,GTF2IRD2,NCF1,TAOK2,UGT1A1,UGT1A10,UGT1A4,UGT1A8,PYGO2,SHC1,ZNF813,CD160,ECE1,LBX2,PARP10,RNFT1,RPS6KB1,STC2,YLPM1,DPRX,PRG3,SERPINB11,SETDB2,ALOX5,DNAJB6,SP7,CD44,LARP4,ADAM8,SLC5A3,CLDN4,RPS6KA5,ZNF484,ZNF93,CSNK1A1,HUS1,MIR346,PRDM2,CXCL17,NOS1,ZNF44,GCKR,MED15,RGS14,ACTN4,MC1R,STK4,TCF25,DRAM1,ZNF282,EPHB2,SLC1A1,EDRF1,TMEFF2,BCO2,IL18,MFSD8,UBE2K,HCAR1,HCAR2,DIS3,DLC1,PPARA,PPP1R10,ARID5B,EPB41L4B,SLC25A33,SMARCAL1,CPEB4,GPSM3,MOB3B,NOTCH4,ZMYND8,ZNF366,CRX,PAX6,PRKCZ,ZC3HAV1,CD96,FAM20C,ADORA2A,GRIN2B,PPP1CC,RABGEF1,CCNG2,CPEB1,OTUD7B,NIPBL,TMIGD2,YEATS4,COL28A1,PHF20,ZNF143,ANGPT1,BRF1,TBL1X,EYA3,FHL2,LRRK1,MBTD1,OSBPL8,ATF6,ZBTB5,ZNF708,IGF2BP3,GCNT2,ETS1,PPP1CA,MTF2,RBMS3,BTN3A2,TAGLN3,CDK5RAP1,CNTN1,NCOR2,PRKCQ,BRAF,CSRNP1,HDAC2,HTR2C,IMPACT,MIR1912,PHKG2,SRCAP,POU3F3,NOTO,PPP1R14A,SPINT2,MBNL3,ZKSCAN5,CD300A,ELF2,PHIP,PPP2CA,SKP1,ANGPT4,RPL23,SH3BP4,RFC5,ATXN1,SP100,ZNF347,ZNF415,ANXA2,TRIM29,PARN,ATP6V0A1,PRMT7,DONSON,ADCY1,MADD,NUMB,RBM42,SYK,CNOT1,RBM39,WWTR1,GTF2H5,ASB1,NFXL1,ZBTB38,BRCA2,ATF7,DVL2,MORC1,MTRF1,YBX1,ANKRD13A,PTPRJ,SLC10A1,TAF1,EIF4E3,TICAM1,TANK,THAP3,CADM1,HSF1,MAX,SAP130,DHFR,EZH1,ETF1,MIR663B,NRF1,SRPK2,SASH1,NCOA2,GBP5,WDR18,EEFSEC,QRICH1,VAV3,ZP3,CHD6,RNF2,ZNF554,MOB3A,GNA12,SH2D3A,AKTIP,MYEF2,INPP5F,AXL,TRIM37,CD109,MET,TBC1D10A,ZNF461,CAMK2D,SNX1,TIMELESS,UCHL5,BRD9,MALT1,SERPINE3,SETD3,TRA2B,KDM2B,MLXIPL,CCT3,NEDD4L,PHB,PTPRC,EDA,ZNF555,CTCF,SH3GL2,TYRO3,NR6A1,RNF213,SPON2,TRAF3IP2,TRIM60,ATF6B,CREB1,RGMB,TNXB,TRAP1,AFF4,CLNK,EBF4,ZNF511,BMP4,ABLIM3,CPNE1
GO:0010647	positive regulation of cell communication	1.8299827271647618e-7	*PRDX2,GPC5,NRXN1,PRKCI,MAP4K4,PDCL,SLC9A1,ADCY8,PDE8A,SEMA3A,CHERP,WWC1,C12ORF49,NOS1AP,MAPRE2,PTGIS,PRLR,PAGR1,NTRK3,CBL,ARNT,SEMA5A,FLT3,GRM5,PLCE1,MAP2K5,KITLG,LRP2,PIK3CD,ROBO1,HTR2B,TENM1,TRAF6,ROBO2,ITPKB,SLC24A2,ITGB1,HSP90AA1,PSMB7,CCDC22,AKAP13,DGKI,LAMA2,PTPN11,MGAT5,CACNB2,ADORA1,ADAMTS3,IKBKB,ERBB4,ADRA1D,MIER1,CDON,NTRK2,EP300,RNF220,PDGFB,RIMS1,TNIK,MID1,STK39,INSR,TCF7L2,USP34,PAK1,LITAF,MAP3K4,ZDHHC13,BDNF,TRIM13,LCK,ECT2,VAMP7,ERN2,RIT2,DEPDC1B,CASS4,FYN,ARNTL,NF1,PLCB1,RTN4,RTN4R,RWDD3,CHI3L1,NDRG4,BMP2K,NRG1,BID,MAP2K1,FNIP1,ROR2,DCN,ZDHHC3,SLC39A14,CDH13,CAPRIN2,CCBE1,JAK2,FBXW7,CLSTN2,SYT1,NPSR1,MLLT3,LAMTOR3,CYFIP2,WNT11,NOX4,SIAH1,PRKCD,TAB2,ACVR2A,CD4,NUP93,PIBF1,CRADD,F2RL1,AAK1,TP73,DISC1,KANK1,MTDH,BMPER,TNFRSF10B,DUSP22,NAIP,YTHDF1,FGF10,IQCJ-SCHIP1,SH3RF3,LOXL3,SMURF2,EPHA4,PRKCA,AUTS2,CNR1,TNFSF11,CTNNB1,PARK2,FCGR2B,IGF1R,PPARG,AXIN1,DLG5,IL18R1,P2RY10,ANKRD17,APOL3,FGF1,MYOCD,TRIM5,AJUBA,SORBS1,TRIM8,CSPG4,GRM1,FLT4,SFRP1,CNTN6,SMAD3,CUX2,SHANK1,NEDD4,TAB1,CDC73,APP,GSX2,PDGFRA,NOX1,YAP1,NLGN2,TNFRSF19,SOX2,DRD1,CHUK,FRS2,S100A12,PRKD1,SHANK2,EREG,RAF1,RASGRF2,CARD16,CASP1,PTPN1,BMPR2,BMPR1A,NLGN1,SPAG9,EVC,FGF2,SEMA4D,DOK6,ADCYAP1R1,DKK2,PRKCG,EEF1E1,EEF1E1-BLOC1S5,GAS8,DVL3,ATF3,SHOC2,AGO3,TERF2IP,WDR59,WNT3,CCL14,CCL15,NLGN3,SCUBE2,PAFAH1B1,PRDM15,HIP1,AKAP6,NR2C1,NEO1,TNKS,SCUBE1,DAB2,WNK1,RELN,NEK10,RSPO2,GUCY1A2,PELI1,IQGAP1,MAP3K7,ZNF423,TRIM22,MACF1,ALK,SLC8A2,STAU1,UBE2V1,XDH,SNX5,CAMTA1,P2RY8,TFRC,WWOX,SYT12,GFRAL,GPC3,PSMB2,TSPAN6,CLEC16A,GHR,SMOC2,PLCG2,ROCK1,C1QTNF1,CAPN10,GPR35,PTPN2,NTRK1,TRIM44,SLC39A10,ANK3,JAG2,CACNG8,VW*

			F,NETO1,ARHGEF3,SPI1,CHRNB4,MCU,CTNNA1,TRDN,LY86,BCAR3,RBPMS,MAP3K13,PTK7,SNCA,BMPR1B,CACNG2,GRM4,ZEB2,RC3H1,WNT7B,KMT2D,RPTOR,KCTD13,SLC4A8,IGF1,HTT,MAPK1,PTEN,SPRED2,BMP7,PUM1,LRRK2,ZRANB1,TMEM108,PKD2,SHANK3,PDGFC,WNT7A,SIK3,BMP6,ANO1,SH3RF2,SOS1,RAPGEF2,SMO,UBR5,GRIN1,RHOA,ROR1,RQCD1,GSK3B,ACTA2,NECAB2,CD27,BOK,SULF1,NACC2,AGT,PTK2,RIMS2,MAP3K5,CREBBP,NCF1,TAOK2,SHC1,LBX2,SCEL,CD44,ADAM8,BCL2L14,CXCL17,RGS14,ACTN4,MC1R,STK4,EPHB2,SLC1A1,WLS,IL18,UBE2K,HCAR2,PRKCZ,ZC3HAV1,ADORA2A,GRIN2B,ANGPT1,TBL1X,LRRK1,OSBPL8,ATF6,GCNT2,PPP1CA,CCL22,BRAF,HTR2C,TNR,CD300A,PHIP,MAOA,RPL23,ADCY1,MADD,SYK,GRIK2,MYRIP,CACNG3,DVL2,PTPRJ,TICAM1,SASH1,CAV2,AXL,MET,UCHL5,ANKRD6,MALT1,PHB,PTPRC,EDA,GPR89A,TSPAN5,TRAF3IP2,CREB1,BMP4,CPNE1
GO:1902532	negative regulation of intracellular signal transduction	1.8506001684204953e-7	*PDE4D,WWC1,ASH1L,WWC3,MARVELD3,PTPRR,DEPDC5,EZR,TNFAIP8L1,MECOM,DNAJA3,ITGB1,CCDC22,ARHGAP24,ESR1,LITAF,FBXW11,NPRL3,UBR2,ARHGAP42,NPLOC4,DMD,MKRN2,ARNTL,NF1,PAQR3,PIK3R2,RANBP9,BDKRB2,BID,FNIP1,MAD1L1,TMBIM6,VGLL4,PPP2CB,RFFL,LINC00473,ITCH,PDE2A,TRIM59,PRKCD,F2RL1,SLIT2,KANK1,ZNF675,HOMER2,RASA4,RASA4B,KCTD10,EPHA4,RORA,PARK2,SOD2,IGF1R,PPARG,DLG5,NOL3,AJUBA,CHEK2,NF2,SFRP1,ITPR1,TXNDC12,HEG1,SESN1,RCAN1,STAT1,DGKG,SHANK2,PTPN1,PDE11A,ATF3,DEPTOR,PAFAH1B1,PRDM15,RASAL1,DAB2,PKHD1,UBR1,ARHGAP12,XDH,ATP2B4,RNF34,TSPAN6,HDAC1,HIPK3,PTPN2,EIF3A,MUC1,ARHGAP44,SPI1,STK38,OTUD3,SH3BP1,MAGI2,ZMYND11,RASA1,KCTD13,PTEN,SPRED2,BMP7,RASA2,LRRK2,LILRB4,ARHGAP25,UACA,SH3RF2,VRK3,RHOA,SYNGAP1,CUL3,GSK3B,FAM49B,DUSP26,AGT,PRKAA2,NDRG2,CD44,PLEKHA1,RGS14,MARK3,EPHB2,DLC1,PPARA,PPP1R10,PHLPP1,RABGEF1,OTUD7B,FHL2,TBC1D10C,CD300A,PPP2CA,SH3BP4,CIT,PTPRJ,TANK,CGNL1,MET,PHB,PTPRC,TRAP1,PRAP1,BMP4,CPNE1*
GO:0008219	cell death	1.8959902496145583e-7	*PRDX2,PRKCI,MAP4K4,DNMT1,TMBIM4,SLC9A1,PDE8A,SEMA3A,IL31RA,GRID2,RPS6KA2,PTGFR,DOCK1,LATS2,NOX5,ANP32A,PTGIS,TJP1,UNC5C,PRLR,NTRK3,CBL,EGLN2,SEMA5A,FLT3,STAT5B,MAP2K5,KITLG,LRP2,PIK3CD,OLFM1,MEGF10,TNFAIP8L1,HTR2B,MECOM,LMNA,TRAF6,ROBO2,ITPKB,DNAJA3,OXR1,GRK5,ITGB1,HSP90AA1,ELMO2,TSC22D3,OMA1,THRB,ITGB3BP,TCIRG1,HDAC6,DDB1,ADORA1,IKBKB,ERBB4,GBE1,MRE11A,HIF3A,CECR2,ESR1,NTRK2,EP300,BRMS1,DAP3,FMN2,UNC5D,PRUNE2,TCF7L2,ZNF443,PAK1,EGLN3,BDNF,TFAP2A,KCNMA1,PEG3,TRIM13,LCK,MDM4,SHC4,ECT2,SNX6,ERN2,C1D,TOX3,GSN,FYN,NF1,MGMT,SNCB,RTN4,DPEP1,CHI3L1,NTN1,NRG1,ARHGAP10,CDK19,BDKRB2,DOCK8,BID,FNIP1,TMBIM6,PPP2CB,WDR92,CSRNP3,RBM5,ZDHHC3,FHIT,COPS5,NCKAP1,ANO6,CERKL,NEUROD1,TMEM117,RFFL,JAK2,FBXW7,ITCH,BCL11B,TEX11,ANXA4,CYFIP2,WNT11,KREMEN1,SIAH1,PRKCD,TGFB1,SGK1,DUS2,CRADD,SLIT3,SLIT2,TP73,HP,HPR,SAP18,RRAS2,BLID,MTDH,FANK1,SMAD6,ETV6,TFAP2D,BCL3,DAPK2,TNFRSF10B,NAIP,FGF10,SH3RF3,CAPN3,PRKCA,CNR1,MAGEA4,MAG,PIP5KL1,UFL1,CTNNB1,PARK2,SOD2,FCGR2B,IGF1R,PPARG,AXIN1,DLG5,RYR2,CDK11A,CDK11B,DRAXIN,NR4A3,NOL3,MYOCD,KIR3DL2,CHEK2,HCK,CAPN2,PPP2R2B,DCC,NF2,FLT4,SGMS1,HDAC4,PAX2,SFRP1,FOXO3,BCL2L13,SMAD3,ITPR1,EVA1A,GRIK5,TXNDC12,CDC73,APP,SLK,NOX1,YAP1,TNFRSF19,VDAC1,EYA2,ANGPTL4,LRP5,CHST11,TMEM14A,FRS2,PALB2,MMP2,DCUN1D3,PRKD1,STAT1,USP53,PLAGL1,TCTN3,ATF2,GRAMD4,RAF1,ACOX2,CARD16,CASP1,PTPN1,BMPR2,CAMK1D,BTBD10,CERS3,FGF2,PPM1F,SEMA4D,RORC,ADCYAP1R1,YME1L1,RILPL1,PLEKHO2,SLC5A8,RARB,PRKCG,NCOA1,AREL1,EEF1E1,TXNDC5,EIF2B5,EIF4G1,ATF3,PAWR,DEPTOR,P4HB,CNTFR,COL4A3,HIP1,TRPC5,GLI2,DAB2,PKHD1,XKR4,SIN3A,PELI1,PPP2R5C,CFDP1,MAP3K7,DDX47,ALK,INPP5D,SRA1,TOP1,PAX7,PACRG,PAK3,ADNP,BCL7C,CELF1,RNF34,TFRC,WWOX,MEF2A,CACNA1A,GFRAL,EYA1,PTGER3,BRINP1,TRPV1,RNF216,GHR,MNAT1,HDAC1,TRIM24,PLCG2,ROCK1,HIPK3,RYBP,CAPN10,CUL2,PTPN2,NTRK1,PKN2,TMIGD1,SLC39A10,EPHB1,AXIN2,SARM1,MUC1,JAG2,AP2B1,SPI1,EPHA7,CTNNA1,AIFM2,MST1,ERCC3,SNCA,BMPR1B,GRM4,USP42,HEY2,CHMP3,ZMYND11,CAST,DFFA,RASA1,MITF,SRSF6,HIPK1,NUGGC,BTK,IGF1,CTNNBL1,HTT,MAPK1,PTEN,BMP7,LRRK2,OSGIN1,EBAG9,BAG6,ITGAM,WNT7A,ZBED6,NLRP1,UACA,RABEP1,RHOT1,GPI,SH3RF2,PRMT2,RAPGEF2,SMO,MARK4,PSEN2,TNFRSF11B,JADE1,RHOA,SYNGAP1,CUL3,SH3KBP1,HSPD1,GSK3B,DNAJC3,EEF2K,CD27,BOK,RNF144B,SULF1,KRT8,NACC2,TNFRSF8,AGT,PRKAA2,ADAR,ZMPSTE24,PTK2,FBXO10,SHB,MAP3K5,DLX1,NCF1,TAOK2,SHC1,CD160,RPS6KB1,DNAJB6,CD44,ADAM8,BCL2L14,ACTN4,STK4,ZFAND6,DRAM1,SPEF1,SLC1A1,SIGMAR1,HCAR2,DLC1,PPARA,PPP1R10,STEAP3,PHLPP1,MAGI3,CPEB4,PRKCZ,ADORA2A,GRIN2B,ANGPT1,PACS2,EYA3,FHL2,ATF6,ETS1,PPP1CA,GRIA4,STPG1,ATG3,PRKCQ,BRAF,CSRNP1,HDAC2,IMPACT,TNS4,POU3F3,HIGD2A,PHIP,PPP2CA,ANGPT4,ARHGEF6,SP100,MADD,SYK,GRIK2,CIT,BRCA2,HRK,TICAM1,CADM1,HSF1,MAX,INPP5A,SRPK2,ANKRD13C,QRICH1,VAV3,AKTIP,NUAK2,AXL,TNFSF9,MET,CAMK2D,MALT1,TRIM69,KDM2B,THEM4,PHB,PTPRC,TYRO3,ADCY10,TRAF3IP2,TRAP1,CLNK,EDNRA,GNGT1,PRAP1,BMP4,CASP12*
GO:0043087	regulation of GTPase activity	1.914748534269687e-7	*MAP4K4,TIAM2,MAPRE2,KALRN,NTRK3,CDKL5,VAV2,ITGB1,ARHGAP24,RGS6,DGKI,DOCK10,EPHA1,ABR,NTRK2,ARHGAP6,ECT2,ARHGAP42,NF1,RTN4R,BCR,DOCK8,RAPGEF6,SBF2,RGS8,RCC2,DENND1A,ARAP2,DOCK9,WNT11,SIPA1L3,F2RL1,BCAS3,CORO1C,RABGAP1L,RASA4,RASA4B,EPHA4,ARHGAP22,GARNL3,RG*

			S10,AJUBA,TBC1D14,RAB3GAP2,TBCK,SFRP1,ARHGAP29,PREX1,NEDD9,SEMA4D,PLXNA2,DVL3,EPHB3,MYO9A,CCL14,CCL15,PAFAH1B1,RASAL1,WNK1,DOCK11,IQGAP1,ARHGAP12,PRKG1,RGS7,CCPG1,EPHA5,TBC1D5,FGD1,SNX9,GAPVD1,NTRK1,ARHGAP44,BCAR3,RAPGEF3,SH3BP1,TBC1D9,RASA1,TBC1D16,RASA2,LRRK2,ARHGAP25,EFNA5,SGSM1,RAPGEF2,SYNGAP1,GSK3B,RDX,PTK2,IQGAP2,ARHGAP15,FGD3,FGD4,RGS14,SIPA1L2,ARHGAP11A,GRTP1,TBC1D10C,CCL22,SH3BP4,SRGAP2,RAP1GAP2,DVL2,ASAP1,RALGAPA1,VAV3,CAV2,MET,TBC1D10A
GO:0045944	positive regulation of transcription by RNA polymerase II	2.1460061047753803e-7	*LDB2,SLC9A1,PBX3,CBFB,ASH1L,STOX2,PAGR1,ARNT,STAT5B,CASK,MAP2K5,SP3,HDGF,CHD7,MECOM,TRAF6,NHLH1,GFI1B,RUNX1,HMGN3,NRIP1,THRB,EFCAB7,IKBKB,ZNF609,BRD8,KAT6B,ESR1,MAML3,CDON,EP300,CELA1,ZNF76,HNF4G,TCF7L2,LITAF,ESRRB,TBR1,TFAP2A,PEG3,MEIS1,ZNF148,TFDP2,SLC30A9,TOX3,KAT7,RBM14,SATB2,RIT2,MKRN2,ARNTL,ARID4B,PIK3R2,UBP1,PPP3R1,HDAC5,CSRNP3,NFIA,RNF4,DCN,CDH13,CREBRF,COPS5,CAPRIN2,NEUROD1,JAK2,SKAP1,MLIP,BCL11B,PKNOX1,KLF15,RPRD1B,CCDC62,ACVR2A,RUNX2,TGFB1,NFATC3,F2RL1,BCAS3,TP73,CLOCK,TCF12,ETV6,TFAP2D,BCL3,SBNO2,FGF10,SMYD3,LUM,RORA,AUTS2,TNFSF11,PPP3CA,NFYB,KLF12,CTNNB1,PARK2,SMARCC1,PPARG,MTF1,SREBF2,FGF1,NPAT,NR4A3,FOXK2,MYOCD,PRDM16,MEF2B,HDAC4,PAX2,MED13,ZNF395,FOXO3,NFIB,SMAD3,WWP2,ARNT2,EBF3,CASZ1,ESRRG,HOXD3,HOXD4,NFATC1,CDC73,APP,SSBP3,YAP1,LRP5,SOX2,TEAD1,ZNF521,ARID3A,CHUK,ESR2,PRKD1,STAT1,ST18,ETV5,PLAGL1,HNF4A,ZBTB7C,ATF2,POU2F2,TCF3,RAF1,BMPR2,BMPR1A,IKZF4,CDK12,MYB,FGF2,ZNF71,BACH1,DDX58,RARB,NCOA1,LMO7,DVL3,TCF20,ATF3,EBF2,MAML2,RFX2,PRDM15,GLI2,TNKS,ERCC1,GLIS3,DAB2,MYSM1,SIN3A,RUVBL2,GMEB1,SP1,TEAD4,HOXC13,ADIRF,OVOL2,NFATC2,ASXL3,TET1,CAMTA1,CCPG1,ARID4A,WWOX,MEF2A,DCAF6,EYA1,HOXB3,HOXB4,HOXB5,TFEB,ACTR2,HDAC1,PAXIP1,EGR2,RNF10,AP3B1,KPNA6,EPCAM,MUC1,AP3D1,ZNF836,SMARCA2,SPI1,GLIS1,BMPR1B,ZEB2,FOXJ3,HEY2,KMT2D,CREM,KMT2C,MACC1,MITF,NFIX,IGF1,MLXIP,BMP7,PCBP2,ZNF780B,PKD2,NCOA3,WNT7A,CRTC3,ARNTL2,KAT6A,ZNF197,BMP6,ATRX,SMO,TCF4,SOX30,TFE3,GRIN1,ONECUT2,TFEC,STAT6,CDK13,SUPT4H1,PBX1,CREBBP,DLX1,SP7,RPS6KA5,ZNF484,PRDM2,NOS1,MC1R,IL18,PPARA,NOTCH4,CRX,PAX6,NIPBL,ZNF143,TBL1X,ATF6,ETS1,MTF2,CSRNP1,HDAC2,POU3F3,PHIP,SP100,WWTR1,ZBTB38,ATF7,DVL2,YBX1,THAP3,HSF1,MAX,EZH1,NRF1,NCOA2,CHD6,MET,SETD3,MLXIPL,NR6A1,ATF6B,CREB1,BMP4,ABLIM3*
GO:0051247	positive regulation of protein metabolic process	2.526835798449016e-7	*POLDIP3,NRXN1,ASPH,SLCO3A1,DNMT1,S1PR2,C6ORF89,ADCY8,PDE8A,IL31RA,PRDM12,NRG3,NOS1AP,PRKAG2,PRLR,NTRK3,ARNT,EGLN2,FLT3,ENPP2,GRM5,MAP2K5,KITLG,LRP2,EZR,ROBO1,TOM1L1,HTR2B,TENM1,LMNA,TRAF6,DNAJA3,ABI1,HSP90AA1,CDC6,KNDC1,CCDC22,AKAP13,DAB1,PTPN11,HDAC6,DDB1,ADORA1,ERBB4,MRE11A,CUL4B,CDON,NTRK2,ZYG11B,FNTA,PDGFB,TNIK,CCND3,BRMS1,CHFR,INSR,ASB5,TCF7L2,CAB39,PAK1,FBXW11,MAP3K4,RNF144A,EGLN3,SAMD4A,BDNF,LCK,ECT2,ERN2,ATG10,LARP4B,USP13,KAT7,GSN,CASS4,FYN,MKRN2,ARNTL,PLCB1,RWDD3,CHI3L1,RANBP9,NRG1,SH2D3C,BID,MAP2K1,FNIP1,MYH9,VGLL4,CSNK2A3,JDP2,ROR2,CREBRF,MAD2L2,CAPRIN2,CCNYL1,CCBE1,JAK2,FBXW7,OAZ2,ITCH,CYFIP2,WNT11,MTA1,NOX4,PRKCD,TAB2,ACVR2A,CD4,TGFB1,SPSB4,PIBF1,BTRC,CRADD,DNAJB2,DISC1,BLID,ZFAND2A,CLN6,BCL3,TNFRSF10B,DOCK3,YTHDF1,FGF10,SMYD3,FANCI,CAPN3,SMURF2,EPHA4,AUTS2,CD6,TNFSF11,CTNNB1,PARK2,PPARG,NGRN,AXIN1,LEPR,FGF1,AJUBA,CHEK2,SPDYA,RAB3GAP2,CSPG4,FLT4,HDAC4,TRABD2B,BCL2L13,SMAD3,RNFT2,WWP2,NEDD4,TAB1,APP,DIP2B,ARIH1,SH3D19,BORA,MTCP1,PRICKLE1,S100A12,DCUN1D3,PRKD1,ST18,EREG,CCNY,ATF2,GRAMD4,RAF1,CASP1,PTPN1,BMPR2,BMPR1A,PIK3R3,MYB,FGF2,PPM1F,NEDD9,AGBL4,SEMA4D,PTAFR,JARID2,DDX58,AREL1,DVL3,EIF2B5,EIF4G1,RBX1,CKS1B,PAWR,TERF2IP,IDE,COL4A3,HIP1,TRPC5,UBA2,DAB2,CACUL1,DLG3,WNK1,RELN,NEK10,RUVBL2,PELI1,IQGAP1,MAP3K7,SP1,SLC8A2,SLC35A4,XDH,ATP2B4,NSF,TET1,CAMTA1,ADNP,TFRC,DCUN1D5,GPC3,GHR,MNAT1,SNX9,PLCG2,PAXIP1,NTRK1,SLC39A10,AXIN2,MUC1,SNX33,DNMT3B,EPHA7,ATG14,BCAR3,RBPMS,MAP3K13,RAPGEF3,SNCA,BMPR1B,MAGI2,USP50,EIF3E,RNF19B,PRKAG1,ADTRP,PLD1,OPRD1,RPTOR,IGF1,MAPK1,PTEN,BMP7,LRRK2,UNC119,BAG6,PKD2,EFNA5,DAZL,PDGFC,PPP2R3C,WNT7A,NLRP1,UACA,BMP6,MYCBP2,SH3RF2,RAPGEF2,VRK3,GRIN1,ARRDC4,RHOA,SPRTN,RQCD1,CUL3,HSPD1,GSK3B,PRR16,DNAJC3,RDX,BOK,RNF144B,AGT,PRKAA2,PTK2,TEC,MAP3K5,NCF1,TAOK2,RNFT1,RPS6KB1,CD44,LARP4,ADAM8,CLDN4,RPS6KA5,CSNK1A1,NOS1,STK4,SLC1A1,IL18,UBE2K,DLC1,MOB3B,PRKCZ,GRIN2B,NIPBL,ANGPT1,LRRK1,OSBPL8,MTF2,CDK5RAP1,CNTN1,BRAF,HDAC2,IMPACT,CD300A,PPP2CA,SKP1,ANGPT4,MADD,SYK,DVL2,PTPRJ,TAF1,TICAM1,TANK,HSF1,SASH1,MOB3A,GNA12,SH2D3A,AKTIP,TBC1D10A,SNX1,MALT1,NEDD4L,PHB,PTPRC,BMP4*
GO:0045860	positive regulation of protein kinase activity	2.714679556835429e-7	*NRXN1,ADCY8,NRG3,PRKAG2,PRLR,NTRK3,FLT3,GRM5,MAP2K5,KITLG,ROBO1,TOM1L1,HTR2B,TENM1,TRAF6,ABI1,HSP90AA1,CDC6,AKAP13,DAB1,ADORA1,PDGFB,CCND3,INSR,CAB39,PAK1,MAP3K4,ECT2,ERN2,CASS4,CHI3L1,NRG1,MAP2K1,ROR2,CCNYL1,JAK2,FBXW7,WNT11,NOX4,PRKCD,TAB2,CD4,TGFB1,PIBF1,TNFRSF10B,DOCK3,EPHA4,TNFSF11,AXIN1,FGF1,AJUBA,SPDYA,TAB1,BORA,MTCP1,S100A12,EREG,CCNY,PTPN1,BMPR2,BMPR1A,FGF2,NEDD9,DVL3,CKS1B,CACUL1,DLG3,WNK1,RELN,NEK10,IQGAP1,MAP3K7,SLC8A2,ATP2B4,ADNP,GH*

			R,MNAT1,SNX9,AXIN2,MAP3K13,SNCA,BMPR1B,PRKAG1,RPTOR,IGF1,LRRK2,UNC119,PKD2,EFNA5,PDGFC,PPP2R3C,RAPGEF2,RHOA,AGT,PTK2,MAP3K5,NCF1,TAOK2,ADAM8,STK4,SLC1A1,IL18,MOB3B,PRKCZ,ANGPT1,OSBPL8,CD300A,PPP2CA,ANGPT4,MADD,SYK,DVL2,SASH1,MOB3A,MALT1,PHB,PTPRC
GO:0031401	positive regulation of protein modification process	2.8435263415698623e-7	NRXN1,SLCO3A1,DNMT1,S1PR2,C6ORF89,ADCY8,PDE8A,IL31RA,PRDM12,NRG3,NOS1AP,PRKAG2,PRLR,NTRK3,ARNT,FLT3,ENPP2,GRM5,MAP2K5,KITLG,ROBO1,TOM1L1,HTR2B,TENM1,LMNA,TRAF6,DNAJA3,ABI1,HSP90AA1,CDC6,KNDC1,AKAP13,DAB1,PTPN11,HDAC6,ADORA1,ERBB4,MRE11A,CDON,NTRK2,FNTA,PDGFB,TNIK,CCND3,BRMS1,CHFR,INSR,CAB39,PAK1,MAP3K4,BDNF,ECT2,ERN2,ATG10,KAT7,CASS4,FYN,ARNTL,RWDD3,CHI3L1,NRG1,SH2D3C,MAP2K1,FNIP1,JDP2,ROR2,MAD2L2,CAPRIN2,CCNYL1,JAK2,FBXW7,WNT11,MTA1,NOX4,PRKCD,TAB2,ACVR2A,CD4,TGFB1,SPSB4,PIBF1,BTRC,DNAJB2,TNFRSF10B,DOCK3,FGF10,SMYD3,FANCI,EPHA4,AUTS2,CD6,TNFSF11,CTNNB1,PARK2,PPARG,AXIN1,LEPR,FGF1,AJUBA,CHEK2,SPDYA,RAB3GAP2,CSPG4,FLT4,HDAC4,TRABD2B,TAB1,APP,DIP2B,BORA,MTCP1,PRICKLE1,S100A12,DCUN1D3,PRKD1,EREG,CCNY,ATF2,RAF1,PTPN1,BMPR2,BMPR1A,PIK3R3,MYB,FGF2,NEDD9,SEMA4D,JARID2,DVL3,EIF4G1,RBX1,CKS1B,TERF2IP,TRPC5,UBA2,DAB2,CACUL1,DLG3,WNK1,RELN,NEK10,RUVBL2,PELI1,IQGAP1,MAP3K7,SLC8A2,ATP2B4,TET1,CAMTA1,ADNP,TFRC,DCUN1D5,GHR,MNAT1,SNX9,PLCG2,PAXIP1,NTRK1,SLC39A10,AXIN2,MUC1,DNMT3B,EPHA7,ATG14,BCAR3,RBPMS,MAP3K13,RAPGEF3,SNCA,BMPR1B,MAGI2,PRKAG1,ADTRP,OPRD1,RPTOR,IGF1,MAPK1,PTEN,BMP7,LRRK2,UNC119,PKD2,EFNA5,PDGFC,PPP2R3C,BMP6,MYCBP2,RAPGEF2,VRK3,ARRDC4,RHOA,SPRTN,RQCD1,CUL3,AGT,PRKAA2,PTK2,TEC,MAP3K5,NCF1,TAOK2,CD44,ADAM8,RPS6KA5,NOS1,STK4,SLC1A1,IL18,UBE2K,DLC1,MOB3B,PRKCZ,NIPBL,ANGPT1,LRRK1,OSBPL8,MTF2,CNTN1,BRAF,HDAC2,CD300A,PPP2CA,SKP1,ANGPT4,MADD,SYK,DVL2,PTPRJ,TICAM1,TANK,HSF1,SASH1,MOB3A,GNA12,SH2D3A,AKTIP,MALT1,PHB,PTPRC,BMP4
GO:0006366	transcription by RNA polymerase II	3.1315305993541517e-7	LDB2,DNMT1,HLX,SLC9A1,PBX3,MED13L,TRPS1,CBFB,ZNF823,PRDM12,MED26,WWC1,ASH1L,SCAF8,STOX2,WWC3,PAGR1,HIVEP3,NPAS3,ARNT,STAT5B,TOX,ZNF566,CASK,MAP2K5,ZNF536,SP3,HDGF,EZR,IKZF2,CHD7,MECOM,TRAF6,GTF2E2,DNAJA3,NHLH1,PSMB7,TSC22D3,GFI1B,RUNX1,HMGN3,NRIP1,THRB,EFCAB7,DACH1,ZNF569,ORC2,MYT1,IKBKB,ZNF609,BRD8,KAT6B,HIF3A,SNIP1,ESR1,MIER1,PCBP3,MAML3,CDON,EP300,CELA1,TENM2,ZNF76,ZNF471,CCND3,ZNF19,ZNF23,BRMS1,ZNF605,HNF4G,PRKAR1A,H2AFY2,TCF7L2,JMJD1C,ZNF443,ZNF490,ZNF564,ZNF709,ZNF799,LITAF,ESRRB,TBR1,TFAP2A,PEG3,ZIM2,TGIF2,MEIS1,VRTN,MDM4,ZNF148,MTA3,TFDP2,SLC30A9,TOX3,KAT7,ZNF667,RBM14,MED12L,SATB2,RIT2,HIRA,MKRN2,ARNTL,ARID4B,PIK3R2,UBP1,MDFI,FNIP1,VAX2,TMBIM6,PPP3R1,HDAC5,CSRNP3,ZNF692,NFIA,RNF4,JDP2,DCN,CDH13,CREBRF,SOX13,COPS5,MAD2L2,TLE6,CAPRIN2,ZNF418,PHF20L1,ZNF286A,FOXN3,NEUROD1,JAK2,SKAP1,MLIP,BCL11B,PKNOX1,TSHZ2,TCF7,PDE2A,KLF15,TBX15,ANXA4,MTA1,KLF8,LCOR,RPRD1B,CCDC62,SOX6,ACVR2A,RUNX2,TGFB1,NSD1,ZHX2,PKNOX2,NFATC3,F2RL1,BCAS3,CC2D1B,TP73,SAP18,ZBTB22,MTDH,MYT1L,ZNF398,CLOCK,TCF12,ZNF675,ETV6,PHRF1,TFAP2D,BCL3,DUSP22,SBNO2,FGF10,POLR2J2,SMYD3,LUM,SMURF2,RORA,HIVEP2,AUTS2,TNFSF11,PPP3CA,NFYB,MAGEA4,KLF12,GATAD2B,TRAK1,CTNNB1,PARK2,SOD2,DACH2,METTL13,SMARCC1,KLF17,PPARG,AXIN1,MTF1,CBX5,BRIP1,SREBF2,FGF1,NPAT,NR4A3,FOXK2,MYOCD,PER2,AJUBA,ZNF626,ZNF737,SUPT3H,PRDM16,BRMS1L,ZBTB8A,ZBTB8B,CTDP1,BAZ1B,MEF2B,HDAC4,PAX2,SNAPC4,MED13,ZNF395,FOXO3,NFIB,SP4,SMAD3,CUX2,WWP2,ARNT2,ZNF662,ZNF777,EBF3,RNF168,CASZ1,MIER3,NEDD4,ESRRG,HOXD3,HOXD4,ZNF114,KTI12,NFATC1,CDC73,APP,SSBP3,GSX2,YAP1,LRP5,ZNF787,SOX2,SETD2,TEAD1,ZNF653,ZNF521,ARID3A,ZNF761,CHUK,ZNF584,ESR2,GTF2F2,PRKD1,STAT1,ST18,ETV5,RHOXF2B,TAF3,PLAGL1,HNF4A,ZBTB7C,ATF2,POU2F2,TCF3,ZNF730,RAF1,BMPR2,BMPR1A,ZKSCAN1,IKZF4,CDK12,TAF8,MYB,FGF2,ZNF71,POU6F2,BACH1,MXD3,GLI4,ZFP41,SEMA4D,NFX1,RORC,ELP3,PTAFR,SP140,SP140L,RHOXF2,JARID2,DDX58,RARB,SPEN,SIN3B,NCOA1,EHMT1,LMO7,DVL3,POLR2H,TCF20,ATF3,PAWR,EBF2,MAML2,TSG101,CRYM,RFX2,ZNF322,SUFU,MAGEA11,PRDM15,ZNF670,ZNF695,SOHLH2,ZNF354C,ZNF704,NR2C1,GLI2,TNKS,ERCC1,GLIS3,WDTC1,ZNF664,DAB2,MYSM1,SIN3A,RUVBL2,GMEB1,ZNF423,SP1,TEAD4,HOXC13,SRA1,ADIRF,OVOL2,NFATC2,PAX7,ATP2B4,ACTL6B,ASXL3,TET1,CAMTA1,CCPG1,ADNP,ARID4A,CDK6,PHF2,WWOX,MEF2A,DCAF6,SATB1,PSMB2,EYA1,GATAD2A,TRPV1,HOXB3,HOXB4,HOXB5,HOXB6,TFEB,MNAT1,ACTR2,HDAC1,RECQL5,TRIM24,PAXIP1,ZNF713,EGR2,RNF10,RYBP,AP3B1,PTPN2,KPNA6,EPCAM,ZNF425,MUC1,BCOR,AP3D1,ZNF41,ZNF383,ZNF616,ZNF836,SMARCA2,CUX1,SPI1,DNMT3B,FOXP2,GLIS1,JAZF1,CREB5,ERCC3,SNCA,BMPR1B,ZEB2,FOXJ3,HEY2,ZMYND11,KMT2D,CREM,KMT2C,MACC1,MITF,NFIX,CBFA2T2,IGF1,MLXIP,ATF7IP,BMP7,MXI1,SOX5,CIR1,PCBP2,ZNF780A,ZNF780B,ZBTB20,PKD2,ZNF652,HSF2BP,RIPPLY1,RCOR3,TADA2A,NCOA3,ZNF146,ZNF565,WNT7A,ZBED6,BRWD3,CRTC3,ARNTL2,KAT6A,ZKSCAN7,ZNF197,ZNF660,BMP6,NFE2L1,WDR43,ZNF30,ATRX,IKZF1,SMO,TCF4,SOX30,TFE3,GRIN1,RHOA,ONECUT2,CUL3,TFEC,STAT6,RBBP8,CDK13,DUSP26,ZNF362,NACC2,SUPT4H1,ZNF354A,HDGFRP3,POLR2F,STAT2,GTF2E1,PBX1,TCFL5,CREBBP,DLX1,GTF2IRD2,ZNF813,LBX2,DPRX,SP7,RPS6KA5,ZNF484,ZNF93,PRDM2,NOS1,ZNF44,MED15,MC1R,TCF25,ZNF282,IL18,PPARA,ARID5B,SMARCAL1,TAF2,NOTCH4,ZN

			F366,CRX,PAX6,OTUD7B,NIPBL,PHF20,ZNF143,TBL1X,FHL2,ATF6,ZBTB5,ZNF708,ETS1,MTF2,TAGLN3,NCOR2,CSRNP1,HDAC2,IMPACT,SRCAP,POU3F3,NOTO,ZKSCAN5,ELF2,PHIP,RPL23,ATXN1,SP100,ZNF347,ZNF415,TRIM29,CNOT1,WWTR1,GTF2H5,NFXL1,ZBTB38,ATF7,DVL2,YBX1,TAF1,THAP3,SNAPC3,HSF1,MAX,SAP130,EZH1,NRF1,NCOA2,CHD6,RNF2,ZNF554,MYEF2,TBP,TRIM37,MET,ZNF461,CAMK2D,TIMELESS,BRD9,SETD3,KDM2B,MLXIPL,PHB,ZNF555,CTCF,NR6A1,ATF6B,CREB1,EBF4,ZNF511,BMP4,ABLIM3
GO:0045892	negative regulation of transcription, DNA-templated	3.141972944783672e-7	*LDB2,DNMT1,TRPS1,CBFB,PRDM12,WWC1,SCAF8,WWC3,ZNF566,MAP2K5,ZNF536,SP3,HDGF,EZR,MECOM,TRAF6,DNAJA3,GFI1B,PTPRK,RUNX1,NRIP1,THRB,DACH1,ORC2,HDAC6,PEX14,KAT6B,ESR1,MIER1,PCBP3,EP300,CELA1,TENM2,PDGFB,CCND3,TOB2,ZNF19,BRMS1,SCML4,RERE,ATP8B1,H2AFY2,TCF7L2,FBXW11,BASP1,TBR1,TFAP2A,PEG3,ZIM2,TGIF2,MDM4,ZNF148,MTA3,SNX6,TFDP2,ERN2,C1D,CENPF,KAT7,SATB2,HIRA,ARNTL,PLCB1,NRG1,MDFI,FNIP1,VAX2,TMBIM6,VGLL4,HDAC5,ZNF692,JDP2,CREBRF,SOX13,MAD2L2,TLE6,FOXN3,UIMC1,MLIP,TCF7,PDE2A,TBX15,WNT11,MTA1,KLF8,LCOR,SOX6,RUNX2,TGFB1,NSD1,ZHX2,BTRC,NFATC3,CDYL2,CC2D1B,SAP18,MTDH,MYT1L,ZNF398,CLOCK,ZNF675,ETV6,BCL3,DUSP22,SBNO2,LOXL3,CAPN3,SMURF2,MAGEA4,KLF12,GATAD2B,CTNNB1,PARK2,METTL13,PPARG,AXIN1,CIPC,CBX5,SREBF2,NPAT,NR4A3,FOXK2,MYOCD,PER2,AJUBA,PRDM16,BRMS1L,HDAC4,PAX2,SFRP1,FOXO3,NFIB,SMAD3,CUX2,WWP2,RNF168,MIER3,NEDD4,CDC73,YAP1,SOX2,PRICKLE1,ZNF653,SFMBT1,ESR2,STAT1,ETV5,TAF3,PLAGL1,HNF4A,EREG,ATF2,TCF3,IKZF4,MYB,BACH1,MXD3,PPM1F,BEND5,SEMA4D,NFX1,RORC,JARID2,PHC2,RARB,SPEN,SIN3B,EHMT1,ATF3,PAWR,TSG101,CRYM,SUFU,MAGEA11,NR2C1,GLI2,GLIS3,WDTC1,DAB2,SIN3A,RUVBL2,ZNF423,TRIM22,OVOL2,NFATC2,ARID4A,CDK6,MEF2A,SATB1,GATAD2A,TRPV1,HOXB3,HOXB4,L3MBTL4,HDAC1,RECQL5,TRIM24,SCMH1,RYBP,PTPN2,ZNF425,MUC1,BCOR,SMARCA2,CUX1,SPI1,DNMT3B,FOXP2,PSPC1,GLIS1,SFMBT2,JAZF1,SMYD1,SNCA,ZEB2,HEY2,ZMYND11,CREM,MITF,NFIX,CBFA2T2,ATF7IP,BMP7,MXI1,CIR1,ZBTB20,LILRB4,KCTD1,RIPPLY1,RCOR3,ZBED6,KAT6A,BMP6,ATRX,IKZF1,PRMT2,SMO,SOX30,CUL3,TFEC,STAT6,RBBP8,DUSP26,NACC2,SUPT4H1,TCFL5,CREBBP,DLX1,SHC1,PARP10,SETDB2,DNAJB6,RPS6KA5,ZNF93,PRDM2,TCF25,ZNF282,PPARA,ARID5B,NOTCH4,ZMYND8,ZNF366,PAX6,OTUD7B,NIPBL,TBL1X,FHL2,MBTD1,ZBTB5,MTF2,TAGLN3,NCOR2,HDAC2,IMPACT,POU3F3,ELF2,RPL23,ATXN1,SP100,TRIM29,CNOT1,WWTR1,ZBTB38,MORC1,YBX1,TAF1,HSF1,MAX,SAP130,EZH1,NCOA2,ZP3,RNF2,ZNF554,TRIM37,TIMELESS,KDM2B,MLXIPL,PHB,ZNF555,CTCF,NR6A1,CREB1,BMP4*
GO:0050803	regulation of synapse structure or activity	3.174259350977586e-7	*NRXN1,NEGR1,GRID2,KALRN,NTRK3,LHFPL4,CDKL5,ROBO2,PTPRO,NTRK2,CDH8,PTPRD,BDNF,AMIGO1,TANC2,FYN,NTN1,DCTN1,FRMPD4,LRFN5,CAPRIN2,ZDHHC15,CLSTN2,IL1RAPL1,CAMK2B,DISC1,SLC17A7,LINGO2,IL1RAPL2,EPHA4,FCGR2B,ARHGAP22,DLG5,DGKB,DNM3,CUX2,ABI2,NEDD4,NRP2,APP,NLGN2,GPC6,SHANK2,NLGN1,CTNNA2,SEMA4D,EIF4G1,EPHB3,NLGN3,PAFAH1B1,SETD5,RELN,PAK3,ADNP,FLRT2,ACTR2,NTRK1,EPHB1,ARHGAP44,EPHA7,SNCA,PTPRS,PTEN,LRRK2,EFNA5,SHANK3,WNT7A,RHOA,SYNGAP1,EEF2K,EPHB2,GRIN2B,SLIT1,GPM6A,SYNDIG1*
GO:0006915	apoptotic process	3.439394278741443e-7	*PRDX2,PRKCI,MAP4K4,DNMT1,TMBIM4,SLC9A1,SEMA3A,IL31RA,GRID2,RPS6KA2,PTGFR,DOCK1,LATS2,NOX5,ANP32A,PTGIS,TJP1,UNC5C,PRLR,CBL,EGLN2,SEMA5A,FLT3,STAT5B,MAP2K5,KITLG,LRP2,PIK3CD,OLFM1,MEGF10,TNFAIP8L1,HTR2B,MECOM,LMNA,TRAF6,ROBO2,ITPKB,DNAJA3,OXR1,GRK5,ITGB1,HSP90AA1,ELMO2,TSC22D3,OMA1,THRB,ITGB3BP,TCIRG1,DDB1,ADORA1,IKBKB,ERBB4,GBE1,MRE11A,HIF3A,CECR2,ESR1,NTRK2,EP300,BRMS1,DAP3,FMN2,UNC5D,PRUNE2,TCF7L2,ZNF443,PAK1,EGLN3,BDNF,TFAP2A,KCNMA1,PEG3,LCK,MDM4,SHC4,ECT2,SNX6,ERN2,C1D,TOX3,GSN,NF1,MGMT,SNCB,RTN4,DPEP1,CHI3L1,NTN1,NRG1,ARHGAP10,CDK19,BDKRB2,DOCK8,BID,FNIP1,TMBIM6,PPP2CB,WDR92,CSRNP3,RBM5,ZDHHC3,FHIT,COPS5,NCKAP1,ANO6,CERKL,NEUROD1,TMEM117,RFFL,JAK2,FBXW7,ITCH,BCL11B,TEX11,ANXA4,CYFIP2,WNT11,KREMEN1,SIAH1,PRKCD,TGFB1,SGK1,CRADD,SLIT3,SLIT2,TP73,SAP18,BLID,MTDH,FANK1,SMAD6,ETV6,TFAP2D,BCL3,DAPK2,TNFRSF10B,NAIP,FGF10,SH3RF3,CAPN3,PRKCA,CNR1,MAGEA4,MAG,PIP5KL1,UFL1,CTNNB1,PARK2,SOD2,IGF1R,PPARG,AXIN1,DLG5,RYR2,CDK11A,CDK11B,DRAXIN,NOL3,MYOCD,CHEK2,HCK,CAPN2,PPP2R2B,DCC,NF2,FLT4,SGMS1,HDAC4,PAX2,SFRP1,FOXO3,BCL2L13,SMAD3,ITPR1,EVA1A,GRIK5,TXNDC12,CDC73,APP,SLK,NOX1,YAP1,TNFRSF19,VDAC1,EYA2,ANGPTL4,LRP5,CHST11,TMEM14A,FRS2,PALB2,MMP2,DCUN1D3,PRKD1,STAT1,USP53,PLAGL1,TCTN3,ATF2,GRAMD4,RAF1,CARD16,CASP1,PTPN1,BMPR2,CAMK1D,PPM1F,SEMA4D,RORC,YME1L1,PLEKHO2,SLC5A8,RARB,PRKCG,NCOA1,AREL1,EEF1E1,TXNDC5,EIF2B5,ATF3,PAWR,DEPTOR,P4HB,CNTFR,COL4A3,HIP1,TRPC5,GLI2,DAB2,PKHD1,XKR4,SIN3A,PPP2R5C,CFDP1,MAP3K7,DDX47,ALK,INPP5D,SRA1,PAX7,PAK3,ADNP,BCL7C,RNF34,TFRC,WWOX,MEF2A,GFRAL,EYA1,TRPV1,RNF216,MNAT1,HDAC1,TRIM24,ROCK1,HIPK3,RYBP,CAPN10,CUL2,PTPN2,NTRK1,PKN2,TMIGD1,SLC39A10,SARM1,MUC1,JAG2,SPI1,EPHA7,CTNNA1,AIFM2,MST1,ERCC3,SNCA,BMPR1B,GRM4,USP42,HEY2,CHMP3,ZMYND11,CAST,DFFA,RASA1,MITF,SRSF6,HIPK1,NUGGC,BTK,IGF1,CTNNBL1,HTT,MAPK1,PTEN,BMP7,LRRK2,OSGIN1,EBAG9,BAG6,ITGAM,WNT7A,NLRP1,UACA,RABEP1,RHOT1,GPI,SH3RF2,PRMT2,RAPGEF2,SMO,PSEN2,TNFRSF11B,JADE1,RHOA,SYNGAP1,CUL3,SH3KBP1,HSPD1,GSK3B,DNAJC3,EEF2K,CD27,BOK,RNF*

			144B,SULF1,KRT8,NACC2,TNFRSF8,AGT,PRKAA2,ADAR,ZMPSTE24,PTK2,FBXO10,SHB,MAP3K5,DLX1,NCF1,TAOK2,SHC1,CD160,RPS6KB1,DNAJB6,CD44,ADAM8,BCL2L14,ACTN4,STK4,ZFAND6,DRAM1,SLC1A1,SIGMAR1,HCAR2,DLC1,PPARA,PPP1R10,STEAP3,PHLPP1,MAGI3,CPEB4,PRKCZ,ADORA2A,ANGPT1,PACS2,EYA3,FHL2,ATF6,ETS1,PPP1CA,GRIA4,STPG1,ATG3,PRKCQ,BRAF,CSRNP1,HDAC2,TNS4,POU3F3,HIGD2A,PHIP,PPP2CA,ANGPT4,ARHGEF6,SP100,MADD,GRIK2,CIT,BRCA2,HRK,TICAM1,CADM1,HSF1,MAX,SRPK2,ANKRD13C,QRICH1,VAV3,AKTIP,NUAK2,AXL,TNFSF9,CAMK2D,MALT1,TRIM69,KDM2B,THEM4,PHB,PTPRC,TYRO3,ADCY10,TRAF3IP2,TRAP1,EDNRA,GNGT1,PRAP1,BMP4,CASP12
GO:0010558	negative regulation of macromolecule biosynthetic process	3.4977862465040943e-7	*ENPP1,LDB2,DNMT1,TRPS1,CBFB,PRDM12,WWC1,SCAF8,WWC3,FTO,ZNF566,MAP2K5,ZNF536,SP3,HDGF,EZR,MECOM,TRAF6,DNAJA3,CDC6,CDAN1,GFI1B,PTPRK,RUNX1,NRIP1,THRB,DACH1,ORC2,HDAC6,PEX14,KAT6B,ESR1,MIER1,PCBP3,TNRC6A,EP300,CELA1,TENM2,PDGFB,CCND3,TOB2,ZNF19,BRMS1,SCML4,RERE,ATP8B1,H2AFY2,TCF7L2,FBXW11,BASP1,TBR1,SAMD4A,TFAP2A,PEG3,ZIM2,TGIF2,MDM4,ZNF148,MTA3,SNX6,TFDP2,ERN2,C1D,CENPF,PDS5A,KAT7,RBM4,SATB2,HIRA,ARNTL,PLCB1,NRG1,MDFI,FNIP1,VAX2,TMBIM6,VGLL4,HDAC5,ZNF692,JDP2,CREBRF,SOX13,MAD2L2,TLE6,CAPRIN2,TERF2,FOXN3,UIMC1,MLIP,TCF7,PDE2A,TBX15,WNT11,MTA1,KLF8,LCOR,SOX6,RUNX2,TGFB1,NSD1,ZHX2,BTRC,NFATC3,CDYL2,CC2D1B,SAP18,MTDH,MYT1L,ZNF398,CLOCK,ZNF675,ETV6,BCL3,DUSP22,HNRNPC,SBNO2,YTHDF1,LOXL3,CAPN3,SMURF2,MAGEA4,KLF12,GATAD2B,CTNNB1,PARK2,METTL13,PPARG,AXIN1,CIPC,CBX5,SREBF2,NPAT,NR4A3,FOXK2,MYOCD,PER2,AJUBA,PRDM16,BRMS1L,HDAC4,PAX2,SFRP1,FOXO3,NFIB,SYNCRIP,SMAD3,CUX2,WWP2,RNF168,DCP1B,MIER3,NEDD4,CDC73,YAP1,TEN1,SOX2,PRICKLE1,ZNF653,SFMBT1,ESR2,STAT1,ETV5,TAF3,PLAGL1,HNF4A,EREG,ATF2,TCF3,CELF4,BMPR2,IKZF4,MYB,BACH1,MXD3,PPM1F,BEND5,SEMA4D,NFX1,RORC,JARID2,PHC2,RARB,SPEN,SIN3B,EHMT1,EIF4G1,ATF3,PAWR,AGO3,TSG101,CRYM,SUFU,MAGEA11,NR2C1,GLI2,TNRC6B,GLIS3,WDTC1,DAB2,BLM,SIN3A,RUVBL2,ZNF423,TRIM22,OVOL2,NFATC2,ARID4A,CDK6,CELF1,MEF2A,XRN1,SATB1,GATAD2A,DIS3L2,TRPV1,HOXB3,HOXB4,L3MBTL4,HDAC1,RECQL5,TRIM24,ROCK1,SCMH1,RYBP,PTPN2,ZNF425,GNL3L,MUC1,BCOR,SMARCA2,CUX1,SPI1,DNMT3B,FOXP2,PSPC1,GLIS1,SFMBT2,JAZF1,ACOT8,SMYD1,SNCA,PNPT1,ZEB2,HEY2,RC3H1,EIF3E,ZMYND11,CREM,MITF,NFIX,CBFA2T2,ATF7IP,BMP7,MXI1,PUM1,CIR1,TMEM59,ZBTB20,LILRB4,KCTD1,RIPPLY1,RCOR3,ZBED6,KAT6A,BMP6,ATRX,IKZF1,PRMT2,SMO,SOX30,RQCD1,CUL3,TFEC,GSK3B,STAT6,RBBP8,DUSP26,NACC2,SUPT4H1,METTL16,EXOSC3,TCFL5,CREBBP,DLX1,SHC1,PARP10,PRG3,SETDB2,DNAJB6,RPS6KA5,ZNF93,PRDM2,TCF25,ZNF282,TMEFF2,DIS3,PPARA,ARID5B,SMARCAL1,CPEB4,NOTCH4,ZMYND8,ZNF366,PAX6,CPEB1,OTUD7B,NIPBL,TBL1X,FHL2,MBTD1,ZBTB5,IGF2BP3,MTF2,TAGLN3,NCOR2,HDAC2,IMPACT,POU3F3,ELF2,RPL23,ATXN1,SP100,TRIM29,PARN,DONSON,CNOT1,WWTR1,ZBTB38,BRCA2,MORC1,YBX1,TAF1,HSF1,MAX,SAP130,DHFR,EZH1,NCOA2,ZP3,RNF2,ZNF554,TRIM37,TIMELESS,KDM2B,MLXIPL,PHB,ZNF555,CTCF,NR6A1,CREB1,BMP4*
GO:1903507	negative regulation of nucleic acid-templated transcription	3.769186363441068e-7	*LDB2,DNMT1,TRPS1,CBFB,PRDM12,WWC1,SCAF8,WWC3,ZNF566,MAP2K5,ZNF536,SP3,HDGF,EZR,MECOM,TRAF6,DNAJA3,GFI1B,PTPRK,RUNX1,NRIP1,THRB,DACH1,ORC2,HDAC6,PEX14,KAT6B,ESR1,MIER1,PCBP3,EP300,CELA1,TENM2,PDGFB,CCND3,TOB2,ZNF19,BRMS1,SCML4,RERE,ATP8B1,H2AFY2,TCF7L2,FBXW11,BASP1,TBR1,TFAP2A,PEG3,ZIM2,TGIF2,MDM4,ZNF148,MTA3,SNX6,TFDP2,ERN2,C1D,CENPF,KAT7,SATB2,HIRA,ARNTL,PLCB1,NRG1,MDFI,FNIP1,VAX2,TMBIM6,VGLL4,HDAC5,ZNF692,JDP2,CREBRF,SOX13,MAD2L2,TLE6,FOXN3,UIMC1,MLIP,TCF7,PDE2A,TBX15,WNT11,MTA1,KLF8,LCOR,SOX6,RUNX2,TGFB1,NSD1,ZHX2,BTRC,NFATC3,CDYL2,CC2D1B,SAP18,MTDH,MYT1L,ZNF398,CLOCK,ZNF675,ETV6,BCL3,DUSP22,SBNO2,LOXL3,CAPN3,SMURF2,MAGEA4,KLF12,GATAD2B,CTNNB1,PARK2,METTL13,PPARG,AXIN1,CIPC,CBX5,SREBF2,NPAT,NR4A3,FOXK2,MYOCD,PER2,AJUBA,PRDM16,BRMS1L,HDAC4,PAX2,SFRP1,FOXO3,NFIB,SMAD3,CUX2,WWP2,RNF168,MIER3,NEDD4,CDC73,YAP1,SOX2,PRICKLE1,ZNF653,SFMBT1,ESR2,STAT1,ETV5,TAF3,PLAGL1,HNF4A,EREG,ATF2,TCF3,IKZF4,MYB,BACH1,MXD3,PPM1F,BEND5,SEMA4D,NFX1,RORC,JARID2,PHC2,RARB,SPEN,SIN3B,EHMT1,ATF3,PAWR,TSG101,CRYM,SUFU,MAGEA11,NR2C1,GLI2,GLIS3,WDTC1,DAB2,SIN3A,RUVBL2,ZNF423,TRIM22,OVOL2,NFATC2,ARID4A,CDK6,MEF2A,SATB1,GATAD2A,TRPV1,HOXB3,HOXB4,L3MBTL4,HDAC1,RECQL5,TRIM24,SCMH1,RYBP,PTPN2,ZNF425,MUC1,BCOR,SMARCA2,CUX1,SPI1,DNMT3B,FOXP2,PSPC1,GLIS1,SFMBT2,JAZF1,SMYD1,SNCA,ZEB2,HEY2,ZMYND11,CREM,MITF,NFIX,CBFA2T2,ATF7IP,BMP7,MXI1,CIR1,ZBTB20,LILRB4,KCTD1,RIPPLY1,RCOR3,ZBED6,KAT6A,BMP6,ATRX,IKZF1,PRMT2,SMO,SOX30,CUL3,TFEC,STAT6,RBBP8,DUSP26,NACC2,SUPT4H1,TCFL5,CREBBP,DLX1,SHC1,PARP10,SETDB2,DNAJB6,RPS6KA5,ZNF93,PRDM2,TCF25,ZNF282,PPARA,ARID5B,NOTCH4,ZMYND8,ZNF366,PAX6,OTUD7B,NIPBL,TBL1X,FHL2,MBTD1,ZBTB5,MTF2,TAGLN3,NCOR2,HDAC2,IMPACT,POU3F3,ELF2,RPL23,ATXN1,SP100,TRIM29,CNOT1,WWTR1,ZBTB38,MORC1,YBX1,TAF1,HSF1,MAX,SAP130,EZH1,NCOA2,ZP3,RNF2,ZNF554,TRIM37,TIMELESS,KDM2B,MLXIPL,PHB,ZNF555,CTCF,NR6A1,CREB1,BMP4*
GO:0016570	histone modification	3.78195656190767e-7	*DNMT1,C6ORF89,PRDM12,SETD4,ASH1L,PAGR1,MECOM,LMNA,HDAC6,DDB1,BRD8,KAT6B,CUL4B,MIER1,KANSL1,EP300,BRMS1,JMJD1C,UBR2,HLCS,MTA3,KAT7,RBM14,ARID4B,BRPF1,HDAC5,JDP2,WDR70,PHF20L1,USP22,JAK2,UIMC1,*

			MTA1,PRKCD,TGFB1,NSD1,KDM4B,CLOCK,TRRAP,SMYD3,PRKCA,AUTS2,UFL1,CTNNB1,OTUB1,MYOCD,PER2,SUPT3H,PRDM16,BRMS1L,BAZ1B,HDAC4,RNF168,CDC73,USP49,KANSL2,EYA2,SETD2,DRD1,KDM6A,PRKD1,ATF2,MYB,PPM1F,SETD1A,JARID2,SIN3B,NCOA1,EHMT1,PADI6,MYSM1,SETD5,SIN3A,RUVBL2,MAP3K7,PADI3,ACTL6B,TET1,ARID4A,PHF2,EYA1,HDAC1,PAXIP1,RYBP,MUC1,BCOR,CARM1,SPI1,DNMT3B,NTMT1,SMYD1,SNCA,KMT2D,KMT2C,TRIP12,ZZZ3,LRRK2,RCOR3,TADA2A,NCOA3,WDR5B,KAT6A,ATRX,PRMT2,UBR5,JADE1,IWS1,PRKAA2,ZMPSTE24,CREBBP,PYGO2,SETDB2,RPS6KA5,PRDM2,NOS1,NIPBL,YEATS4,PHF20,TBL1X,EYA3,MTF2,HDAC2,SRCAP,SKP1,PRMT7,BRCA2,TAF1,EZH1,RNF2,TRIM37,CAMK2D,SETD3,KDM2B,PHB,CTCF
GO:1902679	negative regulation of RNA biosynthetic process	4.517138296614507e-7	*LDB2,DNMT1,TRPS1,CBFB,PRDM12,WWC1,SCAF8,WWC3,ZNF566,MAP2K5,ZNF536,SP3,HDGF,EZR,MECOM,TRAF6,DNAJA3,GFI1B,PTPRK,RUNX1,NRIP1,THRB,DACH1,ORC2,HDAC6,PEX14,KAT6B,ESR1,MIER1,PCBP3,EP300,CELA1,TENM2,PDGFB,CCND3,TOB2,ZNF19,BRMS1,SCML4,RERE,ATP8B1,H2AFY2,TCF7L2,FBXW11,BASP1,TBR1,TFAP2A,PEG3,ZIM2,TGIF2,MDM4,ZNF148,MTA3,SNX6,TFDP2,ERN2,C1D,CENPF,KAT7,SATB2,HIRA,ARNTL,PLCB1,NRG1,MDFI,FNIP1,VAX2,TMBIM6,VGLL4,HDAC5,ZNF692,JDP2,CREBRF,SOX13,MAD2L2,TLE6,FOXN3,UIMC1,MLIP,TCF7,PDE2A,TBX15,WNT11,MTA1,KLF8,LCOR,SOX6,RUNX2,TGFB1,NSD1,ZHX2,BTRC,NFATC3,CDYL2,CC2D1B,SAP18,MTDH,MYT1L,ZNF398,CLOCK,ZNF675,ETV6,BCL3,DUSP22,SBNO2,LOXL3,CAPN3,SMURF2,MAGEA4,KLF12,GATAD2B,CTNNB1,PARK2,METTL13,PPARG,AXIN1,CIPC,CBX5,SREBF2,NPAT,NR4A3,FOXK2,MYOCD,PER2,AJUBA,PRDM16,BRMS1L,HDAC4,PAX2,SFRP1,FOXO3,NFIB,SMAD3,CUX2,WWP2,RNF168,MIER3,NEDD4,CDC73,YAP1,SOX2,PRICKLE1,ZNF653,SFMBT1,ESR2,STAT1,ETV5,TAF3,PLAGL1,HNF4A,EREG,ATF2,TCF3,IKZF4,MYB,BACH1,MXD3,PPM1F,BEND5,SEMA4D,NFX1,RORC,JARID2,PHC2,RARB,SPEN,SIN3B,EHMT1,ATF3,PAWR,TSG101,CRYM,SUFU,MAGEA11,NR2C1,GLI2,GLIS3,WDTC1,DAB2,SIN3A,RUVBL2,ZNF423,TRIM22,OVOL2,NFATC2,ARID4A,CDK6,MEF2A,SATB1,GATAD2A,TRPV1,HOXB3,HOXB4,L3MBTL4,HDAC1,RECQL5,TRIM24,SCMH1,RYBP,PTPN2,ZNF425,MUC1,BCOR,SMARCA2,CUX1,SPI1,DNMT3B,FOXP2,PSPC1,GLIS1,SFMBT2,JAZF1,SMYD1,SNCA,ZEB2,HEY2,ZMYND11,CREM,MITF,NFIX,CBFA2T2,ATF7IP,BMP7,MXI1,CIR1,ZBTB20,LILRB4,KCTD1,RIPPLY1,RCOR3,ZBED6,KAT6A,BMP6,ATRX,IKZF1,PRMT2,SMO,SOX30,CUL3,TFEC,STAT6,RBBP8,DUSP26,NACC2,SUPT4H1,TCFL5,CREBBP,DLX1,SHC1,PARP10,SETDB2,DNAJB6,RPS6KA5,ZNF93,PRDM2,TCF25,ZNF282,PPARA,ARID5B,NOTCH4,ZMYND8,ZNF366,PAX6,OTUD7B,NIPBL,TBL1X,FHL2,MBTD1,ZBTB5,MTF2,TAGLN3,NCOR2,HDAC2,IMPACT,POU3F3,ELF2,RPL23,ATXN1,SP100,TRIM29,CNOT1,WWTR1,ZBTB38,MORC1,YBX1,TAF1,HSF1,MAX,SAP130,EZH1,NCOA2,ZP3,RNF2,ZNF554,TRIM37,TIMELESS,KDM2B,MLXIPL,PHB,ZNF555,CTCF,NR6A1,CREB1,BMP4*
GO:0035249	synaptic transmission, glutamatergic	4.6975232664126435e-7	*NRXN1,GRID2,GRM8,GRM5,CNIH2,DGKI,ADORA1,GRM7,CDH8,GRIK4,NF1,ROR2,SYT1,SLC1A4,GRIK3,DISC1,SLC17A7,CNR1,PARK2,GRM1,GRIK5,NLGN2,DRD1,NLGN1,GRIK1,GRIA2,NLGN3,RELN,ATAD1,NTRK1,UNC13A,CACNG8,CACNG2,GRM4,LRRK2,SHANK3,EXT1,GRID1,ADORA2A,TNR,GRIK2,CACNG3*
GO:0012501	programmed cell death	4.7352894736150707e-7	*PRDX2,PRKCI,MAP4K4,DNMT1,TMBIM4,SLC9A1,SEMA3A,IL31RA,GRID2,RPS6KA2,PTGFR,DOCK1,LATS2,NOX5,ANP32A,PTGIS,TJP1,UNC5C,PRLR,CBL,EGLN2,SEMA5A,FLT3,STAT5B,MAP2K5,KITLG,LRP2,PIK3CD,OLFM1,MEGF10,TNFAIP8L1,HTR2B,MECOM,LMNA,TRAF6,ROBO2,ITPKB,DNAJA3,OXR1,GRK5,ITGB1,HSP90AA1,ELMO2,TSC22D3,OMA1,THRB,ITGB3BP,TCIRG1,HDAC6,DDB1,ADORA1,IKBKB,ERBB4,GBE1,MRE11A,HIF3A,CECR2,ESR1,NTRK2,EP300,BRMS1,DAP3,FMN2,UNC5D,PRUNE2,TCF7L2,ZNF443,PAK1,EGLN3,BDNF,TFAP2A,KCNMA1,PEG3,LCK,MDM4,SHC4,ECT2,SNX6,ERN2,C1D,TOX3,GSN,NF1,MGMT,SNCB,RTN4,DPEP1,CHI3L1,NTN1,NRG1,ARHGAP10,CDK19,BDKRB2,DOCK8,BID,FNIP1,TMBIM6,PPP2CB,WDR92,CSRNP3,RBM5,ZDHHC3,FHIT,COPS5,NCKAP1,ANO6,CERKL,NEUROD1,TMEM117,RFFL,JAK2,FBXW7,ITCH,BCL11B,TEX11,ANXA4,CYFIP2,WNT11,KREMEN1,SIAH1,PRKCD,TGFB1,SGK1,CRADD,SLIT3,SLIT2,TP73,SAP18,BLID,MTDH,FANK1,SMAD6,ETV6,TFAP2D,BCL3,DAPK2,TNFRSF10B,NAIP,FGF10,SH3RF3,CAPN3,PRKCA,CNR1,MAGEA4,MAG,PIP5KL1,UFL1,CTNNB1,PARK2,SOD2,IGF1R,PPARG,AXIN1,DLG5,RYR2,CDK11A,CDK11B,DRAXIN,NOL3,MYOCD,CHEK2,HCK,CAPN2,PPP2R2B,DCC,NF2,FLT4,SGMS1,HDAC4,PAX2,SFRP1,FOXO3,BCL2L13,SMAD3,ITPR1,EVA1A,GRIK5,TXNDC12,CDC73,APP,SLK,NOX1,YAP1,TNFRSF19,VDAC1,EYA2,ANGPTL4,LRP5,CHST11,TMEM14A,FRS2,PALB2,MMP2,DCUN1D3,PRKD1,STAT1,USP53,PLAGL1,TCTN3,ATF2,GRAMD4,RAF1,CARD16,CASP1,PTPN1,BMPR2,CAMK1D,CERS3,FGF2,PPM1F,SEMA4D,RORC,YME1L1,PLEKHO2,SLC5A8,RARB,PRKCG,NCOA1,AREL1,EEF1E1,TXNDC5,EIF2B5,ATF3,PAWR,DEPTOR,P4HB,CNTFR,COL4A3,HIP1,TRPC5,GLI2,DAB2,PKHD1,XKR4,SIN3A,PELI1,PPP2R5C,CFDP1,MAP3K7,DDX47,ALK,INPP5D,SRA1,TOP1,PAX7,PAK3,ADNP,BCL7C,RNF34,TFRC,WWOX,MEF2A,GFRAL,EYA1,TRPV1,RNF216,MNAT1,HDAC1,TRIM24,PLCG2,ROCK1,HIPK3,RYBP,CAPN10,CUL2,PTPN2,NTRK1,PKN2,TMIGD1,SLC39A10,SARM1,MUC1,JAG2,SPI1,EPHA7,CTNNA1,AIFM2,MST1,ERCC3,SNCA,BMPR1B,GRM4,USP42,HEY2,CHMP3,ZMYND11,CAST,DFFA,RASA1,MITF,SRSF6,HIPK1,NUGGC,BTK,IGF1,CTNNBL1,HTT,MAPK1,PTEN,BMP7,LRRK2,OSGIN1,EBAG9,BAG6,ITGAM,WNT7A,NLRP1,UACA,RABEP1,RHOT1,GPI,SH3RF2,PRMT2,RAPGEF2,SMO,MARK4,PSEN2,TNFRSF11B,JADE1,RHOA,SYNGAP1,CUL3,SH3KBP1,HSPD1,GSK3B,DNAJC3,EEF2K,CD27,BOK,RNF144B,SULF1,KRT8,NACC2,TNFRSF8,A*

			GT,PRKAA2,ADAR,ZMPSTE24,PTK2,FBXO10,SHB,MAP3K5,DLX1,NCF1,TAOK2,SHC1,CD160,RPS6KB1,DNAJB6,CD44,ADAM8,BCL2L14,ACTN4,STK4,ZFAND6,DRAM1,SLC1A1,SIGMAR1,HCAR2,DLC1,PPARA,PPP1R10,STEAP3,PHLPP1,MAGI3,CPEB4,PRKCZ,ADORA2A,ANGPT1,PACS2,EYA3,FHL2,ATF6,ETS1,PPP1CA,GRIA4,STPG1,ATG3,PRKCQ,BRAF,CSRNP1,HDAC2,TNS4,POU3F3,HIGD2A,PHIP,PPP2CA,ANGPT4,ARHGEF6,SP100,MADD,SYK,GRIK2,CIT,BRCA2,HRK,TICAM1,CADM1,HSF1,MAX,SRPK2,ANKRD13C,QRICH1,VAV3,AKTIP,NUAK2,AXL,TNFSF9,MET,CAMK2D,MALT1,TRIM69,KDM2B,THEM4,PHB,PTPRC,TYRO3,ADCY10,TRAF3IP2,TRAP1,EDNRA,GNGT1,PRAP1,BMP4,CASP12
GO:0006357	regulation of transcription by RNA polymerase II	4.827639298647801e-7	LDB2,DNMT1,HLX,SLC9A1,PBX3,MED13L,TRPS1,CBFB,ZNF823,PRDM12,MED26,WWC1,ASH1L,SCAF8,STOX2,WWC3,PAGR1,HIVEP3,NPAS3,ARNT,STAT5B,TOX,ZNF566,CASK,MAP2K5,ZNF536,SP3,HDGF,EZR,IKZF2,CHD7,MECOM,TRAF6,DNAJA3,NHLH1,PSMB7,TSC22D3,GFI1B,RUNX1,HMGN3,NRIP1,THRB,EFCAB7,DACH1,ZNF569,ORC2,MYT1,IKBKB,ZNF609,BRD8,KAT6B,HIF3A,SNIP1,ESR1,MIER1,PCBP3,MAML3,CDON,EP300,CELA1,TENM2,ZNF76,ZNF471,CCND3,ZNF19,ZNF23,BRMS1,ZNF605,HNF4G,PRKAR1A,H2AFY2,TCF7L2,JMJD1C,ZNF443,ZNF490,ZNF564,ZNF709,ZNF799,LITAF,ESRRB,TBR1,TFAP2A,PEG3,ZIM2,TGIF2,MEIS1,VRTN,MDM4,ZNF148,MTA3,TFDP2,SLC30A9,TOX3,KAT7,ZNF667,RBM14,MED12L,SATB2,RIT2,HIRA,MKRN2,ARNTL,ARID4B,PIK3R2,UBP1,MDFI,FNIP1,VAX2,TMBIM6,PPP3R1,HDAC5,CSRNP3,ZNF692,NFIA,RNF4,JDP2,DCN,CDH13,CREBRF,SOX13,COPS5,MAD2L2,TLE6,CAPRIN2,ZNF418,PHF20L1,ZNF286A,FOXN3,NEUROD1,JAK2,SKAP1,MLIP,BCL11B,PKNOX1,TSHZ2,TCF7,PDE2A,KLF15,TBX15,ANXA4,MTA1,KLF8,LCOR,RPRD1B,CCDC62,SOX6,ACVR2A,RUNX2,TGFB1,NSD1,ZHX2,PKNOX2,NFATC3,F2RL1,BCAS3,CC2D1B,TP73,SAP18,ZBTB22,MTDH,MYT1L,ZNF398,CLOCK,TCF12,ZNF675,ETV6,TFAP2D,BCL3,DUSP22,SBNO2,FGF10,SMYD3,LUM,SMURF2,RORA,HIVEP2,AUTS2,TNFSF11,PPP3CA,NFYB,MAGEA4,KLF12,GATAD2B,TRAK1,CTNNB1,PARK2,SOD2,DACH2,METTL13,SMARCC1,KLF17,PPARG,MTF1,CBX5,BRIP1,SREBF2,FGF1,NPAT,NR4A3,FOXK2,MYOCD,PER2,AJUBA,ZNF626,ZNF737,SUPT3H,PRDM16,BRMS1L,ZBTB8A,ZBTB8B,CTDP1,BAZ1B,MEF2B,HDAC4,PAX2,MED13,ZNF395,FOXO3,NFIB,SP4,SMAD3,CUX2,WWP2,ARNT2,ZNF662,ZNF777,EBF3,CASZ1,MIER3,NEDD4,ESRRG,HOXD3,HOXD4,ZNF114,KTI12,NFATC1,CDC73,APP,SSBP3,GSX2,YAP1,LRP5,ZNF787,SOX2,TEAD1,ZNF653,ZNF521,ARID3A,ZNF761,CHUK,ZNF584,ESR2,PRKD1,STAT1,ST18,ETV5,RHOXF2B,TAF3,PLAGL1,HNF4A,ZBTB7C,ATF2,POU2F2,TCF3,ZNF730,RAF1,BMPR2,BMPR1A,ZKSCAN1,IKZF4,CDK12,MYB,FGF2,ZNF71,POU6F2,BACH1,MXD3,GLI4,ZFP41,SEMA4D,NFX1,RORC,ELP3,PTAFR,SP140,SP140L,RHOXF2,JARID2,DDX58,RARB,SPEN,SIN3B,NCOA1,EHMT1,LMO7,DVL3,TCF20,ATF3,PAWR,EBF2,MAML2,TSG101,CRYM,RFX2,ZNF322,SUFU,MAGEA11,PRDM15,ZNF670,ZNF695,SOHLH2,ZNF354C,ZNF704,NR2C1,GLI2,TNKS,ERCC1,GLIS3,WDTC1,ZNF664,DAB2,MYSM1,SIN3A,RUVBL2,GMEB1,ZNF423,SP1,TEAD4,HOXC13,SRA1,ADIRF,OVOL2,NFATC2,PAX7,ATP2B4,ACTL6B,ASXL3,TET1,CAMTA1,CCPG1,ADNP,ARID4A,CDK6,PHF2,WWOX,MEF2A,DCAF6,SATB1,PSMB2,EYA1,GATAD2A,TRPV1,HOXB3,HOXB4,HOXB5,HOXB6,TFEB,MNAT1,ACTR2,HDAC1,PAXIP1,ZNF713,EGR2,RNF10,RYBP,AP3B1,PTPN2,KPNA6,EPCAM,ZNF425,MUC1,BCOR,AP3D1,ZNF41,ZNF383,ZNF616,ZNF836,SMARCA2,CUX1,SPI1,DNMT3B,FOXP2,GLIS1,JAZF1,CREB5,SNCA,BMPR1B,ZEB2,FOXJ3,HEY2,KMT2D,CREM,KMT2C,MACC1,MITF,NFIX,CBFA2T2,IGF1,MLXIP,ATF7IP,BMP7,MXI1,SOX5,CIR1,PCBP2,ZNF780A,ZNF780B,ZBTB20,PKD2,ZNF652,RIPPLY1,RCOR3,TADA2A,NCOA3,ZNF146,ZNF565,WNT7A,ZBED6,BRWD3,CRTC3,ARNTL2,KAT6A,ZKSCAN7,ZNF197,ZNF660,BMP6,NFE2L1,ZNF30,ATRX,IKZF1,SMO,TCF4,SOX30,TFE3,GRIN1,RHOA,ONECUT2,CUL3,TFEC,STAT6,RBBP8,CDK13,DUSP26,ZNF362,NACC2,SUPT4H1,ZNF354A,HDGFRP3,STAT2,PBX1,TCFL5,CREBBP,DLX1,GTF2IRD2,ZNF813,LBX2,DPRX,SP7,RPS6KA5,ZNF484,ZNF93,PRDM2,NOS1,ZNF44,MED15,MC1R,TCF25,ZNF282,IL18,PPARA,ARID5B,SMARCAL1,NOTCH4,ZNF366,CRX,PAX6,OTUD7B,NIPBL,PHF20,ZNF143,TBL1X,FHL2,ATF6,ZBTB5,ZNF708,ETS1,MTF2,TAGLN3,NCOR2,CSRNP1,HDAC2,IMPACT,SRCAP,POU3F3,NOTO,ZKSCAN5,ELF2,PHIP,RPL23,ATXN1,SP100,ZNF347,ZNF415,TRIM29,CNOT1,WWTR1,GTF2H5,NFXL1,ZBTB38,ATF7,DVL2,YBX1,TAF1,THAP3,HSF1,MAX,SAP130,EZH1,NRF1,NCOA2,CHD6,RNF2,ZNF554,MYEF2,TRIM37,MET,ZNF461,CAMK2D,TIMELESS,BRD9,SETD3,KDM2B,MLXIPL,PHB,ZNF555,CTCF,NR6A1,ATF6B,CREB1,EBF4,ZNF511,BMP4,ABLIM3
GO:0051253	negative regulation of RNA metabolic process	4.95479208194149e-7	LDB2,DNMT1,TRPS1,CBFB,PRDM12,WWC1,SCAF8,WWC3,ZNF566,MAP2K5,ZNF536,SP3,HDGF,EZR,MECOM,TRAF6,DNAJA3,GFI1B,PTPRK,RUNX1,NRIP1,THRB,DACH1,ORC2,HDAC6,PEX14,KAT6B,ESR1,MIER1,PCBP3,EP300,CELA1,TENM2,PDGFB,CCND3,TOB2,ZNF19,BRMS1,SCML4,RERE,ATP8B1,H2AFY2,TCF7L2,FBXW11,BASP1,TBR1,TFAP2A,PEG3,ZIM2,TGIF2,MDM4,ZNF148,MTA3,SNX6,TFDP2,ERN2,C1D,CENPF,LARP4B,KAT7,SATB2,HIRA,ARNTL,PLCB1,NRG1,MDFI,FNIP1,VAX2,TMBIM6,VGLL4,HDAC5,ZNF692,JDP2,CREBRF,TRDMT1,SOX13,MAD2L2,TLE6,FOXN3,UIMC1,MLIP,TCF7,PDE2A,TBX15,WNT11,MTA1,KLF8,LCOR,SOX6,RUNX2,TGFB1,NSD1,ZHX2,BTRC,NFATC3,CDYL2,CC2D1B,SAP18,MTDH,MYT1L,ZNF398,CLOCK,ZNF675,ETV6,BCL3,DUSP22,HNRNPC,SBNO2,LOXL3,CAPN3,SMURF2,PPP3CA,NSUN2,MAGEA4,KLF12,GATAD2B,CTNNB1,PARK2,METTL13,PPARG,AXIN1,CIPC,CBX5,LRPPRC,SREBF2,NPAT,NR4A3,FOXK2,MYOCD,PER2,AJUBA,PRDM16,BRMS1L,HDAC4,PAX2,SFRP1,FOXO3,NFIB,ZCCHC17,SYNCRIP,SMAD3,C

			UX2,WWP2,RNF168,MIER3,NEDD4,CDC73,YAP1,SOX2,PRICKLE1,ZNF653,SFMBT1,ESR2,STAT1,ETV5,TAF3,PLAGL1,HNF4A,EREG,ATF2,TCF3,CELF4,IKZF4,MYB,BACH1,MXD3,PPM1F,BEND5,SEMA4D,NFX1,RORC,JARID2,PHC2,RARB,SPEN,SIN3B,EHMT1,ATF3,LIN28B,PAWR,TSG101,CRYM,SUFU,MAGEA11,NR2C1,GLI2,GLIS3,WDTC1,DAB2,SIN3A,RUVBL2,ZNF423,TRIM22,OVOL2,NFATC2,MAPKAPK2,ARID4A,CDK6,MEF2A,SATB1,GATAD2A,TRPV1,HOXB3,HOXB4,L3MBTL4,ELAVL4,HDAC1,RECQL5,TRIM24,SCMH1,RYBP,PTPN2,AXIN2,ZNF425,MUC1,BCOR,SMARCA2,CUX1,SPI1,DNMT3B,FOXP2,PSPC1,GLIS1,SFMBT2,JAZF1,SMYD1,SNCA,ZEB2,HEY2,ZMYND11,CREM,MITF,SRSF6,NFIX,CBFA2T2,ATF7IP,BMP7,MXI1,CIR1,ZBTB20,LILRB4,KCTD1,RIPPLY1,RCOR3,DAZL,ZBED6,KAT6A,BMP6,TAF15,ATRX,IKZF1,PRMT2,SMO,SOX30,CUL3,TFEC,STAT6,RBBP8,DUSP26,NACC2,SUPT4H1,METTL16,ZMPSTE24,TCFL5,CREBBP,DLX1,SHC1,PARP10,SETDB2,DNAJB6,RPS6KA5,ZNF93,PRDM2,TCF25,ZNF282,PPARA,ARID5B,NOTCH4,ZMYND8,ZNF366,PAX6,OTUD7B,NIPBL,TBL1X,FHL2,MBTD1,ZBTB5,MTF2,TAGLN3,NCOR2,HDAC2,IMPACT,POU3F3,ELF2,RPL23,ATXN1,SP100,TRIM29,PARN,RBM42,CNOT1,WWTR1,ZBTB38,MORC1,YBX1,TAF1,HSF1,MAX,SAP130,EZH1,NCOA2,ZP3,RNF2,ZNF554,TRIM37,TIMELESS,KDM2B,MLXIPL,PHB,ZNF555,CTCF,NR6A1,TRAF3IP2,CREB1,BMP4
GO:0050807	regulation of synapse organization	5.205375472124855e-7	*NRXN1,NEGR1,GRID2,KALRN,NTRK3,LHFPL4,CDKL5,ROBO2,PTPRO,NTRK2,CDH8,PTPRD,BDNF,AMIGO1,TANC2,FYN,NTN1,DCTN1,FRMPD4,LRFN5,CAPRIN2,ZDHHC15,CLSTN2,IL1RAPL1,CAMK2B,DISC1,LINGO2,IL1RAPL2,EPHA4,FCGR2B,ARHGAP22,DLG5,DGKB,DNM3,CUX2,ABI2,NEDD4,NRP2,APP,NLGN2,GPC6,SHANK2,NLGN1,CTNNA2,SEMA4D,EIF4G1,EPHB3,NLGN3,PAFAH1B1,SETD5,RELN,PAK3,ADNP,FLRT2,ACTR2,NTRK1,EPHB1,ARHGAP44,EPHA7,SNCA,PTPRS,PTEN,LRRK2,EFNA5,SHANK3,WNT7A,RHOA,EEF2K,EPHB2,GRIN2B,SLIT1,GPM6A,SYNDIG1*
GO:0007015	actin filament organization	5.549381679899406e-7	*CLRN1,PRKCI,SLC9A1,CLASP2,PHACTR1,FHOD3,TJP1,SEMA5A,FER,EZR,TENM1,CTNNA3,ABI1,TRIOBP,ELMO2,GAS7,CDC42EP3,XIRP2,EPHA1,LIMK1,FMN2,PAK1,ARHGAP6,DMD,GSN,CASS4,PCDH15,TTN,MAD2L2,TLE6,NCKAP1,JAK2,PLS1,CYFIP2,WNT11,PRKCD,PPM1E,F2RL1,SLIT2,CORO1C,KANK1,KANK4,ENAH,RHPN2,SVIL,MPRIP,PARK2,MYO1D,ADD2,MYO1E,HCK,SORBS1,NF2,SPTBN1,ELN,SFRP1,SSH1,SMAD3,ABI2,SHANK1,ARHGEF18,PREX1,ADD3,MYO1F,SPTBN4,VILL,RND3,CTNNA2,PPM1F,NEDD9,PAWR,HIP1,RUFY3,ARHGAP12,MYO7A,CLASP1,PACSIN1,SPTBN5,PAK3,SSH2,AIF1L,PLS3,CALD1,SNX9,DIAPH2,ACTR2,ROCK1,EPS8,PPP1R9A,MCU,CTNNA1,LIMA1,SHROOM3,FCHSD2,RAPGEF3,PDXP,SH3BP1,RASA1,KCTD13,DPYSL3,ARHGAP25,SHANK3,RHOA,TF,RHOBTB1,SHROOM1,CUL3,SH3KBP1,RDX,FAM49B,ESPNL,IQGAP2,FAM171A1,ALDOA,TMEFF2,DLC1,PHLDB2,SHROOM4,NEBL,BRAF,DIAPH1,CIT,NEB,MYO1A,CGNL1,MET,MICAL3,SPTAN1*
GO:0009967	positive regulation of signal transduction	6.627065755070253e-7	*PRDX2,GPC5,NRXN1,PRKCI,MAP4K4,PDCL,SLC9A1,PDE8A,SEMA3A,CHERP,WWC1,C12ORF49,NOS1AP,MAPRE2,PTGIS,PRLR,PAGR1,NTRK3,CBL,ARNT,SEMA5A,FLT3,GRM5,PLCE1,MAP2K5,KITLG,LRP2,PIK3CD,ROBO1,HTR2B,TENM1,TRAF6,ROBO2,ITPKB,ITGB1,HSP90AA1,PSMB7,CCDC22,AKAP13,DGKI,PTPN11,MGAT5,ADORA1,ADAMTS3,IKBKB,ERBB4,ADRA1D,MIER1,CDON,NTRK2,EP300,RNF220,PDGFB,RIMS1,TNIK,MID1,STK39,INSR,TCF7L2,USP34,PAK1,LITAF,MAP3K4,ZDHHC13,BDNF,TRIM13,LCK,ECT2,ERN2,RIT2,DEPDC1B,CASS4,FYN,ARNTL,NF1,PLCB1,RTN4,RTN4R,RWDD3,CHI3L1,NDRG4,BMP2K,NRG1,BID,MAP2K1,FNIP1,ROR2,DCN,SLC39A14,CDH13,CAPRIN2,CCBE1,JAK2,FBXW7,NPSR1,MLLT3,LAMTOR3,CYFIP2,WNT11,NOX4,SIAH1,PRKCD,TAB2,ACVR2A,CD4,NUP93,PIBF1,CRADD,F2RL1,AAK1,TP73,DISC1,KANK1,MTDH,BMPER,TNFRSF10B,DUSP22,NAIP,FGF10,IQCJ-SCHIP1,SH3RF3,LOXL3,SMURF2,EPHA4,PRKCA,AUTS2,TNFSF11,CTNNB1,PARK2,FCGR2B,IGF1R,PPARG,AXIN1,DLG5,IL18R1,P2RY10,ANKRD17,APOL3,FGF1,MYOCD,TRIM5,AJUBA,SORBS1,TRIM8,CSPG4,GRM1,FLT4,SFRP1,CNTN6,SMAD3,CUX2,SHANK1,NEDD4,TAB1,CDC73,APP,GSX2,PDGFRA,NOX1,YAP1,NLGN2,TNFRSF19,SOX2,CHUK,FRS2,S100A12,PRKD1,EREG,RAF1,CARD16,CASP1,PTPN1,BMPR2,BMPR1A,NLGN1,SPAG9,EVC,FGF2,SEMA4D,DOK6,ADCYAP1R1,DKK2,EEF1E1,EEF1E1-BLOC1S5,GAS8,DVL3,ATF3,SHOC2,AGO3,TERF2IP,WDR59,WNT3,CCL14,CCL15,NLGN3,SCUBE2,PAFAH1B1,PRDM15,HIP1,AKAP6,NR2C1,NEO1,TNKS,SCUBE1,DAB2,WNK1,RELN,NEK10,RSPO2,GUCY1A2,PELI1,IQGAP1,MAP3K7,ZNF423,TRIM22,MACF1,ALK,UBE2V1,XDH,SNX5,CAMTA1,P2RY8,TFRC,WWOX,GFRAL,GPC3,PSMB2,TSPAN6,CLEC16A,GHR,SMOC2,PLCG2,ROCK1,C1QTNF1,GPR35,PTPN2,NTRK1,TRIM44,SLC39A10,JAG2,VWF,NETO1,ARHGEF3,SPI1,CTNNA1,LY86,BCAR3,RBPMS,MAP3K13,PTK7,BMPR1B,GRM4,ZEB2,RC3H1,WNT7B,KMT2D,RPTOR,IGF1,HTT,PTEN,SPRED2,BMP7,PUM1,LRRK2,ZRANB1,TMEM108,PKD2,SHANK3,PDGFC,WNT7A,SIK3,BMP6,SH3RF2,SOS1,RAPGEF2,SMO,UBR5,GRIN1,RHOA,ROR1,RQCD1,ACTA2,NECAB2,CD27,BOK,SULF1,NACC2,AGT,PTK2,RIMS2,MAP3K5,CREBBP,NCF1,TAOK2,SHC1,LBX2,SCEL,CD44,ADAM8,BCL2L14,CXCL17,ACTN4,MC1R,STK4,WLS,IL18,UBE2K,PRKCZ,ZC3HAV1,ADORA2A,ANGPT1,TBL1X,LRRK1,OSBPL8,ATF6,GCNT2,PPP1CA,CCL22,BRAF,HTR2C,CD300A,PHIP,MAOA,RPL23,MADD,SYK,DVL2,PTPRJ,TICAM1,SASH1,CAV2,AXL,MET,UCHL5,ANKRD6,MALT1,PHB,PTPRC,EDA,GPR89A,TSPAN5,TRAF3IP2,BMP4,CPNE1*

GO:0071417	cellular response to organonitrogen compound	7.349087866736742e-7	*ENPP1,PRKCI,PDE4D,DNMT1,SLC9A1,ADCY8,RYR1,STAT5B,GRM5,FER,EZR,SLC26A6,HTR2B,RYR3,GOT1,CDC6,STXBP4,PTPN11,NTRK2,CCND3,INSR,PIP4K2A,AMIGO1,CHRM3,UBR2,SNX6,AP3S1,SLC8A1,FYN,PLCB1,PIK3R2,HDAC5,SLC39A14,IPO5,RGS8,JAK2,PDE2A,KLF15,LAMTOR3,PRKCD,TGFB1,SLIT2,GABRB1,KANK1,CHRM1,GLP2R,EPHA4,CTNNB1,PARK2,FCGR2B,SMARCC1,IGF1R,PPARG,CYBB,BRIP1,RYR2,LEPROT,NR4A3,SORBS1,CAPN2,SFRP1,FOXO3,SSH1,APP,PDGFRA,SESN1,GNG2,DRD1,GLRA2,MMP2,STAT1,HNF4A,ATF2,PTPN1,COL16A1,PIK3R3,ADCY5,PTAFR,PTPRE,SRSF5,IDE,KCNQ1,AKAP6,IRS4,WDTC1,BLM,UBR1,SIN3A,SP1,ALK,SNX5,ATP2B4,MAP1B,COL4A6,CACNA1A,XRN1,TRPV1,GHR,ACTR2,RECQL5,ROCK1,CAPN10,ABCC1,PTPN2,INSRR,HTR1D,PTPRA,CTNNA1,BCAR3,RAB15,RAPGEF3,PDXP,SNCA,HMGCS2,RPTOR,IGF1,MAPK1,LRRK2,GNA14,CHMP5,NSG2,PKD2,PDGFC,GPR21,ANO1,TSHR,PNPLA3,RAPGEF2,GRB14,ITPR2,GABRB3,GSK3B,STAT6,EEF2K,AGT,HRH4,PTK2,PDE4B,SHC1,RPS6KB1,GPR173,EPHB2,SLC1A1,SLC25A33,CPEB4,FBN1,PRKCZ,ATP1A3,CPEB1,OSBPL8,PRKCQ,DIAPH1,HDAC2,HTR2C,IMPACT,PHIP,RPL23,SH3BP4,TAF1,HSF1,MAX,CIB2,CAV2,DENND4C,TIMELESS,EDNRA*
GO:0044271	cellular nitrogen compound biosynthetic process	7.456548810333709e-7	*POLDIP3,ENPP1,PRDX2,LDB2,ASPH,GSS,PRKCI,SLCO3A1,DNMT1,HLX,SLC9A1,PBX3,ADCY8,MED13L,TRPS1,CBFB,PDE8A,ZNF823,IL31RA,PRDM12,MED26,WWC1,ASH1L,NOS1AP,SCAF8,STOX2,PTGIS,PRKAG2,WWC3,PAGR1,ADCY7,HIVEP3,FTO,NPAS3,ARNT,LRRFIP2,STAT5B,TOX,GRM5,ZNF566,FER,CASK,MAP2K5,MAPK10,ZNF536,SP3,HDGF,GTF3C1,EIF4G3,EZR,IKZF2,B3GALT1,CHD7,MECOM,TACC1,TENM1,NADK2,TRAF6,GTF2E2,DNAJA3,NHLH1,HSP90AA1,PSMB7,TSC22D3,ZC4H2,CCDC22,GMDS,GFI1B,PTPRK,RUNX1,ERC1,HMGN3,NRIP1,THRB,EFCAB7,ITGB3BP,DACH1,ZNF569,CCT2,ORC2,HDAC6,SERTAD2,MYT1,IKBKB,PEX14,ERBB4,MRE11A,ZNF609,BRD8,KAT6B,HIF3A,SNIP1,ESR1,MIER1,PCBP3,MAML3,CDON,TNRC6A,EP300,CELA1,TENM2,ZNF76,RNF220,ZNF471,PDGFB,CCND3,TOB2,ZNF19,ZNF23,BRMS1,ZNF605,SCML4,HNF4G,INSR,RERE,PRKAR1A,FUBP1,ATP8B1,H2AFY2,TCF7L2,ST6GALNAC3,JMJD1C,ZNF443,ZNF490,ZNF564,ZNF709,ZNF799,LITAF,FBXW11,ESRRB,MAP3K4,BASP1,SPTSSA,TBR1,SAMD4A,TFAP2A,CDK14,FANCA,PEG3,ZIM2,TGIF2,TGIF2-C20ORF24,MEIS1,VRTN,TRIM13,MDM4,ZNF148,MTA3,SNX6,TFDP2,PTCD3,ERN2,C1D,AFF3,DMD,CENPF,SLC30A9,CERS6,TOX3,LARP4B,USP13,KAT7,ZNF667,RBM14,RBM4,PRPS2,MED12L,SATB2,RIT2,HIRA,DRG2,MKRN2,ARNTL,LINC01138,PLCB1,ARID4B,POLA2,B4GALT6,RWDD3,BRPF1,PIK3R2,NRG1,UBP1,MAP2K1,MDFI,FNIP1,VAX2,TMBIM6,VGLL4,PPP2CB,PPP3R1,HDAC5,CSRNP3,ZNF692,NFIA,RNF4,JDP2,CMKLR1,ROR2,DCN,PRPSAP2,PCBD2,CDH13,CREBRF,SOX13,ACSBG1,COPS5,AK2,MAD2L2,TLE6,SRBD1,CAPRIN2,ZNF418,PHF20L1,BBOX1,PDF,TERF2,ZNF286A,NME7,FOXN3,AK5,NEUROD1,USP22,JAK2,TRAPPC9,OAZ2,SKAP1,UIMC1,ITCH,MLIP,BCL11B,IMPDH1,PKNOX1,MLLT3,TSHZ2,PNPLA1,TCF7,PDE2A,KLF15,TBX15,ANXA4,WNT11,MTA1,KLF8,NOX4,LCOR,RPRD1B,QKI,CCDC62,PRKCD,SOX6,ACVR2A,RUNX2,CD4,TGFB1,BANP,MRPL33,SGK1,PCSK5,NSD1,IGSF1,ZHX2,PKNOX2,ASCC2,BTRC,NFATC3,POLK,F2RL1,BCAS3,CDYL2,CC2D1B,TP73,SAP18,CDKAL1,ZBTB22,ILF2,MTDH,FANK1,MYT1L,SMAD6,BNC2,ZNF398,CLOCK,TCF12,ZNF675,RPL36,ETV6,PHRF1,TFAP2D,BCL3,EIF3L,SND1,DUSP22,HNRNPC,TRRAP,SBNO2,YTHDF1,FGF10,POLR2J2,ADK,SMYD3,LOXL3,ADCY2,CAPN3,LUM,SMURF2,RORA,HIVEP2,HSD17B12,AUTS2,TNFSF11,SMG6,PPP3CA,NFYB,MAGEA4,KLF12,CAMK4,GATAD2B,UFL1,TRAK1,CTNNB1,PARK2,SOD2,DACH2,METTL13,SMARCC1,KLF17,PPARG,IARS2,NGRN,AXIN1,IL18R1,CIPC,MTF1,CBX5,OTC,BRIP1,LRPPRC,SREBF2,AARS2,PFAS,CDK11A,CDK11B,FGF1,NPAT,NR4A3,FOXK2,MYOCD,TRIM5,PER2,AJUBA,ZNF626,ZNF737,CHEK2,SUPT3H,PRDM16,HCK,TRIM8,DIO2,BRMS1L,ZBTB8A,ZBTB8B,CTDP1,BAZ1B,MEF2B,SGMS1,HDAC4,PAX2,PHF5A,CPOX,SECISBP2L,SNAPC4,SFRP1,MED13,ZNF395,FOXO3,TPH2,NFIB,SP4,SYNCRIP,SMAD3,CUX2,WWP2,ARNT2,SBNO1,KRBOX1,ZNF662,ZNF777,EBF3,RNF168,CASZ1,DCP1B,MIER3,NEDD4,ESRRG,HOXD3,HOXD4,ZNF114,KTI12,NFATC1,ST3GAL3,UCK2,CDC73,APP,SSBP3,GSX2,RBM8A,YAP1,ST6GALNAC5,PPCS,TEN1,NVL,PEMT,LRP5,POLR3G,BDH2,ZNF787,SOX2,SETD2,TEAD1,PRICKLE1,ZNF653,ZNF521,ELOVL2,ARID3A,ZNF761,CHUK,SFMBT1,ZNF584,TPH1,ESR2,S100A12,GTF2F2,PRKD1,STAT1,ST18,ETV5,RHOXF2B,SLC6A3,TAF3,PLAGL1,HNF4A,ZBTB7C,TASP1,EREG,ATF2,POU2F2,TCF3,ZNF730,RAF1,CELF4,ABLIM2,ZNF766,CARD16,POLA1,GGT7,BMPR2,CAMK1D,BMPR1A,ZKSCAN1,IDO2,PABPC4,IKZF4,CDK12,TAF8,CAND2,CERS3,DENND4A,MYB,FGF2,ZNF71,POU6F2,BACH1,MXD3,PPM1F,ADCY5,GLI4,ZFP41,BEND5,SEMA4D,NFX1,RORC,ELP3,PTAFR,SP140,SP140L,RHOXF2,JARID2,DDX58,SLC5A8,BRDT,PHC2,RARB,SPEN,SIN3B,NCOA1,EEF1E1,EHMT1,LMO7,DVL3,EIF2B5,EIF4G1,POLR2H,TCF20,ATF3,TCEANC,CKS1B,PAWR,EBF2,AGO3,MAML2,TSG101,TERF2IP,CRYM,RFX2,ZNF322,SUFU,MAGEA11,PRDM15,ZNF670,ZNF695,CCDC169-SOHLH2,SOHLH2,POLE,DDAH1,ZNF354C,TCEA3,PADI6,CMPK1,ZNF704,CERS4,NR2C1,ME1,GLI2,TNKS,WBP2NL,ERCC1,TNRC6B,GLIS3,WDTC1,ZNF664,MRPS28,TPK1,DAB2,BLM,PKHD1,MYSM1,SETD5,SMG5,GTPBP2,RELN,SIN3A,RUVBL2,COMMD6,GUCY1A2,GMEB1,PELI1,SLC25A16,MAP3K7,ZNF423,SP1,TRIM22,ALK,TEAD4,HOXC13,APBB3,SLC35A4,SRA1,UBE2V1,ADIRF,OVOL2,SNX5,NFATC2,PAX7,BNC1,ATP2B4,ACTL6B,ASXL3,PAK3,TET1,CAMTA1,CCPG1,ADNP,ARID4A,C12ORF65,CDK6,PHF2,CELF1,TFRC,EPHA5,WWOX,MEF2A,ADCY9,DCAF6,XRN1,SA*

			TB1,PSMB2,EYA1,GATAD2A,DIS3L2,TRPV1,HOXB3,HOXB4,HOXB5,HOXB6,TFEB,MNAT1,ACTR2,L3MBTL4,ACACA,ELAVL4,HDAC1,RECQL5,SMOC2,TRIM24,PLCG2,ROCK1,SCMH1,MRPS17,PAXIP1,ZNF713,EGR2,HIPK3,RNF10,RYBP,AP3B1,PTPN2,NTRK1,KPNA6,TRIM44,EIF3A,EPCAM,ZNF425,GNL3L,MUC1,PTPN14,BCOR,AP3D1,ZNF41,CARM1,ZNF383,ZNF616,ZNF836,SMARCA2,CUX1,SPI1,DNMT3B,LRRC47,FOXP2,MRPS24,PSPC1,GLIS1,SFMBT2,EIF3H,JAZF1,ABCB7,RBPMS,CHURC1,CREB5,MAP3K13,ERCC3,SMYD1,SNCA,BMPR1B,POLN,PRIM2,PNPT1,ZEB2,FOXJ3,NADK,HEY2,RC3H1,EIF3E,ZMYND11,KMT2D,CREM,KMT2C,PLD1,MACC1,MITF,OPRD1,RPTOR,NFIX,BTK,MLLT1,TROVE2,CBFA2T2,IGF1,MLXIP,ATF7IP,LARS2,MAPK1,PTEN,BMP7,ATXN2,MXI1,PUM1,SOX5,CIR1,PCBP2,ZNF780A,ZNF780B,ATP6V1A,MLYCD,ZBTB20,RFC3,LILRB4,PKD2,ZNF652,HSF2BP,KCTD1,RIPPLY1,NT5E,RCOR3,TADA2A,DAZL,SPTLC3,NCOA3,PKIB,ZNF146,ZNF565,WNT7A,ZBED6,BRWD3,CRTC3,DNAJC1,ARNTL2,ELOVL5,KAT6A,MTIF2,ZKSCAN7,ZNF197,ZNF660,BMP6,TAF15,NFE2L1,WDR43,ZNF30,ATRX,DPH6,IKZF1,PRMT2,ASCC1,MTHFD2L,SMO,RALY,AMPD1,TCF4,SOX30,TFE3,GRIN1,JADE1,RHOA,SPRTN,ROR1,RQCD1,TF,ONECUT2,CUL3,TFEC,PRR16,DNAJC3,STAT6,MLF1,EEF2K,RBBP8,CDK13,ALAS2,DUSP26,MTHFD1L,ZNF362,NACC2,SUPT4H1,ZNF354A,AGT,HDGFRP3,METTL16,ELOVL3,POLH,POLR2F,STAT2,ZMPSTE24,GTF2E1,PPCDC,PBX1,EXOSC3,MAP3K5,TCFL5,CREBBP,DLX1,GTF2IRD2,NCF1,SHC1,ZNF813,ALDOA,LBX2,PARP10,RPS6KB1,DPRX,GUCY2F,PRG3,SETDB2,DNAJB6,SP7,LARP4,ADAM8,MRPS6,RPS6KA5,ZNF484,ZNF93,PRDM2,NOS1,ZNF44,MED15,RGS14,ACTN4,MC1R,TCF25,ZNF282,GATC,SLC1A1,EDRF1,NOTCH4,IL18,TEX12,DIS3,PPARA,ARID5B,SLC25A33,SMARCAL1,TAF2,CPEB4,LDHC,NOTCH4,ZMYND8,ZNF366,CRX,PAX6,PRKCZ,ADORA2A,PAH,CPEB1,OTUD7B,NIPBL,YEATS4,PHF20,ZNF143,BRF1,TBL1X,FHL2,MBTD1,ATF6,ZBTB5,ZNF708,IGF2BP3,ETS1,PPP1CA,MTF2,TAGLN3,CDK5RAP1,NCOR2,PRKCQ,CSRNP1,HDAC2,IMPACT,SRCAP,POU3F3,NOTO,ZKSCAN5,ELF2,MOXD1,PHIP,PPP2CA,SLC26A2,MRPS16,RPL23,RFC5,ATXN1,SP100,ZNF347,ZNF415,REV3L,TRIM29,PARN,FARSB,ADCY1,SYK,CNOT1,WWTR1,GTF2H5,NFXL1,ZBTB38,BRCA2,ATF7,DVL2,MORC1,MTRF1,YBX1,TAF1,EIF4E3,TICAM1,THAP3,SNAPC3,HSF1,MAX,SAP130,DHFR,EZH1,ETF1,NRF1,NCOA2,EEFSEC,QRICH1,ZP3,CHD6,RNF2,ZNF554,MYEF2,TBP,TRIM37,MET,ZNF461,CAMK2D,MRPL1,TIMELESS,BRD9,MALT1,SETD3,KDM2B,MLXIPL,CCT3,PHB,EDA,ZNF555,CTCF,NR6A1,ADCY10,ATF6B,CREB1,LIG1,RGMB,TRAP1,EBF4,SLC25A13,ZNF511,BMP4,ABLIM3
GO:0018212	peptidyl-tyrosine modification	7.632206624740518e-7	IL31RA,PRLR,MVP,NTRK3,CBL,FLT3,ENPP2,GRM5,FER,MAP2K5,KITLG,ABI1,PTPN11,ADORA1,EPHA1,ERBB4,NTRK2,TPST2,PDGFB,INSR,LCK,SNX6,CASS4,FYN,BCR,TTN,NRG1,MAP2K1,ROR2,JAK2,FBXW7,ZFYVE28,TPST1,NOX4,PRKCD,CD4,PIBF1,DUSP22,DOCK3,FGF10,EPHA4,IGF1R,DYRK4,HCK,CSPG4,BAZ1B,NF2,FLT4,SFRP1,ABI2,APP,PDGFRA,IBTK,JAK1,EREG,PTPN1,NEDD9,PEAK1,SEMA4D,EPHB3,TSG101,DLG3,RELN,ALK,ADNP,EPHA5,GHR,PLCG2,HIPK3,PTPN2,INSRR,NTRK1,EPHB1,EPHA10,EPHA7,HIPK1,BTK,IGF1,UNC119,LILRB4,EFNA5,PDGFC,BMP6,ROR1,AGT,PTK2,TEC,LMTK3,NCF1,SHC1,CD44,EPHB2,IL18,PRKCZ,TYK2,ANGPT1,LRRK1,CNTN1,BRAF,HDAC2,CD300A,PPP2CA,ANGPT4,SYK,DVL2,PTPRJ,HSF1,INPP5F,AXL,MET,PTPRC,TYRO3
GO:1901701	cellular response to oxygen-containing compound	8.003977670674823e-7	ENPP1,PRDX2,PRKCI,PDE4D,DNMT1,SLC9A1,ADCY8,PDE8A,RPS6KA2,PTGFR,TJP1,ADCY7,NTRK3,STAT5B,GRM5,FER,EZR,SLC26A6,HTR2B,RYR3,GOT1,TRAF6,OXR1,ERCC6L2,CDC6,PTPRK,HMGN3,NRIP1,STXBP4,PTPN11,HDAC6,ESR1,NTRK2,PDGFB,CCND3,BPI,INSR,PIP4K2A,LITAF,AMIGO1,CHRM3,UBR2,ECT2,SNX6,AP3S1,SLC8A1,FYN,PLCB1,DPEP1,BCR,PIK3R2,CDK19,HDAC5,SLC39A14,IPO5,RGS8,DEFA1B,DEFA3,PTGER2,NEUROD1,GNAQ,JAK2,CLDN1,PDE2A,KLF15,LAMTOR3,WNT11,NOX4,LCOR,CCDC62,PRKCD,TGFB1,SGK1,PTPRN2,SLIT2,KANK1,MTDH,CHRM1,SBNO2,SMYD3,GLP2R,ADCY2,EPHA4,RORA,PRKCA,CD6,PPP3CA,CTNNB1,PARK2,SOD2,FCGR2B,SMARCC1,IGF1R,PPARG,CYBB,BRIP1,RYR2,LEPROT,RGS10,NR4A3,TRIM5,CHEK2,HCK,SORBS1,CAPN2,PAX2,SFRP1,FOXO3,SSH1,GRIK5,CDC73,APP,PDGFRA,YAP1,RAB11FIP5,SESN1,LRP5,SLC9B2,GNG2,DRD1,GLRA2,CHUK,MMP2,ESR2,PRKD1,STAT1,HNF4A,RAF1,CARD16,CASP1,PTPN1,COL16A1,PIK3R3,ADCY5,RORC,PTAFR,PTPRE,SRSF5,PAWR,IDE,KCNQ1,WNT3,TRPA1,AKAP6,IRS4,WDTC1,BLM,UBR1,SIN3A,RUVBL2,SP1,ALK,SNX5,ATP2B4,MAP1B,COL4A6,SCARB1,EPHA5,CACNA1A,XRN1,ADRBK1,BRINP1,SHPK,TRPV1,GHR,ACTR2,ACACA,RECQL5,TRIM24,PLCG2,ROCK1,CAPN10,ABCC1,PTPN2,INSRR,ZFAND1,HTR1D,SPI1,PTPRA,CTNNA1,LY86,BCAR3,RAB15,RAPGEF3,PDXP,PTK7,SNCA,WNT7B,HMGCS2,ABCA12,NUGGC,RPTOR,BTK,IGF1,MAPK1,BMP7,LRRK2,GNA14,ZBTB20,CHMP5,NSG2,PKD2,EFNA5,PDGFC,NCOA3,ZBED6,NME8,GPR21,BMP6,ANO1,TSHR,PDCD1LG2,PNPLA3,RAPGEF2,KCNC2,SMO,CPT1A,GRB14,RHOA,ITPR2,GSK3B,STAT6,EEF2K,AGT,PRKAA2,BRINP3,HRH4,PTK2,PHEX,PDE4B,MAP3K5,NCF1,SLC29A1,UGT1A1,SHC1,RPS6KB1,GPR173,PLEKHA1,SCNN1B,EPHB2,SLC1A1,SIGMAR1,IL18,CALCRL,SLC25A33,CPEB4,FBN1,PRKCZ,ATP1A3,CPEB1,OSBPL8,PRKCQ,HDAC2,HTR2C,IMPACT,PHIP,RPL23,SH3BP4,ADCY1,TAF1,TICAM1,HSF1,MAX,DHFR,SASH1,CIB2,CAV2,AXL,DENND4C,MET,TIMELESS,MALT1,TRA2B,MLXIPL,SPON2,CREB1,TRAP1,EDNRA
GO:0009890	negative regulation of	8.052992097771361e-7	ENPP1,LDB2,PRMT3,DNMT1,TRPS1,CBFB,PRDM12,WWC1,SCAF8,PTGIS,WWC3,FTO,ZNF566,MAP2K5,ZNF536,SP3,HDGF,EZR,MALRD1,MECOM,TRAF6,DNAJA3,CDC6,CDAN1,GFI1B,PTPRK,RUNX1,NRIP1,THRB,DACH1,ORC2,HDAC6,PEX14,K

	biosynthetic process		*AT6B,ESR1,MIER1,PCBP3,TNRC6A,EP300,CELA1,TENM2,PDGFB,CCND3,TOB2,ZNF19,BRMS1,SCML4,RERE,ATP8B1,H2AFY2,TCF7L2,FBXW11,BASP1,TBR1,SAMD4A,TFAP2A,PEG3,ZIM2,TGIF2,MDM4,ZNF148,MTA3,SNX6,TFDP2,ERN2,C1D,CENPF,PDS5A,KAT7,RBM4,SATB2,HIRA,FYN,ARNTL,PLCB1,NRG1,MDFI,FNIP1,VAX2,TMBIM6,VGLL4,HDAC5,ZNF692,JDP2,CREBRF,SOX13,MAD2L2,TLE6,CAPRIN2,TERF2,FOXN3,FBXW7,UIMC1,MLIP,TCF7,PDE2A,TBX15,WNT11,MTA1,KLF8,LCOR,SOX6,RUNX2,TGFB1,NSD1,PIBF1,ZHX2,BTRC,NFATC3,CDYL2,CC2D1B,SAP18,MTDH,MYT1L,ZNF398,CLOCK,ZNF675,ETV6,BCL3,DUSP22,HNRNPC,SBNO2,YTHDF1,LOXL3,CAPN3,SMURF2,MAGEA4,KLF12,GATAD2B,CTNNB1,PARK2,METTL13,PPARG,AXIN1,CIPC,CBX5,SREBF2,LEPR,NPAT,NR4A3,FOXK2,MYOCD,PER2,AJUBA,PRDM16,BRMS1L,HDAC4,PAX2,SFRP1,FOXO3,NFIB,SYNCRIP,SMAD3,CUX2,WWP2,RNF168,DCP1B,MIER3,NEDD4,CDC73,YAP1,TEN1,SOX2,PRICKLE1,ZNF653,ERLIN1,SFMBT1,ESR2,STAT1,ETV5,TAF3,PLAGL1,HNF4A,EREG,ATF2,TCF3,CELF4,BMPR2,IKZF4,MYB,BACH1,MXD3,PPM1F,BEND5,SEMA4D,NFX1,RORC,JARID2,PHC2,RARB,SPEN,SIN3B,EHMT1,EIF4G1,ATF3,PAWR,AGO3,TSG101,CCDC3,CRYM,SUFU,MAGEA11,NR2C1,GLI2,TNRC6B,GLIS3,WDTC1,DAB2,BLM,SIN3A,RUVBL2,ZNF423,TRIM22,OVOL2,NFATC2,ATP2B4,ASXL3,ARID4A,CDK6,CELF1,MEF2A,XRN1,SATB1,GATAD2A,DIS3L2,TRPV1,HOXB3,HOXB4,L3MBTL4,HDAC1,RECQL5,TRIM24,ROCK1,SCMH1,RYBP,PTPN2,ZNF425,GNL3L,MUC1,BCOR,SMARCA2,CUX1,SPI1,DNMT3B,FOXP2,PSPC1,GLIS1,SFMBT2,JAZF1,ABCB7,ACOT8,SMYD1,SNCA,PNPT1,ZEB2,HEY2,RC3H1,EIF3E,ZMYND11,CREM,MITF,NFIX,CBFA2T2,ATF7IP,BMP7,MXI1,PUM1,CIR1,TMEM59,ZBTB20,LILRB4,KCTD1,RIPPLY1,RCOR3,ZBED6,KAT6A,BMP6,ATRX,IKZF1,PRMT2,RAPGEF2,SMO,SOX30,RHOA,RQCD1,CUL3,TFEC,GSK3B,STAT6,RBBP8,DUSP26,NACC2,SUPT4H1,METTL16,EXOSC3,TCFL5,CREBBP,DLX1,SHC1,PARP10,PRG3,SETDB2,DNAJB6,RPS6KA5,ZNF93,PRDM2,TCF25,ZNF282,TMEFF2,DIS3,PPARA,ARID5B,SMARCAL1,CPEB4,NOTCH4,ZMYND8,ZNF366,PAX6,CPEB1,OTUD7B,NIPBL,TBL1X,FHL2,MBTD1,ZBTB5,IGF2BP3,MTF2,TAGLN3,NCOR2,HDAC2,IMPACT,POU3F3,ELF2,RPL23,ATXN1,SP100,TRIM29,PARN,DONSON,CNOT1,WWTR1,ZBTB38,BRCA2,MORC1,YBX1,TAF1,HSF1,MAX,SAP130,DHFR,EZH1,NCOA2,ZP3,RNF2,ZNF554,TRIM37,TIMELESS,KDM2B,MLXIPL,PHB,ZNF555,CTCF,NR6A1,CREB1,TRAP1,BMP4*
GO:1901700	response to oxygen-containing compound	8.341691144024979e-7	*ENPP1,PRDX2,ASPH,PRKCI,PDE4D,DNMT1,SLC9A1,ADCY8,PDE8A,RPS6KA2,PTGFR,CLDN18,TJP1,ADCY7,NTRK3,CBL,STAT5B,GRM5,FER,EZR,SLC26A6,HTR2B,RYR3,GOT1,TRAF6,OXR1,ERCC6L2,CDC6,PTPRK,ABCC8,HMGN3,NRIP1,STXBP4,PTPN11,HDAC6,ESR1,NTRK2,PDGFB,CACNA1B,CCND3,USP46,BPI,INSR,TCF7L2,PIP4K2A,LITAF,AMIGO1,KCNMA1,CHRM3,UBR2,HLCS,ECT2,SNX6,AP3S1,DMD,KAT7,SLC8A1,FYN,PLCB1,MGMT,DPEP1,APOD,BCR,PIK3R2,CDK19,BDKRB1,TMBIM6,PPP2CB,HDAC5,SLC39A14,IPO5,MAN1A1,RGS8,DEFA1B,DEFA3,F5,PTGER2,NEUROD1,GNAQ,JAK2,CLDN1,PDE2A,KLF15,LAMTOR3,WNT11,NOX4,LCOR,CCDC62,PRKCD,TAB2,TGFB1,SGK1,PTPRN2,SLIT3,SLIT2,GABRB1,HP,KANK1,MTDH,CHRM1,SMAD6,BCKDHB,HOMER2,SBNO2,FGF10,SMYD3,GLP2R,ADCY2,EPHA4,RORA,PRKCA,CNR1,CD6,PPP3CA,UFL1,CTNNB1,PARK2,SOD2,FCGR2B,SMARCC1,IGF1R,PPARG,CYBB,OTC,BRIP1,RYR2,LEPROT,RGS10,NR4A3,TRIM5,CHEK2,HCK,SORBS1,CAPN2,DIO2,PAX2,SFRP1,FOXO3,SSH1,GRIK5,IFNAR1,SLC6A1,CDC73,APP,PDGFRA,YAP1,RAB11FIP5,SESN1,RNLS,ALPL,LRP5,SLC9B2,GNG2,DRD1,GLRA2,CHUK,MMP2,ESR2,PRKD1,STAT1,SLC6A3,HNF4A,STRA6,EREG,MAPKAPK3,ATF2,RAF1,CARD16,CASP1,PTPN1,GGT7,COL16A1,PIK3R3,ADCY5,RORC,PTAFR,ADCYAP1R1,EIF2B5,PTPRE,RBX1,SRSF5,PAWR,IDE,KCNQ1,WNT3,COL4A3,TRPA1,AKAP6,EDEM3,IRS4,ME1,ERCC1,WDTC1,BLM,UBR1,SIN3A,RUVBL2,PELI1,SP1,ALK,SNX5,RGS7,ATP2B4,P2RX6,ADNP,MAPKAPK2,MAP1B,COL4A6,SCARB1,TYR,TFRC,EPHA5,CACNA1A,XRN1,ADRBK1,BRINP1,SHPK,TRPV1,GHR,ACTR2,ACACA,ELAVL4,RECQL5,C2,TRIM24,PLCG2,ROCK1,EPS8,EGR2,CAPN10,ABCC1,PTPN2,INSRR,NTRK1,ZFAND1,MAN1B1,SARM1,CARM1,SRD5A2,HTR1D,SPI1,PTPRA,CTNNA1,LY86,BCAR3,ILDR2,RAB15,RAPGEF3,SDK1,PDXP,PTK7,SNCA,PNPT1,CNGA3,WNT7B,HMGCS2,ABCA12,SRSF6,NUGGC,RPTOR,BTK,DSG1,IGF1,MAPK1,BMP7,PIK3C3,LRRK2,GNA14,ZBTB20,CHMP5,NSG2,PKD2,EFNA5,PDGFC,NCOA3,WNT7A,ZBED6,NLRP1,NME8,GLDC,GPR21,BMP6,ANO1,GPI,TSHR,EXT1,PDCD1LG2,PNPLA3,RAPGEF2,KCNC2,SMO,CPT1A,GRB14,GRIN1,RHOA,GRIN3A,ITPR2,GSK3B,STAT6,EEF2K,CD27,KRT8,AGT,PRKAA2,STAT2,BRINP3,HRH4,PTK2,PHEX,PDE4B,MAP3K5,NCF1,SLC29A1,UGT1A1,SHC1,RPS6KB1,STC2,GPR173,CLDN4,NOS1,GCKR,PLEKHA1,SCNN1B,EPHB2,SLC1A1,SIGMAR1,IL18,PPARA,CALCRL,SLC25A33,CPEB4,FBN1,PRKCZ,ATP1A3,CD96,GRIN2B,CPEB1,OSBPL8,NCOR2,PRKCQ,HDAC2,HTR2C,IMPACT,PHIP,RPL23,SH3BP4,SP100,ADCY1,SLC10A1,TAF1,TICAM1,HSF1,MAX,DHFR,SASH1,NCOA2,CIB2,CAV2,AXL,DENND4C,MET,TIMELESS,MALT1,TRA2B,MLXIPL,SPON2,CREB1,TRAP1,EDNRA,HADHA*
GO:0098660	inorganic ion transmembrane transport	8.746559836790789e-7	*SLC39A11,ASPH,PDE4D,SLC9A1,FAM155A,CHERP,CACHD1,RYR1,NOS1AP,NOX5,DPP6,CLCN1,UTRN,ATP2B2,KCNQ5,GRM5,KCNIP4,ANK2,SLC9A9,CHD7,SLC26A6,HTR2B,RYR3,SLC24A2,ITGB1,SLC24A3,ABCC8,GABRA3,TCIRG1,KCNS3,NIPAL2,NETO2,CACNB2,KCNJ16,DPP10,CACNA1B,STK39,CAB39,ZDHHC13,SLC20A2,SLC4A10,CATSPER2,AMIGO1,KCNMA1,KCNRG,LCK,TMC2,SLC12A8,SLC6A16,RHCE,MICU3,DMD,SLC8A1,JPH2,FYN,SLC5A6,ANKH,CACNA1E,SLC4A5,KCNN3,CNNM2,BDKRB1,KCNIP1,STIM1,NALCN,SLC39A14,CATSPER3,FGF14,CLDN16,ANO6,KCNB2,NPSR1,SCN4A,TMC1,SLC9A7,KCNC4,CACNA1H,TGFB1,KEL,JPH3,GAB*

			RR3,GABRB1,KCNJ3,CACNA1D,CACNA2D3,ANO4,XPR1,ANO3,KCNH1,SLC17A7,KCNH7,CAPN3,VMP1,ANO2,COX8A,TRPM1,KCNJ12,RYR2,KCNA6,NOL3,CACNA1C,SCN3B,ITPR1,WWP2,NEDD4,SLC4A4,SLC6A1,KCNK1,VDAC1,IBTK,SLC9B2,DRD1,GLRA2,PRKD1,SLC6A3,FXYD2,FXYD6,KCNG4,FGF2,PTAFR,MRS2,SLC9B1,ATP6V0B,KCNQ1,TRPA1,TRPM3,AKAP6,TRPC5,WNK1,KCNQ2,SLC47A1,SLC8A2,SLC9C1,TPCN1,AHNAK,RGS7,ATP2B4,ATP6V0A2,KCNAB2,GABRG3,CACNA1A,STOM,TRPV1,PLCG2,GPR35,SLC39A10,ANK3,KCND3,CACNG8,SLC9C2,NETO1,MCU,TRDN,GABRA6,STAC,TRPC6,ABCB7,SLC41A2,SNCA,CACNG2,CNGA3,SLC4A8,HTT,SCN8A,ATP6V1A,TMCO1,PKD2,ANO1,GABRR2,KCNC2,SLC6A14,PSEN2,GRIN1,GRIN3A,ITPR2,GABRB3,SCN9A,PDE4B,COX5A,TRPC4AP,KCNJ15,SLC5A10,PKD1L1,SLC25A18,COX5B,NOS1,ACTN4,SCNN1B,COX6A1,SLC1A1,XCR1,LRRC52,MCOLN1,ATP1A3,GRIN2B,DIAPH1,HTR2C,TMEM163,UQCR10,SLC26A2,ANXA2,KCND2,ATP6V0A1,SCN1A,CACNG3,ATP2B3,GPM6A,KCNQ3,SESTD1,CAMK2D,KCNJ6,NEDD4L,PTPRC,EDNRA
GO:0031327	negative regulation of cellular biosynthetic process	9.290959210856593e-7	ENPP1,LDB2,PRMT3,DNMT1,TRPS1,CBFB,PRDM12,WWC1,SCAF8,PTGIS,WWC3,FTO,ZNF566,MAP2K5,ZNF536,SP3,HDGF,EZR,MALRD1,MECOM,TRAF6,DNAJA3,CDC6,CDAN1,GFI1B,PTPRK,RUNX1,NRIP1,THRB,DACH1,ORC2,HDAC6,PEX14,KAT6B,ESR1,MIER1,PCBP3,TNRC6A,EP300,CELA1,TENM2,PDGFB,CCND3,TOB2,ZNF19,BRMS1,SCML4,RERE,ATP8B1,H2AFY2,TCF7L2,FBXW11,BASP1,TBR1,SAMD4A,TFAP2A,PEG3,ZIM2,TGIF2,MDM4,ZNF148,MTA3,SNX6,TFDP2,ERN2,C1D,CENPF,PDS5A,KAT7,RBM4,SATB2,HIRA,FYN,ARNTL,PLCB1,NRG1,MDFI,FNIP1,VAX2,TMBIM6,VGLL4,HDAC5,ZNF692,JDP2,CREBRF,SOX13,MAD2L2,TLE6,CAPRIN2,TERF2,FOXN3,FBXW7,UIMC1,MLIP,TCF7,PDE2A,TBX15,WNT11,MTA1,KLF8,LCOR,SOX6,RUNX2,TGFB1,NSD1,PIBF1,ZHX2,BTRC,NFATC3,CDYL2,CC2D1B,SAP18,MTDH,MYT1L,ZNF398,CLOCK,ZNF675,ETV6,BCL3,DUSP22,HNRNPC,SBNO2,YTHDF1,LOXL3,CAPN3,SMURF2,MAGEA4,KLF12,GATAD2B,CTNNB1,PARK2,METTL13,PPARG,AXIN1,CIPC,CBX5,SREBF2,LEPR,NPAT,NR4A3,FOXK2,MYOCD,PER2,AJUBA,PRDM16,BRMS1L,HDAC4,PAX2,SFRP1,FOXO3,NFIB,SYNCRIP,SMAD3,CUX2,WWP2,RNF168,DCP1B,MIER3,NEDD4,CDC73,YAP1,TEN1,SOX2,PRICKLE1,ZNF653,ERLIN1,SFMBT1,ESR2,STAT1,ETV5,TAF3,PLAGL1,HNF4A,EREG,ATF2,TCF3,CELF4,BMPR2,IKZF4,MYB,BACH1,MXD3,PPM1F,BEND5,SEMA4D,NFX1,RORC,JARID2,PHC2,RARB,SPEN,SIN3B,EHMT1,EIF4G1,ATF3,PAWR,AGO3,TSG101,CRYM,SUFU,MAGEA11,NR2C1,GLI2,TNRC6B,GLIS3,WDTC1,DAB2,BLM,SIN3A,RUVBL2,ZNF423,TRIM22,OVOL2,NFATC2,ATP2B4,ARID4A,CDK6,CELF1,MEF2A,XRN1,SATB1,GATAD2A,DIS3L2,TRPV1,HOXB3,HOXB4,L3MBTL4,HDAC1,RECQL5,TRIM24,ROCK1,SCMH1,RYBP,PTPN2,ZNF425,GNL3L,MUC1,BCOR,SMARCA2,CUX1,SPI1,DNMT3B,FOXP2,PSPC1,GLIS1,SFMBT2,JAZF1,ACOT8,SMYD1,SNCA,PNPT1,ZEB2,HEY2,RC3H1,EIF3E,ZMYND11,CREM,MITF,NFIX,CBFA2T2,ATF7IP,BMP7,MXI1,PUM1,CIR1,TMEM59,ZBTB20,LILRB4,KCTD1,RIPPLY1,RCOR3,ZBED6,KAT6A,BMP6,ATRX,IKZF1,PRMT2,RAPGEF2,SMO,SOX30,RQCD1,CUL3,TFEC,GSK3B,STAT6,RBBP8,DUSP26,NACC2,SUPT4H1,METTL16,EXOSC3,TCFL5,CREBBP,DLX1,SHC1,PARP10,PRG3,SETDB2,DNAJB6,RPS6KA5,ZNF93,PRDM2,TCF25,ZNF282,DIS3,PPARA,ARID5B,SMARCAL1,CPEB4,NOTCH4,ZMYND8,ZNF366,PAX6,CPEB1,OTUD7B,NIPBL,TBL1X,FHL2,MBTD1,ZBTB5,IGF2BP3,MTF2,TAGLN3,NCOR2,HDAC2,IMPACT,POU3F3,ELF2,RPL23,ATXN1,SP100,TRIM29,PARN,DONSON,CNOT1,WWTR1,ZBTB38,BRCA2,MORC1,YBX1,TAF1,HSF1,MAX,SAP130,DHFR,EZH1,NCOA2,ZP3,RNF2,ZNF554,TRIM37,TIMELESS,KDM2B,MLXIPL,PHB,ZNF555,CTCF,NR6A1,CREB1,BMP4
GO:0007215	glutamate receptor signaling pathway	0.000001032077867945 8714	GRID2,GRM8,GRM5,GRM7,GRIK4,FYN,PLCB1,DAGLA,GNAQ,GRIK3,HOMER2,TRPM1,GRM1,GRIK5,APP,GRIA3,GRIK1,GRIA2,GRM4,SHANK3,GRIN1,GRIN3A,NECAB2,GRID1,SLC1A1,CPEB4,GRIN2B,GRIA4,GRIK2
GO:0031345	negative regulation of cell projection organization	0.0000010475697692432059	NRXN1,SEMA3A,SEMA3D,SEMA5A,ARHGAP24,DAB1,PTPRO,DENND5A,RIT2,FYN,FAT3,RTN4,RTN4R,PAQR3,NTN1,KREMEN1,PRKCD,SEMA5B,SLIT2,KANK1,EPHA4,PPP3CA,MAG,DRAXIN,DCC,DNM3,DIP2B,YAP1,FSTL4,PTPRG,KIF24,SEMA6D,NLGN1,CRMP1,SEMA4D,SPOCK1,WNT3,PAFAH1B1,TRPC5,RUFY3,DAB2,MPHOSPH9,PTPN9,TRIM46,CARM1,ARHGAP44,EPHA7,PTPRS,RTN4RL1,CBFA2T2,DPYSL3,PTEN,LRRK2,MAP4,RAPGEF2,RHOA,SYNGAP1,EPHB2,GRIN2B,MAP2,HDAC2,TNR,CDKL3,SLIT1,INPP5F,SEMA3C
GO:0032535	regulation of cellular component size	0.0000010601863290315574	CLASP2,SEMA3A,SEMA3D,FHOD3,SEMA5A,FER,OLFM1,EZR,CDKL5,TENM1,VAV2,TRIOBP,DSCAM,CDC42EP3,GOLGA4,LIMK1,CDH4,BDNF,KCNMA1,SLC12A8,GSN,RTN4,RTN4R,NTN1,NCKAP1,ANO6,PLS1,CYFIP2,PRKCD,SEMA5B,KEL,F2RL1,SLIT2,DISC1,KANK1,KANK4,SVIL,MAG,CDHR2,ADD2,XK,DRAXIN,HCK,DCC,SPTBN1,ELN,SSH1,ADD3,DIP2B,FSTL4,ISLR2,SPTBN4,VILL,SEMA6D,BMPR2,DPYSL2,RAB11A,SEMA4D,AP2M1,DEPTOR,WNT3,PAFAH1B1,TRPC5,RUFY3,WDTC1,SIN3A,MACF1,SPTBN5,PAK3,SSH2,ADNP,MAP1B,GNB3,SNX9,EPS8,TRIM46,EPHA7,LIMA1,FCHSD2,MAP3K13,PDXP,SH3BP1,WNT7B,PTPRS,RASA1,RPTOR,PTEN,EFNA5,SHANK3,WNT7A,RHOA,GSK3B,PRR16,RDX,FAM49B,PPP1CA,MAP2,TNR,CDKL3,SLIT1,CIT,RAP1GAP2,NEB,VAV3,SPTAN1,SEMA3C,CREB1
GO:0009056	catabolic process	0.0000010834063829147746	NT5C1B,ENPP1,PRDX2,GPC5,PDE4D,PDE7B,PDE8A,ALDH4A1,RNASET2,GK5,CALR3,SLC25A17,FBXL2,PRKAG2,HPSE2,FTO,DCAF12,ACOXL,CBL,ARNT,EGLN2,ENPP2,PLCE1,SAMHD1,USP32,CHI3L2,LRP2,DEPDC5,ADPGK,EZR,TOM1L1,HTR2B,PSMD1,GOT1,LPO,INPP4B,ITGB1,HSP90AA1,PSMB7,CCDC22,AMDHD1,OM

			A1,DNAJB14,PDE4A,TCIRG1,HDAC6,DDB1,ADORA1,GPRASP1,ADAMTS3,TEX264,CUL4B,CECR2,ACAD11,FBXL18,TNRC6A,EP300,CELA1,ZYG11B,FBXL7,USP46,CHFR,INSR,FMN2,ASB5,PIP4K2A,USP34,FBXW11,RNF144A,SAMD4A,FUT8,NPRL3,TRIM13,UBR2,MDM4,ENTPD5,SNX6,PIPOX,ACOX1,ERN2,NPLOC4,ATG10,LARP4B,USP13,PAFAH1B2,FYN,MKRN2,ARNTL,ADAMTS9,SMG7,PLCB1,DMGDH,SLC25A21,FBXO21,DPEP1,NEIL2,VTA1,CHI3L1,PIK3R2,BLVRB,NRG1,DAGLA,TMBIM6,VGLL4,PPP2CB,ALDH3B2,RNF4,CSNK2A3,UBE3D,SBF2,DCN,SCFD1,CREBRF,TRDMT1,FHIT,ENTPD1,EXOC4,MAD2L2,MAN1A1,FBXO9,RFFL,USP22,FBXW7,LINC00473,OAZ2,ITCH,USP12,ABCC2,LZTS1,SMG1,DPYS,IDNK,PNPLA1,PDE2A,SPSB1,HADHB,MTA1,WDFY4,SIAH1,ERCC8,PRKCD,TAB2,BANP,PLCXD3,SPSB4,MMP28,TRIM65,ZHX2,ASCC2,BTRC,BCAS3,DNAJB2,RNF133,RNF148,HP,MGAM,DISC1,ZFAND2A,CPT1C,CLN6,MTDH,MMP26,CLOCK,PLCB4,BCKDHB,SYNJ2,NELL1,TIMP2,DAPK2,SND1,HNRNPC,YTHDF1,FBXL17,KCTD10,CAPN3,VMP1,SMURF2,EPHA4,PRKCA,CNR1,SMG6,NSUN2,HBE1,UBQLN3,UBQLNL,LIX1L,WDFY3,UFL1,CTNNB1,PARK2,SMARCC1,AXIN1,CPQ,OTC,LRPPRC,RSPRY1,SREBF2,LEPR,FOXK2,WDR45B,TRIM5,CHEK2,UBQLN4,PPP1CB,TBC1D14,RAB3GAP2,CAPN2,TRIM8,DIO2,MRC2,MMP16,HDAC4,FOXO3,TRHDE,ZCCHC17,SNX14,SYNCRIP,SMAD3,RNFT2,ITPR1,EVA1A,WWP2,CYP4F11,WIPI1,VPS39,RNF168,PSAP,DCP1B,NEDD4,PLA2G4C,OGDHL,SLC4A4,SEC61B,APP,USP49,RBM8A,ARIH1,AMFR,SESN1,VDAC1,SH3D19,LRP5,BDH2,ACBD5,ADAMTS2,PRICKLE1,ERLIN1,MMP2,NUDT10,PRKD1,HEXA,SLC6A3,VPS4A,EPHX1,ACOX2,PTPN1,ADAMTS12,PSMD11,GGT7,USP33,IDO2,PABPC4,EPG5,FGF2,RAB24,UBE2R2,AGBL4,CDC27,HIBADH,YME1L1,KLHL3,PIK3C2B,PRKCG,AREL1,OC90,UCHL3,EIF4G1,PSMD2,RBX1,ANAPC5,FBXO45,LIN28B,TECPR2,AKR1D1,ATP6V0B,STT3B,AGO3,ACAA1,FBXL20,TSG101,DESI1,CRYM,IDE,SUFU,ZFYVE1,PAFAH1B1,PNLIPRP1,ADAMTS14,ATG4C,DDAH1,EDEM3,PXDNL,TNRC6B,ALDH6A1,MGLL,DAB2,UBR1,CACUL1,SMG5,PSMD7,PELI1,PPP2R5C,SPATA18,MAP3K7,TRIM22,ZNRF1,ALK,GSTM3,FASTKD5,TPCN1,XDH,SNX5,RBBP6,ATP2B4,NSF,TMEM2,TET1,DYX1C1,MAPKAPK2,UBE3C,SCARB1,ATP6V0A2,CELF1,RNF34,UVRAG,RBKS,DROSHA,GPC3,XRN1,TBC1D5,PSMB2,SCOC,DIS3L2,RNF216,TFEB,CLEC16A,SNX9,ELAVL4,TRIM24,PLCG2,ROCK1,RYBP,CUL2,ABCC1,MAP1A,HYKK,MAN1B1,AXIN2,SARM1,TMEM199,FBXO39,NUDT5,SNX33,SRD5A2,CD84,VPS16,FBXW4,ATG14,EIF3H,ACOT8,RENBP,PDXP,SNCA,USP42,PNPT1,USP50,RC3H1,CHMP3,EIF3E,RNF103,RNF103-CHMP3,RNF19B,PRKAG1,PSMF1,SULT2A1,TRIP12,DFFA,ADTRP,PLD1,RPTOR,VPS41,ZNRF3,KCTD13,IGF1,PLA2G4E,HTT,PTEN,MIB1,PIK3C3,PUM1,PCBP2,LRRK2,ATP6V1A,MLYCD,TMEM59,ZRANB1,ALDOC,ZBTB20,BAG6,SLC44A1,GALT,BLVRA,SEL1L2,NT5E,DAZL,MTMR3,PEPD,CRTC3,GLDC,PLCL2,TAF15,MYCBP2,GPI,SH3RF2,EXT1,N4BP1,PNPLA3,ENPP3,GET4,ACADSB,CPT1A,UBE2H,BPGM,DECR1,RQCD1,CUL3,PCCA,EPM2A,GSK3B,PLD2,DNAJC3,RDX,UPF2,BOK,RNF144B,RNF43,METTL16,PRKAA2,CUL9,ZMPSTE24,PTK2,ARMC8,RNF121,SNX3,PDE4B,EXOSC3,FBXO10,SEC16B,TRPC4AP,XYLB,UGT1A1,UGT1A4,ALDOA,ECE1,RNFT1,CD44,ADAM8,CSNK1A1,NOS1,PEX7,DRAM1,BCO2,MFSD8,UBE2K,HCAR1,HCAR2,DIS3,FBXL13,PPARA,ABCD3,MCOLN1,PNPLA6,CYP2C18,CYP2C19,RNF150,ZC3HAV1,PAH,PLCXD2,OTUD7B,UPB1,PACS2,TBL1X,ATF6,NT5M,SLCO2B1,HACL1,PPP1CA,FBXO31,ATG3,PHKG2,C18ORF25,MOXD1,SKP1,MAOA,RPL23,SH3BP4,ACAN,VTI1A,ANXA2,PARN,ATP6V0A1,WIPI2,CNOT1,WWTR1,CYP39A1,ALDH8A1,YBX1,TAF1,TICAM1,HSF1,POP1,ETF1,INPP5A,PLEKHM1,ADAMTS7,GNA12,UGGT1,MYEF2,INPP5F,MET,SNX1,UCHL5,MALT1,PFKP,MLXIPL,TRAPPC8,FBXL4,GNS,NEDD4L,PHB,RNF213,TRAF3IP2,SGTB,HADHA
GO:0030900	forebrain development	0.0000013202907348467394	TACC2,SEMA3A,NRG3,PHACTR1,ZSWIM6,SEMA5A,TOX,LRP2,ROBO1,CHD7,TACC1,ROBO2,DAB1,ERBB4,CDON,NTRK2,HOOK3,KIRREL3,SLC4A10,TBR1,DMD,SLC8A1,SATB2,FYN,NF1,PLCB1,RTN4,RTN4R,CCDC141,NRG1,VAX2,NEUROD1,TRAPPC9,BCL11B,RPGRIP1L,SLIT2,DISC1,DCLK1,FGF10,CNTNAP2,CTNNB1,IGF1R,DRAXIN,NIN,DCT,NF2,ARL13B,NFIB,NRP2,BTBD3,APP,GSX2,SOX2,SETD2,DRD1,FRS2,SYNE2,SLC6A3,BMPR1A,FGF2,RARB,EIF2B5,EPHB3,FBXO45,LAMB1,PAFAH1B1,GLI2,RELN,SIN3A,ALK,PRKG1,ATP2B4,CDK6,CELF1,EPHA5,ELAVL4,HDAC1,SRD5A2,FOXP2,WNT7B,PTPRS,RTN4RL1,PTEN,LRRK2,TMEM108,ITGAM,SHANK3,WNT7A,EXT1,ATRX,RAPGEF2,KCNC2,SMO,SUN1,RHOA,NDE1,GSK3B,DLX1,EPHB2,DLC1,PAX6,POU3F3,TNR,SRGAP2,NUMB,SLIT1,EZH1,AXL,UCHL5,TRA2B,KDM2B,TYRO3,CREB1,BMP4
GO:0060627	regulation of vesicle-mediated transport	0.0000013333199338948809	NRXN1,CLASP2,CBL,RAB4B,RAB4B-EGLN2,FER,CASK,EZR,NRXN3,DGKI,CACNB2,STX18,INSR,VAMP7,RIT2,BCR,NDRG4,BMP2K,NRG1,MAP2K1,SCFD1,DOCK2,CDH13,RAB27A,RAB3C,ANO6,SYT1,ATP9A,MKLN1,CACNA1H,IL1RAPL1,TGFB1,F2RL1,AAK1,CADPS2,SYT13,C12ORF4,SLC17A7,STXBP5,CNR1,PPP3CA,PARK2,FCGR2B,PPARG,PRKAR1B,SCAMP5,ANXA13,HCK,RIMS4,ANKFY1,GRIK5,SYT3,BET1L,DRD1,PRKD1,VPS4A,PTPN1,CAMK1D,NLGN1,RAB11A,MCTP1,PTAFR,ABCA13,PRKCG,AP2M1,FBXL20,TSG101,KIF3A,HIP1,BTBD9,STON2,DAB2,CLASP1,NSF,PACSIN1,ATAD1,SCARB1,SYT12,GPC3,SGIP1,TBC1D5,C2,PLCG2,ROCK1,TUB,SH3GL3,AP2B1,ARHGAP44,SNX33,CD84,SPI1,RAB15,SNCA,MAGI2,SYT9,CHMP3,RAB5B,PLD1,SLC4A8,PLA2G4E,MAPK1,MIB1,ATXN2,LRRK2,UNC119,ITGAM,WNT7A,STXBP5L,SYT17,DNAJC6,GRIN3A,TF,GSK3B,RDX,SYN1,EEF2K,NECAB2,SYT7,SNX3,RIMS2,CD160,CPLX2,PACSIN2,SYTL4,ADORA2A,RABGEF1,ANGPT1,CADPS,ATG3,BRAF,CD300A,ANXA2,RAB27B,AD

			CY1,NUMB,SYK,ANKRD13A,PTPRJ,ZP3,INPP5F,AXL,EHD2,NEDD4L,PTPRC,STXBP6
GO:0106027	neuron projection organization	0.000001504053735831304	*PRMT3,KALRN,DOCK10,HDAC6,INSR,TANC2,FYN,DCTN1,CAPRIN2,ZDHHC15,PLS1,CAMK2B,CTNND2,EPHA4,FCGR2B,IGF1R,DNM3,CUX2,ABI2,SHANK1,APP,NLGN1,EPHB3,PAFAH1B1,RELN,PAK3,ACTR2,MAP1A,EPHB1,ARHGAP44,PTEN,LRRK2,SHANK3,WNT7A,GSK3B,EEF2K,EPHB2,ATP1A3,GRIN2B,TANC1*
GO:0060078	regulation of postsynaptic membrane potential	0.000001631576534738205	*NRXN1,S1PR2,GRID2,NLGN4X,RGS7BP,GRM5,DGKI,GABRA3,ADORA1,RIMS1,GRIK4,GRIK3,GABRB1,CHRM1,PPP3CA,GRM1,CUX2,GRIK5,SHANK1,APP,NLGN2,GLRA2,CELF4,NLGN1,GRIK1,NLGN3,RELN,SLC8A2,P2RX6,GABRG3,TRPV1,NETO1,CHRNB4,GABRA6,SNCA,PTEN,LRRK2,TMEM108,SHANK3,WNT7A,GABRR2,GRIN1,GRIN3A,GSK3B,RIMS2,SLC29A1,GRID1,PRKCZ,ADORA2A,GRIN2B,KCND2,GRIK2*
GO:0045934	negative regulation of nucleobase-containing compound metabolic process	0.000001658658236168582 4	*LDB2,DNMT1,TRPS1,CBFB,PRDM12,WWC1,SCAF8,WWC3,ZNF566,MAP2K5,ZNF536,SP3,HDGF,EZR,MECOM,TRAF6,DNAJA3,GFI1B,PTPRK,RUNX1,NRIP1,THRB,DACH1,ORC2,HDAC6,PEX14,KAT6B,ESR1,MIER1,PCBP3,EP300,CELA1,TENM2,PDGFB,CCND3,TOB2,ZNF19,BRMS1,SCML4,RERE,ATP8B1,H2AFY2,TCF7L2,FBXW11,BASP1,TBR1,TFAP2A,PEG3,ZIM2,TGIF2,MDM4,ZNF148,MTA3,SNX6,TFDP2,ERN2,C1D,CENPF,LARP4B,KAT7,SATB2,HIRA,ARNTL,PLCB1,NRG1,MDFI,FNIP1,VAX2,TMBIM6,VGLL4,HDAC5,ZNF692,JDP2,CREBRF,TRDMT1,SOX13,AUNIP,MAD2L2,TLE6,TERF2,SLX1B,FOXN3,UIMC1,MLIP,TCF7,PDE2A,TBX15,WNT11,MTA1,KLF8,LCOR,SOX6,RUNX2,TGFB1,NSD1,ZHX2,BTRC,NFATC3,CDYL2,CC2D1B,SAP18,MTDH,MYT1L,ZNF398,CLOCK,ZNF675,ETV6,BCL3,DUSP22,HNRNPC,SBNO2,LOXL3,CAPN3,SMURF2,SMG6,PPP3CA,NSUN2,MAGEA4,KLF12,GATAD2B,CTNNB1,PARK2,METTL13,PPARG,AXIN1,OTUB1,CIPC,CBX5,LRPPRC,SREBF2,NPAT,NR4A3,FOXK2,MYOCD,PER2,AJUBA,UBQLN4,PRDM16,BRMS1L,HDAC4,PAX2,SFRP1,FOXO3,NFIB,ZCCHC17,SYNCRIP,SMAD3,CUX2,WWP2,RNF168,MIER3,NEDD4,CDC73,YAP1,TEN1,SOX2,PRICKLE1,ZNF653,SFMBT1,ESR2,STAT1,ETV5,TAF3,PLAGL1,HNF4A,EREG,ATF2,TCF3,CELF4,BMPR2,IKZF4,MYB,BACH1,MXD3,PPM1F,BEND5,SEMA4D,NFX1,RORC,JARID2,PHC2,RARB,SPEN,SIN3B,EHMT1,ATF3,LIN28B,PAWR,TSG101,TERF2IP,CRYM,SUFU,MAGEA11,NR2C1,GLI2,TNKS,ERCC1,GLIS3,WDTC1,DAB2,BLM,SIN3A,RUVBL2,ZNF423,TRIM22,OVOL2,NFATC2,MAPKAPK2,ARID4A,CDK6,MEF2A,XRN1,SATB1,GATAD2A,TRPV1,HOXB3,HOXB4,L3MBTL4,ELAVL4,HDAC1,RECQL5,TRIM24,SCMH1,RYBP,PTPN2,AXIN2,ZNF425,GNL3L,MUC1,BCOR,SMARCA2,CUX1,SPI1,DNMT3B,FOXP2,PSPC1,GLIS1,SFMBT2,JAZF1,SMYD1,SNCA,ZEB2,HEY2,ZMYND11,CREM,TRIP12,DFFA,MITF,SRSF6,NFIX,CBFA2T2,ATF7IP,BMP7,MXI1,CIR1,ZBTB20,LILRB4,KCTD1,RIPPLY1,RCOR3,DAZL,ZBED6,KAT6A,BMP6,TAF15,ATRX,IKZF1,PRMT2,SMO,SOX30,UBR5,CUL3,TFEC,STAT6,RBBP8,DUSP26,NACC2,SUPT4H1,METTL16,ZMPSTE24,TCFL5,CREBBP,DLX1,SHC1,PARP10,SETDB2,DNAJB6,RPS6KA5,ZNF93,PRDM2,TCF25,ZNF282,PPARA,ARID5B,NOTCH4,ZMYND8,ZNF366,PAX6,OTUD7B,NIPBL,TBL1X,FHL2,MBTD1,ZBTB5,MTF2,TAGLN3,NCOR2,HDAC2,IMPACT,POU3F3,ELF2,RPL23,ATXN1,SP100,TRIM29,PARN,RBM42,CNOT1,WWTR1,ZBTB38,MORC1,YBX1,TAF1,HSF1,MAX,SAP130,EZH1,NCOA2,ZP3,RNF2,ZNF554,TRIM37,TIMELESS,KDM2B,MLXIPL,PHB,ZNF555,CTCF,NR6A1,TRAF3IP2,CREB1,BMP4*
GO:0045597	positive regulation of cell differentiation	0.000001761727120597338	*PRKCI,S1PR2,HLX,CBFB,DOCK1,TIAM2,KALRN,ARNT,SEMA5A,STAT5B,TOX,GRM5,KITLG,LRP2,OLFM1,ROBO1,CDKL5,TRAF6,ROBO2,ITPKB,TRIOBP,ZC4H2,DSCAM,RUNX1,DAB1,GOLGA4,LIMK1,CDON,NTRK2,H2AFY2,TCF7L2,CDH4,PTPRD,BDNF,AMIGO1,TGIF2,TENM4,ECT2,DMD,KAT7,RBM4,CASS4,ARNTL,ADAMTS9,PLCB1,NTN1,NRG1,MAP2K1,CMKLR1,ROR2,SOX13,CAPRIN2,BOC,NEUROD1,JAK2,IL1RAPL1,CAMK2B,SOX6,ACVR2A,RUNX2,CD4,TGFB1,SLIT2,TP73,DISC1,TCF12,NELL1,SUCO,ANKRD54,EPHA4,PRKCA,TNFSF11,MAG,UFL1,CTNNB1,SOD2,PPARG,MSR1,NIN,DCT,MYOCD,NF2,PAX2,SFRP1,FOXO3,SMAD3,CUX2,PREX1,HOXD3,GSX2,YAP1,LRP5,ISLR2,SLC9B2,SOX2,TPH1,PRKD1,STAT1,PARP6,ZBTB7C,TCF3,BMPR2,BMPR1A,SPAG9,RAB11A,MYB,FGF2,NEDD9,SEMA4D,PLXNA2,SPEN,NCOA1,BLOC1S5,EIF4G1,CCDC3,WNT3,P4HB,SCUBE2,PAFAH1B1,AKAP6,TRPC5,GLI2,RUFY3,DAB2,RELN,SIN3A,MACF1,INPP5D,ADIRF,OVOL2,BNC1,PAK3,ADNP,MAP1B,BRINP1,HOXB4,GHR,ACTR2,HDAC1,EGR2,AP3B1,AXIN2,SH3GL3,AP3D1,CARM1,CUX1,MAP3K13,SMYD1,BMPR1B,ZEB2,WNT7B,BTK,IGF1,BMP7,SOX5,LILRB4,EFNA5,SHANK3,NCOA3,PPP2R3C,BMP6,RAPGEF2,SMO,TCF4,TFE3,RHOA,NFKBID,GSK3B,EEF2K,CD27,AGT,BLOC1S6,FBN2,BRINP3,MAP3K5,DLX1,SP7,ADAM8,CDS1,RGS14,STK4,EPHB2,IL18,PAX6,PRKCZ,FAM20C,PPP1CC,GCNT2,ETS1,VWC2,FBXO31,BRAF,HDAC2,HTR2C,IMPACT,CDKL3,NUMB,SYK,WWTR1,HSF1,SULT2B1,AXL,TNFSF9,WIF1,MALT1,SETD3,PTPRC,CREB1,BMP4,CPNE1*
GO:0006950	response to stress	0.000001884070091849298 3	*PRDX2,F8,GSS,GP6,MAP4K4,SLC9A1,C6ORF89,LY75,ADCY8,PDE8A,CLASP2,SEMA3A,IL31RA,RNASET2,SETD4,PTGFR,CALR3,C12ORF49,KIF22,PMS1,RYR1,ASH1L,NREP,PTGIS,PRKAG2,PAGR1,ADCY7,FTO,NTRK3,CBL,ARNT,EGLN2,RAD51B,SUSD4,STAT5B,SAMHD1,FER,CASK,MARVELD3,MAP2K5,MAPK10,PIK3CD,DEPDC5,ZSWIM7,SH2D1A,EZR,XRCC4,SLC26A6,ITGB6,MECOM,LMNA,LPO,TRAF6,DNAJA3,OXR1,VAV2,ULK4,ERCC6L2,ITGB1,HSP90AA1,PSMB7,TSC22D3,ARHGAP24,PTPRK,OMA1,ABCC8,DGKI,DNAJB14,STXBP4,IL17RB,TCIRG1,PTPN11,HDAC6,DDB1,ADORA1,MSRA,IKBKB,MRE11A,TLK1,TEX264,HIF3A,CUL4B,ESR1,TNRC6A,EP300,CELA1,PDGFB,TNIK,CDH8,USP46,MID1,ALOX5AP,BPI,STK39,FMN2,PLLP,JMJD1C,CAB39,PAK1,MAP3K4,EGLN3,ATRN,TTC4,FANCA,KCNMA1,FUT8,NPR*

			L3,TRIM13,LCK,MDM4,IL5RA,ECT2,ZNF148,VAMP7,ERN2,CDC45,LSP1,SHFM1,MB,NPLOC4,DMD,ATG10,SLC30A9,CERS6,PDS5A,USP13,KAT7,SLC8A1,GSN,RBM14,RBM4,MACROD2,FYN,MKRN2,ARNTL,NF1,NCF2,PLCB1,DST,MGMT,PPP4R2,RTN4,DPEP1,DGKH,APOD,NEIL2,RTN4R,BCR,RWDD3,CHI3L1,PIK3R2,NRG1,SH2D3C,CDK19,BDKRB1,BDKRB2,BID,MAP2K1,FNIP1,MYH9,LRFN5,DAGLA,TMBIM6,PPP2CB,VWA1,HDAC5,CMKLR1,TSPAN8,ROR2,SCFD1,CREBRF,WDR70,ENTPD1,AUNIP,COPS5,FGF14,DMC1,MAD2L2,MAN1A1,RAB27A,DEFA1B,DEFA3,F5,TERF2,PTGER2,SLX1B,FOXN3,ANO6,GNAQ,FBXO9,TMEM117,RAD51D,HERC5,JAK2,FAM168A,FBXW7,SMC3,UIMC1,ITCH,MLIP,SMG1,CLDN1,PDE2A,KLF15,TPST1,TRIM59,MTA1,KREMEN1,IMMP2L,CLPB,NOX4,ERCC8,PRKCD,CD4,TGFB1,SGK1,ASCC2,NFATC3,POLK,CRADD,F2RL1,BCAS3,C9ORF47,DNAJB2,CELSR1,TP73,ITIH4,HP,HPR,KANK1,XPR1,MMP26,SMAD6,CLOCK,ZNF675,BCL3,TNFRSF10B,DUSP22,NAIP,TRRAP,ANKFN1,SBNO2,FGF10,PPL,SH3RF3,LOXL3,FANCI,CAPN3,EPHA4,RORA,PRKCA,CNR1,CD6,TNFSF11,PPP3CA,UBQLN3,MAG,CAMK4,UFL1,CTNNB1,PARK2,SOD2,FCGR2B,HSPA6,IGF1R,PPARG,AXIN1,MACROD1,OTUB1,IL18R1,IL1RL1,MTF1,CBX5,ANKRD17,CYBB,APOL3,SCAMP5,BRIP1,SREBF2,TDP1,RYR2,FGF1,PROS1,NR4A3,FOXK2,WDR45B,NOL3,SCARA3,MYOCD,TRIM5,PER2,KIR2DL4,KIR3DL1,KIR3DL2,AJUBA,CHEK2,UBQLN4,SPDYA,HCK,CAPN2,TRIM8,DIO2,BAZ1B,FLT4,HDAC4,PAX2,SFRP1,ST8SIA1,FOXO3,DGKB,SYNCRIP,SMAD3,RNFT2,ITPR1,ARNT2,ACKR2,CYP4F11,WIPI1,RNF168,C1S,IFNAR1,NEDD4,PLA2G4C,TXNDC12,SEC61B,APP,PDGFRA,NOX1,YAP1,AMFR,SESN1,CDK3,TNFRSF19,EYA2,RNLS,JAK1,ANGPTL4,POLR3G,SOX2,SETD2,DEFB118,RCAN1,ECSIT,DRD1,CHUK,ERLIN1,PALB2,MMP2,TPH1,S100A12,PRKD1,STAT1,DGKG,ETV5,HNF4A,SHPRH,EREG,MYOF,ATF2,RAF1,RASGRF2,ACOX2,CARD16,CASP1,PTPN1,ADAMTS12,POLA1,GGT7,BMPR2,USP33,CAMK1D,BMPR1A,PABPC4,MORC2,FGF2,BACH1,TICRR,ADCY5,AGBL4,NFX1,HELQ,MCTP1,PTAFR,ADCYAP1R1,SP140,DDX58,CHCHD6,PIK3C2B,PRKCG,AREL1,EEF1E1,EEF1E1-BLOC1S5,ALKBH3,ABCF3,EIF2B5,EIF4G1,RBX1,ATF3,FBXO45,STT3B,PAWR,DLEC1,FBXL20,VCL,TERF2IP,ANKS4B,WDR59,P4HB,CCL14,CCL15,ZFYVE1,PAFAH1B1,POLE,TRPA1,DDAH1,NMUR2,EDEM3,SCUBE1,ERCC1,PXDNL,MGLL,C9,BLM,RELN,SIN3A,RUVBL2,ABCB1,CSF3R,PPP2R5C,SPATA18,MAP3K7,SP1,TRIM22,MACF1,SLC8A2,GSTM3,STAU1,CLASP1,DSC2,EEPD1,SLC35A4,UBE2V1,XDH,NFATC2,RBBP6,CHD1L,PRKG1,RGS7,ATP2B4,PACRG,APEX2,PAK3,MASP1,MAPKAPK2,MAP1B,SCARB1,CDK6,CELF1,RNF34,TFRC,UVRAG,NFRKB,DCUN1D5,DROSHA,GFRAL,TBC1D5,PSMB2,EYA1,PTGER3,BRINP1,SHPK,TRPV1,RNF216,TSPAN6,TFEB,CLEC16A,MNAT1,ACTR2,ELAVL4,RECQL5,C2,CFB,SMOC2,ADIPOR2,PLCG2,C1QTNF1,PAXIP1,HIPK3,ABCC1,AP3B1,PTPN2,NTRK1,CRISP3,KPNA6,TRIM44,ACO2,EPHB1,MAN1B1,AXIN2,SARM1,MUC1,VWF,CD84,COCH,RAD51C,SPI1,DGKK,CTNNA1,AIFM2,PSPC1,ATG14,LRRC8C,LRRC8D,LY86,STAC,RBPMS,MAP3K13,MST1,ERCC3,PTK7,CHID1,SNCA,BMPR1B,POLN,PNPT1,HEY2,RNF103,RNF103-CHMP3,RNF19B,WNT7B,HMGCS2,PTPRS,ZMYND11,PRKAG1,TRIP12,ADTRP,CSRP1,SRSF6,OPRD1,HIPK1,RPTOR,RTN4RL1,BTK,VPS41,IGF1,STX8,DPYSL3,MAPK1,PTEN,SPRED2,IFFO1,BMP7,PIK3C3,PUM1,PCBP2,PRCP,LRRK2,MLYCD,RFC3,BAG6,CHAF1B,LILRB4,TMCO1,IPCEF1,ITGAM,MSRB3,PKD2,HSF2BP,SHANK3,SEL1L2,NT5E,SERPINA3,MTMR3,CUEDC2,WNT7A,SNAP23,NLRP1,NME8,KAT6A,BMP6,NFE2L1,ANO1,GPI,SH3RF2,EXT1,N4BP1,ATRX,SERINC5,ASCC1,ENPP3,GET4,BLOC1S3,UBR5,RHOA,SPRTN,MCM3,TF,CUL3,TFEC,HSPD1,ITPR2,NFKBID,GSK3B,DNAJC3,STAT6,ACTA2,EEF2K,RBBP8,ALAS2,CD27,SCN9A,BOK,KRT8,NACC2,AGT,POLH,PRKAA2,SYT7,ADAR,BLOC1S6,STAT2,ZMPSTE24,HRH4,PTK2,RNF121,TEC,GAP43,MAP3K5,CREBBP,NCF1,SLC29A1,TAOK2,UGT1A1,SHC1,NDUFS2,CD160,PARP10,RNFT1,RPS6KB1,STC2,ITGAL,ALOX5,DNAJB6,CD44,ADAM8,CLDN4,RPS6KA5,HUS1,CXCL17,NOS1,PLEKHA1,RGS14,ACTN4,MC1R,SCNN1B,EPHB2,SLC1A1,SIGMAR1,TMEFF2,XCR1,IL18,TEX12,UBE2K,DNAJC7,PPARA,PPP1R10,CALCRL,EPB41L4B,SMARCAL1,PHLPP1,MAGI3,CPEB4,GPSM3,NOTCH4,SYTL4,PAX6,PRKCZ,ZC3HAV1,CD96,ADORA2A,RABGEF1,CPEB1,PHLDB2,TYK2,KIN,NIPBL,EYA3,ATF6,ETS1,PPP1CA,CFI,RBMS3,CCL22,FBXO31,PRKCQ,BRAF,HDAC2,HTR2C,IMPACT,TNR,UBE2E2,ANGPT4,ARHGEF6,RFC5,SP100,REV3L,TRIM29,KCND2,CR2,DONSON,WIPI2,ADCY1,SYK,GRIK2,GTF2H5,ZBTB38,BRCA2,MORC1,YBX1,TAF1,TICAM1,TANK,CADM1,HSF1,MAX,DHFR,SRPK2,SASH1,GBP5,MCM8,QRICH1,VAV3,ZP3,CHD6,GNA12,SH2D3A,UGGT1,MYEF2,INPP5F,NUAK2,AXL,CD109,MET,CCR3,RAD9B,TIMELESS,UCHL5,ANKRD6,IFI44L,MALT1,ZDHHC11,INO80C,PHB,PTPRC,EXD2,TYRO3,RNF213,SPON2,TRAF3IP2,TRIM60,ATF6B,LIG1,SGTB,TRAP1,CLNK,EDNRA,GNGT1,PRAP1,BMP4,CASP12
GO:0018108	peptidyl-tyrosine phosphorylation	0.000002071237298497375	*IL31RA,PRLR,MVP,NTRK3,CBL,FLT3,ENPP2,GRM5,FER,MAP2K5,KITLG,ABI1,PTPN11,ADORA1,EPHA1,ERBB4,NTRK2,PDGFB,INSR,LCK,SNX6,CASS4,FYN,BCR,TTN,NRG1,MAP2K1,ROR2,JAK2,FBXW7,ZFYVE28,NOX4,PRKCD,CD4,PIBF1,DUSP22,DOCK3,FGF10,EPHA4,IGF1R,DYRK4,HCK,CSPG4,BAZ1B,NF2,FLT4,SFRP1,ABI2,APP,PDGFRA,IBTK,JAK1,EREG,PTPN1,NEDD9,PEAK1,SEMA4D,EPHB3,TSG101,DLG3,RELN,ALK,ADNP,EPHA5,GHR,PLCG2,HIPK3,PTPN2,INSRR,NTRK1,EPHB1,EPHA10,EPHA7,HIPK1,BTK,IGF1,UNC119,LILRB4,EFNA5,PDGFC,BMP6,ROR1,AGT,PTK2,TEC,LMTK3,NCF1,SHC1,CD44,EPHB2,IL18,PRKCZ,TYK2,ANGPT1,LRRK1,CNTN1,BRAF,HDAC2,CD300A,PPP2CA,ANGPT4,SYK,DVL2,PTPRJ,HSF1,INPP5F,AXL,MET,PTPRC,TYRO3*

GO:0098655	cation transmembrane transport	0.000002086808302990367	*SLC39A11,NRXN1,ASPH,PDE4D,SLC9A1,FAM155A,CHERP,CACHD1,RYR1,NOS1AP,NOX5,DPP6,UTRN,ATP2B2,KCNQ5,KCNIP4,ANK2,SLC9A9,CHD7,HTR2B,RYR3,SLC24A2,ITGB1,SLC24A3,CNIH2,CNGB1,ABCC8,TCIRG1,KCNS3,NIPAL2,NETO2,CACNB2,KCNJ16,DPP10,CACNA1B,STK39,CAB39,ZDHHC13,SLC20A2,SLC4A10,CATSPER2,AMIGO1,KCNMA1,KCNRG,LCK,TMC2,SLC12A8,SLC6A16,RHCE,MICU3,DMD,SLC30A9,SLC8A1,JPH2,FYN,SHISA9,CACNA1E,SLC4A5,KCNN3,CNNM2,SLC43A2,BDKRB1,KCNIP1,STIM1,NALCN,SLC39A14,CATSPER3,FGF14,CLDN16,ANO6,KCNB2,NPSR1,SCN4A,TMC1,SLC1A4,SLC9A7,KCNC4,CACNA1H,TGFB1,KEL,JPH3,KCNJ3,CACNA1D,CACNA2D3,KCNH1,SLC17A7,KCNH7,CAPN3,VMP1,COX8A,TMEM63C,TRPM1,KCNJ12,RYR2,KCNA6,NOL3,CACNA1C,SCN3B,ITPR1,WWP2,SHANK1,NEDD4,SLC4A4,SLC6A1,KCNK1,APP,NLGN2,VDAC1,IBTK,SLC9B2,DRD1,PRKD1,SLC6A3,FXYD2,FXYD6,KCNG4,RASGRF2,NLGN1,FGF2,MRS2,SLC9B1,ATP6V0B,KCNQ1,NLGN3,TRPA1,TRPM3,AKAP6,TRPC5,WNK1,RELN,KCNQ2,SLC47A1,SLC8A2,SLC9C1,TPCN1,AHNAK,RGS7,ATP2B4,P2RX6,ATP6V0A2,KCNAB2,CACNA1A,STOM,TRPV1,PLCG2,GPR35,SLC39A10,ANK3,ATP13A3,KCND3,CACNG8,SLC9C2,NETO1,MCU,TRDN,STAC,TRPC6,ABCB7,SLC41A2,ATP13A5,SNCA,CACNG2,CNGA3,SLC4A8,HTT,SLC30A7,SCN8A,ATP6V1A,CNIH3,TMCO1,PKD2,SHANK3,ANO1,KCNC2,SLC6A14,PSEN2,GRIN1,GRIN3A,ITPR2,SCN9A,AGT,PEX5L,PDE4B,COX5A,TRPC4AP,KCNJ15,SLC5A10,PKD1L1,SLC25A18,COX5B,NOS1,ACTN4,SCN1B,COX6A1,EPHB2,SLC1A1,XCR1,LRRC52,MCOLN1,ATP1A3,GRIN2B,SLC25A26,DIAPH1,HTR2C,ATP8A1,TMEM163,UQCR10,ANXA2,KCND2,ATP6V0A1,SCN1A,CACNG3,ATP2B3,GPM6A,KCNQ3,SESTD1,CAMK2D,KCNJ6,NEDD4L,PTPRC,SHISA6,EDNRA*
GO:0061387	regulation of extent of cell growth	0.0000022048186478104547	*CLASP2,SEMA3A,SEMA3D,SEMA5A,OLFM1,CDKL5,DSCAM,GOLGA4,LIMK1,CDH4,BDNF,RTN4,RTN4R,NTN1,SEMA5B,DISC1,MAG,DRAXIN,DCC,DIP2B,FSTL4,ISLR2,SEMA6D,BMPR2,DPYSL2,RAB11A,SEMA4D,WNT3,PAFAH1B1,TRPC5,RUFY3,SIN3A,MACF1,ADNP,MAP1B,TRIM46,EPHA7,MAP3K13,PTPRS,EFNA5,GSK3B,MAP2,TNR,CDKL3,SLIT1,SEMA3C*
GO:0099504	synaptic vesicle cycle	0.0000022782236359409803	*NRXN1,NLGN4X,CASK,NRXN3,PCDH17,DGKI,ERC2,CACNB2,RIMS1,SNCB,SYN2,DENND1A,SYN3,SYT1,CADPS2,ITSN2,SYNJ2,SLC17A7,STXBP5,CNR1,CTNNB1,PRKAR1B,RIMS4,DNM3,GRIK5,NLGN2,DRD1,NLGN1,ABCA13,PRKCG,AP2M1,FBXL20,NLGN3,BTBD9,STON2,PACSIN1,SYT12,ROCK1,AP3B1,UNC13A,AP3D1,SNCA,SYT9,PLD1,SLC4A8,PTEN,LRRK2,WNT7A,SNAP23,STXBP5L,DNAJC6,GRIN3A,GSK3B,PLD2,SYN1,SYT7,BLOC1S6,RIMS2,CPLX2,ADORA2A,CADPS,BRAF,RAB27B,ITSN1,ADCY1,SYNDIG1*
GO:0035295	tube development	0.000002598375225818028	*ADAMTS16,NRXN1,HLX,CLDN18,NOX5,PTGIS,TJP1,SEMA5A,ENPP2,MAP2K5,LRP2,PIK3CD,SP3,NRXN3,ROBO1,CHD7,ITGB6,TRAF6,ROBO2,VAV2,ITGB1,KIF26B,ARHGAP24,RUNX1,ABCC8,THRB,EPHA1,HIF3A,CECR2,ESR1,MYO18B,NPHP3,NTRK2,EP300,CELA1,RNF220,PAK1,BASP1,NPRL3,PHACTR4,MEIS1,SGCD,CRISPLD2,ADAMTS9,NF1,RTN4,RXFP1,APOD,CHI3L1,NDRG4,NTN1,UBP1,MAP2K1,STIM1,MYH9,HDAC5,DCN,CDH13,PLXDC1,CCBE1,FBXW7,PKNOX1,WNT11,QKI,TGFB1,PCSK5,BTRC,BCAS3,RPGRIP1L,CELSR1,SLIT2,MTDH,SMAD6,BMPER,PARVA,ADAM12,FGF10,GREB1L,LOXL3,EPHA4,RORA,PRKCA,CTNNB1,ARHGAP22,PPARG,DLG5,CYBB,OTC,BRIP1,RYR2,LEPR,FGF1,MYOCD,MYO1E,CSMD1,CSPG4,FLT4,PAX2,SFRP1,ARL13B,NFIB,IFT122,SMAD3,PTPRM,NRP2,TAB1,SSBP3,PDGFRA,NOX1,YAP1,HEG1,JAK1,ANGPTL4,LRP5,SETD2,ADAMTS2,PRICKLE1,VASH2,MMP2,KDM6A,PRKD1,STAT1,STRA6,EREG,ATF2,ADAMTS12,BMPR2,BMPR1A,TSPAN12,PIK3R3,FGF2,ISM1,PLXNA2,KLHL3,RARB,CHRD,DVL3,EPHB3,CLIC4,KCNQ1,RHOJ,SUFU,COL4A3,DDAH1,GLI2,PKHD1,RSPO2,APOLD1,SP1,LUZP1,XDH,OVOL2,ATP2B4,TMEM2,GPR161,GPC3,CALD1,EYA1,HOXB3,SMOC2,ADIPOR2,ROCK1,PAXIP1,AP3B1,EPHB1,EPCAM,PIFO,SPI1,EPHA7,SHROOM3,RAPGEF3,MST1,PTK7,HEY2,WNT7B,HMGCS2,ADTRP,ABCA12,RASA1,SRSF6,HIPK1,CBFA2T2,MAPK1,PTEN,MIB1,BMP7,PRCP,BAG6,PKD2,SHANK3,PDGFC,AMOTL1,WNT7A,SOS1,EXT1,ATRX,RAPGEF2,SMO,RHOA,PTPRB,SULF1,MTHFD1L,AGT,PTK2,PHEX,PBX1,SHB,SHC1,CD160,ECE1,SETDB2,ALOX5,ADAM8,THSD7A,CXCL17,STK4,EPHB2,SLC1A1,IL18,DLC1,CALCRL,NOTCH4,PAX6,COL22A1,NIPBL,TMIGD2,ANGPT1,ETS1,PPP1CA,POU3F3,NOTO,SPINT2,ANGPT4,SP100,ANXA2,SYK,WWTR1,DVL2,SRPK2,SASH1,VAV3,MET,CCR3,TIMELESS,KDM2B,SEMA3C,EDA,RNF213,VPS52,CREB1,EDNRA,BMP4*
GO:0099003	vesicle-mediated transport in synapse	0.000002909024579054 3765	*NRXN1,NLGN4X,CASK,NRXN3,PCDH17,DGKI,ERC2,CACNB2,RIMS1,SNCB,NRG1,SYN2,DENND1A,SYN3,SYT1,CADPS2,ITSN2,SYNJ2,SLC17A7,STXBP5,CNR1,CTNNB1,PRKAR1B,RIMS4,DNM3,GRIK5,NLGN2,DRD1,NLGN1,ABCA13,PRKCG,AP2M1,FBXL20,NLGN3,BTBD9,STON2,PACSIN1,ATAD1,SYT12,ROCK1,AP3B1,UNC13A,AP3D1,AP2B1,SNCA,SYT9,PLD1,SLC4A8,PTEN,LRRK2,WNT7A,SNAP23,STXBP5L,DNAJC6,GRIN3A,GSK3B,PLD2,SYN1,SYT7,BLOC1S6,RIMS2,CPLX2,ADORA2A,CADPS,BRAF,RAB27B,ITSN1,ADCY1,NUMB,SYNDIG1*
GO:1901698	response to nitrogen compound	0.000002910908613575 4625	*ENPP1,ASPH,PRKCI,PDE4D,DNMT1,SLC9A1,ADCY8,CALR3,RYR1,FLT3,STAT5B,GRM5,FER,EZR,SLC26A6,HTR2B,RYR3,GOT1,ITGB1,CDC6,ABCC8,DNAJB14,STXBP4,PTPN11,NTRK2,CACNA1B,CCND3,INSR,PIP4K2A,AMIGO1,CHRM3,TRIM13,UBR2,HLCS,SNX6,RFTN1,AP3S1,NPLOC4,USP13,KAT7,SLC8A1,FYN,PLCB1,MGMT,DPEP1,PIK3R2,TMBIM6,HDAC5,SLC39A14,CDH13,TRDMT1,IPO5,MAN1A1,RGS8,GNAQ,JAK2,PDE2A,KLF15,LAMTOR3,PRKCD,TGFB1,DNAJB2,SLIT2,TP73,GABRB1,KANK1,CHRM1,BCKDHB,HOMER2,GLP2R,EPHA4,CNR1,PPP3CA,UFL1,CTN*

			NB1,PARK2,FCGR2B,SMARCC1,IGF1R,PPARG,CYBB,OTC,BRIP1,SREBF2,RYR2,LEPROT,RGS10,NR4A3,SORBS1,CAPN2,SFRP1,FOXO3,SSH1,RNFT2,SLC6A1,SEC61B,APP,PDGFRA,AMFR,SESN1,RNLS,GNG2,DRD1,GLRA2,ERLIN1,MMP2,STAT1,SLC6A3,HNF4A,EREG,ATF2,PTPN1,COL16A1,PIK3R3,EPG5,ADCY5,PTAFR,DDX58,PRKCG,EIF2B5,PTPRE,SRSF5,STT3B,IDE,KCNQ1,AKAP6,EDEM3,IRS4,WDTC1,BLM,UBR1,SIN3A,PELI1,SP1,ALK,GSTM3,SNX5,RGS7,ATP2B4,P2RX6,MAP1B,COL4A6,TYR,CACNA1A,XRN1,ADRBK1,PSMB2,TRPV1,GHR,ACTR2,ELAVL4,RECQL5,ROCK1,EGR2,CAPN10,ABCC1,PTPN2,INSRR,MAN1B1,CARM1,SRD5A2,HTR1D,PTPRA,CTNNA1,BCAR3,RAB15,RAPGEF3,SDK1,PDXP,SNCA,PNPT1,RNF103,RNF103-CHMP3,CNGA3,HMGCS2,ADTRP,SRSF6,RPTOR,IGF1,MAPK1,BMP7,PIK3C3,LRRK2,GNA14,CHMP5,BAG6,NSG2,PKD2,SEL1L2,PDGFC,NLRP1,GLDC,GPR21,ANO1,GPI,TSHR,EXT1,ATRX,PNPLA3,RAPGEF2,KCNC2,GET4,PRKAR2B,CPT1A,GRB14,GRIN1,RHOA,ITPR2,GABRB3,GSK3B,STAT6,EEF2K,AGT,STAT2,HRH4,PTK2,PHEX,RNF121,PDE4B,MAP3K5,SHC1,RNFT1,RPS6KB1,STC2,GPR173,EPHB2,SLC1A1,PPARA,SLC25A33,CPEB4,FBN1,PRKCZ,ATP1A3,ADORA2A,CPEB1,OSBPL8,NCOR2,PRKCQ,DIAPH1,HDAC2,HTR2C,IMPACT,PHIP,RPL23,SH3BP4,SYK,TAF1,TICAM1,HSF1,MAX,DHFR,CIB2,CAV2,UGGT1,DENND4C,TIMELESS,CREB1,SGTB,EDNRA,HADHA
GO:0042391	regulation of membrane potential	0.0000031094323943519157	NRXN1,S1PR2,SLC9A1,GRID2,NOS1AP,NLGN4X,CLCN1,RGS7BP,GRM5,ANK2,CTNNA3,CNIH2,CNGB1,DGKI,GABRA3,CACNB2,ADORA1,NTRK2,RIMS1,KCNMA1,GRIK4,DMD,SLC8A1,BID,NALCN,DCN,GNAQ,SCN4A,CACNA1H,GRIK3,GABRR3,GABRB1,KCNJ3,CACNA1D,CHRM1,KCNH1,KCNH7,CNR1,PPP3CA,PIP5KL1,PARK2,SOD2,RYR2,CACNA1C,SCN3B,GRM1,RIMS4,CUX2,WWP2,GRIK5,SHANK1,NEDD4,SLC4A4,KCNK1,APP,NLGN2,DRD1,GLRA2,USP53,CELF4,NLGN1,PPA2,GRIK1,KCNQ1,NLGN3,AKAP6,TRPC5,RELN,SLC8A2,DSC2,P2RX6,KCNAB2,GABRG3,TRPV1,GPR35,ANK3,KCND3,NETO1,CHRNB4,TRDN,GABRA6,SNCA,CACNG2,OPRD1,PKP2,SLC4A8,PTEN,SCN8A,LRRK2,GNA14,TMEM108,SHANK3,PPP2R3C,WNT7A,GABRR2,KCNC2,GRIN1,GRIN3A,FAM19A4,GABRB3,GSK3B,SCN9A,BOK,ZMPSTE24,RIMS2,SLC29A1,GRID1,BCO2,SLC25A33,PRKCZ,ATP1A3,ADORA2A,GRIN2B,KCTD7,KCND2,GRIK2,SCN1A,KCNQ3,CAMK2D,NEDD4L
GO:0032270	positive regulation of cellular protein metabolic process	0.000003158672030717569	POLDIP3,NRXN1,ASPH,SLCO3A1,DNMT1,S1PR2,C6ORF89,ADCY8,PDE8A,IL31RA,PRDM12,NRG3,NOS1AP,PRKAG2,PRLR,NTRK3,ARNT,FLT3,ENPP2,GRM5,MAP2K5,KITLG,LRP2,EZR,ROBO1,TOM1L1,HTR2B,TENM1,LMNA,TRAF6,DNAJA3,ABI1,HSP90AA1,CDC6,KNDC1,CCDC22,AKAP13,DAB1,PTPN11,HDAC6,ADORA1,ERBB4,MRE11A,CDON,NTRK2,ZYG11B,FNTA,PDGFB,TNIK,CCND3,BRMS1,CHFR,INSR,CAB39,PAK1,FBXW11,MAP3K4,RNF144A,EGLN3,SAMD4A,BDNF,LCK,ECT2,ERN2,ATG10,LARP4B,USP13,KAT7,GSN,CASS4,FYN,MKRN2,ARNTL,RWDD3,CHI3L1,NRG1,SH2D3C,BID,MAP2K1,FNIP1,MYH9,VGLL4,JDP2,ROR2,MAD2L2,CAPRIN2,CCNYL1,CCBE1,JAK2,FBXW7,CYFIP2,WNT11,MTA1,NOX4,PRKCD,TAB2,ACVR2A,CD4,TGFB1,SPSB4,PIBF1,BTRC,CRADD,DNAJB2,DISC1,BLID,ZFAND2A,CLN6,BCL3,TNFRSF10B,DOCK3,YTHDF1,FGF10,SMYD3,FANCI,CAPN3,EPHA4,AUTS2,CD6,TNFSF11,CTNNB1,PARK2,PPARG,NGRN,AXIN1,LEPR,FGF1,AJUBA,CHEK2,SPDYA,RAB3GAP2,CSPG4,FLT4,HDAC4,TRABD2B,BCL2L13,SMAD3,RNFT2,TAB1,APP,DIP2B,ARIH1,SH3D19,BORA,MTCP1,PRICKLE1,S100A12,DCUN1D3,PRKD1,ST18,EREG,CCNY,ATF2,GRAMD4,RAF1,CASP1,PTPN1,BMPR2,BMPR1A,PIK3R3,MYB,FGF2,PPM1F,NEDD9,AGBL4,SEMA4D,PTAFR,JARID2,DVL3,EIF2B5,EIF4G1,RBX1,CKS1B,TERF2IP,COL4A3,HIP1,TRPC5,UBA2,DAB2,CACUL1,DLG3,WNK1,RELN,NEK10,RUVBL2,PELI1,IQGAP1,MAP3K7,SLC8A2,SLC35A4,XDH,ATP2B4,TET1,CAMTA1,ADNP,TFRC,DCUN1D5,GHR,MNAT1,SNX9,PLCG2,PAXIP1,NTRK1,SLC39A10,AXIN2,MUC1,SNX33,DNMT3B,EPHA7,ATG14,BCAR3,RBPMS,MAP3K13,RAPGEF3,SNCA,BMPR1B,MAGI2,USP50,EIF3E,RNF19B,PRKAG1,ADTRP,PLD1,OPRD1,RPTOR,IGF1,MAPK1,PTEN,BMP7,LRRK2,UNC119,BAG6,PKD2,EFNA5,DAZL,PDGFC,PPP2R3C,WNT7A,NLRP1,UACA,BMP6,MYCBP2,SH3RF2,RAPGEF2,VRK3,GRIN1,ARRDC4,RHOA,SPRTN,RQCD1,CUL3,HSPD1,GSK3B,PRR16,DNAJC3,RDX,BOK,RNF144B,AGT,PRKAA2,PTK2,TEC,MAP3K5,NCF1,TAOK2,RNFT1,RPS6KB1,CD44,LARP4,ADAM8,CLDN4,RPS6KA5,CSNK1A1,NOS1,STK4,SLC1A1,IL18,UBE2K,DLC1,MOB3B,PRKCZ,GRIN2B,NIPBL,ANGPT1,LRRK1,OSBPL8,MTF2,CDK5RAP1,CNTN1,BRAF,HDAC2,IMPACT,CD300A,PPP2CA,SKP1,ANGPT4,MADD,SYK,DVL2,PTPRJ,TAF1,TICAM1,TANK,HSF1,SASH1,MOB3A,GNA12,SH2D3A,AKTIP,TBC1D10A,MALT1,PHB,PTPRC,BMP4
GO:0040008	regulation of growth	0.0000036788085155378387	ENPP1,HLX,SLC9A1,CLASP2,SEMA3A,WWC1,NRG3,LATS2,SEMA3D,WWC3,FTO,EGLN2,SEMA5A,STAT5B,PLCE1,MAP2K5,OLFM1,EZR,CHD7,CDKL5,DSCAM,RUNX1,IL17RB,GOLGA4,SERTAD2,ERBB4,LIMK1,BRD8,ATP8A2,RIMS1,INSR,CDH4,BASP1,BDNF,ATRN,RFTN1,PLCB1,RTN4,RTN4R,NTN1,NRG1,BDKRB1,VGLL4,CSNK2A3,MAD2L2,CAPRIN2,SYT1,ITCH,PLS1,WNT11,SEMA5B,TGFB1,SGK1,DNAJB2,SLIT3,SLIT2,TP73,DISC1,ITSN2,ENOX2,CAPN3,GNG4,MAG,PARK2,CDHR2,PPARG,CDK11A,CDK11B,DRAXIN,MYOCD,CPNE6,BRMS1L,DCC,CTDP1,SFRP1,MUC12,SMAD3,SYT3,CDC73,APP,DIP2B,YAP1,FSTL4,ISLR2,SPTBN4,ESR2,DCUN1D3,CPNE9,SLC6A3,HNF4A,SEMA6D,BMPR2,DPYSL2,BMPR1A,SPAG9,RAB11A,FGF2,PPM1F,NEDD9,SEMA4D,JARID2,SPOCK1,EIF4G1,TSG101,WNT3,PAFAH1B1,AKAP6,RASAL1,TRPC5,RUFY3,DAB2,SIN3A,RUVBL2,MACF1,ADNP,MAP1B,CELF1,DCUN1D5,GPC3,GHR,UNC13A,TRIM46,SMARCA2,EPHA7,MAP3K13,PNPT1,HEY2,PTPRS,TLL2,RPTOR,IGF1,PTEN,OSGIN1,EBAG9,EFNA5,GPR21,TSHR,SMO,SYT17,J

			ADE1,RHOA,EPM2A,GSK3B,AGT,ZMPSTE24,RIMS2,GAP43,TAOK2,SHC1,RPS6KB1,STC2,STK4,PPARA,SLC25A33,NIPBL,YEATS4,MAP2,PRKCQ,TNR,CDKL3,PPP2CA,SH3BP4,SLIT1,PTPRJ,ZP3,CAMK2D,KDM2B,SEMA3C,NEDD4L,PHB,CREB1,BMP4
GO:0044057	regulation of system process	0.000004277252362901933	NRXN1,ASPH,PDE4D,S1PR2,SLC9A1,PBX3,SEMA3A,NOS1AP,EPB41,NLGN4X,FTO,ATP2B2,PLCE1,ANK2,RYR3,LMNA,CTNNA3,CNIH2,ABCC8,THRB,PTPN11,CACNB2,ADORA1,ADRA1D,PTPRO,PDGFB,RIMS1,STK39,KCNMA1,CHRM3,TENM4,ARHGAP42,DMD,SLC8A1,PPP1R12B,SHISA9,BDKRB2,NEUROD1,JAK2,KCNB2,MLIP,NPSR1,SCN4A,FIG4,CACNA1H,CAMK2B,SGK1,DLGAP1,F2RL1,KCNJ3,CACNA1D,FGF10,PRKCA,PPP3CA,MAG,PPARG,CELF2,KCNJ12,RYR2,NR4A3,NOL3,MYOCD,PER2,CACNA1C,SCN3B,GRM1,CTDP1,HDAC4,FOXO3,SMAD3,CUX2,ITPR1,SHANK1,DOCK4,APP,GSX2,NLGN2,RNLS,TNNI3K,SPTBN4,MMP2,CELF4,BMPR2,NLGN1,PTAFR,JARID2,KCNQ1,NLGN3,NMUR2,AKAP6,MGLL,DAB2,WNK1,RELN,SLC8A2,DSC2,SNX5,PRKG1,ATP2B4,MEF2A,ADRBK1,PTGER3,TRPV1,ROCK1,C1QTNF1,EGR2,RNF10,GPR35,PARD3,KCND3,NETO1,CHRNB4,TRDN,HEY2,OPRD1,PKP2,IGF1,PTEN,LRRK2,TMEM108,SHANK3,WNT7A,BMP6,GRIN1,RHOA,FAM19A4,ABCG8,AGT,ZMPSTE24,PDE4B,RIMS2,ECE1,RPS6KB1,NOS1,SLC1A1,PPARA,CALCRL,PRKCZ,ATP1A3,ADORA2A,HTR2C,TNR,ATP2B3,DLGAP2,CAMK2D,SETD3,SHISA6,EDNRA,PRAP1
GO:1901699	cellular response to nitrogen compound	0.000004530793193534254	ENPP1,PRKCI,PDE4D,DNMT1,SLC9A1,ADCY8,RYR1,STAT5B,GRM5,FER,EZR,SLC26A6,HTR2B,RYR3,GOT1,CDC6,STXBP4,PTPN11,NTRK2,CCND3,INSR,PIP4K2A,AMIGO1,CHRM3,UBR2,SNX6,AP3S1,SLC8A1,FYN,PLCB1,DPEP1,PIK3R2,HDAC5,SLC39A14,IPO5,RGS8,GNAQ,JAK2,PDE2A,KLF15,LAMTOR3,PRKCD,TGFB1,SLIT2,GABRB1,KANK1,CHRM1,GLP2R,EPHA4,CTNNB1,PARK2,FCGR2B,SMARCC1,IGF1R,PPARG,CYBB,BRIP1,RYR2,LEPROT,RGS10,NR4A3,SORBS1,CAPN2,SFRP1,FOXO3,SSH1,APP,PDGFRA,SESN1,GNG2,DRD1,GLRA2,MMP2,STAT1,HNF4A,ATF2,PTPN1,COL16A1,PIK3R3,EPG5,ADCY5,PTAFR,DDX58,PTPRE,SRSF5,IDE,KCNQ1,AKAP6,IRS4,WDTC1,BLM,UBR1,SIN3A,SP1,ALK,SNX5,ATP2B4,MAP1B,COL4A6,CACNA1A,XRN1,ADRBK1,TRPV1,GHR,ACTR2,RECQL5,ROCK1,CAPN10,ABCC1,PTPN2,INSRR,HTR1D,PTPRA,CTNNA1,BCAR3,RAB15,RAPGEF3,PDXP,SNCA,HMGCS2,RPTOR,IGF1,MAPK1,LRRK2,GNA14,CHMP5,NSG2,PKD2,PDGFC,GPR21,ANO1,TSHR,ATRX,PNPLA3,RAPGEF2,KCNC2,GRB14,ITPR2,GABRB3,GSK3B,STAT6,EEF2K,AGT,HRH4,PTK2,PDE4B,MAP3K5,SHC1,RPS6KB1,GPR173,EPHB2,SLC1A1,SLC25A33,CPEB4,FBN1,PRKCZ,ATP1A3,CPEB1,OSBPL8,PRKCQ,DIAPH1,HDAC2,HTR2C,IMPACT,PHIP,RPL23,SH3BP4,TAF1,HSF1,MAX,CIB2,CAV2,DENND4C,TIMELESS,EDNRA
GO:0001558	regulation of cell growth	0.0000053666599794870605	ENPP1,SLC9A1,CLASP2,SEMA3A,NRG3,SEMA3D,EGLN2,SEMA5A,PLCE1,MAP2K5,OLFM1,CDKL5,DSCAM,IL17RB,GOLGA4,SERTAD2,LIMK1,RIMS1,CDH4,BDNF,RTN4,RTN4R,NTN1,NRG1,BDKRB1,VGLL4,CSNK2A3,MAD2L2,CAPRIN2,SYT1,ITCH,WNT11,SEMA5B,TGFB1,SGK1,DNAJB2,SLIT3,SLIT2,DISC1,ITSN2,GNG4,MAG,PARK2,CDHR2,PPARG,CDK11A,CDK11B,DRAXIN,MYOCD,CPNE6,DCC,CTDP1,SFRP1,MUC12,SMAD3,SYT3,CDC73,DIP2B,FSTL4,ISLR2,ESR2,DCUN1D3,CPNE9,HNF4A,SEMA6D,BMPR2,DPYSL2,SPAG9,RAB11A,SEMA4D,SPOCK1,EIF4G1,TSG101,WNT3,PAFAH1B1,AKAP6,RASAL1,TRPC5,RUFY3,DAB2,SIN3A,MACF1,ADNP,MAP1B,DCUN1D5,UNC13A,TRIM46,SMARCA2,EPHA7,MAP3K13,PTPRS,RPTOR,IGF1,OSGIN1,EBAG9,EFNA5,SYT17,JADE1,RHOA,EPM2A,GSK3B,AGT,RIMS2,TAOK2,PPARA,SLC25A33,MAP2,PRKCQ,TNR,CDKL3,PPP2CA,SH3BP4,SLIT1,PTPRJ,CAMK2D,KDM2B,SEMA3C,NEDD4L,PHB
GO:0051656	establishment of organelle localization	0.000005367668966323385	CLASP2,KIF22,LTV1,CBL,FER,PIK3CD,EZR,KIF5C,ITGB1,CNIH2,KLHL12,TCIRG1,HDAC6,PEX14,GBF1,HOOK3,FMN2,FBXW11,VAMP7,AP3S1,CENPF,TANC2,NTN1,DCTN1,MYH9,MAD1L1,PARD3B,LIN7A,COPS5,EXOC4,TLE6,RAB27A,CROCC,DYNC1I1,ATP9A,CENPC,PIBF1,C12ORF4,CLMN,ANKFN1,FGF10,TRAK1,CTNNB1,PARK2,MYO1D,LRPPRC,NR4A3,RIOK2,MYO1E,AP3B2,SPG11,RAB6A,WIPI1,KPNB1,MYO1F,SYNE2,SYBU,VPS4A,NLGN1,RAB11A,AGBL4,BLOC1S5,TSG101,PAFAH1B1,KIF3A,PKHD1,EML4,FAM91A1,MYO7A,CLASP1,SYNE3,MAP1B,MARK1,ACTR2,AP3B1,PARD3,TRIM46,AP3D1,KIF3C,CD84,SNCA,BTK,HTT,NUP88,LRRK2,SPAG5,CHMP5,SNAP23,MAP4,RHOT1,SUN1,LSG1,BLOC1S3,NDE1,CUL3,BLOC1S6,LAT2,EXOC6B,COPG2,SEC16B,CPLX2,ARFGAP3,TBC1D23,ACTN4,GPSM2,PAX6,PRKCZ,RABGEF1,ARHGAP21,MAP2,CD300A,RAB27B,SYK,SLIT1,MYO1A,MLPH,CLNK
GO:0060429	epithelium development	0.0000054718834348512265	CLRN1,ADAMTS16,LDB2,PDE4D,S1PR2,CLASP2,SEMA3A,IL31RA,ASTN2,TJP1,PRLR,SPRR2D,RAD51B,STAT5B,LRP2,PIK3CD,EZR,ROBO1,CHD7,TRAF6,ESRP1,ROBO2,ABI1,TRIOBP,PSMB7,KIF26B,ARHGAP24,THRB,IKBKB,ERBB4,CECR2,ESR1,NPHP3,PTPRO,EP300,RNF220,PDGFB,H2AFY2,LAMA3,PAK1,BASP1,STRC,TFAP2A,PHACTR4,DMD,ARNTL,NF1,PLCB1,MGMT,PCDH15,ARID4B,RTN4,BCR,NDRG4,NTN1,SLC4A5,NRG1,MAP2K1,ROR2,NEUROD1,ACTL8,JAK2,BCL11B,TMC1,CLDN1,MLLT3,PNPLA1,PLS1,PDE2A,KLF15,ANXA4,WNT11,SIPA1L3,TGFB1,BTRC,F2RL1,C9ORF47,RPGRIP1L,CELSR1,SLIT2,CDH23,SMAD6,CLOCK,BMPER,FGF10,GREB1L,PPL,LOXL3,SMURF2,EPHA4,TNFSF11,NSUN2,CTNNB1,CDHR2,PPARG,DLG5,BFSP1,RYR2,FGF1,AJUBA,MYO1E,CSMD1,NF2,PAX2,SFRP1,ARL13B,NFIB,IFT122,SMAD3,ABI2,PSAP,VANGL1,SSBP3,YAP1,HEG1,TNFRSF19,VDAC1,LRP5,BDH2,SETD2,GPC6,PRICKLE1,FRS2,PALB2,MMP2,KDM6A,STAT1,HNF4A,STRA6,EREG,ADAMTS12,BMPR2,BMPR1A,CERS3,FGF2,PLXNA2,FNDC3A,RILPL1,KLHL3,

			RARB,CHRD,DVL3,TSG101,VCL,CLIC4,MYO9A,KCNQ1,WNT3,SUFU,PAFAH1B1,GLI2,SCUBE1,DAB2,PKHD1,USH2A,MYSM1,DLG3,RSPO2,IQGAP1,APOLD1,LUZP1,ARHGAP12,MYO7A,GSTM3,CLASP1,HOXC13,XDH,OVOL2,RBBP6,GPR161,ARID4A,CDK6,GPC3,PSMB2,EYA1,HOXB4,HOXB5,HDAC1,ROCK1,AP3B1,NTRK1,PTPRQ,AXIN2,EPCAM,JAG2,PIFO,EPHA7,SHROOM3,RAPGEF3,SH3BP1,MST1,KAZN,ERCC3,PTK7,MAGI2,HEY2,HYDIN,WNT7B,PTPRS,ABCA12,SRSF6,ZNRF3,CBFA2T2,MAPK1,PTEN,MIB1,SPRED2,BMP7,LHFPL5,ALDOC,PKD2,SHANK3,RIPPLY1,NCOA3,WNT7A,ZBED6,BMP6,SOS1,EXT1,ATRX,PRICKLE2,RAPGEF2,SMO,CPT1A,RHOA,ROR1,ONECUT2,GSK3B,RDX,STAT6,ACTA2,SULF1,MTHFD1L,KRT8,AGT,ZMPSTE24,PBX1,TRPC4AP,PYGO2,ECE1,LBX2,SCEL,SETDB2,CD44,STK4,SPEF1,TMEFF2,DLC1,NOTCH4,PAX6,FAM20C,COL22A1,PHLDB2,PPP1CA,HDAC2,POU3F3,NOTO,SPINT2,CAMSAP3,WWTR1,BRCA2,DVL2,SULT2B1,CD109,MET,TIMELESS,ANKRD6,KDM2B,SEMA3C,EDA,CREB1,EDNRA,BMP4
GO:0003013	circulatory system process	0.000005517834708969571	*ADAMTS16,ASPH,SLCO3A1,PDE4D,SLC9A1,SEMA3A,RPS6KA2,NOS1AP,TJP1,ATP2B2,LRP2,ANK2,CHD7,HTR2B,RYR3,CTNNA3,SLC24A3,SLC22A8,AKAP13,ABCC8,THRB,CACNB2,ADORA1,ADRA1D,PTPRO,PDGFB,STK39,INSR,KCNMA1,CHRM3,NAV2,ARHGAP42,SGCD,DMD,SLC8A1,GSN,FYN,SLC5A6,BCR,TTN,SLC4A5,BDKRB1,BDKRB2,F5,JAK2,ABCC2,SLC1A4,PDE2A,IMMP2L,NOX4,CACNA1H,SGK1,PCSK5,KEL,F2RL1,SLIT2,KCNJ3,CACNA1D,SLC16A1,CNR1,SOD2,PPARG,CELF2,KCNJ12,RYR2,LEPR,PER2,CACNA1C,SCN3B,HDAC4,ELN,TRHDE,SMAD3,ITPR1,SLC16A2,DOCK4,SLC4A4,SLC6A1,NOX1,YAP1,HEG1,PPCS,RNLS,TNNI3K,LRP5,SPTBN4,DRD1,MMP2,STAT1,MYOF,SLC16A12,BMPR2,PTAFR,KCNQ1,COL4A3,DDAH1,WNK1,ABCB1,SLC22A3,SLC8A2,KLK2,DSC2,SNX5,PRKG1,ATP2B4,TFRC,MEF2A,ADRBK1,GNB3,TRPV1,ROCK1,ABCC1,KCND3,HTR1D,LPA,TRDN,RENBP,HEY2,PKP2,SGCZ,SLC4A8,PRCP,SLC44A1,BMP6,EXT1,ABCG2,RHOA,ACTA2,AGT,ZMPSTE24,PDE4B,SLC29A1,ECE1,SLC5A3,NOS1,PLVAP,SCNN1B,SLC1A1,PPARA,ATP1A3,ADORA2A,ANGPT1,SLCO2B1,ATP8A1,WWTR1,SCN1A,PTPRJ,ATP2B3,TAC3,SLC13A3,GNA12,CAMK2D,NEDD4L,SH3GL2,EDNRA*
GO:0001934	positive regulation of protein phosphorylation	0.000005554692100462 4	*NRXN1,SLCO3A1,S1PR2,ADCY8,PDE8A,IL31RA,NRG3,PRKAG2,PRLR,NTRK3,FLT3,ENPP2,GRM5,MAP2K5,KITLG,ROBO1,TOM1L1,HTR2B,TENM1,TRAF6,ABI1,HSP90AA1,CDC6,KNDC1,AKAP13,DAB1,PTPN11,HDAC6,ADORA1,ERBB4,MRE11A,CDON,NTRK2,PDGFB,TNIK,CCND3,INSR,CAB39,PAK1,MAP3K4,BDNF,ECT2,ERN2,CASS4,FYN,CHI3L1,NRG1,SH2D3C,MAP2K1,FNIP1,ROR2,MAD2L2,CAPRIN2,CCNYL1,JAK2,FBXW7,WNT11,NOX4,PRKCD,TAB2,ACVR2A,CD4,TGFB1,PIBF1,TNFRSF10B,DOCK3,FGF10,SMYD3,EPHA4,CD6,TNFSF11,PPARG,AXIN1,LEPR,FGF1,AJUBA,CHEK2,SPDYA,CSPG4,FLT4,TAB1,APP,BORA,MTCP1,S100A12,PRKD1,EREG,CCNY,ATF2,RAF1,PTPN1,BMPR2,BMPR1A,PIK3R3,FGF2,NEDD9,SEMA4D,DVL3,EIF4G1,CKS1B,TERF2IP,TRPC5,DAB2,CACUL1,DLG3,WNK1,RELN,NEK10,IQGAP1,MAP3K7,SLC8A2,ATP2B4,ADNP,TFRC,GHR,MNAT1,SNX9,PLCG2,NTRK1,AXIN2,EPHA7,ATG14,BCAR3,RBPMS,MAP3K13,RAPGEF3,SNCA,BMPR1B,PRKAG1,ADTRP,OPRD1,RPTOR,IGF1,MAPK1,BMP7,LRRK2,UNC119,PKD2,EFNA5,PDGFC,PPP2R3C,BMP6,RAPGEF2,RHOA,RQCD1,AGT,PTK2,TEC,MAP3K5,NCF1,TAOK2,CD44,ADAM8,RPS6KA5,NOS1,STK4,SLC1A1,IL18,UBE2K,MOB3B,PRKCZ,ANGPT1,LRRK1,OSBPL8,CNTN1,BRAF,HDAC2,CD300A,PPP2CA,ANGPT4,MADD,SYK,DVL2,PTPRJ,HSF1,SASH1,MOB3A,SH2D3A,AKTIP,MALT1,PHB,PTPRC,BMP4*
GO:0010977	negative regulation of neuron projection development	0.000005638932724745031	*SEMA3A,SEMA3D,SEMA5A,DAB1,PTPRO,DENND5A,RIT2,FAT3,RTN4,RTN4R,PAQR3,NTN1,KREMEN1,SEMA5B,KANK1,EPHA4,MAG,DRAXIN,DCC,DNM3,DIP2B,FSTL4,PTPRG,SEMA6D,NLGN1,CRMP1,SEMA4D,SPOCK1,WNT3,PAFAH1B1,RUFY3,DAB2,PTPN9,TRIM46,CARM1,EPHA7,PTPRS,RTN4RL1,CBFA2T2,DPYSL3,PTEN,LRRK2,RHOA,SYNGAP1,EPHB2,MAP2,HDAC2,TNR,CDKL3,SLIT1,INPP5F,SEMA3C*
GO:0099173	postsynapse organization	0.0000057880298853352965	*NRXN1,PRMT3,GRID2,NOS1AP,NLGN4X,KALRN,NTRK3,LHFPL4,CDKL5,DNAJA3,DOCK10,HDAC6,FNTA,INSR,PTPRD,TANC2,FYN,FRMPD4,CAPRIN2,ZDHHC15,IL1RAPL1,CAMK2B,CTNND2,EPHA4,FCGR2B,ARHGAP22,IGF1R,DGKB,DNM3,CUX2,ABI2,SHANK1,NRP2,NLGN2,NLGN1,EPHB3,ARHGAP39,NLGN3,PAFAH1B1,DLG3,RELN,PAK3,ACTR2,EPHB1,ARHGAP44,EPHA7,PTPRS,PTEN,LRRK2,TMEM108,SHANK3,WNT7A,SYNGAP1,EEF2K,GAP43,EPHB2,GRIN2B,TANC1,SHISA6*
GO:0030516	regulation of axon extension	0.000006061603927078945	*CLASP2,SEMA3A,SEMA3D,SEMA5A,OLFM1,CDKL5,DSCAM,GOLGA4,LIMK1,CDH4,RTN4,RTN4R,NTN1,SEMA5B,DISC1,MAG,DRAXIN,DIP2B,ISLR2,SEMA6D,BMPR2,DPYSL2,RAB11A,SEMA4D,WNT3,PAFAH1B1,TRPC5,RUFY3,SIN3A,MACF1,ADNP,MAP1B,TRIM46,MAP3K13,PTPRS,GSK3B,MAP2,TNR,CDKL3,SLIT1,SEMA3C*
GO:0008361	regulation of cell size	0.000006180161076969658	*CLASP2,SEMA3A,SEMA3D,SEMA5A,OLFM1,CDKL5,VAV2,DSCAM,GOLGA4,LIMK1,CDH4,BDNF,KCNMA1,SLC12A8,RTN4,RTN4R,NTN1,ANO6,SEMA5B,KEL,DISC1,MAG,XK,DRAXIN,DCC,DIP2B,FSTL4,ISLR2,SEMA6D,BMPR2,DPYSL2,RAB11A,SEMA4D,DEPTOR,WNT3,PAFAH1B1,TRPC5,RUFY3,WDTC1,SIN3A,MACF1,ADNP,MAP1B,GNB3,TRIM46,EPHA7,MAP3K13,PTPRS,RPTOR,PTEN,EFNA5,SHANK3,RHOA,GSK3B,PRR16,RDX,MAP2,TNR,CDKL3,SLIT1,RAP1GAP2,VAV3,SEMA3C,CREB1*
GO:0030155	regulation of cell adhesion	0.000006647728212826715	*PRDX2,B4GALNT2,GP6,MAP4K4,HLX,SLC9A1,CBFB,CLASP2,TENM3,DOCK1,TJP1,PRLR,UTRN,SEMA5A,STAT5B,CASK,MAP2K5,MEGF10,TRAF6,ITPKB,DNAJA3,TRIOBP,KIF26B,DSCAM,RUNX1,DAB1,MYO10,LAMA2,PTPN11,CYTH3,EPHA1,PTPRO,PDGFB,PRKAR1A,LAMA3,ARHGAP6,LCK,PTPRU,DMD,CASS4,FYN,NF1,APOD,EGFLAM,NRG1,DOCK8,MAD1L1,CDH13,SOX13,MAD2L2,RCC2,JAK2,SKAP1,ITCH,MAGI1,APBB1IP,PRKCD,CD4,TGFB1,BCAS3,CORO1C,DISC1,KANK1,DUSP22,LOXL3,EPHA4,HSD17B12,PRKCA,CD6,TNFSF11,PPP3CA,FCGR2B,DLG5,ECM2,*

			NR4A3,PPP1CB,NF2,SFRP1,FOXO3,SMAD3,PREX1,SLK,JAK1,SOX2,MMP2,COL16A1,MYB,PPM1F,NEDD9,PEAK1,SEMA4D,PLXNA2,PTAFR,SPOCK1,LMO7,CHRD,EPHB3,PAWR,VCL,P4HB,GLI2,DAB2,PKHD1,WNK1,PELI1,MACF1,CLASP1,GPM6B,PRKG1,CDK6,TFRC,ROCK1,NID1,C1QTNF1,AP3B1,PTPN2,ANK3,EPCAM,MUC1,JAG2,AP3D1,ADAM19,SPI1,EPHA7,PTPRA,RC3H1,ADTRP,ABCA12,RASA1,IGF1,PTEN,BMP7,LILRB4,EFNA5,TBCD,BMP6,PDCD1LG2,PAG1,TFE3,RHOA,ONECUT2,HSPD1,NFKBID,GSK3B,RDX,FAM49B,CD27,DUSP26,PTK2,CD160,ALOX5,CD44,ADAM8,ACTN4,STK4,EPHB2,IL18,DLC1,PPARA,EPB41L4B,NOTCH4,PRKCZ,ADORA2A,PHLDB2,TMIGD2,ANGPT1,GCNT2,ETS1,VWC2,PRKCQ,BRAF,SPINT2,TNR,CD300A,PPP2CA,CAMSAP3,SYK,FRMD5,PTPRJ,FCHO1,VAV3,ZP3,TNFSF9,MALT1,PTPRC,BMP4
GO:0021537	telencephalon development	0.000007972028193081888	TACC2,SEMA3A,NRG3,PHACTR1,ZSWIM6,ROBO1,CHD7,TACC1,ROBO2,DAB1,ERBB4,CDON,NTRK2,KIRREL3,TBR1,DMD,SLC8A1,NF1,PLCB1,RTN4,RTN4R,CCDC141,NRG1,NEUROD1,TRAPPC9,BCL11B,RPGRIP1L,SLIT2,DISC1,CNTNAP2,CTNNB1,IGF1R,DRAXIN,NIN,NF2,ARL13B,NFIB,BTBD3,GSX2,DRD1,SYNE2,RARB,EIF2B5,EPHB3,FBXO45,LAMB1,PAFAH1B1,RELN,ALK,ATP2B4,CDK6,CELF1,EPHA5,HDAC1,SRD5A2,FOXP2,PTPRS,RTN4RL1,PTEN,LRRK2,TMEM108,SHANK3,EXT1,SMO,SUN1,RHOA,NDE1,GSK3B,DLX1,EPHB2,PAX6,POU3F3,TNR,SRGAP2,NUMB,SLIT1,EZH1,UCHL5,TRA2B,KDM2B,BMP4
GO:0044248	cellular catabolic process	0.000009508047358794415	NT5C1B,ENPP1,PRDX2,PDE4D,PDE7B,PDE8A,ALDH4A1,RNASET2,GK5,CALR3,SLC25A17,FBXL2,FTO,DCAF12,ACOXL,CBL,ARNT,ENPP2,SAMHD1,USP32,LRP2,DEPDC5,EZR,TOM1L1,HTR2B,PSMD1,GOT1,LPO,HSP90AA1,PSMB7,CCDC22,AMDHD1,OMA1,DNAJB14,PDE4A,TCIRG1,HDAC6,DDB1,GPRASP1,TEX264,CUL4B,CECR2,ACAD11,FBXL18,TNRC6A,EP300,CELA1,ZYG11B,FBXL7,USP46,CHFR,INSR,PIP4K2A,USP34,FBXW11,RNF144A,SAMD4A,NPRL3,TRIM13,UBR2,ENTPD5,SNX6,PIPOX,ACOX1,ERN2,NPLOC4,ATG10,LARP4B,USP13,PAFAH1B2,MKRN2,ARNTL,ADAMTS9,SMG7,PLCB1,DMGDH,SLC25A21,FBXO21,DPEP1,NEIL2,VTA1,PIK3R2,BLVRB,DAGLA,TMBIM6,VGLL4,PPP2CB,RNF4,UBE3D,SBF2,DCN,SCFD1,TRDMT1,FHIT,ENTPD1,EXOC4,MAN1A1,FBXO9,RFFL,USP22,FBXW7,LINC00473,ITCH,USP12,ABCC2,LZTS1,SMG1,DPYS,IDNK,PNPLA1,PDE2A,SPSB1,HADHB,MTA1,WDFY4,SIAH1,ERCC8,PRKCD,TAB2,SPSB4,TRIM65,ZHX2,ASCC2,BTRC,BCAS3,DNAJB2,RNF133,RNF148,HP,MGAM,DISC1,ZFAND2A,CPT1C,CLN6,MTDH,CLOCK,BCKDHB,NELL1,TIMP2,DAPK2,SND1,HNRNPC,YTHDF1,FBXL17,KCTD10,VMP1,SMURF2,EPHA4,PRKCA,CNR1,SMG6,NSUN2,HBE1,UBQLN3,UBQLNL,LIX1L,WDFY3,UFL1,CTNNB1,PARK2,SMARCC1,AXIN1,CPQ,OTC,LRPPRC,RSPRY1,SREBF2,LEPR,FOXK2,WDR45B,TRIM5,CHEK2,UBQLN4,PPP1CB,TBC1D14,RAB3GAP2,CAPN2,TRIM8,DIO2,FOXO3,TRHDE,ZCCHC17,SNX14,SYNCRIP,RNFT2,ITPR1,EVA1A,WWP2,CYP4F11,WIPI1,VPS39,RNF168,PSAP,DCP1B,NEDD4,PLA2G4C,SLC4A4,SEC61B,APP,USP49,RBM8A,ARIH1,AMFR,SESN1,VDAC1,SH3D19,BDH2,ACBD5,PRICKLE1,ERLIN1,NUDT10,PRKD1,HEXA,SLC6A3,VPS4A,EPHX1,ACOX2,PTPN1,ADAMTS12,PSMD11,GGT7,USP33,IDO2,PABPC4,EPG5,RAB24,UBE2R2,AGBL4,CDC27,HIBADH,YME1L1,KLHL3,PIK3C2B,PRKCG,AREL1,UCHL3,EIF4G1,PSMD2,RBX1,ANAPC5,FBXO45,LIN28B,TECPR2,AKR1D1,ATP6V0B,STT3B,AGO3,ACAA1,FBXL20,TSG101,DESI1,CRYM,IDE,SUFU,ZFYVE1,ATG4C,DDAH1,EDEM3,PXDNL,TNRC6B,ALDH6A1,MGLL,DAB2,UBR1,CACUL1,SMG5,PSMD7,PELI1,PPP2R5C,SPATA18,MAP3K7,TRIM22,ZNRF1,GSTM3,FASTKD5,TPCN1,XDH,SNX5,RBBP6,ATP2B4,TET1,DYX1C1,MAPKAPK2,UBE3C,SCARB1,ATP6V0A2,CELF1,RNF34,UVRAG,DROSHA,XRN1,TBC1D5,PSMB2,SCOC,DIS3L2,RNF216,TFEB,CLEC16A,SNX9,ELAVL4,PLCG2,ROCK1,RYBP,CUL2,ABCC1,MAP1A,HYKK,MAN1B1,AXIN2,SARM1,TMEM199,FBXO39,NUDT5,SNX33,CD84,VPS16,FBXW4,ATG14,EIF3H,ACOT8,RENBP,PDXP,SNCA,USP42,PNPT1,USP50,RC3H1,CHMP3,EIF3E,RNF103,RNF103-CHMP3,RNF19B,PSMF1,SULT2A1,TRIP12,DFFA,ADTRP,RPTOR,VPS41,ZNRF3,KCTD13,IGF1,PLA2G4E,HTT,PTEN,MIB1,PIK3C3,PUM1,PCBP2,LRRK2,ATP6V1A,MLYCD,TMEM59,ZRANB1,ZBTB20,BAG6,GALT,BLVRA,SEL1L2,NT5E,DAZL,MTMR3,GLDC,TAF15,SH3RF2,N4BP1,PNPLA3,ENPP3,GET4,ACADSB,CPT1A,UBE2H,DECR1,RQCD1,CUL3,PCCA,EPM2A,GSK3B,DNAJC3,RDX,UPF2,BOK,RNF144B,RNF43,METTL16,PRKAA2,CUL9,ZMPSTE24,PTK2,ARMC8,RNF121,PDE4B,EXOSC3,FBXO10,SEC16B,TRPC4AP,XYLB,UGT1A1,UGT1A4,ECE1,RNFT1,ADAM8,CSNK1A1,NOS1,PEX7,DRAM1,BCO2,MFSD8,UBE2K,DIS3,FBXL13,PPARA,ABCD3,MCOLN1,PNPLA6,CYP2C18,CYP2C19,RNF150,ZC3HAV1,PAH,OTUD7B,UPB1,PACS2,TBL1X,ATF6,NT5M,SLCO2B1,HACL1,PPP1CA,FBXO31,ATG3,PHKG2,C18ORF25,MOXD1,SKP1,MAOA,RPL23,SH3BP4,VTI1A,ANXA2,PARN,ATP6V0A1,WIPI2,CNOT1,WWTR1,ALDH8A1,YBX1,TAF1,TICAM1,HSF1,POP1,ETF1,PLEKHM1,ADAMTS7,GNA12,UGGT1,MYEF2,INPP5F,MET,UCHL5,MALT1,MLXIPL,TRAPPC8,FBXL4,GNS,NEDD4L,RNF213,TRAF3IP2,SGTB,HADHA
GO:0060341	regulation of cellular localization	0.00001049571129648865	CD247,GPC5,NRXN1,ASPH,PRKCI,PDE4D,ADCY8,WWC1,CLDN18,CTDSPL2,LATS2,ASTN2,EPB41,FER,ANK2,EZR,CHD7,TENM1,LMNA,ITGB1,CNIH2,PCDH17,PARD6G,HMGN3,STXBP4,EFCAB7,PTPN11,CCT2,ERBB4,GBF1,DPP10,STX18,TCF7L2,BDNF,ECT2,VAMP7,DMD,KAT7,SLC8A1,GSN,RBM4,JPH2,FYN,NF1,RTN4,APOD,NDRG4,PIK3R2,BDKRB1,MAP2K1,MAD1L1,TMBIM6,TMEM30A,SCFD1,CREBRF,IPO5,JAK2,FBXW7,OAZ2,CROCC,NPSR1,ATP9A,PLS1,PRKCD,TGFB1,JPH3,BCAS3,C12ORF4,TTBK2,CHRM1,DCLK1,CAPN3,NSUN2,CTNNB1,PARK2,ANXA13,SREBF2,RYR2,LEPROT,RIOK2,CACNA1C,SORBS1,TRIM8,NF2,SPTBN1,SMAD3,LZTF

			L1,NEDD4,YAP1,RAB11FIP5,NLGN2,NVL,LRP5,SLC9B2,SETD2,GPC6,DRD1,PRKD1,VPS4A,PTPN1,NLGN1,ADCY5,ADCYAP1R1,GAS8,AP2M1,TSG101,SUFU,KIF3A,AKAP6,RUFY3,DAB2,DZIP1,SIN3A,GPM6B,ATP2B4,MAP1B,KCNAB2,TFRC,EPHA5,GPC3,STOM,CAPN10,ZFAND1,MAP1A,ANK3,PTPN9,GNL3L,PTPN14,TRIM46,AP2B1,ARHGAP44,SNX33,CD84,SPI1,CTNNA1,TRDN,STAC,RAPGEF3,SNCA,CACNG2,CHMP3,ABCA12,XPO4,PLA2G4E,STX8,HTT,MAPK1,PTEN,LRRK2,TMEM59,SPAG5,LILRB4,ITGAM,PKD2,EFNA5,SHANK3,ZBED6,ANO1,SMO,MARK4,UBR5,GRIN1,EPM2A,GSK3B,RDX,IWS1,NECAB2,SNX3,SEC16B,SLC5A3,NOS1,EPHB2,SLC1A1,GPSM2,PRKCZ,PPP1CC,RABGEF1,OTUD7B,ANGPT1,EPB41L2,MAP2,DIAPH1,CD300A,SP100,ANXA2,TRIM29,NUP214,NUMB,SYK,WWTR1,ANKRD13A,INPP5F,CAMK2D,EHD2,CCT3,NEDD4L,SHISA6,EDNRA,BMP4,ABLIM3
GO:0007163	establishment or maintenance of cell polarity	0.00001079101727753534	*PRKCI,SLC9A1,CLASP2,WWC1,EZR,LMNA,ITGB1,HSP90AA1,KIF26B,DLG2,PARD6G,TCIRG1,CYTH3,GBF1,FRMD4A,FBXW11,GSN,DST,DCTN1,DOCK8,MYH9,PARD3B,LIN7A,DOCK2,UST,CDC42BPB,WNT11,SIPA1L3,BCAS3,RPGRIP1L,KANK1,ANK1,PARVA,ANKFN1,FGF10,IGF1R,DLG5,RND3,CLIC4,MYO9A,PAFAH1B1,KIF3A,RUFY3,PKHD1,DLG3,CLASP1,MAP1B,ACTR2,ROCK1,EPHB1,PARD3,CTNNA1,SH3BP1,PTK7,WNT7B,HTT,AMOTL1,WNT7A,MAP4,PARVB,RHOA,NDE1,RHOBTB1,GSK3B,FAM49B,PTK2,FRMD4B,GPSM2,PAX6,PRKCZ,SPINT2,CAMSAP3,TRAF3IP2*
GO:0048646	anatomical structure formation involved in morphogenesis	0.00001138403461584622	*NRXN1,RDH13,CLASP2,GRID2,NRG3,NOX5,FHOD3,PTGIS,TJP1,SEMA5A,ENPP2,MAP2K5,LRP2,PIK3CD,SP3,ANK2,OLFM1,NRXN3,ROBO1,ITGB6,TRAF6,ROBO2,VAV2,ABI1,ITGB1,KNDC1,KIF26B,DSCAM,ARHGAP24,KLHL12,AKAP13,RUNX1,ABCC8,TCIRG1,PTPN11,EPHA1,ATP8A2,HIF3A,KLHL6,CECR2,NPHP3,CDON,EP300,CELA1,PRKAR1A,ATP8B1,LAMA3,TBR1,TFAP2A,PHACTR4,MEIS1,TENM4,ADAMTS9,NF1,FAT3,RTN4,APOD,CHI3L1,TTN,UBP1,MAP2K1,STIM1,MYH9,HDAC5,ROR2,DCN,CDH13,PLXDC1,EXOC4,COL12A1,CCBE1,MYPN,FBXW7,PKNOX1,WNT11,FIG4,CACNA1H,TGFB1,BCAS3,CELSR1,SDK2,SLIT2,MTDH,KCNH1,BMPER,BCL3,PARVA,ADAM12,SBNO2,FGF10,CAPN3,RORA,PRKCA,CTNNB1,ARHGAP22,PPARG,AXIN1,CYBB,LEPR,FGF1,PHLDB1,CAPN2,CSPG4,OBSCN,NF2,FLT4,PAX2,SFRP1,NFIB,IFT122,SMAD3,PTPRM,NRP2,CDC73,SSBP3,PDGFRA,NOX1,YAP1,EYA2,JAK1,ANGPTL4,SOX2,SETD2,PRICKLE1,VASH2,FRS2,PALB2,MMP2,KDM6A,PRKD1,STAT1,STRA6,EREG,MYOF,ATF2,BMPR2,BMPR1A,TSPAN12,PIK3R3,FGF2,SH3PXD2A,ISM1,PLXNA2,DVL3,EPHB3,LAMB1,CLIC4,COL11A1,RFX2,WNT3,RHOJ,SUFU,PAFAH1B1,COL4A3,DDAH1,GLI2,ERCC1,RELN,APOLD1,SP1,LUZP1,CLASP1,TEAD4,OVOL2,NFATC2,ATP2B4,TMEM2,TET1,MEF2A,CALD1,EYA1,HOXB3,HDAC1,SMOC2,ADIPOR2,ROCK1,EGR2,EPHB1,AXIN2,PTPN14,SHROOM3,RAPGEF3,SDK1,PTK7,CD53,HEY2,RC3H1,ADTRP,CSRP1,HIPK1,MAPK1,PTEN,MIB1,BMP7,PRCP,MEGF11,SHANK3,RIPPLY1,AMOTL1,WNT7A,GPI,EXT1,SMO,SOX30,RHOA,CUL3,PTPRB,SULF1,MTHFD1L,KRT8,AGT,FBN2,PTK2,SHB,SHC1,CD160,PGM5,ALOX5,ADAM8,THSD7A,CXCL17,NOS1,STK4,EPHB2,SLC1A1,WLS,IL18,DLC1,PPARA,CALCRL,NOTCH4,PAX6,FAM20C,COL22A1,PHLDB2,TMIGD2,ANGPT1,FHL2,ETS1,NEBL,HDAC2,SPINT2,ANGPT4,SP100,ANXA2,SYK,CNOT1,DVL2,NEB,SRPK2,SASH1,VAV3,ZP3,CD109,CCR3,CCDC136,EHD2,KDM2B,SEMA3C,EDA,RNF213,TANC1,EDNRA,BMP4*
GO:0007416	synapse assembly	0.00001297431790621004	*NRXN1,NEGR1,GRID2,NLGN4X,NTRK3,LHFPL4,NRXN3,ROBO2,PCDH17,DSCAM,ERBB4,NTRK2,KIRREL3,PTPRD,CNTN5,BDNF,AMIGO1,NTN1,NRG1,LRFN5,PCDHB16,CLSTN2,IL1RAPL1,SDK2,LINGO2,IL1RAPL2,DLG5,ADD2,DNM3,CUX2,APP,NLGN2,GPC6,DRD1,SHANK2,NLGN1,SEMA4D,EIF4G1,EPHB3,FBXO45,NLGN3,SETD5,ADNP,MAP1B,FLRT2,NTRK1,EPHB1,EPHA7,SDK1,SNCA,PTPRS,PTEN,EFNA5,SHANK3,WNT7A,GABRB3,EEF2K,GAP43,EPHB2,LGI2,SLIT1,GPM6A,SYNDIG1*
GO:0070588	calcium ion transmembrane transport	0.0000154824356572982 9	*ASPH,PDE4D,SLC9A1,FAM155A,CHERP,CACHD1,RYR1,NOS1AP,ATP2B2,ANK2,CHD7,HTR2B,RYR3,SLC24A2,SLC24A3,CACNB2,CACNA1B,CATSPER2,LCK,TMC2,MICU3,DMD,SLC8A1,JPH2,FYN,CACNA1E,BDKRB1,STIM1,NALCN,CATSPER3,ANO6,NPSR1,TMC1,CACNA1H,TGFB1,JPH3,CACNA1D,CACNA2D3,CAPN3,VMP1,TRPM1,RYR2,NOL3,CACNA1C,ITPR1,VDAC1,IBTK,DRD1,PRKD1,FGF2,TRPA1,TRPM3,AKAP6,TRPC5,SLC8A2,TPCN1,AHNAK,ATP2B4,CACNA1A,TRPV1,PLCG2,GPR35,CACNG8,MCU,TRDN,STAC,TRPC6,SNCA,CACNG2,HTT,TMCO1,PKD2,PSEN2,GRIN1,GRIN3A,ITPR2,PDE4B,TRPC4AP,PKD1L1,NOS1,XCR1,MCOLN1,GRIN2B,DIAPH1,HTR2C,ANXA2,CACNG3,ATP2B3,GPM6A,SESTD1,CAMK2D,PTPRC,EDNRA*
GO:0034613	cellular protein localization	0.00001710907023914325	*CD247,DNAH11,ZDHHC14,BLZF1,GPC5,NRXN1,ASPH,PRKCI,CLASP2,GRID2,CLDN18,CTDSPL2,LATS2,ASTN2,EPB41,DPP6,MAPRE2,SEC23B,SNX31,LHFPL4,FAM53A,KCNIP4,TNPO3,HDGF,ANK2,EZR,XRCC4,TOM1L1,TENM1,KIF5C,LMNA,SRP72,NFASC,DNAJA3,ITGB1,HSP90AA1,FAM126A,CNGB1,DLG2,PTPRK,STXBP4,EFCAB7,TCIRG1,PTPN11,CCT2,CACNB2,HDAC6,GOLGA4,ADORA1,IKBKB,PEX14,ERBB4,GBF1,TLK1,ESR1,DPP10,STX18,FNTA,HOOK3,VPS45,RIMS1,TNIK,SNX2,MID1,H2AFY2,TCF7L2,BDNF,ECT2,SNX6,CHML,PTPRU,RFTN1,AP3S1,NPLOC4,DMD,RANBP17,KAT7,GSN,RIT2,FYN,NF1,STX6,RTN4,GOLGA2P5,APOD,BCR,PAQR3,PIK3R2,KLHL21,BID,RAPGEF6,TMEM30A,SYNE1,SCFD2,ZDHHC3,LIN7A,SCFD1,IPO5,MAN1A1,RAB27A,RAB3C,TERF2,TMED6,GOLPH3L,RCC2,ARL3,IPO11,ZDHHC15,JAK2,FBXW7,KCNB2,OAZ2,SKAP1,CROCC,SNAP25-AS1,PLS1,SEPT7,TTC7B,LAMTOR3,KPNA3,WNT11,IFT80,IMMP2L,PRKCD,CD4,TGFB1,BANP,NUP93,PIBF1,BCAS3,CELSR1,DISC1,ZFAND2A,GRPEL2,ANK1,TTBK*

			2,CHRM1,BCL3,DCLK1,RABGAP1L,FGF10,CNTNAP2,CIZ1,CAPN3,SNX8,PPP3CA,TRAK1,CTNNB1,PARK2,RN7SL832P,TSNARE1,SYTL5,ANXA13,SREBF2,RYR2,LEPROT,WDR45B,AJUBA,SCN3B,SORBS1,TBC1D14,RAB3GAP2,TBCK,TRIM8,OBSCN,NF2,SPTBN1,AP3B2,ARL13B,IFT122,RANBP3,SMAD3,RAB6A,WIPI1,MTBP,VPS39,GRIK5,LZTFL1,NEDD4,KPNB1,RAB6C,SEC61B,YAP1,RAB11FIP5,NLGN2,SSR2,NVL,SETD2,ZDHHC6,GPC6,PRICKLE1,SPTBN4,DRD1,PRKD1,TAF3,VPS4A,PITRM1,ATF2,PTPN1,AP1B1,NLGN1,SNX16,TAF8,RAB11A,IPO9,RAB24,XPO6,RILPL1,SORCS2,RAB11FIP3,GAS8,AP2M1,TNPO1,TECPR2,LMBRD1,TSG101,DESI1,VCL,TERF2IP,SUFU,KIF3A,AKAP6,CEP350,PADI6,TNKS,RUFY3,DAB2,TRAM2,DZIP1,DLG3,RELN,SIN3A,RUVBL2,FAM91A1,ZNF423,MACF1,MYO7A,GPM6B,SNX5,ATP2B4,NSF,PACRG,SYNE3,PACSIN1,ATAD1,NXT2,KCNAB2,TFRC,GPC3,STOM,TBC1D5,SNX9,RPGR,ARL5A,ROCK1,EGR2,AP3B1,KPNA6,ZFAND1,MAP1A,ANK3,PTPN9,SNUPN,AXIN2,PARD3,GNL3L,PTPN14,TUB,CACNG8,AP3D1,AP2B1,ARHGAP44,SNX33,SPI1,VPS16,CTNNA1,STAC,RAB15,RAPGEF3,CACNG2,MAGI2,TBC1D9,HEY2,RAB5B,ABCA12,PKP2,TBC1D16,XPO4,VPS41,STX8,IFFO1,BMP7,PIK3C3,NUP88,LRRK2,SEPT6,TMEM59,SPAG5,BAG6,LILRB4,MORC3,ITGAM,SHANK3,MIA2,SGSM1,RABEP1,SH3PXD2B,POM121C,ATRX,SAMM50,RAPGEF2,SMO,GET4,SUN1,MARK4,UBR5,BBS9,SYNGAP1,HSPD1,TSPAN33,EPM2A,GSK3B,RDX,CCDC14,NECAB2,AGT,ADAR,PEX5L,EXOC6B,RIMS2,TOMM5,COPG2,CPE,SEC16B,TIMM44,GTF2IRD2,NCF1,TAOK2,ZDHHC23,ITGAL,DNAJB6,SLC5A3,ARFGAP3,PACSIN2,PEX7,GCKR,STK4,ZFAND6,EPHB2,SLC1A1,WLS,GPSM2,PPP1R10,GRTP1,KPNA4,SYTL4,PAX6,PRKCZ,RABGEF1,WDR83OS,OTUD7B,NIPBL,ANGPT1,PACS2,FRMPD1,OSBPL8,EPB41L2,HACL1,TBC1D10C,ATG3,BRAF,DIAPH1,SIL1,CLTB,EMC3,SKP1,FAM149B1,RPL23,SH3BP4,CAMSAP3,SP100,VTI1A,TRIM29,PIGU,RAB27B,ITSN1,NUP214,WIPI2,NUMB,SYK,WWTR1,GRIK2,MYRIP,SEC31B,BRCA2,CACNG3,DVL2,YBX1,ANKRD13A,GRIP1,ANKRD13C,GBP5,MCM8,MLPH,DENND4C,TBC1D10A,SNX1,ZDHHC11,ZDHHC11B,EHD2,TRAPPC8,CCT3,NEDD4L,RPH3A,CTCF,RAB28,SYNDIG1,TSPAN5,TRAF3IP2,SGTB,SHISA6,EDNRA,BMP4,ABLIM3
GO:0035239	tube morphogenesis	0.000017366977676456807	*ADAMTS16,NRXN1,HLX,NOX5,PTGIS,TJP1,SEMA5A,ENPP2,MAP2K5,LRP2,PIK3CD,NRXN3,ROBO1,CHD7,TRAF6,VAV2,ITGB1,KIF26B,ARHGAP24,RUNX1,ABCC8,EPHA1,HIF3A,CECR2,ESR1,MYO18B,NPHP3,NTRK2,CELA1,PAK1,NPRL3,PHACTR4,MEIS1,SGCD,ADAMTS9,NF1,RTN4,APOD,CHI3L1,NDRG4,NTN1,UBP1,STIM1,MYH9,HDAC5,DCN,CDH13,PLXDC1,CCBE1,FBXW7,PKNOX1,WNT11,QKI,TGFB1,BTRC,BCAS3,CELSR1,SLIT2,MTDH,BMPER,PARVA,ADAM12,FGF10,GREB1L,EPHA4,RORA,PRKCA,CTNNB1,ARHGAP22,PPARG,DLG5,CYBB,RYR2,LEPR,FGF1,MYOCD,MYO1E,CSMD1,CSPG4,FLT4,PAX2,SFRP1,ARL13B,NFIB,IFT122,SMAD3,PTPRM,NRP2,PDGFRA,NOX1,YAP1,HEG1,JAK1,ANGPTL4,LRP5,SETD2,PRICKLE1,VASH2,MMP2,KDM6A,PRKD1,STAT1,STRA6,EREG,ATF2,ADAMTS12,BMPR2,BMPR1A,TSPAN12,PIK3R3,FGF2,ISM1,KLHL3,DVL3,EPHB3,CLIC4,RHOJ,SUFU,COL4A3,DDAH1,GLI2,PKHD1,RSPO2,APOLD1,SP1,LUZP1,XDH,OVOL2,ATP2B4,TMEM2,GPC3,CALD1,EYA1,HOXB3,SMOC2,ADIPOR2,ROCK1,PAXIP1,EPHB1,SPI1,EPHA7,SHROOM3,RAPGEF3,MST1,PTK7,HEY2,WNT7B,ADTRP,RASA1,HIPK1,PTEN,MIB1,BMP7,PRCP,PKD2,SHANK3,AMOTL1,WNT7A,SOS1,EXT1,RAPGEF2,SMO,RHOA,PTPRB,SULF1,MTHFD1L,AGT,PTK2,PBX1,SHB,SHC1,CD160,SETDB2,ALOX5,ADAM8,THSD7A,CXCL17,STK4,EPHB2,SLC1A1,IL18,DLC1,CALCRL,NOTCH4,COL22A1,NIPBL,TMIGD2,ANGPT1,ETS1,PPP1CA,NOTO,SPINT2,ANGPT4,SP100,ANXA2,SYK,DVL2,SRPK2,SASH1,VAV3,MET,CCR3,TIMELESS,KDM2B,EDA,RNF213,EDNRA,BMP4*
GO:0071277	cellular response to calcium ion	0.000019073652876948896	*NRXN1,ASPH,ADCY8,RYR1,RYR3,ITPKB,CPNE4,ALOX5AP,ECT2,DPEP1,SYT1,SYT13,KCNH1,RASA4,RASA4B,CAPN3,CPNE6,SYT3,CPNE9,NLGN1,CLIC4,RASAL1,IQGAP1,MEF2A,SYT12,PLCG2,SYT9,PKD2,SYT17,EEF2K,PRKAA2,SYT7,MCOLN1,BRAF,ADCY1,CAMK2D,CPNE1*
GO:0042330	taxis	0.000019366901840260233	*NRXN1,SEMA3A,NRG3,SEMA3D,MYOT,UNC5C,KALRN,NTRK3,SEMA5A,ENPP2,FER,PIK3CD,NRXN3,ROBO1,KIF5C,ROBO2,NFASC,ELMO2,DSCAM,TRIO,LAMA2,EPHA1,GBF1,PTPRO,EP300,PDGFB,STK39,UNC5D,LAMA3,CDH4,TBR1,BDNF,LSP1,FYN,DPEP1,CCDC141,ADAMTSL1,NTN1,NRG1,MAP2K1,CNTN4,CMKLR1,DOCK2,CDH13,DEFA1B,BOC,ANO6,MYPN,BCL11B,SIAH1,PRKCD,SEMA5B,MMP28,F2RL1,SLIT3,SLIT2,LAMC2,DAPK2,PARVA,YTHDF1,FGF10,ENAH,EPHA4,TNFSF11,DRAXIN,FGF1,DCC,CNTN6,NFIB,SMAD3,PTPRM,ACKR2,NRP2,PREX1,DOCK4,PRTG,APP,PDGFRA,NLGN2,S100A12,PRKD1,DSCAML1,SEMA6D,BMPR2,USP33,DPYSL2,CAMK1D,CRMP1,FGF2,PPM1F,SEMA4D,PLXNA2,PTAFR,EPHB3,WNT3,CCL14,CCL15,CCL15-CCL14,GLI2,NEO1,WNK1,RELN,CSF3R,EPHA5,FLRT2,SMOC2,EGR2,ABCC1,PTPN2,NTRK1,EPHB1,EPHA10,SPI1,EPHA7,MCU,MST1,PTK7,BMPR1B,PLD1,MAPK1,BMP7,EFNA5,MYCBP2,SOS1,EXT1,SMO,RHOA,FAM19A4,EMB,FAM49B,PTK2,PDE4B,GAP43,ECE1,ALOX5,ADAM8,RPS6KA5,CXCL17,TUBB3,EPHB2,XCR1,GPSM3,PAX6,ANGPT1,BIN2,CCL22,PRKCQ,TNR,TMEFF1,SYK,SLIT1,PTPRJ,VAV3,MET,CCR3,SEMA3C,NCAM1,EDNRA,BMP4*
GO:0001944	vasculature development	0.000020908204263850006	*NRXN1,DNMT1,NOX5,PTGIS,TJP1,SEMA5A,ENPP2,MAP2K5,LRP2,PIK3CD,NRXN3,ROBO1,CHD7,ROBO2,VAV2,ITGB1,ARHGAP24,RUNX1,ABCC8,EPHA1,HIF3A,MYO18B,NTRK2,CELA1,PDGFB,TCF7L2,NPRL3,MEIS1,SGCD,ADAMTS9,NF1,RTN4,APOD,CHI3L1,UBP1,MAP2K1,STIM1,MYH9,HDAC5,DCN,CDH13,PLXDC1,CCBE1,FBXW7,PKNOX1,PDE2A,WNT11,IMMP2L,QKI,TGFB1,PCSK5,NFATC3,BCAS3,SLI*

			T2,MTDH,SMAD6,BMPER,PARVA,ADAM12,FGF10,RORA,PRKCA,CTNNB1,SOD2,ARHGAP22,PPARG,ANKRD17,CYBB,LEPR,FGF1,MYOCD,MYO1E,CSPG4,FLT4,SFRP1,PTPRM,NRP2,TAB1,NFATC1,PDGFRA,NOX1,YAP1,HEG1,JAK1,ANGPTL4,LRP5,SETD2,PRICKLE1,VASH2,MMP2,PRKD1,STAT1,STRA6,EREG,ATF2,BMPR2,BMPR1A,TSPAN12,PIK3R3,FGF2,ISM1,EPHB3,CLIC4,ADAMTS6,RHOJ,SUFU,COL4A3,DDAH1,APOLD1,SP1,LUZP1,XDH,OVOL2,NFATC2,ATP2B4,TMEM2,GPC3,CALD1,EYA1,HOXB3,SMOC2,ADIPOR2,ROCK1,PAXIP1,EPHB1,PTPN14,AP2B1,SPI1,RAPGEF3,PTK7,HEY2,WNT7B,ADTRP,RASA1,HIPK1,MAPK1,PTEN,MIB1,BMP7,PRCP,PKD2,AMOTL1,WNT7A,SOS1,RAPGEF2,SMO,RHOA,ACTA2,PTPRB,SULF1,AGT,PTK2,SHB,SHC1,CD160,ALOX5,ADAM8,THSD7A,CXCL17,STK4,EPHB2,SLC1A1,IL18,CALCRL,NOTCH4,PAX6,COL22A1,TMIGD2,ANGPT1,ETS1,ANGPT4,SP100,ANXA2,SYK,SRPK2,SASH1,VAV3,CCR3,SEMA3C,RNF213,EDNRA,BMP4
GO:0051962	positive regulation of nervous system development	0.00002222975229950545	NRXN1,PRKCI,GRID2,TIAM2,KALRN,SEMA5A,GRM5,LRP2,ROBO1,CDKL5,ROBO2,DSCAM,GOLGA4,LIMK1,NTRK2,CDH4,PTPRD,BDNF,AMIGO1,TENM4,NTN1,MAP2K1,CAPRIN2,CLSTN2,IL1RAPL1,CAMK2B,TGFB1,SLIT2,TP73,DISC1,LINGO2,EPHA4,MAG,UFL1,CTNNB1,PPARG,DLG5,NIN,DCT,CUX2,GSX2,NLGN2,ISLR2,PARP6,BMPR2,NLGN1,RAB11A,SEMA4D,PLXNA2,SPEN,EPHB3,WNT3,NLGN3,PAFAH1B1,TRPC5,RUFY3,RELN,MACF1,PAK3,ADNP,MAP1B,FLRT2,ACTR2,HDAC1,EGR2,NTRK1,EPHB1,CUX1,MAP3K13,EFNA5,SHANK3,WNT7A,SMO,EEF2K,RGS14,EPHB2,PAX6,PPP1CC,FBXO31,BRAF,HDAC2,CDKL3,NUMB,SYNDIG1
GO:1902533	positive regulation of intracellular signal transduction	0.00002390588194580805	PRDX2,NRXN1,MAP4K4,SLC9A1,PDE8A,SEMA3A,CHERP,WWC1,NOS1AP,MAPRE2,NTRK3,CBL,SEMA5A,FLT3,GRM5,PLCE1,MAP2K5,KITLG,LRP2,PIK3CD,ROBO1,HTR2B,TENM1,TRAF6,ITPKB,ITGB1,HSP90AA1,CCDC22,AKAP13,DGKI,PTPN11,ADORA1,IKBKB,ERBB4,ADRA1D,MIER1,CDON,NTRK2,EP300,PDGFB,TNIK,MID1,STK39,INSR,TCF7L2,PAK1,LITAF,MAP3K4,ZDHHC13,TRIM13,LCK,ECT2,ERN2,RIT2,CASS4,FYN,PLCB1,RTN4,RTN4R,CHI3L1,NDRG4,NRG1,BID,MAP2K1,FNIP1,ROR2,DCN,CDH13,JAK2,FBXW7,NPSR1,LAMTOR3,WNT11,NOX4,SIAH1,TAB2,CD4,F2RL1,TP73,MTDH,BMPER,TNFRSF10B,DUSP22,NAIP,FGF10,IQCJ-SCHIP1,SH3RF3,EPHA4,PRKCA,AUTS2,TNFSF11,CTNNB1,PARK2,FCGR2B,IGF1R,AXIN1,DLG5,IL18R1,P2RY10,ANKRD17,APOL3,FGF1,TRIM5,AJUBA,TRIM8,CSPG4,GRM1,FLT4,NEDD4,TAB1,APP,PDGFRA,NOX1,TNFRSF19,SOX2,CHUK,FRS2,S100A12,PRKD1,RAF1,CARD16,CASP1,PTPN1,NLGN1,SPAG9,FGF2,SEMA4D,DOK6,ADCYAP1R1,EEF1E1,EEF1E1-BLOC1S5,DVL3,SHOC2,AGO3,TERF2IP,WDR59,CCL14,CCL15,HIP1,AKAP6,RELN,NEK10,GUCY1A2,PELI1,IQGAP1,MAP3K7,TRIM22,ALK,UBE2V1,XDH,CAMTA1,P2RY8,TFRC,GFRAL,TSPAN6,CLEC16A,GHR,PLCG2,ROCK1,C1QTNF1,GPR35,PTPN2,NTRK1,TRIM44,VWF,ARHGEF3,SPI1,BCAR3,MAP3K13,GRM4,RC3H1,WNT7B,RPTOR,IGF1,PTEN,SPRED2,PUM1,LRRK2,PDGFC,WNT7A,SIK3,SH3RF2,SOS1,RAPGEF2,RHOA,ROR1,ACTA2,NECAB2,CD27,BOK,NACC2,AGT,PTK2,MAP3K5,NCF1,TAOK2,SHC1,CD44,ADAM8,CXCL17,ACTN4,MC1R,WLS,IL18,PRKCZ,ZC3HAV1,ANGPT1,LRRK1,OSBPL8,GCNT2,CCL22,BRAF,HTR2C,RPL23,MADD,SYK,DVL2,PTPRJ,TICAM1,SASH1,CAV2,AXL,MET,ANKRD6,MALT1,PHB,PTPRC,EDA,GPR89A,TRAF3IP2,BMP4,CPNE1
GO:0090066	regulation of anatomical structure size	0.00002456615226402257	CLASP2,SEMA3A,SEMA3D,FHOD3,SEMA5A,FER,OLFM1,EZR,HTR2B,CDKL5,TENM1,VAV2,TRIOBP,DSCAM,CDC42EP3,GOLGA4,ADORA1,ADRA1D,LIMK1,CDH4,BDNF,KCNMA1,CHRM3,SLC12A8,ARHGAP42,SLC8A1,GSN,RTN4,RTN4R,NTN1,BDKRB2,NCKAP1,ANO6,PLS1,CYFIP2,PRKCD,SEMA5B,KEL,F2RL1,SLIT2,DISC1,KANK1,KANK4,SVIL,MAG,SOD2,CDHR2,ADD2,XK,DRAXIN,PER2,HCK,DCC,PAX2,SPTBN1,ELN,SSH1,DOCK4,ADD3,DIP2B,FSTL4,ISLR2,SPTBN4,DRD1,VILL,MMP2,SEMA6D,BMPR2,DPYSL2,RAB11A,SEMA4D,AP2M1,DEPTOR,WNT3,PAFAH1B1,TRPC5,RUFY3,WDTC1,SIN3A,MACF1,PRKG1,SPTBN5,PAK3,SSH2,ADNP,MAP1B,GNB3,SNX9,ROCK1,EPS8,TRIM46,HTR1D,EPHA7,LIMA1,FCHSD2,MAP3K13,PDXP,SH3BP1,WNT7B,PTPRS,RASA1,RPTOR,PTEN,LRRK2,EFNA5,SHANK3,WNT7A,EXT1,RHOA,GSK3B,PRR16,RDX,ACTA2,FAM49B,AGT,ECE1,NOS1,SCNN1B,ADORA2A,PPP1CA,MAP2,TNR,CDKL3,SLIT1,CIT,RAP1GAP2,NEB,VAV3,SPTAN1,SEMA3C,CREB1,EDNRA
GO:0031400	negative regulation of protein modification process	0.000025775177842836137	ENPP1,PRMT3,PDE4D,DNMT1,LATS2,PRKAG2,MVP,NTRK3,CBL,DNAJA3,OXR1,MGAT5,EPHA1,IKBKB,LIMK1,PMEPA1,PTPRO,PRKAR1A,SNX6,DMD,SLC8A1,FYN,NF1,PAQR3,BDKRB1,BDKRB2,FRY,PTPRT,IPO5,MAD2L2,GNAQ,ZFYVE28,KLF15,PRKCD,PPM1E,PIBF1,DNAJB2,SLIT2,CORO1C,KDM4B,SMAD6,ZNF675,DUSP22,SMYD3,CAPN3,PIP5KL1,UFL1,CTNNB1,PARK2,PPARG,PRKAR1B,OTUB1,PRKAR2A,MYOCD,PER2,NF2,SFRP1,HEG1,IBTK,LRP5,DCUN1D3,PTPN1,SPAG9,PPM1F,SEMA4D,JARID2,PRKCG,EIF4G1,DEPTOR,TSG101,TERF2IP,PTPN13,LDLRAD4,SIN3A,XDH,ATP2B4,ROCK1,HIPK3,PTPN2,TRIM44,PARD3,GNL3L,BCOR,SPI1,DNMT3B,ATG14,STK38,SNCA,TRIP12,MLLT1,PTEN,SPRED2,BMP7,LRRK2,TMEM59,LILRB4,PKIB,SH3RF2,N4BP1,PRKAR2B,UBR5,EPM2A,GSK3B,DNAJC3,PTPRB,AGT,PRKAA2,ADAR,PARP10,RGS14,EPHB2,PAX6,PRKCZ,ADORA2A,RABGEF1,ANGPT1,LRRK1,MTF2,CDK5RAP1,HDAC2,IMPACT,CD300A,PPP2CA,RPL23,WWTR1,PTPRJ,TAF1,INPP5F,CD109,MLXIPL,PTPRC,SH3GL2,BMP4
GO:0098662	inorganic cation transmembrane transport	0.000027754139752920966	SLC39A11,ASPH,PDE4D,SLC9A1,FAM155A,CHERP,CACHD1,RYR1,NOS1AP,NOX5,DPP6,UTRN,ATP2B2,KCNQ5,KCNIP4,ANK2,SLC9A9,CHD7,HTR2B,RYR3,SLC24A2,ITGB1,SLC24A3,ABCC8,TCIRG1,KCNS3,NIPAL2,NETO2,CACNB2,KCNJ16,DPP10,CACNA1B,STK39,CAB39,ZDHHC13,SLC20A2,SLC4A10,CATSPER2,AMIGO1,KCN

			MA1,KCNRG,LCK,TMC2,SLC12A8,SLC6A16,RHCE,MICU3,DMD,SLC8A1,JPH2,FYN,CACNA1E,SLC4A5,KCNN3,CNNM2,BDKRB1,KCNIP1,STIM1,NALCN,SLC39A14,CATSPER3,FGF14,CLDN16,ANO6,KCNB2,NPSR1,SCN4A,TMC1,SLC9A7,KCNC4,CACNA1H,TGFB1,KEL,JPH3,KCNJ3,CACNA1D,CACNA2D3,KCNH1,SLC17A7,KCNH7,CAPN3,VMP1,COX8A,TRPM1,KCNJ12,RYR2,KCNA6,NOL3,CACNA1C,SCN3B,ITPR1,WWP2,NEDD4,SLC4A4,SLC6A1,KCNK1,VDAC1,IBTK,SLC9B2,DRD1,PRKD1,SLC6A3,FXYD2,FXYD6,KCNG4,FGF2,MRS2,SLC9B1,ATP6V0B,KCNQ1,TRPA1,TRPM3,AKAP6,TRPC5,WNK1,KCNQ2,SLC47A1,SLC8A2,SLC9C1,TPCN1,AHNAK,RGS7,ATP2B4,ATP6V0A2,KCNAB2,CACNA1A,STOM,TRPV1,PLCG2,GPR35,SLC39A10,ANK3,KCND3,CACNG8,SLC9C2,NETO1,MCU,TRDN,STAC,TRPC6,ABCB7,SLC41A2,SNCA,CACNG2,CNGA3,SLC4A8,HTT,SCN8A,ATP6V1A,TMCO1,PKD2,KCNC2,SLC6A14,PSEN2,GRIN1,GRIN3A,ITPR2,SCN9A,PDE4B,COX5A,TRPC4AP,KCNJ15,SLC5A10,PKD1L1,SLC25A18,COX5B,NOS1,ACTN4,SCNN1B,COX6A1,SLC1A1,XCR1,LRRC52,MCOLN1,ATP1A3,GRIN2B,DIAPH1,HTR2C,TMEM163,UQCR10,ANXA2,KCND2,ATP6V0A1,SCN1A,CACNG3,ATP2B3,GPM6A,KCNQ3,SESTD1,CAMK2D,KCNJ6,NEDD4L,PTPRC,EDNRA
GO:0001568	blood vessel development	0.000028548811821188038	NRXN1,NOX5,PTGIS,TJP1,SEMA5A,ENPP2,MAP2K5,LRP2,PIK3CD,NRXN3,ROBO1,CHD7,ROBO2,VAV2,ITGB1,ARHGAP24,RUNX1,ABCC8,EPHA1,HIF3A,MYO18B,NTRK2,CELA1,PDGFB,TCF7L2,NPRL3,MEIS1,SGCD,ADAMTS9,NF1,RTN4,APOD,CHI3L1,UBP1,MAP2K1,STIM1,MYH9,HDAC5,DCN,CDH13,PLXDC1,CCBE1,FBXW7,PKNOX1,PDE2A,WNT11,QKI,TGFB1,PCSK5,BCAS3,SLIT2,MTDH,SMAD6,BMPER,PARVA,ADAM12,FGF10,RORA,PRKCA,CTNNB1,ARHGAP22,PPARG,ANKRD17,CYBB,LEPR,FGF1,MYOCD,MYO1E,CSPG4,FLT4,SFRP1,PTPRM,NRP2,TAB1,PDGFRA,NOX1,YAP1,HEG1,JAK1,ANGPTL4,LRP5,SETD2,PRICKLE1,VASH2,MMP2,PRKD1,STAT1,STRA6,EREG,ATF2,BMPR2,BMPR1A,TSPAN12,PIK3R3,FGF2,ISM1,EPHB3,CLIC4,ADAMTS6,RHOJ,SUFU,COL4A3,DDAH1,APOLD1,SP1,LUZP1,XDH,OVOL2,ATP2B4,TMEM2,GPC3,CALD1,EYA1,HOXB3,SMOC2,ADIPOR2,ROCK1,PAXIP1,EPHB1,AP2B1,SPI1,RAPGEF3,PTK7,HEY2,WNT7B,ADTRP,RASA1,HIPK1,MAPK1,PTEN,MIB1,BMP7,PRCP,PKD2,AMOTL1,WNT7A,SOS1,RAPGEF2,SMO,RHOA,ACTA2,PTPRB,SULF1,AGT,PTK2,SHB,SHC1,CD160,ALOX5,ADAM8,THSD7A,CXCL17,STK4,EPHB2,SLC1A1,IL18,CALCRL,NOTCH4,PAX6,COL22A1,TMIGD2,ANGPT1,ETS1,ANGPT4,SP100,ANXA2,SYK,SRPK2,SASH1,VAV3,CCR3,SEMA3C,RNF213,EDNRA,BMP4
GO:0010941	regulation of cell death	0.000029811335691531447	PRDX2,PRKCI,MAP4K4,DNMT1,TMBIM4,SLC9A1,PDE8A,IL31RA,GRID2,RPS6KA2,PTGFR,LATS2,ANP32A,PTGIS,TJP1,UNC5C,PRLR,NTRK3,CBL,EGLN2,SEMA5A,FLT3,STAT5B,MAP2K5,KITLG,LRP2,PIK3CD,OLFM1,TNFAIP8L1,HTR2B,MECOM,LMNA,TRAF6,ITPKB,DNAJA3,OXR1,GRK5,ITGB1,HSP90AA1,TSC22D3,OMA1,HDAC6,DDB1,ADORA1,IKBKB,ERBB4,GBE1,MRE11A,ESR1,NTRK2,BRMS1,FMN2,TCF7L2,EGLN3,BDNF,TFAP2A,KCNMA1,TRIM13,LCK,MDM4,ECT2,SNX6,TOX3,GSN,FYN,NF1,MGMT,SNCB,RTN4,DPEP1,NRG1,ARHGAP10,CDK19,BDKRB2,DOCK8,BID,FNIP1,TMBIM6,CSRNP3,RBM5,COPS5,ANO6,CERKL,NEUROD1,RFFL,JAK2,FBXW7,ITCH,BCL11B,TEX11,ANXA4,WNT11,SIAH1,PRKCD,TGFB1,SGK1,DUS2,CRADD,SLIT2,TP73,HP,HPR,SAP18,RRAS2,MTDH,FANK1,SMAD6,TFAP2D,BCL3,DAPK2,TNFRSF10B,NAIP,FGF10,CAPN3,PRKCA,CNR1,MAGEA4,MAG,PIP5KL1,UFL1,CTNNB1,PARK2,SOD2,FCGR2B,IGF1R,PPARG,DLG5,DRAXIN,NR4A3,NOL3,MYOCD,KIR3DL2,CHEK2,HCK,CAPN2,DCC,NF2,FLT4,SGMS1,HDAC4,PAX2,SFRP1,FOXO3,BCL2L13,SMAD3,GRIK5,TXNDC12,CDC73,SLK,NOX1,YAP1,VDAC1,EYA2,ANGPTL4,LRP5,CHST11,TMEM14A,FRS2,PALB2,MMP2,DCUN1D3,PRKD1,STAT1,TCTN3,ATF2,RAF1,ACOX2,CARD16,CASP1,PTPN1,CAMK1D,BTBD10,FGF2,SEMA4D,RORC,ADCYAP1R1,YME1L1,RILPL1,RARB,PRKCG,NCOA1,AREL1,EEF1E1,TXNDC5,EIF2B5,EIF4G1,ATF3,PAWR,DEPTOR,P4HB,CNTFR,HIP1,GLI2,DAB2,PKHD1,SIN3A,PELI1,CFDP1,ALK,INPP5D,SRA1,PAX7,PACRG,PAK3,ADNP,CELF1,RNF34,TFRC,WWOX,GFRAL,EYA1,TRPV1,GHR,MNAT1,HDAC1,TRIM24,PLCG2,ROCK1,HIPK3,RYBP,CAPN10,PTPN2,NTRK1,TMIGD1,SLC39A10,EPHB1,AXIN2,SARM1,MUC1,AP2B1,EPHA7,CTNNA1,AIFM2,MST1,ERCC3,SNCA,BMPR1B,GRM4,USP42,HEY2,ZMYND11,CAST,DFFA,RASA1,MITF,SRSF6,NUGGC,BTK,IGF1,CTNNBL1,HTT,PTEN,BMP7,LRRK2,OSGIN1,BAG6,ITGAM,WNT7A,ZBED6,NLRP1,GPI,SH3RF2,PRMT2,RAPGEF2,SMO,MARK4,PSEN2,RHOA,SYNGAP1,HSPD1,GSK3B,DNAJC3,EEF2K,CD27,BOK,RNF144B,NACC2,TNFRSF8,AGT,PRKAA2,ADAR,PTK2,FBXO10,MAP3K5,DLX1,SHC1,CD160,RPS6KB1,DNAJB6,CD44,ADAM8,BCL2L14,ACTN4,STK4,ZFAND6,SPEF1,SLC1A1,SIGMAR1,HCAR2,DLC1,PPARA,PPP1R10,PHLPP1,CPEB4,PRKCZ,ADORA2A,GRIN2B,ANGPT1,EYA3,FHL2,ATF6,ETS1,PPP1CA,GRIA4,STPG1,PRKCQ,BRAF,HDAC2,IMPACT,POU3F3,HIGD2A,PHIP,ANGPT4,SP100,MADD,SYK,GRIK2,HRK,HSF1,INPP5A,SRPK2,ANKRD13C,QRICH1,VAV3,NUAK2,AXL,TNFSF9,MET,CAMK2D,MALT1,KDM2B,PHB,PTPRC,TYRO3,ADCY10,TRAP1,CLNK,PRAP1,BMP4,CASP12
GO:0070727	cellular macromolecule localization	0.000030847987952146786	CD247,DNAH11,ZDHHC14,BLZF1,GPC5,NRXN1,ASPH,PRKCI,CLASP2,GRID2,CLDN18,CTDSPL2,LATS2,ASTN2,EPB41,DPP6,MAPRE2,SEC23B,SNX31,LHFPL4,FAM53A,KCNIP4,TNPO3,HDGF,ANK2,EZR,XRCC4,TOM1L1,TENM1,KIF5C,LMNA,SRP72,NFASC,DNAJA3,ITGB1,HSP90AA1,FAM126A,CNGB1,DLG2,PTPRK,STXBP4,EFCAB7,TCIRG1,PTPN11,CCT2,CACNB2,HDAC6,GOLGA4,ADORA1,IKBKB,PEX14,ERBB4,GBF1,TLK1,ESR1,DPP10,STX18,FNTA,HOOK3,VPS45,RIMS1,TNIK,SNX2,MID1,H2AFY2,TCF7L2,BDNF,ECT2,SNX6,CHML,PTPRU,RFTN1,AP3S1,NPLOC4,D

			MD,RANBP17,KAT7,GSN,RIT2,FYN,NF1,STX6,RTN4,GOLGA2P5,APOD,BCR,PAQR3,PIK3R2,KLHL21,BID,RAPGEF6,TMEM30A,SYNE1,SCFD2,ZDHHC3,LIN7A,SCFD1,IPO5,MAN1A1,RAB27A,RAB3C,TERF2,TMED6,GOLPH3L,RCC2,ARL3,IPO11,ZDHHC15,JAK2,FBXW7,KCNB2,OAZ2,SKAP1,CROCC,SNAP25-AS1,PLS1,SEPT7,TTC7B,LAMTOR3,KPNA3,WNT11,IFT80,IMMP2L,PRKCD,CD4,TGFB1,BANP,NUP93,PIBF1,BCAS3,CELSR1,DISC1,ZFAND2A,GRPEL2,ANK1,TTBK2,CHRM1,BCL3,DCLK1,RABGAP1L,FGF10,CNTNAP2,CIZ1,CAPN3,SNX8,PPP3CA,TRAK1,CTNNB1,PARK2,RN7SL832P,TSNARE1,SYTL5,ANXA13,SREBF2,RYR2,LEPROT,WDR45B,AJUBA,SCN3B,SORBS1,TBC1D14,RAB3GAP2,TBCK,TRIM8,OBSCN,NF2,SPTBN1,AP3B2,ARL13B,IFT122,RANBP3,SMAD3,RAB6A,WIPI1,MTBP,VPS39,GRIK5,LZTFL1,NEDD4,KPNB1,RAB6C,SEC61B,YAP1,RAB11FIP5,NLGN2,SSR2,NVL,SETD2,ZDHHC6,GPC6,PRICKLE1,SPTBN4,DRD1,PRKD1,TAF3,VPS4A,PITRM1,ATF2,PTPN1,AP1B1,NLGN1,SNX16,TAF8,RAB11A,IPO9,RAB24,XPO6,RILPL1,SORCS2,RAB11FIP3,GAS8,AP2M1,TNPO1,TECPR2,LMBRD1,TSG101,DESI1,VCL,TERF2IP,SUFU,KIF3A,AKAP6,CEP350,PADI6,TNKS,RUFY3,DAB2,TRAM2,DZIP1,DLG3,RELN,SIN3A,RUVBL2,FAM91A1,ZNF423,MACF1,MYO7A,GPM6B,SNX5,ATP2B4,NSF,PACRG,SYNE3,PACSIN1,ATAD1,NXT2,KCNAB2,TFRC,GPC3,STOM,TBC1D5,SNX9,RPGR,ARL5A,ROCK1,EGR2,AP3B1,KPNA6,ZFAND1,MAP1A,ANK3,PTPN9,SNUPN,AXIN2,PARD3,GNL3L,PTPN14,TUB,CACNG8,AP3D1,AP2B1,ARHGAP44,SNX33,SPI1,VPS16,CTNNA1,STAC,RAB15,RAPGEF3,CACNG2,MAGI2,TBC1D9,HEY2,RAB5B,ABCA12,PKP2,TBC1D16,XPO4,VPS41,STX8,IFFO1,BMP7,PIK3C3,NUP88,LRRK2,SEPT6,TMEM59,SPAG5,BAG6,LILRB4,MORC3,ITGAM,SHANK3,MIA2,SGSM1,RABEP1,SH3PXD2B,POM121C,ATRX,SAMM50,RAPGEF2,SMO,GET4,SUN1,MARK4,UBR5,BBS9,SYNGAP1,HSPD1,TSPAN33,EPM2A,GSK3B,RDX,CCDC14,NECAB2,AGT,ADAR,PEX5L,EXOC6B,RIMS2,TOMM5,COPG2,CPE,SEC16B,TIMM44,GTF2IRD2,NCF1,TAOK2,ZDHHC23,ITGAL,DNAJB6,SLC5A3,ARFGAP3,PACSIN2,PEX7,GCKR,STK4,ZFAND6,EPHB2,SLC1A1,WLS,GPSM2,PPP1R10,GRTP1,KPNA4,SYTL4,PAX6,PRKCZ,RABGEF1,WDR83OS,OTUD7B,NIPBL,ANGPT1,PACS2,FRMPD1,OSBPL8,EPB41L2,HACL1,TBC1D10C,ATG3,BRAF,DIAPH1,SIL1,CLTB,EMC3,SKP1,FAM149B1,RPL23,SH3BP4,CAMSAP3,SP100,VTI1A,TRIM29,PIGU,RAB27B,ITSN1,NUP214,WIPI2,NUMB,SYK,WWTR1,GRIK2,MYRIP,SEC31B,BRCA2,CACNG3,DVL2,YBX1,ANKRD13A,GRIP1,ANKRD13C,GBP5,MCM8,MLPH,DENND4C,TBC1D10A,SNX1,ZDHHC11,ZDHHC11B,EHD2,TRAPPC8,CCT3,NEDD4L,RPH3A,CTCF,RAB28,SYNDIG1,TSPAN5,TRAF3IP2,SGTB,SHISA6,EDNRA,BMP4,ABLIM3
GO:0097479	synaptic vesicle localization	0.0000322690523628655 8	NRXN1,KIF5C,CNIH2,PCDH17,AP3S1,LIN7A,SYN2,RAB27A,SYN3,CTNNB1,PARK2,AP3B2,SPG11,NLGN2,NLGN1,BLOC1S5,AP3B1,TRIM46,AP3D1,SNCA,PTEN,LRRK2,BLOC1S3,SYN1,BLOC1S6,MAP2,SYNDIG1
GO:0048598	embryonic morphogenesis	0.0000332133810458620 5	CLRN1,HLX,LMBR1,CLASP2,MAP2K5,LRP2,SP3,MFAP5,CHD7,HTR2B,TRAF6,ITGB1,TRIOBP,ATP8A2,CECR2,NPHP3,CDON,PRKAR1A,LAMA3,STRC,TFAP2A,TGIF2,PHACTR4,TENM4,MYO3B,AFF3,SATB2,HIRA,PCDH15,BCR,NDRG4,NTN1,MDFI,VAX2,ROR2,EXOC4,NEUROD1,COL12A1,LRIG3,PLS1,TBX15,WNT11,ACVR2A,RUNX2,TGFB1,MYO3A,C2ORF49,RPGRIP1L,CELSR1,CDH23,FGF10,CTNNB1,AXIN1,RYR2,NR4A3,CACNA1C,PHLDB1,MMP16,NF2,PAX2,SFRP1,ARL13B,IFT122,SMAD3,HOXD3,HOXD4,CDC73,PDGFRA,YAP1,EYA2,LRP5,SOX2,SETD2,TTC39C,CHST11,PRICKLE1,FRS2,MMP2,KDM6A,HNF4A,STRA6,DSCAML1,BMPR2,BMPR1A,FGF2,RARB,NCOA1,DVL3,LMBRD1,LAMB1,COL11A1,KCNQ1,WNT3,SUFU,GLI2,WNK1,RSPO2,LUZP1,MYO7A,CLASP1,OVOL2,GPC3,EYA1,HOXB3,HOXB4,HOXB5,HOXB6,HDAC1,ROCK1,PTPRQ,JAG2,FBXW4,SHROOM3,PTK7,WNT7B,HIPK1,MAPK1,MIB1,BMP7,LHFPL5,PKD2,SHANK3,WNT7A,GPI,SOS1,TSHR,EXT1,SMO,CUL3,SULF1,MTHFD1L,FBN2,PBX1,CREBBP,ECE1,LBX2,SETDB2,DNAJB6,STK4,EPHB2,WLS,DLC1,SOBP,FBN1,PAX6,PHLDB2,NIPBL,HDAC2,NOTO,SPINT2,DVL2,YBX1,RNF2,GNA12,KDM2B,EDNRA,BMP4
GO:0006935	chemotaxis	0.0000373858500209178	NRXN1,SEMA3A,NRG3,SEMA3D,MYOT,UNC5C,KALRN,NTRK3,SEMA5A,ENPP2,FER,PIK3CD,NRXN3,ROBO1,KIF5C,ROBO2,NFASC,ELMO2,DSCAM,TRIO,LAMA2,EPHA1,GBF1,PTPRO,PDGFB,STK39,UNC5D,LAMA3,CDH4,TBR1,BDNF,LSP1,FYN,DPEP1,CCDC141,ADAMTSL1,NTN1,NRG1,MAP2K1,CNTN4,CMKLR1,DOCK2,CDH13,DEFA1B,BOC,ANO6,MYPN,BCL11B,SIAH1,PRKCD,SEMA5B,MMP28,F2RL1,SLIT3,SLIT2,LAMC2,DAPK2,PARVA,YTHDF1,FGF10,ENAH,EPHA4,TNFSF11,DRAXIN,FGF1,DCC,CNTN6,NFIB,SMAD3,PTPRM,ACKR2,NRP2,PREX1,DOCK4,PRTG,APP,PDGFRA,S100A12,PRKD1,DSCAML1,SEMA6D,BMPR2,USP33,DPYSL2,CAMK1D,CRMP1,FGF2,PPM1F,SEMA4D,PLXNA2,PTAFR,EPHB3,WNT3,CCL14,CCL15,CCL15-CCL14,GLI2,NEO1,WNK1,RELN,CSF3R,EPHA5,FLRT2,SMOC2,EGR2,ABCC1,PTPN2,NTRK1,EPHB1,EPHA10,SPI1,EPHA7,MCU,MST1,PTK7,BMPR1B,PLD1,MAPK1,BMP7,EFNA5,MYCBP2,SOS1,EXT1,SMO,RHOA,FAM19A4,EMB,FAM49B,PTK2,PDE4B,GAP43,ECE1,ALOX5,ADAM8,RPS6KA5,CXCL17,TUBB3,EPHB2,XCR1,GPSM3,PAX6,ANGPT1,BIN2,CCL22,PRKCQ,TNR,TMEFF1,SYK,SLIT1,PTPRJ,VAV3,MET,CCR3,SEMA3C,NCAM1,EDNRA,BMP4
GO:0051336	regulation of hydrolase activity	0.0000379690559295494 46	ASPH,MAP4K4,C6ORF89,TIAM2,MAPRE2,KALRN,NTRK3,MAP2K5,ROBO1,HTR2B,CDKL5,DNAJA3,VAV2,ITGB1,ARHGAP24,RGS6,DGKI,MGAT5,DOCK10,EPHA1,IKBKB,ABR,ESR1,NTRK2,FNTA,EGLN3,ARHGAP6,LCK,ECT2,ARHGAP42,CST2,GSN,FYN,NF1,MGMT,DPEP1,RTN4R,BCR,DOCK8,BID,FNIP1,RAPGEF6,TMBIM6,SBF2

			,ITIH2,SLC39A14,PPP1R42,RGS8,TERF2,RCC2,GNAQ,RFFL,DENND1A,JAK2,ARAP2,DOCK9,CYFIP2,WNT11,SIPA1L3,PRKCD,PCSK5,CRADD,F2RL1,BCAS3,ITIH4,CORO1C,BLID,TIMP2,RABGAP1L,NAIP,RASA4,RASA4B,EPHA4,ARHGAP22,PPARG,GARNL3,LEPR,PROS1,RGS10,NOL3,AJUBA,CSTL1,TBC1D14,RAB3GAP2,TBCK,PAX2,SFRP1,PPP6R2,ARHGAP29,BCL2L13,SMAD3,SIMC1,PSAP,PREX1,APP,PDGFRA,ANGPTL4,SOX2,RCAN1,PRKD1,ST18,GRAMD4,RAF1,CARD16,CASP1,FGF2,PPM1F,NEDD9,SEMA4D,PLXNA2,PTAFR,ADCYAP1R1,SPOCK1,DVL3,EPHB3,MYO9A,CCL14,CCL15,PAFAH1B1,COL4A3,NMUR2,HIP1,RASAL1,WNK1,DOCK11,IQGAP1,ARHGAP12,XDH,PRKG1,RGS7,CCPG1,RNF34,EPHA5,GPC3,TBC1D5,FGD1,SNX9,HDAC1,ROCK1,GAPVD1,NTRK1,SLC39A10,ANXA8L1,ARHGAP44,LPA,EPHA7,BCAR3,RENBP,RAPGEF3,SH3BP1,SNCA,MAGI2,TBC1D9,USP50,PSMF1,CAST,DFFA,RASA1,TBC1D16,HTT,RASA2,LRRK2,ARHGAP25,EFNA5,SERPINA3,SERPINA4,SERPINA5,SGSM1,NLRP1,UACA,GPI,RAPGEF2,VRK3,GRIN1,RHOA,SYNGAP1,HSPD1,EPM2A,GSK3B,RDX,CD27,BOK,AGT,PTK2,MAP3K5,IQGAP2,PCSK6,ARHGAP15,SERPINB11,DNAJB6,CD44,FGD3,CLDN4,FGD4,NOS1,RGS14,SIPA1L2,ARHGAP11A,SLC1A1,DLC1,GRTP1,PRKCZ,ADORA2A,GRIN2B,COL28A1,TBC1D10C,CCL22,SPINT2,CD300A,SH3BP4,ANXA2,SRGAP2,SYK,RAP1GAP2,DVL2,TANK,HSF1,ASAP1,RALGAPA1,VAV3,CAV2,GNA12,CD109,MET,TBC1D10A,CAMK2D,UCHL5,MALT1,SERPINE3,PHB,PTPRC,EDNRA
GO:0060996	dendritic spine development	0.000039071470544910394	PRMT3,LLPH,KALRN,DOCK10,HDAC6,TANC2,CAPRIN2,ZDHHC15,CAMK2B,CTNND2,DISC1,EPHA4,DLG5,DNM3,CUX2,ABI2,SHANK1,FSTL4,NLGN2,NLGN1,EPHB3,PAFAH1B1,RELN,PAK3,ACTR2,EPHB1,ARHGAP44,SDK1,PTPRS,PTEN,LRRK2,SHANK3,WNT7A,GRIN3A,EEF2K,EPHB2,HDAC2,SRGAP2,ASAP1
GO:0003008	system process	0.0000398429022411643 8	CLRN1,DNAH11,ADAMTS16,NRXN1,ASPH,SLCO3A1,PDE4D,PDCL,S1PR2,SLC9A1,PBX3,ADCY8,SPAG16,SEMA3A,GRID2,TAS1R2,RPS6KA2,PRDM12,RYR1,NOS1AP,EPB41,MYOT,NLGN4X,TJP1,GRM8,OR5P2,OR5P3,CLCN1,FTO,UTRN,KALRN,ATP2B2,GRM5,PLCE1,LHFPL4,LRP2,ANK2,EZR,NRXN3,CHD7,SLC26A6,HTR2B,RYR3,LMNA,LPO,NFASC,CTNNA3,SLC24A2,ITGB1,TRIOBP,SLC24A3,CNIH2,SLC22A8,CNGB1,AKAP13,ABCC8,DGKI,THRB,PDE4A,GABRA3,IMPG2,PTPN11,CACNB2,ADORA1,ADRA1D,ATP8A2,GRM7,PTPRO,NTRK2,EP300,PDGFB,RIMS1,STK39,INSR,ATP8B1,HPS1,MYOM1,CNTN5,SLC4A10,TBR1,STRC,BDNF,TFAP2A,KCNMA1,CHRM3,OR2T3,TENM4,PKHD1L1,TMC2,MYO3B,NAV2,ARHGAP42,CST2,SGCD,DMD,SLC8A1,GSN,FYN,NF1,PLCB1,PCDH15,AFF2,SLC5A6,BCR,PPP1R12B,SHISA9,TTN,NDRG4,DCTN1,SLC4A5,BDKRB1,BDKRB2,VAX2,OR6N2,PPIP5K2,LHFPL3,F5,CLDN16,NEUROD1,CCBE1,JAK2,KCNB2,MLIP,NPSR1,ABCC2,SCN4A,RBFOX1,TMC1,SLC1A4,PLS1,PDE2A,KLF15,FIG4,IMMP2L,NOX4,CACNA1H,CAMK2B,SEMA5B,SGK1,PCSK5,MYO3A,KEL,JPH3,DLGAP1,F2RL1,SLIT2,GABRR3,GABRB1,CDH23,OR9A4,KCNJ3,CACNA1D,CLN6,CHRM1,OR51A7,OR51F2,OR51T1,OTOGL,ANKFN1,HOMER2,OTOA,YTHDF1,FGF10,CNTNAP2,RHPN2,SLC16A1,LUM,PRKCA,CNR1,PPP3CA,OR51B2,OR51B4,OR51I1,MAG,CAMK4,PARK2,SOD2,PPARG,IGDCC3,AXIN1,PRKAR1B,TMEM63C,CELF2,TRPM1,KCNJ12,RYR2,LEPR,NR4A3,NOL3,MYOCD,OR52E6,OR52E8,OR52N1,PER2,CACNA1C,SCN3B,MYO1E,CSMD1,GRM1,CTDP1,FLT4,HDAC4,PAX2,DNAH9,ELN,FOXO3,TRHDE,SMAD3,CUX2,ITPR1,SNTB1,GRIK5,SLC16A2,SHANK1,OR14K1,DOCK4,SLC4A4,SLC6A1,APP,GSX2,AOC2,NOX1,YAP1,HEG1,AMFR,RAB11FIP5,PPCS,NLGN2,RNLS,PDE6D,TNNI3K,LRP5,RCAN1,SSPN,SPTBN4,DRD1,GLRA2,MMP2,KDM6A,STAT1,SLC6A3,USP53,SHANK2,STRA6,ABLIM1,MYOF,SLC16A12,CELF4,SRRM4,BMPR2,NLGN1,CTNNA2,POU6F2,OR6C70,ADCY5,PTAFR,JARID2,KLHL3,RAB11FIP3,PRKCG,XPNPEP3,MYO9A,CRYM,COL11A1,KCNQ1,NLGN3,PAFAH1B1,COL4A3,TRPA1,DDAH1,TRPM3,NMUR2,AKAP6,BTBD9,MGLL,DAB2,USH2A,SETD5,WNK1,RELN,NEK10,ABCB1,OR5H2,OR5H6,OR5K4,EYS,SLC22A3,TAS2R41,OR56B1,MYO7A,SLC8A2,KLK2,DSC2,CLIC5,SNX5,OR6C74,PRKG1,ATP2B4,CAMTA1,P2RX6,ATAD1,ADNP,SCARB1,TYR,TFRC,SORCS3,GABRG3,MEF2A,ADRBK1,CALD1,EYA1,PTGER3,BRINP1,GNB3,TRPV1,RPGR,ACTR2,ELAVL4,ROCK1,C1QTNF1,EGR2,RNF10,GPR35,ABCC1,AP3B1,NTRK1,MYBPC2,MAP1A,ANK3,PTPRQ,EPHB1,PDE6A,PARD3,TUB,JAG2,KCND3,CACNG8,NETO1,COCH,HTR1D,LPA,HMCN1,CHRNB4,LIMA1,TRDN,GABRA6,STAC,RENBP,SNCA,CACNG2,HEY2,CNGA3,OPRD1,PKP2,SGCZ,SLC4A8,IGF1,HTT,MAPK1,PTEN,SCN8A,LHFPL5,PRCP,LRRK2,UNC119,SLC44A1,TMEM108,SHANK3,RGS9,SERPINA3,WNT7A,BMP6,MYCBP2,ANO1,GPI,EXT1,GABRR2,ABCG2,PRKAR2B,MYLPF,GRIN1,BBS9,RHOA,OR4M1,OR4N2,SYNGAP1,GRIN3A,ROR1,FAM19A4,EPM2A,GABRB3,GSK3B,ABCG8,ACTA2,SCN9A,SULF1,ZNF354A,AGT,ESPNL,ZMPSTE24,PDE4B,RIMS2,CDC14A,SLC29A1,UGT1A7,ALDOA,ECE1,RPS6KB1,GUCY2F,SLC5A3,CLDN4,NOS1,PLVAP,RGS14,MC1R,SCNN1B,EPHB2,SLC1A1,DTNA,HCAR2,PPARA,CALCRL,SOBP,CRX,PAX6,PRKCZ,ATP1A3,ADORA2A,GRIN2B,NIPBL,ANGPT1,TBL1X,EYA3,ATF6,SHROOM4,SLCO2B1,KIFC3,BRAF,DIAPH1,HDAC2,HTR2C,MYOM3,ATP8A1,POU3F3,TNR,ATXN1,OPN1LW,VTI1A,KCND2,ADCY1,MOGAT2,WWTR1,GRIK2,SCN1A,CACNG3,PTPRJ,ATP2B3,MYO1A,TAC3,SLC13A3,DLGAP2,GNA12,SYNM,INPP5F,TMPRSS3,CAMK2D,SETD3,NEDD4L,PBLD,SH3GL2,TANC1,CREB1,SHISA6,EDNRA,PRAP1,BMP4
GO:0048584	positive regulation of response to stimulus	0.0000424652463190052 4	CD247,PRDX2,GPC5,NRXN1,PRKCI,MAP4K4,PDE4D,PDCL,HLX,SLC9A1,PDE8A,SEMA3A,SETD4,CHERP,WWC1,C12ORF49,NOS1AP,MAPRE2,PTGIS,PRLR,PAGR1,NTRK3,CBL,ARNT,SEMA5A,FLT3,SUSD4,STAT5B,GRM5,PLCE1,MAP2K5,KITLG,LRP2,PIK3CD,SH2D1A,EZR,ROBO1,HTR2B,TENM1,TRAF6,ROBO2,ITPKB,VAV2,IT

			GB1,HSP90AA1,PSMB7,CCDC22,DSCAM,AKAP13,DGKI,PTPN11,MGAT5,HDAC6,ADORA1,ADAMTS3,IKBKB,ERBB4,ADRA1D,LIMK1,KLHL6,MIER1,CDON,NTRK2,EP300,RNF220,PDGFB,RIMS1,TNIK,MID1,ALOX5AP,STK39,INSR,FMN2,TCF7L2,USP34,PAK1,LITAF,MAP3K4,ZDHHC13,BDNF,TRIM13,LCK,ECT2,VAMP7,RFTN1,ERN2,USP13,RBM14,RIT2,DEPDC1B,CASS4,FYN,ARNTL,NF1,PLCB1,MGMT,RTN4,RTN4R,RWDD3,CHI3L1,NDRG4,BMP2K,NRG1,CDK19,BID,MAP2K1,FNIP1,CMKLR1,ROR2,DCN,SLC39A14,CDH13,MAD2L2,CAPRIN2,ANO6,CCBE1,JAK2,FAM168A,FBXW7,SKAP1,UIMC1,NPSR1,CLDN1,MLLT3,PDE2A,LAMTOR3,CYFIP2,WNT11,NOX4,SIAH1,ERCC8,PRKCD,TAB2,ACVR2A,CD4,NUP93,PIBF1,CRADD,F2RL1,AAK1,SLIT2,TP73,DISC1,KANK1,MTDH,CLOCK,BMPER,DAPK2,TNFRSF10B,DUSP22,NAIP,FGF10,IQCJ-SCHIP1,SH3RF3,LOXL3,SMURF2,EPHA4,PRKCA,AUTS2,CNR1,TNFSF11,PPP3CA,CTNNB1,PARK2,FCGR2B,IGF1R,PPARG,AXIN1,DLG5,IL18R1,IL1RL1,P2RY10,ANKRD17,APOL3,FGF1,NR4A3,MYOCD,TRIM5,KIR2DL4,AJUBA,HCK,SORBS1,TRIM8,CSPG4,GRM1,FLT4,SFRP1,FOXO3,CNTN6,SMAD3,RNFT2,CUX2,RNF168,C1S,SHANK1,NEDD4,TAB1,CDC73,APP,GSX2,PDGFRA,NOX1,YAP1,NLGN2,TNFRSF19,EYA2,POLR3G,SOX2,CHUK,FRS2,S100A12,PRKD1,EREG,RAF1,ACOX2,CARD16,CASP1,PTPN1,BMPR2,CAMK1D,BMPR1A,NLGN1,SPAG9,EVC,MYB,FGF2,PPM1F,SEMA4D,DOK6,PTAFR,ADCYAP1R1,DDX58,DKK2,PRKCG,EEF1E1,EEF1E1-BLOC1S5,GAS8,DVL3,EIF2B5,ATF3,SHOC2,PAWR,AGO3,TERF2IP,WDR59,WNT3,CCL14,CCL15,NLGN3,SCUBE2,PAFAH1B1,PRDM15,HIP1,AKAP6,NR2C1,NEO1,TNKS,SCUBE1,C9,DAB2,THEMIS,WNK1,RELN,NEK10,SIN3A,RSPO2,GUCY1A2,PELI1,IQGAP1,MAP3K7,ZNF423,TRIM22,MACF1,ALK,INPP5D,UBE2V1,XDH,SNX5,NFATC2,PAK3,MASP1,CAMTA1,P2RY8,TFRC,WWOX,CR1L,GFRAL,GPC3,PSMB2,EYA1,PTGER3,TSPAN6,CLEC16A,GHR,ACTR2,C2,CFB,SMOC2,PLCG2,ROCK1,C1QTNF1,PAXIP1,GPR35,ABCC1,PTPN2,NTRK1,TRIM44,SLC39A10,JAG2,VWF,NETO1,COCH,ARHGEF3,SPI1,MCU,CTNNA1,PSPC1,LY86,BCAR3,RBPMS,MAP3K13,PTK7,SNCA,BMPR1B,GRM4,ZEB2,RC3H1,WNT7B,KMT2D,RPTOR,BTK,IGF1,HTT,MAPK1,PTEN,SPRED2,BMP7,PUM1,LRRK2,ZRANB1,BAG6,LILRB4,TMEM108,ITGAM,PKD2,SHANK3,PDGFC,WNT7A,PLCL2,SIK3,BMP6,SH3RF2,SOS1,RAPGEF2,SMO,UBR5,GRIN1,RHOA,ROR1,RQCD1,HSPD1,NFKBID,PLD2,STAT6,ACTA2,FAM49B,NECAB2,CD27,BOK,SULF1,NACC2,AGT,PTK2,LAT2,PDE4B,RIMS2,TEC,EXOSC3,MAP3K5,CREBBP,NCF1,TAOK2,SHC1,CD160,LBX2,RNFT1,SCEL,CD44,ADAM8,CLDN4,BCL2L14,CXCL17,PLEKHA1,ACTN4,MC1R,STK4,WLS,IL18,UBE2K,GPSM3,PRKCZ,ZC3HAV1,ADORA2A,RABGEF1,ANGPT1,TBL1X,EYA3,LRRK1,OSBPL8,ATF6,GCNT2,ETS1,PPP1CA,CFI,CCL22,BTN3A2,PRKCQ,BRAF,HTR2C,CD300A,PHIP,MAOA,RPL23,CR2,MADD,SYK,DVL2,PTPRJ,TICAM1,CADM1,SASH1,GBP5,FCHO1,VAV3,ZP3,CAV2,AXL,MET,TIMELESS,UCHL5,ANKRD6,MALT1,PHB,PTPRC,EDA,GPR89A,TSPAN5,TRAF3IP2,CLNK,BMP4,CPNE1
GO:0042592	homeostatic process	0.000044953291188089396	CLRN1,ENPP1,PRDX2,LDB2,ASPH,PDE4D,SLC9A1,FAM155A,ADCY8,IL31RA,PTGFR,CHERP,CLDN18,RYR1,NOX5,PTH2R,KSR2,TJP1,PRLR,FTO,ACOXL,ARNT,EGLN2,FLT3,STAT5B,ATP2B2,GRM5,PLCE1,KITLG,SPNS2,PIK3CD,SP3,ANK2,MALRD1,SLC9A9,CHD7,SLC26A6,HTR2B,ITGB6,RYR3,GOT1,TRAF6,ITPKB,DNAJA3,SLC24A2,ITGB1,SLC24A3,TSC22D3,CCDC22,CNGB1,OMA1,ABCC8,HMGN3,STXBP4,LAMA2,TCIRG1,PTPN11,CACNB2,DOCK10,DDB1,ADORA1,GPRASP2,ADRA1D,CNNM1,LARGE,ESR1,NPHP3,CYB561A3,STK39,INSR,TCF7L2,ESRRB,SLC4A10,CLDN12,KCNMA1,LCK,SLC12A8,ACOX1,LAMC1,MB,SGCD,MICU3,DMD,SLC30A9,KAT7,SLC8A1,JPH2,FYN,ARNTL,NF1,PCDH15,RTN4,BCR,PIK3R2,DCTN1,SLC4A5,CNNM2,BDKRB1,BDKRB2,STIM1,TMBIM6,CMKLR1,DCN,SLC39A14,PTGER2,CLDN16,ANO6,NEUROD1,JAK2,FBXW7,CROCC,NPSR1,BBS12,PKNOX1,CLDN1,SLC9A7,PNPLA1,KLF15,NOX4,ACVR2A,TGFB1,KEL,JPH3,PTPRN2,F2RL1,C9ORF47,DNAJB2,CDH23,DISC1,CLN6,XPR1,TMTC2,ZNF675,ANKRD54,HOMER2,CAPN3,SLC16A1,RORA,PRKCA,CNR1,TNFSF11,PPP3CA,UFL1,CTNNB1,PARK2,SOD2,IGF1R,PPARG,IL18R1,MTF1,P2RY10,TRPM1,OTC,XK,SREBF2,RYR2,LEPR,NR4A3,FOXK2,NOL3,PER2,CACNA1C,SCN3B,PRDM16,CSMD1,DIO2,GRM1,PAX2,MED13,FOXO3,SMAD3,ITPR1,ACKR2,GRIK5,ESRRG,SLC4A4,APP,PDGFRA,NOX1,YAP1,RAB11FIP5,IBTK,ALPL,ANGPTL4,PEMT,LRP5,BDH2,SLC9B2,DRD1,PRKD1,STAT1,HNF4A,RAF1,ACOX2,FGF2,ADCY5,ADCYAP1R1,KLHL3,SLC9B1,EHMT1,EIF4G1,EBF2,VCL,TERF2IP,CLIC4,IDE,KCNQ1,CCL14,CCL15,TRPA1,NMUR2,AKAP6,BTBD9,TRPC5,NEO1,WDTC1,PKHD1,USH2A,WNK1,SIN3A,DOCK11,ZNF423,ALK,SLC8A2,INPP5D,STAU1,SLC9C1,TPCN1,SNX5,ATP2B4,ASGR2,ADNP,ARID4A,SCARB1,CDK6,ATP6V0A2,P2RY8,TFRC,EPHA5,CACNA1A,SGIP1,PTGER3,GNB3,TRPV1,HOXB6,ACACA,TRIM24,ADIPOR2,PLCG2,C1QTNF1,GPR35,AP3B1,PTPN2,MAP1A,SLC39A10,ANK3,TMEM199,ATP13A3,TUB,AP3D1,SLC9C2,SPI1,MCU,LIMA1,TRDN,LRRC8D,ILDR2,TRPC6,ABCB7,ATP13A5,SNCA,RC3H1,WNT7B,ABCA12,SGCZ,SLC4A8,HTT,MAPK1,SLC30A7,PRCP,LRRK2,ATP6V1A,ZBTB20,TMCO1,PKD2,EFNA5,SHANK3,PDGFC,SERPINA3,STEAP4,PPP2R3C,ZBED6,CRTC3,PLCL2,GPR21,RHOT1,BMP6,ANO1,GPI,SOS1,TSHR,EXT1,IKZF1,PNPLA3,SMO,RALY,STXBP5L,TNFRSF11B,BPGM,DECR1,TFE3,GRIN1,TF,ITPR2,ABCG8,STAT6,ALAS2,BOK,AGT,ELOVL3,PRKAA2,ADAR,FGGY,HRH4,HEPHL1,PDE4B,CREBBP,SLC29A1,ALDOA,STC2,ALOX5,ADAM8,CLDN4,NOS1,GCKR,SCNN1B,SLC1A1,XCR1,IL18,UBE2K,HCAR2,MCOLN1,STEAP3,PAX6,ATP1A3,ADORA2A,GRIN2B,KCTD7,ANGPT1,PACS2,LRRK1,GCNT2,ETS1,DIAPH1,HTR2C,POU3F3,ATP6V0A1,SYK,WWTR1,CYP39A1,GRIK2,U

			BAP2L,ATP2B3,POTEE,HSF1,PLEKHM1,CIB2,CAV2,POC1B,CA12,TMPRSS3,AXL,MET,CAMK2D,CCR3,NBEA,TRA2B,MLXIPL,NEDD4L,PTPRC,GPR89A,PBLD,SH3GL2,TYRO3,TRAF3IP2,EDNRA,BMP4
GO:0043410	positive regulation of MAPK cascade	0.000062198648338095886	PRDX2,NRXN1,PDE8A,SEMA3A,WWC1,NTRK3,FLT3,GRM5,PLCE1,MAP2K5,KITLG,ROBO1,HTR2B,TENM1,TRAF6,AKAP13,PTPN11,ADORA1,ERBB4,ADRA1D,CDON,NTRK2,PDGFB,TNIK,MID1,STK39,INSR,PAK1,MAP3K4,ERN2,RIT2,PLCB1,CHI3L1,NDRG4,NRG1,MAP2K1,ROR2,JAK2,FBXW7,NPSR1,LAMTOR3,NOX4,CD4,F2RL1,TP73,BMPER,DUSP22,NAIP,FGF10,SH3RF3,EPHA4,PRKCA,TNFSF11,CTNNB1,FCGR2B,IGF1R,AXIN1,FGF1,TRIM5,AJUBA,CSPG4,GRM1,FLT4,TAB1,APP,PDGFRA,NOX1,TNFRSF19,SOX2,FRS2,S100A12,RAF1,PTPN1,SPAG9,FGF2,DOK6,DVL3,CCL14,CCL15,NEK10,IQGAP1,MAP3K7,ALK,XDH,GFRAL,GHR,PLCG2,ROCK1,C1QTNF1,NTRK1,SPI1,BCAR3,MAP3K13,GRM4,WNT7B,IGF1,PTEN,LRRK2,PDGFC,WNT7A,SH3RF2,RAPGEF2,ROR1,ACTA2,NECAB2,CD27,MAP3K5,NCF1,TAOK2,SHC1,CD44,ADAM8,CXCL17,PRKCZ,ANGPT1,GCNT2,CCL22,BRAF,HTR2C,MADD,SYK,DVL2,PTPRJ,SASH1,CAV2,ANKRD6,PHB,PTPRC,BMP4
GO:0098815	modulation of excitatory postsynaptic potential	0.000062200821729818777	NRXN1,S1PR2,NLGN4X,RIMS1,CUX2,SHANK1,APP,NLGN2,CELF4,NLGN1,NLGN3,RELN,SLC8A2,NETO1,PTEN,LRRK2,TMEM108,SHANK3,WNT7A,GRIN1,RIMS2,PRKCZ
GO:0050773	regulation of dendrite development	0.000062929493630172219	KALRN,CDKL5,KNDC1,TNIK,PTPRD,FAT3,CAPRIN2,ZDHHC15,LZTS1,IL1RAPL1,CAMK2B,EPHA4,PPP3CA,DCC,CUX2,NEDD4,PARP6,DGKG,CAMK1D,SEMA4D,PAFAH1B1,TRPC5,RELN,ALK,PACSIN1,PAK3,MARK1,ACTR2,ELAVL4,SARM1,CARM1,CUX1,BMP7,RAPGEF2,RHOA,GSK3B,EEF2K,FBXO31,CDKL3,NEDD4L
GO:0071900	regulation of protein serine/threonine kinase activity	0.0000647515615724251	NRXN1,LATS2,PRKAG2,NTRK3,FLT3,MAP2K5,KITLG,ROBO1,HTR2B,TENM1,TRAF6,CDC6,AKAP13,PDGFB,CCND3,INSR,PRKAR1A,CAB39,PAK1,MAP3K4,ERN2,SLC8A1,NF1,PAQR3,MAP2K1,ROR2,IPO5,CCNYL1,HERC5,NOX4,PRKCD,TGFB1,ZNF675,EPHA4,TNFSF11,PPARG,AXIN1,PRKAR1B,FGF1,PRKAR2A,MYOCD,AJUBA,SPDYA,SFRP1,TAB1,CCNI2,HEG1,LRP5,MTCP1,S100A12,CCNY,PTPN1,BMPR2,BMPR1A,CDK12,FGF2,DVL3,CKS1B,TSG101,DAB2,BLM,NEK10,MAP3K7,SLC8A2,ATP2B4,UVRAG,GHR,MNAT1,HIPK3,STK38,MAP3K13,SNCA,BMPR1B,PRKAG1,RPTOR,HTT,PTEN,BMP7,LRRK2,PKD2,PDGFC,PKIB,RAPGEF2,PRKAR2B,RHOA,AGT,CCNJL,MAP3K5,TAOK2,ADAM8,RGS14,STK4,CCNG2,CDK5RAP1,CD300A,PPP2CA,SYK,DVL2,PTPRJ,SASH1,PTPRC,TNXB,BMP4
GO:0001933	negative regulation of protein phosphorylation	0.00008559168800343762	ENPP1,PDE4D,LATS2,PRKAG2,MVP,NTRK3,CBL,DNAJA3,EPHA1,PMEPA1,PTPRO,PRKAR1A,SNX6,DMD,SLC8A1,NF1,PAQR3,BDKRB1,BDKRB2,PTPRT,IPO5,GNAQ,ZFYVE28,PRKCD,PPM1E,PIBF1,SLIT2,CORO1C,SMAD6,ZNF675,DUSP22,SMYD3,PIP5KL1,PARK2,PPARG,PRKAR1B,PRKAR2A,MYOCD,NF2,SFRP1,HEG1,IBTK,LRP5,PTPN1,SPAG9,PPM1F,SEMA4D,EIF4G1,DEPTOR,TSG101,TERF2IP,PTPN13,LDLRAD4,XDH,HIPK3,PTPN2,PARD3,ATG14,STK38,SNCA,MLLT1,PTEN,SPRED2,BMP7,LRRK2,LILRB4,PKIB,PRKAR2B,EPM2A,DNAJC3,PTPRB,AGT,ADAR,RGS14,EPHB2,PAX6,PRKCZ,ADORA2A,RABGEF1,ANGPT1,LRRK1,CDK5RAP1,IMPACT,CD300A,PPP2CA,WWTR1,PTPRJ,INPP5F,CD109,MLXIPL,PTPRC,SH3GL2,BMP4
GO:0051648	vesicle localization	0.00010458657547414298	NRXN1,CLASP2,KIF5C,CNIH2,PCDH17,KLHL12,TCIRG1,GBF1,FBXW11,AP3S1,TANC2,LIN7A,SYN2,COPS5,EXOC4,RAB27A,SYN3,DYNC1I1,ATP9A,TRAK1,CTNNB1,PARK2,MYO1D,MYO1E,AP3B2,SPG11,WIPI1,MYO1F,NLGN2,VPS4A,NLGN1,RAB11A,BLOC1S5,TSG101,PAFAH1B1,KIF3A,FAM91A1,MYO7A,CLASP1,AP3B1,TRIM46,AP3D1,SNCA,HTT,PTEN,LRRK2,SNAP23,BLOC1S3,NDE1,CUL3,SYN1,BLOC1S6,EXOC6B,SEC16B,ARFGAP3,TBC1D23,ACTN4,PRKCZ,MAP2,RAB27B,MYO1A,MLPH,SYNDIG1
GO:0006897	endocytosis	0.00011482592643451503	ENPP1,HOOK2,LY75,DOCK1,LRP1B,NLGN4X,CBL,RAB4B,RAB4B-EGLN2,ENPP2,LRP2,ANK2,EZR,MEGF10,HTR2B,ITGB1,SNX2,INSR,VAMP7,TMPRSS15,GSN,RIT2,SNCB,BMP2K,NRG1,MYH9,DOCK2,CDH13,ANO6,DENND1A,SYT1,ATP9A,MKLN1,F2RL1,AAK1,ZFYVE9,ITSN2,SYNJ2,RABGAP1L,SLC17A7,LOXL3,CD6,PPP3CA,FCGR2B,PPARG,MSR1,SCAMP5,SCARA3,MYO1E,MRC2,ANKFY1,DNM3,ACKR2,NEDD4,APP,NLGN2,LRP5,SLC9B2,PRKD1,MAPKAPK3,PTPN1,USP33,DPYSL2,NLGN1,ATP9B,MCTP1,HHIPL1,EEA1,ABCA13,AP2M1,LMBRD1,TSG101,RHOJ,NLGN3,KIF3A,HIP1,BTBD9,ENTHD1,STON2,DAB2,XKR4,ARHGAP12,TPCN1,SNX5,PACSIN1,ASGR2,ATAD1,MAPKAPK2,SCARB1,TFRC,FNBP1,GPC3,SGIP1,ADRBK1,TBC1D5,GHR,PRSS12,SNX9,PLCG2,ROCK1,GAPVD1,TUB,SH3GL3,CACNG8,AP2B1,HEATR5A,SNX33,FCHSD2,SH3BP1,SNCA,CACNG2,MAGI2,RAB5B,MAPK1,MIB1,ATXN2,PIK3C3,LRRK2,UNC119,LILRB4,TMEM108,ITGAM,ARHGAP25,MIA2,RABEP1,DNAJC6,TF,RHOBTB1,ESYT2,SH3KBP1,EEF2K,NECAB2,PACSIN2,CALCRL,RABGEF1,GPR107,ANGPT1,CFI,BIN2,CLTB,CD300A,SH3BP4,ANXA2,RAB27B,ITSN1,NUMB,SYK,CACNG3,ANKRD13A,FCHO1,CAV2,EPS15,INPP5F,TMPRSS3,AXL,REPS2,SNX1,EHD2,NEDD4L,SH3GL2,SPON2
GO:0043067	regulation of programmed cell death	0.00012320216908970203	PRDX2,PRKCI,MAP4K4,DNMT1,TMBIM4,SLC9A1,IL31RA,GRID2,RPS6KA2,PTGFR,LATS2,ANP32A,PTGIS,TJP1,UNC5C,PRLR,CBL,EGLN2,SEMA5A,FLT3,STAT5B,MAP2K5,KITLG,LRP2,PIK3CD,OLFM1,TNFAIP8L1,HTR2B,MECOM,LMNA,TRAF6,ITPKB,DNAJA3,OXR1,GRK5,ITGB1,HSP90AA1,TSC22D3,OMA1,HDAC6,DDB1,ADORA1,IKBKB,ERBB4,GBE1,MRE11A,ESR1,NTRK2,BRMS1,FMN2,TCF7L2,EGLN3,BDNF,TFAP2A,KCNMA1,LCK,MDM4,ECT2,SNX6,TOX3,GSN,NF1,MGMT,SNCB,RTN4,DPEP1,NRG1,ARHGAP10,CDK19,BDKRB2,DOCK8,BID,FNIP1,TMBIM6,CSRNP3

			,RBM5,COPS5,ANO6,CERKL,NEUROD1,RFFL,JAK2,FBXW7,ITCH,BCL11B,TEX11,ANXA4,WNT11,SIAH1,PRKCD,TGFB1,SGK1,CRADD,SLIT2,TP73,SAP18,MTDH,FANK1,SMAD6,TFAP2D,BCL3,DAPK2,TNFRSF10B,NAIP,FGF10,CAPN3,PRKCA,CNR1,MAGEA4,MAG,PIP5KL1,UFL1,CTNNB1,PARK2,SOD2,IGF1R,PPARG,DLG5,DRAXIN,NOL3,MYOCD,CHEK2,HCK,CAPN2,NF2,FLT4,SGMS1,HDAC4,PAX2,SFRP1,FOXO3,BCL2L13,SMAD3,GRIK5,TXNDC12,CDC73,SLK,NOX1,YAP1,VDAC1,EYA2,ANGPTL4,LRP5,CHST11,TMEM14A,FRS2,PALB2,MMP2,DCUN1D3,STAT1,TCTN3,ATF2,RAF1,CARD16,CASP1,PTPN1,CAMK1D,FGF2,SEMA4D,RORC,YME1L1,RARB,PRKCG,NCOA1,AREL1,EEF1E1,TXNDC5,EIF2B5,ATF3,PAWR,DEPTOR,P4HB,CNTFR,HIP1,GLI2,DAB2,PKHD1,SIN3A,PELI1,CFDP1,ALK,INPP5D,SRA1,PAX7,PAK3,ADNP,RNF34,TFRC,WWOX,GFRAL,EYA1,TRPV1,MNAT1,HDAC1,TRIM24,PLCG2,ROCK1,HIPK3,RYBP,CAPN10,PTPN2,NTRK1,TMIGD1,SLC39A10,SARM1,MUC1,EPHA7,CTNNA1,AIFM2,MST1,ERCC3,SNCA,BMPR1B,GRM4,USP42,HEY2,ZMYND11,CAST,DFFA,RASA1,MITF,SRSF6,NUGGC,BTK,IGF1,CTNNBL1,HTT,PTEN,BMP7,LRRK2,OSGIN1,BAG6,ITGAM,WNT7A,NLRP1,GPI,SH3RF2,PRMT2,RAPGEF2,SMO,MARK4,PSEN2,RHOA,SYNGAP1,HSPD1,GSK3B,DNAJC3,EEF2K,CD27,BOK,RNF144B,NACC2,TNFRSF8,AGT,PRKAA2,ADAR,PTK2,FBXO10,MAP3K5,DLX1,SHC1,CD160,RPS6KB1,DNAJB6,CD44,ADAM8,BCL2L14,ACTN4,STK4,ZFAND6,SLC1A1,SIGMAR1,HCAR2,DLC1,PPARA,PPP1R10,PHLPP1,CPEB4,PRKCZ,ADORA2A,ANGPT1,EYA3,FHL2,ATF6,ETS1,PPP1CA,GRIA4,STPG1,PRKCQ,BRAF,HDAC2,POU3F3,HIGD2A,PHIP,ANGPT4,SP100,MADD,SYK,GRIK2,HRK,HSF1,SRPK2,ANKRD13C,QRICH1,VAV3,NUAK2,AXL,TNFSF9,MET,CAMK2D,MALT1,KDM2B,PHB,PTPRC,TYRO3,ADCY10,TRAP1,PRAP1,BMP4,CASP12
GO:0060997	dendritic spine morphogenesis	0.00012716804280684296	*PRMT3,KALRN,DOCK10,HDAC6,TANC2,CAPRIN2,ZDHHC15,CAMK2B,CTNND2,EPHA4,DNM3,CUX2,ABI2,SHANK1,NLGN1,EPHB3,PAFAH1B1,RELN,PAK3,ACTR2,EPHB1,ARHGAP44,PTEN,LRRK2,SHANK3,WNT7A,EEF2K,EPHB2*
GO:0009628	response to abiotic stimulus	0.00012737757235583446	*NRXN1,RDH13,SLC9A1,PRDM12,RYR1,PTGIS,TJP1,CBL,ARNT,EGLN2,MARVELD3,MAPK10,XRCC4,HTR2B,ITGB6,CAMKMT,LMNA,NMT2,DNAJA3,SLC24A2,ITGB1,HSP90AA1,PSMB7,TSC22D3,CNGB1,PTPRK,ABCC8,DDB1,ADORA1,ATP8A2,HIF3A,CUL4B,EP300,CDH8,STK39,FMN2,CAB39,MAP3K4,EGLN3,SLC4A10,STRC,KCNMA1,TRIM13,MDM4,ECT2,TMC2,MB,DMD,SLC8A1,GSN,RBM4,FYN,NF1,MGMT,PCDH15,RTN4,RWDD3,CHI3L1,TTN,NDRG4,BDKRB1,BDKRB2,STRBP,TMBIM6,SCFD1,GNAQ,FBXW7,UIMC1,TMC1,CLDN1,PDE2A,WNT11,MTA1,CLPB,ERCC8,PRKCD,SEMA5B,TGFB1,POLK,CRADD,DISC1,KCNJ3,CLOCK,ANO3,BCL3,TNFRSF10B,GPR52,CNTNAP2,FBXL17,PPL,CAPN3,EPHA4,RORA,MAG,CTNNB1,SOD2,HSPA6,IGF1R,PPARG,TRPM1,CYBB,BRIP1,RYR2,FGF1,NOL3,DCT,SCARA3,MYOCD,PER2,AJUBA,CHEK2,PPP1CB,CAPN2,DIO2,GRM1,HDAC4,SFRP1,ST8SIA1,FOXO3,SMAD3,ITPR1,ARNT2,RNF168,NEDD4,APP,NOX1,YAP1,RAB11FIP5,ANGPTL4,DRD1,MMP2,DCUN1D3,STAT1,USP53,STRA6,ATF2,RAF1,CARD16,CASP1,BACH1,TICRR,PTAFR,EIF2B5,COL11A1,KCNQ1,P4HB,TRPA1,DDAH1,TRPM3,ERCC1,BLM,RUVBL2,ABCB1,EYS,RGS7,ATP2B4,MAPKAPK2,MAP1B,ATP6V0A2,TYR,RNF34,TFRC,PSMB2,EYA1,TRPV1,PAXIP1,INSRR,NTRK1,PTPRQ,EPHB1,TMEM199,NETO1,LRRC8C,LRRC8D,STAC,MST1,ERCC3,HMGCS2,OPRD1,TROVE2,IGF1,HTT,PTEN,BMP7,LHFPL5,ATP6V1A,UNC119,PKD2,RGS9,BMP6,ANO1,GPI,EXT1,N4BP1,KCNC2,GRIN1,RHOA,SPRTN,SYNGAP1,TFEC,HSPD1,ITPR2,GSK3B,DNAJC3,EEF2K,ALAS2,KRT8,TNFRSF8,AGT,POLH,ZMPSTE24,PTK2,CREBBP,SLC29A1,NDUFS2,RPS6KB1,STC2,GUCY2F,SCEL,DNAJB6,ADAM8,CDS1,HUS1,PKD1L1,NOS1,RGS14,ACTN4,MC1R,SLC1A1,DNAJC7,PPARA,MCOLN1,CPEB4,PPP1CC,CPEB1,NIPBL,EYA3,PPP1CA,BRAF,HDAC2,IMPACT,ANGPT4,OPN1LW,KCND2,GRIK2,GTF2H5,SCN1A,BRCA2,TAF1,TANK,HSF1,RAD9B,TIMELESS,PTPRC,TANC1,EDNRA,GNGT1,PRAP1*
GO:0048041	focal adhesion assembly	0.00012751511458915365	*MAP4K4,SLC9A1,CLASP2,PTPRK,ARHGAP6,APOD,BCR,RCC2,BCAS3,CORO1C,DUSP22,AJUBA,SORBS1,SFRP1,SMAD3,SLK,COL16A1,PPM1F,PEAK1,VCL,MACF1,CLASP1,GPM6B,ROCK1,PTPRA,PTEN,EFNA5,RHOA,PTK2,TAOK2,DLC1,PHLDB2,THSD1,CAMSAP3,PTPRJ*
GO:0007160	cell-matrix adhesion	0.00014112583670349441	*MAP4K4,SLC9A1,CLASP2,UTRN,CASK,ITGB6,ITGB1,PTPRK,EPHA1,STRC,ARHGAP6,DMD,ADAMTS9,NF1,APOD,BCR,CDH13,RCC2,SKAP1,MKLN1,BCAS3,CORO1C,DISC1,DUSP22,OTOA,CTNNB1,ECM2,AJUBA,SORBS1,NF2,SFRP1,SMAD3,HOXD3,SLK,DEFB118,ADAMTS12,COL16A1,PPM1F,PEAK1,ITGA11,VCL,PKHD1,MACF1,CLASP1,GPM6B,CDK6,ROCK1,NID1,PTPRA,COL13A1,RASA1,PTEN,EFNA5,EPDR1,RHOA,ONECUT2,GSK3B,ITGBL1,PTK2,TAOK2,ITGAL,CD44,DLC1,PRKCZ,CD96,PHLDB2,THSD1,CAMSAP3,PTPRJ,EDA,TNXB*
GO:0040013	negative regulation of locomotion	0.00015018909556330709	*S1PR2,CLASP2,SEMA3A,NRG3,SEMA3D,SEMA5A,GRM5,MARVELD3,MAP2K5,PTPRR,ROBO1,ROBO2,SRGAP3,MCC,SRGAP2B,PTPRK,ABCC8,DACH1,ADORA1,EPHA1,PTPRO,PTPRU,ADAMTS9,NF1,PLCB1,DPEP1,APOD,BCR,NDRG4,NRG1,HDAC5,DCN,PTPRT,WNT11,SEMA5B,MMP28,SLIT2,CORO1C,KANK1,DUSP22,EPHA4,PIP5KL1,PPARG,DLG5,MYOCD,NF2,SFRP1,FOXO3,PTPRM,NAV3,PTPRG,SEMA6D,BMPR1A,FGF2,SEMA4D,MCTP1,CHRD,VCL,CLIC4,WNT3,LDLRAD4,CLASP1,PRKG1,ATP2B4,PTPN2,CTNNA1,MAGI2,ADTRP,MITF,DPYSL3,PTEN,RHOA,SULF1,GPR173,TMEFF2,DLC1,ZMYND8,ADORA2A,RABGEF1,PHLDB2,OSBPL8,BRAF,SPINT2,CD300A,ANGPT4,SP100,SRGAP2,SLIT1,FRMD5,PTPRJ,GNA12,SEMA3C*
GO:00	regulation of	0.00015403	*ENPP1,PRDX2,PRKCI,SLCO3A1,ADCY8,PTGIS,FER,MAP2K5,MAPK10,TRAF6,DN*

51090	DNA-binding transcription factor activity	610390542913	*AJA3,ZC4H2,CCDC22,ERC1,IKBKB,PEX14,ESR1,EP300,RNF220,BRMS1,TCF7L2,FANCA,TRIM13,RWDD3,MDFI,PPP2CB,HDAC5,CMKLR1,COPS5,MAD2L2,NEUROD1,JAK2,TRAPPC9,ITCH,ANXA4,TGFB1,SGK1,BTRC,MTDH,FANK1,CLOCK,ZNF675,BCL3,CAPN3,TNFSF11,PPP3CA,UFL1,CTNNB1,PPARG,IL18R1,MYOCD,TRIM5,HCK,TRIM8,HDAC4,MED13,SMAD3,WWP2,APP,LRP5,CHUK,ESR2,S100A12,PRKD1,TAF3,ATF2,TCF3,CARD16,CAMK1D,DDX58,TERF2IP,SUFU,PKHD1,RELN,COMMD6,PELI1,MAP3K7,TRIM22,ALK,UBE2V1,TFRC,EPHA5,PLCG2,PAXIP1,NTRK1,SPI1,MAP3K13,OPRD1,BTK,PTEN,BMP7,PKD2,CRTC3,PRMT2,SMO,ROR1,AGT,PBX1,PARP10,ADAM8,RPS6KA5,IL18,ARID5B,PRKCZ,PRKCQ,HDAC2,SP100,ADCY1,SYK,DVL2,TAF1,TICAM1,HSF1,RNF2,TRIM37,MALT1,EDA*
GO:0097061	dendritic spine organization	0.00015586653587455876	*PRMT3,KALRN,DOCK10,HDAC6,INSR,TANC2,FYN,CAPRIN2,ZDHHC15,CAMK2B,CTNND2,EPHA4,FCGR2B,IGF1R,DNM3,CUX2,ABI2,SHANK1,NLGN1,EPHB3,PAFAH1B1,RELN,PAK3,ACTR2,EPHB1,ARHGAP44,PTEN,LRRK2,SHANK3,WNT7A,EEF2K,EPHB2,GRIN2B,TANC1*
GO:0090109	regulation of cell-substrate junction assembly	0.00017205284174314254	*MAP4K4,SLC9A1,CLASP2,ARHGAP6,APOD,RCC2,BCAS3,CORO1C,DUSP22,SFRP1,SMAD3,SLK,COL16A1,PPM1F,PEAK1,VCL,MACF1,CLASP1,GPM6B,ROCK1,PTPRA,PTEN,EFNA5,RHOA,PTK2,DLC1,PHLDB2,CAMSAP3,PTPRJ*
GO:0051893	regulation of focal adhesion assembly	0.00017205284174314254	*MAP4K4,SLC9A1,CLASP2,ARHGAP6,APOD,RCC2,BCAS3,CORO1C,DUSP22,SFRP1,SMAD3,SLK,COL16A1,PPM1F,PEAK1,VCL,MACF1,CLASP1,GPM6B,ROCK1,PTPRA,PTEN,EFNA5,RHOA,PTK2,DLC1,PHLDB2,CAMSAP3,PTPRJ*
GO:1901575	organic substance catabolic process	0.00017387638520622464	*NT5C1B,ENPP1,GPC5,PDE4D,PDE7B,PDE8A,ALDH4A1,RNASET2,GK5,CALR3,SLC25A17,FBXL2,PRKAG2,HPSE2,FTO,DCAF12,ACOXL,CBL,ARNT,EGLN2,ENPP2,PLCE1,SAMHD1,USP32,CHI3L2,LRP2,ADPGK,EZR,TOM1L1,PSMD1,GOT1,INPP4B,HSP90AA1,PSMB7,CCDC22,AMDHD1,OMA1,DNAJB14,PDE4A,TCIRG1,HDAC6,DDB1,ADORA1,GPRASP1,CUL4B,CECR2,ACAD11,FBXL18,TNRC6A,EP300,CELA1,ZYG11B,FBXL7,USP46,CHFR,INSR,FMN2,ASB5,USP34,FBXW11,RNF144A,SAMD4A,FUT8,TRIM13,UBR2,MDM4,ENTPD5,PIPOX,ACOX1,ERN2,NPLOC4,LARP4B,USP13,PAFAH1B2,FYN,MKRN2,ARNTL,ADAMTS9,SMG7,PLCB1,DMGDH,SLC25A21,FBXO21,DPEP1,NEIL2,CHI3L1,BLVRB,NRG1,DAGLA,VGLL4,PPP2CB,ALDH3B2,RNF4,CSNK2A3,UBE3D,CREBRF,TRDMT1,FHIT,ENTPD1,MAD2L2,MAN1A1,FBXO9,RFFL,USP22,FBXW7,LINC00473,OAZ2,ITCH,USP12,ABCC2,SMG1,DPYS,IDNK,PNPLA1,PDE2A,SPSB1,HADHB,MTA1,SIAH1,ERCC8,PRKCD,BANP,PLCXD3,SPSB4,ZHX2,ASCC2,BTRC,DNAJB2,RNF133,RNF148,MGAM,DISC1,ZFAND2A,CPT1C,CLN6,CLOCK,PLCB4,BCKDHB,SYNJ2,NELL1,TIMP2,SND1,HNRNPC,YTHDF1,FBXL17,KCTD10,CAPN3,SMURF2,EPHA4,PRKCA,CNR1,SMG6,NSUN2,UBQLN3,UBQLNL,UFL1,CTNNB1,PARK2,SMARCC1,AXIN1,CPQ,OTC,LRPPRC,RSPRY1,FOXK2,CHEK2,UBQLN4,PPP1CB,CAPN2,DIO2,HDAC4,TRHDE,ZCCHC17,SYNCRIP,SMAD3,RNFT2,WWP2,CYP4F11,RNF168,DCP1B,NEDD4,PLA2G4C,OGDHL,SLC4A4,SEC61B,APP,USP49,RBM8A,ARIH1,AMFR,SH3D19,LRP5,BDH2,PRICKLE1,ERLIN1,NUDT10,HEXA,SLC6A3,VPS4A,ACOX2,PTPN1,ADAMTS12,PSMD11,GGT7,USP33,IDO2,PABPC4,FGF2,UBE2R2,AGBL4,CDC27,HIBADH,YME1L1,KLHL3,PRKCG,AREL1,OC90,UCHL3,PSMD2,RBX1,ANAPC5,FBXO45,LIN28B,AKR1D1,STT3B,AGO3,ACAA1,FBXL20,TSG101,DESI1,CRYM,IDE,SUFU,PAFAH1B1,PNLIPRP1,DDAH1,EDEM3,TNRC6B,ALDH6A1,MGLL,DAB2,UBR1,CACUL1,SMG5,PSMD7,PELI1,PPP2R5C,SPATA18,ZNRF1,ALK,FASTKD5,XDH,SNX5,RBBP6,ATP2B4,NSF,TMEM2,TET1,DYX1C1,MAPKAPK2,UBE3C,SCARB1,CELF1,RNF34,UVRAG,RBKS,DROSHA,GPC3,XRN1,PSMB2,DIS3L2,RNF216,SNX9,ELAVL4,TRIM24,PLCG2,ROCK1,RYBP,CUL2,ABCC1,MAP1A,HYKK,MAN1B1,AXIN2,SARM1,TMEM199,FBXO39,NUDT5,SNX33,SRD5A2,FBXW4,EIF3H,ACOT8,RENBP,PDXP,SNCA,USP42,PNPT1,USP50,RC3H1,EIF3E,RNF103,RNF103-CHMP3,RNF19B,PRKAG1,PSMF1,SULT2A1,TRIP12,DFFA,ADTRP,PLD1,ZNRF3,KCTD13,IGF1,PLA2G4E,PTEN,MIB1,PUM1,PCBP2,LRRK2,MLYCD,ZRANB1,ALDOC,ZBTB20,BAG6,SLC44A1,GALT,BLVRA,SEL1L2,NT5E,DAZL,CRTC3,GLDC,PLCL2,TAF15,MYCBP2,GPI,SH3RF2,EXT1,N4BP1,PNPLA3,ENPP3,GET4,ACADSB,CPT1A,UBE2H,BPGM,DECR1,RQCD1,CUL3,PCCA,EPM2A,GSK3B,PLD2,DNAJC3,RDX,UPF2,RNF144B,RNF43,METTL16,PRKAA2,CUL9,ZMPSTE24,PTK2,ARMC8,RNF121,SNX3,PDE4B,EXOSC3,FBXO10,TRPC4AP,XYLB,UGT1A1,UGT1A4,ALDOA,ECE1,RNFT1,CD44,ADAM8,CSNK1A1,NOS1,PEX7,BCO2,MFSD8,UBE2K,HCAR1,HCAR2,DIS3,FBXL13,PPARA,ABCD3,PNPLA6,RNF150,ZC3HAV1,PAH,PLCXD2,OTUD7B,UPB1,TBL1X,NT5M,SLCO2B1,HACL1,PPP1CA,FBXO31,PHKG2,C18ORF25,MOXD1,SKP1,MAOA,RPL23,ACAN,ANXA2,PARN,CNOT1,WWTR1,CYP39A1,ALDH8A1,YBX1,TAF1,HSF1,POP1,ETF1,INPP5A,ADAMTS7,GNA12,UGGT1,MYEF2,INPP5F,SNX1,UCHL5,MALT1,PFKP,MLXIPL,FBXL4,GNS,NEDD4L,PHB,RNF213,TRAF3IP2,SGTB,HADHA*
GO:0030111	regulation of Wnt signaling pathway	0.00017738967820380387	*GPC5,LATS2,SEMA5A,PSMB7,MCC,NPHP3,PTPRO,RNF220,TCF7L2,USP34,CDK14,PTPRU,DEPDC1B,ARNTL,MDFI,VGLL4,ROR2,SOX13,MAD2L2,TLE6,CAPRIN2,CCNYL1,GNAQ,MLLT3,KLF15,WNT11,IFT80,KREMEN1,BTRC,CTNND2,DISC1,KANK1,FGF10,SMURF2,PARK2,AXIN1,DRAXIN,BICC1,TRABD2B,SFRP1,FOXO3,SMAD3,NFATC1,CDC73,APP,YAP1,AMFR,SOX2,PRICKLE1,CCNY,TMEM237,FGF2,DKK2,RBX1,WNT3,PRDM15,TNKS,DAB2,WNK1,RUVBL2,RSPO2,MACF1,WWOX,GPC3,PSMB2,HDAC1,INVS,AXIN2,PTK7,ZEB2,ZNRF3,LRRK2,ZRANB1,SOX30,UBR5,JA*

			DE1,GSK3B,SULF1,RNF43,SNX3,APCDD1L,LBX2,SCEL,CSNK1A1,STK4,SPEF1,WLS,TBL1X,LRRK1,PPP1CA,RBMS3,PPP2CA,WWTR1,WIF1,ANKRD6,EDA,RNF213,SHISA6
GO:0007017	microtubule-based process	0.0001776694844416840 2	DNAH11,HOOK2,TACC2,SPAG16,PDE4DIP,CLASP2,KIF22,ASH1L,TUBA1C,IQCG,MAPRE2,FER,STAG2,EZR,TACC1,KIF5C,LMNA,ULK4,ITGB1,CNIH2,KIF26B,DLG2,IFT43,TTLL5,PARD6G,HDAC6,TBCEL,KIF18B,PEX14,DNAH2,NPHP3,DNAI2,HOOK3,MID1,FMN2,PAK1,FBXW11,CATSPER2,AP3S1,LRRC49,STARD9,RBM14,DST,RANBP9,DCTN1,MYH9,WDR92,NAV1,RNF4,PARD3B,LRGUK,CATSPER3,AUNIP,TLE6,CCNYL1,TERF2,ARL3,KIF2A,ICK,TTLL8,RANBP1,SMC3,CROCC,BBS12,DNAH14,KIF21A,DYNC1I1,PIBF1,BCAS3,DISC1,CEP135,TTBK2,SPAG17,ANKFN1,FGF10,SLC16A1,TRAK1,CTNNB1,AXIN1,DNAH10,LRPPRC,NIN,DNHD1,TUBGCP6,HAUS4,CHEK2,PHLDB1,DNAH9,HEPACAM2,AP3B2,SPG11,IFT122,RAB6A,C10ORF90,KPNB1,NAV3,RAB6C,APP,SLK,BORA,SLC9B2,SETD2,KIF24,SYNE2,SYBU,USP33,VBP1,RAB11A,CRMP1,AGBL4,ARMC2,SLC9B1,BLOC1S5,GAS8,PAFAH1B1,KIF3A,CEP350,TTLL7,TNKS,PKHD1,DZIP1,NEK10,EML4,MACF1,CLASP1,SLC9C1,TTLL9,ATP2B4,DYX1C1,MAP1B,MARK1,UVRAG,ZNF207,RPGR,ACTR2,ROCK1,AP3B1,MAP1A,NCKAP5,PARD3,TRIM46,TUB,DNAH12,TEKT1,AP3D1,KIF3C,TRDN,NTMT1,MST1,SNCA,HAUS3,CHMP3,HYDIN,HTT,TTLL4,SPAG5,CHMP5,IFT81,TMEM108,PKD2,EFNA5,STMN4,PPP2R3C,TBCD,MAP4,NME8,RHOT1,SIK3,ATRX,SUN1,BLOC1S3,MARK4,RHOA,NDE1,GSK3B,HDGFRP3,PRKAA2,BLOC1S6,CUL9,TPPP2,CDC14A,COPG2,CEP70,KIF6,RGS14,TUBB3,MARK3,SPEF1,TTLL11,GPSM2,LDHC,PAX6,PRKCZ,STAG1,PHLDB2,KLC3,ARHGAP21,MAP2,KIFC3,DIAPH1,CAPN6,CAMSAP3,RAB27B,SRGAP2,BRCA2,DNAH3,SKA2,POC1B,TRIM37,MET,ADCY10,RASSF8,CCDC170
GO:0050769	positive regulation of neurogenesis	0.0001806251649832645	PRKCI,TIAM2,KALRN,SEMA5A,GRM5,LRP2,ROBO1,CDKL5,ROBO2,DSCAM,GOLGA4,LIMK1,NTRK2,CDH4,PTPRD,BDNF,AMIGO1,TENM4,NTN1,MAP2K1,CAPRIN2,IL1RAPL1,CAMK2B,TGFB1,SLIT2,TP73,DISC1,EPHA4,MAG,UFL1,CTNNB1,PPARG,NIN,DCT,CUX2,GSX2,ISLR2,PARP6,BMPR2,RAB11A,SEMA4D,PLXNA2,SPEN,WNT3,PAFAH1B1,TRPC5,RUFY3,RELN,MACF1,PAK3,ADNP,MAP1B,ACTR2,HDAC1,EGR2,CUX1,MAP3K13,EFNA5,SHANK3,SMO,EEF2K,RGS14,EPHB2,PAX6,PPP1CC,FBXO31,BRAF,HDAC2,CDKL3,NUMB
GO:0006816	calcium ion transport	0.0002283604071373016 2	ASPH,PDE4D,SLC9A1,FAM155A,CHERP,CACHD1,RYR1,NOS1AP,EPB41,ATP2B2,CASK,ANK2,CHD7,HTR2B,RYR3,SLC24A2,SLC24A3,CACNB2,PDGFB,CACNA1B,CATSPER2,LCK,TMC2,MICU3,DMD,SLC8A1,JPH2,FYN,CACNA1E,BDKRB1,STIM1,NALCN,TMBIM6,CATSPER3,ANO6,NPSR1,TMC1,CACNA1H,CAMK2B,CD4,TGFB1,JPH3,CDH23,CACNA1D,CACNA2D3,HOMER2,CAPN3,VMP1,PPP3CA,CTNNB1,TRPM1,RYR2,NOL3,CACNA1C,ITPR1,VDAC1,IBTK,DRD1,PRKD1,FGF2,ADCYAP1R1,TRPA1,TRPM3,NMUR2,AKAP6,TRPC5,SLC8A2,TPCN1,AHNAK,ATP2B4,CACNA1A,TRPV1,PLCG2,GPR35,CACNG8,CD84,MCU,TRDN,STAC,TRPC6,SNCA,CACNG2,OPRD1,HTT,TMCO1,PKD2,PSEN2,GRIN1,RHOA,GRIN3A,ITPR2,EPM2A,AGT,ZMPSTE24,PDE4B,TRPC4AP,STC2,PKD1L1,NOS1,XCR1,CALCRL,MCOLN1,ADORA2A,GRIN2B,DIAPH1,HTR2C,ANXA2,CACNG3,ATP2B3,GPM6A,SESTD1,CAMK2D,PTPRC,EDNRA
GO:0051963	regulation of synapse assembly	0.0002294341223788804 5	NRXN1,NEGR1,GRID2,NTRK3,LHFPL4,ROBO2,NTRK2,PTPRD,BDNF,AMIGO1,NTN1,LRFN5,CLSTN2,IL1RAPL1,LINGO2,IL1RAPL2,DLG5,CUX2,APP,NLGN2,GPC6,NLGN1,SEMA4D,EIF4G1,EPHB3,NLGN3,SETD5,ADNP,FLRT2,NTRK1,EPHB1,EPHA7,SNCA,PTPRS,EFNA5,WNT7A,EEF2K,EPHB2,SLIT1,SYNDIG1
GO:0052696	flavonoid glucuronidation	0.0002619779187481073	UGT1A1,UGT1A10,UGT1A3,UGT1A4,UGT1A5,UGT1A6,UGT1A7,UGT1A8,UGT1A9
GO:0032870	cellular response to hormone stimulus	0.0002686045599300005	ENPP1,PRKCI,SLC9A1,ADCY8,PTGFR,LATS2,PRLR,PAGR1,EGLN2,FLT3,STAT5B,FER,GOT1,ROBO2,CDC6,THRB,STXBP4,PTPN11,HDAC6,BRD8,ESR1,PMEPA1,EP300,CCND3,INSR,PIP4K2A,PAK1,ESRRB,AP3S1,ARNTL,PLCB1,RXFP1,PIK3R2,HDAC5,SLC39A14,DEFA1B,DEFA3,PTGER2,JAK2,CACNA1H,PRKCD,TGFB1,SGK1,BCAS3,SLIT3,SLIT2,KANK1,RXFP2,CLOCK,SMYD3,GLP2R,UFL1,SMARCC1,IGF1R,PPARG,BRIP1,LEPR,LEPROT,NR4A3,SORBS1,HDAC4,SFRP1,FOXO3,NEDD4,ESRRG,YAP1,GNG2,ESR2,STAT1,HNF4A,PTPN1,PIK3R3,RARB,NCOA1,PTPRE,SRSF5,IDE,NR2C1,IRS4,WDTC1,DAB2,SP1,SRA1,SNX5,DYX1C1,MAP1B,GHR,ACACA,HDAC1,TRIM24,ADIPOR2,ROCK1,CAPN10,PTPN2,INSRR,CARM1,PTPRA,BCAR3,RAB15,HMGCS2,KMT2D,ADTRP,BMP7,PRCP,EFNA5,NCOA3,GPR21,TSHR,PNPLA3,PRMT2,UBR5,GRB14,RHOA,RQCD1,GSK3B,RDX,STAT6,EEF2K,AGT,PRKAA2,PTK2,PHEX,UGT1A1,SHC1,RPS6KB1,GPR173,SCNN1B,PPARA,SLC25A33,ZNF366,FBN1,PRKCZ,ATP1A3,CPEB1,OSBPL8,NCOR2,PRKCQ,PHIP,CNOT1,UCN2,HSF1,MAX,NCOA2,CAV2,DENND4C,PHB,EDNRA,BMP4
GO:0001952	regulation of cell-matrix adhesion	0.0002899177349073299	MAP4K4,SLC9A1,CLASP2,UTRN,CASK,EPHA1,ARHGAP6,DMD,NF1,APOD,CDH13,RCC2,SKAP1,BCAS3,CORO1C,DISC1,DUSP22,NF2,SFRP1,SMAD3,SLK,COL16A1,PPM1F,PEAK1,VCL,PKHD1,MACF1,CLASP1,GPM6B,CDK6,ROCK1,PTPRA,RASA1,PTEN,EFNA5,RHOA,ONECUT2,GSK3B,PTK2,DLC1,PRKCZ,PHLDB2,CAMSAP3,PTPRJ
GO:0010721	negative regulation of cell	0.000291578269351287	SEMA3A,CLDN18,SEMA3D,NTRK3,SEMA5A,DAB1,ABCC8,HOOK3,NF1,RTN4,RTN4R,NTN1,RCC2,FBXW7,SEMA5B,TGFB1,C9ORF47,CORO1C,KANK1,PPP3CA,MAG,CTNNB1,DRAXIN,DCC,PRTG,DIP2B,FSTL4,SEMA6D,BMPR1A,SEMA4D,WNT3,TR

	development	17	*PC5,RUFY3,BRINP1,TRPV1,RNF10,TRIM46,EPHA7,CTNNA1,PTPRS,IGF1,PTEN,BMP7,EFNA5,WNT7A,RAPGEF2,SYNGAP1,GSK3B,DLX1,ACTN4,EPHB2,FBN1,PAX6,MAP2,TNR,CDKL3,SLIT1,SEMA3C*
GO:0048514	blood vessel morphogenesis	0.000297080598688160 3	*NRXN1,NOX5,PTGIS,TJP1,SEMA5A,ENPP2,MAP2K5,LRP2,PIK3CD,NRXN3,ROBO1,CHD7,VAV2,ITGB1,ARHGAP24,RUNX1,ABCC8,EPHA1,HIF3A,MYO18B,NTRK2,CELA1,NPRL3,MEIS1,SGCD,ADAMTS9,NF1,RTN4,APOD,CHI3L1,UBP1,STIM1,MYH9,HDAC5,DCN,CDH13,PLXDC1,CCBE1,FBXW7,PKNOX1,WNT11,QKI,TGFB1,BCAS3,SLIT2,MTDH,BMPER,PARVA,ADAM12,FGF10,RORA,PRKCA,CTNNB1,ARHGAP22,PPARG,CYBB,LEPR,FGF1,MYOCD,MYO1E,CSPG4,FLT4,SFRP1,PTPRM,NRP2,PDGFRA,NOX1,YAP1,HEG1,JAK1,ANGPTL4,LRP5,SETD2,VASH2,MMP2,PRKD1,STAT1,STRA6,EREG,ATF2,BMPR2,BMPR1A,TSPAN12,PIK3R3,FGF2,ISM1,EPHB3,CLIC4,RHOJ,COL4A3,DDAH1,APOLD1,SP1,XDH,OVOL2,ATP2B4,TMEM2,CALD1,EYA1,HOXB3,SMOC2,ADIPOR2,ROCK1,PAXIP1,EPHB1,SPI1,RAPGEF3,HEY2,WNT7B,ADTRP,RASA1,HIPK1,PTEN,PRCP,PKD2,AMOTL1,WNT7A,SOS1,RAPGEF2,SMO,RHOA,PTPRB,SULF1,AGT,PTK2,SHB,SHC1,CD160,ALOX5,ADAM8,THSD7A,CXCL17,STK4,EPHB2,SLC1A1,IL18,CALCRL,NOTCH4,COL22A1,TMIGD2,ANGPT1,ETS1,ANGPT4,SP100,ANXA2,SYK,SRPK2,SASH1,VAV3,CCR3,RNF213,EDNRA,BMP4*
GO:0032880	regulation of protein localization	0.000302359775656283 3	*CD247,GPC5,NRXN1,ASPH,PRKCI,ADCY8,CLDN18,CTDSPL2,LATS2,ASTN2,EPB41,EZR,TENM1,LMNA,ITGB1,ABCC8,HMGN3,STXBP4,EFCAB7,TCIRG1,PTPN11,CCT2,HDAC6,ERBB4,GBF1,DPP10,FRMD4A,TCF7L2,MYOM1,BDNF,ECT2,KAT7,GSN,FYN,ARNTL,NF1,STX6,RTN4,APOD,TTN,ICA1,PIK3R2,TMEM30A,SCFD1,CREBRF,IPO5,GOLPH3L,NEUROD1,JAK2,FBXW7,OAZ2,CROCC,PLS1,PRKCD,TGFB1,F2RL1,BCAS3,DNAJB2,AAK1,CEP135,TTBK2,CHRM1,CLOCK,DCLK1,RABGAP1L,STXBP5,SLC16A1,CNR1,PPP3CA,UFL1,CTNNB1,PARK2,PPARG,ANXA13,SREBF2,LEPROT,TRIM5,PER2,SORBS1,TRIM8,NF2,SPTBN1,SFRP1,SMAD3,WWP2,LZTFL1,YAP1,RAB11FIP5,NLGN2,BORA,NVL,LRP5,SLC9B2,SETD2,GPC6,SERGEF,PRKD1,HNF4A,VPS4A,PTPN1,PPM1F,ADCY5,RAB11FIP3,GAS8,AP2M1,TSG101,VCL,SUFU,KIF3A,RUFY3,DAB2,DZIP1,SIN3A,TRIM22,APBB3,GPM6B,ATP2B4,KCNAB2,TFRC,EPHA5,GPC3,STOM,GAPVD1,CAPN10,ZFAND1,MAP1A,ANK3,PTPN9,GNL3L,PTPN14,AP2B1,ARHGAP44,SNX33,SPI1,MCU,CTNNA1,STAC,RAPGEF3,CACNG2,SYT9,ADTRP,ABCA12,XPO4,IGF1,STX8,PIK3C3,LRRK2,TMEM59,LILRB4,ITGAM,EFNA5,SHANK3,WNT7A,ZBED6,DNAJC1,BMP6,MYCBP2,ANO1,SMO,MARK4,STXBP5L,CPT1A,UBR5,EPM2A,GSK3B,RDX,NECAB2,PRKAA2,SYT7,SNX3,SEC16B,ALOX5,DNAJB6,ADAM8,SLC5A3,EPHB2,SLC1A1,WLS,GPSM2,HCAR2,SYTL4,PRKCZ,ADORA2A,OTUD7B,ANGPT1,EPB41L2,SP100,TRIM29,NUMB,WWTR1,MYRIP,ANKRD13A,CCT3,NEDD4L,SHISA6,EDNRA,BMP4,ABLIM3*
GO:0042981	regulation of apoptotic process	0.000323480384009351 6	*PRDX2,PRKCI,MAP4K4,DNMT1,TMBIM4,SLC9A1,IL31RA,GRID2,RPS6KA2,PTGFR,LATS2,ANP32A,PTGIS,TJP1,UNC5C,PRLR,CBL,EGLN2,SEMA5A,FLT3,STAT5B,MAP2K5,KITLG,LRP2,PIK3CD,OLFM1,TNFAIP8L1,HTR2B,LMNA,TRAF6,ITPKB,DNAJA3,OXR1,GRK5,ITGB1,HSP90AA1,TSC22D3,OMA1,DDB1,ADORA1,IKBKB,ERBB4,GBE1,MRE11A,ESR1,NTRK2,BRMS1,FMN2,TCF7L2,EGLN3,BDNF,TFAP2A,KCNMA1,LCK,MDM4,ECT2,SNX6,TOX3,GSN,NF1,MGMT,SNCB,RTN4,DPEP1,NRG1,ARHGAP10,CDK19,BDKRB2,DOCK8,BID,FNIP1,TMBIM6,CSRNP3,RBM5,COPS5,ANO6,CERKL,NEUROD1,RFFL,JAK2,FBXW7,ITCH,BCL11B,TEX11,ANXA4,WNT11,SIAH1,PRKCD,TGFB1,SGK1,CRADD,SLIT2,TP73,SAP18,MTDH,FANK1,SMAD6,TFAP2D,BCL3,DAPK2,TNFRSF10B,NAIP,FGF10,CAPN3,PRKCA,CNR1,MAGEA4,MAG,PIP5KL1,UFL1,CTNNB1,PARK2,SOD2,IGF1R,PPARG,DLG5,DRAXIN,NOL3,MYOCD,CHEK2,HCK,CAPN2,NF2,FLT4,SGMS1,HDAC4,PAX2,SFRP1,FOXO3,BCL2L13,SMAD3,GRIK5,TXNDC12,CDC73,SLK,NOX1,YAP1,VDAC1,EYA2,ANGPTL4,LRP5,CHST11,TMEM14A,FRS2,PALB2,MMP2,DCUN1D3,STAT1,TCTN3,ATF2,RAF1,CARD16,CASP1,PTPN1,CAMK1D,SEMA4D,RORC,YME1L1,RARB,PRKCG,NCOA1,AREL1,EEF1E1,TXNDC5,EIF2B5,ATF3,PAWR,DEPTOR,P4HB,CNTFR,HIP1,GLI2,DAB2,PKHD1,SIN3A,CFDP1,ALK,INPP5D,SRA1,PAX7,PAK3,ADNP,RNF34,TFRC,WWOX,GFRAL,EYA1,TRPV1,MNAT1,HDAC1,TRIM24,ROCK1,HIPK3,RYBP,CAPN10,PTPN2,NTRK1,TMIGD1,SLC39A10,SARM1,MUC1,EPHA7,CTNNA1,AIFM2,MST1,ERCC3,SNCA,BMPR1B,GRM4,USP42,HEY2,ZMYND11,CAST,DFFA,RASA1,MITF,SRSF6,NUGGC,BTK,IGF1,CTNNBL1,HTT,PTEN,BMP7,LRRK2,OSGIN1,BAG6,ITGAM,WNT7A,NLRP1,GPI,SH3RF2,PRMT2,RAPGEF2,SMO,PSEN2,RHOA,SYNGAP1,HSPD1,GSK3B,DNAJC3,EEF2K,CD27,BOK,RNF144B,NACC2,TNFRSF8,AGT,PRKAA2,ADAR,PTK2,FBXO10,MAP3K5,DLX1,SHC1,CD160,RPS6KB1,DNAJB6,CD44,ADAM8,BCL2L14,ACTN4,STK4,ZFAND6,SLC1A1,SIGMAR1,HCAR2,DLC1,PPARA,PPP1R10,PHLPP1,CPEB4,PRKCZ,ADORA2A,ANGPT1,EYA3,FHL2,ATF6,ETS1,PPP1CA,GRIA4,STPG1,PRKCQ,BRAF,HDAC2,POU3F3,HIGD2A,PHIP,ANGPT4,SP100,MADD,GRIK2,HRK,HSF1,SRPK2,ANKRD13C,QRICH1,VAV3,NUAK2,AXL,TNFSF9,CAMK2D,MALT1,KDM2B,PHB,PTPRC,TYRO3,ADCY10,TRAP1,PRAP1,BMP4,CASP12*
GO:0007612	learning	0.000325544254189820 7	*NRXN1,NLGN4X,KALRN,GRM5,NRXN3,SLC24A2,ITGB1,ABCC8,DGKI,NTRK2,INSR,TBR1,FYN,NF1,PLCB1,NDRG4,JPH3,YTHDF1,CNTNAP2,PARK2,CSMD1,SHANK1,SLC6A1,APP,DRD1,SHANK2,STRA6,NLGN3,RELN,SLC8A2,ATAD1,SORCS3,ACTR2,ELAVL4,MAP1A,NETO1,HTT,SHANK3,PRKAR2B,GRIN1,SYNGAP1,EPM2A,AGT,RGS14,EPHB2,SLC1A1,BRAF,ATP8A1,TNR,ATXN1,TANC1*
GO:0007044	cell-substrate junction assembly	0.000330605159766999	*MAP4K4,SLC9A1,CLASP2,PTPRK,ARHGAP6,LAMC1,DST,APOD,BCR,RCC2,BCAS3,CORO1C,DUSP22,AJUBA,SORBS1,SFRP1,SMAD3,SLK,COL16A1,PPM1F,PEAK1,VCL,MACF1,CLASP1,GPM6B,ROCK1,PTPRA,PTEN,EFNA5,RHOA,PTK2,TAOK2,D*

		4	*LC1,PHLDB2,THSD1,CAMSAP3,PTPRJ*
GO:0150116	regulation of cell-substrate junction organization	0.0003342141386794542 5	*MAP4K4,SLC9A1,CLASP2,MAPRE2,ARHGAP6,APOD,RCC2,BCAS3,CORO1C,DUSP22,SFRP1,SMAD3,SLK,COL16A1,PPM1F,PEAK1,VCL,MACF1,CLASP1,GPM6B,ROCK1,PTPRA,PTEN,EFNA5,RHOA,PTK2,DLC1,PHLDB2,CAMSAP3,PTPRJ*
GO:0080134	regulation of response to stress	0.00036938857953736 48	*PRDX2,MAP4K4,ADCY8,PDE8A,CLASP2,SEMA3A,SETD4,C12ORF49,ASH1L,PTGIS,SUSD4,STAT5B,SAMHD1,CASK,MARVELD3,SH2D1A,EZR,MECOM,LMNA,TRAF6,DNAJA3,OXR1,ULK4,ITGB1,HSP90AA1,PSMB7,ABCC8,PTPN11,HDAC6,ADORA1,IKBKB,ESR1,EP300,PDGFB,TNIK,MID1,ALOX5AP,STK39,FMN2,PAK1,MAP3K4,FANCA,FUT8,VAMP7,NPLOC4,USP13,KAT7,RBM14,FYN,MKRN2,ARNTL,PLCB1,MGMT,PPP4R2,RTN4R,BCR,CDK19,BDKRB2,BID,MAP2K1,LRFN5,DAGLA,TMBIM6,TSPAN8,CREBRF,AUNIP,COPS5,MAD2L2,TERF2,ANO6,HERC5,JAK2,FAM168A,FBXW7,UIMC1,ITCH,MLIP,SMG1,CLDN1,PDE2A,KREMEN1,ERCC8,PRKCD,F2RL1,KANK1,MMP26,CLOCK,ZNF675,DUSP22,NAIP,SBNO2,FGF10,SH3RF3,EPHA4,RORA,CNR1,TNFSF11,PPP3CA,UFL1,CTNNB1,PARK2,SOD2,FCGR2B,IGF1R,PPARG,AXIN1,OTUB1,IL1RL1,PROS1,NR4A3,NOL3,TRIM5,KIR2DL4,AJUBA,CHEK2,UBQLN4,HCK,FLT4,SFRP1,FOXO3,SMAD3,RNFT2,RNF168,TXNDC12,APP,PDGFRA,NOX1,AMFR,SESN1,TNFRSF19,EYA2,POLR3G,SETD2,S100A12,STAT1,EREG,ACOX2,CASP1,PTPN1,ADAMTS12,GGT7,BMPR1A,FGF2,ADCY5,MCTP1,DDX58,PRKCG,AREL1,EEF1E1,EEF1E1-BLOC1S5,EIF4G1,PAWR,TERF2IP,P4HB,PAFAH1B1,DDAH1,ERCC1,MGLL,SIN3A,ABCB1,MAP3K7,CLASP1,UBE2V1,XDH,PRKG1,ATP2B4,PAK3,MAPKAPK2,CDK6,CELF1,DROSHA,PSMB2,EYA1,PTGER3,SHPK,RNF216,TSPAN6,ACTR2,RECQL5,SMOC2,PLCG2,C1QTNF1,PAXIP1,HIPK3,ABCC1,PTPN2,TRIM44,EPHB1,AXIN2,MUC1,COCH,SPI1,AIFM2,PSPC1,SNCA,PNPT1,WNT7B,PTPRS,ZMYND11,TRIP12,ADTRP,SRSF6,RTN4RL1,BTK,IGF1,MAPK1,PTEN,SPRED2,BMP7,PCBP2,LRRK2,BAG6,SHANK3,NT5E,WNT7A,NLRP1,SH3RF2,N4BP1,ENPP3,UBR5,GSK3B,ACTA2,CD27,BOK,NACC2,AGT,POLH,ADAR,STAT2,ZMPSTE24,PTK2,MAP3K5,CREBBP,NCF1,TAOK2,CD160,RNFT1,ALOX5,DNAJB6,CD44,ADAM8,CLDN4,CXCL17,EPHB2,IL18,UBE2K,DNAJC7,PPARA,PPP1R10,CALCRL,PHLPP1,MAGI3,GPSM3,CD96,ADORA2A,RABGEF1,PHLDB2,EYA3,ATF6,ETS1,BRAF,TNR,SYK,GRIK2,YBX1,CADM1,HSF1,DHFR,SASH1,GBP5,ZP3,INPP5F,CD109,MET,TIMELESS,ANKRD6,ZDHHC11,PTPRC,TYRO3,TRAF3IP2,ATF6B,TRAP1,CLNK,BMP4,CASP12*
GO:0009950	dorsal/ventral axis specification	0.00041274450850826 93	*MDFI,VAX2,SMAD6,CTNNB1,AXIN1,SFRP1,BMPR1A,WNT3,AXIN2,PAX6*
GO:0051169	nuclear transport	0.0004177520173433269 4	*POLDIP3,CTDSPL2,ANP32A,LTV1,FAM53A,TNPO3,LMNA,AKAP13,EFCAB7,PTPN11,TCF7L2,ECT2,SHFM1,RANBP17,RBM4,NF1,SMG7,APOD,PIK3R2,IPO5,NEUROD1,IPO11,JAK2,SMG1,KPNA3,PRKCD,TGFB1,NUP93,BCL3,ANKRD54,SMG6,PPP3CA,NSUN2,AXIN1,RIOK2,RANBP3,SMAD3,NEDD4,KPNB1,THOC3,SETD2,PRICKLE1,DRD1,PRKD1,ATF2,IPO9,XPO6,TNPO1,DESI1,SUFU,SMG5,NXT2,EGR2,KPNA6,SNUPN,PTPN14,RAPGEF3,RSRC1,PRKAG1,XPO4,NUP88,LRRK2,ZC3H11A,POM121C,SMO,LSG1,UBR5,EPM2A,GSK3B,IWS1,UPF2,AGT,ADAR,GCKR,RGS14,STK4,PPP1R10,KPNA4,PPP1CC,ANGPT1,RPL23,ATXN1,SP100,NUP214,SYK,MALT1,NDC1,TRAF3IP2,BMP4*
GO:0006913	nucleocytoplasmic transport	0.0004177520173433269 4	*POLDIP3,CTDSPL2,ANP32A,LTV1,FAM53A,TNPO3,LMNA,AKAP13,EFCAB7,PTPN11,TCF7L2,ECT2,SHFM1,RANBP17,RBM4,NF1,SMG7,APOD,PIK3R2,IPO5,NEUROD1,IPO11,JAK2,SMG1,KPNA3,PRKCD,TGFB1,NUP93,BCL3,ANKRD54,SMG6,PPP3CA,NSUN2,AXIN1,RIOK2,RANBP3,SMAD3,NEDD4,KPNB1,THOC3,SETD2,PRICKLE1,DRD1,PRKD1,ATF2,IPO9,XPO6,TNPO1,DESI1,SUFU,SMG5,NXT2,EGR2,KPNA6,SNUPN,PTPN14,RAPGEF3,RSRC1,PRKAG1,XPO4,NUP88,LRRK2,ZC3H11A,POM121C,SMO,LSG1,UBR5,EPM2A,GSK3B,IWS1,UPF2,AGT,ADAR,GCKR,RGS14,STK4,PPP1R10,KPNA4,PPP1CC,ANGPT1,RPL23,ATXN1,SP100,NUP214,SYK,MALT1,NDC1,TRAF3IP2,BMP4*
GO:0007611	learning or memory	0.0004840266134284725 5	*DNAH11,NRXN1,ADCY8,NLGN4X,KALRN,GRM5,NRXN3,SLC24A2,ITGB1,ABCC8,DGKI,NTRK2,EP300,INSR,TBR1,BDNF,FYN,NF1,PLCB1,AFF2,NDRG4,SGK1,JPH3,YTHDF1,CNTNAP2,CNR1,CAMK4,PARK2,PRKAR1B,CSMD1,CUX2,SHANK1,SLC6A1,APP,AMFR,RCAN1,DRD1,SHANK2,STRA6,PRKCG,NLGN3,PAFAH1B1,BTBD9,RELN,SLC8A2,ATAD1,ADNP,SORCS3,BRINP1,ACTR2,ELAVL4,EGR2,NTRK1,MAP1A,NETO1,HTT,MAPK1,PTEN,SHANK3,GPI,PRKAR2B,GRIN1,SYNGAP1,EPM2A,AGT,RPS6KB1,RGS14,EPHB2,SLC1A1,PRKCZ,GRIN2B,BRAF,ATP8A1,TNR,ATXN1,ADCY1,TANC1,CREB1*
GO:0009605	response to external stimulus	0.00048647925092885 47	*PRDX2,NRXN1,SLC9A1,ADCY8,CLASP2,SEMA3A,IL31RA,GRID2,RNASET2,PRDM12,SETD4,PTGFR,NRG3,ASH1L,SEMA3D,MYOT,PTGIS,PRKAG2,TJP1,UNC5C,PRLR,KALRN,NTRK3,CBL,SEMA5A,SUSD4,STAT5B,ENPP2,SAMHD1,FER,CASK,PIK3CD,NAALADL2,DEPDC5,SH2D1A,NRXN3,ROBO1,CHD7,SLC26A6,ITGB6,CAMKMT,KIF5C,LPO,NMT2,TRAF6,ROBO2,NFASC,DNAJA3,SLC24A2,HSP90AA1,ELMO2,PSMB7,DSCAM,CNGB1,TRIO,OMA1,ABCC8,STXBP4,LAMA2,PTPN11,ADORA1,EPHA1,IKBKB,GBF1,ATP8A2,KLHL6,ESR1,PTPRO,TNRC6A,EP300,PDGFB,USP46,ALOX5AP,BPI,STK39,UNC5D,LAMA3,CDH4,PAK1,LITAF,TBR1,STRC,BDNF,TTC4,FANCA,NPRL3,TRIM13,HLCS,LCK,TMC2,VAMP7,LSP1,NPLOC4,DMD,SLC8A1,GSN,RBM14,RBM4,MACROD2,FYN,MKRN2,ADAMTS9,NCF2,MGMT,PCDH15,DPEP1,*

			RTN4R,BCR,CHI3L1,CCDC141,TTN,ADAMTSL1,NTN1,NRG1,CDK19,BDKRB1,STRBP,MAP2K1,FNIP1,LRFN5,DAGLA,CNTN4,CMKLR1,TSPAN8,DOCK2,CDH13,RAB27A,DEFA1B,DEFA3,F5,PTGER2,BOC,ANO6,GNAQ,FBXO9,HERC5,JAK2,MYPN,ITCH,NPSR1,BCL11B,TMC1,CLDN1,B3GALT5,PDE2A,WNT11,TRIM59,MTA1,KREMEN1,WDFY4,CLPB,SIAH1,PRKCD,TAB2,SEMA5B,CD4,TGFB1,MMP28,CRADD,F2RL1,BCAS3,SLIT3,SLIT2,HP,LAMC2,MTDH,XPR1,MMP26,SMAD6,CLOCK,ANO3,BCKDHB,BCL3,DCLK1,DAPK2,TNFRSF10B,PARVA,GPR52,NAIP,SBNO2,YTHDF1,FGF10,CNTNAP2,FBXL17,ENAH,PPL,CAPN3,SLC16A1,EPHA4,RORA,PRKCA,CNR1,CD6,TNFSF11,MAG,UFL1,CTNNB1,PARK2,SOD2,FCGR2B,IGF1R,PPARG,IL1RL1,TRPM1,ANKRD17,CYBB,OTC,BRIP1,SREBF2,RYR2,DRAXIN,FGF1,PROS1,FOXK2,WDR45B,MYOCD,TRIM5,PER2,KIR2DL4,KIR3DL1,CSMD1,PPP1CB,HCK,CAPN2,TRIM8,DIO2,DCC,HDAC4,PAX2,SFRP1,FOXO3,DGKB,TPH2,CNTN6,NFIB,SYNCRIP,SMAD3,PTPRM,ACKR2,WIPI1,C1S,SHANK1,IFNAR1,NRP2,PREX1,DOCK4,PRTG,CDC73,APP,PDGFRA,SESN1,NLGN2,ALPL,JAK1,POLR3G,SETD2,DEFB118,ECSIT,DRD1,CHUK,MMP2,S100A12,PRKD1,STAT1,SLC6A3,USP53,SHANK2,STRA6,EREG,MAPKAPK3,DSCAML1,SEMA6D,ATF2,POU2F2,RAF1,CARD16,CASP1,PTPN1,ADAMTS12,GGT7,BMPR2,USP33,DPYSL2,CAMK1D,CTNNA2,CRMP1,FGF2,PPM1F,ADCY5,AGBL4,SEMA4D,PLXNA2,PTAFR,DDX58,PIK3C2B,PRKCG,NCOA1,AREL1,BLOC1S5,ABCF3,EIF4G1,EPHB3,ATF3,WDR59,COL11A1,KCNQ1,WNT3,FKBP5,CCL14,CCL15,CCL15-CCL14,ZFYVE1,TRPA1,TRPM3,NMUR2,GLI2,NEO1,ERCC1,MGLL,C9,WNK1,RELN,SIN3A,CSF3R,EYS,PELI1,SLC22A3,MAP3K7,TRIM22,ALK,CLASP1,DSC2,PRKG1,PAK3,MASP1,ZPLD1,ADNP,MAPKAPK2,MAP1B,SCARB1,CDK6,CELF1,TYR,TFRC,EPHA5,DROSHA,GFRAL,GPC3,SGIP1,TBC1D5,PSMB2,PTGER3,SHPK,TRPV1,RNF216,TSPAN6,TFEB,CLEC16A,GHR,FLRT2,ACTR2,C2,CFB,SMOC2,TRIM24,PLCG2,C1QTNF1,EGR2,ABCC1,PTPN2,NTRK1,CRISP3,TRIM44,PTPRQ,EPHB1,EPHA10,SARM1,AP3D1,SRD5A2,CD84,COCH,HMCN1,SPI1,EPHA7,MCU,PSPC1,TRDN,ATG14,LY86,SHROOM3,MST1,PTK7,CHID1,SNCA,BMPR1B,CACNG2,RNF19B,HMGCS2,PTPRS,ZMYND11,PRKAG1,ADTRP,PLD1,NUGGC,RPTOR,RTN4RL1,BTK,VPS41,IGF1,STX8,HTT,MAPK1,PTEN,BMP7,PIK3C3,PUM1,LHFPL5,PCBP2,LRRK2,UNC119,CHMP5,ITGAM,PKD2,EFNA5,NT5E,MTMR3,PPP2R3C,NLRP1,ARNTL2,BMP6,MYCBP2,ANO1,GPI,SOS1,EXT1,N4BP1,PDCD1LG2,SERINC5,SMO,ENPP3,BLOC1S3,CPT1A,TNFRSF11B,RHOA,GRIN3A,TF,FAM19A4,ABCG8,DNAJC3,EMB,ACTA2,FAM49B,KRT8,TNFRSF8,AGT,PRKAA2,ADAR,BLOC1S6,STAT2,ZMPSTE24,PTK2,PHEX,SNX3,PDE4B,GAP43,MAP3K5,CREBBP,NCF1,UGT1A1,SHC1,CD160,ECE1,RPS6KB1,STC2,GUCY2F,SCEL,ALOX5,ADAM8,RPS6KA5,CDS1,MUC7,PKD1L1,CXCL17,NOS1,SCNN1B,TUBB3,EPHB2,SLC1A1,XCR1,IL18,UBE2K,PPARA,CALCRL,PHLPP1,CPEB4,GPSM3,PAX6,ZC3HAV1,CD96,ADORA2A,PPP1CC,RABGEF1,PHLDB2,TYK2,ANGPT1,ETS1,PPP1CA,CFI,BIN2,CCL22,PRKCQ,BRAF,HDAC2,HTR2C,IMPACT,TNR,SP100,OPN1LW,TRIM29,CR2,TMEFF1,WIPI2,SYK,GRIK2,SCN1A,SLIT1,PTPRJ,SLC10A1,TICAM1,GPM6A,UCN2,CADM1,HSF1,MAX,SRPK2,SASH1,GBP5,VAV3,ZP3,INPP5F,NUAK2,AXL,CD109,MET,CCR3,IFI44L,MALT1,ZDHHC11,SEMA3C,PHB,PTPRC,TYRO3,RNF213,SPON2,NCAM1,TRAF3IP2,TRIM60,CLNK,EDNRA,GNGT1,BMP4,CASP12
GO:0051966	regulation of synaptic transmission, glutamatergic	0.0004956892592549212	*NRXN1,GRM8,GRM5,DGKI,ADORA1,GRM7,ROR2,SYT1,GRIK3,DISC1,CNR1,GRM1,NLGN2,DRD1,NLGN1,GRIK1,NLGN3,RELN,ATAD1,NTRK1,UNC13A,CACNG8,CACNG2,GRM4,LRRK2,SHANK3,ADORA2A,TNR,GRIK2,CACNG3*
GO:0006814	sodium ion transport	0.0005301771604536596	*SLC9A1,UTRN,SLC38A11,SLC9A9,SLC24A2,NKAIN2,SLC24A3,NKAIN3,NETO2,STK39,SLC20A2,SLC4A10,SLC6A16,DMD,SLC8A1,SLC5A6,SLC4A5,NALCN,CATSPER3,FGF14,ANO6,SCN4A,SLC5A9,SLC9A7,CACNA1H,TGFB1,SGK1,SLC17A7,NDUFA9,SCN3B,NEDD4,SLC4A4,SLC6A1,KCNK1,SLC9B2,SPTBN4,SLC6A3,FXYD2,FXYD6,KLHL3,SLC5A8,SLC9B1,KCNQ1,WNK1,SLC8A2,SLC9C1,TPCN1,ATP2B4,STOM,SLC38A6,ANK3,SLC9C2,NETO1,PKP2,SLC4A8,SCN8A,PKD2,SLC6A14,SCN9A,SLC5A10,SLC5A3,NOS1,ACTN4,SCNN1B,NKAIN1,ATP1A3,CNTN1,SCN1A,SLC10A1,SLC13A3,SLC5A4,CAMK2D,NEDD4L,EDNRA*
GO:0071702	organic substance transport	0.0005460285295360226	*POLDIP3,ZDHHC14,ENPP1,SLC35E3,HOOK2,RTBDN,BLZF1,ASPH,PRKCI,SLCO3A1,ADCY8,CTDSPL2,SLC25A17,LRP1B,ASTN2,SLC14A2,PRKAG2,SLC35F3,MVP,KALRN,PITPNC1,SEC23B,SNX31,RAB4B,RAB4B-EGLN2,FAM53A,CCDC93,SPNS2,LRP2,SLC38A11,TNPO3,ANK2,EZR,TOM1L1,CHD7,SLC26A6,TENM1,KIF5C,LMNA,SRP72,AFM,ITGB1,HSP90AA1,APOL4,CCDC22,DLG2,FLVCR2,ABCC8,ERC1,HMGN3,AFTPH,STXBP4,EFCAB7,TCIRG1,PTPN11,HDAC6,GOLGA4,ADORA1,PEX14,GBF1,TLK1,ATP8A2,OSBPL10,GRM7,NTRK2,FRMD4A,STX18,HOOK3,VPS45,RIMS1,SNX2,INSR,FMN2,ATP8B1,TCF7L2,PIP4K2A,SCAMP4,MYOM1,SLC4A10,GLTP,SFT2D1,ECT2,TMEM144,SNX6,CHML,VAMP7,SPNS3,SLC6A16,RFTN1,SHFM1,AP3S1,NPLOC4,CENPF,RANBP17,ATG10,PRELID2,FYN,ARNTL,ADAMTS9,NF1,SMG7,SLC16A6,LMTK2,STX6,SLC25A21,GOLGA2P5,SLC5A6,APOD,SLC2A14,VTA1,BCR,TTN,ICA1,PIK3R2,SLC4A5,SLC16A10,SLC43A2,BDKRB2,BID,SLC35A2,SLCO1A2,MYH9,TMEM30A,SCFD2,ZDHHC3,LIN7A,SCFD1,PITPNB,CREBRF,EXOC4,IPO5,MAN1A1,RAB27A,MTX3,RAB3C,COG8,TMED6,GOLPH3L,ANO6,ARL3,NEUROD1,IPO11,PARP11,DENND1A,ZDHHC15,JAK2,FBXW7,OAZ2,STARD6,SYT1,SNAP25-*

			AS1,ABCC2,SMG1,RBFOX1,SLC1A4,SLC5A9,ATP9A,PLS1,DENND2A,KLF15,KPNA3,CTAGE6,VPS13B,IMMP2L,QKI,PRKCD,TGFB1,PCSK5,NUP93,PTPRN2,F2RL1,BCAS3,CADPS2,SYT13,ZFAND2A,ABCC11,GRPEL2,ANO4,CHRM1,CLOCK,ANO3,BCL3,RABGAP1L,SLC17A7,STXBP5,SLC16A1,VMP1,SNX8,CNR1,TNFSF11,SMG6,PPP3CA,NSUN2,TRAK1,PARK2,RN7SL832P,TSNARE1,PPARG,MYO1D,MSR1,SYTL5,XK,APOL3,SCAMP5,ANXA13,LRPPRC,SREBF2,LEPROT,NR4A3,AGAP1,PER2,SORBS1,TBC1D14,RAB3GAP2,TBCK,GRM1,SPTBN1,AP3B2,SFRP1,CCDC91,RANBP3,ATP10D,SNX14,SMAD3,RAB6A,WWP2,VPS39,PSAP,SLC16A2,SYT3,NEDD4,KPNB1,SLC4A4,VPS53,SLC6A1,RAB6C,SEC61B,THOC3,BET1L,RBM8A,RAB11FIP5,SFXN5,NLGN2,SSR2,LRP5,SEC22C,SLC9B2,SETD2,ZDHHC6,PRICKLE1,DRD1,RFT1,SERGEF,PRKD1,OSBPL2,SLC6A3,HNF4A,STRA6,VPS4A,PITRM1,SLC16A12,ATF2,RAF1,PTPN1,AP1B1,NLGN1,ATP9B,SNX16,RAB11A,EPG5,IPO9,RAB24,PPM1F,ADCY5,XPO6,ABCA13,RILPL1,SORCS2,RAB11FIP3,SLC5A8,TANGO6,OC90,AP2M1,SLC35D1,TNPO1,TECPR2,TSG101,DESI1,KCNQ1,SLC2A11,SUFU,PAFAH1B1,KIF3A,ATG4C,NMUR2,HIP1,AKAP6,TRPC5,TNKS,RUFY3,DAB2,XKR4,TRAM2,DZIP1,SMG5,ABCB1,SLC22A3,SLC47A1,FAM91A1,PLEKHF2,APOLD1,MACF1,MYO7A,EEPD1,APBB3,SLC35A4,GPM6B,SNX5,NSF,SLCO4A1,NXT2,CEP41,SCARB1,TFRC,EPHA5,MEF2A,SYT12,GPC3,STOM,ADRBK1,TBC1D5,TRPV1,SLC38A6,SNX9,RPGR,ARL5A,C1QTNF1,EGR2,GAPVD1,CAPN10,ABCC1,AP3B1,KPNA6,ZFAND1,MAP1A,ANK3,SNUPN,ATP13A3,PARD3,PTPN14,CACNG8,AP3D1,AP2B1,ARHGAP44,SNX33,LPA,MCU,VPS16,LIMA1,LRRC8C,LRRC8D,ILDR2,ABCB7,FCHSD2,RAB15,RAPGEF3,SLC48A1,SNCA,CACNG2,TBC1D9,PNPT1,OCA2,SYT9,CHMP3,SLC22A10,RAB5B,OSBPL1A,ADTRP,ABCA12,BTK,TBC1D16,XPO4,VPS41,SLC4A8,IGF1,STX8,SLC7A14,ATXN2,PIK3C3,NUP88,LRRK2,UNC119,CHMP5,SLC44A1,ITGAM,EFNA5,MIA2,SERPINA5,ATP8B4,SGSM1,ZBED6,ZC3H11A,SNAP23,DNAJC1,RABEP1,POM121C,BMP6,ANO1,PLIN2,SERINC5,SAMM50,ABCG2,SMO,SLC6A14,LSG1,BLOC1S3,STXBP5L,CPT1A,SYT17,UBR5,ARRDC4,BBS9,ESYT2,HSPD1,EPM2A,GSK3B,ABCG8,EMB,IWS1,UPF2,AGT,SYT7,ADAR,BLOC1S6,PITPNM2,PEX5L,SNX3,EXOC6B,RIMS2,SLC25A51,TOMM5,COPG2,CPE,SEC16B,STOML1,TIMM44,CHKA,SLC25A42,GTF2IRD2,NCF1,SLC29A1,TAOK2,UGT1A3,SLC44A3,RPS6KB1,SLC5A10,ZDHHC23,ALOX5,ADAM8,SLC5A3,ARFGAP3,SLC25A18,MON2,NOS1,PEX7,GCKR,ACTN4,COG4,STK4,ZFAND6,SLC1A1,WLS,SIGMAR1,HCAR2,PPARA,PPP1R10,ABCD3,GRTP1,CALCRL,MCOLN1,STEAP3,SLC25A33,KPNA4,SYTL4,PRKCZ,ADORA2A,RABGEF1,ANGPT1,CADPS,OSBPL8,IGF2BP3,SLC25A26,SLCO2B1,HACL1,TBC1D10C,ATG3,BRAF,HTR2C,SIL1,ATP8A1,CLTB,SLC26A2,RPL23,CAMSAP3,SP100,VTI1A,ANXA2,RAB27B,ITSN1,NUP214,PITPNM3,SYK,MYRIP,RAB2A,SEC31B,SLC44A5,CACNG3,YBX1,GRIP1,SLC10A1,OSBP2,SLC13A3,SLC2A12,HSF1,CNIH4,ATP10B,PLEKHM1,SLC5A4,MLPH,RAB37,AKTIP,EPS15,DENND4C,TBC1D10A,SNX1,ZDHHC11,ZDHHC11B,NDC1,RPH3A,GPR89A,RAB28,SYNDIG1,VPS52,TRAF3IP2,SGTB,EDNRA,PRAP1,SLC25A13,BMP4,ABLIM3
GO:0051058	negative regulation of small GTPase mediated signal transduction	0.000558183475427086 2	ITGB1,ARHGAP24,ARHGAP42,NF1,PPP2CB,SLIT2,KANK1,RASA4,RASA4B,KCTD10,HEG1,RASAL1,ARHGAP12,ARHGAP44,SH3BP1,RASA1,KCTD13,RASA2,ARHGAP25,SYNGAP1,CUL3,FAM49B,EPHB2,DLC1,RABGEF1,CGNL1,MET
GO:0007049	cell cycle	0.000567916923818145 6	MOV10L1,TACC2,C6ORF89,PDE4DIP,CLASP2,TAS1R2,RPS6KA2,KIF22,LATS2,TUBA1C,NOX5,EPB41,MAPRE2,NUDT6,PAGR1,RAD51B,STAT5B,EXD1,MAPK4,PRCC,STAG2,EZR,TOM1L1,CDKL5,MECOM,TACC1,LMNA,SENP5,GRK5,ITGB1,TRIOBP,CDC6,PSMB7,GFI1B,PARD6G,PTPRK,STXBP4,ITGB3BP,DACH1,PTPN11,DDB1,KIF18B,MRE11A,GBF1,TLK1,CUL4B,CECR2,EP300,PDGFB,CCND3,FBXL7,CHFR,INSR,FMN2,FBXW11,ESRRB,CDK14,FANCA,PHACTR4,UBR2,MDM4,ECT2,MTA3,TFDP2,CDC45,STARD9,DMD,CENPF,PDS5A,HFM1,RBM14,ARNTL,PLCB1,BCR,TTN,KLHL21,DCTN1,CDK19,BID,MYH9,MAD1L1,RNF4,CSNK2A3,PARD3B,CDKL2,AUNIP,COPS5,CSPP1,DMC1,IPO5,MAD2L2,TLE6,CCNYL1,TERF2,FOXN3,RCC2,ARL3,KIF2A,RAD51D,RANBP1,HERC5,USP22,YTHDC2,FBXW7,SMC3,UIMC1,CROCC,LZTS1,TEX11,SEPT7,CENPC,RPRD1B,SIAH1,PRKCD,TGFB1,BANP,PIBF1,BTRC,CRADD,TP73,TXNL4B,CEP135,RAB11FIP4,CLOCK,RNF212,SND1,ANKFN1,OIP5,FGF10,FBXL17,FANCI,SVIL,CAPN3,SLC16A1,PRKCA,PPP3CA,NSUN2,MAGEA4,CTNNB1,METTL13,IGF1R,ANKRD17,BRIP1,CDK11A,CDK11B,NIN,NPAT,TUBGCP6,RIOK2,ESCO1,MYOCD,PER2,AJUBA,HAUS4,CHEK2,PPP1CB,SPDYA,CTDP1,BAZ1B,NF2,SMC2,SPTBN1,HEPACAM2,SFRP1,MTBP,C10ORF90,KPNB1,RAB6C,CDC73,APP,CCNI2,CDK3,BORA,LRP5,SOX2,SETD2,DCUN1D3,PLAGL1,VPS4A,EREG,CCNY,ATF2,TCF3,POLA1,USP33,CDK12,RAB11A,MYB,FGF2,BACH1,TICRR,NEDD9,CDC27,RAB11FIP3,BRDT,EIF4G1,ANAPC5,SRSF5,CKS1B,TSG101,C11ORF80,PAFAH1B1,KIF3A,POLE,TNKS,WBP2NL,ERCC1,BLM,PKHD1,CACUL1,NEK10,SIN3A,ABCB1,PPP2R5C,IQGAP1,EML4,CLASP1,SRA1,OVOL2,ATP2B4,APEX2,HORMAD2,CCPG1,CDK6,UVRAG,PSMB2,ZNF207,BRINP1,CDCA3,DIS3L2,MNAT1,SNX9,ACTR2,RECQL5,SMOC2,PLCG2,ROCK1,EPS8,PAXIP1,CUL2,PKN2,ANK3,AXIN2,PARD3,MUC1,SNX33,HMCN1,RAD51C,CUZD1,URGCP,NTMT1,PDXP,ERCC3,HAUS3,PNPT1,MCMBP,CABLES1,FOXJ3,CHMP3,RNF103-CHMP3,ZMYND11,CDKL1,RASA1,NUGGC,RPTOR,IGF1,HTT,MAPK1,PTEN,IFFO1,BMP7,PIK3C3,PUM1,NUP88,SEPT6,SPAG5,UNC119,CHMP5,BAG6,CHAF1B,PKD

			2,HSF2BP,TADA2A,TBCD,ZBED6,MAP4,TTC28,PDCD2L,ATRX,IKZF1,PRMT2,SUN1,MARK4,JADE1,RHOA,MCM3,NDE1,CUL3,EPM2A,RDX,MLF1,RBBP8,CDK13,NACC2,CCNJL,CUL9,ZMPSTE24,BRINP3,PBX1,CDC14A,TAOK2,RPS6KB1,SETDB2,CSNK1A1,HUS1,RGS14,TUBB3,TEX12,GPSM2,PPP1R10,STEAP3,TAF2,PAX6,SPECC1L,SPECC1L-ADORA2A,PPP1CC,STAG1,CCNG2,NIPBL,YEATS4,EPB41L2,ETS1,PPP1CA,FBXO31,CDK5RAP1,UBE2E2,PHIP,CDKL3,PPP2CA,RPL23,DCDC1,OPN1LW,DONSON,NUP214,MADD,CIT,BRCA2,TAF1,PPME1,HSF1,SRPK2,MCM8,RNF2,CAV2,SKA2,POC1B,TRIM37,CENPK,MICAL3,RAD9B,TIMELESS,NDC1,PTPRC,CTCF,LIG1,EDNRA,PRAP1,BMP4
GO:0010810	regulation of cell-substrate adhesion	0.000588226094854464 6	*MAP4K4,SLC9A1,CLASP2,DOCK1,UTRN,CASK,TRIOBP,EPHA1,PTPRO,PDGFB,ARHGAP6,DMD,CASS4,NF1,APOD,EGFLAM,CDH13,RCC2,JAK2,SKAP1,BCAS3,CORO1C,DISC1,KANK1,DUSP22,HSD17B12,ECM2,NF2,SFRP1,SMAD3,PREX1,SLK,COL16A1,PPM1F,NEDD9,PEAK1,SPOCK1,VCL,P4HB,DAB2,PKHD1,MACF1,CLASP1,GPM6B,CDK6,ROCK1,NID1,PTPRA,RASA1,PTEN,EFNA5,TBCD,RHOA,ONECUT2,GSK3B,PTK2,ACTN4,STK4,DLC1,PRKCZ,PHLDB2,GCNT2,VWC2,BRAF,CAMSAP3,PTPRJ*
GO:0042326	negative regulation of phosphorylation	0.000617371533133805 4	*ENPP1,PDE4D,LATS2,PRKAG2,MVP,NTRK3,CBL,DNAJA3,EPHA1,PMEPA1,PTPRO,PRKAR1A,PIP4K2A,SNX6,DMD,SLC8A1,NF1,PAQR3,BDKRB1,BDKRB2,PTPRT,IPO5,GNAQ,ZFYVE28,PRKCD,PPM1E,PIBF1,SLIT2,CORO1C,SMAD6,ZNF675,DUSP22,SMYD3,PIP5KL1,PARK2,PPARG,PRKAR1B,PRKAR2A,MYOCD,AJUBA,NF2,HDAC4,SFRP1,HEG1,IBTK,LRP5,PTPN1,SPAG9,PPM1F,SEMA4D,EIF4G1,DEPTOR,TSG101,TERF2IP,PTPN13,LDLRAD4,XDH,ROCK1,HIPK3,PTPN2,PARD3,ATG14,STK38,SNCA,MLLT1,PTEN,SPRED2,BMP7,LRRK2,LILRB4,PKIB,PRKAR2B,EPM2A,DNAJC3,PTPRB,AGT,ADAR,GCKR,RGS14,EPHB2,PPARA,PAX6,PRKCZ,ADORA2A,RABGEF1,ANGPT1,LRRK1,CDK5RAP1,IMPACT,CD300A,PPP2CA,WWTR1,PTPRJ,INPP5F,CD109,MLXIPL,PTPRC,SH3GL2,BMP4*
GO:0019932	second-messenger-mediated signaling	0.000666464500632526	*ASPH,PDE4D,TMBIM4,SLC9A1,PDE7B,PTGFR,CHERP,NOS1AP,KSR2,GRM5,PLCE1,ANK2,HTR2B,CHRM3,SGCD,DMD,SLC8A1,RIT2,NRG1,PPP3R1,CMKLR1,CDH13,NEUROD1,LINC00473,PDE2A,CD4,NFATC3,HOMER2,ADCY2,TNFSF11,PPP3CA,RYR2,CACNA1C,HDAC4,ITPR1,ACKR2,NFATC1,RCAN1,PDE11A,MCTP1,ADCYAP1R1,DDAH1,NMUR2,AKAP6,GUCY1A2,SLC8A2,NFATC2,PRKG1,ATP2B4,CAMTA1,ADNP,EPHA5,PLCG2,PPP1R9A,RCAN2,MCU,RAPGEF3,BTK,IGF1,HTT,LRRK2,RAPGEF2,KCNC2,GRIN1,ITPR2,GSK3B,MCTP2,AGT,ZMPSTE24,LAT2,PEX5L,GUCY2F,NOS1,XCR1,MCOLN1,GRIN2B,FHL2,TBC1D10C,HTR2C,PPP2CA,ADCY1,SYK,PTPRJ,INPP5A,CAMK2D,CCR3,PTPRC*
GO:0150115	cell-substrate junction organization	0.000675648720332564	*MAP4K4,SLC9A1,CLASP2,MAPRE2,PTPRK,ARHGAP6,LAMC1,DST,APOD,BCR,RCC2,BCAS3,CORO1C,DUSP22,AJUBA,SORBS1,SFRP1,SMAD3,SLK,COL16A1,PPM1F,PEAK1,VCL,MACF1,CLASP1,GPM6B,ROCK1,PTPRA,PTEN,EFNA5,RHOA,PTK2,TAOK2,DLC1,PHLDB2,THSD1,CAMSAP3,PTPRJ*
GO:0046578	regulation of Ras protein signal transduction	0.000753782153548553 8	*MAP4K4,MAPRE2,CBL,PLCE1,KITLG,ROBO1,ITPKB,ITGB1,ARHGAP24,AKAP13,DGKI,CYTH3,GBF1,ARHGAP42,RIT2,NF1,RTN4,RTN4R,BCR,NRG1,RALGPS2,PPP2CB,RALGPS1,DENND1A,F2RL1,KANK1,FGF10,RASA4,RASA4B,ARHGEF28,KCTD10,AUTS2,P2RY10,ARHGEF18,HEG1,RAF1,RASGRF2,DENND4A,SHOC2,RASAL1,P2RY8,EPS8,GPR35,NTRK1,ARHGAP44,ARHGEF3,SH3BP1,RASA1,KCTD13,IGF1,RASA2,SYNGAP1,CUL3,RDX,EPHB2,DLC1,RABGEF1,DENND4B,MADD,DENND4C,MET*
GO:0140352	export from cell	0.000821788140486977 9	*NRXN1,SLC9A1,ADCY8,CLASP2,KALRN,CBL,FER,CASK,PIK3CD,EZR,NRXN3,CHD7,ABCC8,HMGN3,DGKI,STXBP4,ERC2,TCIRG1,PTPN11,CACNB2,ADORA1,GRM7,NTRK2,FRMD4A,RIMS1,TCF7L2,PAK1,MYOM1,VAMP7,SLC8A1,ARNTL,NF1,BCR,TTN,ICA1,SLC16A10,MYH9,SCFD2,LIN7A,SYN2,COPS5,EXOC4,RAB27A,RAB3C,GOLPH3L,ANO6,NEUROD1,JAK2,SYN3,SYT1,ATP9A,CTAGE6,CACNA1H,IL1RAPL1,PCSK5,PTPRN2,F2RL1,CADPS2,SYT13,C12ORF4,ANK1,CLOCK,FGF10,STXBP5,SLC16A1,CNR1,TNFSF11,PPP3CA,PARK2,FCGR2B,PPARG,SYTL5,SCAMP5,NR4A3,PER2,HCK,RIMS4,SFRP1,GRIK5,SLC16A2,SYT3,SLC4A4,RAB11FIP5,NLGN2,SCRN1,LRP5,SLC9B2,DRD1,SERGEF,TPH1,HNF4A,VPS4A,FXYD2,RAF1,NLGN1,RAB11A,ADCY5,MCTP1,PTAFR,RAB11FIP3,PRKCG,TANGO6,TRAPPC11,FBXL20,TSG101,KCNQ1,PAFAH1B1,DAB2,ABCB1,SLC47A1,SLC8A2,CLASP1,APBB3,NSF,EPHA5,SYT12,ADRBK1,TRPV1,PRSS12,C1QTNF1,CAPN10,ABCC1,UNC13A,KCND3,ARHGAP44,CD84,SPI1,CHRNB4,MCU,ILDR2,RAB15,SNCA,GRM4,SYT9,ADTRP,ABCA12,BTK,SLC4A8,IGF1,PIK3C3,LRRK2,ITGAM,EFNA5,MIA2,WNT7A,ZBED6,SNAP23,DNAJC1,BMP6,ANO1,ABCG2,BLOC1S3,STXBP5L,CPT1A,SYT17,GRIN3A,GSK3B,SYN1,MCTP2,AGT,SYT7,BLOC1S6,LAT2,EXOC6B,RIMS2,COPG2,CPE,PCSK6,CD160,ALOX5,CPLX2,ADAM8,ARFGAP3,WLS,HCAR2,STEAP3,SYTL4,ATP1A3,ADORA2A,RABGEF1,CADPS,BRAF,HTR2C,CD300A,RAB27B,ITSN1,ADCY1,SYK,MYRIP,ATP2B3,ZP3,AXL,MICAL3,RPH3A,TYRO3,CREB1,CLNK,STXBP6*
GO:0045596	negative regulation of cell differentiation	0.000857737796425283 4	*ENPP1,PRDX2,DNMT1,HLX,CBFB,SEMA3A,CLDN18,SEMA3D,NTRK3,SEMA5A,STAT5B,ZNF536,ITPKB,ITGB1,TRIO,RUNX1,DAB1,ABCC8,PTPN11,NPHP3,HOOK3,PDGFB,TOB2,ESRRB,BDNF,MEIS1,DMD,GPR171,ARNTL,NF1,RTN4,RTN4R,NTN1,CNTN4,HDAC5,JDP2,MAD2L2,RCC2,FBXW7,BBS12,SOX6,SEMA5B,TGFB1,ZHX2,NFATC3,C9ORF47,TP73,CORO1C,KANK1,SMAD6,ZNF675,FGF10,LOXL3,EPHA4,RORA,PPP3CA,MAG,CTNNB1,SOD2,FCGR2B,PPARG,AXIN1,ANKRD17,ANKRD26,DRAXIN,MYOCD,DCC,CTDP1,HDAC4,SFRP1,FOXO3,SMAD3,NFATC1,PRTG,CD*

			C73,APP,DIP2B,YAP1,FSTL4,LRP5,SOX2,PRICKLE1,FRS2,STAT1,EREG,SEMA6D,ADAMTS12,BMPR2,BMPR1A,CDK12,SPAG9,MYB,SEMA4D,RARB,CHRD,WNT3,SUFU,TRPC5,GLI2,RUFY3,USH2A,LDLRAD4,INPP5D,SRA1,XDH,OVOL2,NFATC2,MARK1,CDK6,BRINP1,TRPV1,RNF10,PTPN2,EPHB1,AXIN2,TRIM46,EPHA7,CTNNA1,GLIS1,HEY2,RC3H1,PTPRS,SRSF6,PKP2,IGF1,MAPK1,PTEN,MIB1,SPRED2,BMP7,LILRB4,EFNA5,WNT7A,ZBED6,IL17RD,RAPGEF2,SMO,RHOA,SYNGAP1,GSK3B,CDK13,PBX1,DLX1,ACTN4,EPHB2,IL18,PPARA,NOTCH4,FBN1,PAX6,MAP2,TNR,MBNL3,CDKL3,PPP2CA,WWTR1,SLIT1,YBX1,ADAMTS7,SEMA3C,BMP4
GO:0051093	negative regulation of developmental process	0.001042586730903488 2	ENPP1,PRDX2,DNMT1,HLX,CBFB,SEMA3A,WWC1,CLDN18,SEMA3D,WWC3,NTRK3,SEMA5A,STAT5B,ZNF536,ROBO2,ITPKB,ITGB1,TRIO,RUNX1,DAB1,OMA1,ABCC8,PTPN11,NPHP3,HOOK3,PDGFB,TOB2,ESRRB,BDNF,MEIS1,DMD,GPR171,ARNTL,ADAMTS9,NF1,FAT3,RTN4,RTN4R,BCR,NTN1,VGLL4,CNTN4,HDAC5,JDP2,DCN,MAD2L2,TERF2,RCC2,FBXW7,BBS12,WNT11,KREMEN1,SOX6,SEMA5B,TGFB1,ZHX2,NFATC3,C9ORF47,TP73,CORO1C,KANK1,SMAD6,ZNF675,FGF10,LOXL3,EPHA4,RORA,PPP3CA,MAG,CTNNB1,PARK2,SOD2,FCGR2B,PPARG,AXIN1,ANKRD17,ANKRD26,DRAXIN,MYOCD,DCC,CTDP1,HDAC4,PAX2,SFRP1,FOXO3,NFIB,DNM3,SMAD3,PTPRM,NFATC1,PRTG,CDC73,APP,DIP2B,YAP1,FSTL4,LRP5,SOX2,PRICKLE1,FRS2,SFMBT1,STAT1,EREG,SEMA6D,ATF2,ADAMTS12,BMPR2,BMPR1A,NLGN1,CDK12,SPAG9,MYB,SEMA4D,ISM1,JARID2,RARB,CHRD,WNT3,SUFU,COL4A3,TRPC5,GLI2,RUFY3,USH2A,LDLRAD4,RSPO2,INPP5D,SRA1,XDH,OVOL2,NFATC2,ATP2B4,MARK1,CDK6,TFRC,BRINP1,TRPV1,ROCK1,RNF10,PTPN2,EPHB1,AXIN2,TRIM46,BCOR,CARM1,SPI1,EPHA7,CTNNA1,GLIS1,RAPGEF3,HEY2,RC3H1,PTPRS,TLL2,SRSF6,PKP2,RTN4RL1,IGF1,MAPK1,PTEN,MIB1,SPRED2,BMP7,LILRB4,EFNA5,WNT7A,ZBED6,IL17RD,RAPGEF2,SMO,TNFRSF11B,RHOA,SYNGAP1,GRIN3A,GSK3B,CDK13,SULF1,AGT,PBX1,DLX1,SHC1,CD160,STC2,ALOX5,ACTN4,STK4,EPHB2,IL18,PPARA,NOTCH4,FBN1,PAX6,ASPN,MAP2,HDAC2,TNR,MBNL3,CDKL3,PPP2CA,ANGPT4,WWTR1,SLIT1,YBX1,ASAP1,ADAMTS7,INPP5F,SEMA3C,BMP4
GO:0048814	regulation of dendrite morphogenesis	0.001066588852612151 7	KALRN,CDKL5,KNDC1,TNIK,PTPRD,CAPRIN2,LZTS1,IL1RAPL1,CAMK2B,EPHA4,PPP3CA,CUX2,NEDD4,PARP6,SEMA4D,PAFAH1B1,TRPC5,RELN,PAK3,ACTR2,SARM1,CUX1,RAPGEF2,GSK3B,EEF2K,FBXO31,CDKL3,NEDD4L
GO:0006508	proteolysis	0.001066975949289003 7	ADAMTS16,ASPH,RPS6KA2,CALR3,FBXL2,CPA6,DPP6,OVCH2,DCAF12,CBL,MAP2K5,USP32,NAALADL2,ROBO1,TOM1L1,PSMD1,SENP5,DNAJA3,PSMB7,PGPEP1L,CCDC22,ADGB,RUNX1,OMA1,DNAJB14,HDAC6,DDB1,ADAMTS3,CUL4B,DPP10,FBXL18,CELA1,ZYG11B,FBXL7,USP46,CAPN14,CHFR,USP34,FBXW11,RNF144A,EGLN3,TRIM13,UBR2,LCK,CST2,NPLOC4,TMPRSS15,USP13,GSN,FYN,MKRN2,ARNTL,ADAMTS9,MGMT,FBXO21,DPEP1,ADAMTS19,TLL1,BID,FNIP1,MYH9,PPP2CB,PCSK2,RNF4,UBE3D,ITIH2,CPB1,FHIT,COPS5,MAN1A1,NLN,FBXO9,RFFL,CCBE1,USP22,JAK2,FBXW7,UIMC1,ITCH,USP12,SPSB1,CYFIP2,CLCA2,MTA1,IMMP2L,SIAH1,ADAM23,ERCC8,PCSK5,SPSB4,MMP28,ASCC2,KEL,BTRC,CRADD,DNAJB2,RNF133,RNF148,ITIH4,HP,HPR,DISC1,BLID,ZFAND2A,CLN6,MMP26,CLOCK,TIMP2,ADAM12,NAIP,TRRAP,FBXL17,KCTD10,CAPN3,SMURF2,EPHA4,UBQLN3,UBQLNL,UFL1,CTNNB1,PARK2,SMARCC1,PPARG,AXIN1,OTUB1,CPQ,RSPRY1,PROS1,NOL3,GLG1,SUPT3H,UBQLN4,CSTL1,CAPN2,ZUFSP,MMP16,PAX2,TRABD2B,SFRP1,TRHDE,USP54,BCL2L13,SMAD3,RNFT2,WWP2,SIMC1,RNF168,C1S,NEDD4,SEC61B,APP,USP49,ARIH1,AMFR,SH3D19,SCRN1,SOX2,ADAMTS2,PRICKLE1,VASH2,ERLIN1,MMP2,ST18,USP53,TASP1,VPS4A,PITRM1,GRAMD4,RAF1,CARD16,CASP1,ADAMTS12,PSMD11,GGT7,USP33,PPM1F,UBE2R2,AGBL4,ADAMTS4,CDC27,YME1L1,KLHL3,PRKCG,SPOCK1,AREL1,UCHL3,ECE2,PSMD2,RBX1,XPNPEP3,ANAPC5,FBXO45,STT3B,FBXL20,ADAM20,ADAM21,TSG101,DESI1,IDE,ADAMTS6,RCE1,SUFU,QPCTL,ADAMTS14,ATG4C,COL4A3,HIP1,EDEM3,DAB2,UBR1,CACUL1,MYSM1,RELN,PSMD7,JOSD1,PELI1,PPP2R5C,ZNRF1,KLK2,TMPRSS12,AGBL1,XDH,RBBP6,MASP1,DYX1C1,UBE3C,RNF34,TFRC,GPC3,PSMB2,RHBDL3,RNF216,PRSS12,SNX9,HDAC1,C2,CFB,ROCK1,RYBP,CAPN10,CUL2,MAP1A,MAN1B1,FBXO39,ANXA8L1,ADAM19,SNX33,FOLH1,LPA,EPHA7,XPNPEP2,CUZD1,FBXW4,EIF3H,RENBP,OTUD3,ADAMTS17,MST1,SNCA,USP42,USP50,RC3H1,RNF103,RNF103-CHMP3,RNF19B,PSMF1,TLL2,CAST,TRIP12,SPPL2A,ZNRF3,KCTD13,PTEN,MIB1,PIK3C3,PCBP2,PRCP,LRRK2,TMEM59,ZRANB1,BAG6,SEL1L2,AMZ2,SERPINA3,SERPINA4,SERPINA5,NLRP1,PEPD,UACA,DNAJC1,GPI,SH3RF2,N4BP1,GET4,PSEN2,UBE2H,GRIN1,ADAM29,RHOA,SPRTN,CPVL,CUL3,HSPD1,EPM2A,GSK3B,DNAJC3,CD27,BOK,RNF144B,RNF43,AGT,CUL9,ZMPSTE24,PTK2,ARMC8,PHEX,RNF121,FBXO10,MAP3K5,CPE,TRPC4AP,PCSK6,ECE1,RNFT1,SERPINB11,DNAJB6,CD44,ADAM8,CLDN4,CSNK1A1,SLC1A1,UBE2K,DLC1,FBXL13,RNF150,ADORA2A,GRIN2B,OTUD7B,COL28A1,TBL1X,CFI,FBXO31,PRKCQ,HDAC2,CAPN6,SPINT2,UQCRC2,C18ORF25,SKP1,RPL23,ACAN,ANXA2,SYK,WWTR1,TAF1,TANK,HSF1,ADAMTS7,BMP1,GNA12,UGGT1,TMPRSS3,AGBL3,CD109,TBC1D10A,UCHL5,MALT1,SERPINE3,FBXL4,NEDD4L,RNF213,SGTB,CASP12,CPNE1
GO:0030705	cytoskeleton-dependent intracellular	0.001124390409335267 1	HOOK2,TUBA1C,KIF5C,CNIH2,DLG2,IFT43,MYO10,HDAC6,PEX14,HOOK3,FBXW11,AP3S1,TANC2,DST,TERF2,ARL3,ICK,BBS12,DYNC1I1,TRAK1,MYO1D,LRPPRC,MYO1E,AP3B2,SPG11,IFT122,RAB6A,APP,MYO1F,SYNE2,SYBU,AGBL4,BLOC1S5,PAFAH1B1,KIF3A,MYO7A,MAP1B,RPGR,AP3B1,MAP1A,TRIM46,TUB,AP3D1,KI

	transport		*F3C,HTT,IFT81,TMEM108,RHOT1,SUN1,BLOC1S3,NDE1,BLOC1S6,COPG2,ACTN4,PRKCZ,KLC3,ARHGAP21,MAP2,CAMSAP3,RAB27B,MYO1A*
GO:0060079	excitatory postsynaptic potential	0.001156091088568804	*NRXN1,S1PR2,GRID2,NLGN4X,DGKI,ADORA1,RIMS1,PPP3CA,CUX2,GRIK5,SHANK1,APP,NLGN2,GLRA2,CELF4,NLGN1,NLGN3,RELN,SLC8A2,P2RX6,TRPV1,NETO1,CHRNB4,SNCA,PTEN,LRRK2,TMEM108,SHANK3,WNT7A,GRIN1,GSK3B,RIMS2,SLC29A1,PRKCZ,ADORA2A,GRIN2B,GRIK2*
GO:0120031	plasma membrane bounded cell projection assembly	0.001163941165677554	*CLRN1,ADAMTS16,NRXN1,S1PR2,SPAG16,IQCG,PLCE1,FER,EZR,CDKL5,TENM1,VAV2,ITGB1,ARHGAP24,IFT43,CDC42EP3,MYO10,HDAC6,GBF1,DNAH2,NPHP3,PTPRO,DNAI2,TENM2,TNIK,ATP8B1,LRRC49,DMD,GSN,PCDH15,NTN1,DCTN1,RAPGEF6,WDR92,LRGUK,CDH13,NCKAP1,RCC2,ANO6,ARL3,ICK,TTLL8,CROCC,SEPT7,IFT80,PRKCD,PIBF1,F2RL1,BCAS3,RPGRIP1L,SLIT2,CORO1C,DISC1,CEP135,KANK1,PTPDC1,TTBK2,SPAG17,PARVA,AUTS2,AJUBA,CSPG4,HDAC4,ARL13B,IFT122,DNM3,ABI2,YAP1,KIF24,TTC39C,SYNE2,TCTN3,ABLIM1,TMEM237,ABLIM2,NLGN1,ARMC2,RILPL1,GAS8,VCL,WDR90,KCNQ1,RFX2,KIF3A,CEP350,PKHD1,DZIP1,DOCK11,ZNF423,DYX1C1,CEP41,MPHOSPH9,AIF1L,FGD1,RPGR,ACTR2,ROCK1,EPS8,TEKT1,ARHGAP44,HYDIN,CDKL1,PLD1,TROVE2,HTT,DPYSL3,SEPT6,IFT81,MAP4,NME8,CEP89,PARVB,RAPGEF2,MARK4,BBS9,ONECUT2,RDX,CDC14A,GAP43,CEP70,FGD3,FGD4,SPEF1,KLC3,ATG3,NOTO,ARHGEF6,FAM149B1,SRGAP2,WWTR1,GPM6A,MYO1A,PLEKHM1,ASAP1,VAV3,POC1B,EHD2,ABLIM3*
GO:0071248	cellular response to metal ion	0.0011806487647240435	*NRXN1,ASPH,ADCY8,RYR1,ADCY7,RYR3,ITPKB,DLG2,CPNE4,ALOX5AP,TFAP2A,ECT2,DPEP1,SYT1,CLDN1,CACNA1H,SYT13,KCNH1,RASA4,RASA4B,CAPN3,PARK2,MTF1,CYBB,CPNE6,TPH2,SYT3,APP,GLRA2,CHUK,CPNE9,NLGN1,CLIC4,RASAL1,IQGAP1,MEF2A,SYT12,PLCG2,ANK3,SNCA,SYT9,MAPK1,LRRK2,PKD2,BMP6,SYT17,TF,EEF2K,PRKAA2,SYT7,NCF1,SLC1A1,MCOLN1,BRAF,ADCY1,A3GALT2,HSF1,CAMK2D,CREB1,CPNE1*
GO:0046907	intracellular transport	0.001198078445928337	*POLDIP3,ZDHHC14,HOOK2,ASPH,PRKCI,SLC9A1,WWC1,CTDSPL2,TUBA1C,ANP32A,SEC23B,LTV1,SNX31,FAM53A,CCDC93,TNPO3,EZR,TOM1L1,TENM1,KIF5C,LMNA,SRP72,HSP90AA1,CNIH2,CCDC22,KLHL12,AKAP13,DLG2,IFT43,MYO10,ERC1,AFTPH,STXBP4,EFCAB7,PTPN11,HDAC6,ADORA1,GPRASP1,PEX14,GBF1,TLK1,STX18,HOOK3,VPS45,RIMS1,SNX2,FMN2,TCF7L2,PIP4K2A,FBXW11,ECT2,SNX6,CHML,VAMP7,RFTN1,SHFM1,AP3S1,NPLOC4,DENND5A,RANBP17,RBM4,TANC2,FYN,NF1,SMG7,DST,LMTK2,STX6,GOLGA2P5,APOD,VTA1,BCR,NDRG4,PIK3R2,NTN1,DCTN1,BID,MAP2K1,TMEM30A,SCFD2,ZDHHC3,SCFD1,CREBRF,IPO5,MAN1A1,RAB27A,TERF2,TMED6,ARL3,NEUROD1,IPO11,ICK,RANBP1,DENND1A,ZDHHC15,JAK2,FBXW7,OAZ2,BBS12,SNAP25-AS1,SMG1,DYNC1I1,ATP9A,DENND2A,KPNA3,CTAGE6,IMMP2L,PRKCD,TGFB1,NUP93,BCAS3,ZFYVE9,CORO1C,ZFAND2A,GRPEL2,ANK1,CLMN,CHRM1,RAB11FIP4,ITSN2,BCL3,DCLK1,ANKRD54,RABGAP1L,SNX8,SMG6,PPP3CA,NSUN2,TRAK1,PARK2,RN7SL832P,TSNARE1,AXIN1,MYO1D,SYTL5,LRPPRC,SREBF2,LEPROT,PROS1,NOL3,RIOK2,MYO1E,TBC1D14,RAB3GAP2,TBCK,ANKFY1,AP3B2,SPG11,CCDC91,IFT122,RANBP3,SMAD3,RAB6A,WIPI1,VPS39,PSAP,NEDD4,KPNB1,VPS53,RAB6C,SEC61B,THOC3,APP,BET1L,MYO1F,EYA2,SSR2,SEC22C,SETD2,ZDHHC6,PRICKLE1,DRD1,TMEM14A,SYNE2,PRKD1,OSBPL2,SYBU,VPS4A,PITRM1,ATF2,PTPN1,AP1B1,NLGN1,SNX16,SPAG9,RAB11A,EPG5,IPO9,RAB24,AGBL4,XPO6,EEA1,RILPL1,SORCS2,RAB11FIP3,BLOC1S5,AP2M1,TNPO1,TRAPPC11,TECPR2,TSG101,DESI1,SUFU,PAFAH1B1,KIF3A,AKAP6,RUFY3,DAB2,TRAM2,DZIP1,SMG5,SLC25A16,FAM91A1,MYO7A,SNX5,NSF,SYNE3,SPTBN5,NXT2,MAP1B,STOM,TBC1D5,CLEC16A,SNX9,RPGR,ARL5A,ACTR2,EGR2,CAPN10,AP3B1,KPNA6,ZFAND1,MAP1A,ANK3,SNUPN,PARD3,PTPN14,TRIM46,TUB,CACNG8,AP3D1,KIF3C,AP2B1,ANXA8L1,ARHGAP44,HEATR5A,SNX33,VPS16,ACAP2,ATG14,RAPGEF3,RSRC1,CACNG2,TBC1D9,CHMP3,RNF103-CHMP3,PRKAG1,RAB5B,ABCA12,TBC1D16,XPO4,VPS41,PLA2G4E,STX8,HTT,MAPK1,PIK3C3,NUP88,LRRK2,SPAG5,CHMP5,IFT81,TMEM108,ITGAM,NSG2,MIA2,SGSM1,TMCC1,ZC3H11A,POM121C,RHOT1,SAMM50,SMO,SUN1,LSG1,BLOC1S3,CPT1A,UBR5,NDE1,CUL3,HSPD1,EPM2A,GSK3B,RDX,IWS1,UPF2,BOK,AGT,SYT7,ADAR,BLOC1S6,ZMPSTE24,PEX5L,SNX3,EXOC6B,RIMS2,TOMM5,COPG2,SEC16B,TIMM44,GTF2IRD2,NCF1,TAOK2,ZDHHC23,ARFGAP3,MON2,PEX7,GCKR,TBC1D23,RGS14,ACTN4,STK4,ZFAND6,SLC1A1,WLS,PPP1R10,ABCD3,GRTP1,KPNA4,SYTL4,PRKCZ,PPP1CC,RABGEF1,KLC3,ANGPT1,HACL1,TBC1D10C,ARHGAP21,MAP2,STPG1,ATG3,SIL1,CLTB,RPL23,ATXN1,CAMSAP3,SP100,VTI1A,ANXA2,RAB27B,ITSN1,NUP214,SYK,SLIT1,MYRIP,RAB2A,SEC31B,CACNG3,GRIP1,ERGIC1,MYO1A,CNIH4,PLEKHM1,HGSNAT,MLPH,AKTIP,EPS15,INPP5F,REPS2,TBC1D10A,SNX1,MALT1,ZDHHC11,ZDHHC11B,EHD2,THEM4,TRAPPC8,NDC1,RPH3A,RAB28,SYNDIG1,VPS52,TRAF3IP2,SGTB,SLC25A13,BMP4,ABLIM3*
GO:0051271	negative regulation of cellular component movement	0.0012407779342219645	*S1PR2,CLASP2,SEMA3A,NRG3,MARVELD3,MAP2K5,PTPRR,ROBO1,CNIH2,SRGAP3,MCC,SRGAP2B,PTPRK,ABCC8,DACH1,ADORA1,EPHA1,PTPRO,PTPRU,ADAMTS9,NF1,PLCB1,DPEP1,APOD,BCR,NDRG4,NRG1,HDAC5,DCN,PTPRT,WNT11,MMP28,SLIT2,CORO1C,KANK1,DUSP22,EPHA4,PIP5KL1,PPARG,DLG5,MYOCD,NF2,SFRP1,FOXO3,PTPRM,NAV3,PTPRG,SEMA6D,BMPR1A,FGF2,MCTP1,CHRD,VCL,CLIC4,PKHD1,LDLRAD4,CLASP1,PRKG1,ATP2B4,CTNNA1,MAGI2,ADTRP,MITF,DPYSL3,PTEN,RHOA,SULF1,GPR173,TMEFF2,DLC1,ZMYND8,RABGEF1,PHLDB2,OSBPL8,BRAF,SPINT2,CD300A,ANGPT4,SP100,SRGAP2,FRMD5,PTPRJ,GNA*

			12
GO:0099175	regulation of postsynapse organization	0.001245078958267091 7	*NRXN1,GRID2,KALRN,NTRK3,CDKL5,PTPRD,TANC2,FYN,CAPRIN2,ZDHHC15,IL1RAPL1,CAMK2B,EPHA4,FCGR2B,ARHGAP22,DGKB,DNM3,CUX2,ABI2,NRP2,NLGN1,PAFAH1B1,RELN,PAK3,ACTR2,ARHGAP44,EPHA7,PTPRS,PTEN,LRRK2,SHANK3,WNT7A,EEF2K,GRIN2B*
GO:0097484	dendrite extension	0.001251952634636137	*LLPH,RIMS1,SYT1,CYFIP2,ITSN2,AUTS2,PARK2,CPNE6,SYT3,CPNE9,SPAG9,RASAL1,UNC13A,TMEM108,SYT17,RIMS2,CDKL3,NEDD4L,SH3GL2*
GO:0007605	sensory perception of sound	0.001266608082961983	*CLRN1,TJP1,ATP2B2,LHFPL4,LRP2,CHD7,TRIOBP,THRB,GRM7,ATP8B1,CNTN5,STRC,TFAP2A,PKHD1L1,TMC2,MYO3B,NAV2,PCDH15,PPIP5K2,LHFPL3,TMC1,MYO3A,CDH23,CACNA1D,OTOGL,HOMER2,OTOA,AXIN1,SPTBN4,USP53,SRRM4,CRYM,COL11A1,KCNQ1,COL4A3,USH2A,MYO7A,CLIC5,EYA1,PTPRQ,TUB,COCH,LHFPL5,ROR1,ZNF354A,ESPNL,CDC14A,SOBP,NIPBL,TBL1X,DIAPH1,MYO1A,TMPRSS3*
GO:0035335	peptidyl-tyrosine dephosphorylation	0.001285422714372689 3	*PTPRR,PTPRK,PTPN11,PTPRO,PTPRD,PTPRU,PTPRT,PTPRN2,PTPDC1,DUSP22,TPTE,SSH1,PTPRM,EYA2,PTPRG,PTPN1,PTPRE,PTPN13,SSH2,EYA1,PTPN2,PTPRQ,PTPN9,PTPN14,PTPRA,PTPRS,PTEN,MTMR3,DNAJC6,EPM2A,PTPRB,DUSP26,CDC14A,ACP1,EYA3,PPP2CA,PTPRJ,RNGTT,PTPRC*
GO:0050954	sensory perception of mechanical stimulus	0.001295074922809662 5	*CLRN1,TJP1,ATP2B2,LHFPL4,LRP2,CHD7,TRIOBP,THRB,GRM7,ATP8B1,CNTN5,STRC,TFAP2A,PKHD1L1,TMC2,MYO3B,NAV2,FYN,PCDH15,PPIP5K2,LHFPL3,TMC1,MYO3A,CDH23,CACNA1D,OTOGL,HOMER2,OTOA,AXIN1,SPTBN4,USP53,SRRM4,CRYM,COL11A1,KCNQ1,COL4A3,TRPA1,USH2A,MYO7A,CLIC5,EYA1,TRPV1,NTRK1,PTPRQ,TUB,COCH,LHFPL5,ROR1,ZNF354A,ESPNL,CDC14A,SOBP,NIPBL,TBL1X,DIAPH1,SCN1A,MYO1A,TMPRSS3*
GO:0050890	cognition	0.001332136916282457	*DNAH11,NRXN1,ADCY8,NLGN4X,KALRN,GRM5,NRXN3,CHD7,SLC24A2,ITGB1,ABCC8,DGKI,ADORA1,NTRK2,EP300,INSR,TBR1,BDNF,FYN,NF1,PLCB1,AFF2,NDRG4,SLC1A4,SGK1,JPH3,CHRM1,YTHDF1,CNTNAP2,CNR1,CAMK4,PARK2,PRKAR1B,CSMD1,CUX2,SHANK1,SLC6A1,APP,AMFR,RCAN1,DRD1,SHANK2,STRA6,PRKCG,NLGN3,PAFAH1B1,BTBD9,SETD5,RELN,SLC8A2,ATAD1,ADNP,SORCS3,BRINP1,ACTR2,ELAVL4,EGR2,NTRK1,MAP1A,NETO1,HTT,MAPK1,PTEN,SHANK3,GPI,PRKAR2B,GRIN1,SYNGAP1,EPM2A,AGT,RPS6KB1,RGS14,EPHB2,SLC1A1,SOBP,PRKCZ,GRIN2B,NIPBL,SHROOM4,BRAF,ATP8A1,TNR,ATXN1,ADCY1,TANC1,CREB1*
GO:0048878	chemical homeostasis	0.001341447578769804 8	*ENPP1,ASPH,PDE4D,SLC9A1,FAM155A,ADCY8,PTGFR,CHERP,RYR1,NOX5,TJP1,ACOXL,ATP2B2,GRM5,PLCE1,ANK2,MALRD1,SLC9A9,CHD7,SLC26A6,HTR2B,ITGB6,RYR3,GOT1,SLC24A2,SLC24A3,CCDC22,ABCC8,HMGN3,STXBP4,TCIRG1,PTPN11,CACNB2,DDB1,ADORA1,ADRA1D,CNNM1,ESR1,CYB561A3,STK39,INSR,TCF7L2,SLC4A10,KCNMA1,LCK,SLC12A8,ACOX1,SGCD,MICU3,DMD,SLC30A9,SLC8A1,JPH2,FYN,RTN4,PIK3R2,SLC4A5,CNNM2,BDKRB1,BDKRB2,STIM1,TMBIM6,CMKLR1,DCN,SLC39A14,PTGER2,CLDN16,NEUROD1,JAK2,FBXW7,NPSR1,CLDN1,SLC9A7,PNPLA1,KLF15,NOX4,TGFB1,KEL,JPH3,PTPRN2,F2RL1,C9ORF47,CDH23,DISC1,CLN6,XPR1,TMTC2,HOMER2,CAPN3,SLC16A1,RORA,CNR1,TNFSF11,PPP3CA,PARK2,SOD2,IGF1R,PPARG,P2RY10,TRPM1,OTC,XK,SREBF2,RYR2,LEPR,FOXK2,NOL3,CACNA1C,SCN3B,CSMD1,GRM1,PAX2,MED13,FOXO3,SMAD3,ITPR1,ACKR2,GRIK5,SLC4A4,APP,PDGFRA,NOX1,RAB11FIP5,IBTK,ANGPTL4,LRP5,BDH2,SLC9B2,DRD1,PRKD1,STAT1,HNF4A,RAF1,ACOX2,FGF2,ADCY5,ADCYAP1R1,KLHL3,SLC9B1,CLIC4,KCNQ1,CCL14,CCL15,TRPA1,NMUR2,AKAP6,BTBD9,TRPC5,NEO1,WDTC1,PKHD1,WNK1,SIN3A,SLC8A2,STAU1,SLC9C1,TPCN1,SNX5,ATP2B4,ASGR2,ADNP,SCARB1,ATP6V0A2,P2RY8,TFRC,EPHA5,CACNA1A,PTGER3,TRPV1,ACACA,TRIM24,ADIPOR2,PLCG2,C1QTNF1,GPR35,AP3B1,PTPN2,SLC39A10,ANK3,TMEM199,ATP13A3,AP3D1,SLC9C2,MCU,LIMA1,TRDN,LRRC8D,TRPC6,ABCB7,ATP13A5,SNCA,WNT7B,ABCA12,SLC4A8,HTT,MAPK1,SLC30A7,PRCP,LRRK2,ATP6V1A,ZBTB20,TMCO1,PKD2,EFNA5,STEAP4,PPP2R3C,ZBED6,GPR21,BMP6,ANO1,GPI,EXT1,PNPLA3,RALY,STXBP5L,GRIN1,TF,ITPR2,ABCG8,ALAS2,BOK,AGT,PRKAA2,HRH4,HEPHL1,SLC29A1,STC2,ALOX5,CLDN4,NOS1,GCKR,SCNN1B,SLC1A1,XCR1,IL18,MCOLN1,STEAP3,PAX6,ATP1A3,ADORA2A,GRIN2B,KCTD7,PACS2,DIAPH1,HTR2C,POU3F3,ATP6V0A1,CYP39A1,GRIK2,ATP2B3,CIB2,CAV2,CA12,TMPRSS3,MET,CAMK2D,CCR3,TRA2B,MLXIPL,NEDD4L,PTPRC,GPR89A,EDNRA*
GO:0008283	cell population proliferation	0.001347291371216850 7	*PRDX2,ASPH,PRKCI,TACC2,DNMT1,S1PR2,HLX,C6ORF89,IL31RA,RPS6KA2,SETD4,PTGFR,CHERP,NOX5,DCHS2,TJP1,NUDT6,HPSE2,PRLR,FTO,NTRK3,ARNT,SEMA5A,FLT3,RAD51B,STAT5B,TOX,FER,CASK,MARVELD3,MAP2K5,KITLG,LRP2,PIK3CD,SAAL1,MEGF10,ROBO1,HTR2B,MECOM,TACC1,TENM1,LMNA,TRAF6,ITPKB,DNAJA3,GRK5,ABI1,ITGB1,CDC6,MCC,PTPRK,RUNX1,ABCC8,STXBP4,TCIRG1,DACH1,ADORA1,EPHA1,ERBB4,ADRA1D,MRE11A,ZNF609,ATP8A2,ESR1,CDON,NTRK2,CELA1,HOOK3,PDGFB,CCND3,TOB2,INSR,RERE,PRKAR1A,TCF7L2,PAK1,ESRRB,EGLN3,TFAP2A,FANCA,MEIS1,TENM4,MDM4,IL5RA,SHC4,MTA3,LAMC1,PTPRU,DMD,USP13,KAT7,FYN,NF1,RTN4,APOD,NDRG4,IFI30,NTN1,NRG1,DOCK8,BID,MAP2K1,FNIP1,MAD1L1,DAGLA,VGLL4,RBM5,CSNK2A3,ROR2,DOCK2,CDH13,PDF,PTGER2,JAK2,FBXW7,ITCH,BCL11B,CLDN1,WNT11,IFT80,NOX4,RPRD1B,PRKCD,ACVR2A,RUNX2,TGFB1,SGK1,BTRC,F2RL1,C9ORF47,DNAJB2,SLIT3,TP73,MUSTN1,DISC1,KANK1,LAMC2,CLMN,CHRM1,SMAD6,ETV6,NELL1,KCNH1,TFAP2D,BMPER,DUSP22,FGF10,GLP2R,EPHA4,RORA,PRKCA,CD6,TNFSF*

			11,UFL1,CTNNB1,SOD2,FCGR2B,IGF1R,PPARG,DLG5,BRIP1,LEPR,FGF1,NR4A3,DCT,MYOCD,PER2,SPDYA,HCK,TBCK,CSPG4,MMP16,NF2,FLT4,HDAC4,PAX2,TPTE,ELN,SFRP1,ST8SIA1,FOXO3,NFIB,SMAD3,PTPRM,MTBP,SLC16A2,NRP2,CDC73,APP,SSBP3,PDGFRA,NOX1,YAP1,CDK3,NLGN2,LRP5,POLR3G,GNG2,SOX2,CHST11,VASH2,FRS2,PALB2,MMP2,PRKD1,STAT1,ST18,HNF4A,ZBTB7C,SHANK2,EREG,ATF2,TCF3,RAF1,BMPR2,BMPR1A,BTBD10,TAF8,FGF2,AGBL4,PTAFR,YME1L1,JARID2,RARB,VIPR1,EEF1E1,GAS8,ALKBH3,CHRD,ATF3,CKS1B,PAWR,AGO3,DLEC1,TSG101,LAMB1,WNT3,CCL14,SCUBE2,PAFAH1B1,CNTFR,COL4A3,DDAH1,TRPC5,GLI2,ERCC1,DAB2,PKHD1,CACUL1,DLG3,ABCB1,PELI1,PPP2R5C,CFDP1,ZNF423,SP1,ALK,INPP5D,XDH,OVOL2,NFATC2,PRKG1,BNC1,TET1,CCPG1,SCARB1,CDK6,CELF1,TYR,TFRC,GPC3,EYA1,DIS3L2,HOXB4,MNAT1,LGI4,HDAC1,TRIM24,RNF10,PTPN2,NTRK1,TMIGD1,SLC39A10,EPHB1,AXIN2,EPCAM,PTPN14,JAG2,CARM1,SMARCA2,CTNNA1,FBXW4,MST1,BMPR1B,MAGI2,OCA2,HEY2,RC3H1,WNT7B,KMT2D,MITF,SRSF6,HIPK1,RPTOR,BTK,ZNRF3,IGF1,MAPK1,PTEN,BMP7,SOX5,LRRK2,OSGIN1,LILRB4,MORC3,IL11RA,PKD2,PDGFC,WNT7A,PLCL2,BMP6,SOS1,TSHR,EXT1,PDCD1LG2,RAPGEF2,SMO,ENPP3,RHOA,VIPR2,NDE1,CUL3,HSPD1,EPM2A,STAT6,CDK13,BOK,SULF1,NACC2,RNF43,TNFRSF8,AGT,STAT2,ZMPSTE24,NDRG2,PTK2,PBX1,MAP3K5,TCFL5,UTP20,PYGO2,SHC1,NDUFS2,PARP10,RPS6KB1,ALOX5,STK4,EPHB2,IL18,DLC1,CALCRL,SLC25A33,PAX6,PRKCZ,ADORA2A,PPP1CC,TMIGD2,ZNF143,ANGPT1,GCNT2,ETS1,PRKCQ,BRAF,HDAC2,POU3F3,SPINT2,CD300A,PHIP,RPL23,SH3BP4,CR2,NUMB,SQLE,SYK,WWTR1,BRCA2,DVL2,PTPRJ,TICAM1,HSF1,SRPK2,SULT2B1,VAV3,ZP3,CAV2,GNA12,POC1B,TNFSF9,CD109,CCR3,MALT1,KDM2B,MLXIPL,PHB,PTPRC,CTCF,EDNRA,BMP4
GO:0030010	establishment of cell polarity	0.001395115708636145 1	*PRKCI,WWC1,ITGB1,HSP90AA1,KIF26B,TCIRG1,CYTH3,GBF1,FRMD4A,FBXW11,GSN,DCTN1,DOCK8,MYH9,PARD3B,DOCK2,UST,SIPA1L3,BCAS3,KANK1,ANKFN1,FGF10,IGF1R,MYO9A,PAFAH1B1,RUFY3,PKHD1,CLASP1,MAP1B,ROCK1,EPHB1,PARD3,SH3BP1,PTK7,HTT,AMOTL1,WNT7A,MAP4,RHOA,NDE1,GSK3B,FAM49B,PTK2,FRMD4B,GPSM2,PAX6,PRKCZ,CAMSAP3,TRAF3IP2*
GO:0051592	response to calcium ion	0.001395115708636145 1	*NRXN1,ASPH,ADCY8,RYR1,RYR3,ITPKB,CPNE4,ALOX5AP,KCNMA1,ECT2,DPEP1,TTN,STIM1,SYT1,SYT13,KCNH1,RASA4,RASA4B,CAPN3,PPP3CA,RYR2,CPNE6,TPH2,SYT3,NEDD4,SLC6A1,CPNE9,NLGN1,CLIC4,RASAL1,IQGAP1,MEF2A,SYT12,MNAT1,PLCG2,CACNG2,SYT9,PKD2,SYT17,EEF2K,PRKAA2,SYT7,MCOLN1,BRAF,ADCY1,ALG2,CAMK2D,SLC25A13,CPNE1*
GO:0097553	calcium ion transmembrane import into cytosol	0.001395115708636145 1	*ASPH,PDE4D,FAM155A,CHERP,RYR1,ANK2,CHD7,HTR2B,RYR3,SLC24A2,LCK,DMD,SLC8A1,JPH2,FYN,BDKRB1,NPSR1,TGFB1,JPH3,CAPN3,RYR2,NOL3,CACNA1C,ITPR1,IBTK,DRD1,PRKD1,FGF2,TRPA1,AKAP6,SLC8A2,TPCN1,ATP2B4,TRPV1,PLCG2,TRDN,SNCA,HTT,PKD2,GRIN1,ITPR2,NOS1,XCR1,MCOLN1,GRIN2B,DIAPH1,HTR2C,CAMK2D,PTPRC*
GO:0050772	positive regulation of axonogenesis	0.001399110067038398 3	*TIAM2,SEMA5A,ROBO1,CDKL5,ROBO2,DSCAM,GOLGA4,LIMK1,NTRK2,CDH4,BDNF,AMIGO1,NTN1,MAP2K1,SLIT2,DISC1,NIN,ISLR2,BMPR2,RAB11A,SEMA4D,PLXNA2,WNT3,PAFAH1B1,TRPC5,RUFY3,MACF1,ADNP,MAP1B,MAP3K13,EFNA5,BRAF*
GO:1904062	regulation of cation transmembrane transport	0.001435862914484249 8	*NRXN1,ASPH,PDE4D,SLC9A1,NOS1AP,DPP6,UTRN,KCNIP4,ANK2,CHD7,ITGB1,CNIH2,ABCC8,TCIRG1,NETO2,CACNB2,DPP10,STK39,CAB39,AMIGO1,KCNRG,TMC2,DMD,SLC8A1,JPH2,FYN,SHISA9,SLC43A2,BDKRB1,KCNIP1,STIM1,FGF14,ANO6,NPSR1,TMC1,TGFB1,KEL,JPH3,CACNA1D,CAPN3,VMP1,RYR2,CACNA1C,SCN3B,WWP2,SHANK1,NEDD4,APP,NLGN2,VDAC1,DRD1,PRKD1,FXYD2,FXYD6,RASGRF2,NLGN1,KCNQ1,NLGN3,AKAP6,WNK1,RELN,AHNAK,RGS7,ATP2B4,KCNAB2,STOM,PLCG2,GPR35,ANK3,CACNG8,NETO1,TRDN,STAC,SNCA,CACNG2,HTT,CNIH3,PKD2,SHANK3,KCNC2,PSEN2,AGT,PDE4B,NOS1,ACTN4,EPHB2,LRRC52,DIAPH1,CACNG3,SESTD1,CAMK2D,NEDD4L,SHISA6,EDNRA*
GO:0019725	cellular homeostasis	0.001462120894144609 8	*ENPP1,PRDX2,ASPH,PDE4D,SLC9A1,FAM155A,ADCY8,PTGFR,CHERP,RYR1,NOX5,TJP1,EGLN2,ATP2B2,GRM5,PLCE1,ANK2,SLC9A9,CHD7,SLC26A6,HTR2B,RYR3,SLC24A2,SLC24A3,CCDC22,ABCC8,HMGN3,STXBP4,TCIRG1,CACNB2,ADORA1,ADRA1D,LARGE,ESR1,CYB561A3,SLC4A10,KCNMA1,LCK,SLC12A8,MICU3,DMD,SLC30A9,SLC8A1,JPH2,FYN,RTN4,PIK3R2,DCTN1,SLC4A5,BDKRB1,BDKRB2,STIM1,TMBIM6,CMKLR1,SLC39A14,PTGER2,CLDN16,ANO6,NEUROD1,JAK2,FBXW7,NPSR1,SLC9A7,KLF15,NOX4,TGFB1,KEL,JPH3,PTPRN2,F2RL1,C9ORF47,DNAJB2,CDH23,DISC1,CLN6,XPR1,CAPN3,CNR1,PPP3CA,PARK2,IGF1R,P2RY10,TRPM1,XK,RYR2,FOXK2,NOL3,CACNA1C,GRM1,PAX2,FOXO3,SMAD3,ITPR1,ACKR2,GRIK5,SLC4A4,APP,PDGFRA,NOX1,RAB11FIP5,IBTK,LRP5,SLC9B2,DRD1,PRKD1,RAF1,FGF2,ADCY5,ADCYAP1R1,SLC9B1,CLIC4,IDE,KCNQ1,CCL14,CCL15,TRPA1,NMUR2,AKAP6,TRPC5,WDTC1,PKHD1,SIN3A,SLC8A2,SLC9C1,TPCN1,ATP2B4,ATP6V0A2,P2RY8,TFRC,EPHA5,CACNA1A,PTGER3,GNB3,TRPV1,PLCG2,C1QTNF1,GPR35,AP3B1,MAP1A,SLC39A10,TMEM199,ATP13A3,AP3D1,SLC9C2,MCU,TRDN,LRRC8D,TRPC6,ABCB7,ATP13A5,SNCA,ABCA12,SGCZ,SLC4A8,HTT,MAPK1,SLC30A7,LRRK2,ATP6V1A,ZBTB20,TMCO1,PKD2,EFNA5,SHANK3,STEAP4,ZBED6,RHOT1,BMP6,ANO1,GRIN1,TF,ITPR2,ALAS2,BOK,AGT,PRKAA2,FGGY,HRH4,HEPHL1,SLC29A1,ALDOA,STC2,NOS1,SLC1A1,XCR1,UBE2K,MCOLN1,ATP1A3,GRIN2B,KCTD7,PACS2,DIAPH1,HTR2C,ATP6V0A1,GRIK2,ATP2B3,CIB2,CAV2,TMPRSS3,CAMK2D,CCR3,TRA2B,MLXIPL,NEDD4L,PTPRC,GPR89A,TYRO3,EDNRA*

GO:0016482	cytosolic transport	0.001521550428299181 7	*HOOK2,EZR,ERC1,GBF1,HOOK3,SNX2,PIP4K2A,SNX6,DENND5A,TANC2,LMTK2,STX6,DCTN1,MAP2K1,ATP9A,DENND2A,SNX8,PARK2,MYO1D,TBC1D14,ANKFY1,CCDC91,RAB6A,WIPI1,VPS53,RAB6C,BET1L,SNX16,SPAG9,EEA1,DAB2,SNX5,TBC1D5,ACTR2,TRIM46,HEATR5A,CHMP3,STX8,MAPK1,PIK3C3,LRRK2,RDX,SYT7,SNX3,MON2,TBC1D23,TBC1D10C,MAP2,VTI1A,AKTIP,EPS15,TBC1D10A,SNX1,VPS52*
GO:0050771	negative regulation of axonogenesis	0.001571578066863775	*SEMA3A,SEMA3D,SEMA5A,DAB1,RTN4,RTN4R,NTN1,SEMA5B,MAG,DRAXIN,DCC,DIP2B,FSTL4,SEMA6D,SEMA4D,WNT3,RUFY3,TRIM46,EPHA7,PTPRS,PTEN,SYNGAP1,EPHB2,MAP2,TNR,CDKL3,SLIT1,SEMA3C*
GO:0007626	locomotory behavior	0.001574136434644052	*NEGR1,PBX3,ADCY8,KALRN,GRM5,CHD7,OXR1,DSCAM,DAB1,SLC4A10,MEIS1,NAV2,PCDH15,ANKH,NRG1,FIG4,MTA1,ASTN1,CELSR1,CDH23,CLN6,GPR52,ANKFN1,EPHA4,PARK2,SOD2,GRM1,SPG11,APP,NLGN2,RCAN1,SPTBN4,DRD1,SLC6A3,SHANK2,ADCY5,PAFAH1B1,BTBD9,RELN,ALK,ELAVL4,LGI4,EPS8,CHRNB4,SNCA,OPRD1,PTEN,PUM1,LRRK2,SHANK3,TSHR,ZMPSTE24,SLC1A1,SOBP,ADORA2A,KLHL1,HTR2C,TNR,KCND2,SCN1A,NCOA2,INPP5F*
GO:0046903	secretion	0.001681635940787544 1	*NRXN1,PRKCI,ADCY8,CLASP2,PRLR,KALRN,CBL,STAT5B,FER,CASK,PIK3CD,EZR,NRXN3,CHD7,SLC26A6,ABCC8,HMGN3,DGKI,STXBP4,ERC2,TCIRG1,PTPN11,CACNB2,ADORA1,ERBB4,GRM7,NTRK2,FRMD4A,RIMS1,STK39,TCF7L2,PAK1,MYOM1,CHRM3,VAMP7,ARNTL,NF1,BCR,TTN,ICA1,SLC4A5,NRG1,SLC16A10,BDKRB2,MYH9,SCFD2,LIN7A,SYN2,COPS5,EXOC4,RAB27A,RAB3C,GOLPH3L,NEUROD1,JAK2,SYN3,SYT1,NPSR1,ATP9A,CTAGE6,CACNA1H,IL1RAPL1,SGK1,PCSK5,PTPRN2,F2RL1,CADPS2,SYT13,C12ORF4,ANK1,CHRM1,CLOCK,FGF10,STXBP5,SLC16A1,CNR1,TNFSF11,PPP3CA,PARK2,FCGR2B,PPARG,SYTL5,SCAMP5,NR4A3,PER2,HCK,RIMS4,SFRP1,GRIK5,SLC16A2,SYT3,SLC6A1,RAB11FIP5,NLGN2,SCRN1,LRP5,SLC9B2,DRD1,SERGEF,TPH1,SLC6A3,HNF4A,VPS4A,RAF1,NLGN1,RAB11A,ADCY5,MCTP1,PTAFR,RAB11FIP3,PRKCG,TANGO6,OC90,TRAPPC11,FBXL20,TSG101,KCNQ1,PAFAH1B1,NMUR2,TPD52,DAB2,WNK1,CLASP1,APBB3,XDH,SNX5,NSF,EPHA5,SYT12,ADRBK1,PTGER3,TRPV1,PRSS12,C1QTNF1,CAPN10,UNC13A,ARHGAP44,CD84,SPI1,CHRNB4,MCU,ILDR2,RAB15,SNCA,GRM4,SYT9,ADTRP,ABCA12,BTK,SLC4A8,IGF1,PIK3C3,LRRK2,ITGAM,EFNA5,MIA2,WNT7A,ZBED6,SNAP23,DNAJC1,BMP6,ANO1,BLOC1S3,STXBP5L,CPT1A,SYT17,GRIN3A,GSK3B,SYN1,MCTP2,AGT,SYT7,BLOC1S6,LAT2,EXOC6B,RIMS2,COPG2,CPE,PCSK6,SLC29A1,UGT1A3,CD160,ALOX5,CPLX2,ADAM8,ARFGAP3,SCNN1B,WLS,HCAR2,STEAP3,SYTL4,ADORA2A,RABGEF1,CADPS,NCOR2,BRAF,HTR2C,CD300A,RAB27B,ITSN1,ADCY1,SYK,MYRIP,SLC10A1,ZP3,AXL,MICAL3,RPH3A,TYRO3,TRAF3IP2,CREB1,CLNK,STXBP6*
GO:0017157	regulation of exocytosis	0.001696108247207584 7	*CLASP2,FER,CASK,NRXN3,DGKI,CACNB2,VAMP7,BCR,RAB27A,RAB3C,SYT1,ATP9A,CACNA1H,IL1RAPL1,F2RL1,CADPS2,SYT13,C12ORF4,STXBP5,CNR1,PARK2,FCGR2B,SCAMP5,RIMS4,SYT3,DRD1,VPS4A,NLGN1,PTAFR,PRKCG,FBXL20,TSG101,CLASP1,NSF,SYT12,CD84,SPI1,RAB15,SNCA,SYT9,SLC4A8,LRRK2,ITGAM,WNT7A,STXBP5L,SYT17,GRIN3A,GSK3B,SYN1,SYT7,RIMS2,CD160,CPLX2,SYTL4,ADORA2A,RABGEF1,CADPS,BRAF,CD300A,RAB27B,ADCY1,SYK,ZP3,STXBP6*
GO:0071902	positive regulation of protein serine/threonine kinase activity	0.001797994753705825 4	*NRXN1,NTRK3,FLT3,MAP2K5,KITLG,ROBO1,HTR2B,TENM1,TRAF6,CDC6,AKAP13,PDGFB,CCND3,INSR,CAB39,PAK1,MAP3K4,ERN2,MAP2K1,ROR2,CCNYL1,NOX4,TGFB1,EPHA4,TNFSF11,AXIN1,FGF1,AJUBA,SPDYA,TAB1,MTCP1,S100A12,CCNY,PTPN1,BMPR2,BMPR1A,FGF2,DVL3,CKS1B,NEK10,MAP3K7,SLC8A2,ATP2B4,GHR,MNAT1,MAP3K13,SNCA,BMPR1B,RPTOR,LRRK2,PKD2,PDGFC,RAPGEF2,RHOA,MAP3K5,TAOK2,ADAM8,STK4,PPP2CA,SYK,DVL2,SASH1,PTPRC*
GO:0060998	regulation of dendritic spine development	0.001811102285685987	*LLPH,KALRN,TANC2,CAPRIN2,CAMK2B,DISC1,DLG5,DNM3,CUX2,SHANK1,FSTL4,NLGN2,NLGN1,PAFAH1B1,RELN,PAK3,ACTR2,SDK1,PTPRS,PTEN,SHANK3,GRIN3A,EEF2K,HDAC2,ASAP1*
GO:0051345	positive regulation of hydrolase activity	0.001828231641024963 2	*ASPH,MAP4K4,C6ORF89,TIAM2,MAPRE2,KALRN,NTRK3,ROBO1,HTR2B,CDKL5,DNAJA3,ITGB1,ARHGAP24,RGS6,DOCK10,EPHA1,ABR,ESR1,FNTA,EGLN3,ARHGAP6,LCK,ECT2,ARHGAP42,GSN,FYN,NF1,RTN4R,BCR,DOCK8,BID,RAPGEF6,RGS8,RCC2,GNAQ,DENND1A,JAK2,ARAP2,DOCK9,CYFIP2,WNT11,SIPA1L3,PRKCD,CRADD,F2RL1,BCAS3,CORO1C,BLID,RABGAP1L,EPHA4,ARHGAP22,PPARG,GARNL3,RGS10,TBC1D14,TBCK,SFRP1,ARHGAP29,BCL2L13,SMAD3,PSAP,PREX1,APP,PDGFRA,PRKD1,ST18,GRAMD4,CASP1,FGF2,PPM1F,NEDD9,SEMA4D,PTAFR,ADCYAP1R1,DVL3,MYO9A,CCL14,CCL15,COL4A3,NMUR2,HIP1,DOCK11,XDH,RGS7,TBC1D5,SNX9,ROCK1,NTRK1,SLC39A10,BCAR3,RAPGEF3,SH3BP1,SNCA,MAGI2,TBC1D9,USP50,RASA1,TBC1D16,LRRK2,ARHGAP25,SGSM1,NLRP1,UACA,RAPGEF2,VRK3,GRIN1,RHOA,HSPD1,GSK3B,BOK,AGT,MAP3K5,CLDN4,RGS14,SIPA1L2,ARHGAP11A,SLC1A1,DLC1,GRTP1,PRKCZ,GRIN2B,TBC1D10C,CCL22,CD300A,SRGAP2,SYK,RAP1GAP2,DVL2,TANK,HSF1,ASAP1,RALGAPA1,CAV2,GNA12,TBC1D10A,MALT1,PHB,PTPRC,EDNRA*
GO:0051235	maintenance of location	0.001845267842842687 4	*ENPP1,ASPH,PDE4D,CHERP,RYR1,FTO,STAT5B,ANK2,CHD7,HTR2B,RYR3,CCDC22,NRIP1,LCK,DMD,SLC8A1,GSN,RIT2,JPH2,BDKRB1,MDFI,MAD1L1,SYNE1,FBXW7,NPSR1,CD4,TGFB1,JPH3,CIZ1,PLIN3,CAPN3,PPARG,MSR1,SREBF2,RYR2,NOL3,CACNA1C,ITPR1,GRIK5,PLA2G4C,IBTK,DRD1,PRKD1,TAF3,TAF8,FGF2,SUFU,ZFYVE1,PAFAH1B1,TRPA1,AKAP6,DZIP1,TPCN1,LTBP1,SCARB1,UVRAG,TRPV1,PLCG2,PTPN2,ANK3,AP3D1,TRDN,SNCA,HTT,SLC30A7,MORC3,PKD2,PLIN2,G*

			ET4,SUN1,CPT1A,ITPR2,FBN2,CDS1,NOS1,XCR1,GPSM2,PPARA,MCOLN1,FBN1,OSBPL8,ARHGAP21,DIAPH1,HTR2C,SKP1,SP100,SQLE,ANKRD13C,CAMK2D,PTPRC,RNF213
GO:0032940	secretion by cell	0.001845842355187936 5	NRXN1,ADCY8,CLASP2,KALRN,CBL,FER,CASK,PIK3CD,EZR,NRXN3,CHD7,ABCC8,HMGN3,DGKI,STXBP4,ERC2,TCIRG1,PTPN11,CACNB2,ADORA1,GRM7,NTRK2,FRMD4A,RIMS1,TCF7L2,PAK1,MYOM1,VAMP7,ARNTL,NF1,BCR,TTN,ICA1,SLC16A10,MYH9,SCFD2,LIN7A,SYN2,COPS5,EXOC4,RAB27A,RAB3C,GOLPH3L,NEUROD1,JAK2,SYN3,SYT1,ATP9A,CTAGE6,CACNA1H,IL1RAPL1,PCSK5,PTPRN2,F2RL1,CADPS2,SYT13,C12ORF4,ANK1,CLOCK,FGF10,STXBP5,SLC16A1,CNR1,TNFSF11,PPP3CA,PARK2,FCGR2B,PPARG,SYTL5,SCAMP5,NR4A3,PER2,HCK,RIMS4,SFRP1,GRIK5,SLC16A2,SYT3,RAB11FIP5,NLGN2,SCRN1,LRP5,SLC9B2,DRD1,SERGEF,TPH1,HNF4A,VPS4A,RAF1,NLGN1,RAB11A,ADCY5,MCTP1,PTAFR,RAB11FIP3,PRKCG,TANGO6,TRAPPC11,FBXL20,TSG101,KCNQ1,PAFAH1B1,DAB2,CLASP1,APBB3,NSF,EPHA5,SYT12,ADRBK1,TRPV1,PRSS12,C1QTNF1,CAPN10,UNC13A,ARHGAP44,CD84,SPI1,CHRNB4,MCU,ILDR2,RAB15,SNCA,GRM4,SYT9,ADTRP,ABCA12,BTK,SLC4A8,IGF1,PIK3C3,LRRK2,ITGAM,EFNA5,MIA2,WNT7A,ZBED6,SNAP23,DNAJC1,BMP6,ANO1,BLOC1S3,STXBP5L,CPT1A,SYT17,GRIN3A,GSK3B,SYN1,MCTP2,AGT,SYT7,BLOC1S6,LAT2,EXOC6B,RIMS2,COPG2,CPE,PCSK6,CD160,ALOX5,CPLX2,ADAM8,ARFGAP3,WLS,HCAR2,STEAP3,SYTL4,ADORA2A,RABGEF1,CADPS,BRAF,HTR2C,CD300A,RAB27B,ITSN1,ADCY1,SYK,MYRIP,ZP3,AXL,MICAL3,RPH3A,TYRO3,CREB1,CLNK,STXBP6
GO:0030336	negative regulation of cell migration	0.001962114240882422 6	S1PR2,CLASP2,SEMA3A,NRG3,MARVELD3,MAP2K5,PTPRR,ROBO1,SRGAP3,MCC,SRGAP2B,PTPRK,ABCC8,DACH1,ADORA1,EPHA1,PTPRU,ADAMTS9,NF1,PLCB1,DPEP1,APOD,BCR,NDRG4,NRG1,HDAC5,DCN,PTPRT,WNT11,MMP28,SLIT2,CORO1C,KANK1,DUSP22,EPHA4,PIP5KL1,PPARG,DLG5,MYOCD,NF2,SFRP1,FOXO3,PTPRM,NAV3,PTPRG,SEMA6D,BMPR1A,FGF2,MCTP1,CHRD,VCL,CLIC4,LDLRAD4,CLASP1,PRKG1,ATP2B4,MAGI2,ADTRP,MITF,DPYSL3,PTEN,RHOA,SULF1,GPR173,TMEFF2,DLC1,ZMYND8,RABGEF1,PHLDB2,OSBPL8,BRAF,CD300A,ANGPT4,SP100,SRGAP2,PTPRJ,GNA12
GO:0098771	inorganic ion homeostasis	0.002009981254232051	ENPP1,ASPH,PDE4D,SLC9A1,FAM155A,ADCY8,PTGFR,CHERP,RYR1,NOX5,ATP2B2,GRM5,PLCE1,ANK2,SLC9A9,CHD7,SLC26A6,HTR2B,RYR3,SLC24A2,SLC24A3,CCDC22,TCIRG1,CACNB2,ADORA1,ADRA1D,CNNM1,ESR1,CYB561A3,SLC4A10,KCNMA1,LCK,SLC12A8,SGCD,MICU3,DMD,SLC30A9,SLC8A1,JPH2,FYN,SLC4A5,CNNM2,BDKRB1,BDKRB2,STIM1,TMBIM6,CMKLR1,SLC39A14,PTGER2,CLDN16,JAK2,NPSR1,SLC9A7,TGFB1,KEL,JPH3,F2RL1,C9ORF47,CDH23,DISC1,CLN6,XPR1,TMTC2,CAPN3,CNR1,TNFSF11,PARK2,SOD2,P2RY10,TRPM1,OTC,XK,RYR2,NOL3,CACNA1C,GRM1,SMAD3,ITPR1,ACKR2,SLC4A4,APP,PDGFRA,NOX1,IBTK,BDH2,DRD1,PRKD1,FGF2,ADCY5,ADCYAP1R1,SLC9B1,CLIC4,KCNQ1,CCL14,CCL15,TRPA1,NMUR2,AKAP6,BTBD9,TRPC5,NEO1,PKHD1,SLC8A2,SLC9C1,TPCN1,SNX5,ATP2B4,ATP6V0A2,P2RY8,TFRC,CACNA1A,PTGER3,TRPV1,TRIM24,PLCG2,C1QTNF1,GPR35,AP3B1,SLC39A10,ANK3,TMEM199,ATP13A3,AP3D1,SLC9C2,MCU,TRDN,TRPC6,ABCB7,ATP13A5,SNCA,SLC4A8,HTT,MAPK1,SLC30A7,LRRK2,ATP6V1A,TMCO1,PKD2,STEAP4,BMP6,EXT1,GRIN1,TF,ITPR2,ALAS2,BOK,AGT,HRH4,HEPHL1,STC2,NOS1,SCNN1B,SLC1A1,XCR1,MCOLN1,STEAP3,ATP1A3,ADORA2A,GRIN2B,KCTD7,PACS2,DIAPH1,HTR2C,ATP6V0A1,GRIK2,ATP2B3,CIB2,CAV2,CA12,TMPRSS3,CAMK2D,CCR3,NEDD4L,PTPRC,GPR89A,EDNRA
GO:0045927	positive regulation of growth	0.002082994443255892	HLX,SLC9A1,SEMA5A,STAT5B,MAP2K5,EZR,CHD7,CDKL5,DSCAM,GOLGA4,ERBB4,LIMK1,ATP8A2,RIMS1,INSR,CDH4,BASP1,BDNF,RFTN1,PLCB1,NTN1,NRG1,CSNK2A3,SYT1,PLS1,DISC1,ITSN2,CAPN3,PARK2,CPNE6,SFRP1,SYT3,YAP1,ISLR2,SPTBN4,CPNE9,SLC6A3,BMPR2,BMPR1A,RAB11A,FGF2,PPM1F,SEMA4D,EIF4G1,WNT3,PAFAH1B1,AKAP6,RASAL1,TRPC5,RUFY3,MACF1,ADNP,MAP1B,CELF1,GHR,UNC13A,MAP3K13,HEY2,RPTOR,IGF1,EFNA5,GPR21,TSHR,SMO,SYT17,RHOA,RIMS2,RPS6KB1,SLC25A33,NIPBL,ZP3,KDM2B,NEDD4L,CREB1
GO:0006470	protein dephosphorylation	0.002289070181355644 3	CTDSPL2,PTPRR,PTPRK,PTPN11,MGAT5,ADORA1,IKBKB,PTPRO,FBXW11,PTPRD,LCK,PPM1L,PTPRU,PPP4R2,PPP2CB,PTPRT,JAK2,RPRD1B,PRKCD,PPM1E,BTRC,PTPRN2,PTPDC1,DAPP1,DUSP22,PPP3CA,PPP1CB,PPP2R2B,CTDP1,TPTE,PPP6R2,SSH1,PTPRM,TAB1,EYA2,PTPRG,RCAN1,LRRC2,PTPN1,PPA2,PPM1F,PTPRE,PTPN13,PPP2R5C,SSH2,CAMTA1,EYA1,ROCK1,PTPN2,SLC39A10,PTPRQ,PTPN9,PTPN14,PTPRA,PDXP,MAGI2,PTPRS,HTT,PTEN,LRRK2,MTMR3,VRK3,DNAJC6,EPM2A,GSK3B,PPP1R16A,PTPRB,DUSP26,CDC14A,DLC1,PDP1,PHLPP1,ACP1,PPP1CC,EYA3,LRRK1,PPP1CA,CD300A,PPP2CA,PTPRJ,RNGTT,GNA12,PTPRC
GO:0009894	regulation of catabolic process	0.002302997521783682 4	FBXL2,PRKAG2,FTO,DCAF12,ARNT,EGLN2,LRP2,DEPDC5,EZR,HTR2B,PSMD1,ITGB1,HSP90AA1,PSMB7,CCDC22,HDAC6,DDB1,ADORA1,CUL4B,TNRC6A,EP300,ZYG11B,CHFR,INSR,FMN2,ASB5,PIP4K2A,RNF144A,SAMD4A,TRIM13,MDM4,ENTPD5,SNX6,LARP4B,USP13,PAFAH1B2,FYN,MKRN2,PIK3R2,NRG1,VGLL4,CSNK2A3,DCN,SCFD1,CREBRF,TRDMT1,FHIT,EXOC4,MAD2L2,FBXW7,OAZ2,ITCH,LZTS1,SMG1,PRKCD,TAB2,BANP,TRIM65,BTRC,DNAJB2,HP,DISC1,ZFAND2A,MTDH,NELL1,TIMP2,DAPK2,HNRNPC,YTHDF1,SMURF2,EPHA4,PRKCA,CNR1,NSUN2,UBQLN3,UFL1,PARK2,SMARCC1,AXIN1,LRPPRC,SREBF2,LEPR,FOXK2,TRIM5,CHEK2,UBQLN4,PPP1CB,TBC1D14,RAB3GAP2,TRIM8,HDAC4,FOXO3,ZCCHC17,SYNCRIP,SMAD3,RNFT2,ITPR1,WWP2,WIPI1,PSAP,DCP1B,NEDD4,SLC4A4,APP,ARIH1,SESN1,VDAC1,SH3D19,PRICKLE1,PRKD1,PTPN1,USP33,PABPC4,AGBL4,PRKCG,AREL1,EIF4G1,PSMD2,RBX1,LIN28B,ATP6V0B,FBXL20,DESI1,IDE,SUFU,TNR

			C6B,DAB2,MAP3K7,TRIM22,ALK,FASTKD5,TPCN1,SNX5,ATP2B4,NSF,DYX1C1,MAPKAPK2,SCARB1,ATP6V0A2,CELF1,UVRAG,GPC3,PSMB2,SCOC,DIS3L2,TFEB,CLEC16A,SNX9,ELAVL4,ROCK1,RYBP,MAP1A,AXIN2,SNX33,EIF3H,SNCA,PNPT1,RC3H1,RNF19B,PRKAG1,PSMF1,DFFA,RPTOR,IGF1,HTT,PTEN,PUM1,LRRK2,ATP6V1A,MLYCD,TMEM59,ZBTB20,BAG6,DAZL,MTMR3,CRTC3,TAF15,MYCBP2,SH3RF2,N4BP1,CPT1A,EPM2A,GSK3B,RDX,BOK,RNF144B,METTL16,PRKAA2,ZMPSTE24,PTK2,SNX3,EXOSC3,RNFT1,ADAM8,CSNK1A1,DRAM1,MFSD8,UBE2K,HCAR1,HCAR2,DIS3,PPARA,ZC3HAV1,ATF6,PPP1CA,PHKG2,RPL23,SH3BP4,ANXA2,PARN,ATP6V0A1,CNOT1,YBX1,TAF1,TICAM1,HSF1,GNA12,MYEF2,MET,SNX1,UCHL5,MLXIPL,NEDD4L,PHB,TRAF3IP2
GO:0055082	cellular chemical homeostasis	0.0023906176567037364	ENPP1,ASPH,PDE4D,SLC9A1,FAM155A,ADCY8,PTGFR,CHERP,RYR1,NOX5,TJP1,ATP2B2,GRM5,PLCE1,ANK2,SLC9A9,CHD7,SLC26A6,HTR2B,RYR3,SLC24A2,SLC24A3,CCDC22,ABCC8,HMGN3,STXBP4,TCIRG1,CACNB2,ADORA1,ADRA1D,ESR1,CYB561A3,SLC4A10,KCNMA1,LCK,MICU3,DMD,SLC30A9,SLC8A1,JPH2,FYN,RTN4,PIK3R2,SLC4A5,BDKRB1,BDKRB2,STIM1,TMBIM6,CMKLR1,SLC39A14,PTGER2,CLDN16,NEUROD1,JAK2,NPSR1,SLC9A7,KLF15,NOX4,TGFB1,KEL,JPH3,PTPRN2,F2RL1,C9ORF47,CDH23,DISC1,CLN6,XPR1,CAPN3,CNR1,PPP3CA,IGF1R,P2RY10,TRPM1,XK,RYR2,FOXK2,NOL3,CACNA1C,GRM1,PAX2,FOXO3,SMAD3,ITPR1,ACKR2,GRIK5,SLC4A4,APP,PDGFRA,NOX1,RAB11FIP5,IBTK,LRP5,SLC9B2,DRD1,PRKD1,RAF1,FGF2,ADCY5,ADCYAP1R1,SLC9B1,CLIC4,KCNQ1,CCL14,CCL15,TRPA1,NMUR2,AKAP6,TRPC5,WDTC1,PKHD1,SIN3A,SLC8A2,SLC9C1,TPCN1,ATP2B4,ATP6V0A2,P2RY8,TFRC,EPHA5,CACNA1A,PTGER3,TRPV1,PLCG2,C1QTNF1,GPR35,AP3B1,SLC39A10,TMEM199,ATP13A3,AP3D1,SLC9C2,MCU,TRDN,LRRC8D,TRPC6,ABCB7,ATP13A5,SNCA,ABCA12,SLC4A8,HTT,MAPK1,SLC30A7,LRRK2,ATP6V1A,ZBTB20,TMCO1,PKD2,EFNA5,STEAP4,ZBED6,BMP6,ANO1,GRIN1,TF,ITPR2,ALAS2,BOK,AGT,PRKAA2,HRH4,HEPHL1,SLC29A1,STC2,NOS1,SLC1A1,XCR1,MCOLN1,ATP1A3,GRIN2B,KCTD7,PACS2,DIAPH1,HTR2C,ATP6V0A1,GRIK2,ATP2B3,CIB2,CAV2,TMPRSS3,CAMK2D,CCR3,TRA2B,MLXIPL,NEDD4L,PTPRC,GPR89A,EDNRA
GO:0001764	neuron migration	0.002413823920021772	SEMA3A,NRG3,ASTN2,PHACTR1,NTRK3,CDKL5,ULK4,DAB1,ZNF609,NTRK2,KIRREL3,UNC5D,POMGNT2,SATB2,FYN,FAT3,NTN1,NRG1,NTNG1,NAV1,ASTN1,CAMK2B,CELSR1,DISC1,DCLK1,AUTS2,CTNNB1,DCC,NRP2,CEP85L,DRD1,CTNNA2,ELP3,SPOCK1,FBXO45,PAFAH1B1,RELN,PRKG1,DYX1C1,MAP1B,MARK1,FLRT2,TRIM46,RAPGEF2,NDE1,GPR173,PEX7,PAX6,NIPBL,FBXO31,SRGAP2,GPM6A,AXL
GO:0010171	body morphogenesis	0.0024700808460089954	ASPH,CLASP2,PTPN11,ANKRD11,CDON,EP300,CRISPLD2,TGFB1,PHLDB1,IFT122,SSBP3,PDGFRA,MMP2,STRA6,WNT3,CLASP1,GPC3,PLEKHA1,ARID5B,PHLDB2,NIPBL,BRAF,CSRNP1
GO:0008038	neuron recognition	0.0024700808460089954	SEMA3A,SEMA5A,ROBO1,ROBO2,DSCAM,AMIGO1,RTN4,CNTN4,MYPN,OPCML,CNTNAP2,EPHA4,CNR1,CNTN6,CRTAC1,APP,DSCAML1,NTM,EPHB3,EXT1,EMB,GAP43,EPHB2
GO:0030198	extracellular matrix organization	0.0025142413530933956	ADAMTS16,CLASP2,HPSE2,MIA,ITGB6,ITGB1,RUNX1,IMPG2,COL14A1,ADAMTS3,CSGALNACT1,LAMC1,CRISPLD2,ADAMTS9,NF1,RXFP1,TLL1,EGFLAM,VWA1,NTNG1,COL12A1,TGFB1,MMP28,COLGALT1,COL23A1,MMP26,COL24A1,SMOC1,BCL3,LOXL3,LUM,HSD17B12,ECM2,PHLDB1,MYO1E,MMP16,ELN,SMAD3,THSD4,APP,PDGFRA,NOX1,ADAMTS2,MMP2,ADAMTS12,COL16A1,ADAMTS4,LAMB1,COL11A1,ADAMTS6,ADAMTS14,COL4A3,COL19A1,COL6A5,CLASP1,GPM6B,COL4A6,FLRT2,SMOC2,NID1,PTPRQ,ADAMTS17,COL13A1,ADAMTSL3,TLL2,ADTRP,SH3PXD2B,EXT1,TNFRSF11B,SULF1,AGT,COL9A1,FMOD,COL21A1,ITGAL,DNAJB6,ADAM8,COL22A1,PHLDB2,COL28A1,SPINT2,TNR,ACAN,ITGAE,ADAMTS7,BMP1,CAV2,COL4A5,TNXB
GO:0006887	exocytosis	0.0025388180537938582	CLASP2,CBL,FER,CASK,PIK3CD,NRXN3,DGKI,ERC2,CACNB2,RIMS1,PAK1,VAMP7,BCR,MYH9,SCFD2,LIN7A,SYN2,COPS5,EXOC4,RAB27A,RAB3C,SYT1,ATP9A,CACNA1H,IL1RAPL1,F2RL1,CADPS2,SYT13,C12ORF4,ANK1,STXBP5,CNR1,PARK2,FCGR2B,SYTL5,SCAMP5,NR4A3,HCK,RIMS4,GRIK5,SYT3,RAB11FIP5,SCRN1,DRD1,TPH1,VPS4A,NLGN1,RAB11A,PTAFR,PRKCG,TRAPPC11,FBXL20,TSG101,CLASP1,NSF,SYT12,PRSS12,UNC13A,ARHGAP44,CD84,SPI1,RAB15,SNCA,SYT9,ABCA12,BTK,SLC4A8,LRRK2,ITGAM,WNT7A,SNAP23,STXBP5L,SYT17,GRIN3A,GSK3B,SYN1,SYT7,BLOC1S6,LAT2,EXOC6B,RIMS2,CD160,CPLX2,SYTL4,ADORA2A,RABGEF1,CADPS,BRAF,CD300A,RAB27B,ITSN1,ADCY1,SYK,ZP3,MICAL3,CLNK,STXBP6
GO:0055080	cation homeostasis	0.00263214359402537	ASPH,PDE4D,SLC9A1,FAM155A,ADCY8,PTGFR,CHERP,RYR1,NOX5,ATP2B2,GRM5,PLCE1,ANK2,SLC9A9,CHD7,SLC26A6,HTR2B,RYR3,SLC24A2,SLC24A3,CCDC22,TCIRG1,CACNB2,ADORA1,ADRA1D,CNNM1,ESR1,CYB561A3,SLC4A10,KCNMA1,LCK,SLC12A8,SGCD,MICU3,DMD,SLC30A9,SLC8A1,JPH2,FYN,SLC4A5,CNNM2,BDKRB1,BDKRB2,STIM1,TMBIM6,CMKLR1,SLC39A14,PTGER2,CLDN16,JAK2,NPSR1,SLC9A7,TGFB1,KEL,JPH3,F2RL1,C9ORF47,CDH23,DISC1,CLN6,TMTC2,CAPN3,CNR1,TNFSF11,PARK2,SOD2,P2RY10,TRPM1,OTC,XK,RYR2,NOL3,CACNA1C,GRM1,SMAD3,ITPR1,ACKR2,SLC4A4,APP,PDGFRA,NOX1,IBTK,BDH2,DRD1,PRKD1,FGF2,ADCY5,ADCYAP1R1,SLC9B1,CLIC4,KCNQ1,CCL14,CCL15,TRPA1,NMUR2,AKAP6,BTBD9,TRPC5,NEO1,PKHD1,SLC8A2,SLC9C1,TPCN1,SNX5,ATP2B4,ATP6V0A2,P2RY8,TFRC,CACNA1A,PTGER3,TRPV1,TRIM24,PLCG2,C1QTNF1,GPR35,AP3B1,SLC39A10,ANK3,TMEM199,ATP13A3,AP3D1,SLC9C2,MCU,TRDN,TRPC6,

			ABCB7,ATP13A5,SNCA,SLC4A8,HTT,MAPK1,SLC30A7,LRRK2,ATP6V1A,TMCO1,PKD2,STEAP4,BMP6,EXT1,GRIN1,TF,ITPR2,ALAS2,BOK,AGT,HRH4,HEPHL1,STC2,NOS1,SCNN1B,SLC1A1,XCR1,MCOLN1,STEAP3,ATP1A3,ADORA2A,GRIN2B,KCTD7,PACS2,DIAPH1,HTR2C,ATP6V0A1,GRIK2,ATP2B3,CIB2,CAV2,TMPRSS3,CAMK2D,CCR3,NEDD4L,PTPRC,GPR89A,EDNRA
GO:0043062	extracellular structure organization	0.002897239891234574 7	*ADAMTS16,CLASP2,HPSE2,MIA,ITGB6,ITGB1,RUNX1,IMPG2,COL14A1,ADAMTS3,CSGALNACT1,LAMC1,CRISPLD2,ADAMTS9,NF1,RXFP1,TLL1,EGFLAM,VWA1,NTNG1,COL12A1,TGFB1,MMP28,COLGALT1,COL23A1,MMP26,COL24A1,SMOC1,BCL3,LOXL3,LUM,HSD17B12,ECM2,PHLDB1,MYO1E,MMP16,ELN,SMAD3,THSD4,APP,PDGFRA,NOX1,ADAMTS2,MMP2,ADAMTS12,COL16A1,ADAMTS4,LAMB1,COL11A1,ADAMTS6,ADAMTS14,COL4A3,COL19A1,COL6A5,CLASP1,GPM6B,COL4A6,FLRT2,SMOC2,NID1,PTPRQ,ADAMTS17,COL13A1,ADAMTSL3,TLL2,ADTRP,SH3PXD2B,EXT1,TNFRSF11B,SULF1,AGT,COL9A1,FMOD,COL21A1,ITGAL,DNAJB6,ADAM8,COL22A1,PHLDB2,COL28A1,SPINT2,TNR,ACAN,ITGAE,ADAMTS7,BMP1,CAV2,COL4A5,TNXB*
GO:2000146	negative regulation of cell motility	0.002907046334069171	*S1PR2,CLASP2,SEMA3A,NRG3,MARVELD3,MAP2K5,PTPRR,ROBO1,SRGAP3,MCC,SRGAP2B,PTPRK,ABCC8,DACH1,ADORA1,EPHA1,PTPRU,ADAMTS9,NF1,PLCB1,DPEP1,APOD,BCR,NDRG4,NRG1,HDAC5,DCN,PTPRT,WNT11,MMP28,SLIT2,CORO1C,KANK1,DUSP22,EPHA4,PIP5KL1,PPARG,DLG5,MYOCD,NF2,SFRP1,FOXO3,PTPRM,NAV3,PTPRG,SEMA6D,BMPR1A,FGF2,MCTP1,CHRD,VCL,CLIC4,LDLRAD4,CLASP1,PRKG1,ATP2B4,CTNNA1,MAGI2,ADTRP,MITF,DPYSL3,PTEN,RHOA,SULF1,GPR173,TMEFF2,DLC1,ZMYND8,RABGEF1,PHLDB2,OSBPL8,BRAF,SPINT2,CD300A,ANGPT4,SP100,SRGAP2,FRMD5,PTPRJ,GNA12*
GO:0000122	negative regulation of transcription by RNA polymerase II	0.002942356523410107	*LDB2,DNMT1,TRPS1,CBFB,PRDM12,WWC1,SCAF8,WWC3,ZNF566,MAP2K5,ZNF536,SP3,HDGF,EZR,TRAF6,DNAJA3,GFI1B,RUNX1,NRIP1,THRB,DACH1,ORC2,ESR1,MIER1,PCBP3,EP300,CELA1,TENM2,CCND3,ZNF19,BRMS1,H2AFY2,TCF7L2,TFAP2A,PEG3,ZIM2,TGIF2,MDM4,ZNF148,MTA3,SATB2,MDFI,FNIP1,VAX2,TMBIM6,HDAC5,ZNF692,JDP2,CREBRF,SOX13,MAD2L2,TLE6,MLIP,PDE2A,TBX15,MTA1,KLF8,LCOR,TGFB1,NSD1,ZHX2,CC2D1B,MTDH,MYT1L,ZNF398,ZNF675,ETV6,DUSP22,SMURF2,MAGEA4,KLF12,GATAD2B,CTNNB1,PARK2,METTL13,PPARG,CBX5,SREBF2,NR4A3,FOXK2,MYOCD,PER2,AJUBA,PRDM16,BRMS1L,HDAC4,FOXO3,SMAD3,CUX2,WWP2,MIER3,NEDD4,CDC73,YAP1,SOX2,ESR2,STAT1,ETV5,TAF3,PLAGL1,ATF2,TCF3,MYB,BACH1,MXD3,SEMA4D,NFX1,RORC,JARID2,RARB,SPEN,SIN3B,EHMT1,ATF3,PAWR,TSG101,CRYM,SUFU,MAGEA11,NR2C1,GLI2,GLIS3,WDTC1,DAB2,SIN3A,OVOL2,NFATC2,CDK6,MEF2A,SATB1,GATAD2A,TRPV1,HOXB3,HOXB4,HDAC1,RYBP,PTPN2,ZNF425,BCOR,SMARCA2,CUX1,SPI1,DNMT3B,FOXP2,GLIS1,JAZF1,SNCA,ZEB2,HEY2,CREM,MITF,NFIX,CBFA2T2,ATF7IP,MXI1,CIR1,ZBTB20,RIPPLY1,ZBED6,BMP6,ATRX,SMO,SOX30,CUL3,TFEC,STAT6,DUSP26,NACC2,SUPT4H1,TCFL5,CREBBP,DLX1,PRDM2,TCF25,ZNF282,PPARA,ARID5B,NOTCH4,ZNF366,PAX6,OTUD7B,NIPBL,TBL1X,FHL2,ZBTB5,MTF2,TAGLN3,NCOR2,HDAC2,IMPACT,RPL23,ATXN1,SP100,TRIM29,CNOT1,WWTR1,YBX1,TAF1,HSF1,MAX,SAP130,EZH1,NCOA2,RNF2,ZNF554,TRIM37,TIMELESS,KDM2B,MLXIPL,PHB,ZNF555,CTCF,NR6A1,BMP4*
GO:0060401	cytosolic calcium ion transport	0.003022379388848272	*ASPH,PDE4D,FAM155A,CHERP,RYR1,ANK2,CHD7,HTR2B,RYR3,SLC24A2,LCK,MICU3,DMD,SLC8A1,JPH2,FYN,BDKRB1,TMBIM6,NPSR1,TGFB1,JPH3,CAPN3,TRPM1,RYR2,NOL3,CACNA1C,ITPR1,VDAC1,IBTK,DRD1,PRKD1,FGF2,ADCYAP1R1,TRPA1,AKAP6,SLC8A2,TPCN1,ATP2B4,TRPV1,PLCG2,MCU,TRDN,SNCA,HTT,PKD2,PSEN2,GRIN1,ITPR2,NOS1,XCR1,MCOLN1,GRIN2B,DIAPH1,HTR2C,ATP2B3,CAMK2D,PTPRC*
GO:0006511	ubiquitin-dependent protein catabolic process	0.003032183952693213	*CALR3,FBXL2,DCAF12,CBL,USP32,TOM1L1,PSMD1,PSMB7,CCDC22,DNAJB14,HDAC6,DDB1,CUL4B,FBXL18,ZYG11B,FBXL7,USP46,CHFR,USP34,FBXW11,RNF144A,TRIM13,UBR2,NPLOC4,USP13,MKRN2,ARNTL,FBXO21,PPP2CB,RNF4,UBE3D,FHIT,MAN1A1,FBXO9,RFFL,USP22,FBXW7,ITCH,USP12,SPSB1,MTA1,SIAH1,ERCC8,SPSB4,ASCC2,BTRC,DNAJB2,RNF133,RNF148,DISC1,ZFAND2A,CLOCK,FBXL17,KCTD10,SMURF2,UBQLN3,UBQLNL,UFL1,CTNNB1,PARK2,SMARCC1,AXIN1,UBQLN4,WWP2,RNF168,NEDD4,SEC61B,USP49,ARIH1,AMFR,PRICKLE1,ERLIN1,VPS4A,PSMD11,USP33,UBE2R2,AGBL4,CDC27,KLHL3,AREL1,UCHL3,PSMD2,RBX1,ANAPC5,FBXO45,STT3B,FBXL20,TSG101,DESI1,SUFU,EDEM3,DAB2,UBR1,CACUL1,PSMD7,PELI1,PPP2R5C,ZNRF1,RBBP6,UBE3C,RNF34,PSMB2,RNF216,RYBP,CUL2,MAP1A,MAN1B1,FBXO39,FBXW4,EIF3H,USP42,USP50,RC3H1,RNF103,RNF103-CHMP3,RNF19B,PSMF1,TRIP12,ZNRF3,KCTD13,PTEN,MIB1,PCBP2,LRRK2,ZRANB1,BAG6,SEL1L2,SH3RF2,N4BP1,UBE2H,CUL3,EPM2A,GSK3B,RNF144B,RNF43,CUL9,PTK2,ARMC8,RNF121,FBXO10,TRPC4AP,CSNK1A1,UBE2K,FBXL13,RNF150,OTUD7B,TBL1X,FBXO31,C18ORF25,SKP1,RPL23,WWTR1,TAF1,GNA12,UCHL5,FBXL4,NEDD4L,RNF213,SGTB*
GO:0030031	cell projection assembly	0.003098570194148197 7	*CLRN1,ADAMTS16,NRXN1,S1PR2,SPAG16,IQCG,PLCE1,FER,EZR,CDKL5,TENM1,VAV2,ITGB1,ARHGAP24,IFT43,CDC42EP3,MYO10,HDAC6,GBF1,DNAH2,NPHP3,PTPRO,DNAI2,TENM2,TNIK,ATP8B1,LRRC49,DMD,GSN,PCDH15,NTN1,DCTN1,RAPGEF6,WDR92,LRGUK,CDH13,NCKAP1,RCC2,ANO6,ARL3,ICK,TTLL8,CROCC,SEPT7,IFT80,PRKCD,PIBF1,F2RL1,BCAS3,RPGRIP1L,SLIT2,CORO1C,DISC1,CEP135,KANK1,PTPDC1,TTBK2,SPAG17,PARVA,AUTS2,AJUBA,CSPG4,HDAC4,ARL13B,IFT122,DNM3,ABI2,YAP1,KIF24,TTC39C,SYNE2,TCTN3,ABLIM1,TMEM237,ABLI*

			M2,NLGN1,ARMC2,RILPL1,GAS8,VCL,WDR90,KCNQ1,RFX2,RHOJ,KIF3A,CEP350,PKHD1,DZIP1,DOCK11,ZNF423,DYX1C1,CEP41,MPHOSPH9,AIF1L,FGD1,RPGR,ACTR2,ROCK1,EPS8,TEKT1,ARHGAP44,HYDIN,CDKL1,PLD1,TROVE2,HTT,DPYSL3,SEPT6,IFT81,MAP4,NME8,CEP89,PARVB,RAPGEF2,MARK4,BBS9,ONECUT2,RDX,CDC14A,GAP43,CEP70,FGD3,FGD4,SPEF1,KLC3,ATG3,NOTO,ARHGEF6,FAM149B1,SRGAP2,WWTR1,GPM6A,MYO1A,PLEKHM1,ASAP1,VAV3,POC1B,EHD2,ABLIM3
GO:1901565	organonitrogen compound catabolic process	0.0031946664693608645	*NT5C1B,GPC5,PDE4D,PDE7B,PDE8A,ALDH4A1,CALR3,FBXL2,HPSE2,DCAF12,CBL,EGLN2,ENPP2,SAMHD1,USP32,CHI3L2,LRP2,EZR,TOM1L1,PSMD1,GOT1,HSP90AA1,PSMB7,CCDC22,AMDHD1,OMA1,DNAJB14,PDE4A,TCIRG1,HDAC6,DDB1,CUL4B,FBXL18,CELA1,ZYG11B,FBXL7,USP46,CHFR,FMN2,ASB5,USP34,FBXW11,RNF144A,TRIM13,UBR2,MDM4,PIPOX,NPLOC4,USP13,FYN,MKRN2,ARNTL,ADAMTS9,DMGDH,SLC25A21,FBXO21,DPEP1,NEIL2,CHI3L1,BLVRB,NRG1,VGLL4,PPP2CB,RNF4,CSNK2A3,UBE3D,CREBRF,FHIT,MAD2L2,MAN1A1,FBXO9,RFFL,USP22,FBXW7,LINC00473,OAZ2,ITCH,USP12,ABCC2,DPYS,PDE2A,SPSB1,MTA1,SIAH1,ERCC8,PRKCD,BANP,SPSB4,ASCC2,BTRC,DNAJB2,RNF133,RNF148,DISC1,ZFAND2A,CLN6,CLOCK,BCKDHB,NELL1,TIMP2,FBXL17,KCTD10,CAPN3,SMURF2,EPHA4,UBQLN3,UBQLNL,UFL1,CTNNB1,PARK2,SMARCC1,AXIN1,CPQ,OTC,RSPRY1,CHEK2,UBQLN4,CAPN2,DIO2,TRHDE,SMAD3,RNFT2,WWP2,RNF168,NEDD4,SEC61B,USP49,ARIH1,AMFR,SH3D19,PRICKLE1,ERLIN1,NUDT10,HEXA,SLC6A3,VPS4A,ADAMTS12,PSMD11,GGT7,USP33,IDO2,FGF2,UBE2R2,AGBL4,CDC27,HIBADH,YME1L1,KLHL3,PRKCG,AREL1,UCHL3,PSMD2,RBX1,ANAPC5,FBXO45,STT3B,FBXL20,TSG101,DESI1,CRYM,IDE,SUFU,DDAH1,EDEM3,ALDH6A1,DAB2,UBR1,CACUL1,PSMD7,PELI1,PPP2R5C,SPATA18,ZNRF1,XDH,SNX5,RBBP6,ATP2B4,NSF,TMEM2,TET1,DYX1C1,UBE3C,SCARB1,RNF34,GPC3,PSMB2,RNF216,SNX9,TRIM24,ROCK1,RYBP,CUL2,ABCC1,MAP1A,HYKK,MAN1B1,SARM1,TMEM199,FBXO39,SNX33,FBXW4,EIF3H,RENBP,PDXP,SNCA,USP42,USP50,RC3H1,RNF103,RNF103-CHMP3,RNF19B,PSMF1,TRIP12,ZNRF3,KCTD13,PTEN,MIB1,PCBP2,LRRK2,MLYCD,ZRANB1,BAG6,SLC44A1,BLVRA,SEL1L2,NT5E,GLDC,MYCBP2,SH3RF2,EXT1,N4BP1,GET4,ACADSB,UBE2H,CUL3,EPM2A,GSK3B,DNAJC3,RDX,RNF144B,RNF43,CUL9,ZMPSTE24,PTK2,ARMC8,RNF121,SNX3,PDE4B,FBXO10,TRPC4AP,UGT1A1,UGT1A4,ECE1,RNFT1,CD44,ADAM8,CSNK1A1,NOS1,MFSD8,UBE2K,FBXL13,RNF150,PAH,OTUD7B,UPB1,TBL1X,NT5M,SLCO2B1,FBXO31,C18ORF25,MOXD1,SKP1,MAOA,RPL23,ACAN,ANXA2,WWTR1,ALDH8A1,TAF1,ADAMTS7,GNA12,UGGT1,SNX1,UCHL5,MALT1,FBXL4,GNS,NEDD4L,PHB,RNF213,SGTB*
GO:0051017	actin filament bundle assembly	0.0032165379068714124	*SLC9A1,CLASP2,PHACTR1,TJP1,EZR,EPHA1,LIMK1,FMN2,PAK1,ARHGAP6,DMD,PLS1,WNT11,PPM1E,RHPN2,PARK2,ADD2,SORBS1,NF2,ELN,SFRP1,SMAD3,SHANK1,ARHGEF18,PPM1F,NEDD9,PAWR,CLASP1,AIF1L,PLS3,CALD1,ROCK1,EPS8,LIMA1,RAPGEF3,PDXP,KCTD13,DPYSL3,SHANK3,RHOA,SHROOM1,CUL3,RDX,ESPNL,FAM171A1,TMEFF2,DLC1,PHLDB2,BRAF,CGNL1,MET*
GO:0051650	establishment of vesicle localization	0.0033369975571252634	*CLASP2,KIF5C,CNIH2,KLHL12,TCIRG1,GBF1,FBXW11,AP3S1,TANC2,LIN7A,COPS5,EXOC4,RAB27A,DYNC1I1,ATP9A,TRAK1,CTNNB1,PARK2,MYO1D,MYO1E,AP3B2,SPG11,WIPI1,MYO1F,VPS4A,NLGN1,RAB11A,BLOC1S5,TSG101,PAFAH1B1,KIF3A,FAM91A1,MYO7A,CLASP1,AP3B1,TRIM46,AP3D1,SNCA,HTT,LRRK2,SNAP23,BLOC1S3,NDE1,CUL3,BLOC1S6,EXOC6B,SEC16B,ARFGAP3,TBC1D23,ACTN4,PRKCZ,MAP2,RAB27B,MYO1A,MLPH*
GO:0009057	macromolecule catabolic process	0.0033607242850286195	*GPC5,RNASET2,CALR3,FBXL2,HPSE2,FTO,DCAF12,CBL,EGLN2,USP32,CHI3L2,LRP2,EZR,TOM1L1,PSMD1,HSP90AA1,PSMB7,CCDC22,OMA1,DNAJB14,TCIRG1,HDAC6,DDB1,GPRASP1,CUL4B,CECR2,FBXL18,TNRC6A,CELA1,ZYG11B,FBXL7,USP46,CHFR,FMN2,ASB5,USP34,FBXW11,RNF144A,SAMD4A,TRIM13,UBR2,MDM4,ERN2,NPLOC4,LARP4B,USP13,FYN,MKRN2,ARNTL,ADAMTS9,SMG7,FBXO21,CHI3L1,NRG1,VGLL4,PPP2CB,RNF4,CSNK2A3,UBE3D,CREBRF,TRDMT1,FHIT,MAD2L2,MAN1A1,FBXO9,RFFL,USP22,FBXW7,OAZ2,ITCH,USP12,SMG1,SPSB1,MTA1,SIAH1,ERCC8,PRKCD,BANP,SPSB4,ZHX2,ASCC2,BTRC,DNAJB2,RNF133,RNF148,MGAM,DISC1,ZFAND2A,CLN6,CLOCK,NELL1,TIMP2,SND1,HNRNPC,YTHDF1,FBXL17,KCTD10,CAPN3,SMURF2,EPHA4,PRKCA,SMG6,NSUN2,UBQLN3,UBQLNL,UFL1,CTNNB1,PARK2,SMARCC1,AXIN1,LRPPRC,RSPRY1,CHEK2,UBQLN4,PPP1CB,CAPN2,ZCCHC17,SYNCRIP,SMAD3,RNFT2,WWP2,RNF168,DCP1B,NEDD4,SEC61B,USP49,RBM8A,ARIH1,AMFR,SH3D19,PRICKLE1,ERLIN1,VPS4A,PTPN1,ADAMTS12,PSMD11,USP33,PABPC4,FGF2,UBE2R2,AGBL4,CDC27,YME1L1,KLHL3,PRKCG,AREL1,UCHL3,PSMD2,RBX1,ANAPC5,FBXO45,LIN28B,STT3B,AGO3,FBXL20,TSG101,DESI1,IDE,SUFU,EDEM3,TNRC6B,DAB2,UBR1,CACUL1,SMG5,PSMD7,PELI1,PPP2R5C,SPATA18,ZNRF1,FASTKD5,SNX5,RBBP6,NSF,TMEM2,DYX1C1,MAPKAPK2,UBE3C,CELF1,RNF34,UVRAG,DROSHA,GPC3,XRN1,PSMB2,DIS3L2,RNF216,SNX9,ELAVL4,TRIM24,ROCK1,RYBP,CUL2,MAP1A,MAN1B1,AXIN2,TMEM199,FBXO39,SNX33,FBXW4,EIF3H,SNCA,USP42,PNPT1,USP50,RC3H1,EIF3E,RNF103,RNF103-CHMP3,RNF19B,PSMF1,TRIP12,DFFA,ZNRF3,KCTD13,PTEN,MIB1,PUM1,PCBP2,LRRK2,ZRANB1,BAG6,SEL1L2,DAZL,TAF15,MYCBP2,SH3RF2,EXT1,N4BP1,GET4,UBE2H,RQCD1,CUL3,EPM2A,GSK3B,DNAJC3,RDX,UPF2,RNF144B,RNF43,METTL16,CUL9,ZMPSTE24,PTK2,ARMC8,RNF121,SNX3,EXOSC3,FBXO10,TRPC4AP,RNFT1,CD44,ADAM8,CSNK1A1,MFSD8,UBE2K,DIS3,FBXL13,RNF150,ZC3HAV1,OT*

			UD7B,TBL1X,PPP1CA,FBXO31,PHKG2,C18ORF25,SKP1,RPL23,ACAN,ANXA2,PARN,CNOT1,WWTR1,YBX1,TAF1,HSF1,POP1,ETF1,ADAMTS7,GNA12,UGGT1,MYEF2,SNX1,UCHL5,MALT1,FBXL4,GNS,NEDD4L,PHB,RNF213,TRAF3IP2,SGTB
GO:0051091	positive regulation of DNA-binding transcription factor activity	0.003406195118475830 6	*PRKCI,SLCO3A1,ADCY8,FER,TRAF6,ZC4H2,ERC1,IKBKB,ESR1,EP300,RNF220,TRIM13,HDAC5,COPS5,NEUROD1,JAK2,TRAPPC9,TGFB1,MTDH,FANK1,CLOCK,CAPN3,TNFSF11,PPP3CA,CTNNB1,PPARG,IL18R1,MYOCD,TRIM5,TRIM8,HDAC4,SMAD3,APP,LRP5,CHUK,ESR2,S100A12,PRKD1,ATF2,TCF3,CARD16,CAMK1D,DDX58,TERF2IP,RELN,MAP3K7,TRIM22,ALK,UBE2V1,TFRC,EPHA5,PLCG2,NTRK1,MAP3K13,OPRD1,BTK,PTEN,CRTC3,SMO,ROR1,AGT,ADAM8,RPS6KA5,IL18,ARID5B,PRKCZ,PRKCQ,SP100,ADCY1,DVL2,TAF1,TICAM1,HSF1,TRIM37,MALT1,EDA*
GO:0043161	proteasome-mediated ubiquitin-dependent protein catabolic process	0.00357928129988292	*CALR3,FBXL2,DCAF12,PSMD1,PSMB7,DNAJB14,DDB1,CUL4B,FBXL18,ZYG11B,FBXL7,CHFR,FBXW11,RNF144A,TRIM13,UBR2,NPLOC4,MKRN2,ARNTL,PPP2CB,RNF4,UBE3D,FHIT,MAN1A1,FBXO9,RFFL,FBXW7,ITCH,SPSB1,MTA1,SIAH1,ERCC8,SPSB4,ASCC2,BTRC,DNAJB2,ZFAND2A,CLOCK,FBXL17,KCTD10,SMURF2,UBQLN3,UFL1,CTNNB1,PARK2,SMARCC1,AXIN1,UBQLN4,WWP2,NEDD4,SEC61B,ARIH1,AMFR,PRICKLE1,ERLIN1,CDC27,AREL1,PSMD2,RBX1,ANAPC5,FBXO45,STT3B,FBXL20,DESI1,EDEM3,DAB2,UBR1,PSMD7,PELI1,PPP2R5C,ZNRF1,RNF34,PSMB2,RNF216,RYBP,CUL2,MAP1A,MAN1B1,FBXO39,FBXW4,EIF3H,RNF103,RNF103-CHMP3,RNF19B,PSMF1,KCTD13,PCBP2,LRRK2,BAG6,SEL1L2,SH3RF2,N4BP1,UBE2H,CUL3,EPM2A,GSK3B,RNF144B,ARMC8,RNF121,TRPC4AP,CSNK1A1,UBE2K,FBXL13,TBL1X,FBXO31,SKP1,WWTR1,TAF1,GNA12,UCHL5,FBXL4,NEDD4L,SGTB*
GO:0035235	ionotropic glutamate receptor signaling pathway	0.003597364461603058 8	*GRID2,GRIK4,GRIK3,GRIK5,APP,GRIA3,GRIK1,GRIA2,GRIN1,GRIN3A,GRID1,CPEB4,GRIN2B,GRIA4,GRIK2*
GO:1990806	ligand-gated ion channel signaling pathway	0.003597364461603058 8	*GRID2,GRIK4,GRIK3,GRIK5,APP,GRIA3,GRIK1,GRIA2,GRIN1,GRIN3A,GRID1,CPEB4,GRIN2B,GRIA4,GRIK2*
GO:2000463	positive regulation of excitatory postsynaptic potential	0.003597364461603058 8	*NRXN1,RIMS1,CUX2,SHANK1,NLGN2,NLGN1,NLGN3,RELN,NETO1,PTEN,SHANK3,WNT7A,GRIN1,RIMS2,PRKCZ*
GO:0007423	sensory organ development	0.003599911311490603	*CLRN1,RCN1,RDH13,PRKCI,PBX3,TENM3,NTRK3,ATP2B2,SP3,MFAP5,CHD7,ESRP1,TRIOBP,DSCAM,TTLL5,THRB,IMPG2,TCIRG1,PTPN11,ATP8A2,CECR2,CDON,NTRK2,ATP8B1,ESRRB,STRC,TFAP2A,TGIF2,PHACTR4,MEIS1,MYO3B,NF1,PCDH15,FAT3,CHRDL1,BCR,NTN1,SLC4A5,VAX2,ROR2,LIN7A,NEUROD1,BCL11B,TMC1,LRIG3,PKNOX1,PLS1,SIPA1L3,TGFB1,MYO3A,ZHX2,RPGRIP1L,CELSR1,SDK2,CDH23,BNC2,SMOC1,BMPER,SLC17A7,FGF10,EPHA4,CTNNB1,BFSP1,CACNA1C,NF2,PAX2,IFT122,SMAD3,PTPRM,NHS,ABI2,PDGFRA,LRP5,SOX2,TTC39C,FRS2,SLC6A3,STRA6,MYH15,CELF4,BMPR2,TSPAN12,FGF2,RARB,OC90,CLIC4,COL11A1,KCNQ1,RHOJ,PAFAH1B1,GLI2,USH2A,MYO7A,HOXC13,ATP2B4,MAPKAPK2,EYA1,HDAC1,PTPRQ,EPHB1,PDE6A,TUB,JAG2,BCAR3,SDK1,PTK7,BMPR1B,HEY2,WNT7B,MITF,HIPK1,MAPK1,SPRED2,BMP7,LHFPL5,MEGF11,WNT7A,SH3PXD2B,BMP6,SOS1,TSHR,BLOC1S3,ROR1,FBN2,PBX1,DLX1,PYGO2,ECE1,EPHB2,SLC1A1,SOBP,FBN1,PAX6,NIPBL,ATF6,HDAC2,DVL2,GPM6A,MAX,KDM2B,EDNRA,GNGT1,BMP4*
GO:0043632	modification-dependent macromolecule catabolic process	0.003721845239737357 7	*CALR3,FBXL2,DCAF12,CBL,USP32,TOM1L1,PSMD1,PSMB7,CCDC22,DNAJB14,HDAC6,DDB1,CUL4B,FBXL18,ZYG11B,FBXL7,USP46,CHFR,USP34,FBXW11,RNF144A,TRIM13,UBR2,NPLOC4,USP13,MKRN2,ARNTL,FBXO21,PPP2CB,RNF4,UBE3D,FHIT,MAN1A1,FBXO9,RFFL,USP22,FBXW7,ITCH,USP12,SPSB1,MTA1,SIAH1,ERCC8,SPSB4,ASCC2,BTRC,DNAJB2,RNF133,RNF148,DISC1,ZFAND2A,CLOCK,FBXL17,KCTD10,SMURF2,UBQLN3,UBQLNL,UFL1,CTNNB1,PARK2,SMARCC1,AXIN1,UBQLN4,WWP2,RNF168,NEDD4,SEC61B,USP49,ARIH1,AMFR,PRICKLE1,ERLIN1,VPS4A,PSMD11,USP33,UBE2R2,AGBL4,CDC27,KLHL3,AREL1,UCHL3,PSMD2,RBX1,ANAPC5,FBXO45,STT3B,FBXL20,TSG101,DESI1,SUFU,EDEM3,DAB2,UBR1,CACUL1,PSMD7,PELI1,PPP2R5C,ZNRF1,RBBP6,UBE3C,RNF34,PSMB2,DIS3L2,RNF216,RYBP,CUL2,MAP1A,MAN1B1,FBXO39,FBXW4,EIF3H,USP42,PNPT1,USP50,RC3H1,RNF103,RNF103-CHMP3,RNF19B,PSMF1,TRIP12,ZNRF3,KCTD13,PTEN,MIB1,PCBP2,LRRK2,ZRANB1,BAG6,SEL1L2,SH3RF2,N4BP1,UBE2H,CUL3,EPM2A,GSK3B,RNF144B,RNF43,CUL9,ZMPSTE24,PTK2,ARMC8,RNF121,EXOSC3,FBXO10,TRPC4AP,CSNK1A1,UBE2K,FBXL13,RNF150,OTUD7B,TBL1X,FBXO31,C18ORF25,SKP1,RPL23,WWTR1,TAF1,GNA12,UCHL5,FBXL4,NEDD4L,RNF213,SGTB*
GO:0045229	external encapsulating structure	0.003833874743387940 3	*ADAMTS16,CLASP2,HPSE2,MIA,ITGB6,ITGB1,RUNX1,IMPG2,COL14A1,ADAMTS3,CSGALNACT1,LAMC1,CRISPLD2,ADAMTS9,NF1,RXFP1,TLL1,EGFLAM,VWA1,NTNG1,COL12A1,TGFB1,MMP28,COLGALT1,COL23A1,MMP26,COL24A1,SMOC1,BCL3,LOXL3,LUM,HSD17B12,ECM2,PHLDB1,MYO1E,MMP16,ELN,SMAD3,THSD4,*

	organization		*APP,PDGFRA,NOX1,ADAMTS2,MMP2,ADAMTS12,COL16A1,ADAMTS4,LAMB1,COL11A1,ADAMTS6,ADAMTS14,COL4A3,COL19A1,COL6A5,CLASP1,GPM6B,COL4A6,FLRT2,SMOC2,NID1,PTPRQ,ADAMTS17,COL13A1,ADAMTSL3,TLL2,ADTRP,SH3PXD2B,EXT1,TNFRSF11B,SULF1,AGT,COL9A1,FMOD,COL21A1,ITGAL,DNAJB6,ADAM8,COL22A1,PHLDB2,COL28A1,SPINT2,TNR,ACAN,ITGAE,ADAMTS7,BMP1,CAV2,COL4A5,TNXB*
GO:0045055	regulated exocytosis	0.003874056089723129 4	*CBL,FER,CASK,PIK3CD,NRXN3,DGKI,ERC2,CACNB2,RIMS1,VAMP7,BCR,MYH9,SYN2,RAB27A,SYT1,CACNA1H,F2RL1,CADPS2,SYT13,C12ORF4,STXBP5,CNR1,FCGR2B,SCAMP5,NR4A3,HCK,RIMS4,GRIK5,SYT3,RAB11FIP5,DRD1,TPH1,NLGN1,PTAFR,PRKCG,FBXL20,SYT12,UNC13A,CD84,SPI1,RAB15,SNCA,SYT9,ABCA12,BTK,SLC4A8,LRRK2,ITGAM,WNT7A,SNAP23,STXBP5L,SYT17,GRIN3A,GSK3B,SYN1,SYT7,BLOC1S6,LAT2,RIMS2,CD160,CPLX2,ADORA2A,RABGEF1,CADPS,BRAF,CD300A,ADCY1,SYK,ZP3,CLNK*
GO:0033043	regulation of organelle organization	0.003882632543495703 3	*ADAMTS16,NRXN1,DNMT1,SLC9A1,PDE4DIP,CLASP2,GRID2,RPS6KA2,FHOD3,MAPRE2,TJP1,NTRK3,SEMA5A,FER,EZR,TOM1L1,CDKL5,TENM1,LMNA,TRIOBP,CDC6,AKAP13,OMA1,CDC42EP3,CCT2,HDAC6,EPHA1,MRE11A,LIMK1,STX18,EP300,PDGFB,MID1,CHFR,INSR,AFAP1,PIP4K2A,PAK1,MAP3K4,PTPRD,NPRL3,ARHGAP6,ECT2,CENPF,GSN,RBM14,PLCB1,DCTN1,BID,MAP2K1,MAD1L1,RNF4,DCN,SCFD1,MAD2L2,NCKAP1,TERF2,SLX1B,ARL3,FBXW7,SYT1,CROCC,SMG1,PDE2A,SEPT7,CYFIP2,WNT11,PRKCD,PPM1E,TGFB1,F2RL1,BCAS3,CELSR1,SLIT2,CEP135,KANK1,TTBK2,HNRNPC,KANK4,IQCJ-SCHIP1,RHPN2,SVIL,SMG6,CTNNB1,PARK2,PPARG,ADD2,SREBF2,NIN,NOL3,RIOK2,PHLDB1,UBQLN4,HCK,TBC1D14,RAB3GAP2,CAPN2,BAZ1B,NF2,SPTBN1,ELN,SFRP1,SSH1,SMAD3,WIPI1,MTBP,ABI2,C10ORF90,GRIK5,SHANK1,ARHGEF18,NAV3,PDGFRA,ADD3,YAP1,TEN1,VDAC1,BORA,LRP5,ZDHHC6,KIF24,SPTBN4,TMEM14A,VILL,SYNE2,RND3,EREG,CTNNA2,MORC2,PPM1F,CDC27,ANAPC5,TSG101,TERF2IP,CLIC4,PAFAH1B1,TNKS,ERCC1,DZIP1,SMG5,SIN3A,IQGAP1,CLASP1,GPM6B,SPTBN5,PAK3,SSH2,MAP1B,MPHOSPH9,TFRC,UVRAG,EPHA5,XRN1,ZNF207,CLEC16A,MNAT1,SNX9,ROCK1,EPS8,MAP1A,AXIN2,GNL3L,ARHGAP44,MCU,LIMA1,FCHSD2,RAPGEF3,PDXP,SH3BP1,SNCA,ZEB2,SYT9,PTPRS,CDKL1,RASA1,IGF1,ATF7IP,HTT,MAPK1,BMP7,LRRK2,SPAG5,CHMP5,EFNA5,SHANK3,STMN4,PKIB,MTMR3,TBCD,MAP4,RHOT1,MYCBP2,ATRX,MARK4,RHOA,RHOBTB1,CUL3,GSK3B,RDX,FAM49B,CDK13,BOK,HDGFRP3,PRKAA2,CUL9,STAT2,PTK2,GAP43,IQGAP2,CEP70,TAOK2,YLPM1,SETDB2,CPLX2,SPEF1,TMEFF2,GPSM2,DLC1,PPP1R10,PHLDB2,MAP2,ATG3,PRKCQ,BRAF,DIAPH1,CAPN6,NOTO,PHIP,CAMSAP3,ANXA2,PARN,CNOT1,CIT,HRK,NEB,ASAP1,CGNL1,CAV2,SKA2,TRIM37,MET,SPTAN1,CCT3,CTCF,RASSF8,PRAP1,BMP4*
GO:0007265	Ras protein signal transduction	0.003905291676344091	*MAP4K4,DNMT1,KSR2,MAPRE2,CBL,RAB4B,RAB4B-EGLN2,PLCE1,KITLG,ROBO1,ITPKB,ITGB1,ARHGAP24,AKAP13,CDC42EP3,DGKI,CYTH3,GBF1,LIMK1,PHACTR4,ARHGAP6,ARHGAP42,RIT2,NF1,RTN4,RTN4R,BCR,NTN1,NRG1,RAPGEF6,RALGPS2,PPP2CB,CDH13,NCKAP1,RALGPS1,DENND1A,F2RL1,CELSR1,RRAS2,KANK1,FGF10,RASA4,RASA4B,ARHGEF28,KCTD10,AUTS2,P2RY10,ARHGAP29,ABI2,ARHGEF18,KPNB1,HEG1,PRKD1,RAF1,RASGRF2,DENND4A,FGF2,SHOC2,RHOJ,RASAL1,P2RY8,ROCK1,EPS8,GPR35,NTRK1,ARHGAP44,ARHGEF3,RAB15,RAPGEF3,SH3BP1,USP50,PLD1,RASA1,KCTD13,IGF1,RASA2,RAB30,SOS1,RAPGEF2,RHOA,SYNGAP1,CUL3,RDX,EPHB2,GPSM2,DLC1,RABGEF1,DENND4B,MADD,GNA12,DENND4C,MET*
GO:0051209	release of sequestered calcium ion into cytosol	0.003910209534301338	*ASPH,PDE4D,CHERP,RYR1,ANK2,CHD7,HTR2B,RYR3,LCK,DMD,SLC8A1,JPH2,BDKRB1,NPSR1,TGFB1,JPH3,CAPN3,RYR2,NOL3,CACNA1C,ITPR1,IBTK,DRD1,PRKD1,FGF2,TRPA1,AKAP6,TPCN1,TRPV1,PLCG2,TRDN,SNCA,HTT,PKD2,ITPR2,NOS1,XCR1,MCOLN1,DIAPH1,HTR2C,CAMK2D,PTPRC*
GO:0051248	negative regulation of protein metabolic process	0.004055997129179171	*ENPP1,PRMT3,PDE4D,DNMT1,LATS2,PRKAG2,MVP,FTO,NTRK3,CBL,MAP2K5,DNAJA3,OXR1,MGAT5,HDAC6,EPHA1,IKBKB,LIMK1,PMEPA1,PTPRO,NTRK2,TNRC6A,FMN2,PRKAR1A,SAMD4A,MDM4,SNX6,CST2,DMD,SLC8A1,RBM4,FYN,NF1,RTN4,DPEP1,APOD,PAQR3,NRG1,BDKRB1,BDKRB2,FRY,FNIP1,ITIH2,FHIT,PTPRT,IPO5,MAD2L2,CAPRIN2,GNAQ,RFFL,ZFYVE28,KLF15,PRKCD,PPM1E,BANP,PIBF1,DNAJB2,SLIT2,ITIH4,CORO1C,KDM4B,SMAD6,ZNF675,NELL1,TIMP2,DUSP22,NAIP,YTHDF1,SMYD3,CAPN3,EPHA4,PIP5KL1,UFL1,CTNNB1,PARK2,SMARCC1,PPARG,AXIN1,PRKAR1B,OTUB1,PROS1,NOL3,PRKAR2A,MYOCD,PER2,GLG1,CSTL1,NF2,PAX2,SFRP1,SYNCRIP,SMAD3,SIMC1,DCP1B,APP,HEG1,IBTK,LRP5,DCUN1D3,RAF1,CELF4,CARD16,PTPN1,VBP1,SPAG9,PPM1F,SEMA4D,JARID2,PRKCG,SPOCK1,EIF4G1,AGO3,DEPTOR,TSG101,TERF2IP,SUFU,PTPN13,COL4A3,TNRC6B,LDLRAD4,SIN3A,XDH,ATP2B4,CELF1,RNF34,GPC3,XRN1,DIS3L2,ROCK1,HIPK3,RYBP,PTPN2,TRIM44,MAP1A,PARD3,GNL3L,BCOR,ANXA8L1,CD84,LPA,SPI1,DNMT3B,ATG14,EIF3H,STK38,ACOT8,RENBP,SNCA,PNPT1,RC3H1,EIF3E,PSMF1,CAST,TRIP12,MLLT1,IGF1,PTEN,SPRED2,BMP7,PUM1,LRRK2,TMEM59,BAG6,LILRB4,SERPINA3,SERPINA4,SERPINA5,PKIB,DNAJC1,MYCBP2,GPI,SH3RF2,N4BP1,PRKAR2B,UBR5,RQCD1,EPM2A,GSK3B,DNAJC3,PTPRB,CD27,AGT,METTL16,PRKAA2,ADAR,SNX3,EXOSC3,PARP10,PRG3,SERPINB11,DNAJB6,CD44,RGS14,EPHB2,DIS3,CPEB4,PAX6,PRKCZ,ADORA2A,RABGEF1,CPEB1,COL28A1,ANGPT1,LRRK1,IGF2BP3,MTF2,CDK5RAP1,HDAC2,IMPACT,SPINT2,CD300A,PPP2CA,RPL23,ANXA2,PARN,CNOT1,WWTR1,YBX1,PTPRJ,TAF1,DHFR,INPP5F,CD109,*

			UCHL5,SERPINE3,MLXIPL,PHB,PTPRC,SH3GL2,BMP4
GO:0001667	ameboidal-type cell migration	0.004414285398574893	*MAP4K4,CLASP2,SEMA3A,DOCK1,SEMA3D,MAPRE2,SEMA5A,ENPP2,FER,MARVELD3,MAP2K5,KITLG,PTPRR,PIK3CD,ROBO1,HTR2B,ITGB1,MCC,PTPN11,HDAC6,ERBB4,PDGFB,PHACTR4,SLC8A1,ADAMTS9,NF1,RTN4,MYH9,HDAC5,DCN,CDH13,RCC2,RFFL,CCBE1,FBXW7,WNT11,SEMA5B,TGFB1,BCAS3,SLIT2,CORO1C,KANK1,BMPER,FGF10,SMURF2,PRKCA,PPARG,FGF1,FLT4,PTPRM,NRP2,LRP5,PTPRG,PRKD1,SEMA6D,BMPR2,PIK3R3,RAB11A,FGF2,PPM1F,SEMA4D,PKN3,RHOJ,PAFAH1B1,IQGAP1,SP1,MACF1,CLASP1,OVOL2,ATP2B4,PAK3,SCARB1,GPC3,SMOC2,PLCG2,ROCK1,PAXIP1,PKN2,SH3BP1,ZEB2,ADTRP,PTEN,BMP7,PRCP,AMOTL1,WNT7A,GPI,SMO,RHOA,ACTA2,AGT,PTK2,ARID5B,EPB41L4B,ANGPT1,ETS1,BRAF,ANGPT4,SP100,FAT2,SRGAP2,RADIL,SASH1,GNA12,MET,SEMA3C,EDNRA,BMP4*
GO:0016043	cellular component organization	0.004639985737408097	*CD247,CLRN1,ENPP1,HOOK2,ADAMTS16,BLZF1,NRXN1,ASPH,PRMT3,PRKCI,MOV10L1,TACC2,MAP4K4,PDCL,DNMT1,S1PR2,LLPH,SLC9A1,NEGR1,ADCY8,SPAG16,PDE4DIP,CLASP2,SEMA3A,GRID2,RPS6KA2,PRDM12,TENM3,DOCK1,CLDN18,KIF22,RYR1,NRG3,ASH1L,NOS1AP,TUBA1C,NOX5,SPESP1,IQCG,TLN2,EPB41,SEMA3D,PHACTR1,MYOT,NREP,SCAF8,TIAM2,FHOD3,MAPRE2,NLGN4X,TJP1,UNC5C,HPSE2,PRLR,UTRN,KALRN,PITPNC1,SEC23B,PHACTR2,NTRK3,CBL,EGLN2,MIA,RAB4B,RAB4B-EGLN2,SEMA5A,RAD51B,PTGES3L,TOX,ENPP2,GRM5,PLCE1,LHFPL4,SAMHD1,FER,RNU6-202P,MARVELD3,MAP2K5,ISCA1,LRP2,ANK2,OLFM1,MFAP5,STAG2,EZR,MEGF10,NRXN3,ROBO1,TOM1L1,CHD7,ITGB6,CDKL5,MECOM,TACC1,TENM1,CAMKMT,RYR3,KIF5C,LMNA,ROBO2,NFASC,CTNNA3,DNAJA3,VAV2,ABI1,ULK4,ITGB1,TRIOBP,HSP90AA1,ELMO2,GAS7,CDC6,PSMB7,PCDH17,KNDC1,ZC4H2,DSCAM,BCORL1,STK38L,CDAN1,CNGB1,TRIO,ARHGAP24,KLHL12,AKAP13,IFT43,TTLL5,GFI1B,PARD6G,PTPRK,RUNX1,DAB1,OMA1,CDC42EP3,MYO10,ABCC8,ERC1,HMGN3,CDH10,ERC2,XIRP2,IMPG2,ITGB3BP,LAMA2,CLVS1,MAST4,COL14A1,IL17RB,TCIRG1,PTPN11,KCNS3,CCT2,CACNB2,DOCK10,HDAC6,GOLGA4,SERTAD2,TBCEL,ADAMTS3,EPHA1,IKBKB,KIF18B,PEX14,ERBB4,CDH12,MRE11A,GBF1,DNAH2,LIMK1,TLK1,BRD8,ATP8A2,RNU6-78P,RAD54L2,TEX264,KAT6B,CUL4B,CECR2,ESR1,NPHP3,RNU6-179P,GRM7,PMEPA1,PTPRO,KANSL1,NTRK2,STX18,DNAI2,EP300,DYM,TENM2,FNTA,HOOK3,RNU1-124P,PDGFB,RIMS1,TNIK,SNX2,KIRREL3,CDH8,CSGALNACT1,MID1,RNU6-1061P,CHFR,ALOX5AP,MSTO1,INSR,FMN2,RERE,PRKAR1A,AFAP1,ATP8B1,UNC5D,H2AFY2,LAMA3,TCF7L2,PIP4K2A,CDH4,HPS1,JMJD1C,PAK1,FBXW11,MAP3K4,PTPRD,CNTN5,TBR1,STRC,BDNF,GLTP,AMIGO1,FANCA,KCNMA1,TGIF2-C20ORF24,NPRL3,FMNL2,PHACTR4,RNU6ATAC27P,TENM4,KCNRG,UBR2,ARHGAP6,MDM4,ECT2,SLC12A8,ZNF148,NAV2,ACOX1,VAMP7,LAMC1,RFTN1,ERN2,CDC45,SHFM1,LRRC49,NPLOC4,PYROXD2,STARD9,DENND5A,DMD,CENPF,RNU6-1269P,CRISPLD2,PDS5A,KAT7,PRELID2,GSN,HFM1,RBM14,SATB2,RIT2,HIRA,TANC2,CASS4,KCTD16,FYN,ADAMTS9,NF1,PLCB1,DST,PCDH15,LMTK2,STX6,ARID4B,SNCB,FAT3,SH3BGR,RTN4,AFF2,RXFP1,GOLGA2P5,APOD,B4GALT6,RTN4R,VTA1,BCR,BRPF1,CCDC141,TTN,NDRG4,BMP2K,PAQR3,COA6,ADAMTSL1,RANBP9,TLL1,NTN1,EGFLAM,DCTN1,SLC4A5,NRG1,ARHGAP10,BDKRB1,BID,FRY,MAP2K1,FNIP1,RAPGEF6,VAX2,MYH9,FRMPD4,MAD1L1,LRFN5,VGLL4,PPP2CB,CNTN4,WDR92,VWA1,HDAC5,RBM5,NTNG1,NAV1,NFIA,RNF4,TMEM30A,CSNK2A3,SYNE1,PARD3B,LRGUK,ROR2,DCN,LIN7A,SCFD1,DOCK2,CDH13,PITPNB,AUNIP,UST,DMC1,PCDHB16,EXOC4,MAD2L2,TLE6,RAB27A,CAPRIN2,MTX3,NCKAP1,TERF2,TMED6,SLX1B,GOLPH3L,CLDN16,RCC2,BOC,ANO6,ARL3,DNAJC11,KIF2A,ICK,RAD51D,TTLL8,PARP11,COL12A1,RANBP1,USP22,ZDHHC15,JAK2,MYPN,FBXW7,KCNB2,SKAP1,SMC3,CLSTN2,UIMC1,SYT1,ITCH,CROCC,RNU1-2,RNU1-4,BBS12,SNAP25-AS1,LZTS1,BCL11B,SMG1,CLDN1,MLLT3,ATP9A,CDC42BPB,PLS1,TEX11,PDE2A,SEPT7,MAGI1,KCNC4,KPNA3,CYFIP2,CENPC,WNT11,IFT80,FIG4,MTA1,KREMEN1,IMMP2L,SIPA1L3,MKLN1,SIAH1,IL1RAPL1,CAMK2B,PRKCD,SEMA5B,PPM1E,TGFB1,BANP,LRCH3,SGK1,PDLIM4,ATL2,NUP93,MMP28,NSD1,PIBF1,KEL,ARMC1,CLDN10,F2RL1,BCAS3,PDZRN3,RPGRIP1L,DNAJB2,CELSR1,SLIT3,AAK1,SDK2,SLIT2,COLGALT1,TP73,SYT13,CDH23,CORO1C,CTNND2,DISC1,CEP135,KANK1,PTPDC1,GRPEL2,ANO4,LAMC2,COL23A1,CLN6,ANK1,CLMN,TTBK2,KDM4B,SPAG17,MMP26,SMAD6,COL24A1,ITSN2,PPFIBP2,ANO3,SMOC1,ETV6,SYNJ2,BCL3,DCLK1,RNF212,EIF3L,LYRM4,PARVA,DUSP22,STRIP1,HNRNPC,RNU6-816P,TRRAP,ANKFN1,SLC17A7,OIP5,LINGO2,YTHDF1,FGF10,CNTNAP2,SMYD3,STXBP5,KANK4,KCTD10,ENAH,IL1RAPL2,PPL,IQCJ-SCHIP1,LOXL3,RHPN2,MAST2,SVIL,CAPN3,SLC16A1,LUM,VMP1,EPHA4,HSD17B12,PRKCA,AUTS2,CNR1,SMG6,PPP3CA,GNG4,MAG,GATAD2B,LIX1L,UFL1,TRAK1,CTNNB1,MPRIP,PARK2,SOD2,RN7SL832P,FCGR2B,ARHGAP22,SMARCC1,TSNARE1,CDHR2,IGF1R,PPARG,NGRN,AXIN1,DLG5,MYO1D,BFSP1,ADD2,MSR1,CELF2,TRPM1,XK,SCAMP5,BRIP1,CENPP,ECM2,SREBF2,KCNJ12,CDK11A,KCNA6,NDUFA9,FBLIM1,CDK11B,DRAXIN,LEPROT,NIN,WDR45B,TUBGCP6,NOL3,RIOK2,ESCO1,MPP6,MYOCD,AJUBA,HAUS4,CPNE6,CHEK2,PHLDB1,KRT6B,UBQLN4,PR*

			DM16,MYO1E,SPDYA,HCK,SORBS1,TBC1D14,RAB3GAP2,CAPN2,TBCK,CSPG4,DCC,ANKFY1,CTDP1,BAZ1B,MMP16,OBSCN,NF2,SMC2,HDAC4,PAX2,DNAH9,SPTBN1,HEPACAM2,ELN,TRABD2B,SPG11,SFRP1,DGKB,ARL13B,CNTN6,NFIB,MUC12,IFT122,DNM3,SSH1,CELF5,ATP10D,SNX14,SMAD3,CUX2,THSD4,PTPRM,WIPI1,MTBP,VPS39,RNF168,EMG1,ABI2,C10ORF90,GRIK5,SHANK1,SYT3,NEDD4,NRP2,ARHGEF18,BTBD3,PREX1,PLA2G4C,CRTAC1,SPAG6,KPNB1,NAV3,SLC6A1,RAB6C,PRTG,CDC73,APP,SSBP3,USP49,PDGFRA,BET1L,SLK,ADD3,DIP2B,KANSL2,NOX1,YAP1,HEG1,MYO1F,TEN1,FSTL4,NLGN2,VDAC1,EYA2,SH3D19,BORA,LRP5,PTPRG,ISLR2,SOX2,SETD2,ZDHHC6,GPC6,KIF24,TTC39C,ADAMTS2,TEAD1,PLEKHA7,SPTBN4,VASH2,DRD1,TMEM14A,RFT1,SFMBT1,VILL,MMP2,ESR2,SYNE2,DCUN1D3,CPNE9,KDM6A,GTF2F2,PRKD1,CELF6,PARP6,OSBPL2,DGKG,SYBU,RND3,HNF4A,TBCA,SHANK2,SHPRH,VPS4A,EREG,TCTN3,ABLIM1,MYOF,DSCAML1,RNU6-167P,PITRM1,SEMA6D,ATF2,TMEM237,KCNG4,RAF1,CELF4,ABLIM2,SMIM20,PTPN1,ADAMTS12,PSMD11,RNU6-923P,BMPR2,USP33,DPYSL2,VBP1,CAMK1D,NLGN1,COL16A1,CTNNA2,ATP9B,IKZF4,TAF8,CAND2,SPAG9,MORC2,RAB11A,CRMP1,EPG5,MYB,LRRC4C,PPM1F,NEDD9,PEAK1,ADAMTS4,SEMA4D,ARMC2,CDC27,HELQ,MCTP1,PLXNA2,POC1A,EEA1,SETD1A,YME1L1,ABCA13,JARID2,RILPL1,BRDT,CHCHD6,PI4KA,PIK3C2B,SPOCK1,BLOC1S5,EHMT1,GAS8,AP2M1,DVL3,EIF4G1,EPHB3,RNU6-229P,ANAPC5,ARHGAP39,FBXO45,TRAPPC11,SRSF5,PAWR,TRMT61B,AGO3,DEPTOR,TSG101,VCL,LAMB1,TERF2IP,WDR90,CLIC4,MYO9A,ANKS4B,COL11A1,KCNQ1,RFX2,WNT3,ADAMTS6,C11ORF80,RHOJ,NLGN3,ZFYVE1,PAFAH1B1,KIF3A,ADAMTS14,COL4A3,TRPA1,TRPM3,HIP1,AKAP6,CEP350,RASAL1,PADI6,BTBD9,TRPC5,TTLL7,GLI2,NEO1,TNKS,WBP2NL,ERCC1,RUFY3,WDTC1,STON2,C9,COL19A1,DAB2,BLM,PKHD1,XKR4,TRAM2,LDLRAD4,MYSM1,SETD5,DZIP1,SMG5,DLG3,RELN,SIN3A,RUVBL2,ABCB1,DOCK11,IQGAP1,TMEM170A,SPATA18,COL6A5,EML4,FAM91A1,ZNF423,MACF1,ARHGAP12,MYO7A,ALK,SLC8A2,STAU1,CLASP1,TOP1,MBOAT1,GPM6B,XDH,NFATC2,CHD1L,PRKG1,TTLL9,NSF,SYNE3,PACSIN1,SPTBN5,ACTL6B,PAK3,HORMAD2,SSH2,TET1,DYX1C1,ATAD1,ADNP,CEP41,ARID4A,MAP1B,C12ORF65,CDC42BPA,COL4A6,MPHOSPH9,SCARB1,MARK1,PHF2,CELF1,AIF1L,TFRC,UVRAG,EPHA5,MEF2A,DHX30,DCUN1D5,GPC3,SGIP1,XRN1,PLS3,SATB1,TBC1D5,PSMB2,ZNF207,CALD1,EYA1,DIS3L2,GNB3,TRPV1,TFEB,CLEC16A,MNAT1,FLRT2,FGD1,SNX9,DIAPH2,RPGR,ACTR2,L3MBTL4,ACACA,ELAVL4,FAM174B,HDAC1,RECQL5,SMOC2,PLCG2,ROCK1,EPS8,NID1,SCMH1,EGR2,CAPN10,GPR35,ABCC1,AP3B1,INSRR,NTRK1,ZFAND1,MAP1A,PKN2,PPP1R9A,EIF3A,ANK3,UNC13A,PTPRQ,EPHB1,EPHA10,PTPN9,AXIN2,NCKAP5,SARM1,TMEM199,PARD3,GNL3L,TRIM46,TUB,SH3GL3,TEKT1,KCND3,BCOR,AP3D1,CARM1,AP2B1,ANXA8L1,NUDT5,ARHGAP44,SNX33,PIFO,SMARCA2,CUX1,RAD51C,SPI1,EPHA7,MCU,RANBP10,PTPRA,VPS16,PPFIA4,CTNNA1,LIMA1,AIFM2,TRDN,ATG14,EIF3H,LRRC8C,LRRC8D,RNU6-979P,SHROOM3,ABCB7,ACOT8,NTMT1,FCHSD2,MAP3K13,RAPGEF3,SDK1,ADAMTS17,PDXP,SH3BP1,RNU2-47P,ERCC3,PTK7,SMYD1,SNCA,BMPR1B,HAUS3,CACNG2,CD53,MAGI2,PNPT1,USP50,ZEB2,MCMBP,FOXJ3,SYT9,HEY2,RC3H1,CHMP3,COL13A1,EIF3E,HYDIN,RNF103-CHMP3,RNU6-640P,WNT7B,PTPRS,ADAMTSL3,ZMYND11,KMT2D,CDKL1,RAB5B,KMT2C,TLL2,CAST,DFFA,ADTRP,ABCA12,CSRP1,PLD1,RASA1,MITF,SRSF6,OPRD1,CABIN1,HIPK1,PKP2,RPTOR,RTN4RL1,SGCZ,DSG1,VPS41,KCTD13,TROVE2,CBFA2T2,IGF1,STX8,ATF7IP,HTT,DPYSL3,MAPK1,PTEN,ARHGEF17,CLDN11,MIB1,BMP7,ATXN2,PIK3C3,LHFPL5,TTLL4,RAB30,LRRK2,OSGIN1,SEPT6,ZRANB1,EBAG9,SPAG5,UNC119,CHMP5,RAPH1,RFC3,BAG6,CHAF1B,IFT81,TMEM108,ITGAM,ARHGAP25,NSG2,PKD2,EFNA5,HSF2BP,SHANK3,KCTD1,STMN4,PDGFC,SERPINA5,AMOTL1,ATP8B4,PKIB,MTMR3,STEAP4,PPP2R3C,TBCD,TMCC1,WNT7A,SNAP23,BRWD3,NLRP1,MAP4,CRTC3,NME8,CEP89,KAT6A,MTIF2,RABEP1,SH3PXD2B,RHOT1,SIK3,BMP6,MYCBP2,SOS1,TSHR,EXT1,ATRX,IKZF1,PARVB,PNPLA3,SAMM50,RAPGEF2,CDH9,KCNC2,RNU6ATAC31P,SMO,GET4,SUN1,BLOC1S3,GEMIN7,MARK4,PSEN2,TCF4,TNFRSF11B,SYT17,SOX30,DNAJC6,GRIN1,JADE1,KCTD8,BBS9,RHOA,SYNGAP1,GRIN3A,MCM3,NDE1,ROR1,TF,ONECUT2,RHOBTB1,ESYT2,SHROOM1,CUL3,SH3KBP1,HSPD1,TSPAN33,EPM2A,GABRB3,GSK3B,ABCG8,PLD2,PRR16,EMB,RDX,SYN1,EEF2K,FAM49B,NECAB2,CDK13,TAPBPL,VAMP1,BOK,SULF1,KRT8,NACC2,SUPT4H1,AGT,HDGFRP3,CDH20,PRKAA2,SYT7,ADAR,BLOC1S6,CUL9,ESPNL,PITPNM2,STAT2,ZMPSTE24,PTK2,TPPP2,GTF2E1,PEX5L,SNX3,EXOC6B,RIMS2,TOMM5,CDC14A,GAP43,RNU6-1056P,SEC16B,CREBBP,TIMM44,IQGAP2,FAM171A1,ARVCF,COL9A1,FMOD,COL21A1,CEP70,GTF2IRD2,TAOK2,PYGO2,SHC1,NDUFS2,ALDOA,ECE1,PARP10,YLPM1,RSBN1,SF3A1,PGM5,SETDB2,ITGAL,CPLX2,DNAJB6,CD44,FGD3,LARP4,ADAM8,ARFGAP3,CLDN4,PACSIN2,RPS6KA5,CDS1,THSD7A,CSNK1A1,HUS1,FGD4,PEX7,TBC1D23,PLEKHA1,RGS14,ACTN4,COG4,STK4,TUBB3,ZFAND6,MARK3,SPEF1,THSD7B,EPHB2,SLC1A1,TTLL11,SIGMAR1,TMEFF2,TEX12,MFSD8,ASF1B,UBE2K,GPSM2,DLC1,PPARA,PPP1R10,ABCD3,MCOLN1,EPB41L4B,SLC25A33,SMARCAL1,CCDC6,PRRC2C,SYTL4,PAX6,PRKCZ,ATP1A3,ADORA2A,SPECC1L,GRIN2B,KCTD7,RABGEF1,STAG1,WDR83OS,COL22A1,PHLDB2,KLC3,KLHL1,NIPBL,YEA

			TS4,COL28A1,PHF20,THSD1,UPB1,ANGPT1,BRF1,PACS2,EYA3,MBTD1,OSBPL8,SHROOM4,EPB41L2,ETS1,HACL1,PPP1CA,ARHGAP21,MAP2,MTF2,BIN2,STPG1,KIFC3,NEBL,FBXO31,ATG3,CNTN1,PRKCQ,LGI2,MDN1,BRAF,DIAPH1,HDAC2,IMPACT,ATP8A1,SRCAP,CAPN6,NOTO,SPINT2,TNR,CLTB,HIGD2A,CD300A,PHIP,CDKL3,EMC3,PPP2CA,ANGPT4,ARHGEF6,DNAJC9,FAM149B1,RPL23,SH3BP4,RFC5,ACAN,CAMSAP3,SP100,VTI1A,ANXA2,CHCHD3,KCND2,PARN,PRMT7,PITPNM3,FARSB,SRGAP2,TMEFF1,WIPI2,ADCY1,NUMB,SQLE,SYK,CNOT1,WWTR1,GTF2H5,SLIT1,FRMD5,CIT,RAB2A,SEC31B,UBAP2L,BRCA2,HRK,RAP1GAP2,DVL2,MORC1,MTRF1,NEB,ANKRD13A,GRIP1,PTPRJ,TAF1,GPRIN3,TICAM1,GPM6A,ITGAE,MYO1A,HSF1,MAX,DHFR,EZH1,ANKRD30BL,ETF1,SRPK2,ATP10B,PLEKHM1,HGSNAT,ASAP1,ADAMTS7,GBP5,MCM8,BMP1,CGNL1,FCHO1,GEMIN6,VAV3,CHD6,EPB41L1,MTFR2,CAV2,SKA2,SYNM,AKTIP,COL4A5,EPS15,POC1B,INPP5F,NUAK2,TBP,AXL,REPS2,TRIM37,CENPK,MET,FMNL1,MICAL3,CAMK2D,SNX1,BRD9,SETD3,INO80C,SPTAN1,TMEM120B,CCDC136,EHD2,KDM2B,SEMA3C,THEM4,TRAPPC8,CCT3,NEDD4L,UQCC1,NDC1,PHB,PTPRC,CTCF,SH3GL2,RNF213,SYNDIG1,NCAM1,TANC1,CREB1,RASSF8,SHISA6,TNXB,CCDC170,EDNRA,PRAP1,STXBP6,BMP4,ABLIM3,CPNE1
GO:0050768	negative regulation of neurogenesis	0.004673716751194455	SEMA3A,SEMA3D,NTRK3,SEMA5A,DAB1,ABCC8,HOOK3,NF1,RTN4,RTN4R,NTN1,SEMA5B,TGFB1,PPP3CA,MAG,CTNNB1,DRAXIN,DCC,PRTG,DIP2B,FSTL4,SEMA6D,BMPR1A,SEMA4D,WNT3,TRPC5,RUFY3,BRINP1,RNF10,TRIM46,EPHA7,CTNNA1,PTPRS,PTEN,BMP7,WNT7A,RAPGEF2,SYNGAP1,DLX1,EPHB2,PAX6,MAP2,TNR,CDKL3,SLIT1,SEMA3C
GO:0060402	calcium ion transport into cytosol	0.004690374422254974	ASPH,PDE4D,FAM155A,CHERP,RYR1,ANK2,CHD7,HTR2B,RYR3,SLC24A2,LCK,DMD,SLC8A1,JPH2,FYN,BDKRB1,TMBIM6,NPSR1,TGFB1,JPH3,CAPN3,TRPM1,RYR2,NOL3,CACNA1C,ITPR1,IBTK,DRD1,PRKD1,FGF2,ADCYAP1R1,TRPA1,AKAP6,SLC8A2,TPCN1,ATP2B4,TRPV1,PLCG2,TRDN,SNCA,HTT,PKD2,GRIN1,ITPR2,NOS1,XCR1,MCOLN1,GRIN2B,DIAPH1,HTR2C,CAMK2D,PTPRC
GO:0051283	negative regulation of sequestering of calcium ion	0.004993451616723576	ASPH,PDE4D,CHERP,RYR1,ANK2,CHD7,HTR2B,RYR3,LCK,DMD,SLC8A1,JPH2,BDKRB1,NPSR1,TGFB1,JPH3,CAPN3,RYR2,NOL3,CACNA1C,ITPR1,IBTK,DRD1,PRKD1,FGF2,TRPA1,AKAP6,TPCN1,TRPV1,PLCG2,TRDN,SNCA,HTT,PKD2,ITPR2,NOS1,XCR1,MCOLN1,DIAPH1,HTR2C,CAMK2D,PTPRC
GO:0009725	response to hormone	0.005016413071108112	ENPP1,PRKCI,SLC9A1,ADCY8,PTGFR,LATS2,PRLR,PAGR1,NTRK3,EGLN2,FLT3,STAT5B,FER,LRP2,GOT1,ROBO2,CDC6,ABCC8,THRB,STXBP4,PTPN11,HDAC6,BRD8,ESR1,PMEPA1,EP300,CCND3,INSR,PIP4K2A,CAB39,PAK1,ESRRB,PTPRU,AP3S1,RBM14,ARNTL,PLCB1,RXFP1,PIK3R2,HDAC5,SLC39A14,ACSBG1,DEFA1B,DEFA3,PTGER2,JAK2,CLDN1,KLF15,CACNA1H,PRKCD,TGFB1,SGK1,BCAS3,SLIT3,SLIT2,GABRB1,KANK1,SMAD6,RXFP2,CLOCK,BCKDHB,TIMP2,SMYD3,GLP2R,UFL1,SMARCC1,IGF1R,PPARG,CYBB,OTC,BRIP1,LEPR,LEPROT,NR4A3,SORBS1,HDAC4,SFRP1,FOXO3,TPH2,NEDD4,ESRRG,YAP1,ALPL,GNG2,MMP2,ESR2,STAT1,HNF4A,EREG,PTPN1,PIK3R3,PTAFR,RARB,NCOA1,EIF2B5,PTPRE,SRSF5,IDE,KCNQ1,NR2C1,IRS4,ME1,WDTC1,DAB2,SP1,GSTM3,SRA1,SNX5,DYX1C1,MAP1B,TRPV1,GHR,ACACA,HDAC1,C2,TRIM24,ADIPOR2,ROCK1,EGR2,CAPN10,PTPN2,INSR,CARM1,SRD5A2,PTPRA,CTNNA1,BCAR3,RAB15,PNPT1,CNGA3,WNT7B,HMGCS2,KMT2D,ADTRP,SRSF6,DSG1,BMP7,PRCP,EFNA5,NCOA3,WNT7A,GPR21,BMP6,GPI,TSHR,PNPLA3,PRMT2,TNFRSF11B,SOX30,UBR5,GRB14,RHOA,RQCD1,GSK3B,RDX,STAT6,EEF2K,AGT,PRKAA2,STAT2,PTK2,PHEX,UGT1A1,SHC1,RPS6KB1,STC2,GPR173,CLDN4,NOS1,SCNN1B,PPARA,SLC25A33,ZNF366,FBN1,PRKCZ,ATP1A3,CPEB1,FHL2,OSBPL8,NCOR2,PRKCQ,PHIP,CNOT1,UCN2,HSF1,MAX,NCOA2,CAV2,DENND4C,PHB,CREB1,EDNRA,HADHA,BMP4
GO:0071407	cellular response to organic cyclic compound	0.005175783314051368	PDE4D,SLC9A1,ADCY8,RYR1,PAGR1,EGLN2,FLT3,EZR,SLC26A6,HTR2B,RYR3,NRIP1,HDAC6,ESR1,PMEPA1,EP300,PDGFB,PAK1,ESRRB,CHRM3,SLC8A1,ARNTL,PLCB1,MGMT,RGS8,DEFA1B,DEFA3,JAK2,PDE2A,LCOR,CCDC62,TGFB1,SGK1,BTRC,GABRB1,CHRM1,CLOCK,SMYD3,ADCY2,SLC16A1,RORA,UFL1,CTNNB1,PARK2,IGF1R,PPARG,MSR1,RYR2,NR4A3,CHEK2,SFRP1,FOXO3,SSH1,NEDD4,ESRRG,APP,YAP1,ALPL,GNG2,DRD1,MMP2,ESR2,STAT1,EPG5,ADCY5,RORC,PTAFR,DDX58,KCNQ1,AKAP6,NR2C1,GLI2,DAB2,BLM,SIN3A,RUVBL2,ALK,ATP2B4,PAK3,DYX1C1,XRN1,TRPV1,ACTR2,HDAC1,RECQL5,TRIM24,AXIN2,CARM1,HTR1D,CTNNA1,RAPGEF3,PDXP,SNCA,HMGCS2,KMT2D,ADTRP,MAPK1,BMP7,LRRK2,GNA14,NSG2,PKD2,EFNA5,NCOA3,PNPLA3,PRMT2,RAPGEF2,SMO,UBR5,RHOA,RQCD1,ITPR2,GABRB3,GSK3B,EEF2K,HRH4,PHEX,PDE4B,UGT1A1,RPS6KB1,SCNN1B,SLC1A1,IL18,PPARA,ZNF366,ATP1A3,NCOR2,DIAPH1,HDAC2,HTR2C,IMPACT,ADCY1,CNOT1,TAF1,HSF1,CIB2,CAV2,PHB,BMP4
GO:0071705	nitrogen compound transport	0.005297014773613552	POLDIP3,ZDHHC14,SLC35E3,HOOK2,RTBDN,BLZF1,ASPH,PRKCI,ADCY8,CTDSPL2,SLC25A17,LRP1B,ASTN2,SLC14A2,SLC35F3,MVP,KALRN,SEC23B,SNX31,RAB4B,RAB4B-EGLN2,FAM53A,CCDC93,LRP2,SLC38A11,TNPO3,ANK2,EZR,TOM1L1,CHD7,SLC26A6,TENM1,KIF5C,LMNA,SRP72,AFM,ITGB1,HSP90AA1,CCDC22,DLG2,FLVCR2,ABCC8,ERC1,HMGN3,AFTPH,STXBP4,EFCAB7,TCIRG1,PTPN11,HDAC6,GOLGA4,ADORA1,PEX14,GBF1,TLK1,ATP8A2,GRM7,NTRK2,FRMD4A,STX18,HOOK3,VPS45,RIMS1,SNX2,FMN2,ATP8B1,TCF7L2,SCAMP4,MYOM1,GLTP,SFT2D1,ECT2,SNX6,CHML,VAMP7,SLC6A16,RFTN1,SHFM1,AP3S1,NPLOC4,RHCE,CENPF,RANBP17,ATG10,FYN,ARNTL,ADAMTS9,NF1,SMG7,LMTK2,STX6,GOLGA2P5,SLC5A6,A

			POD,VTA1,BCR,TTN,ICA1,PIK3R2,SLC16A10,SLC43A2,BID,SLC35A2,MYH9,TMEM30A,SCFD2,ZDHHC3,LIN7A,SCFD1,CREBRF,EXOC4,IPO5,MAN1A1,RAB27A,MTX3,RAB3C,COG8,TMED6,GOLPH3L,ARL3,NEUROD1,IPO11,PARP11,DENND1A,ZDHHC15,JAK2,FBXW7,OAZ2,SYT1,SNAP25-AS1,ABCC2,SMG1,RBFOX1,SLC1A4,DENND2A,KPNA3,CTAGE6,VPS13B,IMMP2L,QKI,PRKCD,TGFB1,PCSK5,NUP93,PTPRN2,F2RL1,BCAS3,CADPS2,SYT13,ZFAND2A,ABCC11,GRPEL2,CHRM1,CLOCK,BCL3,RABGAP1L,SLC17A7,STXBP5,SLC16A1,VMP1,SNX8,CNR1,TNFSF11,SMG6,PPP3CA,NSUN2,TRAK1,PARK2,RN7SL832P,TSNARE1,PPARG,MYO1D,MSR1,SYTL5,XK,SCAMP5,ANXA13,LRPPRC,SREBF2,LEPROT,AGAP1,PER2,TBC1D14,RAB3GAP2,TBCK,GRM1,SPTBN1,AP3B2,SFRP1,CCDC91,RANBP3,SNX14,SMAD3,RAB6A,WWP2,VPS39,PSAP,SLC16A2,SYT3,NEDD4,KPNB1,VPS53,SLC6A1,RAB6C,SEC61B,THOC3,BET1L,RBM8A,RAB11FIP5,SFXN5,NLGN2,SSR2,LRP5,SEC22C,SLC9B2,SETD2,ZDHHC6,PRICKLE1,DRD1,SERGEF,PRKD1,SLC6A3,HNF4A,VPS4A,PITRM1,SLC16A12,ATF2,RAF1,PTPN1,AP1B1,NLGN1,SNX16,RAB11A,EPG5,IPO9,RAB24,PPM1F,ADCY5,XPO6,ABCA13,RILPL1,SORCS2,RAB11FIP3,TANGO6,AP2M1,SLC35D1,TNPO1,TECPR2,TSG101,DESI1,SUFU,PAFAH1B1,KIF3A,ATG4C,HIP1,AKAP6,TNKS,RUFY3,DAB2,TRAM2,DZIP1,SMG5,ABCB1,SLC22A3,SLC47A1,FAM91A1,PLEKHF2,MACF1,MYO7A,APBB3,SLC35A4,GPM6B,SNX5,NSF,NXT2,CEP41,TFRC,EPHA5,SYT12,STOM,ADRBK1,TBC1D5,TRPV1,SLC38A6,SNX9,RPGR,ARL5A,EGR2,GAPVD1,CAPN10,ABCC1,AP3B1,KPNA6,ZFAND1,MAP1A,ANK3,SNUPN,ATP13A3,PARD3,PTPN14,CACNG8,AP3D1,AP2B1,ARHGAP44,SNX33,MCU,VPS16,LRRC8C,LRRC8D,ILDR2,ABCB7,FCHSD2,RAB15,RAPGEF3,SLC48A1,SNCA,CACNG2,TBC1D9,PNPT1,OCA2,SYT9,CHMP3,RAB5B,ADTRP,ABCA12,BTK,TBC1D16,XPO4,VPS41,IGF1,STX8,SLC7A14,ATXN2,PIK3C3,NUP88,LRRK2,UNC119,CHMP5,SLC44A1,ITGAM,EFNA5,MIA2,SGSM1,ZBED6,ZC3H11A,SNAP23,DNAJC1,RABEP1,POM121C,BMP6,ANO1,SERINC5,SAMM50,ABCG2,SMO,SLC6A14,LSG1,BLOC1S3,STXBP5L,CPT1A,SYT17,UBR5,ARRDC4,BBS9,HSPD1,EPM2A,GSK3B,IWS1,UPF2,AGT,SYT7,ADAR,BLOC1S6,PEX5L,SNX3,EXOC6B,RIMS2,SLC25A51,TOMM5,COPG2,CPE,SEC16B,TIMM44,SLC25A42,GTF2IRD2,NCF1,SLC29A1,TAOK2,SLC44A3,ZDHHC23,ALOX5,ADAM8,ARFGAP3,SLC25A18,MON2,NOS1,PEX7,GCKR,ACTN4,COG4,STK4,ZFAND6,SLC1A1,WLS,HCAR2,PPP1R10,GRTP1,CALCRL,MCOLN1,STEAP3,SLC25A33,KPNA4,SYTL4,PRKCZ,ADORA2A,RABGEF1,ANGPT1,CADPS,IGF2BP3,HACL1,TBC1D10C,ATG3,HTR2C,SIL1,ATP8A1,CLTB,RPL23,CAMSAP3,SP100,VTI1A,RAB27B,ITSN1,NUP214,SYK,MYRIP,RAB2A,SEC31B,SLC44A5,CACNG3,YBX1,GRIP1,SLC13A3,HSF1,CNIH4,PLEKHM1,MLPH,RAB37,AKTIP,EPS15,DENND4C,TBC1D10A,SNX1,ZDHHC11,ZDHHC11B,NDC1,RPH3A,GPR89A,RAB28,SYNDIG1,VPS52,TRAF3IP2,SGTB,EDNRA,SLC25A13,BMP4,ABLIM3
GO:0019941	modification-dependent protein catabolic process	0.005348162606580545	CALR3,FBXL2,DCAF12,CBL,USP32,TOM1L1,PSMD1,PSMB7,CCDC22,DNAJB14,HDAC6,DDB1,CUL4B,FBXL18,ZYG11B,FBXL7,USP46,CHFR,USP34,FBXW11,RNF144A,TRIM13,UBR2,NPLOC4,USP13,MKRN2,ARNTL,FBXO21,PPP2CB,RNF4,UBE3D,FHIT,MAN1A1,FBXO9,RFFL,USP22,FBXW7,ITCH,USP12,SPSB1,MTA1,SIAH1,ERCC8,SPSB4,ASCC2,BTRC,DNAJB2,RNF133,RNF148,DISC1,ZFAND2A,CLOCK,FBXL17,KCTD10,SMURF2,UBQLN3,UBQLNL,UFL1,CTNNB1,PARK2,SMARCC1,AXIN1,UBQLN4,WWP2,RNF168,NEDD4,SEC61B,USP49,ARIH1,AMFR,PRICKLE1,ERLIN1,VPS4A,PSMD11,USP33,UBE2R2,AGBL4,CDC27,KLHL3,AREL1,UCHL3,PSMD2,RBX1,ANAPC5,FBXO45,STT3B,FBXL20,TSG101,DESI1,SUFU,EDEM3,DAB2,UBR1,CACUL1,PSMD7,PELI1,PPP2R5C,ZNRF1,RBBP6,UBE3C,RNF34,PSMB2,RNF216,RYBP,CUL2,MAP1A,MAN1B1,FBXO39,FBXW4,EIF3H,USP42,USP50,RC3H1,RNF103,RNF103-CHMP3,RNF19B,PSMF1,TRIP12,ZNRF3,KCTD13,PTEN,MIB1,PCBP2,LRRK2,ZRANB1,BAG6,SEL1L2,SH3RF2,N4BP1,UBE2H,CUL3,EPM2A,GSK3B,RNF144B,RNF43,CUL9,ZMPSTE24,PTK2,ARMC8,RNF121,FBXO10,TRPC4AP,CSNK1A1,UBE2K,FBXL13,RNF150,OTUD7B,TBL1X,FBXO31,C18ORF25,SKP1,RPL23,WWTR1,TAF1,GNA12,UCHL5,FBXL4,NEDD4L,RNF213,SGTB
GO:0010976	positive regulation of neuron projection development	0.0054930095969547175	PRKCI,NEGR1,TENM3,KALRN,NTRK3,TOX,ATP8A2,NTRK2,EP300,BDNF,AMIGO1,DMD,RIT2,FYN,NDRG4,TMEM30A,ROR2,CAPRIN2,ZDHHC15,FIG4,CAMK2B,DISC1,CNR1,IGF1R,CUX2,PRKD1,CAMK1D,PAFAH1B1,RELN,ALK,PACSIN1,PAK3,ADNP,ACTR2,ELAVL4,NTRK1,PTK7,MAGI2,CBFA2T2,DPYSL3,BMP7,RAPGEF2,ROR1,EEF2K,AGT,SNX3,CNTN1,BRAF
GO:0060070	canonical Wnt signaling pathway	0.0055009063507237990	GPC5,LATS2,SEMA5A,PSMB7,MCC,NPHP3,PTPRO,RNF220,TCF7L2,USP34,CDK14,PTPRU,ARNTL,ROR2,SOX13,MAD2L2,TLE6,CAPRIN2,CCNYL1,GNAQ,MLLT3,TCF7,WNT11,KREMEN1,BTRC,CTNND2,DISC1,KANK1,FGF10,SMURF2,CTNNB1,PARK2,AXIN1,DRAXIN,BICC1,SFRP1,FOXO3,SMAD3,YAP1,AMFR,LRP5,SOX2,PRICKLE1,KDM6A,CCNY,FGF2,DKK2,DVL3,RBX1,WNT3,PRDM15,TNKS,DAB2,WNK1,RUVBL2,RSPO2,GPC3,PSMB2,HDAC1,INVS,AXIN2,PTK7,ZEB2,WNT7B,MITF,ZNRF3,PTEN,LRRK2,WNT7A,EXT1,UBR5,JADE1,GSK3B,PYGO2,SCEL,CSNK1A1,STK4,WLS,TBL1X,LRRK1,PPP1CA,RBMS3,WWTR1,DVL2,ANKRD6,EDA,SHISA6,EDNRA
GO:0071241	cellular response to inorganic substance	0.0055278367759909850	NRXN1,ASPH,ADCY8,RYR1,ADCY7,RYR3,ITPKB,DLG2,CPNE4,ALOX5AP,TFAP2A,ECT2,DPEP1,SYT1,CLDN1,CACNA1H,SYT13,KCNH1,RASA4,RASA4B,CAPN3,PARK2,MTF1,CYBB,CPNE6,TPH2,SYT3,APP,GLRA2,CHUK,CPNE9,NLGN1,CLIC4,RASAL1,BLM,IQGAP1,MEF2A,SYT12,PLCG2,ANK3,SNCA,SYT9,MAPK1,LRRK2,PKD2,BMP6,ATRX,KCNC2,SYT17,TF,EEF2K,PRKAA2,SYT7,NCF1,SLC1A1,MCOLN1,BRAF,ADCY1,A3GALT2,HSF1,CAMK2D,TIMELESS,ADCY10,CREB1,CPNE1

GO:0051961	negative regulation of nervous system development	0.005676431933021912	*SEMA3A,SEMA3D,NTRK3,SEMA5A,ROBO2,DAB1,ABCC8,HOOK3,NF1,RTN4,RTN4R,NTN1,SEMA5B,TGFB1,PPP3CA,MAG,CTNNB1,DRAXIN,DCC,PRTG,DIP2B,FSTL4,SEMA6D,BMPR1A,SEMA4D,WNT3,TRPC5,RUFY3,BRINP1,RNF10,TRIM46,EPHA7,CTNNA1,PTPRS,PTEN,BMP7,WNT7A,RAPGEF2,SYNGAP1,DLX1,EPHB2,PAX6,MAP2,TNR,CDKL3,SLIT1,SEMA3C*
GO:0002064	epithelial cell development	0.005693893000948697	*PDE4D,S1PR2,TJP1,EZR,IKBKB,ESR1,PDGFB,DMD,ARNTL,PLCB1,ARID4B,SLC4A5,BCL11B,CLDN1,PDE2A,SIPA1L3,TGFB1,F2RL1,C9ORF47,CLOCK,BFSP1,MYO1E,ABI2,YAP1,HEG1,FRS2,HNF4A,FNDC3A,RILPL1,RARB,VCL,CLIC4,PKHD1,IQGAP1,GSTM3,ARID4A,CDK6,ROCK1,NTRK1,SHROOM3,RAPGEF3,MAGI2,HYDIN,WNT7B,PTPRS,ABCA12,WNT7A,BMP6,ATRX,RAPGEF2,SMO,ONECUT2,GSK3B,RDX,ACTA2,NOTCH4,PAX6,COL22A1,SPINT2,MET,EDNRA,BMP4*
GO:0030335	positive regulation of cell migration	0.005694753297601329	*MAP4K4,CLASP2,SEMA3A,DOCK1,SEMA3D,MAPRE2,TJP1,NTRK3,SEMA5A,ENPP2,FER,KITLG,PIK3CD,ITGB1,MGAT5,HDAC6,EPHA1,ZNF609,PDGFB,STK39,INSR,PAK1,SLC8A1,CASS4,RTN4,BDKRB1,DOCK8,CMKLR1,ROR2,CDH13,CD99,ANO6,CCBE1,JAK2,CLDN1,WNT11,SEMA5B,TGFB1,F2RL1,BCAS3,RRAS2,LAMC2,DAPK2,FGF10,SMURF2,EPHA4,PRKCA,PPP3CA,SOD2,IGF1R,FGF1,NR4A3,FLT4,HDAC4,SMAD3,NRP2,DOCK4,APP,PDGFRA,DRD1,MMP2,SYNE2,PRKD1,SEMA6D,BMPR2,CAMK1D,PIK3R3,SPAG9,RAB11A,FGF2,PPM1F,NEDD9,SEMA4D,ELP3,PTAFR,LAMB1,RHOJ,RUFY3,DAB2,WNK1,RELN,SP1,CLASP1,PAK3,SSH2,SCARB1,SMOC2,PLCG2,SPI1,MCU,IGF1,MAPK1,BMP7,PDGFC,WNT7A,GPI,SH3RF2,RAPGEF2,SMO,RHOA,ONECUT2,RDX,ACTA2,AGT,PTK2,RPS6KB1,ADAM8,CLDN4,CXCL17,PLVAP,ACTN4,STK4,EPHB2,EPB41L4B,GPSM3,NIPBL,ANGPT1,GCNT2,ETS1,FBXO31,DIAPH1,ATP8A1,ANGPT4,NUMB,SASH1,ZP3,MET,SEMA3C,PTPRC,BMP4*
GO:0032147	activation of protein kinase activity	0.005847860514869213	*ADCY8,NRG3,PRKAG2,PRLR,NTRK3,TOM1L1,TRAF6,PDGFB,INSR,CAB39,ECT2,CHI3L1,NRG1,MAP2K1,JAK2,WNT11,PRKCD,PIBF1,TNFRSF10B,AXIN1,FGF1,SPDYA,BORA,WNK1,MAP3K7,ADNP,GHR,RPTOR,IGF1,EFNA5,PDGFC,PPP2R3C,AGT,TAOK2,SLC1A1,IL18,MOB3B,PRKCZ,ANGPT1,OSBPL8,CD300A,ANGPT4,MADD,MOB3A,MALT1,PHB*
GO:0010035	response to inorganic substance	0.0058526765512353736	*PRDX2,NRXN1,ASPH,GSS,ADCY8,PDE8A,RYR1,ADCY7,RYR3,ITPKB,DLG2,ABCC8,TCIRG1,HDAC6,CPNE4,ALOX5AP,TFAP2A,KCNMA1,ECT2,DMD,SLC8A1,FYN,PLCB1,DPEP1,TTN,STIM1,PPP2CB,SYT1,CLDN1,CACNA1H,PRKCD,SYT13,HP,KCNH1,RASA4,RASA4B,CAPN3,PPP3CA,PARK2,SOD2,MTF1,CYBB,OTC,RYR2,NR4A3,CPNE6,CAPN2,PAX2,CPOX,FOXO3,TPH2,ARL13B,SYT3,NEDD4,SLC6A1,APP,GLRA2,CHUK,MMP2,CPNE9,STAT1,SLC6A3,ATF2,NLGN1,EIF2B5,PAWR,CLIC4,TRPA1,RASAL1,ERCC1,BLM,IQGAP1,ATP2B4,ADNP,MAP1B,TFRC,MEF2A,SYT12,MNAT1,PLCG2,NTRK1,ZFAND1,ANK3,SNCA,CACNG2,SYT9,CNGA3,HMGCS2,MAPK1,BMP7,LRRK2,PKD2,BMP6,GPI,ATRX,KCNC2,TNFRSF11B,SYT17,TF,STAT6,EEF2K,KRT8,PRKAA2,SYT7,PHEX,MAP3K5,NCF1,PLEKHA1,SLC1A1,SIGMAR1,MCOLN1,ASPN,PPP1CA,BRAF,HDAC2,IMPACT,PPP2CA,ADCY1,ALG2,A3GALT2,HSF1,DHFR,AXL,MET,CAMK2D,TIMELESS,NEDD4L,ADCY10,CREB1,TRAP1,SLC25A13,CPNE1*
GO:0061572	actin filament bundle organization	0.006015593797572798	*SLC9A1,CLASP2,PHACTR1,TJP1,EZR,EPHA1,LIMK1,FMN2,PAK1,ARHGAP6,DMD,PLS1,WNT11,PPM1E,RHPN2,PARK2,ADD2,SORBS1,NF2,ELN,SFRP1,SMAD3,SHANK1,ARHGEF18,PPM1F,NEDD9,PAWR,CLASP1,AIF1L,PLS3,CALD1,ROCK1,EPS8,LIMA1,RAPGEF3,PDXP,KCTD13,DPYSL3,SHANK3,RHOA,SHROOM1,CUL3,RDX,ESPNL,FAM171A1,TMEFF2,DLC1,PHLDB2,BRAF,CGNL1,MET*
GO:0019722	calcium-mediated signaling	0.006078061859355667	*ASPH,TMBIM4,SLC9A1,PTGFR,CHERP,KSR2,GRM5,PLCE1,ANK2,HTR2B,CHRM3,SGCD,DMD,SLC8A1,RIT2,NRG1,PPP3R1,CMKLR1,CDH13,CD4,NFATC3,HOMER2,TNFSF11,PPP3CA,RYR2,CACNA1C,HDAC4,ITPR1,ACKR2,NFATC1,RCAN1,MCTP1,NMUR2,AKAP6,SLC8A2,NFATC2,ATP2B4,CAMTA1,PLCG2,PPP1R9A,RCAN2,MCU,BTK,IGF1,HTT,LRRK2,GRIN1,GSK3B,MCTP2,ZMPSTE24,LAT2,XCR1,MCOLN1,GRIN2B,FHL2,TBC1D10C,SYK,PTPRJ,CAMK2D,CCR3,PTPRC*
GO:0046777	protein autophosphorylation	0.006100906979035428	*ENPP1,MVP,FLT3,FER,TOM1L1,CDKL5,GRK5,EPHA1,ERBB4,MRE11A,NTRK2,PDGFB,TNIK,STK39,INSR,PAK1,FYN,LMTK2,NRG1,JAK2,SMG1,CAMK2B,MYO3A,AAK1,DAPK2,EPHA4,CAMK4,IGF1R,RIOK2,CHEK2,HCK,OBSCN,FLT4,PDGFRA,SLK,PRKD1,MAPKAPK3,CDK12,PEAK1,PRKCG,EPHB3,NEK10,ALK,MAPKAPK2,TRIM24,INSRR,NTRK1,EPHB1,EPHA7,MAP3K13,BTK,LRRK2,PDGFC,GSK3B,EEF2K,PTK2,TEC,TAOK2,STK4,MARK3,IMPACT,SYK,TAF1,CAMK2D,PTPRC,TYRO3*
GO:0050801	ion homeostasis	0.006262594556675063	*ENPP1,ASPH,PDE4D,SLC9A1,FAM155A,ADCY8,PTGFR,CHERP,RYR1,NOX5,ATP2B2,GRM5,PLCE1,ANK2,SLC9A9,CHD7,SLC26A6,HTR2B,RYR3,SLC24A2,SLC24A3,CCDC22,TCIRG1,CACNB2,ADORA1,ADRA1D,CNNM1,ESR1,CYB561A3,STK39,SLC4A10,KCNMA1,LCK,SLC12A8,SGCD,MICU3,DMD,SLC30A9,SLC8A1,JPH2,FYN,SLC4A5,CNNM2,BDKRB1,BDKRB2,STIM1,TMBIM6,CMKLR1,DCN,SLC39A14,PTGER2,CLDN16,JAK2,NPSR1,SLC9A7,TGFB1,KEL,JPH3,F2RL1,C9ORF47,CDH23,DISC1,CLN6,XPR1,TMTC2,CAPN3,CNR1,TNFSF11,PARK2,SOD2,P2RY10,TRPM1,OTC,XK,RYR2,NOL3,CACNA1C,SCN3B,GRM1,SMAD3,ITPR1,ACKR2,SLC4A4,APP,PDGFRA,NOX1,IBTK,BDH2,DRD1,PRKD1,FGF2,ADCY5,ADCYAP1R1,KLHL3,SLC9B1,CLIC4,KCNQ1,CCL14,CCL15,TRPA1,NMUR2,AKAP6,BTBD9,TRPC5,NEO1,PKHD1,WNK1,SLC8A2,SLC9C1,TPCN1,SNX5,ATP2B4,ATP6V0A2,P2RY8,TFRC,CACNA1A,PTGER3,TRPV1,TRIM24,PLCG2,C1QTNF1,GPR35,AP3B1,SLC39A10,ANK3,TMEM199,ATP13A3,AP3D1,SLC9C2,MCU,TRDN,TRPC6,ABCB7,ATP13A5,SNCA,SLC4A8,HTT,MAPK1,SLC30A7,LRRK2,ATP6V1A,TMCO1,PKD2,STEAP4,PPP2R3C,BMP6,EXT1,GRIN1,TF,ITPR2,ALAS2,BOK,AGT,HRH4,HEPHL1,STC2,NOS1,SCNN1B,SLC1*

			A1,XCR1,MCOLN1,STEAP3,ATP1A3,ADORA2A,GRIN2B,KCTD7,PACS2,DIAPH1,HTR2C,ATP6V0A1,GRIK2,ATP2B3,CIB2,CAV2,CA12,TMPRSS3,CAMK2D,CCR3,NEDD4L,PTPRC,GPR89A,EDNRA
GO:0010970	transport along microtubule	0.006282844384639668	KIF5C,CNIH2,DLG2,IFT43,HDAC6,PEX14,FBXW11,AP3S1,DST,TERF2,ARL3,ICK,BBS12,DYNC1I1,TRAK1,LRPPRC,AP3B2,SPG11,IFT122,RAB6A,APP,SYNE2,SYBU,AGBL4,BLOC1S5,PAFAH1B1,KIF3A,MAP1B,RPGR,AP3B1,MAP1A,TRIM46,TUB,AP3D1,KIF3C,HTT,IFT81,TMEM108,RHOT1,SUN1,BLOC1S3,NDE1,BLOC1S6,COPG2,PRKCZ,KLC3,ARHGAP21,MAP2,CAMSAP3,RAB27B
GO:0099565	chemical synaptic transmission, postsynaptic	0.0062892445022191615	NRXN1,S1PR2,GRID2,NLGN4X,DGKI,ADORA1,RIMS1,PPP3CA,CUX2,GRIK5,SHANK1,APP,NLGN2,GLRA2,CELF4,NLGN1,NLGN3,RELN,SLC8A2,P2RX6,TRPV1,NETO1,CHRNB4,SNCA,PTEN,LRRK2,TMEM108,SHANK3,WNT7A,GRIN1,GSK3B,RIMS2,SLC29A1,PRKCZ,ADORA2A,GRIN2B,GRIK2
GO:0022402	cell cycle process	0.006412268643671339	MOV10L1,TACC2,PDE4DIP,CLASP2,TAS1R2,RPS6KA2,KIF22,LATS2,NOX5,MAPRE2,PAGR1,RAD51B,STAG2,EZR,TOM1L1,TACC1,LMNA,ITGB1,CDC6,PSMB7,GFI1B,PARD6G,STXBP4,DACH1,PTPN11,DDB1,KIF18B,MRE11A,CUL4B,CECR2,PDGFB,CCND3,FBXL7,CHFR,INSR,FMN2,FBXW11,ESRRB,CDK14,FANCA,UBR2,ECT2,MTA3,CDC45,STARD9,CENPF,PDS5A,HFM1,RBM14,PLCB1,TTN,KLHL21,DCTN1,BID,MYH9,MAD1L1,RNF4,AUNIP,CSPP1,DMC1,MAD2L2,TLE6,TERF2,FOXN3,RCC2,ARL3,KIF2A,RAD51D,RANBP1,YTHDC2,FBXW7,SMC3,UIMC1,CROCC,LZTS1,TEX11,SEPT7,CENPC,RPRD1B,TGFB1,PIBF1,CRADD,CEP135,RAB11FIP4,CLOCK,RNF212,SND1,ANKFN1,FGF10,FBXL17,SVIL,CAPN3,SLC16A1,PRKCA,PPP3CA,NSUN2,CTNNB1,METTL13,IGF1R,ANKRD17,BRIP1,NIN,NPAT,TUBGCP6,RIOK2,ESCO1,HAUS4,CHEK2,SPDYA,CTDP1,BAZ1B,SMC2,SPTBN1,HEPACAM2,SFRP1,MTBP,C10ORF90,KPNB1,RAB6C,CDC73,APP,CCNI2,CDK3,BORA,LRP5,SOX2,SETD2,DCUN1D3,VPS4A,EREG,CCNY,ATF2,TCF3,POLA1,USP33,RAB11A,BACH1,TICRR,CDC27,RAB11FIP3,BRDT,EIF4G1,ANAPC5,CKS1B,C11ORF80,PAFAH1B1,KIF3A,POLE,TNKS,ERCC1,BLM,PKHD1,CACUL1,NEK10,SIN3A,ABCB1,PPP2R5C,EML4,CLASP1,ATP2B4,HORMAD2,CDK6,UVRAG,PSMB2,ZNF207,DIS3L2,MNAT1,SNX9,ACTR2,RECQL5,PLCG2,ROCK1,EPS8,PAXIP1,CUL2,PKN2,ANK3,AXIN2,MUC1,SNX33,RAD51C,NTMT1,PDXP,ERCC3,HAUS3,MCMBP,FOXJ3,CHMP3,RNF103-CHMP3,RASA1,NUGGC,RPTOR,IGF1,HTT,PTEN,IFFO1,BMP7,PIK3C3,SEPT6,SPAG5,UNC119,CHMP5,BAG6,PKD2,HSF2BP,MAP4,ATRX,PRMT2,SUN1,MARK4,JADE1,RHOA,MCM3,NDE1,CUL3,RDX,MLF1,RBBP8,CDK13,NACC2,CCNJL,CUL9,ZMPSTE24,PBX1,CDC14A,TAOK2,RPS6KB1,HUS1,RGS14,TEX12,GPSM2,PPP1R10,TAF2,PAX6,STAG1,CCNG2,NIPBL,FBXO31,UBE2E2,PHIP,PPP2CA,RPL23,DCDC1,OPN1LW,DONSON,CIT,BRCA2,TAF1,PPME1,CAV2,POC1B,TRIM37,CENPK,RAD9B,TIMELESS,NDC1,PTPRC,CTCF,LIG1,EDNRA,PRAP1,BMP4
GO:0010720	positive regulation of cell development	0.006913978040697399	PRKCI,S1PR2,DOCK1,TIAM2,KALRN,SEMA5A,GRM5,LRP2,ROBO1,CDKL5,ROBO2,TRIOBP,DSCAM,GOLGA4,LIMK1,NTRK2,CDH4,PTPRD,BDNF,AMIGO1,TENM4,CASS4,NTN1,MAP2K1,CAPRIN2,IL1RAPL1,CAMK2B,TGFB1,SLIT2,TP73,DISC1,EPHA4,TNFSF11,MAG,UFL1,CTNNB1,PPARG,NIN,DCT,CUX2,PREX1,GSX2,ISLR2,SLC9B2,PARP6,BMPR2,RAB11A,NEDD9,SEMA4D,PLXNA2,SPEN,WNT3,P4HB,PAFAH1B1,TRPC5,RUFY3,DAB2,RELN,MACF1,BNC1,PAK3,ADNP,MAP1B,ACTR2,HDAC1,EGR2,CUX1,MAP3K13,EFNA5,SHANK3,SMO,EEF2K,RGS14,EPHB2,PAX6,PPP1CC,FBXO31,BRAF,HDAC2,CDKL3,NUMB,BMP4
GO:0021955	central nervous system neuron axonogenesis	0.007033243889546942	HSP90AA1,B4GALT6,SLIT2,DCLK1,EPHA4,DRAXIN,NIN,DCC,NFIB,SPTBN4,EPHB3,FBXO45,PAFAH1B1,GLI2,EPHB1,PTEN,MYCBP2,EPHB2
GO:0021952	central nervous system projection neuron axonogenesis	0.007165737758036799	SLIT2,DCLK1,EPHA4,DRAXIN,NIN,DCC,NFIB,SPTBN4,EPHB3,FBXO45,PAFAH1B1,GLI2,EPHB1,MYCBP2,EPHB2
GO:0090596	sensory organ morphogenesis	0.007213831489460682	CLRN1,RDH13,PRKCI,TENM3,SP3,MFAP5,CHD7,TRIOBP,DSCAM,THRB,IMPG2,ATP8A2,CDON,NTRK2,STRC,TFAP2A,PHACTR4,MEIS1,MYO3B,NF1,PCDH15,FAT3,BCR,NTN1,VAX2,ROR2,LRIG3,PLS1,MYO3A,ZHX2,RPGRIP1L,CELSR1,SDK2,CDH23,FGF10,CTNNB1,PAX2,IFT122,PTPRM,ABI2,LRP5,TTC39C,FRS2,STRA6,TSPAN12,FGF2,RARB,COL11A1,KCNQ1,GLI2,MYO7A,HOXC13,EYA1,HDAC1,PTPRQ,EPHB1,BCAR3,SDK1,PTK7,HIPK1,MAPK1,BMP7,LHFPL5,MEGF11,TSHR,FBN2,EPHB2,SLC1A1,SOBP,FBN1,PAX6,NIPBL,HDAC2,DVL2,KDM2B,EDNRA,GNGT1,BMP4
GO:0031644	regulation of nervous system process	0.007291452950090203	NRXN1,S1PR2,SLC9A1,NLGN4X,CNIH2,ADORA1,RIMS1,TENM4,SHISA9,FIG4,DLGAP1,MAG,GRM1,CUX2,SHANK1,APP,NLGN2,CELF4,NLGN1,PTAFR,NLGN3,NMUR2,MGLL,RELN,SLC8A2,EGR2,RNF10,GPR35,PARD3,NETO1,CHRNB4,OPRD1,PTEN,LRRK2,TMEM108,SHANK3,WNT7A,GRIN1,FAM19A4,AGT,RIMS2,PRKCZ,HTR2C,TNR,DLGAP2,SHISA6
GO:0035637	multicellular organismal signaling	0.007368608215352249	PDE4D,SLC9A1,CLCN1,ATP2B2,ANK2,RYR3,NFASC,CTNNA3,CACNB2,NTRK2,SLC8A1,SCN4A,CACNA1H,KCNJ3,CACNA1D,OTOA,CNTNAP2,MAG,RYR2,CACNA1C,SCN3B,ITPR1,TNNI3K,SPTBN4,DRD1,KCNQ1,PAFAH1B1,SLC8A2,DSC2,ATP2B4,MEF2A,GPR35,ANK3,KCND3,CACNG8,CHRNB4,TRDN,CACNG2,PKP2,SCN8A,SCN9A,AGT,ZMPSTE24,ATP1A3,TNR,KCND2,GRIK2,SCN1A,CACNG3,ATP2B3,CAMK2D

GO:0048568	embryonic organ development	0.007509634808484994 5	CLRN1,HLX,PBX3,ARNT,KITLG,SP3,MFAP5,CHD7,TRIOBP,ATP8A2,NPHP3,PDGFB,STRC,TFAP2A,PHACTR4,MYO3B,SATB2,PCDH15,BCR,NDRG4,NTN1,MAP2K1,MDFI,VAX2,ROR2,NEUROD1,LRIG3,PLS1,TBX15,WNT11,RUNX2,TGFB1,PCSK5,MYO3A,CELSR1,CDH23,FGF10,CTNNB1,RYR2,MMP16,PAX2,ARL13B,IFT122,SMAD3,HOXD3,HOXD4,PDGFRA,YAP1,SETD2,TTC39C,CHST11,TEAD1,VASH2,FRS2,PALB2,KDM6A,STRA6,DSCAML1,BMPR1A,RARB,NCOA1,COL11A1,KCNQ1,SUFU,POLE,GLI2,ERCC1,MYO7A,TEAD4,OVOL2,RBBP6,EYA1,HOXB3,HOXB4,HOXB5,HOXB6,TFEB,PTPRQ,PIFO,ERCC3,PTK7,HEY2,COL13A1,WNT7B,HIPK1,MAPK1,MIB1,BMP7,LHFPL5,PKD2,PDGFC,TSHR,SMO,MTHFD1L,KRT8,FBN2,PBX1,ECE1,SETDB2,DNAJB6,TBC1D23,STK4,EPHB2,SOBP,FBN1,PAX6,NIPBL,NOTO,SPINT2,DVL2,TRA2B,KDM2B,VPS52,EDNRA,BMP4
GO:0044257	cellular protein catabolic process	0.007770362068260999 6	CALR3,FBXL2,DCAF12,CBL,USP32,LRP2,EZR,TOM1L1,PSMD1,HSP90AA1,PSMB7,CCDC22,OMA1,DNAJB14,TCIRG1,HDAC6,DDB1,CUL4B,FBXL18,ZYG11B,FBXL7,USP46,CHFR,USP34,FBXW11,RNF144A,TRIM13,UBR2,NPLOC4,USP13,MKRN2,ARNTL,FBXO21,VGLL4,PPP2CB,RNF4,UBE3D,FHIT,MAN1A1,FBXO9,RFFL,USP22,FBXW7,ITCH,USP12,SPSB1,MTA1,SIAH1,ERCC8,SPSB4,ASCC2,BTRC,DNAJB2,RNF133,RNF148,DISC1,ZFAND2A,CLOCK,NELL1,FBXL17,KCTD10,SMURF2,EPHA4,UBQLN3,UBQLNL,UFL1,CTNNB1,PARK2,SMARCC1,AXIN1,RSPRY1,CHEK2,UBQLN4,CAPN2,RNFT2,WWP2,RNF168,NEDD4,SEC61B,USP49,ARIH1,AMFR,PRICKLE1,ERLIN1,VPS4A,ADAMTS12,PSMD11,USP33,UBE2R2,AGBL4,CDC27,YME1L1,KLHL3,PRKCG,AREL1,UCHL3,PSMD2,RBX1,ANAPC5,FBXO45,STT3B,FBXL20,TSG101,DESI1,IDE,SUFU,EDEM3,DAB2,UBR1,CACUL1,PSMD7,PELI1,PPP2R5C,SPATA18,ZNRF1,SNX5,RBBP6,DYX1C1,UBE3C,RNF34,PSMB2,RNF216,RYBP,CUL2,MAP1A,MAN1B1,TMEM199,FBXO39,FBXW4,EIF3H,USP42,USP50,RC3H1,RNF103,RNF103-CHMP3,RNF19B,PSMF1,TRIP12,ZNRF3,KCTD13,PTEN,MIB1,PCBP2,LRRK2,ZRANB1,BAG6,SEL1L2,SH3RF2,N4BP1,GET4,UBE2H,CUL3,EPM2A,GSK3B,DNAJC3,RDX,RNF144B,RNF43,CUL9,ZMPSTE24,PTK2,ARMC8,RNF121,FBXO10,TRPC4AP,RNFT1,CSNK1A1,MFSD8,UBE2K,FBXL13,RNF150,OTUD7B,TBL1X,FBXO31,C18ORF25,SKP1,RPL23,ANXA2,WWTR1,TAF1,ADAMTS7,GNA12,UGGT1,UCHL5,MALT1,FBXL4,NEDD4L,RNF213,SGTB
GO:0044089	positive regulation of cellular component biogenesis	0.00782297440145894	CLRN1,LDB2,NRXN1,MAP4K4,DNMT1,PDE4DIP,CLASP2,GRID2,PLCE1,FER,TENM1,HSP90AA1,CDC42EP3,EPHA1,LIMK1,ESR1,NTRK2,STX18,TENM2,PIP4K2A,PAK1,PTPRD,BDNF,AMIGO1,DCTN1,NRG1,BID,FNIP1,NCKAP1,SLX1B,ARL3,SKAP1,CLSTN2,CROCC,CLDN1,SEPT7,WNT11,IL1RAPL1,PPM1E,F2RL1,BCAS3,CEP135,LINGO2,CNTNAP2,PRKCA,AUTS2,DLG5,NIN,AJUBA,PHLDB1,HCK,RAB3GAP2,NF2,HDAC4,TRABD2B,SFRP1,DNM3,SMAD3,CUX2,WIPI1,ABI2,NAV3,NLGN2,VPS4A,NLGN1,COL16A1,MORC2,PPM1F,SEMA4D,EIF4G1,EPHB3,TSG101,NLGN3,ERCC1,DZIP1,SIN3A,DOCK11,CLASP1,ADNP,MAP1B,TFRC,FLRT2,SNX9,ACTR2,PLCG2,ROCK1,EPS8,NTRK1,EPHB1,GNL3L,FCHSD2,RAPGEF3,SNCA,USP50,ATF7IP,HTT,DPYSL3,BMP7,SPAG5,EFNA5,WNT7A,MARK4,RHOA,GSK3B,EEF2K,AGT,TPPP2,SETDB2,EPHB2,GPSM2,PHLDB2,BRAF,SYK,CNOT1,HRK,PTPRJ,GPM6A,HSF1,PLEKHM1,ASAP1,GBP5,MET,SYNDIG1
GO:0016197	endosomal transport	0.00789463007743374	CCDC93,CCDC22,ERC1,GBF1,SNX2,SNX6,DENND5A,LMTK2,STX6,VTA1,NDRG4,DCTN1,DENND1A,ATP9A,DENND2A,ZFYVE9,CORO1C,RAB11FIP4,ITSN2,DCLK1,SNX8,PARK2,LEPROT,TBC1D14,ANKFY1,RAB6A,VPS39,VPS53,RAB6C,BET1L,VPS4A,SPAG9,RAB11A,EPG5,EEA1,RAB11FIP3,SNX5,TBC1D5,CLEC16A,SNX9,AP3B1,AP3D1,ANXA8L1,ARHGAP44,HEATR5A,SNX33,VPS16,ACAP2,CHMP3,PLA2G4E,LRRK2,CHMP5,NSG2,TMCC1,SNX3,TBC1D23,TBC1D10C,VTI1A,ITSN1,GRIP1,EPS15,INPP5F,REPS2,TBC1D10A,SNX1,EHD2,VPS52
GO:0043547	positive regulation of GTPase activity	0.007997289396086845	MAP4K4,TIAM2,MAPRE2,KALRN,NTRK3,CDKL5,ITGB1,ARHGAP24,RGS6,DOCK10,EPHA1,ABR,ARHGAP6,ECT2,ARHGAP42,NF1,RTN4R,BCR,DOCK8,RAPGEF6,RGS8,RCC2,DENND1A,ARAP2,DOCK9,WNT11,SIPA1L3,F2RL1,BCAS3,CORO1C,RABGAP1L,ARHGAP22,GARNL3,RGS10,TBC1D14,TBCK,SFRP1,ARHGAP29,PREX1,NEDD9,SEMA4D,DVL3,MYO9A,CCL14,CCL15,DOCK11,RGS7,TBC1D5,SNX9,NTRK1,BCAR3,RAPGEF3,SH3BP1,TBC1D9,RASA1,TBC1D16,ARHGAP25,SGSM1,RAPGEF2,GSK3B,RGS14,SIPA1L2,ARHGAP11A,GRTP1,TBC1D10C,CCL22,SRGAP2,RAP1GAP2,DVL2,ASAP1,RALGAPA1,CAV2,TBC1D10A
GO:0051282	regulation of sequestering of calcium ion	0.00804153832201548	ASPH,PDE4D,CHERP,RYR1,ANK2,CHD7,HTR2B,RYR3,LCK,DMD,SLC8A1,JPH2,BDKRB1,NPSR1,TGFB1,JPH3,CAPN3,RYR2,NOL3,CACNA1C,ITPR1,IBTK,DRD1,PRKD1,FGF2,TRPA1,AKAP6,TPCN1,TRPV1,PLCG2,TRDN,SNCA,HTT,PKD2,ITPR2,NOS1,XCR1,MCOLN1,DIAPH1,HTR2C,CAMK2D,PTPRC
GO:0099111	microtubule-based transport	0.008265212654689139	DNAH11,SPAG16,KIF5C,CNIH2,DLG2,IFT43,HDAC6,PEX14,NPHP3,FBXW11,AP3S1,DST,TERF2,ARL3,ICK,BBS12,DYNC1I1,SPAG17,TRAK1,LRPPRC,DNAH9,AP3B2,SPG11,IFT122,RAB6A,APP,SYNE2,SYBU,AGBL4,BLOC1S5,GAS8,PAFAH1B1,KIF3A,NEK10,DYX1C1,MAP1B,RPGR,AP3B1,MAP1A,TRIM46,TUB,AP3D1,KIF3C,HTT,IFT81,TMEM108,RHOT1,SUN1,BLOC1S3,NDE1,BLOC1S6,COPG2,PRKCZ,KLC3,ARHGAP21,MAP2,CAMSAP3,RAB27B,ADCY10
GO:0009611	response to wounding	0.008918780009036788	PRDX2,F8,GP6,C6ORF89,CLASP2,NREP,NTRK3,CASK,ITGB6,VAV2,ITGB1,ARHGAP24,ABCC8,DGKI,PDGFB,PLLP,JMJD1C,PAK1,LCK,NF1,DST,DGKH,APOD,RTN4R,NRG1,MAP2K1,MYH9,TSPAN8,ENTPD1,RAB27A,F5,ANO6,GNAQ,JAK2,CLDN1,KREMEN1,PRKCD,TGFB1,F2RL1,CELSR1,KANK1,FGF10,PPL,EPHA4,PRKCA,PPP3CA,MAG,SOD2,IGF1R,PPARG,FGF1,PROS1,NOL3,AJUBA,DGKB,SMAD3,CYP4

			F11,PDGFRA,YAP1,SOX2,MMP2,DGKG,HNF4A,EREG,MYOF,RAF1,PABPC4,FGF2,VCL,SCUBE1,MACF1,CLASP1,PRKG1,MAP1B,SCARB1,SMOC2,ADIPOR2,PLCG2,C1QTNF1,AP3B1,NTRK1,SARM1,VWF,DGKK,CTNNA1,PTK7,PTPRS,ADTRP,CSRP1,SRSF6,RTN4RL1,IGF1,DPYSL3,PTEN,PRCP,WNT7A,EXT1,BLOC1S3,RHOA,ACTA2,SYT7,BLOC1S6,PTK2,TEC,GAP43,MAP3K5,RPS6KB1,ALOX5,CD44,CLDN4,EPHB2,SLC1A1,TMEFF2,PPARA,EPB41L4B,NOTCH4,SYTL4,PAX6,ADORA2A,PHLDB2,PRKCQ,BRAF,TNR,SYK,MAX,DHFR,VAV3,GNA12,INPP5F,AXL,CD109,TYRO3,EDNRA
GO:0098742	cell-cell adhesion via plasma-membrane adhesion molecules	0.009064370920897916	NRXN1,GRID2,TENM3,CLDN18,MYOT,DCHS2,PCDH9,MAP2K5,ROBO1,TENM1,ROBO2,ITGB1,PCDH17,DSCAM,DAB1,CDH10,CDH12,TENM2,KIRREL3,CDH8,UNC5D,CDH4,PTPRD,AMIGO1,CLDN12,PCDH7,TENM4,PCDH15,FAT3,NRG1,LRFN5,CNTN4,NTNG1,CDH13,PTPRT,PCDHB16,CLDN16,MYPN,CLSTN2,CLDN1,IL1RAPL1,CLDN10,CELSR1,SDK2,CDH23,CD6,MAG,CDHR2,CADM3,AJUBA,CNTN6,CDH26,PTPRM,GPC6,DSCAML1,NLGN1,LRRC4C,WNK1,DSC2,CD84,HMCN1,SDK1,PTPRS,PCDH11X,DSG1,CLDN11,ITGAM,EFNA5,CDH9,EMB,CDH20,ARVCF,ITGAL,CLDN4,PCDH10,FAT2,CADM1
GO:0080135	regulation of cellular response to stress	0.010042714140805048	MAP4K4,ADCY8,PDE8A,SEMA3A,C12ORF49,MARVELD3,EZR,MECOM,TRAF6,OXR1,ULK4,HDAC6,EP300,TNIK,MID1,STK39,FMN2,MAP3K4,FUT8,USP13,KAT7,FYN,ARNTL,PLCB1,MGMT,PPP4R2,RTN4R,BDKRB2,BID,MAP2K1,TMBIM6,CREBRF,AUNIP,COPS5,MAD2L2,TERF2,FAM168A,FBXW7,UIMC1,ITCH,SMG1,KREMEN1,ERCC8,PRKCD,F2RL1,ZNF675,DUSP22,NAIP,FGF10,SH3RF3,EPHA4,TNFSF11,UFL1,CTNNB1,PARK2,SOD2,FCGR2B,IGF1R,AXIN1,OTUB1,NR4A3,NOL3,KIR2DL4,AJUBA,CHEK2,UBQLN4,FLT4,SFRP1,FOXO3,RNFT2,RNF168,TXNDC12,APP,NOX1,AMFR,TNFRSF19,EYA2,SETD2,PTPN1,BMPR1A,PRKCG,EEF1E1,EEF1E1-BLOC1S5,EIF4G1,PAWR,TERF2IP,P4HB,PAFAH1B1,DDAH1,ERCC1,UBE2V1,XDH,MAPKAPK2,CDK6,EYA1,ACTR2,RECQL5,PAXIP1,HIPK3,PTPN2,EPHB1,AXIN2,MUC1,SPI1,AIFM2,PNPT1,WNT7B,PTPRS,ZMYND11,TRIP12,RTN4RL1,MAPK1,PTEN,SPRED2,BMP7,LRRK2,BAG6,WNT7A,SH3RF2,UBR5,GSK3B,CD27,BOK,NACC2,POLH,ZMPSTE24,MAP3K5,CREBBP,NCF1,TAOK2,RNFT1,ALOX5,DNAJB6,CD44,DNAJC7,PPP1R10,PHLPP1,MAGI3,EYA3,ATF6,BRAF,TNR,GRIK2,YBX1,HSF1,DHFR,SASH1,INPP5F,MET,TIMELESS,ANKRD6,ATF6B,TRAP1,BMP4
GO:0099643	signal release from synapse	0.010344813069971034	NRXN1,CASK,NRXN3,DGKI,ERC2,CACNB2,RIMS1,NF1,LIN7A,SYN2,SYN3,SYT1,PTPRN2,CADPS2,STXBP5,CNR1,PARK2,RIMS4,GRIK5,DRD1,NLGN1,MCTP1,PRKCG,FBXL20,SYT12,UNC13A,CHRNB4,SNCA,GRM4,SYT9,SLC4A8,LRRK2,WNT7A,SNAP23,STXBP5L,GRIN3A,GSK3B,SYN1,MCTP2,SYT7,BLOC1S6,RIMS2,CPLX2,ADORA2A,CADPS,BRAF,ADCY1,RPH3A
GO:0007269	neurotransmitter secretion	0.010344813069971034	NRXN1,CASK,NRXN3,DGKI,ERC2,CACNB2,RIMS1,NF1,LIN7A,SYN2,SYN3,SYT1,PTPRN2,CADPS2,STXBP5,CNR1,PARK2,RIMS4,GRIK5,DRD1,NLGN1,MCTP1,PRKCG,FBXL20,SYT12,UNC13A,CHRNB4,SNCA,GRM4,SYT9,SLC4A8,LRRK2,WNT7A,SNAP23,STXBP5L,GRIN3A,GSK3B,SYN1,MCTP2,SYT7,BLOC1S6,RIMS2,CPLX2,ADORA2A,CADPS,BRAF,ADCY1,RPH3A
GO:0052697	xenobiotic glucuronidation	0.010356391806710448	UGT1A1,UGT1A10,UGT1A3,UGT1A4,UGT1A5,UGT1A6,UGT1A7,UGT1A8,UGT1A9
GO:0048562	embryonic organ morphogenesis	0.010527199224424747	CLRN1,HLX,SP3,MFAP5,CHD7,TRIOBP,ATP8A2,NPHP3,STRC,TFAP2A,PHACTR4,MYO3B,SATB2,PCDH15,BCR,NDRG4,NTN1,MDFI,VAX2,ROR2,NEUROD1,LRIG3,PLS1,TBX15,WNT11,RUNX2,MYO3A,CELSR1,CDH23,FGF10,CTNNB1,RYR2,MMP16,PAX2,ARL13B,SMAD3,HOXD3,HOXD4,PDGFRA,YAP1,SETD2,TTC39C,CHST11,FRS2,KDM6A,STRA6,DSCAML1,RARB,COL11A1,KCNQ1,SUFU,GLI2,MYO7A,OVOL2,EYA1,HOXB3,HOXB4,HOXB5,HOXB6,PTPRQ,PTK7,HIPK1,MAPK1,MIB1,BMP7,LHFPL5,PKD2,TSHR,SMO,MTHFD1L,FBN2,SETDB2,EPHB2,SOBP,FBN1,PAX6,NIPBL,NOTO,DVL2,KDM2B,EDNRA,BMP4
GO:1903305	regulation of regulated secretory pathway	0.010787434714635686	FER,CASK,NRXN3,DGKI,CACNB2,VAMP7,BCR,RAB27A,SYT1,CACNA1H,F2RL1,SYT13,C12ORF4,STXBP5,CNR1,FCGR2B,SCAMP5,RIMS4,SYT3,DRD1,NLGN1,PTAFR,PRKCG,FBXL20,SYT12,CD84,SPI1,RAB15,SYT9,SLC4A8,LRRK2,ITGAM,WNT7A,STXBP5L,SYT17,GRIN3A,GSK3B,SYN1,SYT7,CD160,ADORA2A,RABGEF1,BRAF,CD300A,ADCY1,SYK,ZP3
GO:0048008	platelet-derived growth factor receptor signaling pathway	0.01079594913283391	CLASP2,CBL,FER,PTPN11,PDGFB,APOD,NDRG4,JAK2,NR4A3,MYOCD,MYO1E,CSPG4,PDGFRA,PTPN1,HIP1,IQGAP1,PTPN2,SNCA,PDGFC,PLEKHA1,RGS14,ARID5B,CSRNP1,PTPRJ
GO:0044265	cellular macromolecule catabolic process	0.011577847285066655	RNASET2,CALR3,FBXL2,FTO,DCAF12,CBL,USP32,LRP2,EZR,TOM1L1,PSMD1,HSP90AA1,PSMB7,CCDC22,OMA1,DNAJB14,TCIRG1,HDAC6,DDB1,CUL4B,CECR2,FBXL18,TNRC6A,CELA1,ZYG11B,FBXL7,USP46,CHFR,USP34,FBXW11,RNF144A,SAMD4A,TRIM13,UBR2,ERN2,NPLOC4,LARP4B,USP13,MKRN2,ARNTL,ADAMTS9,SMG7,FBXO21,VGLL4,PPP2CB,RNF4,UBE3D,TRDMT1,FHIT,MAN1A1,FBXO9,RFFL,USP22,FBXW7,ITCH,USP12,SMG1,SPSB1,MTA1,SIAH1,ERCC8,PRKCD,SPSB4,ZHX2,ASCC2,BTRC,DNAJB2,RNF133,RNF148,DISC1,ZFAND2A,CLN6,CLOCK,NELL1,SND1,HNRNPC,YTHDF1,FBXL17,KCTD10,SMURF2,EPHA4,PRKCA,SMG6,NSUN2,UBQLN3,UBQLNL,UFL1,CTNNB1,PARK2,SMARCC1,AXIN1,LRPPRC,RSPRY1,CHEK2,UBQLN4,CAPN2,ZCCHC17,SYNCRIP,RNFT2,WWP2,RNF168,DCP1B,NEDD4,SEC61B,USP49,RBM8A,ARIH1,AMFR,PRICKLE1,ERLIN1,VPS4A,ADAMTS12,PS

			MD11,USP33,PABPC4,UBE2R2,AGBL4,CDC27,YME1L1,KLHL3,PRKCG,AREL1,UCHL3,PSMD2,RBX1,ANAPC5,FBXO45,LIN28B,STT3B,AGO3,FBXL20,TSG101,DESI1,IDE,SUFU,EDEM3,TNRC6B,DAB2,UBR1,CACUL1,SMG5,PSMD7,PELI1,PPP2R5C,SPATA18,ZNRF1,FASTKD5,SNX5,RBBP6,DYX1C1,MAPKAPK2,UBE3C,CELF1,RNF34,DROSHA,XRN1,PSMB2,DIS3L2,RNF216,ELAVL4,ROCK1,RYBP,CUL2,MAP1A,MAN1B1,AXIN2,TMEM199,FBXO39,FBXW4,EIF3H,USP42,PNPT1,USP50,RC3H1,EIF3E,RNF103,RNF103-CHMP3,RNF19B,PSMF1,TRIP12,DFFA,ZNRF3,KCTD13,PTEN,MIB1,PUM1,PCBP2,LRRK2,ZRANB1,BAG6,SEL1L2,DAZL,TAF15,SH3RF2,N4BP1,GET4,UBE2H,RQCD1,CUL3,EPM2A,GSK3B,DNAJC3,RDX,UPF2,RNF144B,RNF43,METTL16,CUL9,ZMPSTE24,PTK2,ARMC8,RNF121,EXOSC3,FBXO10,TRPC4AP,RNFT1,CSNK1A1,MFSD8,UBE2K,DIS3,FBXL13,RNF150,ZC3HAV1,OTUD7B,TBL1X,FBXO31,C18ORF25,SKP1,RPL23,ANXA2,PARN,CNOT1,WWTR1,YBX1,TAF1,HSF1,POP1,ETF1,ADAMTS7,GNA12,UGGT1,MYEF2,UCHL5,MALT1,FBXL4,NEDD4L,RNF213,TRAF3IP2,SGTB
GO:0030163	protein catabolic process	0.01181587989688001	CALR3,FBXL2,DCAF12,CBL,EGLN2,USP32,LRP2,EZR,TOM1L1,PSMD1,HSP90AA1,PSMB7,CCDC22,OMA1,DNAJB14,TCIRG1,HDAC6,DDB1,CUL4B,FBXL18,CELA1,ZYG11B,FBXL7,USP46,CHFR,FMN2,ASB5,USP34,FBXW11,RNF144A,TRIM13,UBR2,MDM4,NPLOC4,USP13,FYN,MKRN2,ARNTL,ADAMTS9,FBXO21,NRG1,VGLL4,PPP2CB,RNF4,CSNK2A3,UBE3D,CREBRF,FHIT,MAD2L2,MAN1A1,FBXO9,RFFL,USP22,FBXW7,OAZ2,ITCH,USP12,SPSB1,MTA1,SIAH1,ERCC8,BANP,SPSB4,ASCC2,BTRC,DNAJB2,RNF133,RNF148,DISC1,ZFAND2A,CLN6,CLOCK,NELL1,TIMP2,FBXL17,KCTD10,CAPN3,SMURF2,EPHA4,UBQLN3,UBQLNL,UFL1,CTNNB1,PARK2,SMARCC1,AXIN1,RSPRY1,CHEK2,UBQLN4,CAPN2,SMAD3,RNFT2,WWP2,RNF168,NEDD4,SEC61B,USP49,ARIH1,AMFR,SH3D19,PRICKLE1,ERLIN1,VPS4A,ADAMTS12,PSMD11,USP33,UBE2R2,AGBL4,CDC27,YME1L1,KLHL3,PRKCG,AREL1,UCHL3,PSMD2,RBX1,ANAPC5,FBXO45,STT3B,FBXL20,TSG101,DESI1,IDE,SUFU,EDEM3,DAB2,UBR1,CACUL1,PSMD7,PELI1,PPP2R5C,SPATA18,ZNRF1,SNX5,RBBP6,NSF,DYX1C1,UBE3C,RNF34,GPC3,PSMB2,RNF216,SNX9,TRIM24,ROCK1,RYBP,CUL2,MAP1A,MAN1B1,TMEM199,FBXO39,SNX33,FBXW4,EIF3H,SNCA,USP42,USP50,RC3H1,RNF103,RNF103-CHMP3,RNF19B,PSMF1,TRIP12,ZNRF3,KCTD13,PTEN,MIB1,PCBP2,LRRK2,ZRANB1,BAG6,SEL1L2,MYCBP2,SH3RF2,EXT1,N4BP1,GET4,UBE2H,CUL3,EPM2A,GSK3B,DNAJC3,RDX,RNF144B,RNF43,CUL9,ZMPSTE24,PTK2,ARMC8,RNF121,SNX3,FBXO10,TRPC4AP,RNFT1,ADAM8,CSNK1A1,MFSD8,UBE2K,FBXL13,RNF150,OTUD7B,TBL1X,FBXO31,C18ORF25,SKP1,RPL23,ANXA2,WWTR1,TAF1,ADAMTS7,GNA12,UGGT1,SNX1,UCHL5,MALT1,FBXL4,NEDD4L,PHB,RNF213,SGTB
GO:0030003	cellular cation homeostasis	0.01316887807474172	ASPH,PDE4D,SLC9A1,FAM155A,ADCY8,PTGFR,CHERP,RYR1,NOX5,ATP2B2,GRM5,PLCE1,ANK2,SLC9A9,CHD7,SLC26A6,HTR2B,RYR3,SLC24A2,SLC24A3,CCDC22,TCIRG1,CACNB2,ADORA1,ADRA1D,ESR1,CYB561A3,SLC4A10,KCNMA1,LCK,MICU3,DMD,SLC30A9,SLC8A1,JPH2,FYN,SLC4A5,BDKRB1,BDKRB2,STIM1,TMBIM6,CMKLR1,SLC39A14,PTGER2,CLDN16,JAK2,NPSR1,SLC9A7,TGFB1,KEL,JPH3,F2RL1,C9ORF47,CDH23,DISC1,CLN6,CAPN3,CNR1,P2RY10,TRPM1,XK,RYR2,NOL3,CACNA1C,GRM1,SMAD3,ITPR1,ACKR2,SLC4A4,APP,PDGFRA,NOX1,IBTK,DRD1,PRKD1,FGF2,ADCY5,ADCYAP1R1,SLC9B1,CLIC4,CCL14,CCL15,TRPA1,NMUR2,AKAP6,TRPC5,PKHD1,SLC8A2,SLC9C1,TPCN1,ATP2B4,ATP6V0A2,P2RY8,TFRC,CACNA1A,PTGER3,TRPV1,PLCG2,C1QTNF1,GPR35,AP3B1,SLC39A10,TMEM199,ATP13A3,AP3D1,SLC9C2,MCU,TRDN,TRPC6,ABCB7,ATP13A5,SNCA,SLC4A8,HTT,MAPK1,SLC30A7,LRRK2,ATP6V1A,TMCO1,PKD2,STEAP4,BMP6,GRIN1,TF,ITPR2,ALAS2,BOK,AGT,HRH4,HEPHL1,STC2,NOS1,SLC1A1,XCR1,MCOLN1,ATP1A3,GRIN2B,KCTD7,PACS2,DIAPH1,HTR2C,ATP6V0A1,GRIK2,ATP2B3,CIB2,CAV2,TMPRSS3,CAMK2D,CCR3,NEDD4L,PTPRC,GPR89A,EDNRA
GO:0006873	cellular ion homeostasis	0.013187395614661104	ENPP1,ASPH,PDE4D,SLC9A1,FAM155A,ADCY8,PTGFR,CHERP,RYR1,NOX5,ATP2B2,GRM5,PLCE1,ANK2,SLC9A9,CHD7,SLC26A6,HTR2B,RYR3,SLC24A2,SLC24A3,CCDC22,TCIRG1,CACNB2,ADORA1,ADRA1D,ESR1,CYB561A3,SLC4A10,KCNMA1,LCK,MICU3,DMD,SLC30A9,SLC8A1,JPH2,FYN,SLC4A5,BDKRB1,BDKRB2,STIM1,TMBIM6,CMKLR1,SLC39A14,PTGER2,CLDN16,JAK2,NPSR1,SLC9A7,TGFB1,KEL,JPH3,F2RL1,C9ORF47,CDH23,DISC1,CLN6,XPR1,CAPN3,CNR1,P2RY10,TRPM1,XK,RYR2,NOL3,CACNA1C,GRM1,SMAD3,ITPR1,ACKR2,SLC4A4,APP,PDGFRA,NOX1,IBTK,DRD1,PRKD1,FGF2,ADCY5,ADCYAP1R1,SLC9B1,CLIC4,KCNQ1,CCL14,CCL15,TRPA1,NMUR2,AKAP6,TRPC5,PKHD1,SLC8A2,SLC9C1,TPCN1,ATP2B4,ATP6V0A2,P2RY8,TFRC,CACNA1A,PTGER3,TRPV1,PLCG2,C1QTNF1,GPR35,AP3B1,SLC39A10,TMEM199,ATP13A3,AP3D1,SLC9C2,MCU,TRDN,TRPC6,ABCB7,ATP13A5,SNCA,SLC4A8,HTT,MAPK1,SLC30A7,LRRK2,ATP6V1A,TMCO1,PKD2,STEAP4,BMP6,GRIN1,TF,ITPR2,ALAS2,BOK,AGT,HRH4,HEPHL1,STC2,NOS1,SLC1A1,XCR1,MCOLN1,ATP1A3,GRIN2B,KCTD7,PACS2,DIAPH1,HTR2C,ATP6V0A1,GRIK2,ATP2B3,CIB2,CAV2,TMPRSS3,CAMK2D,CCR3,NEDD4L,PTPRC,GPR89A,EDNRA
GO:0032984	protein-containing complex disassembly	0.013787681461715011	ASPH,CLASP2,SCAF8,SEMA5A,TRIOBP,HDAC6,KIF18B,PEX14,MID1,INSR,GSN,VTA1,KIF2A,F2RL1,TTBK2,SVIL,VMP1,LIX1L,SMARCC1,IGF1R,ADD2,UBQLN4,SPTBN1,SNX14,NAV3,ADD3,KIF24,SPTBN4,VILL,VPS4A,EPG5,TSG101,CLASP1,NSF,SPTBN5,MAP1B,C12ORF65,UVRAG,CLEC16A,EPS8,ZFAND1,MAP1A,NCKAP5,VPS16,LIMA1,ATG14,PDXP,SH3BP1,CHMP3,CHMP5,STMN4,DNAJC6,GSK3B,RDX,HDGFRP3,ZMPSTE24,SPEF1,MFSD8,MCOLN1,PPP1CA,RPL23,CAMSAP3,MTRF1,ETF1,MICAL3,SPTAN1

GO:0033135	regulation of peptidyl-serine phosphorylation	0.013849320158932915	*NRXN1,PDE4D,NTRK3,TENM1,HSP90AA1,HDAC6,NTRK2,PAK1,BDNF,DMD,PAQR3,SH2D3C,BDKRB2,FNIP1,MAD2L2,CAPRIN2,NSD1,SMYD3,AXIN1,APP,MTCP1,SPTBN4,PRKD1,RAF1,PPM1F,EIF4G1,TERF2IP,ATP2B4,TFRC,BCAR3,RAPGEF3,SNCA,OPRD1,RPTOR,PTEN,PLCL2,RQCD1,EPM2A,CD44,NOS1,STK4,ANGPT1,BRAF,SH2D3A,INPP5F,MLXIPL*
GO:1901653	cellular response to peptide	0.014374425665783605	*ENPP1,PRKCI,SLC9A1,ADCY8,STAT5B,GRM5,FER,GOT1,CDC6,STXBP4,PTPN11,CCND3,INSR,PIP4K2A,SNX6,AP3S1,FYN,PLCB1,PIK3R2,HDAC5,SLC39A14,JAK2,KLF15,PRKCD,TGFB1,KANK1,GLP2R,EPHA4,FCGR2B,SMARCC1,IGF1R,PPARG,BRIP1,LEPROT,NR4A3,SORBS1,FOXO3,APP,STAT1,HNF4A,PTPN1,PIK3R3,PTPRE,SRSF5,IDE,IRS4,WDTC1,SP1,SNX5,MAP1B,CACNA1A,GHR,ROCK1,CAPN10,ABCC1,PTPN2,INSRR,PTPRA,BCAR3,RAB15,HMGCS2,IGF1,CHMP5,GPR21,ANO1,TSHR,PNPLA3,GRB14,GSK3B,STAT6,EEF2K,AGT,PTK2,SHC1,RPS6KB1,GPR173,EPHB2,SLC25A33,FBN1,PRKCZ,ATP1A3,CPEB1,OSBPL8,PRKCQ,PHIP,RPL23,HSF1,MAX,CAV2,DENND4C,TIMELESS,EDNRA*
GO:0035725	sodium ion transmembrane transport	0.014376244917853437	*SLC9A1,UTRN,SLC9A9,SLC24A2,SLC24A3,NETO2,STK39,SLC20A2,SLC4A10,SLC6A16,DMD,SLC8A1,SLC4A5,NALCN,FGF14,ANO6,SCN4A,SLC9A7,CACNA1H,SLC17A7,SCN3B,NEDD4,SLC4A4,SLC6A1,KCNK1,SLC9B2,SLC6A3,FXYD2,FXYD6,SLC9B1,KCNQ1,WNK1,SLC8A2,SLC9C1,TPCN1,ATP2B4,STOM,ANK3,SLC9C2,NETO1,SLC4A8,SCN8A,PKD2,SLC6A14,SCN9A,SLC5A10,NOS1,ACTN4,SCNN1B,ATP1A3,SCN1A,CAMK2D,NEDD4L*
GO:0055065	metal ion homeostasis	0.014416386025001475	*ASPH,PDE4D,SLC9A1,FAM155A,ADCY8,PTGFR,CHERP,RYR1,NOX5,ATP2B2,GRM5,PLCE1,ANK2,CHD7,HTR2B,RYR3,SLC24A2,SLC24A3,CCDC22,TCIRG1,CACNB2,ADORA1,ADRA1D,CNNM1,ESR1,CYB561A3,KCNMA1,LCK,SLC12A8,SGCD,MICU3,DMD,SLC30A9,SLC8A1,JPH2,FYN,CNNM2,BDKRB1,BDKRB2,STIM1,TMBIM6,CMKLR1,SLC39A14,PTGER2,CLDN16,JAK2,NPSR1,TGFB1,KEL,JPH3,F2RL1,C9ORF47,CDH23,DISC1,TMTC2,CAPN3,CNR1,TNFSF11,PARK2,SOD2,P2RY10,TRPM1,XK,RYR2,NOL3,CACNA1C,GRM1,SMAD3,ITPR1,ACKR2,APP,PDGFRA,IBTK,BDH2,DRD1,PRKD1,FGF2,ADCY5,ADCYAP1R1,KCNQ1,CCL14,CCL15,TRPA1,NMUR2,AKAP6,BTBD9,TRPC5,NEO1,PKHD1,SLC8A2,TPCN1,SNX5,ATP2B4,ATP6V0A2,P2RY8,TFRC,CACNA1A,PTGER3,TRPV1,TRIM24,PLCG2,C1QTNF1,GPR35,AP3B1,SLC39A10,ANK3,TMEM199,ATP13A3,AP3D1,MCU,TRDN,TRPC6,ABCB7,ATP13A5,SNCA,HTT,SLC30A7,ATP6V1A,TMCO1,PKD2,STEAP4,BMP6,EXT1,GRIN1,TF,ITPR2,ALAS2,BOK,AGT,HRH4,HEPHL1,STC2,NOS1,SCNN1B,SLC1A1,XCR1,MCOLN1,STEAP3,ATP1A3,ADORA2A,GRIN2B,KCTD7,PACS2,DIAPH1,HTR2C,GRIK2,ATP2B3,CIB2,CAV2,TMPRSS3,CAMK2D,CCR3,NEDD4L,PTPRC,EDNRA*
GO:0032269	negative regulation of cellular protein metabolic process	0.014623110253370554	*ENPP1,PRMT3,PDE4D,DNMT1,LATS2,PRKAG2,MVP,FTO,NTRK3,CBL,MAP2K5,DNAJA3,OXR1,MGAT5,HDAC6,EPHA1,IKBKB,LIMK1,PMEPA1,PTPRO,TNRC6A,PRKAR1A,SAMD4A,SNX6,CST2,DMD,SLC8A1,RBM4,FYN,NF1,DPEP1,PAQR3,BDKRB1,BDKRB2,FRY,FNIP1,ITIH2,FHIT,PTPRT,IPO5,MAD2L2,CAPRIN2,GNAQ,RFFL,ZFYVE28,KLF15,PRKCD,PPM1E,PIBF1,DNAJB2,SLIT2,ITIH4,CORO1C,KDM4B,SMAD6,ZNF675,NELL1,TIMP2,DUSP22,NAIP,YTHDF1,SMYD3,CAPN3,EPHA4,PIP5KL1,UFL1,CTNNB1,PARK2,SMARCC1,PPARG,PRKAR1B,OTUB1,PROS1,NOL3,PRKAR2A,MYOCD,PER2,GLG1,CSTL1,NF2,PAX2,SFRP1,SYNCRIP,SIMC1,DCP1B,APP,HEG1,IBTK,LRP5,DCUN1D3,RAF1,CELF4,CARD16,PTPN1,SPAG9,PPM1F,SEMA4D,JARID2,PRKCG,SPOCK1,EIF4G1,AGO3,DEPTOR,TSG101,TERF2IP,SUFU,PTPN13,COL4A3,TNRC6B,LDLRAD4,SIN3A,XDH,ATP2B4,CELF1,RNF34,GPC3,XRN1,DIS3L2,ROCK1,HIPK3,RYBP,PTPN2,TRIM44,MAP1A,PARD3,GNL3L,BCOR,ANXA8L1,LPA,SPI1,DNMT3B,ATG14,EIF3H,STK38,RENBP,SNCA,PNPT1,RC3H1,EIF3E,PSMF1,CAST,TRIP12,MLLT1,PTEN,SPRED2,BMP7,PUM1,LRRK2,TMEM59,BAG6,LILRB4,SERPINA3,SERPINA4,SERPINA5,PKIB,DNAJC1,GPI,SH3RF2,N4BP1,PRKAR2B,UBR5,RQCD1,EPM2A,GSK3B,DNAJC3,PTPRB,CD27,AGT,METTL16,PRKAA2,ADAR,EXOSC3,PARP10,PRG3,SERPINB11,DNAJB6,CD44,RGS14,EPHB2,DIS3,CPEB4,PAX6,PRKCZ,ADORA2A,RABGEF1,CPEB1,COL28A1,ANGPT1,LRRK1,IGF2BP3,MTF2,CDK5RAP1,HDAC2,IMPACT,SPINT2,CD300A,PPP2CA,RPL23,ANXA2,PARN,CNOT1,WWTR1,YBX1,PTPRJ,TAF1,DHFR,INPP5F,CD109,UCHL5,SERPINE3,MLXIPL,PTPRC,SH3GL2,BMP4*
GO:0014070	response to organic cyclic compound	0.015231390537330275	*ASPH,PDE4D,SLC9A1,ADCY8,PTGFR,RYR1,PAGR1,NTRK3,CBL,EGLN2,FLT3,STAT5B,EZR,SLC26A6,HTR2B,RYR3,GOT1,NRIP1,HDAC6,ESR1,PMEPA1,EP300,PDGFB,PAK1,ESRRB,CHRM3,HLCS,PTPRU,RFTN1,SLC8A1,ARNTL,PLCB1,MGMT,ACSBG1,RGS8,DEFA1B,DEFA3,PTGER2,JAK2,CLDN1,PDE2A,LCOR,CCDC62,TGFB1,SGK1,BTRC,SLIT3,SLIT2,GABRB1,CHRM1,CLOCK,BCKDHB,HOMER2,FGF10,SMYD3,ADCY2,SLC16A1,RORA,CNR1,HBE1,UFL1,CTNNB1,PARK2,SOD2,IGF1R,PPARG,MSR1,CYBB,OTC,RYR2,NR4A3,CHEK2,SFRP1,FOXO3,TPH2,SSH1,NEDD4,ESRRG,SLC6A1,APP,YAP1,RNLS,ALPL,GNG2,DRD1,MMP2,ESR2,STAT1,SLC6A3,ATF2,EPHX1,GGT7,EPG5,ADCY5,RORC,PTAFR,ADCYAP1R1,DDX58,PRKCG,KCNQ1,TRPA1,AKAP6,NR2C1,GLI2,DAB2,BLM,SIN3A,RUVBL2,PELI1,ALK,ATP2B4,PAK3,DYX1C1,P2RX6,MAP1B,TYR,XRN1,PSMB2,TRPV1,GHR,ACTR2,ELAVL4,HDAC1,RECQL5,TRIM24,AXIN2,CARM1,SRD5A2,HTR1D,CTNNA1,RAPGEF3,SDK1,PDXP,SNCA,PNPT1,CNGA3,WNT7B,HMGCS2,KMT2D,ADTRP,DSG1,MAPK1,BMP7,LRRK2,GNA14,NSG2,PKD2,EFNA5,NCOA3,WNT7A,GLDC,BMP6,GPI,PLIN2,PNPLA3,PRMT2,RAPGEF2,KCNC2,SMO,PRKAR2B,CPT1A,SOX30,UBR5,RHOA,RQCD1,ITPR2,GABRB3,GSK3B,EEF2K,AGT,HRH4,PHEX,PDE4B,UGT1A1,RPS6KB1,STC2,CLDN4,SCNN1B,SLC1A1,IL18,PPARA,ABCD3,TAF2,ZNF366,ATP1A3,ADORA2A,NCOR2,DI*

			APH1,HDAC2,HTR2C,IMPACT,ADCY1,CNOT1,SLC10A1,TAF1,TICAM1,HSF1,DHFR,NCOA2,CIB2,CAV2,PHB,BMP4
GO:0072001	renal system development	0.01524042811094845	*ADAMTS16,DCHS2,PLCE1,LRP2,ROBO2,KIF26B,ERBB4,NPHP3,PTPRO,PDGFB,KIRREL3,BASP1,TFAP2A,CENPF,NF1,NFIA,ARL3,KLF15,WNT11,TGFB1,PCSK5,RPGRIP1L,SLIT2,TP73,SMAD6,BMPER,FGF10,GREB1L,EPHA4,CTNNB1,DLG5,FGF1,MYOCD,MYO1E,BICC1,PAX2,SFRP1,SMAD3,PDGFRA,YAP1,STAT1,STRA6,AP1B1,FGF2,KLHL3,RARB,ADAMTS6,COL4A3,GLI2,PKHD1,IQGAP1,GPC3,EYA1,NID1,EPCAM,AP2B1,EPHA7,PTK7,MAGI2,WNT7B,HMGCS2,BMP7,LRRK2,BAG6,PKD2,BMP6,EXT1,SMO,RHOA,ACTA2,SULF1,AGT,ZMPSTE24,PBX1,PYGO2,ARID5B,FBN1,NIPBL,ANGPT1,POU3F3,WWTR1,TRAF3IP2,EDNRA,BMP4*
GO:0043405	regulation of MAP kinase activity	0.015279230407037638	*NTRK3,FLT3,MAP2K5,KITLG,ROBO1,HTR2B,TENM1,TRAF6,AKAP13,PDGFB,INSR,PAK1,MAP3K4,ERN2,NF1,PAQR3,ROR2,NOX4,PRKCD,ZNF675,EPHA4,TNFSF11,PPARG,AXIN1,FGF1,AJUBA,SFRP1,TAB1,S100A12,PTPN1,CDK12,FGF2,DVL3,TSG101,NEK10,MAP3K7,GHR,HIPK3,STK38,MAP3K13,BMP7,LRRK2,PDGFC,AGT,MAP3K5,ADAM8,RGS14,CD300A,SYK,DVL2,PTPRJ,SASH1,PTPRC,TNXB,BMP4*
GO:0001822	kidney development	0.01585272461142216	*ADAMTS16,DCHS2,PLCE1,LRP2,ROBO2,KIF26B,ERBB4,NPHP3,PTPRO,PDGFB,KIRREL3,BASP1,TFAP2A,CENPF,NF1,ARL3,KLF15,WNT11,TGFB1,PCSK5,RPGRIP1L,SLIT2,TP73,SMAD6,BMPER,FGF10,GREB1L,EPHA4,CTNNB1,DLG5,FGF1,MYO1E,BICC1,PAX2,SFRP1,SMAD3,PDGFRA,YAP1,STAT1,STRA6,AP1B1,FGF2,KLHL3,RARB,ADAMTS6,COL4A3,GLI2,PKHD1,IQGAP1,GPC3,EYA1,NID1,EPCAM,AP2B1,EPHA7,PTK7,MAGI2,WNT7B,HMGCS2,BMP7,LRRK2,BAG6,PKD2,BMP6,EXT1,SMO,RHOA,ACTA2,SULF1,AGT,ZMPSTE24,PBX1,PYGO2,ARID5B,FBN1,NIPBL,ANGPT1,POU3F3,WWTR1,TRAF3IP2,EDNRA,BMP4*
GO:0051480	regulation of cytosolic calcium ion concentration	0.017796118171469893	*ASPH,PDE4D,FAM155A,ADCY8,PTGFR,CHERP,RYR1,ATP2B2,GRM5,PLCE1,ANK2,CHD7,HTR2B,RYR3,SLC24A2,CACNB2,ADORA1,ADRA1D,ESR1,LCK,DMD,SLC8A1,JPH2,FYN,BDKRB1,BDKRB2,TMBIM6,CMKLR1,PTGER2,JAK2,NPSR1,TGFB1,JPH3,F2RL1,C9ORF47,CDH23,CAPN3,CNR1,P2RY10,TRPM1,RYR2,NOL3,CACNA1C,GRM1,SMAD3,ITPR1,ACKR2,PDGFRA,IBTK,DRD1,PRKD1,FGF2,ADCY5,ADCYAP1R1,TRPA1,NMUR2,AKAP6,TRPC5,SLC8A2,TPCN1,ATP2B4,P2RY8,CACNA1A,PTGER3,TRPV1,PLCG2,C1QTNF1,GPR35,TRDN,TRPC6,SNCA,HTT,PKD2,GRIN1,ITPR2,BOK,AGT,HRH4,NOS1,XCR1,MCOLN1,GRIN2B,DIAPH1,HTR2C,ATP2B3,CIB2,CAV2,CAMK2D,CCR3,PTPRC,EDNRA*
GO:0030100	regulation of endocytosis	0.01830135858407241	*CBL,RAB4B,RAB4B-EGLN2,INSR,RIT2,BMP2K,NRG1,CDH13,ANO6,MKLN1,F2RL1,AAK1,SLC17A7,PPP3CA,PPARG,SCAMP5,ANKFY1,PRKD1,PTPN1,NLGN1,MCTP1,ABCA13,AP2M1,TSG101,KIF3A,HIP1,BTBD9,STON2,DAB2,PACSIN1,ATAD1,GPC3,SGIP1,TBC1D5,PLCG2,ROCK1,SH3GL3,AP2B1,SNX33,SNCA,MAGI2,RAB5B,MIB1,ATXN2,LRRK2,UNC119,DNAJC6,TF,EEF2K,NECAB2,PACSIN2,RABGEF1,ANGPT1,CD300A,ANXA2,NUMB,SYK,ANKRD13A,AXL,NEDD4L*
GO:0051603	proteolysis involved in cellular protein catabolic process	0.018911001283017596	*CALR3,FBXL2,DCAF12,CBL,USP32,TOM1L1,PSMD1,PSMB7,CCDC22,OMA1,DNAJB14,HDAC6,DDB1,CUL4B,FBXL18,ZYG11B,FBXL7,USP46,CHFR,USP34,FBXW11,RNF144A,TRIM13,UBR2,NPLOC4,USP13,MKRN2,ARNTL,FBXO21,PPP2CB,RNF4,UBE3D,FHIT,MAN1A1,FBXO9,RFFL,USP22,FBXW7,ITCH,USP12,SPSB1,MTA1,SIAH1,ERCC8,SPSB4,ASCC2,BTRC,DNAJB2,RNF133,RNF148,DISC1,ZFAND2A,CLOCK,FBXL17,KCTD10,SMURF2,EPHA4,UBQLN3,UBQLNL,UFL1,CTNNB1,PARK2,SMARCC1,AXIN1,RSPRY1,UBQLN4,CAPN2,RNFT2,WWP2,RNF168,NEDD4,SEC61B,USP49,ARIH1,AMFR,PRICKLE1,ERLIN1,VPS4A,ADAMTS12,PSMD11,USP33,UBE2R2,AGBL4,CDC27,YME1L1,KLHL3,PRKCG,AREL1,UCHL3,PSMD2,RBX1,ANAPC5,FBXO45,STT3B,FBXL20,TSG101,DESI1,IDE,SUFU,EDEM3,DAB2,UBR1,CACUL1,PSMD7,PELI1,PPP2R5C,ZNRF1,RBBP6,DYX1C1,UBE3C,RNF34,PSMB2,RNF216,RYBP,CUL2,MAP1A,MAN1B1,FBXO39,FBXW4,EIF3H,USP42,USP50,RC3H1,RNF103,RNF103-CHMP3,RNF19B,PSMF1,TRIP12,ZNRF3,KCTD13,PTEN,MIB1,PCBP2,LRRK2,ZRANB1,BAG6,SEL1L2,SH3RF2,N4BP1,GET4,UBE2H,CUL3,EPM2A,GSK3B,DNAJC3,RNF144B,RNF43,CUL9,ZMPSTE24,PTK2,ARMC8,RNF121,FBXO10,TRPC4AP,RNFT1,CSNK1A1,UBE2K,FBXL13,RNF150,OTUD7B,TBL1X,FBXO31,C18ORF25,SKP1,RPL23,WWTR1,TAF1,ADAMTS7,GNA12,UGGT1,UCHL5,MALT1,FBXL4,NEDD4L,RNF213,SGTB*
GO:0051272	positive regulation of cellular component movement	0.019847542146210446	*MAP4K4,CLASP2,SEMA3A,DOCK1,SEMA3D,MAPRE2,TJP1,NTRK3,SEMA5A,ENPP2,FER,KITLG,PIK3CD,ITGB1,MGAT5,HDAC6,EPHA1,ZNF609,PDGFB,STK39,INSR,PAK1,SLC8A1,CASS4,RTN4,NTN1,BDKRB1,DOCK8,CMKLR1,ROR2,CDH13,CD99,ANO6,CCBE1,JAK2,CLDN1,WNT11,SEMA5B,TGFB1,F2RL1,BCAS3,RRAS2,LAMC2,DAPK2,FGF10,SMURF2,EPHA4,PRKCA,PPP3CA,SOD2,IGF1R,FGF1,NR4A3,FLT4,HDAC4,SMAD3,NRP2,DOCK4,APP,PDGFRA,DRD1,MMP2,SYNE2,PRKD1,SEMA6D,BMPR2,CAMK1D,PIK3R3,SPAG9,RAB11A,FGF2,PPM1F,NEDD9,SEMA4D,ELP3,PTAFR,LAMB1,RHOJ,KIF3A,RUFY3,DAB2,WNK1,RELN,SP1,CLASP1,PAK3,SSH2,SCARB1,SMOC2,PLCG2,TRIM46,SPI1,MCU,IGF1,MAPK1,BMP7,PDGFC,WNT7A,GPI,SH3RF2,RAPGEF2,SMO,RHOA,TF,ONECUT2,RDX,ACTA2,AGT,PTK2,RPS6KB1,ADAM8,CLDN4,CXCL17,PLVAP,ACTN4,STK4,EPHB2,EPB41L4B,GPSM3,NIPBL,ANGPT1,GCNT2,ETS1,MAP2,FBXO31,DIAPH1,ATP8A1,ANGPT4,NUMB,SASH1,ZP3,MET,SEMA3C,PTPRC,BMP4*
GO:00	sequestering of	0.01987832	*ASPH,PDE4D,CHERP,RYR1,ANK2,CHD7,HTR2B,RYR3,LCK,DMD,SLC8A1,JPH2,BDKRB1,NPSR1,TGFB1,JPH3,CAPN3,RYR2,NOL3,CACNA1C,ITPR1,IBTK,DRD1,PR*

51208	calcium ion	120323495	*KD1,FGF2,TRPA1,AKAP6,TPCN1,TRPV1,PLCG2,TRDN,SNCA,HTT,PKD2,ITPR2,NOS1,XCR1,MCOLN1,DIAPH1,HTR2C,CAMK2D,PTPRC*
GO:0032409	regulation of transporter activity	0.020127320406113758	*NRXN1,ASPH,PDE4D,SLC9A1,NOS1AP,UTRN,GRM5,ANK2,ITGB1,CNIH2,ABCC8,NETO2,CACNB2,STK39,CAB39,AMIGO1,CHRM3,KCNRG,DMD,JPH2,SHISA9,STIM1,FGF14,PRKCD,SGK1,JPH3,CACNA1D,VMP1,PPARG,RYR2,SCN3B,WWP2,SHANK1,NEDD4,APP,NLGN2,FXYD2,FXYD6,RASGRF2,NLGN1,PTAFR,KCNQ1,NLGN3,AKAP6,WNK1,RELN,ABCB1,AHNAK,STOM,PLCG2,GPR35,ANK3,CACNG8,NETO1,TRDN,STAC,TRPC6,SNCA,CACNG2,HTT,CNIH3,PKD2,SHANK3,KCNC2,PDE4B,SLC5A3,NOS1,ACTN4,EPHB2,LRRC52,KCTD7,CACNG3,CAMK2D,NEDD4L,SHISA6,EDNRA*
GO:0072507	divalent inorganic cation homeostasis	0.02015687756827664	*ASPH,PDE4D,FAM155A,ADCY8,PTGFR,CHERP,RYR1,ATP2B2,GRM5,PLCE1,ANK2,CHD7,HTR2B,RYR3,SLC24A2,SLC24A3,TCIRG1,CACNB2,ADORA1,ADRA1D,CNNM1,ESR1,LCK,SGCD,MICU3,DMD,SLC30A9,SLC8A1,JPH2,FYN,CNNM2,BDKRB1,BDKRB2,STIM1,TMBIM6,CMKLR1,SLC39A14,PTGER2,JAK2,NPSR1,TGFB1,KEL,JPH3,F2RL1,C9ORF47,CDH23,DISC1,TMTC2,CAPN3,CNR1,TNFSF11,PARK2,P2RY10,TRPM1,XK,RYR2,NOL3,CACNA1C,GRM1,SMAD3,ITPR1,ACKR2,APP,PDGFRA,IBTK,DRD1,PRKD1,FGF2,ADCY5,ADCYAP1R1,CCL14,CCL15,TRPA1,NMUR2,AKAP6,TRPC5,PKHD1,SLC8A2,TPCN1,ATP2B4,P2RY8,CACNA1A,PTGER3,TRPV1,TRIM24,PLCG2,C1QTNF1,GPR35,AP3B1,SLC39A10,ANK3,ATP13A3,AP3D1,MCU,TRDN,TRPC6,ATP13A5,SNCA,HTT,SLC30A7,TMCO1,PKD2,GRIN1,ITPR2,BOK,AGT,HRH4,STC2,NOS1,SLC1A1,XCR1,MCOLN1,GRIN2B,PACS2,DIAPH1,HTR2C,GRIK2,ATP2B3,CIB2,CAV2,CAMK2D,CCR3,PTPRC,EDNRA*
GO:0050806	positive regulation of synaptic transmission	0.020644997134153176	*NRXN1,ADCY8,SLC24A2,LAMA2,CACNB2,ADORA1,NTRK2,RIMS1,NF1,ROR2,ZDHHC3,CLSTN2,SYT1,YTHDF1,EPHA4,CNR1,APP,NLGN2,DRD1,SHANK2,RASGRF2,NLGN1,PRKCG,NLGN3,RELN,SLC8A2,STAU1,SYT12,NTRK1,CACNG8,SNCA,CACNG2,KCTD13,SLC4A8,MAPK1,PTEN,SHANK3,GSK3B,RIMS2,RGS14,EPHB2,SLC1A1,PRKCZ,ADORA2A,GRIN2B,BRAF,TNR,ADCY1,GRIK2,CACNG3*
GO:0071840	cellular component organization or biogenesis	0.021092900688764793	*CD247,CLRN1,ENPP1,HOOK2,ADAMTS16,BLZF1,LDB2,NRXN1,ASPH,PRMT3,PRKCI,MOV10L1,TACC2,MAP4K4,PDCL,DNMT1,S1PR2,LLPH,SLC9A1,NEGR1,ADCY8,SPAG16,PDE4DIP,CLASP2,SEMA3A,GRID2,RPS6KA2,PRDM12,TENM3,DOCK1,CLDN18,KIF22,RYR1,NRG3,ASH1L,NOS1AP,TUBA1C,NOX5,SPESP1,IQCG,TLN2,EPB41,SEMA3D,PHACTR1,MYOT,NREP,SCAF8,TIAM2,FHOD3,MAPRE2,NLGN4X,TJP1,UNC5C,HPSE2,PRLR,UTRN,KALRN,PITPNC1,SEC23B,LTV1,PHACTR2,MRM1,NTRK3,CBL,EGLN2,MIA,RAB4B,RAB4B-EGLN2,SEMA5A,RAD51B,PTGES3L,TOX,ENPP2,GRM5,PLCE1,LHFPL4,SAMHD1,FER,RNU6-202P,MARVELD3,MAP2K5,ISCA1,LRP2,ANK2,OLFM1,MFAP5,STAG2,EZR,MEGF10,NRXN3,ROBO1,TOM1L1,CHD7,ITGB6,CDKL5,MECOM,TACC1,TENM1,CAMKMT,RYR3,KIF5C,LMNA,ROBO2,NFASC,CTNNA3,DNAJA3,VAV2,ABI1,ULK4,ITGB1,TRIOBP,HSP90AA1,ELMO2,GAS7,CDC6,PSMB7,PCDH17,KNDC1,ZC4H2,DSCAM,BCORL1,STK38L,CDAN1,CNGB1,TRIO,ARHGAP24,KLHL12,AKAP13,IFT43,TTLL5,GFI1B,PARD6G,PTPRK,RUNX1,DAB1,OMA1,CDC42EP3,MYO10,ABCC8,ERC1,HMGN3,CDH10,ERC2,XIRP2,IMPG2,ITGB3BP,LAMA2,CLVS1,MAST4,COL14A1,IL17RB,TCIRG1,PTPN11,KCNS3,CCT2,CACNB2,DOCK10,HDAC6,GOLGA4,SERTAD2,TBCEL,ADAMTS3,EPHA1,IKBKB,KIF18B,PEX14,ERBB4,CDH12,MRE11A,GBF1,DNAH2,LIMK1,TLK1,BRD8,ATP8A2,RNU6-78P,RAD54L2,TEX264,KAT6B,CUL4B,CECR2,ESR1,NPHP3,RNU6-179P,GRM7,PMEPA1,PTPRO,KANSL1,NTRK2,STX18,DNAI2,EP300,DYM,TENM2,FNTA,HOOK3,RNU1-124P,PDGFB,RIMS1,TNIK,SNX2,KIRREL3,CDH8,CSGALNACT1,MID1,RNU6-1061P,CHFR,ALOX5AP,MSTO1,INSR,FMN2,RERE,PRKAR1A,AFAP1,ATP8B1,UNC5D,H2AFY2,LAMA3,TCF7L2,PIP4K2A,CDH4,HPS1,JMJD1C,PAK1,FBXW11,MAP3K4,PTPRD,CNTN5,TBR1,STRC,BDNF,GLTP,AMIGO1,GTPBP10,FANCA,KCNMA1,TGIF2-C20ORF24,NPRL3,FMNL2,PHACTR4,RNU6ATAC27P,TENM4,KCNRG,UBR2,ARHGAP6,MDM4,ECT2,SLC12A8,ZNF148,NAV2,ACOX1,VAMP7,LAMC1,RFTN1,ERN2,CDC45,SHFM1,C1D,LRRC49,NPLOC4,PYROXD2,STARD9,DENND5A,DMD,CENPF,RNU6-1269P,CRISPLD2,PDS5A,KAT7,PRELID2,GSN,HFM1,RBM14,SATB2,RIT2,HIRA,TANC2,CASS4,KCTD16,FYN,ADAMTS9,NF1,PLCB1,DST,PCDH15,LMTK2,STX6,ARID4B,SNCB,FAT3,SH3BGR,RTN4,AFF2,RXFP1,GOLGA2P5,APOD,B4GALT6,RTN4R,VTA1,BCR,BRPF1,CCDC141,TTN,NDRG4,BMP2K,PAQR3,COA6,ADAMTSL1,RANBP9,TLL1,NTN1,EGFLAM,DCTN1,SLC4A5,NRG1,ARHGAP10,BDKRB1,BID,FRY,MAP2K1,FNIP1,RAPGEF6,VAX2,MYH9,FRMPD4,MAD1L1,LRFN5,VGLL4,PPP2CB,CNTN4,WDR92,VWA1,HDAC5,RBM5,NTNG1,NAV1,NFIA,RNF4,TMEM30A,CSNK2A3,SYNE1,PARD3B,LRGUK,ROR2,DCN,LIN7A,SCFD1,DOCK2,CDH13,PITPNB,AUNIP,UST,COPS5,DMC1,PCDHB16,EXOC4,MAD2L2,TLE6,RAB27A,CAPRIN2,MTX3,NCKAP1,TERF2,TMED6,SLX1B,GOLPH3L,CLDN16,RCC2,BOC,ANO6,ARL3,DNAJC11,KIF2A,ICK,RAD51D,TTLL8,PARP11,COL12A1,RANBP1,USP22,ZDHHC15,JAK2,MYPN,FBXW7,KCNB2,SKAP1,SMC3,CLSTN2,UIMC1,SYT1,ITCH,CROCC,RNU1-2,RNU1-4,BBS12,SNAP25-AS1,LZTS1,BCL11B,SMG1,CLDN1,MLLT3,ATP9A,CDC42BPB,PLS1,TEX11,PDE2A,SEPT7,MAGI1,KCNC4,KPNA3,CYFIP2,CENPC,WNT11,IFT80,FIG4,MTA1,KREME*

			N1,IMMP2L,SIPA1L3,MKLN1,SIAH1,IL1RAPL1,CAMK2B,PRKCD,SEMA5B,PPM1E,TGFB1,BANP,LRCH3,SGK1,PDLIM4,ATL2,NUP93,MMP28,NSD1,PIBF1,KEL,ARMC1,CLDN10,F2RL1,BCAS3,PDZRN3,RPGRIP1L,DNAJB2,CELSR1,SLIT3,AAK1,SDK2,SLIT2,COLGALT1,TP73,SYT13,CDH23,CORO1C,CTNND2,DISC1,CEP135,KANK1,PTPDC1,GRPEL2,ANO4,LAMC2,COL23A1,CLN6,ANK1,CLMN,TTBK2,KDM4B,SPAG17,MMP26,SMAD6,COL24A1,ITSN2,PPFIBP2,ANO3,SMOC1,ETV6,SYNJ2,BCL3,DCLK1,RNF212,EIF3L,LYRM4,PARVA,DUSP22,STRIP1,HNRNPC,RNU6-816P,PDCD11,TRRAP,ANKFN1,SLC17A7,OIP5,LINGO2,YTHDF1,FGF10,CNTNAP2,SMYD3,STXBP5,KANK4,KCTD10,ENAH,IL1RAPL2,PPL,IQCJ-SCHIP1,LOXL3,RHPN2,MAST2,SVIL,CAPN3,SLC16A1,LUM,VMP1,EPHA4,HSD17B12,PRKCA,AUTS2,CNR1,SMG6,PPP3CA,GNG4,MAG,GATAD2B,LIX1L,UFL1,TRAK1,CTNNB1,MPRIP,PARK2,SOD2,RN7SL832P,FCGR2B,ARHGAP22,SMARCC1,TSNARE1,CDHR2,IGF1R,PPARG,NGRN,AXIN1,DLG5,MYO1D,BFSP1,ADD2,MSR1,CELF2,TRPM1,XK,SCAMP5,BRIP1,CENPP,ECM2,SREBF2,KCNJ12,CDK11A,KCNA6,NDUFA9,FBLIM1,CDK11B,DRAXIN,LEPROT,NIN,WDR45B,TUBGCP6,NOL3,RIOK2,ESCO1,MPP6,MYOCD,AJUBA,HAUS4,CPNE6,CHEK2,PHLDB1,KRT6B,UBQLN4,PRDM16,MYO1E,SPDYA,HCK,SORBS1,TBC1D14,RAB3GAP2,CAPN2,TBCK,CSPG4,DCC,ANKFY1,CTDP1,BAZ1B,MMP16,OBSCN,NF2,SMC2,HDAC4,PAX2,DNAH9,SPTBN1,HEPACAM2,ELN,TRABD2B,SPG11,SFRP1,DGKB,ARL13B,CNTN6,NFIB,MUC12,IFT122,DDX10,DNM3,SSH1,CELF5,ATP10D,SNX14,SMAD3,CUX2,THSD4,PTPRM,WIPI1,MTBP,VPS39,RNF168,EMG1,ABI2,C10ORF90,GRIK5,SHANK1,SYT3,NEDD4,NRP2,ARHGEF18,BTBD3,PREX1,PLA2G4C,CRTAC1,SPAG6,KPNB1,NAV3,SLC6A1,RAB6C,PRTG,CDC73,APP,SSBP3,USP49,PDGFRA,BET1L,SLK,ADD3,DIP2B,KANSL2,NOX1,YAP1,HEG1,MYO1F,TEN1,FSTL4,NLGN2,VDAC1,EYA2,SH3D19,BORA,NVL,LRP5,PTPRG,ISLR2,SOX2,SETD2,ZDHHC6,GPC6,KIF24,TTC39C,ADAMTS2,TEAD1,PLEKHA7,SPTBN4,VASH2,DRD1,TMEM14A,RFT1,SFMBT1,VILL,MMP2,ESR2,SYNE2,DCUN1D3,ERI2,CPNE9,KDM6A,GTF2F2,PRKD1,CELF6,PARP6,OSBPL2,DGKG,SYBU,RND3,HNF4A,TBCA,SHANK2,SHPRH,VPS4A,EREG,TCTN3,ABLIM1,MYOF,DSCAML1,RNU6-167P,PITRM1,SEMA6D,ATF2,TMEM237,KCNG4,RAF1,CELF4,ABLIM2,SMIM20,PTPN1,ADAMTS12,PSMD11,RNU6-923P,BMPR2,USP33,DPYSL2,VBP1,CAMK1D,NLGN1,COL16A1,CTNNA2,ATP9B,IKZF4,TAF8,CAND2,SPAG9,MORC2,RAB11A,CRMP1,EPG5,MYB,LRRC4C,PPM1F,NEDD9,PEAK1,ADAMTS4,SEMA4D,ARMC2,CDC27,HELQ,MCTP1,PLXNA2,POC1A,EEA1,SETD1A,YME1L1,ABCA13,JARID2,RILPL1,BRDT,CHCHD6,PI4KA,PIK3C2B,SPOCK1,BLOC1S5,EHMT1,GAS8,AP2M1,DVL3,EIF4G1,EPHB3,RNU6-229P,ANAPC5,ARHGAP39,FBXO45,TRAPPC11,SRSF5,PAWR,TRMT61B,AGO3,DEPTOR,TSG101,VCL,LAMB1,TERF2IP,WDR90,CLIC4,MYO9A,ANKS4B,COL11A1,KCNQ1,RFX2,WNT3,ADAMTS6,C11ORF80,RHOJ,NLGN3,ZFYVE1,PAFAH1B1,KIF3A,ADAMTS14,COL4A3,TRPA1,TRPM3,HIP1,AKAP6,CEP350,RASAL1,PADI6,BTBD9,TRPC5,TTLL7,GLI2,NEO1,TNKS,WBP2NL,ERCC1,RUFY3,WDTC1,STON2,C9,COL19A1,DAB2,BLM,PKHD1,XKR4,TRAM2,LDLRAD4,MYSM1,SETD5,DZIP1,SMG5,DLG3,RELN,SIN3A,RUVBL2,ABCB1,DOCK11,IQGAP1,TMEM170A,SPATA18,COL6A5,EML4,FAM91A1,DDX47,ZNF423,MACF1,ARHGAP12,MYO7A,ALK,SLC8A2,STAU1,CLASP1,TOP1,MBOAT1,GPM6B,XDH,NFATC2,CHD1L,PRKG1,TTLL9,NSF,SYNE3,PACSIN1,SPTBN5,ACTL6B,PAK3,HORMAD2,SSH2,TET1,DYX1C1,ATAD1,ADNP,CEP41,ARID4A,MAP1B,C12ORF65,CDC42BPA,COL4A6,MPHOSPH9,SCARB1,MARK1,PHF2,CELF1,AIF1L,TFRC,UVRAG,EPHA5,MEF2A,DHX30,DCUN1D5,DROSHA,GPC3,SGIP1,XRN1,PLS3,SATB1,TBC1D5,PSMB2,ZNF207,CALD1,EYA1,DIS3L2,GNB3,TRPV1,TFEB,CLEC16A,MNAT1,FLRT2,FGD1,SNX9,DIAPH2,RPGR,ACTR2,L3MBTL4,ACACA,ELAVL4,FAM174B,HDAC1,RECQL5,SMOC2,PLCG2,ROCK1,EPS8,NID1,SCMH1,EGR2,CAPN10,GPR35,ABCC1,AP3B1,INSRR,NTRK1,ZFAND1,MAP1A,PKN2,PPP1R9A,EIF3A,ANK3,UNC13A,PTPRQ,EPHB1,EPHA10,PTPN9,AXIN2,NCKAP5,SARM1,TMEM199,PARD3,GNL3L,TRIM46,TUB,SH3GL3,TEKT1,KCND3,BCOR,AP3D1,CARM1,AP2B1,ANXA8L1,NUDT5,ARHGAP44,SNX33,PIFO,SMARCA2,CUX1,RAD51C,SPI1,EPHA7,MCU,RANBP10,PTPRA,VPS16,PPFIA4,CTNNA1,LIMA1,AIFM2,TRDN,ATG14,EIF3H,LRRC8C,LRRC8D,RNU6-979P,SHROOM3,ABCB7,ACOT8,NTMT1,FCHSD2,MAP3K13,RAPGEF3,SDK1,ADAMTS17,PDXP,SH3BP1,RNU2-47P,ERCC3,PTK7,SMYD1,SNCA,BMPR1B,HAUS3,CACNG2,CD53,MAGI2,PNPT1,USP50,ZEB2,MCMBP,FOXJ3,SYT9,HEY2,RC3H1,CHMP3,COL13A1,EIF3E,HYDIN,RNF103-CHMP3,RNU6-640P,WNT7B,PTPRS,ADAMTSL3,ZMYND11,KMT2D,CDKL1,RAB5B,KMT2C,TLL2,CAST,DFFA,ADTRP,ABCA12,CSRP1,PLD1,RASA1,MITF,SRSF6,OPRD1,CABIN1,HIPK1,PKP2,RPTOR,RTN4RL1,SGCZ,DSG1,VPS41,KCTD13,TROVE2,CBFA2T2,IGF1,STX8,ATF7IP,HTT,DPYSL3,MAPK1,PTEN,ARHGEF17,CLDN11,MIB1,BMP7,ATXN2,PIK3C3,LHFPL5,NUP88,TTLL4,RAB30,LRRK2,OSGIN1,SEPT6,ZRANB1,EBAG9,SPAG5,UNC119,CHMP5,RAPH1,RFC3,BAG6,CHAF1B,IFT81,TMEM108,ITGAM,ARHGAP25,NSG2,PKD2,EFNA5,HSF2BP,SHANK3,KCTD1,STMN4,PDGFC,SERPINA5,AMOTL1,ATP8B4,PKIB,MTMR3,STEAP4,PPP2R3C,TBCD,TMCC1,WNT7A,SNAP23,BRWD3,NLRP1,MAP4,CRTC3,NME8,CEP89,KAT6A,MTIF2,RABEP1,SH3PXD2B,RHOT1,SIK3,BMP6,MYCBP2,SOS1,TSHR,WDR43,EXT1,ATRX,IKZF1,PARVB,PNPLA3,SAMM50,RAPGEF2,CDH9,KCNC2,RNU6ATAC31P,SMO,GET4,SUN1,LSG1,BLOC1

			S3,FDXACB1,GEMIN7,MARK4,PSEN2,TCF4,TNFRSF11B,SYT17,SOX30,DNAJC6,GRIN1,JADE1,KCTD8,ARRDC4,BBS9,RHOA,SYNGAP1,GRIN3A,MCM3,NDE1,ROR1,TF,ONECUT2,RHOBTB1,ESYT2,SHROOM1,CUL3,SH3KBP1,HSPD1,TSPAN33,EPM2A,ESF1,GABRB3,GSK3B,ABCG8,PLD2,PRR16,EMB,RDX,SYN1,EEF2K,FAM49B,NECAB2,CDK13,TAPBPL,VAMP1,BOK,SULF1,KRT8,NACC2,SUPT4H1,AGT,HDGFRP3,METTL16,CDH20,PRKAA2,SYT7,ADAR,BLOC1S6,CUL9,ESPNL,PITPNM2,STAT2,ZMPSTE24,PTK2,TPPP2,GTF2E1,PEX5L,SNX3,EXOC6B,RIMS2,EXOSC3,TOMM5,CDC14A,GAP43,RNU6-1056P,SEC16B,CREBBP,TIMM44,IQGAP2,FAM171A1,UTP20,ARVCF,COL9A1,FMOD,COL21A1,CEP70,GTF2IRD2,TAOK2,PYGO2,SHC1,NDUFS2,ALDOA,ECE1,PARP10,YLPM1,RSBN1,SF3A1,PGM5,SETDB2,ITGAL,CPLX2,DNAJB6,CD44,FGD3,LARP4,ADAM8,NOL10,ARFGAP3,CLDN4,PACSIN2,RPS6KA5,CDS1,THSD7A,CSNK1A1,HUS1,FGD4,PEX7,TBC1D23,PLEKHA1,RGS14,ACTN4,COG4,STK4,TUBB3,ZFAND6,MARK3,SPEF1,THSD7B,EPHB2,SLC1A1,TTLL11,SIGMAR1,TMEFF2,TEX12,MFSD8,ASF1B,UBE2K,GPSM2,DIS3,DLC1,PPARA,PPP1R10,ABCD3,MCOLN1,EPB41L4B,SLC25A33,SMARCAL1,CCDC6,PRRC2C,SYTL4,PAX6,PRKCZ,ATP1A3,ADORA2A,SPECC1L,GRIN2B,KCTD7,RABGEF1,STAG1,WDR83OS,COL22A1,PHLDB2,KLC3,KLHL1,NIPBL,YEATS4,COL28A1,PHF20,THSD1,UPB1,ANGPT1,BRF1,PACS2,EYA3,MBTD1,OSBPL8,SHROOM4,EPB41L2,ETS1,HACL1,PPP1CA,ARHGAP21,MAP2,MTF2,BIN2,STPG1,KIFC3,NEBL,FBXO31,ATG3,CNTN1,PRKCQ,LGI2,MDN1,BRAF,DIAPH1,HDAC2,IMPACT,ATP8A1,SRCAP,CAPN6,NOTO,SPINT2,TNR,CLTB,HIGD2A,CD300A,PHIP,CDKL3,EMC3,PPP2CA,ANGPT4,ARHGEF6,DNAJC9,FAM149B1,RPL23,SH3BP4,RFC5,ACAN,CAMSAP3,SP100,VTI1A,ANXA2,CHCHD3,KCND2,PARN,PRMT7,PITPNM3,FARSB,SRGAP2,TMEFF1,WIPI2,ADCY1,NUMB,SQLE,SYK,CNOT1,WWTR1,GTF2H5,SLIT1,FRMD5,CIT,RAB2A,SEC31B,UBAP2L,BRCA2,HRK,RAP1GAP2,DVL2,MORC1,MTRF1,NEB,ANKRD13A,GRIP1,PTPRJ,TAF1,GPRIN3,TICAM1,GPM6A,ITGAE,MYO1A,HSF1,MAX,DHFR,EZH1,ANKRD30BL,ETF1,SRPK2,ATP10B,PLEKHM1,HGSNAT,ASAP1,ADAMTS7,GBP5,MCM8,WDR18,BMP1,BMS1,CGNL1,FCHO1,GEMIN6,VAV3,CHD6,EPB41L1,MTFR2,CAV2,SKA2,SYNM,AKTIP,COL4A5,EPS15,POC1B,INPP5F,NUAK2,TBP,AXL,REPS2,TRIM37,CENPK,MET,FMNL1,MICAL3,CAMK2D,MRPL1,SNX1,BRD9,SETD3,INO80C,SPTAN1,TMEM120B,CCDC136,EHD2,KDM2B,SEMA3C,THEM4,TRAPPC8,CCT3,NEDD4L,UQCC1,NDC1,PHB,PTPRC,CTCF,SH3GL2,RNF213,SYNDIG1,NCAM1,TANC1,CREB1,RASSF8,SHISA6,TNXB,CCDC170,EDNRA,PRAP1,STXBP6,BMP4,ABLIM3,CPNE1
GO:0007162	negative regulation of cell adhesion	0.021241941228215946	PRDX2,B4GALNT2,MAP4K4,HLX,CBFB,CLASP2,SEMA5A,CASK,MAP2K5,DSCAM,RUNX1,DAB1,PTPN11,PTPRO,PRKAR1A,ARHGAP6,NF1,APOD,MAD1L1,CDH13,MAD2L2,RCC2,JAK2,ITCH,PRKCD,TGFB1,BCAS3,CORO1C,KANK1,DUSP22,LOXL3,EPHA4,FCGR2B,DLG5,NF2,MMP2,PPM1F,SEMA4D,PLXNA2,SPOCK1,PAWR,WNK1,PELI1,PRKG1,C1QTNF1,PTPN2,EPCAM,MUC1,SPI1,RC3H1,ADTRP,ABCA12,RASA1,PTEN,LILRB4,EFNA5,TBCD,BMP6,PDCD1LG2,PAG1,RHOA,RDX,PTK2,ACTN4,EPHB2,DLC1,PPARA,NOTCH4,ADORA2A,PHLDB2,ANGPT1,GCNT2,SPINT2,TNR,CD300A,PTPRC,BMP4
GO:0032412	regulation of ion transmembrane transporter activity	0.021571150558703886	NRXN1,ASPH,PDE4D,SLC9A1,NOS1AP,UTRN,GRM5,ANK2,ITGB1,CNIH2,ABCC8,NETO2,CACNB2,STK39,CAB39,AMIGO1,CHRM3,KCNRG,DMD,JPH2,SHISA9,STIM1,FGF14,JPH3,CACNA1D,VMP1,RYR2,SCN3B,WWP2,SHANK1,NEDD4,APP,NLGN2,FXYD2,FXYD6,RASGRF2,NLGN1,PTAFR,KCNQ1,NLGN3,AKAP6,WNK1,RELN,ABCB1,AHNAK,STOM,PLCG2,GPR35,ANK3,CACNG8,NETO1,TRDN,STAC,TRPC6,CACNG2,HTT,CNIH3,PKD2,SHANK3,KCNC2,PDE4B,NOS1,ACTN4,EPHB2,LRRC52,CACNG3,CAMK2D,NEDD4L,SHISA6,EDNRA
GO:0050775	positive regulation of dendrite morphogenesis	0.021643974408295637	KALRN,CDKL5,PTPRD,CAPRIN2,IL1RAPL1,CAMK2B,EPHA4,CUX2,PARP6,PAFAH1B1,RELN,PAK3,ACTR2,CUX1,EEF2K,FBXO31,CDKL3
GO:0042127	regulation of cell population proliferation	0.022294145532670298	ASPH,PRKCI,DNMT1,S1PR2,HLX,IL31RA,RPS6KA2,SETD4,PTGFR,CHERP,TJP1,NUDT6,HPSE2,PRLR,FTO,NTRK3,ARNT,SEMA5A,FLT3,RAD51B,STAT5B,TOX,FER,CASK,MARVELD3,MAP2K5,KITLG,LRP2,PIK3CD,SAAL1,MEGF10,ROBO1,HTR2B,TENM1,LMNA,TRAF6,DNAJA3,GRK5,ABI1,ITGB1,CDC6,MCC,PTPRK,RUNX1,ABCC8,STXBP4,TCIRG1,DACH1,ADORA1,EPHA1,ERBB4,ADRA1D,ZNF609,ATP8A2,ESR1,CDON,NTRK2,CELA1,PDGFB,CCND3,TOB2,INSR,PRKAR1A,TCF7L2,PAK1,EGLN3,TFAP2A,FANCA,MEIS1,MDM4,IL5RA,SHC4,MTA3,LAMC1,PTPRU,DMD,KAT7,NF1,RTN4,APOD,NDRG4,IFI30,NTN1,NRG1,BID,MAP2K1,FNIP1,MAD1L1,VGLL4,RBM5,CSNK2A3,ROR2,CDH13,PDF,PTGER2,JAK2,FBXW7,ITCH,BCL11B,CLDN1,WNT11,IFT80,NOX4,RPRD1B,RUNX2,TGFB1,SGK1,C9ORF47,DNAJB2,SLIT3,TP73,DISC1,LAMC2,CLMN,CHRM1,SMAD6,NELL1,KCNH1,TFAP2D,DUSP22,FGF10,GLP2R,EPHA4,PRKCA,CD6,UFL1,CTNNB1,SOD2,FCGR2B,IGF1R,PPARG,DLG5,BRIP1,FGF1,NR4A3,DCT,MYOCD,PER2,SPDYA,HCK,NF2,FLT4,HDAC4,PAX2,TPTE,ELN,SFRP1,ST8SIA1,FOXO3,NFIB,SMAD3,PTPRM,MTBP,SLC16A2,NRP2,CDC73,APP,SSBP3,PDGFRA,NOX1,YAP1,NLGN2,LRP5,SOX2,CHST11,VASH2,FRS2,MMP2,PRKD1,STAT1,ST18,HNF4A,ZBTB7C,SHANK2,EREG,ATF2,TCF3,RAF1,BMPR2,BMPR1A,FGF2,AGBL4,PTAFR,JARID2,RARB,VIPR1,EEF1E1,GAS8,CHRD,ATF3,PAWR,AGO3,DLEC1,TSG101,LAMB1,WNT3,CCL14,SCUBE2,CNTFR,COL4A3,DDAH1,TRPC5,GLI2,DAB2,PKHD1,CACUL1,DLG3,PELI1,PPP2R5C,CFDP1,ZNF423,SP1,ALK,INPP5D,XDH,OVOL2,NFATC2,PRKG1,BNC1,TET1,CCPG1,CDK6,CELF1,TFRC,

			GPC3,EYA1,DIS3L2,MNAT1,HDAC1,TRIM24,RNF10,PTPN2,NTRK1,TMIGD1,SLC39A10,EPHB1,AXIN2,EPCAM,PTPN14,JAG2,CARM1,SMARCA2,CTNNA1,FBXW4,MST1,BMPR1B,MAGI2,HEY2,RC3H1,KMT2D,MITF,SRSF6,HIPK1,RPTOR,BTK,IGF1,MAPK1,PTEN,BMP7,LRRK2,OSGIN1,LILRB4,MORC3,IL11RA,PKD2,PDGFC,WNT7A,BMP6,SOS1,TSHR,PDCD1LG2,RAPGEF2,SMO,ENPP3,RHOA,VIPR2,CUL3,STAT6,CDK13,BOK,SULF1,NACC2,TNFRSF8,AGT,STAT2,ZMPSTE24,NDRG2,PTK2,PBX1,MAP3K5,TCFL5,UTP20,PYGO2,SHC1,PARP10,RPS6KB1,ALOX5,STK4,EPHB2,IL18,DLC1,CALCRL,SLC25A33,PAX6,PRKCZ,ADORA2A,PPP1CC,TMIGD2,ZNF143,ANGPT1,GCNT2,ETS1,PRKCQ,BRAF,HDAC2,POU3F3,SPINT2,CD300A,PHIP,RPL23,SH3BP4,SQLE,SYK,WWTR1,BRCA2,DVL2,PTPRJ,TICAM1,HSF1,SRPK2,SULT2B1,VAV3,ZP3,CAV2,GNA12,TNFSF9,CD109,CCR3,KDM2B,MLXIPL,PHB,PTPRC,CTCF,BMP4
GO:0045664	regulation of neuron differentiation	0.022639127194291633	NREP,ZNF536,ESRP1,ZC4H2,DAB1,CDON,BDNF,TGIF2,MEIS1,ECT2,DMD,CNTN4,NEUROD1,BCL11B,ZHX2,TP73,TCF12,MAG,SFRP1,FOXO3,CASZ1,HOXD3,APP,SOX2,TCF3,DPYSL2,NLGN1,SPAG9,NCOA1,EIF4G1,TRPC5,GLI2,SIN3A,ALK,MAP1B,EYA1,BRINP1,ROCK1,SH3GL3,MAP3K13,HEY2,MIB1,BMP7,LRRK2,BMP6,TCF4,RHOA,GSK3B,BRINP3,PBX1,DLX1,PAX6,VWC2,CDK5RAP1,IMPACT,BMP4,CPNE1
GO:0031032	actomyosin structure organization	0.022639127194291633	SLC9A1,CLASP2,EPB41,PHACTR1,FHOD3,TJP1,ITGB1,AKAP13,EPHA1,LIMK1,PRKAR1A,PAK1,ARHGAP6,ECT2,TTN,MYH9,MYPN,CDC42BPB,WNT11,PPM1E,RHPN2,CAPN3,SORBS1,OBSCN,NF2,ELN,SFRP1,SMAD3,ARHGEF18,PDGFRA,VPS4A,PPM1F,CLASP1,CDC42BPA,MEF2A,ROCK1,RAPGEF3,CSRP1,KCTD13,RHOA,CUL3,KRT8,FAM171A1,PGM5,TMEFF2,DLC1,EPB41L4B,PHLDB2,EPB41L2,NEBL,BRAF,FRMD5,CIT,NEB,CGNL1,EPB41L1,MET
GO:0014706	striated muscle tissue development	0.02285705307183873	HLX,SLC9A1,RYR1,FHOD3,HIVEP3,NDUFV2,LRP2,MEGF10,CHD7,LMNA,ITGB1,AKAP13,RUNX1,XIRP2,ERBB4,MYO18B,CDON,EP300,PRKAR1A,NPRL3,TENM4,SGCD,DMD,CENPF,SLC8A1,JPH2,ARNTL,ADAMTS9,NF1,TTN,NDRG4,NRG1,VGLL4,NLN,RBFOX1,NOX4,SOX6,TGFB1,KEL,TP73,LRRC10,SVIL,PPP3CA,CTNNB1,XK,RYR2,MYOCD,CTDP1,HDAC4,ELN,SMAD3,PDGFRA,YAP1,HEG1,EYA2,RCAN1,FRS2,MYH15,BMPR1A,FGF2,JARID2,RARB,ATF3,COL11A1,AKAP6,COL19A1,MEF2A,EYA1,EGR2,EPHB1,SMYD1,HEY2,TLL2,PKP2,SGCZ,IGF1,PTEN,BMP7,ALPK3,SMO,MYLPF,RHOA,AGT,ZMPSTE24,RPS6KB1,PGM5,PPARA,FHL2,NEBL,NEB,CAV2,SEMA3C,CREB1,EDNRA,BMP4
GO:0001655	urogenital system development	0.023527106937158437	ADAMTS16,DCHS2,PRLR,PLCE1,LRP2,ROBO2,KIF26B,ERBB4,ESR1,NPHP3,PTPRO,PDGFB,KIRREL3,BASP1,TFAP2A,CENPF,NF1,NFIA,ARL3,KLF15,WNT11,TGFB1,PCSK5,RPGRIP1L,SLIT2,TP73,SMAD6,BMPER,FGF10,GREB1L,EPHA4,CTNNB1,DLG5,FGF1,MYOCD,MYO1E,BICC1,PAX2,SFRP1,SMAD3,PSAP,PDGFRA,YAP1,FRS2,MMP2,STAT1,STRA6,AP1B1,FGF2,KLHL3,RARB,EPHB3,ADAMTS6,COL4A3,GLI2,PKHD1,IQGAP1,GPC3,EYA1,NID1,EPCAM,AP2B1,EPHA7,PTK7,MAGI2,WNT7B,HMGCS2,PTEN,BMP7,LRRK2,BAG6,PKD2,BMP6,EXT1,SMO,RHOA,ACTA2,SULF1,AGT,ZMPSTE24,PBX1,PYGO2,EPHB2,ARID5B,FBN1,NIPBL,ANGPT1,POU3F3,WWTR1,TRAF3IP2,EDNRA,BMP4
GO:2000147	positive regulation of cell motility	0.02363774062200861	MAP4K4,CLASP2,SEMA3A,DOCK1,SEMA3D,MAPRE2,TJP1,NTRK3,SEMA5A,ENPP2,FER,KITLG,PIK3CD,ITGB1,MGAT5,HDAC6,EPHA1,ZNF609,PDGFB,STK39,INSR,PAK1,SLC8A1,CASS4,RTN4,NTN1,BDKRB1,DOCK8,CMKLR1,ROR2,CDH13,CD99,ANO6,CCBE1,JAK2,CLDN1,WNT11,SEMA5B,TGFB1,F2RL1,BCAS3,RRAS2,LAMC2,DAPK2,FGF10,SMURF2,EPHA4,PRKCA,PPP3CA,SOD2,IGF1R,FGF1,NR4A3,FLT4,HDAC4,SMAD3,NRP2,DOCK4,APP,PDGFRA,DRD1,MMP2,SYNE2,PRKD1,SEMA6D,BMPR2,CAMK1D,PIK3R3,SPAG9,RAB11A,FGF2,PPM1F,NEDD9,SEMA4D,ELP3,PTAFR,LAMB1,RHOJ,RUFY3,DAB2,WNK1,RELN,SP1,CLASP1,PAK3,SSH2,SCARB1,SMOC2,PLCG2,SPI1,MCU,IGF1,MAPK1,BMP7,PDGFC,WNT7A,GPI,SH3RF2,RAPGEF2,SMO,RHOA,TF,ONECUT2,RDX,ACTA2,AGT,PTK2,RPS6KB1,ADAM8,CLDN4,CXCL17,PLVAP,ACTN4,STK4,EPHB2,EPB41L4B,GPSM3,NIPBL,ANGPT1,GCNT2,ETS1,FBXO31,DIAPH1,ATP8A1,ANGPT4,NUMB,SASH1,ZP3,MET,SEMA3C,PTPRC,BMP4
GO:0008015	blood circulation	0.024158260024557704	ADAMTS16,ASPH,PDE4D,SLC9A1,SEMA3A,RPS6KA2,NOS1AP,TJP1,ATP2B2,ANK2,CHD7,HTR2B,RYR3,CTNNA3,ABCC8,THRB,CACNB2,ADORA1,ADRA1D,PTPRO,PDGFB,STK39,KCNMA1,CHRM3,NAV2,ARHGAP42,SGCD,DMD,SLC8A1,GSN,BCR,TTN,SLC4A5,BDKRB1,BDKRB2,F5,JAK2,PDE2A,IMMP2L,CACNA1H,SGK1,PCSK5,KEL,F2RL1,SLIT2,KCNJ3,CACNA1D,CNR1,SOD2,PPARG,CELF2,KCNJ12,RYR2,PER2,CACNA1C,SCN3B,HDAC4,ELN,TRHDE,SMAD3,ITPR1,DOCK4,NOX1,RNLS,TNNI3K,LRP5,SPTBN4,DRD1,MMP2,STAT1,MYOF,BMPR2,PTAFR,KCNQ1,COL4A3,DDAH1,WNK1,SLC8A2,KLK2,DSC2,SNX5,PRKG1,ATP2B4,MEF2A,ADRBK1,GNB3,TRPV1,ROCK1,KCND3,HTR1D,LPA,TRDN,RENBP,HEY2,PKP2,SGCZ,PRCP,BMP6,EXT1,RHOA,ACTA2,AGT,ZMPSTE24,PDE4B,ECE1,NOS1,PLVAP,SCNN1B,SLC1A1,PPARA,ATP1A3,ADORA2A,ANGPT1,SCN1A,PTPRJ,ATP2B3,TAC3,GNA12,CAMK2D,NEDD4L,SH3GL2,EDNRA
GO:0016311	dephosphorylation	0.02458994237141267	NT5C1B,NT5C1B-RDH14,CTDSPL2,PTPRR,INPP4B,PTPRK,PTPN11,MGAT5,ADORA1,IKBKB,PTPRO,FBXW11,PTPRD,LCK,PPM1L,PTPRU,SMG7,PPP4R2,PPP2CB,INPP4A,PTPRT,PPP1R42,JAK2,FIG4,RPRD1B,PRKCD,PPM1E,BTRC,PTPRN2,PTPDC1,SYNJ2,DAPP1,DUSP22,SMG6,PPP3CA,PPP1CB,PPP2R2B,CTDP1,TNS3,TPTE,PPP6R2,SSH1,SMAD3,PTPRM,TAB1,EYA2,ALPL,PTPRG,RCAN1,LRRC2,PTPN1,PPA2,PPM1F,SEM

			A4D,PTPRE,PTPN13,SMG5,PPP2R5C,IQGAP1,INPP5D,SSH2,CAMTA1,PHOSPHO2,EYA1,ROCK1,PTPN2,SLC39A10,PTPRQ,PTPN9,PTPN14,PTPRA,PDXP,MAGI2,PTPRS,HTT,PTEN,LRRK2,NT5E,NT5DC4,MTMR3,PPP2R3C,VRK3,DNAJC6,EPM2A,GSK3B,PPP1R16A,PTPRB,DUSP26,CDC14A,DLC1,PDP1,PHLPP1,ACP1,PPP1CC,EYA3,LRRK1,NT5M,PPP1CA,CD300A,PPP2CA,PTPRJ,PFKFB4,INPP5A,RNGTT,GNA12,INPP5F,PTPRC,MTMR12
GO:0001503	ossification	0.02488928295249406	RDH14,ENPP1,CBFB,RYR1,SP3,TRAF6,SLC24A3,RUNX1,TCIRG1,PTPN11,CSGALNACT1,TOB2,TFAP2A,SLC8A1,SATB2,HIRA,NF1,CHRDL1,BMP2K,ANKH,ROR2,ANO6,WNT11,IFT80,KREMEN1,ACVR2A,RUNX2,TGFB1,RRAS2,SMAD6,SMOC1,NELL1,SUCO,SND1,HNRNPC,SBNO2,TNFSF11,UFL1,CTNNB1,PPARG,MRC2,MMP16,HDAC4,SFRP1,SYNCRIP,SMAD3,YAP1,ALPL,LRP5,SOX2,MMP2,TPH1,PRKD1,BMPR2,BMPR1A,FGF2,SEMA4D,ITGA11,CHRD,COL11A1,WNT3,SUFU,SCUBE2,GLI2,RSPO2,GPM6B,ASGR2,CDK6,WWOX,GPC3,EGR2,AXIN2,BCOR,GLIS1,BMPR1B,COL13A1,WNT7B,IGF1,MAPK1,BMP7,BMP6,EXT1,SMO,RHOA,GSK3B,ADAR,FBN2,ZMPSTE24,PTK2,PHEX,PBX1,ALOX5,SP7,PEX7,FAM20C,NIPBL,ASPN,FHL2,SLC26A2,WWTR1,BMP1,PHB,BMP4
GO:0072384	organelle transport along microtubule	0.02763121956947607	CNIH2,HDAC6,PEX14,FBXW11,AP3S1,DYNC1I1,TRAK1,LRPPRC,AP3B2,RAB6A,SYNE2,SYBU,AGBL4,BLOC1S5,PAFAH1B1,KIF3A,MAP1B,AP3B1,TRIM46,AP3D1,KIF3C,HTT,RHOT1,SUN1,BLOC1S3,NDE1,BLOC1S6,COPG2,PRKCZ,ARHGAP21,MAP2
GO:0006836	neurotransmitter transport	0.027748389737845467	NRXN1,CASK,NRXN3,ITGB1,DGKI,ERC2,CACNB2,SV2B,RIMS1,SLC6A16,NF1,ICA1,LIN7A,SYN2,SYN3,SYT1,PTPRN2,CADPS2,SLC17A7,STXBP5,CNR1,PARK2,PER2,RIMS4,GRIK5,SLC6A1,DRD1,SLC6A3,NLGN1,MCTP1,PRKCG,FBXL20,SLC22A3,GPM6B,SYT12,UNC13A,CHRNB4,SNCA,GRM4,SYT9,SLC4A8,LRRK2,WNT7A,SNAP23,STXBP5L,GRIN3A,GSK3B,SYN1,MCTP2,SYT7,BLOC1S6,RIMS2,SLC29A1,CPLX2,NOS1,SLC1A1,ADORA2A,CADPS,BRAF,ADCY1,RPH3A
GO:0048640	negative regulation of developmental growth	0.0286008412721831115	SEMA3A,WWC1,SEMA3D,WWC3,SEMA5A,RTN4,RTN4R,NTN1,VGLL4,SEMA5B,TP73,MAG,DRAXIN,DCC,CTDP1,DIP2B,FSTL4,SEMA6D,SPAG9,SEMA4D,JARID2,WNT3,TRIM46,EPHA7,PTPRS,TLL2,PTEN,STC2,STK4,PPARA,MAP2,TNR,CDKL3,SLIT1,SEMA3C,BMP4
GO:0051301	cell division	0.029311636068705	CLASP2,TAS1R2,LATS2,TUBA1C,NOX5,EPB41,MAPRE2,HDGF,STAG2,HTR2B,TACC1,SENP5,TRIOBP,CDC6,PARD6G,ITGB3BP,KIF18B,CECR2,PDGFB,CCND3,FBXL7,CHFR,FMN2,ESRRB,CDK14,ECT2,CENPF,PDS5A,KLHL21,DCTN1,MYH9,MAD1L1,PARD3B,CSPP1,MAD2L2,TLE6,RCC2,ARL3,KIF2A,SMC3,MLLT3,SEPT7,CENPC,TGFB1,RAB11FIP4,OIP5,SVIL,NSUN2,IGF1R,BRIP1,FGF1,DCT,HAUS4,CHEK2,PPP1CB,CTDP1,SMC2,SPTBN1,HEPACAM2,CDCA4,CDK3,BORA,SETD2,VPS4A,EREG,CCNY,RAB11A,FGF2,NEDD9,CDC27,RAB11FIP3,ANAPC5,CKS1B,TSG101,PAFAH1B1,PRDM15,PADI6,TNKS,BLM,EML4,CLASP1,TOP1,CDK6,UVRAG,ZNF207,CDCA3,DIS3L2,HOXB4,SNX9,ACTR2,RECQL5,ROCK1,PKN2,ANK3,PARD3,SNX33,HMCN1,CUZD1,PDXP,HAUS3,MCMBP,CABLES1,CHMP3,RNF103-CHMP3,RASA1,MACC1,PIK3C3,SOX5,SEPT6,SPAG5,UNC119,CHMP5,PDGFC,NCOA3,WNT7A,MAP4,TTC28,EXT1,MARK4,RHOA,NDE1,CUL3,RBBP8,CDK13,CDC14A,SETDB2,CSNK1A1,RGS14,GPSM2,PAX6,SPECC1L,SPECC1L-ADORA2A,PPP1CC,STAG1,CCNG2,EPB41L2,PPP1CA,POU3F3,DCDC1,OPN1LW,NUMB,WWTR1,CIT,BRCA2,YBX1,SKA2,MICAL3,TIMELESS,LIG1
GO:0048167	regulation of synaptic plasticity	0.02949485383608509	ADCY8,GRID2,GRM5,SLC24A2,ERC1,DGKI,ERC2,ADORA1,NTRK2,RIMS1,SLC4A10,NF1,SHISA9,CNTN4,LZTS1,CAMK2B,JPH3,YTHDF1,EPHA4,APP,DRD1,SHANK2,RASGRF2,MCTP1,SORCS2,PRKCG,RELN,SLC8A2,STAU1,MAP1B,SORCS3,SYT12,EGR2,MAP1A,NETO1,SNCA,MAPK1,PTEN,SHANK3,RAPGEF2,GRIN1,SYNGAP1,GSK3B,AGT,SYT7,RIMS2,CPLX2,RGS14,EPHB2,SLC1A1,PRKCZ,ADORA2A,GRIN2B,BRAF,TNR,ADCY1,GRIK2,SHISA6
GO:0015698	inorganic anion transport	0.0297186259102023	ENPP1,CLCN1,GRM5,SLC26A6,SLC22A8,GABRA3,ATP8B1,SLC20A2,SLC4A10,SLC12A8,SLC5A6,ANKH,SLC4A5,SLC39A14,ANO6,ABCC2,SLC1A4,CLCA2,GABRR3,GABRB1,ABCC11,ANO4,XPR1,ANO3,SLC17A7,ANO2,SLC4A4,SLC6A1,VDAC1,GLRA2,PTAFR,CLIC4,KCNQ1,TG,NMUR2,ABCB1,CLIC5,GABRG3,ABCC1,GABRA6,LRRC8C,LRRC8D,CLCC1,SLC4A8,BEST3,ANO1,ANO8,GABRR2,GABRB3,CLDN4,SLC1A1,SLC26A2,GPR89A
GO:0051494	negative regulation of cytoskeleton organization	0.030022026307138412	CLASP2,FHOD3,TJP1,TRIOBP,HDAC6,MID1,ARHGAP6,GSN,RBM14,PRKCD,SLIT2,KANK1,TTBK2,KANK4,IQCJ-SCHIP1,RHPN2,SVIL,PARK2,ADD2,SPTBN1,SSH1,SHANK1,ARHGEF18,NAV3,ADD3,SPTBN4,VILL,CTNNA2,CLASP1,SPTBN5,SSH2,MAP1B,EPS8,LIMA1,SNCA,SHANK3,TBCD,RDX,FAM49B,HDGFRP3,SPEF1,TMEFF2,DLC1,PHLDB2,MAP2,CAMSAP3,CGNL1,TRIM37,MET,SPTAN1
GO:0045936	negative regulation of phosphate metabolic process	0.031688933773210165	ENPP1,PDE4D,LATS2,PRKAG2,MVP,NTRK3,CBL,DNAJA3,MGAT5,EPHA1,IKBKB,PMEPA1,PTPRO,PDGFB,PRKAR1A,PIP4K2A,SNX6,DMD,SLC8A1,NF1,PAQR3,BDKRB1,BDKRB2,PTPRT,IPO5,GNAQ,ZFYVE28,PRKCD,PPM1E,PIBF1,SLIT2,CORO1C,SMAD6,ZNF675,DUSP22,SMYD3,PIP5KL1,PARK2,PPARG,PRKAR1B,PRKAR2A,MYOCD,AJUBA,NF2,HDAC4,SFRP1,HEG1,IBTK,LRP5,PTPN1,SPAG9,PPM1F,SEMA4D,EIF4G1,DEPTOR,TSG101,TERF2IP,PTPN13,LDLRAD4,IQGAP1,XDH,ROCK1,HIPK3,PTPN2,PARD3,ATG14,STK38,SNCA,MLLT1,PTEN,SPRED2,BMP7,LRRK2,LILRB4,PKIB,PRKAR2B,EPM2A,GSK3B,DNAJC3,PTPRB,AGT,ADAR,GCKR,RGS14,EPHB2,PPARA,PAX6,PRKCZ,ADORA2A,RABGEF1,ANGPT1,LRRK1,CDK5RAP1,IMP

			ACT,CD300A,PPP2CA,WWTR1,PTPRJ,INPP5F,CD109,MLXIPL,PTPRC,SH3GL2,BMP4
GO:0021543	pallium development	0.031733640932039356	TACC2,PHACTR1,ROBO1,TACC1,DAB1,CDON,NTRK2,KIRREL3,TBR1,DMD,NF1,PLCB1,RTN4,CCDC141,NEUROD1,TRAPPC9,SLIT2,DISC1,CNTNAP2,CTNNB1,IGF1R,NF2,BTBD3,DRD1,SYNE2,EIF2B5,FBXO45,LAMB1,PAFAH1B1,RELN,ALK,ATP2B4,CDK6,CELF1,EPHA5,HDAC1,SRD5A2,FOXP2,PTPRS,PTEN,TMEM108,SMO,SUN1,RHOA,NDE1,GSK3B,DLX1,PAX6,POU3F3,SRGAP2,EZH1,TRA2B
GO:0034504	protein localization to nucleus	0.03206003435692837	CLDN18,LATS2,FAM53A,TNPO3,HDGF,LMNA,EFCAB7,CCT2,ECT2,RANBP17,KAT7,FYN,NF1,APOD,PIK3R2,SYNE1,IPO5,IPO11,JAK2,KPNA3,PRKCD,TGFB1,BANP,NUP93,BCL3,DCLK1,CIZ1,PPP3CA,TRIM8,NF2,SMAD3,KPNB1,YAP1,NVL,PRICKLE1,DRD1,PRKD1,TAF3,ATF2,TAF8,IPO9,TNPO1,SUFU,SIN3A,NXT2,TFRC,KPNA6,SNUPN,CTNNA1,BMP7,NUP88,LRRK2,LILRB4,MORC3,POM121C,SMO,SUN1,UBR5,EPM2A,GSK3B,AGT,ADAR,DNAJB6,GCKR,STK4,PPP1R10,KPNA4,OTUD7B,ANGPT1,OSBPL8,SKP1,RPL23,SP100,TRIM29,NUP214,SYK,WWTR1,CCT3,TRAF3IP2,BMP4
GO:0061061	muscle structure development	0.032111420380786765	DNMT1,HLX,SLC9A1,RYR1,FHOD3,HIVEP3,UTRN,LRP2,ANK2,MEGF10,CHD7,LMNA,ITGB1,AKAP13,XIRP2,LAMA2,ZNF609,LARGE,MYO18B,CDON,EP300,PDGFB,PRKAR1A,TCF7L2,BASP1,BDNF,SGCD,DMD,CENPF,SLC8A1,RBM4,HIRA,JPH2,ARNTL,NF1,PLCB1,PLEKHM3,TTN,NRG1,MYH9,HDAC5,SYNE1,NLN,BOC,MYPN,RBFOX1,NOX4,CACNA1H,QKI,SOX6,TGFB1,PDLIM4,KEL,NFATC3,LRRC10,TCF12,KCNH1,PRR14,ADAM12,FGF10,SMYD3,SVIL,CAPN3,RORA,PPP3CA,CTNNB1,SOD2,ANKRD17,XK,RYR2,MYOCD,CAPN2,CTDP1,OBSCN,MEF2B,HDAC4,ELN,SMAD3,NFATC1,PDGFRA,HEG1,PRICKLE1,RCAN1,FRS2,SFMBT1,STRA6,EREG,MYOF,MYH15,BMPR2,BMPR1A,SPAG9,EVC,ITGA11,RARB,ATF3,COL11A1,CNTFR,AKAP6,COL19A1,TEAD4,SRA1,NFATC2,PAX7,MEF2A,EGR2,EPHB1,SMYD1,CD53,HEY2,TLL2,CSRP1,PKP2,SGCZ,IGF1,ALPK3,ZBED6,SMO,MYLPF,RHOA,KRT8,AGT,ZMPSTE24,MAP3K5,LBX2,RPS6KB1,PGM5,NOS1,ACTN4,IL18,PPARA,ARID5B,FHL2,NEBL,MBNL3,NEB,YBX1,CAV2,MYEF2,SETD3,EHD2,TANC1,CREB1,EDNRA,BMP4
GO:0010038	response to metal ion	0.03273249932253092	NRXN1,ASPH,GSS,ADCY8,RYR1,ADCY7,RYR3,ITPKB,DLG2,ABCC8,TCIRG1,CPNE4,ALOX5AP,TFAP2A,KCNMA1,ECT2,DPEP1,TTN,STIM1,PPP2CB,SYT1,CLDN1,CACNA1H,SYT13,KCNH1,RASA4,RASA4B,CAPN3,PPP3CA,PARK2,SOD2,MTF1,CYBB,OTC,RYR2,CPNE6,CPOX,TPH2,ARL13B,SYT3,NEDD4,SLC6A1,APP,GLRA2,CHUK,CPNE9,SLC6A3,NLGN1,EIF2B5,CLIC4,RASAL1,ERCC1,IQGAP1,TFRC,MEF2A,SYT12,MNAT1,PLCG2,ANK3,SNCA,CACNG2,SYT9,CNGA3,HMGCS2,MAPK1,LRRK2,PKD2,BMP6,GPI,KCNC2,TNFRSF11B,SYT17,TF,EEF2K,PRKAA2,SYT7,NCF1,SLC1A1,MCOLN1,PPP1CA,BRAF,PPP2CA,ADCY1,ALG2,A3GALT2,HSF1,CAMK2D,NEDD4L,CREB1,SLC25A13,CPNE1
GO:0001501	skeletal system development	0.03370109896913515	TRPS1,CLDN18,RYR1,ASH1L,SPNS2,SP3,CHD7,ITGB6,KIAA1217,ABI1,AKAP13,RUNX1,PTPN11,ANKRD11,EP300,DYM,CSGALNACT1,PIP4K2A,TFAP2A,MEIS1,SATB2,CHI3L1,ANKH,TLL1,MDFI,CMKLR1,ROR2,SLC39A14,PRPSAP2,FOXN3,FBXW7,TBX15,WNT11,IFT80,SOX6,ACVR2A,RUNX2,TGFB1,PCSK5,MUSTN1,TNFSF11,CTNNB1,GLG1,MMP16,HDAC4,SFRP1,NFIB,SMAD3,HOXD3,HOXD4,PDGFRA,ALPL,LRP5,SLC9B2,SETD2,CHST11,MMP2,DSCAML1,ATF2,ADAMTS12,BMPR2,BMPR1A,EVC,FGF2,ADAMTS4,SEMA4D,RARB,CHRD,MATN3,COL11A1,SUFU,SCUBE2,PAFAH1B1,GLI2,COL19A1,RSPO2,TEAD4,WWOX,PLS3,EYA1,HOXB3,HOXB4,HOXB5,HOXB6,GHR,AXIN2,JAG2,SRD5A2,FBXW4,TTC9,BMPR1B,COL13A1,WNT7B,IGF1,BMP7,SOX5,PDGFC,WNT7A,SH3PXD2B,BMP6,EXT1,TNFRSF11B,RHOA,SULF1,MTHFD1L,FBN2,ZMPSTE24,PHEX,PBX1,DLX1,CD44,PEX7,PLEKHA1,ARID5B,FBN1,FAM20C,NIPBL,LRRK1,MBTD1,CSRNP1,ACAN,ANXA2,ADAMTS7,BMP1,PTPRC,EDNRA,BMP4
GO:0048013	ephrin receptor signaling pathway	0.03420821653599693	KALRN,NTRK3,ANKS1B,PTPN11,EPHA1,PAK1,FYN,ARHGEF28,EPHA4,MMP2,EPHB3,PAK3,EPHA5,NTRK1,EPHB1,EPHA10,EPHA7,RASA1,EFNA5,PTK2,ANKS1A,EPHB2
GO:0030517	negative regulation of axon extension	0.034355608623358735	SEMA3A,SEMA3D,SEMA5A,RTN4,RTN4R,NTN1,SEMA5B,MAG,DRAXIN,DIP2B,SEMA6D,SEMA4D,WNT3,TRIM46,PTPRS,MAP2,TNR,CDKL3,SLIT1,SEMA3C
GO:0000226	microtubule cytoskeleton organization	0.03499323240482612	HOOK2,TACC2,SPAG16,PDE4DIP,CLASP2,TUBA1C,IQCG,MAPRE2,FER,STAG2,EZR,TACC1,LMNA,ULK4,ITGB1,TTLL5,PARD6G,HDAC6,TBCEL,KIF18B,PEX14,DNAH2,DNAI2,HOOK3,MID1,FMN2,PAK1,FBXW11,LRRC49,STARD9,RBM14,DST,RANBP9,DCTN1,MYH9,WDR92,NAV1,RNF4,PARD3B,LRGUK,AUNIP,TLE6,ARL3,KIF2A,RANBP1,SMC3,CROCC,PIBF1,BCAS3,DISC1,CEP135,TTBK2,SPAG17,ANKFN1,FGF10,SLC16A1,CTNNB1,AXIN1,NIN,TUBGCP6,HAUS4,CHEK2,PHLDB1,HEPACAM2,C10ORF90,KPNB1,NAV3,RAB6C,SLK,BORA,SETD2,KIF24,SYNE2,USP33,RAB11A,CRMP1,ARMC2,GAS8,PAFAH1B1,KIF3A,CEP350,TTLL7,TNKS,PKHD1,DZIP1,EML4,CLASP1,TTLL9,DYX1C1,MAP1B,MARK1,UVRAG,ZNF207,ROCK1,MAP1A,NCKAP5,PARD3,TRIM46,TRDN,NTMT1,SNCA,HAUS3,CHMP3,HYDIN,HTT,TTLL4,SPAG5,CHMP5,PKD2,EFNA5,STMN4,PPP2R3C,TBCD,MAP4,SIK3,ATRX,SUN1,MARK4,RHOA,NDE1,GSK3B,HDGFRP3,PRKAA2,CUL9,CDC14A,CEP70,RGS14,TUBB3,MARK3,SPEF1,TTLL11,GPSM2,PAX6,PRKCZ,STAG1,PHLDB2,MAP2,CAPN6,CAMSAP3,SRGAP2,BRCA2,SKA2,POC1B,TRIM37,MET,RASSF8,CCDC170
GO:00	negative	0.03534106	ENPP1,PDE4D,LATS2,PRKAG2,MVP,NTRK3,CBL,DNAJA3,MGAT5,EPHA1,IKBKB,

10563	regulation of phosphorus metabolic process	584073046	PMEPA1,PTPRO,PDGFB,PRKAR1A,PIP4K2A,SNX6,DMD,SLC8A1,NF1,PAQR3,BDKRB1,BDKRB2,PTPRT,IPO5,GNAQ,ZFYVE28,PRKCD,PPM1E,PIBF1,SLIT2,CORO1C,SMAD6,ZNF675,DUSP22,SMYD3,PIP5KL1,PARK2,PPARG,PRKAR1B,PRKAR2A,MYOCD,AJUBA,NF2,HDAC4,SFRP1,HEG1,IBTK,LRP5,PTPN1,SPAG9,PPM1F,SEMA4D,EIF4G1,DEPTOR,TSG101,TERF2IP,PTPN13,LDLRAD4,IQGAP1,XDH,ROCK1,HIPK3,PTPN2,PARD3,ATG14,STK38,SNCA,MLLT1,PTEN,SPRED2,BMP7,LRRK2,LILRB4,PKIB,PRKAR2B,EPM2A,GSK3B,DNAJC3,PTPRB,AGT,ADAR,GCKR,RGS14,EPHB2,PPARA,PAX6,PRKCZ,ADORA2A,RABGEF1,ANGPT1,LRRK1,CDK5RAP1,IMPACT,CD300A,PPP2CA,WWTR1,PTPRJ,INPP5F,CD109,MLXIPL,PTPRC,SH3GL2,BMP4
GO:0051965	positive regulation of synapse assembly	0.03541159703842759	NRXN1,GRID2,NTRK2,PTPRD,BDNF,AMIGO1,CLSTN2,IL1RAPL1,LINGO2,DLG5,CUX2,NLGN2,NLGN1,SEMA4D,EPHB3,NLGN3,ADNP,FLRT2,NTRK1,EPHB1,EFNA5,WNT7A,EEF2K,EPHB2,SYNDIG1
GO:0008360	regulation of cell shape	0.03579220736829987	EPB41,EZR,CDC42EP3,MYO10,FMNL2,FYN,MYH9,SEPT7,PALMD,MKLN1,PARVA,FBLIM1,HCK,ARHGEF18,RND3,SEMA4D,PLXNA2,RHOJ,KIF3A,CFDP1,AKAP2,SYNE3,FGD1,EPS8,DNMBP,COCH,SHROOM3,RASA1,BRWD3,PARVB,RHOA,RHOBTB1,SH3KBP1,RDX,ZMPSTE24,PTK2,FAM171A1,TAOK2,ALDOA,ARHGAP15,FGD3,FGD4,DLC1,DIAPH1,PHIP,GNA12,FMNL1
GO:0007204	positive regulation of cytosolic calcium ion concentration	0.03618245818550352	ASPH,PDE4D,FAM155A,ADCY8,PTGFR,CHERP,RYR1,PLCE1,ANK2,CHD7,HTR2B,RYR3,SLC24A2,CACNB2,ADRA1D,ESR1,LCK,DMD,SLC8A1,JPH2,FYN,BDKRB1,BDKRB2,TMBIM6,CMKLR1,PTGER2,JAK2,NPSR1,TGFB1,JPH3,F2RL1,C9ORF47,CAPN3,CNR1,P2RY10,TRPM1,RYR2,NOL3,CACNA1C,GRM1,ITPR1,ACKR2,PDGFRA,IBTK,DRD1,PRKD1,FGF2,ADCY5,ADCYAP1R1,TRPA1,NMUR2,AKAP6,TRPC5,SLC8A2,TPCN1,ATP2B4,P2RY8,CACNA1A,PTGER3,TRPV1,PLCG2,C1QTNF1,GPR35,TRDN,TRPC6,SNCA,HTT,PKD2,GRIN1,ITPR2,AGT,HRH4,NOS1,XCR1,MCOLN1,GRIN2B,DIAPH1,HTR2C,CIB2,CAMK2D,CCR3,PTPRC,EDNRA
GO:0048511	rhythmic process	0.03651025536168444	ENOX1,STAT5B,MAPK10,ROBO2,NRIP1,DDB1,ADORA1,ESR1,NTRK2,EP300,FBXW11,RBM4,ARNTL,GNAQ,FBXW7,MTA1,SPSB4,BTRC,SLIT3,SLIT2,GABRB1,GPR176,CLOCK,ENOX2,ANKFN1,FBXL17,KCNH7,RORA,IGF1R,PPARG,CIPC,NDUFA9,PER2,PPP1CB,FOXO3,TPH2,PDGFRA,MMP2,TPH1,HNF4A,EREG,NLGN1,RORC,PRKCG,KCNQ1,BTBD9,SIN3A,SP1,TOP1,ADNP,HDAC1,EGR2,NTRK1,PSPC1,BMPR1B,CREM,PTEN,ARNTL2,MYCBP2,GRIN3A,GSK3B,PRKAA2,CREBBP,CLDN4,PLEKHA1,PPARA,PHLPP1,ADORA2A,PPP1CC,PPP1CA,NCOR2,HDAC2,KCND2,ADCY1,NCOA2,ZP3,AXL,TIMELESS,TYRO3,CREB1
GO:0010468	regulation of gene expression	0.036827885213148344	POLDIP3,ENPP1,PRDX2,LDB2,NRXN1,ASPH,PRKCI,MOV10L1,SLCO3A1,PDE4D,DNMT1,HLX,SLC9A1,PBX3,ADCY8,MED13L,TRPS1,CBFB,PDE8A,ZNF823,IL31RA,RPS6KA2,PRDM12,SETD4,PTGFR,MED26,WWC1,ASH1L,NOS1AP,NOX5,SCAF8,STOX2,PTGIS,WWC3,PAGR1,ADCY7,HIVEP3,FTO,NPAS3,UBAP2,NTRK3,ARNT,LRRFIP2,STAT5B,TOX,EXD1,GRM5,ZNF566,FER,CASK,MAP2K5,MAPK10,PIK3CD,ZNF536,SP3,HDGF,ANK2,EIF4G3,OLFM1,EZR,IKZF2,ROBO1,CHD7,HTR2B,ITGB6,MECOM,TACC1,MIR1185-1,TENM1,LMNA,TRAF6,ESRP1,DNAJA3,NHLH1,HSP90AA1,SLC24A3,PSMB7,TSC22D3,ZC4H2,CCDC22,CNGB1,GFI1B,PTPRK,RUNX1,ABCC8,ERC1,HMGN3,NRIP1,THRB,MIR105-2,MIR767,EFCAB7,ITGB3BP,TCIRG1,DACH1,PTPN11,ZNF569,ORC2,HDAC6,SERTAD2,MYT1,IKBKB,PEX14,ERBB4,ZNF609,BRD8,KAT6B,ACTG2,HIF3A,SNIP1,ESR1,MIER1,PCBP3,MAML3,CDON,NTRK2,TNRC6A,EP300,CELA1,TENM2,ZNF76,RNF220,ZNF471,PDGFB,RIMS1,CCND3,TOB2,ZNF19,ZNF23,BRMS1,ZNF605,SCML4,BPI,HNF4G,INSR,RERE,PRKAR1A,FUBP1,ATP8B1,H2AFY2,TCF7L2,JMJD1C,ZNF443,ZNF490,ZNF564,ZNF709,ZNF799,LITAF,FBXW11,ESRRB,MYOM1,MAP3K4,BASP1,RBM20,TBR1,SAMD4A,TFAP2A,CDK14,FANCA,PEG3,ZIM2,TGIF2,TGIF2-C20ORF24,FUT8,MEIS1,VRTN,TRIM13,MDM4,IL5RA,SHC4,ZNF148,MTA3,SNX6,TFDP2,PTCD3,RFTN1,ERN2,C1D,NPLOC4,AFF3,DMD,CENPF,SLC30A9,TOX3,LARP4B,USP13,KAT7,ZNF667,SLC8A1,GSN,KHDRBS2,RBM14,RBM4,MED12L,SATB2,RIT2,HIRA,FYN,MKRN2,ARNTL,NF1,SMG7,PLCB1,ARID4B,RTN4,AFF2,APOD,RWDD3,BRPF1,CHI3L1,TTN,PIK3R2,SLC4A5,NRG1,UBP1,MIR600HG,MAP2K1,MDFI,FNIP1,VAX2,MYH9,TMBIM6,VGLL4,PPP2CB,PPP3R1,HDAC5,CSRNP3,RBM5,ZNF692,NFIA,RNF4,JDP2,CMKLR1,TSPAN8,ROR2,DCN,PCBD2,HNRNPLL,CDH13,CREBRF,TRDMT1,SOX13,COPS5,MAD2L2,TLE6,RAB27A,CAPRIN2,ZNF418,PHF20L1,TERF2,ZNF286A,FOXN3,NEUROD1,CCBE1,USP22,JAK2,TRAPPC9,FBXW7,SKAP1,UIMC1,ITCH,MLIP,ABCC2,BCL11B,SMG1,RBFOX1,PKNOX1,MLLT3,TSHZ2,TCF7,PDE2A,KLF15,TBX15,ANXA4,WNT11,MTA1,KLF8,LCOR,RPRD1B,QKI,CCDC62,PRKCD,SOX6,ACVR2A,RUNX2,CD4,TGFB1,BANP,SGK1,NSD1,IGSF1,PIBF1,ZHX2,PKNOX2,ASCC2,BTRC,NFATC3,MIR153-2,F2RL1,BCAS3,C9ORF47,CDYL2,SLIT3,CC2D1B,MIR218-1,TP73,SAP18,ZBTB22,ILF2,MTDH,FANK1,MYT1L,SMAD6,BNC2,ZNF398,CLOCK,TCF12,ZNF675,ETV6,NELL1,TFAP2D,BCL3,SND1,DUSP22,HNRNPC,TRRAP,HOMER2,SBNO2,YTHDF1,FGF10,SMYD3,LOXL3,MAST2,CAPN3,LUM,SMURF2,RORA,HIVEP2,PRKCA,AUTS2,CD6,TNFSF11,SMG6,PPP3CA,NSUN2,NFYB,MAGEA4,KLF12,CAMK4,GATAD2B,UFL1,TRAK1,CTNNB1,PARK2,SOD2,DACH2,METTL13,FCGR2B,SMARCC1,KLF17,PPARG,NGRN,AXIN1,IL18R1,IL1RL1,CIPC,MTF1,MSR1,CELF2,CBX5,CYBB,SCAMP5,BRIP1,LRPPRC,SREBF2,CDK11A,CDK11B,FGF1,NPAT,

			NR4A3,FOXK2,NOL3,RIOK2,MYOCD,TRIM5,PER2,KIR2DL4,AJUBA,GLG1,ZNF626,ZNF737,CHEK2,SUPT3H,PRDM16,PPP1CB,HCK,CAPN2,TRIM8,DIO2,BRMS1L,ZBTB8A,ZBTB8B,CTDP1,ZUFSP,BAZ1B,FLT4,MEF2B,HDAC4,PAX2,PHF5A,SECISBP2L,SPTBN1,SFRP1,MED13,ZNF395,FOXO3,NFIB,SP4,ZCCHC17,CELF5,SYNCRIP,SMAD3,CUX2,WWP2,ARNT2,SBNO1,KRBOX1,ZNF662,ZNF777,EBF3,RNF168,CASZ1,DCP1B,MIER3,NEDD4,ESRRG,HOXD3,HOXD4,NAV3,ZNF114,KTI12,NFATC1,CDC73,APP,SSBP3,GSX2,PDGFRA,RBM8A,NOX1,YAP1,HEG1,LRP5,POLR3G,ZNF787,SOX2,SETD2,TEAD1,PRICKLE1,ZNF653,ZNF521,DRD1,ARID3A,ZNF761,CHUK,SFMBT1,ZNF584,ESR2,S100A12,KDM6A,PRKD1,STAT1,CELF6,ST18,ETV5,RHOXF2B,TAF3,PLAGL1,HNF4A,ZBTB7C,TASP1,EREG,RBFOX3,ATF2,POU2F2,TCF3,ZNF730,RAF1,CELF4,ZNF766,CARD16,CASP1,SRRM4,POLA1,BMPR2,CAMK1D,BMPR1A,ZKSCAN1,PABPC4,IKZF4,PIK3R3,CDK12,CAND2,MORC2,DENND4A,MYB,FGF2,ZNF71,POU6F2,BACH1,MXD3,PPM1F,GLI4,ZFP41,BEND5,SEMA4D,NFX1,RORC,ELP3,PTAFR,SP140,SP140L,RHOXF2,JARID2,DDX58,BRDT,PHC2,RARB,SPEN,SIN3B,NCOA1,EHMT1,LMO7,DVL3,EIF2B5,EIF4G1,TCF20,ATF3,LIN28B,SRSF5,LINC00461,CKS1B,PAWR,EBF2,AGO3,MAML2,TSG101,CCDC3,TERF2IP,CRYM,KCNQ1,RFX2,WNT3,ZNF322,SUFU,MAGEA11,PTPN13,PRDM15,ZNF670,ZNF695,CCDC169-SOHLH2,SOHLH2,ZNF354C,TCEA3,PADI6,ZNF704,NR2C1,GLI2,TNKS,WBP2NL,ERCC1,TNRC6B,GLIS3,WDTC1,ZNF664,DAB2,BLM,PKHD1,MYSM1,SETD5,SMG5,RELN,SIN3A,RUVBL2,COMMD6,GMEB1,PELI1,IQGAP1,MAP3K7,ZNF423,SP1,TRIM22,ALK,SLC8A2,INPP5D,TEAD4,FASTKD5,HOXC13,APBB3,SLC35A4,SRA1,TOP1,UBE2V1,ADIRF,XDH,AHNAK,LTBP1,OVOL2,SNX5,NFATC2,PAX7,BNC1,ATP2B4,ACTL6B,ASXL3,TET1,CAMTA1,CCPG1,ADNP,MAPKAPK2,ARID4A,MARK1,CDK6,PHF2,CELF1,TFRC,EPHA5,WWOX,MEF2A,MIR1226,DCAF6,DROSHA,XRN1,SATB1,PSMB2,EYA1,GATAD2A,DIS3L2,GNB3,TRPV1,RBM12B,RNF216,HOXB3,HOXB4,HOXB5,HOXB6,MIR10A,TFEB,MNAT1,ACTR2,L3MBTL4,ELAVL4,HDAC1,RECQL5,TRIM24,PLCG2,ROCK1,SCMH1,C1QTNF1,PAXIP1,ZNF713,EGR2,RNF10,RYBP,AP3B1,PTPN2,NTRK1,KPNA6,TRIM44,ANK3,AXIN2,EPCAM,ZNF425,MUC1,PTPN14,BCOR,AP3D1,ZNF41,CARM1,ZNF383,ADAM19,CD84,ZNF616,ZNF836,SMARCA2,CUX1,MIR1302-10,SPI1,DNMT3B,FOXP2,PSPC1,GLIS1,SFMBT2,ATG14,EIF3H,JAZF1,RBPMS,CHURC1,CREB5,MAP3K13,MST1,SMYD1,CHID1,SNCA,BMPR1B,PNPT1,USP50,ZEB2,FOXJ3,HEY2,RC3H1,EIF3E,PTPRS,ZMYND11,KMT2D,PRKAG1,CREM,KMT2C,CAST,ADTRP,PLD1,MACC1,MITF,SRSF6,OPRD1,RPTOR,NFIX,BTK,MIR383,MLLT1,TROVE2,CBFA2T2,IGF1,MLXIP,ATF7IP,MAPK1,PTEN,BMP7,ATXN2,MXI1,PUM1,SOX5,CIR1,PCBP2,ZNF780A,ZNF780B,LRRK2,TMEM59,ZBTB20,LILRB4,PKD2,ZNF652,KCTD1,RIPPLY1,RCOR3,TADA2A,DAZL,NCOA3,ZNF146,ZNF565,CUEDC2,WNT7A,ZBED6,BRWD3,NLRP1,CRTC3,DNAJC1,ARNTL2,KAT6A,MTIF2,ZKSCAN7,ZNF197,ZNF660,BMP6,TAF15,MYCBP2,NFE2L1,GPI,WDR43,ZNF30,N4BP1,PDCD1LG2,ATRX,DPH6,IKZF1,PRMT2,ASCC1,SMO,RALY,TCF4,AFF1,SOX30,TFE3,UBR5,GRIN1,JADE1,RHOA,ROR1,RQCD1,TF,ONECUT2,CUL3,TFEC,HSPD1,NFKBID,EPM2A,GSK3B,PRR16,DNAJC3,RDX,STAT6,ACTA2,MLF1,FAM49B,RBBP8,CDK13,UPF2,DUSP26,SULF1,ZNF362,NACC2,SUPT4H1,TNFRSF8,ZNF354A,AGT,HDGFRP3,METTL16,PRKAA2,ADAR,STAT2,ZMPSTE24,NDRG2,UPK3B,PDE4B,PBX1,EXOSC3,MAP3K5,SEC16B,TCFL5,CREBBP,DLX1,GTF2IRD2,NCF1,SHC1,ZNF813,CD160,LBX2,PARP10,RPS6KB1,STC2,DPRX,PRG3,SETDB2,ALOX5,DNAJB6,SP7,LARP4,ADAM8,RPS6KA5,ZNF484,ZNF93,MIR346,PRDM2,CXCL17,NOS1,ZNF44,MED15,RGS14,ACTN4,MC1R,TCF25,ZNF282,EPHB2,EDRF1,IL18,DIS3,PPARA,ARID5B,EPB41L4B,SMARCAL1,CPEB4,GPSM3,NOTCH4,ZMYND8,ZNF366,CRX,PAX6,PRKCZ,ZC3HAV1,CD96,ADORA2A,PPP1CC,RABGEF1,CPEB1,OTUD7B,NIPBL,TMIGD2,YEATS4,PHF20,ZNF143,ANGPT1,BRF1,TBL1X,FHL2,MBTD1,ATF6,ZBTB5,ZNF708,IGF2BP3,GCNT2,ETS1,PPP1CA,MTF2,RBMS3,BTN3A2,TAGLN3,CDK5RAP1,CNTN1,NCOR2,PRKCQ,BRAF,CSRNP1,HDAC2,IMPACT,MIR1912,SRCAP,POU3F3,NOTO,MBNL3,ZKSCAN5,ELF2,PHIP,PPP2CA,RPL23,ATXN1,SP100,ZNF347,ZNF415,TRIM29,PARN,PRMT7,ADCY1,RBM42,SYK,CNOT1,RBM39,WWTR1,GTF2H5,ASB1,NFXL1,ZBTB38,BRCA2,ATF7,DVL2,MORC1,MTRF1,YBX1,PTPRJ,SLC10A1,TAF1,EIF4E3,TICAM1,THAP3,CADM1,HSF1,MAX,SAP130,DHFR,EZH1,ETF1,MIR663B,NRF1,SRPK2,NCOA2,GBP5,EEFSEC,QRICH1,ZP3,CHD6,RNF2,ZNF554,MYEF2,AXL,TRIM37,MET,ZNF461,CAMK2D,TIMELESS,BRD9,MALT1,SETD3,TRA2B,KDM2B,MLXIPL,PHB,PTPRC,EDA,ZNF555,CTCF,SH3GL2,NR6A1,SPON2,TRAF3IP2,TRIM60,ATF6B,CREB1,RGMB,TRAP1,AFF4,CLNK,EBF4,ZNF511,BMP4,ABLIM3,CPNE1
GO:0048729	tissue morphogenesis	0.03801793211118072	*ADAMTS16,CLASP2,SEMA3A,ASTN2,LRP2,PIK3CD,ROBO1,CHD7,TRAF6,ROBO2,ITGB1,PSMB7,KIF26B,ARHGAP24,XIRP2,ERBB4,ACTG2,CECR2,ESR1,NPHP3,PRKAR1A,LAMA3,PAK1,TFAP2A,PHACTR4,MDM4,PCDH15,TTN,NDRG4,NTN1,NRG1,ROR2,EXOC4,MLLT3,WNT11,TGFB1,BTRC,RPGRIP1L,CELSR1,SLIT2,FGF10,GREB1L,SMURF2,EPHA4,CTNNB1,AXIN1,DLG5,RYR2,FGF1,AJUBA,CSMD1,NF2,PAX2,SFRP1,ARL13B,IFT122,SMAD3,NRP2,VANGL1,YAP1,HEG1,EYA2,LRP5,SETD2,GPC6,PRICKLE1,FRS2,MMP2,KDM6A,STAT1,ADAMTS12,BMPR2,BMPR1A,FGF2,KLHL3,DVL3,VCL,CLIC4,MYO9A,COL11A1,WNT3,SUFU,PAFAH1B1,GLI2,DAB2,PKHD1,DLG3,RSPO2,LUZP1,ARHGAP12,CLASP1,OVOL2,GPC3,PSMB2,EYA1,HOXB4,JAG2,EPHA7,SHROOM3,SH3BP1,MST1,PTK7,MAGI2,HEY2,WNT7B,PKP2,ZNRF3,PTEN,MIB1,BMP7,PKD2,SHANK3,WNT7A,GPI,SOS1,EXT1,PRICKLE2,SMO,MY*

			LPF,RHOA,ROR1,ACTA2,SULF1,MTHFD1L,AGT,PBX1,LBX2,SETDB2,CD44,STK4,SPEF1,WLS,TMEFF2,DLC1,NOTCH4,PHLDB2,PPP1CA,NOTO,SPINT2,CAMSAP3,DVL2,MET,TIMELESS,ANKRD6,KDM2B,SEMA3C,VPS52,EDNRA,BMP4
GO:0050730	regulation of peptidyl-tyrosine phosphorylation	0.03844378220605475	IL31RA,MVP,CBL,FLT3,ENPP2,GRM5,KITLG,ABI1,PTPN11,ADORA1,ERBB4,PDGFB,SNX6,CASS4,FYN,NRG1,JAK2,FBXW7,ZFYVE28,NOX4,PRKCD,CD4,PIBF1,DUSP22,DOCK3,FGF10,EPHA4,CSPG4,NF2,SFRP1,APP,IBTK,EREG,PTPN1,NEDD9,SEMA4D,TSG101,DLG3,RELN,ADNP,GHR,PLCG2,PTPN2,EPHA7,IGF1,UNC119,LILRB4,EFNA5,PDGFC,BMP6,AGT,TEC,NCF1,SHC1,CD44,IL18,PRKCZ,ANGPT1,LRRK1,CNTN1,HDAC2,CD300A,PPP2CA,ANGPT4,SYK,DVL2,PTPRJ,HSF1,INPP5F,PTPRC
GO:0048016	inositol phosphate-mediated signaling	0.03958915128176423	SLC9A1,CHERP,PLCE1,NRG1,PPP3R1,NFATC3,HOMER2,PPP3CA,ITPR1,NFATC1,RCAN1,NMUR2,AKAP6,NFATC2,ATP2B4,CAMTA1,PLCG2,IGF1,ITPR2,GSK3B,FHL2,TBC1D10C,INPP5A
GO:0016567	protein ubiquitination	0.040669570397405	LNX2,PRMT3,CBFB,FBXL2,RNF145,DCAF12,CBL,TRAF6,DNAJA3,HSP90AA1,PSMB7,ZC4H2,KLHL12,KLHL7,HERC4,HDAC6,DDB1,LIMK1,CUL4B,FBXL18,RNF220,FBXL7,CHFR,ASB5,FBXW11,RNF144A,TRIM13,UBR2,FYN,MKRN2,FBXO21,NOSIP,KLHL21,RNF4,UBE3D,WDSUB1,WDR70,MAD2L2,FBXO9,RFFL,HERC5,USP22,FBXW7,UBR7,ITCH,SPSB1,TRIM59,MTA1,SIAH1,ERCC8,SPSB4,BTRC,PDZRN3,DNAJB2,KLHL2,ASB15,RNF133,RNF148,FBXL17,UNKL,KCTD10,SH3RF3,FANCI,SMURF2,UFL1,CTNNB1,PARK2,AXIN1,OTUB1,RSPRY1,TRIM5,PER2,ASB8,TRIM8,RNFT2,WWP2,MTBP,RNF168,C10ORF90,NEDD4,CDC73,ARIH1,AMFR,PRICKLE1,DCUN1D3,SHPRH,CAND2,UBE2R2,NFX1,CDC27,KLHL3,PRKCG,AREL1,LMO7,UCHL3,RBX1,ANAPC5,FBXO45,FBXL20,TSG101,TNKS,WDTC1,UBR1,PELI1,DCAF5,TRIM22,ZNRF1,UBOX5,UBE2V1,RBBP6,HECTD4,MKRN3,UBE3C,RNF34,DCAF6,DCUN1D5,PSMB2,CDCA3,RNF216,TRIM24,PAXIP1,RNF10,RYBP,CUL2,TRIM44,GNL3L,BCOR,FBXW4,RC3H1,RNF103,RNF103-CHMP3,RNF19B,UBE2QL1,TRIP12,ZNRF3,KCTD13,PTEN,MIB1,LRRK2,SEL1L2,MYCBP2,SH3RF2,N4BP1,UBE2H,UBR5,ARRDC4,SPRTN,WSB2,CUL3,EPM2A,RNF144B,RNF43,CUL9,RNF121,DCAF10,FBXO10,TRPC4AP,PARP10,RNFT1,FBXO28,MARCH8,UBE2K,FBXL13,RNF150,ANGPT1,FBXO31,ATG3,UBE2E2,C18ORF25,SKP1,RPL23,WWTR1,ASB1,TAF1,TICAM1,RNF38,SASH1,RNF2,AKTIP,TRIM37,MALT1,TRIM69,KDM2B,NEDD4L,RNF213,TRAF3IP2,TRIM60
GO:0051651	maintenance of location in cell	0.04109168099044619	ASPH,PDE4D,CHERP,RYR1,ANK2,CHD7,HTR2B,RYR3,LCK,DMD,SLC8A1,GSN,RIT2,JPH2,BDKRB1,SYNE1,NPSR1,CD4,TGFB1,JPH3,CIZ1,CAPN3,RYR2,NOL3,CACNA1C,ITPR1,GRIK5,IBTK,DRD1,PRKD1,TAF3,TAF8,FGF2,PAFAH1B1,TRPA1,AKAP6,TPCN1,UVRAG,TRPV1,PLCG2,ANK3,AP3D1,TRDN,SNCA,HTT,SLC30A7,MORC3,PKD2,SUN1,ITPR2,NOS1,XCR1,GPSM2,MCOLN1,ARHGAP21,DIAPH1,HTR2C,SKP1,SP100,ANKRD13C,CAMK2D,PTPRC
GO:0036465	synaptic vesicle recycling	0.04130360682885987	NLGN4X,SNCB,DENND1A,SYT1,ITSN2,SYNJ2,SLC17A7,DNM3,NLGN2,NLGN1,ABCA13,AP2M1,NLGN3,BTBD9,STON2,PACSIN1,ROCK1,AP3B1,AP3D1,SNCA,LRRK2,WNT7A,DNAJC6,PLD2,SYT7,RAB27B,ITSN1
GO:0040017	positive regulation of locomotion	0.04171583879260053	MAP4K4,CLASP2,SEMA3A,DOCK1,SEMA3D,MAPRE2,TJP1,NTRK3,SEMA5A,ENPP2,FER,KITLG,PIK3CD,ITGB1,DSCAM,MGAT5,HDAC6,EPHA1,ZNF609,PDGFB,STK39,INSR,PAK1,SLC8A1,CASS4,RTN4,NTN1,BDKRB1,DOCK8,CMKLR1,ROR2,CDH13,CD99,ANO6,CCBE1,JAK2,CLDN1,WNT11,SEMA5B,TGFB1,F2RL1,BCAS3,SLIT2,RRAS2,LAMC2,DAPK2,FGF10,SMURF2,EPHA4,PRKCA,PPP3CA,SOD2,IGF1R,FGF1,NR4A3,FLT4,HDAC4,SMAD3,NRP2,DOCK4,APP,PDGFRA,DRD1,MMP2,SYNE2,PRKD1,SEMA6D,BMPR2,CAMK1D,PIK3R3,SPAG9,RAB11A,FGF2,PPM1F,NEDD9,SEMA4D,ELP3,PTAFR,LAMB1,RHOJ,RUFY3,DAB2,WNK1,RELN,SP1,CLASP1,PAK3,SSH2,SCARB1,SMOC2,PLCG2,SPI1,MCU,IGF1,MAPK1,BMP7,PDGFC,WNT7A,GPI,SH3RF2,RAPGEF2,SMO,RHOA,TF,ONECUT2,RDX,ACTA2,AGT,PTK2,RPS6KB1,ADAM8,CLDN4,CXCL17,PLVAP,ACTN4,STK4,EPHB2,EPB41L4B,GPSM3,NIPBL,ANGPT1,GCNT2,ETS1,FBXO31,DIAPH1,ATP8A1,ANGPT4,NUMB,SASH1,ZP3,MET,SEMA3C,PTPRC,BMP4
GO:0048017	inositol lipid-mediated signaling	0.04211997218597487	FBXL2,NTRK3,CBL,FLT3,PLCE1,PIK3CD,EZR,HTR2B,ERBB4,NTRK2,PDGFB,INSR,ENTPD5,NF1,PLCB1,PIK3R2,ROR2,DCN,JAK2,F2RL1,PLCB4,KCNH1,IGF1R,TPTE,NEDD4,PDGFRA,FGF2,SEMA4D,PTAFR,PI4KA,PIK3C2B,PTPN13,RELN,NTRK1,PLD1,IGF1,PTEN,PIK3C3,PDGFC,PLCL2,ROR1,PLD2,AGT,PITPNM2,PTK2,NCF1,CD160,RPS6KB1,PLEKHA1,IL18,ANGPT1,INPP5F,TYRO3
GO:0033555	multicellular organismal response to stress	0.043505997523658635	USP46,VWA1,ANKFN1,PPP3CA,CAPN2,DRD1,ADCYAP1R1,PRKCG,EIF4G1,FBXL20,TRPA1,NMUR2,RELN,BRINP1,TRPV1,NTRK1,HMGCS2,PTEN,SHANK3,EXT1,SCN9A,RPS6KB1,NOS1,EPHB2,SLC1A1,HTR2C,GRIK2,MORC1
GO:0043406	positive regulation of MAP kinase activity	0.043600225067030395	NTRK3,FLT3,MAP2K5,KITLG,ROBO1,HTR2B,TENM1,TRAF6,AKAP13,PDGFB,INSR,PAK1,MAP3K4,ERN2,ROR2,NOX4,EPHA4,TNFSF11,AXIN1,FGF1,AJUBA,TAB1,S100A12,PTPN1,FGF2,DVL3,NEK10,MAP3K7,GHR,MAP3K13,LRRK2,PDGFC,MAP3K5,ADAM8,SYK,DVL2,SASH1,PTPRC
GO:0060537	muscle tissue development	0.04376526850198183	HLX,SLC9A1,RYR1,FHOD3,HIVEP3,NDUFV2,LRP2,MEGF10,CHD7,LMNA,ITGB1,AKAP13,RUNX1,XIRP2,ERBB4,MYO18B,CDON,EP300,PRKAR1A,NPRL3,TENM4,S

			GCD,DMD,CENPF,SLC8A1,JPH2,ARNTL,ADAMTS9,NF1,TTN,NDRG4,NRG1,VGLL4,NLN,RBFOX1,NOX4,SOX6,TGFB1,KEL,TP73,LRRC10,SVIL,PPP3CA,CTNNB1,XK,RYR2,MYOCD,CTDP1,HDAC4,ELN,SMAD3,PDGFRA,YAP1,HEG1,EYA2,RCAN1,FRS2,STRA6,MYH15,BMPR1A,FGF2,JARID2,RARB,ATF3,COL11A1,AKAP6,COL19A1,MEF2A,EYA1,EGR2,EPHB1,SMYD1,HEY2,TLL2,CSRP1,PKP2,SGCZ,IGF1,PTEN,BMP7,PKD2,ALPK3,SMO,MYLPF,RHOA,AGT,ZMPSTE24,RPS6KB1,PGM5,PPARA,FHL2,NEBL,NEB,CAV2,SEMA3C,CREB1,EDNRA,BMP4
GO:0001525	angiogenesis	0.04415406183968074	*NRXN1,NOX5,PTGIS,TJP1,SEMA5A,ENPP2,MAP2K5,PIK3CD,NRXN3,ROBO1,VAV2,ITGB1,ARHGAP24,RUNX1,ABCC8,EPHA1,HIF3A,CELA1,MEIS1,ADAMTS9,NF1,RTN4,APOD,CHI3L1,UBP1,STIM1,MYH9,HDAC5,DCN,CDH13,PLXDC1,CCBE1,FBXW7,PKNOX1,BCAS3,SLIT2,MTDH,BMPER,PARVA,ADAM12,FGF10,RORA,PRKCA,CTNNB1,ARHGAP22,PPARG,CYBB,LEPR,FGF1,CSPG4,FLT4,SFRP1,PTPRM,NRP2,PDGFRA,NOX1,JAK1,ANGPTL4,SETD2,VASH2,MMP2,PRKD1,STAT1,EREG,ATF2,TSPAN12,PIK3R3,FGF2,ISM1,EPHB3,CLIC4,RHOJ,COL4A3,DDAH1,APOLD1,SP1,OVOL2,ATP2B4,TMEM2,CALD1,HOXB3,SMOC2,ADIPOR2,ROCK1,EPHB1,RAPGEF3,ADTRP,HIPK1,PTEN,PRCP,AMOTL1,WNT7A,RHOA,PTPRB,SULF1,AGT,PTK2,SHB,SHC1,CD160,ALOX5,ADAM8,THSD7A,CXCL17,STK4,EPHB2,IL18,CALCRL,NOTCH4,COL22A1,TMIGD2,ANGPT1,ETS1,ANGPT4,SP100,ANXA2,SYK,SRPK2,SASH1,VAV3,CCR3,RNF213,EDNRA,BMP4*
GO:0046058	cAMP metabolic process	0.0448792475496112	*PDE4D,PDE7B,ADCY8,PDE8A,ADCY7,PDE4A,LINC00473,ADCY2,ADCY5,ADCY9,PDE4B,ADCY1,ADCY10*
GO:0060828	regulation of canonical Wnt signaling pathway	0.045123654077305245	*GPC5,LATS2,SEMA5A,PSMB7,MCC,NPHP3,PTPRO,RNF220,TCF7L2,USP34,CDK14,PTPRU,ARNTL,ROR2,SOX13,MAD2L2,TLE6,CAPRIN2,CCNYL1,GNAQ,MLLT3,WNT11,KREMEN1,BTRC,CTNND2,KANK1,FGF10,SMURF2,PARK2,AXIN1,DRAXIN,BICC1,SFRP1,FOXO3,SMAD3,YAP1,AMFR,SOX2,PRICKLE1,CCNY,FGF2,DKK2,RBX1,PRDM15,TNKS,DAB2,WNK1,RUVBL2,RSPO2,GPC3,PSMB2,HDAC1,INVS,AXIN2,PTK7,ZEB2,ZNRF3,LRRK2,UBR5,JADE1,GSK3B,SCEL,CSNK1A1,STK4,WLS,TBL1X,LRRK1,PPP1CA,RBMS3,WWTR1,ANKRD6,EDA,SHISA6*
GO:2000311	regulation of AMPA receptor activity	0.04513983810276199	*NRXN1,CNIH2,SHISA9,SHANK1,NLGN2,NLGN1,NLGN3,RELN,CACNG8,CACNG2,CNIH3,SHANK3,CACNG3,SHISA6*
GO:0022898	regulation of transmembrane transporter activity	0.0467114562143887	*NRXN1,ASPH,PDE4D,SLC9A1,NOS1AP,UTRN,GRM5,ANK2,ITGB1,CNIH2,ABCC8,NETO2,CACNB2,STK39,CAB39,AMIGO1,CHRM3,KCNRG,DMD,JPH2,SHISA9,STIM1,FGF14,JPH3,CACNA1D,VMP1,RYR2,SCN3B,WWP2,SHANK1,NEDD4,APP,NLGN2,FXYD2,FXYD6,RASGRF2,NLGN1,PTAFR,KCNQ1,NLGN3,AKAP6,WNK1,RELN,ABCB1,AHNAK,STOM,PLCG2,GPR35,ANK3,CACNG8,NETO1,TRDN,STAC,TRPC6,SNCA,CACNG2,HTT,CNIH3,PKD2,SHANK3,KCNC2,PDE4B,NOS1,ACTN4,EPHB2,LRRC52,CACNG3,CAMK2D,NEDD4L,SHISA6,EDNRA*
GO:0045773	positive regulation of axon extension	0.04850934814871792	*SEMA5A,CDKL5,DSCAM,GOLGA4,LIMK1,CDH4,NTN1,DISC1,ISLR2,BMPR2,RAB11A,PAFAH1B1,TRPC5,RUFY3,MACF1,ADNP,MAP1B,MAP3K13*
GO:0021885	forebrain cell migration	0.0488074020037026	*NRG3,ROBO1,DAB1,RTN4,CCDC141,NRG1,SLIT2,DISC1,CTNNB1,ARL13B,DRD1,SYNE2,FBXO45,LAMB1,PAFAH1B1,RELN,LRRK2,SUN1,RHOA,POU3F3,TNR,SRGAP2,SLIT1,AXL,TYRO3*
GO:0055074	calcium ion homeostasis	0.04934355538392671	*ASPH,PDE4D,FAM155A,ADCY8,PTGFR,CHERP,RYR1,ATP2B2,GRM5,PLCE1,ANK2,CHD7,HTR2B,RYR3,SLC24A2,SLC24A3,TCIRG1,CACNB2,ADORA1,ADRA1D,ESR1,LCK,SGCD,MICU3,DMD,SLC8A1,JPH2,FYN,BDKRB1,BDKRB2,STIM1,TMBIM6,CMKLR1,PTGER2,JAK2,NPSR1,TGFB1,KEL,JPH3,F2RL1,C9ORF47,CDH23,DISC1,TMTC2,CAPN3,CNR1,TNFSF11,P2RY10,TRPM1,XK,RYR2,NOL3,CACNA1C,GRM1,SMAD3,ITPR1,ACKR2,APP,PDGFRA,IBTK,DRD1,PRKD1,FGF2,ADCY5,ADCYAP1R1,CCL14,CCL15,TRPA1,NMUR2,AKAP6,TRPC5,PKHD1,SLC8A2,TPCN1,ATP2B4,P2RY8,CACNA1A,PTGER3,TRPV1,TRIM24,PLCG2,C1QTNF1,GPR35,ATP13A3,MCU,TRDN,TRPC6,ATP13A5,SNCA,HTT,TMCO1,PKD2,GRIN1,ITPR2,BOK,AGT,HRH4,STC2,NOS1,XCR1,MCOLN1,GRIN2B,PACS2,DIAPH1,HTR2C,GRIK2,ATP2B3,CIB2,CAV2,CAMK2D,CCR3,PTPRC,EDNRA*

Table S2. GO associations with Biological Processes (GO Profiler) of top 4920 rDNA-contacting genes shown in Figure 1B.

GO.ID	Description	padj	Genes
GO:0032501	multicellular organismal process	3.0026941554276024e-32	*ISM1,SRGAP2B,MACROD2,CDH4,PIEZO2,ZHX3,PLCB1,DHX35,CMKLR1,DAB1,OMA1,GTF2I,GABRB3,GABRG3,MAPK14,ABCG8,DSCAM,ARID1B,RUNX1,ASIC2,DTNA,OR11H1,SERPINA1,MYO18B,NCAM2,RAB27A,HUNK,HSF2BP,OR4M2,ANK3,DCC,SVEP1,WLS,RBFOX1,TIMP3,PCSK2,AKAP13,FBLN1,ERG,GRIK1,LARGE,SORBS2,SLC24A3,NPHP3,ITGA2,LOXHD1,ABCG1,KCNQ1,DPF3,SUN2,CA10,EPHA6,O*

			R4K15,GRIN3A,NRXN1,SHC3,TMPRSS3,ISX,TNNI3K,DOK5,NRXN3,SLC1A2,RAB5A,SDK2,DOCK2,CTNND2,EYS,TNC,POTEE,MS4A1,OR8U1,PAX7,CHODL,RIMS2,MTPN,SHANK2,GRIA1,NRG3,SMYD2,CELF2,HUS1,PKD1L1,ARNT2,OLFM3,TGFBR3,TRPM3,FSTL4,CDH13,ANK2,LRRC4C,RTN1,ANKH,NXN,KCND3,CSMD1,IGSF3,SYNDIG1,TF,MYH1,SCN8A,KCNE1,BCAN,SDCCAG8,TMEM108,OR4C12,GADD45A,ADAMTSL1,PRDM9,PCP4,ACIN1,SYT17,PLXNA4,FLRT2,GPC3,NRG1,ETS2,PSMB2,SHISA6,HYDIN,IL4R,ALDH1A2,CECR2,LRFN5,RYR3,GGT1,NLN,SUZ12,ASTN2,PLCE1,UNC5D,KSR2,SEMA6D,SPOCK1,DRP2,CTNNA2,SLCO3A1,CCR1,CCR3,CFTR,BBS9,RBFOX2,DTX4,PCTP,TIAM1,PM20D1,COL22A1,AGBL4,LDLRAD4,KIAA1217,RASGRF1,ZBTB20,DPYSL2,MYO3A,SLC6A2,TET3,CBFA2T2,IL1RAPL2,OR4A5,NKD1,TACC2,BTN2A1,ALK,UTRN,SORCS3,SPEF2,ZNF536,MYOM3,FBXO31,KLRF2,RAB7A,CNTN4,ARID5B,PHACTR1,S100B,ROBO2,C2CD3,EPHA3,PRKACB,DCLK1,SLC44A1,COL15A1,AJAP1,OR8K3,AGT,CST2,FSIP2,SHISA9,ATAT1,CHRM5,FBXO32,VTI1A,WDR11,ATP6V0D1,CDH11,RHOJ,RCAN1,CDH2,PARK2,FHL2,FOXN3,LOXL2,MDGA2,DLGAP1,LAMA2,GREB1L,HMCN1,KIR2DL4,OR4K13,CORO2B,ZNF804A,CYBB,NEGR1,OTC,DNAH9,GNB4,OR4M1,OR4N2,SEMA3D,DACH1,NTN1,OR52N5,TXK,GPC6,ANKFN1,NELL1,TRAPPC9,DBH,TSPAN8,PCDH17,SETD3,ZNRF3,FMNL3,KCNMA1,TRIO,APBA2,FTO,HDAC2,HTRA1,LAMA4,MYO9A,SLIT2,TFAP2D,CNTN5,PDCD6,TM4SF1,OR2L13,RPS6KA2,IGF1R,KRT25,MDM2,SLC24A4,SMAD1,DSC3,NOVA1,GALC,OR11G2,OR9Q1,CTNNBL1,RAPGEF5,VCAN,ADAMTS6,GABRG1,PTPRG,PDE9A,OR8K5,TRPC5,DMD,HCN1,SYT1,BASP1,CACNG3,KRT2,SIPA1L3,VCL,NOX5,SPESP1,TEAD1,CELSR1,CSMD3,FGF14,FSHR,GRM1,MBOAT7,FMN2,MAEL,NRIP1,ASTN1,STK24,OPCML,PLAC1,JAKMIP1,WWOX,ATRX,TRPM2,CTNNA3,RNLS,RYR2,OCA2,XRCC4,DIAPH2,KIF3B,NTNG1,OR2M5,TENM4,CAMTA1,CATSPER2,LRRK2,RORB,SLC1A3,STRC,IGFBP7,PIR,KIF26B,NTRK3,RORA,GGT2,PRICKLE2,SLC4A4,ANKS1A,CHD7,OVGP1,RNF165,DISC1,HEXB,TSNAX,EMB,GLIS1,SH3BP1,SOX6,IER2,MECP2,MTMR2,SEMA4D,GSK3B,LGR5,LINGO2,SLC8A1,CDC14A,PRKD1,SERPINA5,SNRK,PLEKHA5,ERCC6,FLVCR1,OGT,TGFB2,ATP1A4,CLSTN2,FMN1,VAV3,AFF3,DACH2,LCE2B,LCE2C,PIWIL4,PLCD3,RIPPLY3,SEMA5A,SLC5A3,SLCO2B1,ACSBG1,BDNF,KRT74,LSAMP,PRKAR1B,PRKCH,RYK,SNTB1,ZDHHC17,KCNC1,PCDH15,POLR3C,DNAAF2,EML1,ABI3BP,SGCD,WASF1,WDR72,GRIN2B,KDM4C,OR6N1,POLE,EXT2,LAMA3,RAG1,RAG2,ABCB5,ANKRD6,NR2F2,PTGFR,SNAP25,NR5A2,PAQR8,PML,PPARGC1A,RNF180,SGCG,SYBU,MYH8,AKAP6,CNTNAP2,FBN1,MED15,PPP4R4,NLRP2,NLRP7,PLXNA2,SNAP23,TAOK3,ACSM3,BCL2,CHMP4C,RAD51B,RXFP2,ZNF675,ARHGAP24,EPHB1,KCNJ3,MOV10L1,SMARCE1,IL12RB2,SIPA1L1,CSGALNACT1,DISP1,LRP2,MMP16,TMEM100,LRRC4,NFIA,SND1,VANGL2,NR4A2,ABCC9,ARHGEF10,EPHA7,RPGR,FYN,GNRHR,KCNE2,PARN,SLC26A7,ADAMTS18,CHN1,NR4A1,SMARCA4,SULT4A1,CD44,HECW1,MYL12A,PMP22,RCVRN,RHBDD1,RNF213,THBS2,FOXO1,PDE4D,PYY,RBBP8,TBXAS1,KANK1,ENPP2,ABCB10,ACTN2,ATF6,ATP8A2,DCDC2,GABRB1,KLF13,MME,PACRG,TNMD,EXOC4,NAV2,GRIK2,IGSF11,LAMB4,ZNF516,CELF4,RAP1GDS1,SERPINB7,TPO,UNC5C,APBB2,KCND2,MYLK3,NAMPT,NTM,SCN11A,SLC1A1,UGCG,ZFPM2,ZNF141,PALLD,RARB,RFX4,ETV6,NLRP12,MTOR,ROR1,GAS7,LDB2,MOV10,RLBP1,CHRM1,DDX21,FAT3,MEGF9,PKP2,BMPER,CCDC88B,ERMN,PTPRD,TIAM2,ZNF521,BBS2,MATN3,OR51B2,OR51I1,PGK1,PLCL1,ZFP64,ARL6IP5,ATP1A1,CEP85L,COL11A1,EPM2A,PLN,CTNNBIP1,EPB41L4B,KLHL1,SOX8,DSG4,IFT88,NLGN4X,OR4K17,OR8J3,PRKCE,GTF2A1L,HELLS,KMT2A,PRKCB,VDR,ADCY8,FAM19A4,FOXO3,IGF2BP3,LRRTM1,MITF,SCMH1,CAPN3,KALRN,DLC1,EDNRB,MSR1,ROBO1,RXFP1,SATB2,TANC2,FAP,FLT1,NASP,NOX4,SGIP1,ZNF148,CDH17,GRHL2,TCF4,COL2A1,MALL,MAP3K7,NEBL,PREX2,TACR3,TRAF3IP2,CBLN4,CR1,EYA4,GABRA2,OR4C15,SEC24B,MDM1,PKHD1,STK3,TGM2,VASH2,FAM9B,KDR,PPHLN1,PRCP,CHL1,EDA,ABCA12,APP,CALCRL,COL18A1,DGKB,NRP1,PTPRM,SHROOM3,LGR4,SERPING1,TENM2,ADTRP,CD58,EGFLAM,IMMP2L,KAT5,LCE6A,THRB,TTC12,KCNK2,SEMA3C,ADAM17,CAMK1D,CYLC2,DIS3L2,TSPEAR,IGF2R,TFPI,ATF2,ELP3,ETS1,NR2C2,EFEMP1,PRTG,ROS1,TGFB1I1,OR10R2,RGS16,RPS6KA5,TCF7L2,AFF2,ITGA11,PARD3,SSPN,THOC2,TRAF3,ZNF830,ABAT,ATRNL1,FRAS1,GRIN2A,GRIP1,DLX6-AS1,HRNR,OPHN1,TRPS1,ZNF568,CASP5,CCDC88A,CD38,DENND5A,RIN2,TUB,ALCAM,CHRDL1,STK36,UPB1,BCL2L1,ADAMTS16,GUCY2F,HDAC4,MYO16,PDE4B,SOX5,SRRM4,DNMT1,GHR,GLRA2,JAM2,MAPRE2,NTRK2,PRKG1,ACSL4,M1AP,A1CF,DNM3,PAPPA,RASGRP1,STARD13,ARR3,CAMK4,RABGEF1,SDK1,SPIN3,SPRED2,GLI3,KAZ

			N,OR5L2,SMCHD1,CACNA1C,CLDN1,KIF16B,OR11L1,SPRR2B,SPRR2E,CERS3,CRB1,DOCK4,RAP1A,SAFB2,SRP54,TGFBR1,USP53,CYSLTR1,KCNJ12,MGLL,PRDX4,RRP7A,ADAM12,DICER1,DYM,EPHA5,TAS2R38,THEMIS,TNR,TOP1,CUX1,KRTAP6-1,SLC6A17,TAS2R1,TENM3,CHST11,CPE,IL16,RAPGEF1,CD96,CHRNA3,CHRNA5,EP300,MORC3,SLC16A7,SYNE1,DSCAML1,HMG20A,TBX3,CACNA2D1,CACNA2D4,CYP7B1,DIO2,SPRR4,TRIOBP,ATXN1,GAB2,GLRA1,PCSK5,SYCP1,TP73,GRID2,NPC1,OR4C5,PIBF1,MORN2,PSMA1,TRHDE,FLNB,PPP1R12B,SPG11,ADORA2A,CAMK2G,EFNA5,EMCN,FAM20A,MERTK,SPRR2G,TAC4,WARS2,BRD1,CACNG2,FAM9A,MAGI2,NF1,RGS7,TFF1,IMPG1,SORL1,BICC1,ELK3,IFT80,IGSF21,OR5K4,TEAD4,TTC8,DMRT1,ASXL1,EIF4E,FRYL,POR,RELN,SLC39A8,SLC9C1,TNFRSF11B,TSHZ1,GRM5,HAPLN1,HDAC9,IL18R1,IL1RL1,KIRREL3,KRTAP4-3,NECAB1,PTPN9,COL12A1,DYNC1H1,LRRTM3,DDC,NAPEPLD,NDRG2,NUMB,RHPN2,SP2,MAP3K13,NPHP4,RALA,TBX15,ADCYAP1R1,CYP39A1,IL1RAP,NAV3,FBXL17,JAG1,MEOX2,PPP3CA,KCNB2,KLF3,KLF7,MYT1L,NEB,PLS1,SH3GL2,SLC7A1,CXADR,DGKI,MAS1,PER1,SLC24A2,WWTR1,KL,C6,CBLB,DOCK10,ELAVL4,MNAT1,PLSCR1,RNF17,SEC63,SLC5A1,CNTN1,DGUOK,GPC4,GRM7,RIMS1,TRIM16,GABPA,GABRA3,KAT7,L3MBTL3,SLC22A2,AGGF1,ASAP1,FHOD3,FREM1,N4BP2L2,NLGN1,P2RX6,PTPRU,AKIRIN2,ARHGAP5,CHST8,CTTNBP2,RNF128,SLC22A3,SSUH2,AK8,MEGF11,NPR3,SYCP2,LHFPL3,LRRK1,MAPK8IP2,TMPRSS11E,CACNA1D,DDX3X,MYH4,OMG,GABRA5,TPCN2,JAK2,SHOX2,SOBP,SPNS2,AHI1,CATSPERB,PSG7,SMAD3,KIDINS220,NOS1,SERPINB12,COL8A1,PLXNB1,RDH10,TCF12,TMTC4,WDR33,AATF,ANHX,CDK1,HDAC7,HIVEP3,ITPK1,LTB,MAGT1,MCM3AP,NBN,PRRC2C,RRH,TAGLN3,TNF,COBL,NHS,OVOL2,PANX1,SNX5,ZFPM1,CHKB,POMC,PPFIA2,PSG1,TANC1,TMC2,ADNP2,ATXN10,BLOC1S6,BMP15,DLG5,PHEX,POU6F2,LGR6,OR11A1,OR5V1,SIM2,ARID2,MLIP,MMP20,PAXBP1,BRINP1,KATNAL1,KCNK10,PRG3,SPAG16,ADORA3,BCL11A,CALD1,CLEC4A,FREM2,HMGA2,MYO6,DOCK1,KIF18A,PCDH19,ANO1,GCLC,QKI,RAB23,EPS8,LTBP1,MBOAT2,TPD52,DAAM2,PABPC1L,SMOC2,SULF1,TRPC4,C5AR1,CISD2,GNAL,LRP4,PIK3R3,WDR5,XYLT1,CNGB3,CNTN6,EFNB2,PIWIL3,TRDN,AMOTL1,HERC1,PHF8,SPATA5,ZNF423,CLSTN1,INSC,SELP,ALPK2,CTNNA1,SCN1A,TENM1,TSHZ3,ABCG2,ARHGEF11,CDKL5,EFHC2,MAMLD1,MYH14,PRKAR1A,TNFRSF19,ARHGAP11B,BPTF,DYNC2H1,GABRR2,MLLT3,MYH13,TMEM150C,CD109,EGF,MIP,PTPN14,SPIRE2,ALDH1A1,CMA1,FOXP4,IL1RAPL1,KIT,POTEF,SLIT3,TAF4B,TNFSF8,UBR2,OR7A5,TRPV4,VWC2,ABCA5,CC2D2A,CPS1,DNAH11,EDAR,EFNB1,PTPRO,RAPGEF2,RCSD1,SLCO4C1,DNAJB6,ESR1,HTR2C,JAK1,MYPN,NCMAP,SEMA3E,UBE3A,FGF13,GABRB2,MARVELD3,NUP155,PLAG1,SGCZ,ARMC2,EPHB2,FNDC3A,KRT76,MECOM,TBCD,TFDP2,TOX,ABCC1,C14ORF39,ERBB4,MAP3K3,MINPP1,RBM19,SLITRK6,SPTA1,TDRKH,FNIP1,LRP5,PTH2R,SNRNP200,TTLL5,MYH15,SLAMF1,ABL2,BSG,CAMP,F11R,FARP2,GAS8,OR4C6,SETD2,ALX4,AP2B1,ARHGAP35,CD2AP,CLDN11,NSUN2,SGMS2,ATP8A1,CCL3,FGF7,GGN,LAMA1,MYO7B,PREX1,SEMA5B,SPRED3,VWF,IL20RA,KIF2A,MCM9,NHLH2,PSG9,SPA17,SULF2,ARF4,MYH9,PTGER3,SLC2A13,SLC39A12,SOX4,P2RX1,PDGFD,PIK3CD,AGTPBP1,ASGR2,ITGA8,KCNE4,PUM1,ARID4A,CNGA4,ENPEP,FAM172A,KDM3A,MYO3B,PDZD7,PLAT,UNC13A,WDPCP,ADCY9,AUTS2,CDH23,DAPL1,FGFR1,MEF2A,OCLN,RYR1,SYNM,UNC13B,ARID4B,DAD1,GLRA3,MAP2,SOST,ASXL3,BPGM,IL23R,KEAP1,NTF3,P2RX7,PLA2G4A,SCUBE1,TEX14,EDDM3A,GP5,AMFR,AR,BCOR,CASP7,FGL2,KIAA1109,PAPSS1,SCAMP5,SUFU,CEP250,EYA1,IFNA8,IL1RL2,N4BP1,RBM4,SRPK2,BDKRB1,BDKRB2,HLA-DRA,ADRA1B,BHLHB9,DGKZ,GNL3,L3MBTL1,PRKAA2,SEMA3A,ANKRD11,CDC73,EPB41L3,JRKL,MARK2,MEIS2,MOGAT2,PDE5A,PROS1,REEP2,SMPX,ZMIZ1,FLT3,PDLIM5,PPARGC1B,TRIM72,ESRRG,FERMT2,GNAO1,HCN4,LTBR,MAP2K5,MARK1,PAPPA2,SCNN1A,SMPD3,TNN,ZC3H13,CLN6,DAB2,GPR55,LCP1,PBX1,RNF207,SRD5A2,STX3,SYT2,ADAM10,BLOC1S5,DAAM1,FGF12,ITGA3,FRY,LGI1,NREP,OR10K2,OR4C46,PLCL2,PPM1B,RFTN1,STAC,LECT2,LRIG1,MYOM1,PIN1,RPS6KA6,THSD7A,TRPM4,CD226,NME8,SCLT1,SHROOM4,ABCC2,BMP6,BTBD9,CHRM3,CIT,CLASP1,EIF4ENIF1,SMTNL2,CNGB1,NCOA2,TGFA,TMOD1,CREM,GAS2,HGF,HGFAC,MYLK,OBP2A,PRPSAP2,SPATA6,CALCR,CAPN7,CENPF,DPY19L2,FLI1,SCUBE3,SLC13A3,SLC26A8,ZBTB16,CACNB2,ELN,NCAM1,NFASC,NOS1AP,INSR,MACF1,PAK1,PDE3A,PSMB7,SKI,TAF7L,AKT3,CHM,CTDP1,ELF1,ENPP1,FMR1,LIMK2,LITAF,PAX3,TESK2,WDR7,ATP5J,FAM126A,FPGS,GABRA1,GOLGA3,GPRIN2,KLF6,PHC2,PRKX,SBF2,ULK4,ABCD1,C

			RMP1,EVC,GABRR3,IDE,MAD1L1,MFNG,RPGRIP1,TDRD6,AIM2,ARHGAP42,CCDC141,CD84,CSF2RB,GLDN,JPH4,PCK1,PDE6B,PPARA,SORT1,TTN,ZFHX2,C1GALT1,COL19A1,CORIN,FHL1,GNA13,MYH7,SLC22A5,TAF1,CHD5,FUT8,PPP2R3C,QRFP,S1PR3,SPRED1,TRPM1,AKR1C2,CHSY1,LCE1F,NTN4,PRDM16,PTPRF,SCN9A,BTRC,GTSF1,HIRA,MYBPC2,PTPRR,RIT2,TECTA,ESRP2,EXT1,IFT81,MDM4,MYH6,NCAPH2,NCOA1,PRKCG,PRLR,SCO2,TYMP,UMOD,ADAMTS5,CBFA2T3,DOCK7,FBXW11,G3BP1,HDAC5,KLHL3,OR51L1,TDGF1,A2M,ADAM28,MYEF2,SLC38A10,SLC40A1,STIM1,TTC39C,ADARB1,KCNC2,MYH11,NDE1,OR4C16,PEMT,POU2AF1,PRKCQ,SCEL,STAT3,TP63,AMOT,BRIP1,CST1,HPN,ISLR2,PAK3,TBL1X,USH2A,BLNK,CASQ2,DHRS2,DHRS7B,DTNBP1,GAK,CRTAC1,DLGAP2,LCE2A,LGALS9,LRP8,MYOM2,NFAT5,OR2T4,TAS1R1,GDA,MAPKAPK2,SLC7A8,TUSC3,UPK3A,APC,BMPR1A,CSN3,DPP4,ITGA1,KIAA0319,NGF,PRKCA,PTGES,SPINT2,UBASH3B,VLDLR,ASB3,KRT75,ABI1,CTSH,DHX9,NCAPG2,RGCC,ACACA,CYB5D2,GABRG2,GPR21,IL6R,LCE4A,MAP2K6,NCALD,SIAH3,SLC16A12,TFIP11,SPTBN4,CLASP2,MAP2K1,SOS1,TPH1,VPS35,C1QTNF3,COL6A3,FGF1,MCOLN3,TBX20,C6ORF106,DCHS2,DGKK,PPP1R9A,RGN,RREB1,TBX18,BZW2
GO:0032502	developmental process	6.754681769376912e-31	*ISM1,SRGAP2B,MACROD2,CDH4,ZHX3,PLCB1,DHX35,CMKLR1,DAB1,OMA1,GTF2I,FMNL2,GABRB3,CDC42EP3,MAPK14,DSCAM,ARID1B,RUNX1,ASIC2,CTBP2,MYO18B,NCAM2,RAB27A,HUNK,HSF2BP,ANK3,DCC,SVEP1,WLS,RBFOX1,PCSK2,AKAP13,FBLN1,ERG,GRIK1,LARGE,SORBS2,SLC24A3,NPHP3,ITGA2,ABCG1,KCNQ1,DPF3,SUN2,NUBPL,CA10,EPHA6,GRIN3A,NRXN1,SHC3,ZRANB1,ISX,DOK5,NRXN3,SLC1A2,SDK2,DOCK2,CTNND2,EYS,TNC,PLEKHB2,POTEE,MS4A1,PAX7,CHODL,RIMS2,MTPN,SHANK2,COMT,NRG3,SMYD2,HUS1,PKD1L1,ARNT2,FGD6,OLFM3,TGFBR3,FSTL4,CDH13,ANK2,LRRC4C,RTN1,ANKH,NXN,CSMD1,RUNX1T1,IGSF3,SYNDIG1,TF,SCN8A,BCAN,SDCCAG8,TMEM108,CHEK2,GADD45A,ADAMTSL1,PCP4,ACIN1,SYT17,PLXNA4,FLRT2,GPC3,NHSL2,NRG1,ETS2,PSMB2,SHISA6,HYDIN,IL4R,ALDH1A2,CECR2,LRFN5,GGT1,NLN,SUZ12,ASTN2,PLCE1,UNC5D,SEMA6D,SPOCK1,DRP2,CTNNA2,CCR1,CCR3,CFTR,BBS9,RBFOX2,TIAM1,RHOC,COL22A1,AGBL4,LDLRAD4,KIAA1217,RASGRF1,DPYSL2,MYO3A,TET3,CBFA2T2,IL1RAPL2,NKD1,TACC2,ALK,UTRN,SPEF2,ZNF536,FBXO31,CNTN4,ARID5B,PHACTR1,S100B,ROBO2,C2CD3,EPHA3,PRKACB,DCLK1,COL15A1,AJAP1,AGT,FSIP2,ATAT1,WDR11,ATP6V0D1,CDH11,RHOJ,RCAN1,CDH2,CDH9,PARK2,FHL2,FOXN3,LOXL2,MDGA2,LAMA2,GREB1L,KIR2DL4,ZNF804A,CYBB,NEGR1,OTC,GNB4,SEMA3D,DACH1,NTN1,KCNH1,ZNF268,TXK,GPC6,NELL1,TRAPPC9,TSPAN8,PCDH17,SETD3,ZNRF3,FMNL3,TRIO,APBA2,FTO,HDAC2,HTRA1,LAMA4,MYO9A,SLIT2,TFAP2D,CDH8,CNTN5,PDCD6,TM4SF1,RPS6KA2,IGF1R,KRT25,MDM2,PALMD,SLC24A4,SMAD1,DSC3,FOXD4L4,GALC,CTNNBL1,RAPGEF5,VCAN,ADAMTS6,PTPRG,CPQ,TRPC5,DMD,HCN1,SYT1,BASP1,KRT2,SIPA1L3,VCL,NOX5,TEAD1,CELSR1,CSMD3,FGF14,FSHR,MBOAT7,FMN2,MAEL,NRIP1,ASTN1,STK24,OPCML,PLAC1,WWOX,ATRX,TRPM2,RYR2,OCA2,XRCC4,DIAPH2,KIF3B,NTNG1,TENM4,UNC13C,CATSPER2,LRRK2,MAPK10,RORB,SLC1A3,STRC,IGFBP7,PIR,KIF26B,NTRK3,RORA,GGT2,PRICKLE2,ANKS1A,CHD7,RNF165,DISC1,HEXB,TSNAX,EMB,GLIS1,SH3BP1,SOX6,IER2,MECP2,MTMR2,SEMA4D,GSK3B,LGR5,LINGO2,SLC8A1,PRKD1,SERPINA5,SNRK,PLEKHA5,ERCC6,FLVCR1,FRMD6,TGFB2,ATP1A4,CLSTN2,FMN1,PRAMEF12,VAV3,AFF3,DACH2,LCE2B,LCE2C,PIWIL4,PLCD3,RIPPLY3,SEMA5A,SLC5A3,ACSBG1,BDNF,KRT74,LSAMP,PRKCH,RIPK4,RYK,ZDHHC17,KCNC1,PCDH15,DNAAF2,EML1,ABI3BP,SGCD,WASF1,WDR72,GRIN2B,KDM4C,POLE,EXT2,LAMA3,RAG1,RAG2,ABCB5,ANKRD6,MREG,NR2F2,SNAP25,NR5A2,PAQR8,PML,PPARGC1A,SGCG,SYBU,ARHGAP12,AKAP6,CNTNAP2,FBN1,MED15,PPP4R4,PLXNA2,TAOK3,BCL2,CDH10,CHMP4C,RAD51B,RALBP1,RXFP2,ZNF675,ARHGAP24,EPHB1,MOV10L1,SMARCE1,CDH12,SIPA1L1,CSGALNACT1,DISP1,LRP2,MMP16,TMEM100,LRRC4,NFIA,SND1,VANGL2,CDH6,NR4A2,ARHGEF10,EPHA7,ZBTB7C,FYN,GNRHR,KCNE2,ADAMTS18,CHN1,NR4A1,SMARCA4,SULT4A1,CD44,FLNC,HECW1,PMP22,RHBDD1,RNF213,THBS2,CASP6,FOXO1,PDE4D,PYY,RBBP8,KANK1,ENPP2,ABCB10,ACTN2,ATF6,ATP8A2,DCDC2,GABRB1,GRK5,KLF13,MME,PACRG,TNMD,EXOC4,NAV2,LAMB4,ZNF516,CELF4,SERPINB7,TPO,UNC5C,MYLK3,NTM,SLC1A1,UGCG,ZFPM2,ZNF141,PALLD,RARB,RFX4,STRIP1,ETV6,MTOR,ROR1,GAS7,LDB2,MOV10,CHRM1,DDX21,FAT3,MEGF9,PKP2,BMPER,ERMN,PTPRD,TIAM2,ZNF521,BBS2,MATN3,PGK1,ZFP64,CEP85L,COL11A1,EPM2A,PLN,CTNNBIP1,KLHL1,SOX8,DSG4,IFT88,NLGN4X,CDH18,EIF2S1,HELLS,KMT2A,PRKCB,VDR,FOXO3,IGF2BP3,LRRTM1,MITF,SCMH1,CAPN3,KALRN,CCDC88C,DLC1*

			,EDNRB,MSI2,MSR1,ROBO1,RXFP1,SATB2,TANC2,FAP,FLT1,NASP,NOX4,ZNF148,CDH17,GRHL2,TCF4,COL2A1,MALL,MAPK9,NEBL,PREX2,TACR3,TRAF3IP2,ZNF277,CBLN4,CR1,EYA4,GABRA2,SEC24B,MDM1,PKHD1,STK3,TGM2,VASH2,FAM9B,KDR,PPHLN1,PRCP,ARHGAP15,CHL1,EDA,NHSL1,ABCA12,APP,CALCRL,COL18A1,NRP1,PTPRM,SHROOM3,ARHGEF18,LGR4,SERPING1,TENM2,ADTRP,EGFLAM,IMMP2L,KAT5,LCE6A,ST7,THRB,TTC12,KCNK2,SEMA3C,ADAM17,CAMK1D,CYLC2,DIS3L2,MAP3K5,PARVG,TSPEAR,IGF2R,ATF2,ELP3,ETS1,NR2C2,EFEMP1,PRTG,ROS1,TGFB1I1,RPS6KA5,TCF7L2,AFF2,BICD1,ITGA11,PARD3,THOC2,ZNF830,ABAT,ATRNL1,FRAS1,GRIN2A,GRIP1,DLX6-AS1,HRNR,OPHN1,TRPS1,ZNF568,CASP5,CCDC88A,CD38,DENND5A,RIN2,TUB,ALCAM,CHRDL1,CNN3,STK36,UPB1,BCL2L1,ADAMTS16,HDAC4,MYO16,SOX5,SRRM4,DNMT1,GHR,JAM2,NTRK2,PRKG1,ACSL4,M1AP,A1CF,DNM3,RASGRP1,STARD13,CAMK4,SDK1,SPRED2,GLI3,KAZN,SMCHD1,CACNA1C,CLDN1,KIF16B,SPRR2B,SPRR2E,CERS3,CRB1,RAP1A,SAFB2,SRP54,TGFBR1,CYSLTR1,PRDX4,RRP7A,ADAM12,DICER1,DYM,EPHA5,THEMIS,TMEM120B,TNR,TOP1,CUX1,KRTAP6-1,PEAK1,SLC6A17,TENM3,CHST11,CPE,RAPGEF1,CHRNA3,EP300,MORC3,SYNE1,DSCAML1,HMG20A,SCFD1,TBX3,WIF1,CYP7B1,DIO2,SPRR4,TRIOBP,ATXN1,GAB2,PCSK5,SYCP1,TP73,GRID2,PARVB,PNPLA3,MORN2,PSMA1,FLNB,SPG11,ADCK1,ADORA2A,CAMK2G,DDX10,EFNA5,EMCN,FAM20A,MERTK,SPRR2G,WARS2,BRD1,FAM9A,MAGI2,NF1,RGS7,TFF1,SORL1,BICC1,ELK3,IFT80,IGSF21,TEAD4,TTC8,DMRT1,ASXL1,EIF4E,FRYL,POR,RELN,SLC9C1,TNFRSF11B,TSHZ1,GRM5,HAPLN1,HDAC9,IL18R1,KIRREL3,KRTAP4-3,NECAB1,PTPN9,COL12A1,LRRTM3,DDC,NAPEPLD,NDRG2,NUMB,SP2,MAP3K13,NPHP4,RALA,TBX15,ADCYAP1R1,IL1RAP,NAV3,ATP10A,EXOC5,FBXL17,JAG1,MEOX2,PPP3CA,KLF3,KLF7,MYT1L,NEB,PLS1,SH3GL2,CAPZB,CXADR,MAS1,WWTR1,KL,C6,CBLB,DOCK10,ELAVL4,GSTA2,MNAT1,RNF17,SEC63,CNTN1,DGUOK,GPC4,GRM7,RIMS1,TRIM16,GABPA,KAT7,L3MBTL3,TMEM135,AGGF1,ASAP1,FHOD3,FREM1,N4BP2L2,NLGN1,PTPRU,AKIRIN2,ARHGAP5,CHST8,CTTNBP2,SH3KBP1,SSUH2,AK8,MEGF11,NPR3,SYCP2,LRRK1,MAPK8IP2,DDX3X,EGFL6,RBL2,OMG,EHF,GABRA5,JAK2,SHOX2,SOBP,SPNS2,AHI1,CATSPERB,SMAD3,XKR4,KIDINS220,MAP2K4,MIAT,NOS1,PTGFRN,SERPINB12,COL8A1,PLXNB1,RDH10,TCF12,WDR33,AATF,ANHX,CDK1,HDAC7,HIVEP3,ITPK1,LTB,MCM3AP,NBN,PRRC2C,TAGLN3,TNF,COBL,NHS,OVOL2,PANX1,ZFPM1,CHKB,PPFIA2,TANC1,ADNP2,ATXN10,BLOC1S6,BMP15,DLG5,PHEX,POU6F2,RBM11,LGR6,SIM2,ARID2,MMP20,PAXBP1,BRINP1,KATNAL1,SPAG16,BCL11A,CALD1,FREM2,HMGA2,MYO6,NEK4,DOCK1,KIF18A,PCDH19,SULT1B1,GCLC,QKI,RAB23,UBE2V1,EPS8,MBOAT2,TPD52,DAAM2,PABPC1L,SMOC2,SULF1,TRPC4,C5AR1,CISD2,LRP4,PIK3R3,WDR5,XYLT1,CNTN6,EFNB2,PIWIL3,AMOTL1,HERC1,PHF8,SPATA5,ZNF423,CLSTN1,INSC,LRRC8C,ALPK2,CTNNA1,TENM1,WDFY2,CDKL5,DMBT1,EFHC2,MAMLD1,MYH14,PRKAR1A,TNFRSF19,ARHGAP11B,BPTF,DYNC2H1,FAM171A1,MLLT3,CD109,EGF,MIP,PTPN14,CMA1,FOXP4,IL1RAPL1,KIT,SLIT3,TAF4B,TNFSF8,UBR2,TRPV4,VWC2,ABCA5,CC2D2A,CPS1,DNAH11,EDAR,EFNB1,PTPRO,RAPGEF2,SLCO4C1,DNAJB6,ESR1,HTR2C,JAK1,MYPN,NCMAP,SEMA3E,UBE3A,DNMBP,FGF13,GABRB2,PLAG1,SGCZ,ARMC2,EPHB2,FNDC3A,KRT76,MECOM,TBCD,TFDP2,TOX,C14ORF39,ERBB4,FER,MAP3K3,MINPP1,RBM19,SLITRK6,SPTA1,TDRKH,FNIP1,LRP5,SNRNP200,TTLL5,XKR7,MYH15,SLAMF1,ABL2,BSG,CAMP,F11R,FARP2,GAS8,SETD2,ALX4,AP2B1,ARHGAP35,CLDN11,NSUN2,SGMS2,CCL3,FGF7,GGN,LAMA1,MYO7B,PREX1,SEMA5B,SPRED3,FGD4,KIF2A,NHLH2,PSG9,SPA17,SULF2,ARF4,MYH9,NSMCE2,PACSIN2,SLC39A12,SOX4,NEDD9,PDGFD,PIK3CD,AGTPBP1,ASGR2,ITGA8,PUM1,ARID4A,ENPEP,FAM172A,KDM3A,MYO3B,PDZD7,UNC13A,WDPCP,ADCY9,AUTS2,CDH23,DAPL1,FGFR1,MEF2A,RYR1,UNC13B,ARID4B,DAD1,MAP2,ASXL3,BPGM,IL23R,ITGB7,KEAP1,NTF3,P2RX7,SCUBE1,AMFR,AR,BCOR,CASP7,FGL2,KIAA1109,PAPSS1,SUFU,EYA1,IFNA8,IL1RL2,RBM4,SRPK2,HLA-DRA,BHLHB9,GNL3,L3MBTL1,NEK5,SEMA3A,ANKRD11,CDC73,EPB41L3,JRKL,MARK2,MEIS2,PDE5A,ZMIZ1,FLT3,PDLIM5,PPARGC1B,TRIM72,FERMT2,HCN4,LTBR,MAP2K5,MARK1,PAPPA2,SMPD3,TNN,ZC3H13,DAB2,GPR55,LCP1,PBX1,RNF207,SRD5A2,STX3,SYT2,ACTN4,ADAM10,BLOC1S5,CCL7,DAAM1,EEF1E1,FGF12,ITGA3,KMO,POU2F3,PRAMEF8,FRY,LGI1,NREP,PLCL2,LECT2,LRIG1,PIN1,RPS6KA6,THSD7A,TRPM4,NME8,SCLT1,SHROOM4,BMP6,CHRM3,CIT,CLASP1,EIF4ENIF1,CFDP1,CNGB1,TGFA,TMOD1,CREM,GAS2,HGF,

			MYLK,PRPSAP2,SPATA6,CALCR,CENPF,DPY19L2,FLI1,SCUBE3,SLC26A8,ZBTB16,ELN,NCAM1,NFASC,INSR,MACF1,PAK1,PDE3A,PSMB7,SKI,TAF7L,AKT3,CTDP1,ELF1,ENPP1,FMR1,LIMK2,PAX3,TESK2,WDR7,ATP5J,FAM126A,FPGS,GABRA1,GOLGA3,GPRIN2,JDP2,KLF6,PHC2,PRKX,SBF2,ULK4,ABCD1,CRMP1,EVC,IDE,MAD1L1,MFNG,RPGRIP1,TDRD6,CCDC141,GLDN,PCK1,PDE6B,PPARA,SORT1,SPIB,TBX22,TTN,ZFHX2,C1GALT1,COL19A1,FHL1,GNA13,MYH7,TAF1,CHD5,FUT8,PPP2R3C,S1PR3,SPRED1,AKR1C2,CHSY1,KRT4,LCE1F,NTN4,PRDM16,PTPRF,RBL1,SCN9A,BTRC,CCDC3,GTSF1,HIRA,PGM5,PTPRR,RIT2,ESRP2,EXT1,IFT81,MDM4,MYH6,NCAPH2,NCOA1,PRKCG,PRLR,SCO2,TYMP,UMOD,ADAMTS5,CBFA2T3,DOCK7,FBXW11,HDAC5,KLHL3,TDGF1,A2M,ADAM28,AKAP2,LYVE1,MYEF2,SLC24A5,SLC38A10,SLC40A1,STIM1,TTC39C,ADARB1,KCNC2,MYH11,NDE1,PEMT,POU2AF1,PRKCQ,SCEL,STAT3,TP63,AMOT,BRIP1,HPN,ISLR2,PAK3,USH2A,BLNK,DHRS2,DHRS7B,DTNBP1,GAK,RAP1B,CRTAC1,LCE2A,LGALS9,LRP8,MYOM2,GDA,MAPKAPK2,UPK3A,APC,BMPR1A,CSN3,ITGA1,KIAA0319,NGF,PRKCA,SPINT2,UBASH3B,VLDLR,ADAMTS20,KRT75,ABI1,CTSH,DHX9,NCAPG2,RGCC,CYB5D2,EDA2R,GABRG2,GPR21,IL6R,LCE4A,MAP2K6,SIAH3,TFIP11,SPTBN4,CLASP2,MAP2K1,PRAMEF7,SOS1,TPH1,VPS35,C1QTNF3,COL6A3,FGF1,MCOLN3,TBX20,DCHS2,PPP1R9A,RGN,RREB1,TBX18,BZW2
GO:0048731	system development	3.137188725360184e-29	*ISM1,SRGAP2B,MACROD2,CDH4,PLCB1,CMKLR1,DAB1,GTF2I,GABRB3,MAPK14,DSCAM,ARID1B,RUNX1,ASIC2,MYO18B,NCAM2,ANK3,DCC,SVEP1,WLS,RBFOX1,PCSK2,AKAP13,GRIK1,LARGE,SORBS2,SLC24A3,NPHP3,ITGA2,KCNQ1,DPF3,SUN2,CA10,EPHA6,GRIN3A,NRXN1,SHC3,ISX,DOK5,NRXN3,SLC1A2,SDK2,DOCK2,CTNND2,TNC,POTEE,MS4A1,PAX7,CHODL,RIMS2,MTPN,SHANK2,NRG3,SMYD2,ARNT2,OLFM3,TGFBR3,FSTL4,CDH13,ANK2,LRRC4C,RTN1,ANKH,NXN,CSMD1,IGSF3,SYNDIG1,TF,SCN8A,BCAN,SDCCAG8,TMEM108,GADD45A,ADAMTSL1,PCP4,ACIN1,SYT17,PLXNA4,FLRT2,GPC3,NRG1,ETS2,PSMB2,HYDIN,IL4R,ALDH1A2,CECR2,LRFN5,NLN,SUZ12,ASTN2,PLCE1,UNC5D,SEMA6D,SPOCK1,DRP2,CTNNA2,CCR1,CCR3,CFTR,RBFOX2,TIAM1,COL22A1,AGBL4,LDLRAD4,KIAA1217,RASGRF1,DPYSL2,MYO3A,CBFA2T2,IL1RAPL2,NKD1,TACC2,ALK,UTRN,SPEF2,ZNF536,FBXO31,CNTN4,ARID5B,PHACTR1,S100B,ROBO2,C2CD3,EPHA3,PRKACB,DCLK1,COL15A1,AJAP1,AGT,ATAT1,WDR11,ATP6V0D1,CDH11,RHOJ,RCAN1,CDH2,PARK2,FHL2,FOXN3,LOXL2,MDGA2,LAMA2,GREB1L,ZNF804A,CYBB,NEGR1,OTC,GNB4,SEMA3D,NTN1,GPC6,NELL1,TRAPPC9,PCDH17,ZNRF3,FMNL3,TRIO,APBA2,FTO,HDAC2,HTRA1,LAMA4,MYO9A,SLIT2,TFAP2D,CNTN5,PDCD6,RPS6KA2,IGF1R,KRT25,MDM2,SLC24A4,SMAD1,GALC,CTNNBL1,RAPGEF5,VCAN,ADAMTS6,PTPRG,TRPC5,DMD,HCN1,SYT1,BASP1,KRT2,SIPA1L3,VCL,NOX5,TEAD1,CELSR1,CSMD3,FGF14,FSHR,MBOAT7,NRIP1,ASTN1,STK24,OPCML,PLAC1,WWOX,ATRX,TRPM2,RYR2,XRCC4,NTNG1,TENM4,LRRK2,RORB,SLC1A3,STRC,PIR,KIF26B,NTRK3,RORA,PRICKLE2,ANKS1A,CHD7,RNF165,DISC1,HEXB,EMB,SOX6,IER2,MECP2,MTMR2,SEMA4D,GSK3B,LGR5,LINGO2,SLC8A1,PRKD1,SNRK,PLEKHA5,ERCC6,FLVCR1,TGFB2,CLSTN2,FMN1,VAV3,LCE2B,LCE2C,PLCD3,RIPPLY3,SEMA5A,SLC5A3,ACSBG1,BDNF,KRT74,LSAMP,PRKCH,RYK,ZDHHC17,KCNC1,PCDH15,EML1,ABI3BP,SGCD,WASF1,WDR72,GRIN2B,POLE,EXT2,LAMA3,RAG1,RAG2,ABCB5,ANKRD6,NR2F2,SNAP25,NR5A2,PML,PPARGC1A,SGCG,SYBU,AKAP6,CNTNAP2,FBN1,PLXNA2,TAOK3,BCL2,RXFP2,ZNF675,ARHGAP24,EPHB1,SMARCE1,SIPA1L1,CSGALNACT1,DISP1,LRP2,MMP16,TMEM100,LRRC4,NFIA,VANGL2,NR4A2,ARHGEF10,EPHA7,FYN,KCNE2,ADAMTS18,CHN1,NR4A1,SMARCA4,SULT4A1,CD44,HECW1,PMP22,RNF213,THBS2,FOXO1,PYY,KANK1,ENPP2,ABCB10,ACTN2,ATF6,ATP8A2,DCDC2,GABRB1,KLF13,MME,TNMD,EXOC4,NAV2,LAMB4,ZNF516,CELF4,SERPINB7,TPO,UNC5C,MYLK3,NTM,SLC1A1,UGCG,ZFPM2,PALLD,RARB,RFX4,ETV6,MTOR,ROR1,GAS7,LDB2,MOV10,CHRM1,FAT3,MEGF9,PKP2,BMPER,PTPRD,TIAM2,ZNF521,BBS2,MATN3,PGK1,ZFP64,CEP85L,COL11A1,EPM2A,PLN,CTNNBIP1,KLHL1,SOX8,DSG4,IFT88,NLGN4X,HELLS,KMT2A,PRKCB,VDR,FOXO3,IGF2BP3,LRRTM1,MITF,CAPN3,KALRN,DLC1,EDNRB,ROBO1,RXFP1,SATB2,TANC2,FAP,FLT1,NASP,NOX4,ZNF148,CDH17,GRHL2,TCF4,COL2A1,MALL,NEBL,PREX2,TRAF3IP2,CBLN4,CR1,EYA4,GABRA2,SEC24B,MDM1,PKHD1,STK3,TGM2,VASH2,KDR,PPHLN1,PRCP,CHL1,EDA,ABCA12,APP,CALCRL,COL18A1,NRP1,PTPRM,SHROOM3,LGR4,TENM2,ADTRP,EGFLAM,IMMP2L,LCE6A,THRB,KCNK2,SEMA3C,ADAM17,CAMK1D,TSPEAR,IGF2R,ATF2,ELP3,ETS1,NR2C2,EFEMP1,PRTG,TGFB1I1,RPS6KA5,TCF7L2,AFF2,ITGA11,PARD3,THOC2,ZNF830,ABAT,ATRNL1,FRAS1,GRIN2A,GRIP1,DLX6-AS1,HRNR,OPHN1,TRPS1,ZNF568,CASP5,CCDC88A,CD38,DENND5*

			A,RIN2,TUB,ALCAM,CHRDL1,STK36,UPB1,BCL2L1,ADAMTS16,HDAC4,MYO16,SOX5,SRRM4,DNMT1,GHR,JAM2,NTRK2,PRKG1,ACSL4,DNM3,RASGRP1,STARD13,CAMK4,SDK1,SPRED2,GLI3,KAZN,SMCHD1,CACNA1C,CLDN1,SPRR2B,SPRR2E,CERS3,CRB1,RAP1A,SAFB2,SRP54,TGFBR1,CYSLTR1,PRDX4,ADAM12,DICER1,DYM,EPHA5,THEMIS,TNR,CUX1,KRTAP6-1,SLC6A17,TENM3,CHST11,CPE,RAPGEF1,CHRNA3,EP300,DSCAML1,HMG20A,TBX3,CYP7B1,SPRR4,TRIOBP,ATXN1,GAB2,PCSK5,TP73,GRID2,PSMA1,FLNB,SPG11,ADORA2A,CAMK2G,EFNA5,EMCN,FAM20A,MERTK,SPRR2G,WARS2,BRD1,MAGI2,NF1,RGS7,SORL1,BICC1,ELK3,IFT80,IGSF21,TEAD4,TTC8,DMRT1,ASXL1,EIF4E,FRYL,POR,RELN,TNFRSF11B,TSHZ1,GRM5,HAPLN1,HDAC9,IL18R1,KIRREL3,PTPN9,LRRTM3,NDRG2,NUMB,MAP3K13,NPHP4,RALA,TBX15,IL1RAP,NAV3,FBXL17,JAG1,MEOX2,PPP3CA,KLF7,MYT1L,NEB,PLS1,SH3GL2,CXADR,MAS1,WWTR1,KL,CBLB,DOCK10,ELAVL4,MNAT1,SEC63,CNTN1,DGUOK,GPC4,GRM7,RIMS1,TRIM16,GABPA,KAT7,L3MBTL3,AGGF1,ASAP1,FHOD3,FREM1,N4BP2L2,NLGN1,PTPRU,ARHGAP5,CHST8,CTTNBP2,SSUH2,AK8,MEGF11,NPR3,SYCP2,LRRK1,MAPK8IP2,OMG,GABRA5,JAK2,SHOX2,SOBP,SPNS2,AHI1,SMAD3,KIDINS220,SERPINB12,COL8A1,PLXNB1,RDH10,TCF12,ANHX,CDK1,HDAC7,HIVEP3,ITPK1,LTB,MCM3AP,NBN,PRRC2C,TAGLN3,TNF,COBL,NHS,OVOL2,ZFPM1,CHKB,PPFIA2,ADNP2,ATXN10,BLOC1S6,BMP15,DLG5,PHEX,POU6F2,LGR6,SIM2,ARID2,MMP20,PAXBP1,BRINP1,BCL11A,CALD1,FREM2,HMGA2,MYO6,DOCK1,KIF18A,PCDH19,QKI,RAB23,MBOAT2,TPD52,DAAM2,SMOC2,SULF1,TRPC4,C5AR1,LRP4,PIK3R3,WDR5,XYLT1,CNTN6,EFNB2,AMOTL1,HERC1,PHF8,SPATA5,ZNF423,CLSTN1,INSC,ALPK2,CTNNA1,TENM1,CDKL5,EFHC2,MAMLD1,MYH14,PRKAR1A,TNFRSF19,ARHGAP11B,BPTF,DYNC2H1,MLLT3,CD109,EGF,MIP,PTPN14,CMA1,FOXP4,IL1RAPL1,KIT,SLIT3,TNFSF8,TRPV4,VWC2,CC2D2A,CPS1,DNAH11,EDAR,EFNB1,PTPRO,RAPGEF2,DNAJB6,ESR1,JAK1,MYPN,NCMAP,SEMA3E,UBE3A,FGF13,GABRB2,PLAG1,SGCZ,EPHB2,FNDC3A,KRT76,MECOM,TBCD,TFDP2,TOX,ERBB4,MAP3K3,MINPP1,SLITRK6,SPTA1,FNIP1,LRP5,TTLL5,MYH15,SLAMF1,ABL2,BSG,CAMP,FARP2,GAS8,SETD2,ALX4,AP2B1,ARHGAP35,CLDN11,NSUN2,SGMS2,CCL3,FGF7,LAMA1,MYO7B,PREX1,SEMA5B,SPRED3,KIF2A,NHLH2,PSG9,SULF2,ARF4,MYH9,SLC39A12,SOX4,PDGFD,PIK3CD,AGTPBP1,ASGR2,ITGA8,ARID4A,ENPEP,FAM172A,MYO3B,PDZD7,UNC13A,WDPCP,AUTS2,CDH23,FGFR1,MEF2A,RYR1,ARID4B,MAP2,ASXL3,BPGM,IL23R,NTF3,P2RX7,SCUBE1,AR,BCOR,CASP7,FGL2,PAPSS1,SUFU,EYA1,IFNA8,IL1RL2,SRPK2,HLA-DRA,BHLHB9,L3MBTL1,SEMA3A,ANKRD11,CDC73,EPB41L3,JRKL,MARK2,MEIS2,ZMIZ1,FLT3,PDLIM5,PPARGC1B,TRIM72,FERMT2,HCN4,LTBR,MAP2K5,MARK1,PAPPA2,SMPD3,TNN,DAB2,GPR55,LCP1,PBX1,RNF207,SRD5A2,STX3,SYT2,ADAM10,BLOC1S5,DAAM1,FGF12,ITGA3,FRY,LGI1,NREP,PLCL2,LECT2,LRIG1,PIN1,RPS6KA6,THSD7A,TRPM4,SCLT1,SHROOM4,BMP6,CHRM3,CIT,CLASP1,EIF4ENIF1,CNGB1,TGFA,TMOD1,GAS2,HGF,MYLK,PRPSAP2,CALCR,CENPF,FLI1,ZBTB16,ELN,NCAM1,NFASC,INSR,MACF1,PAK1,PSMB7,SKI,AKT3,CTDP1,ENPP1,FMR1,LIMK2,PAX3,WDR7,ATP5J,FAM126A,FPGS,GABRA1,GPRIN2,KLF6,PRKX,SBF2,ULK4,ABCD1,CRMP1,EVC,MAD1L1,MFNG,RPGRIP1,CCDC141,GLDN,PCK1,PDE6B,PPARA,TTN,ZFHX2,C1GALT1,COL19A1,FHL1,GNA13,MYH7,TAF1,CHD5,PPP2R3C,SPRED1,CHSY1,LCE1F,NTN4,PTPRF,BTRC,RIT2,ESRP2,EXT1,MDM4,MYH6,NCAPH2,NCOA1,PRKCG,PRLR,SCO2,TYMP,UMOD,ADAMTS5,CBFA2T3,DOCK7,FBXW11,HDAC5,KLHL3,TDGF1,A2M,MYEF2,SLC38A10,SLC40A1,STIM1,TTC39C,ADARB1,KCNC2,MYH11,NDE1,POU2AF1,PRKCQ,SCEL,STAT3,TP63,AMOT,BRIP1,HPN,ISLR2,PAK3,USH2A,BLNK,DHRS2,DHRS7B,DTNBP1,GAK,CRTAC1,LCE2A,LGALS9,LRP8,GDA,MAPKAPK2,UPK3A,APC,BMPR1A,CSN3,ITGA1,KIAA0319,NGF,PRKCA,SPINT2,UBASH3B,VLDLR,KRT75,ABI1,CTSH,NCAPG2,RGCC,CYB5D2,GABRG2,IL6R,LCE4A,MAP2K6,TFIP11,SPTBN4,CLASP2,MAP2K1,SOS1,TPH1,VPS35,COL6A3,FGF1,MCOLN3,TBX20,DCHS2,PPP1R9A,RGN,RREB1,TBX18,BZW2
GO:0048856	anatomical structure development	4.874862724718194e-29	ISM1,SRGAP2B,MACROD2,CDH4,PLCB1,DHX35,CMKLR1,DAB1,OMA1,GTF2I,FMNL2,GABRB3,CDC42EP3,MAPK14,DSCAM,ARID1B,RUNX1,ASIC2,MYO18B,NCAM2,HUNK,ANK3,DCC,SVEP1,WLS,RBFOX1,PCSK2,AKAP13,FBLN1,ERG,GRIK1,LARGE,SORBS2,SLC24A3,NPHP3,ITGA2,KCNQ1,DPF3,SUN2,NUBPL,CA10,EPHA6,GRIN3A,NRXN1,SHC3,ZRANB1,ISX,DOK5,NRXN3,SLC1A2,SDK2,DOCK2,CTNND2,EYS,TNC,POTEE,MS4A1,PAX7,CHODL,RIMS2,MTPN,SHANK2,NRG3,SMYD2,HUS1,PKD1L1,ARNT2,FGD6,OLFM3,TGFBR3,FSTL4,CDH13,ANK2,LRRC4C,RTN1,ANKH,NXN,CSMD1,IGSF3,SYNDIG1,TF,SCN8A,

			BCAN,SDCCAG8,TMEM108,GADD45A,ADAMTSL1,PCP4,ACIN1,SYT17,PLXNA4,FLRT2,GPC3,NRG1,ETS2,PSMB2,HYDIN,IL4R,ALDH1A2,CECR2,LRFN5,NLN,SUZ12,ASTN2,PLCE1,UNC5D,SEMA6D,SPOCK1,DRP2,CTNNA2,CCR1,CCR3,CFTR,RBFOX2,TIAM1,RHOC,COL22A1,AGBL4,LDLRAD4,KIAA1217,RASGRF1,DPYSL2,MYO3A,TET3,CBFA2T2,IL1RAPL2,NKD1,TACC2,ALK,UTRN,SPEF2,ZNF536,FBXO31,CNTN4,ARID5B,PHACTR1,S100B,ROBO2,C2CD3,EPHA3,PRKACB,DCLK1,COL15A1,AJAP1,AGT,FSIP2,ATAT1,WDR11,ATP6V0D1,CDH11,RHOJ,RCAN1,CDH2,CDH9,PARK2,FHL2,FOXN3,LOXL2,MDGA2,LAMA2,GREB1L,ZNF804A,CYBB,NEGR1,OTC,GNB4,SEMA3D,DACH1,NTN1,KCNH1,TXK,GPC6,NELL1,TRAPPC9,PCDH17,SETD3,ZNRF3,FMNL3,TRIO,APBA2,FTO,HDAC2,HTRA1,LAMA4,MYO9A,SLIT2,TFAP2D,CDH8,CNTN5,PDCD6,TM4SF1,RPS6KA2,IGF1R,KRT25,MDM2,PALMD,SLC24A4,SMAD1,DSC3,FOXD4L4,GALC,CTNNBL1,RAPGEF5,VCAN,ADAMTS6,PTPRG,CPQ,TRPC5,DMD,HCN1,SYT1,BASP1,KRT2,SIPA1L3,VCL,NOX5,TEAD1,CELSR1,CSMD3,FGF14,FSHR,MBOAT7,FMN2,MAEL,NRIP1,ASTN1,STK24,OPCML,PLAC1,WWOX,ATRX,TRPM2,RYR2,OCA2,XRCC4,DIAPH2,KIF3B,NTNG1,TENM4,CATSPER2,LRRK2,RORB,SLC1A3,STRC,IGFBP7,PIR,KIF26B,NTRK3,RORA,PRICKLE2,ANKS1A,CHD7,RNF165,DISC1,HEXB,EMB,SH3BP1,SOX6,IER2,MECP2,MTMR2,SEMA4D,GSK3B,LGR5,LINGO2,SLC8A1,PRKD1,SNRK,PLEKHA5,ERCC6,FLVCR1,FRMD6,TGFB2,CLSTN2,FMN1,VAV3,AFF3,DACH2,LCE2B,LCE2C,PLCD3,RIPPLY3,SEMA5A,SLC5A3,ACSBG1,BDNF,KRT74,LSAMP,PRKCH,RIPK4,RYK,ZDHHC17,KCNC1,PCDH15,DNAAF2,EML1,ABI3BP,SGCD,WASF1,WDR72,GRIN2B,KDM4C,POLE,EXT2,LAMA3,RAG1,RAG2,ABCB5,ANKRD6,NR2F2,SNAP25,NR5A2,PAQR8,PML,PPARGC1A,SGCG,SYBU,ARHGAP12,AKAP6,CNTNAP2,FBN1,PPP4R4,PLXNA2,TAOK3,BCL2,CDH10,CHMP4C,RAD51B,RALBP1,RXFP2,ZNF675,ARHGAP24,EPHB1,MOV10L1,SMARCE1,CDH12,SIPA1L1,CSGALNACT1,DISP1,LRP2,MMP16,TMEM100,LRRC4,NFIA,VANGL2,CDH6,NR4A2,ARHGEF10,EPHA7,FYN,GNRHR,KCNE2,ADAMTS18,CHN1,NR4A1,SMARCA4,SULT4A1,CD44,FLNC,HECW1,PMP22,RNF213,THBS2,CASP6,FOXO1,PDE4D,PYY,RBBP8,KANK1,ENPP2,ABCB10,ACTN2,ATF6,ATP8A2,DCDC2,GABRB1,KLF13,MME,PACRG,TNMD,EXOC4,NAV2,LAMB4,ZNF516,CELF4,SERPINB7,TPO,UNC5C,MYLK3,NTM,SLC1A1,UGCG,ZFPM2,ZNF141,PALLD,RARB,RFX4,STRIP1,ETV6,MTOR,ROR1,GAS7,LDB2,MOV10,CHRM1,FAT3,MEGF9,PKP2,BMPER,ERMN,PTPRD,TIAM2,ZNF521,BBS2,MATN3,PGK1,ZFP64,CEP85L,COL11A1,EPM2A,PLN,CTNNBIP1,KLHL1,SOX8,DSG4,IFT88,NLGN4X,CDH18,HELLS,KMT2A,PRKCB,VDR,FOXO3,IGF2BP3,LRRTM1,MITF,SCMH1,CAPN3,KALRN,CCDC88C,DLC1,EDNRB,MSI2,ROBO1,RXFP1,SATB2,TANC2,FAP,FLT1,NASP,NOX4,ZNF148,CDH17,GRHL2,TCF4,COL2A1,MALL,NEBL,PREX2,TRAF3IP2,CBLN4,CR1,EYA4,GABRA2,SEC24B,MDM1,PKHD1,STK3,TGM2,VASH2,FAM9B,KDR,PPHLN1,PRCP,ARHGAP15,CHL1,EDA,ABCA12,APP,CALCRL,COL18A1,NRP1,PTPRM,SHROOM3,ARHGEF18,LGR4,TENM2,ADTRP,EGFLAM,IMMP2L,LCE6A,THRB,TTC12,KCNK2,SEMA3C,ADAM17,CAMK1D,MAP3K5,PARVG,TSPEAR,IGF2R,ATF2,ELP3,ETS1,NR2C2,EFEMP1,PRTG,ROS1,TGFB1I1,RPS6KA5,TCF7L2,AFF2,BICD1,ITGA11,PARD3,THOC2,ZNF830,ABAT,ATRNL1,FRAS1,GRIN2A,GRIP1,DLX6-AS1,HRNR,OPHN1,TRPS1,ZNF568,CASP5,CCDC88A,CD38,DENND5A,RIN2,TUB,ALCAM,CHRDL1,CNN3,STK36,UPB1,BCL2L1,ADAMTS16,HDAC4,MYO16,SOX5,SRRM4,DNMT1,GHR,JAM2,NTRK2,PRKG1,ACSL4,A1CF,DNM3,RASGRP1,STARD13,CAMK4,SDK1,SPRED2,GLI3,KAZN,SMCHD1,CACNA1C,CLDN1,KIF16B,SPRR2B,SPRR2E,CERS3,CRB1,RAP1A,SAFB2,SRP54,TGFBR1,CYSLTR1,PRDX4,RRP7A,ADAM12,DICER1,DYM,EPHA5,THEMIS,TNR,TOP1,CUX1,KRTAP6-1,PEAK1,SLC6A17,TENM3,CHST11,CPE,RAPGEF1,CHRNA3,EP300,MORC3,SYNE1,DSCAML1,HMG20A,SCFD1,TBX3,WIF1,CYP7B1,SPRR4,TRIOBP,ATXN1,GAB2,PCSK5,SYCP1,TP73,GRID2,PARVB,PSMA1,FLNB,SPG11,ADCK1,ADORA2A,CAMK2G,DDX10,EFNA5,EMCN,FAM20A,MERTK,SPRR2G,WARS2,BRD1,FAM9A,MAGI2,NF1,RGS7,SORL1,BICC1,ELK3,IFT80,IGSF21,TEAD4,TTC8,DMRT1,ASXL1,EIF4E,FRYL,POR,RELN,TNFRSF11B,TSHZ1,GRM5,HAPLN1,HDAC9,IL18R1,KIRREL3,NECAB1,PTPN9,COL12A1,LRRTM3,DDC,NDRG2,NUMB,MAP3K13,NPHP4,RALA,TBX15,ADCYAP1R1,IL1RAP,NAV3,ATP10A,EXOC5,FBXL17,JAG1,MEOX2,PPP3CA,KLF3,KLF7,MYT1L,NEB,PLS1,SH3GL2,CAPZB,CXADR,MAS1,WWTR1,KL,C6,CBLB,DOCK10,ELAVL4,GSTA2,MNAT1,RNF17,SEC63,CNTN1,DGUOK,GPC4,GRM7,RIMS1,TRIM16,GABPA,KAT7,L3MBTL3,TMEM135,AGGF1,ASAP1,FHOD3,FREM1,N4BP2L2,NLGN1,PTPRU,AKIRIN2,ARHGAP5,CHST8,CTTNBP2,SH3KBP1,SSUH2,AK8,MEGF11,NPR3,SYCP2,LRRK1,MAPK8IP2,OM

			G,EHF,GABRA5,JAK2,SHOX2,SOBP,SPNS2,AHI1,SMAD3,XKR4,KIDINS220,NOS1,PTGFRN,SERPINB12,COL8A1,PLXNB1,RDH10,TCF12,AATF,ANHX,CDK1,HDAC7,HIVEP3,ITPK1,LTB,MCM3AP,NBN,PRRC2C,TAGLN3,TNF,COBL,NHS,OVOL2,PANX1,ZFPM1,CHKB,PPFIA2,TANC1,ADNP2,ATXN10,BLOC1S6,BMP15,DLG5,PHEX,POU6F2,LGR6,SIM2,ARID2,MMP20,PAXBP1,BRINP1,SPAG16,BCL11A,CALD1,FREM2,HMGA2,MYO6,DOCK1,KIF18A,PCDH19,SULT1B1,QKI,RAB23,EPS8,MBOAT2,TPD52,DAAM2,PABPC1L,SMOC2,SULF1,TRPC4,C5AR1,CISD2,LRP4,PIK3R3,WDR5,XYLT1,CNTN6,EFNB2,AMOTL1,HERC1,PHF8,SPATA5,ZNF423,CLSTN1,INSC,ALPK2,CTNNA1,TENM1,CDKL5,DMBT1,EFHC2,MAMLD1,MYH14,PRKAR1A,TNFRSF19,ARHGAP11B,BPTF,DYNC2H1,FAM171A1,MLLT3,CD109,EGF,MIP,PTPN14,CMA1,FOXP4,IL1RAPL1,KIT,SLIT3,TAF4B,TNFSF8,TRPV4,VWC2,CC2D2A,CPS1,DNAH11,EDAR,EFNB1,PTPRO,RAPGEF2,DNAJB6,ESR1,JAK1,MYPN,NCMAP,SEMA3E,UBE3A,DNMBP,FGF13,GABRB2,PLAG1,SGCZ,ARMC2,EPHB2,FNDC3A,KRT76,MECOM,TBCD,TFDP2,TOX,C14ORF39,ERBB4,FER,MAP3K3,MINPP1,RBM19,SLITRK6,SPTA1,FNIP1,LRP5,TTLL5,XKR7,MYH15,SLAMF1,ABL2,BSG,CAMP,F11R,FARP2,GAS8,SETD2,ALX4,AP2B1,ARHGAP35,CLDN11,NSUN2,SGMS2,CCL3,FGF7,LAMA1,MYO7B,PREX1,SEMA5B,SPRED3,FGD4,KIF2A,NHLH2,PSG9,SULF2,ARF4,MYH9,PACSIN2,SLC39A12,SOX4,NEDD9,PDGFD,PIK3CD,AGTPBP1,ASGR2,ITGA8,ARID4A,ENPEP,FAM172A,KDM3A,MYO3B,PDZD7,UNC13A,WDPCP,ADCY9,AUTS2,CDH23,FGFR1,MEF2A,RYR1,ARID4B,DAD1,MAP2,ASXL3,BPGM,IL23R,ITGB7,KEAP1,NTF3,P2RX7,SCUBE1,AR,BCOR,CASP7,FGL2,KIAA1109,PAPSS1,SUFU,EYA1,IFNA8,IL1RL2,RBM4,SRPK2,HLA-DRA,BHLHB9,L3MBTL1,NEK5,SEMA3A,ANKRD11,CDC73,EPB41L3,JRKL,MARK2,MEIS2,PDE5A,ZMIZ1,FLT3,PDLIM5,PPARGC1B,TRIM72,FERMT2,HCN4,LTBR,MAP2K5,MARK1,PAPPA2,SMPD3,TNN,DAB2,GPR55,LCP1,PBX1,RNF207,SRD5A2,STX3,SYT2,ACTN4,ADAM10,BLOC1S5,CCL7,DAAM1,FGF12,ITGA3,POU2F3,FRY,LGI1,NREP,PLCL2,LECT2,LRIG1,PIN1,RPS6KA6,THSD7A,TRPM4,SCLT1,SHROOM4,BMP6,CHRM3,CIT,CLASP1,EIF4ENIF1,CFDP1,CNGB1,TGFA,TMOD1,GAS2,HGF,MYLK,PRPSAP2,CALCR,CENPF,DPY19L2,FLI1,SLC26A8,ZBTB16,ELN,NCAM1,NFASC,INSR,MACF1,PAK1,PDE3A,PSMB7,SKI,AKT3,CTDP1,ENPP1,FMR1,LIMK2,PAX3,WDR7,ATP5J,FAM126A,FPGS,GABRA1,GPRIN2,KLF6,PRKX,SBF2,ULK4,ABCD1,CRMP1,EVC,IDE,MAD1L1,MFNG,RPGRIP1,CCDC141,GLDN,PCK1,PDE6B,PPARA,SORT1,TTN,ZFHX2,C1GALT1,COL19A1,FHL1,GNA13,MYH7,TAF1,CHD5,FUT8,PPP2R3C,S1PR3,SPRED1,AKR1C2,CHSY1,KRT4,LCE1F,NTN4,PTPRF,SCN9A,BTRC,HIRA,PGM5,PTPRR,RIT2,ESRP2,EXT1,MDM4,MYH6,NCAPH2,NCOA1,PRKCG,PRLR,SCO2,TYMP,UMOD,ADAMTS5,CBFA2T3,DOCK7,FBXW11,HDAC5,KLHL3,TDGF1,A2M,AKAP2,LYVE1,MYEF2,SLC38A10,SLC40A1,STIM1,TTC39C,ADARB1,KCNC2,MYH11,NDE1,PEMT,POU2AF1,PRKCQ,SCEL,STAT3,TP63,AMOT,BRIP1,HPN,ISLR2,PAK3,USH2A,BLNK,DHRS2,DHRS7B,DTNBP1,GAK,RAP1B,CRTAC1,LCE2A,LGALS9,LRP8,MYOM2,GDA,MAPKAPK2,UPK3A,APC,BMPR1A,CSN3,ITGA1,KIAA0319,NGF,PRKCA,SPINT2,UBASH3B,VLDLR,KRT75,ABI1,CTSH,NCAPG2,RGCC,CYB5D2,EDA2R,GABRG2,IL6R,LCE4A,MAP2K6,SIAH3,TFIP11,SPTBN4,CLASP2,MAP2K1,SOS1,TPH1,VPS35,COL6A3,FGF1,MCOLN3,TBX20,DCHS2,PPP1R9A,RGN,RREB1,TBX18,BZW2
GO:0007275	multicellular organism development	6.035311461212255e-28	*ISM1,SRGAP2B,MACROD2,CDH4,PLCB1,DHX35,CMKLR1,DAB1,GTF2I,GABRB3,MAPK14,DSCAM,ARID1B,RUNX1,ASIC2,MYO18B,NCAM2,HUNK,ANK3,DCC,SVEP1,WLS,RBFOX1,PCSK2,AKAP13,FBLN1,ERG,GRIK1,LARGE,SORBS2,SLC24A3,NPHP3,ITGA2,KCNQ1,DPF3,SUN2,CA10,EPHA6,GRIN3A,NRXN1,SHC3,ISX,DOK5,NRXN3,SLC1A2,SDK2,DOCK2,CTNND2,TNC,POTEE,MS4A1,PAX7,CHODL,RIMS2,MTPN,SHANK2,NRG3,SMYD2,HUS1,PKD1L1,ARNT2,OLFM3,TGFBR3,FSTL4,CDH13,ANK2,LRRC4C,RTN1,ANKH,NXN,CSMD1,IGSF3,SYNDIG1,TF,SCN8A,BCAN,SDCCAG8,TMEM108,GADD45A,ADAMTSL1,PCP4,ACIN1,SYT17,PLXNA4,FLRT2,GPC3,NRG1,ETS2,PSMB2,HYDIN,IL4R,ALDH1A2,CECR2,LRFN5,NLN,SUZ12,ASTN2,PLCE1,UNC5D,SEMA6D,SPOCK1,DRP2,CTNNA2,CCR1,CCR3,CFTR,RBFOX2,TIAM1,COL22A1,AGBL4,LDLRAD4,KIAA1217,RASGRF1,DPYSL2,MYO3A,TET3,CBFA2T2,IL1RAPL2,NKD1,TACC2,ALK,UTRN,SPEF2,ZNF536,FBXO31,CNTN4,ARID5B,PHACTR1,S100B,ROBO2,C2CD3,EPHA3,PRKACB,DCLK1,COL15A1,AJAP1,AGT,ATAT1,WDR11,ATP6V0D1,CDH11,RHOJ,RCAN1,CDH2,PARK2,FHL2,FOXN3,LOXL2,MDGA2,LAMA2,GREB1L,ZNF804A,CYBB,NEGR1,OTC,GNB4,SEMA3D,DACH1,NTN1,GPC6,NELL1,TRAPPC9,PCDH17,ZNRF3,FMNL3,TRIO,APBA2,FTO,HDAC2,HTRA1,LAMA4,MYO9A,SLIT2,TFAP2D,CNTN5,PDCD6,TM4SF1,RPS6KA2,IGF1R,KRT25,MDM2,SLC24A4,SMAD1,DSC3,GALC,CTNN*

			BL1,RAPGEF5,VCAN,ADAMTS6,PTPRG,TRPC5,DMD,HCN1,SYT1,BASP1,KRT2,SIPA1L3,VCL,NOX5,TEAD1,CELSR1,CSMD3,FGF14,FSHR,MBOAT7,NRIP1,ASTN1,STK24,OPCML,PLAC1,WWOX,ATRX,TRPM2,RYR2,XRCC4,KIF3B,NTNG1,TENM4,LRRK2,RORB,SLC1A3,STRC,IGFBP7,PIR,KIF26B,NTRK3,RORA,PRICKLE2,ANKS1A,CHD7,RNF165,DISC1,HEXB,EMB,SOX6,IER2,MECP2,MTMR2,SEMA4D,GSK3B,LGR5,LINGO2,SLC8A1,PRKD1,SNRK,PLEKHA5,ERCC6,FLVCR1,TGFB2,CLSTN2,FMN1,VAV3,AFF3,DACH2,LCE2B,LCE2C,PLCD3,RIPPLY3,SEMA5A,SLC5A3,ACSBG1,BDNF,KRT74,LSAMP,PRKCH,RYK,ZDHHC17,KCNC1,PCDH15,DNAAF2,EML1,ABI3BP,SGCD,WASF1,WDR72,GRIN2B,KDM4C,POLE,EXT2,LAMA3,RAG1,RAG2,ABCB5,ANKRD6,NR2F2,SNAP25,NR5A2,PML,PPARGC1A,SGCG,SYBU,AKAP6,CNTNAP2,FBN1,PPP4R4,PLXNA2,TAOK3,BCL2,CHMP4C,RAD51B,RXFP2,ZNF675,ARHGAP24,EPHB1,SMARCE1,SIPA1L1,CSGALNACT1,DISP1,LRP2,MMP16,TMEM100,LRRC4,NFIA,VANGL2,NR4A2,ARHGEF10,EPHA7,FYN,GNRHR,KCNE2,ADAMTS18,CHN1,NR4A1,SMARCA4,SULT4A1,CD44,HECW1,PMP22,RNF213,THBS2,FOXO1,PYY,RBBP8,KANK1,ENPP2,ABCB10,ACTN2,ATF6,ATP8A2,DCDC2,GABRB1,KLF13,MME,TNMD,EXOC4,NAV2,LAMB4,ZNF516,CELF4,SERPINB7,TPO,UNC5C,MYLK3,NTM,SLC1A1,UGCG,ZFPM2,ZNF141,PALLD,RARB,RFX4,ETV6,MTOR,ROR1,GAS7,LDB2,MOV10,CHRM1,FAT3,MEGF9,PKP2,BMPER,PTPRD,TIAM2,ZNF521,BBS2,MATN3,PGK1,ZFP64,CEP85L,COL11A1,EPM2A,PLN,CTNNBIP1,KLHL1,SOX8,DSG4,IFT88,NLGN4X,HELLS,KMT2A,PRKCB,VDR,FOXO3,IGF2BP3,LRRTM1,MITF,SCMH1,CAPN3,KALRN,DLC1,EDNRB,ROBO1,RXFP1,SATB2,TANC2,FAP,FLT1,NASP,NOX4,ZNF148,CDH17,GRHL2,TCF4,COL2A1,MALL,NEBL,PREX2,TRAF3IP2,CBLN4,CR1,EYA4,GABRA2,SEC24B,MDM1,PKHD1,STK3,TGM2,VASH2,KDR,PPHLN1,PRCP,CHL1,EDA,ABCA12,APP,CALCRL,COL18A1,NRP1,PTPRM,SHROOM3,LGR4,TENM2,ADTRP,EGFLAM,IMMP2L,LCE6A,THRB,KCNK2,SEMA3C,ADAM17,CAMK1D,TSPEAR,IGF2R,ATF2,ELP3,ETS1,NR2C2,EFEMP1,PRTG,TGFB1I1,RPS6KA5,TCF7L2,AFF2,ITGA11,PARD3,THOC2,ZNF830,ABAT,ATRNL1,FRAS1,GRIN2A,GRIP1,DLX6-AS1,HRNR,OPHN1,TRPS1,ZNF568,CASP5,CCDC88A,CD38,DENND5A,RIN2,TUB,ALCAM,CHRDL1,STK36,UPB1,BCL2L1,ADAMTS16,HDAC4,MYO16,SOX5,SRRM4,DNMT1,GHR,JAM2,NTRK2,PRKG1,ACSL4,A1CF,DNM3,RASGRP1,STARD13,CAMK4,SDK1,SPRED2,GLI3,KAZN,SMCHD1,CACNA1C,CLDN1,KIF16B,SPRR2B,SPRR2E,CERS3,CRB1,RAP1A,SAFB2,SRP54,TGFBR1,CYSLTR1,PRDX4,RRP7A,ADAM12,DICER1,DYM,EPHA5,THEMIS,TNR,TOP1,CUX1,KRTAP6-1,SLC6A17,TENM3,CHST11,CPE,RAPGEF1,CHRNA3,EP300,MORC3,DSCAML1,HMG20A,TBX3,CYP7B1,SPRR4,TRIOBP,ATXN1,GAB2,PCSK5,TP73,GRID2,PSMA1,FLNB,SPG11,ADORA2A,CAMK2G,EFNA5,EMCN,FAM20A,MERTK,SPRR2G,WARS2,BRD1,MAGI2,NF1,RGS7,SORL1,BICC1,ELK3,IFT80,IGSF21,TEAD4,TTC8,DMRT1,ASXL1,EIF4E,FRYL,POR,RELN,TNFRSF11B,TSHZ1,GRM5,HAPLN1,HDAC9,IL18R1,KIRREL3,NECAB1,PTPN9,COL12A1,LRRTM3,DDC,NDRG2,NUMB,MAP3K13,NPHP4,RALA,TBX15,ADCYAP1R1,IL1RAP,NAV3,FBXL17,JAG1,MEOX2,PPP3CA,KLF3,KLF7,MYT1L,NEB,PLS1,SH3GL2,CXADR,MAS1,WWTR1,KL,C6,CBLB,DOCK10,ELAVL4,MNAT1,SEC63,CNTN1,DGUOK,GPC4,GRM7,RIMS1,TRIM16,GABPA,KAT7,L3MBTL3,AGGF1,ASAP1,FHOD3,FREM1,N4BP2L2,NLGN1,PTPRU,AKIRIN2,ARHGAP5,CHST8,CTTNBP2,SSUH2,AK8,MEGF11,NPR3,SYCP2,LRRK1,MAPK8IP2,OMG,GABRA5,JAK2,SHOX2,SOBP,SPNS2,AHI1,SMAD3,KIDINS220,SERPINB12,COL8A1,PLXNB1,RDH10,TCF12,AATF,ANHX,CDK1,HDAC7,HIVEP3,ITPK1,LTB,MCM3AP,NBN,PRRC2C,TAGLN3,TNF,COBL,NHS,OVOL2,ZFPM1,CHKB,PPFIA2,ADNP2,ATXN10,BLOC1S6,BMP15,DLG5,PHEX,POU6F2,LGR6,SIM2,ARID2,MMP20,PAXBP1,BRINP1,BCL11A,CALD1,FREM2,HMGA2,MYO6,DOCK1,KIF18A,PCDH19,QKI,RAB23,MBOAT2,TPD52,DAAM2,SMOC2,SULF1,TRPC4,C5AR1,CISD2,LRP4,PIK3R3,WDR5,XYLT1,CNTN6,EFNB2,AMOTL1,HERC1,PHF8,SPATA5,ZNF423,CLSTN1,INSC,ALPK2,CTNNA1,TENM1,CDKL5,EFHC2,MAMLD1,MYH14,PRKAR1A,TNFRSF19,ARHGAP11B,BPTF,DYNC2H1,MLLT3,CD109,EGF,MIP,PTPN14,CMA1,FOXP4,IL1RAPL1,KIT,SLIT3,TNFSF8,TRPV4,VWC2,CC2D2A,CPS1,DNAH11,EDAR,EFNB1,PTPRO,RAPGEF2,DNAJB6,ESR1,JAK1,MYPN,NCMAP,SEMA3E,UBE3A,FGF13,GABRB2,PLAG1,SGCZ,EPHB2,FNDC3A,KRT76,MECOM,TBCD,TFDP2,TOX,ERBB4,MAP3K3,MINPP1,RBM19,SLITRK6,SPTA1,FNIP1,LRP5,TTLL5,MYH15,SLAMF1,ABL2,BSG,CAMP,F11R,FARP2,GAS8,SETD2,ALX4,AP2B1,ARHGAP35,CLDN11,NSUN2,SGMS2,CCL3,FGF7,LAMA1,MYO7B,PREX1,SEMA5B,SPRED3,KIF2A,NHLH2,PSG9,SULF2,ARF4,MYH9,SLC39A12,SOX4,PDGFD,PIK3CD,AGTPBP1,ASGR2,ITGA8,ARID4A,ENPEP,FAM172A,MYO3B,PDZD7,UNC

			13A,WDPCP,ADCY9,AUTS2,CDH23,FGFR1,MEF2A,RYR1,ARID4B,DAD1,MAP2,ASXL3,BPGM,IL23R,KEAP1,NTF3,P2RX7,SCUBE1,AR,BCOR,CASP7,FGL2,KIAA1109,PAPSS1,SUFU,EYA1,IFNA8,IL1RL2,SRPK2,HLA-DRA,BHLHB9,L3MBTL1,SEMA3A,ANKRD11,CDC73,EPB41L3,JRKL,MARK2,MEIS2,ZMIZ1,FLT3,PDLIM5,PPARGC1B,TRIM72,FERMT2,HCN4,LTBR,MAP2K5,MARK1,PAPPA2,SMPD3,TNN,DAB2,GPR55,LCP1,PBX1,RNF207,SRD5A2,STX3,SYT2,ADAM10,BLOC1S5,DAAM1,FGF12,ITGA3,FRY,LGI1,NREP,PLCL2,LECT2,LRIG1,PIN1,RPS6KA6,THSD7A,TRPM4,SCLT1,SHROOM4,BMP6,CHRM3,CIT,CLASP1,EIF4ENIF1,CNGB1,TGFA,TMOD1,GAS2,HGF,MYLK,PRPSAP2,CALCR,CENPF,FLI1,ZBTB16,ELN,NCAM1,NFASC,INSR,MACF1,PAK1,PSMB7,SKI,AKT3,CTDP1,ENPP1,FMR1,LIMK2,PAX3,WDR7,ATP5J,FAM126A,FPGS,GABRA1,GPRIN2,KLF6,PRKX,SBF2,ULK4,ABCD1,CRMP1,EVC,IDE,MAD1L1,MFNG,RPGRIP1,CCDC141,GLDN,PCK1,PDE6B,PPARA,TTN,ZFHX2,C1GALT1,COL19A1,FHL1,GNA13,MYH7,TAF1,CHD5,FUT8,PPP2R3C,S1PR3,SPRED1,CHSY1,LCE1F,NTN4,PTPRF,SCN9A,BTRC,HIRA,PTPRR,RIT2,ESRP2,EXT1,MDM4,MYH6,NCAPH2,NCOA1,PRKCG,PRLR,SCO2,TYMP,UMOD,ADAMTS5,CBFA2T3,DOCK7,FBXW11,HDAC5,KLHL3,TDGF1,A2M,MYEF2,SLC38A10,SLC40A1,STIM1,TTC39C,ADARB1,KCNC2,MYH11,NDE1,PEMT,POU2AF1,PRKCQ,SCEL,STAT3,TP63,AMOT,BRIP1,HPN,ISLR2,PAK3,USH2A,BLNK,DHRS2,DHRS7B,DTNBP1,GAK,CRTAC1,LCE2A,LGALS9,LRP8,GDA,MAPKAPK2,UPK3A,APC,BMPR1A,CSN3,ITGA1,KIAA0319,NGF,PRKCA,SPINT2,UBASH3B,VLDLR,KRT75,ABI1,CTSH,NCAPG2,RGCC,CYB5D2,GABRG2,IL6R,LCE4A,MAP2K6,SIAH3,TFIP11,SPTBN4,CLASP2,MAP2K1,SOS1,TPH1,VPS35,COL6A3,FGF1,MCOLN3,TBX20,DCHS2,PPP1R9A,RGN,RREB1,TBX18,BZW2
GO:0065007	biological regulation	5.4881435190474596e-27	*LINC00273,IGHV1OR21-1,ISM1,TASP1,FANK1,CAST,ADCY2,SRGAP2B,TPTE,CDH4,PIEZO2,ZHX3,PLCB1,KMT2C,KHDRBS2,ZNF595,CMKLR1,DAB1,OMA1,DUX4,GTF2I,ZNF717,FMNL2,GABRB3,CDC42EP3,GABRG3,MAPK14,ABCG8,DSCAM,ARID1B,TPTE2,MIR663A,GPHN,RUNX1,ORC3,SH3RF3,ASIC2,CTBP2,DTNA,OR11H1,SERPINA1,TSHZ2,CCNG2,RAB27A,HUNK,CACNA1E,KCNJ6,PCBD2,ANXA8L1,NPY4R,PRIM2,LYPD6,OR4M2,PTPRT,MIR1185-1,MIR1185-2,MIR134,MIR154,MIR300,MIR323B,MIR376C,MIR381,MIR382,MIR485,MIR487A,MIR487B,MIR539,MIR544A,MIR654,MIR655,MIR889,ANK3,IGHV4-31,DTD2,DCC,BCL2L13,IGHV3-64,SVEP1,WLS,RBFOX1,SYN3,TIMP3,ZNF397,ZSCAN30,DUSP22,PCSK2,TTC28,AKAP13,FBLN1,ERG,VPS13D,GRIK1,ZNF292,GPR139,IGHV1OR15-9,LARGE,SORBS2,SLC24A3,IGHV4OR15-8,NPHP3,ITGA2,ABCG1,TMPRSS2,RGS6,KCNQ1,DPF3,SUN2,GABBR2,ATF7IP,EPHA6,OR4K15,TMEM117,VRK2,GRIN3A,FCRL2,NRXN1,SHC3,TMPRSS3,MIR369,MIR410,MIR656,ZRANB1,ISX,TNNI3K,DOK5,SGSM1,NRXN3,SLC1A2,RAB5A,ZNF578,DOCK2,CTNND2,TNC,PCNT,PLEKHB2,POTEE,MS4A1,OR8U1,PAX7,CHODL,RIMS2,MTPN,SHANK2,COMT,GRIA1,NRG3,ZNF732,SMYD2,CELF2,CLEC16A,HUS1,ARNT2,FGD6,SLC9B1,TGFBR3,ANO4,FSTL4,CDH13,RNF152,ANK2,TBC1D22A,LRRC4C,RTN1,ANKH,NXN,KCND3,CSMD1,RUNX1T1,FAM155A,CSNK2A1,PDE4DIP,SYNDIG1,SLC12A1,ZNF678,DPP10,ZNF112,ZNF229,ZNF285,TF,PLGRKT,PRIMA1,ERCC4,SCN8A,KCNE1,PIK3C2B,SAMD4A,SDCCAG8,MIR99A,MIRLET7C,TMEM108,EIF4EBP3,CHEK2,OR4C12,ZNF486,GADD45A,PRDM9,KCNJ15,PCP4,PSMC6,ACIN1,ZNF331,SYT17,PLXNA4,KCNG3,FLRT2,TOX3,GPC3,FBLN5,NRG1,SCARA5,STXBP6,ETS2,PSMB2,SHISA6,RAD51D,RFFL,NPAS3,SPIDR,IL4R,ALDH1A2,LRFN5,RYR3,EWSR1,MCTP1,GGT1,MIR17HG,NLN,ST8SIA1,SUZ12,LCORL,PRMT8,ASTN2,PLCE1,SH3RF2,UNC5D,KSR2,GLP2R,STK38L,SEMA6D,SPOCK1,ABHD17C,CTNNA2,SLCO3A1,CCR1,CCR3,CFTR,RBFOX2,ZNF420,DTX4,MIR551B,ZNF257,MED13L,PCTP,MC2R,TIAM1,PM20D1,RHOC,AGBL4,BEND5,LDLRAD4,DNAJC15,RGS7BP,ZNF91,RASGRF1,ZBTB20,DPYSL2,GRID1,SLC6A2,TET3,ARFIP1,CBFA2T2,IL1RAPL2,OR4A5,NKD1,HPSE2,SCAI,MX1,BTN2A1,ALK,PTPRE,PIK3C3,UTRN,SORCS3,ZNF536,FAM3B,PLCB4,ERC1,FBXO31,KLRF2,RAB7A,CNTN4,RGL2,TAPBP,ARID5B,PHACTR1,S100B,ROBO2,SPOCK3,ZNF366,C2CD3,EPHA3,PRKACB,PTPRN2,CNTNAP4,DCLK1,PXDN,COL15A1,CRNN,AJAP1,LEMD3,OR8K3,AGT,ERC2,PHACTR3,CST2,MAGEB3,SHISA9,ATAT1,AVEN,CHRM5,FBXO32,WDR11,ATP6V0D1,CDH11,RHOJ,RCAN1,CDH2,KLHL25,PAH,PARK2,BANP,FHL2,FOXN3,LGALS14,LOXL2,DLGAP1,LAMA2,CDC5L,KIR2DL1,KIR2DL4,KIR3DL2,OR4K13,BPIFB1,CORO2B,FRMPD4,TRIM51,ZNF804A,CYBB,MCTP2,NEGR1,OTC,PSPC1,BRF1,GNB4,OR4M1,OR4N2,SEMA3D,DACH1,NTN1,OR52N5,TP53I11,TRIM22,TRIM5,KCNH1,U*

			SP25,ZBTB8B,ZNF268,DCUN1D4,TXK,CP,DENND2A,GPC6,RNF185,USP40,USP6,ZNF232,ANKFN1,NELL1,TRAPPC9,DBH,ITSN1,TNS3,TSPAN8,NCF4,PCDH17,PHC1,SETD3,ZNRF3,FMNL3,KCNMA1,RIN3,TRIO,ZNF845,APBA2,FTO,HDAC2,HTRA1,LAMA4,MYO9A,SLIT2,TFAP2D,ZBTB34,CDH8,KCTD1,AHRR,HSA-MIR-490,PDCD6,ZSCAN5C,OR2L13,RPS6KA2,STXBP4,GPC5,LCMT1,IGF1R,MDM2,MX2,PALMD,SLC24A4,SMAD1,DSC3,E2F3,NOVA1,SYT9,ZBED4,FOXD4L4,OR11G2,OR9Q1,TMPRSS6,ZNF567,ZNF850,CTNNBL1,RAPGEF5,CEP97,GABRG1,KCNK17,NR3C2,PTPRG,PXK,PDE9A,CPQ,OR8K5,SACS,TRIM48,TRPC5,DMD,HCN1,KCTD8,STK33,SYT1,BASP1,CACNA2D3,CACNG3,KRT2,SIPA1L3,VCL,NOX5,TEAD1,CELSR1,CSMD3,FGF14,FSHR,GSG1L,GRIA4,GRM1,MBOAT7,FMN2,HNRNPA2B1,MAEL,MORF4L1,NFE2L3,NRIP1,PRKAR2A,SKAP1,SORCS2,INPP5A,STK24,DCDC1,DPP6,WDR83,WWOX,ATRX,ACTR5,FRMD5,PARP16,TRPM2,CNIH3,CTNNA3,RNLS,RYR2,USP16,XRCC4,KIF3B,NTNG1,OR2M5,TENM4,UNC13C,CAMTA1,CATSPER2,DHRS7C,HSPB8,LINC00472,LRRK2,MAPK10,MIR495,MIR543,RORB,SLC1A3,IGFBP7,KCNH5,PIR,ZFP30,ZNF607,KIF26B,NTRK3,NUSAP1,RORA,CD8B,GGT2,ILDR1,IQCJ-SCHIP1,PRICKLE2,SLC4A4,ANKS1A,CHD7,OVGP1,RNF165,ZNF26,DISC1,HEXB,KCNK13,MEMO1,PAN3,TPRG1L,WRAP73,CDCA2,GLIS1,KCTD9,PDXP,SH3BP1,SOX6,IER2,MECP2,MTMR2,SEMA4D,GSK3B,LGR5,LINGO2,SLC8A1,ZNF432,ZNF841,CDC14A,MAGI3,PLCH2,PRKD1,SERPINA4,SERPINA5,SNRK,ERCC6,FLVCR1,FRMD6,OGT,RNF144A,TGFB2,ATP1A4,CLSTN2,FMN1,PRAMEF12,VAV3,ANKS1B,AFF3,ATP9B,C8A,DACH2,DNAJC6,FUT4,PIWIL4,PLCD3,RIPPLY3,S100A11,SEMA5A,SLC5A3,BDNF,LLGL2,PRKAR1B,PRKCH,RBMS3,RIPK4,RYK,ZDHHC17,KCNC1,NBEA,PCDH15,PLCXD3,POLR3C,TOR1AIP1,ABI3BP,EDIL3,SGCD,WASF1,GRIN2B,KDM4C,OR6N1,OTUD7A,CHST9,EXT2,LAMA3,NKAIN2,RAG1,RAG2,SAMD13,SIK2,ZNF433,ABCB5,ANKRD6,ARHGAP8,NR2F2,PLEKHM2,PRR5,PRR5-ARHGAP8,PTGFR,SNAP25,NR5A2,PAQR8,PCBP3,PITPNC1,PML,PPARGC1A,RNF180,TRUB2,USP36,ZNF627,ARHGAP12,CORO2A,AKAP6,CNTNAP2,TNS1,FBN1,MED15,PPP4R4,GRIK3,IGHV3-16,LRFN2,NLRP2,NLRP7,PLXNA2,SNAP23,TAOK3,ZNF610,ACSM3,BCL2,CHMP4C,MXI1,RAD51B,RALBP1,RXFP2,ZNF675,ARHGAP24,EPHB1,KCNJ3,MOV10L1,SMARCE1,IL12RB2,SIPA1L1,SKAP2,SLC30A5,DISP1,KLF12,LRP2,MMP16,NKAIN3,NSF,TMEM100,FRMD4A,ITPR2,LRRC4,NFIA,SND1,VANGL2,CDH6,DEPTOR,NR4A2,ZNF677,ABCC9,ARHGEF10,CYB561A3,EPHA7,MIR648,PSD3,ZBTB7C,DPT,RBAK,RBAK-RBAKDN,RPGR,FYN,GNRHR,KCNE2,LRRC69,PARN,ADAMTS18,CHN1,NR4A1,SMARCA4,CD44,EIF4G3,HECW1,MYL12A,NEU3,PMP22,RCVRN,RHBDD1,RNF213,THBS2,FOXO1,PDE4D,PYY,RBBP8,RNF144B,TBXAS1,KANK1,ATP10B,ENPP2,ABCB10,ACTN2,ARHGEF3,ATF6,ATP8A2,BACH1,CST9L,DCDC2,GABRB1,GRK5,KLF13,MME,PACRG,RNF217,TNMD,CSNK1G3,EXOC4,KCNS3,NAV2,GRIK2,IGSF11,ZNF516,CELF4,KCNIP4,RAP1GDS1,SERPINB7,TPO,UNC5C,APBB2,EIF3E,KCND2,MYLK3,NAMPT,SCN11A,SETD7,SLC1A1,UGCG,ZFPM2,ZNF141,ZNF429,ZSCAN5A,GLIS3,PHF20,RARB,RERG,RFX4,STRIP1,AGAP1,ETV6,GNG4,NLRP12,GRAP2,MFSD12,MTOR,RMI2,ROR1,CADPS,LDB2,MOV10,TMPRSS4,VPS26B,CHRM1,CTDSPL2,DDX21,FAT3,PKP2,UBXN2B,ZNF510,ABCA13,BMPER,CCDC88B,ERMN,PTPRD,RASGEF1B,SCAF8,TIAM2,ZNF521,BBS2,OGFOD1,OR51B2,OR51I1,PGK1,PLCL1,ZFP64,ZNF483,ARL6IP5,ATP1A1,EPM2A,PLN,SLCO1B1,NOL11,ZMAT3,CLYBL,CTNNBIP1,EPB41L4B,EVI5,HEPHL1,SOX8,ZMYND11,DIDO1,DSG4,HAS3,HIVEP2,IFT88,MIR491,NLGN4X,OR4K17,OR8J3,PRKCE,ATP6V1D,EIF2S1,GTF2A1L,HELLS,KMT2A,PRKCB,STON1,VDR,ADCY8,EFHB,FAM19A4,FHL5,FOXO3,IGF2BP3,LRRTM1,MITF,RAB31,SCMH1,SCP2,TRAPPC12,CAPN3,DPH6,ITLN1,KALRN,PJA1,TTLL12,CCDC88C,CREB5,DLC1,EDNRB,MSR1,OASL,ROBO1,RXFP1,SATB2,TANC2,TBC1D4,TDRD3,BNIP3L,FAP,FLT1,NOX4,SGIP1,SLC12A8,STX12,ZNF148,CDH17,CENPV,GRHL2,TCF4,TSC22D3,ZNF573,ZNF720,COL2A1,KCNC4,MALL,MAP3K7,MAPK9,NBAS,NR3C1,PREX2,TACR3,TRAF3IP2,VPS37B,ZNF277,CR1,EYA4,GABRA2,OR4C15,SEC24B,SPON1,ACSL5,MDM1,PKHD1,STK3,SUPT3H,TGM2,VASH2,ZNF585A,KDR,PPHLN1,PRCP,RAB30,ARHGAP15,CASK,CHL1,EDA,IGHV4-28,PCOLCE2,ABCA12,APP,CALCRL,COL18A1,DGKB,NRP1,PTPRM,SHROOM3,UACA,ARHGEF18,ELMO1,LGR4,LINC00473,SERPING1,TENM2,ADTRP,CD58,EGFLAM,KAT5,RANBP10,ST7,THRB,CPEB2,FUBP1,KCNK2,SEMA3C,ZNF667,ZNF98,ADAM17,CAMK1D,DIS3L2,MAP3K5,PKP4,TSPEAR,CDC42BPG,IGF2R,TFPI,ZER1,ATF2,ELP3,ETS1

			,GPR78,GRK6,NR2C2,PEX5L,PIK3R5,SAMSN1,EFEMP1,GOPC,MT1HL1,OIT3,PRTG,ROS1,SMG6,TGFB1I1,TM9SF4,CDK17,DOT1L,KPNB1,OR10R2,PSIP1,RGS16,RPS6KA5,TCF7L2,TNFRSF10B,TRABD2B,ZNF443,ZNF490,ZNF709,ZNF799,ACSM2A,AFF2,BICD1,CYTIP,ITGA11,PARD3,RALGAPA2,TLK2,TRAF3,ZNF830,ABAT,ATRNL1,DOCK9,GRIN2A,GRIP1,MIR183,MIR96,PXT1,RIMS3,SGK2,DLX6-AS1,HRNR,KIFAP3,OPHN1,BCLAF1,PYGL,SGMS1,TRPS1,ZNF568,CASP5,CCDC88A,CD38,DENND5A,FAM83B,KHDRBS3,MOB3B,MRPS27,RIN2,RNF43,SLC30A7,TUB,ZBED5,ALCAM,CHRDL1,NCF2,RNF126,SMG7,STK36,BCL2L1,ADAMTS16,GUCY2F,HDAC4,MYO16,PDE4B,SOX5,SRRM4,TIMP2,BACE2,BCL2L15,DNMT1,GHR,GLRA2,JAM2,MAPRE2,MIR605,NTRK2,PRKG1,ACSL4,FAM83G,A1CF,CDC42BPA,DNM3,GNG2,BRD4,PKNOX2,PTPDC1,RASGRP1,STARD13,ARR3,CAMK4,COMMD1,KCTD7,LY86,RAB11FIP4,RABGEF1,RGL1,SDK1,SPIN3,SPRED2,STARD4,GLI3,OR5L2,SMCHD1,ZNF207,CACNA1C,CLDN1,COG7,KIF16B,MORC2,OR11L1,PPA2,STXBP5,CRB1,DOCK4,RAP1A,SAFB2,TGFBR1,USP53,ZBTB41,CYSLTR1,KCNJ12,MGLL,PRDX4,ADAM12,DICER1,EPHA5,LCP2,TAS2R38,THEMIS,TNR,TOP1,CUX1,NCOA5,PEAK1,TAS2R1,TENM3,CHST11,CPE,IL16,ITGB3BP,MAGEA11,PTTG1IP,RAPGEF1,ZNF729,CD96,CHRNA3,CHRNA5,EP300,MORC3,ELAVL2,HMG20A,SCFD1,TBX3,TTC39B,WIF1,CACNA2D1,CACNA2D4,CYP7B1,DIO2,GRAMD4,PRAMENP,STX8,TRIOBP,ATXN1,BRD7,CLIC6,GAB2,GLRA1,PCSK5,SYCP1,TP73,COL4A6,GRID2,IFNGR2,NPC1,OR4C5,PARVB,PIBF1,PNPLA3,RASGRF2,TRPM7,ARHGEF33,PSMA1,TRHDE,FHIT,FLNB,ONECUT3,PPP1R12B,ADCK1,ADORA2A,ADRBK2,CABIN1,CAMK2G,EFNA5,EMCN,FAM20A,MERTK,RALGPS1,TAC4,BRD1,CACNG2,GUCY2C,MAGI2,NF1,PPP6R2,RALGPS2,RFC3,RGS7,TFF1,USP17L5,ZNF525,ZNF765,KCNQ5,SORL1,STRN3,ZNF615,ARHGAP6,ASB13,BICC1,BID,ELK3,IFT80,OR5K4,TEAD4,TRIM59,TTC8,DMRT1,HTR4,LZTR1,ASXL1,EIF4E,MICU1,POR,RELN,SLC39A8,SLC9C1,TNFRSF11B,TSHZ1,USP3,GRM5,HDAC9,IGHV4-4,IL18R1,IL1RL1,NECAB1,PTPN9,SELE,DYNC1H1,LRRTM3,MGAT5,PLSCR4,NAPEPLD,NDRG2,NUMB,RHPN2,SP2,TNFAIP8,CCDC57,IGHV3OR16-12,MAP3K13,NLK,NPHP4,RALA,TBX15,ADCYAP1R1,CYP39A1,EGLN3,IL1RAP,NAV3,ATP10A,FBXL17,FBXL5,JAG1,MEOX2,PPP3CA,TBC1D3B,GPR176,JRK,KCNB2,KLF3,KLF7,MYT1L,NEB,PLS1,SH3GL2,SLC7A1,SNX25,ATP8B4,CAPZB,CXADR,DGKI,MAS1,PER1,SATB1,SLC24A2,TBC1D5,WWTR1,KL,ANKRD13A,C6,CBLB,DOCK10,ELAVL4,MAGEB2,MNAT1,PLSCR1,ATP9A,CNTN1,DGUOK,DOK6,GPC4,GRM7,RIMS1,TMEM67,TRIM16,WDR59,ZNF736,AHSA1,GABPA,GABRA3,GPSM2,KAT7,L3MBTL3,MAPK4,MIR105-1,MIR105-2,MIR767,SSBP2,STIM2,TMEM135,ZNF540,ZNF571,SLC22A2,AGGF1,ASAP1,BORA,FHOD3,ITGAE,N4BP2L2,NLGN1,P2RX6,PDE1C,PTPRU,THAP7,AKIRIN2,ARHGAP5,BTBD11,CHST8,CTTNBP2,RNF128,RPS20,SH3KBP1,SLC22A3,ZNF562,ENOX2,NPR3,PHACTR2,RAD51AP1,SYCP2,AKAP7,LRRK1,MAPK8IP2,VEPH1,ZNF208,ACOX3,CACNA1D,DDX3X,EGFL6,LRPPRC,NSG1,RBL2,ARAP2,CNOT7,DTD1,GMDS,MAML3,EHF,GABRA5,PDE7A,TPCN2,ZNF622,ARHGAP39,DNMT3B,JAK2,LMO7,SHOX2,SPNS2,AHI1,SMAD3,XKR4,BAIAP2L1,CD300A,KIDINS220,MAP2K4,NOS1,SERPINB12,COL8A1,PLXNB1,RDH10,TCF12,TMTC4,ZNF337,AATF,ANHX,CDK1,DCBLD2,HDAC7,HIVEP3,ITPK1,LTB,MIR320B2,MRGPRG,NBN,NVL,RRH,SLC39A10,TAGLN3,TNF,COBL,LRCH1,OVOL2,PANX1,SNX5,TNIP1,ZFPM1,CSPP1,EIF3A,ILF3,POMC,PPFIA2,TMC2,PRDM15,ADNP2,BLOC1S6,BMP15,DHRS11,DLG5,FCHSD2,INO80,NUDT4,POU6F2,RBM11,SCAF4,TRIM23,AFAP1,LGR6,MAGI1,OR11A1,OR5V1,RXFP4,SIM2,ARID2,MLIP,MMP20,PAXBP1,SHC2,BRINP1,FAM220A,KCNK10,PLCH1,PRG3,SRSF4,STK32B,ADORA3,ARF1,BCL11A,CLEC4A,HMGA2,MYO6,NEK4,POLR1A,TAF1D,UBE2E3,YAF2,ZNF41,ZNF705A,ZNF880,AGO3,DOCK1,GRIA3,IGLC2,IGLL5,KIF18A,MUC5AC,PAPOLA,RUFY1,STK32C,SULT1B1,TSPAN13,ZSWIM7,ANO1,CDC20B,GCLC,KCNA6,PMEPA1,QKI,RAB23,UBE2V1,ZNF664,ZNF728,ZNF879,ACTR8,EPS8,LTBP1,MBOAT2,SP140,SP140L,CYP3A5,DAAM2,L3MBTL4,SLC7A7,SMOC2,SULF1,TRPC4,ANKRD31,C5AR1,CCP110,CISD2,DEF6,GNAL,LRP4,PIK3R3,TSPAN1,WDR5,ARHGEF6,CNGB3,CNTN6,EFNB2,KCNH8,MXD3,MYCT1,PIWIL3,SAE1,TRDN,TRIM41,AMOTL1,HERC1,NET1,PHF8,ZNF423,CDC14B,CLSTN1,INSC,LRRC8D,SELP,ALPK2,CDK8,CHD6,CTNNA1,CUL2,NSG2,SCN1A,TENM1,TSHZ3,WDFY2,ZNF407,ZNF93,ARHGEF11,CDKL5,DMXL1,EFHC2,GRIK4,GSKIP,MAMLD1,MIR765,MYH14,NELL2,PRKAR1A,TNFRSF19,ZNF345,ARHGAP11B,BPTF,DYNC2H1,FAM171A1,GABRR2,KCNH7,MLLT3,MYRIP,SIPA1L2,CD109,EGF,MIP,PTPN14,SEC16B,SPIRE2,ALDH1A1,CMA1,FOXP4,GBE1,IL1RAPL1,KIT,MT

			RR,POTEF,SLIT3,TNFSF8,TNRC6B,UBR2,ZNF600,BLM,CTNNAL1,OR7A5,PIK3C2G,TRPV4,VWC2,ABCA5,C8B,CC2D2A,CPS1,DNAH11,EDAR,EFNB1,KCNAB1,NAPG,PTPRO,RAPGEF2,REG1B,STK32A,ZIM3,DNAJB6,ESR1,HTR2C,JAK1,NCMAP,SEMA3E,SLC30A8,UBE3A,DNMBP,EBAG9,FGF13,GABRB2,GRM4,MARVELD3,NUP155,PLAG1,SGCZ,AKAP10,ANO3,EPHB2,IGHV3-72,MECOM,NDFIP2,RNF4,TBCD,TFDP2,TOX,ABCC1,C14ORF39,DAP,DAPK1,ERBB4,FER,MAML2,MAP3K3,RBM19,SLITRK6,SPTA1,TDRKH,CACNG6,FNIP1,LRP5,MALRD1,PTH2R,RAPGEF6,SHCBP1,XKR7,ZNF439,ZNF507,LACTB,RPS27L,SLAMF1,ABL2,ASAP2,BSG,CAMP,DEPDC5,F11R,FARP2,GAS8,HS1BP3,OR4C6,SETD2,VPS13C,ALX4,AP2B1,ARHGAP35,CD2AP,COMMD7,NSUN2,PTPRK,SGMS2,ATP8A1,CCL3,FGF7,LAMA1,MCUR1,PREX1,RNF138,SEMA5B,SPRED3,TNFAIP8L2,VWF,ZNF189,ARHGAP25,BTBD17,CDKL1,FGD4,GCFC2,GLYR1,GPR158,IL20RA,ITGA9,KIF2A,LRRC70,NHLH2,PSG9,SIAE,SULF2,ARF4,ARFGAP3,EEFSEC,MYH9,NSMCE2,PACSIN2,PTGER3,SLC2A13,SLC39A12,SOX4,ST18,CAB39L,GARNL3,KIF23,NEDD9,P2RX1,PDE1A,PDGFD,PIK3CD,PRR16,RGPD1,SPTB,STOML3,UBE2N,AGTPBP1,ASGR2,DNM3OS,ITGA8,KCNE4,MIR214,PUM1,ARID4A,DDB1,ENPEP,FAM172A,IRAK2,KDM3A,PLAT,SERTAD2,TNKS,UNC13A,WDPCP,ZFP82,ADCY9,AUTS2,BCL7A,CDH23,CDK19,DAPL1,FGFR1,MEF2A,OCLN,PARP15,PDE11A,RGS3,RYR1,TBC1D9,UNC13B,ZCCHC17,ZNF85,ARID4B,DAD1,GLRA3,ITGBL1,MAP2,RASSF3,SLX1B,SOST,ASXL3,BPGM,IL23R,ITGB7,JMY,KEAP1,NTF3,P2RX7,PLA2G4A,RAPGEF4,SCUBE1,SORCS1,TEX14,ARRDC4,BRD9,GP5,RASA3,RCAN2,TOX2,ZDHHC11,ZDHHC9,ZNF544,AMFR,APIP,AR,BCOR,FGL2,KIAA1109,PVT1,RABGAP1L,SCAMP5,SNX30,SUFU,TRERF1,UPK3B,C10ORF90,CEP250,DENND5B,EXOC2,EYA1,IFNA8,IL1RL2,N4BP1,RBM4,SRPK2,BDKRB1,BDKRB2,HLA-DRA,ADRA1B,ARMC9,BHLHB9,DGKZ,GNL3,IFI27L1,L3MBTL1,MIR127,MIR136,MIR431,MIR432,MIR433,NEK5,OGN,PI3,PRKAA2,RLF,SEMA3A,TAX1BP1,THADA,TLE4,ZNF202,ZNF558,ARHGAP10,CDC73,EPB41L3,GNB5,GRIA2,MARK2,MEIS2,PDE5A,PROS1,REEP2,ZMIZ1,ZNF19,ZNF440,ZNF670,ZNF695,FLT3,LDLRAD3,NFIC,PDLIM5,PPARGC1B,PTPRA,SNX7,SRGAP3,TRIM72,ATP6V1H,DYNAP,ESRRG,FERMT2,GNAO1,HCN4,LTBR,MAP2K5,MARK1,PAPPA2,SCNN1A,SMPD3,TNN,ZC3H13,ATP13A3,C9,CALML4,CLN6,DAB2,GPR55,HDAC8,LCP1,PBX1,RNF207,SRD5A2,STX3,SYT2,TCL1B,ACTN4,ADAM10,BLOC1S5,CCL7,DAAM1,DRG2,EEF1E1,EEF1E1-BLOC1S5,ELP4,FGF12,ITGA3,KCNJ16,KMO,POU2F3,PRAMEF8,TXNDC5,FANCB,FRY,LGI1,MT1F,NREP,OR10K2,OR4C46,PLCL2,PPM1B,QRICH1,RFTN1,SSX5,STAC,MYOM1,NUP153,PHF20L1,PIN1,RPS6KA6,SYNPO2,TRPM4,CD226,ERLIN1,GPR141,KRBOX4,PIP5K1B,STK38,TCF20,ZNF674,ABCA8,ABCC2,BMP6,BTBD9,CHRM3,CIT,CIZ1,CLASP1,EIF4ENIF1,GAD2,SMTNL2,CARF,CDYL2,CFDP1,CNGB1,EBF1,NCOA2,TGFA,TMOD1,WDR12,CREM,G3BP2,GAS2,HGF,HGFAC,MYLK,PKP1,POU2F1,PRPSAP2,RALGAPA1,RASSF8,RGS12,ZNF665,ZNF730,CALCR,CAPN7,CENPF,FLI1,MIR489,MIR653,POMT2,SCUBE3,SH3BP5,TLK1,ZBTB16,CACNB2,CNKSR2,CTIF,ELN,KDM4B,MAST4,MIR556,NCAM1,NOS1AP,PI4KA,RALGDS,SNAP29,ZNF57,ARHGAP28,IL18RAP,INSR,LARP4B,MACF1,PAK1,PDE3A,PSMB7,SKI,AKT3,CDC16,CHM,CTDP1,ELF1,ENPP1,FMR1,LIMK2,LITAF,MICU2,MIR361,PAX3,THEM4,CHFR,DKC1,GABRA1,IGHV1-69,JDP2,KLF6,PHC2,PRKX,RRAGC,SBF2,TMTC2,ULK4,ZNF605,ABCD1,BMF,CRMP1,EVC,GABRR3,IDE,IGHV1-46,MAD1L1,MFNG,SAMD8,AIM2,ARHGAP42,CD84,CSF2RB,DENND2D,EPG5,EVC2,JPH4,LRRN2,PCK1,PDE6B,POLD1,PPARA,SORT1,SPIB,TBX22,TNFAIP8L3,TTN,ZFHX2,CLIP1,CORIN,FAM3D,FHL1,GNA13,KATNBL1,MYH7,NUP214,PPP1R13B,SLC22A5,SMARCAD1,TAF1,WDR41,ADH1B,CACNA1A,CHD5,FNIP2,FUT8,PPP2R3C,QRFP,S1PR3,SPRED1,TRPM1,AKR1C2,CHSY1,ESR2,KRT4,NTN4,PRDM16,PTPRF,QSOX1,RBL1,SCN9A,ZNF451,ATP6V1E1,BTRC,C2,CCDC3,CFB,DEFA1B,DEFA3,FBLN2,HIRA,IGHV1-18,MLLT10,MPDZ,OPTN,PPP2R2C,PTPRR,RIT2,ZNF713,ESRP2,EXT1,GNG12,IFT81,MDM4,MYH6,NCOA1,PRKCG,PRLR,SCO2,TYMP,UMOD,ZNF354B,ADAMTS5,CBFA2T3,DOCK7,FBXW11,G3BP1,HDAC5,ICK,INPP4A,KLHL3,OR51L1,PSMD6,TDGF1,WDR70,ZNF493,A2M,AKAP2,LYVE1,MYEF2,PI15,SERPINB8,SLC24A5,SLC40A1,STAT4,STIM1,TREM1,ZNF623,ADARB1,KCNC2,OR4C16,PEMT,POU2AF1,PRKCQ,SCEL,SLC9A9,STAT3,SUSD4,TFEC,TP63,USP7,ZNF721,AMOT,BRIP1,CDKN3,CST1,DOCK3,HPN,ISLR2,PAK3,PIK3R2,STK17B,TBL1X,USH2A,ADH1A,BLNK,CASQ2,DHRS2,DHRS7B,DTNBP1,GAK,RAP1B,TMEM175,ZNF518A,CNNM3,DLGAP2,KSR1,LGALS9,LRP8,NFAT5,O

			R2T4,SNCAIP,TAS1R1,ZNF697,ACSM1,ALPK1,EFCAB1,GTPBP1,IL10RB,MAPKAPK2,SLC7A8,UPK3A,AHNAK2,AOAH,APC,BMPR1A,CKAP5,CSN3,DPP4,ITGA1,KCTD20,KIAA0319,NGF,PPP1R14A,PRKCA,PTGES,SPINT2,UBASH3B,UBE2E2,VLDLR,ADAMTS20,ASB3,DRAM1,GPR75,ITIH6,MTMR4,MTRF1,OSGIN2,PDS5A,PILRB,ABI1,CTSH,DHX9,FCRL5,NCAPG2,RGCC,RPH3AL,SLC9A2,SRGAP1,TRBV6-8,USP18,ACACA,CCL14,CCL15,CCL15-CCL14,CYB5D2,DPH1,EBF4,EDA2R,GABRG2,GPR21,IL6R,MAP2K6,NCALD,RIC8B,SIAH3,SRRD,TFIP11,TMEM123,SPTBN4,CLASP2,MAP2K1,PDE7B,PRAMEF7,RGMB,SOS1,TPH1,VPS35,ZBTB25,ZRANB3,C1QTNF3,CABLES1,COL6A3,FGF1,MCOLN3,NSUN3,PLB1,RAD18,TBX20,ZNF527,ATMIN,ATP6V1E2,C6ORF106,CRADD,DGKK,NLRC5,PPP1R9A,RGN,RREB1,TBX18,VSTM1,ZFP2,ZNF501
GO:0050794	regulation of cellular process	1.3549371650624336e-26	IGHV1OR21-1,TASP1,FANK1,CAST,ADCY2,SRGAP2B,TPTE,CDH4,ZHX3,PLCB1,KMT2C,KHDRBS2,ZNF595,CMKLR1,DAB1,OMA1,DUX4,GTF2I,ZNF717,GABRB3,CDC42EP3,GABRG3,MAPK14,ABCG8,DSCAM,ARID1B,TPTE2,RUNX1,ORC3,SH3RF3,ASIC2,CTBP2,DTNA,OR11H1,SERPINA1,TSHZ2,CCNG2,RAB27A,HUNK,CACNA1E,KCNJ6,PCBD2,ANXA8L1,NPY4R,PRIM2,LYPD6,OR4M2,PTPRT,ANK3,IGHV4-31,DCC,BCL2L13,IGHV3-64,SVEP1,WLS,RBFOX1,SYN3,TIMP3,ZNF397,ZSCAN30,DUSP22,TTC28,AKAP13,FBLN1,ERG,VPS13D,GRIK1,ZNF292,GPR139,IGHV1OR15-9,SORBS2,IGHV4OR15-8,NPHP3,ITGA2,ABCG1,RGS6,KCNQ1,DPF3,SUN2,GABBR2,ATF7IP,EPHA6,OR4K15,TMEM117,VRK2,GRIN3A,FCRL2,NRXN1,SHC3,ZRANB1,ISX,DOK5,NRXN3,SLC1A2,RAB5A,ZNF578,DOCK2,CTNND2,TNC,PCNT,PLEKHB2,MS4A1,OR8U1,PAX7,CHODL,RIMS2,MTPN,SHANK2,GRIA1,NRG3,ZNF732,SMYD2,CELF2,CLEC16A,HUS1,ARNT2,TGFBR3,FSTL4,CDH13,RNF152,ANK2,LRRC4C,RTN1,NXN,KCND3,RUNX1T1,CSNK2A1,PDE4DIP,SYNDIG1,ZNF678,DPP10,ZNF112,ZNF229,ZNF285,TF,PLGRKT,ERCC4,SCN8A,KCNE1,PIK3C2B,SAMD4A,SDCCAG8,TMEM108,EIF4EBP3,CHEK2,OR4C12,ZNF486,GADD45A,PRDM9,KCNJ15,PCP4,PSMC6,ACIN1,ZNF331,SYT17,PLXNA4,KCNG3,FLRT2,TOX3,GPC3,FBLN5,NRG1,STXBP6,ETS2,PSMB2,SHISA6,RAD51D,RFFL,NPAS3,SPIDR,IL4R,ALDH1A2,LRFN5,RYR3,EWSR1,MCTP1,NLN,ST8SIA1,SUZ12,LCORL,ASTN2,PLCE1,SH3RF2,UNC5D,KSR2,GLP2R,STK38L,SEMA6D,SPOCK1,ABHD17C,CTNNA2,SLCO3A1,CCR1,CCR3,CFTR,RBFOX2,ZNF420,DTX4,ZNF257,MED13L,MC2R,TIAM1,PM20D1,RHOC,AGBL4,BEND5,LDLRAD4,DNAJC15,RGS7BP,ZNF91,RASGRF1,ZBTB20,DPYSL2,GRID1,TET3,ARFIP1,CBFA2T2,IL1RAPL2,OR4A5,NKD1,HPSE2,SCAI,MX1,BTN2A1,ALK,PTPRE,PIK3C3,UTRN,SORCS3,ZNF536,FAM3B,PLCB4,ERC1,FBXO31,RAB7A,CNTN4,RGL2,ARID5B,PHACTR1,S100B,ROBO2,SPOCK3,ZNF366,C2CD3,EPHA3,PRKACB,CNTNAP4,DCLK1,PXDN,COL15A1,CRNN,AJAP1,LEMD3,OR8K3,AGT,ERC2,CST2,MAGEB3,SHISA9,ATAT1,AVEN,CHRM5,WDR11,ATP6V0D1,CDH11,RHOJ,RCAN1,CDH2,KLHL25,PARK2,BANP,FHL2,FOXN3,LGALS14,LOXL2,DLGAP1,LAMA2,CDC5L,KIR2DL1,KIR2DL4,KIR3DL2,OR4K13,BPIFB1,CORO2B,FRMPD4,ZNF804A,CYBB,MCTP2,NEGR1,PSPC1,BRF1,GNB4,OR4M1,OR4N2,SEMA3D,DACH1,NTN1,OR52N5,TP53I11,TRIM22,TRIM5,KCNH1,USP25,ZBTB8B,ZNF268,DCUN1D4,TXK,CP,GPC6,RNF185,USP6,ZNF232,ANKFN1,NELL1,TRAPPC9,DBH,ITSN1,TNS3,PCDH17,PHC1,SETD3,ZNRF3,KCNMA1,RIN3,TRIO,ZNF845,APBA2,FTO,HDAC2,HTRA1,LAMA4,MYO9A,SLIT2,TFAP2D,ZBTB34,CDH8,KCTD1,AHRR,PDCD6,ZSCAN5C,OR2L13,RPS6KA2,STXBP4,GPC5,LCMT1,IGF1R,MDM2,MX2,SLC24A4,SMAD1,E2F3,NOVA1,SYT9,ZBED4,FOXD4L4,OR11G2,OR9Q1,TMPRSS6,ZNF567,ZNF850,CTNNBL1,RAPGEF5,CEP97,GABRG1,KCNK17,NR3C2,PTPRG,PXK,PDE9A,OR8K5,SACS,TRPC5,DMD,HCN1,KCTD8,STK33,SYT1,BASP1,CACNA2D3,CACNG3,SIPA1L3,VCL,NOX5,TEAD1,CELSR1,CSMD3,FGF14,FSHR,GSG1L,GRIA4,GRM1,MBOAT7,FMN2,HNRNPA2B1,MAEL,MORF4L1,NFE2L3,NRIP1,PRKAR2A,SKAP1,SORCS2,INPP5A,STK24,DCDC1,DPP6,WDR83,WWOX,ATRX,ACTR5,FRMD5,PARP16,TRPM2,CNIH3,CTNNA3,RYR2,USP16,KIF3B,NTNG1,OR2M5,TENM4,UNC13C,CAMTA1,CATSPER2,DHRS7C,HSPB8,LRRK2,MAPK10,RORB,SLC1A3,IGFBP7,KCNH5,PIR,ZFP30,ZNF607,KIF26B,NTRK3,NUSAP1,RORA,CD8B,ILDR1,IQCJ-SCHIP1,PRICKLE2,SLC4A4,ANKS1A,CHD7,OVGP1,RNF165,ZNF26,DISC1,HEXB,KCNK13,MEMO1,PAN3,TPRG1L,WRAP73,CDCA2,GLIS1,KCTD9,PDXP,SH3BP1,SOX6,IER2,MECP2,MTMR2,SEMA4D,GSK3B,LGR5,LINGO2,SLC8A1,ZNF432,ZNF841,CDC14A,MAGI3,PLCH2,PRKD1,SERPINA4,SERPINA5,SNRK,ERCC6,FRMD6,OGT,RNF144A,TGFB2,ATP1A4,CLSTN2,FMN1,PRAMEF12,VAV3,ANKS1B,AFF3,DACH

			2,DNAJC6,FUT4,PIWIL4,PLCD3,RIPPLY3,S100A11,SEMA5A,SLC5A3,BDNF,LLGL2,PRKAR1B,PRKCH,RBMS3,RIPK4,RYK,ZDHHC17,KCNC1,PLCXD3,POLR3C,ABI3BP,EDIL3,SGCD,WASF1,GRIN2B,KDM4C,OR6N1,OTUD7A,EXT2,LAMA3,RAG1,RAG2,SAMD13,SIK2,ZNF433,ANKRD6,ARHGAP8,NR2F2,PLEKHM2,PRR5,PRR5-ARHGAP8,PTGFR,SNAP25,NR5A2,PAQR8,PCBP3,PITPNC1,PML,PPARGC1A,RNF180,TRUB2,USP36,ZNF627,ARHGAP12,CORO2A,AKAP6,CNTNAP2,TNS1,FBN1,MED15,PPP4R4,GRIK3,IGHV3-16,LRFN2,NLRP2,NLRP7,PLXNA2,TAOK3,ZNF610,BCL2,CHMP4C,MXI1,RAD51B,RALBP1,RXFP2,ZNF675,ARHGAP24,EPHB1,KCNJ3,SMARCE1,IL12RB2,SIPA1L1,SKAP2,SLC30A5,DISP1,KLF12,LRP2,NSF,TMEM100,FRMD4A,ITPR2,LRRC4,NFIA,SND1,VANGL2,CDH6,DEPTOR,NR4A2,ZNF677,ARHGEF10,EPHA7,PSD3,ZBTB7C,DPT,RBAK,RBAK-RBAKDN,FYN,GNRHR,KCNE2,LRRC69,PARN,ADAMTS18,CHN1,NR4A1,SMARCA4,CD44,EIF4G3,HECW1,NEU3,PMP22,RCVRN,RHBDD1,RNF213,THBS2,FOXO1,PDE4D,PYY,RBBP8,RNF144B,KANK1,ENPP2,ABCB10,ACTN2,ARHGEF3,ATF6,ATP8A2,BACH1,CST9L,DCDC2,GABRB1,GRK5,KLF13,MME,PACRG,RNF217,TNMD,CSNK1G3,EXOC4,KCNS3,GRIK2,IGSF11,ZNF516,CELF4,KCNIP4,SERPINB7,UNC5C,APBB2,EIF3E,KCND2,MYLK3,NAMPT,SCN11A,SETD7,SLC1A1,UGCG,ZFPM2,ZNF141,ZNF429,ZSCAN5A,GLIS3,PHF20,RARB,RERG,RFX4,ETV6,GNG4,NLRP12,GRAP2,MFSD12,MTOR,RMI2,ROR1,CADPS,LDB2,MOV10,VPS26B,CHRM1,CTDSPL2,DDX21,FAT3,PKP2,UBXN2B,ZNF510,ABCA13,BMPER,CCDC88B,ERMN,PTPRD,RASGEF1B,SCAF8,TIAM2,ZNF521,BBS2,OGFOD1,OR51B2,OR51I1,PLCL1,ZFP64,ZNF483,ARL6IP5,ATP1A1,EPM2A,PLN,NOL11,CLYBL,CTNNBIP1,EPB41L4B,SOX8,ZMYND11,DIDO1,DSG4,HAS3,HIVEP2,IFT88,NLGN4X,OR4K17,OR8J3,PRKCE,ATP6V1D,EIF2S1,GTF2A1L,HELLS,KMT2A,PRKCB,STON1,VDR,ADCY8,EFHB,FAM19A4,FHL5,FOXO3,IGF2BP3,LRRTM1,MITF,RAB31,SCMH1,SCP2,TRAPPC12,CAPN3,DPH6,ITLN1,KALRN,PJA1,TTLL12,CCDC88C,CREB5,DLC1,EDNRB,MSR1,OASL,ROBO1,RXFP1,SATB2,TANC2,TBC1D4,TDRD3,BNIP3L,FAP,FLT1,NOX4,SGIP1,ZNF148,CDH17,CENPV,GRHL2,TCF4,TSC22D3,ZNF573,ZNF720,COL2A1,KCNC4,MAP3K7,MAPK9,NBAS,NR3C1,PREX2,TACR3,TRAF3IP2,ZNF277,CR1,EYA4,GABRA2,OR4C15,SEC24B,SPON1,ACSL5,MDM1,PKHD1,STK3,SUPT3H,TGM2,VASH2,ZNF585A,KDR,PPHLN1,PRCP,RAB30,ARHGAP15,CASK,CHL1,EDA,IGHV4-28,PCOLCE2,ABCA12,APP,CALCRL,COL18A1,DGKB,NRP1,PTPRM,UACA,ARHGEF18,ELMO1,LGR4,LINC00473,SERPING1,TENM2,ADTRP,EGFLAM,KAT5,RANBP10,ST7,THRB,CPEB2,FUBP1,KCNK2,SEMA3C,ZNF667,ZNF98,ADAM17,CAMK1D,DIS3L2,MAP3K5,PKP4,TSPEAR,CDC42BPG,IGF2R,TFPI,ZER1,ATF2,ELP3,ETS1,GPR78,GRK6,NR2C2,PEX5L,PIK3R5,SAMSN1,EFEMP1,GOPC,PRTG,ROS1,SMG6,TGFB1I1,TM9SF4,CDK17,DOT1L,KPNB1,OR10R2,PSIP1,RGS16,RPS6KA5,TCF7L2,TNFRSF10B,TRABD2B,ZNF443,ZNF490,ZNF709,ZNF799,AFF2,BICD1,CYTIP,ITGA11,PARD3,RALGAPA2,TLK2,TRAF3,ZNF830,ABAT,ATRNL1,DOCK9,GRIN2A,GRIP1,PXT1,RIMS3,SGK2,DLX6-AS1,KIFAP3,OPHN1,BCLAF1,SGMS1,TRPS1,ZNF568,CASP5,CCDC88A,CD38,DENND5A,FAM83B,KHDRBS3,MOB3B,MRPS27,RIN2,RNF43,TUB,ZBED5,ALCAM,CHRDL1,RNF126,SMG7,STK36,BCL2L1,ADAMTS16,GUCY2F,HDAC4,MYO16,PDE4B,SOX5,SRRM4,TIMP2,BACE2,BCL2L15,DNMT1,GHR,GLRA2,JAM2,MAPRE2,NTRK2,PRKG1,ACSL4,FAM83G,CDC42BPA,DNM3,GNG2,BRD4,PKNOX2,PTPDC1,RASGRP1,STARD13,ARR3,CAMK4,COMMD1,LY86,RAB11FIP4,RABGEF1,RGL1,SPIN3,SPRED2,STARD4,GLI3,OR5L2,SMCHD1,ZNF207,CACNA1C,CLDN1,KIF16B,MORC2,OR11L1,STXBP5,DOCK4,RAP1A,SAFB2,TGFBR1,ZBTB41,CYSLTR1,KCNJ12,MGLL,PRDX4,ADAM12,DICER1,EPHA5,LCP2,TAS2R38,THEMIS,TNR,CUX1,NCOA5,PEAK1,TAS2R1,TENM3,CHST11,CPE,IL16,ITGB3BP,MAGEA11,PTTG1IP,RAPGEF1,ZNF729,CHRNA3,CHRNA5,EP300,MORC3,ELAVL2,HMG20A,SCFD1,TBX3,WIF1,CACNA2D1,CACNA2D4,CYP7B1,DIO2,GRAMD4,STX8,TRIOBP,ATXN1,BRD7,CLIC6,GAB2,GLRA1,TP73,COL4A6,GRID2,IFNGR2,NPC1,OR4C5,PIBF1,RASGRF2,PSMA1,TRHDE,FHIT,FLNB,ONECUT3,PPP1R12B,ADCK1,ADORA2A,ADRBK2,CABIN1,CAMK2G,EFNA5,EMCN,FAM20A,MERTK,RALGPS1,TAC4,BRD1,CACNG2,GUCY2C,MAGI2,NF1,PPP6R2,RALGPS2,RFC3,RGS7,TFF1,USP17L5,ZNF525,ZNF765,KCNQ5,SORL1,STRN3,ZNF615,ARHGAP6,ASB13,BICC1,BID,ELK3,IFT80,OR5K4,TEAD4,TRIM59,TTC8,DMRT1,HTR4,LZTR1,ASXL1,EIF4E,MICU1,POR,RELN,SLC39A8,TNFRSF11B,TSHZ1,GRM5,HDAC9,IGHV4-4,IL18R1,IL1RL1,NECAB1,PTPN9,SELE,DYNC1H1,LRRTM3,MGAT5,NAPEPLD,NDRG2,NUMB,RHPN2,SP2,TNFAIP8,CCDC57,IGHV3OR1

			6-12,MAP3K13,NLK,NPHP4,RALA,TBX15,ADCYAP1R1,EGLN3,IL1RAP,NAV3,ATP10A,FBXL17,FBXL5,JAG1,MEOX2,PPP3CA,GPR176,JRK,KCNB2,KLF3,KLF7,MYT1L,NEB,PLS1,SH3GL2,SLC7A1,SNX25,CAPZB,CXADR,DGKI,MAS1,PER1,SATB1,SLC24A2,TBC1D5,WWTR1,KL,ANKRD13A,CBLB,DOCK10,ELAVL4,MAGEB2,MNAT1,PLSCR1,ATP9A,CNTN1,DGUOK,DOK6,GPC4,GRM7,RIMS1,TMEM67,TRIM16,WDR59,ZNF736,GABPA,GABRA3,GPSM2,KAT7,L3MBTL3,MAPK4,SSBP2,STIM2,TMEM135,ZNF540,ZNF571,SLC22A2,AGGF1,ASAP1,BORA,FHOD3,ITGAE,N4BP2L2,NLGN1,P2RX6,PDE1C,PTPRU,THAP7,AKIRIN2,ARHGAP5,BTBD11,CTTNBP2,RNF128,RPS20,SH3KBP1,ZNF562,NPR3,RAD51AP1,SYCP2,AKAP7,LRRK1,MAPK8IP2,VEPH1,ZNF208,CACNA1D,DDX3X,EGFL6,LRPPRC,NSG1,RBL2,ARAP2,CNOT7,GMDS,MAML3,EHF,GABRA5,PDE7A,TPCN2,ZNF622,ARHGAP39,DNMT3B,JAK2,LMO7,SHOX2,SPNS2,AHI1,SMAD3,BAIAP2L1,CD300A,KIDINS220,MAP2K4,NOS1,SERPINB12,COL8A1,PLXNB1,RDH10,TCF12,TMTC4,ZNF337,AATF,ANHX,CDK1,DCBLD2,HDAC7,HIVEP3,ITPK1,LTB,MIR320B2,MRGPRG,NBN,NVL,RRH,SLC39A10,TAGLN3,TNF,COBL,LRCH1,OVOL2,SNX5,TNIP1,ZFPM1,CSPP1,EIF3A,ILF3,POMC,PPFIA2,TMC2,PRDM15,ADNP2,BLOC1S6,BMP15,DLG5,FCHSD2,INO80,NUDT4,POU6F2,RBM11,SCAF4,AFAP1,LGR6,MAGI1,OR11A1,OR5V1,RXFP4,SIM2,ARID2,MLIP,PAXBP1,SHC2,BRINP1,FAM220A,KCNK10,PLCH1,PRG3,SRSF4,STK32B,ADORA3,ARF1,BCL11A,CLEC4A,HMGA2,MYO6,NEK4,POLR1A,TAF1D,YAF2,ZNF41,ZNF705A,ZNF880,AGO3,DOCK1,GRIA3,IGLC2,IGLL5,KIF18A,MUC5AC,PAPOLA,RUFY1,STK32C,TSPAN13,ANO1,CDC20B,GCLC,KCNA6,PMEPA1,QKI,RAB23,UBE2V1,ZNF664,ZNF728,ZNF879,ACTR8,EPS8,LTBP1,MBOAT2,SP140,SP140L,DAAM2,L3MBTL4,SLC7A7,SMOC2,SULF1,ANKRD31,C5AR1,CCP110,CISD2,DEF6,GNAL,LRP4,PIK3R3,WDR5,ARHGEF6,CNGB3,CNTN6,EFNB2,KCNH8,MXD3,PIWIL3,SAE1,TRDN,TRIM41,AMOTL1,HERC1,NET1,PHF8,ZNF423,CDC14B,CLSTN1,INSC,SELP,ALPK2,CDK8,CHD6,CTNNA1,CUL2,NSG2,SCN1A,TENM1,TSHZ3,WDFY2,ZNF407,ZNF93,ARHGEF11,CDKL5,EFHC2,GRIK4,GSKIP,MAMLD1,PRKAR1A,TNFRSF19,ZNF345,ARHGAP11B,BPTF,DYNC2H1,GABRR2,KCNH7,MLLT3,MYRIP,SIPA1L2,CD109,EGF,MIP,PTPN14,SEC16B,SPIRE2,FOXP4,GBE1,IL1RAPL1,KIT,SLIT3,TNFSF8,TNRC6B,UBR2,ZNF600,BLM,CTNNAL1,OR7A5,PIK3C2G,TRPV4,VWC2,ABCA5,CC2D2A,DNAH11,EDAR,EFNB1,KCNAB1,PTPRO,RAPGEF2,REG1B,STK32A,ZIM3,DNAJB6,ESR1,HTR2C,JAK1,NCMAP,SEMA3E,SLC30A8,UBE3A,DNMBP,EBAG9,FGF13,GABRB2,GRM4,MARVELD3,PLAG1,AKAP10,EPHB2,IGHV3-72,MECOM,NDFIP2,RNF4,TBCD,TFDP2,TOX,C14ORF39,DAP,DAPK1,ERBB4,FER,MAML2,MAP3K3,SLITRK6,SPTA1,CACNG6,FNIP1,LRP5,MALRD1,PTH2R,RAPGEF6,SHCBP1,ZNF439,ZNF507,RPS27L,SLAMF1,ABL2,BSG,CAMP,DEPDC5,F11R,FARP2,GAS8,HS1BP3,OR4C6,SETD2,VPS13C,ALX4,AP2B1,ARHGAP35,CD2AP,COMMD7,NSUN2,PTPRK,ATP8A1,CCL3,FGF7,LAMA1,PREX1,RNF138,SEMA5B,SPRED3,TNFAIP8L2,VWF,ZNF189,ARHGAP25,CDKL1,FGD4,GCFC2,GLYR1,GPR158,IL20RA,ITGA9,KIF2A,NHLH2,PSG9,SULF2,ARF4,EEFSEC,MYH9,NSMCE2,PACSIN2,PTGER3,SLC2A13,SLC39A12,SOX4,ST18,CAB39L,GARNL3,KIF23,NEDD9,P2RX1,PDE1A,PDGFD,PIK3CD,PRR16,SPTB,STOML3,UBE2N,AGTPBP1,ASGR2,ITGA8,KCNE4,PUM1,ARID4A,DDB1,ENPEP,FAM172A,IRAK2,KDM3A,PLAT,SERTAD2,TNKS,UNC13A,WDPCP,ZFP82,ADCY9,AUTS2,BCL7A,CDK19,DAPL1,FGFR1,MEF2A,OCLN,PARP15,PDE11A,RGS3,RYR1,UNC13B,ZCCHC17,ZNF85,ARID4B,DAD1,GLRA3,ITGBL1,MAP2,RASSF3,SLX1B,SOST,ASXL3,IL23R,ITGB7,JMY,KEAP1,NTF3,P2RX7,PLA2G4A,RAPGEF4,SCUBE1,SORCS1,TEX14,ARRDC4,BRD9,RASA3,RCAN2,TOX2,ZDHHC9,ZNF544,AMFR,APIP,AR,BCOR,FGL2,KIAA1109,SCAMP5,SNX30,SUFU,TRERF1,C10ORF90,CEP250,EYA1,IFNA8,IL1RL2,N4BP1,RBM4,SRPK2,BDKRB1,BDKRB2,HLA-DRA,ADRA1B,ARMC9,BHLHB9,DGKZ,GNL3,IFI27L1,L3MBTL1,MIR433,NEK5,OGN,PI3,PRKAA2,RLF,SEMA3A,TAX1BP1,THADA,TLE4,ZNF202,ZNF558,ARHGAP10,CDC73,EPB41L3,GNB5,GRIA2,MARK2,MEIS2,PDE5A,PROS1,REEP2,ZMIZ1,ZNF19,ZNF440,ZNF670,ZNF695,FLT3,NFIC,PDLIM5,PPARGC1B,PTPRA,SNX7,SRGAP3,TRIM72,ATP6V1H,DYNAP,ESRRG,FERMT2,GNAO1,HCN4,LTBR,MAP2K5,MARK1,PAPPA2,SMPD3,TNN,CLN6,DAB2,GPR55,HDAC8,LCP1,PBX1,RNF207,STX3,SYT2,TCL1B,ACTN4,ADAM10,BLOC1S5,CCL7,DAAM1,DRG2,EEF1E1,EEF1E1-BLOC1S5,ELP4,FGF12,ITGA3,KCNJ16,KMO,POU2F3,PRAMEF8,TXNDC5,FANCB,FRY,LGI1,NREP,OR10K2,OR4C46,PLCL2,PPM1B,QRICH1,RFTN1,SSX5,STAC,MYOM1,NUP153,PHF20L1,PIN1,RPS6KA6,S

			YNPO2,TRPM4,CD226,ERLIN1,GPR141,KRBOX4,PIP5K1B,STK38,TCF20,ZNF674,BMP6,BTBD9,CHRM3,CIT,CIZ1,CLASP1,EIF4ENIF1,CARF,CDYL2,CFDP1,CNGB1,EBF1,NCOA2,TGFA,TMOD1,WDR12,CREM,G3BP2,GAS2,HGF,MYLK,PKP1,POU2F1,RALGAPA1,RASSF8,RGS12,ZNF665,ZNF730,CALCR,CAPN7,CENPF,FLI1,POMT2,SCUBE3,SH3BP5,TLK1,ZBTB16,CACNB2,CNKSR2,CTIF,ELN,KDM4B,MAST4,NCAM1,NOS1AP,PI4KA,RALGDS,ZNF57,ARHGAP28,IL18RAP,INSR,LARP4B,MACF1,PAK1,PDE3A,PSMB7,SKI,AKT3,CDC16,CHM,CTDP1,ELF1,ENPP1,FMR1,LIMK2,LITAF,PAX3,THEM4,CHFR,DKC1,GABRA1,IGHV1-69,JDP2,KLF6,PHC2,PRKX,RRAGC,ULK4,ZNF605,ABCD1,BMF,CRMP1,EVC,GABRR3,IDE,IGHV1-46,MAD1L1,MFNG,SAMD8,AIM2,ARHGAP42,CD84,CSF2RB,EPG5,EVC2,JPH4,LRRN2,PCK1,PDE6B,PPARA,SORT1,SPIB,TBX22,TNFAIP8L3,TTN,ZFHX2,CLIP1,FAM3D,FHL1,GNA13,KATNBL1,NUP214,PPP1R13B,SMARCAD1,TAF1,WDR41,CACNA1A,CHD5,FNIP2,FUT8,PPP2R3C,QRFP,S1PR3,SPRED1,TRPM1,AKR1C2,CHSY1,ESR2,KRT4,PRDM16,PTPRF,QSOX1,RBL1,SCN9A,ZNF451,ATP6V1E1,BTRC,C2,CCDC3,DEFA1B,DEFA3,FBLN2,HIRA,IGHV1-18,MLLT10,MPDZ,OPTN,PTPRR,RIT2,ZNF713,ESRP2,EXT1,GNG12,IFT81,MDM4,NCOA1,PRKCG,PRLR,TYMP,UMOD,ZNF354B,CBFA2T3,DOCK7,FBXW11,G3BP1,HDAC5,ICK,INPP4A,OR51L1,TDGF1,WDR70,ZNF493,A2M,LYVE1,MYEF2,PI15,SERPINB8,SLC24A5,SLC40A1,STAT4,STIM1,TREM1,ZNF623,ADARB1,KCNC2,OR4C16,POU2AF1,PRKCQ,SCEL,STAT3,TFEC,TP63,USP7,ZNF721,AMOT,BRIP1,CDKN3,CST1,DOCK3,HPN,ISLR2,PAK3,PIK3R2,STK17B,TBL1X,USH2A,BLNK,CASQ2,DHRS2,DHRS7B,DTNBP1,GAK,RAP1B,ZNF518A,DLGAP2,KSR1,LGALS9,LRP8,NFAT5,OR2T4,SNCAIP,TAS1R1,ZNF697,ALPK1,EFCAB1,GTPBP1,IL10RB,MAPKAPK2,AHNAK2,APC,BMPR1A,CKAP5,DPP4,ITGA1,KCTD20,KIAA0319,NGF,PPP1R14A,PRKCA,PTGES,SPINT2,UBASH3B,UBE2E2,VLDLR,ADAMTS20,ASB3,DRAM1,GPR75,ITIH6,MTMR4,MTRF1,OSGIN2,PDS5A,PILRB,ABI1,CTSH,DHX9,FCRL5,NCAPG2,RGCC,RPH3AL,SRGAP1,TRBV6-8,USP18,CCL14,CCL15,CCL15-CCL14,CYB5D2,DPH1,EBF4,EDA2R,GABRG2,GPR21,IL6R,MAP2K6,NCALD,RIC8B,SIAH3,SRRD,TFIP11,SPTBN4,CLASP2,MAP2K1,PDE7B,PRAMEF7,RGMB,SOS1,TPH1,VPS35,ZBTB25,ZRANB3,C1QTNF3,CABLES1,COL6A3,FGF1,MCOLN3,NSUN3,RAD18,TBX20,ZNF527,ATMIN,ATP6V1E2,C6ORF106,CRADD,DGKK,NLRC5,PPP1R9A,RGN,RREB1,TBX18,VSTM1,ZFP2,ZNF501
GO:0009653	anatomical structure morphogenesis	2.905489988194354e-26	*ISM1,CDH4,DAB1,OMA1,GTF2I,FMNL2,CDC42EP3,MAPK14,DSCAM,RUNX1,MYO18B,ANK3,DCC,SVEP1,WLS,AKAP13,FBLN1,NPHP3,ITGA2,KCNQ1,NUBPL,EPHA6,NRXN1,ZRANB1,NRXN3,SDK2,CTNND2,TNC,PAX7,CHODL,RIMS2,MTPN,NRG3,FGD6,OLFM3,TGFBR3,FSTL4,CDH13,ANK2,LRRC4C,CSMD1,SDCCAG8,TMEM108,GADD45A,ADAMTSL1,SYT17,PLXNA4,FLRT2,GPC3,NRG1,ETS2,PSMB2,IL4R,ALDH1A2,CECR2,ASTN2,UNC5D,SEMA6D,CTNNA2,CCR3,CFTR,RBFOX2,TIAM1,RHOC,COL22A1,DPYSL2,MYO3A,NKD1,SPEF2,FBXO31,CNTN4,ARID5B,PHACTR1,S100B,ROBO2,C2CD3,EPHA3,PRKACB,DCLK1,COL15A1,AJAP1,AGT,CDH11,RHOJ,CDH2,CDH9,PARK2,FHL2,FOXN3,LOXL2,LAMA2,GREB1L,CYBB,SEMA3D,NTN1,KCNH1,GPC6,ZNRF3,FMNL3,TRIO,HDAC2,HTRA1,MYO9A,SLIT2,CDH8,PDCD6,TM4SF1,IGF1R,KRT25,MDM2,PALMD,SLC24A4,SMAD1,FOXD4L4,TRPC5,DMD,HCN1,SYT1,BASP1,SIPA1L3,VCL,NOX5,CELSR1,MBOAT7,MAEL,WWOX,ATRX,RYR2,NTNG1,TENM4,LRRK2,RORB,SLC1A3,STRC,KIF26B,RORA,PRICKLE2,CHD7,RNF165,DISC1,HEXB,EMB,SH3BP1,SOX6,MECP2,MTMR2,SEMA4D,GSK3B,LGR5,PRKD1,FLVCR1,FRMD6,TGFB2,FMN1,VAV3,AFF3,PLCD3,SEMA5A,BDNF,RIPK4,RYK,ZDHHC17,PCDH15,SGCD,WASF1,WDR72,KDM4C,EXT2,LAMA3,ANKRD6,NR2F2,NR5A2,PML,ARHGAP12,CNTNAP2,FBN1,PLXNA2,TAOK3,BCL2,CDH10,RALBP1,ARHGAP24,EPHB1,CDH12,SIPA1L1,CSGALNACT1,LRP2,MMP16,TMEM100,VANGL2,CDH6,NR4A2,EPHA7,FYN,CHN1,NR4A1,SULT4A1,CD44,HECW1,PMP22,RNF213,THBS2,KANK1,ENPP2,ACTN2,ATP8A2,DCDC2,TNMD,EXOC4,LAMB4,UNC5C,MYLK3,SLC1A1,ZFPM2,ZNF141,PALLD,RARB,STRIP1,MTOR,ROR1,GAS7,MOV10,FAT3,MEGF9,PKP2,BMPER,ERMN,PTPRD,TIAM2,BBS2,PGK1,COL11A1,CTNNBIP1,SOX8,CDH18,PRKCB,VDR,FOXO3,IGF2BP3,CAPN3,KALRN,CCDC88C,DLC1,ROBO1,RXFP1,SATB2,TANC2,FAP,FLT1,NOX4,GRHL2,COL2A1,NEBL,PREX2,EYA4,SEC24B,PKHD1,STK3,TGM2,VASH2,KDR,PRCP,ARHGAP15,CHL1,EDA,APP,CALCRL,COL18A1,NRP1,PTPRM,SHROOM3,ARHGEF18,LGR4,ADTRP,EGFLAM,THRB,SEMA3C,ADAM17,PARVG,TSPEAR,ATF2,ETS1,EFEMP1,PRTG,TGFB1I1,RPS6KA5,BICD1,PARD3,ATRNL1,FRAS1,GRIP1,OP*

			HN1,ZNF568,RIN2,ALCAM,BCL2L1,ADAMTS16,MYO16,SOX5,GHR,JAM2,NTRK2,DNM3,STARD13,SDK1,GLI3,CACNA1C,KIF16B,CRB1,RAP1A,TGFBR1,CYSLTR1,RRP7A,ADAM12,DICER1,EPHA5,TNR,CUX1,PEAK1,TENM3,CHST11,CPE,CHRNA3,EP300,DSCAML1,SCFD1,TBX3,CYP7B1,TRIOBP,PCSK5,GRID2,PARVB,PSMA1,FLNB,SPG11,ADCK1,ADORA2A,EFNA5,EMCN,FAM20A,MERTK,WARS2,MAGI2,NF1,ELK3,IFT80,TEAD4,TTC8,DMRT1,ASXL1,FRYL,POR,RELN,TNFRSF11B,TSHZ1,HDAC9,KIRREL3,COL12A1,NUMB,MAP3K13,RALA,TBX15,ATP10A,EXOC5,JAG1,MEOX2,PPP3CA,KLF7,NEB,PLS1,SH3GL2,CAPZB,WWTR1,DOCK10,ELAVL4,GPC4,RIMS1,GABPA,TMEM135,AGGF1,FHOD3,FREM1,NLGN1,SH3KBP1,SSUH2,MEGF11,SYCP2,MAPK8IP2,SHOX2,SOBP,AHI1,SMAD3,KIDINS220,NOS1,PTGFRN,COL8A1,PLXNB1,RDH10,HDAC7,TNF,COBL,OVOL2,ZFPM1,PPFIA2,TANC1,DLG5,PHEX,LGR6,ARID2,MMP20,BCL11A,CALD1,FREM2,HMGA2,MYO6,DOCK1,QKI,RAB23,EPS8,TPD52,SMOC2,SULF1,C5AR1,LRP4,PIK3R3,CNTN6,EFNB2,AMOTL1,HERC1,ALPK2,CTNNA1,CDKL5,MYH14,PRKAR1A,DYNC2H1,FAM171A1,MLLT3,CD109,EGF,PTPN14,CMA1,IL1RAPL1,KIT,SLIT3,TRPV4,CC2D2A,DNAH11,EDAR,EFNB1,PTPRO,RAPGEF2,DNAJB6,ESR1,JAK1,MYPN,NCMAP,SEMA3E,UBE3A,DNMBP,FGF13,PLAG1,EPHB2,TBCD,ERBB4,FER,MAP3K3,SLITRK6,SPTA1,LRP5,BSG,CAMP,F11R,SETD2,ALX4,ARHGAP35,CCL3,FGF7,LAMA1,PREX1,SEMA5B,FGD4,SULF2,MYH9,PACSIN2,SLC39A12,SOX4,NEDD9,PIK3CD,AGTPBP1,ITGA8,ENPEP,MYO3B,PDZD7,UNC13A,WDPCP,AUTS2,CDH23,FGFR1,MEF2A,RYR1,MAP2,ASXL3,ITGB7,NTF3,P2RX7,AR,BCOR,SUFU,EYA1,SRPK2,BHLHB9,SEMA3A,ANKRD11,CDC73,EPB41L3,MARK2,MEIS2,ZMIZ1,PDLIM5,PPARGC1B,FERMT2,MAP2K5,PAPPA2,SMPD3,TNN,DAB2,PBX1,RNF207,SYT2,ACTN4,CCL7,DAAM1,ITGA3,FRY,LGI1,LRIG1,THSD7A,BMP6,CLASP1,CFDP1,TGFA,TMOD1,GAS2,HGF,MYLK,FLI1,ZBTB16,ELN,NCAM1,NFASC,INSR,MACF1,PAK1,PSMB7,SKI,AKT3,PAX3,PRKX,CRMP1,MFNG,RPGRIP1,CCDC141,PPARA,TTN,C1GALT1,FHL1,GNA13,MYH7,S1PR3,SPRED1,CHSY1,NTN4,BTRC,HIRA,PGM5,ESRP2,EXT1,MDM4,MYH6,NCOA1,TYMP,ADAMTS5,DOCK7,FBXW11,HDAC5,KLHL3,TDGF1,AKAP2,LYVE1,SLC40A1,STIM1,TTC39C,ADARB1,MYH11,PRKCQ,STAT3,TP63,AMOT,HPN,ISLR2,PAK3,USH2A,DTNBP1,LRP8,MYOM2,UPK3A,BMPR1A,ITGA1,KIAA0319,NGF,PRKCA,SPINT2,VLDLR,ABI1,CTSH,RGCC,SPTBN4,CLASP2,MAP2K1,SOS1,VPS35,FGF1,TBX20,RREB1,TBX18
GO:0051179	localization	8.755032728190287e-24	IGHV1OR21-1,SRGAP2B,TPTE,PIEZO2,PLCB1,CMKLR1,DAB1,FMNL2,GABRB3,ABCC10,GABRG3,MAPK14,ABCG8,TPTE2,TRAPPC8,GPHN,HNRNPA1L2,ASIC2,CTBP2,TMPRSS15,RAB27A,CACNA1E,KCNJ6,ANXA8L1,ANO2,PTPRT,ANK3,IGHV4-31,HEATR5A,DCC,IGHV3-64,WLS,RBFOX1,SYN3,DUSP22,AKAP13,FBLN1,SNX31,VPS13D,GRIK1,IGHV1OR15-9,SLC24A3,IGHV4OR15-8,NPHP3,ITGA2,ABCG1,TMPRSS2,KCNQ1,SUN2,GRIN3A,NRXN1,TMPRSS3,ZRANB1,SGSM1,NRXN3,SLC1A2,RAB5A,DOCK2,PCNT,MS4A1,RIMS2,GRIA1,NRG3,CLEC16A,PKD1L1,DLG2,SLC9B1,TGFBR3,TRPM3,ANO4,CDH13,ANK2,TBC1D22A,ANKH,GRAMD1C,KCND3,LRP1B,FAM155A,SYNDIG1,SLC12A1,DPP10,TF,SCN8A,KCNE1,PIK3C2B,SDCCAG8,CEP41,TMEM108,GADD45A,KCNJ15,SYT17,PLXNA4,KCNG3,FLRT2,GPC3,FBLN5,NRG1,SCARA5,STXBP6,PSMB2,SHISA6,RFFL,SPIDR,IL4R,ENTHD1,CECR2,RYR3,MCTP1,ASTN2,SH3RF2,UNC5D,SEMA6D,SPOCK1,SYT16,ABHD17C,CTNNA2,SLCO3A1,CCR1,CCR3,CFTR,BBS9,RBFOX2,DNAH6,SCFD2,PCTP,TIAM1,PM20D1,RHOC,AGBL4,LDLRAD4,DNAJC15,RASGRF1,DPYSL2,GRID1,SLC6A2,ARFIP1,SLC17A3,NKD1,SCAI,PIK3C3,UTRN,SPEF2,FAM3B,ERC1,FBXO31,KLRF2,RAB7A,TAPBP,ARID5B,PHACTR1,SPOCK3,C2CD3,EPHA3,PTPRN2,DCLK1,SLC44A1,AGT,ATG4C,ERC2,PSTPIP2,FSIP2,SHISA9,CHRM5,VTI1A,WDR11,ATP6V0D1,RHOJ,ZFYVE1,CDH2,PARK2,BANP,LOXL2,LAMA2,RN7SL659P,CORO2B,CYBB,MCTP2,SYTL5,DNAH9,SEMA3D,DACH1,NTN1,TRIM22,TRIM5,KCNH1,SLC22A23,ZNF268,SLC5A4,CP,DENND2A,GPC6,RNF185,USP6,ANKFN1,DBH,ITSN1,NCF4,PCDH17,FMNL3,KCNMA1,RIN3,APBA2,CYB5R2,FTO,LAMA4,SLIT2,TTLL8,PDCD6,STXBP4,GPC5,SLC44A5,IGF1R,MDM2,MX2,SLC24A4,E2F3,NUTF2,SYT9,TMEM241,CCDC91,VCAN,GABRG1,KCNK17,PTPRG,PXK,SLC14A2,TRPC5,DMD,HCN1,SYT1,CACNA2D3,CACNG3,KRT2,VCL,NOX5,CELSR1,FGF14,FSHR,GSG1L,GRIA4,GRM1,MBOAT7,FMN2,HNRNPA2B1,NRIP1,SKAP1,SORCS2,ASTN1,STK24,JAKMIP1,SLC39A11,DPP6,WDR83OS,ATRX,FRMD5,TRPM2,CNIH3,CTNNA3,IPO13,RYR2,OCA2,XRCC4,KIF3B,NTNG1,UNC13C,CATSPER2,DHRS7C,LRRK2,SLC1A3,KCNH5,KXD1,NTRK3,NUSAP1,ILDR1,SLC4A4,ANKS1A,CHD7,DISC1,DPY30,HEXB,KCNK13,WRAP73,EMB,SH3BP1,IE

			R2,MECP2,MTMR2,SEMA4D,FAM160A1,GSK3B,SLC8A1,PRKD1,SERPINA5,LRBA,FLVCR1,FRMD6,TGFB2,ATP1A4,MFSD9,VAV3,ANKS1B,ATP9B,DNAJC6,FUT4,S100A11,SEMA5A,SLC5A3,SLCO2B1,BDNF,LLGL2,PRKAR1B,PRKCH,ZDHHC17,KCNC1,NBEA,DNAAF2,DNAH3,SNX24,TOR1AIP1,WASF1,WDR72,GRIN2B,ZDHHC14,EXT2,LAMA3,NKAIN2,ABCB5,MREG,NR2F2,PLEKHM2,SNAP25,SNAP25-AS1,PITPNC1,PML,STOML1,SYBU,USP36,ARHGAP12,AKAP6,CNTNAP2,TNS1,FBN1,GRIK3,IGHV3-16,PLXNA2,SNAP23,BCL2,CHMP4C,RALBP1,ARHGAP24,EPHB1,KCNJ3,SLC30A5,DISP1,LRP2,NKAIN3,NSF,FRMD4A,ITPR2,LRRC4,VANGL2,CD163,CD163L1,GDAP1,NR4A2,ABCC9,CYB561A3,MICAL3,RPGR,FYN,KCNE2,PARN,SLC26A7,NR4A1,CD44,HECW1,NEU3,RCVRN,RHBDD1,RNF213,PDE4D,KANK1,ATP10B,ENPP2,ABCB10,ACTN2,ATP8A2,DCDC2,GABRB1,PACRG,CSNK1G3,EXOC4,KCNS3,GRIK2,IGSF11,LAMB4,KCNIP4,UNC5C,KCND2,SCN11A,SLC1A1,UGCG,PALLD,AGAP1,NLRP12,MFSD12,MTOR,CADPS,LDB2,TMPRSS4,VPS26B,CHRM1,CTDSPL2,FAT3,MEGF9,PKP2,RANBP17,UBXN2B,ABCA13,BMPER,CCDC88B,SLC35F3,BBS2,HBE1,ARL6IP5,ATP1A1,CEP85L,EPM2A,PLN,SLCO1B1,SLCO1B3,ZMAT3,EPB41L4B,EVI5,EXOC6B,HEPHL1,SOX8,EHBP1,IFT88,NLGN4X,PRKCE,SLC2A9,ATP6V1D,KMT2A,PRKCB,STON1,STON1-GTF2A1L,VDR,ADCY8,EFHB,FAM19A4,FOXO3,IGF2BP3,LRRTM1,MITF,RAB31,SCP2,SLC47A1,TRAPPC12,CAPN3,ITLN1,KALRN,PARD3B,CCDC88C,DLC1,EDNRB,MSR1,ROBO1,SATB2,TANC2,TBC1D4,BNIP3L,FAP,FLT1,NASP,SGIP1,SLC12A8,STX12,CDH17,CENPV,KCNC4,MAP7,MAPK9,NBAS,TACR3,TRAF3IP2,VPS37B,CBLN4,CR1,GABRA2,SEC24B,ACSL5,PKHD1,STK3,TGM2,KDR,PPHLN1,PRCP,CASK,CHL1,IGHV4-28,ABCA12,APP,CALCRL,NRP1,PTPRM,RN7SL674P,SHROOM3,TSNARE1,ARHGEF18,ELMO1,ADTRP,IMMP2L,PRELID2,TTC12,KCNK2,SEMA3C,SLC38A7,ADAM17,CAMK1D,IGF2R,ATF2,ELP3,ETS1,GC,PEX5L,SLC35F4,CEP112,GOPC,ROS1,SMG6,TM9SF4,KPNB1,TCF7L2,BICD1,CCT6B,ITGA11,PARD3,THOC2,ABAT,ATRNL1,FRAS1,GRIN2A,GRIP1,RIMS3,SGK2,KIFAP3,OPHN1,BCLAF1,SAMD9,SLC35F2,CCDC88A,CD38,DENND5A,RIN2,SLC30A7,TUB,NCF2,RNF126,SMG7,STK36,BCL2L1,DNAH8,HDAC4,PDE4B,GHR,GLRA2,JAM2,MAPRE2,NTRK2,PRKG1,ACSL4,MGST1,SLC5A10,A1CF,CDC42BPA,DNM3,RASGRP1,STARD13,TRAPPC10,ARR3,COMMD1,KCTD7,RAB11FIP4,RABGEF1,STARD4,C17ORF75,GLI3,CACNA1C,CLDN1,COG7,KIF16B,NIPA2,STXBP5,CRB1,DOCK4,LAMP5,RAP1A,SRP54,TGFBR1,CYSLTR1,KCNJ12,SVOPL,EPHA5,SLC13A4,TNR,VPS53,CUX1,PEAK1,SLC25A21,SLC6A17,CHST11,CPE,IL16,PTTG1IP,RAPGEF1,STARD5,CHRNA3,CHRNA5,MORC3,RAB2A,SLC16A7,SYNE1,SCFD1,SPAG17,TBX3,TTC39B,CACNA2D1,CACNA2D4,CYP7B1,STX8,ATXN1,CLIC6,GAB2,GLRA1,HBD,PCSK5,SYCP1,GRID2,IFNGR2,NPC1,PIBF1,RASGRF2,SAMM50,TMEM50B,TRPM7,PSMA1,SV2B,VPS39,SPG11,ADORA2A,ADRBK2,CAMK2G,DYNC1I2,EFNA5,MERTK,TAC4,CACNG2,MAGI2,NF1,RGS7,GOT2,GULP1,KCNQ5,SORL1,BID,IFT80,TTC8,DMRT1,MICU1,RELN,SLC39A8,SLC9C1,GRM5,HDAC9,IGHV4-4,KIRREL3,PTPN9,SELE,SNUPN,DYNC1H1,MGAT5,PLSCR4,DDC,NUMB,IGHV3OR16-12,NPHP4,RALA,ADCYAP1R1,NAV3,SLC35A5,SPTSSA,ATP10A,EXOC5,JAG1,MAN1A1,MEOX2,PPP3CA,TBC1D3B,TMEM163,KCNB2,KLF7,PLS1,SH3GL2,SLC7A1,SNX25,ATP8B4,CXADR,DGKI,GNPTAB,PER1,SLC24A2,TBC1D5,TSPAN33,WWTR1,ANKRD13A,CBLB,DOCK10,PLSCR1,SEC63,SLC38A6,SLC5A1,ATP9A,CNTN1,GPC4,GRM7,RIMS1,FAM126B,GABRA3,GPSM2,KAT7,STIM2,SLC22A2,BORA,CEP128,MZT1,NLGN1,P2RX6,PTPRU,SLC35F1,GBP2,RNF128,SH3KBP1,SLC22A3,NPR3,OSBPL5,AKAP7,MAPK8IP2,CACHD1,CACNA1D,DDX3X,LRPPRC,NSG1,VPS37A,GABRA5,TPCN2,JAK2,SLC5A8,SPNS2,AHI1,RPA1,SLC25A48,SMAD3,VPS41,XKR4,CD300A,NOS1,PLXNB1,CDK1,HDAC7,HGSNAT,MAGT1,MCM3AP,NVL,SLC39A10,TNF,LRCH1,OVOL2,PANX1,SNX5,CPT1B,POMC,PPFIA2,TMC2,BLOC1S6,DLG5,FCHSD2,TRIM23,KCNT2,LGR6,ARID2,TMC5,KCNK10,SPAG16,ADORA3,ARF1,MYO6,POLR1A,DOCK1,GRIA3,IGLC2,IGLL5,KIF18A,RUFY1,TSPAN13,ANO1,GCLC,KCNA6,QKI,RAB23,EPS8,LTBP1,NUDCD3,TPD52,PABPC1L,SLC7A7,SMOC2,SULF1,TOMM34,TRPC4,C5AR1,LRP4,PIK3R3,CDS2,CNGB3,EFNB2,KCNH8,RAB24,RSRC1,SAE1,SHFM1,TRDN,AMOTL1,ANO10,NET1,SLC15A1,ZNF423,CLSTN1,INSC,LRRC8C,LRRC8D,SELP,CSE1L,CTNNA1,NSG2,SCN1A,TENM1,ABCG2,CDKL5,DMBT1,EFHC2,GRIK4,WDR35,ARHGAP11B,DYNC2H1,GABRR2,KCNH7,MYRIP,STX16,STX16-NPEPL1,TMEM150C,EGF,MIP,PTPN14,SEC16B,SPIRE2,VPS8,IL1RA

			PL1,KIT,KPNA3,PIK3C2G,TRPV4,ABCA10,ABCA5,ABCA6,CC2D2A,DNAH11,EFNB1,KCNAB1,NAPG,PTPRO,RAPGEF2,SLCO4C1,DNAJB6,ESR1,HTR2C,JAK1,SEMA3E,SLC30A8,UBE3A,FGF13,GABRB2,GRM4,MARVELD3,NUP155,SLC22A15,AKAP10,ANO3,ARMC2,EPHB2,IGHV3-72,NDFIP2,ABCC1,DAPK1,ERBB4,FER,MAP3K3,CACNG6,FLVCR2,IFT43,LRP5,RAPGEF6,XKR7,SLAMF1,ABCC11,ABL2,BSG,F11R,GAS8,SETD2,TMEM50A,VPS13C,AP2B1,ARHGAP35,CD2AP,NSUN2,PTPRK,SV2C,ATP8A1,CCL3,FGF7,LAMA1,MCUR1,MYO7B,PREX1,SEMA5B,ARHGAP25,GPR158,IPO11,ITGA9,KIF2A,MCM9,NHLH2,REEP1,SPA17,ARF4,ARFGAP3,MYH9,PACSIN2,PTGER3,SLC2A13,SLC39A12,SOX4,SPRN,TEKT4,MIPEP,NEDD9,OSCP1,P2RX1,PDGFD,PIK3CD,RGPD1,AGTPBP1,ASGR2,ITGA8,KCNE4,CNGA4,ENPEP,PDZD7,PLAT,SFXN3,TNKS,UNC13A,VPS45,WDPCP,AUTS2,CDH23,FGFR1,MEF2A,OCLN,RYR1,TBC1D9,UNC13B,GLRA3,ITGBL1,MAP2,TTC7B,ITGB7,JMY,KEAP1,NTF3,P2RX7,PLA2G4A,RAPGEF4,SORCS1,ARRDC4,RASA3,ZDHHC11,ZDHHC11B,ZDHHC9,AR,ATG2B,COG1,KIAA1109,PIGK,RABGAP1L,SCAMP5,SNX30,SUFU,CEP250,DENND5B,EXOC2,RBM4,BDKRB1,BDKRB2,AFTPH,DGKZ,GNL3,PRKAA2,SEMA3A,THADA,ANXA11,AQP10,EPB41L3,GNB5,GRIA2,KIF4A,MARK2,MOGAT2,PROS1,REEP2,ZMIZ1,LDLRAD3,SNX7,SRGAP3,TRIM72,VPS16,ATP6V1H,FERMT2,HCN4,MAP2K5,MARK1,SCNN1A,SMPD3,TNN,ATP13A3,DAB2,HDAC8,LCP1,RNF207,STX3,SYT2,ACTN4,ADAM10,BLOC1S5,CCL7,FGF12,ITGA3,KCNJ16,KMO,TXNDC5,KCNN3,LGI1,RFTN1,RNASEH2B-AS1,SLC16A4,STAC,MYOM1,NUP153,PIN1,SYNPO2,TRPM4,NME8,ABCA8,ABCC2,BMP6,BTBD9,CHRM3,CIZ1,CLASP1,EIF4ENIF1,SIDT1,CNGB1,PCDHGA3,TMCO3,G3BP2,HGF,MYLK,SLC38A4,CALCR,CAPN7,CENPF,MYO1B,OSBPL3,RN7SL318P,SLC13A3,SLC26A8,TLK1,ZBTB16,CACNB2,NFASC,NOS1AP,OSBPL10,SNAP29,INSR,MACF1,PAK1,PSMB7,SKI,AKT3,CHM,ENPP1,FMR1,LIMK2,MICU2,THEM4,ATP5J,DKC1,FAM126A,GABRA1,IGHV1-69,MON2,PRKX,RRAGC,ULK4,ABCD1,AMPH,CHKA,GABRR3,IGHV1-46,MAD1L1,AP1G2,CCDC141,CD84,EPG5,ESYT2,JPH4,PPARA,SIL1,SORT1,TNFAIP8L3,TTN,CORIN,FAM3D,FHL1,GNA13,HEATR5B,NUP214,SLC22A5,CACNA1A,FUT8,SPRED1,TRPM1,NTN4,PTPRF,SCN9A,ATP6V1E1,C2,DEFA1B,IGHV1-18,NUP62CL,OPTN,PTPRR,RIT2,EXT1,IFT81,PRKCG,PRLR,UMOD,DOCK7,ELMO2,FBXW11,HDAC5,ICK,KLHL3,TDGF1,GOSR2,LYVE1,SLC24A5,SLC38A10,SLC40A1,STIM1,TREM1,TRPM6,ADARB1,KCNC2,NDE1,PRKCQ,SLC22A10,SLC22A25,SLC9A9,STAT3,USP7,AMOT,DNM1P46,HPN,PAK3,PIK3R2,SLC15A5,USH2A,CASQ2,DTNBP1,GAK,RAP1B,SLC28A3,TMEM175,CNNM3,LGALS9,LRP8,SNCAIP,EFCAB1,MAPKAPK2,SLC7A8,TMEM63A,TUSC3,UPK3A,APC,BMPR1A,CKAP5,CSN3,DPP4,HDLBP,ITGA1,KIAA0319,PRKCA,PTGES,SPINT2,SRP19,UBASH3B,VLDLR,ASB3,EXOC6,CTSH,DHX9,RGCC,RPH3AL,SLC9A2,SRGAP1,CCL14,CCL15,CCL15-CCL14,GABRG2,IL6R,MAP2K6,NCALD,OSBPL1A,SIAH3,SLC16A12,SPTBN4,AP5M1,CLASP2,MAP2K1,SOS1,TPH1,VPS35,C1QTNF3,FGF1,MCOLN3,TBX20,ATP6V1E2,C6ORF106,RGN,RN7SL673P,RREB1
GO:0007155	cell adhesion	1.7027145451160513e-23	*CDH4,PCDH9,DAB1,MAPK14,DSCAM,RUNX1,NCAM2,PTPRT,ANK3,IGSF5,SVEP1,DUSP22,FBLN1,ITGA2,NRXN1,NRXN3,SDK2,CTNND2,TNC,PKD1L1,DLG2,CDH13,LRRC4C,BCAN,ADAMTSL1,PLXNA4,FLRT2,FBLN5,NRG1,STXBP6,IL4R,LRFN5,MUC16,ASTN2,UNC5D,SPOCK1,CTNNA2,CCR1,CCR3,TIAM1,PCDH11X,PCDH7,PDZD2,UTRN,CNTN4,ROBO2,EPHA3,CNTNAP4,COL15A1,CRNN,AJAP1,CDH11,CDH2,CDH9,LOXL2,LAMA2,HMCN1,CORO2B,NEGR1,NTN1,TLN2,GPC6,PCDH17,LAMA4,CDH8,CNTN5,DSC3,VCAN,DMD,CLDN14,VCL,CELSR1,SKAP1,ASTN1,OPCML,CADM2,FRMD5,CTNNA3,CUZD1,NTNG1,TENM4,STRC,IGFBP7,KIF26B,DISC1,EMB,SEMA4D,GSK3B,TGFB2,CLSTN2,FMN1,VAV3,FUT4,S100A11,SEMA5A,LSAMP,PCDH15,ABI3BP,EDIL3,LAMA3,RAG1,RAG2,PML,CNTNAP2,FBN1,PLXNA2,BCL2,CDH10,EPHB1,CDH12,LRRC4,CDH6,EPHA7,DPT,FYN,ADAMTS18,CD44,MYL12A,THBS2,KANK1,ACTN2,IGSF11,LAMB4,CDHR4,NTM,PALLD,FAT3,MEGF9,PKP2,CCDC88B,PTPRD,EPB41L4B,DSG4,NLGN4X,PRKCE,CDH18,FOXO3,PARD3B,DLC1,ROBO1,FAP,CDH17,GRHL2,CR1,SPON1,LPP,PKHD1,TGM2,KDR,CASK,CHL1,EDA,ABCA12,APP,COL18A1,NRP1,PTPRM,TENM2,VSIG10,ADTRP,CD58,EGFLAM,ADAM17,PARVG,PKP4,ETS1,PRTG,TGFB1I1,TM9SF4,CYTIP,ITGA11,PARD3,SSPN,ABAT,ATRNL1,KIFAP3,RIN2,ALCAM,CNN3,CNTN3,JAM2,PRKG1,PPFIBP1,RASGRP1,SDK1,GLI3,CLDN1,CRB1,ADAM12,TNR,PEAK1,TENM3,ITGB3BP,RAPGEF1,CD96,DSCAML1,TRIOBP,C*

			OL4A6,GRID2,PARVB,TRPM7,CD72,ADORA2A,EFNA5,EMCN,MERTK,SPECC1L,CNTNAP5,NF1,ARHGAP6,IGSF21,RELN,SLC39A8,HAPLN1,KIRREL3,SELE,COL12A1,NPHP4,IL1RAP,JAG1,PPP3CA,SLC7A1,CXADR,CBLB,CNTN1,GPC4,AGGF1,FREM1,ITGAE,NLGN1,PTPRU,ARHGAP5,MEGF11,EGFL6,CADM3,OMG,JAK2,LMO7,SMAD3,BAIAP2L1,CD300A,COL8A1,PLXNB1,AATF,TNF,TNIP1,PPFIA2,DLG5,MAGI1,CLEC4A,FREM2,DOCK1,PCDH19,CNTN6,EFNB2,CLSTN1,SELP,CTNNA1,TENM1,PRKAR1A,MIP,IL1RAPL1,KIT,CTNNAL1,TRPV4,VWC2,EFNB1,PTPRO,DNAJB6,JAK1,MYPN,SEMA3E,EPHB2,FNDC3A,TBCD,FER,SPTA1,SLAMF1,ABL2,BSG,F11R,FARP2,CD2AP,CLDN11,PTPRK,LAMA1,PREX1,TNFAIP8L2,VWF,ITGA9,MYH9,SOX4,NEDD9,ITGA8,WDPCP,CDH23,ITGBL1,IL23R,ITGB7,GP5,FGL2,IL1RL2,HLA-DRA,PDE5A,ZMIZ1,PDLIM5,PTPRA,FERMT2,MAP2K5,TNN,DAB2,STX3,ACTN4,ADAM10,ITGA3,CD226,EPDR1,BMP6,CLASP1,CFDP1,PCDHGA1,PCDHGA10,PCDHGA11,PCDHGA12,PCDHGA2,PCDHGA3,PCDHGA4,PCDHGA5,PCDHGA6,PCDHGA7,PCDHGA8,PCDHGA9,PCDHGB1,PCDHGB2,PCDHGB3,PCDHGB4,PCDHGB6,PCDHGB7,PKP1,ZBTB16,NCAM1,NFASC,MACF1,TESK2,PRKX,MAD1L1,CCDC141,CD84,GLDN,LRRN2,PCK1,PPARA,COL19A1,FIBCD1,NTN4,PTPRF,FBLN2,MYBPC2,PGM5,TECTA,EXT1,PRLR,UMOD,ELMO2,LYVE1,SERPINB8,PRKCQ,USH2A,NID2,LGALS9,NFAT5,APC,DPP4,ITGA1,PRKCA,SPINT2,UBASH3B,CNTNAP3,RGCC,CLASP2,RGMB,COL6A3,DCHS2,RREB1
GO:0000902	cell morphogenesis	2.98896707223533e-23	*CDH4,DAB1,FMNL2,CDC42EP3,MAPK14,DSCAM,ANK3,DCC,FBLN1,EPHA6,NRXN1,ZRANB1,NRXN3,CTNND2,CHODL,RIMS2,FGD6,FSTL4,LRRC4C,TMEM108,ADAMTSL1,SYT17,PLXNA4,FLRT2,NRG1,UNC5D,SEMA6D,CTNNA2,RBFOX2,TIAM1,RHOC,COL22A1,DPYSL2,FBXO31,CNTN4,PHACTR1,S100B,ROBO2,EPHA3,DCLK1,COL15A1,CDH11,RHOJ,CDH2,CDH9,PARK2,LAMA2,SEMA3D,NTN1,FMNL3,TRIO,MYO9A,SLIT2,CDH8,IGF1R,PALMD,TRPC5,DMD,SYT1,SIPA1L3,VCL,MAEL,NTNG1,LRRK2,SLC1A3,STRC,RNF165,DISC1,HEXB,EMB,SEMA4D,GSK3B,FRMD6,TGFB2,SEMA5A,BDNF,RYK,ZDHHC17,PCDH15,WASF1,LAMA3,CNTNAP2,PLXNA2,TAOK3,BCL2,CDH10,EPHB1,CDH12,SIPA1L1,LRP2,CDH6,NR4A2,EPHA7,FYN,CHN1,SULT4A1,CD44,HECW1,KANK1,ENPP2,ATP8A2,DCDC2,TNMD,LAMB4,UNC5C,PALLD,STRIP1,GAS7,MOV10,FAT3,MEGF9,ERMN,PTPRD,TIAM2,CDH18,VDR,KALRN,CCDC88C,DLC1,ROBO1,TANC2,NOX4,GRHL2,PREX2,SEC24B,PKHD1,KDR,ARHGAP15,CHL1,APP,COL18A1,NRP1,PTPRM,SHROOM3,ARHGEF18,SEMA3C,PARVG,PRTG,RPS6KA5,PARD3,ATRNL1,GRIP1,OPHN1,ALCAM,MYO16,NTRK2,DNM3,GLI3,CRB1,DICER1,EPHA5,TNR,CUX1,PEAK1,CHRNA3,EP300,DSCAML1,SCFD1,TRIOBP,PARVB,FLNB,SPG11,ADORA2A,EFNA5,MERTK,TTC8,DMRT1,ASXL1,FRYL,RELN,KIRREL3,NUMB,MAP3K13,ATP10A,PPP3CA,KLF7,PLS1,SH3GL2,CAPZB,DOCK10,ELAVL4,RIMS1,NLGN1,SH3KBP1,MAPK8IP2,SHOX2,KIDINS220,PLXNB1,COBL,ZFPM1,PPFIA2,LGR6,BCL11A,DOCK1,EPS8,LRP4,CNTN6,EFNB2,CDKL5,MYH14,FAM171A1,IL1RAPL1,KIT,SLIT3,EFNB1,PTPRO,RAPGEF2,MYPN,SEMA3E,UBE3A,DNMBP,FGF13,EPHB2,TBCD,FER,SLITRK6,SPTA1,BSG,F11R,ARHGAP35,CCL3,LAMA1,PREX1,SEMA5B,FGD4,MYH9,PACSIN2,NEDD9,ITGA8,PDZD7,UNC13A,WDPCP,AUTS2,CDH23,MEF2A,MAP2,ITGB7,NTF3,P2RX7,AR,BHLHB9,SEMA3A,EPB41L3,MARK2,PDLIM5,FERMT2,TNN,DAB2,SYT2,ACTN4,CCL7,FRY,LGI1,CFDP1,GAS2,HGF,NCAM1,NFASC,MACF1,PAK1,CRMP1,CCDC141,GNA13,NTN4,EXT1,DOCK7,AKAP2,ADARB1,PRKCQ,HPN,ISLR2,PAK3,USH2A,DTNBP1,LRP8,UPK3A,ITGA1,KIAA0319,NGF,SPINT2,VLDLR,ABI1,SPTBN4,CLASP2,MAP2K1,SOS1,RREB1*
GO:0022610	biological adhesion	4.556151712352773e-23	*CDH4,PCDH9,DAB1,MAPK14,DSCAM,RUNX1,NCAM2,PTPRT,ANK3,IGSF5,SVEP1,DUSP22,FBLN1,ITGA2,NRXN1,NRXN3,SDK2,CTNND2,TNC,PKD1L1,DLG2,CDH13,LRRC4C,BCAN,ADAMTSL1,PLXNA4,FLRT2,FBLN5,NRG1,STXBP6,IL4R,LRFN5,MUC16,ASTN2,UNC5D,SPOCK1,CTNNA2,CCR1,CCR3,TIAM1,PCDH11X,PCDH7,PDZD2,UTRN,CNTN4,ROBO2,EPHA3,CNTNAP4,COL15A1,CRNN,AJAP1,CDH11,CDH2,CDH9,LOXL2,LAMA2,HMCN1,CORO2B,NEGR1,NTN1,TLN2,GPC6,PCDH17,LAMA4,CDH8,CNTN5,DSC3,VCAN,DMD,CLDN14,VCL,CELSR1,SKAP1,ASTN1,OPCML,CADM2,FRMD5,CTNNA3,CUZD1,NTNG1,TENM4,STRC,IGFBP7,KIF26B,DISC1,EMB,SEMA4D,GSK3B,TGFB2,CLSTN2,FMN1,VAV3,FUT4,S100A11,SEMA5A,LSAMP,PCDH15,ABI3BP,EDIL3,LAMA3,RAG1,RAG2,PML,CNTNAP2,FBN1,PLXNA2,BCL2,CDH10,EPHB1,CDH12,LRRC4,CDH6,EPHA7,DPT,FYN,ADAMTS18,CD44,MYL12A,THBS2,KANK1,ACTN2,IGSF11,LAMB4,CDHR4,NTM,PALLD,FAT3,MEGF9,PKP2,CCDC88B,PTPRD,EPB41L4B,DSG4,NLGN4X,PRKCE,CDH18,FOXO3,PARD3B,DLC1,ROBO1,FAP,CDH17,GRHL2,CR1,SPON1,LPP,PKHD1,TGM2,KDR,CASK,CHL1,EDA,ABCA1*

			2,APP,COL18A1,NRP1,PTPRM,TENM2,VSIG10,ADTRP,CD58,EGFLAM,ADAM17,PARVG,PKP4,ETS1,PRTG,TGFB1I1,TM9SF4,CYTIP,ITGA11,PARD3,SSPN,ABAT,ATRNL1,KIFAP3,RIN2,ALCAM,CNN3,CNTN3,JAM2,PRKG1,PPFIBP1,RASGRP1,SDK1,GLI3,CLDN1,CRB1,ADAM12,TNR,PEAK1,TENM3,ITGB3BP,RAPGEF1,CD96,DSCAML1,TRIOBP,COL4A6,GRID2,PARVB,TRPM7,CD72,ADORA2A,EFNA5,EMCN,MERTK,SPECC1L,CNTNAP5,NF1,ARHGAP6,IGSF21,RELN,SLC39A8,HAPLN1,KIRREL3,SELE,COL12A1,NPHP4,IL1RAP,JAG1,PPP3CA,SLC7A1,CXADR,CBLB,CNTN1,GPC4,AGGF1,FREM1,ITGAE,NLGN1,PTPRU,ARHGAP5,MEGF11,EGFL6,CADM3,OMG,JAK2,LMO7,SMAD3,BAIAP2L1,CD300A,COL8A1,PLXNB1,AATF,TNF,TNIP1,PPFIA2,DLG5,MAGI1,CLEC4A,FREM2,DOCK1,PCDH19,CNTN6,EFNB2,CLSTN1,SELP,CTNNA1,TENM1,PRKAR1A,MIP,IL1RAPL1,KIT,CTNNAL1,TRPV4,VWC2,EFNB1,PTPRO,DNAJB6,JAK1,MYPN,SEMA3E,EPHB2,FNDC3A,TBCD,FER,SPTA1,SLAMF1,ABL2,BSG,F11R,FARP2,CD2AP,CLDN11,PTPRK,LAMA1,PREX1,TNFAIP8L2,VWF,ITGA9,MYH9,SOX4,NEDD9,ITGA8,WDPCP,CDH23,ITGBL1,IL23R,ITGB7,GP5,FGL2,IL1RL2,HLA-DRA,PDE5A,ZMIZ1,PDLIM5,PTPRA,FERMT2,MAP2K5,TNN,DAB2,STX3,ACTN4,ADAM10,ITGA3,CD226,EPDR1,BMP6,CLASP1,CFDP1,PCDHGA1,PCDHGA10,PCDHGA11,PCDHGA12,PCDHGA2,PCDHGA3,PCDHGA4,PCDHGA5,PCDHGA6,PCDHGA7,PCDHGA8,PCDHGA9,PCDHGB1,PCDHGB2,PCDHGB3,PCDHGB4,PCDHGB6,PCDHGB7,PKP1,ZBTB16,NCAM1,NFASC,MACF1,TESK2,PRKX,MAD1L1,CCDC141,CD84,GLDN,LRRN2,PCK1,PPARA,COL19A1,FIBCD1,NTN4,PTPRF,FBLN2,MYBPC2,PGM5,TECTA,EXT1,PRLR,UMOD,ELMO2,LYVE1,SERPINB8,PRKCQ,USH2A,NID2,LGALS9,NFAT5,APC,DPP4,ITGA1,PRKCA,SPINT2,UBASH3B,CNTNAP3,RGCC,CLASP2,RGMB,COL6A3,DCHS2,RREB1
GO:0048666	neuron development	5.2339606769949857e-23	*CDH4,DAB1,DSCAM,RUNX1,NCAM2,ANK3,DCC,KCNQ1,EPHA6,GRIN3A,NRXN1,NRXN3,CTNND2,TNC,CHODL,RIMS2,OLFM3,FSTL4,LRRC4C,TMEM108,ADAMTSL1,SYT17,PLXNA4,FLRT2,CECR2,UNC5D,SEMA6D,SPOCK1,CTNNA2,RBFOX2,TIAM1,AGBL4,RASGRF1,DPYSL2,CBFA2T2,ALK,FBXO31,CNTN4,PHACTR1,S100B,ROBO2,EPHA3,DCLK1,AGT,ATAT1,CDH11,CDH2,PARK2,LAMA2,ZNF804A,NEGR1,SEMA3D,NTN1,TRIO,HDAC2,MYO9A,SLIT2,IGF1R,PTPRG,TRPC5,DMD,HCN1,SYT1,VCL,CSMD3,FSHR,STK24,OPCML,NTNG1,TENM4,LRRK2,RORB,SLC1A3,STRC,NTRK3,ANKS1A,RNF165,DISC1,EMB,MECP2,MTMR2,SEMA4D,GSK3B,PRKD1,ERCC6,TGFB2,SEMA5A,BDNF,RYK,ZDHHC17,PCDH15,WASF1,LAMA3,SNAP25,CNTNAP2,PLXNA2,TAOK3,BCL2,EPHB1,SIPA1L1,LRP2,NR4A2,EPHA7,FYN,CHN1,SULT4A1,HECW1,PMP22,KANK1,ATP8A2,DCDC2,GABRB1,UNC5C,NTM,UGCG,PALLD,ROR1,GAS7,MOV10,FAT3,PTPRD,TIAM2,KLHL1,IFT88,KALRN,EDNRB,ROBO1,TANC2,PREX2,SEC24B,VASH2,CHL1,APP,NRP1,PTPRM,TENM2,THRB,SEMA3C,CAMK1D,PRTG,RPS6KA5,PARD3,THOC2,GRIP1,OPHN1,CCDC88A,CD38,DENND5A,ALCAM,MYO16,SRRM4,NTRK2,PRKG1,DNM3,SDK1,GLI3,CRB1,RAP1A,DICER1,EPHA5,TNR,CUX1,TENM3,RAPGEF1,CHRNA3,EP300,DSCAML1,TRIOBP,GRID2,SPG11,ADORA2A,CAMK2G,EFNA5,MAGI2,TTC8,FRYL,RELN,KIRREL3,PTPN9,NUMB,MAP3K13,NPHP4,PPP3CA,KLF7,MYT1L,PLS1,SH3GL2,DOCK10,ELAVL4,CNTN1,DGUOK,GRM7,RIMS1,ASAP1,NLGN1,MAPK8IP2,OMG,GABRA5,JAK2,SHOX2,AHI1,KIDINS220,PLXNB1,COBL,PPFIA2,ATXN10,BLOC1S6,DLG5,LGR6,BCL11A,LRP4,WDR5,CNTN6,EFNB2,HERC1,CTNNA1,TENM1,CDKL5,EFHC2,IL1RAPL1,SLIT3,TRPV4,EFNB1,PTPRO,RAPGEF2,MYPN,SEMA3E,UBE3A,FGF13,GABRB2,EPHB2,TBCD,TOX,SLITRK6,ABL2,BSG,FARP2,ARHGAP35,LAMA1,SEMA5B,NHLH2,ARF4,SLC39A12,AGTPBP1,PDZD7,UNC13A,WDPCP,AUTS2,CDH23,MEF2A,MAP2,NTF3,BHLHB9,SEMA3A,EPB41L3,MARK2,ZMIZ1,PDLIM5,MARK1,TNN,DAB2,PBX1,STX3,SYT2,BLOC1S5,ITGA3,FRY,LGI1,NREP,SCLT1,CNGB1,HGF,NCAM1,NFASC,MACF1,PAK1,FMR1,GPRIN2,ULK4,CRMP1,RPGRIP1,CCDC141,GLDN,NTN4,PTPRF,RIT2,EXT1,DOCK7,ADARB1,PRKCQ,ISLR2,PAK3,DTNBP1,GAK,CRTAC1,LRP8,ITGA1,KIAA0319,NGF,VLDLR,ABI1,SPTBN4,CLASP2,MAP2K1,SOS1,PPP1R9A*
GO:0065008	regulation of biological quality	5.505074737529906e-23	*CDH4,PIEZO2,CMKLR1,OMA1,FMNL2,GABRB3,CDC42EP3,GABRG3,MAPK14,ABCG8,DSCAM,GPHN,ASIC2,CTBP2,SERPINA1,RAB27A,ANK3,DTD2,DCC,SYN3,PCSK2,FBLN1,GRIK1,LARGE,SLC24A3,NPHP3,ITGA2,ABCG1,KCNQ1,ATF7IP,GRIN3A,NRXN1,TMPRSS3,TNNI3K,NRXN3,SLC1A2,RAB5A,POTEE,MS4A1,RIMS2,MTPN,SHANK2,COMT,GRIA1,FGD6,SLC9B1,TGFBR3,ANO4,FSTL4,ANK2,KCND3,CSMD1,FAM155A,SYNDIG1,SLC12A1,TF,PRIMA1,SCN8A,KCNE1,SAMD4A,TMEM108,CHEK2,PSMC6,ACIN1,PLXNA4,FLRT2,NRG1,SCARA5,PSMB2,SHISA6,IL4R,ALDH1A2,LRFN5,RYR3,MCTP1,PLCE1,KSR2,SEMA6D,ABHD17C,CTNNA2,CCR1,CCR3,CFTR,RBFOX2,PCTP,PM20D1,RH*

			OC,RGS7BP,RASGRF1,ZBTB20,DPYSL2,GRID1,SLC6A2,IL1RAPL2,ALK,SORCS3,FAM3B,ERC1,RAB7A,CNTN4,TAPBP,S100B,ROBO2,PRKACB,PTPRN2,AGT,ERC2,SHISA9,ATP6V0D1,RHOJ,CDH2,PAH,PARK2,LAMA2,CORO2B,FRMPD4,ZNF804A,MCTP2,NEGR1,OTC,SEMA3D,NTN1,KCNH1,TXK,CP,GPC6,USP40,DBH,TSPAN8,FMNL3,KCNMA1,RIN3,APBA2,FTO,LAMA4,SLIT2,CDH8,RPS6KA2,STXBP4,IGF1R,MDM2,PALMD,SLC24A4,SMAD1,DSC3,NOVA1,SYT9,TMPRSS6,GABRG1,KCNK17,PXK,CPQ,TRPC5,DMD,HCN1,SYT1,CACNG3,VCL,NOX5,FSHR,GSG1L,GRM1,SORCS2,TRPM2,CTNNA3,RNLS,RYR2,UNC13C,DHRS7C,LRRK2,SLC1A3,KCNH5,NTRK3,RORA,ILDR1,SLC4A4,CHD7,DISC1,HEXB,KCNK13,PAN3,PDXP,SH3BP1,MECP2,MTMR2,SEMA4D,GSK3B,LINGO2,SLC8A1,PRKD1,ERCC6,FLVCR1,OGT,ATP1A4,CLSTN2,FMN1,VAV3,ATP9B,PIWIL4,SEMA5A,BDNF,RYK,KCNC1,NBEA,PCDH15,SGCD,GRIN2B,CHST9,EXT2,RAG1,RAG2,ABCB5,NR2F2,PTGFR,SNAP25,NR5A2,PML,PPARGC1A,USP36,AKAP6,GRIK3,LRFN2,PLXNA2,SNAP23,ACSM3,BCL2,ZNF675,EPHB1,KCNJ3,SIPA1L1,SLC30A5,DISP1,ITPR2,DEPTOR,CYB561A3,EPHA7,FYN,KCNE2,PARN,ADAMTS18,NR4A1,SMARCA4,MYL12A,THBS2,FOXO1,PDE4D,TBXAS1,KANK1,ATP10B,ABCB10,ACTN2,ATP8A2,GABRB1,KLF13,MME,NAV2,GRIK2,IGSF11,ZNF516,CELF4,RAP1GDS1,TPO,APBB2,KCND2,MYLK3,SCN11A,SLC1A1,UGCG,MTOR,CADPS,LDB2,MOV10,CHRM1,PKP2,ERMN,PTPRD,BBS2,PLCL1,ARL6IP5,ATP1A1,PLN,SLCO1B1,CTNNBIP1,HEPHL1,SOX8,NLGN4X,PRKCE,PRKCB,VDR,ADCY8,FAM19A4,FOXO3,LRRTM1,SCP2,CAPN3,KALRN,CCDC88C,DLC1,EDNRB,TANC2,BNIP3L,FAP,NOX4,SGIP1,SLC12A8,STX12,TSC22D3,COL2A1,MALL,TACR3,TRAF3IP2,GABRA2,PKHD1,STK3,TGM2,KDR,PRCP,ARHGAP15,CASK,ABCA12,APP,DGKB,NRP1,SHROOM3,ARHGEF18,LGR4,SERPING1,ADTRP,THRB,KCNK2,SEMA3C,ADAM17,DIS3L2,TFPI,ATF2,ETS1,MT1HL1,OIT3,TM9SF4,TCF7L2,ACSM2A,ZNF830,ABAT,GRIN2A,GRIP1,RIMS3,HRNR,OPHN1,PYGL,CD38,SLC30A7,TUB,BCL2L1,ADAMTS16,PDE4B,BACE2,GHR,GLRA2,JAM2,NTRK2,PRKG1,ACSL4,A1CF,DNM3,COMMD1,KCTD7,ZNF207,CACNA1C,CLDN1,COG7,PPA2,STXBP5,CRB1,DOCK4,RAP1A,USP53,CYSLTR1,PRDX4,EPHA5,TNR,NCOA5,CPE,CHRNA3,CHRNA5,EP300,MORC3,TBX3,TTC39B,CACNA2D1,CYP7B1,DIO2,TRIOBP,GLRA1,PCSK5,GRID2,NPC1,PARVB,PNPLA3,RASGRF2,TRPM7,PSMA1,TRHDE,ADCK1,ADORA2A,CAMK2G,EFNA5,EMCN,FAM20A,MERTK,TAC4,BRD1,CACNG2,NF1,TFF1,BID,HTR4,ASXL1,MICU1,POR,RELN,SLC39A8,SLC9C1,TNFRSF11B,USP3,GRM5,HDAC9,IL18R1,DYNC1H1,LRRTM3,PLSCR4,NAPEPLD,NUMB,MAP3K13,NLK,NPHP4,ADCYAP1R1,CYP39A1,IL1RAP,ATP10A,FBXL5,PPP3CA,KLF7,NEB,PLS1,SH3GL2,ATP8B4,CAPZB,CXADR,DGKI,SLC24A2,WWTR1,KL,DOCK10,ELAVL4,PLSCR1,ATP9A,GPC4,RIMS1,GABRA3,KAT7,L3MBTL3,STIM2,SLC22A2,FHOD3,NLGN1,P2RX6,CHST8,CTTNBP2,RNF128,SH3KBP1,SLC22A3,NPR3,AKAP7,LRRK1,MAPK8IP2,ACOX3,CACNA1D,DDX3X,NSG1,CNOT7,DTD1,GABRA5,TPCN2,JAK2,SHOX2,SPNS2,SMAD3,XKR4,BAIAP2L1,NOS1,PLXNB1,RDH10,TMTC4,ITPK1,MIR320B2,SLC39A10,TNF,SNX5,ZFPM1,POMC,PPFIA2,BLOC1S6,DHRS11,DLG5,FCHSD2,ARID2,KCNK10,ARF1,BCL11A,SULT1B1,ZSWIM7,ANO1,GCLC,EPS8,CYP3A5,TRPC4,C5AR1,LRP4,TSPAN1,EFNB2,KCNH8,MYCT1,TRDN,ZNF423,CLSTN1,INSC,LRRC8D,SELP,SCN1A,TENM1,TSHZ3,CDKL5,DMXL1,GRIK4,MYH14,NELL2,ARHGAP11B,FAM171A1,GABRR2,KCNH7,MYRIP,ALDH1A1,CMA1,IL1RAPL1,KIT,POTEF,TNRC6B,TRPV4,ABCA5,CPS1,NAPG,PTPRO,RAPGEF2,ESR1,HTR2C,SEMA3E,SLC30A8,UBE3A,DNMBP,FGF13,GABRB2,GRM4,NUP155,SGCZ,ANO3,EPHB2,ABCC1,ERBB4,FER,SLITRK6,SPTA1,LRP5,MALRD1,PTH2R,XKR7,ABL2,BSG,F11R,AP2B1,ARHGAP35,NSUN2,ATP8A1,CCL3,MCUR1,SEMA5B,VWF,FGD4,IL20RA,ARF4,MYH9,PTGER3,SLC39A12,SOX4,P2RX1,PIK3CD,PRR16,SPTB,AGTPBP1,ASGR2,KCNE4,PUM1,ARID4A,DDB1,ENPEP,KDM3A,PLAT,UNC13A,WDPCP,CDH23,OCLN,RYR1,UNC13B,ZCCHC17,DAD1,GLRA3,MAP2,BPGM,P2RX7,PLA2G4A,RAPGEF4,SCUBE1,GP5,RASA3,AR,C10ORF90,BDKRB1,BDKRB2,ADRA1B,BHLHB9,DGKZ,MIR433,PRKAA2,SEMA3A,THADA,CDC73,EPB41L3,MARK2,PROS1,FLT3,PDLIM5,PPARGC1B,ATP6V1H,ESRRG,FERMT2,HCN4,SCNN1A,SMPD3,ATP13A3,CLN6,DAB2,GPR55,HDAC8,RNF207,SRD5A2,STX3,SYT2,ADAM10,CCL7,FGF12,KMO,LGI1,MT1F,PLCL2,PIN1,TRPM4,BMP6,BTBD9,CHRM3,CIT,EIF4ENIF1,GAD2,SMTNL2,CFDP1,CNGB1,TMOD1,GAS2,HGFAC,CALCR,FLI1,CACNB2,ELN,NOS1AP,SNAP29,ARHGAP28,INSR,LARP4B,MACF1,PDE3A,PSMB7,AKT3,ENPP1,FMR1,MICU2,THEM4,CHFR,DKC1,GABRA1,TMTC2,ABCD1,GABRR3,IDE,ARHGAP42,JPH4,PCK1,POLD1,PPARA,CORIN,FAM3D,FHL1,GNA13,MYH7,SLC22A5,TAF1,ADH1B,CACNA1A,PPP2R3C,QRFP,S1PR3,TRPM1,AKR1C2,PRDM16,SCN9A,BTRC,EXT1,MDM4,MYH6,

			PRKCG,PRLR,SCO2,UMOD,ADAMTS5,DOCK7,FBXW11,KLHL3,AKAP2,MYEF2,SLC24A5,SLC40A1,STIM1,KCNC2,PEMT,PRKCQ,SLC9A9,STAT3,TP63,USP7,AMOT,HPN,ISLR2,PAK3,PIK3R2,TBL1X,USH2A,ADH1A,CASQ2,DHRS2,DHRS7B,DTNBP1,RAP1B,TMEM175,CNNM3,LGALS9,LRP8,SNCAIP,ACSM1,MAPKAPK2,SLC7A8,UPK3A,APC,CSN3,DPP4,ITGA1,KIAA0319,NGF,PRKCA,PTGES,UBASH3B,CTSH,DHX9,NCAPG2,RPH3AL,SLC9A2,ACACA,CCL14,CCL15,GABRG2,GPR21,MAP2K6,NCALD,SIAH3,TMEM123,SPTBN4,CLASP2,SOS1,TPH1,VPS35,C1QTNF3,MCOLN3,PLB1,DGKK,RGN,TBX18
GO:0023052	signaling	2.3392838595374863e-22	IGHV1OR21-1,ADCY2,TPTE,PLCB1,CMKLR1,DAB1,GABRB3,CDC42EP3,GABRG3,MAPK14,ABCG8,DSCAM,TPTE2,SH3RF3,ASIC2,CTBP2,DTNA,OR11H1,HUNK,CACNA1E,NPY4R,LYPD6,OR4M2,PTPRT,ANK3,IGHV4-31,DCC,IGHV3-64,SVEP1,WLS,SYN3,TIMP3,DUSP22,AKAP13,FBLN1,ERG,GRIK1,GPR139,IGHV1OR15-9,SORBS2,IGHV4OR15-8,NPHP3,ITGA2,RGS6,KCNQ1,GABBR2,EPHA6,OR4K15,TMEM117,VRK2,GRIN3A,FCRL2,NRXN1,SHC3,ZRANB1,TNNI3K,DOK5,NRXN3,SLC1A2,RAB5A,DOCK2,CTNND2,TNC,PCNT,MS4A1,OR8U1,RIMS2,SHANK2,GRIA1,NRG3,SMYD2,CLEC16A,HUS1,DLG2,TGFBR3,FSTL4,CDH13,RNF152,ANK2,LRRC4C,NXN,KCND3,CSNK2A1,TF,SCN8A,KCNE1,PIK3C2B,TMEM108,CHEK2,OR4C12,GADD45A,PCP4,SYT17,PLXNA4,FLRT2,GPC3,NRG1,PSMB2,SHISA6,RFFL,IL4R,ALDH1A2,RYR3,MCTP1,NLN,PLCE1,SH3RF2,UNC5D,KSR2,GLP2R,STK38L,SEMA6D,DRP2,CCR1,CCR3,CFTR,RBFOX2,DTX4,MC2R,TIAM1,RHOC,LDLRAD4,RGS7BP,RASGRF1,DPYSL2,GRID1,SLC6A2,CBFA2T2,IL1RAPL2,OR4A5,NKD1,SCAI,MX1,BTN2A1,ALK,PTPRE,PIK3C3,SORCS3,ZNF536,FAM3B,PLCB4,ERC1,FBXO31,RAB7A,CNTN4,RGL2,ARID5B,S100B,ROBO2,ZNF366,C2CD3,EPHA3,PRKACB,PTPRN2,CNTNAP4,DCLK1,PXDN,COL15A1,CRNN,LEMD3,OR8K3,AGT,ERC2,SHISA9,CHRM5,WDR11,CDH11,RHOJ,RCAN1,CDH2,PARK2,FHL2,FOXN3,DLGAP1,LAMA2,CDC5L,KIR2DL1,KIR2DL4,OR4K13,BPIFB1,CYBB,MCTP2,GNB4,OR4M1,OR4N2,SEMA3D,NTN1,OR52N5,TRIM22,TRIM5,KCNH1,TXK,GPC6,DBH,ITSN1,TNS3,PCDH17,ZNRF3,RIN3,TRIO,APBA2,HDAC2,HTRA1,MYO9A,SLIT2,CDH8,PDCD6,OR2L13,RPS6KA2,STXBP4,GPC5,LCMT1,IGF1R,MDM2,SLC24A4,SMAD1,SYT9,OR11G2,OR9Q1,TMPRSS6,RAPGEF5,GABRG1,NR3C2,PTPRG,PXK,PDE9A,OR8K5,DMD,KCTD8,STK33,SYT1,CACNG3,SIPA1L3,TEAD1,CELSR1,FGF14,FSHR,GSG1L,GRIA4,GRM1,FMN2,MAEL,PRKAR2A,SKAP1,SORCS2,INPP5A,STK24,DCDC1,WDR83,WWOX,ATRX,PARP16,TRPM2,CNIH3,CTNNA3,RYR2,NTNG1,OR2M5,TENM4,UNC13C,CAMTA1,LRRK2,MAPK10,RORB,SLC1A3,IGFBP7,RIMBP2,NTRK3,RORA,CD8B,ILDR1,IQCJ-SCHIP1,PRICKLE2,ANKS1A,CHD7,RNF165,DISC1,TPRG1L,KCTD9,SH3BP1,MECP2,MTMR2,SEMA4D,GSK3B,LGR5,SLC8A1,MAGI3,PLCH2,PRKD1,SNRK,ERCC6,OGT,TGFB2,ATP1A4,CLSTN2,VAV3,ANKS1B,PLCD3,S100A11,SEMA5A,BDNF,LLGL2,PRKAR1B,PRKCH,RBMS3,RYK,ZDHHC17,PLCXD3,SGCD,WASF1,GRIN2B,KDM4C,OR6N1,OTUD7A,EXT2,LAMA3,SIK2,ANKRD6,ARHGAP8,NR2F2,PRR5,PRR5-ARHGAP8,PTGFR,SNAP25,NR5A2,PAQR8,PITPNC1,PML,ARHGAP12,CORO2A,AKAP6,CNTNAP2,TNS1,FBN1,GRIK3,IGHV3-16,LRFN2,PLXNA2,SNAP23,TAOK3,BCL2,CHMP4C,RALBP1,RXFP2,ZNF675,ARHGAP24,EPHB1,KCNJ3,IL12RB2,SIPA1L1,SKAP2,LRP2,NSF,TMEM100,ITPR2,LRRC4,VANGL2,CDH6,DEPTOR,NR4A2,ABCC9,ARHGEF10,EPHA7,PSD3,FYN,GNRHR,KCNE2,LRRC69,ADAMTS18,CHN1,NR4A1,SMARCA4,CD44,HECW1,NEU3,PMP22,RCVRN,RNF213,FOXO1,PDE4D,PYY,KANK1,ACTN2,ARHGEF3,ATF6,DCDC2,GABRB1,GRK5,MME,TNMD,CSNK1G3,EXOC4,GRIK2,IGSF11,CELF4,UNC5C,APBB2,KCND2,NAMPT,SCN11A,SLC1A1,UGCG,RARB,RERG,RFX4,GNG4,NLRP12,GRAP2,MTOR,ROR1,CADPS,CHRM1,CTDSPL2,DDX21,PKP2,BMPER,PTPRD,RASGEF1B,TIAM2,BBS2,OR51B2,OR51I1,PLCL1,ARL6IP5,ATP1A1,EPM2A,PLN,CTNNBIP1,SOX8,ZMYND11,DIDO1,DSG4,IFT88,NLGN4X,OR4K17,OR8J3,PRKCE,EIF2S1,HELLS,PRKCB,VDR,ADCY8,EFHB,FAM19A4,FOXO3,LRRTM1,MITF,CAPN3,KALRN,PJA1,TTLL12,CCDC88C,DLC1,EDNRB,OASL,ROBO1,RXFP1,BNIP3L,FLT1,NOX4,CDH17,COL2A1,KCNC4,MAP3K7,MAPK9,NR3C1,PREX2,TACR3,TRAF3IP2,CR1,EYA4,GABRA2,OR4C15,ACSL5,PKHD1,STK3,TGM2,KDR,PRCP,RAB30,ARHGAP15,CASK,CHL1,EDA,IGHV4-28,ABCA12,APP,CALCRL,DGKB,NRP1,PTPRM,UACA,ARHGEF18,ELMO1,LGR4,LINC00473,TENM2,ADTRP,KAT5,RANBP10,THRB,KCNK2,SEMA3C,ADAM17,MAP3K5,PKP4,TSPEAR,CDC42BPG,IGF2R,ATF2,GPR78,GRK6,NR2C2,PEX5L,PIK3R5,EFEMP1,ROS1,TGFB1I1,DOT

			1L,KPNB1,OR10R2,RGS16,RPS6KA5,TCF7L2,TNFRSF10B,TRABD2B,BICD1,ITGA11,PARD3,RALGAPA2,TLK2,TRAF3,ZNF830,ABAT,ATRNL1,DOCK9,GRIN2A,GRIP1,RIMS3,SGK2,KIFAP3,OPHN1,BCLAF1,SGMS1,CASP5,CCDC88A,CD38,FAM83B,MOB3B,RIN2,RNF43,TUB,ALCAM,CHRDL1,RNF126,STK36,BCL2L1,GUCY2F,HDAC4,MYO16,PDE4B,DNMT1,GHR,GLRA2,MAPRE2,NTRK2,PRKG1,ACSL4,FAM83G,CDC42BPA,GNG2,BRD4,PTPDC1,RASGRP1,STARD13,ARR3,CAMK4,LY86,RABGEF1,RGL1,SPRED2,GLI3,OR5L2,ZNF207,CACNA1C,KIF16B,OR11L1,STXBP5,CRB1,DOCK4,RAP1A,SAFB2,TGFBR1,CYSLTR1,MGLL,PRDX4,ADAM12,DICER1,EPHA5,LCP2,TAS2R38,THEMIS,TNR,NCOA5,TAS2R1,TENM3,CHST11,CPE,IL16,ITGB3BP,PTTG1IP,RAPGEF1,CHRNA3,CHRNA5,EP300,TBX3,WIF1,CACNA2D1,CYP7B1,GRAMD4,BRD7,GAB2,GLRA1,PCSK5,TP73,COL4A6,GRID2,IFNGR2,OR4C5,PIBF1,RASGRF2,PSMA1,SV2B,TRHDE,FHIT,FLNB,PPP1R12B,SPG11,ADORA2A,ADRBK2,CABIN1,CAMK2G,EFNA5,MERTK,RALGPS1,TAC4,CACNG2,GUCY2C,MAGI2,NF1,RALGPS2,RGS7,TFF1,SORL1,STRN3,ARHGAP6,ASB13,BICC1,BID,CEP89,ELK3,IFT80,OR5K4,TEAD4,TRIM59,DMRT1,HTR4,LZTR1,ASXL1,EIF4E,POR,RELN,TNFRSF11B,GRM5,IGHV4-4,IL18R1,IL1RL1,SELE,MGAT5,NDRG2,RHPN2,IGHV3OR16-12,MAP3K13,NLK,NPHP4,RALA,ADCYAP1R1,IL1RAP,FBXL17,JAG1,PPP3CA,GPR176,JRK,KLF7,SH3GL2,SNX25,CXADR,DGKI,MAS1,PER1,SLC24A2,WWTR1,KL,CBLB,DOCK10,ELAVL4,PLSCR1,CNTN1,DOK6,GPC4,GRM7,RIMS1,TRIM16,WDR59,GABRA3,GPSM2,KAT7,MAPK4,ITGAE,NLGN1,P2RX6,PDE1C,PTPRU,ARHGAP5,BTBD11,RPS20,SH3KBP1,NPR3,AKAP7,LRRK1,MAPK8IP2,VEPH1,CACNA1D,DDX3X,NSG1,ARAP2,CNOT7,GMDS,MAML3,GABRA5,PDE7A,TPCN2,ZNF622,ARHGAP39,JAK2,LMO7,SHOX2,SPNS2,AHI1,SMAD3,BAIAP2L1,CD300A,KIDINS220,MAP2K4,NOS1,PLXNB1,TMTC4,AATF,CDK1,DCBLD2,HDAC7,ITPK1,LTB,MIR320B2,MRGPRG,NBN,RRH,SLC39A10,TNF,OVOL2,PANX1,SNX5,TNIP1,EIF3A,POMC,PRDM15,BLOC1S6,BMP15,DLG5,FCHSD2,NUDT4,PHEX,AFAP1,LGR6,MAGI1,OR11A1,OR5V1,RXFP4,SHC2,KCNK10,PLCH1,STK32B,ADORA3,ARF1,CLEC4A,HMGA2,MYO6,AGO3,DOCK1,GRIA3,IGLC2,IGLL5,MUC5AC,RUFY1,STK32C,ANO1,GCLC,PMEPA1,UBE2V1,EPS8,LTBP1,DAAM2,SMOC2,SULF1,C5AR1,DEF6,GNAL,LRP4,PIK3R3,ARHGEF6,CNGB3,CNTN6,EFNB2,TRDN,AMOTL1,NET1,ZNF423,CDC14B,CLSTN1,SELP,ALPK2,CTNNA1,CUL2,NSG2,SCN1A,TENM1,TSHZ3,ARHGEF11,GRIK4,GSKIP,MYH14,PRKAR1A,TNFRSF19,ARHGAP11B,DYNC2H1,GABRR2,MLLT3,MYRIP,SIPA1L2,CD109,EGF,IL1RAPL1,KIT,SLIT3,TNFSF8,UBR2,BLM,CTNNAL1,OR7A5,PIK3C2G,TRPV4,VWC2,CC2D2A,EDAR,EFNB1,PTPRO,RAPGEF2,STK32A,ESR1,HTR2C,JAK1,SEMA3E,SLC30A8,UBE3A,DNMBP,FGF13,GABRB2,GRM4,MARVELD3,NUP155,AKAP10,EPHB2,IGHV3-72,MECOM,NDFIP2,DAP,DAPK1,ERBB4,FER,MAML2,MAP3K3,FNIP1,LRP5,PTH2R,RAPGEF6,SHCBP1,RPS27L,SLAMF1,ABL2,BSG,DEPDC5,F11R,FARP2,GAS8,OR4C6,ARHGAP35,CD2AP,COMMD7,NSUN2,PTPRK,SV2C,CCL3,FGF7,LAMA1,PREX1,RNF138,SEMA5B,SPRED3,VWF,ARHGAP25,FGD4,GPR158,IL20RA,ITGA9,PSG9,SULF2,MYH9,PACSIN2,PTGER3,SLC39A12,SOX4,ST18,CAB39L,GARNL3,NEDD9,P2RX1,PDE1A,PDGFD,PIK3CD,STOML3,UBE2N,ASGR2,ITGA8,KCNE4,PUM1,DDB1,ENPEP,IRAK2,KDM3A,PLAT,TNKS,UNC13A,WDPCP,ADCY9,AUTS2,DAPL1,FGFR1,MEF2A,PDE11A,RGS3,UNC13B,GLRA3,ITGBL1,RASSF3,SOST,IL23R,ITGB7,JMY,NTF3,P2RX7,PLA2G4A,RAPGEF4,SCUBE1,SORCS1,TEX14,RASA3,RCAN2,ZDHHC9,AMFR,APIP,AR,SUFU,TRERF1,EYA1,IFNA8,IL1RL2,SRPK2,BDKRB1,BDKRB2,ADRA1B,ARMC9,DGKZ,IFI27L1,MIR433,OGN,PRKAA2,SEMA3A,TLE4,ARHGAP10,CDC73,GNB5,GRIA2,MARK2,PDE5A,ZMIZ1,FLT3,PPARGC1B,PTPRA,SRGAP3,TRIM72,ESRRG,FERMT2,GNAO1,HCN4,LTBR,MAP2K5,MARK1,SMPD3,TNN,DAB2,GPR55,LCP1,RNF207,SRD5A2,STX3,SYT2,ACTN4,ADAM10,CCL7,DAAM1,DRG2,EEF1E1,EEF1E1-BLOC1S5,FGF12,ITGA3,KMO,LGI1,NREP,OR10K2,OR4C46,PLCL2,PPM1B,QRICH1,RFTN1,STAC,MYOM1,PIN1,RPS6KA6,TRPM4,CD226,ERLIN1,GPR141,PIP5K1B,STK38,BMP6,BTBD9,CHRM3,CIT,GAD2,CNGB1,TGFA,WDR12,CREM,G3BP2,GAS2,HGF,PKP1,RALGAPA1,RASSF8,RGS12,CALCR,CENPF,SCUBE3,SH3BP5,TLK1,CACNB2,CNKSR2,MAST4,NCAM1,NFASC,NOS1AP,PI4KA,RALGDS,SNAP29,ARHGAP28,IL18RAP,INSR,MACF1,PAK1,PDE3A,PSMB7,SKI,AKT3,CHM,ELF1,ENPP1,FMR1,LITAF,THEM4,CHFR,GABRA1,IGHV1-69,PRKX,RRAGC,ULK4,AMPH,BMF,EVC,GABRR3,IDE,IGHV1-46,MAD1L1,MFNG,AIM2,ARHGAP42,CSF2RB,EPG5,EVC2,JPH4,LRRN2,PDE6B,PPARA,SORT1,TNFAIP8L3,TTN,CORIN,FAM3D,GNA13,P

			PP1R13B,TAF1,CACNA1A,CHD5,FNIP2,FUT8,QRFP,S1PR3,SPRED1,TRPM1,AKR1C2,CHSY1,ESR2,NTN4,PRDM16,PTPRF,SCN9A,ZNF451,BTRC,CCDC3,DEFA1B,DEFA3,IGHV1-18,OPTN,PTPRR,RIT2,EXT1,GNG12,IFT81,MDM4,NCOA1,PRKCG,PRLR,TYMP,UMOD,DOCK7,FBXW11,G3BP1,ICK,INPP4A,OR51L1,TDGF1,STAT4,TREM1,ADARB1,KCNC2,OR4C16,PRKCQ,SCEL,STAT3,TP63,USP7,AMOT,BRIP1,DOCK3,PAK3,PIK3R2,STK17B,TBL1X,BLNK,CASQ2,DTNBP1,RAP1B,DLGAP2,KSR1,LGALS9,LRP8,NFAT5,OR2T4,SNCAIP,TAS1R1,ALPK1,GTPBP1,IL10RB,MAPKAPK2,APC,BMPR1A,ITGA1,KIAA0319,NGF,PRKCA,PTGES,UBASH3B,VLDLR,ADAMTS20,ASB3,GPR75,MTMR4,OSGIN2,PILRB,ABI1,CTSH,FCRL5,NCAPG2,RPH3AL,SRGAP1,TRBV6-8,USP18,CCL14,CCL15,CCL15-CCL14,EDA2R,GABRG2,GPR21,IL6R,MAP2K6,NCALD,RIC8B,SPTBN4,CLASP2,MAP2K1,PDE7B,RGMB,SOS1,VPS35,C1QTNF3,FGF1,TBX20,CRADD,DGKK,NLRC5,PPP1R9A,RGN,RREB1,TBX18,VSTM1
GO:0030030	cell projection organization	3.056298672692924e-22	*CDH4,DAB1,CDC42EP3,DSCAM,NCAM2,ANK3,DCC,NPHP3,ITGA2,KCNQ1,EPHA6,GRIN3A,NRXN1,NRXN3,RAB5A,CTNND2,TNC,PCNT,CHODL,RIMS2,FGD6,FSTL4,CDH13,LRRC4C,SDCCAG8,CEP41,TMEM108,ADAMTSL1,SYT17,PLXNA4,FLRT2,HYDIN,CECR2,PLCE1,UNC5D,SEMA6D,SPOCK1,CTNNA2,BBS9,RBFOX2,TIAM1,RASGRF1,DPYSL2,CBFA2T2,ALK,SPEF2,FBXO31,CNTN4,PHACTR1,S100B,ROBO2,C2CD3,EPHA3,DCLK1,AGT,FSIP2,ATAT1,WDR11,ATP6V0D1,CDH11,RHOJ,CDH2,PARK2,LAMA2,ZNF804A,NEGR1,DNAH9,SEMA3D,NTN1,TRIO,HDAC2,MYO9A,SLIT2,TTLL8,DYNLL2,IGF1R,CEP97,PTPRG,TRPC5,DMD,SYT1,VCL,CSMD3,FSHR,STK24,TRPM2,KIF3B,NTNG1,LRRK2,STRC,NTRK3,RNF165,DISC1,WRAP73,EMB,MECP2,MTMR2,SEMA4D,GSK3B,CDC14A,PRKD1,ERCC6,VAV3,SEMA5A,BDNF,RYK,ZDHHC17,PCDH15,DNAAF2,WASF1,GRIN2B,LAMA3,SNAP25,CNTNAP2,PLXNA2,TAOK3,BCL2,ARHGAP24,EPHB1,SIPA1L1,LRP2,VANGL2,NR4A2,EPHA7,RPGR,FYN,CHN1,SULT4A1,CD44,HECW1,PMP22,KANK1,ENPP2,ACTN2,ATP8A2,DCDC2,UNC5C,PALLD,RFX4,MTOR,ROR1,GAS7,MOV10,FAT3,ERMN,PTPRD,TIAM2,BBS2,KLHL1,IFT88,ATP6V1D,KALRN,ROBO1,TANC2,LRRC49,PREX2,SEC24B,PKHD1,VASH2,CHL1,TMEM138,APP,NRP1,PTPRM,TENM2,TTC12,SEMA3C,CAMK1D,PARVG,PRTG,RPS6KA5,PARD3,GRIP1,KIFAP3,OPHN1,CCDC88A,CD38,DENND5A,TUB,ALCAM,STK36,ADAMTS16,AK7,DNAH8,HDAC4,MYO16,NTRK2,PRKG1,DNM3,PTPDC1,SDK1,GLI3,RAP1A,TGFBR1,DICER1,EPHA5,TNR,CUX1,TENM3,RAPGEF1,CHRNA3,EP300,DSCAML1,SPAG17,TRIOBP,GRID2,PARVB,PIBF1,SPG11,ADORA2A,CAMK2G,EFNA5,MAGI2,CEP89,IFT80,TTC8,FRYL,RELN,KIRREL3,PTPN9,NUMB,CCDC57,MAP3K13,RALA,EXOC5,PPP3CA,KLF7,PLS1,SH3GL2,CAPZB,WWTR1,DOCK10,ELAVL4,CNTN1,DGUOK,GRM7,RIMS1,TMEM67,ASAP1,NLGN1,MAPK8IP2,OMG,JAK2,SHOX2,AHI1,KIDINS220,PLXNB1,COBL,PPFIA2,TANC1,ATXN10,BLOC1S6,DLG5,LGR6,SPAG16,ARF1,BCL11A,RAB23,EPS8,NUDCD3,CCP110,LRP4,WDR5,ARHGEF6,CNTN6,EFNB2,HERC1,ZNF423,CDC14B,CTNNA1,TENM1,CDKL5,EFHC2,WDR35,DYNC2H1,IL1RAPL1,KIT,SLIT3,TRPV4,CC2D2A,EFNB1,PTPRO,RAPGEF2,MYPN,SEMA3E,UBE3A,FGF13,ARMC2,EPHB2,TOX,FER,SLITRK6,IFT43,RAPGEF6,ABL2,BSG,GAS8,ARHGAP35,CD2AP,LAMA1,SEMA5B,CDKL1,FGD4,ARF4,MYH9,PACSIN2,SLC39A12,TEKT4,ITGA8,PDZD7,UNC13A,WDPCP,AUTS2,CDH23,MEF2A,OCLN,MAP2,NTF3,P2RX7,CEP250,ARMC9,BHLHB9,SEMA3A,EPB41L3,MARK2,PDLIM5,MARK1,TNN,DAB2,STX3,SYT2,BLOC1S5,ITGA3,FRY,LGI1,NREP,NME8,SCLT1,HGF,MYLK,SPATA6,NCAM1,NFASC,SNAP29,INSR,MACF1,PAK1,FMR1,LIMK2,GPRIN2,ULK4,ABCD1,CRMP1,RPGRIP1,CCDC141,GLDN,PTPRF,RIT2,EXT1,IFT81,DOCK7,ICK,TTC29,TTC39C,ADARB1,PRKCQ,ISLR2,PAK3,POC1A,DTNBP1,GAK,CRTAC1,LRP8,ALPK1,APC,ITGA1,KIAA0319,NGF,VLDLR,ABI1,SPTBN4,CLASP2,MAP2K1,SOS1,VPS35,ATMIN,PPP1R9A,RREB1*
GO:0007399	nervous system development	3.7064492328167466e-22	*SRGAP2B,MACROD2,CDH4,PLCB1,DAB1,GABRB3,DSCAM,ARID1B,RUNX1,ASIC2,NCAM2,ANK3,DCC,WLS,RBFOX1,PCSK2,GRIK1,NPHP3,KCNQ1,DPF3,SUN2,CA10,EPHA6,GRIN3A,NRXN1,SHC3,ISX,DOK5,NRXN3,SLC1A2,SDK2,CTNND2,TNC,POTEE,CHODL,RIMS2,MTPN,SHANK2,NRG3,ARNT2,OLFM3,FSTL4,ANK2,LRRC4C,RTN1,SYNDIG1,SCN8A,BCAN,SDCCAG8,TMEM108,ADAMTSL1,PCP4,SYT17,PLXNA4,FLRT2,NRG1,HYDIN,ALDH1A2,CECR2,LRFN5,SUZ12,ASTN2,UNC5D,SEMA6D,SPOCK1,DRP2,CTNNA2,RBFOX2,TIAM1,AGBL4,RASGRF1,DPYSL2,CBFA2T2,IL1RAPL2,NKD1,TACC2,ALK,SPEF2,ZNF536,FBXO31,CNTN4,PHACTR1,S100B,ROBO2,C2CD3,EPHA3,PRKACB,DCLK1,AGT,ATAT1,ATP6V0D1,CDH11,CDH2,PARK2,MDGA2,LAMA2,ZNF804A,NEGR1,GNB4,SEMA3D,NTN1,GPC6,NELL1,TRAPPC9,PCDH17,TRIO,APBA2,HDAC2,MYO9A,SLIT2,TFAP2D,CNTN5,IGF1R,SLC*

			24A4,SMAD1,GALC,RAPGEF5,VCAN,PTPRG,TRPC5,DMD,HCN1,SYT1,BASP1,VCL,CELSR1,CSMD3,FGF14,FSHR,MBOAT7,ASTN1,STK24,OPCML,ATRX,NTNG1,TENM4,LRRK2,RORB,SLC1A3,STRC,NTRK3,RORA,ANKS1A,CHD7,RNF165,DISC1,HEXB,EMB,SOX6,IER2,MECP2,MTMR2,SEMA4D,GSK3B,LINGO2,SLC8A1,PRKD1,ERCC6,TGFB2,CLSTN2,SEMA5A,SLC5A3,ACSBG1,BDNF,LSAMP,PRKCH,RYK,ZDHHC17,KCNC1,PCDH15,EML1,WASF1,GRIN2B,LAMA3,NR2F2,SNAP25,SYBU,CNTNAP2,PLXNA2,TAOK3,BCL2,EPHB1,SMARCE1,SIPA1L1,LRP2,LRRC4,NFIA,VANGL2,NR4A2,ARHGEF10,EPHA7,FYN,CHN1,SMARCA4,SULT4A1,HECW1,PMP22,THBS2,KANK1,ATP8A2,DCDC2,GABRB1,MME,NAV2,UNC5C,NTM,SLC1A1,UGCG,PALLD,RARB,RFX4,ETV6,MTOR,ROR1,GAS7,LDB2,MOV10,CHRM1,FAT3,PTPRD,TIAM2,ZNF521,BBS2,CEP85L,EPM2A,KLHL1,SOX8,IFT88,NLGN4X,FOXO3,IGF2BP3,LRRTM1,KALRN,DLC1,EDNRB,ROBO1,SATB2,TANC2,ZNF148,GRHL2,TCF4,COL2A1,MALL,PREX2,CBLN4,GABRA2,SEC24B,STK3,TGM2,VASH2,CHL1,APP,NRP1,PTPRM,SHROOM3,TENM2,IMMP2L,THRB,SEMA3C,CAMK1D,ATF2,ELP3,NR2C2,PRTG,RPS6KA5,AFF2,PARD3,THOC2,ABAT,GRIN2A,GRIP1,DLX6-AS1,OPHN1,CASP5,CCDC88A,CD38,DENND5A,ALCAM,CHRDL1,STK36,HDAC4,MYO16,SOX5,SRRM4,JAM2,NTRK2,PRKG1,ACSL4,DNM3,SDK1,GLI3,CLDN1,CRB1,RAP1A,TGFBR1,DICER1,EPHA5,TNR,CUX1,SLC6A17,TENM3,RAPGEF1,CHRNA3,EP300,DSCAML1,HMG20A,TBX3,TRIOBP,ATXN1,TP73,GRID2,SPG11,ADORA2A,CAMK2G,EFNA5,MERTK,MAGI2,NF1,RGS7,SORL1,IGSF21,TTC8,EIF4E,FRYL,RELN,GRM5,HAPLN1,HDAC9,KIRREL3,PTPN9,LRRTM3,NDRG2,NUMB,MAP3K13,NPHP4,RALA,IL1RAP,NAV3,FBXL17,JAG1,PPP3CA,KLF7,MYT1L,PLS1,SH3GL2,MAS1,DOCK10,ELAVL4,MNAT1,CNTN1,DGUOK,GPC4,GRM7,RIMS1,ASAP1,NLGN1,CHST8,CTTNBP2,AK8,MAPK8IP2,OMG,GABRA5,JAK2,SHOX2,AHI1,KIDINS220,PLXNB1,TCF12,CDK1,ITPK1,TAGLN3,TNF,COBL,OVOL2,PPFIA2,ADNP2,ATXN10,BLOC1S6,DLG5,POU6F2,LGR6,SIM2,BRINP1,BCL11A,MYO6,PCDH19,QKI,DAAM2,SULF1,TRPC4,C5AR1,LRP4,WDR5,CNTN6,EFNB2,HERC1,PHF8,SPATA5,ZNF423,CLSTN1,INSC,CTNNA1,TENM1,CDKL5,EFHC2,ARHGAP11B,BPTF,DYNC2H1,EGF,CMA1,IL1RAPL1,KIT,SLIT3,TRPV4,VWC2,CC2D2A,DNAH11,EFNB1,PTPRO,RAPGEF2,MYPN,NCMAP,SEMA3E,UBE3A,FGF13,GABRB2,PLAG1,EPHB2,TBCD,TOX,ERBB4,SLITRK6,ABL2,BSG,FARP2,GAS8,SETD2,ARHGAP35,CLDN11,CCL3,LAMA1,SEMA5B,KIF2A,NHLH2,SULF2,ARF4,SLC39A12,SOX4,AGTPBP1,ITGA8,PDZD7,UNC13A,WDPCP,AUTS2,CDH23,FGFR1,MEF2A,MAP2,NTF3,SUFU,EYA1,BHLHB9,SEMA3A,EPB41L3,JRKL,MARK2,MEIS2,ZMIZ1,PDLIM5,MARK1,TNN,DAB2,PBX1,SRD5A2,STX3,SYT2,BLOC1S5,FGF12,ITGA3,FRY,LGI1,NREP,LRIG1,PIN1,RPS6KA6,SCLT1,SHROOM4,BMP6,CHRM3,CIT,EIF4ENIF1,CNGB1,HGF,CENPF,ZBTB16,NCAM1,NFASC,MACF1,PAK1,SKI,AKT3,FMR1,PAX3,ATP5J,FAM126A,FPGS,GABRA1,GPRIN2,SBF2,ULK4,ABCD1,CRMP1,RPGRIP1,CCDC141,GLDN,ZFHX2,TAF1,CHD5,NTN4,PTPRF,RIT2,EXT1,NCOA1,PRKCG,TYMP,DOCK7,FBXW11,MYEF2,ADARB1,KCNC2,NDE1,PRKCQ,STAT3,TP63,ISLR2,PAK3,USH2A,DTNBP1,GAK,CRTAC1,LRP8,GDA,APC,BMPR1A,ITGA1,KIAA0319,NGF,SPINT2,VLDLR,ABI1,CYB5D2,GABRG2,SPTBN4,CLASP2,MAP2K1,SOS1,VPS35,MCOLN3,TBX20,PPP1R9A,BZW2
GO:0120036	plasma membrane bounded cell projection organization	4.899552891991801e-22	*CDH4,DAB1,CDC42EP3,DSCAM,NCAM2,ANK3,DCC,NPHP3,KCNQ1,EPHA6,GRIN3A,NRXN1,NRXN3,RAB5A,CTNND2,TNC,PCNT,CHODL,RIMS2,FGD6,FSTL4,CDH13,LRRC4C,SDCCAG8,CEP41,TMEM108,ADAMTSL1,SYT17,PLXNA4,FLRT2,HYDIN,CECR2,PLCE1,UNC5D,SEMA6D,SPOCK1,CTNNA2,BBS9,RBFOX2,TIAM1,RASGRF1,DPYSL2,CBFA2T2,ALK,SPEF2,FBXO31,CNTN4,PHACTR1,S100B,ROBO2,C2CD3,EPHA3,DCLK1,AGT,FSIP2,ATAT1,WDR11,ATP6V0D1,CDH11,CDH2,PARK2,LAMA2,ZNF804A,NEGR1,SEMA3D,NTN1,TRIO,HDAC2,MYO9A,SLIT2,TTLL8,DYNLL2,IGF1R,CEP97,PTPRG,TRPC5,DMD,SYT1,VCL,CSMD3,FSHR,STK24,TRPM2,KIF3B,NTNG1,LRRK2,STRC,NTRK3,RNF165,DISC1,WRAP73,EMB,MECP2,MTMR2,SEMA4D,GSK3B,CDC14A,PRKD1,ERCC6,VAV3,SEMA5A,BDNF,RYK,ZDHHC17,PCDH15,DNAAF2,WASF1,GRIN2B,LAMA3,SNAP25,CNTNAP2,PLXNA2,TAOK3,BCL2,ARHGAP24,EPHB1,SIPA1L1,LRP2,VANGL2,NR4A2,EPHA7,RPGR,FYN,CHN1,SULT4A1,CD44,HECW1,PMP22,KANK1,ENPP2,ACTN2,ATP8A2,DCDC2,UNC5C,PALLD,RFX4,MTOR,ROR1,GAS7,MOV10,FAT3,PTPRD,TIAM2,BBS2,KLHL1,IFT88,ATP6V1D,KALRN,ROBO1,TANC2,LRRC49,PREX2,SEC24B,PKHD1,VASH2,CHL1,TMEM138,APP,NRP1,PTPRM,TENM2,TTC12,SEMA3C,CAMK1D,PRTG,RPS6KA5,PARD3,GRIP1,KIFAP3,OPHN1,CCDC88A,CD38,DENND5A,TUB,ALCAM,STK36,ADAMTS16,DNAH8,HDAC4,MYO16,NTRK2,PRKG1,DNM3,PTPDC1,SDK1,GLI3,RAP1A,TGFBR1,DICER1,EPHA5,TNR,CUX1,TE*

			NM3,RAPGEF1,CHRNA3,EP300,DSCAML1,SPAG17,TRIOBP,GRID2,PARVB,PIBF1,SPG11,ADORA2A,CAMK2G,EFNA5,MAGI2,CEP89,IFT80,TTC8,FRYL,RELN,KIRREL3,PTPN9,NUMB,CCDC57,MAP3K13,RALA,EXOC5,PPP3CA,KLF7,PLS1,SH3GL2,CAPZB,WWTR1,DOCK10,ELAVL4,CNTN1,DGUOK,GRM7,RIMS1,TMEM67,ASAP1,NLGN1,MAPK8IP2,OMG,JAK2,SHOX2,AHI1,KIDINS220,PLXNB1,COBL,PPFIA2,TANC1,ATXN10,BLOC1S6,DLG5,LGR6,SPAG16,ARF1,BCL11A,RAB23,EPS8,NUDCD3,CCP110,LRP4,WDR5,ARHGEF6,CNTN6,EFNB2,HERC1,ZNF423,CDC14B,CTNNA1,TENM1,CDKL5,EFHC2,WDR35,DYNC2H1,IL1RAPL1,KIT,SLIT3,TRPV4,CC2D2A,EFNB1,PTPRO,RAPGEF2,MYPN,SEMA3E,UBE3A,FGF13,ARMC2,EPHB2,TOX,FER,SLITRK6,IFT43,RAPGEF6,ABL2,BSG,GAS8,ARHGAP35,CD2AP,LAMA1,SEMA5B,CDKL1,FGD4,ARF4,MYH9,SLC39A12,TEKT4,PDZD7,UNC13A,WDPCP,AUTS2,CDH23,MEF2A,OCLN,MAP2,NTF3,P2RX7,CEP250,ARMC9,BHLHB9,SEMA3A,EPB41L3,MARK2,PDLIM5,MARK1,TNN,DAB2,STX3,SYT2,BLOC1S5,ITGA3,FRY,LGI1,NREP,NME8,SCLT1,HGF,MYLK,SPATA6,NCAM1,NFASC,SNAP29,INSR,MACF1,PAK1,FMR1,LIMK2,GPRIN2,ULK4,ABCD1,CRMP1,RPGRIP1,CCDC141,GLDN,PTPRF,RIT2,EXT1,IFT81,DOCK7,ICK,TTC29,TTC39C,ADARB1,PRKCQ,ISLR2,PAK3,DTNBP1,GAK,CRTAC1,LRP8,ALPK1,APC,ITGA1,KIAA0319,NGF,VLDLR,ABI1,SPTBN4,CLASP2,MAP2K1,SOS1,VPS35,ATMIN,PPP1R9A,RREB1*
GO:0030182	neuron differentiation	1.0500012374886105e-21	*CDH4,DAB1,DSCAM,RUNX1,NCAM2,ANK3,DCC,KCNQ1,EPHA6,GRIN3A,NRXN1,DOK5,NRXN3,SDK2,CTNND2,TNC,CHODL,RIMS2,MTPN,OLFM3,FSTL4,LRRC4C,RTN1,TMEM108,ADAMTSL1,PCP4,SYT17,PLXNA4,FLRT2,NRG1,ALDH1A2,CECR2,UNC5D,SEMA6D,SPOCK1,CTNNA2,RBFOX2,TIAM1,AGBL4,RASGRF1,DPYSL2,CBFA2T2,NKD1,ALK,ZNF536,FBXO31,CNTN4,PHACTR1,S100B,ROBO2,EPHA3,DCLK1,AGT,ATAT1,CDH11,CDH2,PARK2,MDGA2,LAMA2,ZNF804A,NEGR1,SEMA3D,NTN1,TRAPPC9,TRIO,HDAC2,MYO9A,SLIT2,IGF1R,PTPRG,TRPC5,DMD,HCN1,SYT1,VCL,CSMD3,FSHR,STK24,OPCML,NTNG1,TENM4,LRRK2,RORB,SLC1A3,STRC,NTRK3,RORA,ANKS1A,RNF165,DISC1,EMB,IER2,MECP2,MTMR2,SEMA4D,GSK3B,PRKD1,ERCC6,TGFB2,SEMA5A,BDNF,RYK,ZDHHC17,PCDH15,WASF1,LAMA3,SNAP25,CNTNAP2,PLXNA2,TAOK3,BCL2,EPHB1,SIPA1L1,LRP2,NR4A2,EPHA7,FYN,CHN1,SULT4A1,HECW1,PMP22,KANK1,ATP8A2,DCDC2,GABRB1,UNC5C,NTM,UGCG,PALLD,ROR1,GAS7,MOV10,FAT3,PTPRD,TIAM2,ZNF521,KLHL1,SOX8,IFT88,NLGN4X,FOXO3,KALRN,EDNRB,ROBO1,SATB2,TANC2,TCF4,PREX2,SEC24B,VASH2,CHL1,APP,NRP1,PTPRM,TENM2,THRB,SEMA3C,CAMK1D,PRTG,RPS6KA5,PARD3,THOC2,GRIP1,OPHN1,CCDC88A,CD38,DENND5A,ALCAM,MYO16,SRRM4,NTRK2,PRKG1,ACSL4,DNM3,SDK1,GLI3,CRB1,RAP1A,TGFBR1,DICER1,EPHA5,TNR,CUX1,TENM3,RAPGEF1,CHRNA3,EP300,DSCAML1,HMG20A,TRIOBP,TP73,GRID2,SPG11,ADORA2A,CAMK2G,EFNA5,MAGI2,TTC8,EIF4E,FRYL,RELN,HDAC9,KIRREL3,PTPN9,NUMB,MAP3K13,NPHP4,JAG1,PPP3CA,KLF7,MYT1L,PLS1,SH3GL2,DOCK10,ELAVL4,CNTN1,DGUOK,GRM7,RIMS1,ASAP1,NLGN1,MAPK8IP2,OMG,GABRA5,JAK2,SHOX2,AHI1,KIDINS220,PLXNB1,TCF12,COBL,PPFIA2,ADNP2,ATXN10,BLOC1S6,DLG5,LGR6,BRINP1,BCL11A,MYO6,LRP4,WDR5,CNTN6,EFNB2,HERC1,CTNNA1,TENM1,CDKL5,EFHC2,DYNC2H1,IL1RAPL1,SLIT3,TRPV4,VWC2,EFNB1,PTPRO,RAPGEF2,MYPN,SEMA3E,UBE3A,FGF13,GABRB2,EPHB2,TBCD,TOX,ERBB4,SLITRK6,ABL2,BSG,FARP2,ARHGAP35,LAMA1,SEMA5B,NHLH2,ARF4,SLC39A12,SOX4,AGTPBP1,PDZD7,UNC13A,WDPCP,AUTS2,CDH23,FGFR1,MEF2A,MAP2,NTF3,SUFU,EYA1,BHLHB9,SEMA3A,EPB41L3,MARK2,ZMIZ1,PDLIM5,MARK1,TNN,DAB2,PBX1,STX3,SYT2,BLOC1S5,ITGA3,FRY,LGI1,NREP,PIN1,SCLT1,BMP6,EIF4ENIF1,CNGB1,HGF,NCAM1,NFASC,MACF1,PAK1,FMR1,GPRIN2,ULK4,CRMP1,RPGRIP1,CCDC141,GLDN,ZFHX2,CHD5,NTN4,PTPRF,RIT2,EXT1,NCOA1,DOCK7,MYEF2,ADARB1,PRKCQ,STAT3,ISLR2,PAK3,USH2A,DTNBP1,GAK,CRTAC1,LRP8,ITGA1,KIAA0319,NGF,VLDLR,ABI1,CYB5D2,SPTBN4,CLASP2,MAP2K1,SOS1,MCOLN3,TBX20,PPP1R9A*
GO:0050789	regulation of biological process	1.1533674606218404e-21	*LINC00273,IGHV1OR21-1,ISM1,TASP1,FANK1,CAST,ADCY2,SRGAP2B,TPTE,CDH4,ZHX3,PLCB1,KMT2C,KHDRBS2,ZNF595,CMKLR1,DAB1,OMA1,DUX4,GTF2I,ZNF717,FMNL2,GABRB3,CDC42EP3,GABRG3,MAPK14,ABCG8,DSCAM,ARID1B,TPTE2,MIR663A,RUNX1,ORC3,SH3RF3,ASIC2,CTBP2,DTNA,OR11H1,SERPINA1,TSHZ2,CCNG2,RAB27A,HUNK,CACNA1E,KCNJ6,PCBD2,ANXA8L1,NPY4R,PRIM2,LYPD6,OR4M2,PTPRT,MIR1185-1,MIR1185-2,MIR134,MIR154,MIR300,MIR323B,MIR376C,MIR381,MIR382,MIR485,MIR487A,MIR487B,MIR539,MIR544A,MIR654,MIR655,MIR889,AN*

			K3,IGHV4-31,DCC,BCL2L13,IGHV3-64,SVEP1,WLS,RBFOX1,SYN3,TIMP3,ZNF397,ZSCAN30,DUSP22,TTC28,AKAP13,FBLN1,ERG,VPS13D,GRIK1,ZNF292,GPR139,IGHV1OR15-9,SORBS2,SLC24A3,IGHV4OR15-8,NPHP3,ITGA2,ABCG1,TMPRSS2,RGS6,KCNQ1,DPF3,SUN2,GABBR2,ATF7IP,EPHA6,OR4K15,TMEM117,VRK2,GRIN3A,FCRL2,NRXN1,SHC3,MIR369,MIR410,MIR656,ZRANB1,ISX,TNNI3K,DOK5,NRXN3,SLC1A2,RAB5A,ZNF578,DOCK2,CTNND2,TNC,PCNT,PLEKHB2,MS4A1,OR8U1,PAX7,CHODL,RIMS2,MTPN,SHANK2,GRIA1,NRG3,ZNF732,SMYD2,CELF2,CLEC16A,HUS1,ARNT2,FGD6,TGFBR3,FSTL4,CDH13,RNF152,ANK2,LRRC4C,RTN1,ANKH,NXN,KCND3,RUNX1T1,CSNK2A1,PDE4DIP,SYNDIG1,ZNF678,DPP10,ZNF112,ZNF229,ZNF285,TF,PLGRKT,ERCC4,SCN8A,KCNE1,PIK3C2B,SAMD4A,SDCCAG8,MIR99A,MIRLET7C,TMEM108,EIF4EBP3,CHEK2,OR4C12,ZNF486,GADD45A,PRDM9,KCNJ15,PCP4,PSMC6,ACIN1,ZNF331,SYT17,PLXNA4,KCNG3,FLRT2,TOX3,GPC3,FBLN5,NRG1,STXBP6,ETS2,PSMB2,SHISA6,RAD51D,RFFL,NPAS3,SPIDR,IL4R,ALDH1A2,LRFN5,RYR3,EWSR1,MCTP1,GGT1,MIR17HG,NLN,ST8SIA1,SUZ12,LCORL,ASTN2,PLCE1,SH3RF2,UNC5D,KSR2,GLP2R,STK38L,SEMA6D,SPOCK1,ABHD17C,CTNNA2,SLCO3A1,CCR1,CCR3,CFTR,RBFOX2,ZNF420,DTX4,MIR551B,ZNF257,MED13L,PCTP,MC2R,TIAM1,PM20D1,RHOC,AGBL4,BEND5,LDLRAD4,DNAJC15,RGS7BP,ZNF91,RASGRF1,ZBTB20,DPYSL2,GRID1,TET3,ARFIP1,CBFA2T2,IL1RAPL2,OR4A5,NKD1,HPSE2,SCAI,MX1,BTN2A1,ALK,PTPRE,PIK3C3,UTRN,SORCS3,ZNF536,FAM3B,PLCB4,ERC1,FBXO31,KLRF2,RAB7A,CNTN4,RGL2,TAPBP,ARID5B,PHACTR1,S100B,ROBO2,SPOCK3,ZNF366,C2CD3,EPHA3,PRKACB,CNTNAP4,DCLK1,PXDN,COL15A1,CRNN,AJAP1,LEMD3,OR8K3,AGT,ERC2,CST2,MAGEB3,SHISA9,ATAT1,AVEN,CHRM5,FBXO32,WDR11,ATP6V0D1,CDH11,RHOJ,RCAN1,CDH2,KLHL25,PARK2,BANP,FHL2,FOXN3,LGALS14,LOXL2,DLGAP1,LAMA2,CDC5L,KIR2DL1,KIR2DL4,KIR3DL2,OR4K13,BPIFB1,CORO2B,FRMPD4,TRIM51,ZNF804A,CYBB,MCTP2,NEGR1,PSPC1,BRF1,GNB4,OR4M1,OR4N2,SEMA3D,DACH1,NTN1,OR52N5,TP53I11,TRIM22,TRIM5,KCNH1,USP25,ZBTB8B,ZNF268,DCUN1D4,TXK,CP,GPC6,RNF185,USP6,ZNF232,ANKFN1,NELL1,TRAPPC9,DBH,ITSN1,TNS3,TSPAN8,PCDH17,PHC1,SETD3,ZNRF3,FMNL3,KCNMA1,RIN3,TRIO,ZNF845,APBA2,FTO,HDAC2,HTRA1,LAMA4,MYO9A,SLIT2,TFAP2D,ZBTB34,CDH8,KCTD1,AHRR,HSA-MIR-490,PDCD6,ZSCAN5C,OR2L13,RPS6KA2,STXBP4,GPC5,LCMT1,IGF1R,MDM2,MX2,PALMD,SLC24A4,SMAD1,E2F3,NOVA1,SYT9,ZBED4,FOXD4L4,OR11G2,OR9Q1,TMPRSS6,ZNF567,ZNF850,CTNNBL1,RAPGEF5,CEP97,GABRG1,KCNK17,NR3C2,PTPRG,PXK,PDE9A,OR8K5,SACS,TRIM48,TRPC5,DMD,HCN1,KCTD8,STK33,SYT1,BASP1,CACNA2D3,CACNG3,KRT2,SIPA1L3,VCL,NOX5,TEAD1,CELSR1,CSMD3,FGF14,FSHR,GSG1L,GRIA4,GRM1,MBOAT7,FMN2,HNRNPA2B1,MAEL,MORF4L1,NFE2L3,NRIP1,PRKAR2A,SKAP1,SORCS2,INPP5A,STK24,DCDC1,DPP6,WDR83,WWOX,ATRX,ACTR5,FRMD5,PARP16,TRPM2,CNIH3,CTNNA3,RNLS,RYR2,USP16,KIF3B,NTNG1,OR2M5,TENM4,UNC13C,CAMTA1,CATSPER2,DHRS7C,HSPB8,LINC00472,LRRK2,MAPK10,MIR495,MIR543,RORB,SLC1A3,IGFBP7,KCNH5,PIR,ZFP30,ZNF607,KIF26B,NTRK3,NUSAP1,RORA,CD8B,GGT2,ILDR1,IQCJ-SCHIP1,PRICKLE2,SLC4A4,ANKS1A,CHD7,OVGP1,RNF165,ZNF26,DISC1,HEXB,KCNK13,MEMO1,PAN3,TPRG1L,WRAP73,CDCA2,GLIS1,KCTD9,PDXP,SH3BP1,SOX6,IER2,MECP2,MTMR2,SEMA4D,GSK3B,LGR5,LINGO2,SLC8A1,ZNF432,ZNF841,CDC14A,MAGI3,PLCH2,PRKD1,SERPINA4,SERPINA5,SNRK,ERCC6,FLVCR1,FRMD6,OGT,RNF144A,TGFB2,ATP1A4,CLSTN2,FMN1,PRAMEF12,VAV3,ANKS1B,AFF3,C8A,DACH2,DNAJC6,FUT4,PIWIL4,PLCD3,RIPPLY3,S100A11,SEMA5A,SLC5A3,BDNF,LLGL2,PRKAR1B,PRKCH,RBMS3,RIPK4,RYK,ZDHHC17,KCNC1,PLCXD3,POLR3C,ABI3BP,EDIL3,SGCD,WASF1,GRIN2B,KDM4C,OR6N1,OTUD7A,EXT2,LAMA3,NKAIN2,RAG1,RAG2,SAMD13,SIK2,ZNF433,ANKRD6,ARHGAP8,NR2F2,PLEKHM2,PRR5,PRR5-ARHGAP8,PTGFR,SNAP25,NR5A2,PAQR8,PCBP3,PITPNC1,PML,PPARGC1A,RNF180,TRUB2,USP36,ZNF627,ARHGAP12,CORO2A,AKAP6,CNTNAP2,TNS1,FBN1,MED15,PPP4R4,GRIK3,IGHV3-16,LRFN2,NLRP2,NLRP7,PLXNA2,TAOK3,ZNF610,BCL2,CHMP4C,MXI1,RAD51B,RALBP1,RXFP2,ZNF675,ARHGAP24,EPHB1,KCNJ3,MOV10L1,SMARCE1,IL12RB2,SIPA1L1,SKAP2,SLC30A5,DISP1,KLF12,LRP2,NKAIN3,NSF,TMEM100,FRMD4A,ITPR2,LRRC4,NFIA,SND1,VANGL2,CDH6,DEPTOR,NR4A2,ZNF677,ABCC9,ARHGEF10,EPHA7,MIR648,PSD3,ZBTB7C,DPT,RBAK,RBAK-RBAKDN,FYN,GNRHR,KCNE2,LRRC69,PARN,ADAMTS18,CHN1,NR4

			A1,SMARCA4,CD44,EIF4G3,HECW1,NEU3,PMP22,RCVRN,RHBDD1,RNF213,THBS2,FOXO1,PDE4D,PYY,RBBP8,RNF144B,TBXAS1,KANK1,ENPP2,ABCB10,ACTN2,ARHGEF3,ATF6,ATP8A2,BACH1,CST9L,DCDC2,GABRB1,GRK5,KLF13,MME,PACRG,RNF217,TNMD,CSNK1G3,EXOC4,KCNS3,GRIK2,IGSF11,ZNF516,CELF4,KCNIP4,SERPINB7,UNC5C,APBB2,EIF3E,KCND2,MYLK3,NAMPT,SCN11A,SETD7,SLC1A1,UGCG,ZFPM2,ZNF141,ZNF429,ZSCAN5A,GLIS3,PHF20,RARB,RERG,RFX4,STRIP1,ETV6,GNG4,NLRP12,GRAP2,MFSD12,MTOR,RMI2,ROR1,CADPS,LDB2,MOV10,TMPRSS4,VPS26B,CHRM1,CTDSPL2,DDX21,FAT3,PKP2,UBXN2B,ZNF510,ABCA13,BMPER,CCDC88B,ERMN,PTPRD,RASGEF1B,SCAF8,TIAM2,ZNF521,BBS2,OGFOD1,OR51B2,OR51I1,PGK1,PLCL1,ZFP64,ZNF483,ARL6IP5,ATP1A1,EPM2A,PLN,NOL11,ZMAT3,CLYBL,CTNNBIP1,EPB41L4B,SOX8,ZMYND11,DIDO1,DSG4,HAS3,HIVEP2,IFT88,MIR491,NLGN4X,OR4K17,OR8J3,PRKCE,ATP6V1D,EIF2S1,GTF2A1L,HELLS,KMT2A,PRKCB,STON1,VDR,ADCY8,EFHB,FAM19A4,FHL5,FOXO3,IGF2BP3,LRRTM1,MITF,RAB31,SCMH1,SCP2,TRAPPC12,CAPN3,DPH6,ITLN1,KALRN,PJA1,TTLL12,CCDC88C,CREB5,DLC1,EDNRB,MSR1,OASL,ROBO1,RXFP1,SATB2,TANC2,TBC1D4,TDRD3,BNIP3L,FAP,FLT1,NOX4,SGIP1,ZNF148,CDH17,CENPV,GRHL2,TCF4,TSC22D3,ZNF573,ZNF720,COL2A1,KCNC4,MAP3K7,MAPK9,NBAS,NR3C1,PREX2,TACR3,TRAF3IP2,VPS37B,ZNF277,CR1,EYA4,GABRA2,OR4C15,SEC24B,SPON1,ACSL5,MDM1,PKHD1,STK3,SUPT3H,TGM2,VASH2,ZNF585A,KDR,PPHLN1,PRCP,RAB30,ARHGAP15,CASK,CHL1,EDA,IGHV4-28,PCOLCE2,ABCA12,APP,CALCRL,COL18A1,DGKB,NRP1,PTPRM,SHROOM3,UACA,ARHGEF18,ELMO1,LGR4,LINC00473,SERPING1,TENM2,ADTRP,CD58,EGFLAM,KAT5,RANBP10,ST7,THRB,CPEB2,FUBP1,KCNK2,SEMA3C,ZNF667,ZNF98,ADAM17,CAMK1D,DIS3L2,MAP3K5,PKP4,TSPEAR,CDC42BPG,IGF2R,TFPI,ZER1,ATF2,ELP3,ETS1,GPR78,GRK6,NR2C2,PEX5L,PIK3R5,SAMSN1,EFEMP1,GOPC,PRTG,ROS1,SMG6,TGFB1I1,TM9SF4,CDK17,DOT1L,KPNB1,OR10R2,PSIP1,RGS16,RPS6KA5,TCF7L2,TNFRSF10B,TRABD2B,ZNF443,ZNF490,ZNF709,ZNF799,AFF2,BICD1,CYTIP,ITGA11,PARD3,RALGAPA2,TLK2,TRAF3,ZNF830,ABAT,ATRNL1,DOCK9,GRIN2A,GRIP1,MIR183,MIR96,PXT1,RIMS3,SGK2,DLX6-AS1,KIFAP3,OPHN1,BCLAF1,SGMS1,TRPS1,ZNF568,CASP5,CCDC88A,CD38,DENND5A,FAM83B,KHDRBS3,MOB3B,MRPS27,RIN2,RNF43,TUB,ZBED5,ALCAM,CHRDL1,RNF126,SMG7,STK36,BCL2L1,ADAMTS16,GUCY2F,HDAC4,MYO16,PDE4B,SOX5,SRRM4,TIMP2,BACE2,BCL2L15,DNMT1,GHR,GLRA2,JAM2,MAPRE2,MIR605,NTRK2,PRKG1,ACSL4,FAM83G,A1CF,CDC42BPA,DNM3,GNG2,BRD4,PKNOX2,PTPDC1,RASGRP1,STARD13,ARR3,CAMK4,COMMD1,KCTD7,LY86,RAB11FIP4,RABGEF1,RGL1,SDK1,SPIN3,SPRED2,STARD4,GLI3,OR5L2,SMCHD1,ZNF207,CACNA1C,CLDN1,KIF16B,MORC2,OR11L1,STXBP5,DOCK4,RAP1A,SAFB2,TGFBR1,ZBTB41,CYSLTR1,KCNJ12,MGLL,PRDX4,ADAM12,DICER1,EPHA5,LCP2,TAS2R38,THEMIS,TNR,TOP1,CUX1,NCOA5,PEAK1,TAS2R1,TENM3,CHST11,CPE,IL16,ITGB3BP,MAGEA11,PTTG1IP,RAPGEF1,ZNF729,CD96,CHRNA3,CHRNA5,EP300,MORC3,ELAVL2,HMG20A,SCFD1,TBX3,TTC39B,WIF1,CACNA2D1,CACNA2D4,CYP7B1,DIO2,GRAMD4,PRAMENP,STX8,TRIOBP,ATXN1,BRD7,CLIC6,GAB2,GLRA1,SYCP1,TP73,COL4A6,GRID2,IFNGR2,NPC1,OR4C5,PARVB,PIBF1,RASGRF2,PSMA1,TRHDE,FHIT,FLNB,ONECUT3,PPP1R12B,ADCK1,ADORA2A,ADRBK2,CABIN1,CAMK2G,EFNA5,EMCN,FAM20A,MERTK,RALGPS1,TAC4,BRD1,CACNG2,GUCY2C,MAGI2,NF1,PPP6R2,RALGPS2,RFC3,RGS7,TFF1,USP17L5,ZNF525,ZNF765,KCNQ5,SORL1,STRN3,ZNF615,ARHGAP6,ASB13,BICC1,BID,ELK3,IFT80,OR5K4,TEAD4,TRIM59,TTC8,DMRT1,HTR4,LZTR1,ASXL1,EIF4E,MICU1,POR,RELN,SLC39A8,TNFRSF11B,TSHZ1,GRM5,HDAC9,IGHV4-4,IL18R1,IL1RL1,NECAB1,PTPN9,SELE,DYNC1H1,LRRTM3,MGAT5,NAPEPLD,NDRG2,NUMB,RHPN2,SP2,TNFAIP8,CCDC57,IGHV3OR16-12,MAP3K13,NLK,NPHP4,RALA,TBX15,ADCYAP1R1,EGLN3,IL1RAP,NAV3,ATP10A,FBXL17,FBXL5,JAG1,MEOX2,PPP3CA,GPR176,JRK,KCNB2,KLF3,KLF7,MYT1L,NEB,PLS1,SH3GL2,SLC7A1,SNX25,CAPZB,CXADR,DGKI,MAS1,PER1,SATB1,SLC24A2,TBC1D5,WWTR1,KL,ANKRD13A,C6,CBLB,DOCK10,ELAVL4,MAGEB2,MNAT1,PLSCR1,ATP9A,CNTN1,DGUOK,DOK6,GPC4,GRM7,RIMS1,TMEM67,TRIM16,WDR59,ZNF736,GABPA,GABRA3,GPSM2,KAT7,L3MBTL3,MAPK4,MIR105-1,MIR105-2,MIR767,SSBP2,STIM2,TMEM135,ZNF540,ZNF571,SLC22A2,AGGF1,ASAP1,BORA,FHOD3,ITGAE,N4BP2L2,NLGN1,P2RX6,PDE1C,PTPRU,THAP7,AKIRIN2,ARHGAP5,BTBD11,CTTNBP2,RNF128,RPS20,SH3

			KBP1,ZNF562,ENOX2,NPR3,RAD51AP1,SYCP2,AKAP7,LRRK1,MAPK8IP2,VEPH1,ZNF208,CACNA1D,DDX3X,EGFL6,LRPPRC,NSG1,RBL2,ARAP2,CNOT7,GMDS,MAML3,EHF,GABRA5,PDE7A,TPCN2,ZNF622,ARHGAP39,DNMT3B,JAK2,LMO7,SHOX2,SPNS2,AHI1,SMAD3,BAIAP2L1,CD300A,KIDINS220,MAP2K4,NOS1,SERPINB12,COL8A1,PLXNB1,RDH10,TCF12,TMTC4,ZNF337,AATF,ANHX,CDK1,DCBLD2,HDAC7,HIVEP3,ITPK1,LTB,MIR320B2,MRGPRG,NBN,NVL,RRH,SLC39A10,TAGLN3,TNF,COBL,LRCH1,OVOL2,PANX1,SNX5,TNIP1,ZFPM1,CSPP1,EIF3A,ILF3,POMC,PPFIA2,TMC2,PRDM15,ADNP2,BLOC1S6,BMP15,DLG5,FCHSD2,INO80,NUDT4,POU6F2,RBM11,SCAF4,AFAP1,LGR6,MAGI1,OR11A1,OR5V1,RXFP4,SIM2,ARID2,MLIP,MMP20,PAXBP1,SHC2,BRINP1,FAM220A,KCNK10,PLCH1,PRG3,SRSF4,STK32B,ADORA3,ARF1,BCL11A,CLEC4A,HMGA2,MYO6,NEK4,POLR1A,TAF1D,UBE2E3,YAF2,ZNF41,ZNF705A,ZNF880,AGO3,DOCK1,GRIA3,IGLC2,IGLL5,KIF18A,MUC5AC,PAPOLA,RUFY1,STK32C,TSPAN13,ANO1,CDC20B,GCLC,KCNA6,PMEPA1,QKI,RAB23,UBE2V1,ZNF664,ZNF728,ZNF879,ACTR8,EPS8,LTBP1,MBOAT2,SP140,SP140L,DAAM2,L3MBTL4,SLC7A7,SMOC2,SULF1,ANKRD31,C5AR1,CCP110,CISD2,DEF6,GNAL,LRP4,PIK3R3,WDR5,ARHGEF6,CNGB3,CNTN6,EFNB2,KCNH8,MXD3,PIWIL3,SAE1,TRDN,TRIM41,AMOTL1,HERC1,NET1,PHF8,ZNF423,CDC14B,CLSTN1,INSC,SELP,ALPK2,CDK8,CHD6,CTNNA1,CUL2,NSG2,SCN1A,TENM1,TSHZ3,WDFY2,ZNF407,ZNF93,ARHGEF11,CDKL5,EFHC2,GRIK4,GSKIP,MAMLD1,MIR765,MYH14,PRKAR1A,TNFRSF19,ZNF345,ARHGAP11B,BPTF,DYNC2H1,FAM171A1,GABRR2,KCNH7,MLLT3,MYRIP,SIPA1L2,CD109,EGF,MIP,PTPN14,SEC16B,SPIRE2,ALDH1A1,CMA1,FOXP4,GBE1,IL1RAPL1,KIT,SLIT3,TNFSF8,TNRC6B,UBR2,ZNF600,BLM,CTNNAL1,OR7A5,PIK3C2G,TRPV4,VWC2,ABCA5,C8B,CC2D2A,DNAH11,EDAR,EFNB1,KCNAB1,PTPRO,RAPGEF2,REG1B,STK32A,ZIM3,DNAJB6,ESR1,HTR2C,JAK1,NCMAP,SEMA3E,SLC30A8,UBE3A,DNMBP,EBAG9,FGF13,GABRB2,GRM4,MARVELD3,NUP155,PLAG1,AKAP10,EPHB2,IGHV3-72,MECOM,NDFIP2,RNF4,TBCD,TFDP2,TOX,ABCC1,C14ORF39,DAP,DAPK1,ERBB4,FER,MAML2,MAP3K3,RBM19,SLITRK6,SPTA1,TDRKH,CACNG6,FNIP1,LRP5,MALRD1,PTH2R,RAPGEF6,SHCBP1,ZNF439,ZNF507,LACTB,RPS27L,SLAMF1,ABL2,BSG,CAMP,DEPDC5,F11R,FARP2,GAS8,HS1BP3,OR4C6,SETD2,VPS13C,ALX4,AP2B1,ARHGAP35,CD2AP,COMMD7,NSUN2,PTPRK,SGMS2,ATP8A1,CCL3,FGF7,LAMA1,PREX1,RNF138,SEMA5B,SPRED3,TNFAIP8L2,VWF,ZNF189,ARHGAP25,BTBD17,CDKL1,FGD4,GCFC2,GLYR1,GPR158,IL20RA,ITGA9,KIF2A,LRRC70,NHLH2,PSG9,SIAE,SULF2,ARF4,EEFSEC,MYH9,NSMCE2,PACSIN2,PTGER3,SLC2A13,SLC39A12,SOX4,ST18,CAB39L,GARNL3,KIF23,NEDD9,P2RX1,PDE1A,PDGFD,PIK3CD,PRR16,SPTB,STOML3,UBE2N,AGTPBP1,ASGR2,DNM3OS,ITGA8,KCNE4,MIR214,PUM1,ARID4A,DDB1,ENPEP,FAM172A,IRAK2,KDM3A,PLAT,SERTAD2,TNKS,UNC13A,WDPCP,ZFP82,ADCY9,AUTS2,BCL7A,CDK19,DAPL1,FGFR1,MEF2A,OCLN,PARP15,PDE11A,RGS3,RYR1,UNC13B,ZCCHC17,ZNF85,ARID4B,DAD1,GLRA3,ITGBL1,MAP2,RASSF3,SLX1B,SOST,ASXL3,IL23R,ITGB7,JMY,KEAP1,NTF3,P2RX7,PLA2G4A,RAPGEF4,SCUBE1,SORCS1,TEX14,ARRDC4,BRD9,RASA3,RCAN2,TOX2,ZDHHC11,ZDHHC9,ZNF544,AMFR,APIP,AR,BCOR,FGL2,KIAA1109,PVT1,RABGAP1L,SCAMP5,SNX30,SUFU,TRERF1,UPK3B,C10ORF90,CEP250,DENND5B,EXOC2,EYA1,IFNA8,IL1RL2,N4BP1,RBM4,SRPK2,BDKRB1,BDKRB2,HLA-DRA,ADRA1B,ARMC9,BHLHB9,DGKZ,GNL3,IFI27L1,L3MBTL1,MIR127,MIR136,MIR431,MIR432,MIR433,NEK5,OGN,PI3,PRKAA2,RLF,SEMA3A,TAX1BP1,THADA,TLE4,ZNF202,ZNF558,ARHGAP10,CDC73,EPB41L3,GNB5,GRIA2,MARK2,MEIS2,PDE5A,PROS1,REEP2,ZMIZ1,ZNF19,ZNF440,ZNF670,ZNF695,FLT3,LDLRAD3,NFIC,PDLIM5,PPARGC1B,PTPRA,SNX7,SRGAP3,TRIM72,ATP6V1H,DYNAP,ESRRG,FERMT2,GNAO1,HCN4,LTBR,MAP2K5,MARK1,PAPPA2,SMPD3,TNN,ZC3H13,C9,CLN6,DAB2,GPR55,HDAC8,LCP1,PBX1,RNF207,STX3,SYT2,TCL1B,ACTN4,ADAM10,BLOC1S5,CCL7,DAAM1,DRG2,EEF1E1,EEF1E1-BLOC1S5,ELP4,FGF12,ITGA3,KCNJ16,KMO,POU2F3,PRAMEF8,TXNDC5,FANCB,FRY,LGI1,MT1F,NREP,OR10K2,OR4C46,PLCL2,PPM1B,QRICH1,RFTN1,SSX5,STAC,MYOM1,NUP153,PHF20L1,PIN1,RPS6KA6,SYNPO2,TRPM4,CD226,ERLIN1,GPR141,KRBOX4,PIP5K1B,STK38,TCF20,ZNF674,ABCA8,ABCC2,BMP6,BTBD9,CHRM3,CIT,CIZ1,CLASP1,EIF4ENIF1,SMTNL2,CARF,CDYL2,CFDP1,CNGB1,EBF1,NCOA2,TGFA,TMOD1,WDR12,CREM,G3BP2,GAS2,HGF,MYLK,PKP1,POU2F1,RALGAPA1,RASSF8,RGS12,ZNF665,ZNF730,CALCR,CAPN7,CENPF,FLI1,MIR489,MIR653,POMT2,SCUBE3,SH3BP5,TLK1,ZBTB16,CACNB2,CNKSR2,CTIF,ELN,KDM4B,MAST4,MIR556,NCAM1,NOS1A

			P,PI4KA,RALGDS,ZNF57,ARHGAP28,IL18RAP,INSR,LARP4B,MACF1,PAK1,PDE3A,PSMB7,SKI,AKT3,CDC16,CHM,CTDP1,ELF1,ENPP1,FMR1,LIMK2,LITAF,MIR361,PAX3,THEM4,CHFR,DKC1,GABRA1,IGHV1-69,JDP2,KLF6,PHC2,PRKX,RRAGC,ULK4,ZNF605,ABCD1,BMF,CRMP1,EVC,GABRR3,IDE,IGHV1-46,MAD1L1,MFNG,SAMD8,AIM2,ARHGAP42,CD84,CSF2RB,EPG5,EVC2,JPH4,LRRN2,PCK1,PDE6B,PPARA,SORT1,SPIB,TBX22,TNFAIP8L3,TTN,ZFHX2,CLIP1,CORIN,FAM3D,FHL1,GNA13,KATNBL1,MYH7,NUP214,PPP1R13B,SLC22A5,SMARCAD1,TAF1,WDR41,CACNA1A,CHD5,FNIP2,FUT8,PPP2R3C,QRFP,S1PR3,SPRED1,TRPM1,AKR1C2,CHSY1,ESR2,KRT4,NTN4,PRDM16,PTPRF,QSOX1,RBL1,SCN9A,ZNF451,ATP6V1E1,BTRC,C2,CCDC3,CFB,DEFA1B,DEFA3,FBLN2,HIRA,IGHV1-18,MLLT10,MPDZ,OPTN,PTPRR,RIT2,ZNF713,ESRP2,EXT1,GNG12,IFT81,MDM4,MYH6,NCOA1,PRKCG,PRLR,TYMP,UMOD,ZNF354B,ADAMTS5,CBFA2T3,DOCK7,FBXW11,G3BP1,HDAC5,ICK,INPP4A,OR51L1,TDGF1,WDR70,ZNF493,A2M,AKAP2,LYVE1,MYEF2,PI15,SERPINB8,SLC24A5,SLC40A1,STAT4,STIM1,TREM1,ZNF623,ADARB1,KCNC2,OR4C16,PEMT,POU2AF1,PRKCQ,SCEL,STAT3,SUSD4,TFEC,TP63,USP7,ZNF721,AMOT,BRIP1,CDKN3,CST1,DOCK3,HPN,ISLR2,PAK3,PIK3R2,STK17B,TBL1X,USH2A,BLNK,CASQ2,DHRS2,DHRS7B,DTNBP1,GAK,RAP1B,ZNF518A,DLGAP2,KSR1,LGALS9,LRP8,NFAT5,OR2T4,SNCAIP,TAS1R1,ZNF697,ALPK1,EFCAB1,GTPBP1,IL10RB,MAPKAPK2,AHNAK2,AOAH,APC,BMPR1A,CKAP5,DPP4,ITGA1,KCTD20,KIAA0319,NGF,PPP1R14A,PRKCA,PTGES,SPINT2,UBASH3B,UBE2E2,VLDLR,ADAMTS20,ASB3,DRAM1,GPR75,ITIH6,MTMR4,MTRF1,OSGIN2,PDS5A,PILRB,ABI1,CTSH,DHX9,FCRL5,NCAPG2,RGCC,RPH3AL,SRGAP1,TRBV6-8,USP18,CCL14,CCL15,CCL15-CCL14,CYB5D2,DPH1,EBF4,EDA2R,GABRG2,GPR21,IL6R,MAP2K6,NCALD,RIC8B,SIAH3,SRRD,TFIP11,SPTBN4,CLASP2,MAP2K1,PDE7B,PRAMEF7,RGMB,SOS1,TPH1,VPS35,ZBTB25,ZRANB3,C1QTNF3,CABLES1,COL6A3,FGF1,MCOLN3,NSUN3,PLB1,RAD18,TBX20,ZNF527,ATMIN,ATP6V1E2,C6ORF106,CRADD,DGKK,NLRC5,PPP1R9A,RGN,RREB1,TBX18,VSTM1,ZFP2,ZNF501
GO:0048869	cellular developmental process	2.6778579534354084e-21	CDH4,ZHX3,PLCB1,CMKLR1,DAB1,MAPK14,DSCAM,RUNX1,CTBP2,MYO18B,NCAM2,RAB27A,ANK3,DCC,RBFOX1,AKAP13,FBLN1,ERG,SORBS2,NPHP3,ITGA2,ABCG1,KCNQ1,SUN2,NUBPL,EPHA6,GRIN3A,NRXN1,DOK5,NRXN3,SDK2,DOCK2,CTNND2,TNC,PLEKHB2,MS4A1,CHODL,RIMS2,MTPN,NRG3,OLFM3,TGFBR3,FSTL4,ANK2,LRRC4C,RTN1,NXN,RUNX1T1,TF,SDCCAG8,TMEM108,CHEK2,ADAMTSL1,PCP4,ACIN1,SYT17,PLXNA4,FLRT2,GPC3,NHSL2,NRG1,ETS2,PSMB2,HYDIN,IL4R,ALDH1A2,CECR2,NLN,SUZ12,ASTN2,UNC5D,SEMA6D,SPOCK1,CTNNA2,CCR1,CFTR,BBS9,RBFOX2,TIAM1,COL22A1,AGBL4,LDLRAD4,RASGRF1,DPYSL2,CBFA2T2,NKD1,ALK,SPEF2,ZNF536,FBXO31,CNTN4,ARID5B,PHACTR1,S100B,ROBO2,EPHA3,DCLK1,COL15A1,AJAP1,AGT,FSIP2,ATAT1,CDH11,RCAN1,CDH2,PARK2,FHL2,LOXL2,MDGA2,LAMA2,KIR2DL4,ZNF804A,NEGR1,SEMA3D,NTN1,KCNH1,ZNF268,NELL1,TRAPPC9,SETD3,TRIO,FTO,HDAC2,HTRA1,LAMA4,MYO9A,SLIT2,PDCD6,RPS6KA2,IGF1R,MDM2,SMAD1,FOXD4L4,VCAN,PTPRG,TRPC5,DMD,HCN1,SYT1,BASP1,KRT2,SIPA1L3,VCL,CELSR1,CSMD3,FSHR,MBOAT7,FMN2,MAEL,ASTN1,STK24,OPCML,WWOX,ATRX,TRPM2,OCA2,DIAPH2,NTNG1,TENM4,CATSPER2,LRRK2,MAPK10,RORB,SLC1A3,STRC,PIR,NTRK3,RORA,ANKS1A,CHD7,RNF165,DISC1,HEXB,TSNAX,EMB,GLIS1,SH3BP1,SOX6,IER2,MECP2,MTMR2,SEMA4D,GSK3B,LGR5,SLC8A1,PRKD1,SNRK,ERCC6,FLVCR1,FRMD6,TGFB2,PRAMEF12,LCE2B,LCE2C,PIWIL4,SEMA5A,BDNF,KRT74,PRKCH,RYK,ZDHHC17,PCDH15,EML1,ABI3BP,SGCD,WASF1,KDM4C,EXT2,LAMA3,RAG1,RAG2,ABCB5,MREG,NR2F2,SNAP25,NR5A2,PAQR8,PML,PPARGC1A,AKAP6,CNTNAP2,FBN1,PLXNA2,TAOK3,BCL2,ZNF675,ARHGAP24,EPHB1,MOV10L1,SMARCE1,SIPA1L1,LRP2,TMEM100,SND1,NR4A2,ARHGEF10,EPHA7,ZBTB7C,FYN,CHN1,NR4A1,SULT4A1,CD44,FLNC,HECW1,PMP22,RHBDD1,CASP6,FOXO1,PDE4D,PYY,KANK1,ENPP2,ABCB10,ACTN2,ATP8A2,DCDC2,GABRB1,GRK5,KLF13,MME,PACRG,TNMD,NAV2,LAMB4,ZNF516,CELF4,UNC5C,MYLK3,NTM,SLC1A1,UGCG,ZFPM2,PALLD,RARB,ETV6,MTOR,ROR1,GAS7,MOV10,CHRM1,DDX21,FAT3,MEGF9,PKP2,PTPRD,TIAM2,ZNF521,BBS2,PGK1,ZFP64,CEP85L,COL11A1,EPM2A,CTNNBIP1,KLHL1,SOX8,DSG4,IFT88,NLGN4X,VDR,FOXO3,MITF,CAPN3,KALRN,CCDC88C,EDNRB,MSI2,MSR1,ROBO1,RXFP1,SATB2,TANC2,FLT1,NOX4,CDH17,GRHL2,TCF4,COL2A1,MAPK9,NEBL,PREX2,TRAF3IP2,ZNF277,CR1,EYA4,SEC24B,PKHD1,STK3,TGM2,VASH2,FAM9B,KDR,PPHLN1,CHL1,EDA,NHSL1,ABCA12,APP,COL

			18A1,NRP1,PTPRM,SHROOM3,LGR4,TENM2,KAT5,LCE6A,ST7,THRB,TTC12,SEMA3C,ADAM17,CAMK1D,CYLC2,MAP3K5,PARVG,ATF2,ELP3,ETS1,NR2C2,EFEMP1,PRTG,ROS1,TGFB1I1,RPS6KA5,TCF7L2,ITGA11,PARD3,THOC2,ZNF830,ATRNL1,GRIN2A,GRIP1,HRNR,OPHN1,TRPS1,CCDC88A,CD38,DENND5A,RIN2,ALCAM,CHRDL1,CNN3,BCL2L1,HDAC4,MYO16,SOX5,SRRM4,DNMT1,GHR,JAM2,NTRK2,PRKG1,ACSL4,M1AP,DNM3,RASGRP1,CAMK4,SDK1,SPRED2,GLI3,KAZN,CLDN1,SPRR2B,SPRR2E,CERS3,CRB1,RAP1A,SAFB2,SRP54,TGFBR1,ADAM12,DICER1,EPHA5,THEMIS,TMEM120B,TNR,CUX1,KRTAP6-1,PEAK1,TENM3,CHST11,RAPGEF1,CHRNA3,EP300,MORC3,SYNE1,DSCAML1,HMG20A,TBX3,WIF1,DIO2,SPRR4,TRIOBP,GAB2,SYCP1,TP73,GRID2,PARVB,PNPLA3,MORN2,PSMA1,FLNB,SPG11,ADORA2A,CAMK2G,EFNA5,MERTK,SPRR2G,WARS2,BRD1,FAM9A,MAGI2,NF1,TFF1,SORL1,ELK3,IFT80,TEAD4,TTC8,DMRT1,ASXL1,EIF4E,FRYL,POR,RELN,SLC9C1,GRM5,HDAC9,IL18R1,KIRREL3,PTPN9,COL12A1,NAPEPLD,NDRG2,NUMB,MAP3K13,NPHP4,TBX15,ADCYAP1R1,NAV3,FBXL17,JAG1,PPP3CA,KLF7,MYT1L,NEB,PLS1,SH3GL2,CXADR,WWTR1,DOCK10,ELAVL4,GSTA2,RNF17,CNTN1,DGUOK,GRM7,RIMS1,TRIM16,GABPA,KAT7,L3MBTL3,AGGF1,ASAP1,FHOD3,N4BP2L2,NLGN1,PTPRU,LRRK1,MAPK8IP2,DDX3X,EGFL6,RBL2,OMG,EHF,GABRA5,JAK2,SHOX2,AHI1,CATSPERB,SMAD3,KIDINS220,MAP2K4,MIAT,NOS1,PTGFRN,SERPINB12,COL8A1,PLXNB1,RDH10,TCF12,CDK1,HDAC7,HIVEP3,PRRC2C,TNF,COBL,NHS,OVOL2,PANX1,ZFPM1,PPFIA2,TANC1,ADNP2,ATXN10,BLOC1S6,BMP15,DLG5,POU6F2,RBM11,LGR6,SIM2,BRINP1,SPAG16,BCL11A,HMGA2,MYO6,NEK4,DOCK1,SULT1B1,QKI,UBE2V1,MBOAT2,TPD52,DAAM2,PABPC1L,SULF1,TRPC4,C5AR1,LRP4,WDR5,CNTN6,EFNB2,PIWIL3,HERC1,SPATA5,ZNF423,INSC,LRRC8C,ALPK2,CTNNA1,TENM1,WDFY2,CDKL5,DMBT1,EFHC2,MYH14,PRKAR1A,DYNC2H1,MLLT3,CD109,IL1RAPL1,KIT,SLIT3,TAF4B,TNFSF8,TRPV4,VWC2,ABCA5,CPS1,EDAR,EFNB1,PTPRO,RAPGEF2,SLCO4C1,DNAJB6,ESR1,HTR2C,MYPN,NCMAP,SEMA3E,UBE3A,FGF13,GABRB2,PLAG1,SGCZ,ARMC2,EPHB2,FNDC3A,MECOM,TBCD,TOX,C14ORF39,ERBB4,FER,MAP3K3,SLITRK6,TDRKH,FNIP1,LRP5,SNRNP200,SLAMF1,ABL2,BSG,F11R,FARP2,SETD2,ARHGAP35,NSUN2,CCL3,FGF7,GGN,LAMA1,MYO7B,PREX1,SEMA5B,SPRED3,KIF2A,NHLH2,PSG9,SULF2,ARF4,MYH9,NSMCE2,PACSIN2,SLC39A12,SOX4,NEDD9,PIK3CD,AGTPBP1,ITGA8,PUM1,ARID4A,FAM172A,KDM3A,PDZD7,UNC13A,WDPCP,AUTS2,CDH23,DAPL1,FGFR1,MEF2A,RYR1,ARID4B,MAP2,BPGM,IL23R,ITGB7,KEAP1,NTF3,SCUBE1,AR,FGL2,KIAA1109,SUFU,EYA1,IFNA8,IL1RL2,RBM4,SRPK2,HLA-DRA,BHLHB9,L3MBTL1,NEK5,SEMA3A,CDC73,EPB41L3,MARK2,MEIS2,PDE5A,ZMIZ1,FLT3,PDLIM5,PPARGC1B,TRIM72,FERMT2,LTBR,MARK1,SMPD3,TNN,DAB2,GPR55,PBX1,SRD5A2,STX3,SYT2,ACTN4,BLOC1S5,EEF1E1,ITGA3,PRAMEF8,FRY,LGI1,NREP,PLCL2,PIN1,THSD7A,TRPM4,NME8,SCLT1,BMP6,CIT,CLASP1,EIF4ENIF1,CNGB1,TMOD1,CREM,HGF,SPATA6,CALCR,CENPF,DPY19L2,FLI1,SCUBE3,SLC26A8,ZBTB16,NCAM1,NFASC,MACF1,PAK1,PDE3A,PSMB7,SKI,TAF7L,AKT3,CTDP1,ELF1,ENPP1,FMR1,WDR7,GPRIN2,JDP2,KLF6,PRKX,ULK4,CRMP1,MFNG,RPGRIP1,TDRD6,CCDC141,GLDN,PCK1,PPARA,SORT1,SPIB,TBX22,TTN,ZFHX2,C1GALT1,COL19A1,FHL1,GNA13,CHD5,PPP2R3C,S1PR3,SPRED1,AKR1C2,CHSY1,KRT4,LCE1F,NTN4,PRDM16,PTPRF,RBL1,CCDC3,GTSF1,HIRA,PGM5,RIT2,EXT1,MYH6,NCAPH2,NCOA1,PRLR,TYMP,ADAMTS5,CBFA2T3,DOCK7,FBXW11,HDAC5,TDGF1,A2M,MYEF2,SLC24A5,ADARB1,MYH11,NDE1,POU2AF1,PRKCQ,SCEL,STAT3,TP63,AMOT,BRIP1,HPN,ISLR2,PAK3,USH2A,BLNK,DHRS2,DHRS7B,DTNBP1,GAK,RAP1B,CRTAC1,LCE2A,LGALS9,LRP8,MYOM2,UPK3A,APC,BMPR1A,ITGA1,KIAA0319,NGF,PRKCA,SPINT2,UBASH3B,VLDLR,ADAMTS20,KRT75,ABI1,DHX9,NCAPG2,RGCC,CYB5D2,EDA2R,IL6R,LCE4A,MAP2K6,SPTBN4,CLASP2,MAP2K1,PRAMEF7,SOS1,TPH1,C1QTNF3,FGF1,MCOLN3,TBX20,PPP1R9A,RREB1,TBX18,BZW2
GO:0007154	cell communication	4.164590779479693e-21	*IGHV1OR21-1,ADCY2,TPTE,PLCB1,CMKLR1,DAB1,GABRB3,CDC42EP3,GABRG3,MAPK14,ABCG8,DSCAM,TPTE2,SH3RF3,ASIC2,CTBP2,DTNA,OR11H1,HUNK,CACNA1E,NPY4R,LYPD6,OR4M2,PTPRT,ANK3,IGHV4-31,DCC,IGHV3-64,SVEP1,WLS,SYN3,TIMP3,DUSP22,AKAP13,FBLN1,ERG,GRIK1,GPR139,IGHV1OR15-9,SORBS2,IGHV4OR15-8,NPHP3,ITGA2,RGS6,KCNQ1,GABBR2,EPHA6,OR4K15,TMEM117,VRK2,GRIN3A,FCRL2,NRXN1,SHC3,ZRANB1,TNNI3K,DOK5,NRXN3,SLC1A2,RAB5A,DOCK2,CTNND2,TNC,PCNT,MS4A1,OR8U1,RIMS2,S*

			HANK2,COMT,GRIA1,NRG3,SMYD2,CLEC16A,HUS1,DLG2,TGFBR3,FSTL4,CDH13,RNF152,ANK2,LRRC4C,NXN,CSNK2A1,TF,SCN8A,PIK3C2B,TMEM108,CHEK2,OR4C12,GADD45A,PCP4,SYT17,PLXNA4,FLRT2,GPC3,NRG1,PSMB2,SHISA6,RFFL,IL4R,ALDH1A2,MCTP1,NLN,PLCE1,SH3RF2,UNC5D,KSR2,GLP2R,STK38L,SEMA6D,DRP2,CCR1,CCR3,CFTR,RBFOX2,DTX4,MC2R,TIAM1,RHOC,LDLRAD4,DNAJC15,RGS7BP,RASGRF1,DPYSL2,GRID1,SLC6A2,CBFA2T2,IL1RAPL2,OR4A5,NKD1,SCAI,MX1,BTN2A1,ALK,PTPRE,PIK3C3,SORCS3,ZNF536,FAM3B,PLCB4,ERC1,FBXO31,RAB7A,CNTN4,RGL2,ARID5B,S100B,ROBO2,ZNF366,C2CD3,EPHA3,PRKACB,PTPRN2,CNTNAP4,DCLK1,PXDN,COL15A1,CRNN,LEMD3,OR8K3,AGT,ERC2,SHISA9,CHRM5,WDR11,CDH11,RHOJ,RCAN1,ZFYVE1,CDH2,PARK2,FHL2,FOXN3,DLGAP1,LAMA2,CDC5L,KIR2DL1,KIR2DL4,OR4K13,BPIFB1,CYBB,MCTP2,GNB4,OR4M1,OR4N2,SEMA3D,NTN1,OR52N5,TRIM22,TRIM5,KCNH1,TXK,GPC6,DBH,ITSN1,TNS3,PCDH17,ZNRF3,RIN3,TRIO,APBA2,HDAC2,HTRA1,MYO9A,SLIT2,CDH8,PDCD6,OR2L13,RPS6KA2,STXBP4,GPC5,LCMT1,IGF1R,MDM2,SLC24A4,SMAD1,SYT9,OR11G2,OR9Q1,TMPRSS6,RAPGEF5,GABRG1,NR3C2,PTPRG,PXK,PDE9A,OR8K5,DMD,KCTD8,STK33,SYT1,CACNG3,SIPA1L3,TEAD1,CELSR1,FGF14,FSHR,GSG1L,GRIA4,GRM1,FMN2,MAEL,PRKAR2A,SKAP1,SORCS2,INPP5A,STK24,DCDC1,WDR83,WWOX,ATRX,PARP16,TRPM2,CNIH3,CTNNA3,RYR2,NTNG1,OR2M5,TENM4,UNC13C,CAMTA1,LRRK2,MAPK10,RORB,SLC1A3,IGFBP7,RIMBP2,NTRK3,RORA,CD8B,ILDR1,IQCJ-SCHIP1,PRICKLE2,ANKS1A,CHD7,RNF165,DISC1,TPRG1L,KCTD9,SH3BP1,MECP2,MTMR2,SEMA4D,GSK3B,LGR5,SLC8A1,MAGI3,PLCH2,PRKD1,SNRK,ERCC6,OGT,TGFB2,ATP1A4,CLSTN2,VAV3,ANKS1B,PLCD3,S100A11,SEMA5A,BDNF,LLGL2,PRKAR1B,PRKCH,RBMS3,RYK,ZDHHC17,PLCXD3,SGCD,WASF1,GRIN2B,KDM4C,OR6N1,OTUD7A,EXT2,LAMA3,SIK2,ANKRD6,ARHGAP8,NR2F2,PRR5,PRR5-ARHGAP8,PTGFR,SNAP25,NR5A2,PAQR8,PITPNC1,PML,ARHGAP12,CORO2A,AKAP6,CNTNAP2,TNS1,FBN1,GRIK3,IGHV3-16,LRFN2,PLXNA2,SNAP23,TAOK3,BCL2,CHMP4C,RALBP1,RXFP2,ZNF675,ARHGAP24,EPHB1,KCNJ3,IL12RB2,SIPA1L1,SKAP2,LRP2,TMEM100,ITPR2,LRRC4,VANGL2,CDH6,DEPTOR,GDAP1,NR4A2,ARHGEF10,EPHA7,PSD3,FYN,GNRHR,LRRC69,ADAMTS18,CHN1,NR4A1,SMARCA4,CD44,HECW1,NEU3,PMP22,RCVRN,RNF213,FOXO1,PDE4D,PYY,KANK1,ACTN2,ARHGEF3,ATF6,DCDC2,GABRB1,GRK5,MME,TNMD,CSNK1G3,EXOC4,GRIK2,IGSF11,CELF4,UNC5C,APBB2,KCND2,NAMPT,SCN11A,SLC1A1,UGCG,RARB,RERG,RFX4,GNG4,NLRP12,GRAP2,MTOR,ROR1,CADPS,CHRM1,CTDSPL2,DDX21,PKP2,BMPER,PTPRD,RASGEF1B,TIAM2,BBS2,OR51B2,OR51I1,PLCL1,ARL6IP5,ATP1A1,EPM2A,PLN,CTNNBIP1,SOX8,ZMYND11,DIDO1,DSG4,IFT88,NLGN4X,OR4K17,OR8J3,PRKCE,EIF2S1,HELLS,PRKCB,VDR,ADCY8,EFHB,FAM19A4,FOXO3,LRRTM1,MITF,CAPN3,KALRN,PJA1,TTLL12,CCDC88C,DLC1,EDNRB,OASL,ROBO1,RXFP1,BNIP3L,FLT1,NOX4,CDH17,COL2A1,KCNC4,MAP3K7,MAPK9,NR3C1,PREX2,TACR3,TRAF3IP2,CR1,EYA4,GABRA2,OR4C15,ACSL5,PKHD1,STK3,TGM2,KDR,PRCP,RAB30,ARHGAP15,CASK,CHL1,EDA,IGHV4-28,ABCA12,APP,CALCRL,DGKB,NRP1,PTPRM,UACA,ARHGEF18,ELMO1,LGR4,LINC00473,TENM2,ADTRP,KAT5,RANBP10,THRB,KCNK2,SEMA3C,ADAM17,MAP3K5,PKP4,TSPEAR,CDC42BPG,IGF2R,ATF2,GPR78,GRK6,NR2C2,PEX5L,PIK3R5,EFEMP1,ROS1,TGFB1I1,DOT1L,KPNB1,OR10R2,RGS16,RPS6KA5,TCF7L2,TNFRSF10B,TRABD2B,BICD1,ITGA11,PARD3,RALGAPA2,TLK2,TRAF3,ZNF830,ABAT,ATRNL1,DOCK9,FRAS1,GRIN2A,GRIP1,RIMS3,SGK2,KIFAP3,OPHN1,BCLAF1,SGMS1,CASP5,CCDC88A,CD38,FAM83B,MOB3B,RIN2,RNF43,TUB,ALCAM,CHRDL1,RNF126,STK36,BCL2L1,GUCY2F,HDAC4,MYO16,PDE4B,DNMT1,GHR,GLRA2,MAPRE2,NTRK2,PRKG1,ACSL4,FAM83G,CDC42BPA,GNG2,SNTG1,BRD4,PTPDC1,RASGRP1,STARD13,ARR3,CAMK4,LY86,RABGEF1,RGL1,SPRED2,GLI3,OR5L2,ZNF207,CACNA1C,KIF16B,OR11L1,STXBP5,CRB1,DOCK4,RAP1A,SAFB2,TGFBR1,CYSLTR1,MGLL,PRDX4,ADAM12,DICER1,EPHA5,LCP2,TAS2R38,THEMIS,TNR,NCOA5,TAS2R1,TENM3,CHST11,CPE,IL16,ITGB3BP,PTTG1IP,RAPGEF1,CHRNA3,CHRNA5,EP300,TBX3,WIF1,CACNA2D1,CYP7B1,GRAMD4,BRD7,GAB2,GLRA1,PCSK5,TP73,COL4A6,GRID2,IFNGR2,OR4C5,PIBF1,RASGRF2,PSMA1,SV2B,TRHDE,FHIT,FLNB,PPP1R12B,SPG11,ADORA2A,ADRBK2,CABIN1,CAMK2G,EFNA5,MERTK,RALGPS1,TAC4,CACNG2,GUCY2C,MAGI2,NF1,RALGPS2,RGS7,TFF1,SORL1,STRN3,ARHGAP6,ASB13,BICC1,BID,CEP89,ELK3,IFT80,OR5K4,TEAD4,TRIM59,DMRT1,HTR4,LZTR1,ASXL1,EIF4E,POR,RELN,TNFRSF11B,GRM5,IGHV4-4,IL18R1,IL1RL1,SELE,MGAT5,NDRG2,RHPN2,IGHV3OR16-

			12,MAP3K13,NLK,NPHP4,RALA,ADCYAP1R1,IL1RAP,FBXL17,JAG1,PPP3CA,GPR176,JRK,KLF7,SH3GL2,SNX25,CXADR,DGKI,MAS1,PER1,SLC24A2,WWTR1,KL,CBLB,DOCK10,ELAVL4,PLSCR1,CNTN1,DOK6,GPC4,GRM7,RIMS1,TRIM16,WDR59,GABRA3,GPSM2,KAT7,MAPK4,FREM1,ITGAE,NLGN1,P2RX6,PDE1C,PTPRU,ARHGAP5,BTBD11,RPS20,SH3KBP1,NPR3,AKAP7,LRRK1,MAPK8IP2,VEPH1,CACNA1D,DDX3X,NSG1,ARAP2,CNOT7,GMDS,MAML3,GABRA5,PDE7A,TPCN2,ZNF622,ARHGAP39,JAK2,SHOX2,SPNS2,AHI1,SMAD3,VPS41,BAIAP2L1,CD300A,KIDINS220,MAP2K4,NOS1,PLXNB1,TMTC4,AATF,CDK1,DCBLD2,HDAC7,ITPK1,LTB,MIR320B2,MRGPRG,NBN,RRH,SLC39A10,TNF,OVOL2,PANX1,SNX5,TNIP1,EIF3A,POMC,PRDM15,BLOC1S6,BMP15,DLG5,FCHSD2,NUDT4,PHEX,AFAP1,LGR6,MAGI1,OR11A1,OR5V1,RXFP4,SHC2,KCNK10,PLCH1,STK32B,ADORA3,ARF1,CLEC4A,FREM2,HMGA2,MYO6,AGO3,DOCK1,GRIA3,IGLC2,IGLL5,MUC5AC,RUFY1,STK32C,ANO1,GCLC,PMEPA1,UBE2V1,EPS8,LTBP1,DAAM2,SMOC2,SULF1,C5AR1,DEF6,GNAL,LRP4,PIK3R3,ARHGEF6,CNGB3,CNTN6,EFNB2,TRDN,AMOTL1,NET1,ZNF423,CDC14B,CLSTN1,SELP,ALPK2,CTNNA1,CUL2,NSG2,SCN1A,TENM1,TSHZ3,ARHGEF11,GRIK4,GSKIP,MYH14,PRKAR1A,TNFRSF19,ARHGAP11B,DYNC2H1,GABRR2,MLLT3,MYH13,MYRIP,SIPA1L2,CD109,EGF,IL1RAPL1,KIT,SLIT3,TNFSF8,UBR2,BLM,CTNNAL1,OR7A5,PIK3C2G,TRPV4,VWC2,CC2D2A,EDAR,EFNB1,PTPRO,RAPGEF2,STK32A,ESR1,HTR2C,JAK1,SEMA3E,SLC30A8,UBE3A,DNMBP,FGF13,GABRB2,GRM4,MARVELD3,NUP155,AKAP10,EPHB2,IGHV3-72,MECOM,NDFIP2,DAP,DAPK1,ERBB4,FER,MAML2,MAP3K3,FNIP1,LRP5,PTH2R,RAPGEF6,SHCBP1,RPS27L,SLAMF1,ABL2,BSG,DEPDC5,F11R,FARP2,GAS8,OR4C6,ARHGAP35,CD2AP,COMMD7,NSUN2,PTPRK,SV2C,CCL3,FGF7,LAMA1,PREX1,RNF138,SEMA5B,SPRED3,VWF,ARHGAP25,FGD4,GPR158,IL20RA,ITGA9,PSG9,SULF2,MYH9,PACSIN2,PTGER3,SLC39A12,SOX4,ST18,CAB39L,GARNL3,NEDD9,P2RX1,PDE1A,PDGFD,PIK3CD,STOML3,UBE2N,ASGR2,ITGA8,PUM1,DDB1,ENPEP,IRAK2,KDM3A,PLAT,TNKS,UNC13A,WDPCP,ADCY9,AUTS2,DAPL1,FGFR1,MEF2A,PDE11A,RGS3,UNC13B,GLRA3,ITGBL1,RASSF3,SOST,IL23R,ITGB7,JMY,NTF3,P2RX7,PLA2G4A,RAPGEF4,SCUBE1,SORCS1,TEX14,RASA3,RCAN2,ZDHHC9,AMFR,APIP,AR,SUFU,TRERF1,EYA1,IFNA8,IL1RL2,SRPK2,BDKRB1,BDKRB2,ADRA1B,ARMC9,DGKZ,IFI27L1,MIR433,OGN,PRKAA2,SEMA3A,TLE4,ARHGAP10,CDC73,GNB5,GRIA2,MARK2,PDE5A,ZMIZ1,FLT3,PPARGC1B,PTPRA,SRGAP3,TRIM72,ESRRG,FERMT2,GNAO1,HCN4,LTBR,MAP2K5,MARK1,SMPD3,TNN,DAB2,GPR55,LCP1,RNF207,SRD5A2,STX3,SYT2,ACTN4,ADAM10,CCL7,DAAM1,DRG2,EEF1E1,EEF1E1-BLOC1S5,FGF12,ITGA3,KMO,LGI1,NREP,OR10K2,OR4C46,PLCL2,PPM1B,QRICH1,RFTN1,STAC,MYOM1,PIN1,RPS6KA6,TRPM4,CD226,ERLIN1,GPR141,PIP5K1B,STK38,BMP6,BTBD9,CHRM3,CIT,GAD2,CNGB1,TGFA,WDR12,CREM,G3BP2,GAS2,HGF,PKP1,RALGAPA1,RASSF8,RGS12,CALCR,CENPF,SCUBE3,SH3BP5,TLK1,CACNB2,CNKSR2,MAST4,NCAM1,NFASC,NOS1AP,PI4KA,RALGDS,SNAP29,ARHGAP28,IL18RAP,INSR,MACF1,PAK1,PDE3A,PSMB7,SKI,AKT3,CHM,ELF1,ENPP1,FMR1,LITAF,THEM4,CHFR,GABRA1,IGHV1-69,PRKX,RRAGC,ULK4,AMPH,BMF,EVC,GABRR3,IDE,IGHV1-46,MAD1L1,MFNG,AIM2,ARHGAP42,CSF2RB,EPG5,EVC2,JPH4,LRRN2,PCK1,PDE6B,PPARA,SORT1,TNFAIP8L3,TTN,FAM3D,GNA13,PPP1R13B,TAF1,CACNA1A,CHD5,FNIP2,FUT8,QRFP,S1PR3,SPRED1,TRPM1,AKR1C2,CHSY1,ESR2,NTN4,PRDM16,PTPRF,SCN9A,ZNF451,BTRC,CCDC3,DEFA1B,DEFA3,IGHV1-18,OPTN,PTPRR,RIT2,EXT1,GNG12,IFT81,MDM4,NCOA1,PRKCG,PRLR,TYMP,UMOD,DOCK7,FBXW11,G3BP1,ICK,INPP4A,OR51L1,TDGF1,STAT4,TREM1,ADARB1,KCNC2,OR4C16,PRKCQ,SCEL,STAT3,TP63,USP7,AMOT,BRIP1,DOCK3,PAK3,PIK3R2,STK17B,TBL1X,BLNK,CASQ2,DTNBP1,RAP1B,DLGAP2,KSR1,LGALS9,LRP8,NFAT5,OR2T4,SNCAIP,TAS1R1,ALPK1,GTPBP1,IL10RB,MAPKAPK2,APC,BMPR1A,ITGA1,KIAA0319,NGF,PRKCA,PTGES,UBASH3B,VLDLR,ADAMTS20,ASB3,GPR75,MTMR4,OSGIN2,PILRB,ABI1,CTSH,FCRL5,NCAPG2,RPH3AL,SRGAP1,TRBV6-8,USP18,CCL14,CCL15,CCL15-CCL14,EDA2R,GABRG2,GPR21,IL6R,MAP2K6,NCALD,RIC8B,SPTBN4,CLASP2,MAP2K1,PDE7B,RGMB,SOS1,VPS35,C1QTNF3,FGF1,TBX20,CRADD,DGKK,NLRC5,PPP1R9A,RGN,RREB1,TBX18,VSTM1
GO:0050896	response to stimulus	8.824283106678189e-21	IGHV1OR21-1,ADCY2,TPTE,MACROD2,CDH4,PIEZO2,ZPLD1,PLCB1,CMKLR1,DAB1,OMA1,GTF2I,GABRB3,CDC42EP3,GABRG3,MAPK14,ABCG8,DSCAM,ARID1B,TPTE2,GPHN,SH3RF3,ASIC2,CTBP2,DTNA,OR11H1,SERPINA1,RAB27A,HUNK,NPY4R,HSF2BP,LYPD6,OR4M2,PTPRT,ANK3,IGHV4-31,DCC,IGHV3-

			64,SVEP1,WLS,TIMP3,DUSP22,AKAP13,FBLN1,ERG,GRIK1,GPR139,IGHV1OR15-9,SORBS2,IGHV4OR15-8,NPHP3,ITGA2,ABCG1,RGS6,KCNQ1,GABBR2,EPHA6,OR4K15,TMEM117,VRK2,GRIN3A,FCRL2,NRXN1,SHC3,ZRANB1,DOK5,NRXN3,SLC1A2,RAB5A,DOCK2,CTNND2,EYS,TNC,PCNT,MS4A1,OR8U1,RIMS2,BCKDHB,MTPN,SHANK2,COMT,GRIA1,NRG3,SMYD2,CLEC16A,HUS1,PKD1L1,DLG2,ARNT2,TGFBR3,TRPM3,FSTL4,CDH13,RNF152,ANK2,NXN,GRAMD1C,CSMD1,CSNK2A1,TF,PLGRKT,ERCC4,HLCS,KCNE1,PIK3C2B,TMEM108,ANKHD1,CHEK2,OR4C12,GADD45A,ADAMTSL1,PRDM9,PCP4,PSMC6,SYT17,PLXNA4,FLRT2,GPC3,CHAF1A,FBLN5,NRG1,SCARA5,PSMB2,SHISA6,AOX1,RAD51D,RFFL,SPIDR,IL4R,ALDH1A2,CHCHD6,LRFN5,RYR3,MCTP1,GGT1,NLN,ST8SIA1,PLCE1,REV1,SH3RF2,UNC5D,KYNU,KSR2,GLP2R,STK38L,SEMA6D,CTNNA2,CCR1,CCR3,CFTR,BBS9,RBFOX2,DTX4,MC2R,TIAM1,RHOC,AGBL4,LDLRAD4,DNAJC15,RGS7BP,RASGRF1,ZBTB20,DPYSL2,GRID1,MYO3A,SLC6A2,CBFA2T2,IL1RAPL2,OR4A5,SLC17A3,NKD1,SCAI,MX1,BTN2A1,ALK,PTPRE,PIK3C3,SORCS3,ZNF536,FAM3B,PLCB4,ERC1,PAMR1,FBXO31,KLRF2,RAB7A,CNTN4,RGL2,TAPBP,ARID5B,S100B,ROBO2,ZNF366,B3GALT5,C2CD3,EPHA3,PRKACB,PTPRN2,DCLK1,PXDN,COL15A1,CRNN,AJAP1,LEMD3,OR8K3,AGT,CST2,SHISA9,CHRM5,FBXO32,WDR11,ATP6V0D1,RHOJ,RCAN1,ZFYVE1,CDH2,PARK2,FHL2,FOXN3,LOXL2,DLGAP1,LAMA2,CDC5L,HMCN1,KIR2DL1,KIR2DL3,KIR2DL4,KIR3DL1,KIR3DL2,OR4K13,BPIFB1,CORO2B,TRIM51,CYBB,MCTP2,OTC,PSPC1,GNB4,OR4M1,OR4N2,SEMA3D,DACH1,NTN1,OR52N5,TRIM22,TRIM5,KCNH1,USP25,TXK,GPC6,RNF185,ANKFN1,DBH,FBXO27,ITSN1,TNS3,TSPAN8,ZNRF3,KCNMA1,RIN3,TRIO,FTO,HDAC2,HTRA1,MYO9A,SLIT2,ACSM2B,CDH8,AHRR,PDCD6,OR2L13,RNASET2,RPS6KA2,STXBP4,GPC5,LCMT1,REG4,IGF1R,MDM2,MX2,SLC24A4,SMAD1,SYT9,OR11G2,OR9Q1,TMPRSS6,RAPGEF5,GABRG1,NR3C2,PTPRG,PXK,PDE9A,OR8K5,TRIM48,DMD,HCN1,KCTD8,STK33,SYT1,CACNG3,SIPA1L3,VCL,TEAD1,CELSR1,FGF14,FSHR,GSG1L,GRIA4,GRM1,FMN2,MAEL,MORF4L1,NRIP1,PRKAR2A,SKAP1,SORCS2,INPP5A,STK24,DCDC1,WDR83,WWOX,ATRX,ACTR5,PARP16,TRPM2,CNIH3,RNLS,RYR2,USP16,XRCC4,OR2M5,TENM4,UNC13C,CAMTA1,HSPB8,LRRK2,MAPK10,RORB,SLC1A3,STRC,IGFBP7,NTRK3,RORA,CD8B,GGT2,ILDR1,IQCJ-SCHIP1,PRICKLE2,ANKS1A,CHD7,RNF165,DISC1,EMB,KCTD9,PDXP,SH3BP1,SOX6,IER2,MECP2,MTMR2,SEMA4D,GSK3B,LGR5,SLC8A1,MAGI3,PLCH2,PRKD1,SNRK,ERCC6,OGT,TGFB2,WDR4,ATP1A4,VAV3,ANKS1B,AFF3,C8A,PLCD3,S100A11,SEMA5A,ACSBG1,BDNF,LLGL2,PRKCH,RBMS3,RYK,ZDHHC17,KCNC1,PCDH15,PLCXD3,POLR3C,DNAAF2,DNAJB4,ABI3BP,SGCD,WASF1,GRIN2B,KDM4C,OR6N1,OTUD7A,POLE,EXT2,INIP,LAMA3,RAG1,RAG2,SIK2,ANKRD6,ARHGAP8,NR2F2,PLEKHM2,PRR5,PRR5-ARHGAP8,PTGFR,NR5A2,PAQR8,PITPNC1,PML,PPARGC1A,ARHGAP12,CORO2A,AKAP6,CNTNAP2,TNS1,FBN1,GRIK3,IGHV3-16,NAALADL2,NLRP2,NLRP7,PLXNA2,SNAP23,TAOK3,TRDV3,BCL2,CHMP4C,RAD51B,RALBP1,RXFP2,ZNF675,ARHGAP24,EPHB1,KCNJ3,IL12RB2,SIPA1L1,SKAP2,SLC30A5,LRP2,TMEM100,TRDV1,ITPR2,VANGL2,CD163,CDH6,DEPTOR,GDAP1,NR4A2,ABCC9,ARHGEF10,EPHA7,PSD3,RPGR,FYN,GNRHR,KCNE2,LRRC69,ADAMTS18,CHN1,NR4A1,SMARCA4,CD44,HECW1,MYL12A,NEU3,RCVRN,RHBDD1,RNF213,CASP6,FOXO1,PDE4D,PYY,RBBP8,TBXAS1,KANK1,ENPP2,ABCB10,ACTN2,ARHGEF3,ATF6,ATP8A2,BACH1,CST9L,DCDC2,GABRB1,GRK5,MME,PACRG,TNMD,CSNK1G3,CPNE4,GRIK2,IGSF11,ZNF516,CELF4,TPO,UNC5C,APBB2,KCND2,MYLK3,NAMPT,SCN11A,SETD7,SLC1A1,UGCG,PALLD,RARB,RERG,RFX4,GNG4,NLRP12,GRAP2,MTOR,RMI2,ROR1,MOV10,RLBP1,TMPRSS4,VPS26B,CHRM1,CTDSPL2,DDX21,BMPER,CCDC88B,PTPRD,RASGEF1B,TIAM2,BBS2,HBE1,MCM3,OR51B2,OR51I1,PGK1,PLCL1,ARL6IP5,ATP1A1,COL11A1,EPM2A,PLN,ZMAT3,CTNNBIP1,EPB41L4B,SOX8,ZMYND11,DIDO1,DSG4,IFT88,NLGN4X,OR4K17,OR8J3,PRKCE,EIF2S1,HELLS,PRKCB,VDR,ADCY8,EFHB,FAM19A4,FOXO3,MITF,RAB31,SLC47A1,CAPN3,ITLN1,KALRN,PJA1,TTLL12,CCDC88C,DLC1,EDNRB,MSR1,OASL,ROBO1,RXFP1,SATB2,TBC1D4,BNIP3L,FAP,FLT1,NASP,NOX4,SGIP1,ZNF148,CDH17,DEFB116,INO80D,TSC22D3,COL2A1,MAP3K7,MAP7,MAPK9,NR3C1,PREX2,TACR3,TRAF3IP2,ZNF277,CR1,EYA4,GABRA2,OR4C15,ACSL5,PKHD1,STK3,TGM2,KDR,PRCP,RAB30,RPL30,ARHGAP15,CASK,CHL1,EDA,IGHV4-28,MSRB3,PCOLCE2,ABCA12,APP,CALCRL,DGKB,NRP1,PTPRM,SHROOM3,UACA,ARHGEF18,ELMO1,LGR4,LINC00473,SERPING1,TENM2,ADTRP,CD58,IMMP2L,KAT5,RANBP10,THRB,CPEB2,KCNK2,SEMA3C,ADAM17,CAMK1D,MAP3K5,TSPEAR,CDC42BPG,IGF2R,SAA2

			,SAA2-SAA4,TFPI,ATF2,ETS1,GPR78,GRK6,NR2C2,PEX5L,PIK3R5,SAMSN1,EFEMP1,MT1HL1,PRTG,ROS1,TGFB1I1,TM9SF4,DOT1L,KPNB1,OR10R2,PSIP1,RGS16,RPS6KA5,TCF7L2,TNFRSF10B,TRABD2B,BICD1,ITGA11,PARD3,RALGAPA2,TLK2,TRAF3,ZNF830,ABAT,ATRNL1,DOCK9,GRIN2A,GRIP1,SGK2,KIFAP3,OPHN1,BCLAF1,HSPA4L,TTC4,PYGL,SGMS1,CASP5,CCDC88A,CD38,FAM83B,MOB3B,RIN2,RNF43,TUB,ALCAM,CHRDL1,NCF2,RNF126,STK36,BCL2L1,ABTB2,GUCY2F,HDAC4,MYO16,PDE4B,SOX5,TIMP2,DNMT1,GHR,GLRA2,MAPRE2,NTRK2,PRKG1,CPNE8,FAM83G,MGST1,CDC42BPA,GNG2,PAPPA,BRD4,PTPDC1,RASGRP1,STARD13,ARR3,CAMK4,COMMD1,LY86,RABGEF1,RGL1,SDK1,SPRED2,GLI3,OR5L2,SMCHD1,ZNF207,CACNA1C,CLDN1,KIF16B,MORC2,OR11L1,CRB1,DOCK4,PXDNL,RAP1A,SAFB2,SRP54,TGFBR1,USP53,CYSLTR1,MGLL,PRDX4,ADAM12,DICER1,EPHA5,LCP2,TAS2R38,THEMIS,TNR,TOP1,NCOA5,TAS2R1,TENM3,CHST11,CPE,IL16,ITGB3BP,PTTG1IP,RAPGEF1,CD96,CHAF1B,CHRNA3,CHRNA5,EP300,DSCAML1,SCFD1,TBX3,WIF1,CACNA2D1,CACNA2D4,CYP7B1,DIO2,GRAMD4,IGLV4-69,STX8,BRD7,GAB2,GLRA1,HBD,IGKV1-37,SYCP1,TP73,COL4A6,GRID2,IFNGR2,NPC1,OR4C5,PIBF1,PNPLA3,RASGRF2,CA3,PSMA1,TPH2,TRHDE,FHIT,FLNB,PPP1R12B,ADORA2A,ADRBK2,CABIN1,CAMK2G,EFNA5,FAM20A,MERTK,RALGPS1,TAC4,BRD1,CACNG2,GUCY2C,MAGI2,NF1,RALGPS2,RFC3,RGS7,TFF1,GOT2,SORL1,STRN3,ARHGAP6,ASB13,BICC1,BID,ELK3,IFT80,OR5K4,TEAD4,TRIM59,TTC8,DMRT1,HTR4,LZTR1,ASXL1,CRISP3,EIF4E,MICU1,POR,RELN,TNFRSF11B,USP3,GRM5,HDAC9,IGHV4-4,IGKV1-5,IL18R1,IL1RL1,SELE,MGAT5,PLSCR4,DDC,MXRA5,NAPEPLD,NDRG2,RHPN2,SP2,IGHV3OR16-12,MAP3K13,NLK,NPHP4,RALA,ADCYAP1R1,EGLN3,IL1RAP,DPP8,FBXL17,JAG1,MAN1A1,PPP3CA,GPR176,JRK,KLF3,KLF7,SH3GL2,SNX25,CXADR,DGKI,GLDC,MAS1,PER1,SLC24A2,TBC1D5,WWTR1,KL,C6,CBLB,DOCK10,ELAVL4,GSTA2,MNAT1,PLSCR1,SLC5A1,CNTN1,DOK6,GPC4,GRM7,RIMS1,TMEM67,TRIM16,WDR59,GABPA,GABRA3,GPSM2,KAT7,MAPK4,TMEM135,ITGAE,NLGN1,P2RX6,PDE1C,PTPRU,AKIRIN2,ARHGAP5,BTBD11,GBP2,GBP7,RPS20,SLC22A3,NPR3,RAD51AP1,AKAP7,FOXRED2,LRRK1,MAPK8IP2,MSRA,VEPH1,CACNA1D,DDX3X,NSG1,ARAP2,CNOT7,GMDS,MAML3,OMG,GABRA5,PDE7A,TPCN2,ZNF622,ARHGAP39,JAK2,SHOX2,SPNS2,AHI1,RPA1,SMAD3,VPS41,BAIAP2L1,CD300A,KIDINS220,MAP2K4,NOS1,WDFY4,PLXNB1,TCF12,TMTC4,WDR33,AATF,CDK1,DCBLD2,HDAC7,ITPK1,LTB,MIR320B2,MRGPRG,NBN,RRH,SLC39A10,TNF,LRCH1,OVOL2,PANX1,SNX5,TNIP1,ZFPM1,CPT1B,EIF3A,ILF3,POMC,TANC1,TMC2,PRDM15,ADNP2,BLOC1S6,BMP15,DLG5,INO80,NUDT4,PHEX,RBM11,TRAV24,TRIM23,AFAP1,LGR6,MAGI1,OR11A1,OR5V1,RXFP4,ARID2,MLIP,SHC2,BRINP1,KCNK10,PLCH1,PRG3,SRSF4,STK32B,ADORA3,ARF1,BCL11A,CLEC4A,HMGA2,MYO6,NEK4,AGO3,DOCK1,GRIA3,IGLC2,IGLJ1,IGLL5,KIF18A,MUC5AC,RUFY1,STK32C,SULT1B1,ZSWIM7,ANO1,CERS6,GCLC,PMEPA1,RAB23,UBE2V1,ACTR8,EPS8,LTBP1,SP140,CYP3A5,DAAM2,SMOC2,SULF1,C5AR1,DEF6,GNAL,LRP4,PIK3R3,ARHGEF6,CNGB3,CNTN6,EFNB2,RSRC1,SHFM1,TRDN,TRIM41,AMOTL1,NET1,ZNF423,CDC14B,LRRC8C,LRRC8D,SELP,ALPK2,CHD6,CTNNA1,CUL2,NSG2,SCN1A,TENM1,ABCG2,ARHGEF11,DMBT1,EFHC2,GRIK4,GSKIP,PRKAR1A,TNFRSF19,WDR35,ARHGAP11B,DYNC2H1,GABRR2,MLLT3,MYH13,SIPA1L2,TMEM150C,CD109,EGF,MIP,SPIRE2,ALDH1A1,CMA1,IL1RAPL1,KIT,SLIT3,TNFSF8,UBR2,BLM,CTNNAL1,OR7A5,PIK3C2G,TRPV4,VWC2,C8B,CC2D2A,CPS1,EDAR,EFNB1,PTPRO,RAPGEF2,RCSD1,REG1B,STK32A,DNAJB6,ESR1,HTR2C,JAK1,MYPN,SEMA3E,SLC30A8,UBE3A,DNMBP,FGF13,GABRB2,GRM4,MARVELD3,AKAP10,ANO3,EPHB2,IGHV3-72,MECOM,NDFIP2,RNF4,ABCC1,C14ORF39,DAP,DAPK1,ERBB4,FER,MAML2,MAP3K3,SLITRK6,ARPP21,FNIP1,LRP5,PTH2R,RAPGEF6,SHCBP1,RPS27L,SLAMF1,ABL2,BSG,CAMP,DEPDC5,F11R,FARP2,GAS8,OR4C6,SETD2,VPS13C,ARHGAP35,CD2AP,COMMD7,NSUN2,PTPRK,CCL3,FGF7,GGN,LAMA1,PREX1,RNF138,SEMA5B,SPRED3,TNFAIP8L2,VWF,ARHGAP25,BTBD17,FGD4,GPR158,IL20RA,ITGA9,LRRC70,MCM9,PSG9,SULF2,CYP2E1,MYH9,NSMCE2,PTGER3,SLC39A12,SOX4,ST18,CAB39L,GARNL3,NEDD9,OSCP1,P2RX1,PDE1A,PDGFD,PIK3CD,STOML3,UBE2N,ASGR2,ITGA8,PUM1,CNGA4,DDB1,ENPEP,IRAK2,KDM3A,MYO3B,PDZD7,PLAT,TNKS,UNC13A,WDPCP,ADCY9,AUTS2,CDH23,CDK19,DAPL1,FGFR1,MEF2A,MGME1,OCLN,PDE11A,RGS3,RYR1,UNC13B,DAD1,GLRA3,ITGBL1,RASSF3,SLX1B,SOST,IL23R,ITGB7,JMY,KEAP1,MACROD1,NTF3,P2RX7,PLA2G4A,

			POLD3,RAPGEF4,SCUBE1,SORCS1,TEX14,GP5,RASA3,RCAN2,RNMT,ZDHHC11,ZDHHC9,AMFR,APIP,AR,CASP7,FGL2,GSTM4,SCAMP5,SUFU,TRERF1,CEP250,EYA1,IFNA8,IGKV1-12,IL1RL2,MGST3,N4BP1,RBM4,SRPK2,BDKRB1,BDKRB2,HLA-DRA,ADRA1B,ARMC9,DGKZ,IFI27L1,MIR431,MIR433,OGN,PI3,PRKAA2,SEMA3A,TLE4,ANXA11,AQP10,ARHGAP10,CDC73,GNB5,GRIA2,MARK2,MEIS2,PDE5A,PROS1,ZMIZ1,FLT3,PPARGC1B,PTPRA,SRGAP3,TRIM72,DYNAP,ESRRG,FERMT2,GNAO1,HCN4,LTBR,MAP2K5,MARK1,PAPPA2,SCNN1A,SMPD3,TNN,C9,DAB2,FECH,GPR55,LCP1,SRD5A2,STX3,SYT2,UROS,ACTN4,ADAM10,BLOC1S5,CCL7,DAAM1,DRG2,EEF1E1,EEF1E1-BLOC1S5,FGF12,IGLV1-36,IGLV5-37,ITGA3,KMO,POLN,FANCB,IGKV1D-12,LGI1,MT1F,NREP,OR10K2,OR4C46,PLCL2,PPM1B,QRICH1,RFTN1,STAC,LECT2,MYOM1,PIN1,RPS6KA6,TRPM4,CD226,ERLIN1,GPR141,NME8,PIP5K1B,STK38,BMP6,CHRM3,CIT,CLASP1,CARF,CNGB1,NCOA2,TGFA,WDR12,CREM,G3BP2,GAS2,HGF,HGFAC,MYLK,OBP2A,PKP1,PRB3,RALGAPA1,RASSF8,RGS12,CALCR,CENPF,SCUBE3,SH3BP5,SNN,TLK1,CNKSR2,MAST4,NCAM1,NFASC,NOS1AP,PI4KA,RALGDS,ARHGAP28,IL18RAP,INSR,MACF1,PAK1,PDE3A,PSMB7,SKI,AKT3,CHM,CTDP1,ELF1,ENPP1,FMR1,LITAF,THEM4,CHFR,GABRA1,IGHV1-69,PRKX,RRAGC,ULK4,ABCD1,BMF,CRMP1,EVC,GABRR3,IDE,IGHV1-46,MAD1L1,MFNG,RPGRIP1,AIM2,ARHGAP42,CCDC141,CD84,CSF2RB,EPG5,EVC2,LRRN2,PCK1,PDE6B,POLD1,PPARA,SORT1,TNFAIP8L3,TTN,FAM3D,GNA13,MYH7,PPP1R13B,SLC22A5,SMARCAD1,TAF1,CACNA1A,CHD5,FNIP2,FUT8,PPP2R3C,QRFP,S1PR3,SPRED1,TRPM1,AKR1C2,CHSY1,ESR2,PRDM16,PTPRF,RBL1,SCN9A,ZNF451,BTRC,C2,CCDC3,CFB,DEFA1B,DEFA3,IGHV1-18,OPTN,PTPRR,RIT2,EXT1,GNG12,IFT81,MDM4,MYH6,NCOA1,PRKCG,PRLR,SCO2,TYMP,UMOD,ADAMTS5,CBFA2T3,DOCK7,ELMO2,FBXW11,G3BP1,HDAC5,ICK,INPP4A,OR51L1,TDGF1,UGGT2,WDR70,A2M,LYVE1,MYEF2,SEL1L2,SLC24A5,SLC40A1,STAT4,STIM1,TREM1,TRPM6,ADARB1,KCNC2,OR4C16,POU2AF1,PRKCQ,SCEL,SHPRH,STAT3,SUSD4,TFEC,TP63,USP7,AMOT,BRIP1,CST1,DEFB127,DOCK3,HPN,PAK3,PIK3R2,STK17B,TBL1X,USH2A,BLNK,CASQ2,DHRS2,DTNBP1,RAP1B,UBE2U,DLGAP2,KSR1,LGALS9,LRP8,NFAT5,OR2T4,TAS1R1,ACSM1,ALPK1,GTPBP1,IL10RB,MAPKAPK2,SLC7A8,AOAH,APC,BMPR1A,DPP4,ITGA1,KIAA0319,NGF,PRKCA,PTGES,SPINT2,UBASH3B,UBE2E2,VLDLR,ADAMTS20,ASB3,GBP4,GPR75,MTMR4,OSGIN2,PDS5A,PILRB,ABI1,CTSH,DHX9,FCRL5,NCAPG2,RGCC,RPH3AL,SRGAP1,TRBV6-8,USP18,ACACA,CCL14,CCL15,CCL15-CCL14,EDA2R,GABRG2,GPR21,IL6R,MAP2K6,NCALD,RIC8B,TFIP11,CLASP2,MAP2K1,PDE7B,RGMB,SOS1,TPH1,TRAV6,VPS35,ZRANB3,C1QTNF3,FGF1,RAD18,TBX20,TDP1,ATMIN,C6ORF106,CRADD,DGKK,NLRC5,PPP1R9A,RGN,RREB1,TBX18,VSTM1
GO:0030154	cell differentiation	9.584149747843348e-21	*CDH4,ZHX3,PLCB1,CMKLR1,DAB1,MAPK14,DSCAM,RUNX1,CTBP2,MYO18B,NCAM2,RAB27A,ANK3,DCC,RBFOX1,AKAP13,FBLN1,ERG,SORBS2,NPHP3,ITGA2,ABCG1,KCNQ1,SUN2,EPHA6,GRIN3A,NRXN1,DOK5,NRXN3,SDK2,DOCK2,CTNND2,TNC,PLEKHB2,MS4A1,CHODL,RIMS2,MTPN,NRG3,OLFM3,TGFBR3,FSTL4,ANK2,LRRC4C,RTN1,NXN,RUNX1T1,TF,SDCCAG8,TMEM108,ADAMTSL1,PCP4,ACIN1,SYT17,PLXNA4,FLRT2,GPC3,NHSL2,NRG1,ETS2,PSMB2,HYDIN,IL4R,ALDH1A2,CECR2,NLN,SUZ12,ASTN2,UNC5D,SEMA6D,SPOCK1,CTNNA2,CCR1,CFTR,BBS9,RBFOX2,TIAM1,COL22A1,AGBL4,LDLRAD4,RASGRF1,DPYSL2,CBFA2T2,NKD1,ALK,SPEF2,ZNF536,FBXO31,CNTN4,ARID5B,PHACTR1,S100B,ROBO2,EPHA3,DCLK1,COL15A1,AJAP1,AGT,FSIP2,ATAT1,CDH11,RCAN1,CDH2,PARK2,FHL2,LOXL2,MDGA2,LAMA2,ZNF804A,NEGR1,SEMA3D,NTN1,KCNH1,ZNF268,NELL1,TRAPPC9,SETD3,TRIO,FTO,HDAC2,HTRA1,LAMA4,MYO9A,SLIT2,PDCD6,RPS6KA2,IGF1R,MDM2,SMAD1,FOXD4L4,VCAN,PTPRG,TRPC5,DMD,HCN1,SYT1,BASP1,KRT2,SIPA1L3,VCL,CELSR1,CSMD3,FSHR,MBOAT7,FMN2,MAEL,ASTN1,STK24,OPCML,WWOX,ATRX,TRPM2,OCA2,DIAPH2,NTNG1,TENM4,CATSPER2,LRRK2,RORB,SLC1A3,STRC,PIR,NTRK3,RORA,ANKS1A,CHD7,RNF165,DISC1,HEXB,TSNAX,EMB,GLIS1,SH3BP1,SOX6,IER2,MECP2,MTMR2,SEMA4D,GSK3B,LGR5,SLC8A1,PRKD1,SNRK,ERCC6,FLVCR1,FRMD6,TGFB2,PRAMEF12,LCE2B,LCE2C,PIWIL4,SEMA5A,BDNF,KRT74,PRKCH,RYK,ZDHHC17,PCDH15,EML1,ABI3BP,SGCD,WASF1,KDM4C,EXT2,LAMA3,RAG1,RAG2,ABCB5,MREG,NR2F2,SNAP25,NR5A2,PAQR8,PML,PPARGC1A,AKAP6,CNTNAP2,FBN1,PLXNA2,TAOK3,BCL2,ZNF675,ARHGAP24,EPHB1,MOV10L1,SMARCE1,SIPA1L1,LRP2,TMEM100,SND1,NR4A2,ARHGEF10,EPHA7,ZBTB7C,FYN,CHN1,NR4A1,SULT4A1,FLN*

			C,HECW1,PMP22,RHBDD1,CASP6,FOXO1,PDE4D,PYY,KANK1,ABCB10,ACTN2,ATP8A2,DCDC2,GABRB1,GRK5,KLF13,MME,PACRG,TNMD,NAV2,LAMB4,ZNF516,CELF4,UNC5C,MYLK3,NTM,SLC1A1,UGCG,ZFPM2,PALLD,RARB,ETV6,MTOR,ROR1,GAS7,MOV10,CHRM1,DDX21,FAT3,MEGF9,PKP2,PTPRD,TIAM2,ZNF521,BBS2,PGK1,ZFP64,CEP85L,COL11A1,EPM2A,CTNNBIP1,KLHL1,SOX8,DSG4,IFT88,NLGN4X,VDR,FOXO3,MITF,CAPN3,KALRN,CCDC88C,EDNRB,MSI2,MSR1,ROBO1,RXFP1,SATB2,TANC2,FLT1,NOX4,CDH17,GRHL2,TCF4,COL2A1,MAPK9,NEBL,PREX2,TRAF3IP2,CR1,EYA4,SEC24B,PKHD1,STK3,TGM2,VASH2,FAM9B,KDR,PPHLN1,CHL1,EDA,NHSL1,ABCA12,APP,COL18A1,NRP1,PTPRM,SHROOM3,LGR4,TENM2,LCE6A,ST7,THRB,TTC12,SEMA3C,ADAM17,CAMK1D,CYLC2,MAP3K5,PARVG,ATF2,ELP3,ETS1,NR2C2,EFEMP1,PRTG,ROS1,TGFB1I1,RPS6KA5,TCF7L2,ITGA11,PARD3,THOC2,ZNF830,ATRNL1,GRIN2A,GRIP1,HRNR,OPHN1,TRPS1,CCDC88A,CD38,DENND5A,RIN2,ALCAM,CHRDL1,CNN3,BCL2L1,HDAC4,MYO16,SOX5,SRRM4,DNMT1,GHR,JAM2,NTRK2,PRKG1,ACSL4,M1AP,DNM3,RASGRP1,CAMK4,SDK1,SPRED2,GLI3,KAZN,CLDN1,SPRR2B,SPRR2E,CERS3,CRB1,RAP1A,SAFB2,SRP54,TGFBR1,ADAM12,DICER1,EPHA5,THEMIS,TMEM120B,TNR,CUX1,KRTAP6-1,PEAK1,TENM3,CHST11,RAPGEF1,CHRNA3,EP300,SYNE1,DSCAML1,HMG20A,TBX3,WIF1,DIO2,SPRR4,TRIOBP,GAB2,SYCP1,TP73,GRID2,PARVB,PNPLA3,MORN2,PSMA1,FLNB,SPG11,ADORA2A,CAMK2G,EFNA5,MERTK,SPRR2G,WARS2,BRD1,FAM9A,MAGI2,NF1,TFF1,SORL1,ELK3,IFT80,TEAD4,TTC8,DMRT1,ASXL1,EIF4E,FRYL,POR,RELN,SLC9C1,GRM5,HDAC9,IL18R1,KIRREL3,PTPN9,COL12A1,NAPEPLD,NDRG2,NUMB,MAP3K13,NPHP4,TBX15,ADCYAP1R1,NAV3,FBXL17,JAG1,PPP3CA,KLF7,MYT1L,NEB,PLS1,SH3GL2,CXADR,WWTR1,DOCK10,ELAVL4,GSTA2,RNF17,CNTN1,DGUOK,GRM7,RIMS1,TRIM16,GABPA,KAT7,L3MBTL3,AGGF1,ASAP1,FHOD3,N4BP2L2,NLGN1,PTPRU,LRRK1,MAPK8IP2,DDX3X,EGFL6,RBL2,OMG,EHF,GABRA5,JAK2,SHOX2,AHI1,CATSPERB,SMAD3,KIDINS220,MIAT,NOS1,PTGFRN,SERPINB12,COL8A1,PLXNB1,RDH10,TCF12,CDK1,HDAC7,HIVEP3,PRRC2C,TNF,COBL,NHS,OVOL2,PANX1,ZFPM1,PPFIA2,TANC1,ADNP2,ATXN10,BLOC1S6,BMP15,DLG5,POU6F2,RBM11,LGR6,SIM2,BRINP1,SPAG16,BCL11A,HMGA2,MYO6,DOCK1,SULT1B1,QKI,UBE2V1,MBOAT2,TPD52,DAAM2,PABPC1L,SULF1,TRPC4,C5AR1,LRP4,WDR5,CNTN6,EFNB2,PIWIL3,HERC1,SPATA5,ZNF423,INSC,LRRC8C,ALPK2,CTNNA1,TENM1,WDFY2,CDKL5,DMBT1,EFHC2,PRKAR1A,DYNC2H1,MLLT3,CD109,IL1RAPL1,KIT,SLIT3,TAF4B,TNFSF8,TRPV4,VWC2,ABCA5,CPS1,EDAR,EFNB1,PTPRO,RAPGEF2,SLCO4C1,DNAJB6,ESR1,HTR2C,MYPN,NCMAP,SEMA3E,UBE3A,FGF13,GABRB2,PLAG1,SGCZ,ARMC2,EPHB2,FNDC3A,MECOM,TBCD,TOX,C14ORF39,ERBB4,FER,SLITRK6,TDRKH,FNIP1,LRP5,SNRNP200,SLAMF1,ABL2,BSG,F11R,FARP2,SETD2,ARHGAP35,NSUN2,CCL3,FGF7,GGN,LAMA1,MYO7B,PREX1,SEMA5B,SPRED3,KIF2A,NHLH2,PSG9,SULF2,ARF4,MYH9,SLC39A12,SOX4,NEDD9,PIK3CD,AGTPBP1,ITGA8,PUM1,ARID4A,FAM172A,KDM3A,PDZD7,UNC13A,WDPCP,AUTS2,CDH23,DAPL1,FGFR1,MEF2A,RYR1,ARID4B,MAP2,BPGM,IL23R,ITGB7,KEAP1,NTF3,SCUBE1,AR,FGL2,KIAA1109,SUFU,EYA1,IFNA8,IL1RL2,RBM4,SRPK2,HLA-DRA,BHLHB9,L3MBTL1,NEK5,SEMA3A,CDC73,EPB41L3,MARK2,MEIS2,PDE5A,ZMIZ1,FLT3,PDLIM5,PPARGC1B,TRIM72,FERMT2,LTBR,MARK1,SMPD3,TNN,DAB2,GPR55,PBX1,SRD5A2,STX3,SYT2,ACTN4,BLOC1S5,ITGA3,PRAMEF8,FRY,LGI1,NREP,PLCL2,PIN1,THSD7A,TRPM4,NME8,SCLT1,BMP6,CIT,CLASP1,EIF4ENIF1,CNGB1,TMOD1,CREM,HGF,SPATA6,CALCR,CENPF,DPY19L2,FLI1,SCUBE3,SLC26A8,ZBTB16,NCAM1,NFASC,MACF1,PAK1,PDE3A,PSMB7,SKI,TAF7L,CTDP1,ELF1,ENPP1,FMR1,WDR7,GPRIN2,JDP2,KLF6,PRKX,ULK4,CRMP1,MFNG,RPGRIP1,TDRD6,CCDC141,GLDN,PCK1,PPARA,SORT1,SPIB,TBX22,TTN,ZFHX2,C1GALT1,COL19A1,FHL1,GNA13,CHD5,PPP2R3C,S1PR3,SPRED1,AKR1C2,CHSY1,KRT4,LCE1F,NTN4,PRDM16,PTPRF,RBL1,CCDC3,GTSF1,HIRA,PGM5,RIT2,EXT1,MYH6,NCAPH2,NCOA1,PRLR,TYMP,ADAMTS5,CBFA2T3,DOCK7,FBXW11,HDAC5,TDGF1,A2M,MYEF2,SLC24A5,ADARB1,MYH11,NDE1,POU2AF1,PRKCQ,SCEL,STAT3,TP63,AMOT,BRIP1,HPN,ISLR2,PAK3,USH2A,BLNK,DHRS2,DHRS7B,DTNBP1,GAK,RAP1B,CRTAC1,LCE2A,LGALS9,LRP8,MYOM2,UPK3A,APC,BMPR1A,ITGA1,KIAA0319,NGF,PRKCA,SPINT2,UBASH3B,VLDLR,ADAMTS20,KRT75,ABI1,DHX9,NCAPG2,RGCC,CYB5D2,EDA2R,IL6R,LCE4A,MAP2K6,SPTBN4,CLASP2,MAP2K1,PRAMEF7,SOS1,TPH1,C1QTNF3,FGF1,MCOLN3,TBX20,PPP1R9A,RREB1,TBX18,BZW2
GO:00486	generatio	2.6055691906282532	*CDH4,DAB1,DSCAM,RUNX1,NCAM2,ANK3,DCC,KCNQ1,EPHA6,GR*

99	n of neurons	e-20	*IN3A,NRXN1,DOK5,NRXN3,SDK2,CTNND2,TNC,CHODL,RIMS2,MTPN,NRG3,OLFM3,FSTL4,LRRC4C,RTN1,SDCCAG8,TMEM108,ADAMTSL1,PCP4,SYT17,PLXNA4,FLRT2,NRG1,ALDH1A2,CECR2,ASTN2,UNC5D,SEMA6D,SPOCK1,CTNNA2,RBFOX2,TIAM1,AGBL4,RASGRF1,DPYSL2,CBFA2T2,NKD1,ALK,ZNF536,FBXO31,CNTN4,PHACTR1,S100B,ROBO2,EPHA3,DCLK1,AGT,ATAT1,CDH11,CDH2,PARK2,MDGA2,LAMA2,ZNF804A,NEGR1,SEMA3D,NTN1,TRAPPC9,TRIO,HDAC2,MYO9A,SLIT2,IGF1R,PTPRG,TRPC5,DMD,HCN1,SYT1,VCL,CELSR1,CSMD3,FSHR,ASTN1,STK24,OPCML,NTNG1,TENM4,LRRK2,RORB,SLC1A3,STRC,NTRK3,RORA,ANKS1A,CHD7,RNF165,DISC1,EMB,IER2,MECP2,MTMR2,SEMA4D,GSK3B,PRKD1,ERCC6,TGFB2,SEMA5A,BDNF,PRKCH,RYK,ZDHHC17,PCDH15,EML1,WASF1,LAMA3,NR2F2,SNAP25,CNTNAP2,PLXNA2,TAOK3,BCL2,EPHB1,SIPA1L1,LRP2,NR4A2,EPHA7,FYN,CHN1,SULT4A1,HECW1,PMP22,KANK1,ATP8A2,DCDC2,GABRB1,MME,UNC5C,NTM,UGCG,PALLD,MTOR,ROR1,GAS7,MOV10,FAT3,PTPRD,TIAM2,ZNF521,CEP85L,KLHL1,SOX8,IFT88,NLGN4X,FOXO3,KALRN,EDNRB,ROBO1,SATB2,TANC2,TCF4,PREX2,SEC24B,TGM2,VASH2,CHL1,APP,NRP1,PTPRM,TENM2,THRB,SEMA3C,CAMK1D,ELP3,PRTG,RPS6KA5,PARD3,THOC2,GRIP1,OPHN1,CCDC88A,CD38,DENND5A,ALCAM,MYO16,SOX5,SRRM4,NTRK2,PRKG1,ACSL4,DNM3,SDK1,GLI3,CRB1,RAP1A,TGFBR1,DICER1,EPHA5,TNR,CUX1,TENM3,RAPGEF1,CHRNA3,EP300,DSCAML1,HMG20A,TRIOBP,TP73,GRID2,SPG11,ADORA2A,CAMK2G,EFNA5,MAGI2,NF1,SORL1,TTC8,EIF4E,FRYL,RELN,GRM5,HDAC9,KIRREL3,PTPN9,NUMB,MAP3K13,NPHP4,JAG1,PPP3CA,KLF7,MYT1L,PLS1,SH3GL2,DOCK10,ELAVL4,CNTN1,DGUOK,GRM7,RIMS1,ASAP1,NLGN1,MAPK8IP2,OMG,GABRA5,JAK2,SHOX2,AHI1,KIDINS220,PLXNB1,TCF12,TNF,COBL,PPFIA2,ADNP2,ATXN10,BLOC1S6,DLG5,LGR6,BRINP1,BCL11A,MYO6,DAAM2,LRP4,WDR5,CNTN6,EFNB2,HERC1,CTNNA1,TENM1,CDKL5,EFHC2,DYNC2H1,IL1RAPL1,KIT,SLIT3,TRPV4,VWC2,EFNB1,PTPRO,RAPGEF2,MYPN,SEMA3E,UBE3A,FGF13,GABRB2,PLAG1,EPHB2,TBCD,TOX,ERBB4,SLITRK6,ABL2,BSG,FARP2,ARHGAP35,LAMA1,SEMA5B,NHLH2,ARF4,SLC39A12,SOX4,AGTPBP1,PDZD7,UNC13A,WDPCP,AUTS2,CDH23,FGFR1,MEF2A,MAP2,NTF3,SUFU,EYA1,BHLHB9,SEMA3A,EPB41L3,MARK2,ZMIZ1,PDLIM5,MARK1,TNN,DAB2,PBX1,STX3,SYT2,BLOC1S5,ITGA3,FRY,LGI1,NREP,PIN1,SCLT1,BMP6,CIT,EIF4ENIF1,CNGB1,HGF,NCAM1,NFASC,MACF1,PAK1,SKI,FMR1,GPRIN2,ULK4,CRMP1,RPGRIP1,CCDC141,GLDN,ZFHX2,CHD5,NTN4,PTPRF,RIT2,EXT1,NCOA1,DOCK7,MYEF2,ADARB1,NDE1,PRKCQ,STAT3,ISLR2,PAK3,USH2A,DTNBP1,GAK,CRTAC1,LRP8,BMPR1A,ITGA1,KIAA0319,NGF,VLDLR,ABI1,CYB5D2,SPTBN4,CLASP2,MAP2K1,SOS1,MCOLN3,TBX20,PPP1R9A*
GO:0048468	cell development	3.273795577903622e-20	*CDH4,PLCB1,DAB1,DSCAM,RUNX1,MYO18B,NCAM2,ANK3,DCC,AKAP13,FBLN1,SORBS2,KCNQ1,EPHA6,GRIN3A,NRXN1,NRXN3,CTNND2,TNC,CHODL,RIMS2,OLFM3,TGFBR3,FSTL4,ANK2,LRRC4C,TMEM108,ADAMTSL1,SYT17,PLXNA4,FLRT2,NRG1,HYDIN,ALDH1A2,CECR2,UNC5D,SEMA6D,SPOCK1,CTNNA2,CFTR,RBFOX2,TIAM1,COL22A1,AGBL4,RASGRF1,DPYSL2,CBFA2T2,ALK,SPEF2,FBXO31,CNTN4,ARID5B,PHACTR1,S100B,ROBO2,EPHA3,DCLK1,COL15A1,AGT,FSIP2,ATAT1,CDH11,RCAN1,CDH2,PARK2,FHL2,LAMA2,ZNF804A,NEGR1,SEMA3D,NTN1,TRIO,HDAC2,MYO9A,SLIT2,PDCD6,RPS6KA2,IGF1R,PTPRG,TRPC5,DMD,HCN1,SYT1,KRT2,SIPA1L3,VCL,CSMD3,FSHR,FMN2,STK24,OPCML,ATRX,OCA2,DIAPH2,NTNG1,TENM4,CATSPER2,LRRK2,RORB,SLC1A3,STRC,NTRK3,ANKS1A,CHD7,RNF165,DISC1,HEXB,EMB,MECP2,MTMR2,SEMA4D,GSK3B,LGR5,SLC8A1,PRKD1,ERCC6,FLVCR1,FRMD6,TGFB2,SEMA5A,BDNF,PRKCH,RYK,ZDHHC17,PCDH15,SGCD,WASF1,LAMA3,SNAP25,PAQR8,AKAP6,CNTNAP2,FBN1,PLXNA2,TAOK3,BCL2,EPHB1,MOV10L1,SIPA1L1,LRP2,NR4A2,ARHGEF10,EPHA7,FYN,CHN1,SULT4A1,FLNC,HECW1,PMP22,PDE4D,KANK1,ABCB10,ACTN2,ATP8A2,DCDC2,GABRB1,MME,PACRG,TNMD,LAMB4,CELF4,UNC5C,MYLK3,NTM,UGCG,PALLD,RARB,MTOR,ROR1,GAS7,MOV10,FAT3,MEGF9,PTPRD,TIAM2,BBS2,COL11A1,KLHL1,SOX8,IFT88,FOXO3,CAPN3,KALRN,CCDC88C,EDNRB,MSI2,ROBO1,SATB2,TANC2,GRHL2,NEBL,PREX2,SEC24B,PKHD1,TGM2,VASH2,FAM9B,KDR,CHL1,ABCA12,APP,COL18A1,NRP1,PTPRM,SHROOM3,TENM2,THRB,TTC12,SEMA3C,CAMK1D,PARVG,PRTG,ROS1,RPS6KA5,PARD3,THOC2,ZNF830,ATRNL1,GRIP1,OPHN1,CCDC88A,CD38,DENND5A,ALCAM,BCL2L1,HDAC4,MYO16,SRRM4,JAM2,NTRK2,PRKG1,DNM3,SDK1,GLI3,CLDN1,CRB1,RAP1A,DICER1,EPHA5,TNR,CUX1,PEAK1,TENM3,CHST11,RAPGEF1,CHRNA3,EP300,DSCAML1,TBX3,TRIOBP,SYCP1,TP73,GRID2,PARVB,FLNB,SPG11,ADORA2A,CAMK2G,EFNA5,MERTK,BRD1,FAM9A,MAGI2,NF1,SORL1,TTC8,DMRT1,ASXL1,FRYL,RELN,GRM5,HDAC9,KIRREL3,P*

			TPN9,NUMB,MAP3K13,NPHP4,JAG1,PPP3CA,KLF7,MYT1L,NEB,PLS1,SH3GL2,CXADR,DOCK10,ELAVL4,RNF17,CNTN1,DGUOK,GRM7,RIMS1,L3MBTL3,ASAP1,FHOD3,NLGN1,LRRK1,MAPK8IP2,OMG,GABRA5,JAK2,SHOX2,AHI1,SMAD3,KIDINS220,PLXNB1,RDH10,CDK1,TNF,COBL,OVOL2,PANX1,ZFPM1,PPFIA2,ATXN10,BLOC1S6,BMP15,DLG5,LGR6,BRINP1,SPAG16,BCL11A,DOCK1,QKI,DAAM2,PABPC1L,SULF1,C5AR1,LRP4,WDR5,CNTN6,EFNB2,HERC1,ALPK2,CTNNA1,TENM1,CDKL5,EFHC2,PRKAR1A,IL1RAPL1,KIT,SLIT3,TAF4B,TRPV4,EFNB1,PTPRO,RAPGEF2,ESR1,MYPN,NCMAP,SEMA3E,UBE3A,FGF13,GABRB2,PLAG1,SGCZ,ARMC2,EPHB2,FNDC3A,TBCD,TOX,C14ORF39,ERBB4,FER,SLITRK6,LRP5,ABL2,BSG,F11R,FARP2,SETD2,ARHGAP35,NSUN2,LAMA1,PREX1,SEMA5B,NHLH2,SULF2,ARF4,MYH9,SLC39A12,SOX4,NEDD9,AGTPBP1,ITGA8,ARID4A,FAM172A,KDM3A,PDZD7,UNC13A,WDPCP,AUTS2,CDH23,MEF2A,RYR1,ARID4B,MAP2,BPGM,ITGB7,NTF3,AR,BHLHB9,SEMA3A,EPB41L3,MARK2,PDE5A,ZMIZ1,PDLIM5,FERMT2,MARK1,SMPD3,TNN,DAB2,PBX1,STX3,SYT2,ACTN4,BLOC1S5,ITGA3,FRY,LGI1,NREP,SCLT1,BMP6,CNGB1,TMOD1,HGF,DPY19L2,FLI1,SLC26A8,ZBTB16,NCAM1,NFASC,MACF1,PAK1,PDE3A,SKI,CTDP1,FMR1,GPRIN2,ULK4,CRMP1,RPGRIP1,CCDC141,GLDN,PPARA,TTN,C1GALT1,CHD5,S1PR3,CHSY1,NTN4,PTPRF,PGM5,RIT2,EXT1,MYH6,DOCK7,FBXW11,ADARB1,MYH11,PRKCQ,TP63,BRIP1,ISLR2,PAK3,USH2A,DTNBP1,GAK,RAP1B,CRTAC1,LRP8,MYOM2,BMPR1A,ITGA1,KIAA0319,NGF,SPINT2,VLDLR,ABI1,SPTBN4,CLASP2,MAP2K1,SOS1,TBX20,PPP1R9A,RREB1,TBX18
GO:0031175	neuron projection development	4.399380719061158e-20	*CDH4,DAB1,DSCAM,NCAM2,ANK3,DCC,EPHA6,GRIN3A,NRXN1,NRXN3,CTNND2,TNC,CHODL,RIMS2,FSTL4,LRRC4C,TMEM108,ADAMTSL1,SYT17,PLXNA4,FLRT2,CECR2,UNC5D,SEMA6D,SPOCK1,CTNNA2,RBFOX2,TIAM1,RASGRF1,DPYSL2,CBFA2T2,ALK,FBXO31,CNTN4,PHACTR1,S100B,ROBO2,EPHA3,DCLK1,AGT,CDH11,CDH2,PARK2,LAMA2,ZNF804A,NEGR1,SEMA3D,NTN1,TRIO,HDAC2,MYO9A,SLIT2,IGF1R,PTPRG,TRPC5,DMD,SYT1,VCL,CSMD3,FSHR,STK24,NTNG1,LRRK2,STRC,NTRK3,RNF165,DISC1,EMB,MECP2,SEMA4D,GSK3B,PRKD1,ERCC6,SEMA5A,BDNF,RYK,ZDHHC17,PCDH15,WASF1,LAMA3,SNAP25,CNTNAP2,PLXNA2,TAOK3,BCL2,EPHB1,SIPA1L1,LRP2,NR4A2,EPHA7,FYN,CHN1,SULT4A1,HECW1,PMP22,KANK1,ATP8A2,DCDC2,UNC5C,PALLD,ROR1,GAS7,MOV10,FAT3,PTPRD,TIAM2,KLHL1,IFT88,KALRN,ROBO1,TANC2,PREX2,SEC24B,VASH2,CHL1,APP,NRP1,PTPRM,SEMA3C,CAMK1D,PRTG,RPS6KA5,PARD3,GRIP1,OPHN1,CCDC88A,CD38,DENND5A,ALCAM,MYO16,NTRK2,PRKG1,DNM3,SDK1,GLI3,RAP1A,DICER1,EPHA5,TNR,CUX1,TENM3,RAPGEF1,CHRNA3,EP300,DSCAML1,TRIOBP,GRID2,SPG11,ADORA2A,CAMK2G,EFNA5,MAGI2,TTC8,FRYL,RELN,KIRREL3,PTPN9,NUMB,MAP3K13,PPP3CA,KLF7,PLS1,SH3GL2,DOCK10,ELAVL4,CNTN1,DGUOK,GRM7,RIMS1,ASAP1,NLGN1,MAPK8IP2,OMG,JAK2,SHOX2,KIDINS220,PLXNB1,COBL,PPFIA2,ATXN10,BLOC1S6,DLG5,LGR6,BCL11A,LRP4,WDR5,CNTN6,EFNB2,HERC1,CTNNA1,CDKL5,EFHC2,IL1RAPL1,SLIT3,TRPV4,EFNB1,PTPRO,RAPGEF2,MYPN,SEMA3E,UBE3A,FGF13,EPHB2,TOX,SLITRK6,ABL2,BSG,ARHGAP35,LAMA1,SEMA5B,ARF4,SLC39A12,PDZD7,UNC13A,AUTS2,CDH23,MEF2A,MAP2,NTF3,BHLHB9,SEMA3A,EPB41L3,MARK2,PDLIM5,MARK1,TNN,DAB2,STX3,SYT2,BLOC1S5,ITGA3,FRY,LGI1,NREP,HGF,NCAM1,NFASC,MACF1,PAK1,FMR1,GPRIN2,ULK4,CRMP1,CCDC141,PTPRF,RIT2,EXT1,DOCK7,ADARB1,PRKCQ,ISLR2,PAK3,DTNBP1,GAK,CRTAC1,LRP8,ITGA1,KIAA0319,NGF,VLDLR,ABI1,SPTBN4,CLASP2,MAP2K1,SOS1,PPP1R9A*
GO:0022008	neurogenesis	1.195424277593319e-19	*CDH4,DAB1,DSCAM,RUNX1,NCAM2,ANK3,DCC,KCNQ1,SUN2,EPHA6,GRIN3A,NRXN1,DOK5,NRXN3,SDK2,CTNND2,TNC,CHODL,RIMS2,MTPN,NRG3,OLFM3,FSTL4,LRRC4C,RTN1,SDCCAG8,TMEM108,ADAMTSL1,PCP4,SYT17,PLXNA4,FLRT2,NRG1,ALDH1A2,CECR2,SUZ12,ASTN2,UNC5D,SEMA6D,SPOCK1,CTNNA2,RBFOX2,TIAM1,AGBL4,RASGRF1,DPYSL2,CBFA2T2,NKD1,ALK,ZNF536,FBXO31,CNTN4,PHACTR1,S100B,ROBO2,EPHA3,DCLK1,AGT,ATAT1,CDH11,CDH2,PARK2,MDGA2,LAMA2,ZNF804A,NEGR1,SEMA3D,NTN1,TRAPPC9,TRIO,HDAC2,MYO9A,SLIT2,IGF1R,VCAN,PTPRG,TRPC5,DMD,HCN1,SYT1,VCL,CELSR1,CSMD3,FSHR,MBOAT7,ASTN1,STK24,OPCML,NTNG1,TENM4,LRRK2,RORB,SLC1A3,STRC,NTRK3,RORA,ANKS1A,CHD7,RNF165,DISC1,HEXB,EMB,SOX6,IER2,MECP2,MTMR2,SEMA4D,GSK3B,PRKD1,ERCC6,TGFB2,SEMA5A,BDNF,PRKCH,RYK,ZDHHC17,PCDH15,EML1,WASF1,LAMA3,NR2F2,SNAP25,CNTNAP2,PLXNA2,TAOK3,BCL2,EPHB1,SMARCE1,SIPA1L1,LRP2,NR4A2,ARHGEF10,EPHA7,FYN,CHN1,SULT4A1,HECW1,PMP22,KANK1,ATP8A2,DCDC2,GABRB1,MME,NAV2,UNC5C,NTM,SLC1A1,UGCG,PALLD,RARB,ETV6,MTOR,ROR1,GAS7,MOV10,CHRM1,FAT3,PTPRD,TIAM2,ZNF52*

			1,CEP85L,EPM2A,KLHL1,SOX8,IFT88,NLGN4X,FOXO3,KALRN,EDNRB,ROBO1,SATB2,TANC2,TCF4,PREX2,SEC24B,TGM2,VASH2,CHL1,APP,NRP1,PTPRM,TENM2,THRB,SEMA3C,CAMK1D,ELP3,PRTG,RPS6KA5,PARD3,THOC2,GRIN2A,GRIP1,OPHN1,CCDC88A,CD38,DENND5A,ALCAM,MYO16,SOX5,SRRM4,NTRK2,PRKG1,ACSL4,DNM3,SDK1,GLI3,CRB1,RAP1A,TGFBR1,DICER1,EPHA5,TNR,CUX1,TENM3,RAPGEF1,CHRNA3,EP300,DSCAML1,HMG20A,TRIOBP,TP73,GRID2,SPG11,ADORA2A,CAMK2G,EFNA5,MAGI2,NF1,SORL1,TTC8,EIF4E,FRYL,RELN,GRM5,HDAC9,KIRREL3,PTPN9,NUMB,MAP3K13,NPHP4,NAV3,JAG1,PPP3CA,KLF7,MYT1L,PLS1,SH3GL2,DOCK10,ELAVL4,CNTN1,DGUOK,GRM7,RIMS1,ASAP1,NLGN1,MAPK8IP2,OMG,GABRA5,JAK2,SHOX2,AHI1,KIDINS220,PLXNB1,TCF12,CDK1,TNF,COBL,PPFIA2,ADNP2,ATXN10,BLOC1S6,DLG5,LGR6,BRINP1,BCL11A,MYO6,DAAM2,TRPC4,C5AR1,LRP4,WDR5,CNTN6,EFNB2,HERC1,CTNNA1,TENM1,CDKL5,EFHC2,DYNC2H1,IL1RAPL1,KIT,SLIT3,TRPV4,VWC2,EFNB1,PTPRO,RAPGEF2,MYPN,NCMAP,SEMA3E,UBE3A,FGF13,GABRB2,PLAG1,EPHB2,TBCD,TOX,ERBB4,SLITRK6,ABL2,BSG,FARP2,ARHGAP35,CCL3,LAMA1,SEMA5B,NHLH2,ARF4,SLC39A12,SOX4,AGTPBP1,PDZD7,UNC13A,WDPCP,AUTS2,CDH23,FGFR1,MEF2A,MAP2,NTF3,SUFU,EYA1,BHLHB9,SEMA3A,EPB41L3,MARK2,ZMIZ1,PDLIM5,MARK1,TNN,DAB2,PBX1,STX3,SYT2,BLOC1S5,ITGA3,FRY,LGI1,NREP,PIN1,SCLT1,BMP6,CIT,EIF4ENIF1,CNGB1,HGF,NCAM1,NFASC,MACF1,PAK1,SKI,FMR1,GPRIN2,ULK4,CRMP1,RPGRIP1,CCDC141,GLDN,ZFHX2,CHD5,NTN4,PTPRF,RIT2,EXT1,NCOA1,DOCK7,MYEF2,ADARB1,NDE1,PRKCQ,STAT3,ISLR2,PAK3,USH2A,DTNBP1,GAK,CRTAC1,LRP8,BMPR1A,ITGA1,KIAA0319,NGF,VLDLR,ABI1,CYB5D2,SPTBN4,CLASP2,MAP2K1,SOS1,MCOLN3,TBX20,PPP1R9A*
GO:0034330	cell junction organization	2.519946385294502e-19	*CAST,GABRB3,MAPK14,DSCAM,GPHN,ASIC2,CTBP2,ANK3,SVEP1,DUSP22,ITGA2,NRXN1,NRXN3,SDK2,CTNND2,TNC,SHANK2,ANK2,LRRC4C,SYNDIG1,BCAN,TMEM108,FLRT2,NRG1,SHISA6,LRFN5,DRP2,ABHD17C,CTNNA2,RHOC,IL1RAPL2,ERC1,ROBO2,EPHA3,AGT,ERC2,CDH11,CDH2,CDH9,CORO2B,FRMPD4,ZNF804A,NEGR1,NTN1,TLN2,GPC6,PCDH17,MYO9A,CDH8,CNTN5,IGF1R,CLDN14,VCL,PDZRN3,NTNG1,UNC13C,LRRK2,NTRK3,DISC1,SH3BP1,MECP2,MTMR2,SEMA4D,LINGO2,TGFB2,CLSTN2,FMN1,BDNF,PRKCH,RYK,WASF1,GRIN2B,SYBU,CNTNAP2,TNS1,LRFN2,BCL2,CDH10,EPHB1,CDH12,SIPA1L1,LRRC4,NFIA,CDH6,EPHA7,FYN,THBS2,ACTN2,APBB2,SLC1A1,PKP2,PTPRD,NLGN4X,CDH18,LRRTM1,KALRN,DLC1,TANC2,GRHL2,CBLN4,GABRA2,PKHD1,KDR,APP,DGKB,NRP1,PKP4,PARD3,OPHN1,MAPRE2,NTRK2,DNM3,PPFIBP1,SDK1,CLDN1,FRMPD2,RAP1A,TGFBR1,TNR,PEAK1,RAPGEF1,PPFIBP2,GRID2,EFNA5,CACNG2,ARHGAP6,IGSF21,RELN,GRM5,KIRREL3,LRRTM3,NUMB,NPHP4,IL1RAP,CXADR,DOCK10,GPC4,NLGN1,CTTNBP2,ARHGAP39,SMAD3,PLXNB1,HDAC7,TNF,PPFIA2,TANC1,DLG5,ARF1,C5AR1,LRP4,EFNB2,CLSTN1,CTNNA1,CDKL5,IL1RAPL1,TRPV4,PTPRO,RAPGEF2,SEMA3E,UBE3A,FGF13,GABRB2,MARVELD3,EPHB2,TBCD,ERBB4,SLITRK6,F11R,CLDN11,PTPRK,ARF4,UNC13A,WDPCP,OCLN,UNC13B,BHLHB9,EPB41L3,PDLIM5,PTPRA,FERMT2,ADAM10,ITGA3,PIN1,BMP6,CLASP1,PKP1,CACNB2,CNKSR2,NFASC,NOS1AP,INSR,MACF1,TESK2,GABRA1,PTPRF,MPDZ,EXT1,AMOT,PAK3,DTNBP1,RAP1B,LRP8,APC,PRKCA,GABRG2,CLASP2,VPS35*
GO:0051716	cellular response to stimulus	4.961177232851569e-19	*IGHV1OR21-1,ADCY2,TPTE,MACROD2,PIEZO2,PLCB1,CMKLR1,DAB1,OMA1,GTF2I,GABRB3,CDC42EP3,GABRG3,MAPK14,ABCG8,DSCAM,ARID1B,TPTE2,SH3RF3,ASIC2,CTBP2,DTNA,OR11H1,HUNK,NPY4R,HSF2BP,LYPD6,OR4M2,PTPRT,ANK3,IGHV4-31,DCC,IGHV3-64,SVEP1,WLS,TIMP3,DUSP22,AKAP13,FBLN1,ERG,GRIK1,GPR139,IGHV1OR15-9,SORBS2,IGHV4OR15-8,NPHP3,ITGA2,ABCG1,RGS6,KCNQ1,GABBR2,EPHA6,OR4K15,TMEM117,VRK2,GRIN3A,FCRL2,NRXN1,SHC3,ZRANB1,DOK5,NRXN3,SLC1A2,RAB5A,DOCK2,CTNND2,TNC,PCNT,MS4A1,OR8U1,RIMS2,MTPN,SHANK2,COMT,GRIA1,NRG3,SMYD2,CLEC16A,HUS1,DLG2,TGFBR3,FSTL4,CDH13,RNF152,ANK2,NXN,GRAMD1C,CSNK2A1,TF,ERCC4,KCNE1,PIK3C2B,TMEM108,CHEK2,OR4C12,GADD45A,PRDM9,PCP4,PSMC6,SYT17,PLXNA4,FLRT2,GPC3,CHAF1A,FBLN5,NRG1,SCARA5,PSMB2,SHISA6,AOX1,RAD51D,RFFL,SPIDR,IL4R,ALDH1A2,CHCHD6,RYR3,MCTP1,NLN,ST8SIA1,PLCE1,REV1,SH3RF2,UNC5D,KSR2,GLP2R,STK38L,SEMA6D,CCR1,CCR3,CFTR,RBFOX2,DTX4,MC2R,TIAM1,RHOC,LDLRAD4,DNAJC15,RGS7BP,RASGRF1,ZBTB20,DPYSL2,GRID1,CBFA2T2,IL1RAPL2,OR4A5,NKD1,SCAI,MX1,BTN2A1,ALK,PTPRE,PIK3C3,SORCS3,ZNF536,FAM3B,PLCB4,ERC1,FBXO31,RAB7A,RGL2,ARID5B,S100B,ROBO2,ZNF366,C2CD3,EPHA3,PRKACB,PTPRN2,DCLK1,PXDN,COL15A1,CRNN,LEMD3,OR8K3,AGT,SHI*

			SA9,CHRM5,FBXO32,WDR11,ATP6V0D1,RHOJ,RCAN1,ZFYVE1,CDH2,PARK2,FHL2,FOXN3,DLGAP1,CDC5L,KIR2DL1,KIR2DL4,OR4K13,BPIFB1,CORO2B,CYBB,MCTP2,GNB4,OR4M1,OR4N2,SEMA3D,NTN1,OR52N5,TRIM22,TRIM5,KCNH1,USP25,TXK,GPC6,RNF185,DBH,FBXO27,ITSN1,TNS3,ZNRF3,RIN3,TRIO,FTO,HDAC2,HTRA1,MYO9A,SLIT2,ACSM2B,AHRR,PDCD6,OR2L13,RPS6KA2,STXBP4,GPC5,LCMT1,IGF1R,MDM2,SLC24A4,SMAD1,SYT9,OR11G2,OR9Q1,TMPRSS6,RAPGEF5,GABRG1,NR3C2,PTPRG,PDE9A,OR8K5,DMD,HCN1,KCTD8,STK33,SYT1,CACNG3,SIPA1L3,TEAD1,CELSR1,FGF14,FSHR,GSG1L,GRIA4,GRM1,FMN2,MAEL,MORF4L1,NRIP1,PRKAR2A,SKAP1,SORCS2,INPP5A,STK24,DCDC1,WDR83,WWOX,ATRX,ACTR5,PARP16,TRPM2,CNIH3,RYR2,USP16,XRCC4,OR2M5,TENM4,UNC13C,CAMTA1,HSPB8,LRRK2,MAPK10,RORB,SLC1A3,IGFBP7,NTRK3,RORA,CD8B,ILDR1,IQCJ-SCHIP1,PRICKLE2,ANKS1A,RNF165,DISC1,KCTD9,PDXP,SH3BP1,SOX6,MECP2,MTMR2,SEMA4D,GSK3B,LGR5,SLC8A1,MAGI3,PLCH2,PRKD1,SNRK,ERCC6,OGT,TGFB2,WDR4,ATP1A4,VAV3,ANKS1B,PLCD3,S100A11,SEMA5A,BDNF,LLGL2,PRKCH,RBMS3,RYK,ZDHHC17,PLCXD3,SGCD,WASF1,GRIN2B,KDM4C,OR6N1,OTUD7A,POLE,EXT2,INIP,LAMA3,SIK2,ANKRD6,ARHGAP8,NR2F2,PRR5,PRR5-ARHGAP8,PTGFR,NR5A2,PAQR8,PITPNC1,PML,PPARGC1A,ARHGAP12,CORO2A,AKAP6,TNS1,FBN1,GRIK3,IGHV3-16,NLRP7,PLXNA2,TAOK3,BCL2,CHMP4C,RAD51B,RALBP1,RXFP2,ZNF675,ARHGAP24,EPHB1,IL12RB2,SIPA1L1,SKAP2,LRP2,TMEM100,ITPR2,VANGL2,CDH6,DEPTOR,GDAP1,NR4A2,ARHGEF10,EPHA7,PSD3,FYN,GNRHR,KCNE2,LRRC69,ADAMTS18,CHN1,NR4A1,SMARCA4,CD44,HECW1,NEU3,RCVRN,RHBDD1,RNF213,CASP6,FOXO1,PDE4D,PYY,RBBP8,KANK1,ABCB10,ACTN2,ARHGEF3,ATF6,BACH1,DCDC2,GABRB1,GRK5,MME,PACRG,TNMD,CSNK1G3,CPNE4,GRIK2,IGSF11,CELF4,TPO,UNC5C,APBB2,KCND2,MYLK3,NAMPT,SETD7,SLC1A1,UGCG,RARB,RERG,RFX4,GNG4,NLRP12,GRAP2,MTOR,RMI2,ROR1,VPS26B,CHRM1,CTDSPL2,DDX21,BMPER,PTPRD,RASGEF1B,TIAM2,BBS2,HBE1,MCM3,OR51B2,OR51I1,PGK1,PLCL1,ARL6IP5,ATP1A1,EPM2A,PLN,ZMAT3,CTNNBIP1,SOX8,ZMYND11,DIDO1,DSG4,IFT88,NLGN4X,OR4K17,OR8J3,PRKCE,EIF2S1,HELLS,PRKCB,VDR,ADCY8,EFHB,FAM19A4,FOXO3,MITF,RAB31,CAPN3,KALRN,PJA1,TTLL12,CCDC88C,DLC1,EDNRB,MSR1,OASL,ROBO1,RXFP1,SATB2,TBC1D4,BNIP3L,FLT1,NOX4,CDH17,INO80D,COL2A1,MAP3K7,MAPK9,NR3C1,PREX2,TACR3,TRAF3IP2,ZNF277,CR1,EYA4,GABRA2,OR4C15,ACSL5,PKHD1,STK3,TGM2,KDR,PRCP,RAB30,ARHGAP15,CASK,CHL1,EDA,IGHV4-28,PCOLCE2,ABCA12,APP,CALCRL,DGKB,NRP1,PTPRM,SHROOM3,UACA,ARHGEF18,ELMO1,LGR4,LINC00473,TENM2,ADTRP,CD58,IMMP2L,KAT5,RANBP10,THRB,CPEB2,KCNK2,SEMA3C,ADAM17,CAMK1D,MAP3K5,TSPEAR,CDC42BPG,IGF2R,TFPI,ATF2,GPR78,GRK6,NR2C2,PEX5L,PIK3R5,EFEMP1,MT1HL1,ROS1,TGFB1I1,DOT1L,KPNB1,OR10R2,RGS16,RPS6KA5,TCF7L2,TNFRSF10B,TRABD2B,BICD1,ITGA11,PARD3,RALGAPA2,TLK2,TRAF3,ZNF830,ABAT,ATRNL1,DOCK9,GRIN2A,GRIP1,SGK2,KIFAP3,OPHN1,BCLAF1,SGMS1,CASP5,CCDC88A,CD38,FAM83B,MOB3B,RIN2,RNF43,TUB,ALCAM,CHRDL1,RNF126,STK36,BCL2L1,ABTB2,GUCY2F,HDAC4,MYO16,PDE4B,SOX5,DNMT1,GHR,GLRA2,MAPRE2,NTRK2,PRKG1,CPNE8,FAM83G,MGST1,CDC42BPA,GNG2,BRD4,PTPDC1,RASGRP1,STARD13,ARR3,CAMK4,COMMD1,LY86,RABGEF1,RGL1,SPRED2,GLI3,OR5L2,SMCHD1,ZNF207,CACNA1C,CLDN1,KIF16B,MORC2,OR11L1,CRB1,DOCK4,PXDNL,RAP1A,SAFB2,SRP54,TGFBR1,CYSLTR1,MGLL,PRDX4,ADAM12,DICER1,EPHA5,LCP2,TAS2R38,THEMIS,TNR,NCOA5,TAS2R1,TENM3,CHST11,CPE,IL16,ITGB3BP,PTTG1IP,RAPGEF1,CHAF1B,CHRNA3,CHRNA5,EP300,TBX3,WIF1,CACNA2D1,CYP7B1,GRAMD4,STX8,BRD7,GAB2,GLRA1,HBD,SYCP1,TP73,COL4A6,GRID2,IFNGR2,NPC1,OR4C5,PIBF1,PNPLA3,RASGRF2,PSMA1,TPH2,TRHDE,FHIT,FLNB,PPP1R12B,ADORA2A,ADRBK2,CABIN1,EFNA5,MERTK,RALGPS1,TAC4,CACNG2,GUCY2C,MAGI2,NF1,RALGPS2,RFC3,RGS7,TFF1,SORL1,STRN3,ARHGAP6,ASB13,BICC1,BID,ELK3,IFT80,OR5K4,TEAD4,TRIM59,DMRT1,HTR4,LZTR1,ASXL1,EIF4E,MICU1,POR,RELN,TNFRSF11B,USP3,GRM5,HDAC9,IGHV4-4,IL18R1,IL1RL1,SELE,MGAT5,PLSCR4,DDC,NDRG2,RHPN2,IGHV3OR16-12,MAP3K13,NLK,NPHP4,RALA,ADCYAP1R1,EGLN3,IL1RAP,FBXL17,JAG1,MAN1A1,PPP3CA,GPR176,JRK,KLF3,KLF7,SH3GL2,SNX25,CXADR,DGKI,GLDC,MAS1,PER1,SLC24A2,WWTR1,KL,CBLB,DOCK10,ELAVL4,GSTA2,MNAT1,PLSCR1,CNTN1,DOK6,GPC4,GRM7,RIMS1,TMEM67,TRIM16,WDR59,GABPA,GABRA3,GPSM2,KAT7,MAPK4,IT

			GAE,NLGN1,P2RX6,PDE1C,PTPRU,ARHGAP5,BTBD11,GBP2,GBP7,RPS20,NPR3,RAD51AP1,AKAP7,FOXRED2,LRRK1,MAPK8IP2,MSRA,VEPH1,CACNA1D,DDX3X,NSG1,ARAP2,CNOT7,GMDS,MAML3,OMG,GABRA5,PDE7A,TPCN2,ZNF622,ARHGAP39,JAK2,SHOX2,SPNS2,AHI1,RPA1,SMAD3,VPS41,BAIAP2L1,CD300A,KIDINS220,MAP2K4,NOS1,PLXNB1,TMTC4,WDR33,AATF,CDK1,DCBLD2,HDAC7,ITPK1,LTB,MIR320B2,MRGPRG,NBN,RRH,SLC39A10,TNF,LRCH1,OVOL2,SNX5,TNIP1,EIF3A,POMC,PRDM15,ADNP2,BLOC1S6,BMP15,DLG5,INO80,NUDT4,PHEX,RBM11,AFAP1,LGR6,MAGI1,OR11A1,OR5V1,RXFP4,ARID2,SHC2,BRINP1,KCNK10,PLCH1,STK32B,ADORA3,BCL11A,CLEC4A,HMGA2,MYO6,NEK4,AGO3,DOCK1,GRIA3,IGLC2,IGLL5,KIF18A,MUC5AC,RUFY1,STK32C,SULT1B1,ZSWIM7,ANO1,GCLC,PMEPA1,UBE2V1,ACTR8,EPS8,LTBP1,CYP3A5,DAAM2,SMOC2,SULF1,C5AR1,DEF6,GNAL,LRP4,PIK3R3,ARHGEF6,CNGB3,CNTN6,EFNB2,SHFM1,TRIM41,AMOTL1,NET1,ZNF423,CDC14B,LRRC8C,LRRC8D,SELP,ALPK2,CHD6,CTNNA1,CUL2,NSG2,TENM1,ABCG2,ARHGEF11,EFHC2,GRIK4,GSKIP,PRKAR1A,TNFRSF19,WDR35,ARHGAP11B,DYNC2H1,GABRR2,MLLT3,MYH13,SIPA1L2,TMEM150C,CD109,EGF,SPIRE2,ALDH1A1,CMA1,IL1RAPL1,KIT,SLIT3,TNFSF8,UBR2,BLM,CTNNAL1,OR7A5,PIK3C2G,TRPV4,VWC2,CC2D2A,CPS1,EDAR,EFNB1,PTPRO,RAPGEF2,RCSD1,STK32A,DNAJB6,ESR1,HTR2C,JAK1,SEMA3E,UBE3A,DNMBP,FGF13,GABRB2,GRM4,MARVELD3,AKAP10,EPHB2,IGHV3-72,MECOM,NDFIP2,ABCC1,C14ORF39,DAP,DAPK1,ERBB4,FER,MAML2,MAP3K3,ARPP21,FNIP1,LRP5,PTH2R,RAPGEF6,SHCBP1,RPS27L,SLAMF1,ABL2,BSG,CAMP,DEPDC5,F11R,FARP2,GAS8,OR4C6,SETD2,VPS13C,ARHGAP35,CD2AP,COMMD7,NSUN2,PTPRK,CCL3,FGF7,GGN,LAMA1,PREX1,RNF138,SEMA5B,SPRED3,VWF,ARHGAP25,FGD4,GPR158,IL20RA,ITGA9,MCM9,PSG9,SULF2,CYP2E1,MYH9,NSMCE2,PTGER3,SLC39A12,SOX4,ST18,CAB39L,GARNL3,NEDD9,P2RX1,PDE1A,PDGFD,PIK3CD,STOML3,UBE2N,ASGR2,ITGA8,PUM1,DDB1,ENPEP,IRAK2,KDM3A,PLAT,TNKS,UNC13A,WDPCP,ADCY9,AUTS2,CDK19,DAPL1,FGFR1,MEF2A,MGME1,PDE11A,RGS3,RYR1,UNC13B,GLRA3,ITGBL1,RASSF3,SLX1B,SOST,IL23R,ITGB7,JMY,KEAP1,MACROD1,NTF3,P2RX7,PLA2G4A,POLD3,RAPGEF4,SCUBE1,SORCS1,TEX14,RASA3,RCAN2,RNMT,ZDHHC9,AMFR,APIP,AR,CASP7,GSTM4,SCAMP5,SUFU,TRERF1,EYA1,IFNA8,IL1RL2,MGST3,N4BP1,RBM4,SRPK2,BDKRB1,BDKRB2,ADRA1B,ARMC9,DGKZ,IFI27L1,MIR431,MIR433,OGN,PRKAA2,SEMA3A,TLE4,AQP10,ARHGAP10,CDC73,GNB5,GRIA2,MARK2,PDE5A,ZMIZ1,FLT3,PPARGC1B,PTPRA,SRGAP3,TRIM72,DYNAP,ESRRG,FERMT2,GNAO1,HCN4,LTBR,MAP2K5,MARK1,SMPD3,TNN,DAB2,GPR55,LCP1,SYT2,UROS,ACTN4,ADAM10,BLOC1S5,CCL7,DAAM1,DRG2,EEF1E1,EEF1E1-BLOC1S5,FGF12,ITGA3,KMO,POLN,FANCB,MT1F,NREP,OR10K2,OR4C46,PLCL2,PPM1B,QRICH1,RFTN1,STAC,MYOM1,PIN1,RPS6KA6,TRPM4,CD226,ERLIN1,GPR141,NME8,PIP5K1B,STK38,BMP6,CHRM3,CIT,CARF,CNGB1,NCOA2,TGFA,WDR12,CREM,G3BP2,GAS2,HGF,MYLK,PKP1,RALGAPA1,RASSF8,RGS12,CALCR,CENPF,SCUBE3,SH3BP5,TLK1,CNKSR2,MAST4,NCAM1,NOS1AP,PI4KA,RALGDS,ARHGAP28,IL18RAP,INSR,MACF1,PAK1,PDE3A,PSMB7,SKI,AKT3,CHM,ELF1,ENPP1,FMR1,LITAF,THEM4,CHFR,GABRA1,IGHV1-69,PRKX,RRAGC,ULK4,ABCD1,BMF,EVC,GABRR3,IDE,IGHV1-46,MAD1L1,MFNG,AIM2,ARHGAP42,CSF2RB,EPG5,EVC2,LRRN2,PCK1,PDE6B,POLD1,PPARA,SORT1,TNFAIP8L3,TTN,FAM3D,GNA13,PPP1R13B,SMARCAD1,TAF1,CACNA1A,CHD5,FNIP2,FUT8,QRFP,S1PR3,SPRED1,TRPM1,AKR1C2,CHSY1,ESR2,PRDM16,PTPRF,RBL1,ZNF451,BTRC,CCDC3,DEFA1B,DEFA3,IGHV1-18,OPTN,PTPRR,RIT2,EXT1,GNG12,IFT81,MDM4,NCOA1,PRKCG,PRLR,TYMP,UMOD,DOCK7,ELMO2,FBXW11,G3BP1,HDAC5,ICK,INPP4A,OR51L1,TDGF1,UGGT2,WDR70,MYEF2,SEL1L2,SLC40A1,STAT4,TREM1,KCNC2,OR4C16,PRKCQ,SCEL,SHPRH,STAT3,TFEC,TP63,USP7,AMOT,BRIP1,DOCK3,PAK3,PIK3R2,STK17B,TBL1X,BLNK,CASQ2,DHRS2,DTNBP1,RAP1B,UBE2U,DLGAP2,KSR1,LGALS9,LRP8,NFAT5,OR2T4,TAS1R1,ACSM1,ALPK1,GTPBP1,IL10RB,MAPKAPK2,APC,BMPR1A,DPP4,ITGA1,KIAA0319,NGF,PRKCA,PTGES,SPINT2,UBASH3B,UBE2E2,VLDLR,ADAMTS20,ASB3,GBP4,GPR75,MTMR4,OSGIN2,PDS5A,PILRB,ABI1,CTSH,DHX9,FCRL5,NCAPG2,RGCC,RPH3AL,SRGAP1,TRBV6-8,USP18,ACACA,CCL14,CCL15,CCL15-CCL14,EDA2R,GABRG2,GPR21,IL6R,MAP2K6,NCALD,RIC8B,TFIP11,CLASP2,MAP2K1,PDE7B,RGMB,SOS1,VPS35,ZRANB3,C1QTNF3,FGF1,RAD18,TBX20,TDP1,ATMIN,CRADD,DGKK,NLRC5,PPP1R9A,RGN,RREB1,TBX18,VSTM1
GO:00069	movemen	1.858818928082901e-	SRGAP2B,TPTE,CDH4,PLCB1,CMKLR1,DAB1,FMNL2,DSCAM,TPTE

28	t of cell or subcellular component	18	*2,PTPRT,DCC,DUSP22,FBLN1,NPHP3,ITGA2,KCNQ1,DNAL4,SUN2,EPHA6,NRXN1,ZRANB1,NRXN3,NRG3,DLG2,SLC9B1,TGFBR3,CDH13,ANK2,KCND3,TF,KCNE1,PIK3C2B,SDCCAG8,TMEM108,GADD45A,ADAMTSL1,PLXNA4,FLRT2,GPC3,NRG1,RFFL,HYDIN,MCTP1,ASTN2,SH3RF2,UNC5D,SEMA6D,SPOCK1,CTNNA2,CCR1,CCR3,RBFOX2,DNAH6,TIAM1,RHOC,AGBL4,LDLRAD4,DPYSL2,NKD1,SCAI,SPEF2,FBXO31,CNTN4,ARID5B,PHACTR1,ROBO2,SPOCK3,EPHA3,DCLK1,AGT,PSTPIP2,FSIP2,RHOJ,CDH2,LOXL2,LAMA2,DNAH9,SEMA3D,DACH1,NTN1,ZNF268,GPC6,DBH,FMNL3,RIN3,TRIO,LAMA4,SLIT2,TTLL8,PDCD6,GPC5,IGF1R,VCAN,PTPRG,KRT2,VCL,CELSR1,MBOAT7,FMN2,ASTN1,STK24,FRMD5,TRPM2,CTNNA3,RYR2,KIF3B,NTNG1,CATSPER2,LRRK2,KIF26B,NTRK3,ANKS1A,RNF165,DISC1,HEXB,EMB,SH3BP1,IER2,MECP2,SEMA4D,SLC8A1,PRKD1,FRMD6,TGFB2,ATP1A4,VAV3,FUT4,S100A11,SEMA5A,BDNF,RYK,DNAAF2,DNAH3,SGCD,WASF1,LAMA3,MREG,NR2F2,PML,SYBU,MYH8,TNS1,PLXNA2,BCL2,ARHGAP24,EPHB1,KCNJ3,VANGL2,NR4A2,EPHA7,RPGR,FYN,KCNE2,CHN1,NR4A1,CD44,PDE4D,KANK1,ENPP2,DCDC2,LAMB4,UNC5C,KIF6,PALLD,NLRP12,MTOR,LDB2,FAT3,MEGF9,PKP2,BMPER,BBS2,ATP1A1,CEP85L,PLN,EPB41L4B,SOX8,IFT88,PRKCE,FAM19A4,FOXO3,MITF,KALRN,CCDC88C,DLC1,DYNLRB2,EDNRB,ROBO1,SATB2,FAP,FLT1,CENPV,TACR3,PKHD1,KDR,PRCP,CHL1,APP,NRP1,PTPRM,ELMO1,ADTRP,TTC12,SEMA3C,ADAM17,CAMK1D,ELP3,ETS1,PRTG,KPNB1,RPS6KA5,BICD1,ITGA11,ATRNL1,KIFAP3,OPHN1,CCDC88A,RIN2,TUB,ALCAM,STK36,DNAH8,HDAC4,PDE4B,JAM2,MAPRE2,NTRK2,PRKG1,CDC42BPA,RASGRP1,STARD13,RABGEF1,GLI3,CACNA1C,CLDN1,KIF16B,DOCK4,SRP54,TGFBR1,EPHA5,TNR,PEAK1,IL16,DSCAML1,SPAG17,CACNA2D1,CYP7B1,SPG11,DYNC1I2,EFNA5,MERTK,TAC4,MAGI2,NF1,SORL1,TTC8,DMRT1,RELN,SLC9C1,HDAC9,KIRREL3,SELE,DYNC1H1,MGAT5,NUMB,NPHP4,NAV3,JAG1,MEOX2,PPP3CA,KLF7,CXADR,DOCK10,GPC4,STARD9,PTPRU,SH3KBP1,CACNA1D,LRPPRC,MYH4,JAK2,SPNS2,SMAD3,CD300A,PLXNB1,CDK1,HDAC7,TNF,LRCH1,OVOL2,BLOC1S6,DLG5,LGR6,ARID2,SPAG16,ADORA3,MYO6,DOCK1,KIF18A,DNAH10,EPS8,SMOC2,SULF1,C5AR1,PIK3R3,CNTN6,EFNB2,AMOTL1,NET1,SELP,CTNNA1,SCN1A,CDKL5,EFHC2,MYH14,WDR35,DYNC2H1,EGF,KIT,SLIT3,PIK3C2G,TRPV4,ZBBX,DNAH11,EFNB1,PTPRO,RAPGEF2,MYPN,SEMA3E,FGF13,MARVELD3,NUP155,WIPF2,ARMC2,EPHB2,ABCC1,ERBB4,FER,MAP3K3,IFT43,LRP5,SLAMF1,ABL2,BSG,F11R,GAS8,SETD2,ARHGAP35,CD2AP,PTPRK,ATP8A1,CCL3,FGF7,LAMA1,MYO7B,PREX1,SEMA5B,ITGA9,KIF2A,NHLH2,SPA17,ARF4,MYH9,TEKT4,KIF23,NEDD9,PDGFD,PIK3CD,AGTPBP1,KCNE4,ENPEP,PLAT,WDPCP,AUTS2,FGFR1,ITGBL1,MAP2,ITGB7,JMY,NTF3,BDKRB1,DGKZ,SEMA3A,KIF4A,MARK2,ZMIZ1,SRGAP3,FERMT2,HCN4,MAP2K5,MARK1,SMPD3,TNN,DAB2,LCP1,RNF207,ACTN4,ADAM10,BLOC1S5,CCL7,FGF12,ITGA3,LGI1,PIN1,SYNPO2,TRPM4,EPDR1,NME8,CLASP1,HGF,MYLK,CAPN7,MYO1B,SLC26A8,CACNB2,NCAM1,NFASC,NOS1AP,INSR,MACF1,PAK1,SKI,AKT3,PRKX,ULK4,CRMP1,CCDC141,GNA13,MYH7,FUT8,SPRED1,NTN4,PTPRF,DEFA1B,PTPRR,EXT1,IFT81,MYH6,UMOD,DOCK7,ELMO2,FBXW11,HDAC5,ICK,TDGF1,TTC29,LYVE1,TREM1,ADARB1,NDE1,PRKCQ,STAT3,AMOT,PAK3,USH2A,DTNBP1,LGALS9,LRP8,EFCAB1,APC,BMPR1A,DPP4,ITGA1,KIAA0319,PRKCA,SPINT2,CTSH,RGCC,SRGAP1,CCL14,CCL15,CCL15-CCL14,IL6R,CLASP2,MAP2K1,SOS1,FGF1,TBX20,RGN,RREB1*
GO:0007267	cell-cell signaling	3.719205439249271e-17	*PLCB1,GABRB3,GABRG3,MAPK14,ASIC2,CTBP2,DTNA,CACNA1E,LYPD6,DCC,WLS,SYN3,GRIK1,NPHP3,KCNQ1,GABBR2,GRIN3A,FCRL2,NRXN1,ZRANB1,NRXN3,SLC1A2,RAB5A,CTNND2,TNC,RIMS2,SHANK2,GRIA1,NRG3,DLG2,ANK2,LRRC4C,NXN,CSNK2A1,TMEM108,SYT17,GPC3,NRG1,PSMB2,SHISA6,MCTP1,DRP2,CCR1,CFTR,TIAM1,RASGRF1,GRID1,SLC6A2,NKD1,SORCS3,FAM3B,PLCB4,ERC1,CNTN4,S100B,PTPRN2,CNTNAP4,AGT,ERC2,SHISA9,CHRM5,CDH11,CDH2,PARK2,DLGAP1,LAMA2,MCTP2,GPC6,DBH,PCDH17,ZNRF3,APBA2,CDH8,RPS6KA2,STXBP4,GPC5,SYT9,GABRG1,PXK,SYT1,CACNG3,CELSR1,FGF14,GRM1,SORCS2,WWOX,TRPM2,RYR2,NTNG1,UNC13C,LRRK2,SLC1A3,RIMBP2,ILDR1,PRICKLE2,CHD7,DISC1,TPRG1L,MECP2,MTMR2,GSK3B,LGR5,CLSTN2,SEMA5A,BDNF,PRKAR1B,RBMS3,RYK,GRIN2B,ANKRD6,SNAP25,GRIK3,LRFN2,SNAP23,EPHB1,KCNJ3,SIPA1L1,LRRC4,VANGL2,NR4A2,FYN,NR4A1,SMARCA4,HECW1,PMP22,RNF213,FOXO1,KANK1,DCDC2,GABRB1,GRK5,MME,CSNK1G3,EXOC4,GRIK2,IGSF11,CELF4,KCND2,NAMPT,SLC1A1,GRAP2,ROR1,CADPS,CHRM1,PKP2,PTPRD,PLCL1,EPM2A,CTNNBIP1,NLGN4X,PRKCE,PRKCB,ADCY8,FOXO3,LRRTM1,MITF,KALRN,CCDC88C,EDNRB,KCNC4,GABRA2,STK3,CASK,EDA,ABCA12,APP,DGKB,NRP1,LGR4,PKP4,GRK6,TGFB1I1,TCF7L2,TRABD2B,ABAT,GRIN2A*

			,RIMS3,OPHN1,CCDC88A,CD38,RNF43,GLRA2,NTRK2,ACSL4,GLI3,CACNA1C,STXBP5,CRB1,RAP1A,EPHA5,TNR,CPE,RAPGEF1,CHRNA3,CHRNA5,TBX3,WIF1,CACNA2D1,BRD7,GLRA1,PCSK5,GRID2,RASGRF2,PSMA1,SV2B,TRHDE,SPG11,ADORA2A,CAMK2G,EFNA5,MERTK,CACNG2,MAGI2,NF1,BICC1,CEP89,IFT80,HTR4,EIF4E,RELN,GRM5,NDRG2,NLK,NPHP4,IL1RAP,PPP3CA,GPR176,JRK,KLF7,CXADR,DGKI,SLC24A2,WWTR1,ELAVL4,GPC4,GRM7,RIMS1,GABRA3,NLGN1,P2RX6,PTPRU,SH3KBP1,AKAP7,LRRK1,MAPK8IP2,CACNA1D,DDX3X,NSG1,GABRA5,JAK2,SMAD3,NOS1,LTB,MIR320B2,TNF,PANX1,POMC,PRDM15,BLOC1S6,FCHSD2,PHEX,LGR6,ADORA3,ARF1,HMGA2,ANO1,DAAM2,SULF1,LRP4,EFNB2,AMOTL1,ZNF423,CLSTN1,ALPK2,TSHZ3,GRIK4,GSKIP,GABRR2,MLLT3,MYRIP,EGF,IL1RAPL1,KIT,TNFSF8,TRPV4,EFNB1,PTPRO,RAPGEF2,HTR2C,SLC30A8,FGF13,GABRB2,GRM4,NUP155,EPHB2,LRP5,SV2C,CCL3,FGF7,RNF138,SULF2,PACSIN2,SOX4,P2RX1,DDB1,ENPEP,PLAT,TNKS,UNC13A,UNC13B,GLRA3,SOST,NTF3,P2RX7,PLA2G4A,RAPGEF4,AMFR,AR,ADRA1B,DGKZ,MIR433,PRKAA2,TLE4,CDC73,GRIA2,MARK2,PTPRA,FERMT2,HCN4,MARK1,SMPD3,TNN,DAB2,RNF207,SRD5A2,STX3,SYT2,ADAM10,CCL7,DAAM1,FGF12,ITGA3,KMO,LGI1,PLCL2,PPM1B,PIN1,TRPM4,BMP6,BTBD9,CHRM3,GAD2,HGF,CACNB2,SNAP29,MACF1,PSMB7,SKI,FMR1,GABRA1,AMPH,GABRR3,JPH4,FAM3D,CACNA1A,ESR2,BTRC,RIT2,EXT1,PRKCG,FBXW11,G3BP1,ADARB1,SCEL,TP63,TBL1X,CASQ2,DTNBP1,RAP1B,DLGAP2,LRP8,SNCAIP,APC,NGF,PTGES,RPH3AL,CCL15,GABRG2,MAP2K6,PDE7B,VPS35,C1QTNF3,PPP1R9A,TBX18
GO:0003008	system process	2.7597286123336736e-16	*PIEZO2,PLCB1,GABRB3,GABRG3,ABCG8,ASIC2,DTNA,OR11H1,NCAM2,OR4M2,ANK3,SVEP1,RBFOX1,TIMP3,AKAP13,SORBS2,SLC24A3,ITGA2,LOXHD1,KCNQ1,OR4K15,GRIN3A,NRXN1,TMPRSS3,TNNI3K,NRXN3,SLC1A2,EYS,OR8U1,RIMS2,MTPN,SHANK2,GRIA1,CELF2,TRPM3,ANK2,KCND3,CSMD1,MYH1,SCN8A,KCNE1,TMEM108,OR4C12,SHISA6,RYR3,PLCE1,CTNNA2,SLCO3A1,BBS9,RBFOX2,RASGRF1,MYO3A,OR4A5,UTRN,SORCS3,MYOM3,S100B,PRKACB,SLC44A1,OR8K3,AGT,CST2,SHISA9,CHRM5,FBXO32,VTI1A,RCAN1,PARK2,DLGAP1,HMCN1,OR4K13,CORO2B,DNAH9,OR4M1,OR4N2,OR52N5,ANKFN1,DBH,SETD3,KCNMA1,FTO,HDAC2,MYO9A,SLIT2,CNTN5,OR2L13,RPS6KA2,MDM2,SLC24A4,OR11G2,OR9Q1,GABRG1,PDE9A,OR8K5,DMD,CACNG3,FSHR,GRM1,JAKMIP1,CTNNA3,RNLS,RYR2,OR2M5,TENM4,CAMTA1,LRRK2,RORB,SLC1A3,STRC,SLC4A4,CHD7,HEXB,MECP2,MTMR2,GSK3B,SLC8A1,CDC14A,TGFB2,ATP1A4,SLC5A3,SLCO2B1,BDNF,PRKAR1B,SNTB1,PCDH15,SGCD,GRIN2B,OR6N1,EXT2,RAG1,NR2F2,SNAP25,SGCG,MYH8,AKAP6,CNTNAP2,ACSM3,BCL2,EPHB1,KCNJ3,LRP2,TMEM100,ABCC9,RPGR,FYN,KCNE2,SLC26A7,NR4A1,RCVRN,FOXO1,PDE4D,TBXAS1,ACTN2,ATF6,ATP8A2,DCDC2,GABRB1,MME,NAV2,GRIK2,IGSF11,CELF4,RAP1GDS1,APBB2,KCND2,MYLK3,SCN11A,SLC1A1,UGCG,MTOR,ROR1,RLBP1,CHRM1,PKP2,BBS2,OR51B2,OR51I1,ARL6IP5,ATP1A1,COL11A1,EPM2A,PLN,CTNNBIP1,NLGN4X,OR4K17,OR8J3,GTF2A1L,VDR,ADCY8,FAM19A4,FOXO3,KALRN,EDNRB,NOX4,COL2A1,TACR3,EYA4,GABRA2,OR4C15,PRCP,CHL1,APP,CALCRL,COL18A1,SERPING1,IMMP2L,THRB,KCNK2,TSPEAR,EFEMP1,OR10R2,RGS16,AFF2,PARD3,SSPN,ZNF830,ABAT,GRIN2A,CD38,TUB,ADAMTS16,GUCY2F,HDAC4,PDE4B,SRRM4,GLRA2,JAM2,NTRK2,PRKG1,ARR3,CAMK4,OR5L2,CACNA1C,OR11L1,CRB1,DOCK4,USP53,CYSLTR1,KCNJ12,MGLL,DICER1,TAS2R38,TNR,SLC6A17,TAS2R1,CHRNA3,CHRNA5,EP300,SLC16A7,TBX3,CACNA2D1,CACNA2D4,TRIOBP,ATXN1,GLRA1,PCSK5,GRID2,OR4C5,TRHDE,PPP1R12B,ADORA2A,CAMK2G,TAC4,CACNG2,NF1,TFF1,IMPG1,OR5K4,TTC8,RELN,GRM5,RHPN2,MEOX2,PPP3CA,KCNB2,PLS1,SH3GL2,SLC7A1,CXADR,DGKI,SLC24A2,WWTR1,KL,ELAVL4,SLC5A1,GRM7,RIMS1,GABRA3,SLC22A2,NLGN1,P2RX6,SLC22A3,NPR3,LHFPL3,MAPK8IP2,TMPRSS11E,CACNA1D,MYH4,GABRA5,TPCN2,JAK2,SHOX2,SOBP,SMAD3,NOS1,RDH10,TMTC4,MAGT1,NBN,RRH,TNF,SNX5,POMC,TANC1,TMC2,POU6F2,OR11A1,OR5V1,MLIP,BRINP1,KCNK10,SPAG16,ADORA3,CALD1,MYO6,ANO1,GCLC,SULF1,C5AR1,GNAL,CNGB3,TRDN,HERC1,SCN1A,TSHZ3,ABCG2,ARHGEF11,MYH14,GABRR2,MYH13,TMEM150C,MIP,CMA1,KIT,OR7A5,TRPV4,CPS1,DNAH11,PTPRO,RCSD1,HTR2C,NCMAP,UBE3A,FGF13,GABRB2,NUP155,SGCZ,EPHB2,ABCC1,SLITRK6,LRP5,F11R,OR4C6,ARHGAP35,ATP8A1,CCL3,MYO7B,SEMA5B,SULF2,ARF4,PTGER3,SLC2A13,P2RX1,AGTPBP1,ITGA8,KCNE4,CNGA4,ENPEP,MYO3B,PDZD7,CDH23,DAPL1,MEF2A,OCLN,RYR1,SYNM,UNC13B,GLRA3,NTF3,P2RX7,AMFR,AR,CEP250,EYA1,BDKRB1,BDKRB2,HLA-DRA,ADRA1B,BHLHB9,SEMA3A,MEIS2,MOGAT2,PDE5A,REEP2,SMPX,PDLIM5,TRIM72,FERMT2,GNAO1,HCN4,SCNN1A,CLN6,DAB2,R*

			NF207,FGF12,ITGA3,OR10K2,OR4C46,STAC,LRIG1,MYOM1,TRPM4,SHROOM4,ABCC2,BMP6,BTBD9,CHRM3,SMTNL2,CNGB1,TMOD1,HGF,MYLK,OBP2A,FLI1,SLC13A3,CACNB2,ELN,NFASC,NOS1AP,INSR,PDE3A,CHM,CTDP1,FMR1,PAX3,GABRA1,GABRR3,RPGRIP1,ARHGAP42,JPH4,PDE6B,PPARA,TTN,ZFHX2,CORIN,GNA13,MYH7,SLC22A5,QRFP,TRPM1,SCN9A,MYBPC2,TECTA,EXT1,MYH6,PRKCG,SCO2,TYMP,UMOD,KLHL3,OR51L1,ADARB1,MYH11,OR4C16,AMOT,CST1,HPN,TBL1X,USH2A,CASQ2,DLGAP2,MYOM2,NFAT5,OR2T4,TAS1R1,SLC7A8,TUSC3,ITGA1,NGF,PRKCA,PTGES,VLDLR,ASB3,GABRG2,MAP2K6,NCALD,SLC16A12,SPTBN4,VPS35,C1QTNF3,TBX20,TBX18
GO:0007165	signal transduction	3.208052193240009e-16	*IGHV1OR21-1,ADCY2,TPTE,PLCB1,CMKLR1,DAB1,GABRB3,CDC42EP3,GABRG3,MAPK14,ABCG8,DSCAM,TPTE2,SH3RF3,ASIC2,CTBP2,DTNA,OR11H1,HUNK,NPY4R,LYPD6,OR4M2,PTPRT,ANK3,IGHV4-31,DCC,IGHV3-64,SVEP1,WLS,TIMP3,DUSP22,AKAP13,FBLN1,ERG,GRIK1,GPR139,IGHV1OR15-9,SORBS2,IGHV4OR15-8,NPHP3,ITGA2,RGS6,KCNQ1,GABBR2,EPHA6,OR4K15,TMEM117,VRK2,GRIN3A,FCRL2,NRXN1,SHC3,ZRANB1,DOK5,NRXN3,RAB5A,DOCK2,CTNND2,PCNT,MS4A1,OR8U1,RIMS2,SHANK2,GRIA1,NRG3,SMYD2,CLEC16A,HUS1,TGFBR3,FSTL4,CDH13,RNF152,ANK2,NXN,CSNK2A1,TF,PIK3C2B,TMEM108,CHEK2,OR4C12,GADD45A,PCP4,PLXNA4,FLRT2,GPC3,NRG1,PSMB2,SHISA6,RFFL,IL4R,ALDH1A2,MCTP1,NLN,PLCE1,SH3RF2,UNC5D,KSR2,GLP2R,STK38L,SEMA6D,CCR1,CCR3,RBFOX2,DTX4,MC2R,TIAM1,RHOC,LDLRAD4,RGS7BP,RASGRF1,DPYSL2,GRID1,CBFA2T2,IL1RAPL2,OR4A5,NKD1,SCAI,MX1,BTN2A1,ALK,PTPRE,PIK3C3,SORCS3,ZNF536,FAM3B,PLCB4,ERC1,FBXO31,RAB7A,RGL2,ARID5B,S100B,ROBO2,ZNF366,C2CD3,EPHA3,PRKACB,DCLK1,PXDN,COL15A1,CRNN,LEMD3,OR8K3,AGT,SHISA9,CHRM5,WDR11,RHOJ,RCAN1,CDH2,PARK2,FHL2,FOXN3,DLGAP1,CDC5L,KIR2DL1,KIR2DL4,OR4K13,BPIFB1,CYBB,MCTP2,GNB4,OR4M1,OR4N2,SEMA3D,NTN1,OR52N5,TRIM22,TRIM5,KCNH1,TXK,GPC6,DBH,ITSN1,TNS3,ZNRF3,RIN3,TRIO,HDAC2,HTRA1,MYO9A,SLIT2,PDCD6,OR2L13,RPS6KA2,STXBP4,GPC5,LCMT1,IGF1R,MDM2,SLC24A4,SMAD1,OR11G2,OR9Q1,TMPRSS6,RAPGEF5,GABRG1,NR3C2,PTPRG,PDE9A,OR8K5,DMD,KCTD8,STK33,CACNG3,SIPA1L3,TEAD1,CELSR1,FGF14,FSHR,GSG1L,GRIA4,GRM1,FMN2,MAEL,PRKAR2A,SKAP1,SORCS2,INPP5A,STK24,DCDC1,WDR83,WWOX,ATRX,PARP16,TRPM2,CNIH3,RYR2,OR2M5,TENM4,UNC13C,CAMTA1,LRRK2,MAPK10,RORB,IGFBP7,NTRK3,RORA,CD8B,IQCJ-SCHIP1,PRICKLE2,ANKS1A,RNF165,DISC1,KCTD9,SH3BP1,MECP2,MTMR2,SEMA4D,GSK3B,LGR5,SLC8A1,MAGI3,PLCH2,PRKD1,SNRK,ERCC6,OGT,TGFB2,ATP1A4,VAV3,ANKS1B,PLCD3,S100A11,SEMA5A,BDNF,LLGL2,PRKCH,RBMS3,RYK,ZDHHC17,PLCXD3,SGCD,WASF1,GRIN2B,KDM4C,OR6N1,OTUD7A,EXT2,LAMA3,SIK2,ANKRD6,ARHGAP8,NR2F2,PRR5,PRR5-ARHGAP8,PTGFR,NR5A2,PAQR8,PITPNC1,PML,ARHGAP12,CORO2A,AKAP6,TNS1,FBN1,GRIK3,IGHV3-16,PLXNA2,TAOK3,BCL2,CHMP4C,RALBP1,RXFP2,ZNF675,ARHGAP24,EPHB1,IL12RB2,SIPA1L1,SKAP2,LRP2,TMEM100,ITPR2,VANGL2,CDH6,DEPTOR,NR4A2,ARHGEF10,EPHA7,PSD3,FYN,GNRHR,LRRC69,ADAMTS18,CHN1,NR4A1,SMARCA4,CD44,HECW1,NEU3,RCVRN,RNF213,FOXO1,PDE4D,PYY,KANK1,ACTN2,ARHGEF3,ATF6,DCDC2,GABRB1,GRK5,TNMD,CSNK1G3,GRIK2,IGSF11,CELF4,UNC5C,APBB2,NAMPT,SLC1A1,UGCG,RARB,RERG,RFX4,GNG4,NLRP12,GRAP2,MTOR,ROR1,CHRM1,CTDSPL2,DDX21,BMPER,PTPRD,RASGEF1B,TIAM2,BBS2,OR51B2,OR51I1,PLCL1,ARL6IP5,EPM2A,PLN,CTNNBIP1,SOX8,ZMYND11,DIDO1,DSG4,IFT88,NLGN4X,OR4K17,OR8J3,PRKCE,EIF2S1,HELLS,PRKCB,VDR,ADCY8,EFHB,FAM19A4,FOXO3,MITF,CAPN3,KALRN,PJA1,TTLL12,CCDC88C,DLC1,EDNRB,OASL,ROBO1,RXFP1,BNIP3L,FLT1,NOX4,CDH17,COL2A1,MAP3K7,MAPK9,NR3C1,PREX2,TACR3,TRAF3IP2,CR1,EYA4,GABRA2,OR4C15,ACSL5,PKHD1,STK3,TGM2,KDR,PRCP,RAB30,ARHGAP15,CHL1,EDA,IGHV4-28,APP,CALCRL,DGKB,NRP1,PTPRM,UACA,ARHGEF18,ELMO1,LGR4,LINC00473,TENM2,ADTRP,KAT5,RANBP10,THRB,KCNK2,SEMA3C,ADAM17,MAP3K5,TSPEAR,CDC42BPG,IGF2R,ATF2,GPR78,GRK6,NR2C2,PEX5L,PIK3R5,EFEMP1,ROS1,TGFB1I1,DOT1L,KPNB1,OR10R2,RGS16,RPS6KA5,TCF7L2,TNFRSF10B,TRABD2B,BICD1,ITGA11,PARD3,RALGAPA2,TLK2,TRAF3,ZNF830,ABAT,ATRNL1,DOCK9,GRIN2A,GRIP1,SGK2,KIFAP3,OPHN1,BCLAF1,SGMS1,CASP5,CCDC88A,CD38,FAM83B,MOB3B,RIN2,RNF43,TUB,ALCAM,CHRDL1,RNF126,STK36,BCL2L1,GUCY2F,HDAC4,MYO16,PDE4B,DNMT1,GHR,GLRA2,*

			MAPRE2,NTRK2,PRKG1,FAM83G,CDC42BPA,GNG2,BRD4,PTPDC1,RASGRP1,STARD13,ARR3,CAMK4,LY86,RABGEF1,RGL1,SPRED2,GLI3,OR5L2,ZNF207,CACNA1C,KIF16B,OR11L1,DOCK4,RAP1A,SAFB2,TGFBR1,CYSLTR1,MGLL,PRDX4,ADAM12,DICER1,EPHA5,LCP2,TAS2R38,THEMIS,NCOA5,TAS2R1,TENM3,CHST11,CPE,IL16,ITGB3BP,PTTG1IP,RAPGEF1,CHRNA3,CHRNA5,EP300,WIF1,CYP7B1,GRAMD4,BRD7,GAB2,GLRA1,TP73,COL4A6,GRID2,IFNGR2,OR4C5,PIBF1,RASGRF2,PSMA1,TRHDE,FHIT,FLNB,PPP1R12B,ADORA2A,ADRBK2,CABIN1,EFNA5,MERTK,RALGPS1,TAC4,CACNG2,GUCY2C,MAGI2,NF1,RALGPS2,RGS7,TFF1,SORL1,STRN3,ARHGAP6,ASB13,BICC1,BID,ELK3,IFT80,OR5K4,TEAD4,TRIM59,DMRT1,HTR4,LZTR1,ASXL1,POR,RELN,TNFRSF11B,GRM5,IGHV4-4,IL18R1,IL1RL1,SELE,MGAT5,NDRG2,RHPN2,IGHV3OR16-12,MAP3K13,NLK,NPHP4,RALA,ADCYAP1R1,IL1RAP,FBXL17,JAG1,PPP3CA,GPR176,JRK,SH3GL2,SNX25,DGKI,MAS1,PER1,SLC24A2,WWTR1,KL,CBLB,DOCK10,PLSCR1,CNTN1,DOK6,GPC4,GRM7,RIMS1,TRIM16,WDR59,GABRA3,GPSM2,KAT7,MAPK4,ITGAE,NLGN1,P2RX6,PDE1C,PTPRU,ARHGAP5,BTBD11,RPS20,NPR3,AKAP7,LRRK1,MAPK8IP2,VEPH1,CACNA1D,DDX3X,NSG1,ARAP2,CNOT7,GMDS,MAML3,GABRA5,PDE7A,TPCN2,ZNF622,ARHGAP39,JAK2,SHOX2,SPNS2,AHI1,SMAD3,BAIAP2L1,CD300A,KIDINS220,MAP2K4,NOS1,PLXNB1,TMTC4,AATF,CDK1,DCBLD2,HDAC7,ITPK1,LTB,MRGPRG,NBN,RRH,SLC39A10,TNF,OVOL2,SNX5,TNIP1,EIF3A,POMC,PRDM15,BMP15,DLG5,NUDT4,AFAP1,LGR6,MAGI1,OR11A1,OR5V1,RXFP4,SHC2,KCNK10,PLCH1,STK32B,ADORA3,CLEC4A,HMGA2,MYO6,AGO3,DOCK1,GRIA3,IGLC2,IGLL5,MUC5AC,RUFY1,STK32C,ANO1,GCLC,PMEPA1,UBE2V1,EPS8,LTBP1,DAAM2,SMOC2,SULF1,C5AR1,DEF6,GNAL,LRP4,PIK3R3,ARHGEF6,CNGB3,CNTN6,EFNB2,AMOTL1,NET1,ZNF423,CDC14B,SELP,ALPK2,CTNNA1,CUL2,NSG2,TENM1,ARHGEF11,GRIK4,GSKIP,PRKAR1A,TNFRSF19,ARHGAP11B,DYNC2H1,GABRR2,MLLT3,SIPA1L2,CD109,EGF,IL1RAPL1,KIT,SLIT3,TNFSF8,UBR2,BLM,CTNNAL1,OR7A5,PIK3C2G,TRPV4,VWC2,CC2D2A,EDAR,EFNB1,PTPRO,RAPGEF2,STK32A,ESR1,HTR2C,JAK1,SEMA3E,UBE3A,DNMBP,FGF13,GABRB2,GRM4,MARVELD3,AKAP10,EPHB2,IGHV3-72,MECOM,NDFIP2,DAP,DAPK1,ERBB4,FER,MAML2,MAP3K3,FNIP1,LRP5,PTH2R,RAPGEF6,SHCBP1,RPS27L,SLAMF1,ABL2,BSG,DEPDC5,F11R,FARP2,GAS8,OR4C6,ARHGAP35,CD2AP,COMMD7,NSUN2,PTPRK,CCL3,FGF7,LAMA1,PREX1,RNF138,SEMA5B,SPRED3,VWF,ARHGAP25,FGD4,GPR158,IL20RA,ITGA9,PSG9,SULF2,MYH9,PTGER3,SLC39A12,SOX4,ST18,CAB39L,GARNL3,NEDD9,P2RX1,PDE1A,PDGFD,PIK3CD,STOML3,UBE2N,ASGR2,ITGA8,PUM1,DDB1,ENPEP,IRAK2,KDM3A,PLAT,TNKS,UNC13A,WDPCP,ADCY9,AUTS2,DAPL1,FGFR1,MEF2A,PDE11A,RGS3,UNC13B,GLRA3,ITGBL1,RASSF3,SOST,IL23R,ITGB7,JMY,NTF3,P2RX7,RAPGEF4,SCUBE1,SORCS1,TEX14,RASA3,RCAN2,ZDHHC9,AMFR,APIP,AR,SUFU,TRERF1,EYA1,IFNA8,IL1RL2,SRPK2,BDKRB1,BDKRB2,ADRA1B,ARMC9,DGKZ,IFI27L1,OGN,PRKAA2,SEMA3A,TLE4,ARHGAP10,CDC73,GNB5,GRIA2,MARK2,PDE5A,ZMIZ1,FLT3,PPARGC1B,PTPRA,SRGAP3,TRIM72,ESRRG,FERMT2,GNAO1,LTBR,MAP2K5,MARK1,SMPD3,TNN,DAB2,GPR55,LCP1,ACTN4,ADAM10,CCL7,DAAM1,DRG2,EEF1E1,EEF1E1-BLOC1S5,FGF12,ITGA3,NREP,OR10K2,OR4C46,PLCL2,PPM1B,QRICH1,RFTN1,STAC,MYOM1,PIN1,RPS6KA6,TRPM4,CD226,ERLIN1,GPR141,PIP5K1B,STK38,BMP6,CHRM3,CIT,CNGB1,TGFA,WDR12,CREM,G3BP2,GAS2,HGF,PKP1,RALGAPA1,RASSF8,RGS12,CALCR,CENPF,SCUBE3,SH3BP5,TLK1,CNKSR2,MAST4,NCAM1,NOS1AP,PI4KA,RALGDS,ARHGAP28,IL18RAP,INSR,MACF1,PAK1,PDE3A,PSMB7,SKI,AKT3,CHM,ELF1,ENPP1,FMR1,LITAF,THEM4,CHFR,GABRA1,IGHV1-69,PRKX,RRAGC,ULK4,BMF,EVC,GABRR3,IDE,IGHV1-46,MAD1L1,MFNG,AIM2,ARHGAP42,CSF2RB,EPG5,EVC2,LRRN2,PDE6B,PPARA,SORT1,TNFAIP8L3,TTN,FAM3D,GNA13,PPP1R13B,TAF1,CHD5,FNIP2,FUT8,QRFP,S1PR3,SPRED1,TRPM1,AKR1C2,CHSY1,ESR2,PRDM16,PTPRF,ZNF451,BTRC,CCDC3,DEFA1B,DEFA3,IGHV1-18,OPTN,PTPRR,RIT2,EXT1,GNG12,IFT81,MDM4,NCOA1,PRKCG,PRLR,TYMP,UMOD,DOCK7,FBXW11,G3BP1,ICK,INPP4A,OR51L1,TDGF1,STAT4,TREM1,KCNC2,OR4C16,PRKCQ,SCEL,STAT3,TP63,USP7,AMOT,BRIP1,DOCK3,PAK3,PIK3R2,STK17B,TBL1X,BLNK,CASQ2,DTNBP1,RAP1B,DLGAP2,KSR1,LGALS9,LRP8,NFAT5,OR2T4,TAS1R1,ALPK1,GTPBP1,IL10RB,MAPKAPK2,APC,BMPR1A,ITGA1,KIAA0319,NGF,PRKCA,PTGES,UBASH3B,VLDLR,ADAMTS20,ASB3,GPR75,MTMR4,OSGIN2,PILRB,ABI1,CTSH,FCRL5,NCAPG2,RPH3AL,SRGAP1,TRBV6-8,USP18,CCL14,CCL15,CCL15-CCL14,EDA2R,GABRG2,GPR21,IL6R,MAP2K6,NCALD,RIC8B,CLASP

			2,MAP2K1,PDE7B,RGMB,SOS1,VPS35,C1QTNF3,FGF1,TBX20,CRADD,DGKK,NLRC5,PPP1R9A,RGN,RREB1,TBX18,VSTM1
GO:0099537	trans-synaptic signaling	3.4385090807965705e-16	*PLCB1,GABRB3,GABRG3,ASIC2,CTBP2,DTNA,CACNA1E,DCC,SYN3,GRIK1,GABBR2,GRIN3A,NRXN1,NRXN3,SLC1A2,RAB5A,RIMS2,SHANK2,GRIA1,NRG3,DLG2,LRRC4C,TMEM108,SHISA6,MCTP1,RASGRF1,GRID1,SLC6A2,SORCS3,PLCB4,ERC1,CNTN4,S100B,PTPRN2,CNTNAP4,AGT,ERC2,SHISA9,CHRM5,CDH11,CDH2,PARK2,DLGAP1,LAMA2,MCTP2,DBH,PCDH17,APBA2,CDH8,RPS6KA2,SYT9,GABRG1,PXK,SYT1,CACNG3,GRM1,SORCS2,NTNG1,UNC13C,LRRK2,SLC1A3,RIMBP2,DISC1,TPRG1L,MECP2,MTMR2,GSK3B,CLSTN2,BDNF,PRKAR1B,GRIN2B,SNAP25,GRIK3,LRFN2,SNAP23,EPHB1,SIPA1L1,LRRC4,FYN,NR4A1,PMP22,GABRB1,MME,EXOC4,GRIK2,IGSF11,CELF4,KCND2,SLC1A1,CADPS,CHRM1,PTPRD,PLCL1,NLGN4X,PRKCE,PRKCB,ADCY8,LRRTM1,KCNC4,GABRA2,CASK,APP,DGKB,ABAT,GRIN2A,RIMS3,OPHN1,CD38,GLRA2,NTRK2,STXBP5,RAP1A,TNR,CHRNA3,CHRNA5,GLRA1,GRID2,RASGRF2,SV2B,SPG11,ADORA2A,CACNG2,NF1,CEP89,HTR4,EIF4E,RELN,GRM5,IL1RAP,PPP3CA,GPR176,DGKI,SLC24A2,ELAVL4,GRM7,RIMS1,GABRA3,NLGN1,P2RX6,AKAP7,MAPK8IP2,NSG1,GABRA5,JAK2,NOS1,MIR320B2,TNF,BLOC1S6,FCHSD2,ARF1,CLSTN1,TSHZ3,GRIK4,GABRR2,IL1RAPL1,KIT,RAPGEF2,HTR2C,GABRB2,GRM4,EPHB2,SV2C,PACSIN2,P2RX1,PLAT,UNC13A,UNC13B,GLRA3,NTF3,P2RX7,DGKZ,MIR433,GRIA2,PTPRA,STX3,SYT2,FGF12,KMO,LGI1,PLCL2,BTBD9,CHRM3,GAD2,CACNB2,SNAP29,FMR1,GABRA1,AMPH,GABRR3,JPH4,CACNA1A,RIT2,EXT1,PRKCG,ADARB1,DTNBP1,RAP1B,DLGAP2,LRP8,SNCAIP,NGF,GABRG2,PDE7B,PPP1R9A*
GO:0010646	regulation of cell communication	4.12405945562908e-16	*TPTE,PLCB1,CMKLR1,DAB1,MAPK14,TPTE2,SH3RF3,CTBP2,LYPD6,PTPRT,ANK3,DCC,WLS,SYN3,TIMP3,DUSP22,AKAP13,FBLN1,GRIK1,NPHP3,ITGA2,RGS6,VRK2,GRIN3A,NRXN1,ZRANB1,DOK5,NRXN3,RAB5A,DOCK2,CTNND2,RIMS2,SHANK2,GRIA1,NRG3,SMYD2,CLEC16A,TGFBR3,FSTL4,CDH13,RNF152,ANK2,LRRC4C,NXN,CSNK2A1,TMEM108,CHEK2,GADD45A,GPC3,NRG1,PSMB2,SHISA6,RFFL,MCTP1,PLCE1,SH3RF2,CCR1,CFTR,TIAM1,RHOC,LDLRAD4,RGS7BP,RASGRF1,GRID1,CBFA2T2,NKD1,SCAI,ALK,PTPRE,SORCS3,ZNF536,PLCB4,ERC1,RAB7A,CNTN4,RGL2,S100B,ROBO2,ZNF366,C2CD3,PRKACB,CNTNAP4,PXDN,CRNN,LEMD3,AGT,ERC2,SHISA9,WDR11,CDH11,RCAN1,CDH2,PARK2,FHL2,DLGAP1,LAMA2,BPIFB1,MCTP2,TRIM22,TRIM5,TXK,GPC6,DBH,ITSN1,PCDH17,ZNRF3,TRIO,APBA2,HDAC2,HTRA1,MYO9A,SLIT2,PDCD6,STXBP4,GPC5,LCMT1,IGF1R,MDM2,SLC24A4,SYT9,TMPRSS6,NR3C2,PXK,DMD,KCTD8,SYT1,CACNG3,SIPA1L3,FSHR,GSG1L,GRM1,SORCS2,WDR83,WWOX,TRPM2,CNIH3,RYR2,NTNG1,UNC13C,CAMTA1,LRRK2,SLC1A3,IGFBP7,NTRK3,RORA,ILDR1,IQCJ-SCHIP1,ANKS1A,CHD7,RNF165,DISC1,TPRG1L,SH3BP1,MECP2,MTMR2,SEMA4D,GSK3B,LGR5,SLC8A1,MAGI3,PRKD1,ERCC6,OGT,TGFB2,CLSTN2,VAV3,SEMA5A,BDNF,LLGL2,PRKAR1B,PRKCH,RBMS3,RYK,ZDHHC17,WASF1,GRIN2B,KDM4C,OTUD7A,SIK2,ANKRD6,ARHGAP8,PRR5,SNAP25,PML,ARHGAP12,AKAP6,FBN1,GRIK3,LRFN2,TAOK3,BCL2,RALBP1,ZNF675,ARHGAP24,EPHB1,SIPA1L1,LRP2,TMEM100,LRRC4,DEPTOR,NR4A2,ARHGEF10,EPHA7,PSD3,FYN,CHN1,SMARCA4,CD44,HECW1,NEU3,RNF213,FOXO1,PDE4D,KANK1,ARHGEF3,ATF6,DCDC2,GRK5,MME,TNMD,CSNK1G3,GRIK2,IGSF11,CELF4,SLC1A1,UGCG,RFX4,GNG4,NLRP12,MTOR,ROR1,CTDSPL2,DDX21,BMPER,PTPRD,TIAM2,PLCL1,ARL6IP5,CTNNBIP1,ZMYND11,NLGN4X,PRKCE,HELLS,PRKCB,ADCY8,EFHB,FAM19A4,FOXO3,LRRTM1,CAPN3,KALRN,PJA1,TTLL12,CCDC88C,DLC1,OASL,ROBO1,FLT1,NOX4,COL2A1,MAP3K7,MAPK9,PREX2,TRAF3IP2,EYA4,ACSL5,PKHD1,STK3,TGM2,KDR,PRCP,ARHGAP15,CASK,EDA,ABCA12,APP,DGKB,NRP1,UACA,ARHGEF18,LGR4,LINC00473,ADAM17,MAP3K5,TSPEAR,GRK6,PEX5L,PIK3R5,ROS1,TGFB1I1,DOT1L,RGS16,TCF7L2,TNFRSF10B,TRABD2B,BICD1,RALGAPA2,TRAF3,ABAT,GRIN2A,RIMS3,OPHN1,BCLAF1,SGMS1,CCDC88A,CD38,MOB3B,RNF43,TUB,CHRDL1,RNF126,STK36,BCL2L1,GUCY2F,PDE4B,GHR,MAPRE2,NTRK2,ACSL4,BRD4,RASGRP1,STARD13,ARR3,LY86,RABGEF1,SPRED2,GLI3,STXBP5,RAP1A,SAFB2,TGFBR1,MGLL,EPHA5,TNR,NCOA5,CHST11,PTTG1IP,RAPGEF1,CHRNA3,CHRNA5,EP300,WIF1,CYP7B1,GRAMD4,GLRA1,TP73,GRID2,IFNGR2,PIBF1,RASGRF2,PSMA1,ADORA2A,ADRBK2,EFNA5,RALGPS1,CACNG2,MAGI2,NF1,RALGPS2,RGS7,SORL1,STRN3,ARHGAP6,BICC1,BID,IFT80,TRIM59,DMRT1,LZTR1,ASXL1,EIF4E,POR,RELN,GRM5,IL18R1,MGAT5,NDRG2,MAP3K13,NLK,NPHP4,ADCYAP1R1,FBXL17,JAG1,PPP3CA,JRK,KLF7,SNX25,CXADR,DGKI,PER1,SLC24A2,WWTR1,KL,CBLB,ELAVL4,PLSCR1,DOK6,GPC4,GRM7,RIMS1,TRIM16,WDR59,NLGN1,PTPRU,ARHGAP5,RP*

			S20,AKAP7,LRRK1,MAPK8IP2,VEPH1,DDX3X,NSG1,CNOT7,ZNF622,ARHGAP39,JAK2,SHOX2,SMAD3,BAIAP2L1,CD300A,PLXNB1,AATF,HDAC7,MIR320B2,SLC39A10,TNF,OVOL2,SNX5,TNIP1,EIF3A,POMC,PRDM15,BMP15,DLG5,AFAP1,LGR6,SHC2,ARF1,AGO3,ANO1,GCLC,PMEPA1,UBE2V1,EPS8,LTBP1,DAAM2,SMOC2,SULF1,C5AR1,DEF6,LRP4,CNTN6,TRDN,NET1,ZNF423,CLSTN1,SELP,ALPK2,CTNNA1,TENM1,TSHZ3,ARHGEF11,GRIK4,GSKIP,TNFRSF19,ARHGAP11B,DYNC2H1,MLLT3,MYRIP,SIPA1L2,CD109,EGF,KIT,SLIT3,UBR2,TRPV4,VWC2,EDAR,PTPRO,RAPGEF2,ESR1,HTR2C,SLC30A8,UBE3A,DNMBP,GRM4,MARVELD3,EPHB2,MECOM,NDFIP2,DAPK1,ERBB4,FER,MAP3K3,FNIP1,LRP5,SLAMF1,ABL2,DEPDC5,F11R,GAS8,ARHGAP35,CD2AP,CCL3,PREX1,SPRED3,VWF,ARHGAP25,FGD4,IL20RA,SULF2,PACSIN2,SOX4,GARNL3,P2RX1,PDGFD,PIK3CD,UBE2N,ITGA8,PUM1,IRAK2,PLAT,TNKS,UNC13A,AUTS2,FGFR1,PDE11A,RGS3,UNC13B,SOST,IL23R,NTF3,P2RX7,SCUBE1,RASA3,AMFR,APIP,AR,SUFU,EYA1,IFNA8,BDKRB2,ADRA1B,ARMC9,DGKZ,MIR433,PRKAA2,SEMA3A,TLE4,ARHGAP10,CDC73,PDE5A,ZMIZ1,FLT3,PTPRA,SRGAP3,TRIM72,FERMT2,LTBR,MAP2K5,TNN,DAB2,GPR55,STX3,ACTN4,ADAM10,CCL7,EEF1E1,EEF1E1-BLOC1S5,FGF12,ITGA3,KMO,LGI1,NREP,PLCL2,PPM1B,PIN1,RPS6KA6,TRPM4,CD226,PIP5K1B,STK38,BMP6,BTBD9,CIT,TGFA,GAS2,HGF,RALGAPA1,RGS12,CALCR,SCUBE3,CACNB2,CNKSR2,NCAM1,NOS1AP,ARHGAP28,INSR,MACF1,PAK1,PDE3A,PSMB7,SKI,AKT3,ELF1,ENPP1,FMR1,LITAF,RRAGC,ULK4,EVC,MAD1L1,MFNG,ARHGAP42,JPH4,PPARA,TNFAIP8L3,FAM3D,GNA13,TAF1,CACNA1A,CHD5,SPRED1,AKR1C2,CHSY1,ESR2,PRDM16,ZNF451,BTRC,CCDC3,OPTN,PTPRR,RIT2,IFT81,NCOA1,PRKCG,PRLR,TYMP,FBXW11,G3BP1,TDGF1,PRKCQ,SCEL,STAT3,TP63,USP7,AMOT,DOCK3,PAK3,PIK3R2,TBL1X,CASQ2,DTNBP1,RAP1B,DLGAP2,KSR1,LGALS9,LRP8,NFAT5,SNCAIP,ALPK1,IL10RB,APC,BMPR1A,ITGA1,KIAA0319,NGF,PRKCA,UBASH3B,ADAMTS20,MTMR4,CTSH,NCAPG2,RPH3AL,USP18,CCL14,CCL15,EDA2R,GPR21,IL6R,MAP2K6,RIC8B,MAP2K1,SOS1,VPS35,C1QTNF3,FGF1,TBX20,CRADD,NLRC5,PPP1R9A,RGN,TBX18
GO:0023051	regulation of signaling	5.058954074399642e-16	*TPTE,PLCB1,CMKLR1,DAB1,MAPK14,TPTE2,SH3RF3,CTBP2,LYPD6,PTPRT,DCC,WLS,SYN3,TIMP3,DUSP22,AKAP13,FBLN1,GRIK1,NPHP3,ITGA2,RGS6,VRK2,GRIN3A,NRXN1,ZRANB1,TNNI3K,DOK5,NRXN3,RAB5A,DOCK2,CTNND2,RIMS2,SHANK2,GRIA1,NRG3,SMYD2,CLEC16A,TGFBR3,FSTL4,CDH13,RNF152,ANK2,LRRC4C,NXN,CSNK2A1,TMEM108,CHEK2,GADD45A,GPC3,NRG1,PSMB2,SHISA6,RFFL,RYR3,MCTP1,PLCE1,SH3RF2,CCR1,CFTR,TIAM1,RHOC,LDLRAD4,RGS7BP,RASGRF1,GRID1,CBFA2T2,NKD1,SCAI,ALK,PTPRE,SORCS3,ZNF536,PLCB4,ERC1,RAB7A,CNTN4,RGL2,S100B,ROBO2,ZNF366,C2CD3,PRKACB,CNTNAP4,PXDN,CRNN,LEMD3,AGT,ERC2,SHISA9,WDR11,CDH11,RCAN1,CDH2,PARK2,FHL2,DLGAP1,LAMA2,BPIFB1,MCTP2,TRIM22,TRIM5,TXK,GPC6,DBH,ITSN1,PCDH17,ZNRF3,TRIO,APBA2,HDAC2,HTRA1,MYO9A,SLIT2,PDCD6,STXBP4,GPC5,LCMT1,IGF1R,MDM2,SLC24A4,SYT9,TMPRSS6,NR3C2,PXK,DMD,KCTD8,SYT1,CACNG3,SIPA1L3,FSHR,GSG1L,GRM1,SORCS2,WDR83,WWOX,TRPM2,CNIH3,RYR2,NTNG1,UNC13C,CAMTA1,LRRK2,SLC1A3,IGFBP7,NTRK3,RORA,ILDR1,IQCJ-SCHIP1,ANKS1A,CHD7,RNF165,DISC1,TPRG1L,SH3BP1,MECP2,MTMR2,SEMA4D,GSK3B,LGR5,SLC8A1,MAGI3,PRKD1,ERCC6,OGT,TGFB2,CLSTN2,VAV3,SEMA5A,BDNF,LLGL2,PRKAR1B,PRKCH,RBMS3,RYK,ZDHHC17,WASF1,GRIN2B,KDM4C,OTUD7A,SIK2,ANKRD6,ARHGAP8,PRR5,SNAP25,PML,ARHGAP12,AKAP6,FBN1,GRIK3,LRFN2,TAOK3,BCL2,RALBP1,ZNF675,ARHGAP24,EPHB1,SIPA1L1,LRP2,NSF,TMEM100,LRRC4,DEPTOR,NR4A2,ARHGEF10,EPHA7,PSD3,FYN,CHN1,SMARCA4,CD44,HECW1,NEU3,RNF213,FOXO1,PDE4D,KANK1,ARHGEF3,ATF6,DCDC2,GRK5,MME,TNMD,CSNK1G3,GRIK2,IGSF11,CELF4,SLC1A1,UGCG,RFX4,GNG4,NLRP12,MTOR,ROR1,CTDSPL2,DDX21,BMPER,PTPRD,TIAM2,PLCL1,ARL6IP5,CTNNBIP1,ZMYND11,NLGN4X,PRKCE,HELLS,PRKCB,ADCY8,EFHB,FAM19A4,FOXO3,LRRTM1,CAPN3,KALRN,PJA1,TTLL12,CCDC88C,DLC1,OASL,ROBO1,FLT1,NOX4,COL2A1,MAP3K7,MAPK9,PREX2,TRAF3IP2,EYA4,ACSL5,PKHD1,STK3,TGM2,KDR,PRCP,ARHGAP15,CASK,EDA,ABCA12,APP,DGKB,NRP1,UACA,ARHGEF18,LGR4,LINC00473,ADAM17,MAP3K5,TSPEAR,GRK6,PEX5L,PIK3R5,ROS1,TGFB1I1,DOT1L,RGS16,TCF7L2,TNFRSF10B,TRABD2B,BICD1,RALGAPA2,TRAF3,ABAT,GRIN2A,RIMS3,OPHN1,BCLAF1,SGMS1,CCDC88A,CD38,MOB3B,RNF43,TUB,CHRDL1,RNF126,STK36,BCL2L1,GUCY2F,PDE4B,GHR,MAPRE2,NTRK2,ACSL4,BRD4,RASGRP1,STARD13,ARR3,LY86,RABGEF1,SPRED2,GLI3,KIF16B,STXBP5,RAP1A,SAFB2,TGFBR1,MGLL,EPHA5,TNR,NCOA5,CHST11,PTTG1IP,RAPGEF1,CHRNA3,CHRNA5,EP300,*

			WIF1,CYP7B1,GRAMD4,GLRA1,TP73,GRID2,IFNGR2,PIBF1,RASGRF2,PSMA1,ADORA2A,ADRBK2,EFNA5,RALGPS1,CACNG2,MAGI2,NF1,RALGPS2,RGS7,SORL1,STRN3,ARHGAP6,BICC1,BID,IFT80,TRIM59,DMRT1,LZTR1,ASXL1,EIF4E,POR,RELN,GRM5,IL18R1,MGAT5,NDRG2,MAP3K13,NLK,NPHP4,ADCYAP1R1,FBXL17,JAG1,PPP3CA,JRK,KLF7,SNX25,DGKI,PER1,SLC24A2,WWTR1,KL,CBLB,ELAVL4,PLSCR1,DOK6,GPC4,GRM7,RIMS1,TRIM16,WDR59,NLGN1,PTPRU,ARHGAP5,RPS20,AKAP7,LRRK1,MAPK8IP2,VEPH1,DDX3X,NSG1,CNOT7,ZNF622,ARHGAP39,JAK2,LMO7,SHOX2,SMAD3,BAIAP2L1,CD300A,PLXNB1,AATF,HDAC7,MIR320B2,SLC39A10,TNF,OVOL2,SNX5,TNIP1,EIF3A,POMC,PRDM15,BMP15,DLG5,AFAP1,LGR6,SHC2,ARF1,AGO3,ANO1,GCLC,PMEPA1,UBE2V1,EPS8,LTBP1,DAAM2,SMOC2,SULF1,C5AR1,DEF6,LRP4,CNTN6,NET1,ZNF423,CLSTN1,SELP,ALPK2,CTNNA1,TENM1,TSHZ3,ARHGEF11,GRIK4,GSKIP,TNFRSF19,ARHGAP11B,DYNC2H1,MLLT3,MYRIP,SIPA1L2,CD109,EGF,KIT,SLIT3,UBR2,TRPV4,VWC2,EDAR,PTPRO,RAPGEF2,ESR1,HTR2C,SLC30A8,UBE3A,DNMBP,GRM4,MARVELD3,EPHB2,MECOM,NDFIP2,DAPK1,ERBB4,FER,MAP3K3,FNIP1,LRP5,SLAMF1,ABL2,DEPDC5,F11R,GAS8,ARHGAP35,CD2AP,CCL3,PREX1,SPRED3,VWF,ARHGAP25,FGD4,IL20RA,SULF2,PACSIN2,SOX4,GARNL3,P2RX1,PDGFD,PIK3CD,UBE2N,ITGA8,PUM1,IRAK2,PLAT,TNKS,UNC13A,AUTS2,FGFR1,PDE11A,RGS3,UNC13B,SOST,IL23R,NTF3,P2RX7,SCUBE1,RASA3,AMFR,APIP,AR,SUFU,EYA1,IFNA8,BDKRB2,ADRA1B,ARMC9,DGKZ,MIR433,PRKAA2,SEMA3A,TLE4,ARHGAP10,CDC73,PDE5A,ZMIZ1,FLT3,PTPRA,SRGAP3,TRIM72,FERMT2,LTBR,MAP2K5,TNN,DAB2,GPR55,STX3,ACTN4,ADAM10,CCL7,EEF1E1,EEF1E1-BLOC1S5,FGF12,ITGA3,KMO,LGI1,NREP,PLCL2,PPM1B,PIN1,RPS6KA6,TRPM4,CD226,PIP5K1B,STK38,BMP6,BTBD9,CIT,TGFA,GAS2,HGF,RALGAPA1,RGS12,CALCR,SCUBE3,CACNB2,CNKSR2,NCAM1,NOS1AP,ARHGAP28,INSR,MACF1,PAK1,PDE3A,PSMB7,SKI,AKT3,ELF1,ENPP1,FMR1,LITAF,RRAGC,ULK4,EVC,MAD1L1,MFNG,ARHGAP42,JPH4,PPARA,TNFAIP8L3,CORIN,FAM3D,GNA13,TAF1,CACNA1A,CHD5,SPRED1,AKR1C2,CHSY1,ESR2,PRDM16,ZNF451,BTRC,CCDC3,OPTN,PTPRR,RIT2,IFT81,NCOA1,PRKCG,PRLR,TYMP,FBXW11,G3BP1,TDGF1,PRKCQ,SCEL,STAT3,TP63,USP7,AMOT,DOCK3,PAK3,PIK3R2,TBL1X,DTNBP1,RAP1B,DLGAP2,KSR1,LGALS9,LRP8,NFAT5,SNCAIP,ALPK1,IL10RB,APC,BMPR1A,ITGA1,KIAA0319,NGF,PRKCA,UBASH3B,ADAMTS20,MTMR4,CTSH,NCAPG2,RPH3AL,USP18,CCL14,CCL15,EDA2R,GPR21,IL6R,MAP2K6,RIC8B,MAP2K1,SOS1,VPS35,C1QTNF3,FGF1,TBX20,CRADD,NLRC5,PPP1R9A,RGN,TBX18
GO:0048858	cell projection morphogenesis	8.707774375584276e-16	CDH4,DAB1,DSCAM,ANK3,DCC,EPHA6,NRXN1,NRXN3,CTNND2,CHODL,RIMS2,FSTL4,LRRC4C,TMEM108,ADAMTSL1,SYT17,PLXNA4,FLRT2,UNC5D,SEMA6D,CTNNA2,RBFOX2,TIAM1,DPYSL2,FBXO31,CNTN4,PHACTR1,S100B,ROBO2,EPHA3,DCLK1,CDH11,CDH2,PARK2,LAMA2,SEMA3D,NTN1,TRIO,MYO9A,SLIT2,IGF1R,TRPC5,DMD,SYT1,VCL,NTNG1,LRRK2,RNF165,DISC1,EMB,SEMA4D,GSK3B,SEMA5A,BDNF,RYK,ZDHHC17,WASF1,LAMA3,CNTNAP2,PLXNA2,TAOK3,BCL2,EPHB1,SIPA1L1,LRP2,NR4A2,EPHA7,FYN,CHN1,SULT4A1,CD44,HECW1,KANK1,ENPP2,ATP8A2,DCDC2,UNC5C,PALLD,GAS7,MOV10,PTPRD,TIAM2,KALRN,ROBO1,TANC2,PREX2,CHL1,APP,NRP1,PTPRM,SEMA3C,PRTG,RPS6KA5,PARD3,GRIP1,OPHN1,ALCAM,MYO16,NTRK2,DNM3,GLI3,DICER1,EPHA5,TNR,CUX1,CHRNA3,DSCAML1,SPG11,ADORA2A,EFNA5,TTC8,RELN,KIRREL3,NUMB,MAP3K13,PPP3CA,KLF7,SH3GL2,DOCK10,ELAVL4,RIMS1,NLGN1,MAPK8IP2,SHOX2,KIDINS220,PLXNB1,COBL,PPFIA2,LGR6,BCL11A,LRP4,CNTN6,EFNB2,CDKL5,IL1RAPL1,SLIT3,EFNB1,PTPRO,RAPGEF2,MYPN,SEMA3E,UBE3A,FGF13,EPHB2,SLITRK6,BSG,ARHGAP35,LAMA1,SEMA5B,PACSIN2,UNC13A,AUTS2,MEF2A,MAP2,NTF3,BHLHB9,SEMA3A,EPB41L3,MARK2,PDLIM5,TNN,SYT2,LGI1,NCAM1,NFASC,MACF1,PAK1,CRMP1,CCDC141,EXT1,DOCK7,ADARB1,PRKCQ,ISLR2,PAK3,DTNBP1,LRP8,ITGA1,KIAA0319,NGF,VLDLR,ABI1,SPTBN4,CLASP2,MAP2K1,SOS1,RREB1
GO:0000904	cell morphogenesis involved in differentiation	9.743923830088466e-16	CDH4,DAB1,DSCAM,ANK3,DCC,FBLN1,EPHA6,NRXN1,NRXN3,CTNND2,CHODL,FSTL4,LRRC4C,ADAMTSL1,PLXNA4,FLRT2,UNC5D,SEMA6D,CTNNA2,RBFOX2,TIAM1,COL22A1,DPYSL2,FBXO31,CNTN4,PHACTR1,S100B,ROBO2,EPHA3,DCLK1,COL15A1,CDH11,CDH2,LAMA2,SEMA3D,NTN1,TRIO,SLIT2,IGF1R,TRPC5,SIPA1L3,VCL,NTNG1,LRRK2,SLC1A3,STRC,RNF165,DISC1,EMB,SEMA4D,GSK3B,FRMD6,SEMA5A,BDNF,RYK,ZDHHC17,PCDH15,LAMA3,PLXNA2,BCL2,EPHB1,SIPA1L1,NR4A2,EPHA7,FYN,CHN1,SULT4A1,HECW1,KANK1,ATP8A2,DCDC2,TNMD,LAMB4,UNC5C,PALLD,FAT3,MEGF9,PTPRD,TIAM2,KALRN,CCDC88C,ROBO1,TANC2,GRHL2,PREX2,SEC24B,PKHD1,CHL1,APP,COL18A1,NRP1,PTPRM,SEMA3C,PARVG,PRTG,RPS6

			KA5,PARD3,ATRNL1,OPHN1,ALCAM,NTRK2,DNM3,GLI3,EPHA5,TNR,CUX1,PEAK1,CHRNA3,EP300,DSCAML1,TRIOBP,PARVB,FLNB,SPG11,EFNA5,MERTK,TTC8,RELN,NUMB,MAP3K13,PPP3CA,KLF7,PLS1,DOCK10,ELAVL4,NLGN1,MAPK8IP2,SHOX2,KIDINS220,PLXNB1,COBL,ZFPM1,PPFIA2,LGR6,BCL11A,DOCK1,LRP4,CNTN6,EFNB2,CDKL5,IL1RAPL1,SLIT3,EFNB1,PTPRO,RAPGEF2,MYPN,SEMA3E,UBE3A,FGF13,EPHB2,TBCD,FER,SLITRK6,BSG,ARHGAP35,LAMA1,PREX1,SEMA5B,MYH9,NEDD9,ITGA8,PDZD7,WDPCP,AUTS2,CDH23,MEF2A,MAP2,ITGB7,AR,BHLHB9,SEMA3A,MARK2,PDLIM5,FERMT2,TNN,DAB2,ACTN4,LGI1,NCAM1,NFASC,MACF1,PAK1,CRMP1,CCDC141,NTN4,EXT1,DOCK7,ADARB1,PRKCQ,ISLR2,PAK3,USH2A,DTNBP1,LRP8,KIAA0319,NGF,SPINT2,VLDLR,ABI1,SPTBN4,CLASP2,MAP2K1,SOS1,RREB1
GO:0120039	plasma membrane bounded cell projection morphogenesis	9.767140155615373e-16	CDH4,DAB1,DSCAM,ANK3,DCC,EPHA6,NRXN1,NRXN3,CTNND2,CHODL,RIMS2,FSTL4,LRRC4C,TMEM108,ADAMTSL1,SYT17,PLXNA4,FLRT2,UNC5D,SEMA6D,CTNNA2,RBFOX2,TIAM1,DPYSL2,FBXO31,CNTN4,PHACTR1,S100B,ROBO2,EPHA3,DCLK1,CDH11,CDH2,PARK2,LAMA2,SEMA3D,NTN1,TRIO,MYO9A,SLIT2,IGF1R,TRPC5,DMD,SYT1,VCL,NTNG1,LRRK2,RNF165,DISC1,EMB,SEMA4D,GSK3B,SEMA5A,BDNF,RYK,ZDHHC17,WASF1,LAMA3,CNTNAP2,PLXNA2,TAOK3,BCL2,EPHB1,SIPA1L1,LRP2,NR4A2,EPHA7,FYN,CHN1,SULT4A1,CD44,HECW1,KANK1,ENPP2,ATP8A2,DCDC2,UNC5C,PALLD,GAS7,MOV10,PTPRD,TIAM2,KALRN,ROBO1,TANC2,PREX2,CHL1,APP,NRP1,PTPRM,SEMA3C,PRTG,RPS6KA5,PARD3,GRIP1,OPHN1,ALCAM,MYO16,NTRK2,DNM3,GLI3,DICER1,EPHA5,TNR,CUX1,CHRNA3,DSCAML1,SPG11,ADORA2A,EFNA5,TTC8,RELN,KIRREL3,NUMB,MAP3K13,PPP3CA,KLF7,SH3GL2,DOCK10,ELAVL4,RIMS1,NLGN1,MAPK8IP2,SHOX2,KIDINS220,PLXNB1,COBL,PPFIA2,LGR6,BCL11A,LRP4,CNTN6,EFNB2,CDKL5,IL1RAPL1,SLIT3,EFNB1,PTPRO,RAPGEF2,MYPN,SEMA3E,UBE3A,FGF13,EPHB2,SLITRK6,BSG,ARHGAP35,LAMA1,SEMA5B,UNC13A,AUTS2,MEF2A,MAP2,NTF3,BHLHB9,SEMA3A,EPB41L3,MARK2,PDLIM5,TNN,SYT2,LGI1,NCAM1,NFASC,MACF1,PAK1,CRMP1,CCDC141,EXT1,DOCK7,ADARB1,PRKCQ,ISLR2,PAK3,DTNBP1,LRP8,ITGA1,KIAA0319,NGF,VLDLR,ABI1,SPTBN4,CLASP2,MAP2K1,SOS1,RREB1
GO:0007268	chemical synaptic transmission	9.93949904093769e-16	PLCB1,GABRB3,GABRG3,ASIC2,CTBP2,DTNA,CACNA1E,DCC,SYN3,GRIK1,GABBR2,GRIN3A,NRXN1,NRXN3,SLC1A2,RAB5A,RIMS2,SHANK2,GRIA1,NRG3,DLG2,LRRC4C,TMEM108,SHISA6,MCTP1,RASGRF1,GRID1,SLC6A2,SORCS3,PLCB4,ERC1,CNTN4,S100B,PTPRN2,CNTNAP4,AGT,ERC2,SHISA9,CHRM5,CDH11,CDH2,PARK2,DLGAP1,LAMA2,MCTP2,DBH,PCDH17,APBA2,CDH8,RPS6KA2,SYT9,GABRG1,PXK,SYT1,CACNG3,GRM1,SORCS2,NTNG1,UNC13C,LRRK2,SLC1A3,RIMBP2,DISC1,TPRG1L,MECP2,MTMR2,GSK3B,CLSTN2,BDNF,PRKAR1B,GRIN2B,SNAP25,GRIK3,LRFN2,SNAP23,EPHB1,SIPA1L1,LRRC4,FYN,NR4A1,PMP22,GABRB1,MME,EXOC4,GRIK2,IGSF11,CELF4,KCND2,SLC1A1,CADPS,CHRM1,PTPRD,PLCL1,NLGN4X,PRKCE,PRKCB,ADCY8,LRRTM1,KCNC4,GABRA2,CASK,APP,DGKB,ABAT,GRIN2A,RIMS3,OPHN1,CD38,GLRA2,NTRK2,STXBP5,RAP1A,TNR,CHRNA3,CHRNA5,GLRA1,GRID2,RASGRF2,SV2B,SPG11,ADORA2A,CACNG2,NF1,CEP89,HTR4,EIF4E,RELN,GRM5,PPP3CA,GPR176,DGKI,SLC24A2,ELAVL4,GRM7,RIMS1,GABRA3,NLGN1,P2RX6,AKAP7,MAPK8IP2,NSG1,GABRA5,JAK2,MIR320B2,TNF,BLOC1S6,FCHSD2,ARF1,CLSTN1,TSHZ3,GRIK4,GABRR2,KIT,RAPGEF2,HTR2C,GABRB2,GRM4,EPHB2,SV2C,PACSIN2,P2RX1,PLAT,UNC13A,UNC13B,GLRA3,NTF3,P2RX7,DGKZ,MIR433,GRIA2,PTPRA,STX3,SYT2,FGF12,KMO,LGI1,PLCL2,BTBD9,CHRM3,GAD2,CACNB2,SNAP29,FMR1,GABRA1,AMPH,GABRR3,JPH4,CACNA1A,RIT2,EXT1,PRKCG,ADARB1,DTNBP1,RAP1B,DLGAP2,LRP8,SNCAIP,NGF,GABRG2,PDE7B,PPP1R9A
GO:0098916	anterograde trans-synaptic signaling	9.93949904093769e-16	PLCB1,GABRB3,GABRG3,ASIC2,CTBP2,DTNA,CACNA1E,DCC,SYN3,GRIK1,GABBR2,GRIN3A,NRXN1,NRXN3,SLC1A2,RAB5A,RIMS2,SHANK2,GRIA1,NRG3,DLG2,LRRC4C,TMEM108,SHISA6,MCTP1,RASGRF1,GRID1,SLC6A2,SORCS3,PLCB4,ERC1,CNTN4,S100B,PTPRN2,CNTNAP4,AGT,ERC2,SHISA9,CHRM5,CDH11,CDH2,PARK2,DLGAP1,LAMA2,MCTP2,DBH,PCDH17,APBA2,CDH8,RPS6KA2,SYT9,GABRG1,PXK,SYT1,CACNG3,GRM1,SORCS2,NTNG1,UNC13C,LRRK2,SLC1A3,RIMBP2,DISC1,TPRG1L,MECP2,MTMR2,GSK3B,CLSTN2,BDNF,PRKAR1B,GRIN2B,SNAP25,GRIK3,LRFN2,SNAP23,EPHB1,SIPA1L1,LRRC4,FYN,NR4A1,PMP22,GABRB1,MME,EXOC4,GRIK2,IGSF11,CELF4,KCND2,SLC1A1,CADPS,CHRM1,PTPRD,PLCL1,NLGN4X,PRKCE,PRKCB,ADCY8,LRRTM1,KCNC4,GABRA2,CASK,APP,DGKB,ABAT,GRIN2A,RIMS3,OPHN1,CD38,GLRA2,NTRK2,STXBP5,RAP1A,TNR,CHRNA3,CHRNA5,GLRA1,GRID2,RASGRF2,SV2B,SPG11,ADORA2A,CACNG2,NF1,CEP89,HTR4,EIF4E,RELN,GRM5,PPP3CA,GPR176,DGKI,SLC24

			A2,ELAVL4,GRM7,RIMS1,GABRA3,NLGN1,P2RX6,AKAP7,MAPK8IP2,NSG1,GABRA5,JAK2,MIR320B2,TNF,BLOC1S6,FCHSD2,ARF1,CLSTN1,TSHZ3,GRIK4,GABRR2,KIT,RAPGEF2,HTR2C,GABRB2,GRM4,EPHB2,SV2C,PACSIN2,P2RX1,PLAT,UNC13A,UNC13B,GLRA3,NTF3,P2RX7,DGKZ,MIR433,GRIA2,PTPRA,STX3,SYT2,FGF12,KMO,LGI1,PLCL2,BTBD9,CHRM3,GAD2,CACNB2,SNAP29,FMR1,GABRA1,AMPH,GABRR3,JPH4,CACNA1A,RIT2,EXT1,PRKCG,ADARB1,DTNBP1,RAP1B,DLGAP2,LRP8,SNCAIP,NGF,GABRG2,PDE7B,PPP1R9A
GO:0032989	cellular component morphogenesis	1.5171255874317009e-15	CDH4,DAB1,DSCAM,ANK3,DCC,AKAP13,NUBPL,EPHA6,NRXN1,NRXN3,CTNND2,CHODL,RIMS2,FSTL4,ANK2,LRRC4C,TMEM108,ADAMTSL1,SYT17,PLXNA4,FLRT2,UNC5D,SEMA6D,CTNNA2,RBFOX2,TIAM1,DPYSL2,FBXO31,CNTN4,PHACTR1,S100B,ROBO2,EPHA3,DCLK1,CDH11,CDH2,PARK2,LAMA2,SEMA3D,NTN1,TRIO,MYO9A,SLIT2,IGF1R,TRPC5,DMD,SYT1,VCL,NTNG1,TENM4,LRRK2,RNF165,DISC1,EMB,MTMR2,SEMA4D,GSK3B,SEMA5A,BDNF,RYK,ZDHHC17,WASF1,LAMA3,CNTNAP2,PLXNA2,TAOK3,BCL2,EPHB1,SIPA1L1,LRP2,NR4A2,EPHA7,FYN,CHN1,SULT4A1,CD44,HECW1,PMP22,KANK1,ENPP2,ACTN2,ATP8A2,DCDC2,UNC5C,MYLK3,PALLD,GAS7,MOV10,PTPRD,TIAM2,CAPN3,KALRN,ROBO1,TANC2,NEBL,PREX2,CHL1,APP,NRP1,PTPRM,SEMA3C,PRTG,RPS6KA5,PARD3,GRIP1,OPHN1,ALCAM,BCL2L1,MYO16,NTRK2,DNM3,GLI3,DICER1,EPHA5,TNR,CUX1,CHRNA3,DSCAML1,SPG11,ADORA2A,EFNA5,TTC8,RELN,KIRREL3,NUMB,MAP3K13,PPP3CA,KLF7,NEB,SH3GL2,DOCK10,ELAVL4,RIMS1,FHOD3,NLGN1,MAPK8IP2,SHOX2,KIDINS220,PLXNB1,COBL,PPFIA2,LGR6,BCL11A,LRP4,CNTN6,EFNB2,CDKL5,MYH14,PRKAR1A,IL1RAPL1,SLIT3,EFNB1,PTPRO,RAPGEF2,MYPN,NCMAP,SEMA3E,UBE3A,FGF13,EPHB2,SLITRK6,BSG,ARHGAP35,LAMA1,SEMA5B,PACSIN2,UNC13A,AUTS2,MEF2A,MAP2,NTF3,BHLHB9,SEMA3A,EPB41L3,MARK2,PDLIM5,TNN,SYT2,LGI1,CLASP1,TMOD1,NCAM1,NFASC,MACF1,PAK1,CRMP1,CCDC141,TTN,PGM5,EXT1,MYH6,DOCK7,ADARB1,MYH11,PRKCQ,ISLR2,PAK3,DTNBP1,LRP8,MYOM2,ITGA1,KIAA0319,NGF,VLDLR,ABI1,SPTBN4,CLASP2,MAP2K1,SOS1,RREB1
GO:0048812	neuron projection morphogenesis	1.856412644432481e-15	CDH4,DAB1,DSCAM,ANK3,DCC,EPHA6,NRXN1,NRXN3,CTNND2,CHODL,RIMS2,FSTL4,LRRC4C,TMEM108,ADAMTSL1,SYT17,PLXNA4,FLRT2,UNC5D,SEMA6D,CTNNA2,RBFOX2,TIAM1,DPYSL2,FBXO31,CNTN4,PHACTR1,S100B,ROBO2,EPHA3,DCLK1,CDH11,CDH2,PARK2,LAMA2,SEMA3D,NTN1,TRIO,MYO9A,SLIT2,IGF1R,TRPC5,DMD,SYT1,VCL,NTNG1,LRRK2,RNF165,DISC1,EMB,SEMA4D,GSK3B,SEMA5A,BDNF,RYK,ZDHHC17,WASF1,LAMA3,CNTNAP2,PLXNA2,TAOK3,BCL2,EPHB1,SIPA1L1,LRP2,NR4A2,EPHA7,FYN,CHN1,SULT4A1,HECW1,ATP8A2,DCDC2,UNC5C,PALLD,GAS7,MOV10,PTPRD,TIAM2,KALRN,ROBO1,TANC2,PREX2,CHL1,APP,NRP1,PTPRM,SEMA3C,PRTG,RPS6KA5,PARD3,GRIP1,OPHN1,ALCAM,MYO16,NTRK2,DNM3,GLI3,DICER1,EPHA5,TNR,CUX1,CHRNA3,DSCAML1,SPG11,ADORA2A,EFNA5,TTC8,RELN,KIRREL3,NUMB,MAP3K13,PPP3CA,KLF7,SH3GL2,DOCK10,ELAVL4,RIMS1,NLGN1,MAPK8IP2,SHOX2,KIDINS220,PLXNB1,COBL,PPFIA2,LGR6,BCL11A,LRP4,CNTN6,EFNB2,CDKL5,IL1RAPL1,SLIT3,EFNB1,PTPRO,RAPGEF2,MYPN,SEMA3E,UBE3A,FGF13,EPHB2,SLITRK6,BSG,ARHGAP35,LAMA1,SEMA5B,UNC13A,AUTS2,MEF2A,MAP2,NTF3,BHLHB9,SEMA3A,EPB41L3,MARK2,PDLIM5,TNN,SYT2,LGI1,NCAM1,NFASC,MACF1,PAK1,CRMP1,CCDC141,EXT1,DOCK7,ADARB1,PRKCQ,ISLR2,PAK3,DTNBP1,LRP8,ITGA1,KIAA0319,NGF,VLDLR,ABI1,SPTBN4,CLASP2,MAP2K1,SOS1
GO:0035556	intracellular signal transduction	1.868159150628115e-15	ADCY2,TPTE,PLCB1,CMKLR1,DAB1,CDC42EP3,MAPK14,TPTE2,SH3RF3,HUNK,WLS,TIMP3,DUSP22,AKAP13,FBLN1,RGS6,TMEM117,VRK2,NRXN1,SHC3,DOK5,DOCK2,SHANK2,NRG3,SMYD2,CLEC16A,HUS1,TGFBR3,CDH13,RNF152,ANK2,TF,PIK3C2B,CHEK2,GADD45A,NRG1,PSMB2,RFFL,MCTP1,PLCE1,SH3RF2,KSR2,STK38L,CCR1,CCR3,TIAM1,RHOC,RASGRF1,SCAI,ALK,PIK3C3,PLCB4,ERC1,FBXO31,RGL2,S100B,PRKACB,DCLK1,CRNN,LEMD3,AGT,RHOJ,RCAN1,CDH2,PARK2,FHL2,FOXN3,CDC5L,MCTP2,NTN1,TRIM22,TRIM5,KCNH1,ITSN1,TNS3,TRIO,MYO9A,SLIT2,PDCD6,RPS6KA2,LCMT1,IGF1R,MDM2,SLC24A4,SMAD1,RAPGEF5,NR3C2,PDE9A,DMD,STK33,SIPA1L3,TEAD1,CELSR1,FGF14,FSHR,GRM1,FMN2,MAEL,PRKAR2A,INPP5A,STK24,DCDC1,WDR83,WWOX,ATRX,TRPM2,RYR2,UNC13C,CAMTA1,LRRK2,MAPK10,NTRK3,RORA,IQCJ-SCHIP1,DISC1,KCTD9,SH3BP1,SEMA4D,GSK3B,SLC8A1,MAGI3,PLCH2,PRKD1,SNRK,ERCC6,OGT,TGFB2,VAV3,PLCD3,SEMA5A,PRKCH,RYK,ZDHHC17,SGCD,WASF1,GRIN2B,OTUD7A,SIK2,ANKRD6,ARHGAP8,PRR5,PTGFR,NR5A2,PML,ARHGAP12,CORO2A,AKAP6,TNS1,TAOK3,BCL2,CHMP4C,RALBP1,ZNF675,ARHGAP24,EPHB1,SIPA1L1,LRP2,TMEM100,ITPR2,DEPTOR,ARHGEF10,EPHA7,PSD3,FYN,CHN1,CD44,FOXO1,PDE4D,KANK1,ARHGEF3,DCDC2,GRIK2,APBB2,R

			ERG,NLRP12,GRAP2,MTOR,ROR1,DDX21,BMPER,RASGEF1B,TIAM2,PLCL1,ARL6IP5,PLN,ZMYND11,PRKCE,HELLS,PRKCB,ADCY8,EFHB,FOXO3,CAPN3,KALRN,PJA1,CCDC88C,DLC1,EDNRB,OASL,ROBO1,FLT1,NOX4,MAP3K7,MAPK9,PREX2,TRAF3IP2,PKHD1,STK3,TGM2,KDR,RAB30,ARHGAP15,EDA,APP,DGKB,NRP1,UACA,ARHGEF18,ELMO1,LINC00473,ADTRP,KAT5,MAP3K5,CDC42BPG,ATF2,PEX5L,PIK3R5,ROS1,DOT1L,KPNB1,RPS6KA5,TCF7L2,TNFRSF10B,RALGAPA2,TLK2,TRAF3,ZNF830,DOCK9,GRIN2A,GRIP1,SGK2,OPHN1,BCLAF1,SGMS1,CCDC88A,MOB3B,RIN2,STK36,BCL2L1,GUCY2F,HDAC4,MYO16,DNMT1,GHR,MAPRE2,NTRK2,PRKG1,CDC42BPA,BRD4,RASGRP1,STARD13,CAMK4,RABGEF1,RGL1,SPRED2,ZNF207,CACNA1C,DOCK4,RAP1A,TGFBR1,PRDX4,DICER1,EPHA5,LCP2,PTTG1IP,RAPGEF1,EP300,GAB2,TP73,RASGRF2,PSMA1,FHIT,MERTK,RALGPS1,GUCY2C,MAGI2,NF1,RALGPS2,RGS7,SORL1,ARHGAP6,ASB13,BID,TEAD4,TRIM59,DMRT1,LZTR1,RELN,GRM5,IL18R1,SELE,NDRG2,MAP3K13,NLK,RALA,ADCYAP1R1,PPP3CA,DGKI,MAS1,PER1,WWTR1,KL,CBLB,DOCK10,DOK6,WDR59,GPSM2,KAT7,MAPK4,NLGN1,ARHGAP5,RPS20,NPR3,AKAP7,LRRK1,MAPK8IP2,DDX3X,PDE7A,TPCN2,ZNF622,ARHGAP39,JAK2,SMAD3,CD300A,MAP2K4,NOS1,PLXNB1,CDK1,HDAC7,NBN,TNF,TNIP1,EIF3A,POMC,PRDM15,DLG5,NUDT4,SHC2,PLCH1,STK32B,HMGA2,MYO6,AGO3,DOCK1,MUC5AC,RUFY1,STK32C,UBE2V1,EPS8,C5AR1,DEF6,PIK3R3,ARHGEF6,AMOTL1,NET1,CDC14B,SELP,CUL2,TENM1,ARHGEF11,GSKIP,PRKAR1A,TNFRSF19,ARHGAP11B,SIPA1L2,EGF,KIT,UBR2,BLM,CTNNAL1,PIK3C2G,TRPV4,EDAR,RAPGEF2,STK32A,ESR1,HTR2C,JAK1,UBE3A,DNMBP,FGF13,GRM4,MARVELD3,EPHB2,MECOM,NDFIP2,DAPK1,ERBB4,FER,MAP3K3,FNIP1,RAPGEF6,RPS27L,SLAMF1,ABL2,DEPDC5,F11R,FARP2,ARHGAP35,CD2AP,NSUN2,CCL3,PREX1,SPRED3,VWF,ARHGAP25,FGD4,IL20RA,SOX4,CAB39L,GARNL3,PDGFD,PIK3CD,UBE2N,PUM1,IRAK2,UNC13A,ADCY9,AUTS2,FGFR1,MEF2A,PDE11A,UNC13B,JMY,NTF3,P2RX7,RAPGEF4,TEX14,RASA3,RCAN2,ZDHHC9,APIP,AR,SRPK2,BDKRB2,ADRA1B,DGKZ,PRKAA2,SEMA3A,ARHGAP10,MARK2,PDE5A,FLT3,SRGAP3,FERMT2,LTBR,MAP2K5,MARK1,SMPD3,DAB2,GPR55,LCP1,ACTN4,CCL7,EEF1E1,EEF1E1-BLOC1S5,FGF12,ITGA3,PLCL2,PPM1B,QRICH1,STAC,MYOM1,PIN1,RPS6KA6,TRPM4,PIP5K1B,STK38,CHRM3,CIT,TGFA,G3BP2,HGF,RALGAPA1,CALCR,CENPF,SH3BP5,TLK1,CNKSR2,MAST4,NOS1AP,PI4KA,RALGDS,ARHGAP28,INSR,PAK1,PDE3A,PSMB7,AKT3,CHM,LITAF,THEM4,CHFR,RRAGC,ULK4,MAD1L1,ARHGAP42,PPARA,TNFAIP8L3,TTN,GNA13,PPP1R13B,TAF1,CHD5,FNIP2,SPRED1,AKR1C2,BTRC,OPTN,PTPRR,RIT2,MDM4,PRKCG,DOCK7,FBXW11,G3BP1,ICK,TDGF1,TREM1,KCNC2,PRKCQ,TP63,USP7,AMOT,BRIP1,DOCK3,PAK3,PIK3R2,STK17B,BLNK,CASQ2,RAP1B,KSR1,LGALS9,NFAT5,ALPK1,MAPKAPK2,APC,ITGA1,NGF,PRKCA,ASB3,CTSH,CCL14,CCL15,EDA2R,IL6R,MAP2K6,NCALD,MAP2K1,PDE7B,SOS1,C1QTNF3,FGF1,CRADD,DGKK,NLRC5,PPP1R9A,RGN,RREB1
GO:0032990	cell part morphogenesis	2.187057877545273e-15	*CDH4,DAB1,DSCAM,ANK3,DCC,NUBPL,EPHA6,NRXN1,NRXN3,CTNND2,CHODL,RIMS2,FSTL4,LRRC4C,TMEM108,ADAMTSL1,SYT17,PLXNA4,FLRT2,UNC5D,SEMA6D,CTNNA2,RBFOX2,TIAM1,DPYSL2,FBXO31,CNTN4,PHACTR1,S100B,ROBO2,EPHA3,DCLK1,CDH11,CDH2,PARK2,LAMA2,SEMA3D,NTN1,TRIO,MYO9A,SLIT2,IGF1R,TRPC5,DMD,SYT1,VCL,NTNG1,LRRK2,RNF165,DISC1,EMB,SEMA4D,GSK3B,SEMA5A,BDNF,RYK,ZDHHC17,WASF1,LAMA3,CNTNAP2,PLXNA2,TAOK3,BCL2,EPHB1,SIPA1L1,LRP2,NR4A2,EPHA7,FYN,CHN1,SULT4A1,CD44,HECW1,KANK1,ENPP2,ATP8A2,DCDC2,UNC5C,PALLD,GAS7,MOV10,PTPRD,TIAM2,KALRN,ROBO1,TANC2,PREX2,CHL1,APP,NRP1,PTPRM,SEMA3C,PRTG,RPS6KA5,PARD3,GRIP1,OPHN1,ALCAM,BCL2L1,MYO16,NTRK2,DNM3,GLI3,DICER1,EPHA5,TNR,CUX1,CHRNA3,DSCAML1,SPG11,ADORA2A,EFNA5,TTC8,RELN,KIRREL3,NUMB,MAP3K13,PPP3CA,KLF7,SH3GL2,DOCK10,ELAVL4,RIMS1,NLGN1,MAPK8IP2,SHOX2,KIDINS220,PLXNB1,COBL,PPFIA2,LGR6,BCL11A,LRP4,CNTN6,EFNB2,CDKL5,MYH14,IL1RAPL1,SLIT3,EFNB1,PTPRO,RAPGEF2,MYPN,SEMA3E,UBE3A,FGF13,EPHB2,SLITRK6,BSG,ARHGAP35,LAMA1,SEMA5B,PACSIN2,UNC13A,AUTS2,MEF2A,MAP2,NTF3,BHLHB9,SEMA3A,EPB41L3,MARK2,PDLIM5,TNN,SYT2,LGI1,NCAM1,NFASC,MACF1,PAK1,CRMP1,CCDC141,EXT1,DOCK7,ADARB1,PRKCQ,ISLR2,PAK3,DTNBP1,LRP8,ITGA1,KIAA0319,NGF,VLDLR,ABI1,SPTBN4,CLASP2,MAP2K1,SOS1,RREB1*
GO:0099536	synaptic signaling	4.120312652437524e-15	*PLCB1,GABRB3,GABRG3,ASIC2,CTBP2,DTNA,CACNA1E,DCC,SYN3,GRIK1,GABBR2,GRIN3A,NRXN1,NRXN3,SLC1A2,RAB5A,RIMS2,SHANK2,GRIA1,NRG3,DLG2,LRRC4C,TMEM108,NRG1,SHISA6,MCTP1,DRP2,RASGRF1,GRID1,SLC6A2,SORCS3,PLCB4,ERC1,CNTN4,S100B,*

			PTPRN2,CNTNAP4,AGT,ERC2,SHISA9,CHRM5,CDH11,CDH2,PARK2,DLGAP1,LAMA2,MCTP2,DBH,PCDH17,APBA2,CDH8,RPS6KA2,SYT9,GABRG1,PXK,SYT1,CACNG3,GRM1,SORCS2,NTNG1,UNC13C,LRRK2,SLC1A3,RIMBP2,DISC1,TPRG1L,MECP2,MTMR2,GSK3B,CLSTN2,BDNF,PRKAR1B,GRIN2B,SNAP25,GRIK3,LRFN2,SNAP23,EPHB1,SIPA1L1,LRRC4,FYN,NR4A1,PMP22,GABRB1,MME,EXOC4,GRIK2,IGSF11,CELF4,KCND2,SLC1A1,CADPS,CHRM1,PTPRD,PLCL1,NLGN4X,PRKCE,PRKCB,ADCY8,LRRTM1,KCNC4,GABRA2,CASK,APP,DGKB,ABAT,GRIN2A,RIMS3,OPHN1,CD38,GLRA2,NTRK2,STXBP5,RAP1A,TNR,CHRNA3,CHRNA5,GLRA1,GRID2,RASGRF2,SV2B,SPG11,ADORA2A,CACNG2,NF1,CEP89,HTR4,EIF4E,RELN,GRM5,IL1RAP,PPP3CA,GPR176,DGKI,SLC24A2,ELAVL4,GRM7,RIMS1,GABRA3,NLGN1,P2RX6,AKAP7,MAPK8IP2,NSG1,GABRA5,JAK2,NOS1,MIR320B2,TNF,BLOC1S6,FCHSD2,ARF1,CLSTN1,TSHZ3,GRIK4,GABRR2,IL1RAPL1,KIT,RAPGEF2,HTR2C,GABRB2,GRM4,EPHB2,SV2C,PACSIN2,P2RX1,PLAT,UNC13A,UNC13B,GLRA3,NTF3,P2RX7,DGKZ,MIR433,GRIA2,PTPRA,STX3,SYT2,FGF12,KMO,LGI1,PLCL2,BTBD9,CHRM3,GAD2,CACNB2,SNAP29,FMR1,GABRA1,AMPH,GABRR3,JPH4,CACNA1A,RIT2,EXT1,PRKCG,ADARB1,DTNBP1,RAP1B,DLGAP2,LRP8,SNCAIP,NGF,GABRG2,PDE7B,PPP1R9A
GO:0048667	cell morphogenesis involved in neuron differentiation	9.887159075524767e-15	*CDH4,DAB1,DSCAM,ANK3,DCC,EPHA6,NRXN1,NRXN3,CTNND2,CHODL,FSTL4,LRRC4C,ADAMTSL1,PLXNA4,FLRT2,UNC5D,SEMA6D,CTNNA2,RBFOX2,TIAM1,DPYSL2,FBXO31,CNTN4,PHACTR1,S100B,ROBO2,EPHA3,DCLK1,CDH11,CDH2,LAMA2,SEMA3D,NTN1,TRIO,SLIT2,IGF1R,TRPC5,VCL,NTNG1,LRRK2,SLC1A3,STRC,RNF165,DISC1,EMB,SEMA4D,GSK3B,SEMA5A,BDNF,RYK,ZDHHC17,PCDH15,LAMA3,PLXNA2,BCL2,EPHB1,SIPA1L1,NR4A2,EPHA7,FYN,CHN1,SULT4A1,HECW1,ATP8A2,DCDC2,UNC5C,PALLD,PTPRD,TIAM2,KALRN,ROBO1,TANC2,PREX2,SEC24B,CHL1,APP,NRP1,PTPRM,SEMA3C,PRTG,RPS6KA5,PARD3,OPHN1,ALCAM,NTRK2,DNM3,GLI3,EPHA5,TNR,CUX1,CHRNA3,DSCAML1,TRIOBP,SPG11,EFNA5,TTC8,RELN,NUMB,MAP3K13,PPP3CA,KLF7,PLS1,DOCK10,ELAVL4,NLGN1,MAPK8IP2,SHOX2,KIDINS220,PLXNB1,COBL,PPFIA2,LGR6,BCL11A,LRP4,CNTN6,EFNB2,CDKL5,IL1RAPL1,SLIT3,EFNB1,PTPRO,RAPGEF2,MYPN,SEMA3E,UBE3A,FGF13,EPHB2,TBCD,SLITRK6,BSG,ARHGAP35,LAMA1,SEMA5B,PDZD7,WDPCP,AUTS2,CDH23,MEF2A,MAP2,BHLHB9,SEMA3A,MARK2,PDLIM5,TNN,LGI1,NCAM1,NFASC,MACF1,PAK1,CRMP1,CCDC141,EXT1,DOCK7,ADARB1,PRKCQ,ISLR2,PAK3,DTNBP1,LRP8,KIAA0319,NGF,VLDLR,ABI1,SPTBN4,CLASP2,MAP2K1,SOS1*
GO:0050808	synapse organization	1.3540993275356493e-14	*CAST,GABRB3,MAPK14,DSCAM,GPHN,ASIC2,CTBP2,ANK3,NRXN1,NRXN3,SDK2,CTNND2,TNC,SHANK2,LRRC4C,SYNDIG1,BCAN,TMEM108,FLRT2,NRG1,SHISA6,LRFN5,DRP2,ABHD17C,CTNNA2,IL1RAPL2,ERC1,ROBO2,ERC2,CDH2,FRMPD4,ZNF804A,NEGR1,NTN1,GPC6,PCDH17,CDH8,CNTN5,IGF1R,PDZRN3,NTNG1,UNC13C,LRRK2,NTRK3,DISC1,MECP2,MTMR2,SEMA4D,LINGO2,CLSTN2,BDNF,RYK,WASF1,GRIN2B,SYBU,LRFN2,EPHB1,SIPA1L1,LRRC4,NFIA,EPHA7,FYN,THBS2,APBB2,SLC1A1,PTPRD,NLGN4X,LRRTM1,KALRN,TANC2,CBLN4,GABRA2,APP,DGKB,NRP1,OPHN1,NTRK2,DNM3,PPFIBP1,SDK1,TNR,PPFIBP2,GRID2,EFNA5,CACNG2,IGSF21,RELN,GRM5,KIRREL3,LRRTM3,IL1RAP,DOCK10,GPC4,NLGN1,CTTNBP2,ARHGAP39,PLXNB1,TNF,PPFIA2,TANC1,DLG5,ARF1,C5AR1,LRP4,EFNB2,CLSTN1,CDKL5,IL1RAPL1,PTPRO,SEMA3E,UBE3A,FGF13,GABRB2,EPHB2,ERBB4,SLITRK6,ARF4,UNC13A,UNC13B,BHLHB9,PDLIM5,ADAM10,ITGA3,PIN1,CACNB2,CNKSR2,NFASC,NOS1AP,INSR,GABRA1,PTPRF,PAK3,DTNBP1,LRP8,GABRG2,VPS35*
GO:0050804	modulation of chemical synaptic transmission	1.646999498217122e-14	*PLCB1,DCC,SYN3,GRIK1,GRIN3A,NRXN1,NRXN3,RAB5A,RIMS2,SHANK2,GRIA1,NRG3,LRRC4C,TMEM108,SHISA6,MCTP1,RASGRF1,GRID1,SORCS3,PLCB4,ERC1,CNTN4,S100B,CNTNAP4,AGT,ERC2,SHISA9,CDH11,CDH2,PARK2,DLGAP1,LAMA2,MCTP2,PCDH17,APBA2,PXK,SYT1,CACNG3,GRM1,SORCS2,NTNG1,UNC13C,LRRK2,SLC1A3,DISC1,TPRG1L,MECP2,MTMR2,GSK3B,CLSTN2,BDNF,PRKAR1B,GRIN2B,SNAP25,GRIK3,LRFN2,EPHB1,SIPA1L1,LRRC4,FYN,MME,GRIK2,IGSF11,CELF4,SLC1A1,PTPRD,PLCL1,NLGN4X,PRKCE,PRKCB,ADCY8,LRRTM1,CASK,APP,DGKB,GRIN2A,RIMS3,OPHN1,CD38,NTRK2,STXBP5,RAP1A,TNR,CHRNA3,CHRNA5,GRID2,RASGRF2,ADORA2A,CACNG2,NF1,EIF4E,RELN,GRM5,PPP3CA,DGKI,SLC24A2,ELAVL4,GRM7,RIMS1,NLGN1,AKAP7,MAPK8IP2,NSG1,JAK2,MIR320B2,TNF,ARF1,CLSTN1,TSHZ3,GRIK4,KIT,RAPGEF2,GRM4,EPHB2,PACSIN2,P2RX1,PLAT,UNC13A,NTF3,DGKZ,MIR433,PTPRA,STX3,KMO,LGI1,PLCL2,BTBD9,CACNB2,FMR1,JPH4,CACNA1A,PRKCG,DTNBP1,RAP1B,DLGAP2,LRP8,SNCAIP,NGF,PPP1R9A*
GO:00423	regulation	1.839922818762281e-	*PIEZO2,GABRB3,GABRG3,ASIC2,ANK3,GRIK1,KCNQ1,GRIN3A,NRX*

91	of membrane potential	14	*N1,RIMS2,GRIA1,ANK2,KCND3,SCN8A,KCNE1,TMEM108,CFTR,RGS7BP,GRID1,PARK2,KCNH1,KCNMA1,SLC24A4,GABRG1,KCNK17,PXK,TRPC5,DMD,HCN1,GRM1,CTNNA3,RYR2,LRRK2,KCNH5,SLC4A4,KCNK13,MECP2,MTMR2,GSK3B,SLC8A1,ATP1A4,KCNC1,GRIN2B,ABCB5,AKAP6,GRIK3,BCL2,KCNJ3,KCNE2,ACTN2,GABRB1,GRIK2,IGSF11,CELF4,KCND2,SCN11A,CHRM1,PKP2,ARL6IP5,ATP1A1,PLN,NLGN4X,FAM19A4,GABRA2,KDR,APP,KCNK2,ABAT,GRIN2A,RIMS3,BCL2L1,GLRA2,NTRK2,KCTD7,CACNA1C,PPA2,USP53,CHRNA3,CHRNA5,CACNA2D1,GLRA1,GRID2,ADORA2A,CACNG2,BID,RELN,SLC39A8,GRM5,PPP3CA,CXADR,DGKI,RIMS1,GABRA3,NLGN1,P2RX6,AKAP7,MAPK8IP2,CACNA1D,GABRA5,TNF,KCNK10,GCLC,KCNH8,TRDN,SCN1A,GRIK4,MYH14,GABRR2,KCNH7,FGF13,GABRB2,NUP155,P2RX1,KCNE4,UNC13B,GLRA3,P2RX7,HCN4,RNF207,FGF12,TRPM4,CNGB1,CACNB2,NOS1AP,FMR1,GABRA1,ABCD1,GABRR3,FHL1,PPP2R3C,SCN9A,KCNC2,CASQ2,GABRG2,TBX18*
GO:0099177	regulation of trans-synaptic signaling	2.0522485782775955e-14	*PLCB1,DCC,SYN3,GRIK1,GRIN3A,NRXN1,NRXN3,RAB5A,RIMS2,SHANK2,GRIA1,NRG3,LRRC4C,TMEM108,SHISA6,MCTP1,RASGRF1,GRID1,SORCS3,PLCB4,ERC1,CNTN4,S100B,CNTNAP4,AGT,ERC2,SHISA9,CDH11,CDH2,PARK2,DLGAP1,LAMA2,MCTP2,PCDH17,APBA2,PXK,SYT1,CACNG3,GRM1,SORCS2,NTNG1,UNC13C,LRRK2,SLC1A3,DISC1,TPRG1L,MECP2,MTMR2,GSK3B,CLSTN2,BDNF,PRKAR1B,GRIN2B,SNAP25,GRIK3,LRFN2,EPHB1,SIPA1L1,LRRC4,FYN,MME,GRIK2,IGSF11,CELF4,SLC1A1,PTPRD,PLCL1,NLGN4X,PRKCE,PRKCB,ADCY8,LRRTM1,CASK,APP,DGKB,GRIN2A,RIMS3,OPHN1,CD38,NTRK2,STXBP5,RAP1A,TNR,CHRNA3,CHRNA5,GRID2,RASGRF2,ADORA2A,CACNG2,NF1,EIF4E,RELN,GRM5,PPP3CA,DGKI,SLC24A2,ELAVL4,GRM7,RIMS1,NLGN1,AKAP7,MAPK8IP2,NSG1,JAK2,MIR320B2,TNF,ARF1,CLSTN1,TSHZ3,GRIK4,KIT,RAPGEF2,GRM4,EPHB2,PACSIN2,P2RX1,PLAT,UNC13A,NTF3,DGKZ,MIR433,PTPRA,STX3,KMO,LGI1,PLCL2,BTBD9,CACNB2,FMR1,JPH4,CACNA1A,PRKCG,DTNBP1,RAP1B,DLGAP2,LRP8,SNCAIP,NGF,PPP1R9A*
GO:0040011	locomotion	9.068578831592224e-14	*SRGAP2B,TPTE,CDH4,PLCB1,CMKLR1,DAB1,FMNL2,MAPK14,DSCAM,TPTE2,PTPRT,DCC,DUSP22,FBLN1,ITGA2,SUN2,EPHA6,NRXN1,ZRANB1,NRXN3,DOCK2,NRG3,SLC9B1,TGFBR3,CDH13,TF,PLGRKT,PIK3C2B,SDCCAG8,GADD45A,ADAMTSL1,PLXNA4,FLRT2,GPC3,NRG1,RFFL,MCTP1,ASTN2,SH3RF2,UNC5D,SEMA6D,SPOCK1,CTNNA2,CCR1,CCR3,RBFOX2,DNAH6,TIAM1,RHOC,LDLRAD4,DPYSL2,NKD1,SCAI,SPEF2,FBXO31,CNTN4,ARID5B,PHACTR1,ROBO2,SPOCK3,EPHA3,DCLK1,AGT,PSTPIP2,FSIP2,RHOJ,RCAN1,CDH2,LOXL2,LAMA2,SEMA3D,DACH1,NTN1,ZNF268,GPC6,DBH,FMNL3,RIN3,TRIO,LAMA4,SLIT2,TTLL8,PDCD6,GPC5,IGF1R,VCAN,PTPRG,KRT2,VCL,CELSR1,MBOAT7,ASTN1,STK24,FRMD5,TRPM2,CTNNA3,NTNG1,CATSPER2,LRRK2,NTRK3,ANKS1A,RNF165,DISC1,HEXB,EMB,SH3BP1,IER2,MECP2,SEMA4D,SLC8A1,PRKD1,TGFB2,ATP1A4,VAV3,FUT4,S100A11,SEMA5A,BDNF,RYK,DNAAF2,DNAH3,LAMA3,NR2F2,PML,TNS1,PLXNA2,BCL2,RALBP1,ARHGAP24,EPHB1,VANGL2,NR4A2,EPHA7,FYN,CHN1,NR4A1,CD44,KANK1,ENPP2,DCDC2,LAMB4,UNC5C,PALLD,NLRP12,MTOR,LDB2,CHRM1,FAT3,MEGF9,BMPER,BBS2,CEP85L,EPB41L4B,SOX8,PRKCE,FAM19A4,FOXO3,MITF,KALRN,DLC1,EDNRB,ROBO1,SATB2,FAP,FLT1,CENPV,TACR3,KDR,PRCP,CHL1,APP,NRP1,PTPRM,ELMO1,ADTRP,TTC12,SEMA3C,ADAM17,CAMK1D,ELP3,ETS1,PRTG,RPS6KA5,ITGA11,ATRNL1,GRIN2A,OPHN1,CCDC88A,RIN2,ALCAM,DNAH8,HDAC4,PDE4B,JAM2,MAPRE2,NTRK2,PRKG1,CDC42BPA,STARD13,RABGEF1,GLI3,CLDN1,DOCK4,SRP54,TGFBR1,CYSLTR1,EPHA5,TNR,PEAK1,IL16,EP300,DSCAML1,CYP7B1,ADORA2A,EFNA5,MERTK,TAC4,MAGI2,NF1,SORL1,TTC8,DMRT1,RELN,SLC9C1,GRM5,HDAC9,KIRREL3,SELE,MGAT5,NUMB,NPHP4,RALA,NAV3,JAG1,MEOX2,PPP3CA,KLF7,CXADR,DOCK10,GPC4,PTPRU,SH3KBP1,JAK2,SPNS2,SMAD3,CD300A,PLXNB1,CDK1,HDAC7,TNF,LRCH1,OVOL2,DLG5,LGR6,ARID2,SPAG16,ADORA3,DOCK1,EPS8,SMOC2,SULF1,C5AR1,PIK3R3,CNTN6,EFNB2,AMOTL1,NET1,SELP,CTNNA1,CDKL5,EFHC2,EGF,KIT,SLIT3,PIK3C2G,TRPV4,DNAH11,EFNB1,PTPRO,RAPGEF2,MYPN,SEMA3E,FGF13,MARVELD3,ARMC2,EPHB2,ABCC1,ERBB4,FER,MAP3K3,LRP5,SLAMF1,ABL2,BSG,F11R,GAS8,SETD2,ARHGAP35,CD2AP,PTPRK,ATP8A1,CCL3,FGF7,LAMA1,PREX1,SEMA5B,ITGA9,KIF2A,NHLH2,ARF4,MYH9,TEKT4,NEDD9,PDGFD,PIK3CD,ENPEP,PLAT,WDPCP,AUTS2,FGFR1,ITGBL1,ITGB7,JMY,NTF3,BDKRB1,DGKZ,SEMA3A,MARK2,ZMIZ1,SRGAP3,FERMT2,MAP2K5,MARK1,SMPD3,TNN,CLN6,DAB2,LCP1,STX3,ACTN4,ADAM10,CCL7,ITGA3,LGI1,LECT2,PIN1,SYNPO2,TRPM4,NME8,CLASP1,HGF,MYLK,CAPN7,SLC26A8,NCAM1,NFASC,INSR,MACF1,PAK1,SKI,AKT3,PRKX,ULK4,CRMP1,CCDC141,GNA13,FUT8,SPRED1,NTN4,PTPRF,DEFA1B,PTPRR,EXT1,TYMP,UMOD,DOCK7,EL*

			MO2,HDAC5,TDGF1,LYVE1,TREM1,ADARB1,NDE1,PRKCQ,STAT3,AMOT,PAK3,USH2A,LGALS9,LRP8,EFCAB1,APC,BMPR1A,DPP4,ITGA1,KIAA0319,PRKCA,SPINT2,CTSH,RGCC,SRGAP1,CCL14,CCL15,CCL15-CCL14,IL6R,CLASP2,MAP2K1,SOS1,VPS35,FGF1,TBX20,RGN,RREB1
GO:0048513	animal organ development	2.0260736979837825e-13	*MACROD2,PLCB1,DAB1,MAPK14,DSCAM,RUNX1,MYO18B,DCC,WLS,RBFOX1,AKAP13,LARGE,SORBS2,SLC24A3,NPHP3,ITGA2,KCNQ1,SUN2,CA10,NRXN1,SLC1A2,SDK2,DOCK2,TNC,POTEE,MS4A1,PAX7,CHODL,MTPN,SHANK2,NRG3,SMYD2,ARNT2,OLFM3,TGFBR3,ANK2,ANKH,CSMD1,IGSF3,TF,BCAN,TMEM108,ACIN1,PLXNA4,FLRT2,GPC3,NRG1,PSMB2,HYDIN,IL4R,ALDH1A2,CECR2,NLN,PLCE1,SEMA6D,CTNNA2,CCR1,CFTR,RBFOX2,TIAM1,LDLRAD4,DPYSL2,MYO3A,NKD1,TACC2,ALK,UTRN,SPEF2,CNTN4,ARID5B,PHACTR1,ROBO2,C2CD3,EPHA3,DCLK1,AJAP1,AGT,ATAT1,WDR11,ATP6V0D1,RHOJ,RCAN1,CDH2,FHL2,FOXN3,LOXL2,MDGA2,LAMA2,GREB1L,NEGR1,OTC,GNB4,SEMA3D,NTN1,GPC6,NELL1,TRAPPC9,ZNRF3,FTO,HDAC2,HTRA1,SLIT2,TFAP2D,PDCD6,RPS6KA2,IGF1R,KRT25,MDM2,SLC24A4,SMAD1,ADAMTS6,PTPRG,DMD,HCN1,SYT1,BASP1,KRT2,SIPA1L3,TEAD1,CELSR1,FSHR,MBOAT7,NRIP1,PLAC1,WWOX,ATRX,TRPM2,RYR2,NTNG1,TENM4,LRRK2,RORB,STRC,PIR,KIF26B,NTRK3,RORA,PRICKLE2,CHD7,DISC1,SOX6,MECP2,SEMA4D,GSK3B,LGR5,SLC8A1,SNRK,FLVCR1,TGFB2,FMN1,LCE2B,LCE2C,PLCD3,RIPPLY3,SEMA5A,ACSBG1,KRT74,PRKCH,RYK,KCNC1,PCDH15,EML1,ABI3BP,SGCD,WDR72,GRIN2B,POLE,EXT2,LAMA3,RAG1,RAG2,ABCB5,ANKRD6,NR2F2,NR5A2,PML,PPARGC1A,SGCG,AKAP6,CNTNAP2,FBN1,PLXNA2,BCL2,RXFP2,ZNF675,EPHB1,CSGALNACT1,DISP1,LRP2,MMP16,TMEM100,NFIA,VANGL2,NR4A2,EPHA7,FYN,KCNE2,ADAMTS18,NR4A1,SMARCA4,CD44,ABCB10,ACTN2,ATF6,ATP8A2,KLF13,MME,EXOC4,LAMB4,ZNF516,CELF4,SERPINB7,TPO,UNC5C,MYLK3,SLC1A1,UGCG,ZFPM2,PALLD,RARB,RFX4,ETV6,MTOR,ROR1,LDB2,FAT3,MEGF9,PKP2,BMPER,BBS2,MATN3,ZFP64,COL11A1,PLN,CTNNBIP1,KLHL1,SOX8,DSG4,IFT88,NLGN4X,HELLS,KMT2A,VDR,FOXO3,MITF,CAPN3,KALRN,DLC1,EDNRB,ROBO1,RXFP1,SATB2,FLT1,NASP,NOX4,ZNF148,CDH17,GRHL2,COL2A1,NEBL,TRAF3IP2,CR1,EYA4,SEC24B,MDM1,PKHD1,STK3,TGM2,VASH2,KDR,PPHLN1,EDA,ABCA12,APP,CALCRL,COL18A1,NRP1,PTPRM,LGR4,EGFLAM,IMMP2L,LCE6A,THRB,KCNK2,SEMA3C,ADAM17,TSPEAR,IGF2R,ATF2,ETS1,EFEMP1,TGFB1I1,TCF7L2,AFF2,ITGA11,ZNF830,ABAT,ATRNL1,FRAS1,GRIN2A,DLX6-AS1,HRNR,OPHN1,TRPS1,ZNF568,CASP5,TUB,CHRDL1,STK36,UPB1,BCL2L1,ADAMTS16,HDAC4,MYO16,SOX5,GHR,NTRK2,PRKG1,RASGRP1,CAMK4,SDK1,SPRED2,GLI3,KAZN,SMCHD1,CACNA1C,CLDN1,SPRR2B,SPRR2E,CERS3,CRB1,SAFB2,SRP54,TGFBR1,PRDX4,DYM,EPHA5,THEMIS,TNR,KRTAP6-1,SLC6A17,TENM3,CHST11,CPE,EP300,DSCAML1,TBX3,CYP7B1,SPRR4,TRIOBP,ATXN1,GAB2,PCSK5,TP73,GRID2,PSMA1,FLNB,FAM20A,MERTK,SPRR2G,BRD1,MAGI2,NF1,RGS7,SORL1,BICC1,IFT80,TEAD4,TTC8,DMRT1,ASXL1,POR,RELN,TNFRSF11B,TSHZ1,HDAC9,IL18R1,KIRREL3,NDRG2,NUMB,NPHP4,TBX15,FBXL17,JAG1,MEOX2,PPP3CA,KLF7,NEB,PLS1,CXADR,MAS1,WWTR1,KL,DOCK10,ELAVL4,MNAT1,SEC63,CNTN1,GPC4,TRIM16,GABPA,KAT7,L3MBTL3,FHOD3,FREM1,N4BP2L2,PTPRU,ARHGAP5,CTTNBP2,SSUH2,AK8,MEGF11,SYCP2,LRRK1,GABRA5,JAK2,SHOX2,SOBP,SPNS2,AHI1,SMAD3,SERPINB12,COL8A1,PLXNB1,RDH10,TCF12,ANHX,CDK1,HIVEP3,LTB,PRRC2C,TNF,COBL,NHS,OVOL2,ZFPM1,CHKB,BMP15,DLG5,PHEX,SIM2,ARID2,MMP20,PAXBP1,FREM2,HMGA2,MYO6,DOCK1,KIF18A,PCDH19,RAB23,MBOAT2,TPD52,DAAM2,SULF1,LRP4,XYLT1,EFNB2,HERC1,PHF8,SPATA5,ZNF423,ALPK2,CTNNA1,MAMLD1,MYH14,PRKAR1A,TNFRSF19,ARHGAP11B,BPTF,DYNC2H1,MLLT3,CD109,EGF,MIP,CMA1,FOXP4,KIT,SLIT3,TNFSF8,TRPV4,CC2D2A,CPS1,DNAH11,EDAR,EFNB1,PTPRO,RAPGEF2,DNAJB6,ESR1,SEMA3E,UBE3A,FGF13,GABRB2,PLAG1,SGCZ,EPHB2,FNDC3A,KRT76,MECOM,TFDP2,TOX,ERBB4,MINPP1,SLITRK6,SPTA1,FNIP1,LRP5,TTLL5,MYH15,SLAMF1,BSG,FARP2,GAS8,SETD2,ALX4,AP2B1,ARHGAP35,NSUN2,SGMS2,CCL3,FGF7,LAMA1,MYO7B,PREX1,SEMA5B,SPRED3,NHLH2,PSG9,SULF2,MYH9,SOX4,PDGFD,PIK3CD,AGTPBP1,ASGR2,ITGA8,ARID4A,ENPEP,FAM172A,MYO3B,PDZD7,WDPCP,CDH23,FGFR1,MEF2A,RYR1,ARID4B,ASXL3,BPGM,IL23R,P2RX7,SCUBE1,AR,BCOR,CASP7,FGL2,SUFU,EYA1,IFNA8,IL1RL2,HLA-DRA,L3MBTL1,SEMA3A,ANKRD11,CDC73,MEIS2,ZMIZ1,FLT3,PDLIM5,PPARGC1B,TRIM72,FERMT2,HCN4,LTBR,MAP2K5,MARK1,PAPPA2,SMPD3,DAB2,GPR55,LCP1,PBX1,RNF207,SRD5A2,ADAM10,DAAM1,FGF12,ITGA3,PLCL2,LRIG1,TRPM4,SHROOM4,BMP6,CIT,CLA*

			SP1,TGFA,TMOD1,GAS2,HGF,MYLK,PRPSAP2,CALCR,CENPF,FLI1,ZBTB16,ELN,INSR,PSMB7,SKI,AKT3,CTDP1,ENPP1,LIMK2,PAX3,WDR7,ATP5J,FPGS,KLF6,PRKX,EVC,MAD1L1,MFNG,RPGRIP1,CCDC141,PCK1,PDE6B,PPARA,TTN,ZFHX2,C1GALT1,COL19A1,FHL1,MYH7,TAF1,CHD5,PPP2R3C,SPRED1,CHSY1,LCE1F,NTN4,BTRC,ESRP2,EXT1,MDM4,MYH6,NCAPH2,NCOA1,PRLR,SCO2,UMOD,ADAMTS5,CBFA2T3,DOCK7,FBXW11,HDAC5,KLHL3,TDGF1,A2M,SLC38A10,SLC40A1,STIM1,TTC39C,ADARB1,KCNC2,MYH11,NDE1,POU2AF1,SCEL,STAT3,TP63,BRIP1,HPN,USH2A,BLNK,DHRS2,DHRS7B,DTNBP1,LCE2A,LGALS9,LRP8,MAPKAPK2,UPK3A,BMPR1A,CSN3,PRKCA,SPINT2,UBASH3B,VLDLR,KRT75,ABI1,CTSH,NCAPG2,RGCC,IL6R,LCE4A,MAP2K6,TFIP11,CLASP2,MAP2K1,SOS1,TPH1,COL6A3,FGF1,MCOLN3,TBX20,DCHS2,RGN,RREB1,TBX18
GO:0098742	cell-cell adhesion via plasma-membrane adhesion molecules	7.431719455873718e-13	*CDH4,PCDH9,DAB1,MAPK14,DSCAM,PTPRT,NRXN1,SDK2,CDH13,LRRC4C,NRG1,LRFN5,UNC5D,PCDH11X,PCDH7,CNTN4,ROBO2,CDH11,CDH2,CDH9,HMCN1,GPC6,PCDH17,CDH8,DSC3,CLDN14,CELSR1,NTNG1,TENM4,EMB,TGFB2,CLSTN2,PCDH15,CDH10,CDH12,LRRC4,CDH6,IGSF11,CDHR4,PALLD,FAT3,PTPRD,DSG4,CDH18,ROBO1,CDH17,PTPRM,TENM2,KIFAP3,ALCAM,SDK1,CLDN1,CRB1,TENM3,DSCAML1,GRID2,EFNA5,IGSF21,KIRREL3,SELE,IL1RAP,CXADR,GPC4,NLGN1,CADM3,PCDH19,CNTN6,CLSTN1,SELP,TENM1,IL1RAPL1,MYPN,BSG,CLDN11,CDH23,MAP2K5,PCDHGA1,PCDHGA10,PCDHGA11,PCDHGA12,PCDHGA2,PCDHGA3,PCDHGA4,PCDHGA5,PCDHGA6,PCDHGA7,PCDHGA8,PCDHGA9,PCDHGB1,PCDHGB2,PCDHGB3,PCDHGB4,PCDHGB6,PCDHGB7,CD84,PTPRF,UMOD,DCHS2*
GO:0030029	actin filament-based process	9.535668302169923e-13	*FMNL2,CDC42EP3,AKAP13,SORBS2,KCNQ1,SUN2,DOCK2,MTPN,FGD6,ANK2,KCND3,TF,KCNE1,CTNNA2,RHOC,ARFIP1,UTRN,PHACTR1,EPHA3,PHACTR3,PSTPIP2,RHOJ,PARK2,CORO2B,FRMPD4,SETD3,FMNL3,SLIT2,DMD,CELSR1,FMN2,FRMD5,TRPM2,CTNNA3,RYR2,DIAPH2,NTRK3,PDXP,SH3BP1,FRMD6,FMN1,SEMA5A,LLGL2,PCDH15,SGCD,WASF1,ARHGAP12,MYH8,BCL2,KCNJ3,SIPA1L1,ARHGEF10,MICAL3,KCNE2,FLNC,PDE4D,KANK1,ACTN2,FRMD3,RAP1GDS1,MYLK3,PALLD,STRIP1,MTOR,GAS7,PKP2,ERMN,ATP1A1,PLN,EPB41L4B,KLHL1,EHBP1,PRKCE,CAPN3,CCDC88C,DLC1,NEBL,THSD7B,NRP1,SHROOM3,ARHGEF18,ELMO1,PARVG,CDC42BPG,DIAPH3,OPHN1,CCDC88A,CNN3,PDE4B,PRKG1,CDC42BPA,STARD13,CACNA1C,TGFBR1,EPHA5,CACNA2D1,TRIOBP,PARVB,TRPM7,FLNB,EFNA5,SPECC1L,NF1,ARHGAP6,TTC8,SELE,RHPN2,NPHP4,RALA,NEB,PLS1,CAPZB,CXADR,MPRIP,FHOD3,SH3KBP1,PHACTR2,CACNA1D,MYH4,JAK2,SMAD3,BAIAP2L1,TNF,COBL,FCHSD2,ARF1,CALD1,MYO6,EPS8,DAAM2,AMOTL1,CTNNA1,SCN1A,TENM1,ARHGEF11,MYH14,PRKAR1A,FAM171A1,SPIRE2,KIT,TRPV4,DNAJB6,MYPN,SEMA3E,SPECC1,FGF13,NUP155,WIPF2,FER,SPTA1,ABL2,F11R,FARP2,ARHGAP35,CD2AP,FGF7,MYO7B,PREX1,ARHGAP25,FGD4,MYH9,PACSIN2,NEDD9,SPTB,KCNE4,AUTS2,MEF2A,JMY,NTF3,EPB41L3,PDLIM5,PPARGC1B,FERMT2,HCN4,LCP1,RNF207,ACTN4,DAAM1,FGF12,SYNPO2,THSD7A,TRPM4,EPDR1,SHROOM4,CIT,CLASP1,SMTNL2,TMOD1,GAS2,MYO1B,CACNB2,ELN,NOS1AP,ARHGAP28,PAK1,LIMK2,TESK2,TTN,MYH7,PGM5,MYH6,ELMO2,MYH11,AMOT,PAK3,DTNBP1,MYOM2,ABI1,RGCC,SPTBN4,CLASP2,PPP1R9A*
GO:0032879	regulation of localization	1.1197348698292729e-12	*SRGAP2B,PLCB1,CMKLR1,MAPK14,ABCG8,ASIC2,RAB27A,CACNA1E,KCNJ6,PTPRT,ANK3,WLS,DUSP22,FBLN1,ITGA2,ABCG1,KCNQ1,SUN2,GRIN3A,NRXN1,NRXN3,SLC1A2,RAB5A,DOCK2,PCNT,RIMS2,NRG3,TGFBR3,CDH13,ANK2,KCND3,DPP10,TF,SCN8A,KCNE1,GADD45A,KCNJ15,SYT17,PLXNA4,KCNG3,FLRT2,GPC3,NRG1,STXBP6,SHISA6,RFFL,SPIDR,IL4R,RYR3,MCTP1,ASTN2,SH3RF2,UNC5D,SEMA6D,ABHD17C,CTNNA2,CCR1,CFTR,TIAM1,PM20D1,RHOC,LDLRAD4,RASGRF1,ARFIP1,NKD1,SCAI,PIK3C3,UTRN,FBXO31,RAB7A,PHACTR1,SPOCK3,EPHA3,DCLK1,AGT,SHISA9,RHOJ,CDH2,PARK2,LAMA2,CORO2B,CYBB,MCTP2,SEMA3D,DACH1,NTN1,TRIM22,TRIM5,KCNH1,ZNF268,GPC6,USP6,PCDH17,KCNMA1,RIN3,APBA2,FTO,LAMA4,SLIT2,PDCD6,STXBP4,GPC5,IGF1R,MX2,SYT9,KCNK17,PTPRG,PXK,DMD,HCN1,SYT1,CACNA2D3,CACNG3,VCL,FGF14,GSG1L,STK24,DPP6,FRMD5,TRPM2,CNIH3,CTNNA3,RYR2,NTNG1,CATSPER2,DHRS7C,LRRK2,KCNH5,NTRK3,ILDR1,CHD7,KCNK13,SH3BP1,MECP2,MTMR2,SEMA4D,GSK3B,SLC8A1,PRKD1,TGFB2,DNAJC6,FUT4,S100A11,SEMA5A,SLC5A3,BDNF,LLGL2,PRKAR1B,PRKCH,KCNC1,LAMA3,NKAIN2,NR2F2,PLEKHM2,SNAP25,PML,USP36,AKAP6,PLXNA2,BCL2,KCNJ3,SLC30A5,DISP1,NKAIN3,NSF,FRMD4A,ITPR2,FYN,KCNE2,PARN,HECW1,NEU3,RCVRN,RHBDD1,PDE4D,KANK1,ENPP2,ACTN2,ATP8A2,KCNS3,KCNIP4,UNC5C,KCND2,SCN11A,SLC1A1,MTOR,CADPS,LDB2,CHRM1,CTDSPL2,PKP2,UBXN2B,ABCA13,BMP*

			ER,BBS2,ARL6IP5,ATP1A1,EPM2A,PLN,EPB41L4B,PRKCE,KMT2A,PRKCB,STON1,ADCY8,EFHB,FOXO3,LRRTM1,MITF,RAB31,SCP2,TRAPPC12,CAPN3,ITLN1,KALRN,DLC1,EDNRB,MSR1,ROBO1,TBC1D4,BNIP3L,FLT1,SGIP1,KCNC4,TACR3,SEC24B,ACSL5,PKHD1,TGM2,KDR,PRCP,CASK,ABCA12,APP,NRP1,PTPRM,ADTRP,SEMA3C,ADAM17,CAMK1D,ELP3,ETS1,GOPC,ROS1,TM9SF4,TCF7L2,BICD1,ABAT,RIMS3,SGK2,OPHN1,CCDC88A,CD38,RIN2,TUB,BCL2L1,HDAC4,PDE4B,JAM2,MAPRE2,PRKG1,ACSL4,DNM3,STARD13,COMMD1,KCTD7,RABGEF1,GLI3,CACNA1C,CLDN1,STXBP5,DOCK4,RAP1A,TGFBR1,KCNJ12,EPHA5,IL16,RAPGEF1,CHRNA3,CHRNA5,SCFD1,TTC39B,CACNA2D1,CACNA2D4,STX8,CLIC6,GAB2,SYCP1,IFNGR2,RASGRF2,ADORA2A,CAMK2G,EFNA5,MERTK,TAC4,CACNG2,MAGI2,NF1,RGS7,KCNQ5,SORL1,TTC8,RELN,GRM5,HDAC9,PTPN9,SELE,DYNC1H1,MGAT5,NUMB,RALA,ADCYAP1R1,NAV3,JAG1,MEOX2,PPP3CA,KCNB2,KLF7,PLS1,DGKI,PER1,TBC1D5,WWTR1,ANKRD13A,DOCK10,PLSCR1,ATP9A,CNTN1,GPC4,GRM7,RIMS1,GPSM2,KAT7,STIM2,BORA,NLGN1,PTPRU,AKAP7,MAPK8IP2,CACNA1D,TPCN2,JAK2,AHI1,SMAD3,CD300A,NOS1,PLXNB1,CDK1,HDAC7,NVL,TNF,LRCH1,SNX5,POMC,TMC2,DLG5,LGR6,ARID2,KCNK10,ADORA3,ARF1,MYO6,POLR1A,DOCK1,RUFY1,TSPAN13,ANO1,GCLC,KCNA6,RAB23,SMOC2,SULF1,C5AR1,LRP4,PIK3R3,EFNB2,KCNH8,SAE1,TRDN,AMOTL1,SELP,CTNNA1,SCN1A,TENM1,KCNH7,MYRIP,EGF,PTPN14,SEC16B,IL1RAPL1,KIT,TRPV4,ABCA5,DNAH11,KCNAB1,PTPRO,RAPGEF2,DNAJB6,HTR2C,SEMA3E,SLC30A8,FGF13,MARVELD3,EPHB2,NDFIP2,DAPK1,ERBB4,FER,MAP3K3,CACNG6,LRP5,SLAMF1,ABL2,BSG,GAS8,SETD2,AP2B1,CD2AP,NSUN2,PTPRK,ATP8A1,CCL3,FGF7,LAMA1,SEMA5B,KIF2A,PACSIN2,PTGER3,SOX4,NEDD9,P2RX1,PDGFD,PIK3CD,KCNE4,WDPCP,FGFR1,MEF2A,OCLN,RYR1,UNC13B,MAP2,NTF3,P2RX7,PLA2G4A,RAPGEF4,RASA3,AR,RABGAP1L,SCAMP5,SUFU,CEP250,DENND5B,RBM4,BDKRB1,GNL3,PRKAA2,SEMA3A,THADA,GNB5,REEP2,SRGAP3,FERMT2,HCN4,MAP2K5,SMPD3,TNN,DAB2,HDAC8,LCP1,RNF207,STX3,SYT2,ACTN4,ADAM10,CCL7,FGF12,ITGA3,KCNJ16,KMO,STAC,MYOM1,NUP153,PIN1,SYNPO2,TRPM4,ABCA8,BMP6,BTBD9,CHRM3,CLASP1,HGF,MYLK,CALCR,CAPN7,CACNB2,NOS1AP,INSR,MACF1,PAK1,AKT3,ENPP1,FMR1,LIMK2,DKC1,PRKX,ULK4,MAD1L1,CD84,JPH4,PPARA,TTN,CORIN,FAM3D,FHL1,GNA13,NUP214,CACNA1A,SPRED1,SCN9A,C2,PTPRR,RIT2,PRKCG,UMOD,DOCK7,HDAC5,TDGF1,LYVE1,STIM1,ADARB1,KCNC2,STAT3,USP7,AMOT,PAK3,PIK3R2,CASQ2,DTNBP1,RAP1B,LGALS9,SNCAIP,EFCAB1,APC,BMPR1A,DPP4,PRKCA,PTGES,SPINT2,UBASH3B,CTSH,DHX9,RGCC,RPH3AL,SRGAP1,IL6R,MAP2K6,SIAH3,SPTBN4,CLASP2,MAP2K1,VPS35,C1QTNF3,FGF1,MCOLN3,C6ORF106,RGN,RREB1*
GO:0006811	ion transport	1.519661758667789e-12	*PIEZO2,GABRB3,ABCC10,GABRG3,ASIC2,CACNA1E,KCNJ6,ANO2,ANK3,GRIK1,SLC24A3,KCNQ1,GRIN3A,NRXN1,SLC1A2,MS4A1,GRIA1,PKD1L1,SLC9B1,TRPM3,ANO4,ANK2,ANKH,KCND3,FAM155A,SLC12A1,DPP10,TF,SCN8A,KCNE1,KCNJ15,SYT17,KCNG3,SCARA5,SHISA6,RYR3,SLCO3A1,CCR1,CFTR,PM20D1,RASGRF1,GRID1,SLC6A2,SLC17A3,UTRN,SLC44A1,AGT,SHISA9,CHRM5,ATP6V0D1,PARK2,CYBB,KCNH1,SLC22A23,SLC5A4,CP,KCNMA1,CYB5R2,SLC44A5,SLC24A4,SYT9,GABRG1,KCNK17,PXK,TRPC5,DMD,HCN1,SYT1,CACNA2D3,CACNG3,NOX5,FGF14,GSG1L,GRIA4,GRM1,SLC39A11,DPP6,TRPM2,CNIH3,RYR2,CATSPER2,DHRS7C,SLC1A3,KCNH5,SLC4A4,CHD7,KCNK13,EMB,SLC8A1,PRKD1,FLVCR1,ATP1A4,SLC5A3,SLCO2B1,ZDHHC17,KCNC1,GRIN2B,NKAIN2,SNAP25,PML,AKAP6,GRIK3,SNAP23,BCL2,RALBP1,KCNJ3,SLC30A5,LRP2,NKAIN3,NSF,ITPR2,ABCC9,FYN,KCNE2,SLC26A7,HECW1,RCVRN,PDE4D,ACTN2,GABRB1,KCNS3,GRIK2,KCNIP4,KCND2,SCN11A,SLC1A1,MFSD12,CHRM1,PKP2,SLC35F3,ARL6IP5,ATP1A1,EPM2A,PLN,SLCO1B1,SLCO1B3,HEPHL1,PRKCE,SLC2A9,ATP6V1D,PRKCB,VDR,EFHB,SLC47A1,CAPN3,EDNRB,SLC12A8,KCNC4,CR1,GABRA2,CASK,APP,CALCRL,KCNK2,SLC38A7,PEX5L,GOPC,ROS1,ABAT,GRIN2A,SLC30A7,PDE4B,GLRA2,NTRK2,ACSL4,MGST1,SLC5A10,COMMD1,CACNA1C,NIPA2,CYSLTR1,KCNJ12,SLC13A4,SLC25A21,SLC6A17,IL16,CHRNA3,CHRNA5,SLC16A7,CACNA2D1,CACNA2D4,CLIC6,GLRA1,GRID2,IFNGR2,RASGRF2,TRPM7,ADORA2A,CAMK2G,CACNG2,NF1,RGS7,KCNQ5,MICU1,RELN,SLC39A8,SLC9C1,GRM5,ADCYAP1R1,ATP10A,PPP3CA,TMEM163,KCNB2,SLC7A1,PER1,SLC24A2,SLC38A6,SLC5A1,CNTN1,GRM7,GABRA3,STIM2,SLC22A2,NLGN1,P2RX6,SLC22A3,AKAP7,MAPK8IP2,CACHD1,CACNA1D,GABRA5,TPCN2,SLC5A8,NOS1,MAGT1,SLC39A10,TNF,PANX1,TMC2,KCNT2,TMC5,KCNK10,GRIA3,TSPAN13,ANO1,KCNA6,SLC7A7,TRPC4,CNGB3,KCNH8,TRDN,ANO10,SLC15A1,LRRC8C,LRRC8D,SCN1A,ABCG2,GRIK4,GABRR2,KCNH7,TMEM150C,E*

			GF,TRPV4,KCNAB1,SLCO4C1,HTR2C,SLC30A8,FGF13,GABRB2,SLC22A15,ANO3,EPHB2,NDFIP2,ABCC1,DAPK1,CACNG6,FLVCR2,ABCC11,ATP8A1,CCL3,MCUR1,SLC39A12,P2RX1,KCNE4,CNGA4,SFXN3,CDH23,RYR1,GLRA3,P2RX7,PLA2G4A,RASA3,BDKRB1,BDKRB2,THADA,GNB5,GRIA2,ATP6V1H,HCN4,SCNN1A,ATP13A3,RNF207,SYT2,ACTN4,FGF12,KCNJ16,KMO,KCNN3,SLC16A4,STAC,TRPM4,ABCC2,CHRM3,CNGB1,TMCO3,MYLK,SLC38A4,CALCR,SLC13A3,SLC26A8,CACNB2,NOS1AP,ENPP1,FMR1,MICU2,ATP5J,GABRA1,GABRR3,CD84,JPH4,FHL1,SLC22A5,CACNA1A,TRPM1,SCN9A,ATP6V1E1,UMOD,KLHL3,SLC24A5,SLC38A10,SLC40A1,STIM1,TRPM6,KCNC2,SLC22A10,SLC22A25,SLC9A9,HPN,CASQ2,DTNBP1,SLC28A3,TMEM175,CNNM3,SLC7A8,TMEM63A,TUSC3,PTGES,UBASH3B,SLC9A2,GABRG2,MAP2K6,SLC16A12,SPTBN4,MCOLN3,ATP6V1E2,RGN
GO:0010975	regulation of neuron projection development	2.0042182105554602e-12	*CDH4,DAB1,DSCAM,DCC,CHODL,FSTL4,LRRC4C,PLXNA4,SEMA6D,SPOCK1,CTNNA2,TIAM1,DPYSL2,CBFA2T2,ALK,FBXO31,ROBO2,EPHA3,AGT,CDH2,ZNF804A,NEGR1,SEMA3D,NTN1,HDAC2,SLIT2,IGF1R,PTPRG,TRPC5,DMD,CSMD3,STK24,NTNG1,LRRK2,NTRK3,DISC1,SEMA4D,GSK3B,PRKD1,SEMA5A,BDNF,RYK,SNAP25,PLXNA2,SIPA1L1,EPHA7,FYN,CHN1,HECW1,PMP22,KANK1,ATP8A2,ROR1,FAT3,PTPRD,TIAM2,KALRN,ROBO1,TANC2,NRP1,SEMA3C,CAMK1D,CCDC88A,CD38,DENND5A,NTRK2,DNM3,RAP1A,TNR,CUX1,TENM3,RAPGEF1,CHRNA3,EP300,GRID2,CAMK2G,EFNA5,MAGI2,RELN,PTPN9,MAP3K13,PPP3CA,ELAVL4,CNTN1,DGUOK,NLGN1,SHOX2,KIDINS220,PLXNB1,COBL,PPFIA2,BCL11A,LRP4,EFNB2,CDKL5,EFHC2,IL1RAPL1,TRPV4,PTPRO,RAPGEF2,SEMA3E,UBE3A,FGF13,EPHB2,TOX,ABL2,ARHGAP35,SEMA5B,SLC39A12,MAP2,BHLHB9,SEMA3A,MARK2,PDLIM5,MARK1,DAB2,ITGA3,HGF,MACF1,PAK1,ULK4,CRMP1,PTPRF,RIT2,ISLR2,PAK3,DTNBP1,GAK,LRP8,KIAA0319,NGF,VLDLR,MAP2K1*
GO:0006810	transport	2.550459407950314e-12	*IGHV1OR21-1,PIEZO2,GABRB3,ABCC10,GABRG3,MAPK14,ABCG8,TRAPPC8,HNRNPA1L2,ASIC2,CTBP2,TMPRSS15,RAB27A,CACNA1E,KCNJ6,ANXA8L1,ANO2,ANK3,IGHV4-31,HEATR5A,IGHV3-64,WLS,RBFOX1,SYN3,AKAP13,SNX31,VPS13D,GRIK1,IGHV1OR15-9,SLC24A3,IGHV4OR15-8,NPHP3,ITGA2,ABCG1,TMPRSS2,KCNQ1,SUN2,GRIN3A,NRXN1,TMPRSS3,SGSM1,NRXN3,SLC1A2,RAB5A,DOCK2,PCNT,MS4A1,RIMS2,GRIA1,CLEC16A,PKD1L1,DLG2,SLC9B1,TRPM3,ANO4,CDH13,ANK2,TBC1D22A,ANKH,GRAMD1C,KCND3,LRP1B,FAM155A,SYNDIG1,SLC12A1,DPP10,TF,SCN8A,KCNE1,CEP41,TMEM108,KCNJ15,SYT17,KCNG3,GPC3,FBLN5,NRG1,SCARA5,STXBP6,PSMB2,SHISA6,IL4R,ENTHD1,CECR2,RYR3,MCTP1,ASTN2,SYT16,SLCO3A1,CCR1,CFTR,BBS9,SCFD2,PCTP,PM20D1,AGBL4,DNAJC15,RASGRF1,DPYSL2,GRID1,SLC6A2,ARFIP1,SLC17A3,PIK3C3,UTRN,SPEF2,FAM3B,ERC1,KLRF2,RAB7A,TAPBP,EPHA3,PTPRN2,DCLK1,SLC44A1,AGT,ATG4C,ERC2,SHISA9,CHRM5,VTI1A,WDR11,ATP6V0D1,RHOJ,CDH2,PARK2,LOXL2,RN7SL659P,CYBB,MCTP2,SYTL5,DNAH9,NTN1,KCNH1,SLC22A23,SLC5A4,CP,DENND2A,RNF185,USP6,ITSN1,NCF4,PCDH17,KCNMA1,RIN3,APBA2,CYB5R2,PDCD6,STXBP4,SLC44A5,IGF1R,MX2,SLC24A4,E2F3,NUTF2,SYT9,TMEM241,CCDC91,GABRG1,KCNK17,PXK,SLC14A2,TRPC5,DMD,HCN1,SYT1,CACNA2D3,CACNG3,NOX5,FGF14,FSHR,GSG1L,GRIA4,GRM1,FMN2,HNRNPA2B1,SORCS2,JAKMIP1,SLC39A11,DPP6,TRPM2,CNIH3,IPO13,RYR2,OCA2,KIF3B,UNC13C,CATSPER2,DHRS7C,LRRK2,SLC1A3,KCNH5,KXD1,ILDR1,SLC4A4,CHD7,DPY30,KCNK13,EMB,SH3BP1,MTMR2,GSK3B,SLC8A1,PRKD1,SERPINA5,FLVCR1,TGFB2,ATP1A4,MFSD9,VAV3,ATP9B,DNAJC6,SLC5A3,SLCO2B1,LLGL2,PRKAR1B,ZDHHC17,KCNC1,DNAAF2,SNX24,WASF1,GRIN2B,ZDHHC14,EXT2,NKAIN2,ABCB5,MREG,SNAP25,SNAP25-AS1,PITPNC1,PML,STOML1,SYBU,USP36,ARHGAP12,AKAP6,GRIK3,IGHV3-16,SNAP23,BCL2,CHMP4C,RALBP1,KCNJ3,SLC30A5,DISP1,LRP2,NKAIN3,NSF,FRMD4A,ITPR2,CD163,CD163L1,GDAP1,ABCC9,CYB561A3,MICAL3,RPGR,FYN,KCNE2,SLC26A7,NR4A1,HECW1,NEU3,RCVRN,RHBDD1,PDE4D,ATP10B,ENPP2,ABCB10,ACTN2,ATP8A2,GABRB1,CSNK1G3,EXOC4,KCNS3,GRIK2,KCNIP4,KCND2,SCN11A,SLC1A1,UGCG,AGAP1,MFSD12,CADPS,TMPRSS4,VPS26B,CHRM1,CTDSPL2,PKP2,RANBP17,ABCA13,CCDC88B,SLC35F3,BBS2,HBE1,ARL6IP5,ATP1A1,EPM2A,PLN,SLCO1B1,SLCO1B3,ZMAT3,EVI5,EXOC6B,HEPHL1,EHBP1,IFT88,NLGN4X,PRKCE,SLC2A9,ATP6V1D,KMT2A,PRKCB,STON1,STON1-GTF2A1L,VDR,ADCY8,EFHB,FAM19A4,IGF2BP3,LRRTM1,RAB31,SCP2,SLC47A1,TRAPPC12,CAPN3,ITLN1,KALRN,CCDC88C,EDNRB,MS*

			R1,TANC2,TBC1D4,BNIP3L,NASP,SGIP1,SLC12A8,STX12,CDH17,KCNC4,NBAS,TRAF3IP2,VPS37B,CBLN4,CR1,GABRA2,SEC24B,ACSL5,STK3,TGM2,CASK,IGHV4-28,ABCA12,APP,CALCRL,NRP1,RN7SL674P,TSNARE1,ELMO1,ADTRP,IMMP2L,PRELID2,KCNK2,SLC38A7,CAMK1D,IGF2R,ATF2,GC,PEX5L,SLC35F4,GOPC,ROS1,SMG6,TM9SF4,KPNB1,TCF7L2,BICD1,CCT6B,PARD3,THOC2,ABAT,FRAS1,GRIN2A,GRIP1,RIMS3,SGK2,KIFAP3,OPHN1,BCLAF1,SAMD9,SLC35F2,CCDC88A,CD38,DENND5A,RIN2,SLC30A7,TUB,NCF2,RNF126,SMG7,STK36,BCL2L1,PDE4B,GHR,GLRA2,NTRK2,ACSL4,MGST1,SLC5A10,DNM3,RASGRP1,TRAPPC10,ARR3,COMMD1,KCTD7,RAB11FIP4,RABGEF1,STARD4,C17ORF75,GLI3,CACNA1C,CLDN1,COG7,KIF16B,NIPA2,STXBP5,RAP1A,SRP54,CYSLTR1,KCNJ12,SVOPL,EPHA5,SLC13A4,VPS53,CUX1,SLC25A21,SLC6A17,CPE,IL16,PTTG1IP,RAPGEF1,STARD5,CHRNA3,CHRNA5,RAB2A,SLC16A7,SCFD1,SPAG17,TBX3,TTC39B,CACNA2D1,CACNA2D4,STX8,ATXN1,CLIC6,GAB2,GLRA1,HBD,PCSK5,GRID2,IFNGR2,NPC1,RASGRF2,SAMM50,TMEM50B,TRPM7,PSMA1,SV2B,VPS39,SPG11,ADORA2A,ADRBK2,CAMK2G,DYNC1I2,EFNA5,MERTK,CACNG2,MAGI2,NF1,RGS7,GOT2,GULP1,KCNQ5,SORL1,BID,TTC8,MICU1,RELN,SLC39A8,SLC9C1,GRM5,IGHV4-4,SELE,SNUPN,DYNC1H1,PLSCR4,DDC,NUMB,IGHV3OR16-12,RALA,ADCYAP1R1,SLC35A5,ATP10A,EXOC5,MAN1A1,PPP3CA,TBC1D3B,TMEM163,KCNB2,KLF7,PLS1,SH3GL2,SLC7A1,SNX25,ATP8B4,CXADR,DGKI,GNPTAB,PER1,SLC24A2,TBC1D5,ANKRD13A,CBLB,PLSCR1,SEC63,SLC38A6,SLC5A1,ATP9A,CNTN1,GRM7,RIMS1,GABRA3,STIM2,SLC22A2,NLGN1,P2RX6,SLC35F1,SH3KBP1,SLC22A3,NPR3,OSBPL5,AKAP7,MAPK8IP2,CACHD1,CACNA1D,LRPPRC,NSG1,VPS37A,GABRA5,TPCN2,JAK2,SLC5A8,SPNS2,AHI1,SLC25A48,SMAD3,VPS41,XKR4,CD300A,NOS1,CDK1,HGSNAT,MAGT1,MCM3AP,SLC39A10,TNF,PANX1,SNX5,CPT1B,POMC,PPFIA2,TMC2,BLOC1S6,FCHSD2,TRIM23,KCNT2,TMC5,KCNK10,SPAG16,ADORA3,ARF1,MYO6,DOCK1,GRIA3,IGLC2,IGLL5,KIF18A,RUFY1,TSPAN13,ANO1,GCLC,KCNA6,QKI,RAB23,TPD52,SLC7A7,TOMM34,TRPC4,LRP4,CNGB3,EFNB2,KCNH8,RAB24,RSRC1,SAE1,SHFM1,TRDN,ANO10,SLC15A1,CLSTN1,LRRC8C,LRRC8D,CSE1L,NSG2,SCN1A,TENM1,ABCG2,DMBT1,GRIK4,WDR35,ARHGAP11B,DYNC2H1,GABRR2,KCNH7,MYRIP,STX16,STX16-NPEPL1,TMEM150C,EGF,MIP,PTPN14,SEC16B,SPIRE2,VPS8,IL1RAPL1,KIT,KPNA3,TRPV4,ABCA10,ABCA5,ABCA6,DNAH11,KCNAB1,NAPG,SLCO4C1,HTR2C,SLC30A8,UBE3A,FGF13,GABRB2,GRM4,NUP155,SLC22A15,ANO3,EPHB2,IGHV3-72,NDFIP2,ABCC1,DAPK1,ERBB4,FER,CACNG6,FLVCR2,IFT43,LRP5,XKR7,SLAMF1,ABCC11,ABL2,BSG,F11R,GAS8,SETD2,TMEM50A,VPS13C,AP2B1,CD2AP,NSUN2,SV2C,ATP8A1,CCL3,FGF7,MCUR1,MYO7B,ARHGAP25,IPO11,SPA17,ARF4,ARFGAP3,MYH9,PACSIN2,PTGER3,SLC2A13,SLC39A12,SOX4,SPRN,MIPEP,OSCP1,P2RX1,PIK3CD,RGPD1,AGTPBP1,ASGR2,KCNE4,CNGA4,PLAT,SFXN3,TNKS,UNC13A,VPS45,CDH23,MEF2A,OCLN,RYR1,TBC1D9,UNC13B,GLRA3,MAP2,NTF3,P2RX7,PLA2G4A,RAPGEF4,SORCS1,ARRDC4,RASA3,ZDHHC11,ZDHHC11B,ZDHHC9,ATG2B,COG1,KIAA1109,RABGAP1L,SCAMP5,SNX30,SUFU,DENND5B,EXOC2,RBM4,BDKRB1,BDKRB2,AFTPH,THADA,ANXA11,AQP10,GNB5,GRIA2,KIF4A,MOGAT2,PROS1,REEP2,LDLRAD3,SNX7,TRIM72,VPS16,ATP6V1H,HCN4,SCNN1A,SMPD3,ATP13A3,DAB2,LCP1,RNF207,STX3,SYT2,ACTN4,ADAM10,BLOC1S5,FGF12,KCNJ16,KMO,TXNDC5,KCNN3,RFTN1,RNASEH2B-AS1,SLC16A4,STAC,MYOM1,NUP153,TRPM4,ABCA8,ABCC2,BMP6,BTBD9,CHRM3,CLASP1,EIF4ENIF1,SIDT1,CNGB1,PCDHGA3,TMCO3,G3BP2,MYLK,SLC38A4,CALCR,CENPF,MYO1B,OSBPL3,RN7SL318P,SLC13A3,SLC26A8,TLK1,CACNB2,NOS1AP,OSBPL10,SNAP29,INSR,MACF1,PAK1,PSMB7,CHM,ENPP1,FMR1,MICU2,THEM4,ATP5J,GABRA1,IGHV1-69,MON2,ABCD1,AMPH,CHKA,GABRR3,IGHV1-46,AP1G2,CD84,EPG5,ESYT2,JPH4,PPARA,SIL1,SORT1,TNFAIP8L3,TTN,CORIN,FAM3D,FHL1,HEATR5B,NUP214,SLC22A5,CACNA1A,TRPM1,SCN9A,ATP6V1E1,C2,IGHV1-18,NUP62CL,OPTN,RIT2,EXT1,IFT81,PRKCG,PRLR,UMOD,DOCK7,ELMO2,FBXW11,ICK,KLHL3,GOSR2,SLC24A5,SLC38A10,SLC40A1,STIM1,TRPM6,KCNC2,NDE1,SLC22A10,SLC22A25,SLC9A9,STAT3,USP7,DNM1P46,HPN,PIK3R2,SLC15A5,CASQ2,DTNBP1,GAK,RAP1B,SLC28A3,TMEM175,CNNM3,LGALS9,LRP8,SNCAIP,MAPKAPK2,SLC7A8,TMEM63A,TUSC3,UPK3A,CKAP5,CSN3,HDLBP,PTGES,SRP19,UBASH3B,VLDLR,EXOC6,DHX9,RGCC,RPH3AL,SLC9A2,GABRG2,MAP2K6,NCALD,OSBPL1A,SIAH3,SLC16A12,SPTBN4,AP5M1,CLASP2,MAP2K1,TPH1,VPS35,C1QTNF3,MCOLN3,ATP6V1E2,RGN,RN7SL673P

GO:0034329	cell junction assembly	2.6808689704299873e-12	*GABRB3,DSCAM,ASIC2,DUSP22,ITGA2,NRXN1,NRXN3,SDK2,CTNND2,SHANK2,ANK2,SYNDIG1,FLRT2,NRG1,LRFN5,RHOC,IL1RAPL2,ROBO2,EPHA3,AGT,CDH11,CDH2,CDH9,CORO2B,NEGR1,NTN1,TLN2,GPC6,PCDH17,MYO9A,CDH8,CNTN5,CLDN14,VCL,NTRK3,SH3BP1,MECP2,SEMA4D,LINGO2,CLSTN2,FMN1,BDNF,PRKCH,RYK,CNTNAP2,TNS1,BCL2,CDH10,EPHB1,CDH12,LRRC4,CDH6,EPHA7,THBS2,ACTN2,PKP2,PTPRD,NLGN4X,CDH18,LRRTM1,DLC1,GRHL2,CBLN4,GABRA2,KDR,APP,NRP1,PKP4,PARD3,OPHN1,NTRK2,DNM3,SDK1,CLDN1,FRMPD2,RAP1A,PEAK1,RAPGEF1,GRID2,EFNA5,ARHGAP6,KIRREL3,LRRTM3,NPHP4,IL1RAP,GPC4,NLGN1,SMAD3,PLXNB1,HDAC7,TNF,DLG5,LRP4,EFNB2,CLSTN1,CTNNA1,IL1RAPL1,TRPV4,PTPRO,RAPGEF2,FGF13,GABRB2,MARVELD3,EPHB2,TBCD,ERBB4,SLITRK6,F11R,CLDN11,PTPRK,WDPCP,OCLN,BHLHB9,EPB41L3,PDLIM5,PTPRA,FERMT2,CLASP1,PKP1,MACF1,TESK2,GABRA1,MPDZ,AMOT,RAP1B,APC,PRKCA,GABRG2,CLASP2,VPS35*
GO:0031344	regulation of cell projection organization	3.333890180514201e-12	*CDH4,DAB1,CDC42EP3,DSCAM,DCC,ITGA2,NRXN1,RAB5A,CHODL,FSTL4,LRRC4C,SDCCAG8,PLXNA4,PLCE1,SEMA6D,SPOCK1,CTNNA2,TIAM1,DPYSL2,CBFA2T2,ALK,FBXO31,ROBO2,EPHA3,AGT,CDH2,ZNF804A,NEGR1,SEMA3D,NTN1,HDAC2,MYO9A,SLIT2,IGF1R,CEP97,PTPRG,TRPC5,DMD,CSMD3,STK24,TRPM2,NTNG1,LRRK2,NTRK3,DISC1,WRAP73,SEMA4D,GSK3B,PRKD1,SEMA5A,BDNF,RYK,GRIN2B,SNAP25,PLXNA2,ARHGAP24,SIPA1L1,EPHA7,FYN,CHN1,CD44,HECW1,PMP22,KANK1,ENPP2,ATP8A2,DCDC2,MTOR,ROR1,MOV10,FAT3,ERMN,PTPRD,TIAM2,IFT88,KALRN,ROBO1,TANC2,NRP1,TENM2,SEMA3C,CAMK1D,GRIP1,CCDC88A,CD38,DENND5A,ADAMTS16,HDAC4,NTRK2,DNM3,RAP1A,TGFBR1,TNR,CUX1,TENM3,RAPGEF1,CHRNA3,EP300,GRID2,CAMK2G,EFNA5,MAGI2,RELN,PTPN9,MAP3K13,RALA,PPP3CA,PLS1,CAPZB,ELAVL4,CNTN1,DGUOK,NLGN1,SHOX2,KIDINS220,PLXNB1,COBL,PPFIA2,BCL11A,EPS8,CCP110,LRP4,EFNB2,TENM1,CDKL5,EFHC2,IL1RAPL1,KIT,TRPV4,PTPRO,RAPGEF2,SEMA3E,UBE3A,FGF13,EPHB2,TOX,FER,ABL2,ARHGAP35,SEMA5B,CDKL1,SLC39A12,WDPCP,AUTS2,OCLN,MAP2,P2RX7,BHLHB9,SEMA3A,MARK2,PDLIM5,MARK1,DAB2,ITGA3,HGF,MACF1,PAK1,FMR1,LIMK2,ULK4,CRMP1,PTPRF,RIT2,ISLR2,PAK3,DTNBP1,GAK,LRP8,APC,KIAA0319,NGF,VLDLR,MAP2K1,VPS35,ATMIN,RREB1*
GO:0003013	circulatory system process	1.0535248154043366e-11	*ASIC2,SVEP1,AKAP13,SLC24A3,KCNQ1,TNNI3K,SLC1A2,CELF2,ANK2,KCND3,KCNE1,RYR3,SLCO3A1,SLC44A1,AGT,CORO2B,DBH,KCNMA1,SLIT2,RPS6KA2,MDM2,DMD,FSHR,CTNNA3,RNLS,RYR2,SLC1A3,SLC4A4,CHD7,MECP2,SLC8A1,TGFB2,ATP1A4,SLC5A3,SLCO2B1,SGCD,EXT2,NR2F2,SGCG,ACSM3,KCNJ3,LRP2,ABCC9,FYN,KCNE2,PDE4D,TBXAS1,NAV2,RAP1GDS1,MYLK3,SLC1A1,MTOR,PKP2,ATP1A1,PLN,CTNNBIP1,EDNRB,NOX4,TACR3,PRCP,SERPING1,IMMP2L,THRB,ABAT,CD38,ADAMTS16,HDAC4,PDE4B,PRKG1,CACNA1C,DOCK4,CYSLTR1,KCNJ12,SLC6A17,SLC16A7,CACNA2D1,PCSK5,TRHDE,ADORA2A,TAC4,MEOX2,SH3GL2,SLC7A1,CXADR,WWTR1,KL,SLC5A1,SLC22A2,SLC22A3,NPR3,CACNA1D,JAK2,SHOX2,SMAD3,NOS1,TNF,SNX5,POMC,ADORA3,GCLC,TRDN,SCN1A,ABCG2,CMA1,TRPV4,CPS1,PTPRO,FGF13,NUP155,SGCZ,ABCC1,LRP5,ARHGAP35,ATP8A1,SLC2A13,P2RX1,KCNE4,ENPEP,MEF2A,OCLN,AR,BDKRB1,BDKRB2,ADRA1B,SEMA3A,PDE5A,FERMT2,HCN4,RNF207,FGF12,TRPM4,ABCC2,BMP6,CHRM3,SMTNL2,FLI1,SLC13A3,CACNB2,ELN,NOS1AP,INSR,PDE3A,ARHGAP42,PPARA,TTN,CORIN,GNA13,MYH7,SLC22A5,QRFP,EXT1,MYH6,UMOD,AMOT,CASQ2,SLC7A8,ITGA1,ASB3,MAP2K6,NCALD,SLC16A12,SPTBN4,TBX20,TBX18*
GO:0120035	regulation of plasma membrane bounded cell projection organization	1.1859210347085754e-11	*CDH4,DAB1,CDC42EP3,DSCAM,DCC,NRXN1,RAB5A,CHODL,FSTL4,LRRC4C,SDCCAG8,PLXNA4,PLCE1,SEMA6D,SPOCK1,CTNNA2,TIAM1,DPYSL2,CBFA2T2,ALK,FBXO31,ROBO2,EPHA3,AGT,CDH2,ZNF804A,NEGR1,SEMA3D,NTN1,HDAC2,SLIT2,IGF1R,CEP97,PTPRG,TRPC5,DMD,CSMD3,STK24,TRPM2,NTNG1,LRRK2,NTRK3,DISC1,WRAP73,SEMA4D,GSK3B,PRKD1,SEMA5A,BDNF,RYK,GRIN2B,SNAP25,PLXNA2,ARHGAP24,SIPA1L1,EPHA7,FYN,CHN1,CD44,HECW1,PMP22,KANK1,ENPP2,ATP8A2,DCDC2,MTOR,ROR1,FAT3,PTPRD,TIAM2,IFT88,KALRN,ROBO1,TANC2,NRP1,TENM2,SEMA3C,CAMK1D,CCDC88A,CD38,DENND5A,ADAMTS16,HDAC4,NTRK2,DNM3,RAP1A,TGFBR1,TNR,CUX1,TENM3,RAPGEF1,CHRNA3,EP300,GRID2,CAMK2G,EFNA5,MAGI2,RELN,PTPN9,MAP3K13,RALA,PPP3CA,PLS1,CAPZB,ELAVL4,CNTN1,DGUOK,NLGN1,SHOX2,KIDINS220,PLXNB1,COBL,PPFIA2,BCL11A,EPS8,CCP110,LRP4,EFNB2,TENM1,CDKL5,EFHC2,IL1RAPL1,KIT,TRPV4,PTPRO,RAPGEF2,SEMA3E,UBE3A,FGF13,EPHB2,TOX,FER,ABL2,ARHGAP35,SEMA5B,CDKL1,SLC39A12,WDPCP,AUTS2,OCLN,MAP2,P2RX7,BHLHB9,SEMA3A,MARK2,PDLIM5,MARK1,DAB2,ITGA3,HGF,MACF1,PAK1,FMR1,LIMK2,ULK4,CRMP1,PT*

			PRF,RIT2,ISLR2,PAK3,DTNBP1,GAK,LRP8,APC,KIAA0319,NGF,VLDLR,MAP2K1,VPS35,ATMIN,RREB1
GO:0051234	establishment of localization	1.340984783725342e-11	IGHV1OR21-1,PIEZO2,GABRB3,ABCC10,GABRG3,MAPK14,ABCG8,TRAPPC8,HNRNPA1L2,ASIC2,CTBP2,TMPRSS15,RAB27A,CACNA1E,KCNJ6,ANXA8L1,ANO2,ANK3,IGHV4-31,HEATR5A,IGHV3-64,WLS,RBFOX1,SYN3,AKAP13,SNX31,VPS13D,GRIK1,IGHV1OR15-9,SLC24A3,IGHV4OR15-8,NPHP3,ITGA2,ABCG1,TMPRSS2,KCNQ1,SUN2,GRIN3A,NRXN1,TMPRSS3,SGSM1,NRXN3,SLC1A2,RAB5A,DOCK2,PCNT,MS4A1,RIMS2,GRIA1,CLEC16A,PKD1L1,DLG2,SLC9B1,TRPM3,ANO4,CDH13,ANK2,TBC1D22A,ANKH,GRAMD1C,KCND3,LRP1B,FAM155A,SYNDIG1,SLC12A1,DPP10,TF,SCN8A,KCNE1,CEP41,TMEM108,KCNJ15,SYT17,KCNG3,GPC3,FBLN5,NRG1,SCARA5,STXBP6,PSMB2,SHISA6,SPIDR,IL4R,ENTHD1,CECR2,RYR3,MCTP1,ASTN2,SYT16,SLCO3A1,CCR1,CFTR,BBS9,SCFD2,PCTP,PM20D1,AGBL4,DNAJC15,RASGRF1,DPYSL2,GRID1,SLC6A2,ARFIP1,SLC17A3,PIK3C3,UTRN,SPEF2,FAM3B,ERC1,KLRF2,RAB7A,TAPBP,EPHA3,PTPRN2,DCLK1,SLC44A1,AGT,ATG4C,ERC2,SHISA9,CHRM5,VTI1A,WDR11,ATP6V0D1,RHOJ,CDH2,PARK2,LOXL2,RN7SL659P,CORO2B,CYBB,MCTP2,SYTL5,DNAH9,NTN1,KCNH1,SLC22A23,SLC5A4,CP,DENND2A,RNF185,USP6,ANKFN1,ITSN1,NCF4,PCDH17,KCNMA1,RIN3,APBA2,CYB5R2,PDCD6,STXBP4,SLC44A5,IGF1R,MDM2,MX2,SLC24A4,E2F3,NUTF2,SYT9,TMEM241,CCDC91,GABRG1,KCNK17,PXK,SLC14A2,TRPC5,DMD,HCN1,SYT1,CACNA2D3,CACNG3,NOX5,FGF14,FSHR,GSG1L,GRIA4,GRM1,FMN2,HNRNPA2B1,SORCS2,JAKMIP1,SLC39A11,DPP6,WDR83OS,TRPM2,CNIH3,IPO13,RYR2,OCA2,KIF3B,UNC13C,CATSPER2,DHRS7C,LRRK2,SLC1A3,KCNH5,KXD1,NUSAP1,ILDR1,SLC4A4,CHD7,DPY30,KCNK13,EMB,SH3BP1,MTMR2,GSK3B,SLC8A1,PRKD1,SERPINA5,FLVCR1,TGFB2,ATP1A4,MFSD9,VAV3,ATP9B,DNAJC6,SLC5A3,SLCO2B1,LLGL2,PRKAR1B,ZDHHC17,KCNC1,DNAAF2,SNX24,WASF1,GRIN2B,ZDHHC14,EXT2,NKAIN2,ABCB5,MREG,SNAP25,SNAP25-AS1,PITPNC1,PML,STOML1,SYBU,USP36,ARHGAP12,AKAP6,GRIK3,IGHV3-16,SNAP23,BCL2,CHMP4C,RALBP1,KCNJ3,SLC30A5,DISP1,LRP2,NKAIN3,NSF,FRMD4A,ITPR2,CD163,CD163L1,GDAP1,ABCC9,CYB561A3,MICAL3,RPGR,FYN,KCNE2,SLC26A7,NR4A1,HECW1,NEU3,RCVRN,RHBDD1,PDE4D,ATP10B,ENPP2,ABCB10,ACTN2,ATP8A2,GABRB1,CSNK1G3,EXOC4,KCNS3,GRIK2,KCNIP4,KCND2,SCN11A,SLC1A1,UGCG,AGAP1,MFSD12,CADPS,TMPRSS4,VPS26B,CHRM1,CTDSPL2,PKP2,RANBP17,UBXN2B,ABCA13,CCDC88B,SLC35F3,BBS2,HBE1,ARL6IP5,ATP1A1,EPM2A,PLN,SLCO1B1,SLCO1B3,ZMAT3,EVI5,EXOC6B,HEPHL1,EHBP1,IFT88,NLGN4X,PRKCE,SLC2A9,ATP6V1D,KMT2A,PRKCB,STON1,STON1-GTF2A1L,VDR,ADCY8,EFHB,FAM19A4,IGF2BP3,LRRTM1,RAB31,SCP2,SLC47A1,TRAPPC12,CAPN3,ITLN1,KALRN,PARD3B,CCDC88C,EDNRB,MSR1,TANC2,TBC1D4,BNIP3L,NASP,SGIP1,SLC12A8,STX12,CDH17,KCNC4,NBAS,TRAF3IP2,VPS37B,CBLN4,CR1,GABRA2,SEC24B,ACSL5,PKHD1,STK3,TGM2,CASK,IGHV4-28,ABCA12,APP,CALCRL,NRP1,RN7SL674P,TSNARE1,ELMO1,ADTRP,IMMP2L,PRELID2,KCNK2,SLC38A7,CAMK1D,IGF2R,ATF2,GC,PEX5L,SLC35F4,GOPC,ROS1,SMG6,TM9SF4,KPNB1,TCF7L2,BICD1,CCT6B,PARD3,THOC2,ABAT,FRAS1,GRIN2A,GRIP1,RIMS3,SGK2,KIFAP3,OPHN1,BCLAF1,SAMD9,SLC35F2,CCDC88A,CD38,DENND5A,RIN2,SLC30A7,TUB,NCF2,RNF126,SMG7,STK36,BCL2L1,PDE4B,GHR,GLRA2,NTRK2,ACSL4,MGST1,SLC5A10,DNM3,RASGRP1,TRAPPC10,ARR3,COMMD1,KCTD7,RAB11FIP4,RABGEF1,STARD4,C17ORF75,GLI3,CACNA1C,CLDN1,COG7,KIF16B,NIPA2,STXBP5,LAMP5,RAP1A,SRP54,CYSLTR1,KCNJ12,SVOPL,EPHA5,SLC13A4,VPS53,CUX1,SLC25A21,SLC6A17,CPE,IL16,PTTG1IP,RAPGEF1,STARD5,CHRNA3,CHRNA5,RAB2A,SLC16A7,SCFD1,SPAG17,TBX3,TTC39B,CACNA2D1,CACNA2D4,STX8,ATXN1,CLIC6,GAB2,GLRA1,HBD,PCSK5,GRID2,IFNGR2,NPC1,PIBF1,RASGRF2,SAMM50,TMEM50B,TRPM7,PSMA1,SV2B,VPS39,SPG11,ADORA2A,ADRBK2,CAMK2G,DYNC1I2,EFNA5,MERTK,CACNG2,MAGI2,NF1,RGS7,GOT2,GULP1,KCNQ5,SORL1,BID,TTC8,MICU1,RELN,SLC39A8,SLC9C1,GRM5,IGHV4-4,SELE,SNUPN,DYNC1H1,PLSCR4,DDC,NUMB,IGHV3OR16-12,RALA,ADCYAP1R1,SLC35A5,ATP10A,EXOC5,MAN1A1,PPP3CA,TBC1D3B,TMEM163,KCNB2,KLF7,PLS1,SH3GL2,SLC7A1,SNX25,ATP8B4,CXADR,DGKI,GNPTAB,PER1,SLC24A2,TBC1D5,ANKRD13A,CBLB,PLSCR1,SEC63,SLC38A6,SLC5A1,ATP9A,CNTN1,GRM7,RIMS1,GABRA3,GPSM2,STIM2,SLC22A2,NLGN1,P2RX6,SLC35F1,SH3KBP1,SLC22A3,NPR3,OSBPL5,AKAP7,MAPK8IP2,CACHD1,CACNA1D,LRPP

			RC,NSG1,VPS37A,GABRA5,TPCN2,JAK2,SLC5A8,SPNS2,AHI1,SLC25A48,SMAD3,VPS41,XKR4,CD300A,NOS1,CDK1,HGSNAT,MAGT1,MCM3AP,SLC39A10,TNF,PANX1,SNX5,CPT1B,POMC,PPFIA2,TMC2,BLOC1S6,FCHSD2,TRIM23,KCNT2,TMC5,KCNK10,SPAG16,ADORA3,ARF1,MYO6,DOCK1,GRIA3,IGLC2,IGLL5,KIF18A,RUFY1,TSPAN13,ANO1,GCLC,KCNA6,QKI,RAB23,TPD52,SLC7A7,TOMM34,TRPC4,LRP4,CNGB3,EFNB2,KCNH8,RAB24,RSRC1,SAE1,SHFM1,TRDN,ANO10,SLC15A1,CLSTN1,INSC,LRRC8C,LRRC8D,CSE1L,NSG2,SCN1A,TENM1,ABCG2,DMBT1,GRIK4,WDR35,ARHGAP11B,DYNC2H1,GABRR2,KCNH7,MYRIP,STX16,STX16-NPEPL1,TMEM150C,EGF,MIP,PTPN14,SEC16B,SPIRE2,VPS8,IL1RAPL1,KIT,KPNA3,TRPV4,ABCA10,ABCA5,ABCA6,DNAH11,KCNAB1,NAPG,SLCO4C1,HTR2C,SLC30A8,UBE3A,FGF13,GABRB2,GRM4,NUP155,SLC22A15,ANO3,EPHB2,IGHV3-72,NDFIP2,ABCC1,DAPK1,ERBB4,FER,CACNG6,FLVCR2,IFT43,LRP5,XKR7,SLAMF1,ABCC11,ABL2,BSG,F11R,GAS8,SETD2,TMEM50A,VPS13C,AP2B1,CD2AP,NSUN2,SV2C,ATP8A1,CCL3,FGF7,MCUR1,MYO7B,ARHGAP25,IPO11,REEP1,SPA17,ARF4,ARFGAP3,MYH9,PACSIN2,PTGER3,SLC2A13,SLC39A12,SOX4,SPRN,MIPEP,OSCP1,P2RX1,PIK3CD,RGPD1,AGTPBP1,ASGR2,ITGA8,KCNE4,CNGA4,PDZD7,PLAT,SFXN3,TNKS,UNC13A,VPS45,WDPCP,CDH23,MEF2A,OCLN,RYR1,TBC1D9,UNC13B,GLRA3,MAP2,NTF3,P2RX7,PLA2G4A,RAPGEF4,SORCS1,ARRDC4,RASA3,ZDHHC11,ZDHHC11B,ZDHHC9,ATG2B,COG1,KIAA1109,RABGAP1L,SCAMP5,SNX30,SUFU,DENND5B,EXOC2,RBM4,BDKRB1,BDKRB2,AFTPH,THADA,ANXA11,AQP10,GNB5,GRIA2,KIF4A,MOGAT2,PROS1,REEP2,LDLRAD3,SNX7,TRIM72,VPS16,ATP6V1H,HCN4,MARK1,SCNN1A,SMPD3,ATP13A3,DAB2,LCP1,RNF207,STX3,SYT2,ACTN4,ADAM10,BLOC1S5,FGF12,KCNJ16,KMO,TXNDC5,KCNN3,RFTN1,RNASEH2B-AS1,SLC16A4,STAC,MYOM1,NUP153,TRPM4,ABCA8,ABCC2,BMP6,BTBD9,CHRM3,CLASP1,EIF4ENIF1,SIDT1,CNGB1,PCDHGA3,TMCO3,G3BP2,MYLK,SLC38A4,CALCR,CENPF,MYO1B,OSBPL3,RN7SL318P,SLC13A3,SLC26A8,TLK1,CACNB2,NOS1AP,OSBPL10,SNAP29,INSR,MACF1,PAK1,PSMB7,CHM,ENPP1,FMR1,LIMK2,MICU2,THEM4,ATP5J,DKC1,GABRA1,IGHV1-69,MON2,ABCD1,AMPH,CHKA,GABRR3,IGHV1-46,MAD1L1,AP1G2,CD84,EPG5,ESYT2,JPH4,PPARA,SIL1,SORT1,TNFAIP8L3,TTN,CORIN,FAM3D,FHL1,HEATR5B,NUP214,SLC22A5,CACNA1A,TRPM1,SCN9A,ATP6V1E1,C2,IGHV1-18,NUP62CL,OPTN,RIT2,EXT1,IFT81,PRKCG,PRLR,UMOD,DOCK7,ELMO2,FBXW11,ICK,KLHL3,GOSR2,SLC24A5,SLC38A10,SLC40A1,STIM1,TRPM6,KCNC2,NDE1,SLC22A10,SLC22A25,SLC9A9,STAT3,USP7,DNM1P46,HPN,PIK3R2,SLC15A5,USH2A,CASQ2,DTNBP1,GAK,RAP1B,SLC28A3,TMEM175,CNNM3,LGALS9,LRP8,SNCAIP,MAPKAPK2,SLC7A8,TMEM63A,TUSC3,UPK3A,CKAP5,CSN3,HDLBP,PTGES,SRP19,UBASH3B,VLDLR,EXOC6,DHX9,RGCC,RPH3AL,SLC9A2,GABRG2,MAP2K6,NCALD,OSBPL1A,SIAH3,SLC16A12,SPTBN4,AP5M1,CLASP2,MAP2K1,TPH1,VPS35,C1QTNF3,MCOLN3,ATP6V1E2,RGN,RN7SL673P
GO:0055085	transmembrane transport	1.7185660776443625e-11	PIEZO2,GABRB3,ABCC10,GABRG3,MAPK14,ABCG8,ASIC2,CACNA1E,KCNJ6,ANO2,ANK3,GRIK1,SLC24A3,ABCG1,KCNQ1,GRIN3A,NRXN1,SLC1A2,GRIA1,PKD1L1,SLC9B1,TRPM3,ANO4,ANK2,ANKH,KCND3,FAM155A,SLC12A1,DPP10,SCN8A,KCNE1,KCNJ15,KCNG3,GPC3,SCARA5,PSMB2,SHISA6,RYR3,SLCO3A1,CFTR,PM20D1,DNAJC15,RASGRF1,GRID1,SLC6A2,SLC17A3,UTRN,SLC44A1,AGT,SHISA9,ATP6V0D1,CYBB,KCNH1,SLC22A23,SLC5A4,RNF185,KCNMA1,STXBP4,SLC44A5,SLC24A4,TMEM241,GABRG1,KCNK17,SLC14A2,TRPC5,DMD,HCN1,CACNA2D3,CACNG3,NOX5,FGF14,GSG1L,GRIA4,GRM1,SLC39A11,DPP6,TRPM2,CNIH3,RYR2,OCA2,CATSPER2,DHRS7C,SLC1A3,KCNH5,SLC4A4,CHD7,KCNK13,EMB,SLC8A1,PRKD1,FLVCR1,ATP1A4,MFSD9,SLC5A3,SLCO2B1,ZDHHC17,KCNC1,GRIN2B,ABCB5,SNAP25,AKAP6,GRIK3,BCL2,RALBP1,KCNJ3,SLC30A5,LRP2,ITPR2,ABCC9,CYB561A3,FYN,KCNE2,SLC26A7,HECW1,PDE4D,ABCB10,ACTN2,GABRB1,KCNS3,GRIK2,KCNIP4,KCND2,SCN11A,SLC1A1,MFSD12,ABCA13,ARL6IP5,ATP1A1,EPM2A,PLN,SLCO1B1,SLCO1B3,PRKCE,SLC2A9,ATP6V1D,PRKCB,SLC47A1,CAPN3,ITLN1,EDNRB,SLC12A8,CDH17,KCNC4,GABRA2,ACSL5,ABCA12,APP,KCNK2,SLC38A7,GC,PEX5L,SLC35F4,GOPC,GRIN2A,SLC35F2,SLC30A7,PDE4B,GLRA2,SLC5A10,COMMD1,CACNA1C,NIPA2,SRP54,KCNJ12,SVOPL,SLC13A4,SLC25A21,SLC6A17,CHRNA5,SLC16A7,CACNA2D1,CACNA2D4,CLIC6,GLRA1,GRID2,IFNGR2,RASGRF2,TRPM7,PSMA1,SV2B,CACNG2,RGS7,KCNQ5,MICU1,RELN,SLC39A8,SLC9C1,GRM5,SLC35A5,ATP10A,TMEM163,KCNB2,SLC7A1,SLC24A2,SEC63,SLC38A6,SL

			C5A1,GABRA3,STIM2,SLC22A2,NLGN1,P2RX6,SLC35F1,SLC22A3,AKAP7,MAPK8IP2,CACHD1,CACNA1D,GABRA5,TPCN2,SLC5A8,SPNS2,SLC25A48,NOS1,MAGT1,SLC39A10,TNF,PANX1,CPT1B,TMC2,KCNT2,TMC5,KCNK10,GRIA3,TSPAN13,ANO1,KCNA6,SLC7A7,TRPC4,CNGB3,KCNH8,TRDN,ANO10,SLC15A1,LRRC8C,LRRC8D,SCN1A,ABCG2,GRIK4,GABRR2,KCNH7,TMEM150C,MIP,TRPV4,ABCA10,ABCA5,ABCA6,KCNAB1,SLCO4C1,HTR2C,SLC30A8,FGF13,GABRB2,SLC22A15,ANO3,EPHB2,ABCC1,DAPK1,CACNG6,FLVCR2,ABCC11,SV2C,ATP8A1,CCL3,MCUR1,SLC2A13,SLC39A12,OSCP1,P2RX1,KCNE4,CNGA4,SFXN3,MEF2A,OCLN,RYR1,GLRA3,P2RX7,RASA3,BDKRB1,THADA,AQP10,GNB5,GRIA2,ATP6V1H,HCN4,SCNN1A,ATP13A3,RNF207,ACTN4,FGF12,KCNJ16,KCNN3,SLC16A4,STAC,TRPM4,ABCA8,ABCC2,CHRM3,CNGB1,TMCO3,SLC38A4,CALCR,SLC13A3,SLC26A8,CACNB2,NOS1AP,INSR,PSMB7,ENPP1,FMR1,MICU2,ATP5J,GABRA1,ABCD1,GABRR3,JPH4,SORT1,FHL1,SLC22A5,CACNA1A,TRPM1,SCN9A,ATP6V1E1,SLC24A5,SLC38A10,SLC40A1,STIM1,TRPM6,KCNC2,SLC22A10,SLC22A25,SLC9A9,HPN,SLC15A5,CASQ2,SLC28A3,TMEM175,CNNM3,SLC7A8,TMEM63A,TUSC3,UBASH3B,SLC9A2,GABRG2,SLC16A12,MCOLN3,ATP6V1E2,RGN
GO:0034220	ion transmembrane transport	1.9527491540557424e-11	*PIEZO2,GABRB3,GABRG3,ASIC2,CACNA1E,KCNJ6,ANO2,ANK3,GRIK1,SLC24A3,KCNQ1,GRIN3A,NRXN1,SLC1A2,GRIA1,PKD1L1,SLC9B1,TRPM3,ANO4,ANK2,ANKH,KCND3,FAM155A,SLC12A1,DPP10,SCN8A,KCNE1,KCNJ15,KCNG3,SCARA5,SHISA6,RYR3,CFTR,PM20D1,RASGRF1,GRID1,SLC6A2,SLC17A3,UTRN,AGT,SHISA9,ATP6V0D1,CYBB,KCNH1,KCNMA1,SLC24A4,GABRG1,KCNK17,TRPC5,DMD,HCN1,CACNA2D3,CACNG3,NOX5,FGF14,GSG1L,GRIA4,GRM1,SLC39A11,DPP6,TRPM2,CNIH3,RYR2,CATSPER2,DHRS7C,SLC1A3,KCNH5,SLC4A4,CHD7,KCNK13,EMB,SLC8A1,PRKD1,ATP1A4,ZDHHC17,KCNC1,GRIN2B,SNAP25,AKAP6,GRIK3,KCNJ3,SLC30A5,LRP2,ITPR2,ABCC9,FYN,KCNE2,SLC26A7,HECW1,PDE4D,ACTN2,GABRB1,KCNS3,GRIK2,KCNIP4,KCND2,SCN11A,SLC1A1,MFSD12,ARL6IP5,ATP1A1,EPM2A,PLN,PRKCE,SLC2A9,ATP6V1D,SLC47A1,CAPN3,EDNRB,SLC12A8,KCNC4,GABRA2,APP,KCNK2,SLC38A7,PEX5L,GOPC,GRIN2A,SLC30A7,PDE4B,GLRA2,SLC5A10,COMMD1,CACNA1C,NIPA2,KCNJ12,SLC13A4,SLC25A21,SLC6A17,CHRNA3,CHRNA5,SLC16A7,CACNA2D1,CACNA2D4,CLIC6,GLRA1,GRID2,IFNGR2,RASGRF2,TRPM7,CACNG2,RGS7,KCNQ5,MICU1,RELN,SLC39A8,SLC9C1,GRM5,ATP10A,TMEM163,KCNB2,SLC7A1,SLC24A2,SLC5A1,GABRA3,STIM2,SLC22A2,NLGN1,P2RX6,AKAP7,MAPK8IP2,CACHD1,CACNA1D,GABRA5,TPCN2,SLC5A8,NOS1,MAGT1,SLC39A10,TNF,PANX1,TMC2,KCNT2,TMC5,KCNK10,GRIA3,TSPAN13,ANO1,KCNA6,TRPC4,CNGB3,KCNH8,TRDN,ANO10,SLC15A1,LRRC8C,LRRC8D,SCN1A,GRIK4,GABRR2,KCNH7,TMEM150C,TRPV4,KCNAB1,HTR2C,SLC30A8,FGF13,GABRB2,ANO3,EPHB2,ABCC1,DAPK1,CACNG6,ATP8A1,CCL3,MCUR1,SLC39A12,P2RX1,KCNE4,CNGA4,SFXN3,RYR1,GLRA3,P2RX7,RASA3,BDKRB1,THADA,GNB5,GRIA2,ATP6V1H,HCN4,SCNN1A,ATP13A3,RNF207,ACTN4,FGF12,KCNJ16,KCNN3,STAC,TRPM4,CHRM3,CNGB1,TMCO3,CALCR,SLC13A3,SLC26A8,CACNB2,NOS1AP,FMR1,MICU2,ATP5J,GABRA1,GABRR3,JPH4,FHL1,SLC22A5,CACNA1A,TRPM1,SCN9A,ATP6V1E1,SLC24A5,SLC40A1,STIM1,TRPM6,KCNC2,SLC9A9,HPN,CASQ2,SLC28A3,TMEM175,SLC7A8,TMEM63A,TUSC3,UBASH3B,SLC9A2,GABRG2,SLC16A12,MCOLN3,ATP6V1E2,RGN*
GO:0009888	tissue development	3.1375770929342685e-11	*PLCB1,MAPK14,RUNX1,MYO18B,SVEP1,WLS,RBFOX1,AKAP13,SORBS2,SLC24A3,NPHP3,ITGA2,KCNQ1,TNC,MTPN,TGFBR3,ANKH,CSMD1,PLXNA4,GPC3,NRG1,ETS2,PSMB2,HYDIN,ALDH1A2,CECR2,NLN,ASTN2,SEMA6D,CCR1,CFTR,TIAM1,RHOC,COL22A1,LDLRAD4,CBFA2T2,NKD1,ARID5B,ROBO2,C2CD3,EPHA3,PRKACB,COL15A1,AJAP1,AGT,RCAN1,CDH2,FHL2,LOXL2,LAMA2,GREB1L,SEMA3D,NTN1,GPC6,NELL1,ZNRF3,FTO,HDAC2,MYO9A,SLIT2,PDCD6,KRT25,MDM2,SLC24A4,SMAD1,DMD,BASP1,KRT2,SIPA1L3,VCL,CELSR1,FSHR,ATRX,RYR2,NTNG1,TENM4,STRC,KIF26B,PRICKLE2,CHD7,SH3BP1,SOX6,SEMA4D,GSK3B,LGR5,SLC8A1,FRMD6,TGFB2,FMN1,LCE2B,LCE2C,SEMA5A,KRT74,PRKCH,RIPK4,RYK,PCDH15,SGCD,WDR72,EXT2,LAMA3,ANKRD6,NR2F2,NR5A2,PML,PPARGC1A,SGCG,ARHGAP12,AKAP6,PLXNA2,BCL2,RAD51B,ARHGAP24,EPHB1,CSGALNACT1,LRP2,TMEM100,VANGL2,EPHA7,NR4A1,CD44,CASP6,PDE4D,PYY,ACTN2,TNMD,EXOC4,LAMB4,ZNF516,SERPINB7,UNC5C,MYLK3,UGCG,ZFPM2,PALLD,RARB,MTOR,ROR1,LDB2,MEGF9,PKP2,BMPER,MATN3,PGK1,ZFP64,COL11A1,PLN,CTNNBIP1,SOX8,DSG4,VDR,CCDC88C,DLC1,EDNRB,ROBO1,RXFP1,SATB2,NOX4,GRHL2,COL2A1,NEBL,SEC24B,PKHD1,STK3,TGM2,KDR,PPHLN1,EDA,ABCA12,COL18A1,NRP1,SHROOM3,LGR4,EGFLAM,LCE6A,THRB,KCNK2,SEMA3C,ADAM17,TSPEAR,ATF2,EFEMP1,ROS1,TGFB1I1,TCF7L2,*

			ATRNL1,FRAS1,HRNR,OPHN1,TRPS1,CNN3,ADAMTS16,HDAC4,SOX5,GHR,STARD13,SPRED2,GLI3,KAZN,CLDN1,KIF16B,SPRR2B,SPRR2E,CERS3,RAP1A,SAFB2,TGFBR1,KRTAP6-1,CHST11,RAPGEF1,EP300,TBX3,CYP7B1,SPRR4,TRIOBP,TP73,PSMA1,FLNB,FAM20A,SPRR2G,MAGI2,NF1,SORL1,IFT80,TTC8,DMRT1,ASXL1,POR,HDAC9,COL12A1,RALA,EXOC5,FBXL17,JAG1,MEOX2,PPP3CA,KLF7,NEB,PLS1,CXADR,WWTR1,KL,GSTA2,GPC4,TRIM16,FHOD3,EHF,JAK2,SHOX2,AHI1,SMAD3,COL8A1,RDH10,CDK1,HIVEP3,ITPK1,TNF,COBL,OVOL2,ZFPM1,BMP15,DLG5,PHEX,ARID2,MMP20,FREM2,HMGA2,MYO6,SULT1B1,MBOAT2,SULF1,LRP4,EFNB2,ALPK2,DMBT1,MYH14,PRKAR1A,TNFRSF19,BPTF,MLLT3,CD109,EGF,TRPV4,CC2D2A,CPS1,EDAR,EFNB1,PTPRO,RAPGEF2,ESR1,SEMA3E,SGCZ,FNDC3A,KRT76,TOX,ERBB4,MINPP1,SLITRK6,LRP5,MYH15,BSG,F11R,SETD2,ALX4,ARHGAP35,NSUN2,SGMS2,CCL3,FGF7,LAMA1,SEMA5B,SPRED3,SULF2,SOX4,PDGFD,PIK3CD,ASGR2,ITGA8,ARID4A,FAM172A,PDZD7,WDPCP,CDH23,FGFR1,MEF2A,RYR1,ARID4B,KEAP1,P2RX7,SCUBE1,AR,BCOR,KIAA1109,SUFU,EYA1,SEMA3A,CDC73,PDLIM5,FERMT2,HCN4,MARK1,SMPD3,DAB2,PBX1,RNF207,DAAM1,ITGA3,POU2F3,RPS6KA6,TRPM4,BMP6,CLASP1,TMOD1,HGF,MYLK,CENPF,ZBTB16,ELN,INSR,PAK1,PSMB7,SKI,CTDP1,ENPP1,PRKX,EVC,PCK1,PPARA,TTN,C1GALT1,COL19A1,GNA13,MYH7,S1PR3,SPRED1,AKR1C2,CHSY1,KRT4,LCE1F,NTN4,BTRC,PGM5,ESRP2,EXT1,MDM4,MYH6,PRLR,UMOD,KLHL3,SLC40A1,STIM1,ADARB1,MYH11,SCEL,TP63,HPN,USH2A,DHRS7B,RAP1B,LCE2A,UPK3A,BMPR1A,SPINT2,ABI1,CTSH,RGCC,EDA2R,IL6R,LCE4A,TFIP11,CLASP2,MAP2K1,SOS1,FGF1,MCOLN3,TBX20,RREB1,TBX18
GO:0050793	regulation of developmental process	9.45561093895826e-11	*ISM1,CDH4,ZHX3,PLCB1,CMKLR1,DAB1,OMA1,GTF2I,FMNL2,CDC42EP3,MAPK14,DSCAM,RUNX1,ASIC2,DCC,RBFOX1,FBLN1,NPHP3,ABCG1,GRIN3A,NRXN1,ZRANB1,PLEKHB2,CHODL,RIMS2,MTPN,FGD6,TGFBR3,FSTL4,LRRC4C,ANKH,RUNX1T1,SYNDIG1,GADD45A,PCP4,ACIN1,SYT17,PLXNA4,FLRT2,GPC3,NRG1,PSMB2,IL4R,NLN,SUZ12,SEMA6D,CCR1,CCR3,CFTR,RBFOX2,TIAM1,RHOC,AGBL4,LDLRAD4,DPYSL2,NKD1,ALK,ZNF536,FBXO31,CNTN4,ROBO2,EPHA3,AJAP1,AGT,ATAT1,RHOJ,CDH2,PARK2,LOXL2,LAMA2,KIR2DL4,CYBB,SEMA3D,NTN1,ZNF268,GPC6,NELL1,SETD3,ZNRF3,FMNL3,TRIO,FTO,HDAC2,LAMA4,MYO9A,SLIT2,PDCD6,IGF1R,MDM2,PALMD,SMAD1,TRPC5,DMD,SYT1,BASP1,KRT2,VCL,CELSR1,CSMD3,FSHR,MAEL,STK24,NTNG1,TENM4,LRRK2,RORB,NTRK3,RORA,PRICKLE2,CHD7,DISC1,HEXB,GLIS1,SOX6,MECP2,MTMR2,SEMA4D,GSK3B,LINGO2,SLC8A1,PRKD1,FLVCR1,TGFB2,CLSTN2,PRAMEF12,SEMA5A,BDNF,PRKCH,RYK,ABI3BP,KDM4C,LAMA3,RAG1,RAG2,ANKRD6,PML,PPARGC1A,AKAP6,FBN1,PLXNA2,BCL2,RALBP1,ZNF675,EPHB1,SIPA1L1,LRP2,TMEM100,VANGL2,EPHA7,ZBTB7C,FYN,CHN1,CD44,HECW1,THBS2,FOXO1,KANK1,ENPP2,ABCB10,ATP8A2,KLF13,MME,TNMD,CELF4,MYLK3,ZFPM2,RARB,STRIP1,MTOR,ROR1,MOV10,FAT3,PKP2,BMPER,ERMN,PTPRD,TIAM2,BBS2,PGK1,CTNNBIP1,SOX8,PRKCB,VDR,FOXO3,LRRTM1,MITF,CAPN3,KALRN,DLC1,EDNRB,MSR1,ROBO1,TANC2,FLT1,GRHL2,TCF4,MAPK9,ZNF277,CR1,SEC24B,PKHD1,STK3,TGM2,VASH2,KDR,ARHGAP15,ABCA12,APP,NRP1,PTPRM,SHROOM3,ARHGEF18,LGR4,ST7,KCNK2,SEMA3C,CAMK1D,MAP3K5,ATF2,ETS1,NR2C2,EFEMP1,PRTG,TGFB1I1,TCF7L2,PARD3,GRIP1,TRPS1,RIN2,HDAC4,SOX5,DNMT1,GHR,JAM2,NTRK2,DNM3,RASGRP1,CAMK4,SDK1,SPRED2,GLI3,RAP1A,TGFBR1,CYSLTR1,ADAM12,DICER1,TNR,CUX1,CHRNA3,HMG20A,TBX3,WIF1,TRIOBP,TP73,GRID2,PARVB,PSMA1,ADCK1,EFNA5,BRD1,MAGI2,NF1,SORL1,TEAD4,DMRT1,ASXL1,EIF4E,POR,RELN,TNFRSF11B,GRM5,HDAC9,LRRTM3,NAPEPLD,NUMB,MAP3K13,RALA,IL1RAP,ATP10A,JAG1,PPP3CA,KLF7,PLS1,CAPZB,WWTR1,KL,ELAVL4,GPC4,RIMS1,TRIM16,GABPA,KAT7,TMEM135,AGGF1,ASAP1,N4BP2L2,NLGN1,SH3KBP1,JAK2,SHOX2,AHI1,SMAD3,PLXNB1,TCF12,CDK1,HDAC7,TNF,COBL,OVOL2,ZFPM1,PPFIA2,BLOC1S6,DLG5,MMP20,BRINP1,BCL11A,HMGA2,NEK4,DOCK1,EPS8,MBOAT2,DAAM2,SMOC2,SULF1,C5AR1,LRP4,EFNB2,CLSTN1,ALPK2,CTNNA1,WDFY2,CDKL5,MYH14,FAM171A1,MLLT3,CD109,EGF,CMA1,IL1RAPL1,KIT,TRPV4,VWC2,ABCA5,RAPGEF2,ESR1,HTR2C,JAK1,NCMAP,SEMA3E,UBE3A,DNMBP,FGF13,PLAG1,EPHB2,TOX,ERBB4,MAP3K3,RBM19,SLITRK6,SPTA1,FNIP1,LRP5,CAMP,F11R,ARHGAP35,NSUN2,SGMS2,CCL3,FGF7,LAMA1,PREX1,SEMA5B,SPRED3,FGD4,PSG9,MYH9,SLC39A12,SOX4,NEDD9,PIK3CD,KDM3A,UNC13A,WDPCP,FGFR1,MAP2,IL23R,KEAP1,NTF3,P2RX7,AR,BCOR,FGL2,KIAA1109,SUFU,EYA1,IL1RL2,RBM4,HLA-DRA,BHLHB9,L3MBTL1,NEK5,SEMA3A,CDC73,EPB41L3,MARK2,MEIS2,PDE5A,ZMIZ1,PDLIM5,PPARGC1B,TRIM72,FERMT2,MARK1,S*

			MPD3,TNN,ZC3H13,DAB2,GPR55,PBX1,RNF207,SYT2,ACTN4,ADAM10,BLOC1S5,CCL7,DAAM1,EEF1E1,PRAMEF8,NREP,RPS6KA6,TRPM4,BMP6,CLASP1,EIF4ENIF1,CFDP1,GAS2,HGF,CENPF,SCUBE3,ZBTB16,INSR,MACF1,PAK1,PDE3A,PSMB7,SKI,AKT3,CTDP1,ENPP1,FMR1,JDP2,PRKX,PCK1,PPARA,SORT1,ZFHX2,GNA13,PPP2R3C,S1PR3,SPRED1,NTN4,PTPRF,RBL1,CCDC3,MYH6,NCOA1,PRLR,TYMP,DOCK7,HDAC5,AKAP2,STIM1,STAT3,TP63,AMOT,HPN,ISLR2,PAK3,USH2A,DTNBP1,LGALS9,LRP8,APC,BMPR1A,KIAA0319,NGF,PRKCA,UBASH3B,VLDLR,ADAMTS20,CTSH,RGCC,CYB5D2,GPR21,IL6R,SPTBN4,CLASP2,MAP2K1,PRAMEF7,SOS1,TPH1,VPS35,FGF1,TBX20,RGN,RREB1,TBX18
GO:0051128	regulation of cellular component organization	1.3533079549239339e-10	*CDH4,PLCB1,DAB1,OMA1,CDC42EP3,MAPK14,DSCAM,RUNX1,ASIC2,DCC,DUSP22,AKAP13,VPS13D,ITGA2,ATF7IP,NRXN1,RAB5A,CHODL,RIMS2,MTPN,SHANK2,NRG3,CLEC16A,FSTL4,CDH13,LRRC4C,CSNK2A1,PDE4DIP,SYNDIG1,TF,ERCC4,SDCCAG8,PRDM9,PSMC6,SYT17,PLXNA4,FLRT2,GPC3,FBLN5,NRG1,STXBP6,SPIDR,IL4R,LRFN5,MCTP1,PLCE1,STK38L,SEMA6D,SPOCK1,ABHD17C,CTNNA2,TIAM1,RHOC,LDLRAD4,DNAJC15,DPYSL2,ARFIP1,CBFA2T2,IL1RAPL2,ALK,FBXO31,RAB7A,ROBO2,EPHA3,AGT,ATAT1,CDH2,PARK2,CORO2B,FRMPD4,ZNF804A,NEGR1,SEMA3D,NTN1,GPC6,RIN3,HDAC2,MYO9A,SLIT2,CDH8,RPS6KA2,LCMT1,IGF1R,SYT9,CEP97,PTPRG,SACS,TRPC5,DMD,SYT1,VCL,NOX5,CELSR1,CSMD3,FSHR,GSG1L,HNRNPA2B1,SKAP1,STK24,ATRX,TRPM2,NTNG1,LRRK2,IGFBP7,NTRK3,NUSAP1,IQCJ-SCHIP1,DISC1,PAN3,WRAP73,CDCA2,PDXP,SH3BP1,MECP2,MTMR2,SEMA4D,GSK3B,LINGO2,PRKD1,TGFB2,CLSTN2,FMN1,DNAJC6,SEMA5A,BDNF,PRKCH,RYK,WASF1,GRIN2B,PLEKHM2,SNAP25,PML,PPARGC1A,USP36,AKAP6,CNTNAP2,LRFN2,PLXNA2,BCL2,CHMP4C,RALBP1,ARHGAP24,EPHB1,SIPA1L1,ARHGEF10,EPHA7,FYN,PARN,CHN1,SMARCA4,CD44,HECW1,NEU3,PMP22,THBS2,KANK1,ENPP2,ACTN2,ATP8A2,DCDC2,MYLK3,RERG,GNG4,MTOR,RMI2,ROR1,MOV10,FAT3,ABCA13,ERMN,PTPRD,SCAF8,TIAM2,OGFOD1,EPM2A,CTNNBIP1,HAS3,IFT88,PRKCE,STON1,LRRTM1,RAB31,TRAPPC12,KALRN,DLC1,ROBO1,TANC2,TBC1D4,BNIP3L,FAP,SGIP1,CDH17,CENPV,MAPK9,MDM1,PKHD1,KDR,PPHLN1,APP,DGKB,NRP1,ARHGEF18,TENM2,ADTRP,SEMA3C,ADAM17,CAMK1D,ROS1,SMG6,TRABD2B,BICD1,TLK2,GRIP1,SGK2,OPHN1,CCDC88A,CD38,DENND5A,BCL2L1,ADAMTS16,HDAC4,DNMT1,MAPRE2,NTRK2,ACSL4,DNM3,RABGEF1,ZNF207,CLDN1,MORC2,STXBP5,RAP1A,TGFBR1,EPHA5,TNR,CUX1,PEAK1,TENM3,RAPGEF1,CHRNA3,EP300,SCFD1,TRIOBP,GRID2,ADCK1,CAMK2G,EFNA5,MAGI2,SORL1,ARHGAP6,BID,TTC8,DMRT1,RELN,PTPN9,SELE,DYNC1H1,LRRTM3,NUMB,RHPN2,MAP3K13,NPHP4,RALA,IL1RAP,NAV3,ATP10A,PPP3CA,NEB,PLS1,CAPZB,TBC1D5,ANKRD13A,ELAVL4,MNAT1,CNTN1,DGUOK,GPC4,RIMS1,TMEM67,GPSM2,L3MBTL3,TMEM135,ASAP1,BORA,FHOD3,NLGN1,CTTNBP2,RAD51AP1,DDX3X,SHOX2,AHI1,SMAD3,BAIAP2L1,CD300A,KIDINS220,PLXNB1,DCBLD2,NBN,TNF,COBL,PPFIA2,ADNP2,DLG5,FCHSD2,INO80,SCAF4,AFAP1,ARF1,BCL11A,KIF18A,RUFY1,GCLC,PMEPA1,EPS8,CCP110,LRP4,EFNB2,NET1,PHF8,CLSTN1,SELP,TENM1,ARHGEF11,CDKL5,EFHC2,MLLT3,EGF,IL1RAPL1,KIT,SLIT3,TRPV4,PTPRO,RAPGEF2,DNAJB6,ESR1,SEMA3E,UBE3A,EBAG9,FGF13,EPHB2,RNF4,TBCD,TOX,FER,SLITRK6,SPTA1,FNIP1,LRP5,SLAMF1,ABL2,F11R,FARP2,VPS13C,AP2B1,ARHGAP35,CD2AP,ATP8A1,SEMA5B,CDKL1,ARF4,MYH9,NSMCE2,PACSIN2,SLC39A12,SPTB,SERTAD2,TNKS,UNC13A,WDPCP,AUTS2,OCLN,UNC13B,MAP2,SLX1B,SOST,NTF3,P2RX7,TEX14,AR,KIAA1109,SCAMP5,SNX30,C10ORF90,BDKRB1,BHLHB9,GNL3,L3MBTL1,PRKAA2,SEMA3A,CDC73,EPB41L3,MARK2,PDLIM5,PTPRA,SNX7,FERMT2,MAP2K5,MARK1,PAPPA2,SMPD3,DAB2,HDAC8,LCP1,SYT2,ADAM10,ITGA3,LGI1,PIN1,SYNPO2,BMP6,BTBD9,CIT,CLASP1,TGFA,TMOD1,G3BP2,HGF,RASSF8,CENPF,TLK1,ELN,ARHGAP28,INSR,MACF1,PAK1,PDE3A,CDC16,CTDP1,ENPP1,FMR1,LIMK2,CHFR,DKC1,ULK4,BMF,CRMP1,MAD1L1,PPARA,CLIP1,FHL1,KATNBL1,FNIP2,ESR2,PTPRF,MPDZ,RIT2,G3BP1,PRKCQ,USP7,AMOT,HPN,ISLR2,PAK3,DTNBP1,GAK,RAP1B,LRP8,SNCAIP,APC,CKAP5,DPP4,KIAA0319,NGF,VLDLR,MTRF1,OSGIN2,RGCC,TFIP11,SPTBN4,CLASP2,MAP2K1,VPS35,TBX20,ATMIN,RREB1*
GO:0051239	regulation of multicellular organism	1.9144579307097004e-10	*ISM1,CDH4,PLCB1,CMKLR1,DAB1,OMA1,GTF2I,MAPK14,ABCG8,DSCAM,RUNX1,ASIC2,DCC,FBLN1,NPHP3,ITGA2,KCNQ1,NRXN1,TNNI3K,CHODL,RIMS2,MTPN,CELF2,TGFBR3,FSTL4,ANK2,ANKH,KCND3,SYNDIG1,TF,KCNE1,TMEM108,GADD45A,ACIN1,PLXNA4,FLRT2,NRG1,SHISA6,IL4R,RYR3,PLCE1,KSR2,SEMA6D,CCR1,CCR3,CFTR,RBFOX2,DTX4,PCTP,TIAM1,LDLRAD4,ZBTB20,BTN2A1,FBXO31,*

	al process		KLRF2,ROBO2,AJAP1,AGT,SHISA9,FBXO32,RHOJ,LOXL2,DLGAP1,LAMA2,KIR2DL4,CORO2B,CYBB,SEMA3D,NTN1,TXK,NELL1,DBH,TSPAN8,SETD3,KCNMA1,FTO,HDAC2,LAMA4,SLIT2,PDCD6,IGF1R,MDM2,SMAD1,NOVA1,PTPRG,PDE9A,TRPC5,DMD,BASP1,VCL,NOX5,FSHR,GRM1,MAEL,CTNNA3,RNLS,RYR2,TENM4,LRRK2,NTRK3,RORA,CHD7,DISC1,SH3BP1,SOX6,MECP2,MTMR2,SEMA4D,GSK3B,LINGO2,SLC8A1,PRKD1,FLVCR1,OGT,TGFB2,CLSTN2,SEMA5A,SLC5A3,BDNF,PRKCH,RYK,POLR3C,ABI3BP,LAMA3,RAG1,RAG2,NR2F2,PML,PPARGC1A,AKAP6,FBN1,NLRP2,NLRP7,PLXNA2,BCL2,ZNF675,EPHB1,KCNJ3,IL12RB2,LRP2,TMEM100,NR4A2,ABCC9,EPHA7,KCNE2,ADAMTS18,NR4A1,THBS2,FOXO1,PDE4D,TBXAS1,ENPP2,ABCB10,ATP8A2,KLF13,MME,TNMD,IGSF11,ZNF516,CELF4,SERPINB7,SCN11A,SLC1A1,ZFPM2,RARB,NLRP12,MTOR,DDX21,PKP2,BMPER,CCDC88B,PTPRD,TIAM2,BBS2,PGK1,PLCL1,ATP1A1,PLN,CTNNBIP1,EPB41L4B,SOX8,NLGN4X,PRKCE,PRKCB,VDR,FAM19A4,FOXO3,IGF2BP3,LRRTM1,MITF,CAPN3,KALRN,EDNRB,ROBO1,FAP,FLT1,GRHL2,MAP3K7,TACR3,CR1,STK3,TGM2,VASH2,KDR,PRCP,ABCA12,APP,CALCRL,NRP1,PTPRM,LGR4,SERPING1,ADTRP,CD58,KAT5,THRB,KCNK2,SEMA3C,ADAM17,TFPI,ATF2,ETS1,NR2C2,EFEMP1,PRTG,TGFB1I1,RPS6KA5,TCF7L2,PARD3,TRAF3,ABAT,TRPS1,CD38,RIN2,HDAC4,PDE4B,SOX5,GHR,JAM2,MAPRE2,NTRK2,PRKG1,RASGRP1,STARD13,CAMK4,RABGEF1,SPRED2,GLI3,CACNA1C,DOCK4,TGFBR1,CYSLTR1,KCNJ12,MGLL,PRDX4,ADAM12,DICER1,TNR,CUX1,IL16,CD96,CHRNA3,TBX3,CACNA2D1,DIO2,GLRA1,TP73,GRID2,PIBF1,PPP1R12B,ADORA2A,CAMK2G,EFNA5,MERTK,TAC4,BRD1,NF1,SORL1,TEAD4,DMRT1,POR,RELN,TNFRSF11B,GRM5,HDAC9,IL18R1,IL1RL1,DYNC1H1,LRRTM3,NDRG2,NUMB,MAP3K13,IL1RAP,NAV3,JAG1,MEOX2,PPP3CA,KCNB2,KLF7,PLS1,CXADR,PER1,WWTR1,KL,CBLB,RIMS1,TRIM16,GABPA,KAT7,AGGF1,NLGN1,AKIRIN2,RNF128,NPR3,CACNA1D,DDX3X,JAK2,SHOX2,AHI1,SMAD3,NOS1,PLXNB1,CDK1,HDAC7,LTB,TNF,OVOL2,PANX1,SNX5,ZFPM1,POMC,DLG5,MLIP,MMP20,BRINP1,PRG3,ADORA3,BCL11A,CLEC4A,HMGA2,DOCK1,LTBP1,MBOAT2,DAAM2,SMOC2,SULF1,C5AR1,LRP4,EFNB2,TRDN,ZNF423,CLSTN1,ALPK2,CTNNA1,TSHZ3,CDKL5,CD109,EGF,ALDH1A1,CMA1,IL1RAPL1,KIT,TRPV4,PTPRO,RAPGEF2,ESR1,HTR2C,JAK1,NCMAP,SEMA3E,FGF13,MARVELD3,NUP155,PLAG1,EPHB2,TOX,ERBB4,MAP3K3,RBM19,SLITRK6,PTH2R,SLAMF1,BSG,CAMP,F11R,SETD2,CD2AP,SGMS2,CCL3,FGF7,LAMA1,SEMA5B,SPRED3,IL20RA,PSG9,SULF2,PTGER3,SLC39A12,SOX4,P2RX1,PIK3CD,KCNE4,KDM3A,PLAT,WDPCP,FGFR1,MEF2A,UNC13B,MAP2,SOST,IL23R,KEAP1,P2RX7,AR,BCOR,FGL2,KIAA1109,SCAMP5,IL1RL2,N4BP1,BDKRB2,HLA-DRA,ADRA1B,BHLHB9,L3MBTL1,SEMA3A,CDC73,MEIS2,PDE5A,PROS1,ZMIZ1,PPARGC1B,ESRRG,FERMT2,HCN4,MAP2K5,MARK1,SMPD3,ZC3H13,DAB2,GPR55,PBX1,RNF207,ADAM10,FGF12,ITGA3,PLCL2,PPM1B,RFTN1,RPS6KA6,TRPM4,CD226,BMP6,CHRM3,CLASP1,SMTNL2,HGF,CALCR,CAPN7,ZBTB16,CACNB2,NOS1AP,INSR,MACF1,PDE3A,SKI,AKT3,CTDP1,ELF1,ENPP1,FMR1,LITAF,PRKX,ABCD1,AIM2,ARHGAP42,CD84,JPH4,PCK1,PPARA,ZFHX2,CORIN,MYH7,SLC22A5,PPP2R3C,S1PR3,SPRED1,CHSY1,NTN4,PRDM16,PTPRR,MYH6,PRLR,TYMP,ADAMTS5,DOCK7,G3BP1,HDAC5,TDGF1,STIM1,PEMT,POU2AF1,PRKCQ,STAT3,TP63,AMOT,HPN,ISLR2,PAK3,CASQ2,DHRS7B,DLGAP2,LGALS9,LRP8,MAPKAPK2,APC,BMPR1A,KIAA0319,NGF,PRKCA,PTGES,UBASH3B,ASB3,CTSH,DHX9,RGCC,GPR21,IL6R,MAP2K6,SPTBN4,CLASP2,MAP2K1,SOS1,TPH1,C1QTNF3,FGF1,TBX20,C6ORF106,RGN,RREB1,TBX18
GO:0051674	localization of cell	3.718576471083065e-10	SRGAP2B,TPTE,PLCB1,CMKLR1,DAB1,FMNL2,TPTE2,PTPRT,DCC,DUSP22,FBLN1,ITGA2,SUN2,ZRANB1,NRG3,SLC9B1,TGFBR3,CDH13,TF,PIK3C2B,SDCCAG8,GADD45A,PLXNA4,FLRT2,GPC3,NRG1,RFFL,MCTP1,ASTN2,SH3RF2,UNC5D,SEMA6D,SPOCK1,CTNNA2,CCR1,CCR3,RBFOX2,DNAH6,TIAM1,RHOC,LDLRAD4,NKD1,SCAI,SPEF2,FBXO31,ARID5B,PHACTR1,SPOCK3,EPHA3,DCLK1,AGT,PSTPIP2,FSIP2,RHOJ,CDH2,LOXL2,LAMA2,SEMA3D,DACH1,NTN1,ZNF268,GPC6,DBH,FMNL3,RIN3,LAMA4,SLIT2,TTLL8,PDCD6,GPC5,IGF1R,VCAN,PTPRG,KRT2,VCL,CELSR1,MBOAT7,ASTN1,STK24,FRMD5,TRPM2,CTNNA3,NTNG1,CATSPER2,LRRK2,NTRK3,ANKS1A,DISC1,HEXB,SH3BP1,IER2,MECP2,SEMA4D,SLC8A1,PRKD1,TGFB2,ATP1A4,VAV3,FUT4,S100A11,SEMA5A,DNAAF2,DNAH3,LAMA3,NR2F2,PML,TNS1,PLXNA2,BCL2,ARHGAP24,EPHB1,VANGL2,NR4A2,FYN,NR4A1,CD44,KANK1,ENPP2,DCDC2,LAMB4,UNC5C,PALLD,NLRP12,MTOR,LDB2,FAT3,MEGF9,BMPER,BBS2,CEP85L,EPB41L4B,SOX8,PRKCE,FAM19A4,FOXO3,MITF,DLC1,EDNRB,ROBO1,SATB2,FAP,FLT1,CENPV,TACR3,KDR,PRCP,CHL1,APP,NRP1,PTPRM,ELMO1,ADTRP,TT

			C12,SEMA3C,ADAM17,CAMK1D,ELP3,ETS1,ITGA11,ATRNL1,OPHN1,CCDC88A,RIN2,DNAH8,HDAC4,PDE4B,JAM2,MAPRE2,NTRK2,PRKG1,CDC42BPA,STARD13,RABGEF1,GLI3,CLDN1,DOCK4,SRP54,TGFBR1,TNR,PEAK1,IL16,CYP7B1,MERTK,TAC4,MAGI2,NF1,SORL1,DMRT1,RELN,SLC9C1,HDAC9,KIRREL3,SELE,MGAT5,NUMB,NPHP4,NAV3,JAG1,MEOX2,PPP3CA,CXADR,DOCK10,GPC4,PTPRU,SH3KBP1,JAK2,SPNS2,SMAD3,CD300A,PLXNB1,CDK1,HDAC7,TNF,LRCH1,OVOL2,DLG5,LGR6,ARID2,SPAG16,ADORA3,DOCK1,EPS8,SMOC2,SULF1,C5AR1,PIK3R3,EFNB2,AMOTL1,NET1,SELP,CTNNA1,CDKL5,EFHC2,EGF,KIT,PIK3C2G,TRPV4,DNAH11,EFNB1,PTPRO,RAPGEF2,SEMA3E,FGF13,MARVELD3,ARMC2,EPHB2,ABCC1,ERBB4,FER,MAP3K3,LRP5,SLAMF1,ABL2,BSG,F11R,GAS8,SETD2,ARHGAP35,CD2AP,PTPRK,ATP8A1,CCL3,FGF7,LAMA1,PREX1,SEMA5B,ITGA9,KIF2A,NHLH2,ARF4,MYH9,TEKT4,NEDD9,PDGFD,PIK3CD,ENPEP,PLAT,WDPCP,AUTS2,FGFR1,ITGBL1,ITGB7,JMY,NTF3,BDKRB1,DGKZ,SEMA3A,MARK2,ZMIZ1,SRGAP3,FERMT2,MAP2K5,MARK1,SMPD3,TNN,DAB2,LCP1,ACTN4,ADAM10,CCL7,ITGA3,PIN1,SYNPO2,TRPM4,NME8,CLASP1,HGF,MYLK,CAPN7,SLC26A8,INSR,MACF1,PAK1,SKI,AKT3,PRKX,ULK4,CCDC141,GNA13,FUT8,SPRED1,NTN4,PTPRF,DEFA1B,PTPRR,EXT1,UMOD,DOCK7,ELMO2,HDAC5,TDGF1,LYVE1,TREM1,ADARB1,NDE1,PRKCQ,STAT3,AMOT,PAK3,USH2A,LGALS9,LRP8,EFCAB1,APC,BMPR1A,DPP4,ITGA1,KIAA0319,PRKCA,SPINT2,CTSH,RGCC,SRGAP1,CCL14,CCL15,CCL15-CCL14,IL6R,CLASP2,MAP2K1,SOS1,FGF1,TBX20,RGN,RREB1
GO:0048870	cell motility	3.718576471083065e-10	SRGAP2B,TPTE,PLCB1,CMKLR1,DAB1,FMNL2,TPTE2,PTPRT,DCC,DUSP22,FBLN1,ITGA2,SUN2,ZRANB1,NRG3,SLC9B1,TGFBR3,CDH13,TF,PIK3C2B,SDCCAG8,GADD45A,PLXNA4,FLRT2,GPC3,NRG1,RFFL,MCTP1,ASTN2,SH3RF2,UNC5D,SEMA6D,SPOCK1,CTNNA2,CCR1,CCR3,RBFOX2,DNAH6,TIAM1,RHOC,LDLRAD4,NKD1,SCAI,SPEF2,FBXO31,ARID5B,PHACTR1,SPOCK3,EPHA3,DCLK1,AGT,PSTPIP2,FSIP2,RHOJ,CDH2,LOXL2,LAMA2,SEMA3D,DACH1,NTN1,ZNF268,GPC6,DBH,FMNL3,RIN3,LAMA4,SLIT2,TTLL8,PDCD6,GPC5,IGF1R,VCAN,PTPRG,KRT2,VCL,CELSR1,MBOAT7,ASTN1,STK24,FRMD5,TRPM2,CTNNA3,NTNG1,CATSPER2,LRRK2,NTRK3,ANKS1A,DISC1,HEXB,SH3BP1,IER2,MECP2,SEMA4D,SLC8A1,PRKD1,TGFB2,ATP1A4,VAV3,FUT4,S100A11,SEMA5A,DNAAF2,DNAH3,LAMA3,NR2F2,PML,TNS1,PLXNA2,BCL2,ARHGAP24,EPHB1,VANGL2,NR4A2,FYN,NR4A1,CD44,KANK1,ENPP2,DCDC2,LAMB4,UNC5C,PALLD,NLRP12,MTOR,LDB2,FAT3,MEGF9,BMPER,BBS2,CEP85L,EPB41L4B,SOX8,PRKCE,FAM19A4,FOXO3,MITF,DLC1,EDNRB,ROBO1,SATB2,FAP,FLT1,CENPV,TACR3,KDR,PRCP,CHL1,APP,NRP1,PTPRM,ELMO1,ADTRP,TTC12,SEMA3C,ADAM17,CAMK1D,ELP3,ETS1,ITGA11,ATRNL1,OPHN1,CCDC88A,RIN2,DNAH8,HDAC4,PDE4B,JAM2,MAPRE2,NTRK2,PRKG1,CDC42BPA,STARD13,RABGEF1,GLI3,CLDN1,DOCK4,SRP54,TGFBR1,TNR,PEAK1,IL16,CYP7B1,MERTK,TAC4,MAGI2,NF1,SORL1,DMRT1,RELN,SLC9C1,HDAC9,KIRREL3,SELE,MGAT5,NUMB,NPHP4,NAV3,JAG1,MEOX2,PPP3CA,CXADR,DOCK10,GPC4,PTPRU,SH3KBP1,JAK2,SPNS2,SMAD3,CD300A,PLXNB1,CDK1,HDAC7,TNF,LRCH1,OVOL2,DLG5,LGR6,ARID2,SPAG16,ADORA3,DOCK1,EPS8,SMOC2,SULF1,C5AR1,PIK3R3,EFNB2,AMOTL1,NET1,SELP,CTNNA1,CDKL5,EFHC2,EGF,KIT,PIK3C2G,TRPV4,DNAH11,EFNB1,PTPRO,RAPGEF2,SEMA3E,FGF13,MARVELD3,ARMC2,EPHB2,ABCC1,ERBB4,FER,MAP3K3,LRP5,SLAMF1,ABL2,BSG,F11R,GAS8,SETD2,ARHGAP35,CD2AP,PTPRK,ATP8A1,CCL3,FGF7,LAMA1,PREX1,SEMA5B,ITGA9,KIF2A,NHLH2,ARF4,MYH9,TEKT4,NEDD9,PDGFD,PIK3CD,ENPEP,PLAT,WDPCP,AUTS2,FGFR1,ITGBL1,ITGB7,JMY,NTF3,BDKRB1,DGKZ,SEMA3A,MARK2,ZMIZ1,SRGAP3,FERMT2,MAP2K5,MARK1,SMPD3,TNN,DAB2,LCP1,ACTN4,ADAM10,CCL7,ITGA3,PIN1,SYNPO2,TRPM4,NME8,CLASP1,HGF,MYLK,CAPN7,SLC26A8,INSR,MACF1,PAK1,SKI,AKT3,PRKX,ULK4,CCDC141,GNA13,FUT8,SPRED1,NTN4,PTPRF,DEFA1B,PTPRR,EXT1,UMOD,DOCK7,ELMO2,HDAC5,TDGF1,LYVE1,TREM1,ADARB1,NDE1,PRKCQ,STAT3,AMOT,PAK3,USH2A,LGALS9,LRP8,EFCAB1,APC,BMPR1A,DPP4,ITGA1,KIAA0319,PRKCA,SPINT2,CTSH,RGCC,SRGAP1,CCL14,CCL15,CCL15-CCL14,IL6R,CLASP2,MAP2K1,SOS1,FGF1,TBX20,RGN,RREB1
GO:0048583	regulation of response to stimulus	6.478985427223598e-10	IGHV1OR21-1,TPTE,PLCB1,CMKLR1,DAB1,MAPK14,DSCAM,TPTE2,SH3RF3,CTBP2,LYPD6,PTPRT,IGHV4-31,IGHV3-64,WLS,TIMP3,DUSP22,AKAP13,FBLN1,IGHV1OR15-9,IGHV4OR15-8,NPHP3,ITGA2,RGS6,VRK2,GRIN3A,NRXN1,ZRANB1,DOK5,DOCK2,CTNND2,MS4A1,RIMS2,MTPN,SHANK2,SMYD2,CLEC16A,TGFBR3,FSTL4,CDH13,RNF152,NXN,CSNK2A1,ERCC4,TMEM108,CHEK2,GADD45A,PLXNA4,GPC3,FBLN5,NRG1,PSMB2,SHISA6,RFFL,SPIDR,IL

			4R,LRFN5,MCTP1,GGT1,PLCE1,SH3RF2,SEMA6D,CTNNA2,CCR1,TIAM1,RHOC,LDLRAD4,RGS7BP,RASGRF1,CBFA2T2,NKD1,SCAI,BTN2A1,ALK,PTPRE,ZNF536,RAB7A,RGL2,S100B,ROBO2,ZNF366,C2CD3,PRKACB,PXDN,CRNN,AJAP1,LEMD3,AGT,SHISA9,FBXO32,WDR11,RCAN1,CDH2,PARK2,FHL2,DLGAP1,KIR2DL1,KIR2DL4,BPIFB1,CORO2B,PSPC1,SEMA3D,TRIM22,TRIM5,USP25,TXK,GPC6,RNF185,DBH,ITSN1,TSPAN8,ZNRF3,RIN3,TRIO,HDAC2,HTRA1,MYO9A,SLIT2,PDCD6,GPC5,LCMT1,IGF1R,MDM2,SLC24A4,TMPRSS6,NR3C2,PDE9A,DMD,KCTD8,CACNG3,SIPA1L3,FSHR,GSG1L,GRM1,FMN2,SKAP1,STK24,WDR83,WWOX,TRPM2,CNIH3,CAMTA1,LRRK2,MAPK10,IGFBP7,NTRK3,RORA,CD8B,GGT2,IQCJ-SCHIP1,ANKS1A,RNF165,DISC1,SH3BP1,MTMR2,SEMA4D,GSK3B,LGR5,MAGI3,PRKD1,ERCC6,OGT,TGFB2,VAV3,C8A,SEMA5A,BDNF,LLGL2,PRKCH,RBMS3,RYK,ZDHHC17,POLR3C,WASF1,KDM4C,OTUD7A,RAG1,SIK2,ANKRD6,ARHGAP8,PRR5,PML,ARHGAP12,AKAP6,FBN1,IGHV3-16,TAOK3,BCL2,RALBP1,ZNF675,ARHGAP24,EPHB1,SIPA1L1,LRP2,TMEM100,DEPTOR,NR4A2,ARHGEF10,EPHA7,PSD3,FYN,ADAMTS18,CHN1,SMARCA4,CD44,HECW1,NEU3,RNF213,FOXO1,PDE4D,KANK1,ARHGEF3,ATF6,DCDC2,GRK5,TNMD,CSNK1G3,GRIK2,IGSF11,CELF4,UGCG,RFX4,GNG4,NLRP12,MTOR,RMI2,ROR1,CTDSPL2,DDX21,BMPER,PTPRD,TIAM2,BBS2,ARL6IP5,CTNNBIP1,ZMYND11,NLGN4X,PRKCE,HELLS,PRKCB,ADCY8,EFHB,FAM19A4,FOXO3,CAPN3,KALRN,PJA1,TTLL12,CCDC88C,DLC1,EDNRB,OASL,ROBO1,FAP,FLT1,NOX4,COL2A1,MAP3K7,MAPK9,PREX2,TRAF3IP2,ZNF277,CR1,EYA4,ACSL5,PKHD1,STK3,TGM2,KDR,PRCP,ARHGAP15,CASK,EDA,IGHV4-28,APP,CALCRL,NRP1,UACA,ARHGEF18,LGR4,LINC00473,SERPING1,ADTRP,KCNK2,SEMA3C,ADAM17,CAMK1D,MAP3K5,TSPEAR,TFPI,ETS1,GRK6,PEX5L,PIK3R5,SAMSN1,ROS1,TGFB1I1,DOT1L,RGS16,TCF7L2,TNFRSF10B,TRABD2B,BICD1,RALGAPA2,TRAF3,ABAT,OPHN1,BCLAF1,SGMS1,CASP5,CCDC88A,CD38,MOB3B,RNF43,TUB,CHRDL1,RNF126,STK36,BCL2L1,GUCY2F,HDAC4,PDE4B,GHR,MAPRE2,NTRK2,PRKG1,BRD4,RASGRP1,STARD13,ARR3,LY86,RABGEF1,SPRED2,GLI3,SMCHD1,CLDN1,RAP1A,SAFB2,TGFBR1,MGLL,LCP2,THEMIS,TNR,NCOA5,CHST11,IL16,PTTG1IP,RAPGEF1,CD96,EP300,WIF1,CYP7B1,GRAMD4,GAB2,TP73,GRID2,IFNGR2,PIBF1,RASGRF2,PSMA1,ADORA2A,ADRBK2,CAMK2G,RALGPS1,CACNG2,MAGI2,NF1,RALGPS2,RGS7,SORL1,STRN3,ARHGAP6,BICC1,BID,IFT80,TRIM59,DMRT1,LZTR1,ASXL1,MICU1,POR,RELN,GRM5,IGHV4-4,IL18R1,IL1RL1,SELE,MGAT5,NAPEPLD,NDRG2,IGHV3OR16-12,MAP3K13,NLK,NPHP4,ADCYAP1R1,FBXL17,JAG1,PPP3CA,JRK,KLF7,SNX25,DGKI,MAS1,PER1,WWTR1,KL,C6,CBLB,PLSCR1,DOK6,GPC4,RIMS1,TRIM16,WDR59,KAT7,NLGN1,PTPRU,AKIRIN2,ARHGAP5,RPS20,RAD51AP1,AKAP7,LRRK1,MAPK8IP2,VEPH1,DDX3X,CNOT7,ZNF622,ARHGAP39,JAK2,SHOX2,SPNS2,SMAD3,BAIAP2L1,CD300A,MAP2K4,PLXNB1,AATF,HDAC7,SLC39A10,TNF,OVOL2,SNX5,TNIP1,EIF3A,POMC,PRDM15,BMP15,DLG5,AFAP1,LGR6,ARID2,MLIP,SHC2,HMGA2,NEK4,AGO3,IGLC2,IGLL5,GCLC,PMEPA1,UBE2V1,EPS8,LTBP1,DAAM2,SMOC2,SULF1,C5AR1,DEF6,LRP4,CNTN6,EFNB2,NET1,ZNF423,SELP,ALPK2,CTNNA1,TENM1,ARHGEF11,GSKIP,TNFRSF19,ARHGAP11B,DYNC2H1,MLLT3,SIPA1L2,CD109,EGF,SPIRE2,CMA1,KIT,SLIT3,UBR2,TRPV4,VWC2,C8B,EDAR,PTPRO,RAPGEF2,DNAJB6,ESR1,HTR2C,SEMA3E,UBE3A,DNMBP,GRM4,MARVELD3,EPHB2,IGHV3-72,MECOM,NDFIP2,ABCC1,DAPK1,ERBB4,FER,MAP3K3,FNIP1,SLAMF1,ABL2,DEPDC5,F11R,GAS8,SETD2,VPS13C,ARHGAP35,CD2AP,CCL3,PREX1,SEMA5B,SPRED3,TNFAIP8L2,VWF,ARHGAP25,FGD4,IL20RA,LRRC70,PSG9,SULF2,PTGER3,SOX4,GARNL3,PDGFD,PIK3CD,UBE2N,ITGA8,PUM1,IRAK2,PLAT,TNKS,AUTS2,CDK19,FGFR1,MEF2A,OCLN,PDE11A,RGS3,UNC13B,SOST,IL23R,NTF3,P2RX7,PLA2G4A,SCUBE1,RASA3,ZDHHC11,AMFR,APIP,AR,FGL2,SUFU,EYA1,IFNA8,IL1RL2,N4BP1,BDKRB2,HLA-DRA,ADRA1B,ARMC9,DGKZ,PRKAA2,SEMA3A,TLE4,ARHGAP10,CDC73,PDE5A,PROS1,ZMIZ1,FLT3,SRGAP3,TRIM72,FERMT2,LTBR,MAP2K5,TNN,C9,DAB2,GPR55,STX3,ACTN4,ADAM10,CCL7,EEF1E1,EEF1E1-BLOC1S5,ITGA3,FANCB,NREP,PLCL2,PPM1B,RFTN1,PIN1,RPS6KA6,TRPM4,CD226,PIP5K1B,STK38,BMP6,CIT,CLASP1,TGFA,GAS2,HGF,MYLK,RALGAPA1,RGS12,CALCR,SCUBE3,CNKSR2,NCAM1,NOS1AP,ARHGAP28,IL18RAP,INSR,MACF1,PAK1,PDE3A,PSMB7,SKI,AKT3,CTDP1,ELF1,ENPP1,FMR1,LITAF,IGHV1-69,RRAGC,ULK4,ABCD1,EVC,IGHV1-

			46,MAD1L1,MFNG,AIM2,ARHGAP42,CD84,EPG5,PCK1,PPARA,TNFAIP8L3,GNA13,MYH7,TAF1,CHD5,FUT8,PPP2R3C,SPRED1,AKR1C2,CHSY1,ESR2,PRDM16,PTPRF,RBL1,ZNF451,BTRC,C2,CCDC3,CFB,IGHV1-18,OPTN,PTPRR,RIT2,IFT81,NCOA1,PRKCG,PRLR,FBXW11,G3BP1,TDGF1,A2M,PRKCQ,SCEL,STAT3,SUSD4,TP63,USP7,AMOT,DOCK3,PAK3,PIK3R2,TBL1X,BLNK,DTNBP1,RAP1B,DLGAP2,KSR1,LGALS9,LRP8,NFAT5,ALPK1,IL10RB,MAPKAPK2,AOAH,APC,BMPR1A,DPP4,ITGA1,KIAA0319,NGF,PRKCA,PTGES,UBASH3B,ADAMTS20,MTMR4,CTSH,DHX9,NCAPG2,RGCC,RPH3AL,USP18,CCL14,CCL15,EDA2R,GPR21,IL6R,MAP2K6,RIC8B,TFIP11,CLASP2,MAP2K1,SOS1,VPS35,C1QTNF3,FGF1,TBX20,C6ORF106,CRADD,NLRC5,RGN,RREB1,TBX18
GO:0007156	homophilic cell adhesion via plasma membrane adhesion molecules	6.90978944836597e-10	CDH4,PCDH9,DSCAM,PTPRT,SDK2,CDH13,PCDH11X,PCDH7,CNTN4,ROBO2,CDH11,CDH2,CDH9,HMCN1,PCDH17,CDH8,DSC3,CELSR1,EMB,CLSTN2,PCDH15,CDH10,CDH12,CDH6,IGSF11,CDHR4,PALLD,FAT3,DSG4,CDH18,ROBO1,CDH17,PTPRM,SDK1,TENM3,DSCAML1,IGSF21,KIRREL3,CADM3,PCDH19,CNTN6,CLSTN1,MYPN,BSG,CDH23,PCDHGA1,PCDHGA10,PCDHGA11,PCDHGA12,PCDHGA2,PCDHGA3,PCDHGA4,PCDHGA5,PCDHGA6,PCDHGA7,PCDHGA8,PCDHGA9,PCDHGB1,PCDHGB2,PCDHGB3,PCDHGB4,PCDHGB6,PCDHGB7,CD84,DCHS2
GO:0061564	axon development	7.07462937776087 8e-10	CDH4,DAB1,DSCAM,NCAM2,ANK3,DCC,EPHA6,NRXN1,NRXN3,TNC,CHODL,FSTL4,LRRC4C,ADAMTSL1,PLXNA4,FLRT2,UNC5D,SEMA6D,CTNNA2,TIAM1,DPYSL2,CNTN4,S100B,ROBO2,EPHA3,DCLK1,CDH11,CDH2,LAMA2,SEMA3D,NTN1,TRIO,SLIT2,IGF1R,TRPC5,VCL,STK24,NTNG1,RNF165,DISC1,EMB,SEMA4D,GSK3B,SEMA5A,BDNF,RYK,ZDHHC17,LAMA3,PLXNA2,BCL2,EPHB1,SIPA1L1,NR4A2,EPHA7,FYN,CHN1,ATP8A2,UNC5C,PALLD,TIAM2,KALRN,ROBO1,VASH2,CHL1,APP,NRP1,PTPRM,SEMA3C,PRTG,RPS6KA5,PARD3,OPHN1,ALCAM,NTRK2,GLI3,EPHA5,TNR,DSCAML1,SPG11,EFNA5,TTC8,RELN,NUMB,MAP3K13,KLF7,GRM7,JAK2,SHOX2,PLXNB1,COBL,LGR6,BCL11A,LRP4,CNTN6,EFNB2,CTNNA1,CDKL5,SLIT3,EFNB1,PTPRO,MYPN,SEMA3E,FGF13,EPHB2,SLITRK6,BSG,ARHGAP35,LAMA1,SEMA5B,AUTS2,MAP2,SEMA3A,MARK2,TNN,LGI1,NREP,NCAM1,NFASC,MACF1,PAK1,CRMP1,CCDC141,PTPRF,EXT1,DOCK7,ADARB1,PRKCQ,ISLR2,PAK3,CRTAC1,KIAA0319,NGF,SPTBN4,CLASP2,MAP2K1,SOS1
GO:0048522	positive regulation of cellular process	7.102607949059883e-10	IGHV1OR21-1,TASP1,FANK1,CDH4,ZHX3,PLCB1,KMT2C,CMKLR1,DAB1,OMA1,DUX4,GTF2I,CDC42EP3,MAPK14,DSCAM,ARID1B,RUNX1,SH3RF3,ASIC2,CTBP2,RAB27A,PCBD2,PRIM2,LYPD6,ANK3,IGHV4-31,BCL2L13,IGHV3-64,WLS,TIMP3,DUSP22,AKAP13,FBLN1,ERG,VPS13D,ZNF292,IGHV1OR15-9,IGHV4OR15-8,ITGA2,ABCG1,KCNQ1,DPF3,SUN2,ATF7IP,EPHA6,NRXN1,ZRANB1,DOK5,SLC1A2,RAB5A,TNC,PCNT,CHODL,RIMS2,MTPN,SHANK2,NRG3,CLEC16A,ARNT2,TGFBR3,CDH13,RNF152,ANK2,CSNK2A1,PDE4DIP,SYNDIG1,DPP10,ZNF112,ZNF229,TF,PLGRKT,KCNE1,SAMD4A,TMEM108,CHEK2,GADD45A,PRDM9,PCP4,PSMC6,ACIN1,SYT17,PLXNA4,FLRT2,TOX3,GPC3,NRG1,ETS2,PSMB2,NPAS3,SPIDR,IL4R,ALDH1A2,ST8SIA1,SUZ12,PLCE1,SH3RF2,GLP2R,SEMA6D,ABHD17C,SLCO3A1,CCR1,CCR3,CFTR,TIAM1,RHOC,AGBL4,ZNF91,RASGRF1,ZBTB20,TET3,CBFA2T2,NKD1,HPSE2,ALK,UTRN,FBXO31,RAB7A,RGL2,ARID5B,S100B,ROBO2,EPHA3,CRNN,AGT,ATAT1,RHOJ,CDH2,PARK2,BANP,LGALS14,LOXL2,LAMA2,CDC5L,KIR2DL4,FRMPD4,ZNF804A,NEGR1,BRF1,SEMA3D,NTN1,TRIM22,TRIM5,ZNF268,DCUN1D4,TXK,RNF185,NELL1,SETD3,KCNMA1,ZNF845,FTO,HDAC2,HTRA1,SLIT2,TFAP2D,PDCD6,RPS6KA2,STXBP4,GPC5,IGF1R,MDM2,SMAD1,E2F3,SYT9,ZBED4,TMPRSS6,ZNF850,CTNNBL1,NR3C2,TRPC5,DMD,SYT1,CACNG3,NOX5,TEAD1,FSHR,GRM1,FMN2,HNRNPA2B1,NRIP1,SKAP1,STK24,WWOX,ATRX,FRMD5,PARP16,TRPM2,RYR2,USP16,KIF3B,TENM4,CAMTA1,HSPB8,LRRK2,RORB,SLC1A3,KIF26B,NTRK3,NUSAP1,RORA,ILDR1,IQCJ-SCHIP1,SLC4A4,CHD7,RNF165,DISC1,HEXB,PAN3,WRAP73,CDCA2,GLIS1,PDXP,SOX6,IER2,MECP2,MTMR2,SEMA4D,GSK3B,LGR5,LINGO2,SLC8A1,ZNF841,CDC14A,PRKD1,ERCC6,OGT,RNF144A,TGFB2,CLSTN2,FMN1,PRAMEF12,VAV3,FUT4,S100A11,SEMA5A,SLC5A3,BDNF,PRKCH,RYK,ZDHHC17,KCNC1,ABI3BP,EDIL3,WASF1,GRIN2B,KDM4C,RAG1,ANKRD6,ARHGAP8,NR2F2,PLEKHM2,PRR5,PTGFR,SNAP25,NR5A2,PML,PPARGC1A,RNF180,TRUB2,USP36,AKAP6,CN

			TNAP2,IGHV3-16,NLRP2,PLXNA2,TAOK3,BCL2,RAD51B,RALBP1,EPHB1,SMARCE1,IL12RB2,KLF12,LRP2,NSF,TMEM100,FRMD4A,NFIA,NR4A2,ARHGEF10,EPHA7,ZBTB7C,FYN,KCNE2,PARN,NR4A1,SMARCA4,CD44,NEU3,RCVRN,RHBDD1,THBS2,FOXO1,RNF144B,KANK1,ENPP2,ABCB10,ACTN2,ARHGEF3,ATF6,ATP8A2,BACH1,DCDC2,GRK5,KLF13,MME,RNF217,CSNK1G3,GRIK2,IGSF11,ZNF516,CELF4,SERPINB7,UNC5C,APBB2,EIF3E,MYLK3,NAMPT,SLC1A1,ZFPM2,GLIS3,RARB,RFX4,ETV6,NLRP12,MTOR,ROR1,CADPS,LDB2,MOV10,CHRM1,CTDSPL2,DDX21,UBXN2B,ABCA13,BMPER,CCDC88B,PTPRD,SCAF8,TIAM2,ZNF521,ZFP64,ARL6IP5,EPM2A,NOL11,CLYBL,CTNNBIP1,EPB41L4B,SOX8,HAS3,PRKCE,EIF2S1,GTF2A1L,KMT2A,PRKCB,VDR,ADCY8,FHL5,FOXO3,LRRTM1,MITF,RAB31,SCP2,TRAPPC12,CAPN3,ITLN1,KALRN,CREB5,DLC1,EDNRB,MSR1,OASL,ROBO1,SATB2,TDRD3,BNIP3L,FAP,FLT1,NOX4,SGIP1,ZNF148,CDH17,CENPV,GRHL2,TCF4,MAP3K7,MAPK9,NR3C1,TACR3,TRAF3IP2,CR1,EYA4,SPON1,ACSL5,PKHD1,STK3,SUPT3H,TGM2,VASH2,ZNF585A,KDR,PPHLN1,CASK,EDA,IGHV4-28,PCOLCE2,ABCA12,APP,CALCRL,NRP1,UACA,LGR4,TENM2,ADTRP,EGFLAM,KAT5,THRB,KCNK2,SEMA3C,ADAM17,CAMK1D,DIS3L2,MAP3K5,PKP4,IGF2R,ZER1,ATF2,ELP3,ETS1,NR2C2,PIK3R5,ROS1,TGFB1I1,TM9SF4,DOT1L,PSIP1,RPS6KA5,TCF7L2,TNFRSF10B,TRABD2B,BICD1,PARD3,TRAF3,ABAT,GRIN2A,GRIP1,PXT1,RIMS3,DLX6-AS1,KIFAP3,BCLAF1,CCDC88A,CD38,MOB3B,MRPS27,RIN2,STK36,BCL2L1,HDAC4,SOX5,DNMT1,GHR,JAM2,MAPRE2,NTRK2,ACSL4,DNM3,BRD4,RASGRP1,CAMK4,COMMD1,LY86,SPRED2,STARD4,GLI3,SMCHD1,CLDN1,MORC2,STXBP5,DOCK4,RAP1A,TGFBR1,DICER1,EPHA5,LCP2,TNR,CUX1,TENM3,PTTG1IP,RAPGEF1,ZNF729,CHRNA3,EP300,TBX3,WIF1,CACNA2D1,CYP7B1,GRAMD4,TRIOBP,BRD7,GAB2,TP73,GRID2,IFNGR2,PIBF1,RASGRF2,PSMA1,ONECUT3,ADCK1,ADORA2A,EFNA5,FAM20A,MERTK,TAC4,BRD1,CACNG2,MAGI2,NF1,RFC3,RGS7,SORL1,STRN3,ZNF615,BID,ELK3,TEAD4,DMRT1,ASXL1,EIF4E,POR,RELN,GRM5,HDAC9,IGHV4-4,IL18R1,IL1RL1,PTPN9,SELE,DYNC1H1,LRRTM3,MGAT5,NAPEPLD,NUMB,TNFAIP8,CCDC57,IGHV3OR16-12,MAP3K13,NPHP4,RALA,ADCYAP1R1,EGLN3,IL1RAP,NAV3,ATP10A,FBXL5,JAG1,MEOX2,PPP3CA,JRK,KLF7,PLS1,SLC7A1,DGKI,MAS1,PER1,SLC24A2,TBC1D5,WWTR1,KL,CBLB,ELAVL4,MNAT1,PLSCR1,CNTN1,DOK6,RIMS1,TRIM16,WDR59,GABPA,GPSM2,KAT7,SSBP2,STIM2,SLC22A2,AGGF1,ASAP1,BORA,N4BP2L2,NLGN1,PTPRU,AKIRIN2,RNF128,RPS20,SH3KBP1,RAD51AP1,AKAP7,LRRK1,MAPK8IP2,ZNF208,DDX3X,EGFL6,NSG1,CNOT7,MAML3,EHF,ZNF622,DNMT3B,JAK2,LMO7,SHOX2,AHI1,SMAD3,BAIAP2L1,CD300A,KIDINS220,MAP2K4,NOS1,COL8A1,PLXNB1,RDH10,TCF12,AATF,CDK1,HDAC7,HIVEP3,MIR320B2,NBN,NVL,SLC39A10,TNF,COBL,OVOL2,SNX5,TNIP1,ZFPM1,CSPP1,ILF3,POMC,PRDM15,ADNP2,BLOC1S6,BMP15,DLG5,FCHSD2,INO80,LGR6,MAGI1,ARID2,MLIP,PAXBP1,SHC2,BRINP1,BCL11A,HMGA2,MYO6,NEK4,YAF2,AGO3,DOCK1,IGLC2,IGLL5,ANO1,CDC20B,GCLC,UBE2V1,EPS8,DAAM2,SMOC2,SULF1,ANKRD31,C5AR1,CCP110,LRP4,PIK3R3,WDR5,CNTN6,EFNB2,SAE1,TRDN,NET1,PHF8,ZNF423,CDC14B,CLSTN1,SELP,CHD6,CTNNA1,TENM1,TSHZ3,WDFY2,ZNF407,ARHGEF11,CDKL5,GSKIP,TNFRSF19,ZNF345,BPTF,DYNC2H1,MLLT3,MYRIP,EGF,MIP,SEC16B,SPIRE2,IL1RAPL1,KIT,TNFSF8,TNRC6B,ZNF600,BLM,TRPV4,VWC2,EDAR,EFNB1,RAPGEF2,REG1B,ESR1,HTR2C,JAK1,NCMAP,SEMA3E,SLC30A8,UBE3A,FGF13,GRM4,PLAG1,EPHB2,IGHV3-72,MECOM,NDFIP2,RNF4,TFDP2,TOX,DAP,DAPK1,ERBB4,FER,MAML2,MAP3K3,SLITRK6,SPTA1,FNIP1,LRP5,ZNF507,RPS27L,SLAMF1,ABL2,BSG,CAMP,F11R,GAS8,ALX4,AP2B1,ARHGAP35,CD2AP,ATP8A1,CCL3,FGF7,PREX1,SEMA5B,VWF,GLYR1,IL20RA,NHLH2,SULF2,ARF4,MYH9,NSMCE2,SLC2A13,SOX4,ST18,CAB39L,KIF23,NEDD9,P2RX1,PDGFD,PIK3CD,PRR16,UBE2N,AGTPBP1,ITGA8,PUM1,ARID4A,DDB1,KDM3A,SERTAD2,TNKS,UNC13A,AUTS2,CDK19,FGFR1,MEF2A,OCLN,UNC13B,ARID4B,MAP2,SLX1B,SOST,ASXL3,IL23R,JMY,KEAP1,NTF3,P2RX7,PLA2G4A,SCUBE1,ARRDC4,TOX2,ZNF544,AR,SCAMP5,SNX30,TRERF1,CEP250,EYA1,IFNA8,IL1RL2,RBM4,SRPK2,BDKRB1,HLA-DRA,ADRA1B,ARMC9,BHLHB9,DGKZ,GNL3,MIR433,NEK5,PRKAA2,RLF,SEMA3A,CDC73,MARK2,MEIS2,PDE5A,ZMIZ1,FLT3,NFIC,PPARGC1B,SNX7,DYNAP,ESRRG,FERMT2,LTBR,MAP2K5,SMPD3,CLN6,DAB2,GPR55,HDAC8,LCP1,PBX1,RNF207,STX3,SYT2,TCL1B,ACTN4,ADAM10,BLOC1S5,CCL7,EEF1E1,EEF1E1-

			BLOC1S5,ITGA3,KMO,POU2F3,PRAMEF8,FANCB,LGI1,PPM1B,QRICH1,STAC,MYOM1,PIN1,SYNPO2,TRPM4,CD226,TCF20,BMP6,CIT,CIZ1,CLASP1,EIF4ENIF1,CARF,NCOA2,TGFA,CREM,G3BP2,HGF,MYLK,POU2F1,CALCR,CAPN7,FLI1,POMT2,SCUBE3,ZBTB16,CACNB2,CTIF,NOS1AP,IL18RAP,INSR,LARP4B,MACF1,PAK1,PDE3A,PSMB7,SKI,AKT3,CDC16,ELF1,FMR1,LIMK2,LITAF,PAX3,CHFR,DKC1,IGHV1-69,JDP2,KLF6,RRAGC,ABCD1,BMF,EVC,IGHV1-46,MAD1L1,MFNG,AIM2,PCK1,PPARA,SPIB,TNFAIP8L3,TTN,CLIP1,KATNBL1,TAF1,CHD5,FNIP2,PPP2R3C,S1PR3,SPRED1,AKR1C2,CHSY1,ESR2,PRDM16,ZNF451,BTRC,CCDC3,FBLN2,IGHV1-18,MLLT10,OPTN,RIT2,ESRP2,NCOA1,PRKCG,PRLR,CBFA2T3,DOCK7,FBXW11,G3BP1,HDAC5,TDGF1,ZNF493,LYVE1,SLC40A1,STAT4,STIM1,KCNC2,POU2AF1,PRKCQ,SCEL,STAT3,TFEC,TP63,USP7,ZNF721,AMOT,DOCK3,HPN,ISLR2,PAK3,PIK3R2,STK17B,TBL1X,CASQ2,DTNBP1,RAP1B,KSR1,LGALS9,LRP8,NFAT5,ALPK1,GTPBP1,IL10RB,APC,BMPR1A,CKAP5,DPP4,ITGA1,KCTD20,KIAA0319,NGF,PRKCA,PTGES,UBE2E2,VLDLR,ADAMTS20,PILRB,ABI1,CTSH,DHX9,RGCC,RPH3AL,CCL14,CCL15,CYB5D2,EDA2R,IL6R,MAP2K6,CLASP2,MAP2K1,PRAMEF7,RGMB,SOS1,TPH1,VPS35,C1QTNF3,FGF1,RAD18,TBX20,ATMIN,CRADD,NLRC5,RGN,RREB1
GO:0007409	axonogenesis	7.736782930598098e-10	CDH4,DAB1,DSCAM,ANK3,DCC,EPHA6,NRXN1,NRXN3,CHODL,FSTL4,LRRC4C,ADAMTSL1,PLXNA4,FLRT2,UNC5D,SEMA6D,CTNNA2,TIAM1,DPYSL2,CNTN4,S100B,ROBO2,EPHA3,DCLK1,CDH11,CDH2,LAMA2,SEMA3D,NTN1,TRIO,SLIT2,IGF1R,TRPC5,VCL,NTNG1,RNF165,DISC1,EMB,SEMA4D,GSK3B,SEMA5A,BDNF,RYK,ZDHHC17,LAMA3,PLXNA2,BCL2,EPHB1,SIPA1L1,NR4A2,EPHA7,FYN,CHN1,ATP8A2,UNC5C,PALLD,TIAM2,KALRN,ROBO1,CHL1,APP,NRP1,PTPRM,SEMA3C,PRTG,RPS6KA5,PARD3,OPHN1,ALCAM,NTRK2,GLI3,EPHA5,TNR,DSCAML1,SPG11,EFNA5,TTC8,RELN,NUMB,MAP3K13,KLF7,SHOX2,PLXNB1,COBL,LGR6,BCL11A,LRP4,CNTN6,EFNB2,CDKL5,SLIT3,EFNB1,PTPRO,MYPN,SEMA3E,FGF13,EPHB2,SLITRK6,BSG,ARHGAP35,LAMA1,SEMA5B,AUTS2,MAP2,SEMA3A,MARK2,TNN,LGI1,NCAM1,NFASC,MACF1,PAK1,CRMP1,CCDC141,EXT1,DOCK7,ADARB1,PRKCQ,ISLR2,PAK3,KIAA0319,NGF,SPTBN4,CLASP2,MAP2K1,SOS1
GO:0048518	positive regulation of biological process	7.962146216503805e-10	IGHV1OR21-1,TASP1,FANK1,CDH4,ZHX3,PLCB1,KMT2C,CMKLR1,DAB1,OMA1,DUX4,GTF2I,CDC42EP3,MAPK14,DSCAM,ARID1B,RUNX1,SH3RF3,ASIC2,CTBP2,RAB27A,PCBD2,PRIM2,LYPD6,ANK3,IGHV4-31,BCL2L13,IGHV3-64,WLS,TIMP3,DUSP22,AKAP13,FBLN1,ERG,VPS13D,ZNF292,IGHV1OR15-9,SLC24A3,IGHV4OR15-8,ITGA2,ABCG1,TMPRSS2,KCNQ1,DPF3,SUN2,ATF7IP,EPHA6,NRXN1,ZRANB1,DOK5,SLC1A2,RAB5A,DOCK2,TNC,PCNT,MS4A1,CHODL,RIMS2,MTPN,SHANK2,NRG3,CLEC16A,ARNT2,TGFBR3,CDH13,RNF152,ANK2,CSNK2A1,PDE4DIP,SYNDIG1,DPP10,ZNF112,ZNF229,TF,PLGRKT,KCNE1,SAMD4A,TMEM108,CHEK2,GADD45A,PRDM9,PCP4,PSMC6,ACIN1,SYT17,PLXNA4,FLRT2,TOX3,GPC3,NRG1,ETS2,PSMB2,NPAS3,SPIDR,IL4R,ALDH1A2,ST8SIA1,SUZ12,PLCE1,SH3RF2,KSR2,GLP2R,SEMA6D,ABHD17C,SLCO3A1,CCR1,CCR3,CFTR,TIAM1,RHOC,AGBL4,ZNF91,RASGRF1,ZBTB20,TET3,CBFA2T2,NKD1,HPSE2,BTN2A1,ALK,UTRN,FBXO31,KLRF2,RAB7A,RGL2,ARID5B,S100B,ROBO2,EPHA3,CRNN,AGT,ATAT1,RHOJ,CDH2,PARK2,BANP,LGALS14,LOXL2,LAMA2,CDC5L,KIR2DL4,CORO2B,FRMPD4,ZNF804A,CYBB,NEGR1,PSPC1,BRF1,SEMA3D,NTN1,TRIM22,TRIM5,ZNF268,DCUN1D4,TXK,RNF185,NELL1,DBH,SETD3,KCNMA1,ZNF845,FTO,HDAC2,HTRA1,SLIT2,TFAP2D,PDCD6,RPS6KA2,STXBP4,GPC5,IGF1R,MDM2,SLC24A4,SMAD1,E2F3,SYT9,ZBED4,TMPRSS6,ZNF850,CTNNBL1,NR3C2,PDE9A,TRPC5,DMD,SYT1,BASP1,CACNG3,KRT2,NOX5,TEAD1,FSHR,GRM1,FMN2,HNRNPA2B1,NRIP1,SKAP1,STK24,WWOX,ATRX,FRMD5,PARP16,TRPM2,RYR2,USP16,KIF3B,TENM4,CAMTA1,HSPB8,LRRK2,RORB,SLC1A3,KIF26B,NTRK3,NUSAP1,RORA,ILDR1,IQCJ-SCHIP1,SLC4A4,CHD7,RNF165,DISC1,HEXB,PAN3,WRAP73,CDCA2,GLIS1,PDXP,SOX6,IER2,MECP2,MTMR2,SEMA4D,GSK3B,LGR5,LINGO2,SLC8A1,ZNF841,CDC14A,PRKD1,ERCC6,OGT,RNF144A,TGFB2,CLSTN2,FMN1,PRAMEF12,VAV3,C8A,FUT4,S100A11,SEMA5A,SLC5A3,BDNF,PRKCH,RBMS3,RYK,ZDHHC17,KCNC1,POLR3C,ABI3BP,EDIL3,WASF1,GRIN2B,KDM4C,RAG1,RAG2,ANKRD6,ARHGAP8,NR2F2,PLEKHM2,PRR5,PTGFR,SNAP25,NR5A2,PML,PPARGC1A,RNF180,TRUB2,USP36,AKAP6,CNTNAP2,IGHV3-16,NLRP2,PLXNA2,TAOK3,BCL2,RAD51B,RALBP1,EPHB1,SMARCE1,IL12RB2,KLF12,LRP2,NSF,TMEM100,FRMD4A,NFIA,NR4A2,ARHGE

			F10,EPHA7,ZBTB7C,FYN,KCNE2,PARN,NR4A1,SMARCA4,CD44,HECW1,NEU3,RCVRN,RHBDD1,THBS2,FOXO1,PDE4D,RNF144B,TBXAS1,KANK1,ENPP2,ABCB10,ACTN2,ARHGEF3,ATF6,ATP8A2,BACH1,DCDC2,GRK5,KLF13,MME,RNF217,CSNK1G3,GRIK2,IGSF11,ZNF516,CELF4,SERPINB7,UNC5C,APBB2,EIF3E,MYLK3,NAMPT,SLC1A1,ZFPM2,GLIS3,RARB,RFX4,ETV6,NLRP12,MTOR,ROR1,CADPS,LDB2,MOV10,TMPRSS4,CHRM1,CTDSPL2,DDX21,PKP2,UBXN2B,ABCA13,BMPER,CCDC88B,PTPRD,SCAF8,TIAM2,ZNF521,ZFP64,ARL6IP5,ATP1A1,EPM2A,NOL11,CLYBL,CTNNBIP1,EPB41L4B,SOX8,HAS3,PRKCE,EIF2S1,GTF2A1L,KMT2A,PRKCB,VDR,ADCY8,FHL5,FOXO3,LRRTM1,MITF,RAB31,SCP2,TRAPPC12,CAPN3,ITLN1,KALRN,CREB5,DLC1,EDNRB,MSR1,OASL,ROBO1,SATB2,TDRD3,BNIP3L,FAP,FLT1,NOX4,SGIP1,ZNF148,CDH17,CENPV,GRHL2,TCF4,MAP3K7,MAPK9,NBAS,NR3C1,TACR3,TRAF3IP2,VPS37B,CR1,EYA4,SPON1,ACSL5,PKHD1,STK3,SUPT3H,TGM2,VASH2,ZNF585A,KDR,PPHLN1,CASK,EDA,IGHV4-28,PCOLCE2,ABCA12,APP,CALCRL,NRP1,UACA,LGR4,SERPING1,TENM2,ADTRP,CD58,EGFLAM,KAT5,THRB,FUBP1,KCNK2,SEMA3C,ADAM17,CAMK1D,DIS3L2,MAP3K5,PKP4,IGF2R,ZER1,ATF2,ELP3,ETS1,NR2C2,PIK3R5,ROS1,TGFB1I1,TM9SF4,DOT1L,PSIP1,RPS6KA5,TCF7L2,TNFRSF10B,TRABD2B,BICD1,PARD3,TRAF3,ABAT,GRIN2A,GRIP1,PXT1,RIMS3,SGK2,DLX6-AS1,KIFAP3,BCLAF1,CCDC88A,CD38,MOB3B,MRPS27,RIN2,TUB,STK36,BCL2L1,HDAC4,PDE4B,SOX5,DNMT1,GHR,JAM2,MAPRE2,NTRK2,ACSL4,DNM3,BRD4,RASGRP1,CAMK4,COMMD1,KCTD7,LY86,RABGEF1,SPRED2,STARD4,GLI3,SMCHD1,CLDN1,MORC2,STXBP5,DOCK4,RAP1A,TGFBR1,CYSLTR1,ADAM12,DICER1,EPHA5,LCP2,THEMIS,TNR,CUX1,TENM3,IL16,PTTG1IP,RAPGEF1,ZNF729,CHRNA3,EP300,TBX3,WIF1,CACNA2D1,CYP7B1,DIO2,GRAMD4,TRIOBP,BRD7,GAB2,GLRA1,TP73,GRID2,IFNGR2,PIBF1,RASGRF2,PSMA1,ONECUT3,ADCK1,ADORA2A,EFNA5,FAM20A,MERTK,TAC4,BRD1,CACNG2,MAGI2,NF1,RFC3,RGS7,SORL1,STRN3,ZNF615,BID,ELK3,TEAD4,DMRT1,ASXL1,EIF4E,POR,RELN,GRM5,HDAC9,IGHV4-4,IL18R1,IL1RL1,PTPN9,SELE,DYNC1H1,LRRTM3,MGAT5,NAPEPLD,NUMB,TNFAIP8,CCDC57,IGHV3OR16-12,MAP3K13,NPHP4,RALA,ADCYAP1R1,EGLN3,IL1RAP,NAV3,ATP10A,FBXL5,JAG1,MEOX2,PPP3CA,JRK,KLF7,PLS1,SLC7A1,DGKI,MAS1,PER1,SLC24A2,TBC1D5,WWTR1,KL,C6,CBLB,ELAVL4,MNAT1,PLSCR1,CNTN1,DOK6,RIMS1,TRIM16,WDR59,GABPA,GPSM2,KAT7,SSBP2,STIM2,SLC22A2,AGGF1,ASAP1,BORA,N4BP2L2,NLGN1,PTPRU,AKIRIN2,RNF128,RPS20,SH3KBP1,RAD51AP1,AKAP7,LRRK1,MAPK8IP2,ZNF208,CACNA1D,DDX3X,EGFL6,NSG1,CNOT7,MAML3,EHF,ZNF622,DNMT3B,JAK2,LMO7,SHOX2,AHI1,SMAD3,BAIAP2L1,CD300A,KIDINS220,MAP2K4,NOS1,COL8A1,PLXNB1,RDH10,TCF12,AATF,CDK1,HDAC7,HIVEP3,LTB,MIR320B2,NBN,NVL,SLC39A10,TNF,COBL,OVOL2,PANX1,SNX5,TNIP1,ZFPM1,CSPP1,ILF3,POMC,PRDM15,ADNP2,BLOC1S6,BMP15,DLG5,FCHSD2,INO80,LGR6,MAGI1,RXFP4,ARID2,MLIP,PAXBP1,SHC2,BRINP1,PRG3,BCL11A,HMGA2,MYO6,NEK4,YAF2,AGO3,DOCK1,IGLC2,IGLL5,ANO1,CDC20B,GCLC,QKI,UBE2V1,EPS8,DAAM2,SMOC2,SULF1,ANKRD31,C5AR1,CCP110,LRP4,PIK3R3,WDR5,CNTN6,EFNB2,SAE1,TRDN,NET1,PHF8,ZNF423,CDC14B,CLSTN1,SELP,CHD6,CTNNA1,TENM1,TSHZ3,WDFY2,ZNF407,ARHGEF11,CDKL5,GSKIP,TNFRSF19,ZNF345,BPTF,DYNC2H1,MLLT3,MYRIP,EGF,MIP,SEC16B,SPIRE2,CMA1,IL1RAPL1,KIT,TNFSF8,TNRC6B,ZNF600,BLM,TRPV4,VWC2,ABCA5,C8B,EDAR,EFNB1,RAPGEF2,REG1B,ESR1,HTR2C,JAK1,NCMAP,SEMA3E,SLC30A8,UBE3A,FGF13,GRM4,PLAG1,EPHB2,IGHV3-72,MECOM,NDFIP2,RNF4,TFDP2,TOX,ABCC1,DAP,DAPK1,ERBB4,FER,MAML2,MAP3K3,RBM19,SLITRK6,SPTA1,FNIP1,LRP5,PTH2R,ZNF507,RPS27L,SLAMF1,ABL2,BSG,CAMP,F11R,GAS8,SETD2,ALX4,AP2B1,ARHGAP35,CD2AP,ATP8A1,CCL3,FGF7,PREX1,SEMA5B,VWF,GLYR1,IL20RA,LRRC70,NHLH2,PSG9,SULF2,ARF4,MYH9,NSMCE2,PTGER3,SLC2A13,SLC39A12,SOX4,ST18,CAB39L,KIF23,NEDD9,P2RX1,PDGFD,PIK3CD,PRR16,UBE2N,AGTPBP1,ITGA8,PUM1,ARID4A,DDB1,KDM3A,PLAT,SERTAD2,TNKS,UNC13A,AUTS2,CDK19,FGFR1,MEF2A,OCLN,UNC13B,ARID4B,MAP2,SLX1B,SOST,ASXL3,IL23R,JMY,KEAP1,NTF3,P2RX7,PLA2G4A,SCUBE1,ARRDC4,TOX2,ZNF544,AR,PVT1,SCAMP5,SNX30,TRERF1,CEP250,DENND5B,EYA1,IFNA8,IL1RL2,RBM4,SRPK2,BDKRB1,HLA-DRA,ADRA1B,ARMC9,BHLHB9,DGKZ,GNL3,MIR433,NEK5,PRKAA2,RLF,SEMA3A,CDC73,MARK2,MEIS2,PDE5A,ZMIZ1,FLT3,NFIC,PPARGC1B,SNX7,DYNAP,ESRRG,FERMT2,LTBR,MAP2K5,MARK1,SMPD3,C9,CLN6,DAB2,GPR55,HDAC8,LCP1,PBX1,RNF207,STX3,SYT2,TC

			L1B,ACTN4,ADAM10,BLOC1S5,CCL7,EEF1E1,EEF1E1-BLOC1S5,FGF12,ITGA3,KMO,POU2F3,PRAMEF8,FANCB,LGI1,PLCL2,PPM1B,QRICH1,RFTN1,STAC,MYOM1,PIN1,SYNPO2,TRPM4,CD226,TCF20,ABCA8,BMP6,CHRM3,CIT,CIZ1,CLASP1,EIF4ENIF1,SMTNL2,CARF,CNGB1,NCOA2,TGFA,CREM,G3BP2,HGF,MYLK,PKP1,POU2F1,CALCR,CAPN7,FLI1,POMT2,SCUBE3,ZBTB16,CACNB2,CTIF,NOS1AP,IL18RAP,INSR,LARP4B,MACF1,PAK1,PDE3A,PSMB7,SKI,AKT3,CDC16,ELF1,FMR1,LIMK2,LITAF,PAX3,CHFR,DKC1,IGHV1-69,JDP2,KLF6,RRAGC,ABCD1,BMF,EVC,IDE,IGHV1-46,MAD1L1,MFNG,AIM2,CD84,PCK1,PPARA,SPIB,TNFAIP8L3,TTN,CLIP1,FHL1,KATNBL1,SLC22A5,TAF1,CHD5,FNIP2,PPP2R3C,S1PR3,SPRED1,AKR1C2,CHSY1,ESR2,PRDM16,ZNF451,BTRC,C2,CCDC3,CFB,FBLN2,IGHV1-18,MLLT10,OPTN,RIT2,ESRP2,NCOA1,PRKCG,PRLR,CBFA2T3,DOCK7,FBXW11,G3BP1,HDAC5,TDGF1,ZNF493,A2M,LYVE1,SLC40A1,STAT4,STIM1,ADARB1,KCNC2,PEMT,POU2AF1,PRKCQ,SCEL,STAT3,SUSD4,TFEC,TP63,USP7,ZNF721,AMOT,DOCK3,HPN,ISLR2,PAK3,PIK3R2,STK17B,TBL1X,BLNK,CASQ2,DTNBP1,RAP1B,KSR1,LGALS9,LRP8,NFAT5,ALPK1,GTPBP1,IL10RB,MAPKAPK2,APC,BMPR1A,CKAP5,DPP4,ITGA1,KCTD20,KIAA0319,NGF,PRKCA,PTGES,UBE2E2,VLDLR,ADAMTS20,PILRB,ABI1,CTSH,DHX9,RGCC,RPH3AL,CCL14,CCL15,CYB5D2,EDA2R,GPR21,IL6R,MAP2K6,SPTBN4,CLASP2,MAP2K1,PRAMEF7,RGMB,SOS1,TPH1,VPS35,C1QTNF3,FGF1,PLB1,RAD18,TBX20,ATMIN,CRADD,NLRC5,RGN,RREB1
GO:0016477	cell migration	8.745221378372606e-10	*SRGAP2B,PLCB1,CMKLR1,DAB1,FMNL2,PTPRT,DCC,DUSP22,FBLN1,ITGA2,SUN2,ZRANB1,NRG3,TGFBR3,CDH13,PIK3C2B,SDCCAG8,GADD45A,PLXNA4,FLRT2,GPC3,NRG1,RFFL,MCTP1,ASTN2,SH3RF2,UNC5D,SEMA6D,SPOCK1,CTNNA2,CCR1,CCR3,RBFOX2,TIAM1,RHOC,LDLRAD4,NKD1,SCAI,FBXO31,ARID5B,PHACTR1,EPHA3,DCLK1,AGT,PSTPIP2,RHOJ,CDH2,LOXL2,LAMA2,SEMA3D,DACH1,NTN1,ZNF268,GPC6,DBH,FMNL3,RIN3,LAMA4,SLIT2,PDCD6,GPC5,IGF1R,VCAN,PTPRG,KRT2,VCL,CELSR1,MBOAT7,ASTN1,STK24,FRMD5,TRPM2,CTNNA3,NTNG1,LRRK2,NTRK3,ANKS1A,DISC1,HEXB,SH3BP1,MECP2,SEMA4D,SLC8A1,PRKD1,TGFB2,VAV3,FUT4,S100A11,SEMA5A,LAMA3,NR2F2,PML,TNS1,PLXNA2,BCL2,ARHGAP24,EPHB1,VANGL2,NR4A2,FYN,NR4A1,CD44,KANK1,ENPP2,DCDC2,LAMB4,UNC5C,PALLD,NLRP12,MTOR,LDB2,FAT3,MEGF9,BMPER,CEP85L,EPB41L4B,SOX8,PRKCE,FAM19A4,FOXO3,MITF,DLC1,EDNRB,ROBO1,SATB2,FAP,FLT1,CENPV,KDR,PRCP,CHL1,APP,NRP1,PTPRM,ELMO1,ADTRP,SEMA3C,ADAM17,CAMK1D,ELP3,ETS1,ITGA11,ATRNL1,OPHN1,CCDC88A,RIN2,HDAC4,PDE4B,JAM2,MAPRE2,NTRK2,PRKG1,CDC42BPA,STARD13,RABGEF1,GLI3,CLDN1,DOCK4,SRP54,TGFBR1,TNR,PEAK1,IL16,CYP7B1,MERTK,MAGI2,NF1,SORL1,DMRT1,RELN,HDAC9,KIRREL3,SELE,MGAT5,NUMB,NAV3,JAG1,MEOX2,PPP3CA,CXADR,DOCK10,GPC4,PTPRU,SH3KBP1,JAK2,SPNS2,SMAD3,CD300A,PLXNB1,CDK1,HDAC7,TNF,LRCH1,OVOL2,DLG5,LGR6,ARID2,ADORA3,DOCK1,EPS8,SMOC2,SULF1,C5AR1,PIK3R3,EFNB2,AMOTL1,NET1,SELP,CTNNA1,CDKL5,EGF,KIT,PIK3C2G,TRPV4,EFNB1,PTPRO,RAPGEF2,SEMA3E,FGF13,MARVELD3,EPHB2,ABCC1,ERBB4,FER,MAP3K3,LRP5,SLAMF1,BSG,F11R,SETD2,ARHGAP35,CD2AP,PTPRK,ATP8A1,CCL3,FGF7,LAMA1,PREX1,SEMA5B,ITGA9,KIF2A,NHLH2,ARF4,MYH9,NEDD9,PDGFD,PIK3CD,ENPEP,PLAT,WDPCP,AUTS2,FGFR1,ITGBL1,ITGB7,NTF3,BDKRB1,DGKZ,SEMA3A,MARK2,ZMIZ1,SRGAP3,FERMT2,MAP2K5,MARK1,SMPD3,TNN,DAB2,LCP1,ACTN4,ADAM10,CCL7,ITGA3,SYNPO2,TRPM4,CLASP1,HGF,MYLK,CAPN7,INSR,MACF1,PAK1,AKT3,PRKX,ULK4,CCDC141,GNA13,FUT8,SPRED1,NTN4,PTPRF,DEFA1B,PTPRR,EXT1,UMOD,DOCK7,ELMO2,HDAC5,TDGF1,LYVE1,TREM1,ADARB1,NDE1,PRKCQ,STAT3,AMOT,PAK3,USH2A,LGALS9,LRP8,APC,BMPR1A,DPP4,ITGA1,KIAA0319,PRKCA,CTSH,RGCC,SRGAP1,CCL14,CCL15,CCL15-CCL14,IL6R,CLASP2,SOS1,FGF1,TBX20,RREB1*
GO:0007166	cell surface receptor signaling pathway	9.470270501784736e-10	*IGHV1OR21-1,PLCB1,CMKLR1,DAB1,MAPK14,DSCAM,LYPD6,PTPRT,IGHV4-31,DCC,IGHV3-64,SVEP1,WLS,TIMP3,DUSP22,GRIK1,IGHV1OR15-9,SORBS2,IGHV4OR15-8,NPHP3,ITGA2,EPHA6,VRK2,GRIN3A,FCRL2,NRXN1,SHC3,ZRANB1,DOK5,RAB5A,CTNND2,MS4A1,RIMS2,GRIA1,NRG3,TGFBR3,FSTL4,CDH13,NXN,CSNK2A1,TF,TMEM108,GADD45A,PLXNA4,FLRT2,GPC3,NRG1,PSMB2,SHISA6,RFFL,IL4R,PLCE1,UNC5D,GLP2R,SEMA6D,CCR1,CCR3,DTX4,TIAM1,LDLRAD4,GRID1,CBFA2T2,IL1RAPL2,NKD1,MX1,BTN2A1,ALK,PTPRE,RAB7A,ARID5B,ROBO2,C2CD3,EPHA3,PRKACB,PXDN,LEMD3,AGT,WDR11,CDH2,PARK2,KIR2DL1,SEMA3D,TRIM5,TXK,GPC6,DBH,ZNRF3,TRIO,HDAC2,HTRA1,SLIT2,P*

			DCD6,STXBP4,GPC5,IGF1R,SMAD1,TMPRSS6,PTPRG,CELSR1,FSHR,GRIA4,GRM1,SKAP1,WWOX,LRRK2,MAPK10,NTRK3,RORA,CD8B,PRICKLE2,ANKS1A,RNF165,DISC1,SH3BP1,MECP2,MTMR2,SEMA4D,GSK3B,LGR5,PRKD1,ERCC6,OGT,TGFB2,ATP1A4,VAV3,ANKS1B,SEMA5A,BDNF,LLGL2,PRKCH,RBMS3,RYK,ZDHHC17,WASF1,GRIN2B,LAMA3,SIK2,ANKRD6,PML,FBN1,GRIK3,IGHV3-16,PLXNA2,BCL2,ZNF675,EPHB1,IL12RB2,SIPA1L1,LRP2,TMEM100,VANGL2,CDH6,DEPTOR,NR4A2,EPHA7,FYN,ADAMTS18,CHN1,SMARCA4,CD44,HECW1,NEU3,RNF213,FOXO1,PDE4D,KANK1,DCDC2,GRK5,TNMD,CSNK1G3,GRIK2,IGSF11,CELF4,UNC5C,SLC1A1,UGCG,RFX4,NLRP12,ROR1,CTDSPL2,BMPER,PTPRD,BBS2,EPM2A,PLN,CTNNBIP1,ZMYND11,DSG4,NLGN4X,PRKCE,PRKCB,FOXO3,MITF,KALRN,TTLL12,CCDC88C,EDNRB,OASL,ROBO1,FLT1,CDH17,COL2A1,MAP3K7,MAPK9,TRAF3IP2,CR1,EYA4,ACSL5,STK3,KDR,EDA,IGHV4-28,APP,CALCRL,NRP1,LGR4,RANBP10,SEMA3C,ADAM17,TSPEAR,IGF2R,ATF2,GRK6,EFEMP1,ROS1,TGFB1I1,DOT1L,RPS6KA5,TCF7L2,TNFRSF10B,TRABD2B,ITGA11,TRAF3,ABAT,GRIN2A,CCDC88A,CD38,FAM83B,RNF43,CHRDL1,RNF126,STK36,BCL2L1,GUCY2F,PDE4B,GHR,GLRA2,NTRK2,FAM83G,PTPDC1,LY86,RABGEF1,SPRED2,GLI3,KIF16B,RAP1A,TGFBR1,CYSLTR1,ADAM12,EPHA5,LCP2,THEMIS,NCOA5,CHST11,CPE,RAPGEF1,CHRNA3,CHRNA5,EP300,WIF1,BRD7,GAB2,GLRA1,COL4A6,GRID2,IFNGR2,PIBF1,PSMA1,ADORA2A,CABIN1,EFNA5,MERTK,GUCY2C,MAGI2,NF1,SORL1,BICC1,BID,IFT80,DMRT1,POR,RELN,GRM5,IGHV4-4,IL18R1,IL1RL1,MGAT5,NDRG2,IGHV3OR16-12,NLK,NPHP4,ADCYAP1R1,IL1RAP,FBXL17,JAG1,PPP3CA,JRK,SNX25,DGKI,WWTR1,KL,CBLB,PLSCR1,CNTN1,DOK6,GPC4,GRM7,RIMS1,ITGAE,NLGN1,P2RX6,PTPRU,BTBD11,LRRK1,MAPK8IP2,VEPH1,DDX3X,CNOT7,GMDS,MAML3,JAK2,SHOX2,AHI1,SMAD3,BAIAP2L1,CD300A,KIDINS220,MAP2K4,PLXNB1,LTB,SLC39A10,TNF,OVOL2,SNX5,PRDM15,BMP15,DLG5,LGR6,MAGI1,SHC2,ADORA3,CLEC4A,DOCK1,GRIA3,IGLC2,IGLL5,GCLC,PMEPA1,LTBP1,DAAM2,SMOC2,SULF1,C5AR1,LRP4,PIK3R3,CNTN6,EFNB2,AMOTL1,ZNF423,ALPK2,CTNNA1,GRIK4,GSKIP,TNFRSF19,DYNC2H1,MLLT3,CD109,EGF,KIT,SLIT3,TNFSF8,VWC2,CC2D2A,EFNB1,PTPRO,RAPGEF2,JAK1,SEMA3E,GRM4,EPHB2,IGHV3-72,DAPK1,ERBB4,FER,MAML2,LRP5,PTH2R,SHCBP1,SLAMF1,BSG,FARP2,GAS8,COMMD7,PTPRK,CCL3,FGF7,LAMA1,RNF138,SEMA5B,SPRED3,IL20RA,ITGA9,PSG9,SULF2,MYH9,SOX4,ST18,NEDD9,P2RX1,PDGFD,PIK3CD,UBE2N,ASGR2,ITGA8,DDB1,IRAK2,PLAT,TNKS,WDPCP,FGFR1,UNC13B,GLRA3,ITGBL1,SOST,IL23R,ITGB7,NTF3,P2RX7,SCUBE1,AMFR,AR,SUFU,EYA1,IFNA8,IL1RL2,BDKRB2,ARMC9,DGKZ,PRKAA2,SEMA3A,TLE4,CDC73,GRIA2,MARK2,ZMIZ1,FLT3,PTPRA,TRIM72,FERMT2,LTBR,MAP2K5,MARK1,SMPD3,TNN,DAB2,ACTN4,ADAM10,CCL7,DAAM1,FGF12,ITGA3,NREP,PLCL2,PPM1B,RFTN1,PIN1,TRPM4,CD226,BMP6,TGFA,WDR12,GAS2,HGF,CALCR,SCUBE3,NCAM1,IL18RAP,INSR,MACF1,PAK1,PSMB7,SKI,ELF1,ENPP1,FMR1,IGHV1-69,EVC,IDE,IGHV1-46,MFNG,AIM2,CSF2RB,EVC2,PPARA,SORT1,FUT8,S1PR3,SPRED1,TRPM1,CHSY1,PRDM16,PTPRF,ZNF451,BTRC,CCDC3,IGHV1-18,PTPRR,EXT1,IFT81,PRLR,UMOD,FBXW11,G3BP1,TDGF1,STAT4,PRKCQ,SCEL,STAT3,TP63,PAK3,PIK3R2,TBL1X,BLNK,LRP8,IL10RB,MAPKAPK2,APC,BMPR1A,ITGA1,KIAA0319,NGF,PRKCA,UBASH3B,VLDLR,GPR75,MTMR4,PILRB,ABI1,FCRL5,TRBV6-8,USP18,CCL14,CCL15,EDA2R,GPR21,IL6R,CLASP2,RGMB,SOS1,VPS35,FGF1,TBX20,CRADD,NLRC5,TBX18
GO:0006812	cation transport	1.941362988831853e-9	PIEZO2,ASIC2,CACNA1E,KCNJ6,ANK3,SLC24A3,KCNQ1,GRIN3A,NRXN1,SLC1A2,MS4A1,PKD1L1,SLC9B1,TRPM3,ANK2,KCND3,FAM155A,SLC12A1,DPP10,TF,SCN8A,KCNE1,KCNJ15,SYT17,KCNG3,SCARA5,SHISA6,RYR3,CCR1,PM20D1,RASGRF1,SLC6A2,SLC17A3,UTRN,SLC44A1,AGT,SHISA9,CHRM5,ATP6V0D1,PARK2,KCNH1,SLC5A4,CP,KCNMA1,SLC44A5,SLC24A4,SYT9,KCNK17,TRPC5,DMD,HCN1,SYT1,CACNA2D3,CACNG3,NOX5,FGF14,GSG1L,SLC39A11,DPP6,TRPM2,CNIH3,RYR2,CATSPER2,DHRS7C,SLC1A3,KCNH5,SLC4A4,CHD7,KCNK13,SLC8A1,PRKD1,FLVCR1,ATP1A4,SLC5A3,ZDHHC17,KCNC1,GRIN2B,NKAIN2,SNAP25,PML,AKAP6,SNAP23,BCL2,RALBP1,KCNJ3,SLC30A5,LRP2,NKAIN3,NSF,ITPR2,ABCC9,FYN,KCNE2,HECW1,RCVRN,PDE4D,ACTN2,KCNS3,KCNIP4,KCND2,SCN11A,SLC1A1,MFSD12,PKP2,SLC35F3,ATP1A1,EPM2A,PLN,HEPHL1,PRKCE,SLC2A9,ATP6V1D,PRKCB,VDR,EFHB,SLC47A1,CAPN3,EDNRB,SLC12A8,KCNC4,CASK,APP,CALCRL,KCNK2,SLC38A7,PEX5L,ABAT,GRIN2A,SLC30A7,PDE4B,SLC5A10,COMMD1,CACNA1C,NIPA2,CYSLTR1,KCNJ12,SL

			C13A4,SLC6A17,IL16,CHRNA3,CACNA2D1,CACNA2D4,IFNGR2,RASGRF2,TRPM7,ADORA2A,CAMK2G,CACNG2,RGS7,KCNQ5,MICU1,RELN,SLC39A8,SLC9C1,ADCYAP1R1,PPP3CA,TMEM163,KCNB2,SLC7A1,PER1,SLC24A2,SLC38A6,SLC5A1,CNTN1,STIM2,SLC22A2,NLGN1,P2RX6,SLC22A3,AKAP7,MAPK8IP2,CACHD1,CACNA1D,TPCN2,SLC5A8,NOS1,MAGT1,SLC39A10,PANX1,TMC2,KCNT2,KCNK10,TSPAN13,ANO1,KCNA6,TRPC4,CNGB3,KCNH8,TRDN,ANO10,SLC15A1,SCN1A,KCNH7,EGF,TRPV4,KCNAB1,HTR2C,SLC30A8,FGF13,EPHB2,NDFIP2,DAPK1,CACNG6,FLVCR2,ATP8A1,CCL3,MCUR1,SLC39A12,P2RX1,KCNE4,CNGA4,SFXN3,CDH23,RYR1,P2RX7,RASA3,BDKRB1,THADA,GNB5,ATP6V1H,HCN4,SCNN1A,ATP13A3,RNF207,SYT2,ACTN4,FGF12,KCNJ16,KCNN3,STAC,TRPM4,CNGB1,TMCO3,MYLK,SLC38A4,CALCR,SLC13A3,CACNB2,NOS1AP,FMR1,MICU2,ATP5J,CD84,JPH4,FHL1,SLC22A5,CACNA1A,TRPM1,SCN9A,ATP6V1E1,UMOD,KLHL3,SLC24A5,SLC38A10,SLC40A1,STIM1,TRPM6,KCNC2,SLC9A9,HPN,CASQ2,DTNBP1,SLC28A3,TMEM175,SLC7A8,TMEM63A,TUSC3,UBASH3B,SLC9A2,SPTBN4,MCOLN3,ATP6V1E2,RGN
GO:0009887	animal organ morphogenesis	2.488102334471033e-9	*MAPK14,DSCAM,NPHP3,ITGA2,KCNQ1,SDK2,TNC,NRG3,OLFM3,TGFBR3,CSMD1,FLRT2,GPC3,NRG1,PSMB2,ALDH1A2,CTNNA2,CFTR,TIAM1,MYO3A,NKD1,SPEF2,ARID5B,ROBO2,C2CD3,AJAP1,AGT,CDH2,FHL2,FOXN3,LAMA2,GREB1L,NTN1,GPC6,ZNRF3,HDAC2,HTRA1,SLIT2,MDM2,SLC24A4,HCN1,BASP1,CELSR1,WWOX,RYR2,NTNG1,LRRK2,RORB,STRC,KIF26B,PRICKLE2,CHD7,SOX6,FLVCR1,TGFB2,FMN1,RYK,PCDH15,WDR72,EXT2,LAMA3,ANKRD6,NR5A2,PML,FBN1,BCL2,EPHB1,CSGALNACT1,LRP2,MMP16,TMEM100,VANGL2,ATP8A2,EXOC4,LAMB4,SLC1A1,ZFPM2,RARB,MTOR,ROR1,FAT3,MEGF9,PKP2,BBS2,COL11A1,CTNNBIP1,SOX8,VDR,FOXO3,DLC1,ROBO1,SATB2,GRHL2,COL2A1,SEC24B,PKHD1,TGM2,KDR,EDA,COL18A1,NRP1,PTPRM,LGR4,EGFLAM,THRB,SEMA3C,TSPEAR,ATF2,EFEMP1,ATRNL1,FRAS1,ADAMTS16,SOX5,GHR,NTRK2,SDK1,GLI3,CRB1,TGFBR1,TENM3,CHST11,CPE,EP300,DSCAML1,TBX3,CYP7B1,TRIOBP,PSMA1,FAM20A,MAGI2,NF1,IFT80,TTC8,ASXL1,POR,TNFRSF11B,TSHZ1,TBX15,JAG1,PLS1,WWTR1,GPC4,FREM1,SSUH2,MEGF11,SYCP2,SHOX2,SOBP,AHI1,SMAD3,COL8A1,RDH10,TNF,OVOL2,ZFPM1,DLG5,PHEX,ARID2,MMP20,MYO6,RAB23,SULF1,LRP4,EFNB2,ALPK2,CTNNA1,MLLT3,SLIT3,TRPV4,DNAH11,EDAR,ESR1,PLAG1,EPHB2,ERBB4,SLITRK6,LRP5,BSG,SETD2,ALX4,FGF7,LAMA1,SULF2,SOX4,AGTPBP1,ITGA8,MYO3B,PDZD7,WDPCP,CDH23,FGFR1,RYR1,ASXL3,P2RX7,AR,BCOR,SUFU,EYA1,SEMA3A,ANKRD11,MEIS2,ZMIZ1,PPARGC1B,PAPPA2,SMPD3,DAB2,PBX1,RNF207,DAAM1,LRIG1,BMP6,TGFA,HGF,MYLK,FLI1,ELN,INSR,PSMB7,SKI,AKT3,PAX3,PRKX,RPGRIP1,PPARA,TTN,FHL1,MYH7,CHSY1,NTN4,BTRC,ESRP2,EXT1,MDM4,MYH6,ADAMTS5,FBXW11,KLHL3,SLC40A1,STIM1,TTC39C,ADARB1,STAT3,TP63,HPN,USH2A,BMPR1A,CTSH,MAP2K1,SOS1,FGF1,TBX20,TBX18*
GO:0022603	regulation of anatomical structure morphogenesis	2.5144827158225935e-9	*ISM1,CDH4,DAB1,OMA1,GTF2I,FMNL2,CDC42EP3,DSCAM,RUNX1,DCC,FBLN1,NPHP3,ZRANB1,CHODL,RIMS2,FGD6,FSTL4,LRRC4C,GADD45A,SYT17,PLXNA4,GPC3,PSMB2,SEMA6D,CCR3,TIAM1,RHOC,NKD1,FBXO31,ROBO2,AJAP1,AGT,RHOJ,CDH2,PARK2,CYBB,SEMA3D,NTN1,GPC6,ZNRF3,FMNL3,MYO9A,SLIT2,PDCD6,PALMD,SMAD1,TRPC5,SYT1,CELSR1,NTNG1,TENM4,LRRK2,PRICKLE2,DISC1,HEXB,MECP2,SEMA4D,GSK3B,PRKD1,TGFB2,SEMA5A,BDNF,RYK,ANKRD6,PML,PLXNA2,RALBP1,SIPA1L1,VANGL2,EPHA7,FYN,CHN1,CD44,HECW1,THBS2,KANK1,ENPP2,TNMD,STRIP1,ROR1,MOV10,BMPER,ERMN,PTPRD,TIAM2,PGK1,SOX8,PRKCB,KALRN,DLC1,ROBO1,TANC2,FLT1,SEC24B,PKHD1,VASH2,KDR,ARHGAP15,NRP1,PTPRM,SHROOM3,ARHGEF18,LGR4,SEMA3C,ATF2,ETS1,GRIP1,NTRK2,DNM3,CYSLTR1,ADAM12,TNR,CUX1,CHRNA3,TRIOBP,PARVB,PSMA1,ADCK1,EFNA5,MAGI2,NF1,RELN,TNFRSF11B,MAP3K13,RALA,ATP10A,PPP3CA,CAPZB,GPC4,RIMS1,TMEM135,AGGF1,NLGN1,SH3KBP1,SHOX2,AHI1,PLXNB1,TNF,PPFIA2,BCL11A,HMGA2,DOCK1,EPS8,SMOC2,SULF1,C5AR1,LRP4,EFNB2,CDKL5,MYH14,FAM171A1,MLLT3,EGF,CMA1,IL1RAPL1,KIT,RAPGEF2,ESR1,JAK1,SEMA3E,UBE3A,DNMBP,FGF13,EPHB2,SPTA1,CAMP,F11R,ARHGAP35,CCL3,FGF7,PREX1,SEMA5B,FGD4,MYH9,SLC39A12,NEDD9,PIK3CD,UNC13A,WDPCP,MAP2,AR,BHLHB9,SEMA3A,EPB41L3,MARK2,PDLIM5,FERMT2,DAB2,RNF207,SYT2,ACTN4,CCL7,DAAM1,CLASP1,CFDP1,GAS2,HGF,MACF1,PAK1,PSMB7,AKT3,GNA13,SPRED1,NTN4,AKAP2,STIM1,STAT3,AMOT,HPN,ISLR2,PAK3,DTNBP1,LRP8,BMPR1A,KIAA0319,NGF,PRKCA,CTSH,RGCC,CLASP2,MAP2K1,VPS35,FGF1,RREB1,TBX18*
GO:00485	negative	2.517346107203332e-	*FANK1,CAST,SRGAP2B,TPTE,ZHX3,PLCB1,DAB1,OMA1,DUX4,MAPK14,DSCAM,TPTE2,RUNX1,ASIC2,CTBP2,SERPINA1,ANXA8L1,PTPR*

23	regulation of cellular process	9	*T,ANK3,DCC,TIMP3,ZNF397,DUSP22,FBLN1,NPHP3,ABCG1,RGS6,KCNQ1,DPF3,ATF7IP,NRXN1,PAX7,MTPN,SHANK2,GRIA1,NRG3,SMYD2,CLEC16A,HUS1,TGFBR3,FSTL4,CDH13,RNF152,ANK2,RTN1,NXN,RUNX1T1,CSNK2A1,ERCC4,KCNE1,SAMD4A,EIF4EBP3,CHEK2,GADD45A,PRDM9,ACIN1,PLXNA4,TOX3,GPC3,NRG1,STXBP6,ETS2,PSMB2,SHISA6,RFFL,IL4R,ALDH1A2,LRFN5,RYR3,MCTP1,SUZ12,ASTN2,SH3RF2,SEMA6D,SPOCK1,ABHD17C,CTNNA2,RBFOX2,PM20D1,AGBL4,BEND5,LDLRAD4,DNAJC15,RGS7BP,ZNF91,ZBTB20,ARFIP1,CBFA2T2,NKD1,SCAI,PTPRE,SORCS3,ZNF536,FBXO31,RAB7A,CNTN4,RGL2,ARID5B,ROBO2,SPOCK3,ZNF366,EPHA3,PRKACB,DCLK1,PXDN,AJAP1,LEMD3,AGT,CST2,MAGEB3,AVEN,RCAN1,CDH2,PARK2,FHL2,FOXN3,LOXL2,CDC5L,KIR2DL4,KIR3DL2,BPIFB1,CORO2B,PSPC1,SEMA3D,DACH1,NTN1,TP53I11,TRIM22,USP25,ZNF268,CP,NELL1,PCDH17,PHC1,ZNRF3,RIN3,TRIO,FTO,HDAC2,HTRA1,SLIT2,TFAP2D,ZBTB34,KCTD1,PDCD6,RPS6KA2,LCMT1,IGF1R,MDM2,SLC24A4,SMAD1,E2F3,TMPRSS6,CEP97,PTPRG,SACS,TRPC5,DMD,STK33,BASP1,VCL,GRIA4,FMN2,HNRNPA2B1,MAEL,NFE2L3,NRIP1,PRKAR2A,SORCS2,INPP5A,STK24,WWOX,ATRX,FRMD5,PARP16,TRPM2,RYR2,DHRS7C,LRRK2,RORB,IGFBP7,NTRK3,RORA,IQCJ-SCHIP1,CHD7,OVGP1,PAN3,GLIS1,SH3BP1,SOX6,MECP2,MTMR2,SEMA4D,GSK3B,SLC8A1,PRKD1,SERPINA4,SERPINA5,ERCC6,OGT,TGFB2,PRAMEF12,PIWIL4,RIPPLY3,S100A11,SEMA5A,BDNF,PRKAR1B,PRKCH,RBMS3,RYK,ABI3BP,GRIN2B,KDM4C,OTUD7A,RAG1,RAG2,SAMD13,ANKRD6,NR2F2,PTGFR,PCBP3,PML,PPARGC1A,USP36,ARHGAP12,AKAP6,FBN1,PPP4R4,GRIK3,NLRP7,PLXNA2,TAOK3,BCL2,CHMP4C,MXI1,ZNF675,ARHGAP24,EPHB1,SMARCE1,SKAP2,KLF12,LRP2,FRMD4A,ITPR2,DEPTOR,NR4A2,EPHA7,ZBTB7C,DPT,RBAK,FYN,KCNE2,PARN,ADAMTS18,NR4A1,SMARCA4,CD44,HECW1,NEU3,PMP22,RHBDD1,RNF213,FOXO1,PDE4D,RBBP8,RNF144B,KANK1,ACTN2,ATP8A2,BACH1,CST9L,GRK5,KLF13,PACRG,TNMD,GRIK2,CELF4,SERPINB7,APBB2,EIF3E,SLC1A1,ZFPM2,ZNF141,GLIS3,RARB,RERG,RFX4,ETV6,GNG4,NLRP12,MFSD12,MTOR,RMI2,LDB2,MOV10,CTDSPL2,FAT3,PKP2,UBXN2B,BMPER,PTPRD,SCAF8,ARL6IP5,ATP1A1,EPM2A,PLN,CTNNBIP1,SOX8,ZMYND11,NLGN4X,PRKCE,EIF2S1,HELLS,KMT2A,PRKCB,VDR,ADCY8,FOXO3,IGF2BP3,LRRTM1,MITF,SCMH1,CAPN3,KALRN,TTLL12,CCDC88C,DLC1,EDNRB,ROBO1,SATB2,TBC1D4,BNIP3L,FAP,FLT1,NOX4,ZNF148,GRHL2,TSC22D3,COL2A1,NBAS,NR3C1,TRAF3IP2,CR1,EYA4,SPON1,MDM1,PKHD1,STK3,KDR,PPHLN1,CASK,CHL1,ABCA12,APP,COL18A1,NRP1,PTPRM,UACA,ARHGEF18,LGR4,LINC00473,SERPING1,TENM2,ADTRP,KAT5,THRB,CPEB2,KCNK2,SEMA3C,ADAM17,CAMK1D,DIS3L2,TFPI,ATF2,ETS1,NR2C2,SAMSN1,EFEMP1,GOPC,PRTG,SMG6,TGFB1I1,DOT1L,RGS16,RPS6KA5,TCF7L2,TRABD2B,BICD1,PARD3,TLK2,ZNF830,ABAT,KIFAP3,OPHN1,BCLAF1,TRPS1,ZNF568,CD38,DENND5A,RNF43,CHRDL1,RNF126,BCL2L1,HDAC4,MYO16,PDE4B,TIMP2,BACE2,DNMT1,GHR,JAM2,NTRK2,PRKG1,ACSL4,DNM3,BRD4,STARD13,ARR3,COMMD1,RABGEF1,SPRED2,GLI3,SMCHD1,ZNF207,CACNA1C,RAP1A,TGFBR1,PRDX4,DICER1,TNR,CUX1,NCOA5,CHST11,MAGEA11,PTTG1IP,RAPGEF1,EP300,MORC3,HMG20A,SCFD1,TBX3,WIF1,CYP7B1,GRAMD4,TRIOBP,ATXN1,BRD7,GLRA1,TP73,GRID2,NPC1,PIBF1,PSMA1,FHIT,ADCK1,ADORA2A,ADRBK2,EFNA5,MERTK,MAGI2,NF1,RGS7,TFF1,SORL1,STRN3,ARHGAP6,BICC1,BID,ELK3,IFT80,TRIM59,DMRT1,LZTR1,ASXL1,EIF4E,POR,GRM5,HDAC9,PTPN9,MGAT5,NDRG2,NUMB,RHPN2,SP2,TNFAIP8,NLK,NPHP4,TBX15,ADCYAP1R1,NAV3,JAG1,MEOX2,PPP3CA,KLF3,KLF7,MYT1L,SH3GL2,SNX25,CAPZB,DGKI,MAS1,PER1,SATB1,SLC24A2,WWTR1,ANKRD13A,CBLB,ELAVL4,MAGEB2,MNAT1,ATP9A,DGUOK,GRM7,TMEM67,GABPA,KAT7,L3MBTL3,ZNF540,FHOD3,N4BP2L2,NLGN1,PTPRU,THAP7,AKIRIN2,RPS20,NPR3,SYCP2,LRRK1,MAPK8IP2,VEPH1,DDX3X,LRPPRC,RBL2,CNOT7,GABRA5,TPCN2,DNMT3B,JAK2,SHOX2,AHI1,SMAD3,CD300A,MAP2K4,NOS1,SERPINB12,PLXNB1,ZNF337,AATF,CDK1,DCBLD2,HDAC7,NBN,SLC39A10,TAGLN3,TNF,LRCH1,OVOL2,SNX5,TNIP1,ZFPM1,EIF3A,ILF3,PRDM15,ADNP2,DLG5,SCAF4,SIM2,ARID2,MLIP,BRINP1,FAM220A,PRG3,SRSF4,ADORA3,ARF1,BCL11A,HMGA2,POLR1A,YAF2,ZNF705A,AGO3,GCLC,PMEPA1,RAB23,EPS8,LTBP1,DAAM2,L3MBTL4,SULF1,CCP110,LRP4,WDR5,EFNB2,MXD3,TRDN,HERC1,PHF8,ZNF423,CDC14B,ALPK2,CTNNA1,TENM1,TSHZ3,ZNF93,GSKIP,PRKAR1A,ZNF345,BPTF,MLLT3,CD109,PTPN14,FOXP4,GBE1,IL1RAPL1,KIT,SLIT3,TNRC6B,UBR2,BLM,TRPV4,VWC2,ABCA5,KCNAB1,PTPRO,RAPGEF2,DNAJB6,ESR1,HTR2C,SEMA3E,UBE3A,FGF13,GABRB2,MARVELD3,EPHB2,MECOM,TBCD,TFDP2,DAP,DAPK1,ERBB4,FER,MAP3K3,SPTA1,FNIP1,LRP5,MALRD1,ZNF439,RPS27L,SLAMF1,ABL2,DEPDC5,F11R,GAS8,VPS13C*

			,AP2B1,ARHGAP35,CD2AP,COMMD7,NSUN2,PTPRK,CCL3,SEMA5B,SPRED3,TNFAIP8L2,ARHGAP25,GCFC2,SULF2,ARF4,MYH9,PACSIN2,SOX4,ST18,SPTB,AGTPBP1,KCNE4,PUM1,ARID4A,DDB1,KDM3A,PLAT,SERTAD2,TNKS,BCL7A,DAPL1,MEF2A,OCLN,PARP15,PDE11A,RGS3,RYR1,ZCCHC17,ZNF85,DAD1,MAP2,SLX1B,SOST,NTF3,P2RX7,TEX14,RASA3,AMFR,APIP,AR,BCOR,FGL2,SCAMP5,SUFU,TRERF1,EYA1,N4BP1,RBM4,BDKRB1,BDKRB2,BHLHB9,DGKZ,L3MBTL1,OGN,PI3,PRKAA2,SEMA3A,TAX1BP1,THADA,TLE4,ZNF202,ZNF558,ARHGAP10,CDC73,GNB5,MEIS2,PDE5A,PROS1,ZNF19,ZNF440,NFIC,PPARGC1B,SRGAP3,TRIM72,FERMT2,MAP2K5,MARK1,TNN,DAB2,GPR55,HDAC8,PBX1,ACTN4,ADAM10,EEF1E1,ITGA3,PRAMEF8,TXNDC5,FANCB,FRY,PLCL2,PPM1B,NUP153,PIN1,RPS6KA6,TRPM4,ERLIN1,STK38,ZNF674,BMP6,CIT,CLASP1,EIF4ENIF1,CDYL2,CFDP1,NCOA2,TGFA,TMOD1,CREM,HGF,PKP1,POU2F1,RGS12,CENPF,SH3BP5,ZBTB16,KDM4B,ARHGAP28,LARP4B,PAK1,PDE3A,PSMB7,SKI,AKT3,CTDP1,ELF1,ENPP1,FMR1,LIMK2,LITAF,CHFR,DKC1,JDP2,PHC2,ABCD1,BMF,CRMP1,MAD1L1,ARHGAP42,CD84,JPH4,PPARA,SORT1,TBX22,FAM3D,FHL1,GNA13,PPP1R13B,TAF1,CHD5,FNIP2,S1PR3,SPRED1,ESR2,KRT4,PRDM16,QSOX1,RBL1,ZNF451,BTRC,CCDC3,HIRA,OPTN,PTPRR,RIT2,MDM4,PRKCG,PRLR,UMOD,ZNF354B,CBFA2T3,FBXW11,G3BP1,HDAC5,TDGF1,A2M,PI15,SERPINB8,SLC24A5,SLC40A1,ADARB1,PRKCQ,STAT3,TFEC,TP63,USP7,BRIP1,CDKN3,CST1,HPN,PIK3R2,TBL1X,USH2A,CASQ2,DHRS2,DTNBP1,GAK,RAP1B,LGALS9,MAPKAPK2,APC,BMPR1A,DPP4,ITGA1,KIAA0319,NGF,PRKCA,PTGES,SPINT2,UBASH3B,ADAMTS20,ITIH6,MTMR4,OSGIN2,PDS5A,ABI1,CTSH,DHX9,NCAPG2,RGCC,RPH3AL,SRGAP1,USP18,GPR21,SIAH3,TFIP11,SPTBN4,CLASP2,MAP2K1,PRAMEF7,VPS35,ZBTB25,ZRANB3,C1QTNF3,COL6A3,MCOLN3,RAD18,TBX20,C6ORF106,CRADD,NLRC5,RGN,RREB1,TBX18
GO:0030001	metal ion transport	2.567154702662006e-9	*ASIC2,CACNA1E,KCNJ6,ANK3,SLC24A3,KCNQ1,GRIN3A,MS4A1,PKD1L1,SLC9B1,TRPM3,ANK2,KCND3,FAM155A,SLC12A1,DPP10,TF,SCN8A,KCNE1,KCNJ15,KCNG3,SCARA5,RYR3,CCR1,SLC6A2,SLC17A3,UTRN,AGT,KCNH1,SLC5A4,CP,KCNMA1,SLC24A4,KCNK17,TRPC5,DMD,HCN1,CACNA2D3,CACNG3,NOX5,FGF14,SLC39A11,DPP6,TRPM2,RYR2,CATSPER2,DHRS7C,SLC1A3,KCNH5,SLC4A4,CHD7,KCNK13,SLC8A1,PRKD1,FLVCR1,ATP1A4,SLC5A3,ZDHHC17,KCNC1,GRIN2B,NKAIN2,SNAP25,PML,AKAP6,BCL2,KCNJ3,SLC30A5,LRP2,NKAIN3,NSF,ITPR2,ABCC9,FYN,KCNE2,HECW1,RCVRN,PDE4D,ACTN2,KCNS3,KCNIP4,KCND2,SCN11A,SLC1A1,PKP2,ATP1A1,EPM2A,PLN,HEPHL1,PRKCE,PRKCB,VDR,EFHB,CAPN3,EDNRB,SLC12A8,KCNC4,CASK,CALCRL,KCNK2,SLC38A7,GRIN2A,SLC30A7,PDE4B,SLC5A10,COMMD1,CACNA1C,NIPA2,CYSLTR1,KCNJ12,SLC13A4,SLC6A17,IL16,CACNA2D1,CACNA2D4,TRPM7,ADORA2A,CAMK2G,CACNG2,RGS7,KCNQ5,MICU1,SLC39A8,SLC9C1,ADCYAP1R1,PPP3CA,TMEM163,KCNB2,PER1,SLC24A2,SLC38A6,SLC5A1,CNTN1,STIM2,AKAP7,CACHD1,CACNA1D,TPCN2,SLC5A8,NOS1,MAGT1,SLC39A10,PANX1,TMC2,KCNT2,KCNK10,TSPAN13,KCNA6,TRPC4,KCNH8,TRDN,SCN1A,KCNH7,EGF,TRPV4,KCNAB1,HTR2C,SLC30A8,FGF13,NDFIP2,CACNG6,FLVCR2,CCL3,MCUR1,SLC39A12,P2RX1,KCNE4,CDH23,RYR1,P2RX7,RASA3,BDKRB1,THADA,GNB5,HCN4,SCNN1A,RNF207,ACTN4,FGF12,KCNJ16,KCNN3,STAC,TRPM4,MYLK,SLC38A4,CALCR,SLC13A3,CACNB2,NOS1AP,FMR1,MICU2,CD84,JPH4,FHL1,SLC22A5,CACNA1A,TRPM1,SCN9A,UMOD,KLHL3,SLC24A5,SLC38A10,SLC40A1,STIM1,TRPM6,KCNC2,SLC9A9,HPN,CASQ2,SLC28A3,TMEM175,TUSC3,UBASH3B,SLC9A2,SPTBN4,MCOLN3,RGN*
GO:0003012	muscle system process	4.286339434267508e-9	*DTNA,AKAP13,SORBS2,ITGA2,KCNQ1,TNNI3K,MTPN,ANK2,KCND3,MYH1,KCNE1,PLCE1,UTRN,MYOM3,AGT,FBXO32,SETD3,KCNMA1,HDAC2,PDE9A,DMD,CTNNA3,RYR2,SLC8A1,SNTB1,SGCD,MYH8,AKAP6,KCNJ3,KCNE2,NR4A1,FOXO1,PDE4D,ACTN2,ATP8A2,RAP1GDS1,APBB2,MTOR,PKP2,ATP1A1,PLN,FOXO3,EDNRB,TACR3,CALCRL,SSPN,ABAT,CD38,HDAC4,PDE4B,PRKG1,CACNA1C,DOCK4,KCNJ12,CHRNA3,TBX3,CACNA2D1,GLRA1,PPP1R12B,CAMK2G,PPP3CA,KCNB2,P2RX6,CACNA1D,MYH4,TPCN2,SMAD3,NOS1,TNF,MLIP,CALD1,SULF1,TRDN,SCN1A,ARHGEF11,MYH14,MYH13,KIT,TRPV4,RCSD1,FGF13,NUP155,SULF2,PTGER3,P2RX1,KCNE4,MEF2A,RYR1,SYNM,BDKRB2,ADRA1B,PDE5A,SMPX,PDLIM5,TRIM72,GNAO1,HCN4,RNF207,FGF12,STAC,MYOM1,TRPM4,CHRM3,TMOD1,MYLK,CACNB2,NOS1AP,CTDP1,ARHGAP42,PPARA,TTN,MYH7,MYBPC2,MYH6,SCO2,MYH11,CASQ2,MYOM2,PRKCA,ASB3,MAP2K6,TBX20*
GO:0009966	regulation of signal transducti	7.120171790632891e-9	*TPTE,PLCB1,CMKLR1,DAB1,MAPK14,TPTE2,SH3RF3,CTBP2,LYPD6,PTPRT,WLS,TIMP3,DUSP22,AKAP13,FBLN1,NPHP3,RGS6,VRK2,NRXN1,ZRANB1,DOK5,DOCK2,CTNND2,RIMS2,SHANK2,SMYD2,CLEC16A,TGFBR3,FSTL4,CDH13,RNF152,NXN,CSNK2A1,TMEM108,CH*

	on		*EK2,GADD45A,GPC3,NRG1,PSMB2,SHISA6,RFFL,PLCE1,SH3RF2,CCR1,TIAM1,RHOC,LDLRAD4,RGS7BP,RASGRF1,CBFA2T2,NKD1,SCAI,ALK,PTPRE,ZNF536,RAB7A,RGL2,S100B,ROBO2,ZNF366,C2CD3,PRKACB,PXDN,CRNN,LEMD3,AGT,SHISA9,WDR11,RCAN1,CDH2,PARK2,FHL2,DLGAP1,BPIFB1,TRIM22,TRIM5,TXK,GPC6,DBH,ITSN1,ZNRF3,TRIO,HDAC2,HTRA1,MYO9A,SLIT2,PDCD6,GPC5,LCMT1,IGF1R,MDM2,SLC24A4,TMPRSS6,NR3C2,DMD,KCTD8,CACNG3,SIPA1L3,FSHR,GSG1L,GRM1,WDR83,WWOX,CNIH3,CAMTA1,LRRK2,IGFBP7,NTRK3,RORA,IQCJ-SCHIP1,ANKS1A,RNF165,DISC1,SH3BP1,MTMR2,SEMA4D,GSK3B,LGR5,MAGI3,PRKD1,ERCC6,OGT,TGFB2,VAV3,SEMA5A,BDNF,LLGL2,PRKCH,RBMS3,RYK,ZDHHC17,WASF1,KDM4C,OTUD7A,SIK2,ANKRD6,ARHGAP8,PRR5,PML,ARHGAP12,AKAP6,FBN1,TAOK3,BCL2,RALBP1,ZNF675,ARHGAP24,EPHB1,SIPA1L1,LRP2,TMEM100,DEPTOR,NR4A2,ARHGEF10,EPHA7,PSD3,FYN,CHN1,SMARCA4,CD44,HECW1,NEU3,RNF213,FOXO1,PDE4D,KANK1,ARHGEF3,ATF6,DCDC2,GRK5,TNMD,CSNK1G3,GRIK2,IGSF11,CELF4,UGCG,RFX4,GNG4,NLRP12,MTOR,ROR1,CTDSPL2,DDX21,BMPER,PTPRD,TIAM2,ARL6IP5,CTNNBIP1,ZMYND11,NLGN4X,PRKCE,HELLS,PRKCB,EFHB,FAM19A4,FOXO3,CAPN3,KALRN,PJA1,TTLL12,CCDC88C,DLC1,OASL,ROBO1,FLT1,NOX4,COL2A1,MAP3K7,MAPK9,PREX2,TRAF3IP2,EYA4,ACSL5,PKHD1,STK3,TGM2,KDR,PRCP,ARHGAP15,EDA,APP,NRP1,UACA,ARHGEF18,LGR4,LINC00473,ADAM17,MAP3K5,TSPEAR,GRK6,PEX5L,PIK3R5,ROS1,TGFB1I1,DOT1L,RGS16,TCF7L2,TNFRSF10B,TRABD2B,BICD1,RALGAPA2,TRAF3,ABAT,OPHN1,BCLAF1,SGMS1,CCDC88A,MOB3B,RNF43,TUB,CHRDL1,RNF126,STK36,BCL2L1,GUCY2F,PDE4B,GHR,MAPRE2,NTRK2,BRD4,RASGRP1,STARD13,ARR3,LY86,RABGEF1,SPRED2,GLI3,RAP1A,SAFB2,TGFBR1,MGLL,NCOA5,CHST11,PTTG1IP,RAPGEF1,EP300,WIF1,CYP7B1,GRAMD4,TP73,IFNGR2,PIBF1,RASGRF2,PSMA1,ADORA2A,ADRBK2,RALGPS1,CACNG2,MAGI2,NF1,RALGPS2,RGS7,SORL1,STRN3,ARHGAP6,BICC1,BID,IFT80,TRIM59,DMRT1,LZTR1,ASXL1,POR,RELN,GRM5,IL18R1,MGAT5,NDRG2,MAP3K13,NLK,NPHP4,ADCYAP1R1,FBXL17,JAG1,JRK,SNX25,DGKI,PER1,WWTR1,KL,CBLB,PLSCR1,DOK6,GPC4,RIMS1,TRIM16,WDR59,NLGN1,PTPRU,ARHGAP5,RPS20,AKAP7,LRRK1,MAPK8IP2,VEPH1,DDX3X,CNOT7,ZNF622,ARHGAP39,JAK2,SHOX2,SMAD3,BAIAP2L1,CD300A,PLXNB1,AATF,HDAC7,SLC39A10,TNF,OVOL2,SNX5,TNIP1,EIF3A,POMC,PRDM15,BMP15,DLG5,AFAP1,LGR6,SHC2,AGO3,GCLC,PMEPA1,UBE2V1,EPS8,LTBP1,DAAM2,SMOC2,SULF1,C5AR1,DEF6,LRP4,CNTN6,NET1,ZNF423,SELP,ALPK2,CTNNA1,TENM1,ARHGEF11,GSKIP,TNFRSF19,ARHGAP11B,DYNC2H1,MLLT3,SIPA1L2,CD109,EGF,KIT,SLIT3,UBR2,TRPV4,VWC2,EDAR,PTPRO,RAPGEF2,ESR1,HTR2C,UBE3A,DNMBP,GRM4,MARVELD3,EPHB2,MECOM,NDFIP2,DAPK1,ERBB4,FER,MAP3K3,FNIP1,SLAMF1,ABL2,DEPDC5,F11R,GAS8,ARHGAP35,CD2AP,CCL3,PREX1,SPRED3,VWF,ARHGAP25,FGD4,IL20RA,SULF2,SOX4,GARNL3,PDGFD,PIK3CD,UBE2N,ITGA8,PUM1,IRAK2,TNKS,AUTS2,FGFR1,PDE11A,RGS3,UNC13B,SOST,IL23R,NTF3,P2RX7,SCUBE1,RASA3,AMFR,APIP,AR,SUFU,EYA1,IFNA8,BDKRB2,ADRA1B,ARMC9,DGKZ,PRKAA2,SEMA3A,TLE4,ARHGAP10,CDC73,PDE5A,ZMIZ1,FLT3,SRGAP3,TRIM72,FERMT2,LTBR,MAP2K5,TNN,DAB2,GPR55,ACTN4,ADAM10,CCL7,EEF1E1,EEF1E1-BLOC1S5,ITGA3,NREP,PLCL2,PPM1B,PIN1,RPS6KA6,TRPM4,CD226,PIP5K1B,STK38,BMP6,CIT,TGFA,GAS2,HGF,RALGAPA1,RGS12,CALCR,SCUBE3,CNKSR2,NCAM1,NOS1AP,ARHGAP28,INSR,MACF1,PAK1,PDE3A,PSMB7,SKI,AKT3,ELF1,ENPP1,LITAF,RRAGC,ULK4,EVC,MAD1L1,MFNG,ARHGAP42,PPARA,TNFAIP8L3,GNA13,TAF1,CHD5,SPRED1,AKR1C2,CHSY1,ESR2,PRDM16,ZNF451,BTRC,CCDC3,OPTN,PTPRR,RIT2,IFT81,NCOA1,PRLR,FBXW11,G3BP1,TDGF1,PRKCQ,SCEL,STAT3,TP63,USP7,AMOT,DOCK3,PAK3,PIK3R2,TBL1X,DTNBP1,RAP1B,DLGAP2,KSR1,LGALS9,NFAT5,ALPK1,IL10RB,APC,BMPR1A,ITGA1,KIAA0319,NGF,PRKCA,UBASH3B,ADAMTS20,MTMR4,CTSH,NCAPG2,RPH3AL,USP18,CCL14,CCL15,EDA2R,GPR21,IL6R,MAP2K6,RIC8B,MAP2K1,SOS1,VPS35,C1QTNF3,FGF1,TBX20,CRADD,NLRC5,RGN,TBX18*
GO:0007010	cytoskeleton organization	8.001092646867468e-9	*FMNL2,CDC42EP3,CEP44,ANK3,AKAP13,SORBS2,TTLL2,SUN2,ZRANB1,DOCK2,PCNT,MTPN,FGD6,ANK2,PDE4DIP,TF,SDCCAG8,CHEK2,TUBGCP6,GADD45A,HYDIN,CECR2,PLCE1,CTNNA2,RHOC,DPYSL2,ARFIP1,TACC2,UTRN,SPEF2,PHACTR1,C2CD3,EPHA3,PHACTR3,PSTPIP2,FSIP2,ATAT1,RHOJ,PARK2,CORO2B,FRMPD4,TLN2,ANKFN1,SETD3,FMNL3,SLIT2,KRT25,CEP97,DMD,KRT2,SIPA1L3,CELSR1,FMN2,NCKAP5,ATRX,FRMD5,TRPM2,CTNNA3,DIAPH2,KIF3B,NTRK3,NUSAP1,IQCJ-*

			SCHIP1,DISC1,WRAP73,PDXP,SH3BP1,MECP2,GSK3B,CDC14A,FRMD6,FMN1,SEMA5A,KRT74,LLGL2,PCDH15,DNAAF2,EML1,WASF1,FIGN,TUBB1,ARHGAP12,BCL2,CHMP4C,SIPA1L1,ARHGEF10,MICAL3,FLNC,KANK1,ACTN2,ATP8A2,FRMD3,RAP1GDS1,MYLK3,PALLD,STRIP1,MTOR,GAS7,PKP2,UBXN2B,CCDC88B,ERMN,BBS2,EPB41L4B,KLHL1,EHBP1,PRKCE,CAPN3,PARD3B,CCDC88C,DLC1,LRRC49,MAP7,NEBL,MDM1,PKHD1,THSD7B,NRP1,SHROOM3,ARHGEF18,ELMO1,RANBP10,TTC12,CYLC2,PARVG,TUBAL3,CDC42BPG,DIAPH3,ATF2,KPNB1,BICD1,PARD3,OPHN1,CCDC88A,CNN3,DNAH8,MAPRE2,PRKG1,CDC42BPA,STARD13,NINL,ZNF207,TGFBR1,EPHA5,SPAG17,TRIOBP,PARVB,PIBF1,TRPM7,CCDC170,FLNB,EFNA5,SPECC1L,NF1,ARHGAP6,TTC8,DYNC1H1,RHPN2,CCDC57,NPHP4,RALA,NAV3,NEB,PLS1,CAPZB,CXADR,CCSER2,STARD9,TMEM67,GPSM2,MPRIP,BORA,FHOD3,MZT1,NLGN1,SH3KBP1,PHACTR2,JAK2,SMAD3,BAIAP2L1,PLXNB1,CDK1,TNF,COBL,FCHSD2,INO80,AFAP1,KATNAL1,SPAG16,ARF1,CALD1,MYO6,KIF18A,EPS8,DAAM2,CCP110,LRCH3,TRDN,AMOTL1,CDC14B,INSC,CTNNA1,TENM1,ARHGEF11,EFHC2,MYH14,PRKAR1A,FAM171A1,SPIRE2,KIT,TRPV4,CC2D2A,DNAJB6,MYPN,SEMA3E,SPECC1,FGF13,ARMC2,KRT76,RNF4,TBCD,FER,SPTA1,TTLL5,ABL2,F11R,FARP2,GAS8,SETD2,ARHGAP35,CD2AP,CCL3,FGF7,MYO7B,PREX1,ARHGAP25,FGD4,KIF2A,MYH9,PACSIN2,SLC39A12,KIF23,NEDD9,SPTB,TNKS,WDPCP,AUTS2,MEF2A,OCLN,SYNM,MAP2,JMY,NTF3,P2RX7,C10ORF90,CEP250,PRKAA2,ARHGAP10,EPB41L3,KIF4A,MARK2,PDLIM5,PPARGC1B,FERMT2,MARK1,LCP1,ACTN4,CCL7,DAAM1,TTLL11,SYNPO2,THSD7A,TUBA3C,SHROOM4,CIT,CLASP1,SMTNL2,KATNAL2,TMOD1,GAS2,PKP1,RASSF8,MYO1B,ELN,KATNA1,MAST4,NOS1AP,ARHGAP28,MACF1,PAK1,LIMK2,TESK2,ULK4,CRMP1,HEPACAM2,TTN,CLIP1,KATNBL1,PPP2R3C,KRT4,MPDZ,PGM5,MYH6,DOCK7,ELMO2,FBXW11,MYH11,NDE1,AMOT,PAK3,DTNBP1,MYOM2,APC,CKAP5,ABI1,RGCC,SPTBN4,CLASP2,PPP1R9A
GO:0060078	regulation of postsynaptic membrane potential	2.001783020756635e-8	GABRG3,GRIK1,GRIN3A,NRXN1,RIMS2,GRIA1,TMEM108,RGS7BP,GRID1,GABRG1,GRM1,LRRK2,MECP2,MTMR2,GSK3B,GRIN2B,GRIK3,GABRB1,GRIK2,IGSF11,CELF4,KCND2,CHRM1,NLGN4X,GABRA2,APP,ABAT,GRIN2A,GLRA2,CHRNA3,CHRNA5,GLRA1,GRID2,ADORA2A,RELN,GRM5,PPP3CA,DGKI,RIMS1,GABRA3,NLGN1,P2RX6,MAPK8IP2,GABRA5,GRIK4,GABRR2,GABRB2,P2RX1,UNC13B,GLRA3,P2RX7,GABRA1,GABRG2
GO:0098609	cell-cell adhesion	2.1362456082267446e-8	CDH4,PCDH9,DAB1,MAPK14,DSCAM,RUNX1,NCAM2,PTPRT,ANK3,IGSF5,DUSP22,NRXN1,NRXN3,SDK2,CTNND2,PKD1L1,DLG2,CDH13,LRRC4C,NRG1,STXBP6,IL4R,LRFN5,ASTN2,UNC5D,CTNNA2,PCDH11X,PCDH7,CNTN4,ROBO2,CRNN,CDH11,CDH2,CDH9,HMCN1,NEGR1,NTN1,TLN2,GPC6,PCDH17,CDH8,DSC3,CLDN14,VCL,CELSR1,SKAP1,ASTN1,CTNNA3,NTNG1,TENM4,KIF26B,EMB,SEMA4D,TGFB2,CLSTN2,FUT4,S100A11,PCDH15,LAMA3,RAG1,RAG2,BCL2,CDH10,CDH12,LRRC4,CDH6,EPHA7,FYN,ADAMTS18,CD44,MYL12A,IGSF11,CDHR4,PALLD,FAT3,PKP2,CCDC88B,PTPRD,DSG4,NLGN4X,CDH18,FOXO3,ROBO1,CDH17,CR1,LPP,PKHD1,ABCA12,PTPRM,TENM2,VSIG10,ADTRP,CD58,PKP4,ETS1,ABAT,KIFAP3,ALCAM,CNN3,JAM2,PRKG1,RASGRP1,SDK1,GLI3,CLDN1,CRB1,TNR,TENM3,RAPGEF1,DSCAML1,GRID2,ADORA2A,EFNA5,EMCN,IGSF21,SLC39A8,KIRREL3,SELE,NPHP4,IL1RAP,JAG1,SLC7A1,CXADR,CBLB,GPC4,NLGN1,PTPRU,MEGF11,CADM3,JAK2,BAIAP2L1,CD300A,TNF,TNIP1,DLG5,MAGI1,PCDH19,CNTN6,EFNB2,CLSTN1,SELP,CTNNA1,TENM1,PRKAR1A,IL1RAPL1,KIT,TRPV4,EFNB1,DNAJB6,JAK1,MYPN,FNDC3A,FER,SPTA1,SLAMF1,BSG,F11R,CD2AP,CLDN11,TNFAIP8L2,MYH9,SOX4,ITGA8,CDH23,IL23R,ITGB7,FGL2,IL1RL2,HLA-DRA,PDE5A,ZMIZ1,PDLIM5,MAP2K5,BMP6,PCDHGA1,PCDHGA10,PCDHGA11,PCDHGA12,PCDHGA2,PCDHGA3,PCDHGA4,PCDHGA5,PCDHGA6,PCDHGA7,PCDHGA8,PCDHGA9,PCDHGB1,PCDHGB2,PCDHGB3,PCDHGB4,PCDHGB6,PCDHGB7,PKP1,ZBTB16,MAD1L1,CD84,GLDN,PCK1,PPARA,COL19A1,PTPRF,EXT1,UMOD,ELMO2,SERPINB8,PRKCQ,LGALS9,NFAT5,DPP4,PRKCA,SPINT2,UBASH3B,RGCC,DCHS2
GO:0030036	actin cytoskeleton organization	2.592209845480845e-8	FMNL2,CDC42EP3,AKAP13,SORBS2,DOCK2,MTPN,FGD6,TF,CTNNA2,RHOC,ARFIP1,UTRN,PHACTR1,EPHA3,PHACTR3,PSTPIP2,RHOJ,PARK2,CORO2B,FRMPD4,SETD3,FMNL3,SLIT2,DMD,CELSR1,FMN2,FRMD5,TRPM2,CTNNA3,DIAPH2,NTRK3,PDXP,SH3BP1,FRMD6,FMN1,SEMA5A,LLGL2,PCDH15,WASF1,ARHGAP12,BCL2,SIPA1L1,ARHGEF10,MICAL3,FLNC,KANK1,ACTN2,FRMD3,RAP1GDS1,MYLK3,PALLD,STRIP1,MTOR,GAS7,ERMN,EPB41L4B,KLHL1,EHBP1,PRKC

			E,CAPN3,DLC1,NEBL,THSD7B,NRP1,SHROOM3,ARHGEF18,ELMO1,PARVG,CDC42BPG,DIAPH3,OPHN1,CCDC88A,CNN3,PRKG1,CDC42BPA,STARD13,TGFBR1,EPHA5,TRIOBP,PARVB,TRPM7,FLNB,EFNA5,SPECC1L,NF1,ARHGAP6,TTC8,RHPN2,NPHP4,RALA,NEB,PLS1,CAPZB,CXADR,MPRIP,FHOD3,SH3KBP1,PHACTR2,JAK2,SMAD3,BAIAP2L1,TNF,COBL,FCHSD2,ARF1,CALD1,MYO6,EPS8,DAAM2,AMOTL1,CTNNA1,TENM1,ARHGEF11,MYH14,PRKAR1A,FAM171A1,SPIRE2,KIT,TRPV4,DNAJB6,MYPN,SEMA3E,SPECC1,FER,SPTA1,ABL2,F11R,FARP2,ARHGAP35,CD2AP,FGF7,MYO7B,PREX1,ARHGAP25,FGD4,MYH9,PACSIN2,NEDD9,SPTB,AUTS2,MEF2A,JMY,NTF3,EPB41L3,PDLIM5,PPARGC1B,FERMT2,LCP1,ACTN4,DAAM1,SYNPO2,THSD7A,SHROOM4,CIT,CLASP1,SMTNL2,TMOD1,GAS2,MYO1B,ELN,NOS1AP,ARHGAP28,PAK1,LIMK2,TESK2,TTN,PGM5,MYH6,ELMO2,MYH11,AMOT,PAK3,DTNBP1,MYOM2,ABI1,RGCC,SPTBN4,CLASP2,PPP1R9A
GO:0006936	muscle contraction	2.6653708457207803e-8	DTNA,ITGA2,KCNQ1,TNNI3K,ANK2,KCND3,MYH1,KCNE1,PLCE1,UTRN,MYOM3,AGT,SETD3,KCNMA1,DMD,CTNNA3,RYR2,SLC8A1,SNTB1,SGCD,MYH8,KCNJ3,KCNE2,NR4A1,PDE4D,ACTN2,ATP8A2,RAP1GDS1,APBB2,MTOR,PKP2,ATP1A1,PLN,EDNRB,TACR3,CALCRL,SSPN,ABAT,CD38,HDAC4,PDE4B,PRKG1,CACNA1C,DOCK4,KCNJ12,CHRNA3,TBX3,CACNA2D1,GLRA1,PPP1R12B,KCNB2,P2RX6,CACNA1D,MYH4,TPCN2,NOS1,TNF,CALD1,SULF1,TRDN,SCN1A,ARHGEF11,MYH14,MYH13,KIT,TRPV4,RCSD1,FGF13,NUP155,SULF2,PTGER3,P2RX1,KCNE4,RYR1,SYNM,BDKRB2,ADRA1B,PDE5A,SMPX,GNAO1,HCN4,RNF207,FGF12,STAC,MYOM1,TRPM4,CHRM3,TMOD1,MYLK,CACNB2,NOS1AP,ARHGAP42,TTN,MYH7,MYBPC2,MYH6,MYH11,CASQ2,MYOM2,ASB3,MAP2K6,TBX20
GO:1905114	cell surface receptor signaling pathway involved in cell-cell signaling	3.467115484945542e-8	MAPK14,LYPD6,WLS,NPHP3,NRXN1,ZRANB1,RAB5A,CTNND2,RIMS2,NXN,CSNK2A1,TMEM108,GPC3,PSMB2,SHISA6,TIAM1,NKD1,CDH2,PARK2,GPC6,ZNRF3,GPC5,CELSR1,WWOX,LRRK2,PRICKLE2,DISC1,MECP2,MTMR2,GSK3B,LGR5,SEMA5A,RBMS3,RYK,GRIN2B,ANKRD6,VANGL2,NR4A2,SMARCA4,HECW1,RNF213,FOXO1,KANK1,DCDC2,GRK5,CSNK1G3,GRIK2,IGSF11,CELF4,ROR1,EPM2A,CTNNBIP1,NLGN4X,FOXO3,MITF,CCDC88C,EDNRB,STK3,EDA,APP,LGR4,GRK6,TGFB1I1,TCF7L2,TRABD2B,ABAT,GRIN2A,CCDC88A,RNF43,GLRA2,GLI3,CPE,RAPGEF1,CHRNA3,CHRNA5,WIF1,BRD7,GLRA1,GRID2,PSMA1,ADORA2A,MAGI2,BICC1,IFT80,RELN,NDRG2,NLK,NPHP4,PPP3CA,JRK,DGKI,WWTR1,GPC4,RIMS1,NLGN1,P2RX6,PTPRU,LRRK1,MAPK8IP2,DDX3X,SMAD3,PRDM15,LGR6,DAAM2,SULF1,LRP4,AMOTL1,ZNF423,ALPK2,GSKIP,MLLT3,EGF,PTPRO,LRP5,RNF138,SULF2,SOX4,P2RX1,DDB1,TNKS,UNC13B,GLRA3,SOST,P2RX7,AMFR,PRKAA2,TLE4,CDC73,MARK2,FERMT2,MARK1,TNN,DAB2,DAAM1,ITGA3,PPM1B,PIN1,TRPM4,MACF1,PSMB7,SKI,BTRC,EXT1,FBXW11,G3BP1,SCEL,TBL1X,APC,VPS35,TBX18
GO:0072359	circulatory system development	4.6199376352807536e-8	ISM1,GTF2I,MAPK14,RUNX1,MYO18B,SVEP1,AKAP13,SORBS2,NPHP3,KCNQ1,NRXN1,NRXN3,SMYD2,TGFBR3,CDH13,ANK2,NXN,GADD45A,PLXNA4,FLRT2,GPC3,NRG1,ALDH1A2,PLCE1,CCR3,COL22A1,ROBO2,C2CD3,COL15A1,AGT,WDR11,RHOJ,CDH2,FHL2,LOXL2,GREB1L,CYBB,FMNL3,LAMA4,SLIT2,PDCD6,RPS6KA2,IGF1R,MDM2,SMAD1,ADAMTS6,BASP1,NOX5,RYR2,TENM4,NTRK3,RORA,CHD7,SOX6,MECP2,SLC8A1,PRKD1,FLVCR1,TGFB2,VAV3,PLCD3,RIPPLY3,SEMA5A,ABI3BP,SGCD,NR2F2,PML,SGCG,AKAP6,FBN1,ARHGAP24,EPHB1,LRP2,TMEM100,VANGL2,NR4A1,RNF213,THBS2,FOXO1,ENPP2,ACTN2,TNMD,SERPINB7,MYLK3,SLC1A1,ZFPM2,RARB,MTOR,PKP2,BMPER,PGK1,COL11A1,PLN,PRKCB,DLC1,ROBO1,FAP,FLT1,NOX4,GRHL2,COL2A1,NEBL,TRAF3IP2,SEC24B,STK3,VASH2,KDR,PRCP,CALCRL,COL18A1,NRP1,PTPRM,ADTRP,IMMP2L,KCNK2,SEMA3C,ATF2,ETS1,TCF7L2,RIN2,DNMT1,NTRK2,STARD13,GLI3,CACNA1C,RAP1A,TGFBR1,CYSLTR1,ADAM12,CPE,RAPGEF1,EP300,TBX3,PCSK5,TP73,EMCN,WARS2,NF1,BICC1,ELK3,ASXL1,HDAC9,JAG1,MEOX2,NEB,CXADR,MNAT1,AGGF1,FHOD3,SHOX2,AHI1,SMAD3,COL8A1,CDK1,HDAC7,TNF,OVOL2,ZFPM1,ARID2,CALD1,FREM2,HMGA2,QKI,SMOC2,SULF1,C5AR1,PIK3R3,EFNB2,AMOTL1,ALPK2,PRKAR1A,DYNC2H1,EGF,PTPN14,CMA1,KIT,SLIT3,CC2D2A,DNAH11,RAPGEF2,JAK1,SEMA3E,SGCZ,EPHB2,TFDP2,ERBB4,MAP3K3,LRP5,BSG,CAMP,SETD2,AP2B1,LAMA1,MYH9,SLC39A12,SOX4,PDGFD,PIK3CD,ENPEP,WDPCP,MEF2A,RYR1,SCUBE1,BCOR,CASP7,SUFU,EYA1,SRPK2,ZMIZ1,PDLIM5,HCN4,MAP2K5,RNF207,ADAM10,FGF12,ITGA3,THSD7A,TGFA,HGF,MYLK,ELN,INSR,AKT3,CTDP1,PRKX,PPARA,TTN,C1GALT1,GNA13,MYH7,SPRED1,EXT1,MDM4,MYH6,TYMP,HDAC5,TDGF1,STIM1,MYH11,STAT3,AMOT,BMPR1A,PRKCA,CTSH,RGCC,IL6R,MAP2K1,SOS1,FGF1,TBX20,TBX18
GO:00650	regulation	6.406033633947756e-	FANK1,CAST,PLCB1,CMKLR1,DAB1,MAPK14,CTBP2,SERPINA1,CC

09	of molecular function	8	NG2,ANXA8L1,PRIM2,LYPD6,PTPRT,ANK3,BCL2L13,TIMP3,DUSP22,AKAP13,FBLN1,ITGA2,RGS6,KCNQ1,GABBR2,EPHA6,NRXN1,SGSM1,DOCK2,MTPN,NRG3,FGD6,TGFBR3,ANK2,TBC1D22A,CSNK2A1,ERCC4,KCNE1,GADD45A,PLXNA4,GPC3,NRG1,SHISA6,RFFL,SUZ12,PRMT8,PLCE1,SH3RF2,SPOCK1,SLCO3A1,CFTR,TIAM1,PM20D1,RHOC,RASGRF1,ALK,UTRN,ERC1,RGL2,ARID5B,PHACTR1,SPOCK3,EPHA3,CRNN,AGT,PHACTR3,CST2,SHISA9,RCAN1,PARK2,DLGAP1,TRIM22,TRIM5,DCUN1D4,TXK,DENND2A,USP6,TRAPPC9,ITSN1,NCF4,RIN3,TRIO,HDAC2,MYO9A,SLIT2,PDCD6,IGF1R,SLC24A4,RAPGEF5,PXK,DMD,CACNG3,SIPA1L3,FGF14,FSHR,GSG1L,HNRNPA2B1,PRKAR2A,PARP16,CNIH3,RYR2,XRCC4,LRRK2,MAPK10,NTRK3,DISC1,SH3BP1,SEMA4D,GSK3B,LGR5,SLC8A1,PRKD1,SERPINA4,SERPINA5,ERCC6,TGFB2,VAV3,SLC5A3,BDNF,LLGL2,PRKAR1B,PRKCH,RIPK4,RYK,KCNC1,TOR1AIP1,GRIN2B,RAG1,ARHGAP8,NR2F2,PML,PPARGC1A,RNF180,ARHGAP12,AKAP6,PPP4R4,NLRP2,NLRP7,PLXNA2,TAOK3,BCL2,RALBP1,RXFP2,ZNF675,ARHGAP24,EPHB1,SIPA1L1,MMP16,DEPTOR,NR4A2,ARHGEF10,EPHA7,PSD3,ZBTB7C,RPGR,FYN,KCNE2,PARN,CHN1,NR4A1,SMARCA4,CD44,HECW1,RCVRN,PDE4D,ACTN2,ARHGEF3,CST9L,RAP1GDS1,SERPINB7,EIF3E,SLC1A1,AGAP1,NLRP12,MTOR,ROR1,LDB2,RASGEF1B,TIAM2,PLCL1,ARL6IP5,EPM2A,PLN,CTNNBIP1,EVI5,PRKCE,EIF2S1,KMT2A,PRKCB,VDR,ADCY8,EFHB,FAM19A4,CAPN3,KALRN,CCDC88C,DLC1,EDNRB,OASL,ROBO1,RXFP1,TBC1D4,FLT1,NOX4,GRHL2,MAP3K7,MAPK9,PREX2,CR1,SPON1,PKHD1,STK3,TGM2,KDR,ARHGAP15,EDA,PCOLCE2,APP,NRP1,UACA,ARHGEF18,ELMO1,LGR4,SERPING1,CPEB2,ADAM17,CAMK1D,MAP3K5,PKP4,TFPI,ATF2,ELP3,PIK3R5,GOPC,ROS1,SMG6,DOT1L,RGS16,RPS6KA5,TCF7L2,TNFRSF10B,BICD1,RALGAPA2,TRAF3,DOCK9,GRIN2A,SGK2,OPHN1,CCDC88A,DENND5A,MOB3B,RIN2,NCF2,STK36,HDAC4,PDE4B,TIMP2,GHR,MAPRE2,NTRK2,PRKG1,RASGRP1,STARD13,COMMD1,KCTD7,RABGEF1,RGL1,SPRED2,CACNA1C,STXBP5,DOCK4,RAP1A,TGFBR1,EPHA5,LCP2,RAPGEF1,CHRNA3,EP300,CACNA2D1,GRAMD4,PCSK5,IFNGR2,PIBF1,RASGRF2,ARHGEF33,PPP1R12B,ADORA2A,CABIN1,EFNA5,FAM20A,MERTK,RALGPS1,CACNG2,MAGI2,NF1,PPP6R2,RALGPS2,RFC3,RGS7,SORL1,ARHGAP6,BID,TTC8,POR,RELN,GRM5,HDAC9,IL18R1,SELE,MGAT5,TNFAIP8,MAP3K13,ADCYAP1R1,EGLN3,IL1RAP,PPP3CA,TBC1D3B,DGKI,MAS1,TBC1D5,WWTR1,CBLB,DOCK10,MNAT1,PLSCR1,GRM7,RIMS1,AHSA1,GPSM2,STIM2,SLC22A2,ASAP1,BORA,NLGN1,AKIRIN2,ARHGAP5,RPS20,NPR3,PHACTR2,AKAP7,MAPK8IP2,CACNA1D,DDX3X,RBL2,ARAP2,ZNF622,ARHGAP39,JAK2,SMAD3,CD300A,KIDINS220,NOS1,SERPINB12,PLXNB1,NBN,NVL,SLC39A10,TNF,LRCH1,ZFPM1,TRIM23,ADORA3,ARF1,HMGA2,DOCK1,CDC20B,UBE2V1,C5AR1,DEF6,PIK3R3,ARHGEF6,SAE1,TRDN,TRIM41,HERC1,NET1,CDC14B,TENM1,ARHGEF11,CDKL5,GSKIP,PRKAR1A,ARHGAP11B,SIPA1L2,CD109,EGF,ALDH1A1,KIT,MTRR,BLM,KCNAB1,PTPRO,RAPGEF2,DNAJB6,ESR1,DNMBP,FGF13,EPHB2,NDFIP2,TBCD,C14ORF39,DAP,DAPK1,ERBB4,FER,SPTA1,FNIP1,LRP5,RAPGEF6,RPS27L,SLAMF1,ABL2,ASAP2,DEPDC5,F11R,FARP2,GAS8,ARHGAP35,COMMD7,CCL3,PREX1,ARHGAP25,FGD4,NHLH2,ARF4,ARFGAP3,ST18,CAB39L,GARNL3,NEDD9,P2RX1,PDGFD,RGPD1,UBE2N,KCNE4,IRAK2,TNKS,FGFR1,RGS3,TBC1D9,DAD1,MAP2,IL23R,JMY,KEAP1,NTF3,P2RX7,RAPGEF4,TEX14,ARRDC4,RASA3,RCAN2,AMFR,AR,RABGAP1L,SUFU,DENND5B,DGKZ,NEK5,PI3,TAX1BP1,THADA,ARHGAP10,GNB5,MARK2,PDE5A,PROS1,FLT3,PPARGC1B,SRGAP3,ATP6V1H,DYNAP,FERMT2,MAP2K5,CALML4,DAB2,GPR55,PBX1,RNF207,TCL1B,ACTN4,CCL7,ELP4,FGF12,FRY,PLCL2,STAC,PIN1,SYNPO2,STK38,CHRM3,CIT,CARF,TGFA,G3BP2,HGF,PRPSAP2,RALGAPA1,RGS12,CALCR,SH3BP5,CACNB2,NOS1AP,RALGDS,ARHGAP28,IL18RAP,INSR,PAK1,SKI,CHM,ENPP1,FMR1,LIMK2,DKC1,SBF2,CRMP1,IDE,MFNG,AIM2,ARHGAP42,DENND2D,JPH4,PPARA,SORT1,TNFAIP8L3,TTN,FHL1,GNA13,TAF1,WDR41,PPP2R3C,SPRED1,ESR2,PTPRF,RBL1,BTRC,PPP2R2C,MYH6,PRLR,DOCK7,HDAC5,PSMD6,TDGF1,A2M,PI15,SERPINB8,STIM1,ADARB1,KCNC2,PRKCQ,STAT3,TP63,USP7,AMOT,CDKN3,CST1,DOCK3,PIK3R2,CASQ2,DHRS7B,DTNBP1,DLGAP2,KSR1,LGALS9,LRP8,APC,BMPR1A,ITGA1,NGF,PPP1R14A,SPINT2,UBASH3B,VLDLR,ITIH6,PILRB,ABI1,CTSH,DHX9,NCAPG2,RGCC,SRGAP1,CCL14,CCL15,EDA2R,IL6R,MAP2K6,RIC8B,TFIP11,MAP2K1,SOS1,COL6A3,FGF1,C6ORF106,CRADD,NLRC5,RGN
GO:0016310	phosphorylation	6.804932524827248e-8	DAB1,MAPK14,DSCAM,CCNG2,HUNK,PTPRT,DUSP22,AKAP13,FBLN1,ERG,EPHA6,VRK2,NRXN1,FPGT-TNNI3K,TNNI3K,NRG3,HUS1,AK5,TGFBR3,CSNK2A1,TF,PIK3C2B,CHEK2,GADD45A,NRG1,PLCE1,KSR2,STK38L,SLCO3A1,LDLRAD4,ZB

			TB20,MYO3A,NME7,ALK,PIK3C3,ERC1,EPHA3,PRKACB,DCLK1,AGT,PARK2,TXK,TRIO,HDAC2,SLIT2,RPS6KA2,IGF1R,SMAD1,PXK,TRPC5,DMD,STK33,FSHR,PRKAR2A,STK24,WDR83,PARP16,TPK1,CKMT1B,LRRK2,MAPK10,VRK1,NTRK3,SLC4A4,PAN3,SEMA4D,GSK3B,SLC8A1,PRKD1,SNRK,ERCC6,OGT,TGFB2,VAV3,BDNF,PRKAR1B,PRKCH,RIPK4,RYK,SIK2,NR2F2,PRR5,PML,TAOK3,BCL2,RALBP1,ZNF675,EPHB1,IL12RB2,DEPTOR,EPHA7,FYN,CD44,PDE4D,ENPP2,GRK5,CSNK1G3,MYLK3,SLC1A1,NLRP12,MTOR,ROR1,LDB2,BMPER,PGK1,PLCL1,EPM2A,PRKCE,EIF2S1,PRKCB,ADCY8,ITLN1,KALRN,CCDC88C,EDNRB,ROBO1,FLT1,NOX4,RBKS,MAP3K7,MAPK9,STK3,KDR,CASK,PHKB,APP,DGKB,NRP1,ADTRP,ADAM17,CAMK1D,MAP3K5,CDC42BPG,ATF2,GRK6,PIK3R5,SAMSN1,EFEMP1,ROS1,CDK17,RPS6KA5,TNFRSF10B,PARD3,TLK2,SGK2,SGMS1,CCDC88A,MOB3B,STK36,AK7,GUCY2F,HDAC4,GHR,NTRK2,PRKG1,CDC42BPA,BRD4,RASGRP1,ARR3,CAMK4,RABGEF1,SPRED2,RAP1A,TGFBR1,PRDX4,EPHA5,LCP2,TOP1,PEAK1,CHRNA3,EP300,MORC3,PIBF1,TRPM7,ADCK1,ADORA2A,ADRBK2,CAMK2G,EFNA5,FAM20A,MERTK,GUCY2C,MAGI2,NF1,SORL1,RELN,GRM5,MAP3K13,NLK,SH3GL2,SNX25,DGKI,GNPTAB,MAS1,WWTR1,CBLB,MNAT1,CNTN1,DGUOK,PDXK,MAPK4,BORA,SH3KBP1,AK8,AKAP7,LRRK1,DDX3X,RBL2,CNOT7,ZNF622,JAK2,ADK,CD300A,MAP2K4,NOS1,CDK1,ITPK1,NBN,TNF,CHKB,CHKB-CPT1B,ILF3,BMP15,STK32B,HMGA2,NEK4,STK32C,DYRK4,PMEPA1,C5AR1,LRP4,PIK3R3,GALK2,RSRC1,ALPK2,CDK8,TENM1,WDFY2,CDKL5,GSKIP,PRKAR1A,CD109,EGF,KIT,BLM,PIK3C2G,PTPRO,RAPGEF2,STK32A,JAK1,FGF13,AKAP10,EPHB2,DAPK1,ERBB4,FER,MAP3K3,FNIP1,LRP5,ABL2,CAMP,SGMS2,CCL3,CERK,FGF7,LAMA1,SPRED3,CDKL1,CAB39L,NEDD9,PDGFD,PIK3CD,PGM2L1,IRAK2,MYO3B,PANK3,TNKS,CDK19,FGFR1,OCLN,BPGM,IL23R,NTF3,P2RX7,TEX14,AR,PAPSS1,IFNA8,SRPK2,BDKRB1,BDKRB2,DGKZ,NEK5,PRKAA2,MARK2,PDE5A,FLT3,PPARGC1B,PTPRA,DYNAP,FERMT2,MAP2K5,MARK1,SMPD3,DAB2,TCL1B,ADAM10,PLCL2,PIN1,RPS6KA6,SYNPO2,PIP5K1B,STK38,BMP6,CIT,TGFA,HGF,MYLK,CALCR,SH3BP5,TLK1,MAST4,PI4KA,INSR,PAK1,AKT3,ENPP1,FMR1,LIMK2,TESK2,PRKX,ULK4,CHKA,PCK1,PPARA,TNFAIP8L3,TTN,TAF1,FNIP2,PPP2R3C,SPRED1,RBL1,RIT2,PRKCG,PRLR,UMOD,CBFA2T3,DOCK7,ICK,STK35,TDGF1,ADPGK,IPMK,TRPM6,ADARB1,PRKCQ,STAT3,CDKN3,DOCK3,PAK3,PIK3R2,STK17B,DTNBP1,GAK,KSR1,LRP8,ALPK1,MAPKAPK2,APC,BMPR1A,KCTD20,NGF,PPP1R14A,PRKCA,UBASH3B,VLDLR,PILRB,ABI1,NCAPG2,RGCC,IL6R,MAP2K6,SPTBN4,MAP2K1,FGF1,DGKK,NLRC5,RGN
GO:0044093	positive regulation of molecular function	9.552274688241664e-8	FANK1,DAB1,MAPK14,CTBP2,PRIM2,ANK3,BCL2L13,AKAP13,FBLN1,ITGA2,RGS6,EPHA6,NRXN1,SGSM1,MTPN,NRG3,ANK2,TBC1D22A,NRG1,SLCO3A1,CFTR,TIAM1,RHOC,RASGRF1,ALK,ERC1,ARID5B,EPHA3,CRNN,AGT,PARK2,TRIM22,TRIM5,DCUN1D4,TXK,USP6,TRAPPC9,HDAC2,MYO9A,PDCD6,IGF1R,DMD,CACNG3,SIPA1L3,HNRNPA2B1,PARP16,RYR2,XRCC4,LRRK2,NTRK3,SH3BP1,SEMA4D,GSK3B,PRKD1,ERCC6,TGFB2,VAV3,SLC5A3,BDNF,PRKCH,RIPK4,RYK,KCNC1,TOR1AIP1,GRIN2B,PML,PPARGC1A,AKAP6,NLRP2,TAOK3,BCL2,RALBP1,ARHGAP24,EPHB1,SIPA1L1,NR4A2,ARHGEF10,EPHA7,FYN,KCNE2,PARN,CHN1,SMARCA4,CD44,RCVRN,ACTN2,RAP1GDS1,EIF3E,SLC1A1,NLRP12,MTOR,ROR1,TIAM2,PLCL1,ARL6IP5,EVI5,EIF2S1,KMT2A,PRKCB,VDR,ADCY8,CAPN3,KALRN,DLC1,ROBO1,TBC1D4,FLT1,NOX4,GRHL2,MAP3K7,CR1,SPON1,STK3,TGM2,KDR,EDA,PCOLCE2,APP,NRP1,UACA,ADAM17,CAMK1D,MAP3K5,PKP4,ATF2,PIK3R5,ROS1,RGS16,RPS6KA5,TCF7L2,TNFRSF10B,RALGAPA2,DOCK9,GRIN2A,SGK2,CCDC88A,MOB3B,STK36,HDAC4,GHR,MAPRE2,NTRK2,RASGRP1,KCTD7,CACNA1C,RAP1A,EPHA5,LCP2,RAPGEF1,CHRNA3,EP300,CACNA2D1,GRAMD4,IFNGR2,PIBF1,EFNA5,FAM20A,MERTK,CACNG2,MAGI2,NF1,RFC3,RGS7,SORL1,ARHGAP6,BID,POR,RELN,GRM5,IL18R1,SELE,MAP3K13,ADCYAP1R1,EGLN3,IL1RAP,PPP3CA,TBC1D3B,MAS1,TBC1D5,DOCK10,MNAT1,PLSCR1,AHSA1,STIM2,SLC22A2,ASAP1,BORA,AKIRIN2,NPR3,AKAP7,CACNA1D,DDX3X,ARAP2,ZNF622,JAK2,SMAD3,CD300A,NOS1,PLXNB1,NBN,NVL,SLC39A10,TNF,HMGA2,CDC20B,UBE2V1,C5AR1,TRDN,TRIM41,NET1,CDC14B,TENM1,CDKL5,SIPA1L2,EGF,KIT,RAPGEF2,ESR1,FGF13,EPHB2,DAP,DAPK1,ERBB4,FER,SPTA1,LRP5,RAPGEF6,RPS27L,ABL2,ASAP2,F11R,ARHGAP35,CCL3,PREX1,ARHGAP25,NHLH2,ARF4,ST18,CAB39L,GARNL3,NEDD9,P2RX1,PDGFD,UBE2N,IRAK2,TNKS,FGFR1,TBC1D9,IL23R,JMY,NTF3,P2RX7,ARRDC4,AMFR,AR,RABGAP1L,DGKZ,NEK5,GNB5,MARK2,PDE5A,FLT3,PPARGC1B,DYNAP,FERMT2,MAP2K5,GPR55,RNF207,TCL1B,ACTN4,CCL7,PLCL2,STAC,PIN1,TGFA,RALGAPA1,CACNB2,NOS1AP,IL18RAP,INSR,PAK1,

			SKI,FMR1,DKC1,IDE,MFNG,AIM2,ARHGAP42,TNFAIP8L3,GNA13,TAF1,WDR41,PPP2R3C,ESR2,BTRC,PRLR,DOCK7,HDAC5,TDGF1,STIM1,KCNC2,PRKCQ,STAT3,DOCK3,LGALS9,LRP8,BMPR1A,ITGA1,NGF,VLDLR,PILRB,ABI1,CTSH,DHX9,RGCC,CCL14,CCL15,EDA2R,IL6R,MAP2K6,MAP2K1,FGF1,CRADD,RGN
GO:0098655	cation transmembrane transport	1.0249966991579541e-7	PIEZO2,ASIC2,CACNA1E,KCNJ6,ANK3,SLC24A3,KCNQ1,GRIN3A,NRXN1,SLC1A2,PKD1L1,SLC9B1,TRPM3,ANK2,KCND3,FAM155A,SLC12A1,DPP10,SCN8A,KCNE1,KCNJ15,KCNG3,SCARA5,SHISA6,RYR3,PM20D1,RASGRF1,SLC6A2,SLC17A3,UTRN,AGT,SHISA9,ATP6V0D1,KCNH1,KCNMA1,SLC24A4,KCNK17,TRPC5,DMD,HCN1,CACNA2D3,CACNG3,NOX5,FGF14,GSG1L,SLC39A11,DPP6,TRPM2,CNIH3,RYR2,CATSPER2,DHRS7C,SLC1A3,KCNH5,SLC4A4,CHD7,KCNK13,SLC8A1,PRKD1,ATP1A4,ZDHHC17,KCNC1,GRIN2B,SNAP25,AKAP6,KCNJ3,SLC30A5,ITPR2,ABCC9,FYN,KCNE2,HECW1,PDE4D,ACTN2,KCNS3,KCNIP4,KCND2,SCN11A,SLC1A1,MFSD12,ATP1A1,PLN,PRKCE,SLC2A9,ATP6V1D,SLC47A1,CAPN3,EDNRB,SLC12A8,KCNC4,APP,KCNK2,SLC38A7,PEX5L,GRIN2A,SLC30A7,PDE4B,SLC5A10,COMMD1,CACNA1C,NIPA2,KCNJ12,SLC6A17,CACNA2D1,CACNA2D4,IFNGR2,RASGRF2,TRPM7,CACNG2,RGS7,KCNQ5,MICU1,RELN,SLC39A8,SLC9C1,TMEM163,KCNB2,SLC7A1,SLC24A2,SLC5A1,STIM2,SLC22A2,NLGN1,P2RX6,AKAP7,MAPK8IP2,CACHD1,CACNA1D,TPCN2,NOS1,MAGT1,SLC39A10,PANX1,TMC2,KCNT2,KCNK10,TSPAN13,ANO1,KCNA6,TRPC4,CNGB3,KCNH8,TRDN,ANO10,SLC15A1,SCN1A,KCNH7,TRPV4,KCNAB1,HTR2C,SLC30A8,FGF13,EPHB2,DAPK1,CACNG6,ATP8A1,CCL3,MCUR1,SLC39A12,P2RX1,KCNE4,CNGA4,SFXN3,RYR1,P2RX7,RASA3,BDKRB1,THADA,GNB5,ATP6V1H,HCN4,SCNN1A,ATP13A3,RNF207,ACTN4,FGF12,KCNJ16,KCNN3,STAC,TRPM4,CNGB1,TMCO3,CALCR,CACNB2,NOS1AP,FMR1,MICU2,ATP5J,JPH4,FHL1,SLC22A5,CACNA1A,TRPM1,SCN9A,ATP6V1E1,SLC24A5,SLC40A1,STIM1,TRPM6,KCNC2,SLC9A9,HPN,CASQ2,SLC28A3,TMEM175,SLC7A8,TMEM63A,TUSC3,UBASH3B,SLC9A2,MCOLN3,ATP6V1E2,RGN
GO:0007610	behavior	1.0974276134939049e-7	PLCB1,DAB1,DSCAM,KCNQ1,NRXN1,NRXN3,SLC1A2,SHANK2,GRIA1,ANKH,CSMD1,NRG1,RASGRF1,GRID1,ALK,SORCS3,S100B,CNTNAP4,AGT,RCAN1,PARK2,NEGR1,DACH1,ANKFN1,DBH,PCDH17,APBA2,HDAC2,SLC24A4,CELSR1,FSHR,GRM1,ASTN1,LRRK2,SLC1A3,CHD7,HEXB,MECP2,BDNF,PRKAR1B,PCDH15,GRIN2B,RAG1,SNAP25,RNF180,CNTNAP2,BCL2,NR4A2,FYN,PYY,ATP8A2,MME,NAV2,GRIK2,KCND2,SLC1A1,MTOR,BBS2,ARL6IP5,EPM2A,KLHL1,NLGN4X,PRKCE,ADCY8,LRRTM1,KALRN,SGIP1,PREX2,TACR3,CHL1,APP,THRB,KCNK2,AFF2,ABAT,GRIN2A,HDAC4,NTRK2,CAMK4,SDK1,GLI3,TNR,CHRNA3,CHRNA5,EP300,ATXN1,GLRA1,NPC1,SPG11,ADORA2A,NF1,EIF4E,RELN,GRM5,KIRREL3,NPHP4,GPR176,DGKI,SLC24A2,ELAVL4,NLGN1,MAPK8IP2,GABRA5,SOBP,AHI1,TANC1,RXFP4,BRINP1,KCNK10,EPS8,SCN1A,MYH14,KIT,DNAH11,HTR2C,UBE3A,FGF13,EPHB2,SLITRK6,ABL2,ATP8A1,CCL3,NHLH2,ARF4,AGTPBP1,ITGA8,PUM1,CDH23,NTF3,AMFR,BHLHB9,MEIS2,CLN6,FGF12,ITGA3,BTBD9,NCOA2,TMOD1,INSR,JPH4,PPARA,ZFHX2,QRFP,SCN9A,EXT1,PRKCG,ADARB1,STAT3,DPP4,NGF,VLDLR,GABRG2,SPTBN4,VPS35,MCOLN3
GO:0044057	regulation of system process	1.3372414868934918e-7	ABCG8,ASIC2,ITGA2,KCNQ1,NRXN1,TNNI3K,RIMS2,MTPN,CELF2,ANK2,KCND3,KCNE1,TMEM108,SHISA6,RYR3,PLCE1,AGT,SHISA9,FBXO32,DLGAP1,CORO2B,DBH,SETD3,KCNMA1,FTO,MDM2,PDE9A,DMD,GRM1,CTNNA3,RNLS,RYR2,TENM4,LRRK2,MECP2,MTMR2,SLC8A1,TGFB2,AKAP6,KCNJ3,TMEM100,ABCC9,KCNE2,NR4A1,FOXO1,PDE4D,TBXAS1,IGSF11,CELF4,SCN11A,SLC1A1,PKP2,ATP1A1,PLN,NLGN4X,FAM19A4,FOXO3,EDNRB,TACR3,APP,CALCRL,THRB,PARD3,ABAT,CD38,HDAC4,PDE4B,JAM2,PRKG1,CACNA1C,DOCK4,CYSLTR1,KCNJ12,MGLL,DICER1,TNR,CHRNA3,CACNA2D1,GLRA1,PPP1R12B,ADORA2A,CAMK2G,TAC4,RELN,PPP3CA,KCNB2,CXADR,RIMS1,NLGN1,CACNA1D,JAK2,SHOX2,SMAD3,NOS1,TNF,SNX5,POMC,MLIP,ADORA3,TRDN,TSHZ3,KIT,TRPV4,PTPRO,HTR2C,NCMAP,FGF13,NUP155,CCL3,PTGER3,P2RX1,KCNE4,MEF2A,UNC13B,BDKRB2,ADRA1B,SEMA3A,PDE5A,HCN4,DAB2,RNF207,FGF12,TRPM4,BMP6,CHRM3,SMTNL2,HGF,CACNB2,NOS1AP,CTDP1,FMR1,ARHGAP42,PPARA,ZFHX2,CORIN,MYH7,SLC22A5,MYH6,TYMP,CASQ2,DLGAP2,PRKCA,ASB3,SPTBN4,TBX18
GO:0007411	axon guidance	1.5333534320379302e-7	CDH4,DSCAM,DCC,EPHA6,NRXN1,NRXN3,ADAMTSL1,PLXNA4,FLRT2,UNC5D,SEMA6D,DPYSL2,CNTN4,ROBO2,EPHA3,LAMA2,SEMA3D,NTN1,TRIO,SLIT2,RNF165,EMB,SEMA4D,SEMA5A,BDNF,RYK,LAMA3,PLXNA2,EPHB1,EPHA7,FYN,CHN1,UNC5C,PALLD,KALRN,ROBO1,CHL1,APP,NRP1,PTPRM,SEMA3C,PRTG,RPS6KA5,OPHN1,ALCAM,GLI3,EPHA5,TNR,DSCAML1,EFNA5,TTC8,RELN,KLF7,PLXNB1,LGR6,CNTN6,EFNB2,SLIT3,EFNB1,PTPRO,MYPN,SEMA3E,EPHB2,B

			SG,ARHGAP35,LAMA1,SEMA5B,SEMA3A,LGI1,NCAM1,NFASC,CRMP1,CCDC141,EXT1,PRKCQ,SOS1
GO:0098660	inorganic ion transmembrane transport	1.7635849012260985e-7	GABRB3,GABRG3,ASIC2,CACNA1E,KCNJ6,ANO2,ANK3,SLC24A3,KCNQ1,GRIN3A,PKD1L1,SLC9B1,TRPM3,ANO4,ANK2,ANKH,KCND3,FAM155A,SLC12A1,DPP10,SCN8A,KCNE1,KCNJ15,KCNG3,SCARA5,RYR3,CFTR,PM20D1,SLC6A2,SLC17A3,UTRN,ATP6V0D1,KCNH1,KCNMA1,SLC24A4,GABRG1,KCNK17,TRPC5,DMD,HCN1,CACNA2D3,CACNG3,NOX5,FGF14,SLC39A11,DPP6,TRPM2,RYR2,CATSPER2,DHRS7C,SLC1A3,KCNH5,SLC4A4,CHD7,KCNK13,SLC8A1,PRKD1,ATP1A4,ZDHHC17,KCNC1,GRIN2B,SNAP25,AKAP6,KCNJ3,SLC30A5,ITPR2,ABCC9,FYN,KCNE2,SLC26A7,HECW1,PDE4D,ACTN2,GABRB1,KCNS3,KCNIP4,KCND2,SCN11A,SLC1A1,ATP1A1,PLN,PRKCE,SLC2A9,ATP6V1D,SLC47A1,CAPN3,EDNRB,SLC12A8,KCNC4,GABRA2,KCNK2,GRIN2A,PDE4B,GLRA2,SLC5A10,COMMD1,CACNA1C,NIPA2,KCNJ12,SLC13A4,SLC6A17,CACNA2D1,CACNA2D4,GLRA1,TRPM7,CACNG2,RGS7,KCNQ5,MICU1,SLC39A8,SLC9C1,GRM5,TMEM163,KCNB2,SLC24A2,SLC5A1,GABRA3,STIM2,AKAP7,CACHD1,CACNA1D,GABRA5,TPCN2,NOS1,MAGT1,SLC39A10,PANX1,TMC2,KCNT2,KCNK10,TSPAN13,ANO1,KCNA6,TRPC4,KCNH8,TRDN,SLC15A1,SCN1A,GABRR2,KCNH7,TRPV4,KCNAB1,HTR2C,SLC30A8,FGF13,GABRB2,ANO3,CACNG6,CCL3,MCUR1,SLC39A12,P2RX1,KCNE4,RYR1,GLRA3,P2RX7,RASA3,BDKRB1,THADA,GNB5,ATP6V1H,HCN4,SCNN1A,RNF207,ACTN4,FGF12,KCNJ16,KCNN3,STAC,TRPM4,TMCO3,CALCR,SLC26A8,CACNB2,NOS1AP,FMR1,MICU2,ATP5J,GABRA1,GABRR3,JPH4,FHL1,CACNA1A,TRPM1,SCN9A,ATP6V1E1,SLC24A5,SLC40A1,STIM1,TRPM6,KCNC2,SLC9A9,HPN,CASQ2,SLC28A3,TMEM175,TUSC3,UBASH3B,SLC9A2,GABRG2,MCOLN3,ATP6V1E2,RGN
GO:0097485	neuron projection guidance	1.9291926831979745e-7	CDH4,DSCAM,DCC,EPHA6,NRXN1,NRXN3,ADAMTSL1,PLXNA4,FLRT2,UNC5D,SEMA6D,DPYSL2,CNTN4,ROBO2,EPHA3,LAMA2,SEMA3D,NTN1,TRIO,SLIT2,RNF165,EMB,SEMA4D,SEMA5A,BDNF,RYK,LAMA3,PLXNA2,EPHB1,EPHA7,FYN,CHN1,UNC5C,PALLD,KALRN,ROBO1,CHL1,APP,NRP1,PTPRM,SEMA3C,PRTG,RPS6KA5,OPHN1,ALCAM,GLI3,EPHA5,TNR,DSCAML1,EFNA5,TTC8,RELN,KLF7,PLXNB1,LGR6,CNTN6,EFNB2,SLIT3,EFNB1,PTPRO,MYPN,SEMA3E,EPHB2,BSG,ARHGAP35,LAMA1,SEMA5B,SEMA3A,LGI1,NCAM1,NFASC,CRMP1,CCDC141,EXT1,PRKCQ,SOS1
GO:0071495	cellular response to endogenous stimulus	2.240928330776862e-7	PLCB1,GABRB3,ARID1B,DUSP22,KCNQ1,NRXN1,SLC1A2,TGFBR3,KCNE1,TMEM108,FLRT2,GPC3,SPIDR,RYR3,GLP2R,CFTR,RBFOX2,LDLRAD4,ALK,PTPRE,ROBO2,ZNF366,LEMD3,AGT,CHRM5,FBXO32,PARK2,CYBB,HDAC2,HTRA1,SLIT2,STXBP4,IGF1R,MDM2,SMAD1,TMPRSS6,NR3C2,HCN1,FSHR,WWOX,TRPM2,RYR2,LRRK2,SLC1A3,IGFBP7,NTRK3,RNF165,PDXP,SOX6,GSK3B,LGR5,SLC8A1,OGT,TGFB2,BDNF,WASF1,KDM4C,EXT2,SIK2,PTGFR,NR5A2,PAQR8,PML,AKAP6,FBN1,RXFP2,LRP2,TMEM100,ITPR2,NR4A2,FYN,GNRHR,NR4A1,SMARCA4,CD44,CASP6,FOXO1,PDE4D,KANK1,ACTN2,GABRB1,SLC1A1,UGCG,RARB,MTOR,CHRM1,CTDSPL2,BMPER,BBS2,ATP1A1,DSG4,IFT88,PRKCE,PRKCB,ADCY8,FOXO3,RAB31,RXFP1,TBC1D4,COL2A1,MAP3K7,NR3C1,TGM2,KDR,PRCP,APP,LGR4,ADTRP,THRB,CPEB2,ADAM17,TFPI,ATF2,TGFB1I1,CHRDL1,BCL2L1,HDAC4,PDE4B,SOX5,DNMT1,GHR,GLRA2,NTRK2,FAM83G,GNG2,SPRED2,CLDN1,KIF16B,RAP1A,SAFB2,TGFBR1,NCOA5,CHST11,RAPGEF1,EP300,CACNA2D1,CYP7B1,GLRA1,COL4A6,NPC1,PNPLA3,EFNA5,MAGI2,SORL1,STRN3,HTR4,EIF4E,POR,GRM5,HDAC9,DDC,NLK,KLF3,SH3GL2,SNX25,MAS1,PER1,KL,ELAVL4,GABPA,AKAP7,VEPH1,NSG1,JAK2,SMAD3,BAIAP2L1,KIDINS220,TNF,OVOL2,SNX5,BMP15,PHEX,BCL11A,ANO1,GCLC,PMEPA1,LTBP1,SMOC2,SULF1,GNAL,PIK3R3,TRIM41,ZNF423,CTNNA1,NSG2,CD109,KIT,SLIT3,UBR2,BLM,VWC2,CPS1,RAPGEF2,ESR1,HTR2C,UBE3A,GABRB2,EPHB2,ABCC1,ERBB4,FER,SHCBP1,CAMP,PTPRK,FGF7,SPRED3,PSG9,SULF2,PDGFD,ITGA8,KDM3A,PLAT,FGFR1,RYR1,SOST,NTF3,P2RX7,AR,CASP7,TRERF1,PRKAA2,GNB5,ZMIZ1,FLT3,PPARGC1B,PTPRA,TRIM72,ESRRG,FERMT2,GNAO1,HCN4,SMPD3,DAB2,UROS,FGF12,ITGA3,NREP,PIN1,TRPM4,BMP6,CHRM3,NCOA2,SCUBE3,INSR,PAK1,PDE3A,SKI,ENPP1,RRAGC,IDE,PCK1,PPARA,SORT1,TAF1,CACNA1A,FUT8,SPRED1,AKR1C2,ESR2,PRDM16,ZNF451,DEFA1B,DEFA3,EXT1,NCOA1,PRLR,HDAC5,TDGF1,PRKCQ,STAT3,TP63,BRIP1,PIK3R2,CASQ2,DTNBP1,RAP1B,APC,BMPR1A,NGF,SPINT2,MTMR4,CTSH,ACACA,GABRG2,GPR21,RGMB,VPS35,FGF1,TBX20
GO:0008015	blood circulation	2.2805770968113545e-7	ASIC2,KCNQ1,TNNI3K,CELF2,ANK2,KCND3,KCNE1,RYR3,AGT,CORO2B,DBH,KCNMA1,SLIT2,RPS6KA2,MDM2,DMD,FSHR,CTNNA3,RNLS,RYR2,CHD7,MECP2,SLC8A1,TGFB2,SGCD,EXT2,NR2F2,SGCG,ACSM3,KCNJ3,ABCC9,KCNE2,PDE4D,TBXAS1,NAV2,RAP1GDS1,MYLK3,SLC1A1,MTOR,PKP2,ATP1A1,PLN,CTNNBIP1,EDNRB,TACR3,PR

			CP,SERPING1,IMMP2L,THRB,ABAT,CD38,ADAMTS16,HDAC4,PDE4B,PRKG1,CACNA1C,DOCK4,CYSLTR1,KCNJ12,CACNA2D1,PCSK5,TRHDE,ADORA2A,TAC4,MEOX2,SH3GL2,CXADR,KL,NPR3,CACNA1D,JAK2,SHOX2,SMAD3,NOS1,TNF,SNX5,POMC,ADORA3,GCLC,TRDN,SCN1A,CMA1,TRPV4,CPS1,PTPRO,FGF13,NUP155,SGCZ,LRP5,ARHGAP35,P2RX1,KCNE4,ENPEP,MEF2A,OCLN,AR,BDKRB1,BDKRB2,ADRA1B,SEMA3A,PDE5A,FERMT2,HCN4,RNF207,FGF12,TRPM4,BMP6,CHRM3,SMTNL2,FLI1,CACNB2,ELN,NOS1AP,PDE3A,ARHGAP42,PPARA,TTN,CORIN,GNA13,MYH7,QRFP,EXT1,MYH6,UMOD,AMOT,CASQ2,ITGA1,ASB3,MAP2K6,NCALD,SPTBN4,TBX20,TBX18
GO:0060429	epithelium development	2.5065384292435467e-7	PLCB1,NPHP3,ITGA2,KCNQ1,TNC,CSMD1,PLXNA4,GPC3,NRG1,PSMB2,HYDIN,ALDH1A2,CECR2,ASTN2,TIAM1,RHOC,COL22A1,CBFA2T2,NKD1,ROBO2,C2CD3,PRKACB,COL15A1,AJAP1,AGT,CDH2,GREB1L,NTN1,GPC6,ZNRF3,HDAC2,MYO9A,SLIT2,KRT25,SMAD1,DMD,BASP1,KRT2,SIPA1L3,VCL,CELSR1,FSHR,ATRX,RYR2,STRC,KIF26B,PRICKLE2,CHD7,SH3BP1,GSK3B,LGR5,FRMD6,TGFB2,FMN1,LCE2B,LCE2C,KRT74,PRKCH,RIPK4,RYK,PCDH15,LAMA3,ANKRD6,NR2F2,NR5A2,PML,ARHGAP12,PLXNA2,BCL2,RAD51B,ARHGAP24,LRP2,TMEM100,VANGL2,EPHA7,CD44,CASP6,PDE4D,PYY,TNMD,UGCG,PALLD,RARB,MTOR,ROR1,LDB2,BMPER,PGK1,CTNNBIP1,SOX8,DSG4,VDR,CCDC88C,DLC1,EDNRB,ROBO1,GRHL2,COL2A1,SEC24B,PKHD1,STK3,TGM2,KDR,PPHLN1,EDA,ABCA12,COL18A1,NRP1,SHROOM3,LGR4,LCE6A,THRB,SEMA3C,ADAM17,ROS1,TGFB1I1,FRAS1,HRNR,OPHN1,CNN3,ADAMTS16,STARD13,SPRED2,GLI3,KAZN,CLDN1,SPRR2B,SPRR2E,CERS3,RAP1A,SAFB2,KRTAP6-1,RAPGEF1,EP300,TBX3,CYP7B1,SPRR4,TRIOBP,PSMA1,FLNB,SPRR2G,MAGI2,NF1,TTC8,DMRT1,ASXL1,RALA,EXOC5,JAG1,MEOX2,KLF7,PLS1,WWTR1,GSTA2,GPC4,TRIM16,EHF,JAK2,AHI1,SMAD3,RDH10,CDK1,ITPK1,TNF,COBL,OVOL2,BMP15,DLG5,FREM2,MYO6,SULT1B1,SULF1,LRP4,EFNB2,DMBT1,TNFRSF19,MLLT3,CD109,EGF,CC2D2A,CPS1,EDAR,PTPRO,RAPGEF2,ESR1,SEMA3E,FNDC3A,ERBB4,SLITRK6,LRP5,BSG,F11R,SETD2,ALX4,ARHGAP35,NSUN2,FGF7,LAMA1,SPRED3,SOX4,PIK3CD,ARID4A,PDZD7,WDPCP,CDH23,ARID4B,KEAP1,SCUBE1,AR,KIAA1109,SUFU,EYA1,SEMA3A,FERMT2,DAB2,PBX1,RNF207,DAAM1,BMP6,CLASP1,TMOD1,HGF,PAK1,PSMB7,SKI,PRKX,PCK1,C1GALT1,GNA13,S1PR3,SPRED1,AKR1C2,KRT4,LCE1F,NTN4,BTRC,ESRP2,EXT1,PRLR,UMOD,KLHL3,SLC40A1,SCEL,TP63,USH2A,RAP1B,LCE2A,UPK3A,BMPR1A,SPINT2,ABI1,CTSH,LCE4A,CLASP2,MAP2K1,SOS1,FGF1,MCOLN3,TBX20,RREB1,TBX18
GO:0006793	phosphorus metabolic process	3.9996109801219823e-7	ADCY2,TPTE,PLCB1,DAB1,MAPK14,DSCAM,TPTE2,GPHN,CCNG2,HUNK,PTPRT,DUSP22,AKAP13,FBLN1,ERG,ITGA2,EPHA6,VRK2,NRXN1,FPGT-TNNI3K,TNNI3K,CPPED1,NRG3,ACSS1,HUS1,DLG2,AK5,TGFBR3,CSNK2A1,TF,PIK3C2B,CHEK2,GADD45A,NT5DC1,NRG1,PLCE1,KYNU,KSR2,STK38L,SLCO3A1,PPM1J,LDLRAD4,ZBTB20,FAR2,MYO3A,NME7,ALK,PTPRE,PIK3C3,ERC1,EPHA3,PRKACB,PTPRN2,DCLK1,SLC44A1,AGT,CHRM5,RCAN1,PARK2,PGAP3,ENTPD4,TXK,TNS3,TRIO,HDAC2,SLIT2,DPYD,ACSM2B,RPS6KA2,SLC44A5,IGF1R,SMAD1,PTPRG,PXK,PDE9A,TRPC5,DMD,STK33,FSHR,MBOAT7,PRKAR2A,INPP5A,STK24,LIPI,WDR83,PARP16,TPK1,AMPD3,CAMTA1,CKMT1B,LRRK2,MAPK10,VRK1,ELOVL7,NTRK3,RORA,SLC4A4,HEXB,PAN3,CDCA2,PDXP,MECP2,MTMR2,SEMA4D,GSK3B,SLC8A1,CDC14A,MAGI3,PLCH2,PRKD1,SNRK,ERCC6,OGT,TGFB2,VAV3,DNAJC6,ACSBG1,BDNF,PRKAR1B,PRKCH,RIPK4,RYK,SIK2,NR2F2,PRR5,PML,PPARGC1A,MYH8,PPP4R4,TAOK3,ACSM3,BCL2,RALBP1,ZNF675,EPHB1,IL12RB2,CSGALNACT1,DEPTOR,EPHA7,FYN,CD44,RCVRN,PDE4D,ENPP2,GRK5,CSNK1G3,MYLK3,NAMPT,SLC1A1,NLRP12,MTOR,ROR1,LDB2,CTDSPL2,BMPER,PTPRD,PGK1,PLCL1,ATP1A1,EPM2A,PRKCE,EIF2S1,PRKCB,ADCY8,SCP2,ITLN1,KALRN,CCDC88C,DLC1,EDNRB,ROBO1,FLT1,NOX4,PIGL,RBKS,MAP3K7,MAPK9,EYA4,ACSL5,STK3,KDR,CASK,PHKB,APP,DGKB,NRP1,PTPRM,LINC00473,ADTRP,ADAM17,CAMK1D,MAP3K5,CDC42BPG,ATF2,GRK6,PIK3R5,SAMSN1,EFEMP1,ROS1,SMG6,CDK17,RPS6KA5,TNFRSF10B,ACSM2A,PARD3,TLK2,SGK2,PYGL,SGMS1,CCDC88A,MOB3B,SMG7,STK36,UPB1,AK7,GUCY2F,HDAC4,PDE4B,GHR,NTRK2,PRKG1,ACSL4,CDC42BPA,BRD4,PTPDC1,RASGRP1,ARR3,CAMK4,RABGEF1,SPRED2,PPA2,RAP1A,TGFBR1,PRDX4,EPHA5,LCP2,TOP1,PEAK1,CHRNA3,EP300,MORC3,DUSP23,PIBF1,PNPLA3,RRM1,TRPM7,CA3,FHIT,ADCK1,ADORA2A,ADRBK2,CAMK2G,EFNA5,FAM20A,MERTK,GUCY2C,MAGI2,NF1,PPP6R2,SORL1,RELN,GRM5,PTPN9,MGAT5,ACYP2,NAPEPLD,MAP3K13,NLK,ADCYAP1R1,PPP3CA,SH3GL2,SNX25,DGKI,GNPTAB,MAS1,WWTR1,CBLB,MNAT1,PLSCR1,CNTN1,DGUOK,PDXK,FAM126B,MAPK4,BORA,PTPRU,SH3KBP1,AK8,OSBPL5,AKAP7,LRRK1,

			DDX3X,RBL2,CNOT7,GMDS,PDE7A,ZNF622,JAK2,SLC5A8,ADK,SMAD3,CD300A,MAP2K4,NOS1,CDK1,ITPK1,NBN,SLC39A10,TNF,CHKB,CHKB-CPT1B,ILF3,PIGB,BMP15,NUDT4,PHEX,FAM220A,PLCH1,STK32B,HMGA2,NEK4,STK32C,SULT1B1,DYRK4,NANP,PMEPA1,RAB23,MBOAT2,C5AR1,LRP4,PIK3R3,CDS2,GALK2,RSRC1,CDC14B,ALPK2,CDK8,TENM1,WDFY2,CDKL5,GSKIP,MTMR10,PRKAR1A,CD109,EGF,PTPN14,KIT,BLM,ENTPD3,PIK3C2G,CPS1,PTPRO,RAPGEF2,STK32A,HTR2C,JAK1,FGF13,AKAP10,EPHB2,DAPK1,ERBB4,FER,MAP3K3,MINPP1,FNIP1,LRP5,ABL2,CAMP,PTPRK,SGMS2,CCL3,CERK,FGF7,LAMA1,SPRED3,CDKL1,CAB39L,FAR1,NEDD9,PDGFD,PIK3CD,PGM2L1,IRAK2,MYO3B,PANK3,TNKS,ADCY9,CDK19,FGFR1,OCLN,TTC7B,BPGM,IL23R,NTF3,P2RX7,PLA2G4A,TEX14,AR,PAPSS1,PIGK,EYA1,IFNA8,SRPK2,BDKRB1,BDKRB2,DGKZ,NEK5,PRKAA2,MARK2,PDE5A,FLT3,PPARGC1B,PTPRA,DYNAP,FERMT2,MAP2K5,MARK1,SMPD3,DAB2,TCL1B,ADAM10,KMO,NMNAT2,PGM2,PLCL2,PPM1B,PIN1,RPS6KA6,SYNPO2,IMPDH1,PIP5K1B,STK38,ABCA8,BMP6,CIT,TGFA,HGF,MYLK,PRPSAP2,TMEM68,CALCR,SH3BP5,TLK1,MAST4,OSBPL10,PI4KA,PNPLA7,INSR,PAK1,PTPN21,AKT3,CTDP1,DUSP11,ENPP1,FMR1,LIMK2,TESK2,ATP5J,FAM126A,PRKX,ULK4,ABCD1,CHKA,SAMD8,PCK1,PPARA,THTPA,TNFAIP8L3,TTN,TAF1,FNIP2,FUT8,PPP2R3C,SPRED1,PTPRF,RBL1,BTRC,PPP2R2C,PTPRR,RIT2,MYH6,PRKCG,PRLR,TYMP,UMOD,CBFA2T3,DOCK7,FBXW11,ICK,INPP4A,STK35,TDGF1,UGGT2,ADPGK,IPMK,TRPM6,ADARB1,MOCS2,PEMT,PRKCQ,STAT3,CDKN3,DOCK3,DPM1,MOCS3,PAK3,PIK3R2,STK17B,ACOT1,DHRS7B,DTNBP1,GAK,KSR1,LRP8,ACSM1,ALPK1,GDA,GTPBP1,MAPKAPK2,APC,BMPR1A,ITGA1,KCTD20,NGF,PPP1R14A,PRKCA,UBASH3B,VLDLR,MTMR4,PILRB,ABI1,NCAPG2,RGCC,ACACA,IL6R,MAP2K6,SPTBN4,MAP2K1,PDE7B,DPYS,FGF1,PLB1,DGKK,NLRC5,RGN
GO:0035249	synaptic transmission, glutamatergic	6.105265706788573e-7	*GRIK1,NRXN1,GRID1,CDH2,PARK2,CDH8,SYT1,CACNG3,GRM1,UNC13C,LRRK2,DISC1,TPRG1L,GRIK3,GRIK2,OPHN1,TNR,GRID2,ADORA2A,CACNG2,NF1,RELN,GRM5,DGKI,GRM7,NLGN1,MAPK8IP2,TNF,TSHZ3,GRIK4,GRM4,P2RX1,PLAT,UNC13A,UNC13B,DGKZ,GRIA2,KMO,EXT1,DTNBP1*
GO:0016358	dendrite development	6.411653748901287e-7	*DAB1,DSCAM,DCC,GRIN3A,CTNND2,FSTL4,CTNNA2,RBFOX2,ALK,FBXO31,PHACTR1,DCLK1,NTN1,HDAC2,TRPC5,CSMD3,LRRK2,DISC1,MECP2,SEMA4D,GSK3B,EPHB1,SIPA1L1,FYN,SULT4A1,HECW1,DCDC2,FAT3,PTPRD,KLHL1,KALRN,TANC2,PREX2,APP,NRP1,CAMK1D,GRIP1,PRKG1,DNM3,SDK1,CUX1,CHRNA3,RELN,PPP3CA,KLF7,DOCK10,ELAVL4,ASAP1,NLGN1,MAPK8IP2,KIDINS220,COBL,PPFIA2,DLG5,BCL11A,LRP4,CDKL5,IL1RAPL1,RAPGEF2,UBE3A,EPHB2,ARF4,MEF2A,MAP2,BHLHB9,SEMA3A,PDLIM5,MARK1,FMR1,PAK3,DTNBP1,LRP8,KIAA0319,VLDLR,ABI1*
GO:0006796	phosphate-containing compound metabolic process	6.427850734608992e-7	*ADCY2,TPTE,PLCB1,DAB1,MAPK14,DSCAM,TPTE2,GPHN,CCNG2,HUNK,PTPRT,DUSP22,AKAP13,FBLN1,ERG,ITGA2,EPHA6,VRK2,NRXN1,FPGT-TNNI3K,TNNI3K,CPPED1,NRG3,ACSS1,HUS1,DLG2,AK5,TGFBR3,CSNK2A1,TF,PIK3C2B,CHEK2,GADD45A,NT5DC1,NRG1,PLCE1,KYNU,KSR2,STK38L,SLCO3A1,PPM1J,LDLRAD4,ZBTB20,FAR2,MYO3A,NME7,ALK,PTPRE,PIK3C3,ERC1,EPHA3,PRKACB,PTPRN2,DCLK1,SLC44A1,AGT,CHRM5,RCAN1,PARK2,PGAP3,ENTPD4,TXK,TNS3,TRIO,HDAC2,SLIT2,DPYD,ACSM2B,RPS6KA2,SLC44A5,IGF1R,SMAD1,PTPRG,PXK,PDE9A,TRPC5,DMD,STK33,FSHR,MBOAT7,PRKAR2A,INPP5A,STK24,LIPI,WDR83,PARP16,TPK1,AMPD3,CAMTA1,CKMT1B,LRRK2,MAPK10,VRK1,ELOVL7,NTRK3,RORA,SLC4A4,HEXB,PAN3,CDCA2,PDXP,MECP2,MTMR2,SEMA4D,GSK3B,SLC8A1,CDC14A,MAGI3,PLCH2,PRKD1,SNRK,ERCC6,OGT,TGFB2,VAV3,DNAJC6,ACSBG1,BDNF,PRKAR1B,PRKCH,RIPK4,RYK,SIK2,NR2F2,PRR5,PML,PPARGC1A,MYH8,PPP4R4,TAOK3,ACSM3,BCL2,RALBP1,ZNF675,EPHB1,IL12RB2,DEPTOR,EPHA7,FYN,CD44,RCVRN,PDE4D,ENPP2,GRK5,CSNK1G3,MYLK3,NAMPT,SLC1A1,NLRP12,MTOR,ROR1,LDB2,CTDSPL2,BMPER,PTPRD,PGK1,PLCL1,ATP1A1,EPM2A,PRKCE,EIF2S1,PRKCB,ADCY8,SCP2,ITLN1,KALRN,CCDC88C,DLC1,EDNRB,ROBO1,FLT1,NOX4,PIGL,RBKS,MAP3K7,MAPK9,EYA4,ACSL5,STK3,KDR,CASK,PHKB,APP,DGKB,NRP1,PTPRM,LINC00473,ADTRP,ADAM17,CAMK1D,MAP3K5,CDC42BPG,ATF2,GRK6,PIK3R5,SAMSN1,EFEMP1,ROS1,SMG6,CDK17,RPS6KA5,TNFRSF10B,ACSM2A,PARD3,TLK2,SGK2,PYGL,SGMS1,CCDC88A,MOB3B,SMG7,STK36,UPB1,AK7,GUCY2F,HDAC4,PDE4B,GHR,NTRK2,PRKG1,ACSL4,CDC42BPA,BRD4,PTPDC1,RASGRP1,ARR3,CAMK4,RABGEF1,SPRED2,PPA2,RAP1A,TGFBR1,PRDX4,EPHA5,LCP2,TOP1,PEAK1,CHRNA3,EP300,MORC3,DU*

			SP23,PIBF1,PNPLA3,RRM1,TRPM7,CA3,FHIT,ADCK1,ADORA2A,ADRBK2,CAMK2G,EFNA5,FAM20A,MERTK,GUCY2C,MAGI2,NF1,PPP6R2,SORL1,RELN,GRM5,PTPN9,MGAT5,ACYP2,NAPEPLD,MAP3K13,NLK,ADCYAP1R1,PPP3CA,SH3GL2,SNX25,DGKI,GNPTAB,MAS1,WWTR1,CBLB,MNAT1,PLSCR1,CNTN1,DGUOK,PDXK,FAM126B,MAPK4,BORA,PTPRU,SH3KBP1,AK8,OSBPL5,AKAP7,LRRK1,DDX3X,RBL2,CNOT7,PDE7A,ZNF622,JAK2,SLC5A8,ADK,SMAD3,CD300A,MAP2K4,NOS1,CDK1,ITPK1,NBN,SLC39A10,TNF,CHKB,CHKB-CPT1B,ILF3,PIGB,BMP15,NUDT4,FAM220A,PLCH1,STK32B,HMGA2,NEK4,STK32C,SULT1B1,DYRK4,NANP,PMEPA1,RAB23,MBOAT2,C5AR1,LRP4,PIK3R3,CDS2,GALK2,RSRC1,CDC14B,ALPK2,CDK8,TENM1,WDFY2,CDKL5,GSKIP,MTMR10,PRKAR1A,CD109,EGF,PTPN14,KIT,BLM,ENTPD3,PIK3C2G,CPS1,PTPRO,RAPGEF2,STK32A,HTR2C,JAK1,FGF13,AKAP10,EPHB2,DAPK1,ERBB4,FER,MAP3K3,MINPP1,FNIP1,LRP5,ABL2,CAMP,PTPRK,SGMS2,CCL3,CERK,FGF7,LAMA1,SPRED3,CDKL1,CAB39L,FAR1,NEDD9,PDGFD,PIK3CD,PGM2L1,IRAK2,MYO3B,PANK3,TNKS,ADCY9,CDK19,FGFR1,OCLN,TTC7B,BPGM,IL23R,NTF3,P2RX7,PLA2G4A,TEX14,AR,PAPSS1,PIGK,EYA1,IFNA8,SRPK2,BDKRB1,BDKRB2,DGKZ,NEK5,PRKAA2,MARK2,PDE5A,FLT3,PPARGC1B,PTPRA,DYNAP,FERMT2,MAP2K5,MARK1,SMPD3,DAB2,TCL1B,ADAM10,KMO,NMNAT2,PGM2,PLCL2,PPM1B,PIN1,RPS6KA6,SYNPO2,IMPDH1,PIP5K1B,STK38,ABCA8,BMP6,CIT,TGFA,HGF,MYLK,PRPSAP2,TMEM68,CALCR,SH3BP5,TLK1,MAST4,OSBPL10,PI4KA,PNPLA7,INSR,PAK1,PTPN21,AKT3,CTDP1,DUSP11,ENPP1,FMR1,LIMK2,TESK2,ATP5J,FAM126A,PRKX,ULK4,ABCD1,CHKA,SAMD8,PCK1,PPARA,THTPA,TNFAIP8L3,TTN,TAF1,FNIP2,PPP2R3C,SPRED1,PTPRF,RBL1,BTRC,PPP2R2C,PTPRR,RIT2,MYH6,PRKCG,PRLR,TYMP,UMOD,CBFA2T3,DOCK7,FBXW11,ICK,INPP4A,STK35,TDGF1,ADPGK,IPMK,TRPM6,ADARB1,MOCS2,PEMT,PRKCQ,STAT3,CDKN3,DOCK3,DPM1,MOCS3,PAK3,PIK3R2,STK17B,ACOT1,DHRS7B,DTNBP1,GAK,KSR1,LRP8,ACSM1,ALPK1,GDA,GTPBP1,MAPKAPK2,APC,BMPR1A,ITGA1,KCTD20,NGF,PPP1R14A,PRKCA,UBASH3B,VLDLR,MTMR4,PILRB,ABI1,NCAPG2,RGCC,ACACA,IL6R,MAP2K6,SPTBN4,MAP2K1,PDE7B,DPYS,FGF1,PLB1,DGKK,NLRC5,RGN
GO:0048729	tissue morphogenesis	8.540962567763355e-7	*WLS,NPHP3,ITGA2,TNC,TGFBR3,CSMD1,GPC3,NRG1,PSMB2,ALDH1A2,CECR2,ASTN2,TIAM1,RHOC,NKD1,ROBO2,C2CD3,PRKACB,AJAP1,AGT,GREB1L,NTN1,GPC6,ZNRF3,MYO9A,SLIT2,KRT25,MDM2,SMAD1,VCL,CELSR1,RYR2,KIF26B,PRICKLE2,CHD7,SH3BP1,LGR5,TGFB2,FMN1,RIPK4,RYK,PCDH15,EXT2,LAMA3,ANKRD6,PML,ARHGAP12,BCL2,ARHGAP24,LRP2,TMEM100,VANGL2,EPHA7,CD44,EXOC4,ZFPM2,MTOR,ROR1,PKP2,COL11A1,CTNNBIP1,SOX8,VDR,DLC1,ROBO1,GRHL2,SEC24B,PKHD1,STK3,TGM2,KDR,NRP1,SHROOM3,LGR4,SEMA3C,ADAM17,TGFB1I1,FRAS1,OPHN1,ADAMTS16,STARD13,GLI3,TGFBR1,TBX3,CYP7B1,PSMA1,MAGI2,TTC8,RALA,EXOC5,JAG1,GPC4,SHOX2,AHI1,SMAD3,RDH10,TNF,COBL,OVOL2,ZFPM1,DLG5,FREM2,HMGA2,SULF1,EFNB2,PRKAR1A,MLLT3,EGF,CC2D2A,ESR1,SEMA3E,ERBB4,LRP5,BSG,SETD2,ARHGAP35,FGF7,LAMA1,SOX4,PIK3CD,ITGA8,AR,SUFU,EYA1,SEMA3A,FERMT2,DAB2,PBX1,RNF207,DAAM1,ITGA3,CLASP1,HGF,MYLK,PAK1,PSMB7,SKI,PRKX,TTN,GNA13,MYH7,NTN4,BTRC,ESRP2,EXT1,MDM4,MYH6,KLHL3,ADARB1,TP63,BMPR1A,SPINT2,CTSH,CLASP2,SOS1,FGF1,TBX20,RREB1,TBX18*
GO:0006468	protein phosphorylation	0.000001128493765535311	*DAB1,MAPK14,CCNG2,HUNK,PTPRT,DUSP22,AKAP13,FBLN1,ERG,EPHA6,VRK2,NRXN1,FPGT-TNNI3K,TNNI3K,NRG3,HUS1,TGFBR3,CSNK2A1,CHEK2,GADD45A,NRG1,PLCE1,KSR2,STK38L,SLCO3A1,LDLRAD4,MYO3A,ALK,PIK3C3,ERC1,EPHA3,PRKACB,DCLK1,AGT,PARK2,TXK,TRIO,HDAC2,SLIT2,RPS6KA2,IGF1R,SMAD1,PXK,TRPC5,DMD,STK33,FSHR,PRKAR2A,STK24,PARP16,LRRK2,MAPK10,VRK1,NTRK3,PAN3,SEMA4D,GSK3B,SLC8A1,PRKD1,SNRK,ERCC6,TGFB2,BDNF,PRKAR1B,PRKCH,RIPK4,RYK,SIK2,NR2F2,PRR5,PML,TAOK3,BCL2,RALBP1,ZNF675,EPHB1,IL12RB2,DEPTOR,EPHA7,FYN,CD44,PDE4D,ENPP2,GRK5,CSNK1G3,MYLK3,SLC1A1,NLRP12,MTOR,ROR1,BMPER,PLCL1,EPM2A,PRKCE,EIF2S1,PRKCB,ADCY8,ITLN1,KALRN,CCDC88C,EDNRB,ROBO1,FLT1,NOX4,MAP3K7,MAPK9,STK3,KDR,CASK,PHKB,APP,NRP1,ADTRP,ADAM17,CAMK1D,MAP3K5,CDC42BPG,ATF2,GRK6,PIK3R5,SAMSN1,EFEMP1,ROS1,CDK17,RPS6KA5,TNFRSF10B,PARD3,TLK2,SGK2,CCDC88A,MOB3B,STK36,GUCY2F,GHR,NTRK2,PRKG1,CDC42BPA,BRD4,RASGRP1,ARR3,CAMK4,RABGEF1,SPRED2,RAP1A,TGFBR1,PRDX4,EPHA5,LCP2,TOP1,PEAK1,CHRNA3,MORC3,PIBF1,TRPM7,ADCK1,ADORA2A,ADRBK2,CAMK2G,EFNA5,FAM20A,MERTK,GUCY2C,NF1,SORL1,RELN,GRM5,MAP3K13,NLK,SH3GL2,SNX25,MAS1,WWTR1,CBLB,MNAT1,CNTN1,DGUOK,MAPK4,BORA,LRRK*

			1,DDX3X,CNOT7,JAK2,CD300A,MAP2K4,NOS1,CDK1,NBN,TNF,ILF3,BMP15,STK32B,HMGA2,NEK4,STK32C,DYRK4,PMEPA1,C5AR1,LRP4,PIK3R3,RSRC1,ALPK2,CDK8,TENM1,WDFY2,CDKL5,GSKIP,PRKAR1A,CD109,EGF,KIT,BLM,PTPRO,RAPGEF2,STK32A,JAK1,FGF13,EPHB2,DAPK1,ERBB4,FER,MAP3K3,FNIP1,LRP5,ABL2,CAMP,CCL3,FGF7,LAMA1,SPRED3,CDKL1,CAB39L,NEDD9,PDGFD,PIK3CD,IRAK2,MYO3B,TNKS,CDK19,FGFR1,OCLN,IL23R,NTF3,P2RX7,TEX14,IFNA8,SRPK2,BDKRB1,BDKRB2,NEK5,PRKAA2,MARK2,PDE5A,FLT3,PTPRA,DYNAP,FERMT2,MAP2K5,MARK1,SMPD3,DAB2,TCL1B,ADAM10,PLCL2,PIN1,RPS6KA6,SYNPO2,STK38,BMP6,CIT,TGFA,HGF,MYLK,CALCR,SH3BP5,TLK1,MAST4,INSR,PAK1,AKT3,ENPP1,FMR1,LIMK2,TESK2,PRKX,ULK4,PCK1,TTN,TAF1,FNIP2,PPP2R3C,SPRED1,RIT2,PRKCG,PRLR,UMOD,DOCK7,ICK,STK35,TDGF1,TRPM6,ADARB1,PRKCQ,CDKN3,DOCK3,PAK3,STK17B,DTNBP1,GAK,KSR1,LRP8,ALPK1,MAPKAPK2,APC,BMPR1A,NGF,PRKCA,UBASH3B,VLDLR,PILRB,ABI1,NCAPG2,RGCC,IL6R,MAP2K6,SPTBN4,MAP2K1,FGF1,RGN
GO:0007416	synapse assembly	0.0000012363487349592484	*GABRB3,DSCAM,ASIC2,NRXN1,NRXN3,SDK2,SHANK2,SYNDIG1,FLRT2,NRG1,LRFN5,IL1RAPL2,ROBO2,CDH2,NEGR1,NTN1,GPC6,PCDH17,CNTN5,NTRK3,MECP2,SEMA4D,LINGO2,CLSTN2,BDNF,RYK,EPHB1,LRRC4,EPHA7,THBS2,PTPRD,NLGN4X,LRRTM1,CBLN4,GABRA2,APP,NTRK2,DNM3,SDK1,GRID2,EFNA5,KIRREL3,LRRTM3,IL1RAP,GPC4,NLGN1,PLXNB1,DLG5,LRP4,EFNB2,CLSTN1,IL1RAPL1,FGF13,GABRB2,EPHB2,ERBB4,SLITRK6,BHLHB9,PDLIM5,GABRA1,GABRG2,VPS35*
GO:0009719	response to endogenous stimulus	0.000001260823438787054	*PLCB1,GABRB3,MAPK14,ARID1B,TIMP3,DUSP22,ITGA2,KCNQ1,NRXN1,SLC1A2,BCKDHB,PKD1L1,TGFBR3,CDH13,KCNE1,TMEM108,FLRT2,GPC3,SPIDR,IL4R,RYR3,GLP2R,CFTR,RBFOX2,LDLRAD4,ALK,PTPRE,ROBO2,ZNF366,LEMD3,AGT,CHRM5,FBXO32,PARK2,FHL2,CYBB,OTC,HDAC2,HTRA1,SLIT2,STXBP4,IGF1R,MDM2,SLC24A4,SMAD1,TMPRSS6,NR3C2,HCN1,FSHR,WWOX,TRPM2,RYR2,LRRK2,SLC1A3,IGFBP7,NTRK3,RNF165,PDXP,SOX6,IER2,GSK3B,LGR5,SLC8A1,OGT,TGFB2,ACSBG1,BDNF,KCNC1,WASF1,KDM4C,EXT2,SIK2,PTGFR,NR5A2,PAQR8,PML,AKAP6,FBN1,BCL2,RXFP2,LRP2,TMEM100,ITPR2,NR4A2,FYN,GNRHR,NR4A1,SMARCA4,CD44,CASP6,FOXO1,PDE4D,KANK1,ACTN2,GABRB1,SLC1A1,UGCG,RARB,RERG,MTOR,CHRM1,CTDSPL2,BMPER,BBS2,ATP1A1,DSG4,IFT88,PRKCE,PRKCB,ADCY8,FOXO3,RAB31,EDNRB,RXFP1,TBC1D4,COL2A1,MAP3K7,NR3C1,TGM2,KDR,PRCP,APP,LGR4,ADTRP,THRB,CPEB2,ADAM17,TFPI,ATF2,TGFB1I1,CD38,CHRDL1,BCL2L1,HDAC4,PDE4B,SOX5,TIMP2,DNMT1,GHR,GLRA2,NTRK2,FAM83G,GNG2,PAPPA,SPRED2,GLI3,CLDN1,KIF16B,RAP1A,SAFB2,TGFBR1,NCOA5,CHST11,RAPGEF1,EP300,CACNA2D1,CYP7B1,GLRA1,COL4A6,NPC1,PNPLA3,TPH2,EFNA5,MAGI2,TFF1,SORL1,STRN3,HTR4,EIF4E,POR,TNFRSF11B,GRM5,HDAC9,DDC,MXRA5,NLK,KLF3,SH3GL2,SNX25,MAS1,PER1,KL,ELAVL4,TRIM16,GABPA,PTPRU,AKAP7,VEPH1,NSG1,JAK2,SMAD3,BAIAP2L1,KIDINS220,NOS1,TNF,OVOL2,SNX5,POMC,BMP15,PHEX,SRSF4,BCL11A,ANO1,GCLC,PMEPA1,LTBP1,SMOC2,SULF1,GNAL,PIK3R3,TRIM41,ZNF423,CTNNA1,NSG2,CD109,KIT,SLIT3,UBR2,BLM,TRPV4,VWC2,CPS1,RAPGEF2,REG1B,ESR1,HTR2C,UBE3A,GABRB2,EPHB2,ABCC1,ERBB4,FER,SHCBP1,BSG,CAMP,VPS13C,PTPRK,FGF7,SPRED3,PSG9,SULF2,PDGFD,ITGA8,KDM3A,PLAT,FGFR1,RYR1,SOST,NTF3,P2RX7,AR,CASP7,TRERF1,PRKAA2,GNB5,ZMIZ1,FLT3,PPARGC1B,PTPRA,TRIM72,ESRRG,FERMT2,GNAO1,HCN4,SMPD3,DAB2,SRD5A2,UROS,FGF12,ITGA3,NREP,PIN1,TRPM4,BMP6,CHRM3,NCOA2,CALCR,SCUBE3,INSR,PAK1,PDE3A,SKI,ENPP1,RRAGC,IDE,PCK1,PPARA,SORT1,TAF1,CACNA1A,FUT8,SPRED1,AKR1C2,ESR2,PRDM16,ZNF451,C2,DEFA1B,DEFA3,EXT1,NCOA1,PRLR,HDAC5,TDGF1,A2M,STAT4,KCNC2,PRKCQ,STAT3,TP63,BRIP1,HPN,PIK3R2,CASQ2,DTNBP1,RAP1B,APC,BMPR1A,NGF,SPINT2,MTMR4,CTSH,ACACA,GABRG2,GPR21,RGMB,VPS35,FGF1,TBX20*
GO:0051130	positive regulation of cellular component organization	0.0000018944896193183662	*CDH4,PLCB1,CDC42EP3,MAPK14,DSCAM,RUNX1,ASIC2,VPS13D,ITGA2,ATF7IP,NRXN1,CHODL,PDE4DIP,SYNDIG1,TF,PRDM9,PSMC6,PLXNA4,FLRT2,GPC3,NRG1,SPIDR,IL4R,PLCE1,TIAM1,RHOC,CBFA2T2,ALK,FBXO31,ROBO2,EPHA3,AGT,ATAT1,PARK2,FRMPD4,ZNF804A,NEGR1,NTN1,SLIT2,IGF1R,SYT9,TRPC5,DMD,SYT1,HNRNPA2B1,SKAP1,STK24,ATRX,LRRK2,NTRK3,NUSAP1,DISC1,PAN3,WRAP73,PDXP,MECP2,SEMA4D,GSK3B,LINGO2,PRKD1,TGFB2,CLSTN2,FMN1,SEMA5A,BDNF,WASF1,PLEKHM2,PML,PPARGC1A,CNTNAP2,PLXNA2,RALBP1,EPHB1,ARHGEF10,FYN,PARN,THBS2,ENPP2,ACTN2,ATP8A2,MYLK3,MTOR,ROR1,ABCA13,PTPRD,TIAM2,HAS3,PRKCE,LRRTM1,RAB31,KALRN,ROBO1,SGIP1,CDH17,MAPK9,KDR,PPHLN1,APP,NRP1,TENM2,CAMK1D,TRABD2B,BICD1,GRIP1,CCDC88A,HD*

			AC4,DNMT1,MAPRE2,NTRK2,DNM3,CLDN1,MORC2,RAP1A,TGFBR1,CUX1,TENM3,RAPGEF1,EP300,GRID2,ADCK1,EFNA5,MAGI2,BID,DMRT1,RELN,SELE,DYNC1H1,LRRTM3,MAP3K13,NPHP4,RALA,IL1RAP,NAV3,ATP10A,PPP3CA,TBC1D5,ELAVL4,CNTN1,GPSM2,ASAP1,NLGN1,RAD51AP1,DDX3X,SHOX2,AHI1,SMAD3,BAIAP2L1,KIDINS220,PLXNB1,NBN,TNF,COBL,DLG5,FCHSD2,BCL11A,EPS8,CCP110,LRP4,CLSTN1,TENM1,CDKL5,EGF,IL1RAPL1,KIT,TRPV4,RAPGEF2,ESR1,EPHB2,TOX,FER,SLITRK6,FNIP1,LRP5,ABL2,AP2B1,ARHGAP35,ATP8A1,NSMCE2,TNKS,AUTS2,OCLN,UNC13B,SLX1B,NTF3,P2RX7,AR,SNX30,BHLHB9,GNL3,MARK2,SNX7,FERMT2,SMPD3,DAB2,LCP1,ITGA3,SYNPO2,CLASP1,TGFA,G3BP2,HGF,INSR,MACF1,PAK1,CDC16,FMR1,DKC1,BMF,MAD1L1,CLIP1,KATNBL1,FNIP2,RIT2,G3BP1,PRKCQ,AMOT,ISLR2,PAK3,DTNBP1,RAP1B,LRP8,APC,CKAP5,NGF,VLDLR,RGCC,CLASP2,MAP2K1,VPS35,ATMIN,RREB1
GO:0031346	positive regulation of cell projection organization	0.00000444270598906312	*CDH4,CDC42EP3,DSCAM,ITGA2,CHODL,PLXNA4,PLCE1,TIAM1,CBFA2T2,ALK,FBXO31,ROBO2,EPHA3,AGT,ZNF804A,NEGR1,NTN1,SLIT2,IGF1R,TRPC5,DMD,STK24,NTRK3,DISC1,WRAP73,SEMA4D,PRKD1,SEMA5A,BDNF,PLXNA2,FYN,ENPP2,ATP8A2,MTOR,ROR1,PTPRD,TIAM2,KALRN,ROBO1,NRP1,TENM2,CAMK1D,GRIP1,CCDC88A,HDAC4,NTRK2,DNM3,RAP1A,TGFBR1,CUX1,TENM3,RAPGEF1,EP300,EFNA5,MAGI2,RELN,MAP3K13,RALA,ELAVL4,CNTN1,NLGN1,SHOX2,KIDINS220,PLXNB1,COBL,BCL11A,EPS8,CCP110,TENM1,CDKL5,IL1RAPL1,KIT,RAPGEF2,TOX,ABL2,ARHGAP35,AUTS2,OCLN,P2RX7,BHLHB9,MARK2,ITGA3,HGF,MACF1,FMR1,RIT2,ISLR2,PAK3,LRP8,APC,NGF,VLDLR,MAP2K1,ATMIN,RREB1*
GO:0016192	vesicle-mediated transport	0.000004853822384894521	*IGHV1OR21-1,TRAPPC8,CTBP2,TMPRSS15,RAB27A,ANXA8L1,ANK3,IGHV4-31,HEATR5A,IGHV3-64,SYN3,IGHV1OR15-9,IGHV4OR15-8,ITGA2,TMPRSS2,GRIN3A,NRXN1,TMPRSS3,NRXN3,RAB5A,DOCK2,RIMS2,GRIA1,CLEC16A,CDH13,ANK2,LRP1B,SYNDIG1,TF,TMEM108,SYT17,GPC3,NRG1,SCARA5,STXBP6,IL4R,ENTHD1,CECR2,MCTP1,SYT16,CCR1,CFTR,SCFD2,DPYSL2,ARFIP1,PIK3C3,ERC1,KLRF2,RAB7A,TAPBP,EPHA3,DCLK1,ERC2,VTI1A,WDR11,RHOJ,CDH2,PARK2,LOXL2,SYTL5,DENND2A,USP6,ITSN1,NCF4,PCDH17,RIN3,APBA2,PDCD6,IGF1R,SYT9,CCDC91,SYT1,CACNG3,FSHR,GSG1L,FMN2,CNIH3,KIF3B,UNC13C,LRRK2,KXD1,ILDR1,DPY30,SH3BP1,MTMR2,GSK3B,PRKD1,VAV3,ATP9B,DNAJC6,LLGL2,PRKAR1B,WASF1,SNAP25,ARHGAP12,IGHV3-16,SNAP23,CHMP4C,RALBP1,LRP2,NSF,CD163,CD163L1,MICAL3,FYN,NEU3,ENPP2,ACTN2,CSNK1G3,EXOC4,SLC1A1,CADPS,TMPRSS4,VPS26B,ABCA13,BBS2,EVI5,EXOC6B,EHBP1,NLGN4X,PRKCE,PRKCB,STON1,STON1-GTF2A1L,FAM19A4,LRRTM1,RAB31,TRAPPC12,KALRN,MSR1,TBC1D4,SGIP1,STX12,NBAS,SEC24B,TGM2,CASK,IGHV4-28,ABCA12,APP,CALCRL,NRP1,TSNARE1,ELMO1,CAMK1D,IGF2R,GOPC,TM9SF4,BICD1,GRIP1,RIMS3,OPHN1,SAMD9,DENND5A,RIN2,TUB,NCF2,RNF126,BCL2L1,GHR,DNM3,RASGRP1,TRAPPC10,ARR3,COMMD1,RAB11FIP4,RABGEF1,C17ORF75,COG7,KIF16B,STXBP5,RAP1A,VPS53,CUX1,RAPGEF1,CHRNA5,RAB2A,SCFD1,STX8,GAB2,NPC1,TMEM50B,VPS39,SPG11,ADORA2A,ADRBK2,MERTK,CACNG2,MAGI2,GULP1,SORL1,IGHV4-4,SELE,DDC,NUMB,IGHV3OR16-12,RALA,EXOC5,PPP3CA,SH3GL2,DGKI,TBC1D5,ANKRD13A,PLSCR1,ATP9A,RIMS1,NLGN1,SH3KBP1,OSBPL5,NSG1,TPCN2,AHI1,VPS41,XKR4,CD300A,TNF,SNX5,BLOC1S6,FCHSD2,TRIM23,ARF1,MYO6,DOCK1,IGLC2,IGLL5,RUFY1,LRP4,EFNB2,CLSTN1,NSG2,DMBT1,STX16,STX16-NPEPL1,EGF,SEC16B,SPIRE2,VPS8,IL1RAPL1,KIT,NAPG,SLC30A8,UBE3A,EPHB2,IGHV3-72,FER,LRP5,XKR7,SLAMF1,ABL2,TMEM50A,VPS13C,AP2B1,CD2AP,CCL3,ARHGAP25,ARF4,ARFGAP3,MYH9,PACSIN2,P2RX1,PIK3CD,ASGR2,UNC13A,VPS45,UNC13B,NTF3,P2RX7,RAPGEF4,SORCS1,COG1,KIAA1109,RABGAP1L,SCAMP5,EXOC2,ANXA11,PROS1,LDLRAD3,SNX7,TRIM72,VPS16,ATP6V1H,SMPD3,DAB2,STX3,SYT2,TXNDC5,BTBD9,CLASP1,PCDHGA3,MYO1B,CACNB2,SNAP29,INSR,MACF1,PAK1,CHM,ENPP1,FMR1,IGHV1-69,MON2,AMPH,IGHV1-46,AP1G2,CD84,EPG5,ESYT2,SORT1,HEATR5B,C2,IGHV1-18,OPTN,RIT2,PRKCG,ELMO2,GOSR2,USP7,DTNBP1,GAK,RAP1B,TMEM175,LGALS9,LRP8,MAPKAPK2,VLDLR,EXOC6,RPH3AL,NCALD,OSBPL1A,SPTBN4,AP5M1,CLASP2,MAP2K1,TPH1,VPS35*
GO:0042221	response to chemical	0.000008306606189987014	*ADCY2,CDH4,PLCB1,CMKLR1,GABRB3,MAPK14,ABCG8,DSCAM,ARID1B,GPHN,CTBP2,OR11H1,OR4M2,PTPRT,ANK3,DCC,TIMP3,DUSP22,ITGA2,ABCG1,KCNQ1,EPHA6,OR4K15,VRK2,GRIN3A,NRXN1,DOK5,NRXN3,SLC1A2,DOCK2,OR8U1,BCKDHB,SHANK2,GRIA1,NR*

			G3,DLG2,TGFBR3,CDH13,ANK2,NXN,GRAMD1C,TF,PLGRKT,HLCS,KCNE1,TMEM108,CHEK2,OR4C12,ADAMTSL1,PSMC6,SYT17,PLXNA4,FLRT2,GPC3,FBLN5,NRG1,PSMB2,AOX1,RFFL,SPIDR,IL4R,ALDH1A2,RYR3,GGT1,UNC5D,KYNU,GLP2R,SEMA6D,CCR1,CCR3,CFTR,RBFOX2,LDLRAD4,ZBTB20,DPYSL2,IL1RAPL2,OR4A5,SLC17A3,MX1,ALK,PTPRE,PIK3C3,CNTN4,ARID5B,ROBO2,ZNF366,EPHA3,PTPRN2,PXDN,CRNN,LEMD3,OR8K3,AGT,CST2,CHRM5,FBXO32,ATP6V0D1,PARK2,FHL2,LOXL2,LAMA2,OR4K13,CYBB,OTC,OR4M1,OR4N2,SEMA3D,NTN1,OR52N5,TRIM5,KCNH1,USP25,TXK,RNF185,DBH,FBXO27,KCNMA1,RIN3,TRIO,HDAC2,HTRA1,SLIT2,ACSM2B,AHRR,PDCD6,OR2L13,RPS6KA2,STXBP4,IGF1R,MDM2,MX2,SLC24A4,SMAD1,SYT9,OR11G2,OR9Q1,TMPRSS6,NR3C2,OR8K5,DMD,HCN1,SYT1,FSHR,FMN2,NRIP1,STK24,WDR83,WWOX,ATRX,PARP16,TRPM2,RNLS,RYR2,OR2M5,HSPB8,LRRK2,RORB,SLC1A3,IGFBP7,NTRK3,RORA,GGT2,ILDR1,CHD7,RNF165,EMB,PDXP,SOX6,IER2,SEMA4D,GSK3B,LGR5,SLC8A1,PRKD1,ERCC6,OGT,TGFB2,VAV3,AFF3,SEMA5A,ACSBG1,BDNF,RYK,ZDHHC17,KCNC1,DNAAF2,DNAJB4,WASF1,GRIN2B,KDM4C,OR6N1,EXT2,LAMA3,SIK2,NR2F2,PTGFR,NR5A2,PAQR8,PML,PPARGC1A,AKAP6,FBN1,NLRP7,PLXNA2,BCL2,RALBP1,RXFP2,ZNF675,EPHB1,IL12RB2,SLC30A5,LRP2,TMEM100,ITPR2,GDAP1,NR4A2,ABCC9,EPHA7,FYN,GNRHR,KCNE2,CHN1,NR4A1,SMARCA4,CD44,RHBDD1,CASP6,FOXO1,PDE4D,TBXAS1,KANK1,ENPP2,ABCB10,ACTN2,ATF6,BACH1,GABRB1,MME,PACRG,TNMD,CPNE4,TPO,UNC5C,KCND2,MYLK3,SCN11A,SLC1A1,UGCG,PALLD,RARB,RERG,NLRP12,MTOR,VPS26B,CHRM1,CTDSPL2,DDX21,BMPER,BBS2,HBE1,OR51B2,OR51I1,PGK1,ARL6IP5,ATP1A1,DSG4,IFT88,OR4K17,OR8J3,PRKCE,EIF2S1,HELLS,PRKCB,VDR,ADCY8,FAM19A4,FOXO3,RAB31,SLC47A1,CAPN3,KALRN,TTLL12,EDNRB,MSR1,OASL,ROBO1,RXFP1,SATB2,TBC1D4,BNIP3L,FLT1,NASP,NOX4,COL2A1,MAP3K7,MAPK9,NR3C1,TACR3,TRAF3IP2,ZNF277,OR4C15,TGM2,KDR,PRCP,CASK,CHL1,EDA,PCOLCE2,ABCA12,APP,CALCRL,NRP1,PTPRM,LGR4,ADTRP,CD58,KAT5,THRB,CPEB2,KCNK2,SEMA3C,ADAM17,CAMK1D,MAP3K5,IGF2R,TFPI,ATF2,ETS1,MT1HL1,PRTG,TGFB1I1,OR10R2,RPS6KA5,TCF7L2,TRAF3,ABAT,GRIN2A,OPHN1,BCLAF1,HSPA4L,CD38,ALCAM,CHRDL1,RNF126,BCL2L1,ABTB2,HDAC4,PDE4B,SOX5,TIMP2,DNMT1,GHR,GLRA2,NTRK2,CPNE8,FAM83G,MGST1,GNG2,PAPPA,LY86,RABGEF1,SDK1,SPRED2,GLI3,OR5L2,CLDN1,KIF16B,OR11L1,DOCK4,PXDNL,RAP1A,SAFB2,SRP54,TGFBR1,CYSLTR1,PRDX4,EPHA5,TAS2R38,TNR,TOP1,NCOA5,TAS2R1,CHST11,IL16,RAPGEF1,CD96,CHRNA3,CHRNA5,EP300,DSCAML1,SCFD1,CACNA2D1,CYP7B1,DIO2,STX8,GLRA1,HBD,TP73,COL4A6,IFNGR2,NPC1,OR4C5,PNPLA3,CA3,PSMA1,TPH2,FLNB,ADORA2A,EFNA5,CACNG2,GUCY2C,MAGI2,NF1,RFC3,RGS7,TFF1,GOT2,SORL1,STRN3,OR5K4,TTC8,HTR4,ASXL1,EIF4E,MICU1,POR,RELN,TNFRSF11B,GRM5,HDAC9,IL18R1,IL1RL1,SELE,PLSCR4,DDC,MXRA5,NLK,RALA,ADCYAP1R1,EGLN3,IL1RAP,JAG1,MAN1A1,PPP3CA,KLF3,KLF7,SH3GL2,SNX25,CXADR,GLDC,MAS1,PER1,KL,ELAVL4,GSTA2,MNAT1,PLSCR1,SLC5A1,TMEM67,TRIM16,GABPA,KAT7,TMEM135,NLGN1,P2RX6,PTPRU,AKIRIN2,GBP2,GBP7,AKAP7,FOXRED2,MSRA,VEPH1,DDX3X,NSG1,CNOT7,ZNF622,JAK2,SMAD3,BAIAP2L1,KIDINS220,NOS1,PLXNB1,TMTC4,CDK1,MIR320B2,TNF,LRCH1,OVOL2,PANX1,SNX5,TNIP1,POMC,ADNP2,BMP15,PHEX,RBM11,LGR6,OR11A1,OR5V1,BRINP1,SRSF4,BCL11A,KIF18A,SULT1B1,ANO1,GCLC,PMEPA1,EPS8,LTBP1,CYP3A5,SMOC2,SULF1,C5AR1,GNAL,PIK3R3,CNTN6,EFNB2,RSRC1,TRIM41,NET1,ZNF423,LRRC8C,LRRC8D,SELP,CHD6,CTNNA1,NSG2,ABCG2,DMBT1,EFHC2,GSKIP,TNFRSF19,WDR35,CD109,ALDH1A1,CMA1,IL1RAPL1,KIT,SLIT3,UBR2,BLM,OR7A5,PIK3C2G,TRPV4,VWC2,CPS1,EFNB1,PTPRO,RAPGEF2,RCSD1,REG1B,ESR1,HTR2C,JAK1,MYPN,SEMA3E,SLC30A8,UBE3A,GABRB2,EPHB2,RNF4,ABCC1,DAPK1,ERBB4,FER,LRP5,SHCBP1,SLAMF1,ABL2,BSG,CAMP,OR4C6,SETD2,VPS13C,ARHGAP35,COMMD7,PTPRK,CCL3,FGF7,LAMA1,PREX1,SEMA5B,SPRED3,IL20RA,ITGA9,LRRC70,PSG9,SULF2,CYP2E1,SOX4,ST18,OSCP1,P2RX1,PDGFD,PIK3CD,ITGA8,IRAK2,KDM3A,PLAT,CDK19,DAPL1,FGFR1,MEF2A,RYR1,UNC13B,DAD1,GLRA3,SOST,IL23R,KEAP1,NTF3,P2RX7,PLA2G4A,TEX14,RNMT,AMFR,AR,CASP7,GSTM4,TRERF1,IFNA8,IL1RL2,MGST3,RBM4,BDKRB1,BDKRB2,MIR431,MIR433,PRKAA2,SEMA3A,TLE4,ANXA11,AQP10,CDC73,GNB5,MEIS2,ZMIZ1,FLT3,PPARGC1B,PTPRA,TRIM72,DYNAP,ESRRG,FERMT2,GNAO1,HCN4,MAP2K5,SMPD3,DAB2,SRD5A2,STX3,SYT2,UROS,ACTN4,ADAM10,CCL7,EEF1E1,FGF12,ITGA3,KMO,LGI1,MT1F,NREP,OR10K2,OR4C46,QRICH1,RFTN1,LECT2,PIN1,TRPM4,ERLIN1,NME8,BMP6,CHRM3,CARF,CNGB1,NCOA2,HGF,MYLK,CALCR,CENPF,SCUBE3,SNN,NCAM1,NFASC,IL18RAP,INSR,PAK1,PDE3A,P

			SMB7,SKI,ENPP1,FMR1,LITAF,RRAGC,ABCD1,CRMP1,IDE,AIM2,CCDC141,CSF2RB,EPG5,PCK1,PPARA,SORT1,TTN,SLC22A5,TAF1,CACNA1A,FUT8,SPRED1,AKR1C2,ESR2,PRDM16,SCN9A,ZNF451,BTRC,C2,CCDC3,DEFA1B,DEFA3,OPTN,EXT1,MDM4,NCOA1,PRKCG,PRLR,TYMP,UMOD,ELMO2,HDAC5,OR51L1,TDGF1,UGGT2,A2M,SEL1L2,SLC40A1,STAT4,STIM1,TREM1,TRPM6,KCNC2,OR4C16,PRKCQ,STAT3,TP63,AMOT,BRIP1,CST1,HPN,PAK3,PIK3R2,CASQ2,DHRS2,DTNBP1,RAP1B,LGALS9,LRP8,NFAT5,OR2T4,TAS1R1,ACSM1,IL10RB,MAPKAPK2,SLC7A8,APC,BMPR1A,DPP4,ITGA1,NGF,PRKCA,PTGES,SPINT2,GBP4,GPR75,MTMR4,CTSH,DHX9,RGCC,USP18,ACACA,CCL14,CCL15,CCL15-CCL14,EDA2R,GABRG2,GPR21,IL6R,MAP2K6,MAP2K1,RGMB,SOS1,VPS35,FGF1,TBX20,NLRC5
GO:0050770	regulation of axonogenesis	0.00000837404057340657	CDH4,DAB1,DSCAM,DCC,CHODL,FSTL4,LRRC4C,PLXNA4,SEMA6D,TIAM1,ROBO2,CDH2,SEMA3D,NTN1,SLIT2,TRPC5,DISC1,SEMA4D,GSK3B,SEMA5A,BDNF,RYK,PLXNA2,SIPA1L1,EPHA7,CHN1,TIAM2,ROBO1,NRP1,SEMA3C,NTRK2,TNR,EFNA5,MAP3K13,SHOX2,PLXNB1,BCL11A,LRP4,CDKL5,SEMA3E,FGF13,EPHB2,ARHGAP35,SEMA5B,MAP2,SEMA3A,MARK2,MACF1,PAK1,ISLR2,PAK3,KIAA0319,NGF,MAP2K1
GO:0099003	vesicle-mediated transport in synapse	0.000010598635393112712	CTBP2,SYN3,GRIN3A,NRXN1,NRXN3,RAB5A,RIMS2,SYNDIG1,NRG1,ERC2,CDH2,ITSN1,PCDH17,APBA2,SYT9,SYT1,GSG1L,UNC13C,LRRK2,GSK3B,DNAJC6,PRKAR1B,SNAP25,SNAP23,CADPS,ABCA13,NLGN4X,PRKCB,STON1,CASK,RIMS3,OPHN1,DNM3,STXBP5,RAP1A,CHRNA5,ADORA2A,DDC,NUMB,DGKI,RIMS1,NLGN1,NSG1,BLOC1S6,EFNB2,CLSTN1,AP2B1,P2RX1,UNC13A,UNC13B,P2RX7,RAPGEF4,KIAA1109,STX3,SYT2,BTBD9,CACNB2,SNAP29,FMR1,AMPH,PRKCG,DTNBP1,GAK,RAP1B,VPS35
GO:0007626	locomotory behavior	0.000011503187196032292	DAB1,DSCAM,SHANK2,ANKH,NRG1,ALK,RCAN1,PARK2,NEGR1,ANKFN1,DBH,APBA2,CELSR1,FSHR,GRM1,ASTN1,LRRK2,CHD7,HEXB,MECP2,PCDH15,SNAP25,NR4A2,NAV2,KCND2,SLC1A1,MTOR,KLHL1,PRKCE,ADCY8,LRRTM1,KALRN,PREX2,CHL1,APP,ABAT,TNR,CHRNA3,GLRA1,NPC1,SPG11,ADORA2A,RELN,GRM5,ELAVL4,SOBP,EPS8,SCN1A,HTR2C,UBE3A,SLITRK6,AGTPBP1,PUM1,CDH23,CLN6,FGF12,BTBD9,NCOA2,TMOD1,QRFP,DPP4,SPTBN4,VPS35,MCOLN3
GO:0050807	regulation of synapse organization	0.0000121073575046 7593	MAPK14,ASIC2,NRXN1,SHANK2,SYNDIG1,FLRT2,LRFN5,ABHD17C,CTNNA2,IL1RAPL2,ROBO2,CDH2,FRMPD4,ZNF804A,NEGR1,NTN1,GPC6,CDH8,LRRK2,NTRK3,DISC1,SEMA4D,LINGO2,CLSTN2,BDNF,GRIN2B,LRFN2,EPHB1,SIPA1L1,EPHA7,FYN,THBS2,PTPRD,LRRTM1,KALRN,TANC2,APP,DGKB,NTRK2,DNM3,GRID2,EFNA5,RELN,LRRTM3,IL1RAP,GPC4,NLGN1,CTTNBP2,TNF,PPFIA2,DLG5,LRP4,CLSTN1,CDKL5,IL1RAPL1,PTPRO,UBE3A,EPHB2,SLITRK6,ARF4,BHLHB9,PDLIM5,PAK3,DTNBP1,LRP8,VPS35
GO:0048589	developmental growth	0.000014036656634136342	CDH4,PLCB1,MAPK14,DSCAM,RUNX1,DCC,AKAP13,LARGE,SORBS2,SLC1A2,EYS,RIMS2,MTPN,TGFBR3,FSTL4,TMEM108,SYT17,PLXNA4,NRG1,SEMA6D,DPYSL2,NKD1,ARID5B,DCLK1,AGT,WDR11,PARK2,SEMA3D,NTN1,TXK,APBA2,FTO,SLIT2,SMAD1,CPQ,TRPC5,DMD,SYT1,BASP1,VCL,MAEL,ATRX,TENM4,KIF26B,CHD7,DISC1,SEMA4D,GSK3B,ERCC6,FLVCR1,TGFB2,FMN1,SEMA5A,BDNF,RYK,PCDH15,WASF1,RAG2,AKAP6,BCL2,RAD51B,EPHA7,ATP8A2,ZFPM2,RARB,MTOR,BBS2,NLGN4X,FOXO3,CAPN3,GRHL2,STK3,APP,NRP1,KCNK2,SEMA3C,ATF2,ZNF830,ALCAM,GHR,GLI3,TGFBR1,TNR,CHST11,EP300,TP73,SPG11,EFNA5,MAGI2,IFT80,TTC8,POR,SP2,MAP3K13,PPP3CA,PLS1,SH3GL2,WWTR1,RIMS1,SMAD3,PTGFRN,RDH10,CDK1,NBN,COBL,LGR6,ARID2,BCL11A,LRP4,CDKL5,PRKAR1A,SLIT3,ESR1,SEMA3E,UBE3A,FGF13,PLAG1,ERBB4,SLITRK6,FGF7,SEMA5B,UNC13A,AUTS2,MAP2,AR,SEMA3A,ZMIZ1,PDLIM5,SMPD3,TNN,SYT2,GAS2,INSR,MACF1,CTDP1,EVC,PPARA,EXT1,MYH6,PRLR,ADARB1,STAT3,ISLR2,BMPR1A,KIAA0319,NGF,NCAPG2,GPR21,SPTBN4,CLASP2,SOS1,FGF1,TBX20
GO:0051049	regulation of transport	0.000014061392601604238	MAPK14,ABCG8,ASIC2,RAB27A,CACNA1E,KCNJ6,ANK3,WLS,ITGA2,ABCG1,KCNQ1,GRIN3A,NRXN1,NRXN3,SLC1A2,RAB5A,DOCK2,PCNT,RIMS2,CDH13,ANK2,KCND3,DPP10,TF,SCN8A,KCNE1,KCNJ15,SYT17,KCNG3,GPC3,NRG1,STXBP6,SHISA6,IL4R,MCTP1,CCR1,CFTR,PM20D1,RASGRF1,ARFIP1,PIK3C3,UTRN,RAB7A,EPHA3,AGT,SHISA9,CDH2,PARK2,CYBB,MCTP2,KCNH1,USP6,KCNMA1,RIN3,APBA2,STXBP4,MX2,SYT9,KCNK17,PXK,DMD,HCN1,SYT1,CACNA2D3,CACNG3,FGF14,GSG1L,DPP6,TRPM2,CNIH3,RYR2,CATSPER2,DHRS7C,LRRK2,KCNH5,ILDR1,CHD7,KCNK13,MTMR2,GSK3B,SLC8A1,PRKD1,TGFB2,DNAJC6,LLGL2,PRKAR1B,KCNC1,NKAIN2,SNAP25,PML,USP36,AKAP6,BCL2,KCNJ3,SLC30A5,DISP1,NKAIN3,NSF,FRMD4A,FYN,KCNE2,HECW1,NEU3,RCVRN,RHBDD1,PDE4D,ACTN2,ATP8A2

			,KCNS3,KCNIP4,KCND2,SCN11A,SLC1A1,CADPS,CHRM1,CTDSPL2,PKP2,ABCA13,ARL6IP5,ATP1A1,EPM2A,PLN,PRKCE,KMT2A,PRKCB,STON1,ADCY8,EFHB,LRRTM1,RAB31,SCP2,CAPN3,ITLN1,KALRN,EDNRB,TBC1D4,BNIP3L,SGIP1,KCNC4,SEC24B,ACSL5,TGM2,CASK,ABCA12,APP,NRP1,ADTRP,CAMK1D,GOPC,ROS1,TM9SF4,TCF7L2,BICD1,ABAT,RIMS3,SGK2,OPHN1,CD38,TUB,PDE4B,ACSL4,DNM3,COMMD1,KCTD7,RABGEF1,GLI3,CACNA1C,STXBP5,RAP1A,KCNJ12,EPHA5,IL16,RAPGEF1,CHRNA3,CHRNA5,SCFD1,TTC39B,CACNA2D1,CACNA2D4,CLIC6,GAB2,IFNGR2,RASGRF2,ADORA2A,CAMK2G,EFNA5,MERTK,CACNG2,MAGI2,NF1,RGS7,KCNQ5,SORL1,RELN,GRM5,SELE,DYNC1H1,NUMB,RALA,ADCYAP1R1,PPP3CA,KCNB2,KLF7,DGKI,PER1,TBC1D5,ANKRD13A,PLSCR1,ATP9A,CNTN1,GRM7,RIMS1,STIM2,NLGN1,AKAP7,MAPK8IP2,CACNA1D,TPCN2,JAK2,AHI1,SMAD3,CD300A,NOS1,CDK1,TNF,SNX5,POMC,TMC2,KCNK10,ADORA3,ARF1,MYO6,RUFY1,TSPAN13,ANO1,GCLC,KCNA6,RAB23,EFNB2,KCNH8,SAE1,TRDN,SCN1A,TENM1,KCNH7,MYRIP,EGF,PTPN14,SEC16B,IL1RAPL1,ABCA5,KCNAB1,HTR2C,SLC30A8,FGF13,EPHB2,NDFIP2,DAPK1,FER,CACNG6,LRP5,SLAMF1,ABL2,BSG,SETD2,AP2B1,CD2AP,NSUN2,ATP8A1,CCL3,PACSIN2,PTGER3,SOX4,P2RX1,KCNE4,MEF2A,OCLN,UNC13B,MAP2,NTF3,P2RX7,PLA2G4A,RAPGEF4,SCAMP5,SUFU,DENND5B,RBM4,BDKRB1,THADA,GNB5,REEP2,HCN4,SMPD3,DAB2,LCP1,RNF207,SYT2,ACTN4,FGF12,KCNJ16,KMO,STAC,MYOM1,NUP153,TRPM4,ABCA8,BMP6,BTBD9,CHRM3,CLASP1,MYLK,CALCR,CACNB2,NOS1AP,INSR,ENPP1,FMR1,CD84,JPH4,PPARA,TTN,CORIN,FAM3D,FHL1,NUP214,CACNA1A,SCN9A,C2,RIT2,PRKCG,UMOD,STIM1,KCNC2,USP7,PIK3R2,CASQ2,DTNBP1,RAP1B,LGALS9,SNCAIP,PTGES,UBASH3B,DHX9,RGCC,RPH3AL,MAP2K6,SIAH3,SPTBN4,CLASP2,MAP2K1,VPS35,C1QTNF3,RGN
GO:0043085	positive regulation of catalytic activity	0.00001555612364406294	DAB1,MAPK14,PRIM2,BCL2L13,AKAP13,FBLN1,ITGA2,RGS6,EPHA6,NRXN1,SGSM1,NRG3,TBC1D22A,NRG1,TIAM1,RHOC,RASGRF1,ALK,EPHA3,AGT,DCUN1D4,TXK,USP6,MYO9A,PDCD6,IGF1R,SIPA1L3,HNRNPA2B1,PARP16,XRCC4,LRRK2,NTRK3,SH3BP1,SEMA4D,GSK3B,PRKD1,ERCC6,TGFB2,VAV3,SLC5A3,RYK,GRIN2B,PML,NLRP2,TAOK3,BCL2,RALBP1,ARHGAP24,EPHB1,SIPA1L1,NR4A2,ARHGEF10,EPHA7,FYN,PARN,CHN1,CD44,RCVRN,RAP1GDS1,SLC1A1,NLRP12,MTOR,ROR1,TIAM2,ARL6IP5,EVI5,VDR,ADCY8,KALRN,DLC1,ROBO1,TBC1D4,FLT1,NOX4,GRHL2,MAP3K7,CR1,STK3,TGM2,KDR,PCOLCE2,APP,UACA,ADAM17,MAP3K5,PKP4,PIK3R5,ROS1,RGS16,TNFRSF10B,RALGAPA2,DOCK9,GRIN2A,CCDC88A,MOB3B,GHR,MAPRE2,NTRK2,RASGRP1,CACNA1C,RAP1A,EPHA5,LCP2,RAPGEF1,CHRNA3,GRAMD4,PIBF1,EFNA5,FAM20A,MERTK,MAGI2,NF1,RFC3,RGS7,SORL1,ARHGAP6,BID,POR,RELN,GRM5,SELE,MAP3K13,ADCYAP1R1,EGLN3,TBC1D3B,MAS1,TBC1D5,DOCK10,MNAT1,PLSCR1,SLC22A2,ASAP1,BORA,AKIRIN2,NPR3,CACNA1D,DDX3X,ARAP2,ZNF622,JAK2,SMAD3,CD300A,NOS1,PLXNB1,NBN,NVL,SLC39A10,TNF,HMGA2,CDC20B,C5AR1,NET1,CDC14B,TENM1,CDKL5,SIPA1L2,EGF,KIT,RAPGEF2,ESR1,FGF13,EPHB2,DAP,DAPK1,ERBB4,RAPGEF6,RPS27L,ABL2,ASAP2,F11R,ARHGAP35,CCL3,PREX1,ARHGAP25,ARF4,ST18,CAB39L,GARNL3,NEDD9,P2RX1,PDGFD,UBE2N,TNKS,FGFR1,TBC1D9,IL23R,NTF3,P2RX7,ARRDC4,RABGAP1L,DGKZ,NEK5,GNB5,MARK2,PDE5A,FLT3,PPARGC1B,DYNAP,FERMT2,MAP2K5,GPR55,TCL1B,CCL7,PIN1,TGFA,RALGAPA1,NOS1AP,INSR,PAK1,DKC1,AIM2,ARHGAP42,TNFAIP8L3,GNA13,WDR41,PPP2R3C,BTRC,PRLR,DOCK7,TDGF1,STIM1,PRKCQ,STAT3,DOCK3,LGALS9,LRP8,BMPR1A,ITGA1,VLDLR,PILRB,ABI1,CTSH,DHX9,RGCC,CCL14,CCL15,IL6R,MAP2K6,MAP2K1,FGF1,CRADD,RGN
GO:0048813	dendrite morphogenesis	0.00001651765995126705	DSCAM,CTNND2,CTNNA2,RBFOX2,FBXO31,PHACTR1,DCLK1,TRPC5,LRRK2,SEMA4D,GSK3B,EPHB1,SIPA1L1,FYN,SULT4A1,HECW1,DCDC2,PTPRD,KALRN,TANC2,PREX2,NRP1,DNM3,CUX1,CHRNA3,RELN,PPP3CA,KLF7,DOCK10,ELAVL4,NLGN1,MAPK8IP2,KIDINS220,PPFIA2,LRP4,CDKL5,IL1RAPL1,RAPGEF2,UBE3A,EPHB2,MEF2A,MAP2,BHLHB9,SEMA3A,PDLIM5,PAK3,DTNBP1,LRP8,VLDLR,ABI1
GO:0034765	regulation of ion transmembrane transport	0.000017482929573183124	ASIC2,CACNA1E,KCNJ6,ANK3,KCNQ1,NRXN1,ANK2,KCND3,DPP10,SCN8A,KCNE1,KCNJ15,KCNG3,SHISA6,CFTR,PM20D1,RASGRF1,UTRN,AGT,SHISA9,CYBB,KCNH1,KCNMA1,KCNK17,DMD,HCN1,CACNA2D3,CACNG3,FGF14,GSG1L,DPP6,CNIH3,RYR2,CATSPER2,DHRS7C,KCNH5,CHD7,KCNK13,SLC8A1,PRKD1,KCNC1,AKAP6,KCNJ3,SLC30A5,FYN,KCNE2,HECW1,PDE4D,ACTN2,KCNS3,KCNIP4,KCND2,SCN11A,ARL6IP5,PLN,PRKCE,CAPN3,KCNC4,APP,GOPC,PDE4B,COMMD1,CACNA1C,KCNJ12,CACNA2D1,CACNA2D4,CLIC6,IFNGR2,RASGRF2,CACNG2,RGS7,KCNQ5,RELN,GRM5,KCNB2,STIM2,NLGN1,AKAP7,MAPK8IP2,CACNA1D,TPCN2,NOS1,TNF,TMC2,KCNK10,TSPAN13,KCNA6,KCNH8,TRDN,SCN1A,KCNH7,KCNAB1,FGF13,E

			PHB2,DAPK1,CACNG6,P2RX1,KCNE4,P2RX7,BDKRB1,THADA,GNB5,HCN4,RNF207,ACTN4,FGF12,KCNJ16,STAC,CHRM3,CALCR,CACNB2,NOS1AP,FMR1,JPH4,FHL1,CACNA1A,SCN9A,STIM1,KCNC2,CASQ2,UBASH3B,RGN
GO:0007264	small GTPase mediated signal transduction	0.0000199435797890263	DAB1,CDC42EP3,AKAP13,DOCK2,CDH13,NRG1,PLCE1,KSR2,TIAM1,RHOC,RASGRF1,SCAI,RGL2,RHOJ,NTN1,ITSN1,TRIO,MYO9A,SLIT2,RAPGEF5,SIPA1L3,CELSR1,SH3BP1,PRKD1,OGT,TGFB2,VAV3,WASF1,ARHGAP8,ARHGAP12,RALBP1,ARHGAP24,SIPA1L1,ARHGEF10,PSD3,CHN1,KANK1,ARHGEF3,RERG,GRAP2,RASGEF1B,TIAM2,KALRN,CCDC88C,DLC1,ROBO1,PREX2,TGM2,RAB30,ARHGAP15,NRP1,ARHGEF18,ELMO1,KPNB1,RALGAPA2,DOCK9,OPHN1,CCDC88A,RIN2,DNMT1,MAPRE2,RASGRP1,STARD13,RABGEF1,RGL1,DOCK4,RAP1A,RAPGEF1,RASGRF2,RALGPS1,NF1,RALGPS2,ARHGAP6,LZTR1,RELN,RALA,ADCYAP1R1,DGKI,DOCK10,GPSM2,ARHGAP5,ARHGAP39,DOCK1,RUFY1,EPS8,DEF6,NET1,ARHGEF11,ARHGAP11B,SIPA1L2,CTNNAL1,RAPGEF2,DNMBP,EPHB2,RAPGEF6,ABL2,F11R,FARP2,ARHGAP35,CD2AP,PREX1,ARHGAP25,FGD4,GARNL3,AUTS2,RAPGEF4,RASA3,ARHGAP10,SRGAP3,GPR55,ITGA3,G3BP2,RALGAPA1,RALGDS,ARHGAP28,CHM,RRAGC,ARHGAP42,GNA13,RIT2,DOCK7,G3BP1,AMOT,DOCK3,RAP1B,KSR1,NGF,SOS1,RREB1
GO:0016055	Wnt signaling pathway	0.000022623154442051518	MAPK14,LYPD6,WLS,NPHP3,ZRANB1,RAB5A,CTNND2,NXN,CSNK2A1,GPC3,PSMB2,SHISA6,TIAM1,NKD1,CDH2,PARK2,GPC6,ZNRF3,GPC5,CELSR1,WWOX,LRRK2,PRICKLE2,DISC1,GSK3B,LGR5,SEMA5A,RBMS3,RYK,ANKRD6,VANGL2,NR4A2,SMARCA4,HECW1,RNF213,FOXO1,KANK1,DCDC2,GRK5,CSNK1G3,ROR1,EPM2A,CTNNBIP1,FOXO3,MITF,CCDC88C,EDNRB,STK3,EDA,APP,LGR4,GRK6,TGFB1I1,TCF7L2,TRABD2B,CCDC88A,RNF43,GLI3,CPE,RAPGEF1,WIF1,BRD7,PSMA1,MAGI2,BICC1,IFT80,NDRG2,NLK,NPHP4,JRK,WWTR1,GPC4,PTPRU,LRRK1,DDX3X,SMAD3,PRDM15,LGR6,DAAM2,SULF1,LRP4,AMOTL1,ALPK2,GSKIP,MLLT3,EGF,PTPRO,LRP5,RNF138,SULF2,SOX4,DDB1,TNKS,SOST,AMFR,PRKAA2,TLE4,CDC73,MARK2,FERMT2,MARK1,TNN,DAB2,DAAM1,ITGA3,PPM1B,PIN1,TRPM4,MACF1,PSMB7,SKI,BTRC,EXT1,FBXW11,G3BP1,SCEL,TBL1X,APC,VPS35,TBX18
GO:0198738	cell-cell signaling by wnt	0.000029453380023739894	MAPK14,LYPD6,WLS,NPHP3,ZRANB1,RAB5A,CTNND2,NXN,CSNK2A1,GPC3,PSMB2,SHISA6,TIAM1,NKD1,CDH2,PARK2,GPC6,ZNRF3,GPC5,CELSR1,WWOX,LRRK2,PRICKLE2,DISC1,GSK3B,LGR5,SEMA5A,RBMS3,RYK,ANKRD6,VANGL2,NR4A2,SMARCA4,HECW1,RNF213,FOXO1,KANK1,DCDC2,GRK5,CSNK1G3,ROR1,EPM2A,CTNNBIP1,FOXO3,MITF,CCDC88C,EDNRB,STK3,EDA,APP,LGR4,GRK6,TGFB1I1,TCF7L2,TRABD2B,CCDC88A,RNF43,GLI3,CPE,RAPGEF1,WIF1,BRD7,PSMA1,MAGI2,BICC1,IFT80,NDRG2,NLK,NPHP4,JRK,WWTR1,GPC4,PTPRU,LRRK1,DDX3X,SMAD3,PRDM15,LGR6,DAAM2,SULF1,LRP4,AMOTL1,ALPK2,GSKIP,MLLT3,EGF,PTPRO,LRP5,RNF138,SULF2,SOX4,DDB1,TNKS,SOST,AMFR,PRKAA2,TLE4,CDC73,MARK2,FERMT2,MARK1,TNN,DAB2,DAAM1,ITGA3,PPM1B,PIN1,TRPM4,MACF1,PSMB7,SKI,BTRC,EXT1,FBXW11,G3BP1,SCEL,TBL1X,APC,VPS35,TBX18
GO:0035637	multicellular organismal signaling	0.000029605506304703138	ANK3,ITGA2,KCNQ1,TNNI3K,ANK2,KCND3,SCN8A,KCNE1,RYR3,AGT,CHRM5,CACNG3,CTNNA3,RYR2,SLC8A1,CNTNAP2,KCNJ3,ABCC9,KCNE2,PDE4D,GRIK2,KCND2,SCN11A,PKP2,ATP1A1,PLN,NTRK2,CACNA1C,TNR,CACNA2D1,GLRA1,CACNG2,CXADR,CACNA1D,TRDN,SCN1A,MYH14,NUP155,P2RX1,KCNE4,MEF2A,HCN4,RNF207,FGF12,TRPM4,CACNB2,NFASC,FMR1,CORIN,SCN9A,TYMP,CASQ2,SPTBN4,TBX18
GO:1901888	regulation of cell junction assembly	0.000029941710445904082	ASIC2,DUSP22,NRXN1,SYNDIG1,FLRT2,LRFN5,IL1RAPL2,ROBO2,EPHA3,AGT,NEGR1,NTN1,GPC6,VCL,NTRK3,SEMA4D,LINGO2,CLSTN2,FMN1,BDNF,PRKCH,CNTNAP2,EPHB1,EPHA7,THBS2,PTPRD,LRRTM1,DLC1,KDR,APP,NRP1,NTRK2,CLDN1,RAP1A,PEAK1,RAPGEF1,GRID2,EFNA5,ARHGAP6,LRRTM3,NPHP4,IL1RAP,GPC4,NLGN1,SMAD3,TNF,DLG5,CLSTN1,IL1RAPL1,RAPGEF2,EPHB2,SLITRK6,F11R,WDPCP,BHLHB9,PDLIM5,PTPRA,FERMT2,CLASP1,MACF1,RAP1B,CLASP2,VPS35
GO:0007167	enzyme linked receptor protein signaling pathway	0.0000350627221255 9739	PLCB1,MAPK14,PTPRT,SVEP1,DUSP22,EPHA6,NRXN1,SHC3,DOK5,NRG3,TGFBR3,FSTL4,CDH13,TF,TMEM108,FLRT2,GPC3,NRG1,PLCE1,TIAM1,LDLRAD4,ALK,PTPRE,RAB7A,ARID5B,EPHA3,LEMD3,AGT,TRIO,HTRA1,PDCD6,STXBP4,IGF1R,SMAD1,TMPRSS6,PTPRG,FSHR,NTRK3,CD8B,ANKS1A,RNF165,GSK3B,PRKD1,OGT,TGFB2,ANKS1B,BDNF,RYK,ZDHHC17,WASF1,SIK2,PML,FBN1,EPHB1,SIPA1L1,LRP2,TMEM100,EPHA7,FYN,CHN1,NEU3,FOXO1,KANK1,TNMD,ROR1,CTDSPL2,BMPER,PTPRD,DSG4,PRKCB,KALRN,ROBO1,FLT1,MAP3K7,KDR,NRP1,ADAM17,IGF2R,ATF2,EFEMP1,ROS1,TGFB1I1,RPS6KA5,CCDC88A,FAM83B,CHRDL1,RNF126,GUCY2F,GHR,NTRK2,F

			AM83G,RABGEF1,SPRED2,KIF16B,TGFBR1,EPHA5,LCP2,NCOA5,CHST11,RAPGEF1,CHRNA3,EP300,GAB2,COL4A6,EFNA5,MERTK,GUCY2C,MAGI2,NF1,SORL1,DMRT1,NLK,SNX25,WWTR1,KL,CBLB,DOK6,PTPRU,BTBD11,VEPH1,JAK2,AHI1,SMAD3,BAIAP2L1,OVOL2,SNX5,BMP15,SHC2,PMEPA1,LTBP1,SMOC2,SULF1,PIK3R3,EFNB2,ZNF423,CD109,EGF,KIT,VWC2,EFNB1,RAPGEF2,EPHB2,ERBB4,FER,SHCBP1,PTPRK,FGF7,SPRED3,PSG9,SULF2,NEDD9,PDGFD,PIK3CD,ITGA8,PLAT,FGFR1,SOST,NTF3,AR,BDKRB2,ZMIZ1,FLT3,PTPRA,TRIM72,FERMT2,SMPD3,DAB2,FGF12,ITGA3,NREP,PIN1,BMP6,TGFA,HGF,SCUBE3,INSR,PAK1,SKI,ENPP1,IDE,PPARA,SORT1,FUT8,SPRED1,PRDM16,PTPRF,ZNF451,PTPRR,EXT1,PRLR,TDGF1,PRKCQ,STAT3,PAK3,PIK3R2,BLNK,MAPKAPK2,APC,BMPR1A,ITGA1,KIAA0319,NGF,UBASH3B,MTMR4,PILRB,ABI1,GPR21,CLASP2,RGMB,SOS1,FGF1,TBX20
GO:0001655	urogenital system development	0.000037198480938524965	*NPHP3,TNC,GPC3,ALDH1A2,PLCE1,ARID5B,ROBO2,AGT,GREB1L,SLIT2,SMAD1,ADAMTS6,BASP1,LRRK2,KIF26B,LGR5,TGFB2,FMN1,FBN1,BCL2,LRP2,NFIA,VANGL2,EPHA7,MME,SERPINB7,RARB,BMPER,CTNNBIP1,SOX8,IFT88,HELLS,EDNRB,TRAF3IP2,PKHD1,NRP1,LGR4,FRAS1,ADAMTS16,GLI3,TGFBR1,CYP7B1,PCSK5,TP73,MAGI2,NF1,BICC1,TTC8,ASXL1,KIRREL3,JAG1,WWTR1,AHI1,SMAD3,RDH10,DLG5,FREM2,SULF1,LRP4,EFNB2,DYNC2H1,CC2D2A,PTPRO,ESR1,UBE3A,PLAG1,EPHB2,ERBB4,AP2B1,SULF2,SOX4,PDGFD,ITGA8,ENPEP,WDPCP,AR,EYA1,PBX1,ITGA3,BMP6,CENPF,ZBTB16,PRKX,C1GALT1,EXT1,PRLR,UMOD,KLHL3,TP63,UPK3A,CTSH,IL6R,FGF1,DCHS2,RGN,TBX18*
GO:0043269	regulation of ion transport	0.0000404005790794 4521	*ASIC2,CACNA1E,KCNJ6,ANK3,KCNQ1,NRXN1,ANK2,KCND3,DPP10,TF,SCN8A,KCNE1,KCNJ15,SYT17,KCNG3,SHISA6,CCR1,CFTR,PM20D1,RASGRF1,UTRN,AGT,SHISA9,PARK2,CYBB,KCNH1,KCNMA1,SYT9,KCNK17,PXK,DMD,HCN1,SYT1,CACNA2D3,CACNG3,FGF14,GSG1L,DPP6,CNIH3,RYR2,CATSPER2,DHRS7C,KCNH5,CHD7,KCNK13,SLC8A1,PRKD1,KCNC1,NKAIN2,PML,AKAP6,BCL2,KCNJ3,SLC30A5,NKAIN3,FYN,KCNE2,HECW1,RCVRN,PDE4D,ACTN2,KCNS3,KCNIP4,KCND2,SCN11A,CHRM1,PKP2,ARL6IP5,ATP1A1,PLN,PRKCE,EFHB,CAPN3,KCNC4,CASK,APP,GOPC,ROS1,ABAT,PDE4B,ACSL4,COMMD1,CACNA1C,KCNJ12,IL16,CHRNA3,CACNA2D1,CACNA2D4,CLIC6,IFNGR2,RASGRF2,ADORA2A,CAMK2G,CACNG2,RGS7,KCNQ5,RELN,GRM5,ADCYAP1R1,KCNB2,PER1,CNTN1,GRM7,STIM2,NLGN1,AKAP7,MAPK8IP2,CACNA1D,TPCN2,NOS1,TNF,TMC2,KCNK10,TSPAN13,KCNA6,KCNH8,TRDN,SCN1A,KCNH7,EGF,KCNAB1,FGF13,EPHB2,DAPK1,CACNG6,CCL3,P2RX1,KCNE4,P2RX7,PLA2G4A,BDKRB1,THADA,GNB5,HCN4,RNF207,SYT2,ACTN4,FGF12,KCNJ16,KMO,STAC,CHRM3,MYLK,CALCR,CACNB2,NOS1AP,FMR1,CD84,JPH4,FHL1,CACNA1A,SCN9A,STIM1,KCNC2,CASQ2,DTNBP1,PTGES,UBASH3B,MAP2K6,SPTBN4,RGN*
GO:0050803	regulation of synapse structure or activity	0.0000424749132353 6258	*MAPK14,ASIC2,NRXN1,SHANK2,SYNDIG1,FLRT2,LRFN5,ABHD17C,CTNNA2,IL1RAPL2,ROBO2,CDH2,FRMPD4,ZNF804A,NEGR1,NTN1,GPC6,CDH8,LRRK2,NTRK3,DISC1,SEMA4D,LINGO2,CLSTN2,BDNF,GRIN2B,LRFN2,EPHB1,SIPA1L1,EPHA7,FYN,THBS2,PTPRD,LRRTM1,KALRN,TANC2,APP,DGKB,NTRK2,DNM3,GRID2,EFNA5,RELN,LRRTM3,IL1RAP,GPC4,NLGN1,CTTNBP2,TNF,PPFIA2,DLG5,LRP4,CLSTN1,CDKL5,IL1RAPL1,PTPRO,UBE3A,EPHB2,SLITRK6,ARF4,BHLHB9,PDLIM5,PAK3,DTNBP1,LRP8,VPS35*
GO:0003015	heart process	0.0000451233032051 4802	*AKAP13,KCNQ1,TNNI3K,CELF2,ANK2,KCND3,KCNE1,RYR3,AGT,RPS6KA2,MDM2,DMD,CTNNA3,RNLS,RYR2,SLC8A1,TGFB2,SGCD,EXT2,SGCG,KCNJ3,ABCC9,FYN,KCNE2,PDE4D,SLC1A1,MTOR,PKP2,ATP1A1,PLN,EDNRB,NOX4,TACR3,THRB,HDAC4,PDE4B,CACNA1C,KCNJ12,CACNA2D1,CXADR,WWTR1,CACNA1D,JAK2,SHOX2,NOS1,TNF,ADORA3,TRDN,SCN1A,FGF13,NUP155,SGCZ,KCNE4,MEF2A,ADRA1B,SEMA3A,PDE5A,HCN4,RNF207,FGF12,TRPM4,CACNB2,NOS1AP,TTN,CORIN,MYH7,EXT1,MYH6,CASQ2,ASB3,MAP2K6,SPTBN4,TBX18*
GO:0099173	postsynapse organization	0.0000479935291309 1713	*GPHN,NRXN1,CTNND2,TMEM108,SHISA6,ABHD17C,CDH2,FRMPD4,ZNF804A,IGF1R,LRRK2,NTRK3,MTMR2,GRIN2B,LRFN2,EPHB1,SIPA1L1,LRRC4,EPHA7,FYN,PTPRD,NLGN4X,KALRN,TANC2,DGKB,NRP1,OPHN1,DNM3,GRID2,RELN,IL1RAP,DOCK10,NLGN1,ARHGAP39,PPFIA2,TANC1,ARF1,LRP4,CDKL5,IL1RAPL1,UBE3A,EPHB2,ARF4,BHLHB9,PDLIM5,ADAM10,ITGA3,CNKSR2,NOS1AP,INSR,PAK3,DTNBP1,LRP8,VPS35*
GO:0072001	renal system development	0.0000510223795299 7825	*NPHP3,GPC3,ALDH1A2,PLCE1,ARID5B,ROBO2,AGT,GREB1L,SLIT2,SMAD1,ADAMTS6,BASP1,LRRK2,KIF26B,LGR5,TGFB2,FMN1,FBN1,BCL2,LRP2,NFIA,VANGL2,EPHA7,MME,SERPINB7,RARB,BMPER,CTNNBIP1,SOX8,IFT88,HELLS,EDNRB,TRAF3IP2,PKHD1,NRP1,LGR4,FRAS1,ADAMTS16,GLI3,TGFBR1,PCSK5,TP73,MAGI2,NF1,BICC1,T*

			TC8,ASXL1,KIRREL3,JAG1,WWTR1,AHI1,SMAD3,RDH10,DLG5,FREM2,SULF1,LRP4,EFNB2,DYNC2H1,CC2D2A,PTPRO,ERBB4,AP2B1,SULF2,SOX4,PDGFD,ITGA8,ENPEP,WDPCP,EYA1,PBX1,ITGA3,BMP6,CENPF,ZBTB16,PRKX,C1GALT1,EXT1,UMOD,KLHL3,UPK3A,CTSH,IL6R,FGF1,DCHS2,RGN,TBX18
GO:0048519	negative regulation of biological process	0.00005132781057345126	LINC00273,ISM1,FANK1,CAST,SRGAP2B,TPTE,ZHX3,PLCB1,CMKLR1,DAB1,OMA1,DUX4,GTF2I,MAPK14,ABCG8,DSCAM,TPTE2,MIR663A,RUNX1,ASIC2,CTBP2,SERPINA1,ANXA8L1,PTPRT,MIR1185-1,MIR1185-2,MIR134,MIR154,MIR300,MIR323B,MIR376C,MIR381,MIR382,MIR485,MIR487A,MIR487B,MIR539,MIR544A,MIR654,MIR655,MIR889,ANK3,DCC,TIMP3,ZNF397,DUSP22,FBLN1,SLC24A3,NPHP3,ABCG1,RGS6,KCNQ1,DPF3,ATF7IP,GRIN3A,NRXN1,MIR369,MIR410,MIR656,PAX7,MTPN,SHANK2,GRIA1,NRG3,SMYD2,CLEC16A,HUS1,TGFBR3,FSTL4,CDH13,RNF152,ANK2,RTN1,NXN,RUNX1T1,CSNK2A1,ERCC4,KCNE1,SAMD4A,MIR99A,MIRLET7C,EIF4EBP3,CHEK2,GADD45A,PRDM9,PSMC6,ACIN1,PLXNA4,TOX3,GPC3,NRG1,STXBP6,ETS2,PSMB2,SHISA6,RFFL,IL4R,ALDH1A2,LRFN5,RYR3,MCTP1,MIR17HG,SUZ12,ASTN2,SH3RF2,SEMA6D,SPOCK1,ABHD17C,CTNNA2,CCR1,RBFOX2,MIR551B,PCTP,PM20D1,AGBL4,BEND5,LDLRAD4,DNAJC15,RGS7BP,ZNF91,ZBTB20,ARFIP1,CBFA2T2,NKD1,SCAI,MX1,ALK,PTPRE,SORCS3,ZNF536,FBXO31,RAB7A,CNTN4,RGL2,ARID5B,ROBO2,SPOCK3,ZNF366,EPHA3,PRKACB,DCLK1,PXDN,AJAP1,LEMD3,AGT,CST2,MAGEB3,AVEN,RCAN1,CDH2,PARK2,BANP,FHL2,FOXN3,LOXL2,CDC5L,KIR2DL4,KIR3DL2,BPIFB1,CORO2B,PSPC1,SEMA3D,DACH1,NTN1,TP53I11,TRIM22,TRIM5,USP25,ZNF268,CP,NELL1,TSPAN8,PCDH17,PHC1,ZNRF3,KCNMA1,RIN3,TRIO,FTO,HDAC2,HTRA1,LAMA4,SLIT2,TFAP2D,ZBTB34,KCTD1,HSA-MIR-490,PDCD6,RPS6KA2,LCMT1,IGF1R,MDM2,SLC24A4,SMAD1,E2F3,NOVA1,TMPRSS6,CEP97,PTPRG,PXK,SACS,TRPC5,DMD,STK33,BASP1,VCL,FSHR,GRIA4,FMN2,HNRNPA2B1,MAEL,NFE2L3,NRIP1,PRKAR2A,SORCS2,INPP5A,STK24,WWOX,ATRX,FRMD5,PARP16,TRPM2,RNLS,RYR2,DHRS7C,LINC00472,LRRK2,MIR495,MIR543,RORB,IGFBP7,NTRK3,RORA,IQCJ-SCHIP1,CHD7,OVGP1,PAN3,GLIS1,SH3BP1,SOX6,MECP2,MTMR2,SEMA4D,GSK3B,SLC8A1,PRKD1,SERPINA4,SERPINA5,ERCC6,OGT,TGFB2,PRAMEF12,PIWIL4,RIPPLY3,S100A11,SEMA5A,BDNF,PRKAR1B,PRKCH,RBMS3,RYK,ABI3BP,GRIN2B,KDM4C,OTUD7A,RAG1,RAG2,SAMD13,ANKRD6,NR2F2,PTGFR,PCBP3,PML,PPARGC1A,USP36,ARHGAP12,AKAP6,FBN1,PPP4R4,GRIK3,NLRP7,PLXNA2,TAOK3,BCL2,CHMP4C,MXI1,ZNF675,ARHGAP24,EPHB1,MOV10L1,SMARCE1,SKAP2,KLF12,LRP2,FRMD4A,ITPR2,SND1,DEPTOR,NR4A2,EPHA7,MIR648,ZBTB7C,DPT,RBAK,FYN,KCNE2,PARN,ADAMTS18,NR4A1,SMARCA4,CD44,HECW1,NEU3,PMP22,RHBDD1,RNF213,THBS2,FOXO1,PDE4D,RBBP8,RNF144B,KANK1,ACTN2,ATP8A2,BACH1,CST9L,GRK5,KLF13,PACRG,TNMD,GRIK2,CELF4,SERPINB7,APBB2,EIF3E,SLC1A1,ZFPM2,ZNF141,GLIS3,RARB,RERG,RFX4,ETV6,GNG4,NLRP12,MFSD12,MTOR,RMI2,LDB2,MOV10,TMPRSS4,CTDSPL2,FAT3,PKP2,UBXN2B,BMPER,PTPRD,SCAF8,BBS2,PGK1,PLCL1,ARL6IP5,ATP1A1,EPM2A,PLN,CTNNBIP1,SOX8,ZMYND11,MIR491,NLGN4X,PRKCE,EIF2S1,HELLS,KMT2A,PRKCB,VDR,ADCY8,FOXO3,IGF2BP3,LRRTM1,MITF,SCMH1,CAPN3,KALRN,TTLL12,CCDC88C,DLC1,EDNRB,MSR1,OASL,ROBO1,SATB2,TBC1D4,BNIP3L,FAP,FLT1,NOX4,ZNF148,CENPV,GRHL2,TSC22D3,COL2A1,NBAS,NR3C1,TRAF3IP2,CR1,EYA4,SPON1,MDM1,PKHD1,STK3,KDR,PPHLN1,CASK,CHL1,ABCA12,APP,CALCRL,COL18A1,NRP1,PTPRM,UACA,ARHGEF18,LGR4,LINC00473,SERPING1,TENM2,ADTRP,KAT5,THRB,CPEB2,KCNK2,SEMA3C,ADAM17,CAMK1D,DIS3L2,TFPI,ATF2,ETS1,NR2C2,SAMSN1,EFEMP1,GOPC,PRTG,ROS1,SMG6,TGFB1I1,DOT1L,RGS16,RPS6KA5,TCF7L2,TRABD2B,AFF2,BICD1,PARD3,TLK2,ZNF830,ABAT,GRIN2A,MIR183,MIR96,KIFAP3,OPHN1,BCLAF1,TRPS1,ZNF568,CD38,DENND5A,RNF43,CHRDL1,RNF126,SMG7,BCL2L1,HDAC4,MYO16,PDE4B,TIMP2,BACE2,DNMT1,GHR,JAM2,MIR605,NTRK2,PRKG1,ACSL4,DNM3,BRD4,STARD13,ARR3,COMMD1,RABGEF1,SPRED2,GLI3,SMCHD1,ZNF207,CACNA1C,MORC2,DOCK4,RAP1A,TGFBR1,PRDX4,DICER1,TNR,CUX1,NCOA5,CHST11,MAGEA11,PTTG1IP,RAPGEF1,CD96,EP300,MORC3,HMG20A,SCFD1,TBX3,TTC39B,WIF1,CYP7B1,GRAMD4,PRAMENP,TRIOBP,ATXN1,BRD7,GLRA1,TP73,GRID2,NPC1,PIBF1,PSMA1,FHIT,ADCK1,ADORA2A,ADRBK2,EFNA5,MERTK,TAC4,MAGI2,NF1,RGS7,TFF1,SORL1,STRN3,ARHGAP6,BICC1,BID,ELK3,IFT80,TRIM59,TTC8,DMRT1,LZTR1,ASXL1,EIF4E,POR,TNFRSF11B,GRM5,HDAC9,IL18R1,IL1RL1,PTPN9,MGAT5,NDRG2,NUMB,RHPN2,SP2,TNFAIP8,NLK,NPHP4,TBX15,ADCYAP1R1,NAV3,JAG1,MEOX2,P

			PP3CA,KLF3,KLF7,MYT1L,SH3GL2,SNX25,CAPZB,DGKI,MAS1,PER1,SATB1,SLC24A2,WWTR1,ANKRD13A,CBLB,ELAVL4,MAGEB2,MNAT1,PLSCR1,ATP9A,DGUOK,GRM7,TMEM67,GABPA,KAT7,L3MBTL3,MIR105-1,MIR105-2,MIR767,ZNF540,ASAP1,FHOD3,N4BP2L2,NLGN1,PTPRU,THAP7,AKIRIN2,RNF128,RPS20,NPR3,SYCP2,LRRK1,MAPK8IP2,VEPH1,DDX3X,LRPPRC,RBL2,CNOT7,GABRA5,TPCN2,DNMT3B,JAK2,SHOX2,AHI1,SMAD3,CD300A,MAP2K4,NOS1,SERPINB12,PLXNB1,ZNF337,AATF,CDK1,DCBLD2,HDAC7,MIR320B2,NBN,SLC39A10,TAGLN3,TNF,LRCH1,OVOL2,SNX5,TNIP1,ZFPM1,EIF3A,ILF3,POMC,PRDM15,ADNP2,DLG5,SCAF4,SIM2,ARID2,MLIP,BRINP1,FAM220A,PRG3,SRSF4,ADORA3,ARF1,BCL11A,CLEC4A,HMGA2,POLR1A,YAF2,ZNF705A,AGO3,GCLC,PMEPA1,RAB23,EPS8,LTBP1,DAAM2,L3MBTL4,SULF1,CCP110,LRP4,WDR5,EFNB2,MXD3,PIWIL3,TRDN,HERC1,PHF8,ZNF423,CDC14B,ALPK2,CTNNA1,TENM1,TSHZ3,ZNF93,GSKIP,MIR765,PRKAR1A,ZNF345,BPTF,MLLT3,CD109,EGF,PTPN14,ALDH1A1,FOXP4,GBE1,IL1RAPL1,KIT,SLIT3,TNRC6B,UBR2,BLM,TRPV4,VWC2,ABCA5,KCNAB1,PTPRO,RAPGEF2,DNAJB6,ESR1,HTR2C,SEMA3E,UBE3A,FGF13,GABRB2,MARVELD3,PLAG1,EPHB2,MECOM,NDFIP2,TBCD,TFDP2,DAP,DAPK1,ERBB4,FER,MAP3K3,SPTA1,TDRKH,FNIP1,LRP5,MALRD1,ZNF439,RPS27L,SLAMF1,ABL2,DEPDC5,F11R,GAS8,VPS13C,AP2B1,ARHGAP35,CD2AP,COMMD7,NSUN2,PTPRK,CCL3,SEMA5B,SPRED3,TNFAIP8L2,ARHGAP25,BTBD17,GCFC2,PSG9,SULF2,ARF4,MYH9,PACSIN2,PTGER3,SOX4,ST18,SPTB,AGTPBP1,DNM3OS,KCNE4,MIR214,PUM1,ARID4A,DDB1,FAM172A,KDM3A,PLAT,SERTAD2,TNKS,BCL7A,DAPL1,MEF2A,OCLN,PARP15,PDE11A,RGS3,RYR1,ZCCHC17,ZNF85,DAD1,MAP2,SLX1B,SOST,ASXL3,IL23R,NTF3,P2RX7,TEX14,RASA3,AMFR,APIP,AR,BCOR,FGL2,PVT1,SCAMP5,SUFU,TRERF1,UPK3B,EYA1,N4BP1,RBM4,SRPK2,BDKRB1,BDKRB2,BHLHB9,DGKZ,L3MBTL1,MIR127,MIR136,MIR431,MIR432,MIR433,OGN,PI3,PRKAA2,SEMA3A,TAX1BP1,THADA,TLE4,ZNF202,ZNF558,ARHGAP10,CDC73,GNB5,MEIS2,PDE5A,PROS1,ZNF19,ZNF440,NFIC,PPARGC1B,SRGAP3,TRIM72,FERMT2,MAP2K5,MARK1,SMPD3,TNN,DAB2,GPR55,HDAC8,PBX1,ACTN4,ADAM10,EEF1E1,ITGA3,PRAMEF8,TXNDC5,FANCB,FRY,MT1F,PLCL2,PPM1B,NUP153,PIN1,RPS6KA6,TRPM4,ERLIN1,STK38,ZNF674,ABCC2,BMP6,CIT,CLASP1,EIF4ENIF1,CDYL2,CFDP1,NCOA2,TGFA,TMOD1,CREM,HGF,PKP1,POU2F1,RGS12,CALCR,CENPF,MIR489,MIR653,SH3BP5,ZBTB16,CTIF,KDM4B,MIR556,ARHGAP28,LARP4B,PAK1,PDE3A,PSMB7,SKI,AKT3,CTDP1,ELF1,ENPP1,FMR1,LIMK2,LITAF,MIR361,CHFR,DKC1,JDP2,PHC2,ABCD1,BMF,CRMP1,MAD1L1,ARHGAP42,CD84,JPH4,PPARA,SORT1,TBX22,FAM3D,FHL1,GNA13,PPP1R13B,TAF1,CHD5,FNIP2,S1PR3,SPRED1,CHSY1,ESR2,KRT4,PRDM16,QSOX1,RBL1,ZNF451,BTRC,CCDC3,HIRA,OPTN,PTPRR,RIT2,MDM4,PRKCG,PRLR,UMOD,ZNF354B,ADAMTS5,CBFA2T3,DOCK7,FBXW11,G3BP1,HDAC5,TDGF1,A2M,MYEF2,PI15,SERPINB8,SLC24A5,SLC40A1,ADARB1,PRKCQ,STAT3,SUSD4,TFEC,TP63,USP7,AMOT,BRIP1,CDKN3,CST1,HPN,PIK3R2,TBL1X,USH2A,CASQ2,DHRS2,DTNBP1,GAK,RAP1B,LGALS9,GTPBP1,MAPKAPK2,AOAH,APC,BMPR1A,DPP4,ITGA1,KIAA0319,NGF,PRKCA,PTGES,SPINT2,UBASH3B,ADAMTS20,ITIH6,MTMR4,OSGIN2,PDS5A,ABI1,CTSH,DHX9,NCAPG2,RGCC,RPH3AL,SRGAP1,USP18,GPR21,IL6R,MAP2K6,SIAH3,TFIP11,SPTBN4,CLASP2,MAP2K1,PRAMEF7,TPH1,VPS35,ZBTB25,ZRANB3,C1QTNF3,COL6A3,MCOLN3,RAD18,TBX20,C6ORF106,CRADD,NLRC5,RGN,RREB1,TBX18
GO:0042330	taxis	0.0000598372609790643	CDH4,CMKLR1,MAPK14,DSCAM,DCC,ITGA2,EPHA6,NRXN1,NRXN3,DOCK2,NRG3,CDH13,PLGRKT,ADAMTSL1,PLXNA4,FLRT2,NRG1,UNC5D,SEMA6D,CCR1,CCR3,DPYSL2,CNTN4,ROBO2,EPHA3,LAMA2,SEMA3D,NTN1,RIN3,TRIO,SLIT2,TRPM2,NTRK3,RNF165,EMB,SEMA4D,PRKD1,TGFB2,VAV3,SEMA5A,BDNF,RYK,LAMA3,PLXNA2,RALBP1,EPHB1,EPHA7,FYN,CHN1,NR4A1,ENPP2,UNC5C,PALLD,FAM19A4,KALRN,EDNRB,ROBO1,FLT1,KDR,CHL1,APP,NRP1,PTPRM,SEMA3C,ADAM17,CAMK1D,PRTG,RPS6KA5,OPHN1,ALCAM,PDE4B,GLI3,DOCK4,SRP54,CYSLTR1,EPHA5,TNR,IL16,EP300,DSCAML1,CYP7B1,EFNA5,TTC8,RELN,RALA,KLF7,CXADR,SMAD3,PLXNB1,LGR6,SMOC2,C5AR1,CNTN6,EFNB2,KIT,SLIT3,PIK3C2G,TRPV4,EFNB1,PTPRO,MYPN,SEMA3E,EPHB2,ABCC1,FER,SLAMF1,BSG,ARHGAP35,CCL3,FGF7,LAMA1,PREX1,SEMA5B,ITGA9,PDGFD,PIK3CD,FGFR1,NTF3,SEMA3A,STX3,ADAM10,CCL7,LGI1,LECT2,TRPM4,HGF,NCAM1,NFASC,CRMP1,CCDC141,DEFA1B,EXT1,TYMP,ELMO2,TREM1,PRKCQ,AMOT,LGALS9,DPP4,ITGA1,CCL14,CCL15,CCL15-CCL14,IL6R,MAP2K1,SOS1,FGF1
GO:00069	chemotaxi	0.0000680615217205	CDH4,CMKLR1,MAPK14,DSCAM,DCC,ITGA2,EPHA6,NRXN1,NRXN3,DOCK2,NRG3,CDH13,PLGRKT,ADAMTSL1,PLXNA4,FLRT2,NRG1,

35	s	0448	*UNC5D,SEMA6D,CCR1,CCR3,DPYSL2,CNTN4,ROBO2,EPHA3,LAMA2,SEMA3D,NTN1,RIN3,TRIO,SLIT2,TRPM2,NTRK3,RNF165,EMB,SEMA4D,PRKD1,TGFB2,VAV3,SEMA5A,BDNF,RYK,LAMA3,PLXNA2,RALBP1,EPHB1,EPHA7,FYN,CHN1,NR4A1,ENPP2,UNC5C,PALLD,FAM19A4,KALRN,EDNRB,ROBO1,FLT1,KDR,CHL1,APP,NRP1,PTPRM,SEMA3C,ADAM17,CAMK1D,PRTG,RPS6KA5,OPHN1,ALCAM,PDE4B,GLI3,DOCK4,SRP54,CYSLTR1,EPHA5,TNR,IL16,DSCAML1,CYP7B1,EFNA5,TTC8,RELN,RALA,KLF7,CXADR,SMAD3,PLXNB1,LGR6,SMOC2,C5AR1,CNTN6,EFNB2,KIT,SLIT3,PIK3C2G,TRPV4,EFNB1,PTPRO,MYPN,SEMA3E,EPHB2,ABCC1,FER,SLAMF1,BSG,ARHGAP35,CCL3,FGF7,LAMA1,PREX1,SEMA5B,ITGA9,PDGFD,PIK3CD,FGFR1,NTF3,SEMA3A,STX3,ADAM10,CCL7,LGI1,LECT2,TRPM4,HGF,NCAM1,NFASC,CRMP1,CCDC141,DEFA1B,EXT1,TYMP,ELMO2,TREM1,PRKCQ,AMOT,LGALS9,DPP4,ITGA1,CCL14,CCL15,CCL15-CCL14,IL6R,MAP2K1,SOS1,FGF1*
GO:0040012	regulation of locomotion	0.00007125005461022637	*SRGAP2B,PLCB1,CMKLR1,DSCAM,PTPRT,DUSP22,FBLN1,ITGA2,SUN2,NRG3,CDH13,TF,GADD45A,PLXNA4,FLRT2,NRG1,RFFL,MCTP1,SH3RF2,UNC5D,SEMA6D,CTNNA2,CCR1,TIAM1,RHOC,LDLRAD4,NKD1,SCAI,FBXO31,PHACTR1,ROBO2,SPOCK3,AGT,RHOJ,LAMA2,SEMA3D,DACH1,NTN1,ZNF268,RIN3,LAMA4,SLIT2,PDCD6,IGF1R,PTPRG,VCL,STK24,FRMD5,NTNG1,LRRK2,NTRK3,SH3BP1,MECP2,SEMA4D,SLC8A1,PRKD1,TGFB2,FUT4,S100A11,SEMA5A,RYK,LAMA3,NR2F2,PLXNA2,BCL2,KANK1,ENPP2,UNC5C,MTOR,LDB2,CHRM1,BMPER,BBS2,EPB41L4B,PRKCE,FOXO3,MITF,DLC1,ROBO1,FLT1,TACR3,KDR,PRCP,APP,NRP1,PTPRM,ADTRP,SEMA3C,ADAM17,CAMK1D,ELP3,ETS1,RIN2,HDAC4,JAM2,MAPRE2,PRKG1,STARD13,RABGEF1,CLDN1,DOCK4,TGFBR1,IL16,ADORA2A,TAC4,MAGI2,NF1,SORL1,RELN,GRM5,HDAC9,SELE,MGAT5,NUMB,NAV3,JAG1,MEOX2,PPP3CA,DOCK10,PTPRU,JAK2,SMAD3,CD300A,PLXNB1,HDAC7,TNF,LRCH1,DLG5,LGR6,ARID2,ADORA3,DOCK1,SMOC2,SULF1,C5AR1,PIK3R3,EFNB2,AMOTL1,SELP,CTNNA1,EGF,KIT,TRPV4,PTPRO,RAPGEF2,SEMA3E,MARVELD3,EPHB2,ERBB4,FER,MAP3K3,SLAMF1,ABL2,BSG,PTPRK,ATP8A1,CCL3,FGF7,LAMA1,SEMA5B,KIF2A,NEDD9,PDGFD,PIK3CD,WDPCP,FGFR1,NTF3,BDKRB1,SEMA3A,SRGAP3,FERMT2,MAP2K5,SMPD3,TNN,DAB2,STX3,ACTN4,ADAM10,CCL7,ITGA3,PIN1,SYNPO2,CLASP1,HGF,MYLK,CAPN7,INSR,MACF1,PAK1,AKT3,PRKX,ULK4,GNA13,SPRED1,PTPRR,DOCK7,HDAC5,TDGF1,LYVE1,ADARB1,STAT3,AMOT,PAK3,LGALS9,EFCAB1,APC,BMPR1A,DPP4,PRKCA,SPINT2,CTSH,RGCC,SRGAP1,IL6R,CLASP2,VPS35,FGF1,RGN,RREB1*
GO:0050790	regulation of catalytic activity	0.00007487013475332203	*CAST,PLCB1,DAB1,MAPK14,SERPINA1,CCNG2,ANXA8L1,PRIM2,PTPRT,BCL2L13,TIMP3,DUSP22,AKAP13,FBLN1,ITGA2,RGS6,GABBR2,EPHA6,NRXN1,SGSM1,DOCK2,NRG3,FGD6,TBC1D22A,CSNK2A1,GADD45A,PLXNA4,GPC3,NRG1,RFFL,SUZ12,PLCE1,SH3RF2,SPOCK1,TIAM1,RHOC,RASGRF1,ALK,RGL2,PHACTR1,SPOCK3,EPHA3,AGT,PHACTR3,CST2,RCAN1,PARK2,DCUN1D4,TXK,DENND2A,USP6,ITSN1,NCF4,RIN3,TRIO,MYO9A,SLIT2,PDCD6,IGF1R,RAPGEF5,SIPA1L3,FSHR,HNRNPA2B1,PRKAR2A,PARP16,XRCC4,LRRK2,NTRK3,SH3BP1,SEMA4D,GSK3B,LGR5,SLC8A1,PRKD1,SERPINA4,SERPINA5,ERCC6,TGFB2,VAV3,SLC5A3,LLGL2,PRKAR1B,RYK,GRIN2B,RAG1,ARHGAP8,NR2F2,PML,RNF180,ARHGAP12,PPP4R4,NLRP2,NLRP7,PLXNA2,TAOK3,BCL2,RALBP1,RXFP2,ZNF675,ARHGAP24,EPHB1,SIPA1L1,MMP16,DEPTOR,NR4A2,ARHGEF10,EPHA7,PSD3,RPGR,FYN,PARN,CHN1,NR4A1,CD44,RCVRN,ARHGEF3,CST9L,RAP1GDS1,SERPINB7,SLC1A1,AGAP1,NLRP12,MTOR,ROR1,LDB2,RASGEF1B,TIAM2,ARL6IP5,EPM2A,PLN,EVI5,PRKCE,EIF2S1,VDR,ADCY8,CAPN3,KALRN,CCDC88C,DLC1,EDNRB,OASL,ROBO1,RXFP1,TBC1D4,FLT1,NOX4,GRHL2,MAP3K7,PREX2,CR1,STK3,TGM2,KDR,ARHGAP15,PCOLCE2,APP,NRP1,UACA,ARHGEF18,ELMO1,LGR4,SERPING1,CPEB2,ADAM17,MAP3K5,PKP4,TFPI,ELP3,PIK3R5,ROS1,SMG6,RGS16,TNFRSF10B,BICD1,RALGAPA2,DOCK9,GRIN2A,OPHN1,CCDC88A,DENND5A,MOB3B,RIN2,NCF2,TIMP2,GHR,MAPRE2,NTRK2,PRKG1,RASGRP1,STARD13,RABGEF1,RGL1,SPRED2,CACNA1C,STXBP5,DOCK4,RAP1A,EPHA5,LCP2,RAPGEF1,CHRNA3,GRAMD4,PCSK5,PIBF1,RASGRF2,ARHGEF33,PPP1R12B,ADORA2A,CABIN1,EFNA5,FAM20A,MERTK,RALGPS1,MAGI2,NF1,PPP6R2,RALGPS2,RFC3,RGS7,SORL1,ARHGAP6,BID,TTC8,POR,RELN,GRM5,HDAC9,SELE,MGAT5,TNFAIP8,MAP3K13,ADCYAP1R1,EGLN3,TBC1D3B,DGKI,MAS1,TBC1D5,WWTR1,CBLB,DOCK10,MNAT1,PLSCR1,GRM7,RIMS1,GPSM2,SLC22A2,ASAP1,BORA,AKIRIN2,ARHGAP5,RPS20,NPR3,PHACTR2,CACNA1D,DDX3X,RBL2,ARAP2,ZNF622,ARHGAP39,JAK2,SMAD3,CD300A,KIDINS220,NOS1,SERPINB12,PLXNB1,NBN,NVL,SLC39A10,TNF,LRCH1,TRIM23,ADORA3,ARF1,HMGA2,DOCK1,CDC20B,C5AR1,DEF6,P*

			IK3R3,ARHGEF6,SAE1,HERC1,NET1,CDC14B,TENM1,ARHGEF11,CDKL5,GSKIP,PRKAR1A,ARHGAP11B,SIPA1L2,CD109,EGF,ALDH1A1,KIT,MTRR,BLM,PTPRO,RAPGEF2,DNAJB6,ESR1,DNMBP,FGF13,EPHB2,TBCD,DAP,DAPK1,ERBB4,FNIP1,LRP5,RAPGEF6,RPS27L,SLAMF1,ABL2,ASAP2,DEPDC5,F11R,FARP2,ARHGAP35,CCL3,PREX1,ARHGAP25,FGD4,ARF4,ARFGAP3,ST18,CAB39L,GARNL3,NEDD9,P2RX1,PDGFD,RGPD1,UBE2N,TNKS,FGFR1,RGS3,TBC1D9,DAD1,IL23R,NTF3,P2RX7,RAPGEF4,ARRDC4,RASA3,RCAN2,RABGAP1L,DENND5B,DGKZ,NEK5,PI3,ARHGAP10,GNB5,MARK2,PDE5A,PROS1,FLT3,PPARGC1B,SRGAP3,ATP6V1H,DYNAP,FERMT2,MAP2K5,CALML4,DAB2,GPR55,TCL1B,CCL7,ELP4,FRY,PIN1,SYNPO2,STK38,CIT,TGFA,HGF,PRPSAP2,RALGAPA1,RGS12,SH3BP5,NOS1AP,RALGDS,ARHGAP28,INSR,PAK1,CHM,LIMK2,DKC1,SBF2,AIM2,ARHGAP42,DENND2D,SORT1,TNFAIP8L3,TTN,GNA13,WDR41,PPP2R3C,SPRED1,RBL1,BTRC,PPP2R2C,PRLR,DOCK7,PSMD6,TDGF1,A2M,PI15,SERPINB8,STIM1,ADARB1,PRKCQ,STAT3,TP63,AMOT,CDKN3,CST1,DOCK3,PIK3R2,DTNBP1,KSR1,LGALS9,LRP8,APC,BMPR1A,ITGA1,NGF,PPP1R14A,SPINT2,UBASH3B,VLDLR,ITIH6,PILRB,ABI1,CTSH,DHX9,NCAPG2,RGCC,SRGAP1,CCL14,CCL15,IL6R,MAP2K6,RIC8B,TFIP11,MAP2K1,SOS1,COL6A3,FGF1,CRADD,NLRC5,RGN
GO:0071310	cellular response to organic substance	0.0000799980713282013	ADCY2,PLCB1,CMKLR1,GABRB3,MAPK14,ARID1B,CTBP2,PTPRT,TIMP3,DUSP22,ITGA2,KCNQ1,VRK2,NRXN1,DOK5,SLC1A2,TGFBR3,GRAMD1C,KCNE1,TMEM108,CHEK2,FLRT2,GPC3,PSMB2,RFFL,SPIDR,IL4R,ALDH1A2,RYR3,GLP2R,CCR1,CCR3,CFTR,RBFOX2,LDLRAD4,ZBTB20,IL1RAPL2,MX1,ALK,PTPRE,ARID5B,ROBO2,ZNF366,EPHA3,PTPRN2,PXDN,CRNN,LEMD3,AGT,CHRM5,FBXO32,PARK2,CYBB,TRIM5,TXK,RNF185,HDAC2,HTRA1,SLIT2,PDCD6,RPS6KA2,STXBP4,IGF1R,MDM2,SMAD1,TMPRSS6,NR3C2,DMD,HCN1,FSHR,NRIP1,WWOX,PARP16,TRPM2,RYR2,HSPB8,LRRK2,RORB,SLC1A3,IGFBP7,NTRK3,RORA,ILDR1,RNF165,PDXP,SOX6,GSK3B,LGR5,SLC8A1,PRKD1,OGT,TGFB2,BDNF,ZDHHC17,WASF1,KDM4C,EXT2,SIK2,PTGFR,NR5A2,PAQR8,PML,AKAP6,FBN1,NLRP7,BCL2,RXFP2,ZNF675,IL12RB2,LRP2,TMEM100,ITPR2,GDAP1,NR4A2,FYN,GNRHR,NR4A1,SMARCA4,CD44,RHBDD1,CASP6,FOXO1,PDE4D,KANK1,ABCB10,ACTN2,ATF6,GABRB1,MME,PACRG,TNMD,MYLK3,SLC1A1,UGCG,RARB,NLRP12,MTOR,VPS26B,CHRM1,CTDSPL2,BMPER,BBS2,ARL6IP5,ATP1A1,DSG4,IFT88,PRKCE,EIF2S1,HELLS,PRKCB,VDR,ADCY8,FOXO3,RAB31,TTLL12,EDNRB,MSR1,OASL,ROBO1,RXFP1,SATB2,TBC1D4,FLT1,NOX4,COL2A1,MAP3K7,NR3C1,TRAF3IP2,TGM2,KDR,PRCP,CASK,EDA,PCOLCE2,ABCA12,APP,CALCRL,NRP1,LGR4,ADTRP,CD58,KAT5,THRB,CPEB2,ADAM17,MAP3K5,TFPI,ATF2,TGFB1I1,RPS6KA5,TRAF3,BCLAF1,CHRDL1,RNF126,BCL2L1,HDAC4,PDE4B,SOX5,DNMT1,GHR,GLRA2,NTRK2,FAM83G,MGST1,GNG2,LY86,RABGEF1,SPRED2,CLDN1,KIF16B,RAP1A,SAFB2,TGFBR1,EPHA5,NCOA5,CHST11,RAPGEF1,EP300,CACNA2D1,CYP7B1,STX8,GLRA1,COL4A6,IFNGR2,NPC1,PNPLA3,PSMA1,FLNB,EFNA5,MAGI2,NF1,SORL1,STRN3,HTR4,EIF4E,POR,GRM5,HDAC9,IL18R1,IL1RL1,PLSCR4,DDC,NLK,IL1RAP,PPP3CA,KLF3,KLF7,SH3GL2,SNX25,GLDC,MAS1,PER1,KL,ELAVL4,GABPA,GBP2,GBP7,AKAP7,VEPH1,NSG1,CNOT7,JAK2,SMAD3,BAIAP2L1,KIDINS220,NOS1,TMTC4,MIR320B2,TNF,LRCH1,OVOL2,SNX5,TNIP1,ADNP2,BMP15,PHEX,BRINP1,BCL11A,KIF18A,ANO1,GCLC,PMEPA1,EPS8,LTBP1,SMOC2,SULF1,GNAL,PIK3R3,EFNB2,TRIM41,ZNF423,CTNNA1,NSG2,EFHC2,TNFRSF19,WDR35,CD109,CMA1,IL1RAPL1,KIT,SLIT3,UBR2,BLM,VWC2,CPS1,RAPGEF2,ESR1,HTR2C,JAK1,UBE3A,GABRB2,EPHB2,ABCC1,DAPK1,ERBB4,FER,LRP5,SHCBP1,ABL2,CAMP,COMMD7,PTPRK,CCL3,FGF7,SPRED3,IL20RA,PSG9,SULF2,SOX4,ST18,PDGFD,PIK3CD,ITGA8,IRAK2,KDM3A,PLAT,CDK19,FGFR1,RYR1,UNC13B,SOST,IL23R,KEAP1,NTF3,P2RX7,TEX14,RNMT,AMFR,AR,CASP7,TRERF1,IFNA8,IL1RL2,MIR431,MIR433,PRKAA2,TLE4,CDC73,GNB5,ZMIZ1,FLT3,PPARGC1B,PTPRA,TRIM72,DYNAP,ESRRG,FERMT2,GNAO1,HCN4,MAP2K5,SMPD3,DAB2,UROS,ACTN4,CCL7,EEF1E1,FGF12,ITGA3,KMO,NREP,QRICH1,PIN1,TRPM4,BMP6,CHRM3,NCOA2,HGF,SCUBE3,IL18RAP,INSR,PAK1,PDE3A,PSMB7,SKI,ENPP1,LITAF,RRAGC,IDE,AIM2,CSF2RB,EPG5,PCK1,PPARA,SORT1,TAF1,CACNA1A,FUT8,SPRED1,AKR1C2,ESR2,PRDM16,ZNF451,BTRC,CCDC3,DEFA1B,DEFA3,OPTN,EXT1,NCOA1,PRLR,UMOD,HDAC5,TDGF1,UGGT2,STAT4,PRKCQ,STAT3,TP63,BRIP1,PAK3,PIK3R2,CASQ2,DTNBP1,RAP1B,LGALS9,LRP8,NFAT5,IL10RB,MAPKAPK2,APC,BMPR1A,NGF,PRKCA,SPINT2,GBP4,GPR75,MTMR4,CTSH,DHX9,USP18,ACACA,CCL14,CCL15,EDA2R,GABRG2,GPR21,IL6R,MAP2K6,RGMB,SOS1,VPS35,FGF1,TBX20,NLRC5
GO:00508	nervous	0.0000840790851052	PIEZO2,PLCB1,GABRB3,GABRG3,ASIC2,OR11H1,NCAM2,OR4M2,ANK3,RBFOX1,TIMP3,ITGA2,LOXHD1,KCNQ1,OR4K15,GRIN3A,NRX

77	system process	9409	N1,TMPRSS3,NRXN3,EYS,OR8U1,RIMS2,SHANK2,GRIA1,TRPM3,CSMD1,SCN8A,KCNE1,TMEM108,OR4C12,SHISA6,CTNNA2,BBS9,RBFOX2,RASGRF1,MYO3A,OR4A5,SORCS3,S100B,OR8K3,AGT,CST2,SHISA9,CHRM5,VTI1A,RCAN1,PARK2,DLGAP1,HMCN1,OR4K13,DNAH9,OR4M1,OR4N2,OR52N5,ANKFN1,DBH,MYO9A,CNTN5,OR2L13,RPS6KA2,SLC24A4,OR11G2,OR9Q1,GABRG1,OR8K5,DMD,CACNG3,GRM1,JAKMIP1,OR2M5,TENM4,CAMTA1,LRRK2,RORB,SLC1A3,STRC,CHD7,HEXB,MECP2,MTMR2,GSK3B,CDC14A,BDNF,PRKAR1B,PCDH15,GRIN2B,OR6N1,RAG1,SNAP25,MYH8,CNTNAP2,EPHB1,LRP2,TMEM100,RPGR,FYN,NR4A1,RCVRN,ATF6,ATP8A2,DCDC2,GABRB1,MME,NAV2,GRIK2,IGSF11,CELF4,KCND2,SCN11A,SLC1A1,MTOR,ROR1,RLBP1,CHRM1,BBS2,OR51B2,OR51I1,ARL6IP5,COL11A1,EPM2A,NLGN4X,OR4K17,OR8J3,GTF2A1L,ADCY8,FAM19A4,KALRN,EDNRB,COL2A1,EYA4,GABRA2,OR4C15,CHL1,APP,COL18A1,THRB,KCNK2,TSPEAR,EFEMP1,OR10R2,RGS16,AFF2,PARD3,ABAT,GRIN2A,TUB,GUCY2F,SRRM4,GLRA2,JAM2,NTRK2,ARR3,CAMK4,OR5L2,OR11L1,CRB1,USP53,MGLL,DICER1,TAS2R38,TNR,TAS2R1,CHRNA3,CHRNA5,EP300,CACNA2D4,TRIOBP,ATXN1,GLRA1,GRID2,OR4C5,ADORA2A,TAC4,CACNG2,NF1,IMPG1,OR5K4,TTC8,RELN,GRM5,PPP3CA,DGKI,SLC24A2,ELAVL4,GRM7,RIMS1,GABRA3,NLGN1,P2RX6,LHFPL3,MAPK8IP2,TMPRSS11E,CACNA1D,GABRA5,SOBP,RDH10,TMTC4,MAGT1,NBN,RRH,TNF,TANC1,TMC2,POU6F2,OR11A1,OR5V1,BRINP1,KCNK10,SPAG16,MYO6,ANO1,C5AR1,GNAL,CNGB3,HERC1,SCN1A,TSHZ3,MYH14,GABRR2,TMEM150C,MIP,KIT,OR7A5,DNAH11,RCSD1,HTR2C,NCMAP,UBE3A,FGF13,GABRB2,EPHB2,SLITRK6,OR4C6,ATP8A1,CCL3,MYO7B,SEMA5B,ARF4,P2RX1,AGTPBP1,ITGA8,CNGA4,MYO3B,PDZD7,CDH23,DAPL1,SYNM,UNC13B,GLRA3,NTF3,P2RX7,AMFR,CEP250,EYA1,BDKRB1,HLA-DRA,BHLHB9,MEIS2,REEP2,SCNN1A,CLN6,FGF12,ITGA3,OR10K2,OR4C46,STAC,LRIG1,SHROOM4,BTBD9,CNGB1,HGF,OBP2A,CACNB2,NFASC,INSR,CHM,FMR1,PAX3,GABRA1,GABRR3,RPGRIP1,JPH4,PDE6B,ZFHX2,MYH7,TRPM1,SCN9A,TECTA,PRKCG,TYMP,OR51L1,ADARB1,OR4C16,CST1,HPN,TBL1X,USH2A,DLGAP2,OR2T4,TAS1R1,TUSC3,NGF,PTGES,VLDLR,GABRG2,SPTBN4,VPS35
GO:0031345	negative regulation of cell projection organization	0.0001092666872059904	DAB1,DCC,NRXN1,FSTL4,SEMA6D,SPOCK1,CBFA2T2,SEMA3D,NTN1,HDAC2,SLIT2,CEP97,PTPRG,TRPC5,LRRK2,SEMA4D,SEMA5A,RYK,GRIN2B,ARHGAP24,EPHA7,FYN,PMP22,KANK1,FAT3,NRP1,SEMA3C,CD38,DENND5A,DNM3,TNR,PTPN9,PPP3CA,CAPZB,DGUOK,NLGN1,BCL11A,CCP110,LRP4,EFNB2,TRPV4,PTPRO,RAPGEF2,SEMA3E,UBE3A,FGF13,EPHB2,SEMA5B,MAP2,SEMA3A,DAB2,ITGA3,LIMK2,CRMP1,RIT2,DTNBP1,GAK,KIAA0319
GO:0008038	neuron recognition	0.0001132107754402716	DSCAM,NCAM2,CNTN4,ROBO2,EPHA3,OPCML,EMB,SEMA5A,CNTNAP2,NTM,PALLD,ROBO1,APP,NRP1,DSCAML1,CNTN6,MYPN,EPHB2,BSG,ARHGAP35,SEMA3A,TNN,EXT1,CRTAC1
GO:0010033	response to organic substance	0.0001153762956785529	ADCY2,PLCB1,CMKLR1,GABRB3,MAPK14,ARID1B,CTBP2,PTPRT,TIMP3,DUSP22,ITGA2,ABCG1,KCNQ1,VRK2,GRIN3A,NRXN1,DOK5,SLC1A2,BCKDHB,TGFBR3,CDH13,ANK2,GRAMD1C,HLCS,KCNE1,TMEM108,CHEK2,PSMC6,FLRT2,GPC3,PSMB2,RFFL,SPIDR,IL4R,ALDH1A2,RYR3,GGT1,KYNU,GLP2R,CCR1,CCR3,CFTR,RBFOX2,LDLRAD4,ZBTB20,IL1RAPL2,MX1,ALK,PTPRE,PIK3C3,ARID5B,ROBO2,ZNF366,EPHA3,PTPRN2,PXDN,CRNN,LEMD3,AGT,CHRM5,FBXO32,PARK2,FHL2,CYBB,OTC,TRIM5,USP25,TXK,RNF185,DBH,FBXO27,KCNMA1,HDAC2,HTRA1,SLIT2,PDCD6,RPS6KA2,STXBP4,IGF1R,MDM2,MX2,SLC24A4,SMAD1,TMPRSS6,NR3C2,DMD,HCN1,FSHR,NRIP1,WDR83,WWOX,ATRX,PARP16,TRPM2,RNLS,RYR2,HSPB8,LRRK2,RORB,SLC1A3,IGFBP7,NTRK3,RORA,GGT2,ILDR1,RNF165,PDXP,SOX6,IER2,GSK3B,LGR5,SLC8A1,PRKD1,OGT,TGFB2,AFF3,ACSBG1,BDNF,ZDHHC17,KCNC1,DNAAF2,DNAJB4,WASF1,GRIN2B,KDM4C,EXT2,SIK2,NR2F2,PTGFR,NR5A2,PAQR8,PML,AKAP6,FBN1,NLRP7,BCL2,RXFP2,ZNF675,IL12RB2,LRP2,TMEM100,ITPR2,GDAP1,NR4A2,ABCC9,FYN,GNRHR,NR4A1,SMARCA4,CD44,RHBDD1,CASP6,FOXO1,PDE4D,TBXAS1,KANK1,ABCB10,ACTN2,ATF6,GABRB1,MME,PACRG,TNMD,MYLK3,SLC1A1,UGCG,RARB,RERG,NLRP12,MTOR,VPS26B,CHRM1,CTDSPL2,DDX21,BMPER,BBS2,HBE1,ARL6IP5,ATP1A1,DSG4,IFT88,PRKCE,EIF2S1,HELLS,PRKCB,VDR,ADCY8,FOXO3,RAB31,TTLL12,EDNRB,MSR1,OASL,ROBO1,RXFP1,SATB2,TBC1D4,FLT1,NASP,NOX4,COL2A1,MAP3K7,NR3C1,TACR3,TRAF3IP2,TGM2,KDR,PRCP,CASK,EDA,PCOLCE2,ABCA12,APP,CALCRL,NRP1,LGR4,ADTRP,CD58,KAT5,THRB,CPEB2,ADAM17,MAP3K5,IGF2R,TFPI,ATF2,TGFB1I1,RPS6KA5,TCF7L2,TRAF3,ABAT,GRIN2A,BCLAF1,HSPA4L,CD38,CHRDL1,RNF126,BCL2L1,HDAC4,PDE4B,SOX5,TIMP2,DNMT1,GHR,GLRA2,NTRK2,FAM83G,MGST1,GNG2,PAPPA,LY86,RABGEF1,S

			DK1,SPRED2,GLI3,CLDN1,KIF16B,RAP1A,SAFB2,TGFBR1,EPHA5,NCOA5,CHST11,RAPGEF1,CD96,CHRNA3,EP300,CACNA2D1,CYP7B1,DIO2,STX8,GLRA1,TP73,COL4A6,IFNGR2,NPC1,PNPLA3,CA3,PSMA1,TPH2,FLNB,ADORA2A,EFNA5,MAGI2,NF1,RFC3,RGS7,TFF1,GOT2,SORL1,STRN3,HTR4,ASXL1,EIF4E,POR,TNFRSF11B,GRM5,HDAC9,IL18R1,IL1RL1,SELE,PLSCR4,DDC,MXRA5,NLK,ADCYAP1R1,IL1RAP,JAG1,MAN1A1,PPP3CA,KLF3,KLF7,SH3GL2,SNX25,GLDC,MAS1,PER1,KL,ELAVL4,PLSCR1,TMEM67,TRIM16,GABPA,KAT7,P2RX6,PTPRU,AKIRIN2,GBP2,GBP7,AKAP7,FOXRED2,VEPH1,NSG1,CNOT7,JAK2,SMAD3,BAIAP2L1,KIDINS220,NOS1,TMTC4,CDK1,MIR320B2,TNF,LRCH1,OVOL2,PANX1,SNX5,TNIP1,POMC,ADNP2,BMP15,PHEX,BRINP1,SRSF4,BCL11A,KIF18A,ANO1,GCLC,PMEPA1,EPS8,LTBP1,SMOC2,SULF1,C5AR1,GNAL,PIK3R3,EFNB2,TRIM41,ZNF423,SELP,CTNNA1,NSG2,DMBT1,EFHC2,TNFRSF19,WDR35,CD109,ALDH1A1,CMA1,IL1RAPL1,KIT,SLIT3,UBR2,BLM,TRPV4,VWC2,CPS1,RAPGEF2,REG1B,ESR1,HTR2C,JAK1,SLC30A8,UBE3A,GABRB2,EPHB2,ABCC1,DAPK1,ERBB4,FER,LRP5,SHCBP1,ABL2,BSG,CAMP,SETD2,VPS13C,COMMD7,PTPRK,CCL3,FGF7,SPRED3,IL20RA,LRRC70,PSG9,SULF2,CYP2E1,SOX4,ST18,P2RX1,PDGFD,PIK3CD,ITGA8,IRAK2,KDM3A,PLAT,CDK19,FGFR1,RYR1,UNC13B,GLRA3,SOST,IL23R,KEAP1,NTF3,P2RX7,TEX14,RNMT,AMFR,AR,CASP7,TRERF1,IFNA8,IL1RL2,BDKRB1,MIR431,MIR433,PRKAA2,TLE4,CDC73,GNB5,MEIS2,ZMIZ1,FLT3,PPARGC1B,PTPRA,TRIM72,DYNAP,ESRRG,FERMT2,GNAO1,HCN4,MAP2K5,SMPD3,DAB2,SRD5A2,UROS,ACTN4,ADAM10,CCL7,EEF1E1,FGF12,ITGA3,KMO,NREP,QRICH1,RFTN1,PIN1,TRPM4,ERLIN1,BMP6,CHRM3,NCOA2,HGF,CALCR,SCUBE3,IL18RAP,INSR,PAK1,PDE3A,PSMB7,SKI,ENPP1,FMR1,LITAF,RRAGC,IDE,AIM2,CSF2RB,EPG5,PCK1,PPARA,SORT1,TAF1,CACNA1A,FUT8,SPRED1,AKR1C2,ESR2,PRDM16,ZNF451,BTRC,C2,CCDC3,DEFA1B,DEFA3,OPTN,EXT1,NCOA1,PRKCG,PRLR,UMOD,HDAC5,TDGF1,UGGT2,A2M,SEL1L2,STAT4,KCNC2,PRKCQ,STAT3,TP63,BRIP1,HPN,PAK3,PIK3R2,CASQ2,DTNBP1,RAP1B,LGALS9,LRP8,NFAT5,IL10RB,MAPKAPK2,APC,BMPR1A,NGF,PRKCA,SPINT2,GBP4,GPR75,MTMR4,CTSH,DHX9,USP18,ACACA,CCL14,CCL15,EDA2R,GABRG2,GPR21,IL6R,MAP2K6,RGMB,SOS1,VPS35,FGF1,TBX20,NLRC5
GO:0051056	regulation of small GTPase mediated signal transduction	0.00012333240011992895	*AKAP13,DOCK2,NRG1,PLCE1,TIAM1,RASGRF1,SCAI,RGL2,ITSN1,TRIO,MYO9A,SLIT2,SIPA1L3,SH3BP1,OGT,TGFB2,VAV3,ARHGAP8,ARHGAP12,RALBP1,ARHGAP24,SIPA1L1,ARHGEF10,PSD3,CHN1,KANK1,ARHGEF3,TIAM2,KALRN,DLC1,ROBO1,PREX2,TGM2,ARHGAP15,NRP1,ARHGEF18,RALGAPA2,OPHN1,MAPRE2,RASGRP1,STARD13,RABGEF1,RAPGEF1,RASGRF2,RALGPS1,NF1,RALGPS2,ARHGAP6,LZTR1,RELN,ADCYAP1R1,DGKI,ARHGAP5,ARHGAP39,EPS8,DEF6,NET1,ARHGEF11,ARHGAP11B,SIPA1L2,DNMBP,EPHB2,ABL2,F11R,ARHGAP35,CD2AP,PREX1,ARHGAP25,FGD4,GARNL3,AUTS2,RASA3,ARHGAP10,SRGAP3,GPR55,ITGA3,RALGAPA1,ARHGAP28,ARHGAP42,GNA13,RIT2,AMOT,DOCK3,NGF,SOS1*
GO:0099643	signal release from synapse	0.00012748616221482597	*CTBP2,SYN3,GRIN3A,NRXN1,NRXN3,RAB5A,RIMS2,MCTP1,PTPRN2,ERC2,PARK2,MCTP2,APBA2,SYT9,SYT1,UNC13C,LRRK2,GSK3B,SNAP25,SNAP23,NR4A1,CADPS,PRKCB,CASK,RIMS3,STXBP5,RAP1A,CHRNA3,CHRNA5,ADORA2A,NF1,DGKI,RIMS1,NLGN1,BLOC1S6,GRM4,P2RX1,UNC13A,UNC13B,P2RX7,STX3,SYT2,KMO,CACNB2,SNAP29,FMR1,PRKCG,DTNBP1,RAP1B,SNCAIP*
GO:0007269	neurotransmitter secretion	0.00012748616221482597	*CTBP2,SYN3,GRIN3A,NRXN1,NRXN3,RAB5A,RIMS2,MCTP1,PTPRN2,ERC2,PARK2,MCTP2,APBA2,SYT9,SYT1,UNC13C,LRRK2,GSK3B,SNAP25,SNAP23,NR4A1,CADPS,PRKCB,CASK,RIMS3,STXBP5,RAP1A,CHRNA3,CHRNA5,ADORA2A,NF1,DGKI,RIMS1,NLGN1,BLOC1S6,GRM4,P2RX1,UNC13A,UNC13B,P2RX7,STX3,SYT2,KMO,CACNB2,SNAP29,FMR1,PRKCG,DTNBP1,RAP1B,SNCAIP*
GO:0002009	morphogenesis of an epithelium	0.00013774009007127367	*NPHP3,TNC,CSMD1,GPC3,PSMB2,ALDH1A2,CECR2,ASTN2,TIAM1,RHOC,NKD1,C2CD3,PRKACB,AJAP1,AGT,GREB1L,NTN1,GPC6,ZNRF3,MYO9A,SLIT2,KRT25,VCL,CELSR1,RYR2,KIF26B,PRICKLE2,SH3BP1,LGR5,TGFB2,FMN1,RIPK4,RYK,PCDH15,LAMA3,ANKRD6,PML,ARHGAP12,BCL2,ARHGAP24,LRP2,VANGL2,EPHA7,CD44,MTOR,ROR1,CTNNBIP1,SOX8,VDR,DLC1,GRHL2,SEC24B,PKHD1,STK3,TGM2,KDR,NRP1,SHROOM3,LGR4,SEMA3C,ADAM17,TGFB1I1,FRAS1,OPHN1,ADAMTS16,STARD13,GLI3,TBX3,CYP7B1,PSMA1,MAGI2,TTC8,RALA,EXOC5,JAG1,GPC4,AHI1,SMAD3,RDH10,TNF,COBL,OVOL2,DLG5,FREM2,SULF1,EFNB2,MLLT3,EGF,CC2D2A,ESR1,SEMA3E,ERBB4,LRP5,BSG,SETD2,ARHGAP35,FGF7,LAMA1,SOX4,PIK3CD,AR,SUFU,EYA1,SEMA3A,FERMT2,DAB2,PBX1,RNF207,DAAM1,CLASP1,HGF,PAK1,PSMB7,SKI,PRKX,GNA13,NTN4,BTRC,ESRP2,EXT1,KLHL3,TP63,SPINT2,CTSH,CLASP2,SOS1,FGF1,TBX20,RREB1,TBX18*
GO:00226	regulation	0.0001393230488770	*FMNL2,CDC42EP3,FBLN1,ZRANB1,RIMS2,FGD6,SYT17,PLXNA4,RH*

04	of cell morphogenesis	2676	*OC,FBXO31,RHOJ,PARK2,FMNL3,MYO9A,PALMD,SYT1,NTNG1,HEXB,SEMA4D,PLXNA2,FYN,CD44,KANK1,ENPP2,STRIP1,MOV10,ERMN,PTPRD,KALRN,DLC1,KDR,ARHGAP15,NRP1,SHROOM3,ARHGEF18,GRIP1,CUX1,TRIOBP,PARVB,EFNA5,RELN,MAP3K13,ATP10A,CAPZB,RIMS1,SH3KBP1,PLXNB1,BCL11A,DOCK1,EPS8,CDKL5,MYH14,FAM171A1,IL1RAPL1,KIT,SEMA3E,DNMBP,SPTA1,F11R,ARHGAP35,CCL3,PREX1,FGD4,MYH9,NEDD9,UNC13A,WDPCP,BHLHB9,EPB41L3,MARK2,FERMT2,DAB2,SYT2,ACTN4,CCL7,CFDP1,GAS2,MACF1,GNA13,AKAP2,HPN,PAK3,LRP8,RREB1*
GO:0070887	cellular response to chemical stimulus	0.00014641344677934545	*ADCY2,PLCB1,CMKLR1,GABRB3,MAPK14,ARID1B,CTBP2,PTPRT,ANK3,TIMP3,DUSP22,ITGA2,KCNQ1,VRK2,NRXN1,DOK5,SLC1A2,GRIA1,DLG2,TGFBR3,NXN,GRAMD1C,TF,KCNE1,TMEM108,CHEK2,SYT17,PLXNA4,FLRT2,GPC3,FBLN5,PSMB2,AOX1,RFFL,SPIDR,IL4R,ALDH1A2,RYR3,GLP2R,CCR1,CCR3,CFTR,RBFOX2,LDLRAD4,ZBTB20,IL1RAPL2,MX1,ALK,PTPRE,ARID5B,ROBO2,ZNF366,EPHA3,PTPRN2,PXDN,CRNN,LEMD3,AGT,CHRM5,FBXO32,ATP6V0D1,PARK2,CYBB,NTN1,TRIM5,KCNH1,TXK,RNF185,RIN3,HDAC2,HTRA1,SLIT2,ACSM2B,AHRR,PDCD6,RPS6KA2,STXBP4,IGF1R,MDM2,SMAD1,SYT9,TMPRSS6,NR3C2,DMD,HCN1,SYT1,FSHR,FMN2,NRIP1,STK24,WWOX,ATRX,PARP16,TRPM2,RYR2,HSPB8,LRRK2,RORB,SLC1A3,IGFBP7,NTRK3,RORA,ILDR1,RNF165,PDXP,SOX6,GSK3B,LGR5,SLC8A1,PRKD1,OGT,TGFB2,VAV3,SEMA5A,BDNF,RYK,ZDHHC17,WASF1,KDM4C,EXT2,SIK2,PTGFR,NR5A2,PAQR8,PML,PPARGC1A,AKAP6,FBN1,NLRP7,BCL2,RXFP2,ZNF675,EPHB1,IL12RB2,LRP2,TMEM100,ITPR2,GDAP1,NR4A2,FYN,GNRHR,KCNE2,NR4A1,SMARCA4,CD44,RHBDD1,CASP6,FOXO1,PDE4D,KANK1,ABCB10,ACTN2,ATF6,BACH1,GABRB1,MME,PACRG,TNMD,CPNE4,TPO,UNC5C,KCND2,MYLK3,SLC1A1,UGCG,RARB,NLRP12,MTOR,VPS26B,CHRM1,CTDSPL2,BMPER,BBS2,HBE1,PGK1,ARL6IP5,ATP1A1,DSG4,IFT88,PRKCE,EIF2S1,HELLS,PRKCB,VDR,ADCY8,FAM19A4,FOXO3,RAB31,CAPN3,TTLL12,EDNRB,MSR1,OASL,ROBO1,RXFP1,SATB2,TBC1D4,BNIP3L,FLT1,NOX4,COL2A1,MAP3K7,MAPK9,NR3C1,TRAF3IP2,ZNF277,TGM2,KDR,PRCP,CASK,EDA,PCOLCE2,ABCA12,APP,CALCRL,NRP1,LGR4,ADTRP,CD58,KAT5,THRB,CPEB2,KCNK2,ADAM17,CAMK1D,MAP3K5,TFPI,ATF2,MT1HL1,TGFB1I1,RPS6KA5,TRAF3,BCLAF1,CHRDL1,RNF126,BCL2L1,ABTB2,HDAC4,PDE4B,SOX5,DNMT1,GHR,GLRA2,NTRK2,CPNE8,FAM83G,MGST1,GNG2,LY86,RABGEF1,SPRED2,CLDN1,KIF16B,DOCK4,PXDNL,RAP1A,SAFB2,SRP54,TGFBR1,PRDX4,EPHA5,NCOA5,CHST11,IL16,RAPGEF1,CHRNA3,EP300,CACNA2D1,CYP7B1,STX8,GLRA1,HBD,COL4A6,IFNGR2,NPC1,PNPLA3,PSMA1,TPH2,FLNB,EFNA5,MAGI2,NF1,SORL1,STRN3,HTR4,EIF4E,MICU1,POR,GRM5,HDAC9,IL18R1,IL1RL1,PLSCR4,DDC,NLK,EGLN3,IL1RAP,PPP3CA,KLF3,KLF7,SH3GL2,SNX25,CXADR,GLDC,MAS1,PER1,KL,ELAVL4,GSTA2,GABPA,NLGN1,GBP2,GBP7,AKAP7,MSRA,VEPH1,DDX3X,NSG1,CNOT7,ZNF622,JAK2,SMAD3,BAIAP2L1,KIDINS220,NOS1,TMTC4,CDK1,MIR320B2,TNF,LRCH1,OVOL2,SNX5,TNIP1,ADNP2,BMP15,PHEX,RBM11,BRINP1,BCL11A,KIF18A,SULT1B1,ANO1,GCLC,PMEPA1,EPS8,LTBP1,CYP3A5,SMOC2,SULF1,C5AR1,GNAL,PIK3R3,EFNB2,TRIM41,NET1,ZNF423,LRRC8C,LRRC8D,CHD6,CTNNA1,NSG2,ABCG2,EFHC2,GSKIP,TNFRSF19,WDR35,CD109,ALDH1A1,CMA1,IL1RAPL1,KIT,SLIT3,UBR2,BLM,TRPV4,VWC2,CPS1,PTPRO,RAPGEF2,RCSD1,ESR1,HTR2C,JAK1,UBE3A,GABRB2,EPHB2,ABCC1,DAPK1,ERBB4,FER,LRP5,SHCBP1,SLAMF1,ABL2,BSG,CAMP,COMMD7,PTPRK,CCL3,FGF7,PREX1,SPRED3,IL20RA,ITGA9,PSG9,SULF2,CYP2E1,SOX4,ST18,PDGFD,PIK3CD,ITGA8,IRAK2,KDM3A,PLAT,CDK19,FGFR1,MEF2A,RYR1,UNC13B,SOST,IL23R,KEAP1,NTF3,P2RX7,PLA2G4A,TEX14,RNMT,AMFR,AR,CASP7,GSTM4,TRERF1,IFNA8,IL1RL2,MGST3,BDKRB2,MIR431,MIR433,PRKAA2,TLE4,CDC73,GNB5,ZMIZ1,FLT3,PPARGC1B,PTPRA,TRIM72,DYNAP,ESRRG,FERMT2,GNAO1,HCN4,MAP2K5,SMPD3,DAB2,SYT2,UROS,ACTN4,ADAM10,CCL7,EEF1E1,FGF12,ITGA3,KMO,MT1F,NREP,QRICH1,PIN1,TRPM4,NME8,BMP6,CHRM3,CARF,NCOA2,HGF,MYLK,SCUBE3,IL18RAP,INSR,PAK1,PDE3A,PSMB7,SKI,ENPP1,FMR1,LITAF,RRAGC,ABCD1,IDE,AIM2,CSF2RB,EPG5,PCK1,PPARA,SORT1,TAF1,CACNA1A,FUT8,SPRED1,AKR1C2,ESR2,PRDM16,ZNF451,BTRC,CCDC3,DEFA1B,DEFA3,OPTN,EXT1,MDM4,NCOA1,PRLR,UMOD,ELMO2,HDAC5,TDGF1,UGGT2,SLC40A1,STAT4,TREM1,KCNC2,PRKCQ,STAT3,TP63,BRIP1,PAK3,PIK3R2,CASQ2,DHRS2,DTNBP1,RAP1B,LGALS9,LRP8,NFAT5,ACSM1,IL10RB,MAPKAPK2,APC,BMPR1A,DPP4,ITGA1,NGF,PRKCA,PTGES,SPINT2,GBP4,GPR75,MTMR4,CTSH,DHX9,RGCC,USP18,ACACA,CCL14,CCL15,CCL15-CCL14,EDA2R,GABRG2,GPR21,IL6R,MAP2K6,RGMB,SOS1,VPS35,FGF1,TBX20,NLRC5*

GO:0098662	inorganic cation transmembrane transport	0.00015322865158968937	*ASIC2,CACNA1E,KCNJ6,ANK3,SLC24A3,KCNQ1,GRIN3A,PKD1L1,SLC9B1,TRPM3,ANK2,KCND3,FAM155A,SLC12A1,DPP10,SCN8A,KCNE1,KCNJ15,KCNG3,SCARA5,RYR3,PM20D1,SLC6A2,SLC17A3,UTRN,ATP6V0D1,KCNH1,KCNMA1,SLC24A4,KCNK17,TRPC5,DMD,HCN1,CACNA2D3,CACNG3,NOX5,FGF14,SLC39A11,DPP6,TRPM2,RYR2,CATSPER2,DHRS7C,SLC1A3,KCNH5,SLC4A4,CHD7,KCNK13,SLC8A1,PRKD1,ATP1A4,ZDHHC17,KCNC1,GRIN2B,SNAP25,AKAP6,KCNJ3,SLC30A5,ITPR2,ABCC9,FYN,KCNE2,HECW1,PDE4D,ACTN2,KCNS3,KCNIP4,KCND2,SCN11A,SLC1A1,ATP1A1,PLN,PRKCE,SLC2A9,ATP6V1D,SLC47A1,CAPN3,EDNRB,SLC12A8,KCNC4,KCNK2,GRIN2A,PDE4B,SLC5A10,COMMD1,CACNA1C,NIPA2,KCNJ12,SLC6A17,CACNA2D1,CACNA2D4,TRPM7,CACNG2,RGS7,KCNQ5,MICU1,SLC39A8,SLC9C1,TMEM163,KCNB2,SLC24A2,SLC5A1,STIM2,AKAP7,CACHD1,CACNA1D,TPCN2,NOS1,MAGT1,SLC39A10,PANX1,TMC2,KCNT2,KCNK10,TSPAN13,KCNA6,TRPC4,KCNH8,TRDN,SLC15A1,SCN1A,KCNH7,TRPV4,KCNAB1,HTR2C,SLC30A8,FGF13,CACNG6,CCL3,MCUR1,SLC39A12,P2RX1,KCNE4,RYR1,P2RX7,RASA3,BDKRB1,THADA,GNB5,ATP6V1H,HCN4,SCNN1A,RNF207,ACTN4,FGF12,KCNJ16,KCNN3,STAC,TRPM4,TMCO3,CALCR,CACNB2,NOS1AP,FMR1,MICU2,ATP5J,JPH4,FHL1,CACNA1A,TRPM1,SCN9A,ATP6V1E1,SLC24A5,SLC40A1,STIM1,TRPM6,KCNC2,SLC9A9,HPN,CASQ2,SLC28A3,TMEM175,TUSC3,UBASH3B,SLC9A2,MCOLN3,ATP6V1E2,RGN*
GO:0042592	homeostatic process	0.0001632496472591159	*CMKLR1,OMA1,MAPK14,ABCG8,ANK3,LARGE,SLC24A3,NPHP3,ABCG1,KCNQ1,TMPRSS3,POTEE,MS4A1,GRIA1,SLC9B1,TGFBR3,ANK2,CSMD1,FAM155A,SLC12A1,TF,ACIN1,SCARA5,IL4R,RYR3,PLCE1,KSR2,CCR1,CCR3,CFTR,RBFOX2,PCTP,PM20D1,ZBTB20,SLC6A2,ALK,FAM3B,RAB7A,PRKACB,PTPRN2,AGT,ATP6V0D1,CDH2,PARK2,LAMA2,OTC,CP,DBH,KCNMA1,FTO,LAMA4,STXBP4,IGF1R,SLC24A4,SMAD1,NOVA1,TMPRSS6,TRPC5,DMD,VCL,NOX5,FSHR,GRM1,TRPM2,RYR2,DHRS7C,LRRK2,SLC1A3,RORA,SLC4A4,CHD7,DISC1,HEXB,SLC8A1,PRKD1,ERCC6,FLVCR1,OGT,ATP1A4,PIWIL4,NBEA,PCDH15,SGCD,GRIN2B,EXT2,RAG1,RAG2,PTGFR,NR5A2,PML,PPARGC1A,AKAP6,ACSM3,BCL2,ZNF675,SLC30A5,ITPR2,CYB561A3,FYN,SMARCA4,FOXO1,PDE4D,TBXAS1,ABCB10,KLF13,GRIK2,ZNF516,RAP1GDS1,APBB2,SLC1A1,UGCG,LDB2,BBS2,PLCL1,ATP1A1,PLN,HEPHL1,PRKCE,PRKCB,VDR,ADCY8,FOXO3,CAPN3,EDNRB,NOX4,SGIP1,SLC12A8,TSC22D3,COL2A1,MALL,TRAF3IP2,PKHD1,TGM2,KDR,PRCP,ABCA12,APP,LGR4,ADAM17,ETS1,MT1HL1,OIT3,TM9SF4,TCF7L2,ACSM2A,ZNF830,ABAT,GRIN2A,HRNR,PYGL,CD38,SLC30A7,TUB,PDE4B,BACE2,JAM2,COMMD1,KCTD7,CACNA1C,CLDN1,CRB1,CYSLTR1,PRDX4,EPHA5,NCOA5,TTC39B,CACNA2D1,CYP7B1,DIO2,NPC1,PNPLA3,TRPM7,ADCK1,ADORA2A,EFNA5,EMCN,FAM20A,MERTK,TAC4,BRD1,NF1,TFF1,ASXL1,MICU1,SLC39A8,SLC9C1,TNFRSF11B,GRM5,HDAC9,IL18R1,DYNC1H1,NAPEPLD,NPHP4,ADCYAP1R1,CYP39A1,FBXL5,PPP3CA,KLF7,SH3GL2,CXADR,SLC24A2,WWTR1,KL,DOCK10,KAT7,L3MBTL3,STIM2,NPR3,LRRK1,ACOX3,DDX3X,TPCN2,JAK2,SPNS2,SMAD3,NOS1,TMTC4,MIR320B2,SLC39A10,TNF,SNX5,ZFPM1,POMC,ARID2,ARF1,ANO1,GCLC,TRPC4,C5AR1,MYCT1,TRDN,ZNF423,LRRC8D,DMXL1,NELL2,ALDH1A1,CMA1,KIT,POTEF,TRPV4,ABCA5,CPS1,ESR1,HTR2C,SLC30A8,UBE3A,SGCZ,SPTA1,LRP5,MALRD1,PTH2R,ABL2,BSG,F11R,CCL3,MCUR1,IL20RA,PTGER3,SLC39A12,SOX4,P2RX1,PIK3CD,ASGR2,ARID4A,DDB1,KDM3A,CDH23,OCLN,RYR1,UNC13B,BPGM,P2RX7,RASA3,BDKRB1,BDKRB2,ADRA1B,PRKAA2,THADA,FLT3,PPARGC1B,ATP6V1H,ESRRG,HCN4,SCNN1A,ATP13A3,CLN6,GPR55,CCL7,MT1F,PLCL2,TRPM4,BMP6,BTBD9,CNGB1,CALCR,CACNB2,INSR,AKT3,ENPP1,MICU2,TMTC2,ABCD1,IDE,JPH4,PCK1,POLD1,CORIN,FHL1,GNA13,SLC22A5,CACNA1A,PPP2R3C,S1PR3,TRPM1,PRDM16,EXT1,PRLR,SCO2,UMOD,ADAMTS5,DOCK7,KLHL3,SLC24A5,SLC40A1,STIM1,PEMT,SLC9A9,STAT3,TP63,PIK3R2,USH2A,CASQ2,DHRS7B,TMEM175,CNNM3,LGALS9,ACSM1,SLC7A8,UPK3A,APC,PRKCA,PTGES,UBASH3B,CTSH,NCAPG2,RPH3AL,SLC9A2,ACACA,CCL14,CCL15,GPR21,MAP2K6,SOS1,C1QTNF3,MCOLN3,RGN*
GO:0051270	regulation of cellular component movement	0.0001662145296019042	*SRGAP2B,PLCB1,CMKLR1,PTPRT,DUSP22,FBLN1,ITGA2,SUN2,NRG3,TGFBR3,CDH13,ANK2,TF,GADD45A,PLXNA4,FLRT2,NRG1,RFFL,MCTP1,SH3RF2,UNC5D,SEMA6D,CTNNA2,CCR1,TIAM1,RHOC,LDLRAD4,NKD1,SCAI,FBXO31,PHACTR1,SPOCK3,AGT,RHOJ,LAMA2,SEMA3D,DACH1,NTN1,ZNF268,RIN3,LAMA4,SLIT2,PDCD6,IGF1R,PTPRG,VCL,STK24,FRMD5,CTNNA3,RYR2,NTNG1,NTRK3,SH3BP1,MECP2,SEMA4D,SLC8A1,PRKD1,TGFB2,FUT4,S100A11,SEMA5A,LAMA3,NR2F2,PLXNA2,BCL2,PDE4D,KANK1,ENPP2,UNC5C,MTOR,LDB2,PKP2,BMPER,BBS2,PLN,EPB41L4B,PRKCE,FOXO3,MITF,DLC1,ROBO1,FLT1,TACR3,PKHD1,KDR,PRCP,APP,NRP1,PTPRM,ADTRP,SE*

			MA3C,ADAM17,CAMK1D,ELP3,ETS1,RIN2,HDAC4,PDE4B,JAM2,MAPRE2,PRKG1,STARD13,RABGEF1,CACNA1C,CLDN1,DOCK4,TGFBR1,TAC4,MAGI2,NF1,SORL1,RELN,HDAC9,SELE,MGAT5,NUMB,NAV3,JAG1,MEOX2,PPP3CA,DOCK10,PTPRU,JAK2,SMAD3,CD300A,PLXNB1,HDAC7,TNF,LRCH1,DLG5,LGR6,ARID2,ADORA3,DOCK1,SMOC2,SULF1,C5AR1,PIK3R3,AMOTL1,SELP,CTNNA1,EGF,KIT,TRPV4,DNAH11,PTPRO,RAPGEF2,SEMA3E,FGF13,MARVELD3,EPHB2,ERBB4,FER,MAP3K3,SLAMF1,ABL2,BSG,PTPRK,ATP8A1,CCL3,FGF7,LAMA1,SEMA5B,KIF2A,NEDD9,PDGFD,PIK3CD,WDPCP,FGFR1,MAP2,NTF3,BDKRB1,SEMA3A,SRGAP3,FERMT2,HCN4,MAP2K5,SMPD3,TNN,DAB2,ACTN4,ADAM10,CCL7,ITGA3,PIN1,SYNPO2,TRPM4,CLASP1,HGF,MYLK,CAPN7,INSR,MACF1,PAK1,AKT3,PRKX,ULK4,GNA13,SPRED1,PTPRR,DOCK7,HDAC5,TDGF1,LYVE1,ADARB1,STAT3,AMOT,PAK3,LGALS9,EFCAB1,APC,BMPR1A,DPP4,PRKCA,SPINT2,CTSH,RGCC,SRGAP1,IL6R,CLASP2,FGF1,RGN,RREB1
GO:0007507	heart development	0.00017363405514758798	*MAPK14,RUNX1,MYO18B,AKAP13,SORBS2,NPHP3,KCNQ1,SMYD2,TGFBR3,ANK2,PLXNA4,FLRT2,GPC3,NRG1,ALDH1A2,PLCE1,ROBO2,C2CD3,AGT,WDR11,FHL2,GREB1L,SLIT2,RPS6KA2,IGF1R,MDM2,SMAD1,ADAMTS6,BASP1,RYR2,TENM4,NTRK3,CHD7,SOX6,SLC8A1,TGFB2,RIPPLY3,ABI3BP,SGCD,SGCG,AKAP6,FBN1,LRP2,TMEM100,VANGL2,ACTN2,MYLK3,ZFPM2,RARB,MTOR,PKP2,COL11A1,PLN,DLC1,ROBO1,NOX4,GRHL2,COL2A1,NEBL,TRAF3IP2,SEC24B,STK3,KDR,CALCRL,NRP1,KCNK2,SEMA3C,ATF2,GLI3,CACNA1C,TGFBR1,CPE,EP300,TBX3,PCSK5,TP73,NF1,BICC1,ASXL1,HDAC9,JAG1,NEB,CXADR,MNAT1,FHOD3,SHOX2,AHI1,SMAD3,CDK1,OVOL2,ZFPM1,ARID2,FREM2,EFNB2,ALPK2,PRKAR1A,DYNC2H1,SLIT3,CC2D2A,DNAH11,SGCZ,TFDP2,ERBB4,SETD2,AP2B1,SOX4,MEF2A,RYR1,SCUBE1,BCOR,CASP7,SUFU,EYA1,ZMIZ1,PDLIM5,HCN4,MAP2K5,RNF207,FGF12,ITGA3,ELN,INSR,CTDP1,PPARA,TTN,MYH7,SPRED1,EXT1,MDM4,MYH6,TDGF1,MYH11,BMPR1A,MAP2K1,SOS1,TBX20,TBX18*
GO:0060047	heart contraction	0.00021033078687065517	*KCNQ1,TNNI3K,CELF2,ANK2,KCND3,KCNE1,RYR3,AGT,RPS6KA2,MDM2,DMD,CTNNA3,RNLS,RYR2,SLC8A1,TGFB2,SGCD,EXT2,SGCG,KCNJ3,ABCC9,KCNE2,PDE4D,SLC1A1,MTOR,PKP2,ATP1A1,PLN,EDNRB,TACR3,THRB,HDAC4,PDE4B,CACNA1C,KCNJ12,CACNA2D1,CXADR,CACNA1D,JAK2,SHOX2,NOS1,TNF,ADORA3,TRDN,SCN1A,FGF13,NUP155,SGCZ,KCNE4,MEF2A,ADRA1B,SEMA3A,PDE5A,HCN4,RNF207,FGF12,TRPM4,CACNB2,NOS1AP,TTN,CORIN,MYH7,EXT1,MYH6,CASQ2,ASB3,MAP2K6,SPTBN4,TBX18*
GO:0001505	regulation of neurotransmitter levels	0.00022493485193682112	*CTBP2,SYN3,GRIN3A,NRXN1,NRXN3,SLC1A2,RAB5A,RIMS2,COMT,PRIMA1,MCTP1,SLC6A2,PTPRN2,ERC2,PAH,PARK2,MCTP2,DBH,APBA2,SYT9,SYT1,FSHR,UNC13C,LRRK2,SLC1A3,GSK3B,SNAP25,SNAP23,NR4A1,CADPS,PRKCB,GABRA2,CASK,ABAT,RIMS3,STXBP5,RAP1A,CHRNA3,CHRNA5,ADORA2A,NF1,DGKI,RIMS1,SLC22A2,NLGN1,SLC22A3,NOS1,BLOC1S6,GRM4,P2RX1,AGTPBP1,UNC13A,UNC13B,P2RX7,STX3,SYT2,KMO,GAD2,CACNB2,SNAP29,FMR1,PRKCG,DTNBP1,RAP1B,SNCAIP*
GO:0001822	kidney development	0.00025212172986310036	*NPHP3,GPC3,ALDH1A2,PLCE1,ARID5B,ROBO2,AGT,GREB1L,SLIT2,SMAD1,ADAMTS6,BASP1,LRRK2,KIF26B,TGFB2,FMN1,FBN1,BCL2,LRP2,VANGL2,EPHA7,MME,SERPINB7,RARB,BMPER,CTNNBIP1,SOX8,IFT88,HELLS,EDNRB,TRAF3IP2,PKHD1,NRP1,LGR4,FRAS1,ADAMTS16,GLI3,TGFBR1,PCSK5,TP73,MAGI2,NF1,BICC1,ASXL1,KIRREL3,JAG1,WWTR1,AHI1,SMAD3,RDH10,DLG5,FREM2,SULF1,LRP4,EFNB2,DYNC2H1,CC2D2A,PTPRO,ERBB4,AP2B1,SULF2,SOX4,PDGFD,ITGA8,ENPEP,WDPCP,EYA1,PBX1,ITGA3,BMP6,CENPF,ZBTB16,PRKX,C1GALT1,EXT1,UMOD,KLHL3,UPK3A,CTSH,IL6R,FGF1,DCHS2,RGN*
GO:0048646	anatomical structure formation involved in morphogenesis	0.00026800770696805236	*ISM1,GTF2I,MAPK14,DSCAM,RUNX1,WLS,AKAP13,NPHP3,ITGA2,NRXN1,NRXN3,SDK2,MTPN,NRG3,TGFBR3,CDH13,ANK2,SDCCAG8,GADD45A,ETS2,IL4R,ALDH1A2,CECR2,CCR3,CFTR,COL22A1,NKD1,ROBO2,PRKACB,COL15A1,AGT,RHOJ,FHL2,LOXL2,CYBB,KCNH1,FMNL3,HDAC2,HTRA1,SLIT2,PDCD6,TM4SF1,SLC24A4,SMAD1,NOX5,CELSR1,MBOAT7,TENM4,KIF26B,RORA,MECP2,MTMR2,PRKD1,TGFB2,FMN1,VAV3,PLCD3,SEMA5A,WDR72,KDM4C,EXT2,LAMA3,PML,PLXNA2,ARHGAP24,EPHB1,LRP2,TMEM100,VANGL2,NR4A1,PMP22,RNF213,THBS2,ENPP2,ACTN2,ATP8A2,TNMD,EXOC4,MYLK3,SLC1A1,FAT3,BMPER,PGK1,COL11A1,SOX8,PRKCB,CAPN3,DLC1,ROBO1,FAP,FLT1,GRHL2,COL2A1,NEBL,SEC24B,STK3,TGM2,VASH2,KDR,PRCP,EDA,CALCRL,COL18A1,NRP1,PTPRM,SHROOM3,ADTRP,SEMA3C,ADAM17,ATF2,ETS1,OPHN1,JAM2,STARD13,SDK1,GLI3,KIF16B,CRB1,TGFBR1,CYSLTR1,RRP7A,ADAM12,DICER1,EP300,TBX3,GRID2,EMCN,FAM20A,NF1,ELK3,TEAD4,RELN,HDAC9,COL12A1,RALA,JAG1,MEOX2,NEB,GABPA,AGGF1,FHOD3,MEGF11,AHI1,S*

			MAD3,NOS1,PTGFRN,COL8A1,RDH10,HDAC7,TNF,COBL,OVOL2,ZFPM1,TANC1,MMP20,CALD1,HMGA2,SMOC2,SULF1,C5AR1,PIK3R3,EFNB2,AMOTL1,HERC1,PRKAR1A,CD109,EGF,PTPN14,CMA1,CC2D2A,EDAR,JAK1,MYPN,NCMAP,SEMA3E,EPHB2,MAP3K3,BSG,CAMP,SETD2,ARHGAP35,MYH9,SLC39A12,SOX4,PIK3CD,AGTPBP1,ITGA8,ENPEP,MEF2A,AR,SUFU,EYA1,SRPK2,CDC73,EPB41L3,PPARGC1B,MAP2K5,SMPD3,ITGA3,THSD7A,CLASP1,TGFA,TMOD1,HGF,SKI,AKT3,PRKX,MFNG,PPARA,TTN,C1GALT1,GNA13,SPRED1,PGM5,EXT1,MYH6,TYMP,ADAMTS5,HDAC5,TDGF1,SLC40A1,STIM1,MYH11,STAT3,TP63,AMOT,LRP8,MYOM2,BMPR1A,PRKCA,SPINT2,ABI1,CTSH,RGCC,CLASP2,MAP2K1,FGF1,TBX20,TBX18
GO:0034762	regulation of transmembrane transport	0.0003180976061658 3827	*MAPK14,ASIC2,CACNA1E,KCNJ6,ANK3,KCNQ1,NRXN1,SLC1A2,ANK2,KCND3,DPP10,SCN8A,KCNE1,KCNJ15,KCNG3,GPC3,SHISA6,CFTR,PM20D1,RASGRF1,UTRN,AGT,SHISA9,CYBB,KCNH1,KCNMA1,STXBP4,KCNK17,DMD,HCN1,CACNA2D3,CACNG3,FGF14,GSG1L,DPP6,CNIH3,RYR2,CATSPER2,DHRS7C,KCNH5,CHD7,KCNK13,SLC8A1,PRKD1,KCNC1,AKAP6,BCL2,KCNJ3,SLC30A5,FYN,KCNE2,HECW1,PDE4D,ACTN2,KCNS3,KCNIP4,KCND2,SCN11A,ARL6IP5,PLN,PRKCE,PRKCB,CAPN3,ITLN1,KCNC4,ACSL5,APP,GOPC,PDE4B,COMMD1,CACNA1C,KCNJ12,CACNA2D1,CACNA2D4,CLIC6,IFNGR2,RASGRF2,CACNG2,RGS7,KCNQ5,RELN,GRM5,KCNB2,STIM2,NLGN1,AKAP7,MAPK8IP2,CACNA1D,TPCN2,NOS1,TNF,TMC2,KCNK10,TSPAN13,KCNA6,KCNH8,TRDN,SCN1A,KCNH7,KCNAB1,FGF13,EPHB2,DAPK1,CACNG6,P2RX1,KCNE4,MEF2A,OCLN,P2RX7,BDKRB1,THADA,GNB5,HCN4,RNF207,ACTN4,FGF12,KCNJ16,STAC,CHRM3,CALCR,CACNB2,NOS1AP,INSR,ENPP1,FMR1,JPH4,FHL1,CACNA1A,SCN9A,STIM1,KCNC2,CASQ2,UBASH3B,RGN*
GO:0009605	response to external stimulus	0.0003198031729690 194	*IGHV1OR21-1,MACROD2,CDH4,PIEZO2,ZPLD1,CMKLR1,OMA1,MAPK14,ABCG8,DSCAM,ASIC2,RAB27A,IGHV4-31,DCC,IGHV3-64,IGHV1OR15-9,IGHV4OR15-8,ITGA2,KCNQ1,EPHA6,GRIN3A,NRXN1,NRXN3,SLC1A2,DOCK2,EYS,MS4A1,BCKDHB,MTPN,SHANK2,COMT,NRG3,CLEC16A,PKD1L1,TRPM3,CDH13,RNF152,CSMD1,TF,PLGRKT,HLCS,PIK3C2B,ANKHD1,GADD45A,ADAMTSL1,PLXNA4,FLRT2,GPC3,NRG1,PSMB2,IL4R,ALDH1A2,LRFN5,GGT1,UNC5D,KYNU,SEMA6D,CTNNA2,CCR1,CCR3,AGBL4,DNAJC15,DPYSL2,MX1,ALK,PIK3C3,KLRF2,CNTN4,ROBO2,B3GALT5,EPHA3,DCLK1,AJAP1,AGT,ZFYVE1,CDH2,PARK2,LAMA2,HMCN1,KIR2DL4,KIR3DL1,BPIFB1,TRIM51,CYBB,OTC,PSPC1,SEMA3D,NTN1,TRIM22,TRIM5,TXK,TSPAN8,RIN3,TRIO,HDAC2,HTRA1,SLIT2,RNASET2,STXBP4,REG4,IGF1R,MX2,SLC24A4,TRIM48,DMD,STK24,WDR83,TRPM2,RYR2,LRRK2,SLC1A3,STRC,NTRK3,RORA,GGT2,CHD7,RNF165,EMB,MECP2,SEMA4D,SLC8A1,PRKD1,ERCC6,OGT,TGFB2,VAV3,C8A,SEMA5A,BDNF,RYK,KCNC1,PCDH15,POLR3C,LAMA3,RAG2,SIK2,PLEKHM2,PTGFR,PML,PPARGC1A,CNTNAP2,IGHV3-16,NAALADL2,NLRP2,NLRP7,PLXNA2,TRDV3,BCL2,RALBP1,EPHB1,IL12RB2,TRDV1,GDAP1,NR4A2,ABCC9,EPHA7,FYN,ADAMTS18,CHN1,NR4A1,RCVRN,RNF213,FOXO1,ENPP2,ATP8A2,CST9L,GRIK2,UNC5C,SLC1A1,PALLD,NLRP12,MTOR,MOV10,VPS26B,DDX21,CCDC88B,BBS2,COL11A1,ZMYND11,PRKCE,EIF2S1,VDR,ADCY8,FAM19A4,FOXO3,CAPN3,ITLN1,KALRN,TTLL12,EDNRB,OASL,ROBO1,BNIP3L,FAP,FLT1,SGIP1,DEFB116,MAP3K7,TRAF3IP2,CR1,KDR,RPL30,CASK,CHL1,IGHV4-28,APP,CALCRL,DGKB,NRP1,PTPRM,SHROOM3,LGR4,SERPING1,ADTRP,CD58,KCNK2,SEMA3C,ADAM17,CAMK1D,MAP3K5,TFPI,ATF2,ETS1,PRTG,RPS6KA5,TNFRSF10B,TRAF3,ABAT,GRIN2A,OPHN1,TTC4,PYGL,CASP5,ALCAM,NCF2,BCL2L1,GUCY2F,HDAC4,PDE4B,GHR,PRKG1,BRD4,RASGRP1,LY86,RABGEF1,GLI3,CLDN1,CRB1,DOCK4,SRP54,USP53,CYSLTR1,MGLL,EPHA5,TNR,IL16,CD96,EP300,DSCAML1,CACNA2D4,CYP7B1,DIO2,STX8,GLRA1,GRID2,IFNGR2,CA3,PSMA1,TPH2,FLNB,ADORA2A,EFNA5,FAM20A,TAC4,CACNG2,SORL1,TRIM59,TTC8,HTR4,CRISP3,POR,RELN,TNFRSF11B,IGHV4-4,IL1RL1,SELE,PLSCR4,NAPEPLD,IGHV3OR16-12,RALA,IL1RAP,FBXL17,KLF7,CXADR,MAS1,PER1,SLC24A2,TBC1D5,KL,C6,PLSCR1,WDR59,TMEM135,AKIRIN2,GBP2,GBP7,SLC22A3,DDX3X,CNOT7,JAK2,SMAD3,VPS41,BAIAP2L1,MAP2K4,NOS1,WDFY4,PLXNB1,RRH,TNF,TNIP1,ILF3,POMC,TMC2,BLOC1S6,PHEX,TRAV24,TRIM23,LGR6,ARF1,CLEC4A,HMGA2,IGLC2,IGLL5,ANO1,GCLC,SMOC2,C5AR1,CNTN6,EFNB2,TRDN,TRIM41,SELP,SCN1A,DMBT1,MYH13,TMEM150C,CD109,CMA1,KIT,SLIT3,TNFSF8,PIK3C2G,TRPV4,C8B,CPS1,EFNB1,PTPRO,REG1B,ESR1,HTR2C,JAK1,MYPN,SEMA3E,SLC30A8,ANO3,EPHB2,IGHV3-*

			72,ABCC1,DAP,DAPK1,FER,SLITRK6,FNIP1,SLAMF1,BSG,CAMP,DEPDC5,F11R,SETD2,ARHGAP35,CCL3,FGF7,LAMA1,PREX1,SEMA5B,TNFAIP8L2,BTBD17,ITGA9,CYP2E1,PTGER3,PDGFD,PIK3CD,PUM1,IRAK2,PDZD7,PLAT,CDK19,DAPL1,FGFR1,DAD1,SOST,IL23R,JMY,NTF3,P2RX7,ZDHHC11,FGL2,CEP250,IFNA8,IL1RL2,N4BP1,RBM4,SRPK2,BDKRB1,MIR431,MIR433,PI3,PRKAA2,SEMA3A,CDC73,MEIS2,PROS1,LTBR,C9,SRD5A2,STX3,ADAM10,BLOC1S5,CCL7,KMO,LGI1,PPM1B,LECT2,TRPM4,CD226,BMP6,CLASP1,CNGB1,G3BP2,HGF,PRB3,NCAM1,NFASC,IL18RAP,PAK1,PSMB7,FMR1,LITAF,IGHV1-69,RRAGC,CRMP1,IGHV1-46,AIM2,CCDC141,CD84,CSF2RB,PCK1,PDE6B,PPARA,TTN,SLC22A5,PPP2R3C,TRPM1,CHSY1,PTPRF,C2,CFB,DEFA1B,DEFA3,IGHV1-18,OPTN,EXT1,NCOA1,PRKCG,PRLR,TYMP,UMOD,ADAMTS5,ELMO2,G3BP1,TDGF1,A2M,TREM1,ADARB1,POU2AF1,PRKCQ,SCEL,SUSD4,AMOT,BRIP1,DEFB127,HPN,PAK3,LGALS9,LRP8,ALPK1,IL10RB,MAPKAPK2,AOAH,DPP4,ITGA1,KIAA0319,PRKCA,PTGES,UBASH3B,GBP4,DHX9,USP18,CCL14,CCL15,CCL15-CCL14,IL6R,CLASP2,MAP2K1,SOS1,TRAV6,VPS35,C1QTNF3,FGF1,C6ORF106,CRADD,NLRC5
GO:0040007	growth	0.00035649035660828	*CDH4,PLCB1,MAPK14,DSCAM,RUNX1,DCC,AKAP13,LARGE,SORBS2,SLC1A2,EYS,RIMS2,MTPN,NRG3,TGFBR3,FSTL4,CSNK2A1,TMEM108,SYT17,PLXNA4,GPC3,FBLN5,NRG1,PLCE1,SEMA6D,SPOCK1,DPYSL2,NKD1,ARID5B,DCLK1,AGT,WDR11,PARK2,SEMA3D,NTN1,TXK,APBA2,FTO,SLIT2,SMAD1,CPQ,TRPC5,DMD,SYT1,BASP1,VCL,FSHR,MAEL,MORF4L1,ATRX,TENM4,IGFBP7,KIF26B,CHD7,DISC1,SEMA4D,GSK3B,ERCC6,FLVCR1,TGFB2,FMN1,SEMA5A,BDNF,RYK,PCDH15,WASF1,RAG2,PML,AKAP6,BCL2,RAD51B,EPHA7,SMARCA4,ATP8A2,IGSF11,ZFPM2,RARB,RERG,GNG4,MTOR,TMPRSS4,BBS2,EPM2A,ZMAT3,NLGN4X,FOXO3,CAPN3,GRHL2,STK3,APP,NRP1,KAT5,KCNK2,SEMA3C,ADAM17,ATF2,ROS1,ZNF830,SGK2,CD38,ALCAM,BCL2L1,GHR,ACSL4,GLI3,TGFBR1,TNR,CHST11,EP300,TP73,SPG11,EFNA5,MAGI2,IFT80,TTC8,POR,SP2,MAP3K13,PPP3CA,PLS1,SH3GL2,WWTR1,RIMS1,ENOX2,DDX3X,SMAD3,PTGFRN,RDH10,CDK1,DCBLD2,NBN,COBL,ADNP2,INO80,LGR6,ARID2,BCL11A,HMGA2,UBE2E3,LRP4,NET1,CLSTN1,ARHGEF11,CDKL5,PRKAR1A,SLIT3,ESR1,SEMA3E,UBE3A,EBAG9,FGF13,PLAG1,ERBB4,SLITRK6,FGF7,SEMA5B,NEDD9,SERTAD2,UNC13A,AUTS2,MAP2,AR,KIAA1109,BDKRB1,SEMA3A,CDC73,EPB41L3,ZMIZ1,PDLIM5,MAP2K5,PAPPA2,SMPD3,TNN,DAB2,SYT2,ADAM10,LGI1,MT1F,RFTN1,GAS2,INSR,MACF1,CTDP1,ENPP1,EVC,PPARA,FHL1,ESR2,EXT1,MYH6,PRLR,ADARB1,PRKCQ,STAT3,HPN,ISLR2,BMPR1A,KIAA0319,NGF,OSGIN2,NCAPG2,GPR21,SPTBN4,CLASP2,SOS1,FGF1,TBX20*
GO:0035239	tube morphogenesis	0.0003905032426077029	*ISM1,GTF2I,MAPK14,RUNX1,MYO18B,NPHP3,NRXN1,NRXN3,TGFBR3,CDH13,CSMD1,SDCCAG8,GADD45A,GPC3,CECR2,CCR3,COL22A1,C2CD3,PRKACB,COL15A1,AGT,RHOJ,CDH2,LOXL2,GREB1L,CYBB,NTN1,FMNL3,SLIT2,PDCD6,SMAD1,NOX5,CELSR1,RYR2,KIF26B,RORA,CHD7,MECP2,LGR5,PRKD1,TGFB2,FMN1,VAV3,PLCD3,SEMA5A,SGCD,NR2F2,PML,BCL2,ARHGAP24,EPHB1,LRP2,TMEM100,VANGL2,EPHA7,NR4A1,RNF213,THBS2,ENPP2,TNMD,SLC1A1,ZFPM2,BMPER,PGK1,CTNNBIP1,SOX8,PRKCB,VDR,DLC1,ROBO1,FAP,FLT1,GRHL2,SEC24B,PKHD1,STK3,TGM2,VASH2,KDR,PRCP,EDA,CALCRL,COL18A1,NRP1,PTPRM,SHROOM3,LGR4,ADTRP,ATF2,ETS1,RIN2,ADAMTS16,NTRK2,STARD13,GLI3,RAP1A,TGFBR1,CYSLTR1,ADAM12,TBX3,EMCN,WARS2,NF1,ELK3,HDAC9,RALA,JAG1,MEOX2,AGGF1,SHOX2,AHI1,SMAD3,COL8A1,RDH10,HDAC7,TNF,COBL,OVOL2,DLG5,ARID2,CALD1,HMGA2,QKI,SMOC2,SULF1,C5AR1,PIK3R3,EFNB2,AMOTL1,EGF,CMA1,CC2D2A,EDAR,RAPGEF2,ESR1,JAK1,SEMA3E,EPHB2,MAP3K3,LRP5,BSG,CAMP,SETD2,ARHGAP35,LAMA1,MYH9,SLC39A12,SOX4,PIK3CD,ENPEP,AR,SUFU,EYA1,SRPK2,ZMIZ1,MAP2K5,PBX1,RNF207,THSD7A,TGFA,HGF,MYLK,PAK1,SKI,AKT3,PRKX,C1GALT1,GNA13,SPRED1,BTRC,ESRP2,EXT1,TYMP,HDAC5,KLHL3,TDGF1,STIM1,STAT3,TP63,AMOT,BMPR1A,PRKCA,SPINT2,CTSH,RGCC,SOS1,FGF1,TBX20*
GO:0071805	potassium ion transmembrane transport	0.00040559165545673746	*KCNJ6,ANK3,SLC24A3,KCNQ1,ANK2,KCND3,SLC12A1,DPP10,KCNE1,KCNJ15,KCNG3,KCNH1,KCNMA1,SLC24A4,KCNK17,HCN1,DPP6,SLC1A3,KCNH5,KCNK13,ATP1A4,KCNC1,SNAP25,AKAP6,KCNJ3,ABCC9,KCNE2,ACTN2,KCNS3,KCNIP4,KCND2,ATP1A1,SLC12A8,KCNC4,KCNK2,KCNJ12,RGS7,KCNQ5,SLC9C1,KCNB2,SLC24A2,AKAP7,CACNA1D,KCNT2,KCNK10,KCNA6,KCNH8,KCNH7,KCNAB1,KCNE4,HCN4,RNF207,KCNJ16,KCNN3,NOS1AP,FHL1,SLC24A5,KCNC2,SLC9A9,HPN,CASQ2,TMEM175,SLC9A2*
GO:20001	regulation	0.0004199408360073	*SRGAP2B,PLCB1,CMKLR1,PTPRT,DUSP22,FBLN1,ITGA2,SUN2,NRG3,CDH13,TF,GADD45A,PLXNA4,FLRT2,NRG1,RFFL,MCTP1,SH3R*

45	of cell motility	123	F2,UNC5D,SEMA6D,CTNNA2,CCR1,TIAM1,RHOC,LDLRAD4,NKD1,SCAI,FBXO31,PHACTR1,SPOCK3,AGT,RHOJ,LAMA2,SEMA3D,DACH1,NTN1,ZNF268,RIN3,LAMA4,SLIT2,PDCD6,IGF1R,PTPRG,VCL,STK24,FRMD5,NTNG1,NTRK3,SH3BP1,MECP2,SEMA4D,SLC8A1,PRKD1,TGFB2,FUT4,S100A11,SEMA5A,LAMA3,NR2F2,PLXNA2,BCL2,KANK1,ENPP2,UNC5C,MTOR,LDB2,BMPER,BBS2,EPB41L4B,PRKCE,FOXO3,MITF,DLC1,ROBO1,FLT1,TACR3,KDR,PRCP,APP,NRP1,PTPRM,ADTRP,SEMA3C,ADAM17,CAMK1D,ELP3,ETS1,RIN2,HDAC4,JAM2,MAPRE2,PRKG1,STARD13,RABGEF1,CLDN1,DOCK4,TGFBR1,TAC4,MAGI2,NF1,SORL1,RELN,HDAC9,SELE,MGAT5,NUMB,NAV3,JAG1,MEOX2,PPP3CA,DOCK10,PTPRU,JAK2,SMAD3,CD300A,PLXNB1,HDAC7,TNF,LRCH1,DLG5,LGR6,ARID2,ADORA3,DOCK1,SMOC2,SULF1,C5AR1,PIK3R3,AMOTL1,SELP,CTNNA1,EGF,KIT,TRPV4,RAPGEF2,SEMA3E,MARVELD3,EPHB2,ERBB4,FER,MAP3K3,SLAMF1,ABL2,BSG,PTPRK,ATP8A1,CCL3,FGF7,LAMA1,SEMA5B,KIF2A,NEDD9,PDGFD,PIK3CD,WDPCP,FGFR1,NTF3,BDKRB1,SEMA3A,SRGAP3,FERMT2,MAP2K5,SMPD3,TNN,DAB2,ACTN4,ADAM10,CCL7,ITGA3,PIN1,SYNPO2,CLASP1,HGF,MYLK,CAPN7,INSR,MACF1,PAK1,AKT3,PRKX,ULK4,GNA13,SPRED1,PTPRR,DOCK7,HDAC5,TDGF1,LYVE1,ADARB1,STAT3,AMOT,PAK3,LGALS9,EFCAB1,APC,BMPR1A,DPP4,PRKCA,SPINT2,CTSH,RGCC,SRGAP1,IL6R,CLASP2,FGF1,RGN,RREB1
GO:0035295	tube development	0.00044838776289950265	ISM1,GTF2I,MAPK14,RUNX1,MYO18B,NPHP3,KCNQ1,NRXN1,NRXN3,TGFBR3,CDH13,CSMD1,SDCCAG8,GADD45A,PLXNA4,GPC3,ALDH1A2,CECR2,CCR3,COL22A1,CBFA2T2,ROBO2,C2CD3,PRKACB,COL15A1,AGT,RHOJ,CDH2,LOXL2,GREB1L,CYBB,OTC,NTN1,FMNL3,SLIT2,PDCD6,SMAD1,BASP1,NOX5,CELSR1,ATRX,RYR2,KIF26B,RORA,CHD7,MECP2,LGR5,PRKD1,TGFB2,FMN1,VAV3,PLCD3,SEMA5A,SGCD,NR2F2,PML,PLXNA2,BCL2,ARHGAP24,EPHB1,LRP2,TMEM100,VANGL2,EPHA7,NR4A1,RNF213,THBS2,PYY,ENPP2,MME,TNMD,SLC1A1,ZFPM2,RARB,BMPER,PGK1,CTNNBIP1,SOX8,PRKCB,VDR,DLC1,EDNRB,ROBO1,RXFP1,FAP,FLT1,GRHL2,COL2A1,SEC24B,PKHD1,STK3,TGM2,VASH2,KDR,PRCP,EDA,ABCA12,CALCRL,COL18A1,NRP1,PTPRM,SHROOM3,LGR4,ADTRP,THRB,SEMA3C,ATF2,ETS1,RIN2,ADAMTS16,NTRK2,STARD13,GLI3,RAP1A,TGFBR1,CYSLTR1,ADAM12,EP300,TBX3,PCSK5,EMCN,WARS2,NF1,ELK3,TTC8,ASXL1,HDAC9,RALA,JAG1,MEOX2,WWTR1,AGGF1,SHOX2,AHI1,SMAD3,COL8A1,RDH10,HDAC7,ITPK1,TNF,COBL,OVOL2,DLG5,PHEX,SIM2,ARID2,CALD1,HMGA2,KIF18A,QKI,SMOC2,SULF1,C5AR1,PIK3R3,EFNB2,AMOTL1,EGF,CMA1,KIT,CC2D2A,CPS1,EDAR,RAPGEF2,ESR1,JAK1,SEMA3E,EPHB2,MAP3K3,LRP5,BSG,CAMP,SETD2,ALX4,ARHGAP35,FGF7,LAMA1,MYH9,SLC39A12,SOX4,PIK3CD,ENPEP,AR,SUFU,EYA1,SRPK2,ZMIZ1,MAP2K5,SMPD3,PBX1,RNF207,ITGA3,THSD7A,TGFA,HGF,MYLK,PAK1,SKI,AKT3,PRKX,C1GALT1,GNA13,SPRED1,BTRC,ESRP2,EXT1,TYMP,UMOD,HDAC5,KLHL3,TDGF1,STIM1,STAT3,TP63,AMOT,BRIP1,BMPR1A,PRKCA,SPINT2,CTSH,RGCC,MAP2K1,SOS1,FGF1,TBX20
GO:0030334	regulation of cell migration	0.00045137191200111016	SRGAP2B,PLCB1,CMKLR1,PTPRT,DUSP22,FBLN1,ITGA2,SUN2,NRG3,CDH13,GADD45A,PLXNA4,FLRT2,NRG1,RFFL,MCTP1,SH3RF2,UNC5D,SEMA6D,CTNNA2,CCR1,TIAM1,RHOC,LDLRAD4,NKD1,SCAI,FBXO31,PHACTR1,AGT,RHOJ,LAMA2,SEMA3D,DACH1,NTN1,ZNF268,RIN3,LAMA4,SLIT2,PDCD6,IGF1R,PTPRG,VCL,STK24,FRMD5,NTNG1,NTRK3,SH3BP1,MECP2,SEMA4D,SLC8A1,PRKD1,TGFB2,FUT4,S100A11,SEMA5A,LAMA3,NR2F2,PLXNA2,BCL2,KANK1,ENPP2,UNC5C,MTOR,LDB2,BMPER,EPB41L4B,PRKCE,FOXO3,MITF,DLC1,ROBO1,FLT1,KDR,PRCP,APP,NRP1,PTPRM,ADTRP,SEMA3C,ADAM17,CAMK1D,ELP3,ETS1,RIN2,HDAC4,JAM2,MAPRE2,PRKG1,STARD13,RABGEF1,CLDN1,DOCK4,TGFBR1,MAGI2,NF1,SORL1,RELN,HDAC9,SELE,MGAT5,NUMB,NAV3,JAG1,MEOX2,PPP3CA,DOCK10,PTPRU,JAK2,SMAD3,CD300A,PLXNB1,HDAC7,TNF,LRCH1,DLG5,LGR6,ARID2,ADORA3,DOCK1,SMOC2,SULF1,C5AR1,PIK3R3,AMOTL1,SELP,EGF,KIT,TRPV4,RAPGEF2,SEMA3E,MARVELD3,EPHB2,ERBB4,FER,MAP3K3,SLAMF1,BSG,PTPRK,ATP8A1,CCL3,FGF7,LAMA1,SEMA5B,KIF2A,NEDD9,PDGFD,PIK3CD,WDPCP,FGFR1,NTF3,BDKRB1,SEMA3A,SRGAP3,FERMT2,MAP2K5,SMPD3,TNN,DAB2,ACTN4,ADAM10,CCL7,ITGA3,SYNPO2,CLASP1,HGF,MYLK,CAPN7,INSR,MACF1,PAK1,AKT3,PRKX,ULK4,GNA13,SPRED1,PTPRR,DOCK7,HDAC5,TDGF1,LYVE1,ADARB1,STAT3,AMOT,PAK3,LGALS9,APC,BMPR1A,DPP4,PRKCA,CTSH,RGCC,SRGAP1,IL6R,CLASP2,FGF1,RREB1
GO:0055080	cation homeostasis	0.0004832049327445401	CMKLR1,ANK3,SLC24A3,KCNQ1,TMPRSS3,MS4A1,GRIA1,SLC9B1,ANK2,FAM155A,SLC12A1,TF,SCARA5,RYR3,PLCE1,CCR1,CCR3,CFTR,RAB7A,AGT,ATP6V0D1,PARK2,OTC,CP,KCNMA1,SLC24A4,TMPRSS6,TRPC5,DMD,NOX5,GRM1,TRPM2,RYR2,DHRS7C,LRRK2,SLC1A3,SLC4A4,CHD7,DISC1,HEXB,SLC8A1,PRKD1,FLVCR1,ATP1A4,SGCD,

			GRIN2B,EXT2,PTGFR,PML,AKAP6,BCL2,SLC30A5,ITPR2,CYB561A3,FYN,PDE4D,GRIK2,RAP1GDS1,SLC1A1,ATP1A1,PLN,HEPHL1,PRKCE,PRKCB,VDR,ADCY8,CAPN3,EDNRB,SLC12A8,PKHD1,TGM2,KDR,APP,MT1HL1,TM9SF4,GRIN2A,CD38,SLC30A7,COMMD1,KCTD7,CACNA1C,CYSLTR1,CACNA2D1,TRPM7,ADORA2A,FAM20A,TAC4,MICU1,SLC39A8,SLC9C1,GRM5,ADCYAP1R1,FBXL5,SLC24A2,KL,STIM2,TPCN2,JAK2,SMAD3,NOS1,TMTC4,SLC39A10,SNX5,ARF1,TRPC4,C5AR1,TRDN,DMXL1,TRPV4,ESR1,HTR2C,SLC30A8,UBE3A,ABL2,CCL3,MCUR1,PTGER3,SLC39A12,P2RX1,CDH23,RYR1,P2RX7,RASA3,BDKRB1,BDKRB2,ADRA1B,THADA,ATP6V1H,SCNN1A,ATP13A3,CLN6,GPR55,CCL7,MT1F,TRPM4,BMP6,BTBD9,CALCR,CACNB2,MICU2,TMTC2,JPH4,CORIN,GNA13,CACNA1A,S1PR3,TRPM1,EXT1,SCO2,UMOD,SLC24A5,SLC40A1,STIM1,SLC9A9,CASQ2,TMEM175,CNNM3,SLC7A8,UPK3A,UBASH3B,SLC9A2,CCL14,CCL15,MCOLN3,RGN
GO:0099504	synaptic vesicle cycle	0.0005123731856145241	*CTBP2,SYN3,GRIN3A,NRXN1,NRXN3,RAB5A,RIMS2,SYNDIG1,ERC2,CDH2,ITSN1,PCDH17,APBA2,SYT9,SYT1,UNC13C,LRRK2,GSK3B,DNAJC6,PRKAR1B,SNAP25,SNAP23,CADPS,ABCA13,NLGN4X,PRKCB,STON1,CASK,RIMS3,OPHN1,DNM3,STXBP5,RAP1A,CHRNA5,ADORA2A,DDC,DGKI,RIMS1,NLGN1,BLOC1S6,P2RX1,UNC13A,UNC13B,P2RX7,RAPGEF4,KIAA1109,STX3,SYT2,BTBD9,CACNB2,SNAP29,FMR1,AMPH,PRKCG,DTNBP1,GAK,RAP1B*
GO:0098771	inorganic ion homeostasis	0.0005469476737069162	*CMKLR1,ANK3,SLC24A3,KCNQ1,TMPRSS3,MS4A1,GRIA1,SLC9B1,ANK2,FAM155A,SLC12A1,TF,SCARA5,RYR3,PLCE1,CCR1,CCR3,CFTR,RAB7A,AGT,ATP6V0D1,PARK2,OTC,CP,KCNMA1,SLC24A4,TMPRSS6,TRPC5,DMD,NOX5,GRM1,TRPM2,RYR2,DHRS7C,LRRK2,SLC1A3,SLC4A4,CHD7,DISC1,HEXB,SLC8A1,PRKD1,FLVCR1,ATP1A4,SGCD,GRIN2B,EXT2,PTGFR,PML,AKAP6,BCL2,SLC30A5,ITPR2,CYB561A3,FYN,PDE4D,TBXAS1,GRIK2,RAP1GDS1,SLC1A1,ATP1A1,PLN,HEPHL1,PRKCE,PRKCB,VDR,ADCY8,CAPN3,EDNRB,SLC12A8,PKHD1,TGM2,KDR,APP,MT1HL1,TM9SF4,GRIN2A,CD38,SLC30A7,COMMD1,KCTD7,CACNA1C,CYSLTR1,CACNA2D1,TRPM7,ADORA2A,FAM20A,TAC4,MICU1,SLC39A8,SLC9C1,GRM5,ADCYAP1R1,FBXL5,SLC24A2,KL,STIM2,TPCN2,JAK2,SMAD3,NOS1,TMTC4,SLC39A10,SNX5,ARF1,TRPC4,C5AR1,TRDN,DMXL1,TRPV4,ESR1,HTR2C,SLC30A8,UBE3A,ABL2,CCL3,MCUR1,PTGER3,SLC39A12,P2RX1,CDH23,RYR1,P2RX7,RASA3,BDKRB1,BDKRB2,ADRA1B,THADA,ATP6V1H,SCNN1A,ATP13A3,CLN6,GPR55,CCL7,MT1F,TRPM4,BMP6,BTBD9,CALCR,CACNB2,ENPP1,MICU2,TMTC2,JPH4,CORIN,GNA13,CACNA1A,S1PR3,TRPM1,EXT1,SCO2,UMOD,SLC24A5,SLC40A1,STIM1,SLC9A9,CASQ2,TMEM175,CNNM3,SLC7A8,UPK3A,UBASH3B,SLC9A2,CCL14,CCL15,MCOLN3,RGN*
GO:0018193	peptidyl-amino acid modification	0.0005770296629945985	*KMT2C,AGBL1,MAPK14,DUSP22,TTLL2,EPHA6,VRK2,NRXN1,SMYD2,CSNK2A1,CEP41,CHEK2,GADD45A,PRDM9,NRG1,PRMT8,STK38L,AGBL4,TET3,ALK,SPOCK3,EPHA3,DCLK1,AGT,ATAT1,LOXL2,TXK,SETD3,HDAC2,TTLL8,RPS6KA2,IGF1R,MDM2,TRPC5,DMD,MORF4L1,ATRX,LRRK2,VRK1,NTRK3,DPY30,GALNTL6,MECP2,SEMA4D,GSK3B,PRKD1,ERCC6,OGT,BDNF,PRKCH,RYK,ZDHHC17,MGAT2,KDM4C,ZDHHC14,PML,PPARGC1A,BCL2,EPHB1,IL12RB2,EPHA7,FYN,CD44,PDE4D,ENPP2,CSNK1G3,SETD7,SLC1A1,PHF20,MTOR,ROR1,OGFOD1,PLCL1,EPM2A,PRKCE,KMT2A,PRKCB,CAPN3,DPH6,FLT1,NOX4,MAP3K7,MAPK9,SUPT3H,KDR,APP,NRP1,EGFLAM,KAT5,ZZZ3,ADAM17,CAMK1D,CDC42BPG,ATF2,SAMSN1,EFEMP1,ROS1,DOT1L,RPS6KA5,PARD3,TLK2,SGK2,HDAC4,DNMT1,GHR,NTRK2,CDC42BPA,BRD4,CAMK4,SPRED2,TGFBR1,PRDX4,EPHA5,TOP1,PEAK1,EP300,MORC3,HMG20A,BRD7,PIBF1,EFNA5,MERTK,BRD1,POR,RELN,GRM5,HDAC9,MGAT5,MAP3K13,NLK,EGLN3,PER1,CBLB,CNTN1,TRIM16,KAT7,THAP7,LRRK1,CNOT7,DNMT3B,JAK2,CD300A,MAP2K4,NOS1,CDK1,HDAC7,MAGT1,MCM3AP,TNF,DPY19L1,STK32B,BCL11A,HMGA2,STK32C,DYRK4,LRP4,WDR5,SAE1,STT3A,TENM1,EGF,KIT,STK32A,JAK1,EPHB2,MECOM,ERBB4,FER,FNIP1,TTLL5,ABL2,SETD2,FGF7,GLYR1,NSMCE2,SOX4,NEDD9,PDGFD,AGTPBP1,WDR82,ARID4A,KDM3A,TNKS,AUTS2,FGFR1,ARID4B,DAD1,IL23R,NTF3,ZDHHC11,ZDHHC11B,ZDHHC9,BCOR,EYA1,IFNA8,SRPK2,BDKRB2,GNL3,PRKAA2,RLF,MARK2,ZMIZ1,FLT3,MAP2K5,MARK1,TCL1B,PLCL2,TTLL11,PHF20L1,PIN1,RPS6KA6,TRPM4,STK38,BMP6,CIT,TGFA,HGF,CALCR,DPY19L2,SH3BP5,TLK1,KDM4B,MAST4,NOS1AP,INSR,PAK1,AKT3,TESK2,PRKX,MSL2,PCK1,TTN,TAF1,CHD5,FNIP2,FUT8,KANSL1,SPRED1,PRDM16,ZNF451,NCOA1,PRKCG,PRLR,UMOD,DOCK7,TDGF1,UGGT2,PRKCQ,DOCK3,DPY19L3,LRP8,MAPKAPK2,TUSC3,NGF,PRKCA,ABI1,NCAPG2,DPH1,IL6R,MAP2K6,SPTBN4,MAP2K1,ART4*
GO:00069	organelle	0.0005879964474120	*PLCB1,KMT2C,OMA1,FMNL2,CDC42EP3,ARID1B,TRAPPC8,CEP44,RAB27A,ANXA8L1,HSF2BP,ANK3,AKAP13,VPS13D,SORBS2,NPHP3,*

96	organization	972	*TTLL2,KCNQ1,DPF3,SUN2,NUBPL,ATF7IP,NRXN1,ZRANB1,RAB5A,DOCK2,PCNT,MTPN,SMYD2,CLEC16A,HUS1,FGD6,ANK2,PDE4DIP,TF,ERCC4,PIK3C2B,SDCCAG8,CEP41,TMEM108,CHEK2,TUBGCP6,GADD45A,PRDM9,ACIN1,CHAF1A,STXBP6,RAD51D,HYDIN,CECR2,CHCHD6,CLVS2,SUZ12,PLCE1,CTNNA2,BBS9,RHOC,DNAJC15,DPYSL2,REEP3,TET3,ARFIP1,TACC2,PIK3C3,UTRN,SPEF2,RAB7A,PHACTR1,C2CD3,EPHA3,LEMD3,PHACTR3,PSTPIP2,FSIP2,ATAT1,VTI1A,WDR11,ATP6V0D1,RHOJ,ZFYVE1,CDH2,PARK2,BANP,LOXL2,CORO2B,FRMPD4,TLN2,ANKFN1,SETD3,FMNL3,HDAC2,SLIT2,PWP2,TTLL8,DYNLL2,PDCD6,RPS6KA2,LCMT1,KRT25,SYT9,CEP97,DMD,SYT1,KRT2,SIPA1L3,CELSR1,FSHR,FMN2,HNRNPA2B1,MAEL,MORF4L1,NCKAP5,MRPS11,WDR83OS,ATRX,ACTR5,FRMD5,TRPM2,CTNNA3,USP16,DIAPH2,KIF3B,TOP3A,UNC13C,LRRK2,VRK1,NTRK3,NUSAP1,IQCJ-SCHIP1,CHD7,DISC1,DPY30,HEXB,PAN3,WRAP73,CDCA2,PDXP,SH3BP1,MECP2,GSK3B,CDC14A,PRKD1,ERCC6,FRMD6,OGT,FMN1,VAV3,SEMA5A,KRT74,LLGL2,PCDH15,DNAAF2,EML1,TOR1AIP1,HPS3,WASF1,KDM4C,RAG1,RAG2,FIGN,MREG,PLEKHM2,SNAP25,TUBB1,PML,PPARGC1A,USP36,ARHGAP12,SNAP23,BCL2,CHMP4C,RAD51B,RALBP1,MOV10L1,SMARCE1,SIPA1L1,LRRC4,VANGL2,GDAP1,ARHGEF10,MICAL3,RPGR,PARN,SMARCA4,FLNC,RNF213,KANK1,ATP10B,ACTN2,ATP8A2,DCDC2,FRMD3,NAV2,RAP1GDS1,MYLK3,SETD7,PALLD,PHF20,RFX4,STRIP1,MTOR,RMI2,GAS7,PKP2,UBXN2B,CCDC88B,ERMN,PTPRD,BBS2,MCM3,OGFOD1,EPM2A,CHCHD3,BANF2,EPB41L4B,KLHL1,ZMYND11,EHBP1,IFT88,PRKCE,ATP6V1D,EIF2S1,HELLS,KMT2A,PRKCB,RAB31,SCMH1,SCP2,TRAPPC12,CAPN3,PARD3B,CCDC88C,DLC1,SATB2,TBC1D4,TDRD3,BNIP3L,NASP,STX12,CENPV,LRRC49,MAP7,MAPK9,NEBL,NR3C1,VPS37B,EYA4,SEC24B,MDM1,PKHD1,THSD7B,KDR,PPHLN1,RAB30,TMEM138,NRP1,SHROOM3,TSNARE1,ARHGEF18,ELMO1,IMMP2L,KAT5,RANBP10,TTC12,CYLC2,DIS3L2,PARVG,TUBAL3,CDC42BPG,DIAPH3,ATF2,ETS1,PEX5L,SMG6,DOT1L,KPNB1,TCF7L2,TEP1,AFF2,BICD1,PARD3,TLK2,ZNF830,KIFAP3,OPHN1,SAMD9,CCDC88A,TUB,CNN3,STK36,BCL2L1,ADAMTS16,DNAH8,HDAC4,DNMT1,MAPRE2,PRKG1,M1AP,CDC42BPA,BRD4,PTPDC1,STARD13,NINL,SMCHD1,ZNF207,COG7,MORC2,TGFBR1,RRP7A,DYM,EPHA5,TOP1,ITGB3BP,CHAF1B,EP300,RAB2A,SYNE1,CSRP2BP,HMG20A,SCFD1,SPAG17,STX8,TRIOBP,SYCP1,GRID2,PARVB,PIBF1,PNPLA3,SAMM50,TRPM7,CCDC170,VPS39,FLNB,SPG11,ADCK1,CABIN1,EFNA5,SPECC1L,BRD1,NF1,RFC3,ARHGAP6,BID,CEP89,IFT80,MND1,TTC8,DMRT1,ASXL1,RELN,USP3,HDAC9,DYNC1H1,RHPN2,CCDC57,NPHP4,RALA,IL1RAP,NAV3,EXOC5,NEB,PLS1,ATP8B4,CAPZB,CXADR,GNPTAB,SATB1,WWTR1,CCSER2,MNAT1,STARD9,TMEM67,GPSM2,KAT7,L3MBTL3,MPRIP,TMEM135,ASAP1,BORA,FHOD3,MZT1,NLGN1,SH3KBP1,PHACTR2,RAD51AP1,SYCP2,DDX3X,RBL2,CNOT7,VPS37A,TPCN2,JAK2,AHI1,RPA1,SMAD3,VPS41,BAIAP2L1,PTGFRN,PLXNB1,CDK1,HDAC7,MCM3AP,NBN,PRRC2C,TNF,COBL,ATXN10,BLOC1S6,FCHSD2,INO80,AFAP1,ARID2,KATNAL1,SPAG16,ARF1,CALD1,HMGA2,MYO6,KIF18A,GCLC,RAB23,ACTR8,EPS8,NUDCD3,DAAM2,L3MBTL4,PABPC1L,TOMM34,ANKRD31,CCP110,CISD2,LRCH3,WDR5,CDS2,SAE1,TRDN,AMOTL1,PHF8,TMEM11,ZNF423,CDC14B,INSC,CHD6,CTNNA1,TENM1,ARHGEF11,CDKL5,EFHC2,MYH14,PRKAR1A,WDR35,ARHGAP11B,BPTF,DYNC2H1,FAM171A1,MLLT3,STX16,EGF,SEC16B,SPIRE2,VPS8,KIT,UBR2,BLM,TRPV4,CC2D2A,DNAJB6,ESR1,MYPN,SEMA3E,SPECC1,FGF13,NUP155,ARMC2,KRT76,MECOM,RNF4,TBCD,TOX,C14ORF39,ERBB4,FER,SPTA1,TDRKH,IFT43,LRP5,TTLL5,RPS27L,SLAMF1,ABL2,F11R,FARP2,GAS8,SETD2,VPS13C,ARHGAP35,CD2AP,CCL3,FGF7,MYO7B,PREX1,ARHGAP25,CDKL1,FGD4,KIF2A,MCM9,REEP1,ARF4,ARFGAP3,MYH9,NSMCE2,PACSIN2,SLC39A12,TEKT4,KIF23,MIPEP,NEDD9,SPTB,AGTPBP1,ARID4A,FAM172A,KDM3A,TNKS,UNC13A,WDPCP,AUTS2,MEF2A,MGME1,OCLN,SYNM,UNC13B,ARID4B,MAP2,SLX1B,JMY,NTF3,P2RX7,TEX14,BRD9,ATG2B,BCOR,COG1,HIGD1B,SNX30,C10ORF90,CEP250,EYA1,SRPK2,ARMC9,CENPP,GNL3,L3MBTL1,PRKAA2,ARHGAP10,EPB41L3,KIF4A,MARK2,REEP2,PDLIM5,PPARGC1B,SNX7,VPS16,FERMT2,MARK1,SMPD3,CLN6,HDAC8,LCP1,STX3,ACTN4,BLOC1S5,CCL7,DAAM1,TTLL11,NUP153,PIN1,SYNPO2,THSD7A,TUBA3C,NME8,SCLT1,SHROOM4,CIT,CLASP1,EIF4ENIF1,SMTNL2,COX16,KATNAL2,PCDHGA3,TGFA,TMEM170A,TMOD1,G3BP2,GAS2,HGF,PKP1,RASSF8,SPATA6,CENPF,MYO1B,TLK1,ELN,KATNA1,KDM4B,MAST4,NOS1AP,SNAP29,ARHGAP28,INSR,MACF1,PAK1,PDE3A,SMIM20,AKT3,CDC16,CTDP1,LIMK2,TESK2,THEM4,CHFR,DKC1,ULK4,ABCD1,BMF,CRMP1,HEPACAM2,MAD1L1,RPGRIP1,ESYT2,SORT1,TTN,CLIP1,KATNBL1,SMARCAD1,CH*

			D5,KANSL1,PPP2R3C,KRT4,PRDM16,RBL1,HIRA,MPDZ,OPTN,PGM5,EXT1,IFT81,MYH6,NCAPH2,SCO2,TYMP,UMOD,DOCK7,ELMO2,FBXW11,G3BP1,HDAC5,ICK,TTC29,GOSR2,TTC39C,MYH11,NDE1,PRKCQ,SHPRH,TP63,USP7,AMOT,BRIP1,PAK3,DTNBP1,GAK,TMEM175,ZNF518A,MYOM2,ALPK1,APC,CKAP5,PRKCA,PDS5A,ABI1,DHX9,NCAPG2,RGCC,MDN1,SIAH3,TFIP11,SPTBN4,CLASP2,MAP2K1,VPS35,ZRANB3,ATMIN,GOLGA6D,GOLGA8J,PPP1R9A,ZNF501
GO:0048017	inositol lipid-mediated signaling	0.0006227315114778355	TPTE,PLCB1,TPTE2,PIK3C2B,PLCE1,PIK3C3,PLCB4,RGL2,CRNN,AGT,KCNH1,IGF1R,FSHR,NTRK3,SEMA4D,PLCH2,OGT,TGFB2,PLCD3,PRR5,ROR1,PLCL1,FLT1,PREX2,KDR,PIK3R5,MYO16,NTRK2,RASGRP1,GAB2,NF1,RELN,NPR3,JAK2,PLXNB1,TNF,PLCH1,MUC5AC,SELP,EGF,KIT,PIK3C2G,UBE3A,ERBB4,PDGFD,PIK3CD,FGFR1,FLT3,PLCL2,PIP5K1B,HGF,PI4KA,INSR,TNFAIP8L3,PIK3R2
GO:0048015	phosphatidylinositol-mediated signaling	0.0006851599055189232	TPTE,PLCB1,TPTE2,PIK3C2B,PLCE1,PIK3C3,PLCB4,RGL2,CRNN,AGT,KCNH1,IGF1R,FSHR,NTRK3,SEMA4D,PLCH2,OGT,TGFB2,PLCD3,PRR5,ROR1,PLCL1,FLT1,PREX2,KDR,PIK3R5,MYO16,NTRK2,RASGRP1,GAB2,NF1,RELN,NPR3,JAK2,PLXNB1,TNF,PLCH1,MUC5AC,SELP,EGF,KIT,PIK3C2G,UBE3A,ERBB4,PDGFD,PIK3CD,FGFR1,FLT3,PLCL2,PIP5K1B,HGF,PI4KA,INSR,PIK3R2
GO:0050919	negative chemotaxis	0.0007044960968940587	NRG3,PLXNA4,FLRT2,NRG1,SEMA6D,ROBO2,SEMA3D,NTN1,SLIT2,SEMA4D,SEMA5A,RYK,EPHA7,UNC5C,ROBO1,SEMA3C,LGR6,SLIT3,SEMA3E,SEMA5B,SEMA3A,DPP4
GO:0010977	negative regulation of neuron projection development	0.0007433403169721776	DAB1,DCC,FSTL4,SEMA6D,SPOCK1,CBFA2T2,SEMA3D,NTN1,HDAC2,PTPRG,LRRK2,SEMA4D,SEMA5A,RYK,EPHA7,PMP22,KANK1,FAT3,NRP1,SEMA3C,CD38,DENND5A,DNM3,TNR,PTPN9,DGUOK,NLGN1,BCL11A,LRP4,EFNB2,TRPV4,PTPRO,SEMA3E,UBE3A,FGF13,EPHB2,SEMA5B,MAP2,SEMA3A,DAB2,CRMP1,RIT2,DTNBP1,GAK,KIAA0319
GO:0006836	neurotransmitter transport	0.0007873653154562602	CTBP2,SYN3,GRIN3A,NRXN1,NRXN3,SLC1A2,RAB5A,RIMS2,MCTP1,SLC6A2,PTPRN2,ERC2,PARK2,MCTP2,APBA2,SYT9,SYT1,UNC13C,LRRK2,SLC1A3,GSK3B,SNAP25,SNAP23,NR4A1,SLC1A1,CADPS,PRKCB,GABRA2,CASK,RIMS3,STXBP5,RAP1A,SLC6A17,CHRNA3,CHRNA5,SV2B,ADORA2A,NF1,DDC,DGKI,RIMS1,SLC22A2,NLGN1,SLC22A3,NOS1,BLOC1S6,GRM4,SV2C,P2RX1,UNC13A,UNC13B,P2RX7,STX3,SYT2,KMO,CACNB2,SNAP29,FMR1,PRKCG,DTNBP1,RAP1B,SNCAIP
GO:0002027	regulation of heart rate	0.0008461597099623309	KCNQ1,TNNI3K,ANK2,KCND3,KCNE1,AGT,MDM2,DMD,CTNNA3,RNLS,RYR2,SLC8A1,KCNJ3,KCNE2,PDE4D,SLC1A1,PKP2,PLN,EDNRB,TACR3,CACNA1C,CACNA2D1,CACNA1D,SHOX2,TNF,KCNE4,ADRA1B,SEMA3A,HCN4,TRPM4,CACNB2,NOS1AP,MYH7,MYH6,CASQ2,SPTBN4
GO:0050801	ion homeostasis	0.0008945146795402421	CMKLR1,ANK3,SLC24A3,KCNQ1,TMPRSS3,MS4A1,GRIA1,SLC9B1,ANK2,FAM155A,SLC12A1,TF,SCARA5,RYR3,PLCE1,CCR1,CCR3,CFTR,RAB7A,AGT,ATP6V0D1,PARK2,OTC,CP,KCNMA1,SLC24A4,TMPRSS6,TRPC5,DMD,NOX5,GRM1,TRPM2,RYR2,DHRS7C,LRRK2,SLC1A3,SLC4A4,CHD7,DISC1,HEXB,SLC8A1,PRKD1,FLVCR1,ATP1A4,SGCD,GRIN2B,EXT2,PTGFR,PML,AKAP6,BCL2,SLC30A5,ITPR2,CYB561A3,FYN,PDE4D,TBXAS1,GRIK2,RAP1GDS1,SLC1A1,ATP1A1,PLN,HEPHL1,PRKCE,PRKCB,VDR,ADCY8,CAPN3,EDNRB,SLC12A8,PKHD1,TGM2,KDR,APP,MT1HL1,TM9SF4,GRIN2A,CD38,SLC30A7,COMMD1,KCTD7,CACNA1C,CYSLTR1,CACNA2D1,TRPM7,ADORA2A,FAM20A,TAC4,MICU1,SLC39A8,SLC9C1,GRM5,ADCYAP1R1,FBXL5,SLC24A2,KL,STIM2,TPCN2,JAK2,SMAD3,NOS1,TMTC4,SLC39A10,SNX5,ARF1,GCLC,TRPC4,C5AR1,TRDN,DMXL1,TRPV4,CPS1,ESR1,HTR2C,SLC30A8,UBE3A,ABL2,CCL3,MCUR1,PTGER3,SLC39A12,P2RX1,CDH23,RYR1,P2RX7,RASA3,BDKRB1,BDKRB2,ADRA1B,THADA,ATP6V1H,HCN4,SCNN1A,ATP13A3,CLN6,GPR55,CCL7,MT1F,TRPM4,BMP6,BTBD9,CALCR,CACNB2,ENPP1,MICU2,TMTC2,ABCD1,JPH4,CORIN,FHL1,GNA13,CACNA1A,PPP2R3C,S1PR3,TRPM1,EXT1,SCO2,UMOD,KLHL3,SLC24A5,SLC40A1,STIM1,SLC9A9,CASQ2,TMEM175,CNNM3,SLC7A8,UPK3A,UBASH3B,SLC9A2,CCL14,CCL15,MCOLN3,RGN
GO:0007417	central nervous system development	0.0009309739948361401	MACROD2,PLCB1,DAB1,ASIC2,DCC,WLS,GRIK1,SUN2,CA10,NRXN1,SHC3,SLC1A2,POTEE,MTPN,SHANK2,NRG3,ARNT2,BCAN,TMEM108,PLXNA4,NRG1,HYDIN,ALDH1A2,SUZ12,SEMA6D,SPOCK1,DRP2,CTNNA2,RBFOX2,AGBL4,DPYSL2,IL1RAPL2,TACC2,ALK,SPEF2,CNTN4,PHACTR1,S100B,ROBO2,C2CD3,DCLK1,ATAT1,ATP6V0D1,CDH11,CDH2,PARK2,MDGA2,NEGR1,GNB4,TRAPPC9,TRIO,HDAC2,SLIT2,TFAP2D,IGF1R,SMAD1,VCAN,PTPRG,DMD,SYT1,BASP1,CELSR1,MBOAT7,ATRX,TENM4,LRRK2,NTRK3,RORA,CHD7,DISC1,SOX6,MECP2,GSK3B,SLC8A1,SEMA5A,RYK,KCNC1,EML1,GRIN2B,NR2F2,CNTNAP2,PLXNA2,BCL2,EPHB1,LRP2,NR4A2,EPHA7,FYN,GABRB1,NA

			V2,UNC5C,SLC1A1,RARB,RFX4,MTOR,ROR1,BBS2,KLHL1,SOX8,NLGN4X,FOXO3,KALRN,DLC1,ROBO1,SATB2,ZNF148,GRHL2,COL2A1,SEC24B,STK3,APP,NRP1,IMMP2L,ATF2,ELP3,AFF2,ABAT,GRIN2A,DLX6-AS1,OPHN1,CASP5,STK36,MYO16,NTRK2,PRKG1,GLI3,EPHA5,TNR,SLC6A17,DSCAML1,TBX3,ATXN1,TP73,GRID2,ADORA2A,NF1,RGS7,TTC8,RELN,HAPLN1,KIRREL3,NDRG2,NUMB,PPP3CA,SH3GL2,MAS1,ELAVL4,MNAT1,CNTN1,CHST8,CTTNBP2,AK8,GABRA5,AHI1,TAGLN3,TNF,DLG5,POU6F2,PCDH19,DAAM2,TRPC4,C5AR1,CNTN6,HERC1,PHF8,SPATA5,ZNF423,CTNNA1,ARHGAP11B,BPTF,DYNC2H1,EGF,CMA1,RAPGEF2,UBE3A,FGF13,EPHB2,TOX,ERBB4,GAS8,SETD2,ARHGAP35,NHLH2,SOX4,AGTPBP1,ITGA8,MAP2,SUFU,SEMA3A,JRKL,MEIS2,ZMIZ1,PBX1,SRD5A2,RPS6KA6,SHROOM4,CENPF,ZBTB16,SKI,AKT3,ATP5J,FPGS,CCDC141,ZFHX2,TAF1,CHD5,EXT1,DOCK7,FBXW11,ADARB1,KCNC2,NDE1,STAT3,DTNBP1,LRP8,BMPR1A,VLDLR,SPTBN4,MAP2K1,SOS1,TBX20
GO:0055065	metal ion homeostasis	0.0011035068701208696	*CMKLR1,ANK3,SLC24A3,KCNQ1,TMPRSS3,MS4A1,GRIA1,ANK2,FAM155A,SLC12A1,TF,SCARA5,RYR3,PLCE1,CCR1,CCR3,AGT,ATP6V0D1,PARK2,CP,KCNMA1,SLC24A4,TMPRSS6,TRPC5,DMD,NOX5,GRM1,TRPM2,RYR2,DHRS7C,SLC1A3,CHD7,DISC1,HEXB,SLC8A1,PRKD1,FLVCR1,ATP1A4,SGCD,GRIN2B,EXT2,PTGFR,PML,AKAP6,BCL2,SLC30A5,ITPR2,CYB561A3,FYN,PDE4D,GRIK2,RAP1GDS1,SLC1A1,ATP1A1,PLN,HEPHL1,PRKCE,PRKCB,VDR,ADCY8,CAPN3,EDNRB,SLC12A8,PKHD1,TGM2,KDR,APP,MT1HL1,GRIN2A,CD38,SLC30A7,COMMD1,KCTD7,CACNA1C,CYSLTR1,CACNA2D1,TRPM7,ADORA2A,FAM20A,TAC4,MICU1,SLC39A8,GRM5,ADCYAP1R1,FBXL5,SLC24A2,KL,STIM2,TPCN2,JAK2,SMAD3,NOS1,TMTC4,SLC39A10,SNX5,ARF1,TRPC4,C5AR1,TRDN,TRPV4,ESR1,HTR2C,SLC30A8,ABL2,CCL3,MCUR1,PTGER3,SLC39A12,P2RX1,CDH23,RYR1,P2RX7,RASA3,BDKRB1,BDKRB2,ADRA1B,THADA,SCNN1A,ATP13A3,GPR55,CCL7,MT1F,TRPM4,BMP6,BTBD9,CALCR,CACNB2,MICU2,TMTC2,JPH4,CORIN,GNA13,CACNA1A,S1PR3,TRPM1,EXT1,SCO2,UMOD,SLC24A5,SLC40A1,STIM1,CASQ2,CNNM3,SLC7A8,UPK3A,UBASH3B,CCL14,CCL15,MCOLN3,RGN*
GO:0050954	sensory perception of mechanical stimulus	0.001134780787498328	*PIEZO2,ASIC2,ITGA2,LOXHD1,KCNQ1,TMPRSS3,KCNE1,MYO3A,CNTN5,SLC1A3,STRC,CHD7,HEXB,CDC14A,PCDH15,LRP2,FYN,DCDC2,NAV2,ROR1,COL11A1,COL2A1,EYA4,THRB,TSPEAR,TUB,SRRM4,USP53,TRIOBP,GRM7,LHFPL3,CACNA1D,GABRA5,SOBP,TMTC4,TNF,TMC2,MYO6,SCN1A,MYH14,KIT,GABRB2,SLITRK6,MYO7B,MYO3B,PDZD7,CDH23,EYA1,LRIG1,PAX3,TECTA,HPN,TBL1X,USH2A,SPTBN4*
GO:0007420	brain development	0.0012207569366897086	*MACROD2,PLCB1,DAB1,WLS,SUN2,CA10,NRXN1,SLC1A2,POTEE,MTPN,SHANK2,NRG3,ARNT2,BCAN,TMEM108,PLXNA4,NRG1,HYDIN,ALDH1A2,SEMA6D,CTNNA2,RBFOX2,DPYSL2,TACC2,ALK,SPEF2,CNTN4,PHACTR1,ROBO2,C2CD3,DCLK1,ATAT1,ATP6V0D1,CDH2,NEGR1,GNB4,TRAPPC9,SLIT2,TFAP2D,IGF1R,SMAD1,PTPRG,DMD,SYT1,BASP1,MBOAT7,ATRX,LRRK2,RORA,CHD7,DISC1,SOX6,MECP2,GSK3B,SLC8A1,SEMA5A,RYK,KCNC1,EML1,GRIN2B,NR2F2,CNTNAP2,PLXNA2,BCL2,EPHB1,LRP2,NR4A2,EPHA7,FYN,UNC5C,SLC1A1,RARB,RFX4,BBS2,KLHL1,NLGN4X,FOXO3,DLC1,ROBO1,SATB2,ZNF148,GRHL2,SEC24B,APP,NRP1,IMMP2L,ATF2,AFF2,ABAT,GRIN2A,DLX6-AS1,OPHN1,CASP5,STK36,MYO16,NTRK2,PRKG1,GLI3,EPHA5,TNR,SLC6A17,DSCAML1,TBX3,ATXN1,GRID2,NF1,RGS7,TTC8,RELN,KIRREL3,NDRG2,NUMB,PPP3CA,MAS1,ELAVL4,MNAT1,CNTN1,CTTNBP2,AK8,GABRA5,AHI1,DLG5,PCDH19,HERC1,PHF8,SPATA5,ZNF423,CTNNA1,ARHGAP11B,BPTF,DYNC2H1,EGF,CMA1,RAPGEF2,UBE3A,FGF13,EPHB2,TOX,ERBB4,GAS8,SETD2,ARHGAP35,NHLH2,AGTPBP1,ITGA8,SEMA3A,MEIS2,ZMIZ1,PBX1,SRD5A2,SHROOM4,CENPF,SKI,AKT3,ATP5J,FPGS,CCDC141,ZFHX2,TAF1,CHD5,EXT1,DOCK7,FBXW11,KCNC2,NDE1,DTNBP1,LRP8,BMPR1A,MAP2K1,SOS1*
GO:0050890	cognition	0.001250296541215369	*PLCB1,NRXN1,NRXN3,SHANK2,GRIA1,CSMD1,RASGRF1,SORCS3,S100B,AGT,RCAN1,PARK2,DBH,JAKMIP1,CHD7,MECP2,BDNF,PRKAR1B,GRIN2B,RAG1,SNAP25,CNTNAP2,FYN,MME,SLC1A1,CHRM1,ARL6IP5,EPM2A,NLGN4X,GTF2A1L,ADCY8,KALRN,CHL1,APP,KCNK2,AFF2,GRIN2A,NTRK2,CAMK4,TNR,EP300,ATXN1,NF1,TTC8,RELN,GRM5,DGKI,SLC24A2,ELAVL4,MAPK8IP2,TMPRSS11E,GABRA5,SOBP,MAGT1,TNF,TANC1,BRINP1,KCNK10,C5AR1,KIT,DNAH11,UBE3A,FGF13,EPHB2,ATP8A1,ARF4,ITGA8,NTF3,AMFR,HLA-DRA,BHLHB9,MEIS2,ITGA3,SHROOM4,BTBD9,INSR,JPH4,PRKCG,TUSC3,NGF,VLDLR*
GO:19025	regulation	0.0012784995553090	*TPTE,PLCB1,CMKLR1,MAPK14,TPTE2,SH3RF3,WLS,TIMP3,DUSP22*

31	of intracellular signal transduction	633	*,AKAP13,FBLN1,VRK2,NRXN1,DOK5,DOCK2,SHANK2,SMYD2,CLEC16A,CDH13,RNF152,CHEK2,GADD45A,NRG1,RFFL,PLCE1,SH3RF2,CCR1,TIAM1,RHOC,RASGRF1,SCAI,ALK,RGL2,S100B,CRNN,LEMD3,AGT,RCAN1,CDH2,PARK2,FHL2,TRIM22,TRIM5,ITSN1,TRIO,MYO9A,SLIT2,PDCD6,LCMT1,IGF1R,MDM2,SLC24A4,NR3C2,DMD,SIPA1L3,FSHR,GRM1,WDR83,CAMTA1,LRRK2,NTRK3,RORA,IQCJ-SCHIP1,SH3BP1,SEMA4D,GSK3B,MAGI3,PRKD1,OGT,TGFB2,VAV3,SEMA5A,RYK,ZDHHC17,OTUD7A,ANKRD6,ARHGAP8,PRR5,PML,ARHGAP12,AKAP6,TAOK3,BCL2,RALBP1,ZNF675,ARHGAP24,EPHB1,SIPA1L1,LRP2,TMEM100,DEPTOR,ARHGEF10,EPHA7,PSD3,FYN,CHN1,CD44,FOXO1,PDE4D,KANK1,ARHGEF3,GRIK2,NLRP12,MTOR,ROR1,DDX21,BMPER,TIAM2,ARL6IP5,ZMYND11,PRKCE,HELLS,PRKCB,EFHB,CAPN3,KALRN,PJA1,DLC1,OASL,ROBO1,FLT1,NOX4,MAP3K7,PREX2,TRAF3IP2,PKHD1,STK3,TGM2,KDR,ARHGAP15,EDA,APP,NRP1,UACA,ARHGEF18,LINC00473,MAP3K5,PEX5L,PIK3R5,ROS1,TCF7L2,TNFRSF10B,RALGAPA2,TRAF3,OPHN1,BCLAF1,SGMS1,MOB3B,BCL2L1,GHR,MAPRE2,NTRK2,BRD4,RASGRP1,STARD13,RABGEF1,SPRED2,RAP1A,TGFBR1,PTTG1IP,RAPGEF1,EP300,TP73,RASGRF2,RALGPS1,MAGI2,NF1,RALGPS2,SORL1,ARHGAP6,BID,TRIM59,LZTR1,RELN,GRM5,IL18R1,NDRG2,MAP3K13,ADCYAP1R1,DGKI,PER1,KL,DOK6,WDR59,NLGN1,ARHGAP5,RPS20,AKAP7,LRRK1,MAPK8IP2,DDX3X,ZNF622,ARHGAP39,JAK2,CD300A,PLXNB1,HDAC7,TNF,TNIP1,EIF3A,POMC,PRDM15,DLG5,SHC2,AGO3,UBE2V1,EPS8,C5AR1,DEF6,NET1,SELP,TENM1,ARHGEF11,TNFRSF19,ARHGAP11B,SIPA1L2,EGF,KIT,UBR2,TRPV4,EDAR,RAPGEF2,ESR1,HTR2C,UBE3A,DNMBP,GRM4,MARVELD3,EPHB2,MECOM,NDFIP2,ERBB4,MAP3K3,FNIP1,SLAMF1,ABL2,DEPDC5,F11R,ARHGAP35,CD2AP,CCL3,PREX1,SPRED3,VWF,ARHGAP25,FGD4,IL20RA,GARNL3,PDGFD,PIK3CD,UBE2N,PUM1,AUTS2,FGFR1,PDE11A,NTF3,P2RX7,RASA3,APIP,AR,BDKRB2,ADRA1B,PRKAA2,SEMA3A,ARHGAP10,PDE5A,FLT3,SRGAP3,FERMT2,LTBR,MAP2K5,DAB2,GPR55,ACTN4,CCL7,EEF1E1,EEF1E1-BLOC1S5,ITGA3,PPM1B,PIN1,RPS6KA6,PIP5K1B,STK38,CIT,TGFA,HGF,RALGAPA1,CALCR,NOS1AP,ARHGAP28,INSR,PAK1,PDE3A,AKT3,LITAF,RRAGC,ULK4,MAD1L1,ARHGAP42,PPARA,TNFAIP8L3,GNA13,TAF1,CHD5,SPRED1,AKR1C2,BTRC,OPTN,PTPRR,RIT2,FBXW11,TDGF1,USP7,AMOT,DOCK3,PAK3,PIK3R2,RAP1B,KSR1,LGALS9,NFAT5,ALPK1,ITGA1,NGF,PRKCA,CCL14,CCL15,EDA2R,IL6R,MAP2K6,MAP2K1,SOS1,C1QTNF3,FGF1,RGN*
GO:0051966	regulation of synaptic transmission, glutamatergic	0.0012859686131720396	*GRIK1,NRXN1,CDH2,SYT1,CACNG3,GRM1,LRRK2,DISC1,TPRG1L,GRIK3,GRIK2,OPHN1,TNR,ADORA2A,CACNG2,RELN,GRM5,DGKI,GRM7,NLGN1,MAPK8IP2,TNF,TSHZ3,GRM4,UNC13A,DGKZ,KMO,DTNBP1*
GO:0006897	endocytosis	0.0013627884595334323	*IGHV1OR21-1,TMPRSS15,IGHV4-31,HEATR5A,IGHV3-64,IGHV1OR15-9,IGHV4OR15-8,ITGA2,TMPRSS2,TMPRSS3,RAB5A,DOCK2,GRIA1,CDH13,ANK2,LRP1B,TF,TMEM108,GPC3,NRG1,SCARA5,ENTHD1,MCTP1,DPYSL2,PIK3C3,RAB7A,EPHA3,RHOJ,LOXL2,ITSN1,RIN3,SYT1,CACNG3,GSG1L,LRRK2,ILDR1,SH3BP1,MTMR2,PRKD1,ATP9B,DNAJC6,WASF1,SNAP25,ARHGAP12,IGHV3-16,RALBP1,LRP2,CD163,CD163L1,NEU3,ENPP2,CSNK1G3,TMPRSS4,ABCA13,EHBP1,NLGN4X,STON1,STON1-GTF2A1L,LRRTM1,RAB31,MSR1,SGIP1,IGHV4-28,APP,CALCRL,ELMO1,IGF2R,BICD1,OPHN1,RIN2,TUB,BCL2L1,GHR,DNM3,ARR3,RABGEF1,NPC1,ADRBK2,CACNG2,MAGI2,GULP1,SORL1,IGHV4-4,SELE,NUMB,IGHV3OR16-12,PPP3CA,SH3GL2,TBC1D5,ANKRD13A,ATP9A,NLGN1,SH3KBP1,TPCN2,AHI1,XKR4,CD300A,SNX5,FCHSD2,ARF1,MYO6,DOCK1,IGLC2,IGLL5,RUFY1,LRP4,EFNB2,DMBT1,EGF,UBE3A,IGHV3-72,LRP5,XKR7,ABL2,AP2B1,CD2AP,ARHGAP25,MYH9,PACSIN2,ASGR2,NTF3,KIAA1109,RABGAP1L,SCAMP5,LDLRAD3,ATP6V1H,DAB2,SYT2,BTBD9,INSR,ENPP1,FMR1,IGHV1-69,AMPH,IGHV1-46,AP1G2,ESYT2,SORT1,HEATR5B,IGHV1-18,RIT2,DTNBP1,GAK,LRP8,MAPKAPK2,VLDLR*
GO:0006941	striated muscle contractio	0.0013869882952329794	*DTNA,KCNQ1,TNNI3K,ANK2,KCND3,KCNE1,DMD,CTNNA3,RYR2,SLC8A1,SGCD,MYH8,KCNJ3,KCNE2,NR4A1,PDE4D,ATP8A2,MTOR,PKP2,ATP1A1,PLN,HDAC4,PDE4B,CACNA1C,CACNA2D1,CACNA1D,NOS1,TNF,SCN1A,ARHGEF11,MYH14,TRPV4,RCSD1,FGF13,NUP15*

	n		5,KCNE4,SYNM,ADRA1B,PDE5A,SMPX,HCN4,RNF207,FGF12,STAC,TRPM4,CACNB2,NOS1AP,TTN,MYH7,MYH6,CASQ2,ASB3,MAP2K6
GO:0043087	regulation of GTPase activity	0.0013924722471696908	RGS6,SGSM1,FGD6,TBC1D22A,PLXNA4,TIAM1,RASGRF1,EPHA3,USP6,MYO9A,SIPA1L3,LRRK2,NTRK3,SH3BP1,SEMA4D,GSK3B,VAV3,ARHGAP12,PLXNA2,RALBP1,ARHGAP24,SIPA1L1,ARHGEF10,CHN1,RAP1GDS1,MTOR,TIAM2,EVI5,EIF2S1,KALRN,TBC1D4,TGM2,ARHGAP15,CPEB2,PKP4,RGS16,RALGAPA2,DOCK9,MAPRE2,NTRK2,PRKG1,RASGRP1,RAP1A,EPHA5,RAPGEF1,EFNA5,NF1,RGS7,ARHGAP6,TTC8,TBC1D3B,DGKI,TBC1D5,CBLB,DOCK10,ASAP1,ARAP2,PLXNB1,LRCH1,NET1,CDKL5,SIPA1L2,RAPGEF2,RAPGEF6,ASAP2,F11R,ARHGAP35,CCL3,PREX1,ARHGAP25,FGD4,GARNL3,NEDD9,TBC1D9,NTF3,RASA3,RABGAP1L,GNB5,FERMT2,CCL7,PIN1,RALGAPA1,SBF2,ARHGAP42,WDR41,DOCK7,AMOT,CCL14,CCL15,RGN
GO:0055006	cardiac cell development	0.0014518613120284 37	MYO18B,AKAP13,SORBS2,TGFBR3,AGT,FHL2,SLC8A1,SGCD,AKAP6,ACTN2,MYLK3,MTOR,NEBL,TBX3,JAG1,NEB,CXADR,FHOD3,SHOX2,CDK1,ALPK2,MEF2A,PDLIM5,CTDP1,PPARA,TTN,MYH6,MYH11,BMPR1A,TBX18
GO:0007605	sensory perception of sound	0.0015086788756330345	ASIC2,LOXHD1,KCNQ1,TMPRSS3,KCNE1,MYO3A,CNTN5,SLC1A3,STRC,CHD7,HEXB,CDC14A,PCDH15,LRP2,DCDC2,NAV2,ROR1,COL11A1,COL2A1,EYA4,THRB,TSPEAR,TUB,SRRM4,USP53,TRIOBP,GRM7,LHFPL3,CACNA1D,GABRA5,SOBP,TMTC4,TMC2,MYO6,MYH14,KIT,GABRB2,SLITRK6,MYO7B,MYO3B,PDZD7,CDH23,EYA1,LRIG1,PAX3,TECTA,HPN,TBL1X,USH2A,SPTBN4
GO:2000026	regulation of multicellular organismal development	0.0015172478068950011	ISM1,CDH4,PLCB1,DAB1,GTF2I,MAPK14,DSCAM,RUNX1,ASIC2,DCC,NPHP3,NRXN1,CHODL,TGFBR3,FSTL4,ANKH,SYNDIG1,GADD45A,ACIN1,PLXNA4,FLRT2,NRG1,IL4R,SEMA6D,CCR1,CCR3,CFTR,RBFOX2,TIAM1,FBXO31,ROBO2,AJAP1,AGT,RHOJ,LOXL2,LAMA2,CYBB,SEMA3D,NTN1,NELL1,HDAC2,LAMA4,SLIT2,PDCD6,SMAD1,TRPC5,BASP1,VCL,FSHR,TENM4,NTRK3,CHD7,DISC1,SOX6,MECP2,MTMR2,SEMA4D,LINGO2,SLC8A1,PRKD1,TGFB2,CLSTN2,SEMA5A,BDNF,PRKCH,RYK,LAMA3,RAG1,RAG2,PML,AKAP6,FBN1,PLXNA2,ZNF675,EPHB1,LRP2,TMEM100,EPHA7,THBS2,ENPP2,ABCB10,KLF13,MME,TNMD,ZFPM2,RARB,MTOR,BMPER,PTPRD,TIAM2,PGK1,CTNNBIP1,SOX8,PRKCB,VDR,FOXO3,LRRTM1,MITF,CAPN3,KALRN,ROBO1,FLT1,GRHL2,CR1,TGM2,VASH2,KDR,ABCA12,NRP1,PTPRM,KCNK2,SEMA3C,ATF2,ETS1,NR2C2,EFEMP1,PRTG,PARD3,TRPS1,SOX5,JAM2,NTRK2,RASGRP1,CAMK4,SPRED2,GLI3,TGFBR1,CYSLTR1,ADAM12,DICER1,TNR,CUX1,TBX3,TP73,GRID2,EFNA5,BRD1,NF1,SORL1,POR,RELN,GRM5,LRRTM3,NUMB,MAP3K13,IL1RAP,JAG1,PPP3CA,KLF7,WWTR1,KL,TRIM16,GABPA,KAT7,AGGF1,NLGN1,SHOX2,AHI1,SMAD3,PLXNB1,CDK1,TNF,OVOL2,ZFPM1,DLG5,MMP20,BRINP1,BCL11A,HMGA2,MBOAT2,DAAM2,SMOC2,SULF1,C5AR1,LRP4,CLSTN1,CTNNA1,CDKL5,CD109,EGF,CMA1,IL1RAPL1,KIT,RAPGEF2,JAK1,NCMAP,SEMA3E,FGF13,PLAG1,EPHB2,TOX,ERBB4,RBM19,SLITRK6,CAMP,F11R,SGMS2,CCL3,LAMA1,SEMA5B,SPRED3,PSG9,SLC39A12,SOX4,PIK3CD,WDPCP,MAP2,IL23R,KEAP1,P2RX7,BCOR,FGL2,KIAA1109,IL1RL2,HLA-DRA,BHLHB9,L3MBTL1,SEMA3A,CDC73,MEIS2,ZMIZ1,PPARGC1B,SMPD3,GPR55,ADAM10,RPS6KA6,TRPM4,BMP6,CLASP1,HGF,ZBTB16,INSR,MACF1,SKI,AKT3,CTDP1,ENPP1,PRKX,PCK1,PPARA,PPP2R3C,S1PR3,SPRED1,MYH6,PRLR,TYMP,DOCK7,STIM1,STAT3,TP63,AMOT,ISLR2,PAK3,LGALS9,LRP8,BMPR1A,KIAA0319,NGF,PRKCA,UBASH3B,CTSH,RGCC,CLASP2,MAP2K1,SOS1,FGF1,TBX20,RGN,TBX18
GO:0030111	regulation of Wnt signaling pathway	0.0015828650547260003	MAPK14,LYPD6,WLS,NPHP3,ZRANB1,CTNND2,NXN,CSNK2A1,GPC3,PSMB2,SHISA6,TIAM1,NKD1,CDH2,PARK2,ZNRF3,GPC5,WWOX,LRRK2,DISC1,GSK3B,LGR5,SEMA5A,RBMS3,ANKRD6,SMARCA4,HECW1,RNF213,FOXO1,KANK1,DCDC2,CSNK1G3,CTNNBIP1,FOXO3,CCDC88C,STK3,EDA,APP,LGR4,TCF7L2,TRABD2B,RNF43,GLI3,RAPGEF1,WIF1,PSMA1,BICC1,IFT80,NLK,NPHP4,JRK,WWTR1,PTPRU,LRRK1,DDX3X,SMAD3,PRDM15,LGR6,DAAM2,SULF1,LRP4,ALPK2,GSKIP,MLLT3,EGF,PTPRO,SULF2,SOX4,TNKS,SOST,AMFR,TLE4,CDC73,TNN,DAB2,ITGA3,PPM1B,PIN1,TRPM4,MACF1,PSMB7,SKI,BTRC,G3BP1,SCEL,TBL1X,APC,VPS35,TBX18
GO:0060322	head development	0.0016865915134516 91	MACROD2,PLCB1,DAB1,WLS,SUN2,CA10,NRXN1,SLC1A2,POTEE,MTPN,SHANK2,NRG3,ARNT2,BCAN,TMEM108,PLXNA4,NRG1,HYDIN,ALDH1A2,SEMA6D,CTNNA2,RBFOX2,DPYSL2,TACC2,ALK,SPEF2,CNTN4,ARID5B,PHACTR1,ROBO2,C2CD3,DCLK1,ATAT1,WDR11,ATP6V0D1,CDH2,NEGR1,GNB4,TRAPPC9,SLIT2,TFAP2D,IGF1R,SMAD1,PTPRG,DMD,SYT1,BASP1,MBOAT7,ATRX,LRRK2,RORA,CHD7,DISC1,SOX6,MECP2,GSK3B,SLC8A1,FLVCR1,SEMA5A,RYK,KCNC1,EML1,GRIN2B,NR2F2,CNTNAP2,PLXNA2,BCL2,EPHB1,LRP2,NR4A2,EPH

			A7,FYN,UNC5C,SLC1A1,RARB,RFX4,BBS2,KLHL1,NLGN4X,FOXO3,DLC1,ROBO1,SATB2,ZNF148,GRHL2,COL2A1,SEC24B,APP,NRP1,IMMP2L,ATF2,AFF2,ABAT,GRIN2A,DLX6-AS1,OPHN1,CASP5,STK36,MYO16,NTRK2,PRKG1,GLI3,EPHA5,TNR,SLC6A17,EP300,DSCAML1,TBX3,ATXN1,GRID2,DDX10,NF1,RGS7,TTC8,RELN,KIRREL3,NDRG2,NUMB,PPP3CA,MAS1,ELAVL4,MNAT1,CNTN1,CTTNBP2,AK8,GABRA5,AHI1,DLG5,PCDH19,HERC1,PHF8,SPATA5,ZNF423,CTNNA1,ARHGAP11B,BPTF,DYNC2H1,EGF,CMA1,RAPGEF2,UBE3A,FGF13,EPHB2,TOX,ERBB4,GAS8,SETD2,ARHGAP35,NHLH2,AGTPBP1,ITGA8,SEMA3A,ANKRD11,MEIS2,ZMIZ1,PBX1,SRD5A2,SHROOM4,CENPF,SKI,AKT3,LIMK2,ATP5J,FPGS,CCDC141,ZFHX2,TAF1,CHD5,EXT1,DOCK7,FBXW11,KCNC2,NDE1,DTNBP1,LRP8,BMPR1A,MAP2K1,SOS1
GO:1903522	regulation of blood circulation	0.0019131116961912525	*ASIC2,KCNQ1,TNNI3K,CELF2,ANK2,KCND3,KCNE1,RYR3,AGT,DBH,MDM2,DMD,CTNNA3,RNLS,RYR2,SLC8A1,TGFB2,KCNJ3,ABCC9,KCNE2,PDE4D,TBXAS1,SLC1A1,PKP2,ATP1A1,PLN,EDNRB,TACR3,THRB,CD38,HDAC4,PDE4B,CACNA1C,DOCK4,CYSLTR1,KCNJ12,CACNA2D1,CXADR,CACNA1D,JAK2,SHOX2,NOS1,TNF,ADORA3,TRDN,FGF13,NUP155,P2RX1,KCNE4,MEF2A,BDKRB2,ADRA1B,SEMA3A,PDE5A,HCN4,RNF207,TRPM4,CHRM3,SMTNL2,CACNB2,NOS1AP,ARHGAP42,CORIN,MYH7,MYH6,CASQ2,ASB3,SPTBN4,TBX18*
GO:0043062	extracellular structure organization	0.0022898721202166614	*THSD4,RUNX1,FBLN1,ITGA2,COL23A1,ADAMTSL3,FLRT2,FBLN5,COL22A1,HPSE2,PXDN,COL15A1,AGT,LOXL2,TMPRSS6,ADAMTS6,FSHR,NTNG1,TGFB2,COL24A1,ABI3BP,ADAMTS17,WDR72,CSGALNACT1,MMP16,DPT,ADAMTS18,LAMB4,COL11A1,HAS3,RXFP1,FAP,COL2A1,APP,COL18A1,ADTRP,EGFLAM,ST7,ADAMTS16,TGFBR1,PRDX4,TNR,COL4A6,NF1,IMPG1,SLC39A8,TNFRSF11B,COL12A1,ITGAE,COL21A1,EGFL6,SMAD3,COL8A1,TNF,MMP20,SMOC2,SULF1,CMA1,DNAJB6,ITGA9,SULF2,ITGA8,TEX14,SMPD3,LCP1,ADAM10,ITGA3,CLASP1,GAS2,ELN,COL19A1,NTN4,QSOX1,FBLN2,EXT1,ADAMTS5,MYH11,HPN,NID2,DPP4,SPINT2,ADAMTS20,RGCC,CLASP2*
GO:0001944	vasculature development	0.002348755008938253	*ISM1,GTF2I,MAPK14,RUNX1,MYO18B,SVEP1,NRXN1,NRXN3,TGFBR3,CDH13,GADD45A,GPC3,ALDH1A2,CCR3,COL22A1,ROBO2,COL15A1,AGT,RHOJ,CDH2,LOXL2,CYBB,FMNL3,LAMA4,SLIT2,PDCD6,MDM2,SMAD1,ADAMTS6,NOX5,RORA,CHD7,MECP2,PRKD1,FLVCR1,TGFB2,VAV3,PLCD3,SEMA5A,SGCD,NR2F2,PML,ARHGAP24,EPHB1,LRP2,TMEM100,NR4A1,RNF213,THBS2,FOXO1,ENPP2,TNMD,SERPINB7,SLC1A1,ZFPM2,BMPER,PGK1,PRKCB,ROBO1,FAP,FLT1,SEC24B,VASH2,KDR,PRCP,CALCRL,COL18A1,NRP1,PTPRM,ADTRP,IMMP2L,SEMA3C,ATF2,ETS1,TCF7L2,RIN2,DNMT1,NTRK2,STARD13,GLI3,RAP1A,TGFBR1,CYSLTR1,ADAM12,RAPGEF1,TBX3,PCSK5,EMCN,WARS2,NF1,ELK3,HDAC9,JAG1,MEOX2,AGGF1,COL8A1,HDAC7,TNF,OVOL2,ARID2,CALD1,HMGA2,QKI,SMOC2,SULF1,C5AR1,PIK3R3,EFNB2,AMOTL1,DYNC2H1,EGF,PTPN14,CMA1,KIT,RAPGEF2,JAK1,SEMA3E,EPHB2,MAP3K3,LRP5,BSG,CAMP,SETD2,AP2B1,LAMA1,MYH9,SLC39A12,SOX4,PDGFD,PIK3CD,ENPEP,SUFU,EYA1,SRPK2,ZMIZ1,MAP2K5,ADAM10,THSD7A,TGFA,HGF,MYLK,AKT3,PRKX,C1GALT1,GNA13,SPRED1,TYMP,HDAC5,TDGF1,STIM1,STAT3,AMOT,BMPR1A,PRKCA,CTSH,RGCC,IL6R,MAP2K1,SOS1,FGF1,TBX20*
GO:0051963	regulation of synapse assembly	0.002564567214437317	*ASIC2,NRXN1,SYNDIG1,FLRT2,LRFN5,IL1RAPL2,ROBO2,NEGR1,NTN1,GPC6,NTRK3,SEMA4D,LINGO2,CLSTN2,BDNF,EPHB1,EPHA7,THBS2,PTPRD,LRRTM1,APP,NTRK2,GRID2,EFNA5,LRRTM3,IL1RAP,GPC4,NLGN1,DLG5,CLSTN1,IL1RAPL1,EPHB2,SLITRK6,BHLHB9,PDLIM5,VPS35*
GO:0006464	cellular protein modification process	0.002568625421892404	*TPTE,MACROD2,KMT2C,DAB1,AGBL1,MAPK14,TPTE2,GPHN,SH3RF3,CCNG2,HUNK,PTPRT,DUSP22,AKAP13,FBLN1,ERG,MGAT4C,LARGE,TTLL2,ST6GALNAC3,EPHA6,VRK2,NRXN1,ZRANB1,FPGT-TNNI3K,TNNI3K,CPPED1,NRG3,SMYD2,HUS1,TGFBR3,RNF152,NXN,CSNK2A1,HLCS,CEP41,CHEK2,GADD45A,PRDM9,NRG1,PSMB2,RFFL,ST8SIA1,SUZ12,PRMT8,PLCE1,SH3RF2,KSR2,STK38L,ABHD17C,SLCO3A1,DTX4,PPM1J,AGBL4,LDLRAD4,MYO3A,TET3,ALK,PTPRE,PIK3C3,ERC1,FBXO31,SPOCK3,B3GALT5,EPHA3,PRKACB,PTPRN2,DCLK1,AGT,UBE2R2,ATXN3L,ATAT1,FBXO32,RCAN1,KLHL25,PARK2,PGAP3,LOXL2,TRIM51,FBXL7,TRIM22,TRIM5,TRIM9,USP25,DCUN1D4,TXK,RNF185,USP40,USP6,FBXO27,PHC1,SETD3,ZNRF3,TRIO,ATE1,HDAC2,SLIT2,TTLL8,PDCD6,RPS6KA2,LCMT1,IGF1R,MDM2,SMAD1,STAMBPL1,TTC3,PTPRG,PXK,TRIM48,TRPC5,DMD,STK33,KRT2,FSHR,MORF4L1,PRKAR2A,STK24,PDZRN3,GALNT14,MAN2B1,ATRX,PARP16,TPP2,USP16,CAMTA1,LRRK2,MAPK10,VRK1,NTRK3,RNF165,DPY30,PAN3,CDCA2,GALNTL6,KCTD9,PDXP,MECP2,MTMR2,SEMA4D,GSK3B,SLC8A1,CDC14A,PRKD1,SNRK,G2E3,ERCC6,OGT,RNF144A,TGFB2,DNAJC6,FUT4,BDNF,PRKAR1B,PRKCH,RIPK4,RYK,ZDHHC17,MGAT2,KDM4C,OTUD7A,ZDHHC14,EXT2,RAG*

			1,RAG2,SIK2,MKRN3,NR2F2,PRR5,PML,PPARGC1A,RNF180,USP36,MYH8,PPP4R4,TAOK3,BCL2,RALBP1,ZNF675,EPHB1,IL12RB2,DEPTOR,EPHA7,FYN,CD44,HECW1,RHBDD1,RNF213,FOXO1,PDE4D,RNF144B,ENPP2,GRK5,RNF217,CSNK1G3,MYLK3,SETD7,SLC1A1,UGCG,MAN1C1,PHF20,NLRP12,MTOR,ROR1,UBE2L6,CTDSPL2,BMPER,PTPRD,OGFOD1,PLCL1,EPM2A,PRKCE,EIF2S1,KMT2A,PRKCB,ADCY8,CAPN3,DPH6,ITLN1,KALRN,PJA1,TTLL12,CCDC88C,DLC1,EDNRB,ROBO1,FLT1,NOX4,PIGL,MAP3K7,MAPK9,TRAF3IP2,EYA4,STK3,SUPT3H,TGM2,KDR,CASK,PHKB,RNF182,APP,NRP1,PTPRM,ADTRP,EGFLAM,KAT5,ST3GAL3,ZZZ3,ADAM17,CAMK1D,MAP3K5,CDC42BPG,ATF2,GRK6,PIK3R5,SAMSN1,EFEMP1,ROS1,CDK17,DOT1L,RPS6KA5,TNFRSF10B,TRABD2B,PARD3,TLK2,TRAF3,SGK2,CCDC88A,MOB3B,RNF43,RNF126,STK36,GUCY2F,HDAC4,DNMT1,GHR,NTRK2,PRKG1,CDC42BPA,BRD4,PTPDC1,RASGRP1,ARR3,CAMK4,COMMD1,RABGEF1,SPRED2,COG7,PPA2,SPRR2E,RAP1A,TGFBR1,USP53,PRDX4,EPHA5,LCP2,TOP1,PEAK1,B4GALT3,CPE,PTTG1IP,SUMF1,CHRNA3,EP300,MORC3,HMG20A,DUSP23,GCNT7,TRIOBP,BRD7,NPC1,PIBF1,TRPM7,PSMA1,ADCK1,ADORA2A,ADRBK2,CAMK2G,EFNA5,FAM20A,MERTK,UBE3C,BRD1,GUCY2C,MAGI2,NF1,PPP6R2,USP17L5,SORL1,ASB13,TRIM59,LZTR1,ASXL1,POR,RELN,SLC39A8,USP3,GRM5,HDAC9,PTPN9,ST6GALNAC5,MGAT5,MAP3K13,NLK,EGLN3,FBXL17,FBXL5,MAN1A1,PPP3CA,SH3GL2,SNX25,MAS1,PER1,WWTR1,CBLB,MNAT1,CNTN1,DGUOK,TRIM16,KAT7,MAPK4,BORA,PTPRU,THAP7,RNF128,RPS20,LRRK1,MSRA,DDX3X,CNOT7,PARP8,DNMT3B,JAK2,LMO7,CD300A,MAP2K4,NOS1,TMTC1,TMTC4,CDK1,HDAC7,MAGT1,MCM3AP,NBN,RRH,SLC39A10,TNF,TNIP1,ILF3,PIGB,BMP15,PHEX,TRIM23,DPY19L1,PAXBP1,FAM220A,STK32B,BCL11A,HMGA2,NEK4,UBE2E3,STK32C,CDC20B,DYRK4,GALNT8,GCLC,PMEPA1,UBE2V1,C5AR1,LRP4,PIK3R3,WDR5,RSRC1,SAE1,STT3A,TRIM41,HERC1,PHF8,CDC14B,ALPK2,CDK8,CUL2,TENM1,WDFY2,CDKL5,GSKIP,PRKAR1A,CD109,EGF,PTPN14,KIT,UBR2,BLM,PTPRO,RAPGEF2,STK32A,JAK1,UBE3A,FGF13,EPHB2,MECOM,NDFIP2,RNF4,DAPK1,ERBB4,FER,MAP3K3,MINPP1,RSPRY1,FNIP1,LRP5,TTLL5,ABL2,CAMP,SETD2,PTPRK,CCL3,FGF7,LAMA1,RNF138,SPRED3,CDKL1,GLYR1,ARF4,NSMCE2,SOX4,CAB39L,NEDD9,PDGFD,PIK3CD,UBE2N,AGTPBP1,WDR82,ARID4A,DDB1,IRAK2,KDM3A,MYO3B,PLAT,TNKS,AUTS2,CDK19,FGFR1,OCLN,PARP15,ARID4B,DAD1,IL23R,KEAP1,MACROD1,NTF3,P2RX7,TEX14,ARRDC4,ZDHHC11,ZDHHC11B,ZDHHC9,AMFR,BCOR,PIGK,TRERF1,C10ORF90,EYA1,IFNA8,N4BP1,SRPK2,BDKRB1,BDKRB2,GNL3,NEK5,PI3,PRKAA2,RLF,CDC73,MARK2,PDE5A,ZMIZ1,FLT3,PTPRA,TRIM72,DYNAP,FERMT2,MAP2K5,MARK1,SMPD3,DAB2,HDAC8,TCL1B,ADAM10,FRY,PLCL2,PPM1B,TTLL11,PHF20L1,PIN1,RPS6KA6,SYNPO2,TRPM4,STK38,BMP6,CHRM3,CIT,GALNT5,TGFA,HGF,MYLK,USP31,CALCR,DPY19L2,POMT2,SH3BP5,TLK1,ZBTB16,KDM4B,MAST4,NOS1AP,INSR,PAK1,PSMB7,PTPN21,SKI,AKT3,CDC16,CHM,CTDP1,DUSP11,ENPP1,FMR1,LIMK2,TESK2,CHFR,JDP2,PRKX,TMTC2,ULK4,MSL2,SENP8,PCK1,TTN,C1GALT1,SMARCAD1,ST6GAL2,TAF1,CHD5,FNIP2,FUT8,KANSL1,PPP2R3C,SPRED1,PRDM16,PTPRF,USP29,ZNF451,BTRC,PPP2R2C,PTPRR,RIT2,EXT1,MYH6,NCOA1,PRKCG,PRLR,UMOD,DOCK7,FBXW11,HDAC5,ICK,KLHL3,MTFMT,STK35,TDGF1,UGGT2,WDR70,SEL1L2,TRPM6,ADARB1,FBXL18,PRKCQ,SHPRH,USP7,CDKN3,DOCK3,DPM1,DPY19L3,MOCS3,PAK3,STK17B,TBL1X,DTNBP1,GAK,UBE2U,KSR1,LRP8,ALPK1,MAPKAPK2,TUSC3,APC,BMPR1A,ITGA1,NGF,PRKCA,TULP4,UBASH3B,UBE2E2,VLDLR,ASB3,MTMR4,PILRB,ABI1,NCAPG2,RGCC,USP18,DPH1,IL6R,MAP2K6,SIAH3,SPTBN4,MAP2K1,ALG12,ART4,FGF1,RAD18,USP12,RGN
GO:0036211	protein modification process	0.002568625421892404	*TPTE,MACROD2,KMT2C,DAB1,AGBL1,MAPK14,TPTE2,GPHN,SH3RF3,CCNG2,HUNK,PTPRT,DUSP22,AKAP13,FBLN1,ERG,MGAT4C,LARGE,TTLL2,ST6GALNAC3,EPHA6,VRK2,NRXN1,ZRANB1,FPGT-TNNI3K,TNNI3K,CPPED1,NRG3,SMYD2,HUS1,TGFBR3,RNF152,NXN,CSNK2A1,HLCS,CEP41,CHEK2,GADD45A,PRDM9,NRG1,PSMB2,RFFL,ST8SIA1,SUZ12,PRMT8,PLCE1,SH3RF2,KSR2,STK38L,ABHD17C,SLCO3A1,DTX4,PPM1J,AGBL4,LDLRAD4,MYO3A,TET3,ALK,PTPRE,PIK3C3,ERC1,FBXO31,SPOCK3,B3GALT5,EPHA3,PRKACB,PTPRN2,DCLK1,AGT,UBE2R2,ATXN3L,ATAT1,FBXO32,RCAN1,KLHL25,PARK2,PGAP3,LOXL2,TRIM51,FBXL7,TRIM22,TRIM5,TRIM9,USP25,DCUN1D4,TXK,RNF185,USP40,USP6,FBXO27,PHC1,SETD3,ZNRF3,TRIO,ATE1,HDAC2,SLIT2,TTLL8,PDCD6,RPS6KA2,LCMT1,IGF1R,MDM2,SMAD1,STAMBPL1,TTC3,PTPRG,PXK,TRIM48,TRPC5,DMD,STK33,KRT2,FSHR,MORF4L1,PRKAR2A,STK24,PDZRN3,GALNT14,MAN2B1,ATRX,PARP16,TPP2,USP16,CAMTA1,LRRK2,MAPK10,VRK1,NTRK3,RNF165,DPY30,PAN3,CDCA2,GALNTL6,KCTD9,PDXP,MECP2,*

			MTMR2,SEMA4D,GSK3B,SLC8A1,CDC14A,PRKD1,SNRK,G2E3,ERCC6,OGT,RNF144A,TGFB2,DNAJC6,FUT4,BDNF,PRKAR1B,PRKCH,RIPK4,RYK,ZDHHC17,MGAT2,KDM4C,OTUD7A,ZDHHC14,EXT2,RAG1,RAG2,SIK2,MKRN3,NR2F2,PRR5,PML,PPARGC1A,RNF180,USP36,MYH8,PPP4R4,TAOK3,BCL2,RALBP1,ZNF675,EPHB1,IL12RB2,DEPTOR,EPHA7,FYN,CD44,HECW1,RHBDD1,RNF213,FOXO1,PDE4D,RNF144B,ENPP2,GRK5,RNF217,CSNK1G3,MYLK3,SETD7,SLC1A1,UGCG,MAN1C1,PHF20,NLRP12,MTOR,ROR1,UBE2L6,CTDSPL2,BMPER,PTPRD,OGFOD1,PLCL1,EPM2A,PRKCE,EIF2S1,KMT2A,PRKCB,ADCY8,CAPN3,DPH6,ITLN1,KALRN,PJA1,TTLL12,CCDC88C,DLC1,EDNRB,ROBO1,FLT1,NOX4,PIGL,MAP3K7,MAPK9,TRAF3IP2,EYA4,STK3,SUPT3H,TGM2,KDR,CASK,PHKB,RNF182,APP,NRP1,PTPRM,ADTRP,EGFLAM,KAT5,ST3GAL3,ZZZ3,ADAM17,CAMK1D,MAP3K5,CDC42BPG,ATF2,GRK6,PIK3R5,SAMSN1,EFEMP1,ROS1,CDK17,DOT1L,RPS6KA5,TNFRSF10B,TRABD2B,PARD3,TLK2,TRAF3,SGK2,CCDC88A,MOB3B,RNF43,RNF126,STK36,GUCY2F,HDAC4,DNMT1,GHR,NTRK2,PRKG1,CDC42BPA,BRD4,PTPDC1,RASGRP1,ARR3,CAMK4,COMMD1,RABGEF1,SPRED2,COG7,PPA2,SPRR2E,RAP1A,TGFBR1,USP53,PRDX4,EPHA5,LCP2,TOP1,PEAK1,B4GALT3,CPE,PTTG1IP,SUMF1,CHRNA3,EP300,MORC3,HMG20A,DUSP23,GCNT7,TRIOBP,BRD7,NPC1,PIBF1,TRPM7,PSMA1,ADCK1,ADORA2A,ADRBK2,CAMK2G,EFNA5,FAM20A,MERTK,UBE3C,BRD1,GUCY2C,MAGI2,NF1,PPP6R2,USP17L5,SORL1,ASB13,TRIM59,LZTR1,ASXL1,POR,RELN,SLC39A8,USP3,GRM5,HDAC9,PTPN9,ST6GALNAC5,MGAT5,MAP3K13,NLK,EGLN3,FBXL17,FBXL5,MAN1A1,PPP3CA,SH3GL2,SNX25,MAS1,PER1,WWTR1,CBLB,MNAT1,CNTN1,DGUOK,TRIM16,KAT7,MAPK4,BORA,PTPRU,THAP7,RNF128,RPS20,LRRK1,MSRA,DDX3X,CNOT7,PARP8,DNMT3B,JAK2,LMO7,CD300A,MAP2K4,NOS1,TMTC1,TMTC4,CDK1,HDAC7,MAGT1,MCM3AP,NBN,RRH,SLC39A10,TNF,TNIP1,ILF3,PIGB,BMP15,PHEX,TRIM23,DPY19L1,PAXBP1,FAM220A,STK32B,BCL11A,HMGA2,NEK4,UBE2E3,STK32C,CDC20B,DYRK4,GALNT8,GCLC,PMEPA1,UBE2V1,C5AR1,LRP4,PIK3R3,WDR5,RSRC1,SAE1,STT3A,TRIM41,HERC1,PHF8,CDC14B,ALPK2,CDK8,CUL2,TENM1,WDFY2,CDKL5,GSKIP,PRKAR1A,CD109,EGF,PTPN14,KIT,UBR2,BLM,PTPRO,RAPGEF2,STK32A,JAK1,UBE3A,FGF13,EPHB2,MECOM,NDFIP2,RNF4,DAPK1,ERBB4,FER,MAP3K3,MINPP1,RSPRY1,FNIP1,LRP5,TTLL5,ABL2,CAMP,SETD2,PTPRK,CCL3,FGF7,LAMA1,RNF138,SPRED3,CDKL1,GLYR1,ARF4,NSMCE2,SOX4,CAB39L,NEDD9,PDGFD,PIK3CD,UBE2N,AGTPBP1,WDR82,ARID4A,DDB1,IRAK2,KDM3A,MYO3B,PLAT,TNKS,AUTS2,CDK19,FGFR1,OCLN,PARP15,ARID4B,DAD1,IL23R,KEAP1,MACROD1,NTF3,P2RX7,TEX14,ARRDC4,ZDHHC11,ZDHHC11B,ZDHHC9,AMFR,BCOR,PIGK,TRERF1,C10ORF90,EYA1,IFNA8,N4BP1,SRPK2,BDKRB1,BDKRB2,GNL3,NEK5,PI3,PRKAA2,RLF,CDC73,MARK2,PDE5A,ZMIZ1,FLT3,PTPRA,TRIM72,DYNAP,FERMT2,MAP2K5,MARK1,SMPD3,DAB2,HDAC8,TCL1B,ADAM10,FRY,PLCL2,PPM1B,TTLL11,PHF20L1,PIN1,RPS6KA6,SYNPO2,TRPM4,STK38,BMP6,CHRM3,CIT,GALNT5,TGFA,HGF,MYLK,USP31,CALCR,DPY19L2,POMT2,SH3BP5,TLK1,ZBTB16,KDM4B,MAST4,NOS1AP,INSR,PAK1,PSMB7,PTPN21,SKI,AKT3,CDC16,CHM,CTDP1,DUSP11,ENPP1,FMR1,LIMK2,TESK2,CHFR,JDP2,PRKX,TMTC2,ULK4,MSL2,SENP8,PCK1,TTN,C1GALT1,SMARCAD1,ST6GAL2,TAF1,CHD5,FNIP2,FUT8,KANSL1,PPP2R3C,SPRED1,PRDM16,PTPRF,USP29,ZNF451,BTRC,PPP2R2C,PTPRR,RIT2,EXT1,MYH6,NCOA1,PRKCG,PRLR,UMOD,DOCK7,FBXW11,HDAC5,ICK,KLHL3,MTFMT,STK35,TDGF1,UGGT2,WDR70,SEL1L2,TRPM6,ADARB1,FBXL18,PRKCQ,SHPRH,USP7,CDKN3,DOCK3,DPM1,DPY19L3,MOCS3,PAK3,STK17B,TBL1X,DTNBP1,GAK,UBE2U,KSR1,LRP8,ALPK1,MAPKAPK2,TUSC3,APC,BMPR1A,ITGA1,NGF,PRKCA,TULP4,UBASH3B,UBE2E2,VLDLR,ASB3,MTMR4,PILRB,ABI1,NCAPG2,RGCC,USP18,DPH1,IL6R,MAP2K6,SIAH3,SPTBN4,MAP2K1,ALG12,ART4,FGF1,RAD18,USP12,RGN
GO:0001568	blood vessel development	0.0026199242429430945	ISM1,GTF2I,MAPK14,RUNX1,MYO18B,NRXN1,NRXN3,TGFBR3,CDH13,GADD45A,GPC3,ALDH1A2,CCR3,COL22A1,ROBO2,COL15A1,AGT,RHOJ,CDH2,LOXL2,CYBB,FMNL3,LAMA4,SLIT2,PDCD6,MDM2,SMAD1,ADAMTS6,NOX5,RORA,CHD7,MECP2,PRKD1,FLVCR1,TGFB2,VAV3,PLCD3,SEMA5A,SGCD,NR2F2,PML,ARHGAP24,EPHB1,LRP2,TMEM100,NR4A1,RNF213,THBS2,FOXO1,ENPP2,TNMD,SERPINB7,SLC1A1,ZFPM2,BMPER,PGK1,PRKCB,ROBO1,FAP,FLT1,SEC24B,VASH2,KDR,PRCP,CALCRL,COL18A1,NRP1,PTPRM,ADTRP,SEMA3C,ATF2,ETS1,TCF7L2,RIN2,NTRK2,STARD13,GLI3,RAP1A,TGFBR1,CYSLTR1,ADAM12,RAPGEF1,TBX3,PCSK5,EMCN,WARS2,NF1,ELK3,HDAC9,JAG1,MEOX2,AGGF1,COL8A1,HDAC7,TNF,OVOL2,ARID2,CALD1,HMGA2,QKI,SMOC2,SULF1,C5AR1,PIK3R3,EFNB2,AMOTL1,DYNC2H1,EGF,CMA1,RAPGEF2,JAK1,SEMA3E,EPHB2,MAP3K3,LRP5,

			BSG,CAMP,SETD2,AP2B1,LAMA1,MYH9,SLC39A12,SOX4,PDGFD,PIK3CD,ENPEP,SUFU,EYA1,SRPK2,ZMIZ1,MAP2K5,THSD7A,TGFA,HGF,MYLK,AKT3,PRKX,C1GALT1,GNA13,SPRED1,TYMP,HDAC5,TDGF1,STIM1,STAT3,AMOT,BMPR1A,PRKCA,CTSH,RGCC,IL6R,MAP2K1,SOS1,FGF1,TBX20
GO:0048878	chemical homeostasis	0.0026379091128199686	*CMKLR1,ABCG8,ANK3,SLC24A3,ABCG1,KCNQ1,TMPRSS3,MS4A1,GRIA1,SLC9B1,ANK2,CSMD1,FAM155A,SLC12A1,TF,SCARA5,RYR3,PLCE1,CCR1,CCR3,CFTR,ZBTB20,FAM3B,RAB7A,PRKACB,PTPRN2,AGT,ATP6V0D1,PARK2,OTC,CP,DBH,KCNMA1,STXBP4,IGF1R,SLC24A4,TMPRSS6,TRPC5,DMD,NOX5,FSHR,GRM1,TRPM2,RYR2,DHRS7C,LRRK2,SLC1A3,RORA,SLC4A4,CHD7,DISC1,HEXB,SLC8A1,PRKD1,FLVCR1,ATP1A4,SGCD,GRIN2B,EXT2,PTGFR,NR5A2,PML,PPARGC1A,AKAP6,ACSM3,BCL2,SLC30A5,ITPR2,CYB561A3,FYN,SMARCA4,FOXO1,PDE4D,TBXAS1,GRIK2,RAP1GDS1,SLC1A1,UGCG,ATP1A1,PLN,HEPHL1,PRKCE,PRKCB,VDR,ADCY8,FOXO3,CAPN3,EDNRB,NOX4,SLC12A8,MALL,PKHD1,TGM2,KDR,PRCP,ABCA12,APP,MT1HL1,OIT3,TM9SF4,TCF7L2,ACSM2A,GRIN2A,HRNR,PYGL,CD38,SLC30A7,BACE2,COMMD1,KCTD7,CACNA1C,CLDN1,CYSLTR1,EPHA5,NCOA5,TTC39B,CACNA2D1,CYP7B1,NPC1,PNPLA3,TRPM7,ADCK1,ADORA2A,EFNA5,FAM20A,TAC4,MICU1,SLC39A8,SLC9C1,GRM5,HDAC9,ADCYAP1R1,CYP39A1,FBXL5,PPP3CA,KLF7,SLC24A2,KL,STIM2,ACOX3,DDX3X,TPCN2,JAK2,SMAD3,NOS1,TMTC4,MIR320B2,SLC39A10,SNX5,POMC,ARF1,ANO1,GCLC,TRPC4,C5AR1,TRDN,LRRC8D,DMXL1,CMA1,TRPV4,ABCA5,CPS1,ESR1,HTR2C,SLC30A8,UBE3A,LRP5,MALRD1,ABL2,CCL3,MCUR1,PTGER3,SLC39A12,SOX4,P2RX1,ASGR2,DDB1,CDH23,RYR1,UNC13B,P2RX7,RASA3,BDKRB1,BDKRB2,ADRA1B,PRKAA2,THADA,ATP6V1H,HCN4,SCNN1A,ATP13A3,CLN6,GPR55,CCL7,MT1F,TRPM4,BMP6,BTBD9,CALCR,CACNB2,INSR,ENPP1,MICU2,TMTC2,ABCD1,JPH4,PCK1,POLD1,CORIN,FHL1,GNA13,CACNA1A,PPP2R3C,S1PR3,TRPM1,EXT1,SCO2,UMOD,KLHL3,SLC24A5,SLC40A1,STIM1,SLC9A9,STAT3,TP63,PIK3R2,CASQ2,TMEM175,CNNM3,ACSM1,SLC7A8,UPK3A,UBASH3B,CTSH,RPH3AL,SLC9A2,ACACA,CCL14,CCL15,GPR21,C1QTNF3,MCOLN3,RGN*
GO:0045229	external encapsulating structure organization	0.0030033911568376937	*THSD4,RUNX1,FBLN1,ITGA2,COL23A1,ADAMTSL3,FLRT2,FBLN5,COL22A1,HPSE2,PXDN,COL15A1,AGT,LOXL2,TMPRSS6,ADAMTS6,FSHR,NTNG1,TGFB2,COL24A1,ABI3BP,ADAMTS17,WDR72,CSGALNACT1,MMP16,DPT,ADAMTS18,LAMB4,COL11A1,HAS3,RXFP1,FAP,COL2A1,APP,COL18A1,ADTRP,EGFLAM,ST7,HRNR,ADAMTS16,TGFBR1,PRDX4,TNR,COL4A6,NF1,IMPG1,SLC39A8,TNFRSF11B,COL12A1,ITGAE,COL21A1,EGFL6,SMAD3,COL8A1,TNF,MMP20,SMOC2,SULF1,CMA1,DNAJB6,ITGA9,SULF2,ITGA8,SMPD3,LCP1,ADAM10,ITGA3,CLASP1,GAS2,ELN,COL19A1,NTN4,QSOX1,FBLN2,EXT1,ADAMTS5,MYH11,HPN,NID2,DPP4,SPINT2,ADAMTS20,RGCC,CLASP2*
GO:0016079	synaptic vesicle exocytosis	0.003027017983120912	*CTBP2,GRIN3A,NRXN3,RAB5A,RIMS2,ERC2,APBA2,SYT9,SYT1,UNC13C,LRRK2,GSK3B,SNAP25,SNAP23,CADPS,PRKCB,CASK,RIMS3,STXBP5,RAP1A,CHRNA5,ADORA2A,DGKI,RIMS1,NLGN1,BLOC1S6,P2RX1,UNC13A,UNC13B,P2RX7,STX3,SYT2,CACNB2,SNAP29,FMR1,PRKCG,DTNBP1,RAP1B*
GO:0031589	cell-substrate adhesion	0.003053564880272794	*DUSP22,FBLN1,ITGA2,CDH13,FBLN5,SPOCK1,TIAM1,UTRN,EPHA3,AJAP1,CORO2B,DMD,VCL,SKAP1,NTNG1,STRC,DISC1,GSK3B,FMN1,ABI3BP,EDIL3,BCL2,EPHB1,CD44,KANK1,ACTN2,LAMB4,MEGF9,PRKCE,DLC1,PKHD1,KDR,CASK,EDA,NRP1,EGFLAM,PARVG,ITGA11,ATRNL1,RIN2,PEAK1,CD96,TRIOBP,PARVB,TRPM7,EFNA5,MERTK,NF1,ARHGAP6,JAG1,FREM1,EGFL6,JAK2,SMAD3,COL8A1,PPFIA2,DOCK1,VWC2,PTPRO,SEMA3E,TBCD,FER,PTPRK,PREX1,VWF,NEDD9,ITGA8,WDPCP,ITGBL1,ITGB7,PTPRA,FERMT2,TNN,DAB2,ACTN4,ITGA3,EPDR1,CLASP1,MACF1,TESK2,PRKX,NTN4,FBLN2,TECTA,LYVE1,USH2A,NID2,ITGA1,CLASP2,RREB1*
GO:0007215	glutamate receptor signaling pathway	0.0032903300652445612	*PLCB1,GRIK1,GRIN3A,GRIA1,GRID1,GRIA4,GRM1,GRIN2B,GRIK3,FYN,GRIK2,SLC1A1,APP,GRIN2A,GRID2,GRM5,GRM7,GRIA3,GRIK4,GRM4,GRIA2,FMR1,TRPM1*
GO:0051960	regulation of nervous system development	0.0033936722050743467	*CDH4,DAB1,DSCAM,ASIC2,DCC,NPHP3,NRXN1,CHODL,FSTL4,SYNDIG1,PLXNA4,FLRT2,SEMA6D,TIAM1,FBXO31,ROBO2,SEMA3D,NTN1,HDAC2,SLIT2,TRPC5,TENM4,NTRK3,CHD7,DISC1,MTMR2,SEMA4D,LINGO2,CLSTN2,SEMA5A,BDNF,PRKCH,RYK,PLXNA2,EPHB1,LRP2,EPHA7,THBS2,MME,MTOR,PTPRD,TIAM2,SOX8,LRRTM1,KALRN,ROBO1,TGM2,NRP1,SEMA3C,PRTG,PARD3,JAM2,NTRK2,GLI3,DICER1,TNR,CUX1,TP73,GRID2,EFNA5,NF1,SORL1,RELN,GRM5,LRRTM3,NUMB,MAP3K13,IL1RAP,PPP3CA,NLGN1,SHOX2,PLXNB1,TNF,DLG5,BRINP1,BCL11A,DAAM2,LRP4,CLSTN1,CTNNA1,CDKL5,IL1RAPL1,KIT,RAPGEF2,NCMAP,SEMA3E,FGF13,PLAG1,EPHB2,SLITRK*

			6,SEMA5B,MAP2,BHLHB9,SEMA3A,HGF,MACF1,SKI,TYMP,DOCK7,ISLR2,PAK3,LRP8,BMPR1A,KIAA0319,NGF,MAP2K1
GO:0055013	cardiac muscle cell development	0.003585796063452421	*MYO18B,AKAP13,SORBS2,AGT,FHL2,SLC8A1,SGCD,AKAP6,ACTN2,MYLK3,MTOR,NEBL,TBX3,NEB,CXADR,FHOD3,SHOX2,CDK1,ALPK2,MEF2A,PDLIM5,CTDP1,PPARA,TTN,MYH6,MYH11,BMPR1A,TBX18*
GO:0086001	cardiac muscle cell action potential	0.003585796063452421	*ANK3,KCNQ1,ANK2,KCND3,KCNE1,DMD,CTNNA3,RYR2,SLC8A1,KCNJ3,KCNE2,PKP2,ATP1A1,CACNA1C,CACNA2D1,CXADR,CACNA1D,SCN1A,FGF13,NUP155,KCNE4,HCN4,RNF207,FGF12,TRPM4,CACNB2,NOS1AP,TBX18*
GO:0008016	regulation of heart contraction	0.003612264069926909	*KCNQ1,TNNI3K,CELF2,ANK2,KCND3,KCNE1,RYR3,AGT,MDM2,DMD,CTNNA3,RNLS,RYR2,SLC8A1,TGFB2,KCNJ3,ABCC9,KCNE2,PDE4D,SLC1A1,PKP2,ATP1A1,PLN,EDNRB,TACR3,THRB,HDAC4,PDE4B,CACNA1C,KCNJ12,CACNA2D1,CXADR,CACNA1D,JAK2,SHOX2,NOS1,TNF,ADORA3,TRDN,FGF13,NUP155,KCNE4,MEF2A,ADRA1B,SEMA3A,PDE5A,HCN4,RNF207,TRPM4,CACNB2,NOS1AP,CORIN,MYH7,MYH6,CASQ2,ASB3,SPTBN4,TBX18*
GO:0048588	developmental cell growth	0.003774409372219028	*CDH4,DSCAM,DCC,AKAP13,SORBS2,RIMS2,FSTL4,TMEM108,SYT17,PLXNA4,SEMA6D,DPYSL2,DCLK1,AGT,PARK2,SEMA3D,NTN1,SLIT2,TRPC5,SYT1,VCL,DISC1,SEMA4D,GSK3B,SEMA5A,BDNF,RYK,WASF1,AKAP6,EPHA7,APP,NRP1,SEMA3C,ALCAM,TNR,SPG11,EFNA5,MAP3K13,SH3GL2,RIMS1,COBL,BCL11A,CDKL5,SLIT3,SEMA3E,FGF13,SEMA5B,UNC13A,AUTS2,MAP2,SEMA3A,PDLIM5,TNN,SYT2,MACF1,CTDP1,PPARA,EXT1,ISLR2,KIAA0319,NGF,CLASP2*
GO:0018105	peptidyl-serine phosphorylation	0.0038508227395507937	*MAPK14,VRK2,NRXN1,CSNK2A1,CHEK2,GADD45A,STK38L,DCLK1,RPS6KA2,DMD,LRRK2,VRK1,NTRK3,GSK3B,PRKD1,ERCC6,BDNF,PRKCH,BCL2,CD44,PDE4D,CSNK1G3,SLC1A1,MTOR,PLCL1,EPM2A,PRKCE,PRKCB,MAPK9,APP,CAMK1D,RPS6KA5,TLK2,SGK2,NTRK2,CAMK4,TGFBR1,TOP1,MORC3,MAP3K13,NLK,NOS1,CDK1,TNF,STK32B,HMGA2,STK32C,DYRK4,TENM1,STK32A,FNIP1,TNKS,NTF3,IFNA8,SRPK2,BDKRB2,PRKAA2,MARK2,MARK1,TCL1B,PLCL2,RPS6KA6,STK38,HGF,CALCR,TLK1,MAST4,PAK1,AKT3,PRKX,PCK1,TAF1,FNIP2,PRKCG,DOCK7,TDGF1,PRKCQ,MAPKAPK2,NGF,PRKCA,SPTBN4*
GO:0030198	extracellular matrix organization	0.0039038803118613343	*THSD4,RUNX1,FBLN1,ITGA2,COL23A1,ADAMTSL3,FLRT2,FBLN5,COL22A1,HPSE2,PXDN,COL15A1,AGT,LOXL2,TMPRSS6,ADAMTS6,FSHR,NTNG1,TGFB2,COL24A1,ABI3BP,ADAMTS17,WDR72,CSGALNACT1,MMP16,DPT,ADAMTS18,LAMB4,COL11A1,HAS3,RXFP1,FAP,COL2A1,APP,COL18A1,ADTRP,EGFLAM,ST7,ADAMTS16,TGFBR1,PRDX4,TNR,COL4A6,NF1,IMPG1,SLC39A8,TNFRSF11B,COL12A1,ITGAE,COL21A1,EGFL6,SMAD3,COL8A1,TNF,MMP20,SMOC2,SULF1,CMA1,DNAJB6,ITGA9,SULF2,ITGA8,SMPD3,LCP1,ADAM10,ITGA3,CLASP1,GAS2,ELN,COL19A1,NTN4,QSOX1,FBLN2,EXT1,ADAMTS5,MYH11,HPN,NID2,DPP4,SPINT2,ADAMTS20,RGCC,CLASP2*
GO:0048585	negative regulation of response to stimulus	0.003932303858383546	*DAB1,MAPK14,PTPRT,TIMP3,DUSP22,FBLN1,NPHP3,RGS6,GRIN3A,NRXN1,SHANK2,TGFBR3,FSTL4,RNF152,NXN,CSNK2A1,ERCC4,CHEK2,GPC3,NRG1,PSMB2,SHISA6,RFFL,IL4R,LRFN5,MCTP1,SH3RF2,SEMA6D,CTNNA2,LDLRAD4,RGS7BP,CBFA2T2,NKD1,SCAI,PTPRE,ZNF536,RAB7A,ROBO2,ZNF366,PRKACB,PXDN,AJAP1,LEMD3,AGT,RCAN1,CDH2,PARK2,FHL2,KIR2DL4,BPIFB1,SEMA3D,USP25,TSPAN8,ZNRF3,RIN3,HTRA1,SLIT2,PDCD6,IGF1R,MDM2,SLC24A4,TMPRSS6,DMD,WWOX,LRRK2,RORA,SH3BP1,MTMR2,SEMA4D,GSK3B,ERCC6,TGFB2,SEMA5A,BDNF,RBMS3,RYK,OTUD7A,ANKRD6,ARHGAP12,FBN1,TAOK3,BCL2,ZNF675,ARHGAP24,LRP2,DEPTOR,NR4A2,FYN,ADAMTS18,SMARCA4,CD44,HECW1,RNF213,FOXO1,PDE4D,KANK1,TNMD,CELF4,RFX4,NLRP12,MTOR,RMI2,CTDSPL2,BMPER,PTPRD,BBS2,CTNNBIP1,ZMYND11,NLGN4X,HELLS,PRKCB,ADCY8,FOXO3,TTLL12,CCDC88C,DLC1,ROBO1,FAP,COL2A1,CR1,EYA4,PKHD1,STK3,CASK,CALCRL,NRP1,UACA,LGR4,LINC00473,SERPING1,ADTRP,SEMA3C,ADAM17,TFPI,SAMSN1,TGFBI1I,RGS16,TCF7L2,TRABD2B,BICD1,ABAT,RNF43,CHRDL1,RNF126,BCL2L1,PDE4B,PRKG1,BRD4,ARR3,RABGEF1,SPRED2,GLI3,SMCHD1,TGFBR1,TNR,NCOA5,CHST11,PTTG1IP,RAPGEF1,CD96,WIF1,CYP7B1,GRAMD4,GRID2,PIBF1,PSMA1,ADORA2A,ADRBK2,MAGI2,NF1,RGS7,SORL1,STRN3,BICC1,BID,IFT80,TRIM59,LZTR1,ASXL1,GRM5,IL1RL1,NDRG2,NLK,NPHP4,KLF7,SNX25,PER1,WWTR1,CBLB,PTPRU,MAPK8IP2,VEPH1,DDX3X,CNOT7,SMAD3,CD300A,AATF,HDAC7,TNF,OVOL2,SNX5,TNIP1,EIF3A,PRDM15,DLG5,MLIP,HMGA2,GCLC,PMEPA1,LTBP1,SULF1,LRP4,ALPK2,CTNNA1,MLLT3,CD109,SLIT3,UBR2,VWC2,PTP*

			RO,ESR1,SEMA3E,MARVELD3,EPHB2,MECOM,FER,MAP3K3,FNIP1,SLAMF1,ABL2,DEPDC5,VPS13C,ARHGAP35,CD2AP,SEMA5B,SPRED3,TNFAIP8L2,ARHGAP25,SULF2,PLAT,PDE11A,RGS3,SOST,P2RX7,RASA3,AMFR,AR,FGL2,SUFU,EYA1,BDKRB2,DGKZ,PRKAA2,SEMA3A,TLE4,PROS1,TRIM72,MAP2K5,TNN,DAB2,ITGA3,FANCB,PLCL2,PPM1B,PIN1,RPS6KA6,STK38,CIT,CLASP1,TGFA,HGF,RGS12,PDE3A,PSMB7,SKI,AKT3,ELF1,ENPP1,LITAF,MAD1L1,ARHGAP42,CD84,PPARA,SPRED1,PRDM16,RBL1,ZNF451,BTRC,CCDC3,OPTN,PTPRR,FBXW11,G3BP1,A2M,PRKCQ,SUSD4,TP63,PIK3R2,LGALS9,AOAH,APC,DPP4,ITGA1,KIAA0319,UBASH3B,MTMR4,RPH3AL,USP18,GPR21,TFIP11,CLASP2,VPS35,C1QTNF3,TBX20,C6ORF106,NLRC5,TBX18
GO:1902476	chloride transmembrane transport	0.004523782674922888	*GABRB3,GABRG3,ANO2,ANO4,SLC12A1,CFTR,GABRG1,SLC1A3,SLC26A7,GABRB1,SLC1A1,SLC12A8,GABRA2,GLRA2,GLRA1,GABRA3,GABRA5,ANO1,GABRR2,GABRB2,ANO3,GLRA3,SLC26A8,GABRA1,GABRR3,GABRG2*
GO:0010243	response to organonitrogen compound	0.004707326426909229	*PLCB1,GABRB3,MAPK14,ARID1B,ITGA2,KCNQ1,SLC1A2,BCKDHB,TGFBR3,CDH13,HLCS,KCNE1,PSMC6,PSMB2,SPIDR,RYR3,KYNU,GLP2R,CFTR,ALK,PTPRE,PIK3C3,AGT,CHRM5,PARK2,CYBB,OTC,USP25,RNF185,DBH,FBXO27,HDAC2,SLIT2,STXBP4,IGF1R,MDM2,SLC24A4,HCN1,TRPM2,RNLS,RYR2,LRRK2,SLC1A3,PDXP,GSK3B,SLC8A1,OGT,KCNC1,SIK2,AKAP6,FBN1,ITPR2,NR4A2,ABCC9,FYN,GNRHR,NR4A1,RHBDD1,CASP6,FOXO1,PDE4D,KANK1,ACTN2,GABRB1,SLC1A1,MTOR,CHRM1,PRKCE,PRKCB,ADCY8,FOXO3,RAB31,EDNRB,TBC1D4,TACR3,TGM2,APP,ADTRP,CPEB2,ATF2,ABAT,GRIN2A,BCL2L1,PDE4B,DNMT1,GHR,GLRA2,NTRK2,GNG2,SDK1,RAP1A,NCOA5,RAPGEF1,CACNA2D1,GLRA1,TP73,COL4A6,NPC1,PNPLA3,ADORA2A,RGS7,TFF1,SORL1,HTR4,POR,GRM5,HDAC9,DDC,JAG1,MAN1A1,PPP3CA,KLF3,GLDC,MAS1,PER1,KL,ELAVL4,TMEM67,TRIM16,GABPA,KAT7,P2RX6,AKAP7,FOXRED2,NSG1,JAK2,BAIAP2L1,CDK1,TNF,PANX1,SNX5,POMC,PHEX,SRSF4,BCL11A,ANO1,GCLC,C5AR1,GNAL,PIK3R3,TRIM41,CTNNA1,NSG2,UBR2,BLM,TRPV4,CPS1,RAPGEF2,REG1B,HTR2C,UBE3A,GABRB2,EPHB2,ABCC1,FER,BSG,CAMP,VPS13C,CYP2E1,P2RX1,PDGFD,PLAT,RYR1,GLRA3,P2RX7,AMFR,CASP7,GNB5,FLT3,PPARGC1B,PTPRA,TRIM72,GNAO1,HCN4,SMPD3,SRD5A2,UROS,TRPM4,ERLIN1,CHRM3,CALCR,INSR,PDE3A,ENPP1,RRAGC,IDE,PCK1,PPARA,SORT1,TAF1,CACNA1A,EXT1,PRKCG,UMOD,HDAC5,UGGT2,SEL1L2,STAT4,KCNC2,PRKCQ,STAT3,BRIP1,PIK3R2,CASQ2,DTNBP1,RAP1B,APC,GABRG2,GPR21,VPS35*
GO:0106027	neuron projection organization	0.0049918721516623256	*CTNND2,ZNF804A,IGF1R,LRRK2,MTMR2,GSK3B,GRIN2B,EPHB1,SIPA1L1,FYN,KALRN,TANC2,APP,DNM3,RELN,PLS1,DOCK10,NLGN1,PPFIA2,TANC1,ARF1,UBE3A,EPHB2,BHLHB9,PDLIM5,ITGA3,INSR,ABCD1,PAK3,DTNBP1,LRP8,VPS35*
GO:0010647	positive regulation of cell communication	0.005015821296598484	*PLCB1,SH3RF3,CTBP2,LYPD6,ANK3,WLS,TIMP3,DUSP22,AKAP13,ITGA2,NRXN1,ZRANB1,DOK5,RIMS2,SHANK2,CLEC16A,TGFBR3,CDH13,CSNK2A1,TMEM108,GADD45A,GPC3,NRG1,PSMB2,PLCE1,SH3RF2,CCR1,CFTR,RHOC,RASGRF1,NKD1,ALK,RGL2,S100B,ROBO2,AGT,CDH2,PARK2,LAMA2,TRIM22,TRIM5,TXK,GPC5,IGF1R,NR3C2,SYT1,CACNG3,FSHR,GRM1,WWOX,TRPM2,CAMTA1,LRRK2,SLC1A3,NTRK3,ILDR1,IQCJ-SCHIP1,RNF165,DISC1,MECP2,SEMA4D,GSK3B,LGR5,PRKD1,TGFB2,CLSTN2,SEMA5A,BDNF,PRKCH,RYK,ZDHHC17,WASF1,GRIN2B,ANKRD6,ARHGAP8,PRR5,SNAP25,PML,AKAP6,TAOK3,LRP2,FYN,SMARCA4,CD44,NEU3,KANK1,ARHGEF3,ATF6,DCDC2,MME,CSNK1G3,GRIK2,IGSF11,SLC1A1,NLRP12,ROR1,DDX21,BMPER,ARL6IP5,PRKCE,PRKCB,ADCY8,LRRTM1,OASL,ROBO1,FLT1,NOX4,MAP3K7,MAPK9,TRAF3IP2,STK3,TGM2,KDR,EDA,APP,NRP1,LGR4,ADAM17,MAP3K5,PIK3R5,TGFB1I1,TCF7L2,TNFRSF10B,TRAF3,ABAT,GRIN2A,RIMS3,BCLAF1,CCDC88A,CD38,STK36,GHR,MAPRE2,NTRK2,ACSL4,BRD4,RASGRP1,LY86,SPRED2,RAP1A,TGFBR1,TNR,RAPGEF1,EP300,TP73,PIBF1,RASGRF2,PSMA1,ADORA2A,CACNG2,NF1,SORL1,BID,ASXL1,POR,RELN,GRM5,IL18R1,MGAT5,MAP3K13,ADCYAP1R1,JAG1,JRK,DGKI,SLC24A2,KL,DOK6,RIMS1,TRIM16,WDR59,NLGN1,RPS20,LRRK1,MAPK8IP2,DDX3X,NSG1,ZNF622,JAK2,SHOX2,SMAD3,CD300A,PLXNB1,MIR320B2,SLC39A10,TNF,SNX5,POMC,PRDM15,BMP15,DLG5,LGR6,SHC2,AGO3,ANO1,UBE2V1,DAAM2,SMOC2,SULF1,C5AR1,CNTN6,TRDN,NET1,ZNF423,CLSTN1,SELP,CTNNA1,TENM1,TSHZ3,GSKIP,TNFRSF19,DYNC2H1,MLLT3,MYRIP,EGF,KIT,TRPV4,EDAR,RAPGEF2,HTR2C,SLC30A8,UBE3A,GRM4,EPHB2,NDFIP2,ERBB4,MAP3K3,FNIP1,SLAMF1,F11R,GAS8,CCL3,VWF,IL20RA,SULF2,SOX4,PDGFD,PIK3CD,UBE2N,ITGA8,PUM1,TNKS,AUTS2,FGFR1,IL23R,NTF3,P2RX7,SCUBE1,AR,ADRA1B,ARMC9,MIR433,SEMA3A,*

			CDC73,PDE5A,ZMIZ1,FLT3,FERMT2,LTBR,MAP2K5,DAB2,GPR55,STX3,ACTN4,CCL7,EEF1E1,EEF1E1-BLOC1S5,KMO,LGI1,PPM1B,PIN1,TRPM4,CD226,BMP6,TGFA,HGF,CALCR,SCUBE3,CACNB2,NOS1AP,INSR,MACF1,PAK1,PSMB7,SKI,AKT3,FMR1,LITAF,RRAGC,EVC,MFNG,TNFAIP8L3,CHD5,SPRED1,AKR1C2,CHSY1,RIT2,PRKCG,PRLR,TDGF1,SCEL,STAT3,TP63,TBL1X,DTNBP1,RAP1B,KSR1,LGALS9,NFAT5,ALPK1,IL10RB,BMPR1A,ITGA1,KIAA0319,NGF,PRKCA,ADAMTS20,CTSH,RPH3AL,CCL14,CCL15,EDA2R,IL6R,MAP2K6,MAP2K1,SOS1,VPS35,C1QTNF3,FGF1,TBX20,CRADD,NLRC5
GO:0099560	synaptic membrane adhesion	0.005128724176777235	*MAPK14,NRXN1,LRRC4C,NRG1,LRFN5,GPC6,PCDH17,NTNG1,LRRC4,PTPRD,EFNA5,IL1RAP,GPC4,NLGN1,PTPRF*
GO:0007214	gamma-aminobutyric acid signaling pathway	0.005128724176777235	*GABRB3,GABRG3,GABBR2,GABRG1,GABRB1,PLCL1,GABRA2,GABRA3,GABRA5,GABRR2,GABRB2,PLCL2,GABRA1,GABRR3,GABRG2*
GO:0007169	transmembrane receptor protein tyrosine kinase signaling pathway	0.0052118455571643066	*PLCB1,MAPK14,PTPRT,SVEP1,EPHA6,NRXN1,SHC3,DOK5,NRG3,FSTL4,CDH13,TMEM108,FLRT2,NRG1,PLCE1,TIAM1,ALK,PTPRE,RAB7A,ARID5B,EPHA3,AGT,PDCD6,STXBP4,IGF1R,PTPRG,FSHR,NTRK3,CD8B,ANKS1A,GSK3B,PRKD1,OGT,ANKS1B,BDNF,RYK,ZDHHC17,WASF1,SIK2,EPHB1,SIPA1L1,EPHA7,FYN,CHN1,NEU3,FOXO1,KANK1,TNMD,ROR1,PRKCB,KALRN,ROBO1,FLT1,KDR,NRP1,ADAM17,IGF2R,EFEMP1,ROS1,RPS6KA5,CCDC88A,FAM83B,RNF126,GHR,NTRK2,RABGEF1,KIF16B,EPHA5,LCP2,NCOA5,RAPGEF1,CHRNA3,GAB2,COL4A6,EFNA5,MERTK,NF1,SORL1,KL,CBLB,DOK6,JAK2,AHI1,BAIAP2L1,SNX5,SHC2,SMOC2,SULF1,PIK3R3,EFNB2,EGF,KIT,EFNB1,RAPGEF2,EPHB2,ERBB4,FER,SHCBP1,FGF7,SULF2,NEDD9,PDGFD,PIK3CD,PLAT,FGFR1,NTF3,AR,BDKRB2,FLT3,PTPRA,TRIM72,SMPD3,FGF12,TGFA,HGF,INSR,PAK1,ENPP1,IDE,SORT1,PTPRR,EXT1,PRLR,PRKCQ,STAT3,PAK3,PIK3R2,BLNK,MAPKAPK2,APC,ITGA1,NGF,UBASH3B,PILRB,ABI1,GPR21,CLASP2,SOS1,FGF1*
GO:0051345	positive regulation of hydrolase activity	0.005393920243867876	*BCL2L13,FBLN1,ITGA2,RGS6,SGSM1,TBC1D22A,TIAM1,RHOC,RASGRF1,AGT,TXK,USP6,MYO9A,PDCD6,SIPA1L3,LRRK2,NTRK3,SH3BP1,SEMA4D,GSK3B,PRKD1,GRIN2B,PML,NLRP2,RALBP1,ARHGAP24,SIPA1L1,ARHGEF10,FYN,CHN1,RAP1GDS1,SLC1A1,NLRP12,MTOR,TIAM2,ARL6IP5,EVI5,KALRN,DLC1,ROBO1,TBC1D4,FLT1,CR1,TGM2,PCOLCE2,APP,UACA,MAP3K5,PKP4,RGS16,RALGAPA2,DOCK9,GRIN2A,MAPRE2,RASGRP1,RAP1A,RAPGEF1,GRAMD4,MAGI2,NF1,RGS7,ARHGAP6,BID,SELE,ADCYAP1R1,EGLN3,TBC1D3B,TBC1D5,DOCK10,SLC22A2,ASAP1,AKIRIN2,DDX3X,ARAP2,JAK2,SMAD3,CD300A,PLXNB1,SLC39A10,TNF,C5AR1,NET1,CDKL5,SIPA1L2,KIT,RAPGEF2,ESR1,DAP,DAPK1,RAPGEF6,RPS27L,ABL2,ASAP2,F11R,ARHGAP35,CCL3,PREX1,ARHGAP25,ARF4,ST18,GARNL3,NEDD9,P2RX1,FGFR1,TBC1D9,NTF3,RABGAP1L,NEK5,GNB5,PPARGC1B,FERMT2,GPR55,CCL7,PIN1,RALGAPA1,AIM2,ARHGAP42,GNA13,WDR41,DOCK7,STAT3,LGALS9,ITGA1,CTSH,CCL14,CCL15,CRADD,RGN*
GO:0031323	regulation of cellular metabolic process	0.005441283583880026	*TASP1,FANK1,CAST,ZHX3,PLCB1,KMT2C,KHDRBS2,ZNF595,CMKLR1,DAB1,DUX4,GTF2I,ZNF717,MAPK14,DSCAM,ARID1B,RUNX1,ORC3,CTBP2,SERPINA1,TSHZ2,CCNG2,RAB27A,PCBD2,ANXA8L1,PRIM2,PTPRT,BCL2L13,RBFOX1,TIMP3,ZNF397,ZSCAN30,DUSP22,AKAP13,FBLN1,ERG,VPS13D,ZNF292,ITGA2,ABCG1,DPF3,ATF7IP,EPHA6,NRXN1,ISX,ZNF578,PAX7,MTPN,NRG3,ZNF732,SMYD2,CELF2,CLEC16A,HUS1,ARNT2,CDH13,RNF152,RTN1,NXN,RUNX1T1,CSNK2A1,ZNF678,ZNF112,ZNF229,ZNF285,TF,PLGRKT,ERCC4,SAMD4A,EIF4EBP3,CHEK2,ZNF486,GADD45A,PRDM9,PSMC6,ACIN1,ZNF331,TOX3,GPC3,FBLN5,NRG1,ETS2,PSMB2,RFFL,NPAS3,SPIDR,EWSR1,NLN,SUZ12,LCORL,PLCE1,SH3RF2,SPOCK1,SLCO3A1,RBFOX2,ZNF420,ZNF257,MED13L,AGBL4,BEND5,LDLRAD4,DNAJC15,ZNF91,ZBTB20,TET3,CBFA2T2,SCAI,ALK,ZNF536,ERC1,ARID5B,SPOCK3,ZNF366,EPHA3,CRNN,AGT,CST2,MAGEB3,CHRM5,ATP6V0D1,RCAN1,KLHL25,PARK2,BANP,FHL2,FOXN3,LOXL2,CDC5L,PSPC1,BRF1,DACH1,TRIM22,TRIM5,USP25,ZBTB8B,ZNF268,DCUN1D4,TXK,CP,RNF185,ZNF232,NELL1,TRAPPC9,PHC1,SETD3,RIN3,ZNF845,FTO,HDAC2,SLIT2,TFAP2D,ZBTB34,KCTD1,AHRR,PDCD6,ZSCAN5C,LCMT1,IGF1R,MDM2,SMAD1,E2F3,NOVA1,ZBED4,FOXD4L4,TMPRSS6,ZNF567,ZNF850,NR3C2,TRPC5,DMD,BASP1,NOX5,TEAD1,FSHR,GSG1L,MBOAT7,FMN2,HNRNPA2B1,MAEL,MORF4L1,NFE2L3,NRIP1,PRKAR2A,SKAP1,WWOX,ATRX,ACTR5,PARP16,USP16,CAMTA1,HSPB*

			8,LRRK2,MAPK10,RORB,PIR,ZFP30,ZNF607,NTRK3,RORA,SLC4A4,CHD7,ZNF26,DISC1,HEXB,PAN3,CDCA2,GLIS1,SOX6,IER2,MECP2,MTMR2,SEMA4D,GSK3B,SLC8A1,ZNF432,ZNF841,PRKD1,SERPINA4,SERPINA5,ERCC6,OGT,RNF144A,TGFB2,PRAMEF12,VAV3,AFF3,DACH2,PIWIL4,RIPPLY3,S100A11,SLC5A3,BDNF,PRKAR1B,PRKCH,RIPK4,RYK,POLR3C,GRIN2B,KDM4C,RAG1,SAMD13,ZNF433,NR2F2,PRR5,NR5A2,PCBP3,PML,PPARGC1A,RNF180,TRUB2,USP36,ZNF627,MED15,PPP4R4,NLRP2,NLRP7,TAOK3,ZNF610,BCL2,MXI1,RALBP1,ZNF675,EPHB1,SMARCE1,KLF12,LRP2,NSF,NFIA,SND1,DEPTOR,NR4A2,ZNF677,EPHA7,ZBTB7C,RBAK,RBAK-RBAKDN,FYN,KCNE2,PARN,NR4A1,SMARCA4,CD44,EIF4G3,RCVRN,RHBDD1,FOXO1,PDE4D,RBBP8,RNF144B,ENPP2,ABCB10,ACTN2,ATF6,BACH1,CST9L,KLF13,RNF217,EXOC4,ZNF516,CELF4,SERPINB7,APBB2,EIF3E,NAMPT,SETD7,SLC1A1,ZFPM2,ZNF141,ZNF429,ZSCAN5A,GLIS3,PHF20,RARB,RFX4,ETV6,NLRP12,MFSD12,MTOR,RMI2,ROR1,LDB2,MOV10,VPS26B,ZNF510,BMPER,SCAF8,ZNF521,OGFOD1,PLCL1,ZFP64,ZNF483,ARL6IP5,ATP1A1,EPM2A,NOL11,CLYBL,CTNNBIP1,SOX8,ZMYND11,HAS3,HIVEP2,PRKCE,ATP6V1D,EIF2S1,GTF2A1L,KMT2A,PRKCB,VDR,ADCY8,FHL5,FOXO3,IGF2BP3,LRRTM1,MITF,SCMH1,SCP2,CAPN3,DPH6,ITLN1,CCDC88C,CREB5,DLC1,EDNRB,OASL,ROBO1,SATB2,TDRD3,BNIP3L,FLT1,NOX4,ZNF148,GRHL2,TCF4,TSC22D3,ZNF573,ZNF720,MAP3K7,MAPK9,NBAS,NR3C1,TACR3,TRAF3IP2,CR1,EYA4,SPON1,PKHD1,STK3,SUPT3H,ZNF585A,KDR,PPHLN1,PRCP,CASK,EDA,PCOLCE2,APP,NRP1,UACA,LGR4,SERPING1,TENM2,ADTRP,KAT5,THRB,CPEB2,FUBP1,KCNK2,ZNF667,ZNF98,ADAM17,CAMK1D,DIS3L2,MAP3K5,TFPI,ZER1,ATF2,ELP3,ETS1,NR2C2,PIK3R5,SAMSN1,EFEMP1,ROS1,SMG6,TGFB1I1,CDK17,DOT1L,PSIP1,RPS6KA5,TCF7L2,TNFRSF10B,TRABD2B,ZNF443,ZNF490,ZNF709,ZNF799,AFF2,PARD3,TLK2,TRAF3,ZNF830,ABAT,GRIN2A,DLX6-AS1,OPHN1,BCLAF1,TRPS1,ZNF568,CCDC88A,CD38,KHDRBS3,MOB3B,MRPS27,ZBED5,SMG7,STK36,BCL2L1,HDAC4,SOX5,SRRM4,TIMP2,BACE2,DNMT1,GHR,NTRK2,BRD4,PKNOX2,RASGRP1,ARR3,CAMK4,COMMD1,RABGEF1,SPIN3,SPRED2,STARD4,GLI3,SMCHD1,KIF16B,RAP1A,SAFB2,TGFBR1,ZBTB41,DICER1,EPHA5,LCP2,CUX1,NCOA5,ITGB3BP,MAGEA11,PTTG1IP,ZNF729,CHRNA3,EP300,ELAVL2,HMG20A,SCFD1,TBX3,DIO2,GRAMD4,ATXN1,BRD7,TP73,NPC1,PIBF1,PSMA1,FHIT,ONECUT3,ADORA2A,EFNA5,FAM20A,MERTK,MAGI2,NF1,PPP6R2,RFC3,ZNF525,ZNF765,SORL1,STRN3,ZNF615,BID,ELK3,TEAD4,DMRT1,ASXL1,EIF4E,POR,RELN,SLC39A8,TSHZ1,GRM5,HDAC9,IL18R1,NECAB1,SELE,LRRTM3,MGAT5,NUMB,SP2,TNFAIP8,MAP3K13,NLK,TBX15,ADCYAP1R1,EGLN3,IL1RAP,FBXL5,JAG1,MEOX2,PPP3CA,KLF3,KLF7,MYT1L,SH3GL2,SNX25,MAS1,PER1,SATB1,TBC1D5,WWTR1,ANKRD13A,CBLB,ELAVL4,MAGEB2,MNAT1,PLSCR1,CNTN1,TRIM16,ZNF736,GABPA,KAT7,L3MBTL3,SSBP2,ZNF540,ZNF571,SLC22A2,BORA,N4BP2L2,THAP7,AKIRIN2,RNF128,RPS20,ZNF562,RAD51AP1,LRRK1,ZNF208,DDX3X,LRPPRC,NSG1,RBL2,CNOT7,MAML3,EHF,TPCN2,ZNF622,DNMT3B,JAK2,LMO7,SHOX2,AHI1,SMAD3,CD300A,NOS1,SERPINB12,RDH10,TCF12,ZNF337,AATF,ANHX,CDK1,HDAC7,HIVEP3,NBN,NVL,SLC39A10,TAGLN3,TNF,OVOL2,SNX5,TNIP1,ZFPM1,ILF3,POMC,PRDM15,ADNP2,BMP15,INO80,POU6F2,RBM11,SCAF4,SIM2,ARID2,MLIP,PAXBP1,FAM220A,PRG3,SRSF4,ADORA3,ARF1,BCL11A,HMGA2,MYO6,NEK4,TAF1D,YAF2,ZNF41,ZNF705A,ZNF880,AGO3,PAPOLA,CDC20B,GCLC,PMEPA1,QKI,UBE2V1,ZNF664,ZNF728,ZNF879,ACTR8,SP140,SP140L,L3MBTL4,SLC7A7,SMOC2,ANKRD31,C5AR1,CISD2,LRP4,PIK3R3,WDR5,EFNB2,MXD3,PIWIL3,SAE1,TRIM41,HERC1,PHF8,ZNF423,CDC14B,CHD6,TENM1,TSHZ3,WDFY2,ZNF407,ZNF93,ARHGEF11,GSKIP,MAMLD1,PRKAR1A,ZNF345,BPTF,MLLT3,CD109,EGF,PTPN14,SPIRE2,FOXP4,KIT,TNFSF8,TNRC6B,ZNF600,BLM,TRPV4,PTPRO,RAPGEF2,ZIM3,DNAJB6,ESR1,HTR2C,UBE3A,FGF13,PLAG1,EPHB2,MECOM,NDFIP2,RNF4,TFDP2,TOX,C14ORF39,DAP,DAPK1,ERBB4,FER,MAML2,FNIP1,LRP5,MALRD1,ZNF439,ZNF507,RPS27L,ABL2,CAMP,DEPDC5,SETD2,VPS13C,ALX4,COMMD7,NSUN2,PTPRK,FGF7,SPRED3,ZNF189,GCFC2,GLYR1,NHLH2,ARF4,EEFSEC,MYH9,SLC2A13,SOX4,ST18,CAB39L,NEDD9,P2RX1,PDGFD,PRR16,UBE2N,AGTPBP1,ITGA8,PUM1,ARID4A,DDB1,FAM172A,IRAK2,KDM3A,PLAT,SERTAD2,TNKS,ZFP82,AUTS2,BCL7A,DAPL1,FGFR1,MEF2A,OCLN,PARP15,ZCCHC17,ZNF85,ARID4B,SLX1B,SOST,ASXL3,IL23R,JMY,KEAP1,NTF3,P2RX7,ARRDC4,BRD9,TOX2,ZNF544,AR,BCOR,SNX30,SUFU,TRERF1,EYA1,IFNA8,N4BP1,RBM4,SRPK2,BDKRB1,BDKRB2,DGKZ,GNL3,L3MBTL1,NEK5,PI3,PRKAA2,RLF,TAX1BP1,TLE4,ZNF202,ZNF558,CDC73,MARK2,MEIS2,PDE5A,PROS1,ZMIZ1,ZNF19,ZNF440,ZNF670,

			ZNF695,FLT3,NFIC,PPARGC1B,SNX7,ATP6V1H,DYNAP,ESRRG,FERMT2,MAP2K5,SMPD3,CLN6,DAB2,HDAC8,PBX1,TCL1B,ACTN4,ELP4,POU2F3,PRAMEF8,FANCB,FRY,PLCL2,QRICH1,SSX5,PHF20L1,PIN1,SYNPO2,ERLIN1,KRBOX4,STK38,TCF20,ZNF674,BMP6,CIZ1,EIF4ENIF1,CARF,CDYL2,EBF1,NCOA2,TGFA,CREM,G3BP2,HGF,PKP1,POU2F1,ZNF665,ZNF730,CALCR,CENPF,FLI1,POMT2,SH3BP5,ZBTB16,CTIF,KDM4B,NOS1AP,ZNF57,IL18RAP,INSR,LARP4B,PAK1,PSMB7,SKI,CTDP1,ELF1,ENPP1,FMR1,LIMK2,LITAF,PAX3,CHFR,DKC1,JDP2,KLF6,PHC2,RRAGC,ZNF605,ABCD1,BMF,IDE,SAMD8,AIM2,PCK1,PPARA,SPIB,TBX22,TNFAIP8L3,TTN,ZFHX2,SMARCAD1,TAF1,WDR41,CHD5,FNIP2,PPP2R3C,SPRED1,ESR2,PRDM16,QSOX1,RBL1,ZNF451,ATP6V1E1,BTRC,HIRA,MLLT10,OPTN,RIT2,ZNF713,ESRP2,MDM4,NCOA1,PRKCG,PRLR,ZNF354B,CBFA2T3,DOCK7,FBXW11,HDAC5,TDGF1,WDR70,ZNF493,A2M,MYEF2,PI15,SERPINB8,SLC24A5,SLC40A1,STAT4,ZNF623,ADARB1,POU2AF1,PRKCQ,STAT3,TFEC,TP63,USP7,ZNF721,BRIP1,CDKN3,CST1,DOCK3,HPN,PAK3,PIK3R2,TBL1X,DHRS7B,DTNBP1,ZNF518A,KSR1,LGALS9,LRP8,NFAT5,ZNF697,GTPBP1,IL10RB,MAPKAPK2,AHNAK2,APC,BMPR1A,ITGA1,KCTD20,NGF,PPP1R14A,PRKCA,SPINT2,UBASH3B,VLDLR,DRAM1,ITIH6,MTMR4,MTRF1,PDS5A,PILRB,ABI1,CTSH,DHX9,NCAPG2,RGCC,DPH1,EBF4,EDA2R,IL6R,MAP2K6,SRRD,TFIP11,SPTBN4,MAP2K1,PRAMEF7,RGMB,VPS35,ZBTB25,ZRANB3,C1QTNF3,COL6A3,FGF1,NSUN3,TBX20,ZNF527,ATMIN,ATP6V1E2,CRADD,NLRC5,RGN,RREB1,TBX18,ZFP2,ZNF501
GO:0045595	regulation of cell differentiation	0.00553324177402709	*CDH4,ZHX3,PLCB1,CMKLR1,DAB1,MAPK14,DSCAM,RUNX1,DCC,RBFOX1,FBLN1,NPHP3,ABCG1,PLEKHB2,CHODL,FSTL4,RUNX1T1,PCP4,ACIN1,PLXNA4,NRG1,PSMB2,IL4R,NLN,SUZ12,SEMA6D,CCR1,RBFOX2,TIAM1,LDLRAD4,DPYSL2,ALK,ZNF536,FBXO31,CNTN4,ROBO2,EPHA3,AJAP1,AGT,ATAT1,LOXL2,SEMA3D,NTN1,ZNF268,NELL1,SETD3,TRIO,FTO,HDAC2,SLIT2,MDM2,SMAD1,TRPC5,DMD,VCL,FSHR,TENM4,LRRK2,RORB,NTRK3,RORA,CHD7,DISC1,GLIS1,SOX6,MECP2,SEMA4D,GSK3B,PRKD1,TGFB2,PRAMEF12,SEMA5A,BDNF,PRKCH,RYK,ABI3BP,KDM4C,RAG1,RAG2,AKAP6,FBN1,PLXNA2,BCL2,ZNF675,EPHB1,LRP2,TMEM100,EPHA7,ZBTB7C,FOXO1,KANK1,ABCB10,KLF13,MME,MYLK3,ZFPM2,RARB,MTOR,PKP2,PTPRD,TIAM2,CTNNBIP1,SOX8,VDR,FOXO3,MITF,CAPN3,KALRN,EDNRB,MSR1,ROBO1,GRHL2,TCF4,MAPK9,CR1,STK3,TGM2,KDR,ABCA12,APP,NRP1,ST7,SEMA3C,MAP3K5,ETS1,EFEMP1,PRTG,TGFB1I1,TCF7L2,TRPS1,RIN2,HDAC4,SOX5,DNMT1,GHR,NTRK2,RASGRP1,CAMK4,SPRED2,GLI3,RAP1A,TGFBR1,DICER1,TNR,CUX1,HMG20A,TBX3,WIF1,TRIOBP,TP73,PSMA1,EFNA5,BRD1,NF1,SORL1,ASXL1,EIF4E,POR,RELN,GRM5,HDAC9,NAPEPLD,NUMB,MAP3K13,JAG1,PPP3CA,KLF7,WWTR1,TRIM16,GABPA,KAT7,N4BP2L2,NLGN1,JAK2,SHOX2,AHI1,SMAD3,PLXNB1,TCF12,CDK1,HDAC7,TNF,OVOL2,ZFPM1,BLOC1S6,BRINP1,BCL11A,DOCK1,MBOAT2,DAAM2,LRP4,EFNB2,CTNNA1,WDFY2,CDKL5,CD109,IL1RAPL1,KIT,TRPV4,VWC2,ABCA5,RAPGEF2,HTR2C,SEMA3E,FGF13,PLAG1,EPHB2,TOX,FNIP1,LRP5,F11R,NSUN2,CCL3,PREX1,SEMA5B,SPRED3,PSG9,SOX4,NEDD9,KDM3A,FGFR1,MAP2,IL23R,KEAP1,NTF3,AR,FGL2,KIAA1109,SUFU,EYA1,IL1RL2,RBM4,HLA-DRA,BHLHB9,L3MBTL1,NEK5,SEMA3A,CDC73,MEIS2,PDE5A,ZMIZ1,PPARGC1B,TRIM72,FERMT2,MARK1,TNN,DAB2,GPR55,PBX1,ACTN4,BLOC1S5,PRAMEF8,NREP,TRPM4,BMP6,CLASP1,EIF4ENIF1,HGF,SCUBE3,ZBTB16,MACF1,PDE3A,PSMB7,SKI,CTDP1,ENPP1,JDP2,PRKX,PCK1,PPARA,SORT1,ZFHX2,PPP2R3C,S1PR3,SPRED1,CCDC3,NCOA1,PRLR,DOCK7,HDAC5,STAT3,TP63,HPN,ISLR2,PAK3,USH2A,LGALS9,LRP8,APC,BMPR1A,KIAA0319,NGF,PRKCA,UBASH3B,ADAMTS20,RGCC,CYB5D2,IL6R,CLASP2,MAP2K1,PRAMEF7,SOS1,TPH1,TBX20,RREB1*
GO:0061061	muscle structure development	0.0057168063734673235	*PLCB1,MAPK14,MYO18B,RBFOX1,AKAP13,LARGE,SORBS2,PAX7,CHODL,MTPN,TGFBR3,ANK2,NRG1,IL4R,NLN,NKD1,UTRN,ARID5B,AGT,RCAN1,CDH2,FHL2,LAMA2,KCNH1,SETD3,MDM2,SMAD1,DMD,BASP1,RYR2,RORA,CHD7,RNF165,SOX6,MECP2,SLC8A1,TGFB2,BDNF,SGCD,NR2F2,SGCG,AKAP6,BCL2,EPHB1,DISP1,LRP2,NR4A1,FLNC,ACTN2,MYLK3,ZFPM2,RARB,MTOR,PKP2,COL11A1,SOX8,CAPN3,EDNRB,NOX4,NEBL,MAP3K5,TCF7L2,ITGA11,HDAC4,DNMT1,JAM2,TGFBR1,ADAM12,EP300,SYNE1,TBX3,FLNB,NF1,TEAD4,HDAC9,JAG1,MEOX2,PPP3CA,NEB,CXADR,FHOD3,SHOX2,SMAD3,NOS1,PTGFRN,TCF12,CDK1,HIVEP3,TNF,ZFPM1,CHKB,TANC1,PAXBP1,QKI,EFNB2,ALPK2,MYH14,PRKAR1A,KIT,MYPN,SGCZ,MYH15,ALX4,MYH9,ITGA8,MEF2A,RYR1,RBM4,NEK5,PDLIM5,TRIM72,ACTN4,TMOD1,MYLK,CENPF,ELN,SKI,CTDP1,PAX3,EVC,PPARA,SORT1,TTN,COL19A1,FHL1,MYH7,HIRA,PGM5,MYH6,ADAMTS5,HDAC5,MYEF*

			2,ADARB1,MYH11,MYOM2,BMPR1A,COL6A3,TBX20,TBX18
GO:0030048	actin filament-based movement	0.005832350977187024	KCNQ1,SUN2,ANK2,KCND3,KCNE1,CTNNA3,RYR2,FRMD6,SGCD,MYH8,KCNJ3,KCNE2,PDE4D,PKP2,ATP1A1,PLN,CCDC88C,PDE4B,CACNA1C,CACNA2D1,CACNA1D,MYH4,MYO6,SCN1A,MYH14,FGF13,NUP155,WIPF2,MYO7B,MYH9,KCNE4,HCN4,RNF207,ACTN4,FGF12,TRPM4,EPDR1,MYO1B,CACNB2,NOS1AP,MYH7,MYH6
GO:0006813	potassium ion transport	0.00586354801350348	KCNJ6,ANK3,SLC24A3,KCNQ1,ANK2,KCND3,SLC12A1,DPP10,KCNE1,KCNJ15,KCNG3,KCNH1,KCNMA1,SLC24A4,KCNK17,HCN1,DPP6,SLC1A3,KCNH5,KCNK13,ATP1A4,KCNC1,SNAP25,AKAP6,KCNJ3,NSF,ABCC9,KCNE2,ACTN2,KCNS3,KCNIP4,KCND2,ATP1A1,SLC12A8,KCNC4,KCNK2,KCNJ12,RGS7,KCNQ5,SLC9C1,KCNB2,SLC24A2,AKAP7,CACNA1D,NOS1,KCNT2,KCNK10,KCNA6,KCNH8,KCNH7,KCNAB1,KCNE4,HCN4,RNF207,KCNJ16,KCNN3,NOS1AP,FHL1,SLC24A5,KCNC2,SLC9A9,HPN,CASQ2,TMEM175,SLC9A2
GO:0001508	action potential	0.0066419514702476205	ANK3,KCNQ1,ANK2,KCND3,SCN8A,KCNE1,DMD,CTNNA3,RYR2,SLC8A1,AKAP6,KCNJ3,KCNE2,GRIK2,KCND2,SCN11A,PKP2,ATP1A1,NTRK2,CACNA1C,USP53,CACNA2D1,GLRA1,CXADR,AKAP7,CACNA1D,TNF,SCN1A,MYH14,FGF13,NUP155,P2RX1,KCNE4,HCN4,RNF207,FGF12,TRPM4,CACNB2,NOS1AP,FMR1,SCN9A,KCNC2,TBX18
GO:0099175	regulation of postsynapse organization	0.006754547208698056	NRXN1,ABHD17C,CDH2,ZNF804A,LRRK2,NTRK3,GRIN2B,LRFN2,SIPA1L1,EPHA7,FYN,PTPRD,KALRN,TANC2,DGKB,DNM3,GRID2,RELN,IL1RAP,NLGN1,PPFIA2,CDKL5,IL1RAPL1,UBE3A,ARF4,BHLHB9,PDLIM5,PAK3,DTNBP1,LRP8,VPS35
GO:0001764	neuron migration	0.006764242015739457	DAB1,DCC,NRG3,SDCCAG8,FLRT2,NRG1,ASTN2,UNC5D,SPOCK1,CTNNA2,FBXO31,PHACTR1,DCLK1,NTN1,CELSR1,ASTN1,NTNG1,NTRK3,DISC1,NR2F2,NR4A2,FYN,DCDC2,FAT3,CEP85L,SATB2,CHL1,NRP1,ELP3,NTRK2,PRKG1,RELN,KIRREL3,CDKL5,RAPGEF2,FGF13,AUTS2,FGFR1,SEMA3A,MARK2,ZMIZ1,MARK1,TNN,ITGA3,ULK4,NDE1,STAT3,KIAA0319,TBX20
GO:0051094	positive regulation of developmental process	0.00703691994893597	CDH4,ZHX3,PLCB1,CMKLR1,DAB1,MAPK14,DSCAM,RUNX1,ASIC2,NRXN1,CHODL,RIMS2,TGFBR3,SYNDIG1,PCP4,ACIN1,SYT17,PLXNA4,FLRT2,NRG1,IL4R,CCR1,CCR3,CFTR,TIAM1,ALK,FBXO31,ROBO2,AGT,PARK2,LOXL2,KIR2DL4,CYBB,NTN1,ZNF268,NELL1,SETD3,HDAC2,SLIT2,PDCD6,IGF1R,MDM2,SMAD1,TRPC5,DMD,SYT1,BASP1,KRT2,STK24,TENM4,CHD7,DISC1,SOX6,SEMA4D,GSK3B,LINGO2,SLC8A1,PRKD1,TGFB2,CLSTN2,SEMA5A,BDNF,PRKCH,ABI3BP,RAG1,RAG2,PML,PPARGC1A,AKAP6,PLXNA2,BCL2,RALBP1,EPHB1,LRP2,TMEM100,ZBTB7C,THBS2,ENPP2,ABCB10,ATP8A2,MME,MYLK3,ZFPM2,MTOR,BMPER,PTPRD,TIAM2,CTNNBIP1,SOX8,PRKCB,VDR,FOXO3,LRRTM1,CAPN3,KALRN,MSR1,ROBO1,FLT1,TCF4,MAPK9,CR1,STK3,TGM2,VASH2,KDR,NRP1,LGR4,MAP3K5,ETS1,NR2C2,TGFB1I1,TCF7L2,GRIP1,RIN2,SOX5,GHR,NTRK2,RASGRP1,GLI3,RAP1A,TGFBR1,CYSLTR1,ADAM12,DICER1,CUX1,WIF1,TRIOBP,TP73,GRID2,EFNA5,BRD1,SORL1,TEAD4,DMRT1,POR,RELN,GRM5,LRRTM3,NAPEPLD,NUMB,MAP3K13,RALA,IL1RAP,JAG1,PLS1,WWTR1,KL,ELAVL4,RIMS1,TRIM16,KAT7,AGGF1,NLGN1,JAK2,SHOX2,AHI1,SMAD3,PLXNB1,TCF12,CDK1,TNF,COBL,OVOL2,BLOC1S6,DLG5,BRINP1,BCL11A,HMGA2,DOCK1,SMOC2,C5AR1,EFNB2,CLSTN1,WDFY2,CDKL5,EGF,CMA1,IL1RAPL1,KIT,VWC2,RAPGEF2,HTR2C,JAK1,PLAG1,EPHB2,TOX,ERBB4,RBM19,SLITRK6,LRP5,CAMP,F11R,PREX1,SLC39A12,SOX4,NEDD9,PIK3CD,UNC13A,FGFR1,IL23R,P2RX7,AR,IL1RL2,RBM4,HLA-DRA,BHLHB9,NEK5,SEMA3A,PDE5A,ZMIZ1,PPARGC1B,FERMT2,DAB2,SYT2,BLOC1S5,EEF1E1,TRPM4,BMP6,CLASP1,HGF,SCUBE3,ZBTB16,INSR,MACF1,PDE3A,AKT3,FMR1,PCK1,PPP2R3C,CCDC3,NCOA1,STIM1,STAT3,TP63,AMOT,ISLR2,PAK3,LGALS9,LRP8,BMPR1A,NGF,PRKCA,VLDLR,ADAMTS20,CTSH,RGCC,CYB5D2,GPR21,IL6R,SPTBN4,CLASP2,MAP2K1,TPH1,VPS35,FGF1,TBX20,RREB1
GO:0023056	positive regulation of signaling	0.008555069505963566	PLCB1,SH3RF3,CTBP2,LYPD6,WLS,TIMP3,DUSP22,AKAP13,ITGA2,NRXN1,ZRANB1,DOK5,RIMS2,SHANK2,CLEC16A,TGFBR3,CDH13,CSNK2A1,TMEM108,GADD45A,GPC3,NRG1,PSMB2,PLCE1,SH3RF2,CCR1,CFTR,RHOC,RASGRF1,NKD1,ALK,RGL2,S100B,ROBO2,AGT,CDH2,PARK2,LAMA2,TRIM22,TRIM5,TXK,GPC5,IGF1R,NR3C2,SYT1,CACNG3,FSHR,GRM1,WWOX,TRPM2,CAMTA1,LRRK2,SLC1A3,NTRK3,ILDR1,IQCJ-SCHIP1,RNF165,DISC1,MECP2,SEMA4D,GSK3B,LGR5,PRKD1,TGFB2,CLSTN2,SEMA5A,BDNF,PRKCH,RYK,ZDHHC17,WASF1,GRIN2B,ANKRD6,ARHGAP8,PRR5,SNAP25,PML,AKAP6,TAOK3,LRP2,NSF,FY

			N,SMARCA4,CD44,NEU3,KANK1,ARHGEF3,ATF6,DCDC2,MME,CSNK1G3,GRIK2,IGSF11,SLC1A1,NLRP12,ROR1,DDX21,BMPER,ARL6IP5,PRKCE,PRKCB,ADCY8,LRRTM1,OASL,ROBO1,FLT1,NOX4,MAP3K7,MAPK9,TRAF3IP2,STK3,TGM2,KDR,EDA,APP,NRP1,LGR4,ADAM17,MAP3K5,PIK3R5,TGFB1I1,TCF7L2,TNFRSF10B,TRAF3,ABAT,GRIN2A,RIMS3,BCLAF1,CCDC88A,CD38,STK36,GHR,MAPRE2,NTRK2,ACSL4,BRD4,RASGRP1,LY86,SPRED2,RAP1A,TGFBR1,TNR,RAPGEF1,EP300,TP73,PIBF1,RASGRF2,PSMA1,ADORA2A,CACNG2,NF1,SORL1,BID,ASXL1,POR,RELN,GRM5,IL18R1,MGAT5,MAP3K13,ADCYAP1R1,JAG1,JRK,DGKI,SLC24A2,KL,DOK6,RIMS1,TRIM16,WDR59,NLGN1,RPS20,LRRK1,MAPK8IP2,DDX3X,NSG1,ZNF622,JAK2,SHOX2,SMAD3,CD300A,PLXNB1,MIR320B2,SLC39A10,TNF,SNX5,POMC,PRDM15,BMP15,DLG5,LGR6,SHC2,AGO3,ANO1,UBE2V1,DAAM2,SMOC2,SULF1,C5AR1,CNTN6,NET1,ZNF423,CLSTN1,SELP,CTNNA1,TENM1,TSHZ3,GSKIP,TNFRSF19,DYNC2H1,MLLT3,MYRIP,EGF,KIT,TRPV4,EDAR,RAPGEF2,HTR2C,SLC30A8,UBE3A,GRM4,EPHB2,NDFIP2,ERBB4,MAP3K3,FNIP1,SLAMF1,F11R,GAS8,CCL3,VWF,IL20RA,SULF2,SOX4,PDGFD,PIK3CD,UBE2N,ITGA8,PUM1,TNKS,AUTS2,FGFR1,IL23R,NTF3,P2RX7,SCUBE1,AR,ADRA1B,ARMC9,MIR433,SEMA3A,CDC73,PDE5A,ZMIZ1,FLT3,FERMT2,LTBR,MAP2K5,DAB2,GPR55,STX3,ACTN4,CCL7,EEF1E1,EEF1E1-BLOC1S5,KMO,LGI1,PPM1B,PIN1,TRPM4,CD226,BMP6,TGFA,HGF,CALCR,SCUBE3,CACNB2,NOS1AP,INSR,MACF1,PAK1,PSMB7,SKI,AKT3,FMR1,LITAF,RRAGC,EVC,MFNG,TNFAIP8L3,CHD5,SPRED1,AKR1C2,CHSY1,RIT2,PRKCG,PRLR,TDGF1,SCEL,STAT3,TP63,TBL1X,DTNBP1,RAP1B,KSR1,LGALS9,NFAT5,ALPK1,IL10RB,BMPR1A,ITGA1,KIAA0319,NGF,PRKCA,ADAMTS20,CTSH,RPH3AL,CCL14,CCL15,EDA2R,IL6R,MAP2K6,MAP2K1,SOS1,VPS35,C1QTNF3,FGF1,TBX20,CRADD,NLRC5
GO:0051336	regulation of hydrolase activity	0.0085711246406082 17	CAST,SERPINA1,ANXA8L1,BCL2L13,TIMP3,FBLN1,ITGA2,RGS6,SGSM1,FGD6,TBC1D22A,CSNK2A1,PLXNA4,GPC3,RFFL,SPOCK1,TIAM1,RHOC,RASGRF1,SPOCK3,EPHA3,AGT,CST2,RCAN1,TXK,USP6,MYO9A,PDCD6,SIPA1L3,LRRK2,NTRK3,SH3BP1,SEMA4D,GSK3B,PRKD1,SERPINA4,SERPINA5,TGFB2,VAV3,GRIN2B,RAG1,PML,ARHGAP12,PPP4R4,NLRP2,NLRP7,PLXNA2,RALBP1,ARHGAP24,SIPA1L1,ARHGEF10,EPHA7,FYN,CHN1,NR4A1,CD44,CST9L,RAP1GDS1,SERPINB7,SLC1A1,NLRP12,MTOR,TIAM2,ARL6IP5,EPM2A,EVI5,EIF2S1,KALRN,DLC1,OASL,ROBO1,TBC1D4,FLT1,CR1,TGM2,ARHGAP15,PCOLCE2,APP,UACA,SERPING1,CPEB2,MAP3K5,PKP4,TFPI,RGS16,BICD1,RALGAPA2,DOCK9,GRIN2A,TIMP2,MAPRE2,NTRK2,PRKG1,RASGRP1,RAP1A,EPHA5,RAPGEF1,GRAMD4,PCSK5,ADORA2A,EFNA5,MAGI2,NF1,PPP6R2,RGS7,SORL1,ARHGAP6,BID,TTC8,POR,HDAC9,SELE,MGAT5,TNFAIP8,ADCYAP1R1,EGLN3,TBC1D3B,DGKI,TBC1D5,CBLB,DOCK10,SLC22A2,ASAP1,AKIRIN2,DDX3X,ARAP2,JAK2,SMAD3,CD300A,NOS1,SERPINB12,PLXNB1,SLC39A10,TNF,LRCH1,C5AR1,NET1,CDKL5,SIPA1L2,CD109,KIT,RAPGEF2,DNAJB6,ESR1,DAP,DAPK1,FNIP1,RAPGEF6,RPS27L,ABL2,ASAP2,F11R,ARHGAP35,CCL3,PREX1,ARHGAP25,FGD4,ARF4,ST18,GARNL3,NEDD9,P2RX1,FGFR1,TBC1D9,NTF3,RASA3,RABGAP1L,NEK5,PI3,GNB5,PROS1,PPARGC1B,FERMT2,MAP2K5,GPR55,CCL7,PIN1,HGF,RALGAPA1,SBF2,AIM2,ARHGAP42,SORT1,GNA13,WDR41,DOCK7,A2M,PI15,SERPINB8,STAT3,TP63,AMOT,CST1,LGALS9,ITGA1,NGF,SPINT2,ITIH6,CTSH,CCL14,CCL15,COL6A3,CRADD,RGN
GO:0043412	macromolecule modification	0.0088461618781698 3	TPTE,MACROD2,KMT2C,DAB1,AGBL1,MAPK14,TPTE2,GPHN,SH3RF3,CCNG2,HUNK,PTPRT,DUSP22,AKAP13,FBLN1,ERG,MGAT4C,LARGE,TTLL2,ST6GALNAC3,ATF7IP,EPHA6,VRK2,NRXN1,ZRANB1,FPGT-TNNI3K,TNNI3K,CPPED1,NRG3,SMYD2,HUS1,TGFBR3,RNF152,NXN,CSNK2A1,HLCS,CEP41,CHEK2,GADD45A,PRDM9,NRG1,PSMB2,RFFL,ST8SIA1,SUZ12,PRMT8,PLCE1,SH3RF2,KSR2,STK38L,ABHD17C,SLCO3A1,DTX4,PPM1J,AGBL4,LDLRAD4,MYO3A,TET3,ALK,PTPRE,PIK3C3,ERC1,FBXO31,SPOCK3,B3GALT5,EPHA3,PRKACB,PTPRN2,DCLK1,AGT,UBE2R2,ATXN3L,ATAT1,FBXO32,RCAN1,KLHL25,PARK2,PGAP3,LOXL2,TRIM51,FBXL7,TRIM22,TRIM5,TRIM9,USP25,DCUN1D4,TXK,RNF185,USP40,USP6,FBXO27,PHC1,SETD3,ZNRF3,TRIO,ATE1,FTO,HDAC2,SLIT2,TTLL8,PDCD6,RPS6KA2,LCMT1,IGF1R,MDM2,SMAD1,STAMBPL1,TTC3,PTPRG,PXK,TRIM48,TRPC5,DMD,STK33,KRT2,FSHR,MAEL,MORF4L1,PRKAR2A,STK24,PDZRN3,GALNT14,MAN2B1,ATRX,PARP16,TPP2,USP16,CAMTA1,LRRK2,MAPK10,VRK1,NTRK3,RNF165,DPY30,PAN3,CDCA2,GALNTL6,KCTD9,PDXP,MECP2,MTMR2,SEMA4D,GSK3B,SLC8A1,CDC14A,PRKD1,SNRK,G2E3,ERCC6,OGT,RNF144A,TGFB2,WDR4,DNAJC6,FUT4,PIWIL4,BDNF,PRKAR1B,PRKCH,RIPK4,RYK,ZDHHC17,MGAT2,KDM4C,OTU

			D7A,ZDHHC14,EXT2,RAG1,RAG2,SIK2,MKRN3,NR2F2,PRR5,PML,PPARGC1A,RNF180,TRUB2,USP36,MYH8,DTWD2,PPP4R4,TAOK3,BCL2,RALBP1,ZNF675,EPHB1,MOV10L1,IL12RB2,DEPTOR,EPHA7,FYN,PARN,CD44,HECW1,RHBDD1,RNF213,FOXO1,PDE4D,RNF144B,ENPP2,GRK5,RNF217,TRMT61B,CSNK1G3,CMTR2,MYLK3,SETD7,SLC1A1,UGCG,MAN1C1,PHF20,NLRP12,MTOR,ROR1,UBE2L6,CTDSPL2,BMPER,PTPRD,OGFOD1,PLCL1,CDKAL1,EPM2A,ADARB2,PRKCE,EIF2S1,HELLS,KMT2A,PRKCB,ADCY8,CAPN3,DPH6,ITLN1,KALRN,PJA1,TTLL12,CCDC88C,DLC1,EDNRB,ROBO1,FLT1,NOX4,GRHL2,PIGL,MAP3K7,MAPK9,TRAF3IP2,EYA4,STK3,SUPT3H,TGM2,KDR,CASK,PHKB,RNF182,APP,NRP1,PTPRM,ADTRP,EGFLAM,KAT5,ST3GAL3,ZZZ3,ADAM17,CAMK1D,MAP3K5,CDC42BPG,ATF2,ELP3,GRK6,PIK3R5,SAMSN1,EFEMP1,ROS1,CDK17,DOT1L,RPS6KA5,TNFRSF10B,TRABD2B,PARD3,TLK2,TRAF3,SGK2,CCDC88A,MOB3B,RNF43,RNF126,STK36,GUCY2F,HDAC4,DNMT1,GHR,NTRK2,PRKG1,A1CF,CDC42BPA,BRD4,PTPDC1,RASGRP1,ARR3,CAMK4,COMMD1,RABGEF1,SPRED2,COG7,PPA2,SPRR2E,RAP1A,TGFBR1,USP53,PRDX4,EPHA5,LCP2,TOP1,PEAK1,B4GALT3,CPE,PTTG1IP,SUMF1,CHRNA3,EP300,MORC3,HMG20A,DUSP23,GCNT7,TRIOBP,BRD7,NPC1,PIBF1,TRPM7,PSMA1,ADCK1,ADORA2A,ADRBK2,CAMK2G,EFNA5,FAM20A,MERTK,UBE3C,BRD1,GUCY2C,MAGI2,NF1,PPP6R2,USP17L5,SORL1,ASB13,TRIM59,LZTR1,ASXL1,POR,RELN,SLC39A8,USP3,GRM5,HDAC9,PTPN9,ST6GALNAC5,MGAT5,MAP3K13,NLK,EGLN3,FBXL17,FBXL5,MAN1A1,PPP3CA,SH3GL2,SNX25,MAS1,PER1,WWTR1,CBLB,MNAT1,CNTN1,DGUOK,TRIM16,KAT7,MAPK4,BORA,PTPRU,THAP7,RNF128,RPS20,LRRK1,MSRA,DDX3X,CNOT7,PARP8,DNMT3B,JAK2,LMO7,CD300A,MAP2K4,NOS1,TMTC1,TMTC4,CDK1,HDAC7,MAGT1,MCM3AP,NBN,RRH,SLC39A10,TNF,TNIP1,ILF3,PIGB,BMP15,MRM1,PHEX,TRIM23,DPY19L1,PAXBP1,FAM220A,STK32B,BCL11A,HMGA2,NEK4,UBE2E3,STK32C,CDC20B,DYRK4,GALNT8,GCLC,PMEPA1,UBE2V1,C5AR1,LRP4,PIK3R3,WDR5,RSRC1,SAE1,STT3A,TRIM41,HERC1,PHF8,CDC14B,ALPK2,CDK8,CUL2,TENM1,WDFY2,CDKL5,GSKIP,PRKAR1A,CD109,EGF,PTPN14,KIT,MTRR,UBR2,BLM,PTPRO,RAPGEF2,STK32A,JAK1,UBE3A,FGF13,EPHB2,MECOM,NDFIP2,RNF4,TOX,DAPK1,ERBB4,FER,MAP3K3,MINPP1,RSPRY1,TDRKH,FNIP1,LRP5,TTLL5,ABL2,CAMP,SETD2,NSUN2,PTPRK,CCL3,FGF7,LAMA1,RNF138,SPRED3,CDKL1,GLYR1,ARF4,NSMCE2,SOX4,CAB39L,NEDD9,PDGFD,PIK3CD,UBE2N,AGTPBP1,WDR82,ARID4A,DDB1,IRAK2,KDM3A,MYO3B,PLAT,TNKS,AUTS2,CDK19,FGFR1,OCLN,PARP15,ARID4B,DAD1,IL23R,KEAP1,MACROD1,NTF3,P2RX7,TEX14,ARRDC4,RNMT,ZDHHC11,ZDHHC11B,ZDHHC9,AMFR,BCOR,PIGK,TRERF1,TRMT11,C10ORF90,EYA1,IFNA8,N4BP1,SRPK2,BDKRB1,BDKRB2,GNL3,NEK5,PI3,PRKAA2,RLF,THADA,CDC73,MARK2,PDE5A,ZMIZ1,FLT3,PTPRA,TRIM72,DYNAP,FERMT2,MAP2K5,MARK1,SMPD3,ZC3H13,DAB2,HDAC8,TCL1B,ADAM10,ELP4,FRY,PLCL2,PPM1B,TTLL11,PHF20L1,PIN1,RPS6KA6,SYNPO2,TRPM4,STK38,BMP6,CHRM3,CIT,GALNT5,TGFA,HGF,MYLK,USP31,CALCR,DPY19L2,POMT2,SH3BP5,TLK1,ZBTB16,KDM4B,MAST4,NOS1AP,INSR,PAK1,PSMB7,PTPN21,SKI,AKT3,CDC16,CHM,CTDP1,DUSP11,ENPP1,FMR1,LIMK2,TESK2,TRMU,CHFR,DKC1,JDP2,PRKX,TMTC2,ULK4,MSL2,SENP8,PCK1,TTN,C1GALT1,SMARCAD1,ST6GAL2,TAF1,CHD5,FNIP2,FUT8,KANSL1,PPP2R3C,SPRED1,PRDM16,PTPRF,USP29,ZNF451,BTRC,PPP2R2C,PTPRR,RIT2,EXT1,MYH6,NCOA1,PRKCG,PRLR,UMOD,DOCK7,FBXW11,HDAC5,ICK,KLHL3,MTFMT,STK35,TDGF1,UGGT2,WDR70,SEL1L2,TRPM6,ADARB1,FBXL18,PRKCQ,SHPRH,USP7,CDKN3,DOCK3,DPM1,DPY19L3,MOCS3,PAK3,STK17B,TBL1X,DTNBP1,GAK,UBE2U,KSR1,LRP8,ALPK1,MAPKAPK2,TUSC3,APC,BMPR1A,ITGA1,NGF,PRKCA,TULP4,UBASH3B,UBE2E2,VLDLR,ASB3,MTMR4,PILRB,ABI1,NCAPG2,RGCC,USP18,DPH1,IL6R,MAP2K6,SIAH3,SPTBN4,MAP2K1,ALG12,ART4,FGF1,NSUN3,RAD18,USP12,RGN
GO:0048738	cardiac muscle tissue development	0.008856308224651357	MAPK14,RUNX1,MYO18B,AKAP13,SORBS2,TGFBR3,NRG1,ALDH1A2,AGT,FHL2,SMAD1,RYR2,TENM4,CHD7,SOX6,SLC8A1,TGFB2,SGCD,SGCG,AKAP6,LRP2,ACTN2,MYLK3,ZFPM2,RARB,MTOR,PKP2,COL11A1,PLN,NOX4,NEBL,KCNK2,TGFBR1,TBX3,TP73,NEB,CXADR,FHOD3,SHOX2,CDK1,ZFPM1,ARID2,EFNB2,ALPK2,PRKAR1A,SGCZ,ERBB4,MEF2A,PDLIM5,HCN4,CTDP1,PPARA,TTN,MYH7,MYH6,MYH11,BMPR1A,TBX20,TBX18
GO:0086003	cardiac muscle cell contraction	0.009130163383532277	KCNQ1,ANK2,KCND3,KCNE1,CTNNA3,RYR2,SGCD,KCNJ3,KCNE2,PDE4D,PKP2,ATP1A1,PLN,PDE4B,CACNA1C,CACNA2D1,CACNA1D,SCN1A,FGF13,NUP155,KCNE4,HCN4,RNF207,FGF12,TRPM4,CACNB2,NOS1AP

GO:0090132	epithelium migration	0.009573701307053149	*ITGA2,CDH13,GADD45A,AGT,RHOJ,LOXL2,SLIT2,PDCD6,PTPRG,KRT2,SH3BP1,MECP2,PRKD1,TGFB2,SEMA5A,NR2F2,NR4A1,KANK1,ENPP2,MTOR,BMPER,EPB41L4B,PRKCE,ROBO1,FAP,GRHL2,KDR,PRCP,NRP1,PTPRM,ADTRP,ADAM17,ETS1,RIN2,MAPRE2,STARD13,TGFBR1,NF1,HDAC9,MEOX2,HDAC7,TNF,DOCK1,SMOC2,PIK3R3,EFNB2,EGF,KIT,MARVELD3,MAP3K3,BSG,FGF7,MYH9,PIK3CD,WDPCP,FGFR1,SEMA3A,MAP2K5,ITGA3,CLASP1,CAPN7,MACF1,AKT3,PRKX,SPRED1,PTPRR,HDAC5,TDGF1,AMOT,DPP4,PRKCA,CTSH,RGCC,CLASP2,FGF1,RREB1*
GO:0035235	ionotropic glutamate receptor signaling pathway	0.010003371902679284	*GRIK1,GRIN3A,GRIA1,GRID1,GRIA4,GRIN2B,GRIK3,GRIK2,APP,GRIN2A,GRID2,GRIA3,GRIK4,GRIA2*
GO:1990806	ligand-gated ion channel signaling pathway	0.010003371902679284	*GRIK1,GRIN3A,GRIA1,GRID1,GRIA4,GRIN2B,GRIK3,GRIK2,APP,GRIN2A,GRID2,GRIA3,GRIK4,GRIA2*
GO:0120031	plasma membrane bounded cell projection assembly	0.010471385489500716	*CDC42EP3,NPHP3,KCNQ1,NRXN1,RAB5A,PCNT,FGD6,CDH13,SDCCAG8,CEP41,HYDIN,PLCE1,BBS9,SPEF2,C2CD3,FSIP2,ATAT1,WDR11,ATP6V0D1,NTN1,SLIT2,TTLL8,DYNLL2,CEP97,DMD,VCL,TRPM2,KIF3B,DISC1,WRAP73,CDC14A,VAV3,PCDH15,DNAAF2,ARHGAP24,VANGL2,RPGR,PMP22,KANK1,ACTN2,DCDC2,RFX4,MTOR,BBS2,IFT88,ATP6V1D,LRRC49,PKHD1,TMEM138,NRP1,TENM2,TTC12,OPHN1,CCDC88A,STK36,ADAMTS16,DNAH8,HDAC4,DNM3,PTPDC1,TGFBR1,SPAG17,PARVB,PIBF1,CEP89,IFT80,TTC8,CCDC57,RALA,EXOC5,CAPZB,WWTR1,TMEM67,ASAP1,NLGN1,AHI1,COBL,ATXN10,SPAG16,RAB23,EPS8,NUDCD3,CCP110,ARHGEF6,ZNF423,CDC14B,TENM1,CDKL5,WDR35,DYNC2H1,KIT,CC2D2A,PTPRO,RAPGEF2,ARMC2,FER,IFT43,RAPGEF6,GAS8,ARHGAP35,CD2AP,CDKL1,FGD4,ARF4,TEKT4,WDPCP,AUTS2,OCLN,P2RX7,CEP250,ARMC9,NME8,SCLT1,MYLK,SPATA6,SNAP29,FMR1,LIMK2,RPGRIP1,IFT81,ICK,TTC39C,ALPK1,APC,ATMIN*
GO:0061337	cardiac conduction	0.010833989567407899	*KCNQ1,TNNI3K,ANK2,KCND3,KCNE1,RYR3,AGT,CTNNA3,RYR2,SLC8A1,KCNJ3,ABCC9,KCNE2,PDE4D,PKP2,ATP1A1,PLN,CACNA1C,CACNA2D1,CXADR,CACNA1D,TRDN,NUP155,KCNE4,MEF2A,HCN4,RNF207,TRPM4,CACNB2,CORIN,CASQ2,SPTBN4,TBX18*
GO:0010648	negative regulation of cell communication	0.011270397114651939	*DAB1,MAPK14,PTPRT,TIMP3,DUSP22,FBLN1,NPHP3,RGS6,SHANK2,GRIA1,TGFBR3,FSTL4,RNF152,NXN,CSNK2A1,CHEK2,GPC3,NRG1,PSMB2,SHISA6,RFFL,SH3RF2,LDLRAD4,RGS7BP,CBFA2T2,NKD1,SCAI,PTPRE,SORCS3,ZNF536,RAB7A,ZNF366,PRKACB,PXDN,LEMD3,AGT,RCAN1,CDH2,PARK2,FHL2,BPIFB1,PCDH17,ZNRF3,HTRA1,SLIT2,PDCD6,IGF1R,MDM2,SLC24A4,TMPRSS6,DMD,SORCS2,WWOX,LRRK2,RORA,SH3BP1,MTMR2,GSK3B,TGFB2,BDNF,RBMS3,OTUD7A,ANKRD6,ARHGAP12,FBN1,GRIK3,TAOK3,BCL2,ZNF675,ARHGAP24,LRP2,DEPTOR,NR4A2,SMARCA4,CD44,HECW1,RNF213,FOXO1,PDE4D,KANK1,TNMD,GRIK2,CELF4,RFX4,NLRP12,MTOR,CTDSPL2,BMPER,PTPRD,CTNNBIP1,ZMYND11,NLGN4X,HELLS,PRKCB,ADCY8,FOXO3,KALRN,TTLL12,CCDC88C,DLC1,ROBO1,COL2A1,EYA4,PKHD1,STK3,NRP1,UACA,LGR4,LINC00473,ADAM17,TGFB1I1,RGS16,TCF7L2,TRABD2B,BICD1,CD38,RNF43,CHRDL1,RNF126,BCL2L1,PDE4B,BRD4,ARR3,RABGEF1,SPRED2,GLI3,RAP1A,TGFBR1,TNR,NCOA5,CHST11,PTTG1IP,RAPGEF1,WIF1,CYP7B1,GRAMD4,GLRA1,GRID2,PIBF1,PSMA1,ADRBK2,MAGI2,NF1,RGS7,SORL1,STRN3,BICC1,BID,IFT80,TRIM59,LZTR1,ASXL1,GRM5,NDRG2,NLK,NPHP4,PPP3CA,KLF7,SNX25,DGKI,PER1,SLC24A2,WWTR1,CBLB,PTPRU,MAPK8IP2,VEPH1,DDX3X,CNOT7,SMAD3,CD300A,AATF,HDAC7,TNF,OVOL2,SNX5,TNIP1,EIF3A,PRDM15,DLG5,ARF1,GCLC,PMEPA1,LTBP1,SULF1,LRP4,ALPK2,CTNNA1,MLLT3,CD109,SLIT3,UBR2,VWC2,PTPRO,ESR1,MARVELD3,EPHB2,MECOM,FNIP1,SLAMF1,ABL2,DEPDC5,ARHGAP35,CD2AP,SPRED3,ARHGAP25,SULF2,PDE11A,RGS3,SOST,P2RX7,RASA3,AMFR,AR,SUFU,EYA1,BDKRB2,DGKZ,PRKAA2,TLE4,TRIM72,MAP2K5,TNN,DAB2,ITGA3,PLCL2,PPM1B,PIN1,RPS6KA6,STK38,CIT,TGFA,HGF,RGS12,PDE3A,PSMB7,SKI,ELF1,ENPP1,FMR1,LITAF,MAD1L1,ARHGAP42,PPARA,FAM3D,SPRED1,PRDM16,ZNF451,BTRC,CCDC3,OPTN,PTPRR,FBXW11,G3BP1,PRKCQ,TP63,PIK3R2,RAP1B,APC,ITGA1,UBASH3B,MTMR4,RPH3AL,USP18,GPR21,C1QTNF3,TBX20,NLRC5,TBX18*
GO:00725	divalent	0.0118488444300199	*CMKLR1,ANK3,SLC24A3,MS4A1,GRIA1,ANK2,FAM155A,RYR3,PLCE*

07	inorganic cation homeostasis	15	*1,CCR1,CCR3,AGT,PARK2,SLC24A4,TRPC5,DMD,GRM1,TRPM2,RYR2,DHRS7C,CHD7,DISC1,HEXB,SLC8A1,PRKD1,SGCD,GRIN2B,PTGFR,PML,AKAP6,BCL2,SLC30A5,ITPR2,FYN,PDE4D,GRIK2,RAP1GDS1,SLC1A1,PLN,PRKCE,PRKCB,VDR,ADCY8,CAPN3,EDNRB,PKHD1,TGM2,KDR,APP,MT1HL1,GRIN2A,CD38,SLC30A7,CACNA1C,CYSLTR1,CACNA2D1,TRPM7,FAM20A,TAC4,MICU1,SLC39A8,GRM5,ADCYAP1R1,SLC24A2,KL,STIM2,TPCN2,JAK2,SMAD3,NOS1,TMTC4,SLC39A10,TRPC4,C5AR1,TRDN,TRPV4,ESR1,HTR2C,SLC30A8,ABL2,CCL3,MCUR1,PTGER3,SLC39A12,P2RX1,CDH23,RYR1,P2RX7,RASA3,BDKRB1,BDKRB2,ADRA1B,THADA,ATP13A3,GPR55,CCL7,MT1F,TRPM4,CALCR,CACNB2,MICU2,TMTC2,JPH4,GNA13,CACNA1A,S1PR3,TRPM1,UMOD,SLC24A5,STIM1,CASQ2,CNNM3,UBASH3B,CCL14,CCL15,MCOLN3,RGN*
GO:0010631	epithelial cell migration	0.012446069463704646	*ITGA2,CDH13,GADD45A,AGT,RHOJ,LOXL2,SLIT2,PDCD6,PTPRG,KRT2,SH3BP1,MECP2,PRKD1,TGFB2,SEMA5A,NR2F2,NR4A1,KANK1,ENPP2,MTOR,BMPER,EPB41L4B,PRKCE,ROBO1,FAP,KDR,PRCP,NRP1,PTPRM,ADTRP,ADAM17,ETS1,RIN2,MAPRE2,STARD13,TGFBR1,NF1,HDAC9,MEOX2,HDAC7,TNF,DOCK1,SMOC2,PIK3R3,EFNB2,EGF,KIT,MARVELD3,MAP3K3,BSG,FGF7,MYH9,PIK3CD,WDPCP,FGFR1,SEMA3A,MAP2K5,ITGA3,CLASP1,CAPN7,MACF1,AKT3,PRKX,SPRED1,PTPRR,HDAC5,TDGF1,AMOT,DPP4,PRKCA,CTSH,RGCC,CLASP2,FGF1,RREB1*
GO:0071417	cellular response to organonitrogen compound	0.013299734403707332	*PLCB1,GABRB3,ARID1B,KCNQ1,SLC1A2,KCNE1,SPIDR,RYR3,GLP2R,CFTR,ALK,PTPRE,AGT,CHRM5,PARK2,CYBB,HDAC2,SLIT2,STXBP4,IGF1R,MDM2,HCN1,TRPM2,RYR2,LRRK2,SLC1A3,PDXP,GSK3B,SLC8A1,OGT,SIK2,AKAP6,FBN1,ITPR2,NR4A2,FYN,GNRHR,NR4A1,CASP6,FOXO1,PDE4D,KANK1,ACTN2,GABRB1,SLC1A1,MTOR,CHRM1,PRKCB,ADCY8,FOXO3,RAB31,TBC1D4,TGM2,APP,CPEB2,ATF2,BCL2L1,PDE4B,DNMT1,GHR,GLRA2,NTRK2,GNG2,RAP1A,NCOA5,RAPGEF1,CACNA2D1,GLRA1,COL4A6,PNPLA3,SORL1,HTR4,POR,GRM5,HDAC9,DDC,KLF3,MAS1,KL,GABPA,AKAP7,NSG1,JAK2,BAIAP2L1,TNF,SNX5,BCL11A,ANO1,GCLC,GNAL,PIK3R3,TRIM41,CTNNA1,NSG2,UBR2,BLM,CPS1,RAPGEF2,HTR2C,GABRB2,EPHB2,ABCC1,FER,CAMP,PDGFD,PLAT,RYR1,P2RX7,CASP7,GNB5,PTPRA,TRIM72,GNAO1,HCN4,SMPD3,UROS,TRPM4,CHRM3,INSR,PDE3A,ENPP1,RRAGC,IDE,PCK1,TAF1,CACNA1A,HDAC5,PRKCQ,STAT3,BRIP1,PIK3R2,CASQ2,DTNBP1,RAP1B,APC,GABRG2,GPR21,VPS35*
GO:0023057	negative regulation of signaling	0.013464846832860112	*DAB1,MAPK14,PTPRT,TIMP3,DUSP22,FBLN1,NPHP3,RGS6,SHANK2,GRIA1,TGFBR3,FSTL4,RNF152,NXN,CSNK2A1,CHEK2,GPC3,NRG1,PSMB2,SHISA6,RFFL,SH3RF2,LDLRAD4,RGS7BP,CBFA2T2,NKD1,SCAI,PTPRE,SORCS3,ZNF536,RAB7A,ZNF366,PRKACB,PXDN,LEMD3,AGT,RCAN1,CDH2,PARK2,FHL2,BPIFB1,PCDH17,ZNRF3,HTRA1,SLIT2,PDCD6,IGF1R,MDM2,SLC24A4,TMPRSS6,DMD,SORCS2,WWOX,LRRK2,RORA,SH3BP1,MTMR2,GSK3B,TGFB2,BDNF,RBMS3,OTUD7A,ANKRD6,ARHGAP12,FBN1,GRIK3,TAOK3,BCL2,ZNF675,ARHGAP24,LRP2,DEPTOR,NR4A2,SMARCA4,CD44,HECW1,RNF213,FOXO1,PDE4D,KANK1,TNMD,GRIK2,CELF4,RFX4,NLRP12,MTOR,CTDSPL2,BMPER,PTPRD,CTNNBIP1,ZMYND11,NLGN4X,HELLS,PRKCB,ADCY8,FOXO3,KALRN,TTLL12,CCDC88C,DLC1,ROBO1,COL2A1,EYA4,PKHD1,STK3,NRP1,UACA,LGR4,LINC00473,ADAM17,TGFB1I1,RGS16,TCF7L2,TRABD2B,BICD1,CD38,RNF43,CHRDL1,RNF126,BCL2L1,PDE4B,BRD4,ARR3,RABGEF1,SPRED2,GLI3,RAP1A,TGFBR1,TNR,NCOA5,CHST11,PTTG1IP,RAPGEF1,WIF1,CYP7B1,GRAMD4,GLRA1,GRID2,PIBF1,PSMA1,ADRBK2,MAGI2,NF1,RGS7,SORL1,STRN3,BICC1,BID,IFT80,TRIM59,LZTR1,ASXL1,GRM5,NDRG2,NLK,NPHP4,PPP3CA,KLF7,SNX25,DGKI,PER1,SLC24A2,WWTR1,CBLB,PTPRU,MAPK8IP2,VEPH1,DDX3X,CNOT7,SMAD3,CD300A,AATF,HDAC7,TNF,OVOL2,SNX5,TNIP1,EIF3A,PRDM15,DLG5,ARF1,GCLC,PMEPA1,LTBP1,SULF1,LRP4,ALPK2,CTNNA1,MLLT3,CD109,SLIT3,UBR2,VWC2,PTPRO,ESR1,MARVELD3,EPHB2,MECOM,FNIP1,SLAMF1,ABL2,DEPDC5,ARHGAP35,CD2AP,SPRED3,ARHGAP25,SULF2,PDE11A,RGS3,SOST,P2RX7,RASA3,AMFR,AR,SUFU,EYA1,BDKRB2,DGKZ,PRKAA2,TLE4,TRIM72,MAP2K5,TNN,DAB2,ITGA3,PLCL2,PPM1B,PIN1,RPS6KA6,STK38,CIT,TGFA,HGF,RGS12,PDE3A,PSMB7,SKI,ELF1,ENPP1,FMR1,LITAF,MAD1L1,ARHGAP42,PPARA,FAM3D,SPRED1,PRDM16,ZNF451,BTRC,CCDC3,OPTN,PTPRR,FBXW11,G3BP1,PRKCQ,TP63,PIK3R2,RAP1B,APC,ITGA1,UBASH3B,MTMR4,RPH3AL,USP18,GPR21,C1QTNF3,TBX20,NLRC5,TBX18*
GO:0051641	cellular localization	0.013477210140969332	*DAB1,MAPK14,TRAPPC8,GPHN,CTBP2,RAB27A,ANXA8L1,ANK3,HEATR5A,WLS,SYN3,AKAP13,SNX31,VPS13D,ABCG1,KCNQ1,SUN2,GRIN3A,NRXN1,SGSM1,NRXN3,SLC1A2,RAB5A,DOCK2,PCNT,MS4A1,RIMS2,CLEC16A,DLG2,CDH13,ANK2,TBC1D22A,KCND3,FAM155A,SYNDIG1,DPP10,KCNE1,TMEM108,GPC3,FBLN5,NRG1,STXBP6,SHISA*

			6,SPIDR,IL4R,RYR3,MCTP1,ASTN2,ABHD17C,CFTR,BBS9,SCFD2,AGBL4,DNAJC15,SLC6A2,ARFIP1,PIK3C3,ERC1,KLRF2,RAB7A,C2CD3,EPHA3,PTPRN2,DCLK1,AGT,ERC2,FSIP2,VTI1A,WDR11,ATP6V0D1,CDH2,PARK2,BANP,RN7SL659P,MCTP2,SYTL5,NTN1,DENND2A,GPC6,USP6,ANKFN1,ITSN1,PCDH17,APBA2,PDCD6,STXBP4,GPC5,MDM2,MX2,SLC24A4,E2F3,NUTF2,SYT9,CCDC91,DMD,SYT1,CACNG3,VCL,NOX5,CELSR1,GSG1L,FMN2,HNRNPA2B1,SKAP1,SORCS2,DPP6,WDR83OS,ATRX,TRPM2,IPO13,RYR2,XRCC4,KIF3B,UNC13C,DHRS7C,LRRK2,KXD1,NUSAP1,CHD7,DISC1,DPY30,WRAP73,MTMR2,GSK3B,SLC8A1,PRKD1,FLVCR1,FRMD6,DNAJC6,SLC5A3,BDNF,LLGL2,PRKAR1B,PRKCH,TOR1AIP1,WASF1,WDR72,GRIN2B,ZDHHC14,MREG,PLEKHM2,SNAP25,SNAP25-AS1,PML,SYBU,USP36,AKAP6,CNTNAP2,SNAP23,BCL2,CHMP4C,KCNJ3,NSF,ITPR2,LRRC4,GDAP1,RPGR,FYN,KCNE2,NR4A1,RHBDD1,PDE4D,ABCB10,ACTN2,PACRG,EXOC4,GRIK2,KCNIP4,SLC1A1,MTOR,CADPS,VPS26B,CHRM1,CTDSPL2,PKP2,RANBP17,UBXN2B,ABCA13,CCDC88B,BBS2,ARL6IP5,ATP1A1,EPM2A,PLN,EVI5,EXOC6B,IFT88,NLGN4X,PRKCE,ATP6V1D,PRKCB,STON1,ADCY8,RAB31,SCP2,TRAPPC12,CAPN3,PARD3B,CCDC88C,TANC2,TBC1D4,BNIP3L,STX12,MAP7,TRAF3IP2,VPS37B,SEC24B,PKHD1,STK3,PPHLN1,CASK,ABCA12,APP,NRP1,RN7SL674P,TSNARE1,IMMP2L,IGF2R,ATF2,PEX5L,GOPC,SMG6,TM9SF4,KPNB1,TCF7L2,BICD1,PARD3,THOC2,GRIN2A,GRIP1,RIMS3,KIFAP3,OPHN1,CCDC88A,DENND5A,SLC30A7,TUB,RNF126,SMG7,BCL2L1,MAPRE2,A1CF,DNM3,RASGRP1,TRAPPC10,COMMD1,RAB11FIP4,RABGEF1,STARD4,C17ORF75,GLI3,CACNA1C,COG7,KIF16B,STXBP5,CRB1,LAMP5,RAP1A,SRP54,EPHA5,VPS53,CPE,PTTG1IP,CHRNA3,CHRNA5,MORC3,RAB2A,SYNE1,SCFD1,CACNA2D1,STX8,ATXN1,GAB2,GRID2,NPC1,PIBF1,SAMM50,TMEM50B,VPS39,SPG11,ADORA2A,DYNC1I2,EFNA5,CACNG2,MAGI2,NF1,SORL1,BID,IFT80,TTC8,RELN,SLC39A8,PTPN9,SNUPN,DYNC1H1,DDC,NUMB,NPHP4,RALA,ADCYAP1R1,EXOC5,MAN1A1,PPP3CA,TBC1D3B,KCNB2,KLF7,PLS1,DGKI,GNPTAB,SLC24A2,TBC1D5,TSPAN33,WWTR1,ANKRD13A,CBLB,SEC63,ATP9A,GPC4,RIMS1,FAM126B,GPSM2,KAT7,SLC22A2,NLGN1,PTPRU,GBP2,RNF128,SLC22A3,OSBPL5,CACNA1D,DDX3X,LRPPRC,NSG1,VPS37A,TPCN2,JAK2,AHI1,RPA1,SMAD3,VPS41,CD300A,NOS1,CDK1,HGSNAT,MCM3AP,NVL,TNF,SNX5,CPT1B,PPFIA2,BLOC1S6,TRIM23,ARF1,MYO6,POLR1A,KIF18A,ANO1,GCLC,RAB23,NUDCD3,PABPC1L,TOMM34,LRP4,EFNB2,RAB24,RSRC1,SAE1,SHFM1,TRDN,ZNF423,CLSTN1,INSC,CSE1L,CTNNA1,NSG2,TENM1,WDR35,ARHGAP11B,DYNC2H1,MYRIP,STX16,STX16-NPEPL1,EGF,PTPN14,SEC16B,SPIRE2,KIT,KPNA3,TRPV4,CC2D2A,DNAH11,NAPG,RAPGEF2,DNAJB6,ESR1,HTR2C,SLC30A8,FGF13,GRM4,NUP155,EPHB2,NDFIP2,ERBB4,FER,IFT43,LRP5,RAPGEF6,BSG,F11R,GAS8,SETD2,TMEM50A,VPS13C,AP2B1,CD2AP,NSUN2,PTPRK,CCL3,FGF7,MYO7B,GPR158,IPO11,MCM9,REEP1,ARF4,ARFGAP3,MYH9,PACSIN2,SPRN,MIPEP,P2RX1,PIK3CD,RGPD1,AGTPBP1,KCNE4,PLAT,TNKS,UNC13A,VPS45,MEF2A,OCLN,RYR1,TBC1D9,UNC13B,MAP2,TTC7B,ITGB7,P2RX7,RAPGEF4,RASA3,ZDHHC11,ZDHHC11B,ZDHHC9,AR,KIAA1109,PIGK,RABGAP1L,SUFU,CEP250,EXOC2,RBM4,BDKRB1,AFTPH,GNL3,EPB41L3,KIF4A,MARK2,PROS1,REEP2,SNX7,VPS16,FERMT2,MARK1,SMPD3,DAB2,HDAC8,LCP1,RNF207,STX3,SYT2,ACTN4,ADAM10,BLOC1S5,ITGA3,KMO,LGI1,RFTN1,RNASEH2B-AS1,STAC,NUP153,PIN1,TRPM4,BTBD9,CIZ1,CLASP1,EIF4ENIF1,CNGB1,PCDHGA3,CALCR,CENPF,MYO1B,RN7SL318P,TLK1,ZBTB16,CACNB2,NFASC,NOS1AP,SNAP29,MACF1,CHM,FMR1,LIMK2,THEM4,ATP5J,DKC1,FAM126A,MON2,RRAGC,ABCD1,AMPH,MAD1L1,AP1G2,CD84,EPG5,ESYT2,JPH4,SIL1,SORT1,HEATR5B,NUP214,TRPM1,NUP62CL,OPTN,RIT2,IFT81,PRKCG,UMOD,DOCK7,FBXW11,ICK,NDE1,STAT3,USP7,AMOT,DNM1P46,PIK3R2,CASQ2,DTNBP1,GAK,RAP1B,LGALS9,SNCAIP,APC,SRP19,UBASH3B,ASB3,EXOC6,DHX9,RPH3AL,SIAH3,SPTBN4,AP5M1,CLASP2,MAP2K1,TPH1,VPS35,MCOLN3,C6ORF106,RN7SL673P
GO:0040013	negative regulation of locomotion	0.014274458618863232	SRGAP2B,PLCB1,PTPRT,DUSP22,FBLN1,NRG3,GADD45A,NRG1,MCTP1,SEMA6D,LDLRAD4,SCAI,ROBO2,SPOCK3,SEMA3D,DACH1,RIN3,SLIT2,PTPRG,VCL,STK24,FRMD5,MECP2,SEMA4D,SEMA5A,RYK,NR2F2,BCL2,KANK1,FOXO3,MITF,DLC1,ROBO1,NRP1,PTPRM,ADTRP,SEMA3C,PRKG1,STARD13,RABGEF1,ADORA2A,MAGI2,NF1,GRM5,NAV3,JAG1,MEOX2,PTPRU,CD300A,TNF,LRCH1,DLG5,ARID2,ADORA3,SULF1,CTNNA1,PTPRO,SEMA3E,MARVELD3,PTPRK,SEMA5B,SEMA3A,SRGAP3,MAP2K5,TNN,PIN1,CLASP1,GNA13,SPRED1,PTPRR,HDAC5,ADARB1,STAT3,BMPR1A,DPP4,SPINT2,RGCC,SRGAP1,CLASP2,RGN

GO:0030031	cell projection assembly	0.014378355412506023	*CDC42EP3,NPHP3,KCNQ1,NRXN1,RAB5A,PCNT,FGD6,CDH13,SDCCAG8,CEP41,HYDIN,PLCE1,BBS9,SPEF2,C2CD3,FSIP2,ATAT1,WDR11,ATP6V0D1,RHOJ,NTN1,SLIT2,TTLL8,DYNLL2,CEP97,DMD,VCL,TRPM2,KIF3B,DISC1,WRAP73,CDC14A,VAV3,PCDH15,DNAAF2,ARHGAP24,VANGL2,RPGR,PMP22,KANK1,ACTN2,DCDC2,RFX4,MTOR,BBS2,IFT88,ATP6V1D,LRRC49,PKHD1,TMEM138,NRP1,TENM2,TTC12,PARVG,OPHN1,CCDC88A,STK36,ADAMTS16,DNAH8,HDAC4,DNM3,PTPDC1,TGFBR1,SPAG17,PARVB,PIBF1,CEP89,IFT80,TTC8,CCDC57,RALA,EXOC5,CAPZB,WWTR1,TMEM67,ASAP1,NLGN1,AHI1,COBL,ATXN10,SPAG16,RAB23,EPS8,NUDCD3,CCP110,ARHGEF6,ZNF423,CDC14B,TENM1,CDKL5,WDR35,DYNC2H1,KIT,CC2D2A,PTPRO,RAPGEF2,ARMC2,FER,IFT43,RAPGEF6,GAS8,ARHGAP35,CD2AP,CDKL1,FGD4,ARF4,TEKT4,WDPCP,AUTS2,OCLN,P2RX7,CEP250,ARMC9,NME8,SCLT1,MYLK,SPATA6,SNAP29,FMR1,LIMK2,RPGRIP1,IFT81,ICK,TTC39C,ALPK1,APC,ATMIN*
GO:0086019	cell-cell signaling involved in cardiac conduction	0.0145891139816658 9	*KCNQ1,ANK2,RYR2,KCNJ3,PKP2,CACNA1C,CACNA2D1,CXADR,CACNA1D,NUP155,HCN4,RNF207,TRPM4,CACNB2,CASQ2,TBX18*
GO:0140352	export from cell	0.0155318452772500 8	*CTBP2,RAB27A,WLS,SYN3,ABCG1,KCNQ1,GRIN3A,NRXN1,NRXN3,RAB5A,RIMS2,KCND3,KCNE1,SYT17,STXBP6,IL4R,MCTP1,SYT16,CCR1,CFTR,SCFD2,ARFIP1,SLC17A3,PIK3C3,FAM3B,KLRF2,RAB7A,PTPRN2,AGT,ERC2,PARK2,MCTP2,SYTL5,ITSN1,APBA2,STXBP4,SLC24A4,SYT9,SYT1,TRPM2,UNC13C,LRRK2,ILDR1,SLC4A4,CHD7,GSK3B,SLC8A1,TGFB2,ATP1A4,LLGL2,SNAP25,SNAP23,RALBP1,DISP1,NSF,FRMD4A,MICAL3,KCNE2,NR4A1,EXOC4,CADPS,ATP1A1,EXOC6B,PRKCE,PRKCB,ADCY8,RAB31,SLC47A1,KALRN,EDNRB,CBLN4,CASK,ABCA12,ADTRP,TCF7L2,ABAT,RIMS3,CD38,NTRK2,ACSL4,RASGRP1,RABGEF1,STXBP5,RAP1A,EPHA5,CPE,CHRNA3,CHRNA5,TBX3,GAB2,PCSK5,ADORA2A,CAMK2G,EFNA5,MERTK,NF1,RALA,EXOC5,PPP3CA,KLF7,DGKI,GNPTAB,ATP9A,GRM7,RIMS1,SLC22A2,NLGN1,TPCN2,JAK2,CD300A,TNF,POMC,BLOC1S6,KCNT2,ADORA3,ANO1,ABCG2,MYRIP,IL1RAPL1,KIT,TRPV4,HTR2C,SLC30A8,GRM4,ABCC1,FER,LRP5,BSG,CD2AP,CCL3,FGF7,ARFGAP3,MYH9,SOX4,OSCP1,P2RX1,PIK3CD,KCNE4,UNC13A,UNC13B,P2RX7,PLA2G4A,RAPGEF4,SCAMP5,EXOC2,TRIM72,SMPD3,DAB2,STX3,SYT2,KMO,MYOM1,TRPM4,BMP6,CLASP1,CACNB2,SNAP29,PAK1,FMR1,CD84,TTN,FAM3D,SLC22A5,PRKCG,SLC40A1,DTNBP1,RAP1B,LGALS9,SNCAIP,PTGES,EXOC6,RGCC,RPH3AL,MAP2K6,CLASP2,TPH1,VPS35,C1QTNF3*
GO:0055082	cellular chemical homeostasis	0.015623947446860565	*CMKLR1,SLC24A3,KCNQ1,TMPRSS3,MS4A1,GRIA1,SLC9B1,ANK2,FAM155A,TF,SCARA5,RYR3,PLCE1,CCR1,CCR3,CFTR,ZBTB20,RAB7A,PTPRN2,AGT,ATP6V0D1,CP,KCNMA1,STXBP4,IGF1R,SLC24A4,TMPRSS6,TRPC5,DMD,NOX5,FSHR,GRM1,TRPM2,RYR2,DHRS7C,LRRK2,SLC1A3,SLC4A4,CHD7,DISC1,HEXB,SLC8A1,PRKD1,FLVCR1,ATP1A4,GRIN2B,PTGFR,PML,PPARGC1A,AKAP6,BCL2,SLC30A5,ITPR2,CYB561A3,FYN,SMARCA4,FOXO1,PDE4D,TBXAS1,GRIK2,RAP1GDS1,SLC1A1,ATP1A1,PLN,HEPHL1,PRKCE,PRKCB,VDR,ADCY8,FOXO3,CAPN3,EDNRB,NOX4,PKHD1,TGM2,ABCA12,APP,MT1HL1,TM9SF4,GRIN2A,CD38,SLC30A7,KCTD7,CACNA1C,CYSLTR1,EPHA5,CACNA2D1,TRPM7,EFNA5,TAC4,MICU1,SLC39A8,SLC9C1,GRM5,ADCYAP1R1,PPP3CA,KLF7,SLC24A2,STIM2,TPCN2,JAK2,SMAD3,NOS1,TMTC4,MIR320B2,SLC39A10,ARF1,ANO1,GCLC,TRPC4,C5AR1,TRDN,LRRC8D,DMXL1,CMA1,TRPV4,ESR1,HTR2C,SLC30A8,UBE3A,LRP5,ABL2,CCL3,MCUR1,PTGER3,SLC39A12,SOX4,P2RX1,CDH23,RYR1,UNC13B,P2RX7,RASA3,BDKRB1,BDKRB2,ADRA1B,PRKAA2,THADA,ATP6V1H,ATP13A3,CLN6,GPR55,CCL7,MT1F,TRPM4,BMP6,CALCR,CACNB2,ENPP1,MICU2,JPH4,PCK1,GNA13,CACNA1A,S1PR3,TRPM1,SCO2,UMOD,SLC24A5,SLC40A1,STIM1,SLC9A9,PIK3R2,CASQ2,TMEM175,UBASH3B,SLC9A2,CCL14,CCL15,C1QTNF3,MCOLN3,RGN*
GO:0044087	regulation of cellular component biogenesis	0.0156980135516285 9	*CDC42EP3,ASIC2,DUSP22,ATF7IP,NRXN1,RAB5A,MTPN,PDE4DIP,SYNDIG1,SDCCAG8,PSMC6,FLRT2,NRG1,STXBP6,SPIDR,LRFN5,PLCE1,RHOC,LDLRAD4,DNAJC15,IL1RAPL2,RAB7A,ROBO2,EPHA3,AGT,ATAT1,PARK2,CORO2B,NEGR1,NTN1,GPC6,SLIT2,LCMT1,CEP97,SACS,VCL,SKAP1,TRPM2,LRRK2,NTRK3,PAN3,WRAP73,MECP2,SEMA4D,GSK3B,LINGO2,CLSTN2,FMN1,BDNF,PRKCH,CNTNAP2,CHMP4C,ARHGAP24,EPHB1,ARHGEF10,EPHA7,THBS2,KANK1,DCDC2,MTOR,LDB2,PTPRD,CTNNBIP1,HAS3,IFT88,PRKCE,LRRTM1,TRAPPC12,DLC1,CDH17,MAPK9,MDM1,KDR,PPHLN1,APP,NRP1,ARHG*

			EF18,TENM2,TRABD2B,CCDC88A,ADAMTS16,HDAC4,DNMT1,NTRK2,DNM3,CLDN1,MORC2,STXBP5,RAP1A,TGFBR1,PEAK1,RAPGEF1,EP300,SCFD1,TRIOBP,GRID2,EFNA5,SORL1,ARHGAP6,BID,TTC8,DYNC1H1,LRRTM3,RHPN2,NPHP4,RALA,IL1RAP,NAV3,CAPZB,GPC4,GPSM2,L3MBTL3,ASAP1,FHOD3,NLGN1,DDX3X,SMAD3,BAIAP2L1,TNF,COBL,DLG5,FCHSD2,BCL11A,PMEPA1,EPS8,CCP110,PHF8,CLSTN1,SELP,TENM1,CDKL5,IL1RAPL1,KIT,RAPGEF2,DNAJB6,ESR1,EPHB2,RNF4,TBCD,FER,SLITRK6,SPTA1,FNIP1,F11R,FARP2,ARHGAP35,CDKL1,SLC39A12,SPTB,WDPCP,AUTS2,OCLN,UNC13B,MAP2,SLX1B,SOST,P2RX7,SNX30,C10ORF90,BHLHB9,PRKAA2,PDLIM5,PTPRA,SNX7,FERMT2,SMPD3,LCP1,SYNPO2,CLASP1,TMOD1,G3BP2,ELN,ARHGAP28,MACF1,PAK1,FMR1,LIMK2,BMF,SAMD8,CLIP1,FNIP2,G3BP1,AMOT,PAK3,RAP1B,SNCAIP,APC,CKAP5,PRKCA,RGCC,TFIP11,SPTBN4,CLASP2,VPS35,TBX20,ATMIN
GO:0006816	calcium ion transport	0.015878865787249067	*CACNA1E,SLC24A3,GRIN3A,MS4A1,PKD1L1,TRPM3,ANK2,FAM155A,RYR3,CCR1,AGT,SLC24A4,TRPC5,DMD,CACNA2D3,CACNG3,TRPM2,RYR2,CATSPER2,DHRS7C,CHD7,SLC8A1,PRKD1,GRIN2B,PML,AKAP6,BCL2,ITPR2,FYN,KCNE2,RCVRN,PDE4D,EPM2A,PLN,PRKCE,PRKCB,VDR,EFHB,CAPN3,EDNRB,CASK,CALCRL,GRIN2A,PDE4B,CACNA1C,CYSLTR1,IL16,CACNA2D1,CACNA2D4,TRPM7,ADORA2A,CAMK2G,CACNG2,MICU1,ADCYAP1R1,PPP3CA,SLC24A2,STIM2,CACHD1,CACNA1D,TPCN2,NOS1,PANX1,TMC2,TSPAN13,TRPC4,TRDN,EGF,TRPV4,HTR2C,CACNG6,CCL3,MCUR1,P2RX1,CDH23,RYR1,P2RX7,RASA3,BDKRB1,THADA,GNB5,STAC,TRPM4,MYLK,CALCR,CACNB2,NOS1AP,FMR1,MICU2,CD84,JPH4,CACNA1A,TRPM1,SLC24A5,STIM1,TRPM6,CASQ2,UBASH3B,MCOLN3,RGN*
GO:0001667	ameboidal-type cell migration	0.0161894571142850 9	*ITGA2,CDH13,GADD45A,GPC3,RFFL,SEMA6D,ARID5B,AGT,RHOJ,CDH2,LOXL2,SEMA3D,SLIT2,PDCD6,PTPRG,KRT2,SH3BP1,MECP2,SEMA4D,SLC8A1,PRKD1,TGFB2,SEMA5A,NR2F2,PML,TNS1,NR4A1,KANK1,ENPP2,MTOR,BMPER,EPB41L4B,SOX8,PRKCE,EDNRB,ROBO1,FAP,CENPV,KDR,PRCP,NRP1,PTPRM,ADTRP,SEMA3C,ADAM17,ETS1,RIN2,MAPRE2,STARD13,TGFBR1,NF1,HDAC9,MEOX2,HDAC7,TNF,OVOL2,DOCK1,SMOC2,PIK3R3,EFNB2,AMOTL1,EGF,KIT,EFNB1,SEMA3E,MARVELD3,ERBB4,FER,MAP3K3,LRP5,BSG,FGF7,SEMA5B,MYH9,PIK3CD,WDPCP,FGFR1,ITGB7,SEMA3A,MAP2K5,ITGA3,CLASP1,CAPN7,MACF1,AKT3,PRKX,SPRED1,PTPRR,HDAC5,TDGF1,AMOT,PAK3,DPP4,PRKCA,CTSH,RGCC,CLASP2,FGF1,RREB1*
GO:0070588	calcium ion transmembrane transport	0.016263968027817766	*CACNA1E,SLC24A3,GRIN3A,PKD1L1,TRPM3,ANK2,FAM155A,RYR3,SLC24A4,TRPC5,DMD,CACNA2D3,CACNG3,TRPM2,RYR2,CATSPER2,DHRS7C,CHD7,SLC8A1,PRKD1,GRIN2B,AKAP6,ITPR2,FYN,KCNE2,PDE4D,PLN,PRKCE,CAPN3,EDNRB,GRIN2A,PDE4B,CACNA1C,CACNA2D1,CACNA2D4,TRPM7,CACNG2,MICU1,SLC24A2,STIM2,CACHD1,CACNA1D,TPCN2,NOS1,PANX1,TMC2,TSPAN13,TRPC4,TRDN,TRPV4,HTR2C,CACNG6,CCL3,MCUR1,P2RX1,RYR1,P2RX7,RASA3,BDKRB1,THADA,GNB5,STAC,TRPM4,CALCR,CACNB2,NOS1AP,FMR1,MICU2,JPH4,CACNA1A,TRPM1,SLC24A5,STIM1,TRPM6,CASQ2,UBASH3B,MCOLN3,RGN*
GO:0001763	morphogenesis of a branching structure	0.0168770130653491 1	*CSMD1,GPC3,AGT,GREB1L,SLIT2,CELSR1,LRRK2,PML,BCL2,EPHA7,ERMN,CTNNBIP1,SOX8,VDR,GRHL2,PKHD1,TGM2,KDR,NRP1,LGR4,SEMA3C,ADAMTS16,GLI3,TBX3,MAP3K13,SHOX2,RDH10,TNF,DLG5,BCL11A,SULF1,EGF,ESR1,SEMA3E,LRP5,SETD2,FGF7,LAMA1,AR,EYA1,SEMA3A,PBX1,HGF,PAK1,GNA13,NTN4,BTRC,ESRP2,EXT1,TDGF1,TP63,SPINT2,CTSH,FGF1,TBX20*
GO:0006873	cellular ion homeostasis	0.017577002328586392	*CMKLR1,SLC24A3,KCNQ1,TMPRSS3,MS4A1,GRIA1,SLC9B1,ANK2,FAM155A,TF,SCARA5,RYR3,PLCE1,CCR1,CCR3,CFTR,RAB7A,AGT,ATP6V0D1,CP,KCNMA1,SLC24A4,TMPRSS6,TRPC5,DMD,NOX5,GRM1,TRPM2,RYR2,DHRS7C,LRRK2,SLC1A3,SLC4A4,CHD7,DISC1,HEXB,SLC8A1,PRKD1,FLVCR1,ATP1A4,GRIN2B,PTGFR,PML,AKAP6,BCL2,SLC30A5,ITPR2,CYB561A3,FYN,PDE4D,TBXAS1,GRIK2,RAP1GDS1,SLC1A1,ATP1A1,PLN,HEPHL1,PRKCE,PRKCB,VDR,ADCY8,CAPN3,EDNRB,PKHD1,TGM2,APP,MT1HL1,TM9SF4,GRIN2A,CD38,SLC30A7,KCTD7,CACNA1C,CYSLTR1,CACNA2D1,TRPM7,TAC4,MICU1,SLC39A8,SLC9C1,GRM5,ADCYAP1R1,SLC24A2,STIM2,TPCN2,JAK2,SMAD3,NOS1,TMTC4,SLC39A10,ARF1,TRPC4,C5AR1,TRDN,DMXL1,TRPV4,ESR1,HTR2C,SLC30A8,UBE3A,ABL2,CCL3,MCUR1,PTGER3,SLC39A12,P2RX1,CDH23,RYR1,P2RX7,RASA3,BDKRB1,BDKRB2,ADRA1B,THADA,ATP6V1H,ATP13A3,CLN6,GPR55,CCL7,MT1F,TRPM4,BMP6,CALCR,CACNB2,ENPP1,MICU2,JPH4,GNA13,CACNA1A,S1PR3,TRPM1,SCO2,UMOD,SLC24A5,SLC40A1,STIM1,SLC9A9,CASQ2,TMEM175,UBASH3B,SLC9A2,CCL14,CCL15,MCOLN3,RGN*
GO:0060048	cardiac muscle	0.018507733458641738	*KCNQ1,TNNI3K,ANK2,KCND3,KCNE1,DMD,CTNNA3,RYR2,SLC8A1,SGCD,KCNJ3,KCNE2,PDE4D,MTOR,PKP2,ATP1A1,PLN,HDAC4,PDE4B,CACNA1C,CACNA2D1,CACNA1D,NOS1,SCN1A,FGF13,NUP155,*

	contraction		KCNE4,ADRA1B,PDE5A,HCN4,RNF207,FGF12,TRPM4,CACNB2,NOS1AP,TTN,MYH7,MYH6,CASQ2,ASB3,MAP2K6
GO:0038180	nerve growth factor signaling pathway	0.0194340969273148 1	BDNF,RAP1A,RAPGEF1,MAGI2,KIDINS220,RAPGEF2,NTF3,SORT1,NGF
GO:0030003	cellular cation homeostasis	0.0195166994840728 22	CMKLR1,SLC24A3,TMPRSS3,MS4A1,GRIA1,SLC9B1,ANK2,FAM155A,TF,SCARA5,RYR3,PLCE1,CCR1,CCR3,CFTR,RAB7A,AGT,ATP6V0D1,CP,KCNMA1,SLC24A4,TMPRSS6,TRPC5,DMD,NOX5,GRM1,TRPM2,RYR2,DHRS7C,LRRK2,SLC1A3,SLC4A4,CHD7,DISC1,HEXB,SLC8A1,PRKD1,FLVCR1,ATP1A4,GRIN2B,PTGFR,PML,AKAP6,BCL2,SLC30A5,ITPR2,CYB561A3,FYN,PDE4D,GRIK2,RAP1GDS1,SLC1A1,ATP1A1,PLN,HEPHL1,PRKCE,PRKCB,VDR,ADCY8,CAPN3,EDNRB,PKHD1,TGM2,APP,MT1HL1,TM9SF4,GRIN2A,CD38,SLC30A7,KCTD7,CACNA1C,CYSLTR1,CACNA2D1,TRPM7,TAC4,MICU1,SLC39A8,SLC9C1,GRM5,ADCYAP1R1,SLC24A2,STIM2,TPCN2,JAK2,SMAD3,NOS1,TMTC4,SLC39A10,ARF1,TRPC4,C5AR1,TRDN,DMXL1,TRPV4,ESR1,HTR2C,SLC30A8,UBE3A,ABL2,CCL3,MCUR1,PTGER3,SLC39A12,P2RX1,CDH23,RYR1,P2RX7,RASA3,BDKRB1,BDKRB2,ADRA1B,THADA,ATP6V1H,ATP13A3,CLN6,GPR55,CCL7,MT1F,TRPM4,BMP6,CALCR,CACNB2,MICU2,JPH4,GNA13,CACNA1A,S1PR3,TRPM1,SCO2,UMOD,SLC24A5,SLC40A1,STIM1,SLC9A9,CASQ2,TMEM175,UBASH3B,SLC9A2,CCL14,CCL15,MCOLN3,RGN
GO:0018209	peptidyl-serine modification	0.0203430099852005 55	MAPK14,VRK2,NRXN1,CSNK2A1,CHEK2,GADD45A,STK38L,SPOCK3,DCLK1,RPS6KA2,DMD,LRRK2,VRK1,NTRK3,GSK3B,PRKD1,ERCC6,BDNF,PRKCH,BCL2,CD44,PDE4D,CSNK1G3,SLC1A1,MTOR,PLCL1,EPM2A,PRKCE,PRKCB,MAPK9,APP,EGFLAM,CAMK1D,RPS6KA5,TLK2,SGK2,NTRK2,CAMK4,TGFBR1,TOP1,MORC3,MAP3K13,NLK,NOS1,CDK1,TNF,STK32B,HMGA2,STK32C,DYRK4,TENM1,STK32A,FNIP1,TNKS,NTF3,IFNA8,SRPK2,BDKRB2,PRKAA2,MARK2,MARK1,TCL1B,PLCL2,RPS6KA6,STK38,HGF,CALCR,TLK1,MAST4,PAK1,AKT3,PRKX,PCK1,TAF1,FNIP2,PRKCG,DOCK7,TDGF1,PRKCQ,MAPKAPK2,NGF,PRKCA,SPTBN4
GO:0090130	tissue migration	0.0210431618990188 78	ITGA2,CDH13,GADD45A,AGT,RHOJ,LOXL2,SLIT2,PDCD6,PTPRG,KRT2,SH3BP1,MECP2,PRKD1,TGFB2,SEMA5A,NR2F2,NR4A1,KANK1,ENPP2,MTOR,BMPER,EPB41L4B,PRKCE,ROBO1,FAP,GRHL2,KDR,PRCP,NRP1,PTPRM,ADTRP,ADAM17,ETS1,RIN2,MAPRE2,STARD13,TGFBR1,NF1,HDAC9,MEOX2,HDAC7,TNF,DOCK1,SMOC2,PIK3R3,EFNB2,EGF,KIT,MARVELD3,MAP3K3,BSG,FGF7,MYH9,PIK3CD,WDPCP,FGFR1,SEMA3A,MAP2K5,ITGA3,CLASP1,CAPN7,MACF1,AKT3,PRKX,SPRED1,PTPRR,HDAC5,TDGF1,AMOT,DPP4,PRKCA,CTSH,RGCC,CLASP2,FGF1,RREB1
GO:0070252	actin-mediated cell contraction	0.0214826734724512 14	KCNQ1,ANK2,KCND3,KCNE1,CTNNA3,RYR2,FRMD6,SGCD,MYH8,KCNJ3,KCNE2,PDE4D,PKP2,ATP1A1,PLN,CCDC88C,PDE4B,CACNA1C,CACNA2D1,CACNA1D,MYH4,SCN1A,FGF13,NUP155,KCNE4,HCN4,RNF207,FGF12,TRPM4,EPDR1,CACNB2,NOS1AP,MYH7,MYH6
GO:0071407	cellular response to organic cyclic compound	0.0227109571747092 06	ADCY2,PLCB1,GABRB3,ITGA2,KCNQ1,SLC1A2,GRAMD1C,KCNE1,CHEK2,SPIDR,RYR3,CFTR,RBFOX2,ALK,ZNF366,CHRM5,FBXO32,PARK2,HDAC2,IGF1R,SMAD1,NR3C2,HCN1,FSHR,NRIP1,TRPM2,RYR2,LRRK2,SLC1A3,RORA,PDXP,GSK3B,SLC8A1,KDM4C,PAQR8,AKAP6,ITPR2,GDAP1,SMARCA4,CASP6,PDE4D,GABRB1,SLC1A1,CHRM1,ARL6IP5,ATP1A1,VDR,ADCY8,FOXO3,MSR1,NR3C1,TGM2,APP,ADTRP,KAT5,TFPI,PDE4B,GNG2,RAP1A,SAFB2,RAPGEF1,EP300,CYP7B1,NPC1,PNPLA3,EFNA5,STRN3,HTR4,EIF4E,PER1,GABPA,AKAP7,NSG1,JAK2,TNF,PHEX,KIF18A,GCLC,PMEPA1,GNAL,CTNNA1,NSG2,BLM,CPS1,RAPGEF2,ESR1,HTR2C,UBE3A,GABRB2,CCL3,KDM3A,PLAT,RYR1,P2RX7,AR,CASP7,TRERF1,GNB5,ZMIZ1,FLT3,PPARGC1B,DYNAP,ESRRG,GNAO1,HCN4,DAB2,TRPM4,CHRM3,PAK1,PDE3A,EPG5,PCK1,PPARA,TAF1,ESR2,BTRC,DEFA1B,DEFA3,TP63,PAK3,CASQ2,DTNBP1,RAP1B,LRP8,DHX9,GABRG2,VPS35
GO:1990089	response to nerve growth factor	0.0231552064724701 16	TMEM108,NTRK3,BDNF,KCNC1,WASF1,FOXO3,APP,NTRK2,RAP1A,RAPGEF1,MAGI2,SH3GL2,ELAVL4,KIDINS220,RAPGEF2,UBE3A,NTF3,SORT1,KCNC2,NGF
GO:0048167	regulation of	0.023362510384379	RAB5A,RIMS2,SHANK2,GRIA1,SHISA6,MCTP1,RASGRF1,SORCS3,ERC1,CNTN4,S100B,AGT,ERC2,SHISA9,SORCS2,UNC13C,MECP2,GSK3B,GRIN2B,SNAP25,SIPA1L1,MME,GRIK2,IGSF11,SLC1A1,ADCY8,LR

	synaptic plasticity		*RTM1,APP,GRIN2A,RIMS3,CD38,NTRK2,TNR,GRID2,RASGRF2,ADORA2A,NF1,RELN,GRM5,DGKI,SLC24A2,RIMS1,NSG1,MIR320B2,ARF1,TSHZ3,KIT,RAPGEF2,EPHB2,PLAT,MIR433,STX3,FMR1,JPH4,PRKCG*
GO:0051962	positive regulation of nervous system development	0.023637185790425553	*CDH4,DSCAM,ASIC2,NRXN1,CHODL,SYNDIG1,PLXNA4,FLRT2,TIAM1,FBXO31,ROBO2,NTN1,HDAC2,SLIT2,TRPC5,TENM4,DISC1,SEMA4D,LINGO2,CLSTN2,SEMA5A,BDNF,PRKCH,PLXNA2,EPHB1,LRP2,THBS2,MME,MTOR,PTPRD,TIAM2,SOX8,LRRTM1,KALRN,ROBO1,TGM2,NRP1,NTRK2,GLI3,DICER1,CUX1,TP73,GRID2,EFNA5,RELN,GRM5,LRRTM3,NUMB,MAP3K13,IL1RAP,NLGN1,SHOX2,PLXNB1,TNF,DLG5,BCL11A,CLSTN1,CDKL5,IL1RAPL1,KIT,PLAG1,EPHB2,SLITRK6,BHLHB9,MACF1,ISLR2,PAK3,LRP8,NGF,MAP2K1*
GO:0086002	cardiac muscle cell action potential involved in contraction	0.025849595953408147	*KCNQ1,ANK2,KCND3,KCNE1,CTNNA3,RYR2,KCNJ3,KCNE2,PKP2,ATP1A1,CACNA1C,CACNA2D1,CACNA1D,SCN1A,NUP155,KCNE4,RNF207,FGF12,TRPM4,CACNB2,NOS1AP*
GO:0043547	positive regulation of GTPase activity	0.0264423039106978	*RGS6,SGSM1,TBC1D22A,TIAM1,RASGRF1,USP6,MYO9A,SIPA1L3,NTRK3,SH3BP1,SEMA4D,GSK3B,RALBP1,ARHGAP24,SIPA1L1,ARHGEF10,CHN1,RAP1GDS1,TIAM2,EVI5,KALRN,TBC1D4,TGM2,PKP4,RGS16,RALGAPA2,DOCK9,MAPRE2,RASGRP1,RAP1A,RAPGEF1,NF1,RGS7,ARHGAP6,TBC1D3B,TBC1D5,DOCK10,ASAP1,ARAP2,PLXNB1,NET1,CDKL5,SIPA1L2,RAPGEF2,RAPGEF6,ASAP2,F11R,ARHGAP35,CCL3,PREX1,ARHGAP25,GARNL3,NEDD9,TBC1D9,NTF3,RABGAP1L,GNB5,FERMT2,CCL7,PIN1,RALGAPA1,ARHGAP42,WDR41,DOCK7,CCL14,CCL15,RGN*
GO:0009968	negative regulation of signal transduction	0.027300446423223004	*DAB1,MAPK14,PTPRT,TIMP3,DUSP22,FBLN1,NPHP3,RGS6,SHANK2,TGFBR3,FSTL4,RNF152,NXN,CSNK2A1,CHEK2,GPC3,NRG1,PSMB2,SHISA6,RFFL,SH3RF2,LDLRAD4,RGS7BP,CBFA2T2,NKD1,SCAI,PTPRE,ZNF536,RAB7A,ZNF366,PRKACB,PXDN,LEMD3,AGT,RCAN1,CDH2,PARK2,FHL2,BPIFB1,ZNRF3,HTRA1,SLIT2,PDCD6,IGF1R,MDM2,SLC24A4,TMPRSS6,DMD,WWOX,LRRK2,RORA,SH3BP1,MTMR2,GSK3B,TGFB2,BDNF,RBMS3,OTUD7A,ANKRD6,ARHGAP12,FBN1,TAOK3,BCL2,ZNF675,ARHGAP24,LRP2,DEPTOR,NR4A2,SMARCA4,CD44,HECW1,RNF213,FOXO1,PDE4D,KANK1,TNMD,CELF4,RFX4,NLRP12,MTOR,CTDSPL2,BMPER,PTPRD,CTNNBIP1,ZMYND11,NLGN4X,HELLS,PRKCB,FOXO3,TTLL12,CCDC88C,DLC1,ROBO1,COL2A1,EYA4,PKHD1,STK3,NRP1,UACA,LGR4,LINC00473,ADAM17,TGFB1I1,RGS16,TCF7L2,TRABD2B,BICD1,RNF43,CHRDL1,RNF126,BCL2L1,PDE4B,BRD4,ARR3,RABGEF1,SPRED2,GLI3,TGFBR1,NCOA5,CHST11,PTTG1IP,RAPGEF1,WIF1,CYP7B1,GRAMD4,PIBF1,PSMA1,ADRBK2,MAGI2,NF1,RGS7,SORL1,STRN3,BICC1,BID,IFT80,TRIM59,LZTR1,ASXL1,GRM5,NDRG2,NLK,NPHP4,SNX25,PER1,WWTR1,CBLB,PTPRU,MAPK8IP2,VEPH1,DDX3X,CNOT7,SMAD3,CD300A,AATF,HDAC7,TNF,OVOL2,SNX5,TNIP1,EIF3A,PRDM15,DLG5,GCLC,PMEPA1,LTBP1,SULF1,LRP4,ALPK2,CTNNA1,MLLT3,CD109,SLIT3,UBR2,VWC2,PTPRO,ESR1,MARVELD3,EPHB2,MECOM,FNIP1,SLAMF1,ABL2,DEPDC5,ARHGAP35,CD2AP,SPRED3,ARHGAP25,SULF2,PDE11A,RGS3,SOST,P2RX7,RASA3,AMFR,AR,SUFU,EYA1,BDKRB2,DGKZ,PRKAA2,TLE4,TRIM72,MAP2K5,TNN,DAB2,ITGA3,PLCL2,PPM1B,PIN1,RPS6KA6,STK38,CIT,TGFA,HGF,RGS12,PDE3A,PSMB7,SKI,ELF1,ENPP1,LITAF,MAD1L1,ARHGAP42,PPARA,SPRED1,PRDM16,ZNF451,BTRC,CCDC3,OPTN,PTPRR,FBXW11,G3BP1,PRKCQ,TP63,PIK3R2,APC,ITGA1,UBASH3B,MTMR4,RPH3AL,USP18,GPR21,C1QTNF3,TBX20,NLRC5,TBX18*
GO:0045216	cell-cell junction organization	0.027409275713491728	*SVEP1,CTNND2,ANK2,RHOC,AGT,CDH11,CDH2,CDH9,TLN2,CDH8,CLDN14,VCL,TGFB2,PRKCH,CNTNAP2,CDH10,CDH12,CDH6,PKP2,NLGN4X,CDH18,GRHL2,PKHD1,PKP4,PARD3,CLDN1,FRMPD2,TGFBR1,NUMB,NPHP4,CXADR,SMAD3,HDAC7,TNF,DLG5,EFNB2,CTNNA1,TRPV4,PTPRO,MARVELD3,EPHB2,TBCD,F11R,CLDN11,OCLN,EPB41L3,FERMT2,ADAM10,BMP6,PKP1,MPDZ,EXT1,AMOT,APC,PRKCA*
GO:0010604	positive regulation of macromol	0.0285074117924162 1	*TASP1,FANK1,PLCB1,KMT2C,DAB1,DUX4,GTF2I,MAPK14,ARID1B,RUNX1,CTBP2,RAB27A,PCBD2,PRIM2,ANK3,BCL2L13,AKAP13,FBLN1,ERG,ZNF292,SLC24A3,ITGA2,ABCG1,DPF3,ATF7IP,NRXN1,TNC,MTPN,NRG3,ARNT2,CDH13,ANK2,CSNK2A1,ZNF112,ZNF229,TF,PLGRKT,SAMD4A,CHEK2,PRDM9,PSMC6,TOX3,GPC3,NRG1,ETS2,NP*

	ecule metabolic process		AS3,SPIDR,IL4R,ALDH1A2,SH3RF2,SLCO3A1,AGBL4,ZNF91,ZBTB20,TET3,NKD1,KLRF2,RAB7A,ARID5B,AGT,PARK2,BANP,CDC5L,KIR2DL4,ZNF804A,CYBB,BRF1,TRIM22,TRIM5,ZNF268,DCUN1D4,TXK,RNF185,SETD3,ZNF845,FTO,HDAC2,TFAP2D,PDCD6,RPS6KA2,IGF1R,MDM2,SLC24A4,SMAD1,E2F3,ZBED4,TMPRSS6,ZNF850,TRPC5,NOX5,TEAD1,FMN2,HNRNPA2B1,NRIP1,SKAP1,WWOX,ATRX,PARP16,USP16,CAMTA1,LRRK2,RORB,NTRK3,RORA,CHD7,DISC1,HEXB,PAN3,CDCA2,GLIS1,IER2,MECP2,SEMA4D,GSK3B,ZNF841,PRKD1,ERCC6,OGT,RNF144A,TGFB2,BDNF,RBMS3,POLR3C,GRIN2B,NR2F2,PRR5,PTGFR,NR5A2,PML,PPARGC1A,RNF180,TRUB2,NLRP2,TAOK3,BCL2,RALBP1,SMARCE1,IL12RB2,KLF12,LRP2,NSF,NFIA,NR4A2,EPHA7,ZBTB7C,FYN,KCNE2,PARN,NR4A1,SMARCA4,CD44,HECW1,RHBDD1,FOXO1,PDE4D,RNF144B,ENPP2,ABCB10,ACTN2,ATF6,BACH1,KLF13,RNF217,ZNF516,CELF4,SERPINB7,APBB2,EIF3E,NAMPT,SLC1A1,ZFPM2,GLIS3,RARB,RFX4,ETV6,NLRP12,MTOR,LDB2,MOV10,DDX21,CCDC88B,SCAF8,ZNF521,ZFP64,ARL6IP5,NOL11,EPB41L4B,SOX8,HAS3,GTF2A1L,KMT2A,PRKCB,VDR,ADCY8,FHL5,FOXO3,MITF,CAPN3,ITLN1,CREB5,DLC1,EDNRB,ROBO1,SATB2,TDRD3,FLT1,NOX4,ZNF148,GRHL2,TCF4,MAP3K7,MAPK9,NBAS,NR3C1,TRAF3IP2,CR1,EYA4,SPON1,STK3,SUPT3H,ZNF585A,KDR,CASK,EDA,PCOLCE2,APP,NRP1,UACA,LGR4,ADTRP,CD58,KAT5,THRB,FUBP1,ADAM17,DIS3L2,MAP3K5,ZER1,ATF2,ETS1,NR2C2,PIK3R5,TGFB1I1,DOT1L,PSIP1,RPS6KA5,TCF7L2,TNFRSF10B,TRABD2B,GRIN2A,DLX6-AS1,BCLAF1,CCDC88A,CD38,MOB3B,MRPS27,HDAC4,PDE4B,DNMT1,GHR,NTRK2,BRD4,RASGRP1,CAMK4,COMMD1,GLI3,SMCHD1,RAP1A,TGFBR1,LCP2,IL16,PTTG1IP,ZNF729,CHRNA3,EP300,TBX3,GRAMD4,BRD7,TP73,PIBF1,ONECUT3,EFNA5,FAM20A,MAGI2,RFC3,SORL1,STRN3,ZNF615,BID,ELK3,TEAD4,DMRT1,ASXL1,RELN,GRM5,IL18R1,IL1RL1,SELE,LRRTM3,MAP3K13,EGLN3,IL1RAP,FBXL5,JAG1,MEOX2,PPP3CA,KLF7,MAS1,PER1,TBC1D5,WWTR1,CBLB,ELAVL4,MNAT1,PLSCR1,CNTN1,RIMS1,TRIM16,GABPA,KAT7,SSBP2,SLC22A2,BORA,AKIRIN2,RNF128,RAD51AP1,LRRK1,ZNF208,DDX3X,NSG1,CNOT7,MAML3,EHF,DNMT3B,JAK2,LMO7,AHI1,SMAD3,CD300A,NOS1,TCF12,AATF,CDK1,HIVEP3,LTB,NBN,NVL,SLC39A10,TNF,OVOL2,PANX1,SNX5,TNIP1,ZFPM1,ILF3,POMC,PRDM15,BMP15,INO80,ARID2,MLIP,PAXBP1,PRG3,BCL11A,HMGA2,MYO6,NEK4,YAF2,AGO3,CDC20B,GCLC,QKI,UBE2V1,SMOC2,SULF1,ANKRD31,C5AR1,LRP4,PIK3R3,WDR5,SAE1,PHF8,ZNF423,CDC14B,CHD6,TENM1,WDFY2,ZNF407,ARHGEF11,ZNF345,BPTF,MLLT3,EGF,SEC16B,SPIRE2,KIT,TNFSF8,TNRC6B,ZNF600,BLM,TRPV4,EDAR,RAPGEF2,ESR1,UBE3A,FGF13,PLAG1,EPHB2,MECOM,NDFIP2,RNF4,TFDP2,TOX,DAP,DAPK1,ERBB4,MAML2,FNIP1,LRP5,ZNF507,RPS27L,SLAMF1,BSG,CAMP,SETD2,ALX4,CCL3,FGF7,GLYR1,NHLH2,PSG9,SULF2,ARF4,MYH9,SLC2A13,SOX4,ST18,CAB39L,NEDD9,P2RX1,PDGFD,PIK3CD,PRR16,UBE2N,AGTPBP1,ITGA8,PUM1,ARID4A,DDB1,KDM3A,SERTAD2,TNKS,AUTS2,FGFR1,MEF2A,OCLN,ARID4B,SLX1B,SOST,ASXL3,IL23R,JMY,KEAP1,NTF3,P2RX7,ARRDC4,TOX2,ZNF544,AR,PVT1,SCAMP5,TRERF1,EYA1,IFNA8,IL1RL2,SRPK2,GNL3,NEK5,PRKAA2,RLF,CDC73,MARK2,MEIS2,PDE5A,ZMIZ1,FLT3,NFIC,PPARGC1B,DYNAP,ESRRG,FERMT2,MAP2K5,MARK1,CLN6,DAB2,HDAC8,PBX1,RNF207,TCL1B,ACTN4,ITGA3,POU2F3,FANCB,QRICH1,RFTN1,MYOM1,PIN1,CD226,TCF20,BMP6,CIZ1,EIF4ENIF1,CARF,CNGB1,NCOA2,TGFA,CREM,HGF,PKP1,POU2F1,CALCR,FLI1,POMT2,ZBTB16,CTIF,NOS1AP,INSR,LARP4B,PAK1,SKI,ELF1,FMR1,LIMK2,LITAF,PAX3,CHFR,DKC1,JDP2,KLF6,IDE,AIM2,CD84,PCK1,PPARA,SPIB,TTN,TAF1,FNIP2,PPP2R3C,ESR2,PRDM16,ZNF451,BTRC,MLLT10,RIT2,NCOA1,PRKCG,PRLR,CBFA2T3,DOCK7,FBXW11,G3BP1,HDAC5,TDGF1,ZNF493,SLC40A1,STAT4,POU2AF1,PRKCQ,STAT3,TFEC,TP63,USP7,ZNF721,DOCK3,HPN,PAK3,PIK3R2,TBL1X,BLNK,DTNBP1,LGALS9,LRP8,NFAT5,GTPBP1,MAPKAPK2,APC,BMPR1A,ITGA1,NGF,VLDLR,PILRB,ABI1,CTSH,DHX9,RGCC,IL6R,MAP2K6,MAP2K1,RGMB,VPS35,C1QTNF3,FGF1,TBX20,ATMIN,CRADD,NLRC5,RGN,RREB1
GO:0050772	positive regulation of axonogenesis	0.029396272615810848	CDH4,DSCAM,CHODL,PLXNA4,TIAM1,ROBO2,NTN1,SLIT2,TRPC5,DISC1,SEMA4D,SEMA5A,BDNF,PLXNA2,TIAM2,ROBO1,NRP1,NTRK2,EFNA5,MAP3K13,SHOX2,PLXNB1,BCL11A,CDKL5,MACF1,ISLR2,NGF,MAP2K1
GO:0051965	positive regulation of	0.0303166283397171 3	ASIC2,NRXN1,SYNDIG1,FLRT2,SEMA4D,LINGO2,CLSTN2,BDNF,EPHB1,THBS2,PTPRD,LRRTM1,NTRK2,GRID2,EFNA5,LRRTM3,IL1RAP,NLGN1,DLG5,CLSTN1,IL1RAPL1,EPHB2,SLITRK6,BHLHB9

	synapse assembly		
GO:0003018	vascular process in circulatory system	0.0303261925847173 8	*ASIC2,SLC24A3,SLC1A2,SLCO3A1,SLC44A1,AGT,DBH,KCNMA1,SLIT2,SLC1A3,SLC4A4,SLC8A1,ATP1A4,SLC5A3,SLCO2B1,EXT2,LRP2,ABCC9,TBXAS1,RAP1GDS1,MYLK3,SLC1A1,CTNNBIP1,EDNRB,CD38,PRKG1,DOCK4,CYSLTR1,SLC6A17,SLC16A7,ADORA2A,SH3GL2,SLC7A1,SLC5A1,SLC22A2,SLC22A3,NPR3,NOS1,TNF,GCLC,ABCG2,TRPV4,CPS1,ABCC1,ARHGAP35,ATP8A1,SLC2A13,P2RX1,OCLN,BDKRB2,ADRA1B,FERMT2,TRPM4,ABCC2,BMP6,CHRM3,SMTNL2,SLC13A3,INSR,PDE3A,ARHGAP42,SLC22A5,EXT1,AMOT,SLC7A8,ITGA1,SLC16A12*
GO:0031644	regulation of nervous system process	0.0304927766423725 6	*ITGA2,NRXN1,RIMS2,TMEM108,SHISA6,AGT,SHISA9,DLGAP1,GRM1,TENM4,LRRK2,MTMR2,TMEM100,IGSF11,CELF4,SCN11A,NLGN4X,FAM19A4,EDNRB,APP,PARD3,ABAT,JAM2,MGLL,DICER1,TNR,GLRA1,TAC4,RELN,RIMS1,NLGN1,TNF,HTR2C,NCMAP,CCL3,UNC13B,FGF12,HGF,FMR1,ZFHX2,TYMP,DLGAP2*
GO:0048514	blood vessel morphogenesis	0.0304987721733442 3	*ISM1,GTF2I,MAPK14,RUNX1,MYO18B,NRXN1,NRXN3,TGFBR3,CDH13,GADD45A,CCR3,COL22A1,COL15A1,AGT,RHOJ,CDH2,LOXL2,CYBB,FMNL3,SLIT2,PDCD6,SMAD1,NOX5,RORA,CHD7,MECP2,PRKD1,TGFB2,VAV3,PLCD3,SEMA5A,SGCD,NR2F2,PML,ARHGAP24,EPHB1,LRP2,TMEM100,NR4A1,RNF213,THBS2,ENPP2,TNMD,SLC1A1,ZFPM2,BMPER,PGK1,PRKCB,ROBO1,FAP,FLT1,SEC24B,VASH2,KDR,PRCP,CALCRL,COL18A1,NRP1,PTPRM,ADTRP,ATF2,ETS1,RIN2,NTRK2,STARD13,RAP1A,TGFBR1,CYSLTR1,ADAM12,EMCN,WARS2,NF1,ELK3,HDAC9,JAG1,MEOX2,AGGF1,COL8A1,HDAC7,TNF,OVOL2,ARID2,CALD1,HMGA2,QKI,SMOC2,SULF1,C5AR1,PIK3R3,EFNB2,AMOTL1,EGF,CMA1,RAPGEF2,JAK1,SEMA3E,EPHB2,MAP3K3,LRP5,BSG,CAMP,SETD2,LAMA1,MYH9,SLC39A12,SOX4,PIK3CD,ENPEP,EYA1,SRPK2,ZMIZ1,MAP2K5,THSD7A,TGFA,HGF,MYLK,AKT3,PRKX,C1GALT1,GNA13,SPRED1,TYMP,HDAC5,TDGF1,STIM1,STAT3,AMOT,BMPR1A,PRKCA,CTSH,RGCC,SOS1,FGF1,TBX20*
GO:0038179	neurotrophin signaling pathway	0.0316588016400427 34	*DOK5,TMEM108,AGT,NTRK3,BDNF,ZDHHC17,WASF1,NTRK2,RAP1A,RAPGEF1,MAGI2,KIDINS220,RAPGEF2,NTF3,SORT1,NGF,SOS1*
GO:2001257	regulation of cation channel activity	0.0326226621764117 8	*ANK3,KCNQ1,NRXN1,ANK2,KCNE1,SHISA6,RASGRF1,SHISA9,DMD,CACNG3,FGF14,GSG1L,CNIH3,KCNC1,AKAP6,KCNE2,PDE4D,ACTN2,PLN,APP,PDE4B,CACNA2D1,IFNGR2,RASGRF2,CACNG2,RELN,STIM2,NLGN1,AKAP7,MAPK8IP2,NOS1,TRDN,KCNAB1,FGF13,EPHB2,DAPK1,KCNE4,GNB5,RNF207,FGF12,STAC,CACNB2,NOS1AP,FMR1,JPH4,STIM1,KCNC2,CASQ2*
GO:0019932	second-messenger-mediated signaling	0.0347915849781537 74	*ADCY2,CMKLR1,CDH13,ANK2,NRG1,MCTP1,PLCE1,KSR2,CCR1,CCR3,AGT,RCAN1,FHL2,MCTP2,SLC24A4,PDE9A,DMD,INPP5A,TRPM2,RYR2,CAMTA1,LRRK2,GSK3B,SLC8A1,SGCD,GRIN2B,PTGFR,NR5A2,AKAP6,TMEM100,ITPR2,PDE4D,MTOR,PLN,EFHB,EDNRB,KDR,LINC00473,PEX5L,GRIN2A,GUCY2F,HDAC4,PRKG1,CACNA1C,EPHA5,GRM5,SELE,ADCYAP1R1,PPP3CA,PDE7A,TPCN2,NOS1,TNF,POMC,NUDT4,SELP,RAPGEF2,HTR2C,CCL3,PDE11A,P2RX7,RCAN2,PDE5A,TRPM4,CHRM3,NOS1AP,PDE3A,RIT2,KCNC2,CASQ2,KSR1,NFAT5,NCALD,PDE7B,PPP1R9A,RGN*
GO:0006875	cellular metal ion homeostasis	0.0373847907398231 5	*CMKLR1,SLC24A3,TMPRSS3,MS4A1,GRIA1,ANK2,FAM155A,TF,SCARA5,RYR3,PLCE1,CCR1,CCR3,AGT,ATP6V0D1,CP,KCNMA1,SLC24A4,TMPRSS6,TRPC5,DMD,NOX5,GRM1,TRPM2,RYR2,DHRS7C,SLC1A3,CHD7,DISC1,HEXB,SLC8A1,PRKD1,FLVCR1,ATP1A4,GRIN2B,PTGFR,PML,AKAP6,BCL2,SLC30A5,ITPR2,CYB561A3,FYN,PDE4D,GRIK2,RAP1GDS1,SLC1A1,ATP1A1,PLN,HEPHL1,PRKCE,PRKCB,VDR,ADCY8,CAPN3,EDNRB,PKHD1,TGM2,APP,MT1HL1,GRIN2A,CD38,SLC30A7,KCTD7,CACNA1C,CYSLTR1,CACNA2D1,TRPM7,TAC4,MICU1,SLC39A8,GRM5,ADCYAP1R1,SLC24A2,STIM2,TPCN2,JAK2,SMAD3,NOS1,TMTC4,SLC39A10,ARF1,TRPC4,C5AR1,TRDN,TRPV4,ESR1,HTR2C,SLC30A8,ABL2,CCL3,MCUR1,PTGER3,SLC39A12,P2RX1,CDH23,RYR1,P2RX7,RASA3,BDKRB1,BDKRB2,ADRA1B,THADA,ATP13A3,GPR55,CCL7,MT1F,TRPM4,BMP6,CALCR,CACNB2,MICU2,JPH4,GNA13,CACNA1A,S1PR3,TRPM1,SCO2,UMOD,SLC24A5,SLC40A1,STIM1,CASQ2,UBASH3B,CCL14,CCL15,MCOLN3,RGN*
GO:1901698	response to nitrogen compoun	0.0374921423812464 7	*PLCB1,GABRB3,MAPK14,ARID1B,ITGA2,KCNQ1,SLC1A2,BCKDHB,GRIA1,TGFBR3,CDH13,HLCS,KCNE1,PSMC6,PSMB2,SPIDR,RYR3,KYNU,GLP2R,CFTR,ALK,PTPRE,PIK3C3,AGT,CHRM5,PARK2,CYBB,OTC,USP25,RNF185,DBH,FBXO27,HDAC2,SLIT2,STXBP4,IGF1R,MDM2,SLC24A4,HCN1,ATRX,TRPM2,RNLS,RYR2,LRRK2,SLC1A3,PDXP,*

	d		*GSK3B,SLC8A1,OGT,KCNC1,SIK2,AKAP6,FBN1,ITPR2,NR4A2,ABCC9,FYN,GNRHR,NR4A1,RHBDD1,CASP6,FOXO1,PDE4D,KANK1,ACTN2,GABRB1,SLC1A1,MTOR,CHRM1,DDX21,PRKCE,PRKCB,ADCY8,FOXO3,RAB31,EDNRB,TBC1D4,TACR3,TGM2,APP,ADTRP,CPEB2,MAP3K5,ATF2,ABAT,GRIN2A,BCL2L1,PDE4B,DNMT1,GHR,GLRA2,NTRK2,GNG2,SDK1,RAP1A,NCOA5,RAPGEF1,CHRNA3,CACNA2D1,GLRA1,TP73,COL4A6,NPC1,PNPLA3,ADORA2A,RGS7,TFF1,SORL1,HTR4,POR,GRM5,HDAC9,DDC,JAG1,MAN1A1,PPP3CA,KLF3,GLDC,MAS1,PER1,KL,ELAVL4,TMEM67,TRIM16,GABPA,KAT7,P2RX6,AKAP7,FOXRED2,NSG1,JAK2,BAIAP2L1,CDK1,TNF,PANX1,SNX5,POMC,PHEX,SRSF4,BCL11A,ANO1,GCLC,C5AR1,GNAL,PIK3R3,TRIM41,CTNNA1,NSG2,UBR2,BLM,TRPV4,CPS1,RAPGEF2,REG1B,HTR2C,UBE3A,GABRB2,EPHB2,ABCC1,FER,BSG,CAMP,VPS13C,CYP2E1,P2RX1,PDGFD,PLAT,RYR1,GLRA3,P2RX7,AMFR,CASP7,IFNA8,GNB5,FLT3,PPARGC1B,PTPRA,TRIM72,GNAO1,HCN4,SMPD3,SRD5A2,UROS,RFTN1,TRPM4,ERLIN1,CHRM3,CALCR,INSR,PDE3A,ENPP1,FMR1,RRAGC,IDE,EPG5,PCK1,PPARA,SORT1,TAF1,CACNA1A,EXT1,PRKCG,UMOD,HDAC5,UGGT2,SEL1L2,STAT4,KCNC2,PRKCQ,STAT3,BRIP1,PIK3R2,CASQ2,DTNBP1,RAP1B,APC,DHX9,GABRG2,GPR21,VPS35*
GO:0060560	developmental growth involved in morphogenesis	0.03784132471948144	*CDH4,DSCAM,DCC,RIMS2,FSTL4,TMEM108,SYT17,PLXNA4,SEMA6D,DPYSL2,NKD1,DCLK1,PARK2,SEMA3D,NTN1,SLIT2,TRPC5,SYT1,VCL,KIF26B,DISC1,SEMA4D,GSK3B,FMN1,SEMA5A,BDNF,RYK,WASF1,EPHA7,APP,NRP1,SEMA3C,ALCAM,TNR,SPG11,EFNA5,MAGI2,MAP3K13,SH3GL2,RIMS1,RDH10,COBL,BCL11A,CDKL5,SLIT3,ESR1,SEMA3E,FGF13,SEMA5B,UNC13A,AUTS2,MAP2,SEMA3A,TNN,SYT2,MACF1,EXT1,ISLR2,KIAA0319,NGF,CLASP2,FGF1*
GO:0097061	dendritic spine organization	0.038566354663348845	*CTNND2,ZNF804A,IGF1R,LRRK2,MTMR2,GRIN2B,EPHB1,SIPA1L1,FYN,KALRN,TANC2,DNM3,RELN,DOCK10,NLGN1,PPFIA2,TANC1,ARF1,UBE3A,EPHB2,BHLHB9,PDLIM5,ITGA3,INSR,PAK3,DTNBP1,LRP8,VPS35*
GO:0009893	positive regulation of metabolic process	0.03878016894872124 4	*TASP1,FANK1,PLCB1,KMT2C,CMKLR1,DAB1,OMA1,DUX4,GTF2I,MAPK14,DSCAM,ARID1B,RUNX1,CTBP2,RAB27A,PCBD2,PRIM2,ANK3,BCL2L13,AKAP13,FBLN1,ERG,VPS13D,ZNF292,SLC24A3,ITGA2,ABCG1,DPF3,ATF7IP,EPHA6,NRXN1,TNC,MTPN,NRG3,ARNT2,CDH13,RNF152,ANK2,CSNK2A1,ZNF112,ZNF229,TF,PLGRKT,SAMD4A,CHEK2,GADD45A,PRDM9,PSMC6,TOX3,GPC3,NRG1,ETS2,NPAS3,SPIDR,IL4R,ALDH1A2,SH3RF2,KSR2,SLCO3A1,AGBL4,ZNF91,ZBTB20,TET3,NKD1,ALK,KLRF2,RAB7A,ARID5B,EPHA3,AGT,PARK2,BANP,CDC5L,KIR2DL4,ZNF804A,CYBB,BRF1,TRIM22,TRIM5,ZNF268,DCUN1D4,TXK,RNF185,DBH,SETD3,ZNF845,FTO,HDAC2,TFAP2D,PDCD6,RPS6KA2,IGF1R,MDM2,SLC24A4,SMAD1,E2F3,ZBED4,TMPRSS6,ZNF850,TRPC5,NOX5,TEAD1,FMN2,HNRNPA2B1,NRIP1,SKAP1,WWOX,ATRX,PARP16,USP16,CAMTA1,HSPB8,LRRK2,RORB,NTRK3,RORA,SLC4A4,CHD7,DISC1,HEXB,PAN3,CDCA2,GLIS1,IER2,MECP2,SEMA4D,GSK3B,ZNF841,PRKD1,ERCC6,OGT,RNF144A,TGFB2,VAV3,SLC5A3,BDNF,RBMS3,RYK,POLR3C,GRIN2B,NR2F2,PRR5,PTGFR,NR5A2,PML,PPARGC1A,RNF180,TRUB2,NLRP2,TAOK3,BCL2,RALBP1,EPHB1,SMARCE1,IL12RB2,KLF12,LRP2,NSF,NFIA,NR4A2,EPHA7,ZBTB7C,FYN,KCNE2,PARN,NR4A1,SMARCA4,CD44,HECW1,RCVRN,RHBDD1,FOXO1,PDE4D,RNF144B,ENPP2,ABCB10,ACTN2,ATF6,BACH1,KLF13,RNF217,ZNF516,CELF4,SERPINB7,APBB2,EIF3E,NAMPT,SLC1A1,ZFPM2,GLIS3,RARB,RFX4,ETV6,NLRP12,MTOR,ROR1,LDB2,MOV10,DDX21,CCDC88B,SCAF8,ZNF521,ZFP64,ARL6IP5,EPM2A,NOL11,CLYBL,EPB41L4B,SOX8,HAS3,PRKCE,GTF2A1L,KMT2A,PRKCB,VDR,ADCY8,FHL5,FOXO3,MITF,SCP2,CAPN3,ITLN1,CREB5,DLC1,EDNRB,ROBO1,SATB2,TDRD3,BNIP3L,FLT1,NOX4,ZNF148,GRHL2,TCF4,MAP3K7,MAPK9,NBAS,NR3C1,TRAF3IP2,CR1,EYA4,SPON1,STK3,SUPT3H,ZNF585A,KDR,CASK,EDA,PCOLCE2,APP,NRP1,UACA,LGR4,ADTRP,CD58,KAT5,THRB,FUBP1,ADAM17,CAMK1D,DIS3L2,MAP3K5,ZER1,ATF2,ETS1,NR2C2,PIK3R5,ROS1,TGFB1I1,DOT1L,PSIP1,RPS6KA5,TCF7L2,TNFRSF10B,TRABD2B,ABAT,GRIN2A,DLX6-AS1,BCLAF1,CCDC88A,CD38,MOB3B,MRPS27,HDAC4,PDE4B,DNMT1,GHR,NTRK2,BRD4,RASGRP1,CAMK4,COMMD1,STARD4,GLI3,SMCHD1,RAP1A,TGFBR1,EPHA5,LCP2,IL16,PTTG1IP,ZNF729,CHRNA3,EP300,TBX3,DIO2,GRAMD4,BRD7,TP73,PIBF1,ONECUT3,EFNA5,FAM20A,MERTK,MAGI2,RFC3,SORL1,STRN3,ZNF615,BID,ELK3,TEAD4,DMRT1,ASXL1,POR,RELN,GRM5,IL18R1,IL1RL1,SELE,DYNC1H1,LRRTM3,MAP3K13,ADCYAP1R1,EGLN3,IL1RAP,FBXL5,JAG1,MEOX2,PPP3CA,KLF7,MAS1,PER1,TBC1D5,WWTR1,CBLB,ELAVL4,MNAT1,PLSCR1,CNTN1,RIMS1,TRIM16,GABPA,KAT7,SSBP2,SLC22A2,BO*

			RA,AKIRIN2,RNF128,RAD51AP1,LRRK1,ZNF208,DDX3X,NSG1,CNOT7,MAML3,EHF,ZNF622,DNMT3B,JAK2,LMO7,AHI1,SMAD3,CD300A,NOS1,RDH10,TCF12,AATF,CDK1,HIVEP3,LTB,NBN,NVL,SLC39A10,TNF,OVOL2,PANX1,SNX5,TNIP1,ZFPM1,ILF3,POMC,PRDM15,BMP15,INO80,ARID2,MLIP,PAXBP1,PRG3,BCL11A,HMGA2,MYO6,NEK4,YAF2,AGO3,CDC20B,GCLC,QKI,UBE2V1,SMOC2,SULF1,ANKRD31,C5AR1,LRP4,PIK3R3,WDR5,SAE1,PHF8,ZNF423,CDC14B,CHD6,TENM1,WDFY2,ZNF407,ARHGEF11,ZNF345,BPTF,MLLT3,EGF,SEC16B,SPIRE2,KIT,TNFSF8,TNRC6B,ZNF600,BLM,TRPV4,EDAR,RAPGEF2,ESR1,HTR2C,UBE3A,FGF13,PLAG1,EPHB2,MECOM,NDFIP2,RNF4,TFDP2,TOX,DAP,DAPK1,ERBB4,MAML2,FNIP1,LRP5,PTH2R,ZNF507,RPS27L,SLAMF1,BSG,CAMP,SETD2,ALX4,CCL3,FGF7,GLYR1,NHLH2,PSG9,SULF2,ARF4,MYH9,SLC2A13,SOX4,ST18,CAB39L,NEDD9,P2RX1,PDGFD,PIK3CD,PRR16,UBE2N,AGTPBP1,ITGA8,PUM1,ARID4A,DDB1,KDM3A,SERTAD2,TNKS,AUTS2,FGFR1,MEF2A,OCLN,ARID4B,SLX1B,SOST,ASXL3,IL23R,JMY,KEAP1,NTF3,P2RX7,ARRDC4,TOX2,ZNF544,AR,PVT1,SCAMP5,SNX30,TRERF1,EYA1,IFNA8,IL1RL2,SRPK2,DGKZ,GNL3,NEK5,PRKAA2,RLF,CDC73,MARK2,MEIS2,PDE5A,ZMIZ1,FLT3,NFIC,PPARGC1B,SNX7,DYNAP,ESRRG,FERMT2,MAP2K5,MARK1,CLN6,DAB2,HDAC8,PBX1,RNF207,TCL1B,ACTN4,ITGA3,POU2F3,FANCB,QRICH1,RFTN1,MYOM1,PIN1,CD226,TCF20,BMP6,CIZ1,EIF4ENIF1,CARF,CNGB1,NCOA2,TGFA,CREM,HGF,PKP1,POU2F1,CALCR,FLI1,POMT2,ZBTB16,CTIF,NOS1AP,INSR,LARP4B,PAK1,SKI,ELF1,FMR1,LIMK2,LITAF,PAX3,CHFR,DKC1,JDP2,KLF6,ABCD1,IDE,AIM2,CD84,PCK1,PPARA,SPIB,TNFAIP8L3,TTN,TAF1,FNIP2,PPP2R3C,ESR2,PRDM16,ZNF451,BTRC,CCDC3,MLLT10,OPTN,RIT2,NCOA1,PRKCG,PRLR,CBFA2T3,DOCK7,FBXW11,G3BP1,HDAC5,TDGF1,ZNF493,SLC40A1,STAT4,PEMT,POU2AF1,PRKCQ,STAT3,TFEC,TP63,USP7,ZNF721,DOCK3,HPN,PAK3,PIK3R2,TBL1X,BLNK,DTNBP1,LGALS9,LRP8,NFAT5,GTPBP1,IL10RB,MAPKAPK2,APC,BMPR1A,ITGA1,KCTD20,NGF,VLDLR,PILRB,ABI1,CTSH,DHX9,RGCC,IL6R,MAP2K6,MAP2K1,RGMB,VPS35,C1QTNF3,FGF1,TBX20,ATMIN,CRADD,NLRC5,RGN,RREB1
GO:1901699	cellular response to nitrogen compound	0.0416942859219860 3	*PLCB1,GABRB3,ARID1B,KCNQ1,SLC1A2,GRIA1,KCNE1,SPIDR,RYR3,GLP2R,CFTR,ALK,PTPRE,AGT,CHRM5,PARK2,CYBB,HDAC2,SLIT2,STXBP4,IGF1R,MDM2,HCN1,ATRX,TRPM2,RYR2,LRRK2,SLC1A3,PDXP,GSK3B,SLC8A1,OGT,SIK2,AKAP6,FBN1,ITPR2,NR4A2,FYN,GNRHR,NR4A1,CASP6,FOXO1,PDE4D,KANK1,ACTN2,GABRB1,SLC1A1,MTOR,CHRM1,PRKCB,ADCY8,FOXO3,RAB31,TBC1D4,TGM2,APP,CPEB2,MAP3K5,ATF2,BCL2L1,PDE4B,DNMT1,GHR,GLRA2,NTRK2,GNG2,RAP1A,NCOA5,RAPGEF1,CHRNA3,CACNA2D1,GLRA1,COL4A6,PNPLA3,SORL1,HTR4,POR,GRM5,HDAC9,DDC,KLF3,MAS1,KL,GABPA,AKAP7,NSG1,JAK2,BAIAP2L1,TNF,SNX5,BCL11A,ANO1,GCLC,GNAL,PIK3R3,TRIM41,CTNNA1,NSG2,UBR2,BLM,CPS1,RAPGEF2,HTR2C,GABRB2,EPHB2,ABCC1,FER,CAMP,PDGFD,PLAT,RYR1,P2RX7,CASP7,GNB5,PTPRA,TRIM72,GNAO1,HCN4,SMPD3,UROS,TRPM4,CHRM3,INSR,PDE3A,ENPP1,FMR1,RRAGC,IDE,EPG5,PCK1,TAF1,CACNA1A,HDAC5,KCNC2,PRKCQ,STAT3,BRIP1,PIK3R2,CASQ2,DTNBP1,RAP1B,APC,DHX9,GABRG2,GPR21,VPS35*
GO:0014706	striated muscle tissue development	0.0426173117377300 4	*MAPK14,RUNX1,MYO18B,RBFOX1,AKAP13,SORBS2,MTPN,TGFBR3,NRG1,ALDH1A2,NLN,AGT,RCAN1,FHL2,SMAD1,DMD,RYR2,TENM4,CHD7,SOX6,SLC8A1,TGFB2,SGCD,NR2F2,SGCG,AKAP6,BCL2,EPHB1,LRP2,NR4A1,ACTN2,MYLK3,ZFPM2,RARB,MTOR,PKP2,COL11A1,PLN,SOX8,NOX4,NEBL,KCNK2,SEMA3C,HDAC4,TGFBR1,EP300,TBX3,TP73,FLNB,NF1,HDAC9,MEOX2,PPP3CA,NEB,CXADR,FHOD3,SHOX2,SMAD3,CDK1,HIVEP3,ZFPM1,ARID2,EFNB2,ALPK2,MYH14,PRKAR1A,SGCZ,ERBB4,MYH15,MEF2A,RYR1,EYA1,PDLIM5,HCN4,CENPF,ELN,SKI,CTDP1,PPARA,TTN,COL19A1,MYH7,PGM5,MYH6,MYH11,BMPR1A,TBX20,TBX18*
GO:0000165	MAPK cascade	0.0431239576404639 36	*PLCB1,MAPK14,SH3RF3,TIMP3,DUSP22,AKAP13,FBLN1,VRK2,NRXN1,DOK5,TF,GADD45A,NRG1,PSMB2,PLCE1,SH3RF2,CCR1,ALK,AGT,CDH2,PARK2,TRIM5,IGF1R,SMAD1,DMD,FGF14,FSHR,GRM1,LRRK2,MAPK10,NTRK3,MAGI3,ERCC6,TGFB2,RYK,ZDHHC17,ANKRD6,ARHGAP8,TAOK3,ZNF675,EPHB1,EPHA7,CD44,FOXO1,GRIK2,NLRP12,ROR1,BMPER,ARL6IP5,ZMYND11,PRKCE,ROBO1,FLT1,NOX4,MAP3K7,MAPK9,PKHD1,STK3,KDR,APP,NRP1,MAP3K5,ATF2,PIK3R5,ROS1,TRAF3,GHR,NTRK2,RASGRP1,SPRED2,RAP1A,TGFBR1,RAPGEF1,TP73,PSMA1,NF1,SORL1,GRM5,NDRG2,MAP3K13,NLK,PER1,KL,DOK6,MAPK4,MAPK8IP2,ZNF622,JAK2,SMAD3,CD300A,MAP2K4,CDK1,TNF,TNIP1,EIF3A,PRDM15,SHC2,C5AR1,ARHGEF6,TENM1,TNFRSF19,EGF,KIT,TRPV4,EDAR,RAPGEF2,HTR2C,FGF13,GRM4,MARVELD3,EPHB2,MECOM,ERBB4,MAP3K3,SLAMF1,CCL3,SPRED3,PDGFD,FGFR1,MEF2A,NTF3,P2RX7,ZDHHC9,APIP,AR,ADRA1B,S*

			EMA3A,PDE5A,FLT3,FERMT2,LTBR,MAP2K5,DAB2,GPR55,CCL7,FGF12,PIN1,RPS6KA6,STK38,TGFA,HGF,CALCR,INSR,PAK1,PSMB7,ULK4,TNFAIP8L3,SPRED1,PTPRR,RIT2,TDGF1,PAK3,PIK3R2,RAP1B,KSR1,LGALS9,MAPKAPK2,ITGA1,PRKCA,CTSH,CCL14,CCL15,EDA2R,IL6R,MAP2K6,MAP2K1,FGF1
GO:0030534	adult behavior	0.0446954657231562 2	*DAB1,NRXN1,NRXN3,SLC1A2,SHANK2,ALK,PARK2,DBH,PCDH17,HDAC2,CHD7,MECP2,PCDH15,RNF180,CNTNAP2,NR4A2,SLC1A1,BBS2,KLHL1,NLGN4X,KALRN,PREX2,CHL1,APP,ABAT,SDK1,CHRNA3,CHRNA5,GLRA1,NPC1,EPS8,SCN1A,SLITRK6,AGTPBP1,PUM1,FGF12,BTBD9,TMOD1,PPARA,ZFHX2,GABRG2,SPTBN4*
GO:0043408	regulation of MAPK cascade	0.0486069274398954 06	*PLCB1,SH3RF3,TIMP3,DUSP22,AKAP13,FBLN1,VRK2,NRXN1,DOK5,GADD45A,NRG1,PLCE1,SH3RF2,CCR1,ALK,AGT,CDH2,PARK2,TRIM5,IGF1R,DMD,FSHR,GRM1,LRRK2,NTRK3,MAGI3,TGFB2,RYK,ZDHHC17,ANKRD6,ARHGAP8,TAOK3,ZNF675,EPHB1,EPHA7,CD44,FOXO1,GRIK2,NLRP12,ROR1,BMPER,ARL6IP5,ZMYND11,PRKCE,ROBO1,FLT1,NOX4,MAP3K7,PKHD1,STK3,KDR,APP,NRP1,MAP3K5,PIK3R5,ROS1,TRAF3,GHR,NTRK2,RASGRP1,SPRED2,RAP1A,TGFBR1,RAPGEF1,TP73,NF1,SORL1,GRM5,NDRG2,MAP3K13,PER1,KL,DOK6,MAPK8IP2,ZNF622,JAK2,CD300A,TNF,TNIP1,EIF3A,PRDM15,SHC2,C5AR1,TENM1,TNFRSF19,EGF,KIT,TRPV4,EDAR,RAPGEF2,HTR2C,GRM4,MARVELD3,EPHB2,MECOM,ERBB4,MAP3K3,SLAMF1,CCL3,SPRED3,PDGFD,FGFR1,NTF3,P2RX7,APIP,AR,ADRA1B,SEMA3A,PDE5A,FLT3,FERMT2,LTBR,MAP2K5,DAB2,GPR55,CCL7,PIN1,RPS6KA6,STK38,TGFA,HGF,CALCR,INSR,PAK1,ULK4,TNFAIP8L3,SPRED1,PTPRR,RIT2,TDGF1,PAK3,PIK3R2,RAP1B,KSR1,LGALS9,ITGA1,PRKCA,CCL14,CCL15,EDA2R,IL6R,MAP2K6,MAP2K1,FGF1*
GO:0007157	heterophilic cell-cell adhesion via plasma membrane cell adhesion molecules	0.0489658493405821 5	*CDH4,NRXN1,CDH2,HMCN1,TENM4,PTPRD,TENM2,ALCAM,CRB1,TENM3,GRID2,IGSF21,SELE,CXADR,NLGN1,CADM3,SELP,TENM1,IL1RAPL1,UMOD*

Table S3. The overlap between the top 4920 genes possessing the most frequent DSBs and rDNA-contacting genes (4C). Related to the Venn diagram in Figure 1C.

Names	total	elements
4C DSBs	1772	*DDC8 ELMO2 RIT2 CD44 SAMD4A TRAF3IP2-AS1 PAX7 KCNMA1 C10orf90 PKNOX2 RALYL GPR98 BBOX1 ZNF799 GGT3P PTPRR NCF4 DDB1 SCAF8 EPB41L4B CNGB1 EP300 GPSM2 PRKCG KCNA6 PARN MSRB3 SEMA4D WSCD1 SLC12A8 TNS3 ZFYVE1 EVC MED13L CYP4F59P TEAD1 NFIA C15orf41 SYN3 RPS6KA5 MAPKAPK2 SUGCT STX8 EIF4G3 SLCO2B1 PHF20 CPNE4 SLC8A1 GRK5 SEL1L3 SRRM4 FAM73A RRN3P2 RAPGEF6 ANKRD6 RSU1P2 ISM1 VAT1L FBXO31 RNF4 KIAA1211L FAM19A4 FPGT TRAPPC9 RPS11P6 MAP3K13 MMP16 CAMTA1 PLCL2 BDKRB1 NME8 CHFR TP53TG3C TF RSRC1 TOX3 CHST11 THRB DNAH11 SLC9A9 CDC42BPA B3GALT5 SAMM50 CDC42EP3 TSPAN7 MIR1185-1 FSIP1 GRIK2 HDAC1P2 MAPK10 QKI NCAM1 OTX2-AS1 MAN2B1 AGT KDM4B GNG12 FGF1 CHRM1 GPATCH2 CDH8 KCNQ5 QRICH1 ZBTB20 HEPHL1 ARHGAP15 CCR3 HBG2 PALM2-AKAP2 LRRC53 TMEM161B-AS1 EMR1 ACAD11 NEGR1-IT1 SYTL5 HUS1 DYRK4 ZNF667 TFEC KCNJ6 STRIP1 ZNF709 BLOC1S5-TXNDC5 ZDHHC11B ITSN1 NDRG2 GRIP1 CPVL TLK1 UPK3B ASTN2 FAM177A1 TANC2 ZNF863P DEFA1B DLGAP1 RCAN1 FAM193A TMEM189 SVOPL JAK2 LINC01028 AKAP2 LRP1B ZBTB8OS ABCG8 ARHGAP25 ANKRD20A5P KALRN CCDC60 RAB11FIP4 USH2A SUMF1 NF1 NEGR1 CACNG2 BTBD9 FPGT-TNNI3K VPS16 CCDC144A WIF1 CMKLR1 SLC44A5 MEGF11 SDK2 SPATS2L LINC00882 RNF43 TRHDE ZNF536 PROS1 PARVB PPP6R2 SETBP1 ZBTB7C MAST4 ZNF713 WDR26 NRXN1 DSCAM-IT1 AGBL1 PARK2 ARHGAP10 DPY19L2P2 IL1RAPL1 MAGI1 LAMA3 SLC14A2 BZRAP1-AS1 GSK3B PDXP KIR2DL1 ADAMTS17 PKP2 MTND6P3 NPHP3*

		ADCYAP1R1 ST6GALNAC3 SERPINA4 CTDP1 ADAMTS6 SETD3 DPP6 MIR4713 PRELID2 GPR110 CD109 GRID1 RPS6KA2 SLC22A3 TDP1 GRIN3A LINC00504 SMOC2 NDE1 KCNH1 HLCS ACSS3 ZNF695 DCDC1 CACNA1C LMO7 CDRT4 MAN1A1 PCED1B NLGN4X FSD2 EXOC4 HEATR5A WDR70 EMB CKMT1B PNPLA3 FAM19A5 ANO4 PIK3C2B BBS9 GPR21 CTNNA1 LINC00595 MYO9A CCDC3 FOXN3 NTRK2 ENTHD1 OR5K4 PAK7 CHD7 WDFY3 PSMB7 ANKH MOV10L1 ANKRD20A14P ITGBL1 THSD7A NBEA FAM19A2 GBE1 CLASP1 SNX5 RASGEF1B AFAP1 SNORD115-35 TIAM2 OR51B2 SLX1B-SULT1A4 MYT1L TMPRSS3 SRGAP2B UPB1 IQCJ-SCHIP1 LRRC49 GUCY1B2 TAGLN3 CNTN1 ZNRF3 PALMD PLEKHB2 GLIS3 OR4K6P SP140 ANKRD36 MTATP8P1 HBE1 TFP1 NRG3 LINC00970 PTPRG PLAC1 LINC01094 BTBD11 DIO2 UNC13A SLC5A10 CABIN1 TRIOBP SYNE1 TUBA3FP NCOA1 MRPS31P5 CHEK2 FBXL17 PDZD2 DNAH10 GRIK3 PDE10A CACNB2 NUMB STXBP4 MED15 METTL24 RHOJ ESYT2 BRE ZBTB8B BACH1-IT2 PHEX ZNF730 SOX6 MECOM POTEM POLE SYBU PDE4DIP COL4A6 CATSPERB TRPM3 STK32B RSPRY1 NHS CNTNAP5 SHANK2 LRRC18 SULF1 UBE2E2 SLC39A10 DTD2 SNORA76 KCTD8 SDIM1 CHCHD6 UNC5D HS3ST2 CD84 EVA1A MAGEA11 SH3BP1 ISLR2 NREP GPC3 DMD NANOGP4 C22orf39 ADCY2 ZRANB2-AS2 STAG3L2 TCF20 OTC MRPS6 KCNC2 CFDP1 DLG5 PGM5 RNU6ATAC31P FNDC3B ADAMTS9-AS2 ASTN1 SH3BGR GNG12-AS1 ATRNL1 KIR3DL3 DTX2P1 CHRM3 PREX1 CPE LINC00587 UBE2V1 RFFL CALD1 EIF3E MIR4681 SH3GL2 COL24A1 ANO3 PSPC1 ARHGAP24 TMEM132D COL11A1 EFCAB8 SLC13A3 SPRED2 SLIT2 PITPNC1 TPTE2P6 ITGAE C2orf88 CEP128 FAR1-IT1 TTC4 XKR4 KCNJ16 GPC5-IT1 C9orf3 ROR1 SYNM MAPK4 GLP2R CCDC178 SLC4A4 KAZN ADAMTSL1 ZNF675 EZH2P1 OR4F16 AOX1 DTNA SERPINA5 PHKB KMT2C TRIM5 TMEM110 KCNS3 RPL23AP82 THEMIS SLC5A8 FLVCR2 COL12A1 DRG2 ABCA13 AFF3 EFTUD1P1 PTGER3 IGKJ5 ERBB4 KANK1 KIF26B LPP ATRX TVP23C BID MACF1 SNORD115-48 TRAF3IP2 MEF2C-AS1 CHRDL1 LAMA2 MNAT1 TRIO CAPN3 TFDP2 CTNNBL1 CCDC12 TRMT61B RAD51B PTPRE EPDR1 MYO3B DUSP22 SLX1B FAM95B1 SH3RF3 GTF2E1 ST6GALNAC5 MYOM1 DIAPH2 CRYZL1 DIP2C MAP3K5 NOS1 CLDN11 BMPR1A ACACA MAML2 SPAG16 DSCAML1 SMAD3 CTDSPL2 HTR2C CCDC141 PRKAR2A CACNA1E MAGI2-IT1 TBC1D5 CNTN4 MUC16 MRPL33 GLRA2 BORA PI4KA VWA8 ACBD5 ALDH4A1 TMC2 LINC00511 OR51B8P TNFRSF11B PIBF1 SLCO3A1 NCF2 MRPL48 GABRR2 PIK3C3 CAPN14 SLC9C1 SND1 MPPED1 SNAP25-AS1 CHD6 HMCN1 FGD4 LY86 ZNF670 NDNL2 IFFO2 POTEE EPHA5 FAM24B MACROD2-IT1 TCF12 SGIP1 HIRA CEACAM18 SLC47A1 RNF144A TP73 GRIK4 RBM19 RUNX1 IL1RL1 KIR3DL2 LINC00535 CYP4F31P TSHZ2 ZNF366 ESRRG MTND4P12 PTGFR SNX31 CDR2 FAM171A1 PCNX SCUBE1 TMPO GPR112 SV2B FAM230A NOTCH2NL LINC00894 PEAK1 EYA1 PRR14L MYPN LINC00669 SATB2 ADAMTS19 KIAA1217 MORC3 ANKS1B P2RX6 HSF2BP PLS1 INTS4L2 ANK2 ROCK1P1 LINC00092 ELN STXBP5-AS1 ANO2 ARHGEF3 RHBDL3 SUPT3H MAML3 SLIT3 BAGE2 GRIN2B RBM14-RBM4 PHC2 ROBO1 LRRIQ3 ANKRD30BL GPR141 TPH1 MAD1L1 GPR176 MATN3 ANKRD11 EGFLAM SHPRH FAM155A-IT1 PLD5 RABGAP1 PAK3 DGKB DPYS GARNL3 DUX4L13 UACA LRPPRC DPH6 TNKS TSNAX PRRC2C TUB KLF12 ARHGEF18 SCAMP5 LINC00473 WBSCR17 ANKRD36BP2 OVOL2 USP53 PRUNE2 DOK6 FCHSD2 SGMS1 ADCY8 LINC01122 GRAMD4 DNAJB6 ELP3 ITPR2 GABRR3 BRINP1 C8orf34 MLLT3 OMA1 BCL2L13 CCDC30 ARRDC4 DRG1 WDR7-OT1 KHDRBS2 CPQ TM4SF2 DARC CRISP3 NEB HNRNPA1P7 FGF14 PRICKLE2 CCL15-CCL14 CEP164P1 BCL7C ITGB3BP CACNA2D3 DRD5P1 RNF213 DGKK OR51I1 WDR47 SPESP1 MIR4695 RNU6-1061P SH3RF2 CNTN3 MGAM GLIS1 CACNA1D PRKAA2 ASGR2 ABCG2 PACRG IL1RAPL2 TBL1X LANCL3 SPAG17 ZDHHC11 CHCHD3 SEMA5B ZNF337-AS1 RPS6KA2-IT1 SKAP1 TPRG1 KLHL33 SNX29P2 CDYL2 ST13P15 LRRTM4 PTPRN2 MYO3A AKR1B1P1 LARGE-IT1 UBE2R2 HIVEP2 FAM65B GRIK1 NOL12 LINC00446 SOX5 KIF6 ADH5P3 DSCAM DGKI NLN TBC1D30 ANKFN1 NME7 DNMBP TAF1 SPNS2 NBPF10 TRAPPC8 FGF14-IT1 SDK1 C5orf66 SLC1A1 RNF144B GRM5 ATG4C CCDC33 NTN1 FEM1AP2 C15orf57 ARAP2 SIL1 LINC00937 LDB2 IGF1R FAR1 PKD1L1 NLGN1 TSNARE1 DNAH9 FAM69A SHISA9 SHROOM3 SNRPN MSI2 LRRC7 TEX41 EPM2A MXD3 MON2 CNTNAP2 TNFRSF10B SPECC1L-ADORA2A SHFM1 MAP2 LARP1B LIPE-AS1 AQP4-AS1 CDC73 KCNIP4 SOX2-OT CAMK1D FLRT2 NALCN-AS1 CCDC144NL RAD51L3-RFFL EFCAB6 FBXW11 NOS1AP EFTUD1 PTPRO ZDHHC14 MSRA SYNPR PCDH9 MKRN3 C22orf34 SUFU ARHGEF33 SNORD114-20 DSCR4 NKAIN2 RANBP10 CD96 RBMS3 FAR2P4 OFCC1 RNF219 SMG6 ARHGEF6 INSR CIZ1 ITGA11 WWTR1 GAS7 SNORD17 MDN1 CIT AMOTL1 CLEC16A PEMT ARHGAP6 PHF20L1 KCTD7 ST3GAL3 TTC6 TCF7L2 JAK1

		CHAF1B KIAA1328 Y_RNA LINC00298 MTRF1 RPGR CATSPER2 STARD9 CRTAC1 PCSK5 LINC00537 HS6ST3 EGLN3 CUX1 GRM4 PRR12 MAP2K1 ANK3 TIMP2 CDH12 ZMAT4 UBR2 CCL15 MORC2 ZNF19 TMEM132B CPSF1P1 ANKRD44-IT1 FLG-AS1 ZNF490 ARID4B GMDS LHFPL3 FBXL18 CNIH3 DOCK3 THSD4 SLC9B1 TBCD KPNB1 FOXO3 TRIM59 TTLL8 GPC6 RELN LINC00284 RASGRF2 TRPS1 STK38 ADAMTSL3 EVA1C SNHG14 LINC00871 MYO18B CDH4 GABRA3 KLHL3 TNR ADCY9 DPP10 OCA2 CELF4 CDKAL1 VAV3 INPP5A C17orf51 CNTN6 CDKL1 SCN9A TTC7B APP ATF7IP2 CENPF KCTD9P2 MDM4 PUM1 C10orf11 ARNT2 KCTD1 RNLS SPOCK1 RAB2A ACTN4 HPSE2 PLCE1 TACC2 ADAM12 CYP39A1 PAK1 AMZ2 PPP1R14A ATP9B MITF ASNSP1 IGF2BP3 DEC1 SP140L SPECC1L TMEM110-MUSTN1 LINC00693 LRRN2 ZNF346-IT1 FRY EPS8 LRFN5 CTBP2P8 UTRN CDC14A GPC5 ENOX2 TENM4 AGBL4 MACROD1 CECR2 KPNA3 CASC16 PPP3CA PRR16 GHR SPTSSA GOLGA3 ARHGAP39 WDR59 LRRC8D CEP170 PRDM16 FRMD5 ULK4 RBFOX1 KIR2DL4 TMEM120B PCDH7 CDH10 LRRC4C ZNF605 PPP1R9A PDZRN3 C1orf168 ZNF41 TMEM117 TPTE2P2 DNMT3B ADAMTS16 TASP1 POTEG LINC01090 ZZEF1 DEPDC5 RYR1 LTBP1 RGL1 LRRK2 NKAIN3 SLC25A21 PALM2 CLSTN2 CD300A PKHD1 KIF2A TTLL11 NEBL RARB CBFA2T2 ITPRIPL2 TCF4 ABCC11 NAALADL2 SRD5A2 SPG11 FUBP1 FOCAD PBX1 DPH6-AS1 PRG3 GANC PHACTR1 MLIP SERTAD2 OR5H8P SORCS2 PRIM2 SCN1A STRC DISC1 BMP6 OSBPL10 RALGPS1 ARHGAP42 PMS2P11 PHF21B GUSBP1 MRPS28 SLC35F1 VSTM4 POC1A TMEM170A C21orf49 NTM VTI1A ASAP1 PCBP3 LINC00971 FRMPD4 ATP8B4 COL23A1 LINC00639 DSCR10 FYN KCND3 CCL14 KIR3DP1 ABCC2 IFT43 CSPP1 XRCC4 RABGEF1 LRBA EPHA7 FHIT MARVELD3 NSG2 LINC00842 FAM19A1 RNU2-42P TRABD2B LRP5 NAV2 EFCAB5 CHN2 GAS8 MARCH3 COL19A1 MSR1 AP2B1 SLC30A7 NRG1-IT1 TOX PCDH15 ESR1 ARHGAP12 GABRG3 KCNN3 SGCZ CCDC176 SEL1L2 TNFRSF19 PLCXD3 DENND2A LUZP2 CLN6 PTPN14 GRM1 SCFD1 PDE4D CNTN5 ERC2 THEM4 GNG2 PCDH11X DMGDH PCBD2 RIMS1 ENPP2 PIK3CD L3MBTL4 DOCK4 FHAD1 VPS53 SLC5A3 BLOC1S5 FRMD4A MCTP2 CERS3 EGFEM1P WWOX PTPN9 ABCA12 PCSK2 HUNK RNU6-78P DNM3-IT1 FUT8 SNX29 SNURF SAMD5 BEND5 EPHB1 LPHN3 LINC00478 LSAMP CREM KCNQ1 FHOD3 GREB1L TEAD4 PDE7B ARMC2 TNRC6B SRRM1 SLC38A6 IPO8P1 RAB24 DOCK1 SNORD115-47 MTUS2 CRADD VASH2 ZNF664 TMEM178B BPGM FAM126A EXT1 LINC00276 EFNA5 TMEM68 ENPP7P5 CACNA1C-IT3 TLN2 BPESC1 HDAC4 RNASE13 KLHL1 TRPC5 KIAA1644 AMFR PLCB4 MRM1 ATP9A FTO ADORA2A-AS1 EIF3A ENOX1 B4GALNT3 UNC5C CHI3L2 AKAP6 ACSBG1 POLR2F CSGALNACT1 CSMD2 SORCS3 LINC00189 LINC00299 EML6 POU6F2 TENM3 OPCML LINGO2 WDR7 SNORD114-16 DIAPH2-AS1 DUX4L19 SFI1 MGAT4C RBMX2P1 CEP85L ATF2 PHACTR2 NOX5 RBBP8 KCNJ12 OSBPL1A PIK3R2 ANKRD44 CYP3A43 CCDC91 KAL1 GRID2 CALN1 ZNF423 LRP2 CYBB SEMA6D ZCCHC17 KCNJ3 C2 RALGPS2 FER GLDC MIR548AS SUSD4 CAMK4 GALNT14 TMEM189-UBE2V1 LINC00466 VWC2 GUCY2F ERLIN1 CELF2 SMIM2-AS1 PDXDC1 NTNG1 ADTRP CRMP1 PDE4B DDX10 ZNF804B FBXL7 ARFGAP3 MAPRE2 PCDH17 SGCD TMEM108 DDX59 RIC8B GABRB3 TPTE LRRC20 GRM7 SHROOM4 RAPGEF2 NAV3 SMG7 PRPSAP2 ANKRD13A ZCCHC11 PPA2 MAP3K7 IMMP2L MIPOL1 CENPP DNAH3 PTPRM ATXN1 PRKCQ SSPN KIRREL3 AK5 NCOA2 N4BP1 GALNT8 NRXN3 RASSF8 USP12 RHPN2 RABGAP1L DLC1 IMPDH1 KCNH7 CLDN1 TSNAX-DISC1 ABCC1 WDR83OS KCND2 CDKL5 INPP4A LY86-AS1 PTPRU ATP8A2 AFTPH NHSL2 ADPGK SBF2-AS1 SNTB1 SLC24A3 SOBP ORC4 ZNF252P ZNF207 MPRIP GRIA4 IDE ANKRD32 CERS6 SCMH1 USP32P1 TPH2 BAALC KIAA1598 JDP2 INADL PLEKHA5 MACROD2 TTLL5 IL18R1 SPTBN4 TMTC1 ARMCX4 RGS7BP AUTS2 LPHN2 EPHB2 MTND6P4 PLEKHA6 TMEM56 ESR2 KAT7 MYBPC2 ERC1 SATB1 VPS45 COX10-AS1 EFCAB11 SLC5A4 NRG1-IT2 AGO3 C9 S1PR3 TMTC2 MCTP1 MOB3B WLS ANKS1A RYR3 PRCP RN7SKP86 ZPLD1 PRTG C12orf42 DCHS2 RGMB SOS1 MIR767 CTNND2 EEF1E1-BLOC1S5 COL22A1 TC2N SETD2 KCNQ5-IT1 PACSIN2 SQRDL BAI3 DOCK2 NUP214 DPYSL2 DSCR8 PTPDC1 TRIM22 CDH13 MDGA2 SAMD3 RFC3 VEPH1 FAM129A BASP1 BDNF-AS LRRC8C DACH1 TRDN CALML4 MIR5704 DAB1 RFTN1 GUSBP3 ZNF148 MYH16 SNTG1 SULT1A4 SEC16B SLC16A12 ALK CACHD1 EXOC6B HDAC5 MARK1 BDNF LDLRAD4 SEMA3A BTRC IQCH ACBD6 RBM4 CCDC26 FNIP1 MGAT5 TSPAN8 ATP13A3 STOML1 CADM2 MALRD1 MRPS17 MYH9 LINC01057 SNORD109B BDKRB2 MYEF2 DCLK1 MEF2A ADORA2A MAGI3 STIM1 COL21A1 FAM135B MORN1 GPATCH2L BACH1-IT3 TSC22D3 NRIP1 EEFSEC MROH7 ARID5B SIPA1L2 CCNG2 RCAN2 TENM2 TANC1 MIR105-2 AKNAD1 VPS41 IFT80 C1orf21 LITAF DYM CUZD1 RNF219-AS1 LINC00351 PIK3R3 BRF1 FSTL4 FAM222B KCNB2 MCM3 TENM1 CCDC170

		STK38L LARGE VPS39 GABRB1 ANO1 SGSM1 ST8SIA1 BLM SH3KBP1 FHL2 PSMB2 CADPS PRKAR1A ZRANB1 RGS7 HS6ST2 TFAP2D TTC39C DIO2-AS1 STXBP5 SLC44A1 FCRL2 FAM196B SIPA1L3 RNU6-1269P TTLL11-IT1 GNG4 MRPS22 DRAM1 TSPAN33 LINC00393 KANSL1 NOX4 VWF CES1P2 WDFY4 LINC01154 MEGF9 SNUPN FAM155A DPY19L2 KIR3DL1 TXNDC5 PTPRT SETD5-AS1 CSMD1 ERI2 LINC01076 MANEAL SLC24A2 COL25A1 MTND5P11 SCEL BCOR GRIA3 NTRK3 RXFP1 LINC00457 FBN1 SLC35F3 ANKRD20A12P HYDIN CHKA CTNNA3 TXNDC16 CEP89 DIS3L2 TBX15 BMPER hsa-mir-6723 NOS2P3 LINC00922 MOGAT2 CLASP2 ZNF521 PDE1A TMEM194B LINC01036 CMAHP ATF7IP DUXAP10 OR4H12P MTATP6P1 TMPRSS15 ICK CREB5 THSD7B EFCAB4B HLCS-IT1 C1orf95 RBKS DNAJC6 CPA6 NSUN2 TPD52 U3 DACH2 DEFA3 PTPRK AGBL4-IT1 WBP1L KIR2DS4 DNAH10OS BRD9 SORCS1 DNM3 SYT1 SYNDIG1 FAM13C ASXL3 DHX32 SCAPER CRYBB2P1 DENND5A PNPT1P1 LINC00856 DOCK9 DLG2 EBAG9 PPP1R12B DAB2 FAM101A AFF2 PPARA NCKAP5 PLXNA2 MGLL THAP7-AS1 SCFD2 RNA5-8SP5 RORA PTPRD MYH15 SHISA6 NSF EBF4 ANKRD20A2 NFASC TPT1P5 ATP5O METTL9 LINC00923 MAP2K5 ZNF663P PLCB1 TMEM56-RWDD3 LRRK1 GRIK1-AS2 DNMT1 PRKG1 BRIP1 UBFD1P1 PLXDC2 ELAVL4 CADM3 SBF2 CDH9 KIR2DL3 STXBP6 SNAP23 ANXA8L1 CHST9 WDR25 MXI1 TTC28 MAGI2 NELL1 GALK2 FLT3 ANKRD30B SNX18P15 SRPK2 ABI1 CACNA1A HPCAL1 GALNTL6 KIAA1671 PXDNL EDA DOCK10 LINC00491 CACNG3 TRPM1 TTC34 EPG5 PTH2R CNTNAP3 EEF1E1 FNDC3A ZNF346 TPK1 PRKD1 ATP8A1 CALCRL ATXN8OS POT1-AS1 MICAL3 ADK CELSR1 TNNI3K HDAC2 RANBP17 LINC00271 GBAS ETS1 TVP23C-CDRT4 RPS2P44 TMEM131 BICC1 RYR2 DISC1-IT1 NBPF11 POLN SEMA3D SLX4IP BANP PRLR PTPRA FAT3 SERTM1 SNORA70 FAM189A2 PDE11A TSPAN9 ARID4A KCNC4 TSPAN1 USP32P3 RAB30 TMEM163 CEP41 ATF6 PRKAR1B EYS IPO11 FAM189A1 VCL IQCJ ZSWIM7 DEPTOR SPATA5 NVL BACH1 PPFIBP2 POTEH ROBO2 SMIM20 IFT81 DTX2P1-UPK3BP1-PMS2P11 ZMYND11 CDH23 TTN RGS6 SRGAP3 RNF144A-AS1 EFCAB2 AKAP13 ZNF93 RAD51D EMR4P PDS5A CUL2 RNASET2 NEDD9 CCDC146 CLRN1-AS1 MYRIP SLC39A11 CDK19 ENPP1 PPP2R3C CFB ZNF443 DUX4L14 RIMS2 SYT17 FOXP1 C16orf95 STAC OR4M1 SCN8A FRG1B ADCK3 FDXR RAB27A PNLIPRP1 RALGAPA1 KSR2 DLGAP2 MROH7-TTC4 ARL15 HIVEP3 SPATA13 SEMA5A TUBGCP6 CABLES1 MORN2 GAREM CST2 PRDM15 HEPACAM2 OR4N2 BLOC1S6 LRRC16A LARP4P AGAP1 MKL1 SNORD115-19 DCC PMEPA1 C1orf94 ZZZ3 UBE3C CTNNA2 CCDC92 PEX5L CYB561A3 PARD3B ETV6 LARP4B HGSNAT KIAA0754 LRCH3 FMNL2 KCNJ15 CEP112 PARD3 snoU13 MYOM3 CCDC73 FAM110B SYNPR-AS1 NRG1 CAST TBC1D9 SYT9 FANK1 DHRS4-AS1 LINC00534 VSIG10 RPS20P1 DLEU1 SPINT2 SLC22A10 CASK DENND2D ATP10B NPAS3 SLC35F4 PRKCA MARCH1 FMN2 SEMA3C GRAMD3 DPY19L1 GRIA2 KIAA0825 BCKDHB PAH TP53TG3B ST18 FRMD4B C3orf67 VSTM1 SELO RXFP2 TOP1
DSBs	3148	*KRTCAP2 MMP2 FAM153A C15orf27 ATF6B XDH ABCB7 VPS4A PFKP DECR1 MTHFD2L IFT122 NTRK1 LINC00864 SMIM17 OR51A8P C2orf49 COL4A5 CDK14 SOD2 TMX4 PNPT1 MIER1 CCDC97 XK MET TOB2 RPL4P6 MAX OSBPL8 PROSC KNDC1 RNU1-124P IPO9 PPCS MRPL1 DUS2 TMEM254-AS1 SNX33 PTCD3 CTAGE5 DMC1 WDR34 LHFPL2 CCL22 TMEM180 LUZP1 SLK EIF3LP1 MIR218-1 TRIQK BAZ1B SNX16 BBS12 LRGUK CRHR1-IT1 CD160 TERF2IP FOXK2 ATL2 FGGY CLDN16 Metazoa_SRP LINC01138 MTCYBP3 ALDOC PRDM2 RNF103-CHMP3 C1orf61 SCGB1C1 SUPT4H1 CEP350 SMYD3 ARL14EPL WHSC1 LGI2 SHROOM1 MIR3121 C19orf10 ABR NCOR2 MIR4310 CNGA3 ZDHHC6 MFSD8 PGBD5 CCNY NT5C1B-RDH14 ROR2 RN7SKP57 MAOA ANGPT4 CCDC41 FRS2 ALG2 SYT13 HSPD1 F2RL1 UGT1A12P LCOR RASA4B GPN2 DESI1 RARRES3 C8orf44-SGK3 HS2ST1 SYT12 CSNK1A1P1 SERPINE3 VAX2 ARL13B SPANXA2 MYO15B FAM198A MLF1 C12orf75 TNXB STPG1 COX8A CDCA4 GAPDHP54 RAC1P5 CD6 ACAP2-IT1 POLR2J4 PSMD11 CELA1 USP42 IPP TMEM59 CNOT1 SNX6 ADAM23 SPTBN1 SRP72 UPK3BL IDO2 NUCB1 ARSH ADAM20P1 TTLL4 METTL21A USP32 KDM6A PI4KAP1 PDP1 DAPP1 ATF7 LRRC10 KNOP1P4 UFL1 CD53 RNU6-816P DNAJA3 GLG1 MIR153-2 EPHA4 L2HGDH PLCXD2 AGPS PIN1P1 LZTFL1 KLHL7 SUV420H1 DCUN1D5 IGDCC3 NBPF3 DOCK11 PCSK6 RPRD1B DNHD1 PTPN2 RNU6-999P PBLD NRG4 AHNAK RGS14 RTN4RL1 PPP1R37 ACTG1P1 ZFHX4-AS1 RBPMS DLEC1 ACO2 XCR1 RBM6 PSMF1 C7orf60 SH3PXD2A NRF1 LINC00475 MIR5010 MRPS24 RBBP6 PRDM12 JADE1 SCHIP1 HP BTK FXYD2 UIMC1 PTGES3L-AARSD1 ABCC6P1 KIN CCND2P1 OTOA FSTL5 ZNF146 VMP1 MIA2 ZAN KLHL6 UGT1A13P MLIP-AS1 HOXB5 CASC4 CUL4B TNRC18 RDX DNAJC9 SLC4A5 FBXO46 SVIL CELF5 CAMK2B DCTN1 LIN28B NDUFB8 CADPS2 TMEM248 JPH2 PAQR3 NECAB2 ANKRD26*

		ATP2B2 NBPF16 TRAM2 NIFKP7 PAWR TEX33 MTMR12 NBPF13P ESPNL URGCP-MRPS24 KLHL21 SCRN1 LINC00577 NUGGC TAB2 GPATCH8 HPS1 UCN2 TFE3 ZNF511 POLR3G SNORD116-7 KCTD13 TMEM256-PLSCR3 DCT TANK OVCH2 FBXL20 UGT1A8 FAM120A CNTNAP3B GLDCP1 LAMTOR3 TARSL2 TERF2 CASP1 LBX1-AS1 GTF2H5 PLEKHM3 QRSL1P2 RAB15 PLA2G4C ANKDD1A XPO4 HAUS4 HCAR2 SPAG6 ATP6V0A2 IFNAR1 MKLN1 KIAA0319L SDHD SQLE OPN3 ARL5B-AS1 TAOK2 EHD2 YTHDC2 TBC1D3P1 NCF1 PARD6G PSMD5-AS1 ACTBP7 GRPEL2 TCFL5 FAM157A H2BFWT PPCDC ERMARD NT5E HNRNPA1P5 IL18 SLC16A2 HHIPL1 GK5 OR51N1P OR51B3P GEMIN8P3 ANP32A CLDN10 DNAJC1 FKBP5 KIAA0232 CHCHD5 ERCC8 KIAA0368 SCNN1B ALG9 RNU6-1056P BTBD10 CUL3 RPL5P15 MUC20 SNCA CLIC5 ESRRB MSTO2P SLC48A1 FAM153C GEMIN6 LINC-PINT METTL16 CPNE9 MEF2B CYCTP HS3ST5 RPL17P42 MORC1 CSRNP1 C17orf105 LINC00622 AIF1L SHANK3 CCDC14 EEF2K KANK4 COG8 TRMT2B NR4A3 SPDYA SPTLC3 UNKL OIP5 GPR124 DACT3-AS1 RPS12P10 RUVBL2 OR52N1 ISCA1 NXPE2 COCH SP4 RNU7-87P EIF3H GNGT1 ATG3 KIF5C NDUFV2 GPM6A RN7SL751P CDH20 CA12 MROH5 DDX50 HNRNPA1P49 SATB2-AS1 SLIT1 TNR-IT1 BLVRB PIFO GTPBP2 GALNT10 MORC2-AS1 STK39 ADCY1 OR9A1P UBE2QL1 SMURF2 FAM13A C20orf112 CCDC129 TRPV1 ADAMTS12 C2orf48 AVL9 IL9RP3 ZFYVE9 LINC01140 TCTN3 TBC1D16 TRIM24 CC2D1B FMNL1 SYN2 SEPT7 ASPN MTX1P1 SERPINB11 QSOX2 HFM1 YBX1 ANXA2 HORMAD2 CLNK LGALS2 ABCD3 MTF2 FIG4 SP7 MTND5P18 ADRBK1 FCGR2A C12orf49 FOXP2 NKAIN1 NEIL2 CHCHD3P2 ZNF114 ATP5J2 KMT2D ZNF385C IMPG2 GUSBP5 CRHR1 URGCP ZFAND1 CBWD1 TRAF6 AXL LMNB1 RRN3P3 SPAG5 SLC10A1 CSNK1E BRCA2 PPP5D1 XPO6 C6orf89 C7orf50 CR1L SLC44A3 PTGER2 FAM153B HACL1 LINC00948 EXD1 ADAM29 C15orf26 PKN3 RN7SL180P SCAMP4 TMEM182 UQCR10 APBB3 CLIC4 WDR92 MIR3692 ALOX5AP THUMPD3 RFT1 SLC8A2 TACC1 RAD9B ADHFE1 SLC6A1 RFPL1S SH3PXD2B SAMHD1 ARL17B PAX6 RPSAP52 MYSM1 SIK3-IT1 CDON PKD1P1 PCAT2 FASTKD5 NETO1 GCNT6 ST13P4 ZDHHC3 SESN1 FBXO9 PTPRQ TNPO1 MAPKAPK3 RAB6A C12orf5 CDK13 CHML OC90 BCR CAPN2 DZIP1 SEC22C GPR161 RPL29P2 LRFN1 ACTG1P3 HOXB-AS1 TAS2R62P TRAPPC11 TMEM196 CREB1 FHP1 FRMPD3-AS1 SPATA3 NEO1 MTND6P5 UGT1A7 RASA2 STAG1 ZHX2 MIR4794 KIR2DP1 LINC01102 SOHLH2 KIAA1377 TTLL9 AKR1D1 ITPKB EML4 OBFC1 STAT5B OR52B1P FKBP3 MIR3605 IPCEF1 RN7SKP245 RN7SKP240 BIN2 PRKRIRP8 UBOX5 SNORD115-16 MPP6 KLF17 APOLD1 ARHGAP29 TMEM251 DDX11L9 SCARA3 TOM1L1 CPEB4 C11orf80 FAM206A MOCOS UBE2H BRDT SPATA18 MKL2 CDC45 MBTD1 USP33 PPP1CB RPL13AP2 CCDC136 PAAF1 CCT3 MTHFD1L RBM5 RGS22 CFL1P1 PTCHD3P2 SSR2 CRELD1 AMDHD1 WDR27 CD247 LINC00865 KIAA1239 CDAN1 PKHD1L1 MCC SLC26A2 ATP2B4 RPL7AP60 GUSBP11 P2RY10 STAU1 ADAR HADHB ADIRF STPG2 MAGI1-AS1 KTI12 MAP3K4 MTA1 XRN1 C1orf105 GNG7 RUNX2 SPATA6L PPP2CA FGF10 MBNL3 CROCCP2 CIRH1A KRBOX1 MIR1226 POLH PLEKHA2 LRRC1 ESRP1 PEG3 NADK GALT PAICSP6 CBWD2 CAPSL TYR TPT1-AS1 NPIPB4 CKAP2L POLR2J3 HERC5 MED12L BRMS1L LEKR1 SGK494 C19orf68 TAF8 ADAM8 SLC9C2 CASP1P2 EIF4BP6 MAN2B2 HSPA7 SHPK HAO2 C10orf131 TOMM5 MIR670HG EMR3 OSBP2 ACOXL MIR202HG DAK ZCCHC2 ACAP2 PGPEP1L PPP4R2 KIF3A ACP1 RPS3AP15 HNRNPA1P6 ILDR2 RTBDN NMUR2 DAGLA RAB11FIP5 C20orf166 MPHOSPH9 LINC00607 SH2D4B FAM157B RASA1 RNF10 FAM168A RPL3P3 PCDH9-AS1 ZNF660 ITCH ITCH-IT1 GTF2H2C TANGO6 SBSPON AJUBA GSG1 PDGFRA PTPRJ FRMPD1 TET1 FAM205A GTF2E2 ZNF737 FAM178B MIR555 KCNQ3 NFYB KCND3-IT1 SNX8 HADHA CHURC1 CCBE1 PLA2G16 MUSTN1 TFEB RN7SL75P HDAC1 RN7SL127P MTCP1 MASP1 BLZF1 CENPK CAV2 ASB5 AREL1 SOD2P1 MED26 KDM2B USP50 RIMKLA TRIM46 C17orf78 C14orf142 ADNP UST GSTM5 PDCD1LG2 VPS26BP1 POM121C PMS1 TMED6 RAB28 SLC25A51 ATP2B3 PIGU ALAS2 SFMBT1 TYW1 HIBADH PHBP15 C10orf113 UPF2 CPT1A ZNF823 KIF22 VIPR1-AS1 DCN GSE1 SUCLG2 PRNCR1 S100A12 UBAP2 RN7SL717P RNU6-229P OR52E6 VAMP7 GBP5 HAUS3 POU3F3 IFRD2 COMMD6 TBC1D3P1-DHX40P1 STX6 FAM20C DNAJB14 RNU6-302P ABCC8 LSM3P5 BPI FOXP1-IT1 BNC2 SERPINA3 FBXO39 RN7SKP202 ASS1P1 FBXL4 KIAA1432 MYO1D OR52V1P CAP2P1 BMP2K TBC1D23 POMGNT2 FAM13B WNT7B HEXA SFXN5 NRD1 SETD4 FLT4 MORF4L2P1 SESTD1 RHOBTB1 PRCC SMAP2 RFC5 BRAF AAK1 GMEB1 PMS2P9 EEA1 RN7SL254P ARL5A C22orf43 SNX18P16 ITGAL SLC38A11 MLYCD VWA3B ALPL SMAD6 TMEM206 VRTN ABLIM1 ADAMTS7 LINC00854 RBMS1 UQCRC2 RYBP ATP1B1P1 TPRX2P RFX2 DPY19L1P2 GNB3 ACTR2 SENP5 SNORD114-13 FAM174B KLF17P1 MYB NBPF24 CASS4 TBC1D19

		ERGIC1 RPTOR MAP4 OR52P1P EIF5AP3 SYNJ2-IT1 ZNF554 TNIK HSPA8P14 FAM65A IRS4 KATNBL1P2 COX5A MPPED2 NCF1B SSXP3 SH2D6 PCMTD2 ADD3 KIAA1279 MALT1 AXIN1 ARVCF STARD4-AS1 KANSL2 RENBP NID1 FBXO16 DEPDC1B FCRL6 IQCK NBEAL1 GPR107 HOXC13 FAM115C SNORD116-25 PADI3 SMARCC1 C20orf24 PSMC1P13 CD4 C14orf23 RNA5SP387 STX18-AS1 DGCR9 AGAP5 OR6C70 CLRN1 NUDT10 SH2D1A ACTG1P12 ZNF347 PHBP4 RPS23P7 SUGT1P KIAA2022 AGAP11 PHKG2 SLC39A14 NGRN IL21-AS1 MIR5002 SERBP1P3 FCGR2B BCO2 RPS14P4 CA14 SNIP1 CIB2 STAG2 HRK NFATC1 SCOC RALY LDHAL6DP NUP93 APCDD1L TTLL13 DGKH ZBTB22 CDK12 ELOVL3 ASB8 CHMP5 GALNT13 NEDD4 PPP3R2 ATP6V0B SKP1 PEX14 ZBTB38 PAFAH1B1 CAMSAP3 FBLIM1 ANKRD35 RAPH1 MUC3A HRH4 RAP1GAP2 DOCK8 ARMC1 GOLGA2P5 LINC00969 BTN3A2 PRKCI GAMTP2 DRAXIN RASAL1 RN7SL390P DDAH1 MIER3 EXD2 MIR4293 RPL17P43 PART1 PARVA CYP4F10P JAKMIP2 CERS6-AS1 RNF34 DDX11L10 A3GALT2 FAM102B COA6 CCNI2 PDE6D CCT2 DCAF12 ITPKB-IT1 MIR10A CCSER1 ZNF692 SPRTN HMGN3 KIF18B C12orf76 PAG1 DHDDS APOL4 UBE2CP2 SYN1 EEF1A1P30 RPL7AP66 ZIM2 ALG14 RNF145 FAT2 EFCAB7 ZMYND8 GATAD2A GPRASP2 FANCD2OS N4BP2 PRMT2 ZNF609 SCN4A FAM135A NACC2 OR10J9P ECE2 CCDC102A NMNAT1P1 FAM194B PACS2 MYOT GAPDHP59 SCD5 UBQLNL RHOXF2B LSG1 EMG1 WDR5B ARMC8 RN7SKP253 DCUN1D3 EMC3 SLC7A14 PRKCD DSC2 C7orf76 NPIPA7 IDNK MEF2BNB-MEF2B TRIM65 NPSR1-AS1 ANTXR2 COL4A3 MYO10 CCDC155 OLFM1 MTND5P10 ENAH MID1 PITRM1-AS1 RCC2 RNF103 THSD1 STON2 TRIM37 LRRC25 STOX2 NLGN2 ZNF566 ABCB1 CNR1 DLG3 PLAGL1 RNF2 USP54 MTF1 DNAJC7 RN7SL867P NOL3 ARNTL2 GRM7-AS3 POLA1 ASPH AQPEP HDGFRP3 RRAS2 LEPR SH3BGRL ERN2 NDUFS2 UBR5 GP6 HSD3BP2 SLC22A8 RASA4 GTF2F2 PPP1R42 HPR BLID SOX30 VBP1 CXorf66 SLC25A13 TMEM132C GATSL3 GATS TTLL7 RPAP3 STOM AIM1 GSN EGR2 WWC1 ANP32AP1 TMEM14A GLB1L2 OR11N1P FAM21B SH3GL3 SPEF1 GGPS1 CERKL DBT ITGB6 SRBD1 INVS PDGFB RNF19B KLK2 hsa-mir-6080 SMIM7 TMEM209 KIF21A CLUL1 LZTS1 ADAMTS4 KIAA1244 CD27 HOXB-AS2 RWDD3 CMC1 ADAMTS14 CACUL1 HSPA6 NT5DC4 SHANK1 LIN7A PPP2R5C ANXA2R SYNJ2 SNORD114-21 MLIP-IT1 AMPD1 DENND4A EBF3 ITPR1 NFKBID EMX2OS RPL12P2 SMYD3-IT1 CNN2P1 OXR1 MRC2 SFMBT2 SETD5 PPP1R16A TMEM169 SNX2 TICRR RBX1 ANKRD54 MADD LINC00667 COX7BP1 KCNK1 BLOC1S3 DAPK2 EREG NEUROD1 IL17RB LNX1-AS2 RNFT2 WDR60 THEGL DCAF8L2 TBC1D26 ZSWIM6 SMEK1 CLMN UGT1A4 AACSP1 TMEM183A PYGO2 PPP1R10 OR51T1 CCNJL CAMK1G LACE1 C2orf76 UGT1A6 LHFPL4 NMT2 CASC2 DFNB31 MARCH7 AFM CENPC STAT6 WDR64 C1orf110 SCARB1 ZNF787 ABHD17AP3 RPS20P20 PRKAG2 PELI1 NPIPB3 MST1 ABLIM2 JAZF1 IGFL2 RQCD1 GABRA6 RN7SL832P PLEKHF2 MGMT FAM157C RPL7P31 MYLPF TRBC2 HNRNPA3P12 F8 ARHGAP23 ALG9-IT1 ADAM21 PRR14 MSANTD3-TMEFF1 SIGMAR1 TSPAN5 MIR3151 AMIGO1 SLC9B2 LINC00266-1 RAB4B-EGLN2 ADCY10 FBXL2 CSRNP3 RNU6-202P MVP MTX3 KLHL32 ZMYM1 HNRNPC DNAH2 ZP3 BCAS4 ASB15 NNT-AS1 ENTPD5 ZNF461 ADAM3B NCKAP1 PKN2 XPR1 PSMA2 CLDN12 ITGAM KCNQ1-AS1 C11orf82 OR5P3 TVP23CP1 RTN4 MOB3A PRICKLE2-AS1 PHRF1 GOLGA8M GNL3L EEF1A1P31 GIMAP2 BTBD3 ACTL8 PRKAR2B FBXO21 SLC8A1-AS1 GAPDHP69 P4HA2 DFFA SERGEF SMC2 LATS2 POLR3DP1 PTPN1 LRRC52 PAPD5 EZH1 SAP130 VWDE OR4N1P LSP1 CD59 PLCG2 KIAA0247 LY75-CD302 HTR2B UVRAG PSAP ATP5L SERINC5 INPP4B BMPR2 B3GALNT1 AARSD1 LINC00358 C3orf84 FAM198B NTMT1 SLC5A6 RNFT1 BMS1P4 CNIH2 MTA3 OR9A4 ORC2 MCOLN1 NFIX SLC16A6 OR52H2P AIFM2 PALB2 DIP2B HMGB1P24 BCORL1 MTND1P11 LPA LY75 OR5BH1P COL28A1 LPHN1 SP1 LINC00383 AXIN2 SLC35E4 SRRM3 LRRC72 NCAPD2P1 SYT3 PPME1 FLJ20373 RBM42 RPL23AP6 TNPO3 HTT GRIK5 TPGS2 BRI3 DAZL CYP2C18 SYK IFI44L FREM3 GGT7 ZNF766 TBR1 IQGAP1 MYCBP2 RPSAP6 WIPI1 GTF2IRD2 TMCO4 FDXACB1 ZNF777 KCNQ2 LRRC37A14P LEMD1 SECISBP2L FGF2 PABPC4 FCGR2C DIAPH1 ZNF71 OPRD1 RABGAP1L-IT1 COMMD10 HNRNPA1P26 KLHL12 GPSM3 CAPRIN2 TSG101 STRBP TNS4 FANCI NAV1 PAQR5 EIF2B5-IT1 CBWD3 TMCC1 ACAA1 SMG5 PCDHB16 ANK1 SRD5A3-AS1 PPM1L CASZ1 TEC C1D LDHC C6orf10 USB1 SETDB2 SPNS3 RPEP1 HNF4A PAPL RABEP1 OPN1LW MIR600HG IL21R-AS1 RN7SL620P SLC41A2 IBTK RPL7P42 GSTM3 FCRL4 LOXL3 EIF3L DYNC1I1 NFATC2 ANAPC5 NOMO2 SPON2 ARHGAP44 AKR7A2P1 ARL3 MIR4654 COX5B COPS8P2 MTND2P16 PRDX2 PRMT3 OTOGL GPRASP1 MRS2 ZFAND3 PIN4P1 OTUD7B KIF24 ZSWIM5 UBA2 SUGP2 C1orf226 ANKRD12 ZNF626 ITFG1 C11orf91 LINC00501 C18orf25 USF1P1 ATP13A5 MYO7A TUBBP6 CCDC15 TIMM44 PSMD1 WASH4P

		C2orf70 CSNK2A3 NOX1 C9orf170 TMEM242 TSPAN6 REXO1L1P ZBTB20-AS2 FUCA1P1 SRGNP1 DTHD1 DDX60L MARK4 SNORD116 RNF212 TSSK1B NFX1 TLE6 HERC4 MIR383 OR2T3 MYO1F POLR2J2 RAB11A KLF15 PPP2R2B SNX1 ARHGEF17 WSB2 GBAP1 HNRNPDLP3 TYW1B IL12A-AS1 RBM8A LINGO4 SCUBE2 VANGL1 QSER1 HNRNPLL RADIL SLC35E3 LYRM4 ADIPOR2 DDX58 PGK1P2 NUTM2A-AS1 CES5A TMEFF1 NDUFA6-AS1 USP22 VPS52 GPRIN3 PPM1F PTCD1 MEF2BNB WDR18 RAP2C-AS1 HSD17B12 CCDC85A DPY19L4 NDUFA9 OR5H2 FBXO45 OR52E8 UNC119 TBCK GPM6B LINC01010 CPSF4 MLXIPL UCHL5 PIP5KL1 DYX1C1 PGAM1P10 ARSE SHC4 SREK1 VOPP1 SCARNA24 CCDC22 TCP11X3P RARS LINC00578 OR5P1P STC2 OPCML-IT1 IFIT1P1 STMN4 TCF7 KITLG HDGF KRT6B WWP2 CBX5 SFT2D1 TTC9C ZNF662 TMEM2 TNFSF9 PPFIA4 MAP4K4 GTF3C1 RNU105B LMNA RNU7-52P CPEB2-AS1 NPM1P37 PRICKLE1 INHBA-AS1 LARS2 ZBTB5 SRSF5 RBMS2P1 HIP1 RPA2P3 MCF2L2 WNK1 DLX1 KPNA6 ACOX2 CYP4F11 PCNXL2 B3GALT1 NF2 AK3P3 ZKSCAN7 VTA1 KALP CRISPLD2 MTMR3 PLXDC1 KIF3C CASP12 OR51A7 BMS1P3 NDC1 GOLGA4 IWS1 PTPN13 SFRP1 IDH3B RPS26P11 RPS6KC1 SCML4 ARHGAP22 FXYD6-FXYD2 MIB1 UGT1A3 ATG14 SLC18B1 UGT1A5 PITPNB ADD2 PAPOLB SKA2 TRAP1 HCAR1 DPYSL3 OR3D1P TEN1-CDK3 PRKAG1 ASLP1 SLC16A10 RNY3P2 ELAVL3 HLX PTGIS ZNRF1 PPIL6 NBPF15 LINC00310 ATRN SNCB NADK2 HELQ PSMA2P3 MIR663B HOXB-AS3 HNRNPCP2 RNU6ATAC27P RNGTT WDSUB1 CIPC EIF2B5 PYROXD2 TRMT2B-AS1 UTP20 PDF RPL18P10 CASC14 RNF38 KLHL2 GLTP CSPG4 IGF1 NCF1C POLK ADCY7 DCAF5 RN7SL20P HMGCS2 LPO PEBP4 SH3BP4 TCF25 TNRC18P1 FBXO42 SLC4A8 KRT8P9 FXYD6 ANKS4B GOT1 UGT1A1 YEATS4 SYNGAP1 OAZ2 BET1L RN7SKP92 CSF3R DKK2 HMGB1P3 RHOT1 ELOVL5 SHC1 ARHGAP21 SAAL1 BRD7P6 BACH1-IT1 HOOK3 S1PR2 CLCC1 CYP2C19 SMO CDK11A IQCF2 SIGLEC9 SMC3 GSS NT5M TEKT1 C10orf67 C1orf132 SPDYE7P TRA2B WASIR2 CDKL3 TMEM63C LAIR1 RPS17P2 YTHDF1 ZNF484 SRSF6 MAGI1-IT1 INSRR PTK2 DLEU2L ATAD1 UGT1A10 GLTSCR2 PLIN2 ASCC1 OTUD3 OR14K1 YME1L1 FAM163A TPCN1 MCU FAM115D LAMTOR5P1 ENO1P4 GPR114 DENND1A SBNO2 TRIM29 CDS1 SNORD66 TMEM151B ADAM19 PRPS2 SDHAP2 CELF1 DNAH12 EDNRA EDRF1 PDCL2 NIPBL TUBBP5 RNA5SP393 PKD1P5 LDHB PHF7 RAB5B NBPF8 HNRNPA3P14 BDH2 TRIP12 UQCC1 ATG10 ANKRD18CP CPEB1 VN2R1P ZNF395 CNTFR ABI2 EIF4A1P9 EZR WNT3 ARIH1 LGI4 EPHA1-AS1 POLDIP3 RPL7P18 SNORD116-2 MFAP5 GPR171 C3orf83 WDR20 CDC6 C12orf4 PLD1 CHI3L1 NUP88 TEN1 NOMO1 CD99 RHEBP2 GJA6P DIRC1 MARK3 PRRG1 RPL5P9 ADAMTS2 BRD8 PKIB SPANXA2-OT1 GALNT18 FXN PNPLA6 NOC3L VWA1 AP3B1 IL5RA ZNF425 DHFR PLEKHM1P ASH1L NANOGP7 RSPO2 TPMT SLC35E2 FRMPD3 EML5 FAIM3 TAB1 MEGF10 ACVR2A ECE1 HCK SH3D19 RC3H1-IT1 CAPN6 MRPS35P3 PPL ZFP41 RPS17L CCDC6 NCOA3 SLC25A18 KCTD10 SYTL4 RNU2-47P OR6N2 ALDH6A1 SNTG2 MUC5B CSRP1 OR5H7P RILPL1 NAV2-IT1 COG4 C12orf65 NFRKB FANCA DNAJC11 ZNF354C EEPD1 MEIS1 FAM114A2 CCDC132 AMMECR1 NSD1 LRRC2 NEDD4L TMEM179 CACNA1H ERCC1 CREBBP ITGB1 RBM26 KCNG4 BRINP3 SGK1 DNAJC16 LEF1-AS1 CCDC169-SOHLH2 NAIP SPATA12 BAI1 P2RY8 FNTB KRT8 TMIGD2 DISC1FP1 HNRNPA1P22 MST1P2 SPAG9 SLC39A9 RHCE TAC3 CORO1C TCEANC PLIN3 OR5D17P MSANTD3 FBN3 DGCR5 EPS15 C19orf47 OSTCP5 RGS8 TRPA1 GNS WIPI2 PHOSPHO2 RANBP1 MYL10 BMP7 RNU6-923P MAP1A CTNNB1 ATP6V1A ANKRD17 ZNF362 REPS2 H2AFY2 TGIF2 FCGR3A RNF150 VEZT EPHX4 SLC7A5P2 IMPACT CA15P1 RNF7P1 HNF4G EXOSC3 PSMD2 FCF1P4 ATXN2 IPO5 C19orf81 GCNT2 PGGT1B SYT7 PLEKHO2 ZNF565 NKAPP1 FMO11P CHMP3 TEX10 DPY19L1P1 IGHEP2 FMOD SH2D3C PP13439 BMS1 FAM53A NFATC3 BPIFB6 EPHB3 GRTP1 ZKSCAN1 RPS3AP22 EPSTI1 GAP43 PAPD4 ZNF354A CAND2 TRANK1 DDX10P2 UBE2K RCN1 SF3B2 GXYLT2 CPB1 HIPK3 BCAR3 MIR4435-1HG SREBF2 MFAP3L PITPNM2 ACAN KRT18P33 PPM1E TGFB1 SLC6A16 PGAM1P7 ZNF780B ACKR2 PAX2 LPAL2 PSEN2 SPERT C1orf131 ZBED6 ADRA1D TXNDC12 ASB1 BCL11B TNFRSF8 TMEFF2 RN7SL582P NLGN3 GLCE PLS3 DPRX TPST2 OR4F29 MIA KCNIP1 ZFAND6 SMG1 ZNRF3-AS1 MDFI FAM91A1 CKMT2-AS1 ZDBF2 RAB4B POU2F2 CHD1L MSTO1 TPM3P9 GFRA2 NEK10 STAT1 TBC1D10A KIAA0556 GAPDHP66 LIX1L CARD16 MEGF6 MYOCD TPM3P6 RPH3A C9orf156 RECQL5 EPCAM CXCL17 FAM188B2 SLC26A6 RERE BNC1 P2RY14 PPP1CA ECT2 SLC25A42 GLCCI1 PTPRC COX6A1 MYO1E LNX1-AS1 BEST3 UCHL3 CCDC167 WDR45B C4orf45 KLHDC8A FAM21A CCDC102B INSL6 INPP5F PDZK1IP1 IRAK1BP1 WWTR1-IT1 GNL3LP1 IFFO1 CDC27 UBR1 COL9A1 WDTC1 ZC4H2 SRCAP LUM RNA5SP139 C1S MIR548T ARHGAP32 KPNA4 CD302 PRR13 CGNL1

		CECR1 PFAS RBM34 ADGB SNORD115-5 ZNF44 MTFR2 FTH1P26 ZNF555 PRKCZ FOXJ3 TSPAN12 PLD2 SLC2A14 SASH1 IL17RD PARP12 SYNE3 RNU6-979P ANGPTL4 BTF3L4P2 SSBP3 WBP2NL TBCEL SPPL2A OR51B4 OR51K1P BRPF1 MAPK1 RNF216 TSKS NHLRC2 TNRC6A PPP3R1 RBM45 USP46 PDCL DNAJC5G DCAF6 SPI1 RBM14 NUPL2 C8orf46 SF3A1 TNFSF11 HOXB4 RPGRIP1L OSGIN1 BLVRA CASC15 IKBKB FAM25A BOK TYK2 ENTPD1-AS1 PIP4K2A FCHO1 RPS23P6 OR5AW1P LBX2 SNX9 SLC25A17 RNU1-2 ARHGEF28 UBQLN3 CLCN1 RAB3GAP2 FUNDC2P3 ZNRF3-IT1 RIOK2 PDLIM4 EEF1DP3 STX18 CCPG1 TRDMT1 TMIGD1 BAZ2B EYA3 NSRP1P1 LRRC37B RHOXF2 SLC35A2 NPIPA8 COPG2 AP1B1 LINC00877 SLC35E1 APOD PLEKHG4B VN1R105P FAM154A SYNCRIP WNT11 ELOVL2 RN7SL623P MED13 GPR35 SNORD116-5 ESCO1 PHF2 TAS1R2 TUBA1C COL6A5 ZFYVE28 NSUN6 SNORD116-3 RAI2 TMBIM6 FCGR3B BMP1 CPNE6 EBF2 MRPS33P4 NFE2L1 STIP1 MMP26 XPNPEP3 MTDH ARSG SHB IKZF4 SLC7A11-AS1 RSBN1 GCKR TRPC6 SLC43A2 OR56B1 LIG1 OR2AT1P EHMT1 ZNF616 MRPS16 MACC1 OR51F2 SRCRB4D ZNF415 ZDHHC13 WWC3 ST5 STRA6 EGLN2 NPLOC4 HDGFRP2 CPLX2 DEFB118 CNIH4 RNF133 SLC35D1 COL16A1 POP1 SLC29A1 SHOC2 FBXW7 HHLA1 TFAP2A TIMELESS ARL5AP2 RDH14 PTPLB DNAJB2 CDK11B SH3GL1P1 DPEP1 HTR1D CCDC180 ZNF143 COL6A4P2 HOXD3 TAF3 MAN1B1 SP3 BFSP1 MIR3182 DHRS4 NR2C1 ADAMTS3 OSBPL2 LHFPL5 DCAF10 GLI2 ICA1 MIR1912 ENTPD1 OTUB1 ZNF564 MIR1203 C7orf73 SETD1A HNRNPCP6 NLRP1 FNTA SUCO OR5P2 ATP6V0A1 SRA1 RIPPLY1 SPSB4 PPIP5K2 CHURC1-FNTB LAMC2 CPOX SPSB1 F5 LINC00467 CPNE1 NDRG4 ATP10D LZIC PITRM1 C12orf56 TRAK1 METTL13 FGD3 CARD17 ASPHD2 HMGN2P40 RORC WDR37 BOC NUAK2 RBM39 LINC00944 ANO8 WDR90 PMS2P3 NFXL1 EIF4HP2 RUFY3 VRK3 PCBP2 RMST NHLH1 RPSAP57 DYX1C1-CCPG1 COLGALT1 ZNF418 LARP4 ZMPSTE24 ZNF503-AS2 APEX2 TXNL4B C1orf228 GFRAL CYP51A1P3 TSGA13 RIMS4 RAD54L2 CPT1C MTIF2 U1 THRB-IT1 ZFAND2A TAF15 TPST1 RPS4XP17 PFKFB4 PLVAP PAGR1 KRT8P42 MTND6P11 TXLNB TMPRSS12 PHB R3HDM1 HSDL2 CLTB NXT2 CNNM2 MTND4P16 SEC61B SBNO1 SNAPC3 GNA12 LIMK1 MECR UBE2FP1 HIGD2A CIB4 SGK3 FBXO28 MCMBP AIMP1P1 TSHR EPHA10 SULT2B1 SEPT6 MIR4295 ME1 ALDH7A1P1 CALM1 C9orf147 B4GALNT2 MCM8 PHLDB2 CYFIP2 TEX11 ZUFSP ACOT8 DNAI2 KRT18P26 CCDC148 HEATR2 MRPS36P4 C2orf40 SRRM2 BCAS3 POLA2 FBXL13 NOL10 ERCC6L2 ITIH4 EDEM3 GATC SLC16A1 RANBP9 PIPOX HIF1A-AS1 TMEM256 LINC00526 CDK3 ENPP3 PDE4A SOX2 AOC2 VGLL4 C1QTNF1 SLC6A3 PPIP5K1 GOLPH3L STT3B UBOX5-AS1 ZNF322P1 FNBP1 RN7SL164P ZNF780A EYA2 OR1X5P BCL2L14 ZNF471 MAGEA4 CERS4 ZNF652 UCK2 SCN3B DRD1 IQGAP2 MLXIP HYAL3 CLPB TEX264 DONSON LTV1 SLC30A9 XPNPEP2 PDE6A PVRL1 FAM178A CEP70 MICU3 RNU6-179P ARHGAP11A DNAH14 PTEN MAST2 AFF1 RNU7-123P TBC1D14 ATP1A3 RNF216-IT1 RBFOX3 TICAM1 CA13 UGGT1 SEC23B RDH13 CALR3 SYNE2 C9orf85 NETO2 ODF3 NOSIP SOX13 KEL SSH2 NUP210L CHID1 ALPK3 RAB3C ALOX5 CEP135 LYRM2 HNRNPH1P1 RC3H1 GPR116 DDX11L1 ILF2 SLC25A26 CROCC FARSB CLDN4 GRM8 UGT1A2P CRYM PKD2 ZNF704 SAMD12 ZNF197 TRIM60 PTPN11 ATF3 FBN2 DAP3 KIAA1958 RTN4R LINC00499 ANXA13 DDX47 DVL2 TAPBPL MIR346 KLHL23 ARMCX5 IKZF1 HIF3A SNORD114-15 LPGAT1 FOLH1 BRMS1 SPRR2D UBQLN4 GPR173 MFAP3 PCMT1 VAMP1 RAB37 RBBP4P1 SNORD114-25 LILRB4 ARNT LMBRD1 USP49 SLC25A16 STEAP3 GTF2F2P1 MBOAT1 PLA2G4E MUC1 IGSF1 C2orf27A ARID3A FAM26D WDR43 ACOX1 MTHFSD RPS26P15 MUC7 CLPTM1 SDHCP4 SEC31B IDH2 TAS2R41 H3F3AP4 GPI ASF1B ZNF836 TAF2 ADAMTS9 AGPAT3 TMEM237 CAMK2D MARCH8 TMEM161B CELF6 UFL1-AS1 ADCY5 DHX40P1 FKBP14 HOXB3 NPAT RN7SL719P CSTL1 PRKD3 CMC4 LINC01155 RCOR3 NRP2 DHX30 PTPRB GPR52 RPL7AP33 SPTAN1 ITSN2 PPARG PNMAL2 CACNA1B SUDS3P1 RHOA GET4 IL31RA GFI1B HCAR3 PPIG TTC18 SUN1 MRE11A HOXD4 TTBK2 STX18-IT1 SSX9 CHRNB4 DUSP26 WIPF3 EPB41L1 DNAJC3 MIA-RAB4B SIMC1 C10orf76 KIAA1549L MYT1 FAM133CP VILL HECTD4 GRK5-IT1 C4orf36 ATP5J2-PTCD1 PARP11 PPP1CC PAXIP1 ZC3HAV1 KRT18P61 CCDC138 PDXDC2P CKS1B USP13 FBXW4 NBPF1 LEPROT STEAP4 RN7SKP197 CUEDC2 DNAJC9-AS1 OR6C74 AP2M1 SNAPC4 SLC9A7 CDK6 NPRL3 LRIG3 POC1B-GALNT4 LIMA1 RAB27B CTAGE6 MUC12 MYOF TLL2 LCK AFF4 ZNF76 RAF1 QPCTL AKTIP ARL10 MAPT-AS1 RPL5P13 DLGAP1-AS5 CNRIP1 NAT6 PDE8A NALCN TIMM10B TCEA1P2 RNU2-72P RPEP4 RPS15P5 NUDT6 IQCH-AS1 CLDND1 REV3L PPP2CB PTK7 PHKA1 SEPHS1P4 RBM20 OR51H2P B4GALT6 SARM1 CA15P2 EPRS SGTB PTGES3L CHUK DENND4B SRGAP2 TNPO1P2 SOS1-IT1 ZCCHC7 PEX7 TGIF2-C20orf24 C14orf159 ZC2HC1C ARMC4 NUDT14

		ASCC2 ZNF90P2 RAB11FIP3 PLEKHA7 RAD51C PARP6 C1orf159 SPEN SULT2A1 TMC1 PNPLA1 AK4P1 CDH26 AGPAT4 LINGO1 IQCG TECPR2 SPTBN5 PLEKHA1 LAT2 NR6A1 SLCO1A2 WDR78 PKNOX1 BMP4 ECSIT SIN3A KRT18P31 CCDC147 MC1R AGFG2 RNF168 TRIM69 PDCD2L GALNT7 CCNYL1 SLC5A9 OBSCN KIAA2026 CLCA2 TNFAIP8L1 RPS23P9 RND3 KLC3 CYP2G1P VDAC1 TJP1 NT5C1B KRT18P11 PER2 MIR1305 ASPHD1 RBM12B DSG1 CIR1 OR5H6 PRAP1 TBP KRT16P4 TUBB3 MTBP RBM43P1 ZNF653 ARHGAP19 VAV2 ERCC3 SLC17A7 CDKL2 NRG1-IT3 TADA2A TFRC AK2 PRPF38B KAT6A CYCSP24 C1QTNF9 GRB14 JOSD1 TYRO3 NIPAL2 CSNK1A1 SNX14 CUX2 PHBP13 SLC6A14 ALDOA MST1L MIR3609 DIS3 AP3B2 THAP3 SPATS2 NOMO3 CCDC62 ARNTL EFCAB3 DROSHA ZNF813 CTCF HEG1 SNTB2 INO80C C4orf40 ALDH3B2 FAM149B1 LAMC1 CCND3 ZBTB8A COL13A1 RPRD2 PBX3 MKRN2 ZNF761 GALNT4 C18orf56 CCDC93 TLDC1 ANGPT1 CCDC114 LMTK2 CASKIN1 FAM35DP HSD11B1L FAM131A VIPR2 EPHA1 ETF1 EIF4G1 HHAT HSP90AA1 RPA3-AS1 LRRC47 RANBP3 GYG1P3 TRAPPC13 PHLPP1 SLC4A10 SIAH1 BAG6 PHLDB1 CLOCK SLC35A4 JAG2 KIFC3 ROCK1 RN7SL741P PHF5A SLCO4A1 THOC3 ACYP1 PHACTR4 GAPVD1 RPL21P123 ARHGAP31 MRPL35P3 TMBIM4 ST13P20 CAMKMT MMP28 MOXD1 PHIP RPIA PPP2R2B-IT1 AARS2 FAM103A1 EPB41 CNNM1 TCP11 RNF220 CARM1 MTRNR2L2 ECM2 PTPRS IKZF2 CBFB PDE2A MIR3194 RPL36 AGBL3 SH2D3A STARD6 ZYG11B DVL3 ZDHHC15 ABHD17AP6 ACTL6B DENND2C CRX STXBP5L RPL18P11 ITPKB-AS1 LONRF3 MLLT1 UGT1A9 COPS5 TG ACADSB PRSS12 DGKG CAMTA1-IT1 CDCA3 snoMe28S-Am2634 SP100 MYO1A LAMB1 ANKRD19P SLC2A12 OR51F5P GSX2 HOMER2 KLF8 EPB41L2 CDK5RAP1 DCAF13P2 CFI CYP20A1 GLTSCR1 NFIB SORBS1 ZNF23 GUCY1A2 TBCA PACSIN1 KREMEN1 LRCH2 PDCL3P6 TRIM13 MB RFPL4B CBL RNU6-167P RNU6-640P SHANK2-AS2 CACNG8 KCNAB2 SBK3 MAG ANKRD13C ADORA1 STRCP1 ARHGAP19-SLIT1 NOTO NIN FOSL1P1 ZNF322 TEX22 SLC1A4 FAM46A ZNF286A GRIN1 ANKFY1 C9orf84 SAP18 CMPK1 ALDH8A1 ZKSCAN5 WNT7A FIP1L1 SSH1 C4orf21 RNF148 RIBC2 GNAQ TUBA8P2 TBC1D10C MMAA HOOK2 SUB1P2 LINC00461 PPP4R1L IFI30 TROVE2 PLEKHM1 AUNIP ESF1 EPHX1 EEF1GP1 MBD3L1 GEMIN7 COL14A1 PARP10 GNB2L1 CASC17 RAPGEF3 SLC20A2 JARID2 USP34 ZDHHC23 ZNF584 POU5F1P5 STK4 ABCF3 CHIC2 ITIH2 PCDH10 NCKAP5-IT1 SLC9A1 RNU1-4 KIAA1467 GTPBP10 PAFAH1B2 FGD1 MARC2 PIGS TACC1P1 UBAP2L SMOC1 ADAT3 HOXB6 RPL23AP32 SLC25A33 TMEM234 RCE1 TCIRG1 MRPL37P1 TRIM44 TMCO1 TRRAP ELF2 PTCHD4 AP3D1 CCDC169 KRT18P67 ZNF569 AP3S1 SNX3 CATSPER3 CEACAM7 HYKK BOK-AS1 GOLIM4 HSF1 DST NPSR1 CAB39 LNX2 TMEM144 ZEB2 POLR2H WSCD2 UBE3D MGAT4A JMJD1C EIF4E3 RNU6-928P CADM1 NOTCH4 ZNF708 KLHL29 INPP5D TEX12 KCNRG XAGE3 C6orf136 RGS9 SPATS1 RAB6C RPL23 PCCA CLVS1 YLPM1 GBF1 ZC3H11A TCF3 MAP1B BRWD3 CUL9 STAT2 C6orf201 EDARADD SEPT8 PTAFR ACTG2 CR2 GNA14 MIR4440 CREBRF PDCD11 CRTC3 LLPH LRRFIP2 CYTH3 CHRD PLLP LMTK3 RNF121 MIR1302-10 RPL26P3 PADI6 PROX1-AS1 TMEM30A DENND4C CD46P1 HEY2 MORC4 ETV5 PITPNM3 IARS2 CCDC150 RGS10 CHERP VWA5B2 CDC42BPB CYB561D1 RNMTL1 CFL1P7 CAPN10 CROCCP4 KCTD16 YAP1 PSMD7 CACNA1G-AS1 SIK3 FAM106CP BCL3 TCEA3 SEP15 IPO9-AS1 C3orf35 CFHR3 BMPR1B PDE8B ABLIM3 KRT8P50 NUDT5 SMARCA2 SNORA35 UBP1 XYLB P4HB TLL1 ZNF282 NAA25 RSBN1L-AS1 VIPR1 ADAM20 GPR89A EIF4A2P2 TMEM199 ZNF30 ACTA2 TRPC4AP FKBP9 C17orf61-PLSCR3 FBXO18 C1orf186 SNRNP40 SNORD116-29 CEPT1 GPA33 UBR7 EBLN2 KBTBD12 RNA5SP424 GATAD2B ZNF398 MED28P1 XIRP2 ANXA4 ALKBH3 SEC14L1P1 RPL21P122 DCP1B TTC9 MLPH APBB1IP ONECUT2 CKS1BP7 HAO2-IT1 MYH7B LINC01104 GLI4 JPH3 OGDHL GPR39 CLDN18 APOL3 FAM180A GALNT16 POC1B SMARCAL1 ANO6 KRT8P13 PRMT7 PHKA1-AS1 MAD2L2 ATP8B1 HIPK1 KRT18P46 MAL2 SIN3B TPPP2 MLTK C9orf47 TRIM8 KAT6B SMYD1 FBXO10 ZNF383 PDGFC PEPD LMBR1 C8orf44 DGKZP1 FAM49B ZNF197-AS1 RPL5P14 GALNTL5 NPHP3-AS1 VPS13B FLRT1 HDAC6 FAM106B SLC2A11 LINC01004 IL11RA RPS6KB1 CDHR2
4C	3148	*USP17L28 CASC20 DPY30 MIR4677 CMTR2 ZHX3 GNL3 APBB2 IFNA20P GLT1D1 FRG2 LCORL MIR4461 ALDOAP2 BSN-AS2 FGFR1OP2P1 DLEU7 HS3ST3A1 RDH10 CPXM2 RTN1 KLHL14 DTNBP1 RGN MIR3156-3 C4orf22 BCRP6 ERG GDA PDE1C MREG AK7 INIP MAP3K3 NINL MIR489 RNF185 NR4A2 PIR IGSF3 RRP7A KIAA1199 FIBCD1 SORL1 SNORA46 SLC22A2 LINC00698 ACTG1P4 CNTN4-AS2 ATXN10 POTED DHRS11 ACTR8 CCDC34 TAOK3 KIAA1210 ITGA3 ZNF510 GADL1 EFNB1 PRKCB ACSM3 C5AR1 ZNF623*

		A2M HIGD1B TRPM6 RN7SL872P GTPBP1 DGUOK C1QTNF3 MANEA RNU6-749P ARHGEF26-AS1 OR8J3 PYY KCNC1 STARD5 GPR55 GBP7 DNAJC15 TLK1P1 SIAH3 LINC01121 C2CD3 CD38 ZNF257 LINC00159 RPL26P9 GPR78 GSTA2 GRAMD2 ANKRD20A3 CORO2A ARHGAP42P4 MIR4499 C12orf40 AP4B1-AS1 CDK8 HDAC7 ABCD1P3 PIWIL3 CHST15 CDK1 NLGN1-AS1 N4BP2L2 ZNF232 OPHN1 RPS15AP6 BANF2 FAM90A6P FAM182B KIAA0100 FNDC1 MYH6 CLUHP4 LCMT1 LINC00924 SSXP1 SAA2-SAA4 SLC24A5 BCL2 MIR654 SAMSN1 LINC01047 OR7E25P KIAA1324 OR4M2 C4orf29 WDFY2 THADA COL18A1 PXDN GXYLT1P1 PRAMEF7 FAXC TOMM34 COG1 PWP2 FGF7P2 DNAL4 GSTM4 ANHX ZSCAN5C RCSD1 SLC13A4 EXOC2 RAG1 OR10J6P HPRTP4 HERC2P8 PAQR8 MIR4768 RNU6-966P SNX25 IGSF11 CDK17 GNRHR UNC13B SUZ12P DPYD SARS GAGE2B LAMB4 RN7SL318P PRDX4 LRRTM3 IL6R FUT4 OR4A42P DBH RPLP0P7 OR5G1P TMEM11 RPL7P55 KRT222 UBE2L6 PCCB IPO13 LTBR TDRKH GNB4 AGTPBP1 PRR5 SPRR2B SMTNL2 HNRNPA1P74 SALL4P7 AHI1 IGHVIII-82 BDH2P1 ACTR5 SCN11A HEATR5B FHL1 ATP10A FAM225B NMNAT2 UBE2N SORBS2 IL23R SKAP2 PIGL GDI2P2 HS1BP3 C1orf112 GOT2 GRIK1-AS1 PLSCR4 PPARGC1A MSNP1 GGN KCNK10 PCAT1 CASP5 ETFA RNF182 AHRR BRD4 CYP3A5 PCDHGB4 GTSF1 LINC00313 GPX1P2 GPR158 VRK2 ZNF568 TMEM241 APBA2 TTC3 DUX4L3 EP400NL ELMO1-AS1 STOML3 PLK1S1 C3orf22 ZNF962P CMA1 MIR3118-2 TBC1D3B PREX2 PI15 SNORD11 ELAVL2 RPS20P22 FAM9B TNFAIP8L3 KIF4A WDR16 SNCAIP CSMD3 KLHL28 DLGAP1-AS4 DUX4L15 OR11L1 DNAJB4 NTN4 PECR PCDHGA10 GTF2IP1 EIF2S1 EDDM3B POLR1A LGR5 TMPRSS4 LINC00348 TSHZ1 TM9SF4 BICD1 LRRC16B RAB5A MIR17HG SMCO2 JRK H2AFZP1 FAM131C SNX18P9 RNA5SP470 KRTAP26-1 OTUD7A TPTE2 EMCN-IT2 MIR491 OR8K3 SMAD1-AS2 LPPR4 RPL39P36 PPFIBP1 USP24P1 FAM49A BCL7A FGF12 PCDHGA5 IL20RA ELF1 MIR4439 IGLL5 OR9I3P CASC9 SAFB2 SLC9A2 SULF2 SOST OR52T1P NFAT5 RRAGC TAF1D RN7SL345P IGHVIII-47-1 STRN3 FLI1 PGBD4P7 PLCB1-IT1 GPRIN2 ANKRD40 CCT6B KRT74 FAM90A19P ZFHX2 FBXL5 RNU6-1241P NR2F2-AS1 SNX18P25 ZNF677 MAP2K4 CSRP2BP EPB41L3 CD72 KAT5 MIR361 SRRD MAS1 OR2L13 LAMA1 CDH11 GIMAP6 RPS7P5 ZBTB41 CDK2AP2P1 CDS2 ITGA2 SHANK2-AS1 MME FAM213A GRIN2A RNU1-142P SNORD116-27 MBL1P DTWD2 MALL IL10RB NELL2 MAPK8IP2 VWA3A ALPK1 ARID1B DEFT1P2 FLJ00273 IGHVIII-2-1 C21orf2 WDPCP NIPA2 RNU6-554P KLK9 RN7SKP80 RN7SKP16 KIF9-AS1 VPS37B PCDHGA4 FOXO1 PCDH9-AS2 IGLV5-37 PANK3 GBP4 FAM211A WDR82 AMPD3 PTCSC3 SFXN3 SNORA71 CHDC2 GCLC TUBAP SNORD113 MTCO3P2 RERGL IL18RAP TBPL2 RNU6-164P TRIM16 CDC40 MIR539 PQLC3 ACOX3 GOLGA8J XKR3 CR1 LINC00161 NUP210P1 SNORD116-17 DAAM2 ARHGAP8 ZNF571-AS1 PLXNA4 PTGER4P2 SLC22A5 OR2T4 RN7SL678P KIF23 GRAMD1C ANAPC1P1 ZDHHC17 IGHVIII-26-1 STK24 CNBD1 VPS26B PARVG RNU1-106P SEPT7P4 FEM1AP4 RNU6-438P PDCD6 AMPH CCSER2 BRI3BP BACH1-AS1 NUTF2 COX16 KRTAP10-5 BMF CD177P1 RNU6-1021P KLF3P1 IGHV4-28 EIF4E LINC01162 ATP5A1 SPRR2C BPIFB1 HTR4 PPP1R26P3 RNU6-890P C14orf183 LINC00320 C12orf55 RNU6-1003P KRT72 OGN GIPC2 FMR1 PA2G4P3 C5orf64 RN7SL542P FAM184B ERCC4 FAM83B OR4K15 RNASEH2C NCOR1P1 C9orf153 IRAK2 COMMD1 RNU6-258P ANXA10 MIR765 WDR33 RN7SKP233 PCMTD1P2 RN7SL92P MTCO1P3 SLC30A8 RNF126 DPM1 RXFP4 C5orf51 C19orf18 OCLN RN7SL179P KRT4 MEP1AP1 IGKC AK8 NLK MDM2 LINC00630 BCRP7 TDRD3 MIR544A OVCH1-AS1 NDST3 ABCA6 RN7SL686P POTEC CDHR4 OR5L2 ANKRD30BP2 ZNF285 FARP2 NCAM2 DPY19L2P1 CYSLTR1 PAPD7 MIR154 NR4A1 TMEM175 TIGD4 C14orf37 GLT8D1 CLDN14 RGS17P1 SKIV2L2 SCARNA15 CARM1P1 LINC00273 SERBP1P6 SMARCA4 ARMC9 HERC2P3 MCOLN3 LRP8 RNF152 IGHV1-69 NFE2L3 MINPP1 MEOX2 TTC39B KCNG3 ENPEP PSMD6 TMC5 DUX4L12 KIAA1257 LINC00365 SKI HIGD1AP13 ZNF544 LRRC70 TMEM50B KRTAP13-5P SNORA1 TNFSF8 PDE5A FBLN2 TEKT4 OR5G5P BCKDHA RPS2P48 RNA5SP265 SCLT1 TMEM52B RNU6-141P EIF4ENIF1 UBE2Q2P10 ZNF433 RPL7L1P12 SRGAP1 ABCA11P SLC16A4 PWAR6 CALCR DUSP27 MC2R TBX3 OR8K4P NANOGNBP2 MIR620 ELMO1 MIR4753 RNU6-837P SLC24A4 ABCD1P4 DOCK9-AS2 FBXO47 OIT3 ZBBX SLC9B1P4 LRMP HTRA1 RAD1P2 ZNF585A IFT88 SIGLEC30P CHST12 ARHGAP5 DDX21 ANKRD20A9P KIAA1731 P2RX1 ILDR1 GAS2 DGCR14 TNIP1 PAN3 FSIP2 TOX2 ZBED4 ZNF720 MT1HL1 MTPN ZER1 CLUHP5 FCRL5 MRPS31P4 RNU1-60P FAM9A ZNF474 OGFOD1 OR52X1P QRSL1P3 FAM182A TSPEAR TBC1D22A FILIP1L ZNF559-ZNF177 DET1 FECH SLC7A7 TACR3 LINC01020 VCAN MPTX1 ACTBP8 POTEB2 DNAH8 IGLC2 SLC1A3 MIR3173 RGS12 PCDHGA11 C14orf119 DNAJA1P1 MT1F FGF12-AS1

		RNA5SP438 ASNSP5 LRCH1 RAPGEF5 FAM124A TCL1B FGF13 CDCA2 IGHVIV-44-1 PDIA6 PTGFRN RNU7-119P PRKG1-AS1 NBEAP1 FTCDNL1 RNU6-631P SPIN3 HCN4 OR1I1P CD8B MRPS21 ZNF830 ZNF567 B3GNTL1 ERMN MIR605 IGKV1D-12 SIM2 C12orf36 GABRA5 TRIM48 KIRREL RNA5-8SP3 DOK5 TLK2P1 RNU4-56P TMEM50A STAMBPL1 SH3BP5-AS1 CRYL1 AGMO NOVA1 IGHV3-64 PER1 ANKRD20A4 SMARCAD1 MIR3118-3 RN7SL736P RNMT ZIM3 MYH4 MIR1324 AIFM3 RNU6-449P PCDHGB3 NF1P8 RN7SL714P SLC25A1P5 TMEM135 ARHGAP35 DUX4L2 PRDX2P3 WARS2 TBX22 C16orf3 RNA5SP385 POLD1 MBOAT7 AIG1 ERICH1 IGLJ2 POMC TRDV1 RPL21P41 NLRP12 TLE4 CNNM3 TLK2 ABCC13 SNX18P8 MIR759 GAK TREM1 N4BP2L1 LINC00907 IGHV1OR15-9 MYLK3 CCP110 LRRC37A17P ODF3B CACNG6 RNU2-33P MCM3AP-AS1 ZNF804A WDR83 MIR1254-2 DOCK7 N6AMT2 ABCA5 WRB OR4N3P ZNF734P C15orf54 OR10AK1P PTGER4P3 PCOLCE2 POTEB CDC20B IGLVI-68 LINC01143 LINC00347 SLC26A7 ZNF57 RAPGEF4-AS1 RNASEH2B-AS1 FAM3D OMG ZNF486 RN7SL83P CSNK2A1 BRAFP1 CWF19L2 NRG3-AS1 VN2R17P EXOSC3P1 MRPS18B IGKV1-5 USP17L27 JRKL AKT3 CRB1 ANKRD34C ZNF697 PRED60 RBAK LINC00472 TYMP DIO3OS GULP1 IFNWP4 GPR75 KCNE4 PRAMEF8 ARHGAP11B PCDHGA3 CYP4B1 MIR4529 MTND2P25 ZNF280D GAGE2C FAM230B RHBDD1 OR8K2P IGHV3-76 ALPK2 PSD3 HDLBP MLLT4 HECW1 RNF6P1 MAST4-AS1 CBFA2T3 RAP1GDS1 COG7 IGHVII-31-1 KLF3 DPH1 LCE1F BAIAP2L1 SLC6A6P1 USP29 LINC01013 UGCG MIR3936 STT3A GPHN SLC6A2 SNORA56 ORC3 VWFP1 DMRT1 CHST8 SLC9B1P3 ELP4 RYK IER2 LINC00839 TGFB2 HSPB8 PXT1 TAF4B ATP6V0D1 RLBP1 LZTR1 RAP1A ZNF385D ZNF85 C8A TEX36 SLC15A5 IGHV3-72 IGLVIV-64 REXO1L10P KCNE1 ITLN1 KLF8P1 FAM20A ZSCAN5A LINC00645 SOX4 UGT3A1 IFI27L1 TSPAN3 KRT223P C18orf8 TPP2 NPM1P25 CHSY1 PSG8 RGS16 EXT2 URB1 C2orf91 TCEB3CL ZSCAN30 OR4C46 RTL1 ABL2 TUBAL3 RNU6-58P MECP2 PSMA1 CSN3 TIMP3 ARPP21 MIR4742 RPL23AP12 ABCG1 RGS3 SLC39A12-AS1 ZNF299P RARRES2P6 FAM207CP DUXAP8 PLN DUX4L8 PRDM9 FBXO27 EML1 C14orf182 NAPG MRVI1 PABPC1P12 CCDC39 C1orf101 USP17L6P RERG SLC16A7 FAM192A RNU6-196P MDH1 ZRANB3 MRPL19 NEK4 DCBLD2 TMEM185AP1 SPATA17 THYN1 KATNAL1 CTIF MYO6 OR9G2P AKIRIN2 VCAN-AS1 KIFAP3 MIR127 C1orf63 BTBD17 CSTF3 SPRN POU2AF1 CEACAM22P FAM105B SAMD13 RNF17 DHRS7C PCDHGA7 TRIM72 INSC PRAMEF26 SLC40A1 SYNJ2BP-COX16 ARF4 SNORD115-20 MIR3667 LMF1 TMPRSS11E RNA5SP453 USP16 SNORA40 IGHV4OR15-8 TRAF3 GALNT5 RIPPLY3 COMMD7 PLXNB1 C9orf171 CNGB3 SLC25A15P2 MIRLET7C STAT4 DPYD-AS1 ZFAND4 CYCSP51 OR4C16 PDXK MTND1P12 CDC27P1 LRRC4 ETS2 IGFBP7 GOLGA6D UFD1L CTBP2P4 ITGA1 OVGP1 LINC01136 FAM27B ZNF721 VN1R7P KTN1-AS1 MERTK RN7SL801P SNORD115-36 CORO2B CST1 USP17L30 POC5 RN7SL864P RPL37P4 CCL7 ITGA8 IGKV1-12 RBMS2 TMPRSS6 SERPINA1 LINC00376 TAPBP RN7SL77P ZNF765 IGHV1-14 GPR64 FEM1AP1 ALDH1A2 TAC4 GABRG1 MYO16 MAPK9 ALG12 RPL30P13 CHIAP3 MIR548U ZNF540 LOH12CR1 ISPD CD58 GRHL2 RNU6-1178P IGHVII-30-1 SELE IGHV1OR21-1 BPIFC PSMC6 FAR2 MAGT1 KLF6 ITGB7 CHKB ARHGAP42P5 RNU6-1171P CNN2P12 ZNF595 CDH17 NLRP13 SNORD69 MIR663A ABCA8 PDE9A SLCO1B7 KCNQ1OT1 MAPK14 DYNLL2 RNU2-53P FBXW12 RPL29P30 RNF216P1 ABTB2 MOCS3 MIR4477A GPR126 PPHLN1 CDH18 RNU6-185P SNORD115-21 CHL1 RAPGEF1 VANGL2 AKAP10 SPOCK3 FRMD6 DGKZ UNC79 SPON1 REXO1L8P PLA2G4A RAB23 FAM8A2P ZNF516 VLDLR HSPD1P7 SLC1A2 KRTAP12-2 ZBTB16 SAMD8 REG1B CST9L HPS3 KCNK17 SNORD115 GABRB2 EDNRB MAN1C1 CFTRP1 ATP5S RCVRN ZNF518A NLRP7 CD226 IGLV3-31 STAT3 RNU6-513P CYCSP17 TPCN2 CNN2P4 DMBT1P1 ZNF578 PRR5-ARHGAP8 SNORA51 MARK2P5 RNU6-1266P MYH11 IFNGR2 PKP4 RNASE9 UOX SIPA1L1 RNU6-538P TGFBR1 GBP2 PWRN1 WDR4 TTLL2 RN7SL607P HEXB RPL34P3 EBF1 ZNF209P RPS4XP22 ABI3BP LAMTOR5-AS1 C12orf60 TAS2R1 C5orf34 IGHV1OR15-6 RUNDC3B TRIM51FP ADAMTS20 GNAS-AS1 CDH6 RNA5SP35 PCDHGB2 NDFIP2 AQR IPMK CAMP GABRA2 KRT126P TRAV8-5 DENND5B TNS1 MDM1 SMPX SSUH2 RBL2 MS4A15 CASP7 CSE1L LENG8 DUX4L9 IGHV3-30-2 AATF OR10K2 MRPL50 CHEK2P4 ACSL5 RNU6-352P C1orf87 RN7SL449P DKC1 hsa-mir-4528 HERC2P4 PCDHGA12 PSG1 RNU6-978P TPST2P1 NFIC PPIAP1 SUN2 SNRPGP9 HIAT1 SEPT7P9 LINC00158 FRMPD2 KEAP1 NPM1P31 RPL30 IGSF5 SULT4A1 SHC3 DYNC2H1 MIR1299 SRP19 SOX9-AS1 UROS TRAPPC10 KLHL25 LEMD3 RNF138 IGLVIVOR22-1 DYNLRB2 SLITRK6 STX16-NPEPL1 ABHD17AP5 CHODL TCAIM FOXD4L4 MYO7B OASL KCTD9 EHBP1 OSGIN2 ACTN2 PET112 SRSF4 GABPA POU5F1P3 CAMK2G STAG3L5P TPTE2P4 C6orf3 BRD1 KIF3B SNX7 MIR1290 GLRA3

		SPRED3 VPS8 TMEM100 PSTPIP2 LINC00861 SNORD115-12 NPM1P48 RNF165 ANP32C CASC4P1 FRG2C ZNF831 ZNF429 CUBNP1 HDHD1 CTBP2P5 LYPD6 DHX9 PDIA5 SUCLG1 TMEM67 KCTD20 VDR MIR4452 SLC25A1P2 KRTAP13-6P PRKCE snR65 DTD1 DUSP11 DUX4L7 OR51L1 RPS3AP46 OR4A5 FOXP4 DUX4L10 RN7SKP168 PSG9 ARHGEF11 ELOVL2-AS1 CD163 DPT RGS5 ACSS1 BBS2 LINC00111 CLSTN1 EPHA3 MUC19 MOV10 EDIL3 RPL39P40 TMEM150C TMBIM7P MIR1280 SELK MS4A1 MYH14 IGHV3-16 PILRB CC2D2A DSC3 WDR11 MIR487B RNA5SP269 SNORA22 CCR1 CACNB4 LINC00710 MYOM2 CHM MTND1P2 SDAD1P1 LINC00395 USP6 ZNF732 MIR655 KLF7 AHSA1 MGAT2 USP31 RN7SL673P KCNH8 PYGL TXK CHTF8 FPGS NUBPL OSBPL10-AS1 PRAMENP GXYLT1P2 ZNF354B IL12RB2 RIN3 REG4 USP17L29 IQCA1 UBL3 C8B OR4A12P TCEB3C RNU6-49P RREB1 PABPC1L MTND1P23 MND1 GTF2A1L PABPC1P5 MIR4519 ANKRD30BP3 HERC2P5 RNU6ATAC33P AP1G2 ATP6V1E2 EFHB ITIH6 CCDC53 KDM4C ACSM2A DCUN1D4 SLC9B1P2 CYCSP41 ATP6V1H DHX57 ACSM1 PTPN21 PLAG1 MIR1267 RNA5SP366 SNORA8 PPP6R2P1 SLC12A1 ENTPD4 NPEPL1 THAP7 MAPKAPK5P1 WDR35 CTBP2P7 ZNF841 PAMR1 SNTN RBM11 EPHA6 ABCB10 LHFPL1 RPL31P40 FBXL21 CA10 KRTAP20-3 LGI1 U6 IGLC1 IGHV7-34-1 ENTPD3-AS1 NR5A2 CYP4Z2P TATDN2P3 SPTB WDR72 SNX30 SNORD112 PGAP3 EFNB2 C21orf62 ALDH1A1 HELLS TMEM260 OR11H1 BTF3P14 ARHGAP42P3 OR9Q1 JAM2 ZNF355P CNGA4 CYCSP32 ALX4 GCFC2 CORIN ABCD1 OR4K17 IGHV4-4 SNHG17 RNA5SP125 LINC00458 LINC00221 FAM172A GLYR1 ANKRD29 COL18A1-AS1 DEFB127 PDZRN4 SGMS2 PPAP2B HIGD1AP8 S100A7L2 SNORD45 LINC00930 QTRTD1 LCE6A KIF18A C1DP1 RN7SL743P CBLN4 ADH1A CFTR OR4C15 MIR96 RNU2-27P SDF2 KMT2A RN7SL435P XBP1P1 FAM210A TCEB1P32 ZNF402P MLLT10 C2CD2 TDGF1 ERCC6 LINC00840 IGKV2OR2-2 HSPE1P25 USP17L5 RPS20P5 DPP8 CDC16 ZFP64 EMCN-IT3 DDX51 LINC00349 SLC30A5 LINC01029 IMPG1 RN7SKP99 GLB1L3 WDR5 LINC01053 CST13P FAM90A17P CSNK1G3 MATN2 TMOD1 NHSL1 SNORD115-41 IGHVIII-13-1 COBL CTNNAL1 ZNF607 HTR4-IT1 MCM3AP RNVU1-18 ZNF98 SLC2A3P1 PLCD3 MIR4307 APCDD1L-AS1 CHD5 ME3 SETD7 HAUS8P1 ITGA9 CYP4A22-AS1 RN7SKP96 PARP4P2 RNU6-286P ZMIZ1 DAP RNU6ATAC5P GBA3 RN7SKP238 IGKV2D-10 COL6A3 OVCH1 GPR125 DNMBP-AS1 LYSMD1 RN7SL766P FKSG68 WBP1LP1 MUC5AC PLB1 CLEC4A CKAP5 AIM2 RNU6-724P LINC00911 CARF GTF3C6 GP5 SNX24 OR11A1 SAE1 FRMD6-AS2 SLC25A15P5 ENPP7P10 PTPLAD2 SAMD9 SV2C HNRNPKP3 CACNA2D4 CTSH RGPD1 C14orf144 RCC2P8 MFNG PLEKHM2 ANKRD62P1-PARP4P3 STX16 RNU1-33P LINC00702 CDC5L EFCAB1 CASQ2 LRRC37A7P TAS2R38 MIR431 NF1P3 TTN-AS1 AHNAK2 ACTR3BP3 MRPS31P2 ST7 HS3ST4 TM4SF19 MFSD9 RNU6-1193P ADAMTS5 MTUS2-AS2 KRBOX4 AOAH FBXO32 PCDHGA6 NXPH1 AR DDX3X PHF2P2 RASA3 IGLV4-69 CDKN3 ARHGEF10 RNU6-410P SERPINB12 E2F3 FRG2B TNFAIP8L2 NUDCD1 FGD6 MIR603 LINC00687 SSX5 PPP4R4 DAPK1 DAPL1 RMI2 DAAM1 MROH1 NPC1 PCDHGB1 ZNF600 RPL12L3 SMARCE1 STK17B SUSD1 VRK1 ZNF678 MLLT10P1 CXorf30 MBOAT2 CLIC6 ACSM2B SMEK2 ATMIN CENPM ZNF420 CDK2AP2P2 RBL1 BNIP3P2 C6 GTF2IP2 MTND2P2 KRT23 SERHL FAM21C MAMLD1 SLC25A53 FBLN5 RNU7-174P HAPLN1 MIR3152 CCDC88A RAB7A MIR551B CHKB-CPT1B C5orf17 WDR63 SPEF2 NF1P6 MIR3118-1 CXorf22 RN7SL247P H2AFY ANKRD20A7P SNORD115-11 CEP250 CEACAMP6 TFIP11 GSKIP KRTAP4-12 GRAP2 WASF1 HNRNPA1P71 ANKRD36B U2SURP RNU6-1280P ITPK1 G3BP1 NUP155 FAM214A NT5DC1 DNAJA1P5 RPL7L1P11 CUBNP3 FRAS1 SNORD103B SIDT1 TECTA SNORD19B GNAL ARL2BPP8 TPRG1L BTN2A1 STK32C CACNA2D1 DHX35 LINC00520 ADCK1 G2E3 RPS15AP34 CCDC88B TMCC3 RN7SL143P NFU1P1 PPP1R13B HCN1 TCL6 SAMSN1-AS1 PAPOLA TOP3A FGFR1 CHRM5 CERK NSMCE2 ZNF208 TET3 CASP6 MAGEB3 NOC4L MSANTD2P1 LINC00670 CXADR PTPN20B ATG2B FAM209A DAD1 GGT2 ENTPD3 NASP PIK3R5 PALLD TRAV24 PARP16 DUX4L18 A1CF ASB13 USP32P2 hsa-mir-490 TMCO3 IPO7P2 RHOC CHRNA5 RNU6-1291P FAM63B TSPAN13 SPIRE2 DUX4 FTH1P27 MIR3118-6 BCLAF1 NKD1 SCUBE3 AKR1CL1 C1orf174 MTRR ZNF345 RIPK4 MYL12A CCDC57 C10orf120 TRUB2 LINC00656 RPS15AP3 RASGRF1 CASC8 TRIM41 PIN1 RIN2 SLC38A10 MIR99A SNORD67 RNU6-1132P RNF217 GPR75-ASB3 FAM90A9P LINC00323 DLX6-AS1 SORT1 LLGL2 USP7 MEIS2 SERPING1 STARD13 KL SCP2 USP17L25 ASUN FAM211A-AS1 ALCAM MZT1 INTS4L1 OBP2A TBC1D3P5 AVEN HLA-DRA GUCY2C OR10T1P ZNF331 SLC17A3 MICU1 RAD18 EBPL CNN2P7 ZNF114P1 SLAMF1 VN1R33P DUX4L6 MIR3198-1 UBTFL8 CCDC181 BACE2 MIR548AX KIAA0355 INO80D PCDHGA1 ZNF728 SLC38A4 DYNC1H1 LSM12 PTGES RNU6-316P SLC35A5 MTND2P28 MEG8 MIR1281

		DIDO1 SNORD114-17 CCDC18 FGF14-AS1 KRTAP29-1 AGGF1 DUX4L11 MYH13 WDR12 C1orf141 NR2C2 MYH1 MGST1 FRYL ZNF432 SLC16A14P1 RARRES2P4 STON1 TIAM1 AGGF1P3 PCA3 MMP20 FAM126B KRT43P MIR4533 DDX24 FAM90A10P ATP1A1 LINC01058 MIR487A PGM2L1 OR4C9P LILRP2 SLC39A12 GNAO1 RNU4-59P RPS6KA6 IL1RL2 SNN BCL2L15 LIPI OR4H6P ASAP2 PSMD6-AS2 MAP3K7CL FMN1 ANKRD20A1 DNM1P51 SLC16A1-AS1 MIR485 TMEM212 CHAF1A ANKRD62P1 TRAV6 ZFPM2 RRP7B SEC63 TDRD10 GC PIEZO2 TOR1AIP1 FAM66A ADC SVEP1 TNF LINC00113 USP17L24 PHYKPL PDSS1 LMCD1-AS1 GLDN EDAR EGF NHLH2 LINC00960 CYP1D1P OR7E89P HIATL2 PDGFD OR10V3P FAM3B LRRC42 RIMBP2 TTC12 PRMT8 SELP RNF185-AS1 KIT MEMO1 GAB4 RNU6-617P OR4A9P MAP7 OSBPL3 PPOX ZNF439 SENP8 MIR1911 MIR3687 OR8A2P ZNF177 AGGF1P2 MIP IGKV2-36 GRIA1 POM121L9P ZNF627 MTUS2-AS1 FAM81B LINC00229 SPIDR ARR3 RNU6-55P UBA6-AS1 ANKHD1-EIF4EBP3 SNORD115-23 MIR183 TERF1P5 STK3 ZNF781 ANO10 CNOT7 ESRP2 FSHR POTEKP NBEAP3 LCE2B GAD2 BMP15 C1GALT1 RUNX1T1 SGK2 PIK3C2G SNORD115-40 PSIP1 NOL11 TRPM2 SPRR4 USP18 SMCHD1 LINC00507 MED15P7 S100B NET1 SLCO1B1 DPY19L3 CILP2 ICT1 EDA2R NAP1L6 MIR889 DMXL1 SIK2 KCNAB1 MXRA5 TRPC4 MTND1P31 LRIG1 PRKACB ZNF705A PDE3A TEX14 RRH TSPY26P DRP2 LINC00387 RNU7-176P PDZD7 POR FHL5 KCNMB3P1 GLULP5 DHRS7B TRIM51 RUFY1 LINC00382 TMPRSS4-AS1 IGHV3-41 ATP6V1E1 RPS3AP41 ZNF965P SNORD115-38 ART4 PIGK SLC25A15P4 DCAF8L1 RNPC3 FIGN PI3 OR4C14P ARID2 BROX MARK2P8 GNA13 FAM228B LINC01043 KRTAP19-10P CNKSR2 TRMT11 HMGB3P20 RPS23P5 COL8A1 ISCA1P3 ATXN3L SSBP2 BMS1P18 FAM85B GABRA1 REEP1 FAM90A22P CHMP4C MIR548F1 NR2F2 CTTNBP2 PSG7 IGHVIII-67-3 BSG PARP8 RNU6-725P EFEMP1 DTX4 PIGB RN7SL321P AJAP1 IGSF21 SLC9B1P1 KIAA1109 USP40 IGLVI-38 RASSF8-AS1 MIPEP DUSP23 HRNR IGKJ4 ABCC9 SNAP29 BPIFB4 MRPL42P4 GSG1L HERC1 NR3C2 SERPINA2P MIR134 RNU6-1233P PRSS3P2 SHC2 NCOA5 EMCN RNA5SP478 BLNK DIAPH3 LINC00704 OR8U1 TPTE2P1 PTTG1IP ADARB1 GGT1 VPS35 LINC01134 GPC4 HAS3 SOD1P3 LRRC63 PARP15 UBASH3B RPL18P13 TTC40 RASA3-IT1 PAGE1 TRPM7 SERPINB8 FLT1 PPP2R4 REXO1L9P CDRT1 RN7SKP6 LINC00032 VSTM2B NOL4 LINC00536 GTF2IP3 NXN SNORD103A AAR2 ACTR3B NEK5 ASL C1orf173 PM20D1 KLHDC7A CDC14B CMSS1 PHC1 SLC22A23 PLCH1 NAMPT PCNXL4 PCDHGB7 ABCA10 ZNF493 C14orf39 GLRA1 MIR376A1 STK36 RNU4-24P ZNF717 CTNNBIP1 TEP1 NF1P4 YAF2 WDR95P KIDINS220 SMPD4P2 TBCE CT49 FNIP2 G3BP2 HNRNPA2B1 GADD45A MIR214 MGC4294 UGGT2 STIM2 POTEF SNORD23 RNU6-1327P NF1P1 PLAT FAM201B SNORD54 RNA5SP492 C10ORF68 HS1BP3-IT1 PTPRF SEC24B DNM3OS RNU6-618P PPFIA2 MYEOV ADAM28 IGSF11-AS1 ZNF268 MYLK RN7SKP60 PRIMA1 SH3BP5 MTMR4 MIR648 ANKRD36C NKAIN1P1 TTC39A KRTAP5-8 LYVE1 RNU6-1005P PTPN20A KRTAP20-1 SMAD1 SLC22A15 LST3 LINC00523 ATP1A4 RPL21P6 ANKHD1 VPS37A C2orf43 LINC00571 TTC8 BRD7 FCF1P10 SNORD6 MARK2 OR52N5 ZNF501 TSPEAR-AS1 HMG20A ARL6IP5 TUSC3 TCEB3CL2 ARFIP1 RNU4-60P PAPPA-AS1 PLSCR1 CD163L1 RNA5SP79 ZNF72P TRPV4 ZNF880 C1orf167 EEF1A1P1 ATP5J HDAC8 OR51AB1P SRP54 STARD4 NLRP11 DNAAF2 ADORA3 LINC00563 SH2D7 HYAL4 SP2 SMPD3 PDE7A CYTIP SPTA1 ZNF483 ZNF573 TBX18 PCDHGA8 NTF3 DPP9 RGL2 SNRK NID2 DHRS4L2 TTC29 SUZ12 PDE6B SPA17 ZNF729 LHFP TSPY5P PJA1 IGLVIV-65 FAM76A RNU6-721P DKFZP761J1410 ZFP2 BZW2 ANKRD20A17P IGHVII-40-1 KRTAP6-1 TP53I11 CACNA1C-IT1 KRTAP9-2 VWA8P1 RBFOX2 ZNF507 OR4C2P SNORD116-18 ZNF26 MICU2 PHF8 TFPI USP17L9P ISX MIR323B TDRD6 RAD51AP1 CA3 PQLC1 CYB5R2 GOSR2 DSCR9 ANKRD20A11P LPPR1 LCP2 RMRPP4 ISOC1 SEZ6L RARRES2P9 SLC39A8 PET117 IL4R TMEM123 MIR653 SCO2 C13orf35 TM4SF1 IGHV3-29 CT64 MX1 PAXBP1 PLGRKT RNU6-713P KIAA1211 RPS27L TGFBR3 MCUR1 UMOD SNORA31 ZNF615 GTF2I C16orf80 SLC7A1 ACTR3BP5 ADAM17 USP36 GABRG2 CHRNA3 TMEM186 NUDCD3 CPS1 EGFLAM-AS1 SLC35F2 BNIP3L LCP1 PRKCH PCDHGB6 RFPL4AP7 OR7D1P WBP11P1 OR6N1 KRT25 KATNAL2 C9orf40 PNPLA7 ASXL1 GNG5P5 KRTAP15-1 ELTD1 LCE2C KMO RNU7-114P NUDT4 SNORA70C RNU1-104P PCTP UBE2U NXPE2P1 IGHV7-56 SPRR2G C14orf64 NSG1 RBAK-RBAKDN ANKRD20A8P GABBR2 BCRP1 NSUN7 PIP5K1B IGF2R C21orf90 SIAE RPL3P9 UBE3A TGFB1I1 SNRNP200 TRBV7-5 TESK2 SHOX2 NPY4R MIR381 KLF13 RN7SKP100 NMNAT1P4 MARK2P9 KIRREL3-AS1 POLD3 RHEBP3 ZNF91 SNORD115-10 MORF4L1 POU2F1 APC PMP22 LOXHD1 FAM108A8P ELK3 OLFM3 GPR139 P2RX7 COX5BP6 ADNP2 OR5AK4P GAPDHP67 ZBTB25 INO80 FAM90A21P MARCH11 AMOT MOCS2 LINC00317 CCDC7 HGFAC SNORA57

		LEPREL1 TFF1 FSD1L PPARGC1B STON1-GTF2A1L KDSR BMS1P17 SCAF4 LACTB SYT16 ENPP7P6 MTND5P14 RALBP1 IGHV3-47 RNU6-16P ZCWPW2 HNRNPA1L2 ADARB2 HGF MIR376C C8orf87 REEP2 FAM220BP ACSL4 RNA5SP303 CAPZB IGHV4-31 OR10N1P ZNF850 ST20-MTHFS COMT PDLIM5 XYLT1 ZNF440 ROS1 SNX19P1 KRT75 SLC6A17 RNU1-39P RNA5SP405 ZBED5 TMEM63A MAGEB2 DUX4L16 CKAP4 SLFN12L RNU1-51P L3MBTL1 SNORA16 PGK1 RNASEH1 ERVK3-1 RLF IGLV1-36 MIR656 RPL10P3 NBAS MTND1P17 SPIB MYO1B HPN NBN PANX1 FGL2 ADAMTS18 EHF FRMD3 ZNF189 KLRF2 SLC28A3 RPS15AP1 RNU6-1320P RASSF3 CYCSP6 KRTAP9-3 ACOT1 SNORD116-19 LGR6 PKP1 ZBTB34 RNU6-954P MIR181A1HG TRIM23 SDCCAG8 SYNPO2 EXOC5 MIR381HG NGF OR4Q1P SLCO1B3 CYLC2 FLVCR1 PPM1J NRP1 UBE2E3 OOSP1P2 BCAN AKAP7 TGM2 MIR4535 RPL21P1 ZNF202 ACYP2 MARCH10 GRK6 FAM46B LINC00492 MIR382 PHACTR3 C7orf69 ZNF277 RNA5-8SP7 ADAM5 7SK ANKRD26P1 ZNF879 RPGRIP1 SNX18P4 RNU6-1066P DEFB116 KRTAP4-3 SLC2A13 KRT39 ZNF397 MIR1276 SYT2 LINC00623 LINC00442 RSU1P1 DPP4 DICER1 TEKT4P2 ZNF141 SLC26A8 RNY4P23 CHODL-AS1 AQP10 GGT8P C5orf38 THOC2 TAF1B LINC00898 COL15A1 EVC2 TAF7L GREB1 CREG2 FAM27E3 NUP62CL REEP3 TCAM1P MGST3 MIR320B2 EIF4EBP3 SEMA3E C15orf43 MIR433 DNAH6 DSCR4-IT1 IGHVII-44-2 MRPS11 MAEL NUP153 RNU6-127P RNU6-469P TMEM233 RNA5SP465 RNF180 ZNF665 DUX4L5 NDST4 MIR543 KATNA1 FAM90A23P MRVI1-AS1 MIR556 IFNG-AS1 ANKRD30BP1 CCDC122 TUBA3C FAM216A KIF16B SWT1 PAXIP1-AS2 PTCD2P1 IGLJ1 CDH2 ARF1 FLNC PLCH2 LRRC69 TMEM261 PPIAP22 JAG1 CNN3 LINC01065 PAPPA SERPINB7 EVI5 FAM27A IGHVII-65-1 SYCP1 BMS1P13 RNA5SP61 LINC00879 COX6CP10 AKR1C2 ZNF407 GPALPP1 MIR3118-4 RN7SL373P ASB3 HSPA4L ZNF527 RPL21P11 NUPL1 FAM208B HDAC9 OR7A5 OR5J7P LRRC3-AS1 ELOVL7 CHST13 ANKRD20A18P GOLGA1 LINC00857 VDAC2P1 SEPT7P5 GUCY1B3 SNORD114-19 NR3C1 PPIAP6 MIR300 MAP2K6 FAM108A10P JRKL-AS1 ACTR3BP6 ARHGAP28 MTOR LINC00658 C17orf75 ZNF585B THTPA OR4C5 CYP3A54P HNRNPA1P40 KSR1 RALGAPA2 CBLB RORB KIAA1407 CFL1P6 SNAP25 FBLN1 MTFMT TPTEP1 REG1P JAKMIP1 MIR5582 IGHV1-46 PCMTD1P1 RIMS3 KRT76 RTFDC1 MIR4290 MIR410 CDC42BPG IGHV1-18 ADRA1B LTB LINC01101 OR4A41P NCAPG2 PCAT6 NEU3 ZFPM1 RNU7-145P GDAP1 RPL8P2 RNA5SP222 XKR7 TPTE2P5 DISP1 PRB2 MYCT1 CPT1B KYNU MS4A5 DCST2 GPN3 STK32A CD2AP ZFP30 TNMD SPATA6 CLVS2 CYP7B1 FAM212B TBXAS1 OSBPL9P4 KXD1 USP25 PRKX RN7SL674P ARMC7 SULT1C2P1 SPRED1 H3F3C BMS1P9 PCK1 KBTBD11 NPR3 HBD AP5M1 MIR4743 ADAM10 REV1 GALC FAM220A CNTNAP4 PPP2R2C TTC32 RGCC IGKV1-37 CYP2E1 LRFN2 RNU6-1049P FLNB UBE2Q2P11 RNU6-157P SCAI TULP4 MIR5190 PAPPA2 OR4C12 SNORA32 RNU1-11P CYB5D2 STK33 PGM5P2 NCOR1P3 QRFP TRIM51DP ABCB5 HEATR4 PCDHGA9 JPH4 NAPEPLD MIR3648 CISD2 GCNT7 HRASLS5 SPECC1 RN7SL484P snoZ6 FCF1P9 OR4Q2 GACAT1 C20orf196 NCOR1P2 CYYR1 IL1RAP MTMR10 UBXN2B LINC00559 NEK2P2 RALGDS FAM118A HNRNPA1P58 TRIM9 DEF6 MTAPP2 WRAP73 RPH3AL TRERF1 CENPBD1P1 SNORD115-24 GLI3 LRRTM1 EIF4E2P1 FAM90A7P RNA5SP488 NSUN3 BHLHB9 ILF3 GOPC OR5E1P MIAT POLR3C SGCG DCDC2 NANP DYNC1I2 RN7SKP285 RAB31 AGGF1P4 LGALS14 ZFP82 LINC00418 CPPED1 GAB2 VPS13D ABHD17C ZNF292 FREM2 PRB3 RAPGEF4 SLC7A8 ANKRD31 MYO5BP3 S100A11 EFHC2 DOT1L LHFPL3-AS1 SNORD113-2 SMYD2 TMPRSS2 PVRL2 OR10R2 ONECUT3 ZMAT3 LIMK2 HMGA2 MIR1185-2 TRPM4 ZNF558 LINC00632 MLLT10P2 MX2 C11orf30 FAM227B LAMP5 PSPC1P2 KIAA0040 RN7SL683P KRT2 FERMT2 MIR105-1 DSG4 KATNBL1 CPNE8 PGM2 LINC00521 STX3 M1AP ZC3H13 ZNF610 TAX1BP1 H2BFM KCNK2 KDR PCDHGA2 TBC1D4 OR2M5 OGT MSL2 RNF207 APIP DPF3 HNRNPA1P68 KCNT2 CCDC36 GZMH CDK2AP2P3 OR4K13 SHCBP1 NPHP4 TNC IGHV3-32 DUX4L17 PML ADAM6 RN7SKP5 SACS TTLL12 RFC3P1 DNM1P46 RN7SL552P FAM27E2 FAM83G LINC00609 TRAPPC12 FAP SCARA5 RPS4XP15 OR52U1P LINC01141 TMSB15B ERICH2 OPTN IGKV2OR22-4 RN7SKP85 RN7SL50P PCDHGB8P RAI1 RPS20 SEPP1 CPEB2 LOXL2 LINC01146 AGGF1P1 BPTF ZNF229 RPS10P7 DYNAP RPL7AP28 MTND1P32 PIWIL4 RAP1B MRGPRG ZNF571 WIPF2 RASGRP1 CTBP2P1 SNORD116-16 DIAPH3-AS2 NLRC5 PPM1B PIP5K1P2 KRTAP9-8 FOXRED2 IGHV3-65 FGF7 SLC15A1 DGUOK-AS1 SNORD116-26 FMNL3 PPAPDC1A CYP4Z1 DMBT1 ZDHHC9 USP17L26 NLRP2 CEP97 PCDH19 JMY ZNF451 SRIP1 ARL2BPP5 MIR514A1 STX12 MRPL39 OR11H13P PLCL1 MEG9 RNU6-368P MCM9 RNU6-405P ST7-OT4 CCDC144CP UPK3A TSHZ3 ASIC2 RALA SPCS2P4 IGDCC4 RN7SKP126 PRB1 MGME1 CCDC144B EDDM3A CTXN2 GNPTAB RN7SL327P RPS26P30 OR11K2P ABCC10 RARRES2P1

		COL2A1 RPA1 PPIAP14 MIR4760 NAP1L4P1 AKR1B10P1 KHDRBS3 NECAB1 MYO5BP1 SOX8 TNFAIP8 KIAA0195 MTHFS ABAT SLC22A25 EGFL6 ZNF674 BCL2L1 LGALS9 MIR136 MIR495 CYP1B1-AS1 THBS2 TPO TAS1R1 MRPS27 TNN RNU6-466P TMEM138 TRBV6-8 C9orf131 GTDC1 RNU6-230P TRIM51CP MIR155HG MYH7 ST6GAL2 OR8K5 RNASE11 RNA5SP497 MIR4480 PAPSS1 CRNN LDLRAD3 KCNK13 MTND2P4 SERHL2 DUX4L4 TGFA DDC YME1L1P1 MIR548AL RNU6-614P MS4A4E SNORD114-18 TBX20 HNRNPA1P53 FLNB-AS1 ENPP7P1 SNORA25 POTEH-AS1 OR11G2 MTMR2 STK35 LINC00707 KCNH5 YWHAQP9 RRM1 MFSD12 SYCP2 CLYBL ACIN1 LECT2 OR4C6 MPDZ GNB5 IL16 LINC00972 UBTD2 CCDC88C B4GALT3 POU2F3 IGHV3-60 RNA5SP490 ATAT1 LINC01030 CENPV EWSR1 CTBP2 OSCP1 MYH8 SNORD113-1 ZNF525 NCALD PGM5P1 ZFYVE21 WDR41 CACNA1C-IT2 ZNF337 ADH1B MIR369 SARDH CPA5 AMY2B NCAPH2 KIAA0319 C6orf106 MIR4273 FAM227A FAM221A NCMAP SCNN1A KCNE2 UNC13C CAB39L LINC00378 QSOX1 PCP4 OR5V1 TMEM55A IGHVII-28-1 CCDC171 ACTA2-AS1 MAS1LP1 SLC5A1 SNORA80 SNRPD3 RBMX2P3 FAM114A1 SNORD19 PAX3 EYA4 DEFB122 PARP4P3 LRP4 TMTC4 L3MBTL3 NOVA1-AS1 IGKV2OR22-3 POMT2 IGHV3OR16-12 ZNF736 CLIP1 RNA5SP518 RARRES2P2 GUSBP6 PLAC4 MTND4P14 BCL11A F11R MIR432 RFX4 RPL18AP14 CEACAMP10 CACNA1C-AS2 EIF3FP1 RNU1-117P OR5B19P SNORD115-45 FREM1 SAA2 ATP6V1D ADPGK-AS1 LAMA4 RN7SL659P IGHV4-55 TMEM185A ZNF112 ATE1 RPSAP55 CEP44 MYO5BP2 PVT1 OR8L1P TERF1P1 RNU6-602P SLC2A9 CHN1 UCHL1-AS1 PRAMEF12 RNU6-249P RAG2 LCE2A VPS13C CAPN7 CCL3 RNU6-540P RNU6-458P KDM3A NPL CES1P1 SNORD115-34 LGR4 SULT1B1 ZNF845 RFPL4AP5 TRDC SLC38A7 CYP4F29P RNF128 PXK SLCO4C1 SLC25A48 CP LINC00973 FAM160A1 OLFML1 STAG3L5P-PVRIG2P-PILRB SCNM1 AMD1 LCE4A ZNF562 SPRR2E LRRC3B DNAJA1P4 KRTAP19-3 PGPEP1 CCNYL2 TUBB1 FANCB TP63 ARMCX2 ANXA11 CSF2RB NUSAP1 ANKRD23 CCDC11P1 PCNT MIR3713 IFNA8 TRMU OR4K11P USP3 LINC00940 HBG1 ADRBK2 LINC00353 ZNF622 OSBPL5 DHRS2 SNORD115-39 TRDV3 EXOC6 SNORD115-25

Table S4. GO associations with Biological Processes of 1772 rDNA-contacting genes possessing frequent DSDs. Related to Figure 1.

GO.ID	Description	padj	Genes
GO:0048856	anatomical structure development	2.8104473171044047e-29	*PTPRR,RCAN1,KALRN,MEGF11,RPS6KA2,NDE1,CTNNA1,FOXN3,NTRK2,CLASP1,ZNRF3,PTPRG,SYNE1,FBXL17,DMD,KCNC2,CHRM3,SPRED2,KAZN,ERBB4,LAMA2,TFDP2,NOS1,CNTN4,ROBO1,PRICKLE2,MYO3A,DSCAM,SHROOM3,CIT,TBCD,FRY,NEBL,WWOX,HUNK,FUT8,HDAC4,OPCML,LINGO2,IMMP2L,ATP8A2,AUTS2,WLS,ANKS1A,LDLRAD4,MYEF2,TENM2,PSMB2,RXFP1,ASXL3,NFASC,PLCB1,FLT3,SRPK2,PRKD1,RYR2,VCL,NEDD9,PPP2R3C,SEMA5A,ETV6,PRKCA,IL1RAPL1,ADAMTS6,MYO9A,PLAC1,NHS,CALD1,KANK1,KIF26B,DIAPH2,TCF12,RUNX1,PLS1,NLGN1,CAMK1D,AMOTL1,TCF7L2,RELN,APP,ADAM12,PAK1,UTRN,PPP3CA,RYR1,NTM,TOX,PCDH15,PTPN9,GREB1L,AKAP6,GRM7,DLC1,IDE,SCMH1,SOS1,CTNND2,ALK,BTRC,DCLK1,MEF2A,TFAP2D,DENND5A,MAP2K5,DNMT1,MAGI2,EDA,CELSR1,EYS,ROBO2,AKAP13,HIVEP3,DCC,EP300,SEMA4D,EVC,RPS6KA5,ANKRD6,TRAPPC9,MMP16,TF,DNAH11,QKI,CHRM1,CDH8,ARHGAP15,HUS1,ASTN2,TANC2,JAK2,NEGR1,CMKLR1,SDK2,PARK2,NPHP3,ADCYAP1R1,GRIN3A,SMOC2,CACNA1C,NLGN4X,EXOC4,EMB,ANKH,UPB1,TAGLN3,PALMD,NRG3,NUMB,SOX6,POLE,SULF1,SH3BP1,OTC,ATRNL1,SH3GL2,ARHGAP24,COL11A1,SLIT2,THEMIS,AFF3,ATRX,CHRDL1,TRAF3IP2,RAD51B,BMPR1A,DSCAML1,SMAD3,TNFRSF11B,POTEE,SCUBE1,PEAK1,EYA1,SATB2,ELN,TPH1,MAD1L1,ANKRD11,EGFLAM,TUB,ARHGEF18,OVOL2,DNAJB6,ELP3,BRINP1,OMA1,CPQ,FGF14,ASGR2,IL1RAPL2,SOX5,DNMBP,TAF1,SPNS2,SLC1A1,NTN1,LDB2,EPM2A,MAP2,CDC73,FLRT2,FBXW11,WWTR1,GAS7,PEMT,CATSPER2,PCSK5,CUX1,ANK3,CDH12,ARID4B,TRPS1,MYO18B,CDH4,TNR,ADCY9,CELF4,VAV3,SCN9A,MDM4,ARNT2,SPOCK1,ACTN4,PLCE1,TACC2,EPS8,TENM4,CECR2,GHR,CDH10,LRRC4C,PPP1R9A,PALM2,CLSTN2,CBFA2T2,SRD5A2,STRC,DISC1,LRP5,NAV2,AP2B1,ESR1,ARHGAP12,TNFRSF19,SCFD1,PDE4D,CNTN5,PIK3CD*

			,SLC5A3,BLOC1S5,CERS3,PCSK2,TEAD4,ARMC2,EFNA5,KLHL1,FTO,POU6F2,CEP85L,ATF2,RBBP8,GRID2,ZNF423,CYBB,SEMA6D,NTNG1,ADTRP,CRMP1,GABRB3,PRKCQ,ATXN1,CLDN1,SLC24A3,PLEKHA5,TTLL5,SPTBN4,EPHB2,S1PR3,PRCP,PRTG,COL22A1,DOCK2,DPYSL2,DACH1,MARK1,BDNF,RBM4,FNIP1,ADORA2A,ARID5B,IFT80,DYM,FSTL4,FHL2,TTC39C,NOX4,MEGF9,CSMD1,BCOR,BMPER,SYT1,DAB2,AFF2,PLXNA2,LRRK1,PRKG1,ABI1,CALCRL,HDAC2,ETS1,FAT3,ARID4A,CDH23,TTN,ENPP1,RIMS2,SCN8A,CTNNA2,FMNL2,PARD3,NRG1,SPINT2,FMN2,TOP1,RIT2,CD44,PAX7,CNGB1,PRKCG,TEAD1,NFIA,MAPKAPK2,SLC8A1,SRRM4,ISM1,FBXO31,MAP3K13,PLCL2,CHST11,THRB,CDC42EP3,NCAM1,AGT,FGF1,CCR3,STRIP1,NDRG2,GRIP1,USH2A,NF1,WIF1,ZNF536,PARVB,NRXN1,LAMA3,GSK3B,PKP2,CTDP1,SETD3,CD109,KCNH1,CHD7,PSMB7,MOV10L1,THSD7A,TIAM2,MYT1L,SRGAP2B,CNTN1,UNC13A,TRIOBP,NCOA1,RHOJ,PHEX,MECOM,SYBU,SHANK2,UNC5D,ISLR2,NREP,GPC3,DLG5,CFDP1,PGM5,ASTN1,PREX1,CPE,XKR4,ROR1,ADAMTSL1,ZNF675,COL12A1,MACF1,MNAT1,TRIO,CAPN3,CTNNBL1,MYO3B,MAP3K5,CLDN11,SPAG16,CCDC141,FGD4,EPHA5,HIRA,TP73,RBM19,FAM171A1,MYPN,KIAA1217,MORC3,ANK2,SLIT3,GRIN2B,MATN3,PAK3,PRRC2C,MLLT3,NEB,RNF213,PACRG,SEMA5B,GRIK1,NLN,SDK1,GRM5,IGF1R,PKD1L1,MSI2,CNTNAP2,PTPRO,SUFU,INSR,ITGA11,JAK1,CRTAC1,MAP2K1,FOXO3,GPC6,KLHL3,OCA2,CNTN6,CENPF,MITF,IGF2BP3,LRFN5,AGBL4,ULK4,RBFOX1,ADAMTS16,LRRK2,PKHD1,KIF2A,RARB,TCF4,SPG11,PBX1,PHACTR1,BMP6,ASAP1,FYN,XRCC4,EPHA7,GAS8,COL19A1,SGCZ,PTPN14,RIMS1,ENPP2,ABCA12,EPHB1,LSAMP,KCNQ1,FHOD3,DOCK1,VASH2,BPGM,FAM126A,EXT1,TRPC5,UNC5C,ACSBG1,CSGALNACT1,TENM3,WDR7,NOX5,LRP2,FER,CAMK4,VWC2,DDX10,PCDH17,SGCD,TMEM108,SHROOM4,RAPGEF2,NAV3,PRPSAP2,PTPRM,KIRREL3,NRXN3,CDKL5,PTPRU,SOBP,MACROD2,IL18R1,KAT7,DCHS2,SETD2,PACSIN2,CDH13,MDGA2,BASP1,DAB1,ZNF148,HDAC5,SEMA3A,MYH9,STIM1,NRIP1,TANC1,PIK3R3,TENM1,LARGE,GABRB1,SH3KBP1,PRKAR1A,ZRANB1,RGS7,SIPA1L3,DPY19L2,SCEL,NTRK3,FBN1,HYDIN,TBX15,CLASP2,ZNF521,NSUN2,TPD52,DACH2,DNM3,SYNDIG1,PPARA,PTPRD,RORA,MYH15,BRIP1,ELAVL4,SBF2,CDH9,NELL1,DOCK10,FNDC3A,BICC1,SEMA3D,PRLR,ATF6,SPATA5,SYT17,BLOC1S6,SEMA3C,RXFP2
GO:0009653	anatomical structure morphogenesis	8.274717567097055e-29	KALRN,MEGF11,CTNNA1,FOXN3,NTRK2,CLASP1,ZNRF3,DMD,ERBB4,LAMA2,NOS1,CNTN4,ROBO1,PRICKLE2,MYO3A,DSCAM,SHROOM3,TBCD,FRY,NEBL,WWOX,ATP8A2,AUTS2,WLS,PSMB2,RXFP1,ASXL3,NFASC,SRPK2,PRKD1,RYR2,VCL,NEDD9,SEMA5A,PRKCA,IL1RAPL1,MYO9A,CALD1,KANK1,KIF26B,RUNX1,PLS1,NLGN1,AMOTL1,RELN,APP,ADAM12,PAK1,PPP3CA,RYR1,PCDH15,GREB1L,DLC1,SOS1,CTNND2,BTRC,DCLK1,MEF2A,MAP2K5,MAGI2,EDA,CELSR1,ROBO2,AKAP13,DCC,EP300,SEMA4D,RPS6KA5,ANKRD6,MMP16,DNAH11,QKI,CDH8,ARHGAP15,ASTN2,TANC2,SDK2,PARK2,NPHP3,SMOC2,CACNA1C,EXOC4,EMB,PALMD,NRG3,NUMB,SOX6,SULF1,SH3BP1,ATRNL1,SH3GL2,ARHGAP24,COL11A1,SLIT2,AFF3,ATRX,BMPR1A,DSCAML1,SMAD3,TNFRSF11B,PEAK1,EYA1,SATB2,ELN,ANKRD11,EGFLAM,ARHGEF18,OVOL2,DNAJB6,OMA1,SOX5,DNMBP,SLC1A1,NTN1,MAP2,CDC73,FLRT2,FBXW11,WWTR1,GAS7,PCSK5,CUX1,ANK3,CDH12,MYO18B,CDH4,TNR,VAV3,MDM4,ACTN4,EPS8,TENM4,CECR2,GHR,CDH10,LRRC4C,PALM2,STRC,DISC1,LRP5,ESR1,ARHGAP12,SCFD1,PIK3CD,TEAD4,EFNA5,ATF2,GRID2,CYBB,SEMA6D,NTNG1,ADTRP,CRMP1,PRKCQ,SPTBN4,EPHB2,S1PR3,PRCP,PRTG,COL22A1,DPYSL2,BDNF,ADORA2A,ARID5B,IFT80,FSTL4,FHL2,TTC39C,NOX4,MEGF9,CSMD1,BCOR,BMPER,SYT1,DAB2,PLXNA2,ABI1,CALCRL,HDAC2,ETS1,FAT3,CDH23,TTN,RIMS2,CTNNA2,FMNL2,PARD3,NRG1,SPINT2,CD44,PAX7,ISM1,FBXO31,MAP3K13,CHST11,THRB,CDC42EP3,NCAM1,AGT,FGF1,CCR3,STRIP1,GRIP1,USH2A,NF1,PARVB,NRXN1,LAMA3,GSK3B,PKP2,CD109,KCNH1,CHD7,PSMB7,THSD7A,TIAM2,UNC13A,TRIOBP,NCOA1,RHOJ,PHEX,UNC5D,ISLR2,GPC3,DLG5,CFDP1,PGM5,PREX1,CPE,ROR1,ADAMTSL1,COL12A1,MACF1,TRIO,CAPN3,MYO3B,CCDC141,FGD4,EPHA5,HIRA,FAM171A1,MYPN,ANK2,SLIT3,PAK3,MLLT3,NEB,RNF213,SEMA5B,SDK1,IGF1R,CNTNAP2,PTPRO,SUFU,INSR,JAK1,MAP2K1,FOXO3,GPC6,KLHL3,CNTN6,IGF2BP3,ADAMTS16,LRRK2,PKHD1,RARB,SPG11,PB

			X1,PHACTR1,BMP6,FYN,EPHA7,PTPN14,RIMS1,ENPP2,EPHB1,KCNQ1,FHOD3,DOCK1,VASH2,EXT1,TRPC5,UNC5C,CSGALNACT1,TENM3,NOX5,LRP2,FER,SGCD,TMEM108,RAPGEF2,PTPRM,KIRREL3,NRXN3,CDKL5,SOBP,SETD2,PACSIN2,CDH13,BASP1,DAB1,HDAC5,SEMA3A,MYH9,STIM1,TANC1,PIK3R3,SH3KBP1,PRKAR1A,ZRANB1,SIPA1L3,FBN1,TBX15,CLASP2,TPD52,DNM3,PPARA,PTPRD,RORA,ELAVL4,CDH9,DOCK10,SEMA3D,SYT17,SEMA3C
GO:0032502	developmental process	1.6601799212830661e-28	*PTPRR,RCAN1,KALRN,MEGF11,RPS6KA2,NDE1,CTNNA1,FOXN3,NTRK2,CLASP1,ZNRF3,PTPRG,SYNE1,FBXL17,DMD,KCNC2,CHRM3,SPRED2,KAZN,ERBB4,LAMA2,TFDP2,NOS1,CNTN4,SND1,ROBO1,PRICKLE2,MYO3A,DSCAM,SHROOM3,CIT,TBCD,PUM1,FRY,GOLGA3,NEBL,WWOX,HUNK,FUT8,HDAC4,OPCML,LINGO2,IMMP2L,ATP8A2,AUTS2,WLS,ANKS1A,LDLRAD4,MYEF2,TENM2,PSMB2,RXFP1,ASXL3,NFASC,PLCB1,FLT3,SRPK2,PRKD1,RYR2,VCL,NEDD9,PPP2R3C,SEMA5A,ETV6,PRKCA,IL1RAPL1,ADAMTS6,MYO9A,CCDC3,PLAC1,DIO2,NHS,CALD1,KANK1,KIF26B,DIAPH2,TCF12,RUNX1,PLS1,NLGN1,CAMK1D,AMOTL1,TCF7L2,RELN,APP,ADAM12,PAK1,UTRN,PPP3CA,RYR1,NTM,TOX,PCDH15,PTPN9,CREM,GREB1L,AKAP6,GRM7,DLC1,IDE,SCMH1,SOS1,CTNND2,LRRC8C,ALK,BTRC,DCLK1,MEF2A,TFAP2D,DENND5A,MAP2K5,DNMT1,MAGI2,EDA,CELSR1,EYS,ROBO2,IFT81,AKAP13,HIVEP3,DCC,EP300,SEMA4D,EVC,RPS6KA5,GRK5,ANKRD6,TRAPPC9,MMP16,NME8,TF,DNAH11,QKI,CHRM1,CDH8,ARHGAP15,HUS1,ASTN2,TANC2,JAK2,NEGR1,CMKLR1,SDK2,ZBTB7C,PARK2,NPHP3,ADCYAP1R1,GRIN3A,SMOC2,CACNA1C,NLGN4X,EXOC4,EMB,PNPLA3,ANKH,UPB1,TAGLN3,PALMD,NRG3,NUMB,MED15,SOX6,POLE,SULF1,SH3BP1,OTC,ATRNL1,SH3GL2,ARHGAP24,COL11A1,SLIT2,THEMIS,AFF3,ATRX,CHRDL1,TRAF3IP2,RAD51B,BMPR1A,DSCAML1,SMAD3,TNFRSF11B,SLC9C1,POTEE,SCUBE1,PEAK1,EYA1,SATB2,HSF2BP,ELN,TPH1,MAD1L1,ANKRD11,EGFLAM,TUB,ARHGEF18,OVOL2,DNAJB6,ELP3,BRINP1,OMA1,CPQ,FGF14,GLIS1,ASGR2,IL1RAPL2,SOX5,DNMBP,TAF1,SPNS2,SLC1A1,NTN1,LDB2,EPM2A,MAP2,CDC73,FLRT2,FBXW11,WWTR1,GAS7,PEMT,CATSPER2,PCSK5,CUX1,ANK3,CDH12,ARID4B,TRPS1,MYO18B,CDH4,TNR,ADCY9,CELF4,VAV3,SCN9A,MDM4,ARNT2,SPOCK1,ACTN4,PLCE1,TACC2,EPS8,TENM4,CECR2,GHR,CDH10,LRRC4C,PPP1R9A,PALM2,CLSTN2,CBFA2T2,SRD5A2,STRC,DISC1,LRP5,NAV2,AP2B1,ESR1,ARHGAP12,TNFRSF19,SCFD1,PDE4D,CNTN5,PIK3CD,SLC5A3,BLOC1S5,CERS3,PCSK2,TEAD4,ARMC2,EFNA5,KLHL1,FTO,POU6F2,CEP85L,ATF2,RBBP8,GRID2,ZNF423,CYBB,SEMA6D,NTNG1,ADTRP,CRMP1,GABRB3,PRKCQ,ATXN1,CLDN1,NHSL2,SLC24A3,JDP2,PLEKHA5,TTLL5,SPTBN4,EPHB2,S1PR3,PRCP,PRTG,COL22A1,DOCK2,DPYSL2,DACH1,MARK1,BDNF,RBM4,FNIP1,ADORA2A,ARID5B,IFT80,DYM,FSTL4,FHL2,TTC39C,NOX4,MEGF9,CSMD1,BCOR,BMPER,SYT1,DAB2,AFF2,PLXNA2,LRRK1,PRKG1,ABI1,CALCRL,HDAC2,ETS1,FAT3,ARID4A,CDH23,TTN,ENPP1,RIMS2,SCN8A,MORN2,CTNNA2,FMNL2,PARD3,NRG1,SPINT2,FMN2,TOP1,RIT2,CD44,PAX7,CNGB1,PRKCG,TEAD1,NFIA,MAPKAPK2,SLC8A1,SRRM4,ISM1,FBXO31,MAP3K13,PLCL2,CHST11,THRB,CDC42EP3,MAPK10,NCAM1,AGT,FGF1,CCR3,STRIP1,NDRG2,GRIP1,USH2A,NF1,WIF1,ZNF536,PARVB,NRXN1,LAMA3,GSK3B,PKP2,CTDP1,SETD3,CD109,KCNH1,BBS9,GPR21,CHD7,PSMB7,MOV10L1,THSD7A,TIAM2,MYT1L,SRGAP2B,CNTN1,PLEKHB2,UNC13A,TRIOBP,NCOA1,CHEK2,RHOJ,PHEX,MECOM,SYBU,CATSPERB,SHANK2,UNC5D,ISLR2,NREP,GPC3,DLG5,CFDP1,PGM5,ASTN1,PREX1,CPE,UBE2V1,XKR4,ROR1,ADAMTSL1,ZNF675,SERPINA5,COL12A1,MACF1,MNAT1,TRIO,CAPN3,CTNNBL1,MYO3B,MAP3K5,CLDN11,SPAG16,HTR2C,CCDC141,FGD4,EPHA5,HIRA,TP73,RBM19,FAM171A1,MYPN,KIAA1217,MORC3,ANK2,SLIT3,GRIN2B,PHC2,MATN3,PAK3,TSNAX,PRRC2C,MLLT3,NEB,RNF213,PACRG,SEMA5B,GRIK1,NLN,SDK1,GRM5,IGF1R,PKD1L1,MSI2,CNTNAP2,PTPRO,SUFU,INSR,ITGA11,JAK1,CRTAC1,MAP2K1,UBR2,FOXO3,GPC6,KLHL3,OCA2,CNTN6,CENPF,MITF,IGF2BP3,LRFN5,AGBL4,PRDM16,ULK4,RBFOX1,KIR2DL4,TMEM120B,ADAMTS16,LRRK2,PKHD1,KIF2A,RARB,TCF4,SPG11,PBX1,PHACTR1,BMP6,ASAP1,FYN,XRCC4,EPHA7,GAS8,COL19A1,MSR1,SGCZ,PTPN14,RIMS1,ENPP2,ABCA12,EPHB1,LSAMP,KCNQ1,FHOD3,DOCK1,VASH2,BPGM,FAM126A,EXT1,TRPC5,AMFR,UNC5C,ACSBG1,CSGALNACT1,TENM3,WDR7,NOX*

			5,LRP2,FER,CAMK4,VWC2,DDX10,PCDH17,SGCD,TMEM108,SHROOM4,RAPGEF2,NAV3,PRPSAP2,PTPRM,KIRREL3,NRXN3,CDKL5,PTPRU,SOBP,MACROD2,IL18R1,KAT7,DCHS2,SETD2,PACSIN2,CDH13,MDGA2,BASP1,DAB1,ZNF148,HDAC5,SEMA3A,TSPAN8,MYH9,STIM1,NRIP1,TANC1,PIK3R3,TENM1,LARGE,GABRB1,SH3KBP1,PRKAR1A,ZRANB1,RGS7,SIPA1L3,DPY19L2,SCEL,NTRK3,FBN1,HYDIN,DIS3L2,TBX15,CLASP2,ZNF521,NSUN2,TPD52,DACH2,DNM3,SYNDIG1,PPARA,PTPRD,RORA,MYH15,SHISA6,BRIP1,ELAVL4,SBF2,CDH9,NELL1,DOCK10,EEF1E1,FNDC3A,BICC1,SEMA3D,PRLR,ATF6,SPATA5,SYT17,RAB27A,BLOC1S6,SEMA3C,RXFP2
GO:0032501	multicellular organismal process	5.453193060397004e-28	*PTPRR,CAMTA1,RCAN1,KALRN,CACNG2,MEGF11,PROS1,RPS6KA2,NDE1,CTNNA1,FOXN3,NTRK2,CLASP1,ZNRF3,PTPRG,SYNE1,FBXL17,DMD,KCNC2,CHRM3,SPRED2,KAZN,ERBB4,LAMA2,TFDP2,NOS1,CNTN4,TMC2,SND1,ESRRG,ROBO1,PRICKLE2,MYO3A,DSCAM,SHROOM3,CIT,TBCD,PUM1,FRY,GOLGA3,NEBL,WWOX,HUNK,FUT8,HDAC4,OPCML,LINGO2,IMMP2L,ABCC1,ATP8A2,AUTS2,WLS,ANKS1A,LDLRAD4,MYEF2,TENM2,PSMB2,SLC44A1,RXFP1,ASXL3,MGLL,NFASC,PLCB1,FLT3,SRPK2,TRPM1,PRKD1,RYR2,VCL,PPP2R3C,SEMA5A,ETV6,PRKCA,EPB41L4B,ZBTB20,DLGAP1,BTBD9,TRHDE,IL1RAPL1,ADAMTS6,MYO9A,PLAC1,DIO2,NHS,CALD1,KANK1,KIF26B,DIAPH2,SLCO3A1,TCF12,RUNX1,PLS1,USP53,NLGN1,DNAH9,CAMK1D,AMOTL1,TCF7L2,RELN,APP,ADAM12,PAK1,UTRN,PPP3CA,RYR1,NTM,TOX,PCDH15,GABRG3,PTPN9,CREM,GREB1L,AKAP6,SORCS3,GRM7,SSPN,DLC1,IDE,SCMH1,RYR3,SOS1,CTNND2,RFTN1,ALK,BTRC,DCLK1,MEF2A,KCNB2,TFAP2D,CTNNA3,DENND5A,PPP1R12B,MAP2K5,DNMT1,MAGI2,EDA,CELSR1,EYS,ROBO2,IFT81,AKAP13,HIVEP3,DCC,EP300,PARN,SEMA4D,EVC,RPS6KA5,SLCO2B1,ANKRD6,TRAPPC9,MMP16,BDKRB1,NME8,TF,DNAH11,GRIK2,QKI,CHRM1,HUS1,ASTN2,TANC2,JAK2,ABCG8,NEGR1,CMKLR1,SDK2,PARK2,NPHP3,ADCYAP1R1,GRIN3A,SMOC2,CACNA1C,NLGN4X,EXOC4,EMB,OR5K4,ANKH,TMPRSS3,UPB1,TAGLN3,NRG3,NUMB,MED15,SOX6,POLE,TRPM3,SULF1,CD84,SH3BP1,OTC,ATRNL1,SH3GL2,ARHGAP24,COL11A1,SLC13A3,SLIT2,SYNM,SLC4A4,DTNA,THEMIS,AFF3,ATRX,CHRDL1,TRAF3IP2,RAD51B,BMPR1A,ACACA,DSCAML1,SMAD3,TNFRSF11B,PIBF1,GABRR2,SLC9C1,HMCN1,POTEE,PTGFR,SCUBE1,EYA1,SATB2,HSF2BP,ELN,TPH1,MAD1L1,ANKRD11,EGFLAM,DGKB,TUB,SCAMP5,OVOL2,DNAJB6,ELP3,BRINP1,OMA1,FGF14,DGKK,OR51I1,SPESP1,GLIS1,ASGR2,IL1RAPL2,SOX5,DGKI,TAF1,SPNS2,SLC1A1,NTN1,LDB2,SHISA9,EPM2A,MAP2,CDC73,FLRT2,FBXW11,NOS1AP,CD96,WWTR1,GAS7,PEMT,CATSPER2,PCSK5,CUX1,ANK3,ARID4B,LHFPL3,TRPS1,MYO18B,CDH4,TNR,ADCY9,CELF4,VAV3,SCN9A,MDM4,ARNT2,SPOCK1,PLCE1,TACC2,CYP39A1,EPS8,TENM4,CECR2,GHR,LRRC4C,PPP1R9A,CLSTN2,CBFA2T2,SRD5A2,PRG3,MLIP,STRC,DISC1,ARHGAP42,RABGEF1,LRP5,NAV2,AP2B1,ESR1,TNFRSF19,GRM1,PDE4D,CNTN5,PIK3CD,DOCK4,SLC5A3,BLOC1S5,CERS3,PCSK2,TEAD4,ARMC2,EFNA5,KLHL1,FTO,POU6F2,CEP85L,ATF2,RBBP8,KCNJ12,GRID2,ZNF423,CYBB,SEMA6D,KCNJ3,CELF2,NTNG1,ADTRP,CRMP1,PDE4B,MAPRE2,GABRB3,MAP3K7,PRKCQ,ATXN1,N4BP1,RHPN2,CLDN1,KCND2,SNTB1,SLC24A3,PLEKHA5,TTLL5,SPTBN4,EPHB2,MYBPC2,S1PR3,PRCP,PRTG,COL22A1,DOCK2,DPYSL2,DACH1,MARK1,BDNF,RBM4,FNIP1,ADORA2A,ARID5B,IFT80,DYM,FSTL4,ANO1,FHL2,TTC39C,NOX4,MEGF9,CSMD1,BCOR,BMPER,SYT1,DAB2,AFF2,PLXNA2,LRRK1,PRKG1,SNAP23,ABI1,CACNG3,PTH2R,CALCRL,TNNI3K,HDAC2,ETS1,FAT3,ARID4A,PRKAR1B,CDH23,TTN,ENPP1,RIMS2,SCN8A,KSR2,DLGAP2,MORN2,OR4N2,CTNNA2,PARD3,MYOM3,NRG1,SPINT2,FMN2,TOP1,RIT2,CD44,PAX7,KCNMA1,CNGB1,PRKCG,TEAD1,NFIA,MAPKAPK2,SLC8A1,SRRM4,ISM1,FBXO31,FAM19A4,MAP3K13,PLCL2,CHST11,THRB,NCAM1,AGT,FGF1,CCR3,NDRG2,GRIP1,USH2A,NF1,ZNF536,NRXN1,LAMA3,GSK3B,PKP2,CTDP1,SETD3,CD109,SLC22A3,BBS9,GPR21,CHD7,PSMB7,MOV10L1,THSD7A,SNX5,TIAM2,OR51B2,MYT1L,SRGAP2B,CNTN1,UNC13A,TRIOBP,NCOA1,CACNB2,RHOJ,PHEX,MECOM,SYBU,CATSPERB,SHANK2,UNC5D,ISLR2,NREP,GPC3,DLG5,ASTN1,PREX1,CPE,ROR1,ADAMTSL1,ZNF675,SERPINA5,COL12A1,PTGER3,MACF1,MNAT1,TRIO,CAPN3,CTNNBL1,MYO3B,MYOM1,CLDN11,SPAG16,HTR2C,CCDC141,GLRA2,EPHA5,SGIP1,HIRA,TP73,RBM19,IL1RL1,MYPN,KIAA1217,MORC3,P2*

			RX6,ANK2,SLIT3,GRIN2B,PHC2,MATN3,PAK3,TSNAX,PRRC2C,ADCY8,GABRR3,MLLT3,NEB,RNF213,CACNA1D,PRKAA2,PACRG,ABCG2,TBL1X,SEMA5B,GRIK1,NLN,ANKFN1,SDK1,GRM5,IGF1R,PKD1L1,CNTNAP2,PTPRO,SUFU,INSR,ITGA11,JAK1,RPGR,CRTAC1,MAP2K1,UBR2,FOXO3,GPC6,GABRA3,KLHL3,OCA2,CNTN6,CENPF,RNLS,MITF,IGF2BP3,LRFN5,CDC14A,AGBL4,PRDM16,ULK4,RBFOX1,KIR2DL4,ADAMTS16,LTBP1,LRRK2,PKHD1,KIF2A,RARB,TCF4,SPG11,PBX1,PHACTR1,SCN1A,BMP6,VTI1A,ASAP1,FYN,KCND3,ABCC2,XRCC4,EPHA7,MARVELD3,GAS8,COL19A1,MSR1,SGCZ,CLN6,PTPN14,RIMS1,ENPP2,ABCA12,EPHB1,LSAMP,KCNQ1,FHOD3,DOCK1,VASH2,BPGM,FAM126A,EXT1,TRPC5,AMFR,UNC5C,ACSBG1,CSGALNACT1,TENM3,WDR7,NOX5,LRP2,CAMK4,VWC2,GUCY2F,PCDH17,SGCD,TMEM108,SHROOM4,RAPGEF2,NAV3,PRPSAP2,PTPRM,KIRREL3,NCOA2,NRXN3,CDKL5,PTPRU,SOBP,MACROD2,IL18R1,KAT7,DCHS2,SETD2,CDH13,MDGA2,BASP1,TRDN,DAB1,ZNF148,SLC16A12,HDAC5,SEMA3A,TSPAN8,MYH9,BDKRB2,STIM1,NRIP1,TANC1,LITAF,PIK3R3,TENM1,LARGE,GABRB1,PRKAR1A,RGS7,SIPA1L3,VWF,DPY19L2,SLC24A2,SCEL,NTRK3,FBN1,HYDIN,DIS3L2,TBX15,MOGAT2,CLASP2,ZNF521,NSUN2,TPD52,DACH2,DNM3,SYNDIG1,PPARA,PTPRD,RORA,MYH15,SHISA6,BRIP1,ELAVL4,SBF2,NELL1,DOCK10,FNDC3A,ATP8A1,BICC1,SEMA3D,PRLR,ATF6,SPATA5,SYT17,STAC,OR4M1,RAB27A,CST2,BLOC1S6,SEMA3C,RXFP2
GO:0048731	system development	1.0667112641450368e-27	RCAN1,KALRN,MEGF11,RPS6KA2,NDE1,CTNNA1,FOXN3,NTRK2,CLASP1,ZNRF3,PTPRG,FBXL17,DMD,KCNC2,CHRM3,SPRED2,KAZN,ERBB4,LAMA2,TFDP2,CNTN4,ROBO1,PRICKLE2,MYO3A,DSCAM,SHROOM3,CIT,TBCD,FRY,NEBL,WWOX,HDAC4,OPCML,LINGO2,IMMP2L,ATP8A2,AUTS2,WLS,ANKS1A,LDLRAD4,MYEF2,TENM2,PSMB2,RXFP1,ASXL3,NFASC,PLCB1,FLT3,SRPK2,PRKD1,RYR2,VCL,PPP2R3C,SEMA5A,ETV6,PRKCA,IL1RAPL1,ADAMTS6,MYO9A,PLAC1,NHS,CALD1,KANK1,KIF26B,TCF12,RUNX1,PLS1,NLGN1,CAMK1D,AMOTL1,TCF7L2,RELN,APP,ADAM12,PAK1,UTRN,PPP3CA,RYR1,NTM,TOX,PCDH15,PTPN9,GREB1L,AKAP6,GRM7,DLC1,SOS1,CTNND2,ALK,BTRC,DCLK1,MEF2A,TFAP2D,DENND5A,MAP2K5,DNMT1,MAGI2,EDA,CELSR1,ROBO2,AKAP13,HIVEP3,DCC,EP300,SEMA4D,EVC,RPS6KA5,ANKRD6,TRAPPC9,MMP16,TF,DNAH11,QKI,CHRM1,ASTN2,TANC2,JAK2,NEGR1,CMKLR1,SDK2,PARK2,NPHP3,GRIN3A,SMOC2,CACNA1C,NLGN4X,EXOC4,EMB,ANKH,UPB1,TAGLN3,NRG3,NUMB,SOX6,POLE,SULF1,OTC,ATRNL1,SH3GL2,ARHGAP24,COL11A1,SLIT2,THEMIS,ATRX,CHRDL1,TRAF3IP2,BMPR1A,DSCAML1,SMAD3,TNFRSF11B,POTEE,SCUBE1,EYA1,SATB2,ELN,TPH1,MAD1L1,ANKRD11,EGFLAM,TUB,OVOL2,DNAJB6,ELP3,BRINP1,FGF14,ASGR2,IL1RAPL2,SOX5,TAF1,SPNS2,SLC1A1,NTN1,LDB2,EPM2A,MAP2,CDC73,FLRT2,FBXW11,WWTR1,GAS7,PCSK5,CUX1,ANK3,ARID4B,TRPS1,MYO18B,CDH4,TNR,CELF4,VAV3,MDM4,ARNT2,SPOCK1,PLCE1,TACC2,TENM4,CECR2,GHR,LRRC4C,PPP1R9A,CLSTN2,CBFA2T2,SRD5A2,STRC,DISC1,LRP5,NAV2,AP2B1,ESR1,TNFRSF19,CNTN5,PIK3CD,SLC5A3,BLOC1S5,CERS3,PCSK2,TEAD4,EFNA5,KLHL1,FTO,POU6F2,CEP85L,ATF2,GRID2,ZNF423,CYBB,SEMA6D,NTNG1,ADTRP,CRMP1,GABRB3,PRKCQ,ATXN1,CLDN1,SLC24A3,PLEKHA5,TTLL5,SPTBN4,EPHB2,PRCP,PRTG,COL22A1,DOCK2,DPYSL2,MARK1,BDNF,FNIP1,ADORA2A,ARID5B,IFT80,DYM,FSTL4,FHL2,TTC39C,NOX4,MEGF9,CSMD1,BCOR,BMPER,SYT1,DAB2,AFF2,PLXNA2,LRRK1,PRKG1,ABI1,CALCRL,HDAC2,ETS1,FAT3,ARID4A,CDH23,TTN,ENPP1,RIMS2,SCN8A,CTNNA2,PARD3,NRG1,SPINT2,RIT2,CD44,PAX7,CNGB1,PRKCG,TEAD1,NFIA,MAPKAPK2,SLC8A1,SRRM4,ISM1,FBXO31,MAP3K13,PLCL2,CHST11,THRB,NCAM1,AGT,FGF1,CCR3,NDRG2,GRIP1,USH2A,NF1,ZNF536,NRXN1,LAMA3,GSK3B,PKP2,CTDP1,CD109,CHD7,PSMB7,THSD7A,TIAM2,MYT1L,SRGAP2B,CNTN1,UNC13A,TRIOBP,NCOA1,RHOJ,PHEX,MECOM,SYBU,SHANK2,UNC5D,ISLR2,NREP,GPC3,DLG5,ASTN1,PREX1,CPE,ROR1,ADAMTSL1,ZNF675,MACF1,MNAT1,TRIO,CAPN3,CTNNBL1,MYO3B,CLDN11,CCDC141,EPHA5,TP73,MYPN,KIAA1217,ANK2,SLIT3,GRIN2B,MATN3,PAK3,PRRC2C,MLLT3,NEB,RNF213,SEMA5B,GRIK1,NLN,SDK1,GRM5,IGF1R,CNTNAP2,PTPRO,SUFU,INSR,ITGA11,JAK1,CRTAC1,MAP2K1,FOXO3,GPC6,KLHL3,CNTN6,CENPF,MITF,IGF2BP3,LRFN5,AGBL4,ULK4,RBFOX1,ADAMTS16,LRRK2,PKHD1,KIF2A,RARB,TCF4,SPG11,PBX1,PHACTR1,BMP6,ASAP1,FYN,XRC

			C4,EPHA7,GAS8,COL19A1,SGCZ,PTPN14,RIMS1,ENPP2,ABCA12,EPHB1,LSAMP,KCNQ1,FHOD3,DOCK1,VASH2,BPGM,FAM126A,EXT1,TRPC5,UNC5C,ACSBG1,CSGALNACT1,TENM3,WDR7,NOX5,LRP2,CAMK4,VWC2,PCDH17,SGCD,TMEM108,SHROOM4,RAPGEF2,NAV3,PRPSAP2,PTPRM,KIRREL3,NRXN3,CDKL5,PTPRU,SOBP,MACROD2,IL18R1,KAT7,DCHS2,SETD2,CDH13,MDGA2,BASP1,DAB1,ZNF148,HDAC5,SEMA3A,MYH9,STIM1,NRIP1,PIK3R3,TENM1,LARGE,GABRB1,PRKAR1A,RGS7,SIPA1L3,SCEL,NTRK3,FBN1,HYDIN,TBX15,CLASP2,ZNF521,NSUN2,TPD52,DNM3,SYNDIG1,PPARA,PTPRD,RORA,MYH15,BRIP1,ELAVL4,SBF2,NELL1,DOCK10,FNDC3A,BICC1,SEMA3D,PRLR,ATF6,SPATA5,SYT17,BLOC1S6,SEMA3C,RXFP2
GO:0048666	neuron development	1.8246044514290075e-27	KALRN,CTNNA1,NTRK2,PTPRG,DMD,LAMA2,CNTN4,ROBO1,DSCAM,TBCD,FRY,OPCML,ATP8A2,AUTS2,ANKS1A,TENM2,NFASC,PRKD1,VCL,SEMA5A,IL1RAPL1,MYO9A,KANK1,RUNX1,PLS1,NLGN1,CAMK1D,RELN,APP,PAK1,PPP3CA,NTM,TOX,PCDH15,PTPN9,GRM7,SOS1,CTNND2,ALK,DCLK1,MEF2A,DENND5A,MAGI2,ROBO2,DCC,EP300,SEMA4D,RPS6KA5,TANC2,JAK2,NEGR1,PARK2,GRIN3A,EMB,NUMB,SH3GL2,SLIT2,DSCAML1,NTN1,MAP2,FLRT2,GAS7,CUX1,ANK3,CDH4,TNR,SPOCK1,TENM4,CECR2,LRRC4C,PPP1R9A,CBFA2T2,STRC,DISC1,BLOC1S5,EFNA5,KLHL1,GRID2,SEMA6D,NTNG1,CRMP1,PRKCQ,SPTBN4,EPHB2,PRTG,DPYSL2,MARK1,BDNF,ADORA2A,FSTL4,SYT1,DAB2,PLXNA2,PRKG1,ABI1,HDAC2,FAT3,CDH23,RIMS2,CTNNA2,PARD3,RIT2,CNGB1,SRRM4,FBXO31,MAP3K13,THRB,NCAM1,AGT,GRIP1,NRXN1,LAMA3,GSK3B,TIAM2,MYT1L,CNTN1,UNC13A,TRIOBP,UNC5D,ISLR2,NREP,DLG5,ROR1,ADAMTSL1,MACF1,TRIO,CCDC141,EPHA5,MYPN,SLIT3,PAK3,SEMA5B,SDK1,IGF1R,CNTNAP2,PTPRO,CRTAC1,MAP2K1,CNTN6,AGBL4,ULK4,LRRK2,SPG11,PBX1,PHACTR1,ASAP1,FYN,EPHA7,RIMS1,EPHB1,KCNQ1,VASH2,EXT1,TRPC5,UNC5C,TENM3,LRP2,TMEM108,RAPGEF2,PTPRM,KIRREL3,NRXN3,CDKL5,DAB1,SEMA3A,TENM1,GABRB1,NTRK3,CLASP2,DNM3,PTPRD,ELAVL4,DOCK10,SEMA3D,SYT17,BLOC1S6,SEMA3C
GO:0000902	cell morphogenesis	2.6015624808949217e-27	KALRN,NTRK2,DMD,LAMA2,CNTN4,ROBO1,DSCAM,SHROOM3,TBCD,FRY,ATP8A2,AUTS2,NFASC,VCL,NEDD9,SEMA5A,IL1RAPL1,MYO9A,KANK1,PLS1,NLGN1,RELN,APP,PAK1,PPP3CA,PCDH15,DLC1,SOS1,CTNND2,DCLK1,MEF2A,ROBO2,DCC,EP300,SEMA4D,RPS6KA5,CDH8,ARHGAP15,TANC2,PARK2,EMB,PALMD,NUMB,ATRNL1,SH3GL2,SLIT2,DSCAML1,PEAK1,ARHGEF18,DNMBP,NTN1,MAP2,FLRT2,GAS7,CUX1,ANK3,CDH12,CDH4,TNR,ACTN4,EPS8,CDH10,LRRC4C,PALM2,STRC,DISC1,SCFD1,EFNA5,SEMA6D,NTNG1,CRMP1,PRKCQ,SPTBN4,EPHB2,PRTG,COL22A1,DPYSL2,BDNF,ADORA2A,FSTL4,NOX4,MEGF9,SYT1,DAB2,PLXNA2,ABI1,FAT3,CDH23,RIMS2,CTNNA2,FMNL2,PARD3,NRG1,SPINT2,CD44,FBXO31,MAP3K13,CDC42EP3,NCAM1,STRIP1,GRIP1,USH2A,PARVB,NRXN1,LAMA3,GSK3B,TIAM2,UNC13A,TRIOBP,RHOJ,UNC5D,ISLR2,CFDP1,PREX1,ADAMTSL1,MACF1,TRIO,CCDC141,FGD4,EPHA5,FAM171A1,MYPN,SLIT3,PAK3,SEMA5B,IGF1R,CNTNAP2,PTPRO,MAP2K1,CNTN6,LRRK2,PKHD1,SPG11,PHACTR1,FYN,EPHA7,RIMS1,ENPP2,EPHB1,DOCK1,EXT1,TRPC5,UNC5C,LRP2,FER,TMEM108,RAPGEF2,PTPRM,KIRREL3,NRXN3,CDKL5,PACSIN2,DAB1,SEMA3A,MYH9,SH3KBP1,ZRANB1,SIPA1L3,CLASP2,DNM3,PTPRD,ELAVL4,CDH9,DOCK10,SEMA3D,SYT17,SEMA3C
GO:0031175	neuron projection development	2.672659779950151e-27	KALRN,CTNNA1,NTRK2,PTPRG,DMD,LAMA2,CNTN4,ROBO1,DSCAM,FRY,ATP8A2,AUTS2,NFASC,PRKD1,VCL,SEMA5A,IL1RAPL1,MYO9A,KANK1,PLS1,NLGN1,CAMK1D,RELN,APP,PAK1,PPP3CA,TOX,PCDH15,PTPN9,GRM7,SOS1,CTNND2,ALK,DCLK1,MEF2A,DENND5A,MAGI2,ROBO2,DCC,EP300,SEMA4D,RPS6KA5,TANC2,JAK2,NEGR1,PARK2,GRIN3A,EMB,NUMB,SH3GL2,SLIT2,DSCAML1,NTN1,MAP2,FLRT2,GAS7,CUX1,ANK3,CDH4,TNR,SPOCK1,CECR2,LRRC4C,PPP1R9A,CBFA2T2,STRC,DISC1,BLOC1S5,EFNA5,KLHL1,GRID2,SEMA6D,NTNG1,CRMP1,PRKCQ,SPTBN4,EPHB2,PRTG,DPYSL2,MARK1,BDNF,ADORA2A,FSTL4,SYT1,DAB2,PLXNA2,PRKG1,ABI1,HDAC2,FAT3,CDH23,RIMS2,CTNNA2,PARD3,RIT2,FBXO31,MAP3K13,NCAM1,AGT,GRIP1,NRXN1,LAMA3,GSK3B,TIAM2,CNTN1,UNC13A,TRIOBP,UNC5D,ISLR2,NREP,DLG5,ROR1,ADAMTSL1,MACF1,TRIO,CCDC141,EPHA5,MYPN,SLIT3,PAK3,SEMA5B,SDK1,IGF1R,CNTNAP2,PTPRO,CRTAC1,MAP2K1,CNTN6,ULK4,LRRK2,SPG11,PHACTR1,ASAP1,FYN,EPHA7,RIMS1,EPHB1,VASH2

			,EXT1,TRPC5,UNC5C,TENM3,LRP2,TMEM108,RAPGEF2,PTPRM,KIRREL3,NRXN3,CDKL5,DAB1,SEMA3A,NTRK3,CLASP2,DNM3,PTPRD,ELAVL4,DOCK10,SEMA3D,SYT17,BLOC1S6,SEMA3C
GO:0007275	multicellular organism development	6.489879431906145e-27	*PTPRR,RCAN1,KALRN,MEGF11,RPS6KA2,NDE1,CTNNA1,FOXN3,NTRK2,CLASP1,ZNRF3,PTPRG,FBXL17,DMD,KCNC2,CHRM3,SPRED2,KAZN,ERBB4,LAMA2,TFDP2,CNTN4,ROBO1,PRICKLE2,MYO3A,DSCAM,SHROOM3,CIT,TBCD,FRY,NEBL,WWOX,HUNK,FUT8,HDAC4,OPCML,LINGO2,IMMP2L,ATP8A2,AUTS2,WLS,ANKS1A,LDLRAD4,MYEF2,TENM2,PSMB2,RXFP1,ASXL3,NFASC,PLCB1,FLT3,SRPK2,PRKD1,RYR2,VCL,PPP2R3C,SEMA5A,ETV6,PRKCA,IL1RAPL1,ADAMTS6,MYO9A,PLAC1,NHS,CALD1,KANK1,KIF26B,TCF12,RUNX1,PLS1,NLGN1,CAMK1D,AMOTL1,TCF7L2,RELN,APP,ADAM12,PAK1,UTRN,PPP3CA,RYR1,NTM,TOX,PCDH15,PTPN9,GREB1L,AKAP6,GRM7,DLC1,IDE,SCMH1,SOS1,CTNND2,ALK,BTRC,DCLK1,MEF2A,TFAP2D,DENND5A,MAP2K5,DNMT1,MAGI2,EDA,CELSR1,ROBO2,AKAP13,HIVEP3,DCC,EP300,SEMA4D,EVC,RPS6KA5,ANKRD6,TRAPPC9,MMP16,TF,DNAH11,QKI,CHRM1,HUS1,ASTN2,TANC2,JAK2,NEGR1,CMKLR1,SDK2,PARK2,NPHP3,ADCYAP1R1,GRIN3A,SMOC2,CACNA1C,NLGN4X,EXOC4,EMB,ANKH,UPB1,TAGLN3,NRG3,NUMB,SOX6,POLE,SULF1,OTC,ATRNL1,SH3GL2,ARHGAP24,COL11A1,SLIT2,THEMIS,AFF3,ATRX,CHRDL1,TRAF3IP2,RAD51B,BMPR1A,DSCAML1,SMAD3,TNFRSF11B,POTEE,SCUBE1,EYA1,SATB2,ELN,TPH1,MAD1L1,ANKRD11,EGFLAM,TUB,OVOL2,DNAJB6,ELP3,BRINP1,FGF14,ASGR2,IL1RAPL2,SOX5,TAF1,SPNS2,SLC1A1,NTN1,LDB2,EPM2A,MAP2,CDC73,FLRT2,FBXW11,WWTR1,GAS7,PEMT,PCSK5,CUX1,ANK3,ARID4B,TRPS1,MYO18B,CDH4,TNR,ADCY9,CELF4,VAV3,SCN9A,MDM4,ARNT2,SPOCK1,PLCE1,TACC2,TENM4,CECR2,GHR,LRRC4C,PPP1R9A,CLSTN2,CBFA2T2,SRD5A2,STRC,DISC1,LRP5,NAV2,AP2B1,ESR1,TNFRSF19,CNTN5,PIK3CD,SLC5A3,BLOC1S5,CERS3,PCSK2,TEAD4,EFNA5,KLHL1,FTO,POU6F2,CEP85L,ATF2,RBBP8,GRID2,ZNF423,CYBB,SEMA6D,NTNG1,ADTRP,CRMP1,GABRB3,PRKCQ,ATXN1,CLDN1,SLC24A3,PLEKHA5,TTLL5,SPTBN4,EPHB2,S1PR3,PRCP,PRTG,COL22A1,DOCK2,DPYSL2,DACH1,MARK1,BDNF,FNIP1,ADORA2A,ARID5B,IFT80,DYM,FSTL4,FHL2,TTC39C,NOX4,MEGF9,CSMD1,BCOR,BMPER,SYT1,DAB2,AFF2,PLXNA2,LRRK1,PRKG1,ABI1,CALCRL,HDAC2,ETS1,FAT3,ARID4A,CDH23,TTN,ENPP1,RIMS2,SCN8A,CTNNA2,PARD3,NRG1,SPINT2,TOP1,RIT2,CD44,PAX7,CNGB1,PRKCG,TEAD1,NFIA,MAPKAPK2,SLC8A1,SRRM4,ISM1,FBXO31,MAP3K13,PLCL2,CHST11,THRB,NCAM1,AGT,FGF1,CCR3,NDRG2,GRIP1,USH2A,NF1,ZNF536,NRXN1,LAMA3,GSK3B,PKP2,CTDP1,CD109,CHD7,PSMB7,THSD7A,TIAM2,MYT1L,SRGAP2B,CNTN1,UNC13A,TRIOBP,NCOA1,RHOJ,PHEX,MECOM,SYBU,SHANK2,UNC5D,ISLR2,NREP,GPC3,DLG5,ASTN1,PREX1,CPE,ROR1,ADAMTSL1,ZNF675,COL12A1,MACF1,MNAT1,TRIO,CAPN3,CTNNBL1,MYO3B,CLDN11,CCDC141,EPHA5,HIRA,TP73,RBM19,MYPN,KIAA1217,MORC3,ANK2,SLIT3,GRIN2B,MATN3,PAK3,PRRC2C,MLLT3,NEB,RNF213,SEMA5B,GRIK1,NLN,SDK1,GRM5,IGF1R,PKD1L1,CNTNAP2,PTPRO,SUFU,INSR,ITGA11,JAK1,CRTAC1,MAP2K1,FOXO3,GPC6,KLHL3,CNTN6,CENPF,MITF,IGF2BP3,LRFN5,AGBL4,ULK4,RBFOX1,ADAMTS16,LRRK2,PKHD1,KIF2A,RARB,TCF4,SPG11,PBX1,PHACTR1,BMP6,ASAP1,FYN,XRCC4,EPHA7,GAS8,COL19A1,SGCZ,PTPN14,RIMS1,ENPP2,ABCA12,EPHB1,LSAMP,KCNQ1,FHOD3,DOCK1,VASH2,BPGM,FAM126A,EXT1,TRPC5,UNC5C,ACSBG1,CSGALNACT1,TENM3,WDR7,NOX5,LRP2,CAMK4,VWC2,PCDH17,SGCD,TMEM108,SHROOM4,RAPGEF2,NAV3,PRPSAP2,PTPRM,KIRREL3,NRXN3,CDKL5,PTPRU,SOBP,MACROD2,IL18R1,KAT7,DCHS2,SETD2,CDH13,MDGA2,BASP1,DAB1,ZNF148,HDAC5,SEMA3A,MYH9,STIM1,NRIP1,PIK3R3,TENM1,LARGE,GABRB1,PRKAR1A,RGS7,SIPA1L3,SCEL,NTRK3,FBN1,HYDIN,TBX15,CLASP2,ZNF521,NSUN2,TPD52,DACH2,DNM3,SYNDIG1,PPARA,PTPRD,RORA,MYH15,BRIP1,ELAVL4,SBF2,NELL1,DOCK10,FNDC3A,BICC1,SEMA3D,PRLR,ATF6,SPATA5,SYT17,BLOC1S6,SEMA3C,RXFP2*
GO:0051179	localization	6.748874921249505e-26	*PTPRR,SYN3,SVOPL,LRP1B,KALRN,CACNG2,PROS1,NDE1,CTNNA1,NTRK2,CLASP1,PTPRG,SYNE1,SLC39A10,DMD,KCNC2,CHRM3,ERBB4,LAMA2,NOS1,TMC2,ROBO1,SIL1,SHROOM3,DPP10,GPC5,SORCS2,LRBA,FUT8,HDAC4,ATP9A,ARFGAP3,IMMP2L,ABCC1,ATP8A2,AFTPH,AUTS2,WLS,ANKS1A,LDLRAD4,*

			SGSM1,PSMB2,SLC44A1,SNUPN,PTPRT,NFASC,PLCB1,CACNA1A,TRPM1,PRKD1,RYR2,VCL,NEDD9,SEMA5A,CASK,PRKCA,EPB41L4B,STX8,BTBD9,IL1RAPL1,HBE1,ABCA13,KANK1,TBC1D5,SLCO3A1,GRIK4,PLS1,FCHSD2,NLGN1,DNAH9,KCNIP4,CAMK1D,SMG6,CLEC16A,AMOTL1,TCF7L2,RELN,APP,PAK1,UTRN,PPP3CA,RYR1,GABRG3,ERC2,PTPN9,AKAP6,OSBPL1A,GRM7,RABGAP1L,DLC1,RYR3,SOS1,LRRC8C,RFTN1,DCLK1,MEF2A,KCNB2,FAM155A,CTNNA3,DENND5A,DLG2,MAP2K5,MAGI2,CELSR1,IFT81,AKAP13,DCC,ELMO2,KCNA6,PARN,SEMA4D,SLCO2B1,BDKRB1,NME8,TF,DNAH11,GRIK2,QKI,CHRM1,SYTL5,ZDHHC11B,ITSN1,ASTN2,TANC2,DEFA1B,JAK2,ABCG8,RAB11FIP4,CMKLR1,PARK2,SLC14A2,NPHP3,ADCYAP1R1,DPP6,PRELID2,GRID1,GRIN3A,SMOC2,CACNA1C,MAN1A1,NLGN4X,EXOC4,HEATR5A,EMB,ANO4,PIK3C2B,ENTHD1,ANKH,ITGBL1,NBEA,TMPRSS3,NRG3,SLC5A10,NUMB,TRPM3,SULF1,CD84,SH3BP1,ATRNL1,RFFL,SH3GL2,ARHGAP24,SLC13A3,PITPNC1,SLIT2,CEP128,KCNJ16,SLC4A4,TRIM5,FLVCR2,ATRX,TRAF3IP2,BMPR1A,SMAD3,CACNA1E,BORA,PIBF1,GABRR2,PIK3C3,SLC9C1,SLC47A1,SV2B,PEAK1,SATB2,ANKS1B,ANO2,TPH1,MAD1L1,TNKS,TUB,ARHGEF18,SCAMP5,OVOL2,DNAJB6,ELP3,ARRDC4,FGF14,CACNA2D3,ASGR2,SPAG17,ZDHHC11,SKAP1,PTPRN2,DGKI,SPNS2,SLC1A1,NTN1,LDB2,SHISA9,EPM2A,SHFM1,MAP2,FLRT2,FBXW11,NOS1AP,WWTR1,KCTD7,CATSPER2,PCSK5,CUX1,GRM4,ANK3,CCL15,CNIH3,TTLL8,TNR,VAV3,SCN9A,SPOCK1,ACTN4,RAB2A,EPS8,CECR2,GHR,LRRC8D,SLC25A21,CD300A,ABCC11,DISC1,OSBPL10,ATP8B4,CCL14,IFT43,RABGEF1,NSG2,LRP5,AP2B1,SLC30A7,ESR1,ARHGAP12,KCNN3,DENND2A,GRM1,SCFD1,PDE4D,THEM4,PIK3CD,DOCK4,SLC5A3,BLOC1S5,FRMD4A,ARMC2,RAB24,EFNA5,FTO,CEP85L,ATF2,KCNJ12,PIK3R2,CCDC91,GRID2,ZNF423,CYBB,SEMA6D,KCNJ3,NTNG1,ADTRP,PDE4B,MAPRE2,GABRB3,SMG7,ANKRD13A,PRKCQ,ATXN1,CLDN1,WDR83OS,KCND2,SLC24A3,SPTBN4,EPHB2,ERC1,MCTP1,PRCP,DOCK2,NUP214,DPYSL2,DACH1,MARK1,BDNF,RBM4,ADORA2A,ARID5B,IFT80,ANO1,CADPS,TSPAN33,MEGF9,CHKA,BMPER,ICK,SYT1,DAB2,PLXNA2,NSF,PRKG1,STXBP6,SNAP23,CACNG3,EPG5,CALCRL,MICAL3,RANBP17,ETS1,BANP,FAT3,TMEM163,PRKAR1B,CDH23,TTN,SRGAP3,MYRIP,SLC39A11,ENPP1,RIMS2,SCN8A,AGAP1,CTNNA2,PEX5L,CYB561A3,FMNL2,PARD3,NRG1,TBC1D9,SPINT2,ATP10B,SLC35F4,FMN2,GRIA2,RIT2,CD44,KCNMA1,NCF4,CNGB1,GPSM2,PRKCG,SLC12A8,ZFYVE1,MAPKAPK2,SLC8A1,RAPGEF6,FBXO31,FAM19A4,RSRC1,CHST11,SLC9A9,CDC42BPA,SAMM50,AGT,FGF1,KCNQ5,HEPHL1,CCR3,KCNJ6,GRIP1,TLK1,ARHGAP25,USH2A,NF1,VPS16,SLC44A5,NRXN1,LAMA3,GSK3B,PKP2,SLC22A3,KCNH1,BBS9,CHD7,PSMB7,SNX5,SRGAP2B,CNTN1,UNC13A,GRIK3,CACNB2,STXBP4,RHOJ,ESYT2,SYBU,UNC5D,GPC3,DLG5,ASTN1,PREX1,CPE,ANO3,XKR4,SERPINA5,KCNS3,SLC5A8,PTGER3,BID,MACF1,CAPN3,DUSP22,MYOM1,SPAG16,CTDSPL2,HTR2C,CCDC141,GLRA2,NCF2,SNAP25-AS1,EPHA5,SGIP1,SNX31,MORC3,P2RX6,ANK2,GRIN2B,PAK3,LRPPRC,ADCY8,ITPR2,GABRR3,CCL15-CCL14,RNF213,SH3RF2,CACNA1D,PRKAA2,PACRG,ABCG2,SEMA5B,GRIK1,ANKFN1,TRAPPC8,GRM5,ATG4C,IGF1R,PKD1L1,TSNARE1,MON2,CNTNAP2,PTPRO,ZDHHC14,SUFU,NKAIN2,INSR,CIZ1,ITGA11,JAK1,RPGR,MAP2K1,SLC9B1,KPNB1,FOXO3,GPC6,RASGRF2,GABRA3,KLHL3,OCA2,TTC7B,CENPF,ATP9B,MITF,IGF2BP3,AGBL4,KPNA3,SPTSSA,FRMD5,ULK4,RBFOX1,LTBP1,LRRK2,NKAIN3,PKHD1,KIF2A,SPG11,PHACTR1,SCN1A,BMP6,SLC35F1,VTI1A,FYN,KCND3,ABCC2,XRCC4,MARVELD3,GAS8,MSR1,PTPN14,RIMS1,ENPP2,VPS53,MCTP2,ABCA12,EPHB1,KCNQ1,SLC38A6,DOCK1,FAM126A,EXT1,TRPC5,UNC5C,NOX5,LRP2,C2,FER,PCDH17,TMEM108,TPTE,RAPGEF2,NAV3,DNAH3,PTPRM,KIRREL3,NRXN3,KCNH7,CDKL5,PTPRU,GRIA4,KAT7,VPS45,SLC5A4,SETD2,PACSIN2,TRIM22,CDH13,TRDN,DAB1,SEC16B,SLC16A12,CACHD1,EXOC6B,HDAC5,SEMA3A,MGAT5,ATP13A3,STOML1,MYH9,BDKRB2,STIM1,NRIP1,VPS41,PIK3R3,TENM1,VPS39,GABRB1,SH3KBP1,ZRANB1,RGS7,STXBP5,TXNDC5,SLC24A2,GRIA3,NTRK3,SLC35F3,FBN1,MOGAT2,CLASP2,TMPRSS15,DNAJC6,NSUN2,TPD52,PTPRK,SORCS1,DNM3,SYNDIG1,PPARA,SCFD2,SHISA6,ANXA8L1,DOCK10,ATP8A1,SEMA3D,PRLR,KCNC4,CEP41,IPO11,NVL,SYT17,STAC,RAB27A,BLOC1S6,PARD3B,HGSNAT,CEP112,K

			CNJ15,SYT9,SLC22A10,SEMA3C
GO:0007399	nervous system development	8.235007583494911e-26	KALRN,NDE1,CTNNA1,NTRK2,PTPRG,FBXL17,DMD,KCNC2,CHRM3,ERBB4,LAMA2,CNTN4,ROBO1,DSCAM,SHROOM3,CIT,TBCD,FRY,HDAC4,OPCML,LINGO2,IMMP2L,ATP8A2,AUTS2,WLS,ANKS1A,MYEF2,TENM2,NFASC,PLCB1,PRKD1,VCL,SEMA5A,ETV6,IL1RAPL1,MYO9A,KANK1,TCF12,RUNX1,PLS1,NLGN1,CAMK1D,RELN,APP,PAK1,PPP3CA,NTM,TOX,PCDH15,PTPN9,GRM7,DLC1,SOS1,CTNND2,ALK,DCLK1,MEF2A,TFAP2D,DENND5A,MAGI2,CELSR1,ROBO2,DCC,EP300,SEMA4D,RPS6KA5,TRAPPC9,DNAH11,QKI,CHRM1,ASTN2,TANC2,JAK2,NEGR1,SDK2,PARK2,NPHP3,GRIN3A,NLGN4X,EMB,TAGLN3,NRG3,NUMB,SOX6,SULF1,SH3GL2,SLIT2,ATRX,CHRDL1,BMPR1A,DSCAML1,POTEE,EYA1,SATB2,OVOL2,ELP3,BRINP1,FGF14,IL1RAPL2,SOX5,TAF1,SLC1A1,NTN1,LDB2,EPM2A,MAP2,FLRT2,FBXW11,GAS7,CUX1,ANK3,CDH4,TNR,ARNT2,SPOCK1,TACC2,TENM4,CECR2,LRRC4C,PPP1R9A,CLSTN2,CBFA2T2,SRD5A2,STRC,DISC1,NAV2,CNTN5,SLC5A3,BLOC1S5,PCSK2,EFNA5,KLHL1,POU6F2,CEP85L,ATF2,GRID2,ZNF423,SEMA6D,NTNG1,CRMP1,GABRB3,PRKCQ,ATXN1,CLDN1,SPTBN4,EPHB2,PRTG,DPYSL2,MARK1,BDNF,ADORA2A,FSTL4,SYT1,DAB2,AFF2,PLXNA2,PRKG1,ABI1,HDAC2,FAT3,CDH23,RIMS2,SCN8A,CTNNA2,PARD3,NRG1,SPINT2,RIT2,CNGB1,PRKCG,NFIA,SLC8A1,SRRM4,FBXO31,MAP3K13,THRB,NCAM1,AGT,NDRG2,GRIP1,USH2A,NF1,ZNF536,NRXN1,LAMA3,GSK3B,CHD7,TIAM2,MYT1L,SRGAP2B,CNTN1,UNC13A,TRIOBP,NCOA1,SYBU,SHANK2,UNC5D,ISLR2,NREP,DLG5,ASTN1,ROR1,ADAMTSL1,MACF1,MNAT1,TRIO,CLDN11,CCDC141,EPHA5,TP73,MYPN,ANK2,SLIT3,GRIN2B,PAK3,SEMA5B,GRIK1,SDK1,GRM5,IGF1R,CNTNAP2,PTPRO,SUFU,CRTAC1,MAP2K1,FOXO3,GPC6,CNTN6,CENPF,IGF2BP3,LRFN5,AGBL4,ULK4,RBFOX1,LRRK2,KIF2A,RARB,TCF4,SPG11,PBX1,PHACTR1,BMP6,ASAP1,FYN,EPHA7,GAS8,RIMS1,EPHB1,LSAMP,KCNQ1,VASH2,FAM126A,EXT1,TRPC5,UNC5C,ACSBG1,TENM3,LRP2,VWC2,PCDH17,TMEM108,SHROOM4,RAPGEF2,NAV3,PTPRM,KIRREL3,NRXN3,CDKL5,MACROD2,SETD2,MDGA2,BASP1,DAB1,ZNF148,SEMA3A,TENM1,GABRB1,RGS7,NTRK3,HYDIN,CLASP2,ZNF521,DNM3,SYNDIG1,PTPRD,RORA,ELAVL4,SBF2,NELL1,DOCK10,SEMA3D,SPATA5,SYT17,BLOC1S6,SEMA3C
GO:0030182	neuron differentiation	9.85348178745227e-26	KALRN,CTNNA1,NTRK2,PTPRG,DMD,ERBB4,LAMA2,CNTN4,ROBO1,DSCAM,TBCD,FRY,OPCML,ATP8A2,AUTS2,ANKS1A,MYEF2,TENM2,NFASC,PRKD1,VCL,SEMA5A,IL1RAPL1,MYO9A,KANK1,TCF12,RUNX1,PLS1,NLGN1,CAMK1D,RELN,APP,PAK1,PPP3CA,NTM,TOX,PCDH15,PTPN9,GRM7,SOS1,CTNND2,ALK,DCLK1,MEF2A,DENND5A,MAGI2,ROBO2,DCC,EP300,SEMA4D,RPS6KA5,TRAPPC9,TANC2,JAK2,NEGR1,SDK2,PARK2,GRIN3A,NLGN4X,EMB,NUMB,SH3GL2,SLIT2,DSCAML1,EYA1,SATB2,BRINP1,NTN1,MAP2,FLRT2,GAS7,CUX1,ANK3,CDH4,TNR,SPOCK1,TENM4,CECR2,LRRC4C,PPP1R9A,CBFA2T2,STRC,DISC1,BLOC1S5,EFNA5,KLHL1,GRID2,SEMA6D,NTNG1,CRMP1,PRKCQ,SPTBN4,EPHB2,PRTG,DPYSL2,MARK1,BDNF,ADORA2A,FSTL4,SYT1,DAB2,PLXNA2,PRKG1,ABI1,HDAC2,FAT3,CDH23,RIMS2,CTNNA2,PARD3,NRG1,RIT2,CNGB1,SRRM4,FBXO31,MAP3K13,THRB,NCAM1,AGT,GRIP1,USH2A,ZNF536,NRXN1,LAMA3,GSK3B,TIAM2,MYT1L,CNTN1,UNC13A,TRIOBP,NCOA1,UNC5D,ISLR2,NREP,DLG5,ROR1,ADAMTSL1,MACF1,TRIO,CCDC141,EPHA5,TP73,MYPN,SLIT3,PAK3,SEMA5B,SDK1,IGF1R,CNTNAP2,PTPRO,SUFU,CRTAC1,MAP2K1,FOXO3,CNTN6,AGBL4,ULK4,LRRK2,TCF4,SPG11,PBX1,PHACTR1,BMP6,ASAP1,FYN,EPHA7,RIMS1,EPHB1,KCNQ1,VASH2,EXT1,TRPC5,UNC5C,TENM3,LRP2,VWC2,TMEM108,RAPGEF2,PTPRM,KIRREL3,NRXN3,CDKL5,MDGA2,DAB1,SEMA3A,TENM1,GABRB1,NTRK3,CLASP2,ZNF521,DNM3,PTPRD,RORA,ELAVL4,DOCK10,SEMA3D,SYT17,BLOC1S6,SEMA3C
GO:0030030	cell projection organization	2.429162218137122e-25	KALRN,CTNNA1,NTRK2,PTPRG,DMD,LAMA2,CNTN4,ROBO1,DSCAM,FRY,HDAC4,ATP8A2,AUTS2,TENM2,NFASC,PRKD1,VCL,SEMA5A,IL1RAPL1,MYO9A,KANK1,PLS1,NLGN1,DNAH9,CAMK1D,RELN,APP,PAK1,PPP3CA,TOX,PCDH15,PTPN9,GRM7,SOS1,CTNND2,ALK,DCLK1,MEF2A,DENND5A,MAGI2,ROBO2,IFT81,DCC,EP300,SEMA4D,RPS6KA5,NME8,TANC2,JAK2,NEGR1,PARK2,NPHP3,GRIN3A,EMB,LRRC49,NUMB,SH3GL2,ARHGAP24,SLIT2,DSCAML1,PIBF1,TUB,SPAG17,NTN1,MAP2,FLRT2,ARHGEF6,WWTR1,GAS7,CUX1,ANK3,TTLL8,CDH4,TNR,VAV3,CDKL1,SPOCK1,PLCE1,EPS8,CECR2,LRRC4C,PPP1R9A

			,CBFA2T2,STRC,DISC1,POC1A,IFT43,BLOC1S5,ARMC2,EFNA5,KLHL1,GRID2,ZNF423,SEMA6D,NTNG1,CRMP1,PRKCQ,SPTBN4,EPHB2,PRTG,DPYSL2,PTPDC1,MARK1,BDNF,ADORA2A,IFT80,FSTL4,TTC39C,ICK,SYT1,DAB2,PLXNA2,PRKG1,ABI1,HDAC2,FAT3,CDH23,RIMS2,CTNNA2,PARD3,RIT2,CD44,RAPGEF6,FBXO31,MAP3K13,CDC42EP3,NCAM1,AGT,GRIP1,PARVB,NRXN1,LAMA3,GSK3B,BBS9,TIAM2,CNTN1,UNC13A,TRIOBP,RHOJ,UNC5D,ISLR2,NREP,DLG5,ROR1,ADAMTSL1,MACF1,TRIO,SPAG16,CCDC141,FGD4,EPHA5,MYPN,SLIT3,GRIN2B,PAK3,SEMA5B,SDK1,IGF1R,CNTNAP2,PTPRO,INSR,RPGR,CRTAC1,MAP2K1,CNTN6,CDC14A,ULK4,ADAMTS16,LRRK2,PKHD1,SPG11,PHACTR1,ASAP1,FYN,EPHA7,GAS8,RIMS1,ENPP2,EPHB1,KCNQ1,VASH2,EXT1,TRPC5,UNC5C,TENM3,LRP2,FER,TMEM108,RAPGEF2,PTPRM,KIRREL3,NRXN3,CDKL5,PACSIN2,CDH13,DAB1,SEMA3A,MYH9,TANC1,TENM1,NTRK3,HYDIN,CEP89,CLASP2,DNM3,PTPRD,ELAVL4,DOCK10,SEMA3D,CEP41,SYT17,BLOC1S6,SEMA3C*
GO:0120036	plasma membrane bounded cell projection organization	2.5542332396493547e-25	*KALRN,CTNNA1,NTRK2,PTPRG,DMD,LAMA2,CNTN4,ROBO1,DSCAM,FRY,HDAC4,ATP8A2,AUTS2,TENM2,NFASC,PRKD1,VCL,SEMA5A,IL1RAPL1,MYO9A,KANK1,PLS1,NLGN1,CAMK1D,RELN,APP,PAK1,PPP3CA,TOX,PCDH15,PTPN9,GRM7,SOS1,CTNND2,ALK,DCLK1,MEF2A,DENND5A,MAGI2,ROBO2,IFT81,DCC,EP300,SEMA4D,RPS6KA5,NME8,TANC2,JAK2,NEGR1,PARK2,NPHP3,GRIN3A,EMB,LRRC49,NUMB,SH3GL2,ARHGAP24,SLIT2,DSCAML1,PIBF1,TUB,SPAG17,NTN1,MAP2,FLRT2,ARHGEF6,WWTR1,GAS7,CUX1,ANK3,TTLL8,CDH4,TNR,VAV3,CDKL1,SPOCK1,PLCE1,EPS8,CECR2,LRRC4C,PPP1R9A,CBFA2T2,STRC,DISC1,IFT43,BLOC1S5,ARMC2,EFNA5,KLHL1,GRID2,ZNF423,SEMA6D,NTNG1,CRMP1,PRKCQ,SPTBN4,EPHB2,PRTG,DPYSL2,PTPDC1,MARK1,BDNF,ADORA2A,IFT80,FSTL4,TTC39C,ICK,SYT1,DAB2,PLXNA2,PRKG1,ABI1,HDAC2,FAT3,CDH23,RIMS2,CTNNA2,PARD3,RIT2,CD44,RAPGEF6,FBXO31,MAP3K13,CDC42EP3,NCAM1,AGT,GRIP1,PARVB,NRXN1,LAMA3,GSK3B,BBS9,TIAM2,CNTN1,UNC13A,TRIOBP,UNC5D,ISLR2,NREP,DLG5,ROR1,ADAMTSL1,MACF1,TRIO,SPAG16,CCDC141,FGD4,EPHA5,MYPN,SLIT3,GRIN2B,PAK3,SEMA5B,SDK1,IGF1R,CNTNAP2,PTPRO,INSR,RPGR,CRTAC1,MAP2K1,CNTN6,CDC14A,ULK4,ADAMTS16,LRRK2,PKHD1,SPG11,PHACTR1,ASAP1,FYN,EPHA7,GAS8,RIMS1,ENPP2,EPHB1,KCNQ1,VASH2,EXT1,TRPC5,UNC5C,TENM3,LRP2,FER,TMEM108,RAPGEF2,PTPRM,KIRREL3,NRXN3,CDKL5,CDH13,DAB1,SEMA3A,MYH9,TANC1,TENM1,NTRK3,HYDIN,CEP89,CLASP2,DNM3,PTPRD,ELAVL4,DOCK10,SEMA3D,CEP41,SYT17,BLOC1S6,SEMA3C*
GO:0048468	cell development	3.896146530192573e-25	*RCAN1,KALRN,RPS6KA2,CTNNA1,NTRK2,PTPRG,DMD,ERBB4,LAMA2,CNTN4,ROBO1,DSCAM,SHROOM3,TBCD,FRY,NEBL,HDAC4,OPCML,ATP8A2,AUTS2,ANKS1A,TENM2,NFASC,PLCB1,PRKD1,VCL,NEDD9,SEMA5A,IL1RAPL1,MYO9A,KANK1,DIAPH2,RUNX1,PLS1,NLGN1,CAMK1D,RELN,APP,PAK1,PPP3CA,RYR1,NTM,TOX,PCDH15,PTPN9,AKAP6,GRM7,SOS1,CTNND2,ALK,DCLK1,MEF2A,DENND5A,MAGI2,ROBO2,AKAP13,DCC,EP300,SEMA4D,RPS6KA5,QKI,TANC2,JAK2,NEGR1,PARK2,GRIN3A,EMB,NUMB,SULF1,ATRNL1,SH3GL2,COL11A1,SLIT2,ATRX,BMPR1A,DSCAML1,SMAD3,PEAK1,SATB2,OVOL2,BRINP1,NTN1,MAP2,FLRT2,FBXW11,GAS7,CATSPER2,CUX1,ANK3,ARID4B,MYO18B,CDH4,TNR,CELF4,SPOCK1,ACTN4,TENM4,CECR2,LRRC4C,PPP1R9A,CBFA2T2,STRC,DISC1,LRP5,ESR1,PDE4D,BLOC1S5,ARMC2,EFNA5,KLHL1,GRID2,SEMA6D,NTNG1,CRMP1,PRKCQ,CLDN1,SPTBN4,EPHB2,S1PR3,PRTG,COL22A1,DPYSL2,MARK1,BDNF,ADORA2A,ARID5B,FSTL4,FHL2,MEGF9,SYT1,DAB2,PLXNA2,LRRK1,PRKG1,ABI1,HDAC2,FAT3,ARID4A,CDH23,TTN,RIMS2,CTNNA2,PARD3,NRG1,SPINT2,FMN2,RIT2,CNGB1,SLC8A1,SRRM4,FBXO31,MAP3K13,CHST11,THRB,NCAM1,AGT,GRIP1,USH2A,NF1,PARVB,NRXN1,LAMA3,GSK3B,CTDP1,CHD7,MOV10L1,TIAM2,MYT1L,CNTN1,UNC13A,TRIOBP,UNC5D,ISLR2,NREP,DLG5,PGM5,PREX1,ROR1,ADAMTSL1,MACF1,TRIO,CAPN3,SPAG16,CCDC141,EPHA5,TP73,MYPN,ANK2,SLIT3,PAK3,NEB,PACRG,SEMA5B,SDK1,GRM5,IGF1R,MSI2,CNTNAP2,PTPRO,CRTAC1,MAP2K1,FOXO3,OCA2,CNTN6,AGBL4,ULK4,LRRK2,PKHD1,RARB,SPG11,PBX1,PHACTR1,BMP6,ASAP1,FYN,EPHA7,SGCZ,RIMS1,ABCA12,EPHB1,KCNQ1,FHOD3,DOCK1,VASH2,BPGM,EXT1,TRPC5,UNC5C,TENM3,LRP2,FER,SGCD,TMEM108,RAPGEF2,PTPRM,KIR*

			REL3,NRXN3,CDKL5,SETD2,DAB1,SEMA3A,MYH9,TENM1,GABRB1,PRKAR1A,SIPA1L3,DPY19L2,NTRK3,FBN1,HYDIN,CLASP2,NSUN2,DNM3,PPARA,PTPRD,BRIP1,ELAVL4,DOCK10,FNDC3A,SEMA3D,SYT17,BLOC1S6,SEMA3C
GO:0048699	generation of neurons	5.353251865240808e-25	*KALRN,NDE1,CTNNA1,NTRK2,PTPRG,DMD,ERBB4,LAMA2,CNTN4,ROBO1,DSCAM,CIT,TBCD,FRY,OPCML,ATP8A2,AUTS2,ANKS1A,MYEF2,TENM2,NFASC,PRKD1,VCL,SEMA5A,IL1RAPL1,MYO9A,KANK1,TCF12,RUNX1,PLS1,NLGN1,CAMK1D,RELN,APP,PAK1,PPP3CA,NTM,TOX,PCDH15,PTPN9,GRM7,SOS1,CTNND2,ALK,DCLK1,MEF2A,DENND5A,MAGI2,CELSR1,ROBO2,DCC,EP300,SEMA4D,RPS6KA5,TRAPPC9,ASTN2,TANC2,JAK2,NEGR1,SDK2,PARK2,GRIN3A,NLGN4X,EMB,NRG3,NUMB,SH3GL2,SLIT2,BMPR1A,DSCAML1,EYA1,SATB2,ELP3,BRINP1,SOX5,NTN1,MAP2,FLRT2,GAS7,CUX1,ANK3,CDH4,TNR,SPOCK1,TENM4,CECR2,LRRC4C,PPP1R9A,CBFA2T2,STRC,DISC1,BLOC1S5,EFNA5,KLHL1,CEP85L,GRID2,SEMA6D,NTNG1,CRMP1,PRKCQ,SPTBN4,EPHB2,PRTG,DPYSL2,MARK1,BDNF,ADORA2A,FSTL4,SYT1,DAB2,PLXNA2,PRKG1,ABI1,HDAC2,FAT3,CDH23,RIMS2,CTNNA2,PARD3,NRG1,RIT2,CNGB1,SRRM4,FBXO31,MAP3K13,THRB,NCAM1,AGT,GRIP1,USH2A,NF1,ZNF536,NRXN1,LAMA3,GSK3B,CHD7,TIAM2,MYT1L,CNTN1,UNC13A,TRIOBP,NCOA1,UNC5D,ISLR2,NREP,DLG5,ASTN1,ROR1,ADAMTSL1,MACF1,TRIO,CCDC141,EPHA5,TP73,MYPN,SLIT3,PAK3,SEMA5B,SDK1,GRM5,IGF1R,CNTNAP2,PTPRO,SUFU,CRTAC1,MAP2K1,FOXO3,CNTN6,AGBL4,ULK4,LRRK2,TCF4,SPG11,PBX1,PHACTR1,BMP6,ASAP1,FYN,EPHA7,RIMS1,EPHB1,KCNQ1,VASH2,EXT1,TRPC5,UNC5C,TENM3,LRP2,VWC2,TMEM108,RAPGEF2,PTPRM,KIRREL3,NRXN3,CDKL5,MDGA2,DAB1,SEMA3A,TENM1,GABRB1,NTRK3,CLASP2,ZNF521,DNM3,PTPRD,RORA,ELAVL4,DOCK10,SEMA3D,SYT17,BLOC1S6,SEMA3C*
GO:0023052	signaling	9.049925869302414e-25	*PTPRR,SYN3,CAMTA1,CHFR,RCAN1,KALRN,CACNG2,RNF43,RPS6KA2,CTNNA1,FOXN3,NTRK2,RASGEF1B,ZNRF3,PTPRG,FBXL17,SLC39A10,DMD,KCNC2,CHRM3,SPRED2,ERBB4,LAMA2,NOS1,CNTN4,ESRRG,ROBO1,PRICKLE2,DSCAM,CIT,PUM1,GPC5,WDR59,SORCS2,WWOX,HUNK,FUT8,HDAC4,AUTS2,WLS,ANKS1A,LDLRAD4,TENM2,BLM,PSMB2,PTPRT,RXFP1,MGLL,NFASC,PLCB1,FLT3,SRPK2,CACNA1A,TRPM1,PRKD1,RYR2,NEDD9,SEMA5A,CASK,PRKCA,DLGAP1,BTBD9,TRHDE,IL1RAPL1,MYO9A,CCDC3,ITGAE,KANK1,GRIK4,GPR176,DOK6,FCHSD2,NLGN1,TNFRSF10B,CLEC16A,AMOTL1,TCF7L2,RELN,APP,ADAM12,PAK1,PPP3CA,DEPDC5,GABRG3,ERC2,CREM,AKAP6,SORCS3,GRM7,DLC1,IDE,RYR3,SOS1,CTNND2,RFTN1,ALK,BTRC,DCLK1,MEF2A,CTNNA3,DLG2,PPP1R12B,MAP2K5,DNMT1,MAGI2,EDA,CELSR1,ROBO2,IFT81,AKAP13,DCC,DDB1,EP300,SEMA4D,EVC,RPS6KA5,GRK5,ANKRD6,BDKRB1,TF,GRIK2,GNG12,CHRM1,CDH8,ARHGAP15,HUS1,ITSN1,DEFA1B,JAK2,ABCG8,CMKLR1,MAST4,PARK2,MAGI1,NPHP3,ADCYAP1R1,GRID1,GRIN3A,SMOC2,CACNA1C,NLGN4X,EXOC4,PIK3C2B,OR5K4,ITGBL1,AFAP1,NRG3,COL4A6,SULF1,SH3BP1,ADCY2,ATRNL1,RFFL,SH3GL2,ARHGAP24,PITPNC1,SLIT2,MAPK4,GLP2R,DTNA,TRIM5,THEMIS,DRG2,ATRX,CHRDL1,TRAF3IP2,SH3RF3,BMPR1A,SMAD3,PRKAR2A,CACNA1E,PI4KA,TNFRSF11B,PIBF1,GABRR2,PIK3C3,PTGFR,SCUBE1,SV2B,EYA1,ANKS1B,MAML3,GPR141,MAD1L1,DGKB,TNKS,TUB,ARHGEF18,LINC00473,OVOL2,SGMS1,GRAMD4,FGF14,DGKK,OR5I1,ASGR2,IL1RAPL2,SKAP1,PTPRN2,DGKI,DNMBP,TAF1,SPNS2,SLC1A1,NTN1,SHISA9,EPM2A,CDC73,FLRT2,FBXW11,NOS1AP,RANBP10,RBMS3,ARHGEF6,WWTR1,ARHGAP6,PCSK5,GRM4,ANK3,CCL15,CNIH3,DOCK3,TRIM59,TNR,ADCY9,CELF4,VAV3,SCN9A,MDM4,ACTN4,PLCE1,LRRN2,EPS8,TENM4,GHR,ARHGAP39,LRRC4C,PPP1R9A,RGL1,CLSTN2,CD300A,CBFA2T2,SRD5A2,DISC1,RALGPS1,ARHGAP42,CCL14,RABGEF1,FHIT,NSG2,TRABD2B,LRP5,ESR1,ARHGAP12,TNFRSF19,PLCXD3,GRM1,PDE4D,THEM4,PIK3CD,DOCK4,TEAD4,PDE7B,EFNA5,EIF3A,ATF2,PIK3R2,GRID2,ZNF423,CYBB,SEMA6D,KCNJ3,NTNG1,ADTRP,PDE4B,MAPRE2,RIC8B,GABRB3,MAP3K7,PRKCQ,RASSF8,RHPN2,KCND2,SPTBN4,EPHB2,ERC1,AGO3,S1PR3,MCTP1,MOB3B,PRCP,RGMB,EEF1E1-BLOC1S5,DOCK2,DPYSL2,PTPDC1,MARK1,BDNF,FNIP1,MAGI3,ADORA2A,ARID5B,SIPA1L2,RCAN2,IFT80,FSTL4,ANO1,FHL2,CADPS,NOX4,BMPER,PDE1A,ICK,DEFA3,SYT1,DAB2,PLXNA2,NSF,LRRK1,PRKG1,SNAP23,ABI1,CACNG3,EPG5,PTH2*

			R,CALCRL,TNNI3K,HDAC2,PDE11A,RAB30,PRKAR1B,ZMYND11,TTN,RGS6,SRGAP3,MYRIP,ENPP1,RIMS2,SCN8A,KSR2,DLGAP2,PRDM15,OR4N2,PEX5L,PARD3,NRG1,FMN2,GRIA2,VSTM1,RIT2,CD44,CNGB1,GPSM2,PRKCG,TNS3,TEAD1,MAPKAPK2,SLC8A1,RAPGEF6,FBXO31,FAM19A4,MAP3K13,PLCL2,CHST11,THRB,CDC42BPA,CDC42EP3,MAPK10,NCAM1,AGT,FGF1,QRICH1,CCR3,NDRG2,GRIP1,TLK1,ARHGAP25,NF1,WIF1,ZNF536,NRXN1,ARHGAP10,LAMA3,GSK3B,KIR2DL1,PKP2,CD109,KCNH1,DCDC1,LMO7,GPR21,CHD7,PSMB7,SNX5,TIAM2,OR51B2,IQCJ-SCHIP1,CNTN1,BTBD11,UNC13A,CABIN1,NCOA1,CHEK2,GRIK3,CACNB2,STXBP4,RHOJ,PHEX,MECOM,STK32B,SHANK2,KCTD8,UNC5D,NREP,GPC3,DLG5,PREX1,CPE,UBE2V1,ROR1,ZNF675,PTGER3,BID,MACF1,TRIO,CAPN3,PTPRE,DUSP22,MYOM1,MAP3K5,MAML2,CTDSPL2,HTR2C,GLRA2,FGD4,LY86,EPHA5,TP73,IL1RL1,ZNF366,P2RX6,ANK2,ARHGEF3,SLIT3,GRIN2B,PAK3,GARNL3,UACA,ADCY8,ITPR2,GABRR3,MLLT3,CCL15-CCL14,ITGB3BP,RNF213,SH3RF2,CACNA1D,PRKAA2,TBL1X,SEMA5B,GRIK1,NLN,GRM5,ARAP2,IGF1R,CNTNAP2,PTPRO,SUFU,INSR,ITGA11,JAK1,MAP2K1,UBR2,GMDS,KPNB1,FOXO3,GPC6,RASGRF2,STK38,GABRA3,INPP5A,CNTN6,CENPF,MITF,PRDM16,ULK4,KIR2DL4,TMEM117,LTBP1,LRRK2,PKHD1,RARB,SPG11,SCN1A,BMP6,FYN,KCND3,EPHA7,MARVELD3,GAS8,GNG2,RIMS1,MCTP2,ABCA12,EPHB1,KCNQ1,DOCK1,CRADD,EXT1,AMFR,PLCB4,UNC5C,TENM3,LRP2,RALGPS2,FER,CAMK4,VWC2,GUCY2F,ERLIN1,PCDH17,SGCD,TMEM108,TPTE,RAPGEF2,PTPRM,NRXN3,INPP4A,PTPRU,ZNF207,GRIA4,IL18R1,RGS7BP,ESR2,KAT7,PACSIN2,TRIM22,CDH13,VEPH1,TRDN,DAB1,SEMA3A,MGAT5,MYH9,BDKRB2,LITAF,PIK3R3,TENM1,STK38L,GABRB1,SH3KBP1,PRKAR1A,ZRANB1,RGS7,STXBP5,FCRL2,SIPA1L3,GNG4,VWF,SLC24A2,SCEL,GRIA3,NTRK3,FBN1,CEP89,CLASP2,NSUN2,PTPRK,SORCS1,DOCK9,PPARA,PTPRD,RORA,SHISA6,BRIP1,ELAVL4,DOCK10,EEF1E1,BICC1,SEMA3D,PRLR,PTPRA,KCNC4,ATF6,DEPTOR,CUL2,SYT17,STAC,OR4M1,RALGAPA1,BLOC1S6,PMEPA1,SYT9,SEMA3C,ST18,RXFP2
GO:0022008	neurogenesis	1.0084590143349665e-23	*KALRN,NDE1,CTNNA1,NTRK2,PTPRG,DMD,ERBB4,LAMA2,CNTN4,ROBO1,DSCAM,CIT,TBCD,FRY,OPCML,ATP8A2,AUTS2,ANKS1A,MYEF2,TENM2,NFASC,PRKD1,VCL,SEMA5A,ETV6,IL1RAPL1,MYO9A,KANK1,TCF12,RUNX1,PLS1,NLGN1,CAMK1D,RELN,APP,PAK1,PPP3CA,NTM,TOX,PCDH15,PTPN9,GRM7,SOS1,CTNND2,ALK,DCLK1,MEF2A,DENND5A,MAGI2,CELSR1,ROBO2,DCC,EP300,SEMA4D,RPS6KA5,TRAPPC9,CHRM1,ASTN2,TANC2,JAK2,NEGR1,SDK2,PARK2,GRIN3A,NLGN4X,EMB,NRG3,NUMB,SOX6,SH3GL2,SLIT2,BMPR1A,DSCAML1,EYA1,SATB2,ELP3,BRINP1,SOX5,SLC1A1,NTN1,EPM2A,MAP2,FLRT2,GAS7,CUX1,ANK3,CDH4,TNR,SPOCK1,TENM4,CECR2,LRRC4C,PPP1R9A,CBFA2T2,STRC,DISC1,NAV2,BLOC1S5,EFNA5,KLHL1,CEP85L,GRID2,SEMA6D,NTNG1,CRMP1,PRKCQ,SPTBN4,EPHB2,PRTG,DPYSL2,MARK1,BDNF,ADORA2A,FSTL4,SYT1,DAB2,PLXNA2,PRKG1,ABI1,HDAC2,FAT3,CDH23,RIMS2,CTNNA2,PARD3,NRG1,RIT2,CNGB1,SRRM4,FBXO31,MAP3K13,THRB,NCAM1,AGT,GRIP1,USH2A,NF1,ZNF536,NRXN1,LAMA3,GSK3B,CHD7,TIAM2,MYT1L,CNTN1,UNC13A,TRIOBP,NCOA1,UNC5D,ISLR2,NREP,DLG5,ASTN1,ROR1,ADAMTSL1,MACF1,TRIO,CCDC141,EPHA5,TP73,MYPN,SLIT3,PAK3,SEMA5B,SDK1,GRM5,IGF1R,CNTNAP2,PTPRO,SUFU,CRTAC1,MAP2K1,FOXO3,CNTN6,AGBL4,ULK4,LRRK2,RARB,TCF4,SPG11,PBX1,PHACTR1,BMP6,ASAP1,FYN,EPHA7,RIMS1,EPHB1,KCNQ1,VASH2,EXT1,TRPC5,UNC5C,TENM3,LRP2,VWC2,TMEM108,RAPGEF2,NAV3,PTPRM,KIRREL3,NRXN3,CDKL5,MDGA2,DAB1,SEMA3A,TENM1,GABRB1,NTRK3,CLASP2,ZNF521,DNM3,PTPRD,RORA,ELAVL4,DOCK10,SEMA3D,SYT17,BLOC1S6,SEMA3C*
GO:0007154	cell communication	1.8700979244122224e-23	*PTPRR,SYN3,CAMTA1,CHFR,RCAN1,KALRN,CACNG2,RNF43,RPS6KA2,CTNNA1,FOXN3,NTRK2,RASGEF1B,ZNRF3,PTPRG,FBXL17,SLC39A10,DMD,KCNC2,CHRM3,SPRED2,ERBB4,LAMA2,NOS1,CNTN4,ESRRG,ROBO1,PRICKLE2,DSCAM,CIT,PUM1,GPC5,WDR59,SORCS2,WWOX,HUNK,FUT8,HDAC4,AUTS2,WLS,ANKS1A,LDLRAD4,TENM2,BLM,PSMB2,PTPRT,RXFP1,MGLL,NFASC,PLCB1,FLT3,SRPK2,CACNA1A,TRPM1,PRKD1,RYR2,NEDD9,SEMA5A,CASK,PRKCA,DLGAP1,BTBD9,TRHDE,IL1RAPL1,MYO9A,CCDC3,ITGAE,KANK1,GRIK4,GPR176,DOK6,*

			FCHSD2,NLGN1,TNFRSF10B,CLEC16A,AMOTL1,TCF7L2,RELN,APP,ADAM12,PAK1,PPP3CA,DEPDC5,GABRG3,ERC2,CREM,AKAP6,SORCS3,GRM7,DLC1,IDE,SOS1,CTNND2,RFTN1,ALK,BTRC,DCLK1,MEF2A,CTNNA3,DLG2,PPP1R12B,MAP2K5,DNMT1,MAGI2,EDA,CELSR1,ROBO2,IFT81,AKAP13,DCC,DDB1,EP300,SEMA4D,EVC,RPS6KA5,GRK5,ANKRD6,BDKRB1,TF,GRIK2,GNG12,CHRM1,CDH8,ARHGAP15,HUS1,ITSN1,DEFA1B,JAK2,ABCG8,CMKLR1,MAST4,PARK2,MAGI1,NPHP3,ADCYAP1R1,GRID1,GRIN3A,SMOC2,CACNA1C,NLGN4X,EXOC4,PIK3C2B,OR5K4,ITGBL1,AFAP1,NRG3,COL4A6,SULF1,SH3BP1,ADCY2,ATRNL1,RFFL,SH3GL2,ARHGAP24,PITPNC1,SLIT2,MAPK4,GLP2R,DTNA,TRIM5,THEMIS,DRG2,ATRX,CHRDL1,TRAF3IP2,SH3RF3,BMPR1A,SMAD3,PRKAR2A,CACNA1E,PI4KA,TNFRSF11B,PIBF1,GABRR2,PIK3C3,PTGFR,SCUBE1,SV2B,EYA1,ANKS1B,MAML3,GPR141,MAD1L1,DGKB,TNKS,TUB,ARHGEF18,LINC00473,OVOL2,SGMS1,GRAMD4,FGF14,DGKK,OR5I1,ASGR2,IL1RAPL2,SKAP1,PTPRN2,DGKI,DNMBP,TAF1,SPNS2,SLC1A1,NTN1,SHISA9,EPM2A,CDC73,FLRT2,FBXW11,NOS1AP,RANBP10,RBMS3,ARHGEF6,WWTR1,ARHGAP6,PCSK5,GRM4,ANK3,CCL15,CNIH3,DOCK3,TRIM59,TNR,ADCY9,CELF4,VAV3,SCN9A,MDM4,ACTN4,PLCE1,LRRN2,EPS8,TENM4,GHR,ARHGAP39,LRRC4C,PPP1R9A,RGL1,CLSTN2,CD300A,CBFA2T2,SRD5A2,DISC1,RALGPS1,ARHGAP42,CCL14,RABGEF1,FHIT,NSG2,TRABD2B,LRP5,ESR1,ARHGAP12,TNFRSF19,PLCXD3,GRM1,PDE4D,THEM4,PIK3CD,DOCK4,TEAD4,PDE7B,EFNA5,EIF3A,ATF2,PIK3R2,GRID2,ZNF423,CYBB,SEMA6D,KCNJ3,NTNG1,ADTRP,PDE4B,MAPRE2,RIC8B,GABRB3,MAP3K7,PRKCQ,RASSF8,RHPN2,KCND2,SPTBN4,EPHB2,ERC1,AGO3,S1PR3,MCTP1,MOB3B,PRCP,RGMB,EEF1E1-BLOC1S5,DOCK2,DPYSL2,PTPDC1,MARK1,BDNF,FNIP1,MAGI3,ADORA2A,ARID5B,SIPA1L2,RCAN2,IFT80,FSTL4,ANO1,FHL2,CADPS,NOX4,BMPER,PDE1A,ICK,DEFA3,SYT1,DAB2,PLXNA2,LRRK1,PRKG1,SNAP23,ABI1,CACNG3,EPG5,PTH2R,CALCRL,TNNI3K,HDAC2,PDE11A,RAB30,PRKAR1B,ZMYND11,TTN,RGS6,SRGAP3,MYRIP,ENPP1,RIMS2,SCN8A,KSR2,DLGAP2,PRDM15,OR4N2,PEX5L,PARD3,NRG1,FMN2,GRIA2,VSTM1,RIT2,CD44,CNGB1,GPSM2,PRKCG,TNS3,ZFYVE1,TEAD1,MAPKAPK2,SLC8A1,RAPGEF6,FBXO31,FAM19A4,MAP3K13,PLCL2,CHST11,THRB,CDC42BPA,CDC42EP3,MAPK10,NCAM1,AGT,FGF1,QRICH1,CCR3,NDRG2,GRIP1,TLK1,ARHGAP25,NF1,WIF1,ZNF536,NRXN1,ARHGAP10,LAMA3,GSK3B,KIR2DL1,PKP2,CD109,KCNH1,DCDC1,GPR21,CHD7,PSMB7,SNX5,TIAM2,OR51B2,IQCJ-SCHIP1,CNTN1,BTBD11,UNC13A,CABIN1,NCOA1,CHEK2,GRIK3,CACNB2,STXBP4,RHOJ,PHEX,MECOM,STK32B,SHANK2,KCTD8,UNC5D,NREP,GPC3,DLG5,PREX1,CPE,UBE2V1,ROR1,ZNF675,PTGER3,BID,MACF1,TRIO,CAPN3,PTPRE,DUSP22,MYOM1,MAP3K5,MAML2,CTDSPL2,HTR2C,GLRA2,FGD4,LY86,EPHA5,TP73,IL1RL1,ZNF366,P2RX6,ANK2,ARHGEF3,SLIT3,GRIN2B,PAK3,GARNL3,UACA,ADCY8,ITPR2,GABRR3,MLLT3,CCL15-CCL14,ITGB3BP,RNF213,SH3RF2,CACNA1D,PRKAA2,TBL1X,SEMA5B,GRIK1,NLN,GRM5,ARAP2,IGF1R,CNTNAP2,PTPRO,SUFU,INSR,ITGA11,JAK1,MAP2K1,UBR2,GMDS,KPNB1,FOXO3,GPC6,RASGRF2,STK38,GABRA3,INPP5A,CNTN6,CENPF,MITF,PRDM16,ULK4,KIR2DL4,TMEM117,LTBP1,LRRK2,PKHD1,RARB,SPG11,SCN1A,BMP6,FYN,EPHA7,MARVELD3,GAS8,GNG2,RIMS1,MCTP2,ABCA12,EPHB1,KCNQ1,DOCK1,CRADD,EXT1,AMFR,PLCB4,UNC5C,TENM3,LRP2,RALGPS2,FER,CAMK4,VWC2,GUCY2F,ERLIN1,PCDH17,SGCD,TMEM108,TPTE,RAPGEF2,PTPRM,NRXN3,INPP4A,PTPRU,ZNF207,GRIA4,IL18R1,RGS7BP,ESR2,KAT7,PACSIN2,TRIM22,CDH13,VEPH1,TRDN,DAB1,SNTG1,SEMA3A,MGAT5,MYH9,BDKRB2,VPS41,LITAF,PIK3R3,TENM1,STK38L,GABRB1,SH3KBP1,PRKAR1A,ZRANB1,RGS7,STXBP5,FCRL2,SIPA1L3,GNG4,VWF,SLC24A2,SCEL,GRIA3,NTRK3,FBN1,CEP89,CLASP2,NSUN2,PTPRK,SORCS1,DOCK9,PPARA,PTPRD,RORA,SHISA6,BRIP1,ELAVL4,DOCK10,EEF1E1,BICC1,SEMA3D,PRLR,PTPRA,KCNC4,ATF6,DEPTOR,CUL2,SYT17,STAC,OR4M1,RALGAPA1,BLOC1S6,PMEPA1,SYT9,SEMA3C,ST18,RXFP2
GO:0048858	cell projection morphogenesis	1.148856465605218e-22	*KALRN,NTRK2,DMD,LAMA2,CNTN4,ROBO1,DSCAM,ATP8A2,AUTS2,NFASC,VCL,SEMA5A,IL1RAPL1,MYO9A,KANK1,NLGN1,RELN,APP,PAK1,PPP3CA,SOS1,CTNND2,DCLK1,MEF2A,RO*

			BO2,DCC,SEMA4D,RPS6KA5,TANC2,PARK2,EMB,NUMB,SH3GL2,SLIT2,DSCAML1,NTN1,MAP2,FLRT2,GAS7,CUX1,ANK3,CDH4,TNR,LRRC4C,DISC1,EFNA5,SEMA6D,NTNG1,CRMP1,PRKCQ,SPTBN4,EPHB2,PRTG,DPYSL2,BDNF,ADORA2A,FSTL4,SYT1,PLXNA2,ABI1,RIMS2,CTNNA2,PARD3,CD44,FBXO31,MAP3K13,NCAM1,GRIP1,NRXN1,LAMA3,GSK3B,TIAM2,UNC13A,UNC5D,ISLR2,ADAMTSL1,MACF1,TRIO,CCDC141,EPHA5,MYPN,SLIT3,PAK3,SEMA5B,IGF1R,CNTNAP2,PTPRO,MAP2K1,CNTN6,LRRK2,SPG11,PHACTR1,FYN,EPHA7,RIMS1,ENPP2,EPHB1,EXT1,TRPC5,UNC5C,LRP2,TMEM108,RAPGEF2,PTPRM,KIRREL3,NRXN3,CDKL5,PACSIN2,DAB1,SEMA3A,CLASP2,DNM3,PTPRD,ELAVL4,DOCK10,SEMA3D,SYT17,SEMA3C
GO:0120039	plasma membrane bounded cell projection morphogenesis	2.308477381236667e-22	*KALRN,NTRK2,DMD,LAMA2,CNTN4,ROBO1,DSCAM,ATP8A2,AUTS2,NFASC,VCL,SEMA5A,IL1RAPL1,MYO9A,KANK1,NLGN1,RELN,APP,PAK1,PPP3CA,SOS1,CTNND2,DCLK1,MEF2A,ROBO2,DCC,SEMA4D,RPS6KA5,TANC2,PARK2,EMB,NUMB,SH3GL2,SLIT2,DSCAML1,NTN1,MAP2,FLRT2,GAS7,CUX1,ANK3,CDH4,TNR,LRRC4C,DISC1,EFNA5,SEMA6D,NTNG1,CRMP1,PRKCQ,SPTBN4,EPHB2,PRTG,DPYSL2,BDNF,ADORA2A,FSTL4,SYT1,PLXNA2,ABI1,RIMS2,CTNNA2,PARD3,CD44,FBXO31,MAP3K13,NCAM1,GRIP1,NRXN1,LAMA3,GSK3B,TIAM2,UNC13A,UNC5D,ISLR2,ADAMTSL1,MACF1,TRIO,CCDC141,EPHA5,MYPN,SLIT3,PAK3,SEMA5B,IGF1R,CNTNAP2,PTPRO,MAP2K1,CNTN6,LRRK2,SPG11,PHACTR1,FYN,EPHA7,RIMS1,ENPP2,EPHB1,EXT1,TRPC5,UNC5C,LRP2,TMEM108,RAPGEF2,PTPRM,KIRREL3,NRXN3,CDKL5,DAB1,SEMA3A,CLASP2,DNM3,PTPRD,ELAVL4,DOCK10,SEMA3D,SYT17,SEMA3C*
GO:0032989	cellular component morphogenesis	5.014067577104319e-22	*KALRN,NTRK2,CLASP1,DMD,LAMA2,CNTN4,ROBO1,DSCAM,NEBL,ATP8A2,AUTS2,NFASC,VCL,SEMA5A,IL1RAPL1,MYO9A,KANK1,NLGN1,RELN,APP,PAK1,PPP3CA,SOS1,CTNND2,DCLK1,MEF2A,ROBO2,AKAP13,DCC,SEMA4D,RPS6KA5,TANC2,PARK2,EMB,NUMB,SH3GL2,SLIT2,DSCAML1,NTN1,MAP2,FLRT2,GAS7,CUX1,ANK3,CDH4,TNR,TENM4,LRRC4C,DISC1,EFNA5,SEMA6D,NTNG1,CRMP1,PRKCQ,SPTBN4,EPHB2,PRTG,DPYSL2,BDNF,ADORA2A,FSTL4,SYT1,PLXNA2,ABI1,TTN,RIMS2,CTNNA2,PARD3,CD44,FBXO31,MAP3K13,NCAM1,GRIP1,NRXN1,LAMA3,GSK3B,TIAM2,UNC13A,UNC5D,ISLR2,PGM5,ADAMTSL1,MACF1,TRIO,CAPN3,CCDC141,EPHA5,MYPN,ANK2,SLIT3,PAK3,NEB,SEMA5B,IGF1R,CNTNAP2,PTPRO,MAP2K1,CNTN6,LRRK2,SPG11,PHACTR1,FYN,EPHA7,RIMS1,ENPP2,EPHB1,FHOD3,EXT1,TRPC5,UNC5C,LRP2,TMEM108,RAPGEF2,PTPRM,KIRREL3,NRXN3,CDKL5,PACSIN2,DAB1,SEMA3A,PRKAR1A,CLASP2,DNM3,PTPRD,ELAVL4,DOCK10,SEMA3D,SYT17,SEMA3C*
GO:0048812	neuron projection morphogenesis	1.2173855854391799e-21	*KALRN,NTRK2,DMD,LAMA2,CNTN4,ROBO1,DSCAM,ATP8A2,AUTS2,NFASC,VCL,SEMA5A,IL1RAPL1,MYO9A,NLGN1,RELN,APP,PAK1,PPP3CA,SOS1,CTNND2,DCLK1,MEF2A,ROBO2,DCC,SEMA4D,RPS6KA5,TANC2,PARK2,EMB,NUMB,SH3GL2,SLIT2,DSCAML1,NTN1,MAP2,FLRT2,GAS7,CUX1,ANK3,CDH4,TNR,LRRC4C,DISC1,EFNA5,SEMA6D,NTNG1,CRMP1,PRKCQ,SPTBN4,EPHB2,PRTG,DPYSL2,BDNF,ADORA2A,FSTL4,SYT1,PLXNA2,ABI1,RIMS2,CTNNA2,PARD3,FBXO31,MAP3K13,NCAM1,GRIP1,NRXN1,LAMA3,GSK3B,TIAM2,UNC13A,UNC5D,ISLR2,ADAMTSL1,MACF1,TRIO,CCDC141,EPHA5,MYPN,SLIT3,PAK3,SEMA5B,IGF1R,CNTNAP2,PTPRO,MAP2K1,CNTN6,LRRK2,SPG11,PHACTR1,FYN,EPHA7,RIMS1,EPHB1,EXT1,TRPC5,UNC5C,LRP2,TMEM108,RAPGEF2,PTPRM,KIRREL3,NRXN3,CDKL5,DAB1,SEMA3A,CLASP2,DNM3,PTPRD,ELAVL4,DOCK10,SEMA3D,SYT17,SEMA3C*
GO:0032990	cell part morphogenesis	1.6285813778353558e-21	*KALRN,NTRK2,DMD,LAMA2,CNTN4,ROBO1,DSCAM,ATP8A2,AUTS2,NFASC,VCL,SEMA5A,IL1RAPL1,MYO9A,KANK1,NLGN1,RELN,APP,PAK1,PPP3CA,SOS1,CTNND2,DCLK1,MEF2A,ROBO2,DCC,SEMA4D,RPS6KA5,TANC2,PARK2,EMB,NUMB,SH3GL2,SLIT2,DSCAML1,NTN1,MAP2,FLRT2,GAS7,CUX1,ANK3,CDH4,TNR,LRRC4C,DISC1,EFNA5,SEMA6D,NTNG1,CRMP1,PRKCQ,SPTBN4,EPHB2,PRTG,DPYSL2,BDNF,ADORA2A,FSTL4,SYT1,PLXNA2,ABI1,RIMS2,CTNNA2,PARD3,CD44,FBXO31,MAP3K13,NCAM1,GRIP1,NRXN1,LAMA3,GSK3B,TIAM2,UNC13A,UNC5D,ISLR2,ADAMTSL1,MACF1,TRIO,CCDC141,EPHA5,MYPN,SLIT3,PAK3,SEMA5B,IGF1R,CNTNAP2,PTPRO,MAP2K1,CNTN6,LRRK2,SPG11,PHACTR1,FYN,EPHA7,RIMS1,ENPP2,EPHB1,EXT1,TRPC5,UNC5C,LRP2,TMEM108,RAPGEF2,PTPRM,KIRREL3,NRXN3,CDKL5,PACSIN2,DAB1,SEMA3A,CLASP2,DNM*

			3,PTPRD,ELAVL4,DOCK10,SEMA3D,SYT17,SEMA3C
GO:0006928	movement of cell or subcellular component	6.721282267175965e-21	PTPRR,KALRN,NDE1,CTNNA1,NTRK2,CLASP1,PTPRG,ERBB4,LAMA2,CNTN4,ROBO1,DSCAM,GPC5,FUT8,HDAC4,ABCC1,AUTS2,ANKS1A,LDLRAD4,PTPRT,NFASC,PLCB1,PRKD1,RYR2,VCL,NEDD9,SEMA5A,PRKCA,EPB41L4B,DNAH10,KANK1,KIF26B,DNAH9,CAMK1D,AMOTL1,RELN,APP,PAK1,PPP3CA,DLC1,SOS1,DCLK1,CTNNA3,DLG2,MAP2K5,MAGI2,CELSR1,ROBO2,IFT81,DCC,ELMO2,SEMA4D,RPS6KA5,BDKRB1,NME8,TF,DNAH11,ASTN2,DEFA1B,JAK2,CMKLR1,NPHP3,SMOC2,CACNA1C,EMB,PIK3C2B,ITGBL1,NRG3,NUMB,SULF1,SH3BP1,ATRNL1,RFFL,ARHGAP24,SLIT2,EPDR1,BMPR1A,DSCAML1,SMAD3,SLC9C1,PEAK1,SATB2,TUB,OVOL2,ELP3,SPAG17,SPNS2,NTN1,LDB2,MAP2,FLRT2,FBXW11,NOS1AP,CATSPER2,STARD9,CCL15,TTLL8,CDH4,TNR,VAV3,SPOCK1,ACTN4,EPS8,CD300A,DISC1,CCL14,IFT43,RABGEF1,LRP5,PDE4D,PIK3CD,DOCK4,BLOC1S5,ARMC2,EFNA5,CEP85L,SEMA6D,KCNJ3,NTNG1,ADTRP,CRMP1,PDE4B,MAPRE2,PRKCQ,CLDN1,EPHB2,MCTP1,PRCP,PRTG,DPYSL2,DACH1,MARK1,BDNF,ARID5B,MEGF9,BMPER,ICK,DAB2,PLXNA2,PRKG1,ETS1,FAT3,SRGAP3,CTNNA2,FMNL2,NRG1,SPINT2,FMN2,CD44,SLC8A1,FBXO31,FAM19A4,CDC42BPA,NCAM1,AGT,FGF1,CCR3,USH2A,NF1,NRXN1,LAMA3,PKP2,SRGAP2B,CACNB2,RHOJ,SYBU,UNC5D,GPC3,DLG5,ASTN1,PREX1,ADAMTSL1,MACF1,TRIO,DUSP22,SPAG16,CCDC141,EPHA5,MYPN,ANK2,SLIT3,PAK3,LRPPRC,CCL15-CCL14,SH3RF2,CACNA1D,SEMA5B,KIF6,IGF1R,PTPRO,INSR,ITGA11,RPGR,MAP2K1,SLC9B1,KPNB1,FOXO3,GPC6,CNTN6,MITF,AGBL4,FRMD5,ULK4,LRRK2,PKHD1,KIF2A,SPG11,PHACTR1,SCN1A,FYN,KCND3,EPHA7,MARVELD3,GAS8,ENPP2,EPHB1,KCNQ1,DOCK1,EXT1,UNC5C,FER,SGCD,TMEM108,TPTE,RAPGEF2,NAV3,DNAH3,PTPRM,KIRREL3,NRXN3,CDKL5,PTPRU,SETD2,CDH13,DAB1,HDAC5,SEMA3A,MGAT5,MYH9,PIK3R3,SH3KBP1,ZRANB1,NTRK3,HYDIN,CLASP2,PTPRK,DOCK10,ATP8A1,SEMA3D,BLOC1S6,SEMA3C
GO:0035556	intracellular signal transduction	9.250881172802243e-21	PTPRR,CAMTA1,CHFR,RCAN1,KALRN,RPS6KA2,FOXN3,NTRK2,RASGEF1B,DMD,KCNC2,CHRM3,SPRED2,ERBB4,NOS1,ROBO1,CIT,PUM1,WDR59,WWOX,HUNK,HDAC4,AUTS2,WLS,BLM,PSMB2,PLCB1,FLT3,SRPK2,PRKD1,RYR2,SEMA5A,PRKCA,MYO9A,KANK1,DOK6,NLGN1,TNFRSF10B,CLEC16A,AMOTL1,TCF7L2,RELN,APP,PAK1,PPP3CA,DEPDC5,AKAP6,DLC1,SOS1,ALK,BTRC,DCLK1,MEF2A,MAP2K5,DNMT1,MAGI2,EDA,CELSR1,AKAP13,EP300,SEMA4D,RPS6KA5,ANKRD6,TF,GRIK2,ARHGAP15,HUS1,ITSN1,JAK2,CMKLR1,MAST4,PARK2,ADCYAP1R1,CACNA1C,PIK3C2B,NRG3,SH3BP1,ADCY2,RFFL,ARHGAP24,SLIT2,MAPK4,TRIM5,ATRX,TRAF3IP2,SH3RF3,SMAD3,PRKAR2A,PI4KA,PIK3C3,PTGFR,MAD1L1,DGKB,ARHGEF18,LINC00473,SGMS1,FGF14,DGKK,DGKI,DNMBP,TAF1,NTN1,FBXW11,NOS1AP,ARHGEF6,WWTR1,ARHGAP6,GRM4,CCL15,DOCK3,TRIM59,ADCY9,VAV3,MDM4,ACTN4,PLCE1,EPS8,GHR,ARHGAP39,PPP1R9A,RGL1,CD300A,DISC1,RALGPS1,ARHGAP42,CCL14,RABGEF1,FHIT,ESR1,ARHGAP12,TNFRSF19,GRM1,PDE4D,THEM4,PIK3CD,DOCK4,TEAD4,PDE7B,EIF3A,ATF2,PIK3R2,ADTRP,MAPRE2,MAP3K7,PRKCQ,EPHB2,ERC1,AGO3,MCTP1,MOB3B,EEF1E1-BLOC1S5,DOCK2,MARK1,FNIP1,MAGI3,SIPA1L2,RCAN2,FHL2,NOX4,BMPER,ICK,DAB2,LRRK1,PRKG1,PDE11A,RAB30,ZMYND11,TTN,RGS6,SRGAP3,KSR2,PRDM15,PEX5L,NRG1,FMN2,RIT2,CD44,GPSM2,PRKCG,TNS3,TEAD1,MAPKAPK2,SLC8A1,RAPGEF6,FBXO31,MAP3K13,PLCL2,CDC42BPA,CDC42EP3,MAPK10,AGT,FGF1,QRICH1,CCR3,NDRG2,GRIP1,TLK1,ARHGAP25,NF1,NRXN1,ARHGAP10,GSK3B,KCNH1,DCDC1,PSMB7,TIAM2,IQCJ-SCHIP1,UNC13A,CHEK2,RHOJ,MECOM,STK32B,SHANK2,DLG5,PREX1,UBE2V1,ROR1,ZNF675,BID,TRIO,CAPN3,DUSP22,MYOM1,MAP3K5,HTR2C,FGD4,EPHA5,TP73,ANK2,ARHGEF3,GRIN2B,PAK3,GARNL3,UACA,ADCY8,ITPR2,SH3RF2,PRKAA2,GRM5,IGF1R,INSR,JAK1,MAP2K1,UBR2,KPNB1,FOXO3,RASGRF2,STK38,INPP5A,CENPF,ULK4,TMEM117,LRRK2,PKHD1,FYN,EPHA7,MARVELD3,MCTP2,EPHB1,DOCK1,CRADD,PLCB4,LRP2,RALGPS2,FER,CAMK4,GUCY2F,SGCD,TPTE,RAPGEF2,ZNF207,IL18R1,KAT7,TRIM22,CDH13,DAB1,SEMA3A,BDKRB2,LITAF,PIK3R3,TENM1,STK38L,PRKAR1A,RGS7,SIPA1L3,VWF,NTRK3,NSUN2,DOCK9,PPARA,RORA,BRIP1,DOCK10,EE

			F1E1,DEPTOR,CUL2,STAC,RALGAPA1
GO:0023051	regulation of signaling	1.1266157098725409e-20	PTPRR,SYN3,CAMTA1,RCAN1,KALRN,CACNG2,RNF43,CTNNA1,NTRK2,ZNRF3,FBXL17,SLC39A10,DMD,SPRED2,ERBB4,LAMA2,CNTN4,ROBO1,CIT,PUM1,GPC5,WDR59,SORCS2,WWOX,AUTS2,WLS,ANKS1A,LDLRAD4,PSMB2,PTPRT,MGLL,PLCB1,FLT3,CACNA1A,PRKD1,RYR2,SEMA5A,CASK,PRKCA,DLGAP1,BTBD9,MYO9A,CCDC3,KANK1,GRIK4,DOK6,NLGN1,TNFRSF10B,CLEC16A,TCF7L2,RELN,APP,PAK1,PPP3CA,DEPDC5,ERC2,AKAP6,SORCS3,GRM7,DLC1,RYR3,SOS1,CTNND2,ALK,BTRC,MAP2K5,MAGI2,EDA,ROBO2,IFT81,AKAP13,DCC,EP300,SEMA4D,EVC,GRK5,ANKRD6,GRIK2,ARHGAP15,ITSN1,JAK2,CMKLR1,PARK2,NPHP3,ADCYAP1R1,GRID1,GRIN3A,SMOC2,NLGN4X,AFAP1,NRG3,SULF1,SH3BP1,RFFL,ARHGAP24,SLIT2,TRIM5,CHRDL1,TRAF3IP2,SH3RF3,BMPR1A,SMAD3,PIBF1,SCUBE1,EYA1,MAD1L1,DGKB,TNKS,TUB,ARHGEF18,LINC00473,OVOL2,SGMS1,GRAMD4,DGKI,DNMBP,TAF1,SLC1A1,SHISA9,CDC73,FBXW11,NOS1AP,RBMS3,WWTR1,ARHGAP6,GRM4,CCL15,CNIH3,DOCK3,TRIM59,TNR,CELF4,VAV3,ACTN4,PLCE1,EPS8,GHR,ARHGAP39,LRRC4C,PPP1R9A,CLSTN2,CD300A,CBFA2T2,DISC1,RALGPS1,ARHGAP42,CCL14,RABGEF1,TRABD2B,LRP5,ESR1,ARHGAP12,TNFRSF19,GRM1,PDE4D,PIK3CD,EFNA5,EIF3A,PIK3R2,GRID2,ZNF423,NTNG1,PDE4B,MAPRE2,RIC8B,MAP3K7,PRKCQ,EPHB2,ERC1,AGO3,MCTP1,MOB3B,PRCP,EEF1E1-BLOC1S5,DOCK2,BDNF,FNIP1,MAGI3,ADORA2A,SIPA1L2,IFT80,FSTL4,ANO1,FHL2,NOX4,BMPER,SYT1,DAB2,NSF,LRRK1,CACNG3,TNNI3K,HDAC2,PDE11A,PRKAR1B,ZMYND11,RGS6,SRGAP3,MYRIP,ENPP1,RIMS2,DLGAP2,PRDM15,PEX5L,NRG1,RIT2,CD44,PRKCG,SLC8A1,FAM19A4,MAP3K13,PLCL2,CHST11,NCAM1,AGT,FGF1,NDRG2,ARHGAP25,NF1,WIF1,ZNF536,NRXN1,ARHGAP10,GSK3B,CD109,LMO7,GPR21,CHD7,PSMB7,SNX5,TIAM2,IQCJ-SCHIP1,UNC13A,NCOA1,CHEK2,GRIK3,CACNB2,STXBP4,MECOM,SHANK2,KCTD8,NREP,GPC3,DLG5,PREX1,UBE2V1,ROR1,ZNF675,BID,MACF1,TRIO,CAPN3,PTPRE,DUSP22,MAP3K5,CTDSPL2,HTR2C,FGD4,LY86,EPHA5,TP73,ZNF366,ANK2,ARHGEF3,SLIT3,GRIN2B,PAK3,GARNL3,UACA,ADCY8,MLLT3,RNF213,SH3RF2,PRKAA2,TBL1X,GRIK1,GRM5,IGF1R,PTPRO,SUFU,INSR,MAP2K1,UBR2,FOXO3,GPC6,RASGRF2,STK38,CNTN6,PRDM16,ULK4,LTBP1,LRRK2,PKHD1,BMP6,FYN,EPHA7,MARVELD3,GAS8,RIMS1,MCTP2,ABCA12,EPHB1,CRADD,AMFR,PLCB4,LRP2,RALGPS2,FER,VWC2,GUCY2F,PCDH17,TMEM108,TPTE,RAPGEF2,NRXN3,PTPRU,IL18R1,RGS7BP,ESR2,PACSIN2,TRIM22,CDH13,VEPH1,DAB1,SEMA3A,MGAT5,BDKRB2,LITAF,TENM1,ZRANB1,RGS7,STXBP5,SIPA1L3,GNG4,VWF,SLC24A2,SCEL,NTRK3,FBN1,PPARA,PTPRD,RORA,SHISA6,ELAVL4,EEF1E1,BICC1,PRLR,PTPRA,ATF6,DEPTOR,RALGAPA1,PMEPA1,SYT9
GO:0000904	cell morphogenesis involved in differentiation	1.1768842970770787e-20	KALRN,NTRK2,LAMA2,CNTN4,ROBO1,DSCAM,TBCD,ATP8A2,AUTS2,NFASC,VCL,NEDD9,SEMA5A,IL1RAPL1,KANK1,PLS1,NLGN1,RELN,APP,PAK1,PPP3CA,PCDH15,SOS1,CTNND2,DCLK1,MEF2A,ROBO2,DCC,EP300,SEMA4D,RPS6KA5,TANC2,EMB,NUMB,ATRNL1,SLIT2,DSCAML1,PEAK1,NTN1,MAP2,FLRT2,CUX1,ANK3,CDH4,TNR,ACTN4,LRRC4C,STRC,DISC1,EFNA5,SEMA6D,NTNG1,CRMP1,PRKCQ,SPTBN4,EPHB2,PRTG,COL22A1,DPYSL2,BDNF,FSTL4,MEGF9,DAB2,PLXNA2,ABI1,FAT3,CDH23,CTNNA2,PARD3,SPINT2,FBXO31,MAP3K13,NCAM1,USH2A,PARVB,NRXN1,LAMA3,GSK3B,TIAM2,TRIOBP,UNC5D,ISLR2,PREX1,ADAMTSL1,MACF1,TRIO,CCDC141,EPHA5,MYPN,SLIT3,PAK3,SEMA5B,IGF1R,PTPRO,MAP2K1,CNTN6,LRRK2,PKHD1,SPG11,PHACTR1,FYN,EPHA7,EPHB1,DOCK1,EXT1,TRPC5,UNC5C,FER,RAPGEF2,PTPRM,NRXN3,CDKL5,DAB1,SEMA3A,MYH9,SIPA1L3,CLASP2,DNM3,PTPRD,ELAVL4,DOCK10,SEMA3D,SEMA3C
GO:0065008	regulation of biological quality	1.3344727242752429e-20	SYN3,CHFR,KALRN,CACNG2,PROS1,RPS6KA2,NTRK2,SLC39A10,DMD,KCNC2,CHRM3,ERBB4,LAMA2,NOS1,CNTN4,ESRRG,DSCAM,SHROOM3,CIT,PUM1,SORCS2,TNRC6B,ATP9A,LINGO2,ABCC1,ATP8A2,MYEF2,PSMB2,ATF7IP,FLT3,CACNA1A,TRPM1,PRKD1,RYR2,VCL,PPP2R3C,SEMA5A,CASK,PRKCA,ZBTB20,BTBD9,TRHDE,IL1RAPL1,DIO2,KANK1,GRIK4,PLS1,USP53,FCHSD2,NLGN1,TCF7L2,RELN,APP,PPP3CA,RYR1,PCDH15,GABRG3,ERC2,AKAP6,SORCS3,DLC1,IDE,RYR3,SOS1,ALK,BTRC,FAM155A,CTNNA3,ROBO2,DCC,SAMD4A,C10ORF90,

			DDB1,EP300,PARN,SEMA4D,BDKRB1,TF,GRIK2,CHRM1,CDH8,ARHGAP15,TANC2,JAK2,ABCG8,NEGR1,CMKLR1,PARK2,NPHP3,ADCYAP1R1,GRID1,GRIN3A,CACNA1C,NLGN4X,PNPLA3,ANO4,NBEA,TMPRSS3,PALMD,NUMB,DTD2,SH3BP1,OTC,SH3GL2,SLIT2,SLC4A4,TRAF3IP2,ACACA,SMAD3,TNFRSF11B,GABRR2,SLC9C1,POTEE,PTGFR,SCUBE1,ELN,TPH1,DGKB,TUB,ARHGEF18,OMA1,CPQ,DGKK,ASGR2,IL1RAPL2,PTPRN2,DGKI,DNMBP,TAF1,SPNS2,SLC1A1,NTN1,LDB2,SHISA9,MAP2,CDC73,FLRT2,FBXW11,NOS1AP,WWTR1,PEMT,KCTD7,PCSK5,GRM4,ANK3,CCL15,CDH4,TNR,CELF4,VAV3,SCN9A,MDM4,PLCE1,CYP39A1,EPS8,PRR16,GHR,LRRC8D,PALM2,CLSTN2,SRD5A2,DISC1,ARHGAP42,ATP8B4,CCL14,LRP5,NAV2,AP2B1,SLC30A7,ESR1,GRM1,PDE4D,THEM4,PIK3CD,DOCK4,PCSK2,EFNA5,FTO,ATF2,PIK3R2,GRID2,ZNF423,SEMA6D,KCNJ3,ADTRP,PDE4B,GABRB3,PPA2,PRKCQ,CLDN1,KCND2,SLC24A3,SPTBN4,EPHB2,ERC1,S1PR3,MCTP1,PRCP,DPYSL2,BDNF,MALRD1,ADORA2A,FSTL4,ANO1,CADPS,NOX4,CSMD1,SYT1,DAB2,PLXNA2,LRRK1,PRKG1,SNAP23,CHST9,CACNG3,PTH2R,TNNI3K,ETS1,ARID4A,ZSWIM7,CDH23,MYRIP,ENPP1,RIMS2,SCN8A,KSR2,CTNNA2,CYB561A3,FMNL2,NRG1,ATP10B,KCNMA1,CNGB1,PRKCG,SLC12A8,MAPKAPK2,SLC8A1,FAM19A4,MAP3K13,PLCL2,THRB,SLC9A9,CDC42EP3,AGT,HEPHL1,CCR3,GRIP1,USH2A,NF1,PARVB,NRXN1,PDXP,GSK3B,PKP2,SLC22A3,KCNH1,GPR21,CHD7,PSMB7,SNX5,UNC13A,TRIOBP,CHEK2,GRIK3,CACNB2,STXBP4,RHOJ,SHANK2,ISLR2,DLG5,CFDP1,CPE,ANO3,XKR4,ZNF675,PTGER3,BID,MACF1,CAPN3,HTR2C,GLRA2,FGD4,EPHA5,SGIP1,FAM171A1,MORC3,P2RX6,ANK2,GRIN2B,PAK3,ADCY8,ITPR2,GABRR3,NEB,CACNA1D,PRKAA2,TBL1X,SEMA5B,GRIK1,GRM5,IGF1R,PTPRO,INSR,SLC9B1,FOXO3,GPC6,RASGRF2,GABRA3,KLHL3,RNLS,ATP9B,LRFN5,PRDM16,ADAMTS16,LRRK2,PKHD1,SCN1A,BMP6,FRMPD4,FYN,KCND3,EPHA7,SGCZ,CLN6,RIMS1,MCTP2,ABCA12,EPHB1,KCNQ1,FHOD3,BPGM,EXT1,TRPC5,NOX5,ZCCHC17,FER,SGCD,TMEM108,RAPGEF2,NRXN3,KCNH7,CDKL5,ZNF207,IL18R1,RGS7BP,KAT7,TMTC2,TRDN,SEMA3A,TSPAN8,ATP13A3,MYH9,BDKRB2,STIM1,TSC22D3,TENM1,LARGE,GABRB1,SH3KBP1,STXBP5,VWF,SLC24A2,NTRK3,DIS3L2,CLASP2,NSUN2,DNM3,SYNDIG1,PPARA,PTPRD,RORA,SHISA6,ELAVL4,DOCK10,ATP8A1,SEMA3D,PRLR,TSPAN1,DEPTOR,RAB27A,BLOC1S6,LARP4B,SYT9,SEMA3C,PAH
GO:0010646	regulation of cell communication	2.0992198400134934e-20	PTPRR,SYN3,CAMTA1,RCAN1,KALRN,CACNG2,RNF43,CTNNA1,NTRK2,ZNRF3,FBXL17,SLC39A10,DMD,SPRED2,ERBB4,LAMA2,CNTN4,ROBO1,CIT,PUM1,GPC5,WDR59,SORCS2,WWOX,AUTS2,WLS,ANKS1A,LDLRAD4,PSMB2,PTPRT,MGLL,PLCB1,FLT3,CACNA1A,PRKD1,RYR2,SEMA5A,CASK,PRKCA,DLGAP1,BTBD9,MYO9A,CCDC3,KANK1,GRIK4,DOK6,NLGN1,TNFRSF10B,CLEC16A,TCF7L2,RELN,APP,PAK1,PPP3CA,DEPDC5,ERC2,AKAP6,SORCS3,GRM7,DLC1,SOS1,CTNND2,ALK,BTRC,MAP2K5,MAGI2,EDA,ROBO2,IFT81,AKAP13,DCC,EP300,SEMA4D,EVC,GRK5,ANKRD6,GRIK2,ARHGAP15,ITSN1,JAK2,CMKLR1,PARK2,NPHP3,ADCYAP1R1,GRID1,GRIN3A,SMOC2,NLGN4X,AFAP1,NRG3,SULF1,SH3BP1,RFFL,ARHGAP24,SLIT2,TRIM5,CHRDL1,TRAF3IP2,SH3RF3,BMPR1A,SMAD3,PIBF1,SCUBE1,EYA1,MAD1L1,DGKB,TNKS,TUB,ARHGEF18,LINC00473,OVOL2,SGMS1,GRAMD4,DGKI,DNMBP,TAF1,SLC1A1,SHISA9,CDC73,FBXW11,NOS1AP,RBMS3,WWTR1,ARHGAP6,GRM4,ANK3,CCL15,CNIH3,DOCK3,TRIM59,TNR,CELF4,VAV3,ACTN4,PLCE1,EPS8,GHR,ARHGAP39,LRRC4C,PPP1R9A,CLSTN2,CD300A,CBFA2T2,DISC1,RALGPS1,ARHGAP42,CCL14,RABGEF1,TRABD2B,LRP5,ESR1,ARHGAP12,TNFRSF19,GRM1,PDE4D,PIK3CD,EFNA5,EIF3A,PIK3R2,GRID2,ZNF423,NTNG1,PDE4B,MAPRE2,RIC8B,MAP3K7,PRKCQ,EPHB2,ERC1,AGO3,MCTP1,MOB3B,PRCP,EEF1E1-BLOC1S5,DOCK2,BDNF,FNIP1,MAGI3,ADORA2A,SIPA1L2,IFT80,FSTL4,ANO1,FHL2,NOX4,BMPER,SYT1,DAB2,LRRK1,CACNG3,HDAC2,PDE11A,PRKAR1B,ZMYND11,RGS6,SRGAP3,MYRIP,ENPP1,RIMS2,DLGAP2,PRDM15,PEX5L,NRG1,RIT2,CD44,PRKCG,SLC8A1,FAM19A4,MAP3K13,PLCL2,CHST11,NCAM1,AGT,FGF1,NDRG2,ARHGAP25,NF1,WIF1,ZNF536,NRXN1,ARHGAP10,GSK3B,CD109,GPR21,CHD7,PSMB7,SNX5,TIAM2,IQCJ-SCHIP1,UNC13A,NCOA1,CHEK2,GRIK3,CACNB2,STXBP4,MECOM,SHANK2,KCTD8,NREP,GPC3,DLG5,PREX1,UBE2V1,RO

			R1,ZNF675,BID,MACF1,TRIO,CAPN3,PTPRE,DUSP22,MAP3K5,CTDSPL2,HTR2C,FGD4,LY86,EPHA5,TP73,ZNF366,ANK2,ARHGEF3,SLIT3,GRIN2B,PAK3,GARNL3,UACA,ADCY8,MLLT3,RNF213,SH3RF2,PRKAA2,TBL1X,GRIK1,GRM5,IGF1R,PTPRO,SUFU,INSR,MAP2K1,UBR2,FOXO3,GPC6,RASGRF2,STK38,CNTN6,PRDM16,ULK4,LTBP1,LRRK2,PKHD1,BMP6,FYN,EPHA7,MARVELD3,GAS8,RIMS1,MCTP2,ABCA12,EPHB1,CRADD,AMFR,PLCB4,LRP2,RALGPS2,FER,VWC2,GUCY2F,PCDH17,TMEM108,TPTE,RAPGEF2,NRXN3,PTPRU,IL18R1,RGS7BP,ESR2,PACSIN2,TRIM22,CDH13,VEPH1,TRDN,DAB1,SEMA3A,MGAT5,BDKRB2,LITAF,TENM1,ZRANB1,RGS7,STXBP5,SIPA1L3,GNG4,VWF,SLC24A2,SCEL,NTRK3,FBN1,PPARA,PTPRD,RORA,SHISA6,ELAVL4,EEF1E1,BICC1,PRLR,PTPRA,ATF6,DEPTOR,RALGAPA1,PMEPA1,SYT9
GO:0048667	cell morphogenesis involved in neuron differentiation	7.312475759700864e-20	*KALRN,NTRK2,LAMA2,CNTN4,ROBO1,DSCAM,TBCD,ATP8A2,AUTS2,NFASC,VCL,SEMA5A,IL1RAPL1,PLS1,NLGN1,RELN,APP,PAK1,PPP3CA,PCDH15,SOS1,CTNND2,DCLK1,MEF2A,ROBO2,DCC,SEMA4D,RPS6KA5,TANC2,EMB,NUMB,SLIT2,DSCAML1,NTN1,MAP2,FLRT2,CUX1,ANK3,CDH4,TNR,LRRC4C,STRC,DISC1,EFNA5,SEMA6D,NTNG1,CRMP1,PRKCQ,SPTBN4,EPHB2,PRTG,DPYSL2,BDNF,FSTL4,PLXNA2,ABI1,CDH23,CTNNA2,PARD3,FBXO31,MAP3K13,NCAM1,NRXN1,LAMA3,GSK3B,TIAM2,TRIOBP,UNC5D,ISLR2,ADAMTSL1,MACF1,TRIO,CCDC141,EPHA5,MYPN,SLIT3,PAK3,SEMA5B,IGF1R,PTPRO,MAP2K1,CNTN6,LRRK2,SPG11,PHACTR1,FYN,EPHA7,EPHB1,EXT1,TRPC5,UNC5C,RAPGEF2,PTPRM,NRXN3,CDKL5,DAB1,SEMA3A,CLASP2,DNM3,PTPRD,ELAVL4,DOCK10,SEMA3D,SEMA3C*
GO:0007155	cell adhesion	1.398187025991899e-19	*MEGF11,CTNNA1,CLASP1,DMD,LPP,LAMA2,CNTN4,ROBO1,DSCAM,PCDH9,TBCD,OPCML,TENM2,PTPRT,NFASC,VCL,NEDD9,SEMA5A,CASK,PRKCA,EPB41L4B,IL1RAPL1,PDZD2,ITGAE,KANK1,KIF26B,RUNX1,NLGN1,RELN,APP,ADAM12,UTRN,PPP3CA,NTM,PCDH15,SSPN,DLC1,CTNND2,CADM2,CTNNA3,DLG2,MAP2K5,EDA,CELSR1,ROBO2,ELMO2,SEMA4D,CDH8,ASTN2,JAK2,NEGR1,SDK2,MAGI1,NLGN4X,EMB,ITGBL1,COL4A6,CNTNAP5,CD84,ATRNL1,EPDR1,DSCAML1,SMAD3,HMCN1,PEAK1,MAD1L1,EGFLAM,DNAJB6,SKAP1,NTN1,FLRT2,CD96,ARHGAP6,ANK3,CDH12,CDH4,TNR,VAV3,SPOCK1,ACTN4,LRRN2,TENM4,CDH10,LRRC4C,CLSTN2,CD300A,STRC,DISC1,CNTN5,EFNA5,TLN2,GRID2,NTNG1,ADTRP,PRKCQ,CLDN1,EPHB2,MYBPC2,PRTG,RGMB,ADORA2A,MEGF9,DAB2,PLXNA2,PRKG1,CADM3,STXBP6,ETS1,FAT3,CDH23,CTNNA2,PARD3,NRG1,VSIG10,SPINT2,CD44,NCAM1,CCR3,USH2A,NF1,PARVB,NRXN1,LAMA3,GSK3B,PKP2,LMO7,CNTN1,TRIOBP,UNC5D,DLG5,CFDP1,PGM5,ASTN1,PREX1,ADAMTSL1,COL12A1,MACF1,DUSP22,CLDN11,CCDC141,MUC16,MYPN,ITGB3BP,CNTN3,SDK1,PKD1L1,CNTNAP2,PTPRO,ITGA11,JAK1,FOXO3,GPC6,CNTN6,SPECC1L,LRFN5,FRMD5,PCDH7,PKHD1,BMP6,FYN,EPHA7,COL19A1,PCDH11X,ABCA12,EPHB1,LSAMP,DOCK1,EXT1,TENM3,FER,VWC2,PCDH17,PTPRM,KIRREL3,NRXN3,PTPRU,DCHS2,CDH13,DAB1,MYH9,CUZD1,TENM1,PRKAR1A,VWF,FBN1,CLASP2,PTPRK,PPARA,PTPRD,CDH9,CNTNAP3,FNDC3A,PRLR,PTPRA,PARD3B*
GO:0022610	biological adhesion	2.458888624013524e-19	*MEGF11,CTNNA1,CLASP1,DMD,LPP,LAMA2,CNTN4,ROBO1,DSCAM,PCDH9,TBCD,OPCML,TENM2,PTPRT,NFASC,VCL,NEDD9,SEMA5A,CASK,PRKCA,EPB41L4B,IL1RAPL1,PDZD2,ITGAE,KANK1,KIF26B,RUNX1,NLGN1,RELN,APP,ADAM12,UTRN,PPP3CA,NTM,PCDH15,SSPN,DLC1,CTNND2,CADM2,CTNNA3,DLG2,MAP2K5,EDA,CELSR1,ROBO2,ELMO2,SEMA4D,CDH8,ASTN2,JAK2,NEGR1,SDK2,MAGI1,NLGN4X,EMB,ITGBL1,COL4A6,CNTNAP5,CD84,ATRNL1,EPDR1,DSCAML1,SMAD3,HMCN1,PEAK1,MAD1L1,EGFLAM,DNAJB6,SKAP1,NTN1,FLRT2,CD96,ARHGAP6,ANK3,CDH12,CDH4,TNR,VAV3,SPOCK1,ACTN4,LRRN2,TENM4,CDH10,LRRC4C,CLSTN2,CD300A,STRC,DISC1,CNTN5,EFNA5,TLN2,GRID2,NTNG1,ADTRP,PRKCQ,CLDN1,EPHB2,MYBPC2,PRTG,RGMB,ADORA2A,MEGF9,DAB2,PLXNA2,PRKG1,CADM3,STXBP6,ETS1,FAT3,CDH23,CTNNA2,PARD3,NRG1,VSIG10,SPINT2,CD44,NCAM1,CCR3,USH2A,NF1,PARVB,NRXN1,LAMA3,GSK3B,PKP2,LMO7,CNTN1,TRIOBP,UNC5D,DLG5,CFDP1,PGM5,ASTN1,PREX1,ADAMTSL1,COL12A1,MACF1,DUSP22,CLDN11,CCDC141,MUC16,MYPN,ITGB3BP,CNTN3,SDK1,PKD1L1,CNTNAP2,PTPRO,ITGA11,JAK1,FOXO3,GPC6,CNTN6,SPECC1L,LRFN5,FRMD5,PCDH7,PKHD1,BMP6,FYN,EPHA7,COL19A1,PCDH11X,ABCA12,EPHB1,LSAMP,D*

			OCK1,EXT1,TENM3,FER,VWC2,PCDH17,PTPRM,KIRREL3,NRXN3,PTPRU,DCHS2,CDH13,DAB1,MYH9,CUZD1,TENM1,PRKAR1A,VWF,FBN1,CLASP2,PTPRK,PPARA,PTPRD,CDH9,CNTNAP3,FNDC3A,PRLR,PTPRA,PARD3B
GO:0048869	cellular developmental process	2.822872172343536e-19	*RCAN1,KALRN,RPS6KA2,NDE1,CTNNA1,NTRK2,CLASP1,PTPRG,SYNE1,FBXL17,DMD,SPRED2,KAZN,ERBB4,LAMA2,NOS1,CNTN4,SND1,ROBO1,DSCAM,SHROOM3,CIT,TBCD,PUM1,FRY,NEBL,WWOX,HDAC4,OPCML,ATP8A2,AUTS2,ANKS1A,LDLRAD4,MYEF2,TENM2,PSMB2,RXFP1,NFASC,PLCB1,FLT3,SRPK2,PRKD1,VCL,NEDD9,PPP2R3C,SEMA5A,ETV6,PRKCA,IL1RAPL1,MYO9A,CCDC3,DIO2,NHS,KANK1,DIAPH2,TCF12,RUNX1,PLS1,NLGN1,CAMK1D,TCF7L2,RELN,APP,ADAM12,PAK1,PPP3CA,RYR1,NTM,TOX,PCDH15,PTPN9,CREM,AKAP6,GRM7,SOS1,CTNND2,LRRC8C,ALK,DCLK1,MEF2A,DENND5A,DNMT1,MAGI2,EDA,CELSR1,ROBO2,AKAP13,HIVEP3,DCC,EP300,SEMA4D,RPS6KA5,GRK5,TRAPPC9,NME8,TF,QKI,CHRM1,ASTN2,TANC2,JAK2,NEGR1,CMKLR1,SDK2,ZBTB7C,PARK2,NPHP3,ADCYAP1R1,GRIN3A,NLGN4X,EMB,PNPLA3,NRG3,NUMB,SOX6,SULF1,SH3BP1,ATRNL1,SH3GL2,ARHGAP24,COL11A1,SLIT2,THEMIS,ATRX,CHRDL1,TRAF3IP2,BMPR1A,DSCAML1,SMAD3,SLC9C1,SCUBE1,PEAK1,EYA1,SATB2,TPH1,OVOL2,DNAJB6,ELP3,BRINP1,GLIS1,SOX5,SLC1A1,NTN1,EPM2A,MAP2,CDC73,FLRT2,FBXW11,WWTR1,GAS7,CATSPER2,CUX1,ANK3,ARID4B,TRPS1,MYO18B,CDH4,TNR,CELF4,SPOCK1,ACTN4,TENM4,CECR2,GHR,LRRC4C,PPP1R9A,CBFA2T2,SRD5A2,STRC,DISC1,LRP5,NAV2,ESR1,PDE4D,PIK3CD,BLOC1S5,CERS3,TEAD4,ARMC2,EFNA5,KLHL1,FTO,POU6F2,CEP85L,ATF2,GRID2,ZNF423,SEMA6D,NTNG1,CRMP1,PRKCQ,CLDN1,NHSL2,JDP2,SPTBN4,EPHB2,S1PR3,PRTG,COL22A1,DOCK2,DPYSL2,MARK1,BDNF,RBM4,FNIP1,ADORA2A,ARID5B,IFT80,FSTL4,FHL2,NOX4,MEGF9,SYT1,DAB2,PLXNA2,LRRK1,PRKG1,ABI1,HDAC2,ETS1,FAT3,ARID4A,CDH23,TTN,ENPP1,RIMS2,MORN2,CTNNA2,PARD3,NRG1,SPINT2,FMN2,RIT2,CD44,CNGB1,SLC8A1,SRRM4,FBXO31,MAP3K13,PLCL2,CHST11,THRB,MAPK10,NCAM1,AGT,FGF1,NDRG2,GRIP1,USH2A,NF1,WIF1,ZNF536,PARVB,NRXN1,LAMA3,GSK3B,PKP2,CTDP1,SETD3,CD109,KCNH1,BBS9,CHD7,PSMB7,MOV10L1,THSD7A,TIAM2,MYT1L,CNTN1,PLEKHB2,UNC13A,TRIOBP,NCOA1,CHEK2,MECOM,CATSPERB,UNC5D,ISLR2,NREP,GPC3,DLG5,PGM5,ASTN1,PREX1,UBE2V1,ROR1,ADAMTSL1,ZNF675,COL12A1,MACF1,TRIO,CAPN3,MAP3K5,SPAG16,HTR2C,CCDC141,EPHA5,HIRA,TP73,MYPN,MORC3,ANK2,SLIT3,PAK3,TSNAX,PRRC2C,MLLT3,NEB,PACRG,SEMA5B,NLN,SDK1,GRM5,IGF1R,MSI2,CNTNAP2,PTPRO,SUFU,ITGA11,CRTAC1,MAP2K1,FOXO3,OCA2,CNTN6,CENPF,MITF,AGBL4,PRDM16,ULK4,RBFOX1,KIR2DL4,TMEM120B,LRRK2,PKHD1,KIF2A,RARB,TCF4,SPG11,PBX1,PHACTR1,BMP6,ASAP1,FYN,EPHA7,COL19A1,MSR1,SGCZ,RIMS1,ENPP2,ABCA12,EPHB1,KCNQ1,FHOD3,DOCK1,VASH2,BPGM,EXT1,TRPC5,UNC5C,TENM3,WDR7,LRP2,FER,CAMK4,VWC2,SGCD,TMEM108,RAPGEF2,NAV3,PTPRM,KIRREL3,NRXN3,CDKL5,PTPRU,IL18R1,KAT7,SETD2,PACSIN2,MDGA2,BASP1,DAB1,HDAC5,SEMA3A,MYH9,TANC1,TENM1,GABRB1,PRKAR1A,SIPA1L3,DPY19L2,SCEL,NTRK3,FBN1,HYDIN,TBX15,CLASP2,ZNF521,NSUN2,TPD52,DNM3,PPARA,PTPRD,RORA,BRIP1,ELAVL4,NELL1,DOCK10,EEF1E1,FNDC3A,SEMA3D,PRLR,SPATA5,SYT17,RAB27A,BLOC1S6,SEMA3C*
GO:0007165	signal transduction	4.971582626819945e-19	*PTPRR,CAMTA1,CHFR,RCAN1,KALRN,CACNG2,RNF43,RPS6KA2,CTNNA1,FOXN3,NTRK2,RASGEF1B,ZNRF3,PTPRG,FBXL17,SLC39A10,DMD,KCNC2,CHRM3,SPRED2,ERBB4,NOS1,ESRRG,ROBO1,PRICKLE2,DSCAM,CIT,PUM1,GPC5,WDR59,SORCS2,WWOX,HUNK,FUT8,HDAC4,AUTS2,WLS,ANKS1A,LDLRAD4,TENM2,BLM,PSMB2,PTPRT,RXFP1,MGLL,PLCB1,FLT3,SRPK2,TRPM1,PRKD1,RYR2,NEDD9,SEMA5A,PRKCA,DLGAP1,TRHDE,IL1RAPL1,MYO9A,CCDC3,ITGAE,KANK1,GRIK4,GPR176,DOK6,NLGN1,TNFRSF10B,CLEC16A,AMOTL1,TCF7L2,RELN,APP,ADAM12,PAK1,PPP3CA,DEPDC5,GABRG3,CREM,AKAP6,SORCS3,GRM7,DLC1,IDE,SOS1,CTNND2,RFTN1,ALK,BTRC,DCLK1,MEF2A,PPP1R12B,MAP2K5,DNMT1,MAGI2,EDA,CELSR1,ROBO2,IFT81,AKAP13,DCC,DDB1,EP300,SEMA4D,EVC,RPS6KA5,GRK5,ANKRD6,BDKRB1,TF,GRIK2,GNG12,CHRM1,ARHGAP15,HUS1,ITSN1,DEFA1B,JAK2,ABCG8,CMKLR1,MAST4,PARK2,MAGI1,NPHP3,ADCYAP1R1,GRID1,GRIN3A,SMOC2,CACNA1C,NLGN4X,PIK3C2B,OR5K4,ITGBL1,AFAP1,NRG3,COL4*

			A6,SULF1,SH3BP1,ADCY2,ATRNL1,RFFL,SH3GL2,ARHGAP24,PITPNC1,SLIT2,MAPK4,GLP2R,DTNA,TRIM5,THEMIS,DRG2,ATRX,CHRDL1,TRAF3IP2,SH3RF3,BMPR1A,SMAD3,PRKAR2A,PI4KA,TNFRSF11B,PIBF1,GABRR2,PIK3C3,PTGFR,SCUBE1,EYA1,ANKS1B,MAML3,GPR141,MAD1L1,DGKB,TNKS,TUB,ARHGEF18,LINC00473,OVOL2,SGMS1,GRAMD4,FGF14,DGKK,OR5I1,ASGR2,IL1RAPL2,SKAP1,DGKI,DNMBP,TAF1,SPNS2,SLC1A1,NTN1,SHISA9,EPM2A,CDC73,FLRT2,FBXW11,NOS1AP,RANBP10,RBMS3,ARHGEF6,WWTR1,ARHGAP6,GRM4,ANK3,CCL15,CNIH3,DOCK3,TRIM59,ADCY9,CELF4,VAV3,MDM4,ACTN4,PLCE1,LRRN2,EPS8,TENM4,GHR,ARHGAP39,PPP1R9A,RGL1,CD300A,CBFA2T2,DISC1,RALGPS1,ARHGAP42,CCL14,RABGEF1,FHIT,NSG2,TRABD2B,LRP5,ESR1,ARHGAP12,TNFRSF19,PLCXD3,GRM1,PDE4D,THEM4,PIK3CD,DOCK4,TEAD4,PDE7B,EFNA5,EIF3A,ATF2,PIK3R2,GRID2,ZNF423,CYBB,SEMA6D,ADTRP,PDE4B,MAPRE2,RIC8B,GABRB3,MAP3K7,PRKCQ,RASSF8,RHPN2,EPHB2,ERC1,AGO3,S1PR3,MCTP1,MOB3B,PRCP,RGMB,EEF1E1-BLOC1S5,DOCK2,DPYSL2,PTPDC1,MARK1,BDNF,FNIP1,MAGI3,ADORA2A,ARID5B,SIPA1L2,RCAN2,IFT80,FSTL4,ANO1,FHL2,NOX4,BMPER,PDE1A,ICK,DEFA3,DAB2,PLXNA2,LRRK1,PRKG1,ABI1,CACNG3,EPG5,PTH2R,CALCRL,HDAC2,PDE11A,RAB30,ZMYND11,TTN,RGS6,SRGAP3,ENPP1,RIMS2,KSR2,DLGAP2,PRDM15,OR4N2,PEX5L,PARD3,NRG1,FMN2,GRIA2,VSTM1,RIT2,CD44,CNGB1,GPSM2,PRKCG,TNS3,TEAD1,MAPKAPK2,SLC8A1,RAPGEF6,FBXO31,FAM19A4,MAP3K13,PLCL2,CHST11,THRB,CDC42BPA,CDC42EP3,MAPK10,NCAM1,AGT,FGF1,QRICH1,CCR3,NDRG2,GRIP1,TLK1,ARHGAP25,NF1,WIF1,ZNF536,NRXN1,ARHGAP10,LAMA3,GSK3B,KIR2DL1,CD109,KCNH1,DCDC1,GPR21,PSMB7,SNX5,TIAM2,OR51B2,IQCJ-SCHIP1,CNTN1,BTBD11,UNC13A,CABIN1,NCOA1,CHEK2,GRIK3,STXBP4,RHOJ,MECOM,STK32B,SHANK2,KCTD8,UNC5D,NREP,GPC3,DLG5,PREX1,CPE,UBE2V1,ROR1,ZNF675,PTGER3,BID,MACF1,TRIO,CAPN3,PTPRE,DUSP22,MYOM1,MAP3K5,MAML2,CTDSPL2,HTR2C,GLRA2,FGD4,LY86,EPHA5,TP73,IL1RL1,ZNF366,P2RX6,ANK2,ARHGEF3,SLIT3,GRIN2B,PAK3,GARNL3,UACA,ADCY8,ITPR2,GABRR3,MLLT3,CCL15-CCL14,ITGB3BP,RNF213,SH3RF2,CACNA1D,PRKAA2,TBL1X,SEMA5B,GRIK1,NLN,GRM5,ARAP2,IGF1R,PTPRO,SUFU,INSR,ITGA11,JAK1,MAP2K1,UBR2,GMDS,KPNB1,FOXO3,GPC6,RASGRF2,STK38,GABRA3,INPP5A,CNTN6,CENPF,MITF,PRDM16,ULK4,KIR2DL4,TMEM117,LTBP1,LRRK2,PKHD1,RARB,BMP6,FYN,EPHA7,MARVELD3,GAS8,GNG2,RIMS1,MCTP2,EPHB1,KCNQ1,DOCK1,CRADD,EXT1,AMFR,PLCB4,UNC5C,TENM3,LRP2,RALGPS2,FER,CAMK4,VWC2,GUCY2F,ERLIN1,SGCD,TMEM108,TPTE,RAPGEF2,PTPRM,NRXN3,INPP4A,PTPRU,ZNF207,GRIA4,IL18R1,RGS7BP,ESR2,KAT7,TRIM22,CDH13,VEPH1,DAB1,SEMA3A,MGAT5,MYH9,BDKRB2,LITAF,PIK3R3,TENM1,STK38L,GABRB1,PRKAR1A,ZRANB1,RGS7,FCRL2,SIPA1L3,GNG4,VWF,SLC24A2,SCEL,GRIA3,NTRK3,FBN1,CLASP2,NSUN2,PTPRK,SORCS1,DOCK9,PPARA,PTPRD,RORA,SHISA6,BRIP1,DOCK10,EEF1E1,BICC1,SEMA3D,PRLR,PTPRA,ATF6,DEPTOR,CUL2,STAC,OR4M1,RALGAPA1,PMEPA1,SEMA3C,ST18,RXFP2
GO:0030154	cell differentiation	8.910466573934e-19	*RCAN1,KALRN,RPS6KA2,NDE1,CTNNA1,NTRK2,CLASP1,PTPRG,SYNE1,FBXL17,DMD,SPRED2,KAZN,ERBB4,LAMA2,NOS1,CNTN4,SND1,ROBO1,DSCAM,SHROOM3,CIT,TBCD,PUM1,FRY,NEBL,WWOX,HDAC4,OPCML,ATP8A2,AUTS2,ANKS1A,LDLRAD4,MYEF2,TENM2,PSMB2,RXFP1,NFASC,PLCB1,FLT3,SRPK2,PRKD1,VCL,NEDD9,PPP2R3C,SEMA5A,ETV6,PRKCA,IL1RAPL1,MYO9A,CCDC3,DIO2,NHS,KANK1,DIAPH2,TCF12,RUNX1,PLS1,NLGN1,CAMK1D,TCF7L2,RELN,APP,ADAM12,PAK1,PPP3CA,RYR1,NTM,TOX,PCDH15,PTPN9,CREM,AKAP6,GRM7,SOS1,CTNND2,LRRC8C,ALK,DCLK1,MEF2A,DENND5A,DNMT1,MAGI2,EDA,CELSR1,ROBO2,AKAP13,HIVEP3,DCC,EP300,SEMA4D,RPS6KA5,GRK5,TRAPPC9,NME8,TF,QKI,CHRM1,ASTN2,TANC2,JAK2,NEGR1,CMKLR1,SDK2,ZBTB7C,PARK2,NPHP3,ADCYAP1R1,GRIN3A,NLGN4X,EMB,PNPLA3,NRG3,NUMB,SOX6,SULF1,SH3BP1,ATRNL1,SH3GL2,ARHGAP24,COL11A1,SLIT2,THEMIS,ATRX,CHRDL1,TRAF3IP2,BMPR1A,DSCAML1,SMAD3,SLC9C1,SCUBE1,PEAK1,EYA1,SATB2,TPH1,OVOL2,DNAJB6,ELP3,BRINP1,GLIS1,SOX5,SLC1A1,NTN1,EPM2A,MAP2,CDC73,FLRT2,FBXW11,WWTR1,GAS7,CATSPER2,CUX1,AN*

			K3,ARID4B,TRPS1,MYO18B,CDH4,TNR,CELF4,SPOCK1,ACTN4,TENM4,CECR2,GHR,LRRC4C,PPP1R9A,CBFA2T2,SRD5A2,STRC,DISC1,LRP5,NAV2,ESR1,PDE4D,PIK3CD,BLOC1S5,CERS3,TEAD4,ARMC2,EFNA5,KLHL1,FTO,POU6F2,CEP85L,ATF2,GRID2,ZNF423,SEMA6D,NTNG1,CRMP1,PRKCQ,CLDN1,NHSL2,JDP2,SPTBN4,EPHB2,S1PR3,PRTG,COL22A1,DOCK2,DPYSL2,MARK1,BDNF,RBM4,FNIP1,ADORA2A,ARID5B,IFT80,FSTL4,FHL2,NOX4,MEGF9,SYT1,DAB2,PLXNA2,LRRK1,PRKG1,ABI1,HDAC2,ETS1,FAT3,ARID4A,CDH23,TTN,ENPP1,RIMS2,MORN2,CTNNA2,PARD3,NRG1,SPINT2,FMN2,RIT2,CNGB1,SLC8A1,SRRM4,FBXO31,MAP3K13,PLCL2,CHST11,THRB,NCAM1,AGT,FGF1,NDRG2,GRIP1,USH2A,NF1,WIF1,ZNF536,PARVB,NRXN1,LAMA3,GSK3B,PKP2,CTDP1,SETD3,CD109,KCNH1,BBS9,CHD7,PSMB7,MOV10L1,THSD7A,TIAM2,MYT1L,CNTN1,PLEKHB2,UNC13A,TRIOBP,NCOA1,MECOM,CATSPERB,UNC5D,ISLR2,NREP,GPC3,DLG5,PGM5,ASTN1,PREX1,UBE2V1,ROR1,ADAMTSL1,ZNF675,COL12A1,MACF1,TRIO,CAPN3,MAP3K5,SPAG16,HTR2C,CCDC141,EPHA5,HIRA,TP73,MYPN,ANK2,SLIT3,PAK3,TSNAX,PRRC2C,MLLT3,NEB,PACRG,SEMA5B,NLN,SDK1,GRM5,IGF1R,MSI2,CNTNAP2,PTPRO,SUFU,ITGA11,CRTAC1,MAP2K1,FOXO3,OCA2,CNTN6,CENPF,MITF,AGBL4,PRDM16,ULK4,RBFOX1,TMEM120B,LRRK2,PKHD1,KIF2A,RARB,TCF4,SPG11,PBX1,PHACTR1,BMP6,ASAP1,FYN,EPHA7,COL19A1,MSR1,SGCZ,RIMS1,ABCA12,EPHB1,KCNQ1,FHOD3,DOCK1,VASH2,BPGM,EXT1,TRPC5,UNC5C,TENM3,WDR7,LRP2,FER,CAMK4,VWC2,SGCD,TMEM108,RAPGEF2,NAV3,PTPRM,KIRREL3,NRXN3,CDKL5,PTPRU,IL18R1,KAT7,SETD2,MDGA2,BASP1,DAB1,HDAC5,SEMA3A,MYH9,TANC1,TENM1,GABRB1,PRKAR1A,SIPA1L3,DPY19L2,SCEL,NTRK3,FBN1,HYDIN,TBX15,CLASP2,ZNF521,NSUN2,TPD52,DNM3,PPARA,PTPRD,RORA,BRIP1,ELAVL4,NELL1,DOCK10,FNDC3A,SEMA3D,PRLR,SPATA5,SYT17,RAB27A,BLOC1S6,SEMA3C
GO:0050794	regulation of cellular process	1.569699862793328e-18	*PKNOX2,PTPRR,SYN3,RNF4,CAMTA1,CHFR,KDM4B,RCAN1,KALRN,CACNG2,RNF43,PROS1,RPS6KA2,CTNNA1,FOXN3,NTRK2,CLASP1,RASGEF1B,ZNRF3,GLIS3,PTPRG,FBXL17,SLC39A10,DMD,KCNC2,CHRM3,EIF3E,SPRED2,ERBB4,LAMA2,TFDP2,NOS1,CNTN4,TMC2,SND1,ESRRG,ROBO1,PRICKLE2,DSCAM,CIT,TBCD,DPP10,PUM1,FRY,GPC5,WDR59,SORCS2,PRIM2,WWOX,HUNK,FUT8,TNRC6B,HDAC4,ATP9A,LINGO2,ATP8A2,AUTS2,WLS,ANKS1A,LDLRAD4,MYEF2,TENM2,BLM,PSMB2,PTPRT,RXFP1,ATF7IP,ASXL3,MGLL,PLCB1,FLT3,SRPK2,CACNA1A,TRPM1,PRKD1,RYR2,VCL,PDS5A,NEDD9,PPP2R3C,SEMA5A,ETV6,CASK,PRKCA,EPB41L4B,STX8,ZBTB20,DLGAP1,BTBD9,TRHDE,ZNF713,IL1RAPL1,MYO9A,CCDC3,DIO2,PDE4DIP,ITGAE,ABCA13,KANK1,KIF26B,TBC1D5,SLCO3A1,TCF12,GRIK4,RUNX1,PLS1,GPR176,DOK6,FCHSD2,NLGN1,TNFRSF10B,KCNIP4,CAMK1D,SMG6,CLEC16A,AMOTL1,TCF7L2,MTRF1,RELN,APP,ADAM12,PAK1,UTRN,PPP3CA,DEPDC5,RYR1,TOX,GABRG3,ERC2,PTPN9,CREM,AKAP6,SORCS3,GRM7,DLC1,IDE,SCMH1,RYR3,SOS1,CTNND2,RFTN1,ALK,BTRC,DCLK1,MEF2A,EEFSEC,KCNB2,TFAP2D,CTNNA3,DENND5A,PPP1R12B,MAP2K5,DNMT1,MAGI2,EDA,CELSR1,ROBO2,IFT81,AKAP13,HIVEP3,DCC,SAMD4A,C10ORF90,ZNF799,DDB1,EP300,KCNA6,PARN,SEMA4D,EVC,RPS6KA5,PHF20,GRK5,ANKRD6,TRAPPC9,BDKRB1,TF,DNAH11,GRIK2,QKI,GNG12,CHRM1,CDH8,ARHGAP15,HUS1,ZNF667,TFEC,ITSN1,ASTN2,TANC2,DEFA1B,JAK2,ABCG8,RAB11FIP4,NEGR1,CMKLR1,PPP6R2,ZBTB7C,MAST4,PARK2,MAGI1,NPHP3,ADCYAP1R1,SERPINA4,DPP6,GRID1,GRIN3A,SMOC2,CACNA1C,NLGN4X,EXOC4,PIK3C2B,OR5K4,ITGBL1,GBE1,AFAP1,TAGLN3,SP140,NRG3,NUMB,MED15,SOX6,COL4A6,SULF1,CD84,MAGEA11,SH3BP1,ADCY2,TCF20,ATRNL1,RFFL,SH3GL2,PSPC1,ARHGAP24,PITPNC1,SLIT2,KCNJ16,MAPK4,GLP2R,SLC4A4,DTNA,KMT2C,TRIM5,THEMIS,DRG2,AFF3,ATRX,CHRDL1,TRAF3IP2,RAD51B,SLX1B,SH3RF3,BMPR1A,SMAD3,PRKAR2A,CACNA1E,BORA,PI4KA,TNFRSF11B,PIBF1,GABRR2,PIK3C3,CHD6,KIR3DL2,PTGFR,SCUBE1,PEAK1,EYA1,SATB2,ANKS1B,ELN,MAML3,GPR141,TPH1,MAD1L1,EGFLAM,DGKB,TNKS,TUB,KLF12,ARHGEF18,SCAMP5,LINC00473,OVOL2,SGMS1,GRAMD4,DNAJB6,ELP3,BRINP1,OMA1,BCL2L13,ARRDC4,KHDRBS2,FGF14,CACNA2D3,DGKK,OR51I1,GLIS1,ASGR2,IL1RAPL2,SKAP1,CDYL2,SOX5,DGKI,DNMBP,TAF1,SPNS2,SLC1A1,RNF144B,NTN1,LDB2,SHISA9,EPM2A,MAP2,CDC73,FLRT2,FBXW11,NOS1AP,RANBP10,RB*

			MS3,ARHGEF6,WWTR1,ARHGAP6,PHF20L1,CATSPER2,EGLN3,CUX1,GRM4,ANK3,TIMP2,CCL15,MORC2,ZNF19,ARID4B,CNIH3,DOCK3,TRIM59,TRPS1,CDH4,TNR,ADCY9,CELF4,VAV3,CDKL1,SCN9A,MDM4,ARNT2,SPOCK1,ACTN4,HPSE2,PLCE1,LRRN2,EPS8,TENM4,PRR16,GHR,ARHGAP39,LRRC4C,PPP1R9A,RGL1,CLSTN2,CD300A,CBFA2T2,FUBP1,PRG3,MLIP,SERTAD2,DISC1,RALGPS1,ARHGAP42,CCL14,RABGEF1,FHIT,NSG2,TRABD2B,LRP5,AP2B1,ESR1,ARHGAP12,TNFRSF19,PLCXD3,GRM1,SCFD1,PDE4D,THEM4,PIK3CD,DOCK4,SLC5A3,BLOC1S5,FRMD4A,TEAD4,PDE7B,EFNA5,FTO,EIF3A,POU6F2,ATF2,RBBP8,KCNJ12,PIK3R2,GRID2,ZNF423,CYBB,SEMA6D,KCNJ3,CELF2,NTNG1,ADTRP,CRMP1,PDE4B,MAPRE2,RIC8B,GABRB3,SMG7,ANKRD13A,MAP3K7,PRKCQ,ATXN1,N4BP1,RASSF8,RHPN2,CLDN1,KCND2,JDP2,SPTBN4,EPHB2,ERC1,AGO3,S1PR3,MCTP1,MOB3B,PRCP,PRTG,RGMB,EEF1E1-BLOC1S5,DOCK2,NUP214,DPYSL2,PTPDC1,RFC3,DACH1,MARK1,BDNF,RBM4,FNIP1,MALRD1,MAGI3,ADORA2A,ARID5B,SIPA1L2,RCAN2,IFT80,FSTL4,ANO1,FHL2,CADPS,DRAM1,NOX4,BCOR,BMPER,PDE1A,ICK,DEFA3,BRD9,SYT1,EBAG9,DAB2,AFF2,PLXNA2,NSF,EBF4,LRRK1,PRKG1,STXBP6,ABI1,CACNG3,EPG5,PTH2R,CALCRL,HDAC2,ETS1,BANP,FAT3,PDE11A,ARID4A,RAB30,PRKAR1B,BACH1,ZMYND11,TTN,RGS6,SRGAP3,ZNF93,MYRIP,CDK19,ENPP1,ZNF443,RIMS2,SCN8A,KSR2,DLGAP2,PRDM15,OR4N2,CTNNA2,PEX5L,PARD3,NRG1,CAST,FANK1,SPINT2,NPAS3,FMN2,GRIA2,VSTM1,RIT2,CD44,PAX7,KCNMA1,SCAF8,CNGB1,GPSM2,PRKCG,TNS3,MED13L,TEAD1,NFIA,MAPKAPK2,EIF4G3,SLC8A1,SRRM4,RAPGEF6,FBXO31,FAM19A4,MAP3K13,PLCL2,TOX3,CHST11,THRB,CDC42BPA,CDC42EP3,MAPK10,NCAM1,AGT,FGF1,KCNQ5,QRICH1,CCR3,KCNJ6,ZNF709,NDRG2,GRIP1,TLK1,ARHGAP25,USH2A,NF1,WIF1,ZNF536,NRXN1,ARHGAP10,LAMA3,PDXP,GSK3B,KIR2DL1,PKP2,CTDP1,SETD3,CD109,KCNH1,ZNF695,DCDC1,LMO7,WDR70,GPR21,CHD7,PSMB7,SNX5,TIAM2,OR51B2,MYT1L,SRGAP2B,IQCJ-SCHIP1,CNTN1,PLEKHB2,BTBD11,UNC13A,TRIOBP,CABIN1,NCOA1,CHEK2,GRIK3,CACNB2,STXBP4,RHOJ,ZBTB8B,ZNF730,MECOM,STK32B,SHANK2,UBE2E2,KCTD8,UNC5D,ISLR2,NREP,GPC3,DLG5,CFDP1,PREX1,CPE,UBE2V1,ROR1,ZNF675,SERPINA5,KCNS3,PTGER3,BID,MACF1,MNAT1,TRIO,CAPN3,CTNNBL1,PTPRE,DUSP22,MYOM1,MAP3K5,MAML2,CTDSPL2,HTR2C,GLRA2,FGD4,LY86,ZNF670,EPHA5,SGIP1,HIRA,RNF144A,TP73,IL1RL1,TSHZ2,ZNF366,MORC3,P2RX6,ANK2,ARHGEF3,SUPT3H,SLIT3,GRIN2B,PHC2,PAK3,GARNL3,UACA,LRPPRC,DPH6,ADCY8,ITPR2,GABRR3,MLLT3,NEB,CCL15-CCL14,ITGB3BP,RNF213,SH3RF2,CACNA1D,PRKAA2,PACRG,TBL1X,SEMA5B,HIVEP2,GRIK1,NLN,ANKFN1,GRM5,ARAP2,IGF1R,MXD3,CNTNAP2,PTPRO,SUFU,INSR,CIZ1,ITGA11,JAK1,MAP2K1,UBR2,ZNF490,GMDS,KPNB1,FOXO3,GPC6,RASGRF2,STK38,GABRA3,INPP5A,CNTN6,CENPF,KCTD1,PPP1R14A,MITF,IGF2BP3,SP140L,LRFN5,CDC14A,AGBL4,PRDM16,FRMD5,ULK4,RBFOX1,KIR2DL4,ZNF605,ZNF41,TMEM117,DNMT3B,ADAMTS16,TASP1,LTBP1,LRRK2,PKHD1,KIF2A,RARB,TCF4,PBX1,PHACTR1,SCN1A,BMP6,PCBP3,ASAP1,FRMPD4,FYN,KCND3,CSPP1,EPHA7,MARVELD3,GAS8,MSR1,CLN6,PTPN14,GNG2,PCBD2,RIMS1,ENPP2,L3MBTL4,MCTP2,ABCA12,BEND5,EPHB1,KCNQ1,FHOD3,DOCK1,CRADD,VASH2,ZNF664,EXT1,TRPC5,AMFR,PLCB4,UNC5C,TENM3,NOX5,LRP2,ZCCHC17,C2,RALGPS2,FER,CAMK4,VWC2,GUCY2F,ERLIN1,PCDH17,SGCD,TMEM108,TPTE,RAPGEF2,NAV3,PTPRM,NCOA2,NRXN3,KCNH7,CDKL5,INPP4A,PTPRU,ZNF207,GRIA4,IL18R1,RGS7BP,ESR2,KAT7,SATB1,SETD2,PACSIN2,TRIM22,CDH13,VEPH1,BASP1,TRDN,DAB1,ZNF148,SEC16B,HDAC5,SEMA3A,MGAT5,MYH9,BDKRB2,STIM1,TSC22D3,NRIP1,CCNG2,LITAF,PIK3R3,BRF1,TENM1,STK38L,GABRB1,ST8SIA1,SH3KBP1,PRKAR1A,ZRANB1,RGS7,STXBP5,FCRL2,SIPA1L3,GNG4,VWF,TXNDC5,SLC24A2,SCEL,GRIA3,NTRK3,FBN1,DIS3L2,TBX15,CLASP2,ZNF521,CREB5,DNAJC6,NSUN2,DACH2,PTPRK,SORCS1,DNM3,SYNDIG1,DOCK9,PPARA,PTPRD,RORA,SHISA6,BRIP1,ELAVL4,ANXA8L1,MXI1,TTC28,NELL1,DOCK10,EEF1E1,ATP8A1,BICC1,SEMA3D,PRLR,PTPRA,KCNC4,ATF6,DEPTOR,NVL,RAD51D,CUL2,SYT17,STAC,OR4M1,RAB27A,RALGAPA1,CABLES1,CST2,BLOC1S6,PMEPA1,LARP4B,KCNJ15,SYT9,SEMA3C,ST18,RXFP2

GO:0099537	trans-synaptic signaling	1.8197692279892247e-18	*SYN3,CACNG2,RPS6KA2,NTRK2,CHRM3,LAMA2,NOS1,CNTN4,SORCS2,PLCB1,CACNA1A,CASK,DLGAP1,BTBD9,IL1RAPL1,GRIK4,GPR176,FCHSD2,NLGN1,RELN,APP,PPP3CA,GABRG3,ERC2,SORCS3,GRM7,DLG2,DCC,GRIK2,CHRM1,CDH8,JAK2,PARK2,GRID1,GRIN3A,NLGN4X,EXOC4,NRG3,DTNA,CACNA1E,GABRR2,SV2B,DGKB,PTPRN2,DGKI,SLC1A1,SHISA9,GRM4,TNR,CELF4,LRRC4C,PPP1R9A,CLSTN2,DISC1,GRM1,PDE7B,GRID2,NTNG1,GABRB3,KCND2,EPHB2,ERC1,MCTP1,BDNF,ADORA2A,CADPS,SYT1,SNAP23,CACNG3,PRKAR1B,RIMS2,DLGAP2,GRIA2,RIT2,PRKCG,PLCL2,AGT,NF1,NRXN1,GSK3B,UNC13A,GRIK3,CACNB2,SHANK2,HTR2C,GLRA2,P2RX6,GRIN2B,ADCY8,GABRR3,GRIK1,GRM5,RASGRF2,GABRA3,LRRK2,SPG11,FYN,RIMS1,MCTP2,EPHB1,EXT1,PLCB4,PCDH17,TMEM108,RAPGEF2,NRXN3,PACSIN2,GABRB1,STXBP5,SLC24A2,CEP89,PTPRD,SHISA6,ELAVL4,PTPRA,KCNC4,BLOC1S6,SYT9*
GO:0050896	response to stimulus	6.003120354955477e-18	*PTPRR,RNF4,CAMTA1,CHFR,RCAN1,KALRN,CACNG2,RNF43,PROS1,RPS6KA2,CTNNA1,FOXN3,NTRK2,CLASP1,RASGEF1B,ZNRF3,PTPRG,FBXL17,SLC39A10,DMD,KCNC2,CHRM3,SPRED2,ERBB4,LAMA2,NOS1,CNTN4,TMC2,ESRRG,ROBO1,PRICKLE2,MYO3A,DSCAM,SHROOM3,CIT,PUM1,GPC5,WDR59,SORCS2,WWOX,HUNK,FUT8,HDAC4,IMMP2L,ABCC1,ATP8A2,AUTS2,WLS,ANKS1A,LDLRAD4,MYEF2,TENM2,BLM,PSMB2,PTPRT,RXFP1,MGLL,NFASC,PLCB1,FLT3,SRPK2,CACNA1A,TRPM1,PRKD1,RYR2,VCL,PDS5A,NEDD9,PPP2R3C,SEMA5A,CASK,PRKCA,EPB41L4B,STX8,ZBTB20,DLGAP1,TRHDE,IL1RAPL1,MYO9A,CCDC3,HBE1,DIO2,ITGAE,KANK1,TBC1D5,TCF12,GRIK4,GPR176,USP53,DOK6,NLGN1,TNFRSF10B,CAMK1D,CLEC16A,AMOTL1,TCF7L2,RELN,APP,ADAM12,PAK1,PPP3CA,DEPDC5,RYR1,NAALADL2,PCDH15,GABRG3,CREM,AKAP6,SORCS3,GRM7,DLC1,IDE,RYR3,ZPLD1,SOS1,CTNND2,LRRC8C,RFTN1,ALK,BTRC,DCLK1,MEF2A,DLG2,PPP1R12B,MAP2K5,DNMT1,MAGI2,EDA,CELSR1,EYS,ROBO2,IFT81,AKAP13,DCC,ELMO2,DDB1,EP300,SEMA4D,EVC,RPS6KA5,CPNE4,GRK5,ANKRD6,BDKRB1,NME8,TF,GRIK2,GNG12,CHRM1,CDH8,ARHGAP15,HUS1,TFEC,ITSN1,DEFA1B,JAK2,ABCG8,CMKLR1,MAST4,PARK2,MAGI1,NPHP3,ADCYAP1R1,GRID1,TDP1,GRIN3A,SMOC2,HLCS,CACNA1C,MAN1A1,NLGN4X,EMB,PNPLA3,PIK3C2B,OR5K4,ITGBL1,AFAP1,SP140,NRG3,SOX6,POLE,COL4A6,TRPM3,SULF1,CHCHD6,CD84,SH3BP1,ADCY2,OTC,ATRNL1,RFFL,SH3GL2,PSPC1,ARHGAP24,COL11A1,PITPNC1,SLIT2,MAPK4,GLP2R,AOX1,DTNA,TRIM5,THEMIS,DRG2,AFF3,ATRX,CHRDL1,TRAF3IP2,RAD51B,SLX1B,SH3RF3,BMPR1A,ACACA,DSCAML1,SMAD3,PRKAR2A,PI4KA,TNFRSF11B,PIBF1,GABRR2,PIK3C3,CHD6,HMCN1,SLC47A1,KIR3DL2,PTGFR,SCUBE1,EYA1,SATB2,ANKS1B,HSF2BP,MAML3,GPR141,TPH1,MAD1L1,DGKB,TNKS,TUB,ARHGEF18,SCAMP5,LINC00473,OVOL2,SGMS1,GRAMD4,DNAJB6,BRINP1,OMA1,FGF14,DGKK,OR51I1,ASGR2,IL1RAPL2,ZDHHC11,SKAP1,PTPRN2,SOX5,DGKI,DNMBP,TAF1,SPNS2,SLC1A1,NTN1,SHISA9,EPM2A,SHFM1,CDC73,FLRT2,FBXW11,NOS1AP,RANBP10,CD96,RBMS3,ARHGEF6,WWTR1,ARHGAP6,CHAF1B,EGLN3,GRM4,ANK3,TIMP2,CCL15,MORC2,CNIH3,DOCK3,TRIM59,CDH4,TNR,ADCY9,CELF4,VAV3,SCN9A,MDM4,ARNT2,ACTN4,PLCE1,LRRN2,EPS8,TENM4,MACROD1,GHR,ARHGAP39,LRRC8D,PPP1R9A,RGL1,CD300A,CBFA2T2,SRD5A2,PRG3,MLIP,STRC,DISC1,RALGPS1,ARHGAP42,CCL14,RABGEF1,FHIT,NSG2,TRABD2B,LRP5,ESR1,ARHGAP12,TNFRSF19,PLCXD3,GRM1,SCFD1,PDE4D,THEM4,PIK3CD,DOCK4,BLOC1S5,TEAD4,PDE7B,EFNA5,FTO,EIF3A,ATF2,RBBP8,PIK3R2,GRID2,ZNF423,CYBB,SEMA6D,KCNJ3,SUSD4,ADTRP,CRMP1,PDE4B,MAPRE2,RIC8B,GABRB3,MAP3K7,PRKCQ,N4BP1,RASSF8,RHPN2,CLDN1,KCND2,TPH2,EPHB2,ERC1,AGO3,S1PR3,MCTP1,MOB3B,PRCP,PRTG,RGMB,EEF1E1-BLOC1S5,DOCK2,DPYSL2,PTPDC1,RFC3,DACH1,MARK1,BDNF,RBM4,FNIP1,MAGI3,ADORA2A,ARID5B,SIPA1L2,RCAN2,IFT80,FSTL4,MCM3,ANO1,FHL2,NOX4,WDFY4,KIR3DL1,CSMD1,BMPER,PDE1A,ICK,DEFA3,SYT1,DAB2,PLXNA2,LRRK1,PRKG1,KIR2DL3,SNAP23,ABI1,PXDNL,CACNG3,EPG5,PTH2R,CALCRL,HDAC2,ETS1,PDE11A,RAB30,ZSWIM7,BACH1,ZMYND11,CDH23,TTN,RGS6,SRGAP3,RNASET2,CDK19,ENPP1,CFB,RIMS2,KSR2,DLGAP2,PRDM15,OR4N2,CTNNA2,PEX5L,PARD3,NRG1,SPINT2,FMN2,GRIA2,BCKDHB,VSTM1,TOP1,RIT2,CD44,KCNMA1,CNGB1,GPSM2,PRKCG,MSRB3,TNS3,ZFYVE1,TE*

			AD1,MAPKAPK2,SLC8A1,RAPGEF6,FBXO31,FAM19A4,MAP3K13,PLCL2,RSRC1,CHST11,THRB,CDC42BPA,B3GALT5,CDC42EP3,MAPK10,NCAM1,AGT,FGF1,QRICH1,CCR3,NDRG2,GRIP1,TLK1,ARHGAP25,USH2A,NF1,WIF1,ZNF536,NRXN1,ARHGAP10,LAMA3,PDXP,GSK3B,KIR2DL1,CTDP1,CD109,SLC22A3,KCNH1,DCDC1,WDR70,BBS9,GPR21,CHD7,PSMB7,SNX5,TIAM2,OR51B2,IQCJ-SCHIP1,CNTN1,BTBD11,UNC13A,CABIN1,NCOA1,CHEK2,GRIK3,STXBP4,RHOJ,PHEX,MECOM,STK32B,SHANK2,UBE2E2,KCTD8,UNC5D,NREP,GPC3,DLG5,PREX1,CPE,UBE2V1,ANO3,TTC4,ROR1,ADAMTSL1,ZNF675,PTGER3,BID,MACF1,MNAT1,TRIO,CAPN3,PTPRE,MYO3B,DUSP22,MYOM1,MAP3K5,MAML2,CTDSPL2,HTR2C,CCDC141,GLRA2,NCF2,FGD4,LY86,EPHA5,SGIP1,TP73,IL1RL1,ZNF366,MYPN,P2RX6,ANK2,ARHGEF3,SLIT3,GRIN2B,SHPRH,PAK3,GARNL3,UACA,ADCY8,ITPR2,GABRR3,MLLT3,CRISP3,CCL15-CCL14,ITGB3BP,RNF213,SH3RF2,CACNA1D,PRKAA2,PACRG,ABCG2,TBL1X,SEMA5B,GRIK1,NLN,ANKFN1,SDK1,GRM5,ARAP2,IGF1R,PKD1L1,CNTNAP2,PTPRO,MSRA,SUFU,INSR,ITGA11,JAK1,RPGR,MAP2K1,UBR2,GMDS,KPNB1,FOXO3,GPC6,RASGRF2,STK38,GABRA3,INPP5A,CNTN6,CENPF,RNLS,MITF,LRFN5,AGBL4,PRDM16,ULK4,KIR2DL4,TMEM117,LTBP1,LRRK2,PKHD1,RARB,SCN1A,BMP6,FYN,XRCC4,EPHA7,MARVELD3,GAS8,MSR1,SEL1L2,GNG2,RIMS1,ENPP2,MCTP2,ABCA12,EPHB1,KCNQ1,DOCK1,CRADD,EXT1,AMFR,PLCB4,UNC5C,ACSBG1,TENM3,LRP2,C2,RALGPS2,FER,GLDC,CAMK4,VWC2,GUCY2F,ERLIN1,SGCD,TMEM108,TPTE,RAPGEF2,PTPRM,NCOA2,NRXN3,INPP4A,PTPRU,ZNF207,GRIA4,CERS6,MACROD2,IL18R1,RGS7BP,ESR2,KAT7,C9,SETD2,TRIM22,CDH13,VEPH1,TRDN,DAB1,ZNF148,HDAC5,SEMA3A,MGAT5,TSPAN8,MYH9,BDKRB2,STIM1,TSC22D3,NRIP1,TANC1,VPS41,LITAF,PIK3R3,TENM1,STK38L,GABRB1,ST8SIA1,PRKAR1A,ZRANB1,RGS7,FCRL2,SIPA1L3,GNG4,VWF,SLC24A2,SCEL,GRIA3,NTRK3,FBN1,CLASP2,NSUN2,PTPRK,SORCS1,DOCK9,PPARA,PTPRD,RORA,SHISA6,BRIP1,ELAVL4,DOCK10,EEF1E1,BICC1,POLN,SEMA3D,PRLR,PTPRA,ATF6,DEPTOR,RAD51D,CUL2,SYT17,STAC,OR4M1,RAB27A,RALGAPA1,CST2,BLOC1S6,PMEPA1,SYT9,SEMA3C,ST18,RXFP2
GO:0007268	chemical synaptic transmission	6.739297165854053e-18	SYN3,CACNG2,RPS6KA2,NTRK2,CHRM3,LAMA2,CNTN4,SORCS2,PLCB1,CACNA1A,CASK,DLGAP1,BTBD9,GRIK4,GPR176,FCHSD2,NLGN1,RELN,APP,PPP3CA,GABRG3,ERC2,SORCS3,GRM7,DLG2,DCC,GRIK2,CHRM1,CDH8,JAK2,PARK2,GRID1,GRIN3A,NLGN4X,EXOC4,NRG3,DTNA,CACNA1E,GABRR2,SV2B,DGKB,PTPRN2,DGKI,SLC1A1,SHISA9,GRM4,TNR,CELF4,LRRC4C,PPP1R9A,CLSTN2,DISC1,GRM1,PDE7B,GRID2,NTNG1,GABRB3,KCND2,EPHB2,ERC1,MCTP1,BDNF,ADORA2A,CADPS,SYT1,SNAP23,CACNG3,PRKAR1B,RIMS2,DLGAP2,GRIA2,RIT2,PRKCG,PLCL2,AGT,NF1,NRXN1,GSK3B,UNC13A,GRIK3,CACNB2,SHANK2,HTR2C,GLRA2,P2RX6,GRIN2B,ADCY8,GABRR3,GRIK1,GRM5,RASGRF2,GABRA3,LRRK2,SPG11,FYN,RIMS1,MCTP2,EPHB1,EXT1,PLCB4,PCDH17,TMEM108,RAPGEF2,NRXN3,PACSIN2,GABRB1,STXBP5,SLC24A2,CEP89,PTPRD,SHISA6,ELAVL4,PTPRA,KCNC4,BLOC1S6,SYT9
GO:0098916	anterograde trans-synaptic signaling	6.739297165854053e-18	SYN3,CACNG2,RPS6KA2,NTRK2,CHRM3,LAMA2,CNTN4,SORCS2,PLCB1,CACNA1A,CASK,DLGAP1,BTBD9,GRIK4,GPR176,FCHSD2,NLGN1,RELN,APP,PPP3CA,GABRG3,ERC2,SORCS3,GRM7,DLG2,DCC,GRIK2,CHRM1,CDH8,JAK2,PARK2,GRID1,GRIN3A,NLGN4X,EXOC4,NRG3,DTNA,CACNA1E,GABRR2,SV2B,DGKB,PTPRN2,DGKI,SLC1A1,SHISA9,GRM4,TNR,CELF4,LRRC4C,PPP1R9A,CLSTN2,DISC1,GRM1,PDE7B,GRID2,NTNG1,GABRB3,KCND2,EPHB2,ERC1,MCTP1,BDNF,ADORA2A,CADPS,SYT1,SNAP23,CACNG3,PRKAR1B,RIMS2,DLGAP2,GRIA2,RIT2,PRKCG,PLCL2,AGT,NF1,NRXN1,GSK3B,UNC13A,GRIK3,CACNB2,SHANK2,HTR2C,GLRA2,P2RX6,GRIN2B,ADCY8,GABRR3,GRIK1,GRM5,RASGRF2,GABRA3,LRRK2,SPG11,FYN,RIMS1,MCTP2,EPHB1,EXT1,PLCB4,PCDH17,TMEM108,RAPGEF2,NRXN3,PACSIN2,GABRB1,STXBP5,SLC24A2,CEP89,PTPRD,SHISA6,ELAVL4,PTPRA,KCNC4,BLOC1S6,SYT9
GO:0099536	synaptic signaling	9.846143664789136e-18	SYN3,CACNG2,RPS6KA2,NTRK2,CHRM3,LAMA2,NOS1,CNTN4,SORCS2,PLCB1,CACNA1A,CASK,DLGAP1,BTBD9,IL1RAPL1,GRIK4,GPR176,FCHSD2,NLGN1,RELN,APP,PPP3CA,GABRG3,ERC2,SORCS3,GRM7,DLG2,DCC,GRIK2,CHRM1,CDH8,JAK2,PARK2,GRID1,GRIN3A,NLGN4X,EXOC4,NRG3,DTNA,CACNA1

			E,GABRR2,SV2B,DGKB,PTPRN2,DGKI,SLC1A1,SHISA9,GRM4,TNR,CELF4,LRRC4C,PPP1R9A,CLSTN2,DISC1,GRM1,PDE7B,GRID2,NTNG1,GABRB3,KCND2,EPHB2,ERC1,MCTP1,BDNF,ADORA2A,CADPS,SYT1,SNAP23,CACNG3,PRKAR1B,RIMS2,DLGAP2,NRG1,GRIA2,RIT2,PRKCG,PLCL2,AGT,NF1,NRXN1,GSK3B,UNC13A,GRIK3,CACNB2,SHANK2,HTR2C,GLRA2,P2RX6,GRIN2B,ADCY8,GABRR3,GRIK1,GRM5,RASGRF2,GABRA3,LRRK2,SPG11,FYN,RIMS1,MCTP2,EPHB1,EXT1,PLCB4,PCDH17,TMEM108,RAPGEF2,NRXN3,PACSIN2,GABRB1,STXBP5,SLC24A2,CEP89,PTPRD,SHISA6,ELAVL4,PTPRA,KCNC4,BLOC1S6,SYT9
GO:0032879	regulation of localization	5.162764988302088e-17	PTPRR,KALRN,CACNG2,CTNNA1,CLASP1,PTPRG,DMD,KCNC2,CHRM3,ERBB4,LAMA2,NOS1,TMC2,ROBO1,DPP10,GPC5,HDAC4,ATP9A,ATP8A2,WLS,LDLRAD4,PTPRT,PLCB1,CACNA1A,PRKD1,RYR2,VCL,NEDD9,SEMA5A,CASK,PRKCA,EPB41L4B,STX8,BTBD9,IL1RAPL1,ABCA13,KANK1,TBC1D5,PLS1,NLGN1,KCNIP4,CAMK1D,AMOTL1,TCF7L2,RELN,APP,PAK1,UTRN,PPP3CA,RYR1,PTPN9,AKAP6,GRM7,RABGAP1L,DLC1,RYR3,DCLK1,MEF2A,KCNB2,CTNNA3,MAP2K5,MAGI2,KCNA6,PARN,SEMA4D,BDKRB1,TF,DNAH11,CHRM1,ASTN2,JAK2,ABCG8,CMKLR1,PARK2,ADCYAP1R1,DPP6,GRIN3A,SMOC2,CACNA1C,NRG3,NUMB,SULF1,CD84,SH3BP1,RFFL,SLIT2,KCNJ16,TRIM5,BMPR1A,SMAD3,CACNA1E,BORA,PIK3C3,MAD1L1,TUB,SCAMP5,DNAJB6,ELP3,FGF14,CACNA2D3,DGKI,SLC1A1,NTN1,LDB2,SHISA9,EPM2A,MAP2,FLRT2,NOS1AP,WWTR1,KCTD7,CATSPER2,ANK3,CNIH3,SCN9A,ACTN4,CD300A,RABGEF1,LRP5,AP2B1,SCFD1,PDE4D,PIK3CD,DOCK4,SLC5A3,FRMD4A,EFNA5,FTO,KCNJ12,PIK3R2,CYBB,SEMA6D,KCNJ3,NTNG1,ADTRP,PDE4B,MAPRE2,ANKRD13A,CLDN1,KCND2,SPTBN4,EPHB2,MCTP1,PRCP,DOCK2,NUP214,DACH1,BDNF,RBM4,ADORA2A,ANO1,CADPS,BMPER,SYT1,DAB2,PLXNA2,NSF,PRKG1,STXBP6,CACNG3,ETS1,PRKAR1B,TTN,SRGAP3,MYRIP,ENPP1,RIMS2,SCN8A,CTNNA2,NRG1,SPINT2,RIT2,KCNMA1,GPSM2,PRKCG,SLC8A1,FBXO31,AGT,FGF1,KCNQ5,KCNJ6,NF1,NRXN1,LAMA3,GSK3B,PKP2,KCNH1,CHD7,SNX5,SRGAP2B,CNTN1,CACNB2,STXBP4,RHOJ,UNC5D,GPC3,DLG5,KCNS3,PTGER3,MACF1,CAPN3,DUSP22,MYOM1,CTDSPL2,HTR2C,EPHA5,SGIP1,ANK2,PAK3,ADCY8,ITPR2,SH3RF2,CACNA1D,PRKAA2,SEMA5B,GRM5,IGF1R,PTPRO,SUFU,NKAIN2,INSR,MAP2K1,FOXO3,GPC6,RASGRF2,MITF,FRMD5,ULK4,LRRK2,NKAIN3,PKHD1,KIF2A,PHACTR1,SCN1A,BMP6,FYN,KCND3,MARVELD3,GAS8,MSR1,PTPN14,RIMS1,ENPP2,MCTP2,ABCA12,KCNQ1,DOCK1,UNC5C,C2,FER,PCDH17,RAPGEF2,NAV3,PTPRM,NRXN3,KCNH7,PTPRU,KAT7,SETD2,PACSIN2,TRIM22,CDH13,TRDN,SEC16B,HDAC5,SEMA3A,MGAT5,STIM1,PIK3R3,TENM1,RGS7,STXBP5,NTRK3,CLASP2,DNAJC6,NSUN2,PTPRK,DNM3,PPARA,SHISA6,DOCK10,ATP8A1,SEMA3D,KCNC4,NVL,SYT17,STAC,RAB27A,KCNJ15,SYT9,SEMA3C
GO:0040011	locomotion	5.363109697749856e-17	PTPRR,RCAN1,KALRN,NDE1,CTNNA1,NTRK2,CLASP1,PTPRG,ERBB4,LAMA2,CNTN4,ROBO1,DSCAM,GPC5,FUT8,HDAC4,ABCC1,AUTS2,ANKS1A,LDLRAD4,PTPRT,NFASC,PLCB1,PRKD1,VCL,NEDD9,SEMA5A,PRKCA,EPB41L4B,KANK1,CAMK1D,AMOTL1,RELN,APP,PAK1,PPP3CA,DLC1,SOS1,DCLK1,CTNNA3,MAP2K5,MAGI2,CELSR1,ROBO2,DCC,ELMO2,EP300,SEMA4D,RPS6KA5,BDKRB1,NME8,TF,DNAH11,CHRM1,ASTN2,DEFA1B,JAK2,CMKLR1,SMOC2,EMB,PIK3C2B,ITGBL1,NRG3,NUMB,SULF1,SH3BP1,ATRNL1,RFFL,ARHGAP24,SLIT2,BMPR1A,DSCAML1,SMAD3,SLC9C1,PEAK1,SATB2,OVOL2,ELP3,SPNS2,NTN1,LDB2,FLRT2,CATSPER2,CCL15,TTLL8,CDH4,TNR,VAV3,SPOCK1,ACTN4,EPS8,CD300A,DISC1,CCL14,RABGEF1,LRP5,PIK3CD,DOCK4,ARMC2,EFNA5,CEP85L,SEMA6D,NTNG1,ADTRP,CRMP1,PDE4B,MAPRE2,PRKCQ,CLDN1,EPHB2,MCTP1,PRCP,PRTG,DOCK2,DPYSL2,DACH1,MARK1,BDNF,ADORA2A,ARID5B,MEGF9,BMPER,DAB2,PLXNA2,PRKG1,ETS1,FAT3,SRGAP3,CTNNA2,FMNL2,NRG1,SPINT2,CD44,SLC8A1,FBXO31,FAM19A4,CDC42BPA,NCAM1,AGT,FGF1,CCR3,USH2A,NF1,NRXN1,LAMA3,SRGAP2B,RHOJ,UNC5D,GPC3,DLG5,ASTN1,PREX1,ADAMTSL1,MACF1,TRIO,DUSP22,SPAG16,CCDC141,EPHA5,MYPN,SLIT3,PAK3,CCL15-CCL14,SH3RF2,SEMA5B,GRM5,IGF1R,PTPRO,INSR,ITGA11,MAP2K1,SLC9B1,FOXO3,GPC6,CNTN6,MITF,FRMD5,ULK4,LRRK2,KIF2A,PHACTR1,FYN,EPHA7,MARVELD3,GAS8,CLN6,ENPP2,EPHB1,DOCK1,EXT1,UNC5C,FER,TPTE,RAPGEF2,NAV3,D

			NAH3,PTPRM,KIRREL3,NRXN3,CDKL5,PTPRU,SETD2,CDH13,DAB1,HDAC5,SEMA3A,MGAT5,MYH9,PIK3R3,SH3KBP1,ZRANB1,NTRK3,CLASP2,PTPRK,DOCK10,ATP8A1,SEMA3D,SEMA3C
GO:0034330	cell junction organization	8.071047581535597e-17	*KALRN,CACNG2,CTNNA1,NTRK2,CLASP1,ERBB4,DSCAM,TBCD,LINGO2,NFASC,VCL,PRKCA,IL1RAPL1,MYO9A,NLGN1,RELN,APP,ERC2,DLC1,CTNND2,ROBO2,SEMA4D,CDH8,TANC2,NEGR1,SDK2,NLGN4X,NUMB,SH3BP1,SMAD3,PEAK1,DGKB,IL1RAPL2,SLC1A1,NTN1,FLRT2,NOS1AP,ARHGAP6,ANK3,CDH12,TNR,ARHGAP39,CDH10,LRRC4C,PDZRN3,CLSTN2,DISC1,CNTN5,EFNA5,TLN2,GRID2,NTNG1,MAPRE2,GABRB3,CLDN1,EPHB2,ERC1,BDNF,CTNNA2,PARD3,NRG1,CAST,NFIA,AGT,NRXN1,PKP2,UNC13A,CACNB2,SYBU,SHANK2,DLG5,MACF1,DUSP22,CLDN11,ANK2,GRIN2B,PAK3,SDK1,GRM5,IGF1R,CNTNAP2,PTPRO,INSR,GPC6,LRFN5,LRRK2,PKHD1,BMP6,FRMPD4,FYN,EPHA7,MARVELD3,EPHB1,EXT1,PCDH17,TMEM108,RAPGEF2,KIRREL3,NRXN3,CDKL5,TANC1,NTRK3,CLASP2,PTPRK,DNM3,SYNDIG1,PTPRD,SHISA6,CDH9,DOCK10,PTPRA,PPFIBP2*
GO:0051716	cellular response to stimulus	8.911180223073403e-17	*PTPRR,CAMTA1,CHFR,RCAN1,KALRN,CACNG2,RNF43,RPS6KA2,CTNNA1,FOXN3,NTRK2,RASGEF1B,ZNRF3,PTPRG,FBXL17,SLC39A10,DMD,KCNC2,CHRM3,SPRED2,ERBB4,NOS1,ESRRG,ROBO1,PRICKLE2,DSCAM,SHROOM3,CIT,PUM1,GPC5,WDR59,SORCS2,WWOX,HUNK,FUT8,HDAC4,IMMP2L,ABCC1,AUTS2,WLS,ANKS1A,LDLRAD4,MYEF2,TENM2,BLM,PSMB2,PTPRT,RXFP1,MGLL,PLCB1,FLT3,SRPK2,CACNA1A,TRPM1,PRKD1,RYR2,PDS5A,NEDD9,SEMA5A,CASK,PRKCA,STX8,ZBTB20,DLGAP1,TRHDE,IL1RAPL1,MYO9A,CCDC3,HBE1,ITGAE,KANK1,GRIK4,GPR176,DOK6,NLGN1,TNFRSF10B,CAMK1D,CLEC16A,AMOTL1,TCF7L2,RELN,APP,ADAM12,PAK1,PPP3CA,DEPDC5,RYR1,GABRG3,CREM,AKAP6,SORCS3,GRM7,DLC1,IDE,RYR3,SOS1,CTNND2,LRRC8C,RFTN1,ALK,BTRC,DCLK1,MEF2A,DLG2,PPP1R12B,MAP2K5,DNMT1,MAGI2,EDA,CELSR1,ROBO2,IFT81,AKAP13,DCC,ELMO2,DDB1,EP300,SEMA4D,EVC,RPS6KA5,CPNE4,GRK5,ANKRD6,BDKRB1,NME8,TF,GRIK2,GNG12,CHRM1,ARHGAP15,HUS1,TFEC,ITSN1,DEFA1B,JAK2,ABCG8,CMKLR1,MAST4,PARK2,MAGI1,NPHP3,ADCYAP1R1,GRID1,TDP1,GRIN3A,SMOC2,CACNA1C,MAN1A1,NLGN4X,PNPLA3,PIK3C2B,OR5K4,ITGBL1,AFAP1,NRG3,SOX6,POLE,COL4A6,SULF1,CHCHD6,SH3BP1,ADCY2,ATRNL1,RFFL,SH3GL2,ARHGAP24,PITPNC1,SLIT2,MAPK4,GLP2R,AOX1,DTNA,TRIM5,THEMIS,DRG2,ATRX,CHRDL1,TRAF3IP2,RAD51B,SLX1B,SH3RF3,BMPR1A,ACACA,SMAD3,PRKAR2A,PI4KA,TNFRSF11B,PIBF1,GABRR2,PIK3C3,CHD6,PTGFR,SCUBE1,EYA1,SATB2,ANKS1B,HSF2BP,MAML3,GPR141,MAD1L1,DGKB,TNKS,TUB,ARHGEF18,SCAMP5,LINC00473,OVOL2,SGMS1,GRAMD4,DNAJB6,BRINP1,OMA1,FGF14,DGKK,OR51I1,ASGR2,IL1RAPL2,SKAP1,PTPRN2,SOX5,DGKI,DNMBP,TAF1,SPNS2,SLC1A1,NTN1,SHISA9,EPM2A,SHFM1,CDC73,FLRT2,FBXW11,NOS1AP,RANBP10,RBMS3,ARHGEF6,WWTR1,ARHGAP6,CHAF1B,EGLN3,GRM4,ANK3,CCL15,MORC2,CNIH3,DOCK3,TRIM59,TNR,ADCY9,CELF4,VAV3,MDM4,ACTN4,PLCE1,LRRN2,EPS8,TENM4,MACROD1,GHR,ARHGAP39,LRRC8D,PPP1R9A,RGL1,CD300A,CBFA2T2,DISC1,RALGPS1,ARHGAP42,CCL14,RABGEF1,FHIT,NSG2,TRABD2B,LRP5,ESR1,ARHGAP12,TNFRSF19,PLCXD3,GRM1,PDE4D,THEM4,PIK3CD,DOCK4,BLOC1S5,TEAD4,PDE7B,EFNA5,FTO,EIF3A,ATF2,RBBP8,PIK3R2,GRID2,ZNF423,CYBB,SEMA6D,ADTRP,PDE4B,MAPRE2,RIC8B,GABRB3,MAP3K7,PRKCQ,N4BP1,RASSF8,RHPN2,CLDN1,KCND2,TPH2,EPHB2,ERC1,AGO3,S1PR3,MCTP1,MOB3B,PRCP,RGMB,EEF1E1-BLOC1S5,DOCK2,DPYSL2,PTPDC1,RFC3,MARK1,BDNF,RBM4,FNIP1,MAGI3,ADORA2A,ARID5B,SIPA1L2,RCAN2,IFT80,FSTL4,MCM3,ANO1,FHL2,NOX4,BMPER,PDE1A,ICK,DEFA3,SYT1,DAB2,PLXNA2,LRRK1,PRKG1,ABI1,PXDNL,CACNG3,EPG5,PTH2R,CALCRL,HDAC2,PDE11A,RAB30,ZSWIM7,BACH1,ZMYND11,TTN,RGS6,SRGAP3,CDK19,ENPP1,RIMS2,KSR2,DLGAP2,PRDM15,OR4N2,PEX5L,PARD3,NRG1,SPINT2,FMN2,GRIA2,VSTM1,RIT2,CD44,CNGB1,GPSM2,PRKCG,TNS3,ZFYVE1,TEAD1,MAPKAPK2,SLC8A1,RAPGEF6,FBXO31,FAM19A4,MAP3K13,PLCL2,CHST11,THRB,CDC42BPA,CDC42EP3,MAPK10,NCAM1,AGT,FGF1,QRICH1,CCR3,NDRG2,GRIP1,TLK1,ARHGAP25,NF1,WIF1,ZNF536,NRXN1,ARHGAP10,LAMA3,PDXP,GSK3B,KIR2DL1,CD109,KCNH1,DCDC1,WDR70,GPR21,PSMB7,SN*

			X5,TIAM2,OR51B2,IQCJ-SCHIP1,CNTN1,BTBD11,UNC13A,CABIN1,NCOA1,CHEK2,GRIK3,STXBP4,RHOJ,PHEX,MECOM,STK32B,SHANK2,UBE2E2,KCTD8,UNC5D,NREP,GPC3,DLG5,PREX1,CPE,UBE2V1,ROR1,ZNF675,PTGER3,BID,MACF1,MNAT1,TRIO,CAPN3,PTPRE,DUSP22,MYOM1,MAP3K5,MAML2,CTDSPL2,HTR2C,GLRA2,FGD4,LY86,EPHA5,TP73,IL1RL1,ZNF366,P2RX6,ANK2,ARHGEF3,SLIT3,GRIN2B,SHPRH,PAK3,GARNL3,UACA,ADCY8,ITPR2,GABRR3,MLLT3,CCL15-CCL14,ITGB3BP,RNF213,SH3RF2,CACNA1D,PRKAA2,PACRG,ABCG2,TBL1X,SEMA5B,GRIK1,NLN,GRM5,ARAP2,IGF1R,PTPRO,MSRA,SUFU,INSR,ITGA11,JAK1,MAP2K1,UBR2,GMDS,KPNB1,FOXO3,GPC6,RASGRF2,STK38,GABRA3,INPP5A,CNTN6,CENPF,MITF,PRDM16,ULK4,KIR2DL4,TMEM117,LTBP1,LRRK2,PKHD1,RARB,BMP6,FYN,XRCC4,EPHA7,MARVELD3,GAS8,MSR1,SEL1L2,GNG2,RIMS1,MCTP2,ABCA12,EPHB1,KCNQ1,DOCK1,CRADD,EXT1,AMFR,PLCB4,UNC5C,TENM3,LRP2,RALGPS2,FER,GLDC,CAMK4,VWC2,GUCY2F,ERLIN1,SGCD,TMEM108,TPTE,RAPGEF2,PTPRM,NCOA2,NRXN3,INPP4A,PTPRU,ZNF207,GRIA4,MACROD2,IL18R1,RGS7BP,ESR2,KAT7,SETD2,TRIM22,CDH13,VEPH1,DAB1,HDAC5,SEMA3A,MGAT5,MYH9,BDKRB2,NRIP1,VPS41,LITAF,PIK3R3,TENM1,STK38L,GABRB1,ST8SIA1,PRKAR1A,ZRANB1,RGS7,FCRL2,SIPA1L3,GNG4,VWF,SLC24A2,SCEL,GRIA3,NTRK3,FBN1,CLASP2,NSUN2,PTPRK,SORCS1,DOCK9,PPARA,PTPRD,RORA,SHISA6,BRIP1,ELAVL4,DOCK10,EEF1E1,BICC1,POLN,SEMA3D,PRLR,PTPRA,ATF6,DEPTOR,RAD51D,CUL2,SYT17,STAC,OR4M1,RALGAPA1,BLOC1S6,PMEPA1,SYT9,SEMA3C,ST18,RXFP2
GO:0007267	cell-cell signaling	2.323880651253876e-16	*SYN3,KALRN,CACNG2,RNF43,RPS6KA2,NTRK2,ZNRF3,CHRM3,LAMA2,NOS1,CNTN4,PRICKLE2,GPC5,SORCS2,WWOX,WLS,PSMB2,PLCB1,CACNA1A,RYR2,SEMA5A,CASK,DLGAP1,BTBD9,TRHDE,IL1RAPL1,KANK1,GRIK4,GPR176,FCHSD2,NLGN1,AMOTL1,TCF7L2,RELN,APP,PPP3CA,GABRG3,ERC2,SORCS3,GRM7,CTNND2,BTRC,DLG2,MAGI2,EDA,CELSR1,DCC,DDB1,GRK5,ANKRD6,GRIK2,CHRM1,CDH8,JAK2,PARK2,NPHP3,GRID1,GRIN3A,CACNA1C,NLGN4X,EXOC4,NRG3,SULF1,DTNA,SMAD3,CACNA1E,GABRR2,SV2B,DGKB,TNKS,FGF14,PTPRN2,DGKI,SLC1A1,SHISA9,EPM2A,CDC73,FBXW11,RBMS3,WWTR1,PCSK5,GRM4,CCL15,TNR,CELF4,LRRC4C,PPP1R9A,CLSTN2,SRD5A2,DISC1,TRABD2B,LRP5,GRM1,PDE7B,EFNA5,GRID2,ZNF423,KCNJ3,NTNG1,GABRB3,KCND2,EPHB2,ERC1,MCTP1,MARK1,BDNF,ADORA2A,IFT80,ANO1,CADPS,SYT1,DAB2,LRRK1,SNAP23,CACNG3,PRKAR1B,MYRIP,RIMS2,DLGAP2,PRDM15,NRG1,GRIA2,RIT2,PRKCG,PLCL2,AGT,NDRG2,NF1,WIF1,NRXN1,GSK3B,PKP2,CHD7,PSMB7,UNC13A,GRIK3,CACNB2,STXBP4,PHEX,SHANK2,GPC3,CPE,ROR1,MACF1,HTR2C,GLRA2,EPHA5,P2RX6,ANK2,GRIN2B,ADCY8,GABRR3,MLLT3,RNF213,CACNA1D,PRKAA2,TBL1X,GRIK1,GRM5,PTPRO,FOXO3,GPC6,RASGRF2,GABRA3,MITF,LRRK2,SPG11,BMP6,FYN,RIMS1,MCTP2,ABCA12,EPHB1,KCNQ1,EXT1,AMFR,PLCB4,PCDH17,TMEM108,RAPGEF2,NRXN3,PTPRU,ESR2,PACSIN2,GABRB1,SH3KBP1,ZRANB1,STXBP5,FCRL2,SLC24A2,SCEL,CEP89,PTPRD,SHISA6,ELAVL4,BICC1,PTPRA,KCNC4,SYT17,BLOC1S6,SYT9*
GO:0010975	regulation of neuron projection development	2.915990585030331e-16	*KALRN,NTRK2,PTPRG,DMD,ROBO1,DSCAM,ATP8A2,PRKD1,SEMA5A,IL1RAPL1,KANK1,NLGN1,CAMK1D,RELN,PAK1,PPP3CA,TOX,PTPN9,ALK,DENND5A,MAGI2,ROBO2,DCC,EP300,SEMA4D,TANC2,NEGR1,SLIT2,NTN1,MAP2,CUX1,CDH4,TNR,SPOCK1,LRRC4C,CBFA2T2,DISC1,EFNA5,GRID2,SEMA6D,NTNG1,CRMP1,EPHB2,DPYSL2,MARK1,BDNF,FSTL4,DAB2,PLXNA2,HDAC2,FAT3,CTNNA2,RIT2,FBXO31,MAP3K13,AGT,GSK3B,TIAM2,CNTN1,ISLR2,ROR1,MACF1,PAK3,SEMA5B,IGF1R,PTPRO,MAP2K1,ULK4,LRRK2,FYN,EPHA7,TRPC5,TENM3,RAPGEF2,CDKL5,DAB1,SEMA3A,NTRK3,DNM3,PTPRD,ELAVL4,SEMA3D,SEMA3C*
GO:0061564	axon development	3.5271248657959014e-16	*KALRN,CTNNA1,NTRK2,LAMA2,CNTN4,ROBO1,DSCAM,ATP8A2,AUTS2,NFASC,VCL,SEMA5A,RELN,APP,PAK1,GRM7,SOS1,DCLK1,ROBO2,DCC,SEMA4D,RPS6KA5,JAK2,EMB,NUMB,SLIT2,DSCAML1,NTN1,MAP2,FLRT2,ANK3,CDH4,TNR,LRRC4C,DISC1,EFNA5,SEMA6D,NTNG1,CRMP1,PRKCQ,SPTBN4,EPHB2,PRTG,DPYSL2,BDNF,FSTL4,PLXNA2,CTNNA2,PARD3,MAP3K13,NCAM1,NRXN1,LAMA3,GSK3B,TIAM2,UNC5D,ISLR2,NREP,ADAMTSL1,MACF1,TRIO,CCDC141,EPHA5,MYPN,SLIT3,PAK*

			3,SEMA5B,IGF1R,PTPRO,CRTAC1,MAP2K1,CNTN6,SPG11,FYN,EPHA7,EPHB1,VASH2,EXT1,TRPC5,UNC5C,PTPRM,NRXN3,CDKL5,DAB1,SEMA3A,CLASP2,SEMA3D,SEMA3C
GO:0050804	modulation of chemical synaptic transmission	5.306390590029785e-16	*SYN3,CACNG2,NTRK2,LAMA2,CNTN4,SORCS2,PLCB1,CACNA1A,CASK,DLGAP1,BTBD9,GRIK4,NLGN1,RELN,APP,PPP3CA,ERC2,SORCS3,GRM7,DCC,GRIK2,JAK2,PARK2,GRID1,GRIN3A,NLGN4X,NRG3,DGKB,DGKI,SLC1A1,SHISA9,GRM4,TNR,CELF4,LRRC4C,PPP1R9A,CLSTN2,DISC1,GRM1,GRID2,NTNG1,EPHB2,ERC1,MCTP1,BDNF,ADORA2A,SYT1,CACNG3,PRKAR1B,RIMS2,DLGAP2,PRKCG,PLCL2,AGT,NF1,NRXN1,GSK3B,UNC13A,GRIK3,CACNB2,SHANK2,GRIN2B,ADCY8,GRIK1,GRM5,RASGRF2,LRRK2,FYN,RIMS1,MCTP2,EPHB1,PLCB4,PCDH17,TMEM108,RAPGEF2,NRXN3,PACSIN2,STXBP5,SLC24A2,PTPRD,SHISA6,ELAVL4,PTPRA*
GO:0099177	regulation of trans-synaptic signaling	6.154791180796712e-16	*SYN3,CACNG2,NTRK2,LAMA2,CNTN4,SORCS2,PLCB1,CACNA1A,CASK,DLGAP1,BTBD9,GRIK4,NLGN1,RELN,APP,PPP3CA,ERC2,SORCS3,GRM7,DCC,GRIK2,JAK2,PARK2,GRID1,GRIN3A,NLGN4X,NRG3,DGKB,DGKI,SLC1A1,SHISA9,GRM4,TNR,CELF4,LRRC4C,PPP1R9A,CLSTN2,DISC1,GRM1,GRID2,NTNG1,EPHB2,ERC1,MCTP1,BDNF,ADORA2A,SYT1,CACNG3,PRKAR1B,RIMS2,DLGAP2,PRKCG,PLCL2,AGT,NF1,NRXN1,GSK3B,UNC13A,GRIK3,CACNB2,SHANK2,GRIN2B,ADCY8,GRIK1,GRM5,RASGRF2,LRRK2,FYN,RIMS1,MCTP2,EPHB1,PLCB4,PCDH17,TMEM108,RAPGEF2,NRXN3,PACSIN2,STXBP5,SLC24A2,PTPRD,SHISA6,ELAVL4,PTPRA*
GO:0007409	axonogenesis	9.15840257195621e-16	*KALRN,NTRK2,LAMA2,CNTN4,ROBO1,DSCAM,ATP8A2,AUTS2,NFASC,VCL,SEMA5A,RELN,APP,PAK1,SOS1,DCLK1,ROBO2,DCC,SEMA4D,RPS6KA5,EMB,NUMB,SLIT2,DSCAML1,NTN1,MAP2,FLRT2,ANK3,CDH4,TNR,LRRC4C,DISC1,EFNA5,SEMA6D,NTNG1,CRMP1,PRKCQ,SPTBN4,EPHB2,PRTG,DPYSL2,BDNF,FSTL4,PLXNA2,CTNNA2,PARD3,MAP3K13,NCAM1,NRXN1,LAMA3,GSK3B,TIAM2,UNC5D,ISLR2,ADAMTSL1,MACF1,TRIO,CCDC141,EPHA5,MYPN,SLIT3,PAK3,SEMA5B,IGF1R,PTPRO,MAP2K1,CNTN6,SPG11,FYN,EPHA7,EPHB1,EXT1,TRPC5,UNC5C,PTPRM,NRXN3,CDKL5,DAB1,SEMA3A,CLASP2,SEMA3D,SEMA3C*
GO:0065007	biological regulation	1.872007699333789e-15	*PKNOX2,PTPRR,SYN3,RNF4,CAMTA1,CHFR,KDM4B,RCAN1,KALRN,CACNG2,RNF43,PROS1,RPS6KA2,CTNNA1,FOXN3,NTRK2,CLASP1,RASGEF1B,ZNRF3,GLIS3,PTPRG,FBXL17,SLC39A10,DMD,KCNC2,CHRM3,EIF3E,SPRED2,ERBB4,LAMA2,TFDP2,NOS1,CNTN4,TMC2,SND1,ESRRG,ROBO1,PRICKLE2,DSCAM,SHROOM3,CIT,TBCD,DPP10,PUM1,FRY,GPC5,WDR59,SORCS2,PRIM2,WWOX,HUNK,FUT8,TNRC6B,HDAC4,ATP9A,LINGO2,ARFGAP3,ABCC1,ATP8A2,AUTS2,WLS,ANKS1A,LDLRAD4,MYEF2,TENM2,SGSM1,BLM,PSMB2,PTPRT,RXFP1,ATF7IP,ASXL3,MGLL,PLCB1,FLT3,SRPK2,CACNA1A,TRPM1,PRKD1,RYR2,VCL,PDS5A,NEDD9,PPP2R3C,SEMA5A,ETV6,CASK,PRKCA,EPB41L4B,STX8,ZBTB20,DLGAP1,BTBD9,TRHDE,ZNF713,IL1RAPL1,MYO9A,CCDC3,DIO2,PDE4DIP,ITGAE,ABCA13,KANK1,KIF26B,TBC1D5,SLCO3A1,TCF12,GRIK4,RUNX1,PLS1,GPR176,USP53,DOK6,FCHSD2,NLGN1,TNFRSF10B,KCNIP4,CAMK1D,SMG6,CLEC16A,AMOTL1,TCF7L2,MTRF1,RELN,APP,ADAM12,PAK1,UTRN,PPP3CA,DEPDC5,RYR1,TOX,PCDH15,GABRG3,ERC2,PTPN9,CREM,AKAP6,SORCS3,GRM7,RABGAP1L,DLC1,IDE,SCMH1,RYR3,SOS1,CTNND2,RFTN1,ALK,BTRC,DCLK1,MEF2A,EEFSEC,KCNB2,TFAP2D,FAM155A,CTNNA3,DENND5A,PPP1R12B,MAP2K5,DNMT1,MAGI2,EDA,CELSR1,ROBO2,IFT81,AKAP13,HIVEP3,DCC,SAMD4A,C10ORF90,ZNF799,DDB1,EP300,KCNA6,PARN,SEMA4D,EVC,RPS6KA5,PHF20,GRK5,ANKRD6,TRAPPC9,MMP16,BDKRB1,TF,DNAH11,GRIK2,QKI,GNG12,CHRM1,CDH8,ARHGAP15,HUS1,ZNF667,TFEC,ITSN1,ASTN2,TANC2,DEFA1B,JAK2,ABCG8,RAB11FIP4,NEGR1,CMKLR1,PPP6R2,ZBTB7C,MAST4,PARK2,MAGI1,NPHP3,ADCYAP1R1,SERPINA4,DPP6,GRID1,GRIN3A,SMOC2,CACNA1C,NLGN4X,EXOC4,PNPLA3,ANO4,PIK3C2B,OR5K4,ANKH,ITGBL1,NBEA,GBE1,AFAP1,TMPRSS3,TAGLN3,PALMD,SP140,NRG3,NUMB,MED15,SOX6,COL4A6,SULF1,DTD2,CD84,MAGEA11,SH3BP1,ADCY2,TCF20,OTC,ATRNL1,RFFL,SH3GL2,PSPC1,ARHGAP24,PITPNC1,SLIT2,KCNJ16,MAPK4,GLP2R,SLC4A4,DTNA,KMT2C,TRIM5,THEMIS,DRG2,AFF3,ATRX,CHRDL1,TRAF3IP2,RAD51B,SLX1B,SH3RF3,BMPR1A,ACACA,SMAD3,PRKAR2A,CACNA1E,BORA,PI4KA,TNFRSF11B,PIBF1,GABRR2,PIK3C3,SLC9C1,CHD6,POTEE,KIR3DL2,PTGFR,SCUBE1,PEAK1,EYA1,S*

			ATB2,ANKS1B,ELN,MAML3,GPR141,TPH1,MAD1L1,EGFLAM,DGKB,TNKS,TUB,KLF12,ARHGEF18,SCAMP5,LINC00473,OVOL2,SGMS1,GRAMD4,DNAJB6,ELP3,BRINP1,OMA1,BCL2L13,ARRDC4,KHDRBS2,CPQ,FGF14,CACNA2D3,DGKK,OR51I1,GLIS1,ASGR2,IL1RAPL2,ZDHHC11,SKAP1,CDYL2,PTPRN2,SOX5,DGKI,DNMBP,TAF1,SPNS2,SLC1A1,RNF144B,NTN1,LDB2,SHISA9,EPM2A,MAP2,CDC73,FLRT2,FBXW11,NOS1AP,ARHGEF33,RANBP10,CD96,RBMS3,ARHGEF6,WWTR1,PEMT,ARHGAP6,PHF20L1,KCTD7,CATSPER2,PCSK5,EGLN3,CUX1,GRM4,ANK3,TIMP2,CCL15,MORC2,ZNF19,ARID4B,CNIH3,DOCK3,TRIM59,TRPS1,CDH4,TNR,ADCY9,CELF4,VAV3,CDKL1,SCN9A,MDM4,ARNT2,SPOCK1,ACTN4,HPSE2,PLCE1,CYP39A1,LRRN2,EPS8,TENM4,PRR16,GHR,ARHGAP39,LRRC8D,LRRC4C,PPP1R9A,RGL1,PALM2,CLSTN2,CD300A,CBFA2T2,SRD5A2,FUBP1,PRG3,MLIP,SERTAD2,DISC1,RALGPS1,ARHGAP42,ATP8B4,CCL14,RABGEF1,FHIT,NSG2,TRABD2B,LRP5,NAV2,AP2B1,SLC30A7,ESR1,ARHGAP12,TNFRSF19,PLCXD3,DENND2A,GRM1,SCFD1,PDE4D,THEM4,PIK3CD,DOCK4,SLC5A3,BLOC1S5,FRMD4A,PCSK2,TEAD4,PDE7B,EFNA5,FTO,EIF3A,POU6F2,ATF2,PHACTR2,RBBP8,KCNJ12,PIK3R2,GRID2,ZNF423,CYBB,SEMA6D,KCNJ3,SUSD4,CELF2,NTNG1,ADTRP,CRMP1,PDE4B,MAPRE2,RIC8B,GABRB3,SMG7,ANKRD13A,PPA2,MAP3K7,PRKCQ,ATXN1,N4BP1,RASSF8,RHPN2,CLDN1,KCND2,SLC24A3,JDP2,SPTBN4,EPHB2,ERC1,AGO3,S1PR3,MCTP1,MOB3B,PRCP,PRTG,RGMB,EEF1E1-BLOC1S5,DOCK2,NUP214,DPYSL2,PTPDC1,RFC3,DACH1,CALML4,MARK1,BDNF,RBM4,FNIP1,MALRD1,MAGI3,ADORA2A,ARID5B,SIPA1L2,RCAN2,IFT80,FSTL4,ANO1,FHL2,CADPS,DRAM1,NOX4,CSMD1,BCOR,BMPER,PDE1A,ICK,DEFA3,BRD9,SYT1,EBAG9,DAB2,AFF2,PLXNA2,NSF,EBF4,LRRK1,PRKG1,STXBP6,SNAP23,CHST9,ABI1,CACNG3,EPG5,PTH2R,CALCRL,TNNI3K,HDAC2,ETS1,BANP,FAT3,PDE11A,ARID4A,RAB30,PRKAR1B,ZSWIM7,BACH1,ZMYND11,CDH23,TTN,RGS6,SRGAP3,ZNF93,MYRIP,CDK19,ENPP1,CFB,ZNF443,RIMS2,SCN8A,KSR2,DLGAP2,PRDM15,OR4N2,AGAP1,CTNNA2,PEX5L,CYB561A3,FMNL2,PARD3,NRG1,CAST,TBC1D9,FANK1,SPINT2,ATP10B,NPAS3,FMN2,GRIA2,VSTM1,TOP1,RIT2,CD44,PAX7,KCNMA1,NCF4,SCAF8,CNGB1,GPSM2,PRKCG,SLC12A8,TNS3,MED13L,TEAD1,NFIA,MAPKAPK2,EIF4G3,SLC8A1,SRRM4,RAPGEF6,ISM1,FBXO31,FAM19A4,MAP3K13,PLCL2,TOX3,CHST11,THRB,SLC9A9,CDC42BPA,CDC42EP3,MIR1185-1,MAPK10,NCAM1,AGT,FGF1,KCNQ5,QRICH1,HEPHL1,CCR3,KCNJ6,STRIP1,ZNF709,NDRG2,GRIP1,TLK1,UPK3B,ARHGAP25,USH2A,NF1,WIF1,ZNF536,PARVB,NRXN1,ARHGAP10,LAMA3,PDXP,GSK3B,KIR2DL1,PKP2,CTDP1,SETD3,CD109,SLC22A3,KCNH1,ZNF695,DCDC1,LMO7,WDR70,GPR21,CHD7,PSMB7,MOV10L1,SNX5,TIAM2,OR51B2,MYT1L,SRGAP2B,IQCJ-SCHIP1,CNTN1,PLEKHB2,BTBD11,UNC13A,TRIOBP,CABIN1,NCOA1,CHEK2,GRIK3,CACNB2,STXBP4,RHOJ,ZBTB8B,ZNF730,MECOM,STK32B,SHANK2,UBE2E2,KCTD8,UNC5D,ISLR2,NREP,GPC3,DLG5,CFDP1,PREX1,CPE,UBE2V1,ANO3,XKR4,ROR1,ZNF675,SERPINA5,KCNS3,PTGER3,BID,MACF1,MNAT1,TRIO,CAPN3,CTNNBL1,PTPRE,DUSP22,MYOM1,MAP3K5,MAML2,CTDSPL2,HTR2C,GLRA2,NCF2,FGD4,LY86,ZNF670,EPHA5,SGIP1,HIRA,RNF144A,TP73,RBM19,IL1RL1,TSHZ2,ZNF366,FAM171A1,MORC3,P2RX6,ANK2,ARHGEF3,SUPT3H,SLIT3,GRIN2B,PHC2,PAK3,GARNL3,UACA,LRPPRC,DPH6,ADCY8,ITPR2,GABRR3,MLLT3,NEB,CCL15-CCL14,ITGB3BP,RNF213,SH3RF2,CACNA1D,PRKAA2,PACRG,TBL1X,SEMA5B,HIVEP2,GRIK1,NLN,ANKFN1,SDK1,GRM5,ARAP2,IGF1R,MXD3,CNTNAP2,PTPRO,SUFU,NKAIN2,INSR,CIZ1,ITGA11,JAK1,RPGR,MAP2K1,UBR2,ZNF490,GMDS,SLC9B1,KPNB1,FOXO3,GPC6,RASGRF2,STK38,GABRA3,KLHL3,INPP5A,CNTN6,CENPF,KCTD1,RNLS,PPP1R14A,ATP9B,MITF,IGF2BP3,SP140L,LRFN5,CDC14A,ENOX2,AGBL4,PRDM16,FRMD5,ULK4,RBFOX1,KIR2DL4,ZNF605,ZNF41,TMEM117,DNMT3B,ADAMTS16,TASP1,LTBP1,LRRK2,NKAIN3,PKHD1,KIF2A,RARB,TCF4,PBX1,PHACTR1,SCN1A,BMP6,PCBP3,ASAP1,FRMPD4,FYN,KCND3,ABCC2,CSPP1,XRCC4,EPHA7,MARVELD3,GAS8,MSR1,SGCZ,CLN6,PTPN14,GNG2,PCBD2,RIMS1,ENPP2,L3MBTL4,MCTP2,ABCA12,BEND5,EPHB1,KCNQ1,FHOD3,DOCK1,CRADD,VASH2,ZNF664,BPGM,EXT1,TRPC5,AMFR,PLCB4,UNC5C,TENM3,NOX5,LRP2,ZCCHC17,C2,RALGPS2,FER,CAMK4,V

			WC2,GUCY2F,ERLIN1,PCDH17,SGCD,TMEM108,TPTE,RAPGEF2,NAV3,PRPSAP2,PTPRM,NCOA2,NRXN3,KCNH7,CDKL5,INPP4A,PTPRU,ZNF207,GRIA4,IL18R1,RGS7BP,ESR2,KAT7,SATB1,C9,TMTC2,MIR767,SETD2,PACSIN2,TRIM22,CDH13,VEPH1,BASP1,TRDN,DAB1,ZNF148,SEC16B,HDAC5,SEMA3A,MGAT5,TSPAN8,ATP13A3,MYH9,BDKRB2,STIM1,TSC22D3,NRIP1,CCNG2,MIR105-2,LITAF,PIK3R3,BRF1,TENM1,STK38L,LARGE,GABRB1,ST8SIA1,SH3KBP1,PRKAR1A,ZRANB1,RGS7,STXBP5,FCRL2,SIPA1L3,GNG4,VWF,TXNDC5,SLC24A2,SCEL,GRIA3,NTRK3,FBN1,DIS3L2,TBX15,CLASP2,ZNF521,CREB5,DNAJC6,NSUN2,DACH2,PTPRK,SORCS1,DNM3,SYNDIG1,DOCK9,PPARA,PTPRD,RORA,SHISA6,BRIP1,ELAVL4,SBF2,ANXA8L1,MXI1,TTC28,NELL1,DOCK10,EEF1E1,ATP8A1,BICC1,SEMA3D,PRLR,PTPRA,KCNC4,TSPAN1,ATF6,DEPTOR,NVL,RAD51D,CUL2,SYT17,STAC,OR4M1,RAB27A,RALGAPA1,CABLES1,CST2,BLOC1S6,PMEPA1,LARP4B,KCNJ15,SYT9,DENND2D,SEMA3C,PAH,ST18,RXFP2
GO:0031344	regulation of cell projection organization	3.315190672019998e-14	*KALRN,NTRK2,PTPRG,DMD,ROBO1,DSCAM,HDAC4,ATP8A2,AUTS2,TENM2,PRKD1,SEMA5A,IL1RAPL1,MYO9A,KANK1,PLS1,NLGN1,CAMK1D,RELN,PAK1,PPP3CA,TOX,PTPN9,ALK,DENND5A,MAGI2,ROBO2,DCC,EP300,SEMA4D,TANC2,NEGR1,ARHGAP24,SLIT2,NTN1,MAP2,CUX1,CDH4,TNR,CDKL1,SPOCK1,PLCE1,EPS8,LRRC4C,CBFA2T2,DISC1,EFNA5,GRID2,SEMA6D,NTNG1,CRMP1,EPHB2,DPYSL2,MARK1,BDNF,FSTL4,DAB2,PLXNA2,HDAC2,FAT3,CTNNA2,RIT2,CD44,FBXO31,MAP3K13,CDC42EP3,AGT,GRIP1,NRXN1,GSK3B,TIAM2,CNTN1,ISLR2,ROR1,MACF1,GRIN2B,PAK3,SEMA5B,IGF1R,PTPRO,MAP2K1,ULK4,ADAMTS16,LRRK2,FYN,EPHA7,ENPP2,TRPC5,TENM3,FER,RAPGEF2,CDKL5,DAB1,SEMA3A,TENM1,NTRK3,DNM3,PTPRD,ELAVL4,SEMA3D,SEMA3C*
GO:0050808	synapse organization	3.97600095224306e-14	*KALRN,CACNG2,NTRK2,ERBB4,DSCAM,LINGO2,NFASC,IL1RAPL1,NLGN1,RELN,APP,ERC2,CTNND2,ROBO2,SEMA4D,CDH8,TANC2,NEGR1,SDK2,NLGN4X,DGKB,IL1RAPL2,SLC1A1,NTN1,FLRT2,NOS1AP,ANK3,TNR,ARHGAP39,LRRC4C,PDZRN3,CLSTN2,DISC1,CNTN5,EFNA5,GRID2,NTNG1,GABRB3,EPHB2,ERC1,BDNF,CTNNA2,NRG1,CAST,NFIA,NRXN1,UNC13A,CACNB2,SYBU,SHANK2,DLG5,GRIN2B,PAK3,SDK1,GRM5,IGF1R,PTPRO,INSR,GPC6,LRFN5,LRRK2,FRMPD4,FYN,EPHA7,EPHB1,PCDH17,TMEM108,KIRREL3,NRXN3,CDKL5,TANC1,NTRK3,DNM3,SYNDIG1,PTPRD,SHISA6,DOCK10,PPFIBP2*
GO:0120035	regulation of plasma membrane bounded cell projection organization	4.8166142453068413e-14	*KALRN,NTRK2,PTPRG,DMD,ROBO1,DSCAM,HDAC4,ATP8A2,AUTS2,TENM2,PRKD1,SEMA5A,IL1RAPL1,KANK1,PLS1,NLGN1,CAMK1D,RELN,PAK1,PPP3CA,TOX,PTPN9,ALK,DENND5A,MAGI2,ROBO2,DCC,EP300,SEMA4D,TANC2,NEGR1,ARHGAP24,SLIT2,NTN1,MAP2,CUX1,CDH4,TNR,CDKL1,SPOCK1,PLCE1,EPS8,LRRC4C,CBFA2T2,DISC1,EFNA5,GRID2,SEMA6D,NTNG1,CRMP1,EPHB2,DPYSL2,MARK1,BDNF,FSTL4,DAB2,PLXNA2,HDAC2,FAT3,CTNNA2,RIT2,CD44,FBXO31,MAP3K13,CDC42EP3,AGT,NRXN1,GSK3B,TIAM2,CNTN1,ISLR2,ROR1,MACF1,GRIN2B,PAK3,SEMA5B,IGF1R,PTPRO,MAP2K1,ULK4,ADAMTS16,LRRK2,FYN,EPHA7,ENPP2,TRPC5,TENM3,FER,RAPGEF2,CDKL5,DAB1,SEMA3A,TENM1,NTRK3,DNM3,PTPRD,ELAVL4,SEMA3D,SEMA3C*
GO:0048513	animal organ development	2.7866236288945747e-13	*RCAN1,KALRN,MEGF11,RPS6KA2,NDE1,CTNNA1,FOXN3,NTRK2,CLASP1,ZNRF3,PTPRG,FBXL17,DMD,KCNC2,SPRED2,KAZN,ERBB4,LAMA2,TFDP2,CNTN4,ROBO1,PRICKLE2,MYO3A,DSCAM,CIT,NEBL,WWOX,HDAC4,IMMP2L,ATP8A2,WLS,LDLRAD4,PSMB2,RXFP1,ASXL3,PLCB1,FLT3,RYR2,PPP2R3C,SEMA5A,ETV6,PRKCA,ADAMTS6,PLAC1,NHS,KIF26B,TCF12,RUNX1,PLS1,TCF7L2,RELN,APP,UTRN,PPP3CA,RYR1,TOX,PCDH15,GREB1L,AKAP6,DLC1,SOS1,ALK,BTRC,DCLK1,MEF2A,TFAP2D,MAP2K5,MAGI2,EDA,CELSR1,ROBO2,AKAP13,HIVEP3,DCC,EP300,SEMA4D,EVC,ANKRD6,TRAPPC9,MMP16,TF,DNAH11,JAK2,NEGR1,SDK2,NPHP3,CACNA1C,NLGN4X,EXOC4,ANKH,UPB1,NRG3,NUMB,SOX6,POLE,SULF1,OTC,ATRNL1,COL11A1,SLIT2,THEMIS,ATRX,CHRDL1,TRAF3IP2,BMPR1A,DSCAML1,SMAD3,TNFRSF11B,POTEE,SCUBE1,EYA1,SATB2,ELN,TPH1,MAD1L1,ANKRD11,EGFLAM,TUB,OVOL2,DNAJB6,ASGR2,SOX5,TAF1,SPNS2,SLC1A1,NTN1,LDB2,CDC73,FLRT2,FBXW11,WWTR1,PCSK5,ARID4B,TRPS1,MYO18B,TNR,CELF4,MDM4,ARNT2,PLCE1,TACC2,TENM4,CECR2,GHR,SRD5A2,STRC,DISC1,LRP5,AP2B1,ESR1,TNFRSF19,PIK3CD,CERS3,TEAD4,KLHL1,FTO,ATF2,GRID2,ZNF423,SEMA6D,NTNG1,ATXN1,CLD*

			N1,SLC24A3,TTLL5,EPHB2,DOCK2,DPYSL2,MARK1,FNIP1,ARID5B,IFT80,DYM,FHL2,TTC39C,NOX4,MEGF9,CSMD1,BCOR,BMPER,SYT1,DAB2,AFF2,PLXNA2,LRRK1,PRKG1,ABI1,CALCRL,HDAC2,ETS1,FAT3,ARID4A,CDH23,TTN,ENPP1,CTNNA2,NRG1,SPINT2,CD44,PAX7,TEAD1,NFIA,MAPKAPK2,SLC8A1,PLCL2,CHST11,THRB,AGT,FGF1,NDRG2,USH2A,NF1,NRXN1,LAMA3,GSK3B,PKP2,CTDP1,CD109,CHD7,PSMB7,CNTN1,TRIOBP,NCOA1,RHOJ,PHEX,MECOM,SHANK2,GPC3,DLG5,PREX1,CPE,ROR1,ZNF675,MNAT1,CAPN3,MYO3B,CCDC141,EPHA5,TP73,ANK2,SLIT3,GRIN2B,MATN3,PRRC2C,MLLT3,NEB,SEMA5B,NLN,SDK1,IGF1R,CNTNAP2,PTPRO,SUFU,INSR,ITGA11,MAP2K1,FOXO3,GPC6,KLHL3,CENPF,MITF,RBFOX1,ADAMTS16,LRRK2,PKHD1,RARB,PBX1,PHACTR1,BMP6,FYN,EPHA7,GAS8,COL19A1,SGCZ,ABCA12,EPHB1,KCNQ1,FHOD3,DOCK1,VASH2,BPGM,EXT1,UNC5C,ACSBG1,CSGALNACT1,TENM3,WDR7,LRP2,CAMK4,SGCD,TMEM108,SHROOM4,RAPGEF2,PRPSAP2,PTPRM,KIRREL3,PTPRU,SOBP,MACROD2,IL18R1,KAT7,DCHS2,SETD2,MDGA2,BASP1,DAB1,ZNF148,HDAC5,SEMA3A,MYH9,STIM1,NRIP1,LARGE,PRKAR1A,RGS7,SIPA1L3,SCEL,NTRK3,FBN1,HYDIN,TBX15,CLASP2,NSUN2,TPD52,PPARA,RORA,MYH15,BRIP1,ELAVL4,NELL1,DOCK10,FNDC3A,BICC1,SEMA3D,PRLR,ATF6,SPATA5,SEMA3C,RXFP2
GO:0007411	axon guidance	8.770264966094059e-13	*KALRN,LAMA2,CNTN4,ROBO1,DSCAM,NFASC,SEMA5A,RELN,APP,SOS1,ROBO2,DCC,SEMA4D,RPS6KA5,EMB,SLIT2,DSCAML1,NTN1,FLRT2,CDH4,TNR,EFNA5,SEMA6D,CRMP1,PRKCQ,EPHB2,PRTG,DPYSL2,BDNF,PLXNA2,NCAM1,NRXN1,LAMA3,UNC5D,ADAMTSL1,TRIO,CCDC141,EPHA5,MYPN,SLIT3,SEMA5B,PTPRO,CNTN6,FYN,EPHA7,EPHB1,EXT1,UNC5C,PTPRM,NRXN3,SEMA3A,SEMA3D,SEMA3C*
GO:0097485	neuron projection guidance	1.0649003960512674e-12	*KALRN,LAMA2,CNTN4,ROBO1,DSCAM,NFASC,SEMA5A,RELN,APP,SOS1,ROBO2,DCC,SEMA4D,RPS6KA5,EMB,SLIT2,DSCAML1,NTN1,FLRT2,CDH4,TNR,EFNA5,SEMA6D,CRMP1,PRKCQ,EPHB2,PRTG,DPYSL2,BDNF,PLXNA2,NCAM1,NRXN1,LAMA3,UNC5D,ADAMTSL1,TRIO,CCDC141,EPHA5,MYPN,SLIT3,SEMA5B,PTPRO,CNTN6,FYN,EPHA7,EPHB1,EXT1,UNC5C,PTPRM,NRXN3,SEMA3A,SEMA3D,SEMA3C*
GO:0022603	regulation of anatomical structure morphogenesis	1.1770541418892323e-12	*KALRN,NTRK2,CLASP1,ZNRF3,ROBO1,PRICKLE2,DSCAM,SHROOM3,PSMB2,PRKD1,NEDD9,SEMA5A,PRKCA,IL1RAPL1,MYO9A,KANK1,RUNX1,NLGN1,RELN,ADAM12,PAK1,PPP3CA,DLC1,MAGI2,CELSR1,ROBO2,DCC,SEMA4D,ANKRD6,ARHGAP15,TANC2,PARK2,NPHP3,SMOC2,PALMD,SULF1,SLIT2,BMPR1A,TNFRSF11B,ARHGEF18,OMA1,DNMBP,NTN1,MAP2,CUX1,CDH4,TNR,ACTN4,EPS8,TENM4,LRRC4C,PALM2,DISC1,ESR1,PIK3CD,EFNA5,ATF2,CYBB,SEMA6D,NTNG1,EPHB2,BDNF,FSTL4,BMPER,SYT1,DAB2,PLXNA2,ETS1,RIMS2,FMNL2,CD44,ISM1,FBXO31,MAP3K13,CDC42EP3,AGT,FGF1,CCR3,STRIP1,GRIP1,NF1,PARVB,GSK3B,PSMB7,TIAM2,UNC13A,TRIOBP,RHOJ,ISLR2,GPC3,CFDP1,PREX1,ROR1,MACF1,FGD4,FAM171A1,PAK3,MLLT3,SEMA5B,JAK1,MAP2K1,GPC6,LRRK2,PKHD1,FYN,EPHA7,RIMS1,ENPP2,DOCK1,VASH2,TRPC5,RAPGEF2,PTPRM,CDKL5,DAB1,SEMA3A,MYH9,STIM1,SH3KBP1,ZRANB1,CLASP2,DNM3,PTPRD,SEMA3D,SYT17,SEMA3C*
GO:0009966	regulation of signal transduction	1.3876223354480998e-12	*PTPRR,CAMTA1,RCAN1,KALRN,CACNG2,RNF43,CTNNA1,NTRK2,ZNRF3,FBXL17,SLC39A10,DMD,SPRED2,ERBB4,ROBO1,CIT,PUM1,GPC5,WDR59,WWOX,AUTS2,WLS,ANKS1A,LDLRAD4,PSMB2,PTPRT,MGLL,PLCB1,FLT3,PRKD1,SEMA5A,PRKCA,DLGAP1,MYO9A,CCDC3,KANK1,DOK6,NLGN1,TNFRSF10B,CLEC16A,TCF7L2,RELN,APP,PAK1,DEPDC5,AKAP6,DLC1,SOS1,CTNND2,ALK,BTRC,MAP2K5,MAGI2,EDA,ROBO2,IFT81,AKAP13,EP300,SEMA4D,EVC,GRK5,ANKRD6,GRIK2,ARHGAP15,ITSN1,JAK2,CMKLR1,PARK2,NPHP3,ADCYAP1R1,SMOC2,NLGN4X,AFAP1,SULF1,SH3BP1,RFFL,ARHGAP24,SLIT2,TRIM5,CHRDL1,TRAF3IP2,SH3RF3,BMPR1A,SMAD3,PIBF1,SCUBE1,EYA1,MAD1L1,TNKS,TUB,ARHGEF18,LINC00473,OVOL2,SGMS1,GRAMD4,DGKI,DNMBP,TAF1,SHISA9,CDC73,FBXW11,NOS1AP,RBMS3,WWTR1,ARHGAP6,GRM4,CCL15,CNIH3,DOCK3,TRIM59,CELF4,VAV3,ACTN4,PLCE1,EPS8,GHR,ARHGAP39,CD300A,CBFA2T2,DISC1,RALGPS1,ARHGAP42,CCL14,RABGEF1,TRABD2B,ESR1,ARHGAP12,TNFRSF19,GRM1,PDE4D,PIK3CD,EIF3A,PIK3R2,ZNF423,PDE4B,MAPRE2,RIC8B,MAP3K7,PRKCQ,EPHB2,AGO3,MOB3B,PRCP,EEF1E1-BLOC1S5,DOCK2,BDNF,FNIP1,MAGI3,ADORA2A,SIPA1L2,IFT80,FSTL4,FHL2,NOX4,BMPER,DAB2,LRRK1,CACNG3,HDAC*

			2,PDE11A,ZMYND11,RGS6,SRGAP3,ENPP1,RIMS2,DLGAP2,PRDM15,PEX5L,NRG1,RIT2,CD44,FAM19A4,MAP3K13,PLCL2,CHST11,NCAM1,AGT,FGF1,NDRG2,ARHGAP25,NF1,WIF1,ZNF536,NRXN1,ARHGAP10,GSK3B,CD109,GPR21,PSMB7,SNX5,TIAM2,IQCJ-SCHIP1,NCOA1,CHEK2,MECOM,SHANK2,KCTD8,NREP,GPC3,DLG5,PREX1,UBE2V1,ROR1,ZNF675,BID,MACF1,TRIO,CAPN3,PTPRE,DUSP22,MAP3K5,CTDSPL2,HTR2C,FGD4,LY86,TP73,ZNF366,ARHGEF3,SLIT3,PAK3,GARNL3,UACA,MLLT3,RNF213,SH3RF2,PRKAA2,TBL1X,GRM5,IGF1R,PTPRO,SUFU,INSR,MAP2K1,UBR2,FOXO3,GPC6,RASGRF2,STK38,CNTN6,PRDM16,ULK4,LTBP1,LRRK2,PKHD1,BMP6,FYN,EPHA7,MARVELD3,GAS8,RIMS1,EPHB1,CRADD,AMFR,LRP2,RALGPS2,FER,VWC2,GUCY2F,TMEM108,TPTE,RAPGEF2,PTPRU,IL18R1,RGS7BP,ESR2,TRIM22,CDH13,VEPH1,DAB1,SEMA3A,MGAT5,BDKRB2,LITAF,TENM1,ZRANB1,RGS7,SIPA1L3,GNG4,VWF,SCEL,NTRK3,FBN1,PPARA,PTPRD,RORA,SHISA6,EEF1E1,BICC1,PRLR,ATF6,DEPTOR,RALGAPA1,PMEPA1
GO:0016477	cell migration	1.5939334474642237e-12	*PTPRR,NDE1,CTNNA1,NTRK2,CLASP1,PTPRG,ERBB4,LAMA2,ROBO1,GPC5,FUT8,HDAC4,ABCC1,AUTS2,ANKS1A,LDLRAD4,PTPRT,PLCB1,PRKD1,VCL,NEDD9,SEMA5A,PRKCA,EPB41L4B,KANK1,CAMK1D,AMOTL1,RELN,APP,PAK1,PPP3CA,DLC1,SOS1,DCLK1,CTNNA3,MAP2K5,MAGI2,CELSR1,DCC,ELMO2,SEMA4D,BDKRB1,ASTN2,DEFA1B,JAK2,CMKLR1,SMOC2,PIK3C2B,ITGBL1,NRG3,NUMB,SULF1,SH3BP1,ATRNL1,RFFL,ARHGAP24,SLIT2,BMPR1A,SMAD3,PEAK1,SATB2,OVOL2,ELP3,SPNS2,NTN1,LDB2,FLRT2,CCL15,TNR,VAV3,SPOCK1,ACTN4,EPS8,CD300A,DISC1,CCL14,RABGEF1,LRP5,PIK3CD,DOCK4,CEP85L,SEMA6D,NTNG1,ADTRP,PDE4B,MAPRE2,PRKCQ,CLDN1,EPHB2,MCTP1,PRCP,DACH1,MARK1,ARID5B,MEGF9,BMPER,DAB2,PLXNA2,PRKG1,ETS1,FAT3,SRGAP3,CTNNA2,FMNL2,NRG1,CD44,SLC8A1,FBXO31,FAM19A4,CDC42BPA,AGT,FGF1,CCR3,USH2A,NF1,LAMA3,SRGAP2B,RHOJ,UNC5D,GPC3,DLG5,ASTN1,PREX1,MACF1,DUSP22,CCDC141,PAK3,CCL15-CCL14,SH3RF2,SEMA5B,IGF1R,PTPRO,INSR,ITGA11,FOXO3,GPC6,MITF,FRMD5,ULK4,LRRK2,KIF2A,PHACTR1,FYN,MARVELD3,ENPP2,EPHB1,DOCK1,EXT1,UNC5C,FER,RAPGEF2,NAV3,PTPRM,KIRREL3,CDKL5,PTPRU,SETD2,CDH13,DAB1,HDAC5,SEMA3A,MGAT5,MYH9,PIK3R3,SH3KBP1,ZRANB1,NTRK3,CLASP2,PTPRK,DOCK10,ATP8A1,SEMA3D,SEMA3C*
GO:0006810	transport	4.778602412249502e-12	*SYN3,SVOPL,LRP1B,KALRN,CACNG2,PROS1,NDE1,NTRK2,CLASP1,SLC39A10,DMD,KCNC2,CHRM3,ERBB4,NOS1,TMC2,SIL1,DPP10,SORCS2,ATP9A,ARFGAP3,IMMP2L,ABCC1,ATP8A2,AFTPH,WLS,SGSM1,PSMB2,SLC44A1,SNUPN,CACNA1A,TRPM1,PRKD1,RYR2,CASK,STX8,BTBD9,IL1RAPL1,HBE1,ABCA13,TBC1D5,SLCO3A1,GRIK4,PLS1,FCHSD2,NLGN1,DNAH9,KCNIP4,CAMK1D,SMG6,CLEC16A,TCF7L2,RELN,APP,PAK1,UTRN,PPP3CA,RYR1,GABRG3,ERC2,AKAP6,OSBPL1A,GRM7,RABGAP1L,RYR3,LRRC8C,RFTN1,DCLK1,MEF2A,KCNB2,FAM155A,DENND5A,DLG2,MAGI2,IFT81,AKAP13,ELMO2,KCNA6,SLCO2B1,BDKRB1,TF,DNAH11,GRIK2,QKI,CHRM1,SYTL5,ZDHHC11B,ITSN1,ASTN2,TANC2,JAK2,ABCG8,RAB11FIP4,PARK2,SLC14A2,NPHP3,ADCYAP1R1,DPP6,PRELID2,GRID1,GRIN3A,CACNA1C,MAN1A1,NLGN4X,EXOC4,HEATR5A,EMB,ANO4,ENTHD1,ANKH,TMPRSS3,SLC5A10,NUMB,TRPM3,CD84,SH3BP1,SH3GL2,SLC13A3,PITPNC1,KCNJ16,SLC4A4,FLVCR2,TRAF3IP2,SMAD3,CACNA1E,GABRR2,PIK3C3,SLC9C1,SLC47A1,SV2B,ANO2,TPH1,TNKS,TUB,SCAMP5,ARRDC4,FGF14,CACNA2D3,ASGR2,SPAG17,ZDHHC11,PTPRN2,DGKI,SPNS2,SLC1A1,NTN1,SHISA9,EPM2A,SHFM1,MAP2,FBXW11,NOS1AP,KCTD7,CATSPER2,PCSK5,CUX1,GRM4,ANK3,CNIH3,VAV3,SCN9A,ACTN4,RAB2A,CECR2,GHR,LRRC8D,SLC25A21,CD300A,ABCC11,OSBPL10,ATP8B4,IFT43,RABGEF1,NSG2,LRP5,AP2B1,SLC30A7,ARHGAP12,KCNN3,DENND2A,GRM1,SCFD1,PDE4D,THEM4,PIK3CD,SLC5A3,BLOC1S5,FRMD4A,RAB24,EFNA5,ATF2,KCNJ12,PIK3R2,CCDC91,GRID2,CYBB,KCNJ3,ADTRP,PDE4B,GABRB3,SMG7,ANKRD13A,ATXN1,CLDN1,KCND2,SLC24A3,SPTBN4,EPHB2,ERC1,MCTP1,DOCK2,NUP214,DPYSL2,RBM4,ADORA2A,ANO1,CADPS,CHKA,ICK,SYT1,DAB2,NSF,STXBP6,SNAP23,CACNG3,EPG5,CALCRL,MICAL3,RANBP17,TMEM163,PRKAR1B,CDH23,TTN,MYRIP,SLC39A11,ENPP1,RIMS2,SCN8A,AGAP1,PEX5L,CYB561A3,PARD3,NRG1,TBC1D9,ATP10B,SLC35F*

			4,FMN2,GRIA2,RIT2,KCNMA1,NCF4,CNGB1,PRKCG,SLC12A8,MAPKAPK2,SLC8A1,FAM19A4,RSRC1,SLC9A9,SAMM50,AGT,KCNQ5,HEPHL1,KCNJ6,GRIP1,TLK1,ARHGAP25,NF1,VPS16,SLC44A5,NRXN1,GSK3B,PKP2,SLC22A3,KCNH1,BBS9,CHD7,PSMB7,SNX5,CNTN1,UNC13A,GRIK3,CACNB2,STXBP4,RHOJ,ESYT2,SYBU,GPC3,CPE,ANO3,XKR4,SERPINA5,KCNS3,SLC5A8,PTGER3,BID,MACF1,CAPN3,MYOM1,SPAG16,CTDSPL2,HTR2C,GLRA2,NCF2,SNAP25-AS1,EPHA5,SGIP1,SNX31,P2RX6,ANK2,GRIN2B,LRPPRC,ADCY8,ITPR2,GABRR3,CACNA1D,ABCG2,GRIK1,TRAPPC8,GRM5,ATG4C,IGF1R,PKD1L1,TSNARE1,MON2,ZDHHC14,SUFU,NKAIN2,INSR,RPGR,MAP2K1,SLC9B1,KPNB1,RASGRF2,GABRA3,KLHL3,OCA2,CENPF,ATP9B,IGF2BP3,AGBL4,KPNA3,RBFOX1,LRRK2,NKAIN3,SPG11,SCN1A,BMP6,SLC35F1,VTI1A,FYN,KCND3,ABCC2,GAS8,MSR1,PTPN14,RIMS1,ENPP2,VPS53,MCTP2,ABCA12,KCNQ1,SLC38A6,DOCK1,EXT1,TRPC5,NOX5,LRP2,C2,FER,PCDH17,TMEM108,NRXN3,KCNH7,GRIA4,VPS45,SLC5A4,SETD2,PACSIN2,CDH13,TRDN,SEC16B,SLC16A12,CACHD1,EXOC6B,ATP13A3,STOML1,MYH9,BDKRB2,STIM1,VPS41,TENM1,VPS39,GABRB1,SH3KBP1,RGS7,STXBP5,TXNDC5,SLC24A2,GRIA3,SLC35F3,MOGAT2,CLASP2,TMPRSS15,DNAJC6,NSUN2,TPD52,SORCS1,DNM3,SYNDIG1,PPARA,SCFD2,SHISA6,ANXA8L1,ATP8A1,PRLR,KCNC4,CEP41,IPO11,SYT17,STAC,RAB27A,BLOC1S6,HGSNAT,KCNJ15,SYT9,SLC22A10
GO:0051674	localization of cell	8.461361433069228e-12	*PTPRR,NDE1,CTNNA1,NTRK2,CLASP1,PTPRG,ERBB4,LAMA2,ROBO1,GPC5,FUT8,HDAC4,ABCC1,AUTS2,ANKS1A,LDLRAD4,PTPRT,PLCB1,PRKD1,VCL,NEDD9,SEMA5A,PRKCA,EPB41L4B,KANK1,CAMK1D,AMOTL1,RELN,APP,PAK1,PPP3CA,DLC1,SOS1,DCLK1,CTNNA3,MAP2K5,MAGI2,CELSR1,DCC,ELMO2,SEMA4D,BDKRB1,NME8,TF,DNAH11,ASTN2,DEFA1B,JAK2,CMKLR1,SMOC2,PIK3C2B,ITGBL1,NRG3,NUMB,SULF1,SH3BP1,ATRNL1,RFFL,ARHGAP24,SLIT2,BMPR1A,SMAD3,SLC9C1,PEAK1,SATB2,OVOL2,ELP3,SPNS2,NTN1,LDB2,FLRT2,CATSPER2,CCL15,TTLL8,TNR,VAV3,SPOCK1,ACTN4,EPS8,CD300A,DISC1,CCL14,RABGEF1,LRP5,PIK3CD,DOCK4,ARMC2,CEP85L,SEMA6D,NTNG1,ADTRP,PDE4B,MAPRE2,PRKCQ,CLDN1,EPHB2,MCTP1,PRCP,DACH1,MARK1,ARID5B,MEGF9,BMPER,DAB2,PLXNA2,PRKG1,ETS1,FAT3,SRGAP3,CTNNA2,FMNL2,NRG1,SPINT2,CD44,SLC8A1,FBXO31,FAM19A4,CDC42BPA,AGT,FGF1,CCR3,USH2A,NF1,LAMA3,SRGAP2B,RHOJ,UNC5D,GPC3,DLG5,ASTN1,PREX1,MACF1,DUSP22,SPAG16,CCDC141,PAK3,CCL15-CCL14,SH3RF2,SEMA5B,IGF1R,PTPRO,INSR,ITGA11,MAP2K1,SLC9B1,FOXO3,GPC6,MITF,FRMD5,ULK4,LRRK2,KIF2A,PHACTR1,FYN,MARVELD3,GAS8,ENPP2,EPHB1,DOCK1,EXT1,UNC5C,FER,TPTE,RAPGEF2,NAV3,DNAH3,PTPRM,KIRREL3,CDKL5,PTPRU,SETD2,CDH13,DAB1,HDAC5,SEMA3A,MGAT5,MYH9,PIK3R3,SH3KBP1,ZRANB1,NTRK3,CLASP2,PTPRK,DOCK10,ATP8A1,SEMA3D,SEMA3C*
GO:0048870	cell motility	8.461361433069228e-12	*PTPRR,NDE1,CTNNA1,NTRK2,CLASP1,PTPRG,ERBB4,LAMA2,ROBO1,GPC5,FUT8,HDAC4,ABCC1,AUTS2,ANKS1A,LDLRAD4,PTPRT,PLCB1,PRKD1,VCL,NEDD9,SEMA5A,PRKCA,EPB41L4B,KANK1,CAMK1D,AMOTL1,RELN,APP,PAK1,PPP3CA,DLC1,SOS1,DCLK1,CTNNA3,MAP2K5,MAGI2,CELSR1,DCC,ELMO2,SEMA4D,BDKRB1,NME8,TF,DNAH11,ASTN2,DEFA1B,JAK2,CMKLR1,SMOC2,PIK3C2B,ITGBL1,NRG3,NUMB,SULF1,SH3BP1,ATRNL1,RFFL,ARHGAP24,SLIT2,BMPR1A,SMAD3,SLC9C1,PEAK1,SATB2,OVOL2,ELP3,SPNS2,NTN1,LDB2,FLRT2,CATSPER2,CCL15,TTLL8,TNR,VAV3,SPOCK1,ACTN4,EPS8,CD300A,DISC1,CCL14,RABGEF1,LRP5,PIK3CD,DOCK4,ARMC2,CEP85L,SEMA6D,NTNG1,ADTRP,PDE4B,MAPRE2,PRKCQ,CLDN1,EPHB2,MCTP1,PRCP,DACH1,MARK1,ARID5B,MEGF9,BMPER,DAB2,PLXNA2,PRKG1,ETS1,FAT3,SRGAP3,CTNNA2,FMNL2,NRG1,SPINT2,CD44,SLC8A1,FBXO31,FAM19A4,CDC42BPA,AGT,FGF1,CCR3,USH2A,NF1,LAMA3,SRGAP2B,RHOJ,UNC5D,GPC3,DLG5,ASTN1,PREX1,MACF1,DUSP22,SPAG16,CCDC141,PAK3,CCL15-CCL14,SH3RF2,SEMA5B,IGF1R,PTPRO,INSR,ITGA11,MAP2K1,SLC9B1,FOXO3,GPC6,MITF,FRMD5,ULK4,LRRK2,KIF2A,PHACTR1,FYN,MARVELD3,GAS8,ENPP2,EPHB1,DOCK1,EXT1,UNC5C,FER,TPTE,RAPGEF2,NAV3,DNAH3,PTPRM,KIRREL3,CDKL5,PTPRU,SETD2,CDH13,DAB1,HDAC5,SEMA3A,MGAT5,MYH9,PIK3R3,SH3KBP1,ZRANB1,NTRK3,CLASP2,PTPRK,DO*

			CK10,ATP8A1,SEMA3D,SEMA3C
GO:0050793	regulation of developmental process	9.733825197335442e-12	*KALRN,CTNNA1,NTRK2,CLASP1,ZNRF3,DMD,SPRED2,ERBB4,LAMA2,CNTN4,ROBO1,PRICKLE2,DSCAM,SHROOM3,HDAC4,LINGO2,ATP8A2,LDLRAD4,PSMB2,PLCB1,PRKD1,VCL,NEDD9,PPP2R3C,SEMA5A,PRKCA,IL1RAPL1,MYO9A,CCDC3,KANK1,TCF12,RUNX1,PLS1,NLGN1,CAMK1D,TCF7L2,RELN,APP,ADAM12,PAK1,PPP3CA,TOX,AKAP6,DLC1,SOS1,ALK,DNMT1,MAGI2,CELSR1,ROBO2,DCC,SEMA4D,ANKRD6,ARHGAP15,TANC2,JAK2,CMKLR1,ZBTB7C,PARK2,NPHP3,GRIN3A,SMOC2,ANKH,PALMD,NUMB,SOX6,SULF1,SLIT2,BMPR1A,SMAD3,TNFRSF11B,EYA1,TPH1,ARHGEF18,OVOL2,BRINP1,OMA1,GLIS1,SOX5,DNMBP,NTN1,MAP2,CDC73,FLRT2,WWTR1,CUX1,TRPS1,CDH4,TNR,CELF4,ACTN4,EPS8,TENM4,GHR,LRRC4C,PALM2,CLSTN2,DISC1,LRP5,ESR1,PIK3CD,BLOC1S5,TEAD4,EFNA5,FTO,ATF2,GRID2,CYBB,SEMA6D,NTNG1,JDP2,SPTBN4,EPHB2,S1PR3,PRTG,DPYSL2,MARK1,BDNF,RBM4,FNIP1,FSTL4,BCOR,BMPER,SYT1,DAB2,PLXNA2,HDAC2,ETS1,FAT3,ENPP1,RIMS2,FMNL2,PARD3,NRG1,CD44,SLC8A1,ISM1,FBXO31,MAP3K13,CDC42EP3,AGT,FGF1,CCR3,STRIP1,GRIP1,USH2A,NF1,WIF1,ZNF536,PARVB,NRXN1,LAMA3,GSK3B,PKP2,CTDP1,SETD3,CD109,GPR21,CHD7,PSMB7,TIAM2,PLEKHB2,UNC13A,TRIOBP,NCOA1,RHOJ,ISLR2,NREP,GPC3,DLG5,CFDP1,PREX1,ROR1,ZNF675,MACF1,TRIO,CAPN3,MAP3K5,HTR2C,FGD4,TP73,RBM19,FAM171A1,PAK3,MLLT3,SEMA5B,NLN,SDK1,GRM5,IGF1R,SUFU,INSR,JAK1,MAP2K1,FOXO3,GPC6,CENPF,MITF,AGBL4,RBFOX1,KIR2DL4,LRRK2,PKHD1,RARB,TCF4,PBX1,BMP6,ASAP1,FYN,EPHA7,MSR1,RIMS1,ENPP2,ABCA12,EPHB1,DOCK1,VASH2,TRPC5,LRP2,CAMK4,VWC2,RAPGEF2,PTPRM,CDKL5,KAT7,BASP1,DAB1,HDAC5,SEMA3A,MYH9,STIM1,SH3KBP1,ZRANB1,NTRK3,FBN1,CLASP2,NSUN2,DNM3,SYNDIG1,PPARA,PTPRD,RORA,ELAVL4,NELL1,EEF1E1,SEMA3D,PRLR,SYT17,BLOC1S6,SEMA3C*
GO:0051234	establishment of localization	1.631991051290535e-11	*SYN3,SVOPL,LRP1B,KALRN,CACNG2,PROS1,NDE1,NTRK2,CLASP1,SLC39A10,DMD,KCNC2,CHRM3,ERBB4,NOS1,TMC2,SIL1,DPP10,SORCS2,ATP9A,ARFGAP3,IMMP2L,ABCC1,ATP8A2,AFTPH,WLS,SGSM1,PSMB2,SLC44A1,SNUPN,CACNA1A,TRPM1,PRKD1,RYR2,CASK,STX8,BTBD9,IL1RAPL1,HBE1,ABCA13,TBC1D5,SLCO3A1,GRIK4,PLS1,FCHSD2,NLGN1,DNAH9,KCNIP4,CAMK1D,SMG6,CLEC16A,TCF7L2,RELN,APP,PAK1,UTRN,PPP3CA,RYR1,GABRG3,ERC2,AKAP6,OSBPL1A,GRM7,RABGAP1L,RYR3,LRRC8C,RFTN1,DCLK1,MEF2A,KCNB2,FAM155A,DENND5A,DLG2,MAGI2,IFT81,AKAP13,ELMO2,KCNA6,SLCO2B1,BDKRB1,TF,DNAH11,GRIK2,QKI,CHRM1,SYTL5,ZDHHC11B,ITSN1,ASTN2,TANC2,JAK2,ABCG8,RAB11FIP4,PARK2,SLC14A2,NPHP3,ADCYAP1R1,DPP6,PRELID2,GRID1,GRIN3A,CACNA1C,MAN1A1,NLGN4X,EXOC4,HEATR5A,EMB,ANO4,ENTHD1,ANKH,TMPRSS3,SLC5A10,NUMB,TRPM3,CD84,SH3BP1,SH3GL2,SLC13A3,PITPNC1,KCNJ16,SLC4A4,FLVCR2,TRAF3IP2,SMAD3,CACNA1E,PIBF1,GABRR2,PIK3C3,SLC9C1,SLC47A1,SV2B,ANO2,TPH1,MAD1L1,TNKS,TUB,SCAMP5,ARRDC4,FGF14,CACNA2D3,ASGR2,SPAG17,ZDHHC11,PTPRN2,DGKI,SPNS2,SLC1A1,NTN1,SHISA9,EPM2A,SHFM1,MAP2,FBXW11,NOS1AP,KCTD7,CATSPER2,PCSK5,CUX1,GRM4,ANK3,CNIH3,VAV3,SCN9A,ACTN4,RAB2A,CECR2,GHR,LRRC8D,SLC25A21,CD300A,ABCC11,OSBPL10,ATP8B4,IFT43,RABGEF1,NSG2,LRP5,AP2B1,SLC30A7,ARHGAP12,KCNN3,DENND2A,GRM1,SCFD1,PDE4D,THEM4,PIK3CD,SLC5A3,BLOC1S5,FRMD4A,RAB24,EFNA5,ATF2,KCNJ12,PIK3R2,CCDC91,GRID2,CYBB,KCNJ3,ADTRP,PDE4B,GABRB3,SMG7,ANKRD13A,ATXN1,CLDN1,WDR83OS,KCND2,SLC24A3,SPTBN4,EPHB2,ERC1,MCTP1,DOCK2,NUP214,DPYSL2,MARK1,RBM4,ADORA2A,ANO1,CADPS,CHKA,ICK,SYT1,DAB2,NSF,STXBP6,SNAP23,CACNG3,EPG5,CALCRL,MICAL3,RANBP17,TMEM163,PRKAR1B,CDH23,TTN,MYRIP,SLC39A11,ENPP1,RIMS2,SCN8A,AGAP1,PEX5L,CYB561A3,PARD3,NRG1,TBC1D9,ATP10B,SLC35F4,FMN2,GRIA2,RIT2,KCNMA1,NCF4,CNGB1,GPSM2,PRKCG,SLC12A8,MAPKAPK2,SLC8A1,FAM19A4,RSRC1,SLC9A9,SAMM50,AGT,KCNQ5,HEPHL1,KCNJ6,GRIP1,TLK1,ARHGAP25,USH2A,NF1,VPS16,SLC44A5,NRXN1,GSK3B,PKP2,SLC22A3,KCNH1,BBS9,CHD7,PSMB7,SNX5,CNTN1,UNC13A,GRIK3,CACNB2,STXBP4,RHOJ,ESYT2,SYBU,GPC3,CPE,ANO3,XKR4,SERPINA5,KCNS3,SLC5A8,PTGER3,BID,MACF1,CAPN3,MYOM1,SPAG16,CTDSPL2,HTR2C,GLRA2,NCF2,SNAP25-*

			AS1,EPHA5,SGIP1,SNX31,P2RX6,ANK2,GRIN2B,LRPPRC,ADCY8,ITPR2,GABRR3,CACNA1D,ABCG2,GRIK1,ANKFN1,TRAPPC8,GRM5,ATG4C,IGF1R,PKD1L1,TSNARE1,MON2,ZDHHC14,SUFU,NKAIN2,INSR,RPGR,MAP2K1,SLC9B1,KPNB1,RASGRF2,GABRA3,KLHL3,OCA2,CENPF,ATP9B,IGF2BP3,AGBL4,KPNA3,RBFOX1,LRRK2,NKAIN3,PKHD1,SPG11,SCN1A,BMP6,SLC35F1,VTI1A,FYN,KCND3,ABCC2,GAS8,MSR1,PTPN14,RIMS1,ENPP2,VPS53,MCTP2,ABCA12,KCNQ1,SLC38A6,DOCK1,EXT1,TRPC5,NOX5,LRP2,C2,FER,PCDH17,TMEM108,NRXN3,KCNH7,GRIA4,VPS45,SLC5A4,SETD2,PACSIN2,CDH13,TRDN,SEC16B,SLC16A12,CACHD1,EXOC6B,ATP13A3,STOML1,MYH9,BDKRB2,STIM1,VPS41,TENM1,VPS39,GABRB1,SH3KBP1,RGS7,STXBP5,TXNDC5,SLC24A2,GRIA3,SLC35F3,MOGAT2,CLASP2,TMPRSS15,DNAJC6,NSUN2,TPD52,SORCS1,DNM3,SYNDIG1,PPARA,SCFD2,SHISA6,ANXA8L1,ATP8A1,PRLR,KCNC4,CEP41,IPO11,SYT17,STAC,RAB27A,BLOC1S6,PARD3B,HGSNAT,KCNJ15,SYT9,SLC22A10
GO:0050789	regulation of biological process	2.041283298452881e-11	*PKNOX2,PTPRR,SYN3,RNF4,CAMTA1,CHFR,KDM4B,RCAN1,KALRN,CACNG2,RNF43,PROS1,RPS6KA2,CTNNA1,FOXN3,NTRK2,CLASP1,RASGEF1B,ZNRF3,GLIS3,PTPRG,FBXL17,SLC39A10,DMD,KCNC2,CHRM3,EIF3E,SPRED2,ERBB4,LAMA2,TFDP2,NOS1,CNTN4,TMC2,SND1,ESRRG,ROBO1,PRICKLE2,DSCAM,SHROOM3,CIT,TBCD,DPP10,PUM1,FRY,GPC5,WDR59,SORCS2,PRIM2,WWOX,HUNK,FUT8,TNRC6B,HDAC4,ATP9A,LINGO2,ABCC1,ATP8A2,AUTS2,WLS,ANKS1A,LDLRAD4,MYEF2,TENM2,BLM,PSMB2,PTPRT,RXFP1,ATF7IP,ASXL3,MGLL,PLCB1,FLT3,SRPK2,CACNA1A,TRPM1,PRKD1,RYR2,VCL,PDS5A,NEDD9,PPP2R3C,SEMA5A,ETV6,CASK,PRKCA,EPB41L4B,STX8,ZBTB20,DLGAP1,BTBD9,TRHDE,ZNF713,IL1RAPL1,MYO9A,CCDC3,DIO2,PDE4DIP,ITGAE,ABCA13,KANK1,KIF26B,TBC1D5,SLCO3A1,TCF12,GRIK4,RUNX1,PLS1,GPR176,DOK6,FCHSD2,NLGN1,TNFRSF10B,KCNIP4,CAMK1D,SMG6,CLEC16A,AMOTL1,TCF7L2,MTRF1,RELN,APP,ADAM12,PAK1,UTRN,PPP3CA,DEPDC5,RYR1,TOX,GABRG3,ERC2,PTPN9,CREM,AKAP6,SORCS3,GRM7,RABGAP1L,DLC1,IDE,SCMH1,RYR3,SOS1,CTND2,RFTN1,ALK,BTRC,DCLK1,MEF2A,EEFSEC,KCNB2,TFAP2D,CTNNA3,DENND5A,PPP1R12B,MAP2K5,DNMT1,MAGI2,EDA,CELSR1,ROBO2,IFT81,AKAP13,HIVEP3,DCC,SAMD4A,C10ORF90,ZNF799,DDB1,EP300,KCNA6,PARN,SEMA4D,EVC,RPS6KA5,PHF20,GRK5,ANKRD6,TRAPPC9,BDKRB1,TF,DNAH11,GRIK2,QKI,GNG12,CHRM1,CDH8,ARHGAP15,HUS1,ZNF667,TFEC,ITSN1,ASTN2,TANC2,DEFA1B,JAK2,ABCG8,RAB11FIP4,NEGR1,CMKLR1,PPP6R2,ZBTB7C,MAST4,PARK2,MAGI1,NPHP3,ADCYAP1R1,SERPINA4,DPP6,GRID1,GRIN3A,SMOC2,CACNA1C,NLGN4X,EXOC4,PIK3C2B,OR5K4,ANKH,ITGBL1,GBE1,AFAP1,TAGLN3,PALMD,SP140,NRG3,NUMB,MED15,SOX6,COL4A6,SULF1,CD84,MAGEA11,SH3BP1,ADCY2,TCF20,ATRNL1,RFFL,SH3GL2,PSPC1,ARHGAP24,PITPNC1,SLIT2,KCNJ16,MAPK4,GLP2R,SLC4A4,DTNA,KMT2C,TRIM5,THEMIS,DRG2,AFF3,ATRX,CHRDL1,TRAF3IP2,RAD51B,SLX1B,SH3RF3,BMPR1A,SMAD3,PRKAR2A,CACNA1E,BORA,PI4KA,TNFRSF11B,PIBF1,GABRR2,PIK3C3,CHD6,KIR3DL2,PTGFR,SCUBE1,PEAK1,EYA1,SATB2,ANKS1B,ELN,MAML3,GPR141,TPH1,MAD1L1,EGFLAM,DGKB,TNKS,TUB,KLF12,ARHGEF18,SCAMP5,LINC00473,OVOL2,SGMS1,GRAMD4,DNAJB6,ELP3,BRINP1,OMA1,BCL2L13,ARRDC4,KHDRBS2,FGF14,CACNA2D3,DGKK,OR5I1,GLIS1,ASGR2,IL1RAPL2,ZDHHC11,SKAP1,CDYL2,SOX5,DGKI,DNMBP,TAF1,SPNS2,SLC1A1,RNF144B,NTN1,LDB2,SHISA9,EPM2A,MAP2,CDC73,FLRT2,FBXW11,NOS1AP,RANBP10,CD96,RBMS3,ARHGEF6,WWTR1,PEMT,ARHGAP6,PHF20L1,KCTD7,CATSPER2,EGLN3,CUX1,GRM4,ANK3,TIMP2,CCL15,MORC2,ZNF19,ARID4B,CNIH3,DOCK3,TRIM59,TRPS1,CDH4,TNR,ADCY9,CELF4,VAV3,CDKL1,SCN9A,MDM4,ARNT2,SPOCK1,ACTN4,HPSE2,PLCE1,LRRN2,EPS8,TENM4,PRR16,GHR,ARHGAP39,LRRC4C,PPP1R9A,RGL1,PALM2,CLSTN2,CD300A,CBFA2T2,FUBP1,PRG3,MLIP,SERTAD2,DISC1,RALGPS1,ARHGAP42,CCL14,RABGEF1,FHIT,NSG2,TRABD2B,LRP5,AP2B1,ESR1,ARHGAP12,TNFRSF19,PLCXD3,GRM1,SCFD1,PDE4D,THEM4,PIK3CD,DOCK4,SLC5A3,BLOC1S5,FRMD4A,TEAD4,PDE7B,EFNA5,FTO,EIF3A,POU6F2,ATF2,RBBP8,KCNJ12,PIK3R2,GRID2,ZNF423,CYBB,SEMA6D,KCNJ3,SUSD4,CELF2,NTNG1,ADTRP,CRMP1,PDE4B,MAPRE2,RIC8B,GABRB3,SMG7,ANKRD13A,MAP3K7,PRKCQ,ATXN1,N4BP1,RASSF8,RHPN2,CLDN1,KCND2,SLC24A*

			3,JDP2,SPTBN4,EPHB2,ERC1,AGO3,S1PR3,MCTP1,MOB3B,PRCP,PRTG,RGMB,EEF1E1-BLOC1S5,DOCK2,NUP214,DPYSL2,PTPDC1,RFC3,DACH1,MARK1,BDNF,RBM4,FNIP1,MALRD1,MAGI3,ADORA2A,ARID5B,SIPA1L2,RCAN2,IFT80,FSTL4,ANO1,FHL2,CADPS,DRAM1,NOX4,BCOR,BMPER,PDE1A,ICK,DEFA3,BRD9,SYT1,EBAG9,DAB2,AFF2,PLXNA2,NSF,EBF4,LRRK1,PRKG1,STXBP6,ABI1,CACNG3,EPG5,PTH2R,CALCRL,TNNI3K,HDAC2,ETS1,BANP,FAT3,PDE11A,ARID4A,RAB30,PRKAR1B,BACH1,ZMYND11,TTN,RGS6,SRGAP3,ZNF93,MYRIP,CDK19,ENPP1,CFB,ZNF443,RIMS2,SCN8A,KSR2,DLGAP2,PRDM15,OR4N2,CTNNA2,PEX5L,FMNL2,PARD3,NRG1,CAST,FANK1,SPINT2,NPAS3,FMN2,GRIA2,VSTM1,TOP1,RIT2,CD44,PAX7,KCNMA1,SCAF8,CNGB1,GPSM2,PRKCG,TNS3,MED13L,TEAD1,NFIA,MAPKAPK2,EIF4G3,SLC8A1,SRRM4,RAPGEF6,ISM1,FBXO31,FAM19A4,MAP3K13,PLCL2,TOX3,CHST11,THRB,CDC42BPA,CDC42EP3,MIR1185-1,MAPK10,NCAM1,AGT,FGF1,KCNQ5,QRICH1,CCR3,KCNJ6,STRIP1,ZNF709,NDRG2,GRIP1,TLK1,UPK3B,ARHGAP25,USH2A,NF1,WIF1,ZNF536,PARVB,NRXN1,ARHGAP10,LAMA3,PDXP,GSK3B,KIR2DL1,PKP2,CTDP1,SETD3,CD109,KCNH1,ZNF695,DCDC1,LMO7,WDR70,GPR21,CHD7,PSMB7,MOV10L1,SNX5,TIAM2,OR51B2,MYT1L,SRGAP2B,IQCJ-SCHIP1,CNTN1,PLEKHB2,BTBD11,UNC13A,TRIOBP,CABIN1,NCOA1,CHEK2,GRIK3,CACNB2,STXBP4,RHOJ,ZBTB8B,ZNF730,MECOM,STK32B,SHANK2,UBE2E2,KCTD8,UNC5D,ISLR2,NREP,GPC3,DLG5,CFDP1,PREX1,CPE,UBE2V1,ROR1,ZNF675,SERPINA5,KCNS3,PTGER3,BID,MACF1,MNAT1,TRIO,CAPN3,CTNNBL1,PTPRE,DUSP22,MYOM1,MAP3K5,MAML2,CTDSPL2,HTR2C,GLRA2,FGD4,LY86,ZNF670,EPHA5,SGIP1,HIRA,RNF144A,TP73,RBM19,IL1RL1,TSHZ2,ZNF366,FAM171A1,MORC3,P2RX6,ANK2,ARHGEF3,SUPT3H,SLIT3,GRIN2B,PHC2,PAK3,GARNL3,UACA,LRPPRC,DPH6,ADCY8,ITPR2,GABRR3,MLLT3,NEB,CCL15-CCL14,ITGB3BP,RNF213,SH3RF2,CACNA1D,PRKAA2,PACRG,TBL1X,SEMA5B,HIVEP2,GRIK1,NLN,ANKFN1,SDK1,GRM5,ARAP2,IGF1R,MXD3,CNTNAP2,PTPRO,SUFU,NKAIN2,INSR,CIZ1,ITGA11,JAK1,MAP2K1,UBR2,ZNF490,GMDS,KPNB1,FOXO3,GPC6,RASGRF2,STK38,GABRA3,INPP5A,CNTN6,CENPF,KCTD1,RNLS,PPP1R14A,MITF,IGF2BP3,SP140L,LRFN5,CDC14A,ENOX2,AGBL4,PRDM16,FRMD5,ULK4,RBFOX1,KIR2DL4,ZNF605,ZNF41,TMEM117,DNMT3B,ADAMTS16,TASP1,LTBP1,LRRK2,NKAIN3,PKHD1,KIF2A,RARB,TCF4,PBX1,PHACTR1,SCN1A,BMP6,PCBP3,ASAP1,FRMPD4,FYN,KCND3,ABCC2,CSPP1,EPHA7,MARVELD3,GAS8,MSR1,CLN6,PTPN14,GNG2,PCBD2,RIMS1,ENPP2,L3MBTL4,MCTP2,ABCA12,BEND5,EPHB1,KCNQ1,FHOD3,DOCK1,CRADD,VASH2,ZNF664,EXT1,TRPC5,AMFR,PLCB4,UNC5C,TENM3,NOX5,LRP2,ZCCHC17,C2,RALGPS2,FER,CAMK4,VWC2,GUCY2F,ERLIN1,PCDH17,SGCD,TMEM108,TPTE,RAPGEF2,NAV3,PTPRM,NCOA2,NRXN3,KCNH7,CDKL5,INPP4A,PTPRU,ZNF207,GRIA4,IL18R1,RGS7BP,ESR2,KAT7,SATB1,C9,MIR767,SETD2,PACSIN2,TRIM22,CDH13,VEPH1,BASP1,TRDN,DAB1,ZNF148,SEC16B,HDAC5,SEMA3A,MGAT5,TSPAN8,MYH9,BDKRB2,STIM1,TSC22D3,NRIP1,CCNG2,MIR105-2,LITAF,PIK3R3,BRF1,TENM1,STK38L,GABRB1,ST8SIA1,SH3KBP1,PRKAR1A,ZRANB1,RGS7,STXBP5,FCRL2,SIPA1L3,GNG4,VWF,TXNDC5,SLC24A2,SCEL,GRIA3,NTRK3,FBN1,DIS3L2,TBX15,CLASP2,ZNF521,CREB5,DNAJC6,NSUN2,DACH2,PTPRK,SORCS1,DNM3,SYNDIG1,DOCK9,PPARA,PTPRD,RORA,SHISA6,BRIP1,ELAVL4,ANXA8L1,MXI1,TTC28,NELL1,DOCK10,EEF1E1,ATP8A1,BICC1,SEMA3D,PRLR,PTPRA,KCNC4,ATF6,DEPTOR,NVL,RAD51D,CUL2,SYT17,STAC,OR4M1,RAB27A,RALGAPA1,CABLES1,CST2,BLOC1S6,PMEPA1,LARP4B,KCNJ15,SYT9,SEMA3C,ST18,RXFP2
GO:0003008	system process	3.045677491154454e-11	CAMTA1,RCAN1,KALRN,CACNG2,RPS6KA2,NTRK2,DMD,CHRM3,NOS1,TMC2,MYO3A,HDAC4,IMMP2L,ABCC1,ATP8A2,SLC44A1,MGLL,NFASC,PLCB1,TRPM1,RYR2,PRKCA,DLGAP1,BTBD9,TRHDE,MYO9A,CALD1,SLCO3A1,PLS1,USP53,NLGN1,DNAH9,RELN,APP,UTRN,PPP3CA,RYR1,PCDH15,GABRG3,AKAP6,SORCS3,GRM7,SSPN,RYR3,MEF2A,KCNB2,CTNNA3,PPP1R12B,EYS,AKAP13,EP300,SLCO2B1,BDKRB1,DNAH11,GRIK2,CHRM1,JAK2,ABCG8,PARK2,GRIN3A,CACNA1C,NLGN4X,OR5K4,TMPRSS3,TRPM3,SULF1,SH3GL2,COL11A1,SLC13A3,SLIT2,SYNM,SLC4A4,DTNA,SMAD3,GABRR2,HMCN1,EYA1,ELN,T

			UB,BRINP1,OR5I1,DGKI,SLC1A1,SHISA9,EPM2A,NOS1AP,WWTR1,PCSK5,ANK3,LHFPL3,TNR,CELF4,SCN9A,PLCE1,TENM4,MLIP,STRC,ARHGAP42,LRP5,NAV2,GRM1,PDE4D,CNTN5,DOCK4,SLC5A3,FTO,POU6F2,KCNJ12,GRID2,KCNJ3,CELF2,PDE4B,GABRB3,ATXN1,RHPN2,KCND2,SNTB1,SLC24A3,SPTBN4,EPHB2,MYBPC2,PRCP,BDNF,ADORA2A,ANO1,NOX4,CSMD1,DAB2,AFF2,PRKG1,CACNG3,CALCRL,TNNI3K,HDAC2,PRKAR1B,CDH23,TTN,RIMS2,SCN8A,DLGAP2,OR4N2,CTNNA2,PARD3,MYOM3,KCNMA1,CNGB1,PRKCG,SLC8A1,SRRM4,FAM19A4,THRB,AGT,USH2A,NF1,NRXN1,GSK3B,PKP2,CTDP1,SETD3,SLC22A3,BBS9,CHD7,SNX5,OR51B2,TRIOBP,CACNB2,SHANK2,ROR1,PTGER3,MYO3B,MYOM1,SPAG16,HTR2C,GLRA2,P2RX6,ANK2,GRIN2B,ADCY8,GABRR3,CACNA1D,ABCG2,TBL1X,SEMA5B,ANKFN1,GRM5,CNTNAP2,PTPRO,INSR,RPGR,FOXO3,GABRA3,KLHL3,RNLS,CDC14A,RBFOX1,ADAMTS16,LRRK2,SCN1A,BMP6,VTI1A,FYN,KCND3,ABCC2,SGCZ,CLN6,RIMS1,EPHB1,KCNQ1,EXT1,AMFR,LRP2,CAMK4,GUCY2F,SGCD,TMEM108,SHROOM4,NRXN3,SOBP,TRDN,SLC16A12,SEMA3A,BDKRB2,TANC1,GABRB1,SLC24A2,MOGAT2,PPARA,SHISA6,ELAVL4,ATP8A1,ATF6,STAC,OR4M1,CST2
GO:0051128	regulation of cellular component organization	3.5340212021058717e-11	*RNF4,CHFR,KALRN,RPS6KA2,NTRK2,CLASP1,PTPRG,DMD,ROBO1,DSCAM,CIT,TBCD,HDAC4,LINGO2,ATP8A2,AUTS2,LDLRAD4,TENM2,ATF7IP,PLCB1,PRKD1,VCL,SEMA5A,BTBD9,IL1RAPL1,MYO9A,PDE4DIP,ABCA13,KANK1,TBC1D5,RUNX1,PLS1,FCHSD2,NLGN1,CAMK1D,SMG6,CLEC16A,MTRF1,RELN,APP,PAK1,PPP3CA,TOX,PTPN9,AKAP6,DLC1,ALK,DENND5A,MAP2K5,DNMT1,MAGI2,CELSR1,ROBO2,AKAP13,DCC,C10ORF90,EP300,PARN,SEMA4D,BDKRB1,TF,CDH8,TANC2,NEGR1,PARK2,AFAP1,NRG3,NUMB,SH3BP1,ARHGAP24,SLIT2,ATRX,SLX1B,SMAD3,BORA,PEAK1,ELN,MAD1L1,DGKB,TNKS,ARHGEF18,SCAMP5,DNAJB6,OMA1,IL1RAPL2,SKAP1,NTN1,EPM2A,MAP2,CDC73,FLRT2,ARHGAP6,CUX1,MORC2,CDH4,TNR,CDKL1,SPOCK1,PLCE1,EPS8,LRRC4C,CLSTN2,CD300A,CBFA2T2,SERTAD2,DISC1,RABGEF1,TRABD2B,LRP5,AP2B1,ESR1,SCFD1,EFNA5,GRID2,SEMA6D,NTNG1,ADTRP,CRMP1,MAPRE2,ANKRD13A,PRKCQ,RASSF8,RHPN2,CLDN1,SPTBN4,EPHB2,MCTP1,DPYSL2,MARK1,BDNF,FNIP1,FSTL4,SYT1,EBAG9,DAB2,PLXNA2,STXBP6,HDAC2,FAT3,ENPP1,RIMS2,CTNNA2,NRG1,RIT2,CD44,SCAF8,GPSM2,FBXO31,MAP3K13,CDC42EP3,AGT,GRIP1,TLK1,NRXN1,PDXP,GSK3B,CTDP1,TIAM2,IQCJ-SCHIP1,CNTN1,UNC13A,TRIOBP,SHANK2,ISLR2,GPC3,DLG5,ROR1,BID,MACF1,MNAT1,DUSP22,EPHA5,SGIP1,SLIT3,GRIN2B,PAK3,MLLT3,NEB,PRKAA2,SEMA5B,IGF1R,CNTNAP2,PTPRO,INSR,MAP2K1,GPC6,CENPF,LRFN5,ULK4,ADAMTS16,LRRK2,PKHD1,BMP6,ASAP1,FRMPD4,FYN,EPHA7,RIMS1,ENPP2,EPHB1,FHOD3,TRPC5,TENM3,NOX5,FER,RAPGEF2,NAV3,CDKL5,ZNF207,ESR2,PACSIN2,CDH13,DAB1,SEMA3A,MYH9,TENM1,STK38L,STXBP5,GNG4,NTRK3,CLASP2,DNAJC6,DNM3,SYNDIG1,PPARA,PTPRD,ELAVL4,ATP8A1,SEMA3D,PTPRA,SYT17,PMEPA1,SYT9,SEMA3C*
GO:0007166	cell surface receptor signaling pathway	3.872573074122261e-11	*PTPRR,KALRN,RNF43,CTNNA1,NTRK2,ZNRF3,PTPRG,FBXL17,SLC39A10,SPRED2,ERBB4,ROBO1,PRICKLE2,DSCAM,GPC5,WWOX,FUT8,WLS,ANKS1A,LDLRAD4,PSMB2,PTPRT,PLCB1,FLT3,TRPM1,PRKD1,NEDD9,SEMA5A,PRKCA,CCDC3,ITGAE,KANK1,GRIK4,DOK6,NLGN1,TNFRSF10B,AMOTL1,TCF7L2,RELN,APP,ADAM12,PAK1,PPP3CA,GRM7,IDE,SOS1,CTNND2,RFTN1,ALK,BTRC,MAP2K5,MAGI2,EDA,CELSR1,ROBO2,IFT81,DCC,DDB1,EP300,SEMA4D,EVC,RPS6KA5,GRK5,ANKRD6,TF,GRIK2,JAK2,CMKLR1,PARK2,MAGI1,NPHP3,ADCYAP1R1,GRID1,GRIN3A,SMOC2,NLGN4X,ITGBL1,NRG3,COL4A6,SULF1,SH3BP1,RFFL,SLIT2,GLP2R,TRIM5,THEMIS,CHRDL1,TRAF3IP2,BMPR1A,SMAD3,PIBF1,SCUBE1,EYA1,ANKS1B,MAML3,TNKS,OVOL2,ASGR2,IL1RAPL2,SKAP1,DGKI,SLC1A1,EPM2A,CDC73,FLRT2,FBXW11,RANBP10,RBMS3,WWTR1,GRM4,CCL15,CELF4,VAV3,ACTN4,PLCE1,GHR,CD300A,CBFA2T2,DISC1,CCL14,RABGEF1,TRABD2B,LRP5,TNFRSF19,GRM1,PDE4D,PIK3CD,EFNA5,ATF2,PIK3R2,GRID2,ZNF423,SEMA6D,PDE4B,MAP3K7,PRKCQ,EPHB2,S1PR3,RGMB,PTPDC1,MARK1,BDNF,ADORA2A,ARID5B,IFT80,FSTL4,BMPER,DAB2,PLXNA2,LRRK1,ABI1,PTH2R,CALCRL,HDAC2,ZMYND11,ENPP1,RIMS2,PRDM15,NRG1,GRIA2,CD44,MAPKAPK2,PLCL2,CHST11,MAPK10,NCAM1,AGT,FGF1,CCR3,NDRG2,NF1,WIF1,NRXN1,LAMA3,GSK3B,KIR2DL1,CD109,GPR21,PSMB7,SNX5,CNTN1,BTBD11,CAB*

			IN1,GRIK3,STXBP4,UNC5D,NREP,GPC3,DLG5,CPE,ROR1,ZNF675,BID,MACF1,TRIO,PTPRE,DUSP22,MAML2,CTDSPL2,GLRA2,LY86,EPHA5,IL1RL1,P2RX6,SLIT3,GRIN2B,PAK3,MLLT3,RNF213,PRKAA2,TBL1X,SEMA5B,GRIK1,GRM5,IGF1R,PTPRO,SUFU,INSR,ITGA11,JAK1,GMDS,FOXO3,GPC6,CNTN6,MITF,PRDM16,LTBP1,LRRK2,BMP6,FYN,EPHA7,GAS8,RIMS1,EPHB1,DOCK1,CRADD,EXT1,AMFR,UNC5C,LRP2,FER,VWC2,GUCY2F,TMEM108,RAPGEF2,PTPRU,GRIA4,IL18R1,CDH13,VEPH1,DAB1,SEMA3A,MGAT5,MYH9,BDKRB2,PIK3R3,ZRANB1,FCRL2,SCEL,GRIA3,NTRK3,FBN1,CLASP2,PTPRK,PPARA,PTPRD,RORA,SHISA6,BICC1,SEMA3D,PRLR,PTPRA,DEPTOR,PMEPA1,SEMA3C,ST18
GO:0048523	negative regulation of cellular process	6.057869325301883e-11	*PTPRR,CHFR,KDM4B,RCAN1,KALRN,RNF43,PROS1,RPS6KA2,CTNNA1,FOXN3,NTRK2,CLASP1,ZNRF3,GLIS3,PTPRG,SLC39A10,DMD,EIF3E,SPRED2,ERBB4,TFDP2,NOS1,CNTN4,ROBO1,DSCAM,CIT,TBCD,PUM1,FRY,SORCS2,WWOX,TNRC6B,HDAC4,ATP9A,ATP8A2,LDLRAD4,TENM2,BLM,PSMB2,PTPRT,ATF7IP,PLCB1,PRKD1,RYR2,VCL,PDS5A,SEMA5A,ETV6,CASK,PRKCA,ZBTB20,IL1RAPL1,CCDC3,KANK1,RUNX1,NLGN1,CAMK1D,SMG6,CLEC16A,TCF7L2,APP,PAK1,PPP3CA,DEPDC5,RYR1,PTPN9,CREM,AKAP6,SORCS3,GRM7,DLC1,SCMH1,RYR3,BTRC,DCLK1,MEF2A,TFAP2D,DENND5A,MAP2K5,DNMT1,MAGI2,ROBO2,DCC,SAMD4A,DDB1,EP300,PARN,SEMA4D,RPS6KA5,GRK5,ANKRD6,BDKRB1,GRIK2,HUS1,TFEC,ASTN2,JAK2,ZBTB7C,PARK2,NPHP3,ADCYAP1R1,SERPINA4,CACNA1C,NLGN4X,GBE1,TAGLN3,NRG3,NUMB,SOX6,SULF1,CD84,MAGEA11,SH3BP1,RFFL,SH3GL2,PSPC1,ARHGAP24,SLIT2,ATRX,CHRDL1,TRAF3IP2,SLX1B,BMPR1A,SMAD3,PRKAR2A,PIBF1,KIR3DL2,PTGFR,EYA1,SATB2,MAD1L1,TNKS,KLF12,ARHGEF18,SCAMP5,LINC00473,OVOL2,GRAMD4,DNAJB6,BRINP1,OMA1,GLIS1,CDYL2,DGKI,TAF1,SLC1A1,RNF144B,NTN1,LDB2,EPM2A,MAP2,CDC73,FBXW11,RBMS3,WWTR1,ARHGAP6,CUX1,ANK3,TIMP2,ZNF19,TRIM59,TRPS1,TNR,CELF4,MDM4,SPOCK1,ACTN4,EPS8,GHR,CD300A,CBFA2T2,PRG3,MLIP,SERTAD2,ARHGAP42,RABGEF1,FHIT,TRABD2B,LRP5,AP2B1,ESR1,ARHGAP12,SCFD1,PDE4D,FRMD4A,EFNA5,FTO,EIF3A,ATF2,RBBP8,PIK3R2,GRID2,ZNF423,SEMA6D,ADTRP,CRMP1,PDE4B,ANKRD13A,PRKCQ,ATXN1,N4BP1,RHPN2,JDP2,SPTBN4,EPHB2,AGO3,S1PR3,MCTP1,PRTG,DACH1,MARK1,BDNF,RBM4,FNIP1,MALRD1,ADORA2A,ARID5B,IFT80,FSTL4,FHL2,NOX4,BCOR,BMPER,DAB2,PLXNA2,LRRK1,PRKG1,STXBP6,ABI1,HDAC2,ETS1,FAT3,PDE11A,ARID4A,PRKAR1B,BACH1,ZMYND11,RGS6,SRGAP3,ZNF93,ENPP1,PRDM15,CTNNA2,PARD3,NRG1,CAST,FANK1,SPINT2,FMN2,RIT2,CD44,PAX7,SCAF8,PRKCG,MAPKAPK2,SLC8A1,FBXO31,PLCL2,TOX3,CHST11,THRB,AGT,NDRG2,ARHGAP25,USH2A,NF1,WIF1,ZNF536,NRXN1,ARHGAP10,GSK3B,PKP2,CTDP1,CD109,GPR21,CHD7,PSMB7,SNX5,MYT1L,SRGAP2B,IQCJ-SCHIP1,TRIOBP,CHEK2,GRIK3,MECOM,SHANK2,GPC3,DLG5,CFDP1,ZNF675,SERPINA5,BID,MNAT1,TRIO,CAPN3,PTPRE,DUSP22,CTDSPL2,HTR2C,HIRA,TP73,ZNF366,MORC3,ANK2,SLIT3,GRIN2B,PHC2,UACA,LRPPRC,ADCY8,ITPR2,MLLT3,RNF213,SH3RF2,PRKAA2,PACRG,TBL1X,SEMA5B,GRM5,IGF1R,MXD3,PTPRO,SUFU,MAP2K1,UBR2,FOXO3,STK38,INPP5A,CENPF,KCTD1,MITF,IGF2BP3,LRFN5,AGBL4,PRDM16,FRMD5,KIR2DL4,DNMT3B,LTBP1,LRRK2,PKHD1,RARB,PBX1,BMP6,PCBP3,FYN,EPHA7,MARVELD3,GAS8,PTPN14,L3MBTL4,ABCA12,BEND5,EPHB1,KCNQ1,FHOD3,CRADD,TRPC5,AMFR,LRP2,ZCCHC17,FER,VWC2,ERLIN1,PCDH17,TPTE,RAPGEF2,NAV3,PTPRM,NCOA2,PTPRU,ZNF207,GRIA4,RGS7BP,ESR2,KAT7,SATB1,PACSIN2,TRIM22,CDH13,VEPH1,BASP1,TRDN,DAB1,ZNF148,HDAC5,SEMA3A,MGAT5,MYH9,BDKRB2,TSC22D3,NRIP1,LITAF,TENM1,PRKAR1A,RGS7,GNG4,TXNDC5,SLC24A2,NTRK3,FBN1,DIS3L2,TBX15,CLASP2,NSUN2,PTPRK,DNM3,PPARA,PTPRD,RORA,SHISA6,BRIP1,ELAVL4,ANXA8L1,MXI1,NELL1,EEF1E1,BICC1,SEMA3D,PRLR,DEPTOR,CST2,PMEPA1,LARP4B,SEMA3C,ST18*
GO:0006811	ion transport	1.9971670822590222e-10	*CACNG2,NTRK2,SLC39A10,DMD,KCNC2,CHRM3,NOS1,TMC2,DPP10,ABCC1,SLC44A1,CACNA1A,TRPM1,PRKD1,RYR2,CASK,SLCO3A1,GRIK4,NLGN1,KCNIP4,RELN,APP,UTRN,PPP3CA,RYR1,GABRG3,AKAP6,GRM7,RYR3,LRRC8C,KCNB2,FAM155A,KCNA6,SLCO2B1,BDKRB1,TF,GRIK2,CHRM1,PARK2,ADCYAP1R1,DPP6,GRID1,GRIN3A,CACNA1C,EMB,ANO4,ANKH,SL*

			C5A10,TRPM3,CD84,SLC13A3,KCNJ16,SLC4A4,FLVCR2,CACNA1E,GABRR2,SLC9C1,SLC47A1,ANO2,FGF14,CACNA2D3,SLC1A1,SHISA9,EPM2A,NOS1AP,CATSPER2,ANK3,CNIH3,SCN9A,ACTN4,LRRC8D,SLC25A21,ABCC11,SLC30A7,KCNN3,GRM1,PDE4D,SLC5A3,KCNJ12,GRID2,CYBB,KCNJ3,PDE4B,GABRB3,KCND2,SLC24A3,SPTBN4,EPHB2,ADORA2A,ANO1,SYT1,NSF,SNAP23,CACNG3,CALCRL,TMEM163,CDH23,SLC39A11,ENPP1,SCN8A,PEX5L,GRIA2,KCNMA1,CNGB1,SLC12A8,SLC8A1,SLC9A9,AGT,KCNQ5,HEPHL1,KCNJ6,NF1,SLC44A5,NRXN1,PKP2,SLC22A3,KCNH1,CHD7,CNTN1,GRIK3,CACNB2,ANO3,KCNS3,SLC5A8,CAPN3,HTR2C,GLRA2,P2RX6,ANK2,GRIN2B,ITPR2,GABRR3,CACNA1D,ABCG2,GRIK1,GRM5,PKD1L1,NKAIN2,SLC9B1,RASGRF2,GABRA3,KLHL3,NKAIN3,SCN1A,FYN,KCND3,ABCC2,KCNQ1,SLC38A6,TRPC5,NOX5,LRP2,KCNH7,GRIA4,SLC5A4,TRDN,SLC16A12,CACHD1,ATP13A3,BDKRB2,STIM1,GABRB1,RGS7,SLC24A2,GRIA3,SLC35F3,SHISA6,ATP8A1,KCNC4,SYT17,STAC,KCNJ15,SYT9,SLC22A10
GO:0042391	regulation of membrane potential	3.5935751236556554e-10	*CACNG2,NTRK2,DMD,KCNC2,RYR2,PPP2R3C,GRIK4,USP53,NLGN1,RELN,APP,PPP3CA,GABRG3,AKAP6,CTNNA3,GRIK2,CHRM1,PARK2,GRID1,GRIN3A,CACNA1C,NLGN4X,SLC4A4,GABRR2,DGKI,NOS1AP,KCTD7,ANK3,CELF4,SCN9A,GRM1,GRID2,KCNJ3,GABRB3,PPA2,KCND2,ADORA2A,RIMS2,SCN8A,KCNMA1,CNGB1,SLC8A1,FAM19A4,NRXN1,GSK3B,PKP2,KCNH1,GRIK3,CACNB2,BID,GLRA2,P2RX6,ANK2,GRIN2B,GABRR3,CACNA1D,GRIK1,GRM5,GABRA3,LRRK2,SCN1A,KCND3,RIMS1,KCNQ1,TRPC5,TMEM108,KCNH7,RGS7BP,TRDN,GABRB1*
GO:0034329	cell junction assembly	4.050451144742697e-10	*CTNNA1,NTRK2,CLASP1,ERBB4,DSCAM,TBCD,LINGO2,VCL,PRKCA,IL1RAPL1,MYO9A,NLGN1,APP,DLC1,CTNND2,ROBO2,SEMA4D,CDH8,NEGR1,SDK2,NLGN4X,SH3BP1,SMAD3,PEAK1,IL1RAPL2,NTN1,FLRT2,ARHGAP6,CDH12,CDH10,CLSTN2,CNTN5,EFNA5,TLN2,GRID2,GABRB3,CLDN1,EPHB2,BDNF,PARD3,NRG1,AGT,NRXN1,PKP2,SHANK2,DLG5,MACF1,DUSP22,CLDN11,ANK2,SDK1,CNTNAP2,PTPRO,GPC6,LRFN5,EPHA7,MARVELD3,EPHB1,PCDH17,RAPGEF2,KIRREL3,NRXN3,NTRK3,CLASP2,PTPRK,DNM3,SYNDIG1,PTPRD,CDH9,PTPRA*
GO:0016358	dendrite development	4.118725889941315e-10	*KALRN,DSCAM,IL1RAPL1,NLGN1,CAMK1D,RELN,APP,PPP3CA,CTNND2,ALK,DCLK1,MEF2A,DCC,SEMA4D,TANC2,GRIN3A,NTN1,MAP2,CUX1,DISC1,KLHL1,EPHB2,MARK1,FSTL4,PRKG1,ABI1,HDAC2,FAT3,CTNNA2,FBXO31,GRIP1,GSK3B,DLG5,PAK3,SDK1,LRRK2,PHACTR1,ASAP1,FYN,EPHB1,TRPC5,RAPGEF2,CDKL5,DAB1,SEMA3A,DNM3,PTPRD,ELAVL4,DOCK10*
GO:0048588	developmental cell growth	6.029965092138808e-10	*DSCAM,AUTS2,VCL,SEMA5A,APP,AKAP6,DCLK1,AKAP13,DCC,SEMA4D,PARK2,SH3GL2,SLIT2,NTN1,MAP2,CDH4,TNR,DISC1,EFNA5,SEMA6D,DPYSL2,BDNF,FSTL4,SYT1,RIMS2,MAP3K13,AGT,GSK3B,CTDP1,UNC13A,ISLR2,MACF1,SLIT3,SEMA5B,SPG11,EPHA7,RIMS1,EXT1,TRPC5,TMEM108,CDKL5,SEMA3A,CLASP2,PPARA,SEMA3D,SYT17,SEMA3C*
GO:0048583	regulation of response to stimulus	7.581794750919801e-10	*PTPRR,CAMTA1,RCAN1,KALRN,CACNG2,RNF43,PROS1,CTNNA1,NTRK2,CLASP1,ZNRF3,FBXL17,SLC39A10,DMD,SPRED2,ERBB4,ROBO1,DSCAM,CIT,PUM1,GPC5,WDR59,WWOX,FUT8,HDAC4,ABCC1,AUTS2,WLS,ANKS1A,LDLRAD4,PSMB2,PTPRT,MGLL,PLCB1,FLT3,PRKD1,PPP2R3C,SEMA5A,CASK,PRKCA,DLGAP1,MYO9A,CCDC3,KANK1,DOK6,NLGN1,TNFRSF10B,CAMK1D,CLEC16A,TCF7L2,RELN,APP,PAK1,PPP3CA,DEPDC5,AKAP6,DLC1,SOS1,CTNND2,RFTN1,ALK,BTRC,MEF2A,MAP2K5,MAGI2,EDA,ROBO2,IFT81,AKAP13,EP300,SEMA4D,EVC,GRK5,ANKRD6,GRIK2,ARHGAP15,ITSN1,JAK2,CMKLR1,PARK2,NPHP3,ADCYAP1R1,GRIN3A,SMOC2,NLGN4X,AFAP1,SULF1,CD84,SH3BP1,RFFL,PSPC1,ARHGAP24,SLIT2,TRIM5,THEMIS,CHRDL1,TRAF3IP2,SH3RF3,BMPR1A,SMAD3,PIBF1,SCUBE1,EYA1,MAD1L1,TNKS,TUB,ARHGEF18,LINC00473,OVOL2,SGMS1,GRAMD4,DNAJB6,ZDHHC11,SKAP1,DGKI,DNMBP,TAF1,SPNS2,SHISA9,CDC73,FBXW11,NOS1AP,CD96,RBMS3,WWTR1,ARHGAP6,GRM4,CCL15,CNIH3,DOCK3,TRIM59,TNR,CELF4,VAV3,ACTN4,PLCE1,EPS8,GHR,ARHGAP39,CD300A,CBFA2T2,MLIP,DISC1,RALGPS1,ARHGAP42,CCL14,RABGEF1,TRABD2B,ESR1,ARHGAP12,TNFRSF19,GRM1,PDE4D,PIK3CD,EIF3A,PIK3R2,GRID2,ZNF423,SEMA6D,SUSD4,ADTRP,PDE4B,MAPRE2,RIC8B,MAP3K7,PRKCQ,N4BP1,CLDN1,EPHB2,AGO3,MCTP1,MOB3B,PRCP,EEF1E1-BLOC1S5,DOCK2,BDNF,FNIP1,MAGI3,ADORA2A,SIPA1L2,IF*

			T80,FSTL4,FHL2,NOX4,BMPER,DAB2,LRRK1,PRKG1,CACNG3,EPG5,CALCRL,HDAC2,ETS1,PDE11A,ZMYND11,RGS6,SRGAP3,CDK19,ENPP1,CFB,RIMS2,DLGAP2,PRDM15,CTNNA2,PEX5L,NRG1,FMN2,RIT2,CD44,PRKCG,MAPKAPK2,FAM19A4,MAP3K13,PLCL2,CHST11,MAPK10,NCAM1,AGT,FGF1,NDRG2,ARHGAP25,NF1,WIF1,ZNF536,NRXN1,ARHGAP10,GSK3B,KIR2DL1,CTDP1,CD109,GPR21,PSMB7,SNX5,TIAM2,IQCJ-SCHIP1,NCOA1,CHEK2,MECOM,SHANK2,KCTD8,NREP,GPC3,DLG5,PREX1,UBE2V1,ROR1,ZNF675,PTGER3,BID,MACF1,TRIO,CAPN3,PTPRE,DUSP22,MAP3K5,CTDSPL2,HTR2C,FGD4,LY86,TP73,IL1RL1,ZNF366,ARHGEF3,SLIT3,PAK3,GARNL3,UACA,ADCY8,MLLT3,RNF213,SH3RF2,PRKAA2,TBL1X,SEMA5B,GRM5,IGF1R,PTPRO,SUFU,INSR,MAP2K1,UBR2,FOXO3,GPC6,RASGRF2,STK38,CNTN6,LRFN5,PRDM16,ULK4,KIR2DL4,LTBP1,LRRK2,PKHD1,BMP6,FYN,EPHA7,MARVELD3,GAS8,RIMS1,EPHB1,CRADD,AMFR,LRP2,C2,RALGPS2,FER,VWC2,GUCY2F,TMEM108,TPTE,RAPGEF2,PTPRU,IL18R1,RGS7BP,ESR2,KAT7,C9,SETD2,TRIM22,CDH13,VEPH1,DAB1,SEMA3A,MGAT5,TSPAN8,BDKRB2,LITAF,TENM1,ZRANB1,RGS7,SIPA1L3,GNG4,VWF,SCEL,NTRK3,FBN1,CLASP2,PPARA,PTPRD,RORA,SHISA6,EEF1E1,BICC1,SEMA3D,PRLR,ATF6,DEPTOR,RALGAPA1,PMEPA1,SEMA3C
GO:0048589	developmental growth	1.6478305122533505e-9	*DMD,ERBB4,DSCAM,ATP8A2,AUTS2,PLCB1,VCL,SEMA5A,KIF26B,RUNX1,PLS1,APP,PPP3CA,PCDH15,AKAP6,SOS1,DCLK1,MAGI2,EYS,AKAP13,DCC,EP300,SEMA4D,EVC,PARK2,NLGN4X,SH3GL2,SLIT2,ATRX,RAD51B,BMPR1A,SMAD3,CPQ,NTN1,MAP2,WWTR1,CDH4,TNR,TENM4,GHR,DISC1,ESR1,EFNA5,FTO,ATF2,SEMA6D,SPTBN4,DPYSL2,BDNF,ARID5B,IFT80,FSTL4,SYT1,RIMS2,NRG1,MAP3K13,CHST11,AGT,FGF1,GSK3B,CTDP1,GPR21,CHD7,UNC13A,ISLR2,MACF1,CAPN3,TP73,SLIT3,SEMA5B,INSR,FOXO3,RARB,SPG11,EPHA7,RIMS1,EXT1,TRPC5,TMEM108,CDKL5,BASP1,SEMA3A,LARGE,PRKAR1A,CLASP2,PPARA,SEMA3D,PRLR,SYT17,SEMA3C*
GO:0007417	central nervous system development	2.926695146612616e-9	*KALRN,NDE1,CTNNA1,NTRK2,PTPRG,DMD,KCNC2,ERBB4,CNTN4,ROBO1,IMMP2L,WLS,PLCB1,SEMA5A,RELN,APP,PPP3CA,TOX,DLC1,SOS1,ALK,DCLK1,TFAP2D,CELSR1,ROBO2,DCC,TRAPPC9,NEGR1,PARK2,NLGN4X,TAGLN3,NRG3,NUMB,SOX6,SH3GL2,SLIT2,ATRX,BMPR1A,DSCAML1,POTEE,SATB2,ELP3,IL1RAPL2,TAF1,SLC1A1,MAP2,FBXW11,TNR,ARNT2,SPOCK1,TACC2,TENM4,SRD5A2,DISC1,NAV2,KLHL1,POU6F2,ATF2,GRID2,ZNF423,SEMA6D,ATXN1,SPTBN4,EPHB2,DPYSL2,ADORA2A,SYT1,AFF2,PLXNA2,PRKG1,HDAC2,CTNNA2,NRG1,SLC8A1,NDRG2,NF1,NRXN1,GSK3B,CHD7,CNTN1,SHANK2,DLG5,ROR1,MNAT1,TRIO,CCDC141,EPHA5,TP73,GRIN2B,GRIK1,IGF1R,CNTNAP2,SUFU,MAP2K1,FOXO3,CNTN6,CENPF,AGBL4,LRRK2,RARB,PBX1,PHACTR1,FYN,EPHA7,GAS8,EPHB1,EXT1,UNC5C,LRP2,TMEM108,SHROOM4,RAPGEF2,KIRREL3,MACROD2,SETD2,MDGA2,BASP1,DAB1,ZNF148,SEMA3A,GABRB1,RGS7,NTRK3,HYDIN,RORA,ELAVL4,SPATA5*
GO:0034220	ion transmembrane transport	4.842278782010806e-9	*CACNG2,SLC39A10,DMD,KCNC2,CHRM3,NOS1,TMC2,DPP10,ABCC1,CACNA1A,TRPM1,PRKD1,RYR2,GRIK4,NLGN1,KCNIP4,RELN,APP,UTRN,RYR1,GABRG3,AKAP6,RYR3,LRRC8C,KCNB2,FAM155A,KCNA6,BDKRB1,GRIK2,DPP6,GRID1,GRIN3A,CACNA1C,EMB,ANO4,ANKH,SLC5A10,TRPM3,SLC13A3,KCNJ16,SLC4A4,CACNA1E,GABRR2,SLC9C1,SLC47A1,ANO2,FGF14,CACNA2D3,SLC1A1,SHISA9,EPM2A,NOS1AP,CATSPER2,ANK3,CNIH3,SCN9A,ACTN4,LRRC8D,SLC25A21,SLC30A7,KCNN3,GRM1,PDE4D,KCNJ12,GRID2,CYBB,KCNJ3,PDE4B,GABRB3,KCND2,SLC24A3,EPHB2,ANO1,CACNG3,TMEM163,SLC39A11,SCN8A,PEX5L,GRIA2,KCNMA1,CNGB1,SLC12A8,SLC8A1,SLC9A9,AGT,KCNQ5,KCNJ6,NRXN1,KCNH1,CHD7,GRIK3,CACNB2,ANO3,KCNS3,SLC5A8,CAPN3,HTR2C,GLRA2,P2RX6,ANK2,GRIN2B,ITPR2,GABRR3,CACNA1D,GRIK1,GRM5,PKD1L1,SLC9B1,RASGRF2,GABRA3,SCN1A,FYN,KCND3,KCNQ1,TRPC5,NOX5,LRP2,KCNH7,GRIA4,TRDN,SLC16A12,CACHD1,ATP13A3,STIM1,GABRB1,RGS7,SLC24A2,GRIA3,SHISA6,ATP8A1,KCNC4,STAC,KCNJ15*
GO:0098742	cell-cell adhesion via plasma-membrane adhesion molecules	5.6648719098345475e-9	*CNTN4,ROBO1,DSCAM,PCDH9,TENM2,PTPRT,IL1RAPL1,NLGN1,PCDH15,MAP2K5,CELSR1,ROBO2,CDH8,SDK2,EMB,CD84,DSCAML1,HMCN1,CDH12,CDH4,TENM4,CDH10,LRRC4C,CLSTN2,EFNA5,GRID2,NTNG1,CLDN1,CADM3,FAT3,CDH23,NRG1,NRXN1,UNC5D,CLDN11,MYPN,SDK1,GPC6,CNTN6,LRFN5,PCDH7,PCDH11X,TENM3,PCDH17,PTPRM,KIRREL3,DC*

			HS2,CDH13,DAB1,TENM1,PTPRD,CDH9
GO:0007420	brain development	7.488381674353073e-9	*NDE1,CTNNA1,NTRK2,PTPRG,DMD,KCNC2,ERBB4,CNTN4,ROBO1,IMMP2L,WLS,PLCB1,SEMA5A,RELN,APP,PPP3CA,TOX,DLC1,SOS1,ALK,DCLK1,TFAP2D,ROBO2,TRAPPC9,NEGR1,NLGN4X,NRG3,NUMB,SOX6,SLIT2,ATRX,BMPR1A,DSCAML1,POTEE,SATB2,TAF1,SLC1A1,FBXW11,TNR,ARNT2,TACC2,SRD5A2,DISC1,KLHL1,ATF2,GRID2,ZNF423,SEMA6D,ATXN1,EPHB2,DPYSL2,SYT1,AFF2,PLXNA2,PRKG1,CTNNA2,NRG1,SLC8A1,NDRG2,NF1,NRXN1,GSK3B,CHD7,CNTN1,SHANK2,DLG5,MNAT1,CCDC141,EPHA5,GRIN2B,IGF1R,CNTNAP2,MAP2K1,FOXO3,CENPF,LRRK2,RARB,PBX1,PHACTR1,FYN,EPHA7,GAS8,EPHB1,EXT1,UNC5C,LRP2,TMEM108,SHROOM4,RAPGEF2,KIRREL3,MACROD2,SETD2,BASP1,DAB1,ZNF148,SEMA3A,RGS7,HYDIN,RORA,ELAVL4,SPATA5*
GO:0030029	actin filament-based process	9.074992674171492e-9	*CTNNA1,CLASP1,DMD,SHROOM3,CIT,NEBL,AUTS2,RYR2,NEDD9,SEMA5A,EPB41L4B,CALD1,KANK1,DIAPH2,PLS1,FCHSD2,AMOTL1,PAK1,UTRN,PCDH15,DLC1,MEF2A,CTNNA3,CELSR1,AKAP13,ELMO2,TF,JAK2,PARK2,CACNA1C,SH3BP1,SLIT2,EPDR1,SMAD3,ELN,ARHGEF18,DNAJB6,NOS1AP,GAS7,ARHGAP6,ACTN4,EPS8,PPP1R9A,ARHGAP12,PDE4D,EFNA5,KLHL1,PHACTR2,KCNJ3,PDE4B,RHPN2,SPTBN4,DOCK2,THSD7B,PRKG1,ABI1,MICAL3,TTN,CTNNA2,FMNL2,FMN2,CDC42BPA,CDC42EP3,STRIP1,ARHGAP25,NF1,PARVB,PDXP,PKP2,SETD3,THSD7A,TRIOBP,CACNB2,RHOJ,PGM5,PREX1,CAPN3,FGD4,EPHA5,FAM171A1,MYPN,ANK2,PAK3,NEB,CACNA1D,SPECC1L,FRMD5,PHACTR1,SCN1A,FRMPD4,KCND3,KCNQ1,FHOD3,FER,SGCD,SHROOM4,MPRIP,PACSIN2,MYH9,TENM1,SH3KBP1,PRKAR1A,NTRK3,CLASP2*
GO:0060322	head development	9.86223318935456e-9	*NDE1,CTNNA1,NTRK2,PTPRG,DMD,KCNC2,ERBB4,CNTN4,ROBO1,IMMP2L,WLS,PLCB1,SEMA5A,RELN,APP,PPP3CA,TOX,DLC1,SOS1,ALK,DCLK1,TFAP2D,ROBO2,EP300,TRAPPC9,NEGR1,NLGN4X,NRG3,NUMB,SOX6,SLIT2,ATRX,BMPR1A,DSCAML1,POTEE,SATB2,ANKRD11,TAF1,SLC1A1,FBXW11,TNR,ARNT2,TACC2,SRD5A2,DISC1,KLHL1,ATF2,GRID2,ZNF423,SEMA6D,ATXN1,EPHB2,DPYSL2,ARID5B,SYT1,AFF2,PLXNA2,PRKG1,CTNNA2,NRG1,SLC8A1,NDRG2,NF1,NRXN1,GSK3B,CHD7,CNTN1,SHANK2,DLG5,MNAT1,CCDC141,EPHA5,GRIN2B,IGF1R,CNTNAP2,MAP2K1,FOXO3,CENPF,LRRK2,RARB,PBX1,PHACTR1,FYN,EPHA7,GAS8,EPHB1,EXT1,UNC5C,LRP2,DDX10,TMEM108,SHROOM4,RAPGEF2,KIRREL3,MACROD2,SETD2,BASP1,DAB1,ZNF148,SEMA3A,RGS7,HYDIN,RORA,ELAVL4,SPATA5*
GO:0031346	positive regulation of cell projection organization	1.1645214773932055e-8	*KALRN,NTRK2,DMD,ROBO1,DSCAM,HDAC4,ATP8A2,AUTS2,TENM2,PRKD1,SEMA5A,IL1RAPL1,NLGN1,CAMK1D,RELN,TOX,ALK,MAGI2,ROBO2,EP300,SEMA4D,NEGR1,SLIT2,NTN1,CUX1,CDH4,PLCE1,EPS8,CBFA2T2,DISC1,EFNA5,BDNF,PLXNA2,RIT2,FBXO31,MAP3K13,CDC42EP3,AGT,GRIP1,TIAM2,CNTN1,ISLR2,ROR1,MACF1,PAK3,IGF1R,MAP2K1,FYN,ENPP2,TRPC5,TENM3,RAPGEF2,CDKL5,TENM1,NTRK3,DNM3,PTPRD,ELAVL4*
GO:0007610	behavior	1.2817825913589694e-8	*RCAN1,KALRN,NTRK2,DSCAM,PUM1,HDAC4,ATP8A2,PLCB1,BTBD9,GPR176,NLGN1,RELN,APP,PCDH15,SORCS3,ALK,CELSR1,EP300,DNAH11,GRIK2,NEGR1,PARK2,GRID1,NLGN4X,ANKH,BRINP1,DGKI,SLC1A1,EPM2A,TNR,SCN9A,EPS8,NAV2,GRM1,KLHL1,ATXN1,KCND2,SPTBN4,EPHB2,DACH1,BDNF,ADORA2A,CSMD1,AFF2,HDAC2,PRKAR1B,CDH23,NRG1,PRKCG,THRB,AGT,NF1,NRXN1,CHD7,SHANK2,ASTN1,HTR2C,SGIP1,GRIN2B,ADCY8,ANKFN1,SDK1,GRM5,CNTNAP2,INSR,LRRK2,SPG11,SCN1A,FYN,CLN6,KCNQ1,EXT1,AMFR,CAMK4,PCDH17,KIRREL3,NCOA2,NRXN3,SOBP,DAB1,TANC1,SLC24A2,PPARA,ELAVL4,ATP8A1*
GO:0051239	regulation of multicellular organismal process	1.438145891981453e-8	*PTPRR,KALRN,PROS1,CTNNA1,NTRK2,CLASP1,PTPRG,DMD,CHRM3,SPRED2,ERBB4,LAMA2,NOS1,ESRRG,ROBO1,DSCAM,HDAC4,LINGO2,ATP8A2,LDLRAD4,MGLL,PLCB1,PRKD1,RYR2,VCL,PPP2R3C,SEMA5A,PRKCA,EPB41L4B,ZBTB20,DLGAP1,IL1RAPL1,DIO2,RUNX1,PLS1,NLGN1,TCF7L2,RELN,APP,ADAM12,PPP3CA,TOX,AKAP6,RYR3,SOS1,RFTN1,MEF2A,KCNB2,CTNNA3,PPP1R12B,MAP2K5,ROBO2,DCC,SEMA4D,RPS6KA5,TF,JAK2,ABCG8,CMKLR1,NPHP3,SMOC2,CACNA1C,NLGN4X,ANKH,NUMB,SOX6,SULF1,CD84,SH3BP1,SLIT2,BMPR1A,SMAD3,TNFRSF11B,PIBF1,TPH1,SCAMP5,OVOL2,BRINP1,OMA1,SOX5,SLC1A1,NTN1,SHISA9,MAP2,CDC73,FLRT2,NOS1AP,CD96,WWTR1,PEMT,CUX1,TRPS1,CDH4,TNR,CELF4,PLCE1,TE*

			NM4,GHR,CLSTN2,PRG3,MLIP,DISC1,ARHGAP42,RABGEF1,ESR1,GRM1,PDE4D,PIK3CD,DOCK4,SLC5A3,TEAD4,EFNA5,FTO,ATF2,KCNJ12,GRID2,ZNF423,CYBB,SEMA6D,KCNJ3,CELF2,ADTRP,PDE4B,MAPRE2,MAP3K7,PRKCQ,N4BP1,SPTBN4,EPHB2,S1PR3,PRCP,PRTG,MARK1,BDNF,ADORA2A,FSTL4,BCOR,BMPER,DAB2,PLXNA2,PRKG1,PTH2R,CALCRL,TNNI3K,HDAC2,ETS1,ENPP1,RIMS2,KSR2,DLGAP2,PARD3,NRG1,KCNMA1,MAPKAPK2,SLC8A1,ISM1,FBXO31,FAM19A4,MAP3K13,PLCL2,THRB,AGT,FGF1,CCR3,NDRG2,NF1,NRXN1,LAMA3,GSK3B,PKP2,CTDP1,SETD3,CD109,GPR21,CHD7,SNX5,TIAM2,CACNB2,RHOJ,ISLR2,DLG5,ZNF675,PTGER3,MACF1,CAPN3,HTR2C,TP73,RBM19,IL1RL1,ANK2,PAK3,CACNA1D,SEMA5B,GRM5,IGF1R,PTPRO,INSR,JAK1,MAP2K1,FOXO3,RNLS,MITF,IGF2BP3,PRDM16,KIR2DL4,LTBP1,LRRK2,RARB,PBX1,BMP6,KCND3,EPHA7,MARVELD3,RIMS1,ENPP2,ABCA12,EPHB1,KCNQ1,DOCK1,VASH2,TRPC5,NOX5,LRP2,CAMK4,TMEM108,RAPGEF2,NAV3,PTPRM,CDKL5,IL18R1,KAT7,SETD2,BASP1,TRDN,DAB1,HDAC5,SEMA3A,TSPAN8,BDKRB2,STIM1,LITAF,NTRK3,FBN1,CLASP2,SYNDIG1,PPARA,PTPRD,RORA,SHISA6,NELL1,SEMA3D,PRLR,SEMA3C
GO:0009887	animal organ morphogenesis	1.643903232450228e-8	MEGF11,CTNNA1,FOXN3,NTRK2,ZNRF3,ERBB4,LAMA2,ROBO1,PRICKLE2,MYO3A,DSCAM,WWOX,ATP8A2,PSMB2,ASXL3,RYR2,KIF26B,PLS1,RYR1,PCDH15,GREB1L,DLC1,SOS1,BTRC,MAGI2,EDA,CELSR1,ROBO2,EP300,ANKRD6,MMP16,DNAH11,SDK2,NPHP3,EXOC4,NRG3,SOX6,SULF1,ATRNL1,COL11A1,SLIT2,BMPR1A,DSCAML1,SMAD3,TNFRSF11B,EYA1,SATB2,ELN,ANKRD11,EGFLAM,OVOL2,SOX5,SLC1A1,NTN1,FLRT2,FBXW11,WWTR1,MDM4,GHR,STRC,LRP5,ESR1,ATF2,NTNG1,EPHB2,ARID5B,IFT80,FHL2,TTC39C,MEGF9,CSMD1,BCOR,DAB2,HDAC2,FAT3,CDH23,TTN,CTNNA2,NRG1,CHST11,THRB,AGT,FGF1,USH2A,NF1,LAMA3,PKP2,CHD7,PSMB7,TRIOBP,PHEX,GPC3,DLG5,CPE,ROR1,MYO3B,SLIT3,MLLT3,SDK1,SUFU,INSR,MAP2K1,FOXO3,GPC6,KLHL3,ADAMTS16,LRRK2,PKHD1,RARB,PBX1,BMP6,EPHB1,KCNQ1,EXT1,CSGALNACT1,TENM3,LRP2,PTPRM,SOBP,SETD2,BASP1,SEMA3A,STIM1,FBN1,TBX15,PPARA,SEMA3C
GO:0065009	regulation of molecular function	2.2565265927508617e-8	RCAN1,KALRN,CACNG2,PROS1,NTRK2,RASGEF1B,SLC39A10,DMD,KCNC2,CHRM3,EIF3E,SPRED2,ERBB4,NOS1,ROBO1,CIT,TBCD,FRY,PRIM2,HDAC4,ARFGAP3,SGSM1,BLM,PTPRT,RXFP1,PLCB1,FLT3,PRKD1,RYR2,NEDD9,PPP2R3C,DLGAP1,MYO9A,TBC1D5,SLCO3A1,NLGN1,TNFRSF10B,CAMK1D,SMG6,TCF7L2,RELN,APP,PAK1,UTRN,PPP3CA,DEPDC5,AKAP6,GRM7,RABGAP1L,DLC1,IDE,SOS1,ALK,BTRC,DENND5A,PPP1R12B,MAP2K5,MAGI2,EDA,AKAP13,EP300,PARN,SEMA4D,RPS6KA5,TRAPPC9,MMP16,ARHGAP15,ITSN1,JAK2,CMKLR1,PPP6R2,ZBTB7C,PARK2,ADCYAP1R1,SERPINA4,CACNA1C,NRG3,SH3BP1,RFFL,ARHGAP24,SLIT2,TRIM5,BMPR1A,SMAD3,PRKAR2A,BORA,PIBF1,TNKS,ARHGEF18,GRAMD4,DNAJB6,ELP3,BCL2L13,ARRDC4,FGF14,DGKI,DNMBP,TAF1,SLC1A1,LDB2,SHISA9,EPM2A,MAP2,NOS1AP,ARHGEF33,ARHGEF6,WWTR1,ARHGAP6,KCTD7,PCSK5,EGLN3,ANK3,TIMP2,CCL15,CNIH3,DOCK3,VAV3,SPOCK1,ACTN4,PLCE1,GHR,ARHGAP39,RGL1,CD300A,DISC1,RALGPS1,ARHGAP42,CCL14,RABGEF1,LRP5,ESR1,ARHGAP12,DENND2A,PDE4D,DOCK4,SLC5A3,EFNA5,ATF2,PHACTR2,PIK3R2,CRMP1,PDE4B,MAPRE2,RIC8B,MAP3K7,PRKCQ,EPHB2,ERC1,MOB3B,DOCK2,RFC3,CALML4,BDNF,FNIP1,ADORA2A,ARID5B,SIPA1L2,RCAN2,NOX4,DAB2,PLXNA2,PRKG1,ABI1,CACNG3,HDAC2,PRKAR1B,TTN,RGS6,SRGAP3,ENPP1,DLGAP2,AGAP1,NRG1,CAST,TBC1D9,FANK1,SPINT2,CD44,NCF4,GPSM2,SLC8A1,RAPGEF6,FAM19A4,MAP3K13,PLCL2,MAPK10,AGT,FGF1,ARHGAP25,NF1,NRXN1,ARHGAP10,GSK3B,CD109,TIAM2,CABIN1,CACNB2,GPC3,PREX1,UBE2V1,ROR1,ZNF675,SERPINA5,BID,MNAT1,TRIO,CAPN3,DUSP22,MAP3K5,NCF2,FGD4,EPHA5,ANK2,ARHGEF3,GRIN2B,GARNL3,UACA,ADCY8,SH3RF2,CACNA1D,GRM5,ARAP2,IGF1R,PTPRO,SUFU,INSR,RPGR,MAP2K1,RASGRF2,STK38,PPP1R14A,LRRK2,PKHD1,PBX1,PHACTR1,ASAP1,FYN,XRCC4,EPHA7,GAS8,RIMS1,EPHB1,KCNQ1,DOCK1,CRADD,AMFR,RALGPS2,FER,RAPGEF2,PRPSAP2,CDKL5,IL18R1,ESR2,TRIM22,TRDN,DAB1,HDAC5,MGAT5,STIM1,CCNG2,PIK3R3,TENM1,PRKAR1A,RGS7,STXBP5,SIPA1L3,NTRK3,DOCK9,PPARA,SHISA6,SBF2,ANXA8L1,DOCK10,PRLR,DEPTOR,NVL,STAC,RALGAPA1,CST2,DENND2D,ST18,RXFP2

GO:0060078	regulation of postsynaptic membrane potential	2.268071529237618e-8	*GRIK4,NLGN1,RELN,APP,PPP3CA,GABRG3,GRIK2,CHRM1,GRID1,GRIN3A,NLGN4X,GABRR2,DGKI,CELF4,GRM1,GRID2,KCND2,ADORA2A,RIMS2,NRXN1,GSK3B,GRIK3,GLRA2,P2RX6,GRIN2B,GRIK1,GRM5,GABRA3,LRRK2,RIMS1,TMEM108,RGS7BP,GABRB1*
GO:0060560	developmental growth involved in morphogenesis	2.7632519709869887e-8	*DSCAM,AUTS2,VCL,SEMA5A,KIF26B,APP,DCLK1,MAGI2,DCC,SEMA4D,PARK2,SH3GL2,SLIT2,NTN1,MAP2,CDH4,TNR,DISC1,ESR1,EFNA5,SEMA6D,DPYSL2,BDNF,FSTL4,SYT1,RIMS2,MAP3K13,FGF1,GSK3B,UNC13A,ISLR2,MACF1,SLIT3,SEMA5B,SPG11,EPHA7,RIMS1,EXT1,TRPC5,TMEM108,CDKL5,SEMA3A,CLASP2,SEMA3D,SYT17,SEMA3C*
GO:0051270	regulation of cellular component movement	2.917353930696346e-8	*PTPRR,CTNNA1,CLASP1,PTPRG,ERBB4,LAMA2,ROBO1,HDAC4,LDLRAD4,PTPRT,PLCB1,PRKD1,RYR2,VCL,NEDD9,SEMA5A,PRKCA,EPB41L4B,KANK1,CAMK1D,AMOTL1,RELN,APP,PAK1,PPP3CA,DLC1,CTNNA3,MAP2K5,MAGI2,SEMA4D,BDKRB1,TF,DNAH11,JAK2,CMKLR1,SMOC2,CACNA1C,NRG3,NUMB,SULF1,SH3BP1,RFFL,SLIT2,BMPR1A,SMAD3,ELP3,NTN1,LDB2,MAP2,FLRT2,ACTN4,CD300A,RABGEF1,PDE4D,PIK3CD,DOCK4,SEMA6D,NTNG1,ADTRP,PDE4B,MAPRE2,CLDN1,EPHB2,MCTP1,PRCP,DACH1,BMPER,DAB2,PLXNA2,PRKG1,ETS1,SRGAP3,CTNNA2,NRG1,SPINT2,SLC8A1,FBXO31,AGT,FGF1,NF1,LAMA3,PKP2,SRGAP2B,RHOJ,UNC5D,DLG5,MACF1,DUSP22,ANK2,PAK3,SH3RF2,SEMA5B,IGF1R,PTPRO,INSR,FOXO3,MITF,FRMD5,ULK4,PKHD1,KIF2A,PHACTR1,MARVELD3,ENPP2,DOCK1,UNC5C,FER,RAPGEF2,NAV3,PTPRM,PTPRU,CDH13,HDAC5,SEMA3A,MGAT5,PIK3R3,NTRK3,CLASP2,PTPRK,DOCK10,ATP8A1,SEMA3D,SEMA3C*
GO:0050770	regulation of axonogenesis	3.228348544236253e-8	*NTRK2,ROBO1,DSCAM,SEMA5A,PAK1,ROBO2,DCC,SEMA4D,SLIT2,NTN1,MAP2,CDH4,TNR,LRRC4C,DISC1,EFNA5,SEMA6D,EPHB2,BDNF,FSTL4,PLXNA2,MAP3K13,GSK3B,TIAM2,ISLR2,MACF1,PAK3,SEMA5B,MAP2K1,EPHA7,TRPC5,CDKL5,DAB1,SEMA3A,SEMA3D,SEMA3C*
GO:1902531	regulation of intracellular signal transduction	3.421447813340021e-8	*PTPRR,CAMTA1,RCAN1,KALRN,NTRK2,DMD,SPRED2,ERBB4,ROBO1,CIT,PUM1,WDR59,AUTS2,WLS,PLCB1,FLT3,PRKD1,SEMA5A,PRKCA,MYO9A,KANK1,DOK6,NLGN1,TNFRSF10B,CLEC16A,TCF7L2,RELN,APP,PAK1,DEPDC5,AKAP6,DLC1,SOS1,ALK,BTRC,MAP2K5,MAGI2,EDA,AKAP13,EP300,SEMA4D,ANKRD6,GRIK2,ARHGAP15,ITSN1,JAK2,CMKLR1,PARK2,ADCYAP1R1,SH3BP1,RFFL,ARHGAP24,SLIT2,TRIM5,TRAF3IP2,SH3RF3,MAD1L1,ARHGEF18,LINC00473,SGMS1,DGKI,DNMBP,TAF1,FBXW11,NOS1AP,ARHGAP6,GRM4,CCL15,DOCK3,TRIM59,VAV3,ACTN4,PLCE1,EPS8,GHR,ARHGAP39,CD300A,RALGPS1,ARHGAP42,CCL14,RABGEF1,ESR1,ARHGAP12,TNFRSF19,GRM1,PDE4D,PIK3CD,EIF3A,PIK3R2,MAPRE2,MAP3K7,EPHB2,AGO3,MOB3B,EEF1E1-BLOC1S5,DOCK2,FNIP1,MAGI3,SIPA1L2,FHL2,NOX4,BMPER,DAB2,LRRK1,PDE11A,ZMYND11,SRGAP3,PRDM15,PEX5L,NRG1,RIT2,CD44,MAP3K13,AGT,FGF1,NDRG2,ARHGAP25,NF1,NRXN1,ARHGAP10,GSK3B,TIAM2,IQCJ-SCHIP1,CHEK2,MECOM,SHANK2,DLG5,PREX1,UBE2V1,ROR1,ZNF675,BID,TRIO,CAPN3,DUSP22,MAP3K5,HTR2C,FGD4,TP73,ARHGEF3,PAK3,GARNL3,UACA,SH3RF2,PRKAA2,GRM5,IGF1R,INSR,MAP2K1,UBR2,RASGRF2,STK38,ULK4,LRRK2,PKHD1,FYN,EPHA7,MARVELD3,EPHB1,LRP2,RALGPS2,TPTE,RAPGEF2,IL18R1,TRIM22,CDH13,SEMA3A,BDKRB2,LITAF,TENM1,SIPA1L3,VWF,NTRK3,PPARA,RORA,EEF1E1,DEPTOR,RALGAPA1*
GO:0007010	cytoskeleton organization	4.0795294062238045e-8	*RNF4,NDE1,CTNNA1,CLASP1,DMD,SHROOM3,CIT,TBCD,NEBL,ATP8A2,AUTS2,NEDD9,PPP2R3C,SEMA5A,EPB41L4B,PDE4DIP,CALD1,KANK1,DIAPH2,PLS1,FCHSD2,NLGN1,AMOTL1,PAK1,UTRN,PCDH15,DLC1,MEF2A,CTNNA3,CELSR1,AKAP13,ELMO2,C10ORF90,TF,JAK2,MAST4,PARK2,AFAP1,LRRC49,SH3BP1,SLIT2,SYNM,ATRX,SMAD3,BORA,PIBF1,ELN,TNKS,ARHGEF18,DNAJB6,SPAG17,MAP2,FBXW11,NOS1AP,RANBP10,GAS7,ARHGAP6,STARD9,ANK3,ACTN4,PLCE1,TACC2,EPS8,CECR2,PPP1R9A,TTLL11,DISC1,ARHGAP12,ARMC2,EFNA5,TLN2,KLHL1,ATF2,PHACTR2,CRMP1,MAPRE2,RASSF8,RHPN2,TTLL5,SPTBN4,DOCK2,DPYSL2,MARK1,CCDC170,THSD7B,PRKG1,ABI1,MICAL3,TTN,HEPACAM2,CTNNA2,LRCH3,FMNL2,PARD3,FMN2,GPSM2,CDC42BPA,CDC42EP3,STRIP1,ARHGAP25,NF1,PARVB,ARHGAP10,PDXP,GSK3B,PKP2,SETD3,THSD7A,IQCJ-SCHIP1,TRIOBP,CHEK2,RHOJ,PGM5,PREX1,MACF1,CAPN3,*

			SPAG16,FGD4,EPHA5,FAM171A1,MYPN,ANK2,PAK3,NEB,PRKAA2,ANKFN1,KPNB1,SPECC1L,CDC14A,FRMD5,ULK4,PKHD1,KIF2A,PHACTR1,FRMPD4,GAS8,FHOD3,FER,SHROOM4,NAV3,ZNF207,MPRIP,SETD2,PACSIN2,TRDN,MYH9,TENM1,SH3KBP1,PRKAR1A,ZRANB1,SIPA1L3,NTRK3,HYDIN,CLASP2,NCKAP5,TUBGCP6,PARD3B
GO:0035249	synaptic transmission, glutamatergic	4.1906435641287666e-8	CACNG2,GRIK4,NLGN1,RELN,GRM7,GRIK2,CDH8,PARK2,GRID1,DGKI,GRM4,TNR,DISC1,GRM1,GRID2,ADORA2A,SYT1,CACNG3,GRIA2,NF1,NRXN1,UNC13A,GRIK3,GRIK1,GRM5,LRRK2,EXT1
GO:0040012	regulation of locomotion	7.141623348828757e-8	PTPRR,CTNNA1,CLASP1,PTPRG,ERBB4,LAMA2,ROBO1,DSCAM,HDAC4,LDLRAD4,PTPRT,PLCB1,PRKD1,VCL,NEDD9,SEMA5A,PRKCA,EPB41L4B,KANK1,CAMK1D,AMOTL1,RELN,APP,PAK1,PPP3CA,DLC1,MAP2K5,MAGI2,ROBO2,SEMA4D,BDKRB1,TF,CHRM1,JAK2,CMKLR1,SMOC2,NRG3,NUMB,SULF1,SH3BP1,RFFL,SLIT2,BMPR1A,SMAD3,ELP3,NTN1,LDB2,FLRT2,ACTN4,CD300A,RABGEF1,PIK3CD,DOCK4,SEMA6D,NTNG1,ADTRP,MAPRE2,CLDN1,EPHB2,MCTP1,PRCP,DACH1,ADORA2A,BMPER,DAB2,PLXNA2,PRKG1,ETS1,SRGAP3,CTNNA2,NRG1,SPINT2,SLC8A1,FBXO31,AGT,FGF1,NF1,LAMA3,SRGAP2B,RHOJ,UNC5D,DLG5,MACF1,DUSP22,PAK3,SH3RF2,SEMA5B,GRM5,IGF1R,PTPRO,INSR,FOXO3,MITF,FRMD5,ULK4,LRRK2,KIF2A,PHACTR1,MARVELD3,ENPP2,DOCK1,UNC5C,FER,RAPGEF2,NAV3,PTPRM,PTPRU,CDH13,HDAC5,SEMA3A,MGAT5,PIK3R3,NTRK3,CLASP2,PTPRK,DOCK10,ATP8A1,SEMA3D,SEMA3C
GO:0055085	transmembrane transport	7.285944385429484e-8	SVOPL,CACNG2,SLC39A10,DMD,KCNC2,CHRM3,NOS1,TMC2,DPP10,ABCC1,PSMB2,SLC44A1,CACNA1A,TRPM1,PRKD1,RYR2,ABCA13,SLCO3A1,GRIK4,NLGN1,KCNIP4,RELN,APP,UTRN,RYR1,GABRG3,AKAP6,RYR3,LRRC8C,MEF2A,KCNB2,FAM155A,KCNA6,SLCO2B1,BDKRB1,GRIK2,ABCG8,SLC14A2,DPP6,GRID1,GRIN3A,CACNA1C,EMB,ANO4,ANKH,SLC5A10,TRPM3,SLC13A3,KCNJ16,SLC4A4,FLVCR2,CACNA1E,GABRR2,SLC9C1,SLC47A1,SV2B,ANO2,FGF14,CACNA2D3,SPNS2,SLC1A1,SHISA9,EPM2A,NOS1AP,CATSPER2,ANK3,CNIH3,SCN9A,ACTN4,LRRC8D,SLC25A21,ABCC11,SLC30A7,KCNN3,GRM1,PDE4D,SLC5A3,KCNJ12,GRID2,CYBB,KCNJ3,PDE4B,GABRB3,KCND2,SLC24A3,EPHB2,ANO1,CACNG3,TMEM163,SLC39A11,ENPP1,SCN8A,PEX5L,CYB561A3,SLC35F4,GRIA2,KCNMA1,CNGB1,SLC12A8,SLC8A1,SLC9A9,AGT,KCNQ5,KCNJ6,SLC44A5,NRXN1,SLC22A3,KCNH1,CHD7,PSMB7,GRIK3,CACNB2,STXBP4,GPC3,ANO3,KCNS3,SLC5A8,CAPN3,HTR2C,GLRA2,P2RX6,ANK2,GRIN2B,ITPR2,GABRR3,CACNA1D,ABCG2,GRIK1,GRM5,PKD1L1,INSR,SLC9B1,RASGRF2,GABRA3,OCA2,SCN1A,SLC35F1,FYN,KCND3,ABCC2,ABCA12,KCNQ1,SLC38A6,TRPC5,NOX5,LRP2,KCNH7,GRIA4,SLC5A4,TRDN,SLC16A12,CACHD1,ATP13A3,STIM1,GABRB1,RGS7,SLC24A2,GRIA3,SHISA6,ATP8A1,KCNC4,STAC,KCNJ15,SLC22A10
GO:0016310	phosphorylation	7.46249768796078e-8	KALRN,RPS6KA2,NTRK2,DMD,SPRED2,PHKB,ERBB4,NOS1,ROBO1,MYO3A,DSCAM,CIT,HUNK,HDAC4,LDLRAD4,BLM,PTPRT,FLT3,SRPK2,PRKD1,ADK,NEDD9,PPP2R3C,CASK,PRKCA,ZBTB20,SLCO3A1,TNFRSF10B,CAMK1D,RELN,APP,PAK1,ALK,DCLK1,MAP2K5,MAGI2,AKAP13,EP300,SEMA4D,RPS6KA5,GRK5,BDKRB1,TF,HUS1,JAK2,FPGT-TNNI3K,MAST4,PARK2,CKMT1B,PIK3C2B,NRG3,SH3GL2,SLIT2,MAPK4,SLC4A4,BMPR1A,PRKAR2A,BORA,PI4KA,PIBF1,PIK3C3,PEAK1,DGKB,TNKS,SGMS1,DGKK,DGKI,TAF1,SLC1A1,LDB2,EPM2A,WWTR1,DOCK3,VAV3,CDKL1,PLCE1,GHR,CD300A,RABGEF1,LRP5,PDE4D,PIK3CD,EFNA5,ATF2,PIK3R2,ADTRP,MAP3K7,PRKCQ,SPTBN4,EPHB2,ERC1,MOB3B,MARK1,BDNF,FNIP1,ADORA2A,NOX4,CHKA,BMPER,ICK,DAB2,LRRK1,PRKG1,GALK2,ABI1,TNNI3K,HDAC2,PRKAR1B,TTN,CDK19,ENPP1,KSR2,PARD3,NRG1,TOP1,RIT2,CD44,PRKCG,MAPKAPK2,SLC8A1,MAP3K13,PLCL2,RSRC1,CDC42BPA,MAPK10,AGT,FGF1,DYRK4,TLK1,NF1,NRXN1,GSK3B,CD109,CNTN1,CHEK2,STK32B,ROR1,ZNF675,MNAT1,TRIO,MYO3B,DUSP22,MAP3K5,EPHA5,MORC3,PAK3,ADCY8,PRKAA2,NME7,GRM5,IGF1R,PTPRO,INSR,JAK1,MAP2K1,STK38,PPP1R14A,ULK4,LRRK2,BMP6,FYN,EPHA7,ENPP2,EPHB1,BPGM,TRPC5,FER,CAMK4,GUCY2F,RAPGEF2,AK5,CDKL5,ADPGK,DAB1,BDKRB2,CCNG2,PIK3R3,TENM1,STK38L,SH3KBP1,PRKAR1A,NTRK3,RBKS,PPARA,TPK1,PRLR,PTPRA,DEPTOR,PMEPA1
GO:000721	glutamate	8.737918782854802e-8	PLCB1,TRPM1,GRIK4,APP,GRM7,GRIK2,GRID1,GRIN3A,SLC1

5	receptor signaling pathway		*A1,GRM4,GRM1,GRID2,GRIA2,GRIK3,GRIN2B,GRIK1,GRM5,FYN,GRIA4,GRIA3*
GO:0098609	cell-cell adhesion	9.664244271603916e-8	*MEGF11,CTNNA1,LPP,CNTN4,ROBO1,DSCAM,PCDH9,TENM2,PTPRT,VCL,PRKCA,IL1RAPL1,KIF26B,RUNX1,NLGN1,PCDH15,CTNND2,CTNNA3,DLG2,MAP2K5,CELSR1,ROBO2,ELMO2,SEMA4D,CDH8,ASTN2,JAK2,NEGR1,SDK2,MAGI1,NLGN4X,EMB,CD84,DSCAML1,HMCN1,MAD1L1,DNAJB6,SKAP1,NTN1,ANK3,CDH12,CDH4,TNR,TENM4,CDH10,LRRC4C,CLSTN2,CD300A,EFNA5,TLN2,GRID2,NTNG1,ADTRP,PRKCQ,CLDN1,ADORA2A,PRKG1,CADM3,STXBP6,ETS1,FAT3,CDH23,CTNNA2,NRG1,VSIG10,SPINT2,CD44,NRXN1,LAMA3,PKP2,UNC5D,DLG5,ASTN1,DUSP22,CLDN11,MYPN,SDK1,PKD1L1,JAK1,FOXO3,GPC6,CNTN6,LRFN5,PCDH7,PKHD1,BMP6,FYN,EPHA7,COL19A1,PCDH11X,ABCA12,EXT1,TENM3,FER,PCDH17,PTPRM,KIRREL3,NRXN3,PTPRU,DCHS2,CDH13,DAB1,MYH9,TENM1,PRKAR1A,PPARA,PTPRD,CDH9,FNDC3A*
GO:0044057	regulation of system process	1.1478955513807303e-7	*DMD,CHRM3,NOS1,HDAC4,MGLL,RYR2,PRKCA,DLGAP1,NLGN1,RELN,APP,PPP3CA,AKAP6,RYR3,MEF2A,KCNB2,CTNNA3,PPP1R12B,JAK2,ABCG8,CACNA1C,NLGN4X,SMAD3,SLC1A1,SHISA9,NOS1AP,TNR,CELF4,PLCE1,TENM4,MLIP,ARHGAP42,GRM1,PDE4D,DOCK4,FTO,KCNJ12,KCNJ3,CELF2,PDE4B,SPTBN4,ADORA2A,DAB2,PRKG1,CALCRL,TNNI3K,RIMS2,DLGAP2,PARD3,KCNMA1,SLC8A1,FAM19A4,THRB,AGT,NRXN1,PKP2,CTDP1,SETD3,SNX5,CACNB2,PTGER3,HTR2C,ANK2,CACNA1D,PTPRO,FOXO3,RNLS,LRRK2,BMP6,KCND3,RIMS1,KCNQ1,TMEM108,TRDN,SEMA3A,BDKRB2,PPARA,SHISA6*
GO:0030334	regulation of cell migration	1.4536541896750415e-7	*PTPRR,CLASP1,PTPRG,ERBB4,LAMA2,ROBO1,HDAC4,LDLRAD4,PTPRT,PLCB1,PRKD1,VCL,NEDD9,SEMA5A,PRKCA,EPB41L4B,KANK1,CAMK1D,AMOTL1,RELN,APP,PAK1,PPP3CA,DLC1,MAP2K5,MAGI2,SEMA4D,BDKRB1,JAK2,CMKLR1,SMOC2,NRG3,NUMB,SULF1,SH3BP1,RFFL,SLIT2,BMPR1A,SMAD3,ELP3,NTN1,LDB2,FLRT2,ACTN4,CD300A,RABGEF1,PIK3CD,DOCK4,SEMA6D,NTNG1,ADTRP,MAPRE2,CLDN1,EPHB2,MCTP1,PRCP,DACH1,BMPER,DAB2,PLXNA2,PRKG1,ETS1,SRGAP3,CTNNA2,NRG1,SLC8A1,FBXO31,AGT,FGF1,NF1,LAMA3,SRGAP2B,RHOJ,UNC5D,DLG5,MACF1,DUSP22,PAK3,SH3RF2,SEMA5B,IGF1R,INSR,FOXO3,MITF,FRMD5,ULK4,KIF2A,PHACTR1,MARVELD3,ENPP2,DOCK1,UNC5C,FER,RAPGEF2,NAV3,PTPRM,PTPRU,CDH13,HDAC5,SEMA3A,MGAT5,PIK3R3,NTRK3,CLASP2,PTPRK,DOCK10,ATP8A1,SEMA3D,SEMA3C*
GO:1905114	cell surface receptor signaling pathway involved in cell-cell signaling	1.7173450780089923e-7	*RNF43,ZNRF3,PRICKLE2,GPC5,WWOX,WLS,PSMB2,SEMA5A,KANK1,NLGN1,AMOTL1,TCF7L2,RELN,APP,PPP3CA,CTNND2,BTRC,MAGI2,EDA,CELSR1,DDB1,GRK5,ANKRD6,GRIK2,PARK2,NPHP3,NLGN4X,SULF1,SMAD3,TNKS,DGKI,EPM2A,CDC73,FBXW11,RBMS3,WWTR1,CELF4,DISC1,TRABD2B,LRP5,GRID2,ZNF423,MARK1,ADORA2A,IFT80,DAB2,LRRK1,RIMS2,PRDM15,NDRG2,WIF1,NRXN1,GSK3B,PSMB7,GPC3,CPE,ROR1,MACF1,GLRA2,P2RX6,GRIN2B,MLLT3,RNF213,PRKAA2,TBL1X,PTPRO,FOXO3,GPC6,MITF,LRRK2,RIMS1,EXT1,AMFR,TMEM108,PTPRU,ZRANB1,SCEL,SHISA6,BICC1*
GO:0007416	synapse assembly	2.1204954553807277e-7	*NTRK2,ERBB4,DSCAM,LINGO2,IL1RAPL1,NLGN1,APP,ROBO2,SEMA4D,NEGR1,SDK2,NLGN4X,IL1RAPL2,NTN1,FLRT2,CLSTN2,CNTN5,EFNA5,GRID2,GABRB3,EPHB2,BDNF,NRG1,NRXN1,SHANK2,DLG5,SDK1,GPC6,LRFN5,EPHA7,EPHB1,PCDH17,KIRREL3,NRXN3,NTRK3,DNM3,SYNDIG1,PTPRD*
GO:0030001	metal ion transport	2.5960834383853885e-7	*CACNG2,SLC39A10,DMD,KCNC2,NOS1,TMC2,DPP10,CACNA1A,TRPM1,PRKD1,RYR2,CASK,KCNIP4,UTRN,PPP3CA,RYR1,AKAP6,RYR3,KCNB2,FAM155A,KCNA6,BDKRB1,TF,ADCYAP1R1,DPP6,GRIN3A,CACNA1C,SLC5A10,TRPM3,CD84,SLC13A3,KCNJ16,SLC4A4,FLVCR2,CACNA1E,SLC9C1,FGF14,CACNA2D3,SLC1A1,EPM2A,NOS1AP,CATSPER2,ANK3,SCN9A,ACTN4,SLC30A7,KCNN3,PDE4D,SLC5A3,KCNJ12,KCNJ3,PDE4B,KCND2,SLC24A3,SPTBN4,ADORA2A,NSF,CACNG3,CALCRL,TMEM163,CDH23,SLC39A11,SCN8A,KCNMA1,SLC12A8,SLC8A1,SLC9A9,AGT,KCNQ5,HEPHL1,KCNJ6,PKP2,KCNH1,CHD7,CNTN1,CACNB2,KCNS3,SLC5A8,CAPN3,HTR2C,ANK2,GRIN2B,ITPR2,CACNA1D,PKD1L1,NKAIN2,SLC9B1,KLHL3,NKAIN3,SCN1A,FYN,KCND3,KCNQ1,SLC38A6,TRPC5,NOX5,LRP2,KCNH7,SLC5A4,TRDN,CACHD1,STIM1,RGS7,SLC24A2,KCNC4,STAC,KCNJ15*
GO:0072359	circulatory system development	2.8958316246836534e-7	*RPS6KA2,NTRK2,ERBB4,TFDP2,ROBO1,NEBL,IMMP2L,SRPK2,PRKD1,RYR2,SEMA5A,PRKCA,ADAMTS6,CALD1,RUNX1,AM*

			OTL1,TCF7L2,ADAM12,RYR1,GREB1L,AKAP6,DLC1,SOS1,MEF2A,MAP2K5,DNMT1,ROBO2,AKAP13,EP300,DNAH11,QKI,NPHP3,SMOC2,CACNA1C,SOX6,SULF1,ARHGAP24,COL11A1,SLIT2,TRAF3IP2,BMPR1A,SMAD3,SCUBE1,EYA1,ELN,OVOL2,SLC1A1,FLRT2,PCSK5,MYO18B,VAV3,MDM4,PLCE1,TENM4,LRP5,AP2B1,PIK3CD,ATF2,CYBB,ADTRP,EPHB2,PRCP,COL22A1,FHL2,NOX4,BCOR,BMPER,CALCRL,ETS1,TTN,NRG1,SLC8A1,ISM1,AGT,FGF1,CCR3,NF1,NRXN1,PKP2,CTDP1,CHD7,THSD7A,RHOJ,GPC3,CPE,MNAT1,TP73,ANK2,SLIT3,NEB,RNF213,IGF1R,SUFU,INSR,JAK1,MAP2K1,RARB,SGCZ,PTPN14,ENPP2,EPHB1,KCNQ1,FHOD3,VASH2,EXT1,NOX5,LRP2,SGCD,RAPGEF2,PTPRM,NRXN3,SETD2,CDH13,BASP1,HDAC5,MYH9,STIM1,PIK3R3,PRKAR1A,NTRK3,FBN1,PPARA,RORA,BICC1,SEMA3C
GO:0031345	negative regulation of cell projection organization	2.975548175120156e-7	*PTPRG,SEMA5A,KANK1,NLGN1,PPP3CA,PTPN9,DENND5A,DCC,SEMA4D,ARHGAP24,SLIT2,NTN1,MAP2,TNR,SPOCK1,CBFA2T2,SEMA6D,CRMP1,EPHB2,FSTL4,DAB2,HDAC2,FAT3,RIT2,NRXN1,GRIN2B,SEMA5B,PTPRO,LRRK2,FYN,EPHA7,TRPC5,RAPGEF2,DAB1,SEMA3A,DNM3,SEMA3D,SEMA3C*
GO:0050807	regulation of synapse organization	2.9796143599124435e-7	*KALRN,NTRK2,LINGO2,IL1RAPL1,NLGN1,RELN,APP,ROBO2,SEMA4D,CDH8,TANC2,NEGR1,DGKB,IL1RAPL2,NTN1,FLRT2,CLSTN2,DISC1,EFNA5,GRID2,EPHB2,BDNF,CTNNA2,NRXN1,SHANK2,DLG5,GRIN2B,PAK3,PTPRO,GPC6,LRFN5,LRRK2,FRMPD4,FYN,EPHA7,EPHB1,CDKL5,NTRK3,DNM3,SYNDIG1,PTPRD*
GO:0006812	cation transport	3.3615294166086747e-7	*CACNG2,SLC39A10,DMD,KCNC2,NOS1,TMC2,DPP10,SLC44A1,CACNA1A,TRPM1,PRKD1,RYR2,CASK,NLGN1,KCNIP4,RELN,APP,UTRN,PPP3CA,RYR1,AKAP6,RYR3,KCNB2,FAM155A,KCNA6,BDKRB1,TF,PARK2,ADCYAP1R1,DPP6,GRIN3A,CACNA1C,SLC5A10,TRPM3,CD84,SLC13A3,KCNJ16,SLC4A4,FLVCR2,CACNA1E,SLC9C1,SLC47A1,FGF14,CACNA2D3,SLC1A1,SHISA9,EPM2A,NOS1AP,CATSPER2,ANK3,CNIH3,SCN9A,ACTN4,SLC30A7,KCNN3,PDE4D,SLC5A3,KCNJ12,KCNJ3,PDE4B,KCND2,SLC24A3,SPTBN4,EPHB2,ADORA2A,ANO1,SYT1,NSF,SNAP23,CACNG3,CALCRL,TMEM163,CDH23,SLC39A11,SCN8A,PEX5L,KCNMA1,CNGB1,SLC12A8,SLC8A1,SLC9A9,AGT,KCNQ5,HEPHL1,KCNJ6,SLC44A5,NRXN1,PKP2,SLC22A3,KCNH1,CHD7,CNTN1,CACNB2,KCNS3,SLC5A8,CAPN3,HTR2C,P2RX6,ANK2,GRIN2B,ITPR2,CACNA1D,PKD1L1,NKAIN2,SLC9B1,RASGRF2,KLHL3,NKAIN3,SCN1A,FYN,KCND3,KCNQ1,SLC38A6,TRPC5,NOX5,LRP2,KCNH7,SLC5A4,TRDN,CACHD1,ATP13A3,STIM1,RGS7,SLC24A2,SLC35F3,SHISA6,ATP8A1,KCNC4,SYT17,STAC,KCNJ15,SYT9*
GO:0051960	regulation of nervous system development	5.45170422278097e-7	*KALRN,CTNNA1,NTRK2,ROBO1,DSCAM,LINGO2,SEMA5A,IL1RAPL1,NLGN1,RELN,PPP3CA,ROBO2,DCC,SEMA4D,NPHP3,NUMB,SLIT2,BMPR1A,BRINP1,NTN1,MAP2,FLRT2,CUX1,CDH4,TNR,TENM4,CLSTN2,DISC1,EFNA5,GRID2,SEMA6D,EPHB2,PRTG,BDNF,FSTL4,PLXNA2,HDAC2,PARD3,FBXO31,MAP3K13,NF1,NRXN1,CHD7,TIAM2,ISLR2,DLG5,MACF1,TP73,PAK3,SEMA5B,GRM5,MAP2K1,EPHA7,EPHB1,TRPC5,LRP2,RAPGEF2,CDKL5,DAB1,SEMA3A,NTRK3,SYNDIG1,PTPRD,SEMA3D,SEMA3C*
GO:0003013	circulatory system process	6.108562396535734e-7	*RPS6KA2,DMD,CHRM3,NOS1,HDAC4,IMMP2L,ABCC1,SLC44A1,RYR2,TRHDE,SLCO3A1,RYR3,MEF2A,CTNNA3,AKAP13,SLCO2B1,BDKRB1,JAK2,CACNA1C,SH3GL2,SLC13A3,SLIT2,SLC4A4,SMAD3,ELN,SLC1A1,NOS1AP,WWTR1,PCSK5,ARHGAP42,LRP5,NAV2,PDE4D,DOCK4,SLC5A3,KCNJ12,KCNJ3,CELF2,PDE4B,SLC24A3,SPTBN4,PRCP,ADORA2A,NOX4,PRKG1,TNNI3K,TTN,KCNMA1,SLC8A1,THRB,AGT,PKP2,SLC22A3,CHD7,SNX5,CACNB2,ANK2,CACNA1D,ABCG2,PTPRO,INSR,RNLS,ADAMTS16,SCN1A,BMP6,FYN,KCND3,ABCC2,SGCZ,KCNQ1,EXT1,LRP2,SGCD,TRDN,SLC16A12,SEMA3A,BDKRB2,PPARA,ATP8A1*
GO:2000145	regulation of cell motility	7.265625282608431e-7	*PTPRR,CTNNA1,CLASP1,PTPRG,ERBB4,LAMA2,ROBO1,HDAC4,LDLRAD4,PTPRT,PLCB1,PRKD1,VCL,NEDD9,SEMA5A,PRKCA,EPB41L4B,KANK1,CAMK1D,AMOTL1,RELN,APP,PAK1,PPP3CA,DLC1,MAP2K5,MAGI2,SEMA4D,BDKRB1,TF,JAK2,CMKLR1,SMOC2,NRG3,NUMB,SULF1,SH3BP1,RFFL,SLIT2,BMPR1A,SMAD3,ELP3,NTN1,LDB2,FLRT2,ACTN4,CD300A,RABGEF1,PIK3CD,DOCK4,SEMA6D,NTNG1,ADTRP,MAPRE2,CLDN1,EPHB2,MCTP1,PRCP,DACH1,BMPER,DAB2,PLXNA2,PRKG1,ETS1,SRGAP3,CTNNA2,NRG1,SPINT2,SLC8A1,FBXO31,AGT,FGF1,NF1,LAMA3,SRGAP2B,RHOJ,UNC5D,DLG5,MACF1,DUSP2*

			2,PAK3,SH3RF2,SEMA5B,IGF1R,INSR,FOXO3,MITF,FRMD5,ULK4,KIF2A,PHACTR1,MARVELD3,ENPP2,DOCK1,UNC5C,FER,RAPGEF2,NAV3,PTPRM,PTPRU,CDH13,HDAC5,SEMA3A,MGAT5,PIK3R3,NTRK3,CLASP2,PTPRK,DOCK10,ATP8A1,SEMA3D,SEMA3C
GO:0044093	positive regulation of molecular function	7.340397221177449e-7	*KALRN,CACNG2,NTRK2,SLC39A10,DMD,KCNC2,EIF3E,ERBB4,NOS1,ROBO1,PRIM2,HDAC4,SGSM1,FLT3,PRKD1,RYR2,NEDD9,PPP2R3C,MYO9A,TBC1D5,SLCO3A1,TNFRSF10B,CAMK1D,TCF7L2,RELN,APP,PAK1,PPP3CA,AKAP6,RABGAP1L,DLC1,IDE,ALK,BTRC,MAP2K5,MAGI2,EDA,AKAP13,EP300,PARN,SEMA4D,RPS6KA5,TRAPPC9,JAK2,PARK2,ADCYAP1R1,CACNA1C,NRG3,SH3BP1,ARHGAP24,TRIM5,BMPR1A,SMAD3,BORA,PIBF1,TNKS,GRAMD4,BCL2L13,ARRDC4,TAF1,SLC1A1,NOS1AP,ARHGAP6,KCTD7,EGLN3,ANK3,CCL15,DOCK3,VAV3,ACTN4,GHR,CD300A,ARHGAP42,CCL14,LRP5,ESR1,SLC5A3,EFNA5,ATF2,MAPRE2,MAP3K7,PRKCQ,EPHB2,ERC1,MOB3B,RFC3,BDNF,ARID5B,SIPA1L2,NOX4,ABI1,CACNG3,HDAC2,RGS6,NRG1,TBC1D9,FANK1,CD44,RAPGEF6,MAP3K13,PLCL2,AGT,FGF1,ARHGAP25,NF1,NRXN1,GSK3B,TIAM2,CACNB2,PREX1,UBE2V1,ROR1,BID,MNAT1,CAPN3,MAP3K5,EPHA5,ANK2,GRIN2B,GARNL3,UACA,ADCY8,CACNA1D,GRM5,ARAP2,IGF1R,INSR,MAP2K1,LRRK2,ASAP1,FYN,XRCC4,EPHA7,EPHB1,CRADD,AMFR,FER,RAPGEF2,CDKL5,IL18R1,ESR2,TRIM22,TRDN,DAB1,HDAC5,STIM1,TENM1,RGS7,SIPA1L3,NTRK3,DOCK9,DOCK10,PRLR,NVL,STAC,RALGAPA1,ST18*
GO:0050803	regulation of synapse structure or activity	7.403288536401147e-7	*KALRN,NTRK2,LINGO2,IL1RAPL1,NLGN1,RELN,APP,ROBO2,SEMA4D,CDH8,TANC2,NEGR1,DGKB,IL1RAPL2,NTN1,FLRT2,CLSTN2,DISC1,EFNA5,GRID2,EPHB2,BDNF,CTNNA2,NRXN1,SHANK2,DLG5,GRIN2B,PAK3,PTPRO,GPC6,LRFN5,LRRK2,FRMPD4,FYN,EPHA7,EPHB1,CDKL5,NTRK3,DNM3,SYNDIG1,PTPRD*
GO:0022604	regulation of cell morphogenesis	7.465126352460029e-7	*KALRN,SHROOM3,NEDD9,IL1RAPL1,MYO9A,KANK1,RELN,DLC1,SEMA4D,ARHGAP15,PARK2,PALMD,ARHGEF18,DNMBP,CUX1,ACTN4,EPS8,PALM2,EFNA5,NTNG1,SYT1,DAB2,PLXNA2,RIMS2,FMNL2,CD44,FBXO31,MAP3K13,CDC42EP3,STRIP1,GRIP1,PARVB,UNC13A,TRIOBP,RHOJ,CFDP1,PREX1,MACF1,FGD4,FAM171A1,PAK3,FYN,RIMS1,ENPP2,DOCK1,CDKL5,MYH9,SH3KBP1,ZRANB1,PTPRD,SYT17*
GO:0051049	regulation of transport	8.901054324934562e-7	*KALRN,CACNG2,CLASP1,DMD,KCNC2,CHRM3,NOS1,TMC2,DPP10,ATP9A,ATP8A2,WLS,CACNA1A,PRKD1,RYR2,CASK,BTBD9,IL1RAPL1,ABCA13,TBC1D5,NLGN1,KCNIP4,CAMK1D,TCF7L2,RELN,APP,UTRN,PPP3CA,AKAP6,GRM7,MEF2A,KCNB2,MAGI2,KCNA6,BDKRB1,TF,CHRM1,JAK2,ABCG8,PARK2,ADCYAP1R1,DPP6,GRIN3A,CACNA1C,NUMB,CD84,KCNJ16,SMAD3,CACNA1E,PIK3C3,TUB,SCAMP5,FGF14,CACNA2D3,DGKI,SLC1A1,SHISA9,EPM2A,MAP2,NOS1AP,KCTD7,CATSPER2,ANK3,CNIH3,SCN9A,ACTN4,CD300A,RABGEF1,LRP5,AP2B1,SCFD1,PDE4D,FRMD4A,EFNA5,KCNJ12,PIK3R2,CYBB,KCNJ3,ADTRP,PDE4B,ANKRD13A,KCND2,SPTBN4,EPHB2,MCTP1,DOCK2,NUP214,RBM4,ADORA2A,ANO1,CADPS,SYT1,DAB2,NSF,STXBP6,CACNG3,PRKAR1B,TTN,MYRIP,ENPP1,RIMS2,SCN8A,NRG1,RIT2,KCNMA1,PRKCG,SLC8A1,AGT,KCNQ5,KCNJ6,NF1,NRXN1,GSK3B,PKP2,KCNH1,CHD7,SNX5,CNTN1,CACNB2,STXBP4,GPC3,KCNS3,PTGER3,CAPN3,MYOM1,CTDSPL2,HTR2C,EPHA5,SGIP1,ANK2,ADCY8,CACNA1D,GRM5,SUFU,NKAIN2,INSR,MAP2K1,RASGRF2,LRRK2,NKAIN3,SCN1A,BMP6,FYN,KCND3,PTPN14,RIMS1,MCTP2,ABCA12,KCNQ1,C2,FER,NRXN3,KCNH7,SETD2,PACSIN2,CDH13,TRDN,SEC16B,STIM1,TENM1,RGS7,STXBP5,CLASP2,DNAJC6,NSUN2,DNM3,PPARA,SHISA6,ATP8A1,KCNC4,SYT17,STAC,RAB27A,KCNJ15,SYT9*
GO:0007626	locomotory behavior	0.000001017640304598238	*RCAN1,KALRN,DSCAM,PUM1,BTBD9,RELN,APP,PCDH15,ALK,CELSR1,NEGR1,PARK2,ANKH,SLC1A1,TNR,EPS8,NAV2,GRM1,KLHL1,KCND2,SPTBN4,ADORA2A,CDH23,NRG1,CHD7,SHANK2,ASTN1,HTR2C,ADCY8,ANKFN1,GRM5,LRRK2,SPG11,SCN1A,CLN6,NCOA2,SOBP,DAB1,ELAVL4*
GO:1901888	regulation of cell junction assembly	0.000001017640304598238	*NTRK2,CLASP1,LINGO2,VCL,IL1RAPL1,NLGN1,APP,DLC1,ROBO2,SEMA4D,NEGR1,SMAD3,PEAK1,IL1RAPL2,NTN1,FLRT2,ARHGAP6,CLSTN2,EFNA5,GRID2,CLDN1,EPHB2,BDNF,AGT,NRXN1,DLG5,MACF1,DUSP22,CNTNAP2,GPC6,LRFN5,EPHA7,EPHB1,RAPGEF2,NTRK3,CLASP2,SYNDIG1,PTPRD,PTPRA*
GO:0009888	tissue development	0.0000010238335558079029	*RCAN1,CLASP1,ZNRF3,FBXL17,DMD,SPRED2,KAZN,ERBB4,LAMA2,ROBO1,PRICKLE2,SHROOM3,NEBL,HDAC4,WLS,LDLRAD4,PSMB2,RXFP1,PLCB1,RYR2,VCL,SEMA5A,MYO9A,KIF26*

			B,RUNX1,PLS1,TCF7L2,PAK1,PPP3CA,RYR1,TOX,PCDH15,GREB1L,AKAP6,DLC1,SOS1,BTRC,MEF2A,MAGI2,EDA,CELSR1,ROBO2,AKAP13,HIVEP3,EP300,SEMA4D,EVC,ANKRD6,ASTN2,JAK2,NPHP3,EXOC4,ANKH,SOX6,SULF1,SH3BP1,ATRNL1,ARHGAP24,COL11A1,SLIT2,ATRX,RAD51B,BMPR1A,SMAD3,SCUBE1,EYA1,SATB2,ELN,EGFLAM,OVOL2,ASGR2,SOX5,NTN1,LDB2,CDC73,WWTR1,ARID4B,TRPS1,MYO18B,MDM4,TENM4,CECR2,GHR,CBFA2T2,STRC,LRP5,ESR1,ARHGAP12,TNFRSF19,PDE4D,PIK3CD,CERS3,FTO,ATF2,SEMA6D,NTNG1,CLDN1,SLC24A3,S1PR3,COL22A1,MARK1,ARID5B,IFT80,FHL2,NOX4,MEGF9,CSMD1,BCOR,BMPER,DAB2,PLXNA2,ABI1,HDAC2,ARID4A,CDH23,TTN,ENPP1,NRG1,SPINT2,CD44,SLC8A1,CHST11,THRB,AGT,FGF1,USH2A,NF1,LAMA3,GSK3B,PKP2,CTDP1,CD109,CHD7,PSMB7,TRIOBP,PHEX,GPC3,DLG5,PGM5,ROR1,COL12A1,TP73,MATN3,MLLT3,NEB,SEMA5B,NLN,PTPRO,SUFU,INSR,MAP2K1,GPC6,KLHL3,CENPF,RBFOX1,ADAMTS16,PKHD1,RARB,PBX1,BMP6,EPHA7,COL19A1,SGCZ,ABCA12,EPHB1,KCNQ1,FHOD3,EXT1,UNC5C,CSGALNACT1,LRP2,SGCD,RAPGEF2,SETD2,BASP1,SEMA3A,STIM1,PRKAR1A,SIPA1L3,SCEL,HYDIN,CLASP2,NSUN2,PPARA,MYH15,NELL1,FNDC3A,SEMA3D,PRLR,SEMA3C
GO:0006793	phosphorus metabolic process	0.000001108861804959673	*PTPRR,CAMTA1,RCAN1,KALRN,RPS6KA2,NTRK2,PTPRG,SLC39A10,DMD,SPRED2,PHKB,ERBB4,NOS1,ROBO1,MYO3A,DSCAM,CIT,HUNK,FUT8,HDAC4,LDLRAD4,BLM,SLC44A1,PTPRT,PLCB1,FLT3,SRPK2,PRKD1,ADK,NEDD9,PPP2R3C,CASK,PRKCA,ZBTB20,SLCO3A1,TNFRSF10B,CAMK1D,SMG6,RELN,APP,PAK1,PPP3CA,PTPN9,DLC1,ALK,BTRC,DCLK1,DLG2,MAP2K5,MAGI2,AKAP13,EP300,SEMA4D,RPS6KA5,GRK5,BDKRB1,TF,HUS1,JAK2,FPGT-TNNI3K,PPP6R2,MAST4,PARK2,ADCYAP1R1,CKMT1B,PNPLA3,PIK3C2B,UPB1,NRG3,ADCY2,SH3GL2,SLIT2,MAPK4,SLC44,BMPR1A,ACACA,SMAD3,PRKAR2A,BORA,PI4KA,PIBF1,PIK3C3,PEAK1,EYA1,DGKB,DPYS,TNKS,LINC00473,SGMS1,DGKK,PTPRN2,DGKI,TAF1,SLC1A1,LDB2,EPM2A,FBXW11,WWTR1,PEMT,DOCK3,ADCY9,VAV3,CDKL1,PLCE1,GHR,CD300A,OSBPL10,RABGEF1,FHIT,LRP5,PDE4D,PIK3CD,PDE7B,EFNA5,ATF2,PIK3R2,ADTRP,PDE4B,SMG7,PPA2,MAP3K7,PRKCQ,IMPDH1,SPTBN4,EPHB2,ERC1,MOB3B,PTPDC1,MARK1,BDNF,FNIP1,MAGI3,ADORA2A,NOX4,CHKA,BMPER,ICK,DAB2,LRRK1,PRKG1,GALK2,ABI1,TNNI3K,HDAC2,PRKAR1B,TTN,CDK19,ENPP1,KSR2,PARD3,NRG1,TOP1,RIT2,CD44,PRKCG,TNS3,MAPKAPK2,SLC8A1,MAP3K13,PLCL2,RSRC1,CDC42BPA,MAPK10,AGT,FGF1,DYRK4,TLK1,NF1,SLC44A5,NRXN1,PDXP,GSK3B,CTDP1,CD109,CNTN1,CHEK2,PHEX,STK32B,ROR1,ZNF675,SLC5A8,MNAT1,TRIO,PTPRE,MYO3B,DUSP22,MAP3K5,CTDSPL2,HTR2C,EPHA5,MORC3,PAK3,ADCY8,PRKAA2,NME7,GRM5,IGF1R,FAR1,PTPRO,INSR,JAK1,MAP2K1,GMDS,STK38,INPP5A,TTC7B,PPP1R14A,CDC14A,ULK4,LRRK2,BMP6,FYN,EPHA7,PTPN14,ENPP2,EPHB1,BPGM,FAM126A,TMEM68,TRPC5,ACSBG1,CSGALNACT1,FER,CAMK4,GUCY2F,TPTE,RAPGEF2,PRPSAP2,PTPRM,AK5,CDKL5,INPP4A,PTPRU,ADPGK,DAB1,MGAT5,BDKRB2,CCNG2,PIK3R3,TENM1,STK38L,SH3KBP1,PRKAR1A,NTRK3,RBKS,DNAJC6,PTPRK,PPARA,PTPRD,RORA,TPK1,PRLR,PTPRA,DEPTOR,PMEPA1*
GO:0034765	regulation of ion transmembrane transport	0.000001110243143885119	*CACNG2,DMD,KCNC2,CHRM3,NOS1,TMC2,DPP10,CACNA1A,PRKD1,RYR2,NLGN1,KCNIP4,RELN,APP,UTRN,AKAP6,KCNB2,KCNA6,BDKRB1,DPP6,CACNA1C,KCNJ16,CACNA1E,FGF14,CACNA2D3,SHISA9,NOS1AP,CATSPER2,ANK3,CNIH3,SCN9A,ACTN4,PDE4D,KCNJ12,CYBB,KCNJ3,PDE4B,KCND2,EPHB2,CACNG3,SCN8A,KCNMA1,SLC8A1,AGT,KCNQ5,KCNJ6,NRXN1,KCNH1,CHD7,CACNB2,KCNS3,CAPN3,ANK2,CACNA1D,GRM5,RASGRF2,SCN1A,FYN,KCND3,KCNQ1,KCNH7,TRDN,STIM1,RGS7,SHISA6,KCNC4,STAC,KCNJ15*
GO:0030036	actin cytoskeleton organization	0.0000011241071495772008	*CTNNA1,CLASP1,DMD,SHROOM3,CIT,NEBL,AUTS2,NEDD9,SEMA5A,EPB41L4B,CALD1,KANK1,DIAPH2,PLS1,FCHSD2,AMOTL1,PAK1,UTRN,PCDH15,DLC1,MEF2A,CTNNA3,CELSR1,AKAP13,ELMO2,TF,JAK2,PARK2,SH3BP1,SLIT2,SMAD3,ELN,ARHGEF18,DNAJB6,NOS1AP,GAS7,ARHGAP6,ACTN4,EPS8,PPP1R9A,ARHGAP12,EFNA5,KLHL1,PHACTR2,RHPN2,SPTBN4,DOCK2,THSD7B,PRKG1,ABI1,MICAL3,TTN,CTNNA2,FMNL2,FMN2,CDC42BPA,CDC42EP3,STRIP1,ARHGAP25,NF1,PARVB,PDXP,SETD3,THSD7A,TRIOBP,RHOJ,PGM5,PREX1,CAPN3,FGD4,EPHA5,FAM171A1,MYPN,PAK3,NEB,SPECC1L,FRMD5,P*

			HACTR1,FRMPD4,FHOD3,FER,SHROOM4,MPRIP,PACSIN2,MYH9,TENM1,SH3KBP1,PRKAR1A,NTRK3,CLASP2
GO:0042330	taxis	0.000001131038380730454	*KALRN,LAMA2,CNTN4,ROBO1,DSCAM,ABCC1,NFASC,PRKD1,SEMA5A,CAMK1D,RELN,APP,SOS1,ROBO2,DCC,ELMO2,EP300,SEMA4D,RPS6KA5,DEFA1B,CMKLR1,SMOC2,EMB,NRG3,SLIT2,DSCAML1,SMAD3,NTN1,FLRT2,CCL15,CDH4,TNR,VAV3,CCL14,PIK3CD,DOCK4,EFNA5,SEMA6D,CRMP1,PDE4B,PRKCQ,EPHB2,PRTG,DOCK2,DPYSL2,BDNF,PLXNA2,NRG1,FAM19A4,NCAM1,FGF1,CCR3,NRXN1,LAMA3,UNC5D,PREX1,ADAMTSL1,TRIO,CCDC141,EPHA5,MYPN,SLIT3,CCL15-CCL14,SEMA5B,PTPRO,MAP2K1,CNTN6,FYN,EPHA7,ENPP2,EPHB1,EXT1,UNC5C,FER,PTPRM,NRXN3,CDH13,SEMA3A,NTRK3,SEMA3D,SEMA3C*
GO:0007507	heart development	0.000001203095883187068	*RPS6KA2,ERBB4,TFDP2,ROBO1,NEBL,RYR2,ADAMTS6,RUNX1,RYR1,GREB1L,AKAP6,DLC1,SOS1,MEF2A,MAP2K5,ROBO2,AKAP13,EP300,DNAH11,NPHP3,CACNA1C,SOX6,COL11A1,SLIT2,TRAF3IP2,BMPR1A,SMAD3,SCUBE1,EYA1,ELN,OVOL2,FLRT2,PCSK5,MYO18B,MDM4,PLCE1,TENM4,AP2B1,ATF2,FHL2,NOX4,BCOR,CALCRL,TTN,NRG1,SLC8A1,AGT,NF1,PKP2,CTDP1,CHD7,GPC3,CPE,MNAT1,TP73,ANK2,SLIT3,NEB,IGF1R,SUFU,INSR,MAP2K1,RARB,SGCZ,KCNQ1,FHOD3,EXT1,LRP2,SGCD,SETD2,BASP1,PRKAR1A,NTRK3,FBN1,PPARA,BICC1,SEMA3C*
GO:0048638	regulation of developmental growth	0.00000127904878657 98075	*ERBB4,DSCAM,ATP8A2,PLCB1,SEMA5A,RUNX1,PLS1,APP,AKAP6,DCC,SEMA4D,PARK2,BMPR1A,NTN1,MAP2,CDH4,TNR,GHR,DISC1,EFNA5,FTO,SEMA6D,SPTBN4,DPYSL2,BDNF,FSTL4,SYT1,RIMS2,NRG1,MAP3K13,GSK3B,CTDP1,GPR21,CHD7,UNC13A,ISLR2,MACF1,CAPN3,TP73,SEMA5B,INSR,EPHA7,RIMS1,TRPC5,CDKL5,BASP1,SEMA3A,CLASP2,PPARA,SEMA3D,SYT17,SEMA3C*
GO:0006468	protein phosphorylation	0.00000158271804114 2778	*KALRN,RPS6KA2,NTRK2,DMD,SPRED2,PHKB,ERBB4,NOS1,ROBO1,MYO3A,CIT,HUNK,LDLRAD4,BLM,PTPRT,FLT3,SRPK2,PRKD1,NEDD9,PPP2R3C,CASK,PRKCA,SLCO3A1,TNFRSF10B,CAMK1D,RELN,APP,PAK1,ALK,DCLK1,MAP2K5,AKAP13,SEMA4D,RPS6KA5,GRK5,BDKRB1,HUS1,JAK2,FPGT-TNNI3K,MAST4,PARK2,NRG3,SH3GL2,SLIT2,MAPK4,BMPR1A,PRKAR2A,BORA,PIBF1,PIK3C3,PEAK1,TNKS,TAF1,SLC1A1,EPM2A,WWTR1,DOCK3,CDKL1,PLCE1,GHR,CD300A,RABGEF1,LRP5,PDE4D,PIK3CD,EFNA5,ATF2,ADTRP,MAP3K7,PRKCQ,SPTBN4,EPHB2,ERC1,MOB3B,MARK1,BDNF,FNIP1,ADORA2A,NOX4,BMPER,ICK,DAB2,LRRK1,PRKG1,ABI1,TNNI3K,HDAC2,PRKAR1B,TTN,CDK19,ENPP1,KSR2,PARD3,NRG1,TOP1,RIT2,CD44,PRKCG,MAPKAPK2,SLC8A1,MAP3K13,PLCL2,RSRC1,CDC42BPA,MAPK10,AGT,FGF1,DYRK4,TLK1,NF1,NRXN1,GSK3B,CD109,CNTN1,CHEK2,STK32B,ROR1,ZNF675,MNAT1,TRIO,MYO3B,DUSP22,MAP3K5,EPHA5,MORC3,PAK3,ADCY8,PRKAA2,GRM5,IGF1R,PTPRO,INSR,JAK1,MAP2K1,STK38,ULK4,LRRK2,BMP6,FYN,EPHA7,ENPP2,EPHB1,TRPC5,FER,CAMK4,GUCY2F,RAPGEF2,CDKL5,DAB1,BDKRB2,CCNG2,PIK3R3,TENM1,STK38L,PRKAR1A,NTRK3,PRLR,PTPRA,DEPTOR,PMEPA1*
GO:0006935	chemotaxis	0.00000192453227683 04965	*KALRN,LAMA2,CNTN4,ROBO1,DSCAM,ABCC1,NFASC,PRKD1,SEMA5A,CAMK1D,RELN,APP,SOS1,ROBO2,DCC,ELMO2,SEMA4D,RPS6KA5,DEFA1B,CMKLR1,SMOC2,EMB,NRG3,SLIT2,DSCAML1,SMAD3,NTN1,FLRT2,CCL15,CDH4,TNR,VAV3,CCL14,PIK3CD,DOCK4,EFNA5,SEMA6D,CRMP1,PDE4B,PRKCQ,EPHB2,PRTG,DOCK2,DPYSL2,BDNF,PLXNA2,NRG1,FAM19A4,NCAM1,FGF1,CCR3,NRXN1,LAMA3,UNC5D,PREX1,ADAMTSL1,TRIO,CCDC141,EPHA5,MYPN,SLIT3,CCL15-CCL14,SEMA5B,PTPRO,MAP2K1,CNTN6,FYN,EPHA7,ENPP2,EPHB1,EXT1,UNC5C,FER,PTPRM,NRXN3,CDH13,SEMA3A,NTRK3,SEMA3D,SEMA3C*
GO:0001764	neuron migration	0.00000254174226654 18685	*NDE1,NTRK2,AUTS2,RELN,DCLK1,CELSR1,DCC,ASTN2,NRG3,SATB2,ELP3,NTN1,FLRT2,SPOCK1,DISC1,CEP85L,NTNG1,MARK1,PRKG1,FAT3,CTNNA2,NRG1,FBXO31,UNC5D,ASTN1,ULK4,PHACTR1,FYN,RAPGEF2,KIRREL3,CDKL5,DAB1,SEMA3A,NTRK3*
GO:0051130	positive regulation of cellular component	0.00000264422448645 12887	*KALRN,NTRK2,CLASP1,DMD,ROBO1,DSCAM,HDAC4,LINGO2,ATP8A2,AUTS2,TENM2,ATF7IP,PLCB1,PRKD1,SEMA5A,IL1RAPL1,PDE4DIP,ABCA13,TBC1D5,RUNX1,FCHSD2,NLGN1,CAMK1D,RELN,APP,PAK1,PPP3CA,TOX,ALK,DNMT1,MAGI2,ROBO2,EP300,PARN,SEMA4D,TF,NEGR1,PARK2,SLIT2,ATRX,SLX1B,SMAD3,MAD1L1,TNKS,SKAP1,NTN1,FLRT2,CUX1,MOR*

	organization		*C2,CDH4,PLCE1,EPS8,CLSTN2,CBFA2T2,DISC1,TRABD2B,LRP5,AP2B1,ESR1,EFNA5,GRID2,MAPRE2,PRKCQ,CLDN1,EPHB2,BDNF,FNIP1,SYT1,DAB2,PLXNA2,NRG1,RIT2,GPSM2,FBXO31,MAP3K13,CDC42EP3,AGT,GRIP1,NRXN1,PDXP,GSK3B,TIAM2,CNTN1,ISLR2,GPC3,DLG5,ROR1,BID,MACF1,SGIP1,PAK3,IGF1R,CNTNAP2,INSR,MAP2K1,LRRK2,ASAP1,FRMPD4,FYN,ENPP2,EPHB1,TRPC5,TENM3,FER,RAPGEF2,NAV3,CDKL5,TENM1,NTRK3,CLASP2,DNM3,SYNDIG1,PTPRD,ELAVL4,ATP8A1,SYT9*
GO:1990138	neuron projection extension	0.0000028849691265730546	*DSCAM,AUTS2,VCL,SEMA5A,DCLK1,SEMA4D,PARK2,SH3GL2,SLIT2,NTN1,MAP2,CDH4,TNR,DISC1,SEMA6D,DPYSL2,SYT1,RIMS2,MAP3K13,GSK3B,UNC13A,ISLR2,MACF1,SLIT3,SEMA5B,SPG11,RIMS1,TRPC5,TMEM108,CDKL5,SEMA3A,CLASP2,SEMA3D,SYT17,SEMA3C*
GO:0051641	cellular localization	0.0000032033576552889889	*SYN3,CACNG2,PROS1,NDE1,CTNNA1,CLASP1,SYNE1,DMD,ERBB4,NOS1,SIL1,DPP10,GPC5,SORCS2,ATP9A,ARFGAP3,IMMP2L,AFTPH,WLS,SGSM1,SNUPN,NFASC,TRPM1,PRKD1,RYR2,VCL,CASK,STX8,BTBD9,ABCA13,TBC1D5,PLS1,NLGN1,KCNIP4,SMG6,CLEC16A,TCF7L2,RELN,APP,PPP3CA,RYR1,ERC2,PTPN9,AKAP6,RABGAP1L,RYR3,RFTN1,DCLK1,MEF2A,KCNB2,FAM155A,DENND5A,DLG2,MAGI2,CELSR1,IFT81,AKAP13,BDKRB1,DNAH11,GRIK2,CHRM1,SYTL5,ZDHHC11B,ITSN1,ASTN2,TANC2,JAK2,RAB11FIP4,PARK2,ADCYAP1R1,DPP6,GRIN3A,CACNA1C,MAN1A1,NLGN4X,EXOC4,HEATR5A,NUMB,CD84,ATRX,TRAF3IP2,SMAD3,PIBF1,PIK3C3,TPH1,MAD1L1,TNKS,TUB,DNAJB6,ZDHHC11,SKAP1,PTPRN2,DGKI,SLC1A1,NTN1,EPM2A,SHFM1,MAP2,FBXW11,NOS1AP,WWTR1,GRM4,ANK3,ACTN4,RAB2A,CD300A,DISC1,IFT43,RABGEF1,NSG2,LRP5,AP2B1,SLC30A7,ESR1,DENND2A,SCFD1,PDE4D,THEM4,PIK3CD,SLC5A3,BLOC1S5,RAB24,EFNA5,ATF2,PIK3R2,CCDC91,GRID2,ZNF423,KCNJ3,MAPRE2,SMG7,ANKRD13A,ATXN1,WDR83OS,SPTBN4,EPHB2,ERC1,MCTP1,DOCK2,NUP214,MARK1,BDNF,RBM4,ADORA2A,IFT80,ANO1,CADPS,TSPAN33,ICK,SYT1,DAB2,NSF,STXBP6,SNAP23,CACNG3,EPG5,RANBP17,BANP,PRKAR1B,MYRIP,RIMS2,PEX5L,PARD3,NRG1,TBC1D9,FMN2,RIT2,CNGB1,GPSM2,PRKCG,SLC8A1,RAPGEF6,RSRC1,SAMM50,AGT,GRIP1,TLK1,NF1,VPS16,NRXN1,GSK3B,PKP2,SLC22A3,BBS9,CHD7,SNX5,UNC13A,CACNB2,STXBP4,ESYT2,SYBU,GPC3,CPE,BID,MACF1,CAPN3,CTDSPL2,HTR2C,SNAP25-AS1,EPHA5,SNX31,MORC3,ANK2,GRIN2B,LRPPRC,ADCY8,ITPR2,CACNA1D,PACRG,ANKFN1,TRAPPC8,TSNARE1,MON2,CNTNAP2,ZDHHC14,SUFU,CIZ1,RPGR,MAP2K1,KPNB1,GPC6,TTC7B,CENPF,AGBL4,KPNA3,LRRK2,PKHD1,SPG11,VTI1A,FYN,KCND3,XRCC4,GAS8,PTPN14,RIMS1,VPS53,MCTP2,ABCA12,KCNQ1,FAM126A,NOX5,FER,PCDH17,TMEM108,RAPGEF2,NRXN3,PTPRU,KAT7,VPS45,SETD2,PACSIN2,CDH13,TRDN,DAB1,SEC16B,EXOC6B,MYH9,VPS41,TENM1,VPS39,STXBP5,SLC24A2,CLASP2,DNAJC6,NSUN2,PTPRK,DNM3,SYNDIG1,SCFD2,SHISA6,ANXA8L1,IPO11,NVL,STAC,RAB27A,BLOC1S6,PARD3B,HGSNAT,SYT9*
GO:0040013	negative regulation of locomotion	0.0000033620606219298 95	*PTPRR,CTNNA1,CLASP1,PTPRG,ROBO1,LDLRAD4,PTPRT,PLCB1,VCL,SEMA5A,KANK1,DLC1,MAP2K5,MAGI2,ROBO2,SEMA4D,NRG3,SULF1,SLIT2,BMPR1A,CD300A,RABGEF1,SEMA6D,ADTRP,MCTP1,DACH1,ADORA2A,PRKG1,SRGAP3,NRG1,SPINT2,NF1,SRGAP2B,DLG5,DUSP22,SEMA5B,GRM5,PTPRO,FOXO3,MITF,FRMD5,MARVELD3,NAV3,PTPRM,PTPRU,HDAC5,SEMA3A,CLASP2,PTPRK,SEMA3D,SEMA3C*
GO:0006796	phosphate-containing compound metabolic process	0.0000034928817386548 47	*PTPRR,CAMTA1,RCAN1,KALRN,RPS6KA2,NTRK2,PTPRG,SLC39A10,DMD,SPRED2,PHKB,ERBB4,NOS1,ROBO1,MYO3A,DSCAM,CIT,HUNK,HDAC4,LDLRAD4,BLM,SLC44A1,PTPRT,PLCB1,FLT3,SRPK2,PRKD1,ADK,NEDD9,PPP2R3C,CASK,PRKCA,ZBTB20,SLCO3A1,TNFRSF10B,CAMK1D,SMG6,RELN,APP,PAK1,PPP3CA,PTPN9,DLC1,ALK,BTRC,DCLK1,DLG2,MAP2K5,MAGI2,AKAP13,EP300,SEMA4D,RPS6KA5,GRK5,BDKRB1,TF,HUS1,JAK2,FPGT-TNNI3K,PPP6R2,MAST4,PARK2,ADCYAP1R1,CKMT1B,PNPLA3,PIK3C2B,UPB1,NRG3,ADCY2,SH3GL2,SLIT2,MAPK4,SLC4A4,BMPR1A,ACACA,SMAD3,PRKAR2A,BORA,PI4KA,PIBF1,PIK3C3,PEAK1,EYA1,DGKB,DPYS,TNKS,LINC00473,SGMS1,DGKK,PTPRN2,DGKI,TAF1,SLC1A1,LDB2,EPM2A,FBXW11,WWTR1,PEMT,DOCK3,ADCY9,VAV3,CDKL1,PLCE1,GHR,CD300A,OSBPL10,RABGEF1,FHIT,LRP5,PDE4D,PIK3CD,PDE7B,EFNA5,ATF2,PIK3R2,ADTRP,PDE4B,SMG7,PPA2,MAP3K7,PRKCQ,IM*

			PDH1,SPTBN4,EPHB2,ERC1,MOB3B,PTPDC1,MARK1,BDNF,FNIP1,MAGI3,ADORA2A,NOX4,CHKA,BMPER,ICK,DAB2,LRRK1,PRKG1,GALK2,ABI1,TNNI3K,HDAC2,PRKAR1B,TTN,CDK19,ENPP1,KSR2,PARD3,NRG1,TOP1,RIT2,CD44,PRKCG,TNS3,MAPKAPK2,SLC8A1,MAP3K13,PLCL2,RSRC1,CDC42BPA,MAPK10,AGT,FGF1,DYRK4,TLK1,NF1,SLC44A5,NRXN1,PDXP,GSK3B,CTDP1,CD109,CNTN1,CHEK2,STK32B,ROR1,ZNF675,SLC5A8,MNAT1,TRIO,PTPRE,MYO3B,DUSP22,MAP3K5,CTDSPL2,HTR2C,EPHA5,MORC3,PAK3,ADCY8,PRKAA2,NME7,GRM5,IGF1R,FAR1,PTPRO,INSR,JAK1,MAP2K1,STK38,INPP5A,TTC7B,PPP1R14A,CDC14A,ULK4,LRRK2,BMP6,FYN,EPHA7,PTPN14,ENPP2,EPHB1,BPGM,FAM126A,TMEM68,TRPC5,ACSBG1,FER,CAMK4,GUCY2F,TPTE,RAPGEF2,PRPSAP2,PTPRM,AK5,CDKL5,INPP4A,PTPRU,ADPGK,DAB1,MGAT5,BDKRB2,CCNG2,PIK3R3,TENM1,STK38L,SH3KBP1,PRKAR1A,NTRK3,RBKS,DNAJC6,PTPRK,PPARA,PTPRD,RORA,TPK1,PRLR,PTPRA,DEPTOR,PMEPA1
GO:0048813	dendrite morphogenesis	0.000003695176890072222	*KALRN,DSCAM,IL1RAPL1,NLGN1,RELN,PPP3CA,CTNND2,DCLK1,MEF2A,SEMA4D,TANC2,MAP2,CUX1,EPHB2,ABI1,CTNNA2,FBXO31,GSK3B,PAK3,LRRK2,PHACTR1,FYN,EPHB1,TRPC5,RAPGEF2,CDKL5,SEMA3A,DNM3,PTPRD,ELAVL4,DOCK10*
GO:0050919	negative chemotaxis	0.000003865144005096112	*ROBO1,SEMA5A,ROBO2,SEMA4D,NRG3,SLIT2,NTN1,FLRT2,SEMA6D,NRG1,SLIT3,SEMA5B,EPHA7,UNC5C,SEMA3A,SEMA3D,SEMA3C*
GO:0048585	negative regulation of response to stimulus	0.000004489614104293961	*PTPRR,RCAN1,RNF43,PROS1,CTNNA1,CLASP1,ZNRF3,DMD,SPRED2,ROBO1,CIT,WWOX,LDLRAD4,PSMB2,PTPRT,SEMA5A,CASK,CCDC3,KANK1,TCF7L2,DEPDC5,DLC1,BTRC,MAP2K5,MAGI2,ROBO2,SEMA4D,ANKRD6,PARK2,NPHP3,GRIN3A,NLGN4X,SULF1,CD84,SH3BP1,RFFL,ARHGAP24,SLIT2,CHRDL1,SMAD3,PIBF1,EYA1,MAD1L1,LINC00473,OVOL2,GRAMD4,FBXW11,CD96,RBMS3,WWTR1,TRIM59,TNR,CELF4,CD300A,CBFA2T2,MLIP,ARHGAP42,RABGEF1,TRABD2B,ESR1,ARHGAP12,PDE4D,EIF3A,PIK3R2,GRID2,SEMA6D,SUSD4,ADTRP,PDE4B,PRKCQ,EPHB2,MCTP1,BDNF,FNIP1,ADORA2A,IFT80,FSTL4,FHL2,BMPER,DAB2,PRKG1,CALCRL,PDE11A,ZMYND11,RGS6,ENPP1,PRDM15,CTNNA2,NRG1,CD44,PLCL2,CHST11,AGT,NDRG2,ARHGAP25,NF1,WIF1,ZNF536,NRXN1,GSK3B,CD109,GPR21,PSMB7,SNX5,CHEK2,MECOM,SHANK2,GPC3,DLG5,ZNF675,BID,PTPRE,DUSP22,CTDSPL2,IL1RL1,ZNF366,SLIT3,UACA,ADCY8,MLLT3,RNF213,SH3RF2,PRKAA2,SEMA5B,GRM5,IGF1R,PTPRO,SUFU,UBR2,FOXO3,STK38,LRFN5,PRDM16,KIR2DL4,LTBP1,LRRK2,PKHD1,FYN,MARVELD3,AMFR,LRP2,FER,VWC2,PTPRU,RGS7BP,VEPH1,DAB1,SEMA3A,TSPAN8,BDKRB2,LITAF,RGS7,FBN1,CLASP2,PPARA,PTPRD,RORA,SHISA6,BICC1,SEMA3D,DEPTOR,PMEPA1,SEMA3C*
GO:0010977	negative regulation of neuron projection development	0.000005228516271026542	*PTPRG,SEMA5A,KANK1,NLGN1,PTPN9,DENND5A,DCC,SEMA4D,NTN1,MAP2,TNR,SPOCK1,CBFA2T2,SEMA6D,CRMP1,EPHB2,FSTL4,DAB2,HDAC2,FAT3,RIT2,SEMA5B,PTPRO,LRRK2,EPHA7,DAB1,SEMA3A,DNM3,SEMA3D,SEMA3C*
GO:0071495	cellular response to endogenous stimulus	0.000005304772065810202	*CTNNA1,NTRK2,CHRM3,SPRED2,ERBB4,ESRRG,WWOX,FUT8,HDAC4,ABCC1,LDLRAD4,BLM,RXFP1,PLCB1,FLT3,CACNA1A,RYR2,KANK1,APP,PAK1,RYR1,AKAP6,IDE,RYR3,ALK,DNMT1,MAGI2,ROBO2,EP300,CHRM1,DEFA1B,JAK2,PARK2,SMOC2,PNPLA3,SOX6,COL4A6,SULF1,SH3GL2,SLIT2,GLP2R,CHRDL1,BMPR1A,ACACA,SMAD3,PTGFR,OVOL2,SOX5,TAF1,SLC1A1,FLRT2,GHR,NSG2,ESR1,PDE4D,EFNA5,ATF2,PIK3R2,ZNF423,CYBB,ADTRP,PDE4B,GABRB3,MAP3K7,PRKCQ,CLDN1,EPHB2,PRCP,RGMB,BDNF,ANO1,BMPER,DEFA3,DAB2,HDAC2,ENPP1,SPINT2,CD44,SLC8A1,CHST11,THRB,AGT,FGF1,NRXN1,PDXP,GSK3B,CD109,GPR21,SNX5,NCOA1,STXBP4,PHEX,NREP,GPC3,PTPRE,DUSP22,CTDSPL2,HTR2C,GLRA2,ZNF366,SLIT3,ADCY8,ITPR2,PRKAA2,GRM5,IGF1R,INSR,UBR2,FOXO3,PRDM16,LTBP1,LRRK2,RARB,BMP6,FYN,GNG2,KCNQ1,EXT1,LRP2,FER,VWC2,TMEM108,RAPGEF2,NCOA2,ESR2,VEPH1,HDAC5,PIK3R3,GABRB1,NTRK3,FBN1,PTPRK,PPARA,BRIP1,ELAVL4,PRLR,PTPRA,PMEPA1,RXFP2*
GO:0010648	negative regulation of cell communication	0.000005367425653729619	*PTPRR,RCAN1,KALRN,RNF43,CTNNA1,ZNRF3,DMD,SPRED2,ROBO1,CIT,SORCS2,WWOX,LDLRAD4,PSMB2,PTPRT,CCDC3,KANK1,TCF7L2,PPP3CA,DEPDC5,SORCS3,DLC1,BTRC,MAP2K5,MAGI2,ANKRD6,GRIK2,PARK2,NPHP3,NLGN4X,SULF1,SH3BP1,RFFL,ARHGAP24,SLIT2,CHRDL1,SMAD3,PIBF1,EYA1,M*

			AD1L1,LINC00473,OVOL2,GRAMD4,DGKI,FBXW11,RBMS3,WWTR1,TRIM59,TNR,CELF4,CD300A,CBFA2T2,ARHGAP42,RABGEF1,TRABD2B,ESR1,ARHGAP12,PDE4D,EIF3A,PIK3R2,GRID2,PDE4B,PRKCQ,EPHB2,BDNF,FNIP1,IFT80,FSTL4,FHL2,BMPER,DAB2,PDE11A,ZMYND11,RGS6,ENPP1,PRDM15,NRG1,CD44,PLCL2,CHST11,AGT,NDRG2,ARHGAP25,NF1,WIF1,ZNF536,GSK3B,CD109,GPR21,PSMB7,SNX5,CHEK2,GRIK3,MECOM,SHANK2,GPC3,DLG5,ZNF675,BID,PTPRE,DUSP22,CTDSPL2,ZNF366,SLIT3,UACA,ADCY8,MLLT3,RNF213,SH3RF2,PRKAA2,GRM5,IGF1R,PTPRO,SUFU,UBR2,FOXO3,STK38,PRDM16,LTBP1,LRRK2,PKHD1,MARVELD3,AMFR,LRP2,VWC2,PCDH17,PTPRU,RGS7BP,VEPH1,DAB1,BDKRB2,LITAF,RGS7,SLC24A2,FBN1,PPARA,PTPRD,RORA,SHISA6,BICC1,DEPTOR,PMEPA1
GO:0003012	muscle system process	0.000005624343376078194	*DMD,CHRM3,NOS1,HDAC4,ATP8A2,RYR2,PRKCA,CALD1,UTRN,PPP3CA,RYR1,AKAP6,SSPN,MEF2A,KCNB2,CTNNA3,PPP1R12B,AKAP13,CACNA1C,SULF1,SYNM,DTNA,SMAD3,NOS1AP,PLCE1,MLIP,ARHGAP42,PDE4D,DOCK4,KCNJ12,KCNJ3,PDE4B,SNTB1,MYBPC2,PRKG1,CALCRL,TNNI3K,HDAC2,TTN,MYOM3,KCNMA1,SLC8A1,AGT,PKP2,CTDP1,SETD3,CACNB2,PTGER3,MYOM1,P2RX6,ANK2,CACNA1D,FOXO3,SCN1A,KCND3,KCNQ1,SGCD,TRDN,BDKRB2,PPARA,STAC*
GO:0023057	negative regulation of signaling	0.000006217900831890311	*PTPRR,RCAN1,KALRN,RNF43,CTNNA1,ZNRF3,DMD,SPRED2,ROBO1,CIT,SORCS2,WWOX,LDLRAD4,PSMB2,PTPRT,CCDC3,KANK1,TCF7L2,PPP3CA,DEPDC5,SORCS3,DLC1,BTRC,MAP2K5,MAGI2,ANKRD6,GRIK2,PARK2,NPHP3,NLGN4X,SULF1,SH3BP1,RFFL,ARHGAP24,SLIT2,CHRDL1,SMAD3,PIBF1,EYA1,MAD1L1,LINC00473,OVOL2,GRAMD4,DGKI,FBXW11,RBMS3,WWTR1,TRIM59,TNR,CELF4,CD300A,CBFA2T2,ARHGAP42,RABGEF1,TRABD2B,ESR1,ARHGAP12,PDE4D,EIF3A,PIK3R2,GRID2,PDE4B,PRKCQ,EPHB2,BDNF,FNIP1,IFT80,FSTL4,FHL2,BMPER,DAB2,PDE11A,ZMYND11,RGS6,ENPP1,PRDM15,NRG1,CD44,PLCL2,CHST11,AGT,NDRG2,ARHGAP25,NF1,WIF1,ZNF536,GSK3B,CD109,GPR21,PSMB7,SNX5,CHEK2,GRIK3,MECOM,SHANK2,GPC3,DLG5,ZNF675,BID,PTPRE,DUSP22,CTDSPL2,ZNF366,SLIT3,UACA,ADCY8,MLLT3,RNF213,SH3RF2,PRKAA2,GRM5,IGF1R,PTPRO,SUFU,UBR2,FOXO3,STK38,PRDM16,LTBP1,LRRK2,PKHD1,MARVELD3,AMFR,LRP2,VWC2,PCDH17,PTPRU,RGS7BP,VEPH1,DAB1,BDKRB2,LITAF,RGS7,SLC24A2,FBN1,PPARA,PTPRD,RORA,SHISA6,BICC1,DEPTOR,PMEPA1*
GO:0050790	regulation of catalytic activity	0.000008108963368195502	*RCAN1,KALRN,PROS1,NTRK2,RASGEF1B,SLC39A10,SPRED2,ERBB4,NOS1,ROBO1,CIT,TBCD,FRY,PRIM2,ARFGAP3,SGSM1,BLM,PTPRT,RXFP1,PLCB1,FLT3,PRKD1,NEDD9,PPP2R3C,MYO9A,TBC1D5,TNFRSF10B,SMG6,RELN,APP,PAK1,DEPDC5,GRM7,RABGAP1L,DLC1,SOS1,ALK,BTRC,DENND5A,PPP1R12B,MAP2K5,MAGI2,AKAP13,PARN,SEMA4D,MMP16,ARHGAP15,ITSN1,JAK2,PPP6R2,PARK2,ADCYAP1R1,SERPINA4,CACNA1C,NRG3,SH3BP1,RFFL,ARHGAP24,SLIT2,BMPR1A,SMAD3,PRKAR2A,BORA,PIBF1,TNKS,ARHGEF18,GRAMD4,DNAJB6,ELP3,BCL2L13,ARRDC4,DGKI,DNMBP,SLC1A1,LDB2,EPM2A,NOS1AP,ARHGEF33,ARHGEF6,WWTR1,ARHGAP6,PCSK5,EGLN3,TIMP2,CCL15,DOCK3,VAV3,SPOCK1,PLCE1,GHR,ARHGAP39,RGL1,CD300A,RALGPS1,ARHGAP42,CCL14,RABGEF1,LRP5,ESR1,ARHGAP12,DENND2A,DOCK4,SLC5A3,EFNA5,PHACTR2,PIK3R2,MAPRE2,RIC8B,MAP3K7,PRKCQ,EPHB2,MOB3B,DOCK2,RFC3,CALML4,FNIP1,ADORA2A,SIPA1L2,RCAN2,NOX4,DAB2,PLXNA2,PRKG1,ABI1,PRKAR1B,TTN,RGS6,SRGAP3,AGAP1,NRG1,CAST,TBC1D9,SPINT2,CD44,NCF4,GPSM2,SLC8A1,RAPGEF6,MAP3K13,AGT,FGF1,ARHGAP25,NF1,NRXN1,ARHGAP10,GSK3B,CD109,TIAM2,CABIN1,GPC3,PREX1,ROR1,ZNF675,SERPINA5,BID,MNAT1,TRIO,CAPN3,DUSP22,MAP3K5,NCF2,FGD4,EPHA5,ARHGEF3,GRIN2B,GARNL3,UACA,ADCY8,SH3RF2,CACNA1D,GRM5,ARAP2,IGF1R,PTPRO,INSR,RPGR,MAP2K1,RASGRF2,STK38,PPP1R14A,LRRK2,PHACTR1,ASAP1,FYN,XRCC4,EPHA7,RIMS1,EPHB1,DOCK1,CRADD,RALGPS2,RAPGEF2,PRPSAP2,CDKL5,DAB1,MGAT5,STIM1,CCNG2,PIK3R3,TENM1,PRKAR1A,RGS7,STXBP5,SIPA1L3,NTRK3,DOCK9,SBF2,ANXA8L1,DOCK10,PRLR,DEPTOR,NVL,RALGAPA1,CST2,DENND2D,ST18,RXFP2*
GO:0060284	regulation of cell development	0.000008278150569994721	*KALRN,CTNNA1,NTRK2,ROBO1,DSCAM,HDAC4,PLCB1,VCL,NEDD9,SEMA5A,IL1RAPL1,KANK1,RELN,PPP3CA,ROBO2,DCC,SEMA4D,NUMB,SLIT2,BMPR1A,BRINP1,NTN1,MAP2,CUX1,*

			CDH4,TNR,ACTN4,TENM4,DISC1,EFNA5,SEMA6D,EPHB2,S1PR3,PRTG,BDNF,FSTL4,DAB2,PLXNA2,HDAC2,FBXO31,MAP3K13,NF1,GSK3B,CHD7,TIAM2,TRIOBP,ISLR2,PREX1,MACF1,TP73,PAK3,SEMA5B,GRM5,MAP2K1,LRRK2,EPHA7,DOCK1,TRPC5,LRP2,RAPGEF2,CDKL5,DAB1,SEMA3A,NTRK3,FBN1,PTPRD,SEMA3D,SEMA3C
GO:0048639	positive regulation of developmental growth	0.00000844295750432991	ERBB4,DSCAM,ATP8A2,PLCB1,SEMA5A,PLS1,AKAP6,SEMA4D,PARK2,BMPR1A,NTN1,CDH4,GHR,DISC1,EFNA5,SPTBN4,BDNF,SYT1,RIMS2,NRG1,MAP3K13,GPR21,CHD7,UNC13A,ISLR2,MACF1,CAPN3,INSR,RIMS1,TRPC5,CDKL5,BASP1,SYT17
GO:0050767	regulation of neurogenesis	0.00001048248922717083 1	KALRN,CTNNA1,NTRK2,ROBO1,DSCAM,SEMA5A,IL1RAPL1,RELN,PPP3CA,ROBO2,DCC,SEMA4D,NUMB,SLIT2,BMPR1A,BRINP1,NTN1,MAP2,CUX1,CDH4,TNR,TENM4,DISC1,EFNA5,SEMA6D,EPHB2,PRTG,BDNF,FSTL4,PLXNA2,HDAC2,FBXO31,MAP3K13,NF1,CHD7,TIAM2,ISLR2,MACF1,TP73,PAK3,SEMA5B,GRM5,MAP2K1,EPHA7,TRPC5,LRP2,RAPGEF2,CDKL5,DAB1,SEMA3A,NTRK3,PTPRD,SEMA3D,SEMA3C
GO:0040007	growth	0.00001064009072841949 8	DMD,ERBB4,DSCAM,ATP8A2,AUTS2,PLCB1,VCL,NEDD9,SEMA5A,KIF26B,RUNX1,PLS1,APP,PPP3CA,PCDH15,AKAP6,SOS1,RFTN1,DCLK1,MAP2K5,MAGI2,EYS,AKAP13,DCC,EP300,SEMA4D,EVC,BDKRB1,PARK2,NLGN4X,NRG3,SH3GL2,SLIT2,ATRX,RAD51B,BMPR1A,SMAD3,CPQ,NTN1,EPM2A,MAP2,CDC73,WWTR1,CDH4,TNR,SPOCK1,PLCE1,TENM4,GHR,SERTAD2,DISC1,ESR1,EFNA5,FTO,ATF2,SEMA6D,PRKCQ,SPTBN4,DPYSL2,BDNF,ARID5B,IFT80,FSTL4,SYT1,EBAG9,DAB2,ENPP1,RIMS2,NRG1,MAP3K13,CHST11,AGT,FGF1,GSK3B,CTDP1,GPR21,CHD7,UNC13A,ISLR2,GPC3,MACF1,CAPN3,TP73,SLIT3,SEMA5B,INSR,FOXO3,ENOX2,RARB,SPG11,EPHA7,RIMS1,EXT1,TRPC5,TMEM108,CDKL5,ESR2,BASP1,SEMA3A,LARGE,PRKAR1A,GNG4,CLASP2,PPARA,SEMA3D,PRLR,SYT17,SEMA3C
GO:0008038	neuron recognition	0.00001194315546574817 7	CNTN4,ROBO1,DSCAM,OPCML,SEMA5A,APP,NTM,ROBO2,EMB,DSCAML1,EPHB2,MYPN,CNTNAP2,CRTAC1,CNTN6,EXT1,SEMA3A
GO:0051963	regulation of synapse assembly	0.00001460109651889783	NTRK2,LINGO2,IL1RAPL1,NLGN1,APP,ROBO2,SEMA4D,NEGR1,IL1RAPL2,NTN1,FLRT2,CLSTN2,EFNA5,GRID2,EPHB2,BDNF,NRXN1,DLG5,GPC6,LRFN5,EPHA7,EPHB1,NTRK3,SYNDIG1,PTPRD
GO:0048518	positive regulation of biological process	0.00001511277598830105 3	RNF4,CAMTA1,CHFR,KALRN,CACNG2,RPS6KA2,CTNNA1,NTRK2,CLASP1,GLIS3,SLC39A10,DMD,KCNC2,CHRM3,EIF3E,SPRED2,ERBB4,LAMA2,TFDP2,NOS1,ESRRG,ROBO1,DSCAM,CIT,DPP10,PUM1,GPC5,WDR59,PRIM2,WWOX,TNRC6B,HDAC4,LINGO2,ABCC1,ATP8A2,AUTS2,WLS,TENM2,BLM,PSMB2,ATF7IP,ASXL3,PLCB1,FLT3,SRPK2,PRKD1,RYR2,NEDD9,PPP2R3C,SEMA5A,ETV6,CASK,PRKCA,EPB41L4B,ZBTB20,IL1RAPL1,CCDC3,DIO2,PDE4DIP,ABCA13,KANK1,KIF26B,TBC1D5,SLCO3A1,TCF12,RUNX1,PLS1,DOK6,FCHSD2,NLGN1,TNFRSF10B,CAMK1D,CLEC16A,TCF7L2,RELN,APP,ADAM12,PAK1,UTRN,PPP3CA,TOX,PTPN9,CREM,AKAP6,DLC1,IDE,SOS1,RFTN1,ALK,BTRC,MEF2A,TFAP2D,MAP2K5,DNMT1,MAGI2,EDA,ROBO2,AKAP13,HIVEP3,SAMD4A,DDB1,EP300,PARN,SEMA4D,EVC,RPS6KA5,GRK5,ANKRD6,BDKRB1,TF,GRIK2,QKI,CHRM1,TFEC,JAK2,NEGR1,CMKLR1,ZBTB7C,PARK2,MAGI1,ADCYAP1R1,SMOC2,NRG3,NUMB,SOX6,SULF1,CD84,TCF20,PSPC1,SLIT2,GLP2R,SLC4A4,KMT2C,TRIM5,THEMIS,ATRX,TRAF3IP2,RAD51B,SLX1B,SH3RF3,BMPR1A,SMAD3,BORA,PIBF1,CHD6,PTGFR,SCUBE1,EYA1,SATB2,MAML3,TPH1,MAD1L1,EGFLAM,TNKS,TUB,KLF12,SCAMP5,OVOL2,GRAMD4,ELP3,BRINP1,OMA1,BCL2L13,ARRDC4,GLIS1,SKAP1,SOX5,DGKI,TAF1,SLC1A1,RNF144B,NTN1,LDB2,EPM2A,MAP2,CDC73,FLRT2,FBXW11,NOS1AP,RBMS3,WWTR1,PEMT,KCTD7,EGLN3,CUX1,GRM4,ANK3,CCL15,MORC2,ARID4B,DOCK3,CDH4,TNR,CELF4,VAV3,ARNT2,ACTN4,HPSE2,PLCE1,EPS8,TENM4,PRR16,GHR,CLSTN2,CD300A,CBFA2T2,FUBP1,PRG3,MLIP,SERTAD2,DISC1,CCL14,RABGEF1,TRABD2B,LRP5,AP2B1,ESR1,TNFRSF19,GRM1,PDE4D,PIK3CD,DOCK4,SLC5A3,BLOC1S5,FRMD4A,TEAD4,EFNA5,FTO,ATF2,PIK3R2,GRID2,ZNF423,CYBB,SEMA6D,SUSD4,ADTRP,PDE4B,MAPRE2,MAP3K7,PRKCQ,CLDN1,SLC24A3,JDP2,SPTBN4,EPHB2,AGO3,S1PR3,MOB3B,RGMB,EEF1E1-BLOC1S5,DOCK2,RFC3,MARK1,BDNF,RBM4,FNIP1,ADORA2A,ARID5B,ANO1,CADPS,NOX4,BMPER,SYT1,DAB2,PLXNA2,NSF,LRRK1,ABI1,CACNG3,PTH2R,CALCRL,HDAC2,ETS1,BANP,

			ARID4A,BACH1,TTN,MYRIP,CDK19,CFB,RIMS2,KSR2,PRDM15,PARD3,NRG1,FANK1,NPAS3,FMN2,RIT2,CD44,KCNMA1,SCAF8,CNGB1,GPSM2,PRKCG,TEAD1,NFIA,MAPKAPK2,SLC8A1,FBXO31,MAP3K13,PLCL2,TOX3,THRB,CDC42EP3,AGT,FGF1,QRICH1,CCR3,GRIP1,NF1,WIF1,NRXN1,PDXP,GSK3B,PKP2,SETD3,LMO7,GPR21,CHD7,PSMB7,SNX5,TIAM2,IQCJ-SCHIP1,CNTN1,UNC13A,TRIOBP,NCOA1,CHEK2,CACNB2,STXBP4,RHOJ,MECOM,SHANK2,UBE2E2,ISLR2,GPC3,DLG5,PREX1,UBE2V1,ROR1,PTGER3,BID,MACF1,MNAT1,CAPN3,CTNNBL1,DUSP22,MYOM1,MAP3K5,MAML2,CTDSPL2,HTR2C,LY86,EPHA5,SGIP1,RNF144A,TP73,RBM19,IL1RL1,ANK2,ARHGEF3,SUPT3H,GRIN2B,PAK3,UACA,ADCY8,MLLT3,SH3RF2,CACNA1D,PRKAA2,TBL1X,SEMA5B,GRM5,IGF1R,CNTNAP2,INSR,CIZ1,JAK1,MAP2K1,FOXO3,RASGRF2,CNTN6,MITF,CDC14A,AGBL4,PRDM16,FRMD5,KIR2DL4,DNMT3B,TASP1,LRRK2,PKHD1,RARB,TCF4,PBX1,BMP6,ASAP1,FRMPD4,FYN,CSPP1,EPHA7,GAS8,MSR1,CLN6,PCBD2,RIMS1,ENPP2,ABCA12,EPHB1,KCNQ1,DOCK1,CRADD,VASH2,TRPC5,UNC5C,TENM3,NOX5,LRP2,C2,FER,CAMK4,VWC2,TMEM108,RAPGEF2,NAV3,NCOA2,CDKL5,PTPRU,IL18R1,ESR2,KAT7,C9,SETD2,TRIM22,CDH13,BASP1,TRDN,DAB1,ZNF148,SEC16B,HDAC5,SEMA3A,MGAT5,MYH9,STIM1,NRIP1,LITAF,PIK3R3,BRF1,TENM1,ST8SIA1,SH3KBP1,ZRANB1,RGS7,STXBP5,VWF,SLC24A2,SCEL,NTRK3,DIS3L2,CLASP2,ZNF521,CREB5,DNM3,SYNDIG1,PPARA,PTPRD,RORA,ELAVL4,NELL1,EEF1E1,ATP8A1,SEMA3D,PRLR,ATF6,NVL,SYT17,STAC,RAB27A,BLOC1S6,LARP4B,SYT9,SEMA3C,ST18
GO:0043269	regulation of ion transport	0.00001609553253336263	*CACNG2,DMD,KCNC2,CHRM3,NOS1,TMC2,DPP10,CACNA1A,PRKD1,RYR2,CASK,NLGN1,KCNIP4,RELN,APP,UTRN,AKAP6,GRM7,KCNB2,KCNA6,BDKRB1,TF,CHRM1,PARK2,ADCYAP1R1,DPP6,CACNA1C,CD84,KCNJ16,CACNA1E,FGF14,CACNA2D3,SHISA9,NOS1AP,CATSPER2,ANK3,CNIH3,SCN9A,ACTN4,PDE4D,KCNJ12,CYBB,KCNJ3,PDE4B,KCND2,SPTBN4,EPHB2,ADORA2A,SYT1,CACNG3,SCN8A,KCNMA1,SLC8A1,AGT,KCNQ5,KCNJ6,NRXN1,PKP2,KCNH1,CHD7,CNTN1,CACNB2,KCNS3,CAPN3,ANK2,CACNA1D,GRM5,NKAIN2,RASGRF2,NKAIN3,SCN1A,FYN,KCND3,KCNQ1,KCNH7,TRDN,STIM1,RGS7,SHISA6,KCNC4,SYT17,STAC,KCNJ15,SYT9*
GO:0061387	regulation of extent of cell growth	0.000016288232300552435	*DSCAM,SEMA5A,DCC,SEMA4D,NTN1,MAP2,CDH4,TNR,DISC1,EFNA5,SEMA6D,DPYSL2,BDNF,FSTL4,MAP3K13,GSK3B,ISLR2,MACF1,SEMA5B,EPHA7,TRPC5,CDKL5,SEMA3A,CLASP2,SEMA3D,SEMA3C*
GO:0034762	regulation of transmembrane transport	0.000016359975314171887	*CACNG2,DMD,KCNC2,CHRM3,NOS1,TMC2,DPP10,CACNA1A,PRKD1,RYR2,NLGN1,KCNIP4,RELN,APP,UTRN,AKAP6,MEF2A,KCNB2,KCNA6,BDKRB1,DPP6,CACNA1C,KCNJ16,CACNA1E,FGF14,CACNA2D3,SHISA9,NOS1AP,CATSPER2,ANK3,CNIH3,SCN9A,ACTN4,PDE4D,KCNJ12,CYBB,KCNJ3,PDE4B,KCND2,EPHB2,CACNG3,ENPP1,SCN8A,KCNMA1,SLC8A1,AGT,KCNQ5,KCNJ6,NRXN1,KCNH1,CHD7,CACNB2,STXBP4,GPC3,KCNS3,CAPN3,ANK2,CACNA1D,GRM5,INSR,RASGRF2,SCN1A,FYN,KCND3,KCNQ1,KCNH7,TRDN,STIM1,RGS7,SHISA6,KCNC4,STAC,KCNJ15*
GO:0007156	homophilic cell adhesion via plasma membrane adhesion molecules	0.000018712887497688143	*CNTN4,ROBO1,DSCAM,PCDH9,PTPRT,PCDH15,CELSR1,ROBO2,CDH8,SDK2,EMB,CD84,DSCAML1,HMCN1,CDH12,CDH4,CDH10,CLSTN2,CADM3,FAT3,CDH23,MYPN,SDK1,CNTN6,PCDH7,PCDH11X,TENM3,PCDH17,PTPRM,KIRREL3,DCHS2,CDH13,CDH9*
GO:0007167	enzyme linked receptor protein signaling pathway	0.000020900933600968875	*PTPRR,KALRN,NTRK2,PTPRG,SPRED2,ERBB4,ROBO1,FUT8,ANKS1A,LDLRAD4,PTPRT,PLCB1,FLT3,PRKD1,NEDD9,KANK1,DOK6,PAK1,IDE,SOS1,ALK,MAGI2,EP300,RPS6KA5,TF,JAK2,SMOC2,NRG3,COL4A6,SULF1,CHRDL1,BMPR1A,SMAD3,ANKS1B,OVOL2,FLRT2,WWTR1,PLCE1,GHR,RABGEF1,PIK3CD,EFNA5,ATF2,PIK3R2,ZNF423,MAP3K7,PRKCQ,EPHB2,RGMB,BDNF,ARID5B,FSTL4,BMPER,DAB2,ABI1,ENPP1,NRG1,MAPKAPK2,CHST11,AGT,FGF1,NF1,NRXN1,GSK3B,CD109,GPR21,SNX5,BTBD11,STXBP4,NREP,GPC3,ROR1,TRIO,PTPRE,DUSP22,CTDSPL2,EPHA5,PAK3,IGF1R,INSR,PRDM16,LTBP1,BMP6,FYN,EPHA7,EPHB1,EXT1,LRP2,FER,VWC2,GUCY2F,TMEM108,RAPGEF2,PTPRU,CDH13,VEPH1,BDKRB2,PIK3R3,NTRK3,FBN1,CLASP2,PTPRK,PPARA,PTPRD,PRLR,PTPRA,PMEPA1*
GO:000726	small GTPase	0.00002390402545423	*KALRN,RASGEF1B,ROBO1,AUTS2,PRKD1,MYO9A,KANK1,RE*

4	mediated signal transduction	209	*LN,DLC1,SOS1,DNMT1,CELSR1,AKAP13,ARHGAP15,ITSN1,ADCYAP1R1,SH3BP1,ARHGAP24,SLIT2,ARHGEF18,DGKI,DNMBP,NTN1,ARHGAP6,DOCK3,VAV3,PLCE1,EPS8,ARHGAP39,RGL1,RALGPS1,ARHGAP42,RABGEF1,ARHGAP12,DOCK4,MAPRE2,EPHB2,DOCK2,SIPA1L2,RAB30,SRGAP3,KSR2,NRG1,RIT2,GPSM2,RAPGEF6,CDC42EP3,ARHGAP25,NF1,ARHGAP10,TIAM2,RHOJ,PREX1,TRIO,FGD4,ARHGEF3,GARNL3,KPNB1,RASGRF2,DOCK1,RALGPS2,RAPGEF2,CDH13,DAB1,SIPA1L3,DOCK9,DOCK10,RALGAPA1*
GO:0051056	regulation of small GTPase mediated signal transduction	0.000029576779762262236	*KALRN,ROBO1,AUTS2,MYO9A,KANK1,RELN,DLC1,SOS1,AKAP13,ARHGAP15,ITSN1,ADCYAP1R1,SH3BP1,ARHGAP24,SLIT2,ARHGEF18,DGKI,DNMBP,ARHGAP6,DOCK3,VAV3,PLCE1,EPS8,ARHGAP39,RALGPS1,ARHGAP42,RABGEF1,ARHGAP12,MAPRE2,EPHB2,DOCK2,SIPA1L2,SRGAP3,NRG1,RIT2,ARHGAP25,NF1,ARHGAP10,TIAM2,PREX1,TRIO,FGD4,ARHGEF3,GARNL3,RASGRF2,RALGPS2,SIPA1L3,RALGAPA1*
GO:0035235	ionotropic glutamate receptor signaling pathway	0.000030306218439870617	*GRIK4,APP,GRIK2,GRID1,GRIN3A,GRID2,GRIA2,GRIK3,GRIN2B,GRIK1,GRIA4,GRIA3*
GO:1990806	ligand-gated ion channel signaling pathway	0.000030306218439870617	*GRIK4,APP,GRIK2,GRID1,GRIN3A,GRID2,GRIA2,GRIK3,GRIN2B,GRIK1,GRIA4,GRIA3*
GO:0045595	regulation of cell differentiation	0.000041419434568640226	*KALRN,CTNNA1,NTRK2,CLASP1,DMD,SPRED2,CNTN4,ROBO1,DSCAM,HDAC4,LDLRAD4,PSMB2,PLCB1,PRKD1,VCL,NEDD9,PPP2R3C,SEMA5A,PRKCA,IL1RAPL1,CCDC3,KANK1,TCF12,RUNX1,NLGN1,TCF7L2,RELN,APP,PPP3CA,TOX,AKAP6,SOS1,ALK,DNMT1,ROBO2,DCC,SEMA4D,JAK2,CMKLR1,ZBTB7C,NPHP3,NUMB,SOX6,SLIT2,BMPR1A,SMAD3,EYA1,TPH1,OVOL2,BRINP1,GLIS1,SOX5,NTN1,MAP2,CDC73,WWTR1,CUX1,TRPS1,CDH4,TNR,ACTN4,TENM4,GHR,DISC1,LRP5,BLOC1S5,EFNA5,FTO,SEMA6D,JDP2,EPHB2,S1PR3,PRTG,DPYSL2,MARK1,BDNF,RBM4,FNIP1,FSTL4,DAB2,PLXNA2,HDAC2,ETS1,ENPP1,NRG1,FBXO31,MAP3K13,AGT,USH2A,NF1,WIF1,ZNF536,GSK3B,PKP2,CTDP1,SETD3,CD109,CHD7,PSMB7,TIAM2,PLEKHB2,TRIOBP,NCOA1,ISLR2,NREP,PREX1,ZNF675,MACF1,TRIO,CAPN3,MAP3K5,HTR2C,TP73,PAK3,SEMA5B,NLN,GRM5,SUFU,MAP2K1,FOXO3,MITF,RBFOX1,LRRK2,RARB,TCF4,PBX1,BMP6,EPHA7,MSR1,ABCA12,EPHB1,DOCK1,TRPC5,LRP2,CAMK4,VWC2,RAPGEF2,CDKL5,KAT7,DAB1,HDAC5,SEMA3A,NTRK3,FBN1,CLASP2,NSUN2,PPARA,PTPRD,RORA,NELL1,SEMA3D,PRLR,BLOC1S6,SEMA3C*
GO:0009968	negative regulation of signal transduction	0.00004238671534933587	*PTPRR,RCAN1,RNF43,CTNNA1,ZNRF3,DMD,SPRED2,ROBO1,CIT,WWOX,LDLRAD4,PSMB2,PTPRT,CCDC3,KANK1,TCF7L2,DEPDC5,DLC1,BTRC,MAP2K5,MAGI2,ANKRD6,PARK2,NPHP3,NLGN4X,SULF1,SH3BP1,RFFL,ARHGAP24,SLIT2,CHRDL1,SMAD3,PIBF1,EYA1,MAD1L1,LINC00473,OVOL2,GRAMD4,FBXW11,RBMS3,WWTR1,TRIM59,CELF4,CD300A,CBFA2T2,ARHGAP42,RABGEF1,TRABD2B,ESR1,ARHGAP12,PDE4D,EIF3A,PIK3R2,PDE4B,PRKCQ,EPHB2,BDNF,FNIP1,IFT80,FSTL4,FHL2,BMPER,DAB2,PDE11A,ZMYND11,RGS6,ENPP1,PRDM15,NRG1,CD44,PLCL2,CHST11,AGT,NDRG2,ARHGAP25,NF1,WIF1,ZNF536,GSK3B,CD109,GPR21,PSMB7,SNX5,CHEK2,MECOM,SHANK2,GPC3,DLG5,ZNF675,BID,PTPRE,DUSP22,CTDSPL2,ZNF366,SLIT3,UACA,MLLT3,RNF213,SH3RF2,PRKAA2,GRM5,IGF1R,PTPRO,SUFU,UBR2,FOXO3,STK38,PRDM16,LTBP1,LRRK2,PKHD1,MARVELD3,AMFR,LRP2,VWC2,PTPRU,RGS7BP,VEPH1,DAB1,BDKRB2,LITAF,RGS7,FBN1,PPARA,PTPRD,RORA,SHISA6,BICC1,DEPTOR,PMEPA1*
GO:0016043	cellular component organization	0.00004252387777744616	*RNF4,CHFR,KDM4B,KALRN,CACNG2,RPS6KA2,NDE1,CTNNA1,NTRK2,CLASP1,PTPRG,SYNE1,DMD,KCNC2,EIF3E,ERBB4,LAMA2,CNTN4,ROBO1,ANKRD30BL,DSCAM,SHROOM3,MDN1,CIT,TBCD,FRY,NEBL,HDAC4,ATP9A,LINGO2,ARFGAP3,IMMP2L,ABCC1,ATP8A2,AUTS2,LDLRAD4,TENM2,BLM,PSMB2,RXFP1,ATF7IP,NFASC,PLCB1,SRPK2,TRPM1,PRKD1,VCL,PDS5A,NEDD9,PPP2R3C,SEMA5A,ETV6,PRKCA,EPB41L4B,STX8,BTBD9,IL1RAPL1,ADAMTS6,MYO9A,PDE4DIP,CALD1,ITGAE,ABCA13,KANK1,DIAPH2,TBC1D5,RUNX1,PLS1,FCHSD2,NLGN1,DNAH9,CAMK1D,SMG6,CLEC16A,AMOTL1,TCF7L2,MTRF1,RELN,APP,PAK1,UTRN,PPP3CA,RYR1,TOX,PCDH15,ERC2,PTPN9,AKAP6,GRM7,DLC1,SCMH1,RYR3,SOS1,CTNND2,LRRC8*

			C,RFTN1,ALK,DCLK1,MEF2A,KCNB2,CTNNA3,DENND5A,MAP2K5,DNMT1,MAGI2,CELSR1,ROBO2,IFT81,AKAP13,DCC,ELMO2,C10ORF90,EP300,KCNA6,PARN,SEMA4D,RPS6KA5,PHF20,MMP16,BDKRB1,NME8,TF,CDH8,HUS1,TANC2,JAK2,ABCG8,NEGR1,SDK2,MAST4,PARK2,MAGI1,NPHP3,PRELID2,GRIN3A,SMOC2,NLGN4X,EXOC4,EMB,PNPLA3,ANO4,PIK3C2B,AFAP1,UPB1,LRRC49,NRG3,NUMB,COL4A6,TRPM3,SULF1,CHCHD6,SH3BP1,RNU6ATAC31P,SH3GL2,COL24A1,ARHGAP24,COL11A1,PITPNC1,SLIT2,SYNM,KMT2C,ATRX,RAD51B,SLX1B,GTF2E1,ACACA,DSCAML1,SMAD3,BORA,PI4KA,TNFRSF11B,PIBF1,PIK3C3,CHD6,PEAK1,EYA1,SATB2,HSF2BP,ELN,MAD1L1,EGFLAM,DGKB,TNKS,TUB,ARHGEF18,SCAMP5,DNAJB6,OMA1,SPESP1,IL1RAPL2,SPAG17,SKAP1,TAF1,SLC1A1,NTN1,EPM2A,SHFM1,MAP2,CDC73,FLRT2,FBXW11,NOS1AP,RANBP10,ARHGEF6,WWTR1,GAS7,ARHGAP6,KCTD7,CHAF1B,STARD9,CUX1,ANK3,CDH12,MORC2,ARID4B,THSD4,TTLL8,CDH4,TNR,CELF4,VAV3,CDKL1,MDM4,SPOCK1,ACTN4,RAB2A,HPSE2,PLCE1,TACC2,EPS8,TENM4,CECR2,PRR16,ARHGAP39,LRRC8D,CDH10,LRRC4C,PPP1R9A,PDZRN3,CLSTN2,CD300A,TTLL11,CBFA2T2,SERTAD2,STRC,DISC1,POC1A,ATP8B4,IFT43,RABGEF1,NSG2,TRABD2B,LRP5,NAV2,AP2B1,ESR1,ARHGAP12,SCFD1,CNTN5,THEM4,BLOC1S5,RNU6-78P,ARMC2,EFNA5,TLN2,KLHL1,EIF3A,ATF2,PHACTR2,KCNJ12,GRID2,ZNF423,SEMA6D,CELF2,NTNG1,ADTRP,CRMP1,MAPRE2,GABRB3,ANKRD13A,PRKCQ,RASSF8,RHPN2,CLDN1,WDR83OS,KCND2,TTLL5,SPTBN4,EPHB2,ERC1,AGO3,MCTP1,PRTG,COL22A1,DOCK2,DPYSL2,PTPDC1,RFC3,MARK1,BDNF,FNIP1,ADORA2A,IFT80,DYM,FSTL4,MCM3,CCDC170,TTC39C,RNU6-1269P,TSPAN33,BCOR,ICK,THSD7B,BRD9,SYT1,EBAG9,DAB2,AFF2,PLXNA2,NSF,PRKG1,STXBP6,SNAP23,ABI1,EPG5,MICAL3,HDAC2,ETS1,BANP,FAT3,ARID4A,RAB30,ZMYND11,CDH23,TTN,ENPP1,RIMS2,HEPACAM2,CTNNA2,PEX5L,LRCH3,FMNL2,PARD3,NRG1,CAST,SPINT2,ATP10B,FMN2,TOP1,RIT2,CD44,KCNMA1,SCAF8,CNGB1,GPSM2,SLC12A8,ZFYVE1,TEAD1,NFIA,RAPGEF6,FBXO31,MAP3K13,CDC42BPA,CDC42EP3,SAMM50,NCAM1,AGT,STRIP1,GRIP1,TLK1,ARHGAP25,NF1,VPS16,PARVB,NRXN1,ARHGAP10,LAMA3,PDXP,GSK3B,ADAMTS17,PKP2,CTDP1,SETD3,BBS9,CHD7,PSMB7,MOV10L1,THSD7A,TIAM2,IQCJ-SCHIP1,CNTN1,UNC13A,TRIOBP,CABIN1,CHEK2,CACNB2,RHOJ,ESYT2,MECOM,SYBU,SHANK2,KCTD8,UNC5D,ISLR2,NREP,GPC3,DLG5,PGM5,SH3BGR,PREX1,ANO3,XKR4,ROR1,ADAMTSL1,SERPINA5,KCNS3,COL12A1,BID,MACF1,MNAT1,TRIO,CAPN3,TRMT61B,DUSP22,CLDN11,SPAG16,CCDC141,SNAP25-AS1,FGD4,EPHA5,SGIP1,HIRA,TP73,FAM171A1,MYPN,ANK2,SLIT3,GRIN2B,SHPRH,PAK3,PRRC2C,ADCY8,MLLT3,NEB,ITGB3BP,RNF213,RNU6-1061P,PRKAA2,CHCHD3,SEMA5B,ANKFN1,TRAPPC8,SDK1,GRM5,IGF1R,TSNARE1,CNTNAP2,PTPRO,INSR,RPGR,CRTAC1,MAP2K1,UBR2,KPNB1,GPC6,ADAMTSL3,CNTN6,CENPF,KCTD1,ATP9B,MITF,SPECC1L,LRFN5,CDC14A,KPNA3,PRDM16,FRMD5,ULK4,TMEM120B,ADAMTS16,LRRK2,PKHD1,KIF2A,TCF4,SPG11,PHACTR1,BMP6,TMEM170A,VTI1A,ASAP1,FRMPD4,COL23A1,FYN,KCND3,EPHA7,MARVELD3,GAS8,COL19A1,MSR1,SGCZ,CLN6,RIMS1,ENPP2,L3MBTL4,ABCA12,EPHB1,KCNQ1,FHOD3,DOCK1,VASH2,EXT1,TRPC5,UNC5C,CSGALNACT1,TENM3,NOX5,LRP2,FER,PCDH17,TMEM108,SHROOM4,RAPGEF2,NAV3,CENPP,PTPRM,KIRREL3,NRXN3,CDKL5,ZNF207,MPRIP,ESR2,KAT7,SATB1,C9,SETD2,PACSIN2,CDH13,TRDN,DAB1,ZNF148,SEC16B,EXOC6B,HDAC5,SEMA3A,MYH9,COL21A1,TANC1,VPS41,BRF1,TENM1,STK38L,VPS39,SH3KBP1,PRKAR1A,ZRANB1,STXBP5,SIPA1L3,GNG4,KANSL1,NTRK3,HYDIN,CEP89,DIS3L2,CLASP2,DNAJC6,PTPRK,DNM3,SYNDIG1,PPARA,NCKAP5,PTPRD,SHISA6,BRIP1,ELAVL4,CDH9,ANXA8L1,DOCK10,ATP8A1,SEMA3D,PRLR,PTPRA,KCNC4,CEP41,DEPTOR,PPFIBP2,SMIM20,RAD51D,SYT17,RAB27A,TUBGCP6,BLOC1S6,PMEPA1,PARD3B,HGSNAT,SYT9,SEMA3C
GO:0016192	vesicle-mediated transport	0.00005184243417283613	*SYN3,LRP1B,KALRN,CACNG2,PROS1,CLASP1,ATP9A,ARFGAP3,PRKD1,CASK,STX8,BTBD9,IL1RAPL1,ABCA13,TBC1D5,FCHSD2,NLGN1,CAMK1D,CLEC16A,APP,PAK1,PPP3CA,ERC2,OSBPL1A,RABGAP1L,DCLK1,DENND5A,MAGI2,ELMO2,TF,SYT*

			L5,ITSN1,RAB11FIP4,PARK2,GRIN3A,NLGN4X,EXOC4,HEATR5A,ENTHD1,TMPRSS3,NUMB,CD84,SH3BP1,SH3GL2,PIK3C3,TPH1,TUB,SCAMP5,ASGR2,DGKI,SLC1A1,CUX1,ANK3,CNIH3,VAV3,RAB2A,CECR2,GHR,CD300A,RABGEF1,NSG2,LRP5,AP2B1,ARHGAP12,DENND2A,SCFD1,PIK3CD,CCDC91,ANKRD13A,SPTBN4,EPHB2,ERC1,MCTP1,DOCK2,DPYSL2,ADORA2A,CADPS,SYT1,DAB2,NSF,STXBP6,SNAP23,CACNG3,EPG5,CALCRL,MICAL3,PRKAR1B,ENPP1,RIMS2,NRG1,FMN2,RIT2,NCF4,PRKCG,MAPKAPK2,FAM19A4,GRIP1,ARHGAP25,VPS16,NRXN1,GSK3B,SNX5,UNC13A,CACNB2,RHOJ,ESYT2,GPC3,XKR4,MACF1,NCF2,SGIP1,ANK2,TRAPPC8,IGF1R,TSNARE1,MON2,INSR,MAP2K1,ATP9B,LRRK2,SPG11,VTI1A,FYN,MSR1,RIMS1,ENPP2,VPS53,ABCA12,DOCK1,LRP2,C2,FER,PCDH17,TMEM108,NRXN3,VPS45,PACSIN2,CDH13,SEC16B,EXOC6B,MYH9,VPS41,VPS39,SH3KBP1,STXBP5,TXNDC5,CLASP2,TMPRSS15,DNAJC6,SORCS1,DNM3,SYNDIG1,SCFD2,ANXA8L1,SYT17,RAB27A,BLOC1S6,SYT9
GO:0048522	positive regulation of cellular process	0.000052087801237933156	*RNF4,CAMTA1,CHFR,KALRN,CACNG2,RPS6KA2,CTNNA1,NTRK2,CLASP1,GLIS3,SLC39A10,DMD,KCNC2,EIF3E,SPRED2,ERBB4,LAMA2,TFDP2,NOS1,ESRRG,ROBO1,DSCAM,CIT,DPP10,PUM1,GPC5,WDR59,PRIM2,WWOX,TNRC6B,HDAC4,LINGO2,ATP8A2,AUTS2,WLS,TENM2,BLM,PSMB2,ATF7IP,ASXL3,PLCB1,FLT3,SRPK2,PRKD1,RYR2,NEDD9,PPP2R3C,SEMA5A,ETV6,CASK,PRKCA,EPB41L4B,ZBTB20,IL1RAPL1,CCDC3,PDE4DIP,ABCA13,KANK1,KIF26B,TBC1D5,SLCO3A1,TCF12,RUNX1,PLS1,DOK6,FCHSD2,NLGN1,TNFRSF10B,CAMK1D,CLEC16A,TCF7L2,RELN,APP,PAK1,UTRN,PPP3CA,TOX,PTPN9,CREM,AKAP6,DLC1,SOS1,ALK,BTRC,MEF2A,TFAP2D,MAP2K5,DNMT1,MAGI2,EDA,ROBO2,AKAP13,HIVEP3,SAMD4A,DDB1,EP300,PARN,SEMA4D,EVC,RPS6KA5,GRK5,ANKRD6,BDKRB1,TF,GRIK2,CHRM1,TFEC,JAK2,NEGR1,CMKLR1,ZBTB7C,PARK2,MAGI1,ADCYAP1R1,SMOC2,NRG3,NUMB,SOX6,SULF1,TCF20,SLIT2,GLP2R,SLC4A4,KMT2C,TRIM5,ATRX,TRAF3IP2,RAD51B,SLX1B,SH3RF3,BMPR1A,SMAD3,BORA,PIBF1,CHD6,PTGFR,SCUBE1,EYA1,SATB2,MAML3,TPH1,MAD1L1,EGFLAM,TNKS,KLF12,SCAMP5,OVOL2,GRAMD4,ELP3,BRINP1,OMA1,BCL2L13,ARRDC4,GLIS1,SKAP1,SOX5,DGKI,TAF1,SLC1A1,RNF144B,NTN1,LDB2,EPM2A,MAP2,CDC73,FLRT2,FBXW11,NOS1AP,WWTR1,EGLN3,CUX1,GRM4,ANK3,CCL15,MORC2,ARID4B,DOCK3,CDH4,TNR,CELF4,VAV3,ARNT2,ACTN4,HPSE2,PLCE1,EPS8,TENM4,PRR16,GHR,CLSTN2,CD300A,CBFA2T2,MLIP,SERTAD2,DISC1,CCL14,TRABD2B,LRP5,AP2B1,ESR1,TNFRSF19,GRM1,PIK3CD,DOCK4,SLC5A3,BLOC1S5,FRMD4A,TEAD4,EFNA5,FTO,ATF2,PIK3R2,GRID2,ZNF423,SEMA6D,ADTRP,MAPRE2,MAP3K7,PRKCQ,CLDN1,JDP2,EPHB2,AGO3,S1PR3,MOB3B,RGMB,EEF1E1-BLOC1S5,RFC3,BDNF,RBM4,FNIP1,ADORA2A,ARID5B,ANO1,CADPS,NOX4,BMPER,SYT1,DAB2,PLXNA2,NSF,LRRK1,ABI1,CACNG3,CALCRL,HDAC2,ETS1,BANP,ARID4A,BACH1,TTN,MYRIP,CDK19,RIMS2,PRDM15,PARD3,NRG1,FANK1,NPAS3,FMN2,RIT2,CD44,KCNMA1,SCAF8,GPSM2,PRKCG,TEAD1,NFIA,SLC8A1,FBXO31,MAP3K13,TOX3,THRB,CDC42EP3,AGT,FGF1,QRICH1,CCR3,GRIP1,NF1,WIF1,NRXN1,PDXP,GSK3B,SETD3,LMO7,CHD7,PSMB7,SNX5,TIAM2,IQCJ-SCHIP1,CNTN1,UNC13A,TRIOBP,NCOA1,CHEK2,CACNB2,STXBP4,RHOJ,MECOM,SHANK2,UBE2E2,ISLR2,GPC3,DLG5,PREX1,UBE2V1,ROR1,BID,MACF1,MNAT1,CAPN3,CTNNBL1,DUSP22,MYOM1,MAP3K5,MAML2,CTDSPL2,HTR2C,LY86,EPHA5,SGIP1,RNF144A,TP73,IL1RL1,ANK2,ARHGEF3,SUPT3H,GRIN2B,PAK3,UACA,ADCY8,MLLT3,SH3RF2,PRKAA2,TBL1X,SEMA5B,GRM5,IGF1R,CNTNAP2,INSR,CIZ1,JAK1,MAP2K1,FOXO3,RASGRF2,CNTN6,MITF,CDC14A,AGBL4,PRDM16,FRMD5,KIR2DL4,DNMT3B,TASP1,LRRK2,PKHD1,RARB,TCF4,PBX1,BMP6,ASAP1,FRMPD4,FYN,CSPP1,EPHA7,GAS8,MSR1,CLN6,PCBD2,RIMS1,ENPP2,ABCA12,EPHB1,KCNQ1,DOCK1,CRADD,VASH2,TRPC5,UNC5C,TENM3,NOX5,LRP2,FER,CAMK4,VWC2,TMEM108,RAPGEF2,NAV3,NCOA2,CDKL5,PTPRU,IL18R1,ESR2,KAT7,TRIM22,CDH13,TRDN,DAB1,ZNF148,SEC16B,HDAC5,SEMA3A,MGAT5,MYH9,STIM1,NRIP1,LITAF,PIK3R3,BRF1,TENM1,ST8SIA1,SH3KBP1,ZRANB1,RGS7,STXBP5,VWF,SLC24A2,SCEL,NTRK3,DIS3L2,CLASP2,ZNF521,CREB5,DNM3,SYNDIG1,PPARA,PTPRD,RORA,ELAVL4,NELL1,EEF1E1,ATP8A1,SEMA3D,PRLR,ATF6,NVL,SYT17,STAC,RAB27A,BLOC1S6,LARP4B,SY*

			T9,SEMA3C,ST18
GO:0048675	axon extension	0.00005366719202137563	*DSCAM,AUTS2,VCL,SEMA5A,DCLK1,SEMA4D,SLIT2,NTN1,MAP2,CDH4,TNR,DISC1,SEMA6D,DPYSL2,MAP3K13,GSK3B,ISLR2,MACF1,SLIT3,SEMA5B,SPG11,TRPC5,CDKL5,SEMA3A,CLASP2,SEMA3D,SEMA3C*
GO:0019932	second-messenger-mediated signaling	0.000054268338886679884	*CAMTA1,RCAN1,DMD,KCNC2,CHRM3,NOS1,HDAC4,RYR2,PPP3CA,AKAP6,CMKLR1,ADCYAP1R1,CACNA1C,ADCY2,PTGFR,LINC00473,NOS1AP,PLCE1,PPP1R9A,PDE4D,PDE7B,MCTP1,RCAN2,FHL2,PRKG1,PDE11A,KSR2,PEX5L,NRG1,RIT2,SLC8A1,AGT,CCR3,GSK3B,HTR2C,EPHA5,ANK2,GRIN2B,ITPR2,GRM5,INPP5A,LRRK2,MCTP2,GUCY2F,SGCD,RAPGEF2,CDH13*
GO:0051129	negative regulation of cellular component organization	0.00006382926630697231	*RPS6KA2,CLASP1,PTPRG,TBCD,LDLRAD4,PRKD1,SEMA5A,KANK1,NLGN1,SMG6,CLEC16A,PPP3CA,PTPN9,DLC1,DENND5A,ROBO2,DCC,EP300,SEMA4D,PARK2,ARHGAP24,SLIT2,ATRX,SLX1B,MAD1L1,TNKS,ARHGEF18,SCAMP5,DNAJB6,OMA1,NTN1,MAP2,ARHGAP6,TNR,SPOCK1,EPS8,CD300A,CBFA2T2,RABGEF1,SCFD1,SEMA6D,ADTRP,CRMP1,ANKRD13A,RHPN2,SPTBN4,EPHB2,MCTP1,FSTL4,DAB2,HDAC2,FAT3,CTNNA2,RIT2,SCAF8,NRXN1,GSK3B,IQCJ-SCHIP1,TRIOBP,MNAT1,DUSP22,GRIN2B,SEMA5B,PTPRO,CENPF,LRRK2,BMP6,FYN,EPHA7,FHOD3,TRPC5,RAPGEF2,NAV3,ZNF207,PACSIN2,DAB1,SEMA3A,CLASP2,DNM3,SEMA3D,PMEPA1,SEMA3C*
GO:0018193	peptidyl-amino acid modification	0.00007723226533102889	*KDM4B,RPS6KA2,NTRK2,DMD,SPRED2,ERBB4,NOS1,CIT,FUT8,HDAC4,AUTS2,FLT3,SRPK2,PRKD1,NEDD9,PRKCA,CAMK1D,RELN,APP,PAK1,ALK,DCLK1,MAP2K5,DNMT1,EP300,SEMA4D,RPS6KA5,PHF20,ZDHHC11B,JAK2,MAST4,KMT2C,ATRX,PIBF1,PEAK1,EYA1,EGFLAM,TNKS,ZDHHC11,TAF1,SLC1A1,EPM2A,NOS1AP,PHF20L1,EGLN3,ARID4B,DOCK3,TTLL8,GHR,CD300A,TTLL11,PDE4D,EFNA5,ATF2,MAP3K7,PRKCQ,TTLL5,SPTBN4,EPHB2,MARK1,BDNF,FNIP1,NOX4,BCOR,LRRK1,ABI1,HDAC2,ARID4A,TTN,ZZZ3,PARD3,NRG1,DPY19L1,TOP1,CD44,PRKCG,MAPKAPK2,MAP3K13,PLCL2,CDC42BPA,AGT,DYRK4,TLK1,NRXN1,AGBL1,GSK3B,SETD3,CNTN1,NCOA1,CHEK2,MECOM,STK32B,ROR1,CAPN3,DUSP22,EPHA5,MORC3,SUPT3H,DPH6,PRKAA2,GRM5,IGF1R,ZDHHC14,INSR,JAK1,MAP2K1,STK38,AGBL4,PRDM16,DNMT3B,LRRK2,BMP6,FYN,EPHA7,ENPP2,EPHB1,TRPC5,FER,CAMK4,KAT7,SETD2,MGAT5,BDKRB2,TENM1,STK38L,KANSL1,DPY19L2,NTRK3,GALNTL6,PRLR,CEP41*
GO:0006936	muscle contraction	0.00009118025472704744	*DMD,CHRM3,NOS1,HDAC4,ATP8A2,RYR2,CALD1,UTRN,RYR1,SSPN,KCNB2,CTNNA3,PPP1R12B,CACNA1C,SULF1,SYNM,DTNA,NOS1AP,PLCE1,ARHGAP42,PDE4D,DOCK4,KCNJ12,KCNJ3,PDE4B,SNTB1,MYBPC2,PRKG1,CALCRL,TNNI3K,TTN,MYOM3,KCNMA1,SLC8A1,AGT,PKP2,SETD3,CACNB2,PTGER3,MYOM1,P2RX6,ANK2,CACNA1D,SCN1A,KCND3,KCNQ1,SGCD,TRDN,BDKRB2,STAC*
GO:0051094	positive regulation of developmental process	0.00009302133120573333	*KALRN,NTRK2,CLASP1,DMD,ERBB4,ROBO1,DSCAM,LINGO2,ATP8A2,PLCB1,PRKD1,NEDD9,PPP2R3C,SEMA5A,PRKCA,IL1RAPL1,CCDC3,TCF12,RUNX1,PLS1,NLGN1,TCF7L2,RELN,ADAM12,TOX,AKAP6,ALK,ROBO2,SEMA4D,JAK2,CMKLR1,ZBTB7C,PARK2,SMOC2,NUMB,SOX6,SLIT2,BMPR1A,SMAD3,TPH1,OVOL2,BRINP1,SOX5,NTN1,FLRT2,WWTR1,CUX1,CDH4,TENM4,GHR,CLSTN2,DISC1,LRP5,PIK3CD,BLOC1S5,TEAD4,EFNA5,GRID2,CYBB,SPTBN4,EPHB2,BDNF,RBM4,BMPER,SYT1,DAB2,PLXNA2,HDAC2,ETS1,RIMS2,NRG1,SLC8A1,FBXO31,MAP3K13,AGT,FGF1,CCR3,GRIP1,WIF1,NRXN1,GSK3B,SETD3,GPR21,CHD7,TIAM2,UNC13A,TRIOBP,NCOA1,ISLR2,DLG5,PREX1,MACF1,CAPN3,MAP3K5,HTR2C,TP73,RBM19,PAK3,GRM5,IGF1R,INSR,JAK1,MAP2K1,FOXO3,KIR2DL4,TCF4,BMP6,MSR1,RIMS1,ENPP2,EPHB1,DOCK1,VASH2,TRPC5,LRP2,VWC2,RAPGEF2,CDKL5,KAT7,BASP1,DAB1,SEMA3A,STIM1,CLASP2,SYNDIG1,PTPRD,ELAVL4,NELL1,EEF1E1,SYT17,BLOC1S6*
GO:0051962	positive regulation of nervous system development	0.00010182996120075196	*KALRN,NTRK2,ROBO1,DSCAM,LINGO2,SEMA5A,IL1RAPL1,NLGN1,RELN,ROBO2,SEMA4D,NUMB,SLIT2,NTN1,FLRT2,CUX1,CDH4,TENM4,CLSTN2,DISC1,EFNA5,GRID2,EPHB2,BDNF,PLXNA2,HDAC2,FBXO31,MAP3K13,NRXN1,TIAM2,ISLR2,DLG5,MACF1,TP73,PAK3,GRM5,MAP2K1,EPHB1,TRPC5,LRP2,CDKL5,SYNDIG1,PTPRD*
GO:0009719	response to endogenous stimulus	0.0001081769703948843	*CTNNA1,NTRK2,KCNC2,CHRM3,SPRED2,ERBB4,NOS1,ESRRG,WWOX,FUT8,HDAC4,ABCC1,LDLRAD4,BLM,RXFP1,PLCB1,FLT3,CACNA1A,RYR2,KANK1,APP,PAK1,RYR1,AKAP6,IDE,RYR3,ALK,DNMT1,MAGI2,ROBO2,EP300,CHRM1,DEFA1B,JAK2,*

			PARK2,SMOC2,PNPLA3,SOX6,COL4A6,SULF1,OTC,SH3GL2,SLIT2,GLP2R,CHRDL1,BMPR1A,ACACA,SMAD3,TNFRSF11B,PTGFR,OVOL2,SOX5,TAF1,SLC1A1,FLRT2,TIMP2,GHR,SRD5A2,NSG2,ESR1,PDE4D,EFNA5,ATF2,PIK3R2,ZNF423,CYBB,ADTRP,PDE4B,GABRB3,MAP3K7,PRKCQ,CLDN1,TPH2,EPHB2,PRCP,RGMB,BDNF,ANO1,FHL2,BMPER,DEFA3,DAB2,HDAC2,ENPP1,SPINT2,BCKDHB,CD44,SLC8A1,CHST11,THRB,AGT,FGF1,NRXN1,PDXP,GSK3B,CD109,GPR21,SNX5,NCOA1,STXBP4,PHEX,NREP,GPC3,PTPRE,DUSP22,CTDSPL2,HTR2C,GLRA2,ZNF366,SLIT3,ADCY8,ITPR2,PRKAA2,GRM5,IGF1R,PKD1L1,INSR,UBR2,FOXO3,PRDM16,LTBP1,LRRK2,RARB,BMP6,FYN,GNG2,KCNQ1,EXT1,ACSBG1,LRP2,C2,FER,VWC2,TMEM108,RAPGEF2,NCOA2,PTPRU,ESR2,CDH13,VEPH1,HDAC5,PIK3R3,GABRB1,NTRK3,FBN1,PTPRK,PPARA,BRIP1,ELAVL4,PRLR,PTPRA,PMEPA1,RXFP2
GO:0051966	regulation of synaptic transmission, glutamatergic	0.00011124616256071918	*CACNG2,NLGN1,RELN,GRM7,GRIK2,DGKI,GRM4,TNR,DISC1,GRM1,ADORA2A,SYT1,CACNG3,NRXN1,UNC13A,GRIK3,GRIK1,GRM5,LRRK2*
GO:0016055	Wnt signaling pathway	0.00011930185028276244	*RNF43,ZNRF3,PRICKLE2,GPC5,WWOX,WLS,PSMB2,SEMA5A,KANK1,AMOTL1,TCF7L2,APP,CTNND2,BTRC,MAGI2,EDA,CELSR1,DDB1,GRK5,ANKRD6,PARK2,NPHP3,SULF1,SMAD3,TNKS,EPM2A,CDC73,FBXW11,RBMS3,WWTR1,DISC1,TRABD2B,LRP5,MARK1,IFT80,DAB2,LRRK1,PRDM15,NDRG2,WIF1,GSK3B,PSMB7,GPC3,CPE,ROR1,MACF1,MLLT3,RNF213,PRKAA2,TBL1X,PTPRO,FOXO3,GPC6,MITF,LRRK2,EXT1,AMFR,PTPRU,ZRANB1,SCEL,SHISA6,BICC1*
GO:0198738	cell-cell signaling by wnt	0.00013968246661504277	*RNF43,ZNRF3,PRICKLE2,GPC5,WWOX,WLS,PSMB2,SEMA5A,KANK1,AMOTL1,TCF7L2,APP,CTNND2,BTRC,MAGI2,EDA,CELSR1,DDB1,GRK5,ANKRD6,PARK2,NPHP3,SULF1,SMAD3,TNKS,EPM2A,CDC73,FBXW11,RBMS3,WWTR1,DISC1,TRABD2B,LRP5,MARK1,IFT80,DAB2,LRRK1,PRDM15,NDRG2,WIF1,GSK3B,PSMB7,GPC3,CPE,ROR1,MACF1,MLLT3,RNF213,PRKAA2,TBL1X,PTPRO,FOXO3,GPC6,MITF,LRRK2,EXT1,AMFR,PTPRU,ZRANB1,SCEL,SHISA6,BICC1*
GO:0003015	heart process	0.00015443590130572083	*RPS6KA2,DMD,NOS1,HDAC4,RYR2,RYR3,MEF2A,CTNNA3,AKAP13,JAK2,CACNA1C,SLC1A1,NOS1AP,WWTR1,PDE4D,KCNJ12,KCNJ3,CELF2,PDE4B,SPTBN4,NOX4,TNNI3K,TTN,SLC8A1,THRB,AGT,PKP2,CACNB2,ANK2,CACNA1D,RNLS,SCN1A,FYN,KCND3,SGCZ,KCNQ1,EXT1,SGCD,TRDN,SEMA3A*
GO:0048519	negative regulation of biological process	0.00016306385082705713	*PTPRR,CHFR,KDM4B,RCAN1,KALRN,RNF43,PROS1,RPS6KA2,CTNNA1,FOXN3,NTRK2,CLASP1,ZNRF3,GLIS3,PTPRG,SLC39A10,DMD,EIF3E,SPRED2,ERBB4,TFDP2,NOS1,CNTN4,SND1,ROBO1,DSCAM,CIT,TBCD,PUM1,FRY,SORCS2,WWOX,TNRC6B,HDAC4,ATP9A,ATP8A2,LDLRAD4,MYEF2,TENM2,BLM,PSMB2,PTPRT,ATF7IP,ASXL3,PLCB1,SRPK2,PRKD1,RYR2,VCL,PDS5A,SEMA5A,ETV6,CASK,PRKCA,ZBTB20,IL1RAPL1,CCDC3,KANK1,RUNX1,NLGN1,CAMK1D,SMG6,CLEC16A,TCF7L2,APP,PAK1,PPP3CA,DEPDC5,RYR1,PTPN9,CREM,AKAP6,SORCS3,GRM7,DLC1,SCMH1,RYR3,ALK,BTRC,DCLK1,MEF2A,TFAP2D,DENND5A,MAP2K5,DNMT1,MAGI2,ROBO2,DCC,SAMD4A,DDB1,EP300,PARN,SEMA4D,RPS6KA5,GRK5,ANKRD6,BDKRB1,GRIK2,HUS1,TFEC,ASTN2,JAK2,ABCG8,CMKLR1,ZBTB7C,PARK2,NPHP3,ADCYAP1R1,SERPINA4,GRIN3A,CACNA1C,NLGN4X,GBE1,TAGLN3,NRG3,NUMB,SOX6,SULF1,CD84,MAGEA11,SH3BP1,RFFL,SH3GL2,PSPC1,ARHGAP24,SLIT2,TRIM5,ATRX,CHRDL1,TRAF3IP2,SLX1B,BMPR1A,SMAD3,PRKAR2A,TNFRSF11B,PIBF1,KIR3DL2,PTGFR,EYA1,SATB2,TPH1,MAD1L1,TNKS,KLF12,ARHGEF18,SCAMP5,LINC00473,OVOL2,GRAMD4,DNAJB6,BRINP1,OMA1,GLIS1,CDYL2,DGKI,TAF1,SLC1A1,RNF144B,NTN1,LDB2,EPM2A,MAP2,CDC73,FBXW11,CD96,RBMS3,WWTR1,ARHGAP6,CUX1,ANK3,TIMP2,MORC2,ZNF19,TRIM59,TRPS1,TNR,CELF4,MDM4,SPOCK1,ACTN4,EPS8,GHR,CD300A,CBFA2T2,PRG3,MLIP,SERTAD2,ARHGAP42,RABGEF1,FHIT,TRABD2B,LRP5,AP2B1,ESR1,ARHGAP12,SCFD1,PDE4D,DOCK4,FRMD4A,EFNA5,FTO,EIF3A,ATF2,RBBP8,PIK3R2,GRID2,ZNF423,SEMA6D,SUSD4,ADTRP,CRMP1,PDE4B,SMG7,ANKRD13A,PRKCQ,ATXN1,N4BP1,RHPN2,SLC24A3,JDP2,SPTBN4,EPHB2,AGO3,S1PR3,MCTP1,PRTG,DACH1,MARK1,BDNF,RBM4,FNIP1,MALRD1,ADORA2A,ARID5B,IFT80,FSTL4,FHL2,NOX4,BCOR,BMPER,DAB2,AFF2,PLXNA2,LRRK1,PRKG1,STXBP6,ABI1,CALCRL,HDAC2,ETS1,BANP,FAT3,PDE11A,ARID4A*

			,PRKAR1B,BACH1,ZMYND11,RGS6,SRGAP3,ZNF93,ENPP1,PRDM15,CTNNA2,PARD3,NRG1,CAST,FANK1,SPINT2,FMN2,RIT2,CD44,PAX7,KCNMA1,SCAF8,PRKCG,MAPKAPK2,SLC8A1,ISM1,FBXO31,PLCL2,TOX3,CHST11,THRB,MIR1185-1,AGT,NDRG2,UPK3B,ARHGAP25,USH2A,NF1,WIF1,ZNF536,NRXN1,ARHGAP10,GSK3B,PKP2,CTDP1,CD109,GPR21,CHD7,PSMB7,MOV10L1,SNX5,MYT1L,SRGAP2B,IQCJ-SCHIP1,TRIOBP,CHEK2,GRIK3,MECOM,SHANK2,GPC3,DLG5,CFDP1,ZNF675,SERPINA5,PTGER3,BID,MNAT1,TRIO,CAPN3,PTPRE,DUSP22,CTDSPL2,HTR2C,HIRA,TP73,IL1RL1,ZNF366,MORC3,ANK2,SLIT3,GRIN2B,PHC2,UACA,LRPPRC,ADCY8,ITPR2,MLLT3,RNF213,SH3RF2,PRKAA2,PACRG,TBL1X,SEMA5B,GRM5,IGF1R,MXD3,PTPRO,SUFU,MAP2K1,UBR2,FOXO3,STK38,INPP5A,CENPF,KCTD1,RNLS,MITF,IGF2BP3,LRFN5,AGBL4,PRDM16,FRMD5,KIR2DL4,DNMT3B,LTBP1,LRRK2,PKHD1,RARB,PBX1,BMP6,PCBP3,ASAP1,FYN,ABCC2,EPHA7,MARVELD3,GAS8,MSR1,PTPN14,L3MBTL4,ABCA12,BEND5,EPHB1,KCNQ1,FHOD3,CRADD,TRPC5,AMFR,LRP2,ZCCHC17,FER,VWC2,ERLIN1,PCDH17,TPTE,RAPGEF2,NAV3,PTPRM,NCOA2,PTPRU,ZNF207,GRIA4,IL18R1,RGS7BP,ESR2,KAT7,SATB1,MIR767,PACSIN2,TRIM22,CDH13,VEPH1,BASP1,TRDN,DAB1,ZNF148,HDAC5,SEMA3A,MGAT5,TSPAN8,MYH9,BDKRB2,TSC22D3,NRIP1,MIR105-2,LITAF,TENM1,PRKAR1A,RGS7,GNG4,TXNDC5,SLC24A2,NTRK3,FBN1,DIS3L2,TBX15,CLASP2,NSUN2,PTPRK,DNM3,PPARA,PTPRD,RORA,SHISA6,BRIP1,ELAVL4,ANXA8L1,MXI1,NELL1,EEF1E1,BICC1,SEMA3D,PRLR,DEPTOR,CST2,PMEPA1,LARP4B,SEMA3C,ST18
GO:0061061	muscle structure development	0.00017262248641926526	RCAN1,SYNE1,DMD,LAMA2,NOS1,NEBL,HDAC4,MYEF2,PLCB1,RYR2,TCF12,TCF7L2,ADAM12,UTRN,PPP3CA,RYR1,AKAP6,MEF2A,DNMT1,AKAP13,HIVEP3,EP300,EVC,QKI,SOX6,COL11A1,BMPR1A,SMAD3,ELN,MYO18B,ACTN4,TEAD4,BDNF,RBM4,ARID5B,FHL2,NOX4,TTN,NRG1,PAX7,SLC8A1,AGT,NF1,PKP2,CTDP1,SETD3,KCNH1,CHD7,PGM5,CAPN3,MAP3K5,HIRA,MYPN,ANK2,NEB,NLN,ITGA11,CENPF,RBFOX1,RARB,COL19A1,SGCZ,EPHB1,FHOD3,LRP2,SGCD,BASP1,HDAC5,MYH9,TANC1,LARGE,PRKAR1A,PPARA,RORA,MYH15
GO:0016049	cell growth	0.0001792946717193678	DSCAM,AUTS2,VCL,SEMA5A,APP,AKAP6,DCLK1,MAP2K5,AKAP13,DCC,SEMA4D,BDKRB1,PARK2,NRG3,SH3GL2,SLIT2,SMAD3,NTN1,EPM2A,MAP2,CDC73,CDH4,TNR,SPOCK1,PLCE1,SERTAD2,DISC1,EFNA5,SEMA6D,PRKCQ,DPYSL2,BDNF,FSTL4,SYT1,EBAG9,DAB2,ENPP1,RIMS2,NRG1,MAP3K13,AGT,GSK3B,CTDP1,UNC13A,ISLR2,MACF1,SLIT3,SEMA5B,SPG11,EPHA7,RIMS1,EXT1,TRPC5,TMEM108,CDKL5,ESR2,SEMA3A,GNG4,CLASP2,PPARA,SEMA3D,SYT17,SEMA3C
GO:0098655	cation transmembrane transport	0.00020147557776425954	CACNG2,SLC39A10,DMD,KCNC2,NOS1,TMC2,DPP10,CACNA1A,TRPM1,PRKD1,RYR2,NLGN1,KCNIP4,RELN,APP,UTRN,RYR1,AKAP6,RYR3,KCNB2,FAM155A,KCNA6,BDKRB1,DPP6,GRIN3A,CACNA1C,SLC5A10,TRPM3,KCNJ16,SLC4A4,CACNA1E,SLC9C1,SLC47A1,FGF14,CACNA2D3,SLC1A1,SHISA9,NOS1AP,CATSPER2,ANK3,CNIH3,SCN9A,ACTN4,SLC30A7,KCNN3,PDE4D,KCNJ12,KCNJ3,PDE4B,KCND2,SLC24A3,EPHB2,ANO1,CACNG3,TMEM163,SLC39A11,SCN8A,PEX5L,KCNMA1,CNGB1,SLC12A8,SLC8A1,SLC9A9,AGT,KCNQ5,KCNJ6,NRXN1,KCNH1,CHD7,CACNB2,KCNS3,CAPN3,HTR2C,P2RX6,ANK2,GRIN2B,ITPR2,CACNA1D,PKD1L1,SLC9B1,RASGRF2,SCN1A,FYN,KCND3,KCNQ1,TRPC5,NOX5,KCNH7,TRDN,CACHD1,ATP13A3,STIM1,RGS7,SLC24A2,SHISA6,ATP8A1,KCNC4,STAC,KCNJ15
GO:0098660	inorganic ion transmembrane transport	0.00020669639127415994	CACNG2,SLC39A10,DMD,KCNC2,NOS1,TMC2,DPP10,CACNA1A,TRPM1,PRKD1,RYR2,KCNIP4,UTRN,RYR1,GABRG3,AKAP6,RYR3,KCNB2,FAM155A,KCNA6,BDKRB1,DPP6,GRIN3A,CACNA1C,ANO4,ANKH,SLC5A10,TRPM3,KCNJ16,SLC4A4,CACNA1E,GABRR2,SLC9C1,SLC47A1,ANO2,FGF14,CACNA2D3,SLC1A1,NOS1AP,CATSPER2,ANK3,SCN9A,ACTN4,KCNN3,PDE4D,KCNJ12,KCNJ3,PDE4B,GABRB3,KCND2,SLC24A3,ANO1,CACNG3,TMEM163,SLC39A11,SCN8A,KCNMA1,SLC12A8,SLC8A1,SLC9A9,KCNQ5,KCNJ6,KCNH1,CHD7,CACNB2,ANO3,KCNS3,CAPN3,HTR2C,GLRA2,ANK2,GRIN2B,ITPR2,GABRR3,CACNA1D,GRM5,PKD1L1,SLC9B1,GABRA3,SCN1A,FYN,KCND3,KCNQ1,TRPC5,NOX5,KCNH7,TRDN,CACHD1,STIM1,GABRB1,RGS7,SLC24A2,KCNC4,STAC,KCNJ15
GO:005077	positive	0.00022997084938020	NTRK2,ROBO1,DSCAM,SEMA5A,ROBO2,SEMA4D,SLIT2,NTN1,CDH4,DISC1,EFNA5,BDNF,PLXNA2,MAP3K13,TIAM2,ISLR2,

2	regulation of axonogenesis	083	*MACF1,MAP2K1,TRPC5,CDKL5*
GO:0051271	negative regulation of cellular component movement	0.0002680210136547245	*PTPRR,CTNNA1,CLASP1,PTPRG,ROBO1,LDLRAD4,PTPRT,PLCB1,VCL,KANK1,DLC1,MAP2K5,MAGI2,NRG3,SULF1,SLIT2,BMPR1A,CD300A,RABGEF1,SEMA6D,ADTRP,MCTP1,DACH1,PRKG1,SRGAP3,NRG1,SPINT2,NF1,SRGAP2B,DLG5,DUSP22,PTPRO,FOXO3,MITF,FRMD5,PKHD1,MARVELD3,NAV3,PTPRM,PTPRU,HDAC5,SEMA3A,CLASP2,PTPRK*
GO:0035637	multicellular organismal signaling	0.00030880773024659873	*CACNG2,NTRK2,NFASC,RYR2,RYR3,MEF2A,CTNNA3,GRIK2,CACNA1C,ANK3,TNR,SCN9A,PDE4D,KCNJ3,KCND2,SPTBN4,CACNG3,TNNI3K,SCN8A,SLC8A1,AGT,PKP2,CACNB2,ANK2,CACNA1D,CNTNAP2,SCN1A,KCND3,KCNQ1,TRDN*
GO:0007611	learning or memory	0.00031164784454738056	*RCAN1,KALRN,NTRK2,PLCB1,BTBD9,RELN,APP,SORCS3,EP300,DNAH11,PARK2,NLGN4X,BRINP1,DGKI,SLC1A1,EPM2A,TNR,ATXN1,EPHB2,BDNF,CSMD1,AFF2,PRKAR1B,PRKCG,AGT,NF1,NRXN1,SHANK2,GRIN2B,ADCY8,GRM5,CNTNAP2,INSR,FYN,AMFR,CAMK4,NRXN3,TANC1,SLC24A2,ELAVL4,ATP8A1*
GO:0050890	cognition	0.00039878042841103804	*RCAN1,KALRN,NTRK2,PLCB1,BTBD9,RELN,APP,SORCS3,EP300,DNAH11,CHRM1,PARK2,NLGN4X,BRINP1,DGKI,SLC1A1,EPM2A,TNR,ATXN1,EPHB2,BDNF,CSMD1,AFF2,PRKAR1B,PRKCG,AGT,NF1,NRXN1,CHD7,SHANK2,GRIN2B,ADCY8,GRM5,CNTNAP2,INSR,FYN,AMFR,CAMK4,SHROOM4,NRXN3,SOBP,TANC1,SLC24A2,ELAVL4,ATP8A1*
GO:0099173	postsynapse organization	0.00041007487651736587	*KALRN,IL1RAPL1,NLGN1,RELN,CTNND2,TANC2,NLGN4X,DGKB,NOS1AP,ARHGAP39,GRID2,EPHB2,NRXN1,GRIN2B,PAK3,IGF1R,INSR,LRRK2,FRMPD4,FYN,EPHA7,EPHB1,TMEM108,CDKL5,TANC1,NTRK3,DNM3,PTPRD,SHISA6,DOCK10*
GO:0071840	cellular component organization or biogenesis	0.0004193357220421231	*RNF4,CHFR,KDM4B,KALRN,CACNG2,RPS6KA2,NDE1,CTNNA1,NTRK2,CLASP1,PTPRG,SYNE1,DMD,KCNC2,EIF3E,ERBB4,LAMA2,CNTN4,ROBO1,ANKRD30BL,DSCAM,SHROOM3,MDN1,CIT,TBCD,FRY,NEBL,HDAC4,ATP9A,LINGO2,ARFGAP3,IMMP2L,ABCC1,ATP8A2,AUTS2,LDLRAD4,TENM2,BLM,PSMB2,RXFP1,ATF7IP,NFASC,PLCB1,SRPK2,TRPM1,PRKD1,VCL,PDS5A,NEDD9,PPP2R3C,SEMA5A,ETV6,PRKCA,EPB41L4B,STX8,BTBD9,IL1RAPL1,ADAMTS6,MYO9A,PDE4DIP,CALD1,ITGAE,ABCA13,KANK1,DIAPH2,TBC1D5,RUNX1,PLS1,FCHSD2,NLGN1,DNAH9,CAMK1D,SMG6,CLEC16A,AMOTL1,TCF7L2,MTRF1,RELN,APP,PAK1,UTRN,PPP3CA,RYR1,TOX,PCDH15,ERC2,PTPN9,AKAP6,GRM7,DLC1,SCMH1,RYR3,SOS1,CTNND2,LRRC8C,RFTN1,ALK,DCLK1,MEF2A,KCNB2,CTNNA3,DENND5A,MAP2K5,DNMT1,MAGI2,CELSR1,ROBO2,IFT81,AKAP13,DCC,ELMO2,C10ORF90,EP300,KCNA6,PARN,SEMA4D,RPS6KA5,PHF20,MMP16,BDKRB1,NME8,TF,CDH8,HUS1,TANC2,JAK2,ABCG8,NEGR1,SDK2,MAST4,PARK2,MAGI1,NPHP3,PRELID2,GRIN3A,SMOC2,NLGN4X,EXOC4,EMB,PNPLA3,ANO4,PIK3C2B,AFAP1,UPB1,LRRC49,NRG3,NUMB,COL4A6,TRPM3,SULF1,CHCHD6,SH3BP1,RNU6ATAC31P,SH3GL2,COL24A1,ARHGAP24,COL11A1,PITPNC1,SLIT2,SYNM,KMT2C,ATRX,RAD51B,SLX1B,GTF2E1,ACACA,DSCAML1,SMAD3,BORA,PI4KA,TNFRSF11B,PIBF1,PIK3C3,CHD6,PEAK1,EYA1,SATB2,HSF2BP,ELN,MAD1L1,EGFLAM,DGKB,TNKS,TUB,ARHGEF18,SCAMP5,DNAJB6,OMA1,ARRDC4,SPESP1,IL1RAPL2,SPAG17,SKAP1,TAF1,SLC1A1,NTN1,LDB2,EPM2A,SHFM1,MAP2,CDC73,FLRT2,FBXW11,NOS1AP,RANBP10,ARHGEF6,WWTR1,GAS7,ARHGAP6,KCTD7,CHAF1B,STARD9,CUX1,ANK3,CDH12,MORC2,ARID4B,THSD4,TTLL8,CDH4,TNR,CELF4,VAV3,CDKL1,MDM4,SPOCK1,ACTN4,RAB2A,HPSE2,PLCE1,TACC2,EPS8,TENM4,CECR2,PRR16,ARHGAP39,LRRC8D,CDH10,LRRC4C,PPP1R9A,PDZRN3,CLSTN2,CD300A,TTLL11,CBFA2T2,SERTAD2,STRC,DISC1,POC1A,ATP8B4,IFT43,RABGEF1,NSG2,TRABD2B,LRP5,NAV2,AP2B1,ESR1,ARHGAP12,SCFD1,CNTN5,THEM4,BLOC1S5,RNU6-78P,ARMC2,EFNA5,TLN2,KLHL1,MRM1,EIF3A,ATF2,PHACTR2,KCNJ12,GRID2,ZNF423,SEMA6D,CELF2,NTNG1,ADTRP,CRMP1,MAPRE2,GABRB3,ANKRD13A,PRKCQ,RASSF8,RHPN2,CLDN1,WDR83OS,KCND2,TTLL5,SPTBN4,EPHB2,ERC1,AGO3,MCTP1,PRTG,COL22A1,DOCK2,DPYSL2,PTPDC1,RFC3,MARK1,BDNF,FNIP1,ADORA2A,IFT80,DYM,FSTL4,MCM3,CCDC170,TTC39C,RNU6-1269P,TSPAN33,ERI2,BCOR,ICK,THSD7B,BRD9,SYT1,EBAG9,DAB2,AFF2,PLXNA2,NSF,PRKG1,STXBP6,SNAP23,ABI1,EPG5,MICAL3,HDAC2,ETS1,BANP,FAT3,ARID4A,RAB30,ZMYND11,CDH23,TTN,ENPP1,RIMS2,HEPACAM2,CTNNA2,PEX5L,LRCH*

			3,FMNL2,PARD3,NRG1,CAST,SPINT2,ATP10B,FMN2,TOP1,RIT2,CD44,KCNMA1,SCAF8,CNGB1,GPSM2,SLC12A8,ZFYVE1,TEAD1,NFIA,RAPGEF6,FBXO31,MAP3K13,CDC42BPA,CDC42EP3,SAMM50,NCAM1,AGT,STRIP1,GRIP1,TLK1,ARHGAP25,NF1,VPS16,PARVB,NRXN1,ARHGAP10,LAMA3,PDXP,GSK3B,ADAMTS17,PKP2,CTDP1,SETD3,BBS9,CHD7,PSMB7,MOV10L1,THSD7A,TIAM2,IQCJ-SCHIP1,CNTN1,UNC13A,TRIOBP,CABIN1,CHEK2,CACNB2,RHOJ,ESYT2,MECOM,SYBU,SHANK2,KCTD8,UNC5D,ISLR2,NREP,GPC3,DLG5,PGM5,SH3BGR,PREX1,ANO3,XKR4,ROR1,ADAMTSL1,SERPINA5,KCNS3,COL12A1,BID,MACF1,MNAT1,TRIO,CAPN3,TRMT61B,DUSP22,CLDN11,SPAG16,CCDC141,SNAP25-AS1,FGD4,EPHA5,SGIP1,HIRA,TP73,FAM171A1,MYPN,ANK2,SLIT3,GRIN2B,SHPRH,PAK3,PRRC2C,ADCY8,MLLT3,NEB,ITGB3BP,RNF213,RNU6-1061P,PRKAA2,CHCHD3,SEMA5B,ANKFN1,TRAPPC8,SDK1,GRM5,IGF1R,TSNARE1,CNTNAP2,PTPRO,INSR,RPGR,CRTAC1,MAP2K1,UBR2,KPNB1,GPC6,ADAMTSL3,CNTN6,CENPF,KCTD1,ATP9B,MITF,SPECC1L,LRFN5,CDC14A,KPNA3,PRDM16,FRMD5,ULK4,TMEM120B,ADAMTS16,LRRK2,PKHD1,KIF2A,TCF4,SPG11,PHACTR1,BMP6,TMEM170A,VTI1A,ASAP1,FRMPD4,COL23A1,FYN,KCND3,EPHA7,MARVELD3,GAS8,COL19A1,MSR1,SGCZ,CLN6,RIMS1,ENPP2,L3MBTL4,ABCA12,EPHB1,KCNQ1,FHOD3,DOCK1,VASH2,EXT1,TRPC5,UNC5C,CSGALNACT1,TENM3,NOX5,LRP2,FER,DDX10,PCDH17,TMEM108,SHROOM4,RAPGEF2,NAV3,CENPP,PTPRM,KIRREL3,NRXN3,CDKL5,ZNF207,MPRIP,ESR2,KAT7,SATB1,C9,SETD2,PACSIN2,CDH13,TRDN,DAB1,ZNF148,SEC16B,EXOC6B,HDAC5,SEMA3A,MYH9,COL21A1,TANC1,VPS41,BRF1,TENM1,STK38L,VPS39,SH3KBP1,PRKAR1A,ZRANB1,STXBP5,SIPA1L3,GNG4,KANSL1,NTRK3,HYDIN,CEP89,DIS3L2,CLASP2,DNAJC6,PTPRK,DNM3,SYNDIG1,PPARA,NCKAP5,PTPRD,SHISA6,BRIP1,ELAVL4,CDH9,ANXA8L1,DOCK10,ATP8A1,SEMA3D,PRLR,PTPRA,KCNC4,CEP41,DEPTOR,NVL,PPFIBP2,SMIM20,RAD51D,SYT17,RAB27A,TUBGCP6,BLOC1S6,PMEPA1,PARD3B,HGSNAT,SYT9,SEMA3C
GO:0007612	learning	0.0005033078560886979	*KALRN,NTRK2,PLCB1,RELN,APP,SORCS3,PARK2,NLGN4X,DGKI,SLC1A1,EPM2A,TNR,ATXN1,EPHB2,CSMD1,AGT,NF1,NRXN1,SHANK2,GRM5,CNTNAP2,INSR,FYN,NRXN3,TANC1,SLC24A2,ELAVL4,ATP8A1*
GO:0043085	positive regulation of catalytic activity	0.0006983658973497254	*KALRN,NTRK2,SLC39A10,ERBB4,NOS1,ROBO1,PRIM2,SGSM1,FLT3,PRKD1,NEDD9,PPP2R3C,MYO9A,TBC1D5,TNFRSF10B,RELN,APP,PAK1,RABGAP1L,DLC1,ALK,BTRC,MAP2K5,MAGI2,AKAP13,PARN,SEMA4D,JAK2,ADCYAP1R1,CACNA1C,NRG3,SH3BP1,ARHGAP24,BMPR1A,SMAD3,BORA,PIBF1,TNKS,GRAMD4,BCL2L13,ARRDC4,SLC1A1,NOS1AP,ARHGAP6,EGLN3,CCL15,DOCK3,VAV3,GHR,CD300A,ARHGAP42,CCL14,ESR1,SLC5A3,EFNA5,MAPRE2,MAP3K7,PRKCQ,EPHB2,MOB3B,RFC3,SIPA1L2,NOX4,ABI1,RGS6,NRG1,TBC1D9,CD44,RAPGEF6,MAP3K13,AGT,FGF1,ARHGAP25,NF1,NRXN1,GSK3B,TIAM2,PREX1,ROR1,BID,MNAT1,MAP3K5,EPHA5,GRIN2B,GARNL3,UACA,ADCY8,CACNA1D,GRM5,ARAP2,IGF1R,INSR,MAP2K1,LRRK2,ASAP1,FYN,XRCC4,EPHA7,EPHB1,CRADD,RAPGEF2,CDKL5,DAB1,STIM1,TENM1,RGS7,SIPA1L3,NTRK3,DOCK9,DOCK10,PRLR,NVL,RALGAPA1,ST18*
GO:0099003	vesicle-mediated transport in synapse	0.0007506061414041525	*SYN3,CASK,BTBD9,ABCA13,NLGN1,ERC2,ITSN1,GRIN3A,NLGN4X,NUMB,DGKI,AP2B1,ADORA2A,CADPS,SYT1,SNAP23,PRKAR1B,RIMS2,NRG1,PRKCG,NRXN1,GSK3B,UNC13A,CACNB2,LRRK2,RIMS1,PCDH17,NRXN3,STXBP5,DNAJC6,DNM3,SYNDIG1,BLOC1S6,SYT9*
GO:0099643	signal release from synapse	0.000779906275079349	*SYN3,CASK,NLGN1,ERC2,PARK2,GRIN3A,PTPRN2,DGKI,GRM4,MCTP1,ADORA2A,CADPS,SYT1,SNAP23,RIMS2,PRKCG,NF1,NRXN1,GSK3B,UNC13A,CACNB2,LRRK2,RIMS1,MCTP2,NRXN3,STXBP5,BLOC1S6,SYT9*
GO:0007269	neurotransmitter secretion	0.000779906275079349	*SYN3,CASK,NLGN1,ERC2,PARK2,GRIN3A,PTPRN2,DGKI,GRM4,MCTP1,ADORA2A,CADPS,SYT1,SNAP23,RIMS2,PRKCG,NF1,NRXN1,GSK3B,UNC13A,CACNB2,LRRK2,RIMS1,MCTP2,NRXN3,STXBP5,BLOC1S6,SYT9*
GO:0050877	nervous system process	0.0008203400021974756	*CAMTA1,RCAN1,KALRN,CACNG2,RPS6KA2,NTRK2,DMD,TMC2,MYO3A,ATP8A2,MGLL,NFASC,PLCB1,TRPM1,DLGAP1,BTBD9,MYO9A,USP53,NLGN1,DNAH9,RELN,APP,PPP3CA,PCDH15,GABRG3,SORCS3,GRM7,EYS,EP300,BDKRB1,DNAH11,G*

			RIK2,CHRM1,PARK2,GRIN3A,NLGN4X,OR5K4,TMPRSS3,TRPM3,COL11A1,SYNM,GABRR2,HMCN1,EYA1,TUB,BRINP1,OR51I1,DGKI,SLC1A1,SHISA9,EPM2A,ANK3,LHFPL3,TNR,CELF4,SCN9A,TENM4,STRC,NAV2,GRM1,CNTN5,POU6F2,GRID2,GABRB3,ATXN1,KCND2,SPTBN4,EPHB2,BDNF,ADORA2A,ANO1,CSMD1,AFF2,CACNG3,PRKAR1B,CDH23,RIMS2,SCN8A,DLGAP2,OR4N2,CTNNA2,PARD3,CNGB1,PRKCG,SRRM4,FAM19A4,THRB,AGT,USH2A,NF1,NRXN1,GSK3B,BBS9,CHD7,OR51B2,TRIOBP,CACNB2,SHANK2,ROR1,MYO3B,SPAG16,HTR2C,GLRA2,P2RX6,GRIN2B,ADCY8,GABRR3,CACNA1D,TBL1X,SEMA5B,ANKFN1,GRM5,CNTNAP2,INSR,RPGR,GABRA3,CDC14A,RBFOX1,LRRK2,SCN1A,VTI1A,FYN,CLN6,RIMS1,EPHB1,KCNQ1,AMFR,LRP2,CAMK4,GUCY2F,TMEM108,SHROOM4,NRXN3,SOBP,TANC1,GABRB1,SLC24A2,SHISA6,ELAVL4,ATP8A1,ATF6,STAC,OR4M1,CST2
GO:0009605	response to external stimulus	0.0008691037581459868	KALRN,CACNG2,PROS1,CLASP1,FBXL17,DMD,LAMA2,NOS1,CNTN4,TMC2,ROBO1,DSCAM,SHROOM3,PUM1,WDR59,HDAC4,ABCC1,ATP8A2,PSMB2,MGLL,NFASC,SRPK2,TRPM1,PRKD1,RYR2,PPP2R3C,SEMA5A,CASK,PRKCA,STX8,DIO2,TBC1D5,USP53,TNFRSF10B,CAMK1D,CLEC16A,RELN,APP,PAK1,DEPDC5,NAALADL2,PCDH15,ZPLD1,SOS1,ALK,DCLK1,EYS,ROBO2,DCC,ELMO2,EP300,SEMA4D,RPS6KA5,BDKRB1,TF,GRIK2,DEFA1B,JAK2,ABCG8,CMKLR1,PARK2,GRIN3A,SMOC2,HLCS,EMB,PIK3C2B,NRG3,TRPM3,CD84,OTC,PSPC1,COL11A1,SLIT2,TRIM5,TRAF3IP2,DSCAML1,SMAD3,TNFRSF11B,PIK3C3,HMCN1,PTGFR,DGKB,OMA1,ZDHHC11,SLC1A1,NTN1,CDC73,FLRT2,CD96,CCL15,TRIM59,CDH4,TNR,VAV3,GHR,SRD5A2,STRC,CCL14,RABGEF1,ESR1,PIK3CD,DOCK4,BLOC1S5,EFNA5,ATF2,GRID2,CYBB,SEMA6D,SUSD4,ADTRP,CRMP1,PDE4B,MAP3K7,PRKCQ,N4BP1,CLDN1,TPH2,EPHB2,PRTG,DOCK2,DPYSL2,BDNF,RBM4,FNIP1,ADORA2A,ANO1,WDFY4,KIR3DL1,CSMD1,DEFA3,PLXNA2,PRKG1,CALCRL,HDAC2,ETS1,ZMYND11,TTN,RNASET2,CDK19,CFB,CTNNA2,NRG1,BCKDHB,CNGB1,PRKCG,ZFYVE1,MAPKAPK2,SLC8A1,FAM19A4,B3GALT5,NCAM1,AGT,FGF1,CCR3,NRXN1,LAMA3,CD109,SLC22A3,CHD7,PSMB7,NCOA1,STXBP4,PHEX,SHANK2,UNC5D,GPC3,PREX1,ANO3,TTC4,ADAMTSL1,PTGER3,TRIO,CAPN3,MAP3K5,HTR2C,CCDC141,NCF2,LY86,EPHA5,SGIP1,IL1RL1,MYPN,SLIT3,PAK3,ADCY8,CRISP3,CCL15-CCL14,RNF213,PRKAA2,SEMA5B,IGF1R,PKD1L1,CNTNAP2,PTPRO,JAK1,MAP2K1,FOXO3,CNTN6,LRFN5,AGBL4,KIR2DL4,LRRK2,SCN1A,BMP6,FYN,EPHA7,ENPP2,EPHB1,KCNQ1,CRADD,EXT1,UNC5C,C2,FER,GUCY2F,PTPRM,NRXN3,MACROD2,C9,SETD2,TRIM22,CDH13,TRDN,SEMA3A,TSPAN8,VPS41,LITAF,SLC24A2,SCEL,NTRK3,CLASP2,PPARA,RORA,BRIP1,SEMA3D,PRLR,RAB27A,BLOC1S6,SEMA3C
GO:2000146	negative regulation of cell motility	0.0008759749586632649	PTPRR,CTNNA1,CLASP1,PTPRG,ROBO1,LDLRAD4,PTPRT,PLCB1,VCL,KANK1,DLC1,MAP2K5,MAGI2,NRG3,SULF1,SLIT2,BMPR1A,CD300A,RABGEF1,SEMA6D,ADTRP,MCTP1,DACH1,PRKG1,SRGAP3,NRG1,SPINT2,NF1,SRGAP2B,DLG5,DUSP22,FOXO3,MITF,FRMD5,MARVELD3,NAV3,PTPRM,PTPRU,HDAC5,SEMA3A,CLASP2,PTPRK
GO:0031589	cell-substrate adhesion	0.0009831341474848504	CLASP1,DMD,TBCD,VCL,NEDD9,CASK,KANK1,UTRN,DLC1,EDA,JAK2,ITGBL1,ATRNL1,EPDR1,SMAD3,PEAK1,EGFLAM,SKAP1,CD96,ARHGAP6,SPOCK1,ACTN4,STRC,DISC1,EFNA5,NTNG1,MEGF9,DAB2,CD44,USH2A,NF1,PARVB,GSK3B,TRIOBP,PREX1,MACF1,DUSP22,PTPRO,ITGA11,PKHD1,EPHB1,DOCK1,FER,VWC2,CDH13,VWF,CLASP2,PTPRK,PTPRA
GO:0019220	regulation of phosphate metabolic process	0.0011678068257716006	CAMTA1,RCAN1,NTRK2,SLC39A10,DMD,SPRED2,ERBB4,NOS1,ROBO1,DSCAM,HDAC4,LDLRAD4,BLM,PTPRT,FLT3,PRKD1,NEDD9,PPP2R3C,ZBTB20,SLCO3A1,TNFRSF10B,SMG6,RELN,APP,PAK1,DLC1,ALK,MAP2K5,MAGI2,AKAP13,EP300,SEMA4D,RPS6KA5,BDKRB1,TF,HUS1,JAK2,PPP6R2,PARK2,ADCYAP1R1,NRG3,SH3GL2,SLIT2,SLC4A4,BMPR1A,SMAD3,PRKAR2A,BORA,PIBF1,SLC1A1,LDB2,EPM2A,WWTR1,DOCK3,VAV3,PLCE1,GHR,CD300A,RABGEF1,LRP5,PDE4D,EFNA5,ATF2,PIK3R2,ADTRP,SMG7,MAP3K7,SPTBN4,EPHB2,MOB3B,BDNF,FNIP1,ADORA2A,NOX4,BMPER,DAB2,LRRK1,ABI1,HDAC2,PRKAR1B,TTN,ENPP1,PARD3,NRG1,RIT2,CD44,SLC8A1,MAP3K13,PLCL2,AGT,FGF1,NF1,NRXN1,GSK3B,CD109,CNTN1,CHEK2,ROR1,ZNF675,MNAT1,DUSP22,MAP3K5,HTR2C,EPHA5,ADCY8,PRKAA2,GRM5,IGF1R,PTPRO,INSR,MAP2K1,STK38,PPP1R14A,LRRK2,BMP6,FYN,EPHA7,ENPP2,EPHB1,TRPC5,FER,RAP

			GEF2,DAB1,MGAT5,BDKRB2,CCNG2,PIK3R3,TENM1,PRKAR1A,NTRK3,PPARA,PRLR,DEPTOR,PMEPA1
GO:0051174	regulation of phosphorus metabolic process	0.001266450731600063	*CAMTA1,RCAN1,NTRK2,SLC39A10,DMD,SPRED2,ERBB4,NOS1,ROBO1,DSCAM,HDAC4,LDLRAD4,BLM,PTPRT,FLT3,PRKD1,NEDD9,PPP2R3C,ZBTB20,SLCO3A1,TNFRSF10B,SMG6,RELN,APP,PAK1,DLC1,ALK,MAP2K5,MAGI2,AKAP13,EP300,SEMA4D,RPS6KA5,BDKRB1,TF,HUS1,JAK2,PPP6R2,PARK2,ADCYAP1R1,NRG3,SH3GL2,SLIT2,SLC4A4,BMPR1A,SMAD3,PRKAR2A,BORA,PIBF1,SLC1A1,LDB2,EPM2A,WWTR1,DOCK3,VAV3,PLCE1,GHR,CD300A,RABGEF1,LRP5,PDE4D,EFNA5,ATF2,PIK3R2,ADTRP,SMG7,MAP3K7,SPTBN4,EPHB2,MOB3B,BDNF,FNIP1,ADORA2A,NOX4,BMPER,DAB2,LRRK1,ABI1,HDAC2,PRKAR1B,TTN,ENPP1,PARD3,NRG1,RIT2,CD44,SLC8A1,MAP3K13,PLCL2,AGT,FGF1,NF1,NRXN1,GSK3B,CD109,CNTN1,CHEK2,ROR1,ZNF675,MNAT1,DUSP22,MAP3K5,HTR2C,EPHA5,ADCY8,PRKAA2,GRM5,IGF1R,PTPRO,INSR,MAP2K1,STK38,PPP1R14A,LRRK2,BMP6,FYN,EPHA7,ENPP2,EPHB1,TRPC5,FER,RAPGEF2,DAB1,MGAT5,BDKRB2,CCNG2,PIK3R3,TENM1,PRKAR1A,NTRK3,PPARA,PRLR,DEPTOR,PMEPA1*
GO:0030516	regulation of axon extension	0.0015862969529097657	*DSCAM,SEMA5A,SEMA4D,NTN1,MAP2,CDH4,TNR,DISC1,SEMA6D,DPYSL2,MAP3K13,GSK3B,ISLR2,MACF1,SEMA5B,TRPC5,CDKL5,SEMA3A,CLASP2,SEMA3D,SEMA3C*
GO:0008361	regulation of cell size	0.0017310025697472098	*DSCAM,SEMA5A,DCC,SEMA4D,NTN1,MAP2,CDH4,TNR,VAV3,PRR16,DISC1,EFNA5,SEMA6D,DPYSL2,BDNF,FSTL4,KCNMA1,SLC12A8,MAP3K13,GSK3B,ISLR2,MACF1,SEMA5B,EPHA7,TRPC5,CDKL5,SEMA3A,CLASP2,SEMA3D,DEPTOR,SEMA3C*
GO:2000026	regulation of multicellular organismal development	0.0018746118142414666	*KALRN,CTNNA1,NTRK2,CLASP1,SPRED2,ERBB4,LAMA2,ROBO1,DSCAM,LINGO2,PLCB1,PRKD1,VCL,PPP2R3C,SEMA5A,PRKCA,IL1RAPL1,RUNX1,NLGN1,RELN,ADAM12,PPP3CA,TOX,AKAP6,SOS1,ROBO2,DCC,SEMA4D,NPHP3,SMOC2,ANKH,NUMB,SOX6,SULF1,SLIT2,BMPR1A,SMAD3,OVOL2,BRINP1,SOX5,NTN1,MAP2,CDC73,FLRT2,WWTR1,CUX1,TRPS1,CDH4,TNR,TENM4,CLSTN2,DISC1,PIK3CD,EFNA5,ATF2,GRID2,CYBB,SEMA6D,EPHB2,S1PR3,PRTG,BDNF,FSTL4,BCOR,BMPER,PLXNA2,HDAC2,ETS1,ENPP1,PARD3,NRG1,SLC8A1,ISM1,FBXO31,MAP3K13,AGT,FGF1,CCR3,NF1,NRXN1,LAMA3,CTDP1,CD109,CHD7,TIAM2,RHOJ,ISLR2,DLG5,ZNF675,MACF1,CAPN3,TP73,RBM19,PAK3,SEMA5B,GRM5,INSR,JAK1,MAP2K1,FOXO3,MITF,RARB,BMP6,EPHA7,ENPP2,ABCA12,EPHB1,VASH2,TRPC5,LRP2,CAMK4,RAPGEF2,PTPRM,CDKL5,KAT7,BASP1,DAB1,SEMA3A,STIM1,NTRK3,FBN1,CLASP2,SYNDIG1,PPARA,PTPRD,NELL1,SEMA3D,PRLR,SEMA3C*
GO:0001558	regulation of cell growth	0.0019690793746625283	*DSCAM,SEMA5A,AKAP6,MAP2K5,DCC,SEMA4D,BDKRB1,PARK2,NRG3,SLIT2,SMAD3,NTN1,EPM2A,MAP2,CDC73,CDH4,TNR,SPOCK1,PLCE1,SERTAD2,DISC1,EFNA5,SEMA6D,PRKCQ,DPYSL2,BDNF,FSTL4,SYT1,EBAG9,DAB2,ENPP1,RIMS2,NRG1,MAP3K13,AGT,GSK3B,CTDP1,UNC13A,ISLR2,MACF1,SLIT3,SEMA5B,EPHA7,RIMS1,TRPC5,CDKL5,ESR2,SEMA3A,GNG4,CLASP2,PPARA,SEMA3D,SYT17,SEMA3C*
GO:0010721	negative regulation of cell development	0.0020141552723614868	*CTNNA1,SEMA5A,KANK1,PPP3CA,DCC,SEMA4D,BMPR1A,BRINP1,NTN1,MAP2,TNR,ACTN4,EFNA5,SEMA6D,EPHB2,S1PR3,PRTG,FSTL4,NF1,GSK3B,SEMA5B,EPHA7,TRPC5,RAPGEF2,DAB1,SEMA3A,NTRK3,FBN1,SEMA3D,SEMA3C*
GO:0018105	peptidyl-serine phosphorylation	0.002149641726870372	*RPS6KA2,NTRK2,DMD,NOS1,SRPK2,PRKD1,PRKCA,CAMK1D,APP,PAK1,DCLK1,RPS6KA5,MAST4,TNKS,TAF1,SLC1A1,EPM2A,PDE4D,PRKCQ,SPTBN4,MARK1,BDNF,FNIP1,TOP1,CD44,PRKCG,MAPKAPK2,MAP3K13,PLCL2,DYRK4,TLK1,NRXN1,GSK3B,CHEK2,STK32B,MORC3,PRKAA2,STK38,LRRK2,CAMK4,BDKRB2,TENM1,STK38L,NTRK3*
GO:0032535	regulation of cellular component size	0.0021823552466816625	*DSCAM,CIT,SEMA5A,KANK1,PLS1,FCHSD2,DCC,SEMA4D,SH3BP1,SLIT2,ELN,NTN1,MAP2,CDH4,TNR,VAV3,EPS8,PRR16,DISC1,EFNA5,SEMA6D,SPTBN4,DPYSL2,BDNF,FSTL4,KCNMA1,SLC12A8,MAP3K13,CDC42EP3,PDXP,GSK3B,TRIOBP,ISLR2,MACF1,PAK3,NEB,SEMA5B,EPHA7,FHOD3,TRPC5,FER,CDKL5,SEMA3A,TENM1,CLASP2,SEMA3D,DEPTOR,SEMA3C*
GO:0036211	protein modification process	0.0025701495463070433	*PTPRR,RNF4,CAMTA1,CHFR,KDM4B,RCAN1,KALRN,RNF43,RPS6KA2,NTRK2,ZNRF3,PTPRG,FBXL17,SLC39A10,DMD,CHRM3,SPRED2,PHKB,ERBB4,NOS1,ROBO1,MYO3A,CIT,FRY,HUNK,FUT8,HDAC4,AUTS2,LDLRAD4,BLM,PSMB2,PTPRT,FLT3,SRPK2,PRKD1,NEDD9,PPP2R3C,CASK,PRKCA,SLCO3A1,USP53,TNFRSF10B,CAMK1D,RELN,APP,PAK1,PPP3CA,PTPN9,MGAT4C,GALNT14,DLC1,ALK,BTRC,DCLK1,MAP2K5,DNMT1,MAGI2,AKAP13,C10ORF90,DDB1,EP300,SEMA4D,RPS6KA5,P*

			HF20,GRK5,BDKRB1,MAN2B1,HUS1,ZDHHC11B,JAK2,FPGT-TNNI3K,PPP6R2,MAST4,PARK2,HLCS,MAN1A1,NRG3,RFFL,SH3GL2,SLIT2,MAPK4,KMT2C,TRIM5,ATRX,TRAF3IP2,SH3RF3,BMPR1A,PRKAR2A,BORA,PIBF1,PIK3C3,PEAK1,EYA1,EGFLAM,TNKS,ARRDC4,ZDHHC11,PTPRN2,UBE2R2,TAF1,SLC1A1,RNF144B,EPM2A,CDC73,FBXW11,NOS1AP,MKRN3,WWTR1,PHF20L1,ST3GAL3,EGLN3,ARID4B,DOCK3,TRIM59,TTLL8,CDKL1,PLCE1,MACROD1,GHR,PDZRN3,CD300A,TTLL11,RABGEF1,TRABD2B,LRP5,PDE4D,PIK3CD,EFNA5,ATF2,ADTRP,FBXL7,PPA2,MAP3K7,PRKCQ,N4BP1,GALNT8,USP12,JDP2,TTLL5,SPTBN4,TMTC1,EPHB2,ERC1,MOB3B,PTPDC1,MARK1,BDNF,FNIP1,ADORA2A,NOX4,BCOR,BMPER,ICK,DAB2,LRRK1,PRKG1,ABI1,TNNI3K,HDAC2,ARID4A,PRKAR1B,TTN,CDK19,ENPP1,KSR2,ZZZ3,PARD3,NRG1,DPY19L1,TOP1,RIT2,CD44,PRKCG,MAPKAPK2,SLC8A1,FBXO31,MAP3K13,PLCL2,RSRC1,CDC42BPA,B3GALT5,MAPK10,AGT,FGF1,DYRK4,TLK1,SUMF1,NF1,NRXN1,AGBL1,PDXP,GSK3B,ST6GALNAC3,CTDP1,SETD3,CD109,LMO7,WDR70,PSMB7,CNTN1,TRIOBP,NCOA1,CHEK2,PHEX,MECOM,STK32B,RSPRY1,UBE2E2,CPE,UBE2V1,ROR1,ZNF675,MNAT1,TRIO,CAPN3,PTPRE,MYO3B,DUSP22,ST6GALNAC5,MAP3K5,CTDSPL2,EPHA5,RNF144A,MORC3,SUPT3H,SHPRH,PAK3,DPH6,ADCY8,RNF213,SH3RF2,PRKAA2,TBL1X,GRM5,IGF1R,PTPRO,ZDHHC14,MSRA,INSR,JAK1,MAP2K1,UBR2,FBXL18,STK38,KLHL3,CDC14A,AGBL4,PRDM16,ULK4,DNMT3B,LRRK2,BMP6,FYN,EPHA7,SEL1L2,PTPN14,ENPP2,EPHB1,EXT1,TRPC5,AMFR,FER,CAMK4,GUCY2F,TPTE,RAPGEF2,PTPRM,CDKL5,PTPRU,MACROD2,KAT7,TMTC2,SETD2,TRIM22,DAB1,HDAC5,MGAT5,BDKRB2,CCNG2,PIK3R3,TENM1,STK38L,LARGE,ST8SIA1,PRKAR1A,ZRANB1,KANSL1,DPY19L2,NTRK3,DNAJC6,PTPRK,PTPRD,GALNTL6,PRLR,PTPRA,CEP41,DEPTOR,CUL2,PMEPA1,UBE3C*
GO:0006464	cellular protein modification process	0.0025701495463070433	*PTPRR,RNF4,CAMTA1,CHFR,KDM4B,RCAN1,KALRN,RNF43,RPS6KA2,NTRK2,ZNRF3,PTPRG,FBXL17,SLC39A10,DMD,CHRM3,SPRED2,PHKB,ERBB4,NOS1,ROBO1,MYO3A,CIT,FRY,HUNK,FUT8,HDAC4,AUTS2,LDLRAD4,BLM,PSMB2,PTPRT,FLT3,SRPK2,PRKD1,NEDD9,PPP2R3C,CASK,PRKCA,SLCO3A1,USP53,TNFRSF10B,CAMK1D,RELN,APP,PAK1,PPP3CA,PTPN9,MGAT4C,GALNT14,DLC1,ALK,BTRC,DCLK1,MAP2K5,DNMT1,MAGI2,AKAP13,C10ORF90,DDB1,EP300,SEMA4D,RPS6KA5,PHF20,GRK5,BDKRB1,MAN2B1,HUS1,ZDHHC11B,JAK2,FPGT-TNNI3K,PPP6R2,MAST4,PARK2,HLCS,MAN1A1,NRG3,RFFL,SH3GL2,SLIT2,MAPK4,KMT2C,TRIM5,ATRX,TRAF3IP2,SH3RF3,BMPR1A,PRKAR2A,BORA,PIBF1,PIK3C3,PEAK1,EYA1,EGFLAM,TNKS,ARRDC4,ZDHHC11,PTPRN2,UBE2R2,TAF1,SLC1A1,RNF144B,EPM2A,CDC73,FBXW11,NOS1AP,MKRN3,WWTR1,PHF20L1,ST3GAL3,EGLN3,ARID4B,DOCK3,TRIM59,TTLL8,CDKL1,PLCE1,MACROD1,GHR,PDZRN3,CD300A,TTLL11,RABGEF1,TRABD2B,LRP5,PDE4D,PIK3CD,EFNA5,ATF2,ADTRP,FBXL7,PPA2,MAP3K7,PRKCQ,N4BP1,GALNT8,USP12,JDP2,TTLL5,SPTBN4,TMTC1,EPHB2,ERC1,MOB3B,PTPDC1,MARK1,BDNF,FNIP1,ADORA2A,NOX4,BCOR,BMPER,ICK,DAB2,LRRK1,PRKG1,ABI1,TNNI3K,HDAC2,ARID4A,PRKAR1B,TTN,CDK19,ENPP1,KSR2,ZZZ3,PARD3,NRG1,DPY19L1,TOP1,RIT2,CD44,PRKCG,MAPKAPK2,SLC8A1,FBXO31,MAP3K13,PLCL2,RSRC1,CDC42BPA,B3GALT5,MAPK10,AGT,FGF1,DYRK4,TLK1,SUMF1,NF1,NRXN1,AGBL1,PDXP,GSK3B,ST6GALNAC3,CTDP1,SETD3,CD109,LMO7,WDR70,PSMB7,CNTN1,TRIOBP,NCOA1,CHEK2,PHEX,MECOM,STK32B,RSPRY1,UBE2E2,CPE,UBE2V1,ROR1,ZNF675,MNAT1,TRIO,CAPN3,PTPRE,MYO3B,DUSP22,ST6GALNAC5,MAP3K5,CTDSPL2,EPHA5,RNF144A,MORC3,SUPT3H,SHPRH,PAK3,DPH6,ADCY8,RNF213,SH3RF2,PRKAA2,TBL1X,GRM5,IGF1R,PTPRO,ZDHHC14,MSRA,INSR,JAK1,MAP2K1,UBR2,FBXL18,STK38,KLHL3,CDC14A,AGBL4,PRDM16,ULK4,DNMT3B,LRRK2,BMP6,FYN,EPHA7,SEL1L2,PTPN14,ENPP2,EPHB1,EXT1,TRPC5,AMFR,FER,CAMK4,GUCY2F,TPTE,RAPGEF2,PTPRM,CDKL5,PTPRU,MACROD2,KAT7,TMTC2,SETD2,TRIM22,DAB1,HDAC5,MGAT5,BDKRB2,CCNG2,PIK3R3,TENM1,STK38L,LARGE,ST8SIA1,PRKAR1A,ZRANB1,KANSL1,DPY19L2,NTRK3,DNAJC6,PTPRK,PTPRD,GALNTL6,PRLR,PTPRA,CEP41,DEPTOR,CUL2,PMEPA1,UBE3C*
GO:0014706	striated muscle tissue	0.0026477294894996484	*RCAN1,DMD,ERBB4,NEBL,HDAC4,RYR2,RUNX1,PPP3CA,RYR1,AKAP6,MEF2A,AKAP13,HIVEP3,EP300,SOX6,COL11A1,BMPR1A,SMAD3,EYA1,ELN,MYO18B,TENM4,FHL2,NOX4,TTN,NR*

	development		*G1,SLC8A1,AGT,NF1,PKP2,CTDP1,CHD7,PGM5,TP73,NEB,NLN,CENPF,RBFOX1,RARB,COL19A1,SGCZ,EPHB1,FHOD3,LRP2,SGCD,PRKAR1A,PPARA,MYH15,SEMA3C*
GO:0097435	supramolecular fiber organization	0.002759489867528371	*NDE1,CTNNA1,CLASP1,DMD,SHROOM3,CIT,TBCD,NEBL,NEDD9,SEMA5A,PDE4DIP,CALD1,KANK1,DIAPH2,PLS1,FCHSD2,APP,PAK1,PCDH15,DLC1,MEF2A,CTNNA3,AKAP13,ELMO2,TF,JAK2,PARK2,COL4A6,SH3BP1,COL24A1,COL11A1,SLIT2,SMAD3,ELN,ARHGEF18,DNAJB6,MAP2,GAS7,ARHGAP6,THSD4,EPS8,PPP1R9A,ARHGAP12,RHPN2,SPTBN4,ABI1,MICAL3,TTN,CTNNA2,FMN2,CDC42EP3,ARHGAP25,NF1,PDXP,PKP2,TRIOBP,PGM5,PREX1,COL12A1,BID,CAPN3,FAM171A1,MYPN,PAK3,NEB,KPNB1,KIF2A,PHACTR1,FHOD3,EXT1,FER,SHROOM4,NAV3,ZNF207,MPRIP,TRDN,COL21A1,TENM1,SH3KBP1,PRKAR1A,CLASP2,NCKAP5,TUBGCP6*
GO:0042325	regulation of phosphorylation	0.002770498368311414	*NTRK2,DMD,SPRED2,ERBB4,NOS1,ROBO1,DSCAM,HDAC4,LDLRAD4,BLM,PTPRT,FLT3,PRKD1,NEDD9,PPP2R3C,ZBTB20,SLCO3A1,TNFRSF10B,RELN,APP,PAK1,ALK,MAP2K5,AKAP13,EP300,SEMA4D,RPS6KA5,BDKRB1,TF,HUS1,JAK2,PARK2,NRG3,SH3GL2,SLIT2,SLC4A4,BMPR1A,PRKAR2A,BORA,PIBF1,SLC1A1,LDB2,EPM2A,WWTR1,DOCK3,VAV3,PLCE1,GHR,CD300A,RABGEF1,LRP5,PDE4D,EFNA5,ATF2,PIK3R2,ADTRP,MAP3K7,SPTBN4,EPHB2,MOB3B,BDNF,FNIP1,ADORA2A,NOX4,BMPER,DAB2,LRRK1,ABI1,HDAC2,PRKAR1B,TTN,ENPP1,PARD3,NRG1,RIT2,CD44,SLC8A1,MAP3K13,PLCL2,AGT,FGF1,NF1,NRXN1,CD109,CNTN1,CHEK2,ROR1,ZNF675,MNAT1,DUSP22,MAP3K5,EPHA5,ADCY8,PRKAA2,GRM5,IGF1R,PTPRO,INSR,MAP2K1,STK38,PPP1R14A,LRRK2,BMP6,FYN,EPHA7,ENPP2,EPHB1,TRPC5,FER,RAPGEF2,DAB1,BDKRB2,CCNG2,PIK3R3,TENM1,PRKAR1A,NTRK3,PPARA,PRLR,DEPTOR,PMEPA1*
GO:0099504	synaptic vesicle cycle	0.0028042356790028903	*SYN3,CASK,BTBD9,ABCA13,NLGN1,ERC2,ITSN1,GRIN3A,NLGN4X,DGKI,ADORA2A,CADPS,SYT1,SNAP23,PRKAR1B,RIMS2,PRKCG,NRXN1,GSK3B,UNC13A,CACNB2,LRRK2,RIMS1,PCDH17,NRXN3,STXBP5,DNAJC6,DNM3,SYNDIG1,BLOC1S6,SYT9*
GO:0060047	heart contraction	0.00342793272505754	*RPS6KA2,DMD,NOS1,HDAC4,RYR2,RYR3,MEF2A,CTNNA3,JAK2,CACNA1C,SLC1A1,NOS1AP,PDE4D,KCNJ12,KCNJ3,CELF2,PDE4B,SPTBN4,TNNI3K,TTN,SLC8A1,THRB,AGT,PKP2,CACNB2,ANK2,CACNA1D,RNLS,SCN1A,KCND3,SGCZ,KCNQ1,EXT1,SGCD,TRDN,SEMA3A*
GO:0030336	negative regulation of cell migration	0.0034362485470634503	*PTPRR,CLASP1,PTPRG,ROBO1,LDLRAD4,PTPRT,PLCB1,VCL,KANK1,DLC1,MAP2K5,MAGI2,NRG3,SULF1,SLIT2,BMPR1A,CD300A,RABGEF1,SEMA6D,ADTRP,MCTP1,DACH1,PRKG1,SRGAP3,NRG1,NF1,SRGAP2B,DLG5,DUSP22,FOXO3,MITF,MARVELD3,NAV3,PTPRM,PTPRU,HDAC5,SEMA3A,CLASP2,PTPRK*
GO:0007605	sensory perception of sound	0.003473454100594395	*TMC2,MYO3A,USP53,PCDH15,GRM7,TMPRSS3,COL11A1,EYA1,TUB,LHFPL3,STRC,NAV2,CNTN5,SPTBN4,CDH23,SRRM4,THRB,USH2A,CHD7,TRIOBP,ROR1,MYO3B,CACNA1D,TBL1X,CDC14A,KCNQ1,LRP2,SOBP*
GO:0051965	positive regulation of synapse assembly	0.004281182355153101	*NTRK2,LINGO2,IL1RAPL1,NLGN1,SEMA4D,FLRT2,CLSTN2,EFNA5,GRID2,EPHB2,BDNF,NRXN1,DLG5,EPHB1,SYNDIG1,PTPRD*
GO:0050954	sensory perception of mechanical stimulus	0.004706996963250193	*TMC2,MYO3A,USP53,PCDH15,GRM7,TMPRSS3,COL11A1,EYA1,TUB,LHFPL3,STRC,NAV2,CNTN5,SPTBN4,CDH23,SRRM4,THRB,USH2A,CHD7,TRIOBP,ROR1,MYO3B,CACNA1D,TBL1X,CDC14A,SCN1A,FYN,KCNQ1,LRP2,SOBP*
GO:0033036	macromolecule localization	0.004820921533930604	*LRP1B,CACNG2,CTNNA1,SYNE1,DMD,ERBB4,SIL1,SHROOM3,DPP10,GPC5,SORCS2,LRBA,ATP9A,ARFGAP3,IMMP2L,ABCC1,ATP8A2,AFTPH,WLS,SGSM1,SNUPN,NFASC,PRKD1,RYR2,VCL,STX8,ABCA13,TBC1D5,SLCO3A1,PLS1,FCHSD2,NLGN1,KCNIP4,SMG6,TCF7L2,RELN,PPP3CA,PTPN9,AKAP6,OSBPL1A,RABGAP1L,RFTN1,DCLK1,KCNB2,DLG2,MAGI2,CELSR1,PARN,SLCO2B1,DNAH11,GRIK2,QKI,CHRM1,SYTL5,ZDHHC11B,ITSN1,ASTN2,JAK2,ABCG8,PARK2,DPP6,PRELID2,MAN1A1,EXOC4,HEATR5A,ANO4,NBEA,NUMB,PITPNC1,CEP128,TRIM5,ATRX,TRAF3IP2,SMAD3,BORA,PIBF1,PIK3C3,MAD1L1,TNKS,TUB,ARHGEF18,SCAMP5,DNAJB6,ARRDC4,ZDHHC11,SKAP1,PTPRN2,SPNS2,SLC1A1,EPM2A,SHFM1,WWTR1,PCSK5,ANK3,ACTN4,RAB2A,ABCC11,DISC1,OSBPL10,ATP8B4,RABGEF1,LRP5,AP2B1,ESR1,DENND2A,SCFD1,SLC5A3,FRMD4A,RAB24,EFNA5,FTO,ATF2,PIK3R2,CCDC91,GRID2,ZNF423,ADTRP,MAPRE2,SMG7,ANKRD13A,WDR83OS,SPTBN4,EPHB2,ERC1,N*

			UP214,BDNF,ADORA2A,IFT80,ANO1,CADPS,TSPAN33,CHKA,DAB2,NSF,SNAP23,CACNG3,CALCRL,RANBP17,BANP,TTN,MYRIP,ENPP1,RIMS2,AGAP1,PEX5L,PARD3,TBC1D9,ATP10B,FMN2,RIT2,CNGB1,GPSM2,ZFYVE1,RAPGEF6,CHST11,SAMM50,AGT,GRIP1,TLK1,USH2A,NF1,VPS16,NRXN1,GSK3B,PKP2,BBS9,SNX5,CACNB2,STXBP4,ESYT2,GPC3,DLG5,CPE,ANO3,XKR4,SERPINA5,SLC5A8,BID,MACF1,CAPN3,MYOM1,CTDSPL2,SNAP25-AS1,EPHA5,SNX31,MORC3,ANK2,LRPPRC,ADCY8,RNF213,PRKAA2,PACRG,TRAPPC8,ATG4C,TSNARE1,MON2,CNTNAP2,ZDHHC14,SUFU,CIZ1,JAK1,RPGR,KPNB1,GPC6,TTC7B,CENPF,ATP9B,IGF2BP3,KPNA3,SPTSSA,RBFOX1,LTBP1,LRRK2,BMP6,VTI1A,FYN,ABCC2,XRCC4,MARVELD3,GAS8,MSR1,PTPN14,RIMS1,VPS53,ABCA12,KCNQ1,FAM126A,TRPC5,LRP2,RAPGEF2,PTPRU,KAT7,VPS45,SETD2,PACSIN2,TRIM22,SEC16B,EXOC6B,STOML1,MYH9,BDKRB2,NRIP1,VPS41,TENM1,VPS39,STXBP5,FBN1,CLASP2,NSUN2,PTPRK,SYNDIG1,PPARA,SCFD2,SHISA6,ATP8A1,CEP41,IPO11,NVL,STAC,RAB27A,BLOC1S6,PARD3B,SYT9
GO:0030900	forebrain development	0.005023504364134767	*NDE1,NTRK2,DMD,KCNC2,ERBB4,ROBO1,PLCB1,SEMA5A,RELN,APP,TOX,DLC1,ALK,DCLK1,ROBO2,TRAPPC9,NRG3,NUMB,SLIT2,ATRX,BMPR1A,SATB2,TNR,TACC2,SRD5A2,DISC1,EPHB2,PRKG1,NRG1,SLC8A1,NF1,GSK3B,CHD7,CCDC141,EPHA5,IGF1R,CNTNAP2,LRRK2,RARB,PHACTR1,FYN,EXT1,LRP2,TMEM108,RAPGEF2,KIRREL3,SETD2,DAB1,SEMA3A,ELAVL4*
GO:0051649	establishment of localization in cell	0.005408825567414865	*SYN3,CACNG2,PROS1,NDE1,CLASP1,DMD,NOS1,SIL1,SORCS2,ATP9A,ARFGAP3,IMMP2L,AFTPH,WLS,SGSM1,SNUPN,TRPM1,PRKD1,RYR2,CASK,STX8,BTBD9,ABCA13,TBC1D5,NLGN1,SMG6,CLEC16A,TCF7L2,APP,PPP3CA,RYR1,ERC2,AKAP6,RABGAP1L,RYR3,RFTN1,DCLK1,FAM155A,DENND5A,DLG2,IFT81,AKAP13,BDKRB1,CHRM1,SYTL5,ZDHHC11B,ITSN1,TANC2,JAK2,RAB11FIP4,PARK2,ADCYAP1R1,GRIN3A,CACNA1C,MAN1A1,NLGN4X,EXOC4,HEATR5A,NUMB,CD84,TRAF3IP2,SMAD3,PIBF1,PIK3C3,TPH1,MAD1L1,TUB,ZDHHC11,PTPRN2,DGKI,SLC1A1,NTN1,EPM2A,SHFM1,MAP2,FBXW11,NOS1AP,GRM4,ANK3,ACTN4,RAB2A,CD300A,IFT43,RABGEF1,NSG2,LRP5,AP2B1,DENND2A,SCFD1,PDE4D,THEM4,PIK3CD,BLOC1S5,RAB24,EFNA5,ATF2,PIK3R2,CCDC91,KCNJ3,SMG7,ATXN1,ERC1,MCTP1,NUP214,RBM4,ADORA2A,ANO1,CADPS,ICK,SYT1,DAB2,NSF,SNAP23,CACNG3,EPG5,RANBP17,PRKAR1B,MYRIP,RIMS2,PEX5L,PARD3,NRG1,TBC1D9,FMN2,GPSM2,PRKCG,SLC8A1,RSRC1,SAMM50,AGT,GRIP1,TLK1,NF1,VPS16,NRXN1,GSK3B,SLC22A3,CHD7,SNX5,UNC13A,CACNB2,STXBP4,SYBU,BID,CAPN3,CTDSPL2,HTR2C,SNAP25-AS1,EPHA5,SNX31,ANK2,GRIN2B,LRPPRC,ADCY8,ITPR2,CACNA1D,ANKFN1,TRAPPC8,TSNARE1,MON2,ZDHHC14,SUFU,RPGR,MAP2K1,KPNB1,CENPF,AGBL4,KPNA3,LRRK2,PKHD1,SPG11,VTI1A,FYN,KCND3,PTPN14,RIMS1,VPS53,MCTP2,ABCA12,KCNQ1,NOX5,FER,PCDH17,TMEM108,NRXN3,VPS45,SETD2,TRDN,SEC16B,EXOC6B,MYH9,VPS41,TENM1,VPS39,STXBP5,SLC24A2,CLASP2,DNAJC6,NSUN2,DNM3,SYNDIG1,SCFD2,ANXA8L1,IPO11,RAB27A,BLOC1S6,PARD3B,HGSNAT,SYT9*
GO:0008015	blood circulation	0.005428462822176292	*RPS6KA2,DMD,CHRM3,NOS1,HDAC4,IMMP2L,RYR2,TRHDE,RYR3,MEF2A,CTNNA3,BDKRB1,JAK2,CACNA1C,SH3GL2,SLIT2,SMAD3,ELN,SLC1A1,NOS1AP,PCSK5,ARHGAP42,LRP5,NAV2,PDE4D,DOCK4,KCNJ12,KCNJ3,CELF2,PDE4B,SPTBN4,PRCP,ADORA2A,PRKG1,TNNI3K,TTN,KCNMA1,SLC8A1,THRB,AGT,PKP2,CHD7,SNX5,CACNB2,ANK2,CACNA1D,PTPRO,RNLS,ADAMTS16,SCN1A,BMP6,KCND3,SGCZ,KCNQ1,EXT1,SGCD,TRDN,SEMA3A,BDKRB2,PPARA*
GO:0010810	regulation of cell-substrate adhesion	0.005429401247442195	*CLASP1,DMD,TBCD,VCL,NEDD9,CASK,KANK1,UTRN,DLC1,JAK2,SMAD3,PEAK1,EGFLAM,SKAP1,ARHGAP6,SPOCK1,ACTN4,DISC1,EFNA5,DAB2,NF1,GSK3B,TRIOBP,PREX1,MACF1,DUSP22,PTPRO,PKHD1,DOCK1,VWC2,CDH13,CLASP2,PTPRA*
GO:0040008	regulation of growth	0.005501643438971647	*ERBB4,DSCAM,ATP8A2,PLCB1,NEDD9,SEMA5A,RUNX1,PLS1,APP,AKAP6,RFTN1,MAP2K5,DCC,SEMA4D,BDKRB1,PARK2,NRG3,SLIT2,BMPR1A,SMAD3,NTN1,EPM2A,MAP2,CDC73,CDH4,TNR,SPOCK1,PLCE1,GHR,SERTAD2,DISC1,EFNA5,FTO,SEMA6D,PRKCQ,SPTBN4,DPYSL2,BDNF,FSTL4,SYT1,EBAG9,DAB2,ENPP1,RIMS2,NRG1,MAP3K13,AGT,GSK3B,CTDP1,GPR21,CHD7,UNC13A,ISLR2,GPC3,MACF1,CAPN3,TP73,SLIT3,SEMA5B,INSR,ENOX2,EPHA7,RIMS1,TRPC5,CDKL5,ESR2,BASP1,SE*

			MA3A,GNG4,CLASP2,PPARA,SEMA3D,SYT17,SEMA3C
GO:0090066	regulation of anatomical structure size	0.005532875677244043	*CHRM3,NOS1,DSCAM,CIT,SEMA5A,KANK1,PLS1,FCHSD2,DCC,SEMA4D,SH3BP1,SLIT2,ELN,NTN1,MAP2,CDH4,TNR,VAV3,EPS8,PRR16,DISC1,ARHGAP42,DOCK4,EFNA5,SEMA6D,SPTBN4,DPYSL2,BDNF,ADORA2A,FSTL4,PRKG1,KCNMA1,SLC12A8,SLC8A1,MAP3K13,CDC42EP3,AGT,PDXP,GSK3B,TRIOBP,ISLR2,MACF1,PAK3,NEB,SEMA5B,LRRK2,EPHA7,FHOD3,EXT1,TRPC5,FER,CDKL5,SEMA3A,BDKRB2,TENM1,CLASP2,SEMA3D,DEPTOR,SEMA3C*
GO:0010976	positive regulation of neuron projection development	0.005944461333866873	*KALRN,NTRK2,DMD,ATP8A2,PRKD1,CAMK1D,RELN,TOX,ALK,MAGI2,EP300,NEGR1,CBFA2T2,DISC1,BDNF,RIT2,AGT,CNTN1,ROR1,PAK3,IGF1R,FYN,TENM3,RAPGEF2,NTRK3,ELAVL4*
GO:0042692	muscle cell differentiation	0.006031043263734672	*RCAN1,SYNE1,DMD,NOS1,NEBL,HDAC4,MYEF2,ADAM12,PPP3CA,RYR1,AKAP6,MEF2A,DNMT1,AKAP13,QKI,SOX6,BMPR1A,MYO18B,ACTN4,BDNF,RBM4,FHL2,NOX4,TTN,NRG1,SLC8A1,AGT,CTDP1,SETD3,KCNH1,PGM5,CAPN3,HIRA,MYPN,ANK2,NEB,NLN,RARB,SGCZ,FHOD3,SGCD,HDAC5,MYH9,TANC1,PRKAR1A,PPARA,RORA*
GO:0050769	positive regulation of neurogenesis	0.00615950964713794	*KALRN,NTRK2,ROBO1,DSCAM,SEMA5A,IL1RAPL1,RELN,ROBO2,SEMA4D,NUMB,SLIT2,NTN1,CUX1,CDH4,TENM4,DISC1,EFNA5,EPHB2,BDNF,PLXNA2,HDAC2,FBXO31,MAP3K13,TIAM2,ISLR2,MACF1,TP73,PAK3,GRM5,MAP2K1,TRPC5,LRP2,CDKL5,PTPRD*
GO:0018209	peptidyl-serine modification	0.006206800883649313	*RPS6KA2,NTRK2,DMD,NOS1,SRPK2,PRKD1,PRKCA,CAMK1D,APP,PAK1,DCLK1,RPS6KA5,MAST4,EGFLAM,TNKS,TAF1,SLC1A1,EPM2A,PDE4D,PRKCQ,SPTBN4,MARK1,BDNF,FNIP1,TOP1,CD44,PRKCG,MAPKAPK2,MAP3K13,PLCL2,DYRK4,TLK1,NRXN1,GSK3B,CHEK2,STK32B,MORC3,PRKAA2,STK38,LRRK2,CAMK4,BDKRB2,TENM1,STK38L,NTRK3*
GO:0010647	positive regulation of cell communication	0.007281608920345459	*CAMTA1,CACNG2,CTNNA1,NTRK2,SLC39A10,SPRED2,ERBB4,LAMA2,ROBO1,PUM1,GPC5,WDR59,WWOX,AUTS2,WLS,PSMB2,PLCB1,FLT3,PRKD1,SEMA5A,PRKCA,KANK1,DOK6,NLGN1,TNFRSF10B,CLEC16A,TCF7L2,RELN,APP,PAK1,AKAP6,SOS1,ALK,MAP2K5,EDA,ROBO2,AKAP13,EP300,SEMA4D,EVC,ANKRD6,GRIK2,JAK2,PARK2,ADCYAP1R1,SMOC2,SULF1,TRIM5,TRAF3IP2,SH3RF3,BMPR1A,SMAD3,PIBF1,SCUBE1,TNKS,DGKI,SLC1A1,CDC73,NOS1AP,GRM4,ANK3,CCL15,TNR,ACTN4,PLCE1,GHR,CLSTN2,CD300A,DISC1,CCL14,TNFRSF19,GRM1,PIK3CD,ZNF423,MAPRE2,MAP3K7,EPHB2,AGO3,EEF1E1-BLOC1S5,BDNF,FNIP1,ADORA2A,ANO1,NOX4,BMPER,SYT1,DAB2,LRRK1,CACNG3,MYRIP,RIMS2,PRDM15,NRG1,RIT2,CD44,PRKCG,MAP3K13,AGT,FGF1,NF1,NRXN1,GSK3B,PSMB7,SNX5,IQCJ-SCHIP1,CACNB2,SHANK2,GPC3,DLG5,UBE2V1,ROR1,BID,MACF1,DUSP22,MAP3K5,HTR2C,LY86,TP73,ARHGEF3,GRIN2B,ADCY8,MLLT3,SH3RF2,TBL1X,GRM5,IGF1R,INSR,MAP2K1,RASGRF2,CNTN6,LRRK2,BMP6,FYN,GAS8,RIMS1,CRADD,LRP2,TMEM108,RAPGEF2,IL18R1,TRIM22,CDH13,TRDN,SEMA3A,MGAT5,LITAF,TENM1,ZRANB1,VWF,SLC24A2,SCEL,NTRK3,EEF1E1,PRLR,ATF6*
GO:0106027	neuron projection organization	0.00797788508466299	*KALRN,PLS1,NLGN1,RELN,APP,CTNND2,TANC2,EPHB2,GSK3B,GRIN2B,PAK3,IGF1R,INSR,LRRK2,FYN,EPHB1,TANC1,DNM3,DOCK10*
GO:0043087	regulation of GTPase activity	0.008078532150326353	*KALRN,NTRK2,SGSM1,NEDD9,MYO9A,TBC1D5,RABGAP1L,SEMA4D,ARHGAP15,SH3BP1,ARHGAP24,DGKI,ARHGAP6,CCL15,VAV3,ARHGAP42,CCL14,ARHGAP12,EFNA5,MAPRE2,SIPA1L2,PLXNA2,PRKG1,RGS6,TBC1D9,RAPGEF6,ARHGAP25,NF1,GSK3B,TIAM2,PREX1,FGD4,EPHA5,GARNL3,ARAP2,LRRK2,ASAP1,RAPGEF2,CDKL5,RGS7,SIPA1L3,NTRK3,DOCK9,SBF2,DOCK10,RALGAPA1*
GO:0099560	synaptic membrane adhesion	0.009696795918603751	*NLGN1,LRRC4C,EFNA5,NTNG1,NRG1,NRXN1,GPC6,LRFN5,PCDH17,PTPRD*
GO:0071310	cellular response to organic substance	0.009832641258657127	*RPS6KA2,CTNNA1,NTRK2,DMD,CHRM3,SPRED2,ERBB4,NOS1,ESRRG,ROBO1,WWOX,FUT8,HDAC4,ABCC1,LDLRAD4,BLM,PSMB2,PTPRT,RXFP1,PLCB1,FLT3,CACNA1A,PRKD1,RYR2,CASK,PRKCA,STX8,ZBTB20,IL1RAPL1,CCDC3,KANK1,APP,PAK1,PPP3CA,RYR1,AKAP6,IDE,RYR3,SOS1,ALK,BTRC,MAP2K5,DNMT1,MAGI2,EDA,ROBO2,EP300,RPS6KA5,CHRM1,DEFA1B,JAK2,CMKLR1,PARK2,SMOC2,PNPLA3,SOX6,COL4A6,SULF1,*

			ADCY2,RFFL,SH3GL2,SLIT2,GLP2R,TRIM5,CHRDL1,TRAF3IP2,BMPR1A,ACACA,SMAD3,PTGFR,SATB2,OVOL2,BRINP1,IL1RAPL2,PTPRN2,SOX5,TAF1,SLC1A1,CDC73,FLRT2,CCL15,ACTN4,EPS8,GHR,CCL14,RABGEF1,NSG2,LRP5,ESR1,TNFRSF19,PDE4D,PIK3CD,EFNA5,ATF2,PIK3R2,ZNF423,CYBB,ADTRP,PDE4B,GABRB3,MAP3K7,PRKCQ,CLDN1,EPHB2,PRCP,RGMB,BDNF,ARID5B,ANO1,NOX4,BMPER,DEFA3,DAB2,EPG5,CALCRL,HDAC2,CDK19,ENPP1,SPINT2,CD44,MAPKAPK2,SLC8A1,CHST11,THRB,AGT,FGF1,QRICH1,CCR3,NF1,NRXN1,PDXP,GSK3B,CD109,GPR21,PSMB7,SNX5,NCOA1,CHEK2,STXBP4,PHEX,NREP,GPC3,ZNF675,PTPRE,DUSP22,MAP3K5,CTDSPL2,HTR2C,GLRA2,LY86,EPHA5,IL1RL1,ZNF366,SLIT3,PAK3,ADCY8,ITPR2,PRKAA2,PACRG,GRM5,IGF1R,INSR,JAK1,UBR2,FOXO3,PRDM16,LTBP1,LRRK2,RARB,BMP6,FYN,MSR1,GNG2,ABCA12,KCNQ1,EXT1,AMFR,LRP2,FER,GLDC,VWC2,TMEM108,RAPGEF2,NCOA2,IL18R1,ESR2,VEPH1,HDAC5,NRIP1,LITAF,PIK3R3,GABRB1,NTRK3,FBN1,PTPRK,PPARA,RORA,BRIP1,ELAVL4,EEF1E1,PRLR,PTPRA,ATF6,PMEPA1,ST18,RXFP2
GO:0042221	response to chemical	0.010260328462075152	*RNF4,KALRN,CACNG2,RPS6KA2,CTNNA1,NTRK2,DMD,KCNC2,CHRM3,SPRED2,ERBB4,LAMA2,NOS1,CNTN4,ESRRG,ROBO1,DSCAM,WWOX,FUT8,HDAC4,ABCC1,LDLRAD4,BLM,PSMB2,PTPRT,RXFP1,NFASC,PLCB1,FLT3,CACNA1A,PRKD1,RYR2,SEMA5A,CASK,PRKCA,STX8,ZBTB20,IL1RAPL1,CCDC3,HBE1,DIO2,KANK1,NLGN1,CAMK1D,TCF7L2,RELN,APP,PAK1,PPP3CA,RYR1,AKAP6,IDE,RYR3,SOS1,LRRC8C,RFTN1,ALK,BTRC,MEF2A,DLG2,MAP2K5,DNMT1,MAGI2,EDA,ROBO2,DCC,ELMO2,EP300,SEMA4D,RPS6KA5,CPNE4,BDKRB1,NME8,TF,CHRM1,DEFA1B,JAK2,ABCG8,CMKLR1,PARK2,ADCYAP1R1,GRIN3A,SMOC2,HLCS,MAN1A1,EMB,PNPLA3,OR5K4,NRG3,SOX6,COL4A6,SULF1,ADCY2,OTC,RFFL,SH3GL2,SLIT2,GLP2R,AOX1,TRIM5,AFF3,ATRX,CHRDL1,TRAF3IP2,BMPR1A,ACACA,DSCAML1,SMAD3,TNFRSF11B,PIK3C3,CHD6,SLC47A1,PTGFR,SATB2,OVOL2,BRINP1,OR5I1,IL1RAPL2,PTPRN2,SOX5,TAF1,SLC1A1,NTN1,CDC73,FLRT2,CD96,EGLN3,ANK3,TIMP2,CCL15,CDH4,TNR,VAV3,SCN9A,MDM4,ACTN4,EPS8,GHR,LRRC8D,SRD5A2,CCL14,RABGEF1,NSG2,LRP5,ESR1,TNFRSF19,SCFD1,PDE4D,PIK3CD,DOCK4,EFNA5,ATF2,PIK3R2,ZNF423,CYBB,SEMA6D,ADTRP,CRMP1,PDE4B,GABRB3,MAP3K7,PRKCQ,CLDN1,KCND2,TPH2,EPHB2,PRCP,PRTG,RGMB,DOCK2,DPYSL2,RFC3,BDNF,RBM4,ADORA2A,ARID5B,ANO1,FHL2,NOX4,BMPER,DEFA3,SYT1,DAB2,PLXNA2,PXDNL,EPG5,CALCRL,HDAC2,ETS1,BACH1,TTN,CDK19,ENPP1,OR4N2,NRG1,SPINT2,FMN2,BCKDHB,TOP1,CD44,KCNMA1,CNGB1,PRKCG,MAPKAPK2,SLC8A1,FAM19A4,RSRC1,CHST11,THRB,NCAM1,AGT,FGF1,QRICH1,CCR3,NF1,NRXN1,LAMA3,PDXP,GSK3B,CD109,KCNH1,GPR21,CHD7,PSMB7,SNX5,OR51B2,NCOA1,CHEK2,STXBP4,PHEX,SHANK2,UNC5D,NREP,GPC3,PREX1,ADAMTSL1,ZNF675,MNAT1,TRIO,CAPN3,PTPRE,DUSP22,MAP3K5,CTDSPL2,HTR2C,CCDC141,GLRA2,LY86,EPHA5,TP73,IL1RL1,ZNF366,MYPN,P2RX6,ANK2,SLIT3,GRIN2B,PAK3,ADCY8,ITPR2,CCL15-CCL14,PRKAA2,PACRG,ABCG2,SEMA5B,SDK1,GRM5,IGF1R,PTPRO,MSRA,INSR,JAK1,MAP2K1,UBR2,FOXO3,CNTN6,CENPF,RNLS,PRDM16,LTBP1,LRRK2,RARB,BMP6,FYN,EPHA7,MSR1,SEL1L2,GNG2,ENPP2,ABCA12,EPHB1,KCNQ1,EXT1,AMFR,UNC5C,ACSBG1,LRP2,C2,FER,GLDC,VWC2,ERLIN1,TMEM108,RAPGEF2,PTPRM,NCOA2,NRXN3,PTPRU,IL18R1,ESR2,KAT7,SETD2,CDH13,VEPH1,HDAC5,SEMA3A,BDKRB2,STIM1,NRIP1,LITAF,PIK3R3,GABRB1,RGS7,NTRK3,FBN1,PTPRK,PPARA,RORA,BRIP1,ELAVL4,EEF1E1,SEMA3D,PRLR,PTPRA,ATF6,SYT17,OR4M1,CST2,PMEPA1,SYT9,SEMA3C,ST18,RXFP2*
GO:0048167	regulation of synaptic plasticity	0.010810155322410057	*NTRK2,CNTN4,SORCS2,RELN,APP,ERC2,SORCS3,GRIK2,DGKI,SLC1A1,SHISA9,TNR,GRID2,EPHB2,ERC1,MCTP1,ADORA2A,RIMS2,PRKCG,AGT,NF1,GSK3B,SHANK2,GRIN2B,ADCY8,GRM5,RASGRF2,RIMS1,RAPGEF2,SLC24A2,SHISA6*
GO:0002027	regulation of heart rate	0.010868876393925796	*DMD,RYR2,CTNNA3,CACNA1C,SLC1A1,NOS1AP,PDE4D,KCNJ3,SPTBN4,TNNI3K,SLC8A1,AGT,PKP2,CACNB2,ANK2,CACNA1D,RNLS,KCND3,KCNQ1,SEMA3A*
GO:0048729	tissue morphogenesis	0.010939967149414292	*CLASP1,ZNRF3,ERBB4,ROBO1,PRICKLE2,SHROOM3,WLS,PSMB2,RYR2,VCL,MYO9A,KIF26B,PAK1,PCDH15,GREB1L,DLC1,SOS1,BTRC,MAGI2,CELSR1,ROBO2,ANKRD6,ASTN2,NPHP3,EXOC4,SULF1,SH3BP1,ARHGAP24,COL11A1,SLIT2,BMPR1A,SMAD3,EYA1,OVOL2,NTN1,MDM4,CECR2,LRP5,ESR1,ARHGA*

			P12,PIK3CD,CSMD1,DAB2,TTN,NRG1,SPINT2,CD44,AGT,FGF1,LAMA3,PKP2,CHD7,PSMB7,GPC3,DLG5,ROR1,MLLT3,SUFU,GPC6,KLHL3,ADAMTS16,PKHD1,PBX1,EPHA7,EXT1,LRP2,SETD2,SEMA3A,PRKAR1A,CLASP2,SEMA3C
GO:0007158	neuron cell-cell adhesion	0.011186049792641546	CNTN4,NLGN1,ASTN2,NLGN4X,TNR,NRXN1,ASTN1,NRXN3
GO:0007169	transmembrane receptor protein tyrosine kinase signaling pathway	0.011294508177385275	PTPRR,KALRN,NTRK2,PTPRG,ERBB4,ROBO1,ANKS1A,PTPRT,PLCB1,FLT3,PRKD1,NEDD9,KANK1,DOK6,PAK1,IDE,SOS1,ALK,RPS6KA5,JAK2,SMOC2,NRG3,COL4A6,SULF1,ANKS1B,FLRT2,PLCE1,GHR,RABGEF1,PIK3CD,EFNA5,PIK3R2,PRKCQ,EPHB2,BDNF,ARID5B,FSTL4,ABI1,ENPP1,NRG1,MAPKAPK2,AGT,FGF1,NF1,NRXN1,GSK3B,GPR21,SNX5,STXBP4,ROR1,PTPRE,EPHA5,PAK3,IGF1R,INSR,FYN,EPHA7,EPHB1,EXT1,FER,TMEM108,RAPGEF2,CDH13,BDKRB2,PIK3R3,NTRK3,CLASP2,PRLR,PTPRA
GO:0051961	negative regulation of nervous system development	0.011507547969828386	CTNNA1,SEMA5A,PPP3CA,ROBO2,DCC,SEMA4D,BMPR1A,BRINP1,NTN1,MAP2,TNR,SEMA6D,EPHB2,PRTG,FSTL4,NF1,SEMA5B,EPHA7,TRPC5,RAPGEF2,DAB1,SEMA3A,NTRK3,SEMA3D,SEMA3C
GO:0060537	muscle tissue development	0.012111160103670844	RCAN1,DMD,ERBB4,NEBL,HDAC4,RYR2,RUNX1,PPP3CA,RYR1,AKAP6,MEF2A,AKAP13,HIVEP3,EP300,SOX6,COL11A1,BMPR1A,SMAD3,EYA1,ELN,MYO18B,TENM4,FHL2,NOX4,TTN,NRG1,SLC8A1,AGT,NF1,PKP2,CTDP1,CHD7,PGM5,TP73,NEB,NLN,CENPF,RBFOX1,RARB,COL19A1,SGCZ,EPHB1,FHOD3,LRP2,SGCD,PRKAR1A,PPARA,MYH15,SEMA3C
GO:0007015	actin filament organization	0.012484133321440664	CTNNA1,CLASP1,DMD,SHROOM3,CIT,NEBL,NEDD9,SEMA5A,CALD1,KANK1,DIAPH2,PLS1,FCHSD2,PAK1,PCDH15,DLC1,CTNNA3,ELMO2,TF,JAK2,PARK2,SH3BP1,SLIT2,SMAD3,ELN,ARHGEF18,GAS7,ARHGAP6,EPS8,PPP1R9A,ARHGAP12,RHPN2,SPTBN4,ABI1,MICAL3,TTN,CTNNA2,FMN2,CDC42EP3,ARHGAP25,PDXP,TRIOBP,PREX1,FAM171A1,PAK3,NEB,PHACTR1,FHOD3,FER,SHROOM4,MPRIP,TENM1,SH3KBP1,CLASP2
GO:0072001	renal system development	0.01273692097350113	ERBB4,ADAMTS6,KIF26B,GREB1L,MAGI2,ROBO2,NPHP3,SULF1,SLIT2,TRAF3IP2,SMAD3,EYA1,WWTR1,PCSK5,PLCE1,AP2B1,ARID5B,BMPER,NFIA,AGT,FGF1,NF1,GPC3,DLG5,TP73,PTPRO,KLHL3,CENPF,ADAMTS16,LRRK2,PKHD1,RARB,PBX1,BMP6,EPHA7,EXT1,LRP2,KIRREL3,DCHS2,BASP1,FBN1,BICC1
[truncated: 79,634 more chars]
