# Supplementary material for: Genes Possessing the Most Frequent DNA DSBs Are Highly Associated with Development and Cancers, and Essentially Overlap with the rDNA-Contacting Genes
Source: Int J Mol Sci. 2022 Jun 28;23(13):7201. doi: 10.3390/ijms23137201 (PMC9266645; doi:10.3390/ijms23137201)
Supplement: Supplementary file 1 [file ijms-23-07201-s001.zip › Table S6.pdf]

Gene name	Gene ID	DSBs, reads	Expression, TPM
OR4F16	ENSG00000185097	107634	0
RDH14	ENSG00000240857	79824.5	17.34
NT5C1B	ENSG00000185013	79824.5	0.255
NT5C1B-RDH14	ENSG00000250741	79824.5	0.075
CD247	ENSG00000198821	69650.5	0.145
SUCLG2	ENSG00000172340	64157.5	29.5
POLDIP3	ENSG00000100227	58379	71.18
LNX2	ENSG00000139517	56897.5	6.975
FAM188B2	ENSG00000214237	55227	0.16
CLRN1-AS1	ENSG00000239265	55227	0.115
CLRN1	ENSG00000163646	55227	0.025
DNAH11	ENSG00000105877	54513	5.51
ZDHHC14	ENSG00000175048	51911	8.01
MIR3692	ENSG00000266617	51911	0
RIC8B	ENSG00000111785	51588	13.7
SLC39A11	ENSG00000133195	50818.5	8.565
RPAP3	ENSG00000005175	49163.5	48.38
RCN1	ENSG00000049449	48254.5	131.885
ENPP1	ENSG00000197594	47473	15.82
RN7SKP245	ENSG00000200895	47473	0.895
ZNF804B	ENSG00000182348	46382.5	0.015
SLC35E3	ENSG00000175782	46031.5	9.82
PRDX2	ENSG00000167815	44406	366.04
HOOK2	ENSG00000095066	44406	15.125
RTBDN	ENSG00000132026	44406	0.72
ADAMTS16	ENSG00000145536	43793.5	0
CCDC26	ENSG00000229140	43015	0
BLZF1	ENSG00000117475	42235.5	16.78
LDB2	ENSG00000169744	42129.5	0.025
GPC5	ENSG00000179399	41765.5	1.075
GPC5-IT1	ENSG00000236240	41765.5	0
MIR548AS	ENSG00000263741	41765.5	0
NRXN1	ENSG00000179915	41617	0.04
ASPH	ENSG00000198363	41602.5	51.64
B4GALNT2	ENSG00000167080	40848.5	0
F8	ENSG00000185010	40125	2.16
EEF1A1P31	ENSG00000237859	40125	0.055
PRMT3	ENSG00000185238	39324.5	33.275
GSS	ENSG00000100983	39297	30.035
RDH13	ENSG00000160439	38754	24.415
GP6	ENSG00000088053	38754	0.265
PRKCI	ENSG00000163558	37486	34.64
MOV10L1	ENSG00000073146	37361	0.065
WDR20	ENSG00000140153	36691	15.39
TACC2	ENSG00000138162	36289.5	5.785
SLCO3A1	ENSG00000176463	35662.5	0.235

EFCAB6	ENSG00000186976	35371.5	1.515
MAP4K4	ENSG00000071054	35071	61.865
FLJ20373	ENSG00000233404	35071	0.14
PDE4D	ENSG00000113448	34838	4.455
COPS8P2	ENSG00000214552	34276.5	0.15
FAM19A1	ENSG00000183662	34276.5	0.1
DISC1FP1	ENSG00000261645	33883.5	0
PDCL	ENSG00000136940	33834.5	19.065
DNMT1	ENSG00000130816	33612	260.19
S1PR2	ENSG00000267534	33612	4.355
TTC9C	ENSG00000162222	33559.5	43.795
HLX	ENSG00000136630	33530.5	14.225
LLPH	ENSG00000139233	32938.5	37.205
TMBIM4	ENSG00000228144	32938.5	13.265
SLC9A1	ENSG00000090020	32457.5	18.16
PDE7B	ENSG00000171408	32308	0.32
ANTXR2	ENSG00000163297	32182.5	9.24
R3HDM1	ENSG00000048991	32145.5	74.22
FAM155A	ENSG00000204442	32015	0.015
FAM155A-IT1	ENSG00000227248	32015	0
C6orf89	ENSG00000198663	31645	22.92
NEGR1	ENSG00000172260	31375.5	0.035
NEGR1-IT1	ENSG00000228853	31375.5	0
PBX3	ENSG00000167081	31115.5	27.01
CD302	ENSG00000241399	30790	13.935
LY75	ENSG00000054219	30790	0.02
LY75-CD302	ENSG00000248672	30790	0
ADCY8	ENSG00000155897	30760	0
MGAT4C	ENSG00000182050	30728	0
MED13L	ENSG00000123066	30505.5	20.075
SPAG16	ENSG00000144451	30158.5	13.515
LMBR1	ENSG00000105983	30010	65.73
FAM65B	ENSG00000111913	29996.5	0.315
ASS1P1	ENSG00000220517	29996.5	0
TRPS1	ENSG00000104447	29947.5	9.18
CBFB	ENSG00000067955	29884.5	60.965
PDE8A	ENSG00000073417	29809	21.555
PDE4DIP	ENSG00000178104	29658.5	47.57
ZNF823	ENSG00000197933	29640	3.53
LINC00948	ENSG00000227877	29620.5	0.15
CLASP2	ENSG00000163539	29486	64.325
SEMA3A	ENSG00000075213	29366	2.87
IL31RA	ENSG00000164509	29351.5	0.02
LSAMP	ENSG00000185565	28894	1.21
RN7SL582P	ENSG00000241156	28894	0
GRID2	ENSG00000152208	28877	1.045
IFFO2	ENSG00000169991	28791	8.16

ALDH4A1	ENSG00000159423	28791	7.655
MIR4695	ENSG00000265606	28791	0
TAS1R2	ENSG00000179002	28791	0
RNASET2	ENSG00000026297	28670	35.505
RPS6KA2	ENSG00000071242	28670	8.88
RPS6KA2-IT1	ENSG00000232082	28670	0.115
PRDM12	ENSG00000130711	28644.5	0.325
SETD4	ENSG00000185917	28492	21.285
LINC00923	ENSG00000251209	28485.5	0.125
GK5	ENSG00000175066	28403.5	14.885
PTGFR	ENSG00000122420	28382	2.595
TENM3	ENSG00000218336	28349.5	15.885
MIR1305	ENSG00000221227	28349.5	0
ANKRD36BP2	ENSG00000230006	28282	0.52
CHERP	ENSG00000085872	28140.5	94.825
SLC35E1	ENSG00000127526	28140.5	55.455
SMIM7	ENSG00000214046	28140.5	40.93
MED26	ENSG00000105085	28140.5	12.52
CALR3	ENSG00000269058	28140.5	0.1
C2orf40	ENSG00000119147	28097.5	0.11
DOCK1	ENSG00000150760	28082.5	13.37
WWC1	ENSG00000113645	28048.5	21.345
CLDN18	ENSG00000066405	27990.5	0.275
C12orf49	ENSG00000111412	27883	22.54
KIF22	ENSG00000079616	27670	80.22
ANXA2R	ENSG00000177721	27470.5	0.25
CTDSPL2	ENSG00000137770	27415	36.01
SLC25A17	ENSG00000100372	27186.5	47.64
PMS1	ENSG00000064933	27173.5	19.535
CACHD1	ENSG00000158966	27062	20.93
MIR4794	ENSG00000264470	27062	0
FBXL2	ENSG00000153558	27018.5	8.75
RYR1	ENSG00000196218	26908.5	0.34
NRG3	ENSG00000185737	26779.5	0
ASH1L	ENSG00000116539	26748	30.6
MIR555	ENSG00000207720	26748	0
C1orf226	ENSG00000239887	26524.5	7.23
NOS1AP	ENSG00000198929	26524.5	4.785
MIR4654	ENSG00000266144	26524.5	0
LATS2	ENSG00000150457	26447	4.59
CPA6	ENSG00000165078	26363.5	0
TUBA1C	ENSG00000167553	26341.5	470.615
LRP1B	ENSG00000168702	26274.5	3.045
TBC1D19	ENSG00000109680	26201.5	3.38
SPESP1	ENSG00000258484	26084	0.21
NOX5	ENSG00000255346	26084	0.08
ANP32A	ENSG00000140350	26010	412.92

PCED1B	ENSG00000179715	25902.5	0
IQCG	ENSG00000114473	25771	7.195
ASTN2	ENSG00000148219	25509	1.63
TLN2	ENSG00000171914	25233	6.775
EPB41	ENSG00000159023	25150	144.37
SEMA3D	ENSG00000153993	25007.5	4.45
PHACTR1	ENSG00000112137	25004.5	2.77
MYOT	ENSG00000120729	24834.5	1.165
DPP6	ENSG00000130226	24712	0
PTH2R	ENSG00000144407	24652.5	0.025
RNF145	ENSG00000145860	24597	40.11
NREP	ENSG00000134986	24540.5	21.07
RN7SKP57	ENSG00000253057	24540.5	0
SCAF8	ENSG00000213079	24374	40.86
TIAM2	ENSG00000146426	24374	8.485
NNT-AS1	ENSG00000248092	24267	9.505
SLC14A2	ENSG00000132874	24250	0
STOX2	ENSG00000173320	24227	8.945
FHOD3	ENSG00000134775	24215	7.275
KSR2	ENSG00000171435	24197	1.27
MAPRE2	ENSG00000166974	24167	88.04
NLGN4X	ENSG00000146938	24109.5	0
PTGIS	ENSG00000124212	24062.5	0.135
PRKAG2	ENSG00000106617	24060.5	19.265
DCHS2	ENSG00000197410	24023	0.77
WWC3	ENSG00000047644	23888.5	0.01
TJP1	ENSG00000104067	23825	37.515
GRM8	ENSG00000179603	23701.5	0.125
LINC01076	ENSG00000225105	23603	0
UNC5C	ENSG00000182168	23586	9.115
ZSWIM6	ENSG00000130449	23548	13.43
NUDT6	ENSG00000170917	23488	5.58
ARL5AP2	ENSG00000236818	23479	0
HPSE2	ENSG00000172987	23479	0
OR5P1P	ENSG00000166408	23277	0
OR5P2	ENSG00000183303	23277	0
OR5P3	ENSG00000182334	23277	0
OVCH2	ENSG00000183378	23277	0
CLCN1	ENSG00000188037	23262.5	0.04
SLC35F3	ENSG00000183780	23087.5	0.135
LINC00467	ENSG00000153363	23053	14.62
PRLR	ENSG00000113494	23045	0.04
PAGR1	ENSG00000185928	23005	19.265
MVP	ENSG00000013364	23005	2.885
ADCY7	ENSG00000121281	22999.5	3.215
HIVEP3	ENSG00000127124	22970	2.185
FTO	ENSG00000140718	22909.5	42.58

MARCH7	ENSG00000136536	22756.5	52.145
NPAS3	ENSG00000151322	22719.5	0.56
UTRN	ENSG00000152818	22543.5	25.645
RGS7BP	ENSG00000186479	22508.5	0.015
KALRN	ENSG00000160145	22461	2.985
MIR5002	ENSG00000266383	22461	0
UBAP2	ENSG00000137073	22284	57.38
PITPNC1	ENSG00000154217	22265.5	10.13
RNF219-AS1	ENSG00000234377	22251.5	1.49
LINC00446	ENSG00000229249	22251.5	0
SRGNP1	ENSG00000271386	22251.5	0
SEC23B	ENSG00000101310	22184	59.25
PCDH9	ENSG00000184226	22178	31.25
RNU7-87P	ENSG00000238500	22178	0
LTV1	ENSG00000135521	22113.5	92.22
PHACTR2	ENSG00000112419	22113.5	29.2
DCAF12	ENSG00000198876	22093.5	40.455
MRM1	ENSG00000129282	21976.5	4.695
WIPF3	ENSG00000122574	21933	2.65
ENOX1	ENSG00000120658	21718	3.805
SNX31	ENSG00000174226	21571.5	0
ACOXL	ENSG00000153093	21541.5	0.3
RPL5P9	ENSG00000236330	21541.5	0.3
SPRR2D	ENSG00000163216	21541	0
NTRK3	ENSG00000140538	21525.5	0.03
CBL	ENSG00000110395	21478	15.72
ARNT	ENSG00000143437	21300.5	31.525
ATRNL1	ENSG00000107518	21299	4.02
PLEKHG4B	ENSG00000153404	21225	0.195
VWDE	ENSG00000146530	21132.5	2.11
EGLN2	ENSG00000269858	21108	39.065
RAB4B	ENSG00000167578	21108	6.065
MIA	ENSG00000261857	21108	0.115
CYP2G1P	ENSG00000130612	21108	0.025
MIA-RAB4B	ENSG00000268975	21108	0
RAB4B-EGLN2	ENSG00000171570	21108	0
GCNT6	ENSG00000205318	21068.5	0.085
SEMA5A	ENSG00000112902	21046	0
LRRFIP2	ENSG00000093167	21014	38.425
UBE2FP1	ENSG00000224080	21014	1.965
FLT3	ENSG00000122025	20985	0.205
IQCH	ENSG00000103599	20942	4.91
IQCH-AS1	ENSG00000259673	20942	1.51
CHN2	ENSG00000106069	20831.5	4.44
NANOGP4	ENSG00000237065	20831.5	0.495
LINC00639	ENSG00000259070	20719.5	0
RAD51B	ENSG00000182185	20715.5	10.455

AARSD1	ENSG00000266967	20615.5	35.745
PTGES3L	ENSG00000267060	20615.5	2.695
PTGES3L-AARSD1	ENSG00000108825	20615.5	0
SUSD4	ENSG00000143502	20506	1.935
STAT5B	ENSG00000173757	20366.5	18.655
TOX	ENSG00000198846	20328	0.105
ENPP2	ENSG00000136960	20303	2.06
TMEM248	ENSG00000106609	20293	52.115
ATP2B2	ENSG00000157087	20281	0.075
KRT18P33	ENSG00000214533	20251.5	0
KCNQ5	ENSG00000185760	20241	5.835
KCNQ5-IT1	ENSG00000233844	20241	0.59
PGAM1P10	ENSG00000220515	20241	0.515
KNOP1P4	ENSG00000232422	20241	0.255
EXD1	ENSG00000178997	20233	0.085
GRM5	ENSG00000168959	20212.5	0.02
NOC3L	ENSG00000173145	20193	35.14
PLCE1	ENSG00000138193	20193	4.225
LHFPL4	ENSG00000156959	20014	0.09
SAMHD1	ENSG00000101347	19997	28.91
FAM53A	ENSG00000174137	19937.5	2.1
ZNF566	ENSG00000186017	19934.5	5.075
FER	ENSG00000151422	19711	10.465
LINC00971	ENSG00000242641	19709	0
CASK	ENSG00000147044	19674.5	18.52
RNU6-202P	ENSG00000251807	19674.5	0
MARVELD3	ENSG00000140832	19657.5	1.17
MAPK4	ENSG00000141639	19653.5	1.055
MAP2K5	ENSG00000137764	19651.5	18.435
HNRNPA1P5	ENSG00000259512	19651.5	0.075
ISCA1	ENSG00000135070	19637.5	26
CCDC93	ENSG00000125633	19623	42.84
SPATA5	ENSG00000145375	19617	9.53
KITLG	ENSG00000049130	19600.5	8.35
KCNIP4	ENSG00000185774	19572	1.175
USP32	ENSG00000170832	19558.5	23.47
MAPK10	ENSG00000109339	19518.5	1.66
LINC00944	ENSG00000256128	19484.5	0
PTPRR	ENSG00000153233	19475.5	0.03
SPNS2	ENSG00000183018	19219.5	5.33
CEP128	ENSG00000100629	19196	15.92
NDUFV2	ENSG00000178127	19144	55.48
ANKRD12	ENSG00000101745	19144	13.665
DENND2D	ENSG00000162777	18986	0.175
CHI3L2	ENSG00000064886	18986	0
CCDC92	ENSG00000119242	18913	23.845
DNAH10OS	ENSG00000250091	18913	0.7

LRP2	ENSG00000081479	18896	8.28
PIK3CD	ENSG00000171608	18837	4.105
ANKRD35	ENSG00000198483	18823.5	0
NAALADL2	ENSG00000177694	18802.5	0.305
ZNF536	ENSG00000198597	18723.5	4.64
SLC38A11	ENSG00000169507	18652	0.175
SP3	ENSG00000172845	18642	29.825
DEPDC5	ENSG00000100150	18589	14.5
RN7SL20P	ENSG00000242251	18589	0.25
TNPO3	ENSG00000064419	18572	47.945
CAMK1G	ENSG00000008118	18519.5	0
HDGF	ENSG00000143321	18472	365.16
PRCC	ENSG00000143294	18472	216.59
C6orf201	ENSG00000185689	18398.5	0.895
GTF3C1	ENSG00000077235	18331.5	92.71
ZSWIM7	ENSG00000214941	18331.5	14.715
ANK2	ENSG00000145362	18271.5	11.6
EIF4G3	ENSG00000075151	18251	109.385
GRM7-AS3	ENSG00000226258	18248	0.67
SAAL1	ENSG00000166788	18235.5	47.595
OLFM1	ENSG00000130558	18202	1.02
MFAP5	ENSG00000197614	18090.5	0.05
BAZ2B	ENSG00000123636	18065	17.19
STAG2	ENSG00000101972	17937	117.565
SH2D1A	ENSG00000183918	17937	0.1
ADPGK	ENSG00000159322	17887	33.33
EZR	ENSG00000092820	17828	85.24
IKZF2	ENSG00000030419	17800.5	1.595
MEGF10	ENSG00000145794	17744.5	1.185
NRXN3	ENSG00000021645	17636	14.645
SEP15	ENSG00000183291	17581	117.25
XRCC4	ENSG00000152422	17460	8.65
OFCC1	ENSG00000181355	17425	0
B3GALT1	ENSG00000172318	17390.5	1.105
ROBO1	ENSG00000169855	17227.5	75.265
RN7SL751P	ENSG00000240964	17227.5	3.285
MALRD1	ENSG00000204740	17203	0.12
MTND2P16	ENSG00000230818	17203	0
SLC9A9	ENSG00000181804	17199.5	3.445
ST13P15	ENSG00000243759	17199.5	0
TOM1L1	ENSG00000141198	17113.5	37.725
C19orf10	ENSG00000074842	17085	79.39
TNFAIP8L1	ENSG00000185361	17085	3.975
CHD7	ENSG00000171316	17083.5	58.09
SLC26A6	ENSG00000225697	17070.5	22.605
PSMD1	ENSG00000173692	17033.5	97.83
HTR2B	ENSG00000135914	17033.5	0.48

ITGB6	ENSG00000115221	16992.5	0
CDKL5	ENSG00000008086	16937	3.49
GJA6P	ENSG00000236187	16937	0.06
MECOM	ENSG00000085276	16820	19.955
ERMARD	ENSG00000130023	16792.5	7.975
TACC1	ENSG00000147526	16749.5	13.69
RN7SL717P	ENSG00000240302	16652.5	0
MIR1185-1	ENSG00000221525	16570.5	0
TENM1	ENSG00000009694	16567	5.53
CAMKMT	ENSG00000143919	16478	14.38
Y_RNA	ENSG00000200344	16471.5	1.095
RYR3	ENSG00000198838	16471.5	0.9
TMEM132C	ENSG00000181234	16470.5	0
NADK2	ENSG00000152620	16439.5	19.975
KIF5C	ENSG00000168280	16397.5	9.27
LMNA	ENSG00000160789	16310	41.175
SRP72	ENSG00000174780	16273.5	133.99
NUCB1	ENSG00000104805	16239.5	63.275
GOT1	ENSG00000120053	16203.5	44.815
LPO	ENSG00000167419	16169.5	0.02
KIAA1217	ENSG00000120549	16108	2.27
NMT2	ENSG00000152465	15989	29.125
INPP4B	ENSG00000109452	15859.5	0.995
RPL5P13	ENSG00000242134	15859.5	0
AFM	ENSG00000079557	15730	0
TRAF6	ENSG00000175104	15696.5	9.985
ESRP1	ENSG00000104413	15660.5	0.285
ROBO2	ENSG00000185008	15625.5	3.975
NFASC	ENSG00000163531	15511.5	1.55
TTC18	ENSG00000156042	15505	1.34
TRBC2	ENSG00000211772	15504	0
SENP5	ENSG00000119231	15468	20.325
GTF2E2	ENSG00000197265	15467	22.35
ITPKB	ENSG00000143772	15443.5	2.63
ITPKB-IT1	ENSG00000228382	15443.5	0
CTNNA3	ENSG00000183230	15293.5	0.285
SCD5	ENSG00000145284	15288.5	13.72
DNAJA3	ENSG00000103423	15186	65.95
OXR1	ENSG00000164830	15172	14.35
SLC24A2	ENSG00000155886	15153	0.115
VAT1L	ENSG00000171724	15050.5	0.03
NKAIN2	ENSG00000188580	15044.5	0.275
CCDC102A	ENSG00000135736	14999.5	7.635
TMEM196	ENSG00000173452	14950.5	0
VAV2	ENSG00000160293	14902	9.685
GRK5	ENSG00000198873	14877	6.94
GRK5-IT1	ENSG00000228485	14877	0.515

MIR4681	ENSG00000265719	14877	0
ABI1	ENSG00000136754	14871.5	33.675
ULK4	ENSG00000168038	14869.5	3.535
ERCC6L2	ENSG00000182150	14853	33.195
C4orf45	ENSG00000164123	14824.5	0.17
NHLH1	ENSG00000171786	14778.5	0.865
PLA2G16	ENSG00000176485	14774.5	7.625
ITGB1	ENSG00000150093	14769	90.73
NOL12	ENSG00000100101	14727	48.86
TRIOBP	ENSG00000100106	14727	15.26
HSP90AA1	ENSG00000080824	14717.5	760.58
APOL4	ENSG00000100336	14641	0.24
SLC24A3	ENSG00000185052	14619.5	0
ELMO2	ENSG00000062598	14580	21.62
ZNF663P	ENSG00000215452	14580	0.04
GAS7	ENSG00000007237	14539.5	2.26
CDC6	ENSG00000094804	14470.5	59.125
MTND5P18	ENSG00000227860	14437.5	0
PSMB7	ENSG00000136930	14428	172.84
TSC22D3	ENSG00000157514	14427	131.88
CNIH2	ENSG00000174871	14425.5	5.165
THUMPD3	ENSG00000134077	14412	64.66
SETD5-AS1	ENSG00000206573	14412	32.175
SRGAP3	ENSG00000196220	14412	3.335
MCC	ENSG00000171444	14319	10.655
TSSK1B	ENSG00000212122	14319	0.05
NKAIN3	ENSG00000185942	14297	0.165
TXNDC16	ENSG00000087301	14292	8.87
PCDH17	ENSG00000118946	14270.5	3.49
KNDC1	ENSG00000171798	14235	1.48
CCDC85A	ENSG00000055813	14206.5	0.155
ZC4H2	ENSG00000126970	14184	16.66
KIF26B	ENSG00000162849	14145	2.38
PGPEP1L	ENSG00000183571	14136	0
FOXP1	ENSG00000114861	14100.5	48.39
FOXP1-IT1	ENSG00000242094	14100.5	0.625
CCDC22	ENSG00000101997	13980.5	17.37
SLC22A8	ENSG00000149452	13976.5	0.025
KIAA1211L	ENSG00000196872	13953.5	0.87
ADGB	ENSG00000118492	13899	0.145
DSCAM	ENSG00000171587	13884.5	0
DSCAM-IT1	ENSG00000233756	13884.5	0
BCORL1	ENSG00000085185	13877.5	32.83
MRPL48	ENSG00000175581	13870	67.685
MOCOS	ENSG00000075643	13825.5	10.54
FAM126A	ENSG00000122591	13772	24.96
STK38L	ENSG00000211455	13739.5	34.02

SRGAP2B	ENSG00000196369	13719	72.63
CDAN1	ENSG00000140326	13699	6.5
GMDS	ENSG00000112699	13662	22.79
AMDHD1	ENSG00000139344	13648.5	0.28
SAMD12	ENSG00000177570	13611.5	1.96
CNGB1	ENSG00000070729	13551	0
TRIO	ENSG00000038382	13516.5	36.7
ARHGAP24	ENSG00000138639	13514.5	3.805
KLHL12	ENSG00000117153	13504	18.95
AKAP13	ENSG00000170776	13455.5	24.345
DLG2	ENSG00000150672	13455.5	0.675
HNRNPCP6	ENSG00000213305	13455.5	0.31
LDHAL6DP	ENSG00000250257	13455.5	0
TTLL5	ENSG00000119685	13426	37.01
IFT43	ENSG00000119650	13426	15.62
FLVCR2	ENSG00000119686	13426	1.435
RNA5SP387	ENSG00000201096	13426	0
C20orf112	ENSG00000197183	13409	6.22
DDX59	ENSG00000118197	13366.5	15.775
STPG2	ENSG00000163116	13356	0.015
GFI1B	ENSG00000165702	13315.5	0.105
PDE1A	ENSG00000115252	13309	1.07
PARD6G	ENSG00000178184	13280	5.05
PTPRK	ENSG00000152894	13274	19.065
RUNX1	ENSG00000159216	13253.5	1.695
EZH2P1	ENSG00000231300	13253.5	0
RPS20P1	ENSG00000229761	13253.5	0
OMA1	ENSG00000162600	13251.5	45.9
DAB1	ENSG00000173406	13251.5	1.145
HNRNPA1P6	ENSG00000229887	13251.5	0
RPS26P15	ENSG00000223416	13251.5	0
CDC42EP3	ENSG00000163171	13218.5	8.985
SREK1	ENSG00000153914	13215	74.71
MYO10	ENSG00000145555	13181.5	17.535
ABCC8	ENSG00000006071	13169	1.53
SDHCP4	ENSG00000255035	13169	0.545
ERC1	ENSG00000082805	13158	20.97
MMAA	ENSG00000151611	13158	1.85
C17orf51	ENSG00000212719	13139.5	32.165
PDZD2	ENSG00000133401	13124	2.865
RPL5P14	ENSG00000243914	13124	0
TPT1P5	ENSG00000214108	13124	0
HMGN3	ENSG00000118418	13121.5	101.71
AFTPH	ENSG00000119844	13097.5	12.595
NCF4	ENSG00000100365	13002	0
CDH10	ENSG00000040731	12981	0.41
NRIP1	ENSG00000180530	12961	7.195

RGS6	ENSG00000182732	12951.5	0.03
DGKI	ENSG00000157680	12949	0.13
DNAJB14	ENSG00000164031	12935	14.805
ANKS1B	ENSG00000185046	12920	0.735
THRB	ENSG00000151090	12919.5	6.065
THRB-IT1	ENSG00000224822	12919.5	0.355
STXBP4	ENSG00000166263	12879	14.26
NUPL2	ENSG00000136243	12862	56.16
KLHL7	ENSG00000122550	12862	16.3
AK3P3	ENSG00000230042	12862	2.155
PDE4A	ENSG00000065989	12858.5	5.85
ERC2	ENSG00000187672	12821.5	6.295
GABRA3	ENSG00000011677	12756	0.285
MIR105-2	ENSG00000207818	12756	0
MIR767	ENSG00000211583	12756	0
XIRP2	ENSG00000163092	12695	0.02
IMPG2	ENSG00000081148	12692.5	0.08
ITGB3BP	ENSG00000142856	12666.5	77.32
EFCAB7	ENSG00000203965	12666.5	10.555
DLEU2L	ENSG00000116652	12666.5	1.215
RNU7-123P	ENSG00000251720	12666.5	0
LAMA2	ENSG00000196569	12597	0.21
CLVS1	ENSG00000177182	12594	0.125
GFRA2	ENSG00000168546	12581	0.225
MAST4	ENSG00000069020	12555.5	4.29
GAMTP2	ENSG00000231910	12545	0
LINC00457	ENSG00000225179	12545	0
COL14A1	ENSG00000187955	12530	4.475
IL17RB	ENSG00000056736	12490	8.645
TCIRG1	ENSG00000110719	12449	7.085
DACH1	ENSG00000165659	12368.5	28.665
PTPN11	ENSG00000179295	12344.5	67.14
ZNF569	ENSG00000196437	12344	5.055
KCNS3	ENSG00000170745	12305.5	0.54
KIAA0232	ENSG00000170871	12271	10.98
NIPAL2	ENSG00000104361	12260.5	2.14
NETO2	ENSG00000171208	12252	40.355
RBM19	ENSG00000122965	12251	32.985
FAM46A	ENSG00000112773	12217.5	12.345
HERC4	ENSG00000148634	12216	15.055
POU5F1P5	ENSG00000236375	12216	0.895
MGAT5	ENSG00000152127	12213.5	20.405
CCT2	ENSG00000166226	12190	273.435
CACNB2	ENSG00000165995	12164.5	3.955
DOCK10	ENSG00000135905	12158.5	0.12
ORC2	ENSG00000115942	12156.5	64.65
CYTH3	ENSG00000008256	12106	17.27

HDAC6	ENSG00000094631	12075	42.035
GOLGA4	ENSG00000144674	12033.5	49.915
TCEA1P2	ENSG00000230409	12033.5	28.54
MTND6P3	ENSG00000254132	12000	8.26
KIAA0825	ENSG00000185261	12000	0.23
SERTAD2	ENSG00000179833	11954	16.535
CCDC147	ENSG00000120051	11943	0.24
DDB1	ENSG00000167986	11830.5	162.625
AQP4-AS1	ENSG00000260372	11800.5	0
MYT1	ENSG00000196132	11721	1.115
SV2B	ENSG00000185518	11703	0.015
TBCEL	ENSG00000154114	11682.5	10.895
ADORA1	ENSG00000163485	11671.5	1.36
MSRA	ENSG00000175806	11663.5	5.4
GPRASP2	ENSG00000158301	11635.5	20.79
ARMCX5	ENSG00000125962	11635.5	15.335
GPRASP1	ENSG00000198932	11635.5	1.11
ADAMTS3	ENSG00000156140	11635	3.71
EPHA1	ENSG00000146904	11626	0.44
IKBKB	ENSG00000104365	11593	27.025
KIF18B	ENSG00000186185	11540	47.76
PEX14	ENSG00000142655	11529	23.34
ERBB4	ENSG00000178568	11506.5	1.255
CDH12	ENSG00000154162	11505	0.55
GBE1	ENSG00000114480	11415	6.885
ADRA1D	ENSG00000171873	11411	0.045
CNNM1	ENSG00000119946	11400.5	3.58
SORCS1	ENSG00000108018	11399.5	0.855
DPY19L2	ENSG00000177990	11348	3.105
MRE11A	ENSG00000020922	11342	16.455
GBF1	ENSG00000107862	11329	45.71
RPL29P2	ENSG00000240480	11326	0.15
DNAH2	ENSG00000183914	11326	0.11
CCDC178	ENSG00000166960	11311.5	0.435
LIMK1	ENSG00000106683	11292.5	25.94
ZNF609	ENSG00000180357	11292	17.31
TLK1	ENSG00000198586	11258	36.795
RALYL	ENSG00000184672	11160.5	0
NXPE2	ENSG00000204361	11134	0.08
BRD8	ENSG00000112983	11130	87.42
ATP8A2	ENSG00000132932	11115	0.39
RN7SL741P	ENSG00000242381	11115	0
RNU6-78P	ENSG00000199767	11115	0
TEX264	ENSG00000164081	11105.5	55.155
RAD54L2	ENSG00000164080	11105.5	30.785
KAT6B	ENSG00000156650	11083.5	17.555
ABR	ENSG00000159842	11071.5	39.835

ACTG2	ENSG00000163017	11003.5	0
HIF3A	ENSG00000124440	10998	0.1
OSBPL10	ENSG00000144645	10981.5	6.1
NIFKP7	ENSG00000251590	10981.5	0
SNIP1	ENSG00000163877	10966.5	10.69
CUL4B	ENSG00000158290	10947.5	65.51
IGKJ5	ENSG00000211593	10943.5	0
KLHL6	ENSG00000172578	10936	0.095
LARGE	ENSG00000133424	10900.5	12.135
LARGE-IT1	ENSG00000232081	10900.5	0.825
SNORA76	ENSG00000252904	10900.5	0.69
CECR2	ENSG00000099954	10862	17.385
ESR1	ENSG00000091831	10812.5	0.17
KCNJ16	ENSG00000153822	10784.5	0.015
LINC01028	ENSG00000267603	10784.5	0
MYO18B	ENSG00000133454	10775.5	0.01
ACAD11	ENSG00000240303	10751.5	25.585
NPHP3	ENSG00000113971	10751.5	13.37
SCAPER	ENSG00000140386	10735	14.445
NUP210L	ENSG00000143552	10692	0.345
RNU6-179P	ENSG00000200220	10692	0
GRM7	ENSG00000196277	10691.5	0.395
MIER1	ENSG00000198160	10679	36.895
UGT1A12P	ENSG00000228949	10647	0
PCBP3	ENSG00000183570	10646.5	3.09
CASC17	ENSG00000260785	10640.5	0
PMEPA1	ENSG00000124225	10608.5	4.725
ANKRD11	ENSG00000167522	10573.5	73.285
SF3B2	ENSG00000087365	10554.5	200.675
MAML3	ENSG00000196782	10550.5	3.06
RN7SKP253	ENSG00000252233	10550.5	0
DPP10	ENSG00000175497	10534	0
CRYBB2P1	ENSG00000100058	10511.5	58.2
PTPRO	ENSG00000151490	10464.5	0.42
CDON	ENSG00000064309	10462	17.05
KANSL1	ENSG00000120071	10450	32.23
FBXL18	ENSG00000155034	10418	14.15
NTRK2	ENSG00000148053	10377.5	0.53
DAK	ENSG00000149476	10336.5	29.99
CYB561A3	ENSG00000162144	10336.5	22.665
FAM129A	ENSG00000135842	10326.5	3.02
TNRC6A	ENSG00000090905	10301	54.435
STX18	ENSG00000168818	10278	33.77
FRMD4A	ENSG00000151474	10278	8.08
MIR4293	ENSG00000266321	10278	0
STX18-IT1	ENSG00000248221	10278	0
DNAI2	ENSG00000171595	10251	0.025

GPN2	ENSG00000142751	10198.5	35.74
EP300	ENSG00000100393	10195.5	37.915
CELA1	ENSG00000139610	10159	0
CBWD2	ENSG00000136682	10138	32.985
ZYG11B	ENSG00000162378	10128.25	16.335
MEGF9	ENSG00000106780	10109.5	5.895
DYM	ENSG00000141627	10038.5	27.815
PDXDC2P	ENSG00000196696	9998	10.685
TENM2	ENSG00000145934	9961	0.015
GAREM	ENSG00000141441	9956	12.64
HNRNPA1P7	ENSG00000215492	9956	1.375
ZNF76	ENSG00000065029	9948	18.25
RNF220	ENSG00000187147	9924.5	76.035
CNTNAP3B	ENSG00000154529	9908.5	2.49
MCF2L2	ENSG00000053524	9897.5	0.785
RGS22	ENSG00000132554	9875	0
ZNF471	ENSG00000196263	9841.5	0.535
CPNE4	ENSG00000196353	9832.5	0.04
MIR5704	ENSG00000265859	9832.5	0
FNTA	ENSG00000168522	9831	44.425
HOOK3	ENSG00000168172	9831	33.93
TPST2	ENSG00000128294	9831	14.775
RNU1-124P	ENSG00000200731	9831	0.63
VPS45	ENSG00000136631	9793	33.51
PDGFB	ENSG00000100311	9791.5	0
CACNA1B	ENSG00000148408	9788	0.51
KRT18P67	ENSG00000233425	9780	0
FKBP9	ENSG00000122642	9763	16.87
AVL9	ENSG00000105778	9763	14.2
DPY19L1P1	ENSG00000229358	9763	1.29
DPY19L1P2	ENSG00000231952	9763	0.255
RIMS1	ENSG00000079841	9760.5	0.475
TNIK	ENSG00000154310	9758	22.96
CCND3	ENSG00000112576	9754	46.42
GALNTL6	ENSG00000174473	9730	3.05
FBXL7	ENSG00000183580	9713	0.04
hsa-mir-6723	ENSG00000237973	9711.5	9018.955
TMEM206	ENSG00000065600	9707.5	25.085
SNX2	ENSG00000205302	9675.5	68.68
KIRREL3	ENSG00000149571	9674.5	0.59
CDH8	ENSG00000150394	9652.5	0.755
USP46	ENSG00000109189	9605.5	20.14
CSGALNACT1	ENSG00000147408	9582	0.73
CAPN14	ENSG00000214711	9578.5	0.03
TOB2	ENSG00000183864	9559	27.235
MID1	ENSG00000101871	9537	98.01
ZNF23	ENSG00000167377	9511	6.575

ZNF19	ENSG00000157429	9511	2.045
RNU6-1061P	ENSG00000252339	9511	0
BRMS1	ENSG00000174744	9484	34.21
RPL7AP66	ENSG00000175886	9463	4.58
LINC00669	ENSG00000267374	9463	3.985
GOLGA3	ENSG00000090615	9461	39.495
CHFR	ENSG00000072609	9461	16.3
ZNF605	ENSG00000196458	9461	3.36
ALOX5AP	ENSG00000132965	9426.5	0
SCML4	ENSG00000146285	9412.5	0.025
TMEM254-AS1	ENSG00000230091	9388.5	0.22
BPI	ENSG00000101425	9371.5	0
STK39	ENSG00000198648	9352	11.615
BCAS4	ENSG00000124243	9339.5	7.925
DAP3	ENSG00000132676	9322.5	156
MSTO1	ENSG00000125459	9322.5	56.965
MSTO2P	ENSG00000203761	9322.5	9.575
HNF4G	ENSG00000164749	9318.5	2.425
INSR	ENSG00000171105	9233.5	10.595
FMN2	ENSG00000155816	9212	5.35
ADH5P3	ENSG00000223694	9212	0.17
RERE	ENSG00000142599	9209.5	46.555
PRKAR1A	ENSG00000108946	9202.5	144.495
ARSG	ENSG00000141337	9202.5	6.15
ASB5	ENSG00000164122	9164.5	0.21
AFAP1	ENSG00000196526	9136.5	8.02
ATP5L	ENSG00000167283	9113.5	319.275
FUBP1	ENSG00000162613	9103	178.665
ATP8B1	ENSG00000081923	9100	1.71
UNC5D	ENSG00000156687	9057.5	0
PRUNE2	ENSG00000106772	9044	4.17
PLEKHM1P	ENSG00000214176	9040	9.9
hsa-mir-6080	ENSG00000215769	9040	4.415
FBXO18	ENSG00000134452	9032.5	37.47
H2AFY2	ENSG00000099284	9029	44.785
PLLP	ENSG00000102934	9008	3
LAMA3	ENSG00000053747	8983	8.005
TCF7L2	ENSG00000148737	8975	34.495
PIP4K2A	ENSG00000150867	8967	29.91
ST6GALNAC3	ENSG00000184005	8965	3.8
CDH4	ENSG00000179242	8957	0.33
HPS1	ENSG00000107521	8953	27.08
JMJD1C	ENSG00000171988	8939	12.18
CAB39	ENSG00000135932	8916	30.62
USP34	ENSG00000115464	8911.5	48.885
ZNF490	ENSG00000188033	8876	4.105
ZNF564	ENSG00000249709	8876	4.09

ZNF799	ENSG00000196466	8876	2.355
ZNF443	ENSG00000180855	8876	1.825
ZNF709	ENSG00000242852	8876	1.005
PGK1P2	ENSG00000213290	8876	0.17
PAK1	ENSG00000149269	8855	39.33
LITAF	ENSG00000189067	8853.5	25.245
FBXW11	ENSG00000072803	8839.5	39.92
GLB1L2	ENSG00000149328	8806	19.05
RSBN1L-AS1	ENSG00000214293	8788	16.09
SCAMP4	ENSG00000227500	8777	58.77
ADAT3	ENSG00000213638	8777	14.57
ESRRB	ENSG00000119715	8734	3.2
MYOM1	ENSG00000101605	8727	0.305
MAP3K4	ENSG00000085511	8723.5	20.39
ZDHHC13	ENSG00000177054	8711.5	12.905
LRRC37B	ENSG00000185158	8698.5	14.885
SH3GL1P1	ENSG00000266777	8698.5	2.885
PTPRD	ENSG00000153707	8696	2.625
RNF144A	ENSG00000151692	8668	5.02
CNTN5	ENSG00000149972	8648.5	0.015
RPA2P3	ENSG00000255547	8648.5	0
BASP1	ENSG00000176788	8644.5	23.655
DCAF13P2	ENSG00000250816	8644.5	0.12
SPTSSA	ENSG00000165389	8643	9.21
EGLN3	ENSG00000129521	8643	8.975
TC2N	ENSG00000165929	8586.5	7.49
CATSPERB	ENSG00000133962	8586.5	0.655
NANOGP7	ENSG00000258774	8586.5	0
RBM20	ENSG00000203867	8577	7.92
PCNX	ENSG00000100731	8575	23.92
SLC20A2	ENSG00000168575	8573.5	30.44
GIMAP2	ENSG00000106560	8542.5	0.12
TBR1	ENSG00000136535	8539	0.205
SLC4A10	ENSG00000144290	8539	0.065
KRT18P46	ENSG00000235716	8539	0
MUC3A	ENSG00000169894	8516.5	2.285
PPIP5K1	ENSG00000168781	8511.5	20.315
CATSPER2	ENSG00000166762	8492.5	4.075
STRC	ENSG00000242866	8492.5	1.35
STRCP1	ENSG00000166763	8492.5	0.03
SAMD4A	ENSG00000020577	8477.5	6.205
BDNF-AS	ENSG00000245573	8474.5	0.355
BDNF	ENSG00000176697	8474.5	0.03
TFAP2A	ENSG00000137203	8434.5	59.655
GLTP	ENSG00000139433	8431.5	34.58
ATRN	ENSG00000088812	8430	12.475
TTC4	ENSG00000243725	8426.5	53.525

MROH7	ENSG00000184313	8426.5	0.065
MROH7-TTC4	ENSG00000271723	8426.5	0.055
AMIGO1	ENSG00000181754	8416	3.545
GTPBP10	ENSG00000105793	8398	25.525
CLDN12	ENSG00000157224	8398	21.505
CDK14	ENSG00000058091	8398	8.465
TVP23CP1	ENSG00000223584	8398	0.205
FANCA	ENSG00000187741	8392.5	64.835
SFT2D1	ENSG00000198818	8392	41.695
HNRNPA1P49	ENSG00000233231	8392	3.11
KCNMA1	ENSG00000156113	8384	0.445
PVRL1	ENSG00000110400	8364.5	10.94
PEG3	ENSG00000198300	8347.5	0
ZIM2	ENSG00000269699	8347.5	0
CCDC132	ENSG00000004766	8342	15.21
VSTM4	ENSG00000165633	8340	1.105
CHRM3	ENSG00000133019	8337.5	1.63
C20orf24	ENSG00000101084	8335	66.59
TGIF2	ENSG00000118707	8335	41.7
TGIF2-C20orf24	ENSG00000259399	8335	13.13
POMGNT2	ENSG00000144647	8320.5	18.79
B4GALNT3	ENSG00000139044	8317	12.6
FUT8	ENSG00000033170	8315.5	13.12
OR2T3	ENSG00000196539	8315.5	0
NPRL3	ENSG00000103148	8292.5	32.245
PCDH7	ENSG00000169851	8288	26.335
TP53TG3C	ENSG00000205457	8287	0.02
TP53TG3B	ENSG00000261509	8287	0
OR52B1P	ENSG00000180909	8278	0
FMNL2	ENSG00000157827	8276.5	13.275
EGFEM1P	ENSG00000206120	8261	5.155
PHACTR4	ENSG00000204138	8223	43.035
RNU6ATAC27P	ENSG00000221216	8223	5.13
MEIS1	ENSG00000143995	8210	23.9
TENM4	ENSG00000149256	8205.5	2.54
GRIK4	ENSG00000149403	8203	0.655
LHFPL2	ENSG00000145685	8198	8.76
SUB1P2	ENSG00000258737	8196	0.275
VRTN	ENSG00000133980	8196	0
GUSBP1	ENSG00000183666	8171.5	18.14
TRIM13	ENSG00000204977	8143.5	13.515
KCNRG	ENSG00000198553	8143.5	2.66
RASGEF1B	ENSG00000138670	8127	6.405
UBR2	ENSG00000024048	8107	19.675
HLCS	ENSG00000159267	8105.5	14.965
HLCS-IT1	ENSG00000237646	8105.5	0.255
C10orf67	ENSG00000179133	8075.5	0.25

PKHD1L1	ENSG00000205038	8066.5	0.005
ARHGAP6	ENSG00000047648	8032.5	0.37
C1orf228	ENSG00000198520	8031	0.45
LCK	ENSG00000182866	8029	0.07
MEF2C-AS1	ENSG00000248309	8015	0.99
PLXDC2	ENSG00000120594	8002	5.135
MDM4	ENSG00000198625	8001.5	26.68
CFHR3	ENSG00000116785	7990	0.675
SEPHS1P4	ENSG00000230146	7981	0
EDARADD	ENSG00000186197	7963	7.305
IL5RA	ENSG00000091181	7946.5	0
TMEM182	ENSG00000170417	7933.5	3.98
ENTPD5	ENSG00000187097	7921	12.9
SHC4	ENSG00000185634	7909	0.175
ECT2	ENSG00000114346	7871	60.235
TMC2	ENSG00000149488	7857.5	0.045
TMEM144	ENSG00000164124	7855	4.805
FAM135B	ENSG00000147724	7844.5	0
ZNF148	ENSG00000163848	7793	21.815
SLC12A8	ENSG00000221955	7793	2.22
MYO3B	ENSG00000071909	7785.5	0
MTA3	ENSG00000057935	7775	36.71
SNX6	ENSG00000129515	7772.5	56.315
PIPOX	ENSG00000179761	7755	0.395
TFDP2	ENSG00000114126	7734	50.29
NAV2	ENSG00000166833	7703	1.36
MIR4713	ENSG00000266593	7703	0
NAV2-IT1	ENSG00000255270	7703	0
CHML	ENSG00000203668	7686.5	19.78
OPN3	ENSG00000054277	7686.5	7.58
ACOX1	ENSG00000161533	7675.5	27.595
LINC00475	ENSG00000225511	7612	0
VAMP7	ENSG00000124333	7598.5	38.055
LINC01090	ENSG00000231689	7596	0
LAMC1	ENSG00000135862	7583.5	108.51
PPM1L	ENSG00000163590	7544	7.775
SPNS3	ENSG00000182557	7541.5	0
WDR25	ENSG00000176473	7529.5	8.72
RN7SKP92	ENSG00000253075	7529.5	0
ARHGAP42	ENSG00000165895	7518	4.06
SLC6A16	ENSG00000063127	7494	0.945
CST2	ENSG00000170369	7490.5	0
PTCD3	ENSG00000132300	7487	113.54
FAM19A2	ENSG00000198673	7478	1.435
KLF17P1	ENSG00000258316	7478	0.22
ZCCHC7	ENSG00000147905	7473	23.235
FBXO42	ENSG00000037637	7472	13.06

PTPRU	ENSG00000060656	7454	25.62
RFTN1	ENSG00000131378	7451.5	6.29
ERN2	ENSG00000134398	7449	0.25
CDC45	ENSG00000093009	7433	81.275
LSP1	ENSG00000130592	7422	0.125
SHFM1	ENSG00000127922	7412	125.295
C7orf76	ENSG00000197851	7412	0
C1D	ENSG00000197223	7406	15.07
MB	ENSG00000198125	7398	0.075
AP3S1	ENSG00000177879	7377.5	58.05
LRRC49	ENSG00000137821	7375	8.795
KRT8P9	ENSG00000259470	7375	0.045
NPLOC4	ENSG00000182446	7373	83
AFF3	ENSG00000144218	7366.5	7.485
TMPRSS15	ENSG00000154646	7353	0
RHCE	ENSG00000188672	7348.5	0.105
CRHR1-IT1	ENSG00000204650	7346	42.67
CRHR1	ENSG00000120088	7346	0.9
PYROXD2	ENSG00000119943	7323.5	1.785
EPSTI1	ENSG00000133106	7318.5	1.75
CA15P2	ENSG00000234757	7318.5	0.145
CA15P1	ENSG00000241527	7311.5	0.635
STARD9	ENSG00000159433	7278	3.955
CCDC144NL	ENSG00000205212	7277.5	0.045
ABHD17AP6	ENSG00000226981	7277.5	0
SGCD	ENSG00000170624	7275.5	0
CYP4F59P	ENSG00000233244	7259.5	0
LY86-AS1	ENSG00000216863	7205	0
DENND5A	ENSG00000184014	7185.5	54.26
MICU3	ENSG00000155970	7159	4.17
DMD	ENSG00000198947	7158	6.945
CENPF	ENSG00000117724	7151.5	77.365
ABHD17AP3	ENSG00000250536	7151.5	0.47
RANBP17	ENSG00000204764	7145	10.775
RN7SL623P	ENSG00000244501	7145	0.23
snoU13	ENSG00000239166	7145	0
ATG10	ENSG00000152348	7123	4.76
LRRN2	ENSG00000170382	7122	1.73
SLC30A9	ENSG00000014824	7118	50.69
ATP1B1P1	ENSG00000249212	7118	0.84
FAM95B1	ENSG00000223839	7109.5	2.605
ANKRD20A2	ENSG00000183148	7109.5	0.435
RNU6-1269P	ENSG00000212441	7109.5	0
CERS6	ENSG00000172292	7101.5	18.115
CRISPLD2	ENSG00000103196	7097.5	0.395
IFRD2	ENSG00000214706	7094.5	90.97
NAT6	ENSG00000243477	7094.5	11.515

HYAL3	ENSG00000186792	7094.5	7.795
TOX3	ENSG00000103460	7084	1.725
LINC00864	ENSG00000228055	7056	0
PDS5A	ENSG00000121892	7050.5	55.805
LARP4B	ENSG00000107929	7043	73.42
USP13	ENSG00000058056	7033.5	19.045
KAT7	ENSG00000136504	7010	49.045
WDR7	ENSG00000091157	7000.5	12.395
WDR7-OT1	ENSG00000267225	7000.5	0.225
ZNF667	ENSG00000198046	6998	0
SLC8A1	ENSG00000183023	6995	7.515
TMEM132B	ENSG00000139364	6977	0.065
PRELID2	ENSG00000186314	6969	7.715
GSN	ENSG00000148180	6946	7.57
HFM1	ENSG00000162669	6940.5	2.255
SLC7A11-AS1	ENSG00000250033	6930	0.315
KHDRBS2	ENSG00000112232	6921	0
TPT1-AS1	ENSG00000170919	6920.5	10.01
RBM4	ENSG00000173933	6916.5	137.54
RBM14	ENSG00000239306	6916.5	99.3
RBM14-RBM4	ENSG00000248643	6916.5	3.435
RNMTL1	ENSG00000171861	6903	27.27
PRPS2	ENSG00000101911	6888.5	44.85
TMEM178B	ENSG00000261115	6888.5	7.37
MED12L	ENSG00000144893	6868.5	12.415
GPR171	ENSG00000174946	6868.5	1.155
SATB2	ENSG00000119042	6830.5	32.435
EFTUD1P1	ENSG00000259404	6827.5	3.01
PAFAH1B2	ENSG00000168092	6815.5	78.28
ACTG1P1	ENSG00000178631	6806.5	0.34
KIAA1244	ENSG00000112379	6800.5	2.915
RIT2	ENSG00000152214	6770.5	0
HIRA	ENSG00000100084	6762	41.785
C22orf39	ENSG00000242259	6762	23.495
C4orf40	ENSG00000187533	6722	0.02
MACROD2	ENSG00000172264	6715.5	3.09
MACROD2-IT1	ENSG00000227927	6715.5	0.23
AIMP1P1	ENSG00000234187	6715.5	0.2
EMR3	ENSG00000131355	6715	0.03
IL21-AS1	ENSG00000227145	6709.5	0
TANC2	ENSG00000170921	6708.5	16.305
JPH2	ENSG00000149596	6706	0.03
DRG2	ENSG00000108591	6705	34.86
DEPDC1B	ENSG00000035499	6696	22.61
CASS4	ENSG00000087589	6673.5	0.195
EEF1DP3	ENSG00000229715	6669.5	0.43
KCTD16	ENSG00000183775	6648.5	0.275

FYN	ENSG00000010810	6643	83.745
MKRN2	ENSG00000075975	6641	33.52
C3orf83	ENSG00000225526	6641	1.44
ARNTL	ENSG00000133794	6635	13.085
ADAMTS9	ENSG00000163638	6620	3.33
LINC01138	ENSG00000215863	6608	12.445
NF1	ENSG00000196712	6602	23.5
AK4P1	ENSG00000263535	6602	16.365
TSPAN9	ENSG00000011105	6596	7.81
SMG7	ENSG00000116698	6577.5	68.94
NCF2	ENSG00000116701	6577.5	0.395
PLCB1	ENSG00000182621	6573.5	7.84
RNU105B	ENSG00000201348	6573.5	0.375
SLC16A6	ENSG00000108932	6554	1.21
DST	ENSG00000151914	6553.5	42.3
MGMT	ENSG00000170430	6550.5	0
DMGDH	ENSG00000132837	6528	0.425
PCDH15	ENSG00000150275	6505	0.05
LMTK2	ENSG00000164715	6499.5	12.96
STX6	ENSG00000135823	6491	22.43
RBM34	ENSG00000188739	6475.5	111.795
ARID4B	ENSG00000054267	6475.5	55.51
SNCB	ENSG00000074317	6475	4.74
SLC25A21	ENSG00000183032	6470	2.5
PPP4R2	ENSG00000163605	6462	60.86
GXYLT2	ENSG00000172986	6462	14.7
EBLN2	ENSG00000255423	6462	5.74
FAT3	ENSG00000165323	6446	2.125
SH3BGR	ENSG00000185437	6437.5	3.915
RTN4	ENSG00000115310	6403	93.445
AFF2	ENSG00000155966	6388.5	0.125
FBXO21	ENSG00000135108	6383	31.605
RXFP1	ENSG00000171509	6376.5	0
MAL2	ENSG00000147676	6371.5	0
DPEP1	ENSG00000015413	6356	0.145
CHRDL1	ENSG00000101938	6326.5	1.345
GOLGA2P5	ENSG00000238105	6320.75	4.36
SLC5A6	ENSG00000138074	6317.5	46.035
POLA2	ENSG00000014138	6310	139.225
DGKH	ENSG00000102780	6309	5.305
FHP1	ENSG00000234213	6309	0
APOD	ENSG00000189058	6306	0.085
SLC2A14	ENSG00000173262	6303	0.11
NEIL2	ENSG00000154328	6293.5	5.53
HS3ST2	ENSG00000122254	6273	0
KIAA1598	ENSG00000187164	6272.5	26.23
B4GALT6	ENSG00000118276	6251.5	27.535

CASC15	ENSG00000272168	6251.5	0.03
CASC14	ENSG00000260455	6251.5	0
RN7SKP240	ENSG00000222515	6251.5	0
ADAMTS19	ENSG00000145808	6243	2.11
ASPHD2	ENSG00000128203	6240	5.865
KIAA1549L	ENSG00000110427	6232	1.72
RTN4R	ENSG00000040608	6224	3.54
PLEKHM3	ENSG00000178385	6215	6.26
VTA1	ENSG00000009844	6211	63.125
VWA3B	ENSG00000168658	6209.5	0
BCR	ENSG00000186716	6203	81.515
PPP1R12B	ENSG00000077157	6196.5	13.01
NOSIP	ENSG00000142546	6192	62.465
RWDD3	ENSG00000122481	6178	10.395
TMEM56	ENSG00000152078	6178	3.305
TMEM56-RWDD3	ENSG00000271092	6178	0.595
BRPF1	ENSG00000156983	6175	28.54
SHISA9	ENSG00000237515	6169.5	0.07
CHI3L1	ENSG00000133048	6157.5	0
TTN	ENSG00000155657	6115	1.19
CCDC141	ENSG00000163492	6115	0.44
LINGO1	ENSG00000169783	6109.5	18.01
NDRG4	ENSG00000103034	6096	15.515
ICA1	ENSG00000003147	6077	6.105
NOMO1	ENSG00000103512	6075.5	145.695
PAQR3	ENSG00000163291	6030	29.54
BMP2K	ENSG00000138756	6030	15.695
RN7SL127P	ENSG00000242175	6030	0
PIK3R2	ENSG00000105647	6024.5	57.725
IFI30	ENSG00000216490	6024.5	28.845
ANKH	ENSG00000154122	6017	18.79
RBBP4P1	ENSG00000249485	6017	1.44
COA6	ENSG00000168275	5988.5	57.215
CACNA1E	ENSG00000198216	5985	0.015
BLVRB	ENSG00000090013	5983	15.335
ADAMTSL1	ENSG00000178031	5957.5	0.15
RANBP9	ENSG00000010017	5939	33.775
TPGS2	ENSG00000134779	5936.5	215.815
TLL1	ENSG00000038295	5927	1.14
AQPEP	ENSG00000172901	5924	0.355
ARL14EPL	ENSG00000268223	5924	0
C12orf5	ENSG00000078237	5921	18.535
KLHL21	ENSG00000162413	5905	21.755
AIM1	ENSG00000112297	5868.5	0.455
NTN1	ENSG00000065320	5854.5	10.835
NOMO2	ENSG00000185164	5834	89.655
EGFLAM	ENSG00000164318	5831	0.755

DCTN1	ENSG00000204843	5826	137.03
SLC4A5	ENSG00000188687	5826	1.07
KRT18P26	ENSG00000229798	5826	0.1
NOMO3	ENSG00000103226	5820	109.72
PPIG	ENSG00000138398	5818.5	78.755
NRG1	ENSG00000157168	5811.5	0.025
NRG1-IT1	ENSG00000253974	5811.5	0
NRG1-IT2	ENSG00000253222	5811.5	0
NRG1-IT3	ENSG00000254049	5811.5	0
ARHGAP10	ENSG00000071205	5808.5	8.945
RN7SL254P	ENSG00000240014	5808.5	0
KCNN3	ENSG00000143603	5806	0.38
SMAP2	ENSG00000084070	5776	36.235
SH2D3C	ENSG00000095370	5756	0.315
ACYP1	ENSG00000119640	5749	12.955
GPR98	ENSG00000164199	5730.5	3.9
CNNM2	ENSG00000148842	5721.5	10.81
KCNQ1-AS1	ENSG00000229414	5721.5	0
C1orf168	ENSG00000187889	5711.5	0.845
CDK19	ENSG00000155111	5700	12.05
ZZEF1	ENSG00000074755	5695	22.425
SLC16A10	ENSG00000112394	5682	17.235
SLC43A2	ENSG00000167703	5674	6.44
UBP1	ENSG00000153560	5663.5	104.915
BDKRB2	ENSG00000168398	5654.5	0.06
BDKRB1	ENSG00000100739	5654.5	0
DOCK8	ENSG00000107099	5639.5	1.465
BID	ENSG00000015475	5636.5	25.175
STRBP	ENSG00000165209	5630	36.92
MIR600HG	ENSG00000236901	5630	5.785
FRY	ENSG00000073910	5626	0.88
MAP2K1	ENSG00000169032	5622	60.05
KCNIP1	ENSG00000182132	5612	0.2
SLC35A2	ENSG00000102100	5607.5	21.08
MDFI	ENSG00000112559	5582	0
SLCO1A2	ENSG00000084453	5564	0.035
RAPGEF6	ENSG00000158987	5539	14.34
FNIP1	ENSG00000217128	5539	10.08
C3orf67	ENSG00000163689	5536.5	7.145
STIM1	ENSG00000167323	5525.5	17.81
VAX2	ENSG00000116035	5523.5	7.255
NALCN	ENSG00000102452	5521	0.165
RALGPS2	ENSG00000116191	5512.5	24.45
MYH9	ENSG00000100345	5491	72.145
FRMPD4	ENSG00000169933	5454.5	0.02
MAD1L1	ENSG00000002822	5451.5	23.59
RPS17P2	ENSG00000197575	5428.5	0

LRFN5	ENSG00000165379	5425.5	1.755
DAGLA	ENSG00000134780	5411.5	2.88
GALNT16	ENSG00000100626	5402	9.395
TMBIM6	ENSG00000139644	5399.5	344.735
PLEKHA5	ENSG00000052126	5393.5	16.855
VGLL4	ENSG00000144560	5386	25.44
PPP2CB	ENSG00000104695	5381.5	31.36
CNTN4	ENSG00000144619	5369	0
PPP3R1	ENSG00000221823	5359	52.9
WDR92	ENSG00000243667	5359	10.05
ZCCHC2	ENSG00000141664	5357	12.975
HDGFRP2	ENSG00000167674	5356	97.125
GPR110	ENSG00000153292	5342.5	0.06
VWA1	ENSG00000179403	5306.5	7.785
HDAC5	ENSG00000108840	5285	23.425
PRR14L	ENSG00000183530	5265.5	49.65
CSRNP3	ENSG00000178662	5263	1.23
RBM5	ENSG00000003756	5262	161.295
RBM6	ENSG00000004534	5262	71.265
NTNG1	ENSG00000162631	5256.5	0.5
PCSK2	ENSG00000125851	5254	0.025
LINC00607	ENSG00000235770	5244.5	0.03
VSTM1	ENSG00000189068	5229.5	0
C9orf170	ENSG00000204446	5224.5	0
ALDH3B2	ENSG00000132746	5221	0.14
NAV1	ENSG00000134369	5219.5	11.415
IPO9-AS1	ENSG00000231871	5219.5	5.275
MTUS2	ENSG00000132938	5196	0.26
GAPDHP69	ENSG00000223460	5196	0.065
ZNF692	ENSG00000171163	5175.5	32.065
SUGT1P	ENSG00000226823	5163.5	0.315
OR6N2	ENSG00000188340	5162	0
NFIA	ENSG00000162599	5158	27.355
RNF4	ENSG00000063978	5148.5	48.705
FAM193A	ENSG00000125386	5148.5	23.67
TMEM30A	ENSG00000112697	5148.5	19.035
GALNT18	ENSG00000110328	5144	6.52
CSNK2A3	ENSG00000254598	5144	2.085
SYNE1	ENSG00000131018	5143	8.535
UBE3D	ENSG00000118420	5108	16.46
JDP2	ENSG00000140044	5097.5	5.61
WDSUB1	ENSG00000196151	5081.5	6.045
CMKLR1	ENSG00000174600	5070	0
SBF2	ENSG00000133812	5065	18.14
INPP4A	ENSG00000040933	5060	17.58
PPIP5K2	ENSG00000145725	5054	52.585
ITIH2	ENSG00000151655	5048	0.21

CSMD2	ENSG00000121904	5034	0.01
MTATP6P1	ENSG00000248527	5032.5	19593.04
MTATP8P1	ENSG00000240409	5032.5	15045.975
PARD3B	ENSG00000116117	5018.5	4.35
CDKL2	ENSG00000138769	5006	2.48
TSPAN8	ENSG00000127324	5005.5	0.08
ZCCHC11	ENSG00000134744	4994.5	59.615
LRGUK	ENSG00000155530	4993	0.4
ROR2	ENSG00000169071	4972.5	5.045
CPB1	ENSG00000153002	4971	0.015
SCFD2	ENSG00000184178	4960	18.01
TTC6	ENSG00000139865	4956	0.53
ZDHHC3	ENSG00000163812	4954.5	55.35
DCN	ENSG00000011465	4954.5	0.12
SLC39A14	ENSG00000104635	4907	34.655
PRPSAP2	ENSG00000141127	4903.5	44.195
LIN7A	ENSG00000111052	4902	7.805
SCFD1	ENSG00000092108	4900.5	59.54
KLHDC8A	ENSG00000162873	4895.5	0.185
MTND6P4	ENSG00000249119	4893.5	1821.69
PCBD2	ENSG00000132570	4893.5	4.72
CATSPER3	ENSG00000152705	4893.5	0.735
SYN2	ENSG00000157152	4831.5	3.875
ACTG1P12	ENSG00000226642	4831.5	0.6
KALP	ENSG00000241859	4817.5	0
HNRNPLL	ENSG00000143889	4810.5	33.155
DOCK2	ENSG00000134516	4803	0.01
CDH13	ENSG00000140945	4782	0
MIR3182	ENSG00000263785	4782	0
PITPNB	ENSG00000180957	4780.5	122.815
FAM21B	ENSG00000152726	4773	0.14
PLXDC1	ENSG00000161381	4771	0.6
EPRS	ENSG00000136628	4765.5	158.565
CREBRF	ENSG00000164463	4764.5	4.015
TRDMT1	ENSG00000107614	4756.5	9.435
SOX13	ENSG00000143842	4742	13.895
FHIT	ENSG00000189283	4741	6.135
U3	ENSG00000199666	4741	0
NBPF10	ENSG00000163386	4716.5	19.825
NOTCH2NL	ENSG00000213240	4716.5	18.84
WDR70	ENSG00000082068	4704	33.05
PTPRT	ENSG00000196090	4701.5	0
ENTPD1-AS1	ENSG00000226688	4700.5	21.81
ENTPD1	ENSG00000138185	4700.5	8.615
C10orf131	ENSG00000173088	4700.5	1.075
ACSBG1	ENSG00000103740	4699	0.545
AUNIP	ENSG00000127423	4692	21.3

UST	ENSG00000111962	4688	10.15
COPS5	ENSG00000121022	4686.5	46.94
CSPP1	ENSG00000104218	4686.5	23.68
PPP1R42	ENSG00000178125	4686.5	0.12
EIF4BP6	ENSG00000197258	4684	7.11
LHFPL3	ENSG00000187416	4684	0.02
FGF14	ENSG00000102466	4639.5	0
FGF14-IT1	ENSG00000243319	4639.5	0
DMC1	ENSG00000100206	4637	13.125
PCDHB16	ENSG00000196963	4632.5	0.055
TEX33	ENSG00000185264	4630.5	0
AK2	ENSG00000004455	4617.5	359.675
FAM198B	ENSG00000164125	4595.5	0.225
IPO5	ENSG00000065150	4574	137.29
EXOC4	ENSG00000131558	4574	26.22
ARHGAP32	ENSG00000134909	4563	9.27
MAD2L2	ENSG00000116670	4560	75.45
MAN1A1	ENSG00000111885	4557.5	17.395
TLE6	ENSG00000104953	4554	0.7
RAB27A	ENSG00000069974	4539.5	2.36
GALK2	ENSG00000156958	4537.5	11.58
SRBD1	ENSG00000068784	4532.5	13.44
RGS8	ENSG00000135824	4529	0
CAPRIN2	ENSG00000110888	4520	9.075
MTX3	ENSG00000177034	4513.5	12.89
CCNYL1	ENSG00000163249	4511.5	14.745
ZNF418	ENSG00000196724	4506	0.185
NLN	ENSG00000123213	4469.5	28.195
NCKAP1	ENSG00000061676	4467.5	46.99
PRR12	ENSG00000126464	4456	22.1
FAM163A	ENSG00000143340	4452	0.485
PHF20L1	ENSG00000129292	4445.5	28.97
BBOX1	ENSG00000129151	4443	0.6
DEFA1B	ENSG00000240247	4430.5	0
DEFA3	ENSG00000239839	4430.5	0
ZNF503-AS2	ENSG00000237149	4426	30.445
CD99	ENSG00000002586	4377.5	157.74
RAB3C	ENSG00000152932	4345	0.83
RPL5P15	ENSG00000240412	4345	0
F5	ENSG00000198734	4338	0.035
LINC01104	ENSG00000232084	4318.5	0
TERF2	ENSG00000132604	4308.5	56.275
COG8	ENSG00000213380	4308.5	26.775
PDF	ENSG00000258429	4308.5	24.23
TMED6	ENSG00000157315	4308.5	3.375
GUSBP3	ENSG00000253203	4306.5	6.985
PTGER2	ENSG00000125384	4296.5	1.235

FAM102B	ENSG00000162636	4290.5	7.78
RALGPS1	ENSG00000136828	4277	7.055
SNX29P2	ENSG00000198106	4276	8.505
SULT1A4	ENSG00000213648	4276	8.035
SLX1B	ENSG00000181625	4276	4.435
SLX1B-SULT1A4	ENSG00000260280	4276	0
ZNF90P2	ENSG00000233366	4272.5	0
ZNF286A	ENSG00000187607	4258.5	29.07
TBC1D26	ENSG00000214946	4258.5	0.145
STIP1	ENSG00000168439	4251	355.645
NME7	ENSG00000143156	4248.5	31.54
SUDS3P1	ENSG00000249129	4246.5	3.215
GOLPH3L	ENSG00000143457	4244.5	36.48
CLDN16	ENSG00000113946	4244	0.02
USP32P3	ENSG00000189423	4243	0.98
FAM106B	ENSG00000205217	4243	0.42
FOXN3	ENSG00000053254	4238	17.385
EFCAB11	ENSG00000140025	4238	7.175
CAP2P1	ENSG00000259151	4238	0.18
RCC2	ENSG00000179051	4205	142.915
BOC	ENSG00000144857	4192	9.615
ANO6	ENSG00000177119	4164	12.705
DNAJC11	ENSG00000007923	4155.5	63.41
ARL3	ENSG00000138175	4155.5	8.47
AK5	ENSG00000154027	4148	1.825
FAM205A	ENSG00000205108	4146.5	0.08
CERKL	ENSG00000188452	4146	1.765
NEUROD1	ENSG00000162992	4146	0.02
TRANK1	ENSG00000168016	4134	0.02
GNAQ	ENSG00000156052	4116	23.9
KIF2A	ENSG00000068796	4113.5	71.315
IPO11	ENSG00000086200	4113.5	38.425
FBXO9	ENSG00000112146	4110	122.815
ICK	ENSG00000112144	4110	7.165
C15orf27	ENSG00000169758	4110	3.45
TMEM117	ENSG00000139173	4106	4.255
RAD51D	ENSG00000185379	4101.5	8.1
RFFL	ENSG00000092871	4101.5	4.445
RAD51L3-RFFL	ENSG00000267618	4101.5	0.235
ACTL8	ENSG00000117148	4101	0.395
TTLL8	ENSG00000138892	4098.5	0
PARP11	ENSG00000111224	4097.5	4.845
COL12A1	ENSG00000111799	4096	0.21
RANBP1	ENSG00000099901	4095	629.82
SMEK1	ENSG00000100796	4089.5	83.475
CCBE1	ENSG00000183287	4073	1.88
DENND1A	ENSG00000119522	4069.5	19.095

USP22	ENSG00000124422	4058	175.415
HERC5	ENSG00000138646	4058	11.605
ZDHHC15	ENSG00000102383	4055	0.035
JAK2	ENSG00000096968	4048	1.985
MTND1P11	ENSG00000228166	4048	0.52
IGHEP2	ENSG00000254017	4048	0.15
MTND6P5	ENSG00000227730	4048	0
MYPN	ENSG00000138347	4046.5	0.09
RN7SKP202	ENSG00000222371	4046.5	0
YTHDC2	ENSG00000047188	4046	19.47
FAM168A	ENSG00000054965	4038	26.3
TRAPPC9	ENSG00000167632	4038	8.95
TRIQK	ENSG00000205133	4030.5	11.085
FBXW7	ENSG00000109670	4023	12.875
PDE10A	ENSG00000112541	4017.5	4.225
LINC00473	ENSG00000223414	4017.5	0.245
SDIM1	ENSG00000256391	4017.5	0
KCNB2	ENSG00000182674	4013.5	3.185
OAZ2	ENSG00000180304	4011.5	61.38
SYN3	ENSG00000185666	4009.5	2.675
SKAP1	ENSG00000141293	4008	0.38
MIR1203	ENSG00000221739	4008	0
UBR7	ENSG00000012963	4004.5	29.905
C14orf142	ENSG00000170270	4004.5	12.355
TMEM251	ENSG00000153485	4004.5	9.385
SMC3	ENSG00000108055	4003	73.91
CLSTN2	ENSG00000158258	3987.5	0.29
STARD6	ENSG00000174448	3980.5	0
UIMC1	ENSG00000087206	3979	35.04
SYT1	ENSG00000067715	3976.5	5.905
ITCH	ENSG00000078747	3970	27.67
ITCH-IT1	ENSG00000231795	3970	0.38
FAM154A	ENSG00000155875	3952	0.07
KIAA1671	ENSG00000197077	3945.5	15.46
MLIP	ENSG00000146147	3944.5	0.06
MLIP-AS1	ENSG00000235050	3944.5	0
MLIP-IT1	ENSG00000236996	3944.5	0
RNU1-2	ENSG00000207005	3937	29.45
RNU1-4	ENSG00000207389	3937	29.45
CROCC	ENSG00000058453	3937	15.425
MST1L	ENSG00000186715	3937	0.58
U1	ENSG00000233421	3937	0.535
CROCCP4	ENSG00000227684	3937	0.135
USP12	ENSG00000152484	3928.5	11.99
NPSR1-AS1	ENSG00000197085	3904.5	0.195
NPSR1	ENSG00000187258	3904.5	0.045
NCAPD2P1	ENSG00000230356	3904.5	0

RPL7P31	ENSG00000235379	3904.5	0
ZFYVE28	ENSG00000159733	3887.5	2.55
BBS12	ENSG00000181004	3885	2.005
SNAP25-AS1	ENSG00000227906	3883	0.02
RPL23AP6	ENSG00000214835	3883	0
LINC00511	ENSG00000227036	3874.5	0.93
ABCC2	ENSG00000023839	3871	0.49
FAM157B	ENSG00000233013	3870.75	0.28
BAI3	ENSG00000135298	3858	2.245
LZTS1	ENSG00000061337	3856	0.53
BCL11B	ENSG00000127152	3845.5	1.385
SMG1	ENSG00000157106	3820.5	45.905
DNAH14	ENSG00000185842	3820	52.34
IMPDH1	ENSG00000106348	3812	79.495
SCN4A	ENSG00000007314	3811	0.78
VOPP1	ENSG00000154978	3800	22.415
RBFOX1	ENSG00000078328	3792.5	0
snoMe28S-Am2634	ENSG00000251844	3792.5	0
MYH7B	ENSG00000078814	3787.5	0.7
TMC1	ENSG00000165091	3779.5	0
SLC1A4	ENSG00000115902	3774	6.75
LINC00535	ENSG00000246662	3769	0.165
LRIG3	ENSG00000139263	3768.5	20.57
PKNOX1	ENSG00000160199	3764.5	22.675
KIF21A	ENSG00000139116	3764	20.735
C3orf35	ENSG00000198590	3760	0.92
DYNC1I1	ENSG00000158560	3729.5	1.075
C10orf11	ENSG00000148655	3725	1.07
DTHD1	ENSG00000197057	3687	0
CLDN1	ENSG00000163347	3685.5	0.495
HUNK	ENSG00000142149	3674.5	4.065
B3GALT5	ENSG00000183778	3674	0.005
MLLT3	ENSG00000171843	3671	33.4
SLC5A9	ENSG00000117834	3657	0.31
TSHZ2	ENSG00000182463	3653.5	2.86
EMX2OS	ENSG00000229847	3652.5	4.03
ATP9A	ENSG00000054793	3646.5	10.1
ARAP2	ENSG00000047365	3636.5	2.565
SLC9A7	ENSG00000065923	3631	8.55
PGAM1P7	ENSG00000213997	3631	1.055
DPYS	ENSG00000147647	3631	0.055
IDNK	ENSG00000148057	3626.5	2.63
CDC42BPB	ENSG00000198752	3615	31.745
PNPLA1	ENSG00000180316	3610.5	0.08
HIF1A-AS1	ENSG00000258777	3607	0.115
PHKA1	ENSG00000067177	3605	16.53
PHKA1-AS1	ENSG00000231944	3605	0.99

PLS1	ENSG00000120756	3599.5	18.155
TEX11	ENSG00000120498	3585	0.02
DOCK9	ENSG00000088387	3575.5	17.395
DHDDS	ENSG00000117682	3573	23.6
DENND2A	ENSG00000146966	3572.5	1.76
HNRNPDLP3	ENSG00000236185	3571	0
NBPF16	ENSG00000203827	3568.25	0.09
SH2D6	ENSG00000152292	3565	0.34
TCF7	ENSG00000081059	3552	17.01
LINC01094	ENSG00000251442	3551	0
CA13	ENSG00000185015	3550.5	3.35
NBPF15	ENSG00000243452	3532	33.065
PDE2A	ENSG00000186642	3527	0.68
MSI2	ENSG00000153944	3526	59.12
SEPT7	ENSG00000122545	3517	108.585
KLF15	ENSG00000163884	3512.5	9.135
SPSB1	ENSG00000171621	3505.5	3.545
TBX15	ENSG00000092607	3505	0.105
ANXA4	ENSG00000196975	3504.5	12.605
TTC7B	ENSG00000165914	3503.5	24.07
FUCA1P1	ENSG00000237053	3499.5	0
LAMTOR3	ENSG00000109270	3489.5	25.25
MST1P2	ENSG00000186301	3489.5	0.93
MAGI1	ENSG00000151276	3485	12.48
MAGI1-AS1	ENSG00000240175	3485	0.665
MAGI1-IT1	ENSG00000272610	3485	0.55
KCNC4	ENSG00000116396	3480	7.295
KPNA3	ENSG00000102753	3466.5	42.72
CYFIP2	ENSG00000055163	3461.5	34.15
FAM115C	ENSG00000170379	3457.5	0.365
FAM115D	ENSG00000159860	3457.5	0.115
CTAGE6	ENSG00000271321	3457.5	0
PAICSP6	ENSG00000241136	3457.5	0
CENPC	ENSG00000145241	3456.5	16.725
WNT11	ENSG00000085741	3456	3.525
PALMD	ENSG00000099260	3454	0.535
AMMECR1	ENSG00000101935	3452.5	16.32
TPST1	ENSG00000169902	3452	13.305
ARHGAP23	ENSG00000225485	3450.5	0.68
HADHB	ENSG00000138029	3449.5	66.305
IFT80	ENSG00000068885	3446.5	21.595
TRIM59	ENSG00000213186	3446.5	17.02
FAM230A	ENSG00000188280	3446	0.145
APBB1IP	ENSG00000077420	3444	0
VPS13B	ENSG00000132549	3439.5	8.04
CLCA2	ENSG00000137975	3435	0
FIG4	ENSG00000112367	3420	10.135

CYB561D1	ENSG00000174151	3402	4.8
MTA1	ENSG00000182979	3398.5	147.06
TEX22	ENSG00000226174	3398.5	0.48
PCNXL2	ENSG00000135749	3390.5	21.82
KREMEN1	ENSG00000183762	3385	17.47
IMMP2L	ENSG00000184903	3383.5	3.88
NRG4	ENSG00000169752	3371.5	8.97
LRRC25	ENSG00000175489	3370	0.065
KLF8	ENSG00000102349	3369	0.125
ASTN1	ENSG00000152092	3365	0.02
LRRC18	ENSG00000165383	3362	0
WDFY4	ENSG00000128815	3362	0
CLPB	ENSG00000162129	3355	64.055
FAM196B	ENSG00000204767	3352	0.11
NOX4	ENSG00000086991	3344.5	0.015
EIF3LP1	ENSG00000258501	3337	0
LCOR	ENSG00000196233	3314	37.365
SIPA1L3	ENSG00000105738	3308	26.97
RPRD1B	ENSG00000101413	3283	36.645
MKLN1	ENSG00000128585	3277	27.89
WDR64	ENSG00000162843	3274	0.27
CACNA1H	ENSG00000196557	3272	8.06
SIAH1	ENSG00000196470	3271.5	13.48
QKI	ENSG00000112531	3260.5	105.265
IL1RAPL1	ENSG00000169306	3258	0
CCDC62	ENSG00000130783	3256.5	0.695
TMEM194B	ENSG00000189362	3254	2.555
CAMK2B	ENSG00000058404	3243.5	2.11
LINC00894	ENSG00000235703	3242.5	7.805
USP32P1	ENSG00000188933	3237.5	2.295
CCDC144A	ENSG00000170160	3237.5	0.795
FAM106CP	ENSG00000266486	3237.5	0
RN7SL620P	ENSG00000265680	3237.5	0
ADAM23	ENSG00000114948	3233	7.11
ERCC8	ENSG00000049167	3231	11.34
GNL3LP1	ENSG00000215032	3231	1.75
PRKCD	ENSG00000163932	3230	21.88
SOX6	ENSG00000110693	3222.5	6.81
TAB2	ENSG00000055208	3203	59.325
ACVR2A	ENSG00000121989	3201.5	9.495
MTND4P12	ENSG00000247627	3196.833333	5423.44
MTND5P11	ENSG00000248923	3196.833333	683.11
SLC35F1	ENSG00000196376	3188.25	10.24
RUNX2	ENSG00000124813	3179	1.08
HAO2	ENSG00000116882	3174.5	0.115
HAO2-IT1	ENSG00000230921	3174.5	0
SEMA5B	ENSG00000082684	3173	0.455

PPM1E	ENSG00000175175	3168	7.96
CD4	ENSG00000010610	3168	0.605
CCDC97	ENSG00000142039	3160.5	40.46
TGFB1	ENSG00000105329	3160.5	23.44
BANP	ENSG00000172530	3157.5	16.86
LRCH3	ENSG00000186001	3147	34.625
MRPL33	ENSG00000243147	3132.5	66.34
BRE	ENSG00000158019	3132.5	51.14
FAM157C	ENSG00000260528	3129.5	7.905
PLCXD3	ENSG00000182836	3122	0
SGK1	ENSG00000118515	3108.5	4.16
KRT8P42	ENSG00000220378	3108.5	0
TPRG1	ENSG00000188001	3108	0.235
RRN3P2	ENSG00000103472	3098	0.495
PCSK5	ENSG00000099139	3089.5	4.255
P4HA2	ENSG00000072682	3086	12.18
PDLIM4	ENSG00000131435	3086	8.44
MROH5	ENSG00000226807	3085.5	0
ATL2	ENSG00000119787	3081	38.44
SPSB4	ENSG00000175093	3071.5	5.72
KIAA1958	ENSG00000165185	3067	12.05
NUP93	ENSG00000102900	3044.5	90.025
MYO3A	ENSG00000095777	3040.5	2.82
LRRTM4	ENSG00000176204	3019	2.5
MMP28	ENSG00000129270	3012	0
TRIM65	ENSG00000141569	3007.5	37.2
NSD1	ENSG00000165671	3001.5	69.415
IGSF1	ENSG00000147255	2996	0.015
OR11N1P	ENSG00000185903	2996	0
OR5AW1P	ENSG00000229082	2996	0
OR5BH1P	ENSG00000242610	2996	0
PIBF1	ENSG00000083535	2980	20.79
ZHX2	ENSG00000178764	2965.5	11.575
PKNOX2	ENSG00000165495	2949	8.045
ASCC2	ENSG00000100325	2944	63.92
KEL	ENSG00000197993	2935.5	0.35
BTRC	ENSG00000166167	2925.5	16.535
NFATC3	ENSG00000072736	2919	37.98
DUS2	ENSG00000167264	2919	18.685
JPH3	ENSG00000154118	2904	6.24
POLK	ENSG00000122008	2901.5	13.395
CRADD	ENSG00000169372	2898	9.035
DLGAP1	ENSG00000170579	2893.5	0.3
DLGAP1-AS5	ENSG00000261520	2893.5	0
GAPDHP66	ENSG00000250933	2893.5	0
PTPRN2	ENSG00000155093	2891	0.015
MIR153-2	ENSG00000207960	2891	0

ARMC1	ENSG00000104442	2890.5	25.88
CLDN10	ENSG00000134873	2880	0.605
F2RL1	ENSG00000164251	2879	7.47
BCAS3	ENSG00000141376	2874.5	24.84
KRT18P61	ENSG00000267083	2874.5	0.245
S1PR3	ENSG00000213694	2872	4.325
C9orf47	ENSG00000186354	2872	0.325
GRIK3	ENSG00000163873	2863	0.02
RAP2C-AS1	ENSG00000232160	2859	1.365
C2orf49	ENSG00000135974	2856	19.87
CDYL2	ENSG00000166446	2850.5	0.27
GTF2H2C	ENSG00000183474	2846.5	78.555
PDZRN3	ENSG00000121440	2840	6.465
RPGRIP1L	ENSG00000103494	2826.5	11.5
DNAJB2	ENSG00000135924	2825.5	13.965
CELSR1	ENSG00000075275	2822	8.76
SLIT3	ENSG00000184347	2821	0.99
AAK1	ENSG00000115977	2817.5	19.93
SPATS2L	ENSG00000196141	2804.5	37.53
KLHL2	ENSG00000109466	2798	9.855
HS6ST2	ENSG00000171004	2792.5	29.4
CC2D1B	ENSG00000154222	2774.5	23.145
SDK2	ENSG00000069188	2774.5	10.61
ZFYVE9	ENSG00000157077	2774.5	9.875
PDCL3P6	ENSG00000224255	2774.5	0.705
DIRC1	ENSG00000174325	2766	0
SBSPON	ENSG00000164764	2763.5	0.82
SLIT2	ENSG00000145147	2754.5	96.795
MIR218-1	ENSG00000207732	2754.5	0
ASB15	ENSG00000146809	2752.5	0.025
COLGALT1	ENSG00000130309	2747.5	73.595
TP73	ENSG00000078900	2723.5	5.42
GABRR3	ENSG00000183185	2719.5	0.045
CADPS2	ENSG00000081803	2711.5	6.185
RNF148	ENSG00000235631	2711.5	0.455
RNF133	ENSG00000188050	2711.5	0.045
TMEM110	ENSG00000213533	2706.5	30.755
TMEM110-MUSTN1	ENSG00000248592	2706.5	4.31
ITIH4	ENSG00000055955	2706.5	3.275
MUSTN1	ENSG00000272573	2706.5	0.335
GABRB1	ENSG00000163288	2701	0
SYT13	ENSG00000019505	2699.5	0.095
NBPF24	ENSG00000203836	2686	9.42
LINC00276	ENSG00000230448	2657.5	0.145
FAM157A	ENSG00000236438	2656.5	1.1
HHAT	ENSG00000054392	2651.5	1.915
CDH23	ENSG00000107736	2644.5	2.845

TXNL4B	ENSG00000140830	2639.5	12.49
GPR176	ENSG00000166073	2639.5	5.35
FSIP1	ENSG00000150667	2639.5	0.58
HPR	ENSG00000261701	2639.5	0.57
HP	ENSG00000257017	2639.5	0.265
CORO1C	ENSG00000110880	2634.5	86.02
SAP18	ENSG00000150459	2633	194.83
RRAS2	ENSG00000133818	2629	30.085
QSOX2	ENSG00000165661	2627.5	21.065
CDKAL1	ENSG00000145996	2621	8.64
CTNND2	ENSG00000169862	2621	0.245
NBPF11	ENSG00000152042	2618	13.59
ZBTB22	ENSG00000236104	2615.5	7.59
C12orf4	ENSG00000047621	2604	18.145
SRRM2	ENSG00000167978	2601	463.01
WDR34	ENSG00000119333	2597.5	63.555
MGAM	ENSG00000257335	2596.5	0.125
OR9A4	ENSG00000258083	2596.5	0
GPATCH2	ENSG00000092978	2593	11.625
ILF2	ENSG00000143621	2587	314.03
TSNAX	ENSG00000116918	2583.5	35.09
DISC1	ENSG00000162946	2583.5	3.36
DISC1-IT1	ENSG00000226758	2583.5	0.335
TSNAX-DISC1	ENSG00000270106	2583.5	0.11
BLID	ENSG00000259571	2580.5	0
KCNJ3	ENSG00000162989	2579	0.525
CEP135	ENSG00000174799	2578	17.96
KANK1	ENSG00000107104	2563.5	8.28
CACNA1D	ENSG00000157388	2556	1.145
ZFAND2A	ENSG00000178381	2554.5	9.68
ABCC11	ENSG00000121270	2553	0.69
CIB4	ENSG00000157884	2550.5	0
CCDC12	ENSG00000160799	2548	46.11
LRRC10	ENSG00000198812	2545.5	0
TEX41	ENSG00000226674	2542	0.19
CPT1C	ENSG00000169169	2537	7.905
PTPDC1	ENSG00000158079	2536.5	6.32
CYCSP24	ENSG00000227666	2536.5	1.765
EML6	ENSG00000214595	2530.5	2.2
CACNA2D3	ENSG00000157445	2521.5	3.8
RPS15P5	ENSG00000229806	2521.5	0.41
RPL12P2	ENSG00000216412	2507	0
GRPEL2	ENSG00000164284	2499.5	25.08
ANO4	ENSG00000151572	2499	1.865
LAMC2	ENSG00000058085	2494.5	0.38
TNRC18	ENSG00000182095	2493	37.71
COL23A1	ENSG00000050767	2486	0.525

CLN6	ENSG00000128973	2479.5	72.945
CALML4	ENSG00000129007	2479.5	22.34
HMGN2P40	ENSG00000260602	2479.5	0
TMTC1	ENSG00000133687	2474	7.07
MTDH	ENSG00000147649	2462.5	186.995
ANK1	ENSG00000029534	2460	1.32
CLMN	ENSG00000165959	2458.75	4.685
TTBK2	ENSG00000128881	2458	6.565
KRT8P50	ENSG00000260799	2458	0.135
FANK1	ENSG00000203780	2455	0.35
XPR1	ENSG00000143324	2446.666667	29.745
MYT1L	ENSG00000186487	2445	0.14
LARP1B	ENSG00000138709	2437.5	30.78
FOSL1P1	ENSG00000248394	2437.5	1.325
OPCML	ENSG00000183715	2435.5	0.225
OPCML-IT1	ENSG00000254896	2435.5	0
FSTL5	ENSG00000168843	2434	0.09
KDM4B	ENSG00000127663	2427	21.64
SPAG17	ENSG00000155761	2418	1.13
GLTSCR2	ENSG00000105373	2412	108.73
CHRM1	ENSG00000168539	2409.5	0
NBPF13P	ENSG00000227242	2405.5	0.285
RAB11FIP4	ENSG00000131242	2396.5	10.18
INTS4L2	ENSG00000232270	2396	0.125
OR51A7	ENSG00000176895	2394.5	0.065
MMP26	ENSG00000167346	2394.5	0
OR51A8P	ENSG00000197984	2394.5	0
OR51F2	ENSG00000176925	2394.5	0
OR51F5P	ENSG00000272634	2394.5	0
OR51H2P	ENSG00000198217	2394.5	0
OR51N1P	ENSG00000176951	2394.5	0
OR51T1	ENSG00000176900	2394.5	0
SMAD6	ENSG00000137834	2385	32.555
LINC00854	ENSG00000236383	2378.5	4.115
RXFP2	ENSG00000133105	2374	0
ZNF398	ENSG00000197024	2372.5	34.005
BNC2	ENSG00000173068	2372.5	2.235
LRRC20	ENSG00000172731	2369.5	9.115
TMTC2	ENSG00000179104	2355.5	3.885
COL24A1	ENSG00000171502	2347.5	1.725
CLOCK	ENSG00000134852	2340	13.84
ITSN2	ENSG00000198399	2339	19.76
CHST9	ENSG00000154080	2325	0.385
TCF12	ENSG00000140262	2323	23.415
ZNF675	ENSG00000197372	2322	10.75
PPFIBP2	ENSG00000166387	2310.5	2.49
ANO3	ENSG00000134343	2304.5	0.01

MARCH3	ENSG00000173926	2298	6.2
SMOC1	ENSG00000198732	2296.5	21.305
RPL36	ENSG00000130255	2292	1089.15
HSD11B1L	ENSG00000167733	2292	5.45
OTOGL	ENSG00000165899	2279	0
PLCB4	ENSG00000101333	2277	4.115
BCKDHB	ENSG00000083123	2270	12.725
ETV6	ENSG00000139083	2269	3.275
SYNJ2	ENSG00000078269	2268	9.205
SYNJ2-IT1	ENSG00000233496	2268	0.11
NELL1	ENSG00000165973	2264	0.15
MORN1	ENSG00000116151	2262	9.78
SUCO	ENSG00000094975	2259	20.94
PHRF1	ENSG00000070047	2250	22.07
KCNH1	ENSG00000143473	2239.5	0.06
IPO8P1	ENSG00000224668	2239.5	0.025
PRR14	ENSG00000156858	2235.5	96.34
ARMCX4	ENSG00000196440	2229.5	2.265
ENOX2	ENSG00000165675	2221.5	12.885
DACT3-AS1	ENSG00000245598	2219.5	0.37
TFAP2D	ENSG00000008197	2219.5	0
BMPER	ENSG00000164619	2214.5	1.52
TIMP2	ENSG00000035862	2205	14.655
DDC8	ENSG00000178404	2205	0.695
BCL3	ENSG00000069399	2199	1.85
FAM19A5	ENSG00000219438	2195	0
DCLK1	ENSG00000133083	2190.5	1.4
RNF212	ENSG00000178222	2181	0
EIF3L	ENSG00000100129	2178.5	335.98
ANKRD54	ENSG00000100124	2178.5	27.395
PCAT2	ENSG00000253264	2169.5	0
LYRM4	ENSG00000214113	2158	23.085
POTEG	ENSG00000222036	2157	0
OR4F29	ENSG00000235249	2153	0
LUZP2	ENSG00000187398	2143.5	0.07
TNFRSF10B	ENSG00000120889	2142.5	20.445
DAPK2	ENSG00000035664	2142.5	1.055
PARVA	ENSG00000197702	2142	11.48
SND1	ENSG00000197157	2140.5	115.495
DAPP1	ENSG00000070190	2139.5	0.025
POTEM	ENSG00000187537	2136	0.055
ADAM12	ENSG00000148848	2135.5	0.055
CASC4	ENSG00000166734	2122.5	42.69
DUSP22	ENSG00000112679	2118.5	24.67
RABGAP1L	ENSG00000152061	2106.5	25.505
GPR52	ENSG00000203737	2106.5	0.72
RABGAP1L-IT1	ENSG00000223525	2106.5	0.09

NAIP	ENSG00000249437	2105.5	7.945
STRIP1	ENSG00000143093	2102	30.93
HNRNPC	ENSG00000092199	2098.5	980.72
POTEH	ENSG00000198062	2095	0
RNU6-816P	ENSG00000212216	2095	0
PDCD11	ENSG00000148843	2086.5	82.675
TRRAP	ENSG00000196367	2085.5	41.625
MIR3609	ENSG00000266019	2085.5	7.635
MIR4435-1HG	ENSG00000172965	2076	9.83
RPS14P4	ENSG00000226928	2076	0
ANKFN1	ENSG00000153930	2075	0.315
TXLNB	ENSG00000164440	2072	0.45
HOMER2	ENSG00000103942	2069	32.335
FAM103A1	ENSG00000169612	2069	13.86
SLC17A7	ENSG00000104888	2063	1.205
FAM153B	ENSG00000182230	2053.5	0.16
NUTM2A-AS1	ENSG00000223482	2053	39.23
DOCK3	ENSG00000088538	2052.5	5.6
OIP5	ENSG00000104147	2048.5	17.56
KIAA1239	ENSG00000174145	2045.5	0.065
SBNO2	ENSG00000064932	2044	21.97
OTOA	ENSG00000155719	2044	0.035
LINGO2	ENSG00000174482	2041.5	0.02
YTHDF1	ENSG00000149658	2034	39.38
FGF10	ENSG00000070193	2021.5	0
CNTNAP2	ENSG00000174469	2020	9.88
CIZ1	ENSG00000148337	2010	162.905
FBXL17	ENSG00000145743	2007.5	5.08
POLR2J3	ENSG00000168255	1994	225.27
UPK3BL	ENSG00000267368	1994	10.315
POLR2J2	ENSG00000228049	1994	5.175
RASA4	ENSG00000105808	1994	3.065
RASA4B	ENSG00000170667	1994	1.74
ARHGEF28	ENSG00000214944	1993	1.97
TSKS	ENSG00000126467	1984.5	0.215
ADK	ENSG00000156110	1979.5	29.425
MRPL35P3	ENSG00000226253	1979.5	2.965
RPSAP6	ENSG00000214629	1979.5	0.7
POLR3DP1	ENSG00000214626	1979.5	0.375
SMYD3	ENSG00000185420	1978	16.88
SMYD3-IT1	ENSG00000230184	1978	0.19
UNKL	ENSG00000059145	1977	33.055
STXBP5	ENSG00000164506	1975	10.655
KCNH7	ENSG00000184611	1972	0.05
FANCD2OS	ENSG00000163705	1971	2.135
USB1	ENSG00000103005	1960	54.86
KANK4	ENSG00000132854	1960	0.08

KCTD10	ENSG00000110906	1953	39.55
ENAH	ENSG00000154380	1949.5	200.005
FAM153A	ENSG00000170074	1949.5	0.12
GREB1L	ENSG00000141449	1940	24.73
KCTD9P2	ENSG00000181819	1939.5	0.14
IL1RAPL2	ENSG00000189108	1939.5	0
TUBBP6	ENSG00000235207	1936.5	0.05
PPL	ENSG00000118898	1930.5	1.005
IQCJ-SCHIP1	ENSG00000250588	1922	7.82
SCHIP1	ENSG00000151967	1922	6.71
IQCJ	ENSG00000214216	1922	0
SH3RF3	ENSG00000172985	1911	9.05
PLIN3	ENSG00000105355	1910.5	23.77
LOXL3	ENSG00000115318	1904	4.855
C15orf41	ENSG00000186073	1894	8.655
LARP4P	ENSG00000261315	1894	0.33
CSNK1A1P1	ENSG00000223518	1894	0.175
C22orf34	ENSG00000188511	1888	0
GLCCI1	ENSG00000106415	1887	18.155
RHPN2	ENSG00000131941	1887	15.58
RPA3-AS1	ENSG00000219545	1887	15.47
H3F3AP4	ENSG00000235655	1883.5	670.805
MAST2	ENSG00000086015	1882.5	21.155
FANCI	ENSG00000140525	1873	85.12
SVIL	ENSG00000197321	1873	3.735
GLP2R	ENSG00000065325	1871.5	0
ADCY2	ENSG00000078295	1868	0.035
GANC	ENSG00000214013	1864.5	5.355
CAPN3	ENSG00000092529	1864.5	0.64
SLC16A1	ENSG00000155380	1859	142.805
AKR7A2P1	ENSG00000229020	1859	1.045
LUM	ENSG00000139329	1858	0.995
IQCF2	ENSG00000184345	1851	0.115
VMP1	ENSG00000062716	1843.5	63.915
ZAN	ENSG00000146839	1838.5	0.015
SMURF2	ENSG00000108854	1835	21.58
EPHA4	ENSG00000116106	1831	24.205
RORA	ENSG00000069667	1830.5	5.89
AGPS	ENSG00000018510	1822	28.345
PTCHD4	ENSG00000244694	1818	0.06
HSD17B12	ENSG00000149084	1814	100.73
HIVEP2	ENSG00000010818	1814	5.55
MIR670HG	ENSG00000235661	1814	0
SNX8	ENSG00000106266	1812	23.655
PRKCA	ENSG00000154229	1811.5	11.43
RNU6-928P	ENSG00000252685	1811.5	0
SNX29	ENSG00000048471	1807.5	7.005

RPS23P6	ENSG00000241641	1807.5	0
AUTS2	ENSG00000158321	1804	88.17
CNR1	ENSG00000118432	1800.5	0.215
CD6	ENSG00000013725	1791.5	0
ANO2	ENSG00000047617	1791	0.24
NHLRC2	ENSG00000196865	1784	10.785
TNFSF11	ENSG00000120659	1780.5	0.03
SMG6	ENSG00000070366	1778	18.665
SYNPR	ENSG00000163630	1772.5	0.095
SYNPR-AS1	ENSG00000241359	1772.5	0
PPP3CA	ENSG00000138814	1768	37.48
NSUN2	ENSG00000037474	1763.5	82.525
HBE1	ENSG00000213931	1762.5	0.115
HBG2	ENSG00000196565	1762.5	0.085
OR52V1P	ENSG00000249633	1762.5	0.07
OR51B2	ENSG00000184881	1762.5	0
OR51B3P	ENSG00000223735	1762.5	0
OR51B4	ENSG00000183251	1762.5	0
OR51B8P	ENSG00000236359	1762.5	0
OR51I1	ENSG00000167359	1762.5	0
OR51K1P	ENSG00000248424	1762.5	0
OR52H2P	ENSG00000248553	1762.5	0
UBQLN3	ENSG00000175520	1762.5	0
UBQLNL	ENSG00000175518	1762.5	0
NFYB	ENSG00000120837	1760.5	27.02
GNG4	ENSG00000168243	1760.5	15.625
MAGEA4	ENSG00000147381	1757.5	0
TPTE2P6	ENSG00000205822	1755	0.375
MAG	ENSG00000105695	1751.5	0
KLF12	ENSG00000118922	1748.5	18.7
CAMK4	ENSG00000152495	1746.5	5.735
SLC8A1-AS1	ENSG00000227028	1744.5	1.945
GATAD2B	ENSG00000143614	1744	31.475
PIP5KL1	ENSG00000167103	1742.25	1.375
ZNF863P	ENSG00000236538	1740	0
LIX1L	ENSG00000152022	1737.5	21.93
WDFY3	ENSG00000163625	1735	25.84
POLR2J4	ENSG00000214783	1735	5.045
UFL1	ENSG00000014123	1729	10.48
TRAK1	ENSG00000182606	1727	23.07
IDH2	ENSG00000182054	1725	36.055
TMX4	ENSG00000125827	1715	16.46
LACE1	ENSG00000135537	1703	6.48
CTNNB1	ENSG00000168036	1700.5	213.9
CXorf66	ENSG00000203933	1700	0
MPRIP	ENSG00000133030	1688.5	108.62
PARK2	ENSG00000185345	1686.5	0.375

SOD2	ENSG00000112096	1684	96.41
HNRNPH1P1	ENSG00000220305	1684	2.82
DACH2	ENSG00000126733	1683	0.705
GEMIN8P3	ENSG00000223772	1683	0
METTL13	ENSG00000010165	1681.5	37.985
C1orf21	ENSG00000116667	1681.5	19.405
RN7SL832P	ENSG00000243819	1681.5	1.485
FCGR2A	ENSG00000143226	1678.5	1
HSPA7	ENSG00000225217	1678.5	0.095
HSPA6	ENSG00000173110	1678.5	0.045
FCGR2B	ENSG00000072694	1678.5	0.03
FCGR2C	ENSG00000244682	1678.5	0
FCGR3A	ENSG00000203747	1678.5	0
FCGR3B	ENSG00000162747	1678.5	0
RPS23P9	ENSG00000231586	1678.5	0
ARHGAP22	ENSG00000128805	1677	10.05
SMARCC1	ENSG00000173473	1666.5	149.755
TSNARE1	ENSG00000171045	1659	5.32
KLHL32	ENSG00000186231	1658	0.955
KLF17	ENSG00000171872	1654.5	0
CDHR2	ENSG00000074276	1653	1.305
IGF1R	ENSG00000140443	1652	30.77
PPARG	ENSG00000132170	1634.5	16.78
IGDCC3	ENSG00000174498	1633.5	4.14
IARS2	ENSG00000067704	1627.5	98.03
NGRN	ENSG00000182768	1624.5	73.835
TTLL13	ENSG00000213471	1624.5	0.355
AXIN1	ENSG00000103126	1624	21.44
GARNL3	ENSG00000136895	1620	12.54
PRKAR1B	ENSG00000188191	1616.5	16.975
DNAH10	ENSG00000197653	1615	1.36
COX8A	ENSG00000176340	1610	282.145
OTUB1	ENSG00000167770	1610	96.57
MACROD1	ENSG00000133315	1610	24.035
PDZK1IP1	ENSG00000162366	1609.5	0
DLG5	ENSG00000151208	1608.5	21.58
CADM3	ENSG00000162706	1608.5	0
DARC	ENSG00000213088	1608.5	0
LINC00856	ENSG00000230417	1586	0.155
LINC00595	ENSG00000227136	1586	0
CCSER1	ENSG00000184305	1585.5	3.195
IL18R1	ENSG00000115604	1574	1.51
IL1RL1	ENSG00000115602	1574	0
MYO1D	ENSG00000176658	1572	14.41
BFSP1	ENSG00000125864	1567.5	2.285
ADD2	ENSG00000075340	1566	23.285
CIPC	ENSG00000198894	1565.25	15.015

TMEM63C	ENSG00000165548	1565.25	2.715
MTF1	ENSG00000188786	1555.5	7.95
MRPS33P4	ENSG00000234821	1553.5	0
CPQ	ENSG00000104324	1550	1.87
MSR1	ENSG00000038945	1545	0.14
CELF2	ENSG00000048740	1542	6.38
CBX5	ENSG00000094916	1540	68.395
RN7SL390P	ENSG00000241785	1540	0.26
P2RY10	ENSG00000078589	1528.5	0
TRPM1	ENSG00000134160	1525	0.04
LPAL2	ENSG00000213071	1523	0.185
ANKRD17	ENSG00000132466	1522.5	71.76
TSPAN7	ENSG00000156298	1521	11.55
PRRG1	ENSG00000130962	1521	10.515
XK	ENSG00000047597	1521	1.7
SYTL5	ENSG00000147041	1521	1.635
CYBB	ENSG00000165168	1521	0.24
LANCL3	ENSG00000147036	1521	0.13
OTC	ENSG00000036473	1521	0.04
TM4SF2	ENSG00000250349	1521	0
CASKIN1	ENSG00000167971	1519	3.415
DGCR5	ENSG00000237517	1517	3.345
DGCR9	ENSG00000273032	1517	0.64
APOL3	ENSG00000128284	1514.5	0
SCAMP5	ENSG00000198794	1508	9.705
BRIP1	ENSG00000136492	1507	17.29
SPATA6L	ENSG00000106686	1506.5	0.82
ANXA13	ENSG00000104537	1502	0
ANKRD26	ENSG00000107890	1501	13.77
CENPP	ENSG00000188312	1496.5	4.57
ECM2	ENSG00000106823	1496.5	0.96
LRPPRC	ENSG00000138095	1496	151.78
SREBF2	ENSG00000198911	1495.5	44.335
RSPRY1	ENSG00000159579	1495.5	29.915
AARS2	ENSG00000124608	1494	20.525
TMEM151B	ENSG00000178233	1494	0.445
SPATS1	ENSG00000249481	1494	0
WDR27	ENSG00000184465	1492.5	34.03
LINC01010	ENSG00000236700	1491.5	0
PSMA2P3	ENSG00000227890	1484.5	0.095
PFAS	ENSG00000178921	1483.5	54.12
TDP1	ENSG00000042088	1475.5	25.58
KCNJ12	ENSG00000184185	1474	4.62
RYR2	ENSG00000198626	1473.5	0.33
ANKRD20A5P	ENSG00000186481	1467.5	4.785
CDK11A	ENSG00000008128	1465.5	50.46
SLC35E2	ENSG00000215790	1465.5	5.775

NDUFA9	ENSG00000139180	1461	62.945
DYRK4	ENSG00000010219	1461	26.07
GALNT8	ENSG00000130035	1461	0.24
KCNA6	ENSG00000151079	1461	0.16
FBLIM1	ENSG00000162458	1460	12.74
IPP	ENSG00000197429	1459	5.885
LINC00499	ENSG00000251372	1455.5	0
ADCK3	ENSG00000163050	1454.5	53.6
CDK11B	ENSG00000248333	1453	38.615
DRAXIN	ENSG00000162490	1452.5	0.035
LEPROT	ENSG00000213625	1448.5	17.735
LEPR	ENSG00000116678	1448.5	6.205
C2orf70	ENSG00000173557	1448.5	0
FGF1	ENSG00000113578	1445	0
RPS12P10	ENSG00000225147	1445	0
NIN	ENSG00000100503	1438	38.19
PROS1	ENSG00000184500	1437.5	7.845
FLRT1	ENSG00000126500	1433.5	1.075
NALCN-AS1	ENSG00000233009	1429.5	0
RGS10	ENSG00000148908	1426.5	21.63
NPAT	ENSG00000149308	1421.5	24.685
NR4A3	ENSG00000119508	1418.5	1.285
LINC00922	ENSG00000261742	1418.5	0
TIMM10B	ENSG00000132286	1417	21.57
DNHD1	ENSG00000179532	1417	6.595
FOXK2	ENSG00000141568	1416	80.195
WDR45B	ENSG00000141580	1416	68.51
LEKR1	ENSG00000197980	1411.5	0.545
WBSCR17	ENSG00000185274	1411	0
TUBGCP6	ENSG00000128159	1408.5	24.215
SELO	ENSG00000073169	1408.5	16.3
LINC00298	ENSG00000235665	1408.5	0
LINC00299	ENSG00000236790	1408.5	0
NOL3	ENSG00000140939	1408	6.775
CES5A	ENSG00000159398	1408	0
XAGE3	ENSG00000171402	1405	0
CASC2	ENSG00000177640	1401	3.21
DCT	ENSG00000080166	1401	0.095
PRKAR2A	ENSG00000114302	1398	106.355
RIOK2	ENSG00000058729	1393.5	36.14
AGAP1	ENSG00000157985	1393	17.075
ESCO1	ENSG00000141446	1391	22.985
SCARA3	ENSG00000168077	1383.5	2.95
TTC34	ENSG00000215912	1382	0.4
MPP6	ENSG00000105926	1377.5	23.7
MYOCD	ENSG00000141052	1372.5	0
TRIM5	ENSG00000132256	1371	2.765

OR52E6	ENSG00000205409	1371	0
OR52E8	ENSG00000183269	1371	0
OR52N1	ENSG00000181001	1371	0
PER2	ENSG00000132326	1369.5	3.175
KIR2DL1	ENSG00000125498	1368.5	0
KIR2DL3	ENSG00000243772	1368.5	0
KIR2DL4	ENSG00000189013	1368.5	0
KIR2DP1	ENSG00000242473	1368.5	0
KIR2DS4	ENSG00000221957	1368.5	0
KIR3DL1	ENSG00000167633	1368.5	0
KIR3DL2	ENSG00000240403	1368.5	0
KIR3DL3	ENSG00000242019	1368.5	0
KIR3DP1	ENSG00000264257	1368.5	0
HAUS4	ENSG00000092036	1365	18.8
AJUBA	ENSG00000129474	1365	5.42
CACNA1C	ENSG00000151067	1361	1.405
CACNA1C-IT3	ENSG00000256721	1361	0.105
CPNE6	ENSG00000100884	1358.5	0.13
SPATS2	ENSG00000123352	1358	38.685
GLG1	ENSG00000090863	1357.5	41.42
ZNF737	ENSG00000237440	1357	0.475
ZNF626	ENSG00000188171	1357	0
SCN3B	ENSG00000166257	1353.5	0.595
CHEK2	ENSG00000183765	1353	37.01
SUPT3H	ENSG00000196284	1348.5	8.34
PHLDB1	ENSG00000019144	1345.5	11.8
KRT6B	ENSG00000185479	1344.5	0.025
UBQLN4	ENSG00000160803	1340.5	68.735
PRDM16	ENSG00000142611	1339	7.295
MYO1E	ENSG00000157483	1337.5	18.935
ASB8	ENSG00000177981	1330	24.595
CSMD1	ENSG00000183117	1330	0.01
PPP1CB	ENSG00000213639	1329.5	63.575
SPDYA	ENSG00000163806	1329.5	2.95
HCK	ENSG00000101336	1327	0
CMAHP	ENSG00000168405	1326.5	0
SLC35E4	ENSG00000100036	1324	3.675
CSTL1	ENSG00000125823	1324	0
SORBS1	ENSG00000095637	1322.5	2.82
TBC1D14	ENSG00000132405	1321	36.35
AGPAT4	ENSG00000026652	1320	15.235
FEM1AP2	ENSG00000265296	1319	0
RAB3GAP2	ENSG00000118873	1314.5	30
CAPN2	ENSG00000162909	1314	61.56
FAM73A	ENSG00000180488	1312	12.3
NSRP1P1	ENSG00000235613	1312	1.29
HSD3BP2	ENSG00000203858	1311	0.1

TBCK	ENSG00000145348	1310	11.335
TRIM8	ENSG00000171206	1308.5	22.985
REXO1L1P	ENSG00000205176	1308	0.165
MANEAL	ENSG00000185090	1307.5	40.6
DIO2-AS1	ENSG00000258766	1304.5	0.195
DIO2	ENSG00000211448	1304.5	0.05
LPHN1	ENSG00000072071	1299.5	43.815
RPRD2	ENSG00000163125	1296.5	39.895
ADAMTS9-AS2	ENSG00000241684	1295	0.345
MRC2	ENSG00000011028	1290.5	12.775
PPP2R2B	ENSG00000156475	1288.5	0
PPP2R2B-IT1	ENSG00000249553	1288.5	0
CSPG4	ENSG00000173546	1285	1.15
GRM1	ENSG00000152822	1283.5	0.085
FUNDC2P3	ENSG00000187103	1283.5	0
BRMS1L	ENSG00000100916	1281	21.76
RIMS4	ENSG00000101098	1281	10.205
AGFG2	ENSG00000106351	1280	10.105
DCC	ENSG00000187323	1278.5	0
ZBTB8A	ENSG00000160062	1276.75	9.17
ZBTB8B	ENSG00000273274	1276.75	1.33
ARHGAP31	ENSG00000031081	1272	2.65
P2RY14	ENSG00000174944	1271.5	1.115
ANKFY1	ENSG00000185722	1266.5	40.67
CTDP1	ENSG00000060069	1265	10.865
ZUFSP	ENSG00000153975	1263	16.795
HS3ST5	ENSG00000249853	1262	0.365
BAZ1B	ENSG00000009954	1260.5	87.88
MMP16	ENSG00000156103	1259.5	1.56
OBSCN	ENSG00000154358	1258.5	4.055
RGL1	ENSG00000143344	1256	2.615
NF2	ENSG00000186575	1255.5	52.785
RPEP4	ENSG00000233010	1255.5	1.42
RARRES3	ENSG00000133321	1255.5	0
FLT4	ENSG00000037280	1255	29.175
MEF2BNB	ENSG00000254901	1246.5	23.595
MEF2BNB-MEF2B	ENSG00000064489	1246.5	3.805
MEF2B	ENSG00000213999	1246.5	1.435
SGMS1	ENSG00000198964	1246	8.225
SMC2	ENSG00000136824	1242	43.57
BICC1	ENSG00000122870	1236	3.885
FAM133CP	ENSG00000183055	1236	0.56
LINC00937	ENSG00000226091	1234	2.54
ENPP7P5	ENSG00000256589	1234	0.19
HDAC4	ENSG00000068024	1233.5	7.82
MIR4440	ENSG00000266109	1233.5	0
PAX2	ENSG00000075891	1232	3.15

PHF5A	ENSG00000100410	1229	68.115
TNS3	ENSG00000136205	1225.5	3.265
CPOX	ENSG00000080819	1222	57.925
CLDND1	ENSG00000080822	1222	56.185
DNAH9	ENSG00000007174	1220	0.04
SECISBP2L	ENSG00000138593	1213	16.72
SPTBN1	ENSG00000115306	1210	56.85
RPL23AP32	ENSG00000237887	1210	0.44
BAGE2	ENSG00000187172	1207.5	0.1
TPTE	ENSG00000166157	1207.5	0
HEPACAM2	ENSG00000188175	1203	0.07
ELN	ENSG00000049540	1202	0.025
CDCA4	ENSG00000170779	1198	33.125
SMIM2-AS1	ENSG00000227258	1196	1.72
TRABD2B	ENSG00000269113	1195	1.045
SNAPC4	ENSG00000165684	1194.5	16.8
OBFC1	ENSG00000107960	1194	6.49
NBPF1	ENSG00000219481	1191	55.37
AP3B2	ENSG00000103723	1191	1.695
SPG11	ENSG00000104133	1190.5	25.285
SFRP1	ENSG00000104332	1189.5	1.24
MED13	ENSG00000108510	1189	36.045
ST8SIA1	ENSG00000111728	1189	0.95
SPERT	ENSG00000174015	1189	0
PPP6R2	ENSG00000100239	1188	32.87
DRG1	ENSG00000185721	1185	146.62
SFI1	ENSG00000198089	1185	34.42
ZNF395	ENSG00000186918	1183.75	14.09
FBXO16	ENSG00000214050	1183.75	7.06
FOXO3	ENSG00000118689	1181	31.185
DGKB	ENSG00000136267	1179	0.105
TRHDE	ENSG00000072657	1177	1.03
CHCHD3P2	ENSG00000257723	1177	0.105
TPH2	ENSG00000139287	1177	0.09
USP54	ENSG00000166348	1169	25.32
ARL13B	ENSG00000169379	1166.5	21.365
CNTN6	ENSG00000134115	1165.5	0
CCDC91	ENSG00000123106	1164.5	19.985
NFIB	ENSG00000147862	1164.5	11.245
MUC12	ENSG00000205277	1163.5	0.02
SP4	ENSG00000105866	1161.5	6.75
ARHGAP29	ENSG00000137962	1160.5	8.67
ZCCHC17	ENSG00000121766	1159	55.71
IFT122	ENSG00000163913	1157.5	12.17
RANBP3	ENSG00000031823	1155.5	93.71
DDX10	ENSG00000178105	1155.5	42.825
BCL2L13	ENSG00000099968	1155	48.075

COX10-AS1	ENSG00000236088	1154.5	6.915
DNM3	ENSG00000197959	1152	3.735
DNM3-IT1	ENSG00000233540	1152	0.345
WHSC1	ENSG00000109685	1149.5	91.675
CDH26	ENSG00000124215	1147	1.33
SSH1	ENSG00000084112	1145.5	12.68
CROCCP2	ENSG00000215908	1144	21.675
CELF5	ENSG00000161082	1144	1.225
ATP10D	ENSG00000145246	1143.5	5.37
SYNCRIP	ENSG00000135316	1141.5	158.77
SNX14	ENSG00000135317	1141.5	31.55
SMAD3	ENSG00000166949	1139.5	17.245
TYW1B	ENSG00000254184	1139.5	3.345
RNFT2	ENSG00000135119	1138.5	10.82
CUX2	ENSG00000111249	1137.5	2.785
ITPR1	ENSG00000150995	1137	5.05
RAB6A	ENSG00000175582	1136.5	47.955
EVA1A	ENSG00000115363	1134.5	2.135
THSD4	ENSG00000187720	1134	0.76
PTPRM	ENSG00000173482	1133	4.615
WWP2	ENSG00000198373	1132.5	18.025
ARNT2	ENSG00000172379	1130.5	6.395
ARL15	ENSG00000185305	1126.5	18.205
SSXP3	ENSG00000225198	1126.5	0
SBNO1	ENSG00000139697	1125.5	40.055
FAM198A	ENSG00000144649	1123	1.6
KRBOX1	ENSG00000273291	1123	0.425
ACKR2	ENSG00000144648	1123	0.29
HNRNPA1P22	ENSG00000236459	1123	0.135
ZNF662	ENSG00000182983	1123	0.05
CYP4F11	ENSG00000171903	1122.5	0.02
NHS	ENSG00000188158	1122	1.92
ZNF777	ENSG00000196453	1121	24.765
SIMC1	ENSG00000170085	1119.5	55.465
WIPI1	ENSG00000070540	1117	6.4
LINC00693	ENSG00000228214	1117	2.265
EBF3	ENSG00000108001	1114	9.505
MTBP	ENSG00000172167	1113.5	11.265
SNTB1	ENSG00000172164	1113.5	2.945
VPS39	ENSG00000166887	1111.5	21.615
RNF168	ENSG00000163961	1111	15.935
EMG1	ENSG00000126749	1110	60.975
C1S	ENSG00000182326	1110	0.8
CASZ1	ENSG00000130940	1108	6.25
ABI2	ENSG00000138443	1105	69.245
GRIK5	ENSG00000105737	1104.5	0.73
C10orf90	ENSG00000154493	1104.5	0

C20orf166	ENSG00000174407	1099	0
PSAP	ENSG00000197746	1098.5	127.105
SLC16A2	ENSG00000147100	1098	10.625
LZTFL1	ENSG00000163818	1097	26.33
SYT3	ENSG00000213023	1095	3.115
SHANK1	ENSG00000161681	1095	1.855
IFNAR1	ENSG00000142166	1093.5	31.08
USF1P1	ENSG00000226501	1093.5	0.255
DCP1B	ENSG00000151065	1092	16.72
OR14K1	ENSG00000153230	1091	0
OR3D1P	ENSG00000230411	1091	0
MIER3	ENSG00000155545	1088.5	17.05
ZNF346	ENSG00000113761	1088	52.215
NRP2	ENSG00000118257	1088	10.345
ZNF346-IT1	ENSG00000251666	1088	4.335
NEDD4	ENSG00000069869	1088	3.545
ANKRD30B	ENSG00000180777	1086.5	0.035
ARHGEF18	ENSG00000104880	1084.5	25.685
TMEM161B-AS1	ENSG00000247828	1084	15.36
RPS3AP22	ENSG00000240083	1084	0
SNORA70	ENSG00000252969	1084	0
VANGL1	ENSG00000173218	1082	19.455
SVOPL	ENSG00000157703	1081	0.34
BTBD3	ENSG00000132640	1076.5	32.57
PRPF38B	ENSG00000134186	1076	73.855
MRPS35P3	ENSG00000270192	1072.5	0
C7orf50	ENSG00000146540	1072	49.48
PREX1	ENSG00000124126	1072	1.89
PLA2G4C	ENSG00000105499	1065.5	2.185
DOCK4	ENSG00000128512	1063.5	8.945
CRTAC1	ENSG00000095713	1063	0.445
C1orf132	ENSG00000203709	1062.5	0.745
ABCC6P1	ENSG00000256340	1062	1.77
ESRRG	ENSG00000196482	1062	1.475
INHBA-AS1	ENSG00000224116	1061	0
SPAG6	ENSG00000077327	1059.5	0.105
HOXD3	ENSG00000128652	1056.5	7.58
HOXD4	ENSG00000170166	1056.5	4.82
SLC35F4	ENSG00000151812	1056	0.19
OGDHL	ENSG00000197444	1054	5.465
KPNB1	ENSG00000108424	1051.5	351.085
NAV3	ENSG00000067798	1049.5	0.56
SLC4A4	ENSG00000080493	1049	1.165
C19orf68	ENSG00000185453	1048.5	6.535
ZNF114	ENSG00000178150	1048.5	4.89
TAB1	ENSG00000100324	1048	25.645
GALNT14	ENSG00000158089	1048	0.89

VPS53	ENSG00000141252	1047.5	52.995
RPS4XP17	ENSG00000244097	1047.5	1.14
SLC6A1	ENSG00000157103	1046.5	0.435
TXNDC12	ENSG00000117862	1045.5	51.035
KTI12	ENSG00000198841	1045.5	10.52
NFATC1	ENSG00000131196	1044	8.455
RAB6C	ENSG00000222014	1044	0.76
SEC61B	ENSG00000106803	1037	91.355
ST3GAL3	ENSG00000126091	1034	9.39
SPATA3	ENSG00000173699	1031.5	0
KCNK1	ENSG00000135750	1030	8.885
PRTG	ENSG00000166450	1029	7.925
UCK2	ENSG00000143179	1028	99.52
CDC73	ENSG00000134371	1027.5	23.59
ZNF252P	ENSG00000196922	1023.5	11.55
THOC3	ENSG00000051596	1022	71.94
APP	ENSG00000142192	1021.5	177.485
PCDH9-AS1	ENSG00000234527	1021.5	0
SSBP3	ENSG00000157216	1020.5	80.375
C1orf105	ENSG00000180999	1020.5	0.145
INADL	ENSG00000132849	1019.25	12.51
LAMTOR5P1	ENSG00000236646	1019.25	0.54
RN7SL180P	ENSG00000242860	1019.25	0
USP49	ENSG00000164663	1019	6.22
FIP1L1	ENSG00000145216	1018	75.35
PDGFRA	ENSG00000134853	1018	0.635
MORF4L2P1	ENSG00000250147	1018	0.15
GSX2	ENSG00000180613	1018	0
LNX1-AS1	ENSG00000250930	1018	0
LNX1-AS2	ENSG00000248494	1018	0
AOC2	ENSG00000131480	1016	3.255
SLK	ENSG00000065613	1014	38.565
BET1L	ENSG00000177951	1014	22.79
ODF3	ENSG00000177947	1014	0.11
FAM178A	ENSG00000119906	1013	20.665
FCRL4	ENSG00000163518	1013	0
CEP85L	ENSG00000111860	1011.5	8.265
ADD3	ENSG00000148700	1011	42.03
RBM8A	ENSG00000131795	1010	211.51
CCNI2	ENSG00000205089	1008	1.445
DIP2B	ENSG00000066084	1006.5	17.34
KANSL2	ENSG00000139620	1005	29.27
NOX1	ENSG00000007952	1005	0.54
HNRNPA1P26	ENSG00000239345	1005	0.06
ARIH1	ENSG00000166233	1003.5	83.45
YAP1	ENSG00000137693	1003.5	24.84
HEG1	ENSG00000173706	1002	9.255

LINC01036	ENSG00000236030	998.5	0.445
BPIFB6	ENSG00000167104	998.5	0
AMFR	ENSG00000159461	996.5	55.375
ST6GALNAC5	ENSG00000117069	995.5	1.495
RAB11FIP5	ENSG00000135631	993.5	21.535
SFXN5	ENSG00000144040	993.5	21.43
SESN1	ENSG00000080546	992.5	13.165
MYO1F	ENSG00000142347	992.5	4.88
PPCS	ENSG00000127125	991.5	24.49
CCDC30	ENSG00000186409	991.5	2.85
TEN1	ENSG00000257949	990.5	7.56
CDK3	ENSG00000250506	990.5	2.26
TEN1-CDK3	ENSG00000261408	990.5	1.285
NLGN2	ENSG00000169992	990	32.56
FSTL4	ENSG00000053108	990	0.38
TNFRSF19	ENSG00000127863	989	3.005
VDAC1	ENSG00000213585	988.5	275.265
EYA2	ENSG00000064655	985	4.58
GAPDHP54	ENSG00000226769	985	0
SSR2	ENSG00000163479	984.5	200.155
SH3D19	ENSG00000109686	983.5	35.55
WASH4P	ENSG00000234769	983	18.155
WASIR2	ENSG00000231439	983	1.68
IL9RP3	ENSG00000226942	983	0.95
BORA	ENSG00000136122	982.5	27.305
PHF7	ENSG00000010318	982	11.33
RNLS	ENSG00000184719	979	0.08
NPIPB4	ENSG00000185864	978	85.035
IBTK	ENSG00000005700	976.5	20.155
SCRN1	ENSG00000136193	976	42.85
SBK3	ENSG00000231274	975.5	0.14
NVL	ENSG00000143748	975	79.67
JAK1	ENSG00000162434	974	40.38
ALPL	ENSG00000162551	974	2.46
PDE6D	ENSG00000156973	972	27.46
ANGPTL4	ENSG00000167772	970.5	1.765
FPGT	ENSG00000254685	969	9.69
FPGT-TNNI3K	ENSG00000259030	969	0.03
LRRC53	ENSG00000162621	969	0
TNNI3K	ENSG00000116783	969	0
PEMT	ENSG00000133027	968.5	24.27
LRP5	ENSG00000162337	966	28.08
CMC4	ENSG00000182712	964.5	23.495
MTCP1	ENSG00000214827	964.5	5.225
POLR3G	ENSG00000113356	964	27.59
LINC00969	ENSG00000242086	964	24.055
MUC20	ENSG00000176945	964	0.845

SDHAP2	ENSG00000215837	964	0
SEC22C	ENSG00000093183	963	37.22
PTPRG	ENSG00000144724	962	16.82
BDH2	ENSG00000164039	960.5	18.96
SLC9B2	ENSG00000164038	960.5	13.615
ISLR2	ENSG00000167178	960.5	0.125
ZNF787	ENSG00000142409	959	29.27
SLX4IP	ENSG00000149346	959	3.87
SOX2	ENSG00000181449	958.5	5.305
GNG2	ENSG00000186469	958.5	1.495
SOX2-OT	ENSG00000242808	958.5	0.24
SETD2	ENSG00000181555	955.5	42.59
ZDHHC6	ENSG00000023041	955.5	34.96
PLAC1	ENSG00000170965	954	7.24
GPC6	ENSG00000183098	951	7.51
KIF24	ENSG00000186638	951	7.315
TTC39C	ENSG00000168234	950	13.975
CHST11	ENSG00000171310	949.5	8.635
ACBD5	ENSG00000107897	949.5	7.05
ADAMTS2	ENSG00000087116	949.5	2.115
TEAD1	ENSG00000187079	949	19.98
RCAN1	ENSG00000159200	946	15.455
PRICKLE1	ENSG00000139174	946	8.64
DEFB118	ENSG00000131068	946	0
NDNL2	ENSG00000185115	944	17.17
GUSBP11	ENSG00000228315	944	11.615
ASLP1	ENSG00000244723	944	3.865
FAM189A1	ENSG00000104059	944	2.29
C22orf43	ENSG00000189269	944	0.525
PLEKHA7	ENSG00000166689	943.5	4.165
SSPN	ENSG00000123096	943	2.005
ECSIT	ENSG00000130159	942	34.45
ZNF653	ENSG00000161914	942	9.18
ELAVL3	ENSG00000196361	942	0.015
EPHX4	ENSG00000172031	940.5	2.83
SPTBN4	ENSG00000160460	940.5	2.035
VASH2	ENSG00000143494	938.5	9.395
ZRANB2-AS2	ENSG00000229956	938.5	0.31
GRIA3	ENSG00000125675	938.5	0
ZNF521	ENSG00000198795	936	7.165
PAQR5	ENSG00000137819	935.5	4.815
ELOVL2	ENSG00000197977	935	8.68
DRD1	ENSG00000184845	934	0
TMEM14A	ENSG00000096092	931.5	11.225
GLRA2	ENSG00000101958	930	0.36
ARID3A	ENSG00000116017	929.5	31.6
EFCAB8	ENSG00000215529	929	0

ZNF761	ENSG00000160336	927	5.7
TPM3P9	ENSG00000241015	927	4.325
CHUK	ENSG00000213341	925.5	29.995
ERLIN1	ENSG00000107566	925.5	21.655
FRS2	ENSG00000166225	925.5	14.38
TMEM256	ENSG00000205544	924	60.485
TMEM256-PLSCR3	ENSG00000187838	924	11.16
C17orf61-PLSCR3	ENSG00000262481	924	1.195
CCDC73	ENSG00000186714	923.5	0.37
PALB2	ENSG00000083093	922	12.98
RFT1	ENSG00000163933	920	33.88
SFMBT1	ENSG00000163935	920	11.535
SERBP1P3	ENSG00000242142	920	0.315
VILL	ENSG00000136059	919.5	2.245
MMP2	ENSG00000087245	919	1.645
ZNF584	ENSG00000171574	918.5	10.35
LINC00534	ENSG00000253394	918	0
SERGEF	ENSG00000129158	917.5	28.095
TPH1	ENSG00000129167	917.5	0.555
SYNE2	ENSG00000054654	916.5	12.485
ESR2	ENSG00000140009	916.5	1.185
S100A12	ENSG00000163221	916	0
ERI2	ENSG00000196678	911.5	18.58
MPPED2	ENSG00000066382	911.5	5.73
DCUN1D3	ENSG00000188215	911.5	4.34
NUDT10	ENSG00000122824	911	11.595
TARSL2	ENSG00000185418	910	21.26
CPNE9	ENSG00000144550	909.5	0.105
KDM6A	ENSG00000147050	909	31.57
GTF2F2	ENSG00000188342	908	26.24
PRKD1	ENSG00000184304	908	6.325
C9orf85	ENSG00000155621	907.5	12.985
STAT1	ENSG00000115415	906.5	31.365
NAA25	ENSG00000111300	906	57.325
PARP6	ENSG00000137817	905	44.96
HEXA	ENSG00000213614	905	42.63
ST18	ENSG00000147488	905	0.105
CELF6	ENSG00000273025	905	0.01
PAK7	ENSG00000101349	904.5	0.025
LRRIQ3	ENSG00000162620	904	2.12
OSBPL2	ENSG00000130703	903	16.665
BRD7P6	ENSG00000235289	902.5	0
SYBU	ENSG00000147642	900.5	8.12
ETV5	ENSG00000244405	900.5	0.45
DGKG	ENSG00000058866	900.5	0.27
LEF1-AS1	ENSG00000232021	899.5	0.59
RHOXF2B	ENSG00000203989	899.5	0.33

RND3	ENSG00000115963	897	21.91
NBPF8	ENSG00000162825	897	0
SLC6A3	ENSG00000142319	896	0.015
USP53	ENSG00000145390	895	22.595
TAF3	ENSG00000165632	894	13.125
MTHFSD	ENSG00000103248	893.5	15.745
MORN2	ENSG00000188010	892.5	16.68
ARHGEF33	ENSG00000214694	892.5	0.395
PLAGL1	ENSG00000118495	891.5	42.325
HNF4A	ENSG00000101076	885.5	0
ZBTB7C	ENSG00000184828	885	1.245
TBCA	ENSG00000171530	884	305.48
LRRC2	ENSG00000163827	884	0.065
TASP1	ENSG00000089123	883.5	9.585
TMEM183A	ENSG00000163444	881.5	76.225
SHANK2	ENSG00000162105	881.5	3.625
SHPRH	ENSG00000146414	879.5	20.67
KIAA2026	ENSG00000183354	879	10.455
CNTNAP3	ENSG00000106714	878.5	5.1
STRA6	ENSG00000137868	874	0.495
CCDC33	ENSG00000140481	874	0
VPS4A	ENSG00000132612	873.5	73.995
CIRH1A	ENSG00000141076	873.5	65.315
SNTB2	ENSG00000168807	873.5	14.815
EREG	ENSG00000124882	871	0.015
TCTN3	ENSG00000119977	870.5	27.085
CCNY	ENSG00000108100	870.5	25.97
ABLIM1	ENSG00000099204	870	33.26
MAPKAPK3	ENSG00000114738	868.5	50.75
NPIPA7	ENSG00000183889	868.5	14.405
PKD1P1	ENSG00000244257	868.5	12.265
MYOF	ENSG00000138119	868.5	0.89
MYH15	ENSG00000144821	867.75	0.37
FXYD6	ENSG00000137726	867.5	11.295
DSCAML1	ENSG00000177103	867.5	0.01
FXYD2	ENSG00000137731	867.5	0
FXYD6-FXYD2	ENSG00000255245	867.5	0
RNU6-167P	ENSG00000207019	867.5	0
PITRM1	ENSG00000107959	867	65.325
SEMA6D	ENSG00000137872	867	4.53
PITRM1-AS1	ENSG00000237399	867	1.32
SLC16A12	ENSG00000152779	866.5	0
RBFOX3	ENSG00000167281	866	1.72
ATF2	ENSG00000115966	865	36.16
POU2F2	ENSG00000028277	865	1.19
TCF3	ENSG00000071564	864.5	89.195
ANKRD32	ENSG00000133302	864.5	17.675

ZNF730	ENSG00000183850	864	0
GRAMD4	ENSG00000075240	863.5	13.75
TMEM237	ENSG00000155755	862.5	39.48
ENO1P4	ENSG00000241790	862.5	0.635
KCNG4	ENSG00000168418	862	0
RAF1	ENSG00000132155	861.5	79.915
CELF4	ENSG00000101489	861	2.5
PKD1P5	ENSG00000254681	860	20.875
NPIPA8	ENSG00000214940	860	0
RASGRF2	ENSG00000113319	859	2.385
KIAA1432	ENSG00000107036	858.5	9.875
FAM194B	ENSG00000165837	858.5	0.405
GPR116	ENSG00000069122	857.5	0
EPHX1	ENSG00000143819	857	45.475
CCDC148	ENSG00000153237	857	0.54
ABLIM2	ENSG00000163995	856.25	0.9
SMIM20	ENSG00000250317	855.5	9.69
ZNF766	ENSG00000196214	855.5	5.74
ACOX2	ENSG00000168306	855	0.295
CARD16	ENSG00000204397	854	0
CARD17	ENSG00000255221	854	0
CASP1	ENSG00000137752	854	0
CASP1P2	ENSG00000254750	854	0
PTPN1	ENSG00000196396	853.5	49.785
PSMD11	ENSG00000108671	852.5	80.79
ADAMTS12	ENSG00000151388	852.5	0.065
RNU6-923P	ENSG00000201623	852.5	0
SRRM4	ENSG00000139767	852.5	0
POLA1	ENSG00000101868	849.5	30.455
GGT7	ENSG00000131067	848	21.575
USP33	ENSG00000077254	847	51.49
BMPR2	ENSG00000204217	847	10.95
VBP1	ENSG00000155959	846	82.53
DPYSL2	ENSG00000092964	846	16.955
CAMK1D	ENSG00000183049	844.5	3.5
DGKZP1	ENSG00000179611	844.5	0.805
LINC00284	ENSG00000233725	844.5	0.145
LINC01057	ENSG00000224081	842.5	1.635
KATNBL1P2	ENSG00000232857	842.5	0
BMPR1A	ENSG00000107779	841.5	14.07
ZKSCAN1	ENSG00000106261	841	25
CBWD3	ENSG00000196873	841	14.29
TMEM131	ENSG00000075568	839.5	20.505
AP1B1	ENSG00000100280	839	82.735
PABPC4	ENSG00000090621	836	199.425
IDO2	ENSG00000188676	836	0.075
TMEM179	ENSG00000258986	835	0.795

TSPAN12	ENSG00000106025	833.5	12.455
NLGN1	ENSG00000169760	832.5	5.43
TLDC1	ENSG00000140950	829.5	9.12
BTBD10	ENSG00000148925	829	14.875
COL16A1	ENSG00000084636	826.5	0.22
CTNNA2	ENSG00000066032	826	0
ATP9B	ENSG00000166377	825	7.195
IKZF4	ENSG00000123411	825	4.105
PIK3R3	ENSG00000117461	824.5	20.485
ATXN8OS	ENSG00000230223	824.5	0.055
CDK12	ENSG00000167258	824	64.85
TAF8	ENSG00000137413	823.5	23.29
SNX16	ENSG00000104497	823.5	2.015
CAND2	ENSG00000144712	822.5	14.24
CERS3	ENSG00000154227	822	0.065
SPAG9	ENSG00000008294	821.5	78.835
MORC2	ENSG00000133422	820.5	23.62
GUCY1B2	ENSG00000123201	820.5	1.57
MORC2-AS1	ENSG00000235989	820.5	0.685
RAB11A	ENSG00000103769	820	178.375
DENND4A	ENSG00000174485	820	17.12
EVC	ENSG00000072840	819	7.3
CRMP1	ENSG00000072832	819	4.31
PDE11A	ENSG00000128655	818.5	0.685
CYCTP	ENSG00000249909	818.5	0
SNX18P16	ENSG00000233218	816	2.27
SNX18P15	ENSG00000225015	816	1.4
ANKRD20A12P	ENSG00000242676	816	0
IPO9	ENSG00000198700	815.5	75.57
EPG5	ENSG00000152223	815.5	5.275
MYB	ENSG00000118513	815.5	2.87
ZNF71	ENSG00000197951	814.5	4.125
FGF2	ENSG00000138685	814.5	3.695
PPA2	ENSG00000138777	813	76.855
POU6F2	ENSG00000106536	813	0.815
LRRC4C	ENSG00000148948	813	0.015
BACH1	ENSG00000156273	812.5	14.555
BACH1-IT1	ENSG00000248476	812.5	1.645
BACH1-IT2	ENSG00000228817	812.5	1.52
BACH1-IT3	ENSG00000234293	812.5	0.845
GRIK1	ENSG00000171189	812.5	0.16
GRIK1-AS2	ENSG00000183653	812.5	0
LINC00189	ENSG00000215533	812.5	0
OR6C70	ENSG00000184954	812	0.065
RAB24	ENSG00000169228	811.5	21.96
MXD3	ENSG00000213347	811.5	13.025
PPM1F	ENSG00000100034	811	46.435

UBE2R2	ENSG00000107341	811	37.06
TICRR	ENSG00000140534	809.5	32.52
DNAJC16	ENSG00000116138	809	12.465
ADCY5	ENSG00000173175	808.5	1.465
GLI4	ENSG00000250571	808	17.28
ZFP41	ENSG00000181638	808	11.175
NEDD9	ENSG00000111859	806.5	0.69
KIAA1279	ENSG00000198954	805.5	30.745
MED28P1	ENSG00000214875	805.5	0.81
BEND5	ENSG00000162373	805.5	0.25
AGBL4	ENSG00000186094	805.5	0.14
AGBL4-IT1	ENSG00000225623	805.5	0
PEAK1	ENSG00000173517	805	11.33
SH3PXD2A	ENSG00000107957	804.5	28.63
SEMA4D	ENSG00000187764	804.5	12.06
ADAMTS4	ENSG00000158859	804.5	0.075
ISM1	ENSG00000101230	804	0.45
SEPT8	ENSG00000164402	803.5	52.545
FBN3	ENSG00000142449	803.5	0.08
ARMC2	ENSG00000118690	803	5.495
DSCR4	ENSG00000184029	802.5	0.08
NFX1	ENSG00000086102	802	34.895
CDC27	ENSG00000004897	801	123.78
HELQ	ENSG00000163312	797	10.755
DOK6	ENSG00000206052	797	0.145
LINGO4	ENSG00000213171	796.5	0
RORC	ENSG00000143365	796.5	0
XPO6	ENSG00000169180	796	119.54
MCTP1	ENSG00000175471	796	2.565
RPL7P18	ENSG00000243806	796	0
ELP3	ENSG00000134014	795.5	29.165
PLXNA2	ENSG00000076356	795.5	2.505
PTAFR	ENSG00000169403	795	0.405
POC1A	ENSG00000164087	794.5	16.82
HHIPL1	ENSG00000182218	794	0.015
PRICKLE2-AS1	ENSG00000241572	794	0
HIBADH	ENSG00000106049	793	28.055
ADCYAP1R1	ENSG00000078549	793	2.125
FNDC3A	ENSG00000102531	792	30.315
EEA1	ENSG00000102189	792	19.18
SP140L	ENSG00000185404	792	1.11
SP140	ENSG00000079263	792	0.105
RNY3P2	ENSG00000199788	792	0
SETD1A	ENSG00000099381	791.5	48.67
YME1L1	ENSG00000136758	791	80.67
NKAPP1	ENSG00000233382	791	3.54
EEF1A1P30	ENSG00000237757	791	0.56

RHOXF2	ENSG00000131721	791	0
KIAA1644	ENSG00000138944	790.5	2.065
CYP3A43	ENSG00000021461	790	0.215
JARID2	ENSG00000008083	789	18.03
ABCA13	ENSG00000179869	789	0.06
RILPL1	ENSG00000188026	788.75	7.355
PLEKHO2	ENSG00000241839	787.5	2.735
ANKDD1A	ENSG00000166839	787.5	2.375
KLHL3	ENSG00000146021	787.5	1.115
DDX58	ENSG00000107201	787	1.485
C1orf94	ENSG00000142698	787	0.025
WDR60	ENSG00000126870	786.5	27.425
MTND5P10	ENSG00000271687	786.5	4.425
MRS2	ENSG00000124532	786	34.045
DKK2	ENSG00000155011	786	0.155
RAC1P5	ENSG00000213522	786	0.125
SORCS2	ENSG00000184985	784.5	0.295
RAB11FIP3	ENSG00000090565	784	25
LINC00478	ENSG00000215386	782.5	1.53
SLC5A8	ENSG00000256870	782	0
PHC2	ENSG00000134686	781.5	50.255
CHCHD6	ENSG00000159685	781.5	19.515
MIR3605	ENSG00000266239	781.5	2.315
BRDT	ENSG00000137948	781.5	0
ITGA11	ENSG00000137809	781	0.44
PI4KA	ENSG00000241973	780	65.445
RARB	ENSG00000077092	780	7.945
CFL1P7	ENSG00000213877	780	0.28
SPEN	ENSG00000065526	779.5	173.66
PIK3C2B	ENSG00000133056	779	11.24
PRKCG	ENSG00000126583	779	0.62
SIN3B	ENSG00000127511	778.5	39.15
SLC9B1	ENSG00000164037	776.5	0.63
LINC00504	ENSG00000248360	776.5	0.39
VIPR1	ENSG00000114812	776	6.39
VIPR1-AS1	ENSG00000232354	776	1.54
NCOA1	ENSG00000084676	775.5	19.77
SPOCK1	ENSG00000152377	775	0.435
PPP5D1	ENSG00000230510	774.5	1.275
PNMAL2	ENSG00000204851	774.5	0
TXNDC5	ENSG00000239264	774	121.5
EEF1E1	ENSG00000124802	774	63.42
AREL1	ENSG00000119682	774	35.395
BLOC1S5	ENSG00000188428	774	8.34
EEF1E1-BLOC1S5	ENSG00000265818	774	7.3
BLOC1S5-TXNDC5	ENSG00000259040	774	0
TANGO6	ENSG00000103047	773	12.26

CACNA1G-AS1	ENSG00000250107	773	0
GRIA2	ENSG00000120251	772.5	0.21
EHMT1	ENSG00000181090	772	48.4
GAS8	ENSG00000141013	772	15.535
HHLA1	ENSG00000132297	771.5	0.165
OC90	ENSG00000258417	771.5	0.015
UCHL3	ENSG00000118939	770	102.325
LMO7	ENSG00000136153	770	17.395
DDX11L10	ENSG00000233614	770	0
ALKBH3	ENSG00000166199	769.5	29.4
SEC14L1P1	ENSG00000213693	769.5	1.42
NTM	ENSG00000182667	769	0.1
EIF4G1	ENSG00000114867	768.5	545.57
PSMD2	ENSG00000175166	768.5	175.855
AP2M1	ENSG00000161203	768.5	156.695
POLR2H	ENSG00000163882	768.5	80.975
EIF2B5	ENSG00000145191	768.5	34.15
DVL3	ENSG00000161202	768.5	28.455
ABCF3	ENSG00000161204	768.5	24.01
ECE2	ENSG00000145194	768.5	5.225
SNORD66	ENSG00000212158	768.5	4.365
FAM131A	ENSG00000175182	768.5	3.315
EPHB3	ENSG00000182580	768.5	1.605
CHRD	ENSG00000090539	768.5	0.92
VWA5B2	ENSG00000145198	768.5	0.84
EIF2B5-IT1	ENSG00000227509	768.5	0
RBX1	ENSG00000100387	768	33.305
XPNPEP3	ENSG00000196236	768	22.89
TCF20	ENSG00000100207	768	19.715
PTPRE	ENSG00000132334	768	0.67
EFCAB3	ENSG00000172421	768	0.04
RNU7-52P	ENSG00000251981	768	0
RPS23P7	ENSG00000239527	768	0
SLC35D1	ENSG00000116704	767.5	11.01
RNU6-229P	ENSG00000252423	767.5	0
ANAPC5	ENSG00000089053	766.5	166.775
TNPO1	ENSG00000083312	766.5	113.77
ARHGAP39	ENSG00000147799	766.5	10.92
ATF3	ENSG00000162772	766	13.54
TMPO	ENSG00000120802	763.5	210.895
TRAPPC11	ENSG00000168538	763.5	18.86
FBXO45	ENSG00000174013	763.5	11.51
MUC16	ENSG00000181143	763.5	0.005
LIN28B	ENSG00000187772	763	35.74
TECPR2	ENSG00000196663	762.5	8.435
TCEANC	ENSG00000176896	762.5	2.9
ATP6V0B	ENSG00000117410	761.5	122.585

SHOC2	ENSG00000108061	761.5	42.795
AKR1D1	ENSG00000122787	761.5	0.055
SATB2-AS1	ENSG00000225953	761	2.63
MATN3	ENSG00000132031	761	0.475
CEP170	ENSG00000143702	760.5	73.195
ZBTB8OS	ENSG00000176261	759.5	69.27
SRSF5	ENSG00000100650	759	354.48
STT3B	ENSG00000163527	759	97.585
LMBRD1	ENSG00000168216	759	11.55
NPM1P37	ENSG00000219085	759	0.705
LINC00461	ENSG00000245526	758.5	0.99
CKS1B	ENSG00000173207	758	133.19
PAWR	ENSG00000177425	758	66.275
TRMT61B	ENSG00000171103	758	16.025
EBF2	ENSG00000221818	757	1.71
AGO3	ENSG00000126070	756.5	17.07
ACAA1	ENSG00000060971	756	34.695
DEPTOR	ENSG00000155792	756	2.115
DLEC1	ENSG00000008226	756	0.445
PKN3	ENSG00000160447	755.5	19.64
FBXL20	ENSG00000108306	755	8.58
ADAM21	ENSG00000139985	754	0.155
ADAM20	ENSG00000134007	754	0.04
ADAM20P1	ENSG00000259158	754	0.04
TSG101	ENSG00000074319	753.5	78.805
MAML2	ENSG00000184384	753.5	1.715
DESI1	ENSG00000100418	751.5	91.54
VCL	ENSG00000035403	751	55.015
CCDC3	ENSG00000151468	751	5.75
TERF2IP	ENSG00000166848	750	59.39
LAMB1	ENSG00000091136	750	29.175
WDR90	ENSG00000161996	749.5	35.265
CLIC4	ENSG00000169504	749	55.44
MYO9A	ENSG00000066933	748.5	19.17
DCAF8L2	ENSG00000189186	748.5	0
WDR59	ENSG00000103091	747.5	54.995
IDE	ENSG00000119912	747.5	37.79
CRYM	ENSG00000103316	747.5	0.9
ANKS4B	ENSG00000175311	747.5	0.02
LINC01154	ENSG00000257477	747.5	0
LINC00865	ENSG00000232229	746	0
RFX2	ENSG00000087903	745	11.385
KLHL29	ENSG00000119771	745	8.645
WNT3	ENSG00000108379	745	2.375
KCNQ1	ENSG00000053918	745	1.49
CEP164P1	ENSG00000226937	745	0.295
COL11A1	ENSG00000060718	745	0.01

RSU1P2	ENSG00000232554	745	0
SOD2P1	ENSG00000234257	745	0
SUGP2	ENSG00000064607	744.5	61.2
FKBP5	ENSG00000096060	744.5	32.875
ADAMTS6	ENSG00000049192	744	0.505
RPEP1	ENSG00000250732	744	0
CBWD1	ENSG00000172785	743.5	47.2
SLC2A11	ENSG00000133460	743	10.77
RCE1	ENSG00000173653	741.5	29.605
C11orf80	ENSG00000173715	741.5	9.135
ZNF322	ENSG00000181315	741	16.835
B3GALNT1	ENSG00000169255	741	0
RHOJ	ENSG00000126785	740.5	0.155
P4HB	ENSG00000185624	740	492.165
LINC00667	ENSG00000263753	740	0.04
LINC00526	ENSG00000264575	740	0
CCL14	ENSG00000213494	739.5	0
CCL15	ENSG00000267596	739.5	0
CCL15-CCL14	ENSG00000161574	739.5	0
C9orf3	ENSG00000148120	739	11.705
SUFU	ENSG00000107882	738	17.225
TG	ENSG00000042832	738	0.28
MAGEA11	ENSG00000185247	737.5	0.235
C8orf34	ENSG00000165084	737	5.61
SUMF1	ENSG00000144455	737	5.36
GBAP1	ENSG00000160766	737	4.35
PNPT1P1	ENSG00000229241	737	0.085
ANKRD18CP	ENSG00000159712	736.5	2.93
ZNF322P1	ENSG00000188801	736.5	2.445
AACSP1	ENSG00000250420	736.5	0
PTPN13	ENSG00000163629	735	29.305
NLGN3	ENSG00000196338	735	0.87
ATP5J2	ENSG00000241468	734.5	617.95
CPSF4	ENSG00000160917	734.5	41.06
PTCD1	ENSG00000106246	734.5	10.945
ATP5J2-PTCD1	ENSG00000248919	734.5	6.875
ZFYVE1	ENSG00000165861	734	6.185
SCUBE2	ENSG00000175356	734	0.64
PAFAH1B1	ENSG00000007168	733.5	93.88
QPCTL	ENSG00000011478	733.5	20.87
SMIM17	ENSG00000268182	733.5	0.115
KIF3A	ENSG00000131437	733	27.2
PNLIPRP1	ENSG00000187021	733	0.455
ZNF695	ENSG00000197472	732	8.26
PRDM15	ENSG00000141956	732	8.06
ZNF670	ENSG00000135747	732	2.67
ADAMTS14	ENSG00000138316	731.5	0.06

LBX1-AS1	ENSG00000227128	731	8.99
SOHLH2	ENSG00000120669	731	0.03
CCDC169	ENSG00000242715	731	0
CCDC169-SOHLH2	ENSG00000250709	731	0
POLE	ENSG00000177084	730.5	73.16
FAM13A	ENSG00000138640	730.5	18.77
ATG4C	ENSG00000125703	730	20.045
CNTFR	ENSG00000122756	730	3.64
COL4A3	ENSG00000169031	729	0.495
TRPA1	ENSG00000104321	728.5	1.28
RPS20P20	ENSG00000241942	728.5	0
DDAH1	ENSG00000153904	728	24.01
TRPM3	ENSG00000083067	726.5	0.175
FAM69A	ENSG00000154511	726	6.03
LINC01004	ENSG00000228393	726	3.04
ZNF354C	ENSG00000177932	725.5	0.01
NMUR2	ENSG00000132911	725.5	0
HIP1	ENSG00000127946	725	17.67
CEP350	ENSG00000135837	723	32.17
AKAP6	ENSG00000151320	723	1.615
RASAL1	ENSG00000111344	723	0.375
TSPAN1	ENSG00000117472	722.5	0
TCEA3	ENSG00000204219	721.5	0.68
PADI6	ENSG00000256049	721	0
FDXR	ENSG00000161513	720	8.64
CMPK1	ENSG00000162368	719.5	51.85
ZNF704	ENSG00000164684	719.5	14.06
CKS1BP7	ENSG00000254331	719.5	0
NR2C1	ENSG00000120798	719	58.48
CERS4	ENSG00000090661	719	11.29
BTBD9	ENSG00000183826	719	5.495
TRPC5	ENSG00000072315	719	0.03
UBA2	ENSG00000126261	718.5	174.22
IRS4	ENSG00000133124	718	370.48
ME1	ENSG00000065833	718	18.555
EDEM3	ENSG00000116406	718	17.825
TTLL7	ENSG00000137941	718	8.785
IGFL2	ENSG00000204866	717.5	0.075
ENTHD1	ENSG00000176177	716.5	0
NEO1	ENSG00000067141	716	31.13
TNKS	ENSG00000173273	716	13.915
GLI2	ENSG00000074047	716	8.7
SCUBE1	ENSG00000159307	715.5	1.125
WBP2NL	ENSG00000183066	715.5	0.185
ERCC1	ENSG00000012061	715	41.165
LPP	ENSG00000145012	714.5	9.55
RUFY3	ENSG00000018189	714	38.13

TNRC6B	ENSG00000100354	714	27.195
PXDNL	ENSG00000147485	714	0.085
GLIS3	ENSG00000107249	713.5	1.93
GPR39	ENSG00000183840	712.5	0.29
GLCE	ENSG00000138604	712.25	17.21
WDTC1	ENSG00000142784	712	23.125
SAMD3	ENSG00000164483	712	0.19
ALDH6A1	ENSG00000119711	711.5	15.78
CCDC176	ENSG00000119636	711.5	2.805
ZNF664	ENSG00000179195	711	91.47
FAM101A	ENSG00000178882	711	0.18
TPD52	ENSG00000076554	710.5	52.32
MRPS28	ENSG00000147586	710.5	35.89
STON2	ENSG00000140022	710.5	7.835
CCDC102B	ENSG00000150636	710.5	0.945
TPK1	ENSG00000196511	710	1.575
MGLL	ENSG00000074416	710	0.975
DAB2	ENSG00000153071	709.5	2.76
COL19A1	ENSG00000082293	709.5	0.665
C9	ENSG00000113600	709.5	0
THEMIS	ENSG00000172673	709.5	0
BLM	ENSG00000197299	708.5	35.41
XKR4	ENSG00000206579	708	0.015
PKHD1	ENSG00000170927	708	0
TRAM2	ENSG00000065308	707.5	7.945
USH2A	ENSG00000042781	707.5	0.045
SNORD116	ENSG00000202498	707.5	0
UBR1	ENSG00000159459	706.5	16.36
SETD5	ENSG00000168137	706	134.645
CACUL1	ENSG00000151893	706	38.695
MYSM1	ENSG00000162601	706	33.9
C7orf60	ENSG00000164603	706	6.345
LDLRAD4	ENSG00000168675	706	2.36
DZIP1	ENSG00000134874	705.5	31.505
SMG5	ENSG00000198952	705	92.695
CALM1	ENSG00000198668	704.5	229.595
WNK1	ENSG00000060237	703.5	102.19
DLG3	ENSG00000082458	703.5	32.02
GTPBP2	ENSG00000172432	703	32.61
RELN	ENSG00000189056	703	0.445
RN7SKP86	ENSG00000222386	703	0
NEK10	ENSG00000163491	702.5	3.01
SUGCT	ENSG00000175600	702	6.22
CALN1	ENSG00000183166	701	0.03
SIN3A	ENSG00000169375	700.5	61.45
PLEKHA6	ENSG00000143850	700.5	1.345
RUVBL2	ENSG00000183207	700	111.03

TMEM68	ENSG00000167904	700	12.86
MAN2B2	ENSG00000013288	699.5	7.15
ABCB1	ENSG00000085563	699.5	6.875
LINC00871	ENSG00000258700	699.5	0
TYW1	ENSG00000198874	699	14.35
PSMD7	ENSG00000103035	698.5	95.09
RSPO2	ENSG00000147655	698	0.27
OR5H2	ENSG00000197938	698	0
OR5H6	ENSG00000230301	698	0
OR5H7P	ENSG00000187900	698	0
OR5H8P	ENSG00000232535	698	0
OR5K4	ENSG00000196098	698	0
UBFD1P1	ENSG00000244653	698	0
COMMD6	ENSG00000188243	697.5	58.945
JOSD1	ENSG00000100221	697.5	37.735
GUCY1A2	ENSG00000152402	697.5	0.32
KCNQ2	ENSG00000075043	697	20.19
CSF3R	ENSG00000119535	697	0.02
EYS	ENSG00000188107	696	0.415
GMEB1	ENSG00000162419	695.5	16.73
DOCK11	ENSG00000147251	695.5	11.755
PELI1	ENSG00000197329	695.5	8.255
SCARNA24	ENSG00000252777	695.5	0
SLC22A3	ENSG00000146477	695.5	0
PPP2R5C	ENSG00000078304	695	120.26
SLC25A16	ENSG00000122912	694.5	13.995
CRELD1	ENSG00000163703	694	12.66
IQGAP1	ENSG00000140575	693.5	95.265
CFDP1	ENSG00000153774	693.5	71.49
TMEM170A	ENSG00000166822	693.5	15.43
SPATA18	ENSG00000163071	693	1.595
COL6A5	ENSG00000172752	692.75	0.055
SLC47A1	ENSG00000142494	692.5	10.255
RPL17P43	ENSG00000228331	692.5	0.19
DCAF5	ENSG00000139990	692	26.185
EML4	ENSG00000143924	691.5	46.03
FAM91A1	ENSG00000176853	691.5	30.87
MAP3K7	ENSG00000135341	691.5	24.345
PLEKHF2	ENSG00000175895	691.5	6.495
LINC00092	ENSG00000225194	691.5	0.425
DDX47	ENSG00000213782	691	57.665
ZNF423	ENSG00000102935	691	2.36
APOLD1	ENSG00000178878	691	1.66
LINC00271	ENSG00000231028	691	0.385
ADAM3B	ENSG00000260089	691	0.15
EPHA1-AS1	ENSG00000229153	691	0.035
TAS2R41	ENSG00000221855	691	0

TAS2R62P	ENSG00000234066	691	0
SP1	ENSG00000185591	690	42.83
TRIM22	ENSG00000132274	689.5	0.33
OR52P1P	ENSG00000181109	689.5	0
OR56B1	ENSG00000181023	689.5	0
LUZP1	ENSG00000169641	689	19.945
MACF1	ENSG00000127603	688	72.08
ZNRF1	ENSG00000186187	688	14.805
KIAA0754	ENSG00000255103	688	10.59
EMR1	ENSG00000174837	688	0.075
ARHGAP12	ENSG00000165322	687.5	9.96
MYO7A	ENSG00000137474	687	1.61
C1orf131	ENSG00000143633	686.5	67.945
SLC8A2	ENSG00000118160	686.5	0.32
ALK	ENSG00000171094	686.5	0.125
GSTM3	ENSG00000134202	686	33.885
GSTM5	ENSG00000134201	686	0.12
KLK2	ENSG00000167751	685.5	0.12
INPP5D	ENSG00000168918	685.5	0
STAU1	ENSG00000124214	685	97.945
TMPRSS12	ENSG00000186452	685	0.02
TEAD4	ENSG00000197905	684.5	35.755
CLASP1	ENSG00000074054	684.5	20.005
DSC2	ENSG00000134755	684.5	13.88
EEPD1	ENSG00000122547	684.5	4.13
FASTKD5	ENSG00000215251	684	21.565
HOXC13	ENSG00000123364	684	11.97
UBOX5	ENSG00000185019	684	3.685
UBOX5-AS1	ENSG00000235958	684	0.725
SLC9C1	ENSG00000172139	683.5	0.035
SLC35A4	ENSG00000176087	681.5	55.295
SRA1	ENSG00000213523	681.5	36.5
AKAP2	ENSG00000241978	681.5	13.635
APBB3	ENSG00000113108	681.5	7.31
PALM2-AKAP2	ENSG00000157654	681.5	2.99
PALM2	ENSG00000243444	681.5	0.835
TOP1	ENSG00000198900	681	128.78
FBXO46	ENSG00000177051	680.75	22.655
CHCHD5	ENSG00000125611	680	16.62
MUC5B	ENSG00000117983	680	0.02
AGBL1	ENSG00000166748	680	0
UBE2V1	ENSG00000244687	679.5	170.1
TMEM189	ENSG00000240849	679.5	41.165
TMEM189-UBE2V1	ENSG00000124208	679.5	1.685
LINC01155	ENSG00000232593	679	3.905
MBOAT1	ENSG00000172197	679	2.29
LINC01122	ENSG00000233723	679	0.095

CCDC167	ENSG00000198937	678	37.695
TPCN1	ENSG00000186815	678	13.755
PADI3	ENSG00000142619	678	0
NBEAL1	ENSG00000144426	677.5	8.29
GPM6B	ENSG00000046653	677	9.88
KIAA1328	ENSG00000150477	676.5	8.51
AGAP11	ENSG00000151303	676.5	0.83
CLIC5	ENSG00000112782	676.5	0.36
BMS1P3	ENSG00000240089	676.5	0.02
ADIRF	ENSG00000148671	676.5	0
FAM25A	ENSG00000188100	676.5	0
XDH	ENSG00000158125	676.5	0
SNX5	ENSG00000089006	676	245.695
SNORD17	ENSG00000212232	676	47.08
DHX32	ENSG00000089876	676	16.415
LTBP1	ENSG00000049323	676	12.185
AHNAK	ENSG00000124942	676	7.655
OVOL2	ENSG00000125850	676	0
RNU2-42P	ENSG00000222629	676	0
RBBP6	ENSG00000122257	675.5	75.76
NFATC2	ENSG00000101096	675.5	0.605
MIR3194	ENSG00000266761	675.5	0
CHD1L	ENSG00000131778	675	54.175
PRKG1	ENSG00000185532	675	4.51
OR6C74	ENSG00000197706	675	0
PAX7	ENSG00000009709	674	0.765
RGS7	ENSG00000182901	674	0.74
PRKRIRP8	ENSG00000223963	674	0.03
BNC1	ENSG00000169594	673.5	0.945
STARD4-AS1	ENSG00000246859	673.5	0.58
TTLL9	ENSG00000131044	673	0.23
NSF	ENSG00000073969	672.5	48.37
ATP2B4	ENSG00000058668	672.5	25.575
ANP32AP1	ENSG00000259516	672.5	12.44
SYNE3	ENSG00000176438	672	0.845
PACRG	ENSG00000112530	672	0.555
PARP12	ENSG00000059378	672	0.37
GALNTL5	ENSG00000106648	671.5	0.175
C19orf81	ENSG00000235034	671	5.77
APEX2	ENSG00000169188	670.5	24.57
PACSIN1	ENSG00000124507	670.5	0.49
SPTBN5	ENSG00000137877	670.5	0.135
MIR4310	ENSG00000264850	670.5	0
RNA5SP393	ENSG00000200293	670.5	0
TMEM2	ENSG00000135048	670	12.675
ASXL3	ENSG00000141431	669.5	3.915
COX7BP1	ENSG00000243453	669.5	3.58

ACTL6B	ENSG00000077080	669.5	0.08
PAK3	ENSG00000077264	669	2.605
FCF1P4	ENSG00000226112	669	0
NDUFA6-AS1	ENSG00000237037	668.5	5.205
MASP1	ENSG00000127241	668.5	0.13
SSH2	ENSG00000141298	668	16.965
SLCO4A1	ENSG00000101187	668	11.49
RPL21P123	ENSG00000240531	668	0.175
HORMAD2	ENSG00000176635	668	0
TET1	ENSG00000138336	667.5	12.58
ZPLD1	ENSG00000170044	667.5	0.085
HECTD4	ENSG00000173064	667	21.695
RPL7AP60	ENSG00000213152	667	1.055
MKRN3	ENSG00000179455	666.5	0.96
CAMTA1	ENSG00000171735	666	102.45
KLHL23	ENSG00000213160	666	38.47
CCPG1	ENSG00000260916	666	15.71
PHOSPHO2	ENSG00000144362	666	6.655
THAP7-AS1	ENSG00000230513	666	3.77
DYX1C1	ENSG00000256061	666	3.125
TUBA3FP	ENSG00000161149	666	1.705
DYX1C1-CCPG1	ENSG00000261771	666	1.21
PTCHD3P2	ENSG00000231550	666	0.625
P2RX6	ENSG00000099957	666	0.225
CAMTA1-IT1	ENSG00000237402	666	0
GSE1	ENSG00000131149	665.5	55.855
ATAD1	ENSG00000138138	665.5	42.14
CFL1P1	ENSG00000223820	665.5	1.255
ASGR2	ENSG00000161944	665.5	0.055
ADNP	ENSG00000101126	665	69.875
GPR161	ENSG00000143147	665	6.985
MAPKAPK2	ENSG00000162889	664.5	40.985
NXT2	ENSG00000101888	664.5	11.31
CEP41	ENSG00000106477	663	13.785
ROCK1P1	ENSG00000263006	663	6.16
MAP1B	ENSG00000131711	662.5	26.23
ARID4A	ENSG00000032219	662.5	18.81
UBE3C	ENSG00000009335	662	91.75
DIP2C	ENSG00000151240	662	5.11
MPHOSPH9	ENSG00000051825	660.5	61.195
SCARB1	ENSG00000073060	660.5	60.65
CDC42BPA	ENSG00000143776	660.5	32.555
C12orf65	ENSG00000130921	660.5	19.695
COL4A6	ENSG00000197565	660.5	4.365
MEGF6	ENSG00000162591	660.5	1.46
MARK1	ENSG00000116141	660	13.3
HDAC1P2	ENSG00000233012	660	2.135

CDK6	ENSG00000105810	659	30.53
BCL7C	ENSG00000099385	658.5	33.545
CCDC41	ENSG00000173588	658	20.855
ATP6V0A2	ENSG00000185344	658	17.7
PHF2	ENSG00000197724	658	12.515
RBMS2P1	ENSG00000213250	658	1.41
CES1P2	ENSG00000260765	658	0
KCNAB2	ENSG00000069424	657.5	4.125
CELF1	ENSG00000149187	657	112.065
MKL1	ENSG00000196588	657	22.34
RPL4P6	ENSG00000230071	657	1.35
TYR	ENSG00000077498	656.5	0.045
P2RY8	ENSG00000182162	656.5	0
TFRC	ENSG00000072274	656	77.17
RNF34	ENSG00000170633	656	57.61
AIF1L	ENSG00000126878	656	21.99
UVRAG	ENSG00000198382	656	8.7
WWOX	ENSG00000186153	655.5	18.035
EPHA5	ENSG00000145242	655.5	1.15
PDE8B	ENSG00000113231	655.5	0.765
SORCS3	ENSG00000156395	655.5	0.01
ALDH7A1P1	ENSG00000251400	655.5	0
FTH1P26	ENSG00000218213	655.5	0
LSM3P5	ENSG00000260014	655.5	0
GABRG3	ENSG00000182256	655	0
MEF2A	ENSG00000068305	654.5	29.355
ALG14	ENSG00000172339	654.5	16.07
PEBP4	ENSG00000134020	654.5	0
SYT12	ENSG00000173227	654	0.32
TMEM209	ENSG00000146842	653.5	33.2
CR1L	ENSG00000197721	653	0.45
OR1X5P	ENSG00000214351	653	0.07
CD46P1	ENSG00000244703	653	0
DHX30	ENSG00000132153	652	110.275
ADCY9	ENSG00000162104	652	16.425
RBKS	ENSG00000171174	652	1.2
CACNA1A	ENSG00000141837	652	0
MIR1226	ENSG00000221585	652	0
NFRKB	ENSG00000170322	651.5	39.975
DROSHA	ENSG00000113360	651	102.3
DCUN1D5	ENSG00000137692	651	78.465
DCAF6	ENSG00000143164	651	54.685
FNBP1	ENSG00000187239	651	28.15
GPC3	ENSG00000147257	650.5	50.21
GFRAL	ENSG00000187871	650.5	0.035
SGIP1	ENSG00000118473	650.5	0.035
STOM	ENSG00000148175	650	23.41

MARCH1	ENSG00000145416	649.5	1.59
XRN1	ENSG00000114127	649	24.215
ADRBK1	ENSG00000173020	648.5	46.455
PLS3	ENSG00000102024	648	440.165
SATB1	ENSG00000182568	648	19.435
TBC1D5	ENSG00000131374	648	8.92
ZNF207	ENSG00000010244	647	161.2
PSMB2	ENSG00000126067	647	114.26
RHBDL3	ENSG00000141314	647	2.54
GATAD2A	ENSG00000167491	646.5	138.66
CALD1	ENSG00000122786	646.5	50.23
SCOC	ENSG00000153130	646.5	25.72
EYA1	ENSG00000104313	646.5	9.12
PTGER3	ENSG00000050628	646.5	0.05
GOLGA8M	ENSG00000188626	646	0.4
BRINP1	ENSG00000078725	646	0
RN7SL719P	ENSG00000241075	646	0
CDCA3	ENSG00000111665	645.5	61.93
DIS3L2	ENSG00000144535	645.5	14.41
GNB3	ENSG00000111664	645.5	6.33
GYG1P3	ENSG00000231095	645.5	0
TRPV1	ENSG00000196689	645	4.31
SHPK	ENSG00000262304	645	2.06
TSPAN6	ENSG00000000003	644.5	40.335
RNF216	ENSG00000011275	644.5	33.47
RBM12B	ENSG00000183808	644.5	24.805
RNF216-IT1	ENSG00000237738	644.5	0.84
RPSAP52	ENSG00000241749	644	0.075
HOXB6	ENSG00000108511	643.5	70.43
HOXB3	ENSG00000120093	643.5	50.605
HOXB-AS3	ENSG00000233101	643.5	42.915
ZFAND3	ENSG00000156639	643.5	37.94
HOXB5	ENSG00000120075	643.5	20.2
HOXB4	ENSG00000182742	643.5	7.88
TFEB	ENSG00000112561	643.5	3.745
HOXB-AS2	ENSG00000239552	643.5	2.805
MIR10A	ENSG00000257178	643.5	2.11
HOXB-AS1	ENSG00000230148	643.5	1.475
MNAT1	ENSG00000020426	642.5	57.31
LZIC	ENSG00000162441	642.5	22.645
CLEC16A	ENSG00000038532	642.5	19.33
SLC38A6	ENSG00000139974	642.5	8.39
GHR	ENSG00000112964	642.5	1.025
JAKMIP2	ENSG00000176049	642	0.435
FLRT2	ENSG00000185070	642	0.01
SNX9	ENSG00000130340	641.5	18.9
FGD1	ENSG00000102302	641.5	17.045

PRSS12	ENSG00000164099	641.5	7.08
RPGR	ENSG00000156313	641	14.815
DIAPH2	ENSG00000147202	641	10.72
DUX4L13	ENSG00000258617	641	0
DUX4L14	ENSG00000258514	641	0
ARL5A	ENSG00000162980	640.5	42.945
ACTR2	ENSG00000138071	640	110.51
L3MBTL4	ENSG00000154655	640	0.745
TMEM169	ENSG00000163449	640	0.57
ACACA	ENSG00000132142	639	43.405
FAM174B	ENSG00000185442	639	3.825
HMGB1P24	ENSG00000254982	639	0.955
ELAVL4	ENSG00000162374	639	0.04
LGI4	ENSG00000153902	639	0.025
HDAC1	ENSG00000116478	638.5	204.22
HS6ST3	ENSG00000185352	638.5	1.66
RN7SL164P	ENSG00000242614	638.5	0.475
HSDL2	ENSG00000119471	638	36.51
RECQL5	ENSG00000108469	638	21.425
INVS	ENSG00000119509	638	12.395
C9orf147	ENSG00000230185	638	2.715
RN7SL75P	ENSG00000239908	638	0.525
TRIM24	ENSG00000122779	637.5	58.57
CFB	ENSG00000243649	637.5	3.05
C2	ENSG00000166278	637.5	1.365
SMOC2	ENSG00000112562	637.5	0.02
ADIPOR2	ENSG00000006831	637	36.48
PLCG2	ENSG00000197943	637	4.805
ROCK1	ENSG00000067900	636.5	23.64
NID1	ENSG00000116962	636	65.145
SCMH1	ENSG00000010803	636	29.82
EPS8	ENSG00000151491	636	13.935
GBAS	ENSG00000146729	635.5	69.59
HNRNPCP2	ENSG00000204253	635.5	66.145
PAXIP1	ENSG00000157212	635.5	36.56
LINC00491	ENSG00000250682	635.5	5.2
ZNF713	ENSG00000178665	635.5	3.97
MRPS17	ENSG00000249773	635.5	3.255
C2orf88	ENSG00000187699	635.5	2.795
C1QTNF1	ENSG00000173918	635.5	2.03
RNF10	ENSG00000022840	635	156.76
HIPK3	ENSG00000110422	635	13.895
RYBP	ENSG00000163602	635	12.235
EGR2	ENSG00000122877	635	0.065
GAPVD1	ENSG00000165219	634.5	46.46
C6orf10	ENSG00000204296	634.5	0
CAPN10	ENSG00000142330	633.5	12.105

GPR35	ENSG00000178623	633.5	0.62
CUL2	ENSG00000108094	633	26.58
AP3B1	ENSG00000132842	632.5	37.14
ABCC1	ENSG00000103222	632.5	21.28
GNB2L1	ENSG00000204628	632	2427.27
FLG-AS1	ENSG00000237975	632	0
TMEM132D	ENSG00000151952	632	0
PTPN2	ENSG00000175354	631.5	30.63
LRBA	ENSG00000198589	631.5	29.13
NTRK1	ENSG00000198400	631	0.515
INSRR	ENSG00000027644	631	0.05
CRISP3	ENSG00000096006	630.5	0
KPNA6	ENSG00000025800	630	56.12
TRIM44	ENSG00000166326	630	23.23
ZFAND1	ENSG00000104231	629.5	25.5
MYBPC2	ENSG00000086967	629.5	0.34
PKN2	ENSG00000065243	629	51.14
MAP1A	ENSG00000166963	629	10.675
PPP1R9A	ENSG00000158528	629	4.29
TMIGD1	ENSG00000182271	629	0
SLC39A10	ENSG00000196950	628.5	25.71
EIF3A	ENSG00000107581	628	184.115
CERS6-AS1	ENSG00000227617	628	4.845
ANK3	ENSG00000151150	627.5	13.575
DNMBP	ENSG00000107554	627.5	5.665
ACO2	ENSG00000100412	627	56.765
HYKK	ENSG00000188266	627	19.26
UNC13A	ENSG00000130477	627	0.195
PTPRQ	ENSG00000139304	626	0.045
MAN1B1	ENSG00000177239	625.5	21.195
EPHB1	ENSG00000154928	625.5	1.715
FAM13C	ENSG00000148541	625.5	1.25
PDE6A	ENSG00000132915	625.5	0.325
PRNCR1	ENSG00000224722	625.5	0
EPHA10	ENSG00000183317	625	1.585
PAPD4	ENSG00000164329	624.5	40.51
SNUPN	ENSG00000169371	624.5	27.435
PTPN9	ENSG00000169410	624.5	21.16
TMEM199	ENSG00000244045	624	14.065
SARM1	ENSG00000004139	624	10.65
CEP112	ENSG00000154240	624	8.505
AXIN2	ENSG00000168646	624	8.315
NCKAP5	ENSG00000176771	624	1.54
SNTG2	ENSG00000172554	624	1.25
NCKAP5-IT1	ENSG00000232474	624	0
ATP13A3	ENSG00000133657	623.5	29.52
PARD3	ENSG00000148498	623.5	19.605

ZNF425	ENSG00000204947	623.5	4.2
EPCAM	ENSG00000119888	623.5	1.115
RCAN2	ENSG00000172348	623.5	0.77
KRTCAP2	ENSG00000163463	623	290.25
GNL3L	ENSG00000130119	623	59.03
TUB	ENSG00000166402	623	24.18
PTPN14	ENSG00000152104	623	15.11
MUC1	ENSG00000185499	623	4.67
TRIM46	ENSG00000163462	623	2.21
RHEBP2	ENSG00000226964	622.5	5.595
FAM35DP	ENSG00000189014	622.5	0.6
ARL17B	ENSG00000228696	622	25.31
KIAA1377	ENSG00000110318	622	1.965
SH3GL3	ENSG00000140600	621.5	9.4
DNAH12	ENSG00000174844	621.5	0.46
C3orf84	ENSG00000236980	621.5	0
FBXO39	ENSG00000177294	621.5	0
RNF7P1	ENSG00000239831	621.5	0
TEKT1	ENSG00000167858	621.5	0
RARS	ENSG00000113643	621	130.06
JAG2	ENSG00000184916	621	13.22
NUDT14	ENSG00000183828	621	10.6
KCND3	ENSG00000171385	621	0.14
KCND3-IT1	ENSG00000232558	621	0
BCOR	ENSG00000183337	620.5	135.01
CACNG8	ENSG00000142408	620.5	3.215
RPS11P6	ENSG00000243024	620.5	0.325
C12orf56	ENSG00000185306	620.5	0.04
AP3D1	ENSG00000065000	620	120.53
ZNF41	ENSG00000147124	620	6.855
LINC00466	ENSG00000224209	620	0.06
KIF3C	ENSG00000084731	619.5	8.99
MGAT4A	ENSG00000071073	619.5	3.99
CARM1	ENSG00000142453	619	105.845
KIAA0368	ENSG00000136813	619	85.04
SLC9C2	ENSG00000162753	619	0.045
VWF	ENSG00000110799	619	0.005
LDHB	ENSG00000111716	618.5	868.94
RBM26	ENSG00000139746	618.5	90.07
AP2B1	ENSG00000006125	618.5	65.31
ZNF383	ENSG00000188283	618.5	3.375
RPL17P42	ENSG00000239520	618.5	0.24
ITPKB-AS1	ENSG00000228548	618.5	0
NETO1	ENSG00000166342	618.5	0
PGBD5	ENSG00000177614	618	6.06
NUDT5	ENSG00000165609	617.5	101.9
LINC00842	ENSG00000223477	617.5	0.02

ANXA8L1	ENSG00000150165	617.5	0
HEATR5A	ENSG00000129493	617	18.11
DTD2	ENSG00000129480	617	16.76
ARHGAP44	ENSG00000006740	617	8.575
SNX33	ENSG00000173548	617	5.56
ADAM19	ENSG00000135074	617	2.265
SRD5A2	ENSG00000049319	616.5	0
PIFO	ENSG00000173947	616	0.675
LGALS2	ENSG00000100079	616	0.225
LINC-PINT	ENSG00000231721	615.5	3.14
CA14	ENSG00000118298	615	1.1
FOLH1	ENSG00000086205	615	0.48
COCH	ENSG00000100473	614.5	39.29
ZNF616	ENSG00000204611	614.5	5.665
ZNF836	ENSG00000196267	614.5	1.21
HTR1D	ENSG00000179546	614.5	0.84
CD84	ENSG00000066294	614.5	0.035
CNTNAP5	ENSG00000155052	614.5	0
LPA	ENSG00000198670	614.5	0
SMARCA2	ENSG00000080503	614	12.87
RAD51C	ENSG00000108384	613	68.465
CUX1	ENSG00000257923	613	26.785
ARHGEF3	ENSG00000163947	613	9.065
HMCN1	ENSG00000143341	613	1.52
SPI1	ENSG00000066336	613	0.6
SPATA12	ENSG00000186451	613	0.33
MIR1302-10	ENSG00000243485	613	0
LRRC47	ENSG00000130764	612.5	48.44
DNMT3B	ENSG00000088305	612.5	20.185
EPHA7	ENSG00000135333	612.5	13.305
EFCAB4B	ENSG00000130038	612.5	5.81
DGKK	ENSG00000204466	612.5	0.055
MCU	ENSG00000156026	612	19.91
RANBP10	ENSG00000141084	612	14.475
FOXP2	ENSG00000128573	612	10.45
CHRNB4	ENSG00000117971	612	0.395
RPL18P11	ENSG00000213307	612	0
XPNPEP2	ENSG00000122121	612	0
PTPRA	ENSG00000132670	611.5	47.85
VPS16	ENSG00000215305	611.5	17.51
FAM24B	ENSG00000213185	611.5	0.705
ADORA2A-AS1	ENSG00000178803	611.5	0.51
CUZD1	ENSG00000138161	611.5	0.135
AGPAT3	ENSG00000160216	611	46.48
PPFIA4	ENSG00000143847	611	2.85
CCDC114	ENSG00000105479	611	0.37
CTNNA1	ENSG00000044115	610.5	140.625

LIMA1	ENSG00000050405	610.5	32.97
RN7SL867P	ENSG00000265361	610.5	0
MRPS24	ENSG00000062582	610	103.52
PSPC1	ENSG00000121390	610	50.605
URGCP	ENSG00000106608	610	15.85
FBXW4	ENSG00000107829	610	12.655
LPHN3	ENSG00000150471	610	8.78
AIFM2	ENSG00000042286	610	3.175
BOK-AS1	ENSG00000234235	610	0
URGCP-MRPS24	ENSG00000270617	610	0
ACAP2	ENSG00000114331	609.5	21.87
ACAP2-IT1	ENSG00000229325	609.5	0.56
SFMBT2	ENSG00000198879	609	13.49
GLIS1	ENSG00000174332	609	0.725
TRDN	ENSG00000186439	609	0.05
ATG14	ENSG00000126775	608.5	7.915
EIF3H	ENSG00000147677	608	138.25
LRRC8D	ENSG00000171492	608	15.435
LRCH2	ENSG00000130224	608	10.57
LRRC8C	ENSG00000171488	608	5.97
STAC	ENSG00000144681	608	0.465
GABRA6	ENSG00000145863	608	0
LY86	ENSG00000112799	608	0
SNORA35	ENSG00000208839	608	0
BCAR3	ENSG00000137936	607.5	6.07
JAZF1	ENSG00000153814	607	5.89
RNU6-979P	ENSG00000206623	607	0
ILDR2	ENSG00000143195	606.5	2.935
FAM189A2	ENSG00000135063	606.5	1.27
OR10J9P	ENSG00000249197	606.5	0.065
ARSH	ENSG00000205667	606.5	0
STK38	ENSG00000112079	606	19.535
SHROOM3	ENSG00000138771	606	6.11
TRPC6	ENSG00000137672	606	0.265
CYP4F10P	ENSG00000267241	606	0
ABCB7	ENSG00000131269	605.5	26.59
LAIR1	ENSG00000167613	605.5	0.025
LINC00577	ENSG00000203809	605.5	0
ACOT8	ENSG00000101473	605	18.76
RBPMS	ENSG00000157110	605	14.24
SLC41A2	ENSG00000136052	604	6.77
RENBP	ENSG00000102032	604	1.665
NTMT1	ENSG00000148335	603	65.42
CREB5	ENSG00000146592	602.5	32.83
CHURC1	ENSG00000258289	602.5	31.74
MAP3K13	ENSG00000073803	602.5	16.345
FNTB	ENSG00000257365	602.5	16.32

FCHSD2	ENSG00000137478	602.5	15.98
RAB15	ENSG00000139998	602.5	11.965
OTUD3	ENSG00000169914	602.5	10.82
CHURC1-FNTB	ENSG00000125954	602.5	9.41
SDK1	ENSG00000146555	602.5	4.86
SLC48A1	ENSG00000211584	602.5	3.68
RAPGEF3	ENSG00000079337	602.5	0.5
RBMX2P1	ENSG00000226406	602.5	0.08
PDXP	ENSG00000241360	601.5	60.225
SH3BP1	ENSG00000100092	601.5	18
ADAMTS17	ENSG00000140470	601.5	4.865
ZNF385C	ENSG00000187595	601	1.02
LINC00622	ENSG00000260941	601	0.32
MST1	ENSG00000173531	600.5	7.85
GPA33	ENSG00000143167	600.5	0.025
RNU2-47P	ENSG00000222581	600.5	0
KAZN	ENSG00000189337	600	12.565
EFCAB5	ENSG00000176927	600	0.71
FAM178B	ENSG00000168754	600	0.23
ERCC3	ENSG00000163161	599.5	52.39
RSRC1	ENSG00000174891	599.5	45.32
PTK7	ENSG00000112655	599.5	30.17
TTC9	ENSG00000133985	599.5	1.72
ATP13A5	ENSG00000187527	599.5	0.025
SMYD1	ENSG00000115593	599.5	0
CHID1	ENSG00000177830	599	55.585
SNCA	ENSG00000145335	599	22.215
N4BP2	ENSG00000078177	599	5.66
NHSL2	ENSG00000204131	599	1.14
RPS26P11	ENSG00000196933	599	0
PI4KAP1	ENSG00000215513	598.5	39.02
FAM222B	ENSG00000173065	598.5	18.875
HAUS3	ENSG00000214367	598.5	14.335
POLN	ENSG00000130997	598.5	7.925
BMPR1B	ENSG00000138696	598.5	4.595
C4orf21	ENSG00000138658	598	12.125
WDR78	ENSG00000152763	598	3.015
GRM4	ENSG00000124493	598	0.36
CD53	ENSG00000143119	598	0.305
CACNG2	ENSG00000166862	598	0
PRIM2	ENSG00000146143	597.5	14.195
USP42	ENSG00000106346	597.5	13.815
TBC1D9	ENSG00000109436	597.5	9.95
MAGI2	ENSG00000187391	597.5	0.97
DIAPH2-AS1	ENSG00000236256	597.5	0.325
TNRC18P1	ENSG00000249661	597.5	0.32
MAGI2-IT1	ENSG00000235751	597.5	0.23

PNPT1	ENSG00000138035	597	36.47
ZEB2	ENSG00000169554	597	14.105
EFCAB2	ENSG00000203666	597	7.925
CECR1	ENSG00000093072	597	0.265
USP50	ENSG00000170236	597	0.215
RNU6-999P	ENSG00000251754	597	0
MCMBP	ENSG00000197771	596.5	73.66
DHRS4	ENSG00000157326	596.5	19.935
TMEM234	ENSG00000160055	596.5	15.51
DHRS4-AS1	ENSG00000215256	596.5	7.9
RAI2	ENSG00000131831	596.5	1.795
NADK	ENSG00000008130	596	45.87
FOXJ3	ENSG00000198815	596	35.315
CABLES1	ENSG00000134508	596	35.045
OCA2	ENSG00000104044	596	1.905
SLC39A9	ENSG00000029364	595.5	31.725
SYT9	ENSG00000170743	595.5	0.29
RC3H1	ENSG00000135870	595	19.24
FNDC3B	ENSG00000075420	595	19.065
HEY2	ENSG00000135547	595	5.72
RC3H1-IT1	ENSG00000236535	595	1.665
EIF3E	ENSG00000104408	594.5	340.015
CHMP3	ENSG00000115561	594.5	47.37
RNF103	ENSG00000239305	594.5	11.21
COL13A1	ENSG00000197467	594.5	2.68
RNF103-CHMP3	ENSG00000249884	594.5	0.35
HYDIN	ENSG00000157423	594.5	0.225
RNU6-640P	ENSG00000200563	594.5	0
RNF19B	ENSG00000116514	594	14.715
WNT7B	ENSG00000188064	594	0.155
CCND2P1	ENSG00000256847	594	0
CNGA3	ENSG00000144191	594	0
SLC22A10	ENSG00000184999	594	0
PTPRS	ENSG00000105426	593.5	36.93
C6orf136	ENSG00000204564	593.5	22.735
HMGCS2	ENSG00000134240	593.5	0.035
ZMYND11	ENSG00000015171	593	22.255
ADAMTSL3	ENSG00000156218	593	0.505
TUBA8P2	ENSG00000261665	593	0
KMT2D	ENSG00000167548	592.75	50.815
PRKAG1	ENSG00000181929	592.75	43.32
PSMF1	ENSG00000125818	592.5	183.89
RAB5B	ENSG00000111540	592.5	38.17
CREM	ENSG00000095794	592.5	12.01
CDKL1	ENSG00000100490	592.5	8.83
ACTG1P3	ENSG00000215388	592.5	1.25
OSBPL1A	ENSG00000141447	592	34.185

KMT2C	ENSG00000055609	591.5	32.25
TLL2	ENSG00000095587	591.5	0.9
HSPA8P14	ENSG00000257539	591.5	0.415
SULT2A1	ENSG00000105398	591.5	0.375
UBE2QL1	ENSG00000215218	591.5	0.125
TRIP12	ENSG00000153827	591	142.54
CAST	ENSG00000153113	591	75.95
GPR124	ENSG00000020181	591	4.375
PLEKHA2	ENSG00000169499	591	2.355
WDR47	ENSG00000085433	590.5	11.02
DFFA	ENSG00000160049	590	97.09
GGPS1	ENSG00000152904	589.5	68.305
SPPL2A	ENSG00000138600	589.5	20.875
ADTRP	ENSG00000111863	589	0.03
CEACAM18	ENSG00000213822	589	0
GNG7	ENSG00000176533	588.5	8.37
ABCA12	ENSG00000144452	588.5	0.07
RASA1	ENSG00000145715	588	15.63
PLD1	ENSG00000075651	588	4.16
CSRP1	ENSG00000159176	588	0.61
PP13439	ENSG00000183657	588	0.21
SRSF6	ENSG00000124193	587.5	178.455
CLCC1	ENSG00000121940	587.5	48.99
MITF	ENSG00000187098	587.5	5.305
AKNAD1	ENSG00000162641	587.5	0.025
MACC1	ENSG00000183742	587.5	0.005
OPRD1	ENSG00000116329	587	0.63
CABIN1	ENSG00000099991	586.75	47.38
RBM45	ENSG00000155636	586.5	8.4
CPEB2-AS1	ENSG00000247624	586.5	0.035
HIPK1	ENSG00000163349	586	33.84
PKP2	ENSG00000057294	586	13.275
NUGGC	ENSG00000189233	586	0.03
RPTOR	ENSG00000141564	585.5	25.415
RTN4RL1	ENSG00000185924	585.5	1.65
CSNK1E	ENSG00000213923	585	431.995
NFIX	ENSG00000008441	585	12.455
BTK	ENSG00000010671	584.5	0.35
DPH6-AS1	ENSG00000248079	584.5	0.22
MIR383	ENSG00000199127	584.5	0
SGCZ	ENSG00000185053	584.5	0
SIGLEC9	ENSG00000129450	584.5	0
TBC1D16	ENSG00000167291	584	17.98
XPO4	ENSG00000132953	584	16.34
PCDH11X	ENSG00000102290	584	0.015
KRT18P11	ENSG00000215089	584	0
MIR202HG	ENSG00000166917	584	0

VPS41	ENSG00000006715	583.5	28.235
ZNRF3	ENSG00000183579	583.5	12.53
ZNRF3-IT1	ENSG00000235786	583.5	0.485
FAIM3	ENSG00000162894	583.5	0.07
DSG1	ENSG00000134760	583.5	0
TROVE2	ENSG00000116747	583	62.815
ZZZ3	ENSG00000036549	583	52.065
MLLT1	ENSG00000130382	583	47.57
KCTD13	ENSG00000174943	583	21.545
SLC4A8	ENSG00000050438	583	3.435
ASPHD1	ENSG00000174939	583	2.55
MLXIP	ENSG00000175727	582.5	54.31
CBFA2T2	ENSG00000078699	582.5	10.91
IGF1	ENSG00000017427	582.5	0.055
PLA2G4E	ENSG00000188089	582.5	0.015
STX8	ENSG00000170310	582	23.76
ANKRD36	ENSG00000135976	581.5	15.47
HTT	ENSG00000197386	581	64.15
CTNNBL1	ENSG00000132792	581	33.245
ATF7IP	ENSG00000171681	581	31.445
CADM2	ENSG00000175161	581	3.685
DPYSL3	ENSG00000113657	580.5	0.62
MAPK1	ENSG00000100030	580	96.48
LARS2	ENSG00000011376	580	42.21
PTEN	ENSG00000171862	580	35.885
RPS3AP15	ENSG00000244674	580	0.1
MIB1	ENSG00000101752	579.5	24.93
SPRED2	ENSG00000198369	579.5	8.81
ARHGEF17	ENSG00000110237	579.5	4.195
CLDN11	ENSG00000013297	579.5	0.32
SLC7A14	ENSG00000013293	579.5	0.03
KRT8P13	ENSG00000241416	579.5	0
IFFO1	ENSG00000010295	579	21.71
SLC30A7	ENSG00000162695	579	16.29
BMP7	ENSG00000101144	578.5	35.595
SCN8A	ENSG00000196876	578.5	2.09
PCMTD2	ENSG00000203880	578	36.755
LINC00266-1	ENSG00000149656	578	0.47
PUM1	ENSG00000134644	577.5	96.085
ATXN2	ENSG00000204842	577.5	83.865
MXI1	ENSG00000119950	577.5	33.74
PIK3C3	ENSG00000078142	577.5	27.275
RASA2	ENSG00000155903	577.5	7.005
SOX5	ENSG00000134532	577.5	3.06
MYH16	ENSG00000002079	577.5	0.615
PCBP2	ENSG00000197111	577	686.745
PRR13	ENSG00000205352	577	192.63

TTLL4	ENSG00000135912	577	78.71
NUP88	ENSG00000108559	577	76.735
CIR1	ENSG00000138433	577	23.52
LHFPL5	ENSG00000197753	577	0
PRCP	ENSG00000137509	576.5	35.17
C11orf82	ENSG00000165490	576.5	15.895
MLTK	ENSG00000091436	576.5	13.98
ZNF780A	ENSG00000197782	576.5	5.96
ZNF780B	ENSG00000128000	576.5	4.85
RAB30	ENSG00000137502	576.5	3.175
SBF2-AS1	ENSG00000246273	576.5	2.71
LRRK2	ENSG00000188906	576	0.635
ATP6V1A	ENSG00000114573	575.5	79.205
SEPT6	ENSG00000125354	575.5	57.185
C19orf47	ENSG00000160392	575.5	21.615
RIBC2	ENSG00000128408	575.5	4.39
MLYCD	ENSG00000103150	575.5	4.295
OSGIN1	ENSG00000140961	575.5	0.87
TMEM59	ENSG00000116209	575	67.235
ZRANB1	ENSG00000019995	575	16.79
TMEM242	ENSG00000215712	575	6.8
MEGF11	ENSG00000157890	575	4.585
C1orf61	ENSG00000125462	575	3.455
GNA14	ENSG00000156049	575	0.375
SPAG5	ENSG00000076382	574.5	65.685
PIGS	ENSG00000087111	574.5	63.115
ACBD6	ENSG00000135847	574.5	47.155
UNC119	ENSG00000109103	574.5	22.325
EBAG9	ENSG00000147654	574.5	18.115
SGK494	ENSG00000167524	574.5	6.805
ALDOC	ENSG00000109107	574.5	3.015
ZBTB20	ENSG00000181722	574.5	1.82
ZBTB20-AS2	ENSG00000241295	574.5	0.185
MIR3121	ENSG00000265435	574.5	0
RFC3	ENSG00000133119	574	58.815
CHMP5	ENSG00000086065	574	37.625
RAPH1	ENSG00000173166	574	11.32
CNIH3	ENSG00000143786	574	8.07
AKR1B1P1	ENSG00000229991	574	0
BAG6	ENSG00000204463	573.5	206.73
CHAF1B	ENSG00000159259	573.5	45.23
MORC3	ENSG00000159256	573.5	16.645
SLC44A1	ENSG00000070214	573.5	14
LILRB4	ENSG00000186818	573.5	0.065
VN1R105P	ENSG00000242856	573.5	0
TMCO1	ENSG00000143183	573	111.75
IFT81	ENSG00000122970	573	21.895

SUV420H1	ENSG00000110066	573	20.09
TMEM108	ENSG00000144868	573	5.92
IPCEF1	ENSG00000074706	572.5	0.685
ITGAM	ENSG00000169896	572.5	0
GALT	ENSG00000213930	572	23.64
ZNF652	ENSG00000198740	572	16.455
PKD2	ENSG00000118762	572	16.085
MSRB3	ENSG00000174099	572	6.93
IL11RA	ENSG00000137070	572	6.09
ARHGAP25	ENSG00000163219	572	0.36
NSG2	ENSG00000170091	572	0.12
SRRM1	ENSG00000133226	571.5	202.06
EFNA5	ENSG00000184349	571.5	28.445
BLVRA	ENSG00000106605	571.5	20.125
PAPD5	ENSG00000121274	571.5	18.84
SHANK3	ENSG00000251322	571.5	7.65
ARMC4	ENSG00000169126	571.5	3.05
HSF2BP	ENSG00000160207	571.5	1.225
KCTD1	ENSG00000134504	571	17.42
STMN4	ENSG00000015592	571	0
MORC4	ENSG00000133131	570.5	19.8
ANKRD20A14P	ENSG00000232745	570.5	4.455
RIPPLY1	ENSG00000147223	570.5	0
SEL1L2	ENSG00000101251	570.5	0
RCOR3	ENSG00000117625	570	34.475
CTAGE5	ENSG00000150527	570	11.285
CCDC15	ENSG00000149548	570	4.03
NT5E	ENSG00000135318	570	0.51
MIA2	ENSG00000150526	570	0
IDH3B	ENSG00000101365	569.5	89.075
TADA2A	ENSG00000108264	569.5	19.93
CKAP2L	ENSG00000169607	569.25	25.03
NT5DC4	ENSG00000144130	569.25	0.035
DAZL	ENSG00000092345	569	0
AMZ2	ENSG00000196704	568.5	86.125
LIPE-AS1	ENSG00000213904	568.5	5.17
BTBD11	ENSG00000151136	568.5	3.07
RGS9	ENSG00000108370	568	0.415
PDGFC	ENSG00000145431	567.5	8.52
TMCO4	ENSG00000162542	567.5	4.23
SPTLC3	ENSG00000172296	567.5	0.24
ALPK3	ENSG00000136383	567.5	0.14
AOX1	ENSG00000138356	567.5	0.035
SERPINA5	ENSG00000188488	567.5	0.03
SERPINA3	ENSG00000196136	567.5	0
SERPINA4	ENSG00000100665	567.5	0
AMOTL1	ENSG00000166025	567	22.585

FAM13B	ENSG00000031003	567	21.37
NCOA3	ENSG00000124151	567	13.36
ATP8B4	ENSG00000104043	567	0.045
PKIB	ENSG00000135549	566.5	3.49
BEST3	ENSG00000127325	566.5	0.065
SSX9	ENSG00000204648	566.5	0
ZNF146	ENSG00000167635	566	83.415
PROSC	ENSG00000147471	566	25.815
MTMR3	ENSG00000100330	566	22.59
ZNF565	ENSG00000196357	566	1.65
ZNRF3-AS1	ENSG00000177993	566	0.855
CNN2P1	ENSG00000227201	566	0.63
HS2ST1	ENSG00000153936	565	26.545
SGSM1	ENSG00000167037	565	1.295
LINC01140	ENSG00000267272	565	0.265
FAM114A2	ENSG00000055147	564.5	13.83
MRPS31P5	ENSG00000243406	564.5	3.48
STEAP4	ENSG00000127954	564.5	0.07
TPTE2P2	ENSG00000272281	564.5	0.06
ZC3H11A	ENSG00000058673	564	87.01
TBCD	ENSG00000141556	564	41.21
FAM177A1	ENSG00000151327	564	25.815
PPP2R3C	ENSG00000092020	564	23.275
CUEDC2	ENSG00000107874	564	22.895
TMCC1	ENSG00000172765	564	11.435
ZBED6	ENSG00000257315	564	6.26
WNT7A	ENSG00000154764	564	0.015
SNAP23	ENSG00000092531	563.75	63.545
PEPD	ENSG00000124299	563.5	39.44
UACA	ENSG00000137831	563.5	26.935
BRWD3	ENSG00000165288	563.5	16.23
RPS6KC1	ENSG00000136643	563.5	11.44
NLRP1	ENSG00000091592	563.5	6.89
MAP4	ENSG00000047849	563	111.025
LPGAT1	ENSG00000123684	563	31.87
FAM206A	ENSG00000119328	563	17.685
VPS26BP1	ENSG00000224895	563	0.85
CRTC3	ENSG00000140577	562.5	35.215
DNAJC1	ENSG00000136770	562.5	24.24
AGAP5	ENSG00000172650	562	12.575
EPDR1	ENSG00000086289	562	5.67
BMS1P4	ENSG00000242338	562	2.215
GPR141	ENSG00000187037	562	0
NME8	ENSG00000086288	562	0
ELOVL5	ENSG00000012660	561.5	74.265
GLDC	ENSG00000178445	561.5	17.735
CEP89	ENSG00000121289	561.5	13.81

ARNTL2	ENSG00000029153	561.5	11.1
PLCL2	ENSG00000154822	561.5	7.005
WDR5B	ENSG00000196981	561.5	2.865
NOS2P3	ENSG00000230528	561.5	0.36
SPANXA2	ENSG00000203926	561.5	0
SPANXA2-OT1	ENSG00000226574	561.5	0
MTIF2	ENSG00000085760	561	52.955
KAT6A	ENSG00000083168	561	25.64
LONRF3	ENSG00000175556	561	14.84
MPPED1	ENSG00000186732	561	1.045
FRMPD3	ENSG00000147234	561	0.835
KLHL33	ENSG00000185271	561	0
RABEP1	ENSG00000029725	560.5	60.55
RABGAP1	ENSG00000011454	560.5	27.375
SH3PXD2B	ENSG00000174705	560.5	16.43
TTC28	ENSG00000100154	560.5	13.4
THEGL	ENSG00000249693	560.5	0.53
GPR21	ENSG00000188394	560.5	0.215
GLDCP1	ENSG00000248590	560.5	0.035
PDCL2	ENSG00000163440	560.5	0
POM121C	ENSG00000135213	560	84.36
SIK3	ENSG00000160584	560	16.615
RHOT1	ENSG00000126858	560	11.965
SIK3-IT1	ENSG00000231865	560	0.53
ZNF197	ENSG00000186448	559.5	11.97
ZKSCAN7	ENSG00000196345	559.5	0.925
ZNF660	ENSG00000144792	559.5	0.74
ZFHX4-AS1	ENSG00000253661	559.5	0.68
DNAJC5G	ENSG00000163793	559.5	0.39
ZNF197-AS1	ENSG00000233509	559.5	0.32
LINC00501	ENSG00000203645	559.5	0
TAF15	ENSG00000172660	559	132.975
MRPS22	ENSG00000175110	559	77.65
BMP6	ENSG00000153162	559	3.89
BPESC1	ENSG00000232416	559	0.015
NFE2L1	ENSG00000082641	558	74.85
MYCBP2	ENSG00000005810	558	69.1
GPI	ENSG00000105220	557.5	308.86
PDCD2L	ENSG00000126249	557.5	36.455
ANO1	ENSG00000131620	557.5	0.34
WDR43	ENSG00000163811	557	165.775
SNRNP40	ENSG00000060688	557	99.16
SOS1	ENSG00000115904	557	38.32
ANO8	ENSG00000074855	557	7.02
IL17RD	ENSG00000144730	557	4.055
ZNF30	ENSG00000168661	557	3.475
SOS1-IT1	ENSG00000229692	557	3.41

SH3RF2	ENSG00000156463	557	0.97
TSHR	ENSG00000165409	557	0.265
NMNAT1P1	ENSG00000259167	557	0
EXT1	ENSG00000182197	556.5	22.12
N4BP1	ENSG00000102921	556	31.555
DPY19L4	ENSG00000156162	556	20.145
C21orf49	ENSG00000205930	556	0.89
RPS2P44	ENSG00000243609	556	0.46
PDCD1LG2	ENSG00000197646	556	0.03
NPIPB3	ENSG00000169246	555.5	68.53
ATRX	ENSG00000085224	555.5	39.57
PLIN2	ENSG00000147872	555.5	25.69
SLC7A5P2	ENSG00000258186	555.5	8.575
DPH6	ENSG00000134146	555.5	7.795
SERINC5	ENSG00000164300	555.5	5.78
PRICKLE2	ENSG00000163637	555.5	0.585
SAMM50	ENSG00000100347	555	45.535
PARVB	ENSG00000188677	555	26.9
PNPLA3	ENSG00000100344	555	13.125
GABRR2	ENSG00000111886	555	0.44
IKZF1	ENSG00000185811	555	0.09
PAPL	ENSG00000183760	555	0.02
PRMT2	ENSG00000160310	554.5	52.225
RAPGEF2	ENSG00000109756	554.5	25.355
SGK3	ENSG00000104205	554.5	11.43
C8orf44	ENSG00000213865	554.5	2.51
C8orf44-SGK3	ENSG00000270024	554.5	0.955
RPL23AP82	ENSG00000184319	554	37.465
ASCC1	ENSG00000138303	554	30.69
SMO	ENSG00000128602	553.5	24.07
MTHFD2L	ENSG00000163738	553.5	9.86
LRRC1	ENSG00000137269	553.5	7.995
ABCG2	ENSG00000118777	553.5	1.19
CDH9	ENSG00000113100	553.5	0.125
KCNC2	ENSG00000166006	553.5	0.02
RNU6ATAC31P	ENSG00000221440	553.5	0
VRK3	ENSG00000105053	553	30.755
SLC6A14	ENSG00000087916	553	0
RALY	ENSG00000125970	552.5	71.815
FAR1	ENSG00000197601	552.5	62.22
FAR1-IT1	ENSG00000254791	552.5	3.58
SUN1	ENSG00000164828	552	63.575
GET4	ENSG00000239857	552	31.155
ENPP3	ENSG00000154269	552	0.435
AMPD1	ENSG00000116748	552	0.05
LSG1	ENSG00000041802	551.5	35.62
TMEM180	ENSG00000138111	551.5	17.395

GEMIN7	ENSG00000142252	551	43.8
MARK4	ENSG00000007047	551	22.955
ALG9	ENSG00000086848	551	20.63
ACADSB	ENSG00000196177	551	17.78
PPP1R37	ENSG00000104866	551	17.03
BLOC1S3	ENSG00000189114	551	11.67
PRKAR2B	ENSG00000005249	551	11.205
FDXACB1	ENSG00000255561	551	2.95
STXBP5L	ENSG00000145087	551	2.63
EIF5AP3	ENSG00000267346	551	0.735
ALG9-IT1	ENSG00000254450	551	0.375
DPY19L1	ENSG00000173852	550.5	19.495
PSEN2	ENSG00000143801	550	26.555
TCF4	ENSG00000196628	550	19.33
CPT1A	ENSG00000110090	550	13.44
MYLPF	ENSG00000180209	550	0.635
Metazoa_SRP	ENSG00000272096	550	0
TNFRSF11B	ENSG00000164761	550	0
PAG1	ENSG00000076641	549.5	11.355
MBD3L1	ENSG00000170948	549.5	0
UBE2H	ENSG00000186591	549	30.3
MFAP3	ENSG00000037749	549	23.005
GALNT10	ENSG00000164574	549	11.115
ITPRIPL2	ENSG00000205730	549	9.9
SYT17	ENSG00000103528	549	2.085
BPGM	ENSG00000172331	548.5	21.28
AFF1	ENSG00000172493	548.5	20.055
DECR1	ENSG00000104325	548.5	18.095
CYP20A1	ENSG00000119004	548.5	16.82
SOX30	ENSG00000039600	548.5	0.185
UBR5	ENSG00000104517	548	61.935
TFE3	ENSG00000068323	548	22.885
DNAJC6	ENSG00000116675	548	11.52
TCP11X3P	ENSG00000251525	548	0
JADE1	ENSG00000077684	547.5	20.67
CKMT2-AS1	ENSG00000247572	547.5	9.295
GRB14	ENSG00000115290	547.5	5
GRIN1	ENSG00000176884	547.5	1.74
KCTD8	ENSG00000183783	547.5	0.17
KRT16P4	ENSG00000229526	547.5	0
TNPO1P2	ENSG00000230662	547.5	0
RHOA	ENSG00000067560	547	476.52
SPRTN	ENSG00000010072	547	57.35
WSB2	ENSG00000176871	547	31.12
CMC1	ENSG00000187118	547	17.35
ARRDC4	ENSG00000140450	547	9.68
BBS9	ENSG00000122507	547	9.48

SAMD5	ENSG00000203727	547	3.505
DDX11L1	ENSG00000223972	547	0.12
ADAM29	ENSG00000168594	547	0
SYNGAP1	ENSG00000197283	546.5	9.97
SRD5A3-AS1	ENSG00000249700	546.5	2.025
VIPR2	ENSG00000106018	546.5	0.22
C1orf186	ENSG00000196533	546.5	0.205
C9orf84	ENSG00000165181	546.5	0.135
OR4N2	ENSG00000176294	546.5	0.13
OR4H12P	ENSG00000176312	546.5	0
OR4M1	ENSG00000176299	546.5	0
OR4N1P	ENSG00000258564	546.5	0
MCM3	ENSG00000112118	546	149.355
RQCD1	ENSG00000144580	546	66.88
NDE1	ENSG00000072864	546	41.525
ROR1	ENSG00000185483	546	6.92
GRIN3A	ENSG00000198785	546	0.305
TF	ENSG00000091513	546	0.215
TFP1	ENSG00000242337	546	0.04
PPP3R2	ENSG00000188386	546	0.02
GPR112	ENSG00000156920	546	0
DLEU1	ENSG00000176124	545.5	22.665
ONECUT2	ENSG00000119547	545.5	8.56
RPL18P10	ENSG00000214359	545.5	1.735
ST13P4	ENSG00000232150	545.5	0.955
RHOBTB1	ENSG00000072422	545	6.02
RNU2-72P	ENSG00000223107	545	0
ESYT2	ENSG00000117868	544.5	33.67
SHROOM1	ENSG00000164403	544.5	8.385
FAM19A4	ENSG00000163377	544.5	0.025
CUL3	ENSG00000036257	544	61.365
CKMT1B	ENSG00000237289	544	58.485
CPVL	ENSG00000106066	544	32.3
SH3KBP1	ENSG00000147010	544	19.085
TFEC	ENSG00000105967	544	0.01
HSPD1	ENSG00000144381	543.5	676.745
TSPAN33	ENSG00000158457	543.5	10.535
ATF7IP2	ENSG00000166669	543.5	9.325
ITPR2	ENSG00000123104	543.5	8.255
PCCA	ENSG00000175198	543.5	7.695
NFKBID	ENSG00000167604	543.5	7.165
GSK3B	ENSG00000082701	543	45.875
ESF1	ENSG00000089048	543	31.025
GABRB3	ENSG00000166206	543	17.635
EPM2A	ENSG00000112425	543	1.51
PLD2	ENSG00000129219	542.5	15.58
MFAP3L	ENSG00000198948	542.5	5.175

PRR16	ENSG00000184838	542.5	0.945
ABCG8	ENSG00000143921	542.5	0.025
BRI3	ENSG00000164713	542	63.345
EMB	ENSG00000170571	542	15.06
DNAJC3	ENSG00000102580	542	12.52
MTCYBP3	ENSG00000232373	542	0.22
RDX	ENSG00000137710	541.5	127.26
GNG12	ENSG00000172380	541.5	27.17
STAT6	ENSG00000166888	541.5	19.995
C17orf78	ENSG00000167230	541.5	0.26
ACTA2	ENSG00000107796	541	1.94
SYN1	ENSG00000008056	541	0.97
GRAMD3	ENSG00000155324	541	0.89
MLF1	ENSG00000178053	540.5	31.005
SHANK2-AS2	ENSG00000236262	540.5	0
FAM49B	ENSG00000153310	540	66.75
RBBP8	ENSG00000101773	540	55.13
IWS1	ENSG00000163166	540	45.065
PPP1R16A	ENSG00000160972	540	18.655
EEF2K	ENSG00000103319	540	18.34
PTPRB	ENSG00000127329	540	0.535
DDX11L9	ENSG00000248472	540	0.205
C1orf110	ENSG00000185860	540	0.07
QRSL1P2	ENSG00000227613	540	0
LINC00882	ENSG00000242759	539.5	0.375
MTND4P16	ENSG00000241787	539.5	0.345
CCDC14	ENSG00000175455	539	63.31
CHIC2	ENSG00000109220	539	11.69
NECAB2	ENSG00000103154	539	1.935
CDK13	ENSG00000065883	538.5	28.865
PGGT1B	ENSG00000164219	538.5	18.435
UPF2	ENSG00000151461	538	30.035
POT1-AS1	ENSG00000224897	538	0.485
EEF1GP1	ENSG00000186676	538	0.145
ALAS2	ENSG00000158578	538	0.13
PLD5	ENSG00000180287	538	0
STK32B	ENSG00000152953	538	0
VAMP1	ENSG00000139190	537.5	11.115
TAPBPL	ENSG00000139192	537.5	2.38
SCN9A	ENSG00000169432	537.5	1.375
DUSP26	ENSG00000133878	537.5	0.27
CD27	ENSG00000139193	537.5	0.19
ITGBL1	ENSG00000198542	537.5	0
MCTP2	ENSG00000140563	537.5	0
KIAA0556	ENSG00000047578	537	11.495
SULF1	ENSG00000137573	537	8.275
RNF144B	ENSG00000137393	537	1.58

BOK	ENSG00000176720	537	0.72
LINC00358	ENSG00000229578	537	0
MTHFD1L	ENSG00000120254	536.5	62.645
ZNF362	ENSG00000160094	536.5	30.605
RNU6-302P	ENSG00000202119	536.5	1.04
VN2R1P	ENSG00000174930	536.5	0
SUPT4H1	ENSG00000213246	536	97.905
ZNF354A	ENSG00000169131	536	13.345
NACC2	ENSG00000148411	536	12.12
KRT8	ENSG00000170421	536	7.245
RNF43	ENSG00000108375	536	4.595
BZRAP1-AS1	ENSG00000265148	536	0.34
TNFRSF8	ENSG00000120949	536	0.15
RPL21P122	ENSG00000241185	536	0.145
PMS2P3	ENSG00000127957	535.75	6.83
HDGFRP3	ENSG00000166503	535	66.845
METTL16	ENSG00000127804	535	35.985
KIAA0247	ENSG00000100647	535	4.19
AGT	ENSG00000135744	535	0
FRMPD3-AS1	ENSG00000227610	535	0
C15orf57	ENSG00000128891	534.5	13.91
POLH	ENSG00000170734	534.5	11.88
PRKAA2	ENSG00000162409	534.5	10.68
SYT7	ENSG00000011347	534.5	2.16
CCNJL	ENSG00000135083	534.5	2.115
CDH20	ENSG00000101542	534.5	0.02
ELOVL3	ENSG00000119915	534.5	0
POLR2F	ENSG00000100142	534	149.8
ADAR	ENSG00000160710	534	116.655
WDR26	ENSG00000162923	534	96.555
FBN2	ENSG00000138829	534	25.72
BLOC1S6	ENSG00000104164	534	24.42
CUL9	ENSG00000112659	534	11.275
SQRDL	ENSG00000137767	534	0.74
ZMPSTE24	ENSG00000084073	533.5	41.13
STAT2	ENSG00000170581	533.5	20.625
ESPNL	ENSG00000144488	533.5	3.435
PITPNM2	ENSG00000090975	533.5	2.095
PTK2	ENSG00000169398	533	62.335
NDRG2	ENSG00000165795	533	18.095
BRINP3	ENSG00000162670	533	4.43
DTX2P1	ENSG00000186704	533	3.495
LRRC7	ENSG00000033122	533	2.575
FGGY	ENSG00000172456	533	2.34
CCDC146	ENSG00000135205	533	1.345
DTX2P1-UPK3BP1-PI	ENSG00000265479	533	1.22
UPK3B	ENSG00000243566	533	0.77

METTL24	ENSG00000053328	533	0.45
HRH4	ENSG00000134489	533	0.14
PIN1P1	ENSG00000229359	533	0
PMS2P11	ENSG00000241350	533	0
PMS2P9	ENSG00000233448	533	0
RNASE13	ENSG00000206150	533	0
SCGB1C1	ENSG00000188076	533	0
TPPP2	ENSG00000179636	533	0
SNX3	ENSG00000112335	532.5	143.56
ARMC8	ENSG00000114098	532.5	28.075
RNF121	ENSG00000137522	532.5	24.89
PPCDC	ENSG00000138621	532.5	24.8
GTF2E1	ENSG00000153767	532.5	13.545
LAT2	ENSG00000086730	532.5	1.86
PEX5L	ENSG00000114757	532.5	0.36
PHEX	ENSG00000102174	532.5	0.215
IL12A-AS1	ENSG00000244040	532.5	0.025
HEPHL1	ENSG00000181333	532.5	0.01
EXOC6B	ENSG00000144036	532	12.33
RIMS2	ENSG00000176406	532	0.78
PDE4B	ENSG00000184588	532	0.715
GUSBP5	ENSG00000236296	532	0.28
FREM3	ENSG00000183090	532	0.02
COX5A	ENSG00000178741	531.5	296.1
PBX1	ENSG00000185630	531.5	38.73
TEC	ENSG00000135605	531.5	1.77
TCP11	ENSG00000124678	531.5	0.07
TOMM5	ENSG00000175768	531	133.525
FAM120A	ENSG00000048828	531	101.985
EXOSC3	ENSG00000107371	531	64.21
SHB	ENSG00000107338	531	21.23
FAM65A	ENSG00000039523	531	19.475
SLC25A51	ENSG00000122696	531	18.69
DCAF10	ENSG00000122741	531	17.64
FBXO10	ENSG00000147912	531	13.16
VEPH1	ENSG00000197415	531	0.12
SPDYE7P	ENSG00000179994	531	0
COPG2	ENSG00000158623	530.5	41.97
CDC14A	ENSG00000079335	530.5	6.2
MAP3K5	ENSG00000197442	530.5	4.965
GAP43	ENSG00000172020	530.5	1.03
RNU6-1056P	ENSG00000206723	530.5	0
TSGA13	ENSG00000213265	530.5	0
CPE	ENSG00000109472	530	44.38
TRPC4AP	ENSG00000100991	530	43.41
TCFL5	ENSG00000101190	530	16.88
SEC16B	ENSG00000120341	530	0.125

TIMM44	ENSG00000104980	529.5	103.06
CREBBP	ENSG00000005339	529.5	59.88
STOML1	ENSG00000067221	529.5	5.305
PRKD3	ENSG00000115825	529	48.745
IQGAP2	ENSG00000145703	529	13.095
LINC01102	ENSG00000235597	529	0
UTP20	ENSG00000120800	528.5	25.41
XYLB	ENSG00000093217	528.5	24.375
FAM171A1	ENSG00000148468	528.5	19.97
LMTK3	ENSG00000142235	528.5	1.745
LRRC72	ENSG00000205858	528.5	0
PCSK6	ENSG00000140479	528	45.75
DLX1	ENSG00000144355	528	24.7
ARVCF	ENSG00000099889	528	8.615
ACSS3	ENSG00000111058	528	2.52
APCDD1L	ENSG00000198768	528	0.255
INSL6	ENSG00000120210	528	0.24
COL9A1	ENSG00000112280	528	0.16
FMOD	ENSG00000122176	528	0.02
DDX10P2	ENSG00000230360	527.5	0.15
VEZT	ENSG00000028203	527	42.015
CHKA	ENSG00000110721	527	29.215
SLC25A42	ENSG00000181035	527	7.54
COL21A1	ENSG00000124749	527	0.94
SLC29A1	ENSG00000112759	526.5	56.435
TAOK2	ENSG00000149930	526.5	45.485
STAG3L2	ENSG00000160828	526.5	24.92
CEP70	ENSG00000114107	526.5	20.935
GTF2IRD2	ENSG00000196275	526.5	4.915
PHBP15	ENSG00000232210	526.5	2.26
UGT1A13P	ENSG00000234143	526.5	0.085
UGT1A10	ENSG00000242515	526.5	0.03
DUX4L19	ENSG00000258991	526.5	0
NCF1	ENSG00000158517	526.5	0
UGT1A1	ENSG00000242366	526.5	0
UGT1A2P	ENSG00000228445	526.5	0
UGT1A3	ENSG00000243135	526.5	0
UGT1A4	ENSG00000244474	526.5	0
UGT1A5	ENSG00000240224	526.5	0
UGT1A6	ENSG00000167165	526.5	0
UGT1A7	ENSG00000244122	526.5	0
UGT1A8	ENSG00000241635	526.5	0
UGT1A9	ENSG00000241119	526.5	0
SHC1	ENSG00000160691	526	34.45
PYGO2	ENSG00000163348	526	33.355
C2orf76	ENSG00000186132	526	9.075
STXBP5-AS1	ENSG00000233452	526	0.22

NDUFS2	ENSG00000158864	525.5	124.495
SLC44A3	ENSG00000143036	525.5	2.88
TPM3P6	ENSG00000250731	525.5	0.705
ZNF813	ENSG00000198346	525.5	0.07
ALDOA	ENSG00000149925	525	1097.925
PDXDC1	ENSG00000179889	525	69.395
YLPM1	ENSG00000119596	525	65.18
RPS6KB1	ENSG00000108443	525	43.525
STC2	ENSG00000113739	525	27.055
ECE1	ENSG00000117298	525	25.095
RNFT1	ENSG00000189050	525	19.285
ZDHHC23	ENSG00000184307	525	8.945
LBX2	ENSG00000179528	525	3.37
UBE2CP2	ENSG00000265939	525	1.31
CD160	ENSG00000117281	525	1.245
PARP10	ENSG00000178685	525	1.02
DHX40P1	ENSG00000266992	525	0.345
DSCR8	ENSG00000198054	525	0.155
KCNJ15	ENSG00000157551	525	0.005
ARHGAP15	ENSG00000075884	525	0
DSCR10	ENSG00000233316	525	0
MTND6P11	ENSG00000226839	525	0
SLC5A10	ENSG00000154025	525	0
TBC1D3P1	ENSG00000238283	525	0
TBC1D3P1-DHX40P1	ENSG00000267104	525	0
SF3A1	ENSG00000099995	524.5	336.195
NRD1	ENSG00000078618	524.5	77.68
RSBN1	ENSG00000081019	524.5	16.245
KIF6	ENSG00000164627	524.5	2.885
C2orf27A	ENSG00000197927	524.5	2.255
DPRX	ENSG00000204595	524.5	0
GALNT13	ENSG00000144278	524	9.465
SETDB2	ENSG00000136169	524	4.935
BAALC	ENSG00000164929	524	1.12
PHBP4	ENSG00000230251	524	0.66
PGM5	ENSG00000154330	524	0.565
GPR173	ENSG00000184194	524	0
GUCY2F	ENSG00000101890	524	0
MIR3151	ENSG00000265657	524	0
PRG3	ENSG00000156575	524	0
SCEL	ENSG00000136155	524	0
SERPINB11	ENSG00000206072	524	0
ANKS1A	ENSG00000064999	523.5	17.145
ITGAL	ENSG00000005844	523.5	0.195
CAPSL	ENSG00000152611	523.5	0
DNAJB6	ENSG00000105993	523	165.245
LMNB1	ENSG00000113368	523	135.215

FBXO28	ENSG00000143756	523	38.72
MDGA2	ENSG00000139915	523	3.08
CPLX2	ENSG00000145920	523	1.155
ALOX5	ENSG00000012779	523	0.54
RPL13AP2	ENSG00000244053	523	0.255
SP7	ENSG00000170374	523	0
LARP4	ENSG00000161813	522.5	55.22
TEX10	ENSG00000136891	522.5	37.31
LRFN1	ENSG00000128011	522.5	15.525
FGD3	ENSG00000127084	522.5	2.225
CD44	ENSG00000026508	522.5	0.18
NOL10	ENSG00000115761	522	43.07
MRPS6	ENSG00000243927	522	34.17
FKBP14	ENSG00000106080	522	17.945
DBT	ENSG00000137992	522	10.765
SLC5A3	ENSG00000272962	522	6.715
ADAM8	ENSG00000151651	522	1.645
LINC00310	ENSG00000227456	522	0.785
NCF1C	ENSG00000165178	522	0.09
PACSIN2	ENSG00000100266	521.5	33.965
ARFGAP3	ENSG00000242247	521.5	31.6
ANKRD19P	ENSG00000187984	521.5	26.385
RPS6KA5	ENSG00000100784	521.5	8.625
ZNF484	ENSG00000127081	521.5	5.675
SNHG14	ENSG00000224078	521.5	0.555
CLDN4	ENSG00000189143	521.5	0.315
SNURF	ENSG00000273173	521.5	0.08
SNORD109B	ENSG00000239169	521.5	0
SNORD115-16	ENSG00000200757	521.5	0
SNORD115-19	ENSG00000199968	521.5	0
SNORD115-35	ENSG00000201992	521.5	0
SNORD115-47	ENSG00000212528	521.5	0
SNORD115-48	ENSG00000201634	521.5	0
SNORD115-5	ENSG00000200503	521.5	0
SNORD116-2	ENSG00000207001	521.5	0
SNORD116-25	ENSG00000252326	521.5	0
SNORD116-29	ENSG00000207245	521.5	0
SNORD116-3	ENSG00000207014	521.5	0
SNORD116-5	ENSG00000207191	521.5	0
SNORD116-7	ENSG00000207133	521.5	0
SNRPN	ENSG00000128739	521.5	0
STX18-AS1	ENSG00000247708	521	6.96
CDS1	ENSG00000163624	521	6.85
C14orf23	ENSG00000186960	521	0.705
MTX1P1	ENSG00000236675	521	0.7
THSD7A	ENSG00000005108	521	0.35
BCL2L14	ENSG00000121380	521	0.345

MUC7	ENSG00000171195	521	0.115
ZNF93	ENSG00000184635	521	0.1
CSNK1A1	ENSG00000113712	520.5	193.34
HUS1	ENSG00000136273	520.5	22.95
PRDM2	ENSG00000116731	520.5	21.355
GRID1	ENSG00000182771	520.5	2.605
SLC25A18	ENSG00000182902	520.5	0.975
PKD1L1	ENSG00000158683	520.5	0.135
MIR346	ENSG00000199104	520.5	0
TACC1P1	ENSG00000224920	520.5	0
UFL1-AS1	ENSG00000233797	520.5	0
COX5B	ENSG00000135940	520	180.295
HPCAL1	ENSG00000115756	520	19.545
MON2	ENSG00000061987	520	18.685
PEX7	ENSG00000112357	520	6.755
ZNF44	ENSG00000197857	520	2.19
FGD4	ENSG00000139132	520	1.745
CXCL17	ENSG00000189377	520	0.635
NOS1	ENSG00000089250	520	0.275
FCRL6	ENSG00000181036	520	0
TBC1D23	ENSG00000036054	519.5	20.095
ZSWIM5	ENSG00000162415	519.5	4.345
GCKR	ENSG00000084734	519.5	0.095
OSTCP5	ENSG00000234315	519.5	0
MED15	ENSG00000099917	519	53.225
PLEKHA1	ENSG00000107679	519	17.495
RGS14	ENSG00000169220	519	13.985
C10orf76	ENSG00000120029	519	12.015
TVP23C	ENSG00000175106	519	11.59
CDRT4	ENSG00000239704	519	9.465
SIPA1L2	ENSG00000116991	519	8.275
TVP23C-CDRT4	ENSG00000259024	519	0.625
PLVAP	ENSG00000130300	519	0.275
LRRC37A14P	ENSG00000215771	519	0.165
ACTN4	ENSG00000130402	518.5	175.825
TCF25	ENSG00000141002	518.5	104.94
ZFAND6	ENSG00000086666	518.5	56.035
ARHGAP11A	ENSG00000198826	518.5	51.285
STK4	ENSG00000101109	518.5	33.675
COG4	ENSG00000103051	518.5	17.375
C14orf159	ENSG00000133943	518.5	10.22
MC1R	ENSG00000258839	518.5	3.52
TUBB3	ENSG00000198211	518.5	1.445
SCNN1B	ENSG00000168447	518.5	0.11
ZNF282	ENSG00000170265	518	56.07
DRAM1	ENSG00000136048	518	1.1
MARK3	ENSG00000075413	517.5	60.155

SEL1L3	ENSG00000091490	517.5	6.655
SPEF1	ENSG00000101222	517.5	0.635
THSD7B	ENSG00000144229	517.5	0
FXN	ENSG00000165060	517	63.34
METTL9	ENSG00000197006	517	47.39
CASC16	ENSG00000249231	516.5	0
COX6A1	ENSG00000111775	516	371.22
WLS	ENSG00000116729	516	89.445
GATC	ENSG00000257218	516	52.135
TTLL11	ENSG00000175764	516	8.225
EPHB2	ENSG00000133216	516	7.44
SLC1A1	ENSG00000106688	516	5.44
CTBP2P8	ENSG00000234383	516	1.64
GNG12-AS1	ENSG00000232284	516	1.37
TTLL11-IT1	ENSG00000237548	516	0.18
SIGMAR1	ENSG00000147955	515.5	217.825
EDRF1	ENSG00000107938	515.5	32.07
MARCH8	ENSG00000165406	515.5	13.735
TMEFF2	ENSG00000144339	515.5	0.99
XCR1	ENSG00000173578	515.5	0.09
NCF1B	ENSG00000182487	515.5	0
CLPTM1	ENSG00000104853	515	112.14
PCMT1	ENSG00000120265	515	109.64
SDHD	ENSG00000204370	515	50.81
QSER1	ENSG00000060749	515	49.14
ZDBF2	ENSG00000204186	515	7.94
FRMD4B	ENSG00000114541	515	4.335
C2orf48	ENSG00000163009	515	1.865
BCO2	ENSG00000197580	515	1.145
IL18	ENSG00000150782	515	0.27
TEX12	ENSG00000150783	515	0.085
RBM43P1	ENSG00000239263	515	0.06
MRPS36P4	ENSG00000255231	515	0
RPL7P42	ENSG00000241825	515	0
DTNA	ENSG00000134769	514.5	31.21
MFSD8	ENSG00000164073	514.5	10.1
NKAIN1	ENSG00000084628	514.5	3.22
FAM26D	ENSG00000164451	514.5	0
LRRC52	ENSG00000162763	514.5	0
UBE2K	ENSG00000078140	514	81.235
ASF1B	ENSG00000105011	514	59.84
TPMT	ENSG00000137364	514	10.505
GPSM2	ENSG00000121957	513.75	20.115
DNAJC7	ENSG00000168259	513.5	157.15
C1orf159	ENSG00000131591	513.5	23.255
HCAR1	ENSG00000196917	513.5	0.19
HCAR2	ENSG00000182782	513.5	0.045

HCAR3	ENSG00000255398	513.5	0.045
PPP1R10	ENSG00000204569	513	96.64
DIS3	ENSG00000083520	513	96.63
DLC1	ENSG00000164741	513	23.83
PPARA	ENSG00000186951	513	7.115
BAI1	ENSG00000181790	513	3.47
FBXL13	ENSG00000161040	513	0.74
ABCD3	ENSG00000117528	512.5	41.91
PDP1	ENSG00000164951	512.5	11.005
ARID5B	ENSG00000150347	512.5	9.345
GRTP1	ENSG00000139835	512.5	6.06
LINC00537	ENSG00000232815	512.5	2.36
PNPLA6	ENSG00000032444	512	30.33
STEAP3	ENSG00000115107	512	20.52
MCOLN1	ENSG00000090674	512	17.59
CALCRL	ENSG00000064989	512	0.53
IL21R-AS1	ENSG00000259954	512	0.095
GAPDHP59	ENSG00000231739	512	0
TAF2	ENSG00000064313	511.5	44.12
SMARCAL1	ENSG00000138375	511.5	19.815
SLC25A33	ENSG00000171612	511.5	9.525
EPB41L4B	ENSG00000095203	511.5	4.45
PHLPP1	ENSG00000081913	511	13.04
TBC1D30	ENSG00000111490	511	4.005
PART1	ENSG00000152931	511	0.02
PRRC2C	ENSG00000117523	510.5	191.71
KPNA4	ENSG00000186432	510.5	77.495
CCDC6	ENSG00000108091	510.5	29.965
MAGI3	ENSG00000081026	510.5	18.38
PPIL6	ENSG00000185250	510.5	2.515
ZMYND8	ENSG00000101040	510	22.55
SOBP	ENSG00000112320	510	11.935
CPEB4	ENSG00000113742	510	9.675
LRRC16A	ENSG00000079691	510	6.16
LDHC	ENSG00000166796	510	3.9
MOB3B	ENSG00000120162	510	3.8
SYTL4	ENSG00000102362	510	2.93
NOTCH4	ENSG00000204301	510	0.565
GPSM3	ENSG00000213654	510	0.215
RN7SKP197	ENSG00000252886	510	0
ZNF366	ENSG00000178175	510	0
ACP1	ENSG00000143727	509.5	144.92
RNF150	ENSG00000170153	509.5	7.815
FBN1	ENSG00000166147	509.5	4.505
CRX	ENSG00000105392	509.5	0.115
H2BFWT	ENSG00000123569	509.5	0.07
TPRX2P	ENSG00000259009	509.5	0.065

CYP2C19	ENSG00000165841	509.5	0.05
CYP2C18	ENSG00000108242	509.5	0
PAX6	ENSG00000007372	509	42.335
ZC3HAV1	ENSG00000105939	509	38.01
PRKCZ	ENSG00000067606	509	20.045
WDR37	ENSG00000047056	509	8.07
EMR4P	ENSG00000268758	509	0
ATP1A3	ENSG00000105409	508.5	10.29
FAM20C	ENSG00000177706	508.5	6.14
SH2D4B	ENSG00000178217	508.5	0.02
CD96	ENSG00000153283	508.5	0
SPECC1L	ENSG00000100014	508	56.705
GPATCH8	ENSG00000186566	508	39.415
TUBBP5	ENSG00000159247	508	9.805
EML5	ENSG00000165521	508	8.97
ADORA2A	ENSG00000128271	508	3.71
SPECC1L-ADORA2A	ENSG00000258555	508	1.835
RMST	ENSG00000255794	508	0
RBMS1	ENSG00000153250	507.75	25.57
PPP1CC	ENSG00000186298	507.5	219.125
WDR83OS	ENSG00000105583	507.5	88.085
MAN2B1	ENSG00000104774	507.5	35.72
STAG1	ENSG00000118007	507.5	25.015
KCTD7	ENSG00000243335	507.5	19.095
RABGEF1	ENSG00000154710	507.5	8.745
PAH	ENSG00000171759	507.5	0.015
GRIN2B	ENSG00000273079	507.5	0
RPS17L	ENSG00000182774	507	1635.125
TYK2	ENSG00000105397	507	67.465
CCNG2	ENSG00000138764	507	16.55
TMEM161B	ENSG00000164180	507	14.455
VWA8	ENSG00000102763	507	10.805
PHLDB2	ENSG00000144824	507	5.46
CPEB1	ENSG00000214575	507	2.87
PLCXD2	ENSG00000240891	507	2.27
COL6A4P2	ENSG00000228252	507	0.47
COL22A1	ENSG00000169436	507	0.115
OTUD7B	ENSG00000163113	506.5	16.255
CCDC180	ENSG00000197816	506.5	5.15
CCDC60	ENSG00000183273	506.5	0
DENND4B	ENSG00000198837	506	39.095
YEATS4	ENSG00000127337	506	35.63
NIPBL	ENSG00000164190	506	32.195
GPR107	ENSG00000148358	506	19.145
KIN	ENSG00000151657	506	15.92
KLC3	ENSG00000104892	506	12.25
C1orf95	ENSG00000203685	506	1.245

KRT18P31	ENSG00000249850	506	0.535
TMIGD2	ENSG00000167664	506	0.05
CNTN3	ENSG00000113805	506	0
KLHL1	ENSG00000150361	506	0
PSMC1P13	ENSG00000232314	506	0
PHF20	ENSG00000025293	505.5	23.17
ZNF143	ENSG00000166478	505.5	20.42
PCDH10	ENSG00000138650	505.5	16.565
CNRIP1	ENSG00000119865	505.5	8.73
THSD1	ENSG00000136114	505.5	1.125
KIAA2022	ENSG00000050030	505.5	0.265
UPB1	ENSG00000100024	505.5	0.15
COL28A1	ENSG00000215018	505.5	0.035
DDX50	ENSG00000107625	505	38.315
ASPN	ENSG00000106819	505	0.525
RFPL4B	ENSG00000251258	505	0
TBL1X	ENSG00000101849	504.5	31.85
PACS2	ENSG00000179364	504.5	23.185
BRF1	ENSG00000185024	504.5	21.805
ANGPT1	ENSG00000154188	504.5	0.2
CADPS	ENSG00000163618	504.5	0.16
EYA3	ENSG00000158161	504	46.88
CCDC150	ENSG00000144395	504	34.66
OSBPL8	ENSG00000091039	504	29.945
TRMT2B	ENSG00000188917	504	16.825
MBTD1	ENSG00000011258	504	12.67
FHL2	ENSG00000115641	504	9.955
FRMPD1	ENSG00000070601	504	0.72
LRRK1	ENSG00000154237	504	0.67
C17orf105	ENSG00000231256	504	0.05
FAM153C	ENSG00000204677	504	0
TRMT2B-AS1	ENSG00000225839	504	0
WSCD2	ENSG00000075035	504	0
C12orf75	ENSG00000235162	503.5	71.96
ATF6	ENSG00000118217	503.5	18.02
ITFG1	ENSG00000129636	503.5	16.365
ZBTB5	ENSG00000168795	503.5	10.59
ZNF708	ENSG00000182141	503.5	8.945
RNA5SP424	ENSG00000212290	503.5	0.515
SHROOM4	ENSG00000158352	503.5	0.195
LINC00393	ENSG00000224853	503.5	0
OR4K6P	ENSG00000228304	503.5	0
IGF2BP3	ENSG00000136231	503	49.22
C12orf76	ENSG00000174456	503	5.45
EPB41L2	ENSG00000079819	502.5	107.19
SLC25A26	ENSG00000144741	502.5	16.16
L2HGDH	ENSG00000087299	502.5	14.52

NT5M	ENSG00000205309	502.5	2.46
PROX1-AS1	ENSG00000230461	502.5	0.44
RPL26P3	ENSG00000240179	502.5	0.295
SLCO2B1	ENSG00000137491	502.5	0.19
OR2AT1P	ENSG00000254475	502.5	0
COL25A1	ENSG00000188517	502	10.025
GCNT2	ENSG00000111846	502	2.755
PPP1CA	ENSG00000172531	501.5	134.965
HACL1	ENSG00000131373	501.5	21.775
ETS1	ENSG00000134954	501.5	1.59
TBC1D10C	ENSG00000175463	501.5	0.715
RNF144A-AS1	ENSG00000228203	501.5	0.535
VWC2	ENSG00000188730	501.5	0.01
MTF2	ENSG00000143033	501	68.18
ARHGAP21	ENSG00000107863	501	27.775
RBMS3	ENSG00000144642	501	2.49
MAP2	ENSG00000078018	501	0.73
GRIA4	ENSG00000152578	501	0.16
CFI	ENSG00000205403	501	0.155
OR5D17P	ENSG00000181837	501	0
CDR2	ENSG00000140743	500.5	27.285
STPG1	ENSG00000001460	500.5	9.015
FAM110B	ENSG00000169122	500.5	6.33
RRN3P3	ENSG00000257122	500.5	3.415
CCL22	ENSG00000102962	500.5	0.06
BIN2	ENSG00000110934	500.5	0.03
LINC00578	ENSG00000228221	500.5	0
RPIA	ENSG00000153574	500	20.86
FRG1B	ENSG00000149531	500	16.405
BTN3A2	ENSG00000186470	500	7.685
NEBL	ENSG00000078114	500	5.95
KIFC3	ENSG00000140859	500	3.75
C10orf113	ENSG00000204683	500	0.115
TAGLN3	ENSG00000144834	500	0.07
DDX60L	ENSG00000181381	500	0.035
FBXO31	ENSG00000103264	499.5	24.495
C16orf95	ENSG00000260456	499.5	3.51
ATG3	ENSG00000144848	499	89.83
NCOR2	ENSG00000196498	499	60.88
CDK5RAP1	ENSG00000101391	499	34.19
CNTN1	ENSG00000018236	499	8.42
ANKRD44	ENSG00000065413	499	5.55
ADHFE1	ENSG00000147576	499	2.705
PRKCQ	ENSG00000065675	499	2.66
C8orf46	ENSG00000169085	499	0.81
ANKRD44-IT1	ENSG00000236977	499	0.415
MDN1	ENSG00000112159	498.5	35.63

LGI2	ENSG00000153012	498.5	1.3
DPY19L2P2	ENSG00000170629	498.5	0.54
NPHP3-AS1	ENSG00000248724	498.5	0.12
HDAC2	ENSG00000196591	498	230.72
DIAPH1	ENSG00000131504	498	68.685
IMPACT	ENSG00000154059	498	25.995
SIL1	ENSG00000120725	498	22.9
BRAF	ENSG00000157764	498	22
DFNB31	ENSG00000095397	498	11.03
CSRNP1	ENSG00000144655	498	3.135
LINC00351	ENSG00000226317	498	1.195
HTR2C	ENSG00000147246	498	0.095
MYOM3	ENSG00000142661	498	0.025
MIR1912	ENSG00000222321	498	0
TNS4	ENSG00000131746	498	0
IQCK	ENSG00000174628	497.75	5.45
SRCAP	ENSG00000080603	497.5	123.405
FKBP3	ENSG00000100442	497.5	108.445
PHKG2	ENSG00000156873	497.5	64.49
ATP8A1	ENSG00000124406	497.5	4.365
LPHN2	ENSG00000117114	497	39.205
POU3F3	ENSG00000198914	497	6.855
EVA1C	ENSG00000166979	497	2.705
CAPN6	ENSG00000077274	497	0.105
ST13P20	ENSG00000215875	497	0.06
HNRNPA3P14	ENSG00000224494	497	0
SPINT2	ENSG00000167642	496.5	37.625
PPP1R14A	ENSG00000167641	496.5	21.07
ZMYM1	ENSG00000197056	496.5	20.42
NOTO	ENSG00000214513	496.5	0.07
TNR	ENSG00000116147	496.5	0.03
TNR-IT1	ENSG00000235628	496.5	0
UBE2E2	ENSG00000182247	496	26.615
MBNL3	ENSG00000076770	496	14
ZKSCAN5	ENSG00000196652	496	12.72
WSCD1	ENSG00000179314	496	8.285
SRRM3	ENSG00000177679	496	2.1
TMEM163	ENSG00000152128	496	1.065
HIGD2A	ENSG00000146066	495.75	141.21
CLTB	ENSG00000175416	495.75	55.825
ARL10	ENSG00000175414	495.75	14.54
UQCR10	ENSG00000184076	495.5	190.53
UQCRC2	ENSG00000140740	495.5	174.435
ELF2	ENSG00000109381	495	31.75
PHIP	ENSG00000146247	495	27.65
C18orf25	ENSG00000152242	495	15.2
MOXD1	ENSG00000079931	495	12.155

IRAK1BP1	ENSG00000146243	495	5.74
CD300A	ENSG00000167851	495	0
SKP1	ENSG00000113558	494	150.07
PPP2CA	ENSG00000113575	494	116.545
EMC3	ENSG00000125037	494	75.845
SLC26A2	ENSG00000155850	494	7.7
CDKL3	ENSG00000006837	494	2.31
GPR114	ENSG00000159618	494	0
RPL23	ENSG00000125691	493.5	952.875
MRPS16	ENSG00000182180	493.5	70.76
DNAJC9	ENSG00000213551	493.5	40.95
FAM149B1	ENSG00000138286	493.5	19.11
SH3BP4	ENSG00000130147	493.5	11.24
PPP4R1L	ENSG00000124224	493.5	5.06
ARHGEF6	ENSG00000129675	493.5	5.03
MAOA	ENSG00000189221	493.5	2.115
EIF4A2P2	ENSG00000227382	493.5	0.89
ANGPT4	ENSG00000101280	493.5	0
RFC5	ENSG00000111445	493	93.585
EFTUD1	ENSG00000140598	492.5	27.71
DUXAP10	ENSG00000257227	492.5	24.605
CAMSAP3	ENSG00000076826	492.5	14.195
ATXN1	ENSG00000124788	492.5	10.65
DCDC1	ENSG00000170959	492.5	0.36
SP100	ENSG00000067066	492.5	0.225
ZNF347	ENSG00000197937	492.5	0.135
ACAN	ENSG00000157766	492.5	0.13
HMGB1P3	ENSG00000250011	492.5	0.105
ZNF415	ENSG00000170954	492.5	0
HEATR2	ENSG00000164818	492	60.095
REV3L	ENSG00000009413	492	18.005
VTI1A	ENSG00000151532	492	15.735
MIR4295	ENSG00000264763	492	1.665
OPN1LW	ENSG00000102076	492	0
CHCHD3	ENSG00000106554	491.5	104.76
ANXA2	ENSG00000182718	491.5	57.58
MIPOL1	ENSG00000151338	491.5	8.39
FAT2	ENSG00000086570	491.5	0.23
SNTG1	ENSG00000147481	491.5	0.135
TRIM29	ENSG00000137699	491.5	0
PARN	ENSG00000140694	491	37.405
KCND2	ENSG00000184408	491	0
ATP6V0A1	ENSG00000033627	490.5	65.065
PRMT7	ENSG00000132600	490.5	49.02
PIGU	ENSG00000101464	490.5	25.535
RAB27B	ENSG00000041353	490.5	0.135
MIR5010	ENSG00000265611	490.5	0

RPSAP57	ENSG00000267524	490.5	0
ATP5O	ENSG00000241837	490	207.61
NUP214	ENSG00000126883	490	50.28
DONSON	ENSG00000159147	490	46.55
ITSN1	ENSG00000205726	490	36.555
CRYZL1	ENSG00000205758	490	30.545
SPATA13	ENSG00000182957	490	6.005
CR2	ENSG00000117322	490	0.98
FCRL2	ENSG00000132704	490	0.055
C1QTNF9	ENSG00000240654	490	0.04
C18orf56	ENSG00000176912	489.5	1.8
PITPNM3	ENSG00000091622	489.5	1.305
CLUL1	ENSG00000079101	489.5	1.245
FARSB	ENSG00000116120	489	79.34
WIPI2	ENSG00000157954	489	56.535
SRGAP2	ENSG00000163486	489	41.385
MSANTD3	ENSG00000066697	489	26.39
TMEFF1	ENSG00000241697	489	7.705
MSANTD3-TMEFF1	ENSG00000251349	489	0
RBM42	ENSG00000126254	488.5	81.89
SQLE	ENSG00000104549	488.5	68.205
NUMB	ENSG00000133961	488.5	35.55
PTPLB	ENSG00000206527	488.5	29.6
MADD	ENSG00000110514	488.5	26.07
SLC18B1	ENSG00000146409	488.5	24.09
ALG2	ENSG00000119523	488.5	18.71
SYK	ENSG00000165025	488.5	6.695
ADCY1	ENSG00000164742	488.5	5.13
FMO11P	ENSG00000232148	488.5	0
RBM39	ENSG00000131051	488	315.32
CNOT1	ENSG00000125107	488	255.465
WWTR1	ENSG00000018408	488	16.045
WWTR1-IT1	ENSG00000241985	488	0.3
LINC00587	ENSG00000204250	488	0
MOGAT2	ENSG00000166391	488	0
CD59	ENSG00000085063	487.5	48.84
ARHGAP19	ENSG00000213390	487.5	15.675
ARHGAP19-SLIT1	ENSG00000269891	487.5	7.485
GTF2H5	ENSG00000272047	487.5	4.825
CYP39A1	ENSG00000146233	487.5	2.58
SLIT1	ENSG00000187122	487.5	0.725
GRIK2	ENSG00000164418	487.5	0.71
C11orf91	ENSG00000205177	487.5	0.495
KBTBD12	ENSG00000187715	487.5	0.045
SCN1A	ENSG00000144285	487.5	0.01
EIF4A1P9	ENSG00000262953	487.5	0
RNA5SP139	ENSG00000201210	487.5	0

RNF219	ENSG00000152193	487	29.8
NFXL1	ENSG00000170448	487	13.845
ASB1	ENSG00000065802	487	12.22
FRMD5	ENSG00000171877	487	3.43
PIN4P1	ENSG00000227973	487	2.18
ACTBP7	ENSG00000185607	487	0.34
CIT	ENSG00000122966	486.5	26.135
GPATCH2L	ENSG00000089916	486.5	18.13
MARC2	ENSG00000117791	486.5	18.075
UBAP2L	ENSG00000143569	486	257.71
RAB2A	ENSG00000104388	486	88.055
ZBTB38	ENSG00000177311	486	12.405
RADIL	ENSG00000157927	486	8.715
SEC31B	ENSG00000075826	486	7.535
SLC44A5	ENSG00000137968	486	4.32
MYO15B	ENSG00000266714	486	4.21
MYRIP	ENSG00000170011	486	0.35
NDUFB8	ENSG00000255339	486	0.3
PAPOLB	ENSG00000218823	486	0.26
BRCA2	ENSG00000139618	485.5	23.27
RAP1GAP2	ENSG00000132359	485.5	17.85
HRK	ENSG00000135116	485.5	2.985
SERTM1	ENSG00000180440	485.5	0.995
IFIT1P1	ENSG00000215515	485.5	0.69
HNRNPA3P12	ENSG00000219102	485.5	0.195
YBX1	ENSG00000065978	485	957.19
DVL2	ENSG00000004975	485	41.99
ATF7	ENSG00000170653	485	20.73
MTRF1	ENSG00000120662	485	10.95
COMMD10	ENSG00000145781	485	7.745
NEB	ENSG00000183091	485	2.955
FHAD1	ENSG00000142621	485	2.705
ALDH8A1	ENSG00000118514	485	1.135
DNAH3	ENSG00000158486	485	0.335
CACNG3	ENSG00000006116	485	0
MORC1	ENSG00000114487	485	0
ANKRD13A	ENSG00000076513	484.5	32.94
KIAA0319L	ENSG00000142687	484.5	32.805
TAF1	ENSG00000147133	484.5	24.385
GRIP1	ENSG00000155974	484.5	7.365
PTPRJ	ENSG00000149177	484.5	3.7
SLC10A1	ENSG00000100652	484.5	0.335
PPME1	ENSG00000214517	484	49.045
CCDC138	ENSG00000163006	484	41.735
SH3BGRL	ENSG00000131171	484	33.21
EIF4E3	ENSG00000163412	484	8.855
GPRIN3	ENSG00000185477	484	4.075

TICAM1	ENSG00000127666	484	2.595
ATP2B3	ENSG00000067842	484	0.405
TANK	ENSG00000136560	483.5	26.46
RNF38	ENSG00000137075	483.5	26.315
FOCAD	ENSG00000188352	483.5	25.345
THAP3	ENSG00000041988	483.5	17.69
MKL2	ENSG00000186260	483.5	16.215
ERGIC1	ENSG00000113719	483	67.795
OSBP2	ENSG00000184792	483	5.49
GPM6A	ENSG00000150625	483	5.145
PFKFB4	ENSG00000114268	483	4.16
RIMKLA	ENSG00000177181	483	2.37
EIF4HP2	ENSG00000237977	483	1.445
UCN2	ENSG00000145040	483	0.515
ITGAE	ENSG00000083457	482.5	32.25
GALNT7	ENSG00000109586	482.5	15.725
SNAPC3	ENSG00000164975	482.5	14.97
NSUN6	ENSG00000241058	482.5	12.555
ARL5B-AS1	ENSG00000152487	482.5	0.625
MYO1A	ENSG00000166866	482.5	0.265
TAC3	ENSG00000166863	482.5	0.115
MIR548T	ENSG00000221296	482.5	0
PLEKHB2	ENSG00000115762	482	35.17
ST5	ENSG00000166444	482	17.805
SLC13A3	ENSG00000158296	482	5.74
SLC2A12	ENSG00000146411	482	1.685
A3GALT2	ENSG00000184389	482	0.125
CYP4F31P	ENSG00000178206	482	0
FAR2P4	ENSG00000231431	482	0
POTEE	ENSG00000188219	482	0
MAX	ENSG00000125952	481.5	87.605
HSF1	ENSG00000185122	481.5	81.515
CADM1	ENSG00000182985	481.5	40.9
SAP130	ENSG00000136715	481.5	40.44
FAM21A	ENSG00000099290	481.5	31.47
DRD5P1	ENSG00000184303	481.5	0.07
MTRNR2L2	ENSG00000271043	481	7534.3
DHFR	ENSG00000228716	481	175.12
CNIH4	ENSG00000143771	481	80.29
POP1	ENSG00000104356	481	28.345
EZH1	ENSG00000108799	481	15.67
WBP1L	ENSG00000166272	481	10.99
MIR663B	ENSG00000221288	480.5	614.805
ETF1	ENSG00000120705	480.5	113.19
SRPK2	ENSG00000135250	480.5	35.835
NRF1	ENSG00000106459	480.5	17.98
RNGTT	ENSG00000111880	480.5	16.685

INPP5A	ENSG00000068383	480.5	15.725
PAAF1	ENSG00000175575	480.5	12.705
ANKRD30BL	ENSG00000163046	480.5	1.26
ASNSP1	ENSG00000248498	480.5	0.67
RNA5-8SP5	ENSG00000202174	480.5	0
ANKRD13C	ENSG00000118454	480	31.975
PLEKHM1	ENSG00000225190	480	14.655
SASH1	ENSG00000111961	480	5.445
CYP51A1P3	ENSG00000215223	480	0.085
ATP10B	ENSG00000118322	480	0.06
CEACAM7	ENSG00000007306	480	0
C9orf156	ENSG00000136932	479.5	17.355
HGSNAT	ENSG00000165102	479.5	12.905
KIAA1467	ENSG00000084444	479.5	11.405
DENND2C	ENSG00000175984	479.5	5.55
GSG1	ENSG00000111305	479.5	0.495
CCDC129	ENSG00000180347	479.5	0.04
ASAP1	ENSG00000153317	479	38.955
NCOA2	ENSG00000140396	479	19.985
CEPT1	ENSG00000134255	478.5	38.82
RALGAPA1	ENSG00000174373	478.5	22.705
CPSF1P1	ENSG00000214076	478.5	3.445
ADAMTS7	ENSG00000136378	478.5	2.015
SLC5A4	ENSG00000100191	478.5	0.92
LINC00383	ENSG00000237534	478.5	0
WDR18	ENSG00000065268	478	57.53
MCM8	ENSG00000125885	478	30.625
SULT2B1	ENSG00000088002	478	0.495
GBP5	ENSG00000154451	478	0.06
GATS	ENSG00000239521	477.75	9.61
QRICH1	ENSG00000198218	477.5	98.495
GEMIN6	ENSG00000152147	477.5	35.105
BMS1	ENSG00000165733	477.5	33.57
EEFSEC	ENSG00000132394	477.5	18.235
FCHO1	ENSG00000130475	477.5	7.58
BMP1	ENSG00000168487	477.5	5.765
VAV3	ENSG00000134215	477.5	2.675
ZP3	ENSG00000188372	477.5	1.665
CGNL1	ENSG00000128849	477.5	1.645
SRCRB4D	ENSG00000146700	477.5	0.72
CHD6	ENSG00000124177	477	22.325
KCNQ3	ENSG00000184156	477	0.005
DLGAP2	ENSG00000198010	477	0
MLPH	ENSG00000115648	477	0
RNF2	ENSG00000121481	476.5	54
EPB41L1	ENSG00000088367	476.5	26.255
MTFR2	ENSG00000146410	476.5	18.625

CIB2	ENSG00000136425	476.5	17.28
ZNF554	ENSG00000172006	476.5	2.285
PSMD5-AS1	ENSG00000226752	476.5	0.305
DNAJC9-AS1	ENSG00000236756	476.5	0.215
RAB37	ENSG00000172794	476.5	0.075
RPL3P3	ENSG00000243388	476.5	0.05
DEC1	ENSG00000173077	476.5	0.025
OTX2-AS1	ENSG00000248550	476.5	0
MOB3A	ENSG00000172081	476	11.71
KAL1	ENSG00000011201	476	0
SNORD114-13	ENSG00000201247	476	0
SNORD114-15	ENSG00000201557	476	0
SNORD114-16	ENSG00000199914	476	0
SNORD114-20	ENSG00000202048	476	0
SNORD114-21	ENSG00000272344	476	0
SNORD114-25	ENSG00000200612	476	0
CAV2	ENSG00000105971	475.75	8.44
SKA2	ENSG00000182628	475.5	78.3
UGGT1	ENSG00000136731	475.5	41.915
GNA12	ENSG00000146535	475.5	21.965
NBPF3	ENSG00000142794	475.5	14.19
C5orf66	ENSG00000224186	475.5	6.905
SH2D3A	ENSG00000125731	475.5	3.13
SYNM	ENSG00000182253	475.5	2.65
ZC2HC1C	ENSG00000119703	475.5	2.285
C15orf26	ENSG00000156206	475.5	0
ZMAT4	ENSG00000165061	475.5	0
EPS15	ENSG00000085832	475	33.965
MYEF2	ENSG00000104177	475	24.865
COL4A5	ENSG00000188153	475	15.285
AKTIP	ENSG00000166971	475	12.55
POC1B	ENSG00000139323	475	10.44
POC1B-GALNT4	ENSG00000259075	475	2.48
GALNT4	ENSG00000257594	475	1.83
TBP	ENSG00000112592	474.5	30.97
INPP5F	ENSG00000198825	474.5	5.2
CA12	ENSG00000074410	474.5	3.115
NUAK2	ENSG00000163545	474.5	0.525
LEMD1	ENSG00000186007	474.5	0
TMPRSS3	ENSG00000160183	474.5	0
RPL7AP33	ENSG00000254200	474	0.225
AXL	ENSG00000167601	474	0.15
ORC4	ENSG00000115947	473.5	45.645
TRIM37	ENSG00000108395	473.5	33.92
TNFSF9	ENSG00000125657	473.5	4.77
AGBL3	ENSG00000146856	473.5	3.37
REPS2	ENSG00000169891	473.5	1.18

WIF1	ENSG00000156076	473.5	0.32
LINC00970	ENSG00000203601	473.5	0.055
C12orf42	ENSG00000179088	473.5	0
CENPK	ENSG00000123219	473	45.39
MECR	ENSG00000116353	473	42.815
DENND4C	ENSG00000137145	473	20.135
TRAPPC13	ENSG00000113597	473	17.845
MET	ENSG00000105976	473	17.24
SESTD1	ENSG00000187231	473	17.14
TBC1D10A	ENSG00000099992	473	7.62
GATSL3	ENSG00000239282	473	3.21
CD109	ENSG00000156535	473	1.925
MYL10	ENSG00000106436	473	0
MICAL3	ENSG00000243156	472.5	33.97
ZNF461	ENSG00000197808	472.5	4.06
ZNF337-AS1	ENSG00000213742	472.5	3.05
FMNL1	ENSG00000184922	472.5	2.54
ARSE	ENSG00000157399	472.5	0.44
FSD2	ENSG00000186628	472.5	0.315
UCHL5	ENSG00000116750	472	134.735
TIMELESS	ENSG00000111602	472	56.64
MRPL1	ENSG00000169288	472	50.165
SNX1	ENSG00000028528	472	43.165
CAMK2D	ENSG00000145349	472	16.2
VSIG10	ENSG00000176834	472	12.58
PHF21B	ENSG00000056487	472	5.42
NBEA	ENSG00000172915	472	4.495
PHBP13	ENSG00000234835	472	1.105
RAD9B	ENSG00000151164	472	0.92
OR9A1P	ENSG00000237621	472	0.205
CCDC155	ENSG00000161609	472	0.03
KCNJ6	ENSG00000157542	472	0.005
CCR3	ENSG00000183625	472	0
TRA2B	ENSG00000136527	471.5	249.795
GOLIM4	ENSG00000173905	471.5	60.075
PFKP	ENSG00000067057	471.5	50.54
SETD3	ENSG00000183576	471.5	38.28
LYRM2	ENSG00000083099	471.5	32.58
BRD9	ENSG00000028310	471.5	26.865
ZDHHC11	ENSG00000188818	471.5	10.62
ANKRD6	ENSG00000135299	471.5	9.965
MALT1	ENSG00000172175	471.5	7.01
TRIM69	ENSG00000185880	471.5	5.045
ZDHHC11B	ENSG00000206077	471.5	4.01
SERPINE3	ENSG00000253309	471.5	1.52
IFI44L	ENSG00000137959	471.5	0.355
MRPL37P1	ENSG00000266946	471.5	0.14

SPTAN1	ENSG00000197694	471	59.765
INO80C	ENSG00000153391	471	48.39
TMEM120B	ENSG00000188735	471	25.93
PSMA2	ENSG00000256646	471	10.995
TRAPPC8	ENSG00000153339	470.5	45.91
KDM2B	ENSG00000089094	470.5	28.41
THEM4	ENSG00000159445	470.5	17.73
CCDC136	ENSG00000128596	470.5	15.335
GLTSCR1	ENSG00000063169	470.5	11.445
MLXIPL	ENSG00000009950	470.5	10.31
SEMA3C	ENSG00000075223	470.5	6.405
EHD2	ENSG00000024422	470.5	2.38
CCT3	ENSG00000163468	470	644.795
UQCC1	ENSG00000101019	470	45.265
GNS	ENSG00000135677	470	33.65
PHKB	ENSG00000102893	470	19.64
NEDD4L	ENSG00000049759	470	11.495
FBXL4	ENSG00000112234	470	6.98
MAPT-AS1	ENSG00000264589	470	0.16
RFPL1S	ENSG00000225465	470	0.15
PHB	ENSG00000167085	469.5	228.415
NDC1	ENSG00000058804	469.5	63.76
PTPRC	ENSG00000081237	469.5	0.145
RPH3A	ENSG00000089169	469.5	0
GPR89A	ENSG00000117262	469	30.48
ZNF555	ENSG00000186300	469	4
EDA	ENSG00000158813	469	2.815
GGT3P	ENSG00000197421	469	0
CTCF	ENSG00000102974	468.5	50.995
FAM135A	ENSG00000082269	468.5	17.57
EXD2	ENSG00000081177	468.5	13.475
RAB28	ENSG00000157869	468.5	12.82
PBLD	ENSG00000108187	468.5	4.68
SH3GL2	ENSG00000107295	468.5	1.89
TYRO3	ENSG00000092445	468.25	12.045
VPS52	ENSG00000223501	468	34.84
RNF213	ENSG00000173821	468	23.895
MTMR12	ENSG00000150712	468	17.485
NR6A1	ENSG00000148200	468	11.985
TSPAN5	ENSG00000168785	468	9.735
SPON2	ENSG00000159674	468	2.075
LINC00877	ENSG00000241163	468	0.41
SYNDIG1	ENSG00000101463	468	0
BTF3L4P2	ENSG00000213189	467.5	48.595
NCAM1	ENSG00000149294	467.5	19.695
TANC1	ENSG00000115183	467.5	11.675
TRAF3IP2	ENSG00000056972	467.5	9.635

TRAF3IP2-AS1	ENSG00000231889	467.5	8.77
ADCY10	ENSG00000143199	467.5	0.19
TRIM60	ENSG00000176979	467.5	0
TRAP1	ENSG00000126602	467	210.155
LIG1	ENSG00000105486	467	57.65
CREB1	ENSG00000118260	467	55.69
ATF6B	ENSG00000213676	467	43.84
SGTB	ENSG00000197860	467	18.01
METTL21A	ENSG00000144401	467	15.99
RGMB	ENSG00000174136	467	13.835
RASSF8	ENSG00000123094	467	13.54
C4orf36	ENSG00000163633	467	1.43
SETBP1	ENSG00000152217	467	0.665
TNXB	ENSG00000168477	467	0.55
SHISA6	ENSG00000188803	467	0
HADHA	ENSG00000084754	466.5	85.6
ZNF511	ENSG00000198546	466.5	62.845
AFF4	ENSG00000072364	466.5	49.015
SLC25A13	ENSG00000004864	466.5	42.255
EBF4	ENSG00000088881	466.5	6.435
EDNRA	ENSG00000151617	466.5	3.41
STXBP6	ENSG00000168952	466.5	1.815
GNGT1	ENSG00000127928	466.5	1.48
CCDC170	ENSG00000120262	466.5	0.59
GTF2F2P1	ENSG00000251254	466.5	0.29
PRAP1	ENSG00000165828	466.5	0.095
CLNK	ENSG00000109684	466.5	0
BMP4	ENSG00000125378	466.25	7.19
CPNE1	ENSG00000214078	466	73.82
C7orf73	ENSG00000243317	466	47.88
ABLIM3	ENSG00000173210	466	0.285
CASP12	ENSG00000204403	466	0.09
FAM180A	ENSG00000189320	466	0.04
