# Supplementary material for: Genes Possessing the Most Frequent DNA DSBs Are Highly Associated with Development and Cancers, and Essentially Overlap with the rDNA-Contacting Genes
Source: Int J Mol Sci. 2022 Jun 28;23(13):7201. doi: 10.3390/ijms23137201 (PMC9266645; doi:10.3390/ijms23137201)
Supplement: Supplementary file 1 [file ijms-23-07201-s001.zip › Table S8.pdf]

DSB point	TSS database	*z*	Correlation	*p* -value	Pairs	*z*	Correlation	*p* -value	Pairs	*z*	Correlation	*p* -value	Pairs
		100 kbp mean				Q1				Q1 + Q2 + Q3			
start	EPD.bi	-1.267		0.20510	87	2.979	strong corr	0.00289	41	10.683	strong corr	$<10^{-6}$	174
end	EPD.bi	-1.446		0.14828	85	3.144	strong corr	0.00166	36	9.038	strong corr	$<10^{-6}$	155
middle	EPD.bi	-1.866		0.06202	83	2.802	strong corr	0.00507	38	9.590	strong corr	$<10^{-6}$	161
start	EPD.uni	-6.248	strong anti	$<10^{-6}$	3848	-4.967	strong anti	$<10^{-6}$	8729	1.172		0.24118	10184
end	EPD.uni	-6.189	strong anti	$<10^{-6}$	3851	-5.013	strong anti	$<10^{-6}$	8727	1.372		0.16994	10177
middle	EPD.uni	-6.278	strong anti	$<10^{-6}$	3849	-5.168	strong anti	$<10^{-6}$	8730	0.700		0.48390	10185
start	GENCODE.bi	-2.739	strong anti	0.00617	115	4.488	strong corr	$<10^{-6}$	49	10.710	strong corr	$<10^{-6}$	208
end	GENCODE.bi	-2.675	strong anti	0.00747	113	4.569	strong corr	$<10^{-6}$	50	12.248	strong corr	$<10^{-6}$	208
middle	GENCODE.bi	-3.082	strong anti	0.00205	113	3.137	strong corr	0.00171	43	11.395	strong corr	$<10^{-6}$	207
start	GENCODE.uni	-4.711	strong anti	$<10^{-6}$	3501	-4.262	strong anti	0.00002	6529	1.653		0.09832	7337
end	GENCODE.uni	-4.753	strong anti	$<10^{-6}$	3505	-4.460	strong anti	$<10^{-6}$	6538	1.935		0.05305	7334
middle	GENCODE.uni	-4.768	strong anti	$<10^{-6}$	3504	-4.510	strong anti	$<10^{-6}$	6533	1.844		0.06520	7342
start	RefSeq.bi	-2.455	anti	0.01408	139	5.127	strong corr	$<10^{-6}$	43	12.987	strong corr	$<10^{-6}$	244
end	RefSeq.bi	-2.237	anti	0.02529	139	5.119	strong corr	$<10^{-6}$	45	13.653	strong corr	$<10^{-6}$	244
mid	RefSeq.bi	-2.581	strong anti	0.00984	137	4.078	strong corr	0.00005	39	13.825	strong corr	$<10^{-6}$	248
start	RefSeq.uni	-4.640	strong anti	$<10^{-6}$	3872	-5.136	strong anti	$<10^{-6}$	7935	1.497		0.13444	9035
end	RefSeq.uni	-4.608	strong anti	$<10^{-6}$	3874	-5.131	strong anti	$<10^{-6}$	7940	1.538		0.12402	9040
middle	RefSeq.uni	-4.619	strong anti	$<10^{-6}$	3875	-5.192	strong anti	$<10^{-6}$	7939	1.338		0.18086	9044

100kbp mean: DSB subset with the alignment depth threshold corresponding to the mean domain (the stretches between DSB) length 100kbp.

Q1: DSB top quartile (25%) by alignment depth subset.

Q1+Q2+Q3: DSB top three quartiles (75%) by alignment depth subset.

Red background: anticorrelation, points tends to be as far as possible from each other.

Green background: correlation, points tends to be as close as possible to each other.
